# Safety, tolerability, and immunogenicity of influenza vaccination with a high-density microarray patch: Results from a randomized, controlled phase I clinical trial

**DOI:** 10.1371/journal.pmed.1003024

**Published:** 2020-03-17

**Authors:** Angus H. Forster, Katey Witham, Alexandra C. I. Depelsenaire, Margaret Veitch, James W. Wells, Adam Wheatley, Melinda Pryor, Jason D. Lickliter, Barbara Francis, Steve Rockman, Jesse Bodle, Peter Treasure, Julian Hickling, Germain J. P. Fernando

**Affiliations:** 1 Vaxxas Pty Ltd, Brisbane, Queensland, Australia; 2 The University of Queensland Diamantina Institute, Faculty of Medicine, The University of Queensland, TRI, Brisbane, Queensland, Australia; 3 Department of Microbiology and Immunology, University of Melbourne, at The Peter Doherty Institute for Infection and Immunity, Melbourne, Victoria, Australia; 4 360biolabs, Melbourne, Victoria, Australia; 5 Nucleus Network Pty Ltd, Melbourne, Victoria, Australia; 6 Avance Clinical Pty Ltd, Thebarton, South Australia, Australia; 7 Seqirus Pty Ltd, Parkville, Victoria, Australia; 8 Peter Treasure Statistical Services Ltd, Kings Lynn, United Kingdom; 9 Working in Tandem Ltd, Cambridge, United Kingdom; 10 The University of Queensland, School of Chemistry & Molecular Biosciences, Faculty of Science, Brisbane, Queensland, Australia; Epicentre, FRANCE

## Abstract

**Background:**

The Vaxxas high-density microarray patch (HD-MAP) consists of a high density of microprojections coated with vaccine for delivery into the skin. Microarray patches (MAPs) offer the possibility of improved vaccine thermostability as well as the potential to be safer, more acceptable, easier to use, and more cost-effective for the administration of vaccines than injection by needle and syringe (N&S). Here, we report a phase I trial using the Vaxxas HD-MAP to deliver a monovalent influenza vaccine that was to the best of our knowledge the first clinical trial to evaluate the safety, tolerability, and immunogenicity of lower doses of influenza vaccine delivered by MAPs.

**Methods and findings:**

HD-MAPs were coated with a monovalent, split inactivated influenza virus vaccine containing A/Singapore/GP1908/2015 H1N1 haemagglutinin (HA). Between February 2018 and March 2018, 60 healthy adults (age 18–35 years) in Melbourne, Australia were enrolled into part A of the study and vaccinated with either: HD-MAPs delivering 15 μg of A/Singapore/GP1908/2015 H1N1 HA antigen (A-Sing) to the volar forearm (FA); uncoated HD-MAPs; intramuscular (IM) injection of commercially available quadrivalent influenza vaccine (QIV) containing A/Singapore/GP1908/2015 H1N1 HA (15 μg/dose); or IM injection of H1N1 HA antigen (15 μg/dose). After 22 days’ follow-up and assessment of the safety data, a further 150 healthy adults were enrolled and randomly assigned to 1 of 9 treatment groups. Participants (20 per group) were vaccinated with HD-MAPs delivering doses of 15, 10, 5, 2.5, or 0 μg of HA to the FA or 15 μg HA to the upper arm (UA), or IM injection of QIV. The primary objectives of the study were safety and tolerability. Secondary objectives were to assess the immunogenicity of the influenza vaccine delivered by HD-MAP. Primary and secondary objectives were assessed for up to 60 days post-vaccination. Clinical staff and participants were blind as to which HD-MAP treatment was administered and to administration of IM-QIV-15 or IM-A/Sing-15. All laboratory investigators were blind to treatment and participant allocation. Two further groups in part B (5 participants per group), not included in the main safety and immunological analysis, received HD-MAPs delivering 15 μg HA or uncoated HD-MAPs applied to the forearm. Biopsies were taken on days 1 and 4 for analysis of the cellular composition from the HD-MAP application sites.

The vaccine coated onto HD-MAPs was antigenically stable when stored at 40°C for at least 12 months. HD-MAP vaccination was safe and well tolerated; any systemic or local adverse events (AEs) were mild or moderate. Observed systemic AEs were mostly headache or myalgia, and local AEs were application-site reactions, usually erythema. HD-MAP administration of 2.5 μg HA induced haemagglutination inhibition (HAI) and microneutralisation (MN) titres that were not significantly different to those induced by 15 μg HA injected IM (IM-QIV-15). HD-MAP delivery resulted in enhanced humoral responses compared with IM injection with higher HAI geometric mean titres (GMTs) at day 8 in the MAP-UA-15 (GMT 242.5, 95% CI 133.2–441.5), MAP-FA-15 (GMT 218.6, 95% CI 111.9–427.0), and MAP-FA-10 (GMT 437.1, 95% CI 254.3–751.3) groups compared with IM-QIV-15 (GMT 82.8, 95% CI 42.4–161.8), *p =* 0.02, *p =* 0.04, *p <* 0.001 for MAP-UA-15, MAP-FA-15, and MAP-FA-10, respectively. Higher titres were also observed at day 22 in the MAP-FA-10 (GMT 485.0, 95% CI 301.5–780.2, *p =* 0.001) and MAP-UA-15 (367.6, 95% CI 197.9–682.7, *p =* 0.02) groups compared with the IM-QIV-15 group (GMT 139.3, 95% CI 79.3–244.5). Results from a panel of exploratory immunoassays (antibody-dependent cellular cytotoxicity, CD4^+^ T-cell cytokine production, memory B cell (MBC) activation, and recognition of non-vaccine strains) indicated that, overall, Vaxxas HD-MAP delivery induced immune responses that were similar to, or higher than, those induced by IM injection of QIV. The small group sizes and use of a monovalent influenza vaccine were limitations of the study.

**Conclusions:**

Influenza vaccine coated onto the HD-MAP was stable stored at temperatures up to 40°C. Vaccination using the HD-MAP was safe and well tolerated and resulted in immune responses that were similar to or significantly enhanced compared with IM injection. Using the HD-MAP, a 2.5 μg dose (1/6 of the standard dose) induced HAI and MN titres similar to those induced by 15 μg HA injected IM.

**Trial registration:**

Australian New Zealand Clinical Trials Registry (ANZCTR.org.au), trial ID 108 ACTRN12618000112268/U1111-1207-3550.

## Abstract 2

In a randomized, phase I clinical trial, Angus Forster and colleagues investigate the safety, tolerability, and immunogenicity associated with the use of a high-density microarray patch to deliver the influenza vaccine.

## Author summary

**Why was this study done?:**

- Microarray patches (MAPs) offer several advantages over injection by needle and syringe for administration of vaccines, including ease of use, greater acceptability, and vaccine thermostability.
- Preclinical studies with several different vaccines have shown that MAPs can induce comparable immune responses to injected vaccine but use less antigen (so-called ‘dose-sparing’).
- However, the dose-sparing potential of MAPs has not yet been evaluated or demonstrated in humans. This study was, to the best of our knowledge, the first clinical trial to evaluate the immunogenicity of lower doses of vaccine delivered by MAPs.

**What did the authors do and find?:**

- A randomised, partially blinded, placebo-controlled phase I clinical trial was conducted using a MAP with a high density of microprojections (HD-MAP) to deliver an influenza vaccine. Clinical staff and participants were blind as to which HD-MAP treatment was administered. All laboratory investigators were blind to treatment and participant allocation. The primary objectives of the trial were to assess safety and tolerability. The immunogenicity of the MAP-delivered vaccine was assessed as a secondary objective, using a number of different assays.
- Using the standard assay for assessing antibody responses to influenza vaccines (the HAI assay), HD-MAP-delivered vaccine at one-sixth of the standard dose induced a similar immune response to the full-dose of injected vaccine. When the full-dose was delivered by HD-MAP, higher titres were seen and at earlier time-points compared with the standard, injected influenza vaccine.
- Other immune parameters measured were at least as potent following HD-MAP delivery compared with the injected vaccine.

**What do these findings mean?:**

- Our results suggest that influenza vaccine delivered by HD-MAP was safe and well tolerated, with evidence to suggest that dose-sparing and enhanced immune responses are induced following HD-MAP delivery. The potential benefits of the technology suggest that further research and development of HD-MAPs is warranted.

## Introduction

Microarray patches (MAPs) are being developed by a number of parties as an alternative method for vaccine delivery [1–7]. MAPs have several potential advantages compared with injection by needle and syringe (N&S), including improved thermostability, ease of use and a reduced need for skilled healthcare workers for administration, greater acceptability by healthcare workers and recipients, avoidance of needle-stick injuries, and avoidance of the need for reconstitution [8,9]. In addition, in preclinical studies with a range of vaccine types, MAPs have been shown to enable dose-sparing, i.e., the induction of immune responses comparable to those obtained with injected vaccines but with lower doses of antigen [10–17]. These results are attributed to the targeted delivery of vaccine by MAPs to the dermis and epidermis of the skin, both of which are rich in the antigen-presenting cells required to initiate immune responses [10].

Clinical trials have been performed with several types of MAP including those with solid microprojections such as the Nanopatch [2,19] and MAPs with dissolving microprojections [3,7]. The Vaxxas high-density MAP (HD-MAP) differs from other MAPs in that it has a high-density array of solid microprojections (thousands per cm^2^) formed from medical grade polymer, and vaccine antigens are dispensed onto the tips of the projections and dried. In the appropriate formulation, vaccines coated onto MAPs have improved thermostability compared with the standard liquid formulation [18]. Initial clinical trials found the original Nanopatch to be safe, well tolerated, preferred by recipients to N&S, and able to induce immune responses at least as potent as intramuscular (IM) injection [2,19]. The Nanopatch MAPs used in these initial clinical trials were formed from dry-etched monocrystalline silicon and also had a high density of microprojections. However, HD-MAPs as used in this study, are prefabricated by injection-moulding of polymer, which means that large-scale commercial manufacture will be less expensive and able to meet seasonal influenza product release timelines.

In this report, we describe the first clinical trial to evaluate the safety and tolerability of Vaxxas HD-MAPs fabricated from polymer; this is also, to the best of our knowledge, the first clinical trial with any MAP to evaluate the immunogenicity of doses lower than the standard IM dose delivered by MAP. We used a monovalent influenza vaccine as a model antigen to assess the safety and tolerability of HD-MAPs as primary objectives of the study. In addition, serological and cell-mediated immune responses induced by HD-MAP delivery, compared with IM injection, were analysed as a secondary objective.

## Methods

### Trial participants and study design

The study was approved by the Bellberry Human Research Ethics Committee (Adelaide,Australia) and conducted in accordance with the Australian National Health and Medical Research Council’s National Statement of Ethical Conduct in Human Research (2007; incorporating all updates as at May 2015). Written informed consent was obtained from all participants. The trial was registered with Australian New Zealand Clinical Trials Registry (ANZCTR.org.au), trial ID ACTRN12618000112268/U1111-1207-3550. This study is reported as per the Consolidated Standards of Reporting Trials (CONSORT) guidelines (S1 Checklist).

The study was a two-part, randomised, partially double-blind, placebo-controlled trial conducted at Nucleus Network Pty Ltd (Melbourne, Australia). Clinical staff and participants were blind as to which MAP treatment was administered and to administration of IM-QIV-15 or IM-A/Sin-15. All laboratory investigators were blind to treatment and participant allocation. The primary objective was to measure the safety and tolerability of A/Singapore/GP1908/2015 H1N1 (A/Sing) monovalent vaccine delivered by HD- MAP in comparison to an uncoated HD-MAP and IM injection of a quadrivalent seasonal influenza vaccine (QIV) delivering approximately the same dose of A/Sing HA protein. Exploratory outcomes were to evaluate the immune responses to HD-MAP application to the forearm with A/Sing at 4 dose levels in comparison to IM administration of A/Sing at the standard 15 μg HA per dose per strain and to assess further measures of immune response through additional assays and assessment of the local skin response via punch biopsy of the HD-MAP application sites.

Because the primary objective was to assess safety and tolerability, the sample size was not based on any formal statistical calculations; this is typical of phase I vaccination studies. However, for this study, the 20 participants in a group would have an 80% probability of showing at least one adverse event if the true rate of that event is more than 8%, and, over the 160 participants receiving any MAP, there was an 80% probability of showing at least one adverse event if the true rate of that event is more than 1%. Randomisation was predetermined, and sealed participant-specific code break envelopes were produced by the statistician responsible for preparing the randomisation.

Part A of the trial was a ‘lead-in’ phase to indicate whether the safety, tolerability, and immunogenicity results seen with the HD-MAP were consistent with the results obtained previously with the silicon Nanopatch [2] before expanding into the larger study (part B).

**Fig 1:**
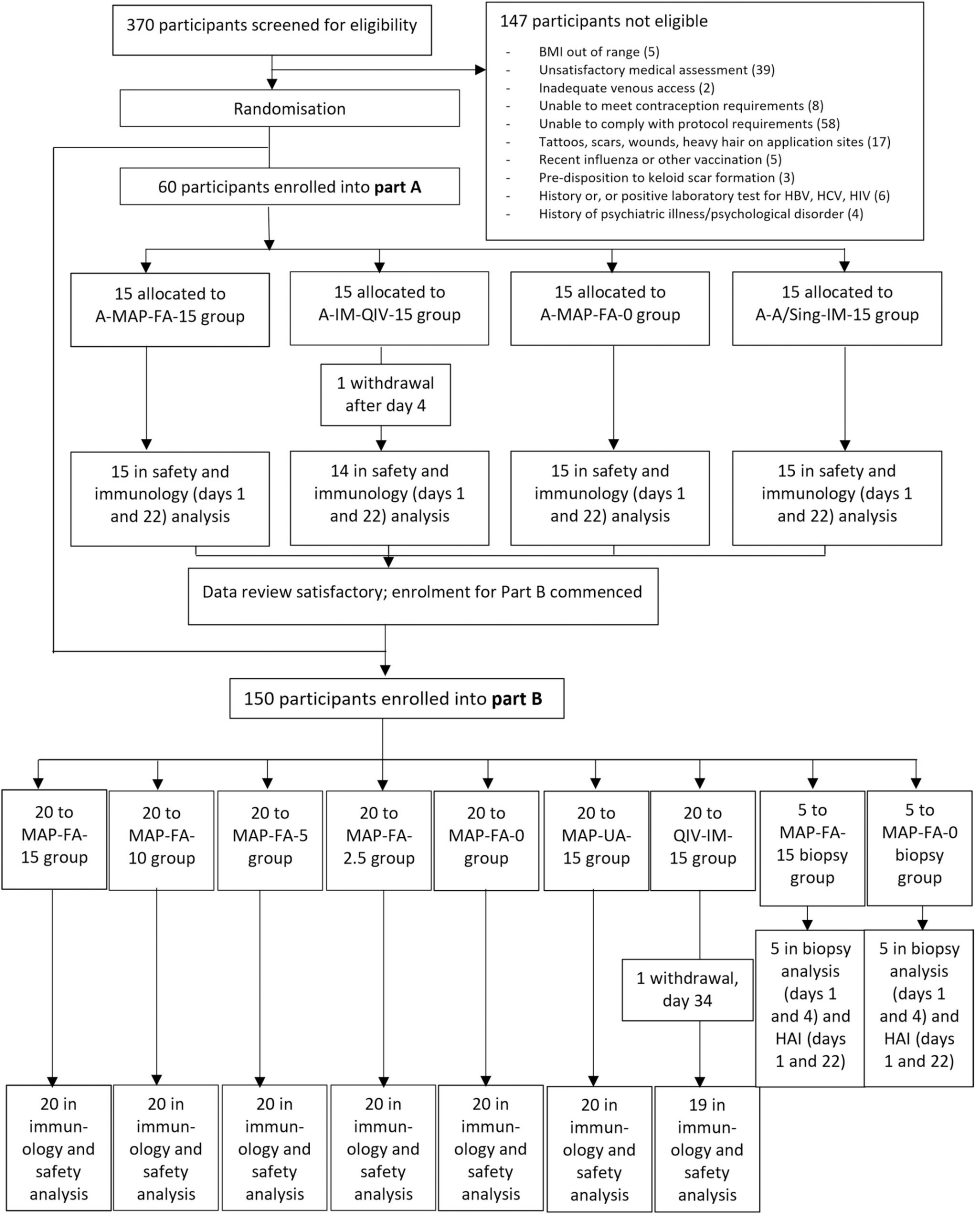
Trial profile. Randomisation and flow of participants in parts A and B of the study. Participants in part A were vaccinated with either A/Singapore/GP1908/2015 H1N1 delivered by HD-MAP (A-MAP-FA-15), IM injection of Afluria quadrivalent vaccine (A-IM-QIV-15), uncoated HD-MAP (A-MAP-FA-0), or A/Singapore/GP1908/2015 H1N1 monovalent pooled harvest injected IM (IM-SIN-15). Participants in part B were vaccinated with A/Singapore/GP1908/2015 H1N1 at 15, 10, 5, or 2.5 **μ**g HA/dose delivered by HD-MAPs applied to the volar forearm (MAP-FA-15, MAP-FA-10, MAP-FA-5, MAP-FA-2.5), uncoated HD-MAPs (MAP-FA-0), A/Singapore/GP1908/2015 H1N1 at 15 **μ**g HA/dose delivered by HD-MAP applied to the upper arm (MAP-UA-15), or injected IM with Afluria quadrivalent vaccine (IM-QIV-15). HA, haemagglutinin; HD-MAP, high-density microarray patch; IM, intramuscular.

Healthy males and females (nonpregnant and non-nursing) aged 18 to 50 years with a BMI in the range of 18 to 30 kg/m^2^ (*N =* 60) were recruited from the panel of volunteers at Nucleus Network, screened, and randomly allocated into 1 of 4 vaccination groups for part A of the trial. Participants (*N =* 150) were randomly allocated into 1 of 9 vaccination groups for part B (Fig 1). Participants were not screened or selected for on the basis of anti-H1N1 HAI titre. The demographic profile of the participants is provided in Table 1.

**Table 1:** Participant demographics.

**PART A**	**A-MAP-FA-15**	**A-IM-QIV-15**	**A-MAP-FA-0**	**A-IM-A/Sin-15**					
**Number**	15	15	15	15					
**Age, mean (SD)**	26.7 (8.0)	26.9 (7.1)	26.9 (6.6)	25.3 (6.8)					
**Age, range (years)**	18–44	20–42	19–42	18–38					
**Female, number (%)**	12 (80)	10 (67)	10 (67)	10 (67)					
**Male, number (%)**	3 (20)	5 (33)	5 (33)	3(33)					
**BMI (kg/m**^**2**^**), mean (SD)**	22.8 (2.9)	24.1 (2.4)	23.9 (3.9)	23.1 (3.0)					
**Race, number (%)**									
**Asian**	1 (7)	1 (7)	4 (27)	6 (40)					
**Black or African American**	1 (7)	0 (0)	0 (0)	1 (7)					
**White**	13 (87)	13 (87)	10 (67)	8 (53)					
**White, American Indian, or Alaskan Native**	0 (0)	0 (0)	1 (7)	0 (0)					
**White, Asian**	0 (0)	1 (7)	0 (0)	0 (0)					
**PART B**	**MAP-FA-15**	**MAP-FA-10**	**MAP-FA-5**	**MAP-FA-2.5**	**MAP-FA-0**	**MAP-UA-15**	**IM-QIV-15**	**MAP-FA-15-bio**	**MAP-FA-0-bio**
**Number**	20	20	20	20	20	20	20	5	5
**Age, mean (SD)**	27.4 (7.4)	26.1 (6.1)	26.5 (6.7)	24.7 (5.4)	26.2 (7.1)	27.4 (9.6)	24.2 (3.4)	39.6 (8.1)	29.6 (4.9)
**Age, range (years)**	18–43	18–40	19–45	18–36	18–43	19–48	19–32	31–50	24–37
**Female, number (%)**	13 (65)	16 (80)	11 (55)	14 (70)	13 (65)	11 (55)	14 (70)	4 (80)	3 (60)
**Male, number (%)**	7 (35)	4 (20)	9 (45)	6 (30)	7 (35)	9 (45)	6 (30)	1 (20)	2 (40)
**BMI (kg/m**^**2**^**), mean (SD)**	24.1 (2.6)	23.0 (3.4)	23.2 (3.5)	22.8 (2.5)	23.0 (3.0)	24.0 (3.1)	23.4 (3.1)	22.7 (2.6)	23.7 (2.1)
**Race, number (%)**									
**Asian**	3 (15)	7 (35)	8 (40)	5 (25)	5 (25)	4 (20)	6 (30)	0 (0)	0 (0)
**Asian, Aboriginal/Torres Strait Islander**	0 (0)	1 (5)	0 (0)	0 (0)	0 (0)	0 (0)	0 (0)	0 (0)	0 (0)
**Asian, Native Hawaiian or Pacific Islander**	0 (0)	0 (0)	0 (0)	0 (0)	0 (0)	1 (5)	0 (0)	0 (0)	0 (0)
**Black or African American**	0 (0)	0 (0)	0 (0)	0 (0)	0 (0)	1 (5)	0 (0)	0 (0)	0 (0)
**Other mixed race**	0 (0)	0 (0)	0 (0)	0 (0)	0 (0)	1 (5)	1 (5)	0 (0)	0 (0)
**White**	17 (85)	11 (55)	12 (60)	14 (70)	15 (75)	10 (50)	13 (65)	5 (100)	5 (100)
**White, Aboriginal/Torres Strait Islander**	0 (0)	0 (0)	0 (0)	0 (0)	0 (0)	1 (5)	0 (0)	0 (0)	0 (0)
**White, Asian**	0 (0)	1 (5)	0 (0)	1 (5)	0 (0)	1 (5)	0 (0)	0 (0)	0 (0)
**White, Black or African American, Asian**	0 (0)	0 (0)	0 (0)	0 (0)	0 (0)	1 (5)	0 (0)	0 (0)	0 (0)

**Abbreviations:** A/Sing, A/Singapore/GP1908/2015 H1N1; bio, biopsy; BMI, body mass index; FA, forearm; IM, intramuscular; MAP, microarray patch; QIV, quadrivalent influenza vaccine; UA, upper arm

The 4 treatment groups in part A were 15 μg HA per dose administered by HD-MAP applied to the volar surface of the forearm (A-MAP-FA-15), QIV (15 μg A/Sing HA per dose) injected IM into the deltoid muscle (A-IM-QIV-15), uncoated HD-MAPs applied to the volar surface of the forearm (A-MAP-FA-0), and monovalent purified harvest (MPH) containing 15 μg A/Sing HA injected IM (A-IM-A/Sin-15). The treatment groups for part B were HD-MAPs applied to the volar surface of the forearm delivering 15 (MAP-FA-15), 10 (MAP-FA-10), 5 (MAP-FA-5), or 2.5 μg HA (MAP-FA-2.5), uncoated HD-MAPs applied to the volar forearm (MAP-FA-0), HD-MAPs delivering 15 μg HA applied to the upper arm over the deltoid muscle (MAP-UA-15), and QIV (15 μg A/Sing HA per dose) injected IM (IM-QIV-15).

Two further groups (5 participants per group) were included in part B. These participants provided additional informed consent during screening and received HD-MAPs delivering 15 μg (MAP-FA-15-bio) or uncoated HD-MAPs (MAP-FA-0-bio) applied to the forearm. Biopsies were taken on days 1 and 4 for analysis of the cellular composition from the HD-MAP application sites. Results of these analyses will be presented elsewhere. These 10 participants were not included in the main safety and immunological analysis.

### Vaccines

Split influenza A/Sing MPH was supplied by Seqirus Pty Ltd (Australia). Sulfobutyl ether (β) cyclodextrin (SBECD) (Ligand, San Diego, CA) was added at a ratio of 4 to 1 (% w/w) HA protein to preserve antigen potency during coating and storage.

Influenza vaccines Afluria Quadrivalent 2017–2018 (lot XF 33608 expiry 30 June 2018) and Afluria Quad 2018 (lot 49601–00901 expiry February 2019) (Seqirus Pty Ltd) were used as IM controls in parts A and B of the trial, respectively. They contained A/Singapore/GP1908/2015 (A/Michigan/45/2015 [H1N1-like]); A/Hong Kong/4801/2014 (NYMC X-263B) (Afluria Quadrivalent 2017–2108) or A/Singapore/INFIMH-16-0019/2016 [H3N2-like] (Afluria Quad 2018); B/Phuket/3073/2013; and B/Brisbane/46/2015 [B/Brisbane/60/2008-like], all at a nominal 15 μg HA/dose.

### HD-MAP manufacture

HD-MAPs were manufactured by injection moulding of a polymer to produce HD-MAPs of 10 × 10 mm with approximately 3,136 projections per patch. Each projection was approximately 250 μm high, 120 μm wide at the base, and had a sharp point of < 25 μm. Vaccine was aseptically applied to the tips of gamma-irradiated (≥25 kGy; Steritech, Australia) HD-MAPs using a ‘Direct-jet’ process (Vaxxas Pty Ltd, Australia) that deposits individual droplets onto the tip of each projection.

HD-MAPs were produced to deliver 2 different doses of A/Sing, 2.5 μg and 5.0 μg (referred to as 2.5 μg and 5 μg HD-MAPs), as well as uncoated (placebo) HD-MAPs. The doses cited throughout this report refer to the estimated delivered dose. Preparatory studies using ex vivo and in vivo pig-skin assays determined the delivery efficiency of this MAP-vaccine combination to be approximately 50%; therefore, to deliver a 2.5 μg dose, 5 μg MPH was loaded onto each MAP. Following coating, HD-MAPs were placed into aluminium MediCan containers (Amcor, UK), foil-sealed, and stored at 2 to 8°C with desiccant until use. The antigen-coated HD-MAPs were used within 6 months of manufacture.

### Vaccination procedure

All HD-MAP participants in part A and part B had 3 HD-MAPs applied. Doses of 15, 10, and 5 μg HA per participant were achieved by applying three, two, or one 5 μg HD-MAPs plus 0, 1, or 2 uncoated HD-MAPs. Participants in the 2.5-μg HA group received a single 2.5-μg HD-MAP and 2 uncoated HD-MAPs. Although this approach means that the area of exposure to antigen varied, it had several advantages. This strategy simplified the manufacturing and product release procedures, because only 3 types of HD-MAP (5 μg, 2.5 μg, and uncoated) were required. It also greatly improved the precision of the 5, 10, and 15 μg doses and enabled within-participant comparison of the local skin response of antigen-loaded compared with uncoated HD-MAPs.

Participants were vaccinated on day 1, with HD-MAPs applied to the nondominant arm (where possible). Application sites were selected to be free from scarring, tattoos, skin conditions, sunburn, and heavy hair. The application process used was the same as described previously [2].The area for application was marked, swabbed using a 70% ethanol swab, and photographed. HD-MAPs were applied using a separate spring-powered applicator that generated an application speed of 20 m/s and left on the skin for 2 min before being removed. All applications were performed by trained study team members.

Participants were monitored by clinic safety assessment visits at days 1, 2 (part B only), 4, 8, 22, and 61 and phone calls at days 2 (part A only), 3, 36, and 50. On day 1, all vaccination sites were assessed at prevaccination, 10 min, 1 h, and 2 h after HD-MAP or IM administration. Photographs of the treatment sites were taken at every clinic review. Skin reactions were assessed for erythema and oedema, with scores combined to generate a skin irritation index (SII) as described previously [2,19]. Induration, tenderness, bruising, skin flaking, visibility, itching, and bleeding were also assessed. Pain scores were collected at 1 and 10 min, 1 and 2 h after the patch removal, and on subsequent visits, using a visual analogue scale with 0 = no pain; 5 = moderate pain; 10 = worst pain possible.

### Serology assays

Haemagglutination inhibition (HAI) assays were run on blinded serum samples collected on days 1, (prevaccination), 4, 8, 22, and 61 (360biolabs Pty Ltd, Australia). Samples were treated with receptor destroying enzyme (Denka Seiken Co Ltd, Japan) and adsorbed to washed, packed turkey red blood cells (TRBCs) (for 60 min at room temperature (RT). TRBCs were diluted to 1% v/v in PBS prior to testing. Two-fold serum dilutions starting from 1:5 were prepared, and 4 HA Units/25 μL of A/Singapore/GP1908/2015 virus (WHO Collaborating Centre, Australia) were added to each test well and incubated for 60 min at RT. TRBCs (25 μl, 0.5% v/v) were added and incubated for a further 60 min at RT. The HAI titre was the reciprocal of the highest dilution of the sera that completely inhibited agglutination of TRBC by the virus.

Microneutralisation (MN) assays (360biolabs Pty Ltd) were conducted on serum samples collected on days 1 and 22 as described by Wagner and colleagues [20], with the exception that 0.5% bovine serum albumin was used instead of 1% bovine serum albumin in the virus diluent. Briefly, samples were heat inactivated at 56°C for 30 min. Two-fold serum dilutions starting from 1:20 were prepared and 100 TCID_50_ of A/Singapore/GP1908/2009 virus (WHO Collaborating Centre, Australia) were added to each test. Prevention of infection of MDCK cells by the virus was tested using enzyme-linked immunosorbent assay (ELISA) detection of influenza nucleoprotein.

Antibodies capable of mediating antibody-dependent cellular cytotoxicity (ADCC) were assayed using an ELISA that detected the ability of immobilized A/Sing MPH-specific antibodies to cross-link soluble recombinant FcγRIIIA receptor dimers [21]. The assays were conducted at the Department of Microbiology and Immunology, University of Melbourne, Australia. Serum samples collected on days 1 and 22 from participants in groups MAP-FA-0, MAP-FA-15, MAP-UA-15, and IM-QIV-15 were tested. Briefly, 96-well Nunc Maxisorp plates (Thermofisher Scientific, Waltham, MA) were coated for 16 h at 4°C with 50 ng of A/Singapore/GP1908/2015 HA in PBS, washed with PBS + 0.05% Tween20 (PBST), and blocked with SuperBlock (Thermofisher Scientific) before addition of duplicate serially diluted serum samples (1:20–1:43,740). Plates were incubated at 37°C for 1 h then washed using PBST. An FcγRIIIA Val158 ectodomain biotin dimer (0.1 μg/mL) was added and incubated at 37°C for 1 h then washed using PBST. Antibody-FcγRIIIA complexes were detected using a 1:10,000 dilution of streptavidin-HRP (Thermofisher Scientific) and development with 3,3',5,5'-tetramethylbenzidine substrate (Sigma-Aldrich, St. Louis, MO). The reaction was stopped with 0.16M H_2_SO_4_, and absorbance was measured at 450 nm. Serum concentrations giving half-maximal signal (EC_50_) were determined using a fitted curve (4 parameter log regression) and GraphPad Prism (GraphPad Software, San Diego, CA, www.graphpad.com).

### Salivary IgA

Saliva samples were collected from participants in the MAP-FA-0, MAP-FA-15, MAP-UA-15, and IM-QIV-15 groups on days 1, 4, 8, and 22. Participants chewed on the cotton swab of a Salivette saliva collector (Sarstedt, France) for approximately 1 min. Following centrifugation, the supernatant (saliva) was stored at −80°C. Influenza specific immunoglobulin A (IgA) was detected by ELISA; assays were carried out at Vaxxas Pty Ltd. Specifically, saliva samples serially diluted in 4 mg/mL BSA in PBS (PBSA) were added to Nunc Maxisorp plates (Thermofisher Scientific) previously coated overnight with A/Singapore/GP1908/2015 HA MPH (60 μl per well at 2 μg/ml) and blocked with PBSA. The presence of A/Sing HA specific IgA was detected using HRP-conjugated goat anti-human polyclonal IgA (PA1-74395, Thermofisher Scientific) and ABTS substrate (Sera-Care, Milford, MA). The reaction was stopped with 1% SDS, and absorbance was measured at 405 nm.

### MBCs

Peripheral blood mononuclear cells (PBMCs) were collected and cryopreserved from participants in the MAP-FA-0, MAP-FA-15, MAP-UA-15, and IM-QIV-15 groups on days 1 and 22 and stored in liquid nitrogen until use. Assays were carried out at the Department of Microbiology and Immunology, University of Melbourne. Recombinant HA proteins for use as flow cytometry probes for A/Michigan/45/2015, A/New Caledonia/20/1999, and the stabilised H1N1 stem domain were derived as previously reported [22]. HA-specific B cells were identified within cryopreserved human PBMC by co-staining with HA probes conjugated to SA-PE, SA-APC, or SA-Ax488 (Thermofisher Scientific). Monoclonal antibodies for surface staining included CD19-ECD (J3-119) (Beckman Coulter, Indianapolis, IN), IgM-BUV395 (G20-127), CD21-BUV737 (B-ly4), IgD-Cy7PE (IA6-2), IgG-BV786 (G18-145) (BD Biosciences, USA), CD14-BV510 (M5E2), CD3-BV510 (OKT3), CD8a-BV510 (RPA-T8), CD16-BV510 (3G8), CD10-BV510 (HI10a), CD27-BV605 (O323) (Biolegend, San Diego, CA), and IgA-Vio450 (REA1014) (Miltenyi, Auburn, CA). Background B cells interacting with streptavidin were excluded by staining with SA-BV510 (BD Biosciences, San Jose, CA). Cell viability was assessed using Aqua Live/Dead amine-reactive dye (Thermofisher Scientific). Samples were collected using a BD Fortessa configured to detect 18 fluorochromes, and analysis was performed using FlowJo software version 9.5.2 (Becton Dickinson, Ashland, OR).

### Flow cytometry of T cells

Cytokine production by CD4^+^ and CD8^+^ T cells was assessed using a modification of the method described by Landry and colleagues [23]. Assays were carried out at the University of Queensland Diamantina Institute, Faculty of Medicine, Australia. PBMC were thawed, plated out at 1.5 × 10^6^ per well, and rested for 6 h. After washing, the PBMC were stimulated with either A/Sing MPH for 20 h (20 μg/ml) or for 6 h with a pool of overlapping synthetic peptides (17 amino acids long overlapping by 11 amino acids, 5 μg/ml) spanning the A/Sing HA sequence (Mimotopes Pty Ltd, Australia). Media only and PMA/ionomycin were used as negative and positive controls, respectively. Golgi blockers (monensin and brefeldin A) were added 5 h before the end of incubation. Cells were labelled with surface stains Live/Dead Aqua (for viability), anti-CD3 BV785, anti-CD4 FITC, and anti CD8 APC/Cy7 (all from Biolegend), and then fixed, permeabilised, and labelled with anti-interferon (IFN)-γ Ax647, anti-tumour necrosis factor (TNF)-α BV421, and anti-interleukin (IL)-2 PE (all from Biolegend). Samples were analysed on a Becton Dickinson LSR Fortessa X20 within 24 h of the last wash step. Approximately 500,000 events were acquired, and the raw data were analysed initially using FlowJo (to obtain percentage positive values for each cytokine) before using SPICE (http://exon.niaid.nih.gov/spice) software to analyse background-subtracted values.

### Thermostability

A/Sing-coated HD-MAPs were stored at 2 to 8°C, 25°C ± 2°C/60% ± 5% relative humidity (RH) and 40°C ± 2°C/60% ± 5% RH for 12 months. At the designated timepoints, the coating was eluted from the HD-MAPs in 1 mL elution buffer (0.041% w/w Hypromellose (Shin-Etsu Chemical Co Ltd, Japan), 0.0295% w/w trehalose dihydrate (Sigma Aldrich Corp., St. Louis, MO)) using water bath sonication at 20 to 28°C, and the potency of HA was determined by enzyme immunoassay [24].

### Statistical analysis

The increases in HAI titres and MN titres were compared between groups using both parametric (Student t test) and nonparametric (exact Mann-Witney test) methods. The significance level was assumed to be *p* < 0.05 (two tailed). Results from the parametric analysis are presented in the main text, tables, and figures. Results from the nonparametric analyses of HAI and MN titres are included in S1 Table and S2 Table. The proportions of participants seroprotected or seroconverted were compared between groups using a Pearson's chi-square test with continuity correction (SAS version 9.4, SAS Institute Inc., Cary, NC). As this was an early phase study, no adjustments were made for multiplicity for any of the analyses, including immunogenicity assays. For the ADCC and MBC response assays, all groups were compared using Kruskal Wallis nonparametric tests (with no corrections for multiple comparisons) and Dunn’s multiple comparison post-tests. Within-group comparisons of cytokine production by CD4^+^ cells at day 1 and day 22 were made using the Wilcoxon rank sum test (GraphPad Software).

## Results

### Stability of A/Sing HA on HD-MAPs at elevated temperatures

**Fig 2:**
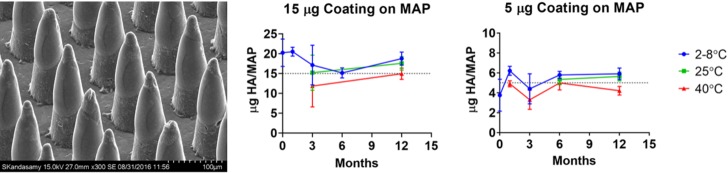
Coating and stability of A/Sing HA on HD-MAPs. The left-hand panel shows a scanning electron micrograph of HD-MAPs coated with A/Sing MPH. The coated vaccine is visible on the tips and top half of the projections. The graphs show stability of A/Sing HA on HD-MAPs coated with either 5 μg or 15 μg HA (in A/Sing MPH) indicated by the dotted line. Antigen-coated HD-MAPs were stored at 2 to 8°C, 25°C or 40°C at 60% ± 5% RH for 1, 3, 6, or 12 months. At each time point, antigen was eluted from the HD-MAP and the HA content determined by enzyme immunoassay. A/Sing, A/Singapore/GP1908/2015 H1N1; HA, haemagglutinin; HD-MAP, high-density microarray patch; RH, relative humidity.

To obtain stability data ahead of the clinical product manufacture, a GLP stability study was performed testing a 5 μg and 15 μg HA A/Sing loading on HD-MAPs. This loading range was selected to bracket the range of A/Sing HA loadings intended for use in the clinic. The A/Sing HA antigen coated at 5 μg or 15 μg per HD-MAP was stable when stored at 2 to 8°C, 25°C, or 40°C for at least 12 months (Fig 2).

### Participants and study procedures

Between 23 Feb 2018 and 23 March 2018, 60 participants (out of 370 screened) were enrolled into part A of the study and vaccinated as described above (Fig 1). The safety data and HAI responses from part A participants at days 1 and 22 (S1 Fig) were assessed by a panel of experts and judged to be acceptable and consistent with results obtained in the previous study with silicon Nanopatches [2], and the study proceeded to part B. For part B, 150 participants were enrolled between 20 April 2018 and 26 June 2018, randomly assigned to 1 of 9 treatment groups and vaccinated as described above (Fig 1).

### Summary of adverse events

For both part A and part B of the study there were no deaths, serious adverse events (SAEs), adverse events (AEs) with a severe intensity, or participant withdrawals due to AEs. No clinically significant events were observed with respect to clinical laboratory tests, vital signs, or oral temperature.

A total of 60 treatment emergent adverse events (TEAEs) were reported for part A in 37 participants (62%; Table 2). Of these, 31 events in 20 participants (33%) were considered possibly, probably, or definitely related to study treatment. Two of the treatment-related (TR)-TEAEs were of moderate severity. Both were headaches, one in the A-MAP-FA-0 group and one in the A-IM-QIV-15 group.

**Table 2:** TEAEs.

	Number (%) participants with TEAEs							
**PART A**	**MAP-FA-15 (*N* = 15)**	**IM-QIV-15 (*N* = 14)**	**MAP-FA-0 (*N* = 15)**	**IM-A/Sing-15 (*N* = 15)**	**Total**			
**TEAEs**	10 (67)	13 (87)	9 (60)	5 (33)	37 (62)			
**TR-TEAEs**	6 (40)	8 (53)	3 (20)	3 (20)	20 (33)			
**Mild TR-TEAEs**	6 (40)	7 (50)	2 (13)	3 (20)	18 (31)			
**Moderate TR-TEAEs**	0 (0)	1 (7)	1 (7)	0 (0)	2 (3)			
**Severe TR-TEAEs**	0 (0)	0 (0)	0 (0)	0 (0)	0 (0)			
**PART B**	**MAP-FA-15 (*N* = 20)**	**MAP-FA-10 (*N* = 20)**	**MAP-FA-5 (*N* = 20)**	**MAP-FA-2.5 (*N* = 20)**	**MAP-FA-0 (*N* = 20)**	**MAP-UA-15 (*N* = 20)**	**IM-QIV-15 (*N* = 20)**	**Total**
**TEAEs**	18 (90)	18 (90)	18 (90)	18 (90)	12 (60)	18 (90)	9 (45)	111 (79)
**TR-TEAEs**	18 (90)	17 (85)	16 (80)	15 (75)	3 (15)	18 (90)	2 (10)	89 (64)
**Mild TR-TEAEs**	18 (90)	15 (75)	15 (75)	12 (60)	3 (15)	18 (90)	2 (10)	83 (69)
**Moderate TR-TEAEs**	0 (0)	2 (10)	1 (5)	3 (15)	0 (0)	0 (0)	0 (0)	6 (4)
**Severe TR-TEAEs**	0 (0)	0 (0)	0 (0)	0 (0)	0 (0)	0 (0)	0 (0)	0 (0)

Number and severity of TR-AEs by treatment group for parts A and B, excluding biopsy groups.

**Abbreviations:** A/Sing, A/Singapore/GP1908/2015 H1N1; FA, forearm; IM, intramuscular; MAP, microarray patch; QIV, quadrivalent influenza vaccine; TEAE, treatment emergent adverse event; TR-AE, treatment-related adverse event; TR-TEAE, treatment related treatment emergent adverse event; UA, upper arm

In Part B nonbiopsy groups, a total of 235 TEAEs were reported in 111 participants (79%). Of these, 158 were considered related to study treatment; 6 were of moderate severity, and the remainder were mild. There were 3 moderate TR-TEAEs in the MAP-FA-2.5 group: headache, myalgia, and application site pruritis. There was 1 moderate event (headache) in the MAP-FA-5 group and 2 moderate events (headache and cervical lymphadenopathy) in the MAP-FA-10 group (Table 2). Of the 158 TR-TEAEs, 149 were related to the local skin response of the HD-MAP or IM injection. A total of 14 TEAEs were reported in the 2 biopsy groups with 8 considered related to study treatment. All these TR-TEAEs were of mild severity.

### Resolution of skin responses at the HD-MAP application site

A summary of application and injection site TEAEs is presented in Table 3. No cases of surface bleeding were noted in any of the 480 patch applications performed across the study. The frequency of local skin reactions was higher with A/Sing-coated HD-MAPs compared with uncoated HD-MAPs, but there was no apparent difference in skin responses at the 2 delivered dose variants of A/Sing-coated HD-MAP. There was a low incidence of tenderness and induration around the application site, with higher prevalence in the A/Sing HD-MAP groups. Itching scores were low and most frequent at day 4 with more itching reported for the A/Sing-coated HD-MAP applications than uncoated. One participant reported a moderate AE (itching) on day 1 for all 3 application sites (1 × 2.5 μg HD-MAP, 2 × uncoated HD-MAP) from 10 min to 1 h after application. Any skin flaking that was observed at the HD-MAP application sites peaked at day 8 and was similar between the active HD-MAP dose variants and the FA and UA sites, with the uncoated HD-MAPs exhibiting less skin flaking.

**Table 3:** Application and injection site TEAEs.

	Number (%) participants with local TEAEs							
**PART A**	**MAP-FA-15 (*N* = 15)**	**IM-QIV-15 (*N* = 14)**	**MAP-FA-0 (*N* = 15)**	**IM-A/Sing-15 (*N* = 15)**	**Total**			
**Application site visibility**	4 (27)	0 (0)	2 (13)	0 (0)	6 (10)			
**Application site erythema**	3 (20)	0 (0)	3 (20)	0 (0)	6 (10)			
**Application site exfoliation**	0 (0)	0 (0)	0 (0)	0 (0)	0 (0)			
**Application site oedema**	2 (13)	0 (0)	0 (0)	0 (0)	2 (3)			
**Application site pruritis**	1 (7)	0 (0)	1 (7)	0 (0)	2 (3)			
**Application site reaction**	1 (7)	0 (0)	0 (0)	0 (0)	1 (2)			
**Injection site discomfort**	0 (0)	0 (0)	0 (0)	0 (0)	0 (0)			
**Injection site pain**	0 (0)	0 (0)	0 (0)	2 (13)	2 (3)			
**Injection site pruritis**	0 (0)	0 (0)	0 (0)	1 (7)	1 (2)			
**PART B**	**MAP-FA-15 (*N* = 20)**	**MAP-FA-10 (*N* = 20)**	**MAP-FA-5 (*N* = 20)**	**MAP-FA-2.5 (*N* = 20)**	**MAP-FA-0 (*N* = 20)**	**MAP-UA-15 (*N* = 20)**	**IM-QIV-15 (*N* = 20)**	**Total**
**Application site visibility**	10 (50)	12 (60)	10 (50)	10 (50)	1 (5)	7 (35)	0 (0)	50 (36)
**Application site erythema**	10 (50)	7 (35)	9 (45)	7 (35)	0 (0)	9 (45)	0 (0)	42 (30)
**Application site exfoliation**	0 (0)	0 (0)	0 (0)	0 (0)	0 (0)	1 (5)	0 (0)	1 (1)
**Application site oedema**	1 (5)	0 (0)	1 (5)	3 (15)	0 (0)	1 (5)	0 (0)	6 (4)
**Application site pruritis**	0 (0)	0 (0)	1 (5)	1 (5)*	0 (0)	1 (5)	1 (5)	4 (3)
**Application site reaction**	4 (20)	6 (30)	3 (15)	4 (20)	0 (0)	5 (25)	0 (0)	22 (16)
**Injection site discomfort**	0 (0)	0 (0)	0 (0)	0 (0)	0 (0)	0 (0)	1 (5)	1 (1)
**Injection site pain**	0 (0)	0 (0)	0 (0)	0 (0)	0 (0)	0 (0)	1 (5)	1 (1)
**Injection site pruritis**	0 (0)	0 (0)	0 (0)	0 (0)	0 (0)	0 (0)	0 (0)	0 (0)

Number and severity of application and injection site TEAEs at all time points by treatment group for parts A and B, excluding biopsy groups. All events were mild, with the exception of *, which indicates a moderate event.

**Abbreviations:** A-Sing, A/Singapore/GP1908/2015 H1N1; FA, forearm; IM, intramuscular; MAP, microarray patch; QIV, quadrivalent influenza vaccine; TEAE, treatment emergent adverse event; UA, upper arm

**Fig 3:**
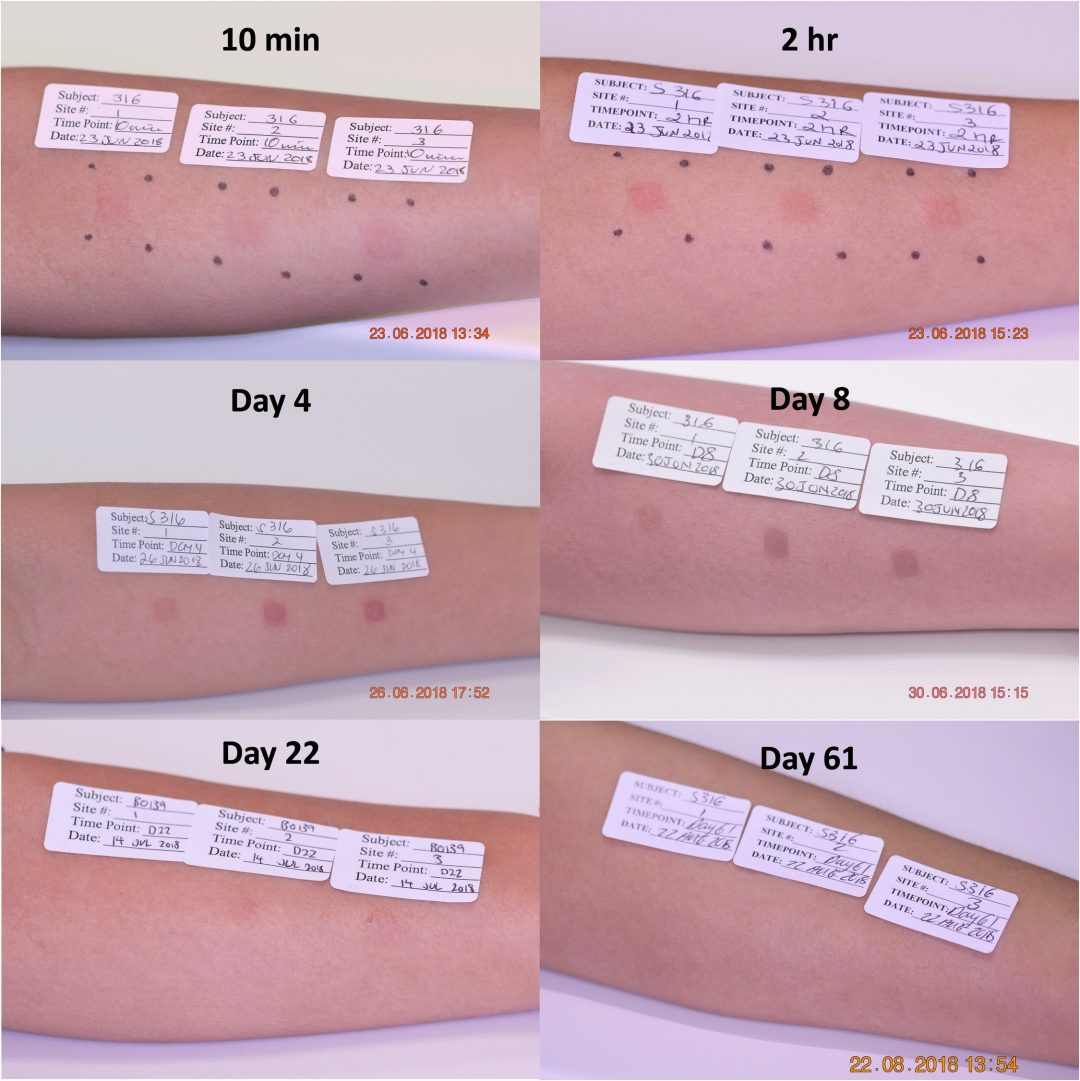
Representative images of skin reactions over time at HD-MAP application sites (single participant (S316/B0139), MAP-FA-10 group). Photographs show 3 HD-MAPs applied to adjacent sites on the forearm; site 1 (nearest the elbow crease) is the uncoated patch, and sites 2 and 3 are applications of the A/Sing coated HD-MAPs delivering 5 **μ**g HA into the skin per patch. Timepoints at 1 h and 24 h are not shown in the image sequence. Photo credit Nucleus Network Pty Ltd. A/Sing, A/Singapore/GP1908/2015 H1N1; FA, forearm; HA, haemagglutinin; HD-MAP, high-density microarray patch; MAP, microarray patch.

A typical example of application site reaction and resolution following application of two 5 μg and one uncoated HD-MAP to the forearm of a single participant is shown in Fig 3. The initial skin response over 2 h was similar between the active and uncoated HD-MAPs, with a defined erythema and occasional petechiae and oedema directly under all application sites. At days 4 and 8, there was less erythema at the uncoated HD-MAP application site compared with the active HD-MAP sites. At day 22, reactions to the uncoated HD-MAP were undetectable but were still just detectable in this participant at the active HD-MAP application site.

**Fig 4:**
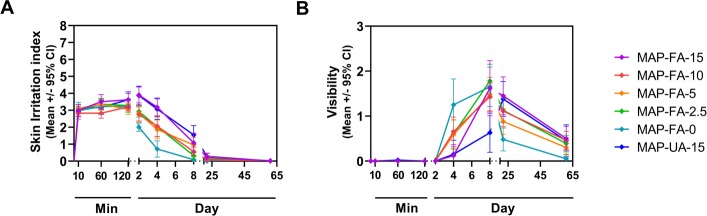
Time course of injection site reaction resolution. (A) SII, scale 0 to 8. (B) visibility (i.e., colouration at the application site), scale 0 to 3. Symbols represent mean scores ± 95% confidence intervals. Day, study day (HD-MAP applied on day 1); HD-MAP, high-density microarray patch; Min, minutes after HD-MAP application; SII, skin irritation index.

Average erythema scores for all 3 HD-MAP variants (5, 2.5, and 0 μg HA) were similar over the first 2 h of response, but erythema following A/Sing coated HD-MAP application peaked between 24 and 72 h for both the FA and UA sites, before resolving significantly by day 8. The peak in erythema was reflected by the SII scores (erythema and oedema scores combined), which followed the same trend (Fig 4A). Approximately half of the active A/Sing coated HD-MAP application sites remained slightly visible at day 61, whereas only 2 participants showed any visual evidence of application with an uncoated HD-MAP at this time point (Fig 4B).

**Fig 5:**
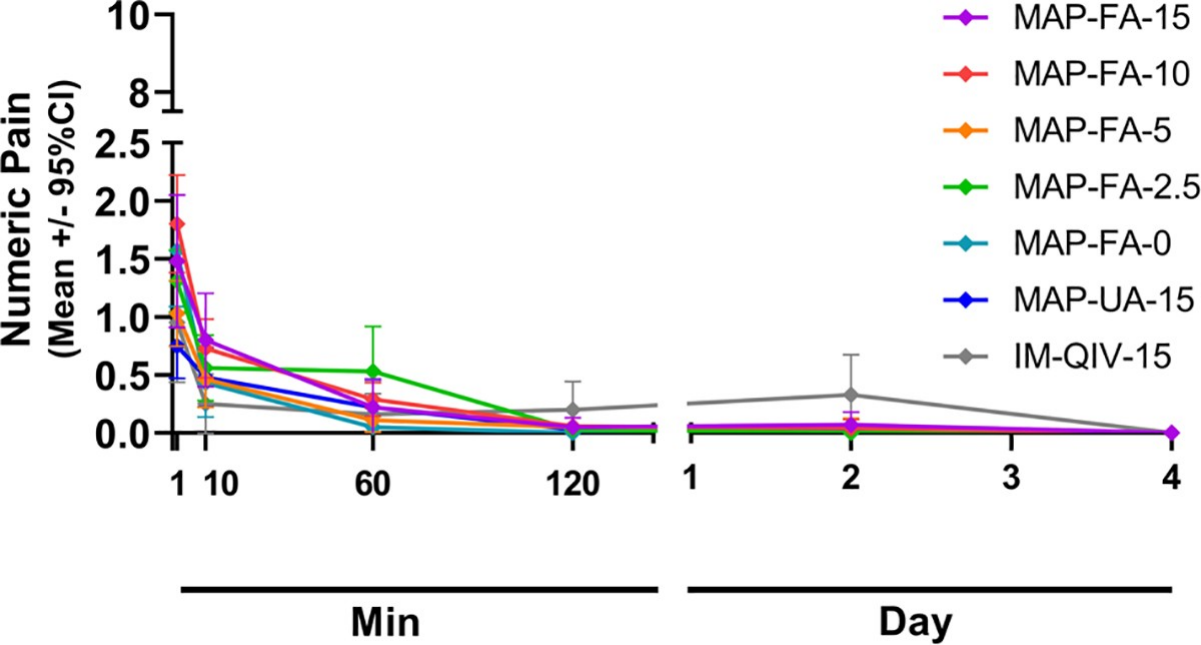
Self-reported pain scores using a visual analogue scale from 0 (no pain) to 10 (worst pain imaginable). Symbols represent mean scores ± 95% confidence intervals. Day, study day (HD-MAP applied on day 1); HD-MAP, high-density microarray patch; Min, minutes after HD-MAP application.

Self-reported pain scores after HD-MAP application or IM injection were very low overall (Fig 5). The highest scores for pain were experienced at the 1-min post-treatment application assessment (i.e., prior to HD-MAP removal).

### Serum antibody responses following vaccination

**Fig 6:**
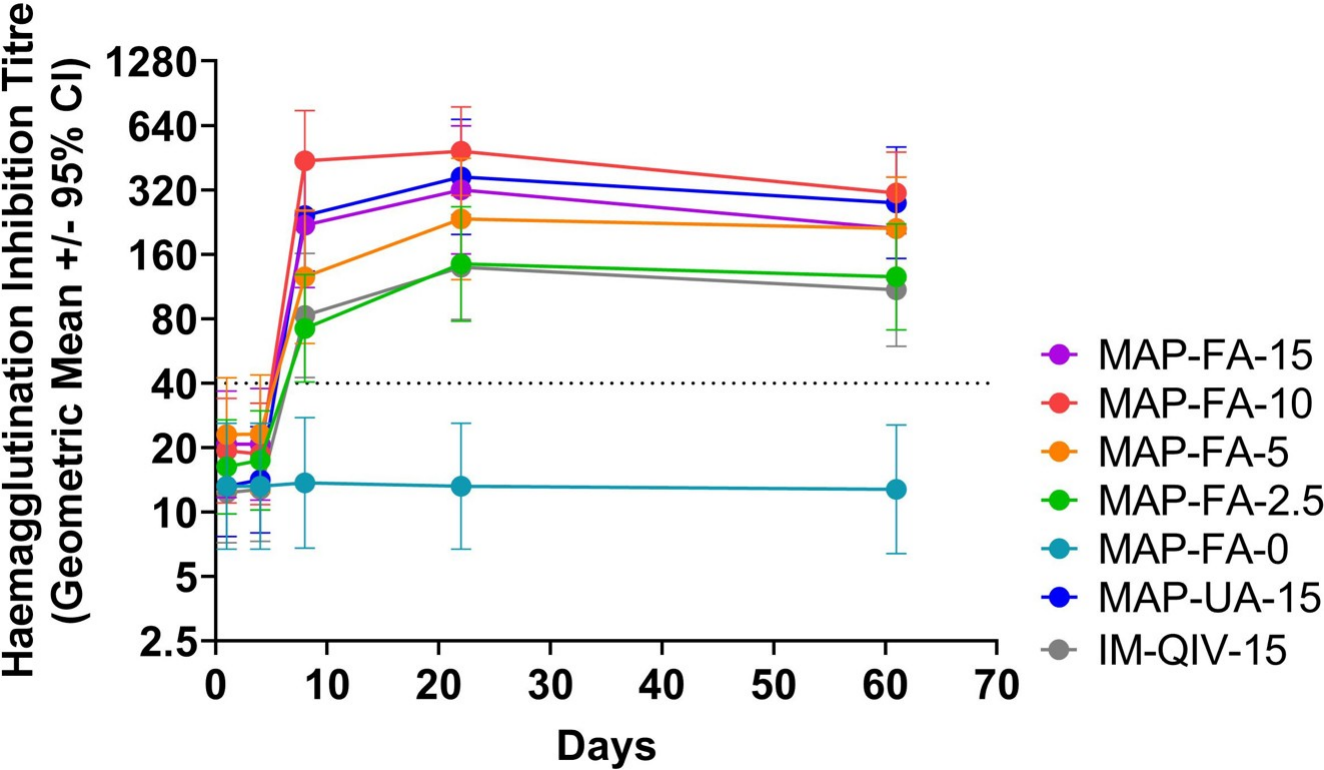
HAI titres for participants in part B at study days 1 (prevaccination), 4, 8, 22, and 61. Participants were vaccinated with A/Singapore/GP1908/2015 H1N1 at 15, 10, 5, or 2.5 μg HA/dose delivered by HD-MAPs applied to the volar forearm (MAP-FA-15, MAP-FA-10, MAP-FA-5, MAP-FA-2.5); uncoated HD-MAPs (MAP-FA-0); A/Singapore/GP1908/2015 H1N1 at 15 μg HA/dose delivered by HD-MAP applied to the upper arm (MAP-UA-15); or injected IM as a component of the Afluria quadrivalent vaccine (IM-QIV-15). Symbols represent the GMTs and the error bars show the 95% confidence intervals. GMT, geometric mean titre; HAI, HA inhibition; HD-MAP, high-density microarray patch; IM, intramuscular.

The geometric mean titres (GMTs) of HAI antibodies at days 1, 4, 8, 22, and 61 for participants in part B are shown in Fig 6. There was no increase in HAI titre in participants receiving uncoated HD-MAPs. In participants receiving the vaccine, either by HD-MAP or IM, titres did not increase above baseline at day 4, but at day 8 the GMTs were significantly higher in the MAP-FA-10 (GMT 437.1, 95% CI 254.3–751.3 *p <* 0.001), MAP-UA-15 (GMT 242.5, 95% CI 133.2–441.5, *p =* 0.02), and MAP-FA-15 (GMT 218.6, 95% CI 111.9–427.0, *p =* 0.04) compared with the IM-QIV-15 group (GMT 82.8, 95% CI 42.4–161.8). Titres continued to increase in all active groups until day 22 and remained significantly higher in the MAP-FA-10 (GMT 485.0 95% CI 301.5–780.2, *p =* 0.001) and MAP-UA-15 groups (GMT 367.6, 95% CI 197.9–682.7, *p =* 0.02) compared with the IM-QIV-15 group (GMT 139.3, 95% CI 79.3–244.5). HAI GMTs were also significantly higher at day 61 in the MAP-FA-10 (GMT 309.1, 95% CI 199.1–479.9 *p =* 0.01) and MAP-UA-15 (GMT 278.6, 95% CI 152.7–508.1, *p =* 0.03) compared with the IM-QIV-15 group (GMT 109.3, 95% CI 59.4–200.9).

The GMT in the MAP-FA-2.5 group (participants that received one-sixth the standard dose of HA) was not significantly different from the GMT in the IM-QIV-15 group at day 4 (GMT 17.4, 95% CI 10.2–29.7 compared with GMT 12.7, 95% CI 7.3–22.3, *p =* 0.40), day 8 (GMT 72.1, 95% CI 40.4–128.7 compared with GMT 82.8, 95% CI 42.4–161.8, *p =* 0.74), day 22 (GMT 144.2, 95% CI 77.9–267.0 compared with GMT 139.3, 95% CI 79.3–244.5, *p =* 0.93), or day 61 (GMT 125.5, 95% CI 71.0–221.9 compared with GMT 109.3, 95% CI 59.4–200.9, *p =* 0.73) after vaccination. Furthermore, at day 22 the HAI GMTs were similar in the MAP-FA-15 (GMT 320, 95% CI 161–638) and MAP-UA-15 (GMT 368, 95% CI 198–683) groups, indicating that the site of HD-MAP application did not appear to affect the subsequent antibody response.

The HAI GMTs, seroprotection, and seroconversion rates and fold-increase in GMT titres for all time points are shown in Table 4. All treatment groups that received active vaccine met the previous Committee for Medicinal Products for Human Use (CHMP) criteria from day 8 onwards [25]. The fold-increases in GMT at day 8 were significantly higher in the MAP-FA-10 (22.6, 95% CI 10.9–47.1, *p =* 0.007) and MAP-UA-15 groups (18.4, 95% CI 10.3–32.9, *p =* 0.01) compared with the IM-QIV-15 group (6.7, 95% CI 4.1–11.1) indicating a more rapid antibody response compared with IM injection. The fold-changes from baseline remained significantly higher in the MAP-UA-15 group at days 22 (27.9, 95% CI 15.0–51.7, *p =* 0.02) and days 61 (21.1, 95% CI 12.0–37.0, *p =* 0.03) than the IM-QIV-15 group at day 22 (11.3, 95% CI 6.8–18.8), and day 61 (8.9, 95% CI 5.1–15.4). An analysis of the HAI data using nonparametric methods and presenting median titres, median fold increase and nonparametric CIs is presented in S1 Table. The *P* values obtained from the Mann-Witney test (S1 Table) were very similar to those obtained from the *t* test.

**Table 4:** HAI responses to vaccination in terms of seroprotection, seroconversion, and fold-increase in GMT above prevaccination levels for part B.

Day	Responses	MAP-FA-15	MAP-FA-10	MAP-FA-5	MAP-FA-2.5	MAP-FA-0	MAP-UA-15	IM-QIV-15
**Day 1**	GMT (95% CI)	20.7 (11.7–36.7)	19.3 (11.0–33.9)	23.0 (12.5–42.3)	16.2 (9.8–26.9)	13.2 (6.7–26.0)	13.2 (7.7–22.7)	12.3 (7.2–21.1)
	Seroprotection; N (%)	10/20 (50)	9/20 (45)	8/20 (40)	8/20 (40)	6/20 (30)	6/20 (30)	7/20 (35)
		*p =* 0.52	*p =* 0.75	*p =* 1.00	*p =* 1.00		*p =* 1.00	
**Day 4**	GMT (95% CI)	20.7 (11.4–37.7)	18.7 (10.8–32.2)	23.1 (12.3–43.7)	17.4 (10.2–29.7)	13.2 (6.7–26.0)	14.1 (8.0–25.0)	12.7 (7.3–22.3)
		*p =* 0.22	*p =* 0.31	*p =* 0.15	*p =* 0.40		*p =* 0.79	
	Seroprotection; *N* (%)	10/20 (50)	9/20 (45)	7/19 (37)	8/20 (40)	6/20 (30)	7/20 (35)	7/20 (35)
		*p =* 0.52	*p =* 0.75	*p =* 1.00	*p =* 1.00		*p =* 1.00	
	Seroconversion; *N* (%)	0/20 (0)	0/20 (0)	0/19 (0)	0/20 (0)	0/20 (0)	0/20 (0)	0/20 (0)
		N/A	N/A	N/A	N/A		N/A	
	GMT fold-increase (95% CI)	1.0 (0.9–1.1)	1.0 (0.91.0)	1.0 (1.0–1.1)	1.1 (1.0–1.2)	1.0 (1.0–1.0)	1.1 (1.0–1.2)	1.0 (1.0–1.1)
		*p =* 0.57	*p =* 0.17	*p =* 0.97	*p =* 0.56		*p =* 0.56	
**Day 8**	GMT (95% CI)	218.6 (111.9–427.0)	437.1 (254.3751.3)	125.5 (61.4–256.9)	72.1 (40.4–128.7)	13.7 (6.8–27.6)	242.5 (133.2–441.5)	82.8 (42.4–161.8)
		*p =* 0.04*	*p <* 0.001**	*p =* 38	*p =* 0.74		*p =* 0.02 *	
	Seroprotection; *N* (%)	**19/20 (95)**	**20/20 (100)**	**17/20 (85)**	**18/20 (90)**	6/20 (30)	**18/20 (90)**	**17/20 (85)**
		*p =* 0.60	*p =* 0.23	*p =* 1.00	*p =* 1.00		*p =* 1.00	
	Seroconversion; *N* (%)	**17/20 (85)**	**18/20 (90)**	**12/20 (60)**	**11/20 (55)**	0/20 (0)	**18/20 (90)**	**15/20 (75)**
		*p =* 0.69	*p =* 0.41	*p =* 0.50	*p =* 0.32		*p =* 0.41	
	GMT fold-increase (95% CI)	**10.6 (4.8–23.3)**	**22.6 (10.9–47.1)**	**5.5 (3.0, 10.0)**	**4.4 (2.8–7.0)**	1.0 (1.0–1.1)	**18.4 (10.3–32.9)**	**6.7 (4.1–11.1)**
		*p =* 0.32	*p =* 0.007**	*p =* 0.59	*p =* 0.21		*p =* 0.001**	
**Day 22**	GMT (95% CI)	320.0 (160.5–638.1)	485.0 (301.5–780.2)	234.3 (121.9–450.0)	144.2 (77.9–267.0)	13.2 (6.7–26.0)	367.6 (197.9–682.7)	139.3 (79.3–244.5)
		*p =* 0.58	*p =* 0.001**	*p =* 0.21	*p =* 0.93		*p =* 0.02*	
	Seroprotection; *N* (%)	**19/20 (95)**	**20/20 (100)**	**18/20 (90)**	**18/20 (90)**	6/20 (30)	**19/20 (95)**	**17/20 (85)**
		*p =* 0.60	*p =* 0.23	*p =* 1.00	*p =* 1.00		*p =* 0.60	
	Seroconversion; *N* (%)	**17/20 (85)**	**18/20 (90)**	**14/20 (70)**	**16/20 (80)**	0/20 (0)	**18/20 (90)**	**15/20 (75)**
		*p =* 0.69	*p =* 0.41	*p =* 1.00	*p =* 1.00		*p =* 0.41	
	GMT fold-increase (95% CI)	**15.5 (6.7–35.7)**	**25.1 (13.4–46.9)**	**10.2 (5.1–20.4)**	**8.9 (5.0–15.8)**	1.0 (1.0–1.0)	**27.9 (15.0–51.7)**	**11.3 (6.8–18.8)**
		*p =* 0.51	*p =* 0.05*	*p =* 0.80	*p =* 0.51		*p =* 0.02*	
**Day 61**	GMT (95% CI)	211.1 (121.7–366.3)	309.1 (199.1–479.9)	211.1 (121.7–366.3)	125.5 (71.0–221.9)	12.7 (6.4–25.5)	278.6 (152.7–508.1)	109.3 (59.4–200.9)
		*p =* 0.10	*p =* 0.001**	*p =* 0.10	*p =* 0.73		*p =* 0.03*	
	Seroprotection; *N* (%)	**19/20 (95)**	**20/20 (100)**	**19/20 (95)**	**18/20 (90)**	6/20 (30)	**19/20 (95)**	**17/20 (85)**
		*p =* 0.60	*p =* 0.23	*p =* 0.60	*p =* 1.00		*p =* 0.60	
	Seroconversion; *N* (%)	**16/20 (80)**	**18/20 (90)**	**14/20 (70)**	**15/20 (75)**	0/20 (0)	**18/20 (90)**	**13/20 (65)**
		*p =* 0.48	*p =* 0.13	*p =* 1.00	*p =* 0.73		*p =* 0.13	
	GMT fold-increase (95% CI)	**10.2 (5.1–20.5)**	**16.0 (9.6–26.8)**	**9.2 (4.9–17.4)**	**7.7 (4.4–13.6)**	1.0 (0.9–1.0)	**21.1 (12.0–37.0)**	**8.9 (5.1–15.4)**
		*p =* 0.75	*p =* 0.11	*p =* 0.93	*p =* 0.71		*p =* 0.03*	

Text in bold indicates that the previous Committee for Medicinal Products for Human Use (CHMP) threshold for the criterion has been met.

*p < 0.05

**p < 0.01 compared to the IM-QIV-15 group by Student *t* test (fold increase and GMTs) and using a Pearson's chi-square test with continuity correction for proportion of participants seroconverted or seroprotected.

N/A (not applicable) indicates no participants seroconverted in this group at this time point.

**Abbreviations:** FA, forearm; GMT, geometric mean titre; HAI, HA inhibition; IM, intramuscular; MAP, microarray patch; QIV, quadrivalent influenza vaccine; UA, upper arm

The HAI titres observed in part A participants (S1 Fig) receiving vaccine delivered by HD-MAP were not significantly different from those in the corresponding treatment group in part B at any time point. A-MAP-FA-15 compared with MAP-FA-15 at day 4 (GMT 14.4, 95% CI 7.0–29.8 compared with GMT 20.7, 95% CI 11.4–37.7, *p =* 0.42), day 8 (GMT 335.9, 95% CI 134.7–833.8 compared with GMT 218.6, 95% CI 111.9–427, *p =* 0.42), day 22 (GMT 422.2, 95% CI 191.3–932.2 compared with GMT 320.0 95% CI 160.5–638.1, *p =* 0.58), and day 61 (GMT 278.6 95% CI 123.9–626.5 compared with GMT 211.1, 95% CI 121.7–366.3, *p =* 0.54) suggesting consistency of delivery and antibody induction. The GMTs induced by IM-QIV-15 in parts A and B were also not significantly different at day 4 (A-IM-QIV-15 GMT 27.7, 95% CI 15.2–50.2 compared with IM-QIV-15 GMT 12.7, 95% CI 7.3–22.3, *p =* 0.57), day 8 (A-IM-QIV-15 GMT 152.4, 95% CI 79.0–293.4 compared with IM-QIV-15 GMT 82.8, 95% CI 42.4–161.8, *p =* 0.19), and day 22 (A-IM-QIV-15 GMT 261.7, 95% CI 162.1–425.1 compared with IM-QIV-15 GMT 139.3, 95% CI 79.3–244.5, *p =* 0.10) but were higher in part A at day 61 (A-IM-QIV-15 GMT 261.7, 95% CI 166.4–414.1 compared with IM-QIV-15 GMT 109.3, 95% CI 59.4–200.9, *p =* 0.03).

MN assays were carried out on serum samples from all part B participants at day 1 (prevaccination) and day 22. As with the HAI antibodies, there was an increase in titre from day 1 to day 22 in all treatment groups that received the vaccine (S2 Fig). The MN titres at day 22 in the MAP-FA-15 (GMT 11,362, 95% CI 6,492–19,884), MAP-FA-10 (GMT 18,458, 95% CI 11,359–29,992) and MAP-UA-15 (GMT 13,219, 95% CI 7,096–24,626) groups were significantly higher than the IM-QIV-15 group (GMT 3,880, 95% CI 1,924–7,824) (*p =* 0.02, *p =* 0.001, and *p =* 0.01, respectively). As with the HAI results, the MN GMTs at day 22 in the MAP-FA-2.5 (one-sixth dose) (GMT 5,301, 95% CI 2,509–11,196) and IM-QIV-15 (GMT 3,880, 95% CI 1,924–7,824) groups were similar (S2 Fig).

**Fig 7:**
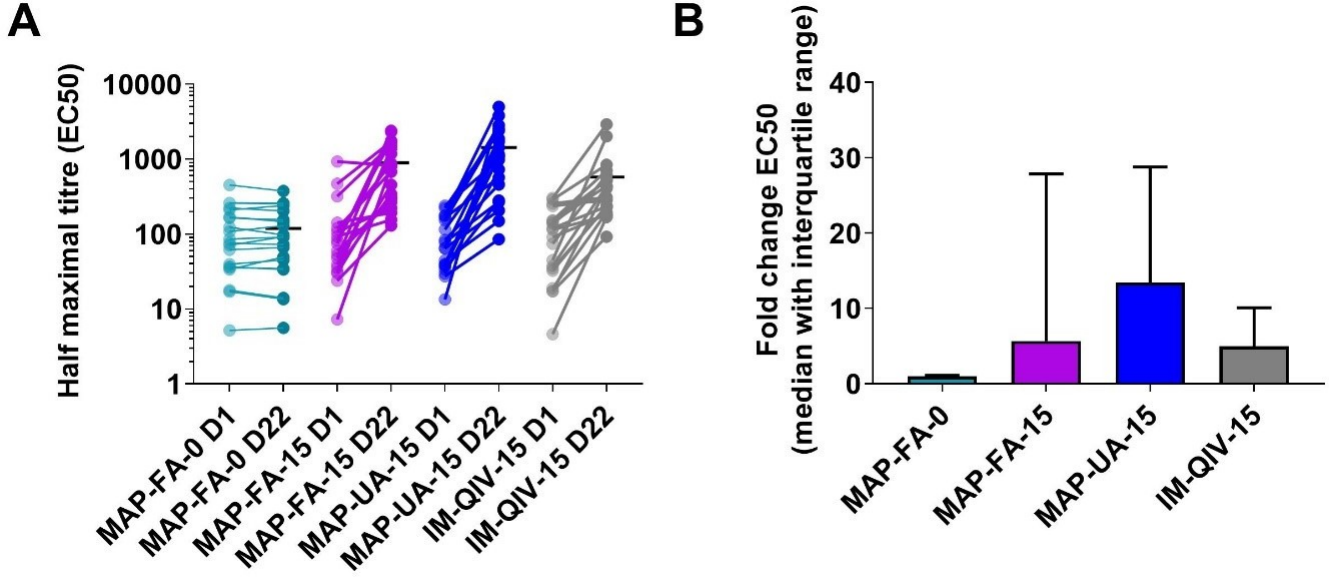
HA-specific FcR-binding antibodies. Antibodies specific for A/Singapore/GP1908/2015 monovalent purified harvest that engage with dimeric, soluble recombinant FcγRIII were measured by ELISA. (A) Midpoint ELISA titres. (B) Fold-change in midpoint titres, day 22 versus day 1. Symbols represent individual responses before (D1) and after (D22) immunisation, in which horizontal lines indicate the mean response (A); columns with error bars represent the median with interquartile ranges (B). ELISA, enzyme-linked immunosorbent assay; FcR, Fc receptor; HA, haemagglutinin.

Titres of HA-specific Fc receptor (FcR) -binding antibodies capable of mediating ADCC were assayed at days 1 and 22. The midpoint titres increased significantly following vaccine delivery in the MAP-FA-15 (day 22 = 893.6, 95% CI 550.7–1,236, *p <* 0.001), MAP-UA-15 (day 22 = 1,433, 95% CI 814.3–2,052, *p <* 0.001) and IM-QIV-15 (day 22 = 576.8, 95% CI 252.9–900.7, *p =* 0.002) groups but not in the MAP-FA-0 group (day 22 = 118.9, 95% CI 71.62–166.2, *p >* 0.99; Fig 7A). There was no significant difference between the midpoint titres at day 22 in these 3 active groups (MAP-FA-15 compared with IM-QIV-15, *p >* 0.99; MAP-UA-15 compared with IM-QIV-15, *p >* 0.99; MAP-FA-15 compared with MAP-UA-15, *p >* 0.99) nor was there a difference when the results were expressed as fold-change from baseline because of the degree of intragroup variation (Fig 7B).

### Assessment of salivary IgA responses

Influenza-specific IgA in saliva was assayed by ELISA in samples taken at days 1, 4, 8, and 22 from participants in groups MAP-FA-0, MAP-FA-15, MAP-UA-15, and IM-QIV-15. Statistical analysis was not performed because of some saliva samples being incomplete and large sample variation. However, there was an apparent increase in IgA between day 4 and day 8 in the MAP-FA-15 and MAP-UA-15 groups, with a 1.92- and 1.57-fold increase, respectively, at day 8 compared with day 1 (S3 Fig). There was no corresponding increase in the MAP-FA-0 (1.01-fold) group, and a 1.22-fold increase in the IM-QIV-15 groups at the same time point. IgA titres had returned to near-baseline levels at day 22.

### Frequency and specificity of influenza-specific MBCs

**Fig 8:**
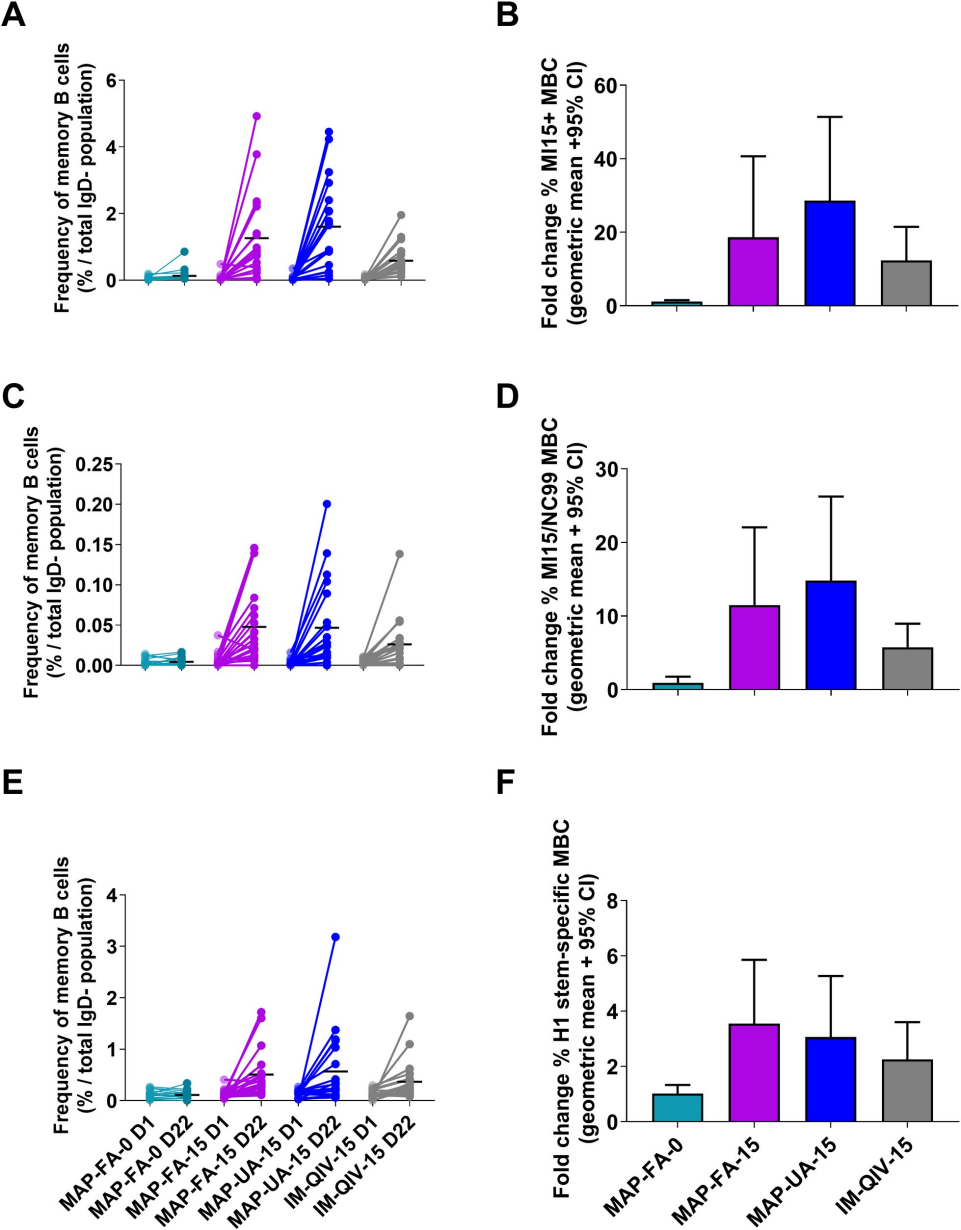
MBC frequencies pre- and postvaccination. The frequencies of HA-specific MBC were assessed in cryopreserved PBMC samples by flow cytometry. Samples were gated for live, CD19+, IgD negative B cells, and specificity determined based upon binding to A/Michigan/2015 probes alone or in combination with A/New Caledonia/1999 or a stabilised H1N1 stem probes. (A and B) A/Michigan/2015 H1N1; (C and D) A/New Caledonia/1999 and A/Michigan/2015 H1N1 cross-reactive MBC; (E and F) H1 stem. Results are expressed as the frequency of probe-binding cells at days 1 and 22 (A, C, and E) with symbols representing individual responses before (D1) and after (D22) immunisation, and horizontal lines indicating the mean response; fold-change at day 22 compared with baseline (B, D, and F). Columns represent the median fold change; error bars represent the median with interquartile ranges. HA, haemagglutinin; MBC, memory B cell; PBMC, peripheral blood mononuclear cell.

A flow cytometry–based assay using fluorescently labelled recombinant HA probes was used to assess the frequency and specificity of HA-specific B cells following immunisation [26]. Frequencies of memory B cells (MBCs) binding a HA-Michigan probe (antigenically matched to A/Singapore/GP1908/2015) increased significantly from day 1 to day 22, following immunisation with either MAP FA-15 (0.641% to 1.262%, *p <* 0.001), MAP-UA-15 (0.055% to 1.609%, *p <* 0.001), or QIV (0.047% to 0.465%, *p <* 0.001) but not in the MAP-FA-0 placebo group (0.051% to 0.131%, *p >* 0.99). The frequencies of HA-Michigan-specific MBCs at day 22 were not significantly different in the 3 vaccine groups however (MAP-FA-15 compared with IM-QIV-15, *p >* 0.99; MAP-UA-15 compared with IM-QIV-15, *p >* 0.99; MAP-FA-15 compared with MAP-UA-15, *p >* 0.99; Fig 8A and Fig 8B). Using binding to a A/New Caledonia/99 probe to assess H1N1 cross-reactivity, we found only a small proportion of the A/Michigan/15-binding cells displayed cross-reactive recognition of A/New Caledonia/99 HA. There were significant increases in frequency in these cells between day 1 and day 22 in the MAP-FA-15 (0.005% to 0.048%, *p <* 0.001) and MAP-UA-15 (0.002% to 0.047%, *p <* 0.001) groups, and an increase that did not reach statistical significance in the IM-QIV-15 groups (0.004% to 0.026%, *p =* 0.05), but again, there were no differences in the MBC frequency in the vaccine groups at day 22 (MAP-FA-15 compared with IM-QIV-15, *p >* 0.99; MAP-UA-15 compared with IM-QIV-15, *p >* 0.99; MAP-FA-15 compared with MAP-UA-15, *p >* 0.99; Fig 8C and Fig 8D). A similar pattern was seen with cross-reactive B cells binding an HA-stalk domain probe; there was a significant expansion from day 1 to day 22 in the MAP FA-15 (0.126% to 0.507%, *p =* 0.01) and IM-QIV-15 (0.128% to 0.368%, *p =* 0.05) groups. The expansion in the MAP-UA-15 groups did not achieve significance, probably because of intragroup variability (0.128% to 0.569%, *p =* 0.17). There was no difference in stalk-reactive MBC frequencies between the active HD-MAP and IM groups at day 22 (MAP-FA-15 compared with IM-QIV-15, *p >* 0.99; MAP-UA-15 compared with IM-QIV-15, *p >* 0.99; MAP-FA-15 compared with MAP-UA-15, *p >* 0.99; Fig 8E and Fig 8F).

### Flow-cytometric analysis of influenza-specific polyfunctional T cells

T-cell responses were assessed by analysing the frequencies of influenza-specific CD4^+^ and CD8^+^ T producing IFN-γ, IL-2, and TNF-α in PBMC harvested on days 1 and 22 from participants in groups MAP-FA-0, MAP-FA-15, MAP-UA-15, and IM-QIV-15. PBMC were stimulated with either A/Sing MPH or overlapping peptides spanning the A/Sing HA sequence.

**Fig 9:**
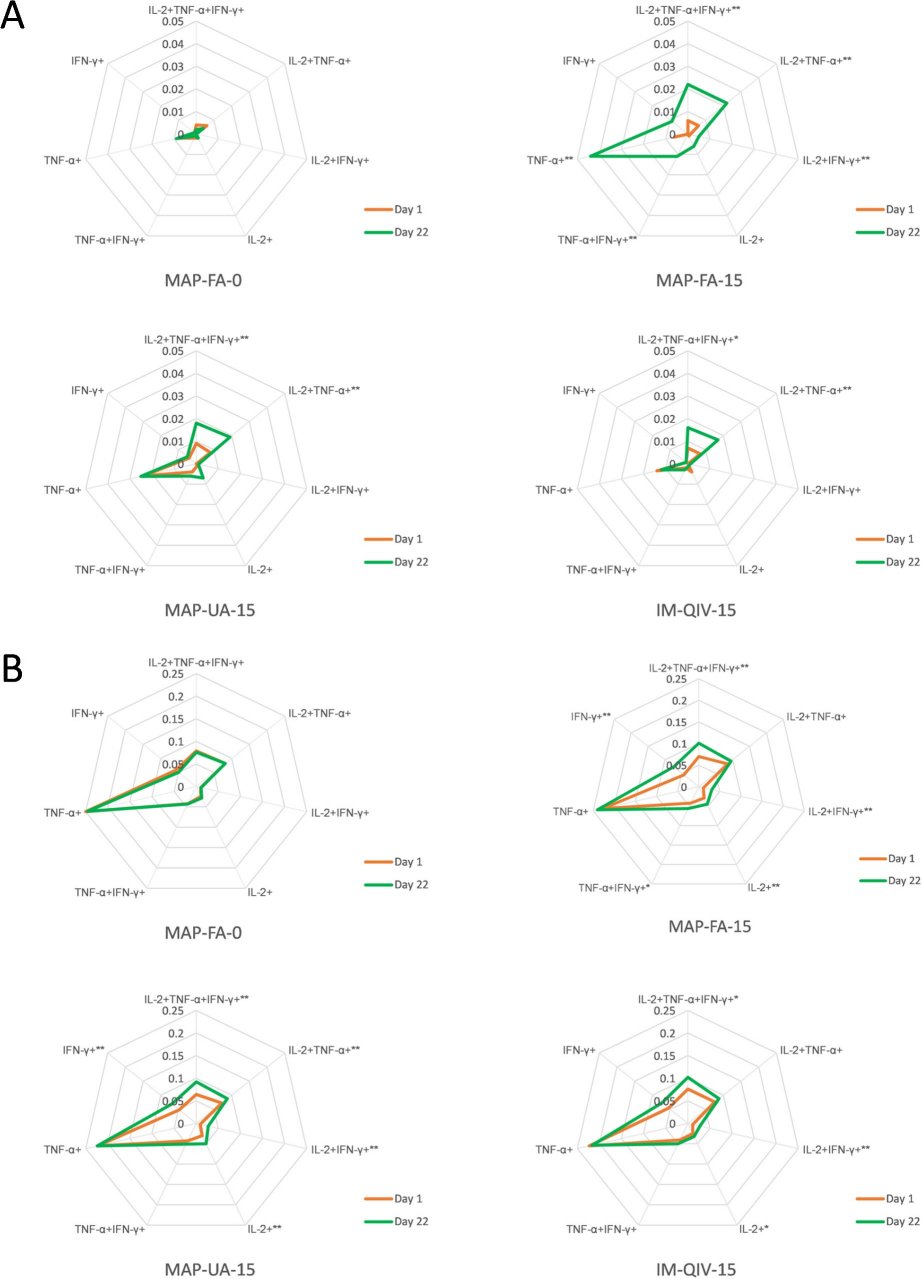
**Frequency of CD4**^**+**^ **cells producing IL-2, TNF-α, or IFN-γ following stimulation of PBMC with (A) overlapping peptides spanning A/Sing HA (5 μg/ml) or (B) A/Sing MPH (20 μg/ml).** Cells were labelled with Live/Dead Aqua for viability; CD3, CD4, and CD8; permeabilised; and subsequently labelled with anti-IFN-γ, anti-TNF-α, and anti-IL-2. Approximately 500,000 events were acquired using a Becton Dickinson LSR Fortessa X20 and data analysed using SPICE software. Day 1 versus day 22 comparisons were made using the Wilcoxon rank sum test: **p < 0.01, *p < 0.05. HA, haemagglutinin; IFN, interferon; IL, interleukinMPH, monovalent purified harvest; PBMC, peripheral blood mononuclear cell; TNF, tumour necrosis factor.

There was an increase in the overall frequency of CD4^+^ cells producing IFN-γ, IL-2, or TNF-α following in vitro stimulation with the peptides at day 22 compared to day 1 in the MAP-FA-15, MAP-UA-15, and IM-QIV-15 groups but not the MAP-FA-0 group (Fig 9A). In particular, there was a significant increase in the abundance of polyfunctional CD4^+^ T cells expressing all 3 cytokines (IFN-γ, TNF-α, and IL-2) at day 22 compared with day 1 for the MAP-FA-15 (0.006% to 0.022%, *p <* 0.001, 95% CI −0.00011 to 0.0002), MAP-UA-15 (0.009% to 0.018%, *p =* 0.005, 95% CI −0.00007 to 0.0002), and IM-QIV-15 (0.007% to 0.016%, *p =* 0.01, 95% CI −0.00011 to 0.00025) groups. There were no statistically significant differences in the proportions of CD4^+^ T cells producing any of the cytokine combinations at day 22 when the MAP-FA-15, MAP-UA-15, and IM-QIV-15 groups were compared with one another.

The overall frequency of cytokine-producing CD4^+^ cells pre- and postvaccination was greater following stimulation with A/Sing MPH compared with the overlapping peptides (Fig 9B), presumably because of the greater number of epitopes present within the MPH preparation. A/Sing MPH stimulation appeared to induce more CD4^+^ cells producing TNF-α alone compared with peptide stimulation. There were not, however, any statistically significant differences in the proportions of CD4^+^ T cells producing each of the cytokine combinations at day 22 when the MAP-FA-15, MAP-UA-15, and IM-QIV-15 groups were compared with one another (*p >* 0.05 for all comparisons).

Day 1 and day 22 CD8^+^ T-cell responses to the peptide pools and A/Sing MPH were also measured but were weak in comparison with the CD4^+^ responses. The weak CD8^+^ T-cell responses were not surprising considering that the nature of antigen used for restimulation (inactivated, split A/Sing MPH, and 17 amino acid peptides) favoured stimulation and detection of CD4^+^ T cells

## Discussion

This phase I clinical trial demonstrated that HD-MAPs delivering a monovalent influenza vaccine were well tolerated and induced immune responses that were similar to or significantly enhanced compared with IM injection. The study was the first clinical trial with Vaxxas HD-MAPs fabricated from polymer, rather than silicon Nanopatches, as used in our previous studies [2,19]. In addition, the trial reported here was, to our knowledge, the first clinical study with any MAP to evaluate the potential for dose-sparing, a phenomenon that has been tested extensively in preclinical models [10–17] but, until now, has not been demonstrated in the clinic.

The HD-MAPs were found to be safe and well tolerated, and the safety and reactogenicity profiles of the HD-MAPs were very similar to those observed with the silicon Nanopatches using a similar H1N1 antigen, A/California/7/2009 [2,19]. The observation that erythema was still present 7 days after vaccination is also consistent with intradermal (ID) delivery of influenza vaccines [27]. The acceptability of the HD-MAP was not assessed in this study, but our previous studies found that the majority of participants preferred the Nanopatch to IM injection with N&S [2,19], and similar results have also been obtained with other MAP systems [7,28].

Assessment of the immune response was a secondary objective in the study. In terms of the proportion of participants seroprotected and seroconverting, the HAI responses induced by HD-MAP delivery in this study were similar to those seen previously with N&S ID injection of inactivated influenza vaccine (IIVs) [27,29–33]. However, the more rapid antibody response seen with HD-MAP delivery as indicated by higher HAI titres at the early day 8 time point have not, to the best of our knowledge, been seen with ID injection of IIVs unless the ID injection site was pretreated with the topical adjuvant Imiquimod, a toll-like receptor 7 agonist [34]. Achieving higher titres sooner after vaccination by using the HD-MAP to deliver seasonal influenza or travel vaccines would be beneficial for vaccine recipients and would be particularly valuable if it was shown to apply to vaccines against pandemic influenza strains and vaccines used in outbreak response.

We conducted several exploratory assays to define whether differences exist in the immune response induced by HD-MAP delivery compared with IM injection. These assays were only conducted on samples from participants in the groups that received the 15 μg or 0 μg doses of HA, so no data were collected on whether or not similar responses would have been seen in these assays with the lower doses of the HD-MAP-delivered vaccine. Overall, HD-MAP delivery of inactivated split influenza vaccine gave comparable albeit increased levels of response in all immunological parameters tested, indicating a broad engagement of the immune system by the Vaxxas HD-MAP.

Influenza vaccines that induce more broadly protective and longer lasting immunity than current seasonal vaccines are needed to limit the consequences of epidemic and pandemic influenza [35,36]. Studies have suggested that ADCC-mediating antibodies recognize epitopes that are more conserved than those bound by neutralising antibodies and might contribute to protection against heterologous strains [37,38]. In our study, the induction of antibodies with ADCC-inducing potential followed a similar pattern of response to the HAI and MN data, with slightly higher titres being observed in groups vaccinated with the HD-MAP compared with the IM injection. The frequencies of B cells recognizing HA-stalk and a historic H1N1 HA probes also increased to a similar extent following IM or HD-MAP vaccination.

CD4^+^ T-cell responses to influenza can have broad specificity and contribute to protection by several mechanisms including providing help for high-affinity neutralising antibody responses; recruiting effector cells; directly lysing infected cells and producing antiviral cytokines such as IFN-γ, and providing long-lived, cross-reactive memory [39]. Our observation that the frequency of CD4^+^ cells responding to the HA peptide pools in participants vaccinated by HD-MAP was at least as high as that in participants vaccinated IM is consistent with the overall pattern of responses to HD-MAP vaccination being as good or enhanced compared with IM injection.

Induction of mucosal immune responses by influenza vaccines would be advantageous, because these responses could control or limit virus replication in the respiratory tract [40]. ID or MAP immunisation has been shown to induce mucosal responses to nonlive vaccines including influenza in mice and swine [17,41–43]. In humans, however, ID delivery of IIVs did not induce superior IgA responses compared with IM [44]. In contrast, superior IgA responses were induced in a phase I trial of *trans*-cutaneous delivery of live-attenuated measles vaccine [45]. We observed a small increase in salivary IgA on day 8 following HD-MAP delivery of the A/Sing antigen but not following IM injection of QIV. Given the method of collection, however, we cannot rule out the possibility that the saliva was contaminated with crevicular fluid, and as such, might reflect responses in the serum, rather than mucosal responses.

In contrast to our previous study using silicon Nanopatches [2], in which application to the forearm appeared to be marginally more immunogenic, no differences in the HAI or MN responses were found following HD-MAP application to the UA or volar surface of the forearm, suggesting that either application site could be used in the future.

The small group sizes limited the statistical power of the study. However, even with these numbers, several Vaxxas HD-MAP groups showed statistically higher responses at days 8, 22, and 61 after vaccination compared with IM injection, and the one-sixth dose induced antibody levels that were similar to those seen with the full-dose IM. At this stage, we do not understand the influence of prior immunity to H1N1 on immunological responses. This will be further investigated by a larger trial that will include H3 and B strains delivered by the HD-MAP. Lower doses than 2.5 μg were not tested in the study and, a further reduction in delivered HA dose may be achievable: in preclinical studies, 1/100th or 10th doses of flu vaccine delivered by Nanopatch induced a similar antibody response to IM and ID injection, respectively [10]. However, the development of Intanza 9 μg, ID seasonal influenza vaccine found that higher doses were required to achieve noninferiority, compared to responses seen in early clinical studies [46]. Full analysis of the dose-sparing potential of HD-MAPs with influenza vaccines would involve clinical testing of all reduced dose levels of the same antigen delivered by IM and HD-MAP, but such a large study was beyond the scope of this phase I study that was the first clinical use of the Vaxxas polymer HD-MAP. Finally, the fact that local skin reactions to uncoated or vaccine-coated HD-MAPs were different could have compromised the blinding of the study. However, all laboratory staff were completely blinded to this information throughout the trial.

The Vaxxas HD-MAP product under test here was a developmental version of the intended commercial product and as such has more components and a more complex administration process. However, all the parameters associated with the actual application of the HD-MAP (such as contact points with the skin, the force generated by the applicator, size of the MAP, etc.) were the same in this device as in the proposed final commercial product. The final product design is an integrated single use, autodisabling, and disposable applicator, and this will be used in the next clinical study. This design will reduce the overall system cost of the product for which, in the influenza market at least, needs to be competitive to a prefilled N&S. A prototype version of the commercial device has been evaluated in an end-user study conducted in Benin, Nepal, and Vietnam and was found to be very acceptable to immunisation managers, healthcare workers, community health volunteers, and caregivers [47]. The demonstration in this study that Vaxxas HD-MAPs fabricated from polymer rather than silicon can be manufactured and used for vaccine delivery is a significant advance. Polymer is preferred to silicon for the final commercial embodiment of the device because HD-MAPs will be significantly less expensive per unit than silicon Nanopatches and will be more amenable to large-scale manufacture—a major challenge for all MAP developers. Finally, future versions of the device and future trials will evaluate delivery of the standard dose (15 μg HA per strain) by a single HD-MAP.

Influenza causes significant morbidity and mortality in adults over 65 years of age, and strategies to improve vaccine coverage, immunogenicity, and effectiveness in this age group are required [48]. Currently IIVs for this population require chemical adjuvants such as MF59 or high doses of antigen (such as 60 μg HA per strain per dose present in HD Fluzone) to achieve satisfactory immune responses [48,49]. The enhanced immunogenicity seen with MAP delivery indicates that HD-MAPs may provide an alternative approach; however, whether any differences seen in the immune responses induced by HD-MAPs compared with IM injection are clinically relevant will require large-scale studies with clinical endpoints.

Furthermore, the exceptional thermostability of the influenza vaccine observed in this study compared with standard formulations [50] would eliminate dependence on the cold-chain and reduce vaccine wastage due to cold-chain excursions. A more stable vaccine would also remove the need to overload the patch to compensate for loss of potency during the shelf life of the vaccine.

If the dose-sparing observed with an H1N1 strain in this trial is also observed with other influenza strains, use of the Vaxxas HD-MAP could increase the number of vaccine doses that can be produced from the primary manufacturing facility in a season, or in a pandemic, because the amount of antigen required per dose would be reduced. Global capacity for seasonal influenza production declined between 2013 and 2015, largely because of the switch from TIV to QIV formulations [51], and pandemic vaccine production capacity is dependent on the implementation of dose-sparing strategies [51]. Dose-sparing and rapid onset of protective immunity would also be a very valuable attribute for many other vaccines of global health importance such as inactivated poliovirus vaccine or yellow fever vaccine for which use is limited by chronic supply constraints [52–54], or by cost, such as rabies vaccine [55]. These vaccines are often needed most in low-resource settings where other key attributes of the Vaxxas HD-MAP, such as thermostability, ease of use, acceptability, and the avoidance of reconstitution would also be very beneficial [47].

In summary, this was the first clinical trial using Vaxxas HD-MAPs fabricated from polymer, coated with an influenza vaccine. The HD-MAP appeared to be safe and well tolerated. Doses of 2.5, 5, 10, and 15 μg HA induced HAI and MN responses as high or higher than 15 μg HA injected IM. This is to the best of our knowledge the first clinical demonstration of dose-sparing for vaccine delivery using a MAP technology. Future clinical studies will evaluate the acceptability and immunogenicity of QIVs delivered by Vaxxas HD-MAP in adults and older adults and examine the immunogenicity of vaccines against potential pandemic strains of influenza. Clinical trials using Vaxxas HD-MAPs are also warranted to determine whether the dose-sparing seen in this trial is seen with other types of vaccines.

## Supporting information

S1 Checklist CONSORT (DOC)

S1 Fig HAI titres for part A. HAI titres at days 1 (prevaccination), 4, 8, 22, and 61 for participants in part A following vaccination with either A/Singapore/GP1908/2015 H1N1 delivered by HD-MAP (A-MAP-FA-15) or injected IM as a component of Afluria quadrivalent vaccine (A-IM-QIV-15), uncoated HD-MAP (A-MAP-FA-0), or A/Singapore/GP1908/2015 H1N1 monovalent pooled harvest injected IM (IM-SIN-15). Symbols represent the GMTs and error bars show the 95% confidence intervals. The dotted line indicates the HAI titre (1:40) regarded as correlating with protection. FA, forearm; GMT, geometric mean titre; HAI, HA inhibition; HD-MAP, high-density microarray patch; IM, intramuscular; QIV, quadrivalent influenza vaccine
(TIF)

S2 Fig Microneutralisation titres, part B. Microneutralisation titres at day 1 (prevaccination) and day 22 for participants in part B following vaccination with A/Singapore/GP1908/2015 H1N1 at 15, 10, 5, or 2.5 μg HA/dose delivered by HD-MAPs applied to the volar forearm (MAP-FA-15, MAP-FA-10, MAP-FA-5, MAP-FA-2.5), uncoated HD-MAPs (MAP-FA-0), A/Singapore/GP1908/2015 H1N1 at 15 μg HA/dose delivered by HD-MAP applied to the upper arm (MAP-UA-15), or injected IM as a component of Afluria quadrivalent vaccine (IM-QIV-15). Columns represent the GMTs, symbols represent the titres from individual participants, and the error bars show the 95% confidence intervals. FA, forearm; GMT, geometric mean titre; HA, haemagglutinin; HD-MAP, high-density microarray patch; IM, intramuscular; QIV, quadrivalent influenza vaccine; UA, upper arm
(TIF)

S3 Fig Influenza-specific IgA titres in saliva samples. Participants were vaccinated with either 15 μg of A/Singapore/GP1908/2015 H1N1 delivered by HD-MAP to either the volar forearm (MAP-FA-15) or upper arm (MAP-UA-15), or injected IM as a component of Afluria quadrivalent vaccine (IM-QIV-15) or uncoated HD-MAPs (MAP-FA-0). Four time points were measured: prevaccination (day 1), day 4, 8, and 22. The absorbance values per group for each time point were averaged and compared against day 1, and the fold-change compared with prevaccination (day 1) were plotted. Symbols represent the means from all participants per group, and the error bars show the 95% confidence intervals. Statistical analysis was not performed because of some saliva samples being incomplete and large sample variation. FA, forearm; HD-MAP, high-density microarray patch; IgA, immunoglobulin A; IM, intramuscular; QIV, quadrivalent influenza vaccine; UA, upper arm
(TIF)

S1 Table HAI responses, part B, nonparametric analysis. HAI responses to vaccination in terms of median titre, seroprotection, seroconversion, and fold-increase in median titre above prevaccination levels for part B. Part B participants were vaccinated with A/Singapore/GP1908/2015 H1N1 at 15, 10, 5, or 2.5 μg HA/dose delivered by HD-MAPs applied to the volar forearm (MAP-FA-15, MAP-FA-10, MAP-FA-5, MAP-FA-2.5), uncoated HD-MAPs (MAP-FA-0), A/Singapore/GP1908/2015 H1N1 at 15 μg HA/dose delivered by HD-MAP applied to the upper arm (MAP-UA-15), or injected IM as a component of the Afluria quadrivalent vaccine (IM-QIV-15). Exact nonparametric CIs are shown in parentheses. *p < 0.05; **p < 0.01 compared to the IM-QIV-15 group by Exact Mann Witney Test (median titre and median fold increase). Pearson's chi-square test with continuity correction was used to compare proportion of participants seroconverted or seroprotected. FA, forearm; HA, haemagglutinin; HAI, HA inhibition; HD-MAP, high-density microarray patch; IM, intramuscular; QIV, quadrivalent influenza vaccine; UA, upper arm.
(DOCX)

S2 Table Microneutralisation responses, part B, nonparametric analysis. Microneutralisation responses (median titres) at days 1 and 22 for part B. Part B participants were vaccinated with A/Singapore/GP1908/2015 H1N1 at 15, 10, 5, or 2.5 μg HA/dose delivered by HD-MAPs applied to the volar forearm (MAP-FA-15, MAP-FA-10, MAP-FA-5, MAP-FA-2.5), uncoated HD-MAPs (MAP-FA-0), A/Singapore/GP1908/2015 H1N1 at 15 μg HA/dose delivered by HD-MAP applied to the upper arm (MAP-UA-15), or injected IM as a component of the Afluria quadrivalent vaccine (IM-QIV-15). Exact nonparametric CIs are shown in parentheses. *p < 0.05; **p < 0.01 compared to the IM-QIV-15 group by Exact Mann Witney Test. FA, forearm; HA, haemagglutinin; HD-MAP, high-density microarray patch; IM, intramuscular; QIV, quadrivalent influenza vaccine; UA, upper arm
(DOCX)

S1 Text Clinical trial protocol. (PDF)

S1 Data Data listing. Data 1: Informed Consent; Data 2: Analysis Sets; Data 3: Demographics; Data 4: Study Drug Administration; Data 5: Skin Hardness Assessment; Data 6: Immunogenicity (ELISA IgG); Data 7: Immunogenicity (ADCC); Data 8: Immunogenicity (MBCs); Data 9: Immunogenicity (CMI); Data 10: Immunogenicity (Mucosal IgA); Data 11: Immunogenicity Results (HAI, MN); Data 12: TEAE–CRF Data only; Data 13: TEAEs–MedDRA Coding; Data 15: Treatment Site Tolerability Assessment; Data 16: Numeric Pain Intensity; Data 17: SII; Data 18: Application Site Status at End of Study; Data 19: Individual Treatment Site Tolerability Assessment; Data 20: Numeric Pain Intensity by Treatment; Data 21: Individual SII by Treatment. ADCC, antibody-dependent cellular cytotoxicity; CMI, cell-mediated immunity; CRF, case report form; ELISA, enzyme-linked immunosorbent assay; HAI, haemagglutination inhibition; IgA, immunoglobulin A; IgG, immunoglobulin G; MBC, memory B cell; MedDRA, medical dictionary for regulatory activities; MN, microneutralisation; SII, Skin Irritation Index; TEA, treatment emergent adverse event.
(ZIP)

## Abbreviations

ADCC: antibody-dependent cellular cytotoxicity
AE: adverse event
A/Sing: A/Singapore/GP1908/2015 H1N1
EC_50_: half-maximal signal
ELISA: enzyme-linked immunosorbent assay
FA: forearm
FcR: Fc receptor
GMT: geometric mean titre
HA: haemagglutinin
HAI: haemagglutination inhibition
HD-MAP: high-density microarray patch
ID: intradermal
IFN: interferon
Ig: immunoglobulin
IIV: inactivated influenza vaccine
IL: interleukin
IM: intramuscular
MAP: microarray patch
MBC: memory B cell
MN: microneutralisation
MPH: monovalent purified harvest
N&S: needle and syringe
PBS: Phosphate-buffered saline
PBST: Phosphate-buffered saline Tween20 PBMC, peripheral blood mononuclear cell
QIV: quadrivalent influenza vaccine
RH: relative humidity
RT: room temperature
SAE: serious adverse event
SII: skin irritation index
TEAE: treatment emergent adverse event
TRBC: turkey red blood cell
TR-TEAE: treatment-related TEAE
TNF: tumour necrosis factor
UA: upper arm
