# Supplementary figures and images for: Safety, tolerability, and immunogenicity of influenza vaccination with a high-density microarray patch: Results from a randomized, controlled phase I clinical trial
Source: PLoS Med. 2020 Mar 17;17(3):e1003024. doi: 10.1371/journal.pmed.1003024 (PMC7077342; doi:10.1371/journal.pmed.1003024)

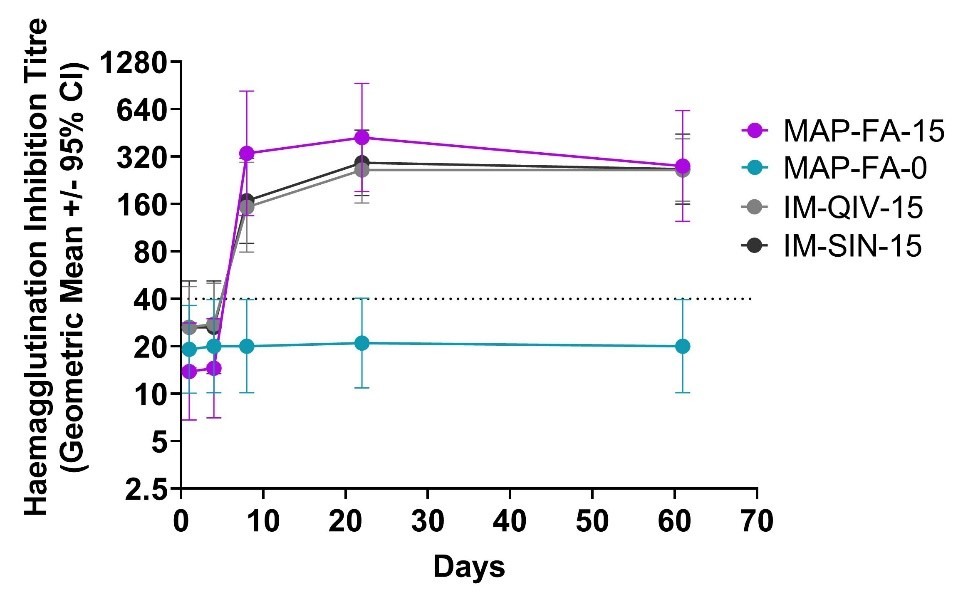

Supplement: S1 Fig — HAI titres at days 1 (prevaccination), 4, 8, 22, and 61 for participants in part A following vaccination with either A/Singapore/GP1908/2015 H1N1 delivered by HD-MAP (A-MAP-FA-15) or injected IM as a component of Afluria quadrivalent vaccine (A-IM-QIV-15), uncoated HD-MAP (A-MAP-FA-0), or A/Singapore/GP1908/2015 H1N1 monovalent pooled harvest injected IM (IM-SIN-15). Symbols represent the GMTs and error bars show the 95% confidence intervals. The dotted line indicates the HAI titre (1:40) regarded as correlating with protection. FA, forearm; GMT, geometric mean titre; HAI, HA inhibition; HD-MAP, high-density microarray patch; IM, intramuscular; QIV, quadrivalent influenza vaccine (TIF) [file pmed.1003024.s002.tif]

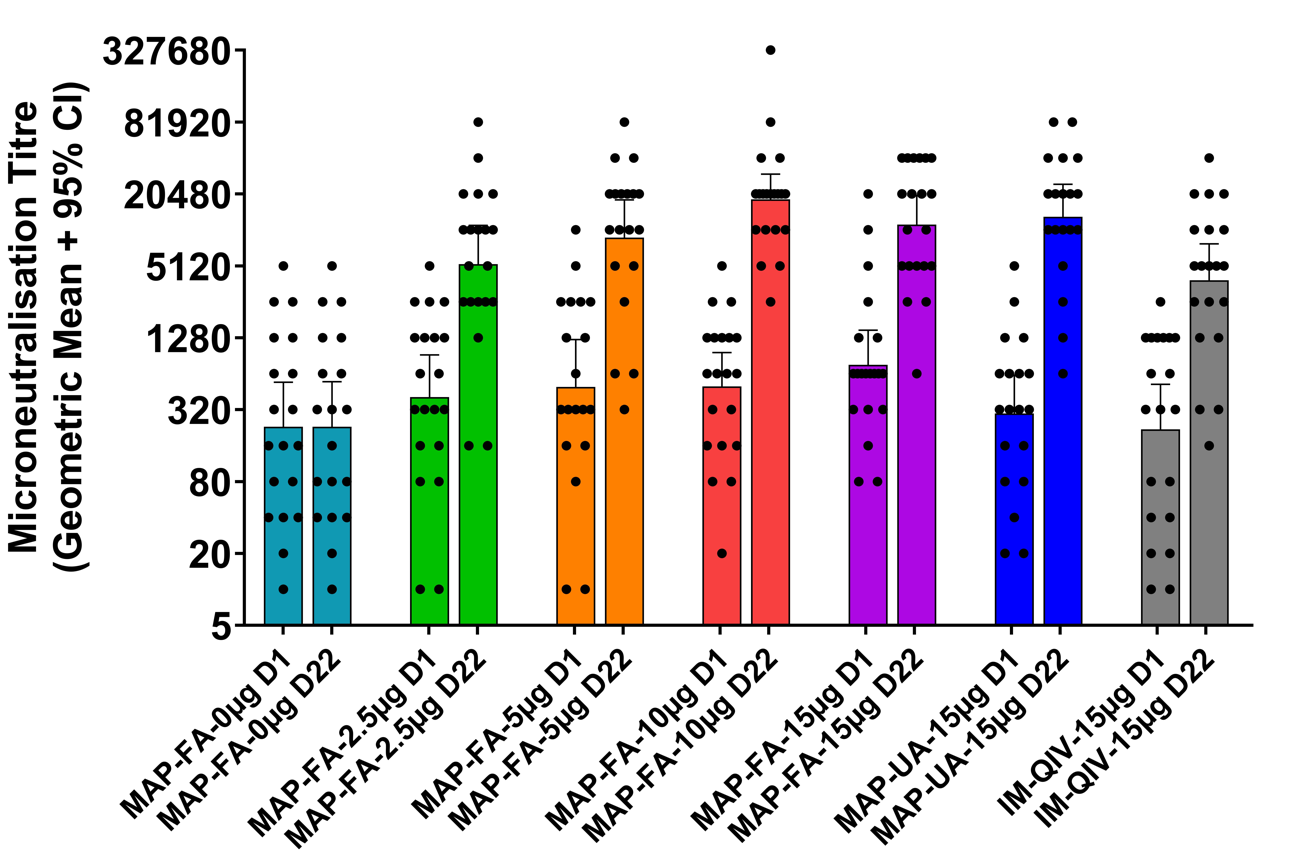

Supplement: S2 Fig — Microneutralisation titres at day 1 (prevaccination) and day 22 for participants in part B following vaccination with A/Singapore/GP1908/2015 H1N1 at 15, 10, 5, or 2.5 μg HA/dose delivered by HD-MAPs applied to the volar forearm (MAP-FA-15, MAP-FA-10, MAP-FA-5, MAP-FA-2.5), uncoated HD-MAPs (MAP-FA-0), A/Singapore/GP1908/2015 H1N1 at 15 μg HA/dose delivered by HD-MAP applied to the upper arm (MAP-UA-15), or injected IM as a component of Afluria quadrivalent vaccine (IM-QIV-15). Columns represent the GMTs, symbols represent the titres from individual participants, and the error bars show the 95% confidence intervals. FA, forearm; GMT, geometric mean titre; HA, haemagglutinin; HD-MAP, high-density microarray patch; IM, intramuscular; QIV, quadrivalent influenza vaccine; UA, upper arm (TIF) [file pmed.1003024.s003.tif]

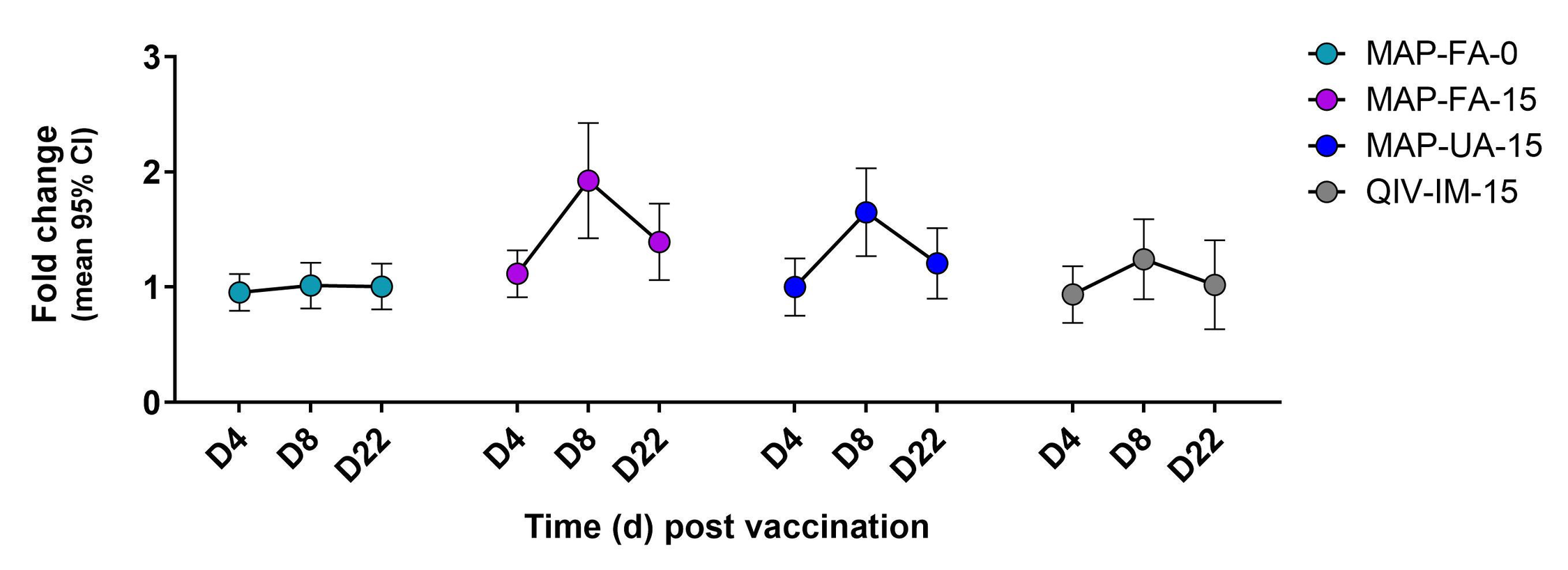

Supplement: S3 Fig — Participants were vaccinated with either 15 μg of A/Singapore/GP1908/2015 H1N1 delivered by HD-MAP to either the volar forearm (MAP-FA-15) or upper arm (MAP-UA-15), or injected IM as a component of Afluria quadrivalent vaccine (IM-QIV-15) or uncoated HD-MAPs (MAP-FA-0). Four time points were measured: prevaccination (day 1), day 4, 8, and 22. The absorbance values per group for each time point were averaged and compared against day 1, and the fold-change compared with prevaccination (day 1) were plotted. Symbols represent the means from all participants per group, and the error bars show the 95% confidence intervals. Statistical analysis was not performed because of some saliva samples being incomplete and large sample variation. FA, forearm; HD-MAP, high-density microarray patch; IgA, immunoglobulin A; IM, intramuscular; QIV, quadrivalent influenza vaccine; UA, upper arm (TIF) [file pmed.1003024.s004.tif]
