# Supplementary material for: Safety, tolerability, and immunogenicity of influenza vaccination with a high-density microarray patch: Results from a randomized, controlled phase I clinical trial
Source: PLoS Med. 2020 Mar 17;17(3):e1003024. doi: 10.1371/journal.pmed.1003024 (PMC7077342; doi:10.1371/journal.pmed.1003024)
Supplement: S1 Table — HAI responses to vaccination in terms of median titre, seroprotection, seroconversion, and fold-increase in median titre above prevaccination levels for part B. Part B participants were vaccinated with A/Singapore/GP1908/2015 H1N1 at 15, 10, 5, or 2.5 μg HA/dose delivered by HD-MAPs applied to the volar forearm (MAP-FA-15, MAP-FA-10, MAP-FA-5, MAP-FA-2.5), uncoated HD-MAPs (MAP-FA-0), A/Singapore/GP1908/2015 H1N1 at 15 μg HA/dose delivered by HD-MAP applied to the upper arm (MAP-UA-15), or injected IM as a component of the Afluria quadrivalent vaccine (IM-QIV-15). Exact nonparametric CIs are shown in parentheses. *p < 0.05; **p < 0.01 compared to the IM-QIV-15 group by Exact Mann Witney Test (median titre and median fold increase). Pearson's chi-square test with continuity correction was used to compare proportion of participants seroconverted or seroprotected. FA, forearm; HA, haemagglutinin; HAI, HA inhibition; HD-MAP, high-density microarray patch; IM, intramuscular; QIV, quadrivalent influenza vaccine; UA, upper arm. (DOCX) [file pmed.1003024.s005.docx]

**S1 Table. Haemagglutination inhibition responses, part B, non-parametric analysis.**

|  |  | **MAP-FA-15** | **MAP-FA-10** | **MAP-FA-5** | **MAP-FA-2.5** | **MAP-FA-0** | **MAP-UA-15** | **IM-QIV-15** |
| --- | --- | --- | --- | --- | --- | --- | --- | --- |
| **Day 1** | Median titre (95% CI) | 28 (10–40) | 20 (5–40) | 20 (10–40) | 20 (10–40) | 14 (5–40) | 14 (5–40) | 14 (5­40) |
|  | Seroprotection; N (%) | 10/20 (50) | 9/20 (45) | 8/20 (40) | 8/20 (40) | 6/20 (30) | 6/20 (30) | 7/20 (35) |
| **Day 4** | Median titre (95% CI) | 28 (10–40) | 20 (5–40) | 20 (20–80) | 20 (10–40) | 14 (5, 40) | 14 (5–40) | 14 (5­40) |
|  |  | p = 0.24 | p = 0.33 | p = 0.23 | p = 0.46 |  | p = 0.81 |  |
|  | Seroprotection; N (%) | 10/20 (50) | 9/20 (45) | 7/19 (37) | 8/20 (40) | 6/20 (30) | 7/20 (35) | 7/20 (35) |
|  | Seroconversion; N (%) | 0/20 (0) | 0/20 (0) | 0/19 (0) | 0/20 (0) | 0/20 (0) | 0/20 (0) | 0/20 (0) |
|  | Median fold-increase (95% CI) | 1 (1–1) | 1 (1–1) | 1 (1–1) | 1 (1–1) | 1 (1, 1) | 1 (1­1) | 1 (1, 1) |
|  |  | p = 1.00 | p = 0.51 | p = 1.00 | p = 1.00 |  | p = 1.00 |  |
| **Day 8** | Median titre (95% CI) | 226 (160–640) | 320 (320–640) | 160 (80–320) | 80 (40–160) | 14 (5–40) | 320 (160­640) | 160 (80­160) |
|  |  | p = 0.02* | p < 0.001** | p = 0.23 | p = 0.56 |  | p = 0.01* |  |
|  | Seroprotection; N (%) | **19/20 (95)** | **20/20 (100)** | **17/20 (85)** | **18/20 (90)** | 6/20 (30) | **18/20 (90)** | **17/20 (85)** |
|  | Seroconversion; N (%) | **17/20 (85)** | **18/20 (90)** | **12/20 (60)** | **11/20 (55)** | 0/20 (0) | **18/20 (90)** | **15/20 (75)** |
|  | Median fold-increase (95% CI) | 8 (4–32) | 23 (8–64) | 4 (2–16) | 4 (2–8) | 1 (1, 1) | 16 (8­32) | 6 (4­16) |
|  |  | p = 0.34 | p = 0.01* | p = 0.57 | p = 02 |  | p = 0.02* |  |
| **Day 22** | Median titre (95% CI) | 320 (160–640) | 453 (320–640) | 320 (160–640) | 226 (80–320) | 14 (5–40) | 640 (160­1280) | 160 (80­320) |
|  |  | p = 0.04* | p = 0.002** | p = 0.18 | p = 85 |  | p = 0.01* |  |
|  | Seroprotection; N (%) | **19/20 (95)** | **20/20 (100)** | **18/20 (90)** | **18/20 (90)** | 6/20 (30) | **19/20 (95)** | **17/20 (85)** |
|  | Seroconversion; N (%) | **17/20 (85)** | **18/20 (90)** | **14/20 (70)** | **16/20 (80)** | 0/20 (0) | **18/20 (90)** | **15/20 (75)** |
|  | Median fold-increase (95% CI) | 16 (8–32) | 32 (16–64) | 8 (4–32) | 8 (4–32) | 1 (1–1) | 32 (16­64) | 8 (8­16) |
|  |  | p = 0.43 | p = 0.04* | p = 0.91 | p = 0.49 |  | p = 0.02* |  |
| **Day 61** | Median titre (95% CI) | 226 (160–320) | 320 (160–640) | 226 (160–640) | 160 (80–320) | 14 (3–40) | 320 (160­640) | 113 (40­320) |
|  |  | p = 0.12 | p = 0.01* | p = 0.10 | p = 0.69 |  | p = 0.03* |  |
|  | Seroprotection; N (%) | **19/20 (95)** | **20/20 (100)** | **19/20 (95)** | **18/20 (90)** | 6/20 (30) | **19/20 (95)** | **17/20 (85)** |
|  | Seroconversion; N (%) | **16/20 (80)** | **18/20 (90)** | **14/20 (70)** | **15/20 (75)** | 0/20 (0) | **18/20 (90)** | **13/20 (65)** |
|  | Median fold-increase (95% CI) | 16 (4–16) | 16 (16–16) | 11 (4–16) | 6 (4–32) | 1 (1–1) | 16 (16­32) | 8 (4­16) |
|  |  | p = 0.66 | p = 0.07 | p = 0.82 | p = 0.73 |  | p = 0.02* |  |
