# Supplementary material for: Safety, tolerability, and immunogenicity of influenza vaccination with a high-density microarray patch: Results from a randomized, controlled phase I clinical trial
Source: PLoS Med. 2020 Mar 17;17(3):e1003024. doi: 10.1371/journal.pmed.1003024 (PMC7077342; doi:10.1371/journal.pmed.1003024)
Supplement: S2 Table — Microneutralisation responses (median titres) at days 1 and 22 for part B. Part B participants were vaccinated with A/Singapore/GP1908/2015 H1N1 at 15, 10, 5, or 2.5 μg HA/dose delivered by HD-MAPs applied to the volar forearm (MAP-FA-15, MAP-FA-10, MAP-FA-5, MAP-FA-2.5), uncoated HD-MAPs (MAP-FA-0), A/Singapore/GP1908/2015 H1N1 at 15 μg HA/dose delivered by HD-MAP applied to the upper arm (MAP-UA-15), or injected IM as a component of the Afluria quadrivalent vaccine (IM-QIV-15). Exact nonparametric CIs are shown in parentheses. *p < 0.05; **p < 0.01 compared to the IM-QIV-15 group by Exact Mann Witney Test. FA, forearm; HA, haemagglutinin; HD-MAP, high-density microarray patch; IM, intramuscular; QIV, quadrivalent influenza vaccine; UA, upper arm (DOCX) [file pmed.1003024.s006.docx]

**S2 Table. Microneutralization responses, part B, non-parametric analysis**.

|  |  | **MAP-FA-15** | **MAP-FA-10** | **MAP-FA-5** | **MAP-FA-2.5** | **MAP-FA-0** | **MAP-UA-15** | **IM-QIV-15** |
| --- | --- | --- | --- | --- | --- | --- | --- | --- |
| **Day 1** | Median titre (95% CI) | 640 (320–1280) | 640 (160–1280) | 320 (320–2560) | 453 (160–1280) | 160 (80–1280) | 320 (160–640) | 320 (40–1280) |
| **Day 22** | Median titre (95% CI) | 14482 (512–40960) | 20480 (10240–20480) | 10240 (5120–20480) | 7241 (2560–10240) | 320 (80–1280) | 20480 (10240–40960) | 5120 (2560–10240) |
|  |  | p = 0.02* | p = 0.001** | p = 0.06 | p = 0.54 |  | p = 0.008** |  |
