# Supplementary material for: Safety, tolerability, and immunogenicity of influenza vaccination with a high-density microarray patch: Results from a randomized, controlled phase I clinical trial
Source: PLoS Med. 2020 Mar 17;17(3):e1003024. doi: 10.1371/journal.pmed.1003024 (PMC7077342; doi:10.1371/journal.pmed.1003024)
Supplement: S1 Text — (PDF) [file pmed.1003024.s007.pdf]

Commercial in Confidence

STUDY PROTOCOL

PROTOCOL TITLE:	***First-in-human study with micro-projection array patches coated with inactivated split influenza virus haemagglutinin (HA) from A/Singapore/GP1908/2015 (A/Michigan/45/2015(H1N1)-like) vaccine.***
PROTOCOL NUMBER:	***SP-1207-022***
INVESTIGATIONAL PRODUCT NAME:	***1. A/Singapore coated micro-projection array patch and Applicator***<br>***2. Afluria® Quadrivalent influenza vaccine***<br>***3. A/Singapore intramuscular injection***
DOSES:	***Intradermal administration using a micro-projection array patch and Applicator: 0, 2.5, 5, 10 or 15 micrograms of A/Singapore/GP1908/2015 HA protein***<br>***Intramuscular injection: 15 micrograms of A/Singapore/GP1908/2015 HA protein (as Afluria® Quadrivalent and as A/Singapore)***
STUDY DESIGN:	***Two-part randomised, double-blind, placebo-controlled study in healthy subjects, with 4 parallel treatment groups in Part A and 9 parallel treatment groups in Part B***
SPONSOR:	***Vaxxas Pty Ltd***<br>***Suite 13.02, Level 13, 179 Elizabeth Street, Sydney, NSW 2000***
SPONSOR REPRESENTATIVE:	***Dr Angus Forster***<br>***Vaxxas Pty Limited***<br>***Translational Research Institute***<br>***37 Kent Street***<br>***Woolloongabba, QLD 4102***<br>***AUSTRALIA***
PRINCIPAL INVESTIGATOR:	***Dr Jason Lickliter, MBBS, PhD, FRACP***<br>***Medical Director, Nucleus Network Pty Ltd***<br>***The Burnet Tower, 5th Floor***<br>***89 Commercial Rd***<br>***Melbourne, VIC 3004***<br>***AUSTRALIA***

CONFIDENTIAL

Information contained in this protocol is confidential and must not be disclosed, other than to those directly involved in the review and conduct of the study, without written approval from Vaxxas Pty Limited. However, this information can be provided to subjects in order to obtain consent.

Document:	***Protocol Date:***	***Approval Date:***
FINAL	*19 December 2017.*	*19 December 2017*
Amendment 1	....	....
Amendment 2	....	....
Amendment 3	....	....

Commercial in Confidence

STATEMENT OF CONFIDENTIALITY

Property of Vaxxas Pty Ltd

May not be published, divulged or otherwise disseminated to others without written approval from the sponsor.

This study will be conducted in accordance to the principles of Good Clinical Practice as described by the International Conference on Harmonisation guidelines, including the archiving of essential documents. Guidelines in the National Statement on Ethical Conduct in Human Research ratified by the National Health & Medical Research Council and Australian Vice Chancellors' Committee will be observed.

TABLE OF CONTENTS

STUDY PROTOCOL 1

STATEMENT OF CONFIDENTIALITY 2

TABLE OF CONTENTS 3

1 LIST OF ABBREVIATIONS 7

2 STUDY INFORMATION 8

3. SIGNATURE PAGES 9

Sponsor's Signature 9

Investigator's Statement and Signature: 9

Principal Investigator: 9

4 SUMMARY 10

4.1 Study Summary 10

5 INTRODUCTION 17

5.1 Background Information 17

5.2 Investigational Product 18

5.3 Non-clinical Studies 19

5.4 Previous Clinical Studies 20

5.4.1 Safety study of MAP without vaccine 20

5.4.2 Supportive clinical data from silicon Nanopatches 20

5.5 Summary of potential risks and benefits 21

6 STUDY OBJECTIVES AND ENDPOINTS 24

6.1 Study Objectives 24

Primary Objective: 24

Exploratory Objectives: 24

6.2 Study Endpoints 24

6.2.1 Primary Safety Endpoints 24

6.2.2 Secondary Endpoints 24

6.2.3 Exploratory Endpoints 24

7 STUDY PLAN 25

7.1 Overall Study Design 25

7.2 Rationale for Study Design and Treatment Regimens 27

7.3 Duration of Study 28

7.4 Treatment Site Examination Prior to MAP application / Intramuscular injection 28

7.5 MAP Application and Removal 28

7.6 Intramuscular Injection 29

7.7 Treatment Site Examination after Application or Intramuscular Injection 29

7.8 Examination of Treatment Site at Each Visit 29

7.9 Photography of Application / Administration Site 29

7.10 Skin Assessment - Durometer 29

7.11 Pain scores ... 29
7.12 Investigation of the MAP Performance ... 29
7.13 Measurement of Immunogenicity ... 29
7.14 Saliva IgA - Part B only ... 30
7.15 Peripheral Blood Mononuclear Cells (PBMCs) for Immunoassay - Part B only ... 30
7.16 Biopsy (punch) - Part B only groups (B1 and B2) ... 30
8 STUDY POPULATION ... 31
8.1 Number and Identification of Subjects ... 31
8.2 Sample Size ... 31
8.3 Inclusion Criteria ... 31
8.4 Exclusion Criteria ... 32
8.5 Screening and Selection ... 33
8.6 Subject Withdrawal Criteria ... 33
9 STUDY PROCEDURES AND ASSESSMENTS ... 35
9.1 Study Procedures for all Cohorts ... 35
9.1.1 Volume of Blood Collected ... 35
9.2 Study procedure by Visits ... 35
9.2.1 Screening and Enrolment Phase ... 35
9.2.2 Treatment Phase ... 36
9.3 Subject Restrictions and Concomitant Medications ... 39
9.3.1 Subject Restrictions ... 39
9.3.2 Concomitant Medications ... 39
9.4 Early Withdrawal Visit. ... 39
9.5 Subject Discontinuation (Lost to Follow-up) ... 40
9.6 Safety and Tolerability Assessments ... 40
9.6.1 Clinical Safety Measurements ... 40
9.6.2 Clinical Laboratory Tests ... 40
9.6.3 Treatment Site Tolerability Assessment ... 40
10 MANAGEMENT OF DRUG AND DEVICES ... 41
10.1 Investigational Product – MAP, Skin conditioning ring and Applicator ... 41
10.3 Packaging and Labelling ... 41
10.4 Dispensing and Accountability of drugs and devices ... 42
10.5 Study Drugs and Devices Handling and Storage ... 42
11 ADVERSE EVENTS ... 43
11.1 Definition of an Adverse Event ... 43
11.2 Definition of a Serious Adverse Event ... 44
11.3 Clinical Laboratory Abnormalities and Other Abnormal Assessments as AEs and SAEs ... 44
11.4 Time Period, Frequency, and Method of Detecting AEs and SAEs ... 45
11.5 Recording of AEs and SAEs ... 45

11.6 Evaluating AEs and SAEs 45
11.6.1 Assessment of severity 45
11.6.2 Assessment of causality 46
11.6.3 Halt Criteria 46
11.7 Follow-up of AEs and SAEs 47
11.8 Reporting of SAEs 48
11.8.1 Completion and transmission of the SAE reports to the Sponsor 48
11.8.2 Regulatory Reporting Requirements for SAEs 48
11.9 Post-study AEs and SAEs 48
12 DATA AND STATISTICAL ANALYSIS 50
12.1 Data collection and Processing 50
12.1.1 Data collection 50
12.1.2 Data Processing 50
12.2 Statistical Analysis 50
12.2.2 Subject Number Allocation 50
12.2.3 Populations to be analysed 50
12.2.4 Data analysis 50
12.2.5 Subject Accountability 51
12.2.6 Protocol Deviations 51
12.2.7 Population Sub-group Analyses 51
12.3 Statistical Methods 51
12.4 Safety Analysis 51
12.4.1 Adverse Events 51
12.4.2 Laboratory Parameters 51
12.4.3 Vital Signs 51
12.4.4 Physical examination 52
12.4.5 Local and Systemic Reactions 52
13 ETHICAL CONSIDERATIONS 53
13.1 Good Clinical Practice 53
13.2 Regulatory Approval 53
13.3 Human Research Ethics Committee Approval 53
13.4 Ethical Considerations 53
13.5 Written Informed Consent 53
13.6 Emergency Contact with Investigator 54
13.7 Clinical Laboratory Certification and Reference Ranges 54
13.8 Protocol Amendments 54
13.9 Termination of the Study 54
14 REPORTS AND PUBLICATIONS 55
14.1 Reports to the HREC 55

14.2 Publications 55
15 STUDY ADMINISTRATION 56
15.1 Quality Assurance 56
15.2 Adherence to the Protocol 56
15.3 Records Retention 56
16 FINANCE, INDEMNITY, AND CONFIDENTIALITY 58
16.1 Ownership 58
16.2 Confidentiality 58
18 APPENDICES 59
18.1 APPENDIX 1: Procedure for the MAP Application and skin hardness assessment 60
18.2 APPENDIX 2: Schedule of Assessments – Part A and Part B 62
18.3 APPENDIX 3: Clinical Laboratory Tests 63
18.4 APPENDIX 4: Treatment Site Tolerability Assessment 64
18.5 APPENDIX 5: Numeric Pain Intensity Scale 66
18.6 APPENDIX 6: Skin irritation index Scoring System for each treatment application site* 67
18.7 APPENDIX 7: Photography 68
18.8 APPENDIX 8: Immunogenicity Assays 69

1 LIST OF ABBREVIATIONS

Abbreviation	*Description*
A/Sing	*A/Singapore/GP1908/2015 (A/Michigan/45/2015 (H1N1)-like) virus*
AE	*Adverse Event*
Beta-HCG	*Beta Human Chorionic Gonadotropin*
BMI	*Body Mass Index (weight in kg divided by height in m^2)*
CAPD	*Vaxxas Clinical Applicator, disposable*
cGMP	*Current Good Manufacturing Practice*
CoA	*Certificate of Analysis*
CRF	*Case Report Form*
CTN	*Clinical Trial Notification*
EOS	*End of Study*
FDA	*US Food and Drug Administration*
FPS	*Finished Product Specification*
GCP	*Good Clinical Practice*
GLP	*Good Laboratory Practice*
HA	*Haemagglutinin protein*
HIV	*Human Immunodeficiency virus*
Hep B	*Hepatitis B*
Hep C	*Hepatitis C*
HI	*Haemagglutination inhibition*
HREC	*Human Research Ethics Committee*
ICH	*International Conference on Harmonisation of Technical Requirements for Registration of Pharmaceuticals for Human Use*
IM	*Intramuscular*
MNT	*Microneutralisation assay*
N&S	*Needle and Syringe*
SAE	*Serious Adverse Event*
SOPs	*Standard Operating Procedures*
TEAE	*Treatment Emergent Adverse Event*
TGA	*Therapeutic Goods Administration*

2 STUDY INFORMATION

SPONSOR:	*Vaxxas Pty Limited<br>Suite 13.02, Level 13<br>179 Elizabeth Street<br>Sydney, NSW 2000<br>AUSTRALIA*
SPONSOR REPRESENTATIVE:	*Dr Angus Forster<br>Vaxxas Pty Limited<br>Translational Research Institute<br>37 Kent Street<br>Woolloongabba, QLD 4102<br>AUSTRALIA<br>Phone: +61 7 3443 7838<br>Mobile: +61 435 599 890<br>E-mail: aforster@vaxxas.com*
INVESTIGATOR:	*Dr Jason Lickliter, MBBS, PhD, FRACP<br>Medical Director, Nucleus Network Pty Ltd<br>The Burnet Tower, 5th Floor<br>89 Commercial Rd<br>Melbourne, VIC 3004<br>AUSTRALIA<br>Phone: +61 3 9076 8900<br>Email: j.lickliter@nucleusnetwork.com.au*
MEDICAL MONITOR:	*Professor Guy Ludbrook<br>Anaesthesia, 3G Room 395<br>Royal Adelaide Hospital<br>Port Rd<br>Adelaide, SA 5000<br>AUSTRALIA<br>Phone: +61 8 7074 1258<br>Mobile: +61 413 817 901<br>Email: guy.ludbrook@sa.gov.au*
Clinical Research Organisation (CRO):	*CPR Pharma Services Pty Ltd<br>28 Dalgleish Street<br>Thebarton, SA 5031<br>AUSTRALIA<br>Phone: +61 8 8159 6255*
STATISTICAL ANALYSIS AND REPORTING:	*CPR Pharma Services Pty Ltd<br>28 Dalgleish Street<br>Thebarton, SA 5031<br>AUSTRALIA<br>Phone: +61 8 8159 6255*
CLINICAL FACILITY:	*Nucleus Network Pty Ltd<br>The Burnet Tower, 5th Floor<br>89 Commercial Rd<br>Melbourne, VIC 3004<br>AUSTRALIA<br>Phone: +61 3 9076 8900*

3. SIGNATURE PAGES

Sponsor's Signature

Protocol Title:	***A First-in-human study with micro-projection array patches coated with inactivated split influenza virus haemagglutinin from A/Singapore/GP1908/2015 (A/Michigan/45/2015(H1N1)-like) vaccine.***
Protocol Number:	***SP-1207-022***

This clinical trial protocol has been reviewed and approved by the Sponsor.

19 Dec 2017

Dr Angus Forster *Date*

Vaxxas Pty Ltd

Investigator's Statement and Signature:

I have read and understood the information in this protocol and agree to conduct the trial according to the protocol (subject to any amendments). Any changes in procedure will only be made if necessary to protect the safety, rights or welfare of subjects.

I agree to conduct in person or to supervise the trial.

I agree to ensure that all that assist me in the conduct of the study are aware of their obligations.

Principal Investigator:

19 DEC 2017

Signature *Date*

Dr Jason Lickliter, MBBS, PhD, FRACP
Medical Director, Nucleus Network Pty Ltd

Commercial in Confidence

4 SUMMARY

4.1 Study Summary

**Sponsor Name and Address:**	Vaxxas Pty Limited<br>Suite 13.02, Level 13<br>179 Elizabeth Street<br>Sydney, New South Wales 2000<br>AUSTRALIA
**Study Title:**	**First-in-human study with micro-projection array patches coated with inactivated split influenza virus haemagglutinin from A/Singapore/GP1908/2015 (A/Michigan/45/2015(H1N1)-like) vaccine.**
**Protocol Number:**	SP-1207-022
**Study Population:**	Healthy male and non-pregnant, non-nursing female subjects aged 18-50 years
**Indication:**	Prevention of influenza infection
**Development Phase:**	Phase I
**Objectives of the Study:**	Primary Objective:<br>• To evaluate the safety and tolerability of A/Singapore/GP1908/2015 antigen delivered by the micro-projection array patch (MAP) in comparison to an uncoated MAP and intramuscular administration of both a quadrivalent seasonal influenza vaccine and a monovalent vaccine delivering approximately the same dose of A/Singapore/GP1908/2015 (A/Sing) HA protein.<br>Exploratory Objectives:<br>• To evaluate the immune responses to MAP application to the forearm with A/Singapore/GP1908/2015 (A/Sing) at 4 dose levels (0, 2.5, 5, 10 and 15 micrograms) in comparison to IM administration of the standard 15 microgram HA dose per strain.<br>• To evaluate the skin penetration performance of applied A/Sing MAPs at two sites of application (forearm and upper arm).<br>• In a subset of subjects, to assess further measures of immune response through additional assays or assessment of the local skin response via punch biopsy at the micro-projection array application sites.
**Study Design:**	Two-part randomised, double-blind, placebo-controlled study in healthy subjects, with 4 parallel treatment groups in Part A and 9 parallel treatment groups in Part B
**Safety Procedures**	Blood pressure, oral temperature, heart rate, respiratory rate, biochemistry, haematology, pregnancy testing for females, serology (human immunodeficiency virus (HIV), Hepatitis B and C), adverse event monitoring and local tolerability and pain assessment of the micro-projection array patch application site and intramuscular injection site.
**Investigational Product and mode of administration**	The A/Sing coated MAP is an aseptically produced 1cm² polymer patch with ~3176 micro-projections coated with A/Sing, sulfobutyl ether (β) cyclodextrin and phosphate buffered saline.<br>Uncoated MAP is a terminally sterilised 1 cm² polymer patch with ~3176 micro-projections.<br>The patch delivery device is a hand-held mechanical, spring loaded, disposable applicator (CAPD) calibrated at a specific speed of 20 m/s.
**Number of Centres:**	1

**Planned number of subjects:**	Two hundred and ten (210) healthy subjects, of whom at least 30% are male, and at least 30% are female.<br>**Part A:** Sixty (60) healthy subjects (4 groups of 15)<br>**Part B:** One hundred and fifty (150) healthy subjects (7 groups of 20, plus 2 groups of 5 with punch biopsies performed)
**Eligibility Criteria:**	**Inclusion Criteria**<br>Subjects must meet all of the following criteria to be eligible for participation in this study:<br>1. Aged 18-50 years (inclusive).<br>2. Subject has a Body Mass Index (BMI) within the range 18.0–30.0 kg/m²<br>3. Satisfactory medical assessment, with no clinically significant or relevant abnormalities in medical history, physical examination, vital signs and laboratory evaluation (haematology or biochemistry)<br>4. Adequate venous access in their left or right arms to allow collection of a number of blood samples.<br>5. Females of childbearing potential and males should either be sexually inactive (abstinent) for 14 days prior to screening and throughout the study or be using one of the following acceptable birth control methods:<br>i. Surgically sterile (hysterectomy and/or bilateral oophorectomy);<br>ii. Surgically sterile (bilateral tubal ligation with surgery at least 6 months prior to study initiation);<br>iii. IUD in place for at least 3 months;<br>iv. Stable hormonal contraceptive for at least 3 months prior to study through completion of study;<br>v. Surgical sterilization (vasectomy) for male participants or for female participant's partner at least 6 months prior to study<br>vi. Condom for male participant together with effective contraception for their female partner.<br>6. Postmenopausal women must have had at least 12 months since their last menstrual period<br>7. Subject is able to communicate effectively with study personnel and is considered reliable, willing and cooperative in terms of compliance with the protocol requirements.<br>8. Subject is able and willing to provide written, personally signed and dated informed consent to participate in the study.<br>**Exclusion Criteria**<br>Subjects meeting any of the following criteria will not be eligible for participation in this study:<br>1. Subject with birthmarks, tattoos, wounds, scars, moles, blemishes, heavy hair or other skin conditions (such as eczema) on forearms and upper arm regions (both arms) which could reasonably obscure application site reactions.<br>2. Subject with known chronic spontaneous urticaria / dermographism<br>3. Known anaphylactic hypersensitivity to a previous influenza vaccination or to eggs, neomycin, polymyxin B sulphate or any of the constituents or trace residues of the study vaccine.<br>4. Has received an influenza vaccine or has been diagnosed by a doctor as having influenza in the last 12 months.<br>5. Known history of Guillain-Barré syndrome.

	6. Recent vaccination (within 30 days prior to enrolment) with any vaccine.<br>7. Known predisposition to keloid scar formation.<br>8. History of granulomatous diseases (especially sarcoidosis and granuloma annulare).<br>9. History of clinically significant gastrointestinal, hepatic, renal, cardiovascular, dermatological, immunological, respiratory, endocrine, oncological, neurological, metabolic, psychiatric disease or haematological disorders.<br>10. History of malignancy, other than non-melanoma skin cancer.<br>11. An active medical condition (which is deemed as clinically significant) that is under evaluation or treatment, or a recent illness, a chronic illness, an autoimmune disease or had major surgery within the last year.<br>12. History of Hepatitis B, Hepatitis C or HIV infection or clinical laboratory serology is positive for Hepatitis B surface antigen, Hepatitis C or HIV antibodies.<br>13. History of abnormal bleeding tendencies or thrombophlebitis unrelated to venepuncture or intravenous cannulation.<br>14. Receiving chronic treatment with immune-suppressive therapy (asthma inhalers and topical corticosteroids are permitted). All medications will be documented and reviewed for acceptance by the Investigator or a medically qualified nominee.<br>15. History of any psychiatric illness or psychological disorder which may impair the ability to provide written informed consent or participate in the study.<br>16. Subject has donated blood or plasma or clinically significant blood loss within 60 days prior to screening visit.<br>17. Subject is pregnant or breast-feeding.<br>18. A history of alcohol or drug abuse in the last 12 months or current alcohol consumption is >4 standard drinks (or equivalent) per day.<br>19. Use of any prescription medication (except for contraceptives) within 7 days, unless approved by the PI. All medications will be documented and reviewed for acceptance by the Investigator or a medically qualified nominee.<br>20. Use of any investigational drug or device within 30 days or 5 half-lives of the drug, whichever is longer, prior to the Day 1.<br>21. Previous exposure to the Nanopatch and its applicator as a participant in previous clinical studies.
**Treatment Groups:**	**<u>Pilot Study (Part A):</u>**<br><br>There will be four treatment groups in the pilot study (Part A):<br><br>**Group A1**: 15 subjects receive 3 x A/Sing MAPs on volar forearm delivering a total of 15 µg of A/Sing Haemagglutinin (HA) protein.<br><br>**Group A2**: 15 subjects receive intramuscular injection to the deltoid muscle delivering a total of **15 µg** of A/Sing Haemagglutinin (HA) protein in a 0.5 mL quadrivalent influenza vaccine injection (Afluria® Quadrivalent).<br><br>**Group A3**: 15 subjects receive 3 x uncoated MAPs (placebo) on volar forearm.<br><br>**Group A4**: 15 subjects receive intramuscular injection to the deltoid muscle delivering a total of 15 µg of A/Sing Haemagglutinin (HA) protein in a 0.5 mL monovalent influenza antigen injection.

	Immunogenicity (Day 22) and safety results from Part A will be assessed and a decision taken to continue to Part B.<br><br>**<u>Main Study (Part B):</u>**<br><br>There will be 9 treatment groups:<br><br>**Group B1:** 5 subjects receive 3 x A/Sing MAPs on volar forearm delivering a total of **15 µg** of A/Sing Haemagglutinin (HA) protein. Punch biopsies are performed at the application sites at defined time-points.<br><br>**Group B2** 5 subjects receive 3 x uncoated MAPs on volar forearm. Punch biopsies are performed at the application sites a defined time-points.<br><br>**Group B3:** 20 subjects receive 3 x A/Sing MAPs on volar forearm delivering a total of **15 µg** of A/Sing Haemagglutinin (HA) protein.<br><br>**Group B4:** 20 subjects receive 2 x A/Sing MAPs delivering a total of **10 µg** of A/Sing Haemagglutinin (HA) protein and 1 x uncoated MAP on volar forearm.<br><br>**Group B5:** 20 subjects receive 1 x A/Sing MAP delivering a total of **5 µg** of A/Sing Haemagglutinin (HA) protein and 2 x uncoated MAPs on volar forearm.<br><br>**Group B6:** 20 subjects receive 1 x A/Sing MAP delivering a total of **2.5 µg** of A/Sing Haemagglutinin (HA) protein and 2 x uncoated MAPs on volar forearm.<br><br>**Group B7:** 20 subjects receive 3 x uncoated MAPs on volar forearm.<br><br>**Group B8:** 20 subjects receive 3 x A/Sing MAPs on the upper arm to the skin overlaying the deltoid muscle delivering a total of **15 µg** of A/Sing Haemagglutinin (HA) protein.<br><br>**Group B9:** 20 subjects receive intramuscular injection to the deltoid muscle delivering a total of **15 µg** of A/Sing Haemagglutinin (HA) protein in a 0.5 mL quadrivalent influenza vaccine injection (Afluria Quadrivalent).
**Study Procedures:**	Potential study subjects will attend for a screening visit. All Subjects will be asked to provide Informed consent prior to any study assessment. Subjects will be assessed for study eligibility including a complete physical examination, height, weight, vital signs. Blood will be taken for Haematology and Biochemistry safety parameters and Serology for Hep B, Hep C and HIV. Subjects will have their medical history, demographic and concomitant medication information recorded. Females will be tested for pregnancy.<br><br>Potential subjects who meet all the eligibility criteria during screening will be admitted on Day 1 (Visit 1) for randomisation into the study upon confirmation of ongoing eligibility. The subjects' medical history and current medications will be reviewed along with their continued eligibility for the study. Prior to treatment, blood samples will be collected for haematology and biochemistry, vital signs will be measured and females will undergo a urine pregnancy test. For all subjects, a 15 mL blood sample will be collected for immunogenicity assays. For subjects in Part B (non biopsy), a saliva sample and an additional, separate, 40 mL blood sample will be collected for exploratory immunogenicity assays. The application/ injection site will be examined and photographed (baseline). For subjects in Part B (biopsy), two 4mm Punch biopsies will be performed on the non-treatment forearm.<br><br>As part of the MAP application process skin hardness values will be taken with and without the skin conditioning ring for each application site. The hardness values are obtained using a non invasive

	durometer. Following treatment administration on Day 1: pain will be assessed at 1 minute, 10 mins, 1 hr and 2 hr post application. The application/injection site will be photographed and assessed at 10 minutes, 1 hr and 2 hr post application/injection for any local site reactions (tolerability and skin irritability). Vital signs will be measured at 2 hr post application. Subjects will be discharged from the unit after all assessments have been completed and a 2 hour observation period has elapsed.<br><br>On Day 2 (24 hr post treatment) for subjects in Part A, a follow up phone call will be made to subjects to ask about adverse events or changes in concurrent medication.<br><br>On Day 2 (24 hr post treatment) for subjects in Part B, subjects will return to the clinic for review of any local site reactions (tolerability and skin irritability) and for further application/injection site photographs to be taken.<br><br>On Day 3 (48 hr post treatment), a follow up phone call will be made to all subjects to ask about adverse events or changes in concurrent medication<br><br>On Day 4 (3 days post treatment), subjects will return to the clinic for review of any local site reactions (tolerability and skin irritability), vital signs measurement, and for further application/injection site photographs to be taken. Two 4mm punch biopsies will be performed for subjects assigned to a punch biopsy group. The punch biopsies will be taken from within the area of patch application from 2 of 3 application sites. For subjects in Part A and Part B a 15 mL blood sample will be taken for immunogenicity analysis. For subjects in Part B (non biopsy) a saliva sample will be collected.<br><br>On Day 8 (+/- 1 day), subjects will return to the clinic for review of any local site reactions (tolerability and skin irritability), vital signs measurement, and for further application/injection site photographs to be taken. For subjects in Part A and B a 15 mL blood sample will be taken for immunogenicity analysis. For subjects in Part B (non biopsy) a saliva sample will be collected.<br><br>On Day 22 (+/- 1 day) subjects will return to the clinic to review any local site reactions (tolerability and skin irritability) and for further application/injection site photographs to be taken. Blood samples will be collected for haematology and biochemistry, vital signs will be measured and females will undergo a urine pregnancy test. For subjects in Part A and B, a 15 mL blood sample will be taken for immunogenicity analysis. For subjects in Part B (non biopsy), a saliva sample will be collected, and also a separate 40 mL blood sample will be taken for exploratory immunogenicity analysis.<br><br>On Day 36 (+/- 2 days) and Day 50 (+/- 2 days), a phone call will be made to subjects to ask about local skin reaction or changes on concurrent medication.<br><br>On Day 61 (+/- 4 days), subjects will return for an End of Study visit, to review any local site reactions (tolerability and skin irritability), vital signs measurement, and for further application/injection site photographs to be taken. Females will undergo a urine pregnancy test. For subjects in Part A and B a 15 mL blood sample will be taken for immunogenicity analysis.<br><br>Subjects will be provided with a memory aid and thermometer on Day 1 to take home and record adverse events for the 6 days after treatment. Memory aid cards will be reviewed by the study staff at each subsequent visit, and collected on Day 8.
**Study Duration:**	For each subject the study duration is 60 days (excluding Screening).

<table>
<tr><td>Immune Response Assessments:</td><td>Measurement of immune response is a secondary objective of the study.

The primary assay for immune response to influenza vaccination is the Haemagglutination Inhibition (HI) assay of serum samples. This will be run at 360biolabs (Melbourne).

In addition, the following assays are intended to be performed on a subset of subjects and time-points:
• Enzyme linked immunosorbent assay (ELISA) for immunoglobulin G (IgG) in serum.
• Microneutralization (MNT) in serum.
• ELISA for mucosal IgA in saliva.
• Antibody dependent cellular cytotoxicity (ADCC) in serum.
• Cell mediated immunity (CMI) in peripheral blood mononuclear cells (PBMCs).
• Memory B cells in PBMCs
• Assays on biopsy samples for local skin response:
    o Flow cytometry
    o Immunohistochemistry.</td></tr>
<tr><td>Additional analysis for skin penetration</td><td>Assessment of MAP skin penetration performance is a secondary objective of the study.

To support this analysis skin hardness values will be collected from each application site immediately prior to patch application. 2 values will be taken from each intended application site.

Applied MAPs are collected for analysis of penetration performance.
• Uncoated MAPs are analysed using scanning electron microscopy
• A/Sing MAPs are analysed using scanning electron microscopy and / or total protein content (UV).</td></tr>
<tr><td>Safety Parameters:</td><td>Adverse events (AEs), physical examination, local injection site reactions and vital signs will be recorded for each subject.</td></tr>
<tr><td>Statistical Analyses:</td><td>Safety endpoints have been identified as being of main interest in this study. Study investigations will be exploratory and conclusions based on the complete set of subject evidence.

Treatment site reaction, pain scores, and skin irritation index will be summarised at each assessment time by treatment group. Comparisons between sites of application and between MAP and intramuscular injection administration and active (A/Sing) versus placebo administrations will be analysed using appropriate statistical tests.

Descriptive statistics of demographics (age, height and weight at screening, race and ethnic origin) will be presented by cohort and overall. Medical history information collected at screening will be listed. All adverse events will be listed by subject and will include details of the onset date and time, duration, severity, causality and treatment/medications administered.

Laboratory parameters will be listed over the scheduled visits by subject number and treatment cohort. Vital signs (blood pressure, heart rate, orall temperature and respiratory rate) which are outside of the normal reference range and clinically significant will be listed in tables.

Physical examination data will be listed in tables.

Local reactions will be listed in tables and summarised by treatment group.</td></tr>
</table>

	Immunogenicity data will be listed in tables and summarised by treatment group.<br><br>Concomitant medications will be entered as Trade names and coded using the WHO DD according to the ATC codes. All medications will be listed by subject, start and stop date and time of dose, route of administration and reason for administration.

5 INTRODUCTION

5.1 Background Information

Vaxxas Pty Ltd is developing a novel micro-projection array patch (MAP) approach for the administration of vaccines (refer to the current Investigator's Brochure). The MAP consists of a 1 cm^2 polymer patch with a dense array of micro-projections on the skin-facing surface. The MAP applicator is a hand-held spring powered device designed to reliably and reproducibly apply the MAP to the skin. The MAP and the applicator are supplied separately for use in combination. The MAP and applicator are used for one application only. This delivery system enables the micro-projections to breach the stratum corneum of the skin and penetrate into the epidermis and upper dermis to a depth of around 80 to 120 microns. MAPs will be applied by trained personnel to the skin of two anatomical sites of healthy subjects, the upper volar forearm (FA) and the outer aspect overlying the deltoid muscle of the upper arm (UA). The MAP remains on the skin of the subjects for two minutes and is then removed by appropriately trained study staff. This clinical study is a critical step in the development of the Vaxxas MAP vaccine delivery system and will test the safety, tolerability and performance of MAPs coated with a single influenza vaccine antigen (A/Singapore/GP1908/2015 (A/Michigan/45/2015 (H1N1)-like)) at equivalent and lower doses than the standard intramuscular needle and syringe dose. Uncoated MAPs will be applied as a placebo comparator. The safety and immune responses will be compared to intramuscular injection of an equivalent A/Singapore/GP1908/2015 HA protein dose (Afluria® Quadrivalent 2017) and also intramuscular injection of the monovalent A/Singapore antigen. The Afluria Quadrivalent 2017 also contains three additional antigens: A/Hong Kong/4801.2014 (NYMC X-263B) (A/Hong Kong/4801/2014(H3N2)-like 15 µg HA per dose, B/Phuket/3073/2013 (B/Phuket/3073/2013-like) 15 µg HA per dose and B/Brisbane/46/2015 (B/Brisbane/60/2008)-like 15 µg HA per dose. The A/Singapore/GP1908/2015 (A/Sing) antigen used in the manufacture of the active MAPs and the monovalent intramuscular comparator has been supplied to Vaxxas from a licenced Australian manufacturer of influenza vaccines (Seqirus PTY LTD,) to a cGMP standard.

The Afluria Quadrivalent vaccine (0.5 mL prefilled syringes) being used in the study is a registered US product, manufactured by Seqirus, Australia and is therefore an investigational product. The A/Singapore monovalent intramuscular product is an investigational product and injections will be prepared at Nucleus Network prior to administration. The safety, tolerability and immunogenicity performance of a previous version of the MAP technology called the Nanopatch has been previously assessed in two clinical studies conducted at Q-Pharm Pty Ltd (Brisbane, Australia), involving a total of 79 healthy subjects (SP-1201-050 and SP-1201-059), including in combination with the influenza antigen A/California/7/2009 (H1N1)-like. The major changes in this study are the material of construction of the MAP, which is now made from polymer and the use of a different H1N1 influenza antigen. No serious adverse events related to treatment were recorded during previous studies with application of Nanopatches to both the volar forearm and upper arm. Nanopatch application was well tolerated and generated immune responses that were similar to those induced by the same dose delivered intramuscularly with a needle and syringe (further detail provided in this document and Investigator's Brochure).

The uncoated MAP is terminally sterilised post manufacture using gamma irradiation (25 kGy). However, the A/Sing coated MAP cannot be terminally sterilised as this would significantly degrade the active HA protein. Therefore, the A/Sing MAP is manufactured under stringent aseptic manufacturing conditions. All clinical MAP products are released into the clinical study following successful Quality Control (QC) testing against pre-defined Finished Product Specifications (FPS), which include sterility and endotoxin testing.

Potential Advantages of the Vaxxas MAP delivery system for vaccination

The Vaxxas MAP technology offers several potential advantages over conventional needle and syringe (N&S) delivery of vaccines via intramuscular (IM), intradermal (ID) and subcutaneous (SC) routes:

- Improved immunogenicity through targeted vaccine delivery to skin immune cells. This could facilitate dose-sparing of supply-constrained / expensive vaccine antigens.

- Eliminating the need for vaccine cold chain (2 to 8 ºC) for transportation and storage due to the likely improved thermostability of the dry coating of vaccine of the MAP.
- Removing the need to reconstitute lyophilised vaccines to create a liquid suspension that can be injected with N&S.
- Increased ease of administration and also avoiding the need to use a N&S sharp. This can facilitate use by vaccinators who have lower levels of training or experience. This can be especially beneficial in high-throughput and emergency vaccination scenarios.
- Improved acceptability compared with N&S, especially in people with a fear / anxiety of needles.
- Improved overall safety, because of the ease of use the MAP product, avoidance of reconstitution errors and prevention of needle-stick injuries.

5.2 Investigational Product

The investigational vaccine delivery system consists of a polymer MAP coated with a formulated A/Sing or an uncoated (bare polymer) MAP. The MAP device is manufactured from liquid crystal polymer by injection moulding (Cyrus Technologies, Singapore).

The MAP, (before deposition of coating) to be used in this study is produced by injection moulding with a liquid crystal polymer (LCP – Vectra S540). The MAP is manufactured by Cyrus Technology (Singapore) using a quality accredited (ISO13485) cleanroom facility. The choice of the LCP material is based on the ability of the LCP to form the micro-projections, the hardness of the polymer to enable effective skin penetration, appropriate polymer ductility, material compatibility with gamma irradiation and the biocompatibility of the plastic when in contact with the skin / tissue.

The skin-facing aspect of MAP has dense array of micro-projections. Each micro-projection is approximately 250 µm in length, 120 µm in width at the base and tapers to a sharp point of less than 25 µm. Each 1 cm² MAP contains approximately 3176 micro-projections. As the MAP is applied to the skin, the micro-projections penetrate into the epidermis and dermis to an average depth of around 100 to 120 µm.

The individual 1 cm x 1 cm squares contain an embedded magnet (The Aussie Magnet Company Pty Ltd, Australia) to allow the MAP to be picked up and held by the applicator prior to administration. Prior to coating or packaging, MAPs are terminally sterilised by gamma irradiation (≥ 25kGy Steritech, Australia).

The A/Sing MAP is coated with a vaccine formulation using a dispensing technology developed by Vaxxas called direct-jet. Direct-jet enables individual droplets to be dispensed onto micro-projections to build up the vaccine dose to be delivered. This highly controlled process is much improved compared to the approach taken to coat vaccine onto the silicon Nanopatch in the previous vaccine clinical study, SP-1201-059.

The A/Sing vaccine is supplied to Vaxxas with a Certificate of Analysis from Seqirus Pty Ltd. The antigen is provided sterile in individually sealed vessels and stored at 2 to 8 °C before use. The manufacturing process for coating the A/Sing MAP is performed according to GMP for aseptic products (GMP Annex 1 PIC/S 2017). The aseptic process has been validated by conducting three full batch process simulation runs with product tested for sterility.

To ensure the antigen retains potency during coating and on subsequent storage a stabilizing excipient is added to the antigen material. Sulfobutyl ether (β) cyclodextrin (SBECD) is added at a ratio of 4 to 1 part (w/w%) HA protein. The SBECD is purchased to a cGMP quality from CyDex Pharmaceuticals Inc and is sold under the trade name Captisol®. Captisol is used in the manufacture of six FDA approved products including Pfizer's VFEND® IV and Onyx Pharmaceutical's Kyprolis® - IV infusion. To prepare the products, a solution of SBECD is prepared in sterile water for injection (Pfizer), and triple filtered using 0.22 µm sterile filters (Sartorius, Australia). The sterile A/Sing antigen is added to the sterilised SBECD solution and coated onto the MAP micro-projections by evaporatively drying down the

dispensed solution under the laminar flow work station (LFWS). The antigen is received as a suspension of particles in phosphate buffered saline and therefore the final solid formulation of antigen on the MAP consists of HA protein, inactive protein (from the A/Sing bulk), SBECD and buffer salts. The coated MAP is then immediately sealed into the product pack (section 3.1.3), removed from the cleanroom and stored at 2 to 8 °C. Two different dose strengths of A/Sing MAP will be produced for the study:

- A/Sing MAP delivering 2.5 µg HA into the skin
- A/Sing MAP delivering 5 µg HA into the skin

The uncoated MAPs are sealed into the product pack in the cleanroom under the LFWS. The packed product is then sterilised by gamma irradiation (≥ 25 kGy).

The MAP applicator device (CAPD) is a hand-held spring powered device designed to reliably and reproducibly apply the MAP to the skin and is produced by Romar Engineering (Australia) under grade D cleanroom conditions. The MAP applicator utilises a mechanical force, generated by a spring, to accelerate the MAP to a sufficiently high speed over a short distance (<5 mm) for the dense array of micro-projections to breach the skin. To compress the spring a simple loading jig is required and each applicator is supplied together with a separate loading jig. The CAPD is for single use and disables automatically. The CAPD uses a magnet to attach, position and retain the MAP. The CAPD is used in conjunction with a skin conditioning ring (Vaxxas). The skin conditioning ring contacts the skin around the area of MAP administration. Approximately 30 Newtons of down force is required to actuate the skin ring, resulting in a pre-conditioning of the skin for MAP administration. The skin contacting component of the skin conditioning ring is made from stainless steel 316 and can be sanitised with a medical wipe between subjects.

5.3 Non-clinical Studies

The active and placebo-coated MAP products proposed for use in this clinical study have been tested in pig and rat models.

In preclinical studies in mice with seasonal influenza vaccine, the Nanopatch induced potent Haemagglutination-inhibition (HI) titres equal to those obtained with IM administration of a 100-fold higher dose of vaccine. In preclinical studies in pigs with seasonal influenza vaccine, the Nanopatch induced HI titres believed to be protective with doses comparable to those administered IM in humans.

Using the polymer MAP and the direct-jet coating approach, immunogenicity studies in rats with two influenza antigens (A/California/7/2009 (H1N1)-like and A/Sing) have been performed by Vaxxas Pty Ltd. Immune responses with the MAP were the same as response with the silicon Nanopatch at an equivalent delivered dose, across a range of doses. Two-minutes of residence time (or “wear time”) has been shown to deliver the required vaccine dose consistently into pig skin [SR-1207-013].

Vaxxas has assessed the local tolerability and safety of A/Sing and A/Cal MAP application to pigs and rats. There have been no noted safety events (including local reactogenicity) linked to MAP application in any of these studies. In the rat, no adverse events have been recorded following immunogenicity studies up to 21 days post vaccination.

A repeat dose GLP toxicology study in rats has been performed at Charles River Laboratories (Scotland). Briefly, rats from three groups received either a repeat dose of 15 µg A/Sing HA via MAP to the skin (flank), a repeat dose of the liquid form of the vaccine (Afluria-Quad® 2017) via IM injection, or skin preparation and uncoated patch application with IM injection of sterile saline (non-vaccine control) into the gluteal muscles. All rats received repeat treatment on study days 0 and 23. Blood samples were collected from all animals on day 25 for assessment of immune response. All animals were terminated at day 25 (3 days after repeat dosing). The formal study report is pending, but the data has been reviewed by Vaxxas in an interim study report (D-1207-017). The interim report concluded that

A/Sing MAP administration to rats did not seem to affect body weight, food consumption, haematology and coagulation or clinical chemistry compared to controls and intramuscular injection of Afluria-Quad. There was good resolution of application sites between doses 1 and 2 for A/Sing MAP with mild erythema following application reducing over a two to three day period. Histology analysis of application sites showed an increase in epidermal hyperplasia, crusts and skin layer turnover for the A/Sing MAPs compared to uncoated MAP sites.

One concern with the use of solid microneedle array technologies is that the micro-projections might break off in the skin on application or removal. With the polymer MAP, to date there has been no evidence to indicate the presence of polymer micro-projection fragments in the skin following MAP application to pigs and rats. The mechanical properties of the LCP polymer used to manufacture the MAP have been selected to ensure that the mode of micro-projection failure is to bend and not break.

5.4 Previous Clinical Studies

There has been no previous human experience with vaccine-coated MAPs.

5.4.1 Safety study of MAP without vaccine

There is an ongoing Investigator led clinical study using the MAP without vaccine to test the skin response and engagement performance of MAPs to the skin of the upper arm and forearm. This small study (12 subjects) is being performed at Department of Clinical and Experimental Medicine, Faculty of Health Sciences, Linköping University, Sweden, sponsored by Professor Christopher Anderson under local HREC approval [SP-1207-014]. All applications and follow-ups in the study have been completed and no adverse events or withdrawals indicated to Vaxxas.

In general, the skin response with both uncoated and placebo-excipient coated MAPs were similar to applications seen with silicon Nanopatches in SP-1201-050 and SP-1201-059. There were a few examples of pin-prink or “punctate” bleeding the study and a slight oedema was also noted at the application sites of some applications.

One female subject with urticaria was entered into the study by the Investigator. Following application of both the uncoated and placebo-excipient coated MAPs to the upper arm a more pronounced oedema response was noted along with some blushing of the skin around the MAP application area. There was no difference noted in the skin response to uncoated or excipient coated MAP. The investigator decided to treat this response with oral anti-histamine tablets. The skin response was not associated with any pain or itching and resolved quickly after anti-histamine treatment. Following treatment with anti-histamine the forearm applications were performed with a noted reduction in skin response.

The MAPs were assessed for quality of skin engagement and the integrity of the micro-projections to skin application. Overall penetration depth into skin was between 80 to 100 microns based on the removal of placebo-excipient coating from the microprojection.

5.4.2 Supportive clinical data from silicon Nanopatches

The silicon Nanopatch vaccine-delivery system has been tested in two clinical studies [SP-1201-050 and SP-1201-059]. SP-1201-050 tested the safety, tolerability and acceptability of application of silicon Nanopatches to deltoid and volar forearm using uncoated (silicon) and placebo-excipient coated silicon Nanopatches. This was followed by clinical study SP-1201-059 in which the safety, tolerability, acceptability and immunogenicity of Nanopatches coated with A/California/7/2009 (H1N1)-like virus antigen from the 2016 seasonal influenza vaccination was assessed. These studies were the first clinical studies to use a high density micro-projection array to delivery vaccine to the skin with a dynamic (high velocity) application. The delivery method, with a short patch wear time of 2 minutes, was shown to be safe, tolerable and acceptable by the subjects and induced immune responses at least as good as those induced by IM injection of the registered product at the standard 15 µg HA dose.

In study SP-1201-050, no serious AEs were reported. Five adverse events were reported: of these, three were considered unlikely to be related to treatment and two were not related to treatment. No clinically significant events were observed with respect to clinical laboratory tests or vital signs.

In study SP-1201-059, one serious AE (not related to treatment) was reported (spontaneous termination of pregnancy). 40 treatment emergent adverse events (TEAEs) were reported by 30 subjects. Of the subjects receiving A/Cal HA protein, 22 of 46 (48%) reported at least one TEAE (IM vaccine 5 out of 15; NP Deltoid 9 of 15; NP Forearm 8 of 16) while of those receiving placebo, 8 of 15 (57%) reported at least one TEAE. With one exception, all TEAEs were mild or moderate, and of those considered to be related to treatment; 3 subjects reported headaches (IM group), and in the NP Forearm and NP Deltoid (upper arm) groups, combined, 1 subjected reported axillary pain, 1 subject reported a headache, 1 subject reported myalgia and 2 subjects reported upper respiratory tract infections. No clinically significant events were observed with respect to clinical laboratory tests, vital signs or oral temperature. Only one severe AE was reported (soft tissue injury because of a bicycle accident, subject withdrew from study) and was not related to treatment. All other AEs were classified as mild or moderate.

With all Nanopatch applications (uncoated, placebo excipient-coated and A/Cal-coated), the skin reaction to Nanopatch application comprised an initial erythema directly under the Nanopatch application site, which was in-line with results from pre-clinical testing. Most of the skin reactions to application were no longer detectable on the skin at day 28 after application although the resolution of skin response was prolonged with the addition of the vaccine (antigen). With the addition of A/California, the erythema peaked at day 3 before fading between day 7 and day 28 post application.

In study SP-1201-059 HI titres against A/Cal were used as the primary measure of immunogenicity. Before vaccination, 32 out of the 60 subjects (53%) had an HI titre value of ≥ 1:40, regarded as seroprotective. There was no increase in HI titre in any of the placebo groups following vaccination while there was a significant increase in the geometric mean (GMT) HI titre for each of the vaccinated groups at day 7 and day 21 post vaccination with A/Cal antigen. There was no difference in the GMTs at day 21 between any of the vaccinated groups (NP or IM).

As part of SP-1201-059, a qualitative assessment of the delivery of vaccine (A/Cal) coating into the skin was performed for every Nanopatch applied. This analysis was performed using SEM in combination with back-scattered electron imaging, which enables detection of differences in atomic number between the carbon containing coating and the underlying silicon of the Nanopatch. SEM analysis showed that, a more consistent vaccine delivery was achieved into the skin of the forearm than the upper arm (deltoid).

5.5 Summary of potential risks and benefits

Any vaccine can cause an anaphylactic reaction, particularly those produced in eggs that might contain egg-derived protein. Such reactions are, however, rare. Clinicians should be trained and equipped to manage anaphylactic reactions when this vaccine is given. Adrenaline should always be ready for immediate use whenever any vaccination is given.

The risk of side effects and serious adverse events associated with use of the A/Sing MAP vaccine-delivery system is considered to be very low. There are however, several potential and theoretical risks for this product because it is a first-in-human application and includes a vaccine that is produced from chicken eggs.

The systemic adverse events from administration of the monovalent A/Sing MAP are expected to be qualitatively similar to those of the IM quadrivalent vaccine containing the same A/Singapore. Listed AEs associated with influenza vaccination to adults (≥ 18 to < 65 years), delivered as a quadrivalent vaccine IM (refer to the Afluria Quadrivalent Product Information), are:

Local: pain, swelling/lump, redness

Systemic: myalgia (muscle ache), headache, malaise, nausea, chills, vomiting, fever.

The following adverse events have been spontaneously reported during post-approval used of Fluvax® vaccine and are in addition to the events observed during clinical trials. There are no post-marketing data available for Afluria Quadrivalent vaccine. The events below are reported according to System Organ class:

Blood and Lymphatic System Disorders: Thrombocytopenia.

Immune System Disorders: Allergic or immediate hypersensitivity reactions including anaphylactic shock.

Nervous System Disorders: Neuralgia, paraesthesia and convulsions, encephalomyelitis, neuritis or neuropathy and Guillain-Barré syndrome.

Vascular Disorders: Vasculitis which may be associated with transient renal involvement.

Skin and Subcutaneous Tissue Disorders: Pruritus, urticaria and rash

General Disorders and Administration Site Conditions: Cellulitis and large injection site swelling, influenza-like illness.

Other risks include irritation or delayed hypersensitivity reactions, infection, slight bruising or scarring at the site of administration. Subjects should be advised to contact the clinic if they suspect the administration site has become infected or there is some unexpected allergic or other reaction.

Clinical studies SP-1201-050 and SP-1201-059 showed that even without vaccine, the site of patch administration site will be pink to red immediately after application and the colouration might persist for up to 28 days. By 2 weeks, in most cases, the application site was difficult to detect. Thus, this reaction is likely to resolve with time and without treatment.

The application might also cause local swelling and induration. If required, local oedema could be treated with oral antihistamines. Bruising may also occur, but has not been detected in previous clinical studies.

Immediate, transient pin-prick bleeding at the administration site was seen occasionally in animal studies and in humans. If pin-prick bleeding or seepage is noted immediately following MAP removal from the skin, a non-irritant adhesive skin dressing should be offered to cover the application site.

There is a potential risk of skin infection from the MAP, due skin penetration carrying skin-resident micro-organisms into deeper layers of skin tissue and causing infection. For this reason, before Nanopatch / MAP application, the site is wiped using a disinfecting / cleaning wipe..

There is also a possible risk that the polymer micro-projections of the MAP may break-off in the skin. There has be no evidence of micro-projections breaking off in the skin following applications to both pigs and humans. The polymer (LCP) has been chosen to prevent the risk of breakage on the basis of its physico-chemical properties and is biocompatible. All applied silicon Nanopatches were inspected after skin application using scanning electron microscopy as part of clinical studies SP-1201-050 and SP-1201-059. The micro-projections remained intact.

All Subjects must be informed that the application site is highly likely to appear pink or red immediately after MAP removal from the skin. Participants must be informed that this redness may increase over the first 24 to 72 hours post removal before fading and that any residual marking at the application site is highly likely to fade over a short-time frame, probably 2 to 4 weeks. Subjects must also be informed that itching and shedding of skin at the application are likely to occur up to 7 to 10 days post removal.

The anatomical sites for application were selected based on standard vaccination sites in adults. The upper arm (deltoid region) is routinely used for intramuscular (IM) and subcutaneous (SC) vaccine

administration, while the volar forearm is routinely used for intradermal (ID) delivery. The MAP will be delivered to both sites in separate study groups. During the screening procedures, the MAP application process will be demonstrated to the subject.

6 STUDY OBJECTIVES AND ENDPOINTS

6.1 Study Objectives

PRIMARY OBJECTIVE:

- To evaluate the safety and tolerability of A/Singapore/GP1908/2015 antigen delivered by the micro-projection array patch (MAP) in comparison to an uncoated MAP and intramuscular administration of both a quadrivalent seasonal influenza vaccine and a monovalent vaccine delivering approximately the same dose of A/Singapore/GP1908/2015 (A/Sing) HA protein.

EXPLORATORY OBJECTIVES:

- To evaluate the immune responses to MAP application to the forearm with A/Singapore/GP1908/2015 (A/Sing) at 4 dose levels (0, 2.5, 5, 10 and 15 micrograms) in comparison to IM administration of the standard 15 microgram HA dose per strain.
- To evaluate the skin penetration performance of applied A/Sing MAPs at two sites of application (forearm and upper arm).
- To assess further measures of immune response through additional assays and assessment of the local skin response via punch biopsy of the micro-projection array application sites.

6.2 Study Endpoints

6.2.1 Primary Safety Endpoints

- Number of subjects with SAEs deemed possibly, probably or definitely related to treatment administration
- Number of subjects with AEs deemed possibly, probably or definitely related to treatment administration
- Clinical laboratory samples (biochemistry, haematology, urinalysis) at screening and Day 22
- Local skin response (erythema / oedema); photo imaging, standardized scoring; at 10 min, 30 min, 1 hr, 2 hr post-dose on Day 1; Day 2 (Part B only); Days 4, 8, 22 and 61. Any local skin responses still present at Day 61 will be followed up to resolution, until the condition stabilizes, or until the subject is lost to follow-up.

6.2.2 Secondary Endpoints

- Immunogenicity (HI assay) at Days 1 (pre-dose), 4, 8, 22 and 61
- Acceptability assessment by pain scores using Visual Analogue Scale at 1 min, 10 min, 30 min, 1 hr, 2 hr post-dose on Day 1; Day 2 (Part B only); Days 4, 8, 22 and 61.
- Skin assessment with application site hardness using non-invasive durometer
- Post-application analysis of delivered dose from MAP to skin using Scanning Electron Microscopy (SEM) and / or residual protein.

6.2.3 Exploratory Endpoints

- Immunogenicity assays (mucosal, PBMC) at Days 1 (pre-dose), 8 and 22 for Part B, groups B3, B7, B8, B9. Note, samples (saliva and blood) will be collected from all non biopsy subjects in Part B.
- Skin cellular response to MAP (biopsy – histochemical analysis and flow cytometry) at Days 1 (pre-dose) and 4.

7 STUDY PLAN

7.1 Overall Study Design

This single centre, randomised study will be conducted in two parts, in a total of 210 healthy adults aged 18-50 years old.

Part A of the study is a pilot study of four (4) treatment groups of 15 subjects, for a total of 60 subjects. Subjects will be randomised to receive treatment on Day 1 as follows:

- **Group A1**: 15 subjects receive 3 x A/Sing MAPs on volar forearm delivering a total of 15 µg of A/Sing Haemagglutinin (HA) protein.
- **Group A2**: 15 subjects receive intramuscular injection to the deltoid muscle delivering a total of **15 µg** of A/Sing Haemagglutinin (HA) protein in a 0.5 mL quadrivalent influenza vaccine injection (Afluria® Quadrivalent).
- **Group A3**: 15 subjects receive 3 x uncoated MAPs (placebo) on volar forearm.
- **Group A4**: 15 subjects receive intramuscular injection to the deltoid muscle delivering a total of 15 µg of A/Sing Haemagglutinin (HA) protein in a 0.5 mL monovalent influenza antigen injection.

In Part A, clinical staff and subjects will be blind to treatment for MAPs (A/Sing MAPs or uncoated MAPs) and for IM injections (A/Sing or Afluria Quadrivalent).

All 30 subjects in groups A2 and A4 need to be vaccinated on the same day – the IM (A/Sing) injections (group A4) must be prepared and administered on the day of use.

Part B of the study will proceed following review of safety and immunogenicity results from Part A. There will be 9 treatment groups, with 7 non-biopsy groups of 20 subjects each, and 2 biopsy groups of 5 subjects each, for a total of 150 subjects.

Ten subjects will be randomised to a biopsy group (5 subjects per group), as follows:

- **Group B1:** 5 subjects receive 3 x A/Sing MAPs on volar forearm delivering a total of **15 µg** of A/Sing Haemagglutinin (HA) protein. Punch biopsies are performed at the application sites at defined time-points.
- **Group B2:** 5 subjects receive 3 x uncoated MAPs on volar forearm. Punch biopsies are performed at the application sites a defined time-points.

One hundred and forty subjects will be randomised to a non- biopsy group (20 subjects per group), as follows:

- **Group B3:** 20 subjects receive 3 x A/Sing MAPs on volar forearm delivering a total of **15 µg** of A/Sing Haemagglutinin (HA) protein.
- **Group B4:** 20 subjects receive 2 x A/Sing MAPs delivering a total of **10 µg** of A/Sing Haemagglutinin (HA) protein and 1 x uncoated MAP on volar forearm.
- **Group B5:** 20 subjects receive 1 x A/Sing MAP delivering a total of **5 µg** of A/Sing Haemagglutinin (HA) protein and 2 x uncoated MAPs on volar forearm.
- **Group B6:** 20 subjects receive 1 x A/Sing MAP delivering a total of **2.5 µg** of A/Sing Haemagglutinin (HA) protein and 2 x uncoated MAPs on volar forearm.
- **Group B7:** 20 subjects receive 3 x uncoated MAPs on volar forearm.
- **Group B8:** 20 subjects receive 3 x A/Sing MAPs on the upper arm to the skin overlaying the deltoid muscle delivering a total of **15 µg** of A/Sing Haemagglutinin (HA) protein.
- **Group B9:** 20 subjects receive intramuscular injection to the deltoid muscle delivering a total of **15 µg** of A/Sing Haemagglutinin (HA) protein in a 0.5 mL quadrivalent influenza vaccine injection (Afluria Quadrivalent).

In Part B, clinical staff and subjects will be blind to treatment for MAPs applied to the volar forearm (A/Sing MAPs or uncoated MAPs).

Prior to each MAP application or intramuscular administration, the treatment administration sites will be identified, examined and marked with indelible ink and photographed. Blood will be collected for safety assessments and measurement of immune response (baseline at Day 1) and vital signs collected. The process for using the applicator and applying the MAP will be demonstrated to all subjects before treatment. Treatment sites will be examined/assessed and photographed after the event as set out in the schedule of events. All subjects will remain at the study site for at least 2 hours following the treatment for safety observation.

Following treatment administration on Day 1, post-dose assessments will include pain scores, application/injection site assessment for any local site reactions (tolerability and skin irritability), photography and vital signs. Subjects will be discharged from the unit after all assessments have been completed and a 2 hour observation period has elapsed.

On Day 2 (24 hr post treatment) for subjects in Part A, and on Day 3 (48 hr post treatment) for all subjects, follow-up phone calls will be made to ask about adverse events or changes in concurrent medication.

On Day 2 (24 hr post treatment), subjects in Part B will return to the clinic for review of any local site reactions (tolerability and skin irritability) and for further application/injection site photographs to be taken.

On Day 4 (3 days post treatment), subjects will return to the clinic for review of any local site reactions (tolerability and skin irritability), vital signs measurement, and for further application/injection site photographs to be taken. All subjects will have a 15 mL blood sample taken for immunogenicity analysis. For subjects in Part B (non biopsy) a saliva sample will be collected.

On Day 8 (+/- 1 day), subjects will return to the clinic for review of any local site reactions (tolerability and skin irritability), vital signs measurement, and for further application/injection site photographs to be taken. All subjects will have a 15 mL blood sample taken for immunogenicity analysis. For subjects in Part B (non biopsy) a saliva sample will be collected.

On Day 22 (+/- 1 day) subjects will return to the clinic to review any local site reactions (tolerability and skin irritability) and for further application/injection site photographs to be taken. Blood samples will be collected for haematology and biochemistry, vital signs will be measured and females will undergo a urine pregnancy test. All subjects will have a 15 mL blood sample taken for immunogenicity analysis. For subjects in Part B (non biopsy), a saliva sample will be collected, and also a separate 40 mL blood sample will be taken for exploratory immunogenicity analysis.

On Day 36 (+/- 2 days) and Day 50 (+/- 2 days), a phone call will be made to subjects to ask about local skin reaction or changes on concurrent medication.

On Day 61 (+/- 4 days), subjects will return for an End of Study visit, to review any local site reactions (tolerability and skin irritability), vital signs measurement, and for further application/injection site photographs to be taken. Females will undergo a urine pregnancy test. All subjects will have a 15 mL blood sample taken for immunogenicity analysis. For subjects where the applications sites are still visible, fortnightly phone calls will be made until the site(s) have visually resolved. Punch biopsies will be performed on Days 1 (pre-dose) and Day 4 for subjects in biopsy groups in Part B. Two 4mm punch biopsies will be performed on each day. The punch biopsies on Day 4 will be taken from within the area of patch application from 2 of 3 application sites.

Subjects will be provided with a memory aid and thermometer on Day 1 to take home and record adverse events for the 6 days after treatment. Memory aid cards will be reviewed by the study staff at each subsequent visit, and collected on Day 8.

Procedures at all visits will be performed according to Appendix 2 – Schedule of Assessments.

After the A/Sing and uncoated MAPs have been removed from subjects, they will be analysed to investigate skin penetration performance using scanning electron microscopy and for active A/Sing MAPs, residual antigen content using total protein analysis.

Subjects will be informed to contact the clinic during the course of the study if they have any concerns at all regarding their health and wellbeing.

If required by the Investigator, subjects from any of the treatment groups may attend the clinical unit for unscheduled visits to allow assessment and treatment of any adverse events they may experience.

7.2 Rationale for Study Design and Treatment Regimens

This study is designed to test the hypothesis that MAP application to the skin using a small number of healthy adult subjects with a well characterised influenza vaccine antigen (A/Singapore/GP1908/2015) results in comparable safety / local skin reaction to conventional intramuscular vaccination. This study represents the first time that polymer MAPs with an active vaccine will be applied to humans. Therefore, this study will assess both systemic and the local reaction to application of the A/Sing MAP delivering a 15 µg HA intramuscular-equivalent dose with the application of three MAPs, in comparison to uncoated MAPs, intramuscular injection with Afluria Quadrivalent (delivering 15 µg A/Sing HA) and intramuscular injection with 15 µg A/Sing HA (as a monovalent antigen). The local skin response will be monitored for up to 60 days. On-site clinic assessments will be performed up to 2 hours post application and at 1, 3, 7, 21 and 60 days (if required) post application. Phone calls will be made at Day 36 and Day 50.

It is expected that local reactions at the MAP application site will occur as a normal consequence of the application. Local, systemic, and clinical laboratory toxicity will be graded using scales according to Appendix 4 and 5. To understand the skin response at the MAP application site and the role that the antigen has on the skin response, in two groups punch biopsies will be performed pre-application and at day 1 (pre-treatment) and 3 days post application (day 4). Analysis of biopsies will be via immuno-histochemical staining and flow cytometry.

This study will also provide information of the immune responses to MAP vaccination of humans over a range of dose levels; the standard intramuscular influenza antigen dose of 15 µg of HA and lower doses of 10, 5, and 2.5 µg HA delivered by MAP at the forearm site. Immune responses at the standard IM dose will be compared between MAP application to the forearm, MAP application to the upper arm, intramuscular injection with Afluria Quadrivalent (quadrivalent vaccine) and intramuscular injection with A/Sing (monovalent). In all subjects blood samples will be collected pre vaccination (day 1) and at 3, 7,21 and 60 days post application in order to compare the kinetics of the immune response. All subjects will have serum tested for functional A/Sing antibody generation via the haemagglutination assay and subjects in Part A will have pre-dose (Day 1) and Day 22 samples also tested by ELISA. Subjects in Part B (non biopsy) will have Day 1, 8 and 22 samples also tested by Microneutralization assay. In order to better understand the immune response to MAP skin vaccination, and whether the response differs to intramuscular delivery, further exploratory assays will be performed on serum samples, peripheral blood mononuclear cells (PBMCs) and saliva samples on selected subjects. All subjects in Part B (non biopsy) will have saliva samples collected at pre-vaccination (day 1), days 4, 8 and 22. All subjects in Part B (non biopsy) will have an additional 40 mL blood samples taken at pre-vaccination (day 1) and day 22 for separation of PBMCs.

The A/Sing antigen has been selected for this study because it was used in the 2017 seasonal influenza product and has been tested pre-clinically in Vaxxas in rats and pigs. Use of a single antigen allows the same safety and performance questions to be addressed as for a multivalent vaccine, but simplifies the antigen supply, manufacture and analytical testing of the clinical product as well as the measurement of the immune response.

7.3 Duration of Study

This study will be conducted over 60 days (excluding the time required for screening the subjects). Potential subjects will be required to attend the clinical facility for a screening medical evaluation. After the administrations on Day 1, each subject will be observed for a period of 2 hours post treatment. The subjects will then return to the clinical facility for safety assessment and examination on Days 2 (Part B only), 4, 8, 22 and Day 61.

7.4 Treatment Site Examination Prior to MAP application / Intramuscular injection

Prior to treatment, the upper arm (deltoid) and forearm regions of the arm must be examined. For the volar forearm, the site of the application must be an area of skin between ~ 4 and 15 cm below the elbow joint. For the deltoid region, the treatment site should avoid the tip of the shoulder and be over the deltoid muscle of the upper arm.

The skin at the site must be free of blemishes, scars, heavy hair, moles, skin conditions, sores or tattoos which might interfere with the detection of local reactions. If possible, the non-dominant arm of the subject should be used for administration of treatment. Pre-dose (baseline) photographs of the application area must be taken. Any blood samples should also be taken from the alternate arm, where possible along with pre-dose (Day 1) biopsy samples. The treatment site must be marked prior to application with indelible ink. For MAP applications, the Investigator should choose a different site at least 3.5 cm away from the previous site. On the forearm, the subsequent applications should be laterally located from the previous site (i.e. down the arm), for the upper arm these may be either radially or laterally applied.

7.5 MAP Application and Removal

This procedure will only be carried out by a trained study team member (trained by Vaxxas personnel). During subject screening, application of the MAP and taking a skin hardness value will be demonstrated to the subjects. The application site is swabbed using a medical wipe before applying the MAP to the skin. For each application, an investigational MAP, applicator, skin conditioning ring, durometer (hardness meter) and applicator loading jig will be required. All of these components will be supplied by Vaxxas. Before loading the MAP into the applicator, the applicator must be primed used the supplied loading jig. The safety mechanism must be disengaged before priming and then re-engaged after priming. The jig functions to compress the applicator spring to a point where it locks in place ready for use. The safety mechanism prevents accidental actuation of the applicator and release of the MAP. All MAPs must be allowed to equilibrate to room temperature prior to opening the primary pack (removing the foil seal). The MAP is loaded into the applicator by pushing down the applicator directly into the open can and between the MAP and yellow ring. The MAP is attached and held in place to the applicator via magnets. The loaded applicator must be held perpendicular to and just touching the skin of the application site. The safety mechanism is disengaged and the button of the applicator pressed to apply the MAP. The MAP applicator and skin conditioning ring are then immediately removed from the skin. The MAP will be left on the skin for two minutes. All applications must be given with the applicator held vertical to the skin (at 90°) with the arm supported. To enable hardness values to be obtained from the upper arm, applications to this site must be performed with the subject lying on their side with their treatment arm resting and relaxed against the side of their body.

After two minutes the MAP is removed by study staff using gloved fingers, by gently tensioning the skin around the patch with one hand, whilst pulling the MAP directly up and away from the skin with the other hand.

Following removal, the MAP should to be placed in a container (supplied by Vaxxas) and the container labelled ready for further analysis.

7.6 Intramuscular Injection

This procedure will only be carried out by trained site staff and will follow the supplied instructions for use for the influenza vaccine product. The administration site is swabbed using a medical wipe before injection. Prefilled syringes will be allowed to equilibrate to room temperature prior to injection.

7.7 Treatment Site Examination after Application or Intramuscular Injection

After the MAP has been removed and the intramuscular injection administered, the treatment site must be examined for local reactions: redness, bruising, tenderness, induration, itching and flaking (see Appendix 4) and the site photographed as per section 7.9. The appearance of the treatment site will be assessed and recorded as per Schedule 4. If erythema, bruising or induration around the treatment site is present, the maximum diameter will be recorded. The Skin Irritation Index system to assess the skin irritation should also be completed (see Appendix 6). Evidence of oedema, bleeding, or treatment site seepage should also be recorded. Any visual evidence for bleeding or seepage must be confirmed by lightly dabbing the skin with a clean tissue. Abnormal observations or symptoms associated with the treatment site that develop or worsen should be recorded and clearly indicate the location at the application site (e.g., “treatment site erythema” rather than “erythema”). Subjects will be asked to assess the treatment site pain using a numerical / pictorial pain intensity scale (see Appendix 5).

7.8 Examination of Treatment Site at Each Visit

During the follow up visits, the treatment site(s) must be assessed for local reactions; redness, bruising, induration, itching, skin coloration and flaking (see Appendix 4) and the site photographed as per section 7.9. The appearance of the treatment site will be assessed and recorded as per Appendix 4. If erythema, bruising or induration around the site is present, the maximum diameter will be recorded. The Skin Irritation Index to assess the skin irritation should also be completed (see Appendix 6). It is expected that local reactions will occur up to 48 hr after the application as a normal consequence of the immune response (e.g., recruitment of inflammatory cells).

7.9 Photography of Application / Administration Site

Photography is mandatory and is to be performed as per the schedule of assessments (Appendix 2). Photography will be performed as set out in Appendix 7.

7.10 Skin Assessment - Durometer

Skin assessment by application site hardness measured using non-invasive durometer (Appendix 1)

7.11 Pain scores

Subjects will be asked to assess the treatment site pain using a numerical / pictorial pain intensity scale (see Appendix 5) at each visit, according to the schedule of events (Appendix 2).

7.12 Investigation of the MAP Performance

Following removal from the skin, the MAPs will be placed in well plates, held securely in-place by custom inserts, sealed with tape and then labelled. Samples will then be sealed in plastic zip-lock bags and sent to Vaxxas for analysis (detailed instructions will be provided in the laboratory manual). The skin penetration performance of the MAPs will be analysed by Scanning Electron Microscopy (SEM) for uncoated MAPs and for A/Sing MAPs by SEM and / or residual antigen content by total protein analysis.

7.13 Measurement of Immunogenicity

Blood samples will be taken for measurement of immune response as per the schedule of assessments (Appendix 2). Titres of anti-A/Singapore/GP1908/2015 antibodies will be measured in serum samples using a Haemagglutination-inhibition assay (HIA) for all subjects at all immunogenicity time points (Appendix 8).

For subjects in Part A serum antibodies will also be measured by ELISA.

For subjects in Part B (non biopsy) serum samples will be used for microneutralisation analysis of day 1 and day 22 samples.

For subjects in Part B (groups B3, B7, B8, B9 only) serum samples will be used for Antibody dependent cell mediated cytotoxicity analysis of day 1 and day 22 samples.

Blood samples (approximately 15mL) will be collected by venepuncture or cannula/butterfly needle for serum into collection tubes without anti-coagulant. The actual times for collection of the blood samples will be recorded on each participant's source documents.

7.14 Saliva IgA - Part B only

Saliva (mouth wash) samples for mucosal immunogenicity will be collected for all groups in Part B (non biopsy) as per the schedule of assessments (Appendix 2) at pre-dose (Day 1), Days 4, 8 and 22.

Subjects will be asked to drink a glass of water and then to chew on a saliva swab collector for 45 – 60 seconds.

Saliva samples will be tested by ELISA for subjects from groups B3, B7, B8, B9 only.

7.15 Peripheral Blood Mononuclear Cells (PBMCs) for Immunoassay - Part B only

Blood samples for PBMC collection (40 mL) will be collected into 5 x 8.5 mL ACD blood collection tubes for all groups in Part B (non biopsy) as per the scheduled of assessments (Appendix 2) at Day 1 and Day 22.

Analysis of samples (for groups B3, B7, B8, B9 only) will be performed using a memory B cell assay and intracellular cytokine staining (ICS) for cell mediated immunity assessment.

7.16 Biopsy (punch) - Part B only groups (B1 and B2)

For Part B groups B1 and B2, 4mm punch biopsies will be performed as per the schedule of assessments (Appendix 2) at pre-dose (Day 1) and Day 4. On Day 1, the biopsies should be performed on the non treatment forearm, with each biopsy separated by greater than 3 cm. On Day 4, two biopsies should be taken from MAP application sites, ideally from the 1st and 3rd application (due to spacing). The excised biopsies will be placed into separate vials.

Biopsy samples will be analysed by histology and flow cytometry.

8 STUDY POPULATION

8.1 Number and Identification of Subjects

For this study, healthy male and non-pregnant, non-lactating female adult subjects between the ages of 18-50 years will be enrolled.

At least 30% of the subjects (63 of 210) are to be male, and at least 30% of the subjects (63 of 210) are to be female.

Prior to acceptance, subjects will be identified by a study-specific subject number with the configuration Snnn [where n is a number allocated sequentially at screening (i.e. S001, S002 etc.)]. This number will be used until Day 1, where the subjects will be randomised A0001 to A0060 for Part A and B0001 to B0150 for Part B. Initial Screening will be performed after informed consent has been taken. Each potential subject must meet all of the inclusion and none of the exclusion criteria as detailed in Sections 8.3 and 8.4 in order to qualify for admission into the study. A list identifying the subjects, by treatment allocation numbers and initials will be kept in the study file.

Subjects in Part A will not be replaced following treatment.

Subjects in Part B will be replaced if they drop out from the study following first treatment of the first Nanopatch (Day 1) and prior to the immunogenicity blood sample on Day 22.

8.2 Sample Size

A total of 210 healthy subjects are planned to be included in the study. The sample size was not based on any formal statistical calculations. It is proposed that 60 subjects in Part A and 150 subjects in Part B will provide sufficient data to fulfil the objectives of the study.

8.3 Inclusion Criteria

1. Aged 18-50 years (inclusive).
2. Subject has a Body Mass Index (BMI) within the range 18.0–30.0 kg/m^2
3. Satisfactory medical assessment, with no clinically significant or relevant abnormalities in medical history, physical examination, vital signs and laboratory evaluation (haematology or biochemistry)
4. Adequate venous access in their left or right arms to allow collection of a number of blood samples.
5. Females of childbearing potential and males should either be sexually inactive (abstinent) for 14 days prior to screening and throughout the study or be using one of the following acceptable birth control methods:
    i. Surgically sterile (hysterectomy and/or bilateral oophorectomy);
    ii. Surgically sterile (bilateral tubal ligation with surgery at least 6 months prior to study initiation);
    iii. IUD in place for at least 3 months;
    iv. Stable hormonal contraceptive for at least 3 months prior to study through completion of study;
    v. Surgical sterilization (vasectomy) for male participants or for female participant's partner at least 6 months prior to study
    vi. Condom for male participant together with effective contraception for their female partner.
6. Postmenopausal women must have had at least 12 months since their last menstrual period.
7. Subject is able to communicate effectively with study personnel and is considered reliable, willing and cooperative in terms of compliance with the protocol requirements.

8. Subject is able and willing to provide written, personally signed and dated informed consent to participate in the study.

8.4 Exclusion Criteria

Subjects meeting any of the following criteria will not be eligible for participation in this study:

1. Subject with birthmarks, tattoos, wounds, scars, moles, blemishes, heavy hair or other skin conditions (such as eczema) on forearms or upper arm regions (both arms) which could reasonably obscure application site reactions.
2. Subject has known chronic spontaneous urticaria / dermographism.
3. Known anaphylactic hypersensitivity to a previous influenza vaccination or allergy to eggs, neomycin, polymyxin B sulphate, or any of the constituents or trace residues of the study vaccine.
4. Has received an influenza vaccine or has been diagnosed by a doctor as having influenza in the last 12 months.
5. Known history of Guillain-Barré syndrome.
6. Recent vaccination (30 days prior to enrolment) with any vaccine.
7. Known predisposition to keloid scar formation.
8. History of granulomatous diseases (especially sarcoidosis or granuloma annulare).
9. History of clinically significant gastrointestinal, hepatic, renal, cardiovascular, dermatological, immunological, respiratory, endocrine, oncological, neurological, metabolic, psychiatric disease or haematological disorders.
10. History of malignancy, other than non-melanoma skin cancer.
11. An active medical condition (which is deemed as clinically significant) that is under evaluation or treatment, or a recent illness, a chronic illness, an autoimmune disease or had major surgery within the last year.
12. History of Hepatitis B, Hepatitis C or HIV infection or clinical laboratory serology is positive for Hepatitis B surface antigen, Hepatitis C or HIV antibodies.
13. History of abnormal bleeding tendencies or thrombophlebitis unrelated to venepuncture or intravenous cannulation.
14. Receiving chronic treatment with immune-suppressive therapy (asthma inhalers and topical corticosteroids are permitted). All medications will be documented and reviewed for acceptance by the Investigator or a medically qualified nominee.
15. History of any psychiatric illness or psychological disorder which may impair the ability to provide written informed consent or participate in the study.
16. Subject has donated blood or plasma or clinically significant blood loss within 60 days prior to screening visit.
17. Subject is pregnant or breast-feeding.
18. A history of alcohol or drug abuse in the last 12 months or current alcohol consumption is >4 standard drinks (or equivalent) per day.
19. Use of any prescription medication within 7 days (except for contraceptives), unless approved by the PI. All medications will be documented and reviewed for acceptance by the Investigator or a medically qualified nominee.
20. Use of any investigational drug or device within 30 days or 5 half-lives of the drug, whichever is longer, prior to the Day 1.
21. Previous exposure to the Nanopatch and its applicator as a participant in previous clinical studies.

8.5 Screening and Selection

Screening and selection of suitable subjects will be performed up to 28 days before study treatment to determine that they meet all of the Inclusion Criteria and none of the Exclusion Criteria specified in Section 8.

Potential subjects for the study will be pre-screened by telephone in the first instance by asking them a series of questions based on inclusion/exclusion criteria to determine if they might be eligible to be included in the study.

If the potential subject finds it difficult to understand or answer the questions then he/she will not have met Inclusion Criteria number 6. If the subject does not wish to answer any particular question, even after the rationale for the question has been explained, then he/she will have not have met Inclusion Criteria number 6. Prior to the conduct of any screening procedures, the Investigator or a medically qualified nominee will explain the aims of the study including the risks and benefits involved and the fact that their participation is voluntary. Each potential subject will confirm their understanding and agreement by giving written informed consent for their involvement in the study in the presence of the Investigator or a medically qualified nominee who will also sign and date the Participant Information Sheet/Informed Consent Form. Each potential subject will be given a copy of their signed Participant Information Sheet/Informed Consent Form.

The screening, which will be assessed by the Investigator, will include details of demography, medical history, drug history (including alcohol use) and a physical examination. During the medical interview, the MAP application process will be demonstrated to the potential subject. Clinical laboratory and serology tests will be performed on each potential subject as detailed in Appendix 3. The results will be assessed by the Investigator before study enrolment. Any deviation(s) in laboratory values, which are deemed clinically significant by the Investigator (i.e. in the opinion of the Investigator would jeopardise the safety of the subjects or impact on the validity of the study results), will result in exclusion of that potential subject. Potential subjects will be counselled by the Investigator or medically qualified nominee, concerning the blood tests for Hep B surface antigen, Hep C and HIV antibodies.

8.6 Subject Withdrawal Criteria

In accordance with the Declaration of Helsinki and its revisions, subjects will be informed that they have the right to withdraw from the study at any time and are not obliged to state their reasons. Additionally, the Principal Investigator or Co-Investigator may withdraw a subject at any time if he / she consider this to be in the subject's best interest.

During the course of the study, subjects may be discontinued for the reasons including:

- Protocol violations;
- Serious intercurrent illness;
- AEs which, in the judgement of the Principal Investigator or Co-Investigator and subject to discussion with the Sponsor’s medical advisor, justifies discontinuation;
- Non-compliance with study requirements;
- Withdrawal of consent by the subject;

The reasons for withdrawal will be recorded on the case report form (CRF) along with details of adverse reactions and any necessary medical treatment. The subject will be encouraged to remain available for follow-up medical monitoring. Before release from the study, the assessments listed in Section 9.2 will be performed.

The Sponsor will be notified as soon as possible of any subject withdrawals. In the event that a study subject decides to withdraw from the study for any reason other than the occurrence of an AE, the Investigator will make every effort to perform the assessments listed in Section 9.2 at the soonest opportunity.

If a subject withdraws from Part A of the study following treatment on Day 1 they will not be replaced.

If a subject from Part B withdraws prior to the Day 22 blood sample for immunogenicity, the subject will be replaced. If a subject withdraws after the Day 22 blood sample they will not be replaced.

9 STUDY PROCEDURES AND ASSESSMENTS

All procedures will be carried out in accordance with the relevant Clinical Site's Standard Operating Procedures (SOPs) unless otherwise agreed with the Sponsor.

The schedule of assessments is presented in Appendix 2.

9.1 Study Procedures for all Cohorts

The three groups (Part A, Part B with biopsy, Part B with no biopsy) will follow similar procedures and assessments as outlined below, and in accordance with the study plan in Section 7, and the study schedule in Appendix 2.

Subjects with clinically significant symptoms or abnormal physical findings will be followed up until the abnormal finding has resolved or until further care is no longer required. Any abnormal clinical laboratory tests must be followed by the Investigator to resolution, until the condition stabilizes, or until the subject is lost to follow-up. At the time of the subject's release, a brief summary statement will be provided by the Investigator assessing the course of treatment and significant events.

9.1.1 Volume of Blood Collected

The volume of blood collected from each subject for planned assessments over the course of the study is:

- Part A: 150 mL
  75 mL safety clinical laboratory assessments (3 x 25 mL) +
  75 mL immune serum (5 x 15 mL)
- Part B, groups B1, B2: 150 mL
  75 mL safety clinical laboratory assessments (3 x 25 mL) +
  75 mL immune serum (5 x 15 mL)
- Part B, groups B3, B4, B5, B6, B7, B8, B9: 230 mL
  75 mL safety clinical laboratory assessments (3 x 25 mL) +
  75 mL immune serum (5 x 15 mL) +
  80 mL PBMCs (2 x 40 mL)

9.2 Study procedure by Visits

9.2.1 Screening and Enrolment Phase

Prior to performing study procedures, the Investigator or medically qualified nominee will obtain written informed consent from the subject, as described in Section 13.5. The screening phase will include all assessments required to ensure compliance with the criteria for Inclusion and Exclusion (Section 8) and will take place during the 28 days prior to Visit 1 (Day 1).

All Screening procedures can be performed at a single clinic visit or may be performed at a sequence of visits if more practical and convenient for subjects and the site. All observations will be recorded in the CRF as applying to Screening although exact dates of evaluations will be recorded as appropriate. The following procedures and evaluations will be performed/ recorded at Screening:

- Medical history, including current and on-going concurrent medications (including history of Flu vaccination and diagnosis of Influenza infections in the past year).
- Demographic information;
- Drug history including alcohol consumption;
- Physical examination;

- Height and weight;
- Treatment sites on forearms and upper arm examined for suitability
- Demonstration of MAP application process.
- Vital signs (Blood pressure, heart rate, oral temperature and respiratory rate), refer to section 9.6.1;
- Serology for HIV-1, Hep B sAg, Hep C;
- Baseline safety clinical laboratory studies (Biochemistry and Haematology) (see Appendix 3);
- Serum beta-HCG (female subjects only);

9.2.2 Treatment Phase

Subjects fulfilling all the criteria for inclusion and none of the criteria for exclusion will enter the treatment phase and undergo the following treatments, evaluations and procedures.

Day 1 Pre-dose, All Groups

- Review of Inclusion and Exclusion criteria;
- Review of medical history and concurrent medications since screening visit;
- Vital sign evaluation (refer to section 9.6.1);
- Collect safety clinical laboratory blood samples (Haematology and Biochemistry) (Appendix 3);
- Collect blood sample for serum for baseline immune response (Appendix 8);
- Collect saliva samples for baseline immune response (Part B, non biopsy) (Appendix 8);
- Collect blood sample for PBMCs for immunoassay (Part B, non biopsy) (Appendix 8);
- Urine beta-HCG (female subjects only)
- **Part B Biopsy groups only**: Perform punch biopsies on non-treatment forearm;

Day 1 Pre-treatment, All MAP Groups:

- Baseline examination of the treatment site(s). Grading of any reaction for redness, bruising, induration, itching and flaking (see Appendix 4) and the Skin Irritation Index for skin irritation (see Appendix 6);
- Indelible ink will be used to mark the site of treatment on the forearm or upper arm. It is preferable to use the non-dominant arm for all treatments (volar forearm or upper arm);
- Planned treatment region will be digitally photographed (see Appendix 7). The photograph file will be recorded with the subject number, visit type, date and time.

Day 1 Treatment, All Groups:

- For the MAP treatment groups, the skin conditioning ring will be applied and two values obtained for skin hardness using the durometer (Appendix 1). One value is obtained without actuating the skin conditioning ring and then a second value is taking whilst pressing down on the ring. One MAP will be applied using a new applicator and associated jig, skin conditioning ring and left on the skin for 2 minutes. Following removal, the same process as above is repeated for the second and third MAP applications.
- For the IM injection treatment groups, investigational products will be delivered by injection to the deltoid muscle

Day 1 Post treatment, All Groups:

- Observation for approximately 2 hours after treatment in accordance to Schedule of Assessments.

- Vital signs evaluation (blood pressure, heart rate, oral temperature and respiratory rate) at 2 hours after application;
- Review of spontaneously reported AEs;
- Review of concomitant medications taken;
- Examination of the treatment site(s) at 10 min, 1 hour and 2 hours post-treatment. Grading of any reaction for redness, bruising, induration, tenderness, itching and flaking (see Appendix 4), pain scores (see Appendix 5) and the Skin Irritation Index for skin irritation (see Appendix 6). Evidence of oedema, pin prick bleeding or seepage will also be recorded;
- Digital photographs will be taken of the treatment sites at 10 min, 1 hour and 2 hours post-treatment, according to the schedule of events (Appendix 2). The photograph file will be recorded with the subject number, visit type, date and time (refer to Appendix 7);
- Prior to discharge, subjects will be issued with memory aids and thermometers, and instructions given for recording any local reactions, adverse events or changes on medication for the following 6 days.

Day 2 Phone Call, Part A only:

- Review of spontaneously reported AEs, local and systemic reactions.
- Review of concomitant medications taken since last contact;

Day 2 Clinic Visit, Part B only:

- Review of spontaneously reported AEs, local and systemic reactions.
- Review of concomitant medications taken since last contact;
- Vital signs evaluation (blood pressure, heart rate, oral temperature and respiratory rate) at approximately 24 hours after treatment;
- Examination of the treatment site(s) at approximately 24 hours after treatment. Grading of any reaction for redness, bruising, induration, tenderness, itching and flaking (see Appendix 4), pain scores (see Appendix 5) and the Skin Irritation Index for skin irritation (see Appendix 6). Evidence of oedema, pin prick bleeding or seepage will also be recorded;
- Digital photographs will be taken of the treatment sites at approximately 24 hours after treatment, according to the schedule of events (Appendix 2). The photograph file will be recorded with the subject number, visit type, date and time (refer to Appendix 7);

Day 3 Phone Call, All Groups:

- Review of spontaneously reported AEs, local and systemic reactions.
- Review of concomitant medications taken since last contact;

Day 4 Clinic Visit, All Groups:

- Review of spontaneously reported AEs, local and systemic reactions.
- Review of concomitant medications taken since last contact;
- Review of memory aids;
- Vital signs evaluation (blood pressure, heart rate, oral temperature and respiratory rate);
- Collection of blood sample(s) for serum for immunogenicity assessment (Appendix 8);
- Collection of saliva sample for IgA assessment (Part B, non-biopsy);
- Examination of the treatment site(s). Grading of any reaction for redness, bruising, induration, tenderness itching, and flaking (see Appendix 4), pain scores (see Appendix 5), and the Skin

Irritation Index for skin irritation (see Appendix 6). Evidence of oedema, pin prick bleeding or seepage will also be recorded;

- Digital photographs will be taken of the treatment sites. The photograph file will be recorded with the subject number, visit type, date and time (refer to Appendix 7);
- **Part B Biopsy groups only:** Perform punch biopsies within the site of MAP application.

Day 8 Clinic Visit (± 1 day), All Groups:

- Review of spontaneously reported AEs, local and systemic reactions.
- Review of concomitant medications taken since last contact;
- Review and collection of memory aids;
- Vital signs evaluation (blood pressure, heart rate, oral temperature and respiratory rate);
- Collect blood sample for serum for immunogenicity assessment (Appendix 8);
- Collect saliva sample for IgA assessment (Part B non biopsy) (Appendix 8);
- Examination of the treatment site(s). Grading of any reaction for redness, bruising, induration itching, and flaking (see Appendix 4), pain scores (see Appendix 5) and the Skin Irritation Index for skin irritation (see Appendix 6). Evidence of oedema, pin prick bleeding or seepage will also be recorded;
- Digital photographs will be taken of the treatment sites. The photograph file will be recorded with the subject number, visit type, date and time (refer to Appendix 7).

Day 22 Clinic Visit (± 1 day), All Groups:

- Review of spontaneously reported AEs, local and systemic reactions.
- Review concomitant medications taken since last contact;
- Vital signs evaluation (blood pressure, heart rate, oral temperature and respiratory rate);
- Collect blood sample for serum for immunogenicity assessment;
- Collect safety clinical laboratory blood samples (Haematology and Biochemistry) (Appendix 3);
- Collect saliva samples for baseline immune response (Part B, non biopsy) (Appendix 8);
- Collect blood sample for PBMCs for immunoassay (Part B, non biopsy) (Appendix 8);
- Examination of the treatment site(s). Grading of any reaction for redness, bruising, induration, tenderness, itching, and flaking (see Appendix 4), pain scores (see Appendix 5) and the Skin Irritation Index for skin irritation (see Appendix 6). Evidence of oedema, pin prick bleeding or seepage will also be recorded;
- Digital photographs will be taken of the treatment sites. The photograph file will be recorded with the subject number, visit type, date and time (refer to Appendix 7);
- Urine beta-HCG (female subjects only).

Day 36 Phone Call (± 2 days), All Groups:

- Review, local reactions (if required).
- Review of concomitant medications taken since last contact;

Day 50 Phone Call (± 2 days), All Groups:

- Review local reactions (if required).
- Review of concomitant medications taken since last contact;

Day 61 Clinic Visit (± 4 days), All Groups:

- Review of local reactions (if required).
- Review concomitant medications taken since last contact;
- Vital signs evaluation (blood pressure, heart rate, oral temperature and respiratory rate);
- Collect blood sample for serum for immunogenicity assessment;
- Examination of the treatment site(s). Grading of any reaction for redness, bruising, induration, tenderness, itching, and flaking (see Appendix 4), pain scores (see Appendix 5) and the Skin Irritation Index for skin irritation (see Appendix 6). Evidence of oedema, pin prick bleeding or seepage will also be recorded;
- Digital photographs will be taken of the treatment sites. The photograph file will be recorded with the subject number, visit type, date and time (refer to Appendix 7);
- Urine beta-HCG (female subjects only).

Any treatment site reactions still present at Day 61 will be followed up to resolution with fortnightly phone calls, until the condition stabilizes, or until the subject is lost to follow-up.

9.3 Subject Restrictions and Concomitant Medications

9.3.1 Subject Restrictions

Confinement: Subjects will remain at the study site for at least 2 hours following MAP application or intramuscular injection.

Contraception: Women of childbearing potential must have a negative pregnancy test prior to treatment and should avoid becoming pregnant over the duration of the study. Urine pregnancy test will also be performed on Day 22 and Day 61.

Exercise: Subjects should refrain from any strenuous physical activity, weight training, aerobics, football, swimming, gym session etc for 24 hours prior to any treatment and up until Day 4 visit.

Smoking: Subjects will not be permitted to smoke whilst inside the study site.

Treatment sites: Subjects should not apply any creams or lotions to the application / administration site(s) for the duration of the study. Subjects should follow their normal bathing routines, but must avoid scrubbing or exfoliating the area of application for duration of the study. Subjects will be instructed to inform the Investigator of the details of any topical creams or lotions applied to the site(s).

9.3.2 Concomitant Medications

Subjects should refrain from the use of prescription medication (excluding contraception) for 7 days prior to Day 1. Subjects will be permitted to take paracetamol, non-steroidal anti-inflammatory drugs (NSAIDs), or antihistamines as required during the study period. Subjects will be instructed to inform the Investigator of the details (name, indication, dose and dates of administration) if they are required to take any medication and the details will be recorded in the subject's CRF. Subjects should not take any Aspirin or medications known to thin blood i.e. Warfarin, during the study.

Any medication taken by a subject during the course of the study, up to the Day 61 (End of Study) visit, and the reason for its use, will be documented in the source document and on the appropriate CRF. For medications commenced after Day 22, the reason for use may not necessarily be an AE, unless the indication satisfies the criteria for a reportable AE during this period.

9.4 Early Withdrawal Visit.

When a subject withdraws or is withdrawn from the study prior to Day 22 the Investigator should attempt to complete the evaluations scheduled for the EOS visit when the evaluations and procedures are consistent with acceptable medical management of the subject.

9.5 Subject Discontinuation (Lost to Follow-up)

In the event that a subject elects not to return to the clinic for the Final Study Visit, the Investigator must make every effort to contact the subject to review all AEs. In the event that a subject drops out of the study at any time, the reason for discontinuation must be fully documented in the source documents and the CRF. The site personnel will document the AEs and any other assessments in the source documents and will make every effort to complete all required end of study assessments.

9.6 Safety and Tolerability Assessments

9.6.1 Clinical Safety Measurements

Vital Signs: The physical condition (blood pressure, heart rate, oral temperature and respiratory rate) of the subjects will be measured prior to the blood draw and treatment, and prior to exiting the unit. All vital signs will be taken after the subject has been seated for at least 3 minutes.

Prior to the study treatment, if a subject's vital signs are out of range, they should be repeated. If vital signs continue to be abnormal and are considered clinically significant by the Investigator, then subjects should not have the treatment administered. Vital signs may be repeated at the discretion of the Investigator.

9.6.2 Clinical Laboratory Tests

Clinical laboratory tests, as outlined in Appendix 3, will be performed on each subject within 28 days prior to the first treatment to establish baseline data and eligibility for enrolment. Out of range laboratory results may be repeated at the discretion of the Investigator. For each subject blood samples will be collected at two time points during the study, at Screening and Day 22, for safety review.

The total volume of blood collected for safety monitoring from each subject during the study will be approximately 50mL.

9.6.3 Treatment Site Tolerability Assessment

In order to assess the treatment site for reactions, the tolerability of the treatment will be assessed and documented at the time points present in the schedule of events (Appendix 2). The Investigator or designee will evaluate the local tolerability, skin irritation using the Skin Irritation Index (Appendix 6) and subjects will be asked to report and record any pain at the treatment site (Appendix 5).

The following parameters will be evaluated (see Appendices 4, 5 and 6):

- Redness of the application site and around the site
- Bruising around the site
- Induration around the site
- Tenderness
- Itching
- Flaking
- Application site definition / visibility (MAP groups only)
- Pin-prick bleeding
- Seepage
- Pain

Any local reaction or skin irritation ongoing at 2 hours post-dose, and evaluated as moderate or severe, will be classified as an adverse event (including a pain score > 4). Within this period, other local events may be classified as an adverse event if the investigator considers these to be of clinical concern.

A digital photograph of the treatment site on the arm will be taken at the time points set out in the schedule of events (Appendix 2).

10 MANAGEMENT OF DRUG AND DEVICES

10.1 Investigational Product – MAP, Skin conditioning ring and Applicator

The single use A/Sing MAPs are packaged in individual foil sealed aluminium cans. These must be stored at 2 to 8^0C (do not freeze) and left in the packaging provided prior to use. Before use the sealed can must be allowed to come to ambient temperature prior to opening.

The single use uncoated MAPs are also packaged in individual foil sealed aluminium cans. These must be stored below 30^0C (do not freeze) and left in the packaging provided prior to use.

When not in use the single use investigational MAP applicators and loading jigs must also be stored in the supplied boxes at the same ambient temperature as listed above.

When not in use the skin conditioning rings must be stored in supplied boxes at the same ambient temperature as listed above.

The Afluria® Quadrivalent intramuscular injections delivering 15 µg HA A/Singapore/GP1908/2015 protein in 0.5 mL with the addition of 3 other HA strains are supplied as packaged prefilled syringes. These must be stored according to the product label at 2 to 8^0C (do not freeze) and left in the packaging prior to use.

The A/Sing intramuscular injections deliver 15 µg HA A/Singapore/GP1908/2015 protein in 0.5 mL. These must be stored at 2 to 8^0C (do not freeze) and left in the packaging prior to use.

10.3 Packaging and Labelling

The A/Sing MAPs for use in this clinical study are manufactured according to Good Manufacturing Practices (GMP) by Vaxxas and are produced using aseptic manufacturing. Endotoxin limits as per parenteral injectable products in the USP are applied to A/Sing and uncoated MAPs. Sterility testing is included in the finished product specifications (FPS). The uncoated MAPs are manufactured according to GMP are terminally sterilised by gamma irradiation at ≥ 25 kGy (Steritech, Australia). The A/Sing MAP product cannot be gamma irradiated as this will denature the A/Sing HA protein component.

A/Sing MAPs are coated with a sterile solution of A/Sing antigen (supplied to Vaxxas by Seqirus Pty Ltd and manufactured to GMP), sulfobutyl ether (β) cyclodextrin (Captisol®) and phosphate saline buffer and the dispensed solution is then dried onto the micro-projections. The excipients are purchased to GMP quality and are known to be used in approved medical products for injection and/or ocular use. The Applicator will be provided by Vaxxas Pty Ltd and is manufactured and assembled by Romar (Australia). The skin conditioning rings are manufactured and supplied by Vaxxas Pty Ltd. The skin contacting face of the skin ring is made from stainless steel grade 316 and is wiped down with a sanitizing medical wipe between subjects.

The MAPs will be supplied to the study site after receipt of required documents in accordance with the Sponsor authorised release procedures. Clinical trial labels will comply with Annex 13 of the Australian Code of Good Manufacturing Practice for Medicinal Products – Manufacture of Investigational Medicinal Products.

Afluria Quadrivalent vaccine have been imported from the USA (FDA approved product) and is therefore labelled accordingly. The vaccines were imported and stored by Pharmaceutical Packaging Professionals (PPP, Melbourne) ahead of supplying to the clinical trial site.

A/Sing intramuscular vaccines are prepared on the day use at the dispensing pharmacy at Nucleus Network) from bulk A/Sing supplied to cGMP by Seqirus diluted with sterile Dulbecco's phosphate buffered saline (dPBS) (Alanza Inc, Canada).

10.4 Dispensing and Accountability of drugs and devices

The MAPs, skin conditioning rings, applicators and intramuscular vaccines are supplied for use only in this clinical study and should not be used for any other purpose.

The Investigator is responsible for the study product accountability, reconciliation and record maintenance. The Investigator or designated site staff must maintain the study accountability records throughout the course of the study. This person(s) will document the number of MAPs, Applicators, A/Singapore vaccines and Afluria Quadrivalent prefilled syringes received from Vaxxas or PPP and the amount dispensed and/or administered to subjects.

Drugs and devices will be received at the study site labelled and ready for use. The A/Sing intramuscular vaccinations will be prepared from supplied antigen on the day of use. The MAPs and Afluria Quadrivalent prefilled syringes will be dispensed for this study by the pharmacist or nominee of the clinical trial facility. The study inventory must be available for inspection by the study monitor during the study.

In Part A, all 30 subjects in groups A2 and A4 need to be vaccinated on the same day – the IM (A/Sing) injections (group A4) must be prepared and administered on the day of use.

Unused study drugs and devices will be collected at the end of the study by the Investigator or designee and returned to the Sponsor. These may only be destroyed under instructions by the Sponsor, but only once the study is closed out.

10.5 Study Drugs and Devices Handling and Storage

Only subjects enrolled in the study may receive the study MAPs, Afluria Quadrivalent prefilled syringes or A/Sing IM injections. Only trained site staff may supply or administer the study drugs and devices. All supplies of the study drugs and devices will be stored in a secure area with access limited to the Investigator and authorised staff. Site-specific SOPs will be followed for the receipt, handling and accountability of the study drugs and devices.

11 ADVERSE EVENTS

The Investigator and site staff are responsible for detection, recording and reporting of events that meet the criteria and definition of an AE or SAE.

All adverse events will be recorded from the first treatment until the Day 22 visit. After Day 22 up to the Day 61 end of study visit, AEs will only be recorded if they are SAEs, medically attended events, or clinically significant events.

All AEs reported during the study will be recorded in the subject’s CRF. The nature, time of onset, duration and severity will be documented, together with the Investigator’s opinion of the relationship to MAP application or IM administration. Adverse event definitions, assignment of severity and causality and procedures for reporting of SAEs are defined in Section 11 and Appendix 4.

11.1 Definition of an Adverse Event

An AE is “Any untoward medical occurrence in a patient, or clinical investigation subject, administered a pharmaceutical product and which does not necessarily have a causal relationship with this treatment”.

An AE can therefore be any unfavourable and unintended sign (including an abnormal laboratory finding, for example), symptom, or disease (new or exacerbated) temporally associated with the use of a medicinal product, whether or not considered related to the medicinal product.

Examples of an AE include:

Exacerbation of a chronic or intermittent pre-existing condition including either an increase in frequency or intensity of the condition.

- A new condition detected or diagnosed after study drug administration even though it may have been present prior to the start of the study;
- Signs, symptoms, or clinical sequelae of a suspected overdose of either study drug or a concurrent medication (“overdose” per se, should not be reported as an AE/SAE); and
- Pre- or post-treatment events that occur as a result of protocol-mandated procedures (e.g. invasive protocol-defined procedures, modification of a subject’s previous drug treatment regimen).

An AE does not include:

- Medical or surgical procedures (e.g. colonoscopy, biopsy). The medical condition that leads to the procedure is an AE;
- Social or convenience hospital admissions where an untoward medical occurrence did not occur; and
- Day to day fluctuations of pre-existing disease or conditions present or detected at the start of the study that do not worsen.

Signs or symptoms include local symptoms (i.e. erythema, redness, induration, tenderness, bruising, oedema, itching and pain, and/or fever) that occur during the observation periods after the MAP application or IM administration will be recorded on memory aid for 6 days following study treatment. Any local reaction or skin irritation ongoing at 2 hours post-dose, and evaluated as moderate or severe, will be classified as an adverse event.

Subjects will also report any local and systemic AEs that occur after they have been discharged and within 7 days after each application, as well as serious adverse events that occur at any time during the trial. Room will be available on each memory aid to record signs or symptoms in the 6 days following administration of study treatment. Concomitant medications will also be recorded in the memory aid.

The subject will be properly instructed on the documentation of AEs occurring after treatment and reporting requirements. Clinically significant signs and symptoms reported in the memory aid which are assessed as being an adverse event by the study staff and/or investigator will be entered on the AE/SAE CRF.

11.2 Definition of a Serious Adverse Event

A SAE is any untoward medical occurrence that, at any dose:

- Results in death;
- Is life threatening; the term life threatening refers to an event that begins after injection (e.g., anaphylaxis, vasovagal reaction, or hyperventilation), in which the subject was at risk of death at the time of the event. It does not refer to an event, which hypothetically might have caused death, if it were more severe;
- Requires hospitalisation or prolongation of an existing hospitalisation. In general, hospitalisation signifies that the subject has been detained (usually involving at least an overnight stay) at the hospital or emergency ward for observation and/or treatment that would not have been appropriate in the physician's office or outpatient setting. Complications that occur during hospitalisation are AEs. If a complication prolongs hospitalisation or fulfils any other serious criteria, the event is serious. When in doubt as to whether hospitalisation occurred or was necessary, the AE should be considered serious;
- Results in disability/incapacity. The term disability means a substantial disruption of a person's ability to conduct normal life functions. This definition is not intended to include experiences of relatively minor medical significance such as uncomplicated headache, nausea, vomiting, diarrhoea, influenza like symptoms, and accidental trauma (e.g. sprained ankle) which may interfere or prevent everyday life functions but do not constitute a substantial disruption; and
- Results in a congenital anomaly/birth defect.

Medical or scientific judgement should be exercised in deciding whether reporting is appropriate in other situations, such as important medical events that might have been associated with the use of the vaccine and may not be immediately life threatening or result in death or hospitalisation but may jeopardise the subject or may require medical or surgical intervention to prevent one of the other outcomes listed in the above definition. These should also be considered serious. Examples of such events are invasive or malignant cancers, intensive treatment in an emergency room or at home for allergic bronchospasm, blood dyscrasias or convulsions that do not result in hospitalisation.

If any subject becomes pregnant while enrolled in the study, the Investigator must notify the Sponsor or designee by telephone within 24 hours of learning about the pregnancy. Within the following 2 weeks, the Investigator must complete the Pregnancy Notification Form provided by the Sponsor or designee. The Investigator must diligently follow the subject until delivery or termination of the pregnancy, providing necessary updated information to the Sponsor or designee using the Pregnancy Notification Form. Information on the status of the mother and the child will be forwarded to the Sponsor or designee using the Pregnancy Notification Form. Generally, follow-up will occur within 6 to 8 weeks following the estimated delivery date. Any premature termination of the pregnancy will also be reported on this form.

Although pregnancy occurring in a clinical study is not considered to be an AE or SAE, any pregnancy complication or elective termination of a pregnancy for medical reasons will be recorded as an AE or SAE and will be followed as such. A spontaneous abortion is always considered to be an SAE.

11.3 Clinical Laboratory Abnormalities and Other Abnormal Assessments as AEs and SAEs

Abnormal laboratory findings or other abnormal assessments (e.g. vital signs) that are judged by the Investigator as clinically significant will be recorded as AEs or SAEs if they meet the definition of an AE, as defined in Section 11.1, or SAE as defined in Section 11.2. Clinically significant abnormal laboratory findings or other abnormal assessments that are detected during the study and significantly worsen following the start of the study will be reported as AEs or SAEs.

The Investigator will exercise his or her medical and scientific judgment in deciding whether an abnormal laboratory finding or other abnormal assessment is clinically significant.

All clinically significant abnormal laboratory results or assessments will be followed until they resolve (return to normal or baseline values) or stabilize, or until they are judged by the Investigator to be no longer clinically significant.

11.4 Time Period, Frequency, and Method of Detecting AEs and SAEs

At each visit after treatment, the Investigator or designee will review the subject's health. As a consistent method of soliciting AEs, the subject should be asked a non-leading question such as: "How do you feel?" Adverse events will be solicited at check-in (Day 1) and during the period of confinement after the first treatment. At each AE check/visit during the period of the study, AEs will be solicited and evaluated by the Investigator and recorded.

Any pre-existing conditions or signs and/or symptoms present in a subject prior to the start of the study (i.e., before on-study informed consent obtained) should be recorded as Medical History.

Any baseline signs and symptoms present at the time prior to the first treatment will be documented as medical history. If a baseline sign or symptom worsens following treatment, then this will be recorded as an AE.

All AEs occurring after the first treatment and on or before the Day 22 visit must be reported. After Day 22 up to the Day 61 end of study visit, AEs will only be recorded if they are SAEs, medically attend events, or clinically significant events. The study for each subject will be concluded once the blood results from the last visit have been analysed and the results are within clinical laboratory values. All AEs must be recorded irrespective of whether they are considered treatment related or not.

Any AEs already documented at a previous AE check/visit and designated as ongoing, should be reviewed at subsequent visits as necessary. If these have resolved, this should be documented.

11.5 Recording of AEs and SAEs

When an AE/SAE occurs, it is the responsibility of the Investigator to review all documentation (e.g. hospital progress notes, laboratory, diagnostic reports and memory aids) relative to the event. The Investigator will then record all relevant information regarding an AE/SAE in the eCRF. It is not acceptable for the Investigator to send photocopies of the subject's medical records to the Sponsor in lieu of completion of the appropriate AE/SAE entries in the eCRF. However, there may be instances when the Sponsor requests copies of medical records for certain cases. In this instance, all subject identifiers will be blinded on the copies of the medical records prior to submission to the Sponsor.

The Investigator will attempt to establish a diagnosis of the event based on signs, symptoms, and/or other clinical information. In such cases, the diagnosis should be documented as the AE/SAE and not the individual signs/symptoms.

11.6 Evaluating AEs and SAEs

11.6.1 Assessment of severity

The Investigator will make an assessment of intensity for each AE and SAE reported during the study. The assessment will be based on the Investigator's clinical judgement. The severity of each AE and SAE recorded in the CRF should be assigned to one of the following categories based on the extent to which an AE affects the subject's daily activities:

Mild (Grade 1):	Normal activities unaltered or annoying, but tolerable
Moderate (Grade 2):	Normal activities altered or require intervention
Severe (Grade 3):	Unable to undertake normal activities or incapacitated
Potentially Life Threatening (Grade 4):	The subject is at risk of death at the time of the event
Fatal (Grade 5):	The event results in death

An AE that is assessed as severe should not be confused with a SAE. Severity is a category utilised for rating the intensity of an event; and both AEs and SAEs can be assessed as severe. An event is defined as 'serious.' when it meets one of the predefined outcomes as described in Section 11.2.

11.6.2 Assessment of causality

The Investigator is obligated to assess the relationship between the treatment and the occurrence of each AE/SAE. The Investigator will use clinical judgement to determine the relationship. Alternative causes, such as natural history of the underlying diseases, concomitant therapy, other risk factors, and the temporal relationship of the event to the treatment will be considered and investigated. The Investigator will also consult the IB in the determination of his/her assessment. All AEs will be assessed into one of the following categories according to the relationship to the treatment as determined by the Investigator or medically qualified delegate:

Not related: Event for which sufficient evidence exists to conclude that the aetiology is unrelated to study product.

Unlikely related: Although sufficient evidence does not exist to conclude that the aetiology is unrelated to study product based on the temporal relationship between the event and the administration of the study product and/or the subject's medical condition or other therapies, it is unlikely that the event is related to study product.

Possibly related: There is some temporal relationship between the event and the administration of the study product, and the event is unlikely to be explained by the subject's medical condition or other therapies.

Probably related: The temporal relationship between the event and administration of the study product is suggestive and the event is unlikely explained by the subject's medical condition or other therapies.

Definitely related: The event follows reasonable temporal sequence from administration of the study product, follows a known or suspected response pattern to the product, is confirmed by improvement upon stopping the product and reappears upon repeated exposure, if that occurs.

There may be situations when a SAE has occurred and the Investigator has minimal information to include in the initial report to the Sponsor. However, it is very important that the Investigator always make an assessment of causality for every event prior to transmission of the SAE CRF to the Sponsor. The Investigator may change his/her opinion of causality in light of follow-up information, amending the SAE CRF accordingly. The causality assessment is one of the criteria used when determining regulatory reporting requirements.

11.6.3 Halt Criteria

Following the completion of Part A, a data review committee will convene to decide continuation to Part B of the study, based on the Part A immunogenicity and safety/tolerability data, or as required in the event of any of the halt/pause criteria listed below. This committee will include:

- Dr Angus Forster (Vaxxas Pty Ltd)

- Dr Julian Hickling (immunologist, Working in Tandem, UK)
- Dr Jason Lickliter (Principal Investigator)
- Prof. Ian Frazer, University of Queensland
- Prof Guy Ludbrook (Medical Monitor)

Criteria for an enrolment and vaccination halt and immediate consultation (within 24 hours) with the medical monitor are

- One subject experiences a serious adverse event which is probably or definitely related to the investigational vaccine(s), or two or more subjects experience similar serious adverse events which are possibly related to the investigational vaccine.
- There is a death assessed as possibly, probably or definitely related to the investigational vaccine.
- Administration site ulceration, sterile abscess or necrosis associated with vaccine administration occurs.
- A severe allergic reaction such as laryngospasm, bronchospasm or anaphylaxis associated with vaccine administration occurs.

Criteria for an enrolment pause and consultation with the full data review committee are:

- Two or more subjects experience a severe (Grade 3 or greater) vaccine-related subjective systemic reaction which is more than 1 day in duration and is confirmed by study personnel.
- One subject develops a Grade 4 adverse event that is judged to be possibly, probably or definitely related to the vaccination.
- Grade 2 adverse events that persists for more than 7 days that is possibly, probably or definitely related to the vaccination.
- Disabling tenderness and soreness of the arm lasting more than 24 hour.
- Elevated Skin Irritation Index score of 8 (oedema + erythema).

11.7 Follow-up of AEs and SAEs

After the initial AE/SAE report, the Investigator is required to proactively follow each subject and provide further information to the Sponsor on the subject's condition.

All AEs and SAEs documented at a previous visit/contact and are designated as ongoing, will be reviewed at subsequent visits/contacts.

All AEs and SAEs will be followed until resolution, until the condition stabilises, until the event is otherwise explained, or until the subject is lost to follow-up. Once resolved, the appropriate AE/SAE CRF page(s) will be updated. The Investigator will ensure that follow-up includes any supplemental investigations as may be indicated to elucidate the nature and/or causality of the AE or SAE. This may include additional laboratory tests or investigations, histopathology examinations, or other health care professionals.

The Sponsor may request that the Investigator perform or arrange for the conduct of supplemental measurements and/or evaluations to elucidate as fully as possible the nature and/or causality of the AE or SAE. The Investigator is obligated to assist. If a subject dies during participation in the study or during a recognised follow-up period, the Sponsor will be provided with a copy of any post-mortem findings, including histopathology (where possible).

New or updated information will be recorded on the originally completed SAE CRF, with all changes signed and dated by the Investigator. The updated SAE CRF should be resent to the Sponsor within the time frames outlined in Section 11.8.

11.8 Reporting of SAEs

11.8.1 Completion and transmission of the SAE reports to the Sponsor

Any clinical adverse event that is serious (as defined in Section 11.2) occurring during the course of the study, irrespective of the treatment received by the subject, must be reported to the Sponsor, or Sponsor's safety representative within 24 hours of the Investigator or designee becoming aware of the situation.

The SAE report form will always be completed as thoroughly as possible with all available details of the event, signed by the Investigator (or designee), and forwarded to the Sponsor, or Sponsor's safety representative within the designated time frames. If the Investigator does not have all information regarding a SAE, he/she will not wait to receive additional information before notifying the Sponsor, or Sponsor's safety representative of the event and completing the form. The form will be updated when additional information is received.

The Investigator will always provide an assessment of causality at the time of the initial report as described in Section 11.6.

Scanning and emailing the SAE report form is the preferred method to transmit this information to the Sponsor's safety representative. In the absence of scanning equipment, notification by telephone is acceptable, with a copy of the SAE report form sent by overnight mail. Initial notification via the telephone does not replace the need for the Investigator to complete and sign the SAE report form within the time frames outlined above.

The Sponsor will provide a contact for SAE receipt, email address, telephone numbers, and mailing addresses. Any event that in the opinion of the Investigator may be of immediate or potential concern for the subject's health or well-being will be reported to the Sponsor emergency contact listed below.

Sponsor Emergency Contact

Caron Hookway
Vaxxas Pty Ltd
TRI, 37 Kent Road
Woolloongabba, QLD 4102
AUSTRALIA

Telephone: +61 (0) 451 991 367
Email: caron.hookway@vaxxas.com

Investigator

Dr Jason Lickliter, MBBS, PhD, FRACP
Medical Director, Nucleus Network Pty Ltd
The Burnet Tower, 5th Floor
89 Commercial Rd
Melbourne, VIC 3004
AUSTRALIA

Phone: +61 (0) 3 9076 8900
Email: j.lickliter@nucleusnetwork.com.au

11.8.2 Regulatory Reporting Requirements for SAEs

The Investigator will promptly report all SAEs to the Sponsor in accordance with the procedures detailed in Section 11.8.1. Prompt notification of SAEs by the Investigator to the appropriate Sponsor contact for SAE receipt is essential so that legal obligations and ethical responsibilities towards the safety of other subjects are met. The Sponsor will comply with the Australian regulatory requirements related to the reporting of SAEs to the TGA. The Investigator will report SAEs to the Human Research Ethics Committee (HREC) within 72 hours of the Investigator or designee becoming aware of the SAE (or earlier if required by local HREC procedures).

11.9 Post-study AEs and SAEs

A post-study AE/SAE is defined as any event that occurs outside of the AE/SAE detection period defined in Section 11. The Investigator is not obligated to actively seek AEs or SAEs in former study subjects. However, if the Investigator learns of any SAE, including a death, at any time after a subject

has completed the study, and he/she considers the event reasonably related to the treatment the Investigator will promptly notify the Sponsor.

Investigators should promptly notify the Sponsor, or designee, if they become aware of a former study participant who is one of the parents of a subsequently conceived child with a congenital anomaly (see Section 11.2 – pregnancy information).

12 DATA AND STATISTICAL ANALYSIS

12.1 Data collection and Processing

12.1.1 Data collection

Subject data will be entered into an electronic case report form (eCRF) and will be substantiated by source documents at the clinical site. Information recorded in the subject memory aids will be assessed by the Investigator or designee at the next clinic visit, and all clinically significant signs and symptoms reported in the diary will be entered in the eCRF, and on an SAE report form, if required.

12.1.2 Data Processing

Data processing will be managed by CPR Pharma Services Pty Ltd in accordance with the Data Management Plan.

12.2 Statistical Analysis

12.2.2 Subject Number Allocation

Subjects will be identified at screening by a subject number starting at S001. A randomisation number will be given on Day 1 when the subjects are confirmed at meeting eligibility criteria. Each potential subject must meet all of the inclusion and none of the exclusion criteria as detailed in Sections 8.3 and 8.4 in order to qualify for admission into the study. A list identifying the subjects, by subject numbers and initials will be kept in the study file.

12.2.3 Populations to be analysed

The safety population will consist of all subjects who received treatment, this population being used in presenting baseline and safety data.

The biopsy population will consist of all subjects who received treatment and have at least one post-dose biopsy. In summary tables, results for subjects in the biopsy population will not be pooled with those from non-biopsy subjects receiving the same treatment, but will be summarised separately.

The immunogenicity population consists of all non-biopsy subjects who received treatment and have a Day 22 sample of serum for HI assay.

The immunogenicity population consists of a subset of subjects (Part B non biopsy) who received treatment and have a Day 22 sample of serum for microneutralisation assay.

The exploratory immunogenicity populations consists only of subjects in groups B3, B7, B8, B9 who received treatment and have a Day 22 sample (saliva and / or PBMCs and / or serum for ADCC assay).

12.2.4 Data analysis

All collected subject information will be included within subject listings and summarized in tables by treatment (route/site, dose level, biopsy).

Any deviation from the protocol specified analysis will be described within the clinical study report with the reason for the deviation being documented.

Treatment site reaction, pain scores, and skin irritation index will be summarised at each assessment time by treatment group. Comparisons between sites of application and between MAP and intramuscular injection administration and active (A/Sing) versus placebo administrations will be analysed using appropriate statistical tests.

Descriptive statistics of demographics (age, height and weight at screening, race and ethnic origin) will be presented by cohort and overall. Medical history information collected at screening will be listed. All adverse events will be listed by subject and will include details of the onset date and time, duration, severity, causality and treatment/medications administered.

Laboratory parameters will be listed over the scheduled visits by subject number and treatment cohort. Laboratory parameters which are outside of the normal reference range and clinically significant will be listed. Vital signs (blood pressure, heart rate, oral temperature and respiratory rate) will be listed by subject and summarised in tables by treatment group.

Physical examination data will be listed by subject.

Local reactions will be listed by subject and summarised in tables by treatment group.

Concomitant medications will be entered as Trade names and coded using the WHO DD according to the ATC codes. All medications will be listed by subject, start and stop date and time, end date and time, dose, route of administration and reason for administration.

12.2.5 Subject Accountability

The number of subjects enrolled will be summarized by treatment group and overall. In addition, the number of subjects completing /not completing all visits up to and including Visit 5 will be presented along with the primary reason for withdrawal from the study.

12.2.6 Protocol Deviations

Protocol deviations will be listed by subject and treatment cohort.

12.2.7 Population Sub-group Analyses

No formal sub-group analyses are planned to be performed.

12.3 Statistical Methods

Demographic and clinical measurements will be summarised by frequencies for categorical variables and by mean, standard deviation and range for continuous variables.

No statistical testing between groups for safety endpoints is planned to be performed. Safety endpoints have been identified as being of main interest in this study. Study investigations will be exploratory and conclusions based on the complete set of subject evidence.

Immune responses post-treatment will be compared within and between groups. Skin irritation index scores in response to vaccination or application statistical tests. Confidence intervals and p-values will be presented for two-tailed tests, where significance is assumed for $p<0.05$. Analyses will be performed using SAS version 9.3 or higher.

12.4 Safety Analysis

12.4.1 Adverse Events

All adverse events will be listed by subjects and will include details of the onset date and time, duration, severity, causality and treatment/medications administered. The incidence and frequency of AEs, and SAEs, will be summarized by treatment group according to System Organ Class (SOC) and Preferred Term (PT). Summaries of Treatment-Emergent Adverse Events (TEAEs) will also be presented by severity and relationship.

12.4.2 Laboratory Parameters

Laboratory parameters (haematology, biochemistry and other laboratory parameters as specified in Appendix 3) will be listed over the scheduled visits by subject number and treatment group. Laboratory values outside the normal range will be classified as clinically or non-clinically significant by the investigator.

12.4.3 Vital Signs

Vital signs (blood pressure, heart rate, oral temperature and respiratory rate) will be listed by subject and summarised in tables by treatment group.

12.4.4 Physical examination

Physical examination data will be listed by subject.

12.4.5 Local and Systemic Reactions

Local and systemic reactions (treatment site tolerability assessment, treatment site pain, skin irritation index, daily temperature) will be listed by subject and summarised in tables by treatment group.

13 ETHICAL CONSIDERATIONS

The following conditions will be met.

13.1 Good Clinical Practice

The Investigator will ensure that this study is conducted in accordance with the protocol and in full conformity with the current revision of the 1964 Declaration of Helsinki (as adopted in the Australian National Statement on Ethical Conduct in Research Involving Humans). This study will be conducted in accordance with Good Clinical Practice (GCP) requirements described in the Note for Guidance on Good Clinical Practice (CPMP/ICH/135/95) annotated with Australian TGA comments. Before the first subject is enrolled in the study, all ethical and legal requirements must be met.

13.2 Regulatory Approval

The requirements for the conduct of clinical trials in accordance with the Clinical Trial Notification (CTN) scheme of the TGA will be met before commencement of this study.

13.3 Human Research Ethics Committee Approval

Prior to initiation of the study, the written HREC approval of the protocol and Participant Information Sheet/Informed Consent Forms based on the ICH Principles of GCP will be received. This approval will be typed on the Institutional letterhead and will refer to the Participant Information Sheet/Informed Consent Forms and to the study by title and protocol number given by the Sponsor on page one of the protocol. A copy of the signed and dated letter of approval will be provided to the clinical trial site and the Sponsor prior to study commencement. Any written information and/or advertisements to be used for subject recruitment will be approved by the HREC prior to use. A list of the HREC members, will be requested before study initiation.

The HREC, following written agreement from the Sponsor will approve protocol modifications that may affect subject safety or the scientific integrity of the study. The clinical facility will maintain records of all correspondence with the approving HREC.

13.4 Ethical Considerations

This study will be carried out in accordance with the Principles of ICH GCP (as adopted in Australia) which builds upon the ethical codes contained in the Declaration of Helsinki and the Australian National Statement on Ethical Conduct in Research Involving Humans.

The Sponsor agrees to abide by the Medicines Australia Guidelines for the "Compensation for Injury Resulting from Participation in a Company-Sponsored Clinical Trial" (16 January 2004). Compensation will only be provided on the understanding that the provision of compensation does not amount to an admission of legal liability, and is subject to the proposed recipient signing a full and complete release of Vaxxas Pty Limited from all claims, damages and costs.

13.5 Written Informed Consent

Informed consent will be obtained before the subject can participate in the study and will be approved by the HREC. It is the responsibility of the Investigator to obtain a written informed consent from each individual participating in this study after adequate explanation of the aims, methods, objectives, and potential hazards of the study. The Investigator must also explain to the subjects that they are completely free to refuse to enter the study or to withdraw from it at any time. Appropriate forms for documenting a written consent will be provided by the Investigator.

All eligible subjects will have the pre-study medical evaluation and study explained by the Investigator or nominee. They will receive a full explanation, in lay terms, of the aims of the study, the discomfort, risks and benefits in taking part, as well as insurance and other procedures for compensation in case of injury. It will be pointed out that they can withdraw from the study at any time without prejudice. Each subject will confirm their understanding and agreement by giving written informed consent for

participation in the study. The subject will be given a copy of the signed Participant Information Sheet/Informed Consent Form to retain.

13.6 Emergency Contact with Investigator

Suitable arrangements will be made for subjects to make contact with the Investigator or a medically qualified nominee in the event of an emergency. This contact information will be documented in the Participant Information Sheet retained by the subject. Subjects will be provided with a Subject Trial Card with relevant Investigator and study staff contact details.

13.7 Clinical Laboratory Certification and Reference Ranges

Before commencement of this study, the Investigator, or nominee, will obtain a copy of the certification form, with certification number and expiration date for all clinical laboratories used in the study. Reference ranges for each clinical laboratory test used in this study will be obtained from the appropriate laboratory, which will perform the test for the study.

13.8 Protocol Amendments

Administrative amendments to the protocol will be classed as amendment of typographical errors, clarifications of confusing wording, and other minor modifications including but not limited to name, address, and contact information changes that have no impact on the safety of the subject or the science of the study. Administrative amendments will be submitted to the HREC for information only. The Sponsor will ensure that acknowledgement is received and filed. Otherwise, an amendment will be classed as a substantial amendment and will be submitted to the appropriate Regulatory Authorities and the HREC for approval.

13.9 Termination of the Study

The Sponsor reserves the right to discontinue or suspend the study at any time. Reasons will be provided in the event of this happening. The Investigator reserves the right to discontinue the study for safety reasons at any time in collaboration with the Sponsor. The HREC will be notified promptly and provided the reason for the termination or suspension by the Sponsor or the Investigator.

14 REPORTS AND PUBLICATIONS

14.1 Reports to the HREC

Upon completion of the study, the Investigator will provide the HREC with a summary of study participation and SAEs.

14.2 Publications

No publication of the results shall take place without the Sponsor's express consent.

15 STUDY ADMINISTRATION

15.1 Quality Assurance

To ensure compliance with ICH GCP and the Sponsor may conduct a quality assurance audit. Regulatory agencies may also conduct a regulatory inspection of this study. Such audits/inspections can occur at any time during or after completion of the study. If an audit or inspection occurs, the Investigator and the clinical trial site agree to allow the auditor/inspector direct access to all relevant documents and allocate his/her time and the time of his/her staff to the auditor/inspector to discuss findings and any relevant issues. The Investigator also agrees to provide direct access to all relevant documents and allocate his/her time and the time of his/her staff to personnel contracted to perform routine study monitoring.

Completed CRFs will be reviewed by the Study Monitor for the study to ensure data accuracy, completeness, and consistency. Any discrepancies found during the CRF review are to be clarified by the Investigator (or his/her designated staff). This includes CRF reviews at the site by the Sponsor or its designee, or during quality assurance review of the data or audits.

An explanation must be documented for any missing data. The Investigator must sign and date a declaration on the CRF attesting to his/her responsibility for the quality of all data recorded, and that the data represents a complete and accurate record of each subject's participation in the study.

15.2 Adherence to the Protocol

Investigator undertakes to adopt all reasonable measures to record data in accordance with the protocol. Under practical working conditions, however, some minor variations may occur due to circumstances beyond the control of the Investigator. All such deviations will be documented in the study records as a protocol deviation, together with the reason for their occurrence; where appropriate, deviations will be detailed in the clinical study report.

15.3 Records Retention

Following closure of the study, the Investigator must maintain all site study records in a safe and secure location. The records must be maintained to allow easy and timely retrieval, when needed (e.g. for audit or inspection) and whenever feasible, to allow any subsequent review of data in conjunction with assessment of the facility, supporting systems and staff. When permitted by local institutional policy, some or all of these records can be maintained in a format other than hard copy (e.g. microfiche, scanned, electronic; however, caution needs to be exercised before such action is taken. The Investigator must assure that all reproductions are legible and are a true and accurate copy of the original and meet accessibility and retrieval standards, including re-generating a hardcopy, if required. Furthermore, the Investigator must ensure there is an acceptable back-up of these reproductions and that an acceptable quality control process exists for making these reproductions.

The retention period will be 15 years in accordance with TGA recommendations.

The material to be stored shall meet the "Essential Documents" requirements of ICH GCP (as adopted by the TGA), which include, but are not limited to, the following examples:

- IB or applicable product information;
- Signed and dated copy of the final study protocol and any amendments;
- Signed and dated letter of HREC approval, letter of constitution of the HREC and copies of any other correspondence relevant to the study with the HREC or regulatory authorities;
- The HREC approved Participant Information Sheet/Informed Consent Form;
- Current curriculum vitae (signed and dated) of the Investigator and co-workers with major responsibilities in the trial;
- Blank CRF;
- Signed subject informed consent forms;

- Laboratory reference ranges (signed and dated);
- A printout of the completed eClinical Trial Notification (CTN) Application Form;
- The Final Study Report; and
- Clinical raw data including the Medical Source Data Forms, all clinical laboratory report forms, study vaccines accountability forms, and dispensing records, etc.
- Subject CRFs (extracted as subject casebooks from the eCRF)

16 FINANCE, INDEMNITY, AND CONFIDENTIALITY

The conduct of the study is subject to financial agreements between Vaxxas Pty Limited, the Investigator and the clinical trial site. Vaxxas Pty Limited has taken out insurance to cover its obligations under both the indemnity and the Medicines Australia compensation guidelines for injury to subjects involved in the study.

16.1 Ownership

All information provided by the Sponsor and all data and information generated as part of the study (other than a subject's medical records) is the sole property of the Sponsor.

All rights, title and interests in any inventions, know-how or other intellectual property rights which are conceived or reduced to practice during the course of, or as a result of, the study are the sole property of the Sponsor and are hereby assigned to the Sponsor.

If a written contract is executed for the conduct of the study, which includes ownership provisions inconsistent with this statement, ownership provisions in the written contract shall apply rather than this statement.

16.2 Confidentiality

All information provided by the Sponsor and all data and information generated as part of the study (other than a subject's medical records) will be kept confidential by the Investigator and will not be used for any purpose other than conducting the study. These restrictions do not apply to: (1) information which becomes publicly available through no fault of the Investigator or site staff; (2) information which is necessary to disclose in confidence to the HREC solely for the evaluation of the study; (3) information which is necessary to disclose in order to provide appropriate medical care to a subject; or (4) study results which may be published.

This study will be listed on the ANZCTR clinical trials registry.

If a written contract is executed for the conduct of the study, which includes confidentiality provisions inconsistent with this statement, the confidentiality provisions in the written contract shall apply rather than this statement.

18 APPENDICES

18.1 Appendix 1: Procedure for MAP applications

18.2 Appendix 2: Schedule of Assessments

18.3 Appendix 3: Clinical Laboratory Tests

18.4 Appendix 4: MAP Application Site Tolerability Assessment

18.5 Appendix 5: Numeric Pain Scores

18.6 Appendix 6: Skin Irritation Index

18.7 Appendix 7: Photography

18.8 Appendix 8: Immunogenicity Assays

18.1 APPENDIX 1: Procedure for the MAP Application and skin hardness assessment

Clinical administration of MAPs

In this study the MAPs will be applied to subjects by a Vaxxas trained and qualified clinical site staff member to the upper volar forearm or upper arm sites. For each application, an investigational MAP, skin conditioning ring, durometer applicator, and loading jig supplied by Vaxxas, will be required. The MAPs and applicators are for single use only. The skin conditioning rings and durometers can be used for different subjects following wiping the skin contact faces with a medical wipe.

Priming the applicator

Before loading the MAP into the MAP applicator, the applicator must be primed using the supplied loading jig. The jig functions to compress the applicator spring to a point where it locks in place ready for use. The safety mechanism prevents accidental release of the MAP.

Loading the MAP into the applicator

Both the A/Sing and uncoated MAPs will be supplied individual packs in foil sealed aluminium cans with a pull-tab. Once the lid is removed, the polymer MAP can be seen in the centre with a yellow ring around it.

The MAP is loaded into the applicator by pushing down the applicator directly into the open can and between the white insert and yellow ring. The MAP is attached and held in place to the applicator via magnets. Before applying the MAP to the skin, the application site is swabbed using a medical wipe.

Skin hardness measurement.

Two skin hardness readings are to be obtained for each MAP application site; both are performed using the skin conditioning ring which helps to guide the durometer to the skin and ensure a reproducible measurement.

Before each measurement the durometer must be wiped with a medical wipe and then set to zero. For the first measurement, the skin conditioning ring is held, lightly in place by the user with no significant downward pressure applied. The durometer is then inserted into the skin ring and gently released to make contact with the skin. A hardness reading is then displayed on the durometer screen and this value recorded. The durometer is then set to zero and the process repeated with the skin conditioning ring under the required application pressure. A second value is obtained and recorded.

Once both measurements are recorded the MAP can be applied at the same site whilst maintaining pressure on the skin conditioning ring.

MAP application and removal from the skin.

The loaded applicator must be held perpendicular and vertical to and just touching the skin of the application site (Figure 1) prior to pressing the top button to actuate the applicator. Subjects receiving the MAP at the upper arm site, must lie down on the side with their treatment arm resting comfortably against their body. This is required to enable skin hardness values to be easily obtained. The MAP applicator and skin conditioning ring are then immediately removed from the skin.

The MAP is left on the skin for two minutes. After two minutes, the MAP is removed by the user using gloved fingers, by gently tensioning the skin around the patch with one hand, whilst pulling the MAP directly up and away from the skin with the other hand. Following removal from the skin the MAP should be carefully placed in the supplied tray and labelled with required subject and site details. The supplied tray contains a custom insert to secure the MAP in place and will prevent damage to the applied MAP before further analysis.

Applicator

Following administration, the applicator and loading jig should be returned to its transport box.

The following images demonstrate marking out the MAP application sites using the template, placing the skin conditioning ring on the first MAP application site, and inserting the durometer (zero'ed) into the skin conditioning ring.

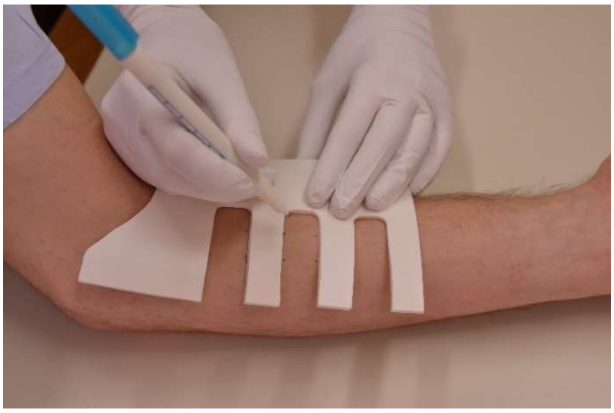

MAP application sites marked out on forearm using template

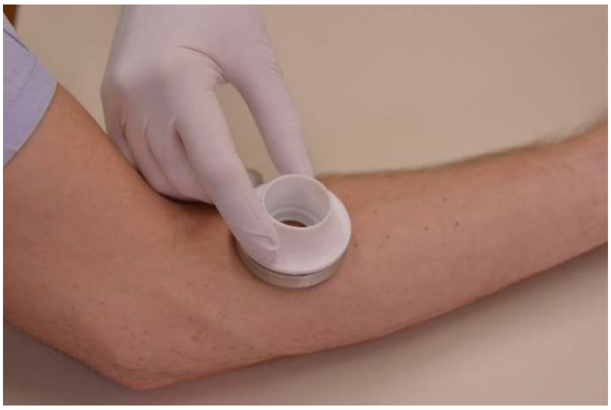

Skin conditioning ring placed on first MAP application site.

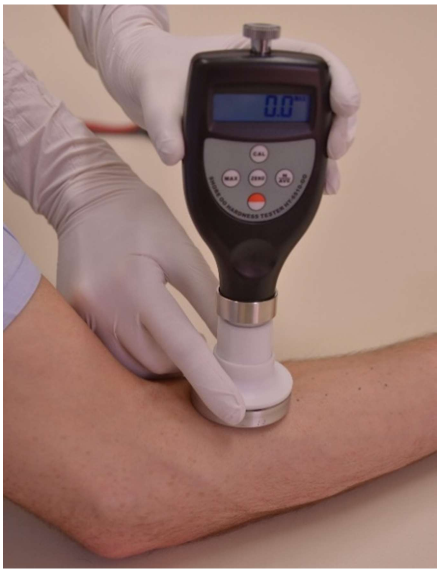

Durometer, zero'ed and inserted into skin conditioning ring.

Figure 1. Demonstration of the durometer for measuring skin hardness and the investigational MAP applicator (CAPD) and skin conditioning ring (SCR) used on the upper volar forearm.

Commercial in Confidence

18.2 APPENDIX 2: Schedule of Assessments – Part A and Part B

Schedule of Assessments (Part A and Part B, with and without punch biopsy

**Phase**	**Screen-ing**	**Study Period**									**EOS/ ET**
**Study Day**	**Day -28 to Day -1**	**Day 1 (visit)**		**Day 2 (Part A phone, Part B visit)**	**Day 3 (phone)**	**Day 4 (visit)**	**Day 8 (± 1) (visit)**	**Day 22 (± 1) (visit)**	**Day 36 (± 2) (phone)**	**Day 50 (± 2) (phone)**	**Day 61 (± 4) (visit)**
**PROCEDURES**		**Pre-dose**	**Dose/ Post-dose**								
Inclusion/Exclusion criteria	X										
Review eligibility / Randomisation		X									
Demography / Informed Consent	X										
Medical history	X	X									
Physical examination	X										
Height / weight / BMI	X										
Vital signs[3]	X	X	X[3]	X[1]		X	X	X			X
Clinical laboratory tests (Haematology and biochemistry)	X	X						X			
Pregnancy test[7] (female subjects)	X[7]	X[7]						X[7]			X[7]
Serology (Hep B sAg, Hep C & HIV)	X										
Immunogenicity blood samples for serum		X				X	X	X			X
Immunogenicity blood samples for PBMCs		X**						X**			
Saliva samples for IgA†		X†				X†	X†	X†			
Biopsy (punch)#		X#				X#					
Skin application site assessment[4]		X	X[4]	X[1]		X	X	X			X
Treatment site photography[5]		X	X[5]	X[1]		X	X	X			X
Pain Scores[6]		X	X[6]	X[1]		X	X	X			X
Study treatment			X[8]								
Issue Memory Aids			X								
Review Memory Aids						X	X				
Telephone Call				X[2]	X				X	X	
Adverse events		X		X	X	X	X	X	X	X	X
Concomitant medications	X	X		X	X	X	X	X	X	X	X

** PBMCs: Part B collect except Groups B1 and B2
Analysis for Groups B3, B7, B8, B9 only

Key: EOS = End of Study,
ET = Early Termination

Punch Biopsy: Part B Groups B1 and B2 only

†Saliva: Part B collect except Groups B1 and B2. Analysis for Groups B3, B7, B8, B9 only

1. Part B only (Day 2, clinic visit)
2. Part A only (Day 2, phone call)
3. Vital signs (Blood pressure, heart rate, oral temperature and respiratory rate). Day 1: pre-application, and 2 hr post-treatment, and all other clinic visits.
4. Treatment site tolerability assessment and Skin Irritation Index. Day 1: pre-application, 10 min, 1 hour and 2 hours post-treatment, and all other clinic visits.
5. Photography to be performed Day 1: pre-dose, 10 min, 1 hour and 2 hours post treatment, and all other clinic visits.
6. Pain assessed at 1 min, 10 min, 1 hour and 2 hours post treatment, and all other clinic visits.
7. Pregnancy test (female subjects only): beta-HCG, serum at screening, urine on Day 1, 22 and 61.
8. MAP application to either volar forearm (Part A, Part B Groups B1 – B7), or upper (Part B Group B8) and intramuscular injection to deltoid (Part B Group B9).

18.3 APPENDIX 3: Clinical Laboratory Tests

Biochemistry
Sodium
Potassium
Chloride
Bicarbonate
Glucose
Urea
Creatinine
Urate
Phosphate
Total Calcium
Calcium (corrected)
Albumin
Globulins
Protein
Total Bilirubin
Direct Bilirubin
Gamma glutamyltransferase (GGT)
Alkaline phosphatase (ALP)
Alanine aminotransferase (ALT)
Aspartate transaminase (AST)

Serology
Hepatitis B surface antigen^
Hepatitis C Virus^
Human Immunodeficiency Virus ^

Haematology
Haemoglobin
Red blood cell count (RBC)
Haematocrit
Mean cell volume (MCV)
Platelets
White cell count & differential
(Neutrophils, lymphocytes, monocytes, eosinophils & basophils)

^ Potential subjects will be counselled prior to testing and if any tests return a positive result.

Each laboratory parameter that lies outside the normal laboratory range will be reviewed by a physician to assess clinical significance.

18.4 APPENDIX 4: Treatment Site Tolerability Assessment

Tolerability assessments will be performed by a member of the clinical staff during outpatient visits. Training in these assessments will be provided by Vaxxas staff. Subjects will be provided with a transparent template to overlay the application site and assess the size of reactions around each treatment area on days 1, 4, 8, 22, and 61. An additional assessment by a physician will be made in the event of a local reaction being evaluated as moderate or severe.

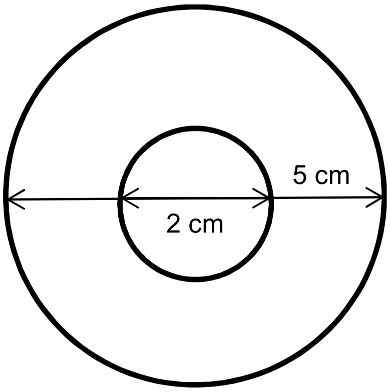

<u>Redness Extent</u>

Grade Description:

0	NONE	No visible redness around treatment area
1	MILD	0 to 2 cm redness around treatment area
2	MODERATE	2 to 5 cm redness around treatment area
3	SEVERE	Greater than 5 cm redness around the treatment area

<u>Bruising Extent</u>

Grade Description:

0	NONE	No visible bruising around treatment area
1	MILD	0 to 2cm bruising around treatment area
2	MODERATE	2 to 5cm bruising around treatment area
3	SEVERE	Greater than 5cm bruising around treatment area

<u>Induration Extent</u>

Grade Description:

0	NONE	No swelling detected around treatment area
1	MILD	Palpable “firmness” only around treatment area
2	MODERATE	< 4 cm swelling around treatment area
3	SEVERE	> 4 cm swelling around treatment area

Itching

Subjects will be asked the degree of itching they are experiencing:

Grade	Description:
0	NONE
1	MILD
2	MODERATE
3	SEVERE

Skin flaking

Subjects will be asked the amount of skin flaking of the treatment area they are experiencing:

Grade	Description:
0	NONE
1	MILD
2	MODERATE
3	SEVERE

Treatment Site Tenderness

Subjects will be asked the degree of tenderness they are experiencing:

Grade	Description:	
0	NONE	
1	MILD	Feel some tenderness, not uncomfortable to touch
2	MODERATE	Uncomfortable but bearable to touch
3	SEVERE	Extremely sensitive to touch

Application Site Definition/Visibility (MAP Sites only)

Subjects will be asked the amount of colouration of the treatment area they are experiencing, following loss of erythema:

Grade	Description:	
0	NONE	
1	MILD	Area slightly visible
2	MODERATE	Area noticeable but not clearly defined
3	SEVERE	Area clearly defined

Other:

MAP sites only: Subjects will be asked if they had any pin prick bleeding or seepage at the application site. Assessment by a study nurse or medical officer will include confirmation by lightly dabbing the site with tissue paper.

18.5 APPENDIX 5: Numeric Pain Intensity Scale

<u>Treatment Site Pain</u>
Subjects will be asked to rate the level of pain on a scale of 0 -10, with 0 being the lowest level and 10 being the maximum pain they experience. Subjects will be asked to circle their response. Assessment of pain will commence after treatment.

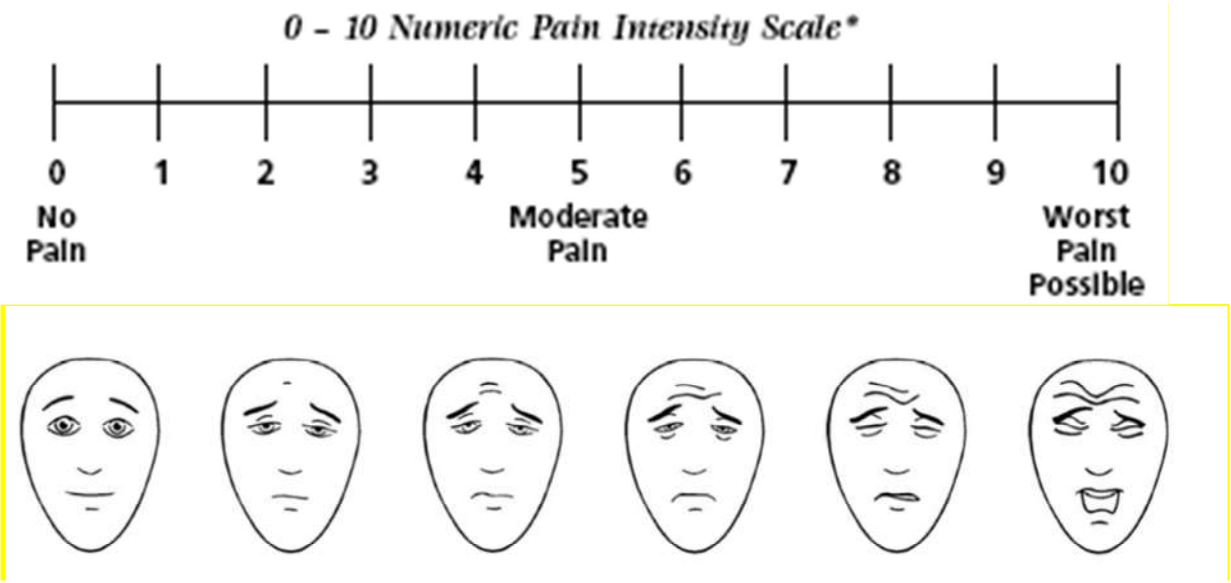

18.6 APPENDIX 6: Skin irritation index Scoring System for each treatment application site*

Skin irritation should be scored according to the following table, with erythema and oedema as separate scores as well as the total irritation index:

**Erythema and eschar formation**	Score
No erythema	0
Very slight erythema (barely perceptible)	1
Well-defined erythema	2
Moderate to severe erythema	3
Severe erythema (beet redness) to slight eschar formation (injuries in depth)	4
**Total possible erythema score**	**4**
**Oedema**	
No oedema	0
Very slight oedema (barely perceptible)	1
Slight oedema (edges of area well defined by definitive raising	2
Moderate oedema (area raised approximately 1mm)	3
Severe oedema (raised more than 1mm and extending beyond area of exposure)	4
**Total possible oedema score**	**4**
**Total possible score for primary irritation = erythema score + oedema score**	**8**

* one site for IM treatments, three sites for MAP treatments

18.7 APPENDIX 7: Photography

All photographs should be taken with a digital camera.

Subject number, subject initials and time point and patch number will be included in each photograph with a label. A close up photograph and far field shot should be taken of each treatment site pre and post application / administration.

All photographs should be taken under consistent lighting conditions (e.g., same location and light source).

In order to avoid shadows, orient the general anatomical location containing the application site or systemic reaction rash parallel to the body of the camera. Avoid distractions in the background.

Centre the application site in the centre of the viewfinder, and and ensure label is visible. To ensure consistency across all photographs, please take note of the way these markers are oriented to the application site/reaction region and the landmarks around it.

18.8 APPENDIX 8: Immunogenicity Assays

Haemagglutination inhibition (HI) assay

This will be the primary immunological readout. The HI assay is accepted as the correlate of protection for inactivated and split seasonal influenza vaccines.

The assay will be run on Day 1 and Day 22 samples from Part A. Following completion of the Day 22 blood collection, samples from Day 1 and Day 22 will be assayed by HI to determine the immune response to treatment before moving to Part B of the study. It is planned that 360biolabs (Melbourne) will run this assay, as for Vaxxas' previous trial.

All samples from part B and part A subjects will be tested for HI antibodies for the main analysis (i.e. Part A Day 1 and Day 22 will be re-run alongside the other timepoints).

ELISA

Vaxxas will use an in-house ELISA to give an initial readout of immunogenicity for Part A as a back-up to the HI assay and main study cohorts. The ELISA assay will be run of Day 1 and Day 22 serum samples and potentially on Day 4 and Day 8 as well.

Microneutralization (MN)

The MN assay measures functional, neutralizing antibodies, and will be used as a secondary serological assay. There is no defined protective titre for this assay, but results from MN assays generally correlate with HI titres, although the MN assay is often more sensitive. It is planned that 360biolabs (Melbourne) will run this assay, as for Vaxxas' previous trial. ***Samples from Day 1 and Day 22, from all Part B non-biopsy subjects will be tested.***

Mucosal IgA

Some preclinical and clinical data that suggest that vaccine delivery by micro-array patches (MAPs), intradermal (ID) injection, or transcutaneous immunization might induce stronger mucosal immune responses than intramuscular (IM) or subcutaneous (SC) injection. Induction of mucosal responses would be a significant differentiator for MAPs compared with injection, and advantageous for many vaccines including influenza. It is planned that Vaxxas will use an in-house ELISA to give an initial readout of influenza specific IgA / total IgA immunogenicity.

Assays for IgA responses following flu vaccination generally use saliva or nasal wash samples; the latter are generally used in studies of live-attenuated influenza vaccines, which are administered intranasally. Vaxxas is proposing to use saliva samples due to ease of collection. It is proposed to run these assays in-house at Vaxxas. ***Samples will be collected from all Part B non-biopsy subjects. The assay will be run only on samples from subjects in Part B Groups B3, B7, B8, B9 (i.e. top dose and uncoated controls) on samples from Day 1, 4, 8 and 22.***

Antibody dependent cellular cytotoxicity (ADCC)

ADCC is of interest because data suggests that antibodies detected by this assay tend to recognize epitopes on the conserved stem of the HA molecule, rather than the variable, globular head; as such they are more likely to cross-react with different strains. ADCC assays will be included as an exploratory assay on a subset of subjects. ***The assay will be performed at the Department of Microbiology and Immunology at the University of Melbourne. The assay will be run on serum from subjects in Part B, groups B3, B7, B8, B9 (i.e. top dose and uncoated controls) on samples from Day 1 and 22.***

T cell-mediated immunity

Historically, there has been more interest in T cell responses, particularly CD8+ T cells, against epitopes in influenza proteins such as NP and M1, rather than HA. However, HA-specific T cell responses are now frequently assayed in phase I trials of subunit or split, HA-based flu vaccines. These assays tend to focus on CD4+ poly-functional T cells (i.e. that produce γ-IFN, IL-2 and TNF-α).

The proposed assay is intracellular cytokine staining (ICS), with the assay planned to be run at the Translational Research Institute, Queensland in laboratory of Dr James Wells (University of Queensland). ***Samples will be collected from all Part B non-biopsy subjects. The assay will be run on PBMCs from subjects in Part B Groups B3, B7, B8, B9 (i.e. top dose and uncoated controls) on samples from Day 1 and 22.***

Memory B cells

HA-specific B cells are required for a rapid response to a secondary exposure to influenza virus. Therefore, we are considering assaying for memory B cells as an exploratory assay at the Department of Microbiology and Immunology at the University of Melbourne. ***Samples will be collected from all Part B non-biopsy subjects. The assay will be run on PBMCs from subjects in Part B Groups B3, B7, B8, B9 (i.e. top dose and uncoated controls) on samples from Day 1 and 22.***

For the T cell mediated and Memory B cell assays, additional blood samples to enable separation of PBMCs will be collected at day 1 and day 22 from all Part B subjects except for groups B1 and B2 (biopsy). PBMC samples will be processed at 360biolabs for subjects from Part B groups B3, B7, B8, B9 (i.e. top dose and uncoated controls).

Punch biopsies of the application site or immunohistochemistry and flow cytometry

We are proposing to take punch biopsies from 10 subjects; five recipients of MAP 15 µg A/Singapore and five recipients of uncoated MAPs (Groups B1 and B2). Biopsies will be taken on day 1 and day 4. Two 4 mm biopsies will be taken at each timepoint for: 1) labelling and image analysis of tissue sections and 2) dispersion of the cells, labelling and analysis by flow cytometry (2 biopsies), to analyse any immune infiltrate into the application site. The markers likely to be used include:

- CD3, CD4, CD8 (T cell subset markers)
- CD45RO (memory and/or activated T cells)
- CD103, CD69 (T_{RM} cells)
- CD20 (B cells)
- H&E staining
- CD1a (Langerhans cells and dermal dendritic cells)
- CD14 (LC, but not dermal dendritic cells)
- CD11c (DC)

Biopsies from Day 1 and Day 4 will be tested. Digestion and labelling of biopsies for cytometry will be carried out by Vaxxas and cytometry will be carried out at the TRI cytometry unit. Labelling of histology sections is planned to be carried out at the QIMR Berghofer Institute.

Commercial in Confidence

Table of Samples and Assays for PART A

Day	Sample taken	For	Volume taken	Assays	Pilot groups (Part A)			
					A1	A2	A3	A4
1	Blood	Serum	15 ml	HI	x	x	x	x
				ELISA	x	x	x	x
4	Blood	Serum	15 ml	HI	x	x	x	x
				ELISA	x	x	x	x
8	Blood	Serum	15 ml	HI	x	x	x	x
				ELISA	x	x	x	x
22	Blood	Serum	15 ml	HI	x	x	x	x
				ELISA	x	x	x	x
61	Blood	Serum	15 ml	HI	x	x	x	x
				ELISA	x	x	x	x

Table of Samples and Assays for PART B

Day	Sample taken	For	Volume taken	Samples collected									Assays	Samples Analysed								
				B1	B2	B3	B4	B5	B6	B7	B8	B9		B1	B2	B3	B4	B5	B6	B7	B8	B9
1	Blood	Serum	15 ml	x	x	x	x	x	x	x	x	x	HI	x	x	x	x	x	x	x	x	x
													MNT			x	x	x	x	x	x	x
													ADCC			x				x	x	x
1	Saliva	Saliva	400-1000 µl (1 x cotton saliva collector)			x	x	x	x	x	x	x	IgA ELISA			x				x	x	x
1	Blood	PBMC	40 ml (5 x 8.5 ml ACT tubes)			x	x	x	x	x	x	x	Memory B cell			x				x	x	x
													CMI ICS			x				x	x	x
1	Punch biopsy	Biopsy	2 x 4 mm biopsies	x	x								Histology	x	x							
				x	x								Flow cytometry	x	x							
4	Blood	Serum	15 ml	x	x	x	x	x	x	x	x	x	HI	x	x	x	x	x	x	x	x	x
4	Saliva	Saliva	400-1000 µl (1 x cotton saliva collector)			x	x	x	x	x	x	x	IgA ELISA			x				x	x	x
4	Punch biopsy	Biopsy	2 x 4 mm biopsies	x	x								Histology	x	x							
				x	x								Flow cytometry	x	x							
8	Blood	Serum	15 ml	x	x	x	x	x	x	x	x	x	HI	x	x	x	x	x	x	x	x	x
8	Saliva	Saliva	400-1,00 µl (1 x cotton saliva collector)			x	x	x	x	x	x	x	IgA ELISA			x				x	x	x
22	Blood	Serum	15 ml	x	x	x	x	x	x	x	x	x	HI	x	x	x	x	x	x	x	x	x
													MNT			x	x	x	x	x	x	x
													ADCC			x				x	x	x
22	Saliva	Saliva	400-1,00 µl (1 x cotton saliva collector)			x	x	x	x	x	x	x	IgA ELISA			x				x	x	x
22	Blood	PBMC	40 ml (5 x 8.5 ml ACT tubes)			x	x	x	x	x	x	x	Memory B cell			x				x	x	x
													CMI ICS			x				x	x	x
61	Blood	Serum	15 ml	x	x	x	x	x	x	x	x	x	HI	x	x	x	x	x	x	x	x	x
