# Supplementary material for: Safety, tolerability, and immunogenicity of influenza vaccination with a high-density microarray patch: Results from a randomized, controlled phase I clinical trial
Source: PLoS Med. 2020 Mar 17;17(3):e1003024. doi: 10.1371/journal.pmed.1003024 (PMC7077342; doi:10.1371/journal.pmed.1003024)
Supplement: S1 Data — Data 1: Informed Consent; Data 2: Analysis Sets; Data 3: Demographics; Data 4: Study Drug Administration; Data 5: Skin Hardness Assessment; Data 6: Immunogenicity (ELISA IgG); Data 7: Immunogenicity (ADCC); Data 8: Immunogenicity (MBCs); Data 9: Immunogenicity (CMI); Data 10: Immunogenicity (Mucosal IgA); Data 11: Immunogenicity Results (HAI, MN); Data 12: TEAE–CRF Data only; Data 13: TEAEs–MedDRA Coding; Data 15: Treatment Site Tolerability Assessment; Data 16: Numeric Pain Intensity; Data 17: SII; Data 18: Application Site Status at End of Study; Data 19: Individual Treatment Site Tolerability Assessment; Data 20: Numeric Pain Intensity by Treatment; Data 21: Individual SII by Treatment. ADCC, antibody-dependent cellular cytotoxicity; CMI, cell-mediated immunity; CRF, case report form; ELISA, enzyme-linked immunosorbent assay; HAI, haemagglutination inhibition; IgA, immunoglobulin A; IgG, immunoglobulin G; MBC, memory B cell; MedDRA, medical dictionary for regulatory activities; MN, microneutralisation; SII, Skin Irritation Index; TEA, treatment emergent adverse event. (ZIP) [file pmed.1003024.s008.zip › S1_Data/clinical supporting/S1_Data1 Informed Consent.pdf]

Listing 16.2.1.1
Informed Consent

Part A

Randomisation Number	Screening Number	Group	Was Informed Consent Obtained?	Date Consent Obtained	Time Consent Obtained	PICF Version	PICF Date	Reason for Subsequent Informed Consent
A0001	S009	Group A3	Yes	27-FEB-2018	13:25	2.0	05-FEB-2018	
A0002	S012	Group A1	Yes	28-FEB-2018	08:31	2.0	05-FEB-2018	
A0003	S001	Group A1	Yes	23-FEB-2018	11:52	2.0	05-FEB-2018	
A0004	S006	Group A3	Yes	26-FEB-2018	13:40	2.0	05-FEB-2018	
A0005	S034	Group A1	Yes	06-MAR-2018	14:07	2.0	05-FEB-2018	
A0006	S018	Group A3	Yes	02-MAR-2018	11:14	2.0	05-FEB-2018	
A0007	S010	Group A1	Yes	27-FEB-2018	15:45	2.0	05-FEB-2018	
A0008	S039	Group A3	Yes	07-MAR-2018	08:46	2.0	05-FEB-2018	
A0009	S017	Group A1	Yes	02-MAR-2018	11:19	2.0	05-FEB-2018	
A0010	S019	Group A3	Yes	02-MAR-2018	13:22	2.0	05-FEB-2018	
A0011	S047	Group A3	Yes	07-MAR-2018	14:16	2.0	05-FEB-2018	
A0012	S023	Group A1	Yes	05-MAR-2018	11:19	2.0	05-FEB-2018	
A0013	S035	Group A1	Yes	06-MAR-2018	14:46	2.0	05-FEB-2018	
A0014	S040	Group A3	Yes	07-MAR-2018	08:38	2.0	05-FEB-2018	
A0015	S022	Group A1	Yes	03-MAR-2018	16:44	2.0	05-FEB-2018	
A0016	S004	Group A3	Yes	24-FEB-2018	13:28	2.0	05-FEB-2018	
A0017	S027	Group A3	Yes	05-MAR-2018	17:33	2.0	05-FEB-2018	

Key: PICF = Participant Informed Consent Form

Administration Site Key: FA = Forearm; UA= Upper arm
Treatments Part A: A1 = A/Sing MAP, 15 ug (FA); A2 = Afluria Quad IM (UA), 15 ug; A3 = Placebo MAP (FA); A4 = A/Sing Mono IM, 15 ug (UA)
Treatments Part B: B1 = A/Sing MAP, 15 ug (FA) with biopsy; B2 = Placebo MAP (FA) with biopsy
B3 = A/Sing MAP, 15 ug (FA); B4 = A/Sing MAP, 10 ug (FA); B5 = A/Sing MAP, 5 ug (FA); B6 = A/Sing MAP, 2.5 ug (FA)
B7 = Placebo MAP (FA); B8 = A/Sing MAP, 15 ug (UA); B9 = A/Sing IM, 15 ug, as 2018 Afluria Quad (UA)

Listing 16.2.1.1
Informed Consent

Part A

Randomisation Number	Screening Number	Group	Was Informed Consent Obtained?	Date Consent Obtained	Time Consent Obtained	PICF Version	PICF Date	Reason for Subsequent Informed Consent
A0018	S028	Group A1	Yes	05-MAR-2018	17:15	2.0	05-FEB-2018	
A0019	S003	Group A3	Yes	24-FEB-2018	08:31	2.0	05-FEB-2018	
A0020	S036	Group A1	Yes	06-MAR-2018	13:45	2.0	05-FEB-2018	
A0021	S042	Group A1	Yes	07-MAR-2018	10:08	2.0	05-FEB-2018	
A0022	S033	Group A3	Yes	06-MAR-2018	12:54	2.0	05-FEB-2018	
A0023	S043	Group A3	Yes	07-MAR-2018	10:48	2.0	05-FEB-2018	
A0024	S048	Group A1	Yes	07-MAR-2018	15:30	2.0	05-FEB-2018	
A0025	S049	Group A3	Yes	08-MAR-2018	08:29	2.0	05-FEB-2018	
A0027	S056	Group A3	Yes	08-MAR-2018	12:31	2.0	05-FEB-2018	
A0028	S061	Group A1	Yes	08-MAR-2018	17:26	2.0	05-FEB-2018	
A0029	S053	Group A1	Yes	08-MAR-2018	11:33	2.0	05-FEB-2018	
A0030	S066	Group A3	Yes	13-MAR-2018	10:57	2.0	05-FEB-2018	
A0031	S068	Group A2	Yes	13-MAR-2018	14:17	2.0	05-FEB-2018	
A0032	S067	Group A4	Yes	13-MAR-2018	12:18	2.0	05-FEB-2018	
A0033	S076	Group A2	Yes	14-MAR-2018	16:50	2.0	05-FEB-2018	
A0034	S074	Group A4	Yes	14-MAR-2018	14:34	2.0	05-FEB-2018	
A0035	S077	Group A4	Yes	14-MAR-2018	17:08	2.0	05-FEB-2018	

Key: PICF = Participant Informed Consent Form

Administration Site Key: FA = Forearm; UA= Upper arm
Treatments Part A: A1 = A/Sing MAP, 15 ug (FA); A2 = Afluria Quad IM (UA), 15 ug; A3 = Placebo MAP (FA); A4 = A/Sing Mono IM, 15 ug (UA)
Treatments Part B: B1 = A/Sing MAP, 15 ug (FA) with biopsy; B2 = Placebo MAP (FA) with biopsy
B3 = A/Sing MAP, 15 ug (FA); B4 = A/Sing MAP, 10 ug (FA); B5 = A/Sing MAP, 5 ug (FA); B6 = A/Sing MAP, 2.5 ug (FA)
B7 = Placebo MAP (FA); B8 = A/Sing MAP, 15 ug (UA); B9 = A/Sing IM, 15 ug, as 2018 Afluria Quad (UA)

Listing 16.2.1.1
Informed Consent

Part A

Randomisation Number	Screening Number	Group	Was Informed Consent Obtained?	Date Consent Obtained	Time Consent Obtained	PICF Version	PICF Date	Reason for Subsequent Informed Consent
A0036	S082	Group A2	Yes	15-MAR-2018	13:39	2.0	05-FEB-2018	
A0037	S079	Group A4	Yes	14-MAR-2018	17:26	2.0	05-FEB-2018	
A0038	S078	Group A2	Yes	14-MAR-2018	17:43	2.0	05-FEB-2018	
A0039	S065	Group A2	Yes	13-MAR-2018	10:05	2.0	05-FEB-2018	
A0040	S016	Group A4	Yes	01-MAR-2018	15:46	2.0	05-FEB-2018	
A0041	S063	Group A4	Yes	09-MAR-2018	15:20	2.0	05-FEB-2018	
A0042	S073	Group A2	Yes	14-MAR-2018	13:20	2.0	05-FEB-2018	
A0043	S059	Group A2	Yes	08-MAR-2018	16:12	2.0	05-FEB-2018	
A0044	S062	Group A4	Yes	09-MAR-2018	09:18	2.0	05-FEB-2018	
A0045	S032	Group A2	Yes	06-MAR-2018	12:53	2.0	05-FEB-2018	
A0046	S072	Group A4	Yes	14-MAR-2018	10:12	2.0	05-FEB-2018	
A0047	S057	Group A2	Yes	08-MAR-2018	12:45	2.0	05-FEB-2018	
A0048	S069	Group A4	Yes	13-MAR-2018	10:30	2.0	05-FEB-2018	
A0049	S055	Group A2	Yes	08-MAR-2018	12:24	2.0	05-FEB-2018	
A0050	S081	Group A4	Yes	15-MAR-2018	10:51	2.0	05-FEB-2018	
A0051	S070	Group A2	Yes	14-MAR-2018	08:12	2.0	05-FEB-2018	
A0052	S051	Group A4	Yes	08-MAR-2018	09:48	2.0	05-FEB-2018	

Key: PICF = Participant Informed Consent Form

Administration Site Key: FA = Forearm; UA= Upper arm
Treatments Part A: A1 = A/Sing MAP, 15 ug (FA); A2 = Afluria Quad IM (UA), 15 ug; A3 = Placebo MAP (FA); A4 = A/Sing Mono IM, 15 ug (UA)
Treatments Part B: B1 = A/Sing MAP, 15 ug (FA) with biopsy; B2 = Placebo MAP (FA) with biopsy
B3 = A/Sing MAP, 15 ug (FA); B4 = A/Sing MAP, 10 ug (FA); B5 = A/Sing MAP, 5 ug (FA); B6 = A/Sing MAP, 2.5 ug (FA)
B7 = Placebo MAP (FA); B8 = A/Sing MAP, 15 ug (UA); B9 = A/Sing IM, 15 ug, as 2018 Afluria Quad (UA)

Listing 16.2.1.1
Informed Consent

Part A

Randomisation Number	Screening Number	Group	Was Informed Consent Obtained?	Date Consent Obtained	Time Consent Obtained	PICF Version	PICF Date	Reason for Subsequent Informed Consent
A0053	S080	Group A4	Yes	15-MAR-2018	09:25	2.0	05-FEB-2018	
A0054	S064	Group A2	Yes	13-MAR-2018	08:57	2.0	05-FEB-2018	
A0055	S086	Group A4	Yes	21-MAR-2018	16:42	2.0	05-FEB-2018	
A0056	S087	Group A2	Yes	21-MAR-2018	17:28	2.0	05-FEB-2018	
A0057	S091	Group A4	Yes	22-MAR-2018	17:35	2.0	05-FEB-2018	
A0058	S092	Group A2	Yes	23-MAR-2018	09:46	2.0	05-FEB-2018	
A0059	S093	Group A2	Yes	23-MAR-2018	16:40	2.0	05-FEB-2018	
A0060	S094	Group A4	Yes	23-MAR-2018	17:46	2.0	05-FEB-2018	
A1026	S011	Group A1	Yes	27-FEB-2018	17:39	2.0	05-FEB-2018	

Key: PICF = Participant Informed Consent Form

Administration Site Key: FA = Forearm; UA= Upper arm
Treatments Part A: A1 = A/Sing MAP, 15 ug (FA); A2 = Afluria Quad IM (UA), 15 ug; A3 = Placebo MAP (FA); A4 = A/Sing Mono IM, 15 ug (UA)
Treatments Part B: B1 = A/Sing MAP, 15 ug (FA) with biopsy; B2 = Placebo MAP (FA) with biopsy
B3 = A/Sing MAP, 15 ug (FA); B4 = A/Sing MAP, 10 ug (FA); B5 = A/Sing MAP, 5 ug (FA); B6 = A/Sing MAP, 2.5 ug (FA)
B7 = Placebo MAP (FA); B8 = A/Sing MAP, 15 ug (UA); B9 = A/Sing IM, 15 ug, as 2018 Afluria Quad (UA)

Listing 16.2.1.1
Informed Consent

Part B

Randomisation Number	Screening Number	Group	Was Informed Consent Obtained?	Date Consent Obtained	Time Consent Obtained	PICF Version	PICF Date	Reason for Subsequent Informed Consent
B0001	S101	Group B1	Yes	23-APR-2018	10:28	2.0	05-FEB-2018	
B0002	S031	Group B2	Yes	11-APR-2018	08:32	2.0	05-FEB-2018	
B0003	S095	Group B2	Yes	10-APR-2018	16:25	2.0	05-FEB-2018	
B0004	S102	Group B1	Yes	23-APR-2018	17:02	2.0	05-FEB-2018	
B0005	S117	Group B1	Yes	27-APR-2018	18:22	2.0	05-FEB-2018	
B0006	S119	Group B2	Yes	30-APR-2018	13:18	2.0	05-FEB-2018	
B0007	S121	Group B2	Yes	02-MAY-2018	09:27	2.0	05-FEB-2018	
B0008	S098	Group B1	Yes	18-APR-2018	08:02	2.0	05-FEB-2018	
B0009	S114	Group B1	Yes	27-APR-2018	11:38	2.0	05-FEB-2018	
B0010	S116	Group B2	Yes	27-APR-2018	14:27	2.0	05-FEB-2018	
B0011	S100	Group B8	Yes	19-APR-2018	17:12	3.0	27-FEB-2018	
B0011		Group B8	Yes	12-MAY-2018	08:17	4.0	24-APR-2018	Informed consent form amendment
B0012	S103	Group B6	Yes	24-APR-2018	10:56	2.0	05-FEB-2018	
B0012		Group B6	Yes	12-MAY-2018	08:36	4.0	24-APR-2018	Informed consent form amendment
B0013	S110	Group B5	Yes	26-APR-2018	11:24	3.0	27-FEB-2018	
B0013		Group B5	Yes	12-MAY-2018	08:32	4.0	24-APR-2018	Informed consent form amendment
B0014	S112	Group B3	Yes	26-APR-2018	17:51	3.0	27-FEB-2018	

Key: PICF = Participant Informed Consent Form

Administration Site Key: FA = Forearm; UA= Upper arm
Treatments Part A: A1 = A/Sing MAP, 15 ug (FA); A2 = Afluria Quad IM (UA), 15 ug; A3 = Placebo MAP (FA); A4 = A/Sing Mono IM, 15 ug (UA)
Treatments Part B: B1 = A/Sing MAP, 15 ug (FA) with biopsy; B2 = Placebo MAP (FA) with biopsy
B3 = A/Sing MAP, 15 ug (FA); B4 = A/Sing MAP, 10 ug (FA); B5 = A/Sing MAP, 5 ug (FA); B6 = A/Sing MAP, 2.5 ug (FA)
B7 = Placebo MAP (FA); B8 = A/Sing MAP, 15 ug (UA); B9 = A/Sing IM, 15 ug, as 2018 Afluria Quad (UA)

Listing 16.2.1.1
Informed Consent

Part B

Randomisation Number	Screening Number	Group	Was Informed Consent Obtained?	Date Consent Obtained	Time Consent Obtained	PICF Version	PICF Date	Reason for Subsequent Informed Consent
B0014		Group B3	Yes	12-MAY-2018	08:43	4.0	24-APR-2018	Informed consent form amendment
B0015	S127	Group B9	Yes	03-MAY-2018	10:26	3.0	27-FEB-2018	
B0015		Group B9	Yes	12-MAY-2018	08:28	4.0	24-APR-2018	Informed consent form amendment
B0016	S118	Group B4	Yes	28-APR-2018	13:26	2.0	05-FEB-2018	
B0016		Group B4	Yes	12-MAY-2018	08:48	4.0	24-APR-2018	Informed consent form amendment
B0017	S128	Group B7	Yes	03-MAY-2018	16:03	3.0	27-FEB-2018	
B0017		Group B7	Yes	12-MAY-2018	08:57	4.0	24-APR-2018	Informed consent form amendment
B0018	S130	Group B9	Yes	03-MAY-2018	17:23	3.0	27-FEB-2018	
B0018		Group B9	Yes	12-MAY-2018	09:03	4.0	24-APR-2018	Informed consent form amendment
B0020	S136	Group B6	Yes	04-MAY-2018	14:43	3.0	27-FEB-2018	
B0020		Group B6	Yes	12-MAY-2018	09:19	4.0	24-APR-2018	Informed consent form amendment
B0021	S134	Group B7	Yes	04-MAY-2018	14:19	3.0	27-FEB-2018	
B0021		Group B7	Yes	12-MAY-2018	09:23	4.0	24-APR-2018	Informed consent form amendment
B0022	S150	Group B4	Yes	08-MAY-2018	11:50	3.0	27-FEB-2018	
B0022		Group B4	Yes	12-MAY-2018	09:02	4.0	24-APR-2018	Informed consent form amendment
B0023	S148	Group B3	Yes	08-MAY-2018	10:44	3.0	27-FEB-2018	
B0023		Group B3	Yes	12-MAY-2018	09:04	4.0	24-APR-2018	Informed consent form amendment

Key: PICF = Participant Informed Consent Form

Administration Site Key: FA = Forearm; UA= Upper arm
Treatments Part A: A1 = A/Sing MAP, 15 ug (FA); A2 = Afluria Quad IM (UA), 15 ug; A3 = Placebo MAP (FA); A4 = A/Sing Mono IM, 15 ug (UA)
Treatments Part B: B1 = A/Sing MAP, 15 ug (FA) with biopsy; B2 = Placebo MAP (FA) with biopsy
B3 = A/Sing MAP, 15 ug (FA); B4 = A/Sing MAP, 10 ug (FA); B5 = A/Sing MAP, 5 ug (FA); B6 = A/Sing MAP, 2.5 ug (FA)
B7 = Placebo MAP (FA); B8 = A/Sing MAP, 15 ug (UA); B9 = A/Sing IM, 15 ug, as 2018 Afluria Quad (UA)

Listing 16.2.1.1
Informed Consent

Part B

Randomisation Number	Screening Number	Group	Was Informed Consent Obtained?	Date Consent Obtained	Time Consent Obtained	PICF Version	PICF Date	Reason for Subsequent Informed Consent
B0024	S137	Group B5	Yes	07-MAY-2018	10:53	3.0	27-FEB-2018	
B0024		Group B5	Yes	12-MAY-2018	08:48	4.0	24-APR-2018	Informed consent form amendment
B0025	S115	Group B8	Yes	27-APR-2018	14:12	3.0	27-FEB-2018	
B0025		Group B8	Yes	12-MAY-2018	08:51	4.0	24-APR-2018	Informed consent form amendment
B0026	S138	Group B9	Yes	07-MAY-2018	12:09	3.0	27-FEB-2018	
B0026		Group B9	Yes	12-MAY-2018	09:29	4.0	24-APR-2018	Informed consent form amendment
B0027	S126	Group B3	Yes	03-MAY-2018	09:43	3.0	27-FEB-2018	
B0027		Group B3	Yes	12-MAY-2018	08:59	4.0	24-APR-2018	Informed consent form amendment
B0028	S105	Group B5	Yes	24-APR-2018	13:35	2.0	05-FEB-2018	
B0028		Group B5	Yes	12-MAY-2018	08:57	4.0	24-APR-2018	Informed consent form amendment
B0029	S151	Group B6	Yes	08-MAY-2018	12:05	3.0	27-FEB-2018	
B0029		Group B6	Yes	12-MAY-2018	08:54	4.0	24-APR-2018	Informed consent form amendment
B0030	S152	Group B7	Yes	08-MAY-2018	16:34	3.0	27-FEB-2018	
B0030		Group B7	Yes	12-MAY-2018	08:46	4.0	24-APR-2018	Informed consent form amendment
B0031	S169	Group B4	Yes	12-MAY-2018	10:25	4.0	24-APR-2018	
B0032	S182	Group B6	Yes	14-MAY-2018	17:32	4.0	24-APR-2018	
B0033	S189	Group B5	Yes	15-MAY-2018	13:30	4.0	24-APR-2018	

Key: PICF = Participant Informed Consent Form

Administration Site Key: FA = Forearm; UA= Upper arm
Treatments Part A: A1 = A/Sing MAP, 15 ug (FA); A2 = Afluria Quad IM (UA), 15 ug; A3 = Placebo MAP (FA); A4 = A/Sing Mono IM, 15 ug (UA)
Treatments Part B: B1 = A/Sing MAP, 15 ug (FA) with biopsy; B2 = Placebo MAP (FA) with biopsy
B3 = A/Sing MAP, 15 ug (FA); B4 = A/Sing MAP, 10 ug (FA); B5 = A/Sing MAP, 5 ug (FA); B6 = A/Sing MAP, 2.5 ug (FA)
B7 = Placebo MAP (FA); B8 = A/Sing MAP, 15 ug (UA); B9 = A/Sing IM, 15 ug, as 2018 Afluria Quad (UA)

Listing 16.2.1.1
Informed Consent

Part B

Randomisation Number	Screening Number	Group	Was Informed Consent Obtained?	Date Consent Obtained	Time Consent Obtained	PICF Version	PICF Date	Reason for Subsequent Informed Consent
B0034	S183	Group B8	Yes	15-MAY-2018	08:24	4.0	24-APR-2018	
B0035	S179	Group B9	Yes	14-MAY-2018	14:30	4.0	24-APR-2018	
B0036	S177	Group B7	Yes	14-MAY-2018	13:23	4.0	24-APR-2018	
B0037	S176	Group B4	Yes	14-MAY-2018	13:06	4.0	24-APR-2018	
B0038	S168	Group B3	Yes	12-MAY-2018	09:35	4.0	24-APR-2018	
B0039	S139	Group B7	Yes	07-MAY-2018	13:58	3.0	27-FEB-2018	
B0039		Group B7	Yes	19-MAY-2018	08:39	4.0	24-APR-2018	Informed consent form amendment
B0040	S167	Group B9	Yes	12-MAY-2018	08:34	4.0	24-APR-2018	
B0041	S146	Group B3	Yes	08-MAY-2018	09:50	3.0	27-FEB-2018	
B0041		Group B3	Yes	19-MAY-2018	08:22	4.0	24-APR-2018	Informed consent form amendment
B0042	S191	Group B5	Yes	15-MAY-2018	16:16	4.0	24-APR-2018	
B0043	S154	Group B8	Yes	09-MAY-2018	17:15	3.0	27-FEB-2018	
B0043		Group B8	Yes	19-MAY-2018	08:14	4.0	24-APR-2018	Informed consent form amendment
B0044	S192	Group B6	Yes	15-MAY-2018	17:38	4.0	24-APR-2018	
B0045	S163	Group B4	Yes	11-MAY-2018	11:43	3.0	27-FEB-2018	
B0045		Group B4	Yes	19-MAY-2018	08:17	4.0	24-APR-2018	Informed consent form amendment
B0046	S162	Group B7	Yes	11-MAY-2018	11:30	3.0	27-FEB-2018	

Key: PICF = Participant Informed Consent Form

Administration Site Key: FA = Forearm; UA= Upper arm
Treatments Part A: A1 = A/Sing MAP, 15 ug (FA); A2 = Afluria Quad IM (UA), 15 ug; A3 = Placebo MAP (FA); A4 = A/Sing Mono IM, 15 ug (UA)
Treatments Part B: B1 = A/Sing MAP, 15 ug (FA) with biopsy; B2 = Placebo MAP (FA) with biopsy
B3 = A/Sing MAP, 15 ug (FA); B4 = A/Sing MAP, 10 ug (FA); B5 = A/Sing MAP, 5 ug (FA); B6 = A/Sing MAP, 2.5 ug (FA)
B7 = Placebo MAP (FA); B8 = A/Sing MAP, 15 ug (UA); B9 = A/Sing IM, 15 ug, as 2018 Afluria Quad (UA)

Listing 16.2.1.1
Informed Consent

Part B

Randomisation Number	Screening Number	Group	Was Informed Consent Obtained?	Date Consent Obtained	Time Consent Obtained	PICF Version	PICF Date	Reason for Subsequent Informed Consent
B0046		Group B7	Yes	19-MAY-2018	08:41	4.0	24-APR-2018	Informed consent form amendment
B0047	S155	Group B9	Yes	10-MAY-2018	09:53	3.0	27-FEB-2018	
B0047		Group B9	Yes	19-MAY-2018	08:17	4.0	24-APR-2018	Informed consent form amendment
B0048	S157	Group B6	Yes	10-MAY-2018	14:07	3.0	27-FEB-2018	
B0048		Group B6	Yes	19-MAY-2018	08:25	4.0	24-APR-2018	Informed consent form amendment
B0049	S156	Group B4	Yes	10-MAY-2018	11:12	3.0	27-FEB-2018	
B0049		Group B4	Yes	19-MAY-2018	08:20	4.0	24-APR-2018	Informed consent form amendment
B0050	S161	Group B8	Yes	11-MAY-2018	10:45	3.0	27-FEB-2018	
B0050		Group B8	Yes	19-MAY-2018	08:22	4.0	24-APR-2018	Informed consent form amendment
B0051	S200	Group B3	Yes	17-MAY-2018	15:26	4.0	24-APR-2018	
B0052	S199	Group B5	Yes	17-MAY-2018	14:24	4.0	24-APR-2018	
B0053	S194	Group B6	Yes	16-MAY-2018	14:36	4.0	24-APR-2018	
B0054	S222	Group B5	Yes	23-MAY-2018	14:13	4.0	24-APR-2018	
B0055	S211	Group B8	Yes	21-MAY-2018	12:10	4.0	24-APR-2018	
B0056	S166	Group B3	Yes	11-MAY-2018	17:49	3.0	27-FEB-2018	
B0056		Group B3	Yes	19-MAY-2018	08:19	4.0	24-APR-2018	Informed consent form amendment
B0057	S217	Group B4	Yes	22-MAY-2018	11:36	4.0	24-APR-2018	

Key: PICF = Participant Informed Consent Form

Administration Site Key: FA = Forearm; UA= Upper arm
Treatments Part A: A1 = A/Sing MAP, 15 ug (FA); A2 = Afluria Quad IM (UA), 15 ug; A3 = Placebo MAP (FA); A4 = A/Sing Mono IM, 15 ug (UA)
Treatments Part B: B1 = A/Sing MAP, 15 ug (FA) with biopsy; B2 = Placebo MAP (FA) with biopsy
B3 = A/Sing MAP, 15 ug (FA); B4 = A/Sing MAP, 10 ug (FA); B5 = A/Sing MAP, 5 ug (FA); B6 = A/Sing MAP, 2.5 ug (FA)
B7 = Placebo MAP (FA); B8 = A/Sing MAP, 15 ug (UA); B9 = A/Sing IM, 15 ug, as 2018 Afluria Quad (UA)

Listing 16.2.1.1
Informed Consent

Part B

Randomisation Number	Screening Number	Group	Was Informed Consent Obtained?	Date Consent Obtained	Time Consent Obtained	PICF Version	PICF Date	Reason for Subsequent Informed Consent
B0058	S216	Group B9	Yes	22-MAY-2018	11:26	4.0	24-APR-2018	
B0059	S212	Group B7	Yes	21-MAY-2018	12:20	4.0	24-APR-2018	
B0060	S188	Group B3	Yes	15-MAY-2018	13:23	4.0	24-APR-2018	
B0061	S203	Group B8	Yes	18-MAY-2018	12:30	4.0	24-APR-2018	
B0062	S190	Group B5	Yes	15-MAY-2018	15:08	4.0	24-APR-2018	
B0063	S218	Group B7	Yes	22-MAY-2018	13:03	4.0	24-APR-2018	
B0064	S196	Group B9	Yes	16-MAY-2018	15:30	4.0	24-APR-2018	
B0065	S210	Group B4	Yes	21-MAY-2018	11:42	4.0	24-APR-2018	
B0066	S224	Group B6	Yes	24-MAY-2018	11:00	4.0	24-APR-2018	
B0067	S184	Group B8	Yes	15-MAY-2018	09:45	4.0	24-APR-2018	
B0068	S153	Group B5	Yes	09-MAY-2018	16:00	3.0	27-FEB-2018	
B0068		Group B5	Yes	25-MAY-2018	08:45	4.0	24-APR-2018	Informed consent form amendment
B0069	S252	Group B7	Yes	29-MAY-2018	15:39	4.0	24-APR-2018	
B0070	S223	Group B9	Yes	24-MAY-2018	11:00	4.0	24-APR-2018	
B0071	S235	Group B4	Yes	28-MAY-2018	13:20	4.0	24-APR-2018	
B0072	S237	Group B3	Yes	28-MAY-2018	13:59	4.0	24-APR-2018	
B0073	S230	Group B6	Yes	26-MAY-2018	10:50	4.0	24-APR-2018	

Key: PICF = Participant Informed Consent Form

Administration Site Key: FA = Forearm; UA= Upper arm
Treatments Part A: A1 = A/Sing MAP, 15 ug (FA); A2 = Afluria Quad IM (UA), 15 ug; A3 = Placebo MAP (FA); A4 = A/Sing Mono IM, 15 ug (UA)
Treatments Part B: B1 = A/Sing MAP, 15 ug (FA) with biopsy; B2 = Placebo MAP (FA) with biopsy
B3 = A/Sing MAP, 15 ug (FA); B4 = A/Sing MAP, 10 ug (FA); B5 = A/Sing MAP, 5 ug (FA); B6 = A/Sing MAP, 2.5 ug (FA)
B7 = Placebo MAP (FA); B8 = A/Sing MAP, 15 ug (UA); B9 = A/Sing IM, 15 ug, as 2018 Afluria Quad (UA)

Listing 16.2.1.1
Informed Consent

Part B

Randomisation Number	Screening Number	Group	Was Informed Consent Obtained?	Date Consent Obtained	Time Consent Obtained	PICF Version	PICF Date	Reason for Subsequent Informed Consent
B0074	S240	Group B9	Yes	28-MAY-2018	15:36	4.0	24-APR-2018	
B0075	S242	Group B3	Yes	28-MAY-2018	17:07	4.0	24-APR-2018	
B0077	S244	Group B4	Yes	29-MAY-2018	09:47	4.0	24-APR-2018	
B0078	S227	Group B8	Yes	25-MAY-2018	11:28	4.0	24-APR-2018	
B0079	S225	Group B6	Yes	24-MAY-2018	10:53	4.0	24-APR-2018	
B0081	S247	Group B6	Yes	29-MAY-2018	13:44	4.0	24-APR-2018	
B0082	S251	Group B3	Yes	29-MAY-2018	15:34	4.0	24-APR-2018	
B0083	S229	Group B8	Yes	26-MAY-2018	11:03	4.0	24-APR-2018	
B0084	S255	Group B5	Yes	29-MAY-2018	17:31	4.0	24-APR-2018	
B0085	S249	Group B4	Yes	29-MAY-2018	14:37	4.0	24-APR-2018	
B0086	S245	Group B9	Yes	29-MAY-2018	12:42	4.0	24-APR-2018	
B0087	S290	Group B7	Yes	06-JUN-2018	08:36	4.0	24-APR-2018	
B0088	S269	Group B9	Yes	04-JUN-2018	15:20	4.0	24-APR-2018	
B0089	S285	Group B5	Yes	05-JUN-2018	14:23	4.0	24-APR-2018	
B0090	S266	Group B6	Yes	04-JUN-2018	12:18	4.0	24-APR-2018	
B0091	S268	Group B8	Yes	04-JUN-2018	14:31	4.0	24-APR-2018	
B0092	S270	Group B4	Yes	04-JUN-2018	15:27	4.0	24-APR-2018	

Key: PICF = Participant Informed Consent Form

Administration Site Key: FA = Forearm; UA= Upper arm
Treatments Part A: A1 = A/Sing MAP, 15 ug (FA); A2 = Afluria Quad IM (UA), 15 ug; A3 = Placebo MAP (FA); A4 = A/Sing Mono IM, 15 ug (UA)
Treatments Part B: B1 = A/Sing MAP, 15 ug (FA) with biopsy; B2 = Placebo MAP (FA) with biopsy
B3 = A/Sing MAP, 15 ug (FA); B4 = A/Sing MAP, 10 ug (FA); B5 = A/Sing MAP, 5 ug (FA); B6 = A/Sing MAP, 2.5 ug (FA)
B7 = Placebo MAP (FA); B8 = A/Sing MAP, 15 ug (UA); B9 = A/Sing IM, 15 ug, as 2018 Afluria Quad (UA)

Listing 16.2.1.1
Informed Consent

Part B

Randomisation Number	Screening Number	Group	Was Informed Consent Obtained?	Date Consent Obtained	Time Consent Obtained	PICF Version	PICF Date	Reason for Subsequent Informed Consent
B0093	S283	Group B7	Yes	05-JUN-2018	14:23	4.0	24-APR-2018	
B0094	S260	Group B3	Yes	31-MAY-2018	17:32	4.0	24-APR-2018	
B0095	S265	Group B9	Yes	04-JUN-2018	08:45	4.0	24-APR-2018	
B0096	S278	Group B6	Yes	04-JUN-2018	17:45	4.0	24-APR-2018	
B0097	S232	Group B5	Yes	26-MAY-2018	14:38	4.0	24-APR-2018	
B0098	S271	Group B7	Yes	04-JUN-2018	15:42	4.0	24-APR-2018	
B0099	S287	Group B4	Yes	05-JUN-2018	15:34	4.0	24-APR-2018	
B0100	S253	Group B8	Yes	29-MAY-2018	15:34	4.0	24-APR-2018	
B0101	S257	Group B3	Yes	30-MAY-2018	16:58	4.0	24-APR-2018	
B0102	S284	Group B8	Yes	05-JUN-2018	14:31	4.0	24-APR-2018	
B0103	S213	Group B5	Yes	21-MAY-2018	13:25	4.0	24-APR-2018	
B0104	S279	Group B3	Yes	05-JUN-2018	08:34	4.0	24-APR-2018	
B0105	S273	Group B9	Yes	04-JUN-2018	16:21	4.0	24-APR-2018	
B0106	S280	Group B4	Yes	05-JUN-2018	10:24	4.0	24-APR-2018	
B0107	S293	Group B6	Yes	06-JUN-2018	12:29	4.0	24-APR-2018	
B0108	S274	Group B7	Yes	04-JUN-2018	16:49	4.0	24-APR-2018	
B0109	S272	Group B6	Yes	04-JUN-2018	15:54	4.0	24-APR-2018	

Key: PICF = Participant Informed Consent Form

Administration Site Key: FA = Forearm; UA= Upper arm
Treatments Part A: A1 = A/Sing MAP, 15 ug (FA); A2 = Afluria Quad IM (UA), 15 ug; A3 = Placebo MAP (FA); A4 = A/Sing Mono IM, 15 ug (UA)
Treatments Part B: B1 = A/Sing MAP, 15 ug (FA) with biopsy; B2 = Placebo MAP (FA) with biopsy
B3 = A/Sing MAP, 15 ug (FA); B4 = A/Sing MAP, 10 ug (FA); B5 = A/Sing MAP, 5 ug (FA); B6 = A/Sing MAP, 2.5 ug (FA)
B7 = Placebo MAP (FA); B8 = A/Sing MAP, 15 ug (UA); B9 = A/Sing IM, 15 ug, as 2018 Afluria Quad (UA)

Listing 16.2.1.1
Informed Consent

Part B

Randomisation Number	Screening Number	Group	Was Informed Consent Obtained?	Date Consent Obtained	Time Consent Obtained	PICF Version	PICF Date	Reason for Subsequent Informed Consent
B0110	S264	Group B3	Yes	04-JUN-2018	08:27	4.0	24-APR-2018	
B0111	S310	Group B8	Yes	13-JUN-2018	09:40	4.0	24-APR-2018	
B0112	S312	Group B4	Yes	13-JUN-2018	11:39	4.0	24-APR-2018	
B0113	S315	Group B5	Yes	13-JUN-2018	14:52	4.0	24-APR-2018	
B0114	S281	Group B7	Yes	05-JUN-2018	11:40	4.0	24-APR-2018	
B0115	S295	Group B9	Yes	06-JUN-2018	17:04	4.0	24-APR-2018	
B0116	S299	Group B7	Yes	07-JUN-2018	15:31	4.0	24-APR-2018	
B0117	S297	Group B9	Yes	07-JUN-2018	09:16	4.0	24-APR-2018	
B0118	S300	Group B6	Yes	07-JUN-2018	16:29	4.0	24-APR-2018	
B0119	S305	Group B4	Yes	12-JUN-2018	13:50	4.0	24-APR-2018	
B0120	S318	Group B3	Yes	13-JUN-2018	15:19	4.0	24-APR-2018	
B0121	S219	Group B8	Yes	23-MAY-2018	11:31	4.0	24-APR-2018	
B0122	S302	Group B5	Yes	08-JUN-2018	15:09	4.0	24-APR-2018	
B0123	S306	Group B6	Yes	12-JUN-2018	14:47	4.0	24-APR-2018	
B0124	S322	Group B4	Yes	13-JUN-2018	17:35	4.0	24-APR-2018	
B0125	S320	Group B7	Yes	13-JUN-2018	16:34	4.0	24-APR-2018	
B0126	S307	Group B3	Yes	12-JUN-2018	15:19	4.0	24-APR-2018	

Key: PICF = Participant Informed Consent Form

Administration Site Key: FA = Forearm; UA= Upper arm
Treatments Part A: A1 = A/Sing MAP, 15 ug (FA); A2 = Afluria Quad IM (UA), 15 ug; A3 = Placebo MAP (FA); A4 = A/Sing Mono IM, 15 ug (UA)
Treatments Part B: B1 = A/Sing MAP, 15 ug (FA) with biopsy; B2 = Placebo MAP (FA) with biopsy
B3 = A/Sing MAP, 15 ug (FA); B4 = A/Sing MAP, 10 ug (FA); B5 = A/Sing MAP, 5 ug (FA); B6 = A/Sing MAP, 2.5 ug (FA)
B7 = Placebo MAP (FA); B8 = A/Sing MAP, 15 ug (UA); B9 = A/Sing IM, 15 ug, as 2018 Afluria Quad (UA)

Listing 16.2.1.1
Informed Consent

Part B

Randomisation Number	Screening Number	Group	Was Informed Consent Obtained?	Date Consent Obtained	Time Consent Obtained	PICF Version	PICF Date	Reason for Subsequent Informed Consent
B0127	S221	Group B9	Yes	23-MAY-2018	12:08	4.0	24-APR-2018	
B0128	S349	Group B5	Yes	20-JUN-2018	14:15	4.0	24-APR-2018	
B0129	S333	Group B8	Yes	19-JUN-2018	09:33	4.0	24-APR-2018	
B0130	S351	Group B5	Yes	21-JUN-2018	13:15	4.0	24-APR-2018	
B0131	S334	Group B8	Yes	19-JUN-2018	08:55	4.0	24-APR-2018	
B0132	S263	Group B6	Yes	02-JUN-2018	15:43	4.0	24-APR-2018	
B0133	S326	Group B4	Yes	14-JUN-2018	14:15	4.0	24-APR-2018	
B0134	S337	Group B3	Yes	19-JUN-2018	13:31	4.0	24-APR-2018	
B0135	S329	Group B7	Yes	15-JUN-2018	14:42	4.0	24-APR-2018	
B0136	S324	Group B9	Yes	14-JUN-2018	09:31	4.0	24-APR-2018	
B0137	S336	Group B8	Yes	19-JUN-2018	10:58	4.0	24-APR-2018	
B0138	S258	Group B7	Yes	31-MAY-2018	14:02	4.0	24-APR-2018	
B0139	S316	Group B4	Yes	13-JUN-2018	14:51	4.0	24-APR-2018	
B0140	S335	Group B9	Yes	19-JUN-2018	08:40	4.0	24-APR-2018	
B0141	S343	Group B3	Yes	20-JUN-2018	08:40	4.0	24-APR-2018	
B0142	S325	Group B5	Yes	14-JUN-2018	09:55	4.0	24-APR-2018	
B0143	S332	Group B6	Yes	18-JUN-2018	16:32	4.0	24-APR-2018	

Key: PICF = Participant Informed Consent Form

Administration Site Key: FA = Forearm; UA= Upper arm
Treatments Part A: A1 = A/Sing MAP, 15 ug (FA); A2 = Afluria Quad IM (UA), 15 ug; A3 = Placebo MAP (FA); A4 = A/Sing Mono IM, 15 ug (UA)
Treatments Part B: B1 = A/Sing MAP, 15 ug (FA) with biopsy; B2 = Placebo MAP (FA) with biopsy
B3 = A/Sing MAP, 15 ug (FA); B4 = A/Sing MAP, 10 ug (FA); B5 = A/Sing MAP, 5 ug (FA); B6 = A/Sing MAP, 2.5 ug (FA)
B7 = Placebo MAP (FA); B8 = A/Sing MAP, 15 ug (UA); B9 = A/Sing IM, 15 ug, as 2018 Afluria Quad (UA)

Listing 16.2.1.1
Informed Consent

Part B

Randomisation Number	Screening Number	Group	Was Informed Consent Obtained?	Date Consent Obtained	Time Consent Obtained	PICF Version	PICF Date	Reason for Subsequent Informed Consent
B0144	S330	Group B6	Yes	18-JUN-2018	13:40	4.0	24-APR-2018	
B0145	S344	Group B3	Yes	20-JUN-2018	10:12	4.0	24-APR-2018	
B0146	S361	Group B7	Yes	26-JUN-2018	13:13	4.0	24-APR-2018	
B0147	S358	Group B9	Yes	26-JUN-2018	12:20	4.0	24-APR-2018	
B0148	S362	Group B5	Yes	26-JUN-2018	14:07	4.0	24-APR-2018	
B0149	S364	Group B8	Yes	26-JUN-2018	14:28	4.0	24-APR-2018	
B0150	S368	Group B4	Yes	26-JUN-2018	15:44	4.0	24-APR-2018	
B1019	S165	Group B8	Yes	11-MAY-2018	14:53	4.0	24-APR-2018	
B1076	S317	Group B5	Yes	13-JUN-2018	15:25	4.0	24-APR-2018	
B1080	S308	Group B7	Yes	12-JUN-2018	16:23	4.0	24-APR-2018	

Key: PICF = Participant Informed Consent Form

Administration Site Key: FA = Forearm; UA= Upper arm
Treatments Part A: A1 = A/Sing MAP, 15 ug (FA); A2 = Afluria Quad IM (UA), 15 ug; A3 = Placebo MAP (FA); A4 = A/Sing Mono IM, 15 ug (UA)
Treatments Part B: B1 = A/Sing MAP, 15 ug (FA) with biopsy; B2 = Placebo MAP (FA) with biopsy
B3 = A/Sing MAP, 15 ug (FA); B4 = A/Sing MAP, 10 ug (FA); B5 = A/Sing MAP, 5 ug (FA); B6 = A/Sing MAP, 2.5 ug (FA)
B7 = Placebo MAP (FA); B8 = A/Sing MAP, 15 ug (UA); B9 = A/Sing IM, 15 ug, as 2018 Afluria Quad (UA)
