# Supplementary material for: Safety, tolerability, and immunogenicity of influenza vaccination with a high-density microarray patch: Results from a randomized, controlled phase I clinical trial
Source: PLoS Med. 2020 Mar 17;17(3):e1003024. doi: 10.1371/journal.pmed.1003024 (PMC7077342; doi:10.1371/journal.pmed.1003024)
Supplement: S1 Data — Data 1: Informed Consent; Data 2: Analysis Sets; Data 3: Demographics; Data 4: Study Drug Administration; Data 5: Skin Hardness Assessment; Data 6: Immunogenicity (ELISA IgG); Data 7: Immunogenicity (ADCC); Data 8: Immunogenicity (MBCs); Data 9: Immunogenicity (CMI); Data 10: Immunogenicity (Mucosal IgA); Data 11: Immunogenicity Results (HAI, MN); Data 12: TEAE–CRF Data only; Data 13: TEAEs–MedDRA Coding; Data 15: Treatment Site Tolerability Assessment; Data 16: Numeric Pain Intensity; Data 17: SII; Data 18: Application Site Status at End of Study; Data 19: Individual Treatment Site Tolerability Assessment; Data 20: Numeric Pain Intensity by Treatment; Data 21: Individual SII by Treatment. ADCC, antibody-dependent cellular cytotoxicity; CMI, cell-mediated immunity; CRF, case report form; ELISA, enzyme-linked immunosorbent assay; HAI, haemagglutination inhibition; IgA, immunoglobulin A; IgG, immunoglobulin G; MBC, memory B cell; MedDRA, medical dictionary for regulatory activities; MN, microneutralisation; SII, Skin Irritation Index; TEA, treatment emergent adverse event. (ZIP) [file pmed.1003024.s008.zip › S1_Data/clinical supporting/S1_Data10 Immunogenicity (Mucosal IgA).pdf]

Listing 16.2.6.7
Immunogenicity Results (Mucosal IgA)

Part B

Randomisation Number	Group	------IgA ELISA Absorbance Data------ Day 1	Day 4	Day 8	Day 22	-IgA ELISA Normalised Absorbance Data- Day 1	Day 4	Day 8	Day 22
B0011	Group B8	0.19	0.16	0.38	0.24	0.22	0.18	0.42	0.27
B0014	Group B3	0.16	0.14	0.43	0.17	0.18	0.16	0.48	0.19
B0015	Group B9	0.02	0.05	0.08	0.10	0.03	0.06	0.09	0.11
B0017	Group B7	0.38	0.25	0.53	0.40	0.42	0.28	0.59	0.45
B0018	Group B9	0.23	0.23	0.44	0.20	0.25	0.26	0.49	0.22
B0021	Group B7	0.28	0.36	0.32	0.37	0.31	0.40	0.36	0.41
B0023	Group B3	0.09	0.16	0.40	0.27	0.10	0.18	0.45	0.30
B0025	Group B8	0.25	0.26	0.30	0.13	0.27	0.28	0.33	0.14
B0026	Group B9	0.46	0.29	0.27	0.60	0.50	0.32	0.29	0.65
B0027	Group B3	0.37	0.12	0.42	0.41	0.41	0.13	0.45	0.45
B0030	Group B7	0.92	0.49	0.45	0.65	1.04	0.55	0.50	0.74
B0034	Group B8	0.38	0.26	0.39	0.20	0.41	0.28	0.43	0.22
B0035	Group B9	0.57	0.32	0.49	0.52	0.62	0.35	0.54	0.57
B0036	Group B7	0.18	0.14	0.13	0.11	0.20	0.15	0.14	0.12
B0038	Group B3	0.53	0.45	0.51	0.51	0.60	0.51	0.58	0.58
B0039	Group B7	0.41	0.53	0.97	0.55	0.46	0.60	1.09	0.63
B0040	Group B9	0.22	0.22	0.20	0.09	0.24	0.23	0.22	0.09
B0041	Group B3	0.49	0.64	0.85	0.32	0.54	0.70	0.93	0.35

Administration Site Key: FA = Forearm; UA= Upper arm
Treatments Part A: A1 = A/Sing MAP, 15 ug (FA); A2 = Afluria Quad IM (UA), 15 ug; A3 = Placebo MAP (FA); A4 = A/Sing Mono IM, 15 ug (UA)
Treatments Part B: B1 = A/Sing MAP, 15 ug (FA) with biopsy; B2 = Placebo MAP (FA) with biopsy
B3 = A/Sing MAP, 15 ug (FA); B4 = A/Sing MAP, 10 ug (FA); B5 = A/Sing MAP, 5 ug (FA); B6 = A/Sing MAP, 2.5 ug (FA)
B7 = Placebo MAP (FA); B8 = A/Sing MAP, 15 ug (UA); B9 = A/Sing IM, 15 ug, as 2018 Afluria Quad (UA)

Listing 16.2.6.7
Immunogenicity Results (Mucosal IgA)

Part B

Randomisation Number	Group	------IgA ELISA Absorbance Data------ Day 1	Day 4	Day 8	Day 22	-IgA ELISA Normalised Absorbance Data- Day 1	Day 4	Day 8	Day 22
B0043	Group B8	0.22	0.20	0.50	0.28	0.24	0.21	0.55	0.31
B0046	Group B7	0.55	0.34	0.55	0.42	0.61	0.37	0.60	0.45
B0047	Group B9	0.38	0.40	0.18	0.19	0.41	0.43	0.20	0.21
B0050	Group B8	0.19	0.30	0.44	0.44	0.20	0.33	0.48	0.48
B0051	Group B3	0.20	0.29	0.31	0.36	0.22	0.31	0.34	0.39
B0055	Group B8	1.14	0.78	0.94	0.65	1.24	0.85	1.03	0.71
B0056	Group B3	0.14	0.21	0.28	0.24	0.16	0.24	0.33	0.28
B0058	Group B9	0.23	0.07	0.59	0.25	0.26	0.07	0.67	0.28
B0059	Group B7	0.41	0.43	0.32	0.31	0.47	0.49	0.37	0.35
B0060	Group B3	0.34	0.59	1.04	0.66	0.39	0.68	1.18	0.75
B0061	Group B8	0.15	0.16	0.20	0.19	0.17	0.18	0.23	0.22
B0063	Group B7	0.23	0.38	0.16	0.21	0.26	0.44	0.19	0.24
B0064	Group B9	1.43	1.33	1.37	1.17	1.63	1.52	1.57	1.34
B0067	Group B8	0.83	0.29	0.33	0.21	0.90	0.31	0.35	0.22
B0069	Group B7	0.61	0.41	0.44	0.36	0.70	0.46	0.51	0.42
B0070	Group B9	0.45	0.28	0.66	0.40	0.49	0.30	0.70	0.43
B0072	Group B3	0.47	0.55	0.36	0.34	0.51	0.59	0.39	0.37
B0074	Group B9	0.83	0.63	0.77	0.48	0.89	0.68	0.83	0.52

Administration Site Key: FA = Forearm; UA= Upper arm
Treatments Part A: A1 = A/Sing MAP, 15 ug (FA); A2 = Afluria Quad IM (UA), 15 ug; A3 = Placebo MAP (FA); A4 = A/Sing Mono IM, 15 ug (UA)
Treatments Part B: B1 = A/Sing MAP, 15 ug (FA) with biopsy; B2 = Placebo MAP (FA) with biopsy
B3 = A/Sing MAP, 15 ug (FA); B4 = A/Sing MAP, 10 ug (FA); B5 = A/Sing MAP, 5 ug (FA); B6 = A/Sing MAP, 2.5 ug (FA)
B7 = Placebo MAP (FA); B8 = A/Sing MAP, 15 ug (UA); B9 = A/Sing IM, 15 ug, as 2018 Afluria Quad (UA)

Listing 16.2.6.7
Immunogenicity Results (Mucosal IgA)

Part B

Randomisation Number	Group	------IgA ELISA Absorbance Data------ Day 1	Day 4	Day 8	Day 22	-IgA ELISA Normalised Absorbance Data- Day 1	Day 4	Day 8	Day 22
B0075	Group B3	0.20	0.35	0.71	0.34	0.21	0.38	0.76	0.37
B0078	Group B8	0.22	0.43	0.60	0.59	0.24	0.46	0.65	0.64
B0082	Group B3	0.46		0.57	0.35	0.48		0.59	0.36
B0083	Group B8	0.45	0.28	1.07	0.71	0.47	0.29	1.11	0.74
B0086	Group B9	0.45	0.54	0.43	0.29	0.48	0.58	0.46	0.32
B0087	Group B7	0.37	0.54	0.47	0.35	0.40	0.58	0.51	0.37
B0088	Group B9	0.57	0.40	1.20	0.44	0.65	0.46	1.36	0.49
B0091	Group B8	0.30	0.56	1.06	0.61	0.34	0.63	1.20	0.69
B0093	Group B7	0.50	0.64	0.33	0.45	0.57	0.72	0.37	0.51
B0094	Group B3	0.10	0.11	0.14	0.11	0.12	0.13	0.16	0.12
B0095	Group B9	1.26	0.35	0.37	0.32	1.36	0.37	0.40	0.35
B0098	Group B7	0.73	0.41	0.65	0.62	0.83	0.46	0.73	0.71
B0100	Group B8	0.95	0.63	0.76	0.85	1.07	0.71	0.86	0.96
B0101	Group B3	0.66	0.58	0.70	0.67	0.75	0.66	0.79	0.75
B0102	Group B8	0.83	0.97	0.74	0.80	0.91	1.06	0.81	0.88
B0104	Group B3	0.19	0.12	0.46	0.40	0.21	0.13	0.50	0.44
B0105	Group B9	0.60	0.37	0.67	0.52	0.66	0.40	0.73	0.57
B0108	Group B7	0.86	0.57	1.04	0.74	0.94	0.62	1.14	0.80

Administration Site Key: FA = Forearm; UA= Upper arm
Treatments Part A: A1 = A/Sing MAP, 15 ug (FA); A2 = Afluria Quad IM (UA), 15 ug; A3 = Placebo MAP (FA); A4 = A/Sing Mono IM, 15 ug (UA)
Treatments Part B: B1 = A/Sing MAP, 15 ug (FA) with biopsy; B2 = Placebo MAP (FA) with biopsy
B3 = A/Sing MAP, 15 ug (FA); B4 = A/Sing MAP, 10 ug (FA); B5 = A/Sing MAP, 5 ug (FA); B6 = A/Sing MAP, 2.5 ug (FA)
B7 = Placebo MAP (FA); B8 = A/Sing MAP, 15 ug (UA); B9 = A/Sing IM, 15 ug, as 2018 Afluria Quad (UA)

Listing 16.2.6.7
Immunogenicity Results (Mucosal IgA)

Part B

Randomisation Number	Group	------IgA ELISA Absorbance Data------ Day 1	Day 4	Day 8	Day 22	-IgA ELISA Normalised Absorbance Data- Day 1	Day 4	Day 8	Day 22
B0110	Group B3	0.87	0.51	0.97	0.64	0.95	0.56	1.06	0.70
B0111	Group B8	0.21	0.10	0.34	0.29	0.23	0.11	0.37	0.32
B0114	Group B7	0.79	0.61	0.75	0.58	0.86	0.66	0.82	0.64
B0115	Group B9	0.43	0.31	0.63	0.42	0.49	0.36	0.72	0.48
B0116	Group B7	0.64	0.83	0.38	0.30	0.73	0.94	0.43	0.34
B0117	Group B9	0.47	0.31	0.30	0.55	0.54	0.35	0.35	0.62
B0120	Group B3	0.14	0.20	0.34	0.19	0.15	0.23	0.39	0.21
B0121	Group B8	0.76	0.57	0.79	0.64	0.79	0.59	0.82	0.67
B0125	Group B7	0.19	0.11	0.13	0.24	0.21	0.12	0.14	0.27
B0126	Group B3	0.26	0.25	0.79	0.57	0.29	0.29	0.90	0.65
B0127	Group B9	0.21	0.18	0.28	0.40	0.22	0.18	0.29	0.42
B0129	Group B8	0.22	0.18	0.34	0.28	0.23	0.20	0.37	0.30
B0131	Group B8	0.37	0.39	0.67	0.39	0.43	0.44	0.76	0.44
B0134	Group B3	0.73	0.77	0.50	0.76	0.73	0.77	0.51	0.77
B0135	Group B7	0.17	0.19	0.15	0.24	0.18	0.20	0.16	0.24
B0136	Group B9	0.31	0.25	0.37	0.25	0.32	0.25	0.37	0.25
B0137	Group B8	0.39	0.23	0.88	0.34	0.39	0.24	0.89	0.35
B0138	Group B7	0.22	0.18	0.30	0.22	0.22	0.18	0.30	0.22

Administration Site Key: FA = Forearm; UA= Upper arm
Treatments Part A: A1 = A/Sing MAP, 15 ug (FA); A2 = Afluria Quad IM (UA), 15 ug; A3 = Placebo MAP (FA); A4 = A/Sing Mono IM, 15 ug (UA)
Treatments Part B: B1 = A/Sing MAP, 15 ug (FA) with biopsy; B2 = Placebo MAP (FA) with biopsy
B3 = A/Sing MAP, 15 ug (FA); B4 = A/Sing MAP, 10 ug (FA); B5 = A/Sing MAP, 5 ug (FA); B6 = A/Sing MAP, 2.5 ug (FA)
B7 = Placebo MAP (FA); B8 = A/Sing MAP, 15 ug (UA); B9 = A/Sing IM, 15 ug, as 2018 Afluria Quad (UA)

Listing 16.2.6.7
Immunogenicity Results (Mucosal IgA)

Part B

Randomisation Number	Group	------IgA ELISA Absorbance Data------ Day 1	Day 4	Day 8	Day 22	-IgA ELISA Normalised Absorbance Data- Day 1	Day 4	Day 8	Day 22
B0140	Group B9	0.55	0.96	0.65	0.39	0.56	0.97	0.66	0.39
B0141	Group B3	0.45	0.44	0.93	1.11	0.47	0.47	0.98	1.17
B0145	Group B3	0.45	0.34	0.38	0.17	0.48	0.36	0.40	0.18
B0146	Group B7	0.51	0.46	0.68	1.23	0.54	0.48	0.72	1.29
B0147	Group B9	0.23	0.48	0.15	0.18	0.24	0.50	0.15	0.19
B0149	Group B8	0.40	0.24	0.26	0.21	0.42	0.25	0.28	0.22
B1019	Group B8	0.44	1.02	1.03	0.88	0.47	1.10	1.11	0.95
B1080	Group B7	0.59	0.65	0.65	0.72	0.62	0.69	0.68	0.76

Administration Site Key: FA = Forearm; UA= Upper arm
Treatments Part A: A1 = A/Sing MAP, 15 ug (FA); A2 = Afluria Quad IM (UA), 15 ug; A3 = Placebo MAP (FA); A4 = A/Sing Mono IM, 15 ug (UA)
Treatments Part B: B1 = A/Sing MAP, 15 ug (FA) with biopsy; B2 = Placebo MAP (FA) with biopsy
B3 = A/Sing MAP, 15 ug (FA); B4 = A/Sing MAP, 10 ug (FA); B5 = A/Sing MAP, 5 ug (FA); B6 = A/Sing MAP, 2.5 ug (FA)
B7 = Placebo MAP (FA); B8 = A/Sing MAP, 15 ug (UA); B9 = A/Sing IM, 15 ug, as 2018 Afluria Quad (UA)
