# Supplementary material for: Safety, tolerability, and immunogenicity of influenza vaccination with a high-density microarray patch: Results from a randomized, controlled phase I clinical trial
Source: PLoS Med. 2020 Mar 17;17(3):e1003024. doi: 10.1371/journal.pmed.1003024 (PMC7077342; doi:10.1371/journal.pmed.1003024)
Supplement: S1 Data — Data 1: Informed Consent; Data 2: Analysis Sets; Data 3: Demographics; Data 4: Study Drug Administration; Data 5: Skin Hardness Assessment; Data 6: Immunogenicity (ELISA IgG); Data 7: Immunogenicity (ADCC); Data 8: Immunogenicity (MBCs); Data 9: Immunogenicity (CMI); Data 10: Immunogenicity (Mucosal IgA); Data 11: Immunogenicity Results (HAI, MN); Data 12: TEAE–CRF Data only; Data 13: TEAEs–MedDRA Coding; Data 15: Treatment Site Tolerability Assessment; Data 16: Numeric Pain Intensity; Data 17: SII; Data 18: Application Site Status at End of Study; Data 19: Individual Treatment Site Tolerability Assessment; Data 20: Numeric Pain Intensity by Treatment; Data 21: Individual SII by Treatment. ADCC, antibody-dependent cellular cytotoxicity; CMI, cell-mediated immunity; CRF, case report form; ELISA, enzyme-linked immunosorbent assay; HAI, haemagglutination inhibition; IgA, immunoglobulin A; IgG, immunoglobulin G; MBC, memory B cell; MedDRA, medical dictionary for regulatory activities; MN, microneutralisation; SII, Skin Irritation Index; TEA, treatment emergent adverse event. (ZIP) [file pmed.1003024.s008.zip › S1_Data/clinical supporting/S1_Data11 Immunogenicity Results (HAI, MNT).pdf]

Listing 16.2.6.8
Immunogenicity Results (HAI, MNT)

Part A

Randomisation Number	Group Treatment	Visit	Collection Date	Test	Virus	Titre	Comment
A0001	Group A3	Day 1 Predose	09MAR2018	HAI	A/Singapore/GP1908/2015	20	
A0001	Group A3	Day 4	12MAR2018	HAI	A/Singapore/GP1908/2015	20	
A0001	Group A3	Day 8	16MAR2018	HAI	A/Singapore/GP1908/2015	20	
A0001	Group A3	Day 22	29MAR2018	HAI	A/Singapore/GP1908/2015	20	
A0001	Group A3	Day 61	06MAY2018	HAI	A/Singapore/GP1908/2015	20	
A0002	Group A1	Day 1 Predose	09MAR2018	HAI	A/Singapore/GP1908/2015	5	
A0002	Group A1	Day 4	12MAR2018	HAI	A/Singapore/GP1908/2015	5	
A0002	Group A1	Day 8	16MAR2018	HAI	A/Singapore/GP1908/2015	1280	
A0002	Group A1	Day 22	30MAR2018	HAI	A/Singapore/GP1908/2015	640	
A0002	Group A1	Day 61	08MAY2018	HAI	A/Singapore/GP1908/2015	320	
A0003	Group A1	Day 1 Predose	09MAR2018	HAI	A/Singapore/GP1908/2015	5	
A0003	Group A1	Day 4	12MAR2018	HAI	A/Singapore/GP1908/2015	5	
A0003	Group A1	Day 8	16MAR2018	HAI	A/Singapore/GP1908/2015	640	
A0003	Group A1	Day 22	30MAR2018	HAI	A/Singapore/GP1908/2015	640	
A0003	Group A1	Day 61	08MAY2018	HAI	A/Singapore/GP1908/2015	320	
A0004	Group A3	Day 1 Predose	09MAR2018	HAI	A/Singapore/GP1908/2015	10	

Administration Site Key: FA = Forearm; UA= Upper arm
Treatments Part A: A1 = A/Sing MAP, 15 ug (FA); A2 = Afluria Quad IM (UA), 15 ug; A3 = Placebo MAP (FA); A4 = A/Sing Mono IM, 15 ug (UA)
Treatments Part B: B1 = A/Sing MAP, 15 ug (FA) with biopsy; B2 = Placebo MAP (FA) with biopsy
B3 = A/Sing MAP, 15 ug (FA); B4 = A/Sing MAP, 10 ug (FA); B5 = A/Sing MAP, 5 ug (FA); B6 = A/Sing MAP, 2.5 ug (FA)
B7 = Placebo MAP (FA); B8 = A/Sing MAP, 15 ug (UA); B9 = A/Sing IM, 15 ug, as 2018 Afluria Quad (UA)

Listing 16.2.6.8
Immunogenicity Results (HAI, MNT)

Part A

Randomisation Number	Group Treatment	Visit	Collection Date	Test	Virus	Titre	Comment
A0004	Group A3	Day 4	12MAR2018	HAI	A/Singapore/GP1908/2015	10	
A0004	Group A3	Day 8	16MAR2018	HAI	A/Singapore/GP1908/2015	10	
A0004	Group A3	Day 22	30MAR2018	HAI	A/Singapore/GP1908/2015	10	
A0004	Group A3	Day 61	08MAY2018	HAI	A/Singapore/GP1908/2015	10	
A0005	Group A1	Day 1 Predose	09MAR2018	HAI	A/Singapore/GP1908/2015	5	
A0005	Group A1	Day 4	12MAR2018	HAI	A/Singapore/GP1908/2015	5	
A0005	Group A1	Day 8	16MAR2018	HAI	A/Singapore/GP1908/2015	20	
A0005	Group A1	Day 22	30MAR2018	HAI	A/Singapore/GP1908/2015	40	
A0005	Group A1	Day 61	08MAY2018	HAI	A/Singapore/GP1908/2015	40	
A0006	Group A3	Day 1 Predose	09MAR2018	HAI	A/Singapore/GP1908/2015	<5	
A0006	Group A3	Day 4	12MAR2018	HAI	A/Singapore/GP1908/2015	<5	
A0006	Group A3	Day 8	16MAR2018	HAI	A/Singapore/GP1908/2015	<5	
A0006	Group A3	Day 22	30MAR2018	HAI	A/Singapore/GP1908/2015	<5	
A0006	Group A3	Day 61	08MAY2018	HAI	A/Singapore/GP1908/2015	<5	
A0007	Group A1	Day 1 Predose	09MAR2018	HAI	A/Singapore/GP1908/2015	20	
A0007	Group A1	Day 4	12MAR2018	HAI	A/Singapore/GP1908/2015	40	

Administration Site Key: FA = Forearm; UA= Upper arm
Treatments Part A: A1 = A/Sing MAP, 15 ug (FA); A2 = Afluria Quad IM (UA), 15 ug; A3 = Placebo MAP (FA); A4 = A/Sing Mono IM, 15 ug (UA)
Treatments Part B: B1 = A/Sing MAP, 15 ug (FA) with biopsy; B2 = Placebo MAP (FA) with biopsy
B3 = A/Sing MAP, 15 ug (FA); B4 = A/Sing MAP, 10 ug (FA); B5 = A/Sing MAP, 5 ug (FA); B6 = A/Sing MAP, 2.5 ug (FA)
B7 = Placebo MAP (FA); B8 = A/Sing MAP, 15 ug (UA); B9 = A/Sing IM, 15 ug, as 2018 Afluria Quad (UA)

Listing 16.2.6.8
Immunogenicity Results (HAI, MNT)

Part A

Randomisation Number	Group Treatment	Visit	Collection Date	Test	Virus	Titre	Comment
A0007	Group A1	Day 8	16MAR2018	HAI	A/Singapore/GP1908/2015	640	
A0007	Group A1	Day 22	29MAR2018	HAI	A/Singapore/GP1908/2015	1280	
A0007	Group A1	Day 61	08MAY2018	HAI	A/Singapore/GP1908/2015	640	
A0008	Group A3	Day 1 Predose	09MAR2018	HAI	A/Singapore/GP1908/2015	40	
A0008	Group A3	Day 4	12MAR2016	HAI	A/Singapore/GP1908/2015	40	
A0008	Group A3	Day 8	16MAR2018	HAI	A/Singapore/GP1908/2015	40	
A0008	Group A3	Day 22	30MAR2018	HAI	A/Singapore/GP1908/2015	40	
A0008	Group A3	Day 61	08MAY2018	HAI	A/Singapore/GP1908/2015	40	
A0009	Group A1	Day 1 Predose	09MAR2018	HAI	A/Singapore/GP1908/2015	160	
A0009	Group A1	Day 4	12MAR2016	HAI	A/Singapore/GP1908/2015	160	
A0009	Group A1	Day 8	16MAR2018	HAI	A/Singapore/GP1908/2015	640	
A0009	Group A1	Day 22	30MAR2018	HAI	A/Singapore/GP1908/2015	640	
A0009	Group A1	Day 61	01MAY2018	HAI	A/Singapore/GP1908/2015	640	
A0010	Group A3	Day 1 Predose	09MAR2018	HAI	A/Singapore/GP1908/2015	20	
A0010	Group A3	Day 4	12MAR2016	HAI	A/Singapore/GP1908/2015	20	
A0010	Group A3	Day 8	16MAR2018	HAI	A/Singapore/GP1908/2015	20	

Administration Site Key: FA = Forearm; UA= Upper arm
Treatments Part A: A1 = A/Sing MAP, 15 ug (FA); A2 = Afluria Quad IM (UA), 15 ug; A3 = Placebo MAP (FA); A4 = A/Sing Mono IM, 15 ug (UA)
Treatments Part B: B1 = A/Sing MAP, 15 ug (FA) with biopsy; B2 = Placebo MAP (FA) with biopsy
B3 = A/Sing MAP, 15 ug (FA); B4 = A/Sing MAP, 10 ug (FA); B5 = A/Sing MAP, 5 ug (FA); B6 = A/Sing MAP, 2.5 ug (FA)
B7 = Placebo MAP (FA); B8 = A/Sing MAP, 15 ug (UA); B9 = A/Sing IM, 15 ug, as 2018 Afluria Quad (UA)

Listing 16.2.6.8
Immunogenicity Results (HAI, MNT)

Part A

Randomisation Number	Group Treatment	Visit	Collection Date	Test	Virus	Titre	Comment
A0010	Group A3	Day 22	30MAR2018	HAI	A/Singapore/GP1908/2015	20	
A0010	Group A3	Day 61	08MAY2018	HAI	A/Singapore/GP1908/2015	20	
A0011	Group A3	Day 1 Predose	09MAR2018	HAI	A/Singapore/GP1908/2015	20	
A0011	Group A3	Day 4	12MAR2016	HAI	A/Singapore/GP1908/2015	20	
A0011	Group A3	Day 8	16MAR2018	HAI	A/Singapore/GP1908/2015	20	
A0011	Group A3	Day 22	30MAR2018	HAI	A/Singapore/GP1908/2015	20	
A0011	Group A3	Day 61	09MAY2018	HAI	A/Singapore/GP1908/2015	20	
A0012	Group A1	Day 1 Predose	09MAR2018	HAI	A/Singapore/GP1908/2015	10	
A0012	Group A1	Day 4	12MAR2016	HAI	A/Singapore/GP1908/2015	10	
A0012	Group A1	Day 8	16MAR2018	HAI	A/Singapore/GP1908/2015	640	
A0012	Group A1	Day 22	20MAR2018	HAI	A/Singapore/GP1908/2015	640	
A0012	Group A1	Day 61	08MAY2018	HAI	A/Singapore/GP1908/2015	320	
A0013	Group A1	Day 1 Predose	09MAR2018	HAI	A/Singapore/GP1908/2015	20	
A0013	Group A1	Day 4	12MAR2016	HAI	A/Singapore/GP1908/2015	20	
A0013	Group A1	Day 8	16MAR2018	HAI	A/Singapore/GP1908/2015	1280	
A0013	Group A1	Day 22	30MAR2018	HAI	A/Singapore/GP1908/2015	1280	

Administration Site Key: FA = Forearm; UA= Upper arm
Treatments Part A: A1 = A/Sing MAP, 15 ug (FA); A2 = Afluria Quad IM (UA), 15 ug; A3 = Placebo MAP (FA); A4 = A/Sing Mono IM, 15 ug (UA)
Treatments Part B: B1 = A/Sing MAP, 15 ug (FA) with biopsy; B2 = Placebo MAP (FA) with biopsy
B3 = A/Sing MAP, 15 ug (FA); B4 = A/Sing MAP, 10 ug (FA); B5 = A/Sing MAP, 5 ug (FA); B6 = A/Sing MAP, 2.5 ug (FA)
B7 = Placebo MAP (FA); B8 = A/Sing MAP, 15 ug (UA); B9 = A/Sing IM, 15 ug, as 2018 Afluria Quad (UA)

Listing 16.2.6.8
Immunogenicity Results (HAI, MNT)

Part A

Randomisation Number	Group Treatment	Visit	Collection Date	Test	Virus	Titre	Comment
A0013	Group A1	Day 61	10MAY2018	HAI	A/Singapore/GP1908/2015	640	
A0014	Group A3	Day 1 Predose	09MAR2018	HAI	A/Singapore/GP1908/2015	20	
A0014	Group A3	Day 4	12MAR2016	HAI	A/Singapore/GP1908/2015	20	
A0014	Group A3	Day 8	16MAR2018	HAI	A/Singapore/GP1908/2015	20	
A0014	Group A3	Day 22	30MAR2018	HAI	A/Singapore/GP1908/2015	20	
A0014	Group A3	Day 61	08MAY2018	HAI	A/Singapore/GP1908/2015	20	
A0015	Group A1	Day 1 Predose	09MAR2018	HAI	A/Singapore/GP1908/2015	80	
A0015	Group A1	Day 4	12MAR2016	HAI	A/Singapore/GP1908/2015	80	
A0015	Group A1	Day 8	16MAR2018	HAI	A/Singapore/GP1908/2015	2560	
A0015	Group A1	Day 22	30MAR2018	HAI	A/Singapore/GP1908/2015	2560	
A0015	Group A1	Day 61	08MAY2018	HAI	A/Singapore/GP1908/2015	2560	
A0016	Group A3	Day 1 Predose	10MAR2018	HAI	A/Singapore/GP1908/2015	5	
A0016	Group A3	Day 4	13MAR2018	HAI	A/Singapore/GP1908/2015	5	
A0016	Group A3	Day 8	17MAR2018	HAI	A/Singapore/GP1908/2015	5	
A0016	Group A3	Day 22	31MAR2018	HAI	A/Singapore/GP1908/2015	5	
A0016	Group A3	Day 61	10MAY2018	HAI	A/Singapore/GP1908/2015	5	

Administration Site Key: FA = Forearm; UA= Upper arm
Treatments Part A: A1 = A/Sing MAP, 15 ug (FA); A2 = Afluria Quad IM (UA), 15 ug; A3 = Placebo MAP (FA); A4 = A/Sing Mono IM, 15 ug (UA)
Treatments Part B: B1 = A/Sing MAP, 15 ug (FA) with biopsy; B2 = Placebo MAP (FA) with biopsy
B3 = A/Sing MAP, 15 ug (FA); B4 = A/Sing MAP, 10 ug (FA); B5 = A/Sing MAP, 5 ug (FA); B6 = A/Sing MAP, 2.5 ug (FA)
B7 = Placebo MAP (FA); B8 = A/Sing MAP, 15 ug (UA); B9 = A/Sing IM, 15 ug, as 2018 Afluria Quad (UA)

Listing 16.2.6.8
Immunogenicity Results (HAI, MNT)

Part A

Randomisation Number	Group Treatment	Visit	Collection Date	Test	Virus	Titre	Comment
A0017	Group A3	Day 1 Predose	10MAR2018	HAI	A/Singapore/GP1908/2015	5	
A0017	Group A3	Day 4	13MAR2018	HAI	A/Singapore/GP1908/2015	5	
A0017	Group A3	Day 8	17MAR2018	HAI	A/Singapore/GP1908/2015	5	
A0017	Group A3	Day 22	31MAR2018	HAI	A/Singapore/GP1908/2015	5	
A0017	Group A3	Day 61	09MAY2018	HAI	A/Singapore/GP1908/2015	5	
A0018	Group A1	Day 1 Predose	10MAR2018	HAI	A/Singapore/GP1908/2015	40	
A0018	Group A1	Day 4	13MAR2018	HAI	A/Singapore/GP1908/2015	40	
A0018	Group A1	Day 8	17MAR2018	HAI	A/Singapore/GP1908/2015	160	
A0018	Group A1	Day 22	31MAR2018	HAI	A/Singapore/GP1908/2015	640	
A0018	Group A1	Day 61	09MAY2018	HAI	A/Singapore/GP1908/2015	640	
A0019	Group A3	Day 1 Predose	10MAR2018	HAI	A/Singapore/GP1908/2015	40	
A0019	Group A3	Day 4	13MAR2018	HAI	A/Singapore/GP1908/2015	40	
A0019	Group A3	Day 8	17MAR2018	HAI	A/Singapore/GP1908/2015	40	
A0019	Group A3	Day 22	31MAR2018	HAI	A/Singapore/GP1908/2015	40	
A0019	Group A3	Day 61	08MAY2018	HAI	A/Singapore/GP1908/2015	40	

Administration Site Key: FA = Forearm; UA= Upper arm
Treatments Part A: A1 = A/Sing MAP, 15 ug (FA); A2 = Afluria Quad IM (UA), 15 ug; A3 = Placebo MAP (FA); A4 = A/Sing Mono IM, 15 ug (UA)
Treatments Part B: B1 = A/Sing MAP, 15 ug (FA) with biopsy; B2 = Placebo MAP (FA) with biopsy
B3 = A/Sing MAP, 15 ug (FA); B4 = A/Sing MAP, 10 ug (FA); B5 = A/Sing MAP, 5 ug (FA); B6 = A/Sing MAP, 2.5 ug (FA)
B7 = Placebo MAP (FA); B8 = A/Sing MAP, 15 ug (UA); B9 = A/Sing IM, 15 ug, as 2018 Afluria Quad (UA)

Listing 16.2.6.8
Immunogenicity Results (HAI, MNT)

Part A

Randomisation Number	Group Treatment	Visit	Collection Date	Test	Virus	Titre	Comment
A0020	Group A1	Day 1 Predose	10MAR2018	HAI	A/Singapore/GP1908/2015	80	
A0020	Group A1	Day 4	13MAR2018	HAI	A/Singapore/GP1908/2015	80	
A0020	Group A1	Day 8	17MAR2018	HAI	A/Singapore/GP1908/2015	160	
A0020	Group A1	Day 22	31MAR2018	HAI	A/Singapore/GP1908/2015	160	
A0020	Group A1	Day 61	09MAY2018	HAI	A/Singapore/GP1908/2015	160	
A0021	Group A1	Day 1 Predose	10MAR2018	HAI	A/Singapore/GP1908/2015	5	
A0021	Group A1	Day 4	13MAR2018	HAI	A/Singapore/GP1908/2015	5	
A0021	Group A1	Day 8	17MAR2018	HAI	A/Singapore/GP1908/2015	5	
A0021	Group A1	Day 22	31MAR2018	HAI	A/Singapore/GP1908/2015	10	
A0021	Group A1	Day 61	10MAY2018	HAI	A/Singapore/GP1908/2015	5	
A0022	Group A3	Day 1 Predose	10MAR2018	HAI	A/Singapore/GP1908/2015	40	
A0022	Group A3	Day 4	13MAR2018	HAI	A/Singapore/GP1908/2015	40	
A0022	Group A3	Day8	17MAR2018	HAI	A/Singapore/GP1908/2015	40	
A0022	Group A3	Day 22	31MAR2018	HAI	A/Singapore/GP1908/2015	40	
A0022	Group A3	Day 61	09MAY2018	HAI	A/Singapore/GP1908/2015	40	
A0023	Group A3	Day 1 Predose	10MAR2018	HAI	A/Singapore/GP1908/2015	80	

Administration Site Key: FA = Forearm; UA= Upper arm
Treatments Part A: A1 = A/Sing MAP, 15 ug (FA); A2 = Afluria Quad IM (UA), 15 ug; A3 = Placebo MAP (FA); A4 = A/Sing Mono IM, 15 ug (UA)
Treatments Part B: B1 = A/Sing MAP, 15 ug (FA) with biopsy; B2 = Placebo MAP (FA) with biopsy
B3 = A/Sing MAP, 15 ug (FA); B4 = A/Sing MAP, 10 ug (FA); B5 = A/Sing MAP, 5 ug (FA); B6 = A/Sing MAP, 2.5 ug (FA)
B7 = Placebo MAP (FA); B8 = A/Sing MAP, 15 ug (UA); B9 = A/Sing IM, 15 ug, as 2018 Afluria Quad (UA)

Listing 16.2.6.8
Immunogenicity Results (HAI, MNT)

Part A

Randomisation Number	Group Treatment	Visit	Collection Date	Test	Virus	Titre	Comment
A0023	Group A3	Day 4	13MAR2018	HAI	A/Singapore/GP1908/2015	160	
A0023	Group A3	Day 8	17MAR2018	HAI	A/Singapore/GP1908/2015	160	
A0023	Group A3	Day 22	31MAR2018	HAI	A/Singapore/GP1908/2015	160	
A0023	Group A3	Day 61	10MAY2018	HAI	A/Singapore/GP1908/2015	160	
A0024	Group A1	Day 1 Predose	10MAR2018	HAI	A/Singapore/GP1908/2015	5	
A0024	Group A1	Day 4	13MAR2018	HAI	A/Singapore/GP1908/2015	5	
A0024	Group A1	Day 8	17MAR2018	HAI	A/Singapore/GP1908/2015	1280	
A0024	Group A1	Day 22	31MAR2018	HAI	A/Singapore/GP1908/2015	1280	
A0024	Group A1	Day 61	09MAY2018	HAI	A/Singapore/GP1908/2015	640	
A0025	Group A3	Day 1 Predose	10MAR2018	HAI	A/Singapore/GP1908/2015	40	
A0025	Group A3	Day 4	13MAR2018	HAI	A/Singapore/GP1908/2015	40	
A0025	Group A3	Day 8	17MAR2018	HAI	A/Singapore/GP1908/2015	40	
A0025	Group A3	Day 22	31MAR2018	HAI	A/Singapore/GP1908/2015	40	
A0025	Group A3	Day 61	09MAY2018	HAI	A/Singapore/GP1908/2015	40	
A0027	Group A3	Day 1 Predose	10MAR2018	HAI	A/Singapore/GP1908/2015	5	
A0027	Group A3	Day 4	13MAR2018	HAI	A/Singapore/GP1908/2015	5	

Administration Site Key: FA = Forearm; UA= Upper arm
Treatments Part A: A1 = A/Sing MAP, 15 ug (FA); A2 = Afluria Quad IM (UA), 15 ug; A3 = Placebo MAP (FA); A4 = A/Sing Mono IM, 15 ug (UA)
Treatments Part B: B1 = A/Sing MAP, 15 ug (FA) with biopsy; B2 = Placebo MAP (FA) with biopsy
B3 = A/Sing MAP, 15 ug (FA); B4 = A/Sing MAP, 10 ug (FA); B5 = A/Sing MAP, 5 ug (FA); B6 = A/Sing MAP, 2.5 ug (FA)
B7 = Placebo MAP (FA); B8 = A/Sing MAP, 15 ug (UA); B9 = A/Sing IM, 15 ug, as 2018 Afluria Quad (UA)

Listing 16.2.6.8
Immunogenicity Results (HAI, MNT)

Part A

Randomisation Number	Group Treatment	Visit	Collection Date	Test	Virus	Titre	Comment
A0027	Group A3	Day 8	17MAR2018	HAI	A/Singapore/GP1908/2015	5	
A0027	Group A3	Day 22	01APR2018	HAI	A/Singapore/GP1908/2015	10	
A0027	Group A3	Day 61	09MAY2018	HAI	A/Singapore/GP1908/2015	5	
A0028	Group A1	Day 1 Predose	10MAR2018	HAI	A/Singapore/GP1908/2015	20	
A0028	Group A1	Day 4	13MAR2018	HAI	A/Singapore/GP1908/2015	20	
A0028	Group A1	Day 8	17MAR2018	HAI	A/Singapore/GP1908/2015	320	
A0028	Group A1	Day 22	31MAR2018	HAI	A/Singapore/GP1908/2015	320	
A0028	Group A1	Day 61	09MAY2018	HAI	A/Singapore/GP1908/2015	160	
A0029	Group A1	Day 1 Predose	10MAR2018	HAI	A/Singapore/GP1908/2015	5	
A0029	Group A1	Day 4	13MAR2018	HAI	A/Singapore/GP1908/2015	5	
A0029	Group A1	Day 8	17MAR2018	HAI	A/Singapore/GP1908/2015	320	
A0029	Group A1	Day 22	31MAR2018	HAI	A/Singapore/GP1908/2015	640	
A0029	Group A1	Day 61	09MAY2018	HAI	A/Singapore/GP1908/2015	640	
A0030	Group A3	Day 1 Predose	16MAR2018	HAI	A/Singapore/GP1908/2015	160	
A0030	Group A3	Day 4	20MAR2018	HAI	A/Singapore/GP1908/2015	160	
A0030	Group A3	Day 8	27MAR2018	HAI	A/Singapore/GP1908/2015	160	

Administration Site Key: FA = Forearm; UA= Upper arm
Treatments Part A: A1 = A/Sing MAP, 15 ug (FA); A2 = Afluria Quad IM (UA), 15 ug; A3 = Placebo MAP (FA); A4 = A/Sing Mono IM, 15 ug (UA)
Treatments Part B: B1 = A/Sing MAP, 15 ug (FA) with biopsy; B2 = Placebo MAP (FA) with biopsy
B3 = A/Sing MAP, 15 ug (FA); B4 = A/Sing MAP, 10 ug (FA); B5 = A/Sing MAP, 5 ug (FA); B6 = A/Sing MAP, 2.5 ug (FA)
B7 = Placebo MAP (FA); B8 = A/Sing MAP, 15 ug (UA); B9 = A/Sing IM, 15 ug, as 2018 Afluria Quad (UA)

Listing 16.2.6.8
Immunogenicity Results (HAI, MNT)

Part A

Randomisation Number	Group Treatment	Visit	Collection Date	Test	Virus	Titre	Comment
A0030	Group A3	Day 22	06APR2018	HAI	A/Singapore/GP1908/2015	160	
A0030	Group A3	Day 61	15MAY2018	HAI	A/Singapore/GP1908/2015	160	
A0031	Group A2	Day 1 Predose	16MAR2018	HAI	A/Singapore/GP1908/2015	20	
A0031	Group A2	Day 4	19MAR2018	HAI	A/Singapore/GP1908/2015	20	
A0031	Group A2	Day 8		HAI	A/Singapore/GP1908/2015	ND	
A0031	Group A2	Day 22		HAI	A/Singapore/GP1908/2015	ND	
A0031	Group A2	Day 61		HAI	A/Singapore/GP1908/2015	ND	
A0032	Group A4	Day 1 Predose	16MAR2018	HAI	A/Singapore/GP1908/2015	160	
A0032	Group A4	Day 4	19MAR2018	HAI	A/Singapore/GP1908/2015	160	
A0032	Group A4	Day 8	23MAR2018	HAI	A/Singapore/GP1908/2015	160	
A0032	Group A4	Day 22	06APR2018	HAI	A/Singapore/GP1908/2015	320	
A0032	Group A4	Day 61	15MAY2018	HAI	A/Singapore/GP1908/2015	160	
A0033	Group A2	Day 1 Predose	16MAR2018	HAI	A/Singapore/GP1908/2015	40	
A0033	Group A2	Day 4	19MAR2018	HAI	A/Singapore/GP1908/2015	40	
A0033	Group A2	Day 8	23MAR2018	HAI	A/Singapore/GP1908/2015	320	
A0033	Group A2	Day 22	06APR2018	HAI	A/Singapore/GP1908/2015	640	

Administration Site Key: FA = Forearm; UA= Upper arm
Treatments Part A: A1 = A/Sing MAP, 15 ug (FA); A2 = Afluria Quad IM (UA), 15 ug; A3 = Placebo MAP (FA); A4 = A/Sing Mono IM, 15 ug (UA)
Treatments Part B: B1 = A/Sing MAP, 15 ug (FA) with biopsy; B2 = Placebo MAP (FA) with biopsy
B3 = A/Sing MAP, 15 ug (FA); B4 = A/Sing MAP, 10 ug (FA); B5 = A/Sing MAP, 5 ug (FA); B6 = A/Sing MAP, 2.5 ug (FA)
B7 = Placebo MAP (FA); B8 = A/Sing MAP, 15 ug (UA); B9 = A/Sing IM, 15 ug, as 2018 Afluria Quad (UA)

Listing 16.2.6.8
Immunogenicity Results (HAI, MNT)

Part A

Randomisation Number	Group Treatment	Visit	Collection Date	Test	Virus	Titre	Comment
A0033	Group A2	Day 61	15MAY2018	HAI	A/Singapore/GP1908/2015	640	
A0034	Group A4	Day 1 Predose	16MAR2018	HAI	A/Singapore/GP1908/2015	20	
A0034	Group A4	Day 4	19MAR2018	HAI	A/Singapore/GP1908/2015	20	
A0034	Group A4	Day 8	23MAR2018	HAI	A/Singapore/GP1908/2015	1280	
A0034	Group A4	Day 22	06APR2018	HAI	A/Singapore/GP1908/2015	1280	
A0034	Group A4	Day 61	17MAY2018	HAI	A/Singapore/GP1908/2015	1280	
A0035	Group A4	Day 1 Predose	16MAR2018	HAI	A/Singapore/GP1908/2015	10	
A0035	Group A4	Day 4	19MAR2018	HAI	A/Singapore/GP1908/2015	10	
A0035	Group A4	Day 8	23MAR2018	HAI	A/Singapore/GP1908/2015	40	
A0035	Group A4	Day 22	08APR2018	HAI	A/Singapore/GP1908/2015	160	
A0035	Group A4	Day 61	15MAY2018	HAI	A/Singapore/GP1908/2015	160	
A0036	Group A2	Day 1 Predose	17MAR2018	HAI	A/Singapore/GP1908/2015	160	
A0036	Group A2	Day 4	21MAR2018	HAI	A/Singapore/GP1908/2015	160	
A0036	Group A2	Day 8	25MAR2018	HAI	A/Singapore/GP1908/2015	320	
A0036	Group A2	Day 22	07APR2018	HAI	A/Singapore/GP1908/2015	320	
A0036	Group A2	Day 61	17MAY2018	HAI	A/Singapore/GP1908/2015	320	

Administration Site Key: FA = Forearm; UA= Upper arm
Treatments Part A: A1 = A/Sing MAP, 15 ug (FA); A2 = Afluria Quad IM (UA), 15 ug; A3 = Placebo MAP (FA); A4 = A/Sing Mono IM, 15 ug (UA)
Treatments Part B: B1 = A/Sing MAP, 15 ug (FA) with biopsy; B2 = Placebo MAP (FA) with biopsy
B3 = A/Sing MAP, 15 ug (FA); B4 = A/Sing MAP, 10 ug (FA); B5 = A/Sing MAP, 5 ug (FA); B6 = A/Sing MAP, 2.5 ug (FA)
B7 = Placebo MAP (FA); B8 = A/Sing MAP, 15 ug (UA); B9 = A/Sing IM, 15 ug, as 2018 Afluria Quad (UA)

Listing 16.2.6.8
Immunogenicity Results (HAI, MNT)

Part A

Randomisation Number	Group Treatment	Visit	Collection Date	Test	Virus	Titre	Comment
A0037	Group A4	Day 1 Predose	17MAR2018	HAI	A/Singapore/GP1908/2015	40	
A0037	Group A4	Day 4	20MAR2018	HAI	A/Singapore/GP1908/2015	40	
A0037	Group A4	Day 8	24MAR2018	HAI	A/Singapore/GP1908/2015	80	
A0037	Group A4	Day 22	07APR2018	HAI	A/Singapore/GP1908/2015	80	
A0037	Group A4	Day 61	17MAY2018	HAI	A/Singapore/GP1908/2015	80	
A0038	Group A2	Day 1 Predose	17MAR2018	HAI	A/Singapore/GP1908/2015	320	
A0038	Group A2	Day 4	20MAR2018	HAI	A/Singapore/GP1908/2015	320	
A0038	Group A2	Day 8	24MAR2018	HAI	A/Singapore/GP1908/2015	320	
A0038	Group A2	Day 22	07APR2018	HAI	A/Singapore/GP1908/2015	320	
A0038	Group A2	Day 61	17MAY2018	HAI	A/Singapore/GP1908/2015	320	
A0039	Group A2	Day 1 Predose	17MAR2018	HAI	A/Singapore/GP1908/2015	40	
A0039	Group A2	Day 4	20MAR2018	HAI	A/Singapore/GP1908/2015	40	
A0039	Group A2	Day 8	24MAR2018	HAI	A/Singapore/GP1908/2015	320	
A0039	Group A2	Day 22	07APR2018	HAI	A/Singapore/GP1908/2015	640	
A0039	Group A2	Day 61	16MAY2018	HAI	A/Singapore/GP1908/2015	640	

Administration Site Key: FA = Forearm; UA= Upper arm
Treatments Part A: A1 = A/Sing MAP, 15 ug (FA); A2 = Afluria Quad IM (UA), 15 ug; A3 = Placebo MAP (FA); A4 = A/Sing Mono IM, 15 ug (UA)
Treatments Part B: B1 = A/Sing MAP, 15 ug (FA) with biopsy; B2 = Placebo MAP (FA) with biopsy
B3 = A/Sing MAP, 15 ug (FA); B4 = A/Sing MAP, 10 ug (FA); B5 = A/Sing MAP, 5 ug (FA); B6 = A/Sing MAP, 2.5 ug (FA)
B7 = Placebo MAP (FA); B8 = A/Sing MAP, 15 ug (UA); B9 = A/Sing IM, 15 ug, as 2018 Afluria Quad (UA)

Listing 16.2.6.8
Immunogenicity Results (HAI, MNT)

Part A

Randomisation Number	Group Treatment	Visit	Collection Date	Test	Virus	Titre	Comment
A0040	Group A4	Day 1 Predose	17MAR2018	HAI	A/Singapore/GP1908/2015	160	
A0040	Group A4	Day 4	20MAR2018	HAI	A/Singapore/GP1908/2015	160	
A0040	Group A4	Day 8	24MAR2018	HAI	A/Singapore/GP1908/2015	320	
A0040	Group A4	Day 22	07APR2018	HAI	A/Singapore/GP1908/2015	320	
A0040	Group A4	Day 61	17MAY2018	HAI	A/Singapore/GP1908/2015	160	
A0041	Group A4	Day 1 Predose	17MAR2018	HAI	A/Singapore/GP1908/2015	80	
A0041	Group A4	Day 4	20MAR2018	HAI	A/Singapore/GP1908/2015	80	
A0041	Group A4	Day 8	26MAR2018	HAI	A/Singapore/GP1908/2015	160	
A0041	Group A4	Day 22	10APR2018	HAI	A/Singapore/GP1908/2015	160	
A0041	Group A4	Day 61	17MAY2018	HAI	A/Singapore/GP1908/2015	160	
A0042	Group A2	Day 1 Predose	17MAR2018	HAI	A/Singapore/GP1908/2015	20	
A0042	Group A2	Day 4	20MAR2018	HAI	A/Singapore/GP1908/2015	20	
A0042	Group A2	Day 8	24MAR2018	HAI	A/Singapore/GP1908/2015	20	
A0042	Group A2	Day 22	07APR2018	HAI	A/Singapore/GP1908/2015	40	
A0042	Group A2	Day 61	16MAY2018	HAI	A/Singapore/GP1908/2015	40	
A0043	Group A2	Day 1 Predose	17MAR2018	HAI	A/Singapore/GP1908/2015	5	

Administration Site Key: FA = Forearm; UA= Upper arm
Treatments Part A: A1 = A/Sing MAP, 15 ug (FA); A2 = Afluria Quad IM (UA), 15 ug; A3 = Placebo MAP (FA); A4 = A/Sing Mono IM, 15 ug (UA)
Treatments Part B: B1 = A/Sing MAP, 15 ug (FA) with biopsy; B2 = Placebo MAP (FA) with biopsy
B3 = A/Sing MAP, 15 ug (FA); B4 = A/Sing MAP, 10 ug (FA); B5 = A/Sing MAP, 5 ug (FA); B6 = A/Sing MAP, 2.5 ug (FA)
B7 = Placebo MAP (FA); B8 = A/Sing MAP, 15 ug (UA); B9 = A/Sing IM, 15 ug, as 2018 Afluria Quad (UA)

Listing 16.2.6.8
Immunogenicity Results (HAI, MNT)

Part A

Randomisation Number	Group Treatment	Visit	Collection Date	Test	Virus	Titre	Comment
A0043	Group A2	Day 4	20MAR2018	HAI	A/Singapore/GP1908/2015	5	
A0043	Group A2	Day 8	24MAR2018	HAI	A/Singapore/GP1908/2015	40	
A0043	Group A2	Day 22	06APR2018	HAI	A/Singapore/GP1908/2015	160	
A0043	Group A2	Day 61	16MAY2018	HAI	A/Singapore/GP1908/2015	320	
A0044	Group A4	Day 1 Predose	17MAR2018	HAI	A/Singapore/GP1908/2015	40	
A0044	Group A4	Day 4	20MAR2018	HAI	A/Singapore/GP1908/2015	40	
A0044	Group A4	Day 8	24MAR2018	HAI	A/Singapore/GP1908/2015	80	
A0044	Group A4	Day 22	07APR2018	HAI	A/Singapore/GP1908/2015	320	
A0044	Group A4	Day 61	16MAY2018	HAI	A/Singapore/GP1908/2015	320	
A0045	Group A2	Day 1 Predose	17MAR2018	HAI	A/Singapore/GP1908/2015	20	
A0045	Group A2	Day 4	20MAR2018	HAI	A/Singapore/GP1908/2015	20	
A0045	Group A2	Day 8	25MAR2018	HAI	A/Singapore/GP1908/2015	640	
A0045	Group A2	Day 22	08APR2018	HAI	A/Singapore/GP1908/2015	640	
A0045	Group A2	Day 61	15MAY2018	HAI	A/Singapore/GP1908/2015	320	
A0046	Group A4	Day 1 Predose	17MAR2018	HAI	A/Singapore/GP1908/2015	<5	
A0046	Group A4	Day 4	20MAR2018	HAI	A/Singapore/GP1908/2015	<5	

Administration Site Key: FA = Forearm; UA= Upper arm
Treatments Part A: A1 = A/Sing MAP, 15 ug (FA); A2 = Afluria Quad IM (UA), 15 ug; A3 = Placebo MAP (FA); A4 = A/Sing Mono IM, 15 ug (UA)
Treatments Part B: B1 = A/Sing MAP, 15 ug (FA) with biopsy; B2 = Placebo MAP (FA) with biopsy
B3 = A/Sing MAP, 15 ug (FA); B4 = A/Sing MAP, 10 ug (FA); B5 = A/Sing MAP, 5 ug (FA); B6 = A/Sing MAP, 2.5 ug (FA)
B7 = Placebo MAP (FA); B8 = A/Sing MAP, 15 ug (UA); B9 = A/Sing IM, 15 ug, as 2018 Afluria Quad (UA)

Listing 16.2.6.8
Immunogenicity Results (HAI, MNT)

Part A

Randomisation Number	Group Treatment	Visit	Collection Date	Test	Virus	Titre	Comment
A0046	Group A4	Day 8	25MAR2018	HAI	A/Singapore/GP1908/2015	1280	
A0046	Group A4	Day 22	07APR2018	HAI	A/Singapore/GP1908/2015	1280	
A0046	Group A4	Day 61	17MAY2018	HAI	A/Singapore/GP1908/2015	1280	
A0047	Group A2	Day 1 Predose	17MAR2018	HAI	A/Singapore/GP1908/2015	10	
A0047	Group A2	Day 4	21MAR2018	HAI	A/Singapore/GP1908/2015	10	
A0047	Group A2	Day 8	25MAR2018	HAI	A/Singapore/GP1908/2015	320	
A0047	Group A2	Day 22	07APR2018	HAI	A/Singapore/GP1908/2015	320	
A0047	Group A2	Day 61	22MAY2018	HAI	A/Singapore/GP1908/2015	320	
A0048	Group A4	Day 1 Predose	17MAR2018	HAI	A/Singapore/GP1908/2015	80	
A0048	Group A4	Day 4	20MAR2018	HAI	A/Singapore/GP1908/2015	80	
A0048	Group A4	Day 8	24MAR2018	HAI	A/Singapore/GP1908/2015	80	
A0048	Group A4	Day 22	06APR2018	HAI	A/Singapore/GP1908/2015	160	
A0048	Group A4	Day 61	16MAY2018	HAI	A/Singapore/GP1908/2015	160	
A0049	Group A2	Day 1 Predose	17MAR2018	HAI	A/Singapore/GP1908/2015	40	
A0049	Group A2	Day 4	20MAR2018	HAI	A/Singapore/GP1908/2015	40	
A0049	Group A2	Day 8	24MAR2018	HAI	A/Singapore/GP1908/2015	80	

Administration Site Key: FA = Forearm; UA= Upper arm
Treatments Part A: A1 = A/Sing MAP, 15 ug (FA); A2 = Afluria Quad IM (UA), 15 ug; A3 = Placebo MAP (FA); A4 = A/Sing Mono IM, 15 ug (UA)
Treatments Part B: B1 = A/Sing MAP, 15 ug (FA) with biopsy; B2 = Placebo MAP (FA) with biopsy
B3 = A/Sing MAP, 15 ug (FA); B4 = A/Sing MAP, 10 ug (FA); B5 = A/Sing MAP, 5 ug (FA); B6 = A/Sing MAP, 2.5 ug (FA)
B7 = Placebo MAP (FA); B8 = A/Sing MAP, 15 ug (UA); B9 = A/Sing IM, 15 ug, as 2018 Afluria Quad (UA)

Listing 16.2.6.8
Immunogenicity Results (HAI, MNT)

Part A

Randomisation Number	Group Treatment	Visit	Collection Date	Test	Virus	Titre	Comment
A0049	Group A2	Day 22	06APR2018	HAI	A/Singapore/GP1908/2015	160	
A0049	Group A2	Day 61	16MAY2018	HAI	A/Singapore/GP1908/2015	160	
A0050	Group A4	Day 1 Predose	17MAR2018	HAI	A/Singapore/GP1908/2015	10	
A0050	Group A4	Day 4	20MAR2018	HAI	A/Singapore/GP1908/2015	10	
A0050	Group A4	Day 8	27MAR2018	HAI	A/Singapore/GP1908/2015	640	
A0050	Group A4	Day 22	07APR2018	HAI	A/Singapore/GP1908/2015	640	
A0050	Group A4	Day 61	16MAY2018	HAI	A/Singapore/GP1908/2015	320	
A0051	Group A2	Day 1 Predose	17MAR2018	HAI	A/Singapore/GP1908/2015	40	
A0051	Group A2	Day 4	20MAR2018	HAI	A/Singapore/GP1908/2015	40	
A0051	Group A2	Day 8	24MAR2018	HAI	A/Singapore/GP1908/2015	80	
A0051	Group A2	Day 22	07APR2018	HAI	A/Singapore/GP1908/2015	320	
A0051	Group A2	Day 61	16MAY2018	HAI	A/Singapore/GP1908/2015	320	
A0052	Group A4	Day 1 Predose	17MAR2018	HAI	A/Singapore/GP1908/2015	40	
A0052	Group A4	Day 4	20MAR2018	HAI	A/Singapore/GP1908/2015	40	
A0052	Group A4	Day 8	24MAR2018	HAI	A/Singapore/GP1908/2015	320	
A0052	Group A4	Day 22	07APR2018	HAI	A/Singapore/GP1908/2015	640	

Administration Site Key: FA = Forearm; UA= Upper arm
Treatments Part A: A1 = A/Sing MAP, 15 ug (FA); A2 = Afluria Quad IM (UA), 15 ug; A3 = Placebo MAP (FA); A4 = A/Sing Mono IM, 15 ug (UA)
Treatments Part B: B1 = A/Sing MAP, 15 ug (FA) with biopsy; B2 = Placebo MAP (FA) with biopsy
B3 = A/Sing MAP, 15 ug (FA); B4 = A/Sing MAP, 10 ug (FA); B5 = A/Sing MAP, 5 ug (FA); B6 = A/Sing MAP, 2.5 ug (FA)
B7 = Placebo MAP (FA); B8 = A/Sing MAP, 15 ug (UA); B9 = A/Sing IM, 15 ug, as 2018 Afluria Quad (UA)

Listing 16.2.6.8
Immunogenicity Results (HAI, MNT)

Part A

Randomisation Number	Group Treatment	Visit	Collection Date	Test	Virus	Titre	Comment
A0052	Group A4	Day 61	16MAY2018	HAI	A/Singapore/GP1908/2015	1280	
A0053	Group A4	Day 1 Predose	17MAR2018	HAI	A/Singapore/GP1908/2015	20	
A0053	Group A4	Day 4	20MAR2018	HAI	A/Singapore/GP1908/2015	20	
A0053	Group A4	Day 8	24MAR2018	HAI	A/Singapore/GP1908/2015	160	
A0053	Group A4	Day 22	09APR2018	HAI	A/Singapore/GP1908/2015	160	
A0053	Group A4	Day 61	16MAY2018	HAI	A/Singapore/GP1908/2015	160	
A0054	Group A2	Day 1 Predose	17MAR2018	HAI	A/Singapore/GP1908/2015	20	
A0054	Group A2	Day 4	20MAR2018	HAI	A/Singapore/GP1908/2015	40	
A0054	Group A2	Day 8	24MAR2018	HAI	A/Singapore/GP1908/2015	320	
A0054	Group A2	Day 22	08APR2018	HAI	A/Singapore/GP1908/2015	320	
A0054	Group A2	Day 61	26MAY2018	HAI	A/Singapore/GP1908/2015	320	
A0055	Group A4	Day 1 Predose	24MAR2018	HAI	A/Singapore/GP1908/2015	10	
A0055	Group A4	Day 4	27MAR2018	HAI	A/Singapore/GP1908/2015	10	
A0055	Group A4	Day 8	31MAR2018	HAI	A/Singapore/GP1908/2015	40	
A0055	Group A4	Day 22	14APR2018	HAI	A/Singapore/GP1908/2015	320	
A0055	Group A4	Day 61	24MAY2018	HAI	A/Singapore/GP1908/2015	320	

Administration Site Key: FA = Forearm; UA= Upper arm
Treatments Part A: A1 = A/Sing MAP, 15 ug (FA); A2 = Afluria Quad IM (UA), 15 ug; A3 = Placebo MAP (FA); A4 = A/Sing Mono IM, 15 ug (UA)
Treatments Part B: B1 = A/Sing MAP, 15 ug (FA) with biopsy; B2 = Placebo MAP (FA) with biopsy
B3 = A/Sing MAP, 15 ug (FA); B4 = A/Sing MAP, 10 ug (FA); B5 = A/Sing MAP, 5 ug (FA); B6 = A/Sing MAP, 2.5 ug (FA)
B7 = Placebo MAP (FA); B8 = A/Sing MAP, 15 ug (UA); B9 = A/Sing IM, 15 ug, as 2018 Afluria Quad (UA)

Listing 16.2.6.8
Immunogenicity Results (HAI, MNT)

Part A

Randomisation Number	Group Treatment	Visit	Collection Date	Test	Virus	Titre	Comment
A0056	Group A2	Day 1 Predose	24MAR2018	HAI	A/Singapore/GP1908/2015	20	
A0056	Group A2	Day 4	27MAR2018	HAI	A/Singapore/GP1908/2015	20	
A0056	Group A2	Day 8	31MAR2018	HAI	A/Singapore/GP1908/2015	320	
A0056	Group A2	Day 22	14APR2018	HAI	A/Singapore/GP1908/2015	640	
A0056	Group A2	Day 61	23MAY2018	HAI	A/Singapore/GP1908/2015	640	
A0057	Group A4	Day 1 Predose	24MAR2018	HAI	A/Singapore/GP1908/2015	40	
A0057	Group A4	Day 4	27MAR2018	HAI	A/Singapore/GP1908/2015	40	
A0057	Group A4	Day 8	02APR2018	HAI	A/Singapore/GP1908/2015	80	
A0057	Group A4	Day 22	14APR2018	HAI	A/Singapore/GP1908/2015	80	
A0057	Group A4	Day 61	23MAY2018	HAI	A/Singapore/GP1908/2015	80	
A0058	Group A2	Day 1 Predose	24MAR2018	HAI	A/Singapore/GP1908/2015	10	
A0058	Group A2	Day 4	27MAR2018	HAI	A/Singapore/GP1908/2015	10	
A0058	Group A2	Day 8	31MAR2018	HAI	A/Singapore/GP1908/2015	160	
A0058	Group A2	Day 22	14APR2018	HAI	A/Singapore/GP1908/2015	160	
A0058	Group A2	Day 61	23MAY2018	HAI	A/Singapore/GP1908/2015	160	

Administration Site Key: FA = Forearm; UA= Upper arm
Treatments Part A: A1 = A/Sing MAP, 15 ug (FA); A2 = Afluria Quad IM (UA), 15 ug; A3 = Placebo MAP (FA); A4 = A/Sing Mono IM, 15 ug (UA)
Treatments Part B: B1 = A/Sing MAP, 15 ug (FA) with biopsy; B2 = Placebo MAP (FA) with biopsy
B3 = A/Sing MAP, 15 ug (FA); B4 = A/Sing MAP, 10 ug (FA); B5 = A/Sing MAP, 5 ug (FA); B6 = A/Sing MAP, 2.5 ug (FA)
B7 = Placebo MAP (FA); B8 = A/Sing MAP, 15 ug (UA); B9 = A/Sing IM, 15 ug, as 2018 Afluria Quad (UA)

Listing 16.2.6.8
Immunogenicity Results (HAI, MNT)

Part A

Randomisation Number	Group Treatment	Visit	Collection Date	Test	Virus	Titre	Comment
A0059	Group A2	Day 1 Predose	24MAR2018	HAI	A/Singapore/GP1908/2015	10	
A0059	Group A2	Day 4	27MAR2018	HAI	A/Singapore/GP1908/2015	10	
A0059	Group A2	Day 8	30MAR2018	HAI	A/Singapore/GP1908/2015	20	
A0059	Group A2	Day 22	17APR2018	HAI	A/Singapore/GP1908/2015	80	
A0059	Group A2	Day 61	24MAY2018	HAI	A/Singapore/GP1908/2015	80	
A0060	Group A4	Day 1 Predose	24MAR2018	HAI	A/Singapore/GP1908/2015	5	
A0060	Group A4	Day 4	27MAR2018	HAI	A/Singapore/GP1908/2015	5	
A0060	Group A4	Day 8	31MAR2018	HAI	A/Singapore/GP1908/2015	80	
A0060	Group A4	Day 22	14APR2018	HAI	A/Singapore/GP1908/2015	320	
A0060	Group A4	Day 61	24MAY2018	HAI	A/Singapore/GP1908/2015	320	
A1026	Group A1	Day 1 Predose	10MAR2018	HAI	A/Singapore/GP1908/2015	<5	
A1026	Group A1	Day 4	13MAR2018	HAI	A/Singapore/GP1908/2015	<5	
A1026	Group A1	Day 8	18MAR2018	HAI	A/Singapore/GP1908/2015	320	
A1026	Group A1	Day 22	31MAR2018	HAI	A/Singapore/GP1908/2015	320	
A1026	Group A1	Day 61	09MAY2018	HAI	A/Singapore/GP1908/2015	160	

Administration Site Key: FA = Forearm; UA= Upper arm
Treatments Part A: A1 = A/Sing MAP, 15 ug (FA); A2 = Afluria Quad IM (UA), 15 ug; A3 = Placebo MAP (FA); A4 = A/Sing Mono IM, 15 ug (UA)
Treatments Part B: B1 = A/Sing MAP, 15 ug (FA) with biopsy; B2 = Placebo MAP (FA) with biopsy
B3 = A/Sing MAP, 15 ug (FA); B4 = A/Sing MAP, 10 ug (FA); B5 = A/Sing MAP, 5 ug (FA); B6 = A/Sing MAP, 2.5 ug (FA)
B7 = Placebo MAP (FA); B8 = A/Sing MAP, 15 ug (UA); B9 = A/Sing IM, 15 ug, as 2018 Afluria Quad (UA)

Listing 16.2.6.8
Immunogenicity Results (HAI, MNT)

Part B

Randomisation Number	Group Treatment	Visit	Collection Date	Test	Virus	Titre	Comment
B0001	Group B1	Day 1 Predose	07MAY2018	HAI	A/Singapore/GP1908/2015	5	
B0001	Group B1	Day 4	10MAY2018	HAI	A/Singapore/GP1908/2015	5	
B0001	Group B1	Day 8	14MAY2018	HAI	A/Singapore/GP1908/2015	20	
B0001	Group B1	Day 22	28MAY2018	HAI	A/Singapore/GP1908/2015	40	
B0001	Group B1	Day 61	06JUL2018	HAI	A/Singapore/GP1908/2015	40	
B0002	Group B2	Day 1 Predose	07MAY2018	HAI	A/Singapore/GP1908/2015	5	
B0002	Group B2	Day 4	10MAY2018	HAI	A/Singapore/GP1908/2015	5	
B0002	Group B2	Day 8	14MAY2018	HAI	A/Singapore/GP1908/2015	5	
B0002	Group B2	Day 22	28MAY2018	HAI	A/Singapore/GP1908/2015	5	
B0002	Group B2	Day 61	06JUL2018	HAI	A/Singapore/GP1908/2015	5	
B0003	Group B2	Day 1 Predose	07MAY2018	HAI	A/Singapore/GP1908/2015	5	
B0003	Group B2	Day 4	10MAY2018	HAI	A/Singapore/GP1908/2015	5	
B0003	Group B2	Day 8	14MAY2018	HAI	A/Singapore/GP1908/2015	5	
B0003	Group B2	Day 22	28MAY2018	HAI	A/Singapore/GP1908/2015	5	
B0003	Group B2	Day 61	06JUL2018	HAI	A/Singapore/GP1908/2015	5	
B0004	Group B1	Day 1 Predose	07MAY2018	HAI	A/Singapore/GP1908/2015	40	

Administration Site Key: FA = Forearm; UA= Upper arm
Treatments Part A: A1 = A/Sing MAP, 15 ug (FA); A2 = Afluria Quad IM (UA), 15 ug; A3 = Placebo MAP (FA); A4 = A/Sing Mono IM, 15 ug (UA)
Treatments Part B: B1 = A/Sing MAP, 15 ug (FA) with biopsy; B2 = Placebo MAP (FA) with biopsy
B3 = A/Sing MAP, 15 ug (FA); B4 = A/Sing MAP, 10 ug (FA); B5 = A/Sing MAP, 5 ug (FA); B6 = A/Sing MAP, 2.5 ug (FA)
B7 = Placebo MAP (FA); B8 = A/Sing MAP, 15 ug (UA); B9 = A/Sing IM, 15 ug, as 2018 Afluria Quad (UA)

Listing 16.2.6.8
Immunogenicity Results (HAI, MNT)

Part B

Randomisation Number	Group Treatment	Visit	Collection Date	Test	Virus	Titre	Comment
B0004	Group B1	Day 4	10MAY2018	HAI	A/Singapore/GP1908/2015	40	
B0004	Group B1	Day 8	14MAY2018	HAI	A/Singapore/GP1908/2015	80	
B0004	Group B1	Day 22	28MAY2018	HAI	A/Singapore/GP1908/2015	80	
B0004	Group B1	Day 61	06JUL2018	HAI	A/Singapore/GP1908/2015	80	
B0005	Group B1	Day 1 Predose	07MAY2018	HAI	A/Singapore/GP1908/2015	10	
B0005	Group B1	Day 4	10MAY2018	HAI	A/Singapore/GP1908/2015	10	
B0005	Group B1	Day 8	14MAY2018	HAI	A/Singapore/GP1908/2015	320	
B0005	Group B1	Day 22	28MAY2018	HAI	A/Singapore/GP1908/2015	320	
B0005	Group B1	Day 61	06JUL2018	HAI	A/Singapore/GP1908/2015	160	
B0006	Group B2	Day 1 Predose	07MAY2018	HAI	A/Singapore/GP1908/2015	5	
B0006	Group B2	Day 4	10MAY2018	HAI	A/Singapore/GP1908/2015	5	
B0006	Group B2	Day 8	14MAY2018	HAI	A/Singapore/GP1908/2015	5	
B0006	Group B2	Day 22	28MAY2018	HAI	A/Singapore/GP1908/2015	5	
B0006	Group B2	Day 61	06JUL2018	HAI	A/Singapore/GP1908/2015	5	
B0007	Group B2	Day 1 Predose	07MAY2018	HAI	A/Singapore/GP1908/2015	5	
B0007	Group B2	Day 4	10MAY2018	HAI	A/Singapore/GP1908/2015	5	

Administration Site Key: FA = Forearm; UA= Upper arm
Treatments Part A: A1 = A/Sing MAP, 15 ug (FA); A2 = Afluria Quad IM (UA), 15 ug; A3 = Placebo MAP (FA); A4 = A/Sing Mono IM, 15 ug (UA)
Treatments Part B: B1 = A/Sing MAP, 15 ug (FA) with biopsy; B2 = Placebo MAP (FA) with biopsy
B3 = A/Sing MAP, 15 ug (FA); B4 = A/Sing MAP, 10 ug (FA); B5 = A/Sing MAP, 5 ug (FA); B6 = A/Sing MAP, 2.5 ug (FA)
B7 = Placebo MAP (FA); B8 = A/Sing MAP, 15 ug (UA); B9 = A/Sing IM, 15 ug, as 2018 Afluria Quad (UA)

Listing 16.2.6.8
Immunogenicity Results (HAI, MNT)

Part B

Randomisation Number	Group Treatment	Visit	Collection Date	Test	Virus	Titre	Comment
B0007	Group B2	Day 8	14MAY2018	HAI	A/Singapore/GP1908/2015	5	
B0007	Group B2	Day 22	28MAY2018	HAI	A/Singapore/GP1908/2015	5	
B0007	Group B2	Day 61	06JUL2018	HAI	A/Singapore/GP1908/2015	5	
B0008	Group B1	Day 1 Predose	07MAY2018	HAI	A/Singapore/GP1908/2015	40	
B0008	Group B1	Day 4	10MAY2018	HAI	A/Singapore/GP1908/2015	40	
B0008	Group B1	Day 8	14MAY2018	HAI	A/Singapore/GP1908/2015	40	
B0008	Group B1	Day 22	28MAY2018	HAI	A/Singapore/GP1908/2015	160	
B0008	Group B1	Day 61	06JUL2018	HAI	A/Singapore/GP1908/2015	160	
B0009	Group B1	Day 1 Predose	07MAY2018	HAI	A/Singapore/GP1908/2015	5	
B0009	Group B1	Day 4	10MAY2018	HAI	A/Singapore/GP1908/2015	5	
B0009	Group B1	Day 8	14MAY2018	HAI	A/Singapore/GP1908/2015	160	
B0009	Group B1	Day 22	28MAY2018	HAI	A/Singapore/GP1908/2015	320	
B0009	Group B1	Day 61	06JUL2018	HAI	A/Singapore/GP1908/2015	160	
B0010	Group B2	Day 1 Predose	07MAY2018	HAI	A/Singapore/GP1908/2015	5	
B0010	Group B2	Day 4	10MAY2018	HAI	A/Singapore/GP1908/2015	5	
B0010	Group B2	Day 8	14MAY2018	HAI	A/Singapore/GP1908/2015	5	

Administration Site Key: FA = Forearm; UA= Upper arm
Treatments Part A: A1 = A/Sing MAP, 15 ug (FA); A2 = Afluria Quad IM (UA), 15 ug; A3 = Placebo MAP (FA); A4 = A/Sing Mono IM, 15 ug (UA)
Treatments Part B: B1 = A/Sing MAP, 15 ug (FA) with biopsy; B2 = Placebo MAP (FA) with biopsy
B3 = A/Sing MAP, 15 ug (FA); B4 = A/Sing MAP, 10 ug (FA); B5 = A/Sing MAP, 5 ug (FA); B6 = A/Sing MAP, 2.5 ug (FA)
B7 = Placebo MAP (FA); B8 = A/Sing MAP, 15 ug (UA); B9 = A/Sing IM, 15 ug, as 2018 Afluria Quad (UA)

Listing 16.2.6.8
Immunogenicity Results (HAI, MNT)

Part B

Randomisation Number	Group Treatment	Visit	Collection Date	Test	Virus	Titre	Comment
B0010	Group B2	Day 22	28MAY2018	HAI	A/Singapore/GP1908/2015	5	
B0010	Group B2	Day 61	06JUL2018	HAI	A/Singapore/GP1908/2015	5	
B0011	Group B8	Day 1 Predose	12MAY2018	HAI	A/Singapore/GP1908/2015	<5	
B0011	Group B8	Day 4	15MAY2018	HAI	A/Singapore/GP1908/2015	<5	
B0011	Group B8	Day 8	19MAY2018	HAI	A/Singapore/GP1908/2015	80	
B0011	Group B8	Day 22	02JUN2018	HAI	A/Singapore/GP1908/2015	80	
B0011	Group B8	Day 61	14JUL2018	HAI	A/Singapore/GP1908/2015	80	
B0011	Group B8	Day 1 Predose	12MAY2018	MNT	A/Singapore/GP1908/2015	20	
B0011	Group B8	Day 22	02JUN2018	MNT	A/Singapore/GP1908/2015	5120	
B0012	Group B6	Day 1 Predose	12MAY2018	HAI	A/Singapore/GP1908/2015	20	
B0012	Group B6	Day 4	15MAY2018	HAI	A/Singapore/GP1908/2015	20	
B0012	Group B6	Day 8	19MAY2018	HAI	A/Singapore/GP1908/2015	40	
B0012	Group B6	Day 22	02JUN2018	HAI	A/Singapore/GP1908/2015	80	
B0012	Group B6	Day 61	11JUL2018	HAI	A/Singapore/GP1908/2015	80	
B0012	Group B6	Day 1 Predose	12MAY2018	MNT	A/Singapore/GP1908/2015	320	
B0012	Group B6	Day 22	02JUN2018	MNT	A/Singapore/GP1908/2015	2560	

Administration Site Key: FA = Forearm; UA= Upper arm
Treatments Part A: A1 = A/Sing MAP, 15 ug (FA); A2 = Afluria Quad IM (UA), 15 ug; A3 = Placebo MAP (FA); A4 = A/Sing Mono IM, 15 ug (UA)
Treatments Part B: B1 = A/Sing MAP, 15 ug (FA) with biopsy; B2 = Placebo MAP (FA) with biopsy
B3 = A/Sing MAP, 15 ug (FA); B4 = A/Sing MAP, 10 ug (FA); B5 = A/Sing MAP, 5 ug (FA); B6 = A/Sing MAP, 2.5 ug (FA)
B7 = Placebo MAP (FA); B8 = A/Sing MAP, 15 ug (UA); B9 = A/Sing IM, 15 ug, as 2018 Afluria Quad (UA)

Listing 16.2.6.8
Immunogenicity Results (HAI, MNT)

Part B

Randomisation Number	Group Treatment	Visit	Collection Date	Test	Virus	Titre	Comment
B0013	Group B5	Day 1 Predose	12MAY2018	HAI	A/Singapore/GP1908/2015	5	
B0013	Group B5	Day 4	15MAY2018	HAI	A/Singapore/GP1908/2015	5	
B0013	Group B5	Day 8	19MAY2018	HAI	A/Singapore/GP1908/2015	10	
B0013	Group B5	Day 22	02JUN2018	HAI	A/Singapore/GP1908/2015	40	
B0013	Group B5	Day 61	11JUL2018	HAI	A/Singapore/GP1908/2015	40	
B0013	Group B5	Day 1 Predose	12MAY2018	MNT	A/Singapore/GP1908/2015	80	
B0013	Group B5	Day 22	02JUN2018	MNT	A/Singapore/GP1908/2015	640	
B0014	Group B3	Day 1 Predose	12MAY2018	HAI	A/Singapore/GP1908/2015	5	
B0014	Group B3	Day 4	15MAY2018	HAI	A/Singapore/GP1908/2015	5	
B0014	Group B3	Day 8	19MAY2018	HAI	A/Singapore/GP1908/2015	320	
B0014	Group B3	Day 22	02JUN2018	HAI	A/Singapore/GP1908/2015	640	
B0014	Group B3	Day 61	12JUL2018	HAI	A/Singapore/GP1908/2015	320	
B0014	Group B3	Day 1 Predose	12MAY2018	MNT	A/Singapore/GP1908/2015	160	
B0014	Group B3	Day 22	02JUN2018	MNT	A/Singapore/GP1908/2015	40960	
B0015	Group B9	Day 1 Predose	12MAY2018	HAI	A/Singapore/GP1908/2015	5	
B0015	Group B9	Day 4	15MAY2018	HAI	A/Singapore/GP1908/2015	5	
B0015	Group B9	Day 8	19MAY2018	HAI	A/Singapore/GP1908/2015	80	

Administration Site Key: FA = Forearm; UA= Upper arm
Treatments Part A: A1 = A/Sing MAP, 15 ug (FA); A2 = Afluria Quad IM (UA), 15 ug; A3 = Placebo MAP (FA); A4 = A/Sing Mono IM, 15 ug (UA)
Treatments Part B: B1 = A/Sing MAP, 15 ug (FA) with biopsy; B2 = Placebo MAP (FA) with biopsy
B3 = A/Sing MAP, 15 ug (FA); B4 = A/Sing MAP, 10 ug (FA); B5 = A/Sing MAP, 5 ug (FA); B6 = A/Sing MAP, 2.5 ug (FA)
B7 = Placebo MAP (FA); B8 = A/Sing MAP, 15 ug (UA); B9 = A/Sing IM, 15 ug, as 2018 Afluria Quad (UA)

Listing 16.2.6.8
Immunogenicity Results (HAI, MNT)

Part B

Randomisation Number	Group Treatment	Visit	Collection Date	Test	Virus	Titre	Comment
B0015	Group B9	Day 22	02JUN2018	HAI	A/Singapore/GP1908/2015	80	
B0015	Group B9	Day 61	11JUL2018	HAI	A/Singapore/GP1908/2015	40	
B0015	Group B9	Day 1 Predose	12MAY2018	MNT	A/Singapore/GP1908/2015	20	
B0015	Group B9	Day 22	02JUN2018	MNT	A/Singapore/GP1908/2015	2560	
B0016	Group B4	Day 1 Predose	12MAY2018	HAI	A/Singapore/GP1908/2015	160	
B0016	Group B4	Day 4	15MAY2018	HAI	A/Singapore/GP1908/2015	160	
B0016	Group B4	Day 8	19MAY2018	HAI	A/Singapore/GP1908/2015	320	
B0016	Group B4	Day 22	02JUN2018	HAI	A/Singapore/GP1908/2015	320	
B0016	Group B4	Day 61	11JUL2018	HAI	A/Singapore/GP1908/2015	320	
B0016	Group B4	Day 1 Predose	12MAY2018	MNT	A/Singapore/GP1908/2015	5120	
B0016	Group B4	Day 22	02JUN2018	MNT	A/Singapore/GP1908/2015	20480	
B0017	Group B7	Day 1 Predose	12MAY2018	HAI	A/Singapore/GP1908/2015	160	
B0017	Group B7	Day 4	15MAY2018	HAI	A/Singapore/GP1908/2015	160	
B0017	Group B7	Day 8	19MAY2018	HAI	A/Singapore/GP1908/2015	160	
B0017	Group B7	Day 22	04JUN2018	HAI	A/Singapore/GP1908/2015	160	
B0017	Group B7	Day 61	07JUL2018	HAI	A/Singapore/GP1908/2015	160	
B0017	Group B7	Day 1 Predose	12MAY2018	MNT	A/Singapore/GP1908/2015	2560	

Administration Site Key: FA = Forearm; UA= Upper arm
Treatments Part A: A1 = A/Sing MAP, 15 ug (FA); A2 = Afluria Quad IM (UA), 15 ug; A3 = Placebo MAP (FA); A4 = A/Sing Mono IM, 15 ug (UA)
Treatments Part B: B1 = A/Sing MAP, 15 ug (FA) with biopsy; B2 = Placebo MAP (FA) with biopsy
B3 = A/Sing MAP, 15 ug (FA); B4 = A/Sing MAP, 10 ug (FA); B5 = A/Sing MAP, 5 ug (FA); B6 = A/Sing MAP, 2.5 ug (FA)
B7 = Placebo MAP (FA); B8 = A/Sing MAP, 15 ug (UA); B9 = A/Sing IM, 15 ug, as 2018 Afluria Quad (UA)

Listing 16.2.6.8
Immunogenicity Results (HAI, MNT)

Part B

Randomisation Number	Group Treatment	Visit	Collection Date	Test	Virus	Titre	Comment
B0017	Group B7	Day 22	04JUN2018	MNT	A/Singapore/GP1908/2015	2560	
B0018	Group B9	Day 1 Predose	12MAY2018	HAI	A/Singapore/GP1908/2015	40	
B0018	Group B9	Day 4	15MAY2018	HAI	A/Singapore/GP1908/2015	40	
B0018	Group B9	Day 8	19MAY2018	HAI	A/Singapore/GP1908/2015	320	
B0018	Group B9	Day 22	02JUN2018	HAI	A/Singapore/GP1908/2015	320	
B0018	Group B9	Day 61	11JUL2018	HAI	A/Singapore/GP1908/2015	160	
B0018	Group B9	Day 1 Predose	12MAY2018	MNT	A/Singapore/GP1908/2015	1280	
B0018	Group B9	Day 22	02JUN2018	MNT	A/Singapore/GP1908/2015	5120	
B0020	Group B6	Day 1 Predose	12MAY2018	HAI	A/Singapore/GP1908/2015	20	
B0020	Group B6	Day 4	15MAY2018	HAI	A/Singapore/GP1908/2015	20	
B0020	Group B6	Day 8	19MAY2018	HAI	A/Singapore/GP1908/2015	160	
B0020	Group B6	Day 22	13JUN2018	HAI	A/Singapore/GP1908/2015	160	
B0020	Group B6	Day 61	11JUL2018	HAI	A/Singapore/GP1908/2015	160	
B0020	Group B6	Day 1 Predose	12MAY2018	MNT	A/Singapore/GP1908/2015	320	
B0020	Group B6	Day 22	13JUN2018	MNT	A/Singapore/GP1908/2015	5120	
B0021	Group B7	Day 1 Predose	12MAY2018	HAI	A/Singapore/GP1908/2015	80	

Administration Site Key: FA = Forearm; UA= Upper arm
Treatments Part A: A1 = A/Sing MAP, 15 ug (FA); A2 = Afluria Quad IM (UA), 15 ug; A3 = Placebo MAP (FA); A4 = A/Sing Mono IM, 15 ug (UA)
Treatments Part B: B1 = A/Sing MAP, 15 ug (FA) with biopsy; B2 = Placebo MAP (FA) with biopsy
B3 = A/Sing MAP, 15 ug (FA); B4 = A/Sing MAP, 10 ug (FA); B5 = A/Sing MAP, 5 ug (FA); B6 = A/Sing MAP, 2.5 ug (FA)
B7 = Placebo MAP (FA); B8 = A/Sing MAP, 15 ug (UA); B9 = A/Sing IM, 15 ug, as 2018 Afluria Quad (UA)

Listing 16.2.6.8
Immunogenicity Results (HAI, MNT)

Part B

Randomisation Number	Group Treatment	Visit	Collection Date	Test	Virus	Titre	Comment
B0021	Group B7	Day 4	15MAY2018	HAI	A/Singapore/GP1908/2015	80	
B0021	Group B7	Day 8	19MAY2018	HAI	A/Singapore/GP1908/2015	80	
B0021	Group B7	Day 22	02JUN2018	HAI	A/Singapore/GP1908/2015	80	
B0021	Group B7	Day 61	11JUL2018	HAI	A/Singapore/GP1908/2015	80	
B0021	Group B7	Day 1 Predose	12MAY2018	MNT	A/Singapore/GP1908/2015	1280	
B0021	Group B7	Day 22	02JUN2018	MNT	A/Singapore/GP1908/2015	1280	
B0022	Group B4	Day 1 Predose	12MAY2018	HAI	A/Singapore/GP1908/2015	10	
B0022	Group B4	Day 4	15MAY2018	HAI	A/Singapore/GP1908/2015	10	
B0022	Group B4	Day 8	19MAY2018	HAI	A/Singapore/GP1908/2015	160	
B0022	Group B4	Day 22	02JUN2018	HAI	A/Singapore/GP1908/2015	160	
B0022	Group B4	Day 61	12JUL2018	HAI	A/Singapore/GP1908/2015	160	
B0022	Group B4	Day 1 Predose	12MAY2018	MNT	A/Singapore/GP1908/2015	320	
B0022	Group B4	Day 22	02JUN2018	MNT	A/Singapore/GP1908/2015	2560	
B0023	Group B3	Day 1 Predose	12MAY2018	HAI	A/Singapore/GP1908/2015	40	
B0023	Group B3	Day 4	15MAY2018	HAI	A/Singapore/GP1908/2015	40	
B0023	Group B3	Day 8	19MAY2018	HAI	A/Singapore/GP1908/2015	1280	
B0023	Group B3	Day 22	02JUN2018	HAI	A/Singapore/GP1908/2015	2560	

Administration Site Key: FA = Forearm; UA= Upper arm
Treatments Part A: A1 = A/Sing MAP, 15 ug (FA); A2 = Afluria Quad IM (UA), 15 ug; A3 = Placebo MAP (FA); A4 = A/Sing Mono IM, 15 ug (UA)
Treatments Part B: B1 = A/Sing MAP, 15 ug (FA) with biopsy; B2 = Placebo MAP (FA) with biopsy
B3 = A/Sing MAP, 15 ug (FA); B4 = A/Sing MAP, 10 ug (FA); B5 = A/Sing MAP, 5 ug (FA); B6 = A/Sing MAP, 2.5 ug (FA)
B7 = Placebo MAP (FA); B8 = A/Sing MAP, 15 ug (UA); B9 = A/Sing IM, 15 ug, as 2018 Afluria Quad (UA)

Listing 16.2.6.8
Immunogenicity Results (HAI, MNT)

Part B

Randomisation Number	Group Treatment	Visit	Collection Date	Test	Virus	Titre	Comment
B0023	Group B3	Day 61	11JUL2018	HAI	A/Singapore/GP1908/2015	1280	
B0023	Group B3	Day 1 Predose	12MAY2018	MNT	A/Singapore/GP1908/2015	640	
B0023	Group B3	Day 22	02JUN2018	MNT	A/Singapore/GP1908/2015	40960	
B0024	Group B5	Day 1 Predose	12MAY2018	HAI	A/Singapore/GP1908/2015	80	
B0024	Group B5	Day 4	15MAY2018	HAI	A/Singapore/GP1908/2015	80	
B0024	Group B5	Day 8	19MAY2018	HAI	A/Singapore/GP1908/2015	320	
B0024	Group B5	Day 22	02JUN2018	HAI	A/Singapore/GP1908/2015	320	
B0024	Group B5	Day 61	12JUL2018	HAI	A/Singapore/GP1908/2015	160	
B0024	Group B5	Day 1 Predose	12MAY2018	MNT	A/Singapore/GP1908/2015	2560	
B0024	Group B5	Day 22	02JUN2018	MNT	A/Singapore/GP1908/2015	20480	
B0025	Group B8	Day 1 Predose	12MAY2018	HAI	A/Singapore/GP1908/2015	80	
B0025	Group B8	Day 4	15MAY2018	HAI	A/Singapore/GP1908/2015	80	
B0025	Group B8	Day 8	19MAY2018	HAI	A/Singapore/GP1908/2015	320	
B0025	Group B8	Day 22	04JUN2018	HAI	A/Singapore/GP1908/2015	160	
B0025	Group B8	Day 61	13JUL2018	HAI	A/Singapore/GP1908/2015	160	
B0025	Group B8	Day 1 Predose	12MAY2018	MNT	A/Singapore/GP1908/2015	5120	
B0025	Group B8	Day 22	04JUN2018	MNT	A/Singapore/GP1908/2015	20480	

Administration Site Key: FA = Forearm; UA= Upper arm
Treatments Part A: A1 = A/Sing MAP, 15 ug (FA); A2 = Afluria Quad IM (UA), 15 ug; A3 = Placebo MAP (FA); A4 = A/Sing Mono IM, 15 ug (UA)
Treatments Part B: B1 = A/Sing MAP, 15 ug (FA) with biopsy; B2 = Placebo MAP (FA) with biopsy
B3 = A/Sing MAP, 15 ug (FA); B4 = A/Sing MAP, 10 ug (FA); B5 = A/Sing MAP, 5 ug (FA); B6 = A/Sing MAP, 2.5 ug (FA)
B7 = Placebo MAP (FA); B8 = A/Sing MAP, 15 ug (UA); B9 = A/Sing IM, 15 ug, as 2018 Afluria Quad (UA)

Listing 16.2.6.8
Immunogenicity Results (HAI, MNT)

Part B

Randomisation Number	Group Treatment	Visit	Collection Date	Test	Virus	Titre	Comment
B0026	Group B9	Day 1 Predose	12MAY2018	HAI	A/Singapore/GP1908/2015	<5	
B0026	Group B9	Day 4	15MAY2018	HAI	A/Singapore/GP1908/2015	<5	
B0026	Group B9	Day 8	19MAY2018	HAI	A/Singapore/GP1908/2015	5	
B0026	Group B9	Day 22	02JUN2018	HAI	A/Singapore/GP1908/2015	10	
B0026	Group B9	Day 61	11JUL2018	HAI	A/Singapore/GP1908/2015	20	
B0026	Group B9	Day 1 Predose	12MAY2018	MNT	A/Singapore/GP1908/2015	40	
B0026	Group B9	Day 22	02JUN2018	MNT	A/Singapore/GP1908/2015	320	
B0027	Group B3	Day 1 Predose	12MAY2018	HAI	A/Singapore/GP1908/2015	10	
B0027	Group B3	Day 4	15MAY2018	HAI	A/Singapore/GP1908/2015	10	
B0027	Group B3	Day 8	19MAY2018	HAI	A/Singapore/GP1908/2015	40	
B0027	Group B3	Day 22	02JUN2018	HAI	A/Singapore/GP1908/2015	160	
B0027	Group B3	Day 61	11JUL2018	HAI	A/Singapore/GP1908/2015	160	
B0027	Group B3	Day 1 Predose	12MAY2018	MNT	A/Singapore/GP1908/2015	80	
B0027	Group B3	Day 22	02JUN2018	MNT	A/Singapore/GP1908/2015	2560	
B0028	Group B5	Day 1 Predose	12MAY2018	HAI	A/Singapore/GP1908/2015	<5	
B0028	Group B5	Day 4	16MAY2018	HAI	A/Singapore/GP1908/2015	<5	

Administration Site Key: FA = Forearm; UA= Upper arm
Treatments Part A: A1 = A/Sing MAP, 15 ug (FA); A2 = Afluria Quad IM (UA), 15 ug; A3 = Placebo MAP (FA); A4 = A/Sing Mono IM, 15 ug (UA)
Treatments Part B: B1 = A/Sing MAP, 15 ug (FA) with biopsy; B2 = Placebo MAP (FA) with biopsy
B3 = A/Sing MAP, 15 ug (FA); B4 = A/Sing MAP, 10 ug (FA); B5 = A/Sing MAP, 5 ug (FA); B6 = A/Sing MAP, 2.5 ug (FA)
B7 = Placebo MAP (FA); B8 = A/Sing MAP, 15 ug (UA); B9 = A/Sing IM, 15 ug, as 2018 Afluria Quad (UA)

Listing 16.2.6.8
Immunogenicity Results (HAI, MNT)

Part B

Randomisation Number	Group Treatment	Visit	Collection Date	Test	Virus	Titre	Comment
B0028	Group B5	Day 8	19MAY2018	HAI	A/Singapore/GP1908/2015	<5	
B0028	Group B5	Day 22	02JUN2018	HAI	A/Singapore/GP1908/2015	10	
B0028	Group B5	Day 61	11JUL2018	HAI	A/Singapore/GP1908/2015	10	
B0028	Group B5	Day 1 Predose	12MAY2018	MNT	A/Singapore/GP1908/2015	<20	
B0028	Group B5	Day 22	02JUN2018	MNT	A/Singapore/GP1908/2015	320	
B0029	Group B6	Day 1 Predose	12MAY2018	HAI	A/Singapore/GP1908/2015	20	
B0029	Group B6	Day 4	15MAY2018	HAI	A/Singapore/GP1908/2015	20	
B0029	Group B6	Day 8	19MAY2018	HAI	A/Singapore/GP1908/2015	40	
B0029	Group B6	Day 22	02JUN2018	HAI	A/Singapore/GP1908/2015	160	
B0029	Group B6	Day 61	11JUL2018	HAI	A/Singapore/GP1908/2015	160	
B0029	Group B6	Day 1 Predose	12MAY2018	MNT	A/Singapore/GP1908/2015	1280	
B0029	Group B6	Day 22	02JUN2018	MNT	A/Singapore/GP1908/2015	10240	
B0030	Group B7	Day 1 Predose	12MAY2018	HAI	A/Singapore/GP1908/2015	80	
B0030	Group B7	Day 4	15MAY2018	HAI	A/Singapore/GP1908/2015	80	
B0030	Group B7	Day 8	19MAY2018	HAI	A/Singapore/GP1908/2015	160	
B0030	Group B7	Day 22	02JUN2018	HAI	A/Singapore/GP1908/2015	80	
B0030	Group B7	Day 61	13JUL2018	HAI	A/Singapore/GP1908/2015	80	

Administration Site Key: FA = Forearm; UA= Upper arm
Treatments Part A: A1 = A/Sing MAP, 15 ug (FA); A2 = Afluria Quad IM (UA), 15 ug; A3 = Placebo MAP (FA); A4 = A/Sing Mono IM, 15 ug (UA)
Treatments Part B: B1 = A/Sing MAP, 15 ug (FA) with biopsy; B2 = Placebo MAP (FA) with biopsy
B3 = A/Sing MAP, 15 ug (FA); B4 = A/Sing MAP, 10 ug (FA); B5 = A/Sing MAP, 5 ug (FA); B6 = A/Sing MAP, 2.5 ug (FA)
B7 = Placebo MAP (FA); B8 = A/Sing MAP, 15 ug (UA); B9 = A/Sing IM, 15 ug, as 2018 Afluria Quad (UA)

Listing 16.2.6.8
Immunogenicity Results (HAI, MNT)

Part B

Randomisation Number	Group Treatment	Visit	Collection Date	Test	Virus	Titre	Comment
B0030	Group B7	Day 1 Predose	12MAY2018	MNT	A/Singapore/GP1908/2015	2560	
B0030	Group B7	Day 22	02JUN2018	MNT	A/Singapore/GP1908/2015	2560	
B0031	Group B4	Day 1 Predose	19MAY2018	HAI	A/Singapore/GP1908/2015	80	
B0031	Group B4	Day 4	22MAY2018	HAI	A/Singapore/GP1908/2015	80	
B0031	Group B4	Day 8	26MAY2018	HAI	A/Singapore/GP1908/2015	320	
B0031	Group B4	Day 22	08JUN2018	HAI	A/Singapore/GP1908/2015	160	
B0031	Group B4	Day 61	18JUL2018	HAI	A/Singapore/GP1908/2015	320	
B0031	Group B4	Day 1 Predose	19MAY2018	MNT	A/Singapore/GP1908/2015	2560	
B0031	Group B4	Day 22	08JUN2018	MNT	A/Singapore/GP1908/2015	20480	
B0032	Group B6	Day 1 Predose	19MAY2018	HAI	A/Singapore/GP1908/2015	10	
B0032	Group B6	Day 4	22MAY2018	HAI	A/Singapore/GP1908/2015	10	
B0032	Group B6	Day 8	26MAY2018	HAI	A/Singapore/GP1908/2015	40	
B0032	Group B6	Day 22	12JUN2018	HAI	A/Singapore/GP1908/2015	320	
B0032	Group B6	Day 61	18JUL2018	HAI	A/Singapore/GP1908/2015	320	
B0032	Group B6	Day 1 Predose	19MAY2018	MNT	A/Singapore/GP1908/2015	320	
B0032	Group B6	Day 22	12JUN2018	MNT	A/Singapore/GP1908/2015	10240	

Administration Site Key: FA = Forearm; UA= Upper arm
Treatments Part A: A1 = A/Sing MAP, 15 ug (FA); A2 = Afluria Quad IM (UA), 15 ug; A3 = Placebo MAP (FA); A4 = A/Sing Mono IM, 15 ug (UA)
Treatments Part B: B1 = A/Sing MAP, 15 ug (FA) with biopsy; B2 = Placebo MAP (FA) with biopsy
B3 = A/Sing MAP, 15 ug (FA); B4 = A/Sing MAP, 10 ug (FA); B5 = A/Sing MAP, 5 ug (FA); B6 = A/Sing MAP, 2.5 ug (FA)
B7 = Placebo MAP (FA); B8 = A/Sing MAP, 15 ug (UA); B9 = A/Sing IM, 15 ug, as 2018 Afluria Quad (UA)

Listing 16.2.6.8
Immunogenicity Results (HAI, MNT)

Part B

Randomisation Number	Group Treatment	Visit	Collection Date	Test	Virus	Titre	Comment
B0033	Group B5	Day 1 Predose	19MAY2018	HAI	A/Singapore/GP1908/2015	160	
B0033	Group B5	Day 4	22MAY2018	HAI	A/Singapore/GP1908/2015	160	
B0033	Group B5	Day 8	26MAY2018	HAI	A/Singapore/GP1908/2015	160	
B0033	Group B5	Day 22	09JUN2018	HAI	A/Singapore/GP1908/2015	160	
B0033	Group B5	Day 61	18JUL2018	HAI	A/Singapore/GP1908/2015	160	
B0033	Group B5	Day 1 Predose	19MAY2018	MNT	A/Singapore/GP1908/2015	10240	
B0033	Group B5	Day 22	09JUN2018	MNT	A/Singapore/GP1908/2015	20480	
B0034	Group B8	Day 1 Predose	19MAY2018	HAI	A/Singapore/GP1908/2015	5	
B0034	Group B8	Day 4	22MAY2018	HAI	A/Singapore/GP1908/2015	5	
B0034	Group B8	Day 8	26MAY2018	HAI	A/Singapore/GP1908/2015	20	
B0034	Group B8	Day 22	09JUN2018	HAI	A/Singapore/GP1908/2015	20	
B0034	Group B8	Day 61	18JUL2018	HAI	A/Singapore/GP1908/2015	20	
B0034	Group B8	Day 1 Predose	19MAY2018	MNT	A/Singapore/GP1908/2015	80	
B0034	Group B8	Day 22	09JUN2018	MNT	A/Singapore/GP1908/2015	640	
B0035	Group B9	Day 1 Predose	19MAY2018	HAI	A/Singapore/GP1908/2015	20	
B0035	Group B9	Day 4	22MAY2018	HAI	A/Singapore/GP1908/2015	20	
B0035	Group B9	Day 8	26MAY2018	HAI	A/Singapore/GP1908/2015	80	

Administration Site Key: FA = Forearm; UA= Upper arm
Treatments Part A: A1 = A/Sing MAP, 15 ug (FA); A2 = Afluria Quad IM (UA), 15 ug; A3 = Placebo MAP (FA); A4 = A/Sing Mono IM, 15 ug (UA)
Treatments Part B: B1 = A/Sing MAP, 15 ug (FA) with biopsy; B2 = Placebo MAP (FA) with biopsy
B3 = A/Sing MAP, 15 ug (FA); B4 = A/Sing MAP, 10 ug (FA); B5 = A/Sing MAP, 5 ug (FA); B6 = A/Sing MAP, 2.5 ug (FA)
B7 = Placebo MAP (FA); B8 = A/Sing MAP, 15 ug (UA); B9 = A/Sing IM, 15 ug, as 2018 Afluria Quad (UA)

Listing 16.2.6.8
Immunogenicity Results (HAI, MNT)

Part B

Randomisation Number	Group Treatment	Visit	Collection Date	Test	Virus	Titre	Comment
B0035	Group B9	Day 22	09JUN2018	HAI	A/Singapore/GP1908/2015	80	
B0035	Group B9	Day 61	18JUL2018	HAI	A/Singapore/GP1908/2015	40	
B0035	Group B9	Day 1 Predose	19MAY2018	MNT	A/Singapore/GP1908/2015	640	
B0035	Group B9	Day 22	09JUN2018	MNT	A/Singapore/GP1908/2015	2560	
B0036	Group B7	Day 1 Predose	19MAY2018	HAI	A/Singapore/GP1908/2015	5	
B0036	Group B7	Day 4	22MAY2018	HAI	A/Singapore/GP1908/2015	5	
B0036	Group B7	Day 8	26MAY2018	HAI	A/Singapore/GP1908/2015	5	
B0036	Group B7	Day 22	09JUN2018	HAI	A/Singapore/GP1908/2015	5	
B0036	Group B7	Day 61	18JUL2018	HAI	A/Singapore/GP1908/2015	5	
B0036	Group B7	Day 1 Predose	19MAY2018	MNT	A/Singapore/GP1908/2015	80	
B0036	Group B7	Day 22	09JUN2018	MNT	A/Singapore/GP1908/2015	80	
B0037	Group B4	Day 1 Predose	19MAY2018	HAI	A/Singapore/GP1908/2015	20	
B0037	Group B4	Day 4	22MAY2018	HAI	A/Singapore/GP1908/2015	20	
B0037	Group B4	Day 8	26MAY2018	HAI	A/Singapore/GP1908/2015	320	
B0037	Group B4	Day 22	09JUN2018	HAI	A/Singapore/GP1908/2015	320	
B0037	Group B4	Day 61	18JUL2018	HAI	A/Singapore/GP1908/2015	320	
B0037	Group B4	Day 1 Predose	19MAY2018	MNT	A/Singapore/GP1908/2015	640	

Administration Site Key: FA = Forearm; UA= Upper arm
Treatments Part A: A1 = A/Sing MAP, 15 ug (FA); A2 = Afluria Quad IM (UA), 15 ug; A3 = Placebo MAP (FA); A4 = A/Sing Mono IM, 15 ug (UA)
Treatments Part B: B1 = A/Sing MAP, 15 ug (FA) with biopsy; B2 = Placebo MAP (FA) with biopsy
B3 = A/Sing MAP, 15 ug (FA); B4 = A/Sing MAP, 10 ug (FA); B5 = A/Sing MAP, 5 ug (FA); B6 = A/Sing MAP, 2.5 ug (FA)
B7 = Placebo MAP (FA); B8 = A/Sing MAP, 15 ug (UA); B9 = A/Sing IM, 15 ug, as 2018 Afluria Quad (UA)

Listing 16.2.6.8
Immunogenicity Results (HAI, MNT)

Part B

Randomisation Number	Group Treatment	Visit	Collection Date	Test	Virus	Titre	Comment
B0037	Group B4	Day 22	09JUN2018	MNT	A/Singapore/GP1908/2015	10240	
B0038	Group B3	Day 1 Predose	19MAY2018	HAI	A/Singapore/GP1908/2015	10	
B0038	Group B3	Day 4	22MAY2018	HAI	A/Singapore/GP1908/2015	10	
B0038	Group B3	Day 8	26MAY2018	HAI	A/Singapore/GP1908/2015	80	
B0038	Group B3	Day 22	09JUN2018	HAI	A/Singapore/GP1908/2015	160	
B0038	Group B3	Day 61	21JUL2018	HAI	A/Singapore/GP1908/2015	80	
B0038	Group B3	Day 1 Predose	19MAY2018	MNT	A/Singapore/GP1908/2015	320	
B0038	Group B3	Day 22	09JUN2018	MNT	A/Singapore/GP1908/2015	5120	
B0039	Group B7	Day 1 Predose	19MAY2018	HAI	A/Singapore/GP1908/2015	20	
B0039	Group B7	Day 4	22MAY2018	HAI	A/Singapore/GP1908/2015	20	
B0039	Group B7	Day 8	26MAY2018	HAI	A/Singapore/GP1908/2015	20	
B0039	Group B7	Day 22	09JUN2018	HAI	A/Singapore/GP1908/2015	20	
B0039	Group B7	Day 61	18JUL2018	HAI	A/Singapore/GP1908/2015	20	
B0039	Group B7	Day 1 Predose	19MAY2018	MNT	A/Singapore/GP1908/2015	640	
B0039	Group B7	Day 22	09JUN2018	MNT	A/Singapore/GP1908/2015	640	
B0040	Group B9	Day 1 Predose	19MAY2018	HAI	A/Singapore/GP1908/2015	5	

Administration Site Key: FA = Forearm; UA= Upper arm
Treatments Part A: A1 = A/Sing MAP, 15 ug (FA); A2 = Afluria Quad IM (UA), 15 ug; A3 = Placebo MAP (FA); A4 = A/Sing Mono IM, 15 ug (UA)
Treatments Part B: B1 = A/Sing MAP, 15 ug (FA) with biopsy; B2 = Placebo MAP (FA) with biopsy
B3 = A/Sing MAP, 15 ug (FA); B4 = A/Sing MAP, 10 ug (FA); B5 = A/Sing MAP, 5 ug (FA); B6 = A/Sing MAP, 2.5 ug (FA)
B7 = Placebo MAP (FA); B8 = A/Sing MAP, 15 ug (UA); B9 = A/Sing IM, 15 ug, as 2018 Afluria Quad (UA)

Listing 16.2.6.8
Immunogenicity Results (HAI, MNT)

Part B

Randomisation Number	Group Treatment	Visit	Collection Date	Test	Virus	Titre	Comment
B0040	Group B9	Day 4	22MAY2018	HAI	A/Singapore/GP1908/2015	5	
B0040	Group B9	Day 8	26MAY2018	HAI	A/Singapore/GP1908/2015	40	
B0040	Group B9	Day 22	09JUN2018	HAI	A/Singapore/GP1908/2015	320	
B0040	Group B9	Day 61	18JUL2018	HAI	A/Singapore/GP1908/2015	320	
B0040	Group B9	Day 1 Predose	19MAY2018	MNT	A/Singapore/GP1908/2015	20	
B0040	Group B9	Day 22	09JUN2018	MNT	A/Singapore/GP1908/2015	10240	
B0041	Group B3	Day 1 Predose	19MAY2018	HAI	A/Singapore/GP1908/2015	320	
B0041	Group B3	Day 4	22MAY2018	HAI	A/Singapore/GP1908/2015	320	
B0041	Group B3	Day 8	26MAY2018	HAI	A/Singapore/GP1908/2015	320	
B0041	Group B3	Day 22	09JUN2018	HAI	A/Singapore/GP1908/2015	320	
B0041	Group B3	Day 61	20JUL2018	HAI	A/Singapore/GP1908/2015	320	
B0041	Group B3	Day 1 Predose	19MAY2018	MNT	A/Singapore/GP1908/2015	20480	
B0041	Group B3	Day 22	09JUN2018	MNT	A/Singapore/GP1908/2015	20480	
B0042	Group B5	Day 1 Predose	19MAY2018	HAI	A/Singapore/GP1908/2015	5	
B0042	Group B5	Day 4	22MAY2018	HAI	A/Singapore/GP1908/2015	5	
B0042	Group B5	Day 8	26MAY2018	HAI	A/Singapore/GP1908/2015	80	
B0042	Group B5	Day 22	09JUN2018	HAI	A/Singapore/GP1908/2015	320	

Administration Site Key: FA = Forearm; UA= Upper arm
Treatments Part A: A1 = A/Sing MAP, 15 ug (FA); A2 = Afluria Quad IM (UA), 15 ug; A3 = Placebo MAP (FA); A4 = A/Sing Mono IM, 15 ug (UA)
Treatments Part B: B1 = A/Sing MAP, 15 ug (FA) with biopsy; B2 = Placebo MAP (FA) with biopsy
B3 = A/Sing MAP, 15 ug (FA); B4 = A/Sing MAP, 10 ug (FA); B5 = A/Sing MAP, 5 ug (FA); B6 = A/Sing MAP, 2.5 ug (FA)
B7 = Placebo MAP (FA); B8 = A/Sing MAP, 15 ug (UA); B9 = A/Sing IM, 15 ug, as 2018 Afluria Quad (UA)

Listing 16.2.6.8
Immunogenicity Results (HAI, MNT)

Part B

Randomisation Number	Group Treatment	Visit	Collection Date	Test	Virus	Titre	Comment
B0042	Group B5	Day 61	18JUL2018	HAI	A/Singapore/GP1908/2015	640	
B0042	Group B5	Day 1 Predose	19MAY2018	MNT	A/Singapore/GP1908/2015	160	
B0042	Group B5	Day 22	09JUN2018	MNT	A/Singapore/GP1908/2015	10240	
B0043	Group B8	Day 1 Predose	19MAY2018	HAI	A/Singapore/GP1908/2015	40	
B0043	Group B8	Day 4	22MAY2018	HAI	A/Singapore/GP1908/2015	40	
B0043	Group B8	Day 8	26MAY2018	HAI	A/Singapore/GP1908/2015	640	
B0043	Group B8	Day 22	09JUN2018	HAI	A/Singapore/GP1908/2015	640	
B0043	Group B8	Day 61	18JUL2018	HAI	A/Singapore/GP1908/2015	640	
B0043	Group B8	Day 1 Predose	19MAY2018	MNT	A/Singapore/GP1908/2015	320	
B0043	Group B8	Day 22	09JUN2018	MNT	A/Singapore/GP1908/2015	20480	
B0044	Group B6	Day 1 Predose	19MAY2018	HAI	A/Singapore/GP1908/2015	<5	
B0044	Group B6	Day 4	22MAY2018	HAI	A/Singapore/GP1908/2015	<5	
B0044	Group B6	Day 8	26MAY2018	HAI	A/Singapore/GP1908/2015	5	
B0044	Group B6	Day 22	09JUN2018	HAI	A/Singapore/GP1908/2015	10	
B0044	Group B6	Day 61	18JUL2018	HAI	A/Singapore/GP1908/2015	10	
B0044	Group B6	Day 1 Predose	19MAY2018	MNT	A/Singapore/GP1908/2015	<20	
B0044	Group B6	Day 22	09JUN2018	MNT	A/Singapore/GP1908/2015	160	

Administration Site Key: FA = Forearm; UA= Upper arm
Treatments Part A: A1 = A/Sing MAP, 15 ug (FA); A2 = Afluria Quad IM (UA), 15 ug; A3 = Placebo MAP (FA); A4 = A/Sing Mono IM, 15 ug (UA)
Treatments Part B: B1 = A/Sing MAP, 15 ug (FA) with biopsy; B2 = Placebo MAP (FA) with biopsy
B3 = A/Sing MAP, 15 ug (FA); B4 = A/Sing MAP, 10 ug (FA); B5 = A/Sing MAP, 5 ug (FA); B6 = A/Sing MAP, 2.5 ug (FA)
B7 = Placebo MAP (FA); B8 = A/Sing MAP, 15 ug (UA); B9 = A/Sing IM, 15 ug, as 2018 Afluria Quad (UA)

Listing 16.2.6.8
Immunogenicity Results (HAI, MNT)

Part B

Randomisation Number	Group Treatment	Visit	Collection Date	Test	Virus	Titre	Comment
B0045	Group B4	Day 1 Predose	19MAY2018	HAI	A/Singapore/GP1908/2015	5	
B0045	Group B4	Day 4	22MAY2018	HAI	A/Singapore/GP1908/2015	5	
B0045	Group B4	Day 8	26MAY2018	HAI	A/Singapore/GP1908/2015	2560	
B0045	Group B4	Day 22	09JUN2018	HAI	A/Singapore/GP1908/2015	1280	
B0045	Group B4	Day 61	18JUL2018	HAI	A/Singapore/GP1908/2015	640	
B0045	Group B4	Day 1 Predose	19MAY2018	MNT	A/Singapore/GP1908/2015	80	
B0045	Group B4	Day 22	09JUN2018	MNT	A/Singapore/GP1908/2015	20480	
B0046	Group B7	Day 1 Predose	19MAY2018	HAI	A/Singapore/GP1908/2015	20	
B0046	Group B7	Day 4	22MAY2018	HAI	A/Singapore/GP1908/2015	20	
B0046	Group B7	Day 8	26MAY2018	HAI	A/Singapore/GP1908/2015	20	
B0046	Group B7	Day 22	12JUN2018	HAI	A/Singapore/GP1908/2015	20	
B0046	Group B7	Day 61	18JUL2018	HAI	A/Singapore/GP1908/2015	20	
B0046	Group B7	Day 1 Predose	19MAY2018	MNT	A/Singapore/GP1908/2015	160	
B0046	Group B7	Day 22	12JUN2018	MNT	A/Singapore/GP1908/2015	160	
B0047	Group B9	Day 1 Predose	19MAY2018	HAI	A/Singapore/GP1908/2015	40	
B0047	Group B9	Day 4	22MAY2018	HAI	A/Singapore/GP1908/2015	40	

Administration Site Key: FA = Forearm; UA= Upper arm
Treatments Part A: A1 = A/Sing MAP, 15 ug (FA); A2 = Afluria Quad IM (UA), 15 ug; A3 = Placebo MAP (FA); A4 = A/Sing Mono IM, 15 ug (UA)
Treatments Part B: B1 = A/Sing MAP, 15 ug (FA) with biopsy; B2 = Placebo MAP (FA) with biopsy
B3 = A/Sing MAP, 15 ug (FA); B4 = A/Sing MAP, 10 ug (FA); B5 = A/Sing MAP, 5 ug (FA); B6 = A/Sing MAP, 2.5 ug (FA)
B7 = Placebo MAP (FA); B8 = A/Sing MAP, 15 ug (UA); B9 = A/Sing IM, 15 ug, as 2018 Afluria Quad (UA)

Listing 16.2.6.8
Immunogenicity Results (HAI, MNT)

Part B

Randomisation Number	Group Treatment	Visit	Collection Date	Test	Virus	Titre	Comment
B0047	Group B9	Day 8	26MAY2018	HAI	A/Singapore/GP1908/2015	160	
B0047	Group B9	Day 22	09JUN2018	HAI	A/Singapore/GP1908/2015	640	
B0047	Group B9	Day 61	18JUL2018	HAI	A/Singapore/GP1908/2015	640	
B0047	Group B9	Day 1 Predose	19MAY2018	MNT	A/Singapore/GP1908/2015	320	
B0047	Group B9	Day 22	09JUN2018	MNT	A/Singapore/GP1908/2015	10240	
B0048	Group B6	Day 1 Predose	19MAY2018	HAI	A/Singapore/GP1908/2015	40	
B0048	Group B6	Day 4	22MAY2018	HAI	A/Singapore/GP1908/2015	40	
B0048	Group B6	Day 8	26MAY2018	HAI	A/Singapore/GP1908/2015	320	
B0048	Group B6	Day 22	09JUN2018	HAI	A/Singapore/GP1908/2015	1280	
B0048	Group B6	Day 61	18JUL2018	HAI	A/Singapore/GP1908/2015	640	
B0048	Group B6	Day 1 Predose	19MAY2018	MNT	A/Singapore/GP1908/2015	1280	
B0048	Group B6	Day 22	09JUN2018	MNT	A/Singapore/GP1908/2015	40960	
B0049	Group B4	Day 1 Predose	19MAY2018	HAI	A/Singapore/GP1908/2015	40	
B0049	Group B4	Day 4	22MAY2018	HAI	A/Singapore/GP1908/2015	40	
B0049	Group B4	Day 8	26MAY2018	HAI	A/Singapore/GP1908/2015	2560	
B0049	Group B4	Day 22	09JUN2018	HAI	A/Singapore/GP1908/2015	1280	
B0049	Group B4	Day 61	18JUL2018	HAI	A/Singapore/GP1908/2015	640	

Administration Site Key: FA = Forearm; UA= Upper arm
Treatments Part A: A1 = A/Sing MAP, 15 ug (FA); A2 = Afluria Quad IM (UA), 15 ug; A3 = Placebo MAP (FA); A4 = A/Sing Mono IM, 15 ug (UA)
Treatments Part B: B1 = A/Sing MAP, 15 ug (FA) with biopsy; B2 = Placebo MAP (FA) with biopsy
B3 = A/Sing MAP, 15 ug (FA); B4 = A/Sing MAP, 10 ug (FA); B5 = A/Sing MAP, 5 ug (FA); B6 = A/Sing MAP, 2.5 ug (FA)
B7 = Placebo MAP (FA); B8 = A/Sing MAP, 15 ug (UA); B9 = A/Sing IM, 15 ug, as 2018 Afluria Quad (UA)

Listing 16.2.6.8
Immunogenicity Results (HAI, MNT)

Part B

Randomisation Number	Group Treatment	Visit	Collection Date	Test	Virus	Titre	Comment
B0049	Group B4	Day 1 Predose	19MAY2018	MNT	A/Singapore/GP1908/2015	1280	
B0049	Group B4	Day 22	09JUN2018	MNT	A/Singapore/GP1908/2015	81920	
B0050	Group B8	Day 1 Predose	19MAY2018	HAI	A/Singapore/GP1908/2015	40	
B0050	Group B8	Day 4	22MAY2018	HAI	A/Singapore/GP1908/2015	80	
B0050	Group B8	Day 8	26MAY2018	HAI	A/Singapore/GP1908/2015	160	
B0050	Group B8	Day 22	09JUN2018	HAI	A/Singapore/GP1908/2015	640	
B0050	Group B8	Day 61	18JUL2018	HAI	A/Singapore/GP1908/2015	640	
B0050	Group B8	Day 1 Predose	19MAY2018	MNT	A/Singapore/GP1908/2015	1280	
B0050	Group B8	Day 22	09JUN2018	MNT	A/Singapore/GP1908/2015	10240	
B0051	Group B3	Day 1 Predose	25MAY2018	HAI	A/Singapore/GP1908/2015	40	
B0051	Group B3	Day 4	28MAY2018	HAI	A/Singapore/GP1908/2015	40	
B0051	Group B3	Day 8	01JUN2018	HAI	A/Singapore/GP1908/2015	160	
B0051	Group B3	Day 22	15JUN2018	HAI	A/Singapore/GP1908/2015	160	
B0051	Group B3	Day 61	24JUL2018	HAI	A/Singapore/GP1908/2015	80	
B0051	Group B3	Day 1 Predose	25MAY2018	MNT	A/Singapore/GP1908/2015	640	
B0051	Group B3	Day 22	15JUN2018	MNT	A/Singapore/GP1908/2015	2560	

Administration Site Key: FA = Forearm; UA= Upper arm
Treatments Part A: A1 = A/Sing MAP, 15 ug (FA); A2 = Afluria Quad IM (UA), 15 ug; A3 = Placebo MAP (FA); A4 = A/Sing Mono IM, 15 ug (UA)
Treatments Part B: B1 = A/Sing MAP, 15 ug (FA) with biopsy; B2 = Placebo MAP (FA) with biopsy
B3 = A/Sing MAP, 15 ug (FA); B4 = A/Sing MAP, 10 ug (FA); B5 = A/Sing MAP, 5 ug (FA); B6 = A/Sing MAP, 2.5 ug (FA)
B7 = Placebo MAP (FA); B8 = A/Sing MAP, 15 ug (UA); B9 = A/Sing IM, 15 ug, as 2018 Afluria Quad (UA)

Listing 16.2.6.8
Immunogenicity Results (HAI, MNT)

Part B

Randomisation Number	Group Treatment	Visit	Collection Date	Test	Virus	Titre	Comment
B0052	Group B5	Day 1 Predose	25MAY2018	HAI	A/Singapore/GP1908/2015	<5	
B0052	Group B5	Day 4	28MAY2018	HAI	A/Singapore/GP1908/2015	<5	
B0052	Group B5	Day 8	01JUN2018	HAI	A/Singapore/GP1908/2015	20	
B0052	Group B5	Day 22	15JUN2018	HAI	A/Singapore/GP1908/2015	20	
B0052	Group B5	Day 61	20JUL2018	HAI	A/Singapore/GP1908/2015	40	
B0052	Group B5	Day 1 Predose	25MAY2018	MNT	A/Singapore/GP1908/2015	<20	
B0052	Group B5	Day 22	15JUN2018	MNT	A/Singapore/GP1908/2015	640	
B0053	Group B6	Day 1 Predose	25MAY2018	HAI	A/Singapore/GP1908/2015	40	
B0053	Group B6	Day 4	28MAY2018	HAI	A/Singapore/GP1908/2015	40	
B0053	Group B6	Day 8	01JUN2018	HAI	A/Singapore/GP1908/2015	320	
B0053	Group B6	Day 22	15JUN2018	HAI	A/Singapore/GP1908/2015	320	
B0053	Group B6	Day 61	25JUL2018	HAI	A/Singapore/GP1908/2015	320	
B0053	Group B6	Day 1 Predose	25MAY2018	MNT	A/Singapore/GP1908/2015	2560	
B0053	Group B6	Day 22	15JUN2018	MNT	A/Singapore/GP1908/2015	20480	
B0054	Group B5	Day 1 Predose	25MAY2018	HAI	A/Singapore/GP1908/2015	20	
B0054	Group B5	Day 4	28MAY2018	HAI	A/Singapore/GP1908/2015	20	
B0054	Group B5	Day 8	01JUN2018	HAI	A/Singapore/GP1908/2015	320	

Administration Site Key: FA = Forearm; UA= Upper arm
Treatments Part A: A1 = A/Sing MAP, 15 ug (FA); A2 = Afluria Quad IM (UA), 15 ug; A3 = Placebo MAP (FA); A4 = A/Sing Mono IM, 15 ug (UA)
Treatments Part B: B1 = A/Sing MAP, 15 ug (FA) with biopsy; B2 = Placebo MAP (FA) with biopsy
B3 = A/Sing MAP, 15 ug (FA); B4 = A/Sing MAP, 10 ug (FA); B5 = A/Sing MAP, 5 ug (FA); B6 = A/Sing MAP, 2.5 ug (FA)
B7 = Placebo MAP (FA); B8 = A/Sing MAP, 15 ug (UA); B9 = A/Sing IM, 15 ug, as 2018 Afluria Quad (UA)

Listing 16.2.6.8
Immunogenicity Results (HAI, MNT)

Part B

Randomisation Number	Group Treatment	Visit	Collection Date	Test	Virus	Titre	Comment
B0054	Group B5	Day 22	15JUN2018	HAI	A/Singapore/GP1908/2015	640	
B0054	Group B5	Day 61	24JUL2018	HAI	A/Singapore/GP1908/2015	320	
B0054	Group B5	Day 1 Predose	25MAY2018	MNT	A/Singapore/GP1908/2015	1280	
B0054	Group B5	Day 22	15JUN2018	MNT	A/Singapore/GP1908/2015	81920	
B0055	Group B8	Day 1 Predose	25MAY2018	HAI	A/Singapore/GP1908/2015	10	
B0055	Group B8	Day 4	28MAY2018	HAI	A/Singapore/GP1908/2015	10	
B0055	Group B8	Day 8	01JUN2018	HAI	A/Singapore/GP1908/2015	160	
B0055	Group B8	Day 22	15JUN2018	HAI	A/Singapore/GP1908/2015	640	
B0055	Group B8	Day 61	24JUL2018	HAI	A/Singapore/GP1908/2015	320	
B0055	Group B8	Day 1 Predose	25MAY2018	MNT	A/Singapore/GP1908/2015	640	
B0055	Group B8	Day 22	15JUN2018	MNT	A/Singapore/GP1908/2015	10240	
B0056	Group B3	Day 1 Predose	25MAY2018	HAI	A/Singapore/GP1908/2015	10	
B0056	Group B3	Day 4	28MAY2018	HAI	A/Singapore/GP1908/2015	10	
B0056	Group B3	Day 8	01JUN2018	HAI	A/Singapore/GP1908/2015	640	
B0056	Group B3	Day 22	15JUN2018	HAI	A/Singapore/GP1908/2015	1280	
B0056	Group B3	Day 61	24JUL2018	HAI	A/Singapore/GP1908/2015	320	
B0056	Group B3	Day 1 Predose	25MAY2018	MNT	A/Singapore/GP1908/2015	320	

Administration Site Key: FA = Forearm; UA= Upper arm
Treatments Part A: A1 = A/Sing MAP, 15 ug (FA); A2 = Afluria Quad IM (UA), 15 ug; A3 = Placebo MAP (FA); A4 = A/Sing Mono IM, 15 ug (UA)
Treatments Part B: B1 = A/Sing MAP, 15 ug (FA) with biopsy; B2 = Placebo MAP (FA) with biopsy
B3 = A/Sing MAP, 15 ug (FA); B4 = A/Sing MAP, 10 ug (FA); B5 = A/Sing MAP, 5 ug (FA); B6 = A/Sing MAP, 2.5 ug (FA)
B7 = Placebo MAP (FA); B8 = A/Sing MAP, 15 ug (UA); B9 = A/Sing IM, 15 ug, as 2018 Afluria Quad (UA)

Listing 16.2.6.8
Immunogenicity Results (HAI, MNT)

Part B

Randomisation Number	Group Treatment	Visit	Collection Date	Test	Virus	Titre	Comment
B0056	Group B3	Day 22	15JUN2018	MNT	A/Singapore/GP1908/2015	40960	
B0057	Group B4	Day 1 Predose	25MAY2018	HAI	A/Singapore/GP1908/2015	20	
B0057	Group B4	Day 4	28MAY2018	HAI	A/Singapore/GP1908/2015	20	
B0057	Group B4	Day 8	01JUN2018	HAI	A/Singapore/GP1908/2015	80	
B0057	Group B4	Day 22	15JUN2018	HAI	A/Singapore/GP1908/2015	160	
B0057	Group B4	Day 61	24JUL2018	HAI	A/Singapore/GP1908/2015	160	
B0057	Group B4	Day 1 Predose	25MAY2018	MNT	A/Singapore/GP1908/2015	1280	
B0057	Group B4	Day 22	15JUN2018	MNT	A/Singapore/GP1908/2015	5120	
B0058	Group B9	Day 1 Predose	25MAY2018	HAI	A/Singapore/GP1908/2015	10	
B0058	Group B9	Day 4	28MAY2018	HAI	A/Singapore/GP1908/2015	10	
B0058	Group B9	Day 8	01JUN2018	HAI	A/Singapore/GP1908/2015	160	
B0058	Group B9	Day 22	16JUN2018	HAI	A/Singapore/GP1908/2015	160	
B0058	Group B9	Day 61	24JUL2018	HAI	A/Singapore/GP1908/2015	320	
B0058	Group B9	Day 1 Predose	25MAY2018	MNT	A/Singapore/GP1908/2015	320	
B0058	Group B9	Day 22	16JUN2018	MNT	A/Singapore/GP1908/2015	20480	
B0059	Group B7	Day 1 Predose	25MAY2018	HAI	A/Singapore/GP1908/2015	40	

Administration Site Key: FA = Forearm; UA= Upper arm
Treatments Part A: A1 = A/Sing MAP, 15 ug (FA); A2 = Afluria Quad IM (UA), 15 ug; A3 = Placebo MAP (FA); A4 = A/Sing Mono IM, 15 ug (UA)
Treatments Part B: B1 = A/Sing MAP, 15 ug (FA) with biopsy; B2 = Placebo MAP (FA) with biopsy
B3 = A/Sing MAP, 15 ug (FA); B4 = A/Sing MAP, 10 ug (FA); B5 = A/Sing MAP, 5 ug (FA); B6 = A/Sing MAP, 2.5 ug (FA)
B7 = Placebo MAP (FA); B8 = A/Sing MAP, 15 ug (UA); B9 = A/Sing IM, 15 ug, as 2018 Afluria Quad (UA)

Listing 16.2.6.8
Immunogenicity Results (HAI, MNT)

Part B

Randomisation Number	Group Treatment	Visit	Collection Date	Test	Virus	Titre	Comment
B0059	Group B7	Day 4	28MAY2018	HAI	A/Singapore/GP1908/2015	40	
B0059	Group B7	Day 8	01JUN2018	HAI	A/Singapore/GP1908/2015	40	
B0059	Group B7	Day 22	15JUN2018	HAI	A/Singapore/GP1908/2015	40	
B0059	Group B7	Day 61	24JUL2018	HAI	A/Singapore/GP1908/2015	40	
B0059	Group B7	Day 1 Predose	25MAY2018	MNT	A/Singapore/GP1908/2015	1280	
B0059	Group B7	Day 22	15JUN2018	MNT	A/Singapore/GP1908/2015	1280	
B0060	Group B3	Day 1 Predose	25MAY2018	HAI	A/Singapore/GP1908/2015	40	
B0060	Group B3	Day 4	28MAY2018	HAI	A/Singapore/GP1908/2015	40	
B0060	Group B3	Day 8	01JUN2018	HAI	A/Singapore/GP1908/2015	320	
B0060	Group B3	Day 22	15JUN2018	HAI	A/Singapore/GP1908/2015	320	
B0060	Group B3	Day 61	24JUL2018	HAI	A/Singapore/GP1908/2015	320	
B0060	Group B3	Day 1 Predose	25MAY2018	MNT	A/Singapore/GP1908/2015	1280	
B0060	Group B3	Day 22	15JUN2018	MNT	A/Singapore/GP1908/2015	20480	
B0061	Group B8	Day 1 Predose	25MAY2018	HAI	A/Singapore/GP1908/2015	20	
B0061	Group B8	Day 4	28MAY2018	HAI	A/Singapore/GP1908/2015	40	
B0061	Group B8	Day 8	01JUN2018	HAI	A/Singapore/GP1908/2015	320	
B0061	Group B8	Day 22	15JUN2018	HAI	A/Singapore/GP1908/2015	320	

Administration Site Key: FA = Forearm; UA= Upper arm
Treatments Part A: A1 = A/Sing MAP, 15 ug (FA); A2 = Afluria Quad IM (UA), 15 ug; A3 = Placebo MAP (FA); A4 = A/Sing Mono IM, 15 ug (UA)
Treatments Part B: B1 = A/Sing MAP, 15 ug (FA) with biopsy; B2 = Placebo MAP (FA) with biopsy
B3 = A/Sing MAP, 15 ug (FA); B4 = A/Sing MAP, 10 ug (FA); B5 = A/Sing MAP, 5 ug (FA); B6 = A/Sing MAP, 2.5 ug (FA)
B7 = Placebo MAP (FA); B8 = A/Sing MAP, 15 ug (UA); B9 = A/Sing IM, 15 ug, as 2018 Afluria Quad (UA)

Listing 16.2.6.8
Immunogenicity Results (HAI, MNT)

Part B

Randomisation Number	Group Treatment	Visit	Collection Date	Test	Virus	Titre	Comment
B0061	Group B8	Day 61	24JUL2018	HAI	A/Singapore/GP1908/2015	320	
B0061	Group B8	Day 1 Predose	25MAY2018	MNT	A/Singapore/GP1908/2015	160	
B0061	Group B8	Day 22	15JUN2018	MNT	A/Singapore/GP1908/2015	10240	
B0062	Group B5	Day 1 Predose	25MAY2018	HAI	A/Singapore/GP1908/2015	20	
B0062	Group B5	Day 4	29MAY2018	HAI	A/Singapore/GP1908/2015	20	
B0062	Group B5	Day 8	01JUN2018	HAI	A/Singapore/GP1908/2015	640	
B0062	Group B5	Day 22	15JUN2018	HAI	A/Singapore/GP1908/2015	640	
B0062	Group B5	Day 61	24JUL2018	HAI	A/Singapore/GP1908/2015	640	
B0062	Group B5	Day 1 Predose	25MAY2018	MNT	A/Singapore/GP1908/2015	320	
B0062	Group B5	Day 22	15JUN2018	MNT	A/Singapore/GP1908/2015	20480	
B0063	Group B7	Day 1 Predose	25MAY2018	HAI	A/Singapore/GP1908/2015	<5	
B0063	Group B7	Day 4	28MAY2018	HAI	A/Singapore/GP1908/2015	<5	
B0063	Group B7	Day 8	01JUN2018	HAI	A/Singapore/GP1908/2015	<5	
B0063	Group B7	Day 22	15JUN2018	HAI	A/Singapore/GP1908/2015	<5	
B0063	Group B7	Day 61	12JUL2018	HAI	A/Singapore/GP1908/2015	<5	
B0063	Group B7	Day 1 Predose	25MAY2018	MNT	A/Singapore/GP1908/2015	<20	
B0063	Group B7	Day 22	15JUN2018	MNT	A/Singapore/GP1908/2015	<20	

Administration Site Key: FA = Forearm; UA= Upper arm
Treatments Part A: A1 = A/Sing MAP, 15 ug (FA); A2 = Afluria Quad IM (UA), 15 ug; A3 = Placebo MAP (FA); A4 = A/Sing Mono IM, 15 ug (UA)
Treatments Part B: B1 = A/Sing MAP, 15 ug (FA) with biopsy; B2 = Placebo MAP (FA) with biopsy
B3 = A/Sing MAP, 15 ug (FA); B4 = A/Sing MAP, 10 ug (FA); B5 = A/Sing MAP, 5 ug (FA); B6 = A/Sing MAP, 2.5 ug (FA)
B7 = Placebo MAP (FA); B8 = A/Sing MAP, 15 ug (UA); B9 = A/Sing IM, 15 ug, as 2018 Afluria Quad (UA)

Listing 16.2.6.8
Immunogenicity Results (HAI, MNT)

Part B

Randomisation Number	Group Treatment	Visit	Collection Date	Test	Virus	Titre	Comment
B0064	Group B9	Day 1 Predose	25MAY2018	HAI	A/Singapore/GP1908/2015	20	
B0064	Group B9	Day 4	28MAY2018	HAI	A/Singapore/GP1908/2015	20	
B0064	Group B9	Day 8	01JUN2018	HAI	A/Singapore/GP1908/2015	160	
B0064	Group B9	Day 22	15JUN2018	HAI	A/Singapore/GP1908/2015	320	
B0064	Group B9	Day 61	24JUL2018	HAI	A/Singapore/GP1908/2015	160	
B0064	Group B9	Day 1 Predose	25MAY2018	MNT	A/Singapore/GP1908/2015	640	
B0064	Group B9	Day 22	15JUN2018	MNT	A/Singapore/GP1908/2015	10240	
B0065	Group B4	Day 1 Predose	25MAY2018	HAI	A/Singapore/GP1908/2015	40	
B0065	Group B4	Day 4	28MAY2018	HAI	A/Singapore/GP1908/2015	40	
B0065	Group B4	Day 8	01JUN2018	HAI	A/Singapore/GP1908/2015	2560	
B0065	Group B4	Day 22	15JUN2018	HAI	A/Singapore/GP1908/2015	2560	
B0065	Group B4	Day 61	24JUL2018	HAI	A/Singapore/GP1908/2015	1280	
B0065	Group B4	Day 1 Predose	25MAY2018	MNT	A/Singapore/GP1908/2015	640	
B0065	Group B4	Day 22	15JUN2018	MNT	A/Singapore/GP1908/2015	40960	
B0066	Group B6	Day 1 Predose	25MAY2018	HAI	A/Singapore/GP1908/2015	80	
B0066	Group B6	Day 4	28MAY2018	HAI	A/Singapore/GP1908/2015	160	

Administration Site Key: FA = Forearm; UA= Upper arm
Treatments Part A: A1 = A/Sing MAP, 15 ug (FA); A2 = Afluria Quad IM (UA), 15 ug; A3 = Placebo MAP (FA); A4 = A/Sing Mono IM, 15 ug (UA)
Treatments Part B: B1 = A/Sing MAP, 15 ug (FA) with biopsy; B2 = Placebo MAP (FA) with biopsy
B3 = A/Sing MAP, 15 ug (FA); B4 = A/Sing MAP, 10 ug (FA); B5 = A/Sing MAP, 5 ug (FA); B6 = A/Sing MAP, 2.5 ug (FA)
B7 = Placebo MAP (FA); B8 = A/Sing MAP, 15 ug (UA); B9 = A/Sing IM, 15 ug, as 2018 Afluria Quad (UA)

Listing 16.2.6.8
Immunogenicity Results (HAI, MNT)

Part B

Randomisation Number	Group Treatment	Visit	Collection Date	Test	Virus	Titre	Comment
B0066	Group B6	Day 8	01JUN2018	HAI	A/Singapore/GP1908/2015	320	
B0066	Group B6	Day 22	15JUN2018	HAI	A/Singapore/GP1908/2015	320	
B0066	Group B6	Day 61	24JUL2018	HAI	A/Singapore/GP1908/2015	320	
B0066	Group B6	Day 1 Predose	25MAY2018	MNT	A/Singapore/GP1908/2015	5120	
B0066	Group B6	Day 22	15JUN2018	MNT	A/Singapore/GP1908/2015	10240	
B0067	Group B8	Day 1 Predose	25MAY2018	HAI	A/Singapore/GP1908/2015	20	
B0067	Group B8	Day 4	28MAY2018	HAI	A/Singapore/GP1908/2015	20	
B0067	Group B8	Day 8	02JUN2018	HAI	A/Singapore/GP1908/2015	320	
B0067	Group B8	Day 22	15JUN2018	HAI	A/Singapore/GP1908/2015	160	
B0067	Group B8	Day 61	25JUL2018	HAI	A/Singapore/GP1908/2015	160	
B0067	Group B8	Day 1 Predose	25MAY2018	MNT	A/Singapore/GP1908/2015	640	
B0067	Group B8	Day 22	15JUN2018	MNT	A/Singapore/GP1908/2015	10240	
B0068	Group B5	Day 1 Predose	25MAY2018	HAI	A/Singapore/GP1908/2015	40	
B0068	Group B5	Day 4		HAI	A/Singapore/GP1908/2015	ND	
B0068	Group B5	Day 8	01JUN2018	HAI	A/Singapore/GP1908/2015	160	
B0068	Group B5	Day 22	16JUN2018	HAI	A/Singapore/GP1908/2015	160	
B0068	Group B5	Day 61	24JUL2018	HAI	A/Singapore/GP1908/2015	160	

Administration Site Key: FA = Forearm; UA= Upper arm
Treatments Part A: A1 = A/Sing MAP, 15 ug (FA); A2 = Afluria Quad IM (UA), 15 ug; A3 = Placebo MAP (FA); A4 = A/Sing Mono IM, 15 ug (UA)
Treatments Part B: B1 = A/Sing MAP, 15 ug (FA) with biopsy; B2 = Placebo MAP (FA) with biopsy
B3 = A/Sing MAP, 15 ug (FA); B4 = A/Sing MAP, 10 ug (FA); B5 = A/Sing MAP, 5 ug (FA); B6 = A/Sing MAP, 2.5 ug (FA)
B7 = Placebo MAP (FA); B8 = A/Sing MAP, 15 ug (UA); B9 = A/Sing IM, 15 ug, as 2018 Afluria Quad (UA)

Listing 16.2.6.8
Immunogenicity Results (HAI, MNT)

Part B

Randomisation Number	Group Treatment	Visit	Collection Date	Test	Virus	Titre	Comment
B0068	Group B5	Day 1 Predose	25MAY2018	MNT	A/Singapore/GP1908/2015	2560	
B0068	Group B5	Day 22	16JUN2018	MNT	A/Singapore/GP1908/2015	10240	
B0069	Group B7	Day 1 Predose	01JUN2018	HAI	A/Singapore/GP1908/2015	10	
B0069	Group B7	Day 4	04JUN2018	HAI	A/Singapore/GP1908/2015	10	
B0069	Group B7	Day 8	09JUN2018	HAI	A/Singapore/GP1908/2015	10	
B0069	Group B7	Day 22	22JUN2018	HAI	A/Singapore/GP1908/2015	10	
B0069	Group B7	Day 61	31JUL2018	HAI	A/Singapore/GP1908/2015	10	
B0069	Group B7	Day 1 Predose	01JUN2018	MNT	A/Singapore/GP1908/2015	320	
B0069	Group B7	Day 22	22JUN2018	MNT	A/Singapore/GP1908/2015	320	
B0070	Group B9	Day 1 Predose	01JUN2018	HAI	A/Singapore/GP1908/2015	20	
B0070	Group B9	Day 4	04JUN2018	HAI	A/Singapore/GP1908/2015	20	
B0070	Group B9	Day 8	08JUN2018	HAI	A/Singapore/GP1908/2015	1280	
B0070	Group B9	Day 22	22JUN2018	HAI	A/Singapore/GP1908/2015	640	
B0070	Group B9	Day 61	04JUL2018	HAI	A/Singapore/GP1908/2015	640	
B0070	Group B9	Day 1 Predose	01JUN2018	MNT	A/Singapore/GP1908/2015	1280	
B0070	Group B9	Day 22	22JUN2018	MNT	A/Singapore/GP1908/2015	40960	

Administration Site Key: FA = Forearm; UA= Upper arm
Treatments Part A: A1 = A/Sing MAP, 15 ug (FA); A2 = Afluria Quad IM (UA), 15 ug; A3 = Placebo MAP (FA); A4 = A/Sing Mono IM, 15 ug (UA)
Treatments Part B: B1 = A/Sing MAP, 15 ug (FA) with biopsy; B2 = Placebo MAP (FA) with biopsy
B3 = A/Sing MAP, 15 ug (FA); B4 = A/Sing MAP, 10 ug (FA); B5 = A/Sing MAP, 5 ug (FA); B6 = A/Sing MAP, 2.5 ug (FA)
B7 = Placebo MAP (FA); B8 = A/Sing MAP, 15 ug (UA); B9 = A/Sing IM, 15 ug, as 2018 Afluria Quad (UA)

Listing 16.2.6.8
Immunogenicity Results (HAI, MNT)

Part B

Randomisation Number	Group Treatment	Visit	Collection Date	Test	Virus	Titre	Comment
B0071	Group B4	Day 1 Predose	01JUN2018	HAI	A/Singapore/GP1908/2015	40	
B0071	Group B4	Day 4	04JUN2018	HAI	A/Singapore/GP1908/2015	40	
B0071	Group B4	Day 8	08JUN2018	HAI	A/Singapore/GP1908/2015	2560	
B0071	Group B4	Day 22	22JUN2018	HAI	A/Singapore/GP1908/2015	2560	
B0071	Group B4	Day 61	31JUL2018	HAI	A/Singapore/GP1908/2015	640	
B0071	Group B4	Day 1 Predose	01JUN2018	MNT	A/Singapore/GP1908/2015	640	
B0071	Group B4	Day 22	22JUN2018	MNT	A/Singapore/GP1908/2015	40960	
B0072	Group B3	Day 1 Predose	01JUN2018	HAI	A/Singapore/GP1908/2015	10	
B0072	Group B3	Day 4	04JUN2018	HAI	A/Singapore/GP1908/2015	10	
B0072	Group B3	Day 8	08JUN2018	HAI	A/Singapore/GP1908/2015	160	
B0072	Group B3	Day 22	22JUN2018	HAI	A/Singapore/GP1908/2015	160	
B0072	Group B3	Day 61	31JUL2018	HAI	A/Singapore/GP1908/2015	160	
B0072	Group B3	Day 1 Predose	01JUN2018	MNT	A/Singapore/GP1908/2015	640	
B0072	Group B3	Day 22	22JUN2018	MNT	A/Singapore/GP1908/2015	5120	
B0073	Group B6	Day 1 Predose	01JUN2018	HAI	A/Singapore/GP1908/2015	80	
B0073	Group B6	Day 4	04JUN2018	HAI	A/Singapore/GP1908/2015	80	
B0073	Group B6	Day 8	08JUN2018	HAI	A/Singapore/GP1908/2015	160	

Administration Site Key: FA = Forearm; UA= Upper arm
Treatments Part A: A1 = A/Sing MAP, 15 ug (FA); A2 = Afluria Quad IM (UA), 15 ug; A3 = Placebo MAP (FA); A4 = A/Sing Mono IM, 15 ug (UA)
Treatments Part B: B1 = A/Sing MAP, 15 ug (FA) with biopsy; B2 = Placebo MAP (FA) with biopsy
B3 = A/Sing MAP, 15 ug (FA); B4 = A/Sing MAP, 10 ug (FA); B5 = A/Sing MAP, 5 ug (FA); B6 = A/Sing MAP, 2.5 ug (FA)
B7 = Placebo MAP (FA); B8 = A/Sing MAP, 15 ug (UA); B9 = A/Sing IM, 15 ug, as 2018 Afluria Quad (UA)

Listing 16.2.6.8
Immunogenicity Results (HAI, MNT)

Part B

Randomisation Number	Group Treatment	Visit	Collection Date	Test	Virus	Titre	Comment
B0073	Group B6	Day 22	22JUN2018	HAI	A/Singapore/GP1908/2015	320	
B0073	Group B6	Day 61	31JUL2018	HAI	A/Singapore/GP1908/2015	160	
B0073	Group B6	Day 1 Predose	01JUN2018	MNT	A/Singapore/GP1908/2015	2560	
B0073	Group B6	Day 22	22JUN2018	MNT	A/Singapore/GP1908/2015	10240	
B0074	Group B9	Day 1 Predose	01JUN2018	HAI	A/Singapore/GP1908/2015	40	
B0074	Group B9	Day 4	04JUN2018	HAI	A/Singapore/GP1908/2015	40	
B0074	Group B9	Day 8	08JUN2018	HAI	A/Singapore/GP1908/2015	160	
B0074	Group B9	Day 22	22JUN2018	HAI	A/Singapore/GP1908/2015	320	
B0074	Group B9	Day 61	31JUL2018	HAI	A/Singapore/GP1908/2015	320	
B0074	Group B9	Day 1 Predose	01JUN2018	MNT	A/Singapore/GP1908/2015	1280	
B0074	Group B9	Day 22	22JUN2018	MNT	A/Singapore/GP1908/2015	20480	
B0075	Group B3	Day 1 Predose	01JUN2018	HAI	A/Singapore/GP1908/2015	40	
B0075	Group B3	Day 4	04JUN2018	HAI	A/Singapore/GP1908/2015	40	
B0075	Group B3	Day 8	08JUN2018	HAI	A/Singapore/GP1908/2015	1280	
B0075	Group B3	Day 22	22JUN2018	HAI	A/Singapore/GP1908/2015	1280	
B0075	Group B3	Day 61	31JUL2018	HAI	A/Singapore/GP1908/2015	640	
B0075	Group B3	Day 1 Predose	01JUN2018	MNT	A/Singapore/GP1908/2015	1280	

Administration Site Key: FA = Forearm; UA= Upper arm
Treatments Part A: A1 = A/Sing MAP, 15 ug (FA); A2 = Afluria Quad IM (UA), 15 ug; A3 = Placebo MAP (FA); A4 = A/Sing Mono IM, 15 ug (UA)
Treatments Part B: B1 = A/Sing MAP, 15 ug (FA) with biopsy; B2 = Placebo MAP (FA) with biopsy
B3 = A/Sing MAP, 15 ug (FA); B4 = A/Sing MAP, 10 ug (FA); B5 = A/Sing MAP, 5 ug (FA); B6 = A/Sing MAP, 2.5 ug (FA)
B7 = Placebo MAP (FA); B8 = A/Sing MAP, 15 ug (UA); B9 = A/Sing IM, 15 ug, as 2018 Afluria Quad (UA)

Listing 16.2.6.8
Immunogenicity Results (HAI, MNT)

Part B

Randomisation Number	Group Treatment	Visit	Collection Date	Test	Virus	Titre	Comment
B0075	Group B3	Day 22	22JUN2018	MNT	A/Singapore/GP1908/2015	40960	
B0077	Group B4	Day 1 Predose	01JUN2018	HAI	A/Singapore/GP1908/2015	5	
B0077	Group B4	Day 4	04JUN2018	HAI	A/Singapore/GP1908/2015	5	
B0077	Group B4	Day 8	08JUN2018	HAI	A/Singapore/GP1908/2015	320	
B0077	Group B4	Day 22	22JUN2018	HAI	A/Singapore/GP1908/2015	320	
B0077	Group B4	Day 61	30JUL2018	HAI	A/Singapore/GP1908/2015	80	
B0077	Group B4	Day 1 Predose	01JUN2018	MNT	A/Singapore/GP1908/2015	320	
B0077	Group B4	Day 22	22JUN2018	MNT	A/Singapore/GP1908/2015	20480	
B0078	Group B8	Day 1 Predose	01JUN2018	HAI	A/Singapore/GP1908/2015	10	
B0078	Group B8	Day 4	04JUN2018	HAI	A/Singapore/GP1908/2015	10	
B0078	Group B8	Day 8	08JUN2018	HAI	A/Singapore/GP1908/2015	320	
B0078	Group B8	Day 22	22JUN2018	HAI	A/Singapore/GP1908/2015	320	
B0078	Group B8	Day 61	06AUG2018	HAI	A/Singapore/GP1908/2015	160	
B0078	Group B8	Day 1 Predose	01JUN2018	MNT	A/Singapore/GP1908/2015	320	
B0078	Group B8	Day 22	22JUN2018	MNT	A/Singapore/GP1908/2015	10240	
B0079	Group B6	Day 1 Predose	01JUN2018	HAI	A/Singapore/GP1908/2015	10	

Administration Site Key: FA = Forearm; UA= Upper arm
Treatments Part A: A1 = A/Sing MAP, 15 ug (FA); A2 = Afluria Quad IM (UA), 15 ug; A3 = Placebo MAP (FA); A4 = A/Sing Mono IM, 15 ug (UA)
Treatments Part B: B1 = A/Sing MAP, 15 ug (FA) with biopsy; B2 = Placebo MAP (FA) with biopsy
B3 = A/Sing MAP, 15 ug (FA); B4 = A/Sing MAP, 10 ug (FA); B5 = A/Sing MAP, 5 ug (FA); B6 = A/Sing MAP, 2.5 ug (FA)
B7 = Placebo MAP (FA); B8 = A/Sing MAP, 15 ug (UA); B9 = A/Sing IM, 15 ug, as 2018 Afluria Quad (UA)

Listing 16.2.6.8
Immunogenicity Results (HAI, MNT)

Part B

Randomisation Number	Group Treatment	Visit	Collection Date	Test	Virus	Titre	Comment
B0079	Group B6	Day 4	04JUN2018	HAI	A/Singapore/GP1908/2015	10	
B0079	Group B6	Day 8	08JUN2018	HAI	A/Singapore/GP1908/2015	320	
B0079	Group B6	Day 22	22JUN2018	HAI	A/Singapore/GP1908/2015	320	
B0079	Group B6	Day 61	31JUL2018	HAI	A/Singapore/GP1908/2015	320	
B0079	Group B6	Day 1 Predose	01JUN2018	MNT	A/Singapore/GP1908/2015	640	
B0079	Group B6	Day 22	22JUN2018	MNT	A/Singapore/GP1908/2015	20480	
B0081	Group B6	Day 1 Predose	01JUN2018	HAI	A/Singapore/GP1908/2015	10	
B0081	Group B6	Day 4	04JUN2018	HAI	A/Singapore/GP1908/2015	20	
B0081	Group B6	Day 8	08JUN2018	HAI	A/Singapore/GP1908/2015	320	
B0081	Group B6	Day 22	23JUN2018	HAI	A/Singapore/GP1908/2015	320	
B0081	Group B6	Day 61	31JUL2018	HAI	A/Singapore/GP1908/2015	320	
B0081	Group B6	Day 1 Predose	01JUN2018	MNT	A/Singapore/GP1908/2015	320	
B0081	Group B6	Day 22	23JUN2018	MNT	A/Singapore/GP1908/2015	81920	
B0082	Group B3	Day 1 Predose	01JUN2018	HAI	A/Singapore/GP1908/2015	10	
B0082	Group B3	Day 4	04JUN2018	HAI	A/Singapore/GP1908/2015	5	
B0082	Group B3	Day 8	08JUN2018	HAI	A/Singapore/GP1908/2015	5	
B0082	Group B3	Day 22	22JUN2018	HAI	A/Singapore/GP1908/2015	5	

Administration Site Key: FA = Forearm; UA= Upper arm
Treatments Part A: A1 = A/Sing MAP, 15 ug (FA); A2 = Afluria Quad IM (UA), 15 ug; A3 = Placebo MAP (FA); A4 = A/Sing Mono IM, 15 ug (UA)
Treatments Part B: B1 = A/Sing MAP, 15 ug (FA) with biopsy; B2 = Placebo MAP (FA) with biopsy
B3 = A/Sing MAP, 15 ug (FA); B4 = A/Sing MAP, 10 ug (FA); B5 = A/Sing MAP, 5 ug (FA); B6 = A/Sing MAP, 2.5 ug (FA)
B7 = Placebo MAP (FA); B8 = A/Sing MAP, 15 ug (UA); B9 = A/Sing IM, 15 ug, as 2018 Afluria Quad (UA)

Listing 16.2.6.8
Immunogenicity Results (HAI, MNT)

Part B

Randomisation Number	Group Treatment	Visit	Collection Date	Test	Virus	Titre	Comment
B0082	Group B3	Day 61	31JUL2018	HAI	A/Singapore/GP1908/2015	10	
B0082	Group B3	Day 1 Predose	01JUN2018	MNT	A/Singapore/GP1908/2015	5120	
B0082	Group B3	Day 22	22JUN2018	MNT	A/Singapore/GP1908/2015	5120	
B0083	Group B8	Day 1 Predose	01JUN2018	HAI	A/Singapore/GP1908/2015	<5	
B0083	Group B8	Day 4	04JUN2018	HAI	A/Singapore/GP1908/2015	<5	
B0083	Group B8	Day 8	08JUN2018	HAI	A/Singapore/GP1908/2015	40	
B0083	Group B8	Day 22	22JUN2018	HAI	A/Singapore/GP1908/2015	80	
B0083	Group B8	Day 61	31JUL2018	HAI	A/Singapore/GP1908/2015	40	
B0083	Group B8	Day 1 Predose	01JUN2018	MNT	A/Singapore/GP1908/2015	320	
B0083	Group B8	Day 22	22JUN2018	MNT	A/Singapore/GP1908/2015	20480	
B0084	Group B5	Day 1 Predose	09JUN2018	HAI	A/Singapore/GP1908/2015	10	
B0084	Group B5	Day 4	12JUN2018	HAI	A/Singapore/GP1908/2015	20	
B0084	Group B5	Day 8	16JUN2018	HAI	A/Singapore/GP1908/2015	40	
B0084	Group B5	Day 22	30JUN2018	HAI	A/Singapore/GP1908/2015	160	
B0084	Group B5	Day 61	08AUG2018	HAI	A/Singapore/GP1908/2015	160	
B0084	Group B5	Day 1 Predose	09JUN2018	MNT	A/Singapore/GP1908/2015	1280	
B0084	Group B5	Day 22	30JUN2018	MNT	A/Singapore/GP1908/2015	10240	

Administration Site Key: FA = Forearm; UA= Upper arm
Treatments Part A: A1 = A/Sing MAP, 15 ug (FA); A2 = Afluria Quad IM (UA), 15 ug; A3 = Placebo MAP (FA); A4 = A/Sing Mono IM, 15 ug (UA)
Treatments Part B: B1 = A/Sing MAP, 15 ug (FA) with biopsy; B2 = Placebo MAP (FA) with biopsy
B3 = A/Sing MAP, 15 ug (FA); B4 = A/Sing MAP, 10 ug (FA); B5 = A/Sing MAP, 5 ug (FA); B6 = A/Sing MAP, 2.5 ug (FA)
B7 = Placebo MAP (FA); B8 = A/Sing MAP, 15 ug (UA); B9 = A/Sing IM, 15 ug, as 2018 Afluria Quad (UA)

Listing 16.2.6.8
Immunogenicity Results (HAI, MNT)

Part B

Randomisation Number	Group Treatment	Visit	Collection Date	Test	Virus	Titre	Comment
B0085	Group B4	Day 1 Predose	09JUN2018	HAI	A/Singapore/GP1908/2015	10	
B0085	Group B4	Day 4	12JUN2018	HAI	A/Singapore/GP1908/2015	10	
B0085	Group B4	Day 8	16JUN2018	HAI	A/Singapore/GP1908/2015	320	
B0085	Group B4	Day 22	30JUN2018	HAI	A/Singapore/GP1908/2015	320	
B0085	Group B4	Day 61	11AUG2018	HAI	A/Singapore/GP1908/2015	160	
B0085	Group B4	Day 1 Predose	09JUN2018	MNT	A/Singapore/GP1908/2015	160	
B0085	Group B4	Day 22	30JUN2018	MNT	A/Singapore/GP1908/2015	10240	
B0086	Group B9	Day 1 Predose	09JUN2018	HAI	A/Singapore/GP1908/2015	<5	
B0086	Group B9	Day 4	12JUN2018	HAI	A/Singapore/GP1908/2015	<5	
B0086	Group B9	Day 8	16JUN2018	HAI	A/Singapore/GP1908/2015	10	
B0086	Group B9	Day 22	02JUL2018	HAI	A/Singapore/GP1908/2015	20	
B0086	Group B9	Day 61	09AUG2018	HAI	A/Singapore/GP1908/2015	10	
B0086	Group B9	Day 1 Predose	09JUN2018	MNT	A/Singapore/GP1908/2015	<20	
B0086	Group B9	Day 22	02JUL2018	MNT	A/Singapore/GP1908/2015	160	
B0087	Group B7	Day 1 Predose	09JUN2018	HAI	A/Singapore/GP1908/2015	5	
B0087	Group B7	Day 4	12JUN2018	HAI	A/Singapore/GP1908/2015	5	

Administration Site Key: FA = Forearm; UA= Upper arm
Treatments Part A: A1 = A/Sing MAP, 15 ug (FA); A2 = Afluria Quad IM (UA), 15 ug; A3 = Placebo MAP (FA); A4 = A/Sing Mono IM, 15 ug (UA)
Treatments Part B: B1 = A/Sing MAP, 15 ug (FA) with biopsy; B2 = Placebo MAP (FA) with biopsy
B3 = A/Sing MAP, 15 ug (FA); B4 = A/Sing MAP, 10 ug (FA); B5 = A/Sing MAP, 5 ug (FA); B6 = A/Sing MAP, 2.5 ug (FA)
B7 = Placebo MAP (FA); B8 = A/Sing MAP, 15 ug (UA); B9 = A/Sing IM, 15 ug, as 2018 Afluria Quad (UA)

Listing 16.2.6.8
Immunogenicity Results (HAI, MNT)

Part B

Randomisation Number	Group Treatment	Visit	Collection Date	Test	Virus	Titre	Comment
B0087	Group B7	Day 8	16JUN2018	HAI	A/Singapore/GP1908/2015	5	
B0087	Group B7	Day 22	03JUL2018	HAI	A/Singapore/GP1908/2015	5	
B0087	Group B7	Day 61	07AUG2018	HAI	A/Singapore/GP1908/2015	5	
B0087	Group B7	Day 1 Predose	09JUN2018	MNT	A/Singapore/GP1908/2015	80	
B0087	Group B7	Day 22	03JUL2018	MNT	A/Singapore/GP1908/2015	40	
B0088	Group B9	Day 1 Predose	09JUN2018	HAI	A/Singapore/GP1908/2015	5	
B0088	Group B9	Day 4	12JUN2018	HAI	A/Singapore/GP1908/2015	5	
B0088	Group B9	Day 8	16JUN2018	HAI	A/Singapore/GP1908/2015	160	
B0088	Group B9	Day 22	30JUN2018	HAI	A/Singapore/GP1908/2015	640	
B0088	Group B9	Day 61	14AUG2018	HAI	A/Singapore/GP1908/2015	640	
B0088	Group B9	Day 1 Predose	09JUN2018	MNT	A/Singapore/GP1908/2015	80	
B0088	Group B9	Day 22	30JUN2018	MNT	A/Singapore/GP1908/2015	20480	
B0089	Group B5	Day 1 Predose	09JUN2018	HAI	A/Singapore/GP1908/2015	80	
B0089	Group B5	Day 4	12JUN2018	HAI	A/Singapore/GP1908/2015	80	
B0089	Group B5	Day 8	16JUN2018	HAI	A/Singapore/GP1908/2015	80	
B0089	Group B5	Day 22	30JUN2018	HAI	A/Singapore/GP1908/2015	80	
B0089	Group B5	Day 61	08AUG2018	HAI	A/Singapore/GP1908/2015	160	

Administration Site Key: FA = Forearm; UA= Upper arm
Treatments Part A: A1 = A/Sing MAP, 15 ug (FA); A2 = Afluria Quad IM (UA), 15 ug; A3 = Placebo MAP (FA); A4 = A/Sing Mono IM, 15 ug (UA)
Treatments Part B: B1 = A/Sing MAP, 15 ug (FA) with biopsy; B2 = Placebo MAP (FA) with biopsy
B3 = A/Sing MAP, 15 ug (FA); B4 = A/Sing MAP, 10 ug (FA); B5 = A/Sing MAP, 5 ug (FA); B6 = A/Sing MAP, 2.5 ug (FA)
B7 = Placebo MAP (FA); B8 = A/Sing MAP, 15 ug (UA); B9 = A/Sing IM, 15 ug, as 2018 Afluria Quad (UA)

Listing 16.2.6.8
Immunogenicity Results (HAI, MNT)

Part B

Randomisation Number	Group Treatment	Visit	Collection Date	Test	Virus	Titre	Comment
B0089	Group B5	Day 1 Predose	09JUN2018	MNT	A/Singapore/GP1908/2015	2560	
B0089	Group B5	Day 22	30JUN2018	MNT	A/Singapore/GP1908/2015	5120	
B0090	Group B6	Day 1 Predose	09JUN2018	HAI	A/Singapore/GP1908/2015	40	
B0090	Group B6	Day 4	12JUN2018	HAI	A/Singapore/GP1908/2015	40	
B0090	Group B6	Day 8	16JUN2018	HAI	A/Singapore/GP1908/2015	80	
B0090	Group B6	Day 22	02JUL2018	HAI	A/Singapore/GP1908/2015	80	
B0090	Group B6	Day 61	08AUG2018	HAI	A/Singapore/GP1908/2015	80	
B0090	Group B6	Day 1 Predose	09JUN2018	MNT	A/Singapore/GP1908/2015	1280	
B0090	Group B6	Day 22	02JUL2018	MNT	A/Singapore/GP1908/2015	2560	
B0091	Group B8	Day 1 Predose	09JUN2018	HAI	A/Singapore/GP1908/2015	<5	
B0091	Group B8	Day 4	12JUN2018	HAI	A/Singapore/GP1908/2015	<5	
B0091	Group B8	Day 8	16JUN2018	HAI	A/Singapore/GP1908/2015	640	
B0091	Group B8	Day 22	30JUN2018	HAI	A/Singapore/GP1908/2015	1280	
B0091	Group B8	Day 61	08AUG2018	HAI	A/Singapore/GP1908/2015	1280	
B0091	Group B8	Day 1 Predose	09JUN2018	MNT	A/Singapore/GP1908/2015	40	
B0091	Group B8	Day 22	30JUN2018	MNT	A/Singapore/GP1908/2015	40960	

Administration Site Key: FA = Forearm; UA= Upper arm
Treatments Part A: A1 = A/Sing MAP, 15 ug (FA); A2 = Afluria Quad IM (UA), 15 ug; A3 = Placebo MAP (FA); A4 = A/Sing Mono IM, 15 ug (UA)
Treatments Part B: B1 = A/Sing MAP, 15 ug (FA) with biopsy; B2 = Placebo MAP (FA) with biopsy
B3 = A/Sing MAP, 15 ug (FA); B4 = A/Sing MAP, 10 ug (FA); B5 = A/Sing MAP, 5 ug (FA); B6 = A/Sing MAP, 2.5 ug (FA)
B7 = Placebo MAP (FA); B8 = A/Sing MAP, 15 ug (UA); B9 = A/Sing IM, 15 ug, as 2018 Afluria Quad (UA)

Listing 16.2.6.8
Immunogenicity Results (HAI, MNT)

Part B

Randomisation Number	Group Treatment	Visit	Collection Date	Test	Virus	Titre	Comment
B0092	Group B4	Day 1 Predose	09JUN2018	HAI	A/Singapore/GP1908/2015	20	
B0092	Group B4	Day 4	12JUN2018	HAI	A/Singapore/GP1908/2015	20	
B0092	Group B4	Day 8	16JUN2018	HAI	A/Singapore/GP1908/2015	640	
B0092	Group B4	Day 22	30JUN2018	HAI	A/Singapore/GP1908/2015	640	
B0092	Group B4	Day 61	02AUG2018	HAI	A/Singapore/GP1908/2015	320	
B0092	Group B4	Day 1 Predose	09JUN2018	MNT	A/Singapore/GP1908/2015	160	
B0092	Group B4	Day 22	30JUN2018	MNT	A/Singapore/GP1908/2015	20480	
B0093	Group B7	Day 1 Predose	09JUN2018	HAI	A/Singapore/GP1908/2015	<5	
B0093	Group B7	Day 4	12JUN2018	HAI	A/Singapore/GP1908/2015	<5	
B0093	Group B7	Day 8	16JUN2018	HAI	A/Singapore/GP1908/2015	<5	
B0093	Group B7	Day 22	30JUN2018	HAI	A/Singapore/GP1908/2015	<5	
B0093	Group B7	Day 61	08AUG2018	HAI	A/Singapore/GP1908/2015	<5	
B0093	Group B7	Day 1 Predose	09JUN2018	MNT	A/Singapore/GP1908/2015	20	
B0093	Group B7	Day 22	30JUN2018	MNT	A/Singapore/GP1908/2015	20	
B0094	Group B3	Day 1 Predose	09JUN2018	HAI	A/Singapore/GP1908/2015	5	
B0094	Group B3	Day 4	12JUN2018	HAI	A/Singapore/GP1908/2015	5	
B0094	Group B3	Day 8	16JUN2018	HAI	A/Singapore/GP1908/2015	80	

Administration Site Key: FA = Forearm; UA= Upper arm
Treatments Part A: A1 = A/Sing MAP, 15 ug (FA); A2 = Afluria Quad IM (UA), 15 ug; A3 = Placebo MAP (FA); A4 = A/Sing Mono IM, 15 ug (UA)
Treatments Part B: B1 = A/Sing MAP, 15 ug (FA) with biopsy; B2 = Placebo MAP (FA) with biopsy
B3 = A/Sing MAP, 15 ug (FA); B4 = A/Sing MAP, 10 ug (FA); B5 = A/Sing MAP, 5 ug (FA); B6 = A/Sing MAP, 2.5 ug (FA)
B7 = Placebo MAP (FA); B8 = A/Sing MAP, 15 ug (UA); B9 = A/Sing IM, 15 ug, as 2018 Afluria Quad (UA)

Listing 16.2.6.8
Immunogenicity Results (HAI, MNT)

Part B

Randomisation Number	Group Treatment	Visit	Collection Date	Test	Virus	Titre	Comment
B0094	Group B3	Day 22	30JUN2018	HAI	A/Singapore/GP1908/2015	80	
B0094	Group B3	Day 61	08AUG2018	HAI	A/Singapore/GP1908/2015	80	
B0094	Group B3	Day 1 Predose	09JUN2018	MNT	A/Singapore/GP1908/2015	320	
B0094	Group B3	Day 22	30JUN2018	MNT	A/Singapore/GP1908/2015	5120	
B0095	Group B9	Day 1 Predose	09JUN2018	HAI	A/Singapore/GP1908/2015	5	
B0095	Group B9	Day 4	12JUN2018	HAI	A/Singapore/GP1908/2015	5	
B0095	Group B9	Day 8	16JUN2018	HAI	A/Singapore/GP1908/2015	40	
B0095	Group B9	Day 22	30JUN2018	HAI	A/Singapore/GP1908/2015	40	
B0095	Group B9	Day 61	08AUG2018	HAI	A/Singapore/GP1908/2015	40	
B0095	Group B9	Day 1 Predose	09JUN2018	MNT	A/Singapore/GP1908/2015	80	
B0095	Group B9	Day 22	30JUN2018	MNT	A/Singapore/GP1908/2015	5120	
B0096	Group B6	Day 1 Predose	09JUN2018	HAI	A/Singapore/GP1908/2015	5	
B0096	Group B6	Day 4	12JUN2018	HAI	A/Singapore/GP1908/2015	5	
B0096	Group B6	Day 8	16JUN2018	HAI	A/Singapore/GP1908/2015	40	
B0096	Group B6	Day 22	30JUN2018	HAI	A/Singapore/GP1908/2015	320	
B0096	Group B6	Day 61	08AUG2018	HAI	A/Singapore/GP1908/2015	160	
B0096	Group B6	Day 1 Predose	09JUN2018	MNT	A/Singapore/GP1908/2015	160	

Administration Site Key: FA = Forearm; UA= Upper arm
Treatments Part A: A1 = A/Sing MAP, 15 ug (FA); A2 = Afluria Quad IM (UA), 15 ug; A3 = Placebo MAP (FA); A4 = A/Sing Mono IM, 15 ug (UA)
Treatments Part B: B1 = A/Sing MAP, 15 ug (FA) with biopsy; B2 = Placebo MAP (FA) with biopsy
B3 = A/Sing MAP, 15 ug (FA); B4 = A/Sing MAP, 10 ug (FA); B5 = A/Sing MAP, 5 ug (FA); B6 = A/Sing MAP, 2.5 ug (FA)
B7 = Placebo MAP (FA); B8 = A/Sing MAP, 15 ug (UA); B9 = A/Sing IM, 15 ug, as 2018 Afluria Quad (UA)

Listing 16.2.6.8
Immunogenicity Results (HAI, MNT)

Part B

Randomisation Number	Group Treatment	Visit	Collection Date	Test	Virus	Titre	Comment
B0096	Group B6	Day 22	30JUN2018	MNT	A/Singapore/GP1908/2015	20480	
B0097	Group B5	Day 1 Predose	09JUN2018	HAI	A/Singapore/GP1908/2015	80	
B0097	Group B5	Day 4	12JUN2018	HAI	A/Singapore/GP1908/2015	80	
B0097	Group B5	Day 8	16JUN2018	HAI	A/Singapore/GP1908/2015	80	
B0097	Group B5	Day 22	30JUN2018	HAI	A/Singapore/GP1908/2015	80	
B0097	Group B5	Day 61	09AUG2018	HAI	A/Singapore/GP1908/2015	80	
B0097	Group B5	Day 1 Predose	09JUN2018	MNT	A/Singapore/GP1908/2015	2560	
B0097	Group B5	Day 22	30JUN2018	MNT	A/Singapore/GP1908/2015	5120	
B0098	Group B7	Day 1 Predose	09JUN2018	HAI	A/Singapore/GP1908/2015	20	
B0098	Group B7	Day 4	12JUN2018	HAI	A/Singapore/GP1908/2015	20	
B0098	Group B7	Day 8	16JUN2018	HAI	A/Singapore/GP1908/2015	20	
B0098	Group B7	Day 22	30JUN2018	HAI	A/Singapore/GP1908/2015	20	
B0098	Group B7	Day 61	07AUG2018	HAI	A/Singapore/GP1908/2015	20	
B0098	Group B7	Day 1 Predose	09JUN2018	MNT	A/Singapore/GP1908/2015	160	
B0098	Group B7	Day 22	30JUN2018	MNT	A/Singapore/GP1908/2015	320	
B0099	Group B4	Day 1 Predose	09JUN2018	HAI	A/Singapore/GP1908/2015	40	

Administration Site Key: FA = Forearm; UA= Upper arm
Treatments Part A: A1 = A/Sing MAP, 15 ug (FA); A2 = Afluria Quad IM (UA), 15 ug; A3 = Placebo MAP (FA); A4 = A/Sing Mono IM, 15 ug (UA)
Treatments Part B: B1 = A/Sing MAP, 15 ug (FA) with biopsy; B2 = Placebo MAP (FA) with biopsy
B3 = A/Sing MAP, 15 ug (FA); B4 = A/Sing MAP, 10 ug (FA); B5 = A/Sing MAP, 5 ug (FA); B6 = A/Sing MAP, 2.5 ug (FA)
B7 = Placebo MAP (FA); B8 = A/Sing MAP, 15 ug (UA); B9 = A/Sing IM, 15 ug, as 2018 Afluria Quad (UA)

Listing 16.2.6.8
Immunogenicity Results (HAI, MNT)

Part B

Randomisation Number	Group Treatment	Visit	Collection Date	Test	Virus	Titre	Comment
B0099	Group B4	Day 4	12JUN2018	HAI	A/Singapore/GP1908/2015	40	
B0099	Group B4	Day 8	16JUN2018	HAI	A/Singapore/GP1908/2015	320	
B0099	Group B4	Day 22	30JUN2018	HAI	A/Singapore/GP1908/2015	640	
B0099	Group B4	Day 61	06AUG2018	HAI	A/Singapore/GP1908/2015	640	
B0099	Group B4	Day 1 Predose	09JUN2018	MNT	A/Singapore/GP1908/2015	1280	
B0099	Group B4	Day 22	30JUN2018	MNT	A/Singapore/GP1908/2015	20480	
B0100	Group B8	Day 1 Predose	09JUN2018	HAI	A/Singapore/GP1908/2015	40	
B0100	Group B8	Day 4	12JUN2018	HAI	A/Singapore/GP1908/2015	40	
B0100	Group B8	Day 8	16JUN2018	HAI	A/Singapore/GP1908/2015	160	
B0100	Group B8	Day 22	30JUN2018	HAI	A/Singapore/GP1908/2015	640	
B0100	Group B8	Day 61	08AUG2018	HAI	A/Singapore/GP1908/2015	640	
B0100	Group B8	Day 1 Predose	09JUN2018	MNT	A/Singapore/GP1908/2015	1280	
B0100	Group B8	Day 22	30JUN2018	MNT	A/Singapore/GP1908/2015	20480	
B0101	Group B3	Day 1 Predose	09JUN2018	HAI	A/Singapore/GP1908/2015	40	
B0101	Group B3	Day 4	12JUN2018	HAI	A/Singapore/GP1908/2015	40	
B0101	Group B3	Day 8	16JUN2018	HAI	A/Singapore/GP1908/2015	40	
B0101	Group B3	Day 22	30JUN2018	HAI	A/Singapore/GP1908/2015	40	

Administration Site Key: FA = Forearm; UA= Upper arm
Treatments Part A: A1 = A/Sing MAP, 15 ug (FA); A2 = Afluria Quad IM (UA), 15 ug; A3 = Placebo MAP (FA); A4 = A/Sing Mono IM, 15 ug (UA)
Treatments Part B: B1 = A/Sing MAP, 15 ug (FA) with biopsy; B2 = Placebo MAP (FA) with biopsy
B3 = A/Sing MAP, 15 ug (FA); B4 = A/Sing MAP, 10 ug (FA); B5 = A/Sing MAP, 5 ug (FA); B6 = A/Sing MAP, 2.5 ug (FA)
B7 = Placebo MAP (FA); B8 = A/Sing MAP, 15 ug (UA); B9 = A/Sing IM, 15 ug, as 2018 Afluria Quad (UA)

Listing 16.2.6.8
Immunogenicity Results (HAI, MNT)

Part B

Randomisation Number	Group Treatment	Visit	Collection Date	Test	Virus	Titre	Comment
B0101	Group B3	Day 61	08AUG2018	HAI	A/Singapore/GP1908/2015	40	
B0101	Group B3	Day 1 Predose	09JUN2018	MNT	A/Singapore/GP1908/2015	640	
B0101	Group B3	Day 22	30JUN2018	MNT	A/Singapore/GP1908/2015	640	
B0102	Group B8	Day 1 Predose	09JUN2018	HAI	A/Singapore/GP1908/2015	10	
B0102	Group B8	Day 4	12JUN2018	HAI	A/Singapore/GP1908/2015	10	
B0102	Group B8	Day 8	16JUN2018	HAI	A/Singapore/GP1908/2015	640	
B0102	Group B8	Day 22	30JUN2018	HAI	A/Singapore/GP1908/2015	1280	
B0102	Group B8	Day 61	08AUG2018	HAI	A/Singapore/GP1908/2015	1280	
B0102	Group B8	Day 1 Predose	09JUN2018	MNT	A/Singapore/GP1908/2015	320	
B0102	Group B8	Day 22	30JUN2018	MNT	A/Singapore/GP1908/2015	81920	
B0103	Group B5	Day 1 Predose	09JUN2018	HAI	A/Singapore/GP1908/2015	320	
B0103	Group B5	Day 4	12JUN2018	HAI	A/Singapore/GP1908/2015	320	
B0103	Group B5	Day 8	16JUN2018	HAI	A/Singapore/GP1908/2015	640	
B0103	Group B5	Day 22	29JUN2018	HAI	A/Singapore/GP1908/2015	640	
B0103	Group B5	Day 61	08AUG2018	HAI	A/Singapore/GP1908/2015	640	
B0103	Group B5	Day 1 Predose	09JUN2018	MNT	A/Singapore/GP1908/2015	5120	
B0103	Group B5	Day 22	29JUN2018	MNT	A/Singapore/GP1908/2015	10240	

Administration Site Key: FA = Forearm; UA= Upper arm
Treatments Part A: A1 = A/Sing MAP, 15 ug (FA); A2 = Afluria Quad IM (UA), 15 ug; A3 = Placebo MAP (FA); A4 = A/Sing Mono IM, 15 ug (UA)
Treatments Part B: B1 = A/Sing MAP, 15 ug (FA) with biopsy; B2 = Placebo MAP (FA) with biopsy
B3 = A/Sing MAP, 15 ug (FA); B4 = A/Sing MAP, 10 ug (FA); B5 = A/Sing MAP, 5 ug (FA); B6 = A/Sing MAP, 2.5 ug (FA)
B7 = Placebo MAP (FA); B8 = A/Sing MAP, 15 ug (UA); B9 = A/Sing IM, 15 ug, as 2018 Afluria Quad (UA)

Listing 16.2.6.8
Immunogenicity Results (HAI, MNT)

Part B

Randomisation Number	Group Treatment	Visit	Collection Date	Test	Virus	Titre	Comment
B0104	Group B3	Day 1 Predose	09JUN2018	HAI	A/Singapore/GP1908/2015	20	
B0104	Group B3	Day 4	12JUN2018	HAI	A/Singapore/GP1908/2015	20	
B0104	Group B3	Day 8	16JUN2018	HAI	A/Singapore/GP1908/2015	640	
B0104	Group B3	Day 22	30JUN2018	HAI	A/Singapore/GP1908/2015	640	
B0104	Group B3	Day 61	10AUG2018	HAI	A/Singapore/GP1908/2015	320	
B0104	Group B3	Day 1 Predose	09JUN2018	MNT	A/Singapore/GP1908/2015	640	
B0104	Group B3	Day 22	30JUN2018	MNT	A/Singapore/GP1908/2015	10240	
B0105	Group B9	Day 1 Predose	09JUN2018	HAI	A/Singapore/GP1908/2015	40	
B0105	Group B9	Day 4	12JUN2018	HAI	A/Singapore/GP1908/2015	80	
B0105	Group B9	Day 8	16JUN2018	HAI	A/Singapore/GP1908/2015	160	
B0105	Group B9	Day 22	30JUN2018	HAI	A/Singapore/GP1908/2015	320	
B0105	Group B9	Day 61	08AUG2018	HAI	A/Singapore/GP1908/2015	320	
B0105	Group B9	Day 1 Predose	09JUN2018	MNT	A/Singapore/GP1908/2015	1280	
B0105	Group B9	Day 22	30JUN2018	MNT	A/Singapore/GP1908/2015	5120	
B0106	Group B4	Day 1 Predose	09JUN2018	HAI	A/Singapore/GP1908/2015	<5	
B0106	Group B4	Day 4	12JUN2018	HAI	A/Singapore/GP1908/2015	<5	

Administration Site Key: FA = Forearm; UA= Upper arm
Treatments Part A: A1 = A/Sing MAP, 15 ug (FA); A2 = Afluria Quad IM (UA), 15 ug; A3 = Placebo MAP (FA); A4 = A/Sing Mono IM, 15 ug (UA)
Treatments Part B: B1 = A/Sing MAP, 15 ug (FA) with biopsy; B2 = Placebo MAP (FA) with biopsy
B3 = A/Sing MAP, 15 ug (FA); B4 = A/Sing MAP, 10 ug (FA); B5 = A/Sing MAP, 5 ug (FA); B6 = A/Sing MAP, 2.5 ug (FA)
B7 = Placebo MAP (FA); B8 = A/Sing MAP, 15 ug (UA); B9 = A/Sing IM, 15 ug, as 2018 Afluria Quad (UA)

Listing 16.2.6.8
Immunogenicity Results (HAI, MNT)

Part B

Randomisation Number	Group Treatment	Visit	Collection Date	Test	Virus	Titre	Comment
B0106	Group B4	Day 8	16JUN2018	HAI	A/Singapore/GP1908/2015	40	
B0106	Group B4	Day 22	30JUN2018	HAI	A/Singapore/GP1908/2015	80	
B0106	Group B4	Day 61	08AUG2018	HAI	A/Singapore/GP1908/2015	40	
B0106	Group B4	Day 1 Predose	09JUN2018	MNT	A/Singapore/GP1908/2015	20	
B0106	Group B4	Day 22	30JUN2018	MNT	A/Singapore/GP1908/2015	5120	
B0107	Group B6	Day 1 Predose	09JUN2018	HAI	A/Singapore/GP1908/2015	40	
B0107	Group B6	Day 4	12JUN2018	HAI	A/Singapore/GP1908/2015	40	
B0107	Group B6	Day 8	16JUN2018	HAI	A/Singapore/GP1908/2015	80	
B0107	Group B6	Day 22	29JUN2018	HAI	A/Singapore/GP1908/2015	160	
B0107	Group B6	Day 61	08AUG2018	HAI	A/Singapore/GP1908/2015	160	
B0107	Group B6	Day 1 Predose	09JUN2018	MNT	A/Singapore/GP1908/2015	640	
B0107	Group B6	Day 22	29JUN2018	MNT	A/Singapore/GP1908/2015	2560	
B0108	Group B7	Day 1 Predose	09JUN2018	HAI	A/Singapore/GP1908/2015	<5	
B0108	Group B7	Day 4	12JUN2018	HAI	A/Singapore/GP1908/2015	<5	
B0108	Group B7	Day 8	16JUN2018	HAI	A/Singapore/GP1908/2015	<5	
B0108	Group B7	Day 22	30JUN2018	HAI	A/Singapore/GP1908/2015	<5	
B0108	Group B7	Day 61	28AUG2018	HAI	A/Singapore/GP1908/2015	<5	

Administration Site Key: FA = Forearm; UA= Upper arm
Treatments Part A: A1 = A/Sing MAP, 15 ug (FA); A2 = Afluria Quad IM (UA), 15 ug; A3 = Placebo MAP (FA); A4 = A/Sing Mono IM, 15 ug (UA)
Treatments Part B: B1 = A/Sing MAP, 15 ug (FA) with biopsy; B2 = Placebo MAP (FA) with biopsy
B3 = A/Sing MAP, 15 ug (FA); B4 = A/Sing MAP, 10 ug (FA); B5 = A/Sing MAP, 5 ug (FA); B6 = A/Sing MAP, 2.5 ug (FA)
B7 = Placebo MAP (FA); B8 = A/Sing MAP, 15 ug (UA); B9 = A/Sing IM, 15 ug, as 2018 Afluria Quad (UA)

Listing 16.2.6.8
Immunogenicity Results (HAI, MNT)

Part B

Randomisation Number	Group Treatment	Visit	Collection Date	Test	Virus	Titre	Comment
B0108	Group B7	Day 1 Predose	09JUN2018	MNT	A/Singapore/GP1908/2015	40	
B0108	Group B7	Day 22	30JUN2018	MNT	A/Singapore/GP1908/2015	40	
B0109	Group B6	Day 1 Predose	16JUN2018	HAI	A/Singapore/GP1908/2015	<5	
B0109	Group B6	Day 4	19JUN2018	HAI	A/Singapore/GP1908/2015	<5	
B0109	Group B6	Day 8	23JUN2018	HAI	A/Singapore/GP1908/2015	5	
B0109	Group B6	Day 22	07JUL2018	HAI	A/Singapore/GP1908/2015	5	
B0109	Group B6	Day 61	15AUG2018	HAI	A/Singapore/GP1908/2015	5	
B0109	Group B6	Day 1 Predose	16JUN2018	MNT	A/Singapore/GP1908/2015	<20	
B0109	Group B6	Day 22	07JUL2018	MNT	A/Singapore/GP1908/2015	160	
B0110	Group B3	Day 1 Predose	16JUN2018	HAI	A/Singapore/GP1908/2015	40	
B0110	Group B3	Day 4	19JUN2018	HAI	A/Singapore/GP1908/2015	40	
B0110	Group B3	Day 8	23JUN2018	HAI	A/Singapore/GP1908/2015	320	
B0110	Group B3	Day 22	07JUL2018	HAI	A/Singapore/GP1908/2015	1280	
B0110	Group B3	Day 61	30AUG2018	HAI	A/Singapore/GP1908/2015	640	
B0110	Group B3	Day 1 Predose	16JUN2018	MNT	A/Singapore/GP1908/2015	640	
B0110	Group B3	Day 22	07JUL2018	MNT	A/Singapore/GP1908/2015	20480	

Administration Site Key: FA = Forearm; UA= Upper arm
Treatments Part A: A1 = A/Sing MAP, 15 ug (FA); A2 = Afluria Quad IM (UA), 15 ug; A3 = Placebo MAP (FA); A4 = A/Sing Mono IM, 15 ug (UA)
Treatments Part B: B1 = A/Sing MAP, 15 ug (FA) with biopsy; B2 = Placebo MAP (FA) with biopsy
B3 = A/Sing MAP, 15 ug (FA); B4 = A/Sing MAP, 10 ug (FA); B5 = A/Sing MAP, 5 ug (FA); B6 = A/Sing MAP, 2.5 ug (FA)
B7 = Placebo MAP (FA); B8 = A/Sing MAP, 15 ug (UA); B9 = A/Sing IM, 15 ug, as 2018 Afluria Quad (UA)

Listing 16.2.6.8
Immunogenicity Results (HAI, MNT)

Part B

Randomisation Number	Group Treatment	Visit	Collection Date	Test	Virus	Titre	Comment
B0111	Group B8	Day 1 Predose	16JUN2018	HAI	A/Singapore/GP1908/2015	5	
B0111	Group B8	Day 4	19JUN2018	HAI	A/Singapore/GP1908/2015	5	
B0111	Group B8	Day 8	23JUN2018	HAI	A/Singapore/GP1908/2015	20	
B0111	Group B8	Day 22	07JUL2018	HAI	A/Singapore/GP1908/2015	40	
B0111	Group B8	Day 61	15AUG2018	HAI	A/Singapore/GP1908/2015	40	
B0111	Group B8	Day 1 Predose	16JUN2018	MNT	A/Singapore/GP1908/2015	80	
B0111	Group B8	Day 22	07JUL2018	MNT	A/Singapore/GP1908/2015	1280	
B0112	Group B4	Day 1 Predose	16JUN2018	HAI	A/Singapore/GP1908/2015	80	
B0112	Group B4	Day 4	19JUN2018	HAI	A/Singapore/GP1908/2015	80	
B0112	Group B4	Day 8	23JUN2018	HAI	A/Singapore/GP1908/2015	160	
B0112	Group B4	Day 22	07JUL2018	HAI	A/Singapore/GP1908/2015	640	
B0112	Group B4	Day 61	15AUG2018	HAI	A/Singapore/GP1908/2015	640	
B0112	Group B4	Day 1 Predose	16JUN2018	MNT	A/Singapore/GP1908/2015	1280	
B0112	Group B4	Day 22	07JUL2018	MNT	A/Singapore/GP1908/2015	20480	
B0113	Group B5	Day 1 Predose	16JUN2018	HAI	A/Singapore/GP1908/2015	10	
B0113	Group B5	Day 4	19JUN2018	HAI	A/Singapore/GP1908/2015	10	
B0113	Group B5	Day 8	23JUN2018	HAI	A/Singapore/GP1908/2015	320	

Administration Site Key: FA = Forearm; UA= Upper arm
Treatments Part A: A1 = A/Sing MAP, 15 ug (FA); A2 = Afluria Quad IM (UA), 15 ug; A3 = Placebo MAP (FA); A4 = A/Sing Mono IM, 15 ug (UA)
Treatments Part B: B1 = A/Sing MAP, 15 ug (FA) with biopsy; B2 = Placebo MAP (FA) with biopsy
B3 = A/Sing MAP, 15 ug (FA); B4 = A/Sing MAP, 10 ug (FA); B5 = A/Sing MAP, 5 ug (FA); B6 = A/Sing MAP, 2.5 ug (FA)
B7 = Placebo MAP (FA); B8 = A/Sing MAP, 15 ug (UA); B9 = A/Sing IM, 15 ug, as 2018 Afluria Quad (UA)

Listing 16.2.6.8
Immunogenicity Results (HAI, MNT)

Part B

Randomisation Number	Group Treatment	Visit	Collection Date	Test	Virus	Titre	Comment
B0113	Group B5	Day 22	07JUL2018	HAI	A/Singapore/GP1908/2015	640	
B0113	Group B5	Day 61	15AUG2018	HAI	A/Singapore/GP1908/2015	320	
B0113	Group B5	Day 1 Predose	16JUN2018	MNT	A/Singapore/GP1908/2015	160	
B0113	Group B5	Day 22	07JUL2018	MNT	A/Singapore/GP1908/2015	20480	
B0114	Group B7	Day 1 Predose	16JUN2018	HAI	A/Singapore/GP1908/2015	<5	
B0114	Group B7	Day 4	19JUN2018	HAI	A/Singapore/GP1908/2015	<5	
B0114	Group B7	Day 8	23JUN2018	HAI	A/Singapore/GP1908/2015	<5	
B0114	Group B7	Day 22	09JUL2018	HAI	A/Singapore/GP1908/2015	<5	
B0114	Group B7	Day 61	15AUG2018	HAI	A/Singapore/GP1908/2015	<5	
B0114	Group B7	Day 1 Predose	16JUN2018	MNT	A/Singapore/GP1908/2015	40	
B0114	Group B7	Day 22	09JUL2018	MNT	A/Singapore/GP1908/2015	40	
B0115	Group B9	Day 1 Predose	16JUN2018	HAI	A/Singapore/GP1908/2015	5	
B0115	Group B9	Day 4	19JUN2018	HAI	A/Singapore/GP1908/2015	5	
B0115	Group B9	Day 8	23JUN2018	HAI	A/Singapore/GP1908/2015	80	
B0115	Group B9	Day 22	07JUL2018	HAI	A/Singapore/GP1908/2015	160	
B0115	Group B9	Day 61	17AUG2018	HAI	A/Singapore/GP1908/2015	80	
B0115	Group B9	Day 1 Predose	16JUN2018	MNT	A/Singapore/GP1908/2015	40	

Administration Site Key: FA = Forearm; UA= Upper arm
Treatments Part A: A1 = A/Sing MAP, 15 ug (FA); A2 = Afluria Quad IM (UA), 15 ug; A3 = Placebo MAP (FA); A4 = A/Sing Mono IM, 15 ug (UA)
Treatments Part B: B1 = A/Sing MAP, 15 ug (FA) with biopsy; B2 = Placebo MAP (FA) with biopsy
B3 = A/Sing MAP, 15 ug (FA); B4 = A/Sing MAP, 10 ug (FA); B5 = A/Sing MAP, 5 ug (FA); B6 = A/Sing MAP, 2.5 ug (FA)
B7 = Placebo MAP (FA); B8 = A/Sing MAP, 15 ug (UA); B9 = A/Sing IM, 15 ug, as 2018 Afluria Quad (UA)

Listing 16.2.6.8
Immunogenicity Results (HAI, MNT)

Part B

Randomisation Number	Group Treatment	Visit	Collection Date	Test	Virus	Titre	Comment
B0115	Group B9	Day 22	07JUL2018	MNT	A/Singapore/GP1908/2015	5120	
B0116	Group B7	Day 1 Predose	16JUN2018	HAI	A/Singapore/GP1908/2015	160	
B0116	Group B7	Day 4	19JUN2018	HAI	A/Singapore/GP1908/2015	160	
B0116	Group B7	Day 8	23JUN2018	HAI	A/Singapore/GP1908/2015	160	
B0116	Group B7	Day 22	07JUL2018	HAI	A/Singapore/GP1908/2015	160	
B0116	Group B7	Day 61	15AUG2018	HAI	A/Singapore/GP1908/2015	160	
B0116	Group B7	Day 1 Predose	16JUN2018	MNT	A/Singapore/GP1908/2015	5120	
B0116	Group B7	Day 22	07JUL2018	MNT	A/Singapore/GP1908/2015	5120	
B0117	Group B9	Day 1 Predose	16JUN2018	HAI	A/Singapore/GP1908/2015	80	
B0117	Group B9	Day 4	19JUN2018	HAI	A/Singapore/GP1908/2015	80	
B0117	Group B9	Day 8	23JUN2018	HAI	A/Singapore/GP1908/2015	160	
B0117	Group B9	Day 22	07JUL2018	HAI	A/Singapore/GP1908/2015	160	
B0117	Group B9	Day 61	16AUG2018	HAI	A/Singapore/GP1908/2015	160	
B0117	Group B9	Day 1 Predose	16JUN2018	MNT	A/Singapore/GP1908/2015	2560	
B0117	Group B9	Day 22	07JUL2018	MNT	A/Singapore/GP1908/2015	2560	
B0118	Group B6	Day 1 Predose	16JUN2018	HAI	A/Singapore/GP1908/2015	10	

Administration Site Key: FA = Forearm; UA= Upper arm
Treatments Part A: A1 = A/Sing MAP, 15 ug (FA); A2 = Afluria Quad IM (UA), 15 ug; A3 = Placebo MAP (FA); A4 = A/Sing Mono IM, 15 ug (UA)
Treatments Part B: B1 = A/Sing MAP, 15 ug (FA) with biopsy; B2 = Placebo MAP (FA) with biopsy
B3 = A/Sing MAP, 15 ug (FA); B4 = A/Sing MAP, 10 ug (FA); B5 = A/Sing MAP, 5 ug (FA); B6 = A/Sing MAP, 2.5 ug (FA)
B7 = Placebo MAP (FA); B8 = A/Sing MAP, 15 ug (UA); B9 = A/Sing IM, 15 ug, as 2018 Afluria Quad (UA)

Listing 16.2.6.8
Immunogenicity Results (HAI, MNT)

Part B

Randomisation Number	Group Treatment	Visit	Collection Date	Test	Virus	Titre	Comment
B0118	Group B6	Day 4	19JUN2018	HAI	A/Singapore/GP1908/2015	10	
B0118	Group B6	Day 8	23JUN2018	HAI	A/Singapore/GP1908/2015	40	
B0118	Group B6	Day 22	07JUL2018	HAI	A/Singapore/GP1908/2015	40	
B0118	Group B6	Day 61	15AUG2018	HAI	A/Singapore/GP1908/2015	40	
B0118	Group B6	Day 1 Predose	16JUN2018	MNT	A/Singapore/GP1908/2015	160	
B0118	Group B6	Day 22	07JUL2018	MNT	A/Singapore/GP1908/2015	2560	
B0119	Group B4	Day 1 Predose	16JUN2018	HAI	A/Singapore/GP1908/2015	5	
B0119	Group B4	Day 4	19JUN2018	HAI	A/Singapore/GP1908/2015	5	
B0119	Group B4	Day 8	26JUN2018	HAI	A/Singapore/GP1908/2015	640	
B0119	Group B4	Day 22	07JUL2018	HAI	A/Singapore/GP1908/2015	320	
B0119	Group B4	Day 61	15AUG2018	HAI	A/Singapore/GP1908/2015	320	
B0119	Group B4	Day 1 Predose	16JUN2018	MNT	A/Singapore/GP1908/2015	80	
B0119	Group B4	Day 22	07JUL2018	MNT	A/Singapore/GP1908/2015	20480	
B0120	Group B3	Day 1 Predose	16JUN2018	HAI	A/Singapore/GP1908/2015	5	
B0120	Group B3	Day 4	19JUN2018	HAI	A/Singapore/GP1908/2015	5	
B0120	Group B3	Day 8	23JUN2018	HAI	A/Singapore/GP1908/2015	2560	
B0120	Group B3	Day 22	07JUL2018	HAI	A/Singapore/GP1908/2015	2560	

Administration Site Key: FA = Forearm; UA= Upper arm
Treatments Part A: A1 = A/Sing MAP, 15 ug (FA); A2 = Afluria Quad IM (UA), 15 ug; A3 = Placebo MAP (FA); A4 = A/Sing Mono IM, 15 ug (UA)
Treatments Part B: B1 = A/Sing MAP, 15 ug (FA) with biopsy; B2 = Placebo MAP (FA) with biopsy
B3 = A/Sing MAP, 15 ug (FA); B4 = A/Sing MAP, 10 ug (FA); B5 = A/Sing MAP, 5 ug (FA); B6 = A/Sing MAP, 2.5 ug (FA)
B7 = Placebo MAP (FA); B8 = A/Sing MAP, 15 ug (UA); B9 = A/Sing IM, 15 ug, as 2018 Afluria Quad (UA)

Listing 16.2.6.8
Immunogenicity Results (HAI, MNT)

Part B

Randomisation Number	Group Treatment	Visit	Collection Date	Test	Virus	Titre	Comment
B0120	Group B3	Day 61	15AUG2018	HAI	A/Singapore/GP1908/2015	1280	
B0120	Group B3	Day 1 Predose	16JUN2018	MNT	A/Singapore/GP1908/2015	640	
B0120	Group B3	Day 22	07JUL2018	MNT	A/Singapore/GP1908/2015	40960	
B0121	Group B8	Day 1 Predose	16JUN2018	HAI	A/Singapore/GP1908/2015	<5	
B0121	Group B8	Day 4	19JUN2018	HAI	A/Singapore/GP1908/2015	<5	
B0121	Group B8	Day 8	23JUN2018	HAI	A/Singapore/GP1908/2015	80	
B0121	Group B8	Day 22	09JUL2018	HAI	A/Singapore/GP1908/2015	160	
B0121	Group B8	Day 61	08AUG2018	HAI	A/Singapore/GP1908/2015	80	
B0121	Group B8	Day 1 Predose	16JUN2018	MNT	A/Singapore/GP1908/2015	20	
B0121	Group B8	Day 22	09JUL2018	MNT	A/Singapore/GP1908/2015	2560	
B0122	Group B5	Day 1 Predose	16JUN2018	HAI	A/Singapore/GP1908/2015	40	
B0122	Group B5	Day 4	19JUN2018	HAI	A/Singapore/GP1908/2015	40	
B0122	Group B5	Day 8	23JUN2018	HAI	A/Singapore/GP1908/2015	160	
B0122	Group B5	Day 22	07JUL2018	HAI	A/Singapore/GP1908/2015	1280	
B0122	Group B5	Day 61	15AUG2018	HAI	A/Singapore/GP1908/2015	640	
B0122	Group B5	Day 1 Predose	16JUN2018	MNT	A/Singapore/GP1908/2015	640	
B0122	Group B5	Day 22	07JUL2018	MNT	A/Singapore/GP1908/2015	20480	

Administration Site Key: FA = Forearm; UA= Upper arm
Treatments Part A: A1 = A/Sing MAP, 15 ug (FA); A2 = Afluria Quad IM (UA), 15 ug; A3 = Placebo MAP (FA); A4 = A/Sing Mono IM, 15 ug (UA)
Treatments Part B: B1 = A/Sing MAP, 15 ug (FA) with biopsy; B2 = Placebo MAP (FA) with biopsy
B3 = A/Sing MAP, 15 ug (FA); B4 = A/Sing MAP, 10 ug (FA); B5 = A/Sing MAP, 5 ug (FA); B6 = A/Sing MAP, 2.5 ug (FA)
B7 = Placebo MAP (FA); B8 = A/Sing MAP, 15 ug (UA); B9 = A/Sing IM, 15 ug, as 2018 Afluria Quad (UA)

Listing 16.2.6.8
Immunogenicity Results (HAI, MNT)

Part B

Randomisation Number	Group Treatment	Visit	Collection Date	Test	Virus	Titre	Comment
B0123	Group B6	Day 1 Predose	16JUN2018	HAI	A/Singapore/GP1908/2015	5	
B0123	Group B6	Day 4	19JUN2018	HAI	A/Singapore/GP1908/2015	5	
B0123	Group B6	Day 8	23JUN2018	HAI	A/Singapore/GP1908/2015	80	
B0123	Group B6	Day 22	06JUL2018	HAI	A/Singapore/GP1908/2015	320	
B0123	Group B6	Day 61	15AUG2018	HAI	A/Singapore/GP1908/2015	320	
B0123	Group B6	Day 1 Predose	16JUN2018	MNT	A/Singapore/GP1908/2015	80	
B0123	Group B6	Day 22	06JUL2018	MNT	A/Singapore/GP1908/2015	5120	
B0124	Group B4	Day 1 Predose	16JUN2018	HAI	A/Singapore/GP1908/2015	5	
B0124	Group B4	Day 4	19JUN2018	HAI	A/Singapore/GP1908/2015	5	
B0124	Group B4	Day 8	23JUN2018	HAI	A/Singapore/GP1908/2015	640	
B0124	Group B4	Day 22	07JUL2018	HAI	A/Singapore/GP1908/2015	640	
B0124	Group B4	Day 61	15AUG2018	HAI	A/Singapore/GP1908/2015	160	
B0124	Group B4	Day 1 Predose	16JUN2018	MNT	A/Singapore/GP1908/2015	160	
B0124	Group B4	Day 22	07JUL2018	MNT	A/Singapore/GP1908/2015	10240	
B0125	Group B7	Day 1 Predose	16JUN2018	HAI	A/Singapore/GP1908/2015	40	
B0125	Group B7	Day 4	19JUN2018	HAI	A/Singapore/GP1908/2015	40	

Administration Site Key: FA = Forearm; UA= Upper arm
Treatments Part A: A1 = A/Sing MAP, 15 ug (FA); A2 = Afluria Quad IM (UA), 15 ug; A3 = Placebo MAP (FA); A4 = A/Sing Mono IM, 15 ug (UA)
Treatments Part B: B1 = A/Sing MAP, 15 ug (FA) with biopsy; B2 = Placebo MAP (FA) with biopsy
B3 = A/Sing MAP, 15 ug (FA); B4 = A/Sing MAP, 10 ug (FA); B5 = A/Sing MAP, 5 ug (FA); B6 = A/Sing MAP, 2.5 ug (FA)
B7 = Placebo MAP (FA); B8 = A/Sing MAP, 15 ug (UA); B9 = A/Sing IM, 15 ug, as 2018 Afluria Quad (UA)

Listing 16.2.6.8
Immunogenicity Results (HAI, MNT)

Part B

Randomisation Number	Group Treatment	Visit	Collection Date	Test	Virus	Titre	Comment
B0125	Group B7	Day 8	22JUN2018	HAI	A/Singapore/GP1908/2015	40	
B0125	Group B7	Day 22	07JUL2018	HAI	A/Singapore/GP1908/2015	40	
B0125	Group B7	Day 61	15AUG2018	HAI	A/Singapore/GP1908/2015	40	
B0125	Group B7	Day 1 Predose	16JUN2018	MNT	A/Singapore/GP1908/2015	640	
B0125	Group B7	Day 22	07JUL2018	MNT	A/Singapore/GP1908/2015	640	
B0126	Group B3	Day 1 Predose	16JUN2018	HAI	A/Singapore/GP1908/2015	40	
B0126	Group B3	Day 4	19JUN2018	HAI	A/Singapore/GP1908/2015	80	
B0126	Group B3	Day 8	23JUN2018	HAI	A/Singapore/GP1908/2015	160	
B0126	Group B3	Day 22	06JUL2018	HAI	A/Singapore/GP1908/2015	320	
B0126	Group B3	Day 61	15AUG2018	HAI	A/Singapore/GP1908/2015	160	
B0126	Group B3	Day 1 Predose	16JUN2018	MNT	A/Singapore/GP1908/2015	10240	
B0126	Group B3	Day 22	06JUL2018	MNT	A/Singapore/GP1908/2015	40960	
B0127	Group B9	Day 1 Predose	16JUN2018	HAI	A/Singapore/GP1908/2015	40	
B0127	Group B9	Day 4	19JUN2018	HAI	A/Singapore/GP1908/2015	40	
B0127	Group B9	Day 8	23JUN2018	HAI	A/Singapore/GP1908/2015	160	
B0127	Group B9	Day 22	13JUL2018	HAI	A/Singapore/GP1908/2015	160	
B0127	Group B9	Day 61	20AUG2018	HAI	A/Singapore/GP1908/2015	80	

Administration Site Key: FA = Forearm; UA= Upper arm
Treatments Part A: A1 = A/Sing MAP, 15 ug (FA); A2 = Afluria Quad IM (UA), 15 ug; A3 = Placebo MAP (FA); A4 = A/Sing Mono IM, 15 ug (UA)
Treatments Part B: B1 = A/Sing MAP, 15 ug (FA) with biopsy; B2 = Placebo MAP (FA) with biopsy
B3 = A/Sing MAP, 15 ug (FA); B4 = A/Sing MAP, 10 ug (FA); B5 = A/Sing MAP, 5 ug (FA); B6 = A/Sing MAP, 2.5 ug (FA)
B7 = Placebo MAP (FA); B8 = A/Sing MAP, 15 ug (UA); B9 = A/Sing IM, 15 ug, as 2018 Afluria Quad (UA)

Listing 16.2.6.8
Immunogenicity Results (HAI, MNT)

Part B

Randomisation Number	Group Treatment	Visit	Collection Date	Test	Virus	Titre	Comment
B0127	Group B9	Day 1 Predose	16JUN2018	MNT	A/Singapore/GP1908/2015	1280	
B0127	Group B9	Day 22	13JUL2018	MNT	A/Singapore/GP1908/2015	5120	
B0128	Group B5	Day 1 Predose	23JUN2018	HAI	A/Singapore/GP1908/2015	20	
B0128	Group B5	Day 4	26JUN2018	HAI	A/Singapore/GP1908/2015	20	
B0128	Group B5	Day 8	30JUN2018	HAI	A/Singapore/GP1908/2015	1280	
B0128	Group B5	Day 22	14JUL2018	HAI	A/Singapore/GP1908/2015	2560	
B0128	Group B5	Day 61	22AUG2018	HAI	A/Singapore/GP1908/2015	1280	
B0128	Group B5	Day 1 Predose	23JUN2018	MNT	A/Singapore/GP1908/2015	320	
B0128	Group B5	Day 22	14JUL2018	MNT	A/Singapore/GP1908/2015	40960	
B0129	Group B8	Day 1 Predose	23JUN2018	HAI	A/Singapore/GP1908/2015	80	
B0129	Group B8	Day 4	26JUN2018	HAI	A/Singapore/GP1908/2015	80	
B0129	Group B8	Day 8	30JUN2018	HAI	A/Singapore/GP1908/2015	640	
B0129	Group B8	Day 22	14JUL2018	HAI	A/Singapore/GP1908/2015	1280	
B0129	Group B8	Day 61	22AUG2018	HAI	A/Singapore/GP1908/2015	1280	
B0129	Group B8	Day 1 Predose	23JUN2018	MNT	A/Singapore/GP1908/2015	2560	
B0129	Group B8	Day 22	14JUL2018	MNT	A/Singapore/GP1908/2015	81920	

Administration Site Key: FA = Forearm; UA= Upper arm
Treatments Part A: A1 = A/Sing MAP, 15 ug (FA); A2 = Afluria Quad IM (UA), 15 ug; A3 = Placebo MAP (FA); A4 = A/Sing Mono IM, 15 ug (UA)
Treatments Part B: B1 = A/Sing MAP, 15 ug (FA) with biopsy; B2 = Placebo MAP (FA) with biopsy
B3 = A/Sing MAP, 15 ug (FA); B4 = A/Sing MAP, 10 ug (FA); B5 = A/Sing MAP, 5 ug (FA); B6 = A/Sing MAP, 2.5 ug (FA)
B7 = Placebo MAP (FA); B8 = A/Sing MAP, 15 ug (UA); B9 = A/Sing IM, 15 ug, as 2018 Afluria Quad (UA)

Listing 16.2.6.8
Immunogenicity Results (HAI, MNT)

Part B

Randomisation Number	Group Treatment	Visit	Collection Date	Test	Virus	Titre	Comment
B0130	Group B5	Day 1 Predose	23JUN2018	HAI	A/Singapore/GP1908/2015	20	
B0130	Group B5	Day 4	26JUN2018	HAI	A/Singapore/GP1908/2015	20	
B0130	Group B5	Day 8	30JUN2018	HAI	A/Singapore/GP1908/2015	320	
B0130	Group B5	Day 22	14JUL2018	HAI	A/Singapore/GP1908/2015	640	
B0130	Group B5	Day 61	22AUG2018	HAI	A/Singapore/GP1908/2015	640	
B0130	Group B5	Day 1 Predose	23JUN2018	MNT	A/Singapore/GP1908/2015	320	
B0130	Group B5	Day 22	14JUL2018	MNT	A/Singapore/GP1908/2015	20480	
B0131	Group B8	Day 1 Predose	23JUN2018	HAI	A/Singapore/GP1908/2015	20	
B0131	Group B8	Day 4	26JUN2018	HAI	A/Singapore/GP1908/2015	20	
B0131	Group B8	Day 8	30JUN2018	HAI	A/Singapore/GP1908/2015	640	
B0131	Group B8	Day 22	14JUL2018	HAI	A/Singapore/GP1908/2015	640	
B0131	Group B8	Day 61	22AUG2018	HAI	A/Singapore/GP1908/2015	320	
B0131	Group B8	Day 1 Predose	23JUN2018	MNT	A/Singapore/GP1908/2015	160	
B0131	Group B8	Day 22	14JUL2018	MNT	A/Singapore/GP1908/2015	20480	
B0132	Group B6	Day 1 Predose	23JUN2018	HAI	A/Singapore/GP1908/2015	5	
B0132	Group B6	Day 4	26JUN2018	HAI	A/Singapore/GP1908/2015	5	
B0132	Group B6	Day 8	30JUN2018	HAI	A/Singapore/GP1908/2015	40	

Administration Site Key: FA = Forearm; UA= Upper arm
Treatments Part A: A1 = A/Sing MAP, 15 ug (FA); A2 = Afluria Quad IM (UA), 15 ug; A3 = Placebo MAP (FA); A4 = A/Sing Mono IM, 15 ug (UA)
Treatments Part B: B1 = A/Sing MAP, 15 ug (FA) with biopsy; B2 = Placebo MAP (FA) with biopsy
B3 = A/Sing MAP, 15 ug (FA); B4 = A/Sing MAP, 10 ug (FA); B5 = A/Sing MAP, 5 ug (FA); B6 = A/Sing MAP, 2.5 ug (FA)
B7 = Placebo MAP (FA); B8 = A/Sing MAP, 15 ug (UA); B9 = A/Sing IM, 15 ug, as 2018 Afluria Quad (UA)

Listing 16.2.6.8
Immunogenicity Results (HAI, MNT)

Part B

Randomisation Number	Group Treatment	Visit	Collection Date	Test	Virus	Titre	Comment
B0132	Group B6	Day 22	14JUL2018	HAI	A/Singapore/GP1908/2015	160	
B0132	Group B6	Day 61	23AUG2018	HAI	A/Singapore/GP1908/2015	160	
B0132	Group B6	Day 1 Predose	23JUN2018	MNT	A/Singapore/GP1908/2015	80	
B0132	Group B6	Day 22	14JUL2018	MNT	A/Singapore/GP1908/2015	1280	
B0133	Group B4	Day 1 Predose	23JUN2018	HAI	A/Singapore/GP1908/2015	40	
B0133	Group B4	Day 4	26JUN2018	HAI	A/Singapore/GP1908/2015	40	
B0133	Group B4	Day 8	30JUN2018	HAI	A/Singapore/GP1908/2015	160	
B0133	Group B4	Day 22	14JUL2018	HAI	A/Singapore/GP1908/2015	160	
B0133	Group B4	Day 61	22AUG2018	HAI	A/Singapore/GP1908/2015	80	
B0133	Group B4	Day 1 Predose	23JUN2018	MNT	A/Singapore/GP1908/2015	1280	
B0133	Group B4	Day 22	14JUL2018	MNT	A/Singapore/GP1908/2015	10240	
B0134	Group B3	Day 1 Predose	23JUN2018	HAI	A/Singapore/GP1908/2015	160	
B0134	Group B3	Day 4	26JUN2018	HAI	A/Singapore/GP1908/2015	160	
B0134	Group B3	Day 8	30JUN2018	HAI	A/Singapore/GP1908/2015	640	
B0134	Group B3	Day 22	14JUL2018	HAI	A/Singapore/GP1908/2015	640	
B0134	Group B3	Day 61	23AUG2018	HAI	A/Singapore/GP1908/2015	640	
B0134	Group B3	Day 1 Predose	23JUN2018	MNT	A/Singapore/GP1908/2015	2560	

Administration Site Key: FA = Forearm; UA= Upper arm
Treatments Part A: A1 = A/Sing MAP, 15 ug (FA); A2 = Afluria Quad IM (UA), 15 ug; A3 = Placebo MAP (FA); A4 = A/Sing Mono IM, 15 ug (UA)
Treatments Part B: B1 = A/Sing MAP, 15 ug (FA) with biopsy; B2 = Placebo MAP (FA) with biopsy
B3 = A/Sing MAP, 15 ug (FA); B4 = A/Sing MAP, 10 ug (FA); B5 = A/Sing MAP, 5 ug (FA); B6 = A/Sing MAP, 2.5 ug (FA)
B7 = Placebo MAP (FA); B8 = A/Sing MAP, 15 ug (UA); B9 = A/Sing IM, 15 ug, as 2018 Afluria Quad (UA)

Listing 16.2.6.8
Immunogenicity Results (HAI, MNT)

Part B

Randomisation Number	Group Treatment	Visit	Collection Date	Test	Virus	Titre	Comment
B0134	Group B3	Day 22	14JUL2018	MNT	A/Singapore/GP1908/2015	10240	
B0135	Group B7	Day 1 Predose	23JUN2018	HAI	A/Singapore/GP1908/2015	20	
B0135	Group B7	Day 4	26JUN2018	HAI	A/Singapore/GP1908/2015	20	
B0135	Group B7	Day 8	30JUN2018	HAI	A/Singapore/GP1908/2015	20	
B0135	Group B7	Day 22	14JUL2018	HAI	A/Singapore/GP1908/2015	20	
B0135	Group B7	Day 61	22AUG2018	HAI	A/Singapore/GP1908/2015	20	
B0135	Group B7	Day 1 Predose	23JUN2018	MNT	A/Singapore/GP1908/2015	320	
B0135	Group B7	Day 22	14JUL2018	MNT	A/Singapore/GP1908/2015	320	
B0136	Group B9	Day 1 Predose	23JUN2018	HAI	A/Singapore/GP1908/2015	5	
B0136	Group B9	Day 4	26JUN2018	HAI	A/Singapore/GP1908/2015	5	
B0136	Group B9	Day 8	30JUN2018	HAI	A/Singapore/GP1908/2015	160	
B0136	Group B9	Day 22	14JUL2018	HAI	A/Singapore/GP1908/2015	160	
B0136	Group B9	Day 61	22AUG2018	HAI	A/Singapore/GP1908/2015	80	
B0136	Group B9	Day 1 Predose	23JUN2018	MNT	A/Singapore/GP1908/2015	320	
B0136	Group B9	Day 22	14JUL2018	MNT	A/Singapore/GP1908/2015	1280	
B0137	Group B8	Day 1 Predose	23JUN2018	HAI	A/Singapore/GP1908/2015	10	

Administration Site Key: FA = Forearm; UA= Upper arm
Treatments Part A: A1 = A/Sing MAP, 15 ug (FA); A2 = Afluria Quad IM (UA), 15 ug; A3 = Placebo MAP (FA); A4 = A/Sing Mono IM, 15 ug (UA)
Treatments Part B: B1 = A/Sing MAP, 15 ug (FA) with biopsy; B2 = Placebo MAP (FA) with biopsy
B3 = A/Sing MAP, 15 ug (FA); B4 = A/Sing MAP, 10 ug (FA); B5 = A/Sing MAP, 5 ug (FA); B6 = A/Sing MAP, 2.5 ug (FA)
B7 = Placebo MAP (FA); B8 = A/Sing MAP, 15 ug (UA); B9 = A/Sing IM, 15 ug, as 2018 Afluria Quad (UA)

Listing 16.2.6.8
Immunogenicity Results (HAI, MNT)

Part B

Randomisation Number	Group Treatment	Visit	Collection Date	Test	Virus	Titre	Comment
B0137	Group B8	Day 4	26JUN2018	HAI	A/Singapore/GP1908/2015	10	
B0137	Group B8	Day 8	30JUN2018	HAI	A/Singapore/GP1908/2015	2560	
B0137	Group B8	Day 22	13JUL2018	HAI	A/Singapore/GP1908/2015	2560	
B0137	Group B8	Day 61	30AUG2018	HAI	A/Singapore/GP1908/2015	640	
B0137	Group B8	Day 1 Predose	23JUN2018	MNT	A/Singapore/GP1908/2015	640	
B0137	Group B8	Day 22	13JUL2018	MNT	A/Singapore/GP1908/2015	40960	
B0138	Group B7	Day 1 Predose	23JUN2018	HAI	A/Singapore/GP1908/2015	5	
B0138	Group B7	Day 4	26JUN2018	HAI	A/Singapore/GP1908/2015	5	
B0138	Group B7	Day 8	30JUN2018	HAI	A/Singapore/GP1908/2015	5	
B0138	Group B7	Day 22	14JUL2018	HAI	A/Singapore/GP1908/2015	5	
B0138	Group B7	Day 61	22AUG2018	HAI	A/Singapore/GP1908/2015	5	
B0138	Group B7	Day 1 Predose	23JUN2018	MNT	A/Singapore/GP1908/2015	160	
B0138	Group B7	Day 22	14JUL2018	MNT	A/Singapore/GP1908/2015	80	
B0139	Group B4	Day 1 Predose	23JUN2018	HAI	A/Singapore/GP1908/2015	5	
B0139	Group B4	Day 4	26JUN2018	HAI	A/Singapore/GP1908/2015	5	
B0139	Group B4	Day 8	30JUN2018	HAI	A/Singapore/GP1908/2015	640	
B0139	Group B4	Day 22	14JUL2018	HAI	A/Singapore/GP1908/2015	640	

Administration Site Key: FA = Forearm; UA= Upper arm
Treatments Part A: A1 = A/Sing MAP, 15 ug (FA); A2 = Afluria Quad IM (UA), 15 ug; A3 = Placebo MAP (FA); A4 = A/Sing Mono IM, 15 ug (UA)
Treatments Part B: B1 = A/Sing MAP, 15 ug (FA) with biopsy; B2 = Placebo MAP (FA) with biopsy
B3 = A/Sing MAP, 15 ug (FA); B4 = A/Sing MAP, 10 ug (FA); B5 = A/Sing MAP, 5 ug (FA); B6 = A/Sing MAP, 2.5 ug (FA)
B7 = Placebo MAP (FA); B8 = A/Sing MAP, 15 ug (UA); B9 = A/Sing IM, 15 ug, as 2018 Afluria Quad (UA)

Listing 16.2.6.8
Immunogenicity Results (HAI, MNT)

Part B

Randomisation Number	Group Treatment	Visit	Collection Date	Test	Virus	Titre	Comment
B0139	Group B4	Day 61	22AUG2018	HAI	A/Singapore/GP1908/2015	640	
B0139	Group B4	Day 1 Predose	23JUN2018	MNT	A/Singapore/GP1908/2015	640	
B0139	Group B4	Day 22	14JUL2018	MNT	A/Singapore/GP1908/2015	20480	
B0140	Group B9	Day 1 Predose	23JUN2018	HAI	A/Singapore/GP1908/2015	40	
B0140	Group B9	Day 4	26JUN2018	HAI	A/Singapore/GP1908/2015	40	
B0140	Group B9	Day 8	01JUL2018	HAI	A/Singapore/GP1908/2015	80	
B0140	Group B9	Day 22	14JUL2018	HAI	A/Singapore/GP1908/2015	80	
B0140	Group B9	Day 61	22AUG2018	HAI	A/Singapore/GP1908/2015	80	
B0140	Group B9	Day 1 Predose	23JUN2018	MNT	A/Singapore/GP1908/2015	1280	
B0140	Group B9	Day 22	14JUL2018	MNT	A/Singapore/GP1908/2015	1280	
B0141	Group B3	Day 1 Predose	23JUN2018	HAI	A/Singapore/GP1908/2015	<5	
B0141	Group B3	Day 4	26JUN2018	HAI	A/Singapore/GP1908/2015	<5	
B0141	Group B3	Day 8	30JUN2018	HAI	A/Singapore/GP1908/2015	160	
B0141	Group B3	Day 22	14JUL2018	HAI	A/Singapore/GP1908/2015	320	
B0141	Group B3	Day 61	23AUG2018	HAI	A/Singapore/GP1908/2015	160	
B0141	Group B3	Day 1 Predose	23JUN2018	MNT	A/Singapore/GP1908/2015	80	
B0141	Group B3	Day 22	14JUL2018	MNT	A/Singapore/GP1908/2015	20480	

Administration Site Key: FA = Forearm; UA= Upper arm
Treatments Part A: A1 = A/Sing MAP, 15 ug (FA); A2 = Afluria Quad IM (UA), 15 ug; A3 = Placebo MAP (FA); A4 = A/Sing Mono IM, 15 ug (UA)
Treatments Part B: B1 = A/Sing MAP, 15 ug (FA) with biopsy; B2 = Placebo MAP (FA) with biopsy
B3 = A/Sing MAP, 15 ug (FA); B4 = A/Sing MAP, 10 ug (FA); B5 = A/Sing MAP, 5 ug (FA); B6 = A/Sing MAP, 2.5 ug (FA)
B7 = Placebo MAP (FA); B8 = A/Sing MAP, 15 ug (UA); B9 = A/Sing IM, 15 ug, as 2018 Afluria Quad (UA)

Listing 16.2.6.8
Immunogenicity Results (HAI, MNT)

Part B

Randomisation Number	Group Treatment	Visit	Collection Date	Test	Virus	Titre	Comment
B0142	Group B5	Day 1 Predose	23JUN2018	HAI	A/Singapore/GP1908/2015	20	
B0142	Group B5	Day 4	26JUN2018	HAI	A/Singapore/GP1908/2015	20	
B0142	Group B5	Day 8	30JUN2018	HAI	A/Singapore/GP1908/2015	40	
B0142	Group B5	Day 22	14JUL2018	HAI	A/Singapore/GP1908/2015	160	
B0142	Group B5	Day 61	25AUG2018	HAI	A/Singapore/GP1908/2015	160	
B0142	Group B5	Day 1 Predose	23JUN2018	MNT	A/Singapore/GP1908/2015	320	
B0142	Group B5	Day 22	14JUL2018	MNT	A/Singapore/GP1908/2015	2560	
B0143	Group B6	Day 1 Predose	23JUN2018	HAI	A/Singapore/GP1908/2015	40	
B0143	Group B6	Day 4	26JUN2018	HAI	A/Singapore/GP1908/2015	40	
B0143	Group B6	Day 8	30JUN2018	HAI	A/Singapore/GP1908/2015	80	
B0143	Group B6	Day 22	14JUL2018	HAI	A/Singapore/GP1908/2015	320	
B0143	Group B6	Day 61	22AUG2018	HAI	A/Singapore/GP1908/2015	160	
B0143	Group B6	Day 1 Predose	23JUN2018	MNT	A/Singapore/GP1908/2015	2560	
B0143	Group B6	Day 22	14JUL2018	MNT	A/Singapore/GP1908/2015	10240	
B0144	Group B6	Day 1 Predose	23JUN2018	HAI	A/Singapore/GP1908/2015	40	
B0144	Group B6	Day 4	26JUN2018	HAI	A/Singapore/GP1908/2015	40	

Administration Site Key: FA = Forearm; UA= Upper arm
Treatments Part A: A1 = A/Sing MAP, 15 ug (FA); A2 = Afluria Quad IM (UA), 15 ug; A3 = Placebo MAP (FA); A4 = A/Sing Mono IM, 15 ug (UA)
Treatments Part B: B1 = A/Sing MAP, 15 ug (FA) with biopsy; B2 = Placebo MAP (FA) with biopsy
B3 = A/Sing MAP, 15 ug (FA); B4 = A/Sing MAP, 10 ug (FA); B5 = A/Sing MAP, 5 ug (FA); B6 = A/Sing MAP, 2.5 ug (FA)
B7 = Placebo MAP (FA); B8 = A/Sing MAP, 15 ug (UA); B9 = A/Sing IM, 15 ug, as 2018 Afluria Quad (UA)

Listing 16.2.6.8
Immunogenicity Results (HAI, MNT)

Part B

Randomisation Number	Group Treatment	Visit	Collection Date	Test	Virus	Titre	Comment
B0144	Group B6	Day 8	30JUN2018	HAI	A/Singapore/GP1908/2015	40	
B0144	Group B6	Day 22	16JUL2018	HAI	A/Singapore/GP1908/2015	40	
B0144	Group B6	Day 61	23AUG2018	HAI	A/Singapore/GP1908/2015	40	
B0144	Group B6	Day 1 Predose	23JUN2018	MNT	A/Singapore/GP1908/2015	1280	
B0144	Group B6	Day 22	16JUL2018	MNT	A/Singapore/GP1908/2015	2560	
B0145	Group B3	Day 1 Predose	23JUN2018	HAI	A/Singapore/GP1908/2015	40	
B0145	Group B3	Day 4	26JUN2018	HAI	A/Singapore/GP1908/2015	40	
B0145	Group B3	Day 8	30JUN2018	HAI	A/Singapore/GP1908/2015	160	
B0145	Group B3	Day 22	14JUL2018	HAI	A/Singapore/GP1908/2015	320	
B0145	Group B3	Day 61	22AUG2018	HAI	A/Singapore/GP1908/2015	160	
B0145	Group B3	Day 1 Predose	23JUN2018	MNT	A/Singapore/GP1908/2015	640	
B0145	Group B3	Day 22	14JUL2018	MNT	A/Singapore/GP1908/2015	5120	
B0146	Group B7	Day 1 Predose	28JUN2018	HAI	A/Singapore/GP1908/2015	<5	
B0146	Group B7	Day 4	01JUL2018	HAI	A/Singapore/GP1908/2015	<5	
B0146	Group B7	Day 8	05JUL2018	HAI	A/Singapore/GP1908/2015	<5	
B0146	Group B7	Day 22	19JUL2018	HAI	A/Singapore/GP1908/2015	<5	
B0146	Group B7	Day 61	28AUG2018	HAI	A/Singapore/GP1908/2015	<5	

Administration Site Key: FA = Forearm; UA= Upper arm
Treatments Part A: A1 = A/Sing MAP, 15 ug (FA); A2 = Afluria Quad IM (UA), 15 ug; A3 = Placebo MAP (FA); A4 = A/Sing Mono IM, 15 ug (UA)
Treatments Part B: B1 = A/Sing MAP, 15 ug (FA) with biopsy; B2 = Placebo MAP (FA) with biopsy
B3 = A/Sing MAP, 15 ug (FA); B4 = A/Sing MAP, 10 ug (FA); B5 = A/Sing MAP, 5 ug (FA); B6 = A/Sing MAP, 2.5 ug (FA)
B7 = Placebo MAP (FA); B8 = A/Sing MAP, 15 ug (UA); B9 = A/Sing IM, 15 ug, as 2018 Afluria Quad (UA)

Listing 16.2.6.8
Immunogenicity Results (HAI, MNT)

Part B

Randomisation Number	Group Treatment	Visit	Collection Date	Test	Virus	Titre	Comment
B0146	Group B7	Day 1 Predose	28JUN2018	MNT	A/Singapore/GP1908/2015	40	
B0146	Group B7	Day 22	19JUL2018	MNT	A/Singapore/GP1908/2015	80	
B0147	Group B9	Day 1 Predose	28JUN2018	HAI	A/Singapore/GP1908/2015	<5	
B0147	Group B9	Day 4	01JUL2018	HAI	A/Singapore/GP1908/2015	<5	
B0147	Group B9	Day 8	05JUL2018	HAI	A/Singapore/GP1908/2015	<5	
B0147	Group B9	Day 22	19JUL2018	HAI	A/Singapore/GP1908/2015	20	
B0147	Group B9	Day 61	27AUG2018	HAI	A/Singapore/GP1908/2015	10	
B0147	Group B9	Day 1 Predose	28JUN2018	MNT	A/Singapore/GP1908/2015	<20	
B0147	Group B9	Day 22	19JUL2018	MNT	A/Singapore/GP1908/2015	320	
B0148	Group B5	Day 1 Predose	28JUN2018	HAI	A/Singapore/GP1908/2015	20	
B0148	Group B5	Day 4	01JUL2018	HAI	A/Singapore/GP1908/2015	20	
B0148	Group B5	Day 8	05JUL2018	HAI	A/Singapore/GP1908/2015	320	
B0148	Group B5	Day 22	18JUL2018	HAI	A/Singapore/GP1908/2015	640	
B0148	Group B5	Day 61	27AUG2018	HAI	A/Singapore/GP1908/2015	320	
B0148	Group B5	Day 1 Predose	28JUN2018	MNT	A/Singapore/GP1908/2015	320	
B0148	Group B5	Day 22	18JUL2018	MNT	A/Singapore/GP1908/2015	40960	

Administration Site Key: FA = Forearm; UA= Upper arm
Treatments Part A: A1 = A/Sing MAP, 15 ug (FA); A2 = Afluria Quad IM (UA), 15 ug; A3 = Placebo MAP (FA); A4 = A/Sing Mono IM, 15 ug (UA)
Treatments Part B: B1 = A/Sing MAP, 15 ug (FA) with biopsy; B2 = Placebo MAP (FA) with biopsy
B3 = A/Sing MAP, 15 ug (FA); B4 = A/Sing MAP, 10 ug (FA); B5 = A/Sing MAP, 5 ug (FA); B6 = A/Sing MAP, 2.5 ug (FA)
B7 = Placebo MAP (FA); B8 = A/Sing MAP, 15 ug (UA); B9 = A/Sing IM, 15 ug, as 2018 Afluria Quad (UA)

Listing 16.2.6.8
Immunogenicity Results (HAI, MNT)

Part B

Randomisation Number	Group Treatment	Visit	Collection Date	Test	Virus	Titre	Comment
B0149	Group B8	Day 1 Predose	28JUN2018	HAI	A/Singapore/GP1908/2015	20	
B0149	Group B8	Day 4	01JUL2018	HAI	A/Singapore/GP1908/2015	20	
B0149	Group B8	Day 8	05JUL2018	HAI	A/Singapore/GP1908/2015	640	
B0149	Group B8	Day 22	19JUL2018	HAI	A/Singapore/GP1908/2015	1280	
B0149	Group B8	Day 61	27AUG2018	HAI	A/Singapore/GP1908/2015	1280	
B0149	Group B8	Day 1 Predose	28JUN2018	MNT	A/Singapore/GP1908/2015	640	
B0149	Group B8	Day 22	19JUL2018	MNT	A/Singapore/GP1908/2015	40960	
B0150	Group B4	Day 1 Predose	28JUN2018	HAI	A/Singapore/GP1908/2015	80	
B0150	Group B4	Day 4	01JUL2018	HAI	A/Singapore/GP1908/2015	40	
B0150	Group B4	Day 8	05JUL2018	HAI	A/Singapore/GP1908/2015	640	
B0150	Group B4	Day 22	19JUL2018	HAI	A/Singapore/GP1908/2015	2560	
B0150	Group B4	Day 61	28AUG2018	HAI	A/Singapore/GP1908/2015	1280	
B0150	Group B4	Day 1 Predose	28JUN2018	MNT	A/Singapore/GP1908/2015	2560	
B0150	Group B4	Day 22	19JUL2018	MNT	A/Singapore/GP1908/2015	327680	
B1019	Group B8	Day 1 Predose	19MAY2018	HAI	A/Singapore/GP1908/2015	40	
B1019	Group B8	Day 4	22MAY2018	HAI	A/Singapore/GP1908/2015	40	
B1019	Group B8	Day 8	28MAY2018	HAI	A/Singapore/GP1908/2015	640	

Administration Site Key: FA = Forearm; UA= Upper arm
Treatments Part A: A1 = A/Sing MAP, 15 ug (FA); A2 = Afluria Quad IM (UA), 15 ug; A3 = Placebo MAP (FA); A4 = A/Sing Mono IM, 15 ug (UA)
Treatments Part B: B1 = A/Sing MAP, 15 ug (FA) with biopsy; B2 = Placebo MAP (FA) with biopsy
B3 = A/Sing MAP, 15 ug (FA); B4 = A/Sing MAP, 10 ug (FA); B5 = A/Sing MAP, 5 ug (FA); B6 = A/Sing MAP, 2.5 ug (FA)
B7 = Placebo MAP (FA); B8 = A/Sing MAP, 15 ug (UA); B9 = A/Sing IM, 15 ug, as 2018 Afluria Quad (UA)

Listing 16.2.6.8
Immunogenicity Results (HAI, MNT)

Part B

Randomisation Number	Group Treatment	Visit	Collection Date	Test	Virus	Titre	Comment
B1019	Group B8	Day 22	13JUN2108	HAI	A/Singapore/GP1908/2015	1280	
B1019	Group B8	Day 61	20JUL2018	HAI	A/Singapore/GP1908/2015	640	
B1019	Group B8	Day 1 Predose	19MAY2018	MNT	A/Singapore/GP1908/2015	1280	
B1019	Group B8	Day 22	13JUN2108	MNT	A/Singapore/GP1908/2015	20480	
B1076	Group B5	Day 1 Predose	16JUN2018	HAI	A/Singapore/GP1908/2015	40	
B1076	Group B5	Day 4	19JUN2018	HAI	A/Singapore/GP1908/2015	40	
B1076	Group B5	Day 8	23JUN2018	HAI	A/Singapore/GP1908/2015	320	
B1076	Group B5	Day 22	07JUL2018	HAI	A/Singapore/GP1908/2015	320	
B1076	Group B5	Day 61	15AUG2018	HAI	A/Singapore/GP1908/2015	320	
B1076	Group B5	Day 1 Predose	16JUN2018	MNT	A/Singapore/GP1908/2015	1280	
B1076	Group B5	Day 22	07JUL2018	MNT	A/Singapore/GP1908/2015	10240	
B1080	Group B7	Day 1 Predose	16JUN2018	HAI	A/Singapore/GP1908/2015	5	
B1080	Group B7	Day 4	19JUN2018	HAI	A/Singapore/GP1908/2015	5	
B1080	Group B7	Day 8	23JUN2018	HAI	A/Singapore/GP1908/2015	5	
B1080	Group B7	Day 22	07JUL2018	HAI	A/Singapore/GP1908/2015	5	
B1080	Group B7	Day 61	15AUG2018	HAI	A/Singapore/GP1908/2015	<5	
B1080	Group B7	Day 1 Predose	16JUN2018	MNT	A/Singapore/GP1908/2015	160	

Administration Site Key: FA = Forearm; UA= Upper arm
Treatments Part A: A1 = A/Sing MAP, 15 ug (FA); A2 = Afluria Quad IM (UA), 15 ug; A3 = Placebo MAP (FA); A4 = A/Sing Mono IM, 15 ug (UA)
Treatments Part B: B1 = A/Sing MAP, 15 ug (FA) with biopsy; B2 = Placebo MAP (FA) with biopsy
B3 = A/Sing MAP, 15 ug (FA); B4 = A/Sing MAP, 10 ug (FA); B5 = A/Sing MAP, 5 ug (FA); B6 = A/Sing MAP, 2.5 ug (FA)
B7 = Placebo MAP (FA); B8 = A/Sing MAP, 15 ug (UA); B9 = A/Sing IM, 15 ug, as 2018 Afluria Quad (UA)

Listing 16.2.6.8
Immunogenicity Results (HAI, MNT)

Part B

Randomisation Number	Group Treatment	Visit	Collection Date	Test	Virus	Titre	Comment
B1080	Group B7	Day 22	07JUL2018	MNT	A/Singapore/GP1908/2015	160	

Administration Site Key: FA = Forearm; UA= Upper arm
Treatments Part A: A1 = A/Sing MAP, 15 ug (FA); A2 = Afluria Quad IM (UA), 15 ug; A3 = Placebo MAP (FA); A4 = A/Sing Mono IM, 15 ug (UA)
Treatments Part B: B1 = A/Sing MAP, 15 ug (FA) with biopsy; B2 = Placebo MAP (FA) with biopsy
B3 = A/Sing MAP, 15 ug (FA); B4 = A/Sing MAP, 10 ug (FA); B5 = A/Sing MAP, 5 ug (FA); B6 = A/Sing MAP, 2.5 ug (FA)
B7 = Placebo MAP (FA); B8 = A/Sing MAP, 15 ug (UA); B9 = A/Sing IM, 15 ug, as 2018 Afluria Quad (UA)
