# Supplementary material for: Safety, tolerability, and immunogenicity of influenza vaccination with a high-density microarray patch: Results from a randomized, controlled phase I clinical trial
Source: PLoS Med. 2020 Mar 17;17(3):e1003024. doi: 10.1371/journal.pmed.1003024 (PMC7077342; doi:10.1371/journal.pmed.1003024)
Supplement: S1 Data — Data 1: Informed Consent; Data 2: Analysis Sets; Data 3: Demographics; Data 4: Study Drug Administration; Data 5: Skin Hardness Assessment; Data 6: Immunogenicity (ELISA IgG); Data 7: Immunogenicity (ADCC); Data 8: Immunogenicity (MBCs); Data 9: Immunogenicity (CMI); Data 10: Immunogenicity (Mucosal IgA); Data 11: Immunogenicity Results (HAI, MN); Data 12: TEAE–CRF Data only; Data 13: TEAEs–MedDRA Coding; Data 15: Treatment Site Tolerability Assessment; Data 16: Numeric Pain Intensity; Data 17: SII; Data 18: Application Site Status at End of Study; Data 19: Individual Treatment Site Tolerability Assessment; Data 20: Numeric Pain Intensity by Treatment; Data 21: Individual SII by Treatment. ADCC, antibody-dependent cellular cytotoxicity; CMI, cell-mediated immunity; CRF, case report form; ELISA, enzyme-linked immunosorbent assay; HAI, haemagglutination inhibition; IgA, immunoglobulin A; IgG, immunoglobulin G; MBC, memory B cell; MedDRA, medical dictionary for regulatory activities; MN, microneutralisation; SII, Skin Irritation Index; TEA, treatment emergent adverse event. (ZIP) [file pmed.1003024.s008.zip › S1_Data/clinical supporting/S1_Data12 TEAE - CRF Data only.pdf]

Listing 16.2.7.1
Adverse Events - CRF Data only

Part A

Randomisation Number	Group	Treatment	AE No.	Adverse Event Verbatim	Onset Date	Onset Time	End Date	End Time	Outcome	Maximum Severity	Relationship	Actions Taken	SAE
A0001	Group A3	Placebo MAP	1	MAP reaction site 1,2,3 Erythema	09-MAR-2018	14:27	16-MAR-2018	11:47	Recovered / Resolved	Mild	Probably Related	None	No
A0002	Group A1	A/Sing MAP, 15 ug	1	Fatigue	10-MAR-2018	10:00	10-MAR-2018	22:00	Recovered / Resolved	Mild	Unlikely Related	None	No
A0003	Group A1	A/Sing MAP, 15 ug		[No Treatment-Emergent Adverse Events Reported]									
A0004	Group A3	Placebo MAP	1	Intermittent Headache	13-MAR-2018	05:40	30-MAR-2018	07:40	Recovered / Resolved	Moderate	Unlikely Related	Concomitant Medication	No
A0004	Group A3	Placebo MAP	2	MAP reaction site 1,2,3 erythema	09-MAR-2018	14:57	16-MAR-2018	15:47	Recovered / Resolved	Mild	Probably Related	None	No
A0005	Group A1	A/Sing MAP, 15 ug	18	MAP Site Reaction 1,2,3 tenderness, itchiness and oedema	12-MAR-2018	13:43	16-MAR-2018	12:42	Recovered / Resolved	Mild	Probably Related	None	No

Administration Site Key: FA = Forearm; UA= Upper arm
Treatments Part A: A1 = A/Sing MAP, 15 ug (FA); A2 = Afluria Quad IM (UA), 15 ug; A3 = Placebo MAP (FA); A4 = A/Sing Mono IM, 15 ug (UA)
Treatments Part B: B1 = A/Sing MAP, 15 ug (FA) with biopsy; B2 = Placebo MAP (FA) with biopsy
B3 = A/Sing MAP, 15 ug (FA); B4 = A/Sing MAP, 10 ug (FA); B5 = A/Sing MAP, 5 ug (FA); B6 = A/Sing MAP, 2.5 ug (FA)
B7 = Placebo MAP (FA); B8 = A/Sing MAP, 15 ug (UA); B9 = A/Sing IM, 15 ug, as 2018 Afluria Quad (UA)

Listing 16.2.7.1
Adverse Events - CRF Data only

Part A

Random-isation Number	Group	Treatment	AE No.	Adverse Event Verbatim	Onset Date	Onset Time	End Date	End Time	Outcome	Maximum Severity	Relation-ship	Actions Taken	SAE
A0006	Group A3	Placebo MAP	10	MAP 1,2,3 visibility	08-MAY-2018	14:42	22-MAY-2018	UK:UK	Recovered / Resolved	Mild	Probably Related	None	No
A0007	Group A1	A/Sing MAP, 15 ug		[No Treatment-Emergent Adverse Events Reported]									
A0008	Group A3	Placebo MAP	7	MAP 2 erythema	09-MAR-2018	16:00	12-MAR-2018	16:23	Recovered / Resolved	Mild	Probably Related	None	No
A0009	Group A1	A/Sing MAP, 15 ug	1	Erythema - Left Upper Back	14-MAR-2018	16:00	24-MAR-2018	12:00	Recovered / Resolved	Mild	Possibly Related	None	No
A0009	Group A1	A/Sing MAP, 15 ug	11	MAP visibility 1,2,3	01-MAY-2018	14:51	12-JUN-2018	UK:UK	Recovered / Resolved	Mild	Probably Related	None	No
A0010	Group A3	Placebo MAP	1	Upper Respiratory Tract Infection	26-MAR-2018	UK:UK	31-MAR-2018	UK:UK	Recovered / Resolved	Mild	Not Related	None	No

Administration Site Key: FA = Forearm; UA= Upper arm
Treatments Part A: A1 = A/Sing MAP, 15 ug (FA); A2 = Afluria Quad IM (UA), 15 ug; A3 = Placebo MAP (FA); A4 = A/Sing Mono IM, 15 ug (UA)
Treatments Part B: B1 = A/Sing MAP, 15 ug (FA) with biopsy; B2 = Placebo MAP (FA) with biopsy
B3 = A/Sing MAP, 15 ug (FA); B4 = A/Sing MAP, 10 ug (FA); B5 = A/Sing MAP, 5 ug (FA); B6 = A/Sing MAP, 2.5 ug (FA)
B7 = Placebo MAP (FA); B8 = A/Sing MAP, 15 ug (UA); B9 = A/Sing IM, 15 ug, as 2018 Afluria Quad (UA)

Listing 16.2.7.1
Adverse Events - CRF Data only

Part A

Randomisation Number	Group	Treatment	AE No.	Adverse Event Verbatim	Onset Date	Onset Time	End Date	End Time	Outcome	Maximum Severity	Relationship	Actions Taken	SAE
A0011	Group A3	Placebo MAP	1	Headache	11-MAR-2018	12:45	11-MAR-2018	UK:UK	Recovered / Resolved	Moderate	Unlikely Related	Concomitant Medication	No
A0012	Group A1	A/Sing MAP, 15 ug	1	Left ankle inversion injury	11-MAR-2018	09:00	14-APR-2018	13:15	Recovered / Resolved	Mild	Not Related	None	No
A0012	Group A1	A/Sing MAP, 15 ug	11	MAP reaction 1,3 erythema	09-MAR-2018	16:18	16-MAR-2018	15:54	Recovered / Resolved	Mild	Probably Related	None	No
A0012	Group A1	A/Sing MAP, 15 ug	12	MAP reaction 1,2 oedema	12-MAR-2018	13:50	16-MAR-2018	15:54	Recovered / Resolved	Mild	Probably Related	None	No
A0013	Group A1	A/Sing MAP, 15 ug	6	MAP 2 reaction Itchiness	16-MAR-2018	15:52	30-MAR-2018	14:40	Recovered / Resolved	Mild	Possibly Related	None	No
A0013	Group A1	A/Sing MAP, 15 ug	12	MAP 1, 2, 3 reaction Erythema	09-MAR-2018	16:23	16-MAR-2018	15:53	Recovered / Resolved	Mild	Probably Related	None	No
A0013	Group A1	A/Sing MAP, 15 ug	13	MAP 1, 2, 3 Visibility	10-MAY-2018	13:25	10-JUN-2018	UK:UK	Recovered / Resolved	Mild	Probably Related	None	No
A0013	Group A1	A/Sing MAP, 15 ug	14	MAP 2 & 3 reaction Oedema	12-MAR-2018	13:19	16-MAR-2018	15:53	Recovered / Resolved	Mild	Probably Related	None	No

Administration Site Key: FA = Forearm; UA= Upper arm
Treatments Part A: A1 = A/Sing MAP, 15 ug (FA); A2 = Afluria Quad IM (UA), 15 ug; A3 = Placebo MAP (FA); A4 = A/Sing Mono IM, 15 ug (UA)
Treatments Part B: B1 = A/Sing MAP, 15 ug (FA) with biopsy; B2 = Placebo MAP (FA) with biopsy
B3 = A/Sing MAP, 15 ug (FA); B4 = A/Sing MAP, 10 ug (FA); B5 = A/Sing MAP, 5 ug (FA); B6 = A/Sing MAP, 2.5 ug (FA)
B7 = Placebo MAP (FA); B8 = A/Sing MAP, 15 ug (UA); B9 = A/Sing IM, 15 ug, as 2018 Afluria Quad (UA)

Listing 16.2.7.1
Adverse Events - CRF Data only

Part A

Random-isation Number	Group	Treatment	AE No.	Adverse Event Verbatim	Onset Date	Onset Time	End Date	End Time	Outcome	Maximum Severity	Relation-ship	Actions Taken	SAE
A0014	Group A3	Placebo MAP		[No Treatment-Emergent Adverse Events Reported]									
A0015	Group A1	A/Sing MAP, 15 ug	1	Headache	11-MAR-2018	00:00	12-MAR-2018	10:30	Recovered / Resolved	Mild	Unlikely Related	None	No
A0015	Group A1	A/Sing MAP, 15 ug	3	MAP 2 and MAP 3 Erythema	09-MAR-2018	16:51	16-MAR-2018	14:03	Recovered / Resolved	Mild	Probably Related	None	No
A0015	Group A1	A/Sing MAP, 15 ug	5	MAP Sites 1,2,3 Visibility	16-MAR-2018	14:03	09-JUN-2018	10:00	Recovered / Resolved	Mild	Definitely Related	None	No
A0016	Group A3	Placebo MAP	7	Headache	10-MAR-2018	15:16	10-MAR-2018	19:00	Recovered / Resolved	Moderate	Possibly Related	Concomitant Medication	No
A0017	Group A3	Placebo MAP		[No Treatment-Emergent Adverse Events Reported]									

Administration Site Key: FA = Forearm; UA= Upper arm
Treatments Part A: A1 = A/Sing MAP, 15 ug (FA); A2 = Afluria Quad IM (UA), 15 ug; A3 = Placebo MAP (FA); A4 = A/Sing Mono IM, 15 ug (UA)
Treatments Part B: B1 = A/Sing MAP, 15 ug (FA) with biopsy; B2 = Placebo MAP (FA) with biopsy
B3 = A/Sing MAP, 15 ug (FA); B4 = A/Sing MAP, 10 ug (FA); B5 = A/Sing MAP, 5 ug (FA); B6 = A/Sing MAP, 2.5 ug (FA)
B7 = Placebo MAP (FA); B8 = A/Sing MAP, 15 ug (UA); B9 = A/Sing IM, 15 ug, as 2018 Afluria Quad (UA)

Listing 16.2.7.1
Adverse Events - CRF Data only

Part A

Random-isation Number	Group	Treatment	AE No.	Adverse Event Verbatim	Onset Date	Onset Time	End Date	End Time	Outcome	Maximum Severity	Relation-ship	Actions Taken	SAE
A0018	Group A1	A/Sing MAP, 15 ug		[No Treatment-Emergent Adverse Events Reported]									
A0019	Group A3	Placebo MAP	10	Phlebitis (venepuncture site)	02-APR-2018	UK:UK	14-APR-2018	UK:UK	Recovered / Resolved	Moderate	Not Related	Concomitant Medication	No
A0020	Group A1	A/Sing MAP, 15 ug	1	Flu-Like Symptoms	23-MAR-2018	08:00	23-MAR-2018	11:59	Recovered / Resolved	Moderate	Not Related	Concomitant Medication	No
A0021	Group A1	A/Sing MAP, 15 ug	1	Headache	10-MAR-2018	15:00	10-MAR-2018	22:00	Recovered / Resolved	Mild	Unlikely Related	None	No
A0022	Group A3	Placebo MAP	1	Pruritus site #3	26-MAR-2018	UK:UK	26-MAR-2018	UK:UK	Recovered / Resolved	Mild	Possibly Related	None	No
A0023	Group A3	Placebo MAP	1	Abdomen Bloating	10-MAR-2018	22:30	10-MAR-2018	23:30	Recovered / Resolved	Mild	Possibly Related	None	No

Administration Site Key: FA = Forearm; UA= Upper arm
Treatments Part A: A1 = A/Sing MAP, 15 ug (FA); A2 = Afluria Quad IM (UA), 15 ug; A3 = Placebo MAP (FA); A4 = A/Sing Mono IM, 15 ug (UA)
Treatments Part B: B1 = A/Sing MAP, 15 ug (FA) with biopsy; B2 = Placebo MAP (FA) with biopsy
B3 = A/Sing MAP, 15 ug (FA); B4 = A/Sing MAP, 10 ug (FA); B5 = A/Sing MAP, 5 ug (FA); B6 = A/Sing MAP, 2.5 ug (FA)
B7 = Placebo MAP (FA); B8 = A/Sing MAP, 15 ug (UA); B9 = A/Sing IM, 15 ug, as 2018 Afluria Quad (UA)

Listing 16.2.7.1
Adverse Events - CRF Data only

Part A

Random-isation Number	Group	Treatment	AE No.	Adverse Event Verbatim	Onset Date	Onset Time	End Date	End Time	Outcome	Maximum Severity	Relation-ship	Actions Taken	SAE
A0023	Group A3	Placebo MAP	2	Intermittent Headaches	12-MAR-2018	17:00	16-MAR-2018	20:30	Recovered / Resolved	Mild	Unlikely Related	None	No
A0023	Group A3	Placebo MAP	3	Abdomen Pain (Intermittent)	10-MAR-2018	22:30	16-MAR-2018	16:10	Recovered / Resolved	Mild	Possibly Related	None	No
A0023	Group A3	Placebo MAP	4	Sore Throat	24-MAR-2018	17:00	14-APR-2018	UK:UK	Recovered / Resolved	Mild	Unlikely Related	None	No
A0023	Group A3	Placebo MAP	14	Diarrhoea	11-MAR-2018	21:30	11-MAR-2018	23:30	Recovered / Resolved	Mild	Possibly Related	None	No
A0023	Group A3	Placebo MAP	15	Drowsiness	16-MAR-2018	07:00	04-APR-2018	UK:UK	Recovered / Resolved	Mild	Unlikely Related	None	No
A0024	Group A1	A/Sing MAP, 15 ug	1	Abdominal discomfort	14-MAR-2018	14:00	15-MAR-2018	12:00	Recovered / Resolved	Mild	Unlikely Related	None	No
A0025	Group A3	Placebo MAP	1	Headache (intermittent)	15-MAR-2018	07:30	17-MAR-2018	10:00	Recovered / Resolved	Moderate	Unlikely Related	Concomitant Medication	No
A0027	Group A3	Placebo MAP	1	Headache	16-MAR-2018	11:00	16-MAR-2018	UK:UK	Recovered / Resolved	Moderate	Unlikely Related	Concomitant Medication	No

Administration Site Key: FA = Forearm; UA= Upper arm
Treatments Part A: A1 = A/Sing MAP, 15 ug (FA); A2 = Afluria Quad IM (UA), 15 ug; A3 = Placebo MAP (FA); A4 = A/Sing Mono IM, 15 ug (UA)
Treatments Part B: B1 = A/Sing MAP, 15 ug (FA) with biopsy; B2 = Placebo MAP (FA) with biopsy
B3 = A/Sing MAP, 15 ug (FA); B4 = A/Sing MAP, 10 ug (FA); B5 = A/Sing MAP, 5 ug (FA); B6 = A/Sing MAP, 2.5 ug (FA)
B7 = Placebo MAP (FA); B8 = A/Sing MAP, 15 ug (UA); B9 = A/Sing IM, 15 ug, as 2018 Afluria Quad (UA)

Listing 16.2.7.1
Adverse Events - CRF Data only

Part A

Random-isation Number	Group	Treatment	AE No.	Adverse Event Verbatim	Onset Date	Onset Time	End Date	End Time	Outcome	Maximum Severity	Relation-ship	Actions Taken	SAE
A0028	Group A1	A/Sing MAP, 15 ug		[No Treatment-Emergent Adverse Events Reported]									
A0029	Group A1	A/Sing MAP, 15 ug	6	MAP visibility site 1,2,3	09-MAY-2018	16:19	UN-Jul-2018	UK:UK	Recovered / Resolved	Mild	Probably Related	None	No
A0030	Group A3	Placebo MAP	1	Upper Respiratory Tract Infection	30-MAR-2018	20:00	12-APR-2018	08:00	Recovered / Resolved	Mild	Not Related	None	No
A0030	Group A3	Placebo MAP	5	MAP site 1,2,3 Visibility	15-MAY-2018	12:25	30-OCT-2018	UK:UK	Recovered / Resolved	Mild	Probably Related	None	No
A0031	Group A2	Afluria Quad IM, 15 ug		[No Treatment-Emergent Adverse Events Reported]									

Administration Site Key: FA = Forearm; UA= Upper arm
Treatments Part A: A1 = A/Sing MAP, 15 ug (FA); A2 = Afluria Quad IM (UA), 15 ug; A3 = Placebo MAP (FA); A4 = A/Sing Mono IM, 15 ug (UA)
Treatments Part B: B1 = A/Sing MAP, 15 ug (FA) with biopsy; B2 = Placebo MAP (FA) with biopsy
B3 = A/Sing MAP, 15 ug (FA); B4 = A/Sing MAP, 10 ug (FA); B5 = A/Sing MAP, 5 ug (FA); B6 = A/Sing MAP, 2.5 ug (FA)
B7 = Placebo MAP (FA); B8 = A/Sing MAP, 15 ug (UA); B9 = A/Sing IM, 15 ug, as 2018 Afluria Quad (UA)

Listing 16.2.7.1
Adverse Events - CRF Data only

Part A

Random-isation Number	Group	Treatment	AE No.	Adverse Event Verbatim	Onset Date	Onset Time	End Date	End Time	Outcome	Maximum Severity	Relation-ship	Actions Taken	SAE
A0032	Group A4	A/Sing Mono IM, 15 ug		[No Treatment-Emergent Adverse Events Reported]									
A0033	Group A2	Afluria Quad IM, 15 ug	1	Headache	17-MAR-2018	07:30	17-MAR-2018	10:00	Recovered / Resolved	Mild	Possibly Related	None	No
A0033	Group A2	Afluria Quad IM, 15 ug	2	Cough	17-MAR-2018	07:30	17-MAR-2018	10:00	Recovered / Resolved	Mild	Not Related	None	No
A0034	Group A4	A/Sing Mono IM, 15 ug		[No Treatment-Emergent Adverse Events Reported]									
A0035	Group A4	A/Sing Mono IM, 15 ug	1	Tenderness at injection site	16-MAR-2018	14:15	16-MAR-2018	UK:UK	Recovered / Resolved	Mild	Probably Related	None	No

Administration Site Key: FA = Forearm; UA= Upper arm
Treatments Part A: A1 = A/Sing MAP, 15 ug (FA); A2 = Afluria Quad IM (UA), 15 ug; A3 = Placebo MAP (FA); A4 = A/Sing Mono IM, 15 ug (UA)
Treatments Part B: B1 = A/Sing MAP, 15 ug (FA) with biopsy; B2 = Placebo MAP (FA) with biopsy
B3 = A/Sing MAP, 15 ug (FA); B4 = A/Sing MAP, 10 ug (FA); B5 = A/Sing MAP, 5 ug (FA); B6 = A/Sing MAP, 2.5 ug (FA)
B7 = Placebo MAP (FA); B8 = A/Sing MAP, 15 ug (UA); B9 = A/Sing IM, 15 ug, as 2018 Afluria Quad (UA)

Listing 16.2.7.1
Adverse Events - CRF Data only

Part A

Randomisation Number	Group	Treatment	AE No.	Adverse Event Verbatim	Onset Date	Onset Time	End Date	End Time	Outcome	Maximum Severity	Relationship	Actions Taken	SAE
A0036	Group A2	Afluria Quad IM, 15 ug		[No Treatment-Emergent Adverse Events Reported]									
A0037	Group A4	A/Sing Mono IM, 15 ug	1	Injection site pain	17-MAR-2018	11:36	20-MAR-2018	08:35	Recovered / Resolved	Mild	Probably Related	None	No
A0037	Group A4	A/Sing Mono IM, 15 ug	2	Pain Left Deltoid (Injection site arm)	18-MAR-2018	15:00	25-MAR-2018	UK:UK	Recovered / Resolved	Mild	Probably Related	None	No
A0038	Group A2	Afluria Quad IM, 15 ug		[No Treatment-Emergent Adverse Events Reported]									
A0039	Group A2	Afluria Quad IM, 15 ug		[No Treatment-Emergent Adverse Events Reported]									

Administration Site Key: FA = Forearm; UA= Upper arm
Treatments Part A: A1 = A/Sing MAP, 15 ug (FA); A2 = Afluria Quad IM (UA), 15 ug; A3 = Placebo MAP (FA); A4 = A/Sing Mono IM, 15 ug (UA)
Treatments Part B: B1 = A/Sing MAP, 15 ug (FA) with biopsy; B2 = Placebo MAP (FA) with biopsy
B3 = A/Sing MAP, 15 ug (FA); B4 = A/Sing MAP, 10 ug (FA); B5 = A/Sing MAP, 5 ug (FA); B6 = A/Sing MAP, 2.5 ug (FA)
B7 = Placebo MAP (FA); B8 = A/Sing MAP, 15 ug (UA); B9 = A/Sing IM, 15 ug, as 2018 Afluria Quad (UA)

Listing 16.2.7.1
Adverse Events - CRF Data only

Part A

Random-isation Number	Group	Treatment	AE No.	Adverse Event Verbatim	Onset Date	Onset Time	End Date	End Time	Outcome	Maximum Severity	Relation-ship	Actions Taken	SAE
A0040	Group A4	A/Sing Mono IM, 15 ug		[No Treatment-Emergent Adverse Events Reported]									
A0041	Group A4	A/Sing Mono IM, 15 ug		[No Treatment-Emergent Adverse Events Reported]									
A0042	Group A2	Afluria Quad IM, 15 ug	1	Upper respiratory tract infections	18-MAR-2018	07:00	21-MAR-2018	09:00	Recovered / Resolved	Mild	Not Related	None	No
A0043	Group A2	Afluria Quad IM, 15 ug	1	Right arm tenderness	19-MAR-2018	23:00	24-MAR-2018	12:20	Recovered / Resolved	Mild	Unlikely Related	None	No
A0043	Group A2	Afluria Quad IM, 15 ug	2	Right Middle Ear Infection	04-APR-2018	18:00	21-APR-2018	UK:UK	Recovered / Resolved	Moderate	Unlikely Related	Concomitant Medication	No

Administration Site Key: FA = Forearm; UA= Upper arm
Treatments Part A: A1 = A/Sing MAP, 15 ug (FA); A2 = Afluria Quad IM (UA), 15 ug; A3 = Placebo MAP (FA); A4 = A/Sing Mono IM, 15 ug (UA)
Treatments Part B: B1 = A/Sing MAP, 15 ug (FA) with biopsy; B2 = Placebo MAP (FA) with biopsy
B3 = A/Sing MAP, 15 ug (FA); B4 = A/Sing MAP, 10 ug (FA); B5 = A/Sing MAP, 5 ug (FA); B6 = A/Sing MAP, 2.5 ug (FA)
B7 = Placebo MAP (FA); B8 = A/Sing MAP, 15 ug (UA); B9 = A/Sing IM, 15 ug, as 2018 Afluria Quad (UA)

Listing 16.2.7.1
Adverse Events - CRF Data only

Part A

Random-isation Number	Group	Treatment	AE No.	Adverse Event Verbatim	Onset Date	Onset Time	End Date	End Time	Outcome	Maximum Severity	Relation-ship	Actions Taken	SAE
A0044	Group A4	A/Sing Mono IM, 15 ug	1	Gastroenteritis	27-MAR-2018	20:00	01-APR-2018	UK:UK	Recovered / Resolved	Mild	Unlikely Related	Concomitant Medication	No
A0045	Group A2	Afluria Quad IM, 15 ug	1	Upper respiratory tract infection	23-MAR-2018	05:30	23-MAR-2018	UK:UK	Recovered / Resolved	Mild	Not Related	None	No
A0046	Group A4	A/Sing Mono IM, 15 ug	1	Vasovagal symptoms	17-MAR-2018	12:20	17-MAR-2018	12:30	Recovered / Resolved	Mild	Not Related	None	No
A0046	Group A4	A/Sing Mono IM, 15 ug	2	Headache	21-MAR-2018	UK:UK	21-MAR-2018	UK:UK	Recovered / Resolved	Moderate	Unlikely Related	Concomitant Medication	No
A0046	Group A4	A/Sing Mono IM, 15 ug	3	Dysmenorrhea	18-MAR-2018	UK:UK	23-MAR-2018	UK:UK	Recovered / Resolved	Moderate	Unlikely Related	Concomitant Medication	No
A0047	Group A2	Afluria Quad IM, 15 ug	1	Headache	17-MAR-2018	17:30	18-MAR-2018	19:00	Recovered / Resolved	Moderate	Possibly Related	Concomitant Medication	No
A0047	Group A2	Afluria Quad IM, 15 ug	2	Fatigue	17-MAR-2018	17:30	18-MAR-2018	UK:UK	Recovered / Resolved	Mild	Possibly Related	None	No
A0048	Group A4	A/Sing Mono IM, 15 ug	2	Upper respiratory tract infection	18-MAR-2018	15:00	30-MAR-2018	12:00	Recovered / Resolved	Mild	Not Related	None	No

Administration Site Key: FA = Forearm; UA= Upper arm
Treatments Part A: A1 = A/Sing MAP, 15 ug (FA); A2 = Afluria Quad IM (UA), 15 ug; A3 = Placebo MAP (FA); A4 = A/Sing Mono IM, 15 ug (UA)
Treatments Part B: B1 = A/Sing MAP, 15 ug (FA) with biopsy; B2 = Placebo MAP (FA) with biopsy
B3 = A/Sing MAP, 15 ug (FA); B4 = A/Sing MAP, 10 ug (FA); B5 = A/Sing MAP, 5 ug (FA); B6 = A/Sing MAP, 2.5 ug (FA)
B7 = Placebo MAP (FA); B8 = A/Sing MAP, 15 ug (UA); B9 = A/Sing IM, 15 ug, as 2018 Afluria Quad (UA)

Listing 16.2.7.1
Adverse Events - CRF Data only

Part A

Randomisation Number	Group	Treatment	AE No.	Adverse Event Verbatim	Onset Date	Onset Time	End Date	End Time	Outcome	Maximum Severity	Relationship	Actions Taken	SAE
A0048	Group A4	A/Sing Mono IM, 15 ug	3	Injection site itchiness	17-MAR-2018	23:00	18-MAR-2018	01:00	Recovered / Resolved	Mild	Definitely Related	None	No
A0049	Group A2	Afluria Quad IM, 15 ug	1	Fatigue	18-MAR-2018	08:30	20-MAR-2018	08:00	Recovered / Resolved	Mild	Possibly Related	None	No
A0049	Group A2	Afluria Quad IM, 15 ug	2	Musculoskeletal aches	18-MAR-2018	08:30	20-MAR-2018	08:00	Recovered / Resolved	Mild	Possibly Related	None	No
A0050	Group A4	A/Sing Mono IM, 15 ug		[No Treatment-Emergent Adverse Events Reported]									
A0051	Group A2	Afluria Quad IM, 15 ug		[No Treatment-Emergent Adverse Events Reported]									

Administration Site Key: FA = Forearm; UA= Upper arm
Treatments Part A: A1 = A/Sing MAP, 15 ug (FA); A2 = Afluria Quad IM (UA), 15 ug; A3 = Placebo MAP (FA); A4 = A/Sing Mono IM, 15 ug (UA)
Treatments Part B: B1 = A/Sing MAP, 15 ug (FA) with biopsy; B2 = Placebo MAP (FA) with biopsy
B3 = A/Sing MAP, 15 ug (FA); B4 = A/Sing MAP, 10 ug (FA); B5 = A/Sing MAP, 5 ug (FA); B6 = A/Sing MAP, 2.5 ug (FA)
B7 = Placebo MAP (FA); B8 = A/Sing MAP, 15 ug (UA); B9 = A/Sing IM, 15 ug, as 2018 Afluria Quad (UA)

Listing 16.2.7.1
Adverse Events - CRF Data only

Part A

Random-isation Number	Group	Treatment	AE No.	Adverse Event Verbatim	Onset Date	Onset Time	End Date	End Time	Outcome	Maximum Severity	Relation-ship	Actions Taken	SAE
A0052	Group A4	A/Sing Mono IM, 15 ug		[No Treatment-Emergent Adverse Events Reported]									
A0053	Group A4	A/Sing Mono IM, 15 ug		[No Treatment-Emergent Adverse Events Reported]									
A0054	Group A2	Afluria Quad IM, 15 ug	1	Shortness of Breath	20-MAR-2018	15:30	20-MAR-2018	16:30	Recovered / Resolved	Mild	Unlikely Related	Other Safety Bloods, ECG, Vital Signs + Oxygen Saturation	No
A0055	Group A4	A/Sing Mono IM, 15 ug		[No Treatment-Emergent Adverse Events Reported]									

Administration Site Key: FA = Forearm; UA= Upper arm
Treatments Part A: A1 = A/Sing MAP, 15 ug (FA); A2 = Afluria Quad IM (UA), 15 ug; A3 = Placebo MAP (FA); A4 = A/Sing Mono IM, 15 ug (UA)
Treatments Part B: B1 = A/Sing MAP, 15 ug (FA) with biopsy; B2 = Placebo MAP (FA) with biopsy
B3 = A/Sing MAP, 15 ug (FA); B4 = A/Sing MAP, 10 ug (FA); B5 = A/Sing MAP, 5 ug (FA); B6 = A/Sing MAP, 2.5 ug (FA)
B7 = Placebo MAP (FA); B8 = A/Sing MAP, 15 ug (UA); B9 = A/Sing IM, 15 ug, as 2018 Afluria Quad (UA)

Listing 16.2.7.1
Adverse Events - CRF Data only

Part A

Randomisation Number	Group	Treatment	AE No.	Adverse Event Verbatim	Onset Date	Onset Time	End Date	End Time	Outcome	Maximum Severity	Relationship	Actions Taken	SAE
A0056	Group A2	Afluria Quad IM, 15 ug		[No Treatment-Emergent Adverse Events Reported]									
A0057	Group A4	A/Sing Mono IM, 15 ug		[No Treatment-Emergent Adverse Events Reported]									
A0058	Group A2	Afluria Quad IM, 15 ug	1	Upper Respiratory Tract Infection	11-MAY-2018	08:00	19-MAY-2018	UK:UK	Recovered / Resolved	Moderate	Not Related	Concomitant Medication	No
A0059	Group A2	Afluria Quad IM, 15 ug	1	Intermittent Headache	28-MAR-2018	16:30	27-APR-2018	21:00	Recovered / Resolved	Mild	Unlikely Related	Concomitant Medication	No
A0060	Group A4	A/Sing Mono IM, 15 ug		[No Treatment-Emergent Adverse Events Reported]									

Administration Site Key: FA = Forearm; UA= Upper arm
Treatments Part A: A1 = A/Sing MAP, 15 ug (FA); A2 = Afluria Quad IM (UA), 15 ug; A3 = Placebo MAP (FA); A4 = A/Sing Mono IM, 15 ug (UA)
Treatments Part B: B1 = A/Sing MAP, 15 ug (FA) with biopsy; B2 = Placebo MAP (FA) with biopsy
B3 = A/Sing MAP, 15 ug (FA); B4 = A/Sing MAP, 10 ug (FA); B5 = A/Sing MAP, 5 ug (FA); B6 = A/Sing MAP, 2.5 ug (FA)
B7 = Placebo MAP (FA); B8 = A/Sing MAP, 15 ug (UA); B9 = A/Sing IM, 15 ug, as 2018 Afluria Quad (UA)

Listing 16.2.7.1
Adverse Events - CRF Data only

Part A

Random-isation Number	Group	Treatment	AE No.	Adverse Event Verbatim	Onset Date	Onset Time	End Date	End Time	Outcome	Maximum Severity	Relation-ship	Actions Taken	SAE
A1026	Group A1	A/Sing MAP, 15 ug		[No Treatment-Emergent Adverse Events Reported]									

Administration Site Key: FA = Forearm; UA= Upper arm
Treatments Part A: A1 = A/Sing MAP, 15 ug (FA); A2 = Afluria Quad IM (UA), 15 ug; A3 = Placebo MAP (FA); A4 = A/Sing Mono IM, 15 ug (UA)
Treatments Part B: B1 = A/Sing MAP, 15 ug (FA) with biopsy; B2 = Placebo MAP (FA) with biopsy
B3 = A/Sing MAP, 15 ug (FA); B4 = A/Sing MAP, 10 ug (FA); B5 = A/Sing MAP, 5 ug (FA); B6 = A/Sing MAP, 2.5 ug (FA)
B7 = Placebo MAP (FA); B8 = A/Sing MAP, 15 ug (UA); B9 = A/Sing IM, 15 ug, as 2018 Afluria Quad (UA)

Listing 16.2.7.1
Adverse Events - CRF Data only

Part B

Random-isation Number	Group	Treatment	AE No.	Adverse Event Verbatim	Onset Date	Onset Time	End Date	End Time	Outcome	Maximum Severity	Relation-ship	Actions Taken	SAE
B0001	Group B1	A/Sing MAP, 15 ug	1	MAP Erythema sites 1, 2 and 3	10-MAY-2018	09:06	UN-Jun-2018	UK:UK	Recovered / Resolved	Mild	Definitely Related	None	No
B0002	Group B2	Placebo MAP	1	Adhesive tapes irritation	07-MAY-2018	11:00	11-MAY-2018	13:17	Recovered / Resolved	Mild	Not Related	None	No
B0003	Group B2	Placebo MAP	1	Biopsy site -1 inflamed scar	28-MAY-2018	UK:UK	UN-Jun-2018	UK:UK	Recovered / Resolved	Mild	Unlikely Related	None	No
B0004	Group B1	A/Sing MAP, 15 ug	1	Erythema MAP sites 1, 2 & 3	10-MAY-2018	14:49	11-JUN-2018	UK:UK	Recovered / Resolved	Mild	Definitely Related	None	No
B0005	Group B1	A/Sing MAP, 15 ug	1	MAP reaction site 1,2,3 Erythema	10-MAY-2018	09:34	06-JUL-2018	17:10	Recovered / Resolved	Mild	Definitely Related	None	No
B0005	Group B1	A/Sing MAP, 15 ug	3	Sore throat	11-MAY-2018	00:00	28-MAY-2018	13:55	Recovered / Resolved	Mild	Unlikely Related	None	No
B0005	Group B1	A/Sing MAP, 15 ug	4	Skin Dysesthesia	20-MAY-2018	18:00	11-JUN-2018	UK:UK	Recovered / Resolved	Mild	Unlikely Related	None	No

Administration Site Key: FA = Forearm; UA= Upper arm
Treatments Part A: A1 = A/Sing MAP, 15 ug (FA); A2 = Afluria Quad IM (UA), 15 ug; A3 = Placebo MAP (FA); A4 = A/Sing Mono IM, 15 ug (UA)
Treatments Part B: B1 = A/Sing MAP, 15 ug (FA) with biopsy; B2 = Placebo MAP (FA) with biopsy
B3 = A/Sing MAP, 15 ug (FA); B4 = A/Sing MAP, 10 ug (FA); B5 = A/Sing MAP, 5 ug (FA); B6 = A/Sing MAP, 2.5 ug (FA)
B7 = Placebo MAP (FA); B8 = A/Sing MAP, 15 ug (UA); B9 = A/Sing IM, 15 ug, as 2018 Afluria Quad (UA)

Listing 16.2.7.1
Adverse Events - CRF Data only

Part B

Random-isation Number	Group	Treatment	AE No.	Adverse Event Verbatim	Onset Date	Onset Time	End Date	End Time	Outcome	Maximum Severity	Relation-ship	Actions Taken	SAE
B0006	Group B2	Placebo MAP	1	Biopsy site pain	07-MAY-2018	12:30	11-JUN-2018	UK:UK	Recovered / Resolved	Mild	Not Related	None	No
B0007	Group B2	Placebo MAP	1	Headache	09-MAY-2018	16:00	17-MAY-2018	14:00	Recovered / Resolved	Mild	Possibly Related	Concomitant Medication	No
B0008	Group B1	A/Sing MAP, 15 ug	1	Nasal congestion	08-MAY-2018	09:00	09-MAY-2018	22:30	Recovered / Resolved	Mild	Possibly Related	None	No
B0008	Group B1	A/Sing MAP, 15 ug	2	MAP 1,2,3 Erythema	10-MAY-2018	09:13	UN-Jun-2018	UK:UK	Recovered / Resolved	Mild	Definitely Related	None	No
B0009	Group B1	A/Sing MAP, 15 ug	1	MAP site 2,3 reaction erythema	10-MAY-2018	09:56	11-JUN-2018	UK:UK	Recovered / Resolved	Mild	Definitely Related	None	No
B0010	Group B2	Placebo MAP	1	Pain on biopsy site - Right arm	07-MAY-2018	16:00	12-MAY-2018	23:00	Recovered / Resolved	Mild	Not Related	Concomitant Medication	No
B0010	Group B2	Placebo MAP	2	Sore throat	12-MAY-2018	07:00	15-MAY-2018	UK:UK	Recovered / Resolved	Mild	Possibly Related	Concomitant Medication	No
B0010	Group B2	Placebo MAP	4	Parasthesia	07-MAY-2018	11:25	06-JUN-2018	11:15	Recovered / Resolved	Mild	Not Related	None	No

Administration Site Key: FA = Forearm; UA= Upper arm
Treatments Part A: A1 = A/Sing MAP, 15 ug (FA); A2 = Afluria Quad IM (UA), 15 ug; A3 = Placebo MAP (FA); A4 = A/Sing Mono IM, 15 ug (UA)
Treatments Part B: B1 = A/Sing MAP, 15 ug (FA) with biopsy; B2 = Placebo MAP (FA) with biopsy
B3 = A/Sing MAP, 15 ug (FA); B4 = A/Sing MAP, 10 ug (FA); B5 = A/Sing MAP, 5 ug (FA); B6 = A/Sing MAP, 2.5 ug (FA)
B7 = Placebo MAP (FA); B8 = A/Sing MAP, 15 ug (UA); B9 = A/Sing IM, 15 ug, as 2018 Afluria Quad (UA)

Listing 16.2.7.1
Adverse Events - CRF Data only

Part B

Randomisation Number	Group	Treatment	AE No.	Adverse Event Verbatim	Onset Date	Onset Time	End Date	End Time	Outcome	Maximum Severity	Relationship	Actions Taken	SAE
B0010	Group B2	Placebo MAP	6	Left Arm Biopsy site pain	08-MAY-2018	UK:UK	11-JUN-2018	UK:UK	Recovered / Resolved	Mild	Unlikely Related	None	No
B0011	Group B8	A/Sing MAP, 15 ug	1	MAP Reaction - Erythema sites 1,2,3	13-MAY-2018	11:01	14-JUL-2018	09:44	Recovered / Resolved	Mild	Definitely Related	None	No
B0011	Group B8	A/Sing MAP, 15 ug	2	MAP site #1 flaking	15-MAY-2018	11:15	14-JUL-2018	09:40	Recovered / Resolved	Mild	Definitely Related	None	No
B0012	Group B6	A/Sing MAP, 2.5 ug	3	MAP 1 Oedema	12-MAY-2018	12:16	19-MAY-2018	13:22	Recovered / Resolved	Mild	Probably Related	None	No
B0012	Group B6	A/Sing MAP, 2.5 ug	4	MAP 1 Erythema	13-MAY-2018	11:29	19-MAY-2018	13:22	Recovered / Resolved	Mild	Probably Related	None	No
B0013	Group B5	A/Sing MAP, 5 ug	1	Upper Respiratory Tract Infection	18-MAY-2018	08:00	11-JUL-2018	08:00	Recovered / Resolved	Mild	Not Related	Concomitant Medication	No
B0013	Group B5	A/Sing MAP, 5 ug	2	Headache	18-MAY-2018	08:00	18-MAY-2018	13:00	Recovered / Resolved	Moderate	Unlikely Related	Concomitant Medication	No
B0013	Group B5	A/Sing MAP, 5 ug	3	Hot Flushes	12-MAY-2018	14:30	13-MAY-2018	15:00	Recovered / Resolved	Mild	Possibly Related	None	No

Administration Site Key: FA = Forearm; UA= Upper arm
Treatments Part A: A1 = A/Sing MAP, 15 ug (FA); A2 = Afluria Quad IM (UA), 15 ug; A3 = Placebo MAP (FA); A4 = A/Sing Mono IM, 15 ug (UA)
Treatments Part B: B1 = A/Sing MAP, 15 ug (FA) with biopsy; B2 = Placebo MAP (FA) with biopsy
B3 = A/Sing MAP, 15 ug (FA); B4 = A/Sing MAP, 10 ug (FA); B5 = A/Sing MAP, 5 ug (FA); B6 = A/Sing MAP, 2.5 ug (FA)
B7 = Placebo MAP (FA); B8 = A/Sing MAP, 15 ug (UA); B9 = A/Sing IM, 15 ug, as 2018 Afluria Quad (UA)

Listing 16.2.7.1
Adverse Events - CRF Data only

Part B

Randomisation Number	Group	Treatment	AE No.	Adverse Event Verbatim	Onset Date	Onset Time	End Date	End Time	Outcome	Maximum Severity	Relationship	Actions Taken	SAE
B0014	Group B3	A/Sing MAP, 15 ug	1	MAP sites 1,2,3 - Visibility	02-JUN-2018	09:33	27-AUG-2018	15:00	Recovered / Resolved	Mild	Definitely Related	None	No
B0015	Group B9	A/Sing IM, 15 ug	1	Upper Respiratory Tract Infection	15-MAY-2018	11:47	20-MAY-2018	09:00	Recovered / Resolved	Mild	Not Related	None	No
B0015	Group B9	A/Sing IM, 15 ug	2	Shingles rash Left torso	18-MAY-2018	18:00	30-JUN-2018	18:00	Recovered / Resolved	Moderate	Unlikely Related	Concomitant Medication	No
B0016	Group B4	A/Sing MAP, 10 ug	3	Headache	26-MAY-2018	UK:UK	26-MAY-2018	UK:UK	Recovered / Resolved	Mild	Not Related	Concomitant Medication	No
B0016	Group B4	A/Sing MAP, 10 ug	4	Cervical lymphadenopathy	29-MAY-2018	23:00	25-JUL-2018	UK:UK	Recovered / Resolved	Moderate	Possibly Related	Concomitant Medication	No
B0016	Group B4	A/Sing MAP, 10 ug	7	MAP reaction - erythema + oedema site 3	15-MAY-2018	08:49	02-JUN-2018	13:05	Recovered / Resolved	Mild	Definitely Related	None	No

Administration Site Key: FA = Forearm; UA= Upper arm
Treatments Part A: A1 = A/Sing MAP, 15 ug (FA); A2 = Afluria Quad IM (UA), 15 ug; A3 = Placebo MAP (FA); A4 = A/Sing Mono IM, 15 ug (UA)
Treatments Part B: B1 = A/Sing MAP, 15 ug (FA) with biopsy; B2 = Placebo MAP (FA) with biopsy
B3 = A/Sing MAP, 15 ug (FA); B4 = A/Sing MAP, 10 ug (FA); B5 = A/Sing MAP, 5 ug (FA); B6 = A/Sing MAP, 2.5 ug (FA)
B7 = Placebo MAP (FA); B8 = A/Sing MAP, 15 ug (UA); B9 = A/Sing IM, 15 ug, as 2018 Afluria Quad (UA)

Listing 16.2.7.1
Adverse Events - CRF Data only

Part B

Randomisation Number	Group	Treatment	AE No.	Adverse Event Verbatim	Onset Date	Onset Time	End Date	End Time	Outcome	Maximum Severity	Relationship	Actions Taken	SAE
B0017	Group B7	Placebo MAP		[No Treatment-Emergent Adverse Events Reported]									
B0018	Group B9	A/Sing IM, 15 ug		[No Treatment-Emergent Adverse Events Reported]									
B0020	Group B6	A/Sing MAP, 2.5 ug		[No Treatment-Emergent Adverse Events Reported]									
B0021	Group B7	Placebo MAP		[No Treatment-Emergent Adverse Events Reported]									

Administration Site Key: FA = Forearm; UA= Upper arm
Treatments Part A: A1 = A/Sing MAP, 15 ug (FA); A2 = Afluria Quad IM (UA), 15 ug; A3 = Placebo MAP (FA); A4 = A/Sing Mono IM, 15 ug (UA)
Treatments Part B: B1 = A/Sing MAP, 15 ug (FA) with biopsy; B2 = Placebo MAP (FA) with biopsy
B3 = A/Sing MAP, 15 ug (FA); B4 = A/Sing MAP, 10 ug (FA); B5 = A/Sing MAP, 5 ug (FA); B6 = A/Sing MAP, 2.5 ug (FA)
B7 = Placebo MAP (FA); B8 = A/Sing MAP, 15 ug (UA); B9 = A/Sing IM, 15 ug, as 2018 Afluria Quad (UA)

Listing 16.2.7.1
Adverse Events - CRF Data only

Part B

Random-isation Number	Group	Treatment	AE No.	Adverse Event Verbatim	Onset Date	Onset Time	End Date	End Time	Outcome	Maximum Severity	Relation-ship	Actions Taken	SAE
B0022	Group B4	A/Sing MAP, 10 ug	1	Tachycardia	12-MAY-2018	15:33	19-MAY-2018	13:12	Recovered / Resolved	Mild	Unlikely Related	None	No
B0022	Group B4	A/Sing MAP, 10 ug	2	Headache	16-MAY-2018	09:45	16-MAY-2018	11:15	Recovered / Resolved	Moderate	Unlikely Related	Concomitant Medication	No
B0022	Group B4	A/Sing MAP, 10 ug	3	MAP visibility sites 1,2,3	12-JUL-2018	11:03	31-AUG-2018	22:29	Recovered / Resolved	Mild	Definitely Related	None	No
B0023	Group B3	A/Sing MAP, 15 ug	1	Dizziness	12-MAY-2018	13:25	12-MAY-2018	13:29	Recovered / Resolved	Mild	Unlikely Related	Other Dose - 2 delayed laid supine for a few minutes, two additional vital sign assessments conducted	No
B0023	Group B3	A/Sing MAP, 15 ug	3	MAP reaction sites 1,2&3 - oedema and erythema	12-MAY-2018	15:39	19-MAY-2018	10:22	Recovered / Resolved	Mild	Definitely Related	None	No

Administration Site Key: FA = Forearm; UA= Upper arm
Treatments Part A: A1 = A/Sing MAP, 15 ug (FA); A2 = Afluria Quad IM (UA), 15 ug; A3 = Placebo MAP (FA); A4 = A/Sing Mono IM, 15 ug (UA)
Treatments Part B: B1 = A/Sing MAP, 15 ug (FA) with biopsy; B2 = Placebo MAP (FA) with biopsy
B3 = A/Sing MAP, 15 ug (FA); B4 = A/Sing MAP, 10 ug (FA); B5 = A/Sing MAP, 5 ug (FA); B6 = A/Sing MAP, 2.5 ug (FA)
B7 = Placebo MAP (FA); B8 = A/Sing MAP, 15 ug (UA); B9 = A/Sing IM, 15 ug, as 2018 Afluria Quad (UA)

Listing 16.2.7.1
Adverse Events - CRF Data only

Part B

Random-isation Number	Group	Treatment	AE No.	Adverse Event Verbatim	Onset Date	Onset Time	End Date	End Time	Outcome	Maximum Severity	Relation-ship	Actions Taken	SAE
B0024	Group B5	A/Sing MAP, 5 ug	1	MAP reaction sites 1,2,3 Erythema, site 3 oedema	12-MAY-2018	14:59	19-MAY-2018	13:25	Recovered / Resolved	Mild	Definitely Related	None	No
B0024	Group B5	A/Sing MAP, 5 ug	4	Map site #3 visibility	12-JUL-2018	14:28	28-AUG-2018	UK:UK	Recovered / Resolved	Mild	Probably Related	None	No
B0025	Group B8	A/Sing MAP, 15 ug	1	Rhinorrhea	13-MAY-2018	UK:UK	16-MAY-2018	07:00	Recovered / Resolved	Mild	Possibly Related	None	No
B0025	Group B8	A/Sing MAP, 15 ug	2	MAP site visibility 1,2,3	13-JUL-2018	13:38	21-AUG-2018	UK:UK	Recovered / Resolved	Mild	Probably Related	None	No
B0026	Group B9	A/Sing IM, 15 ug	1	Headache	31-MAY-2018	20:00	31-MAY-2018	22:00	Recovered / Resolved	Mild	Unlikely Related	None	No
B0027	Group B3	A/Sing MAP, 15 ug	1	MAP sites 1,2,3 - oedema	12-MAY-2018	16:40	02-JUN-2018	16:08	Recovered / Resolved	Mild	Definitely Related	None	No
B0027	Group B3	A/Sing MAP, 15 ug	2	Upper Respiratory Tract Infection	25-MAY-2018	UK:UK	27-MAY-2018	UK:UK	Recovered / Resolved	Mild	Not Related	None	No
B0027	Group B3	A/Sing MAP, 15 ug	3	MAP site 3 Erythema	15-MAY-2018	17:12	22-JUN-2018	16:08	Recovered / Resolved	Mild	Probably Related	None	No

Administration Site Key: FA = Forearm; UA= Upper arm
Treatments Part A: A1 = A/Sing MAP, 15 ug (FA); A2 = Afluria Quad IM (UA), 15 ug; A3 = Placebo MAP (FA); A4 = A/Sing Mono IM, 15 ug (UA)
Treatments Part B: B1 = A/Sing MAP, 15 ug (FA) with biopsy; B2 = Placebo MAP (FA) with biopsy
B3 = A/Sing MAP, 15 ug (FA); B4 = A/Sing MAP, 10 ug (FA); B5 = A/Sing MAP, 5 ug (FA); B6 = A/Sing MAP, 2.5 ug (FA)
B7 = Placebo MAP (FA); B8 = A/Sing MAP, 15 ug (UA); B9 = A/Sing IM, 15 ug, as 2018 Afluria Quad (UA)

Listing 16.2.7.1
Adverse Events - CRF Data only

Part B

Randomisation Number	Group	Treatment	AE No.	Adverse Event Verbatim	Onset Date	Onset Time	End Date	End Time	Outcome	Maximum Severity	Relationship	Actions Taken	SAE
B0027	Group B3	A/Sing MAP, 15 ug	4	MAP Visibility sites 1 and 3	11-JUL-2018	17:20	27-AUG-2018	17:20	Recovered / Resolved	Mild	Definitely Related	None	No
B0028	Group B5	A/Sing MAP, 5 ug	1	Headache	12-MAY-2018	21:00	13-MAY-2018	UK:UK	Recovered / Resolved	Mild	Unlikely Related	None	No
B0028	Group B5	A/Sing MAP, 5 ug	3	MAP site 1 - Erythema	16-MAY-2018	16:38	02-JUN-2018	14:50	Recovered / Resolved	Mild	Definitely Related	None	No
B0029	Group B6	A/Sing MAP, 2.5 ug	2	MAP reaction site 3 erythema and oedema	13-MAY-2018	14:56	19-MAY-2018	14:23	Recovered / Resolved	Mild	Definitely Related	None	No
B0029	Group B6	A/Sing MAP, 2.5 ug	3	Upper Respiratory Tract Infection	29-MAY-2018	14:51	04-JUN-2018	09:00	Recovered / Resolved	Moderate	Not Related	None	No
B0029	Group B6	A/Sing MAP, 2.5 ug	5	MAP site 1,2,3 visibility	11-JUL-2018	14:53	04-OCT-2018	UK:UK	Recovered / Resolved	Mild	Definitely Related	None	No
B0030	Group B7	Placebo MAP		[No Treatment-Emergent Adverse Events Reported]									

Administration Site Key: FA = Forearm; UA= Upper arm
Treatments Part A: A1 = A/Sing MAP, 15 ug (FA); A2 = Afluria Quad IM (UA), 15 ug; A3 = Placebo MAP (FA); A4 = A/Sing Mono IM, 15 ug (UA)
Treatments Part B: B1 = A/Sing MAP, 15 ug (FA) with biopsy; B2 = Placebo MAP (FA) with biopsy
B3 = A/Sing MAP, 15 ug (FA); B4 = A/Sing MAP, 10 ug (FA); B5 = A/Sing MAP, 5 ug (FA); B6 = A/Sing MAP, 2.5 ug (FA)
B7 = Placebo MAP (FA); B8 = A/Sing MAP, 15 ug (UA); B9 = A/Sing IM, 15 ug, as 2018 Afluria Quad (UA)

Listing 16.2.7.1
Adverse Events - CRF Data only

Part B

Randomisation Number	Group	Treatment	AE No.	Adverse Event Verbatim	Onset Date	Onset Time	End Date	End Time	Outcome	Maximum Severity	Relationship	Actions Taken	SAE
B0031	Group B4	A/Sing MAP, 10 ug	5	MAP site 2 & 3 Reaction Erythema and induration	20-MAY-2018	13:19	26-MAY-2018	13:08	Recovered / Resolved	Mild	Definitely Related	None	No
B0032	Group B6	A/Sing MAP, 2.5 ug	1	MAP reaction site 3 erythema	22-MAY-2018	08:15	26-MAY-2018	09:08	Recovered / Resolved	Mild	Probably Related	None	No
B0032	Group B6	A/Sing MAP, 2.5 ug	2	Common cold	25-MAY-2018	20:00	28-MAY-2018	08:00	Recovered / Resolved	Mild	Unlikely Related	None	No
B0033	Group B5	A/Sing MAP, 5 ug	1	MAP reaction site 3 - erythema	20-MAY-2018	11:48	09-JUN-2018	14:00	Recovered / Resolved	Mild	Definitely Related	None	No
B0033	Group B5	A/Sing MAP, 5 ug	2	MAP site 3 visibility	18-JUL-2018	13:40	29-AUG-2018	17:53	Recovered / Resolved	Mild	Probably Related	None	No
B0034	Group B8	A/Sing MAP, 15 ug	2	Patch site reaction - Oedema + Erythema site #1	20-MAY-2018	14:05	20-JUN-2018	11:33	Recovered / Resolved	Mild	Definitely Related	None	No
B0035	Group B9	A/Sing IM, 15 ug	1	Upper Respiratory Tract Infection	31-MAY-2018	12:00	09-JUN-2018	08:00	Recovered / Resolved	Mild	Not Related	Concomitant Medication	No

Administration Site Key: FA = Forearm; UA= Upper arm
Treatments Part A: A1 = A/Sing MAP, 15 ug (FA); A2 = Afluria Quad IM (UA), 15 ug; A3 = Placebo MAP (FA); A4 = A/Sing Mono IM, 15 ug (UA)
Treatments Part B: B1 = A/Sing MAP, 15 ug (FA) with biopsy; B2 = Placebo MAP (FA) with biopsy
B3 = A/Sing MAP, 15 ug (FA); B4 = A/Sing MAP, 10 ug (FA); B5 = A/Sing MAP, 5 ug (FA); B6 = A/Sing MAP, 2.5 ug (FA)
B7 = Placebo MAP (FA); B8 = A/Sing MAP, 15 ug (UA); B9 = A/Sing IM, 15 ug, as 2018 Afluria Quad (UA)

Listing 16.2.7.1
Adverse Events - CRF Data only

Part B

Random-isation Number	Group	Treatment	AE No.	Adverse Event Verbatim	Onset Date	Onset Time	End Date	End Time	Outcome	Maximum Severity	Relation-ship	Actions Taken	SAE
B0036	Group B7	Placebo MAP	1	Nausea	19-MAY-2018	UK:UK	19-MAY-2018	13:00	Recovered / Resolved	Mild	Unlikely Related	None	No
B0037	Group B4	A/Sing MAP, 10 ug	1	Patch sites reaction 2,3 - erythema site	20-MAY-2018	12:02	09-JUN-2018	14:04	Recovered / Resolved	Mild	Definitely Related	None	No
B0037	Group B4	A/Sing MAP, 10 ug	3	Upper Respiratory Tract Infection	20-MAY-2018	08:30	21-MAY-2018	19:30	Recovered / Resolved	Moderate	Not Related	Concomitant Medication	No
B0037	Group B4	A/Sing MAP, 10 ug	4	MAP 2 Erythema extent	22-MAY-2018	12:11	26-MAY-2018	10:55	Recovered / Resolved	Mild	Probably Related	None	No
B0037	Group B4	A/Sing MAP, 10 ug	5	MAP visibility sites 2,3	26-MAY-2018	10:56			Not recovered / Not resolved	Mild	Probably Related	None	No
B0038	Group B3	A/Sing MAP, 15 ug	1	MAP reaction site 1,2,3 erythema	20-MAY-2018	08:24	07-JUL-2018	15:50	Recovered / Resolved	Mild	Definitely Related	None	No
B0038	Group B3	A/Sing MAP, 15 ug	2	Lethargy	21-MAY-2018	12:00	27-MAY-2018	12:00	Recovered / Resolved	Mild	Possibly Related	None	No

Administration Site Key: FA = Forearm; UA= Upper arm
Treatments Part A: A1 = A/Sing MAP, 15 ug (FA); A2 = Afluria Quad IM (UA), 15 ug; A3 = Placebo MAP (FA); A4 = A/Sing Mono IM, 15 ug (UA)
Treatments Part B: B1 = A/Sing MAP, 15 ug (FA) with biopsy; B2 = Placebo MAP (FA) with biopsy
B3 = A/Sing MAP, 15 ug (FA); B4 = A/Sing MAP, 10 ug (FA); B5 = A/Sing MAP, 5 ug (FA); B6 = A/Sing MAP, 2.5 ug (FA)
B7 = Placebo MAP (FA); B8 = A/Sing MAP, 15 ug (UA); B9 = A/Sing IM, 15 ug, as 2018 Afluria Quad (UA)

Listing 16.2.7.1
Adverse Events - CRF Data only

Part B

Randomisation Number	Group	Treatment	AE No.	Adverse Event Verbatim	Onset Date	Onset Time	End Date	End Time	Outcome	Maximum Severity	Relationship	Actions Taken	SAE
B0039	Group B7	Placebo MAP	2	Headache	24-MAY-2018	21:00	25-MAY-2018	12:00	Recovered / Resolved	Moderate	Unlikely Related	Concomitant Medication	No
B0040	Group B9	A/Sing IM, 15 ug		[No Treatment-Emergent Adverse Events Reported]									
B0041	Group B3	A/Sing MAP, 15 ug	1	Musculoskeletal pain Left shoulder	20-MAY-2018	11:00	20-MAY-2018	19:00	Recovered / Resolved	Mild	Unlikely Related	None	No
B0041	Group B3	A/Sing MAP, 15 ug	2	MAP sites 1,2,3 - erythema	20-MAY-2018	13:37	26-MAY-2018	12:36	Recovered / Resolved	Mild	Definitely Related	None	No
B0041	Group B3	A/Sing MAP, 15 ug	3	MAP 1,2,3 visible	20-JUL-2018	15:24			Not recovered / Not resolved	Mild	Definitely Related	None	No
B0042	Group B5	A/Sing MAP, 5 ug	1	Patch site - 3 reaction - Erythema	20-MAY-2018	12:20	07-JUL-2018	18:16	Recovered / Resolved	Mild	Definitely Related	None	No
B0042	Group B5	A/Sing MAP, 5 ug	3	Headache	23-MAY-2018	06:15	09-JUN-2018	UK:UK	Recovered / Resolved	Moderate	Possibly Related	Concomitant Medication	No

Administration Site Key: FA = Forearm; UA= Upper arm
Treatments Part A: A1 = A/Sing MAP, 15 ug (FA); A2 = Afluria Quad IM (UA), 15 ug; A3 = Placebo MAP (FA); A4 = A/Sing Mono IM, 15 ug (UA)
Treatments Part B: B1 = A/Sing MAP, 15 ug (FA) with biopsy; B2 = Placebo MAP (FA) with biopsy
B3 = A/Sing MAP, 15 ug (FA); B4 = A/Sing MAP, 10 ug (FA); B5 = A/Sing MAP, 5 ug (FA); B6 = A/Sing MAP, 2.5 ug (FA)
B7 = Placebo MAP (FA); B8 = A/Sing MAP, 15 ug (UA); B9 = A/Sing IM, 15 ug, as 2018 Afluria Quad (UA)

Listing 16.2.7.1
Adverse Events - CRF Data only

Part B

Randomisation Number	Group	Treatment	AE No.	Adverse Event Verbatim	Onset Date	Onset Time	End Date	End Time	Outcome	Maximum Severity	Relationship	Actions Taken	SAE
B0042	Group B5	A/Sing MAP, 5 ug	5	MAP 3 Visiblity	18-JUL-2018	09:30	05-SEP-2018	09:00	Recovered / Resolved	Mild	Definitely Related	None	No
B0043	Group B8	A/Sing MAP, 15 ug	1	Headache	19-MAY-2018	18:00	19-MAY-2018	19:30	Recovered / Resolved	Mild	Possibly Related	Concomitant Medication	No
B0043	Group B8	A/Sing MAP, 15 ug	2	Left arm bruise	02-JUN-2018	UK:UK	13-JUN-2018	UK:UK	Recovered / Resolved	Mild	Unlikely Related	None	No
B0043	Group B8	A/Sing MAP, 15 ug	3	Myalgia Left deltoid	02-JUN-2018	UK:UK	13-JUN-2018	UK:UK	Recovered / Resolved	Mild	Unlikely Related	None	No
B0043	Group B8	A/Sing MAP, 15 ug	4	MAP Visibility 1,2,3	18-JUL-2018	09:25			Not recovered / Not resolved	Mild	Definitely Related	None	No
B0044	Group B6	A/Sing MAP, 2.5 ug	1	MAP site 3 reaction - erythema and oedema	20-MAY-2018	12:33	07-JUL-2018	18:32	Recovered / Resolved	Mild	Definitely Related	None	No
B0044	Group B6	A/Sing MAP, 2.5 ug	3	MAP #3 visible	18-JUL-2018	13:19			Not recovered / Not resolved	Mild	Definitely Related	None	No

Administration Site Key: FA = Forearm; UA= Upper arm
Treatments Part A: A1 = A/Sing MAP, 15 ug (FA); A2 = Afluria Quad IM (UA), 15 ug; A3 = Placebo MAP (FA); A4 = A/Sing Mono IM, 15 ug (UA)
Treatments Part B: B1 = A/Sing MAP, 15 ug (FA) with biopsy; B2 = Placebo MAP (FA) with biopsy
B3 = A/Sing MAP, 15 ug (FA); B4 = A/Sing MAP, 10 ug (FA); B5 = A/Sing MAP, 5 ug (FA); B6 = A/Sing MAP, 2.5 ug (FA)
B7 = Placebo MAP (FA); B8 = A/Sing MAP, 15 ug (UA); B9 = A/Sing IM, 15 ug, as 2018 Afluria Quad (UA)

Listing 16.2.7.1
Adverse Events - CRF Data only

Part B

Random-isation Number	Group	Treatment	AE No.	Adverse Event Verbatim	Onset Date	Onset Time	End Date	End Time	Outcome	Maximum Severity	Relation-ship	Actions Taken	SAE
B0045	Group B4	A/Sing MAP, 10 ug	2	MAP site #2 reaction oedema and erythema	20-MAY-2018	12:37	22-MAY-2018	10:41	Recovered / Resolved	Mild	Probably Related	None	No
B0045	Group B4	A/Sing MAP, 10 ug	6	MAP site 1,2 visibility	18-JUL-2018	11:51	20-AUG-2018	UK:UK	Recovered / Resolved	Mild	Definitely Related	None	No
B0046	Group B7	Placebo MAP	1	Headache	24-MAY-2018	22:14	25-MAY-2018	00:10	Recovered / Resolved	Mild	Unlikely Related	Concomitant Medication	No
B0047	Group B9	A/Sing IM, 15 ug		[No Treatment-Emergent Adverse Events Reported]									
B0048	Group B6	A/Sing MAP, 2.5 ug	1	MAP reaction site 1 erythema	20-MAY-2018	13:21	26-MAY-2018	10:22	Recovered / Resolved	Mild	Definitely Related	None	No
B0048	Group B6	A/Sing MAP, 2.5 ug	4	MAP visibility site 1	18-JUL-2018	15:55	01-SEP-2018	UK:UK	Recovered / Resolved	Mild	Definitely Related	None	No
B0049	Group B4	A/Sing MAP, 10 ug	1	MAP reaction 2,3 erythema and oedema	20-MAY-2018	14:02	22-MAY-2018	15:10	Recovered / Resolved	Mild	Definitely Related	None	No

Administration Site Key: FA = Forearm; UA= Upper arm
Treatments Part A: A1 = A/Sing MAP, 15 ug (FA); A2 = Afluria Quad IM (UA), 15 ug; A3 = Placebo MAP (FA); A4 = A/Sing Mono IM, 15 ug (UA)
Treatments Part B: B1 = A/Sing MAP, 15 ug (FA) with biopsy; B2 = Placebo MAP (FA) with biopsy
B3 = A/Sing MAP, 15 ug (FA); B4 = A/Sing MAP, 10 ug (FA); B5 = A/Sing MAP, 5 ug (FA); B6 = A/Sing MAP, 2.5 ug (FA)
B7 = Placebo MAP (FA); B8 = A/Sing MAP, 15 ug (UA); B9 = A/Sing IM, 15 ug, as 2018 Afluria Quad (UA)

Listing 16.2.7.1
Adverse Events - CRF Data only

Part B

Random-isation Number	Group	Treatment	AE No.	Adverse Event Verbatim	Onset Date	Onset Time	End Date	End Time	Outcome	Maximum Severity	Relation-ship	Actions Taken	SAE
B0049	Group B4	A/Sing MAP, 10 ug	4	MAP Visibility 1,2,3	18-JUL-2018	10:49			Not recovered / Not resolved	Mild	Definitely Related	None	No
B0050	Group B8	A/Sing MAP, 15 ug	1	MAP Reaction 1,2,3 - Erythema	20-MAY-2018	14:45	18-JUL-2018	14:23	Recovered / Resolved	Mild	Probably Related	None	No
B0051	Group B3	A/Sing MAP, 15 ug		[No Treatment-Emergent Adverse Events Reported]									
B0052	Group B5	A/Sing MAP, 5 ug	1	MAP reaction - Site #1 Erythema, oedema and tenderness	26-MAY-2018	UK:UK	01-JUN-2018	13:55	Recovered / Resolved	Mild	Definitely Related	None	No
B0052	Group B5	A/Sing MAP, 5 ug	2	Left Axilla lymphadenopathy	27-MAY-2018	08:00	28-MAY-2018	07:00	Recovered / Resolved	Mild	Possibly Related	None	No
B0052	Group B5	A/Sing MAP, 5 ug	3	Upper Respiratory Tract Infection	09-JUN-2018	09:00	20-JUN-2018	08:00	Recovered / Resolved	Moderate	Not Related	Concomitant Medication	No

Administration Site Key: FA = Forearm; UA= Upper arm
Treatments Part A: A1 = A/Sing MAP, 15 ug (FA); A2 = Afluria Quad IM (UA), 15 ug; A3 = Placebo MAP (FA); A4 = A/Sing Mono IM, 15 ug (UA)
Treatments Part B: B1 = A/Sing MAP, 15 ug (FA) with biopsy; B2 = Placebo MAP (FA) with biopsy
B3 = A/Sing MAP, 15 ug (FA); B4 = A/Sing MAP, 10 ug (FA); B5 = A/Sing MAP, 5 ug (FA); B6 = A/Sing MAP, 2.5 ug (FA)
B7 = Placebo MAP (FA); B8 = A/Sing MAP, 15 ug (UA); B9 = A/Sing IM, 15 ug, as 2018 Afluria Quad (UA)

Listing 16.2.7.1
Adverse Events - CRF Data only

Part B

Random-isation Number	Group	Treatment	AE No.	Adverse Event Verbatim	Onset Date	Onset Time	End Date	End Time	Outcome	Maximum Severity	Relation-ship	Actions Taken	SAE
B0052	Group B5	A/Sing MAP, 5 ug	4	Headache	01-JUN-2018	23:45	02-JUN-2018	06:00	Recovered / Resolved	Moderate	Not Related	Concomitant Medication	No
B0052	Group B5	A/Sing MAP, 5 ug	5	Patch site 1,2,3 visibility	20-JUL-2018	14:10	18-SEP-2018	UK:UK	Recovered / Resolved	Mild	Definitely Related	None	No
B0053	Group B6	A/Sing MAP, 2.5 ug	1	MAP site 1,2,3 Visibility	25-JUL-2018	12:49	28-AUG-2018	UK:UK	Recovered / Resolved	Mild	Definitely Related	None	No
B0054	Group B5	A/Sing MAP, 5 ug	1	Upper Respiratory Tract Infection	26-MAY-2018	06:12	27-MAY-2018	10:41	Recovered / Resolved	Mild	Not Related	None	No
B0054	Group B5	A/Sing MAP, 5 ug	2	Vasovagal dizziness	15-JUN-2018	08:49	15-JUN-2018	09:04	Recovered / Resolved	Mild	Not Related	Other Trendelenbu rg position, Apple juice 250ml	No
B0055	Group B8	A/Sing MAP, 15 ug	1	Headache	25-MAY-2018	14:15	25-MAY-2018	16:15	Recovered / Resolved	Mild	Possibly Related	None	No
B0055	Group B8	A/Sing MAP, 15 ug	2	Upper Respiratory Tract Infection	27-MAY-2018	10:00	29-MAY-2018	10:00	Recovered / Resolved	Mild	Not Related	None	No

Administration Site Key: FA = Forearm; UA= Upper arm
Treatments Part A: A1 = A/Sing MAP, 15 ug (FA); A2 = Afluria Quad IM (UA), 15 ug; A3 = Placebo MAP (FA); A4 = A/Sing Mono IM, 15 ug (UA)
Treatments Part B: B1 = A/Sing MAP, 15 ug (FA) with biopsy; B2 = Placebo MAP (FA) with biopsy
B3 = A/Sing MAP, 15 ug (FA); B4 = A/Sing MAP, 10 ug (FA); B5 = A/Sing MAP, 5 ug (FA); B6 = A/Sing MAP, 2.5 ug (FA)
B7 = Placebo MAP (FA); B8 = A/Sing MAP, 15 ug (UA); B9 = A/Sing IM, 15 ug, as 2018 Afluria Quad (UA)

Listing 16.2.7.1
Adverse Events - CRF Data only

Part B

Random-isation Number	Group	Treatment	AE No.	Adverse Event Verbatim	Onset Date	Onset Time	End Date	End Time	Outcome	Maximum Severity	Relation-ship	Actions Taken	SAE
B0055	Group B8	A/Sing MAP, 15 ug	3	Tonsillitis	14-JUN-2018	14:00	17-JUN-2018	12:00	Recovered / Resolved	Moderate	Unlikely Related	Concomitant Medication	No
B0056	Group B3	A/Sing MAP, 15 ug	2	MAP site reaction - Erythema 1,2	26-MAY-2018	UK:UK	30-MAY-2018	UK:UK	Recovered / Resolved	Mild	Probably Related	None	No
B0056	Group B3	A/Sing MAP, 15 ug	3	MAP 1,2,3 Visibility	01-JUN-2018	08:50	28-AUG-2018	UK:UK	Recovered / Resolved	Mild	Probably Related	None	No
B0057	Group B4	A/Sing MAP, 10 ug	1	MAP 1,2,3 Visibiity	24-JUL-2018	09:43			Not recovered / Not resolved	Mild	Probably Related	None	No
B0058	Group B9	A/Sing IM, 15 ug	1	Exacerbations of Migraine	06-JUN-2018	13:00	07-JUN-2018	08:00	Recovered / Resolved	Moderate	Unlikely Related	Concomitant Medication	No
B0058	Group B9	A/Sing IM, 15 ug	2	Left arm (IP arm) discomfort	25-MAY-2018	13:00	25-MAY-2018	13:46	Recovered / Resolved	Mild	Probably Related	None	No

Administration Site Key: FA = Forearm; UA= Upper arm
Treatments Part A: A1 = A/Sing MAP, 15 ug (FA); A2 = Afluria Quad IM (UA), 15 ug; A3 = Placebo MAP (FA); A4 = A/Sing Mono IM, 15 ug (UA)
Treatments Part B: B1 = A/Sing MAP, 15 ug (FA) with biopsy; B2 = Placebo MAP (FA) with biopsy
B3 = A/Sing MAP, 15 ug (FA); B4 = A/Sing MAP, 10 ug (FA); B5 = A/Sing MAP, 5 ug (FA); B6 = A/Sing MAP, 2.5 ug (FA)
B7 = Placebo MAP (FA); B8 = A/Sing MAP, 15 ug (UA); B9 = A/Sing IM, 15 ug, as 2018 Afluria Quad (UA)

Listing 16.2.7.1
Adverse Events - CRF Data only

Part B

Random-isation Number	Group	Treatment	AE No.	Adverse Event Verbatim	Onset Date	Onset Time	End Date	End Time	Outcome	Maximum Severity	Relation-ship	Actions Taken	SAE
B0059	Group B7	Placebo MAP		[No Treatment-Emergent Adverse Events Reported]									
B0060	Group B3	A/Sing MAP, 15 ug	5	Bruise to Right elbow	25-MAY-2018	22:00	28-MAY-2018	00:45	Recovered / Resolved	Mild	Unlikely Related	None	No
B0060	Group B3	A/Sing MAP, 15 ug	6	Patch site 1 reaction erythema and oedema	28-MAY-2018	14:39	24-JUL-2018	13:52	Recovered / Resolved	Mild	Definitely Related	None	No
B0061	Group B8	A/Sing MAP, 15 ug	2	MAP sites 1,2 - Erythema	28-MAY-2018	13:37	15-JUN-2018	12:10	Recovered / Resolved	Mild	Definitely Related	None	No
B0062	Group B5	A/Sing MAP, 5 ug		[No Treatment-Emergent Adverse Events Reported]									

Administration Site Key: FA = Forearm; UA= Upper arm
Treatments Part A: A1 = A/Sing MAP, 15 ug (FA); A2 = Afluria Quad IM (UA), 15 ug; A3 = Placebo MAP (FA); A4 = A/Sing Mono IM, 15 ug (UA)
Treatments Part B: B1 = A/Sing MAP, 15 ug (FA) with biopsy; B2 = Placebo MAP (FA) with biopsy
B3 = A/Sing MAP, 15 ug (FA); B4 = A/Sing MAP, 10 ug (FA); B5 = A/Sing MAP, 5 ug (FA); B6 = A/Sing MAP, 2.5 ug (FA)
B7 = Placebo MAP (FA); B8 = A/Sing MAP, 15 ug (UA); B9 = A/Sing IM, 15 ug, as 2018 Afluria Quad (UA)

Listing 16.2.7.1
Adverse Events - CRF Data only

Part B

Random-isation Number	Group	Treatment	AE No.	Adverse Event Verbatim	Onset Date	Onset Time	End Date	End Time	Outcome	Maximum Severity	Relation-ship	Actions Taken	SAE
B0063	Group B7	Placebo MAP		[No Treatment-Emergent Adverse Events Reported]									
B0064	Group B9	A/Sing IM, 15 ug	1	Upper Respiratory Tract Infection	01-JUN-2018	16:30	20-JUN-2018	12:00	Recovered / Resolved	Moderate	Not Related	Concomitant Medication	No
B0065	Group B4	A/Sing MAP, 10 ug	1	Tension Headache	25-MAY-2018	18:00	26-MAY-2018	12:26	Recovered / Resolved	Mild	Possibly Related	None	No
B0065	Group B4	A/Sing MAP, 10 ug	2	Upper Respiratory Tract Infection	08-JUN-2018	08:00	22-JUN-2018	09:00	Recovered / Resolved	Moderate	Not Related	Concomitant Medication	No
B0065	Group B4	A/Sing MAP, 10 ug	3	MAP site 1 & 3 erythema	26-MAY-2018	UK:UK	15-JUN-2018	09:59	Recovered / Resolved	Mild	Probably Related	None	No
B0065	Group B4	A/Sing MAP, 10 ug	4	MAP visibility site 1,2,3	24-JUL-2018	13:54	09-OCT-2018	UK:UK	Recovered / Resolved	Mild	Definitely Related	None	No

Administration Site Key: FA = Forearm; UA= Upper arm
Treatments Part A: A1 = A/Sing MAP, 15 ug (FA); A2 = Afluria Quad IM (UA), 15 ug; A3 = Placebo MAP (FA); A4 = A/Sing Mono IM, 15 ug (UA)
Treatments Part B: B1 = A/Sing MAP, 15 ug (FA) with biopsy; B2 = Placebo MAP (FA) with biopsy
B3 = A/Sing MAP, 15 ug (FA); B4 = A/Sing MAP, 10 ug (FA); B5 = A/Sing MAP, 5 ug (FA); B6 = A/Sing MAP, 2.5 ug (FA)
B7 = Placebo MAP (FA); B8 = A/Sing MAP, 15 ug (UA); B9 = A/Sing IM, 15 ug, as 2018 Afluria Quad (UA)

Listing 16.2.7.1
Adverse Events - CRF Data only

Part B

Random-isation Number	Group	Treatment	AE No.	Adverse Event Verbatim	Onset Date	Onset Time	End Date	End Time	Outcome	Maximum Severity	Relation-ship	Actions Taken	SAE
B0066	Group B6	A/Sing MAP, 2.5 ug		[No Treatment-Emergent Adverse Events Reported]									
B0067	Group B8	A/Sing MAP, 15 ug	1	Itchness - MAP 1,2,3	28-MAY-2018	07:00	31-MAY-2018	UK:UK	Recovered / Resolved	Mild	Definitely Related	None	No
B0068	Group B5	A/Sing MAP, 5 ug	1	MAP site 2 Visibility	24-JUL-2018	17:00	10-SEP-2018	UK:UK	Recovered / Resolved	Mild	Definitely Related	None	No
B0068	Group B5	A/Sing MAP, 5 ug	2	MAP 2 Erythema	01-JUN-2018	09:58	16-JUN-2018	08:13	Recovered / Resolved	Mild	Probably Related	None	No
B0069	Group B7	Placebo MAP	2	Headache	06-JUN-2018	19:00	06-JUN-2018	22:00	Recovered / Resolved	Moderate	Not Related	Concomitant Medication	No
B0070	Group B9	A/Sing IM, 15 ug		[No Treatment-Emergent Adverse Events Reported]									

Administration Site Key: FA = Forearm; UA= Upper arm
Treatments Part A: A1 = A/Sing MAP, 15 ug (FA); A2 = Afluria Quad IM (UA), 15 ug; A3 = Placebo MAP (FA); A4 = A/Sing Mono IM, 15 ug (UA)
Treatments Part B: B1 = A/Sing MAP, 15 ug (FA) with biopsy; B2 = Placebo MAP (FA) with biopsy
B3 = A/Sing MAP, 15 ug (FA); B4 = A/Sing MAP, 10 ug (FA); B5 = A/Sing MAP, 5 ug (FA); B6 = A/Sing MAP, 2.5 ug (FA)
B7 = Placebo MAP (FA); B8 = A/Sing MAP, 15 ug (UA); B9 = A/Sing IM, 15 ug, as 2018 Afluria Quad (UA)

Listing 16.2.7.1
Adverse Events - CRF Data only

Part B

Randomisation Number	Group	Treatment	AE No.	Adverse Event Verbatim	Onset Date	Onset Time	End Date	End Time	Outcome	Maximum Severity	Relation-ship	Actions Taken	SAE
B0071	Group B4	A/Sing MAP, 10 ug	1	MAP reaction site 3 - Erythema	02-JUN-2018	12:33	22-JUN-2018	13:31	Recovered / Resolved	Mild	Definitely Related	None	No
B0072	Group B3	A/Sing MAP, 15 ug	1	Headache	01-JUN-2018	12:40	02-JUN-2018	11:50	Recovered / Resolved	Mild	Possibly Related	None	No
B0072	Group B3	A/Sing MAP, 15 ug	2	MAP reaction sites 1,2,3 - Erythema	02-JUN-2018	12:36	08-JUN-2018	13:46	Recovered / Resolved	Mild	Definitely Related	None	No
B0073	Group B6	A/Sing MAP, 2.5 ug	1	MAP site reaction 2 - Oedema	01-JUN-2018	13:50	08-JUN-2018	12:34	Recovered / Resolved	Mild	Definitely Related	None	No
B0073	Group B6	A/Sing MAP, 2.5 ug	2	MAP reaction sites 1,2,3 - Erythema	02-JUN-2018	12:42	06-JUN-2018	UK:UK	Recovered / Resolved	Mild	Definitely Related	None	No
B0074	Group B9	A/Sing IM, 15 ug		[No Treatment-Emergent Adverse Events Reported]									

Administration Site Key: FA = Forearm; UA= Upper arm
Treatments Part A: A1 = A/Sing MAP, 15 ug (FA); A2 = Afluria Quad IM (UA), 15 ug; A3 = Placebo MAP (FA); A4 = A/Sing Mono IM, 15 ug (UA)
Treatments Part B: B1 = A/Sing MAP, 15 ug (FA) with biopsy; B2 = Placebo MAP (FA) with biopsy
B3 = A/Sing MAP, 15 ug (FA); B4 = A/Sing MAP, 10 ug (FA); B5 = A/Sing MAP, 5 ug (FA); B6 = A/Sing MAP, 2.5 ug (FA)
B7 = Placebo MAP (FA); B8 = A/Sing MAP, 15 ug (UA); B9 = A/Sing IM, 15 ug, as 2018 Afluria Quad (UA)

Listing 16.2.7.1
Adverse Events - CRF Data only

Part B

Random-isation Number	Group	Treatment	AE No.	Adverse Event Verbatim	Onset Date	Onset Time	End Date	End Time	Outcome	Maximum Severity	Relation-ship	Actions Taken	SAE
B0075	Group B3	A/Sing MAP, 15 ug	1	Sore Throat	02-JUN-2018	19:00	03-JUN-2018	10:00	Recovered / Resolved	Mild	Possibly Related	None	No
B0075	Group B3	A/Sing MAP, 15 ug	2	Headache	05-JUN-2018	08:00	05-JUN-2018	12:00	Recovered / Resolved	Mild	Not Related	Concomitant Medication	No
B0075	Group B3	A/Sing MAP, 15 ug	3	MAP 2,3 Visibility	31-JUL-2018	08:27	28-AUG-2018	10:38	Recovered / Resolved	Mild	Probably Related	None	No
B0077	Group B4	A/Sing MAP, 10 ug	1	MAP reaction site 1,3 oedema and erythema	04-JUN-2018	08:45	08-JUN-2018	19:15	Recovered / Resolved	Mild	Definitely Related	None	No
B0078	Group B8	A/Sing MAP, 15 ug	1	MAP 1,2,3 reaction - Erythema	02-JUN-2018	10:22	22-JUN-2018	12:13	Recovered / Resolved	Mild	Definitely Related	None	No
B0078	Group B8	A/Sing MAP, 15 ug	3	MAP reaction - Oedema sites 1,2,3 and site 1 itchiness	04-JUN-2018	11:32	08-JUN-2018	11:00	Recovered / Resolved	Mild	Definitely Related	None	No
B0078	Group B8	A/Sing MAP, 15 ug	4	MAP site 1,2,3 Visibility	06-AUG-2018	10:59	29-OCT-2018	UK:UK	Recovered / Resolved	Mild	Definitely Related	None	No

Administration Site Key: FA = Forearm; UA= Upper arm
Treatments Part A: A1 = A/Sing MAP, 15 ug (FA); A2 = Afluria Quad IM (UA), 15 ug; A3 = Placebo MAP (FA); A4 = A/Sing Mono IM, 15 ug (UA)
Treatments Part B: B1 = A/Sing MAP, 15 ug (FA) with biopsy; B2 = Placebo MAP (FA) with biopsy
B3 = A/Sing MAP, 15 ug (FA); B4 = A/Sing MAP, 10 ug (FA); B5 = A/Sing MAP, 5 ug (FA); B6 = A/Sing MAP, 2.5 ug (FA)
B7 = Placebo MAP (FA); B8 = A/Sing MAP, 15 ug (UA); B9 = A/Sing IM, 15 ug, as 2018 Afluria Quad (UA)

Listing 16.2.7.1
Adverse Events - CRF Data only

Part B

Random-isation Number	Group	Treatment	AE No.	Adverse Event Verbatim	Onset Date	Onset Time	End Date	End Time	Outcome	Maximum Severity	Relation-ship	Actions Taken	SAE
B0079	Group B6	A/Sing MAP, 2.5 ug	1	Rash- Erythema multiforme	02-JUN-2018	09:00	03-JUN-2018	10:00	Recovered / Resolved	Mild	Possibly Related	None	No
B0079	Group B6	A/Sing MAP, 2.5 ug	2	MAP site 2 - redness extension	02-JUN-2018	11:16	22-JUN-2018	12:00	Recovered / Resolved	Mild	Definitely Related	None	No
B0079	Group B6	A/Sing MAP, 2.5 ug	3	Headache	03-JUN-2018	15:00	03-JUN-2018	16:30	Recovered / Resolved	Moderate	Unlikely Related	Concomitant Medication	No
B0079	Group B6	A/Sing MAP, 2.5 ug	4	MAP sites 2,3 -Oedema	01-JUN-2018	14:43	22-JUN-2018	12:02	Recovered / Resolved	Mild	Definitely Related	None	No
B0079	Group B6	A/Sing MAP, 2.5 ug	5	MAP 2 visibility	31-JUL-2018	12:19	22-OCT-2018	UK:UK	Recovered / Resolved	Mild	Probably Related	None	No
B0081	Group B6	A/Sing MAP, 2.5 ug	1	MAP reaction sites 1,2,3 - Erythema and Oedema	01-JUN-2018	14:53	04-JUN-2018	14:21	Recovered / Resolved	Mild	Definitely Related	None	No
B0081	Group B6	A/Sing MAP, 2.5 ug	3	Headache	01-JUN-2018	16:45	01-JUN-2018	17:15	Recovered / Resolved	Moderate	Possibly Related	Concomitant Medication	No
B0081	Group B6	A/Sing MAP, 2.5 ug	4	Headache	08-JUN-2018	11:30	08-JUN-2018	14:30	Recovered / Resolved	Mild	Unlikely Related	Concomitant Medication	No

Administration Site Key: FA = Forearm; UA= Upper arm
Treatments Part A: A1 = A/Sing MAP, 15 ug (FA); A2 = Afluria Quad IM (UA), 15 ug; A3 = Placebo MAP (FA); A4 = A/Sing Mono IM, 15 ug (UA)
Treatments Part B: B1 = A/Sing MAP, 15 ug (FA) with biopsy; B2 = Placebo MAP (FA) with biopsy
B3 = A/Sing MAP, 15 ug (FA); B4 = A/Sing MAP, 10 ug (FA); B5 = A/Sing MAP, 5 ug (FA); B6 = A/Sing MAP, 2.5 ug (FA)
B7 = Placebo MAP (FA); B8 = A/Sing MAP, 15 ug (UA); B9 = A/Sing IM, 15 ug, as 2018 Afluria Quad (UA)

Listing 16.2.7.1
Adverse Events - CRF Data only

Part B

Randomisation Number	Group	Treatment	AE No.	Adverse Event Verbatim	Onset Date	Onset Time	End Date	End Time	Outcome	Maximum Severity	Relationship	Actions Taken	SAE
B0082	Group B3	A/Sing MAP, 15 ug	1	MAP reaction sites 1,2,3 reaction - Erythema & Oedema	02-JUN-2018	11:47	22-JUN-2018	13:47	Recovered / Resolved	Mild	Definitely Related	None	No
B0082	Group B3	A/Sing MAP, 15 ug	3	Chest pain	08-JUN-2018	05:40	08-JUN-2018	15:04	Recovered / Resolved	Moderate	Not Related	Other Unscheduled bloods and vital signs	No
B0082	Group B3	A/Sing MAP, 15 ug	4	MAP site 1,2,3 reaction - visibility	31-JUL-2018	15:19	10-SEP-2018	UK:UK	Recovered / Resolved	Mild	Probably Related	None	No
B0083	Group B8	A/Sing MAP, 15 ug	1	MAP reaction sites 2,3 -Oedema	01-JUN-2018	15:40	08-JUN-2018	14:03	Recovered / Resolved	Mild	Definitely Related	None	No
B0083	Group B8	A/Sing MAP, 15 ug	3	MAP sites 1,2,3 reaction - Erythema	02-JUN-2018	10:00	08-JUN-2018	14:03	Recovered / Resolved	Mild	Definitely Related	None	No
B0083	Group B8	A/Sing MAP, 15 ug	4	MAP site 1,2,3 Visibility	31-JUL-2018	08:33			Not recovered / Not resolved	Mild	Probably Related	None	No

Administration Site Key: FA = Forearm; UA= Upper arm
Treatments Part A: A1 = A/Sing MAP, 15 ug (FA); A2 = Afluria Quad IM (UA), 15 ug; A3 = Placebo MAP (FA); A4 = A/Sing Mono IM, 15 ug (UA)
Treatments Part B: B1 = A/Sing MAP, 15 ug (FA) with biopsy; B2 = Placebo MAP (FA) with biopsy
B3 = A/Sing MAP, 15 ug (FA); B4 = A/Sing MAP, 10 ug (FA); B5 = A/Sing MAP, 5 ug (FA); B6 = A/Sing MAP, 2.5 ug (FA)
B7 = Placebo MAP (FA); B8 = A/Sing MAP, 15 ug (UA); B9 = A/Sing IM, 15 ug, as 2018 Afluria Quad (UA)

Listing 16.2.7.1
Adverse Events - CRF Data only

Part B

Random-isation Number	Group	Treatment	AE No.	Adverse Event Verbatim	Onset Date	Onset Time	End Date	End Time	Outcome	Maximum Severity	Relation-ship	Actions Taken	SAE
B0084	Group B5	A/Sing MAP, 5 ug	1	MAP 3 reaction erythema	10-JUN-2018	12:07	30-JUN-2018	14:22	Recovered / Resolved	Mild	Probably Related	None	No
B0085	Group B4	A/Sing MAP, 10 ug	1	Headache	09-JUN-2018	22:00	10-JUN-2018	08:00	Recovered / Resolved	Moderate	Possibly Related	Concomitant Medication	No
B0085	Group B4	A/Sing MAP, 10 ug	2	Application site erythema -MAP site 2,3	10-JUN-2018	UK:UK	16-JUN-2018	13:30	Recovered / Resolved	Mild	Definitely Related	None	No
B0085	Group B4	A/Sing MAP, 10 ug	3	MAP site 3 visibility	11-AUG-2018	UK:UK	06-SEP-2018	UK:UK	Recovered / Resolved	Mild	Definitely Related	None	No
B0086	Group B9	A/Sing IM, 15 ug		[No Treatment-Emergent Adverse Events Reported]									
B0087	Group B7	Placebo MAP	1	URTI	03-JUL-2018	07:00	28-JUL-2018	14:32	Recovered / Resolved	Moderate	Not Related	Concomitant Medication	No
B0087	Group B7	Placebo MAP	2	Headache	30-JUN-2018	UK:UK	30-JUN-2018	UK:UK	Recovered / Resolved	Mild	Not Related	Concomitant Medication	No

Administration Site Key: FA = Forearm; UA= Upper arm
Treatments Part A: A1 = A/Sing MAP, 15 ug (FA); A2 = Afluria Quad IM (UA), 15 ug; A3 = Placebo MAP (FA); A4 = A/Sing Mono IM, 15 ug (UA)
Treatments Part B: B1 = A/Sing MAP, 15 ug (FA) with biopsy; B2 = Placebo MAP (FA) with biopsy
B3 = A/Sing MAP, 15 ug (FA); B4 = A/Sing MAP, 10 ug (FA); B5 = A/Sing MAP, 5 ug (FA); B6 = A/Sing MAP, 2.5 ug (FA)
B7 = Placebo MAP (FA); B8 = A/Sing MAP, 15 ug (UA); B9 = A/Sing IM, 15 ug, as 2018 Afluria Quad (UA)

Listing 16.2.7.1
Adverse Events - CRF Data only

Part B

Random-isation Number	Group	Treatment	AE No.	Adverse Event Verbatim	Onset Date	Onset Time	End Date	End Time	Outcome	Maximum Severity	Relation-ship	Actions Taken	SAE
B0088	Group B9	A/Sing IM, 15 ug		[No Treatment-Emergent Adverse Events Reported]									
B0089	Group B5	A/Sing MAP, 5 ug	1	MAP 2 Erythema	12-JUN-2018	08:55	14-JUL-2018	UK:UK	Recovered / Resolved	Mild	Probably Related	None	No
B0090	Group B6	A/Sing MAP, 2.5 ug	1	Flu - like symptom	11-JUN-2018	UK:UK	16-JUN-2018	06:00	Recovered / Resolved	Mild	Possibly Related	None	No
B0090	Group B6	A/Sing MAP, 2.5 ug	2	Secondary Hyperalgesia	10-JUN-2018	07:30	10-JUN-2018	08:00	Recovered / Resolved	Mild	Possibly Related	None	No
B0090	Group B6	A/Sing MAP, 2.5 ug	4	MAP itchiness	09-JUN-2018	12:48	09-JUN-2018	14:05	Recovered / Resolved	Moderate	Probably Related	Concomitant Medication	No
B0091	Group B8	A/Sing MAP, 15 ug	1	MAP reaction 1,2,3 Erythema	12-JUN-2018	13:06	16-JUN-2018	10:02	Recovered / Resolved	Mild	Probably Related	None	No
B0091	Group B8	A/Sing MAP, 15 ug	2	Flu like symptom	11-JUN-2018	09:00	11-JUN-2018	17:00	Recovered / Resolved	Mild	Possibly Related	None	No

Administration Site Key: FA = Forearm; UA= Upper arm
Treatments Part A: A1 = A/Sing MAP, 15 ug (FA); A2 = Afluria Quad IM (UA), 15 ug; A3 = Placebo MAP (FA); A4 = A/Sing Mono IM, 15 ug (UA)
Treatments Part B: B1 = A/Sing MAP, 15 ug (FA) with biopsy; B2 = Placebo MAP (FA) with biopsy
B3 = A/Sing MAP, 15 ug (FA); B4 = A/Sing MAP, 10 ug (FA); B5 = A/Sing MAP, 5 ug (FA); B6 = A/Sing MAP, 2.5 ug (FA)
B7 = Placebo MAP (FA); B8 = A/Sing MAP, 15 ug (UA); B9 = A/Sing IM, 15 ug, as 2018 Afluria Quad (UA)

Listing 16.2.7.1
Adverse Events - CRF Data only

Part B

Random-isation Number	Group	Treatment	AE No.	Adverse Event Verbatim	Onset Date	Onset Time	End Date	End Time	Outcome	Maximum Severity	Relation-ship	Actions Taken	SAE
B0091	Group B8	A/Sing MAP, 15 ug	3	Headache	13-JUN-2018	19:36	13-JUN-2018	20:40	Recovered / Resolved	Moderate	Not Related	Concomitant Medication	No
B0091	Group B8	A/Sing MAP, 15 ug	4	MAP sites visibility 1,2,3	08-AUG-2018	10:02			Not recovered / Not resolved	Mild	Definitely Related	None	No
B0092	Group B4	A/Sing MAP, 10 ug	1	MAP 1,3 reaction Erythema	12-JUN-2018	10:16	30-JUN-2018	12:05	Recovered / Resolved	Mild	Probably Related	None	No
B0092	Group B4	A/Sing MAP, 10 ug	2	MAP 1,3 Visibility	02-AUG-2018	08:12	25-SEP-2018	UK:UK	Recovered / Resolved	Mild	Definitely Related	None	No
B0092	Group B4	A/Sing MAP, 10 ug	4	Headache	15-JUN-2018	20:15	15-JUN-2018	21:30	Recovered / Resolved	Moderate	Not Related	Concomitant Medication	No
B0093	Group B7	Placebo MAP		[No Treatment-Emergent Adverse Events Reported]									
B0094	Group B3	A/Sing MAP, 15 ug	1	MAP 1,2,3 - Erythema	10-JUN-2018	13:07	16-JUN-2018	13:51	Recovered / Resolved	Mild	Probably Related	None	No

Administration Site Key: FA = Forearm; UA= Upper arm
Treatments Part A: A1 = A/Sing MAP, 15 ug (FA); A2 = Afluria Quad IM (UA), 15 ug; A3 = Placebo MAP (FA); A4 = A/Sing Mono IM, 15 ug (UA)
Treatments Part B: B1 = A/Sing MAP, 15 ug (FA) with biopsy; B2 = Placebo MAP (FA) with biopsy
B3 = A/Sing MAP, 15 ug (FA); B4 = A/Sing MAP, 10 ug (FA); B5 = A/Sing MAP, 5 ug (FA); B6 = A/Sing MAP, 2.5 ug (FA)
B7 = Placebo MAP (FA); B8 = A/Sing MAP, 15 ug (UA); B9 = A/Sing IM, 15 ug, as 2018 Afluria Quad (UA)

Listing 16.2.7.1
Adverse Events - CRF Data only

Part B

Randomisation Number	Group	Treatment	AE No.	Adverse Event Verbatim	Onset Date	Onset Time	End Date	End Time	Outcome	Maximum Severity	Relationship	Actions Taken	SAE
B0095	Group B9	A/Sing IM, 15 ug	1	Dizziness	13-JUN-2018	21:00	13-JUN-2018	21:05	Recovered / Resolved	Mild	Unlikely Related	None	No
B0096	Group B6	A/Sing MAP, 2.5 ug	1	Headache	09-JUN-2018	22:30	11-JUN-2018	18:00	Recovered / Resolved	Mild	Possibly Related	None	No
B0096	Group B6	A/Sing MAP, 2.5 ug	2	Nausea	09-JUN-2018	23:30	10-JUN-2018	10:30	Recovered / Resolved	Mild	Possibly Related	None	No
B0096	Group B6	A/Sing MAP, 2.5 ug	3	Myalgia	09-JUN-2018	17:00	20-JUN-2018	UK:UK	Recovered / Resolved	Moderate	Probably Related	None	No
B0096	Group B6	A/Sing MAP, 2.5 ug	4	MAP 1 Erythema	12-JUN-2018	12:29	16-JUN-2018	13:35	Recovered / Resolved	Mild	Probably Related	None	No
B0096	Group B6	A/Sing MAP, 2.5 ug	5	MAP 1 visibility	08-AUG-2018	13:48	28-AUG-2018	UK:UK	Recovered / Resolved	Mild	Probably Related	None	No
B0097	Group B5	A/Sing MAP, 5 ug		[No Treatment-Emergent Adverse Events Reported]									

Administration Site Key: FA = Forearm; UA= Upper arm
Treatments Part A: A1 = A/Sing MAP, 15 ug (FA); A2 = Afluria Quad IM (UA), 15 ug; A3 = Placebo MAP (FA); A4 = A/Sing Mono IM, 15 ug (UA)
Treatments Part B: B1 = A/Sing MAP, 15 ug (FA) with biopsy; B2 = Placebo MAP (FA) with biopsy
B3 = A/Sing MAP, 15 ug (FA); B4 = A/Sing MAP, 10 ug (FA); B5 = A/Sing MAP, 5 ug (FA); B6 = A/Sing MAP, 2.5 ug (FA)
B7 = Placebo MAP (FA); B8 = A/Sing MAP, 15 ug (UA); B9 = A/Sing IM, 15 ug, as 2018 Afluria Quad (UA)

Listing 16.2.7.1
Adverse Events - CRF Data only

Part B

Randomisation Number	Group	Treatment	AE No.	Adverse Event Verbatim	Onset Date	Onset Time	End Date	End Time	Outcome	Maximum Severity	Relationship	Actions Taken	SAE
B0098	Group B7	Placebo MAP	1	Nausea	09-JUN-2018	13:40	09-JUN-2018	14:41	Recovered / Resolved	Mild	Possibly Related	None	No
B0099	Group B4	A/Sing MAP, 10 ug	2	URTI	26-JUL-2018	16:00	08-AUG-2018	UK:UK	Recovered / Resolved	Moderate	Not Related	Concomitant Medication	No
B0100	Group B8	A/Sing MAP, 15 ug	1	Headache	09-JUN-2018	16:00	09-JUN-2018	20:30	Recovered / Resolved	Mild	Possibly Related	Concomitant Medication	No
B0100	Group B8	A/Sing MAP, 15 ug	2	MAP site reaction 1,2,3 Erythema and Oedema	10-JUN-2018	13:30	08-AUG-2018	15:23	Recovered / Resolved	Mild	Probably Related	None	No
B0101	Group B3	A/Sing MAP, 15 ug	1	MAP reaction 1,2,3 - Erythema	12-JUN-2018	12:34	30-JUN-2018	13:12	Recovered / Resolved	Mild	Probably Related	None	No
B0101	Group B3	A/Sing MAP, 15 ug	2	Rhinitis	15-JUN-2018	09:00	15-JUN-2018	16:00	Recovered / Resolved	Mild	Unlikely Related	None	No
B0102	Group B8	A/Sing MAP, 15 ug	1	MAP reaction 1,2,3 - Erythema	12-JUN-2018	14:24	23-JUN-2018	11:23	Recovered / Resolved	Mild	Probably Related	None	No

Administration Site Key: FA = Forearm; UA= Upper arm
Treatments Part A: A1 = A/Sing MAP, 15 ug (FA); A2 = Afluria Quad IM (UA), 15 ug; A3 = Placebo MAP (FA); A4 = A/Sing Mono IM, 15 ug (UA)
Treatments Part B: B1 = A/Sing MAP, 15 ug (FA) with biopsy; B2 = Placebo MAP (FA) with biopsy
B3 = A/Sing MAP, 15 ug (FA); B4 = A/Sing MAP, 10 ug (FA); B5 = A/Sing MAP, 5 ug (FA); B6 = A/Sing MAP, 2.5 ug (FA)
B7 = Placebo MAP (FA); B8 = A/Sing MAP, 15 ug (UA); B9 = A/Sing IM, 15 ug, as 2018 Afluria Quad (UA)

Listing 16.2.7.1
Adverse Events - CRF Data only

Part B

Randomisation Number	Group	Treatment	AE No.	Adverse Event Verbatim	Onset Date	Onset Time	End Date	End Time	Outcome	Maximum Severity	Relationship	Actions Taken	SAE
B0103	Group B5	A/Sing MAP, 5 ug	1	Pruritus Left forearm	14-JUN-2018	07:58	29-JUN-2018	08:00	Recovered / Resolved	Mild	Possibly Related	None	No
B0104	Group B3	A/Sing MAP, 15 ug	1	MAP 1,2,3 - Erythema	12-JUN-2018	14:29	16-JUN-2018	16:55	Recovered / Resolved	Mild	Probably Related	None	No
B0104	Group B3	A/Sing MAP, 15 ug	2	Musculoskeletal chest pain	16-JUN-2018	14:00	16-JUN-2018	17:30	Recovered / Resolved	Mild	Unlikely Related	Other ECG performed	No
B0104	Group B3	A/Sing MAP, 15 ug	3	MAP site 1,2,3 reaction visibility	10-AUG-2018	09:30	10-SEP-2018	UK:UK	Recovered / Resolved	Mild	Probably Related	None	No
B0105	Group B9	A/Sing IM, 15 ug	1	Dry Cough	12-JUN-2018	07:00	14-JUN-2018	14:00	Recovered / Resolved	Mild	Possibly Related	None	No
B0105	Group B9	A/Sing IM, 15 ug	2	Myalgia IP arm	10-JUN-2018	UK:UK	13-JUN-2018	08:00	Recovered / Resolved	Mild	Possibly Related	None	No
B0105	Group B9	A/Sing IM, 15 ug	3	Upper Respiratory Tract Infection	29-JUN-2018	08:00	07-JUL-2018	UK:UK	Recovered / Resolved	Mild	Not Related	Concomitant Medication	No
B0106	Group B4	A/Sing MAP, 10 ug	1	Upper Respiratory Tract Infection	13-JUN-2018	08:00	18-JUN-2018	UK:UK	Recovered / Resolved	Mild	Not Related	None	No

Administration Site Key: FA = Forearm; UA= Upper arm
Treatments Part A: A1 = A/Sing MAP, 15 ug (FA); A2 = Afluria Quad IM (UA), 15 ug; A3 = Placebo MAP (FA); A4 = A/Sing Mono IM, 15 ug (UA)
Treatments Part B: B1 = A/Sing MAP, 15 ug (FA) with biopsy; B2 = Placebo MAP (FA) with biopsy
B3 = A/Sing MAP, 15 ug (FA); B4 = A/Sing MAP, 10 ug (FA); B5 = A/Sing MAP, 5 ug (FA); B6 = A/Sing MAP, 2.5 ug (FA)
B7 = Placebo MAP (FA); B8 = A/Sing MAP, 15 ug (UA); B9 = A/Sing IM, 15 ug, as 2018 Afluria Quad (UA)

Listing 16.2.7.1
Adverse Events - CRF Data only

Part B

Random-isation Number	Group	Treatment	AE No.	Adverse Event Verbatim	Onset Date	Onset Time	End Date	End Time	Outcome	Maximum Severity	Relation-ship	Actions Taken	SAE
B0106	Group B4	A/Sing MAP, 10 ug	2	MAP 2,3 - Erythema	12-JUN-2018	12:16	16-JUN-2018	15:52	Recovered / Resolved	Mild	Probably Related	None	No
B0107	Group B6	A/Sing MAP, 2.5 ug	1	MAP reaction site 2,3 - Erythema	12-JUN-2018	11:00	16-JUN-2018	09:58	Recovered / Resolved	Mild	Probably Related	None	No
B0107	Group B6	A/Sing MAP, 2.5 ug	2	MAP sites 1,2,3 visible	08-AUG-2018	17:48	14-SEP-2018	UK:UK	Recovered / Resolved	Mild	Definitely Related	None	No
B0108	Group B7	Placebo MAP		[No Treatment-Emergent Adverse Events Reported]									
B0109	Group B6	A/Sing MAP, 2.5 ug	1	MAP #2 and #3 visibility	15-AUG-2018	UK:UK	28-AUG-2018	UK:UK	Recovered / Resolved	Mild	Definitely Related	None	No
B0110	Group B3	A/Sing MAP, 15 ug	1	MAP 1,2,3 reaction - Erythema	19-JUN-2018	13:18	30-AUG-2018	13:16	Recovered / Resolved	Mild	Probably Related	None	No

Administration Site Key: FA = Forearm; UA= Upper arm
Treatments Part A: A1 = A/Sing MAP, 15 ug (FA); A2 = Afluria Quad IM (UA), 15 ug; A3 = Placebo MAP (FA); A4 = A/Sing Mono IM, 15 ug (UA)
Treatments Part B: B1 = A/Sing MAP, 15 ug (FA) with biopsy; B2 = Placebo MAP (FA) with biopsy
B3 = A/Sing MAP, 15 ug (FA); B4 = A/Sing MAP, 10 ug (FA); B5 = A/Sing MAP, 5 ug (FA); B6 = A/Sing MAP, 2.5 ug (FA)
B7 = Placebo MAP (FA); B8 = A/Sing MAP, 15 ug (UA); B9 = A/Sing IM, 15 ug, as 2018 Afluria Quad (UA)

Listing 16.2.7.1
Adverse Events - CRF Data only

Part B

Random-isation Number	Group	Treatment	AE No.	Adverse Event Verbatim	Onset Date	Onset Time	End Date	End Time	Outcome	Maximum Severity	Relation-ship	Actions Taken	SAE
B0111	Group B8	A/Sing MAP, 15 ug		[No Treatment-Emergent Adverse Events Reported]									
B0112	Group B4	A/Sing MAP, 10 ug	1	MAP reaction sites 2,3 -Erythema	19-JUN-2018	11:55	07-JUL-2018	11:40	Recovered / Resolved	Mild	Definitely Related	None	No
B0112	Group B4	A/Sing MAP, 10 ug	2	MAP sites Visibility 2,3	15-AUG-2018	13:47	13-SEP-2018	UK:UK	Recovered / Resolved	Mild	Definitely Related	None	No
B0113	Group B5	A/Sing MAP, 5 ug	1	Headache	17-JUN-2018	16:00	19-JUN-2018	17:00	Recovered / Resolved	Mild	Unlikely Related	None	No
B0113	Group B5	A/Sing MAP, 5 ug	2	Headache	06-JUL-2018	07:00	06-JUL-2018	09:00	Recovered / Resolved	Mild	Unlikely Related	None	No
B0114	Group B7	Placebo MAP	1	Intermittent Headache	17-JUN-2018	08:00	22-JUN-2018	16:30	Recovered / Resolved	Moderate	Unlikely Related	Concomitant Medication	No
B0114	Group B7	Placebo MAP	3	Insect bite - Right upper arm	16-JUN-2018	20:00	09-JUL-2018	11:28	Recovered / Resolved	Mild	Not Related	None	No

Administration Site Key: FA = Forearm; UA= Upper arm
Treatments Part A: A1 = A/Sing MAP, 15 ug (FA); A2 = Afluria Quad IM (UA), 15 ug; A3 = Placebo MAP (FA); A4 = A/Sing Mono IM, 15 ug (UA)
Treatments Part B: B1 = A/Sing MAP, 15 ug (FA) with biopsy; B2 = Placebo MAP (FA) with biopsy
B3 = A/Sing MAP, 15 ug (FA); B4 = A/Sing MAP, 10 ug (FA); B5 = A/Sing MAP, 5 ug (FA); B6 = A/Sing MAP, 2.5 ug (FA)
B7 = Placebo MAP (FA); B8 = A/Sing MAP, 15 ug (UA); B9 = A/Sing IM, 15 ug, as 2018 Afluria Quad (UA)

Listing 16.2.7.1
Adverse Events - CRF Data only

Part B

Randomisation Number	Group	Treatment	AE No.	Adverse Event Verbatim	Onset Date	Onset Time	End Date	End Time	Outcome	Maximum Severity	Relationship	Actions Taken	SAE
B0115	Group B9	A/Sing IM, 15 ug	1	URTI	02-JUL-2018	UK:UK	06-JUL-2018	UK:UK	Recovered / Resolved	Moderate	Not Related	Concomitant Medication	No
B0116	Group B7	Placebo MAP	1	MAP sites - All three sites - Itchiness	18-JUN-2018	21:30	18-JUN-2018	23:00	Recovered / Resolved	Mild	Definitely Related	None	No
B0116	Group B7	Placebo MAP	2	Headache	20-JUN-2018	10:30	20-JUN-2018	23:30	Recovered / Resolved	Mild	Unlikely Related	None	No
B0116	Group B7	Placebo MAP	3	Headache	05-JUL-2018	10:00	05-JUL-2018	23:00	Recovered / Resolved	Moderate	Not Related	Concomitant Medication	No
B0117	Group B9	A/Sing IM, 15 ug		[No Treatment-Emergent Adverse Events Reported]									
B0118	Group B6	A/Sing MAP, 2.5 ug	1	Vasovagal Syncope	16-JUN-2018	13:02	16-JUN-2018	13:40	Recovered / Resolved	Moderate	Unlikely Related	Other Juice and Museli bar. Dose interrupted	No

Administration Site Key: FA = Forearm; UA= Upper arm
Treatments Part A: A1 = A/Sing MAP, 15 ug (FA); A2 = Afluria Quad IM (UA), 15 ug; A3 = Placebo MAP (FA); A4 = A/Sing Mono IM, 15 ug (UA)
Treatments Part B: B1 = A/Sing MAP, 15 ug (FA) with biopsy; B2 = Placebo MAP (FA) with biopsy
B3 = A/Sing MAP, 15 ug (FA); B4 = A/Sing MAP, 10 ug (FA); B5 = A/Sing MAP, 5 ug (FA); B6 = A/Sing MAP, 2.5 ug (FA)
B7 = Placebo MAP (FA); B8 = A/Sing MAP, 15 ug (UA); B9 = A/Sing IM, 15 ug, as 2018 Afluria Quad (UA)

Listing 16.2.7.1
Adverse Events - CRF Data only

Part B

Randomisation Number	Group	Treatment	AE No.	Adverse Event Verbatim	Onset Date	Onset Time	End Date	End Time	Outcome	Maximum Severity	Relationship	Actions Taken	SAE
B0118	Group B6	A/Sing MAP, 2.5 ug	2	Headache	16-JUN-2018	13:10	05-JUL-2018	20:00	Recovered / Resolved	Moderate	Unlikely Related	Concomitant Medication	No
B0118	Group B6	A/Sing MAP, 2.5 ug	3	Nausea	18-JUN-2018	18:48	18-JUN-2018	19:50	Recovered / Resolved	Mild	Possibly Related	None	No
B0118	Group B6	A/Sing MAP, 2.5 ug	7	MAP site #2 visibility	15-AUG-2018	13:17	09-OCT-2018	UK:UK	Recovered / Resolved	Mild	Definitely Related	None	No
B0119	Group B4	A/Sing MAP, 10 ug	3	Gastroenteritis	21-JUN-2018	10:00	21-JUN-2018	16:00	Recovered / Resolved	Mild	Not Related	None	No
B0119	Group B4	A/Sing MAP, 10 ug	5	MAP 1,3 visibility	15-AUG-2018	14:14	27-AUG-2018	UK:UK	Recovered / Resolved	Mild	Probably Related	None	No
B0120	Group B3	A/Sing MAP, 15 ug	1	MAP site reaction 1,2,3 - Erythema and MAP 2,3 Tenderness	19-JUN-2018	13:38	07-JUL-2018	14:00	Recovered / Resolved	Mild	Definitely Related	None	No
B0120	Group B3	A/Sing MAP, 15 ug	2	MAP site 1,2,3 visibility	07-JUL-2018	14:00	29-AUG-2018	UK:UK	Recovered / Resolved	Mild	Definitely Related	None	No

Administration Site Key: FA = Forearm; UA= Upper arm
Treatments Part A: A1 = A/Sing MAP, 15 ug (FA); A2 = Afluria Quad IM (UA), 15 ug; A3 = Placebo MAP (FA); A4 = A/Sing Mono IM, 15 ug (UA)
Treatments Part B: B1 = A/Sing MAP, 15 ug (FA) with biopsy; B2 = Placebo MAP (FA) with biopsy
B3 = A/Sing MAP, 15 ug (FA); B4 = A/Sing MAP, 10 ug (FA); B5 = A/Sing MAP, 5 ug (FA); B6 = A/Sing MAP, 2.5 ug (FA)
B7 = Placebo MAP (FA); B8 = A/Sing MAP, 15 ug (UA); B9 = A/Sing IM, 15 ug, as 2018 Afluria Quad (UA)

Listing 16.2.7.1
Adverse Events - CRF Data only

Part B

Random-isation Number	Group	Treatment	AE No.	Adverse Event Verbatim	Onset Date	Onset Time	End Date	End Time	Outcome	Maximum Severity	Relation-ship	Actions Taken	SAE
B0121	Group B8	A/Sing MAP, 15 ug	1	MAP 1,2,3 reaction - Erythema, tenderness and induration	19-JUN-2018	14:00	08-AUG-2018	09:40	Recovered / Resolved	Mild	Probably Related	None	No
B0121	Group B8	A/Sing MAP, 15 ug	3	Upper Respiratory Tract Infection	16-JUN-2018	18:00	18-JUN-2018	05:10	Recovered / Resolved	Mild	Not Related	None	No
B0121	Group B8	A/Sing MAP, 15 ug	4	Nausea	18-JUN-2018	05:00	18-JUN-2018	05:10	Recovered / Resolved	Mild	Possibly Related	None	No
B0122	Group B5	A/Sing MAP, 5 ug	1	MAP site 2 - Itching	18-JUN-2018	06:00	21-JUN-2018	UK:UK	Recovered / Resolved	Mild	Definitely Related	None	No
B0122	Group B5	A/Sing MAP, 5 ug	2	MAP reaction site 2 - Erythema, oedema + induration	19-JUN-2018	12:12	12-JUL-2018	09:00	Recovered / Resolved	Mild	Definitely Related	None	No
B0122	Group B5	A/Sing MAP, 5 ug	3	Pharyngitis	22-JUN-2018	06:00	24-JUN-2018	07:00	Recovered / Resolved	Mild	Possibly Related	None	No
B0122	Group B5	A/Sing MAP, 5 ug	4	MAP site 2 reaction Visibility	15-AUG-2018	14:07	28-AUG-2018	16:13	Recovered / Resolved	Mild	Definitely Related	None	No

Administration Site Key: FA = Forearm; UA= Upper arm
Treatments Part A: A1 = A/Sing MAP, 15 ug (FA); A2 = Afluria Quad IM (UA), 15 ug; A3 = Placebo MAP (FA); A4 = A/Sing Mono IM, 15 ug (UA)
Treatments Part B: B1 = A/Sing MAP, 15 ug (FA) with biopsy; B2 = Placebo MAP (FA) with biopsy
B3 = A/Sing MAP, 15 ug (FA); B4 = A/Sing MAP, 10 ug (FA); B5 = A/Sing MAP, 5 ug (FA); B6 = A/Sing MAP, 2.5 ug (FA)
B7 = Placebo MAP (FA); B8 = A/Sing MAP, 15 ug (UA); B9 = A/Sing IM, 15 ug, as 2018 Afluria Quad (UA)

Listing 16.2.7.1
Adverse Events - CRF Data only

Part B

Random-isation Number	Group	Treatment	AE No.	Adverse Event Verbatim	Onset Date	Onset Time	End Date	End Time	Outcome	Maximum Severity	Relation-ship	Actions Taken	SAE
B0123	Group B6	A/Sing MAP, 2.5 ug	1	Headache	17-JUN-2018	11:02	18-JUN-2018	12:30	Recovered / Resolved	Mild	Unlikely Related	None	No
B0123	Group B6	A/Sing MAP, 2.5 ug	3	Upper Respiratory Tract Infection	04-JUL-2018	22:00	08-JUL-2018	09:00	Recovered / Resolved	Mild	Not Related	None	No
B0124	Group B4	A/Sing MAP, 10 ug	1	Headache	16-JUN-2018	14:30	17-JUN-2018	09:00	Recovered / Resolved	Mild	Possibly Related	None	No
B0124	Group B4	A/Sing MAP, 10 ug	2	MAP reaction sites 2,3 - Erythema, oedema, induration	19-JUN-2018	14:40	21-JUL-2018	15:17	Recovered / Resolved	Mild	Definitely Related	None	No
B0124	Group B4	A/Sing MAP, 10 ug	3	Headache	21-JUN-2018	07:00	23-JUN-2018	18:00	Recovered / Resolved	Moderate	Unlikely Related	Concomitant Medication	No
B0124	Group B4	A/Sing MAP, 10 ug	4	Contact dermatitis	25-JUN-2018	06:00	30-JUN-2018	UK:UK	Recovered / Resolved	Mild	Not Related	Concomitant Medication	No
B0124	Group B4	A/Sing MAP, 10 ug	5	MAP visibility site #2 and #3	15-AUG-2018	15:00	10-OCT-2018	UK:UK	Recovered / Resolved	Mild	Definitely Related	None	No

Administration Site Key: FA = Forearm; UA= Upper arm
Treatments Part A: A1 = A/Sing MAP, 15 ug (FA); A2 = Afluria Quad IM (UA), 15 ug; A3 = Placebo MAP (FA); A4 = A/Sing Mono IM, 15 ug (UA)
Treatments Part B: B1 = A/Sing MAP, 15 ug (FA) with biopsy; B2 = Placebo MAP (FA) with biopsy
B3 = A/Sing MAP, 15 ug (FA); B4 = A/Sing MAP, 10 ug (FA); B5 = A/Sing MAP, 5 ug (FA); B6 = A/Sing MAP, 2.5 ug (FA)
B7 = Placebo MAP (FA); B8 = A/Sing MAP, 15 ug (UA); B9 = A/Sing IM, 15 ug, as 2018 Afluria Quad (UA)

Listing 16.2.7.1
Adverse Events - CRF Data only

Part B

Randomisation Number	Group	Treatment	AE No.	Adverse Event Verbatim	Onset Date	Onset Time	End Date	End Time	Outcome	Maximum Severity	Relationship	Actions Taken	SAE
B0125	Group B7	Placebo MAP		[No Treatment-Emergent Adverse Events Reported]									
B0126	Group B3	A/Sing MAP, 15 ug	1	MAP Visibility 1,2,3	15-AUG-2018	15:15	24-AUG-2018	UK:UK	Recovered / Resolved	Mild	Definitely Related	None	No
B0127	Group B9	A/Sing IM, 15 ug		[No Treatment-Emergent Adverse Events Reported]									
B0128	Group B5	A/Sing MAP, 5 ug	1	MAP site reaction site 2 - oedema	26-JUN-2018	12:03	11-AUG-2018	10:00	Recovered / Resolved	Mild	Definitely Related	None	No
B0128	Group B5	A/Sing MAP, 5 ug	2	MAP site 2 Visibility	22-AUG-2018	UK:UK	15-OCT-2018	UK:UK	Recovered / Resolved	Mild	Definitely Related	None	No
B0129	Group B8	A/Sing MAP, 15 ug	1	MAP reaction site 1,2,3 erythema	24-JUN-2018	12:17	22-AUG-2018	11:11	Recovered / Resolved	Mild	Definitely Related	None	No

Administration Site Key: FA = Forearm; UA= Upper arm
Treatments Part A: A1 = A/Sing MAP, 15 ug (FA); A2 = Afluria Quad IM (UA), 15 ug; A3 = Placebo MAP (FA); A4 = A/Sing Mono IM, 15 ug (UA)
Treatments Part B: B1 = A/Sing MAP, 15 ug (FA) with biopsy; B2 = Placebo MAP (FA) with biopsy
B3 = A/Sing MAP, 15 ug (FA); B4 = A/Sing MAP, 10 ug (FA); B5 = A/Sing MAP, 5 ug (FA); B6 = A/Sing MAP, 2.5 ug (FA)
B7 = Placebo MAP (FA); B8 = A/Sing MAP, 15 ug (UA); B9 = A/Sing IM, 15 ug, as 2018 Afluria Quad (UA)

Listing 16.2.7.1
Adverse Events - CRF Data only

Part B

Random-isation Number	Group	Treatment	AE No.	Adverse Event Verbatim	Onset Date	Onset Time	End Date	End Time	Outcome	Maximum Severity	Relation-ship	Actions Taken	SAE
B0129	Group B8	A/Sing MAP, 15 ug	2	MAP reaction site 1,2,3 visibility	22-AUG-2018	11:11	29-OCT-2018	UK:UK	Recovered / Resolved	Mild	Probably Related	None	No
B0130	Group B5	A/Sing MAP, 5 ug	1	MAP site 1 reaction - Erythema	26-JUN-2018	10:13	22-AUG-2018	10:20	Recovered / Resolved	Mild	Probably Related	None	No
B0130	Group B5	A/Sing MAP, 5 ug	2	Upper Respiratory Tract Infection	27-JUN-2018	11:00	29-JUN-2018	09:00	Recovered / Resolved	Moderate	Not Related	Concomitant Medication	No
B0130	Group B5	A/Sing MAP, 5 ug	3	Upper Respiratory Tract Infection	10-JUL-2018	08:00	17-JUL-2018	08:30	Recovered / Resolved	Mild	Not Related	Concomitant Medication	No
B0130	Group B5	A/Sing MAP, 5 ug	4	MAP site 1 visibilty	22-AUG-2018	10:20	01-OCT-2018	UK:UK	Recovered / Resolved	Mild	Probably Related	None	No
B0131	Group B8	A/Sing MAP, 15 ug	1	MAP reaction site 1,2,3 -Erythema extent, erythema, MAP reaction site #2 te	26-JUN-2018	13:36	10-AUG-2018	09:00	Recovered / Resolved	Mild	Probably Related	None	No
B0131	Group B8	A/Sing MAP, 15 ug	2	Right Temporomandibular junction pain	28-JUN-2018	21:40	28-JUN-2018	23:50	Recovered / Resolved	Mild	Unlikely Related	None	No

Administration Site Key: FA = Forearm; UA= Upper arm
Treatments Part A: A1 = A/Sing MAP, 15 ug (FA); A2 = Afluria Quad IM (UA), 15 ug; A3 = Placebo MAP (FA); A4 = A/Sing Mono IM, 15 ug (UA)
Treatments Part B: B1 = A/Sing MAP, 15 ug (FA) with biopsy; B2 = Placebo MAP (FA) with biopsy
B3 = A/Sing MAP, 15 ug (FA); B4 = A/Sing MAP, 10 ug (FA); B5 = A/Sing MAP, 5 ug (FA); B6 = A/Sing MAP, 2.5 ug (FA)
B7 = Placebo MAP (FA); B8 = A/Sing MAP, 15 ug (UA); B9 = A/Sing IM, 15 ug, as 2018 Afluria Quad (UA)

Listing 16.2.7.1
Adverse Events - CRF Data only

Part B

Random-isation Number	Group	Treatment	AE No.	Adverse Event Verbatim	Onset Date	Onset Time	End Date	End Time	Outcome	Maximum Severity	Relation-ship	Actions Taken	SAE
B0132	Group B6	A/Sing MAP, 2.5 ug	1	Nausea	26-JUN-2018	17:30	26-JUN-2018	18:00	Recovered / Resolved	Mild	Not Related	Other Urinalysis performed, Result normal	No
B0133	Group B4	A/Sing MAP, 10 ug		[No Treatment-Emergent Adverse Events Reported]									
B0134	Group B3	A/Sing MAP, 15 ug	1	MAP 2,3 Visibility	23-AUG-2018	09:23	30-AUG-2018	UK:UK	Recovered / Resolved	Mild	Probably Related	None	No
B0135	Group B7	Placebo MAP	1	URTI	24-JUN-2018	09:00	30-JUN-2018	08:00	Recovered / Resolved	Mild	Not Related	None	No
B0136	Group B9	A/Sing IM, 15 ug	1	Cholecystitis	05-JUL-2018	20:00	22-AUG-2018	11:13	Recovered / Resolved	Moderate	Not Related	Concomitant Medication	No

Administration Site Key: FA = Forearm; UA= Upper arm
Treatments Part A: A1 = A/Sing MAP, 15 ug (FA); A2 = Afluria Quad IM (UA), 15 ug; A3 = Placebo MAP (FA); A4 = A/Sing Mono IM, 15 ug (UA)
Treatments Part B: B1 = A/Sing MAP, 15 ug (FA) with biopsy; B2 = Placebo MAP (FA) with biopsy
B3 = A/Sing MAP, 15 ug (FA); B4 = A/Sing MAP, 10 ug (FA); B5 = A/Sing MAP, 5 ug (FA); B6 = A/Sing MAP, 2.5 ug (FA)
B7 = Placebo MAP (FA); B8 = A/Sing MAP, 15 ug (UA); B9 = A/Sing IM, 15 ug, as 2018 Afluria Quad (UA)

Listing 16.2.7.1
Adverse Events - CRF Data only

Part B

Random-isation Number	Group	Treatment	AE No.	Adverse Event Verbatim	Onset Date	Onset Time	End Date	End Time	Outcome	Maximum Severity	Relation-ship	Actions Taken	SAE
B0137	Group B8	A/Sing MAP, 15 ug	1	MAP 1,2,3 - Erythema	26-JUN-2018	10:21	13-JUL-2018	09:00	Recovered / Resolved	Mild	Probably Related	None	No
B0137	Group B8	A/Sing MAP, 15 ug	2	Upper Respiratory Tract Infection	09-JUL-2018	07:00	07-AUG-2018	10:00	Recovered / Resolved	Moderate	Not Related	Concomitant Medication	No
B0138	Group B7	Placebo MAP	2	MAP Visibility 1,2,3	22-AUG-2018	13:26			Not recovered / Not resolved	Mild	Definitely Related	None	No
B0139	Group B4	A/Sing MAP, 10 ug		[No Treatment-Emergent Adverse Events Reported]									
B0140	Group B9	A/Sing IM, 15 ug		[No Treatment-Emergent Adverse Events Reported]									

Administration Site Key: FA = Forearm; UA= Upper arm
Treatments Part A: A1 = A/Sing MAP, 15 ug (FA); A2 = Afluria Quad IM (UA), 15 ug; A3 = Placebo MAP (FA); A4 = A/Sing Mono IM, 15 ug (UA)
Treatments Part B: B1 = A/Sing MAP, 15 ug (FA) with biopsy; B2 = Placebo MAP (FA) with biopsy
B3 = A/Sing MAP, 15 ug (FA); B4 = A/Sing MAP, 10 ug (FA); B5 = A/Sing MAP, 5 ug (FA); B6 = A/Sing MAP, 2.5 ug (FA)
B7 = Placebo MAP (FA); B8 = A/Sing MAP, 15 ug (UA); B9 = A/Sing IM, 15 ug, as 2018 Afluria Quad (UA)

Listing 16.2.7.1
Adverse Events - CRF Data only

Part B

Random-isation Number	Group	Treatment	AE No.	Adverse Event Verbatim	Onset Date	Onset Time	End Date	End Time	Outcome	Maximum Severity	Relation-ship	Actions Taken	SAE
B0141	Group B3	A/Sing MAP, 15 ug		[No Treatment-Emergent Adverse Events Reported]									
B0142	Group B5	A/Sing MAP, 5 ug	1	Medial Tibial Stress Syndrome (Shin Splints)	12-JUL-2018	15:00	11-AUG-2018	09:00	Recovered / Resolved	Moderate	Not Related	Concomitant Medication	No
B0142	Group B5	A/Sing MAP, 5 ug	2	MAP 1 Visibility	25-AUG-2018	11:55	25-SEP-2018	UK:UK	Recovered / Resolved	Mild	Probably Related	None	No
B0143	Group B6	A/Sing MAP, 2.5 ug	1	Headache	23-JUN-2018	14:35	23-JUN-2018	UK:UK	Recovered / Resolved	Mild	Possibly Related	None	No
B0143	Group B6	A/Sing MAP, 2.5 ug	2	MAP site 3 reaction - Erythema Oedema induration	26-JUN-2018	12:08	30-JUN-2018	12:14	Recovered / Resolved	Mild	Definitely Related	None	No
B0143	Group B6	A/Sing MAP, 2.5 ug	3	Exacerbation of Left knee sprain	04-JUL-2018	11:30	17-SEP-2018	UK:UK	Recovered / Resolved	Moderate	Not Related	Concomitant Medication	No

Administration Site Key: FA = Forearm; UA= Upper arm
Treatments Part A: A1 = A/Sing MAP, 15 ug (FA); A2 = Afluria Quad IM (UA), 15 ug; A3 = Placebo MAP (FA); A4 = A/Sing Mono IM, 15 ug (UA)
Treatments Part B: B1 = A/Sing MAP, 15 ug (FA) with biopsy; B2 = Placebo MAP (FA) with biopsy
B3 = A/Sing MAP, 15 ug (FA); B4 = A/Sing MAP, 10 ug (FA); B5 = A/Sing MAP, 5 ug (FA); B6 = A/Sing MAP, 2.5 ug (FA)
B7 = Placebo MAP (FA); B8 = A/Sing MAP, 15 ug (UA); B9 = A/Sing IM, 15 ug, as 2018 Afluria Quad (UA)

Listing 16.2.7.1
Adverse Events - CRF Data only

Part B

Random-isation Number	Group	Treatment	AE No.	Adverse Event Verbatim	Onset Date	Onset Time	End Date	End Time	Outcome	Maximum Severity	Relation-ship	Actions Taken	SAE
B0143	Group B6	A/Sing MAP, 2.5 ug	4	MAP 3 Visibility	22-AUG-2018	13:09			Not recovered / Not resolved	Mild	Probably Related	None	No
B0144	Group B6	A/Sing MAP, 2.5 ug	1	Dizziness	23-JUN-2018	14:03	23-JUN-2018	15:50	Recovered / Resolved	Mild	Unlikely Related	None	No
B0144	Group B6	A/Sing MAP, 2.5 ug	2	Nausea	23-JUN-2018	14:03	23-JUN-2018	15:50	Recovered / Resolved	Mild	Unlikely Related	None	No
B0144	Group B6	A/Sing MAP, 2.5 ug	3	Upper Respiratory Tract Infection	14-JUL-2018	10:00	18-JUL-2018	08:30	Recovered / Resolved	Mild	Not Related	Concomitant Medication	No
B0145	Group B3	A/Sing MAP, 15 ug	1	Nausea	24-JUN-2018	13:00	24-JUN-2018	13:05	Recovered / Resolved	Mild	Unlikely Related	None	No
B0145	Group B3	A/Sing MAP, 15 ug	2	MAP 1,2,3 reaction - Erythema & MAP 2 reaction - Erythema extent	26-JUN-2018	10:44	28-JUL-2018	09:00	Recovered / Resolved	Mild	Probably Related	None	No
B0145	Group B3	A/Sing MAP, 15 ug	3	Headache	27-JUN-2018	17:05	27-JUN-2018	17:10	Recovered / Resolved	Mild	Unlikely Related	None	No

Administration Site Key: FA = Forearm; UA= Upper arm
Treatments Part A: A1 = A/Sing MAP, 15 ug (FA); A2 = Afluria Quad IM (UA), 15 ug; A3 = Placebo MAP (FA); A4 = A/Sing Mono IM, 15 ug (UA)
Treatments Part B: B1 = A/Sing MAP, 15 ug (FA) with biopsy; B2 = Placebo MAP (FA) with biopsy
B3 = A/Sing MAP, 15 ug (FA); B4 = A/Sing MAP, 10 ug (FA); B5 = A/Sing MAP, 5 ug (FA); B6 = A/Sing MAP, 2.5 ug (FA)
B7 = Placebo MAP (FA); B8 = A/Sing MAP, 15 ug (UA); B9 = A/Sing IM, 15 ug, as 2018 Afluria Quad (UA)

Listing 16.2.7.1
Adverse Events - CRF Data only

Part B

Random-isation Number	Group	Treatment	AE No.	Adverse Event Verbatim	Onset Date	Onset Time	End Date	End Time	Outcome	Maximum Severity	Relation-ship	Actions Taken	SAE
B0146	Group B7	Placebo MAP	1	Headache	02-JUL-2018	15:35	02-JUL-2018	16:00	Recovered / Resolved	Mild	Unlikely Related	None	No
B0146	Group B7	Placebo MAP	2	Toothache	02-JUL-2018	15:28	02-JUL-2018	16:00	Recovered / Resolved	Mild	Not Related	None	No
B0147	Group B9	A/Sing IM, 15 ug		[No Treatment-Emergent Adverse Events Reported]									
B0148	Group B5	A/Sing MAP, 5 ug	1	Erythema site 1	28-JUN-2018	22:00	18-JUL-2018	10:20	Recovered / Resolved	Mild	Definitely Related	None	No
B0148	Group B5	A/Sing MAP, 5 ug	2	MAP 1 Visibility	27-AUG-2018	11:20	10-OCT-2018	UK:UK	Recovered / Resolved	Mild	Probably Related	None	No
B0149	Group B8	A/Sing MAP, 15 ug		[No Treatment-Emergent Adverse Events Reported]									

Administration Site Key: FA = Forearm; UA= Upper arm
Treatments Part A: A1 = A/Sing MAP, 15 ug (FA); A2 = Afluria Quad IM (UA), 15 ug; A3 = Placebo MAP (FA); A4 = A/Sing Mono IM, 15 ug (UA)
Treatments Part B: B1 = A/Sing MAP, 15 ug (FA) with biopsy; B2 = Placebo MAP (FA) with biopsy
B3 = A/Sing MAP, 15 ug (FA); B4 = A/Sing MAP, 10 ug (FA); B5 = A/Sing MAP, 5 ug (FA); B6 = A/Sing MAP, 2.5 ug (FA)
B7 = Placebo MAP (FA); B8 = A/Sing MAP, 15 ug (UA); B9 = A/Sing IM, 15 ug, as 2018 Afluria Quad (UA)

Listing 16.2.7.1
Adverse Events - CRF Data only

Part B

Random-isation Number	Group	Treatment	AE No.	Adverse Event Verbatim	Onset Date	Onset Time	End Date	End Time	Outcome	Maximum Severity	Relation-ship	Actions Taken	SAE
B0150	Group B4	A/Sing MAP, 10 ug	4	MAP site 1,2 visibility	28-AUG-2018	13:33	11-OCT-2018	UK:UK	Recovered / Resolved	Mild	Probably Related	None	No
B1019	Group B8	A/Sing MAP, 15 ug	1	MAP visibility 1,2,3	20-JUL-2018	13:49	27-AUG-2018	UK:UK	Recovered / Resolved	Mild	Definitely Related	None	No
B1076	Group B5	A/Sing MAP, 5 ug	1	MAP 1 reaction - Erythema	19-JUN-2018	12:39	23-JUN-2018	10:45	Recovered / Resolved	Mild	Definitely Related	None	No
B1080	Group B7	Placebo MAP	1	Right shoulder sprain	16-JUN-2018	19:00	04-AUG-2018	08:30	Recovered / Resolved	Moderate	Not Related	Concomitant Medication	No

Administration Site Key: FA = Forearm; UA= Upper arm
Treatments Part A: A1 = A/Sing MAP, 15 ug (FA); A2 = Afluria Quad IM (UA), 15 ug; A3 = Placebo MAP (FA); A4 = A/Sing Mono IM, 15 ug (UA)
Treatments Part B: B1 = A/Sing MAP, 15 ug (FA) with biopsy; B2 = Placebo MAP (FA) with biopsy
B3 = A/Sing MAP, 15 ug (FA); B4 = A/Sing MAP, 10 ug (FA); B5 = A/Sing MAP, 5 ug (FA); B6 = A/Sing MAP, 2.5 ug (FA)
B7 = Placebo MAP (FA); B8 = A/Sing MAP, 15 ug (UA); B9 = A/Sing IM, 15 ug, as 2018 Afluria Quad (UA)
