# Supplementary material for: Safety, tolerability, and immunogenicity of influenza vaccination with a high-density microarray patch: Results from a randomized, controlled phase I clinical trial
Source: PLoS Med. 2020 Mar 17;17(3):e1003024. doi: 10.1371/journal.pmed.1003024 (PMC7077342; doi:10.1371/journal.pmed.1003024)
Supplement: S1 Data — Data 1: Informed Consent; Data 2: Analysis Sets; Data 3: Demographics; Data 4: Study Drug Administration; Data 5: Skin Hardness Assessment; Data 6: Immunogenicity (ELISA IgG); Data 7: Immunogenicity (ADCC); Data 8: Immunogenicity (MBCs); Data 9: Immunogenicity (CMI); Data 10: Immunogenicity (Mucosal IgA); Data 11: Immunogenicity Results (HAI, MN); Data 12: TEAE–CRF Data only; Data 13: TEAEs–MedDRA Coding; Data 15: Treatment Site Tolerability Assessment; Data 16: Numeric Pain Intensity; Data 17: SII; Data 18: Application Site Status at End of Study; Data 19: Individual Treatment Site Tolerability Assessment; Data 20: Numeric Pain Intensity by Treatment; Data 21: Individual SII by Treatment. ADCC, antibody-dependent cellular cytotoxicity; CMI, cell-mediated immunity; CRF, case report form; ELISA, enzyme-linked immunosorbent assay; HAI, haemagglutination inhibition; IgA, immunoglobulin A; IgG, immunoglobulin G; MBC, memory B cell; MedDRA, medical dictionary for regulatory activities; MN, microneutralisation; SII, Skin Irritation Index; TEA, treatment emergent adverse event. (ZIP) [file pmed.1003024.s008.zip › S1_Data/clinical supporting/S1_Data13 TEAEs - MedDRA Coding.pdf]

Listing 16.2.7.2
Adverse Events - MedDRA Coding and Calculated Values

Part A

Random-isation Number	Group	Treatment	AE No.	Adverse Event Verbatim	MedDRA System Organ Class	MedDRA Preferred Term	MedDRA Lower Level Term	Duration of AE (days) [C]	Time Since Treatment Admin. (Days) [C]
A0001	Group A3	Placebo MAP	1	MAP reaction site 1,2,3 Erythema	General disorders and administration site conditions	Application site erythema	Application site erythema	6.89	0.10
A0002	Group A1	A/Sing MAP, 15 ug	1	Fatigue	General disorders and administration site conditions	Fatigue	Fatigue	0.50	0.91
A0003	Group A1	A/Sing MAP, 15 ug		[No Treatment-Emergent Adverse Events Reported]					
A0004	Group A3	Placebo MAP	1	Intermittent Headache	Nervous system disorders	Headache	Intermittent headache	17.08	3.72
A0004	Group A3	Placebo MAP	2	MAP reaction site 1,2,3 erythema	General disorders and administration site conditions	Application site erythema	Application site erythema	7.03	0.10

Key: AE = Adverse Event; [C] = Calculated; UK = Unknown
Coded using MedDRA Version 20.01

Administration Site Key: FA = Forearm; UA= Upper arm
Treatments Part A: A1 = A/Sing MAP, 15 ug (FA); A2 = Afluria Quad IM (UA), 15 ug; A3 = Placebo MAP (FA); A4 = A/Sing Mono IM, 15 ug (UA)
Treatments Part B: B1 = A/Sing MAP, 15 ug (FA) with biopsy; B2 = Placebo MAP (FA) with biopsy
B3 = A/Sing MAP, 15 ug (FA); B4 = A/Sing MAP, 10 ug (FA); B5 = A/Sing MAP, 5 ug (FA); B6 = A/Sing MAP, 2.5 ug (FA)
B7 = Placebo MAP (FA); B8 = A/Sing MAP, 15 ug (UA); B9 = A/Sing IM, 15 ug, as 2018 Afluria Quad (UA)

Listing 16.2.7.2
Adverse Events - MedDRA Coding and Calculated Values

Part A

Random-isation Number	Group	Treatment	AE No.	Adverse Event Verbatim	MedDRA System Organ Class	MedDRA Preferred Term	MedDRA Lower Level Term	Duration of AE (days) [C]	Time Since Treatment Admin. (Days) [C]
A0005	Group A1	A/Sing MAP, 15 ug	18	MAP Site Reaction 1,2,3 tenderness, itchiness and oedema	General disorders and administration site conditions	Application site reaction	Application site reaction	3.96	3.04
A0006	Group A3	Placebo MAP	10	MAP 1,2,3 visibility	General disorders and administration site conditions	Application site discolouration	Application site discolouration	~14	60.08
A0007	Group A1	A/Sing MAP, 15 ug		[No Treatment-Emergent Adverse Events Reported]					
A0008	Group A3	Placebo MAP	7	MAP 2 erythema	General disorders and administration site conditions	Application site erythema	Application site erythema	3.02	0.11

Key: AE = Adverse Event; [C] = Calculated; UK = Unknown
Coded using MedDRA Version 20.01

Administration Site Key: FA = Forearm; UA= Upper arm
Treatments Part A: A1 = A/Sing MAP, 15 ug (FA); A2 = Afluria Quad IM (UA), 15 ug; A3 = Placebo MAP (FA); A4 = A/Sing Mono IM, 15 ug (UA)
Treatments Part B: B1 = A/Sing MAP, 15 ug (FA) with biopsy; B2 = Placebo MAP (FA) with biopsy
B3 = A/Sing MAP, 15 ug (FA); B4 = A/Sing MAP, 10 ug (FA); B5 = A/Sing MAP, 5 ug (FA); B6 = A/Sing MAP, 2.5 ug (FA)
B7 = Placebo MAP (FA); B8 = A/Sing MAP, 15 ug (UA); B9 = A/Sing IM, 15 ug, as 2018 Afluria Quad (UA)

Listing 16.2.7.2
Adverse Events - MedDRA Coding and Calculated Values

Part A

Random-isation Number	Group	Treatment	AE No.	Adverse Event Verbatim	MedDRA System Organ Class	MedDRA Preferred Term	MedDRA Lower Level Term	Duration of AE (days) [C]	Time Since Treatment Admin. (Days) [C]
A0009	Group A1	A/Sing MAP, 15 ug	1	Erythema - Left Upper Back	Skin and subcutaneous tissue disorders	Erythema	Erythema	9.83	5.12
A0009	Group A1	A/Sing MAP, 15 ug	11	MAP visibility 1,2,3	General disorders and administration site conditions	Application site discolouration	Application site discolouration	~42	53.07
A0010	Group A3	Placebo MAP	1	Upper Respiratory Tract Infection	Infections and infestations	Upper respiratory tract infection	Upper respiratory tract infection	~5	~17
A0011	Group A3	Placebo MAP	1	Headache	Nervous system disorders	Headache	Headache	<1	1.96
A0012	Group A1	A/Sing MAP, 15 ug	1	Left ankle inversion injury	Injury, poisoning and procedural complications	Ligament sprain	Ankle sprain	34.18	1.80
A0012	Group A1	A/Sing MAP, 15 ug	11	MAP reaction 1,3 erythema	General disorders and administration site conditions	Application site erythema	Application site erythema	6.98	0.10

Key: AE = Adverse Event; [C] = Calculated; UK = Unknown
Coded using MedDRA Version 20.01

Administration Site Key: FA = Forearm; UA= Upper arm
Treatments Part A: A1 = A/Sing MAP, 15 ug (FA); A2 = Afluria Quad IM (UA), 15 ug; A3 = Placebo MAP (FA); A4 = A/Sing Mono IM, 15 ug (UA)
Treatments Part B: B1 = A/Sing MAP, 15 ug (FA) with biopsy; B2 = Placebo MAP (FA) with biopsy
B3 = A/Sing MAP, 15 ug (FA); B4 = A/Sing MAP, 10 ug (FA); B5 = A/Sing MAP, 5 ug (FA); B6 = A/Sing MAP, 2.5 ug (FA)
B7 = Placebo MAP (FA); B8 = A/Sing MAP, 15 ug (UA); B9 = A/Sing IM, 15 ug, as 2018 Afluria Quad (UA)

Listing 16.2.7.2
Adverse Events - MedDRA Coding and Calculated Values

Part A

Random-isation Number	Group	Treatment	AE No.	Adverse Event Verbatim	MedDRA System Organ Class	MedDRA Preferred Term	MedDRA Lower Level Term	Duration of AE (days) [C]	Time Since Treatment Admin. (Days) [C]
A0012	Group A1	A/Sing MAP, 15 ug	12	MAP reaction 1,2 oedema	General disorders and administration site conditions	Application site oedema	Application site oedema	4.09	3.00
A0013	Group A1	A/Sing MAP, 15 ug	6	MAP 2 reaction Itchiness	General disorders and administration site conditions	Application site pruritus	Application site itching	13.95	7.08
A0013	Group A1	A/Sing MAP, 15 ug	12	MAP 1, 2, 3 reaction Erythema	General disorders and administration site conditions	Application site erythema	Application site erythema	6.98	0.10
A0013	Group A1	A/Sing MAP, 15 ug	13	MAP 1, 2, 3 Visibility	General disorders and administration site conditions	Application site discolouration	Application site discolouration	~31	61.98
A0013	Group A1	A/Sing MAP, 15 ug	14	MAP 2 & 3 reaction Oedema	General disorders and administration site conditions	Application site oedema	Application site oedema	4.11	2.97

Key: AE = Adverse Event; [C] = Calculated; UK = Unknown
Coded using MedDRA Version 20.01

Administration Site Key: FA = Forearm; UA= Upper arm
Treatments Part A: A1 = A/Sing MAP, 15 ug (FA); A2 = Afluria Quad IM (UA), 15 ug; A3 = Placebo MAP (FA); A4 = A/Sing Mono IM, 15 ug (UA)
Treatments Part B: B1 = A/Sing MAP, 15 ug (FA) with biopsy; B2 = Placebo MAP (FA) with biopsy
B3 = A/Sing MAP, 15 ug (FA); B4 = A/Sing MAP, 10 ug (FA); B5 = A/Sing MAP, 5 ug (FA); B6 = A/Sing MAP, 2.5 ug (FA)
B7 = Placebo MAP (FA); B8 = A/Sing MAP, 15 ug (UA); B9 = A/Sing IM, 15 ug, as 2018 Afluria Quad (UA)

Listing 16.2.7.2
Adverse Events - MedDRA Coding and Calculated Values

Part A

Random-isation Number	Group	Treatment	AE No.	Adverse Event Verbatim	MedDRA System Organ Class	MedDRA Preferred Term	MedDRA Lower Level Term	Duration of AE (days) [C]	Time Since Treatment Admin. (Days) [C]
A0014	Group A3	Placebo MAP		[No Treatment-Emergent Adverse Events Reported]					
A0015	Group A1	A/Sing MAP, 15 ug	1	Headache	Nervous system disorders	Headache	Headache	1.44	1.40
A0015	Group A1	A/Sing MAP, 15 ug	3	MAP 2 and MAP 3 Erythema	General disorders and administration site conditions	Application site erythema	Application site erythema	6.88	0.11
A0015	Group A1	A/Sing MAP, 15 ug	5	MAP Sites 1,2,3 Visibility	General disorders and administration site conditions	Application site discolouration	Application site discolouration	84.83	6.99
A0016	Group A3	Placebo MAP	7	Headache	Nervous system disorders	Headache	Headache	0.16	0.18

Key: AE = Adverse Event; [C] = Calculated; UK = Unknown
Coded using MedDRA Version 20.01

Administration Site Key: FA = Forearm; UA= Upper arm
Treatments Part A: A1 = A/Sing MAP, 15 ug (FA); A2 = Afluria Quad IM (UA), 15 ug; A3 = Placebo MAP (FA); A4 = A/Sing Mono IM, 15 ug (UA)
Treatments Part B: B1 = A/Sing MAP, 15 ug (FA) with biopsy; B2 = Placebo MAP (FA) with biopsy
B3 = A/Sing MAP, 15 ug (FA); B4 = A/Sing MAP, 10 ug (FA); B5 = A/Sing MAP, 5 ug (FA); B6 = A/Sing MAP, 2.5 ug (FA)
B7 = Placebo MAP (FA); B8 = A/Sing MAP, 15 ug (UA); B9 = A/Sing IM, 15 ug, as 2018 Afluria Quad (UA)

Listing 16.2.7.2
Adverse Events - MedDRA Coding and Calculated Values

Part A

Random-isation Number	Group	Treatment	AE No.	Adverse Event Verbatim	MedDRA System Organ Class	MedDRA Preferred Term	MedDRA Lower Level Term	Duration of AE (days) [C]	Time Since Treatment Admin. (Days) [C]
A0017	Group A3	Placebo MAP		[No Treatment-Emergent Adverse Events Reported]					
A0018	Group A1	A/Sing MAP, 15 ug		[No Treatment-Emergent Adverse Events Reported]					
A0019	Group A3	Placebo MAP	10	Phlebitis (venepuncture site)	General disorders and administration site conditions	Vessel puncture site phlebitis	Venipuncture site phlebitis	~12	~23
A0020	Group A1	A/Sing MAP, 15 ug	1	Flu-Like Symptoms	General disorders and administration site conditions	Influenza like illness	Flu-like symptoms	0.17	12.85

Key: AE = Adverse Event; [C] = Calculated; UK = Unknown
Coded using MedDRA Version 20.01

Administration Site Key: FA = Forearm; UA= Upper arm
Treatments Part A: A1 = A/Sing MAP, 15 ug (FA); A2 = Afluria Quad IM (UA), 15 ug; A3 = Placebo MAP (FA); A4 = A/Sing Mono IM, 15 ug (UA)
Treatments Part B: B1 = A/Sing MAP, 15 ug (FA) with biopsy; B2 = Placebo MAP (FA) with biopsy
B3 = A/Sing MAP, 15 ug (FA); B4 = A/Sing MAP, 10 ug (FA); B5 = A/Sing MAP, 5 ug (FA); B6 = A/Sing MAP, 2.5 ug (FA)
B7 = Placebo MAP (FA); B8 = A/Sing MAP, 15 ug (UA); B9 = A/Sing IM, 15 ug, as 2018 Afluria Quad (UA)

Listing 16.2.7.2
Adverse Events - MedDRA Coding and Calculated Values

Part A

Random-isation Number	Group	Treatment	AE No.	Adverse Event Verbatim	MedDRA System Organ Class	MedDRA Preferred Term	MedDRA Lower Level Term	Duration of AE (days) [C]	Time Since Treatment Admin. (Days) [C]
A0021	Group A1	A/Sing MAP, 15 ug	1	Headache	Nervous system disorders	Headache	Headache	0.29	0.12
A0022	Group A3	Placebo MAP	1	Pruritus site #3	General disorders and administration site conditions	Application site pruritus	Application site pruritus	<1	~16
A0023	Group A3	Placebo MAP	1	Abdomen Bloating	Gastrointestinal disorders	Abdominal distension	Abdominal bloating	0.04	0.44
A0023	Group A3	Placebo MAP	2	Intermittent Headaches	Nervous system disorders	Headache	Intermittent headache	4.15	2.21
A0023	Group A3	Placebo MAP	3	Abdomen Pain (Intermittent)	Gastrointestinal disorders	Abdominal pain	Abdominal pain	5.74	0.44
A0023	Group A3	Placebo MAP	4	Sore Throat	Respiratory, thoracic and mediastinal disorders	Oropharyngeal pain	Sore throat	~21	14.21
A0023	Group A3	Placebo MAP	14	Diarrhoea	Gastrointestinal disorders	Diarrhoea	Diarrhoea	0.08	1.40

Key: AE = Adverse Event; [C] = Calculated; UK = Unknown
Coded using MedDRA Version 20.01

Administration Site Key: FA = Forearm; UA= Upper arm
Treatments Part A: A1 = A/Sing MAP, 15 ug (FA); A2 = Afluria Quad IM (UA), 15 ug; A3 = Placebo MAP (FA); A4 = A/Sing Mono IM, 15 ug (UA)
Treatments Part B: B1 = A/Sing MAP, 15 ug (FA) with biopsy; B2 = Placebo MAP (FA) with biopsy
B3 = A/Sing MAP, 15 ug (FA); B4 = A/Sing MAP, 10 ug (FA); B5 = A/Sing MAP, 5 ug (FA); B6 = A/Sing MAP, 2.5 ug (FA)
B7 = Placebo MAP (FA); B8 = A/Sing MAP, 15 ug (UA); B9 = A/Sing IM, 15 ug, as 2018 Afluria Quad (UA)

Listing 16.2.7.2
Adverse Events - MedDRA Coding and Calculated Values

Part A

Random-isation Number	Group	Treatment	AE No.	Adverse Event Verbatim	MedDRA System Organ Class	MedDRA Preferred Term	MedDRA Lower Level Term	Duration of AE (days) [C]	Time Since Treatment Admin. (Days) [C]
A0023	Group A3	Placebo MAP	15	Drowsiness	Nervous system disorders	Somnolence	Drowsiness	~19	5.79
A0024	Group A1	A/Sing MAP, 15 ug	1	Abdominal discomfort	Gastrointestinal disorders	Abdominal discomfort	Abdominal discomfort	0.92	4.07
A0025	Group A3	Placebo MAP	1	Headache (intermittent)	Nervous system disorders	Headache	Intermittent headache	2.10	4.79
A0027	Group A3	Placebo MAP	1	Headache	Nervous system disorders	Headache	Headache	<1	5.93
A0028	Group A1	A/Sing MAP, 15 ug		[No Treatment-Emergent Adverse Events Reported]					

Key: AE = Adverse Event; [C] = Calculated; UK = Unknown
Coded using MedDRA Version 20.01

Administration Site Key: FA = Forearm; UA= Upper arm
Treatments Part A: A1 = A/Sing MAP, 15 ug (FA); A2 = Afluria Quad IM (UA), 15 ug; A3 = Placebo MAP (FA); A4 = A/Sing Mono IM, 15 ug (UA)
Treatments Part B: B1 = A/Sing MAP, 15 ug (FA) with biopsy; B2 = Placebo MAP (FA) with biopsy
B3 = A/Sing MAP, 15 ug (FA); B4 = A/Sing MAP, 10 ug (FA); B5 = A/Sing MAP, 5 ug (FA); B6 = A/Sing MAP, 2.5 ug (FA)
B7 = Placebo MAP (FA); B8 = A/Sing MAP, 15 ug (UA); B9 = A/Sing IM, 15 ug, as 2018 Afluria Quad (UA)

Listing 16.2.7.2
Adverse Events - MedDRA Coding and Calculated Values

Part A

Random-isation Number	Group	Treatment	AE No.	Adverse Event Verbatim	MedDRA System Organ Class	MedDRA Preferred Term	MedDRA Lower Level Term	Duration of AE (days) [C]	Time Since Treatment Admin. (Days) [C]
A0029	Group A1	A/Sing MAP, 15 ug	6	MAP visibility site 1,2,3	General disorders and administration site conditions	Application site discolouration	Application site discolouration	UK	60.14
A0030	Group A3	Placebo MAP	1	Upper Respiratory Tract Infection	Infections and infestations	Upper respiratory tract infection	Upper respiratory tract infection	12.50	14.33
A0030	Group A3	Placebo MAP	5	MAP site 1,2,3 Visibility	General disorders and administration site conditions	Application site discolouration	Application site discolouration	~168	60.02
A0031	Group A2	Afluria Quad IM, 15 ug		[No Treatment-Emergent Adverse Events Reported]					

Key: AE = Adverse Event; [C] = Calculated; UK = Unknown
Coded using MedDRA Version 20.01

Administration Site Key: FA = Forearm; UA= Upper arm
Treatments Part A: A1 = A/Sing MAP, 15 ug (FA); A2 = Afluria Quad IM (UA), 15 ug; A3 = Placebo MAP (FA); A4 = A/Sing Mono IM, 15 ug (UA)
Treatments Part B: B1 = A/Sing MAP, 15 ug (FA) with biopsy; B2 = Placebo MAP (FA) with biopsy
B3 = A/Sing MAP, 15 ug (FA); B4 = A/Sing MAP, 10 ug (FA); B5 = A/Sing MAP, 5 ug (FA); B6 = A/Sing MAP, 2.5 ug (FA)
B7 = Placebo MAP (FA); B8 = A/Sing MAP, 15 ug (UA); B9 = A/Sing IM, 15 ug, as 2018 Afluria Quad (UA)

Listing 16.2.7.2
Adverse Events - MedDRA Coding and Calculated Values

Part A

Random-isation Number	Group	Treatment	AE No.	Adverse Event Verbatim	MedDRA System Organ Class	MedDRA Preferred Term	MedDRA Lower Level Term	Duration of AE (days) [C]	Time Since Treatment Admin. (Days) [C]
A0032	Group A4	A/Sing Mono IM, 15 ug		[No Treatment-Emergent Adverse Events Reported]					
A0033	Group A2	Afluria Quad IM, 15 ug	1	Headache	Nervous system disorders	Headache	Headache	0.10	0.76
A0033	Group A2	Afluria Quad IM, 15 ug	2	Cough	Respiratory, thoracic and mediastinal disorders	Cough	Cough	0.10	0.76
A0034	Group A4	A/Sing Mono IM, 15 ug		[No Treatment-Emergent Adverse Events Reported]					

Key: AE = Adverse Event; [C] = Calculated; UK = Unknown
Coded using MedDRA Version 20.01

Administration Site Key: FA = Forearm; UA= Upper arm
Treatments Part A: A1 = A/Sing MAP, 15 ug (FA); A2 = Afluria Quad IM (UA), 15 ug; A3 = Placebo MAP (FA); A4 = A/Sing Mono IM, 15 ug (UA)
Treatments Part B: B1 = A/Sing MAP, 15 ug (FA) with biopsy; B2 = Placebo MAP (FA) with biopsy
B3 = A/Sing MAP, 15 ug (FA); B4 = A/Sing MAP, 10 ug (FA); B5 = A/Sing MAP, 5 ug (FA); B6 = A/Sing MAP, 2.5 ug (FA)
B7 = Placebo MAP (FA); B8 = A/Sing MAP, 15 ug (UA); B9 = A/Sing IM, 15 ug, as 2018 Afluria Quad (UA)

Listing 16.2.7.2
Adverse Events - MedDRA Coding and Calculated Values

Part A

Random-isation Number	Group	Treatment	AE No.	Adverse Event Verbatim	MedDRA System Organ Class	MedDRA Preferred Term	MedDRA Lower Level Term	Duration of AE (days) [C]	Time Since Treatment Admin. (Days) [C]
A0035	Group A4	A/Sing Mono IM, 15 ug	1	Tenderness at injection site	General disorders and administration site conditions	Injection site pain	Injection site tenderness	<1	0.02
A0036	Group A2	Afluria Quad IM, 15 ug		[No Treatment-Emergent Adverse Events Reported]					
A0037	Group A4	A/Sing Mono IM, 15 ug	1	Injection site pain	General disorders and administration site conditions	Injection site pain	Injection site pain	2.87	<0.01
A0037	Group A4	A/Sing Mono IM, 15 ug	2	Pain Left Deltoid (Injection site arm)	General disorders and administration site conditions	Injection site pain	Injection site muscle pain	~7	1.14

Key: AE = Adverse Event; [C] = Calculated; UK = Unknown
Coded using MedDRA Version 20.01

Administration Site Key: FA = Forearm; UA= Upper arm
Treatments Part A: A1 = A/Sing MAP, 15 ug (FA); A2 = Afluria Quad IM (UA), 15 ug; A3 = Placebo MAP (FA); A4 = A/Sing Mono IM, 15 ug (UA)
Treatments Part B: B1 = A/Sing MAP, 15 ug (FA) with biopsy; B2 = Placebo MAP (FA) with biopsy
B3 = A/Sing MAP, 15 ug (FA); B4 = A/Sing MAP, 10 ug (FA); B5 = A/Sing MAP, 5 ug (FA); B6 = A/Sing MAP, 2.5 ug (FA)
B7 = Placebo MAP (FA); B8 = A/Sing MAP, 15 ug (UA); B9 = A/Sing IM, 15 ug, as 2018 Afluria Quad (UA)

Listing 16.2.7.2
Adverse Events - MedDRA Coding and Calculated Values

Part A

Random-isation Number	Group	Treatment	AE No.	Adverse Event Verbatim	MedDRA System Organ Class	MedDRA Preferred Term	MedDRA Lower Level Term	Duration of AE (days) [C]	Time Since Treatment Admin. (Days) [C]
A0038	Group A2	Afluria Quad IM, 15 ug		[No Treatment-Emergent Adverse Events Reported]					
A0039	Group A2	Afluria Quad IM, 15 ug		[No Treatment-Emergent Adverse Events Reported]					
A0040	Group A4	A/Sing Mono IM, 15 ug		[No Treatment-Emergent Adverse Events Reported]					

Key: AE = Adverse Event; [C] = Calculated; UK = Unknown
Coded using MedDRA Version 20.01

Administration Site Key: FA = Forearm; UA= Upper arm
Treatments Part A: A1 = A/Sing MAP, 15 ug (FA); A2 = Afluria Quad IM (UA), 15 ug; A3 = Placebo MAP (FA); A4 = A/Sing Mono IM, 15 ug (UA)
Treatments Part B: B1 = A/Sing MAP, 15 ug (FA) with biopsy; B2 = Placebo MAP (FA) with biopsy
B3 = A/Sing MAP, 15 ug (FA); B4 = A/Sing MAP, 10 ug (FA); B5 = A/Sing MAP, 5 ug (FA); B6 = A/Sing MAP, 2.5 ug (FA)
B7 = Placebo MAP (FA); B8 = A/Sing MAP, 15 ug (UA); B9 = A/Sing IM, 15 ug, as 2018 Afluria Quad (UA)

Listing 16.2.7.2
Adverse Events - MedDRA Coding and Calculated Values

Part A

Random-isation Number	Group	Treatment	AE No.	Adverse Event Verbatim	MedDRA System Organ Class	MedDRA Preferred Term	MedDRA Lower Level Term	Duration of AE (days) [C]	Time Since Treatment Admin. (Days) [C]
A0041	Group A4	A/Sing Mono IM, 15 ug		[No Treatment-Emergent Adverse Events Reported]					
A0042	Group A2	Afluria Quad IM, 15 ug	1	Upper respiratory tract infections	Infections and infestations	Upper respiratory tract infection	Upper respiratory tract infection	3.08	0.79
A0043	Group A2	Afluria Quad IM, 15 ug	1	Right arm tenderness	General disorders and administration site conditions	Tenderness	Tenderness	4.56	2.45
A0043	Group A2	Afluria Quad IM, 15 ug	2	Right Middle Ear Infection	Infections and infestations	Otitis media	Middle ear infection	~17	18.25
A0044	Group A4	A/Sing Mono IM, 15 ug	1	Gastroenteritis	Infections and infestations	Gastroenteritis	Gastroenteritis	~5	10.33

Key: AE = Adverse Event; [C] = Calculated; UK = Unknown
Coded using MedDRA Version 20.01

Administration Site Key: FA = Forearm; UA= Upper arm
Treatments Part A: A1 = A/Sing MAP, 15 ug (FA); A2 = Afluria Quad IM (UA), 15 ug; A3 = Placebo MAP (FA); A4 = A/Sing Mono IM, 15 ug (UA)
Treatments Part B: B1 = A/Sing MAP, 15 ug (FA) with biopsy; B2 = Placebo MAP (FA) with biopsy
B3 = A/Sing MAP, 15 ug (FA); B4 = A/Sing MAP, 10 ug (FA); B5 = A/Sing MAP, 5 ug (FA); B6 = A/Sing MAP, 2.5 ug (FA)
B7 = Placebo MAP (FA); B8 = A/Sing MAP, 15 ug (UA); B9 = A/Sing IM, 15 ug, as 2018 Afluria Quad (UA)

Listing 16.2.7.2
Adverse Events - MedDRA Coding and Calculated Values

Part A

Randomisation Number	Group	Treatment	AE No.	Adverse Event Verbatim	MedDRA System Organ Class	MedDRA Preferred Term	MedDRA Lower Level Term	Duration of AE (days) [C]	Time Since Treatment Admin. (Days) [C]
A0045	Group A2	Afluria Quad IM, 15 ug	1	Upper respiratory tract infection	Infections and infestations	Upper respiratory tract infection	Upper respiratory tract infection	<1	5.72
A0046	Group A4	A/Sing Mono IM, 15 ug	1	Vasovagal symptoms	Nervous system disorders	Presyncope	Vasovagal symptoms	0.01	<0.01
A0046	Group A4	A/Sing Mono IM, 15 ug	2	Headache	Nervous system disorders	Headache	Headache	<1	~4
A0046	Group A4	A/Sing Mono IM, 15 ug	3	Dysmenorrhea	Reproductive system and breast disorders	Dysmenorrhoea	Dysmenorrhea	~5	~1
A0047	Group A2	Afluria Quad IM, 15 ug	1	Headache	Nervous system disorders	Headache	Headache	1.06	0.21
A0047	Group A2	Afluria Quad IM, 15 ug	2	Fatigue	General disorders and administration site conditions	Fatigue	Fatigue	~1	0.21
A0048	Group A4	A/Sing Mono IM, 15 ug	2	Upper respiratory tract infection	Infections and infestations	Upper respiratory tract infection	Upper respiratory tract infection	11.88	1.08

Key: AE = Adverse Event; [C] = Calculated; UK = Unknown
Coded using MedDRA Version 20.01

Administration Site Key: FA = Forearm; UA= Upper arm
Treatments Part A: A1 = A/Sing MAP, 15 ug (FA); A2 = Afluria Quad IM (UA), 15 ug; A3 = Placebo MAP (FA); A4 = A/Sing Mono IM, 15 ug (UA)
Treatments Part B: B1 = A/Sing MAP, 15 ug (FA) with biopsy; B2 = Placebo MAP (FA) with biopsy
B3 = A/Sing MAP, 15 ug (FA); B4 = A/Sing MAP, 10 ug (FA); B5 = A/Sing MAP, 5 ug (FA); B6 = A/Sing MAP, 2.5 ug (FA)
B7 = Placebo MAP (FA); B8 = A/Sing MAP, 15 ug (UA); B9 = A/Sing IM, 15 ug, as 2018 Afluria Quad (UA)

Listing 16.2.7.2
Adverse Events - MedDRA Coding and Calculated Values

Part A

Random-isation Number	Group	Treatment	AE No.	Adverse Event Verbatim	MedDRA System Organ Class	MedDRA Preferred Term	MedDRA Lower Level Term	Duration of AE (days) [C]	Time Since Treatment Admin. (Days) [C]
A0048	Group A4	A/Sing Mono IM, 15 ug	3	Injection site itchiness	General disorders and administration site conditions	Injection site pruritus	Injection site itching	0.08	0.42
A0049	Group A2	Afluria Quad IM, 15 ug	1	Fatigue	General disorders and administration site conditions	Fatigue	Fatigue	1.98	0.83
A0049	Group A2	Afluria Quad IM, 15 ug	2	Musculoskeletal aches	Musculoskeletal and connective tissue disorders	Musculoskeletal pain	Musculoskeletal pain	1.98	0.83
A0050	Group A4	A/Sing Mono IM, 15 ug		[No Treatment-Emergent Adverse Events Reported]					

Key: AE = Adverse Event; [C] = Calculated; UK = Unknown
Coded using MedDRA Version 20.01

Administration Site Key: FA = Forearm; UA= Upper arm
Treatments Part A: A1 = A/Sing MAP, 15 ug (FA); A2 = Afluria Quad IM (UA), 15 ug; A3 = Placebo MAP (FA); A4 = A/Sing Mono IM, 15 ug (UA)
Treatments Part B: B1 = A/Sing MAP, 15 ug (FA) with biopsy; B2 = Placebo MAP (FA) with biopsy
B3 = A/Sing MAP, 15 ug (FA); B4 = A/Sing MAP, 10 ug (FA); B5 = A/Sing MAP, 5 ug (FA); B6 = A/Sing MAP, 2.5 ug (FA)
B7 = Placebo MAP (FA); B8 = A/Sing MAP, 15 ug (UA); B9 = A/Sing IM, 15 ug, as 2018 Afluria Quad (UA)

Listing 16.2.7.2
Adverse Events - MedDRA Coding and Calculated Values

Part A

Random-isation Number	Group	Treatment	AE No.	Adverse Event Verbatim	MedDRA System Organ Class	MedDRA Preferred Term	MedDRA Lower Level Term	Duration of AE (days) [C]	Time Since Treatment Admin. (Days) [C]
A0051	Group A2	Afluria Quad IM, 15 ug		[No Treatment-Emergent Adverse Events Reported]					
A0052	Group A4	A/Sing Mono IM, 15 ug		[No Treatment-Emergent Adverse Events Reported]					
A0053	Group A4	A/Sing Mono IM, 15 ug		[No Treatment-Emergent Adverse Events Reported]					
A0054	Group A2	Afluria Quad IM, 15 ug	1	Shortness of Breath	Respiratory, thoracic and mediastinal disorders	Dyspnoea	Shortness of breath	0.04	3.02

Key: AE = Adverse Event; [C] = Calculated; UK = Unknown
Coded using MedDRA Version 20.01

Administration Site Key: FA = Forearm; UA= Upper arm
Treatments Part A: A1 = A/Sing MAP, 15 ug (FA); A2 = Afluria Quad IM (UA), 15 ug; A3 = Placebo MAP (FA); A4 = A/Sing Mono IM, 15 ug (UA)
Treatments Part B: B1 = A/Sing MAP, 15 ug (FA) with biopsy; B2 = Placebo MAP (FA) with biopsy
B3 = A/Sing MAP, 15 ug (FA); B4 = A/Sing MAP, 10 ug (FA); B5 = A/Sing MAP, 5 ug (FA); B6 = A/Sing MAP, 2.5 ug (FA)
B7 = Placebo MAP (FA); B8 = A/Sing MAP, 15 ug (UA); B9 = A/Sing IM, 15 ug, as 2018 Afluria Quad (UA)

Listing 16.2.7.2
Adverse Events - MedDRA Coding and Calculated Values

Part A

Random-isation Number	Group	Treatment	AE No.	Adverse Event Verbatim	MedDRA System Organ Class	MedDRA Preferred Term	MedDRA Lower Level Term	Duration of AE (days) [C]	Time Since Treatment Admin. (Days) [C]
A0055	Group A4	A/Sing Mono IM, 15 ug		[No Treatment-Emergent Adverse Events Reported]					
A0056	Group A2	Afluria Quad IM, 15 ug		[No Treatment-Emergent Adverse Events Reported]					
A0057	Group A4	A/Sing Mono IM, 15 ug		[No Treatment-Emergent Adverse Events Reported]					
A0058	Group A2	Afluria Quad IM, 15 ug	1	Upper Respiratory Tract Infection	Infections and infestations	Upper respiratory tract infection	Upper respiratory tract infection	~8	47.79

Key: AE = Adverse Event; [C] = Calculated; UK = Unknown
Coded using MedDRA Version 20.01

Administration Site Key: FA = Forearm; UA= Upper arm
Treatments Part A: A1 = A/Sing MAP, 15 ug (FA); A2 = Afluria Quad IM (UA), 15 ug; A3 = Placebo MAP (FA); A4 = A/Sing Mono IM, 15 ug (UA)
Treatments Part B: B1 = A/Sing MAP, 15 ug (FA) with biopsy; B2 = Placebo MAP (FA) with biopsy
B3 = A/Sing MAP, 15 ug (FA); B4 = A/Sing MAP, 10 ug (FA); B5 = A/Sing MAP, 5 ug (FA); B6 = A/Sing MAP, 2.5 ug (FA)
B7 = Placebo MAP (FA); B8 = A/Sing MAP, 15 ug (UA); B9 = A/Sing IM, 15 ug, as 2018 Afluria Quad (UA)

Listing 16.2.7.2
Adverse Events - MedDRA Coding and Calculated Values

Part A

Random-isation Number	Group	Treatment	AE No.	Adverse Event Verbatim	MedDRA System Organ Class	MedDRA Preferred Term	MedDRA Lower Level Term	Duration of AE (days) [C]	Time Since Treatment Admin. (Days) [C]
A0059	Group A2	Afluria Quad IM, 15 ug	1	Intermittent Headache	Nervous system disorders	Headache	Intermittent headache	30.19	4.14
A0060	Group A4	A/Sing Mono IM, 15 ug		[No Treatment-Emergent Adverse Events Reported]					
A1026	Group A1	A/Sing MAP, 15 ug		[No Treatment-Emergent Adverse Events Reported]					

Key: AE = Adverse Event; [C] = Calculated; UK = Unknown
Coded using MedDRA Version 20.01

Administration Site Key: FA = Forearm; UA= Upper arm
Treatments Part A: A1 = A/Sing MAP, 15 ug (FA); A2 = Afluria Quad IM (UA), 15 ug; A3 = Placebo MAP (FA); A4 = A/Sing Mono IM, 15 ug (UA)
Treatments Part B: B1 = A/Sing MAP, 15 ug (FA) with biopsy; B2 = Placebo MAP (FA) with biopsy
B3 = A/Sing MAP, 15 ug (FA); B4 = A/Sing MAP, 10 ug (FA); B5 = A/Sing MAP, 5 ug (FA); B6 = A/Sing MAP, 2.5 ug (FA)
B7 = Placebo MAP (FA); B8 = A/Sing MAP, 15 ug (UA); B9 = A/Sing IM, 15 ug, as 2018 Afluria Quad (UA)

Listing 16.2.7.2
Adverse Events - MedDRA Coding and Calculated Values

Part B

Random-isation Number	Group	Treatment	AE No.	Adverse Event Verbatim	MedDRA System Organ Class	MedDRA Preferred Term	MedDRA Lower Level Term	Duration of AE (days) [C]	Time Since Treatment Admin. (Days) [C]
B0001	Group B1	A/Sing MAP, 15 ug	1	MAP Erythema sites 1, 2 and 3	General disorders and administration site conditions	Application site erythema	Application site erythema	UK	2.92
B0002	Group B2	Placebo MAP	1	Adhesive tapes irritation	Skin and subcutaneous tissue disorders	Dermatitis contact	Adhesive plaster sensitivity	4.10	
B0003	Group B2	Placebo MAP	1	Biopsy site -1 inflamed scar	Injury, poisoning and procedural complications	Inflammation of wound	Inflammation of wound	UK	~21
B0004	Group B1	A/Sing MAP, 15 ug	1	Erythema MAP sites 1, 2 & 3	General disorders and administration site conditions	Application site erythema	Application site erythema	~32	3.09
B0005	Group B1	A/Sing MAP, 15 ug	1	MAP reaction site 1,2,3 Erythema	General disorders and administration site conditions	Application site erythema	Application site erythema	57.32	2.91

Key: AE = Adverse Event; [C] = Calculated; UK = Unknown
Coded using MedDRA Version 20.01

Administration Site Key: FA = Forearm; UA= Upper arm
Treatments Part A: A1 = A/Sing MAP, 15 ug (FA); A2 = Afluria Quad IM (UA), 15 ug; A3 = Placebo MAP (FA); A4 = A/Sing Mono IM, 15 ug (UA)
Treatments Part B: B1 = A/Sing MAP, 15 ug (FA) with biopsy; B2 = Placebo MAP (FA) with biopsy
B3 = A/Sing MAP, 15 ug (FA); B4 = A/Sing MAP, 10 ug (FA); B5 = A/Sing MAP, 5 ug (FA); B6 = A/Sing MAP, 2.5 ug (FA)
B7 = Placebo MAP (FA); B8 = A/Sing MAP, 15 ug (UA); B9 = A/Sing IM, 15 ug, as 2018 Afluria Quad (UA)

Listing 16.2.7.2
Adverse Events - MedDRA Coding and Calculated Values

Part B

Random-isation Number	Group	Treatment	AE No.	Adverse Event Verbatim	MedDRA System Organ Class	MedDRA Preferred Term	MedDRA Lower Level Term	Duration of AE (days) [C]	Time Since Treatment Admin. (Days) [C]
B0005	Group B1	A/Sing MAP, 15 ug	3	Sore throat	Respiratory, thoracic and mediastinal disorders	Oropharyngeal pain	Sore throat	17.58	3.51
B0005	Group B1	A/Sing MAP, 15 ug	4	Skin Dysesthesia	Nervous system disorders	Dysaesthesia	Dysesthesia	~22	13.26
B0006	Group B2	Placebo MAP	1	Biopsy site pain	Injury, poisoning and procedural complications	Procedural pain	Pain post biopsy	~35	0.03
B0007	Group B2	Placebo MAP	1	Headache	Nervous system disorders	Headache	Headache	7.92	2.17
B0008	Group B1	A/Sing MAP, 15 ug	1	Nasal congestion	Respiratory, thoracic and mediastinal disorders	Nasal congestion	Nasal congestion	1.56	0.87

Key: AE = Adverse Event; [C] = Calculated; UK = Unknown
Coded using MedDRA Version 20.01

Administration Site Key: FA = Forearm; UA= Upper arm
Treatments Part A: A1 = A/Sing MAP, 15 ug (FA); A2 = Afluria Quad IM (UA), 15 ug; A3 = Placebo MAP (FA); A4 = A/Sing Mono IM, 15 ug (UA)
Treatments Part B: B1 = A/Sing MAP, 15 ug (FA) with biopsy; B2 = Placebo MAP (FA) with biopsy
B3 = A/Sing MAP, 15 ug (FA); B4 = A/Sing MAP, 10 ug (FA); B5 = A/Sing MAP, 5 ug (FA); B6 = A/Sing MAP, 2.5 ug (FA)
B7 = Placebo MAP (FA); B8 = A/Sing MAP, 15 ug (UA); B9 = A/Sing IM, 15 ug, as 2018 Afluria Quad (UA)

Listing 16.2.7.2
Adverse Events - MedDRA Coding and Calculated Values

Part B

Random-isation Number	Group	Treatment	AE No.	Adverse Event Verbatim	MedDRA System Organ Class	MedDRA Preferred Term	MedDRA Lower Level Term	Duration of AE (days) [C]	Time Since Treatment Admin. (Days) [C]
B0008	Group B1	A/Sing MAP, 15 ug	2	MAP 1,2,3 Erythema	General disorders and administration site conditions	Application site erythema	Application site erythema	UK	2.88
B0009	Group B1	A/Sing MAP, 15 ug	1	MAP site 2,3 reaction erythema	General disorders and administration site conditions	Application site erythema	Application site erythema	~32	2.90
B0010	Group B2	Placebo MAP	1	Pain on biopsy site - Right arm	Injury, poisoning and procedural complications	Procedural pain	Pain post biopsy	5.29	0.15
B0010	Group B2	Placebo MAP	2	Sore throat	Respiratory, thoracic and mediastinal disorders	Oropharyngeal pain	Sore throat	~3	4.77
B0010	Group B2	Placebo MAP	4	Parasthesia	Nervous system disorders	Paraesthesia	Paresthesia	29.99	

Key: AE = Adverse Event; [C] = Calculated; UK = Unknown
Coded using MedDRA Version 20.01

Administration Site Key: FA = Forearm; UA= Upper arm
Treatments Part A: A1 = A/Sing MAP, 15 ug (FA); A2 = Afluria Quad IM (UA), 15 ug; A3 = Placebo MAP (FA); A4 = A/Sing Mono IM, 15 ug (UA)
Treatments Part B: B1 = A/Sing MAP, 15 ug (FA) with biopsy; B2 = Placebo MAP (FA) with biopsy
B3 = A/Sing MAP, 15 ug (FA); B4 = A/Sing MAP, 10 ug (FA); B5 = A/Sing MAP, 5 ug (FA); B6 = A/Sing MAP, 2.5 ug (FA)
B7 = Placebo MAP (FA); B8 = A/Sing MAP, 15 ug (UA); B9 = A/Sing IM, 15 ug, as 2018 Afluria Quad (UA)

Listing 16.2.7.2
Adverse Events - MedDRA Coding and Calculated Values

Part B

Random-isation Number	Group	Treatment	AE No.	Adverse Event Verbatim	MedDRA System Organ Class	MedDRA Preferred Term	MedDRA Lower Level Term	Duration of AE (days) [C]	Time Since Treatment Admin. (Days) [C]
B0010	Group B2	Placebo MAP	6	Left Arm Biopsy site pain	Injury, poisoning and procedural complications	Procedural pain	Pain post biopsy	~34	~1
B0011	Group B8	A/Sing MAP, 15 ug	1	MAP Reaction - Erythema sites 1,2,3	General disorders and administration site conditions	Application site erythema	Application site erythema	61.95	0.97
B0011	Group B8	A/Sing MAP, 15 ug	2	MAP site #1 flaking	General disorders and administration site conditions	Application site exfoliation	Application site peeling	59.93	2.98
B0012	Group B6	A/Sing MAP, 2.5 ug	3	MAP 1 Oedema	General disorders and administration site conditions	Application site oedema	Application site oedema	7.05	0.01
B0012	Group B6	A/Sing MAP, 2.5 ug	4	MAP 1 Erythema	General disorders and administration site conditions	Application site erythema	Application site erythema	6.08	0.98

Key: AE = Adverse Event; [C] = Calculated; UK = Unknown
Coded using MedDRA Version 20.01

Administration Site Key: FA = Forearm; UA= Upper arm
Treatments Part A: A1 = A/Sing MAP, 15 ug (FA); A2 = Afluria Quad IM (UA), 15 ug; A3 = Placebo MAP (FA); A4 = A/Sing Mono IM, 15 ug (UA)
Treatments Part B: B1 = A/Sing MAP, 15 ug (FA) with biopsy; B2 = Placebo MAP (FA) with biopsy
B3 = A/Sing MAP, 15 ug (FA); B4 = A/Sing MAP, 10 ug (FA); B5 = A/Sing MAP, 5 ug (FA); B6 = A/Sing MAP, 2.5 ug (FA)
B7 = Placebo MAP (FA); B8 = A/Sing MAP, 15 ug (UA); B9 = A/Sing IM, 15 ug, as 2018 Afluria Quad (UA)

Listing 16.2.7.2
Adverse Events - MedDRA Coding and Calculated Values

Part B

Random-isation Number	Group	Treatment	AE No.	Adverse Event Verbatim	MedDRA System Organ Class	MedDRA Preferred Term	MedDRA Lower Level Term	Duration of AE (days) [C]	Time Since Treatment Admin. (Days) [C]
B0013	Group B5	A/Sing MAP, 5 ug	1	Upper Respiratory Tract Infection	Infections and infestations	Upper respiratory tract infection	Upper respiratory tract infection	54.00	5.83
B0013	Group B5	A/Sing MAP, 5 ug	2	Headache	Nervous system disorders	Headache	Headache	0.21	5.83
B0013	Group B5	A/Sing MAP, 5 ug	3	Hot Flushes	Vascular disorders	Hot flush	Hot flushes	1.02	0.10
B0014	Group B3	A/Sing MAP, 15 ug	1	MAP sites 1,2,3 - Visibility	General disorders and administration site conditions	Application site discolouration	Application site discolouration	86.23	20.88
B0015	Group B9	A/Sing IM, 15 ug	1	Upper Respiratory Tract Infection	Infections and infestations	Upper respiratory tract infection	Upper respiratory tract infection	4.88	3.03
B0015	Group B9	A/Sing IM, 15 ug	2	Shingles rash Left torso	Infections and infestations	Herpes zoster	Shingles	43.00	6.28
B0016	Group B4	A/Sing MAP, 10 ug	3	Headache	Nervous system disorders	Headache	Headache	<1	~14

Key: AE = Adverse Event; [C] = Calculated; UK = Unknown
Coded using MedDRA Version 20.01

Administration Site Key: FA = Forearm; UA= Upper arm
Treatments Part A: A1 = A/Sing MAP, 15 ug (FA); A2 = Afluria Quad IM (UA), 15 ug; A3 = Placebo MAP (FA); A4 = A/Sing Mono IM, 15 ug (UA)
Treatments Part B: B1 = A/Sing MAP, 15 ug (FA) with biopsy; B2 = Placebo MAP (FA) with biopsy
B3 = A/Sing MAP, 15 ug (FA); B4 = A/Sing MAP, 10 ug (FA); B5 = A/Sing MAP, 5 ug (FA); B6 = A/Sing MAP, 2.5 ug (FA)
B7 = Placebo MAP (FA); B8 = A/Sing MAP, 15 ug (UA); B9 = A/Sing IM, 15 ug, as 2018 Afluria Quad (UA)

Listing 16.2.7.2
Adverse Events - MedDRA Coding and Calculated Values

Part B

Random-isation Number	Group	Treatment	AE No.	Adverse Event Verbatim	MedDRA System Organ Class	MedDRA Preferred Term	MedDRA Lower Level Term	Duration of AE (days) [C]	Time Since Treatment Admin. (Days) [C]
B0016	Group B4	A/Sing MAP, 10 ug	4	Cervical lymphadenopathy	Blood and lymphatic system disorders	Lymphadenopathy	Lymphadenopathy cervical	~57	17.44
B0016	Group B4	A/Sing MAP, 10 ug	7	MAP reaction - erythema + oedema site 3	General disorders and administration site conditions	Application site reaction	Application site reaction	18.18	2.85
B0017	Group B7	Placebo MAP		[No Treatment-Emergent Adverse Events Reported]					
B0018	Group B9	A/Sing IM, 15 ug		[No Treatment-Emergent Adverse Events Reported]					

Key: AE = Adverse Event; [C] = Calculated; UK = Unknown
Coded using MedDRA Version 20.01

Administration Site Key: FA = Forearm; UA= Upper arm
Treatments Part A: A1 = A/Sing MAP, 15 ug (FA); A2 = Afluria Quad IM (UA), 15 ug; A3 = Placebo MAP (FA); A4 = A/Sing Mono IM, 15 ug (UA)
Treatments Part B: B1 = A/Sing MAP, 15 ug (FA) with biopsy; B2 = Placebo MAP (FA) with biopsy
B3 = A/Sing MAP, 15 ug (FA); B4 = A/Sing MAP, 10 ug (FA); B5 = A/Sing MAP, 5 ug (FA); B6 = A/Sing MAP, 2.5 ug (FA)
B7 = Placebo MAP (FA); B8 = A/Sing MAP, 15 ug (UA); B9 = A/Sing IM, 15 ug, as 2018 Afluria Quad (UA)

Listing 16.2.7.2
Adverse Events - MedDRA Coding and Calculated Values

Part B

Random-isation Number	Group	Treatment	AE No.	Adverse Event Verbatim	MedDRA System Organ Class	MedDRA Preferred Term	MedDRA Lower Level Term	Duration of AE (days) [C]	Time Since Treatment Admin. (Days) [C]
B0020	Group B6	A/Sing MAP, 2.5 ug		[No Treatment-Emergent Adverse Events Reported]					
B0021	Group B7	Placebo MAP		[No Treatment-Emergent Adverse Events Reported]					
B0022	Group B4	A/Sing MAP, 10 ug	1	Tachycardia	Cardiac disorders	Tachycardia	Tachycardia	6.90	0.10
B0022	Group B4	A/Sing MAP, 10 ug	2	Headache	Nervous system disorders	Headache	Headache	0.06	3.86
B0022	Group B4	A/Sing MAP, 10 ug	3	MAP visibility sites 1,2,3	General disorders and administration site conditions	Application site discolouration	Application site discolouration	50.48	60.91

Key: AE = Adverse Event; [C] = Calculated; UK = Unknown
Coded using MedDRA Version 20.01

Administration Site Key: FA = Forearm; UA= Upper arm
Treatments Part A: A1 = A/Sing MAP, 15 ug (FA); A2 = Afluria Quad IM (UA), 15 ug; A3 = Placebo MAP (FA); A4 = A/Sing Mono IM, 15 ug (UA)
Treatments Part B: B1 = A/Sing MAP, 15 ug (FA) with biopsy; B2 = Placebo MAP (FA) with biopsy
B3 = A/Sing MAP, 15 ug (FA); B4 = A/Sing MAP, 10 ug (FA); B5 = A/Sing MAP, 5 ug (FA); B6 = A/Sing MAP, 2.5 ug (FA)
B7 = Placebo MAP (FA); B8 = A/Sing MAP, 15 ug (UA); B9 = A/Sing IM, 15 ug, as 2018 Afluria Quad (UA)

Listing 16.2.7.2
Adverse Events - MedDRA Coding and Calculated Values

Part B

Random-isation Number	Group	Treatment	AE No.	Adverse Event Verbatim	MedDRA System Organ Class	MedDRA Preferred Term	MedDRA Lower Level Term	Duration of AE (days) [C]	Time Since Treatment Admin. (Days) [C]
B0023	Group B3	A/Sing MAP, 15 ug	1	Dizziness	Nervous system disorders	Dizziness	Dizziness	<0.1	<0.01
B0023	Group B3	A/Sing MAP, 15 ug	3	MAP reaction sites 1,2&3 - oedema and erythema	General disorders and administration site conditions	Application site reaction	Application site reaction	6.78	0.10
B0024	Group B5	A/Sing MAP, 5 ug	1	MAP reaction sites 1,2,3 Erythema, site 3 oedema	General disorders and administration site conditions	Application site reaction	Application site reaction	6.93	0.06
B0024	Group B5	A/Sing MAP, 5 ug	4	Map site #3 visibility	General disorders and administration site conditions	Application site discolouration	Application site discolouration	~47	61.04
B0025	Group B8	A/Sing MAP, 15 ug	1	Rhinorrhea	Respiratory, thoracic and mediastinal disorders	Rhinorrhoea	Rhinorrhea	~3	~1

Key: AE = Adverse Event; [C] = Calculated; UK = Unknown
Coded using MedDRA Version 20.01

Administration Site Key: FA = Forearm; UA= Upper arm
Treatments Part A: A1 = A/Sing MAP, 15 ug (FA); A2 = Afluria Quad IM (UA), 15 ug; A3 = Placebo MAP (FA); A4 = A/Sing Mono IM, 15 ug (UA)
Treatments Part B: B1 = A/Sing MAP, 15 ug (FA) with biopsy; B2 = Placebo MAP (FA) with biopsy
B3 = A/Sing MAP, 15 ug (FA); B4 = A/Sing MAP, 10 ug (FA); B5 = A/Sing MAP, 5 ug (FA); B6 = A/Sing MAP, 2.5 ug (FA)
B7 = Placebo MAP (FA); B8 = A/Sing MAP, 15 ug (UA); B9 = A/Sing IM, 15 ug, as 2018 Afluria Quad (UA)

Listing 16.2.7.2
Adverse Events - MedDRA Coding and Calculated Values

Part B

Random-isation Number	Group	Treatment	AE No.	Adverse Event Verbatim	MedDRA System Organ Class	MedDRA Preferred Term	MedDRA Lower Level Term	Duration of AE (days) [C]	Time Since Treatment Admin. (Days) [C]
B0025	Group B8	A/Sing MAP, 15 ug	2	MAP site visibility 1,2,3	General disorders and administration site conditions	Application site discolouration	Application site discolouration	~39	62.00
B0026	Group B9	A/Sing IM, 15 ug	1	Headache	Nervous system disorders	Headache	Headache	0.08	19.35
B0027	Group B3	A/Sing MAP, 15 ug	1	MAP sites 1,2,3 - oedema	General disorders and administration site conditions	Application site oedema	Application site oedema	20.98	0.09
B0027	Group B3	A/Sing MAP, 15 ug	2	Upper Respiratory Tract Infection	Infections and infestations	Upper respiratory tract infection	Upper respiratory tract infection	~2	~13
B0027	Group B3	A/Sing MAP, 15 ug	3	MAP site 3 Erythema	General disorders and administration site conditions	Application site erythema	Application site erythema	37.96	3.11
B0027	Group B3	A/Sing MAP, 15 ug	4	MAP Visibility sites 1 and 3	General disorders and administration site conditions	Application site discolouration	Application site discolouration	47.00	60.12

Key: AE = Adverse Event; [C] = Calculated; UK = Unknown
Coded using MedDRA Version 20.01

Administration Site Key: FA = Forearm; UA= Upper arm
Treatments Part A: A1 = A/Sing MAP, 15 ug (FA); A2 = Afluria Quad IM (UA), 15 ug; A3 = Placebo MAP (FA); A4 = A/Sing Mono IM, 15 ug (UA)
Treatments Part B: B1 = A/Sing MAP, 15 ug (FA) with biopsy; B2 = Placebo MAP (FA) with biopsy
B3 = A/Sing MAP, 15 ug (FA); B4 = A/Sing MAP, 10 ug (FA); B5 = A/Sing MAP, 5 ug (FA); B6 = A/Sing MAP, 2.5 ug (FA)
B7 = Placebo MAP (FA); B8 = A/Sing MAP, 15 ug (UA); B9 = A/Sing IM, 15 ug, as 2018 Afluria Quad (UA)

Listing 16.2.7.2
Adverse Events - MedDRA Coding and Calculated Values

Part B

Random-isation Number	Group	Treatment	AE No.	Adverse Event Verbatim	MedDRA System Organ Class	MedDRA Preferred Term	MedDRA Lower Level Term	Duration of AE (days) [C]	Time Since Treatment Admin. (Days) [C]
B0028	Group B5	A/Sing MAP, 5 ug	1	Headache	Nervous system disorders	Headache	Headache	~1	0.28
B0028	Group B5	A/Sing MAP, 5 ug	3	MAP site 1 - Erythema	General disorders and administration site conditions	Application site erythema	Application site erythema	16.93	4.10
B0029	Group B6	A/Sing MAP, 2.5 ug	2	MAP reaction site 3 erythema and oedema	General disorders and administration site conditions	Application site reaction	Application site reaction	5.98	1.03
B0029	Group B6	A/Sing MAP, 2.5 ug	3	Upper Respiratory Tract Infection	Infections and infestations	Upper respiratory tract infection	Upper respiratory tract infection	5.76	17.03
B0029	Group B6	A/Sing MAP, 2.5 ug	5	MAP site 1,2,3 visibility	General disorders and administration site conditions	Application site discolouration	Application site discolouration	~85	60.03

Key: AE = Adverse Event; [C] = Calculated; UK = Unknown
Coded using MedDRA Version 20.01

Administration Site Key: FA = Forearm; UA= Upper arm
Treatments Part A: A1 = A/Sing MAP, 15 ug (FA); A2 = Afluria Quad IM (UA), 15 ug; A3 = Placebo MAP (FA); A4 = A/Sing Mono IM, 15 ug (UA)
Treatments Part B: B1 = A/Sing MAP, 15 ug (FA) with biopsy; B2 = Placebo MAP (FA) with biopsy
B3 = A/Sing MAP, 15 ug (FA); B4 = A/Sing MAP, 10 ug (FA); B5 = A/Sing MAP, 5 ug (FA); B6 = A/Sing MAP, 2.5 ug (FA)
B7 = Placebo MAP (FA); B8 = A/Sing MAP, 15 ug (UA); B9 = A/Sing IM, 15 ug, as 2018 Afluria Quad (UA)

Listing 16.2.7.2
Adverse Events - MedDRA Coding and Calculated Values

Part B

Random-isation Number	Group	Treatment	AE No.	Adverse Event Verbatim	MedDRA System Organ Class	MedDRA Preferred Term	MedDRA Lower Level Term	Duration of AE (days) [C]	Time Since Treatment Admin. (Days) [C]
B0030	Group B7	Placebo MAP		[No Treatment-Emergent Adverse Events Reported]					
B0031	Group B4	A/Sing MAP, 10 ug	5	MAP site 2 & 3 Reaction Erythema and induration	General disorders and administration site conditions	Application site reaction	Application site reaction	5.99	1.00
B0032	Group B6	A/Sing MAP, 2.5 ug	1	MAP reaction site 3 erythema	General disorders and administration site conditions	Application site erythema	Application site erythema	4.04	2.86
B0032	Group B6	A/Sing MAP, 2.5 ug	2	Common cold	Infections and infestations	Nasopharyngitis	Common cold	2.50	6.35
B0033	Group B5	A/Sing MAP, 5 ug	1	MAP reaction site 3 - erythema	General disorders and administration site conditions	Application site erythema	Application site erythema	20.09	0.99

Key: AE = Adverse Event; [C] = Calculated; UK = Unknown
Coded using MedDRA Version 20.01

Administration Site Key: FA = Forearm; UA= Upper arm
Treatments Part A: A1 = A/Sing MAP, 15 ug (FA); A2 = Afluria Quad IM (UA), 15 ug; A3 = Placebo MAP (FA); A4 = A/Sing Mono IM, 15 ug (UA)
Treatments Part B: B1 = A/Sing MAP, 15 ug (FA) with biopsy; B2 = Placebo MAP (FA) with biopsy
B3 = A/Sing MAP, 15 ug (FA); B4 = A/Sing MAP, 10 ug (FA); B5 = A/Sing MAP, 5 ug (FA); B6 = A/Sing MAP, 2.5 ug (FA)
B7 = Placebo MAP (FA); B8 = A/Sing MAP, 15 ug (UA); B9 = A/Sing IM, 15 ug, as 2018 Afluria Quad (UA)

Listing 16.2.7.2
Adverse Events - MedDRA Coding and Calculated Values

Part B

Random-isation Number	Group	Treatment	AE No.	Adverse Event Verbatim	MedDRA System Organ Class	MedDRA Preferred Term	MedDRA Lower Level Term	Duration of AE (days) [C]	Time Since Treatment Admin. (Days) [C]
B0033	Group B5	A/Sing MAP, 5 ug	2	MAP site 3 visibility	General disorders and administration site conditions	Application site discolouration	Application site discolouration	42.18	60.07
B0034	Group B8	A/Sing MAP, 15 ug	2	Patch site reaction - Oedema + Erythema site #1	General disorders and administration site conditions	Application site reaction	Application site reaction	30.89	1.00
B0035	Group B9	A/Sing IM, 15 ug	1	Upper Respiratory Tract Infection	Infections and infestations	Upper respiratory tract infection	Upper respiratory tract infection	8.83	12.03
B0036	Group B7	Placebo MAP	1	Nausea	Gastrointestinal disorders	Nausea	Nausea	<1	<1
B0037	Group B4	A/Sing MAP, 10 ug	1	Patch sites reaction 2,3 - erythema site	General disorders and administration site conditions	Application site erythema	Application site erythema	20.08	0.99

Key: AE = Adverse Event; [C] = Calculated; UK = Unknown

Coded using MedDRA Version 20.01

Administration Site Key: FA = Forearm; UA= Upper arm

Treatments Part A: A1 = A/Sing MAP, 15 ug (FA); A2 = Afluria Quad IM (UA), 15 ug; A3 = Placebo MAP (FA); A4 = A/Sing Mono IM, 15 ug (UA)

Treatments Part B: B1 = A/Sing MAP, 15 ug (FA) with biopsy; B2 = Placebo MAP (FA) with biopsy
B3 = A/Sing MAP, 15 ug (FA); B4 = A/Sing MAP, 10 ug (FA); B5 = A/Sing MAP, 5 ug (FA); B6 = A/Sing MAP, 2.5 ug (FA)
B7 = Placebo MAP (FA); B8 = A/Sing MAP, 15 ug (UA); B9 = A/Sing IM, 15 ug, as 2018 Afluria Quad (UA)

Listing 16.2.7.2
Adverse Events - MedDRA Coding and Calculated Values

Part B

Random-isation Number	Group	Treatment	AE No.	Adverse Event Verbatim	MedDRA System Organ Class	MedDRA Preferred Term	MedDRA Lower Level Term	Duration of AE (days) [C]	Time Since Treatment Admin. (Days) [C]
B0037	Group B4	A/Sing MAP, 10 ug	3	Upper Respiratory Tract Infection	Infections and infestations	Upper respiratory tract infection	Upper respiratory tract infection	1.46	0.84
B0037	Group B4	A/Sing MAP, 10 ug	4	MAP 2 Erythema extent	General disorders and administration site conditions	Application site erythema	Application site erythema	3.95	2.99
B0037	Group B4	A/Sing MAP, 10 ug	5	MAP visibility sites 2,3	General disorders and administration site conditions	Application site discolouration	Application site discolouration	UK	6.94
B0038	Group B3	A/Sing MAP, 15 ug	1	MAP reaction site 1,2,3 erythema	General disorders and administration site conditions	Application site erythema	Application site erythema	48.31	0.83
B0038	Group B3	A/Sing MAP, 15 ug	2	Lethargy	Nervous system disorders	Lethargy	Lethargy	6.00	1.98
B0039	Group B7	Placebo MAP	2	Headache	Nervous system disorders	Headache	Headache	0.63	5.30

Key: AE = Adverse Event; [C] = Calculated; UK = Unknown
Coded using MedDRA Version 20.01

Administration Site Key: FA = Forearm; UA= Upper arm
Treatments Part A: A1 = A/Sing MAP, 15 ug (FA); A2 = Afluria Quad IM (UA), 15 ug; A3 = Placebo MAP (FA); A4 = A/Sing Mono IM, 15 ug (UA)
Treatments Part B: B1 = A/Sing MAP, 15 ug (FA) with biopsy; B2 = Placebo MAP (FA) with biopsy
B3 = A/Sing MAP, 15 ug (FA); B4 = A/Sing MAP, 10 ug (FA); B5 = A/Sing MAP, 5 ug (FA); B6 = A/Sing MAP, 2.5 ug (FA)
B7 = Placebo MAP (FA); B8 = A/Sing MAP, 15 ug (UA); B9 = A/Sing IM, 15 ug, as 2018 Afluria Quad (UA)

Listing 16.2.7.2
Adverse Events - MedDRA Coding and Calculated Values

Part B

Random-isation Number	Group	Treatment	AE No.	Adverse Event Verbatim	MedDRA System Organ Class	MedDRA Preferred Term	MedDRA Lower Level Term	Duration of AE (days) [C]	Time Since Treatment Admin. (Days) [C]
B0040	Group B9	A/Sing IM, 15 ug		[No Treatment-Emergent Adverse Events Reported]					
B0041	Group B3	A/Sing MAP, 15 ug	1	Musculoskeletal pain Left shoulder	Musculoskeletal and connective tissue disorders	Musculoskeletal pain	Shoulder pain	0.33	0.93
B0041	Group B3	A/Sing MAP, 15 ug	2	MAP sites 1,2,3 - erythema	General disorders and administration site conditions	Application site erythema	Application site erythema	5.96	1.04
B0041	Group B3	A/Sing MAP, 15 ug	3	MAP 1,2,3 visible	General disorders and administration site conditions	Application site discolouration	Application site discolouration	UK	62.11
B0042	Group B5	A/Sing MAP, 5 ug	1	Patch site - 3 reaction - Erythema	General disorders and administration site conditions	Application site erythema	Application site erythema	48.25	0.98

Key: AE = Adverse Event; [C] = Calculated; UK = Unknown
Coded using MedDRA Version 20.01

Administration Site Key: FA = Forearm; UA= Upper arm
Treatments Part A: A1 = A/Sing MAP, 15 ug (FA); A2 = Afluria Quad IM (UA), 15 ug; A3 = Placebo MAP (FA); A4 = A/Sing Mono IM, 15 ug (UA)
Treatments Part B: B1 = A/Sing MAP, 15 ug (FA) with biopsy; B2 = Placebo MAP (FA) with biopsy
B3 = A/Sing MAP, 15 ug (FA); B4 = A/Sing MAP, 10 ug (FA); B5 = A/Sing MAP, 5 ug (FA); B6 = A/Sing MAP, 2.5 ug (FA)
B7 = Placebo MAP (FA); B8 = A/Sing MAP, 15 ug (UA); B9 = A/Sing IM, 15 ug, as 2018 Afluria Quad (UA)

Listing 16.2.7.2
Adverse Events - MedDRA Coding and Calculated Values

Part B

Randomisation Number	Group	Treatment	AE No.	Adverse Event Verbatim	MedDRA System Organ Class	MedDRA Preferred Term	MedDRA Lower Level Term	Duration of AE (days) [C]	Time Since Treatment Admin. (Days) [C]
B0042	Group B5	A/Sing MAP, 5 ug	3	Headache	Nervous system disorders	Headache	Headache	~17	3.73
B0042	Group B5	A/Sing MAP, 5 ug	5	MAP 3 Visiblity	General disorders and administration site conditions	Application site discolouration	Application site discolouration	48.98	59.86
B0043	Group B8	A/Sing MAP, 15 ug	1	Headache	Nervous system disorders	Headache	Headache	0.06	0.21
B0043	Group B8	A/Sing MAP, 15 ug	2	Left arm bruise	Injury, poisoning and procedural complications	Contusion	Bruising of arm	~11	~14
B0043	Group B8	A/Sing MAP, 15 ug	3	Myalgia Left deltoid	Musculoskeletal and connective tissue disorders	Myalgia	Myalgia	~11	~14
B0043	Group B8	A/Sing MAP, 15 ug	4	MAP Visibility 1,2,3	General disorders and administration site conditions	Application site discolouration	Application site discolouration	UK	59.85

Key: AE = Adverse Event; [C] = Calculated; UK = Unknown
Coded using MedDRA Version 20.01

Administration Site Key: FA = Forearm; UA= Upper arm
Treatments Part A: A1 = A/Sing MAP, 15 ug (FA); A2 = Afluria Quad IM (UA), 15 ug; A3 = Placebo MAP (FA); A4 = A/Sing Mono IM, 15 ug (UA)
Treatments Part B: B1 = A/Sing MAP, 15 ug (FA) with biopsy; B2 = Placebo MAP (FA) with biopsy
B3 = A/Sing MAP, 15 ug (FA); B4 = A/Sing MAP, 10 ug (FA); B5 = A/Sing MAP, 5 ug (FA); B6 = A/Sing MAP, 2.5 ug (FA)
B7 = Placebo MAP (FA); B8 = A/Sing MAP, 15 ug (UA); B9 = A/Sing IM, 15 ug, as 2018 Afluria Quad (UA)

Listing 16.2.7.2
Adverse Events - MedDRA Coding and Calculated Values

Part B

Random-isation Number	Group	Treatment	AE No.	Adverse Event Verbatim	MedDRA System Organ Class	MedDRA Preferred Term	MedDRA Lower Level Term	Duration of AE (days) [C]	Time Since Treatment Admin. (Days) [C]
B0044	Group B6	A/Sing MAP, 2.5 ug	1	MAP site 3 reaction - erythema and oedema	General disorders and administration site conditions	Application site reaction	Application site reaction	48.25	0.97
B0044	Group B6	A/Sing MAP, 2.5 ug	3	MAP #3 visible	General disorders and administration site conditions	Application site discolouration	Application site discolouration	UK	60.01
B0045	Group B4	A/Sing MAP, 10 ug	2	MAP site #2 reaction oedema and erythema	General disorders and administration site conditions	Application site reaction	Application site reaction	1.92	1.03
B0045	Group B4	A/Sing MAP, 10 ug	6	MAP site 1,2 visibility	General disorders and administration site conditions	Application site discolouration	Application site discolouration	~33	60.00
B0046	Group B7	Placebo MAP	1	Headache	Nervous system disorders	Headache	Headache	0.08	5.36

Key: AE = Adverse Event; [C] = Calculated; UK = Unknown
Coded using MedDRA Version 20.01

Administration Site Key: FA = Forearm; UA= Upper arm
Treatments Part A: A1 = A/Sing MAP, 15 ug (FA); A2 = Afluria Quad IM (UA), 15 ug; A3 = Placebo MAP (FA); A4 = A/Sing Mono IM, 15 ug (UA)
Treatments Part B: B1 = A/Sing MAP, 15 ug (FA) with biopsy; B2 = Placebo MAP (FA) with biopsy
B3 = A/Sing MAP, 15 ug (FA); B4 = A/Sing MAP, 10 ug (FA); B5 = A/Sing MAP, 5 ug (FA); B6 = A/Sing MAP, 2.5 ug (FA)
B7 = Placebo MAP (FA); B8 = A/Sing MAP, 15 ug (UA); B9 = A/Sing IM, 15 ug, as 2018 Afluria Quad (UA)

Listing 16.2.7.2
Adverse Events - MedDRA Coding and Calculated Values

Part B

Random-isation Number	Group	Treatment	AE No.	Adverse Event Verbatim	MedDRA System Organ Class	MedDRA Preferred Term	MedDRA Lower Level Term	Duration of AE (days) [C]	Time Since Treatment Admin. (Days) [C]
B0047	Group B9	A/Sing IM, 15 ug		[No Treatment-Emergent Adverse Events Reported]					
B0048	Group B6	A/Sing MAP, 2.5 ug	1	MAP reaction site 1 erythema	General disorders and administration site conditions	Application site erythema	Application site erythema	5.88	0.99
B0048	Group B6	A/Sing MAP, 2.5 ug	4	MAP visibility site 1	General disorders and administration site conditions	Application site discolouration	Application site discolouration	~45	60.09
B0049	Group B4	A/Sing MAP, 10 ug	1	MAP reaction 2,3 erythema and oedema	General disorders and administration site conditions	Application site reaction	Application site reaction	2.05	0.99
B0049	Group B4	A/Sing MAP, 10 ug	4	MAP Visibility 1,2,3	General disorders and administration site conditions	Application site discolouration	Application site discolouration	UK	59.86

Key: AE = Adverse Event; [C] = Calculated; UK = Unknown
Coded using MedDRA Version 20.01

Administration Site Key: FA = Forearm; UA= Upper arm
Treatments Part A: A1 = A/Sing MAP, 15 ug (FA); A2 = Afluria Quad IM (UA), 15 ug; A3 = Placebo MAP (FA); A4 = A/Sing Mono IM, 15 ug (UA)
Treatments Part B: B1 = A/Sing MAP, 15 ug (FA) with biopsy; B2 = Placebo MAP (FA) with biopsy
B3 = A/Sing MAP, 15 ug (FA); B4 = A/Sing MAP, 10 ug (FA); B5 = A/Sing MAP, 5 ug (FA); B6 = A/Sing MAP, 2.5 ug (FA)
B7 = Placebo MAP (FA); B8 = A/Sing MAP, 15 ug (UA); B9 = A/Sing IM, 15 ug, as 2018 Afluria Quad (UA)

Listing 16.2.7.2
Adverse Events - MedDRA Coding and Calculated Values

Part B

Random-isation Number	Group	Treatment	AE No.	Adverse Event Verbatim	MedDRA System Organ Class	MedDRA Preferred Term	MedDRA Lower Level Term	Duration of AE (days) [C]	Time Since Treatment Admin. (Days) [C]
B0050	Group B8	A/Sing MAP, 15 ug	1	MAP Reaction 1,2,3 - Erythema	General disorders and administration site conditions	Application site erythema	Application site erythema	58.98	1.02
B0051	Group B3	A/Sing MAP, 15 ug		[No Treatment-Emergent Adverse Events Reported]					
B0052	Group B5	A/Sing MAP, 5 ug	1	MAP reaction - Site #1 Erythema, oedema and tenderness	General disorders and administration site conditions	Application site reaction	Application site reaction	~6	~1
B0052	Group B5	A/Sing MAP, 5 ug	2	Left Axilla lymphadenopathy	Blood and lymphatic system disorders	Lymphadenopathy	Lymphadenopathy axillary	0.96	1.79
B0052	Group B5	A/Sing MAP, 5 ug	3	Upper Respiratory Tract Infection	Infections and infestations	Upper respiratory tract infection	Upper respiratory tract infection	10.96	14.83

Key: AE = Adverse Event; [C] = Calculated; UK = Unknown
Coded using MedDRA Version 20.01

Administration Site Key: FA = Forearm; UA= Upper arm
Treatments Part A: A1 = A/Sing MAP, 15 ug (FA); A2 = Afluria Quad IM (UA), 15 ug; A3 = Placebo MAP (FA); A4 = A/Sing Mono IM, 15 ug (UA)
Treatments Part B: B1 = A/Sing MAP, 15 ug (FA) with biopsy; B2 = Placebo MAP (FA) with biopsy
B3 = A/Sing MAP, 15 ug (FA); B4 = A/Sing MAP, 10 ug (FA); B5 = A/Sing MAP, 5 ug (FA); B6 = A/Sing MAP, 2.5 ug (FA)
B7 = Placebo MAP (FA); B8 = A/Sing MAP, 15 ug (UA); B9 = A/Sing IM, 15 ug, as 2018 Afluria Quad (UA)

Listing 16.2.7.2
Adverse Events - MedDRA Coding and Calculated Values

Part B

Randomisation Number	Group	Treatment	AE No.	Adverse Event Verbatim	MedDRA System Organ Class	MedDRA Preferred Term	MedDRA Lower Level Term	Duration of AE (days) [C]	Time Since Treatment Admin. (Days) [C]
B0052	Group B5	A/Sing MAP, 5 ug	4	Headache	Nervous system disorders	Headache	Headache	0.26	7.45
B0052	Group B5	A/Sing MAP, 5 ug	5	Patch site 1,2,3 visibility	General disorders and administration site conditions	Application site discolouration	Application site discolouration	~60	56.05
B0053	Group B6	A/Sing MAP, 2.5 ug	1	MAP site 1,2,3 Visibility	General disorders and administration site conditions	Application site discolouration	Application site discolouration	~34	60.99
B0054	Group B5	A/Sing MAP, 5 ug	1	Upper Respiratory Tract Infection	Infections and infestations	Upper respiratory tract infection	Upper respiratory tract infection	1.19	0.70
B0054	Group B5	A/Sing MAP, 5 ug	2	Vasovagal dizziness	Nervous system disorders	Presyncope	Vasovagal symptoms	0.01	20.81
B0055	Group B8	A/Sing MAP, 15 ug	1	Headache	Nervous system disorders	Headache	Headache	0.08	0.03

Key: AE = Adverse Event; [C] = Calculated; UK = Unknown
Coded using MedDRA Version 20.01

Administration Site Key: FA = Forearm; UA= Upper arm
Treatments Part A: A1 = A/Sing MAP, 15 ug (FA); A2 = Afluria Quad IM (UA), 15 ug; A3 = Placebo MAP (FA); A4 = A/Sing Mono IM, 15 ug (UA)
Treatments Part B: B1 = A/Sing MAP, 15 ug (FA) with biopsy; B2 = Placebo MAP (FA) with biopsy
B3 = A/Sing MAP, 15 ug (FA); B4 = A/Sing MAP, 10 ug (FA); B5 = A/Sing MAP, 5 ug (FA); B6 = A/Sing MAP, 2.5 ug (FA)
B7 = Placebo MAP (FA); B8 = A/Sing MAP, 15 ug (UA); B9 = A/Sing IM, 15 ug, as 2018 Afluria Quad (UA)

Listing 16.2.7.2
Adverse Events - MedDRA Coding and Calculated Values

Part B

Random-isation Number	Group	Treatment	AE No.	Adverse Event Verbatim	MedDRA System Organ Class	MedDRA Preferred Term	MedDRA Lower Level Term	Duration of AE (days) [C]	Time Since Treatment Admin. (Days) [C]
B0055	Group B8	A/Sing MAP, 15 ug	2	Upper Respiratory Tract Infection	Infections and infestations	Upper respiratory tract infection	Upper respiratory tract infection	2.00	1.85
B0055	Group B8	A/Sing MAP, 15 ug	3	Tonsillitis	Infections and infestations	Tonsillitis	Tonsillitis	2.92	20.02
B0056	Group B3	A/Sing MAP, 15 ug	2	MAP site reaction - Erythema 1,2	General disorders and administration site conditions	Application site erythema	Application site erythema	~4	~1
B0056	Group B3	A/Sing MAP, 15 ug	3	MAP 1,2,3 Visibility	General disorders and administration site conditions	Application site discolouration	Application site discolouration	~88	6.80
B0057	Group B4	A/Sing MAP, 10 ug	1	MAP 1,2,3 Visibiity	General disorders and administration site conditions	Application site discolouration	Application site discolouration	UK	59.83
B0058	Group B9	A/Sing IM, 15 ug	1	Exacerbations of Migraine	Nervous system disorders	Migraine	Migraine aggravated	0.79	12.02

Key: AE = Adverse Event; [C] = Calculated; UK = Unknown
Coded using MedDRA Version 20.01

Administration Site Key: FA = Forearm; UA= Upper arm
Treatments Part A: A1 = A/Sing MAP, 15 ug (FA); A2 = Afluria Quad IM (UA), 15 ug; A3 = Placebo MAP (FA); A4 = A/Sing Mono IM, 15 ug (UA)
Treatments Part B: B1 = A/Sing MAP, 15 ug (FA) with biopsy; B2 = Placebo MAP (FA) with biopsy
B3 = A/Sing MAP, 15 ug (FA); B4 = A/Sing MAP, 10 ug (FA); B5 = A/Sing MAP, 5 ug (FA); B6 = A/Sing MAP, 2.5 ug (FA)
B7 = Placebo MAP (FA); B8 = A/Sing MAP, 15 ug (UA); B9 = A/Sing IM, 15 ug, as 2018 Afluria Quad (UA)

Listing 16.2.7.2
Adverse Events - MedDRA Coding and Calculated Values

Part B

Random-isation Number	Group	Treatment	AE No.	Adverse Event Verbatim	MedDRA System Organ Class	MedDRA Preferred Term	MedDRA Lower Level Term	Duration of AE (days) [C]	Time Since Treatment Admin. (Days) [C]
B0058	Group B9	A/Sing IM, 15 ug	2	Left arm (IP arm) discomfort	General disorders and administration site conditions	Injection site discomfort	Injection site discomfort	0.03	0.02
B0059	Group B7	Placebo MAP		[No Treatment-Emergent Adverse Events Reported]					
B0060	Group B3	A/Sing MAP, 15 ug	5	Bruise to Right elbow	Injury, poisoning and procedural complications	Contusion	Bruise	2.11	0.33
B0060	Group B3	A/Sing MAP, 15 ug	6	Patch site 1 reaction erythema and oedema	General disorders and administration site conditions	Application site reaction	Application site reaction	56.97	3.03

Key: AE = Adverse Event; [C] = Calculated; UK = Unknown
Coded using MedDRA Version 20.01

Administration Site Key: FA = Forearm; UA= Upper arm
Treatments Part A: A1 = A/Sing MAP, 15 ug (FA); A2 = Afluria Quad IM (UA), 15 ug; A3 = Placebo MAP (FA); A4 = A/Sing Mono IM, 15 ug (UA)
Treatments Part B: B1 = A/Sing MAP, 15 ug (FA) with biopsy; B2 = Placebo MAP (FA) with biopsy
B3 = A/Sing MAP, 15 ug (FA); B4 = A/Sing MAP, 10 ug (FA); B5 = A/Sing MAP, 5 ug (FA); B6 = A/Sing MAP, 2.5 ug (FA)
B7 = Placebo MAP (FA); B8 = A/Sing MAP, 15 ug (UA); B9 = A/Sing IM, 15 ug, as 2018 Afluria Quad (UA)

Listing 16.2.7.2
Adverse Events - MedDRA Coding and Calculated Values

Part B

Random-isation Number	Group	Treatment	AE No.	Adverse Event Verbatim	MedDRA System Organ Class	MedDRA Preferred Term	MedDRA Lower Level Term	Duration of AE (days) [C]	Time Since Treatment Admin. (Days) [C]
B0061	Group B8	A/Sing MAP, 15 ug	2	MAP sites 1,2 - Erythema	General disorders and administration site conditions	Application site erythema	Application site erythema	17.94	2.97
B0062	Group B5	A/Sing MAP, 5 ug		[No Treatment-Emergent Adverse Events Reported]					
B0063	Group B7	Placebo MAP		[No Treatment-Emergent Adverse Events Reported]					
B0064	Group B9	A/Sing IM, 15 ug	1	Upper Respiratory Tract Infection	Infections and infestations	Upper respiratory tract infection	Upper respiratory tract infection	18.81	7.16

Key: AE = Adverse Event; [C] = Calculated; UK = Unknown
Coded using MedDRA Version 20.01

Administration Site Key: FA = Forearm; UA= Upper arm
Treatments Part A: A1 = A/Sing MAP, 15 ug (FA); A2 = Afluria Quad IM (UA), 15 ug; A3 = Placebo MAP (FA); A4 = A/Sing Mono IM, 15 ug (UA)
Treatments Part B: B1 = A/Sing MAP, 15 ug (FA) with biopsy; B2 = Placebo MAP (FA) with biopsy
B3 = A/Sing MAP, 15 ug (FA); B4 = A/Sing MAP, 10 ug (FA); B5 = A/Sing MAP, 5 ug (FA); B6 = A/Sing MAP, 2.5 ug (FA)
B7 = Placebo MAP (FA); B8 = A/Sing MAP, 15 ug (UA); B9 = A/Sing IM, 15 ug, as 2018 Afluria Quad (UA)

Listing 16.2.7.2
Adverse Events - MedDRA Coding and Calculated Values

Part B

Random-isation Number	Group	Treatment	AE No.	Adverse Event Verbatim	MedDRA System Organ Class	MedDRA Preferred Term	MedDRA Lower Level Term	Duration of AE (days) [C]	Time Since Treatment Admin. (Days) [C]
B0065	Group B4	A/Sing MAP, 10 ug	1	Tension Headache	Nervous system disorders	Tension headache	Tension headache	0.77	0.13
B0065	Group B4	A/Sing MAP, 10 ug	2	Upper Respiratory Tract Infection	Infections and infestations	Upper respiratory tract infection	Upper respiratory tract infection	14.04	13.72
B0065	Group B4	A/Sing MAP, 10 ug	3	MAP site 1 & 3 erythema	General disorders and administration site conditions	Application site erythema	Application site erythema	~20	~1
B0065	Group B4	A/Sing MAP, 10 ug	4	MAP visibility site 1,2,3	General disorders and administration site conditions	Application site discolouration	Application site discolouration	~77	59.96
B0066	Group B6	A/Sing MAP, 2.5 ug		[No Treatment-Emergent Adverse Events Reported]					

Key: AE = Adverse Event; [C] = Calculated; UK = Unknown
Coded using MedDRA Version 20.01

Administration Site Key: FA = Forearm; UA= Upper arm
Treatments Part A: A1 = A/Sing MAP, 15 ug (FA); A2 = Afluria Quad IM (UA), 15 ug; A3 = Placebo MAP (FA); A4 = A/Sing Mono IM, 15 ug (UA)
Treatments Part B: B1 = A/Sing MAP, 15 ug (FA) with biopsy; B2 = Placebo MAP (FA) with biopsy
B3 = A/Sing MAP, 15 ug (FA); B4 = A/Sing MAP, 10 ug (FA); B5 = A/Sing MAP, 5 ug (FA); B6 = A/Sing MAP, 2.5 ug (FA)
B7 = Placebo MAP (FA); B8 = A/Sing MAP, 15 ug (UA); B9 = A/Sing IM, 15 ug, as 2018 Afluria Quad (UA)

Listing 16.2.7.2
Adverse Events - MedDRA Coding and Calculated Values

Part B

Random-isation Number	Group	Treatment	AE No.	Adverse Event Verbatim	MedDRA System Organ Class	MedDRA Preferred Term	MedDRA Lower Level Term	Duration of AE (days) [C]	Time Since Treatment Admin. (Days) [C]
B0067	Group B8	A/Sing MAP, 15 ug	1	Itchness - MAP 1,2,3	General disorders and administration site conditions	Application site pruritus	Application site itching	~3	2.66
B0068	Group B5	A/Sing MAP, 5 ug	1	MAP site 2 Visibility	General disorders and administration site conditions	Application site discolouration	Application site discolouration	~48	60.07
B0068	Group B5	A/Sing MAP, 5 ug	2	MAP 2 Erythema	General disorders and administration site conditions	Application site erythema	Application site erythema	14.93	6.78
B0069	Group B7	Placebo MAP	2	Headache	Nervous system disorders	Headache	Headache	0.13	5.29
B0070	Group B9	A/Sing IM, 15 ug		[No Treatment-Emergent Adverse Events Reported]					

Key: AE = Adverse Event; [C] = Calculated; UK = Unknown
Coded using MedDRA Version 20.01

Administration Site Key: FA = Forearm; UA= Upper arm
Treatments Part A: A1 = A/Sing MAP, 15 ug (FA); A2 = Afluria Quad IM (UA), 15 ug; A3 = Placebo MAP (FA); A4 = A/Sing Mono IM, 15 ug (UA)
Treatments Part B: B1 = A/Sing MAP, 15 ug (FA) with biopsy; B2 = Placebo MAP (FA) with biopsy
B3 = A/Sing MAP, 15 ug (FA); B4 = A/Sing MAP, 10 ug (FA); B5 = A/Sing MAP, 5 ug (FA); B6 = A/Sing MAP, 2.5 ug (FA)
B7 = Placebo MAP (FA); B8 = A/Sing MAP, 15 ug (UA); B9 = A/Sing IM, 15 ug, as 2018 Afluria Quad (UA)

Listing 16.2.7.2
Adverse Events - MedDRA Coding and Calculated Values

Part B

Random-isation Number	Group	Treatment	AE No.	Adverse Event Verbatim	MedDRA System Organ Class	MedDRA Preferred Term	MedDRA Lower Level Term	Duration of AE (days) [C]	Time Since Treatment Admin. (Days) [C]
B0071	Group B4	A/Sing MAP, 10 ug	1	MAP reaction site 3 - Erythema	General disorders and administration site conditions	Application site erythema	Application site erythema	20.04	1.04
B0072	Group B3	A/Sing MAP, 15 ug	1	Headache	Nervous system disorders	Headache	Headache	0.97	0.04
B0072	Group B3	A/Sing MAP, 15 ug	2	MAP reaction sites 1,2,3 - Erythema	General disorders and administration site conditions	Application site erythema	Application site erythema	6.05	1.04
B0073	Group B6	A/Sing MAP, 2.5 ug	1	MAP site reaction 2 - Oedema	General disorders and administration site conditions	Application site oedema	Application site oedema	6.95	0.08
B0073	Group B6	A/Sing MAP, 2.5 ug	2	MAP reaction sites 1,2,3 - Erythema	General disorders and administration site conditions	Application site erythema	Application site erythema	~4	1.04

Key: AE = Adverse Event; [C] = Calculated; UK = Unknown
Coded using MedDRA Version 20.01

Administration Site Key: FA = Forearm; UA= Upper arm
Treatments Part A: A1 = A/Sing MAP, 15 ug (FA); A2 = Afluria Quad IM (UA), 15 ug; A3 = Placebo MAP (FA); A4 = A/Sing Mono IM, 15 ug (UA)
Treatments Part B: B1 = A/Sing MAP, 15 ug (FA) with biopsy; B2 = Placebo MAP (FA) with biopsy
B3 = A/Sing MAP, 15 ug (FA); B4 = A/Sing MAP, 10 ug (FA); B5 = A/Sing MAP, 5 ug (FA); B6 = A/Sing MAP, 2.5 ug (FA)
B7 = Placebo MAP (FA); B8 = A/Sing MAP, 15 ug (UA); B9 = A/Sing IM, 15 ug, as 2018 Afluria Quad (UA)

Listing 16.2.7.2
Adverse Events - MedDRA Coding and Calculated Values

Part B

Random-isation Number	Group	Treatment	AE No.	Adverse Event Verbatim	MedDRA System Organ Class	MedDRA Preferred Term	MedDRA Lower Level Term	Duration of AE (days) [C]	Time Since Treatment Admin. (Days) [C]
B0074	Group B9	A/Sing IM, 15 ug		[No Treatment-Emergent Adverse Events Reported]					
B0075	Group B3	A/Sing MAP, 15 ug	1	Sore Throat	Respiratory, thoracic and mediastinal disorders	Oropharyngeal pain	Sore throat	0.63	1.28
B0075	Group B3	A/Sing MAP, 15 ug	2	Headache	Nervous system disorders	Headache	Headache	0.17	3.83
B0075	Group B3	A/Sing MAP, 15 ug	3	MAP 2,3 Visibility	General disorders and administration site conditions	Application site discolouration	Application site discolouration	28.09	59.85
B0077	Group B4	A/Sing MAP, 10 ug	1	MAP reaction site 1,3 oedema and erythema	General disorders and administration site conditions	Application site reaction	Application site reaction	4.44	2.85

Key: AE = Adverse Event; [C] = Calculated; UK = Unknown
Coded using MedDRA Version 20.01

Administration Site Key: FA = Forearm; UA= Upper arm
Treatments Part A: A1 = A/Sing MAP, 15 ug (FA); A2 = Afluria Quad IM (UA), 15 ug; A3 = Placebo MAP (FA); A4 = A/Sing Mono IM, 15 ug (UA)
Treatments Part B: B1 = A/Sing MAP, 15 ug (FA) with biopsy; B2 = Placebo MAP (FA) with biopsy
B3 = A/Sing MAP, 15 ug (FA); B4 = A/Sing MAP, 10 ug (FA); B5 = A/Sing MAP, 5 ug (FA); B6 = A/Sing MAP, 2.5 ug (FA)
B7 = Placebo MAP (FA); B8 = A/Sing MAP, 15 ug (UA); B9 = A/Sing IM, 15 ug, as 2018 Afluria Quad (UA)

Listing 16.2.7.2
Adverse Events - MedDRA Coding and Calculated Values

Part B

Random-isation Number	Group	Treatment	AE No.	Adverse Event Verbatim	MedDRA System Organ Class	MedDRA Preferred Term	MedDRA Lower Level Term	Duration of AE (days) [C]	Time Since Treatment Admin. (Days) [C]
B0078	Group B8	A/Sing MAP, 15 ug	1	MAP 1,2,3 reaction - Erythema	General disorders and administration site conditions	Application site erythema	Application site erythema	20.08	0.91
B0078	Group B8	A/Sing MAP, 15 ug	3	MAP reaction - Oedema sites 1,2,3 and site 1 itchiness	General disorders and administration site conditions	Application site reaction	Application site reaction	3.98	2.96
B0078	Group B8	A/Sing MAP, 15 ug	4	MAP site 1,2,3 Visibility	General disorders and administration site conditions	Application site discolouration	Application site discolouration	~84	65.94
B0079	Group B6	A/Sing MAP, 2.5 ug	1	Rash- Erythema multiforme	Skin and subcutaneous tissue disorders	Erythema multiforme	Erythema multiforme	1.04	0.85
B0079	Group B6	A/Sing MAP, 2.5 ug	2	MAP site 2 - redness extension	General disorders and administration site conditions	Application site erythema	Application site redness	20.03	0.94
B0079	Group B6	A/Sing MAP, 2.5 ug	3	Headache	Nervous system disorders	Headache	Headache	0.06	2.10

Key: AE = Adverse Event; [C] = Calculated; UK = Unknown
Coded using MedDRA Version 20.01

Administration Site Key: FA = Forearm; UA= Upper arm
Treatments Part A: A1 = A/Sing MAP, 15 ug (FA); A2 = Afluria Quad IM (UA), 15 ug; A3 = Placebo MAP (FA); A4 = A/Sing Mono IM, 15 ug (UA)
Treatments Part B: B1 = A/Sing MAP, 15 ug (FA) with biopsy; B2 = Placebo MAP (FA) with biopsy
B3 = A/Sing MAP, 15 ug (FA); B4 = A/Sing MAP, 10 ug (FA); B5 = A/Sing MAP, 5 ug (FA); B6 = A/Sing MAP, 2.5 ug (FA)
B7 = Placebo MAP (FA); B8 = A/Sing MAP, 15 ug (UA); B9 = A/Sing IM, 15 ug, as 2018 Afluria Quad (UA)

Listing 16.2.7.2
Adverse Events - MedDRA Coding and Calculated Values

Part B

Random-isation Number	Group	Treatment	AE No.	Adverse Event Verbatim	MedDRA System Organ Class	MedDRA Preferred Term	MedDRA Lower Level Term	Duration of AE (days) [C]	Time Since Treatment Admin. (Days) [C]
B0079	Group B6	A/Sing MAP, 2.5 ug	4	MAP sites 2,3 -Oedema	General disorders and administration site conditions	Application site oedema	Application site oedema	20.89	0.09
B0079	Group B6	A/Sing MAP, 2.5 ug	5	MAP 2 visibility	General disorders and administration site conditions	Application site discolouration	Application site discolouration	~83	59.99
B0081	Group B6	A/Sing MAP, 2.5 ug	1	MAP reaction sites 1,2,3 - Erythema and Oedema	General disorders and administration site conditions	Application site reaction	Application site reaction	2.98	0.09
B0081	Group B6	A/Sing MAP, 2.5 ug	3	Headache	Nervous system disorders	Headache	Headache	0.02	0.16
B0081	Group B6	A/Sing MAP, 2.5 ug	4	Headache	Nervous system disorders	Headache	Headache	0.13	6.94
B0082	Group B3	A/Sing MAP, 15 ug	1	MAP reaction sites 1,2,3 reaction - Erythema & Oedema	General disorders and administration site conditions	Application site reaction	Application site reaction	20.08	0.94

Key: AE = Adverse Event; [C] = Calculated; UK = Unknown
Coded using MedDRA Version 20.01

Administration Site Key: FA = Forearm; UA= Upper arm
Treatments Part A: A1 = A/Sing MAP, 15 ug (FA); A2 = Afluria Quad IM (UA), 15 ug; A3 = Placebo MAP (FA); A4 = A/Sing Mono IM, 15 ug (UA)
Treatments Part B: B1 = A/Sing MAP, 15 ug (FA) with biopsy; B2 = Placebo MAP (FA) with biopsy
B3 = A/Sing MAP, 15 ug (FA); B4 = A/Sing MAP, 10 ug (FA); B5 = A/Sing MAP, 5 ug (FA); B6 = A/Sing MAP, 2.5 ug (FA)
B7 = Placebo MAP (FA); B8 = A/Sing MAP, 15 ug (UA); B9 = A/Sing IM, 15 ug, as 2018 Afluria Quad (UA)

Listing 16.2.7.2
Adverse Events - MedDRA Coding and Calculated Values

Part B

Randomisation Number	Group	Treatment	AE No.	Adverse Event Verbatim	MedDRA System Organ Class	MedDRA Preferred Term	MedDRA Lower Level Term	Duration of AE (days) [C]	Time Since Treatment Admin. (Days) [C]
B0082	Group B3	A/Sing MAP, 15 ug	3	Chest pain	General disorders and administration site conditions	Chest pain	Chest pain	0.39	6.68
B0082	Group B3	A/Sing MAP, 15 ug	4	MAP site 1,2,3 reaction - visibility	General disorders and administration site conditions	Application site discolouration	Application site discolouration	~41	60.08
B0083	Group B8	A/Sing MAP, 15 ug	1	MAP reaction sites 2,3 -Oedema	General disorders and administration site conditions	Application site oedema	Application site oedema	6.93	0.08
B0083	Group B8	A/Sing MAP, 15 ug	3	MAP sites 1,2,3 reaction - Erythema	General disorders and administration site conditions	Application site erythema	Application site erythema	6.17	0.85
B0083	Group B8	A/Sing MAP, 15 ug	4	MAP site 1,2,3 Visibility	General disorders and administration site conditions	Application site discolouration	Application site discolouration	UK	59.79

Key: AE = Adverse Event; [C] = Calculated; UK = Unknown
Coded using MedDRA Version 20.01

Administration Site Key: FA = Forearm; UA= Upper arm
Treatments Part A: A1 = A/Sing MAP, 15 ug (FA); A2 = Afluria Quad IM (UA), 15 ug; A3 = Placebo MAP (FA); A4 = A/Sing Mono IM, 15 ug (UA)
Treatments Part B: B1 = A/Sing MAP, 15 ug (FA) with biopsy; B2 = Placebo MAP (FA) with biopsy
B3 = A/Sing MAP, 15 ug (FA); B4 = A/Sing MAP, 10 ug (FA); B5 = A/Sing MAP, 5 ug (FA); B6 = A/Sing MAP, 2.5 ug (FA)
B7 = Placebo MAP (FA); B8 = A/Sing MAP, 15 ug (UA); B9 = A/Sing IM, 15 ug, as 2018 Afluria Quad (UA)

Listing 16.2.7.2
Adverse Events - MedDRA Coding and Calculated Values

Part B

Random-isation Number	Group	Treatment	AE No.	Adverse Event Verbatim	MedDRA System Organ Class	MedDRA Preferred Term	MedDRA Lower Level Term	Duration of AE (days) [C]	Time Since Treatment Admin. (Days) [C]
B0084	Group B5	A/Sing MAP, 5 ug	1	MAP 3 reaction erythema	General disorders and administration site conditions	Application site erythema	Application site erythema	20.09	1.01
B0085	Group B4	A/Sing MAP, 10 ug	1	Headache	Nervous system disorders	Headache	Headache	0.42	0.42
B0085	Group B4	A/Sing MAP, 10 ug	2	Application site erythema -MAP site 2,3	General disorders and administration site conditions	Application site erythema	Application site erythema	~6	~1
B0085	Group B4	A/Sing MAP, 10 ug	3	MAP site 3 visibility	General disorders and administration site conditions	Application site discolouration	Application site discolouration	~26	~63
B0086	Group B9	A/Sing IM, 15 ug		[No Treatment-Emergent Adverse Events Reported]					

Key: AE = Adverse Event; [C] = Calculated; UK = Unknown
Coded using MedDRA Version 20.01

Administration Site Key: FA = Forearm; UA= Upper arm
Treatments Part A: A1 = A/Sing MAP, 15 ug (FA); A2 = Afluria Quad IM (UA), 15 ug; A3 = Placebo MAP (FA); A4 = A/Sing Mono IM, 15 ug (UA)
Treatments Part B: B1 = A/Sing MAP, 15 ug (FA) with biopsy; B2 = Placebo MAP (FA) with biopsy
B3 = A/Sing MAP, 15 ug (FA); B4 = A/Sing MAP, 10 ug (FA); B5 = A/Sing MAP, 5 ug (FA); B6 = A/Sing MAP, 2.5 ug (FA)
B7 = Placebo MAP (FA); B8 = A/Sing MAP, 15 ug (UA); B9 = A/Sing IM, 15 ug, as 2018 Afluria Quad (UA)

Listing 16.2.7.2
Adverse Events - MedDRA Coding and Calculated Values

Part B

Random-isation Number	Group	Treatment	AE No.	Adverse Event Verbatim	MedDRA System Organ Class	MedDRA Preferred Term	MedDRA Lower Level Term	Duration of AE (days) [C]	Time Since Treatment Admin. (Days) [C]
B0087	Group B7	Placebo MAP	1	URTI	Infections and infestations	Upper respiratory tract infection	URTI	25.31	23.78
B0087	Group B7	Placebo MAP	2	Headache	Nervous system disorders	Headache	Headache	<1	~21
B0088	Group B9	A/Sing IM, 15 ug		[No Treatment-Emergent Adverse Events Reported]					
B0089	Group B5	A/Sing MAP, 5 ug	1	MAP 2 Erythema	General disorders and administration site conditions	Application site erythema	Application site erythema	~32	2.86
B0090	Group B6	A/Sing MAP, 2.5 ug	1	Flu - like symptom	General disorders and administration site conditions	Influenza like illness	Flu like symptoms	~5	~2

Key: AE = Adverse Event; [C] = Calculated; UK = Unknown
Coded using MedDRA Version 20.01

Administration Site Key: FA = Forearm; UA= Upper arm
Treatments Part A: A1 = A/Sing MAP, 15 ug (FA); A2 = Afluria Quad IM (UA), 15 ug; A3 = Placebo MAP (FA); A4 = A/Sing Mono IM, 15 ug (UA)
Treatments Part B: B1 = A/Sing MAP, 15 ug (FA) with biopsy; B2 = Placebo MAP (FA) with biopsy
B3 = A/Sing MAP, 15 ug (FA); B4 = A/Sing MAP, 10 ug (FA); B5 = A/Sing MAP, 5 ug (FA); B6 = A/Sing MAP, 2.5 ug (FA)
B7 = Placebo MAP (FA); B8 = A/Sing MAP, 15 ug (UA); B9 = A/Sing IM, 15 ug, as 2018 Afluria Quad (UA)

Listing 16.2.7.2
Adverse Events - MedDRA Coding and Calculated Values

Part B

Random-isation Number	Group	Treatment	AE No.	Adverse Event Verbatim	MedDRA System Organ Class	MedDRA Preferred Term	MedDRA Lower Level Term	Duration of AE (days) [C]	Time Since Treatment Admin. (Days) [C]
B0090	Group B6	A/Sing MAP, 2.5 ug	2	Secondary Hyperalgesia	Nervous system disorders	Hyperaesthesia	Hyperalgesia	0.02	0.79
B0090	Group B6	A/Sing MAP, 2.5 ug	4	MAP itchiness	General disorders and administration site conditions	Application site pruritus	Application site itching	0.05	0.01
B0091	Group B8	A/Sing MAP, 15 ug	1	MAP reaction 1,2,3 Erythema	General disorders and administration site conditions	Application site erythema	Application site erythema	3.87	3.02
B0091	Group B8	A/Sing MAP, 15 ug	2	Flu like symptom	General disorders and administration site conditions	Influenza like illness	Flu like symptoms	0.33	1.85
B0091	Group B8	A/Sing MAP, 15 ug	3	Headache	Nervous system disorders	Headache	Headache	0.04	4.29
B0091	Group B8	A/Sing MAP, 15 ug	4	MAP sites visibility 1,2,3	General disorders and administration site conditions	Application site discolouration	Application site discolouration	UK	59.89

Key: AE = Adverse Event; [C] = Calculated; UK = Unknown

Coded using MedDRA Version 20.01

Administration Site Key: FA = Forearm; UA= Upper arm

Treatments Part A: A1 = A/Sing MAP, 15 ug (FA); A2 = Afluria Quad IM (UA), 15 ug; A3 = Placebo MAP (FA); A4 = A/Sing Mono IM, 15 ug (UA)

Treatments Part B: B1 = A/Sing MAP, 15 ug (FA) with biopsy; B2 = Placebo MAP (FA) with biopsy
B3 = A/Sing MAP, 15 ug (FA); B4 = A/Sing MAP, 10 ug (FA); B5 = A/Sing MAP, 5 ug (FA); B6 = A/Sing MAP, 2.5 ug (FA)
B7 = Placebo MAP (FA); B8 = A/Sing MAP, 15 ug (UA); B9 = A/Sing IM, 15 ug, as 2018 Afluria Quad (UA)

Listing 16.2.7.2
Adverse Events - MedDRA Coding and Calculated Values

Part B

Random-isation Number	Group	Treatment	AE No.	Adverse Event Verbatim	MedDRA System Organ Class	MedDRA Preferred Term	MedDRA Lower Level Term	Duration of AE (days) [C]	Time Since Treatment Admin. (Days) [C]
B0092	Group B4	A/Sing MAP, 10 ug	1	MAP 1,3 reaction Erythema	General disorders and administration site conditions	Application site erythema	Application site erythema	18.08	2.89
B0092	Group B4	A/Sing MAP, 10 ug	2	MAP 1,3 Visibility	General disorders and administration site conditions	Application site discolouration	Application site discolouration	~54	53.81
B0092	Group B4	A/Sing MAP, 10 ug	4	Headache	Nervous system disorders	Headache	Headache	0.05	6.31
B0093	Group B7	Placebo MAP		[No Treatment-Emergent Adverse Events Reported]					
B0094	Group B3	A/Sing MAP, 15 ug	1	MAP 1,2,3 - Erythema	General disorders and administration site conditions	Application site erythema	Application site erythema	6.03	1.00

Key: AE = Adverse Event; [C] = Calculated; UK = Unknown
Coded using MedDRA Version 20.01

Administration Site Key: FA = Forearm; UA= Upper arm
Treatments Part A: A1 = A/Sing MAP, 15 ug (FA); A2 = Afluria Quad IM (UA), 15 ug; A3 = Placebo MAP (FA); A4 = A/Sing Mono IM, 15 ug (UA)
Treatments Part B: B1 = A/Sing MAP, 15 ug (FA) with biopsy; B2 = Placebo MAP (FA) with biopsy
B3 = A/Sing MAP, 15 ug (FA); B4 = A/Sing MAP, 10 ug (FA); B5 = A/Sing MAP, 5 ug (FA); B6 = A/Sing MAP, 2.5 ug (FA)
B7 = Placebo MAP (FA); B8 = A/Sing MAP, 15 ug (UA); B9 = A/Sing IM, 15 ug, as 2018 Afluria Quad (UA)

Listing 16.2.7.2
Adverse Events - MedDRA Coding and Calculated Values

Part B

Random-isation Number	Group	Treatment	AE No.	Adverse Event Verbatim	MedDRA System Organ Class	MedDRA Preferred Term	MedDRA Lower Level Term	Duration of AE (days) [C]	Time Since Treatment Admin. (Days) [C]
B0095	Group B9	A/Sing IM, 15 ug	1	Dizziness	Nervous system disorders	Dizziness	Dizziness	<0.1	4.39
B0096	Group B6	A/Sing MAP, 2.5 ug	1	Headache	Nervous system disorders	Headache	Headache	1.81	0.38
B0096	Group B6	A/Sing MAP, 2.5 ug	2	Nausea	Gastrointestinal disorders	Nausea	Nausea	0.46	0.42
B0096	Group B6	A/Sing MAP, 2.5 ug	3	Myalgia	Musculoskeletal and connective tissue disorders	Myalgia	Myalgia	~11	0.15
B0096	Group B6	A/Sing MAP, 2.5 ug	4	MAP 1 Erythema	General disorders and administration site conditions	Application site erythema	Application site erythema	4.05	2.96
B0096	Group B6	A/Sing MAP, 2.5 ug	5	MAP 1 visibility	General disorders and administration site conditions	Application site discolouration	Application site discolouration	~20	60.02

Key: AE = Adverse Event; [C] = Calculated; UK = Unknown
Coded using MedDRA Version 20.01

Administration Site Key: FA = Forearm; UA= Upper arm
Treatments Part A: A1 = A/Sing MAP, 15 ug (FA); A2 = Afluria Quad IM (UA), 15 ug; A3 = Placebo MAP (FA); A4 = A/Sing Mono IM, 15 ug (UA)
Treatments Part B: B1 = A/Sing MAP, 15 ug (FA) with biopsy; B2 = Placebo MAP (FA) with biopsy
B3 = A/Sing MAP, 15 ug (FA); B4 = A/Sing MAP, 10 ug (FA); B5 = A/Sing MAP, 5 ug (FA); B6 = A/Sing MAP, 2.5 ug (FA)
B7 = Placebo MAP (FA); B8 = A/Sing MAP, 15 ug (UA); B9 = A/Sing IM, 15 ug, as 2018 Afluria Quad (UA)

Listing 16.2.7.2
Adverse Events - MedDRA Coding and Calculated Values

Part B

Random-isation Number	Group	Treatment	AE No.	Adverse Event Verbatim	MedDRA System Organ Class	MedDRA Preferred Term	MedDRA Lower Level Term	Duration of AE (days) [C]	Time Since Treatment Admin. (Days) [C]
B0097	Group B5	A/Sing MAP, 5 ug		[No Treatment-Emergent Adverse Events Reported]					
B0098	Group B7	Placebo MAP	1	Nausea	Gastrointestinal disorders	Nausea	Nausea	0.04	<0.01
B0099	Group B4	A/Sing MAP, 10 ug	2	URTI	Infections and infestations	Upper respiratory tract infection	URTI	~13	47.03
B0100	Group B8	A/Sing MAP, 15 ug	1	Headache	Nervous system disorders	Headache	Headache	0.19	0.09
B0100	Group B8	A/Sing MAP, 15 ug	2	MAP site reaction 1,2,3 Erythema and Oedema	General disorders and administration site conditions	Application site reaction	Application site reaction	59.08	0.99

Key: AE = Adverse Event; [C] = Calculated; UK = Unknown
Coded using MedDRA Version 20.01

Administration Site Key: FA = Forearm; UA= Upper arm
Treatments Part A: A1 = A/Sing MAP, 15 ug (FA); A2 = Afluria Quad IM (UA), 15 ug; A3 = Placebo MAP (FA); A4 = A/Sing Mono IM, 15 ug (UA)
Treatments Part B: B1 = A/Sing MAP, 15 ug (FA) with biopsy; B2 = Placebo MAP (FA) with biopsy
B3 = A/Sing MAP, 15 ug (FA); B4 = A/Sing MAP, 10 ug (FA); B5 = A/Sing MAP, 5 ug (FA); B6 = A/Sing MAP, 2.5 ug (FA)
B7 = Placebo MAP (FA); B8 = A/Sing MAP, 15 ug (UA); B9 = A/Sing IM, 15 ug, as 2018 Afluria Quad (UA)

Listing 16.2.7.2
Adverse Events - MedDRA Coding and Calculated Values

Part B

Random-isation Number	Group	Treatment	AE No.	Adverse Event Verbatim	MedDRA System Organ Class	MedDRA Preferred Term	MedDRA Lower Level Term	Duration of AE (days) [C]	Time Since Treatment Admin. (Days) [C]
B0101	Group B3	A/Sing MAP, 15 ug	1	MAP reaction 1,2,3 - Erythema	General disorders and administration site conditions	Application site erythema	Application site erythema	18.03	2.94
B0101	Group B3	A/Sing MAP, 15 ug	2	Rhinitis	Infections and infestations	Rhinitis	Rhinitis	0.29	5.79
B0102	Group B8	A/Sing MAP, 15 ug	1	MAP reaction 1,2,3 - Erythema	General disorders and administration site conditions	Application site erythema	Application site erythema	10.87	3.01
B0103	Group B5	A/Sing MAP, 5 ug	1	Pruritus Left forearm	Skin and subcutaneous tissue disorders	Pruritus	Pruritus	15.00	4.73
B0104	Group B3	A/Sing MAP, 15 ug	1	MAP 1,2,3 - Erythema	General disorders and administration site conditions	Application site erythema	Application site erythema	4.10	3.00

Key: AE = Adverse Event; [C] = Calculated; UK = Unknown
Coded using MedDRA Version 20.01

Administration Site Key: FA = Forearm; UA= Upper arm
Treatments Part A: A1 = A/Sing MAP, 15 ug (FA); A2 = Afluria Quad IM (UA), 15 ug; A3 = Placebo MAP (FA); A4 = A/Sing Mono IM, 15 ug (UA)
Treatments Part B: B1 = A/Sing MAP, 15 ug (FA) with biopsy; B2 = Placebo MAP (FA) with biopsy
B3 = A/Sing MAP, 15 ug (FA); B4 = A/Sing MAP, 10 ug (FA); B5 = A/Sing MAP, 5 ug (FA); B6 = A/Sing MAP, 2.5 ug (FA)
B7 = Placebo MAP (FA); B8 = A/Sing MAP, 15 ug (UA); B9 = A/Sing IM, 15 ug, as 2018 Afluria Quad (UA)

Listing 16.2.7.2
Adverse Events - MedDRA Coding and Calculated Values

Part B

Random-isation Number	Group	Treatment	AE No.	Adverse Event Verbatim	MedDRA System Organ Class	MedDRA Preferred Term	MedDRA Lower Level Term	Duration of AE (days) [C]	Time Since Treatment Admin. (Days) [C]
B0104	Group B3	A/Sing MAP, 15 ug	2	Musculoskeletal chest pain	Musculoskeletal and connective tissue disorders	Musculoskeletal chest pain	Musculoskeletal chest pain	0.15	6.98
B0104	Group B3	A/Sing MAP, 15 ug	3	MAP site 1,2,3 reaction visibility	General disorders and administration site conditions	Application site discolouration	Application site discolouration	~31	61.79
B0105	Group B9	A/Sing IM, 15 ug	1	Dry Cough	Respiratory, thoracic and mediastinal disorders	Cough	Dry cough	2.29	2.81
B0105	Group B9	A/Sing IM, 15 ug	2	Myalgia IP arm	General disorders and administration site conditions	Injection site pain	Injection site muscle pain	~3	~1
B0105	Group B9	A/Sing IM, 15 ug	3	Upper Respiratory Tract Infection	Infections and infestations	Upper respiratory tract infection	Upper respiratory tract infection	~8	19.85
B0106	Group B4	A/Sing MAP, 10 ug	1	Upper Respiratory Tract Infection	Infections and infestations	Upper respiratory tract infection	Upper respiratory tract infection	~5	3.72

Key: AE = Adverse Event; [C] = Calculated; UK = Unknown
Coded using MedDRA Version 20.01

Administration Site Key: FA = Forearm; UA= Upper arm
Treatments Part A: A1 = A/Sing MAP, 15 ug (FA); A2 = Afluria Quad IM (UA), 15 ug; A3 = Placebo MAP (FA); A4 = A/Sing Mono IM, 15 ug (UA)
Treatments Part B: B1 = A/Sing MAP, 15 ug (FA) with biopsy; B2 = Placebo MAP (FA) with biopsy
B3 = A/Sing MAP, 15 ug (FA); B4 = A/Sing MAP, 10 ug (FA); B5 = A/Sing MAP, 5 ug (FA); B6 = A/Sing MAP, 2.5 ug (FA)
B7 = Placebo MAP (FA); B8 = A/Sing MAP, 15 ug (UA); B9 = A/Sing IM, 15 ug, as 2018 Afluria Quad (UA)

Listing 16.2.7.2
Adverse Events - MedDRA Coding and Calculated Values

Part B

Random-isation Number	Group	Treatment	AE No.	Adverse Event Verbatim	MedDRA System Organ Class	MedDRA Preferred Term	MedDRA Lower Level Term	Duration of AE (days) [C]	Time Since Treatment Admin. (Days) [C]
B0106	Group B4	A/Sing MAP, 10 ug	2	MAP 2,3 - Erythema	General disorders and administration site conditions	Application site erythema	Application site erythema	4.15	2.90
B0107	Group B6	A/Sing MAP, 2.5 ug	1	MAP reaction site 2,3 - Erythema	General disorders and administration site conditions	Application site erythema	Application site erythema	3.96	2.84
B0107	Group B6	A/Sing MAP, 2.5 ug	2	MAP sites 1,2,3 visible	General disorders and administration site conditions	Application site discolouration	Application site discolouration	~37	60.12
B0108	Group B7	Placebo MAP		[No Treatment-Emergent Adverse Events Reported]					

Key: AE = Adverse Event; [C] = Calculated; UK = Unknown
Coded using MedDRA Version 20.01

Administration Site Key: FA = Forearm; UA= Upper arm
Treatments Part A: A1 = A/Sing MAP, 15 ug (FA); A2 = Afluria Quad IM (UA), 15 ug; A3 = Placebo MAP (FA); A4 = A/Sing Mono IM, 15 ug (UA)
Treatments Part B: B1 = A/Sing MAP, 15 ug (FA) with biopsy; B2 = Placebo MAP (FA) with biopsy
B3 = A/Sing MAP, 15 ug (FA); B4 = A/Sing MAP, 10 ug (FA); B5 = A/Sing MAP, 5 ug (FA); B6 = A/Sing MAP, 2.5 ug (FA)
B7 = Placebo MAP (FA); B8 = A/Sing MAP, 15 ug (UA); B9 = A/Sing IM, 15 ug, as 2018 Afluria Quad (UA)

Listing 16.2.7.2
Adverse Events - MedDRA Coding and Calculated Values

Part B

Randomisation Number	Group	Treatment	AE No.	Adverse Event Verbatim	MedDRA System Organ Class	MedDRA Preferred Term	MedDRA Lower Level Term	Duration of AE (days) [C]	Time Since Treatment Admin. (Days) [C]
B0109	Group B6	A/Sing MAP, 2.5 ug	1	MAP #2 and #3 visibility	General disorders and administration site conditions	Application site discolouration	Application site discolouration	~13	~60
B0110	Group B3	A/Sing MAP, 15 ug	1	MAP 1,2,3 reaction - Erythema	General disorders and administration site conditions	Application site erythema	Application site erythema	72.00	3.05
B0111	Group B8	A/Sing MAP, 15 ug		[No Treatment-Emergent Adverse Events Reported]					
B0112	Group B4	A/Sing MAP, 10 ug	1	MAP reaction sites 2,3 -Erythema	General disorders and administration site conditions	Application site erythema	Application site erythema	17.99	2.98

Key: AE = Adverse Event; [C] = Calculated; UK = Unknown
Coded using MedDRA Version 20.01

Administration Site Key: FA = Forearm; UA= Upper arm
Treatments Part A: A1 = A/Sing MAP, 15 ug (FA); A2 = Afluria Quad IM (UA), 15 ug; A3 = Placebo MAP (FA); A4 = A/Sing Mono IM, 15 ug (UA)
Treatments Part B: B1 = A/Sing MAP, 15 ug (FA) with biopsy; B2 = Placebo MAP (FA) with biopsy
B3 = A/Sing MAP, 15 ug (FA); B4 = A/Sing MAP, 10 ug (FA); B5 = A/Sing MAP, 5 ug (FA); B6 = A/Sing MAP, 2.5 ug (FA)
B7 = Placebo MAP (FA); B8 = A/Sing MAP, 15 ug (UA); B9 = A/Sing IM, 15 ug, as 2018 Afluria Quad (UA)

Listing 16.2.7.2
Adverse Events - MedDRA Coding and Calculated Values

Part B

Random-isation Number	Group	Treatment	AE No.	Adverse Event Verbatim	MedDRA System Organ Class	MedDRA Preferred Term	MedDRA Lower Level Term	Duration of AE (days) [C]	Time Since Treatment Admin. (Days) [C]
B0112	Group B4	A/Sing MAP, 10 ug	2	MAP sites Visibility 2,3	General disorders and administration site conditions	Application site discolouration	Application site discolouration	~29	60.06
B0113	Group B5	A/Sing MAP, 5 ug	1	Headache	Nervous system disorders	Headache	Headache	2.04	1.15
B0113	Group B5	A/Sing MAP, 5 ug	2	Headache	Nervous system disorders	Headache	Headache	0.08	19.77
B0114	Group B7	Placebo MAP	1	Intermittent Headache	Nervous system disorders	Headache	Intermittent headache	5.35	0.81
B0114	Group B7	Placebo MAP	3	Insect bite - Right upper arm	Injury, poisoning and procedural complications	Arthropod bite	Insect bite NOS	22.64	0.31
B0115	Group B9	A/Sing IM, 15 ug	1	URTI	Infections and infestations	Upper respiratory tract infection	URTI	~4	~16

Key: AE = Adverse Event; [C] = Calculated; UK = Unknown
Coded using MedDRA Version 20.01

Administration Site Key: FA = Forearm; UA= Upper arm
Treatments Part A: A1 = A/Sing MAP, 15 ug (FA); A2 = Afluria Quad IM (UA), 15 ug; A3 = Placebo MAP (FA); A4 = A/Sing Mono IM, 15 ug (UA)
Treatments Part B: B1 = A/Sing MAP, 15 ug (FA) with biopsy; B2 = Placebo MAP (FA) with biopsy
B3 = A/Sing MAP, 15 ug (FA); B4 = A/Sing MAP, 10 ug (FA); B5 = A/Sing MAP, 5 ug (FA); B6 = A/Sing MAP, 2.5 ug (FA)
B7 = Placebo MAP (FA); B8 = A/Sing MAP, 15 ug (UA); B9 = A/Sing IM, 15 ug, as 2018 Afluria Quad (UA)

Listing 16.2.7.2
Adverse Events - MedDRA Coding and Calculated Values

Part B

Random-isation Number	Group	Treatment	AE No.	Adverse Event Verbatim	MedDRA System Organ Class	MedDRA Preferred Term	MedDRA Lower Level Term	Duration of AE (days) [C]	Time Since Treatment Admin. (Days) [C]
B0116	Group B7	Placebo MAP	1	MAP sites - All three sites - Itchiness	General disorders and administration site conditions	Application site pruritus	Application site itching	0.06	2.36
B0116	Group B7	Placebo MAP	2	Headache	Nervous system disorders	Headache	Headache	0.54	3.90
B0116	Group B7	Placebo MAP	3	Headache	Nervous system disorders	Headache	Headache	0.54	18.88
B0117	Group B9	A/Sing IM, 15 ug		[No Treatment-Emergent Adverse Events Reported]					
B0118	Group B6	A/Sing MAP, 2.5 ug	1	Vasovagal Syncope	Nervous system disorders	Syncope	Syncope vasovagal	0.03	<0.01
B0118	Group B6	A/Sing MAP, 2.5 ug	2	Headache	Nervous system disorders	Headache	Headache	19.28	<0.01

Key: AE = Adverse Event; [C] = Calculated; UK = Unknown
Coded using MedDRA Version 20.01

Administration Site Key: FA = Forearm; UA= Upper arm
Treatments Part A: A1 = A/Sing MAP, 15 ug (FA); A2 = Afluria Quad IM (UA), 15 ug; A3 = Placebo MAP (FA); A4 = A/Sing Mono IM, 15 ug (UA)
Treatments Part B: B1 = A/Sing MAP, 15 ug (FA) with biopsy; B2 = Placebo MAP (FA) with biopsy
B3 = A/Sing MAP, 15 ug (FA); B4 = A/Sing MAP, 10 ug (FA); B5 = A/Sing MAP, 5 ug (FA); B6 = A/Sing MAP, 2.5 ug (FA)
B7 = Placebo MAP (FA); B8 = A/Sing MAP, 15 ug (UA); B9 = A/Sing IM, 15 ug, as 2018 Afluria Quad (UA)

Listing 16.2.7.2
Adverse Events - MedDRA Coding and Calculated Values

Part B

Random-isation Number	Group	Treatment	AE No.	Adverse Event Verbatim	MedDRA System Organ Class	MedDRA Preferred Term	MedDRA Lower Level Term	Duration of AE (days) [C]	Time Since Treatment Admin. (Days) [C]
B0118	Group B6	A/Sing MAP, 2.5 ug	3	Nausea	Gastrointestinal disorders	Nausea	Nausea	0.04	2.24
B0118	Group B6	A/Sing MAP, 2.5 ug	7	MAP site #2 visibility	General disorders and administration site conditions	Application site discolouration	Application site discolouration	~55	60.01
B0119	Group B4	A/Sing MAP, 10 ug	3	Gastroenteritis	Infections and infestations	Gastroenteritis	Gastroenteritis	0.25	4.87
B0119	Group B4	A/Sing MAP, 10 ug	5	MAP 1,3 visibility	General disorders and administration site conditions	Application site discolouration	Application site discolouration	~12	60.04
B0120	Group B3	A/Sing MAP, 15 ug	1	MAP site reaction 1,2,3 - Erythema and MAP 2,3 Tenderness	General disorders and administration site conditions	Application site reaction	Application site reaction	18.02	3.01
B0120	Group B3	A/Sing MAP, 15 ug	2	MAP site 1,2,3 visibility	General disorders and administration site conditions	Application site discolouration	Application site discolouration	~53	21.03

Key: AE = Adverse Event; [C] = Calculated; UK = Unknown
Coded using MedDRA Version 20.01

Administration Site Key: FA = Forearm; UA= Upper arm
Treatments Part A: A1 = A/Sing MAP, 15 ug (FA); A2 = Afluria Quad IM (UA), 15 ug; A3 = Placebo MAP (FA); A4 = A/Sing Mono IM, 15 ug (UA)
Treatments Part B: B1 = A/Sing MAP, 15 ug (FA) with biopsy; B2 = Placebo MAP (FA) with biopsy
B3 = A/Sing MAP, 15 ug (FA); B4 = A/Sing MAP, 10 ug (FA); B5 = A/Sing MAP, 5 ug (FA); B6 = A/Sing MAP, 2.5 ug (FA)
B7 = Placebo MAP (FA); B8 = A/Sing MAP, 15 ug (UA); B9 = A/Sing IM, 15 ug, as 2018 Afluria Quad (UA)

Listing 16.2.7.2
Adverse Events - MedDRA Coding and Calculated Values

Part B

Random-isation Number	Group	Treatment	AE No.	Adverse Event Verbatim	MedDRA System Organ Class	MedDRA Preferred Term	MedDRA Lower Level Term	Duration of AE (days) [C]	Time Since Treatment Admin. (Days) [C]
B0121	Group B8	A/Sing MAP, 15 ug	1	MAP 1,2,3 reaction - Erythema, tenderness and induration	General disorders and administration site conditions	Application site reaction	Application site reaction	49.82	3.02
B0121	Group B8	A/Sing MAP, 15 ug	3	Upper Respiratory Tract Infection	Infections and infestations	Upper respiratory tract infection	Upper respiratory tract infection	1.47	0.19
B0121	Group B8	A/Sing MAP, 15 ug	4	Nausea	Gastrointestinal disorders	Nausea	Nausea	0.01	1.65
B0122	Group B5	A/Sing MAP, 5 ug	1	MAP site 2 - Itching	General disorders and administration site conditions	Application site pruritus	Application site itching	~3	1.68
B0122	Group B5	A/Sing MAP, 5 ug	2	MAP reaction site 2 - Erythema, oedema + induration	General disorders and administration site conditions	Application site reaction	Application site reaction	22.87	2.94
B0122	Group B5	A/Sing MAP, 5 ug	3	Pharyngitis	Infections and infestations	Pharyngitis	Pharyngitis	2.04	5.68

Key: AE = Adverse Event; [C] = Calculated; UK = Unknown

Coded using MedDRA Version 20.01

Administration Site Key: FA = Forearm; UA= Upper arm

Treatments Part A: A1 = A/Sing MAP, 15 ug (FA); A2 = Afluria Quad IM (UA), 15 ug; A3 = Placebo MAP (FA); A4 = A/Sing Mono IM, 15 ug (UA)

Treatments Part B: B1 = A/Sing MAP, 15 ug (FA) with biopsy; B2 = Placebo MAP (FA) with biopsy
B3 = A/Sing MAP, 15 ug (FA); B4 = A/Sing MAP, 10 ug (FA); B5 = A/Sing MAP, 5 ug (FA); B6 = A/Sing MAP, 2.5 ug (FA)
B7 = Placebo MAP (FA); B8 = A/Sing MAP, 15 ug (UA); B9 = A/Sing IM, 15 ug, as 2018 Afluria Quad (UA)

Listing 16.2.7.2
Adverse Events - MedDRA Coding and Calculated Values

Part B

Random-isation Number	Group	Treatment	AE No.	Adverse Event Verbatim	MedDRA System Organ Class	MedDRA Preferred Term	MedDRA Lower Level Term	Duration of AE (days) [C]	Time Since Treatment Admin. (Days) [C]
B0122	Group B5	A/Sing MAP, 5 ug	4	MAP site 2 reaction Visibility	General disorders and administration site conditions	Application site discolouration	Application site discolouration	13.09	60.02
B0123	Group B6	A/Sing MAP, 2.5 ug	1	Headache	Nervous system disorders	Headache	Headache	1.06	0.88
B0123	Group B6	A/Sing MAP, 2.5 ug	3	Upper Respiratory Tract Infection	Infections and infestations	Upper respiratory tract infection	Upper respiratory tract infection	3.46	18.34
B0124	Group B4	A/Sing MAP, 10 ug	1	Headache	Nervous system disorders	Headache	Headache	0.77	0.02
B0124	Group B4	A/Sing MAP, 10 ug	2	MAP reaction sites 2,3 - Erythema, oedema, induration	General disorders and administration site conditions	Application site reaction	Application site reaction	32.03	3.03
B0124	Group B4	A/Sing MAP, 10 ug	3	Headache	Nervous system disorders	Headache	Headache	2.46	4.71
B0124	Group B4	A/Sing MAP, 10 ug	4	Contact dermatitis	Skin and subcutaneous tissue disorders	Dermatitis contact	Contact dermatitis	~5	8.67

Key: AE = Adverse Event; [C] = Calculated; UK = Unknown
Coded using MedDRA Version 20.01

Administration Site Key: FA = Forearm; UA= Upper arm
Treatments Part A: A1 = A/Sing MAP, 15 ug (FA); A2 = Afluria Quad IM (UA), 15 ug; A3 = Placebo MAP (FA); A4 = A/Sing Mono IM, 15 ug (UA)
Treatments Part B: B1 = A/Sing MAP, 15 ug (FA) with biopsy; B2 = Placebo MAP (FA) with biopsy
B3 = A/Sing MAP, 15 ug (FA); B4 = A/Sing MAP, 10 ug (FA); B5 = A/Sing MAP, 5 ug (FA); B6 = A/Sing MAP, 2.5 ug (FA)
B7 = Placebo MAP (FA); B8 = A/Sing MAP, 15 ug (UA); B9 = A/Sing IM, 15 ug, as 2018 Afluria Quad (UA)

Listing 16.2.7.2
Adverse Events - MedDRA Coding and Calculated Values

Part B

Random-isation Number	Group	Treatment	AE No.	Adverse Event Verbatim	MedDRA System Organ Class	MedDRA Preferred Term	MedDRA Lower Level Term	Duration of AE (days) [C]	Time Since Treatment Admin. (Days) [C]
B0124	Group B4	A/Sing MAP, 10 ug	5	MAP visibility site #2 and #3	General disorders and administration site conditions	Application site discolouration	Application site discolouration	~56	60.04
B0125	Group B7	Placebo MAP		[No Treatment-Emergent Adverse Events Reported]					
B0126	Group B3	A/Sing MAP, 15 ug	1	MAP Visibility 1,2,3	General disorders and administration site conditions	Application site discolouration	Application site discolouration	~9	60.04
B0127	Group B9	A/Sing IM, 15 ug		[No Treatment-Emergent Adverse Events Reported]					

Key: AE = Adverse Event; [C] = Calculated; UK = Unknown
Coded using MedDRA Version 20.01

Administration Site Key: FA = Forearm; UA= Upper arm
Treatments Part A: A1 = A/Sing MAP, 15 ug (FA); A2 = Afluria Quad IM (UA), 15 ug; A3 = Placebo MAP (FA); A4 = A/Sing Mono IM, 15 ug (UA)
Treatments Part B: B1 = A/Sing MAP, 15 ug (FA) with biopsy; B2 = Placebo MAP (FA) with biopsy
B3 = A/Sing MAP, 15 ug (FA); B4 = A/Sing MAP, 10 ug (FA); B5 = A/Sing MAP, 5 ug (FA); B6 = A/Sing MAP, 2.5 ug (FA)
B7 = Placebo MAP (FA); B8 = A/Sing MAP, 15 ug (UA); B9 = A/Sing IM, 15 ug, as 2018 Afluria Quad (UA)

Listing 16.2.7.2
Adverse Events - MedDRA Coding and Calculated Values

Part B

Random-isation Number	Group	Treatment	AE No.	Adverse Event Verbatim	MedDRA System Organ Class	MedDRA Preferred Term	MedDRA Lower Level Term	Duration of AE (days) [C]	Time Since Treatment Admin. (Days) [C]
B0128	Group B5	A/Sing MAP, 5 ug	1	MAP site reaction site 2 - oedema	General disorders and administration site conditions	Application site oedema	Application site oedema	45.91	3.02
B0128	Group B5	A/Sing MAP, 5 ug	2	MAP site 2 Visibility	General disorders and administration site conditions	Application site discolouration	Application site discolouration	~54	~60
B0129	Group B8	A/Sing MAP, 15 ug	1	MAP reaction site 1,2,3 erythema	General disorders and administration site conditions	Application site erythema	Application site erythema	58.95	1.02
B0129	Group B8	A/Sing MAP, 15 ug	2	MAP reaction site 1,2,3 visibility	General disorders and administration site conditions	Application site discolouration	Application site discolouration	~68	59.97
B0130	Group B5	A/Sing MAP, 5 ug	1	MAP site 1 reaction - Erythema	General disorders and administration site conditions	Application site erythema	Application site erythema	57.00	2.94

Key: AE = Adverse Event; [C] = Calculated; UK = Unknown
Coded using MedDRA Version 20.01

Administration Site Key: FA = Forearm; UA= Upper arm
Treatments Part A: A1 = A/Sing MAP, 15 ug (FA); A2 = Afluria Quad IM (UA), 15 ug; A3 = Placebo MAP (FA); A4 = A/Sing Mono IM, 15 ug (UA)
Treatments Part B: B1 = A/Sing MAP, 15 ug (FA) with biopsy; B2 = Placebo MAP (FA) with biopsy
B3 = A/Sing MAP, 15 ug (FA); B4 = A/Sing MAP, 10 ug (FA); B5 = A/Sing MAP, 5 ug (FA); B6 = A/Sing MAP, 2.5 ug (FA)
B7 = Placebo MAP (FA); B8 = A/Sing MAP, 15 ug (UA); B9 = A/Sing IM, 15 ug, as 2018 Afluria Quad (UA)

Listing 16.2.7.2
Adverse Events - MedDRA Coding and Calculated Values

Part B

Random-isation Number	Group	Treatment	AE No.	Adverse Event Verbatim	MedDRA System Organ Class	MedDRA Preferred Term	MedDRA Lower Level Term	Duration of AE (days) [C]	Time Since Treatment Admin. (Days) [C]
B0130	Group B5	A/Sing MAP, 5 ug	2	Upper Respiratory Tract Infection	Infections and infestations	Upper respiratory tract infection	Upper respiratory tract infection	1.92	3.97
B0130	Group B5	A/Sing MAP, 5 ug	3	Upper Respiratory Tract Infection	Infections and infestations	Upper respiratory tract infection	Upper respiratory tract infection	7.02	16.85
B0130	Group B5	A/Sing MAP, 5 ug	4	MAP site 1 visibilty	General disorders and administration site conditions	Application site discolouration	Application site discolouration	~40	59.94
B0131	Group B8	A/Sing MAP, 15 ug	1	MAP reaction site 1,2,3 -Erythema extent, erythema, MAP reaction site #2 te	General disorders and administration site conditions	Application site reaction	Application site reaction	44.81	3.07
B0131	Group B8	A/Sing MAP, 15 ug	2	Right Temporomandibular junction pain	Musculoskeletal and connective tissue disorders	Temporomandibular joint syndrome	Temporomandibular joint arthralgia	0.09	5.40
B0132	Group B6	A/Sing MAP, 2.5 ug	1	Nausea	Gastrointestinal disorders	Nausea	Nausea	0.02	3.22

Key: AE = Adverse Event; [C] = Calculated; UK = Unknown
Coded using MedDRA Version 20.01

Administration Site Key: FA = Forearm; UA= Upper arm
Treatments Part A: A1 = A/Sing MAP, 15 ug (FA); A2 = Afluria Quad IM (UA), 15 ug; A3 = Placebo MAP (FA); A4 = A/Sing Mono IM, 15 ug (UA)
Treatments Part B: B1 = A/Sing MAP, 15 ug (FA) with biopsy; B2 = Placebo MAP (FA) with biopsy
B3 = A/Sing MAP, 15 ug (FA); B4 = A/Sing MAP, 10 ug (FA); B5 = A/Sing MAP, 5 ug (FA); B6 = A/Sing MAP, 2.5 ug (FA)
B7 = Placebo MAP (FA); B8 = A/Sing MAP, 15 ug (UA); B9 = A/Sing IM, 15 ug, as 2018 Afluria Quad (UA)

Listing 16.2.7.2
Adverse Events - MedDRA Coding and Calculated Values

Part B

Randomisation Number	Group	Treatment	AE No.	Adverse Event Verbatim	MedDRA System Organ Class	MedDRA Preferred Term	MedDRA Lower Level Term	Duration of AE (days) [C]	Time Since Treatment Admin. (Days) [C]
B0133	Group B4	A/Sing MAP, 10 ug		[No Treatment-Emergent Adverse Events Reported]					
B0134	Group B3	A/Sing MAP, 15 ug	1	MAP 2,3 Visibility	General disorders and administration site conditions	Application site discolouration	Application site discolouration	~7	60.87
B0135	Group B7	Placebo MAP	1	URTI	Infections and infestations	Upper respiratory tract infection	URTI	5.96	0.85
B0136	Group B9	A/Sing IM, 15 ug	1	Cholecystitis	Hepatobiliary disorders	Cholecystitis	Cholecystitis	47.63	12.37

Key: AE = Adverse Event; [C] = Calculated; UK = Unknown
Coded using MedDRA Version 20.01

Administration Site Key: FA = Forearm; UA= Upper arm
Treatments Part A: A1 = A/Sing MAP, 15 ug (FA); A2 = Afluria Quad IM (UA), 15 ug; A3 = Placebo MAP (FA); A4 = A/Sing Mono IM, 15 ug (UA)
Treatments Part B: B1 = A/Sing MAP, 15 ug (FA) with biopsy; B2 = Placebo MAP (FA) with biopsy
B3 = A/Sing MAP, 15 ug (FA); B4 = A/Sing MAP, 10 ug (FA); B5 = A/Sing MAP, 5 ug (FA); B6 = A/Sing MAP, 2.5 ug (FA)
B7 = Placebo MAP (FA); B8 = A/Sing MAP, 15 ug (UA); B9 = A/Sing IM, 15 ug, as 2018 Afluria Quad (UA)

Listing 16.2.7.2
Adverse Events - MedDRA Coding and Calculated Values

Part B

Random-isation Number	Group	Treatment	AE No.	Adverse Event Verbatim	MedDRA System Organ Class	MedDRA Preferred Term	MedDRA Lower Level Term	Duration of AE (days) [C]	Time Since Treatment Admin. (Days) [C]
B0137	Group B8	A/Sing MAP, 15 ug	1	MAP 1,2,3 - Erythema	General disorders and administration site conditions	Application site erythema	Application site erythema	16.94	2.90
B0137	Group B8	A/Sing MAP, 15 ug	2	Upper Respiratory Tract Infection	Infections and infestations	Upper respiratory tract infection	Upper respiratory tract infection	29.13	15.76
B0138	Group B7	Placebo MAP	2	MAP Visibility 1,2,3	General disorders and administration site conditions	Application site discolouration	Application site discolouration	UK	60.02
B0139	Group B4	A/Sing MAP, 10 ug		[No Treatment-Emergent Adverse Events Reported]					

Key: AE = Adverse Event; [C] = Calculated; UK = Unknown
Coded using MedDRA Version 20.01

Administration Site Key: FA = Forearm; UA= Upper arm
Treatments Part A: A1 = A/Sing MAP, 15 ug (FA); A2 = Afluria Quad IM (UA), 15 ug; A3 = Placebo MAP (FA); A4 = A/Sing Mono IM, 15 ug (UA)
Treatments Part B: B1 = A/Sing MAP, 15 ug (FA) with biopsy; B2 = Placebo MAP (FA) with biopsy
B3 = A/Sing MAP, 15 ug (FA); B4 = A/Sing MAP, 10 ug (FA); B5 = A/Sing MAP, 5 ug (FA); B6 = A/Sing MAP, 2.5 ug (FA)
B7 = Placebo MAP (FA); B8 = A/Sing MAP, 15 ug (UA); B9 = A/Sing IM, 15 ug, as 2018 Afluria Quad (UA)

Listing 16.2.7.2
Adverse Events - MedDRA Coding and Calculated Values

Part B

Random-isation Number	Group	Treatment	AE No.	Adverse Event Verbatim	MedDRA System Organ Class	MedDRA Preferred Term	MedDRA Lower Level Term	Duration of AE (days) [C]	Time Since Treatment Admin. (Days) [C]
B0140	Group B9	A/Sing IM, 15 ug		[No Treatment-Emergent Adverse Events Reported]					
B0141	Group B3	A/Sing MAP, 15 ug		[No Treatment-Emergent Adverse Events Reported]					
B0142	Group B5	A/Sing MAP, 5 ug	1	Medial Tibial Stress Syndrome (Shin Splints)	Musculoskeletal and connective tissue disorders	Medial tibial stress syndrome	Medial tibial stress syndrome	29.75	19.06
B0142	Group B5	A/Sing MAP, 5 ug	2	MAP 1 Visibility	General disorders and administration site conditions	Application site discolouration	Application site discolouration	~31	62.93

Key: AE = Adverse Event; [C] = Calculated; UK = Unknown
Coded using MedDRA Version 20.01

Administration Site Key: FA = Forearm; UA= Upper arm
Treatments Part A: A1 = A/Sing MAP, 15 ug (FA); A2 = Afluria Quad IM (UA), 15 ug; A3 = Placebo MAP (FA); A4 = A/Sing Mono IM, 15 ug (UA)
Treatments Part B: B1 = A/Sing MAP, 15 ug (FA) with biopsy; B2 = Placebo MAP (FA) with biopsy
B3 = A/Sing MAP, 15 ug (FA); B4 = A/Sing MAP, 10 ug (FA); B5 = A/Sing MAP, 5 ug (FA); B6 = A/Sing MAP, 2.5 ug (FA)
B7 = Placebo MAP (FA); B8 = A/Sing MAP, 15 ug (UA); B9 = A/Sing IM, 15 ug, as 2018 Afluria Quad (UA)

Listing 16.2.7.2
Adverse Events - MedDRA Coding and Calculated Values

Part B

Random-isation Number	Group	Treatment	AE No.	Adverse Event Verbatim	MedDRA System Organ Class	MedDRA Preferred Term	MedDRA Lower Level Term	Duration of AE (days) [C]	Time Since Treatment Admin. (Days) [C]
B0143	Group B6	A/Sing MAP, 2.5 ug	1	Headache	Nervous system disorders	Headache	Headache	<1	0.04
B0143	Group B6	A/Sing MAP, 2.5 ug	2	MAP site 3 reaction - Erythema Oedema induration	General disorders and administration site conditions	Application site reaction	Application site reaction	4.00	2.94
B0143	Group B6	A/Sing MAP, 2.5 ug	3	Exacerbation of Left knee sprain	Injury, poisoning and procedural complications	Ligament sprain	Knee sprain	~75	10.91
B0143	Group B6	A/Sing MAP, 2.5 ug	4	MAP 3 Visibility	General disorders and administration site conditions	Application site discolouration	Application site discolouration	UK	59.98
B0144	Group B6	A/Sing MAP, 2.5 ug	1	Dizziness	Nervous system disorders	Dizziness	Dizziness	0.07	<0.01
B0144	Group B6	A/Sing MAP, 2.5 ug	2	Nausea	Gastrointestinal disorders	Nausea	Nausea	0.07	<0.01
B0144	Group B6	A/Sing MAP, 2.5 ug	3	Upper Respiratory Tract Infection	Infections and infestations	Upper respiratory tract infection	Upper respiratory tract infection	3.94	20.84

Key: AE = Adverse Event; [C] = Calculated; UK = Unknown
Coded using MedDRA Version 20.01

Administration Site Key: FA = Forearm; UA= Upper arm
Treatments Part A: A1 = A/Sing MAP, 15 ug (FA); A2 = Afluria Quad IM (UA), 15 ug; A3 = Placebo MAP (FA); A4 = A/Sing Mono IM, 15 ug (UA)
Treatments Part B: B1 = A/Sing MAP, 15 ug (FA) with biopsy; B2 = Placebo MAP (FA) with biopsy
B3 = A/Sing MAP, 15 ug (FA); B4 = A/Sing MAP, 10 ug (FA); B5 = A/Sing MAP, 5 ug (FA); B6 = A/Sing MAP, 2.5 ug (FA)
B7 = Placebo MAP (FA); B8 = A/Sing MAP, 15 ug (UA); B9 = A/Sing IM, 15 ug, as 2018 Afluria Quad (UA)

Listing 16.2.7.2
Adverse Events - MedDRA Coding and Calculated Values

Part B

Random-isation Number	Group	Treatment	AE No.	Adverse Event Verbatim	MedDRA System Organ Class	MedDRA Preferred Term	MedDRA Lower Level Term	Duration of AE (days) [C]	Time Since Treatment Admin. (Days) [C]
B0145	Group B3	A/Sing MAP, 15 ug	1	Nausea	Gastrointestinal disorders	Nausea	Nausea	<0.1	0.96
B0145	Group B3	A/Sing MAP, 15 ug	2	MAP 1,2,3 reaction - Erythema & MAP 2 reaction - Erythema extent	General disorders and administration site conditions	Application site erythema	Application site erythema	31.93	2.86
B0145	Group B3	A/Sing MAP, 15 ug	3	Headache	Nervous system disorders	Headache	Headache	<0.1	4.13
B0146	Group B7	Placebo MAP	1	Headache	Nervous system disorders	Headache	Headache	0.02	4.18
B0146	Group B7	Placebo MAP	2	Toothache	Gastrointestinal disorders	Toothache	Toothache	0.02	4.18

Key: AE = Adverse Event; [C] = Calculated; UK = Unknown
Coded using MedDRA Version 20.01

Administration Site Key: FA = Forearm; UA= Upper arm
Treatments Part A: A1 = A/Sing MAP, 15 ug (FA); A2 = Afluria Quad IM (UA), 15 ug; A3 = Placebo MAP (FA); A4 = A/Sing Mono IM, 15 ug (UA)
Treatments Part B: B1 = A/Sing MAP, 15 ug (FA) with biopsy; B2 = Placebo MAP (FA) with biopsy
B3 = A/Sing MAP, 15 ug (FA); B4 = A/Sing MAP, 10 ug (FA); B5 = A/Sing MAP, 5 ug (FA); B6 = A/Sing MAP, 2.5 ug (FA)
B7 = Placebo MAP (FA); B8 = A/Sing MAP, 15 ug (UA); B9 = A/Sing IM, 15 ug, as 2018 Afluria Quad (UA)

Listing 16.2.7.2
Adverse Events - MedDRA Coding and Calculated Values

Part B

Random-isation Number	Group	Treatment	AE No.	Adverse Event Verbatim	MedDRA System Organ Class	MedDRA Preferred Term	MedDRA Lower Level Term	Duration of AE (days) [C]	Time Since Treatment Admin. (Days) [C]
B0147	Group B9	A/Sing IM, 15 ug		[No Treatment-Emergent Adverse Events Reported]					
B0148	Group B5	A/Sing MAP, 5 ug	1	Erythema site 1	General disorders and administration site conditions	Application site erythema	Application site erythema	19.51	0.44
B0148	Group B5	A/Sing MAP, 5 ug	2	MAP 1 Visibility	General disorders and administration site conditions	Application site discolouration	Application site discolouration	~44	60.00
B0149	Group B8	A/Sing MAP, 15 ug		[No Treatment-Emergent Adverse Events Reported]					

Key: AE = Adverse Event; [C] = Calculated; UK = Unknown
Coded using MedDRA Version 20.01

Administration Site Key: FA = Forearm; UA= Upper arm
Treatments Part A: A1 = A/Sing MAP, 15 ug (FA); A2 = Afluria Quad IM (UA), 15 ug; A3 = Placebo MAP (FA); A4 = A/Sing Mono IM, 15 ug (UA)
Treatments Part B: B1 = A/Sing MAP, 15 ug (FA) with biopsy; B2 = Placebo MAP (FA) with biopsy
B3 = A/Sing MAP, 15 ug (FA); B4 = A/Sing MAP, 10 ug (FA); B5 = A/Sing MAP, 5 ug (FA); B6 = A/Sing MAP, 2.5 ug (FA)
B7 = Placebo MAP (FA); B8 = A/Sing MAP, 15 ug (UA); B9 = A/Sing IM, 15 ug, as 2018 Afluria Quad (UA)

Listing 16.2.7.2
Adverse Events - MedDRA Coding and Calculated Values

Part B

Random-isation Number	Group	Treatment	AE No.	Adverse Event Verbatim	MedDRA System Organ Class	MedDRA Preferred Term	MedDRA Lower Level Term	Duration of AE (days) [C]	Time Since Treatment Admin. (Days) [C]
B0150	Group B4	A/Sing MAP, 10 ug	4	MAP site 1,2 visibility	General disorders and administration site conditions	Application site discolouration	Application site discolouration	~44	61.08
B1019	Group B8	A/Sing MAP, 15 ug	1	MAP visibility 1,2,3	General disorders and administration site conditions	Application site discolouration	Application site discolouration	~38	62.12
B1076	Group B5	A/Sing MAP, 5 ug	1	MAP 1 reaction - Erythema	General disorders and administration site conditions	Application site erythema	Application site erythema	3.92	3.05
B1080	Group B7	Placebo MAP	1	Right shoulder sprain	Injury, poisoning and procedural complications	Ligament sprain	Shoulder sprain	48.56	0.31

Key: AE = Adverse Event; [C] = Calculated; UK = Unknown
Coded using MedDRA Version 20.01

Administration Site Key: FA = Forearm; UA= Upper arm
Treatments Part A: A1 = A/Sing MAP, 15 ug (FA); A2 = Afluria Quad IM (UA), 15 ug; A3 = Placebo MAP (FA); A4 = A/Sing Mono IM, 15 ug (UA)
Treatments Part B: B1 = A/Sing MAP, 15 ug (FA) with biopsy; B2 = Placebo MAP (FA) with biopsy
B3 = A/Sing MAP, 15 ug (FA); B4 = A/Sing MAP, 10 ug (FA); B5 = A/Sing MAP, 5 ug (FA); B6 = A/Sing MAP, 2.5 ug (FA)
B7 = Placebo MAP (FA); B8 = A/Sing MAP, 15 ug (UA); B9 = A/Sing IM, 15 ug, as 2018 Afluria Quad (UA)
