# Supplementary material for: Safety, tolerability, and immunogenicity of influenza vaccination with a high-density microarray patch: Results from a randomized, controlled phase I clinical trial
Source: PLoS Med. 2020 Mar 17;17(3):e1003024. doi: 10.1371/journal.pmed.1003024 (PMC7077342; doi:10.1371/journal.pmed.1003024)
Supplement: S1 Data — Data 1: Informed Consent; Data 2: Analysis Sets; Data 3: Demographics; Data 4: Study Drug Administration; Data 5: Skin Hardness Assessment; Data 6: Immunogenicity (ELISA IgG); Data 7: Immunogenicity (ADCC); Data 8: Immunogenicity (MBCs); Data 9: Immunogenicity (CMI); Data 10: Immunogenicity (Mucosal IgA); Data 11: Immunogenicity Results (HAI, MN); Data 12: TEAE–CRF Data only; Data 13: TEAEs–MedDRA Coding; Data 15: Treatment Site Tolerability Assessment; Data 16: Numeric Pain Intensity; Data 17: SII; Data 18: Application Site Status at End of Study; Data 19: Individual Treatment Site Tolerability Assessment; Data 20: Numeric Pain Intensity by Treatment; Data 21: Individual SII by Treatment. ADCC, antibody-dependent cellular cytotoxicity; CMI, cell-mediated immunity; CRF, case report form; ELISA, enzyme-linked immunosorbent assay; HAI, haemagglutination inhibition; IgA, immunoglobulin A; IgG, immunoglobulin G; MBC, memory B cell; MedDRA, medical dictionary for regulatory activities; MN, microneutralisation; SII, Skin Irritation Index; TEA, treatment emergent adverse event. (ZIP) [file pmed.1003024.s008.zip › S1_Data/clinical supporting/S1_Data14 Prior and Concomitant Medications.pdf]

Listing 16.2.7.3
Prior and Concomitant Medications - CRF Data Only

Part A

Random-isation Number	No.	Medication Trade/Generic Name	Indication	AE Recorded?	Total Dose	Dose Form	Freq-uency	Route	Start Date	Start Time	End Date/ Ongoing	End Time
A0001		[No prior or concomitant medications recorded]										
A0002	1	Ibuprofen	Dysmenorrhoea	No	400mg	TABLET	ONCE ONLY	ORAL	11-MAR-2018	11:15	11-MAR-2018	11:15
A0002	2	Ibuprofen	Dysmenorrhoea	No	200mg	TABLET	ONCE ONLY	ORAL	12-MAR-2018	14:30	12-MAR-2018	14:30
A0003	1	Maxigesic	Headache	No	500mg/150mg	TABLET	ONCE ONLY	ORAL	01-MAY-2018	19:30	01-MAY-2018	19:30
A0004	1	Paracetamol	Headache	Yes	1000mg	TABLET	AS REQUIRED	ORAL	13-MAR-2018	09:06	16-MAR-2018	09:32
A0005		[No prior or concomitant medications recorded]										
A0006		[No prior or concomitant medications recorded]										

Key: AE = Adverse Event; NA = Not Applicable; UN, UNK = Unknown

Administration Site Key: FA = Forearm; UA= Upper arm
Treatments Part A: A1 = A/Sing MAP, 15 ug (FA); A2 = Afluria Quad IM (UA), 15 ug; A3 = Placebo MAP (FA); A4 = A/Sing Mono IM, 15 ug (UA)
Treatments Part B: B1 = A/Sing MAP, 15 ug (FA) with biopsy; B2 = Placebo MAP (FA) with biopsy
B3 = A/Sing MAP, 15 ug (FA); B4 = A/Sing MAP, 10 ug (FA); B5 = A/Sing MAP, 5 ug (FA); B6 = A/Sing MAP, 2.5 ug (FA)
B7 = Placebo MAP (FA); B8 = A/Sing MAP, 15 ug (UA); B9 = A/Sing IM, 15 ug, as 2018 Afluria Quad (UA)

Listing 16.2.7.3
Prior and Concomitant Medications - CRF Data Only

Part A

Random-isation Number	No.	Medication Trade/Generic Name	Indication	AE Recorded?	Total Dose	Dose Form	Freq-uency	Route	Start Date	Start Time	End Date/ Ongoing	End Time
A0007		[No prior or concomitant medications recorded]										
A0008		[No prior or concomitant medications recorded]										
A0009	1	Fish Oil	Supplement	No	2000IU	CAPSULE	ONCE DAILY	ORAL	01-JAN-2018		Ongoing	
A0010		[No prior or concomitant medications recorded]										
A0011	1	Levonorgestrel/ethinylestradiol	Contraception	No	0.1mg/0.02mg	TABLET	ONCE DAILY	ORAL	UN-UNK-2007		Ongoing	
A0011	2	Nurofen	Headache	No	200mg	TABLET	ONCE ONLY	ORAL	07-APR-2018	19:00	07-APR-2018	19:00
A0011	3	Paracetamol	Headache	Yes	500mg	TABLET	ONCE ONLY	ORAL	11-MAR-2018	12:45	11-MAR-2018	12:45

Key: AE = Adverse Event; NA = Not Applicable; UN, UNK = Unknown

Administration Site Key: FA = Forearm; UA= Upper arm
Treatments Part A: A1 = A/Sing MAP, 15 ug (FA); A2 = Afluria Quad IM (UA), 15 ug; A3 = Placebo MAP (FA); A4 = A/Sing Mono IM, 15 ug (UA)
Treatments Part B: B1 = A/Sing MAP, 15 ug (FA) with biopsy; B2 = Placebo MAP (FA) with biopsy
B3 = A/Sing MAP, 15 ug (FA); B4 = A/Sing MAP, 10 ug (FA); B5 = A/Sing MAP, 5 ug (FA); B6 = A/Sing MAP, 2.5 ug (FA)
B7 = Placebo MAP (FA); B8 = A/Sing MAP, 15 ug (UA); B9 = A/Sing IM, 15 ug, as 2018 Afluria Quad (UA)

Listing 16.2.7.3
Prior and Concomitant Medications - CRF Data Only

Part A

Random-isation Number	No.	Medication Trade/Generic Name	Indication	AE Recorded?	Total Dose	Dose Form	Freq-uency	Route	Start Date	Start Time	End Date/ Ongoing	End Time
A0012	1	Levlen ED	Contraception	No	150mcg/30mcg	TABLET	ONCE DAILY	ORAL	UN-UNK-2013		Ongoing	
A0012	2	Ibuprofen	Headache	No	200mg	TABLET	ONCE ONLY	ORAL	06-MAR-2018		06-MAR-2018	
A0013	1	Levlen	Contraception	No	1 Tablet	TABLET	ONCE DAILY	ORAL	UN-UNK-2016		Ongoing	
A0013	2	Chlorsig	Conjunctivitis	No	5mg/ml	EYE drops	2 TIMES A DAY	OPHTHALMIC	02-MAY-2018	12:00	05-MAY-2018	12:00
A0014		[No prior or concomitant medications recorded]										
A0015	1	Implanon	Contraception	No	1 Device	IMPLANT	Every 3 Years	SUBCUTANEOUS	UN-AUG-2016		Ongoing	
A0016	1	Evra	Contraception	No	150ug	PATCH	3 weeks	TRANSDERMAL	UN-UNK-2016		24-FEB-2018	
A0016	2	Paracetamol	Headache	Yes	1000mg	TABLET	ONCE ONLY	ORAL	10-MAR-2018	15:16	10-MAR-2018	15:16

Key: AE = Adverse Event; NA = Not Applicable; UN, UNK = Unknown

Administration Site Key: FA = Forearm; UA= Upper arm
Treatments Part A: A1 = A/Sing MAP, 15 ug (FA); A2 = Afluria Quad IM (UA), 15 ug; A3 = Placebo MAP (FA); A4 = A/Sing Mono IM, 15 ug (UA)
Treatments Part B: B1 = A/Sing MAP, 15 ug (FA) with biopsy; B2 = Placebo MAP (FA) with biopsy
B3 = A/Sing MAP, 15 ug (FA); B4 = A/Sing MAP, 10 ug (FA); B5 = A/Sing MAP, 5 ug (FA); B6 = A/Sing MAP, 2.5 ug (FA)
B7 = Placebo MAP (FA); B8 = A/Sing MAP, 15 ug (UA); B9 = A/Sing IM, 15 ug, as 2018 Afluria Quad (UA)

Listing 16.2.7.3
Prior and Concomitant Medications - CRF Data Only

Part A

Random-isation Number	No.	Medication Trade/Generic Name	Indication	AE Recorded?	Total Dose	Dose Form	Freq-uency	Route	Start Date	Start Time	End Date/ Ongoing	End Time
A0017		[No prior or concomitant medications recorded]										
A0018	1	Ethinyl estradiol drospirenone	Contraception	No	20ug/3mg	TABLET	ONCE DAILY	ORAL	UN-FEB-2016		Ongoing	
A0018	2	Lemsip (Paracetamol 500mg)	Upper Respiratory Tract Infection	No	500mg	sachet	AS REQUIRED	ORAL	29-MAR-2018		31-MAR-2018	
A0019	1	Flucloxacillin	Phlebitis at venepuncture site	Yes	500mg	TABLET	4 TIMES A DAY	ORAL	04-APR-2018	08:00	10-APR-2018	21:00
A0019	2	Ease a cold +Flu tablets paracetamol/Phenylephrine/	Upper respiratory tract infection	No	1g/10mg	TABLET	3 TIMES A DAY	ORAL	06-MAY-2018		UN-UNK-2018	
A0019	3	Norimin	Contraception	No	1 tablet	TABLET	ONCE DAILY	ORAL	UN-UNK-2016		Ongoing	

Key: AE = Adverse Event; NA = Not Applicable; UN, UNK = Unknown

Administration Site Key: FA = Forearm; UA= Upper arm
Treatments Part A: A1 = A/Sing MAP, 15 ug (FA); A2 = Afluria Quad IM (UA), 15 ug; A3 = Placebo MAP (FA); A4 = A/Sing Mono IM, 15 ug (UA)
Treatments Part B: B1 = A/Sing MAP, 15 ug (FA) with biopsy; B2 = Placebo MAP (FA) with biopsy
B3 = A/Sing MAP, 15 ug (FA); B4 = A/Sing MAP, 10 ug (FA); B5 = A/Sing MAP, 5 ug (FA); B6 = A/Sing MAP, 2.5 ug (FA)
B7 = Placebo MAP (FA); B8 = A/Sing MAP, 15 ug (UA); B9 = A/Sing IM, 15 ug, as 2018 Afluria Quad (UA)

Listing 16.2.7.3
Prior and Concomitant Medications - CRF Data Only

Part A

Randomisation Number	No.	Medication Trade/Generic Name	Indication	AE Recorded?	Total Dose	Dose Form	Frequency	Route	Start Date	Start Time	End Date/ Ongoing	End Time
A0020	1	Panadol	Flu-like symptoms	Yes	1000mg	TABLET	3 TIMES A DAY	ORAL	23-MAR-2018	08:00	23-MAR-2018	19:00
A0021	1	Paracetamol	Unknown	No	1000 mg	TABLET	ONCE ONLY	ORAL	08-MAR-2018	08:00	08-MAR-2018	08:00
A0021	2	Paracetamol	Headache	No	1000mg	TABLET	ONCE ONLY	ORAL	13-APR-2018	10:00	13-APR-2018	10:00
A0022	1	Betadine Gargle Mouthwash	Sore throat and phlegm	No	20mL	1/20 Dilution	3 TIMES A DAY	ORAL	04-APR-2018	16:00	07-APR-2018	21:00
A0023	1	Implanon	Contraception	No	1 Device	IMPLANT	Every 3 Years	SUBCUTANEOUS	UN-SEP-2017		Ongoing	
A0024	1	Panadol	Dysmenorrhoea	No	1000mg	TABLET	2 TIMES A DAY	ORAL	05-MAY-2018	14:00	06-MAY-2018	21:00
A0024	2	Nurofen	Dysmenorrhoea	No	400mg	TABLET	2 TIMES A DAY	ORAL	05-MAY-2018	14:00	06-MAY-2018	21:00
A0024	3	Levlen ED	Contraception	No	1 tablet	TABLET	ONCE DAILY	ORAL	01-FEB-2018		Ongoing	
A0025	1	Implanon	Contraception	No	1 Device	IMPLANT	Every 3 Years	SUBCUTANEOUS	UN-DEC-2016		Ongoing	

Key: AE = Adverse Event; NA = Not Applicable; UN, UNK = Unknown

Administration Site Key: FA = Forearm; UA= Upper arm
Treatments Part A: A1 = A/Sing MAP, 15 ug (FA); A2 = Afluria Quad IM (UA), 15 ug; A3 = Placebo MAP (FA); A4 = A/Sing Mono IM, 15 ug (UA)
Treatments Part B: B1 = A/Sing MAP, 15 ug (FA) with biopsy; B2 = Placebo MAP (FA) with biopsy
B3 = A/Sing MAP, 15 ug (FA); B4 = A/Sing MAP, 10 ug (FA); B5 = A/Sing MAP, 5 ug (FA); B6 = A/Sing MAP, 2.5 ug (FA)
B7 = Placebo MAP (FA); B8 = A/Sing MAP, 15 ug (UA); B9 = A/Sing IM, 15 ug, as 2018 Afluria Quad (UA)

Listing 16.2.7.3
Prior and Concomitant Medications - CRF Data Only

Part A

Random-isation Number	No.	Medication Trade/Generic Name	Indication	AE Recorded?	Total Dose	Dose Form	Freq-uency	Route	Start Date	Start Time	End Date/ Ongoing	End Time
A0025	2	Panadol	Dysmenorrhoea	No	500mg	TABLET	ONCE ONLY	ORAL	02-MAY-2018	14:00	02-MAY-2018	14:00
A0025	3	Panadol	Headache	Yes	1000mg	TABLET	ONCE ONLY	ORAL	15-MAR-2018	07:40	15-MAR-2018	07:40
A0025	4	Panadol	Headache	Yes	1000mg	TABLET	ONCE ONLY	ORAL	17-MAR-2018	09:40	17-MAR-2018	09:40
A0027	1	Paracetamol	Headache	Yes	1gram	TABLET	ONCE ONLY	ORAL	16-MAR-2018	11:00	16-MAR-2018	11:00
A0027	2	Mirena	Contraception	No	1 unit	IUD	5 Yearly	INTRAUTERINE	UN-SEP-2017		Ongoing	
A0028		[No prior or concomitant medications recorded]										
A0029		[No prior or concomitant medications recorded]										
A0030		[No prior or concomitant medications recorded]										

Key: AE = Adverse Event; NA = Not Applicable; UN, UNK = Unknown

Administration Site Key: FA = Forearm; UA= Upper arm
Treatments Part A: A1 = A/Sing MAP, 15 ug (FA); A2 = Afluria Quad IM (UA), 15 ug; A3 = Placebo MAP (FA); A4 = A/Sing Mono IM, 15 ug (UA)
Treatments Part B: B1 = A/Sing MAP, 15 ug (FA) with biopsy; B2 = Placebo MAP (FA) with biopsy
B3 = A/Sing MAP, 15 ug (FA); B4 = A/Sing MAP, 10 ug (FA); B5 = A/Sing MAP, 5 ug (FA); B6 = A/Sing MAP, 2.5 ug (FA)
B7 = Placebo MAP (FA); B8 = A/Sing MAP, 15 ug (UA); B9 = A/Sing IM, 15 ug, as 2018 Afluria Quad (UA)

Listing 16.2.7.3
Prior and Concomitant Medications - CRF Data Only

Part A

Random-isation Number	No.	Medication Trade/Generic Name	Indication	AE Recorded?	Total Dose	Dose Form	Freq-uency	Route	Start Date	Start Time	End Date/ Ongoing	End Time
A0031		[No prior or concomitant medications recorded]										
A0032		[No prior or concomitant medications recorded]										
A0033		[No prior or concomitant medications recorded]										
A0034	1	Estelle-35 ED	Contraception	No	2mg/35ug	TABLET	ONCE DAILY	ORAL	UN-UNK-2014		Ongoing	
A0035	1	Paracetamol	UN	No	1 Tablet	TABLET	AS REQUIRED	ORAL	11-MAR-2018		11-MAR-2018	
A0036	1	Femme 20	Contraception	No	1 Tablet	TABLET	ONCE DAILY	ORAL	UN-NOV-2016		Ongoing	

Key: AE = Adverse Event; NA = Not Applicable; UN, UNK = Unknown

Administration Site Key: FA = Forearm; UA= Upper arm
Treatments Part A: A1 = A/Sing MAP, 15 ug (FA); A2 = Afluria Quad IM (UA), 15 ug; A3 = Placebo MAP (FA); A4 = A/Sing Mono IM, 15 ug (UA)
Treatments Part B: B1 = A/Sing MAP, 15 ug (FA) with biopsy; B2 = Placebo MAP (FA) with biopsy
B3 = A/Sing MAP, 15 ug (FA); B4 = A/Sing MAP, 10 ug (FA); B5 = A/Sing MAP, 5 ug (FA); B6 = A/Sing MAP, 2.5 ug (FA)
B7 = Placebo MAP (FA); B8 = A/Sing MAP, 15 ug (UA); B9 = A/Sing IM, 15 ug, as 2018 Afluria Quad (UA)

Listing 16.2.7.3
Prior and Concomitant Medications - CRF Data Only

Part A

Random-isation Number	No.	Medication Trade/Generic Name	Indication	AE Recorded?	Total Dose	Dose Form	Freq-uency	Route	Start Date	Start Time	End Date/ Ongoing	End Time
A0037		[No prior or concomitant medications recorded]										
A0038	1	Protein powder	Supplement	No	30gm	POWDER	ONCE DAILY	ORAL	UN-UNK-2013		12-MAR-2018	
A0039		[No prior or concomitant medications recorded]										
A0040	1	Evelyn (Levonorgestrel,Ethinyloestradiol)	Contraception	No	150ug/30ug	TABLET	ONCE DAILY	ORAL	01-SEP-2017		Ongoing	
A0041		[No prior or concomitant medications recorded]										

Key: AE = Adverse Event; NA = Not Applicable; UN, UNK = Unknown

Administration Site Key: FA = Forearm; UA= Upper arm
Treatments Part A: A1 = A/Sing MAP, 15 ug (FA); A2 = Afluria Quad IM (UA), 15 ug; A3 = Placebo MAP (FA); A4 = A/Sing Mono IM, 15 ug (UA)
Treatments Part B: B1 = A/Sing MAP, 15 ug (FA) with biopsy; B2 = Placebo MAP (FA) with biopsy
B3 = A/Sing MAP, 15 ug (FA); B4 = A/Sing MAP, 10 ug (FA); B5 = A/Sing MAP, 5 ug (FA); B6 = A/Sing MAP, 2.5 ug (FA)
B7 = Placebo MAP (FA); B8 = A/Sing MAP, 15 ug (UA); B9 = A/Sing IM, 15 ug, as 2018 Afluria Quad (UA)

Listing 16.2.7.3
Prior and Concomitant Medications - CRF Data Only

Part A

Random-isation Number	No.	Medication Trade/Generic Name	Indication	AE Recorded?	Total Dose	Dose Form	Freq-uency	Route	Start Date	Start Time	End Date/ Ongoing	End Time
A0042		[No prior or concomitant medications recorded]										
A0043	1	Roxithromycin	Right Middle Ear Infection	Yes	300mg	TABLET	ONCE DAILY	ORAL	04-APR-2018		08-APR-2018	
A0043	2	Implanon	Contraception	No	1 Device	IMPLANT	Every 3 Years	SUBCUTANEOUS	UN-OCT-2016		Ongoing	
A0044	1	Buscopan	gastroenteriti s	Yes	20mg	TABLET	AS REQUIRED	ORAL	27-MAR-2018		28-MAR-2018	
A0044	2	Paracetamol	gastroenteriti s	Yes	1g	TABLET	AS REQUIRED	ORAL	27-MAR-2018		29-MAR-2018	
A0044	3	Levlen ED	Contraception	No	30mcg/150mc g	TABLET	ONCE DAILY	ORAL	15-JAN-2018		Ongoing	
A0045	1	Depo provera	Contraception	No	150mg/ml	Injection	3 Months	INTRAMUSCULA R	UN-OCT-2017		Ongoing	
A0045	2	Multivitamin	Dietary supplement	No	1 Tablet	TABLET	ONCE DAILY	ORAL	UN-JAN-2018		Ongoing	

Key: AE = Adverse Event; NA = Not Applicable; UN, UNK = Unknown

Administration Site Key: FA = Forearm; UA= Upper arm
Treatments Part A: A1 = A/Sing MAP, 15 ug (FA); A2 = Afluria Quad IM (UA), 15 ug; A3 = Placebo MAP (FA); A4 = A/Sing Mono IM, 15 ug (UA)
Treatments Part B: B1 = A/Sing MAP, 15 ug (FA) with biopsy; B2 = Placebo MAP (FA) with biopsy
B3 = A/Sing MAP, 15 ug (FA); B4 = A/Sing MAP, 10 ug (FA); B5 = A/Sing MAP, 5 ug (FA); B6 = A/Sing MAP, 2.5 ug (FA)
B7 = Placebo MAP (FA); B8 = A/Sing MAP, 15 ug (UA); B9 = A/Sing IM, 15 ug, as 2018 Afluria Quad (UA)

Listing 16.2.7.3
Prior and Concomitant Medications - CRF Data Only

Part A

Randomisation Number	No.	Medication Trade/Generic Name	Indication	AE Recorded?	Total Dose	Dose Form	Frequency	Route	Start Date	Start Time	End Date/ Ongoing	End Time
A0046	1	Telfast	hayfever	No	180mg	TABLET	ONCE ONLY	ORAL	23-MAR-2018	12:00	23-MAR-2018	12:00
A0046	2	Paracetamol	headache	Yes	500mg	TABLET	ONCE ONLY	ORAL	21-MAR-2018	15:30	21-MAR-2018	15:30
A0046	3	Naprogesic	Dysmenorrhoea	Yes	275mg	TABLET	ONCE ONLY	ORAL	18-MAR-2018	18:00	18-MAR-2018	18:00
A0047	1	Paracetamol	Headache	Yes	500mg	TABLET	ONCE ONLY	ORAL	17-MAR-2018		17-MAR-2018	
A0047	2	Ibuprofen	Headache	Yes	200mg	TABLET	ONCE ONLY	ORAL	17-MAR-2018		17-MAR-2018	
A0047	3	Paracetamol	Headache	No	1000mg	TABLET	ONCE ONLY	ORAL	20-APR-2018		20-APR-2018	
A0048	1	Levlen	Contraception	No	0.15mg/0.03 mg	TABLET	ONCE DAILY	ORAL	UN-UNK-2014		Ongoing	
A0049	1	Oral contraceptive pill	Contraception	No	1 TABLET	TABLET	ONCE DAILY	ORAL	UN-OCT-2017		Ongoing	
A0050	1	Yasmin	Contraception	No	1 Tablet	TABLET	ONCE DAILY	ORAL	UN-UNK-2011		Ongoing	
A0050	2	Eltroxin	Hypothyroidism	No	25mcg	TABLET	ONCE DAILY	ORAL	02-MAY-2018	08:00	Ongoing	

Key: AE = Adverse Event; NA = Not Applicable; UN, UNK = Unknown

Administration Site Key: FA = Forearm; UA= Upper arm
Treatments Part A: A1 = A/Sing MAP, 15 ug (FA); A2 = Afluria Quad IM (UA), 15 ug; A3 = Placebo MAP (FA); A4 = A/Sing Mono IM, 15 ug (UA)
Treatments Part B: B1 = A/Sing MAP, 15 ug (FA) with biopsy; B2 = Placebo MAP (FA) with biopsy
B3 = A/Sing MAP, 15 ug (FA); B4 = A/Sing MAP, 10 ug (FA); B5 = A/Sing MAP, 5 ug (FA); B6 = A/Sing MAP, 2.5 ug (FA)
B7 = Placebo MAP (FA); B8 = A/Sing MAP, 15 ug (UA); B9 = A/Sing IM, 15 ug, as 2018 Afluria Quad (UA)

Listing 16.2.7.3
Prior and Concomitant Medications - CRF Data Only

Part A

Randomisation Number	No.	Medication Trade/Generic Name	Indication	AE Recorded?	Total Dose	Dose Form	Frequency	Route	Start Date	Start Time	End Date/ Ongoing	End Time
A0051		[No prior or concomitant medications recorded]										
A0052		[No prior or concomitant medications recorded]										
A0053		[No prior or concomitant medications recorded]										
A0054	1	Vital Strength - Hydroxy Ripped	Nutritional Supplement	No	3 scoops	POWDER	3 TIMES A DAY	ORAL	UN-UNK-2005		Ongoing	
A0054	2	Fish Oil	Supplement	No	5 tablets	TABLET	ONCE DAILY	ORAL	UN-FEB-2018		Ongoing	
A0054	3	Magnesium	Supplement	No	2 tablets	TABLET	ONCE DAILY	ORAL	UN-FEB-2018		Ongoing	
A0055		[No prior or concomitant medications recorded]										

Key: AE = Adverse Event; NA = Not Applicable; UN, UNK = Unknown

Administration Site Key: FA = Forearm; UA= Upper arm
Treatments Part A: A1 = A/Sing MAP, 15 ug (FA); A2 = Afluria Quad IM (UA), 15 ug; A3 = Placebo MAP (FA); A4 = A/Sing Mono IM, 15 ug (UA)
Treatments Part B: B1 = A/Sing MAP, 15 ug (FA) with biopsy; B2 = Placebo MAP (FA) with biopsy
B3 = A/Sing MAP, 15 ug (FA); B4 = A/Sing MAP, 10 ug (FA); B5 = A/Sing MAP, 5 ug (FA); B6 = A/Sing MAP, 2.5 ug (FA)
B7 = Placebo MAP (FA); B8 = A/Sing MAP, 15 ug (UA); B9 = A/Sing IM, 15 ug, as 2018 Afluria Quad (UA)

Listing 16.2.7.3
Prior and Concomitant Medications - CRF Data Only

Part A

Random-isation Number	No.	Medication Trade/Generic Name	Indication	AE Recorded?	Total Dose	Dose Form	Freq-uency	Route	Start Date	Start Time	End Date/ Ongoing	End Time
A0056		[No prior or concomitant medications recorded]										
A0057		[No prior or concomitant medications recorded]										
A0058	1	Ibuprofen	Upper Respiratory Tract Infection	Yes	400mg	TABLET	ONCE DAILY	ORAL	11-MAY-2018	14:00	12-MAY-2018	10:30
A0059	1	Ibuprofen	Headache	Yes	200mg	TABLET	ONCE ONLY	ORAL	22-APR-2018	23:00	22-APR-2018	23:00
A0059	2	Paracetamol	Headache	Yes	500mg	TABLET	ONCE ONLY	ORAL	27-APR-2018	12:00	27-APR-2018	12:00
A0059	3	Copper IUD	Contraception	No	1 device	IUD	Every 10 Years	INTRAUTERINE	UN-JAN-2017		Ongoing	
A0060	1	Ferrograd C	Low Iron	No	325mg	TABLET	ONCE DAILY	ORAL	28-MAR-2018	23:29	30-MAR-2018	22:30

Key: AE = Adverse Event; NA = Not Applicable; UN, UNK = Unknown

Administration Site Key: FA = Forearm; UA= Upper arm
Treatments Part A: A1 = A/Sing MAP, 15 ug (FA); A2 = Afluria Quad IM (UA), 15 ug; A3 = Placebo MAP (FA); A4 = A/Sing Mono IM, 15 ug (UA)
Treatments Part B: B1 = A/Sing MAP, 15 ug (FA) with biopsy; B2 = Placebo MAP (FA) with biopsy
B3 = A/Sing MAP, 15 ug (FA); B4 = A/Sing MAP, 10 ug (FA); B5 = A/Sing MAP, 5 ug (FA); B6 = A/Sing MAP, 2.5 ug (FA)
B7 = Placebo MAP (FA); B8 = A/Sing MAP, 15 ug (UA); B9 = A/Sing IM, 15 ug, as 2018 Afluria Quad (UA)

Listing 16.2.7.3
Prior and Concomitant Medications - CRF Data Only

Part A

Random-isation Number	No.	Medication Trade/Generic Name	Indication	AE Recorded?	Total Dose	Dose Form	Freq-uency	Route	Start Date	Start Time	End Date/ Ongoing	End Time
A1026	1	Aspalgin	Dysmenorrhoea	No	600mg, 16mg	TABLET	ONCE ONLY	ORAL	02-APR-2018	20:00	02-APR-2018	20:00
A1026	2	Laila-35ED	Contraception	No	2mg/35mcg	TABLET	ONCE DAILY	ORAL	UN-JAN-2000		Ongoing	

Key: AE = Adverse Event; NA = Not Applicable; UN, UNK = Unknown

Administration Site Key: FA = Forearm; UA= Upper arm
Treatments Part A: A1 = A/Sing MAP, 15 ug (FA); A2 = Afluria Quad IM (UA), 15 ug; A3 = Placebo MAP (FA); A4 = A/Sing Mono IM, 15 ug (UA)
Treatments Part B: B1 = A/Sing MAP, 15 ug (FA) with biopsy; B2 = Placebo MAP (FA) with biopsy
B3 = A/Sing MAP, 15 ug (FA); B4 = A/Sing MAP, 10 ug (FA); B5 = A/Sing MAP, 5 ug (FA); B6 = A/Sing MAP, 2.5 ug (FA)
B7 = Placebo MAP (FA); B8 = A/Sing MAP, 15 ug (UA); B9 = A/Sing IM, 15 ug, as 2018 Afluria Quad (UA)

Listing 16.2.7.3
Prior and Concomitant Medications - CRF Data Only

Part B

Randomisation Number	No.	Medication Trade/Generic Name	Indication	AE Recorded?	Total Dose	Dose Form	Frequency	Route	Start Date	Start Time	End Date/ Ongoing	End Time
B0001	1	Mirena IUD	Contraception	No	1Device	IUD	5 YEARS	INTRAUTERINE	UN-OCT-2013		Ongoing	
B0001	2	Ibuprofen	Muscle pain	No	400mg	TABLET	2 TIMES A DAY	ORAL	10-JUN-2018	19:00	11-JUN-2018	08:00
B0001	3	1% Lignocaine with adrenaline	Biopsy	No	3 mL	Injection	ONCE ONLY	sub/dermal	07-MAY-2018	09:21	07-MAY-2018	09:21
B0002	1	1% Lignocaine with adrenaline	Biopsy	No	3mL	Injection	ONCE ONLY	SUBDERMAL	07-MAY-2018	09:47	07-MAY-2018	09:47
B0003	1	Estrogen/Progesterone	Contraception	No	1TABLET	TABLET	ONCE DAILY	ORAL	UN-UNK-2008		Ongoing	
B0003	2	1% Lignocaine with adrenaline	Biopsy	No	3 mL	Injection	ONCE ONLY	sub/dermal	07-MAY-2018	09:45	07-MAY-2018	09:45
B0004	1	Estradiol /Dienogest	Contraception	No	30mcg/2mg	TABLET	ONCE DAILY	ORAL	UN-UNK-2016		Ongoing	
B0004	2	1% Lignocaine with adrenaline	Biopsy	No	3 mL	Injection	ONCE ONLY	sub/dermal	07-MAY-2018	10:13	07-MAY-2018	10:13
B0005	1	Norimin EOCP	Contraception	No	1 TABLET	TABLET	ONCE DAILY	ORAL	UN-UNK-2008		Ongoing	

Key: AE = Adverse Event; NA = Not Applicable; UN, UNK = Unknown

Administration Site Key: FA = Forearm; UA= Upper arm
Treatments Part A: A1 = A/Sing MAP, 15 ug (FA); A2 = Afluria Quad IM (UA), 15 ug; A3 = Placebo MAP (FA); A4 = A/Sing Mono IM, 15 ug (UA)
Treatments Part B: B1 = A/Sing MAP, 15 ug (FA) with biopsy; B2 = Placebo MAP (FA) with biopsy
B3 = A/Sing MAP, 15 ug (FA); B4 = A/Sing MAP, 10 ug (FA); B5 = A/Sing MAP, 5 ug (FA); B6 = A/Sing MAP, 2.5 ug (FA)
B7 = Placebo MAP (FA); B8 = A/Sing MAP, 15 ug (UA); B9 = A/Sing IM, 15 ug, as 2018 Afluria Quad (UA)

Listing 16.2.7.3
Prior and Concomitant Medications - CRF Data Only

Part B

Random-isation Number	No.	Medication Trade/Generic Name	Indication	AE Recorded?	Total Dose	Dose Form	Freq-uency	Route	Start Date	Start Time	End Date/ Ongoing	End Time
B0005	2	1% Lignocaine with adrenaline	Biopsy	No	3 mL	Injection	ONCE ONLY	Subdermal	07-MAY-2018	10:20	07-MAY-2018	10:20
B0006	1	Diclofenac	Joint pain	No	50mg	TABLET	2 TIMES A DAY	ORAL	02-JUN-2018		04-JUN-2018	
B0006	2	1% Lignocaine with adrenaline	Biopsy	No	3 mL	Injection	ONCE ONLY	sub/dermal	07-MAY-2018	10:34	07-MAY-2018	10:34
B0007	1	Panadol	Headache	Yes	1000mg	TABLET	2 TIMES A DAY	ORAL	09-MAY-2018	16:30	12-MAY-2018	14:00
B0007	2	1% Lignocaine with adrenaline	Biopsy	No	3 mL	Injection	ONCE ONLY	Subdermal	07-MAY-2018	10:50	07-MAY-2018	10:50
B0007	3	C-OCP	Contraception	No	1 tablet	TABLET	ONCE DAILY	ORAL	UN-UNK-2013		Ongoing	
B0008	1	1% Lignocaine with adrenaline	Biopsy	No	3 mL	Injection	ONCE ONLY	sub/dermal	07-MAY-2018	11:01	07-MAY-2018	11:01
B0009	1	1% Lignocaine with adrenaline	Biopsy	No	3 mL	Injection	ONCE ONLY	sub/dermal	07-MAY-2018	11:18	07-MAY-2018	11:18

Key: AE = Adverse Event; NA = Not Applicable; UN, UNK = Unknown

Administration Site Key: FA = Forearm; UA= Upper arm
Treatments Part A: A1 = A/Sing MAP, 15 ug (FA); A2 = Afluria Quad IM (UA), 15 ug; A3 = Placebo MAP (FA); A4 = A/Sing Mono IM, 15 ug (UA)
Treatments Part B: B1 = A/Sing MAP, 15 ug (FA) with biopsy; B2 = Placebo MAP (FA) with biopsy
B3 = A/Sing MAP, 15 ug (FA); B4 = A/Sing MAP, 10 ug (FA); B5 = A/Sing MAP, 5 ug (FA); B6 = A/Sing MAP, 2.5 ug (FA)
B7 = Placebo MAP (FA); B8 = A/Sing MAP, 15 ug (UA); B9 = A/Sing IM, 15 ug, as 2018 Afluria Quad (UA)

Listing 16.2.7.3
Prior and Concomitant Medications - CRF Data Only

Part B

Randomisation Number	No.	Medication Trade/Generic Name	Indication	AE Recorded?	Total Dose	Dose Form	Frequency	Route	Start Date	Start Time	End Date/ Ongoing	End Time
B0010	1	Paracetamol	Pain at biopsy site	Yes	1000mg	TABLET	ONCE ONLY	ORAL	07-MAY-2018	22:00	07-MAY-2018	22:00
B0010	2	Paracetamol	Pain at biopsy site	Yes	1000mg	TABLET	2 TIMES A DAY	ORAL	08-MAY-2018	12:00	08-MAY-2018	22:00
B0010	3	Paracetamol	Pain at biopsy site	Yes	1000mg	TABLET	ONCE ONLY	ORAL	09-MAY-2018	16:00	09-MAY-2018	16:00
B0010	4	Paracetamol	Pain at biopsy site	Yes	1000mg	TABLET	ONCE ONLY	ORAL	12-MAY-2018	11:15	12-MAY-2018	11:15
B0010	5	Ibuprofen	Pain at biopsy site, Sore throat	Yes	400mg	TABLET	ONCE ONLY	ORAL	12-MAY-2018	11:15	12-MAY-2018	11:15
B0010	6	1% Lignocaine with adrenaline	Biopsy	No	3 mL	Injection	ONCE ONLY	sub/dermal	07-MAY-2018	11:21	07-MAY-2018	11:21
B0011	1	Paracetamol	Headache	No	1000mg	TABLET	ONCE ONLY	ORAL	10-JUN-2018		10-JUN-2018	
B0012	1	Paracetamol	Headache	No	500mg	TABLET	ONCE ONLY	ORAL	12-MAY-2018	21:30	12-MAY-2018	21:30
B0012	2	Ibuprofen	Headache	No	400mg	TABLET	ONCE ONLY	ORAL	26-MAY-2018	11:00	26-MAY-2018	11:00
B0013	1	Paracetamol	Headache	Yes	1000mg	TABLET	ONCE ONLY	ORAL	18-MAY-2018	08:50	18-MAY-2018	08:50

Key: AE = Adverse Event; NA = Not Applicable; UN, UNK = Unknown

Administration Site Key: FA = Forearm; UA= Upper arm
Treatments Part A: A1 = A/Sing MAP, 15 ug (FA); A2 = Afluria Quad IM (UA), 15 ug; A3 = Placebo MAP (FA); A4 = A/Sing Mono IM, 15 ug (UA)
Treatments Part B: B1 = A/Sing MAP, 15 ug (FA) with biopsy; B2 = Placebo MAP (FA) with biopsy
B3 = A/Sing MAP, 15 ug (FA); B4 = A/Sing MAP, 10 ug (FA); B5 = A/Sing MAP, 5 ug (FA); B6 = A/Sing MAP, 2.5 ug (FA)
B7 = Placebo MAP (FA); B8 = A/Sing MAP, 15 ug (UA); B9 = A/Sing IM, 15 ug, as 2018 Afluria Quad (UA)

Listing 16.2.7.3
Prior and Concomitant Medications - CRF Data Only

Part B

Randomisation Number	No.	Medication Trade/Generic Name	Indication	AE Recorded?	Total Dose	Dose Form	Frequency	Route	Start Date	Start Time	End Date/ Ongoing	End Time
B0013	2	Berocca	Upper Respiratory Tract Infection	Yes	1 Tablet	TABLET	ONCE ONLY	ORAL	18-MAY-2018	09:15	18-MAY-2018	09:15
B0013	3	Vitamin B (Mega B)	Dietary Supplement	No	1 Tablet	TABLET	ONCE ONLY	ORAL	25-APR-2018		25-APR-2018	
B0014		[No prior or concomitant medications recorded]										
B0015	1	Famciclovir	Shingles rash	Yes	250mg	TABLET	2 TIMES A DAY	ORAL	19-MAY-2018		26-MAY-2018	
B0016	1	Paracetamol	Headache	Yes	1gm	TABLET	ONCE ONLY	ORAL	26-MAY-2018		26-MAY-2018	
B0016	2	Ibuprofen	Cervical lymphadenopath y	Yes	400mg	TABLET	ONCE ONLY	ORAL	29-MAY-2018	23:30	29-MAY-2018	23:30
B0016	3	Ibuprofen	Cervical lymphadenopath y	Yes	400mg	TABLET	ONCE ONLY	ORAL	03-JUN-2018	20:00	03-JUN-2018	20:00

Key: AE = Adverse Event; NA = Not Applicable; UN, UNK = Unknown

Administration Site Key: FA = Forearm; UA= Upper arm
Treatments Part A: A1 = A/Sing MAP, 15 ug (FA); A2 = Afluria Quad IM (UA), 15 ug; A3 = Placebo MAP (FA); A4 = A/Sing Mono IM, 15 ug (UA)
Treatments Part B: B1 = A/Sing MAP, 15 ug (FA) with biopsy; B2 = Placebo MAP (FA) with biopsy
B3 = A/Sing MAP, 15 ug (FA); B4 = A/Sing MAP, 10 ug (FA); B5 = A/Sing MAP, 5 ug (FA); B6 = A/Sing MAP, 2.5 ug (FA)
B7 = Placebo MAP (FA); B8 = A/Sing MAP, 15 ug (UA); B9 = A/Sing IM, 15 ug, as 2018 Afluria Quad (UA)

Listing 16.2.7.3
Prior and Concomitant Medications - CRF Data Only

Part B

Random-isation Number	No.	Medication Trade/Generic Name	Indication	AE Recorded?	Total Dose	Dose Form	Freq-uency	Route	Start Date	Start Time	End Date/ Ongoing	End Time
B0016	4	Chlorsig	Conjunctivitis	No	5mg/ml	Eye drops	2 drops /Daily 4 times	OPHTHALMIC	10-JUL-2018	21:00	Ongoing	
B0016	5	Livostin	Conjunctivitis	No	0.5mg/ml	Eye drops	Daily 5 times	OPHTHALMIC	09-JUL-2018	09:00	10-JUL-2018	17:00
B0016	6	Codral Day Tablets	Upper Respiratory Tract Infection	No	2 Tablets	TABLET	ONCE DAILY	ORAL	04-JUN-2018		05-JUN-2018	
B0016	7	Codral Night Tablets	Upper Respiratory Tract Infection	No	2 Tablets	TABLET	ONCE DAILY	ORAL	04-JUN-2018		06-JUN-2018	
B0017		[No prior or concomitant medications recorded]										
B0018	1	Rigevidon	Contraception	No	1 TABLET	TABLET	ONCE DAILY	ORAL	UN-SEP-2017		Ongoing	
B0020	1	Hairfinity - Vitamins A, C, B (Biotin)	Supplement	No	1 Capsule	CAPSULE	ONCE DAILY	ORAL	01-APR-2018		01-MAY-2018	

Key: AE = Adverse Event; NA = Not Applicable; UN, UNK = Unknown

Administration Site Key: FA = Forearm; UA= Upper arm
Treatments Part A: A1 = A/Sing MAP, 15 ug (FA); A2 = Afluria Quad IM (UA), 15 ug; A3 = Placebo MAP (FA); A4 = A/Sing Mono IM, 15 ug (UA)
Treatments Part B: B1 = A/Sing MAP, 15 ug (FA) with biopsy; B2 = Placebo MAP (FA) with biopsy
B3 = A/Sing MAP, 15 ug (FA); B4 = A/Sing MAP, 10 ug (FA); B5 = A/Sing MAP, 5 ug (FA); B6 = A/Sing MAP, 2.5 ug (FA)
B7 = Placebo MAP (FA); B8 = A/Sing MAP, 15 ug (UA); B9 = A/Sing IM, 15 ug, as 2018 Afluria Quad (UA)

Listing 16.2.7.3
Prior and Concomitant Medications - CRF Data Only

Part B

Random-isation Number	No.	Medication Trade/Generic Name	Indication	AE Recorded?	Total Dose	Dose Form	Freq-uency	Route	Start Date	Start Time	End Date/ Ongoing	End Time
B0021		[No prior or concomitant medications recorded]										
B0022	1	Nurofen	Headache	Yes	400mg	TABLET	ONCE ONLY	ORAL	16-MAY-2018	09:45	16-MAY-2018	09:45
B0023	1	Implanon	Contraception	No	1 Device	IMPLANT	Every 3 Years	SUBCUTANEOUS	UN-JAN-2017		Ongoing	
B0024	1	Levlen ED	Contraception	No	1 tablet	TABLET	ONCE DAILY	ORAL	13-MAY-2018	23:30	Ongoing	
B0024	2	Paracetamol	Premenstrual syndrome	No	1000mg	TABLET	AS REQUIRED	ORAL	18-MAY-2018	09:30	19-MAY-2018	
B0024	3	Flu Vaccination	Prophylaxis for Influenza	No	0.5mL	Injection	ONCE ONLY	INTRAMUSCULA R	23-JUN-2018	12:00	23-JUN-2018	12:00
B0025	1	Yaz	Contraception	No	1 Tablet	TABLET	ONCE DAILY	ORAL	UN-UNK-2010		Ongoing	

Key: AE = Adverse Event; NA = Not Applicable; UN, UNK = Unknown

Administration Site Key: FA = Forearm; UA= Upper arm
Treatments Part A: A1 = A/Sing MAP, 15 ug (FA); A2 = Afluria Quad IM (UA), 15 ug; A3 = Placebo MAP (FA); A4 = A/Sing Mono IM, 15 ug (UA)
Treatments Part B: B1 = A/Sing MAP, 15 ug (FA) with biopsy; B2 = Placebo MAP (FA) with biopsy
B3 = A/Sing MAP, 15 ug (FA); B4 = A/Sing MAP, 10 ug (FA); B5 = A/Sing MAP, 5 ug (FA); B6 = A/Sing MAP, 2.5 ug (FA)
B7 = Placebo MAP (FA); B8 = A/Sing MAP, 15 ug (UA); B9 = A/Sing IM, 15 ug, as 2018 Afluria Quad (UA)

Listing 16.2.7.3
Prior and Concomitant Medications - CRF Data Only

Part B

Randomisation Number	No.	Medication Trade/Generic Name	Indication	AE Recorded?	Total Dose	Dose Form	Frequency	Route	Start Date	Start Time	End Date/ Ongoing	End Time
B0026		[No prior or concomitant medications recorded]										
B0027	1	Naproxen	Dysmenorrhoea	No	220mg	TABLET	AS REQUIRED	ORAL	UN-UNK-2000		Ongoing	
B0028	1	Copper IUD	Contraception	No	1 Device	IUD	Every 5 years	INTRAUTERINE	UN-NOV-2017		Ongoing	
B0029		[No prior or concomitant medications recorded]										
B0030	1	Mirena	Contraception	No	1 Device	IUD	Every 5 years	INTRAUTERINE	UN-UNK-2014		Ongoing	
B0031		[No prior or concomitant medications recorded]										

Key: AE = Adverse Event; NA = Not Applicable; UN, UNK = Unknown

Administration Site Key: FA = Forearm; UA= Upper arm
Treatments Part A: A1 = A/Sing MAP, 15 ug (FA); A2 = Afluria Quad IM (UA), 15 ug; A3 = Placebo MAP (FA); A4 = A/Sing Mono IM, 15 ug (UA)
Treatments Part B: B1 = A/Sing MAP, 15 ug (FA) with biopsy; B2 = Placebo MAP (FA) with biopsy
B3 = A/Sing MAP, 15 ug (FA); B4 = A/Sing MAP, 10 ug (FA); B5 = A/Sing MAP, 5 ug (FA); B6 = A/Sing MAP, 2.5 ug (FA)
B7 = Placebo MAP (FA); B8 = A/Sing MAP, 15 ug (UA); B9 = A/Sing IM, 15 ug, as 2018 Afluria Quad (UA)

Listing 16.2.7.3
Prior and Concomitant Medications - CRF Data Only

Part B

Randomisation Number	No.	Medication Trade/Generic Name	Indication	AE Recorded?	Total Dose	Dose Form	Frequency	Route	Start Date	Start Time	End Date/ Ongoing	End Time
B0032	1	Vitamin C (Cenovis)	Health supplement	No	2 Tablets	TABLET	ONCE DAILY	ORAL	UN-UNK-2016		Ongoing	
B0032	2	Magnesium (Cenovis)	Health supplement	No	1 Tablet	TABLET	ONCE DAILY	ORAL	UN-UNK-2016		Ongoing	
B0032	3	Fish oil (Cenovis)	Health supplement	No	2 Tablets	TABLET	ONCE DAILY	ORAL	UN-UNK-2016		Ongoing	
B0033	1	Levlen (Oral contraceptive Pill)	Contraception	No	1 Tablet	TABLET	ONCE DAILY	ORAL	UN-UNK-2011		Ongoing	
B0034	1	Paracetamol	Headache	No	1gm	TABLET	AS REQUIRED	ORAL	19-MAY-2018	12:20	19-MAY-2018	
B0034	2	Femiane (Ethinyl estradiol/ Gestodene	Contraception	No	1 tablet	TABLET	ONCE DAILY	ORAL	UN-UNK-2002		Ongoing	
B0035	1	Paracetamol	Upper Respiratory Tract Infection	Yes	1000mg	TABLET	3 TIMES A DAY	ORAL	31-MAY-2018		03-JUN-2018	
B0035	2	Paracetamol	Upper Respiratory Tract Infection	Yes	1000mg	TABLET	2 TIMES A DAY	ORAL	04-JUN-2018		06-JUN-2018	

Key: AE = Adverse Event; NA = Not Applicable; UN, UNK = Unknown

Administration Site Key: FA = Forearm; UA= Upper arm
Treatments Part A: A1 = A/Sing MAP, 15 ug (FA); A2 = Afluria Quad IM (UA), 15 ug; A3 = Placebo MAP (FA); A4 = A/Sing Mono IM, 15 ug (UA)
Treatments Part B: B1 = A/Sing MAP, 15 ug (FA) with biopsy; B2 = Placebo MAP (FA) with biopsy
B3 = A/Sing MAP, 15 ug (FA); B4 = A/Sing MAP, 10 ug (FA); B5 = A/Sing MAP, 5 ug (FA); B6 = A/Sing MAP, 2.5 ug (FA)
B7 = Placebo MAP (FA); B8 = A/Sing MAP, 15 ug (UA); B9 = A/Sing IM, 15 ug, as 2018 Afluria Quad (UA)

Listing 16.2.7.3
Prior and Concomitant Medications - CRF Data Only

Part B

Randomisation Number	No.	Medication Trade/Generic Name	Indication	AE Recorded?	Total Dose	Dose Form	Freq-uency	Route	Start Date	Start Time	End Date/ Ongoing	End Time
B0035	3	Vitamin C	Upper Respiratory Tract Infection	Yes	500mg	TABLET	ONCE DAILY	ORAL	07-JUN-2018	08:00	09-JUN-2018	08:00
B0036		[No prior or concomitant medications recorded]										
B0037	1	Panamax	URTI	Yes	200mg	TABLET	ONCE ONLY	ORAL	20-MAY-2018	09:00	20-MAY-2018	09:00
B0037	2	Ibuprofen	URTI	Yes	400mg	TABLET	ONCE ONLY	ORAL	21-MAY-2018	10:00	21-MAY-2018	10:00
B0037	3	Telfast	URTI	Yes	120mg	TABLET	ONCE ONLY	ORAL	21-MAY-2018	14:00	21-MAY-2018	14:00
B0037	4	Depo Ralovera	Contraception	No	1 Injection	Injection	Every 3 months	INTRAMUSCULAR	UN-UNK-2014		Ongoing	
B0038	1	Inferme 28	Contraception	No	1 Tablet	TABLET	ONCE DAILY	ORAL	UN-UNK-1997		Ongoing	
B0038	2	Fluorometholone	Bilateral blocked tear ducts	No	1mg/mL	Eye drop	3 TIMES A DAY	OPHTHALMIC	30-JUN-2018	09:00	Ongoing	
B0039	1	Paracetamol	Headache	Yes	1330mg	Caplets	ONCE ONLY	ORAL	24-MAY-2018	23:51	24-MAY-2018	23:51

Key: AE = Adverse Event; NA = Not Applicable; UN, UNK = Unknown

Administration Site Key: FA = Forearm; UA= Upper arm
Treatments Part A: A1 = A/Sing MAP, 15 ug (FA); A2 = Afluria Quad IM (UA), 15 ug; A3 = Placebo MAP (FA); A4 = A/Sing Mono IM, 15 ug (UA)
Treatments Part B: B1 = A/Sing MAP, 15 ug (FA) with biopsy; B2 = Placebo MAP (FA) with biopsy
B3 = A/Sing MAP, 15 ug (FA); B4 = A/Sing MAP, 10 ug (FA); B5 = A/Sing MAP, 5 ug (FA); B6 = A/Sing MAP, 2.5 ug (FA)
B7 = Placebo MAP (FA); B8 = A/Sing MAP, 15 ug (UA); B9 = A/Sing IM, 15 ug, as 2018 Afluria Quad (UA)

Listing 16.2.7.3
Prior and Concomitant Medications - CRF Data Only

Part B

Random-isation Number	No.	Medication Trade/Generic Name	Indication	AE Recorded?	Total Dose	Dose Form	Freq-uency	Route	Start Date	Start Time	End Date/ Ongoing	End Time
B0040		[No prior or concomitant medications recorded]										
B0041	1	Panadol	Upper Respiratory Tract Infection	No	1000mg	TABLET	3 TIMES A DAY	ORAL	17-JUL-2018	12:00	20-JUL-2018	12:00
B0041	2	Panadol	Upper Respiratory Tract Infection	No	1000mg	TABLET	ONCE ONLY	ORAL	15-JUL-2018	17:00	15-JUL-2018	17:00
B0042	1	Panadol	Headache	Yes	500mg	TABLET	ONCE ONLY	ORAL	23-MAY-2018	06:15	23-MAY-2018	06:15
B0042	2	Panadol	Headache	Yes	1000mg	TABLET	ONCE ONLY	ORAL	23-MAY-2018	17:35	23-MAY-2018	17:35
B0042	3	Yasmin	Contraception	No	1 Tablet	TABLET	ONCE DAILY	ORAL	UN-UNK-2008		Ongoing	
B0042	4	Ibuprofen	Headache	Yes	400mg	TABLET	ONCE ONLY	ORAL	26-MAY-2018	11:57	26-MAY-2018	11:57
B0043	1	Panadol	Headache	Yes	1gm	TABLET	ONCE ONLY	ORAL	19-MAY-2018	19:00	19-MAY-2018	19:00
B0043	2	Implanon	Contraception	No	1 Device	IMPLANT	Every 3 Years	SUBCUTANEOUS	UN-JAN-2017		Ongoing	

Key: AE = Adverse Event; NA = Not Applicable; UN, UNK = Unknown

Administration Site Key: FA = Forearm; UA= Upper arm
Treatments Part A: A1 = A/Sing MAP, 15 ug (FA); A2 = Afluria Quad IM (UA), 15 ug; A3 = Placebo MAP (FA); A4 = A/Sing Mono IM, 15 ug (UA)
Treatments Part B: B1 = A/Sing MAP, 15 ug (FA) with biopsy; B2 = Placebo MAP (FA) with biopsy
B3 = A/Sing MAP, 15 ug (FA); B4 = A/Sing MAP, 10 ug (FA); B5 = A/Sing MAP, 5 ug (FA); B6 = A/Sing MAP, 2.5 ug (FA)
B7 = Placebo MAP (FA); B8 = A/Sing MAP, 15 ug (UA); B9 = A/Sing IM, 15 ug, as 2018 Afluria Quad (UA)

Listing 16.2.7.3
Prior and Concomitant Medications - CRF Data Only

Part B

Randomisation Number	No.	Medication Trade/Generic Name	Indication	AE Recorded?	Total Dose	Dose Form	Frequency	Route	Start Date	Start Time	End Date/ Ongoing	End Time
B0044		[No prior or concomitant medications recorded]										
B0045	1	Ibuprofen	Sprained Left Ankle	No	400mg	TABLET	AS REQUIRED	ORAL	16-JUN-2018		17-JUN-2018	
B0046	1	Panamax	Headache	Yes	1000mg	TABLET	ONCE ONLY	ORAL	24-MAY-2018	22:15	24-MAY-2018	22:15
B0047	1	Magnesium	Insomnia	No	500mg	TABLET	ONCE DAILY	ORAL	03-MAY-2018	22:30	Ongoing	
B0048	1	Blackmores Bio Zinc	Dietary Supplement	No	1 TABLET	TABLET	ONCE DAILY	ORAL	23-MAY-2018	09:00	Ongoing	
B0048	2	Paracetamol	Upper Respiratory Tract Infection	No	1gm	TABLET	AS REQUIRED	ORAL	15-JUN-2018		15-JUN-2018	
B0048	3	Ibuprofen	Upper Respiratory Tract Infection	No	400mg	TABLET	AS REQUIRED	ORAL	15-JUN-2018		15-JUN-2018	

Key: AE = Adverse Event; NA = Not Applicable; UN, UNK = Unknown

Administration Site Key: FA = Forearm; UA= Upper arm
Treatments Part A: A1 = A/Sing MAP, 15 ug (FA); A2 = Afluria Quad IM (UA), 15 ug; A3 = Placebo MAP (FA); A4 = A/Sing Mono IM, 15 ug (UA)
Treatments Part B: B1 = A/Sing MAP, 15 ug (FA) with biopsy; B2 = Placebo MAP (FA) with biopsy
B3 = A/Sing MAP, 15 ug (FA); B4 = A/Sing MAP, 10 ug (FA); B5 = A/Sing MAP, 5 ug (FA); B6 = A/Sing MAP, 2.5 ug (FA)
B7 = Placebo MAP (FA); B8 = A/Sing MAP, 15 ug (UA); B9 = A/Sing IM, 15 ug, as 2018 Afluria Quad (UA)

Listing 16.2.7.3
Prior and Concomitant Medications - CRF Data Only

Part B

Random-isation Number	No.	Medication Trade/Generic Name	Indication	AE Recorded?	Total Dose	Dose Form	Freq-uency	Route	Start Date	Start Time	End Date/ Ongoing	End Time
B0048	4	Mirena	Dysmenorrhea	No	1 Device	IUD	Every 5 years	INTRAUTERINE	UN-SEP-2016		Ongoing	
B0049		[No prior or concomitant medications recorded]										
B0050		[No prior or concomitant medications recorded]										
B0051	1	Evelyn ED	Contraception	No	150ug/30ug	TABLET	ONCE DAILY	ORAL	10-MAY-2017		Ongoing	
B0051	2	Voltaren	Swollen ankle	No	1 Tablet	TABLET	ONCE ONLY	ORAL	26-JUN-2018		26-JUN-2018	
B0051	3	Sambucol	Cold/Flu	No	2 Tablets	TABLET	ONCE ONLY	ORAL	20-JUL-2018	23:00	20-JUL-2018	23:00
B0051	4	Ibuprofen	Cold/Flu	No	200mg	TABLET	ONCE ONLY	ORAL	20-JUL-2018	23:00	20-JUL-2018	23:00
B0051	5	Vitamin C	Cold/Flu	No	2 Tablets	TABLET	ONCE ONLY	ORAL	20-JUL-2018	23:00	20-JUL-2018	23:00
B0052	1	Paracetamol	Headache	Yes	500mg	TABLET	ONCE ONLY	ORAL	01-JUN-2018	23:45	01-JUN-2018	23:45

Key: AE = Adverse Event; NA = Not Applicable; UN, UNK = Unknown

Administration Site Key: FA = Forearm; UA= Upper arm
Treatments Part A: A1 = A/Sing MAP, 15 ug (FA); A2 = Afluria Quad IM (UA), 15 ug; A3 = Placebo MAP (FA); A4 = A/Sing Mono IM, 15 ug (UA)
Treatments Part B: B1 = A/Sing MAP, 15 ug (FA) with biopsy; B2 = Placebo MAP (FA) with biopsy
B3 = A/Sing MAP, 15 ug (FA); B4 = A/Sing MAP, 10 ug (FA); B5 = A/Sing MAP, 5 ug (FA); B6 = A/Sing MAP, 2.5 ug (FA)
B7 = Placebo MAP (FA); B8 = A/Sing MAP, 15 ug (UA); B9 = A/Sing IM, 15 ug, as 2018 Afluria Quad (UA)

Listing 16.2.7.3
Prior and Concomitant Medications - CRF Data Only

Part B

Random-isation Number	No.	Medication Trade/Generic Name	Indication	AE Recorded?	Total Dose	Dose Form	Freq-uency	Route	Start Date	Start Time	End Date/ Ongoing	End Time
B0052	2	Paracetamol	Upper Respiratory Tract Infection	Yes	1000mg	TABLET	2 TIMES A DAY	ORAL	11-JUN-2018	19:00	12-JUN-2018	17:30
B0052	3	Cold & flu tablet	Upper Respiratory Tract Infection	No	1 Tablet	TABLET	AS REQUIRED	ORAL	08-MAY-2018		08-MAY-2018	
B0052	4	Paracetamol	Upper Respiratory Tract Infection	Yes	1000mg	TABLET	ONCE ONLY	ORAL	12-JUN-2018	23:00	12-JUN-2018	23:00
B0053	1	Diane 35	Facial acne	No	1 Tablet	TABLET	ONCE DAILY	ORAL	UN-UNK-2013		Ongoing	
B0054	1	Estell - 35 ED	Contraception	No	1 Tablet	TABLET	ONCE DAILY	ORAL	UN-UNK-2015		Ongoing	
B0055	1	Nurofen	Tonsillitis	Yes	500mg	TABLET	ONCE ONLY	ORAL	14-JUN-2018	22:00	14-JUN-2018	22:00
B0055	2	Implanon	Contraception	No	1 Device	IMPLANT	Every 3 Years	SUBCUTANEOUS	UN-UNK-2014		Ongoing	

Key: AE = Adverse Event; NA = Not Applicable; UN, UNK = Unknown

Administration Site Key: FA = Forearm; UA= Upper arm
Treatments Part A: A1 = A/Sing MAP, 15 ug (FA); A2 = Afluria Quad IM (UA), 15 ug; A3 = Placebo MAP (FA); A4 = A/Sing Mono IM, 15 ug (UA)
Treatments Part B: B1 = A/Sing MAP, 15 ug (FA) with biopsy; B2 = Placebo MAP (FA) with biopsy
B3 = A/Sing MAP, 15 ug (FA); B4 = A/Sing MAP, 10 ug (FA); B5 = A/Sing MAP, 5 ug (FA); B6 = A/Sing MAP, 2.5 ug (FA)
B7 = Placebo MAP (FA); B8 = A/Sing MAP, 15 ug (UA); B9 = A/Sing IM, 15 ug, as 2018 Afluria Quad (UA)

Listing 16.2.7.3
Prior and Concomitant Medications - CRF Data Only

Part B

Random-isation Number	No.	Medication Trade/Generic Name	Indication	AE Recorded?	Total Dose	Dose Form	Freq-uency	Route	Start Date	Start Time	End Date/ Ongoing	End Time
B0056		[No prior or concomitant medications recorded]										
B0057		[No prior or concomitant medications recorded]										
B0058	1	Aspirin/ Asproclear	Migraine	Yes	150mg	TABLET	1/2 tablet once	ORAL	06-JUN-2018	14:00	06-JUN-2018	14:00
B0059	1	Panadol	Stomach ache	No	500mg	TABLET	ONCE ONLY	ORAL	25-MAY-2018	23:45	25-MAY-2018	23:46
B0059	2	Implanon	Contraception	No	1 Device	IMPLANT	Every 3 Years	SUBCUTANEOUS	UN-FEB-2018		Ongoing	
B0060		[No prior or concomitant medications recorded]										

Key: AE = Adverse Event; NA = Not Applicable; UN, UNK = Unknown

Administration Site Key: FA = Forearm; UA= Upper arm
Treatments Part A: A1 = A/Sing MAP, 15 ug (FA); A2 = Afluria Quad IM (UA), 15 ug; A3 = Placebo MAP (FA); A4 = A/Sing Mono IM, 15 ug (UA)
Treatments Part B: B1 = A/Sing MAP, 15 ug (FA) with biopsy; B2 = Placebo MAP (FA) with biopsy
B3 = A/Sing MAP, 15 ug (FA); B4 = A/Sing MAP, 10 ug (FA); B5 = A/Sing MAP, 5 ug (FA); B6 = A/Sing MAP, 2.5 ug (FA)
B7 = Placebo MAP (FA); B8 = A/Sing MAP, 15 ug (UA); B9 = A/Sing IM, 15 ug, as 2018 Afluria Quad (UA)

Listing 16.2.7.3
Prior and Concomitant Medications - CRF Data Only

Part B

Random-isation Number	No.	Medication Trade/Generic Name	Indication	AE Recorded?	Total Dose	Dose Form	Freq-uency	Route	Start Date	Start Time	End Date/ Ongoing	End Time
B0061		[No prior or concomitant medications recorded]										
B0062		[No prior or concomitant medications recorded]										
B0063		[No prior or concomitant medications recorded]										
B0064	1	Claratyne	Hayfever symptoms	No	10mg	TABLET	ONCE ONLY	ORAL	26-MAY-2018	09:00	26-MAY-2018	09:00
B0064	2	Unknown Antibiotics	Upper Respiratory Tract Infection	Yes	UN	TABLET	4 TIMES A DAY	ORAL	10-JUN-2018	14:00	13-JUN-2018	22:00

Key: AE = Adverse Event; NA = Not Applicable; UN, UNK = Unknown

Administration Site Key: FA = Forearm; UA= Upper arm
Treatments Part A: A1 = A/Sing MAP, 15 ug (FA); A2 = Afluria Quad IM (UA), 15 ug; A3 = Placebo MAP (FA); A4 = A/Sing Mono IM, 15 ug (UA)
Treatments Part B: B1 = A/Sing MAP, 15 ug (FA) with biopsy; B2 = Placebo MAP (FA) with biopsy
B3 = A/Sing MAP, 15 ug (FA); B4 = A/Sing MAP, 10 ug (FA); B5 = A/Sing MAP, 5 ug (FA); B6 = A/Sing MAP, 2.5 ug (FA)
B7 = Placebo MAP (FA); B8 = A/Sing MAP, 15 ug (UA); B9 = A/Sing IM, 15 ug, as 2018 Afluria Quad (UA)

Listing 16.2.7.3
Prior and Concomitant Medications - CRF Data Only

Part B

Random-isation Number	No.	Medication Trade/Generic Name	Indication	AE Recorded?	Total Dose	Dose Form	Freq-uency	Route	Start Date	Start Time	End Date/ Ongoing	End Time
B0065	1	Antibiotics	Upper Respiratory Tract Infection	Yes	UN	TABLET	ONCE DAILY	ORAL	08-JUN-2018	14:00	10-JUN-2018	
B0066	1	Vicks action cold & flu	Upper Respiratory Tract Infection	No	1000mg/10mg	TABLET	2 TIMES A DAY	ORAL	28-JUN-2018	10:00	29-JUN-2018	10:00
B0066	2	Vicks action cold & flu	Upper Respiratory Tract Infection	No	1000mg/20mg	TABLET	ONCE ONLY	ORAL	23-JUL-2018	10:00	23-JUL-2018	10:00
B0066	3	Vicks action cold & flu	Upper Respiratory Tract Infection	No	10mg	TABLET	ONCE ONLY	ORAL	24-JUL-2018	08:00	24-JUL-2018	08:00
B0067		[No prior or concomitant medications recorded]										
B0068	1	Levlen	Contraception	No	1 Tablet	TABLET	ONCE DAILY	ORAL	UN-UNK-2014		Ongoing	
B0068	2	Paracetamol	Headache	No	1gm	TABLET	AS REQUIRED	ORAL	03-MAY-2018		03-MAY-2018	

Key: AE = Adverse Event; NA = Not Applicable; UN, UNK = Unknown

Administration Site Key: FA = Forearm; UA= Upper arm
Treatments Part A: A1 = A/Sing MAP, 15 ug (FA); A2 = Afluria Quad IM (UA), 15 ug; A3 = Placebo MAP (FA); A4 = A/Sing Mono IM, 15 ug (UA)
Treatments Part B: B1 = A/Sing MAP, 15 ug (FA) with biopsy; B2 = Placebo MAP (FA) with biopsy
B3 = A/Sing MAP, 15 ug (FA); B4 = A/Sing MAP, 10 ug (FA); B5 = A/Sing MAP, 5 ug (FA); B6 = A/Sing MAP, 2.5 ug (FA)
B7 = Placebo MAP (FA); B8 = A/Sing MAP, 15 ug (UA); B9 = A/Sing IM, 15 ug, as 2018 Afluria Quad (UA)

Listing 16.2.7.3
Prior and Concomitant Medications - CRF Data Only

Part B

Random-isation Number	No.	Medication Trade/Generic Name	Indication	AE Recorded?	Total Dose	Dose Form	Freq-uency	Route	Start Date	Start Time	End Date/ Ongoing	End Time
B0069	1	Paracetamol	Headache	Yes	1000mg	TABLET	ONCE ONLY	ORAL	06-JUN-2018	21:00	06-JUN-2018	21:00
B0070	1	Triafemi	Contraception	No	1 Tablet	TABLET	ONCE DAILY	ORAL	UN-APR-2017		Ongoing	
B0071	1	Levlen	Contraception	No	1 Tablet	TABLET	ONCE DAILY	ORAL	UN-DEC-2017		Ongoing	
B0072	1	YAZ	Contraception	No	1 Tablet	TABLET	ONCE DAILY	ORAL	UN-MAY-2017		Ongoing	
B0073		[No prior or concomitant medications recorded]										
B0074		[No prior or concomitant medications recorded]										
B0075	1	Panadol	Headache	Yes	500mg	TABLET	ONCE ONLY	ORAL	05-JUN-2018	10:00	05-JUN-2018	10:00

Key: AE = Adverse Event; NA = Not Applicable; UN, UNK = Unknown

Administration Site Key: FA = Forearm; UA= Upper arm
Treatments Part A: A1 = A/Sing MAP, 15 ug (FA); A2 = Afluria Quad IM (UA), 15 ug; A3 = Placebo MAP (FA); A4 = A/Sing Mono IM, 15 ug (UA)
Treatments Part B: B1 = A/Sing MAP, 15 ug (FA) with biopsy; B2 = Placebo MAP (FA) with biopsy
B3 = A/Sing MAP, 15 ug (FA); B4 = A/Sing MAP, 10 ug (FA); B5 = A/Sing MAP, 5 ug (FA); B6 = A/Sing MAP, 2.5 ug (FA)
B7 = Placebo MAP (FA); B8 = A/Sing MAP, 15 ug (UA); B9 = A/Sing IM, 15 ug, as 2018 Afluria Quad (UA)

Listing 16.2.7.3
Prior and Concomitant Medications - CRF Data Only

Part B

Random-isation Number	No.	Medication Trade/Generic Name	Indication	AE Recorded?	Total Dose	Dose Form	Freq-uency	Route	Start Date	Start Time	End Date/ Ongoing	End Time
B0077	1	Paracetamol	Dysmenorrhoea	No	500mg	TABLET	2 TIMES A DAY	ORAL	04-JUN-2018	04:00	05-JUN-2018	15:00
B0077	2	Vitamin A	Supplement	No	1 Tablet	TABLET	ONCE DAILY	ORAL	08-MAY-2018		Ongoing	
B0078		[No prior or concomitant medications recorded]										
B0079	1	Panadol	Headache	Yes	1000mg	TABLET	ONCE ONLY	ORAL	03-JUN-2018	16:15	03-JUN-2018	16:15
B0079	2	Estelle	Contraception	No	1 Tablet	TABLET	ONCE DAILY	ORAL	UN-UNK-2011		Ongoing	
B0079	3	Moxiclav Duo Forte	Tonsillitis	No	875mg/125mg	TABLET	2 TIMES A DAY	ORAL	02-JUL-2018	19:00	07-JUL-2018	19:00
B0081	1	Paracetamol	Headache	Yes	2 Tablets	TABLET	ONCE ONLY	ORAL	01-JUN-2018	16:50	01-JUN-2018	16:50
B0081	2	Panadeine	Headache	No	2 Tablets	TABLET	ONCE ONLY	ORAL	21-JUN-2018	17:30	21-JUN-2018	17:30
B0081	3	Paracetamol	Headache	Yes	1000mg	TABLET	ONCE ONLY	ORAL	08-JUN-2018	12:00	08-JUN-2018	12:00
B0082		[No prior or concomitant medications recorded]										

Key: AE = Adverse Event; NA = Not Applicable; UN, UNK = Unknown

Administration Site Key: FA = Forearm; UA= Upper arm
Treatments Part A: A1 = A/Sing MAP, 15 ug (FA); A2 = Afluria Quad IM (UA), 15 ug; A3 = Placebo MAP (FA); A4 = A/Sing Mono IM, 15 ug (UA)
Treatments Part B: B1 = A/Sing MAP, 15 ug (FA) with biopsy; B2 = Placebo MAP (FA) with biopsy
B3 = A/Sing MAP, 15 ug (FA); B4 = A/Sing MAP, 10 ug (FA); B5 = A/Sing MAP, 5 ug (FA); B6 = A/Sing MAP, 2.5 ug (FA)
B7 = Placebo MAP (FA); B8 = A/Sing MAP, 15 ug (UA); B9 = A/Sing IM, 15 ug, as 2018 Afluria Quad (UA)

Listing 16.2.7.3
Prior and Concomitant Medications - CRF Data Only

Part B

Randomisation Number	No.	Medication Trade/Generic Name	Indication	AE Recorded?	Total Dose	Dose Form	Frequency	Route	Start Date	Start Time	End Date/ Ongoing	End Time
B0083		[No prior or concomitant medications recorded]										
B0084		[No prior or concomitant medications recorded]										
B0085	1	Paracetamol	Headache	Yes	500mg	TABLET	ONCE ONLY	ORAL	09-JUN-2018	22:00	09-JUN-2018	22:00
B0086		[No prior or concomitant medications recorded]										
B0087	1	Paracetamol	Headache	Yes	2 Tablets	TABLET	ONCE ONLY	ORAL	30-JUN-2018	12:00	30-JUN-2018	12:00
B0087	2	Paracetamol	URTI	Yes	2 Tablets	TABLET	3 TIMES A DAY	ORAL	13-JUL-2018	18:00	28-JUL-2018	

Key: AE = Adverse Event; NA = Not Applicable; UN, UNK = Unknown

Administration Site Key: FA = Forearm; UA= Upper arm
Treatments Part A: A1 = A/Sing MAP, 15 ug (FA); A2 = Afluria Quad IM (UA), 15 ug; A3 = Placebo MAP (FA); A4 = A/Sing Mono IM, 15 ug (UA)
Treatments Part B: B1 = A/Sing MAP, 15 ug (FA) with biopsy; B2 = Placebo MAP (FA) with biopsy
B3 = A/Sing MAP, 15 ug (FA); B4 = A/Sing MAP, 10 ug (FA); B5 = A/Sing MAP, 5 ug (FA); B6 = A/Sing MAP, 2.5 ug (FA)
B7 = Placebo MAP (FA); B8 = A/Sing MAP, 15 ug (UA); B9 = A/Sing IM, 15 ug, as 2018 Afluria Quad (UA)

Listing 16.2.7.3
Prior and Concomitant Medications - CRF Data Only

Part B

Random-isation Number	No.	Medication Trade/Generic Name	Indication	AE Recorded?	Total Dose	Dose Form	Freq-uency	Route	Start Date	Start Time	End Date/ Ongoing	End Time
B0088		[No prior or concomitant medications recorded]										
B0089		[No prior or concomitant medications recorded]										
B0090	1	Cetirizine	Pruritis	Yes	10mg	TABLET	ONCE ONLY	ORAL	09-JUN-2018	13:25	09-JUN-2018	13:25
B0090	2	Femme Tab (Combined OCP)	Contraception	No	1 Tablet	TABLET	ONCE DAILY	ORAL	UN-UNK-2015		Ongoing	
B0091	1	Panadol	Headache	Yes	500mg	TABLET	ONCE ONLY	ORAL	13-JUN-2018	19:36	13-JUN-2018	19:36
B0091	2	Yaz	Contraception	No	1 Tablet	TABLET	ONCE DAILY	ORAL	UN-UNK-2008		Ongoing	
B0091	3	Paracetamol	Headache	No	500mg	TABLET	ONCE ONLY	ORAL	13-JUL-2018	10:00	13-JUL-2018	10:00
B0092	1	Paracetamol	Headache	Yes	500mg	TABLET	ONCE ONLY	ORAL	15-JUN-2018	20:30	15-JUN-2018	20:30
B0092	2	Paracetamol	Fever	No	1000mg	TABLET	2 TIMES A DAY	ORAL	18-JUL-2018	07:00	19-JUL-2018	16:30

Key: AE = Adverse Event; NA = Not Applicable; UN, UNK = Unknown

Administration Site Key: FA = Forearm; UA= Upper arm
Treatments Part A: A1 = A/Sing MAP, 15 ug (FA); A2 = Afluria Quad IM (UA), 15 ug; A3 = Placebo MAP (FA); A4 = A/Sing Mono IM, 15 ug (UA)
Treatments Part B: B1 = A/Sing MAP, 15 ug (FA) with biopsy; B2 = Placebo MAP (FA) with biopsy
B3 = A/Sing MAP, 15 ug (FA); B4 = A/Sing MAP, 10 ug (FA); B5 = A/Sing MAP, 5 ug (FA); B6 = A/Sing MAP, 2.5 ug (FA)
B7 = Placebo MAP (FA); B8 = A/Sing MAP, 15 ug (UA); B9 = A/Sing IM, 15 ug, as 2018 Afluria Quad (UA)

Listing 16.2.7.3
Prior and Concomitant Medications - CRF Data Only

Part B

Random-isation Number	No.	Medication Trade/Generic Name	Indication	AE Recorded?	Total Dose	Dose Form	Freq-uency	Route	Start Date	Start Time	End Date/ Ongoing	End Time
B0093	1	Magnesium (Thompsons organic)	Muscle recovery post exercise	No	161mg	TABLET	ONCE DAILY	ORAL	09-MAY-2018		Ongoing	
B0093	2	Ferro F 60	Supplement	No	1 Tablet	TABLET	ONCE DAILY	ORAL	23-JUN-2018		Ongoing	
B0093	3	Paracetamol	Upper respiratory tract infection	No	1g	TABLET	ONCE ONLY	ORAL	10-JUL-2018	11:00	10-JUL-2018	11:00
B0094		[No prior or concomitant medications recorded]										
B0095	1	Vitamin C	Supplement	No	500mg	TABLET	ONCE DAILY	ORAL	UN-APR-2018		Ongoing	
B0096		[No prior or concomitant medications recorded]										
B0097		[No prior or concomitant medications recorded]										

Key: AE = Adverse Event; NA = Not Applicable; UN, UNK = Unknown

Administration Site Key: FA = Forearm; UA= Upper arm
Treatments Part A: A1 = A/Sing MAP, 15 ug (FA); A2 = Afluria Quad IM (UA), 15 ug; A3 = Placebo MAP (FA); A4 = A/Sing Mono IM, 15 ug (UA)
Treatments Part B: B1 = A/Sing MAP, 15 ug (FA) with biopsy; B2 = Placebo MAP (FA) with biopsy
B3 = A/Sing MAP, 15 ug (FA); B4 = A/Sing MAP, 10 ug (FA); B5 = A/Sing MAP, 5 ug (FA); B6 = A/Sing MAP, 2.5 ug (FA)
B7 = Placebo MAP (FA); B8 = A/Sing MAP, 15 ug (UA); B9 = A/Sing IM, 15 ug, as 2018 Afluria Quad (UA)

Listing 16.2.7.3
Prior and Concomitant Medications - CRF Data Only

Part B

Random-isation Number	No.	Medication Trade/Generic Name	Indication	AE Recorded?	Total Dose	Dose Form	Freq-uency	Route	Start Date	Start Time	End Date/ Ongoing	End Time
B0098		[No prior or concomitant medications recorded]										
B0099	1	Cold and Flu tablets	Upper respiratory tract infection	Yes	2 Tablets	TABLET	2 TIMES A DAY	ORAL	27-JUL-2018	22:00	30-JUL-2018	
B0099	2	Nurofen	Dysmenorrhoea	No	2 Gel caps	Gel caps	ONCE ONLY	ORAL	28-JUL-2018	17:30	28-JUL-2018	17:30
B0099	3	Clarithromycin	Upper respiratory tract infection	Yes	250mg	TABLET	2 TIMES A DAY	ORAL	04-AUG-2018	07:00	08-AUG-2018	
B0099	4	Panafcort	Upper respiratory tract infection	Yes	25mg	TABLET	2 TIMES A DAY	ORAL	04-AUG-2018		06-AUG-2018	07:00
B0099	5	Iron	Nutritional supplement	No	2-3 Tablets	TABLET	2-3 daily	ORAL	UN-JAN-2018		Ongoing	
B0099	6	Magnesium	Nutritional supplement	No	1 Tablet	TABLET	Every 2-3 days	ORAL	UN-OCT-2017		Ongoing	

Key: AE = Adverse Event; NA = Not Applicable; UN, UNK = Unknown

Administration Site Key: FA = Forearm; UA= Upper arm
Treatments Part A: A1 = A/Sing MAP, 15 ug (FA); A2 = Afluria Quad IM (UA), 15 ug; A3 = Placebo MAP (FA); A4 = A/Sing Mono IM, 15 ug (UA)
Treatments Part B: B1 = A/Sing MAP, 15 ug (FA) with biopsy; B2 = Placebo MAP (FA) with biopsy
B3 = A/Sing MAP, 15 ug (FA); B4 = A/Sing MAP, 10 ug (FA); B5 = A/Sing MAP, 5 ug (FA); B6 = A/Sing MAP, 2.5 ug (FA)
B7 = Placebo MAP (FA); B8 = A/Sing MAP, 15 ug (UA); B9 = A/Sing IM, 15 ug, as 2018 Afluria Quad (UA)

Listing 16.2.7.3
Prior and Concomitant Medications - CRF Data Only

Part B

Random-isation Number	No.	Medication Trade/Generic Name	Indication	AE Recorded?	Total Dose	Dose Form	Freq-uency	Route	Start Date	Start Time	End Date/ Ongoing	End Time
B0099	7	Vitamin D	Nutritional supplement	No	1 Tablet	TABLET	Every 2-3 days	ORAL	UN-APR-2018		Ongoing	
B0099	8	Mirena IUD	Contraception	No	1 Device	IUD	Every 5 years	INTRAUTERINE	UN-JAN-2018		Ongoing	
B0100	1	Paracetamol	Headache	Yes	500mg	TABLET	ONCE ONLY	ORAL	09-JUN-2018	20:00	09-JUN-2018	20:00
B0100	2	Ginkgo	Supplement	No	1 Tablet	TABLET	ONCE DAILY	ORAL	29-APR-2018		27-MAY-2018	
B0101	1	Levlen ED	Contraception	No	1 Tablet	TABLET	ONCE DAILY	ORAL	UN-UNK-1998		Ongoing	
B0102		[No prior or concomitant medications recorded]										
B0103		[No prior or concomitant medications recorded]										

Key: AE = Adverse Event; NA = Not Applicable; UN, UNK = Unknown

Administration Site Key: FA = Forearm; UA= Upper arm
Treatments Part A: A1 = A/Sing MAP, 15 ug (FA); A2 = Afluria Quad IM (UA), 15 ug; A3 = Placebo MAP (FA); A4 = A/Sing Mono IM, 15 ug (UA)
Treatments Part B: B1 = A/Sing MAP, 15 ug (FA) with biopsy; B2 = Placebo MAP (FA) with biopsy
B3 = A/Sing MAP, 15 ug (FA); B4 = A/Sing MAP, 10 ug (FA); B5 = A/Sing MAP, 5 ug (FA); B6 = A/Sing MAP, 2.5 ug (FA)
B7 = Placebo MAP (FA); B8 = A/Sing MAP, 15 ug (UA); B9 = A/Sing IM, 15 ug, as 2018 Afluria Quad (UA)

Listing 16.2.7.3
Prior and Concomitant Medications - CRF Data Only

Part B

Random-isation Number	No.	Medication Trade/Generic Name	Indication	AE Recorded?	Total Dose	Dose Form	Freq-uency	Route	Start Date	Start Time	End Date/ Ongoing	End Time
B0104	1	Combined contraceptive pill (Levlen)	Contraception	No	0.15mg/0.03 mg	TABLET	ONCE DAILY	ORAL	11-JUN-2018	07:00	Ongoing	
B0104	2	Ethinylestradiol and Levonorgestrel	Contraception	No	150mcg/30mc g	TABLET	ONCE DAILY	ORAL	UN-UNK-2016		Ongoing	
B0105	1	Sudafed	Upper respiratory tract infection	Yes	500mg	TABLET	ONCE ONLY	ORAL	29-JUN-2018	14:00	29-JUN-2018	14:00
B0105	2	Curam Duo Forte	Infected cut on right index finger	No	875mg/125mg	TABLET	2 TIMES A DAY	ORAL	31-JUL-2018	08:00	04-AUG-2018	20:00
B0106	1	Cyproterone acetate / ethinyl oestradiol	Contraception	No	2mg/35mcg	TABLET	ONCE DAILY	ORAL	UN-FEB-2018		Ongoing	
B0106	2	Vegan multivitamin	Supplement	No	1 Capsule	CAPSULE	ONCE DAILY	ORAL	UN-FEB-2018		03-JUN-2018	
B0107	1	Levlen	Contraception	No	150ug/30ug	TABLET	ONCE DAILY	ORAL	UN-OCT-2017		Ongoing	
B0107	2	Usana CellSentials - Vitamin -Oxidant	General health	No	1 Tablet	TABLET	2 TIMES A DAY	ORAL	UN-UNK-2011		03-JUN-2018	

Key: AE = Adverse Event; NA = Not Applicable; UN, UNK = Unknown

Administration Site Key: FA = Forearm; UA= Upper arm
Treatments Part A: A1 = A/Sing MAP, 15 ug (FA); A2 = Afluria Quad IM (UA), 15 ug; A3 = Placebo MAP (FA); A4 = A/Sing Mono IM, 15 ug (UA)
Treatments Part B: B1 = A/Sing MAP, 15 ug (FA) with biopsy; B2 = Placebo MAP (FA) with biopsy
B3 = A/Sing MAP, 15 ug (FA); B4 = A/Sing MAP, 10 ug (FA); B5 = A/Sing MAP, 5 ug (FA); B6 = A/Sing MAP, 2.5 ug (FA)
B7 = Placebo MAP (FA); B8 = A/Sing MAP, 15 ug (UA); B9 = A/Sing IM, 15 ug, as 2018 Afluria Quad (UA)

Listing 16.2.7.3
Prior and Concomitant Medications - CRF Data Only

Part B

Random-isation Number	No.	Medication Trade/Generic Name	Indication	AE Recorded?	Total Dose	Dose Form	Freq-uency	Route	Start Date	Start Time	End Date/ Ongoing	End Time
B0107	3	Usana CellSentials - core in minerals	General health	No	1 Tablet	TABLET	2 TIMES A DAY	ORAL	UN-UNK-2011		03-JUN-2018	
B0108		[No prior or concomitant medications recorded]										
B0109	1	Multivitamin	Nutritional supplement	No	1 Tablet	TABLET	2-3 times per week	ORAL	UN-NOV-2017		Ongoing	
B0110		[No prior or concomitant medications recorded]										
B0111		[No prior or concomitant medications recorded]										

Key: AE = Adverse Event; NA = Not Applicable; UN, UNK = Unknown

Administration Site Key: FA = Forearm; UA= Upper arm
Treatments Part A: A1 = A/Sing MAP, 15 ug (FA); A2 = Afluria Quad IM (UA), 15 ug; A3 = Placebo MAP (FA); A4 = A/Sing Mono IM, 15 ug (UA)
Treatments Part B: B1 = A/Sing MAP, 15 ug (FA) with biopsy; B2 = Placebo MAP (FA) with biopsy
B3 = A/Sing MAP, 15 ug (FA); B4 = A/Sing MAP, 10 ug (FA); B5 = A/Sing MAP, 5 ug (FA); B6 = A/Sing MAP, 2.5 ug (FA)
B7 = Placebo MAP (FA); B8 = A/Sing MAP, 15 ug (UA); B9 = A/Sing IM, 15 ug, as 2018 Afluria Quad (UA)

Listing 16.2.7.3
Prior and Concomitant Medications - CRF Data Only

Part B

Random-isation Number	No.	Medication Trade/Generic Name	Indication	AE Recorded?	Total Dose	Dose Form	Freq-uency	Route	Start Date	Start Time	End Date/ Ongoing	End Time
B0112		[No prior or concomitant medications recorded]										
B0113		[No prior or concomitant medications recorded]										
B0114	1	Paracetamol	Headache	Yes	1000mg	TABLET	ONCE ONLY	ORAL	17-JUN-2018	08:00	17-JUN-2018	08:00
B0114	2	Amoxycillin	Upper Respiratory Tract Infection	No	1g	TABLET	3 TIMES A DAY	ORAL	30-JUL-2018	08:00	15-AUG-2018	
B0115	1	Yasmin	Contraception	No	3mg/0.02mg	TABLET	ONCE DAILY	ORAL	UN-UNK-2012		Ongoing	
B0115	2	Paracetamol	Upper Respiratory Tract Infection	Yes	1000mg	TABLET	3 TIMES A DAY	ORAL	02-JUL-2018	10:00	03-JUL-2018	22:00
B0116	1	Implanon	Contraception	No	1 Device	IMPLANT	Every 3 Years	SUBCUTANEOUS	UN-OCT-2015		Ongoing	

Key: AE = Adverse Event; NA = Not Applicable; UN, UNK = Unknown

Administration Site Key: FA = Forearm; UA= Upper arm
Treatments Part A: A1 = A/Sing MAP, 15 ug (FA); A2 = Afluria Quad IM (UA), 15 ug; A3 = Placebo MAP (FA); A4 = A/Sing Mono IM, 15 ug (UA)
Treatments Part B: B1 = A/Sing MAP, 15 ug (FA) with biopsy; B2 = Placebo MAP (FA) with biopsy
B3 = A/Sing MAP, 15 ug (FA); B4 = A/Sing MAP, 10 ug (FA); B5 = A/Sing MAP, 5 ug (FA); B6 = A/Sing MAP, 2.5 ug (FA)
B7 = Placebo MAP (FA); B8 = A/Sing MAP, 15 ug (UA); B9 = A/Sing IM, 15 ug, as 2018 Afluria Quad (UA)

Listing 16.2.7.3
Prior and Concomitant Medications - CRF Data Only

Part B

Random-isation Number	No.	Medication Trade/Generic Name	Indication	AE Recorded?	Total Dose	Dose Form	Freq-uency	Route	Start Date	Start Time	End Date/ Ongoing	End Time
B0116	2	Ibuprofen	Headache	Yes	400mg	TABLET	2 TIMES A DAY	ORAL	05-JUL-2018	10:30	05-JUL-2018	15:00
B0116	3	Telfast	Hayfever	No	60mg	TABLET	ONCE ONLY	ORAL	11-AUG-2018	10:00	11-AUG-2018	10:00
B0117	1	Magnesium	Supplement	No	1 Tablet	TABLET	Weekly once	ORAL	UN-DEC-2017		Ongoing	
B0117	2	Implanon	Contraception	No	1 device	IMPLANT	Every 3 Years	SUBCUTANEOUS	UN-UNK-2016		Ongoing	
B0118	1	Implanon	Contraception	No	1 Device	IMPLANT	Every 3 Years	SUBCUTANEOUS	08-NOV-2017		Ongoing	
B0118	2	Paracetamol	Headache	Yes	1gm	TABLET	2 TIMES A DAY	ORAL	16-JUN-2018	14:25	16-JUN-2018	
B0118	3	Paracetamol	Upper respiratory tract infection	No	1g	TABLET	ONCE ONLY	ORAL	13-AUG-2018	16:00	13-AUG-2018	16:00
B0118	4	Paracetamol	Headache	Yes	1000mg	TABLET	ONCE ONLY	ORAL	05-JUL-2018	19:00	05-JUL-2018	19:00
B0119		[No prior or concomitant medications recorded]										

Key: AE = Adverse Event; NA = Not Applicable; UN, UNK = Unknown

Administration Site Key: FA = Forearm; UA= Upper arm
Treatments Part A: A1 = A/Sing MAP, 15 ug (FA); A2 = Afluria Quad IM (UA), 15 ug; A3 = Placebo MAP (FA); A4 = A/Sing Mono IM, 15 ug (UA)
Treatments Part B: B1 = A/Sing MAP, 15 ug (FA) with biopsy; B2 = Placebo MAP (FA) with biopsy
B3 = A/Sing MAP, 15 ug (FA); B4 = A/Sing MAP, 10 ug (FA); B5 = A/Sing MAP, 5 ug (FA); B6 = A/Sing MAP, 2.5 ug (FA)
B7 = Placebo MAP (FA); B8 = A/Sing MAP, 15 ug (UA); B9 = A/Sing IM, 15 ug, as 2018 Afluria Quad (UA)

Listing 16.2.7.3
Prior and Concomitant Medications - CRF Data Only

Part B

Random-isation Number	No.	Medication Trade/Generic Name	Indication	AE Recorded?	Total Dose	Dose Form	Freq-uency	Route	Start Date	Start Time	End Date/ Ongoing	End Time
B0120	1	Levlen ED COCP	Contraception	No	1 Tablet	TABLET	ONCE DAILY	ORAL	UN-UNK-2016		Ongoing	
B0121		[No prior or concomitant medications recorded]										
B0122	1	Loette	Contraception	No	100ug/20ug	TABLET	ONCE DAILY	ORAL	UN-UNK-2008		Ongoing	
B0123		[No prior or concomitant medications recorded]										
B0124	1	Microgynon 20	Contraception	No	1 Tablet	TABLET	ONCE DAILY	ORAL	UN-UNK-2004		Ongoing	
B0124	2	Elocon cream 1% Mometasone	Contact dermatitis	Yes	0.1%	CREAM	AS REQUIRED	TOPICAL	25-JUN-2018		25-JUN-2018	
B0124	3	Panadol	Headache	Yes	1000mg	TABLET	ONCE ONLY	ORAL	21-JUN-2018	11:45	21-JUN-2018	11:45
B0125	1	Implanon	Contraception	No	1 Device	IMPLANT	Every 3 years	SUBCUTANEOUS	UN-NOV-2015		Ongoing	

Key: AE = Adverse Event; NA = Not Applicable; UN, UNK = Unknown

Administration Site Key: FA = Forearm; UA= Upper arm
Treatments Part A: A1 = A/Sing MAP, 15 ug (FA); A2 = Afluria Quad IM (UA), 15 ug; A3 = Placebo MAP (FA); A4 = A/Sing Mono IM, 15 ug (UA)
Treatments Part B: B1 = A/Sing MAP, 15 ug (FA) with biopsy; B2 = Placebo MAP (FA) with biopsy
B3 = A/Sing MAP, 15 ug (FA); B4 = A/Sing MAP, 10 ug (FA); B5 = A/Sing MAP, 5 ug (FA); B6 = A/Sing MAP, 2.5 ug (FA)
B7 = Placebo MAP (FA); B8 = A/Sing MAP, 15 ug (UA); B9 = A/Sing IM, 15 ug, as 2018 Afluria Quad (UA)

Listing 16.2.7.3
Prior and Concomitant Medications - CRF Data Only

Part B

Random-isation Number	No.	Medication Trade/Generic Name	Indication	AE Recorded?	Total Dose	Dose Form	Freq-uency	Route	Start Date	Start Time	End Date/ Ongoing	End Time
B0126	1	Vitamin C	Nutritional supplement	No	1 Tablet	TABLET	ONCE DAILY	ORAL	UN-UNK-2016		Ongoing	
B0127	1	Paracetamol	Headache	No	1000mg	TABLET	ONCE ONLY	ORAL	20-MAY-2018		20-MAY-2018	
B0128		[No prior or concomitant medications recorded]										
B0129	1	Ibuprofen	Headache	No	400mg	TABLET	ONCE ONLY	ORAL	07-AUG-2018	14:00	07-AUG-2018	14:00
B0130	1	Panadol	Upper Respiratory Tract Infection	Yes	1000mg	TABLET	ONCE DAILY	ORAL	27-JUN-2018	18:47	28-JUN-2018	10:00
B0130	2	Difflam	Upper Respiratory Tract Infection	Yes	1 Lozenge	TABLET	ONCE ONLY	ORAL	27-JUN-2018	20:00	27-JUN-2018	20:00

Key: AE = Adverse Event; NA = Not Applicable; UN, UNK = Unknown

Administration Site Key: FA = Forearm; UA= Upper arm
Treatments Part A: A1 = A/Sing MAP, 15 ug (FA); A2 = Afluria Quad IM (UA), 15 ug; A3 = Placebo MAP (FA); A4 = A/Sing Mono IM, 15 ug (UA)
Treatments Part B: B1 = A/Sing MAP, 15 ug (FA) with biopsy; B2 = Placebo MAP (FA) with biopsy
B3 = A/Sing MAP, 15 ug (FA); B4 = A/Sing MAP, 10 ug (FA); B5 = A/Sing MAP, 5 ug (FA); B6 = A/Sing MAP, 2.5 ug (FA)
B7 = Placebo MAP (FA); B8 = A/Sing MAP, 15 ug (UA); B9 = A/Sing IM, 15 ug, as 2018 Afluria Quad (UA)

Listing 16.2.7.3
Prior and Concomitant Medications - CRF Data Only

Part B

Random-isation Number	No.	Medication Trade/Generic Name	Indication	AE Recorded?	Total Dose	Dose Form	Freq-uency	Route	Start Date	Start Time	End Date/ Ongoing	End Time
B0130	3	Chesty Forte Bisolvon	Upper Respiratory Tract Infection	No	16mg	TABLET	ONCE ONLY	ORAL	20-AUG-2018	11:00	20-AUG-2018	11:00
B0131		[No prior or concomitant medications recorded]										
B0132	1	Yaz	Contraception	No	1 Tablet	TABLET	ONCE DAILY	ORAL	UN-UNK-2015		Ongoing	
B0133	1	Levonorgestrel and Ethinyl Estradiol	Contraception	No	150ug/30ug	TABLET	ONCE DAILY	ORAL	UN-UNK-2008		Ongoing	
B0134	1	Multivitamin	Nutritional Supplement	No	1 Tablet	TABLET	ONCE DAILY	ORAL	UN-MAR-2018		Ongoing	
B0134	2	Implanon	Contraception	No	1 Device	IMPLANT	Every 3 Years	SUBCUTANEOUS	UN-JAN-2016		Ongoing	
B0135	1	Ethinyl estradiol Cyproterone	Contraception	No	35ug/2mg	TABLET	ONCE DAILY	ORAL	UN-OCT-2017		Ongoing	

Key: AE = Adverse Event; NA = Not Applicable; UN, UNK = Unknown

Administration Site Key: FA = Forearm; UA= Upper arm
Treatments Part A: A1 = A/Sing MAP, 15 ug (FA); A2 = Afluria Quad IM (UA), 15 ug; A3 = Placebo MAP (FA); A4 = A/Sing Mono IM, 15 ug (UA)
Treatments Part B: B1 = A/Sing MAP, 15 ug (FA) with biopsy; B2 = Placebo MAP (FA) with biopsy
B3 = A/Sing MAP, 15 ug (FA); B4 = A/Sing MAP, 10 ug (FA); B5 = A/Sing MAP, 5 ug (FA); B6 = A/Sing MAP, 2.5 ug (FA)
B7 = Placebo MAP (FA); B8 = A/Sing MAP, 15 ug (UA); B9 = A/Sing IM, 15 ug, as 2018 Afluria Quad (UA)

Listing 16.2.7.3
Prior and Concomitant Medications - CRF Data Only

Part B

Randomisation Number	No.	Medication Trade/Generic Name	Indication	AE Recorded?	Total Dose	Dose Form	Frequency	Route	Start Date	Start Time	End Date/ Ongoing	End Time
B0136	1	Oxynorm	Cholecystitis	Yes	5mg	TABLET	ONCE ONLY	ORAL	05-JUL-2018	23:00	05-JUL-2018	23:00
B0136	2	Implanon	Contraception	No	1 Device	IMPLANT	Every 3 Years	SUBCUTANEOUS	UN-UNK-2016		Ongoing	
B0137	1	Progesterone IUD	Contraception	No	1 Device	IUD	Every 5 Years	INTRAUTERINE	UN-MAR-2017		Ongoing	
B0137	2	Guaifenesin Phenylephrine HCl	Upper Respiratory Tract Infection	Yes	100mg/5mg	TABLET	ONCE ONLY	ORAL	09-JUL-2018	22:00	09-JUL-2018	22:00
B0138	1	Panadol	Dysmenorrhoea	No	500mg	TABLET	ONCE ONLY	ORAL	02-JUN-2018	12:17	02-JUN-2018	12:17
B0138	2	Panadol	Headache	No	500mg	TABLET	ONCE ONLY	ORAL	07-JUN-2018	23:30	07-JUN-2018	23:30
B0138	3	Fish oil	Supplement	No	1000mg	TABLET	ONCE DAILY	ORAL	UN-UNK-2012		28-MAY-2018	
B0139	1	Usana health food company - My health pak - (-1) Visionex	Supplement	No	1 Tablet	TABLET	ONCE DAILY	ORAL	UN-UNK-2011		16-JUN-2018	
B0139	2	Procosamine	Supplement	No	2 Tablets	TABLET	ONCE DAILY	ORAL	UN-UNK-2011		16-JUN-2018	
B0139	3	Magnesium	Supplement	No	500mg	TABLET	ONCE DAILY	ORAL	UN-UNK-2011		16-JUN-2018	

Key: AE = Adverse Event; NA = Not Applicable; UN, UNK = Unknown

Administration Site Key: FA = Forearm; UA= Upper arm
Treatments Part A: A1 = A/Sing MAP, 15 ug (FA); A2 = Afluria Quad IM (UA), 15 ug; A3 = Placebo MAP (FA); A4 = A/Sing Mono IM, 15 ug (UA)
Treatments Part B: B1 = A/Sing MAP, 15 ug (FA) with biopsy; B2 = Placebo MAP (FA) with biopsy
B3 = A/Sing MAP, 15 ug (FA); B4 = A/Sing MAP, 10 ug (FA); B5 = A/Sing MAP, 5 ug (FA); B6 = A/Sing MAP, 2.5 ug (FA)
B7 = Placebo MAP (FA); B8 = A/Sing MAP, 15 ug (UA); B9 = A/Sing IM, 15 ug, as 2018 Afluria Quad (UA)

Listing 16.2.7.3
Prior and Concomitant Medications - CRF Data Only

Part B

Random-isation Number	No.	Medication Trade/Generic Name	Indication	AE Recorded?	Total Dose	Dose Form	Freq-uency	Route	Start Date	Start Time	End Date/ Ongoing	End Time
B0139	4	Calcium	Supplement	No	1 Tablet	TABLET	ONCE DAILY	ORAL	UN-UNK-2011		16-JUN-2018	
B0139	5	Vitamin D	Supplement	No	1 Tablet	TABLET	ONCE DAILY	ORAL	UN-UNK-2011		16-JUN-2018	
B0139	6	Hepasil	Supplement	No	1 Tablet	TABLET	ONCE DAILY	ORAL	UN-UNK-2011		16-JUN-2018	
B0139	7	Multivitamins	Supplement	No	1 Tablet	TABLET	ONCE DAILY	ORAL	UN-UNK-2011		16-JUN-2018	
B0139	8	Fish oil	Supplement	No	1 Tablet	TABLET	ONCE DAILY	ORAL	UN-UNK-2011		16-JUN-2018	
B0140	1	Ferrograd C	Iron deficiency	No	1 Tablet	TABLET	ONCE DAILY	ORAL	12-JUN-2018		Ongoing	
B0141		[No prior or concomitant medications recorded]										
B0142	1	Nurofen	Shin splints	Yes	400mg	TABLET	ONCE ONLY	ORAL	12-JUL-2018	15:00	12-JUL-2018	15:00
B0142	2	Penicillin	Bacterial Tonsillitis	No	500mg	TABLET	2 TIMES A DAY	ORAL	24-AUG-2018	19:00	31-AUG-2018	
B0143	1	Hep A vaccine	Prophylaxis for Hep A	No	NA	Injection	ONCE ONLY	INTRAMUSCULA R	21-MAY-2018		21-MAY-2018	
B0143	2	Boostrix Vaccine	Prophylaxis for dTPa	No	NA	Injection	ONCE ONLY	INTRAMUSCULA R	21-MAY-2018		21-MAY-2018	

Key: AE = Adverse Event; NA = Not Applicable; UN, UNK = Unknown

Administration Site Key: FA = Forearm; UA= Upper arm
Treatments Part A: A1 = A/Sing MAP, 15 ug (FA); A2 = Afluria Quad IM (UA), 15 ug; A3 = Placebo MAP (FA); A4 = A/Sing Mono IM, 15 ug (UA)
Treatments Part B: B1 = A/Sing MAP, 15 ug (FA) with biopsy; B2 = Placebo MAP (FA) with biopsy
B3 = A/Sing MAP, 15 ug (FA); B4 = A/Sing MAP, 10 ug (FA); B5 = A/Sing MAP, 5 ug (FA); B6 = A/Sing MAP, 2.5 ug (FA)
B7 = Placebo MAP (FA); B8 = A/Sing MAP, 15 ug (UA); B9 = A/Sing IM, 15 ug, as 2018 Afluria Quad (UA)

Listing 16.2.7.3
Prior and Concomitant Medications - CRF Data Only

Part B

Random-isation Number	No.	Medication Trade/Generic Name	Indication	AE Recorded?	Total Dose	Dose Form	Freq-uency	Route	Start Date	Start Time	End Date/ Ongoing	End Time
B0143	3	MMR Vaccine	Prophylaxis for Measles, mumps and rubella	No	NA	Injection	ONCE ONLY	INTRAMUSCULA R	21-MAY-2018		21-MAY-2018	
B0143	4	Hep B vaccine	Prophylaxis for Hep B	No	NA	Injection	ONCE ONLY	INTRAMUSCULA R	21-MAY-2018		21-MAY-2018	
B0143	5	Ibuprofen	Patellofemoral syndrome	Yes	400mg	TABLET	AS REQUIRED	ORAL	06-JUL-2018	17:00	Ongoing	
B0144	1	Paracetamol	Upper Respiratory Tract Infection symptoms	Yes	2 Tablets	TABLET	ONCE ONLY	ORAL	15-JUL-2018	11:00	15-JUL-2018	11:00
B0144	2	Duro -tuss dry cough	Respiratory Tract Infection symptoms	Yes	5mg	TABLET	AS REQUIRED	ORAL	16-JUL-2018	00:00	18-JUL-2018	
B0145		[No prior or concomitant medications recorded]										

Key: AE = Adverse Event; NA = Not Applicable; UN, UNK = Unknown

Administration Site Key: FA = Forearm; UA= Upper arm
Treatments Part A: A1 = A/Sing MAP, 15 ug (FA); A2 = Afluria Quad IM (UA), 15 ug; A3 = Placebo MAP (FA); A4 = A/Sing Mono IM, 15 ug (UA)
Treatments Part B: B1 = A/Sing MAP, 15 ug (FA) with biopsy; B2 = Placebo MAP (FA) with biopsy
B3 = A/Sing MAP, 15 ug (FA); B4 = A/Sing MAP, 10 ug (FA); B5 = A/Sing MAP, 5 ug (FA); B6 = A/Sing MAP, 2.5 ug (FA)
B7 = Placebo MAP (FA); B8 = A/Sing MAP, 15 ug (UA); B9 = A/Sing IM, 15 ug, as 2018 Afluria Quad (UA)

Listing 16.2.7.3
Prior and Concomitant Medications - CRF Data Only

Part B

Random-isation Number	No.	Medication Trade/Generic Name	Indication	AE Recorded?	Total Dose	Dose Form	Freq-uency	Route	Start Date	Start Time	End Date/ Ongoing	End Time
B0146		[No prior or concomitant medications recorded]										
B0147		[No prior or concomitant medications recorded]										
B0148	1	Vitamin C	Nutritional supplement	No	1000 IU	TABLET	3 times weekly	ORAL	UN-JAN-2018		Ongoing	
B0149		[No prior or concomitant medications recorded]										
B0150	1	Diclofenac	Back Pain	No	50mg	TABLET	ONCE DAILY	ORAL	30-JUL-2018	12:00	01-AUG-2018	
B1019		[No prior or concomitant medications recorded]										

Key: AE = Adverse Event; NA = Not Applicable; UN, UNK = Unknown

Administration Site Key: FA = Forearm; UA= Upper arm
Treatments Part A: A1 = A/Sing MAP, 15 ug (FA); A2 = Afluria Quad IM (UA), 15 ug; A3 = Placebo MAP (FA); A4 = A/Sing Mono IM, 15 ug (UA)
Treatments Part B: B1 = A/Sing MAP, 15 ug (FA) with biopsy; B2 = Placebo MAP (FA) with biopsy
B3 = A/Sing MAP, 15 ug (FA); B4 = A/Sing MAP, 10 ug (FA); B5 = A/Sing MAP, 5 ug (FA); B6 = A/Sing MAP, 2.5 ug (FA)
B7 = Placebo MAP (FA); B8 = A/Sing MAP, 15 ug (UA); B9 = A/Sing IM, 15 ug, as 2018 Afluria Quad (UA)

Listing 16.2.7.3
Prior and Concomitant Medications - CRF Data Only

Part B

Random-isation Number	No.	Medication Trade/Generic Name	Indication	AE Recorded?	Total Dose	Dose Form	Freq-uency	Route	Start Date	Start Time	End Date/ Ongoing	End Time
B1076		[No prior or concomitant medications recorded]										
B1080	1	Diclofenac sandoz	Right shoulder sprain	Yes	50mg	TABLET	ONCE ONLY	ORAL	17-JUN-2018	15:46	17-JUN-2018	15:46
B1080	2	Voltaren	Shoulder sprain	Yes	100mg	TABLET	2 TIMES A DAY	ORAL	02-JUL-2018		04-JUL-2018	

Key: AE = Adverse Event; NA = Not Applicable; UN, UNK = Unknown

Administration Site Key: FA = Forearm; UA= Upper arm
Treatments Part A: A1 = A/Sing MAP, 15 ug (FA); A2 = Afluria Quad IM (UA), 15 ug; A3 = Placebo MAP (FA); A4 = A/Sing Mono IM, 15 ug (UA)
Treatments Part B: B1 = A/Sing MAP, 15 ug (FA) with biopsy; B2 = Placebo MAP (FA) with biopsy
B3 = A/Sing MAP, 15 ug (FA); B4 = A/Sing MAP, 10 ug (FA); B5 = A/Sing MAP, 5 ug (FA); B6 = A/Sing MAP, 2.5 ug (FA)
B7 = Placebo MAP (FA); B8 = A/Sing MAP, 15 ug (UA); B9 = A/Sing IM, 15 ug, as 2018 Afluria Quad (UA)
