# Supplementary material for: Safety, tolerability, and immunogenicity of influenza vaccination with a high-density microarray patch: Results from a randomized, controlled phase I clinical trial
Source: PLoS Med. 2020 Mar 17;17(3):e1003024. doi: 10.1371/journal.pmed.1003024 (PMC7077342; doi:10.1371/journal.pmed.1003024)
Supplement: S1 Data — Data 1: Informed Consent; Data 2: Analysis Sets; Data 3: Demographics; Data 4: Study Drug Administration; Data 5: Skin Hardness Assessment; Data 6: Immunogenicity (ELISA IgG); Data 7: Immunogenicity (ADCC); Data 8: Immunogenicity (MBCs); Data 9: Immunogenicity (CMI); Data 10: Immunogenicity (Mucosal IgA); Data 11: Immunogenicity Results (HAI, MN); Data 12: TEAE–CRF Data only; Data 13: TEAEs–MedDRA Coding; Data 15: Treatment Site Tolerability Assessment; Data 16: Numeric Pain Intensity; Data 17: SII; Data 18: Application Site Status at End of Study; Data 19: Individual Treatment Site Tolerability Assessment; Data 20: Numeric Pain Intensity by Treatment; Data 21: Individual SII by Treatment. ADCC, antibody-dependent cellular cytotoxicity; CMI, cell-mediated immunity; CRF, case report form; ELISA, enzyme-linked immunosorbent assay; HAI, haemagglutination inhibition; IgA, immunoglobulin A; IgG, immunoglobulin G; MBC, memory B cell; MedDRA, medical dictionary for regulatory activities; MN, microneutralisation; SII, Skin Irritation Index; TEA, treatment emergent adverse event. (ZIP) [file pmed.1003024.s008.zip › S1_Data/clinical supporting/S1_Data15 Treatment Site Tolerability Assessment.pdf]

Listing 16.2.9.2
Treatment Site Tolerability Assessment per Site

Part A
Randomisation Number = A0001, Treatment Group = Group A3

Date	Time	Visit	Time Point	Site Number	Treatment for Site	Indura-tion	Redness	Bruising	Itching	Skin Flaking	Tender-ness	Definition/ Visibility	Other#	Unscheduled Reason
09-MAR-2018	11:10	Day 1	Pre-dose	F1	uncoated	0	0	0	0	0	0	0		
09-MAR-2018	11:10	Day 1	Pre-dose	F2	uncoated	0	0	0	0	0	0	0		
09-MAR-2018	11:10	Day 1	Pre-dose	F3	uncoated	0	0	0	0	0	0	0		
09-MAR-2018	12:19	Day 1	10 min	F1	uncoated	0	0	0	0	0	0	0		
09-MAR-2018	12:19	Day 1	10 min	F2	uncoated	0	0	0	0	0	0	0		
09-MAR-2018	12:19	Day 1	10 min	F3	uncoated	0	0	0	0	0	0	0		
09-MAR-2018	13:20	Day 1	1 hour	F1	uncoated	0	0	0	0	0	0	0		
09-MAR-2018	13:20	Day 1	1 hour	F2	uncoated	0	0	0	0	0	0	0		
09-MAR-2018	13:20	Day 1	1 hour	F3	uncoated	0	0	0	0	0	0	0		
09-MAR-2018	14:20	Day 1	2 hour	F1	uncoated	0	0	0	0	0	0	0		
09-MAR-2018	14:20	Day 1	2 hour	F2	uncoated	0	0	0	0	0	0	0		
09-MAR-2018	14:20	Day 1	2 hour	F3	uncoated	0	0	0	0	0	0	0		
12-MAR-2018	11:49	Day 4		F1	uncoated	0	0	0	0	0	0	0		
12-MAR-2018	11:49	Day 4		F2	uncoated	0	0	0	0	0	0	0		
12-MAR-2018	11:49	Day 4		F3	uncoated	0	0	0	0	0	0	0		
16-MAR-2018	11:47	Day 8		F1	uncoated	0	0	0	0	0	0	3		
16-MAR-2018	11:47	Day 8		F2	uncoated	0	0	0	0	0	0	3		
16-MAR-2018	11:47	Day 8		F3	uncoated	0	0	0	0	0	0	3		
29-MAR-2018	12:09	Day 22		F1	uncoated	0	0	0	0	0	0	1		
29-MAR-2018	12:09	Day 22		F2	uncoated	0	0	0	0	0	0	1		

Key: 0= None; 1=Mild; 2=Moderate; 3=Severe; # Other = Pinprick bleeding or seepage

Administration Site Key: FA = Forearm; UA= Upper arm
Treatments Part A: A1 = A/Sing MAP, 15 ug (FA); A2 = Afluria Quad IM (UA), 15 ug; A3 = Placebo MAP (FA); A4 = A/Sing Mono IM, 15 ug (UA)
Treatments Part B: B1 = A/Sing MAP, 15 ug (FA) with biopsy; B2 = Placebo MAP (FA) with biopsy
B3 = A/Sing MAP, 15 ug (FA); B4 = A/Sing MAP, 10 ug (FA); B5 = A/Sing MAP, 5 ug (FA); B6 = A/Sing MAP, 2.5 ug (FA)
B7 = Placebo MAP (FA); B8 = A/Sing MAP, 15 ug (UA); B9 = A/Sing IM, 15 ug, as 2018 Afluria Quad (UA)

Listing 16.2.9.2
Treatment Site Tolerability Assessment per Site

Part A
Randomisation Number = A0001, Treatment Group = Group A3

Date	Time	Visit	Time Point	Site Number	Treatment for Site	Indura-tion	Redness	Bruising	Itching	Skin Flaking	Tender-ness	Definition/ Visibility	Other#	Unscheduled Reason
29-MAR-2018	12:09	Day 22		F3	uncoated	0	0	0	0	0	0	1		
06-MAY-2018	11:50	EOS/ET (Day 61)		F1	uncoated	0	0	0	0	0	0	0		
06-MAY-2018	11:50	EOS/ET (Day 61)		F2	uncoated	0	0	0	0	0	0	0		
06-MAY-2018	11:50	EOS/ET (Day 61)		F3	uncoated	0	0	0	0	0	0	0		

Key: 0= None; 1=Mild; 2=Moderate; 3=Severe; # Other = Pinprick bleeding or seepage

Administration Site Key: FA = Forearm; UA= Upper arm
Treatments Part A: A1 = A/Sing MAP, 15 ug (FA); A2 = Afluria Quad IM (UA), 15 ug; A3 = Placebo MAP (FA); A4 = A/Sing Mono IM, 15 ug (UA)
Treatments Part B: B1 = A/Sing MAP, 15 ug (FA) with biopsy; B2 = Placebo MAP (FA) with biopsy
B3 = A/Sing MAP, 15 ug (FA); B4 = A/Sing MAP, 10 ug (FA); B5 = A/Sing MAP, 5 ug (FA); B6 = A/Sing MAP, 2.5 ug (FA)
B7 = Placebo MAP (FA); B8 = A/Sing MAP, 15 ug (UA); B9 = A/Sing IM, 15 ug, as 2018 Afluria Quad (UA)

Listing 16.2.9.2
Treatment Site Tolerability Assessment per Site

Part A
Randomisation Number = A0002, Treatment Group = Group A1

Date	Time	Visit	Time Point	Site Number	Treatment for Site	Indura-tion	Redness	Bruising	Itching	Skin Flaking	Tender-ness	Definition/ Visibility	Other#	Unscheduled Reason
09-MAR-2018	11:17	Day 1	Pre-dose	F1	5 ug	0	0	0	0	0	0	0		
09-MAR-2018	11:17	Day 1	Pre-dose	F2	5 ug	0	0	0	0	0	0	0		
09-MAR-2018	11:17	Day 1	Pre-dose	F3	5 ug	0	0	0	0	0	0	0		
09-MAR-2018	12:29	Day 1	10 min	F1	5 ug	0	1	0	0	0	0	0		
09-MAR-2018	12:29	Day 1	10 min	F2	5 ug	0	1	0	0	0	0	0		
09-MAR-2018	12:29	Day 1	10 min	F3	5 ug	0	1	0	0	0	0	0		
09-MAR-2018	13:28	Day 1	1 hour	F1	5 ug	1	1	0	0	0	0	0		
09-MAR-2018	13:28	Day 1	1 hour	F2	5 ug	1	1	0	0	0	0	0		
09-MAR-2018	13:28	Day 1	1 hour	F3	5 ug	1	1	0	0	0	0	0		
09-MAR-2018	14:29	Day 1	2 hour	F1	5 ug	0	0	0	0	0	0	0		
09-MAR-2018	14:29	Day 1	2 hour	F2	5 ug	0	0	0	0	0	0	0		
09-MAR-2018	14:29	Day 1	2 hour	F3	5 ug	0	0	0	0	0	0	0		
12-MAR-2018	08:51	Day 4		F1	5 ug	0	0	0	1	0	1	0		
12-MAR-2018	08:51	Day 4		F2	5 ug	0	0	0	1	0	1	0		
12-MAR-2018	08:51	Day 4		F3	5 ug	0	0	0	1	0	1	0		
16-MAR-2018	12:47	Day 8		F1	5 ug	0	0	0	0	2	0	0		
16-MAR-2018	12:47	Day 8		F2	5 ug	0	0	0	0	2	0	0		
16-MAR-2018	12:47	Day 8		F3	5 ug	0	0	0	0	1	0	0		
30-MAR-2018	12:15	Day 22		F1	5 ug	0	0	0	0	0	0	2		
30-MAR-2018	12:15	Day 22		F2	5 ug	0	0	0	0	0	0	2		

Key: 0= None; 1=Mild; 2=Moderate; 3=Severe; # Other = Pinprick bleeding or seepage

Administration Site Key: FA = Forearm; UA= Upper arm
Treatments Part A: A1 = A/Sing MAP, 15 ug (FA); A2 = Afluria Quad IM (UA), 15 ug; A3 = Placebo MAP (FA); A4 = A/Sing Mono IM, 15 ug (UA)
Treatments Part B: B1 = A/Sing MAP, 15 ug (FA) with biopsy; B2 = Placebo MAP (FA) with biopsy
B3 = A/Sing MAP, 15 ug (FA); B4 = A/Sing MAP, 10 ug (FA); B5 = A/Sing MAP, 5 ug (FA); B6 = A/Sing MAP, 2.5 ug (FA)
B7 = Placebo MAP (FA); B8 = A/Sing MAP, 15 ug (UA); B9 = A/Sing IM, 15 ug, as 2018 Afluria Quad (UA)

Listing 16.2.9.2
Treatment Site Tolerability Assessment per Site

Part A
Randomisation Number = A0002, Treatment Group = Group A1

Date	Time	Visit	Time Point	Site Number	Treatment for Site	Indura-tion	Redness	Bruising	Itching	Skin Flaking	Tender-ness	Definition/ Visibility	Other#	Unscheduled Reason
30-MAR-2018	12:15	Day 22		F3	5 ug	0	0	0	0	0	0	3		
08-MAY-2018	12:21	EOS/ET (Day 61)		F1	5 ug	0	0	0	0	0	0	0		
08-MAY-2018	12:21	EOS/ET (Day 61)		F2	5 ug	0	0	0	0	0	0	0		
08-MAY-2018	12:21	EOS/ET (Day 61)		F3	5 ug	0	0	0	0	0	0	0		

Key: 0= None; 1=Mild; 2=Moderate; 3=Severe; # Other = Pinprick bleeding or seepage

Administration Site Key: FA = Forearm; UA= Upper arm
Treatments Part A: A1 = A/Sing MAP, 15 ug (FA); A2 = Afluria Quad IM (UA), 15 ug; A3 = Placebo MAP (FA); A4 = A/Sing Mono IM, 15 ug (UA)
Treatments Part B: B1 = A/Sing MAP, 15 ug (FA) with biopsy; B2 = Placebo MAP (FA) with biopsy
B3 = A/Sing MAP, 15 ug (FA); B4 = A/Sing MAP, 10 ug (FA); B5 = A/Sing MAP, 5 ug (FA); B6 = A/Sing MAP, 2.5 ug (FA)
B7 = Placebo MAP (FA); B8 = A/Sing MAP, 15 ug (UA); B9 = A/Sing IM, 15 ug, as 2018 Afluria Quad (UA)

Listing 16.2.9.2
Treatment Site Tolerability Assessment per Site

Part A
Randomisation Number = A0003, Treatment Group = Group A1

Date	Time	Visit	Time Point	Site Number	Treatment for Site	Indura-tion	Redness	Bruising	Itching	Skin Flaking	Tender-ness	Definition/ Visibility	Other#	Unscheduled Reason
09-MAR-2018	11:15	Day 1	Pre-dose	F1	5 ug	0	0	0	0	0	0	0		
09-MAR-2018	11:15	Day 1	Pre-dose	F2	5 ug	0	0	0	0	0	0	0		
09-MAR-2018	11:15	Day 1	Pre-dose	F3	5 ug	0	0	0	0	0	0	0		
09-MAR-2018	12:38	Day 1	10 min	F1	5 ug	0	1	0	0	0	0	0		
09-MAR-2018	12:38	Day 1	10 min	F2	5 ug	0	1	0	0	0	0	0		
09-MAR-2018	12:38	Day 1	10 min	F3	5 ug	1	1	0	0	0	0	0		
09-MAR-2018	13:38	Day 1	1 hour	F1	5 ug	1	1	0	0	0	0	0		
09-MAR-2018	13:38	Day 1	1 hour	F2	5 ug	1	0	0	0	0	0	0		
09-MAR-2018	13:38	Day 1	1 hour	F3	5 ug	1	0	0	0	0	0	0		
09-MAR-2018	14:38	Day 1	2 hour	F1	5 ug	1	0	0	0	0	0	0		
09-MAR-2018	14:38	Day 1	2 hour	F2	5 ug	1	0	0	0	0	0	0		
09-MAR-2018	14:38	Day 1	2 hour	F3	5 ug	1	0	0	0	0	0	0		
12-MAR-2018	09:40	Day 4		F1	5 ug	0	0	0	0	1	0	0		
12-MAR-2018	09:40	Day 4		F2	5 ug	0	0	0	0	0	0	0		
12-MAR-2018	09:40	Day 4		F3	5 ug	0	0	0	0	0	0	0		
16-MAR-2018	10:36	Day 8		F1	5 ug	0	1	0	0	1	0	0		
16-MAR-2018	10:36	Day 8		F2	5 ug	0	0	0	0	1	0	0		
16-MAR-2018	10:36	Day 8		F3	5 ug	0	0	0	0	1	0	0		
30-MAR-2018	12:09	Day 22		F1	5 ug	0	0	0	0	0	0	0		
30-MAR-2018	12:09	Day 22		F2	5 ug	0	0	0	0	0	0	2		

Key: 0= None; 1=Mild; 2=Moderate; 3=Severe; # Other = Pinprick bleeding or seepage

Administration Site Key: FA = Forearm; UA= Upper arm
Treatments Part A: A1 = A/Sing MAP, 15 ug (FA); A2 = Afluria Quad IM (UA), 15 ug; A3 = Placebo MAP (FA); A4 = A/Sing Mono IM, 15 ug (UA)
Treatments Part B: B1 = A/Sing MAP, 15 ug (FA) with biopsy; B2 = Placebo MAP (FA) with biopsy
B3 = A/Sing MAP, 15 ug (FA); B4 = A/Sing MAP, 10 ug (FA); B5 = A/Sing MAP, 5 ug (FA); B6 = A/Sing MAP, 2.5 ug (FA)
B7 = Placebo MAP (FA); B8 = A/Sing MAP, 15 ug (UA); B9 = A/Sing IM, 15 ug, as 2018 Afluria Quad (UA)

Listing 16.2.9.2
Treatment Site Tolerability Assessment per Site

Part A
Randomisation Number = A0003, Treatment Group = Group A1

Date	Time	Visit	Time Point	Site Number	Treatment for Site	Indura-tion	Redness	Bruising	Itching	Skin Flaking	Tender-ness	Definition/ Visibility	Other#	Unscheduled Reason
30-MAR-2018	12:09	Day 22		F3	5 ug	0	0	0	0	0	0	2		
08-MAY-2018	11:05	EOS/ET (Day 61)		F1	5 ug	0	0	0	0	0	0	0		
08-MAY-2018	11:05	EOS/ET (Day 61)		F2	5 ug	0	0	0	0	0	0	0		
08-MAY-2018	11:05	EOS/ET (Day 61)		F3	5 ug	0	0	0	0	0	0	0		

Key: 0= None; 1=Mild; 2=Moderate; 3=Severe; # Other = Pinprick bleeding or seepage

Administration Site Key: FA = Forearm; UA= Upper arm
Treatments Part A: A1 = A/Sing MAP, 15 ug (FA); A2 = Afluria Quad IM (UA), 15 ug; A3 = Placebo MAP (FA); A4 = A/Sing Mono IM, 15 ug (UA)
Treatments Part B: B1 = A/Sing MAP, 15 ug (FA) with biopsy; B2 = Placebo MAP (FA) with biopsy
B3 = A/Sing MAP, 15 ug (FA); B4 = A/Sing MAP, 10 ug (FA); B5 = A/Sing MAP, 5 ug (FA); B6 = A/Sing MAP, 2.5 ug (FA)
B7 = Placebo MAP (FA); B8 = A/Sing MAP, 15 ug (UA); B9 = A/Sing IM, 15 ug, as 2018 Afluria Quad (UA)

Listing 16.2.9.2
Treatment Site Tolerability Assessment per Site

Part A
Randomisation Number = A0004, Treatment Group = Group A3

Date	Time	Visit	Time Point	Site Number	Treatment for Site	Induration	Redness	Bruising	Itching	Skin Flaking	Tenderness	Definition/ Visibility	Other#	Unscheduled Reason
09-MAR-2018	11:29	Day 1	Pre-dose	F1	uncoated	0	0	0	0	0	0	0		
09-MAR-2018	11:29	Day 1	Pre-dose	F2	uncoated	0	0	0	0	0	0	0		
09-MAR-2018	11:29	Day 1	Pre-dose	F3	uncoated	0	0	0	0	0	0	0		
09-MAR-2018	12:48	Day 1	10 min	F1	uncoated	0	0	0	0	0	1	0		
09-MAR-2018	12:48	Day 1	10 min	F2	uncoated	0	1	0	0	0	1	0		
09-MAR-2018	12:48	Day 1	10 min	F3	uncoated	0	0	0	0	0	1	0		
09-MAR-2018	14:00	Day 1	1 hour	F1	uncoated	0	0	0	0	0	0	0		
09-MAR-2018	14:00	Day 1	1 hour	F2	uncoated	0	0	0	0	0	0	0		
09-MAR-2018	14:00	Day 1	1 hour	F3	uncoated	0	0	0	0	0	0	0		
09-MAR-2018	14:48	Day 1	2 hour	F1	uncoated	0	0	0	0	0	0	0		
09-MAR-2018	14:48	Day 1	2 hour	F2	uncoated	0	0	0	0	0	0	0		
09-MAR-2018	14:48	Day 1	2 hour	F3	uncoated	0	0	0	0	0	0	0		
12-MAR-2018	12:51	Day 4		F1	uncoated	0	0	0	0	0	0	0		
12-MAR-2018	12:51	Day 4		F2	uncoated	0	0	0	0	0	0	0		
12-MAR-2018	12:51	Day 4		F3	uncoated	0	0	0	0	0	0	0		
16-MAR-2018	15:47	Day 8		F1	uncoated	0	0	0	0	0	0	3		
16-MAR-2018	15:47	Day 8		F2	uncoated	0	0	0	0	0	0	3		
16-MAR-2018	15:47	Day 8		F3	uncoated	0	0	0	0	0	0	3		
30-MAR-2018	12:58	Day 22		F1	uncoated	0	0	0	0	0	0	0		
30-MAR-2018	12:58	Day 22		F2	uncoated	0	0	0	0	0	0	0		

Key: 0= None; 1=Mild; 2=Moderate; 3=Severe; # Other = Pinprick bleeding or seepage

Administration Site Key: FA = Forearm; UA= Upper arm
Treatments Part A: A1 = A/Sing MAP, 15 ug (FA); A2 = Afluria Quad IM (UA), 15 ug; A3 = Placebo MAP (FA); A4 = A/Sing Mono IM, 15 ug (UA)
Treatments Part B: B1 = A/Sing MAP, 15 ug (FA) with biopsy; B2 = Placebo MAP (FA) with biopsy
B3 = A/Sing MAP, 15 ug (FA); B4 = A/Sing MAP, 10 ug (FA); B5 = A/Sing MAP, 5 ug (FA); B6 = A/Sing MAP, 2.5 ug (FA)
B7 = Placebo MAP (FA); B8 = A/Sing MAP, 15 ug (UA); B9 = A/Sing IM, 15 ug, as 2018 Afluria Quad (UA)

Listing 16.2.9.2
Treatment Site Tolerability Assessment per Site

Part A
Randomisation Number = A0004, Treatment Group = Group A3

Date	Time	Visit	Time Point	Site Number	Treatment for Site	Indura-tion	Redness	Bruising	Itching	Skin Flaking	Tender-ness	Definition/ Visibility	Other#	Unscheduled Reason
30-MAR-2018	12:58	Day 22		F3	uncoated	0	0	0	0	0	0	1		
08-MAY-2018	12:46	EOS/ET (Day 61)		F1	uncoated	0	0	0	0	0	0	0		
08-MAY-2018	12:46	EOS/ET (Day 61)		F2	uncoated	0	0	0	0	0	0	0		
08-MAY-2018	12:46	EOS/ET (Day 61)		F3	uncoated	0	0	0	0	0	0	0		

Key: 0= None; 1=Mild; 2=Moderate; 3=Severe; # Other = Pinprick bleeding or seepage

Administration Site Key: FA = Forearm; UA= Upper arm
Treatments Part A: A1 = A/Sing MAP, 15 ug (FA); A2 = Afluria Quad IM (UA), 15 ug; A3 = Placebo MAP (FA); A4 = A/Sing Mono IM, 15 ug (UA)
Treatments Part B: B1 = A/Sing MAP, 15 ug (FA) with biopsy; B2 = Placebo MAP (FA) with biopsy
B3 = A/Sing MAP, 15 ug (FA); B4 = A/Sing MAP, 10 ug (FA); B5 = A/Sing MAP, 5 ug (FA); B6 = A/Sing MAP, 2.5 ug (FA)
B7 = Placebo MAP (FA); B8 = A/Sing MAP, 15 ug (UA); B9 = A/Sing IM, 15 ug, as 2018 Afluria Quad (UA)

Listing 16.2.9.2
Treatment Site Tolerability Assessment per Site

Part A
Randomisation Number = A0005, Treatment Group = Group A1

Date	Time	Visit	Time Point	Site Number	Treatment for Site	Induration	Redness	Bruising	Itching	Skin Flaking	Tenderness	Definition/ Visibility	Other#	Unscheduled Reason
09-MAR-2018	11:43	Day 1	Pre-dose	F1	5 ug	0	0	0	0	0	0	0		
09-MAR-2018	11:43	Day 1	Pre-dose	F2	5 ug	0	0	0	0	0	0	0		
09-MAR-2018	11:43	Day 1	Pre-dose	F3	5 ug	0	0	0	0	0	0	0		
09-MAR-2018	13:08	Day 1	10 min	F1	5 ug	0	1	0	0	0	0	0		
09-MAR-2018	13:08	Day 1	10 min	F2	5 ug	1	1	0	0	0	0	0		
09-MAR-2018	13:08	Day 1	10 min	F3	5 ug	0	0	0	0	0	0	0		
09-MAR-2018	14:08	Day 1	1 hour	F1	5 ug	1	1	0	0	0	1	3		
09-MAR-2018	14:08	Day 1	1 hour	F2	5 ug	1	1	0	0	0	1	3		
09-MAR-2018	14:08	Day 1	1 hour	F3	5 ug	1	1	0	0	0	1	0		
09-MAR-2018	15:05	Day 1	2 hour	F1	5 ug	1	0	0	0	0	1	0		
09-MAR-2018	15:05	Day 1	2 hour	F2	5 ug	1	0	0	0	0	1	0		
09-MAR-2018	15:05	Day 1	2 hour	F3	5 ug	1	0	0	0	0	1	0		
12-MAR-2018	13:43	Day 4		F1	5 ug	0	0	0	2	1	2	0		
12-MAR-2018	13:43	Day 4		F2	5 ug	0	1	0	2	1	2	0		
12-MAR-2018	13:43	Day 4		F3	5 ug	0	0	0	2	0	2	0		
16-MAR-2018	12:42	Day 8		F1	5 ug	0	0	0	0	2	0	3		
16-MAR-2018	12:42	Day 8		F2	5 ug	0	0	0	0	1	0	0		
16-MAR-2018	12:42	Day 8		F3	5 ug	0	0	0	0	2	0	0		
30-MAR-2018	13:12	Day 22		F1	5 ug	0	0	0	0	1	0	1		
30-MAR-2018	13:12	Day 22		F2	5 ug	0	0	0	0	1	0	1		

Key: 0= None; 1=Mild; 2=Moderate; 3=Severe; # Other = Pinprick bleeding or seepage

Administration Site Key: FA = Forearm; UA= Upper arm
Treatments Part A: A1 = A/Sing MAP, 15 ug (FA); A2 = Afluria Quad IM (UA), 15 ug; A3 = Placebo MAP (FA); A4 = A/Sing Mono IM, 15 ug (UA)
Treatments Part B: B1 = A/Sing MAP, 15 ug (FA) with biopsy; B2 = Placebo MAP (FA) with biopsy
B3 = A/Sing MAP, 15 ug (FA); B4 = A/Sing MAP, 10 ug (FA); B5 = A/Sing MAP, 5 ug (FA); B6 = A/Sing MAP, 2.5 ug (FA)
B7 = Placebo MAP (FA); B8 = A/Sing MAP, 15 ug (UA); B9 = A/Sing IM, 15 ug, as 2018 Afluria Quad (UA)

Listing 16.2.9.2
Treatment Site Tolerability Assessment per Site

Part A
Randomisation Number = A0005, Treatment Group = Group A1

Date	Time	Visit	Time Point	Site Number	Treatment for Site	Indura-tion	Redness	Bruising	Itching	Skin Flaking	Tender-ness	Definition/ Visibility	Other#	Unscheduled Reason
30-MAR-2018	13:12	Day 22		F3	5 ug	0	0	0	0	1	0	2		
08-MAY-2018	13:19	EOS/ET (Day 61)		F1	5 ug	0	0	0	0	0	0	0		
08-MAY-2018	13:19	EOS/ET (Day 61)		F2	5 ug	0	0	0	0	0	0	0		
08-MAY-2018	13:19	EOS/ET (Day 61)		F3	5 ug	0	0	0	0	0	0	0		

Key: 0= None; 1=Mild; 2=Moderate; 3=Severe; # Other = Pinprick bleeding or seepage

Administration Site Key: FA = Forearm; UA= Upper arm
Treatments Part A: A1 = A/Sing MAP, 15 ug (FA); A2 = Afluria Quad IM (UA), 15 ug; A3 = Placebo MAP (FA); A4 = A/Sing Mono IM, 15 ug (UA)
Treatments Part B: B1 = A/Sing MAP, 15 ug (FA) with biopsy; B2 = Placebo MAP (FA) with biopsy
B3 = A/Sing MAP, 15 ug (FA); B4 = A/Sing MAP, 10 ug (FA); B5 = A/Sing MAP, 5 ug (FA); B6 = A/Sing MAP, 2.5 ug (FA)
B7 = Placebo MAP (FA); B8 = A/Sing MAP, 15 ug (UA); B9 = A/Sing IM, 15 ug, as 2018 Afluria Quad (UA)

Listing 16.2.9.2
Treatment Site Tolerability Assessment per Site

Part A
Randomisation Number = A0006, Treatment Group = Group A3

Date	Time	Visit	Time Point	Site Number	Treatment for Site	Indura-tion	Redness	Bruising	Itching	Skin Flaking	Tender-ness	Definition/ Visibility	Other#	Unscheduled Reason
09-MAR-2018	11:30	Day 1	Pre-dose	F1	uncoated	0	0	0	0	0	0	0		
09-MAR-2018	11:30	Day 1	Pre-dose	F2	uncoated	0	0	0	0	0	0	0		
09-MAR-2018	11:30	Day 1	Pre-dose	F3	uncoated	0	0	0	0	0	0	0		
09-MAR-2018	12:58	Day 1	10 min	F1	uncoated	1	2	0	0	0	1	0		
09-MAR-2018	12:58	Day 1	10 min	F2	uncoated	1	1	0	0	0	1	0		
09-MAR-2018	12:58	Day 1	10 min	F3	uncoated	1	1	0	0	0	1	0		
09-MAR-2018	14:01	Day 1	1 hour	F1	uncoated	0	0	0	0	0	0	0		
09-MAR-2018	14:01	Day 1	1 hour	F2	uncoated	0	0	0	0	0	0	0		
09-MAR-2018	14:01	Day 1	1 hour	F3	uncoated	0	0	0	0	0	0	0		
09-MAR-2018	14:58	Day 1	2 hour	F1	uncoated	0	0	0	0	0	0	0		
09-MAR-2018	14:58	Day 1	2 hour	F2	uncoated	0	0	0	0	0	0	0		
09-MAR-2018	14:58	Day 1	2 hour	F3	uncoated	0	0	0	0	0	0	0		
12-MAR-2018	13:12	Day 4		F1	uncoated	0	0	0	0	0	0	0		
12-MAR-2018	13:12	Day 4		F2	uncoated	0	0	0	0	0	0	0		
12-MAR-2018	13:12	Day 4		F3	uncoated	0	0	0	0	0	0	0		
16-MAR-2018	12:24	Day 8		F1	uncoated	0	0	0	0	0	0	2		
16-MAR-2018	12:24	Day 8		F2	uncoated	0	0	0	0	0	0	2		
16-MAR-2018	12:24	Day 8		F3	uncoated	0	0	0	0	0	0	2		
30-MAR-2018	12:42	Day 22		F1	uncoated	0	0	0	0	0	0	1		
30-MAR-2018	12:42	Day 22		F2	uncoated	0	0	0	0	0	0	1		

Key: 0= None; 1=Mild; 2=Moderate; 3=Severe; # Other = Pinprick bleeding or seepage

Administration Site Key: FA = Forearm; UA= Upper arm
Treatments Part A: A1 = A/Sing MAP, 15 ug (FA); A2 = Afluria Quad IM (UA), 15 ug; A3 = Placebo MAP (FA); A4 = A/Sing Mono IM, 15 ug (UA)
Treatments Part B: B1 = A/Sing MAP, 15 ug (FA) with biopsy; B2 = Placebo MAP (FA) with biopsy
B3 = A/Sing MAP, 15 ug (FA); B4 = A/Sing MAP, 10 ug (FA); B5 = A/Sing MAP, 5 ug (FA); B6 = A/Sing MAP, 2.5 ug (FA)
B7 = Placebo MAP (FA); B8 = A/Sing MAP, 15 ug (UA); B9 = A/Sing IM, 15 ug, as 2018 Afluria Quad (UA)

Listing 16.2.9.2
Treatment Site Tolerability Assessment per Site

Part A
Randomisation Number = A0006, Treatment Group = Group A3

Date	Time	Visit	Time Point	Site Number	Treatment for Site	Indura-tion	Redness	Bruising	Itching	Skin Flaking	Tender-ness	Definition/ Visibility	Other#	Unscheduled Reason
30-MAR-2018	12:42	Day 22		F3	uncoated	0	0	0	0	0	0	1		
08-MAY-2018	14:42	EOS/ET (Day 61)		F1	uncoated	0	0	0	0	0	0	1		
08-MAY-2018	14:42	EOS/ET (Day 61)		F2	uncoated	0	0	0	0	0	0	1		
08-MAY-2018	14:42	EOS/ET (Day 61)		F3	uncoated	0	0	0	0	0	0	1		

Key: 0= None; 1=Mild; 2=Moderate; 3=Severe; # Other = Pinprick bleeding or seepage

Administration Site Key: FA = Forearm; UA= Upper arm
Treatments Part A: A1 = A/Sing MAP, 15 ug (FA); A2 = Afluria Quad IM (UA), 15 ug; A3 = Placebo MAP (FA); A4 = A/Sing Mono IM, 15 ug (UA)
Treatments Part B: B1 = A/Sing MAP, 15 ug (FA) with biopsy; B2 = Placebo MAP (FA) with biopsy
B3 = A/Sing MAP, 15 ug (FA); B4 = A/Sing MAP, 10 ug (FA); B5 = A/Sing MAP, 5 ug (FA); B6 = A/Sing MAP, 2.5 ug (FA)
B7 = Placebo MAP (FA); B8 = A/Sing MAP, 15 ug (UA); B9 = A/Sing IM, 15 ug, as 2018 Afluria Quad (UA)

Listing 16.2.9.2
Treatment Site Tolerability Assessment per Site

Part A
Randomisation Number = A0007, Treatment Group = Group A1

Date	Time	Visit	Time Point	Site Number	Treatment for Site	Indura-tion	Redness	Bruising	Itching	Skin Flaking	Tender-ness	Definition/ Visibility	Other#	Unscheduled Reason
09-MAR-2018	11:45	Day 1	Pre-dose	F1	5 ug	0	0	0	0	0	0	0		
09-MAR-2018	11:45	Day 1	Pre-dose	F2	5 ug	0	0	0	0	0	0	0		
09-MAR-2018	11:45	Day 1	Pre-dose	F3	5 ug	0	0	0	0	0	0	0		
09-MAR-2018	13:18	Day 1	10 min	F1	5 ug	1	1	0	0	0	1	0		
09-MAR-2018	13:18	Day 1	10 min	F2	5 ug	0	0	0	0	0	1	0		
09-MAR-2018	13:18	Day 1	10 min	F3	5 ug	0	1	0	0	0	1	0		
09-MAR-2018	14:12	Day 1	1 hour	F1	5 ug	0	1	0	0	0	0	0		
09-MAR-2018	14:12	Day 1	1 hour	F2	5 ug	1	0	0	0	0	0	0		
09-MAR-2018	14:12	Day 1	1 hour	F3	5 ug	1	1	0	0	0	0	0		
09-MAR-2018	15:33	Day 1	2 hour	F1	5 ug	0	0	0	0	0	0	0		
09-MAR-2018	15:33	Day 1	2 hour	F2	5 ug	0	0	0	0	0	0	0		
09-MAR-2018	15:33	Day 1	2 hour	F3	5 ug	0	0	0	0	0	0	0		
12-MAR-2018	11:18	Day 4		F1	5 ug	0	1	0	0	0	0	0		
12-MAR-2018	11:18	Day 4		F2	5 ug	0	1	0	0	0	0	0		
12-MAR-2018	11:18	Day 4		F3	5 ug	0	1	0	0	0	0	0		
16-MAR-2018	14:47	Day 8		F1	5 ug	0	0	0	0	1	0	0		
16-MAR-2018	14:47	Day 8		F2	5 ug	0	0	0	0	1	0	0		
16-MAR-2018	14:47	Day 8		F3	5 ug	0	0	0	0	1	0	0		
29-MAR-2018	10:49	Day 22		F1	5 ug	0	0	0	0	0	0	0		
29-MAR-2018	10:49	Day 22		F2	5 ug	0	0	0	0	0	0	0		

Key: 0= None; 1=Mild; 2=Moderate; 3=Severe; # Other = Pinprick bleeding or seepage

Administration Site Key: FA = Forearm; UA= Upper arm
Treatments Part A: A1 = A/Sing MAP, 15 ug (FA); A2 = Afluria Quad IM (UA), 15 ug; A3 = Placebo MAP (FA); A4 = A/Sing Mono IM, 15 ug (UA)
Treatments Part B: B1 = A/Sing MAP, 15 ug (FA) with biopsy; B2 = Placebo MAP (FA) with biopsy
B3 = A/Sing MAP, 15 ug (FA); B4 = A/Sing MAP, 10 ug (FA); B5 = A/Sing MAP, 5 ug (FA); B6 = A/Sing MAP, 2.5 ug (FA)
B7 = Placebo MAP (FA); B8 = A/Sing MAP, 15 ug (UA); B9 = A/Sing IM, 15 ug, as 2018 Afluria Quad (UA)

Listing 16.2.9.2
Treatment Site Tolerability Assessment per Site

Part A
Randomisation Number = A0007, Treatment Group = Group A1

Date	Time	Visit	Time Point	Site Number	Treatment for Site	Indura-tion	Redness	Bruising	Itching	Skin Flaking	Tender-ness	Definition/ Visibility	Other#	Unscheduled Reason
29-MAR-2018	10:49	Day 22		F3	5 ug	0	0	0	0	0	0	0		
08-MAY-2018	14:08	EOS/ET (Day 61)		F1	5 ug	0	0	0	0	0	0	0		
08-MAY-2018	14:08	EOS/ET (Day 61)		F2	5 ug	0	0	0	0	0	0	0		
08-MAY-2018	14:08	EOS/ET (Day 61)		F3	5 ug	0	0	0	0	0	0	0		

Key: 0= None; 1=Mild; 2=Moderate; 3=Severe; # Other = Pinprick bleeding or seepage

Administration Site Key: FA = Forearm; UA= Upper arm
Treatments Part A: A1 = A/Sing MAP, 15 ug (FA); A2 = Afluria Quad IM (UA), 15 ug; A3 = Placebo MAP (FA); A4 = A/Sing Mono IM, 15 ug (UA)
Treatments Part B: B1 = A/Sing MAP, 15 ug (FA) with biopsy; B2 = Placebo MAP (FA) with biopsy
B3 = A/Sing MAP, 15 ug (FA); B4 = A/Sing MAP, 10 ug (FA); B5 = A/Sing MAP, 5 ug (FA); B6 = A/Sing MAP, 2.5 ug (FA)
B7 = Placebo MAP (FA); B8 = A/Sing MAP, 15 ug (UA); B9 = A/Sing IM, 15 ug, as 2018 Afluria Quad (UA)

Listing 16.2.9.2
Treatment Site Tolerability Assessment per Site

Part A
Randomisation Number = A0008, Treatment Group = Group A3

Date	Time	Visit	Time Point	Site Number	Treatment for Site	Indura-tion	Redness	Bruising	Itching	Skin Flaking	Tender-ness	Definition/ Visibility	Other#	Unscheduled Reason
09-MAR-2018	12:11	Day 1	Pre-dose	F1	uncoated	0	0	0	0	0	0	0		
09-MAR-2018	12:11	Day 1	Pre-dose	F2	uncoated	0	0	0	0	0	0	0		
09-MAR-2018	12:11	Day 1	Pre-dose	F3	uncoated	0	0	0	0	0	0	0		
09-MAR-2018	13:38	Day 1	10 min	F1	uncoated	0	1	0	1	0	1	0		
09-MAR-2018	13:38	Day 1	10 min	F2	uncoated	1	1	0	0	0	1	0		
09-MAR-2018	13:38	Day 1	10 min	F3	uncoated	1	0	0	1	0	1	0		
09-MAR-2018	14:28	Day 1	1 hour	F1	uncoated	0	1	0	0	0	0	0		
09-MAR-2018	14:28	Day 1	1 hour	F2	uncoated	1	1	0	0	0	0	0		
09-MAR-2018	14:28	Day 1	1 hour	F3	uncoated	1	1	0	0	0	0	0		
09-MAR-2018	15:53	Day 1	2 hour	F1	uncoated	0	0	0	0	0	0	0		
09-MAR-2018	15:53	Day 1	2 hour	F2	uncoated	0	0	0	0	0	0	0		
09-MAR-2018	15:53	Day 1	2 hour	F3	uncoated	0	0	0	0	0	0	0		
12-MAR-2018	16:23	Day 4		F1	uncoated	0	0	0	0	0	0	3		
12-MAR-2018	16:23	Day 4		F2	uncoated	0	0	0	0	0	0	3		
12-MAR-2018	16:23	Day 4		F3	uncoated	0	0	0	0	0	0	3		
16-MAR-2018	08:34	Day 8		F1	uncoated	0	0	0	0	1	0	2		
16-MAR-2018	08:34	Day 8		F2	uncoated	0	0	0	0	0	0	1		
16-MAR-2018	08:34	Day 8		F3	uncoated	0	0	0	0	0	0	1		
30-MAR-2018	09:23	Day 22		F1	uncoated	0	0	0	0	0	0	0		
30-MAR-2018	09:23	Day 22		F2	uncoated	0	0	0	0	0	0	0		

Key: 0= None; 1=Mild; 2=Moderate; 3=Severe; # Other = Pinprick bleeding or seepage

Administration Site Key: FA = Forearm; UA= Upper arm
Treatments Part A: A1 = A/Sing MAP, 15 ug (FA); A2 = Afluria Quad IM (UA), 15 ug; A3 = Placebo MAP (FA); A4 = A/Sing Mono IM, 15 ug (UA)
Treatments Part B: B1 = A/Sing MAP, 15 ug (FA) with biopsy; B2 = Placebo MAP (FA) with biopsy
B3 = A/Sing MAP, 15 ug (FA); B4 = A/Sing MAP, 10 ug (FA); B5 = A/Sing MAP, 5 ug (FA); B6 = A/Sing MAP, 2.5 ug (FA)
B7 = Placebo MAP (FA); B8 = A/Sing MAP, 15 ug (UA); B9 = A/Sing IM, 15 ug, as 2018 Afluria Quad (UA)

Listing 16.2.9.2
Treatment Site Tolerability Assessment per Site

Part A
Randomisation Number = A0008, Treatment Group = Group A3

Date	Time	Visit	Time Point	Site Number	Treatment for Site	Indura- tion	Redness	Bruising	Itching	Skin Flaking	Tender- ness	Definition/ Visibility	Other#	Unscheduled Reason
30-MAR-2018	09:23	Day 22		F3	uncoated	0	0	0	0	0	0	0		
08-MAY-2018	08:49	EOS/ET (Day 61)		F1	uncoated	0	0	0	0	0	0	0		
08-MAY-2018	08:49	EOS/ET (Day 61)		F2	uncoated	0	0	0	0	0	0	0		
08-MAY-2018	08:49	EOS/ET (Day 61)		F3	uncoated	0	0	0	0	0	0	0		

Key: 0= None; 1=Mild; 2=Moderate; 3=Severe; # Other = Pinprick bleeding or seepage

Administration Site Key: FA = Forearm; UA= Upper arm
Treatments Part A: A1 = A/Sing MAP, 15 ug (FA); A2 = Afluria Quad IM (UA), 15 ug; A3 = Placebo MAP (FA); A4 = A/Sing Mono IM, 15 ug (UA)
Treatments Part B: B1 = A/Sing MAP, 15 ug (FA) with biopsy; B2 = Placebo MAP (FA) with biopsy
B3 = A/Sing MAP, 15 ug (FA); B4 = A/Sing MAP, 10 ug (FA); B5 = A/Sing MAP, 5 ug (FA); B6 = A/Sing MAP, 2.5 ug (FA)
B7 = Placebo MAP (FA); B8 = A/Sing MAP, 15 ug (UA); B9 = A/Sing IM, 15 ug, as 2018 Afluria Quad (UA)

Listing 16.2.9.2
Treatment Site Tolerability Assessment per Site

Part A
Randomisation Number = A0009, Treatment Group = Group A1

Date	Time	Visit	Time Point	Site Number	Treatment for Site	Indura-tion	Redness	Bruising	Itching	Skin Flaking	Tender-ness	Definition/ Visibility	Other#	Unscheduled Reason
09-MAR-2018	12:32	Day 1	Pre-dose	F1	5 ug	0	0	0	0	0	0	0		
09-MAR-2018	12:32	Day 1	Pre-dose	F2	5 ug	0	0	0	0	0	0	0		
09-MAR-2018	12:32	Day 1	Pre-dose	F3	5 ug	0	0	0	0	0	0	0		
09-MAR-2018	13:28	Day 1	10 min	F1	5 ug	1	1	0	0	0	1	0		
09-MAR-2018	13:28	Day 1	10 min	F2	5 ug	1	1	0	2	0	1	0		
09-MAR-2018	13:28	Day 1	10 min	F3	5 ug	1	1	0	0	0	1	0		
09-MAR-2018	14:18	Day 1	1 hour	F1	5 ug	1	1	0	1	0	0	0		
09-MAR-2018	14:18	Day 1	1 hour	F2	5 ug	1	1	0	1	0	0	0		
09-MAR-2018	14:18	Day 1	1 hour	F3	5 ug	1	1	0	1	0	0	0		
09-MAR-2018	15:52	Day 1	2 hour	F1	5 ug	0	0	0	0	0	0	0		
09-MAR-2018	15:52	Day 1	2 hour	F2	5 ug	0	0	0	0	0	0	0		
09-MAR-2018	15:52	Day 1	2 hour	F3	5 ug	0	0	0	0	0	0	0		
12-MAR-2018	11:39	Day 4		F1	5 ug	0	0	0	0	0	0	0		
12-MAR-2018	11:39	Day 4		F2	5 ug	0	0	0	0	0	0	0		
12-MAR-2018	11:39	Day 4		F3	5 ug	0	0	0	0	0	0	0		
16-MAR-2018	13:30	Day 8		F1	5 ug	0	0	0	0	1	0	3		
16-MAR-2018	13:30	Day 8		F2	5 ug	0	0	0	0	0	0	3		
16-MAR-2018	13:30	Day 8		F3	5 ug	0	0	0	1	0	0	3		
30-MAR-2018	11:03	Day 22		F1	5 ug	0	1	0	0	0	0	1		
30-MAR-2018	11:03	Day 22		F2	5 ug	0	1	0	0	0	0	1		

Key: 0= None; 1=Mild; 2=Moderate; 3=Severe; # Other = Pinprick bleeding or seepage

Administration Site Key: FA = Forearm; UA= Upper arm
Treatments Part A: A1 = A/Sing MAP, 15 ug (FA); A2 = Afluria Quad IM (UA), 15 ug; A3 = Placebo MAP (FA); A4 = A/Sing Mono IM, 15 ug (UA)
Treatments Part B: B1 = A/Sing MAP, 15 ug (FA) with biopsy; B2 = Placebo MAP (FA) with biopsy
B3 = A/Sing MAP, 15 ug (FA); B4 = A/Sing MAP, 10 ug (FA); B5 = A/Sing MAP, 5 ug (FA); B6 = A/Sing MAP, 2.5 ug (FA)
B7 = Placebo MAP (FA); B8 = A/Sing MAP, 15 ug (UA); B9 = A/Sing IM, 15 ug, as 2018 Afluria Quad (UA)

Listing 16.2.9.2
Treatment Site Tolerability Assessment per Site

Part A
Randomisation Number = A0009, Treatment Group = Group A1

Date	Time	Visit	Time Point	Site Number	Treatment for Site	Indura-tion	Redness	Bruising	Itching	Skin Flaking	Tender-ness	Definition/ Visibility	Other#	Unscheduled Reason
30-MAR-2018	11:03	Day 22		F3	5 ug	0	1	0	0	0	0	1		
01-MAY-2018	14:51	EOS/ET (Day 61)		F1	5 ug	0	0	0	0	0	0	1		
01-MAY-2018	14:51	EOS/ET (Day 61)		F2	5 ug	0	0	0	0	0	0	2		
01-MAY-2018	14:51	EOS/ET (Day 61)		F3	5 ug	0	0	0	0	0	0	2		

Key: 0= None; 1=Mild; 2=Moderate; 3=Severe; # Other = Pinprick bleeding or seepage

Administration Site Key: FA = Forearm; UA= Upper arm
Treatments Part A: A1 = A/Sing MAP, 15 ug (FA); A2 = Afluria Quad IM (UA), 15 ug; A3 = Placebo MAP (FA); A4 = A/Sing Mono IM, 15 ug (UA)
Treatments Part B: B1 = A/Sing MAP, 15 ug (FA) with biopsy; B2 = Placebo MAP (FA) with biopsy
B3 = A/Sing MAP, 15 ug (FA); B4 = A/Sing MAP, 10 ug (FA); B5 = A/Sing MAP, 5 ug (FA); B6 = A/Sing MAP, 2.5 ug (FA)
B7 = Placebo MAP (FA); B8 = A/Sing MAP, 15 ug (UA); B9 = A/Sing IM, 15 ug, as 2018 Afluria Quad (UA)

Listing 16.2.9.2
Treatment Site Tolerability Assessment per Site

Part A
Randomisation Number = A0010,   Treatment Group = Group A3

Date	Time	Visit	Time Point	Site Number	Treatment for Site	Indura-tion	Redness	Bruising	Itching	Skin Flaking	Tender-ness	Definition/ Visibility	Other#	Unscheduled Reason
09-MAR-2018	12:51	Day 1	Pre-dose	F1	uncoated	0	0	0	0	0	0	0		
09-MAR-2018	12:51	Day 1	Pre-dose	F2	uncoated	0	0	0	0	0	0	0		
09-MAR-2018	12:51	Day 1	Pre-dose	F3	uncoated	0	0	0	0	0	0	0		
09-MAR-2018	13:48	Day 1	10 min	F1	uncoated	1	1	0	0	0	2	0		
09-MAR-2018	13:48	Day 1	10 min	F2	uncoated	1	0	0	0	0	2	0		
09-MAR-2018	13:48	Day 1	10 min	F3	uncoated	1	1	0	0	0	2	0		
09-MAR-2018	14:44	Day 1	1 hour	F1	uncoated	0	0	0	0	0	0	0		
09-MAR-2018	14:44	Day 1	1 hour	F2	uncoated	0	0	0	0	0	0	0		
09-MAR-2018	14:44	Day 1	1 hour	F3	uncoated	0	0	0	0	0	0	0		
09-MAR-2018	15:55	Day 1	2 hour	F1	uncoated	0	0	0	0	0	0	0		
09-MAR-2018	15:55	Day 1	2 hour	F2	uncoated	0	0	0	0	0	0	0		
09-MAR-2018	15:55	Day 1	2 hour	F3	uncoated	0	0	0	0	0	0	0		
12-MAR-2018	13:50	Day 4		F1	uncoated	0	0	0	0	0	0	0		
12-MAR-2018	13:50	Day 4		F2	uncoated	0	0	0	0	0	0	0		
12-MAR-2018	13:50	Day 4		F3	uncoated	0	0	0	0	0	0	0		
16-MAR-2018	15:11	Day 8		F1	uncoated	0	0	0	0	0	0	0		
16-MAR-2018	15:11	Day 8		F2	uncoated	0	0	0	0	0	0	3		
16-MAR-2018	15:11	Day 8		F3	uncoated	0	0	0	0	0	0	3		
30-MAR-2018	14:57	Day 22		F1	uncoated	0	0	0	0	0	0	2		
30-MAR-2018	14:57	Day 22		F2	uncoated	0	0	0	0	0	0	2		

Key: 0= None; 1=Mild; 2=Moderate; 3=Severe; # Other = Pinprick bleeding or seepage

Administration Site Key: FA = Forearm; UA= Upper arm
Treatments Part A: A1 = A/Sing MAP, 15 ug (FA); A2 = Afluria Quad IM (UA), 15 ug; A3 = Placebo MAP (FA); A4 = A/Sing Mono IM, 15 ug (UA)
Treatments Part B: B1 = A/Sing MAP, 15 ug (FA) with biopsy; B2 = Placebo MAP (FA) with biopsy
B3 = A/Sing MAP, 15 ug (FA); B4 = A/Sing MAP, 10 ug (FA); B5 = A/Sing MAP, 5 ug (FA); B6 = A/Sing MAP, 2.5 ug (FA)
B7 = Placebo MAP (FA); B8 = A/Sing MAP, 15 ug (UA); B9 = A/Sing IM, 15 ug, as 2018 Afluria Quad (UA)

Listing 16.2.9.2
Treatment Site Tolerability Assessment per Site

Part A
Randomisation Number = A0010, Treatment Group = Group A3

Date	Time	Visit	Time Point	Site Number	Treatment for Site	Indura-tion	Redness	Bruising	Itching	Skin Flaking	Tender-ness	Definition/ Visibility	Other#	Unscheduled Reason
30-MAR-2018	14:57	Day 22		F3	uncoated	0	0	0	0	0	0	2		
08-MAY-2018	12:41	EOS/ET (Day 61)		F1	uncoated	0	0	0	0	0	0	0		
08-MAY-2018	12:41	EOS/ET (Day 61)		F2	uncoated	0	0	0	0	0	0	0		
08-MAY-2018	12:41	EOS/ET (Day 61)		F3	uncoated	0	0	0	0	0	0	0		

Key: 0= None; 1=Mild; 2=Moderate; 3=Severe; # Other = Pinprick bleeding or seepage

Administration Site Key: FA = Forearm; UA= Upper arm
Treatments Part A: A1 = A/Sing MAP, 15 ug (FA); A2 = Afluria Quad IM (UA), 15 ug; A3 = Placebo MAP (FA); A4 = A/Sing Mono IM, 15 ug (UA)
Treatments Part B: B1 = A/Sing MAP, 15 ug (FA) with biopsy; B2 = Placebo MAP (FA) with biopsy
B3 = A/Sing MAP, 15 ug (FA); B4 = A/Sing MAP, 10 ug (FA); B5 = A/Sing MAP, 5 ug (FA); B6 = A/Sing MAP, 2.5 ug (FA)
B7 = Placebo MAP (FA); B8 = A/Sing MAP, 15 ug (UA); B9 = A/Sing IM, 15 ug, as 2018 Afluria Quad (UA)

Listing 16.2.9.2
Treatment Site Tolerability Assessment per Site

Part A
Randomisation Number = A0011, Treatment Group = Group A3

Date	Time	Visit	Time Point	Site Number	Treatment for Site	Induration	Redness	Bruising	Itching	Skin Flaking	Tenderness	Definition/ Visibility	Other#	Unscheduled Reason
09-MAR-2018	13:12	Day 1	Pre-dose	F1	uncoated	0	0	0	0	0	0	0		
09-MAR-2018	13:12	Day 1	Pre-dose	F2	uncoated	0	0	0	0	0	0	0		
09-MAR-2018	13:12	Day 1	Pre-dose	F3	uncoated	0	0	0	0	0	0	0		
09-MAR-2018	13:58	Day 1	10 min	F1	uncoated	0	0	0	0	0	2	0		
09-MAR-2018	13:58	Day 1	10 min	F2	uncoated	0	0	0	0	0	2	0		
09-MAR-2018	13:58	Day 1	10 min	F3	uncoated	0	0	0	0	0	2	0		
09-MAR-2018	15:00	Day 1	1 hour	F1	uncoated	0	0	0	0	0	1	0		
09-MAR-2018	15:00	Day 1	1 hour	F2	uncoated	0	0	0	0	0	1	0		
09-MAR-2018	15:00	Day 1	1 hour	F3	uncoated	0	0	0	0	0	1	0		
09-MAR-2018	15:58	Day 1	2 hour	F1	uncoated	0	0	0	0	0	0	0		
09-MAR-2018	15:58	Day 1	2 hour	F2	uncoated	0	0	0	0	0	0	0		
09-MAR-2018	15:58	Day 1	2 hour	F3	uncoated	0	0	0	0	0	0	0		
12-MAR-2018	14:25	Day 4		F1	uncoated	0	0	0	0	0	0	0		
12-MAR-2018	14:25	Day 4		F2	uncoated	0	0	0	0	0	0	0		
12-MAR-2018	14:25	Day 4		F3	uncoated	0	0	0	0	0	0	0		
16-MAR-2018	14:08	Day 8		F1	uncoated	0	0	0	0	0	0	2		
16-MAR-2018	14:08	Day 8		F2	uncoated	0	0	0	0	0	0	2		
16-MAR-2018	14:08	Day 8		F3	uncoated	0	0	0	0	0	0	2		
30-MAR-2018	14:15	Day 22		F1	uncoated	0	0	0	0	0	0	0		
30-MAR-2018	14:15	Day 22		F2	uncoated	0	0	0	0	0	0	0		

Key: 0= None; 1=Mild; 2=Moderate; 3=Severe; # Other = Pinprick bleeding or seepage

Administration Site Key: FA = Forearm; UA= Upper arm
Treatments Part A: A1 = A/Sing MAP, 15 ug (FA); A2 = Afluria Quad IM (UA), 15 ug; A3 = Placebo MAP (FA); A4 = A/Sing Mono IM, 15 ug (UA)
Treatments Part B: B1 = A/Sing MAP, 15 ug (FA) with biopsy; B2 = Placebo MAP (FA) with biopsy
B3 = A/Sing MAP, 15 ug (FA); B4 = A/Sing MAP, 10 ug (FA); B5 = A/Sing MAP, 5 ug (FA); B6 = A/Sing MAP, 2.5 ug (FA)
B7 = Placebo MAP (FA); B8 = A/Sing MAP, 15 ug (UA); B9 = A/Sing IM, 15 ug, as 2018 Afluria Quad (UA)

Listing 16.2.9.2
Treatment Site Tolerability Assessment per Site

Part A
Randomisation Number = A0011, Treatment Group = Group A3

Date	Time	Visit	Time Point	Site Number	Treatment for Site	Indura-tion	Redness	Bruising	Itching	Skin Flaking	Tender-ness	Definition/ Visibility	Other#	Unscheduled Reason
30-MAR-2018	14:15	Day 22		F3	uncoated	0	0	0	0	0	0	0		
09-MAY-2018	16:48	EOS/ET (Day 61)		F1	uncoated	0	0	0	0	0	0	0		
09-MAY-2018	16:48	EOS/ET (Day 61)		F2	uncoated	0	0	0	0	0	0	0		
09-MAY-2018	16:48	EOS/ET (Day 61)		F3	uncoated	0	0	0	0	0	0	0		

Key: 0= None; 1=Mild; 2=Moderate; 3=Severe; # Other = Pinprick bleeding or seepage

Administration Site Key: FA = Forearm; UA= Upper arm
Treatments Part A: A1 = A/Sing MAP, 15 ug (FA); A2 = Afluria Quad IM (UA), 15 ug; A3 = Placebo MAP (FA); A4 = A/Sing Mono IM, 15 ug (UA)
Treatments Part B: B1 = A/Sing MAP, 15 ug (FA) with biopsy; B2 = Placebo MAP (FA) with biopsy
B3 = A/Sing MAP, 15 ug (FA); B4 = A/Sing MAP, 10 ug (FA); B5 = A/Sing MAP, 5 ug (FA); B6 = A/Sing MAP, 2.5 ug (FA)
B7 = Placebo MAP (FA); B8 = A/Sing MAP, 15 ug (UA); B9 = A/Sing IM, 15 ug, as 2018 Afluria Quad (UA)

Listing 16.2.9.2
Treatment Site Tolerability Assessment per Site

Part A
Randomisation Number = A0012, Treatment Group = Group A1

Date	Time	Visit	Time Point	Site Number	Treatment for Site	Induration	Redness	Bruising	Itching	Skin Flaking	Tenderness	Definition/ Visibility	Other#	Unscheduled Reason
09-MAR-2018	13:33	Day 1	Pre-dose	F1	5 ug	0	0	0	0	0	0	0		
09-MAR-2018	13:33	Day 1	Pre-dose	F2	5 ug	0	0	0	0	0	0	0		
09-MAR-2018	13:33	Day 1	Pre-dose	F3	5 ug	0	0	0	0	0	0	0		
09-MAR-2018	14:09	Day 1	10 min	F1	5 ug	1	1	0	1	0	1	0		
09-MAR-2018	14:09	Day 1	10 min	F2	5 ug	1	1	0	1	0	1	0		
09-MAR-2018	14:09	Day 1	10 min	F3	5 ug	1	1	0	1	0	1	0		
09-MAR-2018	15:11	Day 1	1 hour	F1	5 ug	0	1	0	0	0	1	0		
09-MAR-2018	15:11	Day 1	1 hour	F2	5 ug	0	1	0	0	0	1	0		
09-MAR-2018	15:11	Day 1	1 hour	F3	5 ug	0	1	0	0	0	1	0		
09-MAR-2018	16:15	Day 1	2 hour	F1	5 ug	0	0	0	0	0	1	0		
09-MAR-2018	16:15	Day 1	2 hour	F2	5 ug	0	0	0	0	0	1	0		
09-MAR-2018	16:15	Day 1	2 hour	F3	5 ug	0	0	0	0	0	1	0		
12-MAR-2018	13:49	Day 4		F1	5 ug	0	0	0	0	0	0	0		
12-MAR-2018	13:49	Day 4		F2	5 ug	0	0	0	0	0	0	0		
12-MAR-2018	13:49	Day 4		F3	5 ug	0	0	0	0	0	0	0		
16-MAR-2018	15:04	Day 8		F1	5 ug	0	0	0	1	1	0	3		
16-MAR-2018	15:04	Day 8		F2	5 ug	0	0	0	1	1	0	3		
16-MAR-2018	15:04	Day 8		F3	5 ug	0	0	0	1	1	0	3		
30-MAR-2018	13:21	Day 22		F1	5 ug	0	0	0	0	0	0	2		
30-MAR-2018	13:21	Day 22		F2	5 ug	0	0	0	0	0	0	2		

Key: 0= None; 1=Mild; 2=Moderate; 3=Severe; # Other = Pinprick bleeding or seepage

Administration Site Key: FA = Forearm; UA= Upper arm
Treatments Part A: A1 = A/Sing MAP, 15 ug (FA); A2 = Afluria Quad IM (UA), 15 ug; A3 = Placebo MAP (FA); A4 = A/Sing Mono IM, 15 ug (UA)
Treatments Part B: B1 = A/Sing MAP, 15 ug (FA) with biopsy; B2 = Placebo MAP (FA) with biopsy
B3 = A/Sing MAP, 15 ug (FA); B4 = A/Sing MAP, 10 ug (FA); B5 = A/Sing MAP, 5 ug (FA); B6 = A/Sing MAP, 2.5 ug (FA)
B7 = Placebo MAP (FA); B8 = A/Sing MAP, 15 ug (UA); B9 = A/Sing IM, 15 ug, as 2018 Afluria Quad (UA)

Listing 16.2.9.2
Treatment Site Tolerability Assessment per Site

Part A
Randomisation Number = A0012, Treatment Group = Group A1

Date	Time	Visit	Time Point	Site Number	Treatment for Site	Indura-tion	Redness	Bruising	Itching	Skin Flaking	Tender-ness	Definition/ Visibility	Other#	Unscheduled Reason
30-MAR-2018	13:21	Day 22		F3	5 ug	0	0	0	0	0	0	2		
08-MAY-2018	14:06	EOS/ET (Day 61)		F1	5 ug	0	0	0	0	0	0	0		
08-MAY-2018	14:06	EOS/ET (Day 61)		F2	5 ug	0	0	0	0	0	0	0		
08-MAY-2018	14:06	EOS/ET (Day 61)		F3	5 ug	0	0	0	0	0	0	0		

Key: 0= None; 1=Mild; 2=Moderate; 3=Severe; # Other = Pinprick bleeding or seepage

Administration Site Key: FA = Forearm; UA= Upper arm
Treatments Part A: A1 = A/Sing MAP, 15 ug (FA); A2 = Afluria Quad IM (UA), 15 ug; A3 = Placebo MAP (FA); A4 = A/Sing Mono IM, 15 ug (UA)
Treatments Part B: B1 = A/Sing MAP, 15 ug (FA) with biopsy; B2 = Placebo MAP (FA) with biopsy
B3 = A/Sing MAP, 15 ug (FA); B4 = A/Sing MAP, 10 ug (FA); B5 = A/Sing MAP, 5 ug (FA); B6 = A/Sing MAP, 2.5 ug (FA)
B7 = Placebo MAP (FA); B8 = A/Sing MAP, 15 ug (UA); B9 = A/Sing IM, 15 ug, as 2018 Afluria Quad (UA)

Listing 16.2.9.2
Treatment Site Tolerability Assessment per Site

Part A
Randomisation Number = A0013, Treatment Group = Group A1

Date	Time	Visit	Time Point	Site Number	Treatment for Site	Indura-tion	Redness	Bruising	Itching	Skin Flaking	Tender-ness	Definition/ Visibility	Other#	Unscheduled Reason
09-MAR-2018	13:34	Day 1	Pre-dose	F1	5 ug	0	0	0	0	0	0	0		
09-MAR-2018	13:34	Day 1	Pre-dose	F2	5 ug	0	0	0	0	0	0	0		
09-MAR-2018	13:34	Day 1	Pre-dose	F3	5 ug	0	0	0	0	0	0	0		
09-MAR-2018	14:18	Day 1	10 min	F1	5 ug	1	1	0	1	0	2	0		
09-MAR-2018	14:18	Day 1	10 min	F2	5 ug	1	1	0	1	0	1	0		
09-MAR-2018	14:18	Day 1	10 min	F3	5 ug	1	1	0	1	0	1	0		
09-MAR-2018	15:36	Day 1	1 hour	F1	5 ug	1	0	0	0	0	0	0		
09-MAR-2018	15:36	Day 1	1 hour	F2	5 ug	1	0	0	0	0	0	0		
09-MAR-2018	15:36	Day 1	1 hour	F3	5 ug	1	0	0	0	0	0	0		
09-MAR-2018	16:15	Day 1	2 hour	F1	5 ug	1	0	0	0	0	0	0		
09-MAR-2018	16:15	Day 1	2 hour	F2	5 ug	1	0	0	0	0	0	0		
09-MAR-2018	16:15	Day 1	2 hour	F3	5 ug							0		
12-MAR-2018	13:16	Day 4		F1	5 ug	0	0	0	1	0	1	0		
12-MAR-2018	13:16	Day 4		F2	5 ug	0	0	0	1	0	1	0		
12-MAR-2018	13:16	Day 4		F3	5 ug	0	0	0	1	0	1	0		
16-MAR-2018	15:52	Day 8		F1	5 ug	0	0	0	1	1	0	3		
16-MAR-2018	15:52	Day 8		F2	5 ug	0	0	0	2	2	0	3		
16-MAR-2018	15:52	Day 8		F3	5 ug	0	0	0	1	0	0	3		
30-MAR-2018	14:40	Day 22		F1	5 ug	0	0	0	0	0	0	2		
30-MAR-2018	14:40	Day 22		F2	5 ug	0	0	0	0	0	0	2		

Key: 0= None; 1=Mild; 2=Moderate; 3=Severe; # Other = Pinprick bleeding or seepage

Administration Site Key: FA = Forearm; UA= Upper arm
Treatments Part A: A1 = A/Sing MAP, 15 ug (FA); A2 = Afluria Quad IM (UA), 15 ug; A3 = Placebo MAP (FA); A4 = A/Sing Mono IM, 15 ug (UA)
Treatments Part B: B1 = A/Sing MAP, 15 ug (FA) with biopsy; B2 = Placebo MAP (FA) with biopsy
B3 = A/Sing MAP, 15 ug (FA); B4 = A/Sing MAP, 10 ug (FA); B5 = A/Sing MAP, 5 ug (FA); B6 = A/Sing MAP, 2.5 ug (FA)
B7 = Placebo MAP (FA); B8 = A/Sing MAP, 15 ug (UA); B9 = A/Sing IM, 15 ug, as 2018 Afluria Quad (UA)

Listing 16.2.9.2
Treatment Site Tolerability Assessment per Site

Part A
Randomisation Number = A0013, Treatment Group = Group A1

Date	Time	Visit	Time Point	Site Number	Treatment for Site	Indura-tion	Redness	Bruising	Itching	Skin Flaking	Tender-ness	Definition/ Visibility	Other#	Unscheduled Reason
30-MAR-2018	14:40	Day 22		F3	5 ug	0	0	0	0	0	0	2		
10-MAY-2018	12:26	EOS/ET (Day 61)		F1	5 ug	0	0	0	0	0	0	1		
10-MAY-2018	12:26	EOS/ET (Day 61)		F2	5 ug	0	0	0	0	0	0	1		
10-MAY-2018	12:26	EOS/ET (Day 61)		F3	5 ug	0	0	0	0	0	0	1		

Key: 0= None; 1=Mild; 2=Moderate; 3=Severe; # Other = Pinprick bleeding or seepage

Administration Site Key: FA = Forearm; UA= Upper arm
Treatments Part A: A1 = A/Sing MAP, 15 ug (FA); A2 = Afluria Quad IM (UA), 15 ug; A3 = Placebo MAP (FA); A4 = A/Sing Mono IM, 15 ug (UA)
Treatments Part B: B1 = A/Sing MAP, 15 ug (FA) with biopsy; B2 = Placebo MAP (FA) with biopsy
B3 = A/Sing MAP, 15 ug (FA); B4 = A/Sing MAP, 10 ug (FA); B5 = A/Sing MAP, 5 ug (FA); B6 = A/Sing MAP, 2.5 ug (FA)
B7 = Placebo MAP (FA); B8 = A/Sing MAP, 15 ug (UA); B9 = A/Sing IM, 15 ug, as 2018 Afluria Quad (UA)

Listing 16.2.9.2
Treatment Site Tolerability Assessment per Site

Part A
Randomisation Number = A0014, Treatment Group = Group A3

Date	Time	Visit	Time Point	Site Number	Treatment for Site	Indura-tion	Redness	Bruising	Itching	Skin Flaking	Tender-ness	Definition/ Visibility	Other#	Unscheduled Reason
09-MAR-2018	14:05	Day 1	Pre-dose	F1	uncoated	0	0	0	0	0	0	0		
09-MAR-2018	14:05	Day 1	Pre-dose	F2	uncoated	0	0	0	0	0	0	0		
09-MAR-2018	14:05	Day 1	Pre-dose	F3	uncoated	0	0	0	0	0	0	0		
09-MAR-2018	14:28	Day 1	10 min	F1	uncoated	1	1	0	0	0	2	0		
09-MAR-2018	14:28	Day 1	10 min	F2	uncoated	1	1	0	0	0	2	0		
09-MAR-2018	14:28	Day 1	10 min	F3	uncoated	0	1	0	0	0	2	0		
09-MAR-2018	15:40	Day 1	1 hour	F1	uncoated	0	0	0	0	0	0	0		
09-MAR-2018	15:40	Day 1	1 hour	F2	uncoated	0	0	0	0	0	0	0		
09-MAR-2018	15:40	Day 1	1 hour	F3	uncoated	0	0	0	0	0	0	0		
09-MAR-2018	16:37	Day 1	2 hour	F1	uncoated	0	0	0	0	0	0	0		
09-MAR-2018	16:37	Day 1	2 hour	F2	uncoated	0	0	0	0	0	0	0		
09-MAR-2018	16:37	Day 1	2 hour	F3	uncoated	0	0	0	0	0	0	0		
12-MAR-2018	16:21	Day 4		F1	uncoated	0	0	0	0	0	0	1		
12-MAR-2018	16:21	Day 4		F2	uncoated	0	0	0	0	0	0	1		
12-MAR-2018	16:21	Day 4		F3	uncoated	0	0	0	0	0	0	1		
16-MAR-2018	08:35	Day 8		F1	uncoated	0	0	0	0	0	0	1		
16-MAR-2018	08:35	Day 8		F2	uncoated	0	0	0	0	1	0	2		
16-MAR-2018	08:35	Day 8		F3	uncoated	0	0	0	0	0	0	2		
30-MAR-2018	09:18	Day 22		F1	uncoated	0	0	0	0	0	0	0		
30-MAR-2018	09:18	Day 22		F2	uncoated	0	0	0	0	0	0	0		

Key: 0= None; 1=Mild; 2=Moderate; 3=Severe; # Other = Pinprick bleeding or seepage

Administration Site Key: FA = Forearm; UA= Upper arm
Treatments Part A: A1 = A/Sing MAP, 15 ug (FA); A2 = Afluria Quad IM (UA), 15 ug; A3 = Placebo MAP (FA); A4 = A/Sing Mono IM, 15 ug (UA)
Treatments Part B: B1 = A/Sing MAP, 15 ug (FA) with biopsy; B2 = Placebo MAP (FA) with biopsy
B3 = A/Sing MAP, 15 ug (FA); B4 = A/Sing MAP, 10 ug (FA); B5 = A/Sing MAP, 5 ug (FA); B6 = A/Sing MAP, 2.5 ug (FA)
B7 = Placebo MAP (FA); B8 = A/Sing MAP, 15 ug (UA); B9 = A/Sing IM, 15 ug, as 2018 Afluria Quad (UA)

Listing 16.2.9.2
Treatment Site Tolerability Assessment per Site

Part A
Randomisation Number = A0014, Treatment Group = Group A3

Date	Time	Visit	Time Point	Site Number	Treatment for Site	Indura-tion	Redness	Bruising	Itching	Skin Flaking	Tender-ness	Definition/ Visibility	Other#	Unscheduled Reason
30-MAR-2018	09:18	Day 22		F3	uncoated	0	0	0	0	0	0	0		
08-MAY-2018	09:05	EOS/ET (Day 61)		F1	uncoated	0	0	0	0	0	0	0		
08-MAY-2018	09:05	EOS/ET (Day 61)		F2	uncoated	0	0	0	0	0	0	0		
08-MAY-2018	09:05	EOS/ET (Day 61)		F3	uncoated	0	0	0	0	0	0	0		

Key: 0= None; 1=Mild; 2=Moderate; 3=Severe; # Other = Pinprick bleeding or seepage

Administration Site Key: FA = Forearm; UA= Upper arm
Treatments Part A: A1 = A/Sing MAP, 15 ug (FA); A2 = Afluria Quad IM (UA), 15 ug; A3 = Placebo MAP (FA); A4 = A/Sing Mono IM, 15 ug (UA)
Treatments Part B: B1 = A/Sing MAP, 15 ug (FA) with biopsy; B2 = Placebo MAP (FA) with biopsy
B3 = A/Sing MAP, 15 ug (FA); B4 = A/Sing MAP, 10 ug (FA); B5 = A/Sing MAP, 5 ug (FA); B6 = A/Sing MAP, 2.5 ug (FA)
B7 = Placebo MAP (FA); B8 = A/Sing MAP, 15 ug (UA); B9 = A/Sing IM, 15 ug, as 2018 Afluria Quad (UA)

Listing 16.2.9.2
Treatment Site Tolerability Assessment per Site

Part A
Randomisation Number = A0015, Treatment Group = Group A1

Date	Time	Visit	Time Point	Site Number	Treatment for Site	Indura- tion	Redness	Bruising	Itching	Skin Flaking	Tender- ness	Definition/ Visibility	Other#	Unscheduled Reason
09-MAR-2018	14:13	Day 1	Pre-dose	F1	5 ug	0	0	0	0	0	0	0		
09-MAR-2018	14:13	Day 1	Pre-dose	F2	5 ug	0	0	0	0	0	0	0		
09-MAR-2018	14:13	Day 1	Pre-dose	F3	5 ug	0	0	0	0	0	0	0		
09-MAR-2018	14:38	Day 1	10 min	F1	5 ug	1	1	0	0	0	1	0		
09-MAR-2018	14:38	Day 1	10 min	F2	5 ug	1	1	0	1	0	1	0		
09-MAR-2018	14:38	Day 1	10 min	F3	5 ug	1	1	0	1	0	1	0		
09-MAR-2018	15:43	Day 1	1 hour	F1	5 ug	0	0	0	1	0	0	0		
09-MAR-2018	15:43	Day 1	1 hour	F2	5 ug	0	0	0	1	0	0	0		
09-MAR-2018	15:43	Day 1	1 hour	F3	5 ug	0	0	0	1	0	0	0		
09-MAR-2018	16:39	Day 1	2 hour	F1	5 ug	0	0	0	0	0	0	0		
09-MAR-2018	16:39	Day 1	2 hour	F2	5 ug	0	0	0	0	0	0	0		
09-MAR-2018	16:39	Day 1	2 hour	F3	5 ug	0	0	0	0	0	0	0		
12-MAR-2018	14:16	Day 4		F1	5 ug	0	1	0	0	0	0	0		
12-MAR-2018	14:16	Day 4		F2	5 ug	0	0	0	0	0	0	0		
12-MAR-2018	14:16	Day 4		F3	5 ug	0	0	0	0	0	0	0		
16-MAR-2018	14:03	Day 8		F1	5 ug	0	0	0	0	2	0	2		
16-MAR-2018	14:03	Day 8		F2	5 ug	0	0	0	0	1	0	2		
16-MAR-2018	14:03	Day 8		F3	5 ug	0	0	0	0	2	0	2		
30-MAR-2018	14:16	Day 22		F1	5 ug	0	0	0	0	0	0	2		
30-MAR-2018	14:16	Day 22		F2	5 ug	0	0	0	0	0	0	2		

Key: 0= None; 1=Mild; 2=Moderate; 3=Severe; # Other = Pinprick bleeding or seepage

Administration Site Key: FA = Forearm; UA= Upper arm
Treatments Part A: A1 = A/Sing MAP, 15 ug (FA); A2 = Afluria Quad IM (UA), 15 ug; A3 = Placebo MAP (FA); A4 = A/Sing Mono IM, 15 ug (UA)
Treatments Part B: B1 = A/Sing MAP, 15 ug (FA) with biopsy; B2 = Placebo MAP (FA) with biopsy
B3 = A/Sing MAP, 15 ug (FA); B4 = A/Sing MAP, 10 ug (FA); B5 = A/Sing MAP, 5 ug (FA); B6 = A/Sing MAP, 2.5 ug (FA)
B7 = Placebo MAP (FA); B8 = A/Sing MAP, 15 ug (UA); B9 = A/Sing IM, 15 ug, as 2018 Afluria Quad (UA)

Listing 16.2.9.2
Treatment Site Tolerability Assessment per Site

Part A
Randomisation Number = A0015, Treatment Group = Group A1

Date	Time	Visit	Time Point	Site Number	Treatment for Site	Indura-tion	Redness	Bruising	Itching	Skin Flaking	Tender-ness	Definition/ Visibility	Other#	Unscheduled Reason
30-MAR-2018	14:16	Day 22		F3	5 ug	0	0	0	0	0	0	2		
08-MAY-2018	10:31	EOS/ET (Day 61)		F1	5 ug	0	0	0	0	0	0	1		
08-MAY-2018	10:31	EOS/ET (Day 61)		F2	5 ug	0	0	0	0	0	0	1		
08-MAY-2018	10:31	EOS/ET (Day 61)		F3	5 ug	0	0	0	0	0	0	1		

Key: 0= None; 1=Mild; 2=Moderate; 3=Severe; # Other = Pinprick bleeding or seepage

Administration Site Key: FA = Forearm; UA= Upper arm
Treatments Part A: A1 = A/Sing MAP, 15 ug (FA); A2 = Afluria Quad IM (UA), 15 ug; A3 = Placebo MAP (FA); A4 = A/Sing Mono IM, 15 ug (UA)
Treatments Part B: B1 = A/Sing MAP, 15 ug (FA) with biopsy; B2 = Placebo MAP (FA) with biopsy
B3 = A/Sing MAP, 15 ug (FA); B4 = A/Sing MAP, 10 ug (FA); B5 = A/Sing MAP, 5 ug (FA); B6 = A/Sing MAP, 2.5 ug (FA)
B7 = Placebo MAP (FA); B8 = A/Sing MAP, 15 ug (UA); B9 = A/Sing IM, 15 ug, as 2018 Afluria Quad (UA)

Listing 16.2.9.2
Treatment Site Tolerability Assessment per Site

Part A
Randomisation Number = A0016, Treatment Group = Group A3

Date	Time	Visit	Time Point	Site Number	Treatment for Site	Indura-tion	Redness	Bruising	Itching	Skin Flaking	Tender-ness	Definition/ Visibility	Other#	Unscheduled Reason
10-MAR-2018	10:00	Day 1	Pre-dose	F1	uncoated	0	0	0	0	0	0	0		
10-MAR-2018	10:00	Day 1	Pre-dose	F2	uncoated	0	0	0	0	0	0	0		
10-MAR-2018	10:00	Day 1	Pre-dose	F3	uncoated	0	0	0	0	0	0	0		
10-MAR-2018	11:18	Day 1	10 min	F1	uncoated	1	1	0	0	0	0	0		
10-MAR-2018	11:18	Day 1	10 min	F2	uncoated	1	1	0	0	0	0	0		
10-MAR-2018	11:18	Day 1	10 min	F3	uncoated	0	0	0	0	0	0	0		
10-MAR-2018	12:18	Day 1	1 hour	F1	uncoated	1	1	0	0	0	0	0		
10-MAR-2018	12:18	Day 1	1 hour	F2	uncoated	1	1	0	0	0	0	0		
10-MAR-2018	12:18	Day 1	1 hour	F3	uncoated	1	1	0	0	0	0	0		
10-MAR-2018	13:18	Day 1	2 hour	F1	uncoated	0	0	0	0	0	0	0		
10-MAR-2018	13:18	Day 1	2 hour	F2	uncoated	0	0	0	0	0	0	0		
10-MAR-2018	13:18	Day 1	2 hour	F3	uncoated	0	0	0	0	0	0	0		
13-MAR-2018	11:51	Day 4		F1	uncoated	0	0	0	0	0	0	3		
13-MAR-2018	11:51	Day 4		F2	uncoated	0	0	0	0	0	0	3		
13-MAR-2018	11:51	Day 4		F3	uncoated	0	0	0	0	0	0	3		
17-MAR-2018	11:57	Day 8		F1	uncoated	0	0	0	0	1	0	2		
17-MAR-2018	11:57	Day 8		F2	uncoated	0	0	0	0	1	0	2		
17-MAR-2018	11:57	Day 8		F3	uncoated	0	0	0	0	1	0	2		
31-MAR-2018	10:55	Day 22		F1	uncoated	0	0	0	0	0	0	0		
31-MAR-2018	10:55	Day 22		F2	uncoated	0	0	0	0	0	0	0		

Key: 0= None; 1=Mild; 2=Moderate; 3=Severe; # Other = Pinprick bleeding or seepage

Administration Site Key: FA = Forearm; UA= Upper arm
Treatments Part A: A1 = A/Sing MAP, 15 ug (FA); A2 = Afluria Quad IM (UA), 15 ug; A3 = Placebo MAP (FA); A4 = A/Sing Mono IM, 15 ug (UA)
Treatments Part B: B1 = A/Sing MAP, 15 ug (FA) with biopsy; B2 = Placebo MAP (FA) with biopsy
B3 = A/Sing MAP, 15 ug (FA); B4 = A/Sing MAP, 10 ug (FA); B5 = A/Sing MAP, 5 ug (FA); B6 = A/Sing MAP, 2.5 ug (FA)
B7 = Placebo MAP (FA); B8 = A/Sing MAP, 15 ug (UA); B9 = A/Sing IM, 15 ug, as 2018 Afluria Quad (UA)

Listing 16.2.9.2
Treatment Site Tolerability Assessment per Site

Part A
Randomisation Number = A0016, Treatment Group = Group A3

Date	Time	Visit	Time Point	Site Number	Treatment for Site	Indura-tion	Redness	Bruising	Itching	Skin Flaking	Tender-ness	Definition/ Visibility	Other#	Unscheduled Reason
31-MAR-2018	10:55	Day 22		F3	uncoated	0	0	0	0	0	0	0		
10-MAY-2018	10:53	EOS/ET (Day 61)		F1	uncoated	0	0	0	0	0	0	0		
10-MAY-2018	10:53	EOS/ET (Day 61)		F2	uncoated	0	0	0	0	0	0	0		
10-MAY-2018	10:53	EOS/ET (Day 61)		F3	uncoated	0	0	0	0	0	0	0		

Key: 0= None; 1=Mild; 2=Moderate; 3=Severe; # Other = Pinprick bleeding or seepage

Administration Site Key: FA = Forearm; UA= Upper arm
Treatments Part A: A1 = A/Sing MAP, 15 ug (FA); A2 = Afluria Quad IM (UA), 15 ug; A3 = Placebo MAP (FA); A4 = A/Sing Mono IM, 15 ug (UA)
Treatments Part B: B1 = A/Sing MAP, 15 ug (FA) with biopsy; B2 = Placebo MAP (FA) with biopsy
B3 = A/Sing MAP, 15 ug (FA); B4 = A/Sing MAP, 10 ug (FA); B5 = A/Sing MAP, 5 ug (FA); B6 = A/Sing MAP, 2.5 ug (FA)
B7 = Placebo MAP (FA); B8 = A/Sing MAP, 15 ug (UA); B9 = A/Sing IM, 15 ug, as 2018 Afluria Quad (UA)

Listing 16.2.9.2
Treatment Site Tolerability Assessment per Site

Part A
Randomisation Number = A0017, Treatment Group = Group A3

Date	Time	Visit	Time Point	Site Number	Treatment for Site	Indura-tion	Redness	Bruising	Itching	Skin Flaking	Tender-ness	Definition/ Visibility	Other#	Unscheduled Reason
10-MAR-2018	10:16	Day 1	Pre-dose	F1	uncoated	0	0	0	0	0	0	0		
10-MAR-2018	10:16	Day 1	Pre-dose	F2	uncoated	0	0	0	0	0	0	0		
10-MAR-2018	10:16	Day 1	Pre-dose	F3	uncoated	0	0	0	0	0	0	0		
10-MAR-2018	11:28	Day 1	10 min	F1	uncoated	1	1	0	0	0	0	0		
10-MAR-2018	11:28	Day 1	10 min	F2	uncoated	1	1	0	0	0	0	0		
10-MAR-2018	11:28	Day 1	10 min	F3	uncoated	1	1	0	0	0	0	0		
10-MAR-2018	12:23	Day 1	1 hour	F1	uncoated	1	1	0	0	0	0	0		
10-MAR-2018	12:23	Day 1	1 hour	F2	uncoated	1	1	0	0	0	0	0		
10-MAR-2018	12:23	Day 1	1 hour	F3	uncoated	1	1	0	0	0	0	0		
10-MAR-2018	13:28	Day 1	2 hour	F1	uncoated	0	0	0	0	0	0	0		
10-MAR-2018	13:28	Day 1	2 hour	F2	uncoated	0	0	0	0	0	0	0		
10-MAR-2018	13:28	Day 1	2 hour	F3	uncoated	0	0	0	0	0	0	0		
13-MAR-2018	13:06	Day 4		F1	uncoated	0	0	0	0	0	0	1		
13-MAR-2018	13:06	Day 4		F2	uncoated	0	0	0	0	0	0	3		
13-MAR-2018	13:06	Day 4		F3	uncoated	0	0	0	0	0	0	2		
17-MAR-2018	13:59	Day 8		F1	uncoated	0	0	0	0	1	0	2		
17-MAR-2018	13:59	Day 8		F2	uncoated	0	0	0	0	1	0	2		
17-MAR-2018	13:59	Day 8		F3	uncoated	0	0	0	0	1	0	2		
31-MAR-2018	14:17	Day 22		F1	uncoated	0	0	0	0	0	0	1		
31-MAR-2018	14:17	Day 22		F2	uncoated	0	0	0	0	0	0	1		

Key: 0= None; 1=Mild; 2=Moderate; 3=Severe; # Other = Pinprick bleeding or seepage

Administration Site Key: FA = Forearm; UA= Upper arm
Treatments Part A: A1 = A/Sing MAP, 15 ug (FA); A2 = Afluria Quad IM (UA), 15 ug; A3 = Placebo MAP (FA); A4 = A/Sing Mono IM, 15 ug (UA)
Treatments Part B: B1 = A/Sing MAP, 15 ug (FA) with biopsy; B2 = Placebo MAP (FA) with biopsy
B3 = A/Sing MAP, 15 ug (FA); B4 = A/Sing MAP, 10 ug (FA); B5 = A/Sing MAP, 5 ug (FA); B6 = A/Sing MAP, 2.5 ug (FA)
B7 = Placebo MAP (FA); B8 = A/Sing MAP, 15 ug (UA); B9 = A/Sing IM, 15 ug, as 2018 Afluria Quad (UA)

Listing 16.2.9.2
Treatment Site Tolerability Assessment per Site

Part A
Randomisation Number = A0017, Treatment Group = Group A3

Date	Time	Visit	Time Point	Site Number	Treatment for Site	Indura-tion	Redness	Bruising	Itching	Skin Flaking	Tender-ness	Definition/ Visibility	Other#	Unscheduled Reason
31-MAR-2018	14:17	Day 22		F3	uncoated	0	0	0	0	0	0	1		
09-MAY-2018	12:17	EOS/ET (Day 61)		F1	uncoated	0	0	0	0	0	0	0		
09-MAY-2018	12:17	EOS/ET (Day 61)		F2	uncoated	0	0	0	0	0	0	0		
09-MAY-2018	12:17	EOS/ET (Day 61)		F3	uncoated	0	0	0	0	0	0	0		

Key: 0= None; 1=Mild; 2=Moderate; 3=Severe; # Other = Pinprick bleeding or seepage

Administration Site Key: FA = Forearm; UA= Upper arm
Treatments Part A: A1 = A/Sing MAP, 15 ug (FA); A2 = Afluria Quad IM (UA), 15 ug; A3 = Placebo MAP (FA); A4 = A/Sing Mono IM, 15 ug (UA)
Treatments Part B: B1 = A/Sing MAP, 15 ug (FA) with biopsy; B2 = Placebo MAP (FA) with biopsy
B3 = A/Sing MAP, 15 ug (FA); B4 = A/Sing MAP, 10 ug (FA); B5 = A/Sing MAP, 5 ug (FA); B6 = A/Sing MAP, 2.5 ug (FA)
B7 = Placebo MAP (FA); B8 = A/Sing MAP, 15 ug (UA); B9 = A/Sing IM, 15 ug, as 2018 Afluria Quad (UA)

Listing 16.2.9.2
Treatment Site Tolerability Assessment per Site

Part A
Randomisation Number = A0018, Treatment Group = Group A1

Date	Time	Visit	Time Point	Site Number	Treatment for Site	Indura-tion	Redness	Bruising	Itching	Skin Flaking	Tender-ness	Definition/ Visibility	Other#	Unscheduled Reason
10-MAR-2018	10:20	Day 1	Pre-dose	F1	5 ug	0	0	0	0	0	0	0		
10-MAR-2018	10:20	Day 1	Pre-dose	F2	5 ug	0	0	0	0	0	0	0		
10-MAR-2018	10:20	Day 1	Pre-dose	F3	5 ug	0	0	0	0	0	0	0		
10-MAR-2018	11:38	Day 1	10 min	F1	5 ug	1	1	0	0	0	1	0		
10-MAR-2018	11:38	Day 1	10 min	F2	5 ug	1	1	0	0	0	1	0		
10-MAR-2018	11:38	Day 1	10 min	F3	5 ug	0	0	0	0	0	1	0		
10-MAR-2018	12:27	Day 1	1 hour	F1	5 ug	1	1	0	0	0	0	0		
10-MAR-2018	12:27	Day 1	1 hour	F2	5 ug	1	1	0	0	0	0	0		
10-MAR-2018	12:27	Day 1	1 hour	F3	5 ug	1	1	0	0	0	0	0		
10-MAR-2018	13:38	Day 1	2 hour	F1	5 ug	0	0	0	0	0	0	0		
10-MAR-2018	13:38	Day 1	2 hour	F2	5 ug	0	0	0	0	0	0	0		
10-MAR-2018	13:38	Day 1	2 hour	F3	5 ug	0	0	0	0	0		0		
13-MAR-2018	12:34	Day 4		F1	5 ug	0	0	0	0	0	0	0		
13-MAR-2018	12:34	Day 4		F2	5 ug	0	0	0	0	0	0	0		
13-MAR-2018	12:34	Day 4		F3	5 ug	0	0	0	0	0	0	0		
17-MAR-2018	11:41	Day 8		F1	5 ug	0	0	0	0	1	0	3		
17-MAR-2018	11:41	Day 8		F2	5 ug	0	0	0	0	1	0	3		
17-MAR-2018	11:41	Day 8		F3	5 ug	0	0	0	0	1	0	3		
31-MAR-2018	09:11	Day 22		F1	5 ug	0	0	0	0	0	0	1		
31-MAR-2018	09:11	Day 22		F2	5 ug	0	0	0	0	0	0	1		

Key: 0= None; 1=Mild; 2=Moderate; 3=Severe; # Other = Pinprick bleeding or seepage

Administration Site Key: FA = Forearm; UA= Upper arm
Treatments Part A: A1 = A/Sing MAP, 15 ug (FA); A2 = Afluria Quad IM (UA), 15 ug; A3 = Placebo MAP (FA); A4 = A/Sing Mono IM, 15 ug (UA)
Treatments Part B: B1 = A/Sing MAP, 15 ug (FA) with biopsy; B2 = Placebo MAP (FA) with biopsy
B3 = A/Sing MAP, 15 ug (FA); B4 = A/Sing MAP, 10 ug (FA); B5 = A/Sing MAP, 5 ug (FA); B6 = A/Sing MAP, 2.5 ug (FA)
B7 = Placebo MAP (FA); B8 = A/Sing MAP, 15 ug (UA); B9 = A/Sing IM, 15 ug, as 2018 Afluria Quad (UA)

Listing 16.2.9.2
Treatment Site Tolerability Assessment per Site

Part A
Randomisation Number = A0018, Treatment Group = Group A1

Date	Time	Visit	Time Point	Site Number	Treatment for Site	Indura-tion	Redness	Bruising	Itching	Skin Flaking	Tender-ness	Definition/ Visibility	Other#	Unscheduled Reason
31-MAR-2018	09:11	Day 22		F3	5 ug	0	0	0	0	0	0	1		
09-MAY-2018	17:11	EOS/ET (Day 61)		F1	5 ug	0	0	0	0	0	0	0		
09-MAY-2018	17:11	EOS/ET (Day 61)		F2	5 ug	0	0	0	0	0	0	0		
09-MAY-2018	17:11	EOS/ET (Day 61)		F3	5 ug	0	0	0	0	0	0	0		

Key: 0= None; 1=Mild; 2=Moderate; 3=Severe; # Other = Pinprick bleeding or seepage

Administration Site Key: FA = Forearm; UA= Upper arm
Treatments Part A: A1 = A/Sing MAP, 15 ug (FA); A2 = Afluria Quad IM (UA), 15 ug; A3 = Placebo MAP (FA); A4 = A/Sing Mono IM, 15 ug (UA)
Treatments Part B: B1 = A/Sing MAP, 15 ug (FA) with biopsy; B2 = Placebo MAP (FA) with biopsy
B3 = A/Sing MAP, 15 ug (FA); B4 = A/Sing MAP, 10 ug (FA); B5 = A/Sing MAP, 5 ug (FA); B6 = A/Sing MAP, 2.5 ug (FA)
B7 = Placebo MAP (FA); B8 = A/Sing MAP, 15 ug (UA); B9 = A/Sing IM, 15 ug, as 2018 Afluria Quad (UA)

Listing 16.2.9.2
Treatment Site Tolerability Assessment per Site

Part A
Randomisation Number = A0019, Treatment Group = Group A3

Date	Time	Visit	Time Point	Site Number	Treatment for Site	Indura-tion	Redness	Bruising	Itching	Skin Flaking	Tender-ness	Definition/ Visibility	Other#	Unscheduled Reason
10-MAR-2018	10:44	Day 1	Pre-dose	F1	uncoated	0	0	0	0	0	0	0		
10-MAR-2018	10:44	Day 1	Pre-dose	F2	uncoated	0	0	0	0	0	0	0		
10-MAR-2018	10:44	Day 1	Pre-dose	F3	uncoated	0	0	0	0	0	0	0		
10-MAR-2018	11:48	Day 1	10 min	F1	uncoated	1	1	0	0	0	0	0		
10-MAR-2018	11:48	Day 1	10 min	F2	uncoated	1	1	0	0	0	0	0		
10-MAR-2018	11:48	Day 1	10 min	F3	uncoated	1	1	0	0	0	0	0		
10-MAR-2018	12:38	Day 1	1 hour	F1	uncoated	1	1	0	0	0	0	0		
10-MAR-2018	12:38	Day 1	1 hour	F2	uncoated	1	1	0	0	0	0	0		
10-MAR-2018	12:38	Day 1	1 hour	F3	uncoated	1	1	0	0	0	0	0		
10-MAR-2018	13:48	Day 1	2 hour	F1	uncoated	0	0	0	0	0	0	0		
10-MAR-2018	13:48	Day 1	2 hour	F2	uncoated	0	0	0	0	0	0	0		
10-MAR-2018	13:48	Day 1	2 hour	F3	uncoated	0	0	0	0	0		0		
13-MAR-2018	12:17	Day 4		F1	uncoated	0	0	0	0	0	0	2		
13-MAR-2018	12:17	Day 4		F2	uncoated	0	0	0	0	0	0	2		
13-MAR-2018	12:17	Day 4		F3	uncoated	0	0	0	0	0	0	2		
17-MAR-2018	11:48	Day 8		F1	uncoated	0	0	0	0	0	0	3		
17-MAR-2018	11:48	Day 8		F2	uncoated	0	0	0	0	0	0	2		
17-MAR-2018	11:48	Day 8		F3	uncoated	0	0	0	0	0	0	1		
31-MAR-2018	11:43	Day 22		F1	uncoated	0	0	0	0	0	0	0		
31-MAR-2018	11:43	Day 22		F2	uncoated	0	0	0	0	0	0	0		

Key: 0= None; 1=Mild; 2=Moderate; 3=Severe; # Other = Pinprick bleeding or seepage

Administration Site Key: FA = Forearm; UA= Upper arm
Treatments Part A: A1 = A/Sing MAP, 15 ug (FA); A2 = Afluria Quad IM (UA), 15 ug; A3 = Placebo MAP (FA); A4 = A/Sing Mono IM, 15 ug (UA)
Treatments Part B: B1 = A/Sing MAP, 15 ug (FA) with biopsy; B2 = Placebo MAP (FA) with biopsy
B3 = A/Sing MAP, 15 ug (FA); B4 = A/Sing MAP, 10 ug (FA); B5 = A/Sing MAP, 5 ug (FA); B6 = A/Sing MAP, 2.5 ug (FA)
B7 = Placebo MAP (FA); B8 = A/Sing MAP, 15 ug (UA); B9 = A/Sing IM, 15 ug, as 2018 Afluria Quad (UA)

Listing 16.2.9.2
Treatment Site Tolerability Assessment per Site

Part A
Randomisation Number = A0019, Treatment Group = Group A3

Date	Time	Visit	Time Point	Site Number	Treatment for Site	Indura-tion	Redness	Bruising	Itching	Skin Flaking	Tender-ness	Definition/ Visibility	Other#	Unscheduled Reason
31-MAR-2018	11:43	Day 22		F3	uncoated	0	0	0	0	0	0	0		
08-MAY-2018	12:30	EOS/ET (Day 61)		F1	uncoated	0	0	0	0	0	0	0		
08-MAY-2018	12:30	EOS/ET (Day 61)		F2	uncoated	0	0	0	0	0	0	0		
08-MAY-2018	12:30	EOS/ET (Day 61)		F3	uncoated	0	0	0	0	0	0	0		

Key: 0= None; 1=Mild; 2=Moderate; 3=Severe; # Other = Pinprick bleeding or seepage

Administration Site Key: FA = Forearm; UA= Upper arm
Treatments Part A: A1 = A/Sing MAP, 15 ug (FA); A2 = Afluria Quad IM (UA), 15 ug; A3 = Placebo MAP (FA); A4 = A/Sing Mono IM, 15 ug (UA)
Treatments Part B: B1 = A/Sing MAP, 15 ug (FA) with biopsy; B2 = Placebo MAP (FA) with biopsy
B3 = A/Sing MAP, 15 ug (FA); B4 = A/Sing MAP, 10 ug (FA); B5 = A/Sing MAP, 5 ug (FA); B6 = A/Sing MAP, 2.5 ug (FA)
B7 = Placebo MAP (FA); B8 = A/Sing MAP, 15 ug (UA); B9 = A/Sing IM, 15 ug, as 2018 Afluria Quad (UA)

Listing 16.2.9.2
Treatment Site Tolerability Assessment per Site

Part A
Randomisation Number = A0020, Treatment Group = Group A1

Date	Time	Visit	Time Point	Site Number	Treatment for Site	Indura-tion	Redness	Bruising	Itching	Skin Flaking	Tender-ness	Definition/ Visibility	Other#	Unscheduled Reason
10-MAR-2018	10:25	Day 1	Pre-dose	F1	5 ug	0	0	0	0	0	0	0		
10-MAR-2018	10:25	Day 1	Pre-dose	F2	5 ug	0	0	0	0	0	0	0		
10-MAR-2018	10:25	Day 1	Pre-dose	F3	5 ug	0	0	0	0	0	0	0		
10-MAR-2018	11:58	Day 1	10 min	F1	5 ug	1	1	0	0	0	0	0		
10-MAR-2018	11:58	Day 1	10 min	F2	5 ug	1	1	0	0	0	0	0		
10-MAR-2018	11:58	Day 1	10 min	F3	5 ug	1	1	0	0	0	0	0		
10-MAR-2018	12:48	Day 1	1 hour	F1	5 ug	1	0	0	0	0	0	0		
10-MAR-2018	12:48	Day 1	1 hour	F2	5 ug	1	0	0	0	0	0	0		
10-MAR-2018	12:48	Day 1	1 hour	F3	5 ug	1	1	0	0	0	0	0		
10-MAR-2018	14:08	Day 1	2 hour	F1	5 ug	0	0	0	0	0	0	0		
10-MAR-2018	14:08	Day 1	2 hour	F2	5 ug	1	0	0	0	0	0	0		
10-MAR-2018	14:08	Day 1	2 hour	F3	5 ug	1	0	0	0	0	0	0		
13-MAR-2018	10:34	Day 4		F1	5 ug	0	0	0	1	0	0	0		
13-MAR-2018	10:34	Day 4		F2	5 ug	0	0	0	1	0	0	0		
13-MAR-2018	10:34	Day 4		F3	5 ug	0	0	0	1	0	0	0		
17-MAR-2018	11:45	Day 8		F1	5 ug	0	0	0	0	1	0	3		
17-MAR-2018	11:45	Day 8		F2	5 ug	0	0	0	0	0	0	3		
17-MAR-2018	11:45	Day 8		F3	5 ug	0	0	0	0	0	0	3		
31-MAR-2018	11:46	Day 22		F1	5 ug	0	0	0	0	0	0	0		
31-MAR-2018	11:46	Day 22		F2	5 ug	0	0	0	0	0	0	0		

Key: 0= None; 1=Mild; 2=Moderate; 3=Severe; # Other = Pinprick bleeding or seepage

Administration Site Key: FA = Forearm; UA= Upper arm
Treatments Part A: A1 = A/Sing MAP, 15 ug (FA); A2 = Afluria Quad IM (UA), 15 ug; A3 = Placebo MAP (FA); A4 = A/Sing Mono IM, 15 ug (UA)
Treatments Part B: B1 = A/Sing MAP, 15 ug (FA) with biopsy; B2 = Placebo MAP (FA) with biopsy
B3 = A/Sing MAP, 15 ug (FA); B4 = A/Sing MAP, 10 ug (FA); B5 = A/Sing MAP, 5 ug (FA); B6 = A/Sing MAP, 2.5 ug (FA)
B7 = Placebo MAP (FA); B8 = A/Sing MAP, 15 ug (UA); B9 = A/Sing IM, 15 ug, as 2018 Afluria Quad (UA)

Listing 16.2.9.2
Treatment Site Tolerability Assessment per Site

Part A
Randomisation Number = A0020, Treatment Group = Group A1

Date	Time	Visit	Time Point	Site Number	Treatment for Site	Indura-tion	Redness	Bruising	Itching	Skin Flaking	Tender-ness	Definition/ Visibility	Other#	Unscheduled Reason
31-MAR-2018	11:46	Day 22		F3	5 ug	0	0	0	0	0	0	0		
09-MAY-2018	13:13	EOS/ET (Day 61)		F1	5 ug	0	0	0	0	0	0	0		
09-MAY-2018	13:13	EOS/ET (Day 61)		F2	5 ug	0	0	0	0	0	0	0		
09-MAY-2018	13:13	EOS/ET (Day 61)		F3	5 ug	0	0	0	0	0	0	0		

Key: 0= None; 1=Mild; 2=Moderate; 3=Severe; # Other = Pinprick bleeding or seepage

Administration Site Key: FA = Forearm; UA= Upper arm
Treatments Part A: A1 = A/Sing MAP, 15 ug (FA); A2 = Afluria Quad IM (UA), 15 ug; A3 = Placebo MAP (FA); A4 = A/Sing Mono IM, 15 ug (UA)
Treatments Part B: B1 = A/Sing MAP, 15 ug (FA) with biopsy; B2 = Placebo MAP (FA) with biopsy
B3 = A/Sing MAP, 15 ug (FA); B4 = A/Sing MAP, 10 ug (FA); B5 = A/Sing MAP, 5 ug (FA); B6 = A/Sing MAP, 2.5 ug (FA)
B7 = Placebo MAP (FA); B8 = A/Sing MAP, 15 ug (UA); B9 = A/Sing IM, 15 ug, as 2018 Afluria Quad (UA)

Listing 16.2.9.2
Treatment Site Tolerability Assessment per Site

Part A
Randomisation Number = A0021, Treatment Group = Group A1

Date	Time	Visit	Time Point	Site Number	Treatment for Site	Indura-tion	Redness	Bruising	Itching	Skin Flaking	Tender-ness	Definition/ Visibility	Other#	Unscheduled Reason
10-MAR-2018	10:27	Day 1	Pre-dose	F1	5 ug	0	0	0	0	0	0	0		
10-MAR-2018	10:27	Day 1	Pre-dose	F2	5 ug	0	0	0	0	0	0	0		
10-MAR-2018	10:27	Day 1	Pre-dose	F3	5 ug	0	0	0	0	0	0	0		
10-MAR-2018	12:28	Day 1	10 min	F1	5 ug	1	1	0	0	0	1	0		
10-MAR-2018	12:28	Day 1	10 min	F2	5 ug	1	1	0	1	0	1	0		
10-MAR-2018	12:28	Day 1	10 min	F3	5 ug	1	1	0	1	0	1	0		
10-MAR-2018	13:18	Day 1	1 hour	F1	5 ug	1	1	0	0	0	1	0		
10-MAR-2018	13:18	Day 1	1 hour	F2	5 ug	1	1	0	0	0	1	0		
10-MAR-2018	13:18	Day 1	1 hour	F3	5 ug	1	1	0	0	0	1	0		
10-MAR-2018	14:29	Day 1	2 hour	F1	5 ug	1	0	0	0	0	0	0		
10-MAR-2018	14:29	Day 1	2 hour	F2	5 ug	1	0	0	0	0	0	0		
10-MAR-2018	14:29	Day 1	2 hour	F3	5 ug	1	0	0	0	0	0	0		
13-MAR-2018	14:54	Day 4		F1	5 ug	0	0	0	0	1	0	0		
13-MAR-2018	14:54	Day 4		F2	5 ug	0	0	0	0	0	0	0		
13-MAR-2018	14:54	Day 4		F3	5 ug	0	0	0	0	1	0	0		
17-MAR-2018	13:40	Day 8		F1	5 ug	0	0	0	0	1	0	0		
17-MAR-2018	13:40	Day 8		F2	5 ug	0	0	0	0	1	0	0		
17-MAR-2018	13:40	Day 8		F3	5 ug	0	0	0	0	1	0	0		
31-MAR-2018	13:02	Day 22		F1	5 ug	0	0	0	0	0	0	2		
31-MAR-2018	13:02	Day 22		F2	5 ug	0	0	0	0	0	0	2		

Key: 0= None; 1=Mild; 2=Moderate; 3=Severe; # Other = Pinprick bleeding or seepage

Administration Site Key: FA = Forearm; UA= Upper arm
Treatments Part A: A1 = A/Sing MAP, 15 ug (FA); A2 = Afluria Quad IM (UA), 15 ug; A3 = Placebo MAP (FA); A4 = A/Sing Mono IM, 15 ug (UA)
Treatments Part B: B1 = A/Sing MAP, 15 ug (FA) with biopsy; B2 = Placebo MAP (FA) with biopsy
B3 = A/Sing MAP, 15 ug (FA); B4 = A/Sing MAP, 10 ug (FA); B5 = A/Sing MAP, 5 ug (FA); B6 = A/Sing MAP, 2.5 ug (FA)
B7 = Placebo MAP (FA); B8 = A/Sing MAP, 15 ug (UA); B9 = A/Sing IM, 15 ug, as 2018 Afluria Quad (UA)

Listing 16.2.9.2
Treatment Site Tolerability Assessment per Site

Part A
Randomisation Number = A0021, Treatment Group = Group A1

Date	Time	Visit	Time Point	Site Number	Treatment for Site	Indura- tion	Redness	Bruising	Itching	Skin Flaking	Tender- ness	Definition/ Visibility	Other#	Unscheduled Reason
31-MAR-2018	13:02	Day 22		F3	5 ug	0	0	0	0	0	0	2		
10-MAY-2018	11:45	EOS/ET (Day 61)		F1	5 ug	0	0	0	0	0	0	0		
10-MAY-2018	11:45	EOS/ET (Day 61)		F2	5 ug	0	0	0	0	0	0	0		
10-MAY-2018	11:45	EOS/ET (Day 61)		F3	5 ug	0	0	0	0	0	0	0		

Key: 0= None; 1=Mild; 2=Moderate; 3=Severe; # Other = Pinprick bleeding or seepage

Administration Site Key: FA = Forearm; UA= Upper arm
Treatments Part A: A1 = A/Sing MAP, 15 ug (FA); A2 = Afluria Quad IM (UA), 15 ug; A3 = Placebo MAP (FA); A4 = A/Sing Mono IM, 15 ug (UA)
Treatments Part B: B1 = A/Sing MAP, 15 ug (FA) with biopsy; B2 = Placebo MAP (FA) with biopsy
B3 = A/Sing MAP, 15 ug (FA); B4 = A/Sing MAP, 10 ug (FA); B5 = A/Sing MAP, 5 ug (FA); B6 = A/Sing MAP, 2.5 ug (FA)
B7 = Placebo MAP (FA); B8 = A/Sing MAP, 15 ug (UA); B9 = A/Sing IM, 15 ug, as 2018 Afluria Quad (UA)

Listing 16.2.9.2
Treatment Site Tolerability Assessment per Site

Part A
Randomisation Number = A0022, Treatment Group = Group A3

Date	Time	Visit	Time Point	Site Number	Treatment for Site	Indura- tion	Redness	Bruising	Itching	Skin Flaking	Tender- ness	Definition/ Visibility	Other#	Unscheduled Reason
10-MAR-2018	10:22	Day 1	Pre-dose	F1	uncoated	0	0	0	0	0	0	0		
10-MAR-2018	10:22	Day 1	Pre-dose	F2	uncoated	0	0	0	0	0	0	0		
10-MAR-2018	10:22	Day 1	Pre-dose	F3	uncoated	0	0	0	0	0	0	0		
10-MAR-2018	12:08	Day 1	10 min	F1	uncoated	1	1	0	0	0	2	0		
10-MAR-2018	12:08	Day 1	10 min	F2	uncoated	1	1	0	0	0	1	0		
10-MAR-2018	12:08	Day 1	10 min	F3	uncoated	1	1	0	0	0	0	0		
10-MAR-2018	12:58	Day 1	1 hour	F1	uncoated	1	1	0	0	0	0	0		
10-MAR-2018	12:58	Day 1	1 hour	F2	uncoated	1	1	0	0	0	0	0		
10-MAR-2018	12:58	Day 1	1 hour	F3	uncoated	1	1	0	0	0	0	0		
10-MAR-2018	14:18	Day 1	2 hour	F1	uncoated	0	0	0	0	0	0	0		
10-MAR-2018	14:18	Day 1	2 hour	F2	uncoated	0	0	0	0	0	0	0		
10-MAR-2018	14:18	Day 1	2 hour	F3	uncoated	0	0	0	0	0	0	0		
13-MAR-2018	12:09	Day 4		F1	uncoated	0	0	0	0	0	0	3		
13-MAR-2018	12:09	Day 4		F2	uncoated	0	0	0	0	0	0	3		
13-MAR-2018	12:09	Day 4		F3	uncoated	0	0	0	0	0	0	3		
17-MAR-2018	12:06	Day 8		F1	uncoated	0	0	0	0	0	0	2		
17-MAR-2018	12:06	Day 8		F2	uncoated	0	0	0	0	0	0	3		
17-MAR-2018	12:06	Day 8		F3	uncoated	0	0	0	0	0	0	3		
31-MAR-2018	11:55	Day 22		F1	uncoated	0	0	0	0	0	0	0		
31-MAR-2018	11:55	Day 22		F2	uncoated	0	0	0	0	0	0	0		

Key: 0= None; 1=Mild; 2=Moderate; 3=Severe; # Other = Pinprick bleeding or seepage

Administration Site Key: FA = Forearm; UA= Upper arm
Treatments Part A: A1 = A/Sing MAP, 15 ug (FA); A2 = Afluria Quad IM (UA), 15 ug; A3 = Placebo MAP (FA); A4 = A/Sing Mono IM, 15 ug (UA)
Treatments Part B: B1 = A/Sing MAP, 15 ug (FA) with biopsy; B2 = Placebo MAP (FA) with biopsy
B3 = A/Sing MAP, 15 ug (FA); B4 = A/Sing MAP, 10 ug (FA); B5 = A/Sing MAP, 5 ug (FA); B6 = A/Sing MAP, 2.5 ug (FA)
B7 = Placebo MAP (FA); B8 = A/Sing MAP, 15 ug (UA); B9 = A/Sing IM, 15 ug, as 2018 Afluria Quad (UA)

Listing 16.2.9.2
Treatment Site Tolerability Assessment per Site

Part A
Randomisation Number = A0022, Treatment Group = Group A3

Date	Time	Visit	Time Point	Site Number	Treatment for Site	Indura-tion	Redness	Bruising	Itching	Skin Flaking	Tender-ness	Definition/ Visibility	Other#	Unscheduled Reason
31-MAR-2018	11:55	Day 22		F3	uncoated	0	0	0	0	0	0	0		
09-MAY-2018	13:23	EOS/ET (Day 61)		F1	uncoated	0	0	0	0	0	0	0		
09-MAY-2018	13:23	EOS/ET (Day 61)		F2	uncoated	0	0	0	0	0	0	0		
09-MAY-2018	13:23	EOS/ET (Day 61)		F3	uncoated	0	0	0	0	0	0	0		

Key: 0= None; 1=Mild; 2=Moderate; 3=Severe; # Other = Pinprick bleeding or seepage

Administration Site Key: FA = Forearm; UA= Upper arm
Treatments Part A: A1 = A/Sing MAP, 15 ug (FA); A2 = Afluria Quad IM (UA), 15 ug; A3 = Placebo MAP (FA); A4 = A/Sing Mono IM, 15 ug (UA)
Treatments Part B: B1 = A/Sing MAP, 15 ug (FA) with biopsy; B2 = Placebo MAP (FA) with biopsy
B3 = A/Sing MAP, 15 ug (FA); B4 = A/Sing MAP, 10 ug (FA); B5 = A/Sing MAP, 5 ug (FA); B6 = A/Sing MAP, 2.5 ug (FA)
B7 = Placebo MAP (FA); B8 = A/Sing MAP, 15 ug (UA); B9 = A/Sing IM, 15 ug, as 2018 Afluria Quad (UA)

Listing 16.2.9.2
Treatment Site Tolerability Assessment per Site

Part A
Randomisation Number = A0023, Treatment Group = Group A3

Date	Time	Visit	Time Point	Site Number	Treatment for Site	Indura-tion	Redness	Bruising	Itching	Skin Flaking	Tender-ness	Definition/ Visibility	Other#	Unscheduled Reason
10-MAR-2018	10:41	Day 1	Pre-dose	F1	uncoated	0	0	0	0	0	0	0		
10-MAR-2018	10:41	Day 1	Pre-dose	F2	uncoated	0	0	0	0	0	0	0		
10-MAR-2018	10:41	Day 1	Pre-dose	F3	uncoated	0	0	0	0	0	0	0		
10-MAR-2018	12:18	Day 1	10 min	F1	uncoated	0	0	0	0	0	0	0		
10-MAR-2018	12:18	Day 1	10 min	F2	uncoated	1	1	0	0	0	1	0		
10-MAR-2018	12:18	Day 1	10 min	F3	uncoated	0	0	0	0	0	1	0		
10-MAR-2018	13:08	Day 1	1 hour	F1	uncoated	1	0	0	0	0	1	0		
10-MAR-2018	13:08	Day 1	1 hour	F2	uncoated	1	0	0	0	0	1	0		
10-MAR-2018	13:08	Day 1	1 hour	F3	uncoated	1	1	0	0	0	1	0		
10-MAR-2018	14:28	Day 1	2 hour	F1	uncoated	0	0	0	0	0	0	0		
10-MAR-2018	14:28	Day 1	2 hour	F2	uncoated	0	0	0	0	0	0	0		
10-MAR-2018	14:28	Day 1	2 hour	F3	uncoated	0	0	0	0	0	0	0		
13-MAR-2018	12:36	Day 4		F1	uncoated	0	0	0	0	0	0	2		
13-MAR-2018	12:36	Day 4		F2	uncoated	0	0	0	0	0	0	2		
13-MAR-2018	12:36	Day 4		F3	uncoated	0	0	0	0	0	0	2		
17-MAR-2018	12:01	Day 8		F1	uncoated	0	0	0	0	0	0	2		
17-MAR-2018	12:01	Day 8		F2	uncoated	0	0	0	0	0	0	2		
17-MAR-2018	12:01	Day 8		F3	uncoated	0	0	0	0	0	0	2		
31-MAR-2018	11:50	Day 22		F1	uncoated	0	0	0	0	0	0	0		
31-MAR-2018	11:50	Day 22		F2	uncoated	0	0	0	0	0	0	0		

Key: 0= None; 1=Mild; 2=Moderate; 3=Severe; # Other = Pinprick bleeding or seepage

Administration Site Key: FA = Forearm; UA= Upper arm
Treatments Part A: A1 = A/Sing MAP, 15 ug (FA); A2 = Afluria Quad IM (UA), 15 ug; A3 = Placebo MAP (FA); A4 = A/Sing Mono IM, 15 ug (UA)
Treatments Part B: B1 = A/Sing MAP, 15 ug (FA) with biopsy; B2 = Placebo MAP (FA) with biopsy
B3 = A/Sing MAP, 15 ug (FA); B4 = A/Sing MAP, 10 ug (FA); B5 = A/Sing MAP, 5 ug (FA); B6 = A/Sing MAP, 2.5 ug (FA)
B7 = Placebo MAP (FA); B8 = A/Sing MAP, 15 ug (UA); B9 = A/Sing IM, 15 ug, as 2018 Afluria Quad (UA)

Listing 16.2.9.2
Treatment Site Tolerability Assessment per Site

Part A
Randomisation Number = A0023, Treatment Group = Group A3

Date	Time	Visit	Time Point	Site Number	Treatment for Site	Indura-tion	Redness	Bruising	Itching	Skin Flaking	Tender-ness	Definition/ Visibility	Other#	Unscheduled Reason
31-MAR-2018	11:50	Day 22		F3	uncoated	0	0	0	0	0	0	0		
10-MAY-2018	13:24	EOS/ET (Day 61)		F1	uncoated	0	0	0	0	0	0	0		
10-MAY-2018	13:24	EOS/ET (Day 61)		F2	uncoated	0	0	0	0	0	0	0		
10-MAY-2018	13:24	EOS/ET (Day 61)		F3	uncoated	0	0	0	0	0	0	0		

Key: 0= None; 1=Mild; 2=Moderate; 3=Severe; # Other = Pinprick bleeding or seepage

Administration Site Key: FA = Forearm; UA= Upper arm
Treatments Part A: A1 = A/Sing MAP, 15 ug (FA); A2 = Afluria Quad IM (UA), 15 ug; A3 = Placebo MAP (FA); A4 = A/Sing Mono IM, 15 ug (UA)
Treatments Part B: B1 = A/Sing MAP, 15 ug (FA) with biopsy; B2 = Placebo MAP (FA) with biopsy
B3 = A/Sing MAP, 15 ug (FA); B4 = A/Sing MAP, 10 ug (FA); B5 = A/Sing MAP, 5 ug (FA); B6 = A/Sing MAP, 2.5 ug (FA)
B7 = Placebo MAP (FA); B8 = A/Sing MAP, 15 ug (UA); B9 = A/Sing IM, 15 ug, as 2018 Afluria Quad (UA)

Listing 16.2.9.2
Treatment Site Tolerability Assessment per Site

Part A
Randomisation Number = A0024, Treatment Group = Group A1

Date	Time	Visit	Time Point	Site Number	Treatment for Site	Indura-tion	Redness	Bruising	Itching	Skin Flaking	Tender-ness	Definition/ Visibility	Other#	Unscheduled Reason
10-MAR-2018	10:49	Day 1	Pre-dose	F1	5 ug	0	0	0	0	0	0	0		
10-MAR-2018	10:49	Day 1	Pre-dose	F2	5 ug	0	0	0	0	0	0	0		
10-MAR-2018	10:49	Day 1	Pre-dose	F3	5 ug	0	0	0	0	0	0	0		
10-MAR-2018	12:38	Day 1	10 min	F1	5 ug	1	1	0	0	0	1	0		
10-MAR-2018	12:38	Day 1	10 min	F2	5 ug	1	1	0	0	0	0	0		
10-MAR-2018	12:38	Day 1	10 min	F3	5 ug	1	1	0	0	0	0	0		
10-MAR-2018	13:28	Day 1	1 hour	F1	5 ug	1	0	0	0	0	0	0		
10-MAR-2018	13:28	Day 1	1 hour	F2	5 ug	1	1	0	0	0	0	0		
10-MAR-2018	13:28	Day 1	1 hour	F3	5 ug	1	1	0	0	0	0	0		
10-MAR-2018	14:35	Day 1	2 hour	F1	5 ug	1	0	0	0	0	0	0		
10-MAR-2018	14:35	Day 1	2 hour	F2	5 ug	1	0	0	0	0	0	0		
10-MAR-2018	14:35	Day 1	2 hour	F3	5 ug	1	0	0	0	0	0	0		
13-MAR-2018	13:03	Day 4		F1	5 ug	0	0	0	1	0	0	0		
13-MAR-2018	13:03	Day 4		F2	5 ug	0	0	0	1	0	0	0		
13-MAR-2018	13:03	Day 4		F3	5 ug	0	0	0	0	0	0	0		
17-MAR-2018	12:03	Day 8		F1	5 ug	0	0	0	0	1	0	3		
17-MAR-2018	12:03	Day 8		F2	5 ug	0	0	0	0	1	0	3		
17-MAR-2018	12:03	Day 8		F3	5 ug	0	0	0	0	1	0	3		
31-MAR-2018	11:11	Day 22		F1	5 ug	0	0	0	0	0	0	0		
31-MAR-2018	11:11	Day 22		F2	5 ug	0	0	0	0	0	0	0		

Key: 0= None; 1=Mild; 2=Moderate; 3=Severe; # Other = Pinprick bleeding or seepage

Administration Site Key: FA = Forearm; UA= Upper arm
Treatments Part A: A1 = A/Sing MAP, 15 ug (FA); A2 = Afluria Quad IM (UA), 15 ug; A3 = Placebo MAP (FA); A4 = A/Sing Mono IM, 15 ug (UA)
Treatments Part B: B1 = A/Sing MAP, 15 ug (FA) with biopsy; B2 = Placebo MAP (FA) with biopsy
B3 = A/Sing MAP, 15 ug (FA); B4 = A/Sing MAP, 10 ug (FA); B5 = A/Sing MAP, 5 ug (FA); B6 = A/Sing MAP, 2.5 ug (FA)
B7 = Placebo MAP (FA); B8 = A/Sing MAP, 15 ug (UA); B9 = A/Sing IM, 15 ug, as 2018 Afluria Quad (UA)

Listing 16.2.9.2
Treatment Site Tolerability Assessment per Site

Part A
Randomisation Number = A0024, Treatment Group = Group A1

Date	Time	Visit	Time Point	Site Number	Treatment for Site	Indura-tion	Redness	Bruising	Itching	Skin Flaking	Tender-ness	Definition/ Visibility	Other#	Unscheduled Reason
31-MAR-2018	11:11	Day 22		F3	5 ug	0	0	0	0	0	0	0		
09-MAY-2018	14:42	EOS/ET (Day 61)		F1	5 ug	0	0	0	0	0	0	0		
09-MAY-2018	14:42	EOS/ET (Day 61)		F2	5 ug	0	0	0	0	0	0	0		
09-MAY-2018	14:42	EOS/ET (Day 61)		F3	5 ug	0	0	0	0	0	0	0		

Key: 0= None; 1=Mild; 2=Moderate; 3=Severe; # Other = Pinprick bleeding or seepage

Administration Site Key: FA = Forearm; UA= Upper arm
Treatments Part A: A1 = A/Sing MAP, 15 ug (FA); A2 = Afluria Quad IM (UA), 15 ug; A3 = Placebo MAP (FA); A4 = A/Sing Mono IM, 15 ug (UA)
Treatments Part B: B1 = A/Sing MAP, 15 ug (FA) with biopsy; B2 = Placebo MAP (FA) with biopsy
B3 = A/Sing MAP, 15 ug (FA); B4 = A/Sing MAP, 10 ug (FA); B5 = A/Sing MAP, 5 ug (FA); B6 = A/Sing MAP, 2.5 ug (FA)
B7 = Placebo MAP (FA); B8 = A/Sing MAP, 15 ug (UA); B9 = A/Sing IM, 15 ug, as 2018 Afluria Quad (UA)

Listing 16.2.9.2
Treatment Site Tolerability Assessment per Site

Part A
Randomisation Number = A0025, Treatment Group = Group A3

Date	Time	Visit	Time Point	Site Number	Treatment for Site	Indura-tion	Redness	Bruising	Itching	Skin Flaking	Tender-ness	Definition/ Visibility	Other#	Unscheduled Reason
10-MAR-2018	12:22	Day 1	Pre-dose	F1	uncoated	0	0	0	0	0	0	0		
10-MAR-2018	12:22	Day 1	Pre-dose	F2	uncoated	0	0	0	0	0	0	0		
10-MAR-2018	12:22	Day 1	Pre-dose	F3	uncoated	0	0	0	0	0	0	0		
10-MAR-2018	12:48	Day 1	10 min	F1	uncoated	1	1	0	0	0	2	0		
10-MAR-2018	12:48	Day 1	10 min	F2	uncoated	1	1	0	0	0	2	0		
10-MAR-2018	12:48	Day 1	10 min	F3	uncoated	1	1	0	0	0	2	0		
10-MAR-2018	13:38	Day 1	1 hour	F1	uncoated	1	0	0	0	0	0	0		
10-MAR-2018	13:38	Day 1	1 hour	F2	uncoated	1	1	0	0	0	0	0		
10-MAR-2018	13:38	Day 1	1 hour	F3	uncoated	1	1	0	0	0	0	0		
10-MAR-2018	14:40	Day 1	2 hour	F1	uncoated	0	0	0	0	0	0	0		
10-MAR-2018	14:40	Day 1	2 hour	F2	uncoated	1	0	0	0	0	0	0		
10-MAR-2018	14:40	Day 1	2 hour	F3	uncoated	1	0	0	0	0	0	0		
13-MAR-2018	13:11	Day 4		F1	uncoated	0	0	0	0	0	0	0		
13-MAR-2018	13:11	Day 4		F2	uncoated	0	0	0	0	0	0	0		
13-MAR-2018	13:11	Day 4		F3	uncoated	0	0	0	0	0	0	0		
17-MAR-2018	13:54	Day 8		F1	uncoated	0	0	0	0	0	0	1		
17-MAR-2018	13:54	Day 8		F2	uncoated	0	0	0	0	0	0	1		
17-MAR-2018	13:54	Day 8		F3	uncoated	0	0	0	0	0	0	1		
31-MAR-2018	12:55	Day 22		F1	uncoated	0	0	0	0	0	0	0		
31-MAR-2018	12:55	Day 22		F2	uncoated	0	0	0	0	0	0	0		

Key: 0= None; 1=Mild; 2=Moderate; 3=Severe; # Other = Pinprick bleeding or seepage

Administration Site Key: FA = Forearm; UA= Upper arm
Treatments Part A: A1 = A/Sing MAP, 15 ug (FA); A2 = Afluria Quad IM (UA), 15 ug; A3 = Placebo MAP (FA); A4 = A/Sing Mono IM, 15 ug (UA)
Treatments Part B: B1 = A/Sing MAP, 15 ug (FA) with biopsy; B2 = Placebo MAP (FA) with biopsy
B3 = A/Sing MAP, 15 ug (FA); B4 = A/Sing MAP, 10 ug (FA); B5 = A/Sing MAP, 5 ug (FA); B6 = A/Sing MAP, 2.5 ug (FA)
B7 = Placebo MAP (FA); B8 = A/Sing MAP, 15 ug (UA); B9 = A/Sing IM, 15 ug, as 2018 Afluria Quad (UA)

Listing 16.2.9.2
Treatment Site Tolerability Assessment per Site

Part A
Randomisation Number = A0025, Treatment Group = Group A3

Date	Time	Visit	Time Point	Site Number	Treatment for Site	Indura-tion	Redness	Bruising	Itching	Skin Flaking	Tender-ness	Definition/ Visibility	Other#	Unscheduled Reason
31-MAR-2018	12:55	Day 22		F3	uncoated	0	0	0	0	0	0	0		
09-MAY-2018	11:55	EOS/ET (Day 61)		F1	uncoated	0	0	0	0	0	0	0		
09-MAY-2018	11:55	EOS/ET (Day 61)		F2	uncoated	0	0	0	0	0	0	0		
09-MAY-2018	11:55	EOS/ET (Day 61)		F3	uncoated	0	0	0	0	0	0	0		

Key: 0= None; 1=Mild; 2=Moderate; 3=Severe; # Other = Pinprick bleeding or seepage

Administration Site Key: FA = Forearm; UA= Upper arm
Treatments Part A: A1 = A/Sing MAP, 15 ug (FA); A2 = Afluria Quad IM (UA), 15 ug; A3 = Placebo MAP (FA); A4 = A/Sing Mono IM, 15 ug (UA)
Treatments Part B: B1 = A/Sing MAP, 15 ug (FA) with biopsy; B2 = Placebo MAP (FA) with biopsy
B3 = A/Sing MAP, 15 ug (FA); B4 = A/Sing MAP, 10 ug (FA); B5 = A/Sing MAP, 5 ug (FA); B6 = A/Sing MAP, 2.5 ug (FA)
B7 = Placebo MAP (FA); B8 = A/Sing MAP, 15 ug (UA); B9 = A/Sing IM, 15 ug, as 2018 Afluria Quad (UA)

Listing 16.2.9.2
Treatment Site Tolerability Assessment per Site

Part A
Randomisation Number = A0027, Treatment Group = Group A3

Date	Time	Visit	Time Point	Site Number	Treatment for Site	Indura-tion	Redness	Bruising	Itching	Skin Flaking	Tender-ness	Definition/ Visibility	Other#	Unscheduled Reason
10-MAR-2018	12:30	Day 1	Pre-dose	F1	uncoated									
10-MAR-2018	12:30	Day 1	Pre-dose	F2	uncoated									
10-MAR-2018	12:30	Day 1	Pre-dose	F3	uncoated	0	0	0	0	0	0	0		
10-MAR-2018	12:58	Day 1	10 min	F1	uncoated	1	1	0	0	0	1	0		
10-MAR-2018	12:58	Day 1	10 min	F2	uncoated	1	1	0	0	0	1	0		
10-MAR-2018	12:58	Day 1	10 min	F3	uncoated	1	1	0	0	0	1	0		
10-MAR-2018	13:48	Day 1	1 hour	F1	uncoated	0	0		0	0	0	0		
10-MAR-2018	13:48	Day 1	1 hour	F2	uncoated	1	0		0	0	0	0		
10-MAR-2018	13:48	Day 1	1 hour	F3	uncoated	1	1	0	0	0	0	0		
10-MAR-2018	14:48	Day 1	2 hour	F1	uncoated	0	0	0	0	0	0	0		
10-MAR-2018	14:48	Day 1	2 hour	F2	uncoated	1	0	0	0	0	0	0		
10-MAR-2018	14:48	Day 1	2 hour	F3	uncoated	1	0	0	0	0	0	0		
13-MAR-2018	13:26	Day 4		F1	uncoated	0	0	0	0	0	0	3		
13-MAR-2018	13:26	Day 4		F2	uncoated	0	0	0	0	0	0	3		
13-MAR-2018	13:26	Day 4		F3	uncoated	0	0	0	0	0	0	3		
17-MAR-2018	13:57	Day 8		F1	uncoated	0	0	0	0	0	0	2		
17-MAR-2018	13:57	Day 8		F2	uncoated	0	0	0	0	0	0	2		
17-MAR-2018	13:57	Day 8		F3	uncoated	0	0	0	0	0	0	2		
01-APR-2018	09:00	Day 22		F1	uncoated	0	0	0	0	0	0	1		
01-APR-2018	09:00	Day 22		F2	uncoated	0	0	0	0	0	0	1		

Key: 0= None; 1=Mild; 2=Moderate; 3=Severe; # Other = Pinprick bleeding or seepage

Administration Site Key: FA = Forearm; UA= Upper arm
Treatments Part A: A1 = A/Sing MAP, 15 ug (FA); A2 = Afluria Quad IM (UA), 15 ug; A3 = Placebo MAP (FA); A4 = A/Sing Mono IM, 15 ug (UA)
Treatments Part B: B1 = A/Sing MAP, 15 ug (FA) with biopsy; B2 = Placebo MAP (FA) with biopsy
B3 = A/Sing MAP, 15 ug (FA); B4 = A/Sing MAP, 10 ug (FA); B5 = A/Sing MAP, 5 ug (FA); B6 = A/Sing MAP, 2.5 ug (FA)
B7 = Placebo MAP (FA); B8 = A/Sing MAP, 15 ug (UA); B9 = A/Sing IM, 15 ug, as 2018 Afluria Quad (UA)

Listing 16.2.9.2
Treatment Site Tolerability Assessment per Site

Part A
Randomisation Number = A0027, Treatment Group = Group A3

Date	Time	Visit	Time Point	Site Number	Treatment for Site	Indura-tion	Redness	Bruising	Itching	Skin Flaking	Tender-ness	Definition/ Visibility	Other#	Unscheduled Reason
01-APR-2018	09:00	Day 22		F3	uncoated	0	0	0	0	0	0	1		
09-MAY-2018	12:40	EOS/ET (Day 61)		F1	uncoated	0	0	0	0	0	0	0		
09-MAY-2018	12:40	EOS/ET (Day 61)		F2	uncoated	0	0	0	0	0	0	0		
09-MAY-2018	12:40	EOS/ET (Day 61)		F3	uncoated	0	0	0	0	0	0	0		

Key: 0= None; 1=Mild; 2=Moderate; 3=Severe; # Other = Pinprick bleeding or seepage

Administration Site Key: FA = Forearm; UA= Upper arm
Treatments Part A: A1 = A/Sing MAP, 15 ug (FA); A2 = Afluria Quad IM (UA), 15 ug; A3 = Placebo MAP (FA); A4 = A/Sing Mono IM, 15 ug (UA)
Treatments Part B: B1 = A/Sing MAP, 15 ug (FA) with biopsy; B2 = Placebo MAP (FA) with biopsy
B3 = A/Sing MAP, 15 ug (FA); B4 = A/Sing MAP, 10 ug (FA); B5 = A/Sing MAP, 5 ug (FA); B6 = A/Sing MAP, 2.5 ug (FA)
B7 = Placebo MAP (FA); B8 = A/Sing MAP, 15 ug (UA); B9 = A/Sing IM, 15 ug, as 2018 Afluria Quad (UA)

Listing 16.2.9.2
Treatment Site Tolerability Assessment per Site

Part A
Randomisation Number = A0028, Treatment Group = Group A1

Date	Time	Visit	Time Point	Site Number	Treatment for Site	Indura-tion	Redness	Bruising	Itching	Skin Flaking	Tender-ness	Definition/ Visibility	Other#	Unscheduled Reason
10-MAR-2018	12:43	Day 1	Pre-dose	F1	5 ug	0	0	0	0	0	0	0		
10-MAR-2018	12:43	Day 1	Pre-dose	F2	5 ug	0	0	0	0	0	0	0		
10-MAR-2018	12:43	Day 1	Pre-dose	F3	5 ug	0	0	0	0	0	0	0		
10-MAR-2018	13:08	Day 1	10 min	F1	5 ug	1	1	0	1	0	0	0		
10-MAR-2018	13:08	Day 1	10 min	F2	5 ug	1	1	0	0	0	0	0		
10-MAR-2018	13:08	Day 1	10 min	F3	5 ug	1	1	0	0	0	0	0		
10-MAR-2018	13:58	Day 1	1 hour	F1	5 ug	1	1	0	1	0	0	0		
10-MAR-2018	13:58	Day 1	1 hour	F2	5 ug	1	2	0	1	0	0	0		
10-MAR-2018	13:58	Day 1	1 hour	F3	5 ug	1	2	0	0	0	0	0		
10-MAR-2018	14:58	Day 1	2 hour	F1	5 ug	0	0	0	0	0	0	0		
10-MAR-2018	14:58	Day 1	2 hour	F2	5 ug	1	0	0	0	0	0	0		
10-MAR-2018	14:58	Day 1	2 hour	F3	5 ug	1	0	0	0	0	0	0		
13-MAR-2018	09:01	Day 4		F1	5 ug	0	0	0	1	0	0	3		
13-MAR-2018	09:01	Day 4		F2	5 ug	0	0	0	0	0	0	3		
13-MAR-2018	09:01	Day 4		F3	5 ug	0	0	0	0	0	0	3		
17-MAR-2018	14:02	Day 8		F1	5 ug	0	1	0	0	1	0	3		
17-MAR-2018	14:02	Day 8		F2	5 ug	0	1	0	0	1	0	3		
17-MAR-2018	14:02	Day 8		F3	5 ug	0	1	0	0	1	0	3		
31-MAR-2018	14:40	Day 22		F1	5 ug	0	0	0	0	0	0	1		
31-MAR-2018	14:40	Day 22		F2	5 ug	0	0	0	0	1	0	1		

Key: 0= None; 1=Mild; 2=Moderate; 3=Severe; # Other = Pinprick bleeding or seepage

Administration Site Key: FA = Forearm; UA= Upper arm
Treatments Part A: A1 = A/Sing MAP, 15 ug (FA); A2 = Afluria Quad IM (UA), 15 ug; A3 = Placebo MAP (FA); A4 = A/Sing Mono IM, 15 ug (UA)
Treatments Part B: B1 = A/Sing MAP, 15 ug (FA) with biopsy; B2 = Placebo MAP (FA) with biopsy
B3 = A/Sing MAP, 15 ug (FA); B4 = A/Sing MAP, 10 ug (FA); B5 = A/Sing MAP, 5 ug (FA); B6 = A/Sing MAP, 2.5 ug (FA)
B7 = Placebo MAP (FA); B8 = A/Sing MAP, 15 ug (UA); B9 = A/Sing IM, 15 ug, as 2018 Afluria Quad (UA)

Listing 16.2.9.2
Treatment Site Tolerability Assessment per Site

Part A
Randomisation Number = A0028, Treatment Group = Group A1

Date	Time	Visit	Time Point	Site Number	Treatment for Site	Indura-tion	Redness	Bruising	Itching	Skin Flaking	Tender-ness	Definition/ Visibility	Other#	Unscheduled Reason
31-MAR-2018	14:40	Day 22		F3	5 ug	0	0	0	0	0	0	1		
09-MAY-2018	16:51	EOS/ET (Day 61)		F1	5 ug	0	0	0	0	0	0	0		
09-MAY-2018	16:51	EOS/ET (Day 61)		F2	5 ug	0	0	0	0	0	0	0		
09-MAY-2018	16:51	EOS/ET (Day 61)		F3	5 ug	0	0	0	0	0	0	0		

Key: 0= None; 1=Mild; 2=Moderate; 3=Severe; # Other = Pinprick bleeding or seepage

Administration Site Key: FA = Forearm; UA= Upper arm
Treatments Part A: A1 = A/Sing MAP, 15 ug (FA); A2 = Afluria Quad IM (UA), 15 ug; A3 = Placebo MAP (FA); A4 = A/Sing Mono IM, 15 ug (UA)
Treatments Part B: B1 = A/Sing MAP, 15 ug (FA) with biopsy; B2 = Placebo MAP (FA) with biopsy
B3 = A/Sing MAP, 15 ug (FA); B4 = A/Sing MAP, 10 ug (FA); B5 = A/Sing MAP, 5 ug (FA); B6 = A/Sing MAP, 2.5 ug (FA)
B7 = Placebo MAP (FA); B8 = A/Sing MAP, 15 ug (UA); B9 = A/Sing IM, 15 ug, as 2018 Afluria Quad (UA)

Listing 16.2.9.2
Treatment Site Tolerability Assessment per Site

Part A
Randomisation Number = A0029, Treatment Group = Group A1

Date	Time	Visit	Time Point	Site Number	Treatment for Site	Induration	Redness	Bruising	Itching	Skin Flaking	Tenderness	Definition/ Visibility	Other#	Unscheduled Reason
10-MAR-2018	12:51	Day 1	Pre-dose	F1	5 ug	0	0	0	0	0	0	0		
10-MAR-2018	12:51	Day 1	Pre-dose	F2	5 ug	0	0	0	0	0	0	0		
10-MAR-2018	12:51	Day 1	Pre-dose	F3	5 ug	0	0	0	0	0	0	0		
10-MAR-2018	13:08	Day 1	10 min	F1	5 ug	0	1	0	0	0	1	0		
10-MAR-2018	13:08	Day 1	10 min	F2	5 ug	1	1	0	0	0	1	0		
10-MAR-2018	13:08	Day 1	10 min	F3	5 ug	1	1	0	0	0	1	0		
10-MAR-2018	14:11	Day 1	1 hour	F1	5 ug	1	1	0	0	0	0	0		
10-MAR-2018	14:11	Day 1	1 hour	F2	5 ug	2	1		0	0	0	0		
10-MAR-2018	14:11	Day 1	1 hour	F3	5 ug	2	1	0	0	0	0	0		
10-MAR-2018	15:11	Day 1	2 hour	F1	5 ug	1	1	0	0	0	0	0		
10-MAR-2018	15:11	Day 1	2 hour	F2	5 ug	1	1	0	0	0	0	0		
10-MAR-2018	15:11	Day 1	2 hour	F3	5 ug	1	1	0	0	0	0	0		
13-MAR-2018	16:17	Day 4		F1	5 ug	0	1	0	0	0	0	0		
13-MAR-2018	16:17	Day 4		F2	5 ug	0	1	0	0	0	0	0		
13-MAR-2018	16:17	Day 4		F3	5 ug	0	1	0	0	0	0	0		
17-MAR-2018	15:56	Day 8		F1	5 ug	0	1	0	0	0	0	0		
17-MAR-2018	15:56	Day 8		F2	5 ug	0	1	0	0	0	0	0		
17-MAR-2018	15:56	Day 8		F3	5 ug	0	1	0	0	0	0	0		
31-MAR-2018	13:20	Day 22		F1	5 ug	0	0	0	0	0	0	0		
31-MAR-2018	13:20	Day 22		F2	5 ug	0	0	0	0	0	0	0		

Key: 0= None; 1=Mild; 2=Moderate; 3=Severe; # Other = Pinprick bleeding or seepage

Administration Site Key: FA = Forearm; UA= Upper arm
Treatments Part A: A1 = A/Sing MAP, 15 ug (FA); A2 = Afluria Quad IM (UA), 15 ug; A3 = Placebo MAP (FA); A4 = A/Sing Mono IM, 15 ug (UA)
Treatments Part B: B1 = A/Sing MAP, 15 ug (FA) with biopsy; B2 = Placebo MAP (FA) with biopsy
B3 = A/Sing MAP, 15 ug (FA); B4 = A/Sing MAP, 10 ug (FA); B5 = A/Sing MAP, 5 ug (FA); B6 = A/Sing MAP, 2.5 ug (FA)
B7 = Placebo MAP (FA); B8 = A/Sing MAP, 15 ug (UA); B9 = A/Sing IM, 15 ug, as 2018 Afluria Quad (UA)

Listing 16.2.9.2
Treatment Site Tolerability Assessment per Site

Part A
Randomisation Number = A0029, Treatment Group = Group A1

Date	Time	Visit	Time Point	Site Number	Treatment for Site	Indura-tion	Redness	Bruising	Itching	Skin Flaking	Tender-ness	Definition/ Visibility	Other#	Unscheduled Reason
31-MAR-2018	13:20	Day 22		F3	5 ug	0	0	0	0	0	0	0		
09-MAY-2018	16:19	EOS/ET (Day 61)		F1	5 ug	0	0	0	0	0	0	1		
09-MAY-2018	16:19	EOS/ET (Day 61)		F2	5 ug	0	0	0	0	0	0	1		
09-MAY-2018	16:19	EOS/ET (Day 61)		F3	5 ug	0	0	0	0	0	0	2		

Key: 0= None; 1=Mild; 2=Moderate; 3=Severe; # Other = Pinprick bleeding or seepage

Administration Site Key: FA = Forearm; UA= Upper arm
Treatments Part A: A1 = A/Sing MAP, 15 ug (FA); A2 = Afluria Quad IM (UA), 15 ug; A3 = Placebo MAP (FA); A4 = A/Sing Mono IM, 15 ug (UA)
Treatments Part B: B1 = A/Sing MAP, 15 ug (FA) with biopsy; B2 = Placebo MAP (FA) with biopsy
B3 = A/Sing MAP, 15 ug (FA); B4 = A/Sing MAP, 10 ug (FA); B5 = A/Sing MAP, 5 ug (FA); B6 = A/Sing MAP, 2.5 ug (FA)
B7 = Placebo MAP (FA); B8 = A/Sing MAP, 15 ug (UA); B9 = A/Sing IM, 15 ug, as 2018 Afluria Quad (UA)

Listing 16.2.9.2
Treatment Site Tolerability Assessment per Site

Part A
Randomisation Number = A0030, Treatment Group = Group A3

Date	Time	Visit	Time Point	Site Number	Treatment for Site	Indura-tion	Redness	Bruising	Itching	Skin Flaking	Tender-ness	Definition/ Visibility	Other#	Unscheduled Reason
16-MAR-2018	11:53	Day 1	Pre-dose	F1	uncoated	0	0	0	0	0	0	0		
16-MAR-2018	11:53	Day 1	Pre-dose	F2	uncoated	0	0	0	0	0	0	0		
16-MAR-2018	11:53	Day 1	Pre-dose	F3	uncoated	0	0	0	0	0	0	0		
16-MAR-2018	12:19	Day 1	10 min	F1	uncoated	0	1	0	0	0	0	0		
16-MAR-2018	12:19	Day 1	10 min	F2	uncoated	0	1	0	0	0	1	0		
16-MAR-2018	12:19	Day 1	10 min	F3	uncoated	0	1	0	0	0	1	0		
16-MAR-2018	13:12	Day 1	1 hour	F1	uncoated	0	0	0	0	0	0	0		
16-MAR-2018	13:12	Day 1	1 hour	F2	uncoated	0	0	0	0	0	0	0		
16-MAR-2018	13:12	Day 1	1 hour	F3	uncoated	0	0	0	0	0	0	0		
16-MAR-2018	14:51	Day 1	2 hour	F1	uncoated	0	0	0	0	0	0	0		
16-MAR-2018	14:51	Day 1	2 hour	F2	uncoated	0	0	0	0	0	0	0		
16-MAR-2018	14:51	Day 1	2 hour	F3	uncoated	0	0	0	0	0	0	0		
20-MAR-2018	09:21	Day 4		F1	uncoated	0	0	0	0	0	0	3		
20-MAR-2018	09:21	Day 4		F2	uncoated	0	0	0	0	0	0	3		
20-MAR-2018	09:21	Day 4		F3	uncoated	0	0	0	0	0	0	3		
27-MAR-2018	12:21	Day 8		F1	uncoated	0	0	0	0	0	0	3		
27-MAR-2018	12:21	Day 8		F2	uncoated	0	0	0	0	1	0	3		
27-MAR-2018	12:21	Day 8		F3	uncoated	0	0	0	0	1	0	3		
06-APR-2018	13:01	Day 22		F1	uncoated	0	0	0	0	0	0	3		
06-APR-2018	13:01	Day 22		F2	uncoated	0	0	0	0	0	0	3		

Key: 0= None; 1=Mild; 2=Moderate; 3=Severe; # Other = Pinprick bleeding or seepage

Administration Site Key: FA = Forearm; UA= Upper arm
Treatments Part A: A1 = A/Sing MAP, 15 ug (FA); A2 = Afluria Quad IM (UA), 15 ug; A3 = Placebo MAP (FA); A4 = A/Sing Mono IM, 15 ug (UA)
Treatments Part B: B1 = A/Sing MAP, 15 ug (FA) with biopsy; B2 = Placebo MAP (FA) with biopsy
B3 = A/Sing MAP, 15 ug (FA); B4 = A/Sing MAP, 10 ug (FA); B5 = A/Sing MAP, 5 ug (FA); B6 = A/Sing MAP, 2.5 ug (FA)
B7 = Placebo MAP (FA); B8 = A/Sing MAP, 15 ug (UA); B9 = A/Sing IM, 15 ug, as 2018 Afluria Quad (UA)

Listing 16.2.9.2
Treatment Site Tolerability Assessment per Site

Part A
Randomisation Number = A0030, Treatment Group = Group A3

Date	Time	Visit	Time Point	Site Number	Treatment for Site	Indura-tion	Redness	Bruising	Itching	Skin Flaking	Tender-ness	Definition/ Visibility	Other#	Unscheduled Reason
06-APR-2018	13:01	Day 22		F3	uncoated	0	0	0	0	0	0	3		
15-MAY-2018	12:25	EOS/ET (Day 61)		F1	uncoated	0	0	0	0	0	0	3		
15-MAY-2018	12:25	EOS/ET (Day 61)		F2	uncoated	0	0	0	0	0	0	3		
15-MAY-2018	12:25	EOS/ET (Day 61)		F3	uncoated	0	0	0	0	0	0	3		

Key: 0= None; 1=Mild; 2=Moderate; 3=Severe; # Other = Pinprick bleeding or seepage

Administration Site Key: FA = Forearm; UA= Upper arm
Treatments Part A: A1 = A/Sing MAP, 15 ug (FA); A2 = Afluria Quad IM (UA), 15 ug; A3 = Placebo MAP (FA); A4 = A/Sing Mono IM, 15 ug (UA)
Treatments Part B: B1 = A/Sing MAP, 15 ug (FA) with biopsy; B2 = Placebo MAP (FA) with biopsy
B3 = A/Sing MAP, 15 ug (FA); B4 = A/Sing MAP, 10 ug (FA); B5 = A/Sing MAP, 5 ug (FA); B6 = A/Sing MAP, 2.5 ug (FA)
B7 = Placebo MAP (FA); B8 = A/Sing MAP, 15 ug (UA); B9 = A/Sing IM, 15 ug, as 2018 Afluria Quad (UA)

Listing 16.2.9.2
Treatment Site Tolerability Assessment per Site

Part A
Randomisation Number = A0031, Treatment Group = Group A2

Date	Time	Visit	Time Point	Site Number	Treatment for Site	Indura-tion	Redness	Bruising	Itching	Skin Flaking	Tender-ness	Definition/ Visibility	Other#	Unscheduled Reason
16-MAR-2018	11:38	Day 1	Pre-dose	D1	[IM]	0	0	0	0	0	0	5		
16-MAR-2018	13:10	Day 1	10 min	D1	[IM]	0	0	0	0	0	0	5		
16-MAR-2018	13:56	Day 1	1 hour	D1	[IM]	0	0	0	0	0	0	5		
16-MAR-2018	15:06	Day 1	2 hour	D1	[IM]	0	0	0	0	0	0	5		
19-MAR-2018	13:19	Day 4		D1	[IM]	0	0	0	0	0	0	5		

Key: 0= None; 1=Mild; 2=Moderate; 3=Severe; # Other = Pinprick bleeding or seepage

Administration Site Key: FA = Forearm; UA= Upper arm
Treatments Part A: A1 = A/Sing MAP, 15 ug (FA); A2 = Afluria Quad IM (UA), 15 ug; A3 = Placebo MAP (FA); A4 = A/Sing Mono IM, 15 ug (UA)
Treatments Part B: B1 = A/Sing MAP, 15 ug (FA) with biopsy; B2 = Placebo MAP (FA) with biopsy
B3 = A/Sing MAP, 15 ug (FA); B4 = A/Sing MAP, 10 ug (FA); B5 = A/Sing MAP, 5 ug (FA); B6 = A/Sing MAP, 2.5 ug (FA)
B7 = Placebo MAP (FA); B8 = A/Sing MAP, 15 ug (UA); B9 = A/Sing IM, 15 ug, as 2018 Afluria Quad (UA)

Listing 16.2.9.2
Treatment Site Tolerability Assessment per Site

Part A
Randomisation Number = A0032, Treatment Group = Group A4

Date	Time	Visit	Time Point	Site Number	Treatment for Site	Indura- tion	Redness	Bruising	Itching	Skin Flaking	Tender- ness	Definition/ Visibility	Other#	Unscheduled Reason
16-MAR-2018	11:35	Day 1	Pre-dose	D1	[IM]	0	0	0	0	0	0	5		
16-MAR-2018	13:20	Day 1	10 min	D1	[IM]	0	0	0	0	0	0	5		
16-MAR-2018	14:10	Day 1	1 hour	D1	[IM]	0	0	0	0	0	0	5		
16-MAR-2018	15:16	Day 1	2 hour	D1	[IM]	0	0	0	0	0	0	5		
19-MAR-2018	13:53	Day 4		D1	[IM]	1	1	0	0	0	0	5		
23-MAR-2018	12:59	Day 8		D1	[IM]	0	0	0	0	0	0	5		
06-APR-2018	13:20	Day 22		D1	[IM]	0	0	0	0	0	0	5		
15-MAY-2018	12:12	EOS/ET (Day 61)		D1	[IM]	0	0	0	0	0	0	5		

Key: 0= None; 1=Mild; 2=Moderate; 3=Severe; # Other = Pinprick bleeding or seepage

Administration Site Key: FA = Forearm; UA= Upper arm
Treatments Part A: A1 = A/Sing MAP, 15 ug (FA); A2 = Afluria Quad IM (UA), 15 ug; A3 = Placebo MAP (FA); A4 = A/Sing Mono IM, 15 ug (UA)
Treatments Part B: B1 = A/Sing MAP, 15 ug (FA) with biopsy; B2 = Placebo MAP (FA) with biopsy
B3 = A/Sing MAP, 15 ug (FA); B4 = A/Sing MAP, 10 ug (FA); B5 = A/Sing MAP, 5 ug (FA); B6 = A/Sing MAP, 2.5 ug (FA)
B7 = Placebo MAP (FA); B8 = A/Sing MAP, 15 ug (UA); B9 = A/Sing IM, 15 ug, as 2018 Afluria Quad (UA)

Listing 16.2.9.2
Treatment Site Tolerability Assessment per Site

Part A
Randomisation Number = A0033, Treatment Group = Group A2

Date	Time	Visit	Time Point	Site Number	Treatment for Site	Indura-tion	Redness	Bruising	Itching	Skin Flaking	Tender-ness	Definition/ Visibility	Other#	Unscheduled Reason
16-MAR-2018	11:48	Day 1	Pre-dose	D1	[IM]	0	0	0	0	0	0	5		
16-MAR-2018	13:30	Day 1	10 min	D1	[IM]	0	0	0	0	0	0	5		
16-MAR-2018	14:19	Day 1	1 hour	D1	[IM]	0	0	0	0	0	0	5		
16-MAR-2018	15:26	Day 1	2 hour	D1	[IM]	0	0	0	0	0	0	5		
19-MAR-2018	15:31	Day 4		D1	[IM]	0	0	0	0	0	0	5		
23-MAR-2018	15:35	Day 8		D1	[IM]	0	0	0	0	0	0	5		
06-APR-2018	15:43	Day 22		D1	[IM]	0	0	0	0	0	0	5		
15-MAY-2018	12:13	EOS/ET (Day 61)		D1	[IM]	0	0	0	0	0	0	5		

Key: 0= None; 1=Mild; 2=Moderate; 3=Severe; # Other = Pinprick bleeding or seepage

Administration Site Key: FA = Forearm; UA= Upper arm
Treatments Part A: A1 = A/Sing MAP, 15 ug (FA); A2 = Afluria Quad IM (UA), 15 ug; A3 = Placebo MAP (FA); A4 = A/Sing Mono IM, 15 ug (UA)
Treatments Part B: B1 = A/Sing MAP, 15 ug (FA) with biopsy; B2 = Placebo MAP (FA) with biopsy
B3 = A/Sing MAP, 15 ug (FA); B4 = A/Sing MAP, 10 ug (FA); B5 = A/Sing MAP, 5 ug (FA); B6 = A/Sing MAP, 2.5 ug (FA)
B7 = Placebo MAP (FA); B8 = A/Sing MAP, 15 ug (UA); B9 = A/Sing IM, 15 ug, as 2018 Afluria Quad (UA)

Listing 16.2.9.2
Treatment Site Tolerability Assessment per Site

Part A
Randomisation Number = A0034, Treatment Group = Group A4

Date	Time	Visit	Time Point	Site Number	Treatment for Site	Indura-tion	Redness	Bruising	Itching	Skin Flaking	Tender-ness	Definition/ Visibility	Other#	Unscheduled Reason
16-MAR-2018	11:45	Day 1	Pre-dose	D1	[IM]	0	0	0	0	0	0	5		
16-MAR-2018	13:40	Day 1	10 min	D1	[IM]	0	0	0	0	0	0	5		
16-MAR-2018	14:31	Day 1	1 hour	D1	[IM]	0	0	0	0	0	0	5		
16-MAR-2018	15:36	Day 1	2 hour	D1	[IM]	0	0	0	0	0	0	5		
19-MAR-2018	11:44	Day 4		D1	[IM]	0	0	0	0	0	0	5		
23-MAR-2018	08:39	Day 8		D1	[IM]	0	0	0	0	0	0	5		
06-APR-2018	09:16	Day 22		D1	[IM]	0	0	0	0	0	0	5		
17-MAY-2018	09:15	EOS/ET (Day 61)		D1	[IM]	0	0	0	0	0	0	5		

Key: 0= None; 1=Mild; 2=Moderate; 3=Severe; # Other = Pinprick bleeding or seepage

Administration Site Key: FA = Forearm; UA= Upper arm
Treatments Part A: A1 = A/Sing MAP, 15 ug (FA); A2 = Afluria Quad IM (UA), 15 ug; A3 = Placebo MAP (FA); A4 = A/Sing Mono IM, 15 ug (UA)
Treatments Part B: B1 = A/Sing MAP, 15 ug (FA) with biopsy; B2 = Placebo MAP (FA) with biopsy
B3 = A/Sing MAP, 15 ug (FA); B4 = A/Sing MAP, 10 ug (FA); B5 = A/Sing MAP, 5 ug (FA); B6 = A/Sing MAP, 2.5 ug (FA)
B7 = Placebo MAP (FA); B8 = A/Sing MAP, 15 ug (UA); B9 = A/Sing IM, 15 ug, as 2018 Afluria Quad (UA)

Listing 16.2.9.2

Treatment Site Tolerability Assessment per Site

Part A

Randomisation Number = A0035,   Treatment Group = Group A4

Date	Time	Visit	Time Point	Site Number	Treatment for Site	Induration	Redness	Bruising	Itching	Skin Flaking	Tenderness	Definition/ Visibility	Other#	Unscheduled Reason
16-MAR-2018	11:49	Day 1	Pre-dose	D1	[IM]	0	0	0	0	0	0	5		
16-MAR-2018	13:50	Day 1	10 min	D1	[IM]	0	0	0	0	0	0	5		
16-MAR-2018	14:40	Day 1	1 hour	D1	[IM]	0	0	0	0	0	1	5		
16-MAR-2018	15:46	Day 1	2 hour	D1	[IM]	0	0	0	0	0	0	5		
19-MAR-2018	16:30	Day 4		D1	[IM]	0	0	0	0	0	0	5		
23-MAR-2018	15:33	Day 8		D1	[IM]	0	0	0	0	0	0	5		
08-APR-2018	12:22	Day 22		D1	[IM]	0	0	0	0	0	0	5		
15-MAY-2018	13:51	EOS/ET (Day 61)		D1	[IM]	0	0	0	0	0	0	5		

Key: 0= None; 1=Mild; 2=Moderate; 3=Severe; # Other = Pinprick bleeding or seepage

Administration Site Key: FA = Forearm; UA= Upper arm

Treatments Part A: A1 = A/Sing MAP, 15 ug (FA); A2 = Afluria Quad IM (UA), 15 ug; A3 = Placebo MAP (FA); A4 = A/Sing Mono IM, 15 ug (UA)

Treatments Part B: B1 = A/Sing MAP, 15 ug (FA) with biopsy; B2 = Placebo MAP (FA) with biopsy

B3 = A/Sing MAP, 15 ug (FA); B4 = A/Sing MAP, 10 ug (FA); B5 = A/Sing MAP, 5 ug (FA); B6 = A/Sing MAP, 2.5 ug (FA)

B7 = Placebo MAP (FA); B8 = A/Sing MAP, 15 ug (UA); B9 = A/Sing IM, 15 ug, as 2018 Afluria Quad (UA)

Listing 16.2.9.2
Treatment Site Tolerability Assessment per Site

Part A
Randomisation Number = A0036, Treatment Group = Group A2

Date	Time	Visit	Time Point	Site Number	Treatment for Site	Indura-tion	Redness	Bruising	Itching	Skin Flaking	Tender-ness	Definition/ Visibility	Other#	Unscheduled Reason
17-MAR-2018	10:40	Day 1	Pre-dose	D1	[IM]	0	0	0	0	0	0	5		
17-MAR-2018	11:40	Day 1	10 min	D1	[IM]	0	0	0	0	0	0	5		
17-MAR-2018	12:30	Day 1	1 hour	D1	[IM]	0	0	0	0	0	0	5		
17-MAR-2018	13:35	Day 1	2 hour	D1	[IM]	0	0	0	0	0	0	5		
21-MAR-2018	17:04	Day 4		D1	[IM]	0	0	0	0	0	0	5		
25-MAR-2018	10:37	Day 8		D1	[IM]	0	0	0	0	0	0	5		
07-APR-2018	11:09	Day 22		D1	[IM]	0	0	0	0	0	0	5		
17-MAY-2018	14:06	EOS/ET (Day 61)		D1	[IM]	0	0	0	0	0	0	5		

Key: 0= None; 1=Mild; 2=Moderate; 3=Severe; # Other = Pinprick bleeding or seepage

Administration Site Key: FA = Forearm; UA= Upper arm
Treatments Part A: A1 = A/Sing MAP, 15 ug (FA); A2 = Afluria Quad IM (UA), 15 ug; A3 = Placebo MAP (FA); A4 = A/Sing Mono IM, 15 ug (UA)
Treatments Part B: B1 = A/Sing MAP, 15 ug (FA) with biopsy; B2 = Placebo MAP (FA) with biopsy
B3 = A/Sing MAP, 15 ug (FA); B4 = A/Sing MAP, 10 ug (FA); B5 = A/Sing MAP, 5 ug (FA); B6 = A/Sing MAP, 2.5 ug (FA)
B7 = Placebo MAP (FA); B8 = A/Sing MAP, 15 ug (UA); B9 = A/Sing IM, 15 ug, as 2018 Afluria Quad (UA)

Listing 16.2.9.2
Treatment Site Tolerability Assessment per Site

Part A
Randomisation Number = A0037, Treatment Group = Group A4

Date	Time	Visit	Time Point	Site Number	Treatment for Site	Indura-tion	Redness	Bruising	Itching	Skin Flaking	Tender-ness	Definition/ Visibility	Other#	Unscheduled Reason
17-MAR-2018	10:31	Day 1	Pre-dose	D1	[IM]	0	0	0	0	0	0	5		
17-MAR-2018	11:45	Day 1	10 min	D1	[IM]	0	0	0	0	0	0	5		
17-MAR-2018	12:33	Day 1	1 hour	D1	[IM]	0	0	0	0	0	0	5		
17-MAR-2018	13:40	Day 1	2 hour	D1	[IM]	0	0	0	0	0	0	5		
20-MAR-2018	08:39	Day 4		D1	[IM]	0	0	0	0	0	0	5		
24-MAR-2018	09:36	Day 8		D1	[IM]	0	0	0	0	0	0	5		
07-APR-2018	09:21	Day 22		D1	[IM]	0	0	0	0	0	0	5		
17-MAY-2018	08:53	EOS/ET (Day 61)		D1	[IM]	0	0	0	0	0	0	5		

Key: 0= None; 1=Mild; 2=Moderate; 3=Severe; # Other = Pinprick bleeding or seepage

Administration Site Key: FA = Forearm; UA= Upper arm
Treatments Part A: A1 = A/Sing MAP, 15 ug (FA); A2 = Afluria Quad IM (UA), 15 ug; A3 = Placebo MAP (FA); A4 = A/Sing Mono IM, 15 ug (UA)
Treatments Part B: B1 = A/Sing MAP, 15 ug (FA) with biopsy; B2 = Placebo MAP (FA) with biopsy
B3 = A/Sing MAP, 15 ug (FA); B4 = A/Sing MAP, 10 ug (FA); B5 = A/Sing MAP, 5 ug (FA); B6 = A/Sing MAP, 2.5 ug (FA)
B7 = Placebo MAP (FA); B8 = A/Sing MAP, 15 ug (UA); B9 = A/Sing IM, 15 ug, as 2018 Afluria Quad (UA)

Listing 16.2.9.2
Treatment Site Tolerability Assessment per Site

Part A
Randomisation Number = A0038, Treatment Group = Group A2

Date	Time	Visit	Time Point	Site Number	Treatment for Site	Indura-tion	Redness	Bruising	Itching	Skin Flaking	Tender-ness	Definition/ Visibility	Other#	Unscheduled Reason
17-MAR-2018	10:25	Day 1	Pre-dose	D1	[IM]	0	0	0	0	0	0	5		
17-MAR-2018	11:50	Day 1	10 min	D1	[IM]	0	0	0	0	0	0	5		
17-MAR-2018	12:35	Day 1	1 hour	D1	[IM]	0	0	0	0	0	0	5		
17-MAR-2018	13:45	Day 1	2 hour	D1	[IM]	0	0	0	0	0	0	5		
20-MAR-2018	08:34	Day 4		D1	[IM]	0	0	0	0	0	0	5		
24-MAR-2018	09:34	Day 8		D1	[IM]	0	0	0	0	0	0	5		
07-APR-2018	09:23	Day 22		D1	[IM]	0	0	0	0	0	0	5		
17-MAY-2018	08:52	EOS/ET (Day 61)		D1	[IM]	0	0	0	0	0	0	5		

Key: 0= None; 1=Mild; 2=Moderate; 3=Severe; # Other = Pinprick bleeding or seepage

Administration Site Key: FA = Forearm; UA= Upper arm
Treatments Part A: A1 = A/Sing MAP, 15 ug (FA); A2 = Afluria Quad IM (UA), 15 ug; A3 = Placebo MAP (FA); A4 = A/Sing Mono IM, 15 ug (UA)
Treatments Part B: B1 = A/Sing MAP, 15 ug (FA) with biopsy; B2 = Placebo MAP (FA) with biopsy
B3 = A/Sing MAP, 15 ug (FA); B4 = A/Sing MAP, 10 ug (FA); B5 = A/Sing MAP, 5 ug (FA); B6 = A/Sing MAP, 2.5 ug (FA)
B7 = Placebo MAP (FA); B8 = A/Sing MAP, 15 ug (UA); B9 = A/Sing IM, 15 ug, as 2018 Afluria Quad (UA)

Listing 16.2.9.2
Treatment Site Tolerability Assessment per Site

Part A
Randomisation Number = A0039, Treatment Group = Group A2

Date	Time	Visit	Time Point	Site Number	Treatment for Site	Indura-tion	Redness	Bruising	Itching	Skin Flaking	Tender-ness	Definition/ Visibility	Other#	Unscheduled Reason
17-MAR-2018	10:20	Day 1	Pre-dose	D1	[IM]	0	0	0	0	0	0	5		
17-MAR-2018	11:55	Day 1	10 min	D1	[IM]	0	0	0	0	0	0	5		
17-MAR-2018	12:45	Day 1	1 hour	D1	[IM]	0	0	0	0	0	0	5		
17-MAR-2018	13:51	Day 1	2 hour	D1	[IM]	0	0	0	0	0	0	5		
20-MAR-2018	17:41	Day 4		D1	[IM]	0	0	0	0	0	0	5		
24-MAR-2018	11:38	Day 8		D1	[IM]	0	0	0	0	0	0	5		
07-APR-2018	10:37	Day 22		D1	[IM]	0	0	0	0	0	0	5		
16-MAY-2018	11:13	EOS/ET (Day 61)		D1	[IM]	0	0	0	0	0	0	5		

Key: 0= None; 1=Mild; 2=Moderate; 3=Severe; # Other = Pinprick bleeding or seepage

Administration Site Key: FA = Forearm; UA= Upper arm
Treatments Part A: A1 = A/Sing MAP, 15 ug (FA); A2 = Afluria Quad IM (UA), 15 ug; A3 = Placebo MAP (FA); A4 = A/Sing Mono IM, 15 ug (UA)
Treatments Part B: B1 = A/Sing MAP, 15 ug (FA) with biopsy; B2 = Placebo MAP (FA) with biopsy
B3 = A/Sing MAP, 15 ug (FA); B4 = A/Sing MAP, 10 ug (FA); B5 = A/Sing MAP, 5 ug (FA); B6 = A/Sing MAP, 2.5 ug (FA)
B7 = Placebo MAP (FA); B8 = A/Sing MAP, 15 ug (UA); B9 = A/Sing IM, 15 ug, as 2018 Afluria Quad (UA)

Listing 16.2.9.2
Treatment Site Tolerability Assessment per Site

Part A
Randomisation Number = A0040, Treatment Group = Group A4

Date	Time	Visit	Time Point	Site Number	Treatment for Site	Indura-tion	Redness	Bruising	Itching	Skin Flaking	Tender-ness	Definition/ Visibility	Other#	Unscheduled Reason
17-MAR-2018	11:05	Day 1	Pre-dose	D1	[IM]	0	0	0	0	0	0	5		
17-MAR-2018	12:00	Day 1	10 min	D1	[IM]	0	0	0	0	0	0	5		
17-MAR-2018	12:50	Day 1	1 hour	D1	[IM]	0	0	0	0	0	0	5		
17-MAR-2018	13:55	Day 1	2 hour	D1	[IM]	0	0	0	0	0	0	5		
20-MAR-2018	17:36	Day 4		D1	[IM]	0	0	0	0	0	0	5		
24-MAR-2018	12:29	Day 8		D1	[IM]	0	0	0	0	0	0	5		
07-APR-2018	11:11	Day 22		D1	[IM]	0	0	0	0	0	0	5		
17-MAY-2018	10:52	EOS/ET (Day 61)		D1	[IM]	0	0	0	0	0	0	5		

Key: 0= None; 1=Mild; 2=Moderate; 3=Severe; # Other = Pinprick bleeding or seepage

Administration Site Key: FA = Forearm; UA= Upper arm
Treatments Part A: A1 = A/Sing MAP, 15 ug (FA); A2 = Afluria Quad IM (UA), 15 ug; A3 = Placebo MAP (FA); A4 = A/Sing Mono IM, 15 ug (UA)
Treatments Part B: B1 = A/Sing MAP, 15 ug (FA) with biopsy; B2 = Placebo MAP (FA) with biopsy
B3 = A/Sing MAP, 15 ug (FA); B4 = A/Sing MAP, 10 ug (FA); B5 = A/Sing MAP, 5 ug (FA); B6 = A/Sing MAP, 2.5 ug (FA)
B7 = Placebo MAP (FA); B8 = A/Sing MAP, 15 ug (UA); B9 = A/Sing IM, 15 ug, as 2018 Afluria Quad (UA)

Listing 16.2.9.2
Treatment Site Tolerability Assessment per Site

Part A
Randomisation Number = A0041, Treatment Group = Group A4

Date	Time	Visit	Time Point	Site Number	Treatment for Site	Indura-tion	Redness	Bruising	Itching	Skin Flaking	Tender-ness	Definition/ Visibility	Other#	Unscheduled Reason
17-MAR-2018	11:07	Day 1	Pre-dose	D1	[IM]	0	0	0	0	0	0	5		
17-MAR-2018	12:05	Day 1	10 min	D1	[IM]	0	0	0	0	0	0	5		
17-MAR-2018	12:55	Day 1	1 hour	D1	[IM]	0	0	0	0	0	0	5		
17-MAR-2018	14:01	Day 1	2 hour	D1	[IM]	0	0	0	0	0	0	5		
20-MAR-2018	12:43	Day 4		D1	[IM]	0	0	0	0	0	0	5		
26-MAR-2018	15:08	Day 8		D1	[IM]	0	0	0	0	0	0	5		
10-APR-2018	11:48	Day 22		D1	[IM]	0	0	0	0	0	0	5		
17-MAY-2018	14:58	EOS/ET (Day 61)		D1	[IM]	0	0	0	0	0	0	5		

Key: 0= None; 1=Mild; 2=Moderate; 3=Severe; # Other = Pinprick bleeding or seepage

Administration Site Key: FA = Forearm; UA= Upper arm
Treatments Part A: A1 = A/Sing MAP, 15 ug (FA); A2 = Afluria Quad IM (UA), 15 ug; A3 = Placebo MAP (FA); A4 = A/Sing Mono IM, 15 ug (UA)
Treatments Part B: B1 = A/Sing MAP, 15 ug (FA) with biopsy; B2 = Placebo MAP (FA) with biopsy
B3 = A/Sing MAP, 15 ug (FA); B4 = A/Sing MAP, 10 ug (FA); B5 = A/Sing MAP, 5 ug (FA); B6 = A/Sing MAP, 2.5 ug (FA)
B7 = Placebo MAP (FA); B8 = A/Sing MAP, 15 ug (UA); B9 = A/Sing IM, 15 ug, as 2018 Afluria Quad (UA)

Listing 16.2.9.2
Treatment Site Tolerability Assessment per Site

Part A
Randomisation Number = A0042, Treatment Group = Group A2

Date	Time	Visit	Time Point	Site Number	Treatment for Site	Indura-tion	Redness	Bruising	Itching	Skin Flaking	Tender-ness	Definition/ Visibility	Other#	Unscheduled Reason
17-MAR-2018	11:09	Day 1	Pre-dose	D1	[IM]	0	0	0	0	0	0	5		
17-MAR-2018	12:10	Day 1	10 min	D1	[IM]	0	1	0	0	0	0	5		
17-MAR-2018	13:00	Day 1	1 hour	D1	[IM]	0	0	0	0	0	0	5		
17-MAR-2018	14:05	Day 1	2 hour	D1	[IM]	0	0	0	0	0	0	5		
20-MAR-2018	08:35	Day 4		D1	[IM]	0	0	0	0	0	0	5		
24-MAR-2018	10:58	Day 8		D1	[IM]	0	0	0	0	0	0	5		
07-APR-2018	12:15	Day 22		D1	[IM]	0	0	0	0	0	0	5		
16-MAY-2018	10:08	EOS/ET (Day 61)		D1	[IM]	0	0	0	0	0	0	5		

Key: 0= None; 1=Mild; 2=Moderate; 3=Severe; # Other = Pinprick bleeding or seepage

Administration Site Key: FA = Forearm; UA= Upper arm
Treatments Part A: A1 = A/Sing MAP, 15 ug (FA); A2 = Afluria Quad IM (UA), 15 ug; A3 = Placebo MAP (FA); A4 = A/Sing Mono IM, 15 ug (UA)
Treatments Part B: B1 = A/Sing MAP, 15 ug (FA) with biopsy; B2 = Placebo MAP (FA) with biopsy
B3 = A/Sing MAP, 15 ug (FA); B4 = A/Sing MAP, 10 ug (FA); B5 = A/Sing MAP, 5 ug (FA); B6 = A/Sing MAP, 2.5 ug (FA)
B7 = Placebo MAP (FA); B8 = A/Sing MAP, 15 ug (UA); B9 = A/Sing IM, 15 ug, as 2018 Afluria Quad (UA)

Listing 16.2.9.2
Treatment Site Tolerability Assessment per Site

Part A
Randomisation Number = A0043, Treatment Group = Group A2

Date	Time	Visit	Time Point	Site Number	Treatment for Site	Indura- tion	Redness	Bruising	Itching	Skin Flaking	Tender- ness	Definition/ Visibility	Other#	Unscheduled Reason
17-MAR-2018	11:38	Day 1	Pre-dose	D1	[IM]	0	0	0	0	0	0	5		
17-MAR-2018	12:15	Day 1	10 min	D1	[IM]	0	0	0	0	0	0	5		
17-MAR-2018	13:05	Day 1	1 hour	D1	[IM]	0	0	0	0	0	0	5		
17-MAR-2018	14:10	Day 1	2 hour	D1	[IM]	0	0	0	0	0	0	5		
20-MAR-2018	17:08	Day 4		D1	[IM]	0	0	0	0	0	0	5		
24-MAR-2018	12:47	Day 8		D1	[IM]	0	0	0	0	0	0	5		
06-APR-2018	17:34	Day 22		D1	[IM]	0	0	0	0	0	0	5		
16-MAY-2018	16:48	EOS/ET (Day 61)		D1	[IM]	0	0	0	0	0	0	5		

Key: 0= None; 1=Mild; 2=Moderate; 3=Severe; # Other = Pinprick bleeding or seepage

Administration Site Key: FA = Forearm; UA= Upper arm
Treatments Part A: A1 = A/Sing MAP, 15 ug (FA); A2 = Afluria Quad IM (UA), 15 ug; A3 = Placebo MAP (FA); A4 = A/Sing Mono IM, 15 ug (UA)
Treatments Part B: B1 = A/Sing MAP, 15 ug (FA) with biopsy; B2 = Placebo MAP (FA) with biopsy
B3 = A/Sing MAP, 15 ug (FA); B4 = A/Sing MAP, 10 ug (FA); B5 = A/Sing MAP, 5 ug (FA); B6 = A/Sing MAP, 2.5 ug (FA)
B7 = Placebo MAP (FA); B8 = A/Sing MAP, 15 ug (UA); B9 = A/Sing IM, 15 ug, as 2018 Afluria Quad (UA)

Listing 16.2.9.2
Treatment Site Tolerability Assessment per Site

Part A
Randomisation Number = A0044, Treatment Group = Group A4

Date	Time	Visit	Time Point	Site Number	Treatment for Site	Indura-tion	Redness	Bruising	Itching	Skin Flaking	Tender-ness	Definition/ Visibility	Other#	Unscheduled Reason
17-MAR-2018	11:42	Day 1	Pre-dose	D1	[IM]	0	0	0	0	0	0	5		
17-MAR-2018	12:21	Day 1	10 min	D1	[IM]	0	1	0	0	0	0	5		
17-MAR-2018	13:10	Day 1	1 hour	D1	[IM]	0	0	0	0	0	0	5		
17-MAR-2018	14:14	Day 1	2 hour	D1	[IM]	0	0	0	0	0	0	5		
20-MAR-2018	12:42	Day 4		D1	[IM]	0	0	0	0	0	0	5		
24-MAR-2018	12:48	Day 8		D1	[IM]	0	0	0	0	0	0	5		
07-APR-2018	12:17	Day 22		D1	[IM]	0	0	0	0	0	0	5		
16-MAY-2018	13:47	EOS/ET (Day 61)		D1	[IM]	0	0	0	0	0	0	5		

Key: 0= None; 1=Mild; 2=Moderate; 3=Severe; # Other = Pinprick bleeding or seepage

Administration Site Key: FA = Forearm; UA= Upper arm
Treatments Part A: A1 = A/Sing MAP, 15 ug (FA); A2 = Afluria Quad IM (UA), 15 ug; A3 = Placebo MAP (FA); A4 = A/Sing Mono IM, 15 ug (UA)
Treatments Part B: B1 = A/Sing MAP, 15 ug (FA) with biopsy; B2 = Placebo MAP (FA) with biopsy
B3 = A/Sing MAP, 15 ug (FA); B4 = A/Sing MAP, 10 ug (FA); B5 = A/Sing MAP, 5 ug (FA); B6 = A/Sing MAP, 2.5 ug (FA)
B7 = Placebo MAP (FA); B8 = A/Sing MAP, 15 ug (UA); B9 = A/Sing IM, 15 ug, as 2018 Afluria Quad (UA)

Listing 16.2.9.2
Treatment Site Tolerability Assessment per Site

Part A
Randomisation Number = A0045, Treatment Group = Group A2

Date	Time	Visit	Time Point	Site Number	Treatment for Site	Indura-tion	Redness	Bruising	Itching	Skin Flaking	Tender-ness	Definition/ Visibility	Other#	Unscheduled Reason
17-MAR-2018	11:53	Day 1	Pre-dose	D1	[IM]	0	0	0	0	0	0	5		
17-MAR-2018	12:25	Day 1	10 min	D1	[IM]	0	0	0	0	0	0	5		
17-MAR-2018	13:15	Day 1	1 hour	D1	[IM]	0	0	0	0	0	0	5		
17-MAR-2018	14:26	Day 1	2 hour	D1	[IM]	0	0	0	0	0	0	5		
20-MAR-2018	12:01	Day 4		D1	[IM]	0	0	0	0	0	0	5		
25-MAR-2018	10:28	Day 8		D1	[IM]	0	0	0	0	0	0	5		
08-APR-2018	10:55	Day 22		D1	[IM]	0	0	0	0	0	0	5		
15-MAY-2018	13:08	EOS/ET (Day 61)		D1	[IM]	0	0	0	0	0	0	5		

Key: 0= None; 1=Mild; 2=Moderate; 3=Severe; # Other = Pinprick bleeding or seepage

Administration Site Key: FA = Forearm; UA= Upper arm
Treatments Part A: A1 = A/Sing MAP, 15 ug (FA); A2 = Afluria Quad IM (UA), 15 ug; A3 = Placebo MAP (FA); A4 = A/Sing Mono IM, 15 ug (UA)
Treatments Part B: B1 = A/Sing MAP, 15 ug (FA) with biopsy; B2 = Placebo MAP (FA) with biopsy
B3 = A/Sing MAP, 15 ug (FA); B4 = A/Sing MAP, 10 ug (FA); B5 = A/Sing MAP, 5 ug (FA); B6 = A/Sing MAP, 2.5 ug (FA)
B7 = Placebo MAP (FA); B8 = A/Sing MAP, 15 ug (UA); B9 = A/Sing IM, 15 ug, as 2018 Afluria Quad (UA)

Listing 16.2.9.2
Treatment Site Tolerability Assessment per Site

Part A
Randomisation Number = A0046, Treatment Group = Group A4

Date	Time	Visit	Time Point	Site Number	Treatment for Site	Indura-tion	Redness	Bruising	Itching	Skin Flaking	Tender-ness	Definition/ Visibility	Other#	Unscheduled Reason
17-MAR-2018	11:58	Day 1	Pre-dose	D1	[IM]	0	0	0	0	0	0	5		
17-MAR-2018	12:32	Day 1	10 min	D1	[IM]	0	0	0	0	0	0	5		
17-MAR-2018	13:20	Day 1	1 hour	D1	[IM]	0	0	0	0	0	0	5		
17-MAR-2018	14:27	Day 1	2 hour	D1	[IM]	0	0	0	0	0	0	5		
20-MAR-2018	18:44	Day 4		D1	[IM]	0	0	0	0	0	0	5		
25-MAR-2018	08:47	Day 8		D1	[IM]	0	0	0	0	0	0	5		
07-APR-2018	14:18	Day 22		D1	[IM]	0	0	0	0	0	0	5		
17-MAY-2018	14:23	EOS/ET (Day 61)		D1	[IM]	0	0	0	0	0	0	5		

Key: 0= None; 1=Mild; 2=Moderate; 3=Severe; # Other = Pinprick bleeding or seepage

Administration Site Key: FA = Forearm; UA= Upper arm
Treatments Part A: A1 = A/Sing MAP, 15 ug (FA); A2 = Afluria Quad IM (UA), 15 ug; A3 = Placebo MAP (FA); A4 = A/Sing Mono IM, 15 ug (UA)
Treatments Part B: B1 = A/Sing MAP, 15 ug (FA) with biopsy; B2 = Placebo MAP (FA) with biopsy
B3 = A/Sing MAP, 15 ug (FA); B4 = A/Sing MAP, 10 ug (FA); B5 = A/Sing MAP, 5 ug (FA); B6 = A/Sing MAP, 2.5 ug (FA)
B7 = Placebo MAP (FA); B8 = A/Sing MAP, 15 ug (UA); B9 = A/Sing IM, 15 ug, as 2018 Afluria Quad (UA)

Listing 16.2.9.2
Treatment Site Tolerability Assessment per Site

Part A
Randomisation Number = A0047, Treatment Group = Group A2

Date	Time	Visit	Time Point	Site Number	Treatment for Site	Indura-tion	Redness	Bruising	Itching	Skin Flaking	Tender-ness	Definition/ Visibility	Other#	Unscheduled Reason
17-MAR-2018	12:02	Day 1	Pre-dose	D1	[IM]	0	0	0	0	0	0	5		
17-MAR-2018	12:35	Day 1	10 min	D1	[IM]	0	1	0	0	0	0	5		
17-MAR-2018	13:25	Day 1	1 hour	D1	[IM]	0	0	0	0	0	1	5		
17-MAR-2018	14:29	Day 1	2 hour	D1	[IM]	0	0	0	0	0	0	5		
21-MAR-2018	14:16	Day 4		D1	[IM]	0	0	0	0	0	0	5		
25-MAR-2018	10:26	Day 8		D1	[IM]	0	0	0	0	0	0	5		
07-APR-2018	15:36	Day 22		D1	[IM]	0	0	0	0	0	0	5		
22-MAY-2018	15:07	EOS/ET (Day 61)		D1	[IM]	0	0	0	0	0	0	5		

Key: 0= None; 1=Mild; 2=Moderate; 3=Severe; # Other = Pinprick bleeding or seepage

Administration Site Key: FA = Forearm; UA= Upper arm
Treatments Part A: A1 = A/Sing MAP, 15 ug (FA); A2 = Afluria Quad IM (UA), 15 ug; A3 = Placebo MAP (FA); A4 = A/Sing Mono IM, 15 ug (UA)
Treatments Part B: B1 = A/Sing MAP, 15 ug (FA) with biopsy; B2 = Placebo MAP (FA) with biopsy
B3 = A/Sing MAP, 15 ug (FA); B4 = A/Sing MAP, 10 ug (FA); B5 = A/Sing MAP, 5 ug (FA); B6 = A/Sing MAP, 2.5 ug (FA)
B7 = Placebo MAP (FA); B8 = A/Sing MAP, 15 ug (UA); B9 = A/Sing IM, 15 ug, as 2018 Afluria Quad (UA)

Listing 16.2.9.2
Treatment Site Tolerability Assessment per Site

Part A
Randomisation Number = A0048, Treatment Group = Group A4

Date	Time	Visit	Time Point	Site Number	Treatment for Site	Indura-tion	Redness	Bruising	Itching	Skin Flaking	Tender-ness	Definition/ Visibility	Other#	Unscheduled Reason
17-MAR-2018	12:08	Day 1	Pre-dose	D1	[IM]	0	0	0	0	0	0	5		
17-MAR-2018	13:10	Day 1	10 min	D1	[IM]	0	0	0	0	0	0	5		
17-MAR-2018	14:00	Day 1	1 hour	D1	[IM]	0	0	0	0	0	0	5		
17-MAR-2018	15:04	Day 1	2 hour	D1	[IM]	0	0	0	0	0	0	5		
20-MAR-2018	13:46	Day 4		D1	[IM]	0	0	0	0	0	0	5		
24-MAR-2018	13:14	Day 8		D1	[IM]	0	0	0	0	0	0	5		
06-APR-2018	11:39	Day 22		D1	[IM]	0	0	0	0	0	0	5		
16-MAY-2018	08:39	EOS/ET (Day 61)		D1	[IM]	0	0	0	0	0	0	5		

Key: 0= None; 1=Mild; 2=Moderate; 3=Severe; # Other = Pinprick bleeding or seepage

Administration Site Key: FA = Forearm; UA= Upper arm
Treatments Part A: A1 = A/Sing MAP, 15 ug (FA); A2 = Afluria Quad IM (UA), 15 ug; A3 = Placebo MAP (FA); A4 = A/Sing Mono IM, 15 ug (UA)
Treatments Part B: B1 = A/Sing MAP, 15 ug (FA) with biopsy; B2 = Placebo MAP (FA) with biopsy
B3 = A/Sing MAP, 15 ug (FA); B4 = A/Sing MAP, 10 ug (FA); B5 = A/Sing MAP, 5 ug (FA); B6 = A/Sing MAP, 2.5 ug (FA)
B7 = Placebo MAP (FA); B8 = A/Sing MAP, 15 ug (UA); B9 = A/Sing IM, 15 ug, as 2018 Afluria Quad (UA)

Listing 16.2.9.2
Treatment Site Tolerability Assessment per Site

Part A
Randomisation Number = A0049, Treatment Group = Group A2

Date	Time	Visit	Time Point	Site Number	Treatment for Site	Indura-tion	Redness	Bruising	Itching	Skin Flaking	Tender-ness	Definition/ Visibility	Other#	Unscheduled Reason
17-MAR-2018	12:12	Day 1	Pre-dose	D1	[IM]	0	0	0	0	0	0	5		
17-MAR-2018	12:46	Day 1	10 min	D1	[IM]	0	0	0	0	0	0	5		
17-MAR-2018	13:35	Day 1	1 hour	D1	[IM]	0	0	0	0	0	0	5		
17-MAR-2018	14:35	Day 1	2 hour	D1	[IM]	0	0	0	0	0	0	5		
20-MAR-2018	11:50	Day 4		D1	[IM]	0	0	0	0	0	0	5		
24-MAR-2018	12:05	Day 8		D1	[IM]	0	0	0	0	0	0	5		
06-APR-2018	08:33	Day 22		D1	[IM]	0	0	0	0	0	0	5		
16-MAY-2018	09:37	EOS/ET (Day 61)		D1	[IM]	0	0	0	0	0	0	5		

Key: 0= None; 1=Mild; 2=Moderate; 3=Severe; # Other = Pinprick bleeding or seepage

Administration Site Key: FA = Forearm; UA= Upper arm
Treatments Part A: A1 = A/Sing MAP, 15 ug (FA); A2 = Afluria Quad IM (UA), 15 ug; A3 = Placebo MAP (FA); A4 = A/Sing Mono IM, 15 ug (UA)
Treatments Part B: B1 = A/Sing MAP, 15 ug (FA) with biopsy; B2 = Placebo MAP (FA) with biopsy
B3 = A/Sing MAP, 15 ug (FA); B4 = A/Sing MAP, 10 ug (FA); B5 = A/Sing MAP, 5 ug (FA); B6 = A/Sing MAP, 2.5 ug (FA)
B7 = Placebo MAP (FA); B8 = A/Sing MAP, 15 ug (UA); B9 = A/Sing IM, 15 ug, as 2018 Afluria Quad (UA)

Listing 16.2.9.2
Treatment Site Tolerability Assessment per Site

Part A
Randomisation Number = A0050, Treatment Group = Group A4

Date	Time	Visit	Time Point	Site Number	Treatment for Site	Indura-tion	Redness	Bruising	Itching	Skin Flaking	Tender-ness	Definition/ Visibility	Other#	Unscheduled Reason
17-MAR-2018	12:38	Day 1	Pre-dose	D1	[IM]	0	0	0	0	0	0	5		
17-MAR-2018	12:50	Day 1	10 min	D1	[IM]	0	0	0	0	0	0	5		
17-MAR-2018	13:40	Day 1	1 hour	D1	[IM]	0	0	0	0	0	1	5		
17-MAR-2018	14:40	Day 1	2 hour	D1	[IM]	0	0	0	0	0	1	5		
20-MAR-2018	12:45	Day 4		D1	[IM]	0	0	0	0	0	0	5		
27-MAR-2018	16:17	Day 8		D1	[IM]	0	0	0	0	0	0	5		
07-APR-2018	12:36	Day 22		D1	[IM]	0	0	0	0	0	0	5		
16-MAY-2018	15:55	EOS/ET (Day 61)		D1	[IM]	0	0	0	0	0	0	5		

Key: 0= None; 1=Mild; 2=Moderate; 3=Severe; # Other = Pinprick bleeding or seepage

Administration Site Key: FA = Forearm; UA= Upper arm
Treatments Part A: A1 = A/Sing MAP, 15 ug (FA); A2 = Afluria Quad IM (UA), 15 ug; A3 = Placebo MAP (FA); A4 = A/Sing Mono IM, 15 ug (UA)
Treatments Part B: B1 = A/Sing MAP, 15 ug (FA) with biopsy; B2 = Placebo MAP (FA) with biopsy
B3 = A/Sing MAP, 15 ug (FA); B4 = A/Sing MAP, 10 ug (FA); B5 = A/Sing MAP, 5 ug (FA); B6 = A/Sing MAP, 2.5 ug (FA)
B7 = Placebo MAP (FA); B8 = A/Sing MAP, 15 ug (UA); B9 = A/Sing IM, 15 ug, as 2018 Afluria Quad (UA)

Listing 16.2.9.2
Treatment Site Tolerability Assessment per Site

Part A
Randomisation Number = A0051, Treatment Group = Group A2

Date	Time	Visit	Time Point	Site Number	Treatment for Site	Indura-tion	Redness	Bruising	Itching	Skin Flaking	Tender-ness	Definition/ Visibility	Other#	Unscheduled Reason
17-MAR-2018	12:17	Day 1	Pre-dose	D1	[IM]	0	0	0	0	0	0	5		
17-MAR-2018	12:55	Day 1	10 min	D1	[IM]	0	1	0	0	0	0	5		
17-MAR-2018	13:45	Day 1	1 hour	D1	[IM]	0	0	0	0	0	0	5		
17-MAR-2018	14:43	Day 1	2 hour	D1	[IM]	0	0	0	0	0	0	5		
20-MAR-2018	13:44	Day 4		D1	[IM]	0	0	0	0	0		5		
24-MAR-2018	12:53	Day 8		D1	[IM]	0	0	0	0	0	0	5		
07-APR-2018	12:41	Day 22		D1	[IM]	0	0	0	0	0	0	5		
16-MAY-2018	11:48	EOS/ET (Day 61)		D1	[IM]	0	0	0	0	0	0	5		

Key: 0= None; 1=Mild; 2=Moderate; 3=Severe; # Other = Pinprick bleeding or seepage

Administration Site Key: FA = Forearm; UA= Upper arm
Treatments Part A: A1 = A/Sing MAP, 15 ug (FA); A2 = Afluria Quad IM (UA), 15 ug; A3 = Placebo MAP (FA); A4 = A/Sing Mono IM, 15 ug (UA)
Treatments Part B: B1 = A/Sing MAP, 15 ug (FA) with biopsy; B2 = Placebo MAP (FA) with biopsy
B3 = A/Sing MAP, 15 ug (FA); B4 = A/Sing MAP, 10 ug (FA); B5 = A/Sing MAP, 5 ug (FA); B6 = A/Sing MAP, 2.5 ug (FA)
B7 = Placebo MAP (FA); B8 = A/Sing MAP, 15 ug (UA); B9 = A/Sing IM, 15 ug, as 2018 Afluria Quad (UA)

Listing 16.2.9.2
Treatment Site Tolerability Assessment per Site

Part A
Randomisation Number = A0052, Treatment Group = Group A4

Date	Time	Visit	Time Point	Site Number	Treatment for Site	Indura-tion	Redness	Bruising	Itching	Skin Flaking	Tender-ness	Definition/ Visibility	Other#	Unscheduled Reason
17-MAR-2018	12:04	Day 1	Pre-dose	D1	[IM]	0	0	0	0	0	0	5		
17-MAR-2018	13:01	Day 1	10 min	D1	[IM]	0	0	0	0	0	0	5		
17-MAR-2018	13:50	Day 1	1 hour	D1	[IM]	0	0	0	0	0	0	5		
17-MAR-2018	14:52	Day 1	2 hour	D1	[IM]	0	0	0	0	0	0	5		
20-MAR-2018	13:43	Day 4		D1	[IM]	0	0	0	0	0	0	5		
24-MAR-2018	13:16	Day 8		D1	[IM]	0	0	0	0	0	0	5		
16-MAY-2018	12:30	EOS/ET (Day 61)		D1	[IM]	0	0	0	0	0	0	5		

Key: 0= None; 1=Mild; 2=Moderate; 3=Severe; # Other = Pinprick bleeding or seepage

Administration Site Key: FA = Forearm; UA= Upper arm
Treatments Part A: A1 = A/Sing MAP, 15 ug (FA); A2 = Afluria Quad IM (UA), 15 ug; A3 = Placebo MAP (FA); A4 = A/Sing Mono IM, 15 ug (UA)
Treatments Part B: B1 = A/Sing MAP, 15 ug (FA) with biopsy; B2 = Placebo MAP (FA) with biopsy
B3 = A/Sing MAP, 15 ug (FA); B4 = A/Sing MAP, 10 ug (FA); B5 = A/Sing MAP, 5 ug (FA); B6 = A/Sing MAP, 2.5 ug (FA)
B7 = Placebo MAP (FA); B8 = A/Sing MAP, 15 ug (UA); B9 = A/Sing IM, 15 ug, as 2018 Afluria Quad (UA)

Listing 16.2.9.2
Treatment Site Tolerability Assessment per Site

Part A
Randomisation Number = A0053, Treatment Group = Group A4

Date	Time	Visit	Time Point	Site Number	Treatment for Site	Indura-tion	Redness	Bruising	Itching	Skin Flaking	Tender-ness	Definition/ Visibility	Other#	Unscheduled Reason
17-MAR-2018	12:29	Day 1	Pre-dose	D1	[IM]	0	0	0	0	0	0	5		
17-MAR-2018	13:05	Day 1	10 min	D1	[IM]	0	0	0	0	0	0	5		
17-MAR-2018	13:55	Day 1	1 hour	D1	[IM]	0	0	0	0	0	0	5		
17-MAR-2018	14:58	Day 1	2 hour	D1	[IM]	0	0	0	0	0	0	5		
20-MAR-2018	16:32	Day 4		D1	[IM]	0	0	0	0	0	0	5		
24-MAR-2018	12:48	Day 8		D1	[IM]	0	0	0	0	0	0	5		
07-APR-2018	12:34	Day 22		D1	[IM]	0	0	0	0	0	0	5		
16-MAY-2018	10:35	EOS/ET (Day 61)		D1	[IM]	0	0	0	0	0	0	5		

Key: 0= None; 1=Mild; 2=Moderate; 3=Severe; # Other = Pinprick bleeding or seepage

Administration Site Key: FA = Forearm; UA= Upper arm
Treatments Part A: A1 = A/Sing MAP, 15 ug (FA); A2 = Afluria Quad IM (UA), 15 ug; A3 = Placebo MAP (FA); A4 = A/Sing Mono IM, 15 ug (UA)
Treatments Part B: B1 = A/Sing MAP, 15 ug (FA) with biopsy; B2 = Placebo MAP (FA) with biopsy
B3 = A/Sing MAP, 15 ug (FA); B4 = A/Sing MAP, 10 ug (FA); B5 = A/Sing MAP, 5 ug (FA); B6 = A/Sing MAP, 2.5 ug (FA)
B7 = Placebo MAP (FA); B8 = A/Sing MAP, 15 ug (UA); B9 = A/Sing IM, 15 ug, as 2018 Afluria Quad (UA)

Listing 16.2.9.2
Treatment Site Tolerability Assessment per Site

Part A
Randomisation Number = A0054, Treatment Group = Group A2

Date	Time	Visit	Time Point	Site Number	Treatment for Site	Indura-tion	Redness	Bruising	Itching	Skin Flaking	Tender-ness	Definition/ Visibility	Other#	Unscheduled Reason
17-MAR-2018	14:56	Day 1	Pre-dose	D1	[IM]	0	0	0	0	0	0	5		
17-MAR-2018	15:16	Day 1	10 min	D1	[IM]	1	0	0	0	0	0	5		
17-MAR-2018	16:36	Day 1	1 hour	D1	[IM]	1	0	0	0	0	1	5		
17-MAR-2018	17:12	Day 1	2 hour	D1	[IM]	1	0	0	0	0	1	5		
20-MAR-2018	16:15	Day 4		D1	[IM]	0	0	1	0	0	0	5		
24-MAR-2018	16:21	Day 8		D1	[IM]	0	0	1	0	0	0	5		
08-APR-2018	11:49	Day 22		D1	[IM]	0	0	0	0	0	0	5		
26-MAY-2018	10:05	EOS/ET (Day 61)		D1	[IM]	0	0	0	0	0	0	5		

Key: 0= None; 1=Mild; 2=Moderate; 3=Severe; # Other = Pinprick bleeding or seepage

Administration Site Key: FA = Forearm; UA= Upper arm
Treatments Part A: A1 = A/Sing MAP, 15 ug (FA); A2 = Afluria Quad IM (UA), 15 ug; A3 = Placebo MAP (FA); A4 = A/Sing Mono IM, 15 ug (UA)
Treatments Part B: B1 = A/Sing MAP, 15 ug (FA) with biopsy; B2 = Placebo MAP (FA) with biopsy
B3 = A/Sing MAP, 15 ug (FA); B4 = A/Sing MAP, 10 ug (FA); B5 = A/Sing MAP, 5 ug (FA); B6 = A/Sing MAP, 2.5 ug (FA)
B7 = Placebo MAP (FA); B8 = A/Sing MAP, 15 ug (UA); B9 = A/Sing IM, 15 ug, as 2018 Afluria Quad (UA)

Listing 16.2.9.2
Treatment Site Tolerability Assessment per Site

Part A
Randomisation Number = A0055, Treatment Group = Group A4

Date	Time	Visit	Time Point	Site Number	Treatment for Site	Indura- tion	Redness	Bruising	Itching	Skin Flaking	Tender- ness	Definition/ Visibility	Other#	Unscheduled Reason
24-MAR-2018	11:54	Day 1	Pre-dose	D1	[IM]	0	0	0	0	0	0	5		
24-MAR-2018	12:41	Day 1	10 min	D1	[IM]	0	0	0	0	0	0	5		
24-MAR-2018	13:31	Day 1	1 hour	D1	[IM]	0	0	0	0	0	0	5		
24-MAR-2018	14:36	Day 1	2 hour	D1	[IM]	0	0	0	0	0	0	5		
27-MAR-2018	15:08	Day 4		D1	[IM]	0	0	0	0	0	0	5		
31-MAR-2018	09:55	Day 8		D1	[IM]	0	0	0	0	0	0	5		
14-APR-2018	15:20	Day 22		D1	[IM]	0	0	0	0	0	0	5		
24-MAY-2018	17:07	EOS/ET (Day 61)		D1	[IM]	0	0	0	0	0	0	5		

Key: 0= None; 1=Mild; 2=Moderate; 3=Severe; # Other = Pinprick bleeding or seepage

Administration Site Key: FA = Forearm; UA= Upper arm
Treatments Part A: A1 = A/Sing MAP, 15 ug (FA); A2 = Afluria Quad IM (UA), 15 ug; A3 = Placebo MAP (FA); A4 = A/Sing Mono IM, 15 ug (UA)
Treatments Part B: B1 = A/Sing MAP, 15 ug (FA) with biopsy; B2 = Placebo MAP (FA) with biopsy
B3 = A/Sing MAP, 15 ug (FA); B4 = A/Sing MAP, 10 ug (FA); B5 = A/Sing MAP, 5 ug (FA); B6 = A/Sing MAP, 2.5 ug (FA)
B7 = Placebo MAP (FA); B8 = A/Sing MAP, 15 ug (UA); B9 = A/Sing IM, 15 ug, as 2018 Afluria Quad (UA)

Listing 16.2.9.2
Treatment Site Tolerability Assessment per Site

Part A
Randomisation Number = A0056, Treatment Group = Group A2

Date	Time	Visit	Time Point	Site Number	Treatment for Site	Indura-tion	Redness	Bruising	Itching	Skin Flaking	Tender-ness	Definition/ Visibility	Other#	Unscheduled Reason
24-MAR-2018	12:00	Day 1	Pre-dose	D1	[IM]	0	0	0	0	0	0	5		
24-MAR-2018	12:50	Day 1	10 min	D1	[IM]	0	0	0	0	0	0	5		
24-MAR-2018	13:41	Day 1	1 hour	D1	[IM]	0	0	0	0	0	0	5		
24-MAR-2018	14:45	Day 1	2 hour	D1	[IM]	0	0	0	0	0	0	5		
27-MAR-2018	17:43	Day 4		D1	[IM]	0	0	0	0	0	0	5		
31-MAR-2018	12:39	Day 8		D1	[IM]	0	0	0	0	0	0	5		
14-APR-2018	13:03	Day 22		D1	[IM]	0	0	0	0	0	0	5		
23-MAY-2018	17:24	EOS/ET (Day 61)		D1	[IM]	0	0	0	0	0	0	5		

Key: 0= None; 1=Mild; 2=Moderate; 3=Severe; # Other = Pinprick bleeding or seepage

Administration Site Key: FA = Forearm; UA= Upper arm
Treatments Part A: A1 = A/Sing MAP, 15 ug (FA); A2 = Afluria Quad IM (UA), 15 ug; A3 = Placebo MAP (FA); A4 = A/Sing Mono IM, 15 ug (UA)
Treatments Part B: B1 = A/Sing MAP, 15 ug (FA) with biopsy; B2 = Placebo MAP (FA) with biopsy
B3 = A/Sing MAP, 15 ug (FA); B4 = A/Sing MAP, 10 ug (FA); B5 = A/Sing MAP, 5 ug (FA); B6 = A/Sing MAP, 2.5 ug (FA)
B7 = Placebo MAP (FA); B8 = A/Sing MAP, 15 ug (UA); B9 = A/Sing IM, 15 ug, as 2018 Afluria Quad (UA)

Listing 16.2.9.2
Treatment Site Tolerability Assessment per Site

Part A
Randomisation Number = A0057, Treatment Group = Group A4

Date	Time	Visit	Time Point	Site Number	Treatment for Site	Indura-tion	Redness	Bruising	Itching	Skin Flaking	Tender-ness	Definition/ Visibility	Other#	Unscheduled Reason
24-MAR-2018	12:10	Day 1	Pre-dose	D1	[IM]	0	0	0	0	0	0	5		
24-MAR-2018	13:01	Day 1	10 min	D1	[IM]	0	0	0	0	0	0	5		
24-MAR-2018	13:51	Day 1	1 hour	D1	[IM]	0	0	0	0	0	0	5		
24-MAR-2018	14:56	Day 1	2 hour	D1	[IM]	0	0	0	0	0	0	5		
27-MAR-2018	16:18	Day 4		D1	[IM]	0	0	0	0	0	0	5		
02-APR-2018	11:20	Day 8		D1	[IM]	0	0	0	0	0	0	5		
14-APR-2018	12:35	Day 22		D1	[IM]	0	0	0	0	0	0	5		
23-MAY-2018	09:31	EOS/ET (Day 61)		D1	[IM]	0	0	0	0	0	0	5		

Key: 0= None; 1=Mild; 2=Moderate; 3=Severe; # Other = Pinprick bleeding or seepage

Administration Site Key: FA = Forearm; UA= Upper arm
Treatments Part A: A1 = A/Sing MAP, 15 ug (FA); A2 = Afluria Quad IM (UA), 15 ug; A3 = Placebo MAP (FA); A4 = A/Sing Mono IM, 15 ug (UA)
Treatments Part B: B1 = A/Sing MAP, 15 ug (FA) with biopsy; B2 = Placebo MAP (FA) with biopsy
B3 = A/Sing MAP, 15 ug (FA); B4 = A/Sing MAP, 10 ug (FA); B5 = A/Sing MAP, 5 ug (FA); B6 = A/Sing MAP, 2.5 ug (FA)
B7 = Placebo MAP (FA); B8 = A/Sing MAP, 15 ug (UA); B9 = A/Sing IM, 15 ug, as 2018 Afluria Quad (UA)

Listing 16.2.9.2
Treatment Site Tolerability Assessment per Site

Part A
Randomisation Number = A0058, Treatment Group = Group A2

Date	Time	Visit	Time Point	Site Number	Treatment for Site	Indura-tion	Redness	Bruising	Itching	Skin Flaking	Tender-ness	Definition/ Visibility	Other#	Unscheduled Reason
24-MAR-2018	12:20	Day 1	Pre-dose	D1	[IM]	0	0	0	0	0	0	5		
24-MAR-2018	13:11	Day 1	10 min	D1	[IM]	0	0	0	0	0	0	5		
24-MAR-2018	14:01	Day 1	1 hour	D1	[IM]	0	0	0	0	0	0	5		
24-MAR-2018	15:06	Day 1	2 hour	D1	[IM]	0	0	0	0	0	0	5		
27-MAR-2018	15:09	Day 4		D1	[IM]	0	0	0	0	0	0	5		
31-MAR-2018	11:58	Day 8		D1	[IM]	0	0	0	0	0	0	5		
14-APR-2018	15:19	Day 22		D1	[IM]	0	0	0	0	0	0	5		
23-MAY-2018	10:26	EOS/ET (Day 61)		D1	[IM]	0	0	0	0	0	0	5		

Key: 0= None; 1=Mild; 2=Moderate; 3=Severe; # Other = Pinprick bleeding or seepage

Administration Site Key: FA = Forearm; UA= Upper arm
Treatments Part A: A1 = A/Sing MAP, 15 ug (FA); A2 = Afluria Quad IM (UA), 15 ug; A3 = Placebo MAP (FA); A4 = A/Sing Mono IM, 15 ug (UA)
Treatments Part B: B1 = A/Sing MAP, 15 ug (FA) with biopsy; B2 = Placebo MAP (FA) with biopsy
B3 = A/Sing MAP, 15 ug (FA); B4 = A/Sing MAP, 10 ug (FA); B5 = A/Sing MAP, 5 ug (FA); B6 = A/Sing MAP, 2.5 ug (FA)
B7 = Placebo MAP (FA); B8 = A/Sing MAP, 15 ug (UA); B9 = A/Sing IM, 15 ug, as 2018 Afluria Quad (UA)

Listing 16.2.9.2
Treatment Site Tolerability Assessment per Site

Part A
Randomisation Number = A0059, Treatment Group = Group A2

Date	Time	Visit	Time Point	Site Number	Treatment for Site	Indura-tion	Redness	Bruising	Itching	Skin Flaking	Tender-ness	Definition/ Visibility	Other#	Unscheduled Reason
24-MAR-2018	12:40	Day 1	Pre-dose	D1	[IM]	0	0	0	0	0	0	5		
24-MAR-2018	13:21	Day 1	10 min	D1	[IM]	0	0	0	0	0	0	5		
24-MAR-2018	14:11	Day 1	1 hour	D1	[IM]	0	0	0	0	0	0	5		
24-MAR-2018	15:16	Day 1	2 hour	D1	[IM]	0	0	0	0	0	0	5		
27-MAR-2018	08:24	Day 4		D1	[IM]	0	0	0	0	0	0	5		
30-MAR-2018	13:01	Day 8		D1	[IM]	0	0	0	0	0	0	5		
17-APR-2018	09:10	Day 22		D1	[IM]	0	0	0	0	1	0	5		
24-MAY-2018	13:13	EOS/ET (Day 61)		D1	[IM]	0	0	0	0	0	0	5		

Key: 0= None; 1=Mild; 2=Moderate; 3=Severe; # Other = Pinprick bleeding or seepage

Administration Site Key: FA = Forearm; UA= Upper arm
Treatments Part A: A1 = A/Sing MAP, 15 ug (FA); A2 = Afluria Quad IM (UA), 15 ug; A3 = Placebo MAP (FA); A4 = A/Sing Mono IM, 15 ug (UA)
Treatments Part B: B1 = A/Sing MAP, 15 ug (FA) with biopsy; B2 = Placebo MAP (FA) with biopsy
B3 = A/Sing MAP, 15 ug (FA); B4 = A/Sing MAP, 10 ug (FA); B5 = A/Sing MAP, 5 ug (FA); B6 = A/Sing MAP, 2.5 ug (FA)
B7 = Placebo MAP (FA); B8 = A/Sing MAP, 15 ug (UA); B9 = A/Sing IM, 15 ug, as 2018 Afluria Quad (UA)

Listing 16.2.9.2
Treatment Site Tolerability Assessment per Site

Part A
Randomisation Number = A0060, Treatment Group = Group A4

Date	Time	Visit	Time Point	Site Number	Treatment for Site	Indura-tion	Redness	Bruising	Itching	Skin Flaking	Tender-ness	Definition/ Visibility	Other#	Unscheduled Reason
24-MAR-2018	12:45	Day 1	Pre-dose	D1	[IM]	0	0	0	0	0	0	5		
24-MAR-2018	13:31	Day 1	10 min	D1	[IM]	0	0	0	0	0	0	5		
24-MAR-2018	14:21	Day 1	1 hour	D1	[IM]	0	0	0	0	0	0	5		
24-MAR-2018	15:26	Day 1	2 hour	D1	[IM]	0	0	0	0	0	0	5		
27-MAR-2018	13:42	Day 4		D1	[IM]	0	0	0	0	0	0	5		
31-MAR-2018	13:23	Day 8		D1	[IM]	0	0	0	0	0	0	5		
14-APR-2018	13:45	Day 22		D1	[IM]	0	0	0	0	0	0	5		
24-MAY-2018	13:18	EOS/ET (Day 61)		D1	[IM]	0	0	0	0	0	0	5		

Key: 0= None; 1=Mild; 2=Moderate; 3=Severe; # Other = Pinprick bleeding or seepage

Administration Site Key: FA = Forearm; UA= Upper arm
Treatments Part A: A1 = A/Sing MAP, 15 ug (FA); A2 = Afluria Quad IM (UA), 15 ug; A3 = Placebo MAP (FA); A4 = A/Sing Mono IM, 15 ug (UA)
Treatments Part B: B1 = A/Sing MAP, 15 ug (FA) with biopsy; B2 = Placebo MAP (FA) with biopsy
B3 = A/Sing MAP, 15 ug (FA); B4 = A/Sing MAP, 10 ug (FA); B5 = A/Sing MAP, 5 ug (FA); B6 = A/Sing MAP, 2.5 ug (FA)
B7 = Placebo MAP (FA); B8 = A/Sing MAP, 15 ug (UA); B9 = A/Sing IM, 15 ug, as 2018 Afluria Quad (UA)

Listing 16.2.9.2
Treatment Site Tolerability Assessment per Site

Part A
Randomisation Number = A1026, Treatment Group = Group A1

Date	Time	Visit	Time Point	Site Number	Treatment for Site	Induration	Redness	Bruising	Itching	Skin Flaking	Tenderness	Definition/ Visibility	Other#	Unscheduled Reason
10-MAR-2018	13:51	Day 1	Pre-dose	F1	5 ug	0	0	0	0	0	0	0		
10-MAR-2018	13:51	Day 1	Pre-dose	F2	5 ug	0	0	0	0	0	0	0		
10-MAR-2018	13:51	Day 1	Pre-dose	F3	5 ug	0	0	0	0	0	0	0		
10-MAR-2018	14:18	Day 1	10 min	F1	5 ug	1	1	0	0	0	0	0		
10-MAR-2018	14:18	Day 1	10 min	F2	5 ug	1	1	0	0	0	0	0		
10-MAR-2018	14:18	Day 1	10 min	F3	5 ug	1	1	0	0	0	0	0		
10-MAR-2018	15:07	Day 1	1 hour	F1	5 ug	0	0	0	0	0	0	0		
10-MAR-2018	15:07	Day 1	1 hour	F2	5 ug	1	0	0	0	0	0	0		
10-MAR-2018	15:07	Day 1	1 hour	F3	5 ug	1	0	0	0	0	0	0		
10-MAR-2018	16:08	Day 1	2 hour	F1	5 ug	1	0	0	0	0	0	0		
10-MAR-2018	16:08	Day 1	2 hour	F2	5 ug	1	1	0	0	0	0	0		
10-MAR-2018	16:08	Day 1	2 hour	F3	5 ug	1	0	0	0	0	0	0		
13-MAR-2018	08:12	Day 4		F1	5 ug	0	0	0	0	0	0	0		
13-MAR-2018	08:12	Day 4		F2	5 ug	0	0	0	0	0	0	0		
13-MAR-2018	08:12	Day 4		F3	5 ug	0	0	0	0	0	0	0		
18-MAR-2018	16:01	Day 8		F1	5 ug	0	0	0	0	1	0	3		
18-MAR-2018	16:01	Day 8		F2	5 ug	0	0	0	0	2	0	3		
18-MAR-2018	16:01	Day 8		F3	5 ug	0	0	0	0	2	0	3		
31-MAR-2018	10:28	Day 22		F1	5 ug	0	0	0	0	0	0	0		
31-MAR-2018	10:28	Day 22		F2	5 ug	0	0	0	0	0	0	0		

Key: 0= None; 1=Mild; 2=Moderate; 3=Severe; # Other = Pinprick bleeding or seepage

Administration Site Key: FA = Forearm; UA= Upper arm
Treatments Part A: A1 = A/Sing MAP, 15 ug (FA); A2 = Afluria Quad IM (UA), 15 ug; A3 = Placebo MAP (FA); A4 = A/Sing Mono IM, 15 ug (UA)
Treatments Part B: B1 = A/Sing MAP, 15 ug (FA) with biopsy; B2 = Placebo MAP (FA) with biopsy
B3 = A/Sing MAP, 15 ug (FA); B4 = A/Sing MAP, 10 ug (FA); B5 = A/Sing MAP, 5 ug (FA); B6 = A/Sing MAP, 2.5 ug (FA)
B7 = Placebo MAP (FA); B8 = A/Sing MAP, 15 ug (UA); B9 = A/Sing IM, 15 ug, as 2018 Afluria Quad (UA)

Listing 16.2.9.2
Treatment Site Tolerability Assessment per Site

Part A
Randomisation Number = A1026, Treatment Group = Group A1

Date	Time	Visit	Time Point	Site Number	Treatment for Site	Indura-tion	Redness	Bruising	Itching	Skin Flaking	Tender-ness	Definition/ Visibility	Other#	Unscheduled Reason
31-MAR-2018	10:28	Day 22		F3	5 ug	0	0	0	0	0	0	0		
09-MAY-2018	15:21	EOS/ET (Day 61)		F1	5 ug	0	0	0	0	0	0	0		
09-MAY-2018	15:21	EOS/ET (Day 61)		F2	5 ug	0	0	0	0	0	0	0		
09-MAY-2018	15:21	EOS/ET (Day 61)		F3	5 ug	0	0	0	0	0	0	0		

Key: 0= None; 1=Mild; 2=Moderate; 3=Severe; # Other = Pinprick bleeding or seepage

Administration Site Key: FA = Forearm; UA= Upper arm
Treatments Part A: A1 = A/Sing MAP, 15 ug (FA); A2 = Afluria Quad IM (UA), 15 ug; A3 = Placebo MAP (FA); A4 = A/Sing Mono IM, 15 ug (UA)
Treatments Part B: B1 = A/Sing MAP, 15 ug (FA) with biopsy; B2 = Placebo MAP (FA) with biopsy
B3 = A/Sing MAP, 15 ug (FA); B4 = A/Sing MAP, 10 ug (FA); B5 = A/Sing MAP, 5 ug (FA); B6 = A/Sing MAP, 2.5 ug (FA)
B7 = Placebo MAP (FA); B8 = A/Sing MAP, 15 ug (UA); B9 = A/Sing IM, 15 ug, as 2018 Afluria Quad (UA)

Listing 16.2.9.2
Treatment Site Tolerability Assessment per Site

Part B
Randomisation Number = B0001, Treatment Group = Group B1

Date	Time	Visit	Time Point	Site Number	Treatment for Site	Induration	Redness	Bruising	Itching	Skin Flaking	Tenderness	Definition/ Visibility	Other#	Unscheduled Reason
07-MAY-2018	08:39	Day 1	Pre-dose	F1	5 ug	0	0	0	0	0	0	0		
07-MAY-2018	08:39	Day 1	Pre-dose	F2	5 ug	0	0	0	0	0	0	0		
07-MAY-2018	08:39	Day 1	Pre-dose	F3	5 ug	0	0	0	0	0	0	0		
07-MAY-2018	11:18	Day 1	10 min	F1	5 ug	0	0	0	0	0	0	0		
07-MAY-2018	11:18	Day 1	10 min	F2	5 ug	0	0	0	0	0	0	0		
07-MAY-2018	11:18	Day 1	10 min	F3	5 ug	0	0	0	0	0	0	0		
07-MAY-2018	12:08	Day 1	1 hour	F1	5 ug	0	0	0	0	0	0	0		
07-MAY-2018	12:08	Day 1	1 hour	F2	5 ug	0	0	0	0	0	0	0		
07-MAY-2018	12:08	Day 1	1 hour	F3	5 ug	0	0	0	0	0	0	0		
07-MAY-2018	13:08	Day 1	2 hour	F1	5 ug	0	0	0	0	0	0	0		
07-MAY-2018	13:08	Day 1	2 hour	F2	5 ug	0	0	0	0	0	0	0		
07-MAY-2018	13:08	Day 1	2 hour	F3	5 ug	0	0	0	0	0	0	0		
07-MAY-2018	12:24	Unscheduled		F1	5 ug	0	0	0	0	0	0	0	NA	MO notifies as repeat
07-MAY-2018	12:24	Unscheduled		F2	5 ug	0	0	0	0	0	0	0	NA	MO notifies as repeat
07-MAY-2018	12:24	Unscheduled		F3	5 ug	0	0	0	0	0	0	0	NA	MO notifies as repeat
08-MAY-2018	16:43	Day 2		F1	5 ug	0	0	0	0	0	0	0		
08-MAY-2018	16:43	Day 2		F2	5 ug	0	0	0	0	0	0	0		

Key: 0= None; 1=Mild; 2=Moderate; 3=Severe; # Other = Pinprick bleeding or seepage

Administration Site Key: FA = Forearm; UA= Upper arm
Treatments Part A: A1 = A/Sing MAP, 15 ug (FA); A2 = Afluria Quad IM (UA), 15 ug; A3 = Placebo MAP (FA); A4 = A/Sing Mono IM, 15 ug (UA)
Treatments Part B: B1 = A/Sing MAP, 15 ug (FA) with biopsy; B2 = Placebo MAP (FA) with biopsy
B3 = A/Sing MAP, 15 ug (FA); B4 = A/Sing MAP, 10 ug (FA); B5 = A/Sing MAP, 5 ug (FA); B6 = A/Sing MAP, 2.5 ug (FA)
B7 = Placebo MAP (FA); B8 = A/Sing MAP, 15 ug (UA); B9 = A/Sing IM, 15 ug, as 2018 Afluria Quad (UA)

Listing 16.2.9.2
Treatment Site Tolerability Assessment per Site

Part B
Randomisation Number = B0001,   Treatment Group = Group B1

Date	Time	Visit	Time Point	Site Number	Treatment for Site	Indura-tion	Redness	Bruising	Itching	Skin Flaking	Tender-ness	Definition/ Visibility	Other#	Unscheduled Reason
08-MAY-2018	16:43	Day 2		F3	5 ug	0	0	0	0	0	0	0		
10-MAY-2018	09:03	Day 4		F1	5 ug	0	0	0	0	0	0	0		
10-MAY-2018	09:03	Day 4		F2	5 ug	1	0	0	0	0	0	0		
10-MAY-2018	09:03	Day 4		F3	5 ug	1	0	0	0	0	0	0		
14-MAY-2018	13:19	Day 8		F1	5 ug	0	0	0	0	0	0	0		
14-MAY-2018	13:19	Day 8		F2	5 ug	0	0	0	0	0	0	0		
14-MAY-2018	13:19	Day 8		F3	5 ug	0	0	1	0	0	0	0		
28-MAY-2018	12:16	Day 22		F1	5 ug	0	0	0	0	1	0	0		
28-MAY-2018	12:16	Day 22		F2	5 ug	0	0	0	0	0	0	1		
28-MAY-2018	12:16	Day 22		F3	5 ug	0	1	1	0	1	0	0		
06-JUL-2018	11:22	EOS/ET (Day 61)		F1	5 ug	0	0	0	0	0	0	0		
06-JUL-2018	11:22	EOS/ET (Day 61)		F2	5 ug	0	0	0	0	0	0	0		
06-JUL-2018	11:22	EOS/ET (Day 61)		F3	5 ug	0	0	0	0	0	0	0		

Key: 0= None; 1=Mild; 2=Moderate; 3=Severe; # Other = Pinprick bleeding or seepage

Administration Site Key: FA = Forearm; UA= Upper arm
Treatments Part A: A1 = A/Sing MAP, 15 ug (FA); A2 = Afluria Quad IM (UA), 15 ug; A3 = Placebo MAP (FA); A4 = A/Sing Mono IM, 15 ug (UA)
Treatments Part B: B1 = A/Sing MAP, 15 ug (FA) with biopsy; B2 = Placebo MAP (FA) with biopsy
B3 = A/Sing MAP, 15 ug (FA); B4 = A/Sing MAP, 10 ug (FA); B5 = A/Sing MAP, 5 ug (FA); B6 = A/Sing MAP, 2.5 ug (FA)
B7 = Placebo MAP (FA); B8 = A/Sing MAP, 15 ug (UA); B9 = A/Sing IM, 15 ug, as 2018 Afluria Quad (UA)

Listing 16.2.9.2
Treatment Site Tolerability Assessment per Site

Part B
Randomisation Number = B0002, Treatment Group = Group B2

Date	Time	Visit	Time Point	Site Number	Treatment for Site	Indura-tion	Redness	Bruising	Itching	Skin Flaking	Tender-ness	Definition/ Visibility	Other#	Unscheduled Reason
07-MAY-2018	08:45	Day 1	Pre-dose	F1	uncoated	0	0	0	0	0	0	0	NA	
07-MAY-2018	08:45	Day 1	Pre-dose	F2	uncoated	0	0	0	0	0	0	0	NA	
07-MAY-2018	08:45	Day 1	Pre-dose	F3	uncoated	0	0	0	0	0	0	0	NA	
07-MAY-2018	11:28	Day 1	10 min	F1	uncoated	1	2	0	1	0	0	0	NA	
07-MAY-2018	11:28	Day 1	10 min	F2	uncoated	1	2	0	1	0	0	0	NA	
07-MAY-2018	11:28	Day 1	10 min	F3	uncoated	1	2	0	1	0	0	0	NA	
07-MAY-2018	12:13	Day 1	1 hour	F1	uncoated	0	1	0	0	0	0	0	NA	
07-MAY-2018	12:13	Day 1	1 hour	F2	uncoated	0	2	0	0	0	0	0	NA	
07-MAY-2018	12:13	Day 1	1 hour	F3	uncoated	0	1	0	0	0	0	0	NA	
07-MAY-2018	13:18	Day 1	2 hour	F1	uncoated	0	1	0	0	0	0	0	NA	
07-MAY-2018	13:18	Day 1	2 hour	F2	uncoated	1	1	0	0	0	0	0	NA	
07-MAY-2018	13:18	Day 1	2 hour	F3	uncoated	1	1	0	0	0	0	0	NA	
07-MAY-2018	11:38	Unscheduled		F1	uncoated	1	2	0	2	0	0	0	NA	Medical Officer performed as repeat
07-MAY-2018	11:38	Unscheduled		F2	uncoated	1	2	0	2	0	0	0	NA	Medical Officer performed as repeat

Key: 0= None; 1=Mild; 2=Moderate; 3=Severe; # Other = Pinprick bleeding or seepage

Administration Site Key: FA = Forearm; UA= Upper arm
Treatments Part A: A1 = A/Sing MAP, 15 ug (FA); A2 = Afluria Quad IM (UA), 15 ug; A3 = Placebo MAP (FA); A4 = A/Sing Mono IM, 15 ug (UA)
Treatments Part B: B1 = A/Sing MAP, 15 ug (FA) with biopsy; B2 = Placebo MAP (FA) with biopsy
B3 = A/Sing MAP, 15 ug (FA); B4 = A/Sing MAP, 10 ug (FA); B5 = A/Sing MAP, 5 ug (FA); B6 = A/Sing MAP, 2.5 ug (FA)
B7 = Placebo MAP (FA); B8 = A/Sing MAP, 15 ug (UA); B9 = A/Sing IM, 15 ug, as 2018 Afluria Quad (UA)

Listing 16.2.9.2
Treatment Site Tolerability Assessment per Site

Part B
Randomisation Number = B0002, Treatment Group = Group B2

Date	Time	Visit	Time Point	Site Number	Treatment for Site	Indura-tion	Redness	Bruising	Itching	Skin Flaking	Tender-ness	Definition/ Visibility	Other#	Unscheduled Reason
07-MAY-2018	11:38	Unscheduled		F3	uncoated	1	2	0	2	0	0	0	NA	Medical Officer performed as repeat
07-MAY-2018	12:30	Unscheduled		F2	uncoated	0	2	0	0	0	0	0	NA	Medical Officer performed as repeat
08-MAY-2018	12:04	Day 2		F1	uncoated	0	0	0	0	0	0	0		
08-MAY-2018	12:04	Day 2		F2	uncoated	0	0	0	0	0	0	0		
08-MAY-2018	12:04	Day 2		F3	uncoated	0	0	0	0	0	0	0		
10-MAY-2018	09:25	Day 4		F1	uncoated	0	0	0	0	0	0	0		
10-MAY-2018	09:25	Day 4		F2	uncoated	0	0	0	0	0	0	0		
10-MAY-2018	09:25	Day 4		F3	uncoated	0	0	0	0	0	0	0		
14-MAY-2018	13:25	Day 8		F1	uncoated	1	0	0	0	0	0	0		
14-MAY-2018	13:25	Day 8		F2	uncoated	0	0	0	0	0	0	1		
14-MAY-2018	13:25	Day 8		F3	uncoated	0	0	0	0	0	0	0		
28-MAY-2018	11:32	Day 22		F1	uncoated	1	0	0	0	0	0	0		
28-MAY-2018	11:32	Day 22		F2	uncoated	0	0	0	0	0	0	0		
28-MAY-2018	11:32	Day 22		F3	uncoated	1	0	0	0	0	0	0		

Key: 0= None; 1=Mild; 2=Moderate; 3=Severe; # Other = Pinprick bleeding or seepage

Administration Site Key: FA = Forearm; UA= Upper arm
Treatments Part A: A1 = A/Sing MAP, 15 ug (FA); A2 = Afluria Quad IM (UA), 15 ug; A3 = Placebo MAP (FA); A4 = A/Sing Mono IM, 15 ug (UA)
Treatments Part B: B1 = A/Sing MAP, 15 ug (FA) with biopsy; B2 = Placebo MAP (FA) with biopsy
B3 = A/Sing MAP, 15 ug (FA); B4 = A/Sing MAP, 10 ug (FA); B5 = A/Sing MAP, 5 ug (FA); B6 = A/Sing MAP, 2.5 ug (FA)
B7 = Placebo MAP (FA); B8 = A/Sing MAP, 15 ug (UA); B9 = A/Sing IM, 15 ug, as 2018 Afluria Quad (UA)

Listing 16.2.9.2
Treatment Site Tolerability Assessment per Site

Part B
Randomisation Number = B0002, Treatment Group = Group B2

Date	Time	Visit	Time Point	Site Number	Treatment for Site	Indura-tion	Redness	Bruising	Itching	Skin Flaking	Tender-ness	Definition/ Visibility	Other#	Unscheduled Reason
06-JUL-2018	11:06	EOS/ET (Day 61)		F1	uncoated	0	0	0	0	0	0	0		
06-JUL-2018	11:06	EOS/ET (Day 61)		F2	uncoated	0	0	0	0	0	0	0		
06-JUL-2018	11:06	EOS/ET (Day 61)		F3	uncoated	0	0	0	0	0	0	0		

Key: 0= None; 1=Mild; 2=Moderate; 3=Severe; # Other = Pinprick bleeding or seepage

Administration Site Key: FA = Forearm; UA= Upper arm
Treatments Part A: A1 = A/Sing MAP, 15 ug (FA); A2 = Afluria Quad IM (UA), 15 ug; A3 = Placebo MAP (FA); A4 = A/Sing Mono IM, 15 ug (UA)
Treatments Part B: B1 = A/Sing MAP, 15 ug (FA) with biopsy; B2 = Placebo MAP (FA) with biopsy
B3 = A/Sing MAP, 15 ug (FA); B4 = A/Sing MAP, 10 ug (FA); B5 = A/Sing MAP, 5 ug (FA); B6 = A/Sing MAP, 2.5 ug (FA)
B7 = Placebo MAP (FA); B8 = A/Sing MAP, 15 ug (UA); B9 = A/Sing IM, 15 ug, as 2018 Afluria Quad (UA)

Listing 16.2.9.2
Treatment Site Tolerability Assessment per Site

Part B
Randomisation Number = B0003, Treatment Group = Group B2

Date	Time	Visit	Time Point	Site Number	Treatment for Site	Induration	Redness	Bruising	Itching	Skin Flaking	Tenderness	Definition/ Visibility	Other#	Unscheduled Reason
07-MAY-2018	08:55	Day 1	Pre-dose	F1	uncoated	0	0	0	0	0	0	0		
07-MAY-2018	08:55	Day 1	Pre-dose	F2	uncoated	0	0	0	0	0	0	0		
07-MAY-2018	08:55	Day 1	Pre-dose	F3	uncoated	0	0	0	0	0	0	0		
07-MAY-2018	11:38	Day 1	10 min	F1	uncoated	0	1	0	0	0	0	0		
07-MAY-2018	11:38	Day 1	10 min	F2	uncoated	0	1	0	0	0	0	0		
07-MAY-2018	11:38	Day 1	10 min	F3	uncoated	0	1	0	0	0	0	0		
07-MAY-2018	12:18	Day 1	1 hour	F1	uncoated	0	0	0	0	0	0	0		
07-MAY-2018	12:18	Day 1	1 hour	F2	uncoated	0	0	0	0	0	0	0		
07-MAY-2018	12:18	Day 1	1 hour	F3	uncoated	0	0	0	0	0	0	0		
07-MAY-2018	13:28	Day 1	2 hour	F1	uncoated	0	0	0	0	0	0	0		
07-MAY-2018	13:28	Day 1	2 hour	F2	uncoated	0	0	0	0	0	0	0		
07-MAY-2018	13:28	Day 1	2 hour	F3	uncoated	0	0	0	0	0	0	0		
08-MAY-2018	15:18	Day 2		F1	uncoated	0	0	0	0	0	0	0		
08-MAY-2018	15:18	Day 2		F2	uncoated	0	0	0	0	0	0	0		
08-MAY-2018	15:18	Day 2		F3	uncoated	0	0	0	0	0	0	0		
10-MAY-2018	09:58	Day 4		F1	uncoated	0	0	0	0	0	0	2	NA	
10-MAY-2018	09:58	Day 4		F2	uncoated	0	0	0	0	0	0	2	NA	
10-MAY-2018	09:58	Day 4		F3	uncoated	0	0	0	0	0	0	2	NA	
10-MAY-2018	10:09	Unscheduled		F1	uncoated	0	0	0	0	0	0	2	NA	MO notifies as repeat

Key: 0= None; 1=Mild; 2=Moderate; 3=Severe; # Other = Pinprick bleeding or seepage

Administration Site Key: FA = Forearm; UA= Upper arm
Treatments Part A: A1 = A/Sing MAP, 15 ug (FA); A2 = Afluria Quad IM (UA), 15 ug; A3 = Placebo MAP (FA); A4 = A/Sing Mono IM, 15 ug (UA)
Treatments Part B: B1 = A/Sing MAP, 15 ug (FA) with biopsy; B2 = Placebo MAP (FA) with biopsy
B3 = A/Sing MAP, 15 ug (FA); B4 = A/Sing MAP, 10 ug (FA); B5 = A/Sing MAP, 5 ug (FA); B6 = A/Sing MAP, 2.5 ug (FA)
B7 = Placebo MAP (FA); B8 = A/Sing MAP, 15 ug (UA); B9 = A/Sing IM, 15 ug, as 2018 Afluria Quad (UA)

Listing 16.2.9.2
Treatment Site Tolerability Assessment per Site

Part B
Randomisation Number = B0003, Treatment Group = Group B2

Date	Time	Visit	Time Point	Site Number	Treatment for Site	Indura-tion	Redness	Bruising	Itching	Skin Flaking	Tender-ness	Definition/ Visibility	Other#	Unscheduled Reason
10-MAY-2018	10:09	Unscheduled		F2	uncoated	0	0	0	0	0	0	2	NA	MO notifies as repeat
10-MAY-2018	10:09	Unscheduled		F3	uncoated	0	0	0	0	0	0	2	NA	MO notifies as repeat
14-MAY-2018	15:36	Day 8		F1	uncoated	0	0	0	1	0	0	1		
14-MAY-2018	15:36	Day 8		F2	uncoated	0	0	0	0	0	0	0		
14-MAY-2018	15:36	Day 8		F3	uncoated	0	0	0	0	0	0	0		
28-MAY-2018	15:48	Day 22		F1	uncoated	1	1	0	0	2	0	0		
28-MAY-2018	15:48	Day 22		F2	uncoated	0	0	0	0	0	0	0		
28-MAY-2018	15:48	Day 22		F3	uncoated	0	0	0	0	1	0	0		
06-JUL-2018	14:55	EOS/ET (Day 61)		F1	uncoated	0	0	0	0	0	0	0		
06-JUL-2018	14:55	EOS/ET (Day 61)		F2	uncoated	0	0	0	0	0	0	0		
06-JUL-2018	14:55	EOS/ET (Day 61)		F3	uncoated	0	0	0	0	0	0	0		

Key: 0= None; 1=Mild; 2=Moderate; 3=Severe; # Other = Pinprick bleeding or seepage

Administration Site Key: FA = Forearm; UA= Upper arm
Treatments Part A: A1 = A/Sing MAP, 15 ug (FA); A2 = Afluria Quad IM (UA), 15 ug; A3 = Placebo MAP (FA); A4 = A/Sing Mono IM, 15 ug (UA)
Treatments Part B: B1 = A/Sing MAP, 15 ug (FA) with biopsy; B2 = Placebo MAP (FA) with biopsy
B3 = A/Sing MAP, 15 ug (FA); B4 = A/Sing MAP, 10 ug (FA); B5 = A/Sing MAP, 5 ug (FA); B6 = A/Sing MAP, 2.5 ug (FA)
B7 = Placebo MAP (FA); B8 = A/Sing MAP, 15 ug (UA); B9 = A/Sing IM, 15 ug, as 2018 Afluria Quad (UA)

Listing 16.2.9.2
Treatment Site Tolerability Assessment per Site

Part B
Randomisation Number = B0004, Treatment Group = Group B1

Date	Time	Visit	Time Point	Site Number	Treatment for Site	Indura-tion	Redness	Bruising	Itching	Skin Flaking	Tender-ness	Definition/ Visibility	Other#	Unscheduled Reason
07-MAY-2018	09:05	Day 1	Pre-dose	F1	5 ug	0	0	0	0	0	0	0		
07-MAY-2018	09:05	Day 1	Pre-dose	F2	5 ug	0	0	0	0	0	0	0		
07-MAY-2018	09:05	Day 1	Pre-dose	F3	5 ug	0	0	0	0	0	0	0		
07-MAY-2018	12:59	Day 1	10 min	F1	5 ug	0	1	0	0	0	0	0		
07-MAY-2018	12:59	Day 1	10 min	F2	5 ug	0	1	0	0	0	0	0		
07-MAY-2018	12:59	Day 1	10 min	F3	5 ug	0	1	0	0	0	0	0		
07-MAY-2018	13:49	Day 1	1 hour	F1	5 ug	0	1	0	0	0	1	0		
07-MAY-2018	13:49	Day 1	1 hour	F2	5 ug	0	1	0	0	0	0	0		
07-MAY-2018	13:49	Day 1	1 hour	F3	5 ug	0	1	0	0	0	0	0		
07-MAY-2018	14:49	Day 1	2 hour	F1	5 ug	0	0	0	0	0	1	0		
07-MAY-2018	14:49	Day 1	2 hour	F2	5 ug	0	0	0	0	0	0	0		
07-MAY-2018	14:49	Day 1	2 hour	F3	5 ug	0	0	0	0	0	0	0		
08-MAY-2018	12:35	Day 2		F1	5 ug	0	1	0	1	0	0	0		
08-MAY-2018	12:35	Day 2		F2	5 ug	0	1	0	0	0	0	0		
08-MAY-2018	12:35	Day 2		F3	5 ug	0	1	0	0	0	0	0		
10-MAY-2018	08:49	Day 4		F1	5 ug	1	1	0	0	0	0	0		
10-MAY-2018	08:49	Day 4		F2	5 ug	0	0	0	0	0	0	0		
10-MAY-2018	08:49	Day 4		F3	5 ug	0	1	0	0	0	0	0		
14-MAY-2018	13:49	Day 8		F1	5 ug	0	0	0	1	1	0	1		
14-MAY-2018	13:49	Day 8		F2	5 ug	0	0	0	0	0	0	1		

Key: 0= None; 1=Mild; 2=Moderate; 3=Severe; # Other = Pinprick bleeding or seepage

Administration Site Key: FA = Forearm; UA= Upper arm
Treatments Part A: A1 = A/Sing MAP, 15 ug (FA); A2 = Afluria Quad IM (UA), 15 ug; A3 = Placebo MAP (FA); A4 = A/Sing Mono IM, 15 ug (UA)
Treatments Part B: B1 = A/Sing MAP, 15 ug (FA) with biopsy; B2 = Placebo MAP (FA) with biopsy
B3 = A/Sing MAP, 15 ug (FA); B4 = A/Sing MAP, 10 ug (FA); B5 = A/Sing MAP, 5 ug (FA); B6 = A/Sing MAP, 2.5 ug (FA)
B7 = Placebo MAP (FA); B8 = A/Sing MAP, 15 ug (UA); B9 = A/Sing IM, 15 ug, as 2018 Afluria Quad (UA)

Listing 16.2.9.2
Treatment Site Tolerability Assessment per Site

Part B
Randomisation Number = B0004, Treatment Group = Group B1

Date	Time	Visit	Time Point	Site Number	Treatment for Site	Indura-tion	Redness	Bruising	Itching	Skin Flaking	Tender-ness	Definition/ Visibility	Other#	Unscheduled Reason
14-MAY-2018	13:49	Day 8		F3	5 ug	0	0	2	1	0	0	1		
28-MAY-2018	15:44	Day 22		F1	5 ug	0	0	0	0	1	0	0		
28-MAY-2018	15:44	Day 22		F2	5 ug	0	0	0	0	0	0	2		
28-MAY-2018	15:44	Day 22		F3	5 ug	0	0	0	0	1	0	0		
06-JUL-2018	12:59	EOS/ET (Day 61)		F1	5 ug	0	0	0	0	0	0	0		
06-JUL-2018	12:59	EOS/ET (Day 61)		F2	5 ug	0	0	0	0	0	0	0		
06-JUL-2018	12:59	EOS/ET (Day 61)		F3	5 ug	0	0	0	0	0	0	0		

Key: 0= None; 1=Mild; 2=Moderate; 3=Severe; # Other = Pinprick bleeding or seepage

Administration Site Key: FA = Forearm; UA= Upper arm
Treatments Part A: A1 = A/Sing MAP, 15 ug (FA); A2 = Afluria Quad IM (UA), 15 ug; A3 = Placebo MAP (FA); A4 = A/Sing Mono IM, 15 ug (UA)
Treatments Part B: B1 = A/Sing MAP, 15 ug (FA) with biopsy; B2 = Placebo MAP (FA) with biopsy
B3 = A/Sing MAP, 15 ug (FA); B4 = A/Sing MAP, 10 ug (FA); B5 = A/Sing MAP, 5 ug (FA); B6 = A/Sing MAP, 2.5 ug (FA)
B7 = Placebo MAP (FA); B8 = A/Sing MAP, 15 ug (UA); B9 = A/Sing IM, 15 ug, as 2018 Afluria Quad (UA)

Listing 16.2.9.2
Treatment Site Tolerability Assessment per Site

Part B
Randomisation Number = B0005, Treatment Group = Group B1

Date	Time	Visit	Time Point	Site Number	Treatment for Site	Indura-tion	Redness	Bruising	Itching	Skin Flaking	Tender-ness	Definition/ Visibility	Other#	Unscheduled Reason
07-MAY-2018	09:15	Day 1	Pre-dose	F1	5 ug	0	0	0	0	0	0	0		
07-MAY-2018	09:15	Day 1	Pre-dose	F2	5 ug	0	0	0	0	0	0	0		
07-MAY-2018	09:15	Day 1	Pre-dose	F3	5 ug	0	0	0	0	0	0	0		
07-MAY-2018	11:58	Day 1	10 min	F1	5 ug	0	1	0	0	0	0	0		
07-MAY-2018	11:58	Day 1	10 min	F2	5 ug	0	1	0	0	0	1	0		
07-MAY-2018	11:58	Day 1	10 min	F3	5 ug	0	1	0	0	0	1	0		
07-MAY-2018	12:48	Day 1	1 hour	F1	5 ug	0	0	0	0	0	1	0		
07-MAY-2018	12:48	Day 1	1 hour	F2	5 ug	0	1	0	0	0	1	0		
07-MAY-2018	12:48	Day 1	1 hour	F3	5 ug	0	0	0	0	0	0	0		
07-MAY-2018	13:48	Day 1	2 hour	F1	5 ug	0	0	0	0	0	0	0		
07-MAY-2018	13:48	Day 1	2 hour	F2	5 ug	0	0	0	0	0	0	0		
07-MAY-2018	13:48	Day 1	2 hour	F3	5 ug	0	0	0	0	0	0	0		
08-MAY-2018	17:35	Day 2		F1	5 ug	0	1	0	0	0	0	0		
08-MAY-2018	17:35	Day 2		F2	5 ug	0	1	0	0	0	0	0		
08-MAY-2018	17:35	Day 2		F3	5 ug	0	1	0	0	0	0	0		
10-MAY-2018	09:34	Day 4		F1	5 ug	0	0	0	0	2	0	0		
10-MAY-2018	09:34	Day 4		F2	5 ug	0	0	0	0	1	1	0		
10-MAY-2018	09:34	Day 4		F3	5 ug	0	0	0	0	1	1	0		
14-MAY-2018	17:09	Day 8		F1	5 ug	0	2	0	0	0	1	0		
14-MAY-2018	17:09	Day 8		F2	5 ug	0	0	0	0	1	0	0		

Key: 0= None; 1=Mild; 2=Moderate; 3=Severe; # Other = Pinprick bleeding or seepage

Administration Site Key: FA = Forearm; UA= Upper arm
Treatments Part A: A1 = A/Sing MAP, 15 ug (FA); A2 = Afluria Quad IM (UA), 15 ug; A3 = Placebo MAP (FA); A4 = A/Sing Mono IM, 15 ug (UA)
Treatments Part B: B1 = A/Sing MAP, 15 ug (FA) with biopsy; B2 = Placebo MAP (FA) with biopsy
B3 = A/Sing MAP, 15 ug (FA); B4 = A/Sing MAP, 10 ug (FA); B5 = A/Sing MAP, 5 ug (FA); B6 = A/Sing MAP, 2.5 ug (FA)
B7 = Placebo MAP (FA); B8 = A/Sing MAP, 15 ug (UA); B9 = A/Sing IM, 15 ug, as 2018 Afluria Quad (UA)

Listing 16.2.9.2
Treatment Site Tolerability Assessment per Site

Part B
Randomisation Number = B0005, Treatment Group = Group B1

Date	Time	Visit	Time Point	Site Number	Treatment for Site	Indura-tion	Redness	Bruising	Itching	Skin Flaking	Tender-ness	Definition/ Visibility	Other#	Unscheduled Reason
14-MAY-2018	17:09	Day 8		F3	5 ug	1	2	0	0	0	1	0		
28-MAY-2018	14:23	Day 22		F1	5 ug	1	0	0	0	0	1	0		
28-MAY-2018	14:23	Day 22		F2	5 ug	0	0	0	0	0	0	0		
28-MAY-2018	14:23	Day 22		F3	5 ug	0	0	0	0	0	0	0		
06-JUL-2018	15:00	EOS/ET (Day 61)		F1	5 ug	0	0	0	0	0	0	0		
06-JUL-2018	15:00	EOS/ET (Day 61)		F2	5 ug	0	0	0	0	0	0	0		
06-JUL-2018	15:00	EOS/ET (Day 61)		F3	5 ug	0	0	0	0	0	0	0		

Key: 0= None; 1=Mild; 2=Moderate; 3=Severe; # Other = Pinprick bleeding or seepage

Administration Site Key: FA = Forearm; UA= Upper arm
Treatments Part A: A1 = A/Sing MAP, 15 ug (FA); A2 = Afluria Quad IM (UA), 15 ug; A3 = Placebo MAP (FA); A4 = A/Sing Mono IM, 15 ug (UA)
Treatments Part B: B1 = A/Sing MAP, 15 ug (FA) with biopsy; B2 = Placebo MAP (FA) with biopsy
B3 = A/Sing MAP, 15 ug (FA); B4 = A/Sing MAP, 10 ug (FA); B5 = A/Sing MAP, 5 ug (FA); B6 = A/Sing MAP, 2.5 ug (FA)
B7 = Placebo MAP (FA); B8 = A/Sing MAP, 15 ug (UA); B9 = A/Sing IM, 15 ug, as 2018 Afluria Quad (UA)

Listing 16.2.9.2
Treatment Site Tolerability Assessment per Site

Part B
Randomisation Number = B0006, Treatment Group = Group B2

Date	Time	Visit	Time Point	Site Number	Treatment for Site	Indura-tion	Redness	Bruising	Itching	Skin Flaking	Tender-ness	Definition/ Visibility	Other#	Unscheduled Reason
07-MAY-2018	09:25	Day 1	Pre-dose	F1	uncoated	0	0	0	0	0	0	0		
07-MAY-2018	09:25	Day 1	Pre-dose	F2	uncoated	0	0	0	0	0	0	0		
07-MAY-2018	09:25	Day 1	Pre-dose	F3	uncoated	0	0	0	0	0	0	0		
07-MAY-2018	12:08	Day 1	10 min	F1	uncoated	0	1	0	0	0	0	0		
07-MAY-2018	12:08	Day 1	10 min	F2	uncoated	0	1	0	0	0	0	0		
07-MAY-2018	12:08	Day 1	10 min	F3	uncoated	0	1	0	0	0	0	0		
07-MAY-2018	12:58	Day 1	1 hour	F1	uncoated	0	0	0	0	0	0	0		
07-MAY-2018	12:58	Day 1	1 hour	F2	uncoated	0	0	0	0	0	0	0		
07-MAY-2018	12:58	Day 1	1 hour	F3	uncoated	0	0	0	0	0	0	0		
07-MAY-2018	13:58	Day 1	2 hour	F1	uncoated	0	0	0	0	0	0	0		
07-MAY-2018	13:58	Day 1	2 hour	F2	uncoated	0	0	0	0	0	0	0		
07-MAY-2018	13:58	Day 1	2 hour	F3	uncoated	0	0	0	0	0	0	0		
08-MAY-2018	12:17	Day 2		F1	uncoated	0	0	0	0	0	0	0		
08-MAY-2018	12:17	Day 2		F2	uncoated	0	0	0	0	0	0	0		
08-MAY-2018	12:17	Day 2		F3	uncoated	0	0	0	0	0	0	0		
10-MAY-2018	09:26	Day 4		F1	uncoated	0	0	0	0	0	0	0		
10-MAY-2018	09:26	Day 4		F2	uncoated	0	0	0	0	0	0	0		
10-MAY-2018	09:26	Day 4		F3	uncoated	0	0	0	0	0	0	0		
14-MAY-2018	12:38	Day 8		F1	uncoated	1	1	0	0	0	0	0		
14-MAY-2018	12:38	Day 8		F2	uncoated	0	0	0	0	0	0	0		

Key: 0= None; 1=Mild; 2=Moderate; 3=Severe; # Other = Pinprick bleeding or seepage

Administration Site Key: FA = Forearm; UA= Upper arm
Treatments Part A: A1 = A/Sing MAP, 15 ug (FA); A2 = Afluria Quad IM (UA), 15 ug; A3 = Placebo MAP (FA); A4 = A/Sing Mono IM, 15 ug (UA)
Treatments Part B: B1 = A/Sing MAP, 15 ug (FA) with biopsy; B2 = Placebo MAP (FA) with biopsy
B3 = A/Sing MAP, 15 ug (FA); B4 = A/Sing MAP, 10 ug (FA); B5 = A/Sing MAP, 5 ug (FA); B6 = A/Sing MAP, 2.5 ug (FA)
B7 = Placebo MAP (FA); B8 = A/Sing MAP, 15 ug (UA); B9 = A/Sing IM, 15 ug, as 2018 Afluria Quad (UA)

Listing 16.2.9.2
Treatment Site Tolerability Assessment per Site

Part B
Randomisation Number = B0006, Treatment Group = Group B2

Date	Time	Visit	Time Point	Site Number	Treatment for Site	Indura-tion	Redness	Bruising	Itching	Skin Flaking	Tender-ness	Definition/ Visibility	Other#	Unscheduled Reason
14-MAY-2018	12:38	Day 8		F3	uncoated	1	1	0	0	0	0	0		
28-MAY-2018	11:43	Day 22		F1	uncoated	1	1	0	0	0	0	0		
28-MAY-2018	11:43	Day 22		F2	uncoated	0	0	0	0	0	0	0		
28-MAY-2018	11:43	Day 22		F3	uncoated	1	1	0	0	0	0	0		
06-JUL-2018	12:00	EOS/ET (Day 61)		F1	uncoated	0	0	0	0	0	0	0		
06-JUL-2018	12:00	EOS/ET (Day 61)		F2	uncoated	0	0	0	0	0	0	0		
06-JUL-2018	12:00	EOS/ET (Day 61)		F3	uncoated	0	0	0	0	0	0	0		

Key: 0= None; 1=Mild; 2=Moderate; 3=Severe; # Other = Pinprick bleeding or seepage

Administration Site Key: FA = Forearm; UA= Upper arm
Treatments Part A: A1 = A/Sing MAP, 15 ug (FA); A2 = Afluria Quad IM (UA), 15 ug; A3 = Placebo MAP (FA); A4 = A/Sing Mono IM, 15 ug (UA)
Treatments Part B: B1 = A/Sing MAP, 15 ug (FA) with biopsy; B2 = Placebo MAP (FA) with biopsy
B3 = A/Sing MAP, 15 ug (FA); B4 = A/Sing MAP, 10 ug (FA); B5 = A/Sing MAP, 5 ug (FA); B6 = A/Sing MAP, 2.5 ug (FA)
B7 = Placebo MAP (FA); B8 = A/Sing MAP, 15 ug (UA); B9 = A/Sing IM, 15 ug, as 2018 Afluria Quad (UA)

Listing 16.2.9.2
Treatment Site Tolerability Assessment per Site

Part B
Randomisation Number = B0007, Treatment Group = Group B2

Date	Time	Visit	Time Point	Site Number	Treatment for Site	Induration	Redness	Bruising	Itching	Skin Flaking	Tenderness	Definition/ Visibility	Other#	Unscheduled Reason
07-MAY-2018	09:35	Day 1	Pre-dose	F1	uncoated	0	0	0	0	0	0	0		
07-MAY-2018	09:35	Day 1	Pre-dose	F2	uncoated	0	0	0	0	0	0	0		
07-MAY-2018	09:35	Day 1	Pre-dose	F3	uncoated	0	0	0	0	0	0	0		
07-MAY-2018	12:18	Day 1	10 min	F1	uncoated	0	0	0	0	0	1	0		
07-MAY-2018	12:18	Day 1	10 min	F2	uncoated	0	0	0	0	0	0	0		
07-MAY-2018	12:18	Day 1	10 min	F3	uncoated	0	0	0	0	0	1	0		
07-MAY-2018	13:08	Day 1	1 hour	F1	uncoated	0	0	0	0	0	0	0		
07-MAY-2018	13:08	Day 1	1 hour	F2	uncoated	0	0	0	0	0	0	0		
07-MAY-2018	13:08	Day 1	1 hour	F3	uncoated	0	0	0	0	0	0	0		
07-MAY-2018	14:08	Day 1	2 hour	F1	uncoated	0	0	0	0	0	0	0		
07-MAY-2018	14:08	Day 1	2 hour	F2	uncoated	0	0	0	0	0	0	0		
07-MAY-2018	14:08	Day 1	2 hour	F3	uncoated	0	0	0	0	0	0	0		
08-MAY-2018	12:12	Day 2		F1	uncoated	0	1	0	0	0	0	0		
08-MAY-2018	12:12	Day 2		F2	uncoated	0	1	0	0	0	0	0		
08-MAY-2018	12:12	Day 2		F3	uncoated	0	1	0	0	0	0	0		
10-MAY-2018	09:18	Day 4		F1	uncoated	0	0	0	0	0	0	0		
10-MAY-2018	09:18	Day 4		F2	uncoated	0	0	0	0	0	0	0		
10-MAY-2018	09:18	Day 4		F3	uncoated	0	0	0	0	0	0	0		
14-MAY-2018	12:50	Day 8		F1	uncoated	1	0	0	0	1	0	1		
14-MAY-2018	12:50	Day 8		F2	uncoated	0	0	0	0	0	0	1		

Key: 0= None; 1=Mild; 2=Moderate; 3=Severe; # Other = Pinprick bleeding or seepage

Administration Site Key: FA = Forearm; UA= Upper arm
Treatments Part A: A1 = A/Sing MAP, 15 ug (FA); A2 = Afluria Quad IM (UA), 15 ug; A3 = Placebo MAP (FA); A4 = A/Sing Mono IM, 15 ug (UA)
Treatments Part B: B1 = A/Sing MAP, 15 ug (FA) with biopsy; B2 = Placebo MAP (FA) with biopsy
B3 = A/Sing MAP, 15 ug (FA); B4 = A/Sing MAP, 10 ug (FA); B5 = A/Sing MAP, 5 ug (FA); B6 = A/Sing MAP, 2.5 ug (FA)
B7 = Placebo MAP (FA); B8 = A/Sing MAP, 15 ug (UA); B9 = A/Sing IM, 15 ug, as 2018 Afluria Quad (UA)

Listing 16.2.9.2
Treatment Site Tolerability Assessment per Site

Part B
Randomisation Number = B0007, Treatment Group = Group B2

Date	Time	Visit	Time Point	Site Number	Treatment for Site	Indura-tion	Redness	Bruising	Itching	Skin Flaking	Tender-ness	Definition/ Visibility	Other#	Unscheduled Reason
14-MAY-2018	12:50	Day 8		F3	uncoated	0	0	0	0	1	0	1		
26-MAY-2018	13:01	Day 22		F1	uncoated	0	0	0	0	0	0	0		
26-MAY-2018	13:01	Day 22		F2	uncoated	0	0	0	0	0	0	0		
26-MAY-2018	13:01	Day 22		F3	uncoated	0	0	0	0	0	0	0		
06-JUL-2018	08:50	EOS/ET (Day 61)		F1	uncoated	0	0	0	0	0	0	0		
06-JUL-2018	08:50	EOS/ET (Day 61)		F2	uncoated	0	0	0	0	0	0	0		
06-JUL-2018	08:50	EOS/ET (Day 61)		F3	uncoated	0	0	0	0	0	0	0		

Key: 0= None; 1=Mild; 2=Moderate; 3=Severe; # Other = Pinprick bleeding or seepage

Administration Site Key: FA = Forearm; UA= Upper arm
Treatments Part A: A1 = A/Sing MAP, 15 ug (FA); A2 = Afluria Quad IM (UA), 15 ug; A3 = Placebo MAP (FA); A4 = A/Sing Mono IM, 15 ug (UA)
Treatments Part B: B1 = A/Sing MAP, 15 ug (FA) with biopsy; B2 = Placebo MAP (FA) with biopsy
B3 = A/Sing MAP, 15 ug (FA); B4 = A/Sing MAP, 10 ug (FA); B5 = A/Sing MAP, 5 ug (FA); B6 = A/Sing MAP, 2.5 ug (FA)
B7 = Placebo MAP (FA); B8 = A/Sing MAP, 15 ug (UA); B9 = A/Sing IM, 15 ug, as 2018 Afluria Quad (UA)

Listing 16.2.9.2
Treatment Site Tolerability Assessment per Site

Part B
Randomisation Number = B0008, Treatment Group = Group B1

Date	Time	Visit	Time Point	Site Number	Treatment for Site	Induration	Redness	Bruising	Itching	Skin Flaking	Tenderness	Definition/ Visibility	Other#	Unscheduled Reason
07-MAY-2018	09:49	Day 1	Pre-dose	F1	5 ug	0	0	0	0	0	0	0		
07-MAY-2018	09:49	Day 1	Pre-dose	F2	5 ug	0	0	0	0	0	0	0		
07-MAY-2018	09:49	Day 1	Pre-dose	F3	5 ug	0	0	0	0	0	0	0		
07-MAY-2018	12:28	Day 1	10 min	F1	5 ug	0	1	0	0	0	0	0		
07-MAY-2018	12:28	Day 1	10 min	F2	5 ug	1	2	0	1	0	0	0		
07-MAY-2018	12:28	Day 1	10 min	F3	5 ug	1	2	0	0	0	0	0		
07-MAY-2018	13:18	Day 1	1 hour	F1	5 ug	0	1	0	0	0	0	0		
07-MAY-2018	13:18	Day 1	1 hour	F2	5 ug	0	1	0	0	0	0	0		
07-MAY-2018	13:18	Day 1	1 hour	F3	5 ug	0	1	0	0	0	0	0		
07-MAY-2018	14:18	Day 1	2 hour	F1	5 ug	0	0	0	0	0	0	0		
07-MAY-2018	14:18	Day 1	2 hour	F2	5 ug	1	0	0	0	0	0	0		
07-MAY-2018	14:18	Day 1	2 hour	F3	5 ug	1	0	0	0	0	0	0		
07-MAY-2018	12:37	Unscheduled		F2	5 ug	1	2	0	0	0	0	0	NA	MO notifies as repeat
07-MAY-2018	12:37	Unscheduled		F3	5 ug	1	2	0	0	0	0	0	NA	MO notifies as repeat
08-MAY-2018	12:25	Day 2		F1	5 ug	0	1	0	0	0	0	0		
08-MAY-2018	12:25	Day 2		F2	5 ug	0	1	0	0	0	1	0		
08-MAY-2018	12:25	Day 2		F3	5 ug	0	1	0	0	0	0	0		
10-MAY-2018	09:11	Day 4		F1	5 ug	0	0	0	0	0	0	0		

Key: 0= None; 1=Mild; 2=Moderate; 3=Severe; # Other = Pinprick bleeding or seepage

Administration Site Key: FA = Forearm; UA= Upper arm
Treatments Part A: A1 = A/Sing MAP, 15 ug (FA); A2 = Afluria Quad IM (UA), 15 ug; A3 = Placebo MAP (FA); A4 = A/Sing Mono IM, 15 ug (UA)
Treatments Part B: B1 = A/Sing MAP, 15 ug (FA) with biopsy; B2 = Placebo MAP (FA) with biopsy
B3 = A/Sing MAP, 15 ug (FA); B4 = A/Sing MAP, 10 ug (FA); B5 = A/Sing MAP, 5 ug (FA); B6 = A/Sing MAP, 2.5 ug (FA)
B7 = Placebo MAP (FA); B8 = A/Sing MAP, 15 ug (UA); B9 = A/Sing IM, 15 ug, as 2018 Afluria Quad (UA)

Listing 16.2.9.2
Treatment Site Tolerability Assessment per Site

Part B
Randomisation Number = B0008, Treatment Group = Group B1

Date	Time	Visit	Time Point	Site Number	Treatment for Site	Indura-tion	Redness	Bruising	Itching	Skin Flaking	Tender-ness	Definition/ Visibility	Other#	Unscheduled Reason
10-MAY-2018	09:11	Day 4		F2	5 ug	0	0	0	0	0	0	0		
10-MAY-2018	09:11	Day 4		F3	5 ug	0	0	0	0	0	0	0		
14-MAY-2018	12:59	Day 8		F1	5 ug	0	0	0	0	0	0	0		
14-MAY-2018	12:59	Day 8		F2	5 ug	0	0	0	0	0	0	2		
14-MAY-2018	12:59	Day 8		F3	5 ug	0	0	0	0	0	0	0		
28-MAY-2018	12:38	Day 22		F1	5 ug	0	0	0	0	1	0	0		
28-MAY-2018	12:38	Day 22		F2	5 ug	0	0	0	0	0	0	0		
28-MAY-2018	12:38	Day 22		F3	5 ug	0	0	0	0	0	0	0		
06-JUL-2018	08:25	EOS/ET (Day 61)		F1	5 ug	0	0	0	0	0	0	0		
06-JUL-2018	08:25	EOS/ET (Day 61)		F2	5 ug	0	0	0	0	0	0	0		
06-JUL-2018	08:25	EOS/ET (Day 61)		F3	5 ug	0	0	0	0	0	0	0		

Key: 0= None; 1=Mild; 2=Moderate; 3=Severe; # Other = Pinprick bleeding or seepage

Administration Site Key: FA = Forearm; UA= Upper arm
Treatments Part A: A1 = A/Sing MAP, 15 ug (FA); A2 = Afluria Quad IM (UA), 15 ug; A3 = Placebo MAP (FA); A4 = A/Sing Mono IM, 15 ug (UA)
Treatments Part B: B1 = A/Sing MAP, 15 ug (FA) with biopsy; B2 = Placebo MAP (FA) with biopsy
B3 = A/Sing MAP, 15 ug (FA); B4 = A/Sing MAP, 10 ug (FA); B5 = A/Sing MAP, 5 ug (FA); B6 = A/Sing MAP, 2.5 ug (FA)
B7 = Placebo MAP (FA); B8 = A/Sing MAP, 15 ug (UA); B9 = A/Sing IM, 15 ug, as 2018 Afluria Quad (UA)

Listing 16.2.9.2
Treatment Site Tolerability Assessment per Site

Part B
Randomisation Number = B0009, Treatment Group = Group B1

Date	Time	Visit	Time Point	Site Number	Treatment for Site	Indura-tion	Redness	Bruising	Itching	Skin Flaking	Tender-ness	Definition/ Visibility	Other#	Unscheduled Reason
07-MAY-2018	09:55	Day 1	Pre-dose	F1	5 ug	0	0	0	0	0	0	0		
07-MAY-2018	09:55	Day 1	Pre-dose	F2	5 ug	0	0	0	0	0	0	0		
07-MAY-2018	09:55	Day 1	Pre-dose	F3	5 ug	0	0	0	0	0	0	0		
07-MAY-2018	12:38	Day 1	10 min	F1	5 ug	0	1	0	1	0	0	0		
07-MAY-2018	12:38	Day 1	10 min	F2	5 ug	0	1	0	1	0	0	0		
07-MAY-2018	12:38	Day 1	10 min	F3	5 ug	0	1	0	0	0	0	0		
07-MAY-2018	13:28	Day 1	1 hour	F1	5 ug	0	1	0	0	0	0	0		
07-MAY-2018	13:28	Day 1	1 hour	F2	5 ug	0	1	0	0	0	0	0		
07-MAY-2018	13:28	Day 1	1 hour	F3	5 ug	0	1	0	0	0	0	0		
07-MAY-2018	14:28	Day 1	2 hour	F1	5 ug	0	0	0	0	0	0	0		
07-MAY-2018	14:28	Day 1	2 hour	F2	5 ug	0	0	0	0	0	0	0		
07-MAY-2018	14:28	Day 1	2 hour	F3	5 ug	0	0	0	0	0	0	0		
08-MAY-2018	13:19	Day 2		F1	5 ug	0	1	0	0	0	0	0		
08-MAY-2018	13:19	Day 2		F2	5 ug	0	1	0	0	0	0	0		
08-MAY-2018	13:19	Day 2		F3	5 ug	0	1	0	0	0	0	0		
10-MAY-2018	09:54	Day 4		F1	5 ug	0	1	0	0	2	0	0		
10-MAY-2018	09:54	Day 4		F2	5 ug	0	1	0	1	2	0	0		
10-MAY-2018	09:54	Day 4		F3	5 ug	0	1	0	1	1	0	0		
14-MAY-2018	11:39	Day 8		F1	5 ug	0	0	0	0	2	1	2		
14-MAY-2018	11:39	Day 8		F2	5 ug	0	0	0	0	1	0	2		

Key: 0= None; 1=Mild; 2=Moderate; 3=Severe; # Other = Pinprick bleeding or seepage

Administration Site Key: FA = Forearm; UA= Upper arm
Treatments Part A: A1 = A/Sing MAP, 15 ug (FA); A2 = Afluria Quad IM (UA), 15 ug; A3 = Placebo MAP (FA); A4 = A/Sing Mono IM, 15 ug (UA)
Treatments Part B: B1 = A/Sing MAP, 15 ug (FA) with biopsy; B2 = Placebo MAP (FA) with biopsy
B3 = A/Sing MAP, 15 ug (FA); B4 = A/Sing MAP, 10 ug (FA); B5 = A/Sing MAP, 5 ug (FA); B6 = A/Sing MAP, 2.5 ug (FA)
B7 = Placebo MAP (FA); B8 = A/Sing MAP, 15 ug (UA); B9 = A/Sing IM, 15 ug, as 2018 Afluria Quad (UA)

Listing 16.2.9.2
Treatment Site Tolerability Assessment per Site

Part B
Randomisation Number = B0009, Treatment Group = Group B1

Date	Time	Visit	Time Point	Site Number	Treatment for Site	Indura-tion	Redness	Bruising	Itching	Skin Flaking	Tender-ness	Definition/ Visibility	Other#	Unscheduled Reason
14-MAY-2018	11:39	Day 8		F3	5 ug	0	0	0	0	2	0	1		
14-MAY-2018	11:42	Unscheduled		F1	5 ug					2		2	NA	MO repeat
14-MAY-2018	11:42	Unscheduled		F2	5 ug							2	NA	MO repeat
14-MAY-2018	11:42	Unscheduled		F3	5 ug					2			NA	MO repeat
28-MAY-2018	12:55	Day 22		F1	5 ug	0	0	0	0	1	0	0		
28-MAY-2018	12:55	Day 22		F2	5 ug	0	0	0	0	0	0	0		
28-MAY-2018	12:55	Day 22		F3	5 ug	0	0	0	0	0	0	0		
06-JUL-2018	11:41	EOS/ET (Day 61)		F1	5 ug	0	0	0	0	0	0	0		
06-JUL-2018	11:41	EOS/ET (Day 61)		F2	5 ug	0	0	0	0	0	0	0		
06-JUL-2018	11:41	EOS/ET (Day 61)		F3	5 ug	0	0	0	0	0	0	0		

Key: 0= None; 1=Mild; 2=Moderate; 3=Severe; # Other = Pinprick bleeding or seepage

Administration Site Key: FA = Forearm; UA= Upper arm
Treatments Part A: A1 = A/Sing MAP, 15 ug (FA); A2 = Afluria Quad IM (UA), 15 ug; A3 = Placebo MAP (FA); A4 = A/Sing Mono IM, 15 ug (UA)
Treatments Part B: B1 = A/Sing MAP, 15 ug (FA) with biopsy; B2 = Placebo MAP (FA) with biopsy
B3 = A/Sing MAP, 15 ug (FA); B4 = A/Sing MAP, 10 ug (FA); B5 = A/Sing MAP, 5 ug (FA); B6 = A/Sing MAP, 2.5 ug (FA)
B7 = Placebo MAP (FA); B8 = A/Sing MAP, 15 ug (UA); B9 = A/Sing IM, 15 ug, as 2018 Afluria Quad (UA)

Listing 16.2.9.2
Treatment Site Tolerability Assessment per Site

Part B
Randomisation Number = B0010, Treatment Group = Group B2

Date	Time	Visit	Time Point	Site Number	Treatment for Site	Induration	Redness	Bruising	Itching	Skin Flaking	Tenderness	Definition/ Visibility	Other#	Unscheduled Reason
07-MAY-2018	10:05	Day 1	Pre-dose	F1	uncoated	0	0	0	0	0	0	0		
07-MAY-2018	10:05	Day 1	Pre-dose	F2	uncoated	0	0	0	0	0	0	0		
07-MAY-2018	10:05	Day 1	Pre-dose	F3	uncoated	0	0	0	0	0	0	0		
07-MAY-2018	12:48	Day 1	10 min	F1	uncoated	0	0	0	0	0	1	0		
07-MAY-2018	12:48	Day 1	10 min	F2	uncoated	0	0	0	0	0	0	0		
07-MAY-2018	12:48	Day 1	10 min	F3	uncoated	0	0	0	0	0	0	0		
07-MAY-2018	13:38	Day 1	1 hour	F1	uncoated	0	0	0	0	0	0	0		
07-MAY-2018	13:38	Day 1	1 hour	F2	uncoated	0	0	0	0	0	0	0		
07-MAY-2018	13:38	Day 1	1 hour	F3	uncoated	0	0	0	0	0	0	0		
07-MAY-2018	14:38	Day 1	2 hour	F1	uncoated	0	0	0	0	0	0	0		
07-MAY-2018	14:38	Day 1	2 hour	F2	uncoated	0	0	0	0	0	0	0		
07-MAY-2018	14:38	Day 1	2 hour	F3	uncoated	0	0	0	0	0	0	1		
08-MAY-2018	14:05	Day 2		F1	uncoated	0	0	0	0	0	0	0		
08-MAY-2018	14:05	Day 2		F2	uncoated	0	0	0	0	0	0	0		
08-MAY-2018	14:05	Day 2		F3	uncoated	0	0	0	0	0	0	0		
10-MAY-2018	13:33	Day 4		F1	uncoated	0	0	0	0	0	0	0		
10-MAY-2018	13:33	Day 4		F2	uncoated	0	0	0	0	0	0	0		
10-MAY-2018	13:33	Day 4		F3	uncoated	0	0	0	0	0	0	0		
14-MAY-2018	08:31	Day 8		F1	uncoated	0	0	0	0	0	0	0		
14-MAY-2018	08:31	Day 8		F2	uncoated	0	0	0	0	1	0	2		

Key: 0= None; 1=Mild; 2=Moderate; 3=Severe; # Other = Pinprick bleeding or seepage

Administration Site Key: FA = Forearm; UA= Upper arm
Treatments Part A: A1 = A/Sing MAP, 15 ug (FA); A2 = Afluria Quad IM (UA), 15 ug; A3 = Placebo MAP (FA); A4 = A/Sing Mono IM, 15 ug (UA)
Treatments Part B: B1 = A/Sing MAP, 15 ug (FA) with biopsy; B2 = Placebo MAP (FA) with biopsy
B3 = A/Sing MAP, 15 ug (FA); B4 = A/Sing MAP, 10 ug (FA); B5 = A/Sing MAP, 5 ug (FA); B6 = A/Sing MAP, 2.5 ug (FA)
B7 = Placebo MAP (FA); B8 = A/Sing MAP, 15 ug (UA); B9 = A/Sing IM, 15 ug, as 2018 Afluria Quad (UA)

Listing 16.2.9.2
Treatment Site Tolerability Assessment per Site

Part B
Randomisation Number = B0010, Treatment Group = Group B2

Date	Time	Visit	Time Point	Site Number	Treatment for Site	Indura-tion	Redness	Bruising	Itching	Skin Flaking	Tender-ness	Definition/ Visibility	Other#	Unscheduled Reason
14-MAY-2018	08:31	Day 8		F3	uncoated	0	1	0	0	0	1	0		
28-MAY-2018	09:27	Day 22		F1	uncoated	0	1	0	0	0	1	0		
28-MAY-2018	09:27	Day 22		F2	uncoated	0	0	0	0	0	0	0		
28-MAY-2018	09:27	Day 22		F3	uncoated	0	1	0	0	0	1	0		
06-JUL-2018	11:25	EOS/ET (Day 61)		F1	uncoated	0	0	0	0	0	0	0		
06-JUL-2018	11:25	EOS/ET (Day 61)		F2	uncoated	0	0	0	0	0	0	0		
06-JUL-2018	11:25	EOS/ET (Day 61)		F3	uncoated	0	0	0	0	0	0	0		

Key: 0= None; 1=Mild; 2=Moderate; 3=Severe; # Other = Pinprick bleeding or seepage

Administration Site Key: FA = Forearm; UA= Upper arm
Treatments Part A: A1 = A/Sing MAP, 15 ug (FA); A2 = Afluria Quad IM (UA), 15 ug; A3 = Placebo MAP (FA); A4 = A/Sing Mono IM, 15 ug (UA)
Treatments Part B: B1 = A/Sing MAP, 15 ug (FA) with biopsy; B2 = Placebo MAP (FA) with biopsy
B3 = A/Sing MAP, 15 ug (FA); B4 = A/Sing MAP, 10 ug (FA); B5 = A/Sing MAP, 5 ug (FA); B6 = A/Sing MAP, 2.5 ug (FA)
B7 = Placebo MAP (FA); B8 = A/Sing MAP, 15 ug (UA); B9 = A/Sing IM, 15 ug, as 2018 Afluria Quad (UA)

Listing 16.2.9.2
Treatment Site Tolerability Assessment per Site

Part B
Randomisation Number = B0011, Treatment Group = Group B8

Date	Time	Visit	Time Point	Site Number	Treatment for Site	Induration	Redness	Bruising	Itching	Skin Flaking	Tenderness	Definition/ Visibility	Other#	Unscheduled Reason
12-MAY-2018	11:11	Day 1	Pre-dose	D1	5 ug	0	0	0	0	0	0	0		
12-MAY-2018	11:11	Day 1	Pre-dose	D2	5 ug	0	0	0	0	0	0	0		
12-MAY-2018	11:11	Day 1	Pre-dose	D3	5 ug	0	0	0	0	0	0	0		
12-MAY-2018	12:08	Day 1	10 min	D1	5 ug	0	1	0	0	0	0	0		
12-MAY-2018	12:08	Day 1	10 min	D2	5 ug	0	1	0	0	0	0	0		
12-MAY-2018	12:08	Day 1	10 min	D3	5 ug	0	1	0	0	0	0	0		
12-MAY-2018	12:58	Day 1	1 hour	D1	5 ug	1	1	0	0	0	0	1		
12-MAY-2018	12:58	Day 1	1 hour	D2	5 ug	1	0	0	0	0	0	0		
12-MAY-2018	12:58	Day 1	1 hour	D3	5 ug	1	0	0	0	0	0	0		
12-MAY-2018	13:58	Day 1	2 hour	D1	5 ug	0	0	0	0	0	0	0		
12-MAY-2018	13:58	Day 1	2 hour	D2	5 ug	0	0	0	0	0	0	0		
12-MAY-2018	13:58	Day 1	2 hour	D3	5 ug	0	0	0	0	0	0	0		
13-MAY-2018	10:58	Day 2		D1	5 ug	0	0	0	0	0	0	0		
13-MAY-2018	10:58	Day 2		D2	5 ug	0	0	0	0	0	0	0		
13-MAY-2018	10:58	Day 2		D3	5 ug	0	0	0	0	0	0	0		
15-MAY-2018	11:15	Day 4		D1	5 ug	0	1	0	0	2	0	0		
15-MAY-2018	11:15	Day 4		D2	5 ug	0	1	0	0	2	0	0		
15-MAY-2018	11:15	Day 4		D3	5 ug	0	1	0	0	2	0	0		
19-MAY-2018	13:17	Day 8		D1	5 ug	0	0	0	1	1	0	0		
19-MAY-2018	13:17	Day 8		D2	5 ug	0	0	0	1	1	0	0		

Key: 0= None; 1=Mild; 2=Moderate; 3=Severe; # Other = Pinprick bleeding or seepage

Administration Site Key: FA = Forearm; UA= Upper arm
Treatments Part A: A1 = A/Sing MAP, 15 ug (FA); A2 = Afluria Quad IM (UA), 15 ug; A3 = Placebo MAP (FA); A4 = A/Sing Mono IM, 15 ug (UA)
Treatments Part B: B1 = A/Sing MAP, 15 ug (FA) with biopsy; B2 = Placebo MAP (FA) with biopsy
B3 = A/Sing MAP, 15 ug (FA); B4 = A/Sing MAP, 10 ug (FA); B5 = A/Sing MAP, 5 ug (FA); B6 = A/Sing MAP, 2.5 ug (FA)
B7 = Placebo MAP (FA); B8 = A/Sing MAP, 15 ug (UA); B9 = A/Sing IM, 15 ug, as 2018 Afluria Quad (UA)

Listing 16.2.9.2
Treatment Site Tolerability Assessment per Site

Part B
Randomisation Number = B0011, Treatment Group = Group B8

Date	Time	Visit	Time Point	Site Number	Treatment for Site	Indura-tion	Redness	Bruising	Itching	Skin Flaking	Tender-ness	Definition/ Visibility	Other#	Unscheduled Reason
19-MAY-2018	13:17	Day 8		D3	5 ug	0	0	0	1	1	0	0		
02-JUN-2018	09:08	Day 22		D1	5 ug	0	0	0	0	1	0	2		
02-JUN-2018	09:08	Day 22		D2	5 ug	0	0	0	0	1	0	2		
02-JUN-2018	09:08	Day 22		D3	5 ug	0	0	0	0	1	0	2		
14-JUL-2018	09:44	EOS/ET (Day 61)		D1	5 ug	0	0	0	0	0	0	0		
14-JUL-2018	09:44	EOS/ET (Day 61)		D2	5 ug	0	0	0	0	0	0	0		
14-JUL-2018	09:44	EOS/ET (Day 61)		D3	5 ug	0	0	0	0	0	0	0		

Key: 0= None; 1=Mild; 2=Moderate; 3=Severe; # Other = Pinprick bleeding or seepage

Administration Site Key: FA = Forearm; UA= Upper arm
Treatments Part A: A1 = A/Sing MAP, 15 ug (FA); A2 = Afluria Quad IM (UA), 15 ug; A3 = Placebo MAP (FA); A4 = A/Sing Mono IM, 15 ug (UA)
Treatments Part B: B1 = A/Sing MAP, 15 ug (FA) with biopsy; B2 = Placebo MAP (FA) with biopsy
B3 = A/Sing MAP, 15 ug (FA); B4 = A/Sing MAP, 10 ug (FA); B5 = A/Sing MAP, 5 ug (FA); B6 = A/Sing MAP, 2.5 ug (FA)
B7 = Placebo MAP (FA); B8 = A/Sing MAP, 15 ug (UA); B9 = A/Sing IM, 15 ug, as 2018 Afluria Quad (UA)

Listing 16.2.9.2
Treatment Site Tolerability Assessment per Site

Part B
Randomisation Number = B0012, Treatment Group = Group B6

Date	Time	Visit	Time Point	Site Number	Treatment for Site	Induration	Redness	Bruising	Itching	Skin Flaking	Tenderness	Definition/ Visibility	Other#	Unscheduled Reason
12-MAY-2018	11:26	Day 1	Pre-dose	F1	2.5 ug	0	0	0	0	0	0	0		
12-MAY-2018	11:26	Day 1	Pre-dose	F2	uncoated	0	0	0	0	0	0	0		
12-MAY-2018	11:26	Day 1	Pre-dose	F3	uncoated	0	0	0	0	0	0	0		
12-MAY-2018	12:46	Day 1	10 min	F1	2.5 ug	0	1	0	0	0	0	0		
12-MAY-2018	12:46	Day 1	10 min	F2	uncoated	0	0	0	0	0	1	0		
12-MAY-2018	12:46	Day 1	10 min	F3	uncoated	0	0	0	0	0	1	0		
12-MAY-2018	13:23	Day 1	1 hour	F1	2.5 ug	0	0	0	0	0		0		
12-MAY-2018	13:23	Day 1	1 hour	F2	uncoated	0	0	0	1	0	1	0		
12-MAY-2018	13:23	Day 1	1 hour	F3	uncoated	0	0	0	1	0	1	0		
12-MAY-2018	14:23	Day 1	2 hour	F1	2.5 ug	0	0	0	0	0	0	0		
12-MAY-2018	14:23	Day 1	2 hour	F2	uncoated	0	0	0	0	0	1	0		
12-MAY-2018	14:23	Day 1	2 hour	F3	uncoated	0	0	0	0	0	0	0		
13-MAY-2018	11:26	Day 2		F1	2.5 ug	0	0	0	0	0	0	0		
13-MAY-2018	11:26	Day 2		F2	uncoated	0	0	0	0	0	0	0		
13-MAY-2018	11:26	Day 2		F3	uncoated	0	0	0	0	0	0	0		
15-MAY-2018	12:15	Day 4		F1	2.5 ug	0	0	0	0	0	0	0		
15-MAY-2018	12:15	Day 4		F2	uncoated	0	0	0	0	0	0	0		
15-MAY-2018	12:15	Day 4		F3	uncoated	0	0	0	0	0	0	0		
19-MAY-2018	13:22	Day 8		F1	2.5 ug	0	0	0	0	1	0	1		
19-MAY-2018	13:22	Day 8		F2	uncoated	0	0	0	0	1	0	1		

Key: 0= None; 1=Mild; 2=Moderate; 3=Severe; # Other = Pinprick bleeding or seepage

Administration Site Key: FA = Forearm; UA= Upper arm
Treatments Part A: A1 = A/Sing MAP, 15 ug (FA); A2 = Afluria Quad IM (UA), 15 ug; A3 = Placebo MAP (FA); A4 = A/Sing Mono IM, 15 ug (UA)
Treatments Part B: B1 = A/Sing MAP, 15 ug (FA) with biopsy; B2 = Placebo MAP (FA) with biopsy
B3 = A/Sing MAP, 15 ug (FA); B4 = A/Sing MAP, 10 ug (FA); B5 = A/Sing MAP, 5 ug (FA); B6 = A/Sing MAP, 2.5 ug (FA)
B7 = Placebo MAP (FA); B8 = A/Sing MAP, 15 ug (UA); B9 = A/Sing IM, 15 ug, as 2018 Afluria Quad (UA)

Listing 16.2.9.2
Treatment Site Tolerability Assessment per Site

Part B
Randomisation Number = B0012, Treatment Group = Group B6

Date	Time	Visit	Time Point	Site Number	Treatment for Site	Indura-tion	Redness	Bruising	Itching	Skin Flaking	Tender-ness	Definition/ Visibility	Other#	Unscheduled Reason
19-MAY-2018	13:22	Day 8		F3	uncoated	0	0	0	0	0	0	1		
02-JUN-2018	12:40	Day 22		F1	2.5 ug	0	0	0	0	0	0	2		
02-JUN-2018	12:40	Day 22		F2	uncoated	0	0	0	0	0	0	1		
02-JUN-2018	12:40	Day 22		F3	uncoated	0	0	0	0	0	0	1		
11-JUL-2018	14:45	EOS/ET (Day 61)		F1	2.5 ug	0	0	0	0	0	0	0		
11-JUL-2018	14:45	EOS/ET (Day 61)		F2	uncoated	0	0	0	0	0	0	0		
11-JUL-2018	14:45	EOS/ET (Day 61)		F3	uncoated	0	0	0	0	0	0	0		

Key: 0= None; 1=Mild; 2=Moderate; 3=Severe; # Other = Pinprick bleeding or seepage

Administration Site Key: FA = Forearm; UA= Upper arm
Treatments Part A: A1 = A/Sing MAP, 15 ug (FA); A2 = Afluria Quad IM (UA), 15 ug; A3 = Placebo MAP (FA); A4 = A/Sing Mono IM, 15 ug (UA)
Treatments Part B: B1 = A/Sing MAP, 15 ug (FA) with biopsy; B2 = Placebo MAP (FA) with biopsy
B3 = A/Sing MAP, 15 ug (FA); B4 = A/Sing MAP, 10 ug (FA); B5 = A/Sing MAP, 5 ug (FA); B6 = A/Sing MAP, 2.5 ug (FA)
B7 = Placebo MAP (FA); B8 = A/Sing MAP, 15 ug (UA); B9 = A/Sing IM, 15 ug, as 2018 Afluria Quad (UA)

Listing 16.2.9.2
Treatment Site Tolerability Assessment per Site

Part B
Randomisation Number = B0013, Treatment Group = Group B5

Date	Time	Visit	Time Point	Site Number	Treatment for Site	Indura-tion	Redness	Bruising	Itching	Skin Flaking	Tender-ness	Definition/ Visibility	Other#	Unscheduled Reason
12-MAY-2018	11:32	Day 1	Pre-dose	F1	5 ug	0	0	0	0	0	0	0		
12-MAY-2018	11:32	Day 1	Pre-dose	F2	uncoated	0	0	0	0	0	0	0		
12-MAY-2018	11:32	Day 1	Pre-dose	F3	uncoated	0	0	0	0	0	0	0		
12-MAY-2018	12:28	Day 1	10 min	F1	5 ug	0	1	0	0	0	0	0		
12-MAY-2018	12:28	Day 1	10 min	F2	uncoated	0	1	0	0	0	1	0		
12-MAY-2018	12:28	Day 1	10 min	F3	uncoated	0	0	0	0	0	1	0		
12-MAY-2018	13:18	Day 1	1 hour	F1	5 ug	1	1	0	0	0	0	0		
12-MAY-2018	13:18	Day 1	1 hour	F2	uncoated	1	1	0	0	0	0	0		
12-MAY-2018	13:18	Day 1	1 hour	F3	uncoated	0	0	0	0	0	0	0		
12-MAY-2018	14:18	Day 1	2 hour	F1	5 ug	0	0	0	0	0	0	0		
12-MAY-2018	14:18	Day 1	2 hour	F2	uncoated	0	0	0	0	0	0	0		
12-MAY-2018	14:18	Day 1	2 hour	F3	uncoated	0	0	0	0	0	0	0		
13-MAY-2018	11:34	Day 2		F1	5 ug	0	0	0	0	0	0	0		
13-MAY-2018	11:34	Day 2		F2	uncoated	0	0	0	0	0	0	0		
13-MAY-2018	11:34	Day 2		F3	uncoated	0	0	0	0	0	0	0		
15-MAY-2018	08:30	Day 4		F1	5 ug	0	0	0	0	1	0	0		
15-MAY-2018	08:30	Day 4		F2	uncoated	0	0	0	0	0	0	0		
15-MAY-2018	08:30	Day 4		F3	uncoated	0	0	0	0	0	0	0		
19-MAY-2018	10:32	Day 8		F1	5 ug	0	0	0	0	2	0	0		
19-MAY-2018	10:32	Day 8		F2	uncoated	0	0	0	0	1	0	3		

Key: 0= None; 1=Mild; 2=Moderate; 3=Severe; # Other = Pinprick bleeding or seepage

Administration Site Key: FA = Forearm; UA= Upper arm
Treatments Part A: A1 = A/Sing MAP, 15 ug (FA); A2 = Afluria Quad IM (UA), 15 ug; A3 = Placebo MAP (FA); A4 = A/Sing Mono IM, 15 ug (UA)
Treatments Part B: B1 = A/Sing MAP, 15 ug (FA) with biopsy; B2 = Placebo MAP (FA) with biopsy
B3 = A/Sing MAP, 15 ug (FA); B4 = A/Sing MAP, 10 ug (FA); B5 = A/Sing MAP, 5 ug (FA); B6 = A/Sing MAP, 2.5 ug (FA)
B7 = Placebo MAP (FA); B8 = A/Sing MAP, 15 ug (UA); B9 = A/Sing IM, 15 ug, as 2018 Afluria Quad (UA)

Listing 16.2.9.2
Treatment Site Tolerability Assessment per Site

Part B
Randomisation Number = B0013, Treatment Group = Group B5

Date	Time	Visit	Time Point	Site Number	Treatment for Site	Induration	Redness	Bruising	Itching	Skin Flaking	Tenderness	Definition/ Visibility	Other#	Unscheduled Reason
19-MAY-2018	10:32	Day 8		F3	uncoated	0	0	0	0	0	0	2		
02-JUN-2018	11:27	Day 22		F1	5 ug	0	0	0	0	0	0	1		
02-JUN-2018	11:27	Day 22		F2	uncoated	0	0	0	0	0	0	0		
02-JUN-2018	11:27	Day 22		F3	uncoated	0	0	0	0	0	0	0		
11-JUL-2018	11:15	EOS/ET (Day 61)		F1	5 ug	0	0	0	0	0	0	0		
11-JUL-2018	11:15	EOS/ET (Day 61)		F2	uncoated	0	0	0	0	0	0	0		
11-JUL-2018	11:15	EOS/ET (Day 61)		F3	uncoated	0	0	0	0	0	0	0		

Key: 0= None; 1=Mild; 2=Moderate; 3=Severe; # Other = Pinprick bleeding or seepage

Administration Site Key: FA = Forearm; UA= Upper arm
Treatments Part A: A1 = A/Sing MAP, 15 ug (FA); A2 = Afluria Quad IM (UA), 15 ug; A3 = Placebo MAP (FA); A4 = A/Sing Mono IM, 15 ug (UA)
Treatments Part B: B1 = A/Sing MAP, 15 ug (FA) with biopsy; B2 = Placebo MAP (FA) with biopsy
B3 = A/Sing MAP, 15 ug (FA); B4 = A/Sing MAP, 10 ug (FA); B5 = A/Sing MAP, 5 ug (FA); B6 = A/Sing MAP, 2.5 ug (FA)
B7 = Placebo MAP (FA); B8 = A/Sing MAP, 15 ug (UA); B9 = A/Sing IM, 15 ug, as 2018 Afluria Quad (UA)

Listing 16.2.9.2
Treatment Site Tolerability Assessment per Site

Part B
Randomisation Number = B0014, Treatment Group = Group B3

Date	Time	Visit	Time Point	Site Number	Treatment for Site	Indura-tion	Redness	Bruising	Itching	Skin Flaking	Tender-ness	Definition/ Visibility	Other#	Unscheduled Reason
12-MAY-2018	11:38	Day 1	Pre-dose	F1	5 ug	0	0	0	0	0	0	0		
12-MAY-2018	11:38	Day 1	Pre-dose	F2	5 ug	0	0	0	0	0	0	0		
12-MAY-2018	11:38	Day 1	Pre-dose	F3	5 ug	0	0	0	0	0	0	0		
12-MAY-2018	12:38	Day 1	10 min	F1	5 ug	0	0	0	0	0	1	0		
12-MAY-2018	12:38	Day 1	10 min	F2	5 ug	0	0	0	0	0	1	0		
12-MAY-2018	12:38	Day 1	10 min	F3	5 ug	0	0	0	0	0	1	0		
12-MAY-2018	13:28	Day 1	1 hour	F1	5 ug	0	1	0	0	0	0	0		
12-MAY-2018	13:28	Day 1	1 hour	F2	5 ug	0	0	0	0	0	0	0		
12-MAY-2018	13:28	Day 1	1 hour	F3	5 ug	0	0	0	0	0	0	0		
12-MAY-2018	14:30	Day 1	2 hour	F1	5 ug	0	0	0	0	0	0	0		
12-MAY-2018	14:30	Day 1	2 hour	F2	5 ug	0	0	0	0	0	0	0		
12-MAY-2018	14:30	Day 1	2 hour	F3	5 ug	0	0	0	0	0	0	0		
13-MAY-2018	11:50	Day 2		F1	5 ug	0	0	0	0	0	0	0		
13-MAY-2018	11:50	Day 2		F2	5 ug	0	0	0	0	0	0	0		
13-MAY-2018	11:50	Day 2		F3	5 ug	0	0	0	0	0	0	0		
15-MAY-2018	09:36	Day 4		F1	5 ug	0	0	0	1	0	0	0		
15-MAY-2018	09:36	Day 4		F2	5 ug	0	0	0	1	0	0	0		
15-MAY-2018	09:36	Day 4		F3	5 ug	0	0	0	1	0	0	0		
19-MAY-2018	16:26	Day 8		F1	5 ug	0	0	0	1	1	0	3		
19-MAY-2018	16:26	Day 8		F2	5 ug	0	0	0	1	1	0	2		

Key: 0= None; 1=Mild; 2=Moderate; 3=Severe; # Other = Pinprick bleeding or seepage

Administration Site Key: FA = Forearm; UA= Upper arm
Treatments Part A: A1 = A/Sing MAP, 15 ug (FA); A2 = Afluria Quad IM (UA), 15 ug; A3 = Placebo MAP (FA); A4 = A/Sing Mono IM, 15 ug (UA)
Treatments Part B: B1 = A/Sing MAP, 15 ug (FA) with biopsy; B2 = Placebo MAP (FA) with biopsy
B3 = A/Sing MAP, 15 ug (FA); B4 = A/Sing MAP, 10 ug (FA); B5 = A/Sing MAP, 5 ug (FA); B6 = A/Sing MAP, 2.5 ug (FA)
B7 = Placebo MAP (FA); B8 = A/Sing MAP, 15 ug (UA); B9 = A/Sing IM, 15 ug, as 2018 Afluria Quad (UA)

Listing 16.2.9.2
Treatment Site Tolerability Assessment per Site

Part B
Randomisation Number = B0014, Treatment Group = Group B3

Date	Time	Visit	Time Point	Site Number	Treatment for Site	Indura-tion	Redness	Bruising	Itching	Skin Flaking	Tender-ness	Definition/ Visibility	Other#	Unscheduled Reason
19-MAY-2018	16:26	Day 8		F3	5 ug	0	0	0	1	1	0	2		
02-JUN-2018	09:33	Day 22		F1	5 ug	0	0	0	0	1	0	2		
02-JUN-2018	09:33	Day 22		F2	5 ug	0	0	0	0	1	0	2		
02-JUN-2018	09:33	Day 22		F3	5 ug	0	0	0	0	1	0	2		
12-JUL-2018	11:21	EOS/ET (Day 61)		F1	5 ug	0	0	0	0	0	0	1		
12-JUL-2018	11:21	EOS/ET (Day 61)		F2	5 ug	0	0	0	0	0	0	1		
12-JUL-2018	11:21	EOS/ET (Day 61)		F3	5 ug	0	0	0	0	0	0	1		

Key: 0= None; 1=Mild; 2=Moderate; 3=Severe; # Other = Pinprick bleeding or seepage

Administration Site Key: FA = Forearm; UA= Upper arm
Treatments Part A: A1 = A/Sing MAP, 15 ug (FA); A2 = Afluria Quad IM (UA), 15 ug; A3 = Placebo MAP (FA); A4 = A/Sing Mono IM, 15 ug (UA)
Treatments Part B: B1 = A/Sing MAP, 15 ug (FA) with biopsy; B2 = Placebo MAP (FA) with biopsy
B3 = A/Sing MAP, 15 ug (FA); B4 = A/Sing MAP, 10 ug (FA); B5 = A/Sing MAP, 5 ug (FA); B6 = A/Sing MAP, 2.5 ug (FA)
B7 = Placebo MAP (FA); B8 = A/Sing MAP, 15 ug (UA); B9 = A/Sing IM, 15 ug, as 2018 Afluria Quad (UA)

Listing 16.2.9.2
Treatment Site Tolerability Assessment per Site

Part B
Randomisation Number = B0015, Treatment Group = Group B9

Date	Time	Visit	Time Point	Site Number	Treatment for Site	Induration	Redness	Bruising	Itching	Skin Flaking	Tenderness	Definition/ Visibility	Other#	Unscheduled Reason
12-MAY-2018	10:55	Day 1	Pre-dose	D1	[IM]	0	0	0	0	0	0			
12-MAY-2018	11:21	Day 1	10 min	D1	[IM]	0	1	0	0	0	0			
12-MAY-2018	12:11	Day 1	1 hour	D1	[IM]	0	0	0	0	0	0			
12-MAY-2018	13:31	Day 1	2 hour	D1	[IM]	0	0	0	0	0	0			
13-MAY-2018	11:09	Day 2		D1	[IM]	0	0	0	0	0	1	5		
15-MAY-2018	11:33	Day 4		D1	[IM]	0	0	0	0	0	0	5		
19-MAY-2018	11:06	Day 8		D1	[IM]	0	0	0	0	0	0	5		
02-JUN-2018	12:40	Day 22		D1	[IM]	0	0	0	0	0	0			
11-JUL-2018	09:37	EOS/ET (Day 61)		D1	[IM]	0	0	0	0	0	0	5		

Key: 0= None; 1=Mild; 2=Moderate; 3=Severe; # Other = Pinprick bleeding or seepage

Administration Site Key: FA = Forearm; UA= Upper arm
Treatments Part A: A1 = A/Sing MAP, 15 ug (FA); A2 = Afluria Quad IM (UA), 15 ug; A3 = Placebo MAP (FA); A4 = A/Sing Mono IM, 15 ug (UA)
Treatments Part B: B1 = A/Sing MAP, 15 ug (FA) with biopsy; B2 = Placebo MAP (FA) with biopsy
B3 = A/Sing MAP, 15 ug (FA); B4 = A/Sing MAP, 10 ug (FA); B5 = A/Sing MAP, 5 ug (FA); B6 = A/Sing MAP, 2.5 ug (FA)
B7 = Placebo MAP (FA); B8 = A/Sing MAP, 15 ug (UA); B9 = A/Sing IM, 15 ug, as 2018 Afluria Quad (UA)

Listing 16.2.9.2
Treatment Site Tolerability Assessment per Site

Part B
Randomisation Number = B0016, Treatment Group = Group B4

Date	Time	Visit	Time Point	Site Number	Treatment for Site	Indura-tion	Redness	Bruising	Itching	Skin Flaking	Tender-ness	Definition/ Visibility	Other#	Unscheduled Reason
12-MAY-2018	11:44	Day 1	Pre-dose	F1	uncoated	0	1	0	0	0	0	0		
12-MAY-2018	11:44	Day 1	Pre-dose	F2	5 ug	0	0	0	0	0	0	0		
12-MAY-2018	11:44	Day 1	Pre-dose	F3	5 ug	0	0	0	0	0	0	0		
12-MAY-2018	12:48	Day 1	10 min	F1	uncoated	0	0	0	0	0	0	0		
12-MAY-2018	12:48	Day 1	10 min	F2	5 ug	0	0	0	0	0	1	0		
12-MAY-2018	12:48	Day 1	10 min	F3	5 ug	0	0	0	0	0	1	0		
12-MAY-2018	13:38	Day 1	1 hour	F1	uncoated	0	0	0	0	0	1	0		
12-MAY-2018	13:38	Day 1	1 hour	F2	5 ug	0	0	0	0	0	1	0		
12-MAY-2018	13:38	Day 1	1 hour	F3	5 ug	0	0	0	0	0	1	0		
12-MAY-2018	14:38	Day 1	2 hour	F1	uncoated	0	0	0	0	0	0	0		
12-MAY-2018	14:38	Day 1	2 hour	F2	5 ug	0	0	0	0	0	0	0		
12-MAY-2018	14:38	Day 1	2 hour	F3	5 ug	0	0	0	0	0	0	0		
13-MAY-2018	12:13	Day 2		F1	uncoated	0	0	0	0	0	0	0		
13-MAY-2018	12:13	Day 2		F2	5 ug	0	1	0	0	0	0	0		
13-MAY-2018	12:13	Day 2		F3	5 ug	1	1	0	0	0	1	0		
15-MAY-2018	08:47	Day 4		F1	uncoated	0	0	0	0	0	0	0		
15-MAY-2018	08:47	Day 4		F2	5 ug	0	1	0	0	0	0	0		
15-MAY-2018	08:47	Day 4		F3	5 ug	0	1	0	0	0	0	0		
19-MAY-2018	10:13	Day 8		F1	uncoated	0	0	0	0	0	0	1		
19-MAY-2018	10:13	Day 8		F2	5 ug	0	0	0	0	1	0	3		

Key: 0= None; 1=Mild; 2=Moderate; 3=Severe; # Other = Pinprick bleeding or seepage

Administration Site Key: FA = Forearm; UA= Upper arm
Treatments Part A: A1 = A/Sing MAP, 15 ug (FA); A2 = Afluria Quad IM (UA), 15 ug; A3 = Placebo MAP (FA); A4 = A/Sing Mono IM, 15 ug (UA)
Treatments Part B: B1 = A/Sing MAP, 15 ug (FA) with biopsy; B2 = Placebo MAP (FA) with biopsy
B3 = A/Sing MAP, 15 ug (FA); B4 = A/Sing MAP, 10 ug (FA); B5 = A/Sing MAP, 5 ug (FA); B6 = A/Sing MAP, 2.5 ug (FA)
B7 = Placebo MAP (FA); B8 = A/Sing MAP, 15 ug (UA); B9 = A/Sing IM, 15 ug, as 2018 Afluria Quad (UA)

Listing 16.2.9.2
Treatment Site Tolerability Assessment per Site

Part B
Randomisation Number = B0016, Treatment Group = Group B4

Date	Time	Visit	Time Point	Site Number	Treatment for Site	Indura-tion	Redness	Bruising	Itching	Skin Flaking	Tender-ness	Definition/ Visibility	Other#	Unscheduled Reason
19-MAY-2018	10:13	Day 8		F3	5 ug	0	0	0	0	1	0	3		
19-MAY-2018	10:41	Unscheduled		F1	uncoated	0	0	0	0	0	0	3	NA	MO notifies as repeat
02-JUN-2018	13:05	Day 22		F1	uncoated	0	0	0	0	0	0	0		
02-JUN-2018	13:05	Day 22		F2	5 ug	0	0	0	0	0	0	1		
02-JUN-2018	13:05	Day 22		F3	5 ug	0	0	0	0	0	0	1		
11-JUL-2018	08:30	EOS/ET (Day 61)		F1	uncoated	0	0	0	0	0	0	0		
11-JUL-2018	08:30	EOS/ET (Day 61)		F2	5 ug	0	0	0	0	0	0	0		
11-JUL-2018	08:30	EOS/ET (Day 61)		F3	5 ug	0	0	0	0	0	0	0		

Key: 0= None; 1=Mild; 2=Moderate; 3=Severe; # Other = Pinprick bleeding or seepage

Administration Site Key: FA = Forearm; UA= Upper arm
Treatments Part A: A1 = A/Sing MAP, 15 ug (FA); A2 = Afluria Quad IM (UA), 15 ug; A3 = Placebo MAP (FA); A4 = A/Sing Mono IM, 15 ug (UA)
Treatments Part B: B1 = A/Sing MAP, 15 ug (FA) with biopsy; B2 = Placebo MAP (FA) with biopsy
B3 = A/Sing MAP, 15 ug (FA); B4 = A/Sing MAP, 10 ug (FA); B5 = A/Sing MAP, 5 ug (FA); B6 = A/Sing MAP, 2.5 ug (FA)
B7 = Placebo MAP (FA); B8 = A/Sing MAP, 15 ug (UA); B9 = A/Sing IM, 15 ug, as 2018 Afluria Quad (UA)

Listing 16.2.9.2
Treatment Site Tolerability Assessment per Site

Part B
Randomisation Number = B0017, Treatment Group = Group B7

Date	Time	Visit	Time Point	Site Number	Treatment for Site	Indura-tion	Redness	Bruising	Itching	Skin Flaking	Tender-ness	Definition/ Visibility	Other#	Unscheduled Reason
12-MAY-2018	11:51	Day 1	Pre-dose	F1	uncoated	0	0	0	0	0	0	0		
12-MAY-2018	11:51	Day 1	Pre-dose	F2	uncoated	0	0	0	0	0	0	0		
12-MAY-2018	11:51	Day 1	Pre-dose	F3	uncoated	0	0	0	0	0	0	0		
12-MAY-2018	12:58	Day 1	10 min	F1	uncoated	0	0	0	0	0	0	0		
12-MAY-2018	12:58	Day 1	10 min	F2	uncoated	0	0	0	0	0	0	0		
12-MAY-2018	12:58	Day 1	10 min	F3	uncoated	0	0	0	0	0	0	0		
12-MAY-2018	13:48	Day 1	1 hour	F1	uncoated	0	0	0	0	0	0	0		
12-MAY-2018	13:48	Day 1	1 hour	F2	uncoated	0	0	0	0	0	0	0		
12-MAY-2018	13:48	Day 1	1 hour	F3	uncoated	0	0	0	0	0	0	0		
12-MAY-2018	14:47	Day 1	2 hour	F1	uncoated	0	0	0	0	0	0	0		
12-MAY-2018	14:47	Day 1	2 hour	F2	uncoated	0	0	0	0	0	0	0		
12-MAY-2018	14:47	Day 1	2 hour	F3	uncoated	0	0	0	0	0	0	0		
13-MAY-2018	12:25	Day 2		F1	uncoated	0	0	0	0	0	0	0		
13-MAY-2018	12:25	Day 2		F2	uncoated	0	1	0	0	0	0	0		
13-MAY-2018	12:25	Day 2		F3	uncoated	0	1	0	0	0	0	0		
15-MAY-2018	15:14	Day 4		F1	uncoated	0	0	0	0	0	0	0		
15-MAY-2018	15:14	Day 4		F2	uncoated	0	0	0	0	0	0	0		
15-MAY-2018	15:14	Day 4		F3	uncoated	0	0	0	0	0	0	0		
19-MAY-2018	12:14	Day 8		F1	uncoated	0	0	0	0	1	0	2		
19-MAY-2018	12:14	Day 8		F2	uncoated	0	0	0	0	2	0	2		

Key: 0= None; 1=Mild; 2=Moderate; 3=Severe; # Other = Pinprick bleeding or seepage

Administration Site Key: FA = Forearm; UA= Upper arm
Treatments Part A: A1 = A/Sing MAP, 15 ug (FA); A2 = Afluria Quad IM (UA), 15 ug; A3 = Placebo MAP (FA); A4 = A/Sing Mono IM, 15 ug (UA)
Treatments Part B: B1 = A/Sing MAP, 15 ug (FA) with biopsy; B2 = Placebo MAP (FA) with biopsy
B3 = A/Sing MAP, 15 ug (FA); B4 = A/Sing MAP, 10 ug (FA); B5 = A/Sing MAP, 5 ug (FA); B6 = A/Sing MAP, 2.5 ug (FA)
B7 = Placebo MAP (FA); B8 = A/Sing MAP, 15 ug (UA); B9 = A/Sing IM, 15 ug, as 2018 Afluria Quad (UA)

Listing 16.2.9.2
Treatment Site Tolerability Assessment per Site

Part B
Randomisation Number = B0017, Treatment Group = Group B7

Date	Time	Visit	Time Point	Site Number	Treatment for Site	Indura-tion	Redness	Bruising	Itching	Skin Flaking	Tender-ness	Definition/ Visibility	Other#	Unscheduled Reason
19-MAY-2018	12:14	Day 8		F3	uncoated	0	0	0	0	1	0	2		
04-JUN-2018	11:42	Day 22		F1	uncoated	0	0	0	0	0	0	1		
04-JUN-2018	11:42	Day 22		F2	uncoated	0	0	0	0	0	0	1		
04-JUN-2018	11:42	Day 22		F3	uncoated	0	0	0	0	0	0	1		
07-JUL-2018	11:34	EOS/ET (Day 61)		F1	uncoated	0	0	0	0	0	0	0		
07-JUL-2018	11:34	EOS/ET (Day 61)		F2	uncoated	0	0	0	0	0	0	0		
07-JUL-2018	11:34	EOS/ET (Day 61)		F3	uncoated	0	0	0	0	0	0	0		

Key: 0= None; 1=Mild; 2=Moderate; 3=Severe; # Other = Pinprick bleeding or seepage

Administration Site Key: FA = Forearm; UA= Upper arm
Treatments Part A: A1 = A/Sing MAP, 15 ug (FA); A2 = Afluria Quad IM (UA), 15 ug; A3 = Placebo MAP (FA); A4 = A/Sing Mono IM, 15 ug (UA)
Treatments Part B: B1 = A/Sing MAP, 15 ug (FA) with biopsy; B2 = Placebo MAP (FA) with biopsy
B3 = A/Sing MAP, 15 ug (FA); B4 = A/Sing MAP, 10 ug (FA); B5 = A/Sing MAP, 5 ug (FA); B6 = A/Sing MAP, 2.5 ug (FA)
B7 = Placebo MAP (FA); B8 = A/Sing MAP, 15 ug (UA); B9 = A/Sing IM, 15 ug, as 2018 Afluria Quad (UA)

Listing 16.2.9.2
Treatment Site Tolerability Assessment per Site

Part B
Randomisation Number = B0018, Treatment Group = Group B9

Date	Time	Visit	Time Point	Site Number	Treatment for Site	Indura-tion	Redness	Bruising	Itching	Skin Flaking	Tender-ness	Definition/ Visibility	Other#	Unscheduled Reason
12-MAY-2018	10:57	Day 1	Pre-dose	D1	[IM]	0	0	0	0	0	0			
12-MAY-2018	11:31	Day 1	10 min	D1	[IM]	0	0	0	0	0	1			
12-MAY-2018	12:21	Day 1	1 hour	D1	[IM]	0	0	0	0	0	0			
12-MAY-2018	13:26	Day 1	2 hour	D1	[IM]	0	0	0	0	0	1			
13-MAY-2018	10:51	Day 2		D1	[IM]	0	0	0	0	0	1	5		
15-MAY-2018	16:55	Day 4		D1	[IM]	0	0	0	0	0	0	5		
19-MAY-2018	11:09	Day 8		D1	[IM]	0	0	0	0	0	0	5		
02-JUN-2018	11:24	Day 22		D1	[IM]	0	0	0	0	0	0			
11-JUL-2018	16:47	EOS/ET (Day 61)		D1	[IM]	0	0	0	0	0	0	5		

Key: 0= None; 1=Mild; 2=Moderate; 3=Severe; # Other = Pinprick bleeding or seepage

Administration Site Key: FA = Forearm; UA= Upper arm
Treatments Part A: A1 = A/Sing MAP, 15 ug (FA); A2 = Afluria Quad IM (UA), 15 ug; A3 = Placebo MAP (FA); A4 = A/Sing Mono IM, 15 ug (UA)
Treatments Part B: B1 = A/Sing MAP, 15 ug (FA) with biopsy; B2 = Placebo MAP (FA) with biopsy
B3 = A/Sing MAP, 15 ug (FA); B4 = A/Sing MAP, 10 ug (FA); B5 = A/Sing MAP, 5 ug (FA); B6 = A/Sing MAP, 2.5 ug (FA)
B7 = Placebo MAP (FA); B8 = A/Sing MAP, 15 ug (UA); B9 = A/Sing IM, 15 ug, as 2018 Afluria Quad (UA)

Listing 16.2.9.2
Treatment Site Tolerability Assessment per Site

Part B
Randomisation Number = B0020, Treatment Group = Group B6

Date	Time	Visit	Time Point	Site Number	Treatment for Site	Indura-tion	Redness	Bruising	Itching	Skin Flaking	Tender-ness	Definition/ Visibility	Other#	Unscheduled Reason
12-MAY-2018	12:03	Day 1	Pre-dose	F1	uncoated	0	0	0	0	0	0	0		
12-MAY-2018	12:03	Day 1	Pre-dose	F2	uncoated	0	0	0	0	0	0	0		
12-MAY-2018	12:03	Day 1	Pre-dose	F3	2.5 ug	0	0	0	0	0	0	0		
12-MAY-2018	13:08	Day 1	10 min	F1	uncoated	0	1	0	0	0	0	0		
12-MAY-2018	13:08	Day 1	10 min	F2	uncoated	0	0	0	0	0	1	0		
12-MAY-2018	13:08	Day 1	10 min	F3	2.5 ug	0	0	0	0	0	0	0		
12-MAY-2018	13:58	Day 1	1 hour	F1	uncoated	0	0	0	0	0	1	0		
12-MAY-2018	13:58	Day 1	1 hour	F2	uncoated	1	0	0	0	0	2	0		
12-MAY-2018	13:58	Day 1	1 hour	F3	2.5 ug	1	0	0	0	0	2	0		
12-MAY-2018	14:58	Day 1	2 hour	F1	uncoated	0	0	0	0	0	0	0		
12-MAY-2018	14:58	Day 1	2 hour	F2	uncoated	0	0	0	0	0	0	0		
12-MAY-2018	14:58	Day 1	2 hour	F3	2.5 ug	0	0	0	0	0	0	0		
13-MAY-2018	12:05	Day 2		F1	uncoated	0	0	0	0	0	0	0		
13-MAY-2018	12:05	Day 2		F2	uncoated	0	0	0	0	0	0	0		
13-MAY-2018	12:05	Day 2		F3	2.5 ug	0	1	0	0	0	0	0		
15-MAY-2018	13:04	Day 4		F1	uncoated	0	0	0	0	0	0	3		
15-MAY-2018	13:04	Day 4		F2	uncoated	0	0	0	0	0	0	3		
15-MAY-2018	13:04	Day 4		F3	2.5 ug	0	0	0	0	0	0	0		
19-MAY-2018	16:08	Day 8		F1	uncoated	0	0	0	0	1	0	2		
19-MAY-2018	16:08	Day 8		F2	uncoated	0	0	0	0	1	0	2		

Key: 0= None; 1=Mild; 2=Moderate; 3=Severe; # Other = Pinprick bleeding or seepage

Administration Site Key: FA = Forearm; UA= Upper arm
Treatments Part A: A1 = A/Sing MAP, 15 ug (FA); A2 = Afluria Quad IM (UA), 15 ug; A3 = Placebo MAP (FA); A4 = A/Sing Mono IM, 15 ug (UA)
Treatments Part B: B1 = A/Sing MAP, 15 ug (FA) with biopsy; B2 = Placebo MAP (FA) with biopsy
B3 = A/Sing MAP, 15 ug (FA); B4 = A/Sing MAP, 10 ug (FA); B5 = A/Sing MAP, 5 ug (FA); B6 = A/Sing MAP, 2.5 ug (FA)
B7 = Placebo MAP (FA); B8 = A/Sing MAP, 15 ug (UA); B9 = A/Sing IM, 15 ug, as 2018 Afluria Quad (UA)

Listing 16.2.9.2
Treatment Site Tolerability Assessment per Site

Part B
Randomisation Number = B0020, Treatment Group = Group B6

Date	Time	Visit	Time Point	Site Number	Treatment for Site	Indura-tion	Redness	Bruising	Itching	Skin Flaking	Tender-ness	Definition/ Visibility	Other#	Unscheduled Reason
19-MAY-2018	16:08	Day 8		F3	2.5 ug	0	0	0	0	1	0	3		
19-MAY-2018	16:20	Unscheduled		F1	uncoated	0	0	0	0	1	0	2	NA	MO notifies as repeat
19-MAY-2018	16:20	Unscheduled		F2	uncoated	0	0	0	0	1	0	2	NA	MO notifies as repeat
19-MAY-2018	16:20	Unscheduled		F3	2.5 ug	0	0	0	0	1	0	3	NA	MO notifies as repeat
13-JUN-2018	12:50	Day 22		F1	uncoated	0	0	0	0	0	0	0		
13-JUN-2018	12:50	Day 22		F2	uncoated	0	0	0	0	0	0	1		
13-JUN-2018	12:50	Day 22		F3	2.5 ug	0	0	0	0	1	0	1		
11-JUL-2018	13:29	EOS/ET (Day 61)		F1	uncoated	0	0	0	0	0	0	0		
11-JUL-2018	13:29	EOS/ET (Day 61)		F2	uncoated	0	0	0	0	0	0	0		
11-JUL-2018	13:29	EOS/ET (Day 61)		F3	2.5 ug	0	0	0	0	0	0	0		

Key: 0= None; 1=Mild; 2=Moderate; 3=Severe; # Other = Pinprick bleeding or seepage

Administration Site Key: FA = Forearm; UA= Upper arm
Treatments Part A: A1 = A/Sing MAP, 15 ug (FA); A2 = Afluria Quad IM (UA), 15 ug; A3 = Placebo MAP (FA); A4 = A/Sing Mono IM, 15 ug (UA)
Treatments Part B: B1 = A/Sing MAP, 15 ug (FA) with biopsy; B2 = Placebo MAP (FA) with biopsy
B3 = A/Sing MAP, 15 ug (FA); B4 = A/Sing MAP, 10 ug (FA); B5 = A/Sing MAP, 5 ug (FA); B6 = A/Sing MAP, 2.5 ug (FA)
B7 = Placebo MAP (FA); B8 = A/Sing MAP, 15 ug (UA); B9 = A/Sing IM, 15 ug, as 2018 Afluria Quad (UA)

Listing 16.2.9.2
Treatment Site Tolerability Assessment per Site

Part B
Randomisation Number = B0021, Treatment Group = Group B7

Date	Time	Visit	Time Point	Site Number	Treatment for Site	Indura-tion	Redness	Bruising	Itching	Skin Flaking	Tender-ness	Definition/ Visibility	Other#	Unscheduled Reason
12-MAY-2018	12:12	Day 1	Pre-dose	F1	uncoated	0	0	0	0	0	0	0		
12-MAY-2018	12:12	Day 1	Pre-dose	F2	uncoated	0	0	0	0	0	0	0		
12-MAY-2018	12:12	Day 1	Pre-dose	F3	uncoated	0	0	0	0	0	0	0		
12-MAY-2018	13:18	Day 1	10 min	F1	uncoated	0	1	0	0	0	0	0		
12-MAY-2018	13:18	Day 1	10 min	F2	uncoated	0	1	0	0	0	0	0		
12-MAY-2018	13:18	Day 1	10 min	F3	uncoated	0	0	0	0	0	0	0		
12-MAY-2018	14:08	Day 1	1 hour	F1	uncoated	0	1	0	0	0	0	0		
12-MAY-2018	14:08	Day 1	1 hour	F2	uncoated	0	1	0	0	0	0	0		
12-MAY-2018	14:08	Day 1	1 hour	F3	uncoated	0	0	0	0	0	0	0		
12-MAY-2018	15:08	Day 1	2 hour	F1	uncoated	0	0	0	0	0	0	0		
12-MAY-2018	15:08	Day 1	2 hour	F2	uncoated	0	1	0	0	0	0	0		
12-MAY-2018	15:08	Day 1	2 hour	F3	uncoated	0	1	0	0	0	0	0		
13-MAY-2018	12:38	Day 2		F1	uncoated	0	0	0	0	0	0	0		
13-MAY-2018	12:38	Day 2		F2	uncoated	0	0	0	0	0	0	0		
13-MAY-2018	12:38	Day 2		F3	uncoated	0	0	0	0	0	0	0		
15-MAY-2018	09:58	Day 4		F1	uncoated	0	0	0	0	0	0	0		
15-MAY-2018	09:58	Day 4		F2	uncoated	0	0	0	0	0	0	0		
15-MAY-2018	09:58	Day 4		F3	uncoated	0	0	0	0	0	0	0		
19-MAY-2018	12:04	Day 8		F1	uncoated	0	0	0	0	0	0	2		
19-MAY-2018	12:04	Day 8		F2	uncoated	0	0	0	0	0	0	2		

Key: 0= None; 1=Mild; 2=Moderate; 3=Severe; # Other = Pinprick bleeding or seepage

Administration Site Key: FA = Forearm; UA= Upper arm
Treatments Part A: A1 = A/Sing MAP, 15 ug (FA); A2 = Afluria Quad IM (UA), 15 ug; A3 = Placebo MAP (FA); A4 = A/Sing Mono IM, 15 ug (UA)
Treatments Part B: B1 = A/Sing MAP, 15 ug (FA) with biopsy; B2 = Placebo MAP (FA) with biopsy
B3 = A/Sing MAP, 15 ug (FA); B4 = A/Sing MAP, 10 ug (FA); B5 = A/Sing MAP, 5 ug (FA); B6 = A/Sing MAP, 2.5 ug (FA)
B7 = Placebo MAP (FA); B8 = A/Sing MAP, 15 ug (UA); B9 = A/Sing IM, 15 ug, as 2018 Afluria Quad (UA)

Listing 16.2.9.2
Treatment Site Tolerability Assessment per Site

Part B
Randomisation Number = B0021, Treatment Group = Group B7

Date	Time	Visit	Time Point	Site Number	Treatment for Site	Indura-tion	Redness	Bruising	Itching	Skin Flaking	Tender-ness	Definition/ Visibility	Other#	Unscheduled Reason
19-MAY-2018	12:04	Day 8		F3	uncoated	0	0	0	0	0	0	2		
02-JUN-2018	13:15	Day 22		F1	uncoated	0	0	0	0	0	0	1		
02-JUN-2018	13:15	Day 22		F2	uncoated	0	0	0	0	0	0	1		
02-JUN-2018	13:15	Day 22		F3	uncoated	0	0	0	0	0	0	1		
11-JUL-2018	13:35	EOS/ET (Day 61)		F1	uncoated	0	0	0	0	0	0	0		
11-JUL-2018	13:35	EOS/ET (Day 61)		F2	uncoated	0	0	0	0	0	0	0		
11-JUL-2018	13:35	EOS/ET (Day 61)		F3	uncoated	0	0	0	0	0	0	0		

Key: 0= None; 1=Mild; 2=Moderate; 3=Severe; # Other = Pinprick bleeding or seepage

Administration Site Key: FA = Forearm; UA= Upper arm
Treatments Part A: A1 = A/Sing MAP, 15 ug (FA); A2 = Afluria Quad IM (UA), 15 ug; A3 = Placebo MAP (FA); A4 = A/Sing Mono IM, 15 ug (UA)
Treatments Part B: B1 = A/Sing MAP, 15 ug (FA) with biopsy; B2 = Placebo MAP (FA) with biopsy
B3 = A/Sing MAP, 15 ug (FA); B4 = A/Sing MAP, 10 ug (FA); B5 = A/Sing MAP, 5 ug (FA); B6 = A/Sing MAP, 2.5 ug (FA)
B7 = Placebo MAP (FA); B8 = A/Sing MAP, 15 ug (UA); B9 = A/Sing IM, 15 ug, as 2018 Afluria Quad (UA)

Listing 16.2.9.2
Treatment Site Tolerability Assessment per Site

Part B
Randomisation Number = B0022, Treatment Group = Group B4

Date	Time	Visit	Time Point	Site Number	Treatment for Site	Induration	Redness	Bruising	Itching	Skin Flaking	Tenderness	Definition/ Visibility	Other#	Unscheduled Reason
12-MAY-2018	12:33	Day 1	Pre-dose	F1	uncoated	0	0	0	0	0	0	0		
12-MAY-2018	12:33	Day 1	Pre-dose	F2	5 ug	0	0	0	0	0	0	0		
12-MAY-2018	12:33	Day 1	Pre-dose	F3	5 ug	0	0	0	0	0	0	0		
12-MAY-2018	13:28	Day 1	10 min	F1	uncoated	0	1	0	0	0	0	0		
12-MAY-2018	13:28	Day 1	10 min	F2	5 ug	0	1	0	0	0	0	0		
12-MAY-2018	13:28	Day 1	10 min	F3	5 ug	0	1	0	0	0	0	0		
12-MAY-2018	14:19	Day 1	1 hour	F1	uncoated	0	0	0	0	0	0	0		
12-MAY-2018	14:19	Day 1	1 hour	F2	5 ug	0	1	0	0	0	0	0		
12-MAY-2018	14:19	Day 1	1 hour	F3	5 ug	0	1	0	0	0	0	0		
12-MAY-2018	15:22	Day 1	2 hour	F1	uncoated	0	0	0	0	0	0	0		
12-MAY-2018	15:22	Day 1	2 hour	F2	5 ug	0	0	0	0	0	0	0		
12-MAY-2018	15:22	Day 1	2 hour	F3	5 ug	0	0	0	0	0	0	0		
13-MAY-2018	13:05	Day 2		F1	uncoated	0	0	0	0	0	0	0		
13-MAY-2018	13:05	Day 2		F2	5 ug	0	1	0	0	0	0	0		
13-MAY-2018	13:05	Day 2		F3	5 ug	0	0	0	0	0	0	0		
15-MAY-2018	13:49	Day 4		F1	uncoated	0	0	0	0	0	0	0		
15-MAY-2018	13:49	Day 4		F2	5 ug	0	0	0	0	0	0	0		
15-MAY-2018	13:49	Day 4		F3	5 ug	0	0	0	0	0	0	0		
19-MAY-2018	13:28	Day 8		F1	uncoated	0	0	0	0	0	0	1		
19-MAY-2018	13:28	Day 8		F2	5 ug	0	0	0	0	0	0	2		

Key: 0= None; 1=Mild; 2=Moderate; 3=Severe; # Other = Pinprick bleeding or seepage

Administration Site Key: FA = Forearm; UA= Upper arm
Treatments Part A: A1 = A/Sing MAP, 15 ug (FA); A2 = Afluria Quad IM (UA), 15 ug; A3 = Placebo MAP (FA); A4 = A/Sing Mono IM, 15 ug (UA)
Treatments Part B: B1 = A/Sing MAP, 15 ug (FA) with biopsy; B2 = Placebo MAP (FA) with biopsy
B3 = A/Sing MAP, 15 ug (FA); B4 = A/Sing MAP, 10 ug (FA); B5 = A/Sing MAP, 5 ug (FA); B6 = A/Sing MAP, 2.5 ug (FA)
B7 = Placebo MAP (FA); B8 = A/Sing MAP, 15 ug (UA); B9 = A/Sing IM, 15 ug, as 2018 Afluria Quad (UA)

Listing 16.2.9.2
Treatment Site Tolerability Assessment per Site

Part B
Randomisation Number = B0022, Treatment Group = Group B4

Date	Time	Visit	Time Point	Site Number	Treatment for Site	Indura-tion	Redness	Bruising	Itching	Skin Flaking	Tender-ness	Definition/ Visibility	Other#	Unscheduled Reason
19-MAY-2018	13:28	Day 8		F3	5 ug	0	0	0	0	1	0	2		
19-MAY-2018	13:40	Unscheduled		F1	uncoated	0	0	0	0	0	0	2	NA	MO notifies as repeat
19-MAY-2018	13:40	Unscheduled		F2	5 ug	1	0	0	0	1	0	0	NA	MO notifies as repeat
19-MAY-2018	13:40	Unscheduled		F3	5 ug	1	0	0	0	1	0	0	NA	MO notifies as repeat
02-JUN-2018	13:51	Day 22		F1	uncoated	0	0	0	0	0	0	1		
02-JUN-2018	13:51	Day 22		F2	5 ug	0	0	0	0	0	0	2		
02-JUN-2018	13:51	Day 22		F3	5 ug	0	0	0	0	0	0	2		
12-JUL-2018	11:07	EOS/ET (Day 61)		F1	uncoated	0	0	0	0	0	0	1		
12-JUL-2018	11:07	EOS/ET (Day 61)		F2	5 ug	0	0	0	0	0	0	1		
12-JUL-2018	11:07	EOS/ET (Day 61)		F3	5 ug	0	0	0	0	0	0	1		

Key: 0= None; 1=Mild; 2=Moderate; 3=Severe; # Other = Pinprick bleeding or seepage

Administration Site Key: FA = Forearm; UA= Upper arm
Treatments Part A: A1 = A/Sing MAP, 15 ug (FA); A2 = Afluria Quad IM (UA), 15 ug; A3 = Placebo MAP (FA); A4 = A/Sing Mono IM, 15 ug (UA)
Treatments Part B: B1 = A/Sing MAP, 15 ug (FA) with biopsy; B2 = Placebo MAP (FA) with biopsy
B3 = A/Sing MAP, 15 ug (FA); B4 = A/Sing MAP, 10 ug (FA); B5 = A/Sing MAP, 5 ug (FA); B6 = A/Sing MAP, 2.5 ug (FA)
B7 = Placebo MAP (FA); B8 = A/Sing MAP, 15 ug (UA); B9 = A/Sing IM, 15 ug, as 2018 Afluria Quad (UA)

Listing 16.2.9.2
Treatment Site Tolerability Assessment per Site

Part B
Randomisation Number = B0023, Treatment Group = Group B3

Date	Time	Visit	Time Point	Site Number	Treatment for Site	Induration	Redness	Bruising	Itching	Skin Flaking	Tenderness	Definition/ Visibility	Other#	Unscheduled Reason
12-MAY-2018	12:42	Day 1	Pre-dose	F1	5 ug	0	0	0	0	0	0	0		
12-MAY-2018	12:42	Day 1	Pre-dose	F2	5 ug	0	0	0	0	0	0	0		
12-MAY-2018	12:42	Day 1	Pre-dose	F3	5 ug	0	0	0	0	0	0	0		
12-MAY-2018	13:44	Day 1	10 min	F1	5 ug	0	1	0	0	0	0	0		
12-MAY-2018	13:44	Day 1	10 min	F2	5 ug	0	1	0	1	0	0	0		
12-MAY-2018	13:44	Day 1	10 min	F3	5 ug	0	1	0	1	0	0	0		
12-MAY-2018	14:34	Day 1	1 hour	F1	5 ug	0	1	0	0	0	1	0		
12-MAY-2018	14:34	Day 1	1 hour	F2	5 ug	0	1	0	0	0	0	0		
12-MAY-2018	14:34	Day 1	1 hour	F3	5 ug	0	1	0	0	0	0	0		
12-MAY-2018	15:34	Day 1	2 hour	F1	5 ug	0	0	0	0	0	0	0		
12-MAY-2018	15:34	Day 1	2 hour	F2	5 ug	0	0	0	0	0	0	0		
12-MAY-2018	15:34	Day 1	2 hour	F3	5 ug	0	0	0	0	0	0	0		
12-MAY-2018	14:51	Unscheduled		F1	5 ug	0	1	0	0	0	0	0	NA	MO notifies as repeat
12-MAY-2018	14:51	Unscheduled		F2	5 ug	0	1	0	0	0	0	0	NA	MO notifies as repeat
12-MAY-2018	14:51	Unscheduled		F3	5 ug	0	1	0	0	0	0	0	NA	MO notifies as repeat
12-MAY-2018	15:39	Unscheduled		F1	5 ug	0	0	0	0	0	0	0	NA	MO notifies as repeat

Key: 0= None; 1=Mild; 2=Moderate; 3=Severe; # Other = Pinprick bleeding or seepage

Administration Site Key: FA = Forearm; UA= Upper arm
Treatments Part A: A1 = A/Sing MAP, 15 ug (FA); A2 = Afluria Quad IM (UA), 15 ug; A3 = Placebo MAP (FA); A4 = A/Sing Mono IM, 15 ug (UA)
Treatments Part B: B1 = A/Sing MAP, 15 ug (FA) with biopsy; B2 = Placebo MAP (FA) with biopsy
B3 = A/Sing MAP, 15 ug (FA); B4 = A/Sing MAP, 10 ug (FA); B5 = A/Sing MAP, 5 ug (FA); B6 = A/Sing MAP, 2.5 ug (FA)
B7 = Placebo MAP (FA); B8 = A/Sing MAP, 15 ug (UA); B9 = A/Sing IM, 15 ug, as 2018 Afluria Quad (UA)

Listing 16.2.9.2
Treatment Site Tolerability Assessment per Site

Part B
Randomisation Number = B0023, Treatment Group = Group B3

Date	Time	Visit	Time Point	Site Number	Treatment for Site	Indura-tion	Redness	Bruising	Itching	Skin Flaking	Tender-ness	Definition/ Visibility	Other#	Unscheduled Reason
12-MAY-2018	15:41	Unscheduled		F2	5 ug	0	0	0	0	0	0	0	NA	MO notifies as repeat
12-MAY-2018	15:43	Unscheduled		F3	5 ug	0	0	0	0	0	0	0	NA	MO notifies as repeat
13-MAY-2018	13:23	Day 2		F1	5 ug	0	1	0	1	0	1	0		
13-MAY-2018	13:23	Day 2		F2	5 ug	1	1	0	1	0	1	0		
13-MAY-2018	13:23	Day 2		F3	5 ug	0	1	0	1	0	1	0		
15-MAY-2018	14:31	Day 4		F1	5 ug	0	0	0	0	1	0	0		
15-MAY-2018	14:31	Day 4		F2	5 ug	1	1	0	0	1	0	0		
15-MAY-2018	14:31	Day 4		F3	5 ug	1	1	0	0	1	0	0		
19-MAY-2018	10:22	Day 8		F1	5 ug	0	1	0	0	2	0	0		
19-MAY-2018	10:22	Day 8		F2	5 ug	0	1	0	0	1	0	0		
19-MAY-2018	10:22	Day 8		F3	5 ug	0	0	0	0	1	0	0		
19-MAY-2018	10:25	Unscheduled		F1	5 ug	0	0	0	0	2	0	0	NA	MO notifies as repeat
02-JUN-2018	11:00	Day 22		F1	5 ug	0	0	0	0	0	0	2		
02-JUN-2018	11:00	Day 22		F2	5 ug	0	0	0	0	0	0	2		
02-JUN-2018	11:00	Day 22		F3	5 ug	0	0	0	0	0	0	2		
11-JUL-2018	14:15	EOS/ET (Day 61)		F1	5 ug	0	0	0	0	0	0	0		

Key: 0= None; 1=Mild; 2=Moderate; 3=Severe; # Other = Pinprick bleeding or seepage

Administration Site Key: FA = Forearm; UA= Upper arm
Treatments Part A: A1 = A/Sing MAP, 15 ug (FA); A2 = Afluria Quad IM (UA), 15 ug; A3 = Placebo MAP (FA); A4 = A/Sing Mono IM, 15 ug (UA)
Treatments Part B: B1 = A/Sing MAP, 15 ug (FA) with biopsy; B2 = Placebo MAP (FA) with biopsy
B3 = A/Sing MAP, 15 ug (FA); B4 = A/Sing MAP, 10 ug (FA); B5 = A/Sing MAP, 5 ug (FA); B6 = A/Sing MAP, 2.5 ug (FA)
B7 = Placebo MAP (FA); B8 = A/Sing MAP, 15 ug (UA); B9 = A/Sing IM, 15 ug, as 2018 Afluria Quad (UA)

Listing 16.2.9.2
Treatment Site Tolerability Assessment per Site

Part B
Randomisation Number = B0023, Treatment Group = Group B3

Date	Time	Visit	Time Point	Site Number	Treatment for Site	Indura-tion	Redness	Bruising	Itching	Skin Flaking	Tender-ness	Definition/ Visibility	Other#	Unscheduled Reason
11-JUL-2018	14:15	EOS/ET (Day 61)		F2	5 ug	0	0	0	0	0	0	0		
11-JUL-2018	14:15	EOS/ET (Day 61)		F3	5 ug	0	0	0	0	0	0	0		

Key: 0= None; 1=Mild; 2=Moderate; 3=Severe; # Other = Pinprick bleeding or seepage

Administration Site Key: FA = Forearm; UA= Upper arm
Treatments Part A: A1 = A/Sing MAP, 15 ug (FA); A2 = Afluria Quad IM (UA), 15 ug; A3 = Placebo MAP (FA); A4 = A/Sing Mono IM, 15 ug (UA)
Treatments Part B: B1 = A/Sing MAP, 15 ug (FA) with biopsy; B2 = Placebo MAP (FA) with biopsy
B3 = A/Sing MAP, 15 ug (FA); B4 = A/Sing MAP, 10 ug (FA); B5 = A/Sing MAP, 5 ug (FA); B6 = A/Sing MAP, 2.5 ug (FA)
B7 = Placebo MAP (FA); B8 = A/Sing MAP, 15 ug (UA); B9 = A/Sing IM, 15 ug, as 2018 Afluria Quad (UA)

Listing 16.2.9.2
Treatment Site Tolerability Assessment per Site

Part B
Randomisation Number = B0024, Treatment Group = Group B5

Date	Time	Visit	Time Point	Site Number	Treatment for Site	Indura-tion	Redness	Bruising	Itching	Skin Flaking	Tender-ness	Definition/ Visibility	Other#	Unscheduled Reason
12-MAY-2018	12:52	Day 1	Pre-dose	F1	uncoated	0	0	0	0	0	0	0		
12-MAY-2018	12:52	Day 1	Pre-dose	F2	uncoated	0	0	0	0	0	0	0		
12-MAY-2018	12:52	Day 1	Pre-dose	F3	5 ug	0	0	0	0	0	0	0		
12-MAY-2018	13:54	Day 1	10 min	F1	uncoated	0	0	0	0	0	1	0		
12-MAY-2018	13:54	Day 1	10 min	F2	uncoated	0	0	0	0	0	1	0		
12-MAY-2018	13:54	Day 1	10 min	F3	5 ug	0	0	0	0	0	1	0		
12-MAY-2018	14:48	Day 1	1 hour	F1	uncoated	0	0	0	0	0	0	0		
12-MAY-2018	14:48	Day 1	1 hour	F2	uncoated	0	0	0	0	0	0	0		
12-MAY-2018	14:48	Day 1	1 hour	F3	5 ug	0	0	0	0	0	0	0		
12-MAY-2018	15:44	Day 1	2 hour	F1	uncoated	0	0	0	0	0	0	0		
12-MAY-2018	15:44	Day 1	2 hour	F2	uncoated	0	0	0	0	0	0	0		
12-MAY-2018	15:44	Day 1	2 hour	F3	5 ug	0	0	0	0	0	0	0		
12-MAY-2018	14:59	Unscheduled		F1	uncoated	0	0	0	0	0	0	0	NA	MO notifies as repeat
12-MAY-2018	15:00	Unscheduled		F1	uncoated	0	0	0	0	0	1	0	NA	MO notifies as repeat
12-MAY-2018	15:04	Unscheduled		F3	5 ug	0	1	0	0	0	1	0	NA	MO notifies as repeat
12-MAY-2018	15:50	Unscheduled		F1	uncoated	0	0	0	0	0	0	0	NA	MO notifies as repeat

Key: 0= None; 1=Mild; 2=Moderate; 3=Severe; # Other = Pinprick bleeding or seepage

Administration Site Key: FA = Forearm; UA= Upper arm
Treatments Part A: A1 = A/Sing MAP, 15 ug (FA); A2 = Afluria Quad IM (UA), 15 ug; A3 = Placebo MAP (FA); A4 = A/Sing Mono IM, 15 ug (UA)
Treatments Part B: B1 = A/Sing MAP, 15 ug (FA) with biopsy; B2 = Placebo MAP (FA) with biopsy
B3 = A/Sing MAP, 15 ug (FA); B4 = A/Sing MAP, 10 ug (FA); B5 = A/Sing MAP, 5 ug (FA); B6 = A/Sing MAP, 2.5 ug (FA)
B7 = Placebo MAP (FA); B8 = A/Sing MAP, 15 ug (UA); B9 = A/Sing IM, 15 ug, as 2018 Afluria Quad (UA)

Listing 16.2.9.2
Treatment Site Tolerability Assessment per Site

Part B
Randomisation Number = B0024, Treatment Group = Group B5

Date	Time	Visit	Time Point	Site Number	Treatment for Site	Indura-tion	Redness	Bruising	Itching	Skin Flaking	Tender-ness	Definition/ Visibility	Other#	Unscheduled Reason
12-MAY-2018	15:53	Unscheduled		F2	uncoated	0	0	0	0	0	0	0	NA	MO notifies as repeat
12-MAY-2018	15:55	Unscheduled		F3	5 ug	0	0	0	0	0	0	0	NA	MO notifies as repeat
13-MAY-2018	13:38	Day 2		F1	uncoated	0	0	0	0	0	0	0		
13-MAY-2018	13:38	Day 2		F2	uncoated	0	0	0	0	0	0	0		
13-MAY-2018	13:38	Day 2		F3	5 ug	0	0	0	0	0	1	0		
15-MAY-2018	13:54	Day 4		F1	uncoated	0	0	0	0	0	0	2		
15-MAY-2018	13:54	Day 4		F2	uncoated	0	0	0	0	0	0	2		
15-MAY-2018	13:54	Day 4		F3	5 ug	0	0	0	0	0	0	0		
19-MAY-2018	13:25	Day 8		F1	uncoated	0	0	0	0	0	0	1		
19-MAY-2018	13:25	Day 8		F2	uncoated	0	0	0	0	0	0	1		
19-MAY-2018	13:25	Day 8		F3	5 ug	0	0	0	1	0	0	1		
02-JUN-2018	13:47	Day 22		F1	uncoated	0	0	0	0	0	0	1		
02-JUN-2018	13:47	Day 22		F2	uncoated	0	0	0	0	0	0	1		
02-JUN-2018	13:47	Day 22		F3	5 ug	0	0	0	0	0	0	1		
12-JUL-2018	14:28	EOS/ET (Day 61)		F1	uncoated	0	0	0	0	0	0	0		
12-JUL-2018	14:28	EOS/ET (Day 61)		F2	uncoated	0	0	0	0	0	0	0		
12-JUL-2018	14:28	EOS/ET (Day 61)		F3	5 ug	0	0	0	0	0	0	1		

Key: 0= None; 1=Mild; 2=Moderate; 3=Severe; # Other = Pinprick bleeding or seepage

Administration Site Key: FA = Forearm; UA= Upper arm
Treatments Part A: A1 = A/Sing MAP, 15 ug (FA); A2 = Afluria Quad IM (UA), 15 ug; A3 = Placebo MAP (FA); A4 = A/Sing Mono IM, 15 ug (UA)
Treatments Part B: B1 = A/Sing MAP, 15 ug (FA) with biopsy; B2 = Placebo MAP (FA) with biopsy
B3 = A/Sing MAP, 15 ug (FA); B4 = A/Sing MAP, 10 ug (FA); B5 = A/Sing MAP, 5 ug (FA); B6 = A/Sing MAP, 2.5 ug (FA)
B7 = Placebo MAP (FA); B8 = A/Sing MAP, 15 ug (UA); B9 = A/Sing IM, 15 ug, as 2018 Afluria Quad (UA)

Listing 16.2.9.2
Treatment Site Tolerability Assessment per Site

Part B
Randomisation Number = B0025, Treatment Group = Group B8

Date	Time	Visit	Time Point	Site Number	Treatment for Site	Indura-tion	Redness	Bruising	Itching	Skin Flaking	Tender-ness	Definition/ Visibility	Other#	Unscheduled Reason
12-MAY-2018	13:05	Day 1	Pre-dose	D1	5 ug	0	0	0	0	0	0	0		
12-MAY-2018	13:05	Day 1	Pre-dose	D2	5 ug	0	0	0	0	0	0	0		
12-MAY-2018	13:05	Day 1	Pre-dose	D3	5 ug	0	0	0	0	0	0	0		
12-MAY-2018	13:58	Day 1	10 min	D1	5 ug	0	1	0	0	0	1	0		
12-MAY-2018	13:58	Day 1	10 min	D2	5 ug	0	1	0	0	0	1	0		
12-MAY-2018	13:58	Day 1	10 min	D3	5 ug	0	1	0	0	0	1	0		
12-MAY-2018	14:48	Day 1	1 hour	D1	5 ug	0	1	0	0	0	0	0		
12-MAY-2018	14:48	Day 1	1 hour	D2	5 ug	0	1	0	0	0	0	0		
12-MAY-2018	14:48	Day 1	1 hour	D3	5 ug	0	1	0	0	0	0	0		
12-MAY-2018	15:48	Day 1	2 hour	D1	5 ug	0	0	0	0	0	0	0		
12-MAY-2018	15:48	Day 1	2 hour	D2	5 ug	0	0	0	0	0	0	0		
12-MAY-2018	15:48	Day 1	2 hour	D3	5 ug	0	0	0	0	0	0	0		
13-MAY-2018	12:35	Day 2		D1	5 ug	0	1	0	0	0	0	0		
13-MAY-2018	12:35	Day 2		D2	5 ug	0	1	0	0	0	0	0		
13-MAY-2018	12:35	Day 2		D3	5 ug	0	1	0	0	0	0	0		
15-MAY-2018	16:43	Day 4		D1	5 ug	0	0	0	0	0	0	0		
15-MAY-2018	16:43	Day 4		D2	5 ug	0	0	0	0	0	0	0		
15-MAY-2018	16:43	Day 4		D3	5 ug	0	0	0	0	0	0	0		
19-MAY-2018	12:38	Day 8		D1	5 ug	0	0	0	0	1	0	0		
19-MAY-2018	12:38	Day 8		D2	5 ug	0	0	0	0	1	0	0		

Key: 0= None; 1=Mild; 2=Moderate; 3=Severe; # Other = Pinprick bleeding or seepage

Administration Site Key: FA = Forearm; UA= Upper arm
Treatments Part A: A1 = A/Sing MAP, 15 ug (FA); A2 = Afluria Quad IM (UA), 15 ug; A3 = Placebo MAP (FA); A4 = A/Sing Mono IM, 15 ug (UA)
Treatments Part B: B1 = A/Sing MAP, 15 ug (FA) with biopsy; B2 = Placebo MAP (FA) with biopsy
B3 = A/Sing MAP, 15 ug (FA); B4 = A/Sing MAP, 10 ug (FA); B5 = A/Sing MAP, 5 ug (FA); B6 = A/Sing MAP, 2.5 ug (FA)
B7 = Placebo MAP (FA); B8 = A/Sing MAP, 15 ug (UA); B9 = A/Sing IM, 15 ug, as 2018 Afluria Quad (UA)

Listing 16.2.9.2
Treatment Site Tolerability Assessment per Site

Part B
Randomisation Number = B0025, Treatment Group = Group B8

Date	Time	Visit	Time Point	Site Number	Treatment for Site	Indura-tion	Redness	Bruising	Itching	Skin Flaking	Tender-ness	Definition/ Visibility	Other#	Unscheduled Reason
19-MAY-2018	12:38	Day 8		D3	5 ug	0	0	0	0	1	0	0		
04-JUN-2018	13:10	Day 22		D1	5 ug	0	0	0	0	1	0	0		
04-JUN-2018	13:10	Day 22		D2	5 ug	0	0	0	0	1	0	1		
04-JUN-2018	13:10	Day 22		D3	5 ug	0	0	0	0	1	0	1		
13-JUL-2018	13:38	EOS/ET (Day 61)		D1	5 ug	0	0	0	0	0	0	1		
13-JUL-2018	13:38	EOS/ET (Day 61)		D2	5 ug	0	0	0	0	0	0	1		
13-JUL-2018	13:38	EOS/ET (Day 61)		D3	5 ug	0	0	0	0	0	0	1		

Key: 0= None; 1=Mild; 2=Moderate; 3=Severe; # Other = Pinprick bleeding or seepage

Administration Site Key: FA = Forearm; UA= Upper arm
Treatments Part A: A1 = A/Sing MAP, 15 ug (FA); A2 = Afluria Quad IM (UA), 15 ug; A3 = Placebo MAP (FA); A4 = A/Sing Mono IM, 15 ug (UA)
Treatments Part B: B1 = A/Sing MAP, 15 ug (FA) with biopsy; B2 = Placebo MAP (FA) with biopsy
B3 = A/Sing MAP, 15 ug (FA); B4 = A/Sing MAP, 10 ug (FA); B5 = A/Sing MAP, 5 ug (FA); B6 = A/Sing MAP, 2.5 ug (FA)
B7 = Placebo MAP (FA); B8 = A/Sing MAP, 15 ug (UA); B9 = A/Sing IM, 15 ug, as 2018 Afluria Quad (UA)

Listing 16.2.9.2
Treatment Site Tolerability Assessment per Site

Part B
Randomisation Number = B0026, Treatment Group = Group B9

Date	Time	Visit	Time Point	Site Number	Treatment for Site	Indura-tion	Redness	Bruising	Itching	Skin Flaking	Tender-ness	Definition/ Visibility	Other#	Unscheduled Reason
12-MAY-2018	11:02	Day 1	Pre-dose	D1	[IM]	0	0	0	0	0	0			
12-MAY-2018	11:41	Day 1	10 min	D1	[IM]	0	0	0	0	0	0			
12-MAY-2018	12:31	Day 1	1 hour	D1	[IM]	0	0	0	0	0	0			
12-MAY-2018	13:36	Day 1	2 hour	D1	[IM]	0	0	0	0	0	0			
13-MAY-2018	12:21	Day 2		D1	[IM]	0	0	0	0	0	0			
15-MAY-2018	15:00	Day 4		D1	[IM]	0	0	0	0	0	0			
19-MAY-2018	15:57	Day 8		D1	[IM]	0	0	0	0	0	0			
02-JUN-2018	14:31	Day 22		D1	[IM]	0	0	0	0	0	0			
11-JUL-2018	16:15	EOS/ET (Day 61)		D1	[IM]	0	0	0	0	0	0	5		

Key: 0= None; 1=Mild; 2=Moderate; 3=Severe; # Other = Pinprick bleeding or seepage

Administration Site Key: FA = Forearm; UA= Upper arm
Treatments Part A: A1 = A/Sing MAP, 15 ug (FA); A2 = Afluria Quad IM (UA), 15 ug; A3 = Placebo MAP (FA); A4 = A/Sing Mono IM, 15 ug (UA)
Treatments Part B: B1 = A/Sing MAP, 15 ug (FA) with biopsy; B2 = Placebo MAP (FA) with biopsy
B3 = A/Sing MAP, 15 ug (FA); B4 = A/Sing MAP, 10 ug (FA); B5 = A/Sing MAP, 5 ug (FA); B6 = A/Sing MAP, 2.5 ug (FA)
B7 = Placebo MAP (FA); B8 = A/Sing MAP, 15 ug (UA); B9 = A/Sing IM, 15 ug, as 2018 Afluria Quad (UA)

Listing 16.2.9.2
Treatment Site Tolerability Assessment per Site

Part B
Randomisation Number = B0027, Treatment Group = Group B3

Date	Time	Visit	Time Point	Site Number	Treatment for Site	Indura- tion	Redness	Bruising	Itching	Skin Flaking	Tender- ness	Definition/ Visibility	Other#	Unscheduled Reason
12-MAY-2018	13:18	Day 1	Pre-dose	F1	5 ug	0	0	0	0	0	0	0		
12-MAY-2018	13:18	Day 1	Pre-dose	F2	5 ug	0	0	0	0	0	0	0		
12-MAY-2018	13:18	Day 1	Pre-dose	F3	5 ug	0	0	0	0	0	0	0		
12-MAY-2018	14:48	Day 1	10 min	F1	5 ug	0	0	0	1	0	0	0		
12-MAY-2018	14:48	Day 1	10 min	F2	5 ug	0	0	0	1	0	0	0		
12-MAY-2018	14:48	Day 1	10 min	F3	5 ug	0	0	0	1	0	0	0		
12-MAY-2018	15:38	Day 1	1 hour	F1	5 ug	0	1	0	0	0	1	0		
12-MAY-2018	15:38	Day 1	1 hour	F2	5 ug	0	1	0	0	0	1	0		
12-MAY-2018	15:38	Day 1	1 hour	F3	5 ug	0	1	0	1	0	1	0		
12-MAY-2018	16:40	Day 1	2 hour	F1	5 ug	0	0	0	0	0	0	0		
12-MAY-2018	16:40	Day 1	2 hour	F2	5 ug	0	0	0	0	0	0	0		
12-MAY-2018	16:40	Day 1	2 hour	F3	5 ug	0	0	0	0	0	0	0		
12-MAY-2018	16:42	Unscheduled		F1	5 ug	0	0	0	0	0	1	0	NA	Mo notifies as repeat
12-MAY-2018	16:42	Unscheduled		F2	5 ug	0	0	0	0	0	1	0	NA	Mo notifies as repeat
12-MAY-2018	16:42	Unscheduled		F3	5 ug	0	0	0	0	0	1	0	NA	Mo notifies as repeat
13-MAY-2018	14:00	Day 2		F1	5 ug	0	1	0	0	0	1	0		
13-MAY-2018	14:00	Day 2		F2	5 ug	1	1	0	0	0	1	0		

Key: 0= None; 1=Mild; 2=Moderate; 3=Severe; # Other = Pinprick bleeding or seepage

Administration Site Key: FA = Forearm; UA= Upper arm
Treatments Part A: A1 = A/Sing MAP, 15 ug (FA); A2 = Afluria Quad IM (UA), 15 ug; A3 = Placebo MAP (FA); A4 = A/Sing Mono IM, 15 ug (UA)
Treatments Part B: B1 = A/Sing MAP, 15 ug (FA) with biopsy; B2 = Placebo MAP (FA) with biopsy
B3 = A/Sing MAP, 15 ug (FA); B4 = A/Sing MAP, 10 ug (FA); B5 = A/Sing MAP, 5 ug (FA); B6 = A/Sing MAP, 2.5 ug (FA)
B7 = Placebo MAP (FA); B8 = A/Sing MAP, 15 ug (UA); B9 = A/Sing IM, 15 ug, as 2018 Afluria Quad (UA)

Listing 16.2.9.2
Treatment Site Tolerability Assessment per Site

Part B
Randomisation Number = B0027, Treatment Group = Group B3

Date	Time	Visit	Time Point	Site Number	Treatment for Site	Indura- tion	Redness	Bruising	Itching	Skin Flaking	Tender- ness	Definition/ Visibility	Other#	Unscheduled Reason
13-MAY-2018	14:00	Day 2		F3	5 ug	0	1	0	0	0	1	0		
15-MAY-2018	17:08	Day 4		F1	5 ug	1	1	0	1	0	0	0		
15-MAY-2018	17:08	Day 4		F2	5 ug	1	1	0	2	0	1	0		
15-MAY-2018	17:08	Day 4		F3	5 ug	1	1	0	2	0	1	0		
19-MAY-2018	14:27	Day 8		F1	5 ug	0	0	0	0	1	0	0		
19-MAY-2018	14:27	Day 8		F2	5 ug	0	0	0	1	1	0	0		
19-MAY-2018	14:27	Day 8		F3	5 ug	0	0	0	1	1	0	0		
02-JUN-2018	16:08	Day 22		F1	5 ug	0	0	0	0	0	0	2		
02-JUN-2018	16:08	Day 22		F2	5 ug	0	0	0	0	0	0	2		
02-JUN-2018	16:08	Day 22		F3	5 ug	0	0	0	0	0	0	2		
11-JUL-2018	17:20	EOS/ET (Day 61)		F1	5 ug	0	0	0	0	0	0	1		
11-JUL-2018	17:20	EOS/ET (Day 61)		F2	5 ug	0	0	0	0	0	0	0		
11-JUL-2018	17:20	EOS/ET (Day 61)		F3	5 ug	0	0	0	0	0	0	1		

Key: 0= None; 1=Mild; 2=Moderate; 3=Severe; # Other = Pinprick bleeding or seepage

Administration Site Key: FA = Forearm; UA= Upper arm
Treatments Part A: A1 = A/Sing MAP, 15 ug (FA); A2 = Afluria Quad IM (UA), 15 ug; A3 = Placebo MAP (FA); A4 = A/Sing Mono IM, 15 ug (UA)
Treatments Part B: B1 = A/Sing MAP, 15 ug (FA) with biopsy; B2 = Placebo MAP (FA) with biopsy
B3 = A/Sing MAP, 15 ug (FA); B4 = A/Sing MAP, 10 ug (FA); B5 = A/Sing MAP, 5 ug (FA); B6 = A/Sing MAP, 2.5 ug (FA)
B7 = Placebo MAP (FA); B8 = A/Sing MAP, 15 ug (UA); B9 = A/Sing IM, 15 ug, as 2018 Afluria Quad (UA)

Listing 16.2.9.2
Treatment Site Tolerability Assessment per Site

Part B
Randomisation Number = B0028, Treatment Group = Group B5

Date	Time	Visit	Time Point	Site Number	Treatment for Site	Induration	Redness	Bruising	Itching	Skin Flaking	Tenderness	Definition/ Visibility	Other#	Unscheduled Reason
12-MAY-2018	13:27	Day 1	Pre-dose	F1	5 ug	0	0	0	0	0	0	0		
12-MAY-2018	13:27	Day 1	Pre-dose	F2	uncoated	0	0	0	0	0	0	0		
12-MAY-2018	13:27	Day 1	Pre-dose	F3	uncoated	0	0	0	0	0	0	0		
12-MAY-2018	14:38	Day 1	10 min	F1	5 ug	0	0	0	0	0	0	0		
12-MAY-2018	14:38	Day 1	10 min	F2	uncoated	0	0	0	0	0	0	0		
12-MAY-2018	14:38	Day 1	10 min	F3	uncoated	0	0	0	0	0	0	0		
12-MAY-2018	15:28	Day 1	1 hour	F1	5 ug	0	1	0	0	0	0	0		
12-MAY-2018	15:28	Day 1	1 hour	F2	uncoated	0	1	0	0	0	1	0		
12-MAY-2018	15:28	Day 1	1 hour	F3	uncoated	0	1	0	0	0	0	0		
12-MAY-2018	16:28	Day 1	2 hour	F1	5 ug	0	0	0	0	0	0	0		
12-MAY-2018	16:28	Day 1	2 hour	F2	uncoated	0	0	0	0	0	0	0		
12-MAY-2018	16:28	Day 1	2 hour	F3	uncoated	0	0	0	0	0	0	0		
13-MAY-2018	10:08	Day 2		F1	5 ug	0	0	0	0	0	0	0		
13-MAY-2018	10:08	Day 2		F2	uncoated	0	0	0	0	0	0	0		
13-MAY-2018	10:08	Day 2		F3	uncoated	0	0	0	0	0	0	0		
16-MAY-2018	16:32	Day 4		F1	5 ug	0	0	0	1	2	0	0		
16-MAY-2018	16:32	Day 4		F2	uncoated	0	0	0	0	0	0	1		
16-MAY-2018	16:32	Day 4		F3	uncoated	0	0	0	0	0	0	2		
19-MAY-2018	14:31	Day 8		F1	5 ug	0	0	0	0	3	0	0		
19-MAY-2018	14:31	Day 8		F2	uncoated	0	0	0	0	0	0	2		

Key: 0= None; 1=Mild; 2=Moderate; 3=Severe; # Other = Pinprick bleeding or seepage

Administration Site Key: FA = Forearm; UA= Upper arm
Treatments Part A: A1 = A/Sing MAP, 15 ug (FA); A2 = Afluria Quad IM (UA), 15 ug; A3 = Placebo MAP (FA); A4 = A/Sing Mono IM, 15 ug (UA)
Treatments Part B: B1 = A/Sing MAP, 15 ug (FA) with biopsy; B2 = Placebo MAP (FA) with biopsy
B3 = A/Sing MAP, 15 ug (FA); B4 = A/Sing MAP, 10 ug (FA); B5 = A/Sing MAP, 5 ug (FA); B6 = A/Sing MAP, 2.5 ug (FA)
B7 = Placebo MAP (FA); B8 = A/Sing MAP, 15 ug (UA); B9 = A/Sing IM, 15 ug, as 2018 Afluria Quad (UA)

Listing 16.2.9.2
Treatment Site Tolerability Assessment per Site

Part B
Randomisation Number = B0028, Treatment Group = Group B5

Date	Time	Visit	Time Point	Site Number	Treatment for Site	Indura-tion	Redness	Bruising	Itching	Skin Flaking	Tender-ness	Definition/ Visibility	Other#	Unscheduled Reason
19-MAY-2018	14:31	Day 8		F3	uncoated	0	0	0	0	0	0	2		
02-JUN-2018	14:50	Day 22		F1	5 ug	0	1	0	0	0	0	0		
02-JUN-2018	14:50	Day 22		F2	uncoated	0	0	0	0	0	0	0		
02-JUN-2018	14:50	Day 22		F3	uncoated	0	0	0	0	0	0	0		
11-JUL-2018	14:34	EOS/ET (Day 61)		F1	5 ug	0	0	0	0	0	0	0		
11-JUL-2018	14:34	EOS/ET (Day 61)		F2	uncoated	0	0	0	0	0	0	0		
11-JUL-2018	14:34	EOS/ET (Day 61)		F3	uncoated	0	0	0	0	0	0	0		

Key: 0= None; 1=Mild; 2=Moderate; 3=Severe; # Other = Pinprick bleeding or seepage

Administration Site Key: FA = Forearm; UA= Upper arm
Treatments Part A: A1 = A/Sing MAP, 15 ug (FA); A2 = Afluria Quad IM (UA), 15 ug; A3 = Placebo MAP (FA); A4 = A/Sing Mono IM, 15 ug (UA)
Treatments Part B: B1 = A/Sing MAP, 15 ug (FA) with biopsy; B2 = Placebo MAP (FA) with biopsy
B3 = A/Sing MAP, 15 ug (FA); B4 = A/Sing MAP, 10 ug (FA); B5 = A/Sing MAP, 5 ug (FA); B6 = A/Sing MAP, 2.5 ug (FA)
B7 = Placebo MAP (FA); B8 = A/Sing MAP, 15 ug (UA); B9 = A/Sing IM, 15 ug, as 2018 Afluria Quad (UA)

Listing 16.2.9.2
Treatment Site Tolerability Assessment per Site

Part B
Randomisation Number = B0029, Treatment Group = Group B6

Date	Time	Visit	Time Point	Site Number	Treatment for Site	Indura-tion	Redness	Bruising	Itching	Skin Flaking	Tender-ness	Definition/ Visibility	Other#	Unscheduled Reason
12-MAY-2018	13:46	Day 1	Pre-dose	F1	uncoated	0	0	0	0	0	0	0		
12-MAY-2018	13:46	Day 1	Pre-dose	F2	uncoated	0	0	0	0	0	0	0		
12-MAY-2018	13:46	Day 1	Pre-dose	F3	2.5 ug	0	0	0	0	0	0	0		
12-MAY-2018	14:29	Day 1	10 min	F1	uncoated	0	0	0	0	0	0	0		
12-MAY-2018	14:29	Day 1	10 min	F2	uncoated	0	0	0	1	0	0	0		
12-MAY-2018	14:29	Day 1	10 min	F3	2.5 ug	0	0	0	1	0	0	0		
12-MAY-2018	15:19	Day 1	1 hour	F1	uncoated	1	0	0	0	0	0	0		
12-MAY-2018	15:19	Day 1	1 hour	F2	uncoated	1	0	0	0	0	0	0		
12-MAY-2018	15:19	Day 1	1 hour	F3	2.5 ug	1	0	0	0	0	0	0		
12-MAY-2018	16:21	Day 1	2 hour	F1	uncoated	0	0	0	0	0	0	0		
12-MAY-2018	16:21	Day 1	2 hour	F2	uncoated	0	0	0	0	0	0	0		
12-MAY-2018	16:21	Day 1	2 hour	F3	2.5 ug	0	0	0	0	0	0	0		
13-MAY-2018	13:42	Day 2		F1	uncoated	0	0	0	0	0	0	0		
13-MAY-2018	13:42	Day 2		F2	uncoated	0	0	0	0	0	0	0		
13-MAY-2018	13:42	Day 2		F3	2.5 ug	1	1	0	0	0	1	0		
15-MAY-2018	14:55	Day 4		F1	uncoated	0	0	0	0	0	0	2		
15-MAY-2018	14:55	Day 4		F2	uncoated	0	0	0	0	0	0	2		
15-MAY-2018	14:55	Day 4		F3	2.5 ug	0	1	0	0	1	0	0		
19-MAY-2018	14:23	Day 8		F1	uncoated	0	0	0	0	1	0	3		
19-MAY-2018	14:23	Day 8		F2	uncoated	0	0	0	0	1	0	3		

Key: 0= None; 1=Mild; 2=Moderate; 3=Severe; # Other = Pinprick bleeding or seepage

Administration Site Key: FA = Forearm; UA= Upper arm
Treatments Part A: A1 = A/Sing MAP, 15 ug (FA); A2 = Afluria Quad IM (UA), 15 ug; A3 = Placebo MAP (FA); A4 = A/Sing Mono IM, 15 ug (UA)
Treatments Part B: B1 = A/Sing MAP, 15 ug (FA) with biopsy; B2 = Placebo MAP (FA) with biopsy
B3 = A/Sing MAP, 15 ug (FA); B4 = A/Sing MAP, 10 ug (FA); B5 = A/Sing MAP, 5 ug (FA); B6 = A/Sing MAP, 2.5 ug (FA)
B7 = Placebo MAP (FA); B8 = A/Sing MAP, 15 ug (UA); B9 = A/Sing IM, 15 ug, as 2018 Afluria Quad (UA)

Listing 16.2.9.2
Treatment Site Tolerability Assessment per Site

Part B
Randomisation Number = B0029, Treatment Group = Group B6

Date	Time	Visit	Time Point	Site Number	Treatment for Site	Indura-tion	Redness	Bruising	Itching	Skin Flaking	Tender-ness	Definition/ Visibility	Other#	Unscheduled Reason
19-MAY-2018	14:23	Day 8		F3	2.5 ug	0	0	0	0	3	0	3		
02-JUN-2018	15:59	Day 22		F1	uncoated	0	0	0	0	0	0	0		
02-JUN-2018	15:59	Day 22		F2	uncoated	0	0	0	0	0	0	0		
02-JUN-2018	15:59	Day 22		F3	2.5 ug	0	0	0	0	0	0	2		
11-JUL-2018	14:54	EOS/ET (Day 61)		F1	uncoated	0	0	0	0	0	0	1		
11-JUL-2018	14:54	EOS/ET (Day 61)		F2	uncoated	0	0	0	0	0	0	1		
11-JUL-2018	14:54	EOS/ET (Day 61)		F3	2.5 ug	0	0	0	0	0	0	1		

Key: 0= None; 1=Mild; 2=Moderate; 3=Severe; # Other = Pinprick bleeding or seepage

Administration Site Key: FA = Forearm; UA= Upper arm
Treatments Part A: A1 = A/Sing MAP, 15 ug (FA); A2 = Afluria Quad IM (UA), 15 ug; A3 = Placebo MAP (FA); A4 = A/Sing Mono IM, 15 ug (UA)
Treatments Part B: B1 = A/Sing MAP, 15 ug (FA) with biopsy; B2 = Placebo MAP (FA) with biopsy
B3 = A/Sing MAP, 15 ug (FA); B4 = A/Sing MAP, 10 ug (FA); B5 = A/Sing MAP, 5 ug (FA); B6 = A/Sing MAP, 2.5 ug (FA)
B7 = Placebo MAP (FA); B8 = A/Sing MAP, 15 ug (UA); B9 = A/Sing IM, 15 ug, as 2018 Afluria Quad (UA)

Listing 16.2.9.2
Treatment Site Tolerability Assessment per Site

Part B
Randomisation Number = B0030, Treatment Group = Group B7

Date	Time	Visit	Time Point	Site Number	Treatment for Site	Indura-tion	Redness	Bruising	Itching	Skin Flaking	Tender-ness	Definition/ Visibility	Other#	Unscheduled Reason
12-MAY-2018	13:45	Day 1	Pre-dose	F1	uncoated	0	0	0	0	0	0	0		
12-MAY-2018	13:45	Day 1	Pre-dose	F2	uncoated	0	0	0	0	0	0	0		
12-MAY-2018	13:45	Day 1	Pre-dose	F3	uncoated	0	0	0	0	0	0	0		
12-MAY-2018	14:18	Day 1	10 min	F1	uncoated	0	2	0	0	0	0	0		
12-MAY-2018	14:18	Day 1	10 min	F2	uncoated	0	2	0	0	0	0	0		
12-MAY-2018	14:18	Day 1	10 min	F3	uncoated	0	2	0	0	0	0	0		
12-MAY-2018	15:08	Day 1	1 hour	F1	uncoated	1	2	0	0	0	0	0		
12-MAY-2018	15:08	Day 1	1 hour	F2	uncoated	1	2	0	0	0	0	0		
12-MAY-2018	15:08	Day 1	1 hour	F3	uncoated	1	2	0	0	0	0	0		
12-MAY-2018	16:08	Day 1	2 hour	F1	uncoated	0	0	0	0	0	0	0		
12-MAY-2018	16:08	Day 1	2 hour	F2	uncoated	0	0	0	0	0	0	0		
12-MAY-2018	16:08	Day 1	2 hour	F3	uncoated	0	0	0	0	0	0	0		
12-MAY-2018	14:31	Unscheduled		F1	uncoated	0	2	0	0	0	0	0	NA	MO notifies as repeat
12-MAY-2018	14:45	Unscheduled		F2	uncoated	0	2	0	0	0	0	0	NA	MO notifies as repeat
12-MAY-2018	14:46	Unscheduled		F3	uncoated	0	2	0	0	0	0	0	NA	MO notifies as repeat
12-MAY-2018	15:16	Unscheduled		F1	uncoated	0	0	0	0	0	0	2	NA	MO notifies as repeat

Key: 0= None; 1=Mild; 2=Moderate; 3=Severe; # Other = Pinprick bleeding or seepage

Administration Site Key: FA = Forearm; UA= Upper arm
Treatments Part A: A1 = A/Sing MAP, 15 ug (FA); A2 = Afluria Quad IM (UA), 15 ug; A3 = Placebo MAP (FA); A4 = A/Sing Mono IM, 15 ug (UA)
Treatments Part B: B1 = A/Sing MAP, 15 ug (FA) with biopsy; B2 = Placebo MAP (FA) with biopsy
B3 = A/Sing MAP, 15 ug (FA); B4 = A/Sing MAP, 10 ug (FA); B5 = A/Sing MAP, 5 ug (FA); B6 = A/Sing MAP, 2.5 ug (FA)
B7 = Placebo MAP (FA); B8 = A/Sing MAP, 15 ug (UA); B9 = A/Sing IM, 15 ug, as 2018 Afluria Quad (UA)

Listing 16.2.9.2
Treatment Site Tolerability Assessment per Site

Part B
Randomisation Number = B0030, Treatment Group = Group B7

Date	Time	Visit	Time Point	Site Number	Treatment for Site	Indura-tion	Redness	Bruising	Itching	Skin Flaking	Tender-ness	Definition/ Visibility	Other#	Unscheduled Reason
12-MAY-2018	15:22	Unscheduled		F2	uncoated	1	1	0	0	0	0	2	NA	MO notifies as repeat
12-MAY-2018	15:24	Unscheduled		F3	uncoated	0	1	0	0	0	0	1	NA	MO notifies as repeat
13-MAY-2018	13:48	Day 2		F1	uncoated	0	0	0	0	0	0	0		
13-MAY-2018	13:48	Day 2		F2	uncoated	0	0	0	0	0	0	0		
13-MAY-2018	13:48	Day 2		F3	uncoated	0	0	0	0	0	0	0		
15-MAY-2018	13:59	Day 4		F1	uncoated	0	0	0	0	0	0	2		
15-MAY-2018	13:59	Day 4		F2	uncoated	0	0	0	0	0	0	2		
15-MAY-2018	13:59	Day 4		F3	uncoated	0	0	0	0	0	0	2		
19-MAY-2018	16:41	Day 8		F1	uncoated	0	0	0	0	0	0	2		
19-MAY-2018	16:41	Day 8		F2	uncoated	0	0	0	0	0	0	2		
19-MAY-2018	16:41	Day 8		F3	uncoated	0	0	0	0	0	0	2		
02-JUN-2018	15:26	Day 22		F1	uncoated	0	0	0	0	0	0	2		
02-JUN-2018	15:26	Day 22		F2	uncoated	0	0	0	0	0	0	2		
02-JUN-2018	15:26	Day 22		F3	uncoated	0	0	0	0	0	0	1		
13-JUL-2018	10:47	EOS/ET (Day 61)		F1	uncoated	0	0	0	0	0	0	0		
13-JUL-2018	10:47	EOS/ET (Day 61)		F2	uncoated	0	0	0	0	0	0	0		
13-JUL-2018	10:47	EOS/ET (Day 61)		F3	uncoated	0	0	0	0	0	0	0		

Key: 0= None; 1=Mild; 2=Moderate; 3=Severe; # Other = Pinprick bleeding or seepage

Administration Site Key: FA = Forearm; UA= Upper arm
Treatments Part A: A1 = A/Sing MAP, 15 ug (FA); A2 = Afluria Quad IM (UA), 15 ug; A3 = Placebo MAP (FA); A4 = A/Sing Mono IM, 15 ug (UA)
Treatments Part B: B1 = A/Sing MAP, 15 ug (FA) with biopsy; B2 = Placebo MAP (FA) with biopsy
B3 = A/Sing MAP, 15 ug (FA); B4 = A/Sing MAP, 10 ug (FA); B5 = A/Sing MAP, 5 ug (FA); B6 = A/Sing MAP, 2.5 ug (FA)
B7 = Placebo MAP (FA); B8 = A/Sing MAP, 15 ug (UA); B9 = A/Sing IM, 15 ug, as 2018 Afluria Quad (UA)

Listing 16.2.9.2
Treatment Site Tolerability Assessment per Site

Part B
Randomisation Number = B0031, Treatment Group = Group B4

Date	Time	Visit	Time Point	Site Number	Treatment for Site	Induration	Redness	Bruising	Itching	Skin Flaking	Tenderness	Definition/ Visibility	Other#	Unscheduled Reason
19-MAY-2018	12:59	Day 1	Pre-dose	F1	uncoated	0	0	0	0	0	0	0		
19-MAY-2018	12:59	Day 1	Pre-dose	F2	5 ug	0	0	0	0	0	0	0		
19-MAY-2018	12:59	Day 1	Pre-dose	F3	5 ug	0	0	0	0	0	0	0		
19-MAY-2018	13:38	Day 1	10 min	F1	uncoated	0	0	0	0	0	0	0		
19-MAY-2018	13:38	Day 1	10 min	F2	5 ug	0	2	0	0	0	0	0		
19-MAY-2018	13:38	Day 1	10 min	F3	5 ug	0	2	0	0	0	0	0		
19-MAY-2018	14:28	Day 1	1 hour	F1	uncoated	0	0	0	0	0	0	0		
19-MAY-2018	14:28	Day 1	1 hour	F2	5 ug	0	1	0	0	0	0	0		
19-MAY-2018	14:28	Day 1	1 hour	F3	5 ug	0	1	0	0	0	0	0		
19-MAY-2018	15:28	Day 1	2 hour	F1	uncoated	0	0	0	0	0	0	0		
19-MAY-2018	15:28	Day 1	2 hour	F2	5 ug	0	1	0	0	0	0	0		
19-MAY-2018	15:28	Day 1	2 hour	F3	5 ug	0	1	0	0	0	0	0		
19-MAY-2018	14:15	Unscheduled		F1	uncoated	0	0	0	0	0	0	0	NA	MO notifies as repeat
19-MAY-2018	14:15	Unscheduled		F2	5 ug	0	2	0	0	0	0	0	NA	MO notifies as repeat
19-MAY-2018	14:15	Unscheduled		F3	5 ug	1	2	0	0	0	0	0	NA	MO notifies as repeat
20-MAY-2018	13:10	Day 2		F1	uncoated	0	0	0	0	0	0	0		
20-MAY-2018	13:10	Day 2		F2	5 ug	2	1	0	0	0	0	0		

Key: 0= None; 1=Mild; 2=Moderate; 3=Severe; # Other = Pinprick bleeding or seepage

Administration Site Key: FA = Forearm; UA= Upper arm
Treatments Part A: A1 = A/Sing MAP, 15 ug (FA); A2 = Afluria Quad IM (UA), 15 ug; A3 = Placebo MAP (FA); A4 = A/Sing Mono IM, 15 ug (UA)
Treatments Part B: B1 = A/Sing MAP, 15 ug (FA) with biopsy; B2 = Placebo MAP (FA) with biopsy
B3 = A/Sing MAP, 15 ug (FA); B4 = A/Sing MAP, 10 ug (FA); B5 = A/Sing MAP, 5 ug (FA); B6 = A/Sing MAP, 2.5 ug (FA)
B7 = Placebo MAP (FA); B8 = A/Sing MAP, 15 ug (UA); B9 = A/Sing IM, 15 ug, as 2018 Afluria Quad (UA)

Listing 16.2.9.2
Treatment Site Tolerability Assessment per Site

Part B
Randomisation Number = B0031, Treatment Group = Group B4

Date	Time	Visit	Time Point	Site Number	Treatment for Site	Indura-tion	Redness	Bruising	Itching	Skin Flaking	Tender-ness	Definition/ Visibility	Other#	Unscheduled Reason
20-MAY-2018	13:10	Day 2		F3	5 ug	2	1	0	0	0	0	0		
20-MAY-2018	13:19	Unscheduled		F2	5 ug	2	1	0	0	0	0	0	NA	MO notifies as repeat
20-MAY-2018	13:19	Unscheduled		F3	5 ug	2	1	0	0	0	0	0	NA	MO notifies as repeat
22-MAY-2018	13:08	Day 4		F1	uncoated	0	0	0	0	0	0	0		
22-MAY-2018	13:08	Day 4		F2	5 ug	0	1	0	0	0	0	0		
22-MAY-2018	13:08	Day 4		F3	5 ug	0	1	0	0	0	0	0		
26-MAY-2018	11:07	Day 8		F1	uncoated	0	0	0	0	0	0	1		
26-MAY-2018	11:07	Day 8		F2	5 ug	0	0	0	0	1	0	1		
26-MAY-2018	11:07	Day 8		F3	5 ug	0	0	0	0	1	0	3		
08-JUN-2018	13:52	Day 22		F1	uncoated	0	0	0	0	0	0	1		
08-JUN-2018	13:52	Day 22		F2	5 ug	0	0	0	0	0	0	1		
08-JUN-2018	13:52	Day 22		F3	5 ug	0	0	0	0	0	0	1		
18-JUL-2018	10:35	EOS/ET (Day 61)		F1	uncoated	0	0	0	0	0	0	0		
18-JUL-2018	10:35	EOS/ET (Day 61)		F2	5 ug	0	0	0	0	0	0	0		
18-JUL-2018	10:35	EOS/ET (Day 61)		F3	5 ug	0	0	0	0	0	0	0		

Key: 0= None; 1=Mild; 2=Moderate; 3=Severe; # Other = Pinprick bleeding or seepage

Administration Site Key: FA = Forearm; UA= Upper arm
Treatments Part A: A1 = A/Sing MAP, 15 ug (FA); A2 = Afluria Quad IM (UA), 15 ug; A3 = Placebo MAP (FA); A4 = A/Sing Mono IM, 15 ug (UA)
Treatments Part B: B1 = A/Sing MAP, 15 ug (FA) with biopsy; B2 = Placebo MAP (FA) with biopsy
B3 = A/Sing MAP, 15 ug (FA); B4 = A/Sing MAP, 10 ug (FA); B5 = A/Sing MAP, 5 ug (FA); B6 = A/Sing MAP, 2.5 ug (FA)
B7 = Placebo MAP (FA); B8 = A/Sing MAP, 15 ug (UA); B9 = A/Sing IM, 15 ug, as 2018 Afluria Quad (UA)

Listing 16.2.9.2
Treatment Site Tolerability Assessment per Site

Part B
Randomisation Number = B0032, Treatment Group = Group B6

Date	Time	Visit	Time Point	Site Number	Treatment for Site	Indura-tion	Redness	Bruising	Itching	Skin Flaking	Tender-ness	Definition/ Visibility	Other#	Unscheduled Reason
19-MAY-2018	11:07	Day 1	Pre-dose	F1	uncoated	0	0	0	0	0	0	0		
19-MAY-2018	11:07	Day 1	Pre-dose	F2	uncoated	0	0	0	0	0	0	0		
19-MAY-2018	11:07	Day 1	Pre-dose	F3	2.5 ug	0	0	0	0	0	0	0		
19-MAY-2018	11:58	Day 1	10 min	F1	uncoated	0	0	0	0	0	0	0		
19-MAY-2018	11:58	Day 1	10 min	F2	uncoated	0	0	0	0	0	0	0		
19-MAY-2018	11:58	Day 1	10 min	F3	2.5 ug	0	0	0	0	0	0	0		
19-MAY-2018	12:49	Day 1	1 hour	F1	uncoated	0	0	0	0	0	0	0		
19-MAY-2018	12:49	Day 1	1 hour	F2	uncoated	0	0	0	0	0	0	0		
19-MAY-2018	12:49	Day 1	1 hour	F3	2.5 ug	0	0	0	0	0	0	0		
19-MAY-2018	13:48	Day 1	2 hour	F1	uncoated	0	0	0	0	0	0	0		
19-MAY-2018	13:48	Day 1	2 hour	F2	uncoated	1	0	0	0	0	0	0		
19-MAY-2018	13:48	Day 1	2 hour	F3	2.5 ug	0	0	0	0	0	0	0		
19-MAY-2018	13:07	Unscheduled		F1	uncoated	0	0	0	0	0	0	0	NA	MO notifies as repeat
19-MAY-2018	13:08	Unscheduled		F2	uncoated	0	0	0	0	0	0	0	NA	MO notifies as repeat
20-MAY-2018	11:36	Day 2		F1	uncoated	0	0	0	0	0	0	0		
20-MAY-2018	11:36	Day 2		F2	uncoated	0	0	0	0	0	0	0		
20-MAY-2018	11:36	Day 2		F3	2.5 ug	0	1	0	0	0	0	0		
22-MAY-2018	08:15	Day 4		F1	uncoated	0	0	0	0	0	0	0		

Key: 0= None; 1=Mild; 2=Moderate; 3=Severe; # Other = Pinprick bleeding or seepage

Administration Site Key: FA = Forearm; UA= Upper arm
Treatments Part A: A1 = A/Sing MAP, 15 ug (FA); A2 = Afluria Quad IM (UA), 15 ug; A3 = Placebo MAP (FA); A4 = A/Sing Mono IM, 15 ug (UA)
Treatments Part B: B1 = A/Sing MAP, 15 ug (FA) with biopsy; B2 = Placebo MAP (FA) with biopsy
B3 = A/Sing MAP, 15 ug (FA); B4 = A/Sing MAP, 10 ug (FA); B5 = A/Sing MAP, 5 ug (FA); B6 = A/Sing MAP, 2.5 ug (FA)
B7 = Placebo MAP (FA); B8 = A/Sing MAP, 15 ug (UA); B9 = A/Sing IM, 15 ug, as 2018 Afluria Quad (UA)

Listing 16.2.9.2
Treatment Site Tolerability Assessment per Site

Part B
Randomisation Number = B0032, Treatment Group = Group B6

Date	Time	Visit	Time Point	Site Number	Treatment for Site	Indura-tion	Redness	Bruising	Itching	Skin Flaking	Tender-ness	Definition/ Visibility	Other#	Unscheduled Reason
22-MAY-2018	08:15	Day 4		F2	uncoated	0	0	0	0	0	0	0		
22-MAY-2018	08:15	Day 4		F3	2.5 ug	0	0	0	0	0	0	0		
26-MAY-2018	08:39	Day 8		F1	uncoated	0	0	0	0	0	0	0		
26-MAY-2018	08:39	Day 8		F2	uncoated	0	0	0	0	0	0	0		
26-MAY-2018	08:39	Day 8		F3	2.5 ug	0	0	0	0	0	0	0		
26-MAY-2018	09:03	Unscheduled		F1	uncoated	0	0	0	0	0	0	1	NA	MO notifies as repeat
26-MAY-2018	09:04	Unscheduled		F2	uncoated	0	0	0	0	0	0	1	NA	MO notifies as repeat
26-MAY-2018	09:07	Unscheduled		F2	uncoated	0	0	0	0	0	0	1	NA	MO notifies as repeat
12-JUN-2018	08:23	Day 22		F1	uncoated	0	0	0	0	0	0	0		
12-JUN-2018	08:23	Day 22		F2	uncoated	0	0	0	0	0	0	1		
12-JUN-2018	08:23	Day 22		F3	2.5 ug	0	0	0	0	0	0	1		
18-JUL-2018	08:14	EOS/ET (Day 61)		F1	uncoated	0	0	0	0	0	0	0		
18-JUL-2018	08:14	EOS/ET (Day 61)		F2	uncoated	0	0	0	0	0	0	0		
18-JUL-2018	08:14	EOS/ET (Day 61)		F3	2.5 ug	0	0	0	0	0	0	0		

Key: 0= None; 1=Mild; 2=Moderate; 3=Severe; # Other = Pinprick bleeding or seepage

Administration Site Key: FA = Forearm; UA= Upper arm
Treatments Part A: A1 = A/Sing MAP, 15 ug (FA); A2 = Afluria Quad IM (UA), 15 ug; A3 = Placebo MAP (FA); A4 = A/Sing Mono IM, 15 ug (UA)
Treatments Part B: B1 = A/Sing MAP, 15 ug (FA) with biopsy; B2 = Placebo MAP (FA) with biopsy
B3 = A/Sing MAP, 15 ug (FA); B4 = A/Sing MAP, 10 ug (FA); B5 = A/Sing MAP, 5 ug (FA); B6 = A/Sing MAP, 2.5 ug (FA)
B7 = Placebo MAP (FA); B8 = A/Sing MAP, 15 ug (UA); B9 = A/Sing IM, 15 ug, as 2018 Afluria Quad (UA)

Listing 16.2.9.2
Treatment Site Tolerability Assessment per Site

Part B
Randomisation Number = B0033, Treatment Group = Group B5

Date	Time	Visit	Time Point	Site Number	Treatment for Site	Induration	Redness	Bruising	Itching	Skin Flaking	Tenderness	Definition/ Visibility	Other#	Unscheduled Reason
19-MAY-2018	11:13	Day 1	Pre-dose	F1	uncoated	0	0	0	0	0	0	0		
19-MAY-2018	11:13	Day 1	Pre-dose	F2	uncoated	0	0	0	0	0	0	0		
19-MAY-2018	11:13	Day 1	Pre-dose	F3	5 ug	0	0	0	0	0	0	0		
19-MAY-2018	12:18	Day 1	10 min	F1	uncoated	0	1	1	0	0	2			
19-MAY-2018	12:18	Day 1	10 min	F2	uncoated	0	1	0	0	0	2	0		
19-MAY-2018	12:18	Day 1	10 min	F3	5 ug	0	1	0	0	0	1	0		
19-MAY-2018	13:08	Day 1	1 hour	F1	uncoated	0	0	0	0	0	0	0		
19-MAY-2018	13:08	Day 1	1 hour	F2	uncoated	0	0	0	0	0	0	0		
19-MAY-2018	13:08	Day 1	1 hour	F3	5 ug	0	1	0	0	0	0	0		
19-MAY-2018	14:08	Day 1	2 hour	F1	uncoated	0	0	0	0	0	0	0		
19-MAY-2018	14:08	Day 1	2 hour	F2	uncoated	1	0	0	0	0	0	0		
19-MAY-2018	14:08	Day 1	2 hour	F3	5 ug	1	1	0	0	0	0	0		
19-MAY-2018	12:32	Unscheduled		F1	uncoated	0	0	0	0	0	1	0	NA	MO notifies as repeat
19-MAY-2018	12:33	Unscheduled		F2	uncoated	0	0	0	0	0	1	0	NA	MO notifies as repeat
19-MAY-2018	12:34	Unscheduled		F2	uncoated	0	0	0	0	0	1	0	NA	MO notifies as repeat
19-MAY-2018	13:26	Unscheduled		F1	uncoated	0	0	0	0	0	1	0	NA	MO notifies as repeat

Key: 0= None; 1=Mild; 2=Moderate; 3=Severe; # Other = Pinprick bleeding or seepage

Administration Site Key: FA = Forearm; UA= Upper arm
Treatments Part A: A1 = A/Sing MAP, 15 ug (FA); A2 = Afluria Quad IM (UA), 15 ug; A3 = Placebo MAP (FA); A4 = A/Sing Mono IM, 15 ug (UA)
Treatments Part B: B1 = A/Sing MAP, 15 ug (FA) with biopsy; B2 = Placebo MAP (FA) with biopsy
B3 = A/Sing MAP, 15 ug (FA); B4 = A/Sing MAP, 10 ug (FA); B5 = A/Sing MAP, 5 ug (FA); B6 = A/Sing MAP, 2.5 ug (FA)
B7 = Placebo MAP (FA); B8 = A/Sing MAP, 15 ug (UA); B9 = A/Sing IM, 15 ug, as 2018 Afluria Quad (UA)

Listing 16.2.9.2
Treatment Site Tolerability Assessment per Site

Part B
Randomisation Number = B0033, Treatment Group = Group B5

Date	Time	Visit	Time Point	Site Number	Treatment for Site	Indura-tion	Redness	Bruising	Itching	Skin Flaking	Tender-ness	Definition/ Visibility	Other#	Unscheduled Reason
19-MAY-2018	13:27	Unscheduled		F2	uncoated	0	0	0	0	0	0	0	NA	MO notifies as repeat
19-MAY-2018	13:28	Unscheduled		F3	5 ug	0	1	0	0	0	0	0	NA	MO notifies as repeat
20-MAY-2018	11:46	Day 2		F1	uncoated	0	0	0	0	0	0	0		
20-MAY-2018	11:46	Day 2		F2	uncoated	0	0	0	0	0	0	0		
20-MAY-2018	11:46	Day 2		F3	5 ug	0	1	0	0	0	0	0		
22-MAY-2018	12:14	Day 4		F1	uncoated	0	0	0	0	0	0	0		
22-MAY-2018	12:14	Day 4		F2	uncoated	0	0	0	0	0	0	0		
22-MAY-2018	12:14	Day 4		F3	5 ug	0	1	0	1	0	0	0		
26-MAY-2018	11:13	Day 8		F1	uncoated	0	0	0	0	0	0	0		
26-MAY-2018	11:13	Day 8		F2	uncoated	0	0	0	0	0	0	0		
26-MAY-2018	11:13	Day 8		F3	5 ug	0	0	0	0	0	0	0		
09-JUN-2018	14:00	Day 22		F1	uncoated	0	0	0	0	0	0	0		
09-JUN-2018	14:00	Day 22		F2	uncoated	0	0	0	0	0	0	0		
09-JUN-2018	14:00	Day 22		F3	5 ug	0	0	0	0	0	0	2		
18-JUL-2018	13:40	EOS/ET (Day 61)		F1	uncoated	0	0	0	0	0	0	0		
18-JUL-2018	13:40	EOS/ET (Day 61)		F2	uncoated	0	0	0	0	0	0	0		
18-JUL-2018	13:40	EOS/ET (Day 61)		F3	5 ug	0	0	0	0	0	0	1		

Key: 0= None; 1=Mild; 2=Moderate; 3=Severe; # Other = Pinprick bleeding or seepage

Administration Site Key: FA = Forearm; UA= Upper arm
Treatments Part A: A1 = A/Sing MAP, 15 ug (FA); A2 = Afluria Quad IM (UA), 15 ug; A3 = Placebo MAP (FA); A4 = A/Sing Mono IM, 15 ug (UA)
Treatments Part B: B1 = A/Sing MAP, 15 ug (FA) with biopsy; B2 = Placebo MAP (FA) with biopsy
B3 = A/Sing MAP, 15 ug (FA); B4 = A/Sing MAP, 10 ug (FA); B5 = A/Sing MAP, 5 ug (FA); B6 = A/Sing MAP, 2.5 ug (FA)
B7 = Placebo MAP (FA); B8 = A/Sing MAP, 15 ug (UA); B9 = A/Sing IM, 15 ug, as 2018 Afluria Quad (UA)

Listing 16.2.9.2
Treatment Site Tolerability Assessment per Site

Part B
Randomisation Number = B0034, Treatment Group = Group B8

Date	Time	Visit	Time Point	Site Number	Treatment for Site	Induration	Redness	Bruising	Itching	Skin Flaking	Tenderness	Definition/ Visibility	Other#	Unscheduled Reason
19-MAY-2018	11:22	Day 1	Pre-dose	D1	5 ug	0	0	0	0	0	0	0		
19-MAY-2018	11:22	Day 1	Pre-dose	D2	5 ug	0	0	0	0	0	0	0		
19-MAY-2018	11:22	Day 1	Pre-dose	D3	5 ug	0	0	0	0	0	0	0		
19-MAY-2018	14:18	Day 1	10 min	D1	5 ug	0	1	0	0	0	1	0		
19-MAY-2018	14:18	Day 1	10 min	D2	5 ug	0	0	0	0	0	1	0		
19-MAY-2018	14:18	Day 1	10 min	D3	5 ug	0	0	0	0	0	1	0		
19-MAY-2018	15:08	Day 1	1 hour	D1	5 ug	0	0	0	0	0	0	0		
19-MAY-2018	15:08	Day 1	1 hour	D2	5 ug	0	0	0	0	0	0	0		
19-MAY-2018	15:08	Day 1	1 hour	D3	5 ug	0	0	0	0	0	0	0		
19-MAY-2018	16:08	Day 1	2 hour	D1	5 ug	1	0	0	0	0	0	0		
19-MAY-2018	16:08	Day 1	2 hour	D2	5 ug	1	0	0	0	0	0	0		
19-MAY-2018	16:08	Day 1	2 hour	D3	5 ug	1	0	0	0	0	0	0		
20-MAY-2018	13:52	Day 2		D1	5 ug	1	1	0	0	0	0	0		
20-MAY-2018	13:52	Day 2		D2	5 ug	0	0	0	0	0	0	0		
20-MAY-2018	13:52	Day 2		D3	5 ug	1	1	0	0	0	0	0		
22-MAY-2018	14:54	Day 4		D1	5 ug	0	0	0	0	1	0	0		
22-MAY-2018	14:54	Day 4		D2	5 ug	0	0	0	0	1	0	0		
22-MAY-2018	14:54	Day 4		D3	5 ug	0	0	0	0	1	0	0		
26-MAY-2018	11:33	Day 8		D1	5 ug	0	0	0	0	2	0	2		
26-MAY-2018	11:33	Day 8		D2	5 ug	0	0	0	0	1	0	1		

Key: 0= None; 1=Mild; 2=Moderate; 3=Severe; # Other = Pinprick bleeding or seepage

Administration Site Key: FA = Forearm; UA= Upper arm
Treatments Part A: A1 = A/Sing MAP, 15 ug (FA); A2 = Afluria Quad IM (UA), 15 ug; A3 = Placebo MAP (FA); A4 = A/Sing Mono IM, 15 ug (UA)
Treatments Part B: B1 = A/Sing MAP, 15 ug (FA) with biopsy; B2 = Placebo MAP (FA) with biopsy
B3 = A/Sing MAP, 15 ug (FA); B4 = A/Sing MAP, 10 ug (FA); B5 = A/Sing MAP, 5 ug (FA); B6 = A/Sing MAP, 2.5 ug (FA)
B7 = Placebo MAP (FA); B8 = A/Sing MAP, 15 ug (UA); B9 = A/Sing IM, 15 ug, as 2018 Afluria Quad (UA)

Listing 16.2.9.2
Treatment Site Tolerability Assessment per Site

Part B
Randomisation Number = B0034, Treatment Group = Group B8

Date	Time	Visit	Time Point	Site Number	Treatment for Site	Indura-tion	Redness	Bruising	Itching	Skin Flaking	Tender-ness	Definition/ Visibility	Other#	Unscheduled Reason
26-MAY-2018	11:33	Day 8		D3	5 ug	0	0	0	0	1	0	1		
09-JUN-2018	12:50	Day 22		D1	5 ug	0	0	0	0	0	0	1		
09-JUN-2018	12:50	Day 22		D2	5 ug	0	0	0	0	0	0	1		
09-JUN-2018	12:50	Day 22		D3	5 ug	0	0	0	0	0	0	1		
18-JUL-2018	10:54	EOS/ET (Day 61)		D1	5 ug	0	0	0	0	0	0	0		
18-JUL-2018	10:54	EOS/ET (Day 61)		D2	5 ug	0	0	0	0	0	0	0		
18-JUL-2018	10:54	EOS/ET (Day 61)		D3	5 ug	0	0	0	0	0	0	0		

Key: 0= None; 1=Mild; 2=Moderate; 3=Severe; # Other = Pinprick bleeding or seepage

Administration Site Key: FA = Forearm; UA= Upper arm
Treatments Part A: A1 = A/Sing MAP, 15 ug (FA); A2 = Afluria Quad IM (UA), 15 ug; A3 = Placebo MAP (FA); A4 = A/Sing Mono IM, 15 ug (UA)
Treatments Part B: B1 = A/Sing MAP, 15 ug (FA) with biopsy; B2 = Placebo MAP (FA) with biopsy
B3 = A/Sing MAP, 15 ug (FA); B4 = A/Sing MAP, 10 ug (FA); B5 = A/Sing MAP, 5 ug (FA); B6 = A/Sing MAP, 2.5 ug (FA)
B7 = Placebo MAP (FA); B8 = A/Sing MAP, 15 ug (UA); B9 = A/Sing IM, 15 ug, as 2018 Afluria Quad (UA)

Listing 16.2.9.2
Treatment Site Tolerability Assessment per Site

Part B
Randomisation Number = B0035, Treatment Group = Group B9

Date	Time	Visit	Time Point	Site Number	Treatment for Site	Induration	Redness	Bruising	Itching	Skin Flaking	Tenderness	Definition/ Visibility	Other#	Unscheduled Reason
19-MAY-2018	10:50	Day 1	Pre-dose	D1	[IM]	0	0	0	0	0	0			
19-MAY-2018	11:30	Day 1	10 min	D1	[IM]	0	1	0	0	0	0			
19-MAY-2018	12:21	Day 1	1 hour	D1	[IM]	0	0	0	0	0	0			
19-MAY-2018	13:25	Day 1	2 hour	D1	[IM]	0	1	0	0	0	0			
20-MAY-2018	11:03	Day 2		D1	[IM]	0	0	0	0	0	0			
22-MAY-2018	12:47	Day 4		D1	[IM]	0	0	0	0	0	0			
26-MAY-2018	13:12	Day 8		D1	[IM]	0	0	0	0	0	0			
09-JUN-2018	13:52	Day 22		D1	[IM]	0	0	0	0	0	0			
18-JUL-2018	08:18	EOS/ET (Day 61)		D1	[IM]	0	0	0	0	0	0	5		

Key: 0= None; 1=Mild; 2=Moderate; 3=Severe; # Other = Pinprick bleeding or seepage

Administration Site Key: FA = Forearm; UA= Upper arm
Treatments Part A: A1 = A/Sing MAP, 15 ug (FA); A2 = Afluria Quad IM (UA), 15 ug; A3 = Placebo MAP (FA); A4 = A/Sing Mono IM, 15 ug (UA)
Treatments Part B: B1 = A/Sing MAP, 15 ug (FA) with biopsy; B2 = Placebo MAP (FA) with biopsy
B3 = A/Sing MAP, 15 ug (FA); B4 = A/Sing MAP, 10 ug (FA); B5 = A/Sing MAP, 5 ug (FA); B6 = A/Sing MAP, 2.5 ug (FA)
B7 = Placebo MAP (FA); B8 = A/Sing MAP, 15 ug (UA); B9 = A/Sing IM, 15 ug, as 2018 Afluria Quad (UA)

Listing 16.2.9.2
Treatment Site Tolerability Assessment per Site

Part B
Randomisation Number = B0036, Treatment Group = Group B7

Date	Time	Visit	Time Point	Site Number	Treatment for Site	Indura-tion	Redness	Bruising	Itching	Skin Flaking	Tender-ness	Definition/ Visibility	Other#	Unscheduled Reason
19-MAY-2018	11:30	Day 1	Pre-dose	F1	uncoated	0	0	0	0	0	0	0		
19-MAY-2018	11:30	Day 1	Pre-dose	F2	uncoated	0	0	0	0	0	0	0		
19-MAY-2018	11:30	Day 1	Pre-dose	F3	uncoated	0	0	0	0	0	0	0		
19-MAY-2018	12:30	Day 1	10 min	F1	uncoated	0	0	0	0	0	0	0		
19-MAY-2018	12:30	Day 1	10 min	F2	uncoated	0	0	0	0	0	1	0		
19-MAY-2018	12:30	Day 1	10 min	F3	uncoated	0	0	0	0	0	2	0		
19-MAY-2018	13:20	Day 1	1 hour	F1	uncoated	0	0	0	0	0	0	0		
19-MAY-2018	13:20	Day 1	1 hour	F2	uncoated	0	0	0	0	0	0	0		
19-MAY-2018	13:20	Day 1	1 hour	F3	uncoated	0	0	0	0	0	0	0		
19-MAY-2018	14:20	Day 1	2 hour	F1	uncoated	0	0	0	0	0	0	0		
19-MAY-2018	14:20	Day 1	2 hour	F2	uncoated	0	0	0	0	0	0	0		
19-MAY-2018	14:20	Day 1	2 hour	F3	uncoated	0	0	0	0	0	0	0		
19-MAY-2018	12:54	Unscheduled		F1	uncoated	0	0	0	0	0	0	0	NA	MO notifies as repeat
19-MAY-2018	12:55	Unscheduled		F2	uncoated	0	0	0	0	0	1	0	NA	MO notifies as repeat
19-MAY-2018	12:56	Unscheduled		F3	uncoated	0	0	0	0	0	1	0	NA	MO notifies as repeat
20-MAY-2018	11:40	Day 2		F1	uncoated	0	0	0	0	0	0	0		
20-MAY-2018	11:40	Day 2		F2	uncoated	0	0	0	0	0	0	0		

Key: 0= None; 1=Mild; 2=Moderate; 3=Severe; # Other = Pinprick bleeding or seepage

Administration Site Key: FA = Forearm; UA= Upper arm
Treatments Part A: A1 = A/Sing MAP, 15 ug (FA); A2 = Afluria Quad IM (UA), 15 ug; A3 = Placebo MAP (FA); A4 = A/Sing Mono IM, 15 ug (UA)
Treatments Part B: B1 = A/Sing MAP, 15 ug (FA) with biopsy; B2 = Placebo MAP (FA) with biopsy
B3 = A/Sing MAP, 15 ug (FA); B4 = A/Sing MAP, 10 ug (FA); B5 = A/Sing MAP, 5 ug (FA); B6 = A/Sing MAP, 2.5 ug (FA)
B7 = Placebo MAP (FA); B8 = A/Sing MAP, 15 ug (UA); B9 = A/Sing IM, 15 ug, as 2018 Afluria Quad (UA)

Listing 16.2.9.2
Treatment Site Tolerability Assessment per Site

Part B
Randomisation Number = B0036, Treatment Group = Group B7

Date	Time	Visit	Time Point	Site Number	Treatment for Site	Indura-tion	Redness	Bruising	Itching	Skin Flaking	Tender-ness	Definition/ Visibility	Other#	Unscheduled Reason
20-MAY-2018	11:40	Day 2		F3	uncoated	0	0	0	0	0	0	0		
22-MAY-2018	12:38	Day 4		F1	uncoated	0	0	0	0	0	1	0		
22-MAY-2018	12:38	Day 4		F2	uncoated	0	0	0	0	0	1	0		
22-MAY-2018	12:38	Day 4		F3	uncoated	0	0	0	0	0	1	0		
26-MAY-2018	10:52	Day 8		F1	uncoated	0	0	0	0	0	0	1		
26-MAY-2018	10:52	Day 8		F2	uncoated	0	0	0	0	0	0	1		
26-MAY-2018	10:52	Day 8		F3	uncoated	0	0	0	0	0	0	1		
09-JUN-2018	11:39	Day 22		F1	uncoated	0	0	0	0	0	0	0		
09-JUN-2018	11:39	Day 22		F2	uncoated	0	0	0	0	0	0	0		
09-JUN-2018	11:39	Day 22		F3	uncoated	0	0	0	0	0	0	0		
18-JUL-2018	10:22	EOS/ET (Day 61)		F1	uncoated	0	0	0	0	0	0	0		
18-JUL-2018	10:22	EOS/ET (Day 61)		F2	uncoated	0	0	0	0	0	0	0		
18-JUL-2018	10:22	EOS/ET (Day 61)		F3	uncoated	0	0	0	0	0	0	0		

Key: 0= None; 1=Mild; 2=Moderate; 3=Severe; # Other = Pinprick bleeding or seepage

Administration Site Key: FA = Forearm; UA= Upper arm
Treatments Part A: A1 = A/Sing MAP, 15 ug (FA); A2 = Afluria Quad IM (UA), 15 ug; A3 = Placebo MAP (FA); A4 = A/Sing Mono IM, 15 ug (UA)
Treatments Part B: B1 = A/Sing MAP, 15 ug (FA) with biopsy; B2 = Placebo MAP (FA) with biopsy
B3 = A/Sing MAP, 15 ug (FA); B4 = A/Sing MAP, 10 ug (FA); B5 = A/Sing MAP, 5 ug (FA); B6 = A/Sing MAP, 2.5 ug (FA)
B7 = Placebo MAP (FA); B8 = A/Sing MAP, 15 ug (UA); B9 = A/Sing IM, 15 ug, as 2018 Afluria Quad (UA)

Listing 16.2.9.2
Treatment Site Tolerability Assessment per Site

Part B
Randomisation Number = B0037, Treatment Group = Group B4

Date	Time	Visit	Time Point	Site Number	Treatment for Site	Indura-tion	Redness	Bruising	Itching	Skin Flaking	Tender-ness	Definition/ Visibility	Other#	Unscheduled Reason
19-MAY-2018	11:38	Day 1	Pre-dose	F1	uncoated	0	0	0	0	0	0	0		
19-MAY-2018	11:38	Day 1	Pre-dose	F2	5 ug	0	0	0	0	0	0	0		
19-MAY-2018	11:38	Day 1	Pre-dose	F3	5 ug	0	0	0	0	0	0	0		
19-MAY-2018	12:39	Day 1	10 min	F1	uncoated	0	1	0	0	0	0	0		
19-MAY-2018	12:39	Day 1	10 min	F2	5 ug	0	2	0	0	0	0	0		
19-MAY-2018	12:39	Day 1	10 min	F3	5 ug	0	2	0	0	0	0	0		
19-MAY-2018	13:31	Day 1	1 hour	F1	uncoated	0	1	0	0	0	0	0		
19-MAY-2018	13:31	Day 1	1 hour	F2	5 ug	0	2	0	0	0	0	0		
19-MAY-2018	13:31	Day 1	1 hour	F3	5 ug	0	2	0	0	0	0	0		
19-MAY-2018	14:28	Day 1	2 hour	F1	uncoated	0	0	0	0	0	0	0		
19-MAY-2018	14:28	Day 1	2 hour	F2	5 ug	0	1	0	0	0	0	0		
19-MAY-2018	14:28	Day 1	2 hour	F3	5 ug	0	1	0	0	1	0	0		
19-MAY-2018	13:01	Unscheduled		F1	uncoated	0	1	0	0	0	0	0	NA	MO notifies as repeat
19-MAY-2018	13:02	Unscheduled		F2	5 ug	0	2	0	0	0	0	0	NA	MO notifies as repeat
19-MAY-2018	13:03	Unscheduled		F3	5 ug	0	2	0	0	0	0	0	NA	MO notifies as repeat
19-MAY-2018	13:52	Unscheduled		F1	uncoated	1	1	0	0	0	0	0	NA	MO notifies as repeat

Key: 0= None; 1=Mild; 2=Moderate; 3=Severe; # Other = Pinprick bleeding or seepage

Administration Site Key: FA = Forearm; UA= Upper arm
Treatments Part A: A1 = A/Sing MAP, 15 ug (FA); A2 = Afluria Quad IM (UA), 15 ug; A3 = Placebo MAP (FA); A4 = A/Sing Mono IM, 15 ug (UA)
Treatments Part B: B1 = A/Sing MAP, 15 ug (FA) with biopsy; B2 = Placebo MAP (FA) with biopsy
B3 = A/Sing MAP, 15 ug (FA); B4 = A/Sing MAP, 10 ug (FA); B5 = A/Sing MAP, 5 ug (FA); B6 = A/Sing MAP, 2.5 ug (FA)
B7 = Placebo MAP (FA); B8 = A/Sing MAP, 15 ug (UA); B9 = A/Sing IM, 15 ug, as 2018 Afluria Quad (UA)

Listing 16.2.9.2
Treatment Site Tolerability Assessment per Site

Part B
Randomisation Number = B0037, Treatment Group = Group B4

Date	Time	Visit	Time Point	Site Number	Treatment for Site	Indura-tion	Redness	Bruising	Itching	Skin Flaking	Tender-ness	Definition/ Visibility	Other#	Unscheduled Reason
19-MAY-2018	13:52	Unscheduled		F2	5 ug	1	2	0	0	0	0	0	NA	MO notifies as repeat
19-MAY-2018	13:52	Unscheduled		F3	5 ug	1	2	0	0	0	0	0	NA	MO notifies as repeat
20-MAY-2018	12:00	Day 2		F1	uncoated	0	0	0	0	0	0	0		
20-MAY-2018	12:00	Day 2		F2	5 ug	1	1	0	0	0	0	0		
20-MAY-2018	12:00	Day 2		F3	5 ug	0	1	0	0	0	0	0		
22-MAY-2018	12:11	Day 4		F1	uncoated	0	0	0	0	0	0	2		
22-MAY-2018	12:11	Day 4		F2	5 ug	1	2	0	1	0	0	0		
22-MAY-2018	12:11	Day 4		F3	5 ug	1	1		1	0	0	0		
26-MAY-2018	10:54	Day 8		F1	uncoated	0	0	0	0	0	0	0		
26-MAY-2018	10:54	Day 8		F2	5 ug	0	0	0	0	1				
26-MAY-2018	10:54	Day 8		F3	5 ug	0	0	0	1	1	0	0		
09-JUN-2018	14:04	Day 22		F1	uncoated	0	0	0	0	0	0	0		
09-JUN-2018	14:04	Day 22		F2	5 ug	0	0	0	0	1	0	1		
09-JUN-2018	14:04	Day 22		F3	5 ug	0	0	0	0	1	0	1		
18-JUL-2018	15:17	EOS/ET (Day 61)		F1	uncoated	0	0	0	0	0	0	0		
18-JUL-2018	15:17	EOS/ET (Day 61)		F2	5 ug	0	0	0	0	0	0	1		
18-JUL-2018	15:17	EOS/ET (Day 61)		F3	5 ug	0	0	0	0	0	0	1		

Key: 0= None; 1=Mild; 2=Moderate; 3=Severe; # Other = Pinprick bleeding or seepage

Administration Site Key: FA = Forearm; UA= Upper arm
Treatments Part A: A1 = A/Sing MAP, 15 ug (FA); A2 = Afluria Quad IM (UA), 15 ug; A3 = Placebo MAP (FA); A4 = A/Sing Mono IM, 15 ug (UA)
Treatments Part B: B1 = A/Sing MAP, 15 ug (FA) with biopsy; B2 = Placebo MAP (FA) with biopsy
B3 = A/Sing MAP, 15 ug (FA); B4 = A/Sing MAP, 10 ug (FA); B5 = A/Sing MAP, 5 ug (FA); B6 = A/Sing MAP, 2.5 ug (FA)
B7 = Placebo MAP (FA); B8 = A/Sing MAP, 15 ug (UA); B9 = A/Sing IM, 15 ug, as 2018 Afluria Quad (UA)

Listing 16.2.9.2
Treatment Site Tolerability Assessment per Site

Part B
Randomisation Number = B0038, Treatment Group = Group B3

Date	Time	Visit	Time Point	Site Number	Treatment for Site	Induration	Redness	Bruising	Itching	Skin Flaking	Tenderness	Definition/ Visibility	Other#	Unscheduled Reason
19-MAY-2018	12:04	Day 1	Pre-dose	F1	5 ug	0	0	0	0	0	0	0		
19-MAY-2018	12:04	Day 1	Pre-dose	F2	5 ug	0	0	0	0	0	0	0		
19-MAY-2018	12:04	Day 1	Pre-dose	F3	5 ug	0	0	0	0	0	0	0		
19-MAY-2018	12:58	Day 1	10 min	F1	5 ug	0	1	0	0	0	0	0		
19-MAY-2018	12:58	Day 1	10 min	F2	5 ug	0	1	0	0	0	0	0		
19-MAY-2018	12:58	Day 1	10 min	F3	5 ug	0	1	0	0	0	0	0		
19-MAY-2018	13:46	Day 1	1 hour	F1	5 ug	1	1	0	0	0	0	0		
19-MAY-2018	13:46	Day 1	1 hour	F2	5 ug	1	1	0	0	0	0	0		
19-MAY-2018	13:46	Day 1	1 hour	F3	5 ug	0	1	0	0	0	0	0		
19-MAY-2018	14:41	Day 1	2 hour	F1	5 ug	1	0	0	0	0	0	0		
19-MAY-2018	14:41	Day 1	2 hour	F2	5 ug	0	0	0	0	0	0	0		
19-MAY-2018	14:41	Day 1	2 hour	F3	5 ug	0	0	0	0	0	0	0		
20-MAY-2018	08:23	Day 2		F1	5 ug	1	1	0	0	0	1	0		
20-MAY-2018	08:23	Day 2		F2	5 ug	1	1	0	0	0	0	0		
20-MAY-2018	08:23	Day 2		F3	5 ug	0	0	0	0	0	0	0		
22-MAY-2018	09:02	Day 4		F1	5 ug	1	0	0	0	0	0	0		
22-MAY-2018	09:02	Day 4		F2	5 ug	1	0	0	0	0	0	0		
22-MAY-2018	09:02	Day 4		F3	5 ug	1	0	0	0	0	0	0		
26-MAY-2018	09:21	Day 8		F1	5 ug	0	0	0	0	0	0	0		
26-MAY-2018	09:21	Day 8		F2	5 ug	0	0	0	0	0	0	0		

Key: 0= None; 1=Mild; 2=Moderate; 3=Severe; # Other = Pinprick bleeding or seepage

Administration Site Key: FA = Forearm; UA= Upper arm
Treatments Part A: A1 = A/Sing MAP, 15 ug (FA); A2 = Afluria Quad IM (UA), 15 ug; A3 = Placebo MAP (FA); A4 = A/Sing Mono IM, 15 ug (UA)
Treatments Part B: B1 = A/Sing MAP, 15 ug (FA) with biopsy; B2 = Placebo MAP (FA) with biopsy
B3 = A/Sing MAP, 15 ug (FA); B4 = A/Sing MAP, 10 ug (FA); B5 = A/Sing MAP, 5 ug (FA); B6 = A/Sing MAP, 2.5 ug (FA)
B7 = Placebo MAP (FA); B8 = A/Sing MAP, 15 ug (UA); B9 = A/Sing IM, 15 ug, as 2018 Afluria Quad (UA)

Listing 16.2.9.2
Treatment Site Tolerability Assessment per Site

Part B
Randomisation Number = B0038, Treatment Group = Group B3

Date	Time	Visit	Time Point	Site Number	Treatment for Site	Indura-tion	Redness	Bruising	Itching	Skin Flaking	Tender-ness	Definition/ Visibility	Other#	Unscheduled Reason
26-MAY-2018	09:21	Day 8		F3	5 ug	0	0	0	0	0	0	0		
09-JUN-2018	08:22	Day 22		F1	5 ug	0	0	0	0	1	0	0		
09-JUN-2018	08:22	Day 22		F2	5 ug	0	0	0	0	0	0	0		
09-JUN-2018	08:22	Day 22		F3	5 ug	0	0	0	0	0	0	0		
21-JUL-2018	09:16	EOS/ET (Day 61)		F1	5 ug	0	0	0	0	0	0	0		
21-JUL-2018	09:16	EOS/ET (Day 61)		F2	5 ug	0	0	0	0	0	0	0		
21-JUL-2018	09:16	EOS/ET (Day 61)		F3	5 ug	0	0	0	0	0	0	0		

Key: 0= None; 1=Mild; 2=Moderate; 3=Severe; # Other = Pinprick bleeding or seepage

Administration Site Key: FA = Forearm; UA= Upper arm
Treatments Part A: A1 = A/Sing MAP, 15 ug (FA); A2 = Afluria Quad IM (UA), 15 ug; A3 = Placebo MAP (FA); A4 = A/Sing Mono IM, 15 ug (UA)
Treatments Part B: B1 = A/Sing MAP, 15 ug (FA) with biopsy; B2 = Placebo MAP (FA) with biopsy
B3 = A/Sing MAP, 15 ug (FA); B4 = A/Sing MAP, 10 ug (FA); B5 = A/Sing MAP, 5 ug (FA); B6 = A/Sing MAP, 2.5 ug (FA)
B7 = Placebo MAP (FA); B8 = A/Sing MAP, 15 ug (UA); B9 = A/Sing IM, 15 ug, as 2018 Afluria Quad (UA)

Listing 16.2.9.2
Treatment Site Tolerability Assessment per Site

Part B
Randomisation Number = B0039, Treatment Group = Group B7

Date	Time	Visit	Time Point	Site Number	Treatment for Site	Indura-tion	Redness	Bruising	Itching	Skin Flaking	Tender-ness	Definition/ Visibility	Other#	Unscheduled Reason
19-MAY-2018	12:15	Day 1	Pre-dose	F1	uncoated	0	0	0	0	0	0	0		
19-MAY-2018	12:15	Day 1	Pre-dose	F2	uncoated	0	0	0	0	0	0	0		
19-MAY-2018	12:15	Day 1	Pre-dose	F3	uncoated	0	0	0	0	0	0	0		
19-MAY-2018	14:08	Day 1	10 min	F1	uncoated	0	1	0	0	0	0	0		
19-MAY-2018	14:08	Day 1	10 min	F2	uncoated	0	0	0	0	0	0	0		
19-MAY-2018	14:08	Day 1	10 min	F3	uncoated	0	0	0	0	0	0	0		
19-MAY-2018	14:58	Day 1	1 hour	F1	uncoated	0	0	0	0	0	0	0		
19-MAY-2018	14:58	Day 1	1 hour	F2	uncoated	0	0	0	0	0	0	0		
19-MAY-2018	14:58	Day 1	1 hour	F3	uncoated	0	0	0	0	0	0	0		
19-MAY-2018	15:58	Day 1	2 hour	F1	uncoated	0	0	0	0	0	1	0		
19-MAY-2018	15:58	Day 1	2 hour	F2	uncoated	1	1	0	0	0	1	0		
19-MAY-2018	15:58	Day 1	2 hour	F3	uncoated	0	1	0	0	0	0	0		
20-MAY-2018	14:36	Day 2		F1	uncoated	0	0	0	0	0	0	0		
20-MAY-2018	14:36	Day 2		F2	uncoated	0	0	0	0	0	0	0		
20-MAY-2018	14:36	Day 2		F3	uncoated	0	0	0	0	0	0	0		
22-MAY-2018	12:25	Day 4		F1	uncoated	0	0	0	0	0	0	3		
22-MAY-2018	12:25	Day 4		F2	uncoated	0	0	0	0	0	0	3		
22-MAY-2018	12:25	Day 4		F3	uncoated	0	0	0	0	0	0	3		
26-MAY-2018	15:07	Day 8		F1	uncoated	0	0	0	0	0	0	3		
26-MAY-2018	15:07	Day 8		F2	uncoated	0	0	0	0	0	0	2		

Key: 0= None; 1=Mild; 2=Moderate; 3=Severe; # Other = Pinprick bleeding or seepage

Administration Site Key: FA = Forearm; UA= Upper arm
Treatments Part A: A1 = A/Sing MAP, 15 ug (FA); A2 = Afluria Quad IM (UA), 15 ug; A3 = Placebo MAP (FA); A4 = A/Sing Mono IM, 15 ug (UA)
Treatments Part B: B1 = A/Sing MAP, 15 ug (FA) with biopsy; B2 = Placebo MAP (FA) with biopsy
B3 = A/Sing MAP, 15 ug (FA); B4 = A/Sing MAP, 10 ug (FA); B5 = A/Sing MAP, 5 ug (FA); B6 = A/Sing MAP, 2.5 ug (FA)
B7 = Placebo MAP (FA); B8 = A/Sing MAP, 15 ug (UA); B9 = A/Sing IM, 15 ug, as 2018 Afluria Quad (UA)

Listing 16.2.9.2
Treatment Site Tolerability Assessment per Site

Part B
Randomisation Number = B0039, Treatment Group = Group B7

Date	Time	Visit	Time Point	Site Number	Treatment for Site	Indura-tion	Redness	Bruising	Itching	Skin Flaking	Tender-ness	Definition/ Visibility	Other#	Unscheduled Reason
26-MAY-2018	15:07	Day 8		F3	uncoated	0	0	0	0	0	0	2		
26-MAY-2018	15:23	Unscheduled		F1	uncoated							3	NA	MO notifies as repeat
26-MAY-2018	15:24	Unscheduled		F2	uncoated							2	NA	MO notifies as repeat
26-MAY-2018	15:25	Unscheduled		F3	uncoated							2	NA	MO notifies as repeat
09-JUN-2018	14:01	Day 22		F1	uncoated	0	0	0	0	1	0	0		
09-JUN-2018	14:01	Day 22		F2	uncoated	0	0	0	0	1	0	0		
09-JUN-2018	14:01	Day 22		F3	uncoated	0	0	0	0	0	0	0		
18-JUL-2018	16:59	EOS/ET (Day 61)		F1	uncoated	0	0	0	0	0	0	0		
18-JUL-2018	16:59	EOS/ET (Day 61)		F2	uncoated	0	0	0	0	0	0	0		
18-JUL-2018	16:59	EOS/ET (Day 61)		F3	uncoated	0	0	0	0	0	0	0		

Key: 0= None; 1=Mild; 2=Moderate; 3=Severe; # Other = Pinprick bleeding or seepage

Administration Site Key: FA = Forearm; UA= Upper arm
Treatments Part A: A1 = A/Sing MAP, 15 ug (FA); A2 = Afluria Quad IM (UA), 15 ug; A3 = Placebo MAP (FA); A4 = A/Sing Mono IM, 15 ug (UA)
Treatments Part B: B1 = A/Sing MAP, 15 ug (FA) with biopsy; B2 = Placebo MAP (FA) with biopsy
B3 = A/Sing MAP, 15 ug (FA); B4 = A/Sing MAP, 10 ug (FA); B5 = A/Sing MAP, 5 ug (FA); B6 = A/Sing MAP, 2.5 ug (FA)
B7 = Placebo MAP (FA); B8 = A/Sing MAP, 15 ug (UA); B9 = A/Sing IM, 15 ug, as 2018 Afluria Quad (UA)

Listing 16.2.9.2
Treatment Site Tolerability Assessment per Site

Part B
Randomisation Number = B0040, Treatment Group = Group B9

Date	Time	Visit	Time Point	Site Number	Treatment for Site	Indura-tion	Redness	Bruising	Itching	Skin Flaking	Tender-ness	Definition/ Visibility	Other#	Unscheduled Reason
19-MAY-2018	10:43	Day 1	Pre-dose	D1	[IM]	0	0	0	0	0	0	5		
19-MAY-2018	11:21	Day 1	10 min	D1	[IM]	0	0	0	0	0	0	5		
19-MAY-2018	12:13	Day 1	1 hour	D1	[IM]	0	0	0	0	0	0	5		
19-MAY-2018	13:35	Day 1	2 hour	D1	[IM]	0	0	0	0	0	0	5		
20-MAY-2018	11:05	Day 2		D1	[IM]	0	0	0	0	0	0	5		
22-MAY-2018	08:01	Day 4		D1	[IM]	0	0	0	0	0	0	5		
26-MAY-2018	12:29	Day 8		D1	[IM]	0	0	0	0	0	0	5		
09-JUN-2018	11:42	Day 22		D1	[IM]	0	0	0	0	0	0	5		
18-JUL-2018	08:43	EOS/ET (Day 61)		D1	[IM]	0	0	0	0	0	0	5		

Key: 0= None; 1=Mild; 2=Moderate; 3=Severe; # Other = Pinprick bleeding or seepage

Administration Site Key: FA = Forearm; UA= Upper arm
Treatments Part A: A1 = A/Sing MAP, 15 ug (FA); A2 = Afluria Quad IM (UA), 15 ug; A3 = Placebo MAP (FA); A4 = A/Sing Mono IM, 15 ug (UA)
Treatments Part B: B1 = A/Sing MAP, 15 ug (FA) with biopsy; B2 = Placebo MAP (FA) with biopsy
B3 = A/Sing MAP, 15 ug (FA); B4 = A/Sing MAP, 10 ug (FA); B5 = A/Sing MAP, 5 ug (FA); B6 = A/Sing MAP, 2.5 ug (FA)
B7 = Placebo MAP (FA); B8 = A/Sing MAP, 15 ug (UA); B9 = A/Sing IM, 15 ug, as 2018 Afluria Quad (UA)

Listing 16.2.9.2
Treatment Site Tolerability Assessment per Site

Part B
Randomisation Number = B0041, Treatment Group = Group B3

Date	Time	Visit	Time Point	Site Number	Treatment for Site	Indura-tion	Redness	Bruising	Itching	Skin Flaking	Tender-ness	Definition/ Visibility	Other#	Unscheduled Reason
19-MAY-2018	12:23	Day 1	Pre-dose	F1	5 ug	0	0	0	0	0	0	0		
19-MAY-2018	12:23	Day 1	Pre-dose	F2	5 ug	0	0	0	0	0	0	0		
19-MAY-2018	12:23	Day 1	Pre-dose	F3	5 ug	0	0	0	0	0	0	0		
19-MAY-2018	13:12	Day 1	10 min	F1	5 ug	0	0	0	0	0	1	0		
19-MAY-2018	13:12	Day 1	10 min	F2	5 ug	0	0	0	0	0	1	0		
19-MAY-2018	13:12	Day 1	10 min	F3	5 ug	0	0	0	0	0	1	0		
19-MAY-2018	13:38	Day 1	1 hour	F1	5 ug	0	0	0	0	0	0	0		
19-MAY-2018	13:38	Day 1	1 hour	F2	5 ug	0	0	0	0	0	0	0		
19-MAY-2018	13:38	Day 1	1 hour	F3	5 ug	0	0	0	0	0	0	0		
19-MAY-2018	14:53	Day 1	2 hour	F1	5 ug	0	0	0	1	0	0	0		
19-MAY-2018	14:53	Day 1	2 hour	F2	5 ug	0	0	0	0	0	0	0		
19-MAY-2018	14:53	Day 1	2 hour	F3	5 ug	0	0	0	0	0	0	0		
20-MAY-2018	13:15	Day 2		F1	5 ug	0	1	0	1	0	0	0		
20-MAY-2018	13:15	Day 2		F2	5 ug	0	1	0	1	0	0	0		
20-MAY-2018	13:15	Day 2		F3	5 ug	0	1	0	1	0	0	0		
22-MAY-2018	15:30	Day 4		F1	5 ug	0	0	0	0	0	0	0		
22-MAY-2018	15:30	Day 4		F2	5 ug	1	0	0	0	0	0	0		
22-MAY-2018	15:30	Day 4		F3	5 ug	0	0	0	0	0	0	0		
26-MAY-2018	12:36	Day 8		F1	5 ug	0	0	0	0	0	0	2		
26-MAY-2018	12:36	Day 8		F2	5 ug	0	0	0	0	0	0	2		

Key: 0= None; 1=Mild; 2=Moderate; 3=Severe; # Other = Pinprick bleeding or seepage

Administration Site Key: FA = Forearm; UA= Upper arm
Treatments Part A: A1 = A/Sing MAP, 15 ug (FA); A2 = Afluria Quad IM (UA), 15 ug; A3 = Placebo MAP (FA); A4 = A/Sing Mono IM, 15 ug (UA)
Treatments Part B: B1 = A/Sing MAP, 15 ug (FA) with biopsy; B2 = Placebo MAP (FA) with biopsy
B3 = A/Sing MAP, 15 ug (FA); B4 = A/Sing MAP, 10 ug (FA); B5 = A/Sing MAP, 5 ug (FA); B6 = A/Sing MAP, 2.5 ug (FA)
B7 = Placebo MAP (FA); B8 = A/Sing MAP, 15 ug (UA); B9 = A/Sing IM, 15 ug, as 2018 Afluria Quad (UA)

Listing 16.2.9.2
Treatment Site Tolerability Assessment per Site

Part B
Randomisation Number = B0041, Treatment Group = Group B3

Date	Time	Visit	Time Point	Site Number	Treatment for Site	Indura-tion	Redness	Bruising	Itching	Skin Flaking	Tender-ness	Definition/ Visibility	Other#	Unscheduled Reason
26-MAY-2018	12:36	Day 8		F3	5 ug	0	0	0	0	0	0	1		
09-JUN-2018	15:04	Day 22		F1	5 ug	0	0	0	0	0	0	2		
09-JUN-2018	15:04	Day 22		F2	5 ug	0	0	0	0	0	0	2		
09-JUN-2018	15:04	Day 22		F3	5 ug	0	0	0	0	0	0	2		
20-JUL-2018	15:24	EOS/ET (Day 61)		F1	5 ug	0	0	0	0	0	0	1		
20-JUL-2018	15:24	EOS/ET (Day 61)		F2	5 ug	0	0	0	0	0	0	1		
20-JUL-2018	15:24	EOS/ET (Day 61)		F3	5 ug	0	0	0	0	0	0	1		

Key: 0= None; 1=Mild; 2=Moderate; 3=Severe; # Other = Pinprick bleeding or seepage

Administration Site Key: FA = Forearm; UA= Upper arm
Treatments Part A: A1 = A/Sing MAP, 15 ug (FA); A2 = Afluria Quad IM (UA), 15 ug; A3 = Placebo MAP (FA); A4 = A/Sing Mono IM, 15 ug (UA)
Treatments Part B: B1 = A/Sing MAP, 15 ug (FA) with biopsy; B2 = Placebo MAP (FA) with biopsy
B3 = A/Sing MAP, 15 ug (FA); B4 = A/Sing MAP, 10 ug (FA); B5 = A/Sing MAP, 5 ug (FA); B6 = A/Sing MAP, 2.5 ug (FA)
B7 = Placebo MAP (FA); B8 = A/Sing MAP, 15 ug (UA); B9 = A/Sing IM, 15 ug, as 2018 Afluria Quad (UA)

Listing 16.2.9.2
Treatment Site Tolerability Assessment per Site

Part B
Randomisation Number = B0042, Treatment Group = Group B5

Date	Time	Visit	Time Point	Site Number	Treatment for Site	Indura-tion	Redness	Bruising	Itching	Skin Flaking	Tender-ness	Definition/ Visibility	Other#	Unscheduled Reason
19-MAY-2018	12:30	Day 1	Pre-dose	F1	uncoated	0	0	0	0	0	0	0		
19-MAY-2018	12:30	Day 1	Pre-dose	F2	uncoated	0	0	0	0	0	0	0		
19-MAY-2018	12:30	Day 1	Pre-dose	F3	5 ug	0	0	0	0	0	0	0		
19-MAY-2018	13:13	Day 1	10 min	F1	uncoated	0	0	0	0	0	0	0		
19-MAY-2018	13:13	Day 1	10 min	F2	uncoated	0	0	0	0	0	0	0		
19-MAY-2018	13:13	Day 1	10 min	F3	5 ug	0	0	0	0	0	1	0		
19-MAY-2018	13:58	Day 1	1 hour	F1	uncoated	0	0	0	0	0	0	0		
19-MAY-2018	13:58	Day 1	1 hour	F2	uncoated	0	0	0	0	0	0	0		
19-MAY-2018	13:58	Day 1	1 hour	F3	5 ug	0	0	0	0	0	0	0		
19-MAY-2018	14:58	Day 1	2 hour	F1	uncoated	0	0	0	0	0	0	0		
19-MAY-2018	14:58	Day 1	2 hour	F2	uncoated	0	0	0	0	0	0	0		
19-MAY-2018	14:58	Day 1	2 hour	F3	5 ug	0	0	0	0	0	0	0		
20-MAY-2018	12:16	Day 2		F1	uncoated	0	0	0	0	0	0	0		
20-MAY-2018	12:16	Day 2		F2	uncoated	0	0	0	0	0	0	0		
20-MAY-2018	12:16	Day 2		F3	5 ug	1	1	0	0	0	1	0		
22-MAY-2018	10:02	Day 4		F1	uncoated	0	0	0	0	0	0	2		
22-MAY-2018	10:02	Day 4		F2	uncoated	0	0	0	0	0	0	2		
22-MAY-2018	10:02	Day 4		F3	5 ug	1	1	0	0	0	1	0		
26-MAY-2018	11:42	Day 8		F1	uncoated	0	0	0	0	0	0	2		
26-MAY-2018	11:42	Day 8		F2	uncoated	0	0	0	0	0	0	2		

Key: 0= None; 1=Mild; 2=Moderate; 3=Severe; # Other = Pinprick bleeding or seepage

Administration Site Key: FA = Forearm; UA= Upper arm
Treatments Part A: A1 = A/Sing MAP, 15 ug (FA); A2 = Afluria Quad IM (UA), 15 ug; A3 = Placebo MAP (FA); A4 = A/Sing Mono IM, 15 ug (UA)
Treatments Part B: B1 = A/Sing MAP, 15 ug (FA) with biopsy; B2 = Placebo MAP (FA) with biopsy
B3 = A/Sing MAP, 15 ug (FA); B4 = A/Sing MAP, 10 ug (FA); B5 = A/Sing MAP, 5 ug (FA); B6 = A/Sing MAP, 2.5 ug (FA)
B7 = Placebo MAP (FA); B8 = A/Sing MAP, 15 ug (UA); B9 = A/Sing IM, 15 ug, as 2018 Afluria Quad (UA)

Listing 16.2.9.2
Treatment Site Tolerability Assessment per Site

Part B
Randomisation Number = B0042, Treatment Group = Group B5

Date	Time	Visit	Time Point	Site Number	Treatment for Site	Indura-tion	Redness	Bruising	Itching	Skin Flaking	Tender-ness	Definition/ Visibility	Other#	Unscheduled Reason
26-MAY-2018	11:42	Day 8		F3	5 ug	0	0	0	0	2	0	0		
09-JUN-2018	13:37	Day 22		F1	uncoated	0	0	0	0	0	0	0		
09-JUN-2018	13:37	Day 22		F2	uncoated	0	0	0	0	0	0	0		
09-JUN-2018	13:37	Day 22		F3	5 ug	0	0	0	0	1	0	0		
18-JUL-2018	09:30	EOS/ET (Day 61)		F1	uncoated	0	0	0	0	0	0	0		
18-JUL-2018	09:30	EOS/ET (Day 61)		F2	uncoated	0	0	0	0	0	0	0		
18-JUL-2018	09:30	EOS/ET (Day 61)		F3	5 ug	0	0	0	0	0	0	1		

Key: 0= None; 1=Mild; 2=Moderate; 3=Severe; # Other = Pinprick bleeding or seepage

Administration Site Key: FA = Forearm; UA= Upper arm
Treatments Part A: A1 = A/Sing MAP, 15 ug (FA); A2 = Afluria Quad IM (UA), 15 ug; A3 = Placebo MAP (FA); A4 = A/Sing Mono IM, 15 ug (UA)
Treatments Part B: B1 = A/Sing MAP, 15 ug (FA) with biopsy; B2 = Placebo MAP (FA) with biopsy
B3 = A/Sing MAP, 15 ug (FA); B4 = A/Sing MAP, 10 ug (FA); B5 = A/Sing MAP, 5 ug (FA); B6 = A/Sing MAP, 2.5 ug (FA)
B7 = Placebo MAP (FA); B8 = A/Sing MAP, 15 ug (UA); B9 = A/Sing IM, 15 ug, as 2018 Afluria Quad (UA)

Listing 16.2.9.2
Treatment Site Tolerability Assessment per Site

Part B
Randomisation Number = B0043, Treatment Group = Group B8

Date	Time	Visit	Time Point	Site Number	Treatment for Site	Indura-tion	Redness	Bruising	Itching	Skin Flaking	Tender-ness	Definition/ Visibility	Other#	Unscheduled Reason
19-MAY-2018	12:33	Day 1	Pre-dose	D1	5 ug	0	0	0	0	0	0	0		
19-MAY-2018	12:33	Day 1	Pre-dose	D2	5 ug	0	0	0	0	0	0	0		
19-MAY-2018	12:33	Day 1	Pre-dose	D3	5 ug	0	0	0	0	0	0	0		
19-MAY-2018	13:19	Day 1	10 min	D1	5 ug	0	0	0	0	0	1	0		
19-MAY-2018	13:19	Day 1	10 min	D2	5 ug	0	1	0	0	0	0	0		
19-MAY-2018	13:19	Day 1	10 min	D3	5 ug	0	0	0	0	0	0	0		
19-MAY-2018	14:15	Day 1	1 hour	D1	5 ug	0	0	0	0	0	0	0		
19-MAY-2018	14:15	Day 1	1 hour	D2	5 ug	0	0	0	0	0	0	0		
19-MAY-2018	14:15	Day 1	1 hour	D3	5 ug	0	0	0	0	0	0	0		
19-MAY-2018	15:08	Day 1	2 hour	D1	5 ug	0	0	0	0	0	0	0		
19-MAY-2018	15:08	Day 1	2 hour	D2	5 ug	0	0	0	0	0	1	0		
19-MAY-2018	15:08	Day 1	2 hour	D3	5 ug	0	0	0	0	0	0	0		
20-MAY-2018	12:27	Day 2		D1	5 ug	0	0	0	0	0	0	0		
20-MAY-2018	12:27	Day 2		D2	5 ug	0	0	0	0	0	0	0		
20-MAY-2018	12:27	Day 2		D3	5 ug	0	0	0	0	0	0	0		
22-MAY-2018	14:01	Day 4		D1	5 ug	0	0	0	0	0	0	0		
22-MAY-2018	14:01	Day 4		D2	5 ug	0	0	0	0	0	0	0		
22-MAY-2018	14:01	Day 4		D3	5 ug	0	0	0	0	0	0	0		
26-MAY-2018	08:16	Day 8		D1	5 ug	0	0	0	0	2	0	0		
26-MAY-2018	08:16	Day 8		D2	5 ug	0	0	0	0	2	0	0		

Key: 0= None; 1=Mild; 2=Moderate; 3=Severe; # Other = Pinprick bleeding or seepage

Administration Site Key: FA = Forearm; UA= Upper arm
Treatments Part A: A1 = A/Sing MAP, 15 ug (FA); A2 = Afluria Quad IM (UA), 15 ug; A3 = Placebo MAP (FA); A4 = A/Sing Mono IM, 15 ug (UA)
Treatments Part B: B1 = A/Sing MAP, 15 ug (FA) with biopsy; B2 = Placebo MAP (FA) with biopsy
B3 = A/Sing MAP, 15 ug (FA); B4 = A/Sing MAP, 10 ug (FA); B5 = A/Sing MAP, 5 ug (FA); B6 = A/Sing MAP, 2.5 ug (FA)
B7 = Placebo MAP (FA); B8 = A/Sing MAP, 15 ug (UA); B9 = A/Sing IM, 15 ug, as 2018 Afluria Quad (UA)

Listing 16.2.9.2
Treatment Site Tolerability Assessment per Site

Part B
Randomisation Number = B0043, Treatment Group = Group B8

Date	Time	Visit	Time Point	Site Number	Treatment for Site	Induration	Redness	Bruising	Itching	Skin Flaking	Tenderness	Definition/ Visibility	Other#	Unscheduled Reason
26-MAY-2018	08:16	Day 8		D3	5 ug	0	0	0	0	2	0	0		
09-JUN-2018	10:16	Day 22		D1	5 ug	0	0	0	0	1	0	0		
09-JUN-2018	10:16	Day 22		D2	5 ug	0	0	0	0	1	0	0		
09-JUN-2018	10:16	Day 22		D3	5 ug	0	0	0	0	1	0	0		
18-JUL-2018	09:26	EOS/ET (Day 61)		D1	5 ug	0	0	0	0	0	0	1		
18-JUL-2018	09:26	EOS/ET (Day 61)		D2	5 ug	0	0	0	0	0	0	1		
18-JUL-2018	09:26	EOS/ET (Day 61)		D3	5 ug	0	0	0	0	0	0	1		

Key: 0= None; 1=Mild; 2=Moderate; 3=Severe; # Other = Pinprick bleeding or seepage

Administration Site Key: FA = Forearm; UA= Upper arm
Treatments Part A: A1 = A/Sing MAP, 15 ug (FA); A2 = Afluria Quad IM (UA), 15 ug; A3 = Placebo MAP (FA); A4 = A/Sing Mono IM, 15 ug (UA)
Treatments Part B: B1 = A/Sing MAP, 15 ug (FA) with biopsy; B2 = Placebo MAP (FA) with biopsy
B3 = A/Sing MAP, 15 ug (FA); B4 = A/Sing MAP, 10 ug (FA); B5 = A/Sing MAP, 5 ug (FA); B6 = A/Sing MAP, 2.5 ug (FA)
B7 = Placebo MAP (FA); B8 = A/Sing MAP, 15 ug (UA); B9 = A/Sing IM, 15 ug, as 2018 Afluria Quad (UA)

Listing 16.2.9.2
Treatment Site Tolerability Assessment per Site

Part B
Randomisation Number = B0044, Treatment Group = Group B6

Date	Time	Visit	Time Point	Site Number	Treatment for Site	Indura- tion	Redness	Bruising	Itching	Skin Flaking	Tender- ness	Definition/ Visibility	Other#	Unscheduled Reason
19-MAY-2018	12:44	Day 1	Pre-dose	F1	uncoated	0	0	0	0	0	0	0		
19-MAY-2018	12:44	Day 1	Pre-dose	F2	uncoated	0	0	0	0	0	0	0		
19-MAY-2018	12:44	Day 1	Pre-dose	F3	2.5 ug	0	0	0	0	0	0	0		
19-MAY-2018	13:28	Day 1	10 min	F1	uncoated	0	0	0	0	0	1	0		
19-MAY-2018	13:28	Day 1	10 min	F2	uncoated	0	0	0	0	0	1	0		
19-MAY-2018	13:28	Day 1	10 min	F3	2.5 ug	0	0	0	0	0	1	0		
19-MAY-2018	14:19	Day 1	1 hour	F1	uncoated	0	0	0	0	0	0	0		
19-MAY-2018	14:19	Day 1	1 hour	F2	uncoated	0	0	0	0	0	0	0		
19-MAY-2018	14:19	Day 1	1 hour	F3	2.5 ug	0	0	0	0	0	0	0		
19-MAY-2018	15:18	Day 1	2 hour	F1	uncoated	0	0	0	0	0	1	0		
19-MAY-2018	15:18	Day 1	2 hour	F2	uncoated	0	0	0	0	0	1	0		
19-MAY-2018	15:18	Day 1	2 hour	F3	2.5 ug	0	0	0	0	0	1	0		
20-MAY-2018	12:32	Day 2		F1	uncoated	0	0	0	0	0	0	0		
20-MAY-2018	12:32	Day 2		F2	uncoated	0	0	0	0	0	0	0		
20-MAY-2018	12:32	Day 2		F3	2.5 ug	1	1	0	0	0	1	0		
22-MAY-2018	18:21	Day 4		F1	uncoated	0	0	0	0	0	0	0		
22-MAY-2018	18:21	Day 4		F2	uncoated	0	0	0	0	0	0	0		
22-MAY-2018	18:21	Day 4		F3	2.5 ug	0	1	0	0	0	0	0		
26-MAY-2018	14:45	Day 8		F1	uncoated	0	0	0	0	1	0	0		
26-MAY-2018	14:45	Day 8		F2	uncoated	0	0	0	0	1	0	0		

Key: 0= None; 1=Mild; 2=Moderate; 3=Severe; # Other = Pinprick bleeding or seepage

Administration Site Key: FA = Forearm; UA= Upper arm
Treatments Part A: A1 = A/Sing MAP, 15 ug (FA); A2 = Afluria Quad IM (UA), 15 ug; A3 = Placebo MAP (FA); A4 = A/Sing Mono IM, 15 ug (UA)
Treatments Part B: B1 = A/Sing MAP, 15 ug (FA) with biopsy; B2 = Placebo MAP (FA) with biopsy
B3 = A/Sing MAP, 15 ug (FA); B4 = A/Sing MAP, 10 ug (FA); B5 = A/Sing MAP, 5 ug (FA); B6 = A/Sing MAP, 2.5 ug (FA)
B7 = Placebo MAP (FA); B8 = A/Sing MAP, 15 ug (UA); B9 = A/Sing IM, 15 ug, as 2018 Afluria Quad (UA)

Listing 16.2.9.2
Treatment Site Tolerability Assessment per Site

Part B
Randomisation Number = B0044, Treatment Group = Group B6

Date	Time	Visit	Time Point	Site Number	Treatment for Site	Indura-tion	Redness	Bruising	Itching	Skin Flaking	Tender-ness	Definition/ Visibility	Other#	Unscheduled Reason
26-MAY-2018	14:45	Day 8		F3	2.5 ug	0	0	0	0	1	0	0		
09-JUN-2018	13:47	Day 22		F1	uncoated	0	0	0	0	0	0	0		
09-JUN-2018	13:47	Day 22		F2	uncoated	0	0	0	0	0	0	0		
09-JUN-2018	13:47	Day 22		F3	2.5 ug	0	0	0	0	0	0	0		
18-JUL-2018	13:19	EOS/ET (Day 61)		F1	uncoated	0	0	0	0	0	0	0		
18-JUL-2018	13:19	EOS/ET (Day 61)		F2	uncoated	0	0	0	0	0	0	0		
18-JUL-2018	13:19	EOS/ET (Day 61)		F3	2.5 ug	0	0	0	0	0	0	2		

Key: 0= None; 1=Mild; 2=Moderate; 3=Severe; # Other = Pinprick bleeding or seepage

Administration Site Key: FA = Forearm; UA= Upper arm
Treatments Part A: A1 = A/Sing MAP, 15 ug (FA); A2 = Afluria Quad IM (UA), 15 ug; A3 = Placebo MAP (FA); A4 = A/Sing Mono IM, 15 ug (UA)
Treatments Part B: B1 = A/Sing MAP, 15 ug (FA) with biopsy; B2 = Placebo MAP (FA) with biopsy
B3 = A/Sing MAP, 15 ug (FA); B4 = A/Sing MAP, 10 ug (FA); B5 = A/Sing MAP, 5 ug (FA); B6 = A/Sing MAP, 2.5 ug (FA)
B7 = Placebo MAP (FA); B8 = A/Sing MAP, 15 ug (UA); B9 = A/Sing IM, 15 ug, as 2018 Afluria Quad (UA)

Listing 16.2.9.2
Treatment Site Tolerability Assessment per Site

Part B
Randomisation Number = B0045, Treatment Group = Group B4

Date	Time	Visit	Time Point	Site Number	Treatment for Site	Indura-tion	Redness	Bruising	Itching	Skin Flaking	Tender-ness	Definition/ Visibility	Other#	Unscheduled Reason
19-MAY-2018	10:59	Day 1	Pre-dose	F1	5 ug	0	0	0	0	0	0	0		
19-MAY-2018	10:59	Day 1	Pre-dose	F2	5 ug	0	0	0	0	0	0	0		
19-MAY-2018	10:59	Day 1	Pre-dose	F3	uncoated	0	0	0	0	0	0	0		
19-MAY-2018	12:08	Day 1	10 min	F1	5 ug	0	1	0	0	0	0	0		
19-MAY-2018	12:08	Day 1	10 min	F2	5 ug	0	1	0	0	0	0	0		
19-MAY-2018	12:08	Day 1	10 min	F3	uncoated	0	0	0	0	0	1	0		
19-MAY-2018	12:59	Day 1	1 hour	F1	5 ug	0	0	0	0	0	0	0		
19-MAY-2018	12:59	Day 1	1 hour	F2	5 ug	1	1	0	0	0	0	0		
19-MAY-2018	12:59	Day 1	1 hour	F3	uncoated	0	0	0	0	0	0	0		
19-MAY-2018	13:58	Day 1	2 hour	F1	5 ug	0	0	0	0	0	0	0		
19-MAY-2018	13:58	Day 1	2 hour	F2	5 ug	1	0	0	0	0	0	0		
19-MAY-2018	13:58	Day 1	2 hour	F3	uncoated	0	0	0	0	0	0	0		
19-MAY-2018	13:15	Unscheduled		F1	5 ug	0	0	0	0	0	0	0	NA	MO notifies as repeat
19-MAY-2018	13:16	Unscheduled		F2	5 ug	0	0	0	0	0	1	0	NA	MO notifies as repeat
19-MAY-2018	13:17	Unscheduled		F3	uncoated	0	0	0	0	0	0	0	NA	MO notifies as repeat
20-MAY-2018	12:37	Day 2		F1	5 ug	0	1	0	0	0	0	0		
20-MAY-2018	12:37	Day 2		F2	5 ug	2	1	0	0	0	1	0		

Key: 0= None; 1=Mild; 2=Moderate; 3=Severe; # Other = Pinprick bleeding or seepage

Administration Site Key: FA = Forearm; UA= Upper arm
Treatments Part A: A1 = A/Sing MAP, 15 ug (FA); A2 = Afluria Quad IM (UA), 15 ug; A3 = Placebo MAP (FA); A4 = A/Sing Mono IM, 15 ug (UA)
Treatments Part B: B1 = A/Sing MAP, 15 ug (FA) with biopsy; B2 = Placebo MAP (FA) with biopsy
B3 = A/Sing MAP, 15 ug (FA); B4 = A/Sing MAP, 10 ug (FA); B5 = A/Sing MAP, 5 ug (FA); B6 = A/Sing MAP, 2.5 ug (FA)
B7 = Placebo MAP (FA); B8 = A/Sing MAP, 15 ug (UA); B9 = A/Sing IM, 15 ug, as 2018 Afluria Quad (UA)

Listing 16.2.9.2
Treatment Site Tolerability Assessment per Site

Part B
Randomisation Number = B0045, Treatment Group = Group B4

Date	Time	Visit	Time Point	Site Number	Treatment for Site	Indura-tion	Redness	Bruising	Itching	Skin Flaking	Tender-ness	Definition/ Visibility	Other#	Unscheduled Reason
20-MAY-2018	12:37	Day 2		F3	uncoated	0	0	0	0	0	0	0		
22-MAY-2018	10:41	Day 4		F1	5 ug	0	2	0	0	0	0	0		
22-MAY-2018	10:41	Day 4		F2	5 ug	1	2	0	0	0	0	0		
22-MAY-2018	10:41	Day 4		F3	uncoated	0	0	0	0	0	2	0		
26-MAY-2018	11:47	Day 8		F1	5 ug	0	0	0	0	1	0	1		
26-MAY-2018	11:47	Day 8		F2	5 ug	0	0	0	0	1	0	1		
26-MAY-2018	11:47	Day 8		F3	uncoated	0	0	0	0	0	0	1		
09-JUN-2018	10:17	Day 22		F1	5 ug	0	0	0	0	0	0	0		
09-JUN-2018	10:17	Day 22		F2	5 ug	0	0	0	0	0	0	0		
09-JUN-2018	10:17	Day 22		F3	uncoated	0	0	0	0	0	0	1		
18-JUL-2018	11:49	EOS/ET (Day 61)		F1	5 ug	0	0	0	0	0	0	1		
18-JUL-2018	11:49	EOS/ET (Day 61)		F2	5 ug	0	0	0	0	0	0	1		
18-JUL-2018	11:49	EOS/ET (Day 61)		F3	uncoated	0	0	0	0	0	0	0		

Key: 0= None; 1=Mild; 2=Moderate; 3=Severe; # Other = Pinprick bleeding or seepage

Administration Site Key: FA = Forearm; UA= Upper arm
Treatments Part A: A1 = A/Sing MAP, 15 ug (FA); A2 = Afluria Quad IM (UA), 15 ug; A3 = Placebo MAP (FA); A4 = A/Sing Mono IM, 15 ug (UA)
Treatments Part B: B1 = A/Sing MAP, 15 ug (FA) with biopsy; B2 = Placebo MAP (FA) with biopsy
B3 = A/Sing MAP, 15 ug (FA); B4 = A/Sing MAP, 10 ug (FA); B5 = A/Sing MAP, 5 ug (FA); B6 = A/Sing MAP, 2.5 ug (FA)
B7 = Placebo MAP (FA); B8 = A/Sing MAP, 15 ug (UA); B9 = A/Sing IM, 15 ug, as 2018 Afluria Quad (UA)

Listing 16.2.9.2
Treatment Site Tolerability Assessment per Site

Part B
Randomisation Number = B0046, Treatment Group = Group B7

Date	Time	Visit	Time Point	Site Number	Treatment for Site	Indura-tion	Redness	Bruising	Itching	Skin Flaking	Tender-ness	Definition/ Visibility	Other#	Unscheduled Reason
19-MAY-2018	13:05	Day 1	Pre-dose	F1	uncoated	0	0	0	0	0	0	0		
19-MAY-2018	13:05	Day 1	Pre-dose	F2	uncoated	0	0	0	0	0	0	0		
19-MAY-2018	13:05	Day 1	Pre-dose	F3	uncoated	0	0	0	0	0	0	0		
19-MAY-2018	13:49	Day 1	10 min	F1	uncoated	0	1	0	0	0	1	0		
19-MAY-2018	13:49	Day 1	10 min	F2	uncoated	0	1	0	0	0	1	0		
19-MAY-2018	13:49	Day 1	10 min	F3	uncoated	0	1	0	0	0	1	0		
19-MAY-2018	14:38	Day 1	1 hour	F1	uncoated	0	0	0	0	0	0	0		
19-MAY-2018	14:38	Day 1	1 hour	F2	uncoated	0	0	0	0	0	0	0		
19-MAY-2018	14:38	Day 1	1 hour	F3	uncoated	0	0	0	0	0	0	0		
19-MAY-2018	15:38	Day 1	2 hour	F1	uncoated	0	1	0	0	0	1	0		
19-MAY-2018	15:38	Day 1	2 hour	F2	uncoated	0	1	0	0	0	1	0		
19-MAY-2018	15:38	Day 1	2 hour	F3	uncoated	0	1	0	0	0	1	0		
19-MAY-2018	14:22	Unscheduled		F2	uncoated	0	1	0	0	0	0		NA	MO notifies as repeat
20-MAY-2018	13:29	Day 2		F1	uncoated	0	0	0	0	0	0	0		
20-MAY-2018	13:29	Day 2		F2	uncoated	0	0	0	0	0	0	0		
20-MAY-2018	13:29	Day 2		F3	uncoated	0	0	0	0	0	0	0		
22-MAY-2018	13:45	Day 4		F1	uncoated	0	0	0	0	0	0	0		
22-MAY-2018	13:45	Day 4		F2	uncoated	0	0	0	0	0	0	0		
22-MAY-2018	13:45	Day 4		F3	uncoated	0	0	0	0	0	0	0		

Key: 0= None; 1=Mild; 2=Moderate; 3=Severe; # Other = Pinprick bleeding or seepage

Administration Site Key: FA = Forearm; UA= Upper arm
Treatments Part A: A1 = A/Sing MAP, 15 ug (FA); A2 = Afluria Quad IM (UA), 15 ug; A3 = Placebo MAP (FA); A4 = A/Sing Mono IM, 15 ug (UA)
Treatments Part B: B1 = A/Sing MAP, 15 ug (FA) with biopsy; B2 = Placebo MAP (FA) with biopsy
B3 = A/Sing MAP, 15 ug (FA); B4 = A/Sing MAP, 10 ug (FA); B5 = A/Sing MAP, 5 ug (FA); B6 = A/Sing MAP, 2.5 ug (FA)
B7 = Placebo MAP (FA); B8 = A/Sing MAP, 15 ug (UA); B9 = A/Sing IM, 15 ug, as 2018 Afluria Quad (UA)

Listing 16.2.9.2
Treatment Site Tolerability Assessment per Site

Part B
Randomisation Number = B0046, Treatment Group = Group B7

Date	Time	Visit	Time Point	Site Number	Treatment for Site	Indura-tion	Redness	Bruising	Itching	Skin Flaking	Tender-ness	Definition/ Visibility	Other#	Unscheduled Reason
29-MAY-2018	13:26	Day 8		F1	uncoated	0	0	0	0	0	0	1		
29-MAY-2018	13:26	Day 8		F2	uncoated	0	0	0	0	1	0	1		
29-MAY-2018	13:26	Day 8		F3	uncoated	0	0	0	0	1	0	1		
09-JUN-2018	13:22	Day 22		F1	uncoated	0	0	0	0	0	0	0		
09-JUN-2018	13:22	Day 22		F2	uncoated	0	0	0	0	0	0	0		
09-JUN-2018	13:22	Day 22		F3	uncoated	0	0	0	0	0	0	0		
18-JUL-2018	13:01	EOS/ET (Day 61)		F1	uncoated	0	0	0	0	0	0	0		
18-JUL-2018	13:01	EOS/ET (Day 61)		F2	uncoated	0	0	0	0	0	0	0		
18-JUL-2018	13:01	EOS/ET (Day 61)		F3	uncoated	0	0	0	0	0	0	0		

Key: 0= None; 1=Mild; 2=Moderate; 3=Severe; # Other = Pinprick bleeding or seepage

Administration Site Key: FA = Forearm; UA= Upper arm
Treatments Part A: A1 = A/Sing MAP, 15 ug (FA); A2 = Afluria Quad IM (UA), 15 ug; A3 = Placebo MAP (FA); A4 = A/Sing Mono IM, 15 ug (UA)
Treatments Part B: B1 = A/Sing MAP, 15 ug (FA) with biopsy; B2 = Placebo MAP (FA) with biopsy
B3 = A/Sing MAP, 15 ug (FA); B4 = A/Sing MAP, 10 ug (FA); B5 = A/Sing MAP, 5 ug (FA); B6 = A/Sing MAP, 2.5 ug (FA)
B7 = Placebo MAP (FA); B8 = A/Sing MAP, 15 ug (UA); B9 = A/Sing IM, 15 ug, as 2018 Afluria Quad (UA)

Listing 16.2.9.2
Treatment Site Tolerability Assessment per Site

Part B
Randomisation Number = B0047, Treatment Group = Group B9

Date	Time	Visit	Time Point	Site Number	Treatment for Site	Indura-tion	Redness	Bruising	Itching	Skin Flaking	Tender-ness	Definition/ Visibility	Other#	Unscheduled Reason
19-MAY-2018	10:42	Day 1	Pre-dose	D1	[IM]	0	0	0	0	0	0	5		
19-MAY-2018	11:40	Day 1	10 min	D1	[IM]	0	0	0	0	0	0	5		
19-MAY-2018	12:31	Day 1	1 hour	D1	[IM]	0	0	0	0	0	0	5		
19-MAY-2018	13:42	Day 1	2 hour	D1	[IM]	0	0	0	0	0	0	5		
20-MAY-2018	10:57	Day 2		D1	[IM]	0	0	0	0	0	0	5		
22-MAY-2018	08:24	Day 4		D1	[IM]	0	0	0	0	0	0	5		
26-MAY-2018	12:24	Day 8		D1	[IM]	0	0	0	0	0	0	5		
09-JUN-2018	13:35	Day 22		D1	[IM]	0	0	0	0	0	0	5		
18-JUL-2018	08:43	EOS/ET (Day 61)		D1	[IM]	0	0	0	0	0	0	5		

Key: 0= None; 1=Mild; 2=Moderate; 3=Severe; # Other = Pinprick bleeding or seepage

Administration Site Key: FA = Forearm; UA= Upper arm
Treatments Part A: A1 = A/Sing MAP, 15 ug (FA); A2 = Afluria Quad IM (UA), 15 ug; A3 = Placebo MAP (FA); A4 = A/Sing Mono IM, 15 ug (UA)
Treatments Part B: B1 = A/Sing MAP, 15 ug (FA) with biopsy; B2 = Placebo MAP (FA) with biopsy
B3 = A/Sing MAP, 15 ug (FA); B4 = A/Sing MAP, 10 ug (FA); B5 = A/Sing MAP, 5 ug (FA); B6 = A/Sing MAP, 2.5 ug (FA)
B7 = Placebo MAP (FA); B8 = A/Sing MAP, 15 ug (UA); B9 = A/Sing IM, 15 ug, as 2018 Afluria Quad (UA)

Listing 16.2.9.2
Treatment Site Tolerability Assessment per Site

Part B
Randomisation Number = B0048, Treatment Group = Group B6

Date	Time	Visit	Time Point	Site Number	Treatment for Site	Indura-tion	Redness	Bruising	Itching	Skin Flaking	Tender-ness	Definition/ Visibility	Other#	Unscheduled Reason
19-MAY-2018	12:52	Day 1	Pre-dose	F1	2.5 ug	0	0	0	0	0	0	0		
19-MAY-2018	12:52	Day 1	Pre-dose	F2	uncoated	0	0	0	0	0	0	0		
19-MAY-2018	12:52	Day 1	Pre-dose	F3	uncoated	0	0	0	0	0	0	0		
19-MAY-2018	13:59	Day 1	10 min	F1	2.5 ug	0	1	0	0	0	0	0		
19-MAY-2018	13:59	Day 1	10 min	F2	uncoated	0	0	0	0	0	0	0		
19-MAY-2018	13:59	Day 1	10 min	F3	uncoated	0	0	0	0	0	0	0		
19-MAY-2018	14:48	Day 1	1 hour	F1	2.5 ug	0	0	0	0	0	0	0		
19-MAY-2018	14:48	Day 1	1 hour	F2	uncoated	0	0	0	0	0	0	0		
19-MAY-2018	14:48	Day 1	1 hour	F3	uncoated	0	0	0	0	0	0	0		
19-MAY-2018	15:48	Day 1	2 hour	F1	2.5 ug	0	1	0	0	0	1	0		
19-MAY-2018	15:48	Day 1	2 hour	F2	uncoated	0	1	0	1	0	1	0		
19-MAY-2018	15:48	Day 1	2 hour	F3	uncoated	0	1	0	0	0	1	0		
20-MAY-2018	13:17	Day 2		F1	2.5 ug	1	1	0	0	0	0	0		
20-MAY-2018	13:17	Day 2		F2	uncoated	1	0	0	0	0	0	0		
20-MAY-2018	13:17	Day 2		F3	uncoated	0	0	0	0	0	0	0		
22-MAY-2018	10:49	Day 4		F1	2.5 ug	1	0	0	0	0	0	0		
22-MAY-2018	10:49	Day 4		F2	uncoated	0	0	0	0	0	0	1		
22-MAY-2018	10:49	Day 4		F3	uncoated	0	0	0	0	0	0	1		
26-MAY-2018	10:22	Day 8		F1	2.5 ug	0	0	0	0	1	0	2		
26-MAY-2018	10:22	Day 8		F2	uncoated	0	0	0	0	0	0	0		

Key: 0= None; 1=Mild; 2=Moderate; 3=Severe; # Other = Pinprick bleeding or seepage

Administration Site Key: FA = Forearm; UA= Upper arm
Treatments Part A: A1 = A/Sing MAP, 15 ug (FA); A2 = Afluria Quad IM (UA), 15 ug; A3 = Placebo MAP (FA); A4 = A/Sing Mono IM, 15 ug (UA)
Treatments Part B: B1 = A/Sing MAP, 15 ug (FA) with biopsy; B2 = Placebo MAP (FA) with biopsy
B3 = A/Sing MAP, 15 ug (FA); B4 = A/Sing MAP, 10 ug (FA); B5 = A/Sing MAP, 5 ug (FA); B6 = A/Sing MAP, 2.5 ug (FA)
B7 = Placebo MAP (FA); B8 = A/Sing MAP, 15 ug (UA); B9 = A/Sing IM, 15 ug, as 2018 Afluria Quad (UA)

Listing 16.2.9.2
Treatment Site Tolerability Assessment per Site

Part B
Randomisation Number = B0048, Treatment Group = Group B6

Date	Time	Visit	Time Point	Site Number	Treatment for Site	Indura-tion	Redness	Bruising	Itching	Skin Flaking	Tender-ness	Definition/ Visibility	Other#	Unscheduled Reason
26-MAY-2018	10:22	Day 8		F3	uncoated	0	0	0	0	0	0	0		
09-JUN-2018	12:15	Day 22		F1	2.5 ug	0	0	0	0	1	0	1		
09-JUN-2018	12:15	Day 22		F2	uncoated	0	0	0	0	0	0	0		
09-JUN-2018	12:15	Day 22		F3	uncoated	0	0	0	0	0	0	0		
18-JUL-2018	15:55	EOS/ET (Day 61)		F1	2.5 ug	0	0	0	0	0	0	1		
18-JUL-2018	15:55	EOS/ET (Day 61)		F2	uncoated	0	0	0	0	0	0	0		
18-JUL-2018	15:55	EOS/ET (Day 61)		F3	uncoated	0	0	0	0	0	0	0		

Key: 0= None; 1=Mild; 2=Moderate; 3=Severe; # Other = Pinprick bleeding or seepage

Administration Site Key: FA = Forearm; UA= Upper arm
Treatments Part A: A1 = A/Sing MAP, 15 ug (FA); A2 = Afluria Quad IM (UA), 15 ug; A3 = Placebo MAP (FA); A4 = A/Sing Mono IM, 15 ug (UA)
Treatments Part B: B1 = A/Sing MAP, 15 ug (FA) with biopsy; B2 = Placebo MAP (FA) with biopsy
B3 = A/Sing MAP, 15 ug (FA); B4 = A/Sing MAP, 10 ug (FA); B5 = A/Sing MAP, 5 ug (FA); B6 = A/Sing MAP, 2.5 ug (FA)
B7 = Placebo MAP (FA); B8 = A/Sing MAP, 15 ug (UA); B9 = A/Sing IM, 15 ug, as 2018 Afluria Quad (UA)

Listing 16.2.9.2
Treatment Site Tolerability Assessment per Site

Part B
Randomisation Number = B0049, Treatment Group = Group B4

Date	Time	Visit	Time Point	Site Number	Treatment for Site	Indura-tion	Redness	Bruising	Itching	Skin Flaking	Tender-ness	Definition/ Visibility	Other#	Unscheduled Reason
19-MAY-2018	14:00	Day 1	Pre-dose	F1	uncoated	0	0	0	0	0	0	0		
19-MAY-2018	14:00	Day 1	Pre-dose	F2	5 ug	0	0	0	0	0	0	0		
19-MAY-2018	14:00	Day 1	Pre-dose	F3	5 ug	0	0	0	0	0	0	0		
19-MAY-2018	14:28	Day 1	10 min	F1	uncoated	0	0	0	0	0	1	0		
19-MAY-2018	14:28	Day 1	10 min	F2	5 ug	0	0	0	0	0	1	0		
19-MAY-2018	14:28	Day 1	10 min	F3	5 ug	0	0	0	0	0	0	0		
19-MAY-2018	15:18	Day 1	1 hour	F1	uncoated	0	0	0	0	0	0	0		
19-MAY-2018	15:18	Day 1	1 hour	F2	5 ug	0	1	0	0	0	0	0		
19-MAY-2018	15:18	Day 1	1 hour	F3	5 ug	0	1	0	0	0	0	0		
19-MAY-2018	16:18	Day 1	2 hour	F1	uncoated	0	1	0	0	0	1	0		
19-MAY-2018	16:18	Day 1	2 hour	F2	5 ug	0	1	0	0	0	1	0		
19-MAY-2018	16:18	Day 1	2 hour	F3	5 ug	0	1	0	0	0	0	0		
20-MAY-2018	14:02	Day 2		F1	uncoated	0	0	0	0	0	0	0		
20-MAY-2018	14:02	Day 2		F2	5 ug	1	1	0	0	0	0	0		
20-MAY-2018	14:02	Day 2		F3	5 ug	1	1	0	0	0	0	0		
22-MAY-2018	15:08	Day 4		F1	uncoated	0	0	0	0	1	0	2		
22-MAY-2018	15:08	Day 4		F2	5 ug	0	1	0	1	0	0	2		
22-MAY-2018	15:08	Day 4		F3	5 ug	0	1	0	1	0	0	2		
22-MAY-2018	15:17	Unscheduled		F1	uncoated	0	0	0	0	0	0	2	NA	MO notifies as repeat

Key: 0= None; 1=Mild; 2=Moderate; 3=Severe; # Other = Pinprick bleeding or seepage

Administration Site Key: FA = Forearm; UA= Upper arm
Treatments Part A: A1 = A/Sing MAP, 15 ug (FA); A2 = Afluria Quad IM (UA), 15 ug; A3 = Placebo MAP (FA); A4 = A/Sing Mono IM, 15 ug (UA)
Treatments Part B: B1 = A/Sing MAP, 15 ug (FA) with biopsy; B2 = Placebo MAP (FA) with biopsy
B3 = A/Sing MAP, 15 ug (FA); B4 = A/Sing MAP, 10 ug (FA); B5 = A/Sing MAP, 5 ug (FA); B6 = A/Sing MAP, 2.5 ug (FA)
B7 = Placebo MAP (FA); B8 = A/Sing MAP, 15 ug (UA); B9 = A/Sing IM, 15 ug, as 2018 Afluria Quad (UA)

Listing 16.2.9.2
Treatment Site Tolerability Assessment per Site

Part B
Randomisation Number = B0049, Treatment Group = Group B4

Date	Time	Visit	Time Point	Site Number	Treatment for Site	Indura-tion	Redness	Bruising	Itching	Skin Flaking	Tender-ness	Definition/ Visibility	Other#	Unscheduled Reason
22-MAY-2018	15:17	Unscheduled		F2	5 ug	0	0	0	1	1	0	3	NA	MO notifies as repeat
22-MAY-2018	15:17	Unscheduled		F3	5 ug	0	0	0	1	1	0	3	NA	MO notifies as repeat
26-MAY-2018	10:59	Day 8		F1	uncoated	0	0	0	0	0	0	2		
26-MAY-2018	10:59	Day 8		F2	5 ug	0	0	0	0	2	0	2		
26-MAY-2018	10:59	Day 8		F3	5 ug	0	0	0	0	1	0	2		
09-JUN-2018	12:22	Day 22		F1	uncoated	0	0	0	0	0	0	2		
09-JUN-2018	12:22	Day 22		F2	5 ug	0	0	0	0	1	0	2		
09-JUN-2018	12:22	Day 22		F3	5 ug	0	0	0	0	1	0	3		
18-JUL-2018	10:49	EOS/ET (Day 61)		F1	uncoated	0	0	0	0	0	0	2		
18-JUL-2018	10:49	EOS/ET (Day 61)		F2	5 ug	0	0	0	0	0	0	2		
18-JUL-2018	10:49	EOS/ET (Day 61)		F3	5 ug	0	0	0	0	0	0	2		

Key: 0= None; 1=Mild; 2=Moderate; 3=Severe; # Other = Pinprick bleeding or seepage

Administration Site Key: FA = Forearm; UA= Upper arm
Treatments Part A: A1 = A/Sing MAP, 15 ug (FA); A2 = Afluria Quad IM (UA), 15 ug; A3 = Placebo MAP (FA); A4 = A/Sing Mono IM, 15 ug (UA)
Treatments Part B: B1 = A/Sing MAP, 15 ug (FA) with biopsy; B2 = Placebo MAP (FA) with biopsy
B3 = A/Sing MAP, 15 ug (FA); B4 = A/Sing MAP, 10 ug (FA); B5 = A/Sing MAP, 5 ug (FA); B6 = A/Sing MAP, 2.5 ug (FA)
B7 = Placebo MAP (FA); B8 = A/Sing MAP, 15 ug (UA); B9 = A/Sing IM, 15 ug, as 2018 Afluria Quad (UA)

Listing 16.2.9.2
Treatment Site Tolerability Assessment per Site

Part B
Randomisation Number = B0050, Treatment Group = Group B8

Date	Time	Visit	Time Point	Site Number	Treatment for Site	Induration	Redness	Bruising	Itching	Skin Flaking	Tenderness	Definition/ Visibility	Other#	Unscheduled Reason
19-MAY-2018	13:32	Day 1	Pre-dose	D1	5 ug	0	0	0	0	0	0	0		
19-MAY-2018	13:32	Day 1	Pre-dose	D2	5 ug	0	0	0	0	0	0	0		
19-MAY-2018	13:32	Day 1	Pre-dose	D3	5 ug	0	0	0	0	0	0	0		
19-MAY-2018	14:39	Day 1	10 min	D1	5 ug	0	0	0	0	0	1	0		
19-MAY-2018	14:39	Day 1	10 min	D2	5 ug	0	0	0	0	0	1	0		
19-MAY-2018	14:39	Day 1	10 min	D3	5 ug	0	0	0	0	0	1	0		
19-MAY-2018	15:28	Day 1	1 hour	D1	5 ug	0	0	0	0	0	0	0		
19-MAY-2018	15:28	Day 1	1 hour	D2	5 ug	0	0	0	0	0	0	0		
19-MAY-2018	15:28	Day 1	1 hour	D3	5 ug	0	0	0	0	0	0	0		
19-MAY-2018	16:28	Day 1	2 hour	D1	5 ug	0	0	0	0	0	0	0		
19-MAY-2018	16:28	Day 1	2 hour	D2	5 ug	0	0	0	0	0	0	0		
19-MAY-2018	16:28	Day 1	2 hour	D3	5 ug	0	0	0	0	0	0	0		
20-MAY-2018	14:45	Day 2		D1	5 ug	1	1	0	0	0	1	0		
20-MAY-2018	14:45	Day 2		D2	5 ug	1	1	0	0	0	1	0		
20-MAY-2018	14:45	Day 2		D3	5 ug	2	1	0	0	0	1	0		
20-MAY-2018	15:01	Unscheduled		D3	5 ug	2							NA	MO notifies as repeat
22-MAY-2018	11:48	Day 4		D1	5 ug	0	1	0	0	0	0	0		
22-MAY-2018	11:48	Day 4		D2	5 ug	1	1	0	0	0	0	0		
22-MAY-2018	11:48	Day 4		D3	5 ug	0	1	0	0	0	0	0		

Key: 0= None; 1=Mild; 2=Moderate; 3=Severe; # Other = Pinprick bleeding or seepage

Administration Site Key: FA = Forearm; UA= Upper arm
Treatments Part A: A1 = A/Sing MAP, 15 ug (FA); A2 = Afluria Quad IM (UA), 15 ug; A3 = Placebo MAP (FA); A4 = A/Sing Mono IM, 15 ug (UA)
Treatments Part B: B1 = A/Sing MAP, 15 ug (FA) with biopsy; B2 = Placebo MAP (FA) with biopsy
B3 = A/Sing MAP, 15 ug (FA); B4 = A/Sing MAP, 10 ug (FA); B5 = A/Sing MAP, 5 ug (FA); B6 = A/Sing MAP, 2.5 ug (FA)
B7 = Placebo MAP (FA); B8 = A/Sing MAP, 15 ug (UA); B9 = A/Sing IM, 15 ug, as 2018 Afluria Quad (UA)

Listing 16.2.9.2
Treatment Site Tolerability Assessment per Site

Part B
Randomisation Number = B0050, Treatment Group = Group B8

Date	Time	Visit	Time Point	Site Number	Treatment for Site	Indura-tion	Redness	Bruising	Itching	Skin Flaking	Tender-ness	Definition/ Visibility	Other#	Unscheduled Reason
26-MAY-2018	11:45	Day 8		D1	5 ug	0	0	0	0	0	0	0		
26-MAY-2018	11:45	Day 8		D2	5 ug	0	0	0	0	0	0	0		
26-MAY-2018	11:45	Day 8		D3	5 ug	0	0	0	0	0	0	0		
09-JUN-2018	12:42	Day 22		D1	5 ug	0	0	0	0	1	0	2		
09-JUN-2018	12:42	Day 22		D2	5 ug	0	0	0	0	1	0	2		
09-JUN-2018	12:42	Day 22		D3	5 ug	0	0	0	0	1	0	2		
18-JUL-2018	14:38	EOS/ET (Day 61)		D1	5 ug	0	0	0	0	0	0	0		
18-JUL-2018	14:38	EOS/ET (Day 61)		D2	5 ug	0	0	0	0	0	0	0		
18-JUL-2018	14:38	EOS/ET (Day 61)		D3	5 ug	0	0	0	0	0	0	0		

Key: 0= None; 1=Mild; 2=Moderate; 3=Severe; # Other = Pinprick bleeding or seepage

Administration Site Key: FA = Forearm; UA= Upper arm
Treatments Part A: A1 = A/Sing MAP, 15 ug (FA); A2 = Afluria Quad IM (UA), 15 ug; A3 = Placebo MAP (FA); A4 = A/Sing Mono IM, 15 ug (UA)
Treatments Part B: B1 = A/Sing MAP, 15 ug (FA) with biopsy; B2 = Placebo MAP (FA) with biopsy
B3 = A/Sing MAP, 15 ug (FA); B4 = A/Sing MAP, 10 ug (FA); B5 = A/Sing MAP, 5 ug (FA); B6 = A/Sing MAP, 2.5 ug (FA)
B7 = Placebo MAP (FA); B8 = A/Sing MAP, 15 ug (UA); B9 = A/Sing IM, 15 ug, as 2018 Afluria Quad (UA)

Listing 16.2.9.2
Treatment Site Tolerability Assessment per Site

Part B
Randomisation Number = B0051, Treatment Group = Group B3

Date	Time	Visit	Time Point	Site Number	Treatment for Site	Indura-tion	Redness	Bruising	Itching	Skin Flaking	Tender-ness	Definition/ Visibility	Other#	Unscheduled Reason
25-MAY-2018	10:55	Day 1	Pre-dose	F1	5 ug	0	0	0	0	0	0	0		
25-MAY-2018	10:55	Day 1	Pre-dose	F2	5 ug	0	0	0	0	0	0	0		
25-MAY-2018	10:55	Day 1	Pre-dose	F3	5 ug	0	0	0	0	0	0	0		
25-MAY-2018	13:08	Day 1	10 min	F1	5 ug	0	1	0	0	0	0	0		
25-MAY-2018	13:08	Day 1	10 min	F2	5 ug	0	1	0	0	0	0	0		
25-MAY-2018	13:08	Day 1	10 min	F3	5 ug	0	0	0	0	0	0	0		
25-MAY-2018	13:58	Day 1	1 hour	F1	5 ug	0	1	0	0	0	0	0		
25-MAY-2018	13:58	Day 1	1 hour	F2	5 ug	0	1	0	0	0	0	0		
25-MAY-2018	13:58	Day 1	1 hour	F3	5 ug	0	1	0	0	0	0	0		
25-MAY-2018	14:59	Day 1	2 hour	F1	5 ug	0	0	0	0	0	0	0		
25-MAY-2018	14:59	Day 1	2 hour	F2	5 ug	0	0	0	0	0	0	0		
25-MAY-2018	14:59	Day 1	2 hour	F3	5 ug	0	0	0	0	0	0	0		
26-MAY-2018	10:25	Day 2		F1	5 ug	0	1	0	0	0	0	0		
26-MAY-2018	10:25	Day 2		F2	5 ug	0	1	0	0	0	0	0		
26-MAY-2018	10:25	Day 2		F3	5 ug	0	1	0	0	0	0	0		
26-MAY-2018	10:35	Unscheduled		F1	5 ug	0	0	0	0	0	0	0		MO notifies as repeat
26-MAY-2018	10:36	Unscheduled		F2	5 ug	0	0	0	0	0	0	0		MO notifies as repeat

Key: 0= None; 1=Mild; 2=Moderate; 3=Severe; # Other = Pinprick bleeding or seepage

Administration Site Key: FA = Forearm; UA= Upper arm
Treatments Part A: A1 = A/Sing MAP, 15 ug (FA); A2 = Afluria Quad IM (UA), 15 ug; A3 = Placebo MAP (FA); A4 = A/Sing Mono IM, 15 ug (UA)
Treatments Part B: B1 = A/Sing MAP, 15 ug (FA) with biopsy; B2 = Placebo MAP (FA) with biopsy
B3 = A/Sing MAP, 15 ug (FA); B4 = A/Sing MAP, 10 ug (FA); B5 = A/Sing MAP, 5 ug (FA); B6 = A/Sing MAP, 2.5 ug (FA)
B7 = Placebo MAP (FA); B8 = A/Sing MAP, 15 ug (UA); B9 = A/Sing IM, 15 ug, as 2018 Afluria Quad (UA)

Listing 16.2.9.2
Treatment Site Tolerability Assessment per Site

Part B
Randomisation Number = B0051, Treatment Group = Group B3

Date	Time	Visit	Time Point	Site Number	Treatment for Site	Indura-tion	Redness	Bruising	Itching	Skin Flaking	Tender-ness	Definition/ Visibility	Other#	Unscheduled Reason
26-MAY-2018	10:37	Unscheduled		F3	5 ug	0	0	0	0	0	0	0		MO notifies as repeat
28-MAY-2018	08:52	Day 4		F1	5 ug	0	1	0	1	1	0	0		
28-MAY-2018	08:52	Day 4		F2	5 ug	0	1	0	1	1	0	0		
28-MAY-2018	08:52	Day 4		F3	5 ug	0	1	0	1	1	0	0		
01-JUN-2018	10:46	Day 8		F1	5 ug	0	0	0	1	0	0	0		
01-JUN-2018	10:46	Day 8		F2	5 ug	0	0	0	1	2	0	0		
01-JUN-2018	10:46	Day 8		F3	5 ug	0	0	0	1	2	0	0		
15-JUN-2018	08:10	Day 22		F1	5 ug	0	0	0	0	0	0	1		
15-JUN-2018	08:10	Day 22		F2	5 ug	0	0	0	0	0	0	0		
15-JUN-2018	08:10	Day 22		F3	5 ug	0	0	0	0	0	0	0		
24-JUL-2018	08:38	EOS/ET (Day 61)		F1	5 ug	0	0	0	0	0	0	0		
24-JUL-2018	08:38	EOS/ET (Day 61)		F2	5 ug	0	0	0	0	0	0	0		
24-JUL-2018	08:38	EOS/ET (Day 61)		F3	5 ug	0	0	0	0	0	0	0		

Key: 0= None; 1=Mild; 2=Moderate; 3=Severe; # Other = Pinprick bleeding or seepage

Administration Site Key: FA = Forearm; UA= Upper arm
Treatments Part A: A1 = A/Sing MAP, 15 ug (FA); A2 = Afluria Quad IM (UA), 15 ug; A3 = Placebo MAP (FA); A4 = A/Sing Mono IM, 15 ug (UA)
Treatments Part B: B1 = A/Sing MAP, 15 ug (FA) with biopsy; B2 = Placebo MAP (FA) with biopsy
B3 = A/Sing MAP, 15 ug (FA); B4 = A/Sing MAP, 10 ug (FA); B5 = A/Sing MAP, 5 ug (FA); B6 = A/Sing MAP, 2.5 ug (FA)
B7 = Placebo MAP (FA); B8 = A/Sing MAP, 15 ug (UA); B9 = A/Sing IM, 15 ug, as 2018 Afluria Quad (UA)

Listing 16.2.9.2
Treatment Site Tolerability Assessment per Site

Part B
Randomisation Number = B0052, Treatment Group = Group B5

Date	Time	Visit	Time Point	Site Number	Treatment for Site	Indura-tion	Redness	Bruising	Itching	Skin Flaking	Tender-ness	Definition/ Visibility	Other#	Unscheduled Reason
25-MAY-2018	11:09	Day 1	Pre-dose	F1	5 ug	0	0	0	0	0	0	0		
25-MAY-2018	11:09	Day 1	Pre-dose	F2	uncoated	0	0	0	0	0	0	0		
25-MAY-2018	11:09	Day 1	Pre-dose	F3	uncoated	0	0	0	0	0	0	0		
25-MAY-2018	13:22	Day 1	10 min	F1	5 ug	0	0	0	0	0	1	0		
25-MAY-2018	13:22	Day 1	10 min	F2	uncoated	0	0	0	0	0	0	0		
25-MAY-2018	13:22	Day 1	10 min	F3	uncoated	0	0	0	0	0	0	0		
25-MAY-2018	14:12	Day 1	1 hour	F1	5 ug	0	0	0	0	0	0	0		
25-MAY-2018	14:12	Day 1	1 hour	F2	uncoated	0	0	0	0	0	0	0		
25-MAY-2018	14:12	Day 1	1 hour	F3	uncoated	0	0	0	0	0	1	0		
25-MAY-2018	15:13	Day 1	2 hour	F1	5 ug	0	0	0	0	0	0	0		
25-MAY-2018	15:13	Day 1	2 hour	F2	uncoated	0	0	0	0	0	1	0		
25-MAY-2018	15:13	Day 1	2 hour	F3	uncoated	0	0	0	0	0	1	0		
26-MAY-2018	09:38	Day 2		F1	5 ug	0	0	0	0	0	1	0		
26-MAY-2018	09:38	Day 2		F2	uncoated	0	0	0	0	0	0	0		
26-MAY-2018	09:38	Day 2		F3	uncoated	0	0	0	0	0	1	0		
26-MAY-2018	09:55	Unscheduled		F1	5 ug	0	0	0	0	0	1	0	NA	Mo notifies as repeat
28-MAY-2018	13:08	Day 4		F1	5 ug	0	1	0	0	0	0	0		
28-MAY-2018	13:08	Day 4		F2	uncoated	0	0	0	0	0	0	0		
28-MAY-2018	13:08	Day 4		F3	uncoated	0	0	0	0	0	0	0		

Key: 0= None; 1=Mild; 2=Moderate; 3=Severe; # Other = Pinprick bleeding or seepage

Administration Site Key: FA = Forearm; UA= Upper arm
Treatments Part A: A1 = A/Sing MAP, 15 ug (FA); A2 = Afluria Quad IM (UA), 15 ug; A3 = Placebo MAP (FA); A4 = A/Sing Mono IM, 15 ug (UA)
Treatments Part B: B1 = A/Sing MAP, 15 ug (FA) with biopsy; B2 = Placebo MAP (FA) with biopsy
B3 = A/Sing MAP, 15 ug (FA); B4 = A/Sing MAP, 10 ug (FA); B5 = A/Sing MAP, 5 ug (FA); B6 = A/Sing MAP, 2.5 ug (FA)
B7 = Placebo MAP (FA); B8 = A/Sing MAP, 15 ug (UA); B9 = A/Sing IM, 15 ug, as 2018 Afluria Quad (UA)

Listing 16.2.9.2
Treatment Site Tolerability Assessment per Site

Part B
Randomisation Number = B0052, Treatment Group = Group B5

Date	Time	Visit	Time Point	Site Number	Treatment for Site	Indura-tion	Redness	Bruising	Itching	Skin Flaking	Tender-ness	Definition/ Visibility	Other#	Unscheduled Reason
01-JUN-2018	13:55	Day 8		F1	5 ug	0	0	0	0	3	0	3		
01-JUN-2018	13:55	Day 8		F2	uncoated	0	0	0	0	0	0	2		
01-JUN-2018	13:55	Day 8		F3	uncoated	0	0	0	0	0	0	2		
15-JUN-2018	13:22	Day 22		F1	5 ug	0	0	0	0	2	0	0		
15-JUN-2018	13:22	Day 22		F2	uncoated	0	0	0	0	0	0	2		
15-JUN-2018	13:22	Day 22		F3	uncoated	0	0	0	0	0	0	2		
20-JUL-2018	14:10	EOS/ET (Day 61)		F1	5 ug	0	0	0	0	0	0	2		
20-JUL-2018	14:10	EOS/ET (Day 61)		F2	uncoated	0	0	0	0	0	0	1		
20-JUL-2018	14:10	EOS/ET (Day 61)		F3	uncoated	0	0	0	0	0	0	1		

Key: 0= None; 1=Mild; 2=Moderate; 3=Severe; # Other = Pinprick bleeding or seepage

Administration Site Key: FA = Forearm; UA= Upper arm
Treatments Part A: A1 = A/Sing MAP, 15 ug (FA); A2 = Afluria Quad IM (UA), 15 ug; A3 = Placebo MAP (FA); A4 = A/Sing Mono IM, 15 ug (UA)
Treatments Part B: B1 = A/Sing MAP, 15 ug (FA) with biopsy; B2 = Placebo MAP (FA) with biopsy
B3 = A/Sing MAP, 15 ug (FA); B4 = A/Sing MAP, 10 ug (FA); B5 = A/Sing MAP, 5 ug (FA); B6 = A/Sing MAP, 2.5 ug (FA)
B7 = Placebo MAP (FA); B8 = A/Sing MAP, 15 ug (UA); B9 = A/Sing IM, 15 ug, as 2018 Afluria Quad (UA)

Listing 16.2.9.2
Treatment Site Tolerability Assessment per Site

Part B
Randomisation Number = B0053, Treatment Group = Group B6

Date	Time	Visit	Time Point	Site Number	Treatment for Site	Indura-tion	Redness	Bruising	Itching	Skin Flaking	Tender-ness	Definition/ Visibility	Other#	Unscheduled Reason
25-MAY-2018	11:17	Day 1	Pre-dose	F1	uncoated	0	0	0	0	0	0	0		
25-MAY-2018	11:17	Day 1	Pre-dose	F2	uncoated	0	0	0	0	0	0	0		
25-MAY-2018	11:17	Day 1	Pre-dose	F3	2.5 ug	0	0	0	0	0	0	0		
25-MAY-2018	13:28	Day 1	10 min	F1	uncoated	0	1	0	0	0	0	0		
25-MAY-2018	13:28	Day 1	10 min	F2	uncoated	0	1	0	0	0	0	0		
25-MAY-2018	13:28	Day 1	10 min	F3	2.5 ug	0	1	0	0	0	0	0		
25-MAY-2018	14:18	Day 1	1 hour	F1	uncoated	0	0	0	0	0	1	0		
25-MAY-2018	14:18	Day 1	1 hour	F2	uncoated	0	0	0	0	0	1	0		
25-MAY-2018	14:18	Day 1	1 hour	F3	2.5 ug	0	0	0	0	0	1	0		
25-MAY-2018	15:19	Day 1	2 hour	F1	uncoated	0	0	0	0	0	0	0		
25-MAY-2018	15:19	Day 1	2 hour	F2	uncoated	0	0	0	0	0	0	0		
25-MAY-2018	15:19	Day 1	2 hour	F3	2.5 ug	0	0	0	0	0	0	0		
26-MAY-2018	10:39	Day 2		F1	uncoated	0	0	0	0	0	0	0		
26-MAY-2018	10:39	Day 2		F2	uncoated	0	0	0	0	0	0	0		
26-MAY-2018	10:39	Day 2		F3	2.5 ug	0	0	0	0	0	0	0		
28-MAY-2018	12:30	Day 4		F1	uncoated	0	0	0	0	0	0	0		
28-MAY-2018	12:30	Day 4		F2	uncoated	0	0	0	0	0	0	0		
28-MAY-2018	12:30	Day 4		F3	2.5 ug	0	0	0	1	0	0	0		
01-JUN-2018	11:10	Day 8		F1	uncoated	0	0	0	0	1	0	3		
01-JUN-2018	11:10	Day 8		F2	uncoated	0	0	0	0	1	0	3		

Key: 0= None; 1=Mild; 2=Moderate; 3=Severe; # Other = Pinprick bleeding or seepage

Administration Site Key: FA = Forearm; UA= Upper arm
Treatments Part A: A1 = A/Sing MAP, 15 ug (FA); A2 = Afluria Quad IM (UA), 15 ug; A3 = Placebo MAP (FA); A4 = A/Sing Mono IM, 15 ug (UA)
Treatments Part B: B1 = A/Sing MAP, 15 ug (FA) with biopsy; B2 = Placebo MAP (FA) with biopsy
B3 = A/Sing MAP, 15 ug (FA); B4 = A/Sing MAP, 10 ug (FA); B5 = A/Sing MAP, 5 ug (FA); B6 = A/Sing MAP, 2.5 ug (FA)
B7 = Placebo MAP (FA); B8 = A/Sing MAP, 15 ug (UA); B9 = A/Sing IM, 15 ug, as 2018 Afluria Quad (UA)

Listing 16.2.9.2
Treatment Site Tolerability Assessment per Site

Part B
Randomisation Number = B0053, Treatment Group = Group B6

Date	Time	Visit	Time Point	Site Number	Treatment for Site	Indura-tion	Redness	Bruising	Itching	Skin Flaking	Tender-ness	Definition/ Visibility	Other#	Unscheduled Reason
01-JUN-2018	11:10	Day 8		F3	2.5 ug	0	0	0	1	3	0	3		
15-JUN-2018	13:02	Day 22		F1	uncoated	0	0	0	0	0	0	3		
15-JUN-2018	13:02	Day 22		F2	uncoated	0	0	0	0	0	0	3		
15-JUN-2018	13:02	Day 22		F3	2.5 ug	0	0	0	0	0	0	3		
25-JUL-2018	12:49	EOS/ET (Day 61)		F1	uncoated	0	0	0	0	0	0	1		
25-JUL-2018	12:49	EOS/ET (Day 61)		F2	uncoated	0	0	0	0	0	0	1		
25-JUL-2018	12:49	EOS/ET (Day 61)		F3	2.5 ug	0	0	0	0	0	0	1		

Key: 0= None; 1=Mild; 2=Moderate; 3=Severe; # Other = Pinprick bleeding or seepage

Administration Site Key: FA = Forearm; UA= Upper arm
Treatments Part A: A1 = A/Sing MAP, 15 ug (FA); A2 = Afluria Quad IM (UA), 15 ug; A3 = Placebo MAP (FA); A4 = A/Sing Mono IM, 15 ug (UA)
Treatments Part B: B1 = A/Sing MAP, 15 ug (FA) with biopsy; B2 = Placebo MAP (FA) with biopsy
B3 = A/Sing MAP, 15 ug (FA); B4 = A/Sing MAP, 10 ug (FA); B5 = A/Sing MAP, 5 ug (FA); B6 = A/Sing MAP, 2.5 ug (FA)
B7 = Placebo MAP (FA); B8 = A/Sing MAP, 15 ug (UA); B9 = A/Sing IM, 15 ug, as 2018 Afluria Quad (UA)

Listing 16.2.9.2
Treatment Site Tolerability Assessment per Site

Part B
Randomisation Number = B0054, Treatment Group = Group B5

Date	Time	Visit	Time Point	Site Number	Treatment for Site	Indura-tion	Redness	Bruising	Itching	Skin Flaking	Tender-ness	Definition/ Visibility	Other#	Unscheduled Reason
25-MAY-2018	11:24	Day 1	Pre-dose	F1	uncoated	0	0	0	0	0	0	0		
25-MAY-2018	11:24	Day 1	Pre-dose	F2	uncoated	0	0	0	0	0	0	0		
25-MAY-2018	11:24	Day 1	Pre-dose	F3	5 ug	0	0	0	0	0	0	0		
25-MAY-2018	13:38	Day 1	10 min	F1	uncoated	0	1	0	0	0	0	0		
25-MAY-2018	13:38	Day 1	10 min	F2	uncoated	0	1	0	0	0	0	0		
25-MAY-2018	13:38	Day 1	10 min	F3	5 ug	0	0	0	0	0	0	0		
25-MAY-2018	14:28	Day 1	1 hour	F1	uncoated	0	0	0	0	0	0	0		
25-MAY-2018	14:28	Day 1	1 hour	F2	uncoated	0	0	0	0	0	0	0		
25-MAY-2018	14:28	Day 1	1 hour	F3	5 ug	0	0	0	0	0	0	0		
25-MAY-2018	15:30	Day 1	2 hour	F1	uncoated	0	0	0	0	0	0	0		
25-MAY-2018	15:30	Day 1	2 hour	F2	uncoated	0	0	0	0	0	0	0		
25-MAY-2018	15:30	Day 1	2 hour	F3	5 ug	0	0	0	0	0	0	0		
26-MAY-2018	08:47	Day 2		F1	uncoated	0	0	0	0	0	0	0		
26-MAY-2018	08:47	Day 2		F2	uncoated	0	0	0	0	0	0	0		
26-MAY-2018	08:47	Day 2		F3	5 ug	0	1	0	0	0	0	0		
28-MAY-2018	09:14	Day 4		F1	uncoated	0	0	0	0	0	0	0		
28-MAY-2018	09:14	Day 4		F2	uncoated	0	0	0	0	0	0	0		
28-MAY-2018	09:14	Day 4		F3	5 ug	0	1	0	1	0	0	0		
01-JUN-2018	09:38	Day 8		F1	uncoated	0	0	0	0	0	0	2		
01-JUN-2018	09:38	Day 8		F2	uncoated	0	0	0	0	0	0	2		

Key: 0= None; 1=Mild; 2=Moderate; 3=Severe; # Other = Pinprick bleeding or seepage

Administration Site Key: FA = Forearm; UA= Upper arm
Treatments Part A: A1 = A/Sing MAP, 15 ug (FA); A2 = Afluria Quad IM (UA), 15 ug; A3 = Placebo MAP (FA); A4 = A/Sing Mono IM, 15 ug (UA)
Treatments Part B: B1 = A/Sing MAP, 15 ug (FA) with biopsy; B2 = Placebo MAP (FA) with biopsy
B3 = A/Sing MAP, 15 ug (FA); B4 = A/Sing MAP, 10 ug (FA); B5 = A/Sing MAP, 5 ug (FA); B6 = A/Sing MAP, 2.5 ug (FA)
B7 = Placebo MAP (FA); B8 = A/Sing MAP, 15 ug (UA); B9 = A/Sing IM, 15 ug, as 2018 Afluria Quad (UA)

Listing 16.2.9.2
Treatment Site Tolerability Assessment per Site

Part B
Randomisation Number = B0054, Treatment Group = Group B5

Date	Time	Visit	Time Point	Site Number	Treatment for Site	Indura-tion	Redness	Bruising	Itching	Skin Flaking	Tender-ness	Definition/ Visibility	Other#	Unscheduled Reason
01-JUN-2018	09:38	Day 8		F3	5 ug	0	0	0	0	0	0	2		
15-JUN-2018	08:12	Day 22		F1	uncoated	0	0	0	0	0	0	1		
15-JUN-2018	08:12	Day 22		F2	uncoated	0	0	0	0	0	0	0		
15-JUN-2018	08:12	Day 22		F3	5 ug	0	0	0	0	0	0	2		
24-JUL-2018	08:42	EOS/ET (Day 61)		F1	uncoated	0	0	0	0	0	0	0		
24-JUL-2018	08:42	EOS/ET (Day 61)		F2	uncoated	0	0	0	0	0	0	0		
24-JUL-2018	08:42	EOS/ET (Day 61)		F3	5 ug	0	0	0	0	0	0	0		

Key: 0= None; 1=Mild; 2=Moderate; 3=Severe; # Other = Pinprick bleeding or seepage

Administration Site Key: FA = Forearm; UA= Upper arm
Treatments Part A: A1 = A/Sing MAP, 15 ug (FA); A2 = Afluria Quad IM (UA), 15 ug; A3 = Placebo MAP (FA); A4 = A/Sing Mono IM, 15 ug (UA)
Treatments Part B: B1 = A/Sing MAP, 15 ug (FA) with biopsy; B2 = Placebo MAP (FA) with biopsy
B3 = A/Sing MAP, 15 ug (FA); B4 = A/Sing MAP, 10 ug (FA); B5 = A/Sing MAP, 5 ug (FA); B6 = A/Sing MAP, 2.5 ug (FA)
B7 = Placebo MAP (FA); B8 = A/Sing MAP, 15 ug (UA); B9 = A/Sing IM, 15 ug, as 2018 Afluria Quad (UA)

Listing 16.2.9.2
Treatment Site Tolerability Assessment per Site

Part B
Randomisation Number = B0055, Treatment Group = Group B8

Date	Time	Visit	Time Point	Site Number	Treatment for Site	Indura-tion	Redness	Bruising	Itching	Skin Flaking	Tender-ness	Definition/ Visibility	Other#	Unscheduled Reason
25-MAY-2018	11:35	Day 1	Pre-dose	D1	5 ug	0	0	0	0	0	0	0		
25-MAY-2018	11:35	Day 1	Pre-dose	D2	5 ug	0	0	0	0	0	0	0		
25-MAY-2018	11:35	Day 1	Pre-dose	D3	5 ug	0	0	0	0	0	0	0		
25-MAY-2018	13:48	Day 1	10 min	D1	5 ug	0	1	0	0	0	1	0		
25-MAY-2018	13:48	Day 1	10 min	D2	5 ug	0	1	0	0	0	1	0		
25-MAY-2018	13:48	Day 1	10 min	D3	5 ug	0	1	0	0	0	1	0		
25-MAY-2018	14:38	Day 1	1 hour	D1	5 ug	0	0	0	0	0	1	0		
25-MAY-2018	14:38	Day 1	1 hour	D2	5 ug	0	0	0	0	0	1	0		
25-MAY-2018	14:38	Day 1	1 hour	D3	5 ug	0	0	0	0	0	1	0		
25-MAY-2018	15:39	Day 1	2 hour	D1	5 ug	0	0	0	0	0	0			
25-MAY-2018	15:39	Day 1	2 hour	D2	5 ug	0	0	0	0	0	2	0		
25-MAY-2018	15:39	Day 1	2 hour	D3	5 ug	0	0	0	0	0	2	0		
25-MAY-2018	15:45	Unscheduled		D2	5 ug	0	0	0	0	0	2		NA	MO notifies as repeat
25-MAY-2018	15:45	Unscheduled		D3	5 ug	0	0	0	0	0	2	0	NA	MO notifies as repeat
26-MAY-2018	12:54	Day 2		D1	5 ug	0	1	0	0	0	0	0		
26-MAY-2018	12:54	Day 2		D2	5 ug	0	0	0	0	0	0	0		
26-MAY-2018	12:54	Day 2		D3	5 ug	0	1	0	0	0	0	0		
28-MAY-2018	17:38	Day 4		D1	5 ug	0	0	0	2	0	0	0		

Key: 0= None; 1=Mild; 2=Moderate; 3=Severe; # Other = Pinprick bleeding or seepage

Administration Site Key: FA = Forearm; UA= Upper arm
Treatments Part A: A1 = A/Sing MAP, 15 ug (FA); A2 = Afluria Quad IM (UA), 15 ug; A3 = Placebo MAP (FA); A4 = A/Sing Mono IM, 15 ug (UA)
Treatments Part B: B1 = A/Sing MAP, 15 ug (FA) with biopsy; B2 = Placebo MAP (FA) with biopsy
B3 = A/Sing MAP, 15 ug (FA); B4 = A/Sing MAP, 10 ug (FA); B5 = A/Sing MAP, 5 ug (FA); B6 = A/Sing MAP, 2.5 ug (FA)
B7 = Placebo MAP (FA); B8 = A/Sing MAP, 15 ug (UA); B9 = A/Sing IM, 15 ug, as 2018 Afluria Quad (UA)

Listing 16.2.9.2
Treatment Site Tolerability Assessment per Site

Part B
Randomisation Number = B0055, Treatment Group = Group B8

Date	Time	Visit	Time Point	Site Number	Treatment for Site	Indura-tion	Redness	Bruising	Itching	Skin Flaking	Tender-ness	Definition/ Visibility	Other#	Unscheduled Reason
28-MAY-2018	17:38	Day 4		D2	5 ug	0	0	0	2	0	0	0		
28-MAY-2018	17:38	Day 4		D3	5 ug	0	0	0	2	0	0	0		
01-JUN-2018	14:58	Day 8		D1	5 ug	0	0	0	0	1	0	1		
01-JUN-2018	14:58	Day 8		D2	5 ug	0	0	0	0	2	0	2		
01-JUN-2018	14:58	Day 8		D3	5 ug	0	0	0	0	2	0	2		
15-JUN-2018	13:57	Day 22		D1	5 ug	0	0	0	0	0	0	2		
15-JUN-2018	13:57	Day 22		D2	5 ug	0	0	0	0	0	0	2		
15-JUN-2018	13:57	Day 22		D3	5 ug	0	0	0	0	0	0	2		
24-JUL-2018	15:48	EOS/ET (Day 61)		D1	5 ug	0	0	0	0	0	0	0		
24-JUL-2018	15:48	EOS/ET (Day 61)		D2	5 ug	0	0	0	0	0	0	0		
24-JUL-2018	15:48	EOS/ET (Day 61)		D3	5 ug	0	0	0	0	0	0	0		

Key: 0= None; 1=Mild; 2=Moderate; 3=Severe; # Other = Pinprick bleeding or seepage

Administration Site Key: FA = Forearm; UA= Upper arm
Treatments Part A: A1 = A/Sing MAP, 15 ug (FA); A2 = Afluria Quad IM (UA), 15 ug; A3 = Placebo MAP (FA); A4 = A/Sing Mono IM, 15 ug (UA)
Treatments Part B: B1 = A/Sing MAP, 15 ug (FA) with biopsy; B2 = Placebo MAP (FA) with biopsy
B3 = A/Sing MAP, 15 ug (FA); B4 = A/Sing MAP, 10 ug (FA); B5 = A/Sing MAP, 5 ug (FA); B6 = A/Sing MAP, 2.5 ug (FA)
B7 = Placebo MAP (FA); B8 = A/Sing MAP, 15 ug (UA); B9 = A/Sing IM, 15 ug, as 2018 Afluria Quad (UA)

Listing 16.2.9.2
Treatment Site Tolerability Assessment per Site

Part B
Randomisation Number = B0056, Treatment Group = Group B3

Date	Time	Visit	Time Point	Site Number	Treatment for Site	Induration	Redness	Bruising	Itching	Skin Flaking	Tenderness	Definition/ Visibility	Other#	Unscheduled Reason
25-MAY-2018	11:45	Day 1	Pre-dose	F1	5 ug	0	0	0	0	0	0	0		
25-MAY-2018	11:45	Day 1	Pre-dose	F2	5 ug	0	0	0	0	0	0	0		
25-MAY-2018	11:45	Day 1	Pre-dose	F3	5 ug	0	0	0	0	0	0	0		
25-MAY-2018	13:58	Day 1	10 min	F1	5 ug	0	0	0	0	0	0	0		
25-MAY-2018	13:58	Day 1	10 min	F2	5 ug	0	0	0	0	0	0	0		
25-MAY-2018	13:58	Day 1	10 min	F3	5 ug	0	1	0	0	0	0	0		
25-MAY-2018	14:48	Day 1	1 hour	F1	5 ug	0	0	0	0	0	0	0		
25-MAY-2018	14:48	Day 1	1 hour	F2	5 ug	0	0	0	0	0	0	0		
25-MAY-2018	14:48	Day 1	1 hour	F3	5 ug	0	1	0	0	0	0	0		
25-MAY-2018	15:49	Day 1	2 hour	F1	5 ug	0	0	0	0	0	0	0		
25-MAY-2018	15:49	Day 1	2 hour	F2	5 ug	0	0	0	0	0	0	0		
25-MAY-2018	15:49	Day 1	2 hour	F3	5 ug	0	0	0	0	0	0	0		
26-MAY-2018	12:31	Day 2		F1	5 ug	0	1	0	0	0	1	0		
26-MAY-2018	12:31	Day 2		F2	5 ug	0	1	0	0	0	1	0		
26-MAY-2018	12:31	Day 2		F3	5 ug	0	1	0	0	1	1	0		
28-MAY-2018	14:33	Day 4		F1	5 ug	0	0	0	0	0	0	2		
28-MAY-2018	14:33	Day 4		F2	5 ug	0	0	0	0	1	0	3		
28-MAY-2018	14:33	Day 4		F3	5 ug	0	0	0	0	1	0	2		
01-JUN-2018	08:50	Day 8		F1	5 ug	0	0	0	0	2	0	3		
01-JUN-2018	08:50	Day 8		F2	5 ug	0	0	0	0	2	0	3		

Key: 0= None; 1=Mild; 2=Moderate; 3=Severe; # Other = Pinprick bleeding or seepage

Administration Site Key: FA = Forearm; UA= Upper arm
Treatments Part A: A1 = A/Sing MAP, 15 ug (FA); A2 = Afluria Quad IM (UA), 15 ug; A3 = Placebo MAP (FA); A4 = A/Sing Mono IM, 15 ug (UA)
Treatments Part B: B1 = A/Sing MAP, 15 ug (FA) with biopsy; B2 = Placebo MAP (FA) with biopsy
B3 = A/Sing MAP, 15 ug (FA); B4 = A/Sing MAP, 10 ug (FA); B5 = A/Sing MAP, 5 ug (FA); B6 = A/Sing MAP, 2.5 ug (FA)
B7 = Placebo MAP (FA); B8 = A/Sing MAP, 15 ug (UA); B9 = A/Sing IM, 15 ug, as 2018 Afluria Quad (UA)

Listing 16.2.9.2
Treatment Site Tolerability Assessment per Site

Part B
Randomisation Number = B0056, Treatment Group = Group B3

Date	Time	Visit	Time Point	Site Number	Treatment for Site	Indura-tion	Redness	Bruising	Itching	Skin Flaking	Tender-ness	Definition/ Visibility	Other#	Unscheduled Reason
01-JUN-2018	08:50	Day 8		F3	5 ug	0	0	0	0	1	0	3		
15-JUN-2018	14:24	Day 22		F1	5 ug	0	0	0	0	0	0	2		
15-JUN-2018	14:24	Day 22		F2	5 ug	0	0	0	0	0	0	2		
15-JUN-2018	14:24	Day 22		F3	5 ug	0	0	0	0	0	0	3		
24-JUL-2018	18:19	EOS/ET (Day 61)		F1	5 ug	0	0	0	0	0	0	1		
24-JUL-2018	18:19	EOS/ET (Day 61)		F2	5 ug	0	0	0	0	0	0	1		
24-JUL-2018	18:19	EOS/ET (Day 61)		F3	5 ug	0	0	0	0	0	0	1		

Key: 0= None; 1=Mild; 2=Moderate; 3=Severe; # Other = Pinprick bleeding or seepage

Administration Site Key: FA = Forearm; UA= Upper arm
Treatments Part A: A1 = A/Sing MAP, 15 ug (FA); A2 = Afluria Quad IM (UA), 15 ug; A3 = Placebo MAP (FA); A4 = A/Sing Mono IM, 15 ug (UA)
Treatments Part B: B1 = A/Sing MAP, 15 ug (FA) with biopsy; B2 = Placebo MAP (FA) with biopsy
B3 = A/Sing MAP, 15 ug (FA); B4 = A/Sing MAP, 10 ug (FA); B5 = A/Sing MAP, 5 ug (FA); B6 = A/Sing MAP, 2.5 ug (FA)
B7 = Placebo MAP (FA); B8 = A/Sing MAP, 15 ug (UA); B9 = A/Sing IM, 15 ug, as 2018 Afluria Quad (UA)

Listing 16.2.9.2
Treatment Site Tolerability Assessment per Site

Part B
Randomisation Number = B0057, Treatment Group = Group B4

Date	Time	Visit	Time Point	Site Number	Treatment for Site	Induration	Redness	Bruising	Itching	Skin Flaking	Tenderness	Definition/ Visibility	Other#	Unscheduled Reason
25-MAY-2018	11:50	Day 1	Pre-dose	F1	5 ug	0	0	0	0	0	0	0		
25-MAY-2018	11:50	Day 1	Pre-dose	F2	5 ug	0	0	0	0	0	0	0		
25-MAY-2018	11:50	Day 1	Pre-dose	F3	uncoated	0	0	0	0	0	0	0		
25-MAY-2018	14:08	Day 1	10 min	F1	5 ug	0	0	0	0	0	0	0		
25-MAY-2018	14:08	Day 1	10 min	F2	5 ug	0	0	0	1	0	1	0		
25-MAY-2018	14:08	Day 1	10 min	F3	uncoated	0	0	0	1	0	1	0		
25-MAY-2018	15:00	Day 1	1 hour	F1	5 ug	0	0	0	0	0	0	0		
25-MAY-2018	15:00	Day 1	1 hour	F2	5 ug	0	0	0	0	0	1	0		
25-MAY-2018	15:00	Day 1	1 hour	F3	uncoated	0	0	0	0	0	1	0		
25-MAY-2018	15:59	Day 1	2 hour	F1	5 ug	0	0	0	0	0	0	0		
25-MAY-2018	15:59	Day 1	2 hour	F2	5 ug	0	0	0	1	0	0	0		
25-MAY-2018	15:59	Day 1	2 hour	F3	uncoated	0	0	0	0	0	0	0		
26-MAY-2018	08:26	Day 2		F1	5 ug	0	0	0	0	1	0	0		
26-MAY-2018	08:26	Day 2		F2	5 ug	0	0	0	0	0	0	0		
26-MAY-2018	08:26	Day 2		F3	uncoated	0	0	0	0	0	0	0		
28-MAY-2018	11:52	Day 4		F1	5 ug	0	0	0	0	0	0	0		
28-MAY-2018	11:52	Day 4		F2	5 ug	0	1	0	0	0	0	0		
28-MAY-2018	11:52	Day 4		F3	uncoated	0	1	0	0	0	0	0		
01-JUN-2018	09:27	Day 8		F1	5 ug	0	0	0	0	1	0	2		
01-JUN-2018	09:27	Day 8		F2	5 ug	0	0	0	0	1	0	2		

Key: 0= None; 1=Mild; 2=Moderate; 3=Severe; # Other = Pinprick bleeding or seepage

Administration Site Key: FA = Forearm; UA= Upper arm
Treatments Part A: A1 = A/Sing MAP, 15 ug (FA); A2 = Afluria Quad IM (UA), 15 ug; A3 = Placebo MAP (FA); A4 = A/Sing Mono IM, 15 ug (UA)
Treatments Part B: B1 = A/Sing MAP, 15 ug (FA) with biopsy; B2 = Placebo MAP (FA) with biopsy
B3 = A/Sing MAP, 15 ug (FA); B4 = A/Sing MAP, 10 ug (FA); B5 = A/Sing MAP, 5 ug (FA); B6 = A/Sing MAP, 2.5 ug (FA)
B7 = Placebo MAP (FA); B8 = A/Sing MAP, 15 ug (UA); B9 = A/Sing IM, 15 ug, as 2018 Afluria Quad (UA)

Listing 16.2.9.2
Treatment Site Tolerability Assessment per Site

Part B
Randomisation Number = B0057, Treatment Group = Group B4

Date	Time	Visit	Time Point	Site Number	Treatment for Site	Indura-tion	Redness	Bruising	Itching	Skin Flaking	Tender-ness	Definition/ Visibility	Other#	Unscheduled Reason
01-JUN-2018	09:27	Day 8		F3	uncoated	0	0	0	0	0	0	1		
15-JUN-2018	09:40	Day 22		F1	5 ug	0	0	0	0	0	0	3		
15-JUN-2018	09:40	Day 22		F2	5 ug	0	0	0	0	0	0	3		
15-JUN-2018	09:40	Day 22		F3	uncoated	0	0	0	0	0	0	0		
24-JUL-2018	09:43	EOS/ET (Day 61)		F1	5 ug	0	0	0	0	0	0	2		
24-JUL-2018	09:43	EOS/ET (Day 61)		F2	5 ug	0	0	0	0	0	0	2		
24-JUL-2018	09:43	EOS/ET (Day 61)		F3	uncoated	0	0	0	0	0	0	1		
24-JUL-2018	09:58	Unscheduled		F1	5 ug	0	0	0	0	0	0	1	NA	MO notifies as repeat
24-JUL-2018	09:59	Unscheduled		F2	5 ug	0	0	0	0	0	0	1	NA	MO notifies as repeat
24-JUL-2018	10:00	Unscheduled		F3	uncoated	0	0	0	0	0	0	0	NA	MO notifies as repeat

Key: 0= None; 1=Mild; 2=Moderate; 3=Severe; # Other = Pinprick bleeding or seepage

Administration Site Key: FA = Forearm; UA= Upper arm
Treatments Part A: A1 = A/Sing MAP, 15 ug (FA); A2 = Afluria Quad IM (UA), 15 ug; A3 = Placebo MAP (FA); A4 = A/Sing Mono IM, 15 ug (UA)
Treatments Part B: B1 = A/Sing MAP, 15 ug (FA) with biopsy; B2 = Placebo MAP (FA) with biopsy
B3 = A/Sing MAP, 15 ug (FA); B4 = A/Sing MAP, 10 ug (FA); B5 = A/Sing MAP, 5 ug (FA); B6 = A/Sing MAP, 2.5 ug (FA)
B7 = Placebo MAP (FA); B8 = A/Sing MAP, 15 ug (UA); B9 = A/Sing IM, 15 ug, as 2018 Afluria Quad (UA)

Listing 16.2.9.2
Treatment Site Tolerability Assessment per Site

Part B
Randomisation Number = B0058, Treatment Group = Group B9

Date	Time	Visit	Time Point	Site Number	Treatment for Site	Indura-tion	Redness	Bruising	Itching	Skin Flaking	Tender-ness	Definition/ Visibility	Other#	Unscheduled Reason
25-MAY-2018	10:40	Day 1	Pre-dose	D1	[IM]	0	0	0	0	0	0	5		
25-MAY-2018	12:41	Day 1	10 min	D1	[IM]	0	0	0	0	0	0	5		
25-MAY-2018	13:31	Day 1	1 hour	D1	[IM]	0	0	0	1	0		5		
25-MAY-2018	14:35	Day 1	2 hour	D1	[IM]	0	0	0	0	0	1	5		
28-MAY-2018	17:33	Day 4		D1	[IM]	0	0	0	0	0	0	5		
01-JUN-2018	17:35	Day 8		D1	[IM]	0	0	0	0	0	0			
16-JUN-2018	13:04	Day 22		D1	[IM]	0	0	0	0	0	0	5		
24-JUL-2018	10:23	EOS/ET (Day 61)		D1	[IM]	0	0	0	0	0	0	5		

Key: 0= None; 1=Mild; 2=Moderate; 3=Severe; # Other = Pinprick bleeding or seepage

Administration Site Key: FA = Forearm; UA= Upper arm
Treatments Part A: A1 = A/Sing MAP, 15 ug (FA); A2 = Afluria Quad IM (UA), 15 ug; A3 = Placebo MAP (FA); A4 = A/Sing Mono IM, 15 ug (UA)
Treatments Part B: B1 = A/Sing MAP, 15 ug (FA) with biopsy; B2 = Placebo MAP (FA) with biopsy
B3 = A/Sing MAP, 15 ug (FA); B4 = A/Sing MAP, 10 ug (FA); B5 = A/Sing MAP, 5 ug (FA); B6 = A/Sing MAP, 2.5 ug (FA)
B7 = Placebo MAP (FA); B8 = A/Sing MAP, 15 ug (UA); B9 = A/Sing IM, 15 ug, as 2018 Afluria Quad (UA)

Listing 16.2.9.2
Treatment Site Tolerability Assessment per Site

Part B
Randomisation Number = B0059, Treatment Group = Group B7

Date	Time	Visit	Time Point	Site Number	Treatment for Site	Indura-tion	Redness	Bruising	Itching	Skin Flaking	Tender-ness	Definition/ Visibility	Other#	Unscheduled Reason
25-MAY-2018	12:00	Day 1	Pre-dose	F1	uncoated	0	0	0	0	0	0	0		
25-MAY-2018	12:00	Day 1	Pre-dose	F2	uncoated	0	0	0	0	0	0	0		
25-MAY-2018	12:00	Day 1	Pre-dose	F3	uncoated	0	0	0	0	0	0	0		
25-MAY-2018	14:28	Day 1	10 min	F1	uncoated	0	0	0	0	0	1	0		
25-MAY-2018	14:28	Day 1	10 min	F2	uncoated	0	0	0	0	0	1	0		
25-MAY-2018	14:28	Day 1	10 min	F3	uncoated	0	0	0	0	0	0	0		
25-MAY-2018	15:08	Day 1	1 hour	F1	uncoated	0	0	0	0	0	1	0		
25-MAY-2018	15:08	Day 1	1 hour	F2	uncoated	0	0	0	0	0	1	0		
25-MAY-2018	15:08	Day 1	1 hour	F3	uncoated	0	0	0	0	0	0	0		
25-MAY-2018	16:18	Day 1	2 hour	F1	uncoated	0	0	0	0	0	0	0		
25-MAY-2018	16:18	Day 1	2 hour	F2	uncoated	0	0	0	0	0	0	0		
25-MAY-2018	16:18	Day 1	2 hour	F3	uncoated	0	0	0	0	0	0	0		
26-MAY-2018	11:03	Day 2		F1	uncoated	0	0	0	0	0	0	0		
26-MAY-2018	11:03	Day 2		F2	uncoated	0	0	0	0	0	0	0		
26-MAY-2018	11:03	Day 2		F3	uncoated	0	0	0	0	0	0	0		
28-MAY-2018	14:23	Day 4		F1	uncoated	0	0	0	0	0	0	1		
28-MAY-2018	14:23	Day 4		F2	uncoated	0	0	0	0	0	0	1		
28-MAY-2018	14:23	Day 4		F3	uncoated	0	0	0	0	0	0	1		
01-JUN-2018	13:52	Day 8		F1	uncoated	0	0	0	0	0	0	1		
01-JUN-2018	13:52	Day 8		F2	uncoated	0	0	0	0	0	0	1		

Key: 0= None; 1=Mild; 2=Moderate; 3=Severe; # Other = Pinprick bleeding or seepage

Administration Site Key: FA = Forearm; UA= Upper arm
Treatments Part A: A1 = A/Sing MAP, 15 ug (FA); A2 = Afluria Quad IM (UA), 15 ug; A3 = Placebo MAP (FA); A4 = A/Sing Mono IM, 15 ug (UA)
Treatments Part B: B1 = A/Sing MAP, 15 ug (FA) with biopsy; B2 = Placebo MAP (FA) with biopsy
B3 = A/Sing MAP, 15 ug (FA); B4 = A/Sing MAP, 10 ug (FA); B5 = A/Sing MAP, 5 ug (FA); B6 = A/Sing MAP, 2.5 ug (FA)
B7 = Placebo MAP (FA); B8 = A/Sing MAP, 15 ug (UA); B9 = A/Sing IM, 15 ug, as 2018 Afluria Quad (UA)

Listing 16.2.9.2
Treatment Site Tolerability Assessment per Site

Part B
Randomisation Number = B0059, Treatment Group = Group B7

Date	Time	Visit	Time Point	Site Number	Treatment for Site	Indura-tion	Redness	Bruising	Itching	Skin Flaking	Tender-ness	Definition/ Visibility	Other#	Unscheduled Reason
01-JUN-2018	13:52	Day 8		F3	uncoated	0	0	0	0	0	0	1		
15-JUN-2018	14:57	Day 22		F1	uncoated	0	0	0	0	0	0	0		
15-JUN-2018	14:57	Day 22		F2	uncoated	0	0	0	0	0	0	0		
15-JUN-2018	14:57	Day 22		F3	uncoated	0	0	0	0	0	0	0		
24-JUL-2018	16:22	EOS/ET (Day 61)		F1	uncoated	0	0	0	0	0	0	0		
24-JUL-2018	16:22	EOS/ET (Day 61)		F2	uncoated	0	0	0	0	0	0	0		
24-JUL-2018	16:22	EOS/ET (Day 61)		F3	uncoated	0	0	0	0	0	0	0		

Key: 0= None; 1=Mild; 2=Moderate; 3=Severe; # Other = Pinprick bleeding or seepage

Administration Site Key: FA = Forearm; UA= Upper arm
Treatments Part A: A1 = A/Sing MAP, 15 ug (FA); A2 = Afluria Quad IM (UA), 15 ug; A3 = Placebo MAP (FA); A4 = A/Sing Mono IM, 15 ug (UA)
Treatments Part B: B1 = A/Sing MAP, 15 ug (FA) with biopsy; B2 = Placebo MAP (FA) with biopsy
B3 = A/Sing MAP, 15 ug (FA); B4 = A/Sing MAP, 10 ug (FA); B5 = A/Sing MAP, 5 ug (FA); B6 = A/Sing MAP, 2.5 ug (FA)
B7 = Placebo MAP (FA); B8 = A/Sing MAP, 15 ug (UA); B9 = A/Sing IM, 15 ug, as 2018 Afluria Quad (UA)

Listing 16.2.9.2
Treatment Site Tolerability Assessment per Site

Part B
Randomisation Number = B0060, Treatment Group = Group B3

Date	Time	Visit	Time Point	Site Number	Treatment for Site	Indura-tion	Redness	Bruising	Itching	Skin Flaking	Tender-ness	Definition/ Visibility	Other#	Unscheduled Reason
25-MAY-2018	12:13	Day 1	Pre-dose	F1	5 ug	0	0	0	0	0	0	0		
25-MAY-2018	12:13	Day 1	Pre-dose	F2	5 ug	0	0	0	0	0	0	0		
25-MAY-2018	12:13	Day 1	Pre-dose	F3	5 ug	0	0	0	0	0	0	0		
25-MAY-2018	14:18	Day 1	10 min	F1	5 ug	0	0	0	0	0	0	0		
25-MAY-2018	14:18	Day 1	10 min	F2	5 ug	0	0	0	0	0	1	0		
25-MAY-2018	14:18	Day 1	10 min	F3	5 ug	0	0	0	0	0	1	0		
25-MAY-2018	15:08	Day 1	1 hour	F1	5 ug	0	0	0	0	0	0	0		
25-MAY-2018	15:08	Day 1	1 hour	F2	5 ug	0	0	0	0	0	0	0		
25-MAY-2018	15:08	Day 1	1 hour	F3	5 ug	0	0	0	0	0	0	0		
25-MAY-2018	16:09	Day 1	2 hour	F1	5 ug	0	0	0	0	0	0	0		
25-MAY-2018	16:09	Day 1	2 hour	F2	5 ug	0	0	0	0	0	0	0		
25-MAY-2018	16:09	Day 1	2 hour	F3	5 ug	0	0	0	0	0	0	0		
26-MAY-2018	11:28	Day 2		F1	5 ug	0	1	0	0	0	0	0		
26-MAY-2018	11:28	Day 2		F2	5 ug	0	1	0	0	0	0	0		
26-MAY-2018	11:28	Day 2		F3	5 ug	0	1	0	0	0	0	0		
28-MAY-2018	14:39	Day 4		F1	5 ug	1	1	0	0	0	0	0		
28-MAY-2018	14:39	Day 4		F2	5 ug	0	1	0	0	0	0	0		
28-MAY-2018	14:39	Day 4		F3	5 ug	1	1	0	0	0	0	0		
01-JUN-2018	15:54	Day 8		F1	5 ug	0	0	0	0	2	0	0		
01-JUN-2018	15:54	Day 8		F2	5 ug	0	0	0	0	2	0	2		

Key: 0= None; 1=Mild; 2=Moderate; 3=Severe; # Other = Pinprick bleeding or seepage

Administration Site Key: FA = Forearm; UA= Upper arm
Treatments Part A: A1 = A/Sing MAP, 15 ug (FA); A2 = Afluria Quad IM (UA), 15 ug; A3 = Placebo MAP (FA); A4 = A/Sing Mono IM, 15 ug (UA)
Treatments Part B: B1 = A/Sing MAP, 15 ug (FA) with biopsy; B2 = Placebo MAP (FA) with biopsy
B3 = A/Sing MAP, 15 ug (FA); B4 = A/Sing MAP, 10 ug (FA); B5 = A/Sing MAP, 5 ug (FA); B6 = A/Sing MAP, 2.5 ug (FA)
B7 = Placebo MAP (FA); B8 = A/Sing MAP, 15 ug (UA); B9 = A/Sing IM, 15 ug, as 2018 Afluria Quad (UA)

Listing 16.2.9.2
Treatment Site Tolerability Assessment per Site

Part B
Randomisation Number = B0060, Treatment Group = Group B3

Date	Time	Visit	Time Point	Site Number	Treatment for Site	Indura-tion	Redness	Bruising	Itching	Skin Flaking	Tender-ness	Definition/ Visibility	Other#	Unscheduled Reason
01-JUN-2018	15:54	Day 8		F3	5 ug	0	0	0	0	2	0	2		
15-JUN-2018	12:13	Day 22		F1	5 ug	0	0	0	0	0	0	2		
15-JUN-2018	12:13	Day 22		F2	5 ug	0	0	0	0	0	0	2		
15-JUN-2018	12:13	Day 22		F3	5 ug	0	0	0	0	0	0	2		
24-JUL-2018	13:50	EOS/ET (Day 61)		F1	5 ug	0	0	0	0	0	0	0		
24-JUL-2018	13:50	EOS/ET (Day 61)		F2	5 ug	0	0	0	0	0	0	0		
24-JUL-2018	13:50	EOS/ET (Day 61)		F3	5 ug	0	0	0	0	0	0	0		

Key: 0= None; 1=Mild; 2=Moderate; 3=Severe; # Other = Pinprick bleeding or seepage

Administration Site Key: FA = Forearm; UA= Upper arm
Treatments Part A: A1 = A/Sing MAP, 15 ug (FA); A2 = Afluria Quad IM (UA), 15 ug; A3 = Placebo MAP (FA); A4 = A/Sing Mono IM, 15 ug (UA)
Treatments Part B: B1 = A/Sing MAP, 15 ug (FA) with biopsy; B2 = Placebo MAP (FA) with biopsy
B3 = A/Sing MAP, 15 ug (FA); B4 = A/Sing MAP, 10 ug (FA); B5 = A/Sing MAP, 5 ug (FA); B6 = A/Sing MAP, 2.5 ug (FA)
B7 = Placebo MAP (FA); B8 = A/Sing MAP, 15 ug (UA); B9 = A/Sing IM, 15 ug, as 2018 Afluria Quad (UA)

Listing 16.2.9.2
Treatment Site Tolerability Assessment per Site

Part B
Randomisation Number = B0061, Treatment Group = Group B8

Date	Time	Visit	Time Point	Site Number	Treatment for Site	Indura-tion	Redness	Bruising	Itching	Skin Flaking	Tender-ness	Definition/ Visibility	Other#	Unscheduled Reason
25-MAY-2018	12:22	Day 1	Pre-dose	D1	5 ug	0	0	0	0	0	0	0		
25-MAY-2018	12:22	Day 1	Pre-dose	D2	5 ug	0	0	0	0	0	0	0		
25-MAY-2018	12:22	Day 1	Pre-dose	D3	5 ug	0	0	0	0	0	0	0		
25-MAY-2018	14:38	Day 1	10 min	D1	5 ug	0	1	0	0	0	0	0		
25-MAY-2018	14:38	Day 1	10 min	D2	5 ug	0	1	0	0	0	0	0		
25-MAY-2018	14:38	Day 1	10 min	D3	5 ug	0	1	0	0	0	0	0		
25-MAY-2018	15:28	Day 1	1 hour	D1	5 ug	0	0	0	0	0	0	0		
25-MAY-2018	15:28	Day 1	1 hour	D2	5 ug	0	0	0	0	0	0	0		
25-MAY-2018	15:28	Day 1	1 hour	D3	5 ug	0	0	0	0	0	0	0		
25-MAY-2018	16:28	Day 1	2 hour	D1	5 ug	0	0	0	0	0	0	0		
25-MAY-2018	16:28	Day 1	2 hour	D2	5 ug	0	0	0	0	0	0	0		
25-MAY-2018	16:28	Day 1	2 hour	D3	5 ug	0	1	0	0	0	0	0		
26-MAY-2018	10:46	Day 2		D1	5 ug	0	0	0	0	0	0	0		
26-MAY-2018	10:46	Day 2		D2	5 ug	0	0	0	0	0	0	0		
26-MAY-2018	10:46	Day 2		D3	5 ug	0	0	0	0	0	0	0		
28-MAY-2018	13:36	Day 4		D1	5 ug	0	0	0	0	1	0	0		
28-MAY-2018	13:36	Day 4		D2	5 ug	0	0	0	0	1	0	0		
28-MAY-2018	13:36	Day 4		D3	5 ug	0	1	0	0	1	0	0		
01-JUN-2018	11:45	Day 8		D1	5 ug	0	0	0	0	0	0	0		
01-JUN-2018	11:45	Day 8		D2	5 ug	0	0	0	0	0	0	0		

Key: 0= None; 1=Mild; 2=Moderate; 3=Severe; # Other = Pinprick bleeding or seepage

Administration Site Key: FA = Forearm; UA= Upper arm
Treatments Part A: A1 = A/Sing MAP, 15 ug (FA); A2 = Afluria Quad IM (UA), 15 ug; A3 = Placebo MAP (FA); A4 = A/Sing Mono IM, 15 ug (UA)
Treatments Part B: B1 = A/Sing MAP, 15 ug (FA) with biopsy; B2 = Placebo MAP (FA) with biopsy
B3 = A/Sing MAP, 15 ug (FA); B4 = A/Sing MAP, 10 ug (FA); B5 = A/Sing MAP, 5 ug (FA); B6 = A/Sing MAP, 2.5 ug (FA)
B7 = Placebo MAP (FA); B8 = A/Sing MAP, 15 ug (UA); B9 = A/Sing IM, 15 ug, as 2018 Afluria Quad (UA)

Listing 16.2.9.2
Treatment Site Tolerability Assessment per Site

Part B
Randomisation Number = B0061, Treatment Group = Group B8

Date	Time	Visit	Time Point	Site Number	Treatment for Site	Indura-tion	Redness	Bruising	Itching	Skin Flaking	Tender-ness	Definition/ Visibility	Other#	Unscheduled Reason
01-JUN-2018	11:45	Day 8		D3	5 ug	0	0	0	0	0	0	0		
15-JUN-2018	12:10	Day 22		D1	5 ug	0	0	0	0	0	0	1		
15-JUN-2018	12:10	Day 22		D2	5 ug	0	0	0	0	0	0	1		
15-JUN-2018	12:10	Day 22		D3	5 ug	0	0	0	0	0	0	2		
24-JUL-2018	14:41	EOS/ET (Day 61)		D1	5 ug	0	0	0	0	0	0	0		
24-JUL-2018	14:41	EOS/ET (Day 61)		D2	5 ug	0	0	0	0	0	0	0		
24-JUL-2018	14:41	EOS/ET (Day 61)		D3	5 ug	0	0	0	0	0	0	0		

Key: 0= None; 1=Mild; 2=Moderate; 3=Severe; # Other = Pinprick bleeding or seepage

Administration Site Key: FA = Forearm; UA= Upper arm
Treatments Part A: A1 = A/Sing MAP, 15 ug (FA); A2 = Afluria Quad IM (UA), 15 ug; A3 = Placebo MAP (FA); A4 = A/Sing Mono IM, 15 ug (UA)
Treatments Part B: B1 = A/Sing MAP, 15 ug (FA) with biopsy; B2 = Placebo MAP (FA) with biopsy
B3 = A/Sing MAP, 15 ug (FA); B4 = A/Sing MAP, 10 ug (FA); B5 = A/Sing MAP, 5 ug (FA); B6 = A/Sing MAP, 2.5 ug (FA)
B7 = Placebo MAP (FA); B8 = A/Sing MAP, 15 ug (UA); B9 = A/Sing IM, 15 ug, as 2018 Afluria Quad (UA)

Listing 16.2.9.2
Treatment Site Tolerability Assessment per Site

Part B
Randomisation Number = B0062, Treatment Group = Group B5

Date	Time	Visit	Time Point	Site Number	Treatment for Site	Indura- tion	Redness	Bruising	Itching	Skin Flaking	Tender- ness	Definition/ Visibility	Other#	Unscheduled Reason
25-MAY-2018	12:31	Day 1	Pre-dose	F1	uncoated	0	0	0	0	0	0	0		
25-MAY-2018	12:31	Day 1	Pre-dose	F2	5 ug	0	0	0	0	0	0	0		
25-MAY-2018	12:31	Day 1	Pre-dose	F3	uncoated	0	0	0	0	0	0	0		
25-MAY-2018	14:48	Day 1	10 min	F1	uncoated	0	0	0	0	0	0	0		
25-MAY-2018	14:48	Day 1	10 min	F2	5 ug	0	1	0	0	0	0	0		
25-MAY-2018	14:48	Day 1	10 min	F3	uncoated	0	0	0	0	0	0	0		
25-MAY-2018	15:38	Day 1	1 hour	F1	uncoated	0	0	0	0	0	0	0		
25-MAY-2018	15:38	Day 1	1 hour	F2	5 ug	0	0	0	0	0	0	0		
25-MAY-2018	15:38	Day 1	1 hour	F3	uncoated	0	0	0	0	0	0	0		
25-MAY-2018	16:38	Day 1	2 hour	F1	uncoated	0	0	0	0	0	0	0		
25-MAY-2018	16:38	Day 1	2 hour	F2	5 ug	1	0	0	0	0	0	0		
25-MAY-2018	16:38	Day 1	2 hour	F3	uncoated	1	0	0	0	0	0	0		
26-MAY-2018	11:30	Day 2		F1	uncoated	0	0	0	0	0	0	0		
26-MAY-2018	11:30	Day 2		F2	5 ug	0	0	0	0	0	0	0		
26-MAY-2018	11:30	Day 2		F3	uncoated	0	0	0	0	0	0	0		
29-MAY-2018	14:07	Day 4		F1	uncoated	0	0	0	0	0	0	0		
29-MAY-2018	14:07	Day 4		F2	5 ug	1	0	0	0	0	0	0		
29-MAY-2018	14:07	Day 4		F3	uncoated	0	0	0	0	0	0	0		
01-JUN-2018	12:46	Day 8		F1	uncoated	0	0	0	0	0	0	3		
01-JUN-2018	12:46	Day 8		F2	5 ug	0	0	0	0	0	0	3		

Key: 0= None; 1=Mild; 2=Moderate; 3=Severe; # Other = Pinprick bleeding or seepage

Administration Site Key: FA = Forearm; UA= Upper arm
Treatments Part A: A1 = A/Sing MAP, 15 ug (FA); A2 = Afluria Quad IM (UA), 15 ug; A3 = Placebo MAP (FA); A4 = A/Sing Mono IM, 15 ug (UA)
Treatments Part B: B1 = A/Sing MAP, 15 ug (FA) with biopsy; B2 = Placebo MAP (FA) with biopsy
B3 = A/Sing MAP, 15 ug (FA); B4 = A/Sing MAP, 10 ug (FA); B5 = A/Sing MAP, 5 ug (FA); B6 = A/Sing MAP, 2.5 ug (FA)
B7 = Placebo MAP (FA); B8 = A/Sing MAP, 15 ug (UA); B9 = A/Sing IM, 15 ug, as 2018 Afluria Quad (UA)

Listing 16.2.9.2
Treatment Site Tolerability Assessment per Site

Part B
Randomisation Number = B0062, Treatment Group = Group B5

Date	Time	Visit	Time Point	Site Number	Treatment for Site	Indura-tion	Redness	Bruising	Itching	Skin Flaking	Tender-ness	Definition/ Visibility	Other#	Unscheduled Reason
01-JUN-2018	12:46	Day 8		F3	uncoated	0	0	0	0	0	0	3		
15-JUN-2018	10:59	Day 22		F1	uncoated	0	0	0	0	0	0	2		
15-JUN-2018	10:59	Day 22		F2	5 ug	0	0	0	0	0	0	1		
15-JUN-2018	10:59	Day 22		F3	uncoated	0	0	0	0	0	0	1		
24-JUL-2018	15:51	EOS/ET (Day 61)		F1	uncoated	0	0	0	0	0	0	0		
24-JUL-2018	15:51	EOS/ET (Day 61)		F2	5 ug	0	0	0	0	0	0	0		
24-JUL-2018	15:51	EOS/ET (Day 61)		F3	uncoated	0	0	0	0	0	0	0		

Key: 0= None; 1=Mild; 2=Moderate; 3=Severe; # Other = Pinprick bleeding or seepage

Administration Site Key: FA = Forearm; UA= Upper arm
Treatments Part A: A1 = A/Sing MAP, 15 ug (FA); A2 = Afluria Quad IM (UA), 15 ug; A3 = Placebo MAP (FA); A4 = A/Sing Mono IM, 15 ug (UA)
Treatments Part B: B1 = A/Sing MAP, 15 ug (FA) with biopsy; B2 = Placebo MAP (FA) with biopsy
B3 = A/Sing MAP, 15 ug (FA); B4 = A/Sing MAP, 10 ug (FA); B5 = A/Sing MAP, 5 ug (FA); B6 = A/Sing MAP, 2.5 ug (FA)
B7 = Placebo MAP (FA); B8 = A/Sing MAP, 15 ug (UA); B9 = A/Sing IM, 15 ug, as 2018 Afluria Quad (UA)

Listing 16.2.9.2
Treatment Site Tolerability Assessment per Site

Part B
Randomisation Number = B0063, Treatment Group = Group B7

Date	Time	Visit	Time Point	Site Number	Treatment for Site	Indura-tion	Redness	Bruising	Itching	Skin Flaking	Tender-ness	Definition/ Visibility	Other#	Unscheduled Reason
25-MAY-2018	12:42	Day 1	Pre-dose	F1	uncoated	0	0	0	0	0	0	0		
25-MAY-2018	12:42	Day 1	Pre-dose	F2	uncoated	0	0	0	0	0	0	0		
25-MAY-2018	12:42	Day 1	Pre-dose	F3	uncoated	0	0	0	0	0	0	0		
25-MAY-2018	14:58	Day 1	10 min	F1	uncoated	0	1	0	1	0	0	0		
25-MAY-2018	14:58	Day 1	10 min	F2	uncoated	0	0	0	1	0	0	0		
25-MAY-2018	14:58	Day 1	10 min	F3	uncoated	0	0	0	0	0	0	0		
25-MAY-2018	15:48	Day 1	1 hour	F1	uncoated	0	0	0	0	0	0	0		
25-MAY-2018	15:48	Day 1	1 hour	F2	uncoated	0	0	0	0	0	0	0		
25-MAY-2018	15:48	Day 1	1 hour	F3	uncoated	0	0	0	0	0	0	0		
25-MAY-2018	16:48	Day 1	2 hour	F1	uncoated	0	0	0	0	0	0	0		
25-MAY-2018	16:48	Day 1	2 hour	F2	uncoated	0	0	0	0	0	0	0		
25-MAY-2018	16:48	Day 1	2 hour	F3	uncoated	0	0	0	0	0	0	0		
26-MAY-2018	11:19	Day 2		F1	uncoated	0	0	0	0	0	0	0		
26-MAY-2018	11:19	Day 2		F2	uncoated	0	0	0	0	0	0	0		
26-MAY-2018	11:19	Day 2		F3	uncoated	0	0	0	0	0	0	0		
28-MAY-2018	12:47	Day 4		F1	uncoated	0	0	0	0	0	0	0		
28-MAY-2018	12:47	Day 4		F2	uncoated	0	0	0	0	0	0	0		
28-MAY-2018	12:47	Day 4		F3	uncoated	0	0	0	0	0	0	0		
01-JUN-2018	14:01	Day 8		F1	uncoated	0	0	0	0	1	0	1		
01-JUN-2018	14:01	Day 8		F2	uncoated	0	0	0	0	0	0	1		

Key: 0= None; 1=Mild; 2=Moderate; 3=Severe; # Other = Pinprick bleeding or seepage

Administration Site Key: FA = Forearm; UA= Upper arm
Treatments Part A: A1 = A/Sing MAP, 15 ug (FA); A2 = Afluria Quad IM (UA), 15 ug; A3 = Placebo MAP (FA); A4 = A/Sing Mono IM, 15 ug (UA)
Treatments Part B: B1 = A/Sing MAP, 15 ug (FA) with biopsy; B2 = Placebo MAP (FA) with biopsy
B3 = A/Sing MAP, 15 ug (FA); B4 = A/Sing MAP, 10 ug (FA); B5 = A/Sing MAP, 5 ug (FA); B6 = A/Sing MAP, 2.5 ug (FA)
B7 = Placebo MAP (FA); B8 = A/Sing MAP, 15 ug (UA); B9 = A/Sing IM, 15 ug, as 2018 Afluria Quad (UA)

Listing 16.2.9.2

Treatment Site Tolerability Assessment per Site

Part B

Randomisation Number = B0063, Treatment Group = Group B7

Date	Time	Visit	Time Point	Site Number	Treatment for Site	Indura-tion	Redness	Bruising	Itching	Skin Flaking	Tender-ness	Definition/ Visibility	Other#	Unscheduled Reason
01-JUN-2018	14:01	Day 8		F3	uncoated	0	0	0	0	0	0	1		
15-JUN-2018	13:20	Day 22		F1	uncoated	0	0	0	0	0	0	1		
15-JUN-2018	13:20	Day 22		F2	uncoated	0	0	0	0	0	0	1		
15-JUN-2018	13:20	Day 22		F3	uncoated	0	0	0	0	0	0	1		
12-JUL-2018	14:10	EOS/ET (Day 61)		F1	uncoated	0	0	0	0	0	0	0		
12-JUL-2018	14:10	EOS/ET (Day 61)		F2	uncoated	0	0	0	0	0	0	0		
12-JUL-2018	14:10	EOS/ET (Day 61)		F3	uncoated	0	0	0	0	0	0	0		

Key: 0= None; 1=Mild; 2=Moderate; 3=Severe; # Other = Pinprick bleeding or seepage

Administration Site Key: FA = Forearm; UA= Upper arm

Treatments Part A: A1 = A/Sing MAP, 15 ug (FA); A2 = Afluria Quad IM (UA), 15 ug; A3 = Placebo MAP (FA); A4 = A/Sing Mono IM, 15 ug (UA)

Treatments Part B: B1 = A/Sing MAP, 15 ug (FA) with biopsy; B2 = Placebo MAP (FA) with biopsy

B3 = A/Sing MAP, 15 ug (FA); B4 = A/Sing MAP, 10 ug (FA); B5 = A/Sing MAP, 5 ug (FA); B6 = A/Sing MAP, 2.5 ug (FA)

B7 = Placebo MAP (FA); B8 = A/Sing MAP, 15 ug (UA); B9 = A/Sing IM, 15 ug, as 2018 Afluria Quad (UA)

Listing 16.2.9.2
Treatment Site Tolerability Assessment per Site

Part B
Randomisation Number = B0064, Treatment Group = Group B9

Date	Time	Visit	Time Point	Site Number	Treatment for Site	Indura-tion	Redness	Bruising	Itching	Skin Flaking	Tender-ness	Definition/ Visibility	Other#	Unscheduled Reason
25-MAY-2018	10:42	Day 1	Pre-dose	D1	[IM]	0	0	0	0	0	0	5		
25-MAY-2018	12:51	Day 1	10 min	D1	[IM]	0	0	0	0	0	0	5		
25-MAY-2018		Day 1	1 hour	D1	[IM]	0	0	0	0	0	0	5		
25-MAY-2018	14:45	Day 1	2 hour	D1	[IM]	0	0	0	0	0	0	5		
26-MAY-2018	12:00	Day 2		D1	[IM]	0	0	0	0	0	1	5		
28-MAY-2018	13:29	Day 4		D1	[IM]	0	0	0	0	0	1	5		
01-JUN-2018	09:52	Day 8		D1	[IM]	0	0	0	0	0	0			
15-JUN-2018	10:08	Day 22		D1	[IM]	0	0	0	0	0	0	5		
24-JUL-2018	12:59	EOS/ET (Day 61)		D1	[IM]	0	0	0	0	0	0	5		

Key: 0= None; 1=Mild; 2=Moderate; 3=Severe; # Other = Pinprick bleeding or seepage

Administration Site Key: FA = Forearm; UA= Upper arm
Treatments Part A: A1 = A/Sing MAP, 15 ug (FA); A2 = Afluria Quad IM (UA), 15 ug; A3 = Placebo MAP (FA); A4 = A/Sing Mono IM, 15 ug (UA)
Treatments Part B: B1 = A/Sing MAP, 15 ug (FA) with biopsy; B2 = Placebo MAP (FA) with biopsy
B3 = A/Sing MAP, 15 ug (FA); B4 = A/Sing MAP, 10 ug (FA); B5 = A/Sing MAP, 5 ug (FA); B6 = A/Sing MAP, 2.5 ug (FA)
B7 = Placebo MAP (FA); B8 = A/Sing MAP, 15 ug (UA); B9 = A/Sing IM, 15 ug, as 2018 Afluria Quad (UA)

Listing 16.2.9.2
Treatment Site Tolerability Assessment per Site

Part B
Randomisation Number = B0065, Treatment Group = Group B4

Date	Time	Visit	Time Point	Site Number	Treatment for Site	Induration	Redness	Bruising	Itching	Skin Flaking	Tenderness	Definition/ Visibility	Other#	Unscheduled Reason
25-MAY-2018	12:53	Day 1	Pre-dose	F1	5 ug	0	0	0	0	0	0	0		
25-MAY-2018	12:53	Day 1	Pre-dose	F2	uncoated	0	0	0	0	0	0	0		
25-MAY-2018	12:53	Day 1	Pre-dose	F3	5 ug	0	0	0	0	0	0	0		
25-MAY-2018	15:08	Day 1	10 min	F1	5 ug	0	1	0	0	0	0	0		
25-MAY-2018	15:08	Day 1	10 min	F2	uncoated	0	0	0	0	0	0	0		
25-MAY-2018	15:08	Day 1	10 min	F3	5 ug	0	0	0	0	0	0	0		
25-MAY-2018	15:58	Day 1	1 hour	F1	5 ug	0	0	0	0	0	0	0		
25-MAY-2018	15:58	Day 1	1 hour	F2	uncoated	0	0	0	0	0	1	0		
25-MAY-2018	15:58	Day 1	1 hour	F3	5 ug	0	0	0	0	0	0	0		
25-MAY-2018	16:58	Day 1	2 hour	F1	5 ug	0	0	0	0	0	0	0		
25-MAY-2018	16:58	Day 1	2 hour	F2	uncoated	0	0	0	0	0	1	0		
25-MAY-2018	16:58	Day 1	2 hour	F3	5 ug	0	0	0	0	0	0	0		
26-MAY-2018	12:11	Day 2		F1	5 ug	0	1	0	0	0	1	0		
26-MAY-2018	12:11	Day 2		F2	uncoated	0	0	0	0	0	0	0		
26-MAY-2018	12:11	Day 2		F3	5 ug	0	0	0	0	0	1	0		
26-MAY-2018	12:21	Unscheduled		F1	5 ug	0	1	0	0	0	2	0	NA	MO notifies as repeat
26-MAY-2018	12:22	Unscheduled		F3	5 ug	0	1	0	0	0	1	0	NA	MO notifies as repeat
28-MAY-2018	13:32	Day 4		F1	5 ug	0	1	0	0	1	1	0		

Key: 0= None; 1=Mild; 2=Moderate; 3=Severe; # Other = Pinprick bleeding or seepage

Administration Site Key: FA = Forearm; UA= Upper arm
Treatments Part A: A1 = A/Sing MAP, 15 ug (FA); A2 = Afluria Quad IM (UA), 15 ug; A3 = Placebo MAP (FA); A4 = A/Sing Mono IM, 15 ug (UA)
Treatments Part B: B1 = A/Sing MAP, 15 ug (FA) with biopsy; B2 = Placebo MAP (FA) with biopsy
B3 = A/Sing MAP, 15 ug (FA); B4 = A/Sing MAP, 10 ug (FA); B5 = A/Sing MAP, 5 ug (FA); B6 = A/Sing MAP, 2.5 ug (FA)
B7 = Placebo MAP (FA); B8 = A/Sing MAP, 15 ug (UA); B9 = A/Sing IM, 15 ug, as 2018 Afluria Quad (UA)

Listing 16.2.9.2
Treatment Site Tolerability Assessment per Site

Part B
Randomisation Number = B0065, Treatment Group = Group B4

Date	Time	Visit	Time Point	Site Number	Treatment for Site	Indura-tion	Redness	Bruising	Itching	Skin Flaking	Tender-ness	Definition/ Visibility	Other#	Unscheduled Reason
28-MAY-2018	13:32	Day 4		F2	uncoated	0	0	0	0	0	0	1		
28-MAY-2018	13:32	Day 4		F3	5 ug	0	1	0	0	1	0	0		
01-JUN-2018	09:53	Day 8		F1	5 ug	0	0	0	0	1	0	0		
01-JUN-2018	09:53	Day 8		F2	uncoated	0	0	0	0	0	0	3		
01-JUN-2018	09:53	Day 8		F3	5 ug	0	0	0	0	0	0	3		
15-JUN-2018	09:59	Day 22		F1	5 ug	0	0	0	0	0	0	3		
15-JUN-2018	09:59	Day 22		F2	uncoated	0	0	0	0	0	0	3		
15-JUN-2018	09:59	Day 22		F3	5 ug	0	0	0	0	0	0	3		
24-JUL-2018	13:04	EOS/ET (Day 61)		F1	5 ug	0	0	0	0	0	0	1		
24-JUL-2018	13:04	EOS/ET (Day 61)		F2	uncoated	0	0	0	0	0	0	1		
24-JUL-2018	13:04	EOS/ET (Day 61)		F3	5 ug	0	0	0	0	0	0	1		

Key: 0= None; 1=Mild; 2=Moderate; 3=Severe; # Other = Pinprick bleeding or seepage

Administration Site Key: FA = Forearm; UA= Upper arm
Treatments Part A: A1 = A/Sing MAP, 15 ug (FA); A2 = Afluria Quad IM (UA), 15 ug; A3 = Placebo MAP (FA); A4 = A/Sing Mono IM, 15 ug (UA)
Treatments Part B: B1 = A/Sing MAP, 15 ug (FA) with biopsy; B2 = Placebo MAP (FA) with biopsy
B3 = A/Sing MAP, 15 ug (FA); B4 = A/Sing MAP, 10 ug (FA); B5 = A/Sing MAP, 5 ug (FA); B6 = A/Sing MAP, 2.5 ug (FA)
B7 = Placebo MAP (FA); B8 = A/Sing MAP, 15 ug (UA); B9 = A/Sing IM, 15 ug, as 2018 Afluria Quad (UA)

Listing 16.2.9.2
Treatment Site Tolerability Assessment per Site

Part B
Randomisation Number = B0066, Treatment Group = Group B6

Date	Time	Visit	Time Point	Site Number	Treatment for Site	Indura-tion	Redness	Bruising	Itching	Skin Flaking	Tender-ness	Definition/ Visibility	Other#	Unscheduled Reason
25-MAY-2018	13:53	Day 1	Pre-dose	F1	2.5 ug	0	0	0	0	0	0	0		
25-MAY-2018	13:53	Day 1	Pre-dose	F2	uncoated	0	0	0	0	0	0	0		
25-MAY-2018	13:53	Day 1	Pre-dose	F3	uncoated	0	0	0	0	0	0	0		
25-MAY-2018	15:18	Day 1	10 min	F1	2.5 ug	0	1	0	1	0	1	0		
25-MAY-2018	15:18	Day 1	10 min	F2	uncoated	0	0	0	0	0	1	0		
25-MAY-2018	15:18	Day 1	10 min	F3	uncoated	0	0	0	0	0	0	0		
25-MAY-2018	16:08	Day 1	1 hour	F1	2.5 ug	0	1	0	0	0	0	0		
25-MAY-2018	16:08	Day 1	1 hour	F2	uncoated	0	1	0	0	0	0	0		
25-MAY-2018	16:08	Day 1	1 hour	F3	uncoated	0	1	0	0	0	0	0		
25-MAY-2018	17:08	Day 1	2 hour	F1	2.5 ug	0	0	0	0	0	0	0		
25-MAY-2018	17:08	Day 1	2 hour	F2	uncoated	0	0	0	0	0	0	0		
25-MAY-2018	17:08	Day 1	2 hour	F3	uncoated	0	0	0	0	0	0	0		
26-MAY-2018	10:28	Day 2		F1	2.5 ug	0	1	0	0	0	0	0		
26-MAY-2018	10:28	Day 2		F2	uncoated	0	0	0	0	0	0	0		
26-MAY-2018	10:28	Day 2		F3	uncoated	0	0	0	0	0	0	0		
28-MAY-2018	10:29	Day 4		F1	2.5 ug	0	1	0	0	0	0	0		
28-MAY-2018	10:29	Day 4		F2	uncoated	0	0	0	0	0	0	0		
28-MAY-2018	10:29	Day 4		F3	uncoated	0	0	0	0	0	0	0		
01-JUN-2018	10:20	Day 8		F1	2.5 ug	0	0	0	0	3	0	3		
01-JUN-2018	10:20	Day 8		F2	uncoated	0	0	0	0	0	0	2		

Key: 0= None; 1=Mild; 2=Moderate; 3=Severe; # Other = Pinprick bleeding or seepage

Administration Site Key: FA = Forearm; UA= Upper arm
Treatments Part A: A1 = A/Sing MAP, 15 ug (FA); A2 = Afluria Quad IM (UA), 15 ug; A3 = Placebo MAP (FA); A4 = A/Sing Mono IM, 15 ug (UA)
Treatments Part B: B1 = A/Sing MAP, 15 ug (FA) with biopsy; B2 = Placebo MAP (FA) with biopsy
B3 = A/Sing MAP, 15 ug (FA); B4 = A/Sing MAP, 10 ug (FA); B5 = A/Sing MAP, 5 ug (FA); B6 = A/Sing MAP, 2.5 ug (FA)
B7 = Placebo MAP (FA); B8 = A/Sing MAP, 15 ug (UA); B9 = A/Sing IM, 15 ug, as 2018 Afluria Quad (UA)

Listing 16.2.9.2
Treatment Site Tolerability Assessment per Site

Part B
Randomisation Number = B0066, Treatment Group = Group B6

Date	Time	Visit	Time Point	Site Number	Treatment for Site	Indura-tion	Redness	Bruising	Itching	Skin Flaking	Tender-ness	Definition/ Visibility	Other#	Unscheduled Reason
01-JUN-2018	10:20	Day 8		F3	uncoated	0	0	0	0	0	0	2		
15-JUN-2018	10:56	Day 22		F1	2.5 ug	0	0	0	0	0	0	1		
15-JUN-2018	10:56	Day 22		F2	uncoated	0	0	0	0	0	0	1		
15-JUN-2018	10:56	Day 22		F3	uncoated	0	0	0	0	0	0	1		
24-JUL-2018	10:51	EOS/ET (Day 61)		F1	2.5 ug	0	0	0	0	0	0	0		
24-JUL-2018	10:51	EOS/ET (Day 61)		F2	uncoated	0	0	0	0	0	0	0		
24-JUL-2018	10:51	EOS/ET (Day 61)		F3	uncoated	0	0	0	0	0	0	0		

Key: 0= None; 1=Mild; 2=Moderate; 3=Severe; # Other = Pinprick bleeding or seepage

Administration Site Key: FA = Forearm; UA= Upper arm
Treatments Part A: A1 = A/Sing MAP, 15 ug (FA); A2 = Afluria Quad IM (UA), 15 ug; A3 = Placebo MAP (FA); A4 = A/Sing Mono IM, 15 ug (UA)
Treatments Part B: B1 = A/Sing MAP, 15 ug (FA) with biopsy; B2 = Placebo MAP (FA) with biopsy
B3 = A/Sing MAP, 15 ug (FA); B4 = A/Sing MAP, 10 ug (FA); B5 = A/Sing MAP, 5 ug (FA); B6 = A/Sing MAP, 2.5 ug (FA)
B7 = Placebo MAP (FA); B8 = A/Sing MAP, 15 ug (UA); B9 = A/Sing IM, 15 ug, as 2018 Afluria Quad (UA)

Listing 16.2.9.2
Treatment Site Tolerability Assessment per Site

Part B
Randomisation Number = B0067, Treatment Group = Group B8

Date	Time	Visit	Time Point	Site Number	Treatment for Site	Induration	Redness	Bruising	Itching	Skin Flaking	Tenderness	Definition/ Visibility	Other#	Unscheduled Reason
25-MAY-2018	14:10	Day 1	Pre-dose	D1	5 ug	0	0	0	0	0	0	0		
25-MAY-2018	14:10	Day 1	Pre-dose	D2	5 ug	0	0	0	0	0	0	0		
25-MAY-2018	14:10	Day 1	Pre-dose	D3	5 ug	0	0	0	0	0	0	0		
25-MAY-2018	15:28	Day 1	10 min	D1	5 ug	0	1	0	1	0	0	0		
25-MAY-2018	15:28	Day 1	10 min	D2	5 ug	0	1	0	1	0	1	0		
25-MAY-2018	15:28	Day 1	10 min	D3	5 ug	0	0	0	0	0	0	0		
25-MAY-2018	16:18	Day 1	1 hour	D1	5 ug	0	0	0	0	0	0	0		
25-MAY-2018	16:18	Day 1	1 hour	D2	5 ug	0	0	0	0	0	1	0		
25-MAY-2018	16:18	Day 1	1 hour	D3	5 ug	0	0	0	0	0	1	0		
25-MAY-2018	17:18	Day 1	2 hour	D1	5 ug	0	0	0	0	0	0	0		
25-MAY-2018	17:18	Day 1	2 hour	D2	5 ug	1	0	0	0	0	1	0		
25-MAY-2018	17:18	Day 1	2 hour	D3	5 ug	0	0	0	0	0	1	0		
26-MAY-2018	08:58	Day 2		D1	5 ug	0	1	0	0	0	0	0		
26-MAY-2018	08:58	Day 2		D2	5 ug	0	1	0	0	0	0	0		
26-MAY-2018	08:58	Day 2		D3	5 ug	0	1	0	0	0	0	0		
26-MAY-2018	09:22	Unscheduled		D1	5 ug	0	0	0	0	0	0	0	NA	MO notifies as repeat
26-MAY-2018	09:24	Unscheduled		D2	5 ug	0	1	0	0	0	1	0	NA	MO notifies as repeat

Key: 0= None; 1=Mild; 2=Moderate; 3=Severe; # Other = Pinprick bleeding or seepage

Administration Site Key: FA = Forearm; UA= Upper arm
Treatments Part A: A1 = A/Sing MAP, 15 ug (FA); A2 = Afluria Quad IM (UA), 15 ug; A3 = Placebo MAP (FA); A4 = A/Sing Mono IM, 15 ug (UA)
Treatments Part B: B1 = A/Sing MAP, 15 ug (FA) with biopsy; B2 = Placebo MAP (FA) with biopsy
B3 = A/Sing MAP, 15 ug (FA); B4 = A/Sing MAP, 10 ug (FA); B5 = A/Sing MAP, 5 ug (FA); B6 = A/Sing MAP, 2.5 ug (FA)
B7 = Placebo MAP (FA); B8 = A/Sing MAP, 15 ug (UA); B9 = A/Sing IM, 15 ug, as 2018 Afluria Quad (UA)

Listing 16.2.9.2
Treatment Site Tolerability Assessment per Site

Part B
Randomisation Number = B0067,   Treatment Group = Group B8

Date	Time	Visit	Time Point	Site Number	Treatment for Site	Indura-tion	Redness	Bruising	Itching	Skin Flaking	Tender-ness	Definition/ Visibility	Other#	Unscheduled Reason
26-MAY-2018	09:26	Unscheduled		D3	5 ug	0	1	0	0	0	1	0	NA	MO notifies as repeat
28-MAY-2018	17:10	Day 4		D1	5 ug	0	1	0	1	0	0	0		
28-MAY-2018	17:10	Day 4		D2	5 ug	1	1	0	1	0	1	0		
28-MAY-2018	17:10	Day 4		D3	5 ug	0	1	0	1	0	0	0		
02-JUN-2018	08:36	Day 8		D1	5 ug	0	0	0	0	2	0	2		
02-JUN-2018	08:36	Day 8		D2	5 ug	0	0	0	0	1	0	2		
02-JUN-2018	08:36	Day 8		D3	5 ug	0	0	0	0	1	0	2		
02-JUN-2018	08:55	Unscheduled		D1	5 ug	0	0	0	0	2	0	2	NA	MO notifies as repeat
02-JUN-2018	08:55	Unscheduled		D2	5 ug	0	0	0	0	2	0	2	NA	MO notifies as repeat
15-JUN-2018	14:05	Day 22		D1	5 ug	0	0	0	0	1	0	3		
15-JUN-2018	14:05	Day 22		D2	5 ug	0	0	0	0	1	0	3		
15-JUN-2018	14:05	Day 22		D3	5 ug	0	0	0	0	1	0	3		
25-JUL-2018	17:53	EOS/ET (Day 61)		D1	5 ug	0	0	0	0	0	0	0		
25-JUL-2018	17:53	EOS/ET (Day 61)		D2	5 ug	0	0	0	0	0	0	0		
25-JUL-2018	17:53	EOS/ET (Day 61)		D3	5 ug	0	0	0	0	0	0	0		

Key: 0= None; 1=Mild; 2=Moderate; 3=Severe; # Other = Pinprick bleeding or seepage

Administration Site Key: FA = Forearm; UA= Upper arm
Treatments Part A: A1 = A/Sing MAP, 15 ug (FA); A2 = Afluria Quad IM (UA), 15 ug; A3 = Placebo MAP (FA); A4 = A/Sing Mono IM, 15 ug (UA)
Treatments Part B: B1 = A/Sing MAP, 15 ug (FA) with biopsy; B2 = Placebo MAP (FA) with biopsy
B3 = A/Sing MAP, 15 ug (FA); B4 = A/Sing MAP, 10 ug (FA); B5 = A/Sing MAP, 5 ug (FA); B6 = A/Sing MAP, 2.5 ug (FA)
B7 = Placebo MAP (FA); B8 = A/Sing MAP, 15 ug (UA); B9 = A/Sing IM, 15 ug, as 2018 Afluria Quad (UA)

Listing 16.2.9.2
Treatment Site Tolerability Assessment per Site

Part B
Randomisation Number = B0068, Treatment Group = Group B5

Date	Time	Visit	Time Point	Site Number	Treatment for Site	Indura-tion	Redness	Bruising	Itching	Skin Flaking	Tender-ness	Definition/ Visibility	Other#	Unscheduled Reason
25-MAY-2018	14:20	Day 1	Pre-dose	F1	uncoated	0	0	0	0	0	0	0		
25-MAY-2018	14:20	Day 1	Pre-dose	F2	5 ug	0	0	0	0	0	0	0		
25-MAY-2018	14:20	Day 1	Pre-dose	F3	uncoated	0	0	0	0	0	0	0		
25-MAY-2018	15:36	Day 1	10 min	F1	uncoated	0	0	0	0	0	0	0		
25-MAY-2018	15:36	Day 1	10 min	F2	5 ug	0	0	0	0	0	1	0		
25-MAY-2018	15:36	Day 1	10 min	F3	uncoated	0	0	0	0	0	0	0		
25-MAY-2018	17:00	Day 1	1 hour	F1	uncoated	0	0	0	0	0	0	0		
25-MAY-2018	17:00	Day 1	1 hour	F2	5 ug	0	1	0	0	0	0	0		
25-MAY-2018	17:00	Day 1	1 hour	F3	uncoated	0	1	0	0	0	0	0		
25-MAY-2018	18:00	Day 1	2 hour	F1	uncoated	0	0	0	0	0	0	0		
25-MAY-2018	18:00	Day 1	2 hour	F2	5 ug	0	0	0	0	0	0	0		
25-MAY-2018	18:00	Day 1	2 hour	F3	uncoated	0	0	0	0	0	0	0		
01-JUN-2018	09:58	Day 8		F1	uncoated	0	0	0	0	1	0	1		
01-JUN-2018	09:58	Day 8		F2	5 ug	0	0	0	0	0	0	0		
01-JUN-2018	09:58	Day 8		F3	uncoated	0	0	0	0	1	0	1		
16-JUN-2018	08:13	Day 22		F1	uncoated	0	0	0	0	0	0	0		
16-JUN-2018	08:13	Day 22		F2	5 ug	0	0	0	0	1	0	3		
16-JUN-2018	08:13	Day 22		F3	uncoated	0	0	0	0	0	0	0		
24-JUL-2018	10:44	EOS/ET (Day 61)		F1	uncoated	0	0	0	0	0	0	0		

Key: 0= None; 1=Mild; 2=Moderate; 3=Severe; # Other = Pinprick bleeding or seepage

Administration Site Key: FA = Forearm; UA= Upper arm
Treatments Part A: A1 = A/Sing MAP, 15 ug (FA); A2 = Afluria Quad IM (UA), 15 ug; A3 = Placebo MAP (FA); A4 = A/Sing Mono IM, 15 ug (UA)
Treatments Part B: B1 = A/Sing MAP, 15 ug (FA) with biopsy; B2 = Placebo MAP (FA) with biopsy
B3 = A/Sing MAP, 15 ug (FA); B4 = A/Sing MAP, 10 ug (FA); B5 = A/Sing MAP, 5 ug (FA); B6 = A/Sing MAP, 2.5 ug (FA)
B7 = Placebo MAP (FA); B8 = A/Sing MAP, 15 ug (UA); B9 = A/Sing IM, 15 ug, as 2018 Afluria Quad (UA)

Listing 16.2.9.2
Treatment Site Tolerability Assessment per Site

Part B
Randomisation Number = B0068, Treatment Group = Group B5

Date	Time	Visit	Time Point	Site Number	Treatment for Site	Indura-tion	Redness	Bruising	Itching	Skin Flaking	Tender-ness	Definition/ Visibility	Other#	Unscheduled Reason
24-JUL-2018	10:44	EOS/ET (Day 61)		F2	5 ug	0	0	0	0	0	0	1		
24-JUL-2018	10:44	EOS/ET (Day 61)		F3	uncoated	0	0	0	0	0	0	0		

Key: 0= None; 1=Mild; 2=Moderate; 3=Severe; # Other = Pinprick bleeding or seepage

Administration Site Key: FA = Forearm; UA= Upper arm
Treatments Part A: A1 = A/Sing MAP, 15 ug (FA); A2 = Afluria Quad IM (UA), 15 ug; A3 = Placebo MAP (FA); A4 = A/Sing Mono IM, 15 ug (UA)
Treatments Part B: B1 = A/Sing MAP, 15 ug (FA) with biopsy; B2 = Placebo MAP (FA) with biopsy
B3 = A/Sing MAP, 15 ug (FA); B4 = A/Sing MAP, 10 ug (FA); B5 = A/Sing MAP, 5 ug (FA); B6 = A/Sing MAP, 2.5 ug (FA)
B7 = Placebo MAP (FA); B8 = A/Sing MAP, 15 ug (UA); B9 = A/Sing IM, 15 ug, as 2018 Afluria Quad (UA)

Listing 16.2.9.2
Treatment Site Tolerability Assessment per Site

Part B
Randomisation Number = B0069, Treatment Group = Group B7

Date	Time	Visit	Time Point	Site Number	Treatment for Site	Indura-tion	Redness	Bruising	Itching	Skin Flaking	Tender-ness	Definition/ Visibility	Other#	Unscheduled Reason
01-JUN-2018	11:10	Day 1	Pre-dose	F1	uncoated	0	0	0	0	0	0	0		
01-JUN-2018	11:10	Day 1	Pre-dose	F2	uncoated	0	0	0	0	0	0	0		
01-JUN-2018	11:10	Day 1	Pre-dose	F3	uncoated	0	0	0	0	0	0	0		
01-JUN-2018	12:18	Day 1	10 min	F1	uncoated	0	1	0	0	0	1	0		
01-JUN-2018	12:18	Day 1	10 min	F2	uncoated	0	0	0	0	0	0	0		
01-JUN-2018	12:18	Day 1	10 min	F3	uncoated	0	0	0	0		1	0		
01-JUN-2018	13:08	Day 1	1 hour	F1	uncoated	0	1	0	0	0	0	0		
01-JUN-2018	13:08	Day 1	1 hour	F2	uncoated	0	1	0	0	0	0	0		
01-JUN-2018	13:08	Day 1	1 hour	F3	uncoated	0	1	0	0	0	0	0		
01-JUN-2018	14:02	Day 1	2 hour	F1	uncoated	0	0	0	0	0	0	0		
01-JUN-2018	14:02	Day 1	2 hour	F2	uncoated	0	0	0	0	0	0	0		
01-JUN-2018	14:02	Day 1	2 hour	F3	uncoated	0	0	0	0	0	0	0		
02-JUN-2018	14:35	Day 2		F1	uncoated	0	0	0	0	0	0	0		
02-JUN-2018	14:35	Day 2		F2	uncoated	0	0	0	0	0	0	0		
02-JUN-2018	14:35	Day 2		F3	uncoated	1	0	0	0	0	0	0		
04-JUN-2018	11:03	Day 4		F1	uncoated	0	0	0	0	0	0	0		
04-JUN-2018	11:03	Day 4		F2	uncoated	0	0	0	0	0	0	0		
04-JUN-2018	11:03	Day 4		F3	uncoated	0	0	0	0	0	0	0		
08-JUN-2018	13:54	Day 8		F1	uncoated	0	0	0	0	0	0	2		
08-JUN-2018	13:54	Day 8		F2	uncoated	0	0	0	0	0	0	2		

Key: 0= None; 1=Mild; 2=Moderate; 3=Severe; # Other = Pinprick bleeding or seepage

Administration Site Key: FA = Forearm; UA= Upper arm
Treatments Part A: A1 = A/Sing MAP, 15 ug (FA); A2 = Afluria Quad IM (UA), 15 ug; A3 = Placebo MAP (FA); A4 = A/Sing Mono IM, 15 ug (UA)
Treatments Part B: B1 = A/Sing MAP, 15 ug (FA) with biopsy; B2 = Placebo MAP (FA) with biopsy
B3 = A/Sing MAP, 15 ug (FA); B4 = A/Sing MAP, 10 ug (FA); B5 = A/Sing MAP, 5 ug (FA); B6 = A/Sing MAP, 2.5 ug (FA)
B7 = Placebo MAP (FA); B8 = A/Sing MAP, 15 ug (UA); B9 = A/Sing IM, 15 ug, as 2018 Afluria Quad (UA)

Listing 16.2.9.2
Treatment Site Tolerability Assessment per Site

Part B
Randomisation Number = B0069, Treatment Group = Group B7

Date	Time	Visit	Time Point	Site Number	Treatment for Site	Indura-tion	Redness	Bruising	Itching	Skin Flaking	Tender-ness	Definition/ Visibility	Other#	Unscheduled Reason
08-JUN-2018	13:54	Day 8		F3	uncoated	0	0	0	0	0	0	2		
22-JUN-2018	10:12	Day 22		F1	uncoated	0	0	0	0	0	0	1		
22-JUN-2018	10:12	Day 22		F2	uncoated	0	0	0	0	0	0	1		
22-JUN-2018	10:12	Day 22		F3	uncoated	0	0	0	0	0	0	1		
31-JUL-2018	10:44	EOS/ET (Day 61)		F1	uncoated	0	0	0	0	0	0	0		
31-JUL-2018	10:44	EOS/ET (Day 61)		F2	uncoated	0	0	0	0	0	0	0		
31-JUL-2018	10:44	EOS/ET (Day 61)		F3	uncoated	0	0	0	0	0	0	0		

Key: 0= None; 1=Mild; 2=Moderate; 3=Severe; # Other = Pinprick bleeding or seepage

Administration Site Key: FA = Forearm; UA= Upper arm
Treatments Part A: A1 = A/Sing MAP, 15 ug (FA); A2 = Afluria Quad IM (UA), 15 ug; A3 = Placebo MAP (FA); A4 = A/Sing Mono IM, 15 ug (UA)
Treatments Part B: B1 = A/Sing MAP, 15 ug (FA) with biopsy; B2 = Placebo MAP (FA) with biopsy
B3 = A/Sing MAP, 15 ug (FA); B4 = A/Sing MAP, 10 ug (FA); B5 = A/Sing MAP, 5 ug (FA); B6 = A/Sing MAP, 2.5 ug (FA)
B7 = Placebo MAP (FA); B8 = A/Sing MAP, 15 ug (UA); B9 = A/Sing IM, 15 ug, as 2018 Afluria Quad (UA)

Listing 16.2.9.2
Treatment Site Tolerability Assessment per Site

Part B
Randomisation Number = B0070, Treatment Group = Group B9

Date	Time	Visit	Time Point	Site Number	Treatment for Site	Indura-tion	Redness	Bruising	Itching	Skin Flaking	Tender-ness	Definition/ Visibility	Other#	Unscheduled Reason
01-JUN-2018	10:53	Day 1	Pre-dose	D1	[IM]	0	0	0	0	0	0	5		
01-JUN-2018	11:31	Day 1	10 min	D1	[IM]	0	0	0	0	0	0	5		
01-JUN-2018	12:21	Day 1	1 hour	D1	[IM]	0	0	0	0	0	0	5		
01-JUN-2018	13:22	Day 1	2 hour	D1	[IM]	0	0	0	0	0	0	5		
02-JUN-2018	11:53	Day 2		D1	[IM]	0	0	0	0	0	0	5		
04-JUN-2018	12:22	Day 4		D1	[IM]	0	0	0	0	0	0	5		
08-JUN-2018	10:14	Day 8		D1	[IM]	0	0	0	0	0	0	5		
22-JUN-2018	10:14	Day 22		D1	[IM]	0	0	0	0	0	0	5		
04-JUL-2018	12:02	EOS/ET (Day 61)		D1	[IM]	0	0	0	0	0	0	5		

Key: 0= None; 1=Mild; 2=Moderate; 3=Severe; # Other = Pinprick bleeding or seepage

Administration Site Key: FA = Forearm; UA= Upper arm
Treatments Part A: A1 = A/Sing MAP, 15 ug (FA); A2 = Afluria Quad IM (UA), 15 ug; A3 = Placebo MAP (FA); A4 = A/Sing Mono IM, 15 ug (UA)
Treatments Part B: B1 = A/Sing MAP, 15 ug (FA) with biopsy; B2 = Placebo MAP (FA) with biopsy
B3 = A/Sing MAP, 15 ug (FA); B4 = A/Sing MAP, 10 ug (FA); B5 = A/Sing MAP, 5 ug (FA); B6 = A/Sing MAP, 2.5 ug (FA)
B7 = Placebo MAP (FA); B8 = A/Sing MAP, 15 ug (UA); B9 = A/Sing IM, 15 ug, as 2018 Afluria Quad (UA)

Listing 16.2.9.2
Treatment Site Tolerability Assessment per Site

Part B
Randomisation Number = B0071, Treatment Group = Group B4

Date	Time	Visit	Time Point	Site Number	Treatment for Site	Induration	Redness	Bruising	Itching	Skin Flaking	Tenderness	Definition/ Visibility	Other#	Unscheduled Reason
01-JUN-2018	10:55	Day 1	Pre-dose	F1	5 ug	0	0	0	0	0	0	0		
01-JUN-2018	10:55	Day 1	Pre-dose	F2	uncoated	0	0	0	0	0	0	0		
01-JUN-2018	10:55	Day 1	Pre-dose	F3	5 ug	0	0	0	0	0	0	0		
01-JUN-2018	11:48	Day 1	10 min	F1	5 ug	0	0	0	1	0	0	0		
01-JUN-2018	11:48	Day 1	10 min	F2	uncoated	0	0	0	1	0	0	0		
01-JUN-2018	11:48	Day 1	10 min	F3	5 ug	0	1	0	1	0	0	0		
01-JUN-2018	12:38	Day 1	1 hour	F1	5 ug	0	1	0	0	0	0	0		
01-JUN-2018	12:38	Day 1	1 hour	F2	uncoated	0	1	0	0	0	0	0		
01-JUN-2018	12:38	Day 1	1 hour	F3	5 ug	0	1	0	0	0	0	0		
01-JUN-2018	13:38	Day 1	2 hour	F1	5 ug	0	0	0	0	0	0	0		
01-JUN-2018	13:38	Day 1	2 hour	F2	uncoated	0	0	0	0	0	0	0		
01-JUN-2018	13:38	Day 1	2 hour	F3	5 ug	0	0	0	0	0	0	0		
02-JUN-2018	12:04	Day 2		F1	5 ug	0	1	0	0	0	0	0		
02-JUN-2018	12:04	Day 2		F2	uncoated	0	0	0	0	0	0	0		
02-JUN-2018	12:04	Day 2		F3	5 ug	1	1	0	0	0	1	0		
04-JUN-2018	12:06	Day 4		F1	5 ug	0	0	0	0	0	0	0		
04-JUN-2018	12:06	Day 4		F2	uncoated	0	0	0	0	0	0	2		
04-JUN-2018	12:06	Day 4		F3	5 ug	0	0	0	0	0	0	0		
08-JUN-2018	08:18	Day 8		F1	5 ug	0	0	0	0	1	0	3		
08-JUN-2018	08:18	Day 8		F2	uncoated	0	0	0	0	0	0	2		

Key: 0= None; 1=Mild; 2=Moderate; 3=Severe; # Other = Pinprick bleeding or seepage

Administration Site Key: FA = Forearm; UA= Upper arm
Treatments Part A: A1 = A/Sing MAP, 15 ug (FA); A2 = Afluria Quad IM (UA), 15 ug; A3 = Placebo MAP (FA); A4 = A/Sing Mono IM, 15 ug (UA)
Treatments Part B: B1 = A/Sing MAP, 15 ug (FA) with biopsy; B2 = Placebo MAP (FA) with biopsy
B3 = A/Sing MAP, 15 ug (FA); B4 = A/Sing MAP, 10 ug (FA); B5 = A/Sing MAP, 5 ug (FA); B6 = A/Sing MAP, 2.5 ug (FA)
B7 = Placebo MAP (FA); B8 = A/Sing MAP, 15 ug (UA); B9 = A/Sing IM, 15 ug, as 2018 Afluria Quad (UA)

Listing 16.2.9.2
Treatment Site Tolerability Assessment per Site

Part B
Randomisation Number = B0071, Treatment Group = Group B4

Date	Time	Visit	Time Point	Site Number	Treatment for Site	Indura-tion	Redness	Bruising	Itching	Skin Flaking	Tender-ness	Definition/ Visibility	Other#	Unscheduled Reason
08-JUN-2018	08:18	Day 8		F3	5 ug	0	0	0	0	1	0	0		
22-JUN-2018	13:31	Day 22		F1	5 ug	0	0	0	0	0	0	1		
22-JUN-2018	13:31	Day 22		F2	uncoated	0	0	0	0	0	0	0		
22-JUN-2018	13:31	Day 22		F3	5 ug	0	0	0	0	0	0	1		
31-JUL-2018	09:33	EOS/ET (Day 61)		F1	5 ug	0	0	0	0	0	0	0		
31-JUL-2018	09:33	EOS/ET (Day 61)		F2	uncoated	0	0	0	0	0	0	0		
31-JUL-2018	09:33	EOS/ET (Day 61)		F3	5 ug	0	0	0	0	0	0	0		

Key: 0= None; 1=Mild; 2=Moderate; 3=Severe; # Other = Pinprick bleeding or seepage

Administration Site Key: FA = Forearm; UA= Upper arm
Treatments Part A: A1 = A/Sing MAP, 15 ug (FA); A2 = Afluria Quad IM (UA), 15 ug; A3 = Placebo MAP (FA); A4 = A/Sing Mono IM, 15 ug (UA)
Treatments Part B: B1 = A/Sing MAP, 15 ug (FA) with biopsy; B2 = Placebo MAP (FA) with biopsy
B3 = A/Sing MAP, 15 ug (FA); B4 = A/Sing MAP, 10 ug (FA); B5 = A/Sing MAP, 5 ug (FA); B6 = A/Sing MAP, 2.5 ug (FA)
B7 = Placebo MAP (FA); B8 = A/Sing MAP, 15 ug (UA); B9 = A/Sing IM, 15 ug, as 2018 Afluria Quad (UA)

Listing 16.2.9.2
Treatment Site Tolerability Assessment per Site

Part B
Randomisation Number = B0072, Treatment Group = Group B3

Date	Time	Visit	Time Point	Site Number	Treatment for Site	Induration	Redness	Bruising	Itching	Skin Flaking	Tenderness	Definition/ Visibility	Other#	Unscheduled Reason
01-JUN-2018	11:00	Day 1	Pre-dose	F1	5 ug	0	0	0	0	0	0	0		
01-JUN-2018	11:00	Day 1	Pre-dose	F2	5 ug	0	0	0	0	0	0	0		
01-JUN-2018	11:00	Day 1	Pre-dose	F3	5 ug	0	0	0	0	0	0	0		
01-JUN-2018	11:59	Day 1	10 min	F1	5 ug	0	1	0	1	0	1	0		
01-JUN-2018	11:59	Day 1	10 min	F2	5 ug	0	0	0	1	0	1	0		
01-JUN-2018	11:59	Day 1	10 min	F3	5 ug	0	0	0	1	0	1	0		
01-JUN-2018	12:48	Day 1	1 hour	F1	5 ug	0	1	0	0	0	1	0		
01-JUN-2018	12:48	Day 1	1 hour	F2	5 ug	0	1	0	0	0	1	0		
01-JUN-2018	12:48	Day 1	1 hour	F3	5 ug	0	1	0	0	0	1	0		
01-JUN-2018	13:45	Day 1	2 hour	F1	5 ug	0	0	0	0	0	0	0		
01-JUN-2018	13:45	Day 1	2 hour	F2	5 ug	0	0	0	0	0	0	0		
01-JUN-2018	13:45	Day 1	2 hour	F3	5 ug	0	0	0	0	0	0	0		
02-JUN-2018	12:12	Day 2		F1	5 ug	0	1	0	0	0	1	0		
02-JUN-2018	12:12	Day 2		F2	5 ug	1	1	0	0	0	1	0		
02-JUN-2018	12:12	Day 2		F3	5 ug	1	1	0	0	1	0	0		
04-JUN-2018	12:25	Day 4		F1	5 ug	0	0	0	0	2	0	0		
04-JUN-2018	12:25	Day 4		F2	5 ug	0	0	0	0	2	0	0		
04-JUN-2018	12:25	Day 4		F3	5 ug	0	0	0	0	2	0	0		
08-JUN-2018	13:46	Day 8		F1	5 ug	0	0	0	0	2	0	2		
08-JUN-2018	13:46	Day 8		F2	5 ug	0	0	0	1	2	0	2		

Key: 0= None; 1=Mild; 2=Moderate; 3=Severe; # Other = Pinprick bleeding or seepage

Administration Site Key: FA = Forearm; UA= Upper arm
Treatments Part A: A1 = A/Sing MAP, 15 ug (FA); A2 = Afluria Quad IM (UA), 15 ug; A3 = Placebo MAP (FA); A4 = A/Sing Mono IM, 15 ug (UA)
Treatments Part B: B1 = A/Sing MAP, 15 ug (FA) with biopsy; B2 = Placebo MAP (FA) with biopsy
B3 = A/Sing MAP, 15 ug (FA); B4 = A/Sing MAP, 10 ug (FA); B5 = A/Sing MAP, 5 ug (FA); B6 = A/Sing MAP, 2.5 ug (FA)
B7 = Placebo MAP (FA); B8 = A/Sing MAP, 15 ug (UA); B9 = A/Sing IM, 15 ug, as 2018 Afluria Quad (UA)

Listing 16.2.9.2
Treatment Site Tolerability Assessment per Site

Part B
Randomisation Number = B0072, Treatment Group = Group B3

Date	Time	Visit	Time Point	Site Number	Treatment for Site	Indura-tion	Redness	Bruising	Itching	Skin Flaking	Tender-ness	Definition/ Visibility	Other#	Unscheduled Reason
08-JUN-2018	13:46	Day 8		F3	5 ug	0	0	0	1	2	0	2		
08-JUN-2018	14:05	Unscheduled		F1	5 ug	0	0	0	0	1	0	2	NA	MO notifies as repeat
08-JUN-2018	14:05	Unscheduled		F2	5 ug	0	0	0	1	2	0	0	NA	MO notifies as repeat
08-JUN-2018	14:05	Unscheduled		F3	5 ug	0	0	0	1	2	0	0	NA	MO notifies as repeat
22-JUN-2018	12:36	Day 22		F1	5 ug	0	0	0	0	0	0	3		
22-JUN-2018	12:36	Day 22		F2	5 ug	0	0	0	0	0	0	3		
22-JUN-2018	12:36	Day 22		F3	5 ug	0	0	0	0	0	0	2		
31-JUL-2018	12:13	EOS/ET (Day 61)		F1	5 ug	0	0	0	0	0	0	0		
31-JUL-2018	12:13	EOS/ET (Day 61)		F2	5 ug	0	0	0	0	0	0	0		
31-JUL-2018	12:13	EOS/ET (Day 61)		F3	5 ug	0	0	0	0	0	0	0		

Key: 0= None; 1=Mild; 2=Moderate; 3=Severe; # Other = Pinprick bleeding or seepage

Administration Site Key: FA = Forearm; UA= Upper arm
Treatments Part A: A1 = A/Sing MAP, 15 ug (FA); A2 = Afluria Quad IM (UA), 15 ug; A3 = Placebo MAP (FA); A4 = A/Sing Mono IM, 15 ug (UA)
Treatments Part B: B1 = A/Sing MAP, 15 ug (FA) with biopsy; B2 = Placebo MAP (FA) with biopsy
B3 = A/Sing MAP, 15 ug (FA); B4 = A/Sing MAP, 10 ug (FA); B5 = A/Sing MAP, 5 ug (FA); B6 = A/Sing MAP, 2.5 ug (FA)
B7 = Placebo MAP (FA); B8 = A/Sing MAP, 15 ug (UA); B9 = A/Sing IM, 15 ug, as 2018 Afluria Quad (UA)

Listing 16.2.9.2
Treatment Site Tolerability Assessment per Site

Part B
Randomisation Number = B0073, Treatment Group = Group B6

Date	Time	Visit	Time Point	Site Number	Treatment for Site	Induration	Redness	Bruising	Itching	Skin Flaking	Tenderness	Definition/ Visibility	Other#	Unscheduled Reason
01-JUN-2018	11:04	Day 1	Pre-dose	F1	uncoated	0	0	0	0	0	0	0		
01-JUN-2018	11:04	Day 1	Pre-dose	F2	2.5 ug	0	0	0	0	0	0	0		
01-JUN-2018	11:04	Day 1	Pre-dose	F3	uncoated	0	0	0	0	0	0	0		
01-JUN-2018	12:06	Day 1	10 min	F1	uncoated	0	0	0	1	0	0	0		
01-JUN-2018	12:06	Day 1	10 min	F2	2.5 ug	0	1	0	1	0	0	0		
01-JUN-2018	12:06	Day 1	10 min	F3	uncoated	0	0	0	1	0	0	0		
01-JUN-2018	12:58	Day 1	1 hour	F1	uncoated	0	0	0	0	0	0	0		
01-JUN-2018	12:58	Day 1	1 hour	F2	2.5 ug	0	1	0	1	0	0	0		
01-JUN-2018	12:58	Day 1	1 hour	F3	uncoated	0	1	0	0	0	0	0		
01-JUN-2018	13:50	Day 1	2 hour	F1	uncoated	0	0	0	0	0	0	0		
01-JUN-2018	13:50	Day 1	2 hour	F2	2.5 ug	0	0	0	0	0	0	0		
01-JUN-2018	13:50	Day 1	2 hour	F3	uncoated	0	0	0	0	0	0	0		
02-JUN-2018	11:57	Day 2		F1	uncoated	0	0	0	0	0	0	0		
02-JUN-2018	11:57	Day 2		F2	2.5 ug	1	1	0	0	0	0	0		
02-JUN-2018	11:57	Day 2		F3	uncoated	0	0	0	0	0	0	0		
04-JUN-2018	13:54	Day 4		F1	uncoated	0	0	0	0	1	0	1		
04-JUN-2018	13:54	Day 4		F2	2.5 ug	0	0	0	0	0	0	0		
04-JUN-2018	13:54	Day 4		F3	uncoated	0	0	0	0	0	0	0		
08-JUN-2018	12:30	Day 8		F1	uncoated	0	0	0	0	0	0	3		
08-JUN-2018	12:30	Day 8		F2	2.5 ug	2	0	0	0	0	0	3		

Key: 0= None; 1=Mild; 2=Moderate; 3=Severe; # Other = Pinprick bleeding or seepage

Administration Site Key: FA = Forearm; UA= Upper arm
Treatments Part A: A1 = A/Sing MAP, 15 ug (FA); A2 = Afluria Quad IM (UA), 15 ug; A3 = Placebo MAP (FA); A4 = A/Sing Mono IM, 15 ug (UA)
Treatments Part B: B1 = A/Sing MAP, 15 ug (FA) with biopsy; B2 = Placebo MAP (FA) with biopsy
B3 = A/Sing MAP, 15 ug (FA); B4 = A/Sing MAP, 10 ug (FA); B5 = A/Sing MAP, 5 ug (FA); B6 = A/Sing MAP, 2.5 ug (FA)
B7 = Placebo MAP (FA); B8 = A/Sing MAP, 15 ug (UA); B9 = A/Sing IM, 15 ug, as 2018 Afluria Quad (UA)

Listing 16.2.9.2
Treatment Site Tolerability Assessment per Site

Part B
Randomisation Number = B0073, Treatment Group = Group B6

Date	Time	Visit	Time Point	Site Number	Treatment for Site	Indura-tion	Redness	Bruising	Itching	Skin Flaking	Tender-ness	Definition/ Visibility	Other#	Unscheduled Reason
08-JUN-2018	12:30	Day 8		F3	uncoated	0	0	0	0	0	0	3		
22-JUN-2018	14:20	Day 22		F1	uncoated	0	0	0	0	0	0	1		
22-JUN-2018	14:20	Day 22		F2	2.5 ug	0	0	0	0	0	0	2		
22-JUN-2018	14:20	Day 22		F3	uncoated	0	0	0	0	0	0	1		
31-JUL-2018	11:30	EOS/ET (Day 61)		F1	uncoated	0	0	0	0	0	0	0		
31-JUL-2018	11:30	EOS/ET (Day 61)		F2	2.5 ug	0	0	0	0	0	0	0		
31-JUL-2018	11:30	EOS/ET (Day 61)		F3	uncoated	0	0	0	0	0	0	0		

Key: 0= None; 1=Mild; 2=Moderate; 3=Severe; # Other = Pinprick bleeding or seepage

Administration Site Key: FA = Forearm; UA= Upper arm
Treatments Part A: A1 = A/Sing MAP, 15 ug (FA); A2 = Afluria Quad IM (UA), 15 ug; A3 = Placebo MAP (FA); A4 = A/Sing Mono IM, 15 ug (UA)
Treatments Part B: B1 = A/Sing MAP, 15 ug (FA) with biopsy; B2 = Placebo MAP (FA) with biopsy
B3 = A/Sing MAP, 15 ug (FA); B4 = A/Sing MAP, 10 ug (FA); B5 = A/Sing MAP, 5 ug (FA); B6 = A/Sing MAP, 2.5 ug (FA)
B7 = Placebo MAP (FA); B8 = A/Sing MAP, 15 ug (UA); B9 = A/Sing IM, 15 ug, as 2018 Afluria Quad (UA)

Listing 16.2.9.2
Treatment Site Tolerability Assessment per Site

Part B
Randomisation Number = B0074, Treatment Group = Group B9

Date	Time	Visit	Time Point	Site Number	Treatment for Site	Indura-tion	Redness	Bruising	Itching	Skin Flaking	Tender-ness	Definition/ Visibility	Other#	Unscheduled Reason
01-JUN-2018	10:51	Day 1	Pre-dose	D1	[IM]	0	0	0	0	0	0	5		
01-JUN-2018	11:11	Day 1	10 min	D1	[IM]	0	0	0	0	0	0	5		
01-JUN-2018	12:03	Day 1	1 hour	D1	[IM]	0	0	0	0	0	0	5		
01-JUN-2018	13:08	Day 1	2 hour	D1	[IM]	0	0	0	0	0	0	5		
02-JUN-2018	11:04	Day 2		D1	[IM]	0		0	0	0	0	5		
04-JUN-2018	15:02	Day 4		D1	[IM]	0	0	0	0	0	0	5		
08-JUN-2018	12:08	Day 8		D1	[IM]	0	0	0	0	0	0			
22-JUN-2018	11:56	Day 22		D1	[IM]	0	0	0	0	0	0	5		
31-JUL-2018	16:43	EOS/ET (Day 61)		D1	[IM]	0	0	0	0	0	0	5		

Key: 0= None; 1=Mild; 2=Moderate; 3=Severe; # Other = Pinprick bleeding or seepage

Administration Site Key: FA = Forearm; UA= Upper arm
Treatments Part A: A1 = A/Sing MAP, 15 ug (FA); A2 = Afluria Quad IM (UA), 15 ug; A3 = Placebo MAP (FA); A4 = A/Sing Mono IM, 15 ug (UA)
Treatments Part B: B1 = A/Sing MAP, 15 ug (FA) with biopsy; B2 = Placebo MAP (FA) with biopsy
B3 = A/Sing MAP, 15 ug (FA); B4 = A/Sing MAP, 10 ug (FA); B5 = A/Sing MAP, 5 ug (FA); B6 = A/Sing MAP, 2.5 ug (FA)
B7 = Placebo MAP (FA); B8 = A/Sing MAP, 15 ug (UA); B9 = A/Sing IM, 15 ug, as 2018 Afluria Quad (UA)

Listing 16.2.9.2
Treatment Site Tolerability Assessment per Site

Part B
Randomisation Number = B0075, Treatment Group = Group B3

Date	Time	Visit	Time Point	Site Number	Treatment for Site	Induration	Redness	Bruising	Itching	Skin Flaking	Tenderness	Definition/ Visibility	Other#	Unscheduled Reason
01-JUN-2018	11:17	Day 1	Pre-dose	F1	5 ug	0	0	0	0	0	0	0		
01-JUN-2018	11:17	Day 1	Pre-dose	F2	5 ug	0	0	0	0	0	0	0		
01-JUN-2018	11:17	Day 1	Pre-dose	F3	5 ug	0	0	0	0	0	0	0		
01-JUN-2018	12:27	Day 1	10 min	F1	5 ug	0	0	0	0	0	0	0		
01-JUN-2018	12:27	Day 1	10 min	F2	5 ug	0	0	0	0	0	1	0		
01-JUN-2018	12:27	Day 1	10 min	F3	5 ug	0	0	0	0	0	1	0		
01-JUN-2018	13:18	Day 1	1 hour	F1	5 ug	0	1	0	0	0	0	0		
01-JUN-2018	13:18	Day 1	1 hour	F2	5 ug	0	1	0	0	0	1	0		
01-JUN-2018	13:18	Day 1	1 hour	F3	5 ug	0	1	0	0	0	1	0		
01-JUN-2018	14:02	Day 1	2 hour	F1	5 ug	0	0	0	0	0	0	0		
01-JUN-2018	14:02	Day 1	2 hour	F2	5 ug	0	0	0	0	0	0	0		
01-JUN-2018	14:02	Day 1	2 hour	F3	5 ug	0	0	0	0	0	0	0		
02-JUN-2018	08:28	Day 2		F1	5 ug	0	0	0	0	0	0	0		
02-JUN-2018	08:28	Day 2		F2	5 ug	0	0	0	0	0	1	0		
02-JUN-2018	08:28	Day 2		F3	5 ug	0	0	0	0	0	0	0		
04-JUN-2018	08:51	Day 4		F1	5 ug	0	0	0	0	0	0	0		
04-JUN-2018	08:51	Day 4		F2	5 ug	0	1	0	1	0	0	0		
04-JUN-2018	08:51	Day 4		F3	5 ug	0	1	0	1	0	0	0		
08-JUN-2018	12:39	Day 8		F1	5 ug	0	0	0	0	2	0	2		
08-JUN-2018	12:39	Day 8		F2	5 ug	0	0	0	0	2	0	2		

Key: 0= None; 1=Mild; 2=Moderate; 3=Severe; # Other = Pinprick bleeding or seepage

Administration Site Key: FA = Forearm; UA= Upper arm
Treatments Part A: A1 = A/Sing MAP, 15 ug (FA); A2 = Afluria Quad IM (UA), 15 ug; A3 = Placebo MAP (FA); A4 = A/Sing Mono IM, 15 ug (UA)
Treatments Part B: B1 = A/Sing MAP, 15 ug (FA) with biopsy; B2 = Placebo MAP (FA) with biopsy
B3 = A/Sing MAP, 15 ug (FA); B4 = A/Sing MAP, 10 ug (FA); B5 = A/Sing MAP, 5 ug (FA); B6 = A/Sing MAP, 2.5 ug (FA)
B7 = Placebo MAP (FA); B8 = A/Sing MAP, 15 ug (UA); B9 = A/Sing IM, 15 ug, as 2018 Afluria Quad (UA)

Listing 16.2.9.2
Treatment Site Tolerability Assessment per Site

Part B
Randomisation Number = B0075, Treatment Group = Group B3

Date	Time	Visit	Time Point	Site Number	Treatment for Site	Indura-tion	Redness	Bruising	Itching	Skin Flaking	Tender-ness	Definition/ Visibility	Other#	Unscheduled Reason
08-JUN-2018	12:39	Day 8		F3	5 ug	0	0	0	0	2	0	2		
22-JUN-2018	13:33	Day 22		F1	5 ug	0	0	0	0	0	0	0		
22-JUN-2018	13:33	Day 22		F2	5 ug	0	0	0	0	0	0	0		
22-JUN-2018	13:33	Day 22		F3	5 ug	0	0	0	0	0	0	0		
31-JUL-2018	08:27	EOS/ET (Day 61)		F1	5 ug	0	0	0	0	0	0	0		
31-JUL-2018	08:27	EOS/ET (Day 61)		F2	5 ug	0	0	0	0	0	0	1		
31-JUL-2018	08:27	EOS/ET (Day 61)		F3	5 ug	0	0	0	0	0	0	1		

Key: 0= None; 1=Mild; 2=Moderate; 3=Severe; # Other = Pinprick bleeding or seepage

Administration Site Key: FA = Forearm; UA= Upper arm
Treatments Part A: A1 = A/Sing MAP, 15 ug (FA); A2 = Afluria Quad IM (UA), 15 ug; A3 = Placebo MAP (FA); A4 = A/Sing Mono IM, 15 ug (UA)
Treatments Part B: B1 = A/Sing MAP, 15 ug (FA) with biopsy; B2 = Placebo MAP (FA) with biopsy
B3 = A/Sing MAP, 15 ug (FA); B4 = A/Sing MAP, 10 ug (FA); B5 = A/Sing MAP, 5 ug (FA); B6 = A/Sing MAP, 2.5 ug (FA)
B7 = Placebo MAP (FA); B8 = A/Sing MAP, 15 ug (UA); B9 = A/Sing IM, 15 ug, as 2018 Afluria Quad (UA)

Listing 16.2.9.2
Treatment Site Tolerability Assessment per Site

Part B
Randomisation Number = B0077, Treatment Group = Group B4

Date	Time	Visit	Time Point	Site Number	Treatment for Site	Induration	Redness	Bruising	Itching	Skin Flaking	Tenderness	Definition/ Visibility	Other#	Unscheduled Reason
01-JUN-2018	11:27	Day 1	Pre-dose	F1	5 ug	0	0	0	0	0	0	0		
01-JUN-2018	11:27	Day 1	Pre-dose	F2	uncoated	0	0	0	0	0	0	0		
01-JUN-2018	11:27	Day 1	Pre-dose	F3	5 ug	0	0	0	0	0	0	0		
01-JUN-2018	12:38	Day 1	10 min	F1	5 ug	0	0	0	1	0	1	0		
01-JUN-2018	12:38	Day 1	10 min	F2	uncoated	0	0	0	1	0	1	0		
01-JUN-2018	12:38	Day 1	10 min	F3	5 ug	0	0	0	1	0	1	0		
01-JUN-2018		Day 1	1 hour	F1	5 ug	0	1	0	0	0	1	0		
01-JUN-2018		Day 1	1 hour	F2	uncoated	0	1	0	0	0	1	0		
01-JUN-2018		Day 1	1 hour	F3	5 ug	0	1	0	0	0	0	0		
01-JUN-2018	14:21	Day 1	2 hour	F1	5 ug	0	0	0	0	0	0	0		
01-JUN-2018	14:21	Day 1	2 hour	F2	uncoated	0	0	0	0	0	0	0		
01-JUN-2018	14:21	Day 1	2 hour	F3	5 ug	0	0	0	0	0	0	0		
02-JUN-2018	14:42	Day 2		F1	5 ug	1	1	0	0	0	0	0		
02-JUN-2018	14:42	Day 2		F2	uncoated	1	0	0	0	0	0	0		
02-JUN-2018	14:42	Day 2		F3	5 ug	1	1	0	0	0	0	0		
04-JUN-2018	08:45	Day 4		F1	5 ug	0	0	0	1	1	0	0		
04-JUN-2018	08:45	Day 4		F2	uncoated	0	0	0	0	0	0	0		
04-JUN-2018	08:45	Day 4		F3	5 ug	0	1	0	0	0	0	0		
08-JUN-2018	18:58	Day 8		F1	5 ug	0	1	0	0	1	0	0		
08-JUN-2018	18:58	Day 8		F2	uncoated	0	0	0	0	0	0	2		

Key: 0= None; 1=Mild; 2=Moderate; 3=Severe; # Other = Pinprick bleeding or seepage

Administration Site Key: FA = Forearm; UA= Upper arm
Treatments Part A: A1 = A/Sing MAP, 15 ug (FA); A2 = Afluria Quad IM (UA), 15 ug; A3 = Placebo MAP (FA); A4 = A/Sing Mono IM, 15 ug (UA)
Treatments Part B: B1 = A/Sing MAP, 15 ug (FA) with biopsy; B2 = Placebo MAP (FA) with biopsy
B3 = A/Sing MAP, 15 ug (FA); B4 = A/Sing MAP, 10 ug (FA); B5 = A/Sing MAP, 5 ug (FA); B6 = A/Sing MAP, 2.5 ug (FA)
B7 = Placebo MAP (FA); B8 = A/Sing MAP, 15 ug (UA); B9 = A/Sing IM, 15 ug, as 2018 Afluria Quad (UA)

Listing 16.2.9.2
Treatment Site Tolerability Assessment per Site

Part B
Randomisation Number = B0077, Treatment Group = Group B4

Date	Time	Visit	Time Point	Site Number	Treatment for Site	Indura-tion	Redness	Bruising	Itching	Skin Flaking	Tender-ness	Definition/ Visibility	Other#	Unscheduled Reason
08-JUN-2018	18:58	Day 8		F3	5 ug	0	1	0	0	1	0	0		
08-JUN-2018	19:15	Unscheduled		F1	5 ug	0	0	0	0	1	0	3	NA	MO notifies as repeat
08-JUN-2018	19:15	Unscheduled		F2	uncoated	0	0	0	0	0	0	2	NA	MO notifies as repeat
08-JUN-2018	19:15	Unscheduled		F3	5 ug	0	0	0	0	1	0	3	NA	MO notifies as repeat
22-JUN-2018	14:45	Day 22		F1	5 ug	0	0	0	0	1	0	2		
22-JUN-2018	14:45	Day 22		F2	uncoated	0	0	0	0	1	0	2		
22-JUN-2018	14:45	Day 22		F3	5 ug	0	0	0	0	1	0	2		
22-JUN-2018	14:53	Unscheduled		F1	5 ug	0	0	0	0	1	0	2	NA	MO notifies as repeat
22-JUN-2018	14:53	Unscheduled		F2	uncoated	0	0	0	0	1	0	3	NA	MO notifies as repeat
22-JUN-2018	14:53	Unscheduled		F3	5 ug	0	0	0	0	1	0	2	NA	Mo notifies as repeat
30-JUL-2018	15:31	EOS/ET (Day 61)		F1	5 ug	0	0	0	0	0	0	0		
30-JUL-2018	15:31	EOS/ET (Day 61)		F2	uncoated	0	0	0	0	0	0	0		
30-JUL-2018	15:31	EOS/ET (Day 61)		F3	5 ug	0	0	0	0	0	0	0		

Key: 0= None; 1=Mild; 2=Moderate; 3=Severe; # Other = Pinprick bleeding or seepage

Administration Site Key: FA = Forearm; UA= Upper arm
Treatments Part A: A1 = A/Sing MAP, 15 ug (FA); A2 = Afluria Quad IM (UA), 15 ug; A3 = Placebo MAP (FA); A4 = A/Sing Mono IM, 15 ug (UA)
Treatments Part B: B1 = A/Sing MAP, 15 ug (FA) with biopsy; B2 = Placebo MAP (FA) with biopsy
B3 = A/Sing MAP, 15 ug (FA); B4 = A/Sing MAP, 10 ug (FA); B5 = A/Sing MAP, 5 ug (FA); B6 = A/Sing MAP, 2.5 ug (FA)
B7 = Placebo MAP (FA); B8 = A/Sing MAP, 15 ug (UA); B9 = A/Sing IM, 15 ug, as 2018 Afluria Quad (UA)

Listing 16.2.9.2
Treatment Site Tolerability Assessment per Site

Part B
Randomisation Number = B0078, Treatment Group = Group B8

Date	Time	Visit	Time Point	Site Number	Treatment for Site	Induration	Redness	Bruising	Itching	Skin Flaking	Tenderness	Definition/ Visibility	Other#	Unscheduled Reason
01-JUN-2018	11:37	Day 1	Pre-dose	D1	5 ug	0	0	0	0	0	0	0		
01-JUN-2018	11:37	Day 1	Pre-dose	D2	5 ug	0	0	0	0	0	0	0		
01-JUN-2018	11:37	Day 1	Pre-dose	D3	5 ug	0	0	0	0	0	0	0		
01-JUN-2018	12:47	Day 1	10 min	D1	5 ug	0	0	0	0	0	0	0		
01-JUN-2018	12:47	Day 1	10 min	D2	5 ug	0	0	0	0	0	0	0		
01-JUN-2018	12:47	Day 1	10 min	D3	5 ug	0	0	0	0	0	1	0		
01-JUN-2018	13:38	Day 1	1 hour	D1	5 ug	0	1	0	0	0	0	0		
01-JUN-2018	13:38	Day 1	1 hour	D2	5 ug	0	1	0	0	0	0	0		
01-JUN-2018	13:38	Day 1	1 hour	D3	5 ug	0	1	0	0	0	1	0		
01-JUN-2018	14:30	Day 1	2 hour	D1	5 ug	0	0	0	0	0	0	0		
01-JUN-2018	14:30	Day 1	2 hour	D2	5 ug	0	0	0	0	0	0	0		
01-JUN-2018	14:30	Day 1	2 hour	D3	5 ug	0	0	0	0	0	0	0		
02-JUN-2018	10:22	Day 2		D1	5 ug	0	0	0	0	0	0	0		
02-JUN-2018	10:22	Day 2		D2	5 ug	0	0	0	0	0	0	0		
02-JUN-2018	10:22	Day 2		D3	5 ug	0	0	0	0	0	1	0		
04-JUN-2018	11:32	Day 4		D1	5 ug	0	1	0	1	1	1	0		
04-JUN-2018	11:32	Day 4		D2	5 ug	0	1	0	1	1	1	0		
04-JUN-2018	11:32	Day 4		D3	5 ug	0	1	0	2	1	1	0		
08-JUN-2018	10:59	Day 8		D1	5 ug	0	0	0	0	2	0	3		
08-JUN-2018	10:59	Day 8		D2	5 ug	0	0	0	0	1	0	1		

Key: 0= None; 1=Mild; 2=Moderate; 3=Severe; # Other = Pinprick bleeding or seepage

Administration Site Key: FA = Forearm; UA= Upper arm
Treatments Part A: A1 = A/Sing MAP, 15 ug (FA); A2 = Afluria Quad IM (UA), 15 ug; A3 = Placebo MAP (FA); A4 = A/Sing Mono IM, 15 ug (UA)
Treatments Part B: B1 = A/Sing MAP, 15 ug (FA) with biopsy; B2 = Placebo MAP (FA) with biopsy
B3 = A/Sing MAP, 15 ug (FA); B4 = A/Sing MAP, 10 ug (FA); B5 = A/Sing MAP, 5 ug (FA); B6 = A/Sing MAP, 2.5 ug (FA)
B7 = Placebo MAP (FA); B8 = A/Sing MAP, 15 ug (UA); B9 = A/Sing IM, 15 ug, as 2018 Afluria Quad (UA)

Listing 16.2.9.2
Treatment Site Tolerability Assessment per Site

Part B
Randomisation Number = B0078, Treatment Group = Group B8

Date	Time	Visit	Time Point	Site Number	Treatment for Site	Indura-tion	Redness	Bruising	Itching	Skin Flaking	Tender-ness	Definition/ Visibility	Other#	Unscheduled Reason
08-JUN-2018	10:59	Day 8		D3	5 ug	0	0	0	0	2	0	3		
22-JUN-2018	12:10	Day 22		D1	5 ug	0	0	0	0	0	0	1		
22-JUN-2018	12:10	Day 22		D2	5 ug	0	0	0	0	0	0	1		
22-JUN-2018	12:10	Day 22		D3	5 ug	0	0	0	0	0	0	1		
06-AUG-2018	18:01	EOS/ET (Day 61)		D1	5 ug	0	0	0	0	0	0	3		
06-AUG-2018	18:01	EOS/ET (Day 61)		D2	5 ug	0	0	0	0	0	0	3		
06-AUG-2018	18:01	EOS/ET (Day 61)		D3	5 ug	0	0	0	0	0	0	3		

Key: 0= None; 1=Mild; 2=Moderate; 3=Severe; # Other = Pinprick bleeding or seepage

Administration Site Key: FA = Forearm; UA= Upper arm
Treatments Part A: A1 = A/Sing MAP, 15 ug (FA); A2 = Afluria Quad IM (UA), 15 ug; A3 = Placebo MAP (FA); A4 = A/Sing Mono IM, 15 ug (UA)
Treatments Part B: B1 = A/Sing MAP, 15 ug (FA) with biopsy; B2 = Placebo MAP (FA) with biopsy
B3 = A/Sing MAP, 15 ug (FA); B4 = A/Sing MAP, 10 ug (FA); B5 = A/Sing MAP, 5 ug (FA); B6 = A/Sing MAP, 2.5 ug (FA)
B7 = Placebo MAP (FA); B8 = A/Sing MAP, 15 ug (UA); B9 = A/Sing IM, 15 ug, as 2018 Afluria Quad (UA)

Listing 16.2.9.2
Treatment Site Tolerability Assessment per Site

Part B
Randomisation Number = B0079, Treatment Group = Group B6

Date	Time	Visit	Time Point	Site Number	Treatment for Site	Indura-tion	Redness	Bruising	Itching	Skin Flaking	Tender-ness	Definition/ Visibility	Other#	Unscheduled Reason
01-JUN-2018	11:43	Day 1	Pre-dose	F1	uncoated	0	0	0	0	0	0	0		
01-JUN-2018	11:43	Day 1	Pre-dose	F2	2.5 ug	0	0	0	0	0	0	0		
01-JUN-2018	11:43	Day 1	Pre-dose	F3	uncoated	0	0	0	0	0	0	0		
01-JUN-2018	12:57	Day 1	10 min	F1	uncoated	0	1	0	0	0	0	0		
01-JUN-2018	12:57	Day 1	10 min	F2	2.5 ug	0	1	0	0	0	0	0		
01-JUN-2018	12:57	Day 1	10 min	F3	uncoated	0	1	0	0	0	0	0		
01-JUN-2018	13:48	Day 1	1 hour	F1	uncoated	0	1	0	0	0	0	0		
01-JUN-2018	13:48	Day 1	1 hour	F2	2.5 ug	0	1	0	0	0	0	0		
01-JUN-2018	13:48	Day 1	1 hour	F3	uncoated	0	1	0	0	0	0	0		
01-JUN-2018	14:42	Day 1	2 hour	F1	uncoated	1	0	0	0	0	0	0		
01-JUN-2018	14:42	Day 1	2 hour	F2	2.5 ug	1	0	0	0	0	0	0		
01-JUN-2018	14:42	Day 1	2 hour	F3	uncoated	1	0	0	0	0	0	0		
02-JUN-2018	11:15	Day 2		F1	uncoated	0	1	0	0	0	0	0		
02-JUN-2018	11:15	Day 2		F2	2.5 ug	1	2	0	0	0	0	0		
02-JUN-2018	11:15	Day 2		F3	uncoated	0	1	0	0	0	0	0		
04-JUN-2018	11:23	Day 4		F1	uncoated	0	0	0	1	0	0	0		
04-JUN-2018	11:23	Day 4		F2	2.5 ug	0	1	0	2	0	0	0		
04-JUN-2018	11:23	Day 4		F3	uncoated	0	0	0	1	0	0	0		
08-JUN-2018	11:52	Day 8		F1	uncoated	0	0	0	0	0	0	0		
08-JUN-2018	11:52	Day 8		F2	2.5 ug	1	1	0	0	1	0	0		

Key: 0= None; 1=Mild; 2=Moderate; 3=Severe; # Other = Pinprick bleeding or seepage

Administration Site Key: FA = Forearm; UA= Upper arm
Treatments Part A: A1 = A/Sing MAP, 15 ug (FA); A2 = Afluria Quad IM (UA), 15 ug; A3 = Placebo MAP (FA); A4 = A/Sing Mono IM, 15 ug (UA)
Treatments Part B: B1 = A/Sing MAP, 15 ug (FA) with biopsy; B2 = Placebo MAP (FA) with biopsy
B3 = A/Sing MAP, 15 ug (FA); B4 = A/Sing MAP, 10 ug (FA); B5 = A/Sing MAP, 5 ug (FA); B6 = A/Sing MAP, 2.5 ug (FA)
B7 = Placebo MAP (FA); B8 = A/Sing MAP, 15 ug (UA); B9 = A/Sing IM, 15 ug, as 2018 Afluria Quad (UA)

Listing 16.2.9.2
Treatment Site Tolerability Assessment per Site

Part B
Randomisation Number = B0079, Treatment Group = Group B6

Date	Time	Visit	Time Point	Site Number	Treatment for Site	Induration	Redness	Bruising	Itching	Skin Flaking	Tenderness	Definition/ Visibility	Other#	Unscheduled Reason
08-JUN-2018	11:52	Day 8		F3	uncoated	0	0	0	0	0	0	1		
22-JUN-2018	12:00	Day 22		F1	uncoated	0	0	0	0	0	0	1		
22-JUN-2018	12:00	Day 22		F2	2.5 ug	0	0	0	0	0	0	2		
22-JUN-2018	12:00	Day 22		F3	uncoated	0	0	0	0	0	0	3		
31-JUL-2018	12:19	EOS/ET (Day 61)		F1	uncoated	0	0	0	0	0	0	0		
31-JUL-2018	12:19	EOS/ET (Day 61)		F2	2.5 ug	0	0	0	0	0	0	1		
31-JUL-2018	12:19	EOS/ET (Day 61)		F3	uncoated	0	0	0	0	0	0	0		

Key: 0= None; 1=Mild; 2=Moderate; 3=Severe; # Other = Pinprick bleeding or seepage

Administration Site Key: FA = Forearm; UA= Upper arm
Treatments Part A: A1 = A/Sing MAP, 15 ug (FA); A2 = Afluria Quad IM (UA), 15 ug; A3 = Placebo MAP (FA); A4 = A/Sing Mono IM, 15 ug (UA)
Treatments Part B: B1 = A/Sing MAP, 15 ug (FA) with biopsy; B2 = Placebo MAP (FA) with biopsy
B3 = A/Sing MAP, 15 ug (FA); B4 = A/Sing MAP, 10 ug (FA); B5 = A/Sing MAP, 5 ug (FA); B6 = A/Sing MAP, 2.5 ug (FA)
B7 = Placebo MAP (FA); B8 = A/Sing MAP, 15 ug (UA); B9 = A/Sing IM, 15 ug, as 2018 Afluria Quad (UA)

Listing 16.2.9.2
Treatment Site Tolerability Assessment per Site

Part B
Randomisation Number = B0081, Treatment Group = Group B6

Date	Time	Visit	Time Point	Site Number	Treatment for Site	Indura- tion	Redness	Bruising	Itching	Skin Flaking	Tender- ness	Definition/ Visibility	Other#	Unscheduled Reason
01-JUN-2018	12:00	Day 1	Pre-dose	F1	2.5 ug	0	0	0	0	0	0	0		
01-JUN-2018	12:00	Day 1	Pre-dose	F2	uncoated	0	0	0	0	0	0	0		
01-JUN-2018	12:00	Day 1	Pre-dose	F3	uncoated	0	0	0	0	0	0	0		
01-JUN-2018	13:07	Day 1	10 min	F1	2.5 ug	0	1	0	0	0	1	0		
01-JUN-2018	13:07	Day 1	10 min	F2	uncoated	0	0	0	0	0	1	0		
01-JUN-2018	13:07	Day 1	10 min	F3	uncoated	0	1	0	0	0	1	0		
01-JUN-2018	13:58	Day 1	1 hour	F1	2.5 ug	0	1	0	0	0	1	0		
01-JUN-2018	13:58	Day 1	1 hour	F2	uncoated	0	1	0	0	0	1	0		
01-JUN-2018	13:58	Day 1	1 hour	F3	uncoated	0	1	0	0	0	1	0		
01-JUN-2018	14:51	Day 1	2 hour	F1	2.5 ug	1	0	0	0	0	0	0		
01-JUN-2018	14:51	Day 1	2 hour	F2	uncoated	1	0	0	0	0	0	0		
01-JUN-2018	14:51	Day 1	2 hour	F3	uncoated	1	0	0	0	0	0	0		
02-JUN-2018	13:40	Day 2		F1	2.5 ug	0	1	0	0	0	1	0		
02-JUN-2018	13:40	Day 2		F2	uncoated	0	0	0	0	0	1	0		
02-JUN-2018	13:40	Day 2		F3	uncoated	0	0	0	0	0	1	0		
04-JUN-2018	14:21	Day 4		F1	2.5 ug	0	0	0	0	0	0	2		
04-JUN-2018	14:21	Day 4		F2	uncoated	0	0	0	0	0	0	1		
04-JUN-2018	14:21	Day 4		F3	uncoated	0	0	0	0	0	0	1		
08-JUN-2018	15:15	Day 8		F1	2.5 ug	0	0	0	0	2	0	1		
08-JUN-2018	15:15	Day 8		F2	uncoated	0	0	0	0	0	0	1		

Key: 0= None; 1=Mild; 2=Moderate; 3=Severe; # Other = Pinprick bleeding or seepage

Administration Site Key: FA = Forearm; UA= Upper arm
Treatments Part A: A1 = A/Sing MAP, 15 ug (FA); A2 = Afluria Quad IM (UA), 15 ug; A3 = Placebo MAP (FA); A4 = A/Sing Mono IM, 15 ug (UA)
Treatments Part B: B1 = A/Sing MAP, 15 ug (FA) with biopsy; B2 = Placebo MAP (FA) with biopsy
B3 = A/Sing MAP, 15 ug (FA); B4 = A/Sing MAP, 10 ug (FA); B5 = A/Sing MAP, 5 ug (FA); B6 = A/Sing MAP, 2.5 ug (FA)
B7 = Placebo MAP (FA); B8 = A/Sing MAP, 15 ug (UA); B9 = A/Sing IM, 15 ug, as 2018 Afluria Quad (UA)

Listing 16.2.9.2

Treatment Site Tolerability Assessment per Site

Part B

Randomisation Number = B0081, Treatment Group = Group B6

Date	Time	Visit	Time Point	Site Number	Treatment for Site	Indura-tion	Redness	Bruising	Itching	Skin Flaking	Tender-ness	Definition/ Visibility	Other#	Unscheduled Reason
08-JUN-2018	15:15	Day 8		F3	uncoated	0	0	0	0	0	0	1		
23-JUN-2018	10:06	Day 22		F1	2.5 ug	0	0	0	0	0	0	2		
23-JUN-2018	10:06	Day 22		F2	uncoated	0	0	0	0	0	0	1		
23-JUN-2018	10:06	Day 22		F3	uncoated	0	0	0	0	0	0	1		
31-JUL-2018	13:51	EOS/ET (Day 61)		F1	2.5 ug	0	0	0	0	0	0	0		
31-JUL-2018	13:51	EOS/ET (Day 61)		F2	uncoated	0	0	0	0	0	0	0		
31-JUL-2018	13:51	EOS/ET (Day 61)		F3	uncoated	0	0	0	0	0	0	0		

Key: 0= None; 1=Mild; 2=Moderate; 3=Severe; # Other = Pinprick bleeding or seepage

Administration Site Key: FA = Forearm; UA= Upper arm

Treatments Part A: A1 = A/Sing MAP, 15 ug (FA); A2 = Afluria Quad IM (UA), 15 ug; A3 = Placebo MAP (FA); A4 = A/Sing Mono IM, 15 ug (UA)

Treatments Part B: B1 = A/Sing MAP, 15 ug (FA) with biopsy; B2 = Placebo MAP (FA) with biopsy

B3 = A/Sing MAP, 15 ug (FA); B4 = A/Sing MAP, 10 ug (FA); B5 = A/Sing MAP, 5 ug (FA); B6 = A/Sing MAP, 2.5 ug (FA)

B7 = Placebo MAP (FA); B8 = A/Sing MAP, 15 ug (UA); B9 = A/Sing IM, 15 ug, as 2018 Afluria Quad (UA)

Listing 16.2.9.2
Treatment Site Tolerability Assessment per Site

Part B
Randomisation Number = B0082, Treatment Group = Group B3

Date	Time	Visit	Time Point	Site Number	Treatment for Site	Indura-tion	Redness	Bruising	Itching	Skin Flaking	Tender-ness	Definition/ Visibility	Other#	Unscheduled Reason
01-JUN-2018	13:08	Day 1	Pre-dose	F1	5 ug	0	0	0	0	0	0	0		
01-JUN-2018	13:08	Day 1	Pre-dose	F2	5 ug	0	0	0	0	0	0	0		
01-JUN-2018	13:08	Day 1	Pre-dose	F3	5 ug	0	0	0	0	0	0	0		
01-JUN-2018	13:36	Day 1	10 min	F1	5 ug	0	1	0	1	0	0	0		
01-JUN-2018	13:36	Day 1	10 min	F2	5 ug	0	1	0	0	0	0	0		
01-JUN-2018	13:36	Day 1	10 min	F3	5 ug	0	1	0	0	0	0	0		
01-JUN-2018	14:28	Day 1	1 hour	F1	5 ug	0	1	0	0	0	0	0		
01-JUN-2018	14:28	Day 1	1 hour	F2	5 ug	0	1	0	0	0	0	0		
01-JUN-2018	14:28	Day 1	1 hour	F3	5 ug	0	1	0	0	0	0	0		
01-JUN-2018	15:20	Day 1	2 hour	F1	5 ug	0	0	0	1	0	0	0		
01-JUN-2018	15:20	Day 1	2 hour	F2	5 ug	0	0	0	1	0	0	0		
01-JUN-2018	15:20	Day 1	2 hour	F3	5 ug	0	0	0	1	0	0	0		
02-JUN-2018	11:47	Day 2		F1	5 ug	1	1	0	1	1	0	0		
02-JUN-2018	11:47	Day 2		F2	5 ug	1	1	0	1	1	0	0		
02-JUN-2018	11:47	Day 2		F3	5 ug	1	1	0	1	1	0	0		
04-JUN-2018	16:33	Day 4		F1	5 ug	1	0	0	0	0	0	0		
04-JUN-2018	16:33	Day 4		F2	5 ug	1	0	0	0	1	0	0		
04-JUN-2018	16:33	Day 4		F3	5 ug	1	1	0	0	0	0	0		
08-JUN-2018	14:23	Day 8		F1	5 ug	0	0	0	0	2	0	3		
08-JUN-2018	14:23	Day 8		F2	5 ug	1	1	0	0	2	0	3		

Key: 0= None; 1=Mild; 2=Moderate; 3=Severe; # Other = Pinprick bleeding or seepage

Administration Site Key: FA = Forearm; UA= Upper arm
Treatments Part A: A1 = A/Sing MAP, 15 ug (FA); A2 = Afluria Quad IM (UA), 15 ug; A3 = Placebo MAP (FA); A4 = A/Sing Mono IM, 15 ug (UA)
Treatments Part B: B1 = A/Sing MAP, 15 ug (FA) with biopsy; B2 = Placebo MAP (FA) with biopsy
B3 = A/Sing MAP, 15 ug (FA); B4 = A/Sing MAP, 10 ug (FA); B5 = A/Sing MAP, 5 ug (FA); B6 = A/Sing MAP, 2.5 ug (FA)
B7 = Placebo MAP (FA); B8 = A/Sing MAP, 15 ug (UA); B9 = A/Sing IM, 15 ug, as 2018 Afluria Quad (UA)

Listing 16.2.9.2
Treatment Site Tolerability Assessment per Site

Part B
Randomisation Number = B0082, Treatment Group = Group B3

Date	Time	Visit	Time Point	Site Number	Treatment for Site	Indura-tion	Redness	Bruising	Itching	Skin Flaking	Tender-ness	Definition/ Visibility	Other#	Unscheduled Reason
08-JUN-2018	14:23	Day 8		F3	5 ug	1	1	0	0	2	0	3		
22-JUN-2018	13:45	Day 22		F1	5 ug	0	0	0	0	0		0		
22-JUN-2018	13:45	Day 22		F2	5 ug	0	0	0	0	0	1	0		
22-JUN-2018	13:45	Day 22		F3	5 ug	0	0	0	0	0	0	0		
31-JUL-2018	15:19	EOS/ET (Day 61)		F1	5 ug	0	0	0	0	0	0	1		
31-JUL-2018	15:19	EOS/ET (Day 61)		F2	5 ug	1	0	0	0	0	1	1		
31-JUL-2018	15:19	EOS/ET (Day 61)		F3	5 ug	0	0	0	0	0	0	1		

Key: 0= None; 1=Mild; 2=Moderate; 3=Severe; # Other = Pinprick bleeding or seepage

Administration Site Key: FA = Forearm; UA= Upper arm
Treatments Part A: A1 = A/Sing MAP, 15 ug (FA); A2 = Afluria Quad IM (UA), 15 ug; A3 = Placebo MAP (FA); A4 = A/Sing Mono IM, 15 ug (UA)
Treatments Part B: B1 = A/Sing MAP, 15 ug (FA) with biopsy; B2 = Placebo MAP (FA) with biopsy
B3 = A/Sing MAP, 15 ug (FA); B4 = A/Sing MAP, 10 ug (FA); B5 = A/Sing MAP, 5 ug (FA); B6 = A/Sing MAP, 2.5 ug (FA)
B7 = Placebo MAP (FA); B8 = A/Sing MAP, 15 ug (UA); B9 = A/Sing IM, 15 ug, as 2018 Afluria Quad (UA)

Listing 16.2.9.2
Treatment Site Tolerability Assessment per Site

Part B
Randomisation Number = B0083, Treatment Group = Group B8

Date	Time	Visit	Time Point	Site Number	Treatment for Site	Induration	Redness	Bruising	Itching	Skin Flaking	Tenderness	Definition/ Visibility	Other#	Unscheduled Reason
01-JUN-2018	13:17	Day 1	Pre-dose	D1	5 ug	0	0	0	0	0	0	0		
01-JUN-2018	13:17	Day 1	Pre-dose	D2	5 ug	0	0	0	0	0	0	0		
01-JUN-2018	13:17	Day 1	Pre-dose	D3	5 ug	0	0	0	0	0	0	0		
01-JUN-2018	13:57	Day 1	10 min	D1	5 ug	0	1	0	1	0	0	0		
01-JUN-2018	13:57	Day 1	10 min	D2	5 ug	0	1	0	1	0	0	0		
01-JUN-2018	13:57	Day 1	10 min	D3	5 ug	0	0	0	0	0	0	0		
01-JUN-2018	14:48	Day 1	1 hour	D1	5 ug	0	1	0	0	0	0	0		
01-JUN-2018	14:48	Day 1	1 hour	D2	5 ug	0	1	0	0	0	0	0		
01-JUN-2018	14:48	Day 1	1 hour	D3	5 ug	0	1	0	0	0	1	0		
01-JUN-2018	15:40	Day 1	2 hour	D1	5 ug	0	0	0	0	0	0	0		
01-JUN-2018	15:40	Day 1	2 hour	D2	5 ug	1	0	0	0	0	0	0		
01-JUN-2018	15:40	Day 1	2 hour	D3	5 ug	1	0	0	0	0	0	0		
02-JUN-2018	09:54	Day 2		D1	5 ug	1	1	0	0	0	0	0		
02-JUN-2018	09:54	Day 2		D2	5 ug	1	1	0	0	0	0	0		
02-JUN-2018	09:54	Day 2		D3	5 ug	0	1	0	1	0	0	0		
04-JUN-2018	14:02	Day 4		D1	5 ug	0	0	0	0	0	0	0		
04-JUN-2018	14:02	Day 4		D2	5 ug	0	1	0	0	0	0	0		
04-JUN-2018	14:02	Day 4		D3	5 ug	0	0	0	0	0	0	0		
08-JUN-2018	14:03	Day 8		D1	5 ug	0	0	0	1	1	0	1		
08-JUN-2018	14:03	Day 8		D2	5 ug	0	0	0	0	1	0	1		

Key: 0= None; 1=Mild; 2=Moderate; 3=Severe; # Other = Pinprick bleeding or seepage

Administration Site Key: FA = Forearm; UA= Upper arm
Treatments Part A: A1 = A/Sing MAP, 15 ug (FA); A2 = Afluria Quad IM (UA), 15 ug; A3 = Placebo MAP (FA); A4 = A/Sing Mono IM, 15 ug (UA)
Treatments Part B: B1 = A/Sing MAP, 15 ug (FA) with biopsy; B2 = Placebo MAP (FA) with biopsy
B3 = A/Sing MAP, 15 ug (FA); B4 = A/Sing MAP, 10 ug (FA); B5 = A/Sing MAP, 5 ug (FA); B6 = A/Sing MAP, 2.5 ug (FA)
B7 = Placebo MAP (FA); B8 = A/Sing MAP, 15 ug (UA); B9 = A/Sing IM, 15 ug, as 2018 Afluria Quad (UA)

Listing 16.2.9.2
Treatment Site Tolerability Assessment per Site

Part B
Randomisation Number = B0083, Treatment Group = Group B8

Date	Time	Visit	Time Point	Site Number	Treatment for Site	Indura-tion	Redness	Bruising	Itching	Skin Flaking	Tender-ness	Definition/ Visibility	Other#	Unscheduled Reason
08-JUN-2018	14:03	Day 8		D3	5 ug	0	0	0	0	1	0	1		
22-JUN-2018	14:36	Day 22		D1	5 ug	0	0	0	0	0	0	1		
22-JUN-2018	14:36	Day 22		D2	5 ug	0	0	0	0	0	0	2		
22-JUN-2018	14:36	Day 22		D3	5 ug	0	0	0	0	0	0	2		
31-JUL-2018	08:33	EOS/ET (Day 61)		D1	5 ug	0	0	0	0	0	0	1		
31-JUL-2018	08:33	EOS/ET (Day 61)		D2	5 ug	0	0	0	0	0	0	1		
31-JUL-2018	08:33	EOS/ET (Day 61)		D3	5 ug	0	0	0	0	0	0	1		

Key: 0= None; 1=Mild; 2=Moderate; 3=Severe; # Other = Pinprick bleeding or seepage

Administration Site Key: FA = Forearm; UA= Upper arm
Treatments Part A: A1 = A/Sing MAP, 15 ug (FA); A2 = Afluria Quad IM (UA), 15 ug; A3 = Placebo MAP (FA); A4 = A/Sing Mono IM, 15 ug (UA)
Treatments Part B: B1 = A/Sing MAP, 15 ug (FA) with biopsy; B2 = Placebo MAP (FA) with biopsy
B3 = A/Sing MAP, 15 ug (FA); B4 = A/Sing MAP, 10 ug (FA); B5 = A/Sing MAP, 5 ug (FA); B6 = A/Sing MAP, 2.5 ug (FA)
B7 = Placebo MAP (FA); B8 = A/Sing MAP, 15 ug (UA); B9 = A/Sing IM, 15 ug, as 2018 Afluria Quad (UA)

Listing 16.2.9.2
Treatment Site Tolerability Assessment per Site

Part B
Randomisation Number = B0084, Treatment Group = Group B5

Date	Time	Visit	Time Point	Site Number	Treatment for Site	Indura-tion	Redness	Bruising	Itching	Skin Flaking	Tender-ness	Definition/ Visibility	Other#	Unscheduled Reason
09-JUN-2018	11:10	Day 1	Pre-dose	F1	uncoated	0	0	0	0	0	0	0		
09-JUN-2018	11:10	Day 1	Pre-dose	F2	uncoated	0	0	0	0	0	0	0		
09-JUN-2018	11:10	Day 1	Pre-dose	F3	5 ug	0	0	0	0	0	0	0		
09-JUN-2018	12:08	Day 1	10 min	F1	uncoated	0	0	0	0	0	0	0		
09-JUN-2018	12:08	Day 1	10 min	F2	uncoated	0	0	0	0	0	0	0		
09-JUN-2018	12:08	Day 1	10 min	F3	5 ug	0	0	0	0	0	0	0		
09-JUN-2018	12:58	Day 1	1 hour	F1	uncoated	0	1	0	0	0	0	0		
09-JUN-2018	12:58	Day 1	1 hour	F2	uncoated	0	1	0	0	0	0	0		
09-JUN-2018	12:58	Day 1	1 hour	F3	5 ug	0	1	0	0	0	0	0		
09-JUN-2018	13:58	Day 1	2 hour	F1	uncoated	0	0	0	0	0	0	0		
09-JUN-2018	13:58	Day 1	2 hour	F2	uncoated	0	0	0	0	0	0	0		
09-JUN-2018	13:58	Day 1	2 hour	F3	5 ug	0	0	0	0	0	0	0		
10-JUN-2018	12:04	Day 2		F1	uncoated	0	0	0	0	0	0	0		
10-JUN-2018	12:04	Day 2		F2	uncoated	2	0	0	0	0	0	0		
10-JUN-2018	12:04	Day 2		F3	5 ug	2	0	0	0	0	0	0		
12-JUN-2018	15:19	Day 4		F1	uncoated	0	0	0	0	0	0	2		
12-JUN-2018	15:19	Day 4		F2	uncoated	0	0	0	0	0	0	2		
12-JUN-2018	15:19	Day 4		F3	5 ug	0	0	0	0	0	0	0		
16-JUN-2018	13:10	Day 8		F1	uncoated	0	0	0	0	0	0	1		
16-JUN-2018	13:10	Day 8		F2	uncoated	0	0	0	0	0	0	1		

Key: 0= None; 1=Mild; 2=Moderate; 3=Severe; # Other = Pinprick bleeding or seepage

Administration Site Key: FA = Forearm; UA= Upper arm
Treatments Part A: A1 = A/Sing MAP, 15 ug (FA); A2 = Afluria Quad IM (UA), 15 ug; A3 = Placebo MAP (FA); A4 = A/Sing Mono IM, 15 ug (UA)
Treatments Part B: B1 = A/Sing MAP, 15 ug (FA) with biopsy; B2 = Placebo MAP (FA) with biopsy
B3 = A/Sing MAP, 15 ug (FA); B4 = A/Sing MAP, 10 ug (FA); B5 = A/Sing MAP, 5 ug (FA); B6 = A/Sing MAP, 2.5 ug (FA)
B7 = Placebo MAP (FA); B8 = A/Sing MAP, 15 ug (UA); B9 = A/Sing IM, 15 ug, as 2018 Afluria Quad (UA)

Listing 16.2.9.2
Treatment Site Tolerability Assessment per Site

Part B
Randomisation Number = B0084, Treatment Group = Group B5

Date	Time	Visit	Time Point	Site Number	Treatment for Site	Indura-tion	Redness	Bruising	Itching	Skin Flaking	Tender-ness	Definition/ Visibility	Other#	Unscheduled Reason
16-JUN-2018	13:10	Day 8		F3	5 ug	0	0	0	0	1	0	0		
30-JUN-2018	14:22	Day 22		F1	uncoated	0	0	0	0	0	0	0		
30-JUN-2018	14:22	Day 22		F2	uncoated	0	0	0	0	0	0	0		
30-JUN-2018	14:22	Day 22		F3	5 ug	0	0	0	0	0	0	2		
08-AUG-2018	16:31	EOS/ET (Day 61)		F1	uncoated	0	0	0	0	0	0	0		
08-AUG-2018	16:31	EOS/ET (Day 61)		F2	uncoated	0	0	0	0	0	0	0		
08-AUG-2018	16:31	EOS/ET (Day 61)		F3	5 ug	0	0	0	0	0	0	0		

Key: 0= None; 1=Mild; 2=Moderate; 3=Severe; # Other = Pinprick bleeding or seepage

Administration Site Key: FA = Forearm; UA= Upper arm
Treatments Part A: A1 = A/Sing MAP, 15 ug (FA); A2 = Afluria Quad IM (UA), 15 ug; A3 = Placebo MAP (FA); A4 = A/Sing Mono IM, 15 ug (UA)
Treatments Part B: B1 = A/Sing MAP, 15 ug (FA) with biopsy; B2 = Placebo MAP (FA) with biopsy
B3 = A/Sing MAP, 15 ug (FA); B4 = A/Sing MAP, 10 ug (FA); B5 = A/Sing MAP, 5 ug (FA); B6 = A/Sing MAP, 2.5 ug (FA)
B7 = Placebo MAP (FA); B8 = A/Sing MAP, 15 ug (UA); B9 = A/Sing IM, 15 ug, as 2018 Afluria Quad (UA)

Listing 16.2.9.2
Treatment Site Tolerability Assessment per Site

Part B
Randomisation Number = B0085, Treatment Group = Group B4

Date	Time	Visit	Time Point	Site Number	Treatment for Site	Indura-tion	Redness	Bruising	Itching	Skin Flaking	Tender-ness	Definition/ Visibility	Other#	Unscheduled Reason
09-JUN-2018	11:19	Day 1	Pre-dose	F1	uncoated	0	0	0	0	0	0	0		
09-JUN-2018	11:19	Day 1	Pre-dose	F2	5 ug	0	0	0	0	0	0	0		
09-JUN-2018	11:19	Day 1	Pre-dose	F3	5 ug	0	0	0	0	0	0	0		
09-JUN-2018	12:17	Day 1	10 min	F1	uncoated	0	0	0	0	0	0	0		
09-JUN-2018	12:17	Day 1	10 min	F2	5 ug	0	1	0	0	0	1	0		
09-JUN-2018	12:17	Day 1	10 min	F3	5 ug	0	0	0	0	0	0	0		
09-JUN-2018	13:08	Day 1	1 hour	F1	uncoated	0	1	0	0	0	0	0		
09-JUN-2018	13:08	Day 1	1 hour	F2	5 ug	0	1	0	0	0	1	0		
09-JUN-2018	13:08	Day 1	1 hour	F3	5 ug	0	1	0	0	0	0	0		
09-JUN-2018	14:08	Day 1	2 hour	F1	uncoated	0	0	0	0	0	0	0		
09-JUN-2018	14:08	Day 1	2 hour	F2	5 ug	0	0	0	1	0	0	0		
09-JUN-2018	14:08	Day 1	2 hour	F3	5 ug	0	0	0	0	0	0	0		
10-JUN-2018	12:18	Day 2		F1	uncoated	0	1	0	0	0	0	0		
10-JUN-2018	12:18	Day 2		F2	5 ug	2	0	0	0	0	0	0		
10-JUN-2018	12:18	Day 2		F3	5 ug	1	1	0	0	0	0	0		
12-JUN-2018	16:35	Day 4		F1	uncoated	0	0	0	0	1	0	2		
12-JUN-2018	16:35	Day 4		F2	5 ug	1	1	0	1	1	0	0		
12-JUN-2018	16:35	Day 4		F3	5 ug	0	1	0	1	1	0	0		
16-JUN-2018	13:27	Day 8		F1	uncoated	0	0	0	0	0	0	1		
16-JUN-2018	13:27	Day 8		F2	5 ug	0	0	0	0	1	0	3		

Key: 0= None; 1=Mild; 2=Moderate; 3=Severe; # Other = Pinprick bleeding or seepage

Administration Site Key: FA = Forearm; UA= Upper arm
Treatments Part A: A1 = A/Sing MAP, 15 ug (FA); A2 = Afluria Quad IM (UA), 15 ug; A3 = Placebo MAP (FA); A4 = A/Sing Mono IM, 15 ug (UA)
Treatments Part B: B1 = A/Sing MAP, 15 ug (FA) with biopsy; B2 = Placebo MAP (FA) with biopsy
B3 = A/Sing MAP, 15 ug (FA); B4 = A/Sing MAP, 10 ug (FA); B5 = A/Sing MAP, 5 ug (FA); B6 = A/Sing MAP, 2.5 ug (FA)
B7 = Placebo MAP (FA); B8 = A/Sing MAP, 15 ug (UA); B9 = A/Sing IM, 15 ug, as 2018 Afluria Quad (UA)

Listing 16.2.9.2
Treatment Site Tolerability Assessment per Site

Part B
Randomisation Number = B0085, Treatment Group = Group B4

Date	Time	Visit	Time Point	Site Number	Treatment for Site	Indura-tion	Redness	Bruising	Itching	Skin Flaking	Tender-ness	Definition/ Visibility	Other#	Unscheduled Reason
16-JUN-2018	13:27	Day 8		F3	5 ug	0	0	0	0	1	0	3		
30-JUN-2018	12:31	Day 22		F1	uncoated	0	0	0	0	0	0	1		
30-JUN-2018	12:31	Day 22		F2	5 ug	0	0	0	0	0	0	3		
30-JUN-2018	12:31	Day 22		F3	5 ug	0	0	0	0	0	0	3		
11-AUG-2018	10:33	EOS/ET (Day 61)		F1	uncoated	0	0	0	0	0	0	0		
11-AUG-2018	10:33	EOS/ET (Day 61)		F2	5 ug	0	0	0	0	0	0	0		
11-AUG-2018	10:33	EOS/ET (Day 61)		F3	5 ug	0	0	0	0	0	0	1		

Key: 0= None; 1=Mild; 2=Moderate; 3=Severe; # Other = Pinprick bleeding or seepage

Administration Site Key: FA = Forearm; UA= Upper arm
Treatments Part A: A1 = A/Sing MAP, 15 ug (FA); A2 = Afluria Quad IM (UA), 15 ug; A3 = Placebo MAP (FA); A4 = A/Sing Mono IM, 15 ug (UA)
Treatments Part B: B1 = A/Sing MAP, 15 ug (FA) with biopsy; B2 = Placebo MAP (FA) with biopsy
B3 = A/Sing MAP, 15 ug (FA); B4 = A/Sing MAP, 10 ug (FA); B5 = A/Sing MAP, 5 ug (FA); B6 = A/Sing MAP, 2.5 ug (FA)
B7 = Placebo MAP (FA); B8 = A/Sing MAP, 15 ug (UA); B9 = A/Sing IM, 15 ug, as 2018 Afluria Quad (UA)

Listing 16.2.9.2
Treatment Site Tolerability Assessment per Site

Part B
Randomisation Number = B0086, Treatment Group = Group B9

Date	Time	Visit	Time Point	Site Number	Treatment for Site	Indura-tion	Redness	Bruising	Itching	Skin Flaking	Tender-ness	Definition/ Visibility	Other#	Unscheduled Reason
09-JUN-2018	10:57	Day 1	Pre-dose	D1	[IM]	0	0	0	0	0	0	5		
09-JUN-2018	11:31	Day 1	10 min	D1	[IM]	0	0	0	0	0	0	5		
09-JUN-2018	12:21	Day 1	1 hour	D1	[IM]	0	0	0	0	0	0	5		
09-JUN-2018	13:26	Day 1	2 hour	D1	[IM]	1	1	0	0	0	0	5		
10-JUN-2018	09:28	Day 2		D1	[IM]	1	0	0	0	0	1	5		
12-JUN-2018	14:56	Day 4		D1	[IM]	0	0	0	0	0	0	5		
16-JUN-2018	09:57	Day 8		D1	[IM]	0	0	0	0	0	0	5		
02-JUL-2018	14:21	Day 22		D1	[IM]	0	0	0	0	0	0	5		
09-AUG-2018	13:38	EOS/ET (Day 61)		D1	[IM]	0	0	0	0	0	0	5		

Key: 0= None; 1=Mild; 2=Moderate; 3=Severe; # Other = Pinprick bleeding or seepage

Administration Site Key: FA = Forearm; UA= Upper arm
Treatments Part A: A1 = A/Sing MAP, 15 ug (FA); A2 = Afluria Quad IM (UA), 15 ug; A3 = Placebo MAP (FA); A4 = A/Sing Mono IM, 15 ug (UA)
Treatments Part B: B1 = A/Sing MAP, 15 ug (FA) with biopsy; B2 = Placebo MAP (FA) with biopsy
B3 = A/Sing MAP, 15 ug (FA); B4 = A/Sing MAP, 10 ug (FA); B5 = A/Sing MAP, 5 ug (FA); B6 = A/Sing MAP, 2.5 ug (FA)
B7 = Placebo MAP (FA); B8 = A/Sing MAP, 15 ug (UA); B9 = A/Sing IM, 15 ug, as 2018 Afluria Quad (UA)

Listing 16.2.9.2
Treatment Site Tolerability Assessment per Site

Part B
Randomisation Number = B0087, Treatment Group = Group B7

Date	Time	Visit	Time Point	Site Number	Treatment for Site	Indura-tion	Redness	Bruising	Itching	Skin Flaking	Tender-ness	Definition/ Visibility	Other#	Unscheduled Reason
09-JUN-2018	11:30	Day 1	Pre-dose	F1	uncoated	0	0	0	0	0	0	0		
09-JUN-2018	11:30	Day 1	Pre-dose	F2	uncoated	0	0	0	0	0	0	0		
09-JUN-2018	11:30	Day 1	Pre-dose	F3	uncoated	0	0	0	0	0	0	0		
09-JUN-2018	12:26	Day 1	10 min	F1	uncoated	0	0	0	0	0	0	0		
09-JUN-2018	12:26	Day 1	10 min	F2	uncoated	0	0	0	0	0	0	0		
09-JUN-2018	12:26	Day 1	10 min	F3	uncoated	0	0	0	1	0	0	0		
09-JUN-2018	13:18	Day 1	1 hour	F1	uncoated	0	1	0	0	0	0	0		
09-JUN-2018	13:18	Day 1	1 hour	F2	uncoated	0	1	0	0	0	0	0		
09-JUN-2018	13:18	Day 1	1 hour	F3	uncoated	0	1	0	0	0	0	0		
09-JUN-2018	14:18	Day 1	2 hour	F1	uncoated	0	0	0	0	0	0	0		
09-JUN-2018	14:18	Day 1	2 hour	F2	uncoated	0	0	0	1	0	0	0		
09-JUN-2018	14:18	Day 1	2 hour	F3	uncoated	0	0	0	0	0	0	0		
10-JUN-2018	09:42	Day 2		F1	uncoated	0	0	0	0	0	0	0		
10-JUN-2018	09:42	Day 2		F2	uncoated	0	0	0	0	0	0	0		
10-JUN-2018	09:42	Day 2		F3	uncoated	0	0	0	0	0	0	0		
12-JUN-2018	08:58	Day 4		F1	uncoated	0	0	0	0	0	0	0		
12-JUN-2018	08:58	Day 4		F2	uncoated	0	0	0	0	0	0	3		
12-JUN-2018	08:58	Day 4		F3	uncoated	0	0	0	0	0	0	3		
16-JUN-2018	09:47	Day 8		F1	uncoated	0	0	0	0	0	0	3		
16-JUN-2018	09:47	Day 8		F2	uncoated	0	0	0	0	0	0	3		

Key: 0= None; 1=Mild; 2=Moderate; 3=Severe; # Other = Pinprick bleeding or seepage

Administration Site Key: FA = Forearm; UA= Upper arm
Treatments Part A: A1 = A/Sing MAP, 15 ug (FA); A2 = Afluria Quad IM (UA), 15 ug; A3 = Placebo MAP (FA); A4 = A/Sing Mono IM, 15 ug (UA)
Treatments Part B: B1 = A/Sing MAP, 15 ug (FA) with biopsy; B2 = Placebo MAP (FA) with biopsy
B3 = A/Sing MAP, 15 ug (FA); B4 = A/Sing MAP, 10 ug (FA); B5 = A/Sing MAP, 5 ug (FA); B6 = A/Sing MAP, 2.5 ug (FA)
B7 = Placebo MAP (FA); B8 = A/Sing MAP, 15 ug (UA); B9 = A/Sing IM, 15 ug, as 2018 Afluria Quad (UA)

Listing 16.2.9.2
Treatment Site Tolerability Assessment per Site

Part B
Randomisation Number = B0087, Treatment Group = Group B7

Date	Time	Visit	Time Point	Site Number	Treatment for Site	Indura-tion	Redness	Bruising	Itching	Skin Flaking	Tender-ness	Definition/ Visibility	Other#	Unscheduled Reason
16-JUN-2018	09:47	Day 8		F3	uncoated	0	0	0	0	0	0	3		
03-JUL-2018	09:27	Day 22		F1	uncoated	0	0	0	0	0	0	1		
03-JUL-2018	09:27	Day 22		F2	uncoated	0	0	0	0	0	0	0		
03-JUL-2018	09:27	Day 22		F3	uncoated	0	0	0	0	0	0	0		
07-AUG-2018	09:02	EOS/ET (Day 61)		F1	uncoated	0	0	0	0	0	0	0		
07-AUG-2018	09:02	EOS/ET (Day 61)		F2	uncoated	0	0	0	0	0	0	0		
07-AUG-2018	09:02	EOS/ET (Day 61)		F3	uncoated	0	0	0	0	0	0	0		

Key: 0= None; 1=Mild; 2=Moderate; 3=Severe; # Other = Pinprick bleeding or seepage

Administration Site Key: FA = Forearm; UA= Upper arm
Treatments Part A: A1 = A/Sing MAP, 15 ug (FA); A2 = Afluria Quad IM (UA), 15 ug; A3 = Placebo MAP (FA); A4 = A/Sing Mono IM, 15 ug (UA)
Treatments Part B: B1 = A/Sing MAP, 15 ug (FA) with biopsy; B2 = Placebo MAP (FA) with biopsy
B3 = A/Sing MAP, 15 ug (FA); B4 = A/Sing MAP, 10 ug (FA); B5 = A/Sing MAP, 5 ug (FA); B6 = A/Sing MAP, 2.5 ug (FA)
B7 = Placebo MAP (FA); B8 = A/Sing MAP, 15 ug (UA); B9 = A/Sing IM, 15 ug, as 2018 Afluria Quad (UA)

Listing 16.2.9.2
Treatment Site Tolerability Assessment per Site

Part B
Randomisation Number = B0088, Treatment Group = Group B9

Date	Time	Visit	Time Point	Site Number	Treatment for Site	Indura-tion	Redness	Bruising	Itching	Skin Flaking	Tender-ness	Definition/ Visibility	Other#	Unscheduled Reason
09-JUN-2018	10:52	Day 1	Pre-dose	D1	[IM]	0	0	0	0	0	0	5		
09-JUN-2018	11:21	Day 1	10 min	D1	[IM]	0	1	0	0	0	0	5		
09-JUN-2018	12:11	Day 1	1 hour	D1	[IM]	0	0	0	0	0	0	5		
09-JUN-2018	13:16	Day 1	2 hour	D1	[IM]	1	0	0	0	0	0	5		
10-JUN-2018	11:14	Day 2		D1	[IM]	0	0	0	0	0	0	5		
12-JUN-2018	13:52	Day 4		D1	[IM]	0	0	0	0	0	0	5		
16-JUN-2018	17:27	Day 8		D1	[IM]	0	0	0	0	0	0	5		
30-JUN-2018	13:17	Day 22		D1	[IM]	0	0	0	0	0	0	5		
14-AUG-2018	14:44	EOS/ET (Day 61)		D1	[IM]	0	0	0	0	0	0	5		

Key: 0= None; 1=Mild; 2=Moderate; 3=Severe; # Other = Pinprick bleeding or seepage

Administration Site Key: FA = Forearm; UA= Upper arm
Treatments Part A: A1 = A/Sing MAP, 15 ug (FA); A2 = Afluria Quad IM (UA), 15 ug; A3 = Placebo MAP (FA); A4 = A/Sing Mono IM, 15 ug (UA)
Treatments Part B: B1 = A/Sing MAP, 15 ug (FA) with biopsy; B2 = Placebo MAP (FA) with biopsy
B3 = A/Sing MAP, 15 ug (FA); B4 = A/Sing MAP, 10 ug (FA); B5 = A/Sing MAP, 5 ug (FA); B6 = A/Sing MAP, 2.5 ug (FA)
B7 = Placebo MAP (FA); B8 = A/Sing MAP, 15 ug (UA); B9 = A/Sing IM, 15 ug, as 2018 Afluria Quad (UA)

Listing 16.2.9.2
Treatment Site Tolerability Assessment per Site

Part B
Randomisation Number = B0089, Treatment Group = Group B5

Date	Time	Visit	Time Point	Site Number	Treatment for Site	Induration	Redness	Bruising	Itching	Skin Flaking	Tenderness	Definition/ Visibility	Other#	Unscheduled Reason
09-JUN-2018	11:41	Day 1	Pre-dose	F1	uncoated	0	0	0	0	0	0	0		
09-JUN-2018	11:41	Day 1	Pre-dose	F2	5 ug	0	0	0	0	0	0	0		
09-JUN-2018	11:41	Day 1	Pre-dose	F3	uncoated	0	0	0	0	0	0	0		
09-JUN-2018	12:37	Day 1	10 min	F1	uncoated	0	0	0	0	0	0	0		
09-JUN-2018	12:37	Day 1	10 min	F2	5 ug	1	1	0	0	0	0	0		
09-JUN-2018	12:37	Day 1	10 min	F3	uncoated	0	0	0	0	0	0	0		
09-JUN-2018	13:18	Day 1	1 hour	F1	uncoated	0	1	0	0	0	0	0		
09-JUN-2018	13:18	Day 1	1 hour	F2	5 ug	0	1	0	0	0	0	0		
09-JUN-2018	13:18	Day 1	1 hour	F3	uncoated	0	1	0	0	0	0	0		
09-JUN-2018	14:28	Day 1	2 hour	F1	uncoated	0	0	0	0	0	0	0		
09-JUN-2018	14:28	Day 1	2 hour	F2	5 ug	0	0	0	0	0	0	0		
09-JUN-2018	14:28	Day 1	2 hour	F3	uncoated	0	0	0	0	0	0	0		
10-JUN-2018	10:46	Day 2		F1	uncoated	0	0	0	0	0	0	0		
10-JUN-2018	10:46	Day 2		F2	5 ug	1	0	0	0	0	0	0		
10-JUN-2018	10:46	Day 2		F3	uncoated	0	0	0	0	0	0	0		
12-JUN-2018	08:55	Day 4		F1	uncoated	0	0	0	0	0	0	0		
12-JUN-2018	08:55	Day 4		F2	5 ug	0	0	0	0	0	0	0		
12-JUN-2018	08:55	Day 4		F3	uncoated	0	0	0	0	0	0	0		
16-JUN-2018	12:02	Day 8		F1	uncoated	0	0	0	0	0	0	2		
16-JUN-2018	12:02	Day 8		F2	5 ug	0	0	0	0	0	0	2		

Key: 0= None; 1=Mild; 2=Moderate; 3=Severe; # Other = Pinprick bleeding or seepage

Administration Site Key: FA = Forearm; UA= Upper arm
Treatments Part A: A1 = A/Sing MAP, 15 ug (FA); A2 = Afluria Quad IM (UA), 15 ug; A3 = Placebo MAP (FA); A4 = A/Sing Mono IM, 15 ug (UA)
Treatments Part B: B1 = A/Sing MAP, 15 ug (FA) with biopsy; B2 = Placebo MAP (FA) with biopsy
B3 = A/Sing MAP, 15 ug (FA); B4 = A/Sing MAP, 10 ug (FA); B5 = A/Sing MAP, 5 ug (FA); B6 = A/Sing MAP, 2.5 ug (FA)
B7 = Placebo MAP (FA); B8 = A/Sing MAP, 15 ug (UA); B9 = A/Sing IM, 15 ug, as 2018 Afluria Quad (UA)

Listing 16.2.9.2
Treatment Site Tolerability Assessment per Site

Part B
Randomisation Number = B0089, Treatment Group = Group B5

Date	Time	Visit	Time Point	Site Number	Treatment for Site	Indura- tion	Redness	Bruising	Itching	Skin Flaking	Tender- ness	Definition/ Visibility	Other#	Unscheduled Reason
16-JUN-2018	12:02	Day 8		F3	uncoated	0	0	0	0	0	0	2		
30-JUN-2018	08:22	Day 22		F1	uncoated	0	0	0	0	0	0	1		
30-JUN-2018	08:22	Day 22		F2	5 ug	0	0	0	0	1	0	3		
30-JUN-2018	08:22	Day 22		F3	uncoated	0	0	0	0	0	0	2		
30-JUN-2018	08:30	Unscheduled		F2	5 ug	0	0	0	0	0	0	3	NA	MO notifies as repeat
30-JUN-2018	08:30	Unscheduled		F3	uncoated	0	0	0	0	0	0	2	NA	MO notifies as repeat
08-AUG-2018	12:30	EOS/ET (Day 61)		F1	uncoated	0	0	0	0	0	0	0		
08-AUG-2018	12:30	EOS/ET (Day 61)		F2	5 ug	0	0	0	0	0	0	0		
08-AUG-2018	12:30	EOS/ET (Day 61)		F3	uncoated	0	0	0	0	0	0	0		

Key: 0= None; 1=Mild; 2=Moderate; 3=Severe; # Other = Pinprick bleeding or seepage

Administration Site Key: FA = Forearm; UA= Upper arm
Treatments Part A: A1 = A/Sing MAP, 15 ug (FA); A2 = Afluria Quad IM (UA), 15 ug; A3 = Placebo MAP (FA); A4 = A/Sing Mono IM, 15 ug (UA)
Treatments Part B: B1 = A/Sing MAP, 15 ug (FA) with biopsy; B2 = Placebo MAP (FA) with biopsy
B3 = A/Sing MAP, 15 ug (FA); B4 = A/Sing MAP, 10 ug (FA); B5 = A/Sing MAP, 5 ug (FA); B6 = A/Sing MAP, 2.5 ug (FA)
B7 = Placebo MAP (FA); B8 = A/Sing MAP, 15 ug (UA); B9 = A/Sing IM, 15 ug, as 2018 Afluria Quad (UA)

Listing 16.2.9.2
Treatment Site Tolerability Assessment per Site

Part B
Randomisation Number = B0090, Treatment Group = Group B6

Date	Time	Visit	Time Point	Site Number	Treatment for Site	Indura-tion	Redness	Bruising	Itching	Skin Flaking	Tender-ness	Definition/ Visibility	Other#	Unscheduled Reason
09-JUN-2018	11:50	Day 1	Pre-dose	F1	2.5 ug	0	0	0	0	0	0	0		
09-JUN-2018	11:50	Day 1	Pre-dose	F2	uncoated	0	0	0	0	0	0	0		
09-JUN-2018	11:50	Day 1	Pre-dose	F3	uncoated	0	0	0	0	0	0	0		
09-JUN-2018	12:46	Day 1	10 min	F1	2.5 ug	0	1	0	2	0	0	0		
09-JUN-2018	12:46	Day 1	10 min	F2	uncoated	0	0	0	2	0	1	0		
09-JUN-2018	12:46	Day 1	10 min	F3	uncoated	0	0	0	2	0	0	0		
09-JUN-2018	13:38	Day 1	1 hour	F1	2.5 ug	0	1	0	2	0	0	0		
09-JUN-2018	13:38	Day 1	1 hour	F2	uncoated	0	1	0	2	0	1	0		
09-JUN-2018	13:38	Day 1	1 hour	F3	uncoated	0	1	0	2	0	0	0		
09-JUN-2018	14:38	Day 1	2 hour	F1	2.5 ug	0	0	0	0	0	0	0		
09-JUN-2018	14:38	Day 1	2 hour	F2	uncoated	0	0	0	0	0	1	0		
09-JUN-2018	14:38	Day 1	2 hour	F3	uncoated	0	0	0	0	0	0	0		
09-JUN-2018	12:49	Unscheduled		F1	2.5 ug				2				NA	MO notifies as repeat
09-JUN-2018	12:49	Unscheduled		F2	uncoated				1				NA	MO notifies as repeat
09-JUN-2018	12:49	Unscheduled		F3	uncoated				2				NA	MO notifies as repeat
09-JUN-2018	13:40	Unscheduled		F1	2.5 ug				2				NA	MO notifies as repeat

Key: 0= None; 1=Mild; 2=Moderate; 3=Severe; # Other = Pinprick bleeding or seepage

Administration Site Key: FA = Forearm; UA= Upper arm
Treatments Part A: A1 = A/Sing MAP, 15 ug (FA); A2 = Afluria Quad IM (UA), 15 ug; A3 = Placebo MAP (FA); A4 = A/Sing Mono IM, 15 ug (UA)
Treatments Part B: B1 = A/Sing MAP, 15 ug (FA) with biopsy; B2 = Placebo MAP (FA) with biopsy
B3 = A/Sing MAP, 15 ug (FA); B4 = A/Sing MAP, 10 ug (FA); B5 = A/Sing MAP, 5 ug (FA); B6 = A/Sing MAP, 2.5 ug (FA)
B7 = Placebo MAP (FA); B8 = A/Sing MAP, 15 ug (UA); B9 = A/Sing IM, 15 ug, as 2018 Afluria Quad (UA)

Listing 16.2.9.2
Treatment Site Tolerability Assessment per Site

Part B
Randomisation Number = B0090, Treatment Group = Group B6

Date	Time	Visit	Time Point	Site Number	Treatment for Site	Induration	Redness	Bruising	Itching	Skin Flaking	Tenderness	Definition/ Visibility	Other#	Unscheduled Reason
09-JUN-2018	13:40	Unscheduled		F2	uncoated				2				NA	MO notifies as repeat
09-JUN-2018	13:40	Unscheduled		F3	uncoated				2				NA	MO notifies as repeat
10-JUN-2018	11:47	Day 2		F1	2.5 ug	0	1	0	0	0	1	0		
10-JUN-2018	11:47	Day 2		F2	uncoated	0	0	0	0	0	0	0		
10-JUN-2018	11:47	Day 2		F3	uncoated	0	0	0	0	0	0	0		
12-JUN-2018	10:55	Day 4		F1	2.5 ug	0	0	0	0	0	0	0		
12-JUN-2018	10:55	Day 4		F2	uncoated	0	0	0	0	0	0	2		
12-JUN-2018	10:55	Day 4		F3	uncoated	0	0	0	0	0	1	2		
16-JUN-2018	11:43	Day 8		F1	2.5 ug	0	0	0	0	0	0	2		
16-JUN-2018	11:43	Day 8		F2	uncoated	0	0	0	0	0	0	1		
16-JUN-2018	11:43	Day 8		F3	uncoated	0	0	0	0	0	0	1		
02-JUL-2018	10:36	Day 22		F1	2.5 ug	0	0	0	0	0	0	1		
02-JUL-2018	10:36	Day 22		F2	uncoated	0	0	0	0	0	0	1		
02-JUL-2018	10:36	Day 22		F3	uncoated	0	0	0	0	0	0	0		
08-AUG-2018	10:21	EOS/ET (Day 61)		F1	2.5 ug	0	0	0	0	0	0	0		
08-AUG-2018	10:21	EOS/ET (Day 61)		F2	uncoated	0	0	0	0	0	0	0		
08-AUG-2018	10:21	EOS/ET (Day 61)		F3	uncoated	0	0	0	0	0	0	0		

Key: 0= None; 1=Mild; 2=Moderate; 3=Severe; # Other = Pinprick bleeding or seepage

Administration Site Key: FA = Forearm; UA= Upper arm
Treatments Part A: A1 = A/Sing MAP, 15 ug (FA); A2 = Afluria Quad IM (UA), 15 ug; A3 = Placebo MAP (FA); A4 = A/Sing Mono IM, 15 ug (UA)
Treatments Part B: B1 = A/Sing MAP, 15 ug (FA) with biopsy; B2 = Placebo MAP (FA) with biopsy
B3 = A/Sing MAP, 15 ug (FA); B4 = A/Sing MAP, 10 ug (FA); B5 = A/Sing MAP, 5 ug (FA); B6 = A/Sing MAP, 2.5 ug (FA)
B7 = Placebo MAP (FA); B8 = A/Sing MAP, 15 ug (UA); B9 = A/Sing IM, 15 ug, as 2018 Afluria Quad (UA)

Listing 16.2.9.2
Treatment Site Tolerability Assessment per Site

Part B
Randomisation Number = B0091, Treatment Group = Group B8

Date	Time	Visit	Time Point	Site Number	Treatment for Site	Indura-tion	Redness	Bruising	Itching	Skin Flaking	Tender-ness	Definition/ Visibility	Other#	Unscheduled Reason
09-JUN-2018	12:02	Day 1	Pre-dose	D1	5 ug	0	0	0	0	0	0	0		
09-JUN-2018	12:02	Day 1	Pre-dose	D2	5 ug	0	0	0	0	0	0	0		
09-JUN-2018	12:02	Day 1	Pre-dose	D3	5 ug	0	0	0	0	0	0	0		
09-JUN-2018	12:57	Day 1	10 min	D1	5 ug	0	1	0	0	0	1	0		
09-JUN-2018	12:57	Day 1	10 min	D2	5 ug	0	1	0	0	0	0	0		
09-JUN-2018	12:57	Day 1	10 min	D3	5 ug	0	1	0	0	0	0	0		
09-JUN-2018	13:48	Day 1	1 hour	D1	5 ug	0	1	0	0	0	0	0		
09-JUN-2018	13:48	Day 1	1 hour	D2	5 ug	0	1	0	0	0	0	0		
09-JUN-2018	13:48	Day 1	1 hour	D3	5 ug	0	1	0	0	0	0	0		
09-JUN-2018	14:48	Day 1	2 hour	D1	5 ug	0	0	0	0	0	0	0		
09-JUN-2018	14:48	Day 1	2 hour	D2	5 ug	0	0	0	0	0	0	0		
09-JUN-2018	14:48	Day 1	2 hour	D3	5 ug	0	0	0	0	0	0	0		
10-JUN-2018	14:37	Day 2		D1	5 ug	0	1	0	0	0	0	0		
10-JUN-2018	14:37	Day 2		D2	5 ug	0	1	0	0	0	0	0		
10-JUN-2018	14:37	Day 2		D3	5 ug	0	0	0	0	0	0	0		
12-JUN-2018	13:06	Day 4		D1	5 ug	0	1	0	0	0	0	0		
12-JUN-2018	13:06	Day 4		D2	5 ug	0	1	0	0	0	0	0		
12-JUN-2018	13:06	Day 4		D3	5 ug	0	0	0	0	0	0	0		
16-JUN-2018	10:02	Day 8		D1	5 ug	0	0	0	0	2	0	2		
16-JUN-2018	10:02	Day 8		D2	5 ug	0	0	0	0	2	0	3		

Key: 0= None; 1=Mild; 2=Moderate; 3=Severe; # Other = Pinprick bleeding or seepage

Administration Site Key: FA = Forearm; UA= Upper arm
Treatments Part A: A1 = A/Sing MAP, 15 ug (FA); A2 = Afluria Quad IM (UA), 15 ug; A3 = Placebo MAP (FA); A4 = A/Sing Mono IM, 15 ug (UA)
Treatments Part B: B1 = A/Sing MAP, 15 ug (FA) with biopsy; B2 = Placebo MAP (FA) with biopsy
B3 = A/Sing MAP, 15 ug (FA); B4 = A/Sing MAP, 10 ug (FA); B5 = A/Sing MAP, 5 ug (FA); B6 = A/Sing MAP, 2.5 ug (FA)
B7 = Placebo MAP (FA); B8 = A/Sing MAP, 15 ug (UA); B9 = A/Sing IM, 15 ug, as 2018 Afluria Quad (UA)

Listing 16.2.9.2
Treatment Site Tolerability Assessment per Site

Part B
Randomisation Number = B0091, Treatment Group = Group B8

Date	Time	Visit	Time Point	Site Number	Treatment for Site	Indura-tion	Redness	Bruising	Itching	Skin Flaking	Tender-ness	Definition/ Visibility	Other#	Unscheduled Reason
16-JUN-2018	10:02	Day 8		D3	5 ug	0	0	0	0	3	0	2		
30-JUN-2018	09:35	Day 22		D1	5 ug	0	0	0	0	0	0	1		
30-JUN-2018	09:35	Day 22		D2	5 ug	0	0	0	0	0	0	1		
30-JUN-2018	09:35	Day 22		D3	5 ug	0	0	0	0	0	0	1		
08-AUG-2018	10:05	EOS/ET (Day 61)		D1	5 ug	0	0	0	0	0	0	1		
08-AUG-2018	10:05	EOS/ET (Day 61)		D2	5 ug	0	0	0	0	0	0	1		
08-AUG-2018	10:05	EOS/ET (Day 61)		D3	5 ug	0	0	0	0	0	0	1		

Key: 0= None; 1=Mild; 2=Moderate; 3=Severe; # Other = Pinprick bleeding or seepage

Administration Site Key: FA = Forearm; UA= Upper arm
Treatments Part A: A1 = A/Sing MAP, 15 ug (FA); A2 = Afluria Quad IM (UA), 15 ug; A3 = Placebo MAP (FA); A4 = A/Sing Mono IM, 15 ug (UA)
Treatments Part B: B1 = A/Sing MAP, 15 ug (FA) with biopsy; B2 = Placebo MAP (FA) with biopsy
B3 = A/Sing MAP, 15 ug (FA); B4 = A/Sing MAP, 10 ug (FA); B5 = A/Sing MAP, 5 ug (FA); B6 = A/Sing MAP, 2.5 ug (FA)
B7 = Placebo MAP (FA); B8 = A/Sing MAP, 15 ug (UA); B9 = A/Sing IM, 15 ug, as 2018 Afluria Quad (UA)

Listing 16.2.9.2
Treatment Site Tolerability Assessment per Site

Part B
Randomisation Number = B0092, Treatment Group = Group B4

Date	Time	Visit	Time Point	Site Number	Treatment for Site	Indura-tion	Redness	Bruising	Itching	Skin Flaking	Tender-ness	Definition/ Visibility	Other#	Unscheduled Reason
09-JUN-2018	12:11	Day 1	Pre-dose	F1	5 ug	0	0	0	0	0	0	0		
09-JUN-2018	12:11	Day 1	Pre-dose	F2	uncoated	0	0	0	0	0	0	0		
09-JUN-2018	12:11	Day 1	Pre-dose	F3	5 ug	0	0	0	0	0	0	0		
09-JUN-2018	13:08	Day 1	10 min	F1	5 ug	0	0	0	0	0	0	0		
09-JUN-2018	13:08	Day 1	10 min	F2	uncoated	0	0	0	0	0	0	0		
09-JUN-2018	13:08	Day 1	10 min	F3	5 ug	0	0	0	0	0	1	0		
09-JUN-2018	13:58	Day 1	1 hour	F1	5 ug	0	1	0	0	0	0	0		
09-JUN-2018	13:58	Day 1	1 hour	F2	uncoated	0	1	0	0	0	0	0		
09-JUN-2018	13:58	Day 1	1 hour	F3	5 ug	0	1	0	0	0	1	0		
09-JUN-2018	14:58	Day 1	2 hour	F1	5 ug	0	0	0	0	0	0	0		
09-JUN-2018	14:58	Day 1	2 hour	F2	uncoated	0	0	0	0	0	0	0		
09-JUN-2018	14:58	Day 1	2 hour	F3	5 ug	0	0	0	0	0	1	0		
10-JUN-2018	09:14	Day 2		F1	5 ug	0	0	0	0	0	0	0		
10-JUN-2018	09:14	Day 2		F2	uncoated	0	0	0	0	0	0	0		
10-JUN-2018	09:14	Day 2		F3	5 ug	0	0	0	0	0	0	0		
12-JUN-2018	10:16	Day 4		F1	5 ug	0	0	0	1	0	0	0		
12-JUN-2018	10:16	Day 4		F2	uncoated	0	0	0	0	0	0	2		
12-JUN-2018	10:16	Day 4		F3	5 ug	0	0	0	0	0	0	0		
16-JUN-2018	13:41	Day 8		F1	5 ug	0	0	0	0	1	0	0		
16-JUN-2018	13:41	Day 8		F2	uncoated	0	0	0	0	0	0	1		

Key: 0= None; 1=Mild; 2=Moderate; 3=Severe; # Other = Pinprick bleeding or seepage

Administration Site Key: FA = Forearm; UA= Upper arm
Treatments Part A: A1 = A/Sing MAP, 15 ug (FA); A2 = Afluria Quad IM (UA), 15 ug; A3 = Placebo MAP (FA); A4 = A/Sing Mono IM, 15 ug (UA)
Treatments Part B: B1 = A/Sing MAP, 15 ug (FA) with biopsy; B2 = Placebo MAP (FA) with biopsy
B3 = A/Sing MAP, 15 ug (FA); B4 = A/Sing MAP, 10 ug (FA); B5 = A/Sing MAP, 5 ug (FA); B6 = A/Sing MAP, 2.5 ug (FA)
B7 = Placebo MAP (FA); B8 = A/Sing MAP, 15 ug (UA); B9 = A/Sing IM, 15 ug, as 2018 Afluria Quad (UA)

Listing 16.2.9.2
Treatment Site Tolerability Assessment per Site

Part B
Randomisation Number = B0092, Treatment Group = Group B4

Date	Time	Visit	Time Point	Site Number	Treatment for Site	Indura-tion	Redness	Bruising	Itching	Skin Flaking	Tender-ness	Definition/ Visibility	Other#	Unscheduled Reason
16-JUN-2018	13:41	Day 8		F3	5 ug	0	0	0	0	1	0	2		
30-JUN-2018	12:05	Day 22		F1	5 ug	0	0	0	0	0	0	2		
30-JUN-2018	12:05	Day 22		F2	uncoated	0	0	0	0	0	0			
30-JUN-2018	12:05	Day 22		F3	5 ug	0	0	0	0	0	0	2		
02-AUG-2018	08:12	EOS/ET (Day 61)		F1	5 ug	0	0	0	0	0	0	1		
02-AUG-2018	08:12	EOS/ET (Day 61)		F2	uncoated	0	0	0	0	0	0	0		
02-AUG-2018	08:12	EOS/ET (Day 61)		F3	5 ug	0	0	0	0	0	0	1		

Key: 0= None; 1=Mild; 2=Moderate; 3=Severe; # Other = Pinprick bleeding or seepage

Administration Site Key: FA = Forearm; UA= Upper arm
Treatments Part A: A1 = A/Sing MAP, 15 ug (FA); A2 = Afluria Quad IM (UA), 15 ug; A3 = Placebo MAP (FA); A4 = A/Sing Mono IM, 15 ug (UA)
Treatments Part B: B1 = A/Sing MAP, 15 ug (FA) with biopsy; B2 = Placebo MAP (FA) with biopsy
B3 = A/Sing MAP, 15 ug (FA); B4 = A/Sing MAP, 10 ug (FA); B5 = A/Sing MAP, 5 ug (FA); B6 = A/Sing MAP, 2.5 ug (FA)
B7 = Placebo MAP (FA); B8 = A/Sing MAP, 15 ug (UA); B9 = A/Sing IM, 15 ug, as 2018 Afluria Quad (UA)

Listing 16.2.9.2
Treatment Site Tolerability Assessment per Site

Part B
Randomisation Number = B0093, Treatment Group = Group B7

Date	Time	Visit	Time Point	Site Number	Treatment for Site	Indura-tion	Redness	Bruising	Itching	Skin Flaking	Tender-ness	Definition/ Visibility	Other#	Unscheduled Reason
09-JUN-2018	12:17	Day 1	Pre-dose	F1	uncoated	0	0	0	0	0	0	0		
09-JUN-2018	12:17	Day 1	Pre-dose	F2	uncoated	0	0	0	0	0	0	0		
09-JUN-2018	12:17	Day 1	Pre-dose	F3	uncoated	0	0	0	0	0	0	0		
09-JUN-2018	13:16	Day 1	10 min	F1	uncoated	0	0	0	0	0	0	0		
09-JUN-2018	13:16	Day 1	10 min	F2	uncoated	0	0	0	0	0	0	0		
09-JUN-2018	13:16	Day 1	10 min	F3	uncoated	0	0	0	0	0	0	0		
09-JUN-2018	14:08	Day 1	1 hour	F1	uncoated	0	1	0	0	0	0	0		
09-JUN-2018	14:08	Day 1	1 hour	F2	uncoated	0	1	0	0	0	0	0		
09-JUN-2018	14:08	Day 1	1 hour	F3	uncoated	0	1	0	0	0	0	0		
09-JUN-2018	15:08	Day 1	2 hour	F1	uncoated	0	0	0	0	0	0	0		
09-JUN-2018	15:08	Day 1	2 hour	F2	uncoated	0	0	0	0	0	0	0		
09-JUN-2018	15:08	Day 1	2 hour	F3	uncoated	0	0	0	0	0	0	0		
10-JUN-2018	10:08	Day 2		F1	uncoated	0	0	0	0		0	0		
10-JUN-2018	10:08	Day 2		F2	uncoated	0	0	0	0	0	0	0		
10-JUN-2018	10:08	Day 2		F3	uncoated	1	0	0	0	0	0	0		
12-JUN-2018	09:25	Day 4		F1	uncoated	0	0	0	0	0	0	2		
12-JUN-2018	09:25	Day 4		F2	uncoated	0	0	0	0	0	0	1		
12-JUN-2018	09:25	Day 4		F3	uncoated	0	0	0	0	0	0	1		
16-JUN-2018	09:59	Day 8		F1	uncoated	0	0	0	0	0	0	2		
16-JUN-2018	09:59	Day 8		F2	uncoated	0	0	0	0	0	0	2		

Key: 0= None; 1=Mild; 2=Moderate; 3=Severe; # Other = Pinprick bleeding or seepage

Administration Site Key: FA = Forearm; UA= Upper arm
Treatments Part A: A1 = A/Sing MAP, 15 ug (FA); A2 = Afluria Quad IM (UA), 15 ug; A3 = Placebo MAP (FA); A4 = A/Sing Mono IM, 15 ug (UA)
Treatments Part B: B1 = A/Sing MAP, 15 ug (FA) with biopsy; B2 = Placebo MAP (FA) with biopsy
B3 = A/Sing MAP, 15 ug (FA); B4 = A/Sing MAP, 10 ug (FA); B5 = A/Sing MAP, 5 ug (FA); B6 = A/Sing MAP, 2.5 ug (FA)
B7 = Placebo MAP (FA); B8 = A/Sing MAP, 15 ug (UA); B9 = A/Sing IM, 15 ug, as 2018 Afluria Quad (UA)

Listing 16.2.9.2
Treatment Site Tolerability Assessment per Site

Part B
Randomisation Number = B0093, Treatment Group = Group B7

Date	Time	Visit	Time Point	Site Number	Treatment for Site	Indura- tion	Redness	Bruising	Itching	Skin Flaking	Tender- ness	Definition/ Visibility	Other#	Unscheduled Reason
16-JUN-2018	09:59	Day 8		F3	uncoated	0	0	0	0	0	0	1		
30-JUN-2018	09:05	Day 22		F1	uncoated	0	0	0	0	2	0	1		
30-JUN-2018	09:05	Day 22		F2	uncoated	0	0	0	0	2	0	1		
30-JUN-2018	09:05	Day 22		F3	uncoated	0	0	0	0	2	0	1		
30-JUN-2018	09:14	Unscheduled		F1	uncoated	0	0	0	0	2	0	1	NA	MO notifies as repeat
30-JUN-2018	09:14	Unscheduled		F2	uncoated	0	0	0	0	2	0	1	NA	MO notifies as repeat
30-JUN-2018	09:14	Unscheduled		F3	uncoated	0	0	0	0	1	0	1	NA	MO notifies as repeat
08-AUG-2018	09:27	EOS/ET (Day 61)		F1	uncoated	0	0	0	0	0	0	0		
08-AUG-2018	09:27	EOS/ET (Day 61)		F2	uncoated	0	0	0	0	0	0	0		
08-AUG-2018	09:27	EOS/ET (Day 61)		F3	uncoated	0	0	0	0	0	0	0		

Key: 0= None; 1=Mild; 2=Moderate; 3=Severe; # Other = Pinprick bleeding or seepage

Administration Site Key: FA = Forearm; UA= Upper arm
Treatments Part A: A1 = A/Sing MAP, 15 ug (FA); A2 = Afluria Quad IM (UA), 15 ug; A3 = Placebo MAP (FA); A4 = A/Sing Mono IM, 15 ug (UA)
Treatments Part B: B1 = A/Sing MAP, 15 ug (FA) with biopsy; B2 = Placebo MAP (FA) with biopsy
B3 = A/Sing MAP, 15 ug (FA); B4 = A/Sing MAP, 10 ug (FA); B5 = A/Sing MAP, 5 ug (FA); B6 = A/Sing MAP, 2.5 ug (FA)
B7 = Placebo MAP (FA); B8 = A/Sing MAP, 15 ug (UA); B9 = A/Sing IM, 15 ug, as 2018 Afluria Quad (UA)

Listing 16.2.9.2
Treatment Site Tolerability Assessment per Site

Part B
Randomisation Number = B0094, Treatment Group = Group B3

Date	Time	Visit	Time Point	Site Number	Treatment for Site	Indura-tion	Redness	Bruising	Itching	Skin Flaking	Tender-ness	Definition/ Visibility	Other#	Unscheduled Reason
09-JUN-2018	12:27	Day 1	Pre-dose	F1	5 ug	0	0	0	0	0	0	0		
09-JUN-2018	12:27	Day 1	Pre-dose	F2	5 ug	0	0	0	0	0	0	0		
09-JUN-2018	12:27	Day 1	Pre-dose	F3	5 ug	0	0	0	0	0	0	0		
09-JUN-2018	13:28	Day 1	10 min	F1	5 ug	0	0	0	0	0	0	0		
09-JUN-2018	13:28	Day 1	10 min	F2	5 ug	0	0	0	0	0	0	0		
09-JUN-2018	13:28	Day 1	10 min	F3	5 ug	0	0	0	0	0	0	0		
09-JUN-2018	14:18	Day 1	1 hour	F1	5 ug	0	1	0	0	0	0	0		
09-JUN-2018	14:18	Day 1	1 hour	F2	5 ug	0	1	0	0	0	0	0		
09-JUN-2018	14:18	Day 1	1 hour	F3	5 ug	0	1	0	0	0	0	0		
09-JUN-2018	15:18	Day 1	2 hour	F1	5 ug	0	0	0	0	0	0	0		
09-JUN-2018	15:18	Day 1	2 hour	F2	5 ug	0	0	0	0	0	0	0		
09-JUN-2018	15:18	Day 1	2 hour	F3	5 ug	0	0	0	0	0	0	0		
10-JUN-2018	13:05	Day 2		F1	5 ug	1	0	0	0	0	0	0		
10-JUN-2018	13:05	Day 2		F2	5 ug	1	0	0	0	0	0	0		
10-JUN-2018	13:05	Day 2		F3	5 ug	1	1	0	0	0	0	0		
12-JUN-2018	13:25	Day 4		F1	5 ug	0	0	0	0	0	0	0		
12-JUN-2018	13:25	Day 4		F2	5 ug	0	0	0	0	0	0	0		
12-JUN-2018	13:25	Day 4		F3	5 ug	0	0	0	0	0	0	0		
16-JUN-2018	13:51	Day 8		F1	5 ug	0	0	0	0	1	0	3		
16-JUN-2018	13:51	Day 8		F2	5 ug	0	0	0	0	2	0	3		

Key: 0= None; 1=Mild; 2=Moderate; 3=Severe; # Other = Pinprick bleeding or seepage

Administration Site Key: FA = Forearm; UA= Upper arm
Treatments Part A: A1 = A/Sing MAP, 15 ug (FA); A2 = Afluria Quad IM (UA), 15 ug; A3 = Placebo MAP (FA); A4 = A/Sing Mono IM, 15 ug (UA)
Treatments Part B: B1 = A/Sing MAP, 15 ug (FA) with biopsy; B2 = Placebo MAP (FA) with biopsy
B3 = A/Sing MAP, 15 ug (FA); B4 = A/Sing MAP, 10 ug (FA); B5 = A/Sing MAP, 5 ug (FA); B6 = A/Sing MAP, 2.5 ug (FA)
B7 = Placebo MAP (FA); B8 = A/Sing MAP, 15 ug (UA); B9 = A/Sing IM, 15 ug, as 2018 Afluria Quad (UA)

Listing 16.2.9.2
Treatment Site Tolerability Assessment per Site

Part B
Randomisation Number = B0094, Treatment Group = Group B3

Date	Time	Visit	Time Point	Site Number	Treatment for Site	Indura-tion	Redness	Bruising	Itching	Skin Flaking	Tender-ness	Definition/ Visibility	Other#	Unscheduled Reason
16-JUN-2018	13:51	Day 8		F3	5 ug	0	0	0	0	0	0	3		
30-JUN-2018	13:48	Day 22		F1	5 ug	0	0	0	0	0	0	1		
30-JUN-2018	13:48	Day 22		F2	5 ug	0	0	0	0	0	0	1		
30-JUN-2018	13:48	Day 22		F3	5 ug	0	0	0	0	0	0	1		
08-AUG-2018	10:13	EOS/ET (Day 61)		F1	5 ug	0	0	0	0	0	0	0		
08-AUG-2018	10:13	EOS/ET (Day 61)		F2	5 ug	0	0	0	0	0	0	0		
08-AUG-2018	10:13	EOS/ET (Day 61)		F3	5 ug	0	0	0	0	0	0	0		

Key: 0= None; 1=Mild; 2=Moderate; 3=Severe; # Other = Pinprick bleeding or seepage

Administration Site Key: FA = Forearm; UA= Upper arm
Treatments Part A: A1 = A/Sing MAP, 15 ug (FA); A2 = Afluria Quad IM (UA), 15 ug; A3 = Placebo MAP (FA); A4 = A/Sing Mono IM, 15 ug (UA)
Treatments Part B: B1 = A/Sing MAP, 15 ug (FA) with biopsy; B2 = Placebo MAP (FA) with biopsy
B3 = A/Sing MAP, 15 ug (FA); B4 = A/Sing MAP, 10 ug (FA); B5 = A/Sing MAP, 5 ug (FA); B6 = A/Sing MAP, 2.5 ug (FA)
B7 = Placebo MAP (FA); B8 = A/Sing MAP, 15 ug (UA); B9 = A/Sing IM, 15 ug, as 2018 Afluria Quad (UA)

Listing 16.2.9.2
Treatment Site Tolerability Assessment per Site

Part B
Randomisation Number = B0095, Treatment Group = Group B9

Date	Time	Visit	Time Point	Site Number	Treatment for Site	Indura-tion	Redness	Bruising	Itching	Skin Flaking	Tender-ness	Definition/ Visibility	Other#	Unscheduled Reason
09-JUN-2018	11:05	Day 1	Pre-dose	D1	[IM]	0	0	0	0	0	0	5		
09-JUN-2018	11:51	Day 1	10 min	D1	[IM]	0	0	0	0	0	0	5		
09-JUN-2018	12:41	Day 1	1 hour	D1	[IM]	0	0	0	0	0	0	5		
09-JUN-2018	13:46	Day 1	2 hour	D1	[IM]	1	0	0	0	0	0	5		
10-JUN-2018	13:00	Day 2		D1	[IM]	0	0	0	0	0	1	5		
12-JUN-2018	13:13	Day 4		D1	[IM]	0	0	0	0	0	0	5		
16-JUN-2018	13:23	Day 8		D1	[IM]	0	0	0	0	0	0	5		
30-JUN-2018	13:35	Day 22		D1	[IM]	0	0	0	0	0	0	5		
08-AUG-2018	12:34	EOS/ET (Day 61)		D1	[IM]	0	0	0	0	0	0	5		

Key: 0= None; 1=Mild; 2=Moderate; 3=Severe; # Other = Pinprick bleeding or seepage

Administration Site Key: FA = Forearm; UA= Upper arm
Treatments Part A: A1 = A/Sing MAP, 15 ug (FA); A2 = Afluria Quad IM (UA), 15 ug; A3 = Placebo MAP (FA); A4 = A/Sing Mono IM, 15 ug (UA)
Treatments Part B: B1 = A/Sing MAP, 15 ug (FA) with biopsy; B2 = Placebo MAP (FA) with biopsy
B3 = A/Sing MAP, 15 ug (FA); B4 = A/Sing MAP, 10 ug (FA); B5 = A/Sing MAP, 5 ug (FA); B6 = A/Sing MAP, 2.5 ug (FA)
B7 = Placebo MAP (FA); B8 = A/Sing MAP, 15 ug (UA); B9 = A/Sing IM, 15 ug, as 2018 Afluria Quad (UA)

Listing 16.2.9.2
Treatment Site Tolerability Assessment per Site

Part B
Randomisation Number = B0096, Treatment Group = Group B6

Date	Time	Visit	Time Point	Site Number	Treatment for Site	Indura-tion	Redness	Bruising	Itching	Skin Flaking	Tender-ness	Definition/ Visibility	Other#	Unscheduled Reason
09-JUN-2018	12:42	Day 1	Pre-dose	F1	2.5 ug	0	0	0	0	0	0	0		
09-JUN-2018	12:42	Day 1	Pre-dose	F2	uncoated	0	0	0	0	0	0	0		
09-JUN-2018	12:42	Day 1	Pre-dose	F3	uncoated	0	0	0	0	0	0	0		
09-JUN-2018	13:36	Day 1	10 min	F1	2.5 ug	0	2	0	0	0	0	0		
09-JUN-2018	13:36	Day 1	10 min	F2	uncoated	0	2	0	0	0	0	0		
09-JUN-2018	13:36	Day 1	10 min	F3	uncoated	0	2	0	0	0	0	0		
09-JUN-2018	14:28	Day 1	1 hour	F1	2.5 ug	0	1	0	0	0	0	0		
09-JUN-2018	14:28	Day 1	1 hour	F2	uncoated	0	1	0	0	0	0	0		
09-JUN-2018	14:28	Day 1	1 hour	F3	uncoated	0	1	0	0	0	0	0		
09-JUN-2018	15:28	Day 1	2 hour	F1	2.5 ug	0	0	0	0	0	0	0		
09-JUN-2018	15:28	Day 1	2 hour	F2	uncoated	0	0	0	0	0	0	0		
09-JUN-2018	15:28	Day 1	2 hour	F3	uncoated	0	0	0	0	0	0	0		
09-JUN-2018	13:42	Unscheduled		F1	2.5 ug		2						NA	MO notifies as repeat
09-JUN-2018	13:42	Unscheduled		F2	uncoated		2						NA	MO notifies as repeat
09-JUN-2018	13:42	Unscheduled		F3	uncoated		2						NA	MO notifies as repeat
10-JUN-2018	13:47	Day 2		F1	2.5 ug	0	1	0	0	0	0	0		
10-JUN-2018	13:47	Day 2		F2	uncoated	0	0	0	0	0	0	0		

Key: 0= None; 1=Mild; 2=Moderate; 3=Severe; # Other = Pinprick bleeding or seepage

Administration Site Key: FA = Forearm; UA= Upper arm
Treatments Part A: A1 = A/Sing MAP, 15 ug (FA); A2 = Afluria Quad IM (UA), 15 ug; A3 = Placebo MAP (FA); A4 = A/Sing Mono IM, 15 ug (UA)
Treatments Part B: B1 = A/Sing MAP, 15 ug (FA) with biopsy; B2 = Placebo MAP (FA) with biopsy
B3 = A/Sing MAP, 15 ug (FA); B4 = A/Sing MAP, 10 ug (FA); B5 = A/Sing MAP, 5 ug (FA); B6 = A/Sing MAP, 2.5 ug (FA)
B7 = Placebo MAP (FA); B8 = A/Sing MAP, 15 ug (UA); B9 = A/Sing IM, 15 ug, as 2018 Afluria Quad (UA)

Listing 16.2.9.2
Treatment Site Tolerability Assessment per Site

Part B
Randomisation Number = B0096, Treatment Group = Group B6

Date	Time	Visit	Time Point	Site Number	Treatment for Site	Indura-tion	Redness	Bruising	Itching	Skin Flaking	Tender-ness	Definition/ Visibility	Other#	Unscheduled Reason
10-JUN-2018	13:47	Day 2		F3	uncoated	0	0	0	0	0	0	0		
12-JUN-2018	12:29	Day 4		F1	2.5 ug	0	1	0	0	0	0	0		
12-JUN-2018	12:29	Day 4		F2	uncoated	0	0	0	0	0	0	3		
12-JUN-2018	12:29	Day 4		F3	uncoated	0	0	0	0	0	0	2		
16-JUN-2018	13:34	Day 8		F1	2.5 ug	0	0	0	0	1	0	2		
16-JUN-2018	13:34	Day 8		F2	uncoated	0	0	0	0	0	0	2		
16-JUN-2018	13:34	Day 8		F3	uncoated	0	0	0	0	0	0	2		
30-JUN-2018	13:40	Day 22		F1	2.5 ug	0	0	0	0	2	0	2		
30-JUN-2018	13:40	Day 22		F2	uncoated	0	0	0	0	1	0	1		
30-JUN-2018	13:40	Day 22		F3	uncoated	0	0	0	0	0	0	1		
08-AUG-2018	13:48	EOS/ET (Day 61)		F1	2.5 ug	0	0	0	0	0	0	1		
08-AUG-2018	13:48	EOS/ET (Day 61)		F2	uncoated	0	0	0	0	0	0	0		
08-AUG-2018	13:48	EOS/ET (Day 61)		F3	uncoated	0	0	0	0	0	0	0		

Key: 0= None; 1=Mild; 2=Moderate; 3=Severe; # Other = Pinprick bleeding or seepage

Administration Site Key: FA = Forearm; UA= Upper arm
Treatments Part A: A1 = A/Sing MAP, 15 ug (FA); A2 = Afluria Quad IM (UA), 15 ug; A3 = Placebo MAP (FA); A4 = A/Sing Mono IM, 15 ug (UA)
Treatments Part B: B1 = A/Sing MAP, 15 ug (FA) with biopsy; B2 = Placebo MAP (FA) with biopsy
B3 = A/Sing MAP, 15 ug (FA); B4 = A/Sing MAP, 10 ug (FA); B5 = A/Sing MAP, 5 ug (FA); B6 = A/Sing MAP, 2.5 ug (FA)
B7 = Placebo MAP (FA); B8 = A/Sing MAP, 15 ug (UA); B9 = A/Sing IM, 15 ug, as 2018 Afluria Quad (UA)

Listing 16.2.9.2
Treatment Site Tolerability Assessment per Site

Part B
Randomisation Number = B0097, Treatment Group = Group B5

Date	Time	Visit	Time Point	Site Number	Treatment for Site	Indura-tion	Redness	Bruising	Itching	Skin Flaking	Tender-ness	Definition/ Visibility	Other#	Unscheduled Reason
09-JUN-2018	12:48	Day 1	Pre-dose	F1	5 ug	0	0	0	0	0	0	0		
09-JUN-2018	12:48	Day 1	Pre-dose	F2	uncoated	0	0	0	0	0	0	0		
09-JUN-2018	12:48	Day 1	Pre-dose	F3	uncoated	0	0	0	0	0	0	0		
09-JUN-2018	13:47	Day 1	10 min	F1	5 ug	0	1	0	0	0	0	0		
09-JUN-2018	13:47	Day 1	10 min	F2	uncoated	0	0	0	0	0	0	0		
09-JUN-2018	13:47	Day 1	10 min	F3	uncoated	0	0	0	0	0	0	0		
09-JUN-2018	14:38	Day 1	1 hour	F1	5 ug	0	1	0	0	0	0	0		
09-JUN-2018	14:38	Day 1	1 hour	F2	uncoated	0	1	0	0	0	0	0		
09-JUN-2018	14:38	Day 1	1 hour	F3	uncoated	0	1	0	0	0	0	0		
09-JUN-2018	15:38	Day 1	2 hour	F1	5 ug	0	0	0	0	0	0	0		
09-JUN-2018	15:38	Day 1	2 hour	F2	uncoated	0	0	0	0	0	0	0		
09-JUN-2018	15:38	Day 1	2 hour	F3	uncoated	0	0	0	0	0	0	0		
10-JUN-2018	13:41	Day 2		F1	5 ug	1	1	0	0	0	0	0		
10-JUN-2018	13:41	Day 2		F2	uncoated	1	0	0	0	0	0	0		
10-JUN-2018	13:41	Day 2		F3	uncoated	0	0	0	0	0	0	0		
12-JUN-2018	16:40	Day 4		F1	5 ug	0	1	0	0	0	0	0		
12-JUN-2018	16:40	Day 4		F2	uncoated	1	0	0	0	0	0	0		
12-JUN-2018	16:40	Day 4		F3	uncoated	0	0	0	0	0	0	0		
16-JUN-2018	14:38	Day 8		F1	5 ug	0	0	0	0	0	0	0		
16-JUN-2018	14:38	Day 8		F2	uncoated	0	0	0	0	0	0	3		

Key: 0= None; 1=Mild; 2=Moderate; 3=Severe; # Other = Pinprick bleeding or seepage

Administration Site Key: FA = Forearm; UA= Upper arm
Treatments Part A: A1 = A/Sing MAP, 15 ug (FA); A2 = Afluria Quad IM (UA), 15 ug; A3 = Placebo MAP (FA); A4 = A/Sing Mono IM, 15 ug (UA)
Treatments Part B: B1 = A/Sing MAP, 15 ug (FA) with biopsy; B2 = Placebo MAP (FA) with biopsy
B3 = A/Sing MAP, 15 ug (FA); B4 = A/Sing MAP, 10 ug (FA); B5 = A/Sing MAP, 5 ug (FA); B6 = A/Sing MAP, 2.5 ug (FA)
B7 = Placebo MAP (FA); B8 = A/Sing MAP, 15 ug (UA); B9 = A/Sing IM, 15 ug, as 2018 Afluria Quad (UA)

Listing 16.2.9.2
Treatment Site Tolerability Assessment per Site

Part B
Randomisation Number = B0097, Treatment Group = Group B5

Date	Time	Visit	Time Point	Site Number	Treatment for Site	Indura-tion	Redness	Bruising	Itching	Skin Flaking	Tender-ness	Definition/ Visibility	Other#	Unscheduled Reason
16-JUN-2018	14:38	Day 8		F3	uncoated	0	0	0	0	0	0	3		
30-JUN-2018	14:57	Day 22		F1	5 ug	0	0	0	0	0	0	2		
30-JUN-2018	14:57	Day 22		F2	uncoated	0	0	0	0	0	0	1		
30-JUN-2018	14:57	Day 22		F3	uncoated	0	0	0	0	0	0	1		
09-AUG-2018	17:18	EOS/ET (Day 61)		F1	5 ug	0	0	0	0	0	0	0		
09-AUG-2018	17:18	EOS/ET (Day 61)		F2	uncoated	0	0	0	0	0	0	0		
09-AUG-2018	17:18	EOS/ET (Day 61)		F3	uncoated	0	0	0	0	0	0	0		

Key: 0= None; 1=Mild; 2=Moderate; 3=Severe; # Other = Pinprick bleeding or seepage

Administration Site Key: FA = Forearm; UA= Upper arm
Treatments Part A: A1 = A/Sing MAP, 15 ug (FA); A2 = Afluria Quad IM (UA), 15 ug; A3 = Placebo MAP (FA); A4 = A/Sing Mono IM, 15 ug (UA)
Treatments Part B: B1 = A/Sing MAP, 15 ug (FA) with biopsy; B2 = Placebo MAP (FA) with biopsy
B3 = A/Sing MAP, 15 ug (FA); B4 = A/Sing MAP, 10 ug (FA); B5 = A/Sing MAP, 5 ug (FA); B6 = A/Sing MAP, 2.5 ug (FA)
B7 = Placebo MAP (FA); B8 = A/Sing MAP, 15 ug (UA); B9 = A/Sing IM, 15 ug, as 2018 Afluria Quad (UA)

Listing 16.2.9.2
Treatment Site Tolerability Assessment per Site

Part B
Randomisation Number = B0098, Treatment Group = Group B7

Date	Time	Visit	Time Point	Site Number	Treatment for Site	Indura-tion	Redness	Bruising	Itching	Skin Flaking	Tender-ness	Definition/ Visibility	Other#	Unscheduled Reason
09-JUN-2018	12:57	Day 1	Pre-dose	F1	uncoated	0	0	0	0	0	0	0		
09-JUN-2018	12:57	Day 1	Pre-dose	F2	uncoated	0	0	0	0	0	0	0		
09-JUN-2018	12:57	Day 1	Pre-dose	F3	uncoated	0	0	0	0	0	0	0		
09-JUN-2018	13:57	Day 1	10 min	F1	uncoated	0	0	0	0	0	0	0		
09-JUN-2018	13:57	Day 1	10 min	F2	uncoated	0	0	0	0	0	0	0		
09-JUN-2018	13:57	Day 1	10 min	F3	uncoated	0	0	0	0	0	0	0		
09-JUN-2018	14:48	Day 1	1 hour	F1	uncoated	0	1	0	0	0	1	0		
09-JUN-2018	14:48	Day 1	1 hour	F2	uncoated	0	1	0	0	0	1	0		
09-JUN-2018	14:48	Day 1	1 hour	F3	uncoated	0	1	0	0	0	1	0		
09-JUN-2018	15:48	Day 1	2 hour	F1	uncoated	0	0	0	0	0	0	0		
09-JUN-2018	15:48	Day 1	2 hour	F2	uncoated	0	0	0	0	0	0	0		
09-JUN-2018	15:48	Day 1	2 hour	F3	uncoated	0	0	0	0	0	0	0		
10-JUN-2018	14:03	Day 2		F1	uncoated	0	0	0	0	0	0	0		
10-JUN-2018	14:03	Day 2		F2	uncoated	0	0	0	0	0	0	0		
10-JUN-2018	14:03	Day 2		F3	uncoated	0	0	0	0	0	0	0		
12-JUN-2018	13:53	Day 4		F1	uncoated	0	0	0	0	0	0	3		
12-JUN-2018	13:53	Day 4		F2	uncoated	0	0	0	0	0	0	3		
12-JUN-2018	13:53	Day 4		F3	uncoated	0	0	0	0	0	0	3		
16-JUN-2018	14:17	Day 8		F1	uncoated	0	0	0	0	0	0	1		
16-JUN-2018	14:17	Day 8		F2	uncoated	0	0	0	0	0	0	1		

Key: 0= None; 1=Mild; 2=Moderate; 3=Severe; # Other = Pinprick bleeding or seepage

Administration Site Key: FA = Forearm; UA= Upper arm
Treatments Part A: A1 = A/Sing MAP, 15 ug (FA); A2 = Afluria Quad IM (UA), 15 ug; A3 = Placebo MAP (FA); A4 = A/Sing Mono IM, 15 ug (UA)
Treatments Part B: B1 = A/Sing MAP, 15 ug (FA) with biopsy; B2 = Placebo MAP (FA) with biopsy
B3 = A/Sing MAP, 15 ug (FA); B4 = A/Sing MAP, 10 ug (FA); B5 = A/Sing MAP, 5 ug (FA); B6 = A/Sing MAP, 2.5 ug (FA)
B7 = Placebo MAP (FA); B8 = A/Sing MAP, 15 ug (UA); B9 = A/Sing IM, 15 ug, as 2018 Afluria Quad (UA)

Listing 16.2.9.2
Treatment Site Tolerability Assessment per Site

Part B
Randomisation Number = B0098, Treatment Group = Group B7

Date	Time	Visit	Time Point	Site Number	Treatment for Site	Indura- tion	Redness	Bruising	Itching	Skin Flaking	Tender- ness	Definition/ Visibility	Other#	Unscheduled Reason
16-JUN-2018	14:17	Day 8		F3	uncoated	0	0	0	0	0	0	1		
30-JUN-2018	15:00	Day 22		F1	uncoated	0	0	0	0	0	0	1		
30-JUN-2018	15:00	Day 22		F2	uncoated	0	0	0	0	0	0	1		
30-JUN-2018	15:00	Day 22		F3	uncoated	0	0	0	0	0	0	0		
07-AUG-2018	09:13	EOS/ET (Day 61)		F1	uncoated	0	0	0	0	0	0	0		
07-AUG-2018	09:13	EOS/ET (Day 61)		F2	uncoated	0	0	0	0	0	0	0		
07-AUG-2018	09:13	EOS/ET (Day 61)		F3	uncoated	0	0	0	0	0	0	0		

Key: 0= None; 1=Mild; 2=Moderate; 3=Severe; # Other = Pinprick bleeding or seepage

Administration Site Key: FA = Forearm; UA= Upper arm
Treatments Part A: A1 = A/Sing MAP, 15 ug (FA); A2 = Afluria Quad IM (UA), 15 ug; A3 = Placebo MAP (FA); A4 = A/Sing Mono IM, 15 ug (UA)
Treatments Part B: B1 = A/Sing MAP, 15 ug (FA) with biopsy; B2 = Placebo MAP (FA) with biopsy
B3 = A/Sing MAP, 15 ug (FA); B4 = A/Sing MAP, 10 ug (FA); B5 = A/Sing MAP, 5 ug (FA); B6 = A/Sing MAP, 2.5 ug (FA)
B7 = Placebo MAP (FA); B8 = A/Sing MAP, 15 ug (UA); B9 = A/Sing IM, 15 ug, as 2018 Afluria Quad (UA)

Listing 16.2.9.2
Treatment Site Tolerability Assessment per Site

Part B
Randomisation Number = B0099, Treatment Group = Group B4

Date	Time	Visit	Time Point	Site Number	Treatment for Site	Induration	Redness	Bruising	Itching	Skin Flaking	Tenderness	Definition/ Visibility	Other#	Unscheduled Reason
09-JUN-2018	13:07	Day 1	Pre-dose	F1	5 ug	0	0	0	0	0	0	0		
09-JUN-2018	13:07	Day 1	Pre-dose	F2	5 ug	0	0	0	0	0	0	0		
09-JUN-2018	13:07	Day 1	Pre-dose	F3	uncoated	0	0	0	0	0	0	0		
09-JUN-2018	15:28	Day 1	10 min	F1	5 ug	0	0	0	0	0	0	0		
09-JUN-2018	15:28	Day 1	10 min	F2	5 ug	0	0	0	0	0	0	0		
09-JUN-2018	15:28	Day 1	10 min	F3	uncoated	0	0	0	0	0	0	0		
09-JUN-2018	16:18	Day 1	1 hour	F1	5 ug	0	1	0	0	0	0	0		
09-JUN-2018	16:18	Day 1	1 hour	F2	5 ug	1	1	0	0	0	0	0		
09-JUN-2018	16:18	Day 1	1 hour	F3	uncoated	0	1	0	0	0	0	0		
09-JUN-2018	17:18	Day 1	2 hour	F1	5 ug	0	0	0	0	0	0	0		
09-JUN-2018	17:18	Day 1	2 hour	F2	5 ug	0	0	0	0	0	0	0		
09-JUN-2018	17:18	Day 1	2 hour	F3	uncoated	0	0	0	0	0	0	0		
10-JUN-2018	11:17	Day 2		F1	5 ug	0	1	0	0	0	0	0		
10-JUN-2018	11:17	Day 2		F2	5 ug	0	1	0	0	0	0	0		
10-JUN-2018	11:17	Day 2		F3	uncoated	0	0	0	0	0	0	0		
12-JUN-2018	09:22	Day 4		F1	5 ug	0	0	0	0	0	0	0		
12-JUN-2018	09:22	Day 4		F2	5 ug	0	0	0	0	0	0	0		
12-JUN-2018	09:22	Day 4		F3	uncoated	0	0	0	0	0	0	2		
16-JUN-2018	11:21	Day 8		F1	5 ug	0	0	0	0	0	0	2		
16-JUN-2018	11:21	Day 8		F2	5 ug	0	0	0	0	0	0	3		

Key: 0= None; 1=Mild; 2=Moderate; 3=Severe; # Other = Pinprick bleeding or seepage

Administration Site Key: FA = Forearm; UA= Upper arm
Treatments Part A: A1 = A/Sing MAP, 15 ug (FA); A2 = Afluria Quad IM (UA), 15 ug; A3 = Placebo MAP (FA); A4 = A/Sing Mono IM, 15 ug (UA)
Treatments Part B: B1 = A/Sing MAP, 15 ug (FA) with biopsy; B2 = Placebo MAP (FA) with biopsy
B3 = A/Sing MAP, 15 ug (FA); B4 = A/Sing MAP, 10 ug (FA); B5 = A/Sing MAP, 5 ug (FA); B6 = A/Sing MAP, 2.5 ug (FA)
B7 = Placebo MAP (FA); B8 = A/Sing MAP, 15 ug (UA); B9 = A/Sing IM, 15 ug, as 2018 Afluria Quad (UA)

Listing 16.2.9.2
Treatment Site Tolerability Assessment per Site

Part B
Randomisation Number = B0099, Treatment Group = Group B4

Date	Time	Visit	Time Point	Site Number	Treatment for Site	Indura-tion	Redness	Bruising	Itching	Skin Flaking	Tender-ness	Definition/ Visibility	Other#	Unscheduled Reason
16-JUN-2018	11:21	Day 8		F3	uncoated	0	0	0	0	0	0	1		
16-JUN-2018	11:33	Unscheduled		F1	5 ug							2	NA	MO notifies as repeat
16-JUN-2018	11:34	Unscheduled		F2	5 ug	0	0	0	0	0	0	3	NA	MO notifies as repeat
06-AUG-2018	11:24	EOS/ET (Day 61)		F1	5 ug	0	0	0	0	0	0	0		
06-AUG-2018	11:24	EOS/ET (Day 61)		F2	5 ug	0	0	0	0	0	0	0		
06-AUG-2018	11:24	EOS/ET (Day 61)		F3	uncoated	0	0	0	0	0	0	0		

Key: 0= None; 1=Mild; 2=Moderate; 3=Severe; # Other = Pinprick bleeding or seepage

Administration Site Key: FA = Forearm; UA= Upper arm
Treatments Part A: A1 = A/Sing MAP, 15 ug (FA); A2 = Afluria Quad IM (UA), 15 ug; A3 = Placebo MAP (FA); A4 = A/Sing Mono IM, 15 ug (UA)
Treatments Part B: B1 = A/Sing MAP, 15 ug (FA) with biopsy; B2 = Placebo MAP (FA) with biopsy
B3 = A/Sing MAP, 15 ug (FA); B4 = A/Sing MAP, 10 ug (FA); B5 = A/Sing MAP, 5 ug (FA); B6 = A/Sing MAP, 2.5 ug (FA)
B7 = Placebo MAP (FA); B8 = A/Sing MAP, 15 ug (UA); B9 = A/Sing IM, 15 ug, as 2018 Afluria Quad (UA)

Listing 16.2.9.2
Treatment Site Tolerability Assessment per Site

Part B
Randomisation Number = B0100, Treatment Group = Group B8

Date	Time	Visit	Time Point	Site Number	Treatment for Site	Indura-tion	Redness	Bruising	Itching	Skin Flaking	Tender-ness	Definition/ Visibility	Other#	Unscheduled Reason
09-JUN-2018	13:18	Day 1	Pre-dose	D1	5 ug	0	0	0	0	0	0	0		
09-JUN-2018	13:18	Day 1	Pre-dose	D2	5 ug	0	0	0	0	0	0	0		
09-JUN-2018	13:18	Day 1	Pre-dose	D3	5 ug	0	0	0	0	0	0	0		
09-JUN-2018	14:06	Day 1	10 min	D1	5 ug	0	1	0	0	0	0	0		
09-JUN-2018	14:06	Day 1	10 min	D2	5 ug	0	1	0	0	0	0	0		
09-JUN-2018	14:06	Day 1	10 min	D3	5 ug	0	0	0	0	0	0	0		
09-JUN-2018	14:58	Day 1	1 hour	D1	5 ug	0	1	0	0	0	0	0		
09-JUN-2018	14:58	Day 1	1 hour	D2	5 ug	0	1	0	0	0	1	0		
09-JUN-2018	14:58	Day 1	1 hour	D3	5 ug	0	1	0	0	0	0	0		
09-JUN-2018	15:58	Day 1	2 hour	D1	5 ug	0	0	0	0	0	0	0		
09-JUN-2018	15:58	Day 1	2 hour	D2	5 ug	0	0	0	0	0	0	0		
09-JUN-2018	15:58	Day 1	2 hour	D3	5 ug	0	0	0	0	0	0	0		
10-JUN-2018	13:23	Day 2		D1	5 ug	1	1	0	0	0	1	0		
10-JUN-2018	13:23	Day 2		D2	5 ug	1	2	0	0	0	1	0		
10-JUN-2018	13:23	Day 2		D3	5 ug	1	1	0	0	0	1	0		
12-JUN-2018	17:19	Day 4		D1	5 ug	0	1	0	0	0	0	0		
12-JUN-2018	17:19	Day 4		D2	5 ug	0	1	0	0	0	0	0		
12-JUN-2018	17:19	Day 4		D3	5 ug	0	1	0	0	0	0	0		
16-JUN-2018	16:47	Day 8		D1	5 ug	1	0	0	1	1	0	0		
16-JUN-2018	16:47	Day 8		D2	5 ug	0	0	0	1	1	0	0		

Key: 0= None; 1=Mild; 2=Moderate; 3=Severe; # Other = Pinprick bleeding or seepage

Administration Site Key: FA = Forearm; UA= Upper arm
Treatments Part A: A1 = A/Sing MAP, 15 ug (FA); A2 = Afluria Quad IM (UA), 15 ug; A3 = Placebo MAP (FA); A4 = A/Sing Mono IM, 15 ug (UA)
Treatments Part B: B1 = A/Sing MAP, 15 ug (FA) with biopsy; B2 = Placebo MAP (FA) with biopsy
B3 = A/Sing MAP, 15 ug (FA); B4 = A/Sing MAP, 10 ug (FA); B5 = A/Sing MAP, 5 ug (FA); B6 = A/Sing MAP, 2.5 ug (FA)
B7 = Placebo MAP (FA); B8 = A/Sing MAP, 15 ug (UA); B9 = A/Sing IM, 15 ug, as 2018 Afluria Quad (UA)

Listing 16.2.9.2
Treatment Site Tolerability Assessment per Site

Part B
Randomisation Number = B0100, Treatment Group = Group B8

Date	Time	Visit	Time Point	Site Number	Treatment for Site	Indura-tion	Redness	Bruising	Itching	Skin Flaking	Tender-ness	Definition/ Visibility	Other#	Unscheduled Reason
16-JUN-2018	16:47	Day 8		D3	5 ug	1	0	0	1	1	0	0		
30-JUN-2018	09:22	Day 22		D1	5 ug	0	0	0	0	1	0	2		
30-JUN-2018	09:22	Day 22		D2	5 ug	0	0	0	0	0	0	2		
30-JUN-2018	09:22	Day 22		D3	5 ug	0	0	0	0	0	0	3		
08-AUG-2018	15:23	EOS/ET (Day 61)		D1	5 ug	0	0	0	0	0	0	0		
08-AUG-2018	15:23	EOS/ET (Day 61)		D2	5 ug	0	0	0	0	0	0	0		
08-AUG-2018	15:23	EOS/ET (Day 61)		D3	5 ug	0	0	0	0	0	0	0		

Key: 0= None; 1=Mild; 2=Moderate; 3=Severe; # Other = Pinprick bleeding or seepage

Administration Site Key: FA = Forearm; UA= Upper arm
Treatments Part A: A1 = A/Sing MAP, 15 ug (FA); A2 = Afluria Quad IM (UA), 15 ug; A3 = Placebo MAP (FA); A4 = A/Sing Mono IM, 15 ug (UA)
Treatments Part B: B1 = A/Sing MAP, 15 ug (FA) with biopsy; B2 = Placebo MAP (FA) with biopsy
B3 = A/Sing MAP, 15 ug (FA); B4 = A/Sing MAP, 10 ug (FA); B5 = A/Sing MAP, 5 ug (FA); B6 = A/Sing MAP, 2.5 ug (FA)
B7 = Placebo MAP (FA); B8 = A/Sing MAP, 15 ug (UA); B9 = A/Sing IM, 15 ug, as 2018 Afluria Quad (UA)

Listing 16.2.9.2
Treatment Site Tolerability Assessment per Site

Part B
Randomisation Number = B0101, Treatment Group = Group B3

Date	Time	Visit	Time Point	Site Number	Treatment for Site	Induration	Redness	Bruising	Itching	Skin Flaking	Tenderness	Definition/ Visibility	Other#	Unscheduled Reason
09-JUN-2018	13:28	Day 1	Pre-dose	F1	5 ug	0	0	0	0	0	0	0		
09-JUN-2018	13:28	Day 1	Pre-dose	F2	5 ug	0	0	0	0	0	0	0		
09-JUN-2018	13:28	Day 1	Pre-dose	F3	5 ug	0	0	0	0	0	0	0		
09-JUN-2018	14:19	Day 1	10 min	F1	5 ug	0	0	0	0	0	0	0		
09-JUN-2018	14:19	Day 1	10 min	F2	5 ug	0	1	0	0	0	1	0		
09-JUN-2018	14:19	Day 1	10 min	F3	5 ug	0	0	0	0	0	0	0		
09-JUN-2018	15:08	Day 1	1 hour	F1	5 ug	0	1	0	0	0	0	0		
09-JUN-2018	15:08	Day 1	1 hour	F2	5 ug	0	1	0	0	0	0	0		
09-JUN-2018	15:08	Day 1	1 hour	F3	5 ug	0	1	0	0	0	0	0		
09-JUN-2018	16:09	Day 1	2 hour	F1	5 ug	0	0	0	0	0	0	0		
09-JUN-2018	16:09	Day 1	2 hour	F2	5 ug	0	0	0	0	0	0	0		
09-JUN-2018	16:09	Day 1	2 hour	F3	5 ug	0	0	0	0	0	0	0		
10-JUN-2018	13:59	Day 2		F1	5 ug	0	0	0	0	0	0	0		
10-JUN-2018	13:59	Day 2		F2	5 ug	1	0	0	0	0	0	0		
10-JUN-2018	13:59	Day 2		F3	5 ug	1	0	0	0	0	0	0		
12-JUN-2018	12:34	Day 4		F1	5 ug	0	1	0	1	0	0	0		
12-JUN-2018	12:34	Day 4		F2	5 ug	0	0	0	1	0	1	0		
12-JUN-2018	12:34	Day 4		F3	5 ug	0	0	0	1	0	0	0		
16-JUN-2018	14:25	Day 8		F1	5 ug	0	1	0	0	0	0	0		
16-JUN-2018	14:25	Day 8		F2	5 ug	0	0	0	0	2	0	0		

Key: 0= None; 1=Mild; 2=Moderate; 3=Severe; # Other = Pinprick bleeding or seepage

Administration Site Key: FA = Forearm; UA= Upper arm
Treatments Part A: A1 = A/Sing MAP, 15 ug (FA); A2 = Afluria Quad IM (UA), 15 ug; A3 = Placebo MAP (FA); A4 = A/Sing Mono IM, 15 ug (UA)
Treatments Part B: B1 = A/Sing MAP, 15 ug (FA) with biopsy; B2 = Placebo MAP (FA) with biopsy
B3 = A/Sing MAP, 15 ug (FA); B4 = A/Sing MAP, 10 ug (FA); B5 = A/Sing MAP, 5 ug (FA); B6 = A/Sing MAP, 2.5 ug (FA)
B7 = Placebo MAP (FA); B8 = A/Sing MAP, 15 ug (UA); B9 = A/Sing IM, 15 ug, as 2018 Afluria Quad (UA)

Listing 16.2.9.2
Treatment Site Tolerability Assessment per Site

Part B
Randomisation Number = B0101, Treatment Group = Group B3

Date	Time	Visit	Time Point	Site Number	Treatment for Site	Indura-tion	Redness	Bruising	Itching	Skin Flaking	Tender-ness	Definition/ Visibility	Other#	Unscheduled Reason
16-JUN-2018	14:25	Day 8		F3	5 ug	0	0	0	0	0	0	0		
30-JUN-2018	13:12	Day 22		F1	5 ug	0	0	0	0	0	0	2		
30-JUN-2018	13:12	Day 22		F2	5 ug	0	0	0	1	0	0	2		
30-JUN-2018	13:12	Day 22		F3	5 ug	0	0	0	0	0	0	2		
08-AUG-2018	16:33	EOS/ET (Day 61)		F1	5 ug	0	0	0	0	0	0	0		
08-AUG-2018	16:33	EOS/ET (Day 61)		F2	5 ug	0	0	0	0	0	0	0		
08-AUG-2018	16:33	EOS/ET (Day 61)		F3	5 ug	0	0	0	0	0	0	0		

Key: 0= None; 1=Mild; 2=Moderate; 3=Severe; # Other = Pinprick bleeding or seepage

Administration Site Key: FA = Forearm; UA= Upper arm
Treatments Part A: A1 = A/Sing MAP, 15 ug (FA); A2 = Afluria Quad IM (UA), 15 ug; A3 = Placebo MAP (FA); A4 = A/Sing Mono IM, 15 ug (UA)
Treatments Part B: B1 = A/Sing MAP, 15 ug (FA) with biopsy; B2 = Placebo MAP (FA) with biopsy
B3 = A/Sing MAP, 15 ug (FA); B4 = A/Sing MAP, 10 ug (FA); B5 = A/Sing MAP, 5 ug (FA); B6 = A/Sing MAP, 2.5 ug (FA)
B7 = Placebo MAP (FA); B8 = A/Sing MAP, 15 ug (UA); B9 = A/Sing IM, 15 ug, as 2018 Afluria Quad (UA)

Listing 16.2.9.2
Treatment Site Tolerability Assessment per Site

Part B
Randomisation Number = B0102, Treatment Group = Group B8

Date	Time	Visit	Time Point	Site Number	Treatment for Site	Induration	Redness	Bruising	Itching	Skin Flaking	Tenderness	Definition/ Visibility	Other#	Unscheduled Reason
09-JUN-2018	13:38	Day 1	Pre-dose	D1	5 ug	0	0	0	0	0	0	0		
09-JUN-2018	13:38	Day 1	Pre-dose	D2	5 ug	0	0	0	0	0	0	0		
09-JUN-2018	13:38	Day 1	Pre-dose	D3	5 ug	0	0	0	0	0	0	0		
09-JUN-2018	14:29	Day 1	10 min	D1	5 ug	0	0	0	0	0	0	0		
09-JUN-2018	14:29	Day 1	10 min	D2	5 ug	0	0	0	0	0	0	0		
09-JUN-2018	14:29	Day 1	10 min	D3	5 ug	0	0	0	0	0	0	0		
09-JUN-2018	15:18	Day 1	1 hour	D1	5 ug	0	1	0	0	0	0	0		
09-JUN-2018	15:18	Day 1	1 hour	D2	5 ug	0	1	0	0	0	0	0		
09-JUN-2018	15:18	Day 1	1 hour	D3	5 ug	0	1	0	0	0	0	0		
09-JUN-2018	16:18	Day 1	2 hour	D1	5 ug	0	0	0	0	0	0	0		
09-JUN-2018	16:18	Day 1	2 hour	D2	5 ug	0	0	0	0	0	0	0		
09-JUN-2018	16:18	Day 1	2 hour	D3	5 ug	0	0	0	0	0	0	0		
10-JUN-2018	17:03	Day 2		D1	5 ug	0	1	0	0	0	0	0		
10-JUN-2018	17:03	Day 2		D2	5 ug	0	1	0	0	0	0	0		
10-JUN-2018	17:03	Day 2		D3	5 ug	0	1	0	0	0	0	0		
12-JUN-2018	14:24	Day 4		D1	5 ug	0	1	0	0	0	0	0		
12-JUN-2018	14:24	Day 4		D2	5 ug	0	1	0	0	0	0	0		
12-JUN-2018	14:24	Day 4		D3	5 ug	0	1	0	0	0	0	0		
16-JUN-2018	14:52	Day 8		D1	5 ug	0	0	0	0	1	0	0		
16-JUN-2018	14:52	Day 8		D2	5 ug	0	0	0	0	1	0	0		

Key: 0= None; 1=Mild; 2=Moderate; 3=Severe; # Other = Pinprick bleeding or seepage

Administration Site Key: FA = Forearm; UA= Upper arm
Treatments Part A: A1 = A/Sing MAP, 15 ug (FA); A2 = Afluria Quad IM (UA), 15 ug; A3 = Placebo MAP (FA); A4 = A/Sing Mono IM, 15 ug (UA)
Treatments Part B: B1 = A/Sing MAP, 15 ug (FA) with biopsy; B2 = Placebo MAP (FA) with biopsy
B3 = A/Sing MAP, 15 ug (FA); B4 = A/Sing MAP, 10 ug (FA); B5 = A/Sing MAP, 5 ug (FA); B6 = A/Sing MAP, 2.5 ug (FA)
B7 = Placebo MAP (FA); B8 = A/Sing MAP, 15 ug (UA); B9 = A/Sing IM, 15 ug, as 2018 Afluria Quad (UA)

Listing 16.2.9.2
Treatment Site Tolerability Assessment per Site

Part B
Randomisation Number = B0102, Treatment Group = Group B8

Date	Time	Visit	Time Point	Site Number	Treatment for Site	Indura-tion	Redness	Bruising	Itching	Skin Flaking	Tender-ness	Definition/ Visibility	Other#	Unscheduled Reason
16-JUN-2018	14:52	Day 8		D3	5 ug	0	0	0	0	1	0	0		
30-JUN-2018	11:19	Day 22		D1	5 ug	0	0	0	0	0	0	1		
30-JUN-2018	11:19	Day 22		D2	5 ug	0	0	0	0	0	0	2		
30-JUN-2018	11:19	Day 22		D3	5 ug	0	0	0	0	0	0	2		
08-AUG-2018	16:27	EOS/ET (Day 61)		D1	5 ug	0	0	0	0	0	0	0		
08-AUG-2018	16:27	EOS/ET (Day 61)		D2	5 ug	0	0	0	0	0	0	0		
08-AUG-2018	16:27	EOS/ET (Day 61)		D3	5 ug	0	0	0	0	0	0	0		

Key: 0= None; 1=Mild; 2=Moderate; 3=Severe; # Other = Pinprick bleeding or seepage

Administration Site Key: FA = Forearm; UA= Upper arm
Treatments Part A: A1 = A/Sing MAP, 15 ug (FA); A2 = Afluria Quad IM (UA), 15 ug; A3 = Placebo MAP (FA); A4 = A/Sing Mono IM, 15 ug (UA)
Treatments Part B: B1 = A/Sing MAP, 15 ug (FA) with biopsy; B2 = Placebo MAP (FA) with biopsy
B3 = A/Sing MAP, 15 ug (FA); B4 = A/Sing MAP, 10 ug (FA); B5 = A/Sing MAP, 5 ug (FA); B6 = A/Sing MAP, 2.5 ug (FA)
B7 = Placebo MAP (FA); B8 = A/Sing MAP, 15 ug (UA); B9 = A/Sing IM, 15 ug, as 2018 Afluria Quad (UA)

Listing 16.2.9.2
Treatment Site Tolerability Assessment per Site

Part B
Randomisation Number = B0103, Treatment Group = Group B5

Date	Time	Visit	Time Point	Site Number	Treatment for Site	Indura-tion	Redness	Bruising	Itching	Skin Flaking	Tender-ness	Definition/ Visibility	Other#	Unscheduled Reason
09-JUN-2018	13:50	Day 1	Pre-dose	F1	5 ug	0	0	0	0	0	0	0		
09-JUN-2018	13:50	Day 1	Pre-dose	F2	uncoated	0	0	0	0	0	0	0		
09-JUN-2018	13:50	Day 1	Pre-dose	F3	uncoated	0	0	0	0	0	0	0		
09-JUN-2018	14:37	Day 1	10 min	F1	5 ug	0	1	0	0	0	0	0		
09-JUN-2018	14:37	Day 1	10 min	F2	uncoated	0	1	0	0	0	0	0		
09-JUN-2018	14:37	Day 1	10 min	F3	uncoated	0	1	0	0	0	0	0		
09-JUN-2018	15:28	Day 1	1 hour	F1	5 ug	1	1	0	0	0	0	0		
09-JUN-2018	15:28	Day 1	1 hour	F2	uncoated	0	1	0	0	0	0	0		
09-JUN-2018	15:28	Day 1	1 hour	F3	uncoated	0	1	0	0	0	0	0		
09-JUN-2018	16:28	Day 1	2 hour	F1	5 ug	0	1	0	0	0	0	0		
09-JUN-2018	16:28	Day 1	2 hour	F2	uncoated	0	0	0	0	0	0	0		
09-JUN-2018	16:28	Day 1	2 hour	F3	uncoated	0	0	0	0	0	0	0		
10-JUN-2018	14:10	Day 2		F1	5 ug	0	1	0	0	0	0	0		
10-JUN-2018	14:10	Day 2		F2	uncoated	0	0	0	0	0	0	0		
10-JUN-2018	14:10	Day 2		F3	uncoated	0	0	0	0	0	0	0		
12-JUN-2018	15:36	Day 4		F1	5 ug	0	1	0	0	0	0	0		
12-JUN-2018	15:36	Day 4		F2	uncoated	0	0	0	0	0	0	3		
12-JUN-2018	15:36	Day 4		F3	uncoated	0	0	0	0	0	0	3		
16-JUN-2018	10:44	Day 8		F1	5 ug	0	0	0	1	1	0	3		
16-JUN-2018	10:44	Day 8		F2	uncoated	0	0	0	0	0	0	2		

Key: 0= None; 1=Mild; 2=Moderate; 3=Severe; # Other = Pinprick bleeding or seepage

Administration Site Key: FA = Forearm; UA= Upper arm
Treatments Part A: A1 = A/Sing MAP, 15 ug (FA); A2 = Afluria Quad IM (UA), 15 ug; A3 = Placebo MAP (FA); A4 = A/Sing Mono IM, 15 ug (UA)
Treatments Part B: B1 = A/Sing MAP, 15 ug (FA) with biopsy; B2 = Placebo MAP (FA) with biopsy
B3 = A/Sing MAP, 15 ug (FA); B4 = A/Sing MAP, 10 ug (FA); B5 = A/Sing MAP, 5 ug (FA); B6 = A/Sing MAP, 2.5 ug (FA)
B7 = Placebo MAP (FA); B8 = A/Sing MAP, 15 ug (UA); B9 = A/Sing IM, 15 ug, as 2018 Afluria Quad (UA)

Listing 16.2.9.2
Treatment Site Tolerability Assessment per Site

Part B
Randomisation Number = B0103, Treatment Group = Group B5

Date	Time	Visit	Time Point	Site Number	Treatment for Site	Indura- tion	Redness	Bruising	Itching	Skin Flaking	Tender- ness	Definition/ Visibility	Other#	Unscheduled Reason
16-JUN-2018	10:44	Day 8		F3	uncoated	0	0	0	1	0	0	3		
29-JUN-2018	08:42	Day 22		F1	5 ug	0	0	0	0	0	0	1		
29-JUN-2018	08:42	Day 22		F2	uncoated	0	0	0	0	0	0	1		
29-JUN-2018	08:42	Day 22		F3	uncoated	0	0	0	0	0	0	1		
08-AUG-2018	11:25	EOS/ET (Day 61)		F1	5 ug	0	0	0	0	0	0	0		
08-AUG-2018	11:25	EOS/ET (Day 61)		F2	uncoated	0	0	0	0	0	0	0		
08-AUG-2018	11:25	EOS/ET (Day 61)		F3	uncoated	0	0	0	0	0	0	0		

Key: 0= None; 1=Mild; 2=Moderate; 3=Severe; # Other = Pinprick bleeding or seepage

Administration Site Key: FA = Forearm; UA= Upper arm
Treatments Part A: A1 = A/Sing MAP, 15 ug (FA); A2 = Afluria Quad IM (UA), 15 ug; A3 = Placebo MAP (FA); A4 = A/Sing Mono IM, 15 ug (UA)
Treatments Part B: B1 = A/Sing MAP, 15 ug (FA) with biopsy; B2 = Placebo MAP (FA) with biopsy
B3 = A/Sing MAP, 15 ug (FA); B4 = A/Sing MAP, 10 ug (FA); B5 = A/Sing MAP, 5 ug (FA); B6 = A/Sing MAP, 2.5 ug (FA)
B7 = Placebo MAP (FA); B8 = A/Sing MAP, 15 ug (UA); B9 = A/Sing IM, 15 ug, as 2018 Afluria Quad (UA)

Listing 16.2.9.2
Treatment Site Tolerability Assessment per Site

Part B
Randomisation Number = B0104, Treatment Group = Group B3

Date	Time	Visit	Time Point	Site Number	Treatment for Site	Indura-tion	Redness	Bruising	Itching	Skin Flaking	Tender-ness	Definition/ Visibility	Other#	Unscheduled Reason
09-JUN-2018	13:59	Day 1	Pre-dose	F1	5 ug	0	0	0	0	0	0	0		
09-JUN-2018	13:59	Day 1	Pre-dose	F2	5 ug	0	0	0	0	0	0	0		
09-JUN-2018	13:59	Day 1	Pre-dose	F3	5 ug	0	0	0	0	0	0	0		
09-JUN-2018	14:41	Day 1	10 min	F1	5 ug	0	0	0	0	0	1	0		
09-JUN-2018	14:41	Day 1	10 min	F2	5 ug	0	0	0	0	0	1	0		
09-JUN-2018	14:41	Day 1	10 min	F3	5 ug	0	0	0	0	0	1	0		
09-JUN-2018	15:38	Day 1	1 hour	F1	5 ug	0	1	0	0	0	0	0		
09-JUN-2018	15:38	Day 1	1 hour	F2	5 ug	0	1	0	0	0	0	0		
09-JUN-2018	15:38	Day 1	1 hour	F3	5 ug	0	1	0	0	0	1	0		
09-JUN-2018	16:38	Day 1	2 hour	F1	5 ug	0	0	0	0	0	1	0		
09-JUN-2018	16:38	Day 1	2 hour	F2	5 ug	0	0	0	0	0	0	0		
09-JUN-2018	16:38	Day 1	2 hour	F3	5 ug	0	0	0	0	0	0	0		
10-JUN-2018	15:19	Day 2		F1	5 ug	0	0	0	0	0	1	0		
10-JUN-2018	15:19	Day 2		F2	5 ug	0	0	0	0	0	1	0		
10-JUN-2018	15:19	Day 2		F3	5 ug	0	0	0	0	0	1	0		
12-JUN-2018	14:29	Day 4		F1	5 ug	0	0	0	0	0	0	0		
12-JUN-2018	14:29	Day 4		F2	5 ug	1	1	0	0	0	0	0		
12-JUN-2018	14:29	Day 4		F3	5 ug	1	1	0	0	0	0	0		
16-JUN-2018	16:55	Day 8		F1	5 ug	0	0	0	1	0	0	3		
16-JUN-2018	16:55	Day 8		F2	5 ug	0	0	0	1	0	0	3		

Key: 0= None; 1=Mild; 2=Moderate; 3=Severe; # Other = Pinprick bleeding or seepage

Administration Site Key: FA = Forearm; UA= Upper arm
Treatments Part A: A1 = A/Sing MAP, 15 ug (FA); A2 = Afluria Quad IM (UA), 15 ug; A3 = Placebo MAP (FA); A4 = A/Sing Mono IM, 15 ug (UA)
Treatments Part B: B1 = A/Sing MAP, 15 ug (FA) with biopsy; B2 = Placebo MAP (FA) with biopsy
B3 = A/Sing MAP, 15 ug (FA); B4 = A/Sing MAP, 10 ug (FA); B5 = A/Sing MAP, 5 ug (FA); B6 = A/Sing MAP, 2.5 ug (FA)
B7 = Placebo MAP (FA); B8 = A/Sing MAP, 15 ug (UA); B9 = A/Sing IM, 15 ug, as 2018 Afluria Quad (UA)

Listing 16.2.9.2
Treatment Site Tolerability Assessment per Site

Part B
Randomisation Number = B0104, Treatment Group = Group B3

Date	Time	Visit	Time Point	Site Number	Treatment for Site	Indura-tion	Redness	Bruising	Itching	Skin Flaking	Tender-ness	Definition/ Visibility	Other#	Unscheduled Reason
16-JUN-2018	16:55	Day 8		F3	5 ug	0	0	0	1	0	0	3		
30-JUN-2018	10:26	Day 22		F1	5 ug	0	0	0	0	0	0	1		
30-JUN-2018	10:26	Day 22		F2	5 ug	0	0	0	0	0	0	2		
30-JUN-2018	10:26	Day 22		F3	5 ug	0	0	0	0	0	0	2		
10-AUG-2018	09:30	EOS/ET (Day 61)		F1	5 ug	0	0	0	0	0	0	1		
10-AUG-2018	09:30	EOS/ET (Day 61)		F2	5 ug	0	0	0	0	0	0	1		
10-AUG-2018	09:30	EOS/ET (Day 61)		F3	5 ug	0	0	0	0	0	0	1		

Key: 0= None; 1=Mild; 2=Moderate; 3=Severe; # Other = Pinprick bleeding or seepage

Administration Site Key: FA = Forearm; UA= Upper arm
Treatments Part A: A1 = A/Sing MAP, 15 ug (FA); A2 = Afluria Quad IM (UA), 15 ug; A3 = Placebo MAP (FA); A4 = A/Sing Mono IM, 15 ug (UA)
Treatments Part B: B1 = A/Sing MAP, 15 ug (FA) with biopsy; B2 = Placebo MAP (FA) with biopsy
B3 = A/Sing MAP, 15 ug (FA); B4 = A/Sing MAP, 10 ug (FA); B5 = A/Sing MAP, 5 ug (FA); B6 = A/Sing MAP, 2.5 ug (FA)
B7 = Placebo MAP (FA); B8 = A/Sing MAP, 15 ug (UA); B9 = A/Sing IM, 15 ug, as 2018 Afluria Quad (UA)

Listing 16.2.9.2
Treatment Site Tolerability Assessment per Site

Part B
Randomisation Number = B0105, Treatment Group = Group B9

Date	Time	Visit	Time Point	Site Number	Treatment for Site	Indura-tion	Redness	Bruising	Itching	Skin Flaking	Tender-ness	Definition/ Visibility	Other#	Unscheduled Reason
09-JUN-2018	11:01	Day 1	Pre-dose	D1	[IM]	0	0	0	0	0	0	5		
09-JUN-2018	11:41	Day 1	10 min	D1	[IM]	0	0	0	0	0	0	5		
09-JUN-2018	12:31	Day 1	1 hour	D1	[IM]	0	0	0	0	0	0	5		
09-JUN-2018	13:36	Day 1	2 hour	D1	[IM]	0	0	0	0	0	0	5		
10-JUN-2018	11:42	Day 2		D1	[IM]	0	0	0	0	0	2	5		
10-JUN-2018	11:57	Unscheduled		D1	[IM]	0	0	0	0	0	2	5		MO notifies as repeat
12-JUN-2018	15:53	Day 4		D1	[IM]	0	0	0	0	0	0	5		
16-JUN-2018	11:36	Day 8		D1	[IM]	0	0	0	0	0	0	5		
30-JUN-2018	10:40	Day 22		D1	[IM]	0	0	0	0	0	0	5		
08-AUG-2018	15:17	EOS/ET (Day 61)		D1	[IM]	0	0	0	0	0	0	5		

Key: 0= None; 1=Mild; 2=Moderate; 3=Severe; # Other = Pinprick bleeding or seepage

Administration Site Key: FA = Forearm; UA= Upper arm
Treatments Part A: A1 = A/Sing MAP, 15 ug (FA); A2 = Afluria Quad IM (UA), 15 ug; A3 = Placebo MAP (FA); A4 = A/Sing Mono IM, 15 ug (UA)
Treatments Part B: B1 = A/Sing MAP, 15 ug (FA) with biopsy; B2 = Placebo MAP (FA) with biopsy
B3 = A/Sing MAP, 15 ug (FA); B4 = A/Sing MAP, 10 ug (FA); B5 = A/Sing MAP, 5 ug (FA); B6 = A/Sing MAP, 2.5 ug (FA)
B7 = Placebo MAP (FA); B8 = A/Sing MAP, 15 ug (UA); B9 = A/Sing IM, 15 ug, as 2018 Afluria Quad (UA)

Listing 16.2.9.2
Treatment Site Tolerability Assessment per Site

Part B
Randomisation Number = B0106, Treatment Group = Group B4

Date	Time	Visit	Time Point	Site Number	Treatment for Site	Indura-tion	Redness	Bruising	Itching	Skin Flaking	Tender-ness	Definition/ Visibility	Other#	Unscheduled Reason
09-JUN-2018	14:08	Day 1	Pre-dose	F1	uncoated	0	0	0	0	0	0	0		
09-JUN-2018	14:08	Day 1	Pre-dose	F2	5 ug	0	0	0	0	0	0	0		
09-JUN-2018	14:08	Day 1	Pre-dose	F3	5 ug	0	0	0	0	0	0	0		
09-JUN-2018	14:50	Day 1	10 min	F1	uncoated	1	1	0	0	0	0	0		
09-JUN-2018	14:50	Day 1	10 min	F2	5 ug	1	1	0	0	0	1	0		
09-JUN-2018	14:50	Day 1	10 min	F3	5 ug	1	1	0	0	0	1	0		
09-JUN-2018	15:48	Day 1	1 hour	F1	uncoated									
09-JUN-2018	15:48	Day 1	1 hour	F2	5 ug	0	1	0	0	0	0	0		
09-JUN-2018	15:48	Day 1	1 hour	F3	5 ug	0	1	0	0	0	0	0		
09-JUN-2018	16:48	Day 1	2 hour	F1	uncoated	0	0	0	0	0	0	0		
09-JUN-2018	16:48	Day 1	2 hour	F2	5 ug	0	0	0	0	0	0	0		
09-JUN-2018	16:48	Day 1	2 hour	F3	5 ug	0	0	0	0	0	0	0		
10-JUN-2018	14:30	Day 2		F1	uncoated	0	0	0	0	0	0	0		
10-JUN-2018	14:30	Day 2		F2	5 ug	0	1	0	0	0	0	0		
10-JUN-2018	14:30	Day 2		F3	5 ug	0	1	0	0	0	0	0		
12-JUN-2018	12:16	Day 4		F1	uncoated	0	0	0	0	0	0	3		
12-JUN-2018	12:16	Day 4		F2	5 ug	0	0	0	0	0	0	0		
12-JUN-2018	12:16	Day 4		F3	5 ug	0	0	0	0	0	0	0		
16-JUN-2018	15:52	Day 8		F1	uncoated	0	0	0	0	0	0	2		
16-JUN-2018	15:52	Day 8		F2	5 ug	0	0	0	0	2	0	3		

Key: 0= None; 1=Mild; 2=Moderate; 3=Severe; # Other = Pinprick bleeding or seepage

Administration Site Key: FA = Forearm; UA= Upper arm
Treatments Part A: A1 = A/Sing MAP, 15 ug (FA); A2 = Afluria Quad IM (UA), 15 ug; A3 = Placebo MAP (FA); A4 = A/Sing Mono IM, 15 ug (UA)
Treatments Part B: B1 = A/Sing MAP, 15 ug (FA) with biopsy; B2 = Placebo MAP (FA) with biopsy
B3 = A/Sing MAP, 15 ug (FA); B4 = A/Sing MAP, 10 ug (FA); B5 = A/Sing MAP, 5 ug (FA); B6 = A/Sing MAP, 2.5 ug (FA)
B7 = Placebo MAP (FA); B8 = A/Sing MAP, 15 ug (UA); B9 = A/Sing IM, 15 ug, as 2018 Afluria Quad (UA)

Listing 16.2.9.2
Treatment Site Tolerability Assessment per Site

Part B
Randomisation Number = B0106, Treatment Group = Group B4

Date	Time	Visit	Time Point	Site Number	Treatment for Site	Indura-tion	Redness	Bruising	Itching	Skin Flaking	Tender-ness	Definition/ Visibility	Other#	Unscheduled Reason
16-JUN-2018	15:52	Day 8		F3	5 ug	0	0	0	0	2	0	3		
30-JUN-2018	12:34	Day 22		F1	uncoated	0	0	0	0	0	0	0		
30-JUN-2018	12:34	Day 22		F2	5 ug	0	0	0	0	0	0	2		
30-JUN-2018	12:34	Day 22		F3	5 ug	0	0	0	0	0	0	3		
08-AUG-2018	15:18	EOS/ET (Day 61)		F1	uncoated	0	0	0	0	0	0	0		
08-AUG-2018	15:18	EOS/ET (Day 61)		F2	5 ug	0	0	0	0	0	0	0		
08-AUG-2018	15:18	EOS/ET (Day 61)		F3	5 ug	0	0	0	0	0	0	0		

Key: 0= None; 1=Mild; 2=Moderate; 3=Severe; # Other = Pinprick bleeding or seepage

Administration Site Key: FA = Forearm; UA= Upper arm
Treatments Part A: A1 = A/Sing MAP, 15 ug (FA); A2 = Afluria Quad IM (UA), 15 ug; A3 = Placebo MAP (FA); A4 = A/Sing Mono IM, 15 ug (UA)
Treatments Part B: B1 = A/Sing MAP, 15 ug (FA) with biopsy; B2 = Placebo MAP (FA) with biopsy
B3 = A/Sing MAP, 15 ug (FA); B4 = A/Sing MAP, 10 ug (FA); B5 = A/Sing MAP, 5 ug (FA); B6 = A/Sing MAP, 2.5 ug (FA)
B7 = Placebo MAP (FA); B8 = A/Sing MAP, 15 ug (UA); B9 = A/Sing IM, 15 ug, as 2018 Afluria Quad (UA)

Listing 16.2.9.2
Treatment Site Tolerability Assessment per Site

Part B
Randomisation Number = B0107, Treatment Group = Group B6

Date	Time	Visit	Time Point	Site Number	Treatment for Site	Induration	Redness	Bruising	Itching	Skin Flaking	Tenderness	Definition/ Visibility	Other#	Unscheduled Reason
09-JUN-2018	14:18	Day 1	Pre-dose	F1	uncoated	0	0	0	0	0	0	0		
09-JUN-2018	14:18	Day 1	Pre-dose	F2	uncoated	0	0	0	0	0	0	0		
09-JUN-2018	14:18	Day 1	Pre-dose	F3	2.5 ug	0	0	0	0	0	0	0		
09-JUN-2018	15:08	Day 1	10 min	F1	uncoated	0	0	0	0	0	0	0		
09-JUN-2018	15:08	Day 1	10 min	F2	uncoated	0	1	0	0	0	0	0		
09-JUN-2018	15:08	Day 1	10 min	F3	2.5 ug	0	1	0	0	0	0	0		
09-JUN-2018	15:58	Day 1	1 hour	F1	uncoated	0	1	0	0	0	0	0		
09-JUN-2018	15:58	Day 1	1 hour	F2	uncoated	0	1	0	0	0	0	0		
09-JUN-2018	15:58	Day 1	1 hour	F3	2.5 ug	0	1	0	0	0	0	0		
09-JUN-2018	16:58	Day 1	2 hour	F1	uncoated	0	0	0	0	0	0	0		
09-JUN-2018	16:58	Day 1	2 hour	F2	uncoated	0	0	0	0	0	0	0		
09-JUN-2018	16:58	Day 1	2 hour	F3	2.5 ug	0	0	0	0	0	0	0		
10-JUN-2018	10:03	Day 2		F1	uncoated	0	0	0	0	0	0	0		
10-JUN-2018	10:03	Day 2		F2	uncoated	1	0	0	0	0	0	0		
10-JUN-2018	10:03	Day 2		F3	2.5 ug	1	0	0	0	0	0	0		
12-JUN-2018	11:00	Day 4		F1	uncoated	0	0	0	1	0	0	0		
12-JUN-2018	11:00	Day 4		F2	uncoated	0	0	0	1	0	0	0		
12-JUN-2018	11:00	Day 4		F3	2.5 ug	0	0	0	1	0	0	0		
16-JUN-2018	09:58	Day 8		F1	uncoated	0	0	0	1	0	0	3		
16-JUN-2018	09:58	Day 8		F2	uncoated	0	0	0	0	1	0	3		

Key: 0= None; 1=Mild; 2=Moderate; 3=Severe; # Other = Pinprick bleeding or seepage

Administration Site Key: FA = Forearm; UA= Upper arm
Treatments Part A: A1 = A/Sing MAP, 15 ug (FA); A2 = Afluria Quad IM (UA), 15 ug; A3 = Placebo MAP (FA); A4 = A/Sing Mono IM, 15 ug (UA)
Treatments Part B: B1 = A/Sing MAP, 15 ug (FA) with biopsy; B2 = Placebo MAP (FA) with biopsy
B3 = A/Sing MAP, 15 ug (FA); B4 = A/Sing MAP, 10 ug (FA); B5 = A/Sing MAP, 5 ug (FA); B6 = A/Sing MAP, 2.5 ug (FA)
B7 = Placebo MAP (FA); B8 = A/Sing MAP, 15 ug (UA); B9 = A/Sing IM, 15 ug, as 2018 Afluria Quad (UA)

Listing 16.2.9.2
Treatment Site Tolerability Assessment per Site

Part B
Randomisation Number = B0107, Treatment Group = Group B6

Date	Time	Visit	Time Point	Site Number	Treatment for Site	Indura-tion	Redness	Bruising	Itching	Skin Flaking	Tender-ness	Definition/ Visibility	Other#	Unscheduled Reason
16-JUN-2018	09:58	Day 8		F3	2.5 ug	0	0	0	0	1	0	3		
29-JUN-2018	17:31	Day 22		F1	uncoated	0	0	0	0	0	0	2		
29-JUN-2018	17:31	Day 22		F2	uncoated	0	0	0	0	0	0	2		
29-JUN-2018	17:31	Day 22		F3	2.5 ug	0	0	0	0	0	0	2		
08-AUG-2018	17:42	EOS/ET (Day 61)		F1	uncoated	0	0	0	0	0	0	2		
08-AUG-2018	17:42	EOS/ET (Day 61)		F2	uncoated	0	0	0	0	0	0	2		
08-AUG-2018	17:42	EOS/ET (Day 61)		F3	2.5 ug	0	0	0	0	0	0	1		

Key: 0= None; 1=Mild; 2=Moderate; 3=Severe; # Other = Pinprick bleeding or seepage

Administration Site Key: FA = Forearm; UA= Upper arm
Treatments Part A: A1 = A/Sing MAP, 15 ug (FA); A2 = Afluria Quad IM (UA), 15 ug; A3 = Placebo MAP (FA); A4 = A/Sing Mono IM, 15 ug (UA)
Treatments Part B: B1 = A/Sing MAP, 15 ug (FA) with biopsy; B2 = Placebo MAP (FA) with biopsy
B3 = A/Sing MAP, 15 ug (FA); B4 = A/Sing MAP, 10 ug (FA); B5 = A/Sing MAP, 5 ug (FA); B6 = A/Sing MAP, 2.5 ug (FA)
B7 = Placebo MAP (FA); B8 = A/Sing MAP, 15 ug (UA); B9 = A/Sing IM, 15 ug, as 2018 Afluria Quad (UA)

Listing 16.2.9.2
Treatment Site Tolerability Assessment per Site

Part B
Randomisation Number = B0108, Treatment Group = Group B7

Date	Time	Visit	Time Point	Site Number	Treatment for Site	Indura-tion	Redness	Bruising	Itching	Skin Flaking	Tender-ness	Definition/ Visibility	Other#	Unscheduled Reason
09-JUN-2018	14:26	Day 1	Pre-dose	F1	uncoated	0	0	0	0	0	0	0		
09-JUN-2018	14:26	Day 1	Pre-dose	F2	uncoated	0	0	0	0	0	0	0		
09-JUN-2018	14:26	Day 1	Pre-dose	F3	uncoated	0	0	0	0	0	0	0		
09-JUN-2018	15:13	Day 1	10 min	F1	uncoated	0	1	0	0	0		0		
09-JUN-2018	15:13	Day 1	10 min	F2	uncoated	0	1	0	0	0	1	0		
09-JUN-2018	15:13	Day 1	10 min	F3	uncoated	0	1	0	0	0	1	0		
09-JUN-2018	16:08	Day 1	1 hour	F1	uncoated	0	1	0	0	0	0	0		
09-JUN-2018	16:08	Day 1	1 hour	F2	uncoated	0	1	0	0	0	0	0		
09-JUN-2018	16:08	Day 1	1 hour	F3	uncoated	0	1	0	0	0	0	0		
09-JUN-2018	17:08	Day 1	2 hour	F1	uncoated	0	0	0	0	0	0	0		
09-JUN-2018	17:08	Day 1	2 hour	F2	uncoated	0	0	0	0	0	0	0		
09-JUN-2018	17:08	Day 1	2 hour	F3	uncoated	0	0	0	0	0	0	0		
10-JUN-2018	15:07	Day 2		F1	uncoated	0	0	0	0	0	0	0		
10-JUN-2018	15:07	Day 2		F2	uncoated	0	0	0	0	0	0	0		
10-JUN-2018	15:07	Day 2		F3	uncoated	0	0	0	0	0	0	0		
12-JUN-2018	14:33	Day 4		F1	uncoated	0	0	0	0	0		1		
12-JUN-2018	14:33	Day 4		F2	uncoated	0	0	0	0	0	0	1		
12-JUN-2018	14:33	Day 4		F3	uncoated	0	0	0	0	0	0	1		
16-JUN-2018	08:59	Day 8		F1	uncoated	0	0	0	0	0	0	1		
16-JUN-2018	08:59	Day 8		F2	uncoated	0	0	0	0	0	0	1		

Key: 0= None; 1=Mild; 2=Moderate; 3=Severe; # Other = Pinprick bleeding or seepage

Administration Site Key: FA = Forearm; UA= Upper arm
Treatments Part A: A1 = A/Sing MAP, 15 ug (FA); A2 = Afluria Quad IM (UA), 15 ug; A3 = Placebo MAP (FA); A4 = A/Sing Mono IM, 15 ug (UA)
Treatments Part B: B1 = A/Sing MAP, 15 ug (FA) with biopsy; B2 = Placebo MAP (FA) with biopsy
B3 = A/Sing MAP, 15 ug (FA); B4 = A/Sing MAP, 10 ug (FA); B5 = A/Sing MAP, 5 ug (FA); B6 = A/Sing MAP, 2.5 ug (FA)
B7 = Placebo MAP (FA); B8 = A/Sing MAP, 15 ug (UA); B9 = A/Sing IM, 15 ug, as 2018 Afluria Quad (UA)

Listing 16.2.9.2
Treatment Site Tolerability Assessment per Site

Part B
Randomisation Number = B0108, Treatment Group = Group B7

Date	Time	Visit	Time Point	Site Number	Treatment for Site	Indura-tion	Redness	Bruising	Itching	Skin Flaking	Tender-ness	Definition/ Visibility	Other#	Unscheduled Reason
16-JUN-2018	08:59	Day 8		F3	uncoated	0	0	0	0	0	0	1		
30-JUN-2018	13:30	Day 22		F1	uncoated	0	0	0	0	0	0	0		
30-JUN-2018	13:30	Day 22		F2	uncoated	0	0	0	0	0	0	0		
30-JUN-2018	13:30	Day 22		F3	uncoated	0	0	0	0	0	0	0		
28-AUG-2018	16:25	EOS/ET (Day 61)		F1	uncoated	0	0	0	0	0	0	0		
28-AUG-2018	16:25	EOS/ET (Day 61)		F2	uncoated	0	0	0	0	0	0	0		
28-AUG-2018	16:25	EOS/ET (Day 61)		F3	uncoated	0	0	0	0	0	0	0		

Key: 0= None; 1=Mild; 2=Moderate; 3=Severe; # Other = Pinprick bleeding or seepage

Administration Site Key: FA = Forearm; UA= Upper arm
Treatments Part A: A1 = A/Sing MAP, 15 ug (FA); A2 = Afluria Quad IM (UA), 15 ug; A3 = Placebo MAP (FA); A4 = A/Sing Mono IM, 15 ug (UA)
Treatments Part B: B1 = A/Sing MAP, 15 ug (FA) with biopsy; B2 = Placebo MAP (FA) with biopsy
B3 = A/Sing MAP, 15 ug (FA); B4 = A/Sing MAP, 10 ug (FA); B5 = A/Sing MAP, 5 ug (FA); B6 = A/Sing MAP, 2.5 ug (FA)
B7 = Placebo MAP (FA); B8 = A/Sing MAP, 15 ug (UA); B9 = A/Sing IM, 15 ug, as 2018 Afluria Quad (UA)

Listing 16.2.9.2
Treatment Site Tolerability Assessment per Site

Part B
Randomisation Number = B0109, Treatment Group = Group B6

Date	Time	Visit	Time Point	Site Number	Treatment for Site	Indura-tion	Redness	Bruising	Itching	Skin Flaking	Tender-ness	Definition/ Visibility	Other#	Unscheduled Reason
16-JUN-2018	11:06	Day 1	Pre-dose	F1	uncoated	0	0	0	0	0	0	0		
16-JUN-2018	11:06	Day 1	Pre-dose	F2	2.5 ug	0	0	0	0	0	0	0		
16-JUN-2018	11:06	Day 1	Pre-dose	F3	uncoated	0	0	0	0	0	0	0		
16-JUN-2018	12:08	Day 1	10 min	F1	uncoated	0	1	0	0	0	0	0		
16-JUN-2018	12:08	Day 1	10 min	F2	2.5 ug	0	2	0	0	0	0	0		
16-JUN-2018	12:08	Day 1	10 min	F3	uncoated	0	0	0	0	0	0	0		
16-JUN-2018	12:58	Day 1	1 hour	F1	uncoated	1	1	0	0	0	0	0		
16-JUN-2018	12:58	Day 1	1 hour	F2	2.5 ug	1	1	0	0	0	0	0		
16-JUN-2018	12:58	Day 1	1 hour	F3	uncoated	1	1	0	0	0	0	0		
16-JUN-2018	13:58	Day 1	2 hour	F1	uncoated	1	0	0	0	0	0	0		
16-JUN-2018	13:58	Day 1	2 hour	F2	2.5 ug	2	0	0	0	0	0	0		
16-JUN-2018	13:58	Day 1	2 hour	F3	uncoated	0	0	0	0	0	0	0		
16-JUN-2018	12:58	Unscheduled		F2	2.5 ug		3						NA	MO notifies as repeat
16-JUN-2018	14:05	Unscheduled		F2	2.5 ug	2							NA	MO notifies as repeat
17-JUN-2018	12:02	Day 2		F1	uncoated	0	0	0	0	0	0	0		
17-JUN-2018	12:02	Day 2		F2	2.5 ug	1	1	0	0	0	0	0		
17-JUN-2018	12:02	Day 2		F3	uncoated	0	0	0	0	0	0	0		
19-JUN-2018	15:28	Day 4		F1	uncoated	0	0	0	0	0	0	2		

Key: 0= None; 1=Mild; 2=Moderate; 3=Severe; # Other = Pinprick bleeding or seepage

Administration Site Key: FA = Forearm; UA= Upper arm
Treatments Part A: A1 = A/Sing MAP, 15 ug (FA); A2 = Afluria Quad IM (UA), 15 ug; A3 = Placebo MAP (FA); A4 = A/Sing Mono IM, 15 ug (UA)
Treatments Part B: B1 = A/Sing MAP, 15 ug (FA) with biopsy; B2 = Placebo MAP (FA) with biopsy
B3 = A/Sing MAP, 15 ug (FA); B4 = A/Sing MAP, 10 ug (FA); B5 = A/Sing MAP, 5 ug (FA); B6 = A/Sing MAP, 2.5 ug (FA)
B7 = Placebo MAP (FA); B8 = A/Sing MAP, 15 ug (UA); B9 = A/Sing IM, 15 ug, as 2018 Afluria Quad (UA)

Listing 16.2.9.2
Treatment Site Tolerability Assessment per Site

Part B
Randomisation Number = B0109, Treatment Group = Group B6

Date	Time	Visit	Time Point	Site Number	Treatment for Site	Indura-tion	Redness	Bruising	Itching	Skin Flaking	Tender-ness	Definition/ Visibility	Other#	Unscheduled Reason
19-JUN-2018	15:28	Day 4		F2	2.5 ug	1	0	0	1	0	0	0		
19-JUN-2018	15:28	Day 4		F3	uncoated	0	0	0	0	0	0	2		
23-JUN-2018	09:48	Day 8		F1	uncoated	0	0	0	0	0	0	1		
23-JUN-2018	09:48	Day 8		F2	2.5 ug	0	0	0	0	1	0	3		
23-JUN-2018	09:48	Day 8		F3	uncoated	0	0	0	0	0	0	2		
07-JUL-2018	10:17	Day 22		F1	uncoated	0	0	0	0	0	0	1		
07-JUL-2018	10:17	Day 22		F2	2.5 ug	0	0	0	0	1	0	2		
07-JUL-2018	10:17	Day 22		F3	uncoated	0	0	0	0	0	0	1		
15-AUG-2018	09:54	EOS/ET (Day 61)		F1	uncoated	0	0	0	0	0	0	0		
15-AUG-2018	09:54	EOS/ET (Day 61)		F2	2.5 ug	0	0	0	0	0	0	1		
15-AUG-2018	09:54	EOS/ET (Day 61)		F3	uncoated	0	0	0	0	0	0	1		

Key: 0= None; 1=Mild; 2=Moderate; 3=Severe; # Other = Pinprick bleeding or seepage

Administration Site Key: FA = Forearm; UA= Upper arm
Treatments Part A: A1 = A/Sing MAP, 15 ug (FA); A2 = Afluria Quad IM (UA), 15 ug; A3 = Placebo MAP (FA); A4 = A/Sing Mono IM, 15 ug (UA)
Treatments Part B: B1 = A/Sing MAP, 15 ug (FA) with biopsy; B2 = Placebo MAP (FA) with biopsy
B3 = A/Sing MAP, 15 ug (FA); B4 = A/Sing MAP, 10 ug (FA); B5 = A/Sing MAP, 5 ug (FA); B6 = A/Sing MAP, 2.5 ug (FA)
B7 = Placebo MAP (FA); B8 = A/Sing MAP, 15 ug (UA); B9 = A/Sing IM, 15 ug, as 2018 Afluria Quad (UA)

Listing 16.2.9.2
Treatment Site Tolerability Assessment per Site

Part B
Randomisation Number = B0110, Treatment Group = Group B3

Date	Time	Visit	Time Point	Site Number	Treatment for Site	Indura- tion	Redness	Bruising	Itching	Skin Flaking	Tender- ness	Definition/ Visibility	Other#	Unscheduled Reason
16-JUN-2018	11:18	Day 1	Pre-dose	F1	5 ug	0	0	0	0	0	0	0		
16-JUN-2018	11:18	Day 1	Pre-dose	F2	5 ug	0	0	0	0	0	0	0		
16-JUN-2018	11:18	Day 1	Pre-dose	F3	5 ug	0	0	0	0	0	0	0		
16-JUN-2018	12:18	Day 1	10 min	F1	5 ug	0	0	0	0	0	0	0		
16-JUN-2018	12:18	Day 1	10 min	F2	5 ug	0	1	0	0	0	0	0		
16-JUN-2018	12:18	Day 1	10 min	F3	5 ug	0	1	0	0	0	0	0		
16-JUN-2018	13:08	Day 1	1 hour	F1	5 ug	0	1	0	0	0	0	0		
16-JUN-2018	13:08	Day 1	1 hour	F2	5 ug	0	1	0	0	0	0	0		
16-JUN-2018	13:08	Day 1	1 hour	F3	5 ug	0	1	0	0	0	0	0		
16-JUN-2018	14:08	Day 1	2 hour	F1	5 ug	0	1	0	0	0	0	0		
16-JUN-2018	14:08	Day 1	2 hour	F2	5 ug	0	1	0	0	0	0	0		
16-JUN-2018	14:08	Day 1	2 hour	F3	5 ug	0	1	0	0	0	0	0		
17-JUN-2018	12:46	Day 2		F1	5 ug	1	1	0	0	0	0	0		
17-JUN-2018	12:46	Day 2		F2	5 ug	1	1	0	0	0	0	0		
17-JUN-2018	12:46	Day 2		F3	5 ug	1	1	0	0	0	0	0		
19-JUN-2018	13:18	Day 4		F1	5 ug	0	0	0	1	1	0	0		
19-JUN-2018	13:18	Day 4		F2	5 ug	0	0	0	1	1	0	0		
19-JUN-2018	13:18	Day 4		F3	5 ug	0	0	0	1	1	0	0		
23-JUN-2018	13:54	Day 8		F1	5 ug	0	0	0	0	1	0	0		
23-JUN-2018	13:54	Day 8		F2	5 ug	0	0	0	0	1	0	0		

Key: 0= None; 1=Mild; 2=Moderate; 3=Severe; # Other = Pinprick bleeding or seepage

Administration Site Key: FA = Forearm; UA= Upper arm
Treatments Part A: A1 = A/Sing MAP, 15 ug (FA); A2 = Afluria Quad IM (UA), 15 ug; A3 = Placebo MAP (FA); A4 = A/Sing Mono IM, 15 ug (UA)
Treatments Part B: B1 = A/Sing MAP, 15 ug (FA) with biopsy; B2 = Placebo MAP (FA) with biopsy
B3 = A/Sing MAP, 15 ug (FA); B4 = A/Sing MAP, 10 ug (FA); B5 = A/Sing MAP, 5 ug (FA); B6 = A/Sing MAP, 2.5 ug (FA)
B7 = Placebo MAP (FA); B8 = A/Sing MAP, 15 ug (UA); B9 = A/Sing IM, 15 ug, as 2018 Afluria Quad (UA)

Listing 16.2.9.2
Treatment Site Tolerability Assessment per Site

Part B
Randomisation Number = B0110, Treatment Group = Group B3

Date	Time	Visit	Time Point	Site Number	Treatment for Site	Indura-tion	Redness	Bruising	Itching	Skin Flaking	Tender-ness	Definition/ Visibility	Other#	Unscheduled Reason
23-JUN-2018	13:54	Day 8		F3	5 ug	0	0	0	0	1	0	0		
07-JUL-2018	13:56	Day 22		F1	5 ug	0	0	0	0	0	0	2		
07-JUL-2018	13:56	Day 22		F2	5 ug	0	0	0	0	0	0	2		
07-JUL-2018	13:56	Day 22		F3	5 ug	0	0	0	0	0	0	2		
30-AUG-2018	13:14	EOS/ET (Day 61)		F1	5 ug	0	0	0	0	0	0	0		
30-AUG-2018	13:14	EOS/ET (Day 61)		F2	5 ug	0	0	0	0	0	0	0		
30-AUG-2018	13:14	EOS/ET (Day 61)		F3	5 ug	0	0	0	0	0	0	0		

Key: 0= None; 1=Mild; 2=Moderate; 3=Severe; # Other = Pinprick bleeding or seepage

Administration Site Key: FA = Forearm; UA= Upper arm
Treatments Part A: A1 = A/Sing MAP, 15 ug (FA); A2 = Afluria Quad IM (UA), 15 ug; A3 = Placebo MAP (FA); A4 = A/Sing Mono IM, 15 ug (UA)
Treatments Part B: B1 = A/Sing MAP, 15 ug (FA) with biopsy; B2 = Placebo MAP (FA) with biopsy
B3 = A/Sing MAP, 15 ug (FA); B4 = A/Sing MAP, 10 ug (FA); B5 = A/Sing MAP, 5 ug (FA); B6 = A/Sing MAP, 2.5 ug (FA)
B7 = Placebo MAP (FA); B8 = A/Sing MAP, 15 ug (UA); B9 = A/Sing IM, 15 ug, as 2018 Afluria Quad (UA)

Listing 16.2.9.2
Treatment Site Tolerability Assessment per Site

Part B
Randomisation Number = B0111, Treatment Group = Group B8

Date	Time	Visit	Time Point	Site Number	Treatment for Site	Indura-tion	Redness	Bruising	Itching	Skin Flaking	Tender-ness	Definition/ Visibility	Other#	Unscheduled Reason
16-JUN-2018	11:29	Day 1	Pre-dose	D1	5 ug	0	0	0	0	0	0	0		
16-JUN-2018	11:29	Day 1	Pre-dose	D2	5 ug	0	0	0	0	0	0	0		
16-JUN-2018	11:29	Day 1	Pre-dose	D3	5 ug	0	0	0	0	0	0	0		
16-JUN-2018	12:28	Day 1	10 min	D1	5 ug	0	1	0	0	0	0	0		
16-JUN-2018	12:28	Day 1	10 min	D2	5 ug	0	2	0	0	0	0	0		
16-JUN-2018	12:28	Day 1	10 min	D3	5 ug	0	2	0	0	0	0	0		
16-JUN-2018	13:18	Day 1	1 hour	D1	5 ug	0	1	0	0	0	0	0		
16-JUN-2018	13:18	Day 1	1 hour	D2	5 ug	0	1	0	0	0	0	0		
16-JUN-2018	13:18	Day 1	1 hour	D3	5 ug	0	1	0	0	0	0	0		
16-JUN-2018	14:18	Day 1	2 hour	D1	5 ug	0	1	0	0	0	0	0		
16-JUN-2018	14:18	Day 1	2 hour	D2	5 ug	0	1	0	0	0	0	0		
16-JUN-2018	14:18	Day 1	2 hour	D3	5 ug	0	1	0	0	0	0	0		
17-JUN-2018	12:40	Day 2		D1	5 ug	1	1	0	0	0	0	0		
17-JUN-2018	12:40	Day 2		D2	5 ug	1	1	0	0	0	0	0		
17-JUN-2018	12:40	Day 2		D3	5 ug	1	1	0	0	0	0	0		
19-JUN-2018	15:54	Day 4		D1	5 ug	0	1	0	1	1	0	0		
19-JUN-2018	15:54	Day 4		D2	5 ug	0	1	0	1	1	0	0		
19-JUN-2018	15:54	Day 4		D3	5 ug	0	1	0	1	1	0	0		
23-JUN-2018	13:09	Day 8		D1	5 ug	0	0	0	0	0	0	0		
23-JUN-2018	13:09	Day 8		D2	5 ug	0	0	0	0	0	0	0		

Key: 0= None; 1=Mild; 2=Moderate; 3=Severe; # Other = Pinprick bleeding or seepage

Administration Site Key: FA = Forearm; UA= Upper arm
Treatments Part A: A1 = A/Sing MAP, 15 ug (FA); A2 = Afluria Quad IM (UA), 15 ug; A3 = Placebo MAP (FA); A4 = A/Sing Mono IM, 15 ug (UA)
Treatments Part B: B1 = A/Sing MAP, 15 ug (FA) with biopsy; B2 = Placebo MAP (FA) with biopsy
B3 = A/Sing MAP, 15 ug (FA); B4 = A/Sing MAP, 10 ug (FA); B5 = A/Sing MAP, 5 ug (FA); B6 = A/Sing MAP, 2.5 ug (FA)
B7 = Placebo MAP (FA); B8 = A/Sing MAP, 15 ug (UA); B9 = A/Sing IM, 15 ug, as 2018 Afluria Quad (UA)

Listing 16.2.9.2
Treatment Site Tolerability Assessment per Site

Part B
Randomisation Number = B0111, Treatment Group = Group B8

Date	Time	Visit	Time Point	Site Number	Treatment for Site	Indura-tion	Redness	Bruising	Itching	Skin Flaking	Tender-ness	Definition/ Visibility	Other#	Unscheduled Reason
23-JUN-2018	13:09	Day 8		D3	5 ug	0	0	0	0	0	0	0		
07-JUL-2018	13:05	Day 22		D1	5 ug	0	0	0	0	0	0	0		
07-JUL-2018	13:05	Day 22		D2	5 ug	0	0	0	0	0	0	0		
07-JUL-2018	13:05	Day 22		D3	5 ug	0	0	0	0	0	0	0		
15-AUG-2018	09:42	EOS/ET (Day 61)		D1	5 ug	0	0	0	0	0	0	0		
15-AUG-2018	09:42	EOS/ET (Day 61)		D2	5 ug	0	0	0	0	0	0	0		
15-AUG-2018	09:42	EOS/ET (Day 61)		D3	5 ug	0	0	0	0	0	0	0		

Key: 0= None; 1=Mild; 2=Moderate; 3=Severe; # Other = Pinprick bleeding or seepage

Administration Site Key: FA = Forearm; UA= Upper arm
Treatments Part A: A1 = A/Sing MAP, 15 ug (FA); A2 = Afluria Quad IM (UA), 15 ug; A3 = Placebo MAP (FA); A4 = A/Sing Mono IM, 15 ug (UA)
Treatments Part B: B1 = A/Sing MAP, 15 ug (FA) with biopsy; B2 = Placebo MAP (FA) with biopsy
B3 = A/Sing MAP, 15 ug (FA); B4 = A/Sing MAP, 10 ug (FA); B5 = A/Sing MAP, 5 ug (FA); B6 = A/Sing MAP, 2.5 ug (FA)
B7 = Placebo MAP (FA); B8 = A/Sing MAP, 15 ug (UA); B9 = A/Sing IM, 15 ug, as 2018 Afluria Quad (UA)

Listing 16.2.9.2
Treatment Site Tolerability Assessment per Site

Part B
Randomisation Number = B0112,   Treatment Group = Group B4

Date	Time	Visit	Time Point	Site Number	Treatment for Site	Indura-tion	Redness	Bruising	Itching	Skin Flaking	Tender-ness	Definition/ Visibility	Other#	Unscheduled Reason
16-JUN-2018	11:41	Day 1	Pre-dose	F1	uncoated	0	0	0	0	0	0	0		
16-JUN-2018	11:41	Day 1	Pre-dose	F2	5 ug	0	0	0	0	0	0	0		
16-JUN-2018	11:41	Day 1	Pre-dose	F3	5 ug	0	0	0	0	0	0	0		
16-JUN-2018	12:38	Day 1	10 min	F1	uncoated	0	1	0	0	0	0	0		
16-JUN-2018	12:38	Day 1	10 min	F2	5 ug	0	1	0	0	0	0	0		
16-JUN-2018	12:38	Day 1	10 min	F3	5 ug	0	1	0	0	0	0	0		
16-JUN-2018	13:28	Day 1	1 hour	F1	uncoated	0	1	0	0	0	0	0		
16-JUN-2018	13:28	Day 1	1 hour	F2	5 ug	0	1	0	0	0	0	0		
16-JUN-2018	13:28	Day 1	1 hour	F3	5 ug	0	1	0	0	0	0	0		
16-JUN-2018	14:28	Day 1	2 hour	F1	uncoated	0	1	0	0	0	0	0		
16-JUN-2018	14:28	Day 1	2 hour	F2	5 ug	1	1	0	0	0	0	0		
16-JUN-2018	14:28	Day 1	2 hour	F3	5 ug	0	1	0	0	0	0	0		
17-JUN-2018	13:05	Day 2		F1	uncoated	0	0	0	0	0	1	0		
17-JUN-2018	13:05	Day 2		F2	5 ug	1	1	0	0	0	1	0		
17-JUN-2018	13:05	Day 2		F3	5 ug	1	1	0	0	0	1	0		
19-JUN-2018	11:55	Day 4		F1	uncoated	0	0	0	0	0	0	1		
19-JUN-2018	11:55	Day 4		F2	5 ug	1	1	0	0	1	1	0		
19-JUN-2018	11:55	Day 4		F3	5 ug	1	1	0	1	0	1	0		
23-JUN-2018	15:09	Day 8		F1	uncoated	0	0	0	0	0	0	0		
23-JUN-2018	15:09	Day 8		F2	5 ug	1	0	0	0	1	0	0		

Key: 0= None; 1=Mild; 2=Moderate; 3=Severe; # Other = Pinprick bleeding or seepage

Administration Site Key: FA = Forearm; UA= Upper arm
Treatments Part A: A1 = A/Sing MAP, 15 ug (FA); A2 = Afluria Quad IM (UA), 15 ug; A3 = Placebo MAP (FA); A4 = A/Sing Mono IM, 15 ug (UA)
Treatments Part B: B1 = A/Sing MAP, 15 ug (FA) with biopsy; B2 = Placebo MAP (FA) with biopsy
B3 = A/Sing MAP, 15 ug (FA); B4 = A/Sing MAP, 10 ug (FA); B5 = A/Sing MAP, 5 ug (FA); B6 = A/Sing MAP, 2.5 ug (FA)
B7 = Placebo MAP (FA); B8 = A/Sing MAP, 15 ug (UA); B9 = A/Sing IM, 15 ug, as 2018 Afluria Quad (UA)

Listing 16.2.9.2
Treatment Site Tolerability Assessment per Site

Part B
Randomisation Number = B0112, Treatment Group = Group B4

Date	Time	Visit	Time Point	Site Number	Treatment for Site	Indura-tion	Redness	Bruising	Itching	Skin Flaking	Tender-ness	Definition/ Visibility	Other#	Unscheduled Reason
23-JUN-2018	15:09	Day 8		F3	5 ug	1	0	0	0	0	0	0		
07-JUL-2018	11:44	Day 22		F1	uncoated	0	0	0	0	0	0	0		
07-JUL-2018	11:44	Day 22		F2	5 ug	0	0	0	0	0	0	2		
07-JUL-2018	11:44	Day 22		F3	5 ug	0	0	0	0	0	0	2		
15-AUG-2018	13:47	EOS/ET (Day 61)		F1	uncoated	0	0	0	0	0	0	0		
15-AUG-2018	13:47	EOS/ET (Day 61)		F2	5 ug	0	0	0	0	0	0	1		
15-AUG-2018	13:47	EOS/ET (Day 61)		F3	5 ug	0	0	0	0	0	0	1		

Key: 0= None; 1=Mild; 2=Moderate; 3=Severe; # Other = Pinprick bleeding or seepage

Administration Site Key: FA = Forearm; UA= Upper arm
Treatments Part A: A1 = A/Sing MAP, 15 ug (FA); A2 = Afluria Quad IM (UA), 15 ug; A3 = Placebo MAP (FA); A4 = A/Sing Mono IM, 15 ug (UA)
Treatments Part B: B1 = A/Sing MAP, 15 ug (FA) with biopsy; B2 = Placebo MAP (FA) with biopsy
B3 = A/Sing MAP, 15 ug (FA); B4 = A/Sing MAP, 10 ug (FA); B5 = A/Sing MAP, 5 ug (FA); B6 = A/Sing MAP, 2.5 ug (FA)
B7 = Placebo MAP (FA); B8 = A/Sing MAP, 15 ug (UA); B9 = A/Sing IM, 15 ug, as 2018 Afluria Quad (UA)

Listing 16.2.9.2
Treatment Site Tolerability Assessment per Site

Part B
Randomisation Number = B0113, Treatment Group = Group B5

Date	Time	Visit	Time Point	Site Number	Treatment for Site	Indura-tion	Redness	Bruising	Itching	Skin Flaking	Tender-ness	Definition/ Visibility	Other#	Unscheduled Reason
16-JUN-2018	11:52	Day 1	Pre-dose	F1	uncoated	0	0	0	0	0	0	0		
16-JUN-2018	11:52	Day 1	Pre-dose	F2	5 ug	0	0	0	0	0	0	0		
16-JUN-2018	11:52	Day 1	Pre-dose	F3	uncoated	0	0	0	0	0	0	0		
16-JUN-2018	12:48	Day 1	10 min	F1	uncoated	0	1	0	0	0	0	0		
16-JUN-2018	12:48	Day 1	10 min	F2	5 ug	0	1	0	0	0	0	0		
16-JUN-2018	12:48	Day 1	10 min	F3	uncoated	0	1	0	0	0	0	0		
16-JUN-2018	13:38	Day 1	1 hour	F1	uncoated	0	0	0	0	0	0	0		
16-JUN-2018	13:38	Day 1	1 hour	F2	5 ug	1	0	0	0	0	0	0		
16-JUN-2018	13:38	Day 1	1 hour	F3	uncoated	1	0	0	0	0	0	0		
16-JUN-2018	14:38	Day 1	2 hour	F1	uncoated	0	1	0	0	0	1	0		
16-JUN-2018	14:38	Day 1	2 hour	F2	5 ug	0	1	0	0	0	1	0		
16-JUN-2018	14:38	Day 1	2 hour	F3	uncoated	0	1	0	0	0	1	0		
17-JUN-2018	12:54	Day 2		F1	uncoated	0	0	0	0	0	0	0		
17-JUN-2018	12:54	Day 2		F2	5 ug	1	0	0	0	0	0	0		
17-JUN-2018	12:54	Day 2		F3	uncoated	0	0	0	0	0	0	0		
19-JUN-2018	13:13	Day 4		F1	uncoated	0	0	0	0	0	0	2		
19-JUN-2018	13:13	Day 4		F2	5 ug	0	0	0	0	0	1	3		
19-JUN-2018	13:13	Day 4		F3	uncoated	0	0	0	0	0	0	2		
23-JUN-2018	10:43	Day 8		F1	uncoated	0	0	0	0	0	0	3		
23-JUN-2018	10:43	Day 8		F2	5 ug	0	0	0	0	2	0	3		

Key: 0= None; 1=Mild; 2=Moderate; 3=Severe; # Other = Pinprick bleeding or seepage

Administration Site Key: FA = Forearm; UA= Upper arm
Treatments Part A: A1 = A/Sing MAP, 15 ug (FA); A2 = Afluria Quad IM (UA), 15 ug; A3 = Placebo MAP (FA); A4 = A/Sing Mono IM, 15 ug (UA)
Treatments Part B: B1 = A/Sing MAP, 15 ug (FA) with biopsy; B2 = Placebo MAP (FA) with biopsy
B3 = A/Sing MAP, 15 ug (FA); B4 = A/Sing MAP, 10 ug (FA); B5 = A/Sing MAP, 5 ug (FA); B6 = A/Sing MAP, 2.5 ug (FA)
B7 = Placebo MAP (FA); B8 = A/Sing MAP, 15 ug (UA); B9 = A/Sing IM, 15 ug, as 2018 Afluria Quad (UA)

Listing 16.2.9.2
Treatment Site Tolerability Assessment per Site

Part B
Randomisation Number = B0113, Treatment Group = Group B5

Date	Time	Visit	Time Point	Site Number	Treatment for Site	Indura-tion	Redness	Bruising	Itching	Skin Flaking	Tender-ness	Definition/ Visibility	Other#	Unscheduled Reason
23-JUN-2018	10:43	Day 8		F3	uncoated	0	0	0	0	0	0	3		
07-JUL-2018	12:18	Day 22		F1	uncoated	0	0	0	0	0	0	0		
07-JUL-2018	12:18	Day 22		F2	5 ug	0	0	0	0	1	0	1		
07-JUL-2018	12:18	Day 22		F3	uncoated	0	0	0	0	0	0	0		
15-AUG-2018	12:00	EOS/ET (Day 61)		F1	uncoated	0	0	0	0	0	0	0		
15-AUG-2018	12:00	EOS/ET (Day 61)		F2	5 ug	0	0	0	0	0	0	0		
15-AUG-2018	12:00	EOS/ET (Day 61)		F3	uncoated	0	0	0	0	0	0	0		

Key: 0= None; 1=Mild; 2=Moderate; 3=Severe; # Other = Pinprick bleeding or seepage

Administration Site Key: FA = Forearm; UA= Upper arm
Treatments Part A: A1 = A/Sing MAP, 15 ug (FA); A2 = Afluria Quad IM (UA), 15 ug; A3 = Placebo MAP (FA); A4 = A/Sing Mono IM, 15 ug (UA)
Treatments Part B: B1 = A/Sing MAP, 15 ug (FA) with biopsy; B2 = Placebo MAP (FA) with biopsy
B3 = A/Sing MAP, 15 ug (FA); B4 = A/Sing MAP, 10 ug (FA); B5 = A/Sing MAP, 5 ug (FA); B6 = A/Sing MAP, 2.5 ug (FA)
B7 = Placebo MAP (FA); B8 = A/Sing MAP, 15 ug (UA); B9 = A/Sing IM, 15 ug, as 2018 Afluria Quad (UA)

Listing 16.2.9.2
Treatment Site Tolerability Assessment per Site

Part B
Randomisation Number = B0114, Treatment Group = Group B7

Date	Time	Visit	Time Point	Site Number	Treatment for Site	Indura-tion	Redness	Bruising	Itching	Skin Flaking	Tender-ness	Definition/ Visibility	Other#	Unscheduled Reason
16-JUN-2018	11:58	Day 1	Pre-dose	F1	uncoated	0	0	0	0	0	0	0		
16-JUN-2018	11:58	Day 1	Pre-dose	F2	uncoated	0	0	0	0	0	0	0		
16-JUN-2018	11:58	Day 1	Pre-dose	F3	uncoated	0	0	0	0	0	0	0		
16-JUN-2018	12:58	Day 1	10 min	F1	uncoated	0	1	0	0	0	1	0		
16-JUN-2018	12:58	Day 1	10 min	F2	uncoated	0	1	0	0	0	0	0		
16-JUN-2018	12:58	Day 1	10 min	F3	uncoated	0	1	0	0	0	0	0		
16-JUN-2018	13:48	Day 1	1 hour	F1	uncoated	0	0	0	0	0	0	0		
16-JUN-2018	13:48	Day 1	1 hour	F2	uncoated	1	0	0	0	0	0	0		
16-JUN-2018	13:48	Day 1	1 hour	F3	uncoated	0	0	0	0	0	0			
16-JUN-2018	14:48	Day 1	2 hour	F1	uncoated	0	1	0	0	0	0	0		
16-JUN-2018	14:48	Day 1	2 hour	F2	uncoated	0	1	0	0	0	0	0		
16-JUN-2018	14:48	Day 1	2 hour	F3	uncoated	0	1	0	0	0	0	0		
17-JUN-2018	11:07	Day 2		F1	uncoated	0	0	0	0	0	0	0		
17-JUN-2018	11:07	Day 2		F2	uncoated	0	0	0	0	0	0	0		
17-JUN-2018	11:07	Day 2		F3	uncoated	0	0	0	0	0	0	0		
19-JUN-2018	12:02	Day 4		F1	uncoated	0	0	0	0	0	0	2		
19-JUN-2018	12:02	Day 4		F2	uncoated	0	0	0	0	0	0	0		
19-JUN-2018	12:02	Day 4		F3	uncoated	0	0	0	0	0	0	0		
23-JUN-2018	12:48	Day 8		F1	uncoated	0	0	0	0	0	0	0		
23-JUN-2018	12:48	Day 8		F2	uncoated	0	0	0	0	0	0	0		

Key: 0= None; 1=Mild; 2=Moderate; 3=Severe; # Other = Pinprick bleeding or seepage

Administration Site Key: FA = Forearm; UA= Upper arm
Treatments Part A: A1 = A/Sing MAP, 15 ug (FA); A2 = Afluria Quad IM (UA), 15 ug; A3 = Placebo MAP (FA); A4 = A/Sing Mono IM, 15 ug (UA)
Treatments Part B: B1 = A/Sing MAP, 15 ug (FA) with biopsy; B2 = Placebo MAP (FA) with biopsy
B3 = A/Sing MAP, 15 ug (FA); B4 = A/Sing MAP, 10 ug (FA); B5 = A/Sing MAP, 5 ug (FA); B6 = A/Sing MAP, 2.5 ug (FA)
B7 = Placebo MAP (FA); B8 = A/Sing MAP, 15 ug (UA); B9 = A/Sing IM, 15 ug, as 2018 Afluria Quad (UA)

Listing 16.2.9.2
Treatment Site Tolerability Assessment per Site

Part B
Randomisation Number = B0114, Treatment Group = Group B7

Date	Time	Visit	Time Point	Site Number	Treatment for Site	Indura-tion	Redness	Bruising	Itching	Skin Flaking	Tender-ness	Definition/ Visibility	Other#	Unscheduled Reason
23-JUN-2018	12:48	Day 8		F3	uncoated	0	0	0	0	1	0	0		
09-JUL-2018	11:52	Day 22		F1	uncoated	0	0	0	0	0	0	1		
09-JUL-2018	11:52	Day 22		F2	uncoated	0	0	0	0	0	0	1		
09-JUL-2018	11:52	Day 22		F3	uncoated	0	0	0	0	0	0	1		
15-AUG-2018	13:04	EOS/ET (Day 61)		F1	uncoated	0	0	0	0	0	0	0		
15-AUG-2018	13:04	EOS/ET (Day 61)		F2	uncoated	0	0	0	0	0	0	0		
15-AUG-2018	13:04	EOS/ET (Day 61)		F3	uncoated	0	0	0	0	0	0	0		

Key: 0= None; 1=Mild; 2=Moderate; 3=Severe; # Other = Pinprick bleeding or seepage

Administration Site Key: FA = Forearm; UA= Upper arm
Treatments Part A: A1 = A/Sing MAP, 15 ug (FA); A2 = Afluria Quad IM (UA), 15 ug; A3 = Placebo MAP (FA); A4 = A/Sing Mono IM, 15 ug (UA)
Treatments Part B: B1 = A/Sing MAP, 15 ug (FA) with biopsy; B2 = Placebo MAP (FA) with biopsy
B3 = A/Sing MAP, 15 ug (FA); B4 = A/Sing MAP, 10 ug (FA); B5 = A/Sing MAP, 5 ug (FA); B6 = A/Sing MAP, 2.5 ug (FA)
B7 = Placebo MAP (FA); B8 = A/Sing MAP, 15 ug (UA); B9 = A/Sing IM, 15 ug, as 2018 Afluria Quad (UA)

Listing 16.2.9.2
Treatment Site Tolerability Assessment per Site

Part B
Randomisation Number = B0115, Treatment Group = Group B9

Date	Time	Visit	Time Point	Site Number	Treatment for Site	Indura-tion	Redness	Bruising	Itching	Skin Flaking	Tender-ness	Definition/ Visibility	Other#	Unscheduled Reason
16-JUN-2018	10:27	Day 1	Pre-dose	D1	[IM]	0	0	0	0	0	0	5		
16-JUN-2018	11:10	Day 1	10 min	D1	[IM]	0	1	0	0	0	0	5		
16-JUN-2018	11:57	Day 1	1 hour	D1	[IM]	0	0	0	0	0	0	5		
16-JUN-2018	13:06	Day 1	2 hour	D1	[IM]	0	0	0	0	0	0	5		
17-JUN-2018	11:12	Day 2		D1	[IM]	0	0	0	0	0	0			
19-JUN-2018	11:30	Day 4		D1	[IM]	0	0	0	0	0	0	5		
23-JUN-2018	12:43	Day 8		D1	[IM]	0	0	0	0	0	0	5		
07-JUL-2018	11:51	Day 22		D1	[IM]	0	0	0	0	0	0	5		
17-AUG-2018	19:27	EOS/ET (Day 61)		D1	[IM]	0	0	0	0	0	0	5		

Key: 0= None; 1=Mild; 2=Moderate; 3=Severe; # Other = Pinprick bleeding or seepage

Administration Site Key: FA = Forearm; UA= Upper arm
Treatments Part A: A1 = A/Sing MAP, 15 ug (FA); A2 = Afluria Quad IM (UA), 15 ug; A3 = Placebo MAP (FA); A4 = A/Sing Mono IM, 15 ug (UA)
Treatments Part B: B1 = A/Sing MAP, 15 ug (FA) with biopsy; B2 = Placebo MAP (FA) with biopsy
B3 = A/Sing MAP, 15 ug (FA); B4 = A/Sing MAP, 10 ug (FA); B5 = A/Sing MAP, 5 ug (FA); B6 = A/Sing MAP, 2.5 ug (FA)
B7 = Placebo MAP (FA); B8 = A/Sing MAP, 15 ug (UA); B9 = A/Sing IM, 15 ug, as 2018 Afluria Quad (UA)

Listing 16.2.9.2
Treatment Site Tolerability Assessment per Site

Part B
Randomisation Number = B0116, Treatment Group = Group B7

Date	Time	Visit	Time Point	Site Number	Treatment for Site	Indura-tion	Redness	Bruising	Itching	Skin Flaking	Tender-ness	Definition/ Visibility	Other#	Unscheduled Reason
16-JUN-2018	12:03	Day 1	Pre-dose	F1	uncoated	0	0	0	0	0	0	0		
16-JUN-2018	12:03	Day 1	Pre-dose	F2	uncoated	0	0	0	0	0	0	0		
16-JUN-2018	12:03	Day 1	Pre-dose	F3	uncoated	0	0	0	0	0	0	0		
16-JUN-2018	13:08	Day 1	10 min	F1	uncoated	0	2	0	0	0	1	0		
16-JUN-2018	13:08	Day 1	10 min	F2	uncoated	0	2	0	0	0	1	0		
16-JUN-2018	13:08	Day 1	10 min	F3	uncoated	0	2	0	0	0	1	0		
16-JUN-2018	13:58	Day 1	1 hour	F1	uncoated	1	2	0	0	0	0	0		
16-JUN-2018	13:58	Day 1	1 hour	F2	uncoated	1	2	0	0	0	0	0		
16-JUN-2018	13:58	Day 1	1 hour	F3	uncoated	1	2	0	0	0	0	0		
16-JUN-2018	14:58	Day 1	2 hour	F1	uncoated	0	1	0	0	0	1	0		
16-JUN-2018	14:58	Day 1	2 hour	F2	uncoated	0	1	0	0	0	1	0		
16-JUN-2018	14:58	Day 1	2 hour	F3	uncoated	0	1	0	0	0	1	0		
16-JUN-2018	13:15	Unscheduled		F1	uncoated	1	2						NA	MO notifies as repeat
16-JUN-2018	13:15	Unscheduled		F2	uncoated		2						NA	MO notifies as repeat
16-JUN-2018	13:15	Unscheduled		F3	uncoated		2						NA	MO notifies as repeat
16-JUN-2018	13:58	Unscheduled		F1	uncoated		2						NA	MO notifies as repeat

Key: 0= None; 1=Mild; 2=Moderate; 3=Severe; # Other = Pinprick bleeding or seepage

Administration Site Key: FA = Forearm; UA= Upper arm
Treatments Part A: A1 = A/Sing MAP, 15 ug (FA); A2 = Afluria Quad IM (UA), 15 ug; A3 = Placebo MAP (FA); A4 = A/Sing Mono IM, 15 ug (UA)
Treatments Part B: B1 = A/Sing MAP, 15 ug (FA) with biopsy; B2 = Placebo MAP (FA) with biopsy
B3 = A/Sing MAP, 15 ug (FA); B4 = A/Sing MAP, 10 ug (FA); B5 = A/Sing MAP, 5 ug (FA); B6 = A/Sing MAP, 2.5 ug (FA)
B7 = Placebo MAP (FA); B8 = A/Sing MAP, 15 ug (UA); B9 = A/Sing IM, 15 ug, as 2018 Afluria Quad (UA)

Listing 16.2.9.2
Treatment Site Tolerability Assessment per Site

Part B
Randomisation Number = B0116, Treatment Group = Group B7

Date	Time	Visit	Time Point	Site Number	Treatment for Site	Indura-tion	Redness	Bruising	Itching	Skin Flaking	Tender-ness	Definition/ Visibility	Other#	Unscheduled Reason
16-JUN-2018	13:58	Unscheduled		F2	uncoated		2						NA	MO notifies as repeat
16-JUN-2018	13:58	Unscheduled		F3	uncoated		2						NA	MO notifies as repeat
17-JUN-2018	13:07	Day 2		F1	uncoated	0	0	0	0	0	0	0		
17-JUN-2018	13:07	Day 2		F2	uncoated	0	0	0	0	0	0	0		
17-JUN-2018	13:07	Day 2		F3	uncoated	0	0	0	0	0	0	0		
19-JUN-2018	11:48	Day 4		F1	uncoated	0	0	0	0	0	0	1		
19-JUN-2018	11:48	Day 4		F2	uncoated	0	0	0	0	0	2	0		
19-JUN-2018	11:48	Day 4		F3	uncoated	0	0	0	0	0	2	0		
23-JUN-2018	13:00	Day 8		F1	uncoated	0	0	0	0	1	0	1		
23-JUN-2018	13:00	Day 8		F2	uncoated	0	0	0	0	1	0	1		
23-JUN-2018	13:00	Day 8		F3	uncoated	0	0	0	0	0	0	1		
07-JUL-2018	11:53	Day 22		F1	uncoated	0	0	0	0	0	0	0		
07-JUL-2018	11:53	Day 22		F2	uncoated	0	0	0	0	0	0	0		
07-JUL-2018	11:53	Day 22		F3	uncoated	0	0	0	0	0	0	0		
15-AUG-2018	10:55	EOS/ET (Day 61)		F1	uncoated	0	0	0	0	0	0	0		
15-AUG-2018	10:55	EOS/ET (Day 61)		F2	uncoated	0	0	0	0	0	0	0		
15-AUG-2018	10:55	EOS/ET (Day 61)		F3	uncoated	0	0	0	0	0	0	0		

Key: 0= None; 1=Mild; 2=Moderate; 3=Severe; # Other = Pinprick bleeding or seepage

Administration Site Key: FA = Forearm; UA= Upper arm
Treatments Part A: A1 = A/Sing MAP, 15 ug (FA); A2 = Afluria Quad IM (UA), 15 ug; A3 = Placebo MAP (FA); A4 = A/Sing Mono IM, 15 ug (UA)
Treatments Part B: B1 = A/Sing MAP, 15 ug (FA) with biopsy; B2 = Placebo MAP (FA) with biopsy
B3 = A/Sing MAP, 15 ug (FA); B4 = A/Sing MAP, 10 ug (FA); B5 = A/Sing MAP, 5 ug (FA); B6 = A/Sing MAP, 2.5 ug (FA)
B7 = Placebo MAP (FA); B8 = A/Sing MAP, 15 ug (UA); B9 = A/Sing IM, 15 ug, as 2018 Afluria Quad (UA)

Listing 16.2.9.2
Treatment Site Tolerability Assessment per Site

Part B
Randomisation Number = B0117, Treatment Group = Group B9

Date	Time	Visit	Time Point	Site Number	Treatment for Site	Indura-tion	Redness	Bruising	Itching	Skin Flaking	Tender-ness	Definition/ Visibility	Other#	Unscheduled Reason
16-JUN-2018	10:32	Day 1	Pre-dose	D1	[IM]	0	0	0	0	0	0	5		
16-JUN-2018	11:20	Day 1	10 min	D1	[IM]	0	0	0	0	0	0	5		
16-JUN-2018	12:11	Day 1	1 hour	D1	[IM]	0	1	0	0	0	0	5		
16-JUN-2018	13:18	Day 1	2 hour	D1	[IM]	0	1	0	0	0	1	5		
18-JUN-2018	11:10	Day 2		D1	[IM]	0	0	0	0	0	0	5		
19-JUN-2018	11:31	Day 4		D1	[IM]	0	0	0	0	0	0	5		
23-JUN-2018	11:20	Day 8		D1	[IM]	0	0	0	0	0	0	5		
07-JUL-2018	19:21	Day 22		D1	[IM]	0	0	0	0	0	0	5		
16-AUG-2018	14:48	EOS/ET (Day 61)		D1	[IM]	0	0	0	0	0	0	5		

Key: 0= None; 1=Mild; 2=Moderate; 3=Severe; # Other = Pinprick bleeding or seepage

Administration Site Key: FA = Forearm; UA= Upper arm
Treatments Part A: A1 = A/Sing MAP, 15 ug (FA); A2 = Afluria Quad IM (UA), 15 ug; A3 = Placebo MAP (FA); A4 = A/Sing Mono IM, 15 ug (UA)
Treatments Part B: B1 = A/Sing MAP, 15 ug (FA) with biopsy; B2 = Placebo MAP (FA) with biopsy
B3 = A/Sing MAP, 15 ug (FA); B4 = A/Sing MAP, 10 ug (FA); B5 = A/Sing MAP, 5 ug (FA); B6 = A/Sing MAP, 2.5 ug (FA)
B7 = Placebo MAP (FA); B8 = A/Sing MAP, 15 ug (UA); B9 = A/Sing IM, 15 ug, as 2018 Afluria Quad (UA)

Listing 16.2.9.2
Treatment Site Tolerability Assessment per Site

Part B
Randomisation Number = B0118, Treatment Group = Group B6

Date	Time	Visit	Time Point	Site Number	Treatment for Site	Induration	Redness	Bruising	Itching	Skin Flaking	Tenderness	Definition/ Visibility	Other#	Unscheduled Reason
16-JUN-2018	12:06	Day 1	Pre-dose	F1	uncoated	0	0	0	0	0	0	0		
16-JUN-2018	12:06	Day 1	Pre-dose	F2	2.5 ug	0	0	0	0	0	0	0		
16-JUN-2018	12:06	Day 1	Pre-dose	F3	uncoated	0	0	0	0	0	0	0		
16-JUN-2018	13:13	Day 1	10 min	F1	uncoated	0	0	0	0	0	0	0		
16-JUN-2018	13:13	Day 1	10 min	F2	2.5 ug	0	0	0	0	0	0	0		
16-JUN-2018	13:13	Day 1	10 min	F3	uncoated	0	0	0	0	0	0	0		
16-JUN-2018	14:03	Day 1	1 hour	F1	uncoated	0	0	0	0	0	1	0		
16-JUN-2018	14:03	Day 1	1 hour	F2	2.5 ug		1	0	0	0	1	0		
16-JUN-2018	14:03	Day 1	1 hour	F3	uncoated		1	0	0	0	0	0		
16-JUN-2018	15:03	Day 1	2 hour	F1	uncoated	0	1	0	0	0	1	0		
16-JUN-2018	15:03	Day 1	2 hour	F2	2.5 ug	0	0	0	0	0	1	0		
16-JUN-2018	15:03	Day 1	2 hour	F3	uncoated	0	0	0	0	0	1	0		
17-JUN-2018	10:21	Day 2		F1	uncoated	0	0	0	0	0	0	0		
17-JUN-2018	10:21	Day 2		F2	2.5 ug	1	1	0	0	0	0	0		
17-JUN-2018	10:21	Day 2		F3	uncoated	0	0	0	0	0	0	0		
19-JUN-2018	14:13	Day 4		F1	uncoated	0	0	0	0	0	0	3		
19-JUN-2018	14:13	Day 4		F2	2.5 ug	0	0	0	0	0	0	0		
19-JUN-2018	14:13	Day 4		F3	uncoated	0	0	0	0	0	0	3		
23-JUN-2018	15:16	Day 8		F1	uncoated	0	0	0	0	1	0	1		
23-JUN-2018	15:16	Day 8		F2	2.5 ug	0	1	0	0	3	0	0		

Key: 0= None; 1=Mild; 2=Moderate; 3=Severe; # Other = Pinprick bleeding or seepage

Administration Site Key: FA = Forearm; UA= Upper arm
Treatments Part A: A1 = A/Sing MAP, 15 ug (FA); A2 = Afluria Quad IM (UA), 15 ug; A3 = Placebo MAP (FA); A4 = A/Sing Mono IM, 15 ug (UA)
Treatments Part B: B1 = A/Sing MAP, 15 ug (FA) with biopsy; B2 = Placebo MAP (FA) with biopsy
B3 = A/Sing MAP, 15 ug (FA); B4 = A/Sing MAP, 10 ug (FA); B5 = A/Sing MAP, 5 ug (FA); B6 = A/Sing MAP, 2.5 ug (FA)
B7 = Placebo MAP (FA); B8 = A/Sing MAP, 15 ug (UA); B9 = A/Sing IM, 15 ug, as 2018 Afluria Quad (UA)

Listing 16.2.9.2
Treatment Site Tolerability Assessment per Site

Part B
Randomisation Number = B0118, Treatment Group = Group B6

Date	Time	Visit	Time Point	Site Number	Treatment for Site	Indura-tion	Redness	Bruising	Itching	Skin Flaking	Tender-ness	Definition/ Visibility	Other#	Unscheduled Reason
23-JUN-2018	15:16	Day 8		F3	uncoated	0	0	0	0	1	0	1		
07-JUL-2018	13:17	Day 22		F1	uncoated	0	0	0	0	0	0	1		
07-JUL-2018	13:17	Day 22		F2	2.5 ug	0	0	0	0	1	0	2		
07-JUL-2018	13:17	Day 22		F3	uncoated	0	0	0	0	0	0	0		
07-JUL-2018	13:22	Unscheduled		F2	2.5 ug							2	NA	MO notifies as repeat
15-AUG-2018	13:13	EOS/ET (Day 61)		F1	uncoated	0	0	0	0	0	0	0		
15-AUG-2018	13:13	EOS/ET (Day 61)		F2	2.5 ug	0	0	0	0	0	0	1		
15-AUG-2018	13:13	EOS/ET (Day 61)		F3	uncoated	0	0	0	0	0	0	0		

Key: 0= None; 1=Mild; 2=Moderate; 3=Severe; # Other = Pinprick bleeding or seepage

Administration Site Key: FA = Forearm; UA= Upper arm
Treatments Part A: A1 = A/Sing MAP, 15 ug (FA); A2 = Afluria Quad IM (UA), 15 ug; A3 = Placebo MAP (FA); A4 = A/Sing Mono IM, 15 ug (UA)
Treatments Part B: B1 = A/Sing MAP, 15 ug (FA) with biopsy; B2 = Placebo MAP (FA) with biopsy
B3 = A/Sing MAP, 15 ug (FA); B4 = A/Sing MAP, 10 ug (FA); B5 = A/Sing MAP, 5 ug (FA); B6 = A/Sing MAP, 2.5 ug (FA)
B7 = Placebo MAP (FA); B8 = A/Sing MAP, 15 ug (UA); B9 = A/Sing IM, 15 ug, as 2018 Afluria Quad (UA)

Listing 16.2.9.2
Treatment Site Tolerability Assessment per Site

Part B
Randomisation Number = B0119, Treatment Group = Group B4

Date	Time	Visit	Time Point	Site Number	Treatment for Site	Indura-tion	Redness	Bruising	Itching	Skin Flaking	Tender-ness	Definition/ Visibility	Other#	Unscheduled Reason
16-JUN-2018	12:08	Day 1	Pre-dose	F1	5 ug	0	0	0	0	0	0	0		
16-JUN-2018	12:08	Day 1	Pre-dose	F2	uncoated	0	0	0	0	0	0	0		
16-JUN-2018	12:08	Day 1	Pre-dose	F3	5 ug	0	0	0	0	0	0	0		
16-JUN-2018	13:28	Day 1	10 min	F1	5 ug	0	0	0	0	0	1	0		
16-JUN-2018	13:28	Day 1	10 min	F2	uncoated	0	0	0	0	0	1	0		
16-JUN-2018	13:28	Day 1	10 min	F3	5 ug	0	0	0	0	0	1	0		
16-JUN-2018	14:18	Day 1	1 hour	F1	5 ug	0	0	0	0	0	1	0		
16-JUN-2018	14:18	Day 1	1 hour	F2	uncoated	1	0	0	0	0	1	0		
16-JUN-2018	14:18	Day 1	1 hour	F3	5 ug	1	0	0	0	0	1	0		
16-JUN-2018	15:18	Day 1	2 hour	F1	5 ug	0	1	0	0	0	1	0		
16-JUN-2018	15:18	Day 1	2 hour	F2	uncoated	0	1	0	0	0	1	0		
16-JUN-2018	15:18	Day 1	2 hour	F3	5 ug	0	1	0	0	0	1	0		
17-JUN-2018	13:16	Day 2		F1	5 ug	1	1	0	0	0	0	0		
17-JUN-2018	13:16	Day 2		F2	uncoated	0	0	0	0	0	0	0		
17-JUN-2018	13:16	Day 2		F3	5 ug	1	1	0	1	0	0	0		
19-JUN-2018	15:25	Day 4		F1	5 ug	1	0	0	1	1	0	0		
19-JUN-2018	15:25	Day 4		F2	uncoated	0	0	0	0	0	0	3		
19-JUN-2018	15:25	Day 4		F3	5 ug	0	0	0	1	0	0	0		
26-JUN-2018	15:19	Day 8		F1	5 ug	0	0	0	0	1	0	2		
26-JUN-2018	15:19	Day 8		F2	uncoated	0	0	0	0	0	0	0		

Key: 0= None; 1=Mild; 2=Moderate; 3=Severe; # Other = Pinprick bleeding or seepage

Administration Site Key: FA = Forearm; UA= Upper arm
Treatments Part A: A1 = A/Sing MAP, 15 ug (FA); A2 = Afluria Quad IM (UA), 15 ug; A3 = Placebo MAP (FA); A4 = A/Sing Mono IM, 15 ug (UA)
Treatments Part B: B1 = A/Sing MAP, 15 ug (FA) with biopsy; B2 = Placebo MAP (FA) with biopsy
B3 = A/Sing MAP, 15 ug (FA); B4 = A/Sing MAP, 10 ug (FA); B5 = A/Sing MAP, 5 ug (FA); B6 = A/Sing MAP, 2.5 ug (FA)
B7 = Placebo MAP (FA); B8 = A/Sing MAP, 15 ug (UA); B9 = A/Sing IM, 15 ug, as 2018 Afluria Quad (UA)

Listing 16.2.9.2
Treatment Site Tolerability Assessment per Site

Part B
Randomisation Number = B0119, Treatment Group = Group B4

Date	Time	Visit	Time Point	Site Number	Treatment for Site	Indura-tion	Redness	Bruising	Itching	Skin Flaking	Tender-ness	Definition/ Visibility	Other#	Unscheduled Reason
26-JUN-2018	15:19	Day 8		F3	5 ug	0	0	0	0	0	0	1		
07-JUL-2018	12:07	Day 22		F1	5 ug	0	0	0	0	0	0	1		
07-JUL-2018	12:07	Day 22		F2	uncoated	0	0	0	0	0	0	0		
07-JUL-2018	12:07	Day 22		F3	5 ug	0	0	0	0	1	0	1		
15-AUG-2018	14:14	EOS/ET (Day 61)		F1	5 ug	0	0	0	0	0	0	1		
15-AUG-2018	14:14	EOS/ET (Day 61)		F2	uncoated	0	0	0	0	0	0	0		
15-AUG-2018	14:14	EOS/ET (Day 61)		F3	5 ug	0	0	0	0	0	0	1		

Key: 0= None; 1=Mild; 2=Moderate; 3=Severe; # Other = Pinprick bleeding or seepage

Administration Site Key: FA = Forearm; UA= Upper arm
Treatments Part A: A1 = A/Sing MAP, 15 ug (FA); A2 = Afluria Quad IM (UA), 15 ug; A3 = Placebo MAP (FA); A4 = A/Sing Mono IM, 15 ug (UA)
Treatments Part B: B1 = A/Sing MAP, 15 ug (FA) with biopsy; B2 = Placebo MAP (FA) with biopsy
B3 = A/Sing MAP, 15 ug (FA); B4 = A/Sing MAP, 10 ug (FA); B5 = A/Sing MAP, 5 ug (FA); B6 = A/Sing MAP, 2.5 ug (FA)
B7 = Placebo MAP (FA); B8 = A/Sing MAP, 15 ug (UA); B9 = A/Sing IM, 15 ug, as 2018 Afluria Quad (UA)

Listing 16.2.9.2
Treatment Site Tolerability Assessment per Site

Part B
Randomisation Number = B0120, Treatment Group = Group B3

Date	Time	Visit	Time Point	Site Number	Treatment for Site	Indura-tion	Redness	Bruising	Itching	Skin Flaking	Tender-ness	Definition/ Visibility	Other#	Unscheduled Reason
16-JUN-2018	12:16	Day 1	Pre-dose	F1	5 ug	0	0	0	0	0	0	0		
16-JUN-2018	12:16	Day 1	Pre-dose	F2	5 ug	0	0	0	0	0	0	0		
16-JUN-2018	12:16	Day 1	Pre-dose	F3	5 ug	0	0	0	0	0	0	0		
16-JUN-2018	13:38	Day 1	10 min	F1	5 ug	0	1	0	0	0	1	0		
16-JUN-2018	13:38	Day 1	10 min	F2	5 ug	0	1	0	0	0	1	0		
16-JUN-2018	13:38	Day 1	10 min	F3	5 ug	0	1	0	0	0	1	0		
16-JUN-2018	14:28	Day 1	1 hour	F1	5 ug	1	1	0	0	0	2	0		
16-JUN-2018	14:28	Day 1	1 hour	F2	5 ug	1	1	0	0	0	2	0		
16-JUN-2018	14:28	Day 1	1 hour	F3	5 ug	1	1	0	0	0	2	0		
16-JUN-2018	15:28	Day 1	2 hour	F1	5 ug	0	1	0	0	0	2	0		
16-JUN-2018	15:28	Day 1	2 hour	F2	5 ug	0	1	0	0	0	2	0		
16-JUN-2018	15:28	Day 1	2 hour	F3	5 ug	0	1	0	0	0	2	0		
16-JUN-2018	14:47	Unscheduled		F1	5 ug						1		NA	MO notifies as repeat
16-JUN-2018	14:47	Unscheduled		F2	5 ug						2		NA	MO notifies as repeat
16-JUN-2018	14:47	Unscheduled		F3	5 ug						2		NA	MO notifies as repeat
16-JUN-2018	15:28	Unscheduled		F1	5 ug						2			MO notifies as repeat

Key: 0= None; 1=Mild; 2=Moderate; 3=Severe; # Other = Pinprick bleeding or seepage

Administration Site Key: FA = Forearm; UA= Upper arm
Treatments Part A: A1 = A/Sing MAP, 15 ug (FA); A2 = Afluria Quad IM (UA), 15 ug; A3 = Placebo MAP (FA); A4 = A/Sing Mono IM, 15 ug (UA)
Treatments Part B: B1 = A/Sing MAP, 15 ug (FA) with biopsy; B2 = Placebo MAP (FA) with biopsy
B3 = A/Sing MAP, 15 ug (FA); B4 = A/Sing MAP, 10 ug (FA); B5 = A/Sing MAP, 5 ug (FA); B6 = A/Sing MAP, 2.5 ug (FA)
B7 = Placebo MAP (FA); B8 = A/Sing MAP, 15 ug (UA); B9 = A/Sing IM, 15 ug, as 2018 Afluria Quad (UA)

Listing 16.2.9.2
Treatment Site Tolerability Assessment per Site

Part B
Randomisation Number = B0120, Treatment Group = Group B3

Date	Time	Visit	Time Point	Site Number	Treatment for Site	Indura-tion	Redness	Bruising	Itching	Skin Flaking	Tender-ness	Definition/ Visibility	Other#	Unscheduled Reason
16-JUN-2018	15:28	Unscheduled		F2	5 ug						2			MO notifies as repeat
16-JUN-2018	15:28	Unscheduled		F3	5 ug						2			MO notifies as repeat
17-JUN-2018	13:25	Day 2		F1	5 ug	2	1	0	0	0	0	0		
17-JUN-2018	13:25	Day 2		F2	5 ug	1	1	0	0	0	0	0		
17-JUN-2018	13:25	Day 2		F3	5 ug	1	1	0	0	0	0	0		
19-JUN-2018	13:55	Day 4		F1	5 ug	0	0	0	2	1	0	0		
19-JUN-2018	13:55	Day 4		F2	5 ug	0	0	0	2	1	1	0		
19-JUN-2018	13:55	Day 4		F3	5 ug	0	0	0	0	0	1	0		
23-JUN-2018	14:12	Day 8		F1	5 ug	0	0	0	0	0	0	0		
23-JUN-2018	14:12	Day 8		F2	5 ug	0	0	0	0	0	0	0		
23-JUN-2018	14:12	Day 8		F3	5 ug	0	0	0	0	1	0	0		
07-JUL-2018	14:00	Day 22		F1	5 ug	0	0	0	0	0	0	2		
07-JUL-2018	14:00	Day 22		F2	5 ug	0	0	0	0	0	0	2		
07-JUL-2018	14:00	Day 22		F3	5 ug	0	0	0	0	0	0	2		
15-AUG-2018	13:58	EOS/ET (Day 61)		F1	5 ug	0	0	0	0	0	0	1		
15-AUG-2018	13:58	EOS/ET (Day 61)		F2	5 ug	0	0	0	0	0	0	1		
15-AUG-2018	13:58	EOS/ET (Day 61)		F3	5 ug	0	0	0	0	0	0	1		

Key: 0= None; 1=Mild; 2=Moderate; 3=Severe; # Other = Pinprick bleeding or seepage

Administration Site Key: FA = Forearm; UA= Upper arm
Treatments Part A: A1 = A/Sing MAP, 15 ug (FA); A2 = Afluria Quad IM (UA), 15 ug; A3 = Placebo MAP (FA); A4 = A/Sing Mono IM, 15 ug (UA)
Treatments Part B: B1 = A/Sing MAP, 15 ug (FA) with biopsy; B2 = Placebo MAP (FA) with biopsy
B3 = A/Sing MAP, 15 ug (FA); B4 = A/Sing MAP, 10 ug (FA); B5 = A/Sing MAP, 5 ug (FA); B6 = A/Sing MAP, 2.5 ug (FA)
B7 = Placebo MAP (FA); B8 = A/Sing MAP, 15 ug (UA); B9 = A/Sing IM, 15 ug, as 2018 Afluria Quad (UA)

Listing 16.2.9.2
Treatment Site Tolerability Assessment per Site

Part B
Randomisation Number = B0121, Treatment Group = Group B8

Date	Time	Visit	Time Point	Site Number	Treatment for Site	Induration	Redness	Bruising	Itching	Skin Flaking	Tenderness	Definition/ Visibility	Other#	Unscheduled Reason
16-JUN-2018	12:15	Day 1	Pre-dose	D1	5 ug	0	0	0	0	0	0	0		
16-JUN-2018	12:15	Day 1	Pre-dose	D2	5 ug	0	0	0	0	0	0	0		
16-JUN-2018	12:15	Day 1	Pre-dose	D3	5 ug	0	0	0	0	0	0	0		
16-JUN-2018	13:48	Day 1	10 min	D1	5 ug	0	0	0	0	0	1	0		
16-JUN-2018	13:48	Day 1	10 min	D2	5 ug	0	0	0	0	0	1	0		
16-JUN-2018	13:48	Day 1	10 min	D3	5 ug	0	0	0	0	0	1	0		
16-JUN-2018	14:38	Day 1	1 hour	D1	5 ug	1	1	0	0	0	0	0		
16-JUN-2018	14:38	Day 1	1 hour	D2	5 ug	1	1	0	0	0	1	0		
16-JUN-2018	14:38	Day 1	1 hour	D3	5 ug	1	1	0	0	0	0	0		
16-JUN-2018	15:38	Day 1	2 hour	D1	5 ug	0	1	0	0	0	0	0		
16-JUN-2018	15:38	Day 1	2 hour	D2	5 ug	0	1	0	0	0	1	0		
16-JUN-2018	15:38	Day 1	2 hour	D3	5 ug	0	1	0	0	0	0	0		
17-JUN-2018	10:17	Day 2		D1	5 ug	1	1	0	0	0	1	0		
17-JUN-2018	10:17	Day 2		D2	5 ug	1	1	0	0	0	1	0		
17-JUN-2018	10:17	Day 2		D3	5 ug	1	1	0	0	0	1	0		
19-JUN-2018	14:00	Day 4		D1	5 ug	1	1	0	0	2	1	0		
19-JUN-2018	14:00	Day 4		D2	5 ug	1	1	0	0	2	1	0		
19-JUN-2018	14:00	Day 4		D3	5 ug	1	1	0	0	2	1	0		
23-JUN-2018	15:03	Day 8		D1	5 ug	0	0	0	0	1	0	0		
23-JUN-2018	15:03	Day 8		D2	5 ug	0	0	0	2	1	0	0		

Key: 0= None; 1=Mild; 2=Moderate; 3=Severe; # Other = Pinprick bleeding or seepage

Administration Site Key: FA = Forearm; UA= Upper arm
Treatments Part A: A1 = A/Sing MAP, 15 ug (FA); A2 = Afluria Quad IM (UA), 15 ug; A3 = Placebo MAP (FA); A4 = A/Sing Mono IM, 15 ug (UA)
Treatments Part B: B1 = A/Sing MAP, 15 ug (FA) with biopsy; B2 = Placebo MAP (FA) with biopsy
B3 = A/Sing MAP, 15 ug (FA); B4 = A/Sing MAP, 10 ug (FA); B5 = A/Sing MAP, 5 ug (FA); B6 = A/Sing MAP, 2.5 ug (FA)
B7 = Placebo MAP (FA); B8 = A/Sing MAP, 15 ug (UA); B9 = A/Sing IM, 15 ug, as 2018 Afluria Quad (UA)

Listing 16.2.9.2
Treatment Site Tolerability Assessment per Site

Part B
Randomisation Number = B0121, Treatment Group = Group B8

Date	Time	Visit	Time Point	Site Number	Treatment for Site	Indura-tion	Redness	Bruising	Itching	Skin Flaking	Tender-ness	Definition/ Visibility	Other#	Unscheduled Reason
23-JUN-2018	15:03	Day 8		D3	5 ug	0	0	0	2	1	0	0		
09-JUL-2018	09:51	Day 22		D1	5 ug	0	0	0	0	0	0	0		
09-JUL-2018	09:51	Day 22		D2	5 ug	0	0	0	0	0	0	0		
09-JUL-2018	09:51	Day 22		D3	5 ug	0	0	0	0	0	0	0		
08-AUG-2018	09:40	EOS/ET (Day 61)		D1	5 ug	0	0	0	0	0	0	0		
08-AUG-2018	09:40	EOS/ET (Day 61)		D2	5 ug	0	0	0	0	0	0	0		
08-AUG-2018	09:40	EOS/ET (Day 61)		D3	5 ug	0	0	0	0	0	0	0		

Key: 0= None; 1=Mild; 2=Moderate; 3=Severe; # Other = Pinprick bleeding or seepage

Administration Site Key: FA = Forearm; UA= Upper arm
Treatments Part A: A1 = A/Sing MAP, 15 ug (FA); A2 = Afluria Quad IM (UA), 15 ug; A3 = Placebo MAP (FA); A4 = A/Sing Mono IM, 15 ug (UA)
Treatments Part B: B1 = A/Sing MAP, 15 ug (FA) with biopsy; B2 = Placebo MAP (FA) with biopsy
B3 = A/Sing MAP, 15 ug (FA); B4 = A/Sing MAP, 10 ug (FA); B5 = A/Sing MAP, 5 ug (FA); B6 = A/Sing MAP, 2.5 ug (FA)
B7 = Placebo MAP (FA); B8 = A/Sing MAP, 15 ug (UA); B9 = A/Sing IM, 15 ug, as 2018 Afluria Quad (UA)

Listing 16.2.9.2
Treatment Site Tolerability Assessment per Site

Part B
Randomisation Number = B0122, Treatment Group = Group B5

Date	Time	Visit	Time Point	Site Number	Treatment for Site	Indura-tion	Redness	Bruising	Itching	Skin Flaking	Tender-ness	Definition/ Visibility	Other#	Unscheduled Reason
16-JUN-2018	12:23	Day 1	Pre-dose	F1	uncoated	0	0	0	0	0	0	0		
16-JUN-2018	12:23	Day 1	Pre-dose	F2	5 ug	0	0	0	0	0	0	0		
16-JUN-2018	12:23	Day 1	Pre-dose	F3	uncoated	0	0	0	0	0	0	0		
16-JUN-2018	13:58	Day 1	10 min	F1	uncoated	0	1	0	1	0	0	0		
16-JUN-2018	13:58	Day 1	10 min	F2	5 ug	0	1	0	1	0	0	0		
16-JUN-2018	13:58	Day 1	10 min	F3	uncoated	0	1	0	1	0	0	0		
16-JUN-2018	14:48	Day 1	1 hour	F1	uncoated	0	1	0	0	0	0	0		
16-JUN-2018	14:48	Day 1	1 hour	F2	5 ug	1	1	0	0	0	0	0		
16-JUN-2018	14:48	Day 1	1 hour	F3	uncoated	0	1	0	0	0	0	0		
16-JUN-2018	15:48	Day 1	2 hour	F1	uncoated	0	1	0	0	0	0	0		
16-JUN-2018	15:48	Day 1	2 hour	F2	5 ug	0	1	0	1	0	0	0		
16-JUN-2018	15:48	Day 1	2 hour	F3	uncoated	0	1	0	0	0	0	0		
17-JUN-2018	09:11	Day 2		F1	uncoated	0	0	0	0	0	0	0		
17-JUN-2018	09:11	Day 2		F2	5 ug	1	1	0	1	0	0	0		
17-JUN-2018	09:11	Day 2		F3	uncoated	0	0	0	0	0	0	0		
19-JUN-2018	12:12	Day 4		F1	uncoated	0	0	0	0	0	0	2		
19-JUN-2018	12:12	Day 4		F2	5 ug	2	1	0	2	0	0	0		
19-JUN-2018	12:12	Day 4		F3	uncoated	0	0	0	0	0	0	0		
23-JUN-2018	09:44	Day 8		F1	uncoated	0	0	0	0	0	0	2		
23-JUN-2018	09:44	Day 8		F2	5 ug	0	1	0	0	2	0	0		

Key: 0= None; 1=Mild; 2=Moderate; 3=Severe; # Other = Pinprick bleeding or seepage

Administration Site Key: FA = Forearm; UA= Upper arm
Treatments Part A: A1 = A/Sing MAP, 15 ug (FA); A2 = Afluria Quad IM (UA), 15 ug; A3 = Placebo MAP (FA); A4 = A/Sing Mono IM, 15 ug (UA)
Treatments Part B: B1 = A/Sing MAP, 15 ug (FA) with biopsy; B2 = Placebo MAP (FA) with biopsy
B3 = A/Sing MAP, 15 ug (FA); B4 = A/Sing MAP, 10 ug (FA); B5 = A/Sing MAP, 5 ug (FA); B6 = A/Sing MAP, 2.5 ug (FA)
B7 = Placebo MAP (FA); B8 = A/Sing MAP, 15 ug (UA); B9 = A/Sing IM, 15 ug, as 2018 Afluria Quad (UA)

Listing 16.2.9.2
Treatment Site Tolerability Assessment per Site

Part B
Randomisation Number = B0122, Treatment Group = Group B5

Date	Time	Visit	Time Point	Site Number	Treatment for Site	Indura-tion	Redness	Bruising	Itching	Skin Flaking	Tender-ness	Definition/ Visibility	Other#	Unscheduled Reason
23-JUN-2018	09:44	Day 8		F3	uncoated	0	0	0	0	0	0	2		
07-JUL-2018	09:00	Day 22		F1	uncoated	0	0	0	0	0	0	1		
07-JUL-2018	09:00	Day 22		F2	5 ug	1	0	0	0	1	0	0		
07-JUL-2018	09:00	Day 22		F3	uncoated	0	0	0	0	0	0	1		
15-AUG-2018	14:07	EOS/ET (Day 61)		F1	uncoated	0	0	0	0	0	0	0		
15-AUG-2018	14:07	EOS/ET (Day 61)		F2	5 ug	0	0	0	0	0	0	1		
15-AUG-2018	14:07	EOS/ET (Day 61)		F3	uncoated	0	0	0	0	0	0	0		

Key: 0= None; 1=Mild; 2=Moderate; 3=Severe; # Other = Pinprick bleeding or seepage

Administration Site Key: FA = Forearm; UA= Upper arm
Treatments Part A: A1 = A/Sing MAP, 15 ug (FA); A2 = Afluria Quad IM (UA), 15 ug; A3 = Placebo MAP (FA); A4 = A/Sing Mono IM, 15 ug (UA)
Treatments Part B: B1 = A/Sing MAP, 15 ug (FA) with biopsy; B2 = Placebo MAP (FA) with biopsy
B3 = A/Sing MAP, 15 ug (FA); B4 = A/Sing MAP, 10 ug (FA); B5 = A/Sing MAP, 5 ug (FA); B6 = A/Sing MAP, 2.5 ug (FA)
B7 = Placebo MAP (FA); B8 = A/Sing MAP, 15 ug (UA); B9 = A/Sing IM, 15 ug, as 2018 Afluria Quad (UA)

Listing 16.2.9.2
Treatment Site Tolerability Assessment per Site

Part B
Randomisation Number = B0123, Treatment Group = Group B6

Date	Time	Visit	Time Point	Site Number	Treatment for Site	Indura-tion	Redness	Bruising	Itching	Skin Flaking	Tender-ness	Definition/ Visibility	Other#	Unscheduled Reason
16-JUN-2018	12:27	Day 1	Pre-dose	F1	2.5 ug	0	0	0	0	0	0	0		
16-JUN-2018	12:27	Day 1	Pre-dose	F2	uncoated	0	0	0	0	0	0	0		
16-JUN-2018	12:27	Day 1	Pre-dose	F3	uncoated	0	0	0	0	0	0	0		
16-JUN-2018	14:08	Day 1	10 min	F1	2.5 ug	0	1	0	0	0	1	0		
16-JUN-2018	14:08	Day 1	10 min	F2	uncoated	0	0	0	0	0	1	0		
16-JUN-2018	14:08	Day 1	10 min	F3	uncoated	0	0	0	0	0	0	0		
16-JUN-2018	14:58	Day 1	1 hour	F1	2.5 ug	0	0	0	0	0	1	0		
16-JUN-2018	14:58	Day 1	1 hour	F2	uncoated	0	0	0	0	0	1	0		
16-JUN-2018	14:58	Day 1	1 hour	F3	uncoated	1	0	0	0	0	0	0		
16-JUN-2018	15:58	Day 1	2 hour	F1	2.5 ug	0	1	0	0	0	0	0		
16-JUN-2018	15:58	Day 1	2 hour	F2	uncoated	0	1	0	0	0	0	0		
16-JUN-2018	15:58	Day 1	2 hour	F3	uncoated	0	1	0	0	0	0	0		
17-JUN-2018	10:39	Day 2		F1	2.5 ug	1	0	0	0	0	0	0		
17-JUN-2018	10:39	Day 2		F2	uncoated	0	0	0	0	0	0	0		
17-JUN-2018	10:39	Day 2		F3	uncoated	0	0	0	0	0	0	0		
19-JUN-2018	12:09	Day 4		F1	2.5 ug	0	0	0	0	0	0	0		
19-JUN-2018	12:09	Day 4		F2	uncoated	0	0	0	0	0	0	2		
19-JUN-2018	12:09	Day 4		F3	uncoated	0	0	0	0	0	0	2		
23-JUN-2018	12:06	Day 8		F1	2.5 ug	0	0	0	0	0	0	2		
23-JUN-2018	12:06	Day 8		F2	uncoated	0	0	0	0	0	0	2		

Key: 0= None; 1=Mild; 2=Moderate; 3=Severe; # Other = Pinprick bleeding or seepage

Administration Site Key: FA = Forearm; UA= Upper arm
Treatments Part A: A1 = A/Sing MAP, 15 ug (FA); A2 = Afluria Quad IM (UA), 15 ug; A3 = Placebo MAP (FA); A4 = A/Sing Mono IM, 15 ug (UA)
Treatments Part B: B1 = A/Sing MAP, 15 ug (FA) with biopsy; B2 = Placebo MAP (FA) with biopsy
B3 = A/Sing MAP, 15 ug (FA); B4 = A/Sing MAP, 10 ug (FA); B5 = A/Sing MAP, 5 ug (FA); B6 = A/Sing MAP, 2.5 ug (FA)
B7 = Placebo MAP (FA); B8 = A/Sing MAP, 15 ug (UA); B9 = A/Sing IM, 15 ug, as 2018 Afluria Quad (UA)

Listing 16.2.9.2
Treatment Site Tolerability Assessment per Site

Part B
Randomisation Number = B0123, Treatment Group = Group B6

Date	Time	Visit	Time Point	Site Number	Treatment for Site	Indura-tion	Redness	Bruising	Itching	Skin Flaking	Tender-ness	Definition/ Visibility	Other#	Unscheduled Reason
23-JUN-2018	12:06	Day 8		F3	uncoated	0	0	0	0	0	0	2		
06-JUL-2018	11:07	Day 22		F1	2.5 ug	0	0	0	0	0	0	0		
06-JUL-2018	11:07	Day 22		F2	uncoated	0	0	0	0	0	0	1		
06-JUL-2018	11:07	Day 22		F3	uncoated	0	0	0	0	0	0	1		
15-AUG-2018	11:49	EOS/ET (Day 61)		F1	2.5 ug	0	0	0	0	0	0	0		
15-AUG-2018	11:49	EOS/ET (Day 61)		F2	uncoated	0	0	0	0	0	0	0		
15-AUG-2018	11:49	EOS/ET (Day 61)		F3	uncoated	0	0	0	0	0	0	0		

Key: 0= None; 1=Mild; 2=Moderate; 3=Severe; # Other = Pinprick bleeding or seepage

Administration Site Key: FA = Forearm; UA= Upper arm
Treatments Part A: A1 = A/Sing MAP, 15 ug (FA); A2 = Afluria Quad IM (UA), 15 ug; A3 = Placebo MAP (FA); A4 = A/Sing Mono IM, 15 ug (UA)
Treatments Part B: B1 = A/Sing MAP, 15 ug (FA) with biopsy; B2 = Placebo MAP (FA) with biopsy
B3 = A/Sing MAP, 15 ug (FA); B4 = A/Sing MAP, 10 ug (FA); B5 = A/Sing MAP, 5 ug (FA); B6 = A/Sing MAP, 2.5 ug (FA)
B7 = Placebo MAP (FA); B8 = A/Sing MAP, 15 ug (UA); B9 = A/Sing IM, 15 ug, as 2018 Afluria Quad (UA)

Listing 16.2.9.2
Treatment Site Tolerability Assessment per Site

Part B
Randomisation Number = B0124, Treatment Group = Group B4

Date	Time	Visit	Time Point	Site Number	Treatment for Site	Indura-tion	Redness	Bruising	Itching	Skin Flaking	Tender-ness	Definition/ Visibility	Other#	Unscheduled Reason
16-JUN-2018	12:33	Day 1	Pre-dose	F1	uncoated	0	0	0	0	0	0	0		
16-JUN-2018	12:33	Day 1	Pre-dose	F2	5 ug	0	0	0	0	0	0	0		
16-JUN-2018	12:33	Day 1	Pre-dose	F3	5 ug	0	0	0	0	0	0	0		
16-JUN-2018	14:18	Day 1	10 min	F1	uncoated	0	1	0	0	0	0	0		
16-JUN-2018	14:18	Day 1	10 min	F2	5 ug	0	2	0	0	0	0	0		
16-JUN-2018	14:18	Day 1	10 min	F3	5 ug	0	1	0	0	0	0	0		
16-JUN-2018	15:08	Day 1	1 hour	F1	uncoated	0	1	0	0	0	0	0		
16-JUN-2018	15:08	Day 1	1 hour	F2	5 ug	1	1	0	0	0	0	0		
16-JUN-2018	15:08	Day 1	1 hour	F3	5 ug	1	1	0	0	0	1	0		
16-JUN-2018	16:08	Day 1	2 hour	F1	uncoated	0	1	0	0	0	0	0		
16-JUN-2018	16:08	Day 1	2 hour	F2	5 ug	0	1	0	0	0	1	0		
16-JUN-2018	16:08	Day 1	2 hour	F3	5 ug	0	1	0	0	0	1	0		
16-JUN-2018	14:19	Unscheduled		F2	5 ug		2						NA	MO notifies as repeat
16-JUN-2018	15:08	Unscheduled		F2	5 ug	2							NA	MO notifies as repeat
16-JUN-2018	15:08	Unscheduled		F3	5 ug	2							NA	MO notifies as repeat
17-JUN-2018	11:47	Day 2		F1	uncoated	0	0	0	0	0	0	0		
17-JUN-2018	11:47	Day 2		F2	5 ug	2	1	0	0	0	0	0		

Key: 0= None; 1=Mild; 2=Moderate; 3=Severe; # Other = Pinprick bleeding or seepage

Administration Site Key: FA = Forearm; UA= Upper arm
Treatments Part A: A1 = A/Sing MAP, 15 ug (FA); A2 = Afluria Quad IM (UA), 15 ug; A3 = Placebo MAP (FA); A4 = A/Sing Mono IM, 15 ug (UA)
Treatments Part B: B1 = A/Sing MAP, 15 ug (FA) with biopsy; B2 = Placebo MAP (FA) with biopsy
B3 = A/Sing MAP, 15 ug (FA); B4 = A/Sing MAP, 10 ug (FA); B5 = A/Sing MAP, 5 ug (FA); B6 = A/Sing MAP, 2.5 ug (FA)
B7 = Placebo MAP (FA); B8 = A/Sing MAP, 15 ug (UA); B9 = A/Sing IM, 15 ug, as 2018 Afluria Quad (UA)

Listing 16.2.9.2
Treatment Site Tolerability Assessment per Site

Part B
Randomisation Number = B0124, Treatment Group = Group B4

Date	Time	Visit	Time Point	Site Number	Treatment for Site	Indura-tion	Redness	Bruising	Itching	Skin Flaking	Tender-ness	Definition/ Visibility	Other#	Unscheduled Reason
17-JUN-2018	11:47	Day 2		F3	5 ug	2	1	0	0	0	0	0		
19-JUN-2018	14:46	Day 4		F1	uncoated	0	0	0	0	0	0	1		
19-JUN-2018	14:46	Day 4		F2	5 ug	1	0	0	1	0	0	0		
19-JUN-2018	14:46	Day 4		F3	5 ug	2	1	0	1	0	0	0		
23-JUN-2018	13:42	Day 8		F1	uncoated	0	0	0	0	0	0	1		
23-JUN-2018	13:42	Day 8		F2	5 ug	0	1	0	0	1	0	2		
23-JUN-2018	13:42	Day 8		F3	5 ug	2	0	0	0	0	0	0		
07-JUL-2018	13:08	Day 22		F1	uncoated	0	0	0	0	0	0	0		
07-JUL-2018	13:08	Day 22		F2	5 ug	0	0	0	0	0	0	1		
07-JUL-2018	13:08	Day 22		F3	5 ug	0	0	0	0	0	0	1		
15-AUG-2018	15:00	EOS/ET (Day 61)		F1	uncoated	0	0	0	0	0	0	0		
15-AUG-2018	15:00	EOS/ET (Day 61)		F2	5 ug	0	0	0	0	0	0	1		
15-AUG-2018	15:00	EOS/ET (Day 61)		F3	5 ug	0	0	0	0	0	0	1		

Key: 0= None; 1=Mild; 2=Moderate; 3=Severe; # Other = Pinprick bleeding or seepage

Administration Site Key: FA = Forearm; UA= Upper arm
Treatments Part A: A1 = A/Sing MAP, 15 ug (FA); A2 = Afluria Quad IM (UA), 15 ug; A3 = Placebo MAP (FA); A4 = A/Sing Mono IM, 15 ug (UA)
Treatments Part B: B1 = A/Sing MAP, 15 ug (FA) with biopsy; B2 = Placebo MAP (FA) with biopsy
B3 = A/Sing MAP, 15 ug (FA); B4 = A/Sing MAP, 10 ug (FA); B5 = A/Sing MAP, 5 ug (FA); B6 = A/Sing MAP, 2.5 ug (FA)
B7 = Placebo MAP (FA); B8 = A/Sing MAP, 15 ug (UA); B9 = A/Sing IM, 15 ug, as 2018 Afluria Quad (UA)

Listing 16.2.9.2
Treatment Site Tolerability Assessment per Site

Part B
Randomisation Number = B0125,   Treatment Group = Group B7

Date	Time	Visit	Time Point	Site Number	Treatment for Site	Indura-tion	Redness	Bruising	Itching	Skin Flaking	Tender-ness	Definition/ Visibility	Other#	Unscheduled Reason
16-JUN-2018	12:37	Day 1	Pre-dose	F1	uncoated	0	0	0	0	0	0	0		
16-JUN-2018	12:37	Day 1	Pre-dose	F2	uncoated	0	0	0	0	0	0	0		
16-JUN-2018	12:37	Day 1	Pre-dose	F3	uncoated	0	0	0	0	0	0	0		
16-JUN-2018	14:28	Day 1	10 min	F1	uncoated	0	0	0	0	0	0	0		
16-JUN-2018	14:28	Day 1	10 min	F2	uncoated	0	0	0	0	0	1	0		
16-JUN-2018	14:28	Day 1	10 min	F3	uncoated	0	1	0	0	0	1	0		
16-JUN-2018	15:18	Day 1	1 hour	F1	uncoated	0	1	0	0	0	0	0		
16-JUN-2018	15:18	Day 1	1 hour	F2	uncoated	0	0	0	0	0	0	0		
16-JUN-2018	15:18	Day 1	1 hour	F3	uncoated	0	0	0	0	0	0	0		
16-JUN-2018	16:18	Day 1	2 hour	F1	uncoated	0	0	0	0	0	0	0		
16-JUN-2018	16:18	Day 1	2 hour	F2	uncoated	0	0	0	0	0	0	0		
16-JUN-2018	16:18	Day 1	2 hour	F3	uncoated	0	0	0	0	0	0	0		
17-JUN-2018	13:04	Day 2		F1	uncoated	0	0	0	0	0	0	0		
17-JUN-2018	13:04	Day 2		F2	uncoated	0	0	0	0	0	0	0		
17-JUN-2018	13:04	Day 2		F3	uncoated	0	0	0	0	0	0	0		
19-JUN-2018	15:19	Day 4		F1	uncoated	0	0	0	0	0	0	3		
19-JUN-2018	15:19	Day 4		F2	uncoated	0	0	0	0	0	0	3		
19-JUN-2018	15:19	Day 4		F3	uncoated	0	0	0	0	0	0	3		
22-JUN-2018	14:03	Day 8		F1	uncoated	0	0	0	0	0	0	2		
22-JUN-2018	14:03	Day 8		F2	uncoated	0	0	0	0	1	0	2		

Key: 0= None; 1=Mild; 2=Moderate; 3=Severe; # Other = Pinprick bleeding or seepage

Administration Site Key: FA = Forearm; UA= Upper arm
Treatments Part A: A1 = A/Sing MAP, 15 ug (FA); A2 = Afluria Quad IM (UA), 15 ug; A3 = Placebo MAP (FA); A4 = A/Sing Mono IM, 15 ug (UA)
Treatments Part B: B1 = A/Sing MAP, 15 ug (FA) with biopsy; B2 = Placebo MAP (FA) with biopsy
B3 = A/Sing MAP, 15 ug (FA); B4 = A/Sing MAP, 10 ug (FA); B5 = A/Sing MAP, 5 ug (FA); B6 = A/Sing MAP, 2.5 ug (FA)
B7 = Placebo MAP (FA); B8 = A/Sing MAP, 15 ug (UA); B9 = A/Sing IM, 15 ug, as 2018 Afluria Quad (UA)

Listing 16.2.9.2
Treatment Site Tolerability Assessment per Site

Part B
Randomisation Number = B0125, Treatment Group = Group B7

Date	Time	Visit	Time Point	Site Number	Treatment for Site	Indura-tion	Redness	Bruising	Itching	Skin Flaking	Tender-ness	Definition/ Visibility	Other#	Unscheduled Reason
22-JUN-2018	14:03	Day 8		F3	uncoated	0	0	0	0	1	0	2		
07-JUL-2018	13:52	Day 22		F1	uncoated	0	0	0	0	0	0	0		
07-JUL-2018	13:52	Day 22		F2	uncoated	0	0	0	0	0	0	0		
07-JUL-2018	13:52	Day 22		F3	uncoated	0	0	0	0	0	0	0		
15-AUG-2018	15:47	EOS/ET (Day 61)		F1	uncoated	0	0	0	0	0	0	0		
15-AUG-2018	15:47	EOS/ET (Day 61)		F2	uncoated	0	0	0	0	0	0	0		
15-AUG-2018	15:47	EOS/ET (Day 61)		F3	uncoated	0	0	0	0	0	0	0		

Key: 0= None; 1=Mild; 2=Moderate; 3=Severe; # Other = Pinprick bleeding or seepage

Administration Site Key: FA = Forearm; UA= Upper arm
Treatments Part A: A1 = A/Sing MAP, 15 ug (FA); A2 = Afluria Quad IM (UA), 15 ug; A3 = Placebo MAP (FA); A4 = A/Sing Mono IM, 15 ug (UA)
Treatments Part B: B1 = A/Sing MAP, 15 ug (FA) with biopsy; B2 = Placebo MAP (FA) with biopsy
B3 = A/Sing MAP, 15 ug (FA); B4 = A/Sing MAP, 10 ug (FA); B5 = A/Sing MAP, 5 ug (FA); B6 = A/Sing MAP, 2.5 ug (FA)
B7 = Placebo MAP (FA); B8 = A/Sing MAP, 15 ug (UA); B9 = A/Sing IM, 15 ug, as 2018 Afluria Quad (UA)

Listing 16.2.9.2
Treatment Site Tolerability Assessment per Site

Part B
Randomisation Number = B0126, Treatment Group = Group B3

Date	Time	Visit	Time Point	Site Number	Treatment for Site	Indura-tion	Redness	Bruising	Itching	Skin Flaking	Tender-ness	Definition/ Visibility	Other#	Unscheduled Reason
16-JUN-2018	12:50	Day 1	Pre-dose	F1	5 ug	0	0	0	0	0	0	0		
16-JUN-2018	12:50	Day 1	Pre-dose	F2	5 ug	0	0	0	0	0	0	0		
16-JUN-2018	12:50	Day 1	Pre-dose	F3	5 ug	0	0	0	0	0	0	0		
16-JUN-2018	14:38	Day 1	10 min	F1	5 ug	0	1	0	0	0	0	0		
16-JUN-2018	14:38	Day 1	10 min	F2	5 ug	0	1	0	0	0	0	0		
16-JUN-2018	14:38	Day 1	10 min	F3	5 ug	0	1	0	0	0	0	0		
16-JUN-2018	15:28	Day 1	1 hour	F1	5 ug	1	1	0	0	0	0	0		
16-JUN-2018	15:28	Day 1	1 hour	F2	5 ug	1	1	0	0	0	0	0		
16-JUN-2018	15:28	Day 1	1 hour	F3	5 ug	1	1	0	0	0	0	0		
16-JUN-2018	16:28	Day 1	2 hour	F1	5 ug	0	0	0	0	0	0	0		
16-JUN-2018	16:28	Day 1	2 hour	F2	5 ug	0	0	0	0	0	0	0		
16-JUN-2018	16:28	Day 1	2 hour	F3	5 ug	0	0	0	0	0	0	0		
17-JUN-2018	11:41	Day 2		F1	5 ug	1	0	0	0	0	0	0		
17-JUN-2018	11:41	Day 2		F2	5 ug	1	0	0	0	0	0	0		
17-JUN-2018	11:41	Day 2		F3	5 ug	1	0	0	0	0	0	0		
19-JUN-2018	15:35	Day 4		F1	5 ug	0	0	0	0	0	0	0		
19-JUN-2018	15:35	Day 4		F2	5 ug	0	0	0	0	0	0	0		
19-JUN-2018	15:35	Day 4		F3	5 ug	0	0	0	0	0	0	0		
23-JUN-2018	15:32	Day 8		F1	5 ug	1	0	0	0	0	0	3		
23-JUN-2018	15:32	Day 8		F2	5 ug	1	0	0	0	0	0	0		

Key: 0= None; 1=Mild; 2=Moderate; 3=Severe; # Other = Pinprick bleeding or seepage

Administration Site Key: FA = Forearm; UA= Upper arm
Treatments Part A: A1 = A/Sing MAP, 15 ug (FA); A2 = Afluria Quad IM (UA), 15 ug; A3 = Placebo MAP (FA); A4 = A/Sing Mono IM, 15 ug (UA)
Treatments Part B: B1 = A/Sing MAP, 15 ug (FA) with biopsy; B2 = Placebo MAP (FA) with biopsy
B3 = A/Sing MAP, 15 ug (FA); B4 = A/Sing MAP, 10 ug (FA); B5 = A/Sing MAP, 5 ug (FA); B6 = A/Sing MAP, 2.5 ug (FA)
B7 = Placebo MAP (FA); B8 = A/Sing MAP, 15 ug (UA); B9 = A/Sing IM, 15 ug, as 2018 Afluria Quad (UA)

Listing 16.2.9.2
Treatment Site Tolerability Assessment per Site

Part B
Randomisation Number = B0126, Treatment Group = Group B3

Date	Time	Visit	Time Point	Site Number	Treatment for Site	Indura-tion	Redness	Bruising	Itching	Skin Flaking	Tender-ness	Definition/ Visibility	Other#	Unscheduled Reason
23-JUN-2018	15:32	Day 8		F3	5 ug	1	0	0	0	0	0	3		
06-JUL-2018	14:34	Day 22		F1	5 ug	0	0	0	0	0	0	2		
06-JUL-2018	14:34	Day 22		F2	5 ug	0	0	0	0	1	0	2		
06-JUL-2018	14:34	Day 22		F3	5 ug	0	0	0	0	0	0	2		
15-AUG-2018	15:15	EOS/ET (Day 61)		F1	5 ug	0	0	0	0	0	0	2		
15-AUG-2018	15:15	EOS/ET (Day 61)		F2	5 ug	0	0	0	0	0	0	2		
15-AUG-2018	15:15	EOS/ET (Day 61)		F3	5 ug	0	0	0	0	0	0	2		

Key: 0= None; 1=Mild; 2=Moderate; 3=Severe; # Other = Pinprick bleeding or seepage

Administration Site Key: FA = Forearm; UA= Upper arm
Treatments Part A: A1 = A/Sing MAP, 15 ug (FA); A2 = Afluria Quad IM (UA), 15 ug; A3 = Placebo MAP (FA); A4 = A/Sing Mono IM, 15 ug (UA)
Treatments Part B: B1 = A/Sing MAP, 15 ug (FA) with biopsy; B2 = Placebo MAP (FA) with biopsy
B3 = A/Sing MAP, 15 ug (FA); B4 = A/Sing MAP, 10 ug (FA); B5 = A/Sing MAP, 5 ug (FA); B6 = A/Sing MAP, 2.5 ug (FA)
B7 = Placebo MAP (FA); B8 = A/Sing MAP, 15 ug (UA); B9 = A/Sing IM, 15 ug, as 2018 Afluria Quad (UA)

Listing 16.2.9.2
Treatment Site Tolerability Assessment per Site

Part B
Randomisation Number = B0127, Treatment Group = Group B9

Date	Time	Visit	Time Point	Site Number	Treatment for Site	Indura-tion	Redness	Bruising	Itching	Skin Flaking	Tender-ness	Definition/ Visibility	Other#	Unscheduled Reason
16-JUN-2018	10:36	Day 1	Pre-dose	D1	[IM]	0	0	0	0	0	0	5		
16-JUN-2018	11:30	Day 1	10 min	D1	[IM]	0	1	0	0	0	0	5		
16-JUN-2018	12:21	Day 1	1 hour	D1	[IM]	1	0	0	0	0	0	5		
16-JUN-2018	13:26	Day 1	2 hour	D1	[IM]	1	0	0	0	0	1	5		
17-JUN-2018	11:14	Day 2		D1	[IM]	0	0	0	0	0	1	5		
19-JUN-2018	15:36	Day 4		D1	[IM]	0	1	0	0	0	0	5		
23-JUN-2018	13:03	Day 8		D1	[IM]	0	0	0	0	0	0	5		
13-JUL-2018	14:20	Day 22		D1	[IM]	0	0	0	0	0	0	5		
20-AUG-2018	13:59	EOS/ET (Day 61)		D1	[IM]	0	0	0	0	0	0	5		

Key: 0= None; 1=Mild; 2=Moderate; 3=Severe; # Other = Pinprick bleeding or seepage

Administration Site Key: FA = Forearm; UA= Upper arm
Treatments Part A: A1 = A/Sing MAP, 15 ug (FA); A2 = Afluria Quad IM (UA), 15 ug; A3 = Placebo MAP (FA); A4 = A/Sing Mono IM, 15 ug (UA)
Treatments Part B: B1 = A/Sing MAP, 15 ug (FA) with biopsy; B2 = Placebo MAP (FA) with biopsy
B3 = A/Sing MAP, 15 ug (FA); B4 = A/Sing MAP, 10 ug (FA); B5 = A/Sing MAP, 5 ug (FA); B6 = A/Sing MAP, 2.5 ug (FA)
B7 = Placebo MAP (FA); B8 = A/Sing MAP, 15 ug (UA); B9 = A/Sing IM, 15 ug, as 2018 Afluria Quad (UA)

Listing 16.2.9.2
Treatment Site Tolerability Assessment per Site

Part B
Randomisation Number = B0128, Treatment Group = Group B5

Date	Time	Visit	Time Point	Site Number	Treatment for Site	Indura-tion	Redness	Bruising	Itching	Skin Flaking	Tender-ness	Definition/ Visibility	Other#	Unscheduled Reason
23-JUN-2018	10:47	Day 1	Pre-dose	F1	uncoated	0	0	0	0	0	0	0		
23-JUN-2018	10:47	Day 1	Pre-dose	F2	5 ug	0	0	0	0	0	0	0		
23-JUN-2018	10:47	Day 1	Pre-dose	F3	uncoated	0	0	0	0	0	0	0		
23-JUN-2018	11:48	Day 1	10 min	F1	uncoated	0	0	0	0	0	0	0		
23-JUN-2018	11:48	Day 1	10 min	F2	5 ug	0	0	0	0	0	0	0		
23-JUN-2018	11:48	Day 1	10 min	F3	uncoated	0	0	0	0	0	0	0		
23-JUN-2018	12:38	Day 1	1 hour	F1	uncoated	0	1	0	0	0	0	0		
23-JUN-2018	12:38	Day 1	1 hour	F2	5 ug	1	1	0	1	0	0	0		
23-JUN-2018	12:38	Day 1	1 hour	F3	uncoated	1	1	0	1	0	0	0		
23-JUN-2018	13:38	Day 1	2 hour	F1	uncoated	0	0	0	0	0	1	0		
23-JUN-2018	13:38	Day 1	2 hour	F2	5 ug	0	0	0	0	0	1	0		
23-JUN-2018	13:38	Day 1	2 hour	F3	uncoated	0	0	0	0	0	1	0		
24-JUN-2018	11:58	Day 2		F1	uncoated	0	0	0	0	0	0	0		
24-JUN-2018	11:58	Day 2		F2	5 ug	0	0	0	0	0	0	0		
24-JUN-2018	11:58	Day 2		F3	uncoated	0	0	0	0	0	0	0		
26-JUN-2018	11:59	Day 4		F1	uncoated	0	0	0	0	0	0	0		
26-JUN-2018	11:59	Day 4		F2	5 ug	1	0	0	1	0	0	0		
26-JUN-2018	11:59	Day 4		F3	uncoated	0	0	0	0	0	0	0		
26-JUN-2018	12:08	Unscheduled		F2	5 ug	2	0	0	1	0	0	0	NA	MO notifies as repeat

Key: 0= None; 1=Mild; 2=Moderate; 3=Severe; # Other = Pinprick bleeding or seepage

Administration Site Key: FA = Forearm; UA= Upper arm
Treatments Part A: A1 = A/Sing MAP, 15 ug (FA); A2 = Afluria Quad IM (UA), 15 ug; A3 = Placebo MAP (FA); A4 = A/Sing Mono IM, 15 ug (UA)
Treatments Part B: B1 = A/Sing MAP, 15 ug (FA) with biopsy; B2 = Placebo MAP (FA) with biopsy
B3 = A/Sing MAP, 15 ug (FA); B4 = A/Sing MAP, 10 ug (FA); B5 = A/Sing MAP, 5 ug (FA); B6 = A/Sing MAP, 2.5 ug (FA)
B7 = Placebo MAP (FA); B8 = A/Sing MAP, 15 ug (UA); B9 = A/Sing IM, 15 ug, as 2018 Afluria Quad (UA)

Listing 16.2.9.2
Treatment Site Tolerability Assessment per Site

Part B
Randomisation Number = B0128, Treatment Group = Group B5

Date	Time	Visit	Time Point	Site Number	Treatment for Site	Indura-tion	Redness	Bruising	Itching	Skin Flaking	Tender-ness	Definition/ Visibility	Other#	Unscheduled Reason
30-JUN-2018	12:09	Day 8		F1	uncoated	0	0	0	0	0	0	2		
30-JUN-2018	12:09	Day 8		F2	5 ug	0	0	0	0	2	0	3		
30-JUN-2018	12:09	Day 8		F3	uncoated	0	0	0	0	0	0	2		
14-JUL-2018	11:39	Day 22		F1	uncoated	0	0	0	0	0	0	0		
14-JUL-2018	11:39	Day 22		F2	5 ug	0	0	0	0	0	0	1		
14-JUL-2018	11:39	Day 22		F3	uncoated	0	0	0	0	0	0	0		
22-AUG-2018	11:14	EOS/ET (Day 61)		F1	uncoated	0	0	0	0	0	0	0		
22-AUG-2018	11:14	EOS/ET (Day 61)		F2	5 ug	0	0	0	0	0	0	1		
22-AUG-2018	11:14	EOS/ET (Day 61)		F3	uncoated	0	0	0	0	0	0	0		

Key: 0= None; 1=Mild; 2=Moderate; 3=Severe; # Other = Pinprick bleeding or seepage

Administration Site Key: FA = Forearm; UA= Upper arm
Treatments Part A: A1 = A/Sing MAP, 15 ug (FA); A2 = Afluria Quad IM (UA), 15 ug; A3 = Placebo MAP (FA); A4 = A/Sing Mono IM, 15 ug (UA)
Treatments Part B: B1 = A/Sing MAP, 15 ug (FA) with biopsy; B2 = Placebo MAP (FA) with biopsy
B3 = A/Sing MAP, 15 ug (FA); B4 = A/Sing MAP, 10 ug (FA); B5 = A/Sing MAP, 5 ug (FA); B6 = A/Sing MAP, 2.5 ug (FA)
B7 = Placebo MAP (FA); B8 = A/Sing MAP, 15 ug (UA); B9 = A/Sing IM, 15 ug, as 2018 Afluria Quad (UA)

Listing 16.2.9.2
Treatment Site Tolerability Assessment per Site

Part B
Randomisation Number = B0129, Treatment Group = Group B8

Date	Time	Visit	Time Point	Site Number	Treatment for Site	Indura- tion	Redness	Bruising	Itching	Skin Flaking	Tender- ness	Definition/ Visibility	Other#	Unscheduled Reason
23-JUN-2018	11:47	Day 1	Pre-dose	D1	5 ug	0	0	0	0	0	0	0		
23-JUN-2018	11:47	Day 1	Pre-dose	D2	5 ug	0	0	0	0	0	0	0		
23-JUN-2018	11:47	Day 1	Pre-dose	D3	5 ug	0	0	0	0	0	0	0		
23-JUN-2018	12:08	Day 1	10 min	D1	5 ug	0	1	0	0	0	0	0		
23-JUN-2018	12:08	Day 1	10 min	D2	5 ug	0	1	0	0	0	0	0		
23-JUN-2018	12:08	Day 1	10 min	D3	5 ug	0	1	0	0	0	0	0		
23-JUN-2018	12:58	Day 1	1 hour	D1	5 ug	1	1	0	0	0	0	0		
23-JUN-2018	12:58	Day 1	1 hour	D2	5 ug	1	1	0	0	0	0	0		
23-JUN-2018	12:58	Day 1	1 hour	D3	5 ug	1	1	0	0	0	0	0		
23-JUN-2018	13:58	Day 1	2 hour	D1	5 ug	0	0	0	0	0	0	0		
23-JUN-2018	13:58	Day 1	2 hour	D2	5 ug	0	0	0	0	0	0	0		
23-JUN-2018	13:58	Day 1	2 hour	D3	5 ug	0	0	0	0	0	0	0		
24-JUN-2018	12:11	Day 2		D1	5 ug	1	1	0	1	0	0	0		
24-JUN-2018	12:11	Day 2		D2	5 ug	1	1	0	1	0	0	0		
24-JUN-2018	12:11	Day 2		D3	5 ug	1	1	0	1	0	0	0		
26-JUN-2018	14:11	Day 4		D1	5 ug	0	2	0	0	1		0		
26-JUN-2018	14:11	Day 4		D2	5 ug	0	2	0	1	1	0	0		
26-JUN-2018	14:11	Day 4		D3	5 ug	0	2	0	1	1	0	0		
30-JUN-2018	15:06	Day 8		D1	5 ug	0	1	0	0	1	0	0		
30-JUN-2018	15:06	Day 8		D2	5 ug	0	1	0	1	1	0	0		

Key: 0= None; 1=Mild; 2=Moderate; 3=Severe; # Other = Pinprick bleeding or seepage

Administration Site Key: FA = Forearm; UA= Upper arm
Treatments Part A: A1 = A/Sing MAP, 15 ug (FA); A2 = Afluria Quad IM (UA), 15 ug; A3 = Placebo MAP (FA); A4 = A/Sing Mono IM, 15 ug (UA)
Treatments Part B: B1 = A/Sing MAP, 15 ug (FA) with biopsy; B2 = Placebo MAP (FA) with biopsy
B3 = A/Sing MAP, 15 ug (FA); B4 = A/Sing MAP, 10 ug (FA); B5 = A/Sing MAP, 5 ug (FA); B6 = A/Sing MAP, 2.5 ug (FA)
B7 = Placebo MAP (FA); B8 = A/Sing MAP, 15 ug (UA); B9 = A/Sing IM, 15 ug, as 2018 Afluria Quad (UA)

Listing 16.2.9.2
Treatment Site Tolerability Assessment per Site

Part B
Randomisation Number = B0129, Treatment Group = Group B8

Date	Time	Visit	Time Point	Site Number	Treatment for Site	Indura-tion	Redness	Bruising	Itching	Skin Flaking	Tender-ness	Definition/ Visibility	Other#	Unscheduled Reason
30-JUN-2018	15:06	Day 8		D3	5 ug	0	1	0	1	0	0	0		
14-JUL-2018	14:31	Day 22		D1	5 ug	0	0	0	0	1	0	2		
14-JUL-2018	14:31	Day 22		D2	5 ug	0	0	0	0	1	0	2		
14-JUL-2018	14:31	Day 22		D3	5 ug	0	0	0	0	0	0	2		
22-AUG-2018	11:11	EOS/ET (Day 61)		D1	5 ug	0	0	0	0	0	0	1		
22-AUG-2018	11:11	EOS/ET (Day 61)		D2	5 ug	0	0	0	0	0	0	1		
22-AUG-2018	11:11	EOS/ET (Day 61)		D3	5 ug	0	0	0	0	0	0	1		

Key: 0= None; 1=Mild; 2=Moderate; 3=Severe; # Other = Pinprick bleeding or seepage

Administration Site Key: FA = Forearm; UA= Upper arm
Treatments Part A: A1 = A/Sing MAP, 15 ug (FA); A2 = Afluria Quad IM (UA), 15 ug; A3 = Placebo MAP (FA); A4 = A/Sing Mono IM, 15 ug (UA)
Treatments Part B: B1 = A/Sing MAP, 15 ug (FA) with biopsy; B2 = Placebo MAP (FA) with biopsy
B3 = A/Sing MAP, 15 ug (FA); B4 = A/Sing MAP, 10 ug (FA); B5 = A/Sing MAP, 5 ug (FA); B6 = A/Sing MAP, 2.5 ug (FA)
B7 = Placebo MAP (FA); B8 = A/Sing MAP, 15 ug (UA); B9 = A/Sing IM, 15 ug, as 2018 Afluria Quad (UA)

Listing 16.2.9.2
Treatment Site Tolerability Assessment per Site

Part B
Randomisation Number = B0130, Treatment Group = Group B5

Date	Time	Visit	Time Point	Site Number	Treatment for Site	Indura-tion	Redness	Bruising	Itching	Skin Flaking	Tender-ness	Definition/ Visibility	Other#	Unscheduled Reason
23-JUN-2018	10:54	Day 1	Pre-dose	F1	5 ug	0	0	0	0	0	0	0		
23-JUN-2018	10:54	Day 1	Pre-dose	F2	uncoated	0	0	0	0	0	0	0		
23-JUN-2018	10:54	Day 1	Pre-dose	F3	uncoated	0	0	0	0	0	0	0		
23-JUN-2018	11:58	Day 1	10 min	F1	5 ug	0	1	0	0	0	1	0		
23-JUN-2018	11:58	Day 1	10 min	F2	uncoated	0	1	0	0	0	1	0		
23-JUN-2018	11:58	Day 1	10 min	F3	uncoated	0	1	0	0	0	1	0		
23-JUN-2018	12:48	Day 1	1 hour	F1	5 ug	0	1	0	0	0	1	0		
23-JUN-2018	12:48	Day 1	1 hour	F2	uncoated	1	1	0	0	0	1	0		
23-JUN-2018	12:48	Day 1	1 hour	F3	uncoated	1	1	0	0	0	1	0		
23-JUN-2018	13:48	Day 1	2 hour	F1	5 ug	0	0	0	0	0	0	0		
23-JUN-2018	13:48	Day 1	2 hour	F2	uncoated	0	0	0	0	0	0	0		
23-JUN-2018	13:48	Day 1	2 hour	F3	uncoated	0	0	0	0	0	0	0		
24-JUN-2018	12:05	Day 2		F1	5 ug	0	0	0	0	0	1	0		
24-JUN-2018	12:05	Day 2		F2	uncoated	0	0	0	0	0	1	0		
24-JUN-2018	12:05	Day 2		F3	uncoated	0	0	0	0	0	1	0		
26-JUN-2018	10:13	Day 4		F1	5 ug	0	0	0	0	0	1	0		
26-JUN-2018	10:13	Day 4		F2	uncoated	0	0	0	0	0	0	3		
26-JUN-2018	10:13	Day 4		F3	uncoated	0	0	0	0	0	0	3		
30-JUN-2018	10:02	Day 8		F1	5 ug	0	0	0	0	2	0	0		
30-JUN-2018	10:02	Day 8		F2	uncoated	0	0	0	0	0	0	1		

Key: 0= None; 1=Mild; 2=Moderate; 3=Severe; # Other = Pinprick bleeding or seepage

Administration Site Key: FA = Forearm; UA= Upper arm
Treatments Part A: A1 = A/Sing MAP, 15 ug (FA); A2 = Afluria Quad IM (UA), 15 ug; A3 = Placebo MAP (FA); A4 = A/Sing Mono IM, 15 ug (UA)
Treatments Part B: B1 = A/Sing MAP, 15 ug (FA) with biopsy; B2 = Placebo MAP (FA) with biopsy
B3 = A/Sing MAP, 15 ug (FA); B4 = A/Sing MAP, 10 ug (FA); B5 = A/Sing MAP, 5 ug (FA); B6 = A/Sing MAP, 2.5 ug (FA)
B7 = Placebo MAP (FA); B8 = A/Sing MAP, 15 ug (UA); B9 = A/Sing IM, 15 ug, as 2018 Afluria Quad (UA)

Listing 16.2.9.2
Treatment Site Tolerability Assessment per Site

Part B
Randomisation Number = B0130, Treatment Group = Group B5

Date	Time	Visit	Time Point	Site Number	Treatment for Site	Indura-tion	Redness	Bruising	Itching	Skin Flaking	Tender-ness	Definition/ Visibility	Other#	Unscheduled Reason
30-JUN-2018	10:02	Day 8		F3	uncoated	0	0	0	0	0	0	1		
30-JUN-2018	10:12	Unscheduled		F1	5 ug	0	0	0	0	2	0	0	NA	MO notifies as repeat
14-JUL-2018	12:17	Day 22		F1	5 ug	0	0	0	0	0	0	2		
14-JUL-2018	12:17	Day 22		F2	uncoated	0	0	0	0	0	0	0		
14-JUL-2018	12:17	Day 22		F3	uncoated	0	0	0	0	0	0	1		
22-AUG-2018	10:20	EOS/ET (Day 61)		F1	5 ug	0	0	0	0	0	0	1		
22-AUG-2018	10:20	EOS/ET (Day 61)		F2	uncoated	0	0	0	0	0	0	0		
22-AUG-2018	10:20	EOS/ET (Day 61)		F3	uncoated	0	0	0	0	0	0	0		

Key: 0= None; 1=Mild; 2=Moderate; 3=Severe; # Other = Pinprick bleeding or seepage

Administration Site Key: FA = Forearm; UA= Upper arm
Treatments Part A: A1 = A/Sing MAP, 15 ug (FA); A2 = Afluria Quad IM (UA), 15 ug; A3 = Placebo MAP (FA); A4 = A/Sing Mono IM, 15 ug (UA)
Treatments Part B: B1 = A/Sing MAP, 15 ug (FA) with biopsy; B2 = Placebo MAP (FA) with biopsy
B3 = A/Sing MAP, 15 ug (FA); B4 = A/Sing MAP, 10 ug (FA); B5 = A/Sing MAP, 5 ug (FA); B6 = A/Sing MAP, 2.5 ug (FA)
B7 = Placebo MAP (FA); B8 = A/Sing MAP, 15 ug (UA); B9 = A/Sing IM, 15 ug, as 2018 Afluria Quad (UA)

Listing 16.2.9.2
Treatment Site Tolerability Assessment per Site

Part B
Randomisation Number = B0131,   Treatment Group = Group B8

Date	Time	Visit	Time Point	Site Number	Treatment for Site	Indura-tion	Redness	Bruising	Itching	Skin Flaking	Tender-ness	Definition/ Visibility	Other#	Unscheduled Reason
23-JUN-2018	11:06	Day 1	Pre-dose	D1	5 ug	0	0	0	0	0	0	0		
23-JUN-2018	11:06	Day 1	Pre-dose	D2	5 ug	0	0	0	0	0	0	0		
23-JUN-2018	11:06	Day 1	Pre-dose	D3	5 ug	0	0	0	0	0	0	0		
23-JUN-2018	12:18	Day 1	10 min	D1	5 ug	0	0	0	0	0	0	0		
23-JUN-2018	12:18	Day 1	10 min	D2	5 ug	0	0	0	0	0	1	0		
23-JUN-2018	12:18	Day 1	10 min	D3	5 ug	0	0	0	0	0	0	0		
23-JUN-2018	13:08	Day 1	1 hour	D1	5 ug	0	1	0	0	0	0	0		
23-JUN-2018	13:08	Day 1	1 hour	D2	5 ug	1	1	0	0	0	1	0		
23-JUN-2018	13:08	Day 1	1 hour	D3	5 ug	1	1	0	0	0	0	0		
23-JUN-2018	14:08	Day 1	2 hour	D1	5 ug	0	0	0	0	0	0	0		
23-JUN-2018	14:08	Day 1	2 hour	D2	5 ug	0	0	0	0	0	0	0		
23-JUN-2018	14:08	Day 1	2 hour	D3	5 ug	0	0	0	0	0	0	0		
24-JUN-2018	12:13	Day 2		D1	5 ug	1	1	0	0	0	1	0		
24-JUN-2018	12:13	Day 2		D2	5 ug	0	0	0	0	0	1	0		
24-JUN-2018	12:13	Day 2		D3	5 ug	0	0	0	0	0	1	0		
26-JUN-2018	13:36	Day 4		D1	5 ug	0	1	0	0	1	0	0		
26-JUN-2018	13:36	Day 4		D2	5 ug	0	1	0	1	1	2	0		
26-JUN-2018	13:36	Day 4		D3	5 ug	0	1	0	0	1	1	0		
30-JUN-2018	11:54	Day 8		D1	5 ug	0	0	0	0	0	0	0		
30-JUN-2018	11:54	Day 8		D2	5 ug	0	0	0	0	0	0	0		

Key: 0= None; 1=Mild; 2=Moderate; 3=Severe; # Other = Pinprick bleeding or seepage

Administration Site Key: FA = Forearm; UA= Upper arm
Treatments Part A: A1 = A/Sing MAP, 15 ug (FA); A2 = Afluria Quad IM (UA), 15 ug; A3 = Placebo MAP (FA); A4 = A/Sing Mono IM, 15 ug (UA)
Treatments Part B: B1 = A/Sing MAP, 15 ug (FA) with biopsy; B2 = Placebo MAP (FA) with biopsy
B3 = A/Sing MAP, 15 ug (FA); B4 = A/Sing MAP, 10 ug (FA); B5 = A/Sing MAP, 5 ug (FA); B6 = A/Sing MAP, 2.5 ug (FA)
B7 = Placebo MAP (FA); B8 = A/Sing MAP, 15 ug (UA); B9 = A/Sing IM, 15 ug, as 2018 Afluria Quad (UA)

Listing 16.2.9.2
Treatment Site Tolerability Assessment per Site

Part B
Randomisation Number = B0131, Treatment Group = Group B8

Date	Time	Visit	Time Point	Site Number	Treatment for Site	Indura-tion	Redness	Bruising	Itching	Skin Flaking	Tender-ness	Definition/ Visibility	Other#	Unscheduled Reason
30-JUN-2018	11:54	Day 8		D3	5 ug	0	0	0	0	1	0	0		
14-JUL-2018	11:51	Day 22		D1	5 ug	0	0	0	0	0	0	1		
14-JUL-2018	11:51	Day 22		D2	5 ug	0	0	0	0	0	0	1		
14-JUL-2018	11:51	Day 22		D3	5 ug	0	0	0	0	1	0	1		
22-AUG-2018	16:58	EOS/ET (Day 61)		D1	5 ug	0	0	0	0	0	0	0		
22-AUG-2018	16:58	EOS/ET (Day 61)		D2	5 ug	0	0	0	0	0	0	0		
22-AUG-2018	16:58	EOS/ET (Day 61)		D3	5 ug	0	0	0	0	0	0	0		

Key: 0= None; 1=Mild; 2=Moderate; 3=Severe; # Other = Pinprick bleeding or seepage

Administration Site Key: FA = Forearm; UA= Upper arm
Treatments Part A: A1 = A/Sing MAP, 15 ug (FA); A2 = Afluria Quad IM (UA), 15 ug; A3 = Placebo MAP (FA); A4 = A/Sing Mono IM, 15 ug (UA)
Treatments Part B: B1 = A/Sing MAP, 15 ug (FA) with biopsy; B2 = Placebo MAP (FA) with biopsy
B3 = A/Sing MAP, 15 ug (FA); B4 = A/Sing MAP, 10 ug (FA); B5 = A/Sing MAP, 5 ug (FA); B6 = A/Sing MAP, 2.5 ug (FA)
B7 = Placebo MAP (FA); B8 = A/Sing MAP, 15 ug (UA); B9 = A/Sing IM, 15 ug, as 2018 Afluria Quad (UA)

Listing 16.2.9.2
Treatment Site Tolerability Assessment per Site

Part B
Randomisation Number = B0132, Treatment Group = Group B6

Date	Time	Visit	Time Point	Site Number	Treatment for Site	Induration	Redness	Bruising	Itching	Skin Flaking	Tenderness	Definition/ Visibility	Other#	Unscheduled Reason
23-JUN-2018	11:13	Day 1	Pre-dose	F1	2.5 ug	0	0	0	0	0	0	0		
23-JUN-2018	11:13	Day 1	Pre-dose	F2	uncoated	0	0	0	0	0	0	0		
23-JUN-2018	11:13	Day 1	Pre-dose	F3	uncoated	0	0	0	0	0	0	0		
23-JUN-2018	12:28	Day 1	10 min	F1	2.5 ug	0	1	0	0	0	0	0		
23-JUN-2018	12:28	Day 1	10 min	F2	uncoated	0	1	0	0	0	0	0		
23-JUN-2018	12:28	Day 1	10 min	F3	uncoated	0	1	0	1	0	0	0		
23-JUN-2018	13:18	Day 1	1 hour	F1	2.5 ug	1	1	0	0	0	0	0		
23-JUN-2018	13:18	Day 1	1 hour	F2	uncoated	1	1	0	0	0	0	0		
23-JUN-2018	13:18	Day 1	1 hour	F3	uncoated	1	1	0	0	0	0	0		
23-JUN-2018	14:18	Day 1	2 hour	F1	2.5 ug	0	0	0	0	0	0	0		
23-JUN-2018	14:18	Day 1	2 hour	F2	uncoated	0	0	0	0	0	0	0		
23-JUN-2018	14:18	Day 1	2 hour	F3	uncoated	0	0	0	0	0	0	0		
24-JUN-2018	13:20	Day 2		F1	2.5 ug	0	0	0	0	0	0	0		
24-JUN-2018	13:20	Day 2		F2	uncoated	0	0	0	0	0	0	0		
24-JUN-2018	13:20	Day 2		F3	uncoated	0	0	0	0	0	0	0		
26-JUN-2018	18:11	Day 4		F1	2.5 ug	0	0	0	1	0	0	0		
26-JUN-2018	18:11	Day 4		F2	uncoated	0	0	0	0	0	0	1		
26-JUN-2018	18:11	Day 4		F3	uncoated	0	0	0	0	0	0	1		
30-JUN-2018	11:49	Day 8		F1	2.5 ug	0	0	0	0	1	0	3		
30-JUN-2018	11:49	Day 8		F2	uncoated	0	0	0	0	0	0	3		

Key: 0= None; 1=Mild; 2=Moderate; 3=Severe; # Other = Pinprick bleeding or seepage

Administration Site Key: FA = Forearm; UA= Upper arm
Treatments Part A: A1 = A/Sing MAP, 15 ug (FA); A2 = Afluria Quad IM (UA), 15 ug; A3 = Placebo MAP (FA); A4 = A/Sing Mono IM, 15 ug (UA)
Treatments Part B: B1 = A/Sing MAP, 15 ug (FA) with biopsy; B2 = Placebo MAP (FA) with biopsy
B3 = A/Sing MAP, 15 ug (FA); B4 = A/Sing MAP, 10 ug (FA); B5 = A/Sing MAP, 5 ug (FA); B6 = A/Sing MAP, 2.5 ug (FA)
B7 = Placebo MAP (FA); B8 = A/Sing MAP, 15 ug (UA); B9 = A/Sing IM, 15 ug, as 2018 Afluria Quad (UA)

Listing 16.2.9.2
Treatment Site Tolerability Assessment per Site

Part B
Randomisation Number = B0132, Treatment Group = Group B6

Date	Time	Visit	Time Point	Site Number	Treatment for Site	Indura-tion	Redness	Bruising	Itching	Skin Flaking	Tender-ness	Definition/ Visibility	Other#	Unscheduled Reason
30-JUN-2018	11:49	Day 8		F3	uncoated	0	0	0	0	0	0	3		
14-JUL-2018	10:35	Day 22		F1	2.5 ug	0	0	0	0	0	0	0		
14-JUL-2018	10:35	Day 22		F2	uncoated	0	0	0	0	0	0	0		
14-JUL-2018	10:35	Day 22		F3	uncoated	0	0	0	0	0	0	0		
23-AUG-2018	08:24	EOS/ET (Day 61)		F1	2.5 ug	0	0	0	0	0	0	0		
23-AUG-2018	08:24	EOS/ET (Day 61)		F2	uncoated	0	0	0	0	0	0	0		
23-AUG-2018	08:24	EOS/ET (Day 61)		F3	uncoated	0	0	0	0	0	0	0		

Key: 0= None; 1=Mild; 2=Moderate; 3=Severe; # Other = Pinprick bleeding or seepage

Administration Site Key: FA = Forearm; UA= Upper arm
Treatments Part A: A1 = A/Sing MAP, 15 ug (FA); A2 = Afluria Quad IM (UA), 15 ug; A3 = Placebo MAP (FA); A4 = A/Sing Mono IM, 15 ug (UA)
Treatments Part B: B1 = A/Sing MAP, 15 ug (FA) with biopsy; B2 = Placebo MAP (FA) with biopsy
B3 = A/Sing MAP, 15 ug (FA); B4 = A/Sing MAP, 10 ug (FA); B5 = A/Sing MAP, 5 ug (FA); B6 = A/Sing MAP, 2.5 ug (FA)
B7 = Placebo MAP (FA); B8 = A/Sing MAP, 15 ug (UA); B9 = A/Sing IM, 15 ug, as 2018 Afluria Quad (UA)

Listing 16.2.9.2
Treatment Site Tolerability Assessment per Site

Part B
Randomisation Number = B0133, Treatment Group = Group B4

Date	Time	Visit	Time Point	Site Number	Treatment for Site	Indura-tion	Redness	Bruising	Itching	Skin Flaking	Tender-ness	Definition/ Visibility	Other#	Unscheduled Reason
23-JUN-2018	11:20	Day 1	Pre-dose	F1	uncoated	0	0	0	0	0	0	0		
23-JUN-2018	11:20	Day 1	Pre-dose	F2	5 ug	0	0	0	0	0	0	0		
23-JUN-2018	11:20	Day 1	Pre-dose	F3	5 ug	0	0	0	0	0	0	0		
23-JUN-2018	12:38	Day 1	10 min	F1	uncoated	0	0	0	0	0	0	0		
23-JUN-2018	12:38	Day 1	10 min	F2	5 ug	0	0	0	0	0	0	0		
23-JUN-2018	12:38	Day 1	10 min	F3	5 ug	0	0	0	0	0	0	0		
23-JUN-2018	13:28	Day 1	1 hour	F1	uncoated	1	1	0	0	0	0	0		
23-JUN-2018	13:28	Day 1	1 hour	F2	5 ug	1	1	0	0	0	0	0		
23-JUN-2018	13:28	Day 1	1 hour	F3	5 ug	1	1	0	0	0	0	0		
23-JUN-2018	14:28	Day 1	2 hour	F1	uncoated	0	0	0	0	0	0	0		
23-JUN-2018	14:28	Day 1	2 hour	F2	5 ug	0	0	0	0	0	0	0		
23-JUN-2018	14:28	Day 1	2 hour	F3	5 ug	0	0	0	0	0	0	0		
24-JUN-2018	10:59	Day 2		F1	uncoated	0	0	0	0	0	0	0		
24-JUN-2018	10:59	Day 2		F2	5 ug	1	1	0	0	0	0	0		
24-JUN-2018	10:59	Day 2		F3	5 ug	0	1	0	0	0	0	0		
26-JUN-2018	17:30	Day 4		F1	uncoated	0	0	0	0	0	0	0		
26-JUN-2018	17:30	Day 4		F2	5 ug	0	0	0	1	1	0	0		
26-JUN-2018	17:30	Day 4		F3	5 ug	0	0	0	1	1	0	0		
30-JUN-2018	11:49	Day 8		F1	uncoated	0	0	0	0	1	0	2		
30-JUN-2018	11:49	Day 8		F2	5 ug	0	0	0	0	1	0	0		

Key: 0= None; 1=Mild; 2=Moderate; 3=Severe; # Other = Pinprick bleeding or seepage

Administration Site Key: FA = Forearm; UA= Upper arm
Treatments Part A: A1 = A/Sing MAP, 15 ug (FA); A2 = Afluria Quad IM (UA), 15 ug; A3 = Placebo MAP (FA); A4 = A/Sing Mono IM, 15 ug (UA)
Treatments Part B: B1 = A/Sing MAP, 15 ug (FA) with biopsy; B2 = Placebo MAP (FA) with biopsy
B3 = A/Sing MAP, 15 ug (FA); B4 = A/Sing MAP, 10 ug (FA); B5 = A/Sing MAP, 5 ug (FA); B6 = A/Sing MAP, 2.5 ug (FA)
B7 = Placebo MAP (FA); B8 = A/Sing MAP, 15 ug (UA); B9 = A/Sing IM, 15 ug, as 2018 Afluria Quad (UA)

Listing 16.2.9.2
Treatment Site Tolerability Assessment per Site

Part B
Randomisation Number = B0133, Treatment Group = Group B4

Date	Time	Visit	Time Point	Site Number	Treatment for Site	Indura-tion	Redness	Bruising	Itching	Skin Flaking	Tender-ness	Definition/ Visibility	Other#	Unscheduled Reason
30-JUN-2018	11:49	Day 8		F3	5 ug	0	0	0	0	1	0	0		
14-JUL-2018	12:50	Day 22		F1	uncoated	0	0	0	0	0	0	0		
14-JUL-2018	12:50	Day 22		F2	5 ug	0	0	0	0	0	0	0		
14-JUL-2018	12:50	Day 22		F3	5 ug	0	0	0	0	0	0	0		
22-AUG-2018	14:03	EOS/ET (Day 61)		F1	uncoated	0	0	0	0	0	0	0		
22-AUG-2018	14:03	EOS/ET (Day 61)		F2	5 ug	0	0	0	0	0	0	0		
22-AUG-2018	14:03	EOS/ET (Day 61)		F3	5 ug	0	0	0	0	0	0	0		

Key: 0= None; 1=Mild; 2=Moderate; 3=Severe; # Other = Pinprick bleeding or seepage

Administration Site Key: FA = Forearm; UA= Upper arm
Treatments Part A: A1 = A/Sing MAP, 15 ug (FA); A2 = Afluria Quad IM (UA), 15 ug; A3 = Placebo MAP (FA); A4 = A/Sing Mono IM, 15 ug (UA)
Treatments Part B: B1 = A/Sing MAP, 15 ug (FA) with biopsy; B2 = Placebo MAP (FA) with biopsy
B3 = A/Sing MAP, 15 ug (FA); B4 = A/Sing MAP, 10 ug (FA); B5 = A/Sing MAP, 5 ug (FA); B6 = A/Sing MAP, 2.5 ug (FA)
B7 = Placebo MAP (FA); B8 = A/Sing MAP, 15 ug (UA); B9 = A/Sing IM, 15 ug, as 2018 Afluria Quad (UA)

Listing 16.2.9.2
Treatment Site Tolerability Assessment per Site

Part B
Randomisation Number = B0134, Treatment Group = Group B3

Date	Time	Visit	Time Point	Site Number	Treatment for Site	Indura-tion	Redness	Bruising	Itching	Skin Flaking	Tender-ness	Definition/ Visibility	Other#	Unscheduled Reason
23-JUN-2018	11:23	Day 1	Pre-dose	F1	5 ug	0	0	0	0	0	0	0		
23-JUN-2018	11:23	Day 1	Pre-dose	F2	5 ug	0	0	0	0	0	0	0		
23-JUN-2018	11:23	Day 1	Pre-dose	F3	5 ug	0	0	0	0	0	0	0		
23-JUN-2018	12:48	Day 1	10 min	F1	5 ug	0	2	0	0	0	0	0		
23-JUN-2018	12:48	Day 1	10 min	F2	5 ug	0	1	0	0	0	0	0		
23-JUN-2018	12:48	Day 1	10 min	F3	5 ug	0	2	0	0	0	0	0		
23-JUN-2018	13:38	Day 1	1 hour	F1	5 ug	1	1	0	0	0	0	0		
23-JUN-2018	13:38	Day 1	1 hour	F2	5 ug	1	1	0	0	0	0	0		
23-JUN-2018	13:38	Day 1	1 hour	F3	5 ug	1	1	0	0	0	0	0		
23-JUN-2018	14:38	Day 1	2 hour	F1	5 ug	0	0	0	0	0	0	0		
23-JUN-2018	14:38	Day 1	2 hour	F2	5 ug	0	0	0	0	0	0	0		
23-JUN-2018	14:38	Day 1	2 hour	F3	5 ug	0	0	0	0	0	0	0		
23-JUN-2018	12:50	Unscheduled		F1	5 ug		2						NA	MO notifies as repeat
23-JUN-2018	12:50	Unscheduled		F3	5 ug		2						NA	MO notifies as repeat
24-JUN-2018	10:02	Day 2		F1	5 ug	1	1	0	0	0	0	0		
24-JUN-2018	10:02	Day 2		F2	5 ug	1	1	0	0	0	0	0		
24-JUN-2018	10:02	Day 2		F3	5 ug	1	1	0	0	0	0	0		
26-JUN-2018	11:23	Day 4		F1	5 ug	0	0	0	0	0	0	0		

Key: 0= None; 1=Mild; 2=Moderate; 3=Severe; # Other = Pinprick bleeding or seepage

Administration Site Key: FA = Forearm; UA= Upper arm
Treatments Part A: A1 = A/Sing MAP, 15 ug (FA); A2 = Afluria Quad IM (UA), 15 ug; A3 = Placebo MAP (FA); A4 = A/Sing Mono IM, 15 ug (UA)
Treatments Part B: B1 = A/Sing MAP, 15 ug (FA) with biopsy; B2 = Placebo MAP (FA) with biopsy
B3 = A/Sing MAP, 15 ug (FA); B4 = A/Sing MAP, 10 ug (FA); B5 = A/Sing MAP, 5 ug (FA); B6 = A/Sing MAP, 2.5 ug (FA)
B7 = Placebo MAP (FA); B8 = A/Sing MAP, 15 ug (UA); B9 = A/Sing IM, 15 ug, as 2018 Afluria Quad (UA)

Listing 16.2.9.2
Treatment Site Tolerability Assessment per Site

Part B
Randomisation Number = B0134, Treatment Group = Group B3

Date	Time	Visit	Time Point	Site Number	Treatment for Site	Indura-tion	Redness	Bruising	Itching	Skin Flaking	Tender-ness	Definition/ Visibility	Other#	Unscheduled Reason
26-JUN-2018	11:23	Day 4		F2	5 ug	0	0	0	0	0	0	0		
26-JUN-2018	11:23	Day 4		F3	5 ug	0	0	0	1	0	0	0		
30-JUN-2018	13:45	Day 8		F1	5 ug	0	0	0	0	1	0	3		
30-JUN-2018	13:45	Day 8		F2	5 ug	0	0	0	0	2	0	3		
30-JUN-2018	13:45	Day 8		F3	5 ug	0	0	0	0	2	0	3		
14-JUL-2018	11:46	Day 22		F1	5 ug	0	0	0	0	0	0	1		
14-JUL-2018	11:46	Day 22		F2	5 ug	0	0	0	0	0	0	1		
14-JUL-2018	11:46	Day 22		F3	5 ug	0	0	0	0	0	0	1		
23-AUG-2018	09:23	EOS/ET (Day 61)		F1	5 ug	0	0	0	0	0	0	0		
23-AUG-2018	09:23	EOS/ET (Day 61)		F2	5 ug	0	0	0	0	0	0	1		
23-AUG-2018	09:23	EOS/ET (Day 61)		F3	5 ug	0	0	0	0	0	0	1		

Key: 0= None; 1=Mild; 2=Moderate; 3=Severe; # Other = Pinprick bleeding or seepage

Administration Site Key: FA = Forearm; UA= Upper arm
Treatments Part A: A1 = A/Sing MAP, 15 ug (FA); A2 = Afluria Quad IM (UA), 15 ug; A3 = Placebo MAP (FA); A4 = A/Sing Mono IM, 15 ug (UA)
Treatments Part B: B1 = A/Sing MAP, 15 ug (FA) with biopsy; B2 = Placebo MAP (FA) with biopsy
B3 = A/Sing MAP, 15 ug (FA); B4 = A/Sing MAP, 10 ug (FA); B5 = A/Sing MAP, 5 ug (FA); B6 = A/Sing MAP, 2.5 ug (FA)
B7 = Placebo MAP (FA); B8 = A/Sing MAP, 15 ug (UA); B9 = A/Sing IM, 15 ug, as 2018 Afluria Quad (UA)

Listing 16.2.9.2
Treatment Site Tolerability Assessment per Site

Part B
Randomisation Number = B0135, Treatment Group = Group B7

Date	Time	Visit	Time Point	Site Number	Treatment for Site	Indura-tion	Redness	Bruising	Itching	Skin Flaking	Tender-ness	Definition/ Visibility	Other#	Unscheduled Reason
23-JUN-2018	11:30	Day 1	Pre-dose	F1	uncoated	0	0	0	0	0	0	0		
23-JUN-2018	11:30	Day 1	Pre-dose	F2	uncoated	0	0	0	0	0	0	0		
23-JUN-2018	11:30	Day 1	Pre-dose	F3	uncoated	0	0	0	0	0	0	0		
23-JUN-2018	12:58	Day 1	10 min	F1	uncoated	0	2	0	0	0	0	0		
23-JUN-2018	12:58	Day 1	10 min	F2	uncoated	0	2	0	1	0	0	0		
23-JUN-2018	12:58	Day 1	10 min	F3	uncoated	0	2	0	0	0	0	0		
23-JUN-2018	13:48	Day 1	1 hour	F1	uncoated	1	2	0	0	0	1	0		
23-JUN-2018	13:48	Day 1	1 hour	F2	uncoated	1	2	0	0	0	0	0		
23-JUN-2018	13:48	Day 1	1 hour	F3	uncoated	1	2	0	0	0	0	0		
23-JUN-2018	14:48	Day 1	2 hour	F1	uncoated	1	0		0	0	0	0		
23-JUN-2018	14:48	Day 1	2 hour	F2	uncoated	0	0	1	0	0	0	0		
23-JUN-2018	14:48	Day 1	2 hour	F3	uncoated		1	1	0	0	0	0		
23-JUN-2018	12:59	Unscheduled		F1	uncoated		2						NA	MO notifies as repeat
23-JUN-2018	12:59	Unscheduled		F2	uncoated		2						NA	MO notifies as repeat
23-JUN-2018	12:59	Unscheduled		F3	uncoated		2						NA	MO notifies as repeat
23-JUN-2018	13:50	Unscheduled		F1	uncoated		2						NA	MO notifies as repeat

Key: 0= None; 1=Mild; 2=Moderate; 3=Severe; # Other = Pinprick bleeding or seepage

Administration Site Key: FA = Forearm; UA= Upper arm
Treatments Part A: A1 = A/Sing MAP, 15 ug (FA); A2 = Afluria Quad IM (UA), 15 ug; A3 = Placebo MAP (FA); A4 = A/Sing Mono IM, 15 ug (UA)
Treatments Part B: B1 = A/Sing MAP, 15 ug (FA) with biopsy; B2 = Placebo MAP (FA) with biopsy
B3 = A/Sing MAP, 15 ug (FA); B4 = A/Sing MAP, 10 ug (FA); B5 = A/Sing MAP, 5 ug (FA); B6 = A/Sing MAP, 2.5 ug (FA)
B7 = Placebo MAP (FA); B8 = A/Sing MAP, 15 ug (UA); B9 = A/Sing IM, 15 ug, as 2018 Afluria Quad (UA)

Listing 16.2.9.2
Treatment Site Tolerability Assessment per Site

Part B
Randomisation Number = B0135, Treatment Group = Group B7

Date	Time	Visit	Time Point	Site Number	Treatment for Site	Indura-tion	Redness	Bruising	Itching	Skin Flaking	Tender-ness	Definition/ Visibility	Other#	Unscheduled Reason
23-JUN-2018	13:50	Unscheduled		F2	uncoated		2						NA	MO notifies as repeat
23-JUN-2018	13:50	Unscheduled		F3	uncoated		2						NA	MO notifies as repeat
24-JUN-2018	13:15	Day 2		F1	uncoated	0	0	0	0	0	0	0		
24-JUN-2018	13:15	Day 2		F2	uncoated	0	0	0	0	0	0	0		
24-JUN-2018	13:15	Day 2		F3	uncoated	0	0	0	0	0	0	0		
26-JUN-2018	13:40	Day 4		F1	uncoated	0	0	0	0	0	0	3		
26-JUN-2018	13:40	Day 4		F2	uncoated	0	0	0	0	0	0	3		
26-JUN-2018	13:40	Day 4		F3	uncoated	0	0	0	0	0	0	3		
30-JUN-2018	13:56	Day 8		F1	uncoated	0	0	0	0	1	0	3		
30-JUN-2018	13:56	Day 8		F2	uncoated	0	0	0	0	1	0	3		
30-JUN-2018	13:56	Day 8		F3	uncoated	0	0	0	0	1	0	3		
14-JUL-2018	14:20	Day 22		F1	uncoated	0	0	0	0	0	0	1		
14-JUL-2018	14:20	Day 22		F2	uncoated	0	0	0	0	0	0	1		
14-JUL-2018	14:20	Day 22		F3	uncoated	0	0	0	0	0	0	1		
22-AUG-2018	16:01	EOS/ET (Day 61)		F1	uncoated	0	0	0	0	0	0	0		
22-AUG-2018	16:01	EOS/ET (Day 61)		F2	uncoated	0	0	0	0	0	0	0		
22-AUG-2018	16:01	EOS/ET (Day 61)		F3	uncoated	0	0	0	0	0	0	0		

Key: 0= None; 1=Mild; 2=Moderate; 3=Severe; # Other = Pinprick bleeding or seepage

Administration Site Key: FA = Forearm; UA= Upper arm
Treatments Part A: A1 = A/Sing MAP, 15 ug (FA); A2 = Afluria Quad IM (UA), 15 ug; A3 = Placebo MAP (FA); A4 = A/Sing Mono IM, 15 ug (UA)
Treatments Part B: B1 = A/Sing MAP, 15 ug (FA) with biopsy; B2 = Placebo MAP (FA) with biopsy
B3 = A/Sing MAP, 15 ug (FA); B4 = A/Sing MAP, 10 ug (FA); B5 = A/Sing MAP, 5 ug (FA); B6 = A/Sing MAP, 2.5 ug (FA)
B7 = Placebo MAP (FA); B8 = A/Sing MAP, 15 ug (UA); B9 = A/Sing IM, 15 ug, as 2018 Afluria Quad (UA)

Listing 16.2.9.2
Treatment Site Tolerability Assessment per Site

Part B
Randomisation Number = B0136, Treatment Group = Group B9

Date	Time	Visit	Time Point	Site Number	Treatment for Site	Indura-tion	Redness	Bruising	Itching	Skin Flaking	Tender-ness	Definition/ Visibility	Other#	Unscheduled Reason
23-JUN-2018	10:36	Day 1	Pre-dose	D1	[IM]	0	0	0	0	0	0	5		
23-JUN-2018	11:20	Day 1	10 min	D1	[IM]	0	1	0	0	0	0	5		
23-JUN-2018	12:11	Day 1	1 hour	D1	[IM]	0	0	0	0	0	0	5		
23-JUN-2018	13:13	Day 1	2 hour	D1	[IM]	0	0	0	0	0	0	5		
24-JUN-2018	11:05	Day 2		D1	[IM]	0	0	0	0	0	0	5		
26-JUN-2018	11:21	Day 4		D1	[IM]	0	0	0	0	0	0	5		
30-JUN-2018	15:47	Day 8		D1	[IM]	0	0	0	0	0	0	5		
14-JUL-2018	14:02	Day 22		D1	[IM]	0	0	0	0	0	0	5		
22-AUG-2018	11:23	EOS/ET (Day 61)		D1	[IM]	0	0	0	0	0	0	5		

Key: 0= None; 1=Mild; 2=Moderate; 3=Severe; # Other = Pinprick bleeding or seepage

Administration Site Key: FA = Forearm; UA= Upper arm
Treatments Part A: A1 = A/Sing MAP, 15 ug (FA); A2 = Afluria Quad IM (UA), 15 ug; A3 = Placebo MAP (FA); A4 = A/Sing Mono IM, 15 ug (UA)
Treatments Part B: B1 = A/Sing MAP, 15 ug (FA) with biopsy; B2 = Placebo MAP (FA) with biopsy
B3 = A/Sing MAP, 15 ug (FA); B4 = A/Sing MAP, 10 ug (FA); B5 = A/Sing MAP, 5 ug (FA); B6 = A/Sing MAP, 2.5 ug (FA)
B7 = Placebo MAP (FA); B8 = A/Sing MAP, 15 ug (UA); B9 = A/Sing IM, 15 ug, as 2018 Afluria Quad (UA)

Listing 16.2.9.2
Treatment Site Tolerability Assessment per Site

Part B
Randomisation Number = B0137, Treatment Group = Group B8

Date	Time	Visit	Time Point	Site Number	Treatment for Site	Indura-tion	Redness	Bruising	Itching	Skin Flaking	Tender-ness	Definition/ Visibility	Other#	Unscheduled Reason
23-JUN-2018	11:32	Day 1	Pre-dose	D1	5 ug	0	0	0	0	0	0	0		
23-JUN-2018	11:32	Day 1	Pre-dose	D2	5 ug	0	0	0	0	0	0	0		
23-JUN-2018	11:32	Day 1	Pre-dose	D3	5 ug	0	0	0	0	0	0	0		
23-JUN-2018	13:08	Day 1	10 min	D1	5 ug	0	1	0	0	0	0	0		
23-JUN-2018	13:08	Day 1	10 min	D2	5 ug	0	1	0	0	0	0	0		
23-JUN-2018	13:08	Day 1	10 min	D3	5 ug	0	1	0	0	0	1	0		
23-JUN-2018	13:58	Day 1	1 hour	D1	5 ug	1	1	0	0	0	0	0		
23-JUN-2018	13:58	Day 1	1 hour	D2	5 ug	1	1	0	0	0	0	0		
23-JUN-2018	13:58	Day 1	1 hour	D3	5 ug	1	1	0	0	0	0	0		
23-JUN-2018	14:58	Day 1	2 hour	D1	5 ug	1	1	0	0	0	0	0		
23-JUN-2018	14:58	Day 1	2 hour	D2	5 ug	1	1	0	0	0	0	0		
23-JUN-2018	14:58	Day 1	2 hour	D3	5 ug	1	1	0	0	0	0	0		
24-JUN-2018	08:50	Day 2		D1	5 ug	0	1	0	0	0	0	0		
24-JUN-2018	08:50	Day 2		D2	5 ug	0	1	0	0	0	0	0		
24-JUN-2018	08:50	Day 2		D3	5 ug	0	0	0	0	0	0	0		
26-JUN-2018	10:21	Day 4		D1	5 ug	0	0	0	1	0	0	0		
26-JUN-2018	10:21	Day 4		D2	5 ug	0	0	0	1	0	0	0		
26-JUN-2018	10:21	Day 4		D3	5 ug	0	0	0	1	0	0	0		
30-JUN-2018	12:27	Day 8		D1	5 ug	0	0	0	0	1	0	0		
30-JUN-2018	12:27	Day 8		D2	5 ug	0	0	0	0	1	0	0		

Key: 0= None; 1=Mild; 2=Moderate; 3=Severe; # Other = Pinprick bleeding or seepage

Administration Site Key: FA = Forearm; UA= Upper arm
Treatments Part A: A1 = A/Sing MAP, 15 ug (FA); A2 = Afluria Quad IM (UA), 15 ug; A3 = Placebo MAP (FA); A4 = A/Sing Mono IM, 15 ug (UA)
Treatments Part B: B1 = A/Sing MAP, 15 ug (FA) with biopsy; B2 = Placebo MAP (FA) with biopsy
B3 = A/Sing MAP, 15 ug (FA); B4 = A/Sing MAP, 10 ug (FA); B5 = A/Sing MAP, 5 ug (FA); B6 = A/Sing MAP, 2.5 ug (FA)
B7 = Placebo MAP (FA); B8 = A/Sing MAP, 15 ug (UA); B9 = A/Sing IM, 15 ug, as 2018 Afluria Quad (UA)

Listing 16.2.9.2
Treatment Site Tolerability Assessment per Site

Part B
Randomisation Number = B0137, Treatment Group = Group B8

Date	Time	Visit	Time Point	Site Number	Treatment for Site	Indura-tion	Redness	Bruising	Itching	Skin Flaking	Tender-ness	Definition/ Visibility	Other#	Unscheduled Reason
30-JUN-2018	12:27	Day 8		D3	5 ug	0	0	0	0	1	0	0		
13-JUL-2018	09:33	Day 22		D1	5 ug	0	0	0	0	1	0	2		
13-JUL-2018	09:33	Day 22		D2	5 ug	0	0	0	0	1	0	2		
13-JUL-2018	09:33	Day 22		D3	5 ug	0	0	0	0	1	0	2		
30-AUG-2018	12:16	EOS/ET (Day 61)		D1	5 ug	0	0	0	0	0	0	0		
30-AUG-2018	12:16	EOS/ET (Day 61)		D2	5 ug	0	0	0	0	0	0	0		
30-AUG-2018	12:16	EOS/ET (Day 61)		D3	5 ug	0	0	0	0	0	0	0		

Key: 0= None; 1=Mild; 2=Moderate; 3=Severe; # Other = Pinprick bleeding or seepage

Administration Site Key: FA = Forearm; UA= Upper arm
Treatments Part A: A1 = A/Sing MAP, 15 ug (FA); A2 = Afluria Quad IM (UA), 15 ug; A3 = Placebo MAP (FA); A4 = A/Sing Mono IM, 15 ug (UA)
Treatments Part B: B1 = A/Sing MAP, 15 ug (FA) with biopsy; B2 = Placebo MAP (FA) with biopsy
B3 = A/Sing MAP, 15 ug (FA); B4 = A/Sing MAP, 10 ug (FA); B5 = A/Sing MAP, 5 ug (FA); B6 = A/Sing MAP, 2.5 ug (FA)
B7 = Placebo MAP (FA); B8 = A/Sing MAP, 15 ug (UA); B9 = A/Sing IM, 15 ug, as 2018 Afluria Quad (UA)

Listing 16.2.9.2
Treatment Site Tolerability Assessment per Site

Part B
Randomisation Number = B0138, Treatment Group = Group B7

Date	Time	Visit	Time Point	Site Number	Treatment for Site	Indura-tion	Redness	Bruising	Itching	Skin Flaking	Tender-ness	Definition/ Visibility	Other#	Unscheduled Reason
23-JUN-2018	11:41	Day 1	Pre-dose	F1	uncoated	0	0	0	0	0	0	0		
23-JUN-2018	11:41	Day 1	Pre-dose	F2	uncoated	0	0	0	0	0	0	0		
23-JUN-2018	11:41	Day 1	Pre-dose	F3	uncoated	0	0	0	0	0	0	0		
23-JUN-2018	13:18	Day 1	10 min	F1	uncoated	0	1	0	0	0	1	0		
23-JUN-2018	13:18	Day 1	10 min	F2	uncoated	1	1	0	0	0	0	0		
23-JUN-2018	13:18	Day 1	10 min	F3	uncoated	1	1	0	0	0	0	0		
23-JUN-2018	14:08	Day 1	1 hour	F1	uncoated	0	1	0	0	0	1	0		
23-JUN-2018	14:08	Day 1	1 hour	F2	uncoated	1	1	0	0	0	0	0		
23-JUN-2018	14:08	Day 1	1 hour	F3	uncoated	1	1	0	0	0	0	0		
23-JUN-2018	15:08	Day 1	2 hour	F1	uncoated	0	1	0	0	0	1	0		
23-JUN-2018	15:08	Day 1	2 hour	F2	uncoated	1	1	0	0	0	0	0		
23-JUN-2018	15:08	Day 1	2 hour	F3	uncoated	0	1	0	0	0	0	0		
24-JUN-2018	13:11	Day 2		F1	uncoated	0	0	0	0	0	0	0		
24-JUN-2018	13:11	Day 2		F2	uncoated	0	0	0	0	0	0	0		
24-JUN-2018	13:11	Day 2		F3	uncoated	0	0	0	0	0	0	0		
26-JUN-2018	12:30	Day 4		F1	uncoated	0	0	0	0	0	0	3		
26-JUN-2018	12:30	Day 4		F2	uncoated	0	0	0	0	0	0	3		
26-JUN-2018	12:30	Day 4		F3	uncoated	0	0	0	0	0	0	3		
30-JUN-2018	13:36	Day 8		F1	uncoated	0	0	0	0	0	0	3		
30-JUN-2018	13:36	Day 8		F2	uncoated	0	0	0	0	0	0	3		

Key: 0= None; 1=Mild; 2=Moderate; 3=Severe; # Other = Pinprick bleeding or seepage

Administration Site Key: FA = Forearm; UA= Upper arm
Treatments Part A: A1 = A/Sing MAP, 15 ug (FA); A2 = Afluria Quad IM (UA), 15 ug; A3 = Placebo MAP (FA); A4 = A/Sing Mono IM, 15 ug (UA)
Treatments Part B: B1 = A/Sing MAP, 15 ug (FA) with biopsy; B2 = Placebo MAP (FA) with biopsy
B3 = A/Sing MAP, 15 ug (FA); B4 = A/Sing MAP, 10 ug (FA); B5 = A/Sing MAP, 5 ug (FA); B6 = A/Sing MAP, 2.5 ug (FA)
B7 = Placebo MAP (FA); B8 = A/Sing MAP, 15 ug (UA); B9 = A/Sing IM, 15 ug, as 2018 Afluria Quad (UA)

Listing 16.2.9.2
Treatment Site Tolerability Assessment per Site

Part B
Randomisation Number = B0138, Treatment Group = Group B7

Date	Time	Visit	Time Point	Site Number	Treatment for Site	Indura-tion	Redness	Bruising	Itching	Skin Flaking	Tender-ness	Definition/ Visibility	Other#	Unscheduled Reason
30-JUN-2018	13:36	Day 8		F3	uncoated	0	0	0	0	0	0	3		
14-JUL-2018	10:50	Day 22		F1	uncoated	0	0	0	0	0	0	0		
14-JUL-2018	10:50	Day 22		F2	uncoated	0	0	0	0	0	0	0		
14-JUL-2018	10:50	Day 22		F3	uncoated	0	0	0	0	0	0	0		
22-AUG-2018	13:26	EOS/ET (Day 61)		F1	uncoated	0	0	0	0	0	0	1		
22-AUG-2018	13:26	EOS/ET (Day 61)		F2	uncoated	0	0	0	0	0	0	1		
22-AUG-2018	13:26	EOS/ET (Day 61)		F3	uncoated	0	0	0	0	0	0	1		

Key: 0= None; 1=Mild; 2=Moderate; 3=Severe; # Other = Pinprick bleeding or seepage

Administration Site Key: FA = Forearm; UA= Upper arm
Treatments Part A: A1 = A/Sing MAP, 15 ug (FA); A2 = Afluria Quad IM (UA), 15 ug; A3 = Placebo MAP (FA); A4 = A/Sing Mono IM, 15 ug (UA)
Treatments Part B: B1 = A/Sing MAP, 15 ug (FA) with biopsy; B2 = Placebo MAP (FA) with biopsy
B3 = A/Sing MAP, 15 ug (FA); B4 = A/Sing MAP, 10 ug (FA); B5 = A/Sing MAP, 5 ug (FA); B6 = A/Sing MAP, 2.5 ug (FA)
B7 = Placebo MAP (FA); B8 = A/Sing MAP, 15 ug (UA); B9 = A/Sing IM, 15 ug, as 2018 Afluria Quad (UA)

Listing 16.2.9.2
Treatment Site Tolerability Assessment per Site

Part B
Randomisation Number = B0139, Treatment Group = Group B4

Date	Time	Visit	Time Point	Site Number	Treatment for Site	Indura-tion	Redness	Bruising	Itching	Skin Flaking	Tender-ness	Definition/ Visibility	Other#	Unscheduled Reason
23-JUN-2018	11:50	Day 1	Pre-dose	F1	uncoated	0	0	0	0	0	0	0		
23-JUN-2018	11:50	Day 1	Pre-dose	F2	5 ug	0	0	0	0	0	0	0		
23-JUN-2018	11:50	Day 1	Pre-dose	F3	5 ug	0	0	0	0	0	0	0		
23-JUN-2018	13:28	Day 1	10 min	F1	uncoated	0	1	0	0	0	0	0		
23-JUN-2018	13:28	Day 1	10 min	F2	5 ug	0	1	0	0	0	1	0		
23-JUN-2018	13:28	Day 1	10 min	F3	5 ug	0	1	0	0	0	0	0		
23-JUN-2018	14:18	Day 1	1 hour	F1	uncoated	0	1	0	0	0	0	0		
23-JUN-2018	14:18	Day 1	1 hour	F2	5 ug	0	1	0	0	0	0	0		
23-JUN-2018	14:18	Day 1	1 hour	F3	5 ug	1	1	0	0	0	0	0		
23-JUN-2018	15:18	Day 1	2 hour	F1	uncoated	0	0	0	0	0	0	0		
23-JUN-2018	15:18	Day 1	2 hour	F2	5 ug	0	0	0	0	0	0	0		
23-JUN-2018	15:18	Day 1	2 hour	F3	5 ug	0	0	0	0	0	0	0		
24-JUN-2018	13:40	Day 2		F1	uncoated	0	0	0	0	0	0	1		
24-JUN-2018	13:40	Day 2		F2	5 ug	1	0	0	0	0	0	0		
24-JUN-2018	13:40	Day 2		F3	5 ug	1	0	0	0	0	0	0		
26-JUN-2018	17:58	Day 4		F1	uncoated	0	0	0	0	0	0	0		
26-JUN-2018	17:58	Day 4		F2	5 ug	0	0	0	1	0	0	0		
26-JUN-2018	17:58	Day 4		F3	5 ug	0	0	0	0	0	0	0		
30-JUN-2018	15:03	Day 8		F1	uncoated	0	0	0	0	0	0	2		
30-JUN-2018	15:03	Day 8		F2	5 ug	0	0	0	0	0	0	3		

Key: 0= None; 1=Mild; 2=Moderate; 3=Severe; # Other = Pinprick bleeding or seepage

Administration Site Key: FA = Forearm; UA= Upper arm
Treatments Part A: A1 = A/Sing MAP, 15 ug (FA); A2 = Afluria Quad IM (UA), 15 ug; A3 = Placebo MAP (FA); A4 = A/Sing Mono IM, 15 ug (UA)
Treatments Part B: B1 = A/Sing MAP, 15 ug (FA) with biopsy; B2 = Placebo MAP (FA) with biopsy
B3 = A/Sing MAP, 15 ug (FA); B4 = A/Sing MAP, 10 ug (FA); B5 = A/Sing MAP, 5 ug (FA); B6 = A/Sing MAP, 2.5 ug (FA)
B7 = Placebo MAP (FA); B8 = A/Sing MAP, 15 ug (UA); B9 = A/Sing IM, 15 ug, as 2018 Afluria Quad (UA)

Listing 16.2.9.2
Treatment Site Tolerability Assessment per Site

Part B
Randomisation Number = B0139, Treatment Group = Group B4

Date	Time	Visit	Time Point	Site Number	Treatment for Site	Indura-tion	Redness	Bruising	Itching	Skin Flaking	Tender-ness	Definition/ Visibility	Other#	Unscheduled Reason
30-JUN-2018	15:03	Day 8		F3	5 ug	0	0	0	0	0	0	3		
14-JUL-2018	09:15	Day 22		F1	uncoated	0	0	0	0	0	0	0		
14-JUL-2018	09:15	Day 22		F2	5 ug	0	0	0	0	0	0	1		
14-JUL-2018	09:15	Day 22		F3	5 ug	0	0	0	0	1	0	1		
22-AUG-2018	14:01	EOS/ET (Day 61)		F1	uncoated	0	0	0	0	0	0	0		
22-AUG-2018	14:01	EOS/ET (Day 61)		F2	5 ug	0	0	0	0	0	0	0		
22-AUG-2018	14:01	EOS/ET (Day 61)		F3	5 ug	0	0	0	0	0	0	0		

Key: 0= None; 1=Mild; 2=Moderate; 3=Severe; # Other = Pinprick bleeding or seepage

Administration Site Key: FA = Forearm; UA= Upper arm
Treatments Part A: A1 = A/Sing MAP, 15 ug (FA); A2 = Afluria Quad IM (UA), 15 ug; A3 = Placebo MAP (FA); A4 = A/Sing Mono IM, 15 ug (UA)
Treatments Part B: B1 = A/Sing MAP, 15 ug (FA) with biopsy; B2 = Placebo MAP (FA) with biopsy
B3 = A/Sing MAP, 15 ug (FA); B4 = A/Sing MAP, 10 ug (FA); B5 = A/Sing MAP, 5 ug (FA); B6 = A/Sing MAP, 2.5 ug (FA)
B7 = Placebo MAP (FA); B8 = A/Sing MAP, 15 ug (UA); B9 = A/Sing IM, 15 ug, as 2018 Afluria Quad (UA)

Listing 16.2.9.2
Treatment Site Tolerability Assessment per Site

Part B
Randomisation Number = B0140, Treatment Group = Group B9

Date	Time	Visit	Time Point	Site Number	Treatment for Site	Indura-tion	Redness	Bruising	Itching	Skin Flaking	Tender-ness	Definition/ Visibility	Other#	Unscheduled Reason
23-JUN-2018	10:40	Day 1	Pre-dose	D1	[IM]	0	0	0	0	0	0	5		
23-JUN-2018	11:10	Day 1	10 min	D1	[IM]	0	0	0	0	0	0	5		
23-JUN-2018	12:00	Day 1	1 hour	D1	[IM]	0	0	0	0	0	0	5		
23-JUN-2018	13:08	Day 1	2 hour	D1	[IM]	0	0	0	0	0	0	5		
24-JUN-2018	11:18	Day 2		D1	[IM]	0	0	0	0	0	0	5		
26-JUN-2018	14:18	Day 4		D1	[IM]	0	0	0	0	0	0	5		
01-JUL-2018	14:07	Day 8		D1	[IM]	0	0	0	0	0	0	5		
14-JUL-2018	12:50	Day 22		D1	[IM]	0	0	0	0	0	0	5		
22-AUG-2018	09:14	EOS/ET (Day 61)		D1	[IM]	0	0	0	0	0	0	5		

Key: 0= None; 1=Mild; 2=Moderate; 3=Severe; # Other = Pinprick bleeding or seepage

Administration Site Key: FA = Forearm; UA= Upper arm
Treatments Part A: A1 = A/Sing MAP, 15 ug (FA); A2 = Afluria Quad IM (UA), 15 ug; A3 = Placebo MAP (FA); A4 = A/Sing Mono IM, 15 ug (UA)
Treatments Part B: B1 = A/Sing MAP, 15 ug (FA) with biopsy; B2 = Placebo MAP (FA) with biopsy
B3 = A/Sing MAP, 15 ug (FA); B4 = A/Sing MAP, 10 ug (FA); B5 = A/Sing MAP, 5 ug (FA); B6 = A/Sing MAP, 2.5 ug (FA)
B7 = Placebo MAP (FA); B8 = A/Sing MAP, 15 ug (UA); B9 = A/Sing IM, 15 ug, as 2018 Afluria Quad (UA)

Listing 16.2.9.2
Treatment Site Tolerability Assessment per Site

Part B
Randomisation Number = B0141, Treatment Group = Group B3

Date	Time	Visit	Time Point	Site Number	Treatment for Site	Indura-tion	Redness	Bruising	Itching	Skin Flaking	Tender-ness	Definition/ Visibility	Other#	Unscheduled Reason
23-JUN-2018	11:53	Day 1	Pre-dose	F1	5 ug	0	0	0	0	0	0	0		
23-JUN-2018	11:53	Day 1	Pre-dose	F2	5 ug	0	0	0	0	0	0	0		
23-JUN-2018	11:53	Day 1	Pre-dose	F3	5 ug	0	0	0	0	0	0	0		
23-JUN-2018	13:38	Day 1	10 min	F1	5 ug	0	1	0	0	0	1	0		
23-JUN-2018	13:38	Day 1	10 min	F2	5 ug	0	1	0	0	0	1	0		
23-JUN-2018	13:38	Day 1	10 min	F3	5 ug	0	1	0	0	0	1	0		
23-JUN-2018	14:28	Day 1	1 hour	F1	5 ug	0	1	0	0	0	0	0		
23-JUN-2018	14:28	Day 1	1 hour	F2	5 ug	0	1	0	0	0	0	0		
23-JUN-2018	14:28	Day 1	1 hour	F3	5 ug	1	1	0	0	0	1	0		
23-JUN-2018	15:28	Day 1	2 hour	F1	5 ug	0	0	0	0	0	0	0		
23-JUN-2018	15:28	Day 1	2 hour	F2	5 ug	0	0	0	0	0	0	0		
23-JUN-2018	15:28	Day 1	2 hour	F3	5 ug	0	0	0	0	0	0	0		
24-JUN-2018	13:46	Day 2		F1	5 ug	0	1	0	1	0	0	0		
24-JUN-2018	13:46	Day 2		F2	5 ug	0	1	0	1	0	0	0		
24-JUN-2018	13:46	Day 2		F3	5 ug	0	1	0	1	0	0	0		
26-JUN-2018	14:15	Day 4		F1	5 ug	0	0	0	0	0	0	0		
26-JUN-2018	14:15	Day 4		F2	5 ug	0	0	0	0	0	0	0		
26-JUN-2018	14:15	Day 4		F3	5 ug	0	0	0	0	0	0	0		
30-JUN-2018	13:51	Day 8		F1	5 ug	0	0	0	0	2	0	3		
30-JUN-2018	13:51	Day 8		F2	5 ug	0	0	0	0	1	0	3		

Key: 0= None; 1=Mild; 2=Moderate; 3=Severe; # Other = Pinprick bleeding or seepage

Administration Site Key: FA = Forearm; UA= Upper arm
Treatments Part A: A1 = A/Sing MAP, 15 ug (FA); A2 = Afluria Quad IM (UA), 15 ug; A3 = Placebo MAP (FA); A4 = A/Sing Mono IM, 15 ug (UA)
Treatments Part B: B1 = A/Sing MAP, 15 ug (FA) with biopsy; B2 = Placebo MAP (FA) with biopsy
B3 = A/Sing MAP, 15 ug (FA); B4 = A/Sing MAP, 10 ug (FA); B5 = A/Sing MAP, 5 ug (FA); B6 = A/Sing MAP, 2.5 ug (FA)
B7 = Placebo MAP (FA); B8 = A/Sing MAP, 15 ug (UA); B9 = A/Sing IM, 15 ug, as 2018 Afluria Quad (UA)

Listing 16.2.9.2
Treatment Site Tolerability Assessment per Site

Part B
Randomisation Number = B0141, Treatment Group = Group B3

Date	Time	Visit	Time Point	Site Number	Treatment for Site	Indura-tion	Redness	Bruising	Itching	Skin Flaking	Tender-ness	Definition/ Visibility	Other#	Unscheduled Reason
30-JUN-2018	13:51	Day 8		F3	5 ug	0	0	0	0	1	0	3		
14-JUL-2018	14:31	Day 22		F1	5 ug	0	0	0	0	1	0	2		
14-JUL-2018	14:31	Day 22		F2	5 ug	0	0	0	0	1	0	2		
14-JUL-2018	14:31	Day 22		F3	5 ug	0	0	0	0	1	0	2		
23-AUG-2018	13:51	EOS/ET (Day 61)		F1	5 ug	0	0	0	0	0	0	0		
23-AUG-2018	13:51	EOS/ET (Day 61)		F2	5 ug	0	0	0	0	0	0	0		
23-AUG-2018	13:51	EOS/ET (Day 61)		F3	5 ug	0	0	0	0	0	0	0		

Key: 0= None; 1=Mild; 2=Moderate; 3=Severe; # Other = Pinprick bleeding or seepage

Administration Site Key: FA = Forearm; UA= Upper arm
Treatments Part A: A1 = A/Sing MAP, 15 ug (FA); A2 = Afluria Quad IM (UA), 15 ug; A3 = Placebo MAP (FA); A4 = A/Sing Mono IM, 15 ug (UA)
Treatments Part B: B1 = A/Sing MAP, 15 ug (FA) with biopsy; B2 = Placebo MAP (FA) with biopsy
B3 = A/Sing MAP, 15 ug (FA); B4 = A/Sing MAP, 10 ug (FA); B5 = A/Sing MAP, 5 ug (FA); B6 = A/Sing MAP, 2.5 ug (FA)
B7 = Placebo MAP (FA); B8 = A/Sing MAP, 15 ug (UA); B9 = A/Sing IM, 15 ug, as 2018 Afluria Quad (UA)

Listing 16.2.9.2
Treatment Site Tolerability Assessment per Site

Part B
Randomisation Number = B0142, Treatment Group = Group B5

Date	Time	Visit	Time Point	Site Number	Treatment for Site	Induration	Redness	Bruising	Itching	Skin Flaking	Tenderness	Definition/ Visibility	Other#	Unscheduled Reason
23-JUN-2018	12:02	Day 1	Pre-dose	F1	5 ug	0	0	0	0	0	0	0		
23-JUN-2018	12:02	Day 1	Pre-dose	F2	uncoated	0	0	0	0	0	0	0		
23-JUN-2018	12:02	Day 1	Pre-dose	F3	uncoated	0	0	0	0	0	0	0		
23-JUN-2018	13:48	Day 1	10 min	F1	5 ug	0	0	0	0	0	0	0		
23-JUN-2018	13:48	Day 1	10 min	F2	uncoated	0	0	0	0	0	0	0		
23-JUN-2018	13:48	Day 1	10 min	F3	uncoated	0	0	0	0	0	0	0		
23-JUN-2018	14:38	Day 1	1 hour	F1	5 ug	0	1	0	0	0	0	0		
23-JUN-2018	14:38	Day 1	1 hour	F2	uncoated	0	1	0	0	0	0	0		
23-JUN-2018	14:38	Day 1	1 hour	F3	uncoated	0	1	0	0	0	0	0		
23-JUN-2018	15:38	Day 1	2 hour	F1	5 ug	0	0	0	0	0	0	0		
23-JUN-2018	15:38	Day 1	2 hour	F2	uncoated	0	0	0	0	0	0	0		
23-JUN-2018	15:38	Day 1	2 hour	F3	uncoated	0	0	0	0	0	0	0		
24-JUN-2018	14:09	Day 2		F1	5 ug	0	0	0	1	0	0	0		
24-JUN-2018	14:09	Day 2		F2	uncoated	0	0	0	0	0	0	0		
24-JUN-2018	14:09	Day 2		F3	uncoated	0	0	0	0	0	0	0		
26-JUN-2018	08:49	Day 4		F1	5 ug	0	0	0	1	0	0	0		
26-JUN-2018	08:49	Day 4		F2	uncoated	0	0	0	0	0	0	1		
26-JUN-2018	08:49	Day 4		F3	uncoated	0	0	0	0	0	0	1		
30-JUN-2018	11:11	Day 8		F1	5 ug	0	0	0	0	2	0	0		
30-JUN-2018	11:11	Day 8		F2	uncoated	0	0	0	0	0	0	0		

Key: 0= None; 1=Mild; 2=Moderate; 3=Severe; # Other = Pinprick bleeding or seepage

Administration Site Key: FA = Forearm; UA= Upper arm
Treatments Part A: A1 = A/Sing MAP, 15 ug (FA); A2 = Afluria Quad IM (UA), 15 ug; A3 = Placebo MAP (FA); A4 = A/Sing Mono IM, 15 ug (UA)
Treatments Part B: B1 = A/Sing MAP, 15 ug (FA) with biopsy; B2 = Placebo MAP (FA) with biopsy
B3 = A/Sing MAP, 15 ug (FA); B4 = A/Sing MAP, 10 ug (FA); B5 = A/Sing MAP, 5 ug (FA); B6 = A/Sing MAP, 2.5 ug (FA)
B7 = Placebo MAP (FA); B8 = A/Sing MAP, 15 ug (UA); B9 = A/Sing IM, 15 ug, as 2018 Afluria Quad (UA)

Listing 16.2.9.2
Treatment Site Tolerability Assessment per Site

Part B
Randomisation Number = B0142, Treatment Group = Group B5

Date	Time	Visit	Time Point	Site Number	Treatment for Site	Indura-tion	Redness	Bruising	Itching	Skin Flaking	Tender-ness	Definition/ Visibility	Other#	Unscheduled Reason
30-JUN-2018	11:11	Day 8		F3	uncoated	0	0	0	0	0	0	0		
14-JUL-2018	13:30	Day 22		F1	5 ug	0	0	0	0	0	0	0		
14-JUL-2018	13:30	Day 22		F2	uncoated	0	0	0	0	0	0	0		
14-JUL-2018	13:30	Day 22		F3	uncoated	0	0	0	0	0	0	0		
25-AUG-2018	11:55	EOS/ET (Day 61)		F1	5 ug	0	0	0	0	0	0	1		
25-AUG-2018	11:55	EOS/ET (Day 61)		F2	uncoated	0	0	0	0	0	0	0		
25-AUG-2018	11:55	EOS/ET (Day 61)		F3	uncoated	0	0	0	0	0	0	0		

Key: 0= None; 1=Mild; 2=Moderate; 3=Severe; # Other = Pinprick bleeding or seepage

Administration Site Key: FA = Forearm; UA= Upper arm
Treatments Part A: A1 = A/Sing MAP, 15 ug (FA); A2 = Afluria Quad IM (UA), 15 ug; A3 = Placebo MAP (FA); A4 = A/Sing Mono IM, 15 ug (UA)
Treatments Part B: B1 = A/Sing MAP, 15 ug (FA) with biopsy; B2 = Placebo MAP (FA) with biopsy
B3 = A/Sing MAP, 15 ug (FA); B4 = A/Sing MAP, 10 ug (FA); B5 = A/Sing MAP, 5 ug (FA); B6 = A/Sing MAP, 2.5 ug (FA)
B7 = Placebo MAP (FA); B8 = A/Sing MAP, 15 ug (UA); B9 = A/Sing IM, 15 ug, as 2018 Afluria Quad (UA)

Listing 16.2.9.2
Treatment Site Tolerability Assessment per Site

Part B
Randomisation Number = B0143, Treatment Group = Group B6

Date	Time	Visit	Time Point	Site Number	Treatment for Site	Indura-tion	Redness	Bruising	Itching	Skin Flaking	Tender-ness	Definition/ Visibility	Other#	Unscheduled Reason
23-JUN-2018	12:10	Day 1	Pre-dose	F1	uncoated	0	0	0	0	0	0	0		
23-JUN-2018	12:10	Day 1	Pre-dose	F2	uncoated	0	0	0	0	0	0	0		
23-JUN-2018	12:10	Day 1	Pre-dose	F3	2.5 ug	0	0	0	0	0	0	0		
23-JUN-2018	13:58	Day 1	10 min	F1	uncoated	0	1	0	0	0	0	0		
23-JUN-2018	13:58	Day 1	10 min	F2	uncoated	0	1	0	0	0	0	0		
23-JUN-2018	13:58	Day 1	10 min	F3	2.5 ug	0	1	0	0	0	1	0		
23-JUN-2018	14:48	Day 1	1 hour	F1	uncoated	0	1	0	0	0	0	0		
23-JUN-2018	14:48	Day 1	1 hour	F2	uncoated	0	1	0	0	0	1	0		
23-JUN-2018	14:48	Day 1	1 hour	F3	2.5 ug	0	1	0	0	0	1	0		
23-JUN-2018	15:48	Day 1	2 hour	F1	uncoated	0	0	0	0	0	0	0		
23-JUN-2018	15:48	Day 1	2 hour	F2	uncoated	0	0	0	0	0	0	0		
23-JUN-2018	15:48	Day 1	2 hour	F3	2.5 ug	0	0	0	0	0	1	0		
24-JUN-2018	11:53	Day 2		F1	uncoated	0	0	0	0	0	0	0		
24-JUN-2018	11:53	Day 2		F2	uncoated	0	0	0	0	0	0	0		
24-JUN-2018	11:53	Day 2		F3	2.5 ug	1	0	0	0	0	0	0		
26-JUN-2018	12:08	Day 4		F1	uncoated	0	0	0	0	0	0	0		
26-JUN-2018	12:08	Day 4		F2	uncoated	0	0	0	0	0	0	0		
26-JUN-2018	12:08	Day 4		F3	2.5 ug	1	1	0	0	0	1	0		
26-JUN-2018	12:18	Unscheduled		F3	2.5 ug	2	1	0	0	0	1	0	NA	MO notifies as repeat

Key: 0= None; 1=Mild; 2=Moderate; 3=Severe; # Other = Pinprick bleeding or seepage

Administration Site Key: FA = Forearm; UA= Upper arm
Treatments Part A: A1 = A/Sing MAP, 15 ug (FA); A2 = Afluria Quad IM (UA), 15 ug; A3 = Placebo MAP (FA); A4 = A/Sing Mono IM, 15 ug (UA)
Treatments Part B: B1 = A/Sing MAP, 15 ug (FA) with biopsy; B2 = Placebo MAP (FA) with biopsy
B3 = A/Sing MAP, 15 ug (FA); B4 = A/Sing MAP, 10 ug (FA); B5 = A/Sing MAP, 5 ug (FA); B6 = A/Sing MAP, 2.5 ug (FA)
B7 = Placebo MAP (FA); B8 = A/Sing MAP, 15 ug (UA); B9 = A/Sing IM, 15 ug, as 2018 Afluria Quad (UA)

Listing 16.2.9.2
Treatment Site Tolerability Assessment per Site

Part B
Randomisation Number = B0143, Treatment Group = Group B6

Date	Time	Visit	Time Point	Site Number	Treatment for Site	Indura- tion	Redness	Bruising	Itching	Skin Flaking	Tender- ness	Definition/ Visibility	Other#	Unscheduled Reason
30-JUN-2018	12:14	Day 8		F1	uncoated	0	0	0	0	0	0	3		
30-JUN-2018	12:14	Day 8		F2	uncoated	0	0	0	0	0	0	3		
30-JUN-2018	12:14	Day 8		F3	2.5 ug	0	0	0	0	2	0	3		
14-JUL-2018	13:41	Day 22		F1	uncoated	0	0	0	0	0	0	1		
14-JUL-2018	13:41	Day 22		F2	uncoated	0	0	0	0	0	0	1		
14-JUL-2018	13:41	Day 22		F3	2.5 ug	0	0	0	0	0	0	1		
22-AUG-2018	13:09	EOS/ET (Day 61)		F1	uncoated	0	0	0	0	0	0	0		
22-AUG-2018	13:09	EOS/ET (Day 61)		F2	uncoated	0	0	0	0	0	0	0		
22-AUG-2018	13:09	EOS/ET (Day 61)		F3	2.5 ug	0	0	0	0	0	0	1		

Key: 0= None; 1=Mild; 2=Moderate; 3=Severe; # Other = Pinprick bleeding or seepage

Administration Site Key: FA = Forearm; UA= Upper arm
Treatments Part A: A1 = A/Sing MAP, 15 ug (FA); A2 = Afluria Quad IM (UA), 15 ug; A3 = Placebo MAP (FA); A4 = A/Sing Mono IM, 15 ug (UA)
Treatments Part B: B1 = A/Sing MAP, 15 ug (FA) with biopsy; B2 = Placebo MAP (FA) with biopsy
B3 = A/Sing MAP, 15 ug (FA); B4 = A/Sing MAP, 10 ug (FA); B5 = A/Sing MAP, 5 ug (FA); B6 = A/Sing MAP, 2.5 ug (FA)
B7 = Placebo MAP (FA); B8 = A/Sing MAP, 15 ug (UA); B9 = A/Sing IM, 15 ug, as 2018 Afluria Quad (UA)

Listing 16.2.9.2
Treatment Site Tolerability Assessment per Site

Part B
Randomisation Number = B0144, Treatment Group = Group B6

Date	Time	Visit	Time Point	Site Number	Treatment for Site	Indura-tion	Redness	Bruising	Itching	Skin Flaking	Tender-ness	Definition/ Visibility	Other#	Unscheduled Reason
23-JUN-2018	12:20	Day 1	Pre-dose	F1	2.5 ug	0	0	0	0	0	0	0		
23-JUN-2018	12:20	Day 1	Pre-dose	F2	uncoated	0	0	0	0	0	0	0		
23-JUN-2018	12:20	Day 1	Pre-dose	F3	uncoated	0	0	0	0	0	0	0		
23-JUN-2018	14:08	Day 1	10 min	F1	2.5 ug	0	1	0	0	0	0	0		
23-JUN-2018	14:08	Day 1	10 min	F2	uncoated	0	1	0	1	0	1	0		
23-JUN-2018	14:08	Day 1	10 min	F3	uncoated	0	1	0	0	0	0	0		
23-JUN-2018	14:58	Day 1	1 hour	F1	2.5 ug	1	1	0	0	0	0	0		
23-JUN-2018	14:58	Day 1	1 hour	F2	uncoated	1	1	0	0	0	0	0		
23-JUN-2018	14:58	Day 1	1 hour	F3	uncoated	1	1	0	0	0	0	0		
23-JUN-2018	15:58	Day 1	2 hour	F1	2.5 ug	0	0	0	0	0	0	0		
23-JUN-2018	15:58	Day 1	2 hour	F2	uncoated	0	0	0	0	0	0	0		
23-JUN-2018	15:58	Day 1	2 hour	F3	uncoated	0	0	0	0	0	0	0		
24-JUN-2018	12:22	Day 2		F1	2.5 ug	1	0	0	0	0	1	0		
24-JUN-2018	12:22	Day 2		F2	uncoated	0	0	0	0	0	1	0		
24-JUN-2018	12:22	Day 2		F3	uncoated	0	0	0	0	0	0	0		
26-JUN-2018	12:22	Day 4		F1	2.5 ug	0	0	0	1	0	0	0		
26-JUN-2018	12:22	Day 4		F2	uncoated	0	0	0	0	0	0	2		
26-JUN-2018	12:22	Day 4		F3	uncoated	0	0	0	0	0	0	2		
30-JUN-2018	13:05	Day 8		F1	2.5 ug	0	0	0	0	0	0	0		
30-JUN-2018	13:05	Day 8		F2	uncoated	0	0	0	0	0	0	2		

Key: 0= None; 1=Mild; 2=Moderate; 3=Severe; # Other = Pinprick bleeding or seepage

Administration Site Key: FA = Forearm; UA= Upper arm
Treatments Part A: A1 = A/Sing MAP, 15 ug (FA); A2 = Afluria Quad IM (UA), 15 ug; A3 = Placebo MAP (FA); A4 = A/Sing Mono IM, 15 ug (UA)
Treatments Part B: B1 = A/Sing MAP, 15 ug (FA) with biopsy; B2 = Placebo MAP (FA) with biopsy
B3 = A/Sing MAP, 15 ug (FA); B4 = A/Sing MAP, 10 ug (FA); B5 = A/Sing MAP, 5 ug (FA); B6 = A/Sing MAP, 2.5 ug (FA)
B7 = Placebo MAP (FA); B8 = A/Sing MAP, 15 ug (UA); B9 = A/Sing IM, 15 ug, as 2018 Afluria Quad (UA)

Listing 16.2.9.2
Treatment Site Tolerability Assessment per Site

Part B
Randomisation Number = B0144, Treatment Group = Group B6

Date	Time	Visit	Time Point	Site Number	Treatment for Site	Indura-tion	Redness	Bruising	Itching	Skin Flaking	Tender-ness	Definition/ Visibility	Other#	Unscheduled Reason
30-JUN-2018	13:05	Day 8		F3	uncoated	0	0	0	0	0	0	2		
16-JUL-2018	13:11	Day 22		F1	2.5 ug	0	0	0	0	0	0	0		
16-JUL-2018	13:11	Day 22		F2	uncoated	0	0	0	0	0	0	1		
16-JUL-2018	13:11	Day 22		F3	uncoated	0	0	0	0	0	0	1		
23-AUG-2018	17:04	EOS/ET (Day 61)		F1	2.5 ug	0	0	0	0	0	0	0		
23-AUG-2018	17:04	EOS/ET (Day 61)		F2	uncoated	0	0	0	0	0	0	0		
23-AUG-2018	17:04	EOS/ET (Day 61)		F3	uncoated	0	0	0	0	0	0	0		

Key: 0= None; 1=Mild; 2=Moderate; 3=Severe; # Other = Pinprick bleeding or seepage

Administration Site Key: FA = Forearm; UA= Upper arm
Treatments Part A: A1 = A/Sing MAP, 15 ug (FA); A2 = Afluria Quad IM (UA), 15 ug; A3 = Placebo MAP (FA); A4 = A/Sing Mono IM, 15 ug (UA)
Treatments Part B: B1 = A/Sing MAP, 15 ug (FA) with biopsy; B2 = Placebo MAP (FA) with biopsy
B3 = A/Sing MAP, 15 ug (FA); B4 = A/Sing MAP, 10 ug (FA); B5 = A/Sing MAP, 5 ug (FA); B6 = A/Sing MAP, 2.5 ug (FA)
B7 = Placebo MAP (FA); B8 = A/Sing MAP, 15 ug (UA); B9 = A/Sing IM, 15 ug, as 2018 Afluria Quad (UA)

Listing 16.2.9.2
Treatment Site Tolerability Assessment per Site

Part B
Randomisation Number = B0145, Treatment Group = Group B3

Date	Time	Visit	Time Point	Site Number	Treatment for Site	Indura-tion	Redness	Bruising	Itching	Skin Flaking	Tender-ness	Definition/ Visibility	Other#	Unscheduled Reason
23-JUN-2018	12:31	Day 1	Pre-dose	F1	5 ug	0	0	0	0	0	0	0		
23-JUN-2018	12:31	Day 1	Pre-dose	F2	5 ug	0	0	0	0	0	0	0		
23-JUN-2018	12:31	Day 1	Pre-dose	F3	5 ug	0	0	0	0	0	0	0		
23-JUN-2018	14:18	Day 1	10 min	F1	5 ug	0	1	0	0	0	0	0		
23-JUN-2018	14:18	Day 1	10 min	F2	5 ug	0	1	0	0	0	0	0		
23-JUN-2018	14:18	Day 1	10 min	F3	5 ug	0	1	0	0	0	0	0		
23-JUN-2018	15:08	Day 1	1 hour	F1	5 ug	1	1	0	0	0	0	0		
23-JUN-2018	15:08	Day 1	1 hour	F2	5 ug	1	1	0	0	0	0	0		
23-JUN-2018	15:08	Day 1	1 hour	F3	5 ug	1	1	0	0	0	0	0		
23-JUN-2018	16:08	Day 1	2 hour	F1	5 ug	0	0	0	0	0	0	0		
23-JUN-2018	16:08	Day 1	2 hour	F2	5 ug	0	0	0	0	0	1	0		
23-JUN-2018	16:08	Day 1	2 hour	F3	5 ug	0	0	0	0	0	0	0		
24-JUN-2018	13:54	Day 2		F1	5 ug	0	0	0	0	0	1	0		
24-JUN-2018	13:54	Day 2		F2	5 ug	1	0	0	0	0	1	0		
24-JUN-2018	13:54	Day 2		F3	5 ug	0	0	0	0	0	1	0		
26-JUN-2018	10:44	Day 4		F1	5 ug	0	0	0	0	0	0	0		
26-JUN-2018	10:44	Day 4		F2	5 ug	0	1	0	1	0	0	0		
26-JUN-2018	10:44	Day 4		F3	5 ug	0	0	0	1	0	0	0		
30-JUN-2018	08:16	Day 8		F1	5 ug	0	0	0	0	1	0	3		
30-JUN-2018	08:16	Day 8		F2	5 ug	0	0	0	0	1	0	3		

Key: 0= None; 1=Mild; 2=Moderate; 3=Severe; # Other = Pinprick bleeding or seepage

Administration Site Key: FA = Forearm; UA= Upper arm
Treatments Part A: A1 = A/Sing MAP, 15 ug (FA); A2 = Afluria Quad IM (UA), 15 ug; A3 = Placebo MAP (FA); A4 = A/Sing Mono IM, 15 ug (UA)
Treatments Part B: B1 = A/Sing MAP, 15 ug (FA) with biopsy; B2 = Placebo MAP (FA) with biopsy
B3 = A/Sing MAP, 15 ug (FA); B4 = A/Sing MAP, 10 ug (FA); B5 = A/Sing MAP, 5 ug (FA); B6 = A/Sing MAP, 2.5 ug (FA)
B7 = Placebo MAP (FA); B8 = A/Sing MAP, 15 ug (UA); B9 = A/Sing IM, 15 ug, as 2018 Afluria Quad (UA)

Listing 16.2.9.2
Treatment Site Tolerability Assessment per Site

Part B
Randomisation Number = B0145, Treatment Group = Group B3

Date	Time	Visit	Time Point	Site Number	Treatment for Site	Indura-tion	Redness	Bruising	Itching	Skin Flaking	Tender-ness	Definition/ Visibility	Other#	Unscheduled Reason
30-JUN-2018	08:16	Day 8		F3	5 ug	0	0	0	0	1	0	3		
30-JUN-2018	08:38	Unscheduled		F1	5 ug	0	0	0	0	1	0	3	NA	MO notifies as repeat
30-JUN-2018	08:38	Unscheduled		F2	5 ug	0	0	0	0	1	0	3	NA	MO notifies as repeat
30-JUN-2018	08:38	Unscheduled		F3	5 ug	0	0	0	0	1	0	3	NA	MO notifies as repeat
14-JUL-2018	09:00	Day 22		F1	5 ug	0	0	0	0	0	0	0		
14-JUL-2018	09:00	Day 22		F2	5 ug	0	0	0	0	0	0	0		
14-JUL-2018	09:00	Day 22		F3	5 ug	0	0	0	0	0	0	0		
22-AUG-2018	10:39	EOS/ET (Day 61)		F1	5 ug	0	0	0	0	0	0	0		
22-AUG-2018	10:39	EOS/ET (Day 61)		F2	5 ug	0	0	0	0	0	0	0		
22-AUG-2018	10:39	EOS/ET (Day 61)		F3	5 ug	0	0	0	0	0	0	0		

Key: 0= None; 1=Mild; 2=Moderate; 3=Severe; # Other = Pinprick bleeding or seepage

Administration Site Key: FA = Forearm; UA= Upper arm
Treatments Part A: A1 = A/Sing MAP, 15 ug (FA); A2 = Afluria Quad IM (UA), 15 ug; A3 = Placebo MAP (FA); A4 = A/Sing Mono IM, 15 ug (UA)
Treatments Part B: B1 = A/Sing MAP, 15 ug (FA) with biopsy; B2 = Placebo MAP (FA) with biopsy
B3 = A/Sing MAP, 15 ug (FA); B4 = A/Sing MAP, 10 ug (FA); B5 = A/Sing MAP, 5 ug (FA); B6 = A/Sing MAP, 2.5 ug (FA)
B7 = Placebo MAP (FA); B8 = A/Sing MAP, 15 ug (UA); B9 = A/Sing IM, 15 ug, as 2018 Afluria Quad (UA)

Listing 16.2.9.2
Treatment Site Tolerability Assessment per Site

Part B
Randomisation Number = B0146, Treatment Group = Group B7

Date	Time	Visit	Time Point	Site Number	Treatment for Site	Induration	Redness	Bruising	Itching	Skin Flaking	Tenderness	Definition/ Visibility	Other#	Unscheduled Reason
28-JUN-2018	09:12	Day 1	Pre-dose	F1	uncoated	0	0	0	0	0	0	0		
28-JUN-2018	09:12	Day 1	Pre-dose	F2	uncoated	0	0	0	0	0	0	0		
28-JUN-2018	09:12	Day 1	Pre-dose	F3	uncoated	0	0	0	0	0	0	0		
28-JUN-2018	11:28	Day 1	10 min	F1	uncoated		1	0	1	0	0	0		
28-JUN-2018	11:28	Day 1	10 min	F2	uncoated	1	1	0	1		0	0		
28-JUN-2018	11:28	Day 1	10 min	F3	uncoated	1	1	0	1	0	0	0		
28-JUN-2018	12:18	Day 1	1 hour	F1	uncoated	0	1	0	0	0	1	0		
28-JUN-2018	12:18	Day 1	1 hour	F2	uncoated	0	1	0	0	0	1	0		
28-JUN-2018	12:18	Day 1	1 hour	F3	uncoated	0	1	0	0	0	1	0		
28-JUN-2018	13:18	Day 1	2 hour	F1	uncoated	0	0	0	0	0	1	0		
28-JUN-2018	13:18	Day 1	2 hour	F2	uncoated	1	0	0	0	0	1	0		
28-JUN-2018	13:18	Day 1	2 hour	F3	uncoated	0	0	0	0	0	1	0		
29-JUN-2018	11:21	Day 2		F1	uncoated	0	0	0	0	0	0	0		
29-JUN-2018	11:21	Day 2		F2	uncoated	0	0	0	0	0	0	0		
29-JUN-2018	11:21	Day 2		F3	uncoated	0	0	0	0	0	0	0		
01-JUL-2018	08:17	Day 4		F1	uncoated	0	0	0	0	0	0	0		
01-JUL-2018	08:17	Day 4		F2	uncoated	0	0	0	0	0	0	0		
01-JUL-2018	08:17	Day 4		F3	uncoated	0	0	0	0	0	0	0		
05-JUL-2018	08:44	Day 8		F1	uncoated	0	0	0	0	0	0	3		
05-JUL-2018	08:44	Day 8		F2	uncoated	0	0	0	0	0	0	3		

Key: 0= None; 1=Mild; 2=Moderate; 3=Severe; # Other = Pinprick bleeding or seepage

Administration Site Key: FA = Forearm; UA= Upper arm
Treatments Part A: A1 = A/Sing MAP, 15 ug (FA); A2 = Afluria Quad IM (UA), 15 ug; A3 = Placebo MAP (FA); A4 = A/Sing Mono IM, 15 ug (UA)
Treatments Part B: B1 = A/Sing MAP, 15 ug (FA) with biopsy; B2 = Placebo MAP (FA) with biopsy
B3 = A/Sing MAP, 15 ug (FA); B4 = A/Sing MAP, 10 ug (FA); B5 = A/Sing MAP, 5 ug (FA); B6 = A/Sing MAP, 2.5 ug (FA)
B7 = Placebo MAP (FA); B8 = A/Sing MAP, 15 ug (UA); B9 = A/Sing IM, 15 ug, as 2018 Afluria Quad (UA)

Listing 16.2.9.2
Treatment Site Tolerability Assessment per Site

Part B
Randomisation Number = B0146, Treatment Group = Group B7

Date	Time	Visit	Time Point	Site Number	Treatment for Site	Indura-tion	Redness	Bruising	Itching	Skin Flaking	Tender-ness	Definition/ Visibility	Other#	Unscheduled Reason
05-JUL-2018	08:44	Day 8		F3	uncoated	0	0	0	0	0	0	3		
19-JUL-2018	08:14	Day 22		F1	uncoated	0	0	0	0	0	0	0		
19-JUL-2018	08:14	Day 22		F2	uncoated	0	0	0	0	0	0	0		
19-JUL-2018	08:14	Day 22		F3	uncoated	0	0	0	0	0	0	0		
28-AUG-2018	12:50	EOS/ET (Day 61)		F1	uncoated	0	0	0	0	0	0	0		
28-AUG-2018	12:50	EOS/ET (Day 61)		F2	uncoated	0	0	0	0	0	0	0		
28-AUG-2018	12:50	EOS/ET (Day 61)		F3	uncoated	0	0	0	0	0	0	0		

Key: 0= None; 1=Mild; 2=Moderate; 3=Severe; # Other = Pinprick bleeding or seepage

Administration Site Key: FA = Forearm; UA= Upper arm
Treatments Part A: A1 = A/Sing MAP, 15 ug (FA); A2 = Afluria Quad IM (UA), 15 ug; A3 = Placebo MAP (FA); A4 = A/Sing Mono IM, 15 ug (UA)
Treatments Part B: B1 = A/Sing MAP, 15 ug (FA) with biopsy; B2 = Placebo MAP (FA) with biopsy
B3 = A/Sing MAP, 15 ug (FA); B4 = A/Sing MAP, 10 ug (FA); B5 = A/Sing MAP, 5 ug (FA); B6 = A/Sing MAP, 2.5 ug (FA)
B7 = Placebo MAP (FA); B8 = A/Sing MAP, 15 ug (UA); B9 = A/Sing IM, 15 ug, as 2018 Afluria Quad (UA)

Listing 16.2.9.2
Treatment Site Tolerability Assessment per Site

Part B
Randomisation Number = B0147, Treatment Group = Group B9

Date	Time	Visit	Time Point	Site Number	Treatment for Site	Indura-tion	Redness	Bruising	Itching	Skin Flaking	Tender-ness	Definition/ Visibility	Other#	Unscheduled Reason
28-JUN-2018	09:03	Day 1	Pre-dose	D1	[IM]	0	0	0	0	0	0	5		
28-JUN-2018	11:11	Day 1	10 min	D1	[IM]	0	0	0	0	0	0	5		
28-JUN-2018	12:03	Day 1	1 hour	D1	[IM]	0	0	0	0	0	0	5		
28-JUN-2018	13:05	Day 1	2 hour	D1	[IM]	0	0	0	0	0	0	5		
29-JUN-2018	11:21	Day 2		D1	[IM]	0	0	1	0	0	0	5		
01-JUL-2018	11:45	Day 4		D1	[IM]	0	0	1	0	0	0	5		
05-JUL-2018	11:59	Day 8		D1	[IM]	0	0	2	0	0	0	5		
19-JUL-2018	12:59	Day 22		D1	[IM]	0	0	0	0	0	0	5		
27-AUG-2018	11:44	EOS/ET (Day 61)		D1	[IM]	0	0	0	0	0	0	5		

Key: 0= None; 1=Mild; 2=Moderate; 3=Severe; # Other = Pinprick bleeding or seepage

Administration Site Key: FA = Forearm; UA= Upper arm
Treatments Part A: A1 = A/Sing MAP, 15 ug (FA); A2 = Afluria Quad IM (UA), 15 ug; A3 = Placebo MAP (FA); A4 = A/Sing Mono IM, 15 ug (UA)
Treatments Part B: B1 = A/Sing MAP, 15 ug (FA) with biopsy; B2 = Placebo MAP (FA) with biopsy
B3 = A/Sing MAP, 15 ug (FA); B4 = A/Sing MAP, 10 ug (FA); B5 = A/Sing MAP, 5 ug (FA); B6 = A/Sing MAP, 2.5 ug (FA)
B7 = Placebo MAP (FA); B8 = A/Sing MAP, 15 ug (UA); B9 = A/Sing IM, 15 ug, as 2018 Afluria Quad (UA)

Listing 16.2.9.2
Treatment Site Tolerability Assessment per Site

Part B
Randomisation Number = B0148, Treatment Group = Group B5

Date	Time	Visit	Time Point	Site Number	Treatment for Site	Indura-tion	Redness	Bruising	Itching	Skin Flaking	Tender-ness	Definition/ Visibility	Other#	Unscheduled Reason
28-JUN-2018	09:16	Day 1	Pre-dose	F1	5 ug	0	0	0	0	0	0	0		
28-JUN-2018	09:16	Day 1	Pre-dose	F2	uncoated	0	0	0	0	0	0	0		
28-JUN-2018	09:16	Day 1	Pre-dose	F3	uncoated	0	0	0	0	0	0	0		
28-JUN-2018	11:38	Day 1	10 min	F1	5 ug	1	2	0	0	0	1	0		
28-JUN-2018	11:38	Day 1	10 min	F2	uncoated	1	2	0	0	0	0	0		
28-JUN-2018	11:38	Day 1	10 min	F3	uncoated	1	2	0	0	0	0	0		
28-JUN-2018	12:28	Day 1	1 hour	F1	5 ug	1	1	0	0	0	0	0		
28-JUN-2018	12:28	Day 1	1 hour	F2	uncoated	1	1	0	0	0	0	0		
28-JUN-2018	12:28	Day 1	1 hour	F3	uncoated	1	1	0	0	0	0	0		
28-JUN-2018	13:38	Day 1	2 hour	F1	5 ug	0	0	0	0	0	1	0		
28-JUN-2018	13:38	Day 1	2 hour	F2	uncoated	0	0	0	0	0	0	0		
28-JUN-2018	13:38	Day 1	2 hour	F3	uncoated	0	0	0	0	0	0	0		
29-JUN-2018	09:50	Day 2		F1	5 ug	0	1	0	0	0	2	0		
29-JUN-2018	09:50	Day 2		F2	uncoated	0	0	0	0	0	0	0		
29-JUN-2018	09:50	Day 2		F3	uncoated	0	0	0	0	0	0	0		
01-JUL-2018	09:52	Day 4		F1	5 ug	0	1	0	0	0	0	0		
01-JUL-2018	09:52	Day 4		F2	uncoated	0	0	0	0	0	0	0		
01-JUL-2018	09:52	Day 4		F3	uncoated	0	0	0	0	0	0	0		
01-JUL-2018	10:27	Unscheduled		F1	5 ug	0	1	0	1	0	0	0	NA	MO notifies as repeat

Key: 0= None; 1=Mild; 2=Moderate; 3=Severe; # Other = Pinprick bleeding or seepage

Administration Site Key: FA = Forearm; UA= Upper arm
Treatments Part A: A1 = A/Sing MAP, 15 ug (FA); A2 = Afluria Quad IM (UA), 15 ug; A3 = Placebo MAP (FA); A4 = A/Sing Mono IM, 15 ug (UA)
Treatments Part B: B1 = A/Sing MAP, 15 ug (FA) with biopsy; B2 = Placebo MAP (FA) with biopsy
B3 = A/Sing MAP, 15 ug (FA); B4 = A/Sing MAP, 10 ug (FA); B5 = A/Sing MAP, 5 ug (FA); B6 = A/Sing MAP, 2.5 ug (FA)
B7 = Placebo MAP (FA); B8 = A/Sing MAP, 15 ug (UA); B9 = A/Sing IM, 15 ug, as 2018 Afluria Quad (UA)

Listing 16.2.9.2
Treatment Site Tolerability Assessment per Site

Part B
Randomisation Number = B0148, Treatment Group = Group B5

Date	Time	Visit	Time Point	Site Number	Treatment for Site	Indura-tion	Redness	Bruising	Itching	Skin Flaking	Tender-ness	Definition/ Visibility	Other#	Unscheduled Reason
01-JUL-2018	10:27	Unscheduled		F2	uncoated	0	0	0	0	0	0	2	NA	MO notifies as repeat
01-JUL-2018	10:27	Unscheduled		F3	uncoated	0	0	0	0	0	0	2	NA	MO notifies as repeat
05-JUL-2018	09:39	Day 8		F1	5 ug	0	0	0	1	2	0	0		
05-JUL-2018	09:39	Day 8		F2	uncoated	0	0	0	0	0	0	3		
05-JUL-2018	09:39	Day 8		F3	uncoated	0	0	0	0	0	0	3		
18-JUL-2018	10:18	Day 22		F1	5 ug	0	0	0	0	0	0	2		
18-JUL-2018	10:18	Day 22		F2	uncoated	0	0	0	0	0	0	1		
18-JUL-2018	10:18	Day 22		F3	uncoated	0	0	0	0	0	0	1		
27-AUG-2018	11:20	EOS/ET (Day 61)		F1	5 ug	0	0	0	0	0	0	1		
27-AUG-2018	11:20	EOS/ET (Day 61)		F2	uncoated	0	0	0	0	0	0	0		
27-AUG-2018	11:20	EOS/ET (Day 61)		F3	uncoated	0	0	0	0	0	0	0		

Key: 0= None; 1=Mild; 2=Moderate; 3=Severe; # Other = Pinprick bleeding or seepage

Administration Site Key: FA = Forearm; UA= Upper arm
Treatments Part A: A1 = A/Sing MAP, 15 ug (FA); A2 = Afluria Quad IM (UA), 15 ug; A3 = Placebo MAP (FA); A4 = A/Sing Mono IM, 15 ug (UA)
Treatments Part B: B1 = A/Sing MAP, 15 ug (FA) with biopsy; B2 = Placebo MAP (FA) with biopsy
B3 = A/Sing MAP, 15 ug (FA); B4 = A/Sing MAP, 10 ug (FA); B5 = A/Sing MAP, 5 ug (FA); B6 = A/Sing MAP, 2.5 ug (FA)
B7 = Placebo MAP (FA); B8 = A/Sing MAP, 15 ug (UA); B9 = A/Sing IM, 15 ug, as 2018 Afluria Quad (UA)

Listing 16.2.9.2
Treatment Site Tolerability Assessment per Site

Part B
Randomisation Number = B0149, Treatment Group = Group B8

Date	Time	Visit	Time Point	Site Number	Treatment for Site	Induration	Redness	Bruising	Itching	Skin Flaking	Tenderness	Definition/ Visibility	Other#	Unscheduled Reason
28-JUN-2018	09:34	Day 1	Pre-dose	D1	5 ug	0	0	0	0	0	0	0		
28-JUN-2018	09:34	Day 1	Pre-dose	D2	5 ug	0	0	0	0	0	0	0		
28-JUN-2018	09:34	Day 1	Pre-dose	D3	5 ug	0	0	0	0	0	0	0		
28-JUN-2018	11:48	Day 1	10 min	D1	5 ug	1	1	0	0	0	0	0		
28-JUN-2018	11:48	Day 1	10 min	D2	5 ug	1	1	0	0	0	0	0		
28-JUN-2018	11:48	Day 1	10 min	D3	5 ug	1	1	0	0	0	0	0		
28-JUN-2018	12:38	Day 1	1 hour	D1	5 ug	0	0	0	0	0	0	0		
28-JUN-2018	12:38	Day 1	1 hour	D2	5 ug	0	0	0	0	0	0	0		
28-JUN-2018	12:38	Day 1	1 hour	D3	5 ug	0	1	0	0	0	0	0		
28-JUN-2018	13:39	Day 1	2 hour	D1	5 ug	0	0	0	0	0	0	0		
28-JUN-2018	13:39	Day 1	2 hour	D2	5 ug	1	0	0	0	0	0	0		
28-JUN-2018	13:39	Day 1	2 hour	D3	5 ug	0	0	0	0	0	0	0		
29-JUN-2018	08:57	Day 2		D1	5 ug	0	1	0	0	0	0	0		
29-JUN-2018	08:57	Day 2		D2	5 ug	0	1	0	0	0	0	0		
29-JUN-2018	08:57	Day 2		D3	5 ug	0	1	0	0	0	0	0		
01-JUL-2018	10:30	Day 4		D1	5 ug	0	1	0	0	0	0	0		
01-JUL-2018	10:30	Day 4		D2	5 ug	0	1	0	0	0	0	0		
01-JUL-2018	10:30	Day 4		D3	5 ug	0	1	0	0	0	0	0		
05-JUL-2018	09:50	Day 8		D1	5 ug	0	0	0	0	1	0	0		
05-JUL-2018	09:50	Day 8		D2	5 ug	0	0	0	0	1	0	0		

Key: 0= None; 1=Mild; 2=Moderate; 3=Severe; # Other = Pinprick bleeding or seepage

Administration Site Key: FA = Forearm; UA= Upper arm
Treatments Part A: A1 = A/Sing MAP, 15 ug (FA); A2 = Afluria Quad IM (UA), 15 ug; A3 = Placebo MAP (FA); A4 = A/Sing Mono IM, 15 ug (UA)
Treatments Part B: B1 = A/Sing MAP, 15 ug (FA) with biopsy; B2 = Placebo MAP (FA) with biopsy
B3 = A/Sing MAP, 15 ug (FA); B4 = A/Sing MAP, 10 ug (FA); B5 = A/Sing MAP, 5 ug (FA); B6 = A/Sing MAP, 2.5 ug (FA)
B7 = Placebo MAP (FA); B8 = A/Sing MAP, 15 ug (UA); B9 = A/Sing IM, 15 ug, as 2018 Afluria Quad (UA)

Listing 16.2.9.2
Treatment Site Tolerability Assessment per Site

Part B
Randomisation Number = B0149,   Treatment Group = Group B8

Date	Time	Visit	Time Point	Site Number	Treatment for Site	Indura-tion	Redness	Bruising	Itching	Skin Flaking	Tender-ness	Definition/ Visibility	Other#	Unscheduled Reason
05-JUL-2018	09:50	Day 8		D3	5 ug	0	0	0	0	2	0	0		
19-JUL-2018	11:44	Day 22		D1	5 ug	0	0	0	0	1	0	2		
19-JUL-2018	11:44	Day 22		D2	5 ug	0	0	0	0	1	0	2		
19-JUL-2018	11:44	Day 22		D3	5 ug	0	0	0	0	1	0	2		
27-AUG-2018	12:04	EOS/ET (Day 61)		D1	5 ug	0	0	0	0	0	0	0		
27-AUG-2018	12:04	EOS/ET (Day 61)		D2	5 ug	0	0	0	0	0	0	0		
27-AUG-2018	12:04	EOS/ET (Day 61)		D3	5 ug	0	0	0	0	0	0	0		

Key: 0= None; 1=Mild; 2=Moderate; 3=Severe; # Other = Pinprick bleeding or seepage

Administration Site Key: FA = Forearm; UA= Upper arm
Treatments Part A: A1 = A/Sing MAP, 15 ug (FA); A2 = Afluria Quad IM (UA), 15 ug; A3 = Placebo MAP (FA); A4 = A/Sing Mono IM, 15 ug (UA)
Treatments Part B: B1 = A/Sing MAP, 15 ug (FA) with biopsy; B2 = Placebo MAP (FA) with biopsy
B3 = A/Sing MAP, 15 ug (FA); B4 = A/Sing MAP, 10 ug (FA); B5 = A/Sing MAP, 5 ug (FA); B6 = A/Sing MAP, 2.5 ug (FA)
B7 = Placebo MAP (FA); B8 = A/Sing MAP, 15 ug (UA); B9 = A/Sing IM, 15 ug, as 2018 Afluria Quad (UA)

Listing 16.2.9.2
Treatment Site Tolerability Assessment per Site

Part B
Randomisation Number = B0150, Treatment Group = Group B4

Date	Time	Visit	Time Point	Site Number	Treatment for Site	Indura-tion	Redness	Bruising	Itching	Skin Flaking	Tender-ness	Definition/ Visibility	Other#	Unscheduled Reason
28-JUN-2018	09:40	Day 1	Pre-dose	F1	5 ug	0	0	0	0	0	0	0		
28-JUN-2018	09:40	Day 1	Pre-dose	F2	5 ug	0	0	0	0	0	0	0		
28-JUN-2018	09:40	Day 1	Pre-dose	F3	uncoated	0	0	0	0	0	0	0		
28-JUN-2018	11:58	Day 1	10 min	F1	5 ug	0	1	0	0	0	0	0		
28-JUN-2018	11:58	Day 1	10 min	F2	5 ug	0	1	0	0	0	1			
28-JUN-2018	11:58	Day 1	10 min	F3	uncoated	0	1	0	0	0	0	0		
28-JUN-2018	12:48	Day 1	1 hour	F1	5 ug	0	1	0	0	0	0	0		
28-JUN-2018	12:48	Day 1	1 hour	F2	5 ug	0	1	0	0	0	0	0		
28-JUN-2018	12:48	Day 1	1 hour	F3	uncoated	0	1	0	0	0	0	0		
28-JUN-2018	13:50	Day 1	2 hour	F1	5 ug	0	0	0	0	0	0	0		
28-JUN-2018	13:50	Day 1	2 hour	F2	5 ug	1	0	0	0	0	1	0		
28-JUN-2018	13:50	Day 1	2 hour	F3	uncoated	0	0	0	0	0	0	0		
29-JUN-2018	10:20	Day 2		F1	5 ug	0	1	0	1	0	0	0		
29-JUN-2018	10:20	Day 2		F2	5 ug	0	1	0	1	0	1	0		
29-JUN-2018	10:20	Day 2		F3	uncoated	0	1	0	0	0	0	0		
01-JUL-2018	09:21	Day 4		F1	5 ug	0	0	0	0	1	0	0		
01-JUL-2018	09:21	Day 4		F2	5 ug	0	1	0	0	0	0	0		
01-JUL-2018	09:21	Day 4		F3	uncoated	0	0	0	0	0	0	3		
05-JUL-2018	14:39	Day 8		F1	5 ug	0	0	0	0	1	0	0		
05-JUL-2018	14:39	Day 8		F2	5 ug	0	0	0	0	2	0	0		

Key: 0= None; 1=Mild; 2=Moderate; 3=Severe; # Other = Pinprick bleeding or seepage

Administration Site Key: FA = Forearm; UA= Upper arm
Treatments Part A: A1 = A/Sing MAP, 15 ug (FA); A2 = Afluria Quad IM (UA), 15 ug; A3 = Placebo MAP (FA); A4 = A/Sing Mono IM, 15 ug (UA)
Treatments Part B: B1 = A/Sing MAP, 15 ug (FA) with biopsy; B2 = Placebo MAP (FA) with biopsy
B3 = A/Sing MAP, 15 ug (FA); B4 = A/Sing MAP, 10 ug (FA); B5 = A/Sing MAP, 5 ug (FA); B6 = A/Sing MAP, 2.5 ug (FA)
B7 = Placebo MAP (FA); B8 = A/Sing MAP, 15 ug (UA); B9 = A/Sing IM, 15 ug, as 2018 Afluria Quad (UA)

Listing 16.2.9.2
Treatment Site Tolerability Assessment per Site

Part B
Randomisation Number = B0150, Treatment Group = Group B4

Date	Time	Visit	Time Point	Site Number	Treatment for Site	Indura-tion	Redness	Bruising	Itching	Skin Flaking	Tender-ness	Definition/ Visibility	Other#	Unscheduled Reason
05-JUL-2018	14:39	Day 8		F3	uncoated	0	0	0	0	0	0	3		
19-JUL-2018	15:15	Day 22		F1	5 ug	0	0	0	0	1	0	0		
19-JUL-2018	15:15	Day 22		F2	5 ug	0	0	0	0	1	0	0		
19-JUL-2018	15:15	Day 22		F3	uncoated	0	0	0	0	0	0	1		
28-AUG-2018	13:33	EOS/ET (Day 61)		F1	5 ug	0	0	0	0	0	0	1		
28-AUG-2018	13:33	EOS/ET (Day 61)		F2	5 ug	0	0	0	0	0	0	1		
28-AUG-2018	13:33	EOS/ET (Day 61)		F3	uncoated	0	0	0	0	0	0	0		

Key: 0= None; 1=Mild; 2=Moderate; 3=Severe; # Other = Pinprick bleeding or seepage

Administration Site Key: FA = Forearm; UA= Upper arm
Treatments Part A: A1 = A/Sing MAP, 15 ug (FA); A2 = Afluria Quad IM (UA), 15 ug; A3 = Placebo MAP (FA); A4 = A/Sing Mono IM, 15 ug (UA)
Treatments Part B: B1 = A/Sing MAP, 15 ug (FA) with biopsy; B2 = Placebo MAP (FA) with biopsy
B3 = A/Sing MAP, 15 ug (FA); B4 = A/Sing MAP, 10 ug (FA); B5 = A/Sing MAP, 5 ug (FA); B6 = A/Sing MAP, 2.5 ug (FA)
B7 = Placebo MAP (FA); B8 = A/Sing MAP, 15 ug (UA); B9 = A/Sing IM, 15 ug, as 2018 Afluria Quad (UA)

Listing 16.2.9.2
Treatment Site Tolerability Assessment per Site

Part B
Randomisation Number = B1019, Treatment Group = Group B8

Date	Time	Visit	Time Point	Site Number	Treatment for Site	Indura-tion	Redness	Bruising	Itching	Skin Flaking	Tender-ness	Definition/ Visibility	Other#	Unscheduled Reason
19-MAY-2018	10:30	Day 1	Pre-dose	D1	5 ug	0	0	0	0	0	0	0		
19-MAY-2018	10:30	Day 1	Pre-dose	D2	5 ug	0	0	0	0	0	0	0		
19-MAY-2018	10:30	Day 1	Pre-dose	D3	5 ug	0	0	0	0	0	0	0		
19-MAY-2018	11:19	Day 1	10 min	D1	5 ug	0	0	0	0	0	1	0		
19-MAY-2018	11:19	Day 1	10 min	D2	5 ug	0	0	0	0	0	0	0		
19-MAY-2018	11:19	Day 1	10 min	D3	5 ug	0	0	0	0	0	0	0		
19-MAY-2018	12:09	Day 1	1 hour	D1	5 ug	1	0	0	0	0	0	0		
19-MAY-2018	12:09	Day 1	1 hour	D2	5 ug	1	0	0	0	0	0	0		
19-MAY-2018	12:09	Day 1	1 hour	D3	5 ug	1	0	0	0	0	0	0		
19-MAY-2018	13:22	Day 1	2 hour	D1	5 ug	0	1	0	0	0	0	0		
19-MAY-2018	13:22	Day 1	2 hour	D2	5 ug	0	1	0	0	0	0	0		
19-MAY-2018	13:22	Day 1	2 hour	D3	5 ug	0	1	0	0	0	0	0		
19-MAY-2018	11:34	Unscheduled		D1	5 ug	0	0	0	0	0	0	0	NA	MO notifies as repeat
19-MAY-2018	11:35	Unscheduled		D2	5 ug	0	0	0	0	0	0	0	NA	MO notifies as repeat
19-MAY-2018	11:36	Unscheduled		D3	5 ug	0	0	0	0	0	0	0	NA	MO notifies as repeat
20-MAY-2018	13:43	Day 2		D1	5 ug	0	1	0	0	0	0	0		
20-MAY-2018	13:43	Day 2		D2	5 ug	0	1	0	0	0	0	0		

Key: 0= None; 1=Mild; 2=Moderate; 3=Severe; # Other = Pinprick bleeding or seepage

Administration Site Key: FA = Forearm; UA= Upper arm
Treatments Part A: A1 = A/Sing MAP, 15 ug (FA); A2 = Afluria Quad IM (UA), 15 ug; A3 = Placebo MAP (FA); A4 = A/Sing Mono IM, 15 ug (UA)
Treatments Part B: B1 = A/Sing MAP, 15 ug (FA) with biopsy; B2 = Placebo MAP (FA) with biopsy
B3 = A/Sing MAP, 15 ug (FA); B4 = A/Sing MAP, 10 ug (FA); B5 = A/Sing MAP, 5 ug (FA); B6 = A/Sing MAP, 2.5 ug (FA)
B7 = Placebo MAP (FA); B8 = A/Sing MAP, 15 ug (UA); B9 = A/Sing IM, 15 ug, as 2018 Afluria Quad (UA)

Listing 16.2.9.2
Treatment Site Tolerability Assessment per Site

Part B
Randomisation Number = B1019, Treatment Group = Group B8

Date	Time	Visit	Time Point	Site Number	Treatment for Site	Indura-tion	Redness	Bruising	Itching	Skin Flaking	Tender-ness	Definition/ Visibility	Other#	Unscheduled Reason
20-MAY-2018	13:43	Day 2		D3	5 ug	0	1	0	0	0	0	0		
22-MAY-2018	12:05	Day 4		D1	5 ug	0	0	0	0	0	0	3		
22-MAY-2018	12:05	Day 4		D2	5 ug	0	0	0	0	1	0	3		
22-MAY-2018	12:05	Day 4		D3	5 ug	0	0	0	0	0	0	3		
28-MAY-2018	14:08	Day 8		D1	5 ug	0	0	0	0	0	0	2		
28-MAY-2018	14:08	Day 8		D2	5 ug	0	0	0	0	0	0	2		
28-MAY-2018	14:08	Day 8		D3	5 ug	0	0	0	0	0	0	2		
28-MAY-2018	14:23	Unscheduled		D1	5 ug	0	0	0	0	0	0	2	NA	MO notifies as repeat
28-MAY-2018	14:23	Unscheduled		D2	5 ug	0	0	0	0	0	0	2	NA	MO notifies as repeat
28-MAY-2018	14:23	Unscheduled		D3	5 ug	0	0	0	0	0	0	2	NA	MO notifies as repeat
13-JUN-2018	14:48	Day 22		D1	5 ug	0	0	0	0	0	0	1		
13-JUN-2018	14:48	Day 22		D2	5 ug	0	0	0	0	0	0	1		
13-JUN-2018	14:48	Day 22		D3	5 ug	0	0	0	0	1	0	1		
20-JUL-2018	13:50	EOS/ET (Day 61)		D1	5 ug	0	0	0	0	0	0	1		
20-JUL-2018	13:50	EOS/ET (Day 61)		D2	5 ug	0	0	0	0	0	0	1		
20-JUL-2018	13:50	EOS/ET (Day 61)		D3	5 ug	0	0	0	0	0	0	1		

Key: 0= None; 1=Mild; 2=Moderate; 3=Severe; # Other = Pinprick bleeding or seepage

Administration Site Key: FA = Forearm; UA= Upper arm
Treatments Part A: A1 = A/Sing MAP, 15 ug (FA); A2 = Afluria Quad IM (UA), 15 ug; A3 = Placebo MAP (FA); A4 = A/Sing Mono IM, 15 ug (UA)
Treatments Part B: B1 = A/Sing MAP, 15 ug (FA) with biopsy; B2 = Placebo MAP (FA) with biopsy
B3 = A/Sing MAP, 15 ug (FA); B4 = A/Sing MAP, 10 ug (FA); B5 = A/Sing MAP, 5 ug (FA); B6 = A/Sing MAP, 2.5 ug (FA)
B7 = Placebo MAP (FA); B8 = A/Sing MAP, 15 ug (UA); B9 = A/Sing IM, 15 ug, as 2018 Afluria Quad (UA)

Listing 16.2.9.2
Treatment Site Tolerability Assessment per Site

Part B
Randomisation Number = B1076, Treatment Group = Group B5

Date	Time	Visit	Time Point	Site Number	Treatment for Site	Induration	Redness	Bruising	Itching	Skin Flaking	Tenderness	Definition/ Visibility	Other#	Unscheduled Reason
16-JUN-2018	10:44	Day 1	Pre-dose	F1	5 ug	0	0	0	0	0	0	0		
16-JUN-2018	10:44	Day 1	Pre-dose	F2	uncoated	0	0	0	0	0	0	0		
16-JUN-2018	10:44	Day 1	Pre-dose	F3	uncoated	0	0	0	0	0	0	0		
16-JUN-2018	11:48	Day 1	10 min	F1	5 ug	0	2	0	0	0	0	0		
16-JUN-2018	11:48	Day 1	10 min	F2	uncoated	0	0	0	0	0	0	0		
16-JUN-2018	11:48	Day 1	10 min	F3	uncoated	0	0	0	0	0	0	0		
16-JUN-2018	12:33	Day 1	1 hour	F1	5 ug	1	2	0	0	0	0	0		
16-JUN-2018	12:33	Day 1	1 hour	F2	uncoated	0	0	0	0	0	0	0		
16-JUN-2018	12:33	Day 1	1 hour	F3	uncoated	0	0	0	0	0	0	0		
16-JUN-2018	13:38	Day 1	2 hour	F1	5 ug	2	0	0	0	0	0	0		
16-JUN-2018	13:38	Day 1	2 hour	F2	uncoated	0	0	0	0	0	0	0		
16-JUN-2018	13:38	Day 1	2 hour	F3	uncoated	0	1	0	0	0	0	0		
16-JUN-2018	11:50	Unscheduled		F1	5 ug		2						NA	MO notifies as repeat
16-JUN-2018	12:34	Unscheduled		F1	5 ug		2						NA	MO notifies as repeat
16-JUN-2018	13:46	Unscheduled		F1	5 ug	2							NA	MO notifies as repeat
17-JUN-2018	11:36	Day 2		F1	5 ug	1	1	0	0	0	0	0		
17-JUN-2018	11:36	Day 2		F2	uncoated	0	0	0	0	0	0	0		

Key: 0= None; 1=Mild; 2=Moderate; 3=Severe; # Other = Pinprick bleeding or seepage

Administration Site Key: FA = Forearm; UA= Upper arm
Treatments Part A: A1 = A/Sing MAP, 15 ug (FA); A2 = Afluria Quad IM (UA), 15 ug; A3 = Placebo MAP (FA); A4 = A/Sing Mono IM, 15 ug (UA)
Treatments Part B: B1 = A/Sing MAP, 15 ug (FA) with biopsy; B2 = Placebo MAP (FA) with biopsy
B3 = A/Sing MAP, 15 ug (FA); B4 = A/Sing MAP, 10 ug (FA); B5 = A/Sing MAP, 5 ug (FA); B6 = A/Sing MAP, 2.5 ug (FA)
B7 = Placebo MAP (FA); B8 = A/Sing MAP, 15 ug (UA); B9 = A/Sing IM, 15 ug, as 2018 Afluria Quad (UA)

Listing 16.2.9.2
Treatment Site Tolerability Assessment per Site

Part B
Randomisation Number = B1076, Treatment Group = Group B5

Date	Time	Visit	Time Point	Site Number	Treatment for Site	Indura-tion	Redness	Bruising	Itching	Skin Flaking	Tender-ness	Definition/ Visibility	Other#	Unscheduled Reason
17-JUN-2018	11:36	Day 2		F3	uncoated	0	0	0	0	0	0	0		
19-JUN-2018	12:38	Day 4		F1	5 ug	0	0	0	1	1	0	0		
19-JUN-2018	12:38	Day 4		F2	uncoated	0	0	0	0	0	0	2		
19-JUN-2018	12:38	Day 4		F3	uncoated	0	0	0	0	0	0	2		
23-JUN-2018	10:45	Day 8		F1	5 ug	0	0	0	0	1	0	2		
23-JUN-2018	10:45	Day 8		F2	uncoated	0	0	0	0	1	0	2		
23-JUN-2018	10:45	Day 8		F3	uncoated	0	0	0	0	1	0	2		
07-JUL-2018	10:22	Day 22		F1	5 ug	0	0	0	0	0	0	0		
07-JUL-2018	10:22	Day 22		F2	uncoated	0	0	0	0	0	0	0		
07-JUL-2018	10:22	Day 22		F3	uncoated	0	0	0	0	0	0	0		
15-AUG-2018	13:42	EOS/ET (Day 61)		F1	5 ug	0	0	0	0	0	0	0		
15-AUG-2018	13:42	EOS/ET (Day 61)		F2	uncoated	0	0	0	0	0	0	0		
15-AUG-2018	13:42	EOS/ET (Day 61)		F3	uncoated	0	0	0	0	0	0	0		

Key: 0= None; 1=Mild; 2=Moderate; 3=Severe; # Other = Pinprick bleeding or seepage

Administration Site Key: FA = Forearm; UA= Upper arm
Treatments Part A: A1 = A/Sing MAP, 15 ug (FA); A2 = Afluria Quad IM (UA), 15 ug; A3 = Placebo MAP (FA); A4 = A/Sing Mono IM, 15 ug (UA)
Treatments Part B: B1 = A/Sing MAP, 15 ug (FA) with biopsy; B2 = Placebo MAP (FA) with biopsy
B3 = A/Sing MAP, 15 ug (FA); B4 = A/Sing MAP, 10 ug (FA); B5 = A/Sing MAP, 5 ug (FA); B6 = A/Sing MAP, 2.5 ug (FA)
B7 = Placebo MAP (FA); B8 = A/Sing MAP, 15 ug (UA); B9 = A/Sing IM, 15 ug, as 2018 Afluria Quad (UA)

Listing 16.2.9.2
Treatment Site Tolerability Assessment per Site

Part B
Randomisation Number = B1080, Treatment Group = Group B7

Date	Time	Visit	Time Point	Site Number	Treatment for Site	Indura-tion	Redness	Bruising	Itching	Skin Flaking	Tender-ness	Definition/ Visibility	Other#	Unscheduled Reason
16-JUN-2018	10:55	Day 1	Pre-dose	F1	uncoated	0	0	0	0	0	0	0		
16-JUN-2018	10:55	Day 1	Pre-dose	F2	uncoated	0	0	0	0	0	0	0		
16-JUN-2018	10:55	Day 1	Pre-dose	F3	uncoated	0	0	0	0	0	0	0		
16-JUN-2018	11:58	Day 1	10 min	F1	uncoated	0	0	0	0	0	0	0		
16-JUN-2018	11:58	Day 1	10 min	F2	uncoated	0	0	0	0	0	0	0		
16-JUN-2018	11:58	Day 1	10 min	F3	uncoated	0	0	0	0	0	0	0		
16-JUN-2018	12:49	Day 1	1 hour	F1	uncoated	0	0	0	0	0	0	0		
16-JUN-2018	12:49	Day 1	1 hour	F2	uncoated	0	1	0	0	0	0	0		
16-JUN-2018	12:49	Day 1	1 hour	F3	uncoated	0	1	0	0	0	0	0		
16-JUN-2018	13:48	Day 1	2 hour	F1	uncoated	0	0	0	0	0	0	0		
16-JUN-2018	13:48	Day 1	2 hour	F2	uncoated	1	0		0	0	0	0		
16-JUN-2018	13:48	Day 1	2 hour	F3	uncoated	0	0	0	0	0	0	0		
17-JUN-2018	13:34	Day 2		F1	uncoated	0	0	0	0	0	0	0		
17-JUN-2018	13:34	Day 2		F2	uncoated	0	0	0	0	0	0	0		
17-JUN-2018	13:34	Day 2		F3	uncoated	0	0	0	0	0	0	0		
19-JUN-2018	12:30	Day 4		F1	uncoated	0	0	0	0	0	0	1		
19-JUN-2018	12:30	Day 4		F2	uncoated	0	0	0	0	0	0	2		
19-JUN-2018	12:30	Day 4		F3	uncoated	0	0	0	0	0	0	2		
23-JUN-2018	12:58	Day 8		F1	uncoated	0	0	0	0	0	0	0		
23-JUN-2018	12:58	Day 8		F2	uncoated	0	0	0	0	0	0	0		

Key: 0= None; 1=Mild; 2=Moderate; 3=Severe; # Other = Pinprick bleeding or seepage

Administration Site Key: FA = Forearm; UA= Upper arm
Treatments Part A: A1 = A/Sing MAP, 15 ug (FA); A2 = Afluria Quad IM (UA), 15 ug; A3 = Placebo MAP (FA); A4 = A/Sing Mono IM, 15 ug (UA)
Treatments Part B: B1 = A/Sing MAP, 15 ug (FA) with biopsy; B2 = Placebo MAP (FA) with biopsy
B3 = A/Sing MAP, 15 ug (FA); B4 = A/Sing MAP, 10 ug (FA); B5 = A/Sing MAP, 5 ug (FA); B6 = A/Sing MAP, 2.5 ug (FA)
B7 = Placebo MAP (FA); B8 = A/Sing MAP, 15 ug (UA); B9 = A/Sing IM, 15 ug, as 2018 Afluria Quad (UA)

Listing 16.2.9.2
Treatment Site Tolerability Assessment per Site

Part B
Randomisation Number = B1080, Treatment Group = Group B7

Date	Time	Visit	Time Point	Site Number	Treatment for Site	Indura-tion	Redness	Bruising	Itching	Skin Flaking	Tender-ness	Definition/ Visibility	Other#	Unscheduled Reason
23-JUN-2018	12:58	Day 8		F3	uncoated	0	0	0	0	0	0	0		
07-JUL-2018	13:12	Day 22		F1	uncoated	0	0	0	0	0	0	0		
07-JUL-2018	13:12	Day 22		F2	uncoated	0	0	0	0	0	0	0		
07-JUL-2018	13:12	Day 22		F3	uncoated	0	0	0	0	0	0	0		
15-AUG-2018	09:37	EOS/ET (Day 61)		F1	uncoated	0	0	0	0	0	0	0		
15-AUG-2018	09:37	EOS/ET (Day 61)		F2	uncoated	0	0	0	0	0	0	0		
15-AUG-2018	09:37	EOS/ET (Day 61)		F3	uncoated	0	0	0	0	0	0	0		

Key: 0= None; 1=Mild; 2=Moderate; 3=Severe; # Other = Pinprick bleeding or seepage

Administration Site Key: FA = Forearm; UA= Upper arm
Treatments Part A: A1 = A/Sing MAP, 15 ug (FA); A2 = Afluria Quad IM (UA), 15 ug; A3 = Placebo MAP (FA); A4 = A/Sing Mono IM, 15 ug (UA)
Treatments Part B: B1 = A/Sing MAP, 15 ug (FA) with biopsy; B2 = Placebo MAP (FA) with biopsy
B3 = A/Sing MAP, 15 ug (FA); B4 = A/Sing MAP, 10 ug (FA); B5 = A/Sing MAP, 5 ug (FA); B6 = A/Sing MAP, 2.5 ug (FA)
B7 = Placebo MAP (FA); B8 = A/Sing MAP, 15 ug (UA); B9 = A/Sing IM, 15 ug, as 2018 Afluria Quad (UA)
