# Supplementary material for: Safety, tolerability, and immunogenicity of influenza vaccination with a high-density microarray patch: Results from a randomized, controlled phase I clinical trial
Source: PLoS Med. 2020 Mar 17;17(3):e1003024. doi: 10.1371/journal.pmed.1003024 (PMC7077342; doi:10.1371/journal.pmed.1003024)
Supplement: S1 Data — Data 1: Informed Consent; Data 2: Analysis Sets; Data 3: Demographics; Data 4: Study Drug Administration; Data 5: Skin Hardness Assessment; Data 6: Immunogenicity (ELISA IgG); Data 7: Immunogenicity (ADCC); Data 8: Immunogenicity (MBCs); Data 9: Immunogenicity (CMI); Data 10: Immunogenicity (Mucosal IgA); Data 11: Immunogenicity Results (HAI, MN); Data 12: TEAE–CRF Data only; Data 13: TEAEs–MedDRA Coding; Data 15: Treatment Site Tolerability Assessment; Data 16: Numeric Pain Intensity; Data 17: SII; Data 18: Application Site Status at End of Study; Data 19: Individual Treatment Site Tolerability Assessment; Data 20: Numeric Pain Intensity by Treatment; Data 21: Individual SII by Treatment. ADCC, antibody-dependent cellular cytotoxicity; CMI, cell-mediated immunity; CRF, case report form; ELISA, enzyme-linked immunosorbent assay; HAI, haemagglutination inhibition; IgA, immunoglobulin A; IgG, immunoglobulin G; MBC, memory B cell; MedDRA, medical dictionary for regulatory activities; MN, microneutralisation; SII, Skin Irritation Index; TEA, treatment emergent adverse event. (ZIP) [file pmed.1003024.s008.zip › S1_Data/clinical supporting/S1_Data16 Numeric Pain Intensity.pdf]

Listing 16.2.9.3
Numeric Pain Intensity per Site

Part A

Randomisation Number	Group	Visit	Time Point	Was Pain Assessment Performed?	Site Number	Treatment	Date of assessment	Time of Assessment	Score	Unscheduled Reason
A0001	Group A3	Day 1	1 min	Yes	F1	uncoated	09-MAR-2018	12:01	1	
A0001	Group A3	Day 1	1 min	Yes	F2	uncoated	09-MAR-2018	12:04	1	
A0001	Group A3	Day 1	1 min	Yes	F3	uncoated	09-MAR-2018	12:07	1	
A0001	Group A3	Day 1	10 min	Yes	F1	uncoated	09-MAR-2018	12:10	0	
A0001	Group A3	Day 1	10 min	Yes	F2	uncoated	09-MAR-2018	12:10	0	
A0001	Group A3	Day 1	10 min	Yes	F3	uncoated	09-MAR-2018	12:10	0	
A0001	Group A3	Day 1	1 hour	Yes	F1	uncoated	09-MAR-2018	13:20	0	
A0001	Group A3	Day 1	1 hour	Yes	F2	uncoated	09-MAR-2018	13:20	0	
A0001	Group A3	Day 1	1 hour	Yes	F3	uncoated	09-MAR-2018	13:20	0	
A0001	Group A3	Day 1	2 hour	Yes	F1	uncoated	09-MAR-2018	14:20	0	
A0001	Group A3	Day 1	2 hour	Yes	F2	uncoated	09-MAR-2018	14:20	0	
A0001	Group A3	Day 1	2 hour	Yes	F3	uncoated	09-MAR-2018	14:20	0	
A0001	Group A3	Day 4		Yes	F1	uncoated	12-MAR-2018	11:43	0	
A0001	Group A3	Day 4		Yes	F2	uncoated	12-MAR-2018	11:43	0	
A0001	Group A3	Day 4		Yes	F3	uncoated	12-MAR-2018	11:43	0	
A0001	Group A3	Day 8		Yes	F1	uncoated	16-MAR-2018	11:31	0	
A0001	Group A3	Day 8		Yes	F2	uncoated	16-MAR-2018	11:32	0	
A0001	Group A3	Day 8		Yes	F3	uncoated	16-MAR-2018	11:33	0	
A0001	Group A3	Day 22		Yes	F1	uncoated	29-MAR-2018	11:51	0	
A0001	Group A3	Day 22		Yes	F2	uncoated	29-MAR-2018	11:51	0	
A0001	Group A3	Day 22		Yes	F3	uncoated	29-MAR-2018	11:51	0	

Scale: 0= No Pain; 10= Worst Pain Possible
Administration Site Key: FA = Forearm; UA= Upper arm
Treatments Part A: A1 = A/Sing MAP, 15 ug (FA); A2 = Afluria Quad IM (UA), 15 ug; A3 = Placebo MAP (FA); A4 = A/Sing Mono IM, 15 ug (UA)
Treatments Part B: B1 = A/Sing MAP, 15 ug (FA) with biopsy; B2 = Placebo MAP (FA) with biopsy
B3 = A/Sing MAP, 15 ug (FA); B4 = A/Sing MAP, 10 ug (FA); B5 = A/Sing MAP, 5 ug (FA); B6 = A/Sing MAP, 2.5 ug (FA)
B7 = Placebo MAP (FA); B8 = A/Sing MAP, 15 ug (UA); B9 = A/Sing IM, 15 ug, as 2018 Afluria Quad (UA)

Listing 16.2.9.3
Numeric Pain Intensity per Site

Part A

Randomisation Number	Group	Visit	Time Point	Was Pain Assessment Performed?	Site Number	Treatment	Date of assessment	Time of Assessment	Score	Unscheduled Reason
A0001	Group A3	EOS/ET (Day 61)		Yes	F1	uncoated	06-MAY-2018	11:55	0	
A0001	Group A3	EOS/ET (Day 61)		Yes	F2	uncoated	06-MAY-2018	11:55	0	
A0001	Group A3	EOS/ET (Day 61)		Yes	F3	uncoated	06-MAY-2018	11:55	0	
A0002	Group A1	Day 1	1 min	Yes	F1	5 ug	09-MAR-2018	12:12	1	
A0002	Group A1	Day 1	1 min	Yes	F2	5 ug	09-MAR-2018	12:15	1	
A0002	Group A1	Day 1	1 min	Yes	F3	5 ug	09-MAR-2018	12:18	1	
A0002	Group A1	Day 1	10 min	Yes	F1	5 ug	09-MAR-2018	12:29	1	
A0002	Group A1	Day 1	10 min	Yes	F2	5 ug	09-MAR-2018	12:29	1	
A0002	Group A1	Day 1	10 min	Yes	F3	5 ug	09-MAR-2018	12:29	1	
A0002	Group A1	Day 1	1 hour	Yes	F1	5 ug	09-MAR-2018	13:28	0	
A0002	Group A1	Day 1	1 hour	Yes	F2	5 ug	09-MAR-2018	13:28	0	
A0002	Group A1	Day 1	1 hour	Yes	F3	5 ug	09-MAR-2018	13:28	0	
A0002	Group A1	Day 1	2 hour	Yes	F1	5 ug	09-MAR-2018	14:29	0	
A0002	Group A1	Day 1	2 hour	Yes	F2	5 ug	09-MAR-2018	14:29	0	
A0002	Group A1	Day 1	2 hour	Yes	F3	5 ug	09-MAR-2018	14:29	0	
A0002	Group A1	Day 4		Yes	F1	5 ug	12-MAR-2018	08:35	0	
A0002	Group A1	Day 4		Yes	F2	5 ug	12-MAR-2018	08:35	0	
A0002	Group A1	Day 4		Yes	F3	5 ug	12-MAR-2018	08:35	0	
A0002	Group A1	Day 8		Yes	F1	5 ug	16-MAR-2018	12:30	0	
A0002	Group A1	Day 8		Yes	F2	5 ug	16-MAR-2018	12:30	0	
A0002	Group A1	Day 8		Yes	F3	5 ug	16-MAR-2018	12:30	0	

Scale: 0= No Pain; 10= Worst Pain Possible
Administration Site Key: FA = Forearm; UA= Upper arm
Treatments Part A: A1 = A/Sing MAP, 15 ug (FA); A2 = Afluria Quad IM (UA), 15 ug; A3 = Placebo MAP (FA); A4 = A/Sing Mono IM, 15 ug (UA)
Treatments Part B: B1 = A/Sing MAP, 15 ug (FA) with biopsy; B2 = Placebo MAP (FA) with biopsy
B3 = A/Sing MAP, 15 ug (FA); B4 = A/Sing MAP, 10 ug (FA); B5 = A/Sing MAP, 5 ug (FA); B6 = A/Sing MAP, 2.5 ug (FA)
B7 = Placebo MAP (FA); B8 = A/Sing MAP, 15 ug (UA); B9 = A/Sing IM, 15 ug, as 2018 Afluria Quad (UA)

Listing 16.2.9.3
Numeric Pain Intensity per Site

Part A

Randomisation Number	Group	Visit	Time Point	Was Pain Assessment Performed?	Site Number	Treatment	Date of assessment	Time of Assessment	Score	Unscheduled Reason
A0002	Group A1	Day 22		Yes	F1	5 ug	30-MAR-2018	12:03	0	
A0002	Group A1	Day 22		Yes	F2	5 ug	30-MAR-2018	12:04	0	
A0002	Group A1	Day 22		Yes	F3	5 ug	30-MAR-2018	12:04	0	
A0002	Group A1	EOS/ET (Day 61)		Yes	F1	5 ug	08-MAY-2018	12:09	0	
A0002	Group A1	EOS/ET (Day 61)		Yes	F2	5 ug	08-MAY-2018	12:09	0	
A0002	Group A1	EOS/ET (Day 61)		Yes	F3	5 ug	08-MAY-2018	12:09	0	
A0003	Group A1	Day 1	1 min	Yes	F1	5 ug	09-MAR-2018	12:21	0	
A0003	Group A1	Day 1	1 min	Yes	F2	5 ug	09-MAR-2018	12:24	1	
A0003	Group A1	Day 1	1 min	Yes	F3	5 ug	09-MAR-2018	12:27	1	
A0003	Group A1	Day 1	10 min	Yes	F1	5 ug	09-MAR-2018	12:38	0	
A0003	Group A1	Day 1	10 min	Yes	F2	5 ug	09-MAR-2018	12:38	0	
A0003	Group A1	Day 1	10 min	Yes	F3	5 ug	09-MAR-2018	12:38	0	
A0003	Group A1	Day 1	1 hour	Yes	F1	5 ug	09-MAR-2018	13:38	0	
A0003	Group A1	Day 1	1 hour	Yes	F2	5 ug	09-MAR-2018	13:38	0	
A0003	Group A1	Day 1	1 hour	Yes	F3	5 ug	09-MAR-2018	13:38	0	
A0003	Group A1	Day 1	2 hour	Yes	F1	5 ug	09-MAR-2018	14:38	0	
A0003	Group A1	Day 1	2 hour	Yes	F2	5 ug	09-MAR-2018	14:38	0	
A0003	Group A1	Day 1	2 hour	Yes	F3	5 ug	09-MAR-2018	14:38	0	
A0003	Group A1	Day 4		Yes	F1	5 ug	12-MAR-2018	09:36	0	
A0003	Group A1	Day 4		Yes	F2	5 ug	12-MAR-2018	09:36	0	
A0003	Group A1	Day 4		Yes	F3	5 ug	12-MAR-2018	09:36	0	

Scale: 0= No Pain; 10= Worst Pain Possible
Administration Site Key: FA = Forearm; UA= Upper arm
Treatments Part A: A1 = A/Sing MAP, 15 ug (FA); A2 = Afluria Quad IM (UA), 15 ug; A3 = Placebo MAP (FA); A4 = A/Sing Mono IM, 15 ug (UA)
Treatments Part B: B1 = A/Sing MAP, 15 ug (FA) with biopsy; B2 = Placebo MAP (FA) with biopsy
B3 = A/Sing MAP, 15 ug (FA); B4 = A/Sing MAP, 10 ug (FA); B5 = A/Sing MAP, 5 ug (FA); B6 = A/Sing MAP, 2.5 ug (FA)
B7 = Placebo MAP (FA); B8 = A/Sing MAP, 15 ug (UA); B9 = A/Sing IM, 15 ug, as 2018 Afluria Quad (UA)

Listing 16.2.9.3
Numeric Pain Intensity per Site

Part A

Randomisation Number	Group	Visit	Time Point	Was Pain Assessment Performed?	Site Number	Treatment	Date of assessment	Time of Assessment	Score	Unscheduled Reason
A0003	Group A1	Day 8		Yes	F1	5 ug	16-MAR-2018	10:24	0	
A0003	Group A1	Day 8		Yes	F2	5 ug	16-MAR-2018	10:25	0	
A0003	Group A1	Day 8		Yes	F3	5 ug	16-MAR-2018	10:25	0	
A0003	Group A1	Day 22		Yes	F1	5 ug	30-MAR-2018	11:39	0	
A0003	Group A1	Day 22		Yes	F2	5 ug	30-MAR-2018	11:39	0	
A0003	Group A1	Day 22		Yes	F3	5 ug	30-MAR-2018	11:39	0	
A0003	Group A1	EOS/ET (Day 61)		Yes	F1	5 ug	08-MAY-2018	10:53	0	
A0003	Group A1	EOS/ET (Day 61)		Yes	F2	5 ug	08-MAY-2018	10:53	0	
A0003	Group A1	EOS/ET (Day 61)		Yes	F3	5 ug	08-MAY-2018	10:53	0	
A0004	Group A3	Day 1	1 min	Yes	F1	uncoated	09-MAR-2018	12:31	2	
A0004	Group A3	Day 1	1 min	Yes	F2	uncoated	09-MAR-2018	12:34	2	
A0004	Group A3	Day 1	1 min	Yes	F3	uncoated	09-MAR-2018	12:37	2	
A0004	Group A3	Day 1	10 min	Yes	F1	uncoated	09-MAR-2018	12:48	1	
A0004	Group A3	Day 1	10 min	Yes	F2	uncoated	09-MAR-2018	12:48	1	
A0004	Group A3	Day 1	10 min	Yes	F3	uncoated	09-MAR-2018	12:48	1	
A0004	Group A3	Day 1	1 hour	Yes	F1	uncoated	09-MAR-2018	14:00	0	
A0004	Group A3	Day 1	1 hour	Yes	F2	uncoated	09-MAR-2018	14:00	0	
A0004	Group A3	Day 1	1 hour	Yes	F3	uncoated	09-MAR-2018	14:00	0	
A0004	Group A3	Day 1	2 hour	Yes	F1	uncoated	09-MAR-2018	14:48	0	
A0004	Group A3	Day 1	2 hour	Yes	F2	uncoated	09-MAR-2018	14:48	0	
A0004	Group A3	Day 1	2 hour	Yes	F3	uncoated	09-MAR-2018	14:48	0	

Scale: 0= No Pain; 10= Worst Pain Possible
Administration Site Key: FA = Forearm; UA= Upper arm
Treatments Part A: A1 = A/Sing MAP, 15 ug (FA); A2 = Afluria Quad IM (UA), 15 ug; A3 = Placebo MAP (FA); A4 = A/Sing Mono IM, 15 ug (UA)
Treatments Part B: B1 = A/Sing MAP, 15 ug (FA) with biopsy; B2 = Placebo MAP (FA) with biopsy
B3 = A/Sing MAP, 15 ug (FA); B4 = A/Sing MAP, 10 ug (FA); B5 = A/Sing MAP, 5 ug (FA); B6 = A/Sing MAP, 2.5 ug (FA)
B7 = Placebo MAP (FA); B8 = A/Sing MAP, 15 ug (UA); B9 = A/Sing IM, 15 ug, as 2018 Afluria Quad (UA)

Listing 16.2.9.3
Numeric Pain Intensity per Site

Part A

Randomisation Number	Group	Visit	Time Point	Was Pain Assessment Performed?	Site Number	Treatment	Date of assessment	Time of Assessment	Score	Unscheduled Reason
A0004	Group A3	Day 4		Yes	F1	uncoated	12-MAR-2018	12:28	0	
A0004	Group A3	Day 4		Yes	F2	uncoated	12-MAR-2018	12:28	0	
A0004	Group A3	Day 4		Yes	F3	uncoated	12-MAR-2018	12:28	0	
A0004	Group A3	Day 8		Yes	F1	uncoated	16-MAR-2018	15:24	0	
A0004	Group A3	Day 8		Yes	F2	uncoated	16-MAR-2018	15:24	0	
A0004	Group A3	Day 8		Yes	F3	uncoated	16-MAR-2018	15:24	0	
A0004	Group A3	Day 22		Yes	F1	uncoated	30-MAR-2018	12:33	0	
A0004	Group A3	Day 22		Yes	F2	uncoated	30-MAR-2018	12:33	0	
A0004	Group A3	Day 22		Yes	F3	uncoated	30-MAR-2018	12:33	0	
A0004	Group A3	EOS/ET (Day 61)		Yes	F1	uncoated	08-MAY-2018	12:03	0	
A0004	Group A3	EOS/ET (Day 61)		Yes	F2	uncoated	08-MAY-2018	12:03	0	
A0004	Group A3	EOS/ET (Day 61)		Yes	F3	uncoated	08-MAY-2018	12:03	0	
A0005	Group A1	Day 1	1 min	Yes	F1	5 ug	09-MAR-2018	12:51	0	
A0005	Group A1	Day 1	1 min	Yes	F2	5 ug	09-MAR-2018	12:54	0	
A0005	Group A1	Day 1	1 min	Yes	F3	5 ug	09-MAR-2018	12:57	0	
A0005	Group A1	Day 1	10 min	Yes	F1	5 ug	09-MAR-2018	13:08	0	
A0005	Group A1	Day 1	10 min	Yes	F2	5 ug	09-MAR-2018	13:08	0	
A0005	Group A1	Day 1	10 min	Yes	F3	5 ug	09-MAR-2018	13:08	0	
A0005	Group A1	Day 1	1 hour	Yes	F1	5 ug	09-MAR-2018	14:08	0	
A0005	Group A1	Day 1	1 hour	No	F2	5 ug				
A0005	Group A1	Day 1	1 hour	No	F3	5 ug				

Scale: 0= No Pain; 10= Worst Pain Possible
Administration Site Key: FA = Forearm; UA= Upper arm
Treatments Part A: A1 = A/Sing MAP, 15 ug (FA); A2 = Afluria Quad IM (UA), 15 ug; A3 = Placebo MAP (FA); A4 = A/Sing Mono IM, 15 ug (UA)
Treatments Part B: B1 = A/Sing MAP, 15 ug (FA) with biopsy; B2 = Placebo MAP (FA) with biopsy
B3 = A/Sing MAP, 15 ug (FA); B4 = A/Sing MAP, 10 ug (FA); B5 = A/Sing MAP, 5 ug (FA); B6 = A/Sing MAP, 2.5 ug (FA)
B7 = Placebo MAP (FA); B8 = A/Sing MAP, 15 ug (UA); B9 = A/Sing IM, 15 ug, as 2018 Afluria Quad (UA)

Listing 16.2.9.3
Numeric Pain Intensity per Site

Part A

Randomisation Number	Group	Visit	Time Point	Was Pain Assessment Performed?	Site Number	Treatment	Date of assessment	Time of Assessment	Score	Unscheduled Reason
A0005	Group A1	Day 1	2 hour	Yes	F1	5 ug	09-MAR-2018	15:04	0	
A0005	Group A1	Day 1	2 hour	Yes	F2	5 ug	09-MAR-2018	15:04	0	
A0005	Group A1	Day 1	2 hour	Yes	F3	5 ug	09-MAR-2018	15:04	0	
A0005	Group A1	Day 4		Yes	F1	5 ug	12-MAR-2018	13:08	0	
A0005	Group A1	Day 4		Yes	F2	5 ug	12-MAR-2018	13:08	0	
A0005	Group A1	Day 4		Yes	F3	5 ug	12-MAR-2018	13:08	0	
A0005	Group A1	Day 8		Yes	F1	5 ug	16-MAR-2018	12:37	0	
A0005	Group A1	Day 8		Yes	F2	5 ug	16-MAR-2018	12:37	0	
A0005	Group A1	Day 8		Yes	F3	5 ug	16-MAR-2018	12:37	0	
A0005	Group A1	Day 22		Yes	F1	5 ug	30-MAR-2018	12:57	0	
A0005	Group A1	Day 22		Yes	F2	5 ug	30-MAR-2018	12:57	0	
A0005	Group A1	Day 22		Yes	F3	5 ug	30-MAR-2018	12:57	0	
A0005	Group A1	EOS/ET (Day 61)		Yes	F1	5 ug	08-MAY-2018	13:16	0	
A0005	Group A1	EOS/ET (Day 61)		Yes	F2	5 ug	08-MAY-2018	13:16	0	
A0005	Group A1	EOS/ET (Day 61)		Yes	F3	5 ug	08-MAY-2018	13:16	0	
A0006	Group A3	Day 1	1 min	Yes	F1	uncoated	09-MAR-2018	12:41	0	
A0006	Group A3	Day 1	1 min	Yes	F2	uncoated	09-MAR-2018	12:44	0	
A0006	Group A3	Day 1	1 min	Yes	F3	uncoated	09-MAR-2018	12:47	0	
A0006	Group A3	Day 1	10 min	Yes	F1	uncoated	09-MAR-2018	12:58	1	
A0006	Group A3	Day 1	10 min	Yes	F2	uncoated	09-MAR-2018	12:58	1	
A0006	Group A3	Day 1	10 min	Yes	F3	uncoated	09-MAR-2018	12:58	1	

Scale: 0= No Pain; 10= Worst Pain Possible
Administration Site Key: FA = Forearm; UA= Upper arm
Treatments Part A: A1 = A/Sing MAP, 15 ug (FA); A2 = Afluria Quad IM (UA), 15 ug; A3 = Placebo MAP (FA); A4 = A/Sing Mono IM, 15 ug (UA)
Treatments Part B: B1 = A/Sing MAP, 15 ug (FA) with biopsy; B2 = Placebo MAP (FA) with biopsy
B3 = A/Sing MAP, 15 ug (FA); B4 = A/Sing MAP, 10 ug (FA); B5 = A/Sing MAP, 5 ug (FA); B6 = A/Sing MAP, 2.5 ug (FA)
B7 = Placebo MAP (FA); B8 = A/Sing MAP, 15 ug (UA); B9 = A/Sing IM, 15 ug, as 2018 Afluria Quad (UA)

Listing 16.2.9.3
Numeric Pain Intensity per Site

Part A

Randomisation Number	Group	Visit	Time Point	Was Pain Assessment Performed?	Site Number	Treatment	Date of assessment	Time of Assessment	Score	Unscheduled Reason
A0006	Group A3	Day 1	1 hour	Yes	F1	uncoated	09-MAR-2018	14:01	0	
A0006	Group A3	Day 1	1 hour	Yes	F2	uncoated	09-MAR-2018	14:01	0	
A0006	Group A3	Day 1	1 hour	Yes	F3	uncoated	09-MAR-2018	14:01	0	
A0006	Group A3	Day 1	2 hour	Yes	F1	uncoated	09-MAR-2018	14:58	0	
A0006	Group A3	Day 1	2 hour	Yes	F2	uncoated	09-MAR-2018	14:58	0	
A0006	Group A3	Day 1	2 hour	Yes	F3	uncoated	09-MAR-2018	14:58	0	
A0006	Group A3	Day 4		Yes	F1	uncoated	12-MAR-2018	12:27	0	
A0006	Group A3	Day 4		Yes	F2	uncoated	12-MAR-2018	12:27	0	
A0006	Group A3	Day 4		Yes	F3	uncoated	12-MAR-2018	12:27	0	
A0006	Group A3	Day 8		Yes	F1	uncoated	16-MAR-2018	12:20	0	
A0006	Group A3	Day 8		Yes	F2	uncoated	16-MAR-2018	12:20	0	
A0006	Group A3	Day 8		Yes	F3	uncoated	16-MAR-2018	12:20	0	
A0006	Group A3	Day 22		Yes	F1	uncoated	30-MAR-2018	12:27	0	
A0006	Group A3	Day 22		Yes	F2	uncoated	30-MAR-2018	12:27	0	
A0006	Group A3	Day 22		Yes	F3	uncoated	30-MAR-2018	12:27	0	
A0006	Group A3	EOS/ET (Day 61)		Yes	F1	uncoated	08-MAY-2018	14:26	0	
A0006	Group A3	EOS/ET (Day 61)		Yes	F2	uncoated	08-MAY-2018	14:27	0	
A0006	Group A3	EOS/ET (Day 61)		Yes	F3	uncoated	08-MAY-2018	14:27	0	
A0007	Group A1	Day 1	1 min	Yes	F1	5 ug	09-MAR-2018	13:01	4	
A0007	Group A1	Day 1	1 min	Yes	F2	5 ug	09-MAR-2018	13:04	3	
A0007	Group A1	Day 1	1 min	Yes	F3	5 ug	09-MAR-2018	13:07	3	

Scale: 0= No Pain; 10= Worst Pain Possible
Administration Site Key: FA = Forearm; UA= Upper arm
Treatments Part A: A1 = A/Sing MAP, 15 ug (FA); A2 = Afluria Quad IM (UA), 15 ug; A3 = Placebo MAP (FA); A4 = A/Sing Mono IM, 15 ug (UA)
Treatments Part B: B1 = A/Sing MAP, 15 ug (FA) with biopsy; B2 = Placebo MAP (FA) with biopsy
B3 = A/Sing MAP, 15 ug (FA); B4 = A/Sing MAP, 10 ug (FA); B5 = A/Sing MAP, 5 ug (FA); B6 = A/Sing MAP, 2.5 ug (FA)
B7 = Placebo MAP (FA); B8 = A/Sing MAP, 15 ug (UA); B9 = A/Sing IM, 15 ug, as 2018 Afluria Quad (UA)

Listing 16.2.9.3
Numeric Pain Intensity per Site

Part A

Randomisation Number	Group	Visit	Time Point	Was Pain Assessment Performed?	Site Number	Treatment	Date of assessment	Time of Assessment	Score	Unscheduled Reason
A0007	Group A1	Day 1	10 min	Yes	F1	5 ug	09-MAR-2018	13:18	2	
A0007	Group A1	Day 1	10 min	Yes	F2	5 ug	09-MAR-2018	13:18	2	
A0007	Group A1	Day 1	10 min	Yes	F3	5 ug	09-MAR-2018	13:18	2	
A0007	Group A1	Day 1	1 hour	Yes	F1	5 ug	09-MAR-2018	14:12	0	
A0007	Group A1	Day 1	1 hour	Yes	F2	5 ug	09-MAR-2018	14:12	0	
A0007	Group A1	Day 1	1 hour	Yes	F3	5 ug	09-MAR-2018	14:12	0	
A0007	Group A1	Day 1	2 hour	Yes	F1	5 ug	09-MAR-2018	15:32	0	
A0007	Group A1	Day 1	2 hour	Yes	F2	5 ug	09-MAR-2018	15:32	0	
A0007	Group A1	Day 1	2 hour	Yes	F3	5 ug	09-MAR-2018	15:32	0	
A0007	Group A1	Day 4		Yes	F1	5 ug	12-MAR-2018	10:52	0	
A0007	Group A1	Day 4		Yes	F2	5 ug	12-MAR-2018	10:53	0	
A0007	Group A1	Day 4		Yes	F3	5 ug	12-MAR-2018	10:54	0	
A0007	Group A1	Day 8		Yes	F1	5 ug	16-MAR-2018	14:23	0	
A0007	Group A1	Day 8		Yes	F2	5 ug	16-MAR-2018	14:23	0	
A0007	Group A1	Day 8		Yes	F3	5 ug	16-MAR-2018	14:23	0	
A0007	Group A1	Day 22		Yes	F1	5 ug	29-MAR-2018	10:33	0	
A0007	Group A1	Day 22		Yes	F2	5 ug	29-MAR-2018	10:33	0	
A0007	Group A1	Day 22		Yes	F3	5 ug	29-MAR-2018	10:33	0	
A0007	Group A1	EOS/ET (Day 61)		Yes	F1	5 ug	08-MAY-2018	13:58	0	
A0007	Group A1	EOS/ET (Day 61)		Yes	F2	5 ug	08-MAY-2018	13:58	0	
A0007	Group A1	EOS/ET (Day 61)		Yes	F3	5 ug	08-MAY-2018	13:58	0	

Scale: 0= No Pain; 10= Worst Pain Possible
Administration Site Key: FA = Forearm; UA= Upper arm
Treatments Part A: A1 = A/Sing MAP, 15 ug (FA); A2 = Afluria Quad IM (UA), 15 ug; A3 = Placebo MAP (FA); A4 = A/Sing Mono IM, 15 ug (UA)
Treatments Part B: B1 = A/Sing MAP, 15 ug (FA) with biopsy; B2 = Placebo MAP (FA) with biopsy
B3 = A/Sing MAP, 15 ug (FA); B4 = A/Sing MAP, 10 ug (FA); B5 = A/Sing MAP, 5 ug (FA); B6 = A/Sing MAP, 2.5 ug (FA)
B7 = Placebo MAP (FA); B8 = A/Sing MAP, 15 ug (UA); B9 = A/Sing IM, 15 ug, as 2018 Afluria Quad (UA)

Listing 16.2.9.3
Numeric Pain Intensity per Site

Part A

Randomisation Number	Group	Visit	Time Point	Was Pain Assessment Performed?	Site Number	Treatment	Date of assessment	Time of Assessment	Score	Unscheduled Reason
A0008	Group A3	Day 1	1 min	Yes	F1	uncoated	09-MAR-2018	13:21	3	
A0008	Group A3	Day 1	1 min	Yes	F2	uncoated	09-MAR-2018	13:24	2	
A0008	Group A3	Day 1	1 min	Yes	F3	uncoated	09-MAR-2018	13:27	2	
A0008	Group A3	Day 1	10 min	Yes	F1	uncoated	09-MAR-2018	13:38	2	
A0008	Group A3	Day 1	10 min	Yes	F2	uncoated	09-MAR-2018	13:38	2	
A0008	Group A3	Day 1	10 min	Yes	F3	uncoated	09-MAR-2018	13:38	0	
A0008	Group A3	Day 1	1 hour	Yes	F1	uncoated	09-MAR-2018	14:28	0	
A0008	Group A3	Day 1	1 hour	Yes	F2	uncoated	09-MAR-2018	14:28	0	
A0008	Group A3	Day 1	1 hour	Yes	F3	uncoated	09-MAR-2018	14:28	0	
A0008	Group A3	Day 1	2 hour	Yes	F1	uncoated	09-MAR-2018	15:53	0	
A0008	Group A3	Day 1	2 hour	Yes	F2	uncoated	09-MAR-2018	15:53	0	
A0008	Group A3	Day 1	2 hour	Yes	F3	uncoated	09-MAR-2018	15:53	0	
A0008	Group A3	Day 4		Yes	F1	uncoated	12-MAR-2018	16:17	0	
A0008	Group A3	Day 4		Yes	F2	uncoated	12-MAR-2018	16:17	0	
A0008	Group A3	Day 4		Yes	F3	uncoated	12-MAR-2018	16:17	0	
A0008	Group A3	Day 8		Yes	F1	uncoated	16-MAR-2018	08:24	0	
A0008	Group A3	Day 8		Yes	F2	uncoated	16-MAR-2018	08:24	0	
A0008	Group A3	Day 8		Yes	F3	uncoated	16-MAR-2018	08:24	0	
A0008	Group A3	Day 22		Yes	F1	uncoated	30-MAR-2018	09:00	0	
A0008	Group A3	Day 22		Yes	F2	uncoated	30-MAR-2018	09:00	0	
A0008	Group A3	Day 22		Yes	F3	uncoated	30-MAR-2018	09:00	0	

Scale: 0= No Pain; 10= Worst Pain Possible
Administration Site Key: FA = Forearm; UA= Upper arm
Treatments Part A: A1 = A/Sing MAP, 15 ug (FA); A2 = Afluria Quad IM (UA), 15 ug; A3 = Placebo MAP (FA); A4 = A/Sing Mono IM, 15 ug (UA)
Treatments Part B: B1 = A/Sing MAP, 15 ug (FA) with biopsy; B2 = Placebo MAP (FA) with biopsy
B3 = A/Sing MAP, 15 ug (FA); B4 = A/Sing MAP, 10 ug (FA); B5 = A/Sing MAP, 5 ug (FA); B6 = A/Sing MAP, 2.5 ug (FA)
B7 = Placebo MAP (FA); B8 = A/Sing MAP, 15 ug (UA); B9 = A/Sing IM, 15 ug, as 2018 Afluria Quad (UA)

Listing 16.2.9.3
Numeric Pain Intensity per Site

Part A

Randomisation Number	Group	Visit	Time Point	Was Pain Assessment Performed?	Site Number	Treatment	Date of assessment	Time of Assessment	Score	Unscheduled Reason
A0008	Group A3	EOS/ET (Day 61)		Yes	F1	uncoated	08-MAY-2018	08:37	0	
A0008	Group A3	EOS/ET (Day 61)		Yes	F2	uncoated	08-MAY-2018	08:37	0	
A0008	Group A3	EOS/ET (Day 61)		Yes	F3	uncoated	08-MAY-2018	08:38	0	
A0009	Group A1	Day 1	1 min	Yes	F1	5 ug	09-MAR-2018	13:11	3	
A0009	Group A1	Day 1	1 min	Yes	F2	5 ug	09-MAR-2018	13:14	5	
A0009	Group A1	Day 1	1 min	Yes	F3	5 ug	09-MAR-2018	13:17	6	
A0009	Group A1	Day 1	10 min	Yes	F1	5 ug	09-MAR-2018	13:28	2	
A0009	Group A1	Day 1	10 min	Yes	F2	5 ug	09-MAR-2018	13:28	2	
A0009	Group A1	Day 1	10 min	Yes	F3	5 ug	09-MAR-2018	13:28	2	
A0009	Group A1	Day 1	1 hour	No	F1	5 ug				
A0009	Group A1	Day 1	1 hour	No	F2	5 ug				
A0009	Group A1	Day 1	1 hour	No	F3	5 ug				
A0009	Group A1	Day 1	2 hour	Yes	F1	5 ug	09-MAR-2018	15:52	0	
A0009	Group A1	Day 1	2 hour	Yes	F2	5 ug	09-MAR-2018	15:52	0	
A0009	Group A1	Day 1	2 hour	Yes	F3	5 ug	09-MAR-2018	15:52	0	
A0009	Group A1	Day 4		Yes	F1	5 ug	12-MAR-2018	10:43	0	
A0009	Group A1	Day 4		Yes	F2	5 ug	12-MAR-2018	10:43	0	
A0009	Group A1	Day 4		Yes	F3	5 ug	12-MAR-2018	10:43	1	
A0009	Group A1	Day 8		Yes	F1	5 ug	16-MAR-2018	13:22	0	
A0009	Group A1	Day 8		Yes	F2	5 ug	16-MAR-2018	13:22	0	
A0009	Group A1	Day 8		Yes	F3	5 ug	16-MAR-2018	13:23	0	

Scale: 0= No Pain; 10= Worst Pain Possible
Administration Site Key: FA = Forearm; UA= Upper arm
Treatments Part A: A1 = A/Sing MAP, 15 ug (FA); A2 = Afluria Quad IM (UA), 15 ug; A3 = Placebo MAP (FA); A4 = A/Sing Mono IM, 15 ug (UA)
Treatments Part B: B1 = A/Sing MAP, 15 ug (FA) with biopsy; B2 = Placebo MAP (FA) with biopsy
B3 = A/Sing MAP, 15 ug (FA); B4 = A/Sing MAP, 10 ug (FA); B5 = A/Sing MAP, 5 ug (FA); B6 = A/Sing MAP, 2.5 ug (FA)
B7 = Placebo MAP (FA); B8 = A/Sing MAP, 15 ug (UA); B9 = A/Sing IM, 15 ug, as 2018 Afluria Quad (UA)

Listing 16.2.9.3
Numeric Pain Intensity per Site

Part A

Randomisation Number	Group	Visit	Time Point	Was Pain Assessment Performed?	Site Number	Treatment	Date of assessment	Time of Assessment	Score	Unscheduled Reason
A0009	Group A1	Day 22		Yes	F1	5 ug	30-MAR-2018	10:55	0	
A0009	Group A1	Day 22		Yes	F2	5 ug	30-MAR-2018	10:56	0	
A0009	Group A1	Day 22		Yes	F3	5 ug	30-MAR-2018	10:56	0	
A0009	Group A1	EOS/ET (Day 61)		Yes	F1	5 ug	01-MAY-2018	14:42	0	
A0009	Group A1	EOS/ET (Day 61)		Yes	F2	5 ug	01-MAY-2018	14:42	0	
A0009	Group A1	EOS/ET (Day 61)		Yes	F3	5 ug	01-MAY-2018	14:43	0	
A0010	Group A3	Day 1	1 min	Yes	F1	uncoated	09-MAR-2018	13:31	6	
A0010	Group A3	Day 1	1 min	Yes	F2	uncoated	09-MAR-2018	13:34	5	
A0010	Group A3	Day 1	1 min	Yes	F3	uncoated	09-MAR-2018	13:37	7	
A0010	Group A3	Day 1	10 min	Yes	F1	uncoated	09-MAR-2018	13:48	3	
A0010	Group A3	Day 1	10 min	Yes	F2	uncoated	09-MAR-2018	13:48	3	
A0010	Group A3	Day 1	10 min	Yes	F3	uncoated	09-MAR-2018	13:48	3	
A0010	Group A3	Day 1	1 hour	Yes	F1	uncoated	09-MAR-2018	14:44	0	
A0010	Group A3	Day 1	1 hour	Yes	F2	uncoated	09-MAR-2018	14:44	0	
A0010	Group A3	Day 1	1 hour	Yes	F3	uncoated	09-MAR-2018	14:44	0	
A0010	Group A3	Day 1	2 hour	Yes	F1	uncoated	09-MAR-2018	15:55	0	
A0010	Group A3	Day 1	2 hour	Yes	F2	uncoated	09-MAR-2018	15:55	0	
A0010	Group A3	Day 1	2 hour	Yes	F3	uncoated	09-MAR-2018	15:55	0	
A0010	Group A3	Day 4		Yes	F1	uncoated	12-MAR-2018	13:17	0	
A0010	Group A3	Day 4		Yes	F2	uncoated	12-MAR-2018	13:17	0	
A0010	Group A3	Day 4		Yes	F3	uncoated	12-MAR-2018	13:17	0	

Scale: 0= No Pain; 10= Worst Pain Possible
Administration Site Key: FA = Forearm; UA= Upper arm
Treatments Part A: A1 = A/Sing MAP, 15 ug (FA); A2 = Afluria Quad IM (UA), 15 ug; A3 = Placebo MAP (FA); A4 = A/Sing Mono IM, 15 ug (UA)
Treatments Part B: B1 = A/Sing MAP, 15 ug (FA) with biopsy; B2 = Placebo MAP (FA) with biopsy
B3 = A/Sing MAP, 15 ug (FA); B4 = A/Sing MAP, 10 ug (FA); B5 = A/Sing MAP, 5 ug (FA); B6 = A/Sing MAP, 2.5 ug (FA)
B7 = Placebo MAP (FA); B8 = A/Sing MAP, 15 ug (UA); B9 = A/Sing IM, 15 ug, as 2018 Afluria Quad (UA)

Listing 16.2.9.3
Numeric Pain Intensity per Site

Part A

Randomisation Number	Group	Visit	Time Point	Was Pain Assessment Performed?	Site Number	Treatment	Date of assessment	Time of Assessment	Score	Unscheduled Reason
A0010	Group A3	Day 8		Yes	F1	uncoated	16-MAR-2018	13:52	0	
A0010	Group A3	Day 8		Yes	F2	uncoated	16-MAR-2018	13:52	0	
A0010	Group A3	Day 8		Yes	F3	uncoated	16-MAR-2018	13:55	0	
A0010	Group A3	Day 22		Yes	F1	uncoated	30-MAR-2018	15:26	0	
A0010	Group A3	Day 22		Yes	F2	uncoated	30-MAR-2018	15:26	0	
A0010	Group A3	Day 22		Yes	F3	uncoated	30-MAR-2018	15:26	0	
A0010	Group A3	EOS/ET (Day 61)		Yes	F1	uncoated	08-MAY-2018	12:39	0	
A0010	Group A3	EOS/ET (Day 61)		Yes	F2	uncoated	08-MAY-2018	12:39	0	
A0010	Group A3	EOS/ET (Day 61)		Yes	F3	uncoated	08-MAY-2018	12:39	0	
A0011	Group A3	Day 1	1 min	Yes	F1	uncoated	09-MAR-2018	13:41	1	
A0011	Group A3	Day 1	1 min	Yes	F2	uncoated	09-MAR-2018	13:44	1	
A0011	Group A3	Day 1	1 min	Yes	F3	uncoated	09-MAR-2018	13:47	1	
A0011	Group A3	Day 1	10 min	Yes	F1	uncoated	09-MAR-2018	13:58	1	
A0011	Group A3	Day 1	10 min	Yes	F2	uncoated	09-MAR-2018	13:58	1	
A0011	Group A3	Day 1	10 min	Yes	F3	uncoated	09-MAR-2018	13:58	1	
A0011	Group A3	Day 1	1 hour	Yes	F1	uncoated	09-MAR-2018	15:00	0	
A0011	Group A3	Day 1	1 hour	Yes	F2	uncoated	09-MAR-2018	15:00	0	
A0011	Group A3	Day 1	1 hour	Yes	F3	uncoated	09-MAR-2018	15:00	0	
A0011	Group A3	Day 1	2 hour	Yes	F1	uncoated	09-MAR-2018	15:58	0	
A0011	Group A3	Day 1	2 hour	Yes	F2	uncoated	09-MAR-2018	15:58	0	
A0011	Group A3	Day 1	2 hour	Yes	F3	uncoated	09-MAR-2018	15:58	0	

Scale: 0= No Pain; 10= Worst Pain Possible
Administration Site Key: FA = Forearm; UA= Upper arm
Treatments Part A: A1 = A/Sing MAP, 15 ug (FA); A2 = Afluria Quad IM (UA), 15 ug; A3 = Placebo MAP (FA); A4 = A/Sing Mono IM, 15 ug (UA)
Treatments Part B: B1 = A/Sing MAP, 15 ug (FA) with biopsy; B2 = Placebo MAP (FA) with biopsy
B3 = A/Sing MAP, 15 ug (FA); B4 = A/Sing MAP, 10 ug (FA); B5 = A/Sing MAP, 5 ug (FA); B6 = A/Sing MAP, 2.5 ug (FA)
B7 = Placebo MAP (FA); B8 = A/Sing MAP, 15 ug (UA); B9 = A/Sing IM, 15 ug, as 2018 Afluria Quad (UA)

Listing 16.2.9.3
Numeric Pain Intensity per Site

Part A

Randomisation Number	Group	Visit	Time Point	Was Pain Assessment Performed?	Site Number	Treatment	Date of assessment	Time of Assessment	Score	Unscheduled Reason
A0011	Group A3	Day 4		Yes	F1	uncoated	12-MAR-2018	14:15	0	
A0011	Group A3	Day 4		Yes	F2	uncoated	12-MAR-2018	14:15	0	
A0011	Group A3	Day 4		Yes	F3	uncoated	12-MAR-2018	14:15	0	
A0011	Group A3	Day 8		Yes	F1	uncoated	16-MAR-2018	14:00	0	
A0011	Group A3	Day 8		Yes	F2	uncoated	16-MAR-2018	14:01	0	
A0011	Group A3	Day 8		Yes	F3	uncoated	16-MAR-2018	14:02	0	
A0011	Group A3	Day 22		Yes	F1	uncoated	30-MAR-2018	13:46	0	
A0011	Group A3	Day 22		Yes	F2	uncoated	30-MAR-2018	13:46	0	
A0011	Group A3	Day 22		Yes	F3	uncoated	30-MAR-2018	13:46	0	
A0011	Group A3	EOS/ET (Day 61)		Yes	F1	uncoated	09-MAY-2018	16:31	0	
A0011	Group A3	EOS/ET (Day 61)		Yes	F2	uncoated	09-MAY-2018	16:31	0	
A0011	Group A3	EOS/ET (Day 61)		Yes	F3	uncoated	09-MAY-2018	16:31	0	
A0012	Group A1	Day 1	1 min	Yes	F1	5 ug	09-MAR-2018	13:51	1	
A0012	Group A1	Day 1	1 min	Yes	F2	5 ug	09-MAR-2018	13:54	1	
A0012	Group A1	Day 1	1 min	Yes	F3	5 ug	09-MAR-2018	13:57	1	
A0012	Group A1	Day 1	10 min	Yes	F1	5 ug	09-MAR-2018	14:08	1	
A0012	Group A1	Day 1	10 min	Yes	F2	5 ug	09-MAR-2018	14:08	1	
A0012	Group A1	Day 1	10 min	Yes	F3	5 ug	09-MAR-2018	14:08	1	
A0012	Group A1	Day 1	1 hour	Yes	F1	5 ug	09-MAR-2018	15:10	0	
A0012	Group A1	Day 1	1 hour	Yes	F2	5 ug	09-MAR-2018	15:10	0	
A0012	Group A1	Day 1	1 hour	Yes	F3	5 ug	09-MAR-2018	15:10	0	

Scale: 0= No Pain; 10= Worst Pain Possible
Administration Site Key: FA = Forearm; UA= Upper arm
Treatments Part A: A1 = A/Sing MAP, 15 ug (FA); A2 = Afluria Quad IM (UA), 15 ug; A3 = Placebo MAP (FA); A4 = A/Sing Mono IM, 15 ug (UA)
Treatments Part B: B1 = A/Sing MAP, 15 ug (FA) with biopsy; B2 = Placebo MAP (FA) with biopsy
B3 = A/Sing MAP, 15 ug (FA); B4 = A/Sing MAP, 10 ug (FA); B5 = A/Sing MAP, 5 ug (FA); B6 = A/Sing MAP, 2.5 ug (FA)
B7 = Placebo MAP (FA); B8 = A/Sing MAP, 15 ug (UA); B9 = A/Sing IM, 15 ug, as 2018 Afluria Quad (UA)

Listing 16.2.9.3
Numeric Pain Intensity per Site

Part A

Randomisation Number	Group	Visit	Time Point	Was Pain Assessment Performed?	Site Number	Treatment	Date of assessment	Time of Assessment	Score	Unscheduled Reason
A0012	Group A1	Day 1	2 hour	Yes	F1	5 ug	09-MAR-2018	16:14	0	
A0012	Group A1	Day 1	2 hour	Yes	F2	5 ug	09-MAR-2018	16:14	0	
A0012	Group A1	Day 1	2 hour	Yes	F3	5 ug	09-MAR-2018	16:14	0	
A0012	Group A1	Day 4		Yes	F1	5 ug	12-MAR-2018	12:51	0	
A0012	Group A1	Day 4		Yes	F2	5 ug	12-MAR-2018	12:51	0	
A0012	Group A1	Day 4		Yes	F3	5 ug	12-MAR-2018	12:51	0	
A0012	Group A1	Day 8		Yes	F1	5 ug	16-MAR-2018	15:30	0	
A0012	Group A1	Day 8		Yes	F2	5 ug	16-MAR-2018	15:30	0	
A0012	Group A1	Day 8		Yes	F3	5 ug	16-MAR-2018	15:31	0	
A0012	Group A1	Day 22		Yes	F1	5 ug	30-MAR-2018	13:33	0	
A0012	Group A1	Day 22		Yes	F2	5 ug	30-MAR-2018	13:34	0	
A0012	Group A1	Day 22		Yes	F3	5 ug	30-MAR-2018	13:34	0	
A0012	Group A1	EOS/ET (Day 61)		Yes	F1	5 ug	08-MAY-2018	13:57	0	
A0012	Group A1	EOS/ET (Day 61)		Yes	F2	5 ug	08-MAY-2018	13:57	0	
A0012	Group A1	EOS/ET (Day 61)		Yes	F3	5 ug	08-MAY-2018	13:57	0	
A0013	Group A1	Day 1	1 min	Yes	F1	5 ug	09-MAR-2018	14:01	1	
A0013	Group A1	Day 1	1 min	Yes	F2	5 ug	09-MAR-2018	14:04	2	
A0013	Group A1	Day 1	1 min	Yes	F3	5 ug	09-MAR-2018	14:07	2	
A0013	Group A1	Day 1	10 min	Yes	F1	5 ug	09-MAR-2018	14:16	2	
A0013	Group A1	Day 1	10 min	No	F2	5 ug				
A0013	Group A1	Day 1	10 min	No	F3	5 ug				

Scale: 0= No Pain; 10= Worst Pain Possible
Administration Site Key: FA = Forearm; UA= Upper arm
Treatments Part A: A1 = A/Sing MAP, 15 ug (FA); A2 = Afluria Quad IM (UA), 15 ug; A3 = Placebo MAP (FA); A4 = A/Sing Mono IM, 15 ug (UA)
Treatments Part B: B1 = A/Sing MAP, 15 ug (FA) with biopsy; B2 = Placebo MAP (FA) with biopsy
B3 = A/Sing MAP, 15 ug (FA); B4 = A/Sing MAP, 10 ug (FA); B5 = A/Sing MAP, 5 ug (FA); B6 = A/Sing MAP, 2.5 ug (FA)
B7 = Placebo MAP (FA); B8 = A/Sing MAP, 15 ug (UA); B9 = A/Sing IM, 15 ug, as 2018 Afluria Quad (UA)

Listing 16.2.9.3
Numeric Pain Intensity per Site

Part A

Randomisation Number	Group	Visit	Time Point	Was Pain Assessment Performed?	Site Number	Treatment	Date of assessment	Time of Assessment	Score	Unscheduled Reason
A0013	Group A1	Day 1	1 hour	Yes	F1	5 ug	09-MAR-2018	15:36	0	
A0013	Group A1	Day 1	1 hour	No	F2	5 ug				
A0013	Group A1	Day 1	1 hour	No	F3	5 ug				
A0013	Group A1	Day 1	2 hour	Yes	F1	5 ug	09-MAR-2018	16:17	0	
A0013	Group A1	Day 1	2 hour	No	F2	5 ug				
A0013	Group A1	Day 1	2 hour	No	F3	5 ug				
A0013	Group A1	Day 4		Yes	F1	5 ug	12-MAR-2018	13:10	0	
A0013	Group A1	Day 4		Yes	F2	5 ug	12-MAR-2018	13:11	0	
A0013	Group A1	Day 4		Yes	F3	5 ug	12-MAR-2018	13:11	0	
A0013	Group A1	Day 8		Yes	F1	5 ug	16-MAR-2018	15:30	0	
A0013	Group A1	Day 8		Yes	F2	5 ug	16-MAR-2018	15:31	0	
A0013	Group A1	Day 8		Yes	F3	5 ug	16-MAR-2018	15:31	0	
A0013	Group A1	Day 22		Yes	F1	5 ug	30-MAR-2018	14:33	0	
A0013	Group A1	Day 22		Yes	F2	5 ug	30-MAR-2018	14:33	0	
A0013	Group A1	Day 22		Yes	F3	5 ug	30-MAR-2018	14:33	0	
A0013	Group A1	EOS/ET (Day 61)		Yes	F1	5 ug	10-MAY-2018	12:21	0	
A0013	Group A1	EOS/ET (Day 61)		Yes	F2	5 ug	10-MAY-2018	12:21	0	
A0013	Group A1	EOS/ET (Day 61)		Yes	F3	5 ug	10-MAY-2018	12:21	0	
A0014	Group A3	Day 1	1 min	Yes	F1	uncoated	09-MAR-2018	14:11	3	
A0014	Group A3	Day 1	1 min	Yes	F2	uncoated	09-MAR-2018	14:14	3	
A0014	Group A3	Day 1	1 min	Yes	F3	uncoated	09-MAR-2018	14:17	3	

Scale: 0= No Pain; 10= Worst Pain Possible
Administration Site Key: FA = Forearm; UA= Upper arm
Treatments Part A: A1 = A/Sing MAP, 15 ug (FA); A2 = Afluria Quad IM (UA), 15 ug; A3 = Placebo MAP (FA); A4 = A/Sing Mono IM, 15 ug (UA)
Treatments Part B: B1 = A/Sing MAP, 15 ug (FA) with biopsy; B2 = Placebo MAP (FA) with biopsy
B3 = A/Sing MAP, 15 ug (FA); B4 = A/Sing MAP, 10 ug (FA); B5 = A/Sing MAP, 5 ug (FA); B6 = A/Sing MAP, 2.5 ug (FA)
B7 = Placebo MAP (FA); B8 = A/Sing MAP, 15 ug (UA); B9 = A/Sing IM, 15 ug, as 2018 Afluria Quad (UA)

Listing 16.2.9.3
Numeric Pain Intensity per Site

Part A

Randomisation Number	Group	Visit	Time Point	Was Pain Assessment Performed?	Site Number	Treatment	Date of assessment	Time of Assessment	Score	Unscheduled Reason
A0014	Group A3	Day 1	10 min	Yes	F1	uncoated	09-MAR-2018	14:28	2	
A0014	Group A3	Day 1	10 min	Yes	F2	uncoated	09-MAR-2018	14:28	2	
A0014	Group A3	Day 1	10 min	Yes	F3	uncoated	09-MAR-2018	14:28	2	
A0014	Group A3	Day 1	1 hour	Yes	F1	uncoated	09-MAR-2018	15:39	1	
A0014	Group A3	Day 1	1 hour	Yes	F2	uncoated	09-MAR-2018	15:39	1	
A0014	Group A3	Day 1	1 hour	Yes	F3	uncoated	09-MAR-2018	15:39	1	
A0014	Group A3	Day 1	2 hour	Yes	F1	uncoated	09-MAR-2018	16:37	0	
A0014	Group A3	Day 1	2 hour	Yes	F2	uncoated	09-MAR-2018	16:37	0	
A0014	Group A3	Day 1	2 hour	Yes	F3	uncoated	09-MAR-2018	16:37	0	
A0014	Group A3	Day 4		Yes	F1	uncoated	12-MAR-2018	16:23	0	
A0014	Group A3	Day 4		Yes	F2	uncoated	12-MAR-2018	16:23	0	
A0014	Group A3	Day 4		Yes	F3	uncoated	12-MAR-2018	16:24	0	
A0014	Group A3	Day 8		Yes	F1	uncoated	16-MAR-2018	08:24	0	
A0014	Group A3	Day 8		Yes	F2	uncoated	16-MAR-2018	08:24	0	
A0014	Group A3	Day 8		Yes	F3	uncoated	16-MAR-2018	08:24	0	
A0014	Group A3	Day 22		Yes	F1	uncoated	30-MAR-2018	09:18	0	
A0014	Group A3	Day 22		Yes	F2	uncoated	30-MAR-2018	09:18	0	
A0014	Group A3	Day 22		Yes	F3	uncoated	30-MAR-2018	09:18	0	
A0014	Group A3	EOS/ET (Day 61)		Yes	F1	uncoated	08-MAY-2018	08:35	0	
A0014	Group A3	EOS/ET (Day 61)		Yes	F2	uncoated	08-MAY-2018	08:35	0	
A0014	Group A3	EOS/ET (Day 61)		Yes	F3	uncoated	08-MAY-2018	08:35	0	

Scale: 0= No Pain; 10= Worst Pain Possible
Administration Site Key: FA = Forearm; UA= Upper arm
Treatments Part A: A1 = A/Sing MAP, 15 ug (FA); A2 = Afluria Quad IM (UA), 15 ug; A3 = Placebo MAP (FA); A4 = A/Sing Mono IM, 15 ug (UA)
Treatments Part B: B1 = A/Sing MAP, 15 ug (FA) with biopsy; B2 = Placebo MAP (FA) with biopsy
B3 = A/Sing MAP, 15 ug (FA); B4 = A/Sing MAP, 10 ug (FA); B5 = A/Sing MAP, 5 ug (FA); B6 = A/Sing MAP, 2.5 ug (FA)
B7 = Placebo MAP (FA); B8 = A/Sing MAP, 15 ug (UA); B9 = A/Sing IM, 15 ug, as 2018 Afluria Quad (UA)

Listing 16.2.9.3
Numeric Pain Intensity per Site

Part A

Randomisation Number	Group	Visit	Time Point	Was Pain Assessment Performed?	Site Number	Treatment	Date of assessment	Time of Assessment	Score	Unscheduled Reason
A0015	Group A1	Day 1	1 min	Yes	F1	5 ug	09-MAR-2018	14:20	1	
A0015	Group A1	Day 1	1 min	Yes	F2	5 ug	09-MAR-2018	14:24	1	
A0015	Group A1	Day 1	1 min	Yes	F3	5 ug	09-MAR-2018	14:27	2	
A0015	Group A1	Day 1	10 min	Yes	F1	5 ug	09-MAR-2018	14:38	1	
A0015	Group A1	Day 1	10 min	Yes	F2	5 ug	09-MAR-2018	14:38	1	
A0015	Group A1	Day 1	10 min	Yes	F3	5 ug	09-MAR-2018	14:38	1	
A0015	Group A1	Day 1	1 hour	Yes	F1	5 ug	09-MAR-2018	15:43	0	
A0015	Group A1	Day 1	1 hour	Yes	F2	5 ug	09-MAR-2018	15:43	0	
A0015	Group A1	Day 1	1 hour	Yes	F3	5 ug	09-MAR-2018	15:43	0	
A0015	Group A1	Day 1	2 hour	Yes	F1	5 ug	09-MAR-2018	16:39	0	
A0015	Group A1	Day 1	2 hour	Yes	F2	5 ug	09-MAR-2018	16:39	0	
A0015	Group A1	Day 1	2 hour	Yes	F3	5 ug	09-MAR-2018	16:39	0	
A0015	Group A1	Day 4		Yes	F1	5 ug	12-MAR-2018	14:15	0	
A0015	Group A1	Day 4		Yes	F2	5 ug	12-MAR-2018	14:15	0	
A0015	Group A1	Day 4		Yes	F3	5 ug	12-MAR-2018	14:15	0	
A0015	Group A1	Day 8		Yes	F1	5 ug	16-MAR-2018	13:26	0	
A0015	Group A1	Day 8		Yes	F2	5 ug	16-MAR-2018	13:26	0	
A0015	Group A1	Day 8		Yes	F3	5 ug	16-MAR-2018	13:26	0	
A0015	Group A1	Day 22		Yes	F1	5 ug	30-MAR-2018	14:05	0	
A0015	Group A1	Day 22		Yes	F2	5 ug	30-MAR-2018	14:05	0	
A0015	Group A1	Day 22		Yes	F3	5 ug	30-MAR-2018	14:05	0	

Scale: 0= No Pain; 10= Worst Pain Possible
Administration Site Key: FA = Forearm; UA= Upper arm
Treatments Part A: A1 = A/Sing MAP, 15 ug (FA); A2 = Afluria Quad IM (UA), 15 ug; A3 = Placebo MAP (FA); A4 = A/Sing Mono IM, 15 ug (UA)
Treatments Part B: B1 = A/Sing MAP, 15 ug (FA) with biopsy; B2 = Placebo MAP (FA) with biopsy
B3 = A/Sing MAP, 15 ug (FA); B4 = A/Sing MAP, 10 ug (FA); B5 = A/Sing MAP, 5 ug (FA); B6 = A/Sing MAP, 2.5 ug (FA)
B7 = Placebo MAP (FA); B8 = A/Sing MAP, 15 ug (UA); B9 = A/Sing IM, 15 ug, as 2018 Afluria Quad (UA)

Listing 16.2.9.3
Numeric Pain Intensity per Site

Part A

Randomisation Number	Group	Visit	Time Point	Was Pain Assessment Performed?	Site Number	Treatment	Date of assessment	Time of Assessment	Score	Unscheduled Reason
A0015	Group A1	EOS/ET (Day 61)		Yes	F1	5 ug	08-MAY-2018	10:22	0	
A0015	Group A1	EOS/ET (Day 61)		Yes	F2	5 ug	08-MAY-2018	10:22	0	
A0015	Group A1	EOS/ET (Day 61)		Yes	F3	5 ug	08-MAY-2018	10:22	0	
A0016	Group A3	Day 1	1 min	Yes	F1	uncoated	10-MAR-2018	11:01	1	
A0016	Group A3	Day 1	1 min	Yes	F2	uncoated	10-MAR-2018	11:04	1	
A0016	Group A3	Day 1	1 min	Yes	F3	uncoated	10-MAR-2018	11:07	1	
A0016	Group A3	Day 1	10 min	Yes	F1	uncoated	10-MAR-2018	11:18	0	
A0016	Group A3	Day 1	10 min	Yes	F2	uncoated	10-MAR-2018	11:18	0	
A0016	Group A3	Day 1	10 min	Yes	F3	uncoated	10-MAR-2018	11:18	0	
A0016	Group A3	Day 1	1 hour	Yes	F1	uncoated	10-MAR-2018	12:18	0	
A0016	Group A3	Day 1	1 hour	Yes	F2	uncoated	10-MAR-2018	12:18	0	
A0016	Group A3	Day 1	1 hour	Yes	F3	uncoated	10-MAR-2018	12:18	0	
A0016	Group A3	Day 1	2 hour	Yes	F1	uncoated	10-MAR-2018	13:18	0	
A0016	Group A3	Day 1	2 hour	Yes	F2	uncoated	10-MAR-2018	13:18	0	
A0016	Group A3	Day 1	2 hour	Yes	F3	uncoated	10-MAR-2018	13:18	0	
A0016	Group A3	Day 4		Yes	F1	uncoated	13-MAR-2018	11:54	0	
A0016	Group A3	Day 4		Yes	F2	uncoated	13-MAR-2018	11:54	0	
A0016	Group A3	Day 4		Yes	F3	uncoated	13-MAR-2018	11:54	0	
A0016	Group A3	Day 8		Yes	F1	uncoated	17-MAR-2018	11:29	0	
A0016	Group A3	Day 8		Yes	F2	uncoated	17-MAR-2018	11:29	0	
A0016	Group A3	Day 8		Yes	F3	uncoated	17-MAR-2018	11:29	0	

Scale: 0= No Pain; 10= Worst Pain Possible
Administration Site Key: FA = Forearm; UA= Upper arm
Treatments Part A: A1 = A/Sing MAP, 15 ug (FA); A2 = Afluria Quad IM (UA), 15 ug; A3 = Placebo MAP (FA); A4 = A/Sing Mono IM, 15 ug (UA)
Treatments Part B: B1 = A/Sing MAP, 15 ug (FA) with biopsy; B2 = Placebo MAP (FA) with biopsy
B3 = A/Sing MAP, 15 ug (FA); B4 = A/Sing MAP, 10 ug (FA); B5 = A/Sing MAP, 5 ug (FA); B6 = A/Sing MAP, 2.5 ug (FA)
B7 = Placebo MAP (FA); B8 = A/Sing MAP, 15 ug (UA); B9 = A/Sing IM, 15 ug, as 2018 Afluria Quad (UA)

Listing 16.2.9.3
Numeric Pain Intensity per Site

Part A

Randomisation Number	Group	Visit	Time Point	Was Pain Assessment Performed?	Site Number	Treatment	Date of assessment	Time of Assessment	Score	Unscheduled Reason
A0016	Group A3	Day 22		Yes	F1	uncoated	31-MAR-2018	10:50	0	
A0016	Group A3	Day 22		Yes	F2	uncoated	31-MAR-2018	10:50	0	
A0016	Group A3	Day 22		Yes	F3	uncoated	31-MAR-2018	10:50	0	
A0016	Group A3	EOS/ET (Day 61)		Yes	F1	uncoated	10-MAY-2018	10:42	0	
A0016	Group A3	EOS/ET (Day 61)		Yes	F2	uncoated	10-MAY-2018	10:43	0	
A0016	Group A3	EOS/ET (Day 61)		Yes	F3	uncoated	10-MAY-2018	10:43	0	
A0017	Group A3	Day 1	1 min	Yes	F1	uncoated	10-MAR-2018	11:11	0	
A0017	Group A3	Day 1	1 min	Yes	F2	uncoated	10-MAR-2018	11:14	0	
A0017	Group A3	Day 1	1 min	Yes	F3	uncoated	10-MAR-2018	11:17	0	
A0017	Group A3	Day 1	10 min	Yes	F1	uncoated	10-MAR-2018	11:28	0	
A0017	Group A3	Day 1	10 min	Yes	F2	uncoated	10-MAR-2018	11:28	0	
A0017	Group A3	Day 1	10 min	Yes	F3	uncoated	10-MAR-2018	11:28	0	
A0017	Group A3	Day 1	1 hour	Yes	F1	uncoated	10-MAR-2018	12:23	0	
A0017	Group A3	Day 1	1 hour	Yes	F2	uncoated	10-MAR-2018	12:23	0	
A0017	Group A3	Day 1	1 hour	Yes	F3	uncoated	10-MAR-2018	12:23	0	
A0017	Group A3	Day 1	2 hour	Yes	F1	uncoated	10-MAR-2018	13:28	0	
A0017	Group A3	Day 1	2 hour	Yes	F2	uncoated	10-MAR-2018	13:28	0	
A0017	Group A3	Day 1	2 hour	Yes	F3	uncoated	10-MAR-2018	13:28	0	
A0017	Group A3	Day 4		Yes	F1	uncoated	13-MAR-2018	12:42	0	
A0017	Group A3	Day 4		Yes	F2	uncoated	13-MAR-2018	12:42	0	
A0017	Group A3	Day 4		Yes	F3	uncoated	13-MAR-2018	12:42	0	

Scale: 0= No Pain; 10= Worst Pain Possible
Administration Site Key: FA = Forearm; UA= Upper arm
Treatments Part A: A1 = A/Sing MAP, 15 ug (FA); A2 = Afluria Quad IM (UA), 15 ug; A3 = Placebo MAP (FA); A4 = A/Sing Mono IM, 15 ug (UA)
Treatments Part B: B1 = A/Sing MAP, 15 ug (FA) with biopsy; B2 = Placebo MAP (FA) with biopsy
B3 = A/Sing MAP, 15 ug (FA); B4 = A/Sing MAP, 10 ug (FA); B5 = A/Sing MAP, 5 ug (FA); B6 = A/Sing MAP, 2.5 ug (FA)
B7 = Placebo MAP (FA); B8 = A/Sing MAP, 15 ug (UA); B9 = A/Sing IM, 15 ug, as 2018 Afluria Quad (UA)

Listing 16.2.9.3
Numeric Pain Intensity per Site

Part A

Randomisation Number	Group	Visit	Time Point	Was Pain Assessment Performed?	Site Number	Treatment	Date of assessment	Time of Assessment	Score	Unscheduled Reason
A0017	Group A3	Day 8		Yes	F1	uncoated	17-MAR-2018	13:44	0	
A0017	Group A3	Day 8		Yes	F2	uncoated	17-MAR-2018	13:44	0	
A0017	Group A3	Day 8		Yes	F3	uncoated	17-MAR-2018	13:44	0	
A0017	Group A3	Day 22		Yes	F1	uncoated	31-MAR-2018	14:12	0	
A0017	Group A3	Day 22		Yes	F2	uncoated	31-MAR-2018	14:12	0	
A0017	Group A3	Day 22		Yes	F3	uncoated	31-MAR-2018	14:12	0	
A0017	Group A3	EOS/ET (Day 61)		Yes	F1	uncoated	09-MAY-2018	12:09	0	
A0017	Group A3	EOS/ET (Day 61)		Yes	F2	uncoated	09-MAY-2018	12:09	0	
A0017	Group A3	EOS/ET (Day 61)		Yes	F3	uncoated	09-MAY-2018	12:09	0	
A0018	Group A1	Day 1	1 min	Yes	F1	5 ug	10-MAR-2018	11:21	1	
A0018	Group A1	Day 1	1 min	Yes	F2	5 ug	10-MAR-2018	11:24	1	
A0018	Group A1	Day 1	1 min	Yes	F3	5 ug	10-MAR-2018	11:27	1	
A0018	Group A1	Day 1	10 min	Yes	F1	5 ug	10-MAR-2018	11:38	1	
A0018	Group A1	Day 1	10 min	Yes	F2	5 ug	10-MAR-2018	11:38	1	
A0018	Group A1	Day 1	10 min	Yes	F3	5 ug	10-MAR-2018	11:38	1	
A0018	Group A1	Day 1	1 hour	Yes	F1	5 ug	10-MAR-2018	12:27	0	
A0018	Group A1	Day 1	1 hour	Yes	F2	5 ug	10-MAR-2018	12:27	0	
A0018	Group A1	Day 1	1 hour	Yes	F3	5 ug	10-MAR-2018	12:27	0	
A0018	Group A1	Day 1	2 hour	Yes	F1	5 ug	10-MAR-2018	13:38	0	
A0018	Group A1	Day 1	2 hour	Yes	F2	5 ug	10-MAR-2018	13:38	0	
A0018	Group A1	Day 1	2 hour	Yes	F3	5 ug	10-MAR-2018	13:38	0	

Scale: 0= No Pain; 10= Worst Pain Possible
Administration Site Key: FA = Forearm; UA= Upper arm
Treatments Part A: A1 = A/Sing MAP, 15 ug (FA); A2 = Afluria Quad IM (UA), 15 ug; A3 = Placebo MAP (FA); A4 = A/Sing Mono IM, 15 ug (UA)
Treatments Part B: B1 = A/Sing MAP, 15 ug (FA) with biopsy; B2 = Placebo MAP (FA) with biopsy
B3 = A/Sing MAP, 15 ug (FA); B4 = A/Sing MAP, 10 ug (FA); B5 = A/Sing MAP, 5 ug (FA); B6 = A/Sing MAP, 2.5 ug (FA)
B7 = Placebo MAP (FA); B8 = A/Sing MAP, 15 ug (UA); B9 = A/Sing IM, 15 ug, as 2018 Afluria Quad (UA)

Listing 16.2.9.3
Numeric Pain Intensity per Site

Part A

Randomisation Number	Group	Visit	Time Point	Was Pain Assessment Performed?	Site Number	Treatment	Date of assessment	Time of Assessment	Score	Unscheduled Reason
A0018	Group A1	Day 4		Yes	F1	5 ug	13-MAR-2018	12:17	0	
A0018	Group A1	Day 4		Yes	F2	5 ug	13-MAR-2018	12:18	0	
A0018	Group A1	Day 4		Yes	F3	5 ug	13-MAR-2018	12:18	0	
A0018	Group A1	Day 8		Yes	F1	5 ug	17-MAR-2018	11:16	0	
A0018	Group A1	Day 8		Yes	F2	5 ug	17-MAR-2018	11:16	0	
A0018	Group A1	Day 8		Yes	F3	5 ug	17-MAR-2018	11:16	0	
A0018	Group A1	Day 22		Yes	F1	5 ug	31-MAR-2018	08:58	0	
A0018	Group A1	Day 22		Yes	F2	5 ug	31-MAR-2018	08:58	0	
A0018	Group A1	Day 22		Yes	F3	5 ug	31-MAR-2018	08:59	0	
A0018	Group A1	EOS/ET (Day 61)		Yes	F1	5 ug	09-MAY-2018	17:11	0	
A0018	Group A1	EOS/ET (Day 61)		Yes	F2	5 ug	09-MAY-2018	17:11	0	
A0018	Group A1	EOS/ET (Day 61)		Yes	F3	5 ug	09-MAY-2018	17:11	0	
A0019	Group A3	Day 1	1 min	Yes	F1	uncoated	10-MAR-2018	11:31	0	
A0019	Group A3	Day 1	1 min	Yes	F2	uncoated	10-MAR-2018	11:34	0	
A0019	Group A3	Day 1	1 min	Yes	F3	uncoated	10-MAR-2018	11:37	1	
A0019	Group A3	Day 1	10 min	Yes	F1	uncoated	10-MAR-2018	11:48	0	
A0019	Group A3	Day 1	10 min	Yes	F2	uncoated	10-MAR-2018	11:48	0	
A0019	Group A3	Day 1	10 min	Yes	F3	uncoated	10-MAR-2018	11:48	0	
A0019	Group A3	Day 1	1 hour	Yes	F1	uncoated	10-MAR-2018	12:38	0	
A0019	Group A3	Day 1	1 hour	Yes	F2	uncoated	10-MAR-2018	12:38	0	
A0019	Group A3	Day 1	1 hour	Yes	F3	uncoated	10-MAR-2018	12:38	0	

Scale: 0= No Pain; 10= Worst Pain Possible
Administration Site Key: FA = Forearm; UA= Upper arm
Treatments Part A: A1 = A/Sing MAP, 15 ug (FA); A2 = Afluria Quad IM (UA), 15 ug; A3 = Placebo MAP (FA); A4 = A/Sing Mono IM, 15 ug (UA)
Treatments Part B: B1 = A/Sing MAP, 15 ug (FA) with biopsy; B2 = Placebo MAP (FA) with biopsy
B3 = A/Sing MAP, 15 ug (FA); B4 = A/Sing MAP, 10 ug (FA); B5 = A/Sing MAP, 5 ug (FA); B6 = A/Sing MAP, 2.5 ug (FA)
B7 = Placebo MAP (FA); B8 = A/Sing MAP, 15 ug (UA); B9 = A/Sing IM, 15 ug, as 2018 Afluria Quad (UA)

Listing 16.2.9.3
Numeric Pain Intensity per Site

Part A

Randomisation Number	Group	Visit	Time Point	Was Pain Assessment Performed?	Site Number	Treatment	Date of assessment	Time of Assessment	Score	Unscheduled Reason
A0019	Group A3	Day 1	2 hour	Yes	F1	uncoated	10-MAR-2018	13:48	0	
A0019	Group A3	Day 1	2 hour	Yes	F2	uncoated	10-MAR-2018	13:48	0	
A0019	Group A3	Day 1	2 hour	Yes	F3	uncoated	10-MAR-2018	13:48	0	
A0019	Group A3	Day 4		Yes	F1	uncoated	13-MAR-2018	12:02	0	
A0019	Group A3	Day 4		Yes	F2	uncoated	13-MAR-2018	12:02	0	
A0019	Group A3	Day 4		Yes	F3	uncoated	13-MAR-2018	12:02	0	
A0019	Group A3	Day 8		Yes	F1	uncoated	17-MAR-2018	11:33	0	
A0019	Group A3	Day 8		Yes	F2	uncoated	17-MAR-2018	11:33	0	
A0019	Group A3	Day 8		Yes	F3	uncoated	17-MAR-2018	11:33	0	
A0019	Group A3	Day 22		Yes	F1	uncoated	31-MAR-2018	11:40	0	
A0019	Group A3	Day 22		Yes	F2	uncoated	31-MAR-2018	11:40	0	
A0019	Group A3	Day 22		Yes	F3	uncoated	31-MAR-2018	11:40	0	
A0019	Group A3	EOS/ET (Day 61)		Yes	F1	uncoated	08-MAY-2018	11:56	0	
A0019	Group A3	EOS/ET (Day 61)		Yes	F2	uncoated	08-MAY-2018	11:56	0	
A0019	Group A3	EOS/ET (Day 61)		Yes	F3	uncoated	08-MAY-2018	11:56	0	
A0020	Group A1	Day 1	1 min	Yes	F1	5 ug	10-MAR-2018	11:41	0	
A0020	Group A1	Day 1	1 min	Yes	F2	5 ug	10-MAR-2018	11:44	0	
A0020	Group A1	Day 1	1 min	Yes	F3	5 ug	10-MAR-2018	11:47	0	
A0020	Group A1	Day 1	10 min	Yes	F1	5 ug	10-MAR-2018	11:58	0	
A0020	Group A1	Day 1	10 min	Yes	F2	5 ug	10-MAR-2018	11:58	0	
A0020	Group A1	Day 1	10 min	Yes	F3	5 ug	10-MAR-2018	11:58	0	

Scale: 0= No Pain; 10= Worst Pain Possible
Administration Site Key: FA = Forearm; UA= Upper arm
Treatments Part A: A1 = A/Sing MAP, 15 ug (FA); A2 = Afluria Quad IM (UA), 15 ug; A3 = Placebo MAP (FA); A4 = A/Sing Mono IM, 15 ug (UA)
Treatments Part B: B1 = A/Sing MAP, 15 ug (FA) with biopsy; B2 = Placebo MAP (FA) with biopsy
B3 = A/Sing MAP, 15 ug (FA); B4 = A/Sing MAP, 10 ug (FA); B5 = A/Sing MAP, 5 ug (FA); B6 = A/Sing MAP, 2.5 ug (FA)
B7 = Placebo MAP (FA); B8 = A/Sing MAP, 15 ug (UA); B9 = A/Sing IM, 15 ug, as 2018 Afluria Quad (UA)

Listing 16.2.9.3
Numeric Pain Intensity per Site

Part A

Randomisation Number	Group	Visit	Time Point	Was Pain Assessment Performed?	Site Number	Treatment	Date of assessment	Time of Assessment	Score	Unscheduled Reason
A0020	Group A1	Day 1	1 hour	Yes	F1	5 ug	10-MAR-2018	12:48	0	
A0020	Group A1	Day 1	1 hour	Yes	F2	5 ug	10-MAR-2018	12:48	0	
A0020	Group A1	Day 1	1 hour	Yes	F3	5 ug	10-MAR-2018	12:48	0	
A0020	Group A1	Day 1	2 hour	Yes	F1	5 ug	10-MAR-2018	14:06	0	
A0020	Group A1	Day 1	2 hour	Yes	F2	5 ug	10-MAR-2018	14:06	0	
A0020	Group A1	Day 1	2 hour	Yes	F3	5 ug	10-MAR-2018	14:06	0	
A0020	Group A1	Day 4		Yes	F1	5 ug	13-MAR-2018	10:28	0	
A0020	Group A1	Day 4		Yes	F2	5 ug	13-MAR-2018	10:28	0	
A0020	Group A1	Day 4		Yes	F3	5 ug	13-MAR-2018	10:28	0	
A0020	Group A1	Day 8		Yes	F1	5 ug	17-MAR-2018	11:09	0	
A0020	Group A1	Day 8		Yes	F2	5 ug	17-MAR-2018	11:10	0	
A0020	Group A1	Day 8		Yes	F3	5 ug	17-MAR-2018	11:11	0	
A0020	Group A1	Day 22		Yes	F1	5 ug	31-MAR-2018	11:33	0	
A0020	Group A1	Day 22		Yes	F2	5 ug	31-MAR-2018	11:33	0	
A0020	Group A1	Day 22		Yes	F3	5 ug	31-MAR-2018	11:34	0	
A0020	Group A1	EOS/ET (Day 61)		Yes	F1	5 ug	09-MAY-2018	13:09	0	
A0020	Group A1	EOS/ET (Day 61)		Yes	F2	5 ug	09-MAY-2018	13:10	0	
A0020	Group A1	EOS/ET (Day 61)		Yes	F3	5 ug	09-MAY-2018	13:10	0	
A0021	Group A1	Day 1	1 min	Yes	F1	5 ug	10-MAR-2018	12:11	2	
A0021	Group A1	Day 1	1 min	Yes	F2	5 ug	10-MAR-2018	12:14	1	
A0021	Group A1	Day 1	1 min	Yes	F3	5 ug	10-MAR-2018	12:17	2	

Scale: 0= No Pain; 10= Worst Pain Possible
Administration Site Key: FA = Forearm; UA= Upper arm
Treatments Part A: A1 = A/Sing MAP, 15 ug (FA); A2 = Afluria Quad IM (UA), 15 ug; A3 = Placebo MAP (FA); A4 = A/Sing Mono IM, 15 ug (UA)
Treatments Part B: B1 = A/Sing MAP, 15 ug (FA) with biopsy; B2 = Placebo MAP (FA) with biopsy
B3 = A/Sing MAP, 15 ug (FA); B4 = A/Sing MAP, 10 ug (FA); B5 = A/Sing MAP, 5 ug (FA); B6 = A/Sing MAP, 2.5 ug (FA)
B7 = Placebo MAP (FA); B8 = A/Sing MAP, 15 ug (UA); B9 = A/Sing IM, 15 ug, as 2018 Afluria Quad (UA)

Listing 16.2.9.3
Numeric Pain Intensity per Site

Part A

Randomisation Number	Group	Visit	Time Point	Was Pain Assessment Performed?	Site Number	Treatment	Date of assessment	Time of Assessment	Score	Unscheduled Reason
A0021	Group A1	Day 1	10 min	Yes	F1	5 ug	10-MAR-2018	12:28	1	
A0021	Group A1	Day 1	10 min	Yes	F2	5 ug	10-MAR-2018	12:28	1	
A0021	Group A1	Day 1	10 min	Yes	F3	5 ug	10-MAR-2018	12:28	1	
A0021	Group A1	Day 1	1 hour	Yes	F1	5 ug	10-MAR-2018	13:18	0	
A0021	Group A1	Day 1	1 hour	Yes	F2	5 ug	10-MAR-2018	13:18	0	
A0021	Group A1	Day 1	1 hour	Yes	F3	5 ug	10-MAR-2018	13:18	0	
A0021	Group A1	Day 1	2 hour	Yes	F1	5 ug	10-MAR-2018	14:29	0	
A0021	Group A1	Day 1	2 hour	Yes	F2	5 ug	10-MAR-2018	14:29	0	
A0021	Group A1	Day 1	2 hour	Yes	F3	5 ug	10-MAR-2018	14:29	0	
A0021	Group A1	Day 4		Yes	F1	5 ug	13-MAR-2018	14:48	0	
A0021	Group A1	Day 4		Yes	F2	5 ug	13-MAR-2018	14:48	0	
A0021	Group A1	Day 4		Yes	F3	5 ug	13-MAR-2018	14:48	0	
A0021	Group A1	Day 8		Yes	F1	5 ug	17-MAR-2018	13:20	0	
A0021	Group A1	Day 8		Yes	F2	5 ug	17-MAR-2018	13:20	0	
A0021	Group A1	Day 8		Yes	F3	5 ug	17-MAR-2018	13:20	0	
A0021	Group A1	Day 22		Yes	F1	5 ug	31-MAR-2018	13:01	0	
A0021	Group A1	Day 22		Yes	F2	5 ug	31-MAR-2018	13:01	0	
A0021	Group A1	Day 22		Yes	F3	5 ug	31-MAR-2018	13:01	0	
A0021	Group A1	EOS/ET (Day 61)		Yes	F1	5 ug	10-MAY-2018	11:35	0	
A0021	Group A1	EOS/ET (Day 61)		Yes	F2	5 ug	10-MAY-2018	11:36	0	
A0021	Group A1	EOS/ET (Day 61)		Yes	F3	5 ug	10-MAY-2018	11:36	0	

Scale: 0= No Pain; 10= Worst Pain Possible
Administration Site Key: FA = Forearm; UA= Upper arm
Treatments Part A: A1 = A/Sing MAP, 15 ug (FA); A2 = Afluria Quad IM (UA), 15 ug; A3 = Placebo MAP (FA); A4 = A/Sing Mono IM, 15 ug (UA)
Treatments Part B: B1 = A/Sing MAP, 15 ug (FA) with biopsy; B2 = Placebo MAP (FA) with biopsy
B3 = A/Sing MAP, 15 ug (FA); B4 = A/Sing MAP, 10 ug (FA); B5 = A/Sing MAP, 5 ug (FA); B6 = A/Sing MAP, 2.5 ug (FA)
B7 = Placebo MAP (FA); B8 = A/Sing MAP, 15 ug (UA); B9 = A/Sing IM, 15 ug, as 2018 Afluria Quad (UA)

Listing 16.2.9.3
Numeric Pain Intensity per Site

Part A

Randomisation Number	Group	Visit	Time Point	Was Pain Assessment Performed?	Site Number	Treatment	Date of assessment	Time of Assessment	Score	Unscheduled Reason
A0022	Group A3	Day 1	1 min	Yes	F1	uncoated	10-MAR-2018	11:51	2	
A0022	Group A3	Day 1	1 min	Yes	F2	uncoated	10-MAR-2018	11:54	3	
A0022	Group A3	Day 1	1 min	Yes	F3	uncoated	10-MAR-2018	11:57	1	
A0022	Group A3	Day 1	10 min	Yes	F1	uncoated	10-MAR-2018	12:08	4	
A0022	Group A3	Day 1	10 min	Yes	F2	uncoated	10-MAR-2018	12:08	2	
A0022	Group A3	Day 1	10 min	Yes	F3	uncoated	10-MAR-2018	12:08	0	
A0022	Group A3	Day 1	1 hour	Yes	F1	uncoated	10-MAR-2018	12:58	0	
A0022	Group A3	Day 1	1 hour	Yes	F2	uncoated	10-MAR-2018	12:58	0	
A0022	Group A3	Day 1	1 hour	Yes	F3	uncoated	10-MAR-2018	12:58	0	
A0022	Group A3	Day 1	2 hour	Yes	F1	uncoated	10-MAR-2018	14:18	0	
A0022	Group A3	Day 1	2 hour	Yes	F2	uncoated	10-MAR-2018	14:18	0	
A0022	Group A3	Day 1	2 hour	Yes	F3	uncoated	10-MAR-2018	14:18	0	
A0022	Group A3	Day 4		Yes	F1	uncoated	13-MAR-2018	11:59	0	
A0022	Group A3	Day 4		Yes	F2	uncoated	13-MAR-2018	11:59	0	
A0022	Group A3	Day 4		Yes	F3	uncoated	13-MAR-2018	11:59	0	
A0022	Group A3	Day 8		Yes	F1	uncoated	17-MAR-2018	12:15	0	
A0022	Group A3	Day 8		Yes	F2	uncoated	17-MAR-2018	12:16	0	
A0022	Group A3	Day 8		Yes	F3	uncoated	17-MAR-2018	12:16	0	
A0022	Group A3	Day 22		Yes	F1	uncoated	31-MAR-2018	11:54	0	
A0022	Group A3	Day 22		Yes	F2	uncoated	31-MAR-2018	11:54	0	
A0022	Group A3	Day 22		Yes	F3	uncoated	31-MAR-2018	11:54	0	

Scale: 0= No Pain; 10= Worst Pain Possible
Administration Site Key: FA = Forearm; UA= Upper arm
Treatments Part A: A1 = A/Sing MAP, 15 ug (FA); A2 = Afluria Quad IM (UA), 15 ug; A3 = Placebo MAP (FA); A4 = A/Sing Mono IM, 15 ug (UA)
Treatments Part B: B1 = A/Sing MAP, 15 ug (FA) with biopsy; B2 = Placebo MAP (FA) with biopsy
B3 = A/Sing MAP, 15 ug (FA); B4 = A/Sing MAP, 10 ug (FA); B5 = A/Sing MAP, 5 ug (FA); B6 = A/Sing MAP, 2.5 ug (FA)
B7 = Placebo MAP (FA); B8 = A/Sing MAP, 15 ug (UA); B9 = A/Sing IM, 15 ug, as 2018 Afluria Quad (UA)

Listing 16.2.9.3
Numeric Pain Intensity per Site

Part A

Randomisation Number	Group	Visit	Time Point	Was Pain Assessment Performed?	Site Number	Treatment	Date of assessment	Time of Assessment	Score	Unscheduled Reason
A0022	Group A3	EOS/ET (Day 61)		Yes	F1	uncoated	09-MAY-2018	13:12	0	
A0022	Group A3	EOS/ET (Day 61)		Yes	F2	uncoated	09-MAY-2018	13:12	0	
A0022	Group A3	EOS/ET (Day 61)		Yes	F3	uncoated	09-MAY-2018	13:12	0	
A0023	Group A3	Day 1	1 min	Yes	F1	uncoated	10-MAR-2018	12:01	1	
A0023	Group A3	Day 1	1 min	Yes	F2	uncoated	10-MAR-2018	12:04	3	
A0023	Group A3	Day 1	1 min	Yes	F3	uncoated	10-MAR-2018	12:07	2	
A0023	Group A3	Day 1	10 min	Yes	F1	uncoated	10-MAR-2018	12:15	0	
A0023	Group A3	Day 1	10 min	Yes	F2	uncoated	10-MAR-2018	12:18	1	
A0023	Group A3	Day 1	10 min	Yes	F3	uncoated	10-MAR-2018	12:18	1	
A0023	Group A3	Day 1	1 hour	Yes	F1	uncoated	10-MAR-2018	13:08	0	
A0023	Group A3	Day 1	1 hour	Yes	F2	uncoated	10-MAR-2018	13:08	0	
A0023	Group A3	Day 1	1 hour	Yes	F3	uncoated	10-MAR-2018	13:08	0	
A0023	Group A3	Day 1	2 hour	Yes	F1	uncoated	10-MAR-2018	14:28	0	
A0023	Group A3	Day 1	2 hour	Yes	F2	uncoated	10-MAR-2018	14:28	0	
A0023	Group A3	Day 1	2 hour	Yes	F3	uncoated	10-MAR-2018	14:28	0	
A0023	Group A3	Day 4		Yes	F1	uncoated	13-MAR-2018	12:17	0	
A0023	Group A3	Day 4		Yes	F2	uncoated	13-MAR-2018	12:18	0	
A0023	Group A3	Day 4		Yes	F3	uncoated	13-MAR-2018	12:18	0	
A0023	Group A3	Day 8		Yes	F1	uncoated	17-MAR-2018	11:23	0	
A0023	Group A3	Day 8		Yes	F2	uncoated	17-MAR-2018	11:23	0	
A0023	Group A3	Day 8		Yes	F3	uncoated	17-MAR-2018	11:23	0	

Scale: 0= No Pain; 10= Worst Pain Possible
Administration Site Key: FA = Forearm; UA= Upper arm
Treatments Part A: A1 = A/Sing MAP, 15 ug (FA); A2 = Afluria Quad IM (UA), 15 ug; A3 = Placebo MAP (FA); A4 = A/Sing Mono IM, 15 ug (UA)
Treatments Part B: B1 = A/Sing MAP, 15 ug (FA) with biopsy; B2 = Placebo MAP (FA) with biopsy
B3 = A/Sing MAP, 15 ug (FA); B4 = A/Sing MAP, 10 ug (FA); B5 = A/Sing MAP, 5 ug (FA); B6 = A/Sing MAP, 2.5 ug (FA)
B7 = Placebo MAP (FA); B8 = A/Sing MAP, 15 ug (UA); B9 = A/Sing IM, 15 ug, as 2018 Afluria Quad (UA)

Listing 16.2.9.3
Numeric Pain Intensity per Site

Part A

Randomisation Number	Group	Visit	Time Point	Was Pain Assessment Performed?	Site Number	Treatment	Date of assessment	Time of Assessment	Score	Unscheduled Reason
A0023	Group A3	Day 22		Yes	F1	uncoated	31-MAR-2018	11:51	0	
A0023	Group A3	Day 22		Yes	F2	uncoated	31-MAR-2018	11:51	0	
A0023	Group A3	Day 22		Yes	F3	uncoated	31-MAR-2018	11:51	0	
A0023	Group A3	EOS/ET (Day 61)		Yes	F1	uncoated	10-MAY-2018	13:15	0	
A0023	Group A3	EOS/ET (Day 61)		Yes	F2	uncoated	10-MAY-2018	13:15	0	
A0023	Group A3	EOS/ET (Day 61)		Yes	F3	uncoated	10-MAY-2018	13:15	0	
A0024	Group A1	Day 1	1 min	Yes	F1	5 ug	10-MAR-2018	12:21	2	
A0024	Group A1	Day 1	1 min	Yes	F2	5 ug	10-MAR-2018	12:24	1	
A0024	Group A1	Day 1	1 min	Yes	F3	5 ug	10-MAR-2018	12:27	1	
A0024	Group A1	Day 1	10 min	Yes	F1	5 ug	10-MAR-2018	12:38	1	
A0024	Group A1	Day 1	10 min	Yes	F2	5 ug	10-MAR-2018	12:38	0	
A0024	Group A1	Day 1	10 min	Yes	F3	5 ug	10-MAR-2018	12:38	0	
A0024	Group A1	Day 1	1 hour	Yes	F1	5 ug	10-MAR-2018	13:28	0	
A0024	Group A1	Day 1	1 hour	Yes	F2	5 ug	10-MAR-2018	13:28	0	
A0024	Group A1	Day 1	1 hour	Yes	F3	5 ug	10-MAR-2018	13:28	0	
A0024	Group A1	Day 1	2 hour	Yes	F1	5 ug	10-MAR-2018	14:33	0	
A0024	Group A1	Day 1	2 hour	Yes	F2	5 ug	10-MAR-2018	14:33	0	
A0024	Group A1	Day 1	2 hour	Yes	F3	5 ug	10-MAR-2018	14:33	0	
A0024	Group A1	Day 4		Yes	F1	5 ug	13-MAR-2018	12:49	0	
A0024	Group A1	Day 4		Yes	F2	5 ug	13-MAR-2018	12:49	0	
A0024	Group A1	Day 4		Yes	F3	5 ug	13-MAR-2018	12:49	0	

Scale: 0= No Pain; 10= Worst Pain Possible
Administration Site Key: FA = Forearm; UA= Upper arm
Treatments Part A: A1 = A/Sing MAP, 15 ug (FA); A2 = Afluria Quad IM (UA), 15 ug; A3 = Placebo MAP (FA); A4 = A/Sing Mono IM, 15 ug (UA)
Treatments Part B: B1 = A/Sing MAP, 15 ug (FA) with biopsy; B2 = Placebo MAP (FA) with biopsy
B3 = A/Sing MAP, 15 ug (FA); B4 = A/Sing MAP, 10 ug (FA); B5 = A/Sing MAP, 5 ug (FA); B6 = A/Sing MAP, 2.5 ug (FA)
B7 = Placebo MAP (FA); B8 = A/Sing MAP, 15 ug (UA); B9 = A/Sing IM, 15 ug, as 2018 Afluria Quad (UA)

Listing 16.2.9.3
Numeric Pain Intensity per Site

Part A

Randomisation Number	Group	Visit	Time Point	Was Pain Assessment Performed?	Site Number	Treatment	Date of assessment	Time of Assessment	Score	Unscheduled Reason
A0024	Group A1	Day 8		Yes	F1	5 ug	17-MAR-2018	11:56	0	
A0024	Group A1	Day 8		Yes	F2	5 ug	17-MAR-2018	11:56	0	
A0024	Group A1	Day 8		Yes	F3	5 ug	17-MAR-2018	11:56	0	
A0024	Group A1	Day 22		Yes	F1	5 ug	31-MAR-2018	11:10	0	
A0024	Group A1	Day 22		Yes	F2	5 ug	31-MAR-2018	11:10	0	
A0024	Group A1	Day 22		Yes	F3	5 ug	31-MAR-2018	11:10	0	
A0024	Group A1	EOS/ET (Day 61)		Yes	F1	5 ug	09-MAY-2018	14:28	0	
A0024	Group A1	EOS/ET (Day 61)		Yes	F2	5 ug	09-MAY-2018	14:28	0	
A0024	Group A1	EOS/ET (Day 61)		Yes	F3	5 ug	09-MAY-2018	14:28	0	
A0025	Group A3	Day 1	1 min	Yes	F1	uncoated	10-MAR-2018	12:22	0	
A0025	Group A3	Day 1	1 min	Yes	F2	uncoated	10-MAR-2018	12:22	0	
A0025	Group A3	Day 1	1 min	Yes	F3	uncoated	10-MAR-2018	12:22	0	
A0025	Group A3	Day 1	10 min	Yes	F1	uncoated	10-MAR-2018	12:31	2	
A0025	Group A3	Day 1	10 min	Yes	F2	uncoated	10-MAR-2018	12:34	3	
A0025	Group A3	Day 1	10 min	Yes	F3	uncoated	10-MAR-2018	12:37	3	
A0025	Group A3	Day 1	1 hour	Yes	F1	uncoated	10-MAR-2018	13:38	0	
A0025	Group A3	Day 1	1 hour	Yes	F2	uncoated	10-MAR-2018	13:38	0	
A0025	Group A3	Day 1	1 hour	Yes	F3	uncoated	10-MAR-2018	13:38	0	
A0025	Group A3	Day 1	2 hour	Yes	F1	uncoated	10-MAR-2018	14:39	0	
A0025	Group A3	Day 1	2 hour	Yes	F2	uncoated	10-MAR-2018	14:39	0	
A0025	Group A3	Day 1	2 hour	Yes	F3	uncoated	10-MAR-2018	14:39	0	

Scale: 0= No Pain; 10= Worst Pain Possible
Administration Site Key: FA = Forearm; UA= Upper arm
Treatments Part A: A1 = A/Sing MAP, 15 ug (FA); A2 = Afluria Quad IM (UA), 15 ug; A3 = Placebo MAP (FA); A4 = A/Sing Mono IM, 15 ug (UA)
Treatments Part B: B1 = A/Sing MAP, 15 ug (FA) with biopsy; B2 = Placebo MAP (FA) with biopsy
B3 = A/Sing MAP, 15 ug (FA); B4 = A/Sing MAP, 10 ug (FA); B5 = A/Sing MAP, 5 ug (FA); B6 = A/Sing MAP, 2.5 ug (FA)
B7 = Placebo MAP (FA); B8 = A/Sing MAP, 15 ug (UA); B9 = A/Sing IM, 15 ug, as 2018 Afluria Quad (UA)

Listing 16.2.9.3
Numeric Pain Intensity per Site

Part A

Randomisation Number	Group	Visit	Time Point	Was Pain Assessment Performed?	Site Number	Treatment	Date of assessment	Time of Assessment	Score	Unscheduled Reason
A0025	Group A3	Day 4		Yes	F1	uncoated	13-MAR-2018	13:06	0	
A0025	Group A3	Day 4		Yes	F2	uncoated	13-MAR-2018	13:06	0	
A0025	Group A3	Day 4		Yes	F3	uncoated	13-MAR-2018	13:06	0	
A0025	Group A3	Day 8		Yes	F1	uncoated	17-MAR-2018	13:32	0	
A0025	Group A3	Day 8		Yes	F2	uncoated	17-MAR-2018	13:32	0	
A0025	Group A3	Day 8		Yes	F3	uncoated	17-MAR-2018	13:32	0	
A0025	Group A3	Day 22		Yes	F1	uncoated	31-MAR-2018	12:50	0	
A0025	Group A3	Day 22		Yes	F2	uncoated	31-MAR-2018	12:51	0	
A0025	Group A3	Day 22		Yes	F3	uncoated	31-MAR-2018	12:51	0	
A0025	Group A3	EOS/ET (Day 61)		Yes	F1	uncoated	09-MAY-2018	11:17	0	
A0025	Group A3	EOS/ET (Day 61)		Yes	F2	uncoated	09-MAY-2018	11:18	0	
A0025	Group A3	EOS/ET (Day 61)		No	F3	uncoated				
A0027	Group A3	Day 1	1 min	Yes	F1	uncoated	10-MAR-2018	12:41	1	
A0027	Group A3	Day 1	1 min	Yes	F2	uncoated	10-MAR-2018	12:44	1	
A0027	Group A3	Day 1	1 min	Yes	F3	uncoated	10-MAR-2018	12:47	1	
A0027	Group A3	Day 1	10 min	Yes	F1	uncoated	10-MAR-2018	12:58	1	
A0027	Group A3	Day 1	10 min	Yes	F2	uncoated	10-MAR-2018	12:58	1	
A0027	Group A3	Day 1	10 min	Yes	F3	uncoated	10-MAR-2018	12:58	1	
A0027	Group A3	Day 1	1 hour	Yes	F1	uncoated	10-MAR-2018	13:48	0	
A0027	Group A3	Day 1	1 hour	Yes	F2	uncoated	10-MAR-2018	13:48	0	
A0027	Group A3	Day 1	1 hour	Yes	F3	uncoated	10-MAR-2018	13:48	0	

Scale: 0= No Pain; 10= Worst Pain Possible
Administration Site Key: FA = Forearm; UA= Upper arm
Treatments Part A: A1 = A/Sing MAP, 15 ug (FA); A2 = Afluria Quad IM (UA), 15 ug; A3 = Placebo MAP (FA); A4 = A/Sing Mono IM, 15 ug (UA)
Treatments Part B: B1 = A/Sing MAP, 15 ug (FA) with biopsy; B2 = Placebo MAP (FA) with biopsy
B3 = A/Sing MAP, 15 ug (FA); B4 = A/Sing MAP, 10 ug (FA); B5 = A/Sing MAP, 5 ug (FA); B6 = A/Sing MAP, 2.5 ug (FA)
B7 = Placebo MAP (FA); B8 = A/Sing MAP, 15 ug (UA); B9 = A/Sing IM, 15 ug, as 2018 Afluria Quad (UA)

Listing 16.2.9.3
Numeric Pain Intensity per Site

Part A

Randomisation Number	Group	Visit	Time Point	Was Pain Assessment Performed?	Site Number	Treatment	Date of assessment	Time of Assessment	Score	Unscheduled Reason
A0027	Group A3	Day 1	2 hour	Yes	F1	uncoated	10-MAR-2018	14:48	0	
A0027	Group A3	Day 1	2 hour	Yes	F2	uncoated	10-MAR-2018	14:48	0	
A0027	Group A3	Day 1	2 hour	Yes	F3	uncoated	10-MAR-2018	14:48	0	
A0027	Group A3	Day 4		Yes	F1	uncoated	13-MAR-2018	13:26	0	
A0027	Group A3	Day 4		Yes	F2	uncoated	13-MAR-2018	13:26	0	
A0027	Group A3	Day 4		Yes	F3	uncoated	13-MAR-2018	13:26	0	
A0027	Group A3	Day 8		Yes	F1	uncoated	17-MAR-2018	13:33	0	
A0027	Group A3	Day 8		Yes	F2	uncoated	17-MAR-2018	13:33	0	
A0027	Group A3	Day 8		Yes	F3	uncoated	17-MAR-2018	13:33	0	
A0027	Group A3	Day 22		Yes	F1	uncoated	01-APR-2018	08:26	0	
A0027	Group A3	Day 22		Yes	F2	uncoated	01-APR-2018	08:26	0	
A0027	Group A3	Day 22		Yes	F3	uncoated	01-APR-2018	08:27	0	
A0027	Group A3	EOS/ET (Day 61)		Yes	F1	uncoated	09-MAY-2018	12:31	0	
A0027	Group A3	EOS/ET (Day 61)		Yes	F2	uncoated	09-MAY-2018	12:31	0	
A0027	Group A3	EOS/ET (Day 61)		Yes	F3	uncoated	09-MAY-2018	12:31	0	
A0028	Group A1	Day 1	1 min	Yes	F1	5 ug	10-MAR-2018	12:51	0	
A0028	Group A1	Day 1	1 min	Yes	F2	5 ug	10-MAR-2018	12:54	1	
A0028	Group A1	Day 1	1 min	Yes	F3	5 ug	10-MAR-2018	12:57	1	
A0028	Group A1	Day 1	10 min	Yes	F1	5 ug	10-MAR-2018	13:08	0	
A0028	Group A1	Day 1	10 min	Yes	F2	5 ug	10-MAR-2018	13:08	0	
A0028	Group A1	Day 1	10 min	Yes	F3	5 ug	10-MAR-2018	13:08	0	

Scale: 0= No Pain; 10= Worst Pain Possible
Administration Site Key: FA = Forearm; UA= Upper arm
Treatments Part A: A1 = A/Sing MAP, 15 ug (FA); A2 = Afluria Quad IM (UA), 15 ug; A3 = Placebo MAP (FA); A4 = A/Sing Mono IM, 15 ug (UA)
Treatments Part B: B1 = A/Sing MAP, 15 ug (FA) with biopsy; B2 = Placebo MAP (FA) with biopsy
B3 = A/Sing MAP, 15 ug (FA); B4 = A/Sing MAP, 10 ug (FA); B5 = A/Sing MAP, 5 ug (FA); B6 = A/Sing MAP, 2.5 ug (FA)
B7 = Placebo MAP (FA); B8 = A/Sing MAP, 15 ug (UA); B9 = A/Sing IM, 15 ug, as 2018 Afluria Quad (UA)

Listing 16.2.9.3
Numeric Pain Intensity per Site

Part A

Randomisation Number	Group	Visit	Time Point	Was Pain Assessment Performed?	Site Number	Treatment	Date of assessment	Time of Assessment	Score	Unscheduled Reason
A0028	Group A1	Day 1	1 hour	Yes	F1	5 ug	10-MAR-2018	13:58	0	
A0028	Group A1	Day 1	1 hour	Yes	F2	5 ug	10-MAR-2018	13:58	0	
A0028	Group A1	Day 1	1 hour	Yes	F3	5 ug	10-MAR-2018	13:58	0	
A0028	Group A1	Day 1	2 hour	Yes	F1	5 ug	10-MAR-2018	14:58	0	
A0028	Group A1	Day 1	2 hour	Yes	F2	5 ug	10-MAR-2018	14:58	0	
A0028	Group A1	Day 1	2 hour	Yes	F3	5 ug	10-MAR-2018	14:58	0	
A0028	Group A1	Day 4		Yes	F1	5 ug	13-MAR-2018	08:31	0	
A0028	Group A1	Day 4		Yes	F2	5 ug	13-MAR-2018	08:31	0	
A0028	Group A1	Day 4		Yes	F3	5 ug	13-MAR-2018	08:31	0	
A0028	Group A1	Day 8		Yes	F1	5 ug	17-MAR-2018	13:59	0	
A0028	Group A1	Day 8		Yes	F2	5 ug	17-MAR-2018	13:59	0	
A0028	Group A1	Day 8		Yes	F3	5 ug	17-MAR-2018	13:59	0	
A0028	Group A1	Day 22		Yes	F1	5 ug	31-MAR-2018	14:42	0	
A0028	Group A1	Day 22		Yes	F2	5 ug	31-MAR-2018	14:42	0	
A0028	Group A1	Day 22		Yes	F3	5 ug	31-MAR-2018	14:42	0	
A0028	Group A1	EOS/ET (Day 61)		Yes	F1	5 ug	09-MAY-2018	16:39	0	
A0028	Group A1	EOS/ET (Day 61)		Yes	F2	5 ug	09-MAY-2018	16:39	0	
A0028	Group A1	EOS/ET (Day 61)		Yes	F3	5 ug	09-MAY-2018	16:39	0	
A0029	Group A1	Day 1	1 min	Yes	F1	5 ug	10-MAR-2018	13:01	1	
A0029	Group A1	Day 1	1 min	Yes	F2	5 ug	10-MAR-2018	13:04	1	
A0029	Group A1	Day 1	1 min	Yes	F3	5 ug	10-MAR-2018	13:07	2	

Scale: 0= No Pain; 10= Worst Pain Possible
Administration Site Key: FA = Forearm; UA= Upper arm
Treatments Part A: A1 = A/Sing MAP, 15 ug (FA); A2 = Afluria Quad IM (UA), 15 ug; A3 = Placebo MAP (FA); A4 = A/Sing Mono IM, 15 ug (UA)
Treatments Part B: B1 = A/Sing MAP, 15 ug (FA) with biopsy; B2 = Placebo MAP (FA) with biopsy
B3 = A/Sing MAP, 15 ug (FA); B4 = A/Sing MAP, 10 ug (FA); B5 = A/Sing MAP, 5 ug (FA); B6 = A/Sing MAP, 2.5 ug (FA)
B7 = Placebo MAP (FA); B8 = A/Sing MAP, 15 ug (UA); B9 = A/Sing IM, 15 ug, as 2018 Afluria Quad (UA)

Listing 16.2.9.3
Numeric Pain Intensity per Site

Part A

Randomisation Number	Group	Visit	Time Point	Was Pain Assessment Performed?	Site Number	Treatment	Date of assessment	Time of Assessment	Score	Unscheduled Reason
A0029	Group A1	Day 1	10 min	Yes	F1	5 ug	10-MAR-2018	13:08	1	
A0029	Group A1	Day 1	10 min	Yes	F2	5 ug	10-MAR-2018	13:08	1	
A0029	Group A1	Day 1	10 min	Yes	F3	5 ug	10-MAR-2018	13:08	2	
A0029	Group A1	Day 1	1 hour	Yes	F1	5 ug	10-MAR-2018	14:11	0	
A0029	Group A1	Day 1	1 hour	Yes	F2	5 ug	10-MAR-2018	14:11	0	
A0029	Group A1	Day 1	1 hour	Yes	F3	5 ug	10-MAR-2018	14:11	0	
A0029	Group A1	Day 1	2 hour	Yes	F1	5 ug	10-MAR-2018	15:11	0	
A0029	Group A1	Day 1	2 hour	Yes	F2	5 ug	10-MAR-2018	15:11	0	
A0029	Group A1	Day 1	2 hour	Yes	F3	5 ug	10-MAR-2018	15:11	0	
A0029	Group A1	Day 4		Yes	F1	5 ug	13-MAR-2018	16:04	0	
A0029	Group A1	Day 4		Yes	F2	5 ug	13-MAR-2018	16:04	0	
A0029	Group A1	Day 4		Yes	F3	5 ug	13-MAR-2018	16:04	0	
A0029	Group A1	Day 8		Yes	F1	5 ug	17-MAR-2018	15:42	0	
A0029	Group A1	Day 8		Yes	F2	5 ug	17-MAR-2018	15:42	0	
A0029	Group A1	Day 8		Yes	F3	5 ug	17-MAR-2018	15:42	0	
A0029	Group A1	Day 22		Yes	F1	5 ug	31-MAR-2018	13:24	0	
A0029	Group A1	Day 22		Yes	F2	5 ug	31-MAR-2018	13:24	0	
A0029	Group A1	Day 22		Yes	F3	5 ug	31-MAR-2018	13:24	0	
A0029	Group A1	EOS/ET (Day 61)		Yes	F1	5 ug	09-MAY-2018	16:17	0	
A0029	Group A1	EOS/ET (Day 61)		Yes	F2	5 ug	09-MAY-2018	16:17	0	
A0029	Group A1	EOS/ET (Day 61)		Yes	F3	5 ug	09-MAY-2018	16:17	0	

Scale: 0= No Pain; 10= Worst Pain Possible
Administration Site Key: FA = Forearm; UA= Upper arm
Treatments Part A: A1 = A/Sing MAP, 15 ug (FA); A2 = Afluria Quad IM (UA), 15 ug; A3 = Placebo MAP (FA); A4 = A/Sing Mono IM, 15 ug (UA)
Treatments Part B: B1 = A/Sing MAP, 15 ug (FA) with biopsy; B2 = Placebo MAP (FA) with biopsy
B3 = A/Sing MAP, 15 ug (FA); B4 = A/Sing MAP, 10 ug (FA); B5 = A/Sing MAP, 5 ug (FA); B6 = A/Sing MAP, 2.5 ug (FA)
B7 = Placebo MAP (FA); B8 = A/Sing MAP, 15 ug (UA); B9 = A/Sing IM, 15 ug, as 2018 Afluria Quad (UA)

Listing 16.2.9.3
Numeric Pain Intensity per Site

Part A

Randomisation Number	Group	Visit	Time Point	Was Pain Assessment Performed?	Site Number	Treatment	Date of assessment	Time of Assessment	Score	Unscheduled Reason
A0030	Group A3	Day 1	1 min	Yes	F1	uncoated	16-MAR-2018	12:01	1	
A0030	Group A3	Day 1	1 min	Yes	F2	uncoated	16-MAR-2018	12:04	1	
A0030	Group A3	Day 1	1 min	Yes	F3	uncoated	16-MAR-2018	12:07	1	
A0030	Group A3	Day 1	10 min	Yes	F1	uncoated	16-MAR-2018	12:18	0	
A0030	Group A3	Day 1	10 min	Yes	F2	uncoated	16-MAR-2018	12:18	0	
A0030	Group A3	Day 1	10 min	Yes	F3	uncoated	16-MAR-2018	12:18	0	
A0030	Group A3	Day 1	1 hour	Yes	F1	uncoated	16-MAR-2018	13:12	0	
A0030	Group A3	Day 1	1 hour	Yes	F2	uncoated	16-MAR-2018	13:12	0	
A0030	Group A3	Day 1	1 hour	Yes	F3	uncoated	16-MAR-2018	13:12	0	
A0030	Group A3	Day 1	2 hour	Yes	F1	uncoated	16-MAR-2018	14:08	0	
A0030	Group A3	Day 1	2 hour	Yes	F2	uncoated	16-MAR-2018	14:08	0	
A0030	Group A3	Day 1	2 hour	Yes	F3	uncoated	16-MAR-2018	14:08	0	
A0030	Group A3	Day 4		Yes	F1	uncoated	20-MAR-2018	09:01	0	
A0030	Group A3	Day 4		Yes	F2	uncoated	20-MAR-2018	09:01	0	
A0030	Group A3	Day 4		Yes	F3	uncoated	20-MAR-2018	09:01	0	
A0030	Group A3	Day 8		Yes	F1	uncoated	27-MAR-2018	12:10	0	
A0030	Group A3	Day 8		Yes	F2	uncoated	27-MAR-2018	12:11	0	
A0030	Group A3	Day 8		Yes	F3	uncoated	27-MAR-2018	12:11	0	
A0030	Group A3	Day 22		Yes	F1	uncoated	06-APR-2018	12:59	0	
A0030	Group A3	Day 22		Yes	F2	uncoated	06-APR-2018	12:59	0	
A0030	Group A3	Day 22		Yes	F3	uncoated	06-APR-2018	12:59	0	

Scale: 0= No Pain; 10= Worst Pain Possible
Administration Site Key: FA = Forearm; UA= Upper arm
Treatments Part A: A1 = A/Sing MAP, 15 ug (FA); A2 = Afluria Quad IM (UA), 15 ug; A3 = Placebo MAP (FA); A4 = A/Sing Mono IM, 15 ug (UA)
Treatments Part B: B1 = A/Sing MAP, 15 ug (FA) with biopsy; B2 = Placebo MAP (FA) with biopsy
B3 = A/Sing MAP, 15 ug (FA); B4 = A/Sing MAP, 10 ug (FA); B5 = A/Sing MAP, 5 ug (FA); B6 = A/Sing MAP, 2.5 ug (FA)
B7 = Placebo MAP (FA); B8 = A/Sing MAP, 15 ug (UA); B9 = A/Sing IM, 15 ug, as 2018 Afluria Quad (UA)

Listing 16.2.9.3
Numeric Pain Intensity per Site

Part A

Randomisation Number	Group	Visit	Time Point	Was Pain Assessment Performed?	Site Number	Treatment	Date of assessment	Time of Assessment	Score	Unscheduled Reason
A0030	Group A3	EOS/ET (Day 61)		Yes	F1	uncoated	15-MAY-2018	12:25	0	
A0030	Group A3	EOS/ET (Day 61)		Yes	F2	uncoated	15-MAY-2018	12:25	0	
A0030	Group A3	EOS/ET (Day 61)		Yes	F3	uncoated	15-MAY-2018	12:25	0	
A0031	Group A2	Day 1	1 min	Yes	D1	[IM]	16-MAR-2018	13:07	0	
A0031	Group A2	Day 1	10 min	Yes	D1	[IM]	16-MAR-2018	13:09	0	
A0031	Group A2	Day 1	1 hour	Yes	D1	[IM]	16-MAR-2018	13:56	0	
A0031	Group A2	Day 1	2 hour	Yes	D1	[IM]	16-MAR-2018	15:05	0	
A0031	Group A2	Day 4		Yes	D1	[IM]	19-MAR-2018	13:08	0	
A0032	Group A4	Day 1	1 min	Yes	D1	[IM]	16-MAR-2018	13:10	0	
A0032	Group A4	Day 1	10 min	Yes	D1	[IM]	16-MAR-2018	13:20	2	
A0032	Group A4	Day 1	1 hour	Yes	D1	[IM]	16-MAR-2018	14:05	0	
A0032	Group A4	Day 1	2 hour	Yes	D1	[IM]	16-MAR-2018	15:15	0	
A0032	Group A4	Day 4		Yes	D1	[IM]	19-MAR-2018	13:28	0	
A0032	Group A4	Day 8		Yes	D1	[IM]	23-MAR-2018	12:44	0	
A0032	Group A4	Day 22		Yes	D1	[IM]	06-APR-2018	13:06	0	
A0032	Group A4	EOS/ET (Day 61)		Yes	D1	[IM]	15-MAY-2018	12:04	0	
A0033	Group A2	Day 1	1 min	Yes	D1	[IM]	16-MAR-2018	13:21	1	
A0033	Group A2	Day 1	10 min	Yes	D1	[IM]	16-MAR-2018	13:30	0	
A0033	Group A2	Day 1	1 hour	Yes	D1	[IM]	16-MAR-2018	14:19	0	

Scale: 0= No Pain; 10= Worst Pain Possible
Administration Site Key: FA = Forearm; UA= Upper arm
Treatments Part A: A1 = A/Sing MAP, 15 ug (FA); A2 = Afluria Quad IM (UA), 15 ug; A3 = Placebo MAP (FA); A4 = A/Sing Mono IM, 15 ug (UA)
Treatments Part B: B1 = A/Sing MAP, 15 ug (FA) with biopsy; B2 = Placebo MAP (FA) with biopsy
B3 = A/Sing MAP, 15 ug (FA); B4 = A/Sing MAP, 10 ug (FA); B5 = A/Sing MAP, 5 ug (FA); B6 = A/Sing MAP, 2.5 ug (FA)
B7 = Placebo MAP (FA); B8 = A/Sing MAP, 15 ug (UA); B9 = A/Sing IM, 15 ug, as 2018 Afluria Quad (UA)

Listing 16.2.9.3
Numeric Pain Intensity per Site

Part A

Randomisation Number	Group	Visit	Time Point	Was Pain Assessment Performed?	Site Number	Treatment	Date of assessment	Time of Assessment	Score	Unscheduled Reason
A0033	Group A2	Day 1	2 hour	Yes	D1	[IM]	16-MAR-2018	15:25	0	
A0033	Group A2	Day 4		Yes	D1	[IM]	19-MAR-2018	15:28	0	
A0033	Group A2	Day 8		Yes	D1	[IM]	23-MAR-2018	15:23	0	
A0033	Group A2	Day 22		Yes	D1	[IM]	06-APR-2018	15:35	0	
A0033	Group A2	EOS/ET (Day 61)		Yes	D1	[IM]	15-MAY-2018	12:12	0	
A0034	Group A4	Day 1	1 min	Yes	D1	[IM]	16-MAR-2018	13:31	1	
A0034	Group A4	Day 1	10 min	Yes	D1	[IM]	16-MAR-2018	13:40	0	
A0034	Group A4	Day 1	1 hour	Yes	D1	[IM]	16-MAR-2018	14:30	0	
A0034	Group A4	Day 1	2 hour	Yes	D1	[IM]	16-MAR-2018	15:36	0	
A0034	Group A4	Day 4		Yes	D1	[IM]	19-MAR-2018	11:39	0	
A0034	Group A4	Day 8		Yes	D1	[IM]	23-MAR-2018	08:25	0	
A0034	Group A4	Day 22		Yes	D1	[IM]	06-APR-2018	09:05	0	
A0034	Group A4	EOS/ET (Day 61)		Yes	D1	[IM]	17-MAY-2018	09:14	0	
A0035	Group A4	Day 1	1 min	Yes	D1	[IM]	16-MAR-2018	13:41	1	
A0035	Group A4	Day 1	10 min	Yes	D1	[IM]	16-MAR-2018	13:50	0	
A0035	Group A4	Day 1	1 hour	Yes	D1	[IM]	16-MAR-2018	14:40	0	
A0035	Group A4	Day 1	2 hour	Yes	D1	[IM]	16-MAR-2018	15:45	0	
A0035	Group A4	Day 4		Yes	D1	[IM]	19-MAR-2018	15:56		
A0035	Group A4	Day 8		Yes	D1	[IM]	23-MAR-2018	15:23	0	
A0035	Group A4	Day 22		Yes	D1	[IM]	08-APR-2018	11:55	0	

Scale: 0= No Pain; 10= Worst Pain Possible
Administration Site Key: FA = Forearm; UA= Upper arm
Treatments Part A: A1 = A/Sing MAP, 15 ug (FA); A2 = Afluria Quad IM (UA), 15 ug; A3 = Placebo MAP (FA); A4 = A/Sing Mono IM, 15 ug (UA)
Treatments Part B: B1 = A/Sing MAP, 15 ug (FA) with biopsy; B2 = Placebo MAP (FA) with biopsy
B3 = A/Sing MAP, 15 ug (FA); B4 = A/Sing MAP, 10 ug (FA); B5 = A/Sing MAP, 5 ug (FA); B6 = A/Sing MAP, 2.5 ug (FA)
B7 = Placebo MAP (FA); B8 = A/Sing MAP, 15 ug (UA); B9 = A/Sing IM, 15 ug, as 2018 Afluria Quad (UA)

Listing 16.2.9.3
Numeric Pain Intensity per Site

Part A

Randomisation Number	Group	Visit	Time Point	Was Pain Assessment Performed?	Site Number	Treatment	Date of assessment	Time of Assessment	Score	Unscheduled Reason
A0035	Group A4	EOS/ET (Day 61)		Yes	D1	[IM]	15-MAY-2018	13:27	0	
A0036	Group A2	Day 1	1 min	Yes	D1	[IM]	17-MAR-2018	11:31	0	
A0036	Group A2	Day 1	10 min	Yes	D1	[IM]	17-MAR-2018	11:40	0	
A0036	Group A2	Day 1	1 hour	Yes	D1	[IM]	17-MAR-2018	12:30	0	
A0036	Group A2	Day 1	2 hour	Yes	D1	[IM]	17-MAR-2018	13:45	0	
A0036	Group A2	Day 4		Yes	D1	[IM]	21-MAR-2018	16:23	0	
A0036	Group A2	Day 8		Yes	D1	[IM]	25-MAR-2018	10:08	0	
A0036	Group A2	Day 22		Yes	D1	[IM]	07-APR-2018	11:08	0	
A0036	Group A2	EOS/ET (Day 61)		Yes	D1	[IM]	17-MAY-2018	14:06	0	
A0037	Group A4	Day 1	1 min	Yes	D1	[IM]	17-MAR-2018	11:36	5	
A0037	Group A4	Day 1	10 min	Yes	D1	[IM]	17-MAR-2018	11:45	1	
A0037	Group A4	Day 1	1 hour	Yes	D1	[IM]	17-MAR-2018	12:34	0	
A0037	Group A4	Day 1	2 hour	Yes	D1	[IM]	17-MAR-2018	13:40	0	
A0037	Group A4	Day 4		Yes	D1	[IM]	20-MAR-2018	08:38	0	
A0037	Group A4	Day 8		Yes	D1	[IM]	24-MAR-2018	09:05	0	
A0037	Group A4	Day 22		Yes	D1	[IM]	07-APR-2018	09:20	0	
A0037	Group A4	EOS/ET (Day 61)		Yes	D1	[IM]	17-MAY-2018	08:53	0	
A0038	Group A2	Day 1	1 min	Yes	D1	[IM]	17-MAR-2018	11:41	0	
A0038	Group A2	Day 1	10 min	Yes	D1	[IM]	17-MAR-2018	11:50	0	

Scale: 0= No Pain; 10= Worst Pain Possible
Administration Site Key: FA = Forearm; UA= Upper arm
Treatments Part A: A1 = A/Sing MAP, 15 ug (FA); A2 = Afluria Quad IM (UA), 15 ug; A3 = Placebo MAP (FA); A4 = A/Sing Mono IM, 15 ug (UA)
Treatments Part B: B1 = A/Sing MAP, 15 ug (FA) with biopsy; B2 = Placebo MAP (FA) with biopsy
B3 = A/Sing MAP, 15 ug (FA); B4 = A/Sing MAP, 10 ug (FA); B5 = A/Sing MAP, 5 ug (FA); B6 = A/Sing MAP, 2.5 ug (FA)
B7 = Placebo MAP (FA); B8 = A/Sing MAP, 15 ug (UA); B9 = A/Sing IM, 15 ug, as 2018 Afluria Quad (UA)

Listing 16.2.9.3
Numeric Pain Intensity per Site

Part A

Randomisation Number	Group	Visit	Time Point	Was Pain Assessment Performed?	Site Number	Treatment	Date of assessment	Time of Assessment	Score	Unscheduled Reason
A0038	Group A2	Day 1	1 hour	Yes	D1	[IM]	17-MAR-2018	12:35	0	
A0038	Group A2	Day 1	2 hour	Yes	D1	[IM]	17-MAR-2018	13:44	0	
A0038	Group A2	Day 4		Yes	D1	[IM]	20-MAR-2018	08:33	0	
A0038	Group A2	Day 8		Yes	D1	[IM]	24-MAR-2018	09:15	0	
A0038	Group A2	Day 22		Yes	D1	[IM]	07-APR-2018	09:17	0	
A0038	Group A2	EOS/ET (Day 61)		Yes	D1	[IM]	17-MAY-2018	08:50	0	
A0039	Group A2	Day 1	1 min	Yes	D1	[IM]	17-MAR-2018	11:46	0	
A0039	Group A2	Day 1	10 min	Yes	D1	[IM]	17-MAR-2018	11:55	0	
A0039	Group A2	Day 1	1 hour	Yes	D1	[IM]	17-MAR-2018	12:45	0	
A0039	Group A2	Day 1	2 hour	Yes	D1	[IM]	17-MAR-2018	13:50	0	
A0039	Group A2	Day 4		Yes	D1	[IM]	20-MAR-2018	17:23	0	
A0039	Group A2	Day 8		Yes	D1	[IM]	24-MAR-2018	11:38	0	
A0039	Group A2	Day 22		Yes	D1	[IM]	07-APR-2018	10:02	0	
A0039	Group A2	EOS/ET (Day 61)		Yes	D1	[IM]	16-MAY-2018	11:14	0	
A0040	Group A4	Day 1	1 min	Yes	D1	[IM]	17-MAR-2018	11:51	1	
A0040	Group A4	Day 1	10 min	Yes	D1	[IM]	17-MAR-2018	12:00	0	
A0040	Group A4	Day 1	1 hour	Yes	D1	[IM]	17-MAR-2018	12:50	0	
A0040	Group A4	Day 1	2 hour	Yes	D1	[IM]	17-MAR-2018	13:55	1	
A0040	Group A4	Day 4		Yes	D1	[IM]	20-MAR-2018	17:19	0	
A0040	Group A4	Day 8		Yes	D1	[IM]	24-MAR-2018	12:10	0	

Scale: 0= No Pain; 10= Worst Pain Possible
Administration Site Key: FA = Forearm; UA= Upper arm
Treatments Part A: A1 = A/Sing MAP, 15 ug (FA); A2 = Afluria Quad IM (UA), 15 ug; A3 = Placebo MAP (FA); A4 = A/Sing Mono IM, 15 ug (UA)
Treatments Part B: B1 = A/Sing MAP, 15 ug (FA) with biopsy; B2 = Placebo MAP (FA) with biopsy
B3 = A/Sing MAP, 15 ug (FA); B4 = A/Sing MAP, 10 ug (FA); B5 = A/Sing MAP, 5 ug (FA); B6 = A/Sing MAP, 2.5 ug (FA)
B7 = Placebo MAP (FA); B8 = A/Sing MAP, 15 ug (UA); B9 = A/Sing IM, 15 ug, as 2018 Afluria Quad (UA)

Listing 16.2.9.3
Numeric Pain Intensity per Site

Part A

Randomisation Number	Group	Visit	Time Point	Was Pain Assessment Performed?	Site Number	Treatment	Date of assessment	Time of Assessment	Score	Unscheduled Reason
A0040	Group A4	Day 22		Yes	D1	[IM]	07-APR-2018	11:11	0	
A0040	Group A4	EOS/ET (Day 61)		Yes	D1	[IM]	17-MAY-2018	10:52	0	
A0041	Group A4	Day 1	1 min	Yes	D1	[IM]	17-MAR-2018	11:56	2	
A0041	Group A4	Day 1	10 min	Yes	D1	[IM]	17-MAR-2018	12:05	1	
A0041	Group A4	Day 1	1 hour	Yes	D1	[IM]	17-MAR-2018	12:55	0	
A0041	Group A4	Day 1	2 hour	Yes	D1	[IM]	17-MAR-2018	14:01	0	
A0041	Group A4	Day 4		Yes	D1	[IM]	20-MAR-2018	12:38	0	
A0041	Group A4	Day 8		Yes	D1	[IM]	26-MAR-2018	14:36	0	
A0041	Group A4	Day 22		Yes	D1	[IM]	10-APR-2018	11:42	0	
A0041	Group A4	EOS/ET (Day 61)		Yes	D1	[IM]	17-MAY-2018	14:58	0	
A0042	Group A2	Day 1	1 min	Yes	D1	[IM]	17-MAR-2018	12:01	1	
A0042	Group A2	Day 1	10 min	Yes	D1	[IM]	17-MAR-2018	12:10	0	
A0042	Group A2	Day 1	1 hour	Yes	D1	[IM]	17-MAR-2018	13:00	0	
A0042	Group A2	Day 1	2 hour	Yes	D1	[IM]	17-MAR-2018	14:05	0	
A0042	Group A2	Day 4		Yes	D1	[IM]	20-MAR-2018	08:27	0	
A0042	Group A2	Day 8		Yes	D1	[IM]	24-MAR-2018	10:31	0	
A0042	Group A2	Day 22		Yes	D1	[IM]	07-APR-2018	12:15	0	
A0042	Group A2	EOS/ET (Day 61)		Yes	D1	[IM]	16-MAY-2018	10:00	0	
A0043	Group A2	Day 1	1 min	Yes	D1	[IM]	17-MAR-2018	12:06	0	

Scale: 0= No Pain; 10= Worst Pain Possible
Administration Site Key: FA = Forearm; UA= Upper arm
Treatments Part A: A1 = A/Sing MAP, 15 ug (FA); A2 = Afluria Quad IM (UA), 15 ug; A3 = Placebo MAP (FA); A4 = A/Sing Mono IM, 15 ug (UA)
Treatments Part B: B1 = A/Sing MAP, 15 ug (FA) with biopsy; B2 = Placebo MAP (FA) with biopsy
B3 = A/Sing MAP, 15 ug (FA); B4 = A/Sing MAP, 10 ug (FA); B5 = A/Sing MAP, 5 ug (FA); B6 = A/Sing MAP, 2.5 ug (FA)
B7 = Placebo MAP (FA); B8 = A/Sing MAP, 15 ug (UA); B9 = A/Sing IM, 15 ug, as 2018 Afluria Quad (UA)

Listing 16.2.9.3
Numeric Pain Intensity per Site

Part A

Randomisation Number	Group	Visit	Time Point	Was Pain Assessment Performed?	Site Number	Treatment	Date of assessment	Time of Assessment	Score	Unscheduled Reason
A0043	Group A2	Day 1	10 min	Yes	D1	[IM]	17-MAR-2018	12:15	0	
A0043	Group A2	Day 1	1 hour	Yes	D1	[IM]	17-MAR-2018	13:05	0	
A0043	Group A2	Day 1	2 hour	Yes	D1	[IM]	17-MAR-2018	14:10	0	
A0043	Group A2	Day 4		Yes	D1	[IM]	20-MAR-2018	16:53	1	
A0043	Group A2	Day 8		Yes	D1	[IM]	24-MAR-2018	12:47	0	
A0043	Group A2	Day 22		Yes	D1	[IM]	06-APR-2018	16:37	0	
A0043	Group A2	EOS/ET (Day 61)		Yes	D1	[IM]	16-MAY-2018	16:29	0	
A0044	Group A4	Day 1	1 min	Yes	D1	[IM]	17-MAR-2018	12:11	1	
A0044	Group A4	Day 1	10 min	Yes	D1	[IM]	17-MAR-2018	12:20	0	
A0044	Group A4	Day 1	1 hour	Yes	D1	[IM]	17-MAR-2018	13:10	0	
A0044	Group A4	Day 1	2 hour	Yes	D1	[IM]	17-MAR-2018	14:14	0	
A0044	Group A4	Day 4		Yes	D1	[IM]	20-MAR-2018	12:42	0	
A0044	Group A4	Day 8		Yes	D1	[IM]	24-MAR-2018	12:23	0	
A0044	Group A4	Day 22		Yes	D1	[IM]	07-APR-2018	12:12	0	
A0044	Group A4	EOS/ET (Day 61)		Yes	D1	[IM]	16-MAY-2018	13:36	0	
A0045	Group A2	Day 1	1 min	Yes	D1	[IM]	17-MAR-2018	12:16	0	
A0045	Group A2	Day 1	10 min	Yes	D1	[IM]	17-MAR-2018	12:25	0	
A0045	Group A2	Day 1	1 hour	Yes	D1	[IM]	17-MAR-2018	13:15	0	
A0045	Group A2	Day 1	2 hour	Yes	D1	[IM]	17-MAR-2018	14:25	0	
A0045	Group A2	Day 4		Yes	D1	[IM]	20-MAR-2018	11:58	0	

Scale: 0= No Pain; 10= Worst Pain Possible
Administration Site Key: FA = Forearm; UA= Upper arm
Treatments Part A: A1 = A/Sing MAP, 15 ug (FA); A2 = Afluria Quad IM (UA), 15 ug; A3 = Placebo MAP (FA); A4 = A/Sing Mono IM, 15 ug (UA)
Treatments Part B: B1 = A/Sing MAP, 15 ug (FA) with biopsy; B2 = Placebo MAP (FA) with biopsy
B3 = A/Sing MAP, 15 ug (FA); B4 = A/Sing MAP, 10 ug (FA); B5 = A/Sing MAP, 5 ug (FA); B6 = A/Sing MAP, 2.5 ug (FA)
B7 = Placebo MAP (FA); B8 = A/Sing MAP, 15 ug (UA); B9 = A/Sing IM, 15 ug, as 2018 Afluria Quad (UA)

Listing 16.2.9.3
Numeric Pain Intensity per Site

Part A

Randomisation Number	Group	Visit	Time Point	Was Pain Assessment Performed?	Site Number	Treatment	Date of assessment	Time of Assessment	Score	Unscheduled Reason
A0045	Group A2	Day 8		Yes	D1	[IM]	25-MAR-2018	10:36	0	
A0045	Group A2	Day 22		Yes	D1	[IM]	08-APR-2018	10:35	0	
A0045	Group A2	EOS/ET (Day 61)		Yes	D1	[IM]	15-MAY-2018	13:09	0	
A0046	Group A4	Day 1	1 min	Yes	D1	[IM]	17-MAR-2018	12:21	3	
A0046	Group A4	Day 1	10 min	Yes	D1	[IM]	17-MAR-2018	12:32	0	
A0046	Group A4	Day 1	1 hour	Yes	D1	[IM]	17-MAR-2018	13:20	0	
A0046	Group A4	Day 1	2 hour	Yes	D1	[IM]	17-MAR-2018	14:27	0	
A0046	Group A4	Day 4		Yes	D1	[IM]	20-MAR-2018	18:34	0	
A0046	Group A4	Day 8		Yes	D1	[IM]	25-MAR-2018	08:47	0	
A0046	Group A4	Day 22		Yes	D1	[IM]	07-APR-2018	14:04	0	
A0046	Group A4	EOS/ET (Day 61)		Yes	D1	[IM]	17-MAY-2018	14:23	0	
A0047	Group A2	Day 1	1 min	Yes	D1	[IM]	17-MAR-2018	12:26	1	
A0047	Group A2	Day 1	10 min	Yes	D1	[IM]	17-MAR-2018	12:35	0	
A0047	Group A2	Day 1	1 hour	Yes	D1	[IM]	17-MAR-2018	13:25	0	
A0047	Group A2	Day 1	2 hour	Yes	D1	[IM]	17-MAR-2018	14:29	0	
A0047	Group A2	Day 4		Yes	D1	[IM]	21-MAR-2018	14:15	0	
A0047	Group A2	Day 8		Yes	D1	[IM]	25-MAR-2018	10:24	0	
A0047	Group A2	Day 22		Yes	D1	[IM]	07-APR-2018	15:36	0	
A0047	Group A2	EOS/ET (Day 61)		Yes	D1	[IM]	22-MAY-2018	15:07	0	

Scale: 0= No Pain; 10= Worst Pain Possible
Administration Site Key: FA = Forearm; UA= Upper arm
Treatments Part A: A1 = A/Sing MAP, 15 ug (FA); A2 = Afluria Quad IM (UA), 15 ug; A3 = Placebo MAP (FA); A4 = A/Sing Mono IM, 15 ug (UA)
Treatments Part B: B1 = A/Sing MAP, 15 ug (FA) with biopsy; B2 = Placebo MAP (FA) with biopsy
B3 = A/Sing MAP, 15 ug (FA); B4 = A/Sing MAP, 10 ug (FA); B5 = A/Sing MAP, 5 ug (FA); B6 = A/Sing MAP, 2.5 ug (FA)
B7 = Placebo MAP (FA); B8 = A/Sing MAP, 15 ug (UA); B9 = A/Sing IM, 15 ug, as 2018 Afluria Quad (UA)

Listing 16.2.9.3
Numeric Pain Intensity per Site

Part A

Randomisation Number	Group	Visit	Time Point	Was Pain Assessment Performed?	Site Number	Treatment	Date of assessment	Time of Assessment	Score	Unscheduled Reason
A0048	Group A4	Day 1	1 min	Yes	D1	[IM]	17-MAR-2018	13:01	0	
A0048	Group A4	Day 1	10 min	Yes	D1	[IM]	17-MAR-2018	13:10	0	
A0048	Group A4	Day 1	1 hour	Yes	D1	[IM]	17-MAR-2018	14:00	1	
A0048	Group A4	Day 1	2 hour	Yes	D1	[IM]	17-MAR-2018	15:03	0	
A0048	Group A4	Day 4		Yes	D1	[IM]	20-MAR-2018	14:24	0	
A0048	Group A4	Day 8		Yes	D1	[IM]	24-MAR-2018	13:11	0	
A0048	Group A4	Day 22		Yes	D1	[IM]	06-APR-2018	11:38	0	
A0048	Group A4	EOS/ET (Day 61)		Yes	D1	[IM]	16-MAY-2018	08:39	0	
A0049	Group A2	Day 1	1 min	Yes	D1	[IM]	17-MAR-2018	12:36	1	
A0049	Group A2	Day 1	10 min	Yes	D1	[IM]	17-MAR-2018	12:46	0	
A0049	Group A2	Day 1	1 hour	Yes	D1	[IM]	17-MAR-2018	13:35	0	
A0049	Group A2	Day 1	2 hour	Yes	D1	[IM]	17-MAR-2018	14:35	0	
A0049	Group A2	Day 4		Yes	D1	[IM]	20-MAR-2018	11:39	0	
A0049	Group A2	Day 8		Yes	D1	[IM]	24-MAR-2018	12:05	0	
A0049	Group A2	Day 22		Yes	D1	[IM]	06-APR-2018	08:18	0	
A0049	Group A2	EOS/ET (Day 61)		Yes	D1	[IM]	16-MAY-2018	09:36	0	
A0050	Group A4	Day 1	1 min	Yes	D1	[IM]	17-MAR-2018	12:41	1	
A0050	Group A4	Day 1	10 min	Yes	D1	[IM]	17-MAR-2018	12:50	0	
A0050	Group A4	Day 1	1 hour	Yes	D1	[IM]	17-MAR-2018	13:40	1	
A0050	Group A4	Day 1	2 hour	Yes	D1	[IM]	17-MAR-2018	14:40	1	

Scale: 0= No Pain; 10= Worst Pain Possible
Administration Site Key: FA = Forearm; UA= Upper arm
Treatments Part A: A1 = A/Sing MAP, 15 ug (FA); A2 = Afluria Quad IM (UA), 15 ug; A3 = Placebo MAP (FA); A4 = A/Sing Mono IM, 15 ug (UA)
Treatments Part B: B1 = A/Sing MAP, 15 ug (FA) with biopsy; B2 = Placebo MAP (FA) with biopsy
B3 = A/Sing MAP, 15 ug (FA); B4 = A/Sing MAP, 10 ug (FA); B5 = A/Sing MAP, 5 ug (FA); B6 = A/Sing MAP, 2.5 ug (FA)
B7 = Placebo MAP (FA); B8 = A/Sing MAP, 15 ug (UA); B9 = A/Sing IM, 15 ug, as 2018 Afluria Quad (UA)

Listing 16.2.9.3
Numeric Pain Intensity per Site

Part A

Randomisation Number	Group	Visit	Time Point	Was Pain Assessment Performed?	Site Number	Treatment	Date of assessment	Time of Assessment	Score	Unscheduled Reason
A0050	Group A4	Day 4		Yes	D1	[IM]	20-MAR-2018	12:26	0	
A0050	Group A4	Day 8		Yes	D1	[IM]	27-MAR-2018	16:05	0	
A0050	Group A4	Day 22		Yes	D1	[IM]	07-APR-2018	12:36	0	
A0050	Group A4	EOS/ET (Day 61)		Yes	D1	[IM]	16-MAY-2018	16:08	0	
A0051	Group A2	Day 1	1 min	Yes	D1	[IM]	17-MAR-2018	12:46	2	
A0051	Group A2	Day 1	10 min	Yes	D1	[IM]	17-MAR-2018	12:55	0	
A0051	Group A2	Day 1	1 hour	Yes	D1	[IM]	17-MAR-2018	13:45	0	
A0051	Group A2	Day 1	2 hour	Yes	D1	[IM]	17-MAR-2018	14:43	0	
A0051	Group A2	Day 4		Yes	D1	[IM]	20-MAR-2018	13:42	0	
A0051	Group A2	Day 8		Yes	D1	[IM]	24-MAR-2018	12:45	0	
A0051	Group A2	Day 22		Yes	D1	[IM]	07-APR-2018	12:40	0	
A0051	Group A2	EOS/ET (Day 61)		Yes	D1	[IM]	16-MAY-2018	11:37	0	
A0052	Group A4	Day 1	1 min	Yes	D1	[IM]	17-MAR-2018	12:51	0	
A0052	Group A4	Day 1	10 min	Yes	D1	[IM]	17-MAR-2018	13:01	0	
A0052	Group A4	Day 1	1 hour	Yes	D1	[IM]	17-MAR-2018	13:50	0	
A0052	Group A4	Day 1	2 hour	Yes	D1	[IM]	17-MAR-2018	14:52	0	
A0052	Group A4	Day 4		Yes	D1	[IM]	20-MAR-2018	13:40	0	
A0052	Group A4	Day 8		Yes	D1	[IM]	24-MAR-2018	13:02	0	
A0052	Group A4	Day 22		Yes	D1	[IM]	07-APR-2018	13:12	0	
A0052	Group A4	EOS/ET (Day 61)		Yes	D1	[IM]	16-MAY-2018	12:30	0	

Scale: 0= No Pain; 10= Worst Pain Possible
Administration Site Key: FA = Forearm; UA= Upper arm
Treatments Part A: A1 = A/Sing MAP, 15 ug (FA); A2 = Afluria Quad IM (UA), 15 ug; A3 = Placebo MAP (FA); A4 = A/Sing Mono IM, 15 ug (UA)
Treatments Part B: B1 = A/Sing MAP, 15 ug (FA) with biopsy; B2 = Placebo MAP (FA) with biopsy
B3 = A/Sing MAP, 15 ug (FA); B4 = A/Sing MAP, 10 ug (FA); B5 = A/Sing MAP, 5 ug (FA); B6 = A/Sing MAP, 2.5 ug (FA)
B7 = Placebo MAP (FA); B8 = A/Sing MAP, 15 ug (UA); B9 = A/Sing IM, 15 ug, as 2018 Afluria Quad (UA)

Listing 16.2.9.3
Numeric Pain Intensity per Site

Part A

Randomisation Number	Group	Visit	Time Point	Was Pain Assessment Performed?	Site Number	Treatment	Date of assessment	Time of Assessment	Score	Unscheduled Reason
A0053	Group A4	Day 1	1 min	Yes	D1	[IM]	17-MAR-2018	12:56	2	
A0053	Group A4	Day 1	10 min	Yes	D1	[IM]	17-MAR-2018	13:05	0	
A0053	Group A4	Day 1	1 hour	Yes	D1	[IM]	17-MAR-2018	13:55	0	
A0053	Group A4	Day 1	2 hour	Yes	D1	[IM]	17-MAR-2018	14:58	0	
A0053	Group A4	Day 4		Yes	D1	[IM]	20-MAR-2018	16:13	0	
A0053	Group A4	Day 8		Yes	D1	[IM]	24-MAR-2018	12:48	0	
A0053	Group A4	Day 22		Yes	D1	[IM]	07-APR-2018	12:20	0	
A0053	Group A4	EOS/ET (Day 61)		Yes	D1	[IM]	16-MAY-2018	10:22	0	
A0054	Group A2	Day 1	1 min	Yes	D1	[IM]	17-MAR-2018	15:11	0	
A0054	Group A2	Day 1	10 min	Yes	D1	[IM]	17-MAR-2018	15:16	0	
A0054	Group A2	Day 1	1 hour	Yes	D1	[IM]	17-MAR-2018	16:36	0	
A0054	Group A2	Day 1	2 hour	Yes	D1	[IM]	17-MAR-2018	17:12	0	
A0054	Group A2	Day 4		Yes	D1	[IM]	20-MAR-2018	16:15	0	
A0054	Group A2	Day 8		Yes	D1	[IM]	24-MAR-2018	16:09	0	
A0054	Group A2	Day 22		Yes	D1	[IM]	08-APR-2018	11:40	0	
A0054	Group A2	EOS/ET (Day 61)		Yes	D1	[IM]	26-MAY-2018	10:05	0	
A0055	Group A4	Day 1	1 min	Yes	D1	[IM]	24-MAR-2018	12:31	0	
A0055	Group A4	Day 1	10 min	Yes	D1	[IM]	24-MAR-2018	12:40	0	
A0055	Group A4	Day 1	1 hour	Yes	D1	[IM]	24-MAR-2018	13:30	0	

Scale: 0= No Pain; 10= Worst Pain Possible
Administration Site Key: FA = Forearm; UA= Upper arm
Treatments Part A: A1 = A/Sing MAP, 15 ug (FA); A2 = Afluria Quad IM (UA), 15 ug; A3 = Placebo MAP (FA); A4 = A/Sing Mono IM, 15 ug (UA)
Treatments Part B: B1 = A/Sing MAP, 15 ug (FA) with biopsy; B2 = Placebo MAP (FA) with biopsy
B3 = A/Sing MAP, 15 ug (FA); B4 = A/Sing MAP, 10 ug (FA); B5 = A/Sing MAP, 5 ug (FA); B6 = A/Sing MAP, 2.5 ug (FA)
B7 = Placebo MAP (FA); B8 = A/Sing MAP, 15 ug (UA); B9 = A/Sing IM, 15 ug, as 2018 Afluria Quad (UA)

Listing 16.2.9.3
Numeric Pain Intensity per Site

Part A

Randomisation Number	Group	Visit	Time Point	Was Pain Assessment Performed?	Site Number	Treatment	Date of assessment	Time of Assessment	Score	Unscheduled Reason
A0055	Group A4	Day 1	2 hour	Yes	D1	[IM]	24-MAR-2018	14:35	0	
A0055	Group A4	Day 4		Yes	D1	[IM]	27-MAR-2018	15:04	0	
A0055	Group A4	Day 8		Yes	D1	[IM]	31-MAR-2018	09:48	0	
A0055	Group A4	Day 22		Yes	D1	[IM]	14-APR-2018	15:17	0	
A0055	Group A4	EOS/ET (Day 61)		Yes	D1	[IM]	24-MAY-2018	17:06	0	
A0056	Group A2	Day 1	1 min	Yes	D1	[IM]	24-MAR-2018	12:41	1	
A0056	Group A2	Day 1	10 min	Yes	D1	[IM]	24-MAR-2018	12:50	0	
A0056	Group A2	Day 1	1 hour	Yes	D1	[IM]	24-MAR-2018	13:40	0	
A0056	Group A2	Day 1	2 hour	Yes	D1	[IM]	24-MAR-2018	14:44	0	
A0056	Group A2	Day 4		Yes	D1	[IM]	27-MAR-2018	17:21	0	
A0056	Group A2	Day 8		Yes	D1	[IM]	31-MAR-2018	12:39	0	
A0056	Group A2	Day 22		Yes	D1	[IM]	14-APR-2018	12:49	0	
A0056	Group A2	EOS/ET (Day 61)		Yes	D1	[IM]	23-MAY-2018	17:15	0	
A0057	Group A4	Day 1	1 min	Yes	D1	[IM]	24-MAR-2018	12:51	0	
A0057	Group A4	Day 1	10 min	Yes	D1	[IM]	24-MAR-2018	13:00	3	
A0057	Group A4	Day 1	1 hour	Yes	D1	[IM]	24-MAR-2018	13:50	2	
A0057	Group A4	Day 1	2 hour	Yes	D1	[IM]	24-MAR-2018	14:55	2	
A0057	Group A4	Day 4		Yes	D1	[IM]	27-MAR-2018	16:02	0	
A0057	Group A4	Day 8		Yes	D1	[IM]	02-APR-2018	11:18	0	
A0057	Group A4	Day 22		Yes	D1	[IM]	14-APR-2018	12:19	0	

Scale: 0= No Pain; 10= Worst Pain Possible
Administration Site Key: FA = Forearm; UA= Upper arm
Treatments Part A: A1 = A/Sing MAP, 15 ug (FA); A2 = Afluria Quad IM (UA), 15 ug; A3 = Placebo MAP (FA); A4 = A/Sing Mono IM, 15 ug (UA)
Treatments Part B: B1 = A/Sing MAP, 15 ug (FA) with biopsy; B2 = Placebo MAP (FA) with biopsy
B3 = A/Sing MAP, 15 ug (FA); B4 = A/Sing MAP, 10 ug (FA); B5 = A/Sing MAP, 5 ug (FA); B6 = A/Sing MAP, 2.5 ug (FA)
B7 = Placebo MAP (FA); B8 = A/Sing MAP, 15 ug (UA); B9 = A/Sing IM, 15 ug, as 2018 Afluria Quad (UA)

Listing 16.2.9.3
Numeric Pain Intensity per Site

Part A

Randomisation Number	Group	Visit	Time Point	Was Pain Assessment Performed?	Site Number	Treatment	Date of assessment	Time of Assessment	Score	Unscheduled Reason
A0057	Group A4	EOS/ET (Day 61)		Yes	D1	[IM]	23-MAY-2018	09:24	0	
A0058	Group A2	Day 1	1 min	Yes	D1	[IM]	24-MAR-2018	13:01	0	
A0058	Group A2	Day 1	10 min	Yes	D1	[IM]	24-MAR-2018	13:10	0	
A0058	Group A2	Day 1	1 hour	Yes	D1	[IM]	24-MAR-2018	14:00	0	
A0058	Group A2	Day 1	2 hour	Yes	D1	[IM]	24-MAR-2018	15:05	0	
A0058	Group A2	Day 4		Yes	D1	[IM]	27-MAR-2018	15:01	0	
A0058	Group A2	Day 8		Yes	D1	[IM]	31-MAR-2018	11:54	0	
A0058	Group A2	Day 22		Yes	D1	[IM]	14-APR-2018	14:59	0	
A0058	Group A2	EOS/ET (Day 61)		Yes	D1	[IM]	23-MAY-2018	10:37	0	
A0059	Group A2	Day 1	1 min	Yes	D1	[IM]	24-MAR-2018	13:11	1	
A0059	Group A2	Day 1	10 min	Yes	D1	[IM]	24-MAR-2018	13:20	0	
A0059	Group A2	Day 1	1 hour	Yes	D1	[IM]	24-MAR-2018	14:10	0	
A0059	Group A2	Day 1	2 hour	Yes	D1	[IM]	24-MAR-2018	15:15	0	
A0059	Group A2	Day 4		Yes	D1	[IM]	27-MAR-2018	08:07	0	
A0059	Group A2	Day 8		Yes	D1	[IM]	30-MAR-2018	13:02	0	
A0059	Group A2	Day 22		Yes	D1	[IM]	17-APR-2018	08:54	0	
A0059	Group A2	EOS/ET (Day 61)		Yes	D1	[IM]	24-MAY-2018	13:13	0	
A0060	Group A4	Day 1	1 min	Yes	D1	[IM]	24-MAR-2018	13:21	1	
A0060	Group A4	Day 1	10 min	Yes	D1	[IM]	24-MAR-2018	13:30	0	

Scale: 0= No Pain; 10= Worst Pain Possible
Administration Site Key: FA = Forearm; UA= Upper arm
Treatments Part A: A1 = A/Sing MAP, 15 ug (FA); A2 = Afluria Quad IM (UA), 15 ug; A3 = Placebo MAP (FA); A4 = A/Sing Mono IM, 15 ug (UA)
Treatments Part B: B1 = A/Sing MAP, 15 ug (FA) with biopsy; B2 = Placebo MAP (FA) with biopsy
B3 = A/Sing MAP, 15 ug (FA); B4 = A/Sing MAP, 10 ug (FA); B5 = A/Sing MAP, 5 ug (FA); B6 = A/Sing MAP, 2.5 ug (FA)
B7 = Placebo MAP (FA); B8 = A/Sing MAP, 15 ug (UA); B9 = A/Sing IM, 15 ug, as 2018 Afluria Quad (UA)

Listing 16.2.9.3
Numeric Pain Intensity per Site

Part A

Randomisation Number	Group	Visit	Time Point	Was Pain Assessment Performed?	Site Number	Treatment	Date of assessment	Time of Assessment	Score	Unscheduled Reason
A0060	Group A4	Day 1	1 hour	Yes	D1	[IM]	24-MAR-2018	14:20	0	
A0060	Group A4	Day 1	2 hour	Yes	D1	[IM]	24-MAR-2018	15:25	0	
A0060	Group A4	Day 4		Yes	D1	[IM]	27-MAR-2018	13:25	0	
A0060	Group A4	Day 8		Yes	D1	[IM]	31-MAR-2018	13:22	0	
A0060	Group A4	Day 22		Yes	D1	[IM]	14-APR-2018	13:28	0	
A0060	Group A4	EOS/ET (Day 61)		Yes	D1	[IM]	24-MAY-2018	13:18	0	
A1026	Group A1	Day 1	1 min	Yes	F1	5 ug	10-MAR-2018	14:01	0	
A1026	Group A1	Day 1	1 min	Yes	F2	5 ug	10-MAR-2018	14:04	0	
A1026	Group A1	Day 1	1 min	Yes	F3	5 ug	10-MAR-2018	14:07	0	
A1026	Group A1	Day 1	10 min	Yes	F1	5 ug	10-MAR-2018	14:18	0	
A1026	Group A1	Day 1	10 min	Yes	F2	5 ug	10-MAR-2018	14:18	0	
A1026	Group A1	Day 1	10 min	Yes	F3	5 ug	10-MAR-2018	14:18	0	
A1026	Group A1	Day 1	1 hour	Yes	F1	5 ug	10-MAR-2018	15:07	0	
A1026	Group A1	Day 1	1 hour	Yes	F2	5 ug	10-MAR-2018	15:07	0	
A1026	Group A1	Day 1	1 hour	Yes	F3	5 ug	10-MAR-2018	15:07	0	
A1026	Group A1	Day 1	2 hour	Yes	F1	5 ug	10-MAR-2018	16:08	0	
A1026	Group A1	Day 1	2 hour	Yes	F2	5 ug	10-MAR-2018	16:08	0	
A1026	Group A1	Day 1	2 hour	Yes	F3	5 ug	10-MAR-2018	16:08	0	
A1026	Group A1	Day 4		Yes	F1	5 ug	13-MAR-2018	08:22	0	
A1026	Group A1	Day 4		Yes	F2	5 ug	13-MAR-2018	08:22	0	
A1026	Group A1	Day 4		Yes	F3	5 ug	13-MAR-2018	08:23	0	

Scale: 0= No Pain; 10= Worst Pain Possible
Administration Site Key: FA = Forearm; UA= Upper arm
Treatments Part A: A1 = A/Sing MAP, 15 ug (FA); A2 = Afluria Quad IM (UA), 15 ug; A3 = Placebo MAP (FA); A4 = A/Sing Mono IM, 15 ug (UA)
Treatments Part B: B1 = A/Sing MAP, 15 ug (FA) with biopsy; B2 = Placebo MAP (FA) with biopsy
B3 = A/Sing MAP, 15 ug (FA); B4 = A/Sing MAP, 10 ug (FA); B5 = A/Sing MAP, 5 ug (FA); B6 = A/Sing MAP, 2.5 ug (FA)
B7 = Placebo MAP (FA); B8 = A/Sing MAP, 15 ug (UA); B9 = A/Sing IM, 15 ug, as 2018 Afluria Quad (UA)

Listing 16.2.9.3
Numeric Pain Intensity per Site

Part A

Randomisation Number	Group	Visit	Time Point	Was Pain Assessment Performed?	Site Number	Treatment	Date of assessment	Time of Assessment	Score	Unscheduled Reason
A1026	Group A1	Day 8		Yes	F1	5 ug	18-MAR-2018	16:01	0	
A1026	Group A1	Day 8		Yes	F2	5 ug	18-MAR-2018	16:01	0	
A1026	Group A1	Day 8		Yes	F3	5 ug	18-MAR-2018	16:01	0	
A1026	Group A1	Day 22		Yes	F1	5 ug	31-MAR-2018	10:07	0	
A1026	Group A1	Day 22		Yes	F2	5 ug	31-MAR-2018	10:07	0	
A1026	Group A1	Day 22		Yes	F3	5 ug	31-MAR-2018	10:08	0	
A1026	Group A1	EOS/ET (Day 61)		Yes	F1	5 ug	09-MAY-2018	15:08	0	
A1026	Group A1	EOS/ET (Day 61)		Yes	F2	5 ug	09-MAY-2018	15:08	0	
A1026	Group A1	EOS/ET (Day 61)		Yes	F3	5 ug	09-MAY-2018	15:08	0	

Scale: 0= No Pain; 10= Worst Pain Possible
Administration Site Key: FA = Forearm; UA= Upper arm
Treatments Part A: A1 = A/Sing MAP, 15 ug (FA); A2 = Afluria Quad IM (UA), 15 ug; A3 = Placebo MAP (FA); A4 = A/Sing Mono IM, 15 ug (UA)
Treatments Part B: B1 = A/Sing MAP, 15 ug (FA) with biopsy; B2 = Placebo MAP (FA) with biopsy
B3 = A/Sing MAP, 15 ug (FA); B4 = A/Sing MAP, 10 ug (FA); B5 = A/Sing MAP, 5 ug (FA); B6 = A/Sing MAP, 2.5 ug (FA)
B7 = Placebo MAP (FA); B8 = A/Sing MAP, 15 ug (UA); B9 = A/Sing IM, 15 ug, as 2018 Afluria Quad (UA)

Listing 16.2.9.3
Numeric Pain Intensity per Site

Part B

Randomisation Number	Group	Visit	Time Point	Was Pain Assessment Performed?	Site Number	Treatment	Date of assessment	Time of Assessment	Score	Unscheduled Reason
B0001	Group B1	Day 1	1 min	Yes	F1	5 ug	07-MAY-2018	11:01	3	
B0001	Group B1	Day 1	1 min	Yes	F2	5 ug	07-MAY-2018	11:04	2	
B0001	Group B1	Day 1	1 min	Yes	F3	5 ug	07-MAY-2018	11:07	3	
B0001	Group B1	Day 1	10 min	Yes	F1	5 ug	07-MAY-2018	11:18	1	
B0001	Group B1	Day 1	10 min	Yes	F2	5 ug	07-MAY-2018	11:18	1	
B0001	Group B1	Day 1	10 min	Yes	F3	5 ug	07-MAY-2018	11:18	1	
B0001	Group B1	Day 1	1 hour	Yes	F1	5 ug	07-MAY-2018	12:08	0	
B0001	Group B1	Day 1	1 hour	Yes	F2	5 ug	07-MAY-2018	12:08	0	
B0001	Group B1	Day 1	1 hour	Yes	F3	5 ug	07-MAY-2018	12:08	0	
B0001	Group B1	Day 1	2 hour	Yes	F1	5 ug	07-MAY-2018	13:08	0	
B0001	Group B1	Day 1	2 hour	Yes	F2	5 ug	07-MAY-2018	13:08	0	
B0001	Group B1	Day 1	2 hour	Yes	F3	5 ug	07-MAY-2018	13:08	0	
B0001	Group B1	Day 2		Yes	F1	5 ug	08-MAY-2018	16:43	0	
B0001	Group B1	Day 2		Yes	F2	5 ug	08-MAY-2018	16:43	0	
B0001	Group B1	Day 2		Yes	F3	5 ug	08-MAY-2018	16:43	0	
B0001	Group B1	Day 4		Yes	F1	5 ug	10-MAY-2018	09:02	0	
B0001	Group B1	Day 4		Yes	F2	5 ug	10-MAY-2018	09:02	0	
B0001	Group B1	Day 4		Yes	F3	5 ug	10-MAY-2018	09:02	0	
B0001	Group B1	Day 8		Yes	F1	5 ug	14-MAY-2018	13:01	0	
B0001	Group B1	Day 8		Yes	F2	5 ug	14-MAY-2018	13:01	0	
B0001	Group B1	Day 8		Yes	F3	5 ug	14-MAY-2018	13:02	0	

Scale: 0= No Pain; 10= Worst Pain Possible
Administration Site Key: FA = Forearm; UA= Upper arm
Treatments Part A: A1 = A/Sing MAP, 15 ug (FA); A2 = Afluria Quad IM (UA), 15 ug; A3 = Placebo MAP (FA); A4 = A/Sing Mono IM, 15 ug (UA)
Treatments Part B: B1 = A/Sing MAP, 15 ug (FA) with biopsy; B2 = Placebo MAP (FA) with biopsy
B3 = A/Sing MAP, 15 ug (FA); B4 = A/Sing MAP, 10 ug (FA); B5 = A/Sing MAP, 5 ug (FA); B6 = A/Sing MAP, 2.5 ug (FA)
B7 = Placebo MAP (FA); B8 = A/Sing MAP, 15 ug (UA); B9 = A/Sing IM, 15 ug, as 2018 Afluria Quad (UA)

Listing 16.2.9.3
Numeric Pain Intensity per Site

Part B

Randomisation Number	Group	Visit	Time Point	Was Pain Assessment Performed?	Site Number	Treatment	Date of assessment	Time of Assessment	Score	Unscheduled Reason
B0001	Group B1	Day 22		Yes	F1	5 ug	28-MAY-2018	12:14	0	
B0001	Group B1	Day 22		Yes	F2	5 ug	28-MAY-2018	12:14	0	
B0001	Group B1	Day 22		Yes	F3	5 ug	28-MAY-2018	12:15	0	
B0001	Group B1	EOS/ET (Day 61)		Yes	F1	5 ug	06-JUL-2018	11:22	0	
B0001	Group B1	EOS/ET (Day 61)		Yes	F2	5 ug	06-JUL-2018	11:22	0	
B0001	Group B1	EOS/ET (Day 61)		Yes	F3	5 ug	06-JUL-2018	11:22	0	
B0002	Group B2	Day 1	1 min	Yes	F1	uncoated	07-MAY-2018	11:11	1	
B0002	Group B2	Day 1	1 min	Yes	F2	uncoated	07-MAY-2018	11:14	1	
B0002	Group B2	Day 1	1 min	Yes	F3	uncoated	07-MAY-2018	11:17	1	
B0002	Group B2	Day 1	10 min	Yes	F1	uncoated	07-MAY-2018	11:28	1	
B0002	Group B2	Day 1	10 min	Yes	F2	uncoated	07-MAY-2018	11:28	1	
B0002	Group B2	Day 1	10 min	Yes	F3	uncoated	07-MAY-2018	11:28	1	
B0002	Group B2	Day 1	1 hour	Yes	F1	uncoated	07-MAY-2018	12:13	0	
B0002	Group B2	Day 1	1 hour	Yes	F2	uncoated	07-MAY-2018	12:13	0	
B0002	Group B2	Day 1	1 hour	Yes	F3	uncoated	07-MAY-2018	12:13	0	
B0002	Group B2	Day 1	2 hour	Yes	F1	uncoated	07-MAY-2018	13:18	0	
B0002	Group B2	Day 1	2 hour	Yes	F2	uncoated	07-MAY-2018	13:18	0	
B0002	Group B2	Day 1	2 hour	Yes	F3	uncoated	07-MAY-2018	13:18	0	
B0002	Group B2	Unscheduled		Yes	F1	uncoated	07-MAY-2018	11:38	0	MO notifies as repeat
B0002	Group B2	Unscheduled		Yes	F2	uncoated	07-MAY-2018	11:39	0	MO notifies as repeat
B0002	Group B2	Unscheduled		Yes	F3	uncoated	07-MAY-2018	11:39	0	MO notifies as repeat

Scale: 0= No Pain; 10= Worst Pain Possible
Administration Site Key: FA = Forearm; UA= Upper arm
Treatments Part A: A1 = A/Sing MAP, 15 ug (FA); A2 = Afluria Quad IM (UA), 15 ug; A3 = Placebo MAP (FA); A4 = A/Sing Mono IM, 15 ug (UA)
Treatments Part B: B1 = A/Sing MAP, 15 ug (FA) with biopsy; B2 = Placebo MAP (FA) with biopsy
B3 = A/Sing MAP, 15 ug (FA); B4 = A/Sing MAP, 10 ug (FA); B5 = A/Sing MAP, 5 ug (FA); B6 = A/Sing MAP, 2.5 ug (FA)
B7 = Placebo MAP (FA); B8 = A/Sing MAP, 15 ug (UA); B9 = A/Sing IM, 15 ug, as 2018 Afluria Quad (UA)

Listing 16.2.9.3
Numeric Pain Intensity per Site

Part B

Randomisation Number	Group	Visit	Time Point	Was Pain Assessment Performed?	Site Number	Treatment	Date of assessment	Time of Assessment	Score	Unscheduled Reason
B0002	Group B2	Day 2		Yes	F1	uncoated	08-MAY-2018	11:43	0	
B0002	Group B2	Day 2		Yes	F2	uncoated	08-MAY-2018	11:43	0	
B0002	Group B2	Day 2		Yes	F3	uncoated	08-MAY-2018	11:43	0	
B0002	Group B2	Day 4		Yes	F1	uncoated	10-MAY-2018	09:15	0	
B0002	Group B2	Day 4		Yes	F2	uncoated	10-MAY-2018	09:15	0	
B0002	Group B2	Day 4		Yes	F3	uncoated	10-MAY-2018	09:15	0	
B0002	Group B2	Day 8		Yes	F1	uncoated	14-MAY-2018	13:25	0	
B0002	Group B2	Day 8		Yes	F2	uncoated	14-MAY-2018	13:25	0	
B0002	Group B2	Day 8		Yes	F3	uncoated	14-MAY-2018	13:25	0	
B0002	Group B2	Day 22		Yes	F1	uncoated	28-MAY-2018	11:29	0	
B0002	Group B2	Day 22		Yes	F2	uncoated	28-MAY-2018	11:30	0	
B0002	Group B2	Day 22		Yes	F3	uncoated	28-MAY-2018	11:31	0	
B0002	Group B2	EOS/ET (Day 61)		Yes	F1	uncoated	06-JUL-2018	11:06	0	
B0002	Group B2	EOS/ET (Day 61)		Yes	F2	uncoated	06-JUL-2018	11:06	0	
B0002	Group B2	EOS/ET (Day 61)		Yes	F3	uncoated	06-JUL-2018	11:06	0	
B0003	Group B2	Day 1	1 min	Yes	F1	uncoated	07-MAY-2018	11:21	2	
B0003	Group B2	Day 1	1 min	Yes	F2	uncoated	07-MAY-2018	11:24	3	
B0003	Group B2	Day 1	1 min	Yes	F3	uncoated	07-MAY-2018	11:27	3	
B0003	Group B2	Day 1	10 min	Yes	F1	uncoated	07-MAY-2018	11:38	3	
B0003	Group B2	Day 1	10 min	Yes	F2	uncoated	07-MAY-2018	11:38	2	
B0003	Group B2	Day 1	10 min	Yes	F3	uncoated	07-MAY-2018	11:38	2	

Scale: 0= No Pain; 10= Worst Pain Possible
Administration Site Key: FA = Forearm; UA= Upper arm
Treatments Part A: A1 = A/Sing MAP, 15 ug (FA); A2 = Afluria Quad IM (UA), 15 ug; A3 = Placebo MAP (FA); A4 = A/Sing Mono IM, 15 ug (UA)
Treatments Part B: B1 = A/Sing MAP, 15 ug (FA) with biopsy; B2 = Placebo MAP (FA) with biopsy
B3 = A/Sing MAP, 15 ug (FA); B4 = A/Sing MAP, 10 ug (FA); B5 = A/Sing MAP, 5 ug (FA); B6 = A/Sing MAP, 2.5 ug (FA)
B7 = Placebo MAP (FA); B8 = A/Sing MAP, 15 ug (UA); B9 = A/Sing IM, 15 ug, as 2018 Afluria Quad (UA)

Listing 16.2.9.3
Numeric Pain Intensity per Site

Part B

Randomisation Number	Group	Visit	Time Point	Was Pain Assessment Performed?	Site Number	Treatment	Date of assessment	Time of Assessment	Score	Unscheduled Reason
B0003	Group B2	Day 1	1 hour	Yes	F1	uncoated	07-MAY-2018	12:18	0	
B0003	Group B2	Day 1	1 hour	Yes	F2	uncoated	07-MAY-2018	12:18	0	
B0003	Group B2	Day 1	1 hour	Yes	F3	uncoated	07-MAY-2018	12:18	0	
B0003	Group B2	Day 1	2 hour	Yes	F1	uncoated	07-MAY-2018	13:28	0	
B0003	Group B2	Day 1	2 hour	Yes	F2	uncoated	07-MAY-2018	13:28	0	
B0003	Group B2	Day 1	2 hour	Yes	F3	uncoated	07-MAY-2018	13:28	0	
B0003	Group B2	Day 2		Yes	F1	uncoated	08-MAY-2018	15:15	0	
B0003	Group B2	Day 2		Yes	F2	uncoated	08-MAY-2018	15:15	0	
B0003	Group B2	Day 2		Yes	F3	uncoated	08-MAY-2018	15:15	0	
B0003	Group B2	Day 4		Yes	F1	uncoated	10-MAY-2018	09:47	0	
B0003	Group B2	Day 4		Yes	F2	uncoated	10-MAY-2018	09:47	0	
B0003	Group B2	Day 4		Yes	F3	uncoated	10-MAY-2018	09:47	0	
B0003	Group B2	Day 8		Yes	F1	uncoated	14-MAY-2018	15:36	0	
B0003	Group B2	Day 8		Yes	F2	uncoated	14-MAY-2018	15:36	0	
B0003	Group B2	Day 8		Yes	F3	uncoated	14-MAY-2018	15:36	0	
B0003	Group B2	Day 22		Yes	F1	uncoated	28-MAY-2018	15:45	0	
B0003	Group B2	Day 22		Yes	F2	uncoated	28-MAY-2018	15:45	0	
B0003	Group B2	Day 22		Yes	F3	uncoated	28-MAY-2018	15:45	0	
B0003	Group B2	EOS/ET (Day 61)		Yes	F1	uncoated	06-JUL-2018	14:55	0	
B0003	Group B2	EOS/ET (Day 61)		Yes	F2	uncoated	06-JUL-2018	14:55	0	
B0003	Group B2	EOS/ET (Day 61)		Yes	F3	uncoated	06-JUL-2018	14:55	0	

Scale: 0= No Pain; 10= Worst Pain Possible
Administration Site Key: FA = Forearm; UA= Upper arm
Treatments Part A: A1 = A/Sing MAP, 15 ug (FA); A2 = Afluria Quad IM (UA), 15 ug; A3 = Placebo MAP (FA); A4 = A/Sing Mono IM, 15 ug (UA)
Treatments Part B: B1 = A/Sing MAP, 15 ug (FA) with biopsy; B2 = Placebo MAP (FA) with biopsy
B3 = A/Sing MAP, 15 ug (FA); B4 = A/Sing MAP, 10 ug (FA); B5 = A/Sing MAP, 5 ug (FA); B6 = A/Sing MAP, 2.5 ug (FA)
B7 = Placebo MAP (FA); B8 = A/Sing MAP, 15 ug (UA); B9 = A/Sing IM, 15 ug, as 2018 Afluria Quad (UA)

Listing 16.2.9.3
Numeric Pain Intensity per Site

Part B

Randomisation Number	Group	Visit	Time Point	Was Pain Assessment Performed?	Site Number	Treatment	Date of assessment	Time of Assessment	Score	Unscheduled Reason
B0004	Group B1	Day 1	1 min	Yes	F1	5 ug	07-MAY-2018	12:41	1	
B0004	Group B1	Day 1	1 min	Yes	F2	5 ug	07-MAY-2018	12:45	1	
B0004	Group B1	Day 1	1 min	Yes	F3	5 ug	07-MAY-2018	12:48	1	
B0004	Group B1	Day 1	10 min	Yes	F1	5 ug	07-MAY-2018	12:59	2	
B0004	Group B1	Day 1	10 min	Yes	F2	5 ug	07-MAY-2018	12:59	1	
B0004	Group B1	Day 1	10 min	Yes	F3	5 ug	07-MAY-2018	12:59	1	
B0004	Group B1	Day 1	1 hour	Yes	F1	5 ug	07-MAY-2018	13:49	0	
B0004	Group B1	Day 1	1 hour	Yes	F2	5 ug	07-MAY-2018	13:49	0	
B0004	Group B1	Day 1	1 hour	Yes	F3	5 ug	07-MAY-2018	13:49	0	
B0004	Group B1	Day 1	2 hour	Yes	F1	5 ug	07-MAY-2018	14:49	0	
B0004	Group B1	Day 1	2 hour	Yes	F2	5 ug	07-MAY-2018	14:49	0	
B0004	Group B1	Day 1	2 hour	Yes	F3	5 ug	07-MAY-2018	14:49	0	
B0004	Group B1	Day 2		Yes	F1	5 ug	08-MAY-2018	12:35	1	
B0004	Group B1	Day 2		Yes	F2	5 ug	08-MAY-2018	12:35	1	
B0004	Group B1	Day 2		Yes	F3	5 ug	08-MAY-2018	12:35	1	
B0004	Group B1	Day 4		Yes	F1	5 ug	10-MAY-2018	08:44	0	
B0004	Group B1	Day 4		Yes	F2	5 ug	10-MAY-2018	08:44	0	
B0004	Group B1	Day 4		Yes	F3	5 ug	10-MAY-2018	08:44	0	
B0004	Group B1	Day 8		Yes	F1	5 ug	14-MAY-2018	13:49	0	
B0004	Group B1	Day 8		Yes	F2	5 ug	14-MAY-2018	13:49	0	
B0004	Group B1	Day 8		Yes	F3	5 ug	14-MAY-2018	13:49	0	

Scale: 0= No Pain; 10= Worst Pain Possible
Administration Site Key: FA = Forearm; UA= Upper arm
Treatments Part A: A1 = A/Sing MAP, 15 ug (FA); A2 = Afluria Quad IM (UA), 15 ug; A3 = Placebo MAP (FA); A4 = A/Sing Mono IM, 15 ug (UA)
Treatments Part B: B1 = A/Sing MAP, 15 ug (FA) with biopsy; B2 = Placebo MAP (FA) with biopsy
B3 = A/Sing MAP, 15 ug (FA); B4 = A/Sing MAP, 10 ug (FA); B5 = A/Sing MAP, 5 ug (FA); B6 = A/Sing MAP, 2.5 ug (FA)
B7 = Placebo MAP (FA); B8 = A/Sing MAP, 15 ug (UA); B9 = A/Sing IM, 15 ug, as 2018 Afluria Quad (UA)

Listing 16.2.9.3
Numeric Pain Intensity per Site

Part B

Randomisation Number	Group	Visit	Time Point	Was Pain Assessment Performed?	Site Number	Treatment	Date of assessment	Time of Assessment	Score	Unscheduled Reason
B0004	Group B1	Day 22		Yes	F1	5 ug	28-MAY-2018	15:44	0	
B0004	Group B1	Day 22		Yes	F2	5 ug	28-MAY-2018	15:44	0	
B0004	Group B1	Day 22		Yes	F3	5 ug	28-MAY-2018	15:44	0	
B0004	Group B1	EOS/ET (Day 61)		Yes	F1	5 ug	06-JUL-2018	12:59	0	
B0004	Group B1	EOS/ET (Day 61)		Yes	F2	5 ug	06-JUL-2018	12:59	0	
B0004	Group B1	EOS/ET (Day 61)		Yes	F3	5 ug	06-JUL-2018	12:59	0	
B0005	Group B1	Day 1	1 min	Yes	F1	5 ug	07-MAY-2018	11:41	2	
B0005	Group B1	Day 1	1 min	Yes	F2	5 ug	07-MAY-2018	11:44	1	
B0005	Group B1	Day 1	1 min	Yes	F3	5 ug	07-MAY-2018	11:47	1	
B0005	Group B1	Day 1	10 min	Yes	F1	5 ug	07-MAY-2018	11:58	0	
B0005	Group B1	Day 1	10 min	Yes	F2	5 ug	07-MAY-2018	11:58	1	
B0005	Group B1	Day 1	10 min	Yes	F3	5 ug	07-MAY-2018	11:58	2	
B0005	Group B1	Day 1	1 hour	Yes	F1	5 ug	07-MAY-2018	12:48	0	
B0005	Group B1	Day 1	1 hour	Yes	F2	5 ug	07-MAY-2018	12:48	0	
B0005	Group B1	Day 1	1 hour	Yes	F3	5 ug	07-MAY-2018	12:48	0	
B0005	Group B1	Day 1	2 hour	Yes	F1	5 ug	07-MAY-2018	13:48	0	
B0005	Group B1	Day 1	2 hour	Yes	F2	5 ug	07-MAY-2018	13:48	0	
B0005	Group B1	Day 1	2 hour	Yes	F3	5 ug	07-MAY-2018	13:48	0	
B0005	Group B1	Day 2		Yes	F1	5 ug	08-MAY-2018	16:50	0	
B0005	Group B1	Day 2		Yes	F2	5 ug	08-MAY-2018	16:50	0	
B0005	Group B1	Day 2		Yes	F3	5 ug	08-MAY-2018	16:50	0	

Scale: 0= No Pain; 10= Worst Pain Possible
Administration Site Key: FA = Forearm; UA= Upper arm
Treatments Part A: A1 = A/Sing MAP, 15 ug (FA); A2 = Afluria Quad IM (UA), 15 ug; A3 = Placebo MAP (FA); A4 = A/Sing Mono IM, 15 ug (UA)
Treatments Part B: B1 = A/Sing MAP, 15 ug (FA) with biopsy; B2 = Placebo MAP (FA) with biopsy
B3 = A/Sing MAP, 15 ug (FA); B4 = A/Sing MAP, 10 ug (FA); B5 = A/Sing MAP, 5 ug (FA); B6 = A/Sing MAP, 2.5 ug (FA)
B7 = Placebo MAP (FA); B8 = A/Sing MAP, 15 ug (UA); B9 = A/Sing IM, 15 ug, as 2018 Afluria Quad (UA)

Listing 16.2.9.3
Numeric Pain Intensity per Site

Part B

Randomisation Number	Group	Visit	Time Point	Was Pain Assessment Performed?	Site Number	Treatment	Date of assessment	Time of Assessment	Score	Unscheduled Reason
B0005	Group B1	Day 4		Yes	F1	5 ug	10-MAY-2018	09:33	1	
B0005	Group B1	Day 4		Yes	F2	5 ug	10-MAY-2018	09:33	1	
B0005	Group B1	Day 4		Yes	F3	5 ug	10-MAY-2018	09:33	1	
B0005	Group B1	Day 8		Yes	F1	5 ug	14-MAY-2018	17:08	1	
B0005	Group B1	Day 8		Yes	F2	5 ug	14-MAY-2018	17:08	0	
B0005	Group B1	Day 8		Yes	F3	5 ug	14-MAY-2018	17:08	1	
B0005	Group B1	Day 22		Yes	F1	5 ug	28-MAY-2018	14:23	0	
B0005	Group B1	Day 22		Yes	F2	5 ug	28-MAY-2018	14:23	0	
B0005	Group B1	Day 22		Yes	F3	5 ug	28-MAY-2018	14:23	0	
B0005	Group B1	EOS/ET (Day 61)		Yes	F1	5 ug	06-JUL-2018	15:00	0	
B0005	Group B1	EOS/ET (Day 61)		Yes	F2	5 ug	06-JUL-2018	15:00	0	
B0005	Group B1	EOS/ET (Day 61)		Yes	F3	5 ug	06-JUL-2018	15:00	0	
B0006	Group B2	Day 1	1 min	Yes	F1	uncoated	07-MAY-2018	11:51	1	
B0006	Group B2	Day 1	1 min	Yes	F2	uncoated	07-MAY-2018	11:54	0	
B0006	Group B2	Day 1	1 min	Yes	F3	uncoated	07-MAY-2018	11:57	0	
B0006	Group B2	Day 1	10 min	Yes	F1	uncoated	07-MAY-2018	12:08	1	
B0006	Group B2	Day 1	10 min	Yes	F2	uncoated	07-MAY-2018	12:08	1	
B0006	Group B2	Day 1	10 min	Yes	F3	uncoated	07-MAY-2018	12:08	1	
B0006	Group B2	Day 1	1 hour	Yes	F1	uncoated	07-MAY-2018	12:58	0	
B0006	Group B2	Day 1	1 hour	Yes	F2	uncoated	07-MAY-2018	12:58	0	
B0006	Group B2	Day 1	1 hour	Yes	F3	uncoated	07-MAY-2018	12:58	0	

Scale: 0= No Pain; 10= Worst Pain Possible
Administration Site Key: FA = Forearm; UA= Upper arm
Treatments Part A: A1 = A/Sing MAP, 15 ug (FA); A2 = Afluria Quad IM (UA), 15 ug; A3 = Placebo MAP (FA); A4 = A/Sing Mono IM, 15 ug (UA)
Treatments Part B: B1 = A/Sing MAP, 15 ug (FA) with biopsy; B2 = Placebo MAP (FA) with biopsy
B3 = A/Sing MAP, 15 ug (FA); B4 = A/Sing MAP, 10 ug (FA); B5 = A/Sing MAP, 5 ug (FA); B6 = A/Sing MAP, 2.5 ug (FA)
B7 = Placebo MAP (FA); B8 = A/Sing MAP, 15 ug (UA); B9 = A/Sing IM, 15 ug, as 2018 Afluria Quad (UA)

Listing 16.2.9.3
Numeric Pain Intensity per Site

Part B

Randomisation Number	Group	Visit	Time Point	Was Pain Assessment Performed?	Site Number	Treatment	Date of assessment	Time of Assessment	Score	Unscheduled Reason
B0006	Group B2	Day 1	2 hour	Yes	F1	uncoated	07-MAY-2018	13:58	1	
B0006	Group B2	Day 1	2 hour	Yes	F2	uncoated	07-MAY-2018	13:58	1	
B0006	Group B2	Day 1	2 hour	Yes	F3	uncoated	07-MAY-2018	13:58	1	
B0006	Group B2	Day 2		Yes	F1	uncoated	08-MAY-2018	12:17	0	
B0006	Group B2	Day 2		Yes	F2	uncoated	08-MAY-2018	12:17	0	
B0006	Group B2	Day 2		Yes	F3	uncoated	08-MAY-2018	12:17	0	
B0006	Group B2	Day 4		Yes	F1	uncoated	10-MAY-2018	09:25	0	
B0006	Group B2	Day 4		Yes	F2	uncoated	10-MAY-2018	09:25	0	
B0006	Group B2	Day 4		Yes	F3	uncoated	10-MAY-2018	09:25	0	
B0006	Group B2	Day 8		Yes	F1	uncoated	14-MAY-2018	12:36	3	
B0006	Group B2	Day 8		Yes	F2	uncoated	14-MAY-2018	12:36	3	
B0006	Group B2	Day 8		Yes	F3	uncoated	14-MAY-2018	12:36	3	
B0006	Group B2	Day 22		Yes	F1	uncoated	28-MAY-2018	11:40	0	
B0006	Group B2	Day 22		Yes	F2	uncoated	28-MAY-2018	11:40	0	
B0006	Group B2	Day 22		Yes	F3	uncoated	28-MAY-2018	11:40	0	
B0006	Group B2	EOS/ET (Day 61)		Yes	F1	uncoated	06-JUL-2018	12:00	0	
B0006	Group B2	EOS/ET (Day 61)		Yes	F2	uncoated	06-JUL-2018	12:00	0	
B0006	Group B2	EOS/ET (Day 61)		Yes	F3	uncoated	06-JUL-2018	12:00	0	
B0007	Group B2	Day 1	1 min	Yes	F1	uncoated	07-MAY-2018	12:01	2	
B0007	Group B2	Day 1	1 min	Yes	F2	uncoated	07-MAY-2018	12:04	1	
B0007	Group B2	Day 1	1 min	Yes	F3	uncoated	07-MAY-2018	12:07	2	

Scale: 0= No Pain; 10= Worst Pain Possible
Administration Site Key: FA = Forearm; UA= Upper arm
Treatments Part A: A1 = A/Sing MAP, 15 ug (FA); A2 = Afluria Quad IM (UA), 15 ug; A3 = Placebo MAP (FA); A4 = A/Sing Mono IM, 15 ug (UA)
Treatments Part B: B1 = A/Sing MAP, 15 ug (FA) with biopsy; B2 = Placebo MAP (FA) with biopsy
B3 = A/Sing MAP, 15 ug (FA); B4 = A/Sing MAP, 10 ug (FA); B5 = A/Sing MAP, 5 ug (FA); B6 = A/Sing MAP, 2.5 ug (FA)
B7 = Placebo MAP (FA); B8 = A/Sing MAP, 15 ug (UA); B9 = A/Sing IM, 15 ug, as 2018 Afluria Quad (UA)

Listing 16.2.9.3
Numeric Pain Intensity per Site

Part B

Randomisation Number	Group	Visit	Time Point	Was Pain Assessment Performed?	Site Number	Treatment	Date of assessment	Time of Assessment	Score	Unscheduled Reason
B0007	Group B2	Day 1	10 min	Yes	F1	uncoated	07-MAY-2018	12:18	1	
B0007	Group B2	Day 1	10 min	Yes	F2	uncoated	07-MAY-2018	12:18	0	
B0007	Group B2	Day 1	10 min	Yes	F3	uncoated	07-MAY-2018	12:18	0	
B0007	Group B2	Day 1	1 hour	Yes	F1	uncoated	07-MAY-2018	13:08	0	
B0007	Group B2	Day 1	1 hour	Yes	F2	uncoated	07-MAY-2018	13:08	0	
B0007	Group B2	Day 1	1 hour	Yes	F3	uncoated	07-MAY-2018	13:08	0	
B0007	Group B2	Day 1	2 hour	Yes	F1	uncoated	07-MAY-2018	14:08	0	
B0007	Group B2	Day 1	2 hour	Yes	F2	uncoated	07-MAY-2018	14:08	0	
B0007	Group B2	Day 1	2 hour	Yes	F3	uncoated	07-MAY-2018	14:08	0	
B0007	Group B2	Day 2		Yes	F1	uncoated	08-MAY-2018	12:19	0	
B0007	Group B2	Day 2		Yes	F2	uncoated	08-MAY-2018	12:19	0	
B0007	Group B2	Day 2		Yes	F3	uncoated	08-MAY-2018	12:19		
B0007	Group B2	Day 4		Yes	F1	uncoated	10-MAY-2018	09:13	0	
B0007	Group B2	Day 4		Yes	F2	uncoated	10-MAY-2018	09:13	0	
B0007	Group B2	Day 4		Yes	F3	uncoated	10-MAY-2018	09:13	0	
B0007	Group B2	Day 8		Yes	F1	uncoated	14-MAY-2018	12:50	0	
B0007	Group B2	Day 8		Yes	F2	uncoated	14-MAY-2018	12:50	0	
B0007	Group B2	Day 8		Yes	F3	uncoated	14-MAY-2018	12:50	0	
B0007	Group B2	Day 22		Yes	F1	uncoated	26-MAY-2018	13:01	0	
B0007	Group B2	Day 22		Yes	F2	uncoated	26-MAY-2018	13:01	0	
B0007	Group B2	Day 22		Yes	F3	uncoated	26-MAY-2018	13:01	0	

Scale: 0= No Pain; 10= Worst Pain Possible
Administration Site Key: FA = Forearm; UA= Upper arm
Treatments Part A: A1 = A/Sing MAP, 15 ug (FA); A2 = Afluria Quad IM (UA), 15 ug; A3 = Placebo MAP (FA); A4 = A/Sing Mono IM, 15 ug (UA)
Treatments Part B: B1 = A/Sing MAP, 15 ug (FA) with biopsy; B2 = Placebo MAP (FA) with biopsy
B3 = A/Sing MAP, 15 ug (FA); B4 = A/Sing MAP, 10 ug (FA); B5 = A/Sing MAP, 5 ug (FA); B6 = A/Sing MAP, 2.5 ug (FA)
B7 = Placebo MAP (FA); B8 = A/Sing MAP, 15 ug (UA); B9 = A/Sing IM, 15 ug, as 2018 Afluria Quad (UA)

Listing 16.2.9.3
Numeric Pain Intensity per Site

Part B

Randomisation Number	Group	Visit	Time Point	Was Pain Assessment Performed?	Site Number	Treatment	Date of assessment	Time of Assessment	Score	Unscheduled Reason
B0007	Group B2	EOS/ET (Day 61)		Yes	F1	uncoated	06-JUL-2018	08:50	0	
B0007	Group B2	EOS/ET (Day 61)		Yes	F2	uncoated	06-JUL-2018	08:50	0	
B0007	Group B2	EOS/ET (Day 61)		Yes	F3	uncoated	06-JUL-2018	08:50	0	
B0008	Group B1	Day 1	1 min	Yes	F1	5 ug	07-MAY-2018	12:11	0	
B0008	Group B1	Day 1	1 min	Yes	F2	5 ug	07-MAY-2018	12:14	0	
B0008	Group B1	Day 1	1 min	Yes	F3	5 ug	07-MAY-2018	12:17	0	
B0008	Group B1	Day 1	10 min	Yes	F1	5 ug	07-MAY-2018	12:28	0	
B0008	Group B1	Day 1	10 min	Yes	F2	5 ug	07-MAY-2018	12:28	0	
B0008	Group B1	Day 1	10 min	Yes	F3	5 ug	07-MAY-2018	12:28	0	
B0008	Group B1	Day 1	1 hour	Yes	F1	5 ug	07-MAY-2018	13:18	0	
B0008	Group B1	Day 1	1 hour	Yes	F2	5 ug	07-MAY-2018	13:18	0	
B0008	Group B1	Day 1	1 hour	Yes	F3	5 ug	07-MAY-2018	13:18	0	
B0008	Group B1	Day 1	2 hour	Yes	F1	5 ug	07-MAY-2018	14:18	0	
B0008	Group B1	Day 1	2 hour	Yes	F2	5 ug	07-MAY-2018	14:18	0	
B0008	Group B1	Day 1	2 hour	Yes	F3	5 ug	07-MAY-2018	14:18	0	
B0008	Group B1	Day 2		Yes	F1	5 ug	08-MAY-2018	12:22	0	
B0008	Group B1	Day 2		Yes	F2	5 ug	08-MAY-2018	12:22	0	
B0008	Group B1	Day 2		Yes	F3	5 ug	08-MAY-2018	12:22	0	
B0008	Group B1	Day 4		Yes	F1	5 ug	10-MAY-2018	08:58	0	
B0008	Group B1	Day 4		Yes	F2	5 ug	10-MAY-2018	08:58	0	
B0008	Group B1	Day 4		Yes	F3	5 ug	10-MAY-2018	08:58	0	

Scale: 0= No Pain; 10= Worst Pain Possible
Administration Site Key: FA = Forearm; UA= Upper arm
Treatments Part A: A1 = A/Sing MAP, 15 ug (FA); A2 = Afluria Quad IM (UA), 15 ug; A3 = Placebo MAP (FA); A4 = A/Sing Mono IM, 15 ug (UA)
Treatments Part B: B1 = A/Sing MAP, 15 ug (FA) with biopsy; B2 = Placebo MAP (FA) with biopsy
B3 = A/Sing MAP, 15 ug (FA); B4 = A/Sing MAP, 10 ug (FA); B5 = A/Sing MAP, 5 ug (FA); B6 = A/Sing MAP, 2.5 ug (FA)
B7 = Placebo MAP (FA); B8 = A/Sing MAP, 15 ug (UA); B9 = A/Sing IM, 15 ug, as 2018 Afluria Quad (UA)

Listing 16.2.9.3
Numeric Pain Intensity per Site

Part B

Randomisation Number	Group	Visit	Time Point	Was Pain Assessment Performed?	Site Number	Treatment	Date of assessment	Time of Assessment	Score	Unscheduled Reason
B0008	Group B1	Day 8		Yes	F1	5 ug	14-MAY-2018	12:58	0	
B0008	Group B1	Day 8		Yes	F2	5 ug	14-MAY-2018	12:58	0	
B0008	Group B1	Day 8		Yes	F3	5 ug	14-MAY-2018	12:58	0	
B0008	Group B1	Day 22		Yes	F1	5 ug	28-MAY-2018	12:36	0	
B0008	Group B1	Day 22		Yes	F2	5 ug	28-MAY-2018	12:37	0	
B0008	Group B1	Day 22		Yes	F3	5 ug	28-MAY-2018	12:38	0	
B0008	Group B1	EOS/ET (Day 61)		Yes	F1	5 ug	06-JUL-2018	08:28	0	
B0008	Group B1	EOS/ET (Day 61)		Yes	F2	5 ug	06-JUL-2018	08:28	0	
B0008	Group B1	EOS/ET (Day 61)		Yes	F3	5 ug	06-JUL-2018	08:28	0	
B0009	Group B1	Day 1	1 min	Yes	F1	5 ug	07-MAY-2018	12:21	0	
B0009	Group B1	Day 1	1 min	Yes	F2	5 ug	07-MAY-2018	12:24	1	
B0009	Group B1	Day 1	1 min	Yes	F3	5 ug	07-MAY-2018	12:27	0	
B0009	Group B1	Day 1	10 min	Yes	F1	5 ug	07-MAY-2018	12:38	0	
B0009	Group B1	Day 1	10 min	Yes	F2	5 ug	07-MAY-2018	12:38	1	
B0009	Group B1	Day 1	10 min	Yes	F3	5 ug	07-MAY-2018	12:38	0	
B0009	Group B1	Day 1	1 hour	Yes	F1	5 ug	07-MAY-2018	13:28	0	
B0009	Group B1	Day 1	1 hour	Yes	F2	5 ug	07-MAY-2018	13:28	0	
B0009	Group B1	Day 1	1 hour	Yes	F3	5 ug	07-MAY-2018	13:28	0	
B0009	Group B1	Day 1	2 hour	Yes	F1	5 ug	07-MAY-2018	14:28	0	
B0009	Group B1	Day 1	2 hour	Yes	F2	5 ug	07-MAY-2018	14:28	0	
B0009	Group B1	Day 1	2 hour	Yes	F3	5 ug	07-MAY-2018	14:28	0	

Scale: 0= No Pain; 10= Worst Pain Possible
Administration Site Key: FA = Forearm; UA= Upper arm
Treatments Part A: A1 = A/Sing MAP, 15 ug (FA); A2 = Afluria Quad IM (UA), 15 ug; A3 = Placebo MAP (FA); A4 = A/Sing Mono IM, 15 ug (UA)
Treatments Part B: B1 = A/Sing MAP, 15 ug (FA) with biopsy; B2 = Placebo MAP (FA) with biopsy
B3 = A/Sing MAP, 15 ug (FA); B4 = A/Sing MAP, 10 ug (FA); B5 = A/Sing MAP, 5 ug (FA); B6 = A/Sing MAP, 2.5 ug (FA)
B7 = Placebo MAP (FA); B8 = A/Sing MAP, 15 ug (UA); B9 = A/Sing IM, 15 ug, as 2018 Afluria Quad (UA)

Listing 16.2.9.3
Numeric Pain Intensity per Site

Part B

Randomisation Number	Group	Visit	Time Point	Was Pain Assessment Performed?	Site Number	Treatment	Date of assessment	Time of Assessment	Score	Unscheduled Reason
B0009	Group B1	Day 2		Yes	F1	5 ug	08-MAY-2018	13:17	0	
B0009	Group B1	Day 2		Yes	F2	5 ug	08-MAY-2018	13:17	0	
B0009	Group B1	Day 2		Yes	F3	5 ug	08-MAY-2018	13:17	0	
B0009	Group B1	Day 4		Yes	F1	5 ug	10-MAY-2018	09:53	0	
B0009	Group B1	Day 4		Yes	F2	5 ug	10-MAY-2018	09:53	0	
B0009	Group B1	Day 4		Yes	F3	5 ug	10-MAY-2018	09:53	0	
B0009	Group B1	Day 8		Yes	F1	5 ug	14-MAY-2018	11:36	1	
B0009	Group B1	Day 8		Yes	F2	5 ug	14-MAY-2018	11:36	0	
B0009	Group B1	Day 8		Yes	F3	5 ug	14-MAY-2018	11:36	0	
B0009	Group B1	Day 22		Yes	F1	5 ug	28-MAY-2018	12:54	0	
B0009	Group B1	Day 22		Yes	F2	5 ug	28-MAY-2018	12:54	0	
B0009	Group B1	Day 22		Yes	F3	5 ug	28-MAY-2018	12:54	0	
B0009	Group B1	EOS/ET (Day 61)		Yes	F1	5 ug	06-JUL-2018	11:41	0	
B0009	Group B1	EOS/ET (Day 61)		Yes	F2	5 ug	06-JUL-2018	11:41	0	
B0009	Group B1	EOS/ET (Day 61)		Yes	F3	5 ug	06-JUL-2018	11:41	0	
B0010	Group B2	Day 1	1 min	Yes	F1	uncoated	07-MAY-2018	12:31	1	
B0010	Group B2	Day 1	1 min	Yes	F2	uncoated	07-MAY-2018	12:34	1	
B0010	Group B2	Day 1	1 min	Yes	F3	uncoated	07-MAY-2018	12:37	1	
B0010	Group B2	Day 1	10 min	Yes	F1	uncoated	07-MAY-2018	12:48	1	
B0010	Group B2	Day 1	10 min	Yes	F2	uncoated	07-MAY-2018	12:48	0	
B0010	Group B2	Day 1	10 min	Yes	F3	uncoated	07-MAY-2018	12:48	0	

Scale: 0= No Pain; 10= Worst Pain Possible
Administration Site Key: FA = Forearm; UA= Upper arm
Treatments Part A: A1 = A/Sing MAP, 15 ug (FA); A2 = Afluria Quad IM (UA), 15 ug; A3 = Placebo MAP (FA); A4 = A/Sing Mono IM, 15 ug (UA)
Treatments Part B: B1 = A/Sing MAP, 15 ug (FA) with biopsy; B2 = Placebo MAP (FA) with biopsy
B3 = A/Sing MAP, 15 ug (FA); B4 = A/Sing MAP, 10 ug (FA); B5 = A/Sing MAP, 5 ug (FA); B6 = A/Sing MAP, 2.5 ug (FA)
B7 = Placebo MAP (FA); B8 = A/Sing MAP, 15 ug (UA); B9 = A/Sing IM, 15 ug, as 2018 Afluria Quad (UA)

Listing 16.2.9.3
Numeric Pain Intensity per Site

Part B

Randomisation Number	Group	Visit	Time Point	Was Pain Assessment Performed?	Site Number	Treatment	Date of assessment	Time of Assessment	Score	Unscheduled Reason
B0010	Group B2	Day 1	1 hour	Yes	F1	uncoated	07-MAY-2018	13:38	0	
B0010	Group B2	Day 1	1 hour	Yes	F2	uncoated	07-MAY-2018	13:38	0	
B0010	Group B2	Day 1	1 hour	Yes	F3	uncoated	07-MAY-2018	13:38	0	
B0010	Group B2	Day 1	2 hour	Yes	F1	uncoated	07-MAY-2018	14:38	0	
B0010	Group B2	Day 1	2 hour	Yes	F2	uncoated	07-MAY-2018	14:38	0	
B0010	Group B2	Day 1	2 hour	Yes	F3	uncoated	07-MAY-2018	14:38	0	
B0010	Group B2	Day 2		Yes	F1	uncoated	08-MAY-2018	13:41	0	
B0010	Group B2	Day 2		Yes	F2	uncoated	08-MAY-2018	13:41	0	
B0010	Group B2	Day 2		Yes	F3	uncoated	08-MAY-2018	13:41	0	
B0010	Group B2	Day 4		Yes	F1	uncoated	10-MAY-2018	13:32	0	
B0010	Group B2	Day 4		Yes	F2	uncoated	10-MAY-2018	13:32	0	
B0010	Group B2	Day 4		Yes	F3	uncoated	10-MAY-2018	13:32	0	
B0010	Group B2	Day 8		Yes	F1	uncoated	14-MAY-2018	08:28	1	
B0010	Group B2	Day 8		Yes	F2	uncoated	14-MAY-2018	08:28	0	
B0010	Group B2	Day 8		Yes	F3	uncoated	14-MAY-2018	08:28	1	
B0010	Group B2	Day 22		Yes	F1	uncoated	28-MAY-2018	09:27	2	
B0010	Group B2	Day 22		Yes	F2	uncoated	28-MAY-2018	09:28	0	
B0010	Group B2	Day 22		Yes	F3	uncoated	28-MAY-2018	09:29	2	
B0010	Group B2	EOS/ET (Day 61)		Yes	F1	uncoated	06-JUL-2018	11:25	0	
B0010	Group B2	EOS/ET (Day 61)		Yes	F2	uncoated	06-JUL-2018	11:25	0	
B0010	Group B2	EOS/ET (Day 61)		Yes	F3	uncoated	06-JUL-2018	11:25	0	

Scale: 0= No Pain; 10= Worst Pain Possible
Administration Site Key: FA = Forearm; UA= Upper arm
Treatments Part A: A1 = A/Sing MAP, 15 ug (FA); A2 = Afluria Quad IM (UA), 15 ug; A3 = Placebo MAP (FA); A4 = A/Sing Mono IM, 15 ug (UA)
Treatments Part B: B1 = A/Sing MAP, 15 ug (FA) with biopsy; B2 = Placebo MAP (FA) with biopsy
B3 = A/Sing MAP, 15 ug (FA); B4 = A/Sing MAP, 10 ug (FA); B5 = A/Sing MAP, 5 ug (FA); B6 = A/Sing MAP, 2.5 ug (FA)
B7 = Placebo MAP (FA); B8 = A/Sing MAP, 15 ug (UA); B9 = A/Sing IM, 15 ug, as 2018 Afluria Quad (UA)

Listing 16.2.9.3
Numeric Pain Intensity per Site

Part B

Randomisation Number	Group	Visit	Time Point	Was Pain Assessment Performed?	Site Number	Treatment	Date of assessment	Time of Assessment	Score	Unscheduled Reason
B0011	Group B8	Day 1	1 min	Yes	D1	5 ug	12-MAY-2018	11:51	0	
B0011	Group B8	Day 1	1 min	Yes	D2	5 ug	12-MAY-2018	11:54	1	
B0011	Group B8	Day 1	1 min	Yes	D3	5 ug	12-MAY-2018	11:57	0	
B0011	Group B8	Day 1	10 min	Yes	D1	5 ug	12-MAY-2018	12:08	0	
B0011	Group B8	Day 1	10 min	Yes	D2	5 ug	12-MAY-2018	12:08	0	
B0011	Group B8	Day 1	10 min	Yes	D3	5 ug	12-MAY-2018	12:08	0	
B0011	Group B8	Day 1	1 hour	Yes	D1	5 ug	12-MAY-2018	12:53	0	
B0011	Group B8	Day 1	1 hour	Yes	D2	5 ug	12-MAY-2018	12:53	0	
B0011	Group B8	Day 1	1 hour	Yes	D3	5 ug	12-MAY-2018	12:53	0	
B0011	Group B8	Day 1	2 hour	Yes	D1	5 ug	12-MAY-2018	13:58	0	
B0011	Group B8	Day 1	2 hour	Yes	D2	5 ug	12-MAY-2018	13:58	0	
B0011	Group B8	Day 1	2 hour	Yes	D3	5 ug	12-MAY-2018	13:58	0	
B0011	Group B8	Day 2		Yes	D1	5 ug	13-MAY-2018	10:54	0	
B0011	Group B8	Day 2		Yes	D2	5 ug	13-MAY-2018	10:54	0	
B0011	Group B8	Day 2		Yes	D3	5 ug	13-MAY-2018	10:54	0	
B0011	Group B8	Day 4		Yes	D1	5 ug	15-MAY-2018	11:15	0	
B0011	Group B8	Day 4		Yes	D2	5 ug	15-MAY-2018	11:15	0	
B0011	Group B8	Day 4		Yes	D3	5 ug	15-MAY-2018	11:15	0	
B0011	Group B8	Day 8		Yes	D1	5 ug	19-MAY-2018	12:50	0	
B0011	Group B8	Day 8		Yes	D2	5 ug	19-MAY-2018	12:50	0	
B0011	Group B8	Day 8		Yes	D3	5 ug	19-MAY-2018	12:50	0	

Scale: 0= No Pain; 10= Worst Pain Possible
Administration Site Key: FA = Forearm; UA= Upper arm
Treatments Part A: A1 = A/Sing MAP, 15 ug (FA); A2 = Afluria Quad IM (UA), 15 ug; A3 = Placebo MAP (FA); A4 = A/Sing Mono IM, 15 ug (UA)
Treatments Part B: B1 = A/Sing MAP, 15 ug (FA) with biopsy; B2 = Placebo MAP (FA) with biopsy
B3 = A/Sing MAP, 15 ug (FA); B4 = A/Sing MAP, 10 ug (FA); B5 = A/Sing MAP, 5 ug (FA); B6 = A/Sing MAP, 2.5 ug (FA)
B7 = Placebo MAP (FA); B8 = A/Sing MAP, 15 ug (UA); B9 = A/Sing IM, 15 ug, as 2018 Afluria Quad (UA)

Listing 16.2.9.3
Numeric Pain Intensity per Site

Part B

Randomisation Number	Group	Visit	Time Point	Was Pain Assessment Performed?	Site Number	Treatment	Date of assessment	Time of Assessment	Score	Unscheduled Reason
B0011	Group B8	Day 22		Yes	D1	5 ug	02-JUN-2018	09:06	0	
B0011	Group B8	Day 22		Yes	D2	5 ug	02-JUN-2018	09:06	0	
B0011	Group B8	Day 22		Yes	D3	5 ug	02-JUN-2018	09:06	0	
B0011	Group B8	EOS/ET (Day 61)		Yes	D1	5 ug	14-JUL-2018	09:44	0	
B0011	Group B8	EOS/ET (Day 61)		Yes	D2	5 ug	14-JUL-2018	09:44	0	
B0011	Group B8	EOS/ET (Day 61)		Yes	D3	5 ug	14-JUL-2018	09:44	0	
B0012	Group B6	Day 1	1 min	Yes	F1	2.5 ug	12-MAY-2018	12:01	0	
B0012	Group B6	Day 1	1 min	Yes	F2	uncoated	12-MAY-2018	12:19	3	
B0012	Group B6	Day 1	1 min	Yes	F3	uncoated	12-MAY-2018	12:22	0	
B0012	Group B6	Day 1	10 min	Yes	F1	2.5 ug	12-MAY-2018	12:44	2	
B0012	Group B6	Day 1	10 min	Yes	F2	uncoated	12-MAY-2018	12:44	0	
B0012	Group B6	Day 1	10 min	Yes	F3	uncoated	12-MAY-2018	12:44	0	
B0012	Group B6	Day 1	1 hour	Yes	F1	2.5 ug	12-MAY-2018	13:23	0	
B0012	Group B6	Day 1	1 hour	Yes	F2	uncoated	12-MAY-2018	13:23	1	
B0012	Group B6	Day 1	1 hour	Yes	F3	uncoated	12-MAY-2018	13:23	2	
B0012	Group B6	Day 1	2 hour	Yes	F1	2.5 ug	12-MAY-2018	14:23	0	
B0012	Group B6	Day 1	2 hour	Yes	F2	uncoated	12-MAY-2018	14:23	0	
B0012	Group B6	Day 1	2 hour	Yes	F3	uncoated	12-MAY-2018	14:23	0	

Scale: 0= No Pain; 10= Worst Pain Possible
Administration Site Key: FA = Forearm; UA= Upper arm
Treatments Part A: A1 = A/Sing MAP, 15 ug (FA); A2 = Afluria Quad IM (UA), 15 ug; A3 = Placebo MAP (FA); A4 = A/Sing Mono IM, 15 ug (UA)
Treatments Part B: B1 = A/Sing MAP, 15 ug (FA) with biopsy; B2 = Placebo MAP (FA) with biopsy
B3 = A/Sing MAP, 15 ug (FA); B4 = A/Sing MAP, 10 ug (FA); B5 = A/Sing MAP, 5 ug (FA); B6 = A/Sing MAP, 2.5 ug (FA)
B7 = Placebo MAP (FA); B8 = A/Sing MAP, 15 ug (UA); B9 = A/Sing IM, 15 ug, as 2018 Afluria Quad (UA)

Listing 16.2.9.3
Numeric Pain Intensity per Site

Part B

Randomisation Number	Group	Visit	Time Point	Was Pain Assessment Performed?	Site Number	Treatment	Date of assessment	Time of Assessment	Score	Unscheduled Reason
B0012	Group B6	Unscheduled		Yes	F1	2.5 ug	12-MAY-2018	12:13	2	Initial assessment performed at 12:13 was based on first patch removal
B0012	Group B6	Day 2		Yes	F1	2.5 ug	13-MAY-2018	11:24	0	
B0012	Group B6	Day 2		Yes	F2	uncoated	13-MAY-2018	11:24	0	
B0012	Group B6	Day 2		Yes	F3	uncoated	13-MAY-2018	11:24	0	
B0012	Group B6	Day 4		Yes	F1	2.5 ug	15-MAY-2018	12:15	0	
B0012	Group B6	Day 4		Yes	F2	uncoated	15-MAY-2018	12:15	0	
B0012	Group B6	Day 4		Yes	F3	uncoated	15-MAY-2018	12:15	0	
B0012	Group B6	Day 8		Yes	F1	2.5 ug	19-MAY-2018	13:18	0	
B0012	Group B6	Day 8		Yes	F2	uncoated	19-MAY-2018	13:18	0	
B0012	Group B6	Day 8		Yes	F3	uncoated	19-MAY-2018	13:18	0	
B0012	Group B6	Day 22		Yes	F1	2.5 ug	02-JUN-2018	12:37	0	
B0012	Group B6	Day 22		Yes	F2	uncoated	02-JUN-2018	12:37	0	
B0012	Group B6	Day 22		Yes	F3	uncoated	02-JUN-2018	12:37	0	
B0012	Group B6	EOS/ET (Day 61)		Yes	F1	2.5 ug	11-JUL-2018	14:45	0	
B0012	Group B6	EOS/ET (Day 61)		Yes	F2	uncoated	11-JUL-2018	14:45	0	
B0012	Group B6	EOS/ET (Day 61)		Yes	F3	uncoated	11-JUL-2018	14:45	0	
B0013	Group B5	Day 1	1 min	Yes	F1	5 ug	12-MAY-2018	12:11	1	
B0013	Group B5	Day 1	1 min	Yes	F2	uncoated	12-MAY-2018	12:14	1	

Scale: 0= No Pain; 10= Worst Pain Possible
Administration Site Key: FA = Forearm; UA= Upper arm
Treatments Part A: A1 = A/Sing MAP, 15 ug (FA); A2 = Afluria Quad IM (UA), 15 ug; A3 = Placebo MAP (FA); A4 = A/Sing Mono IM, 15 ug (UA)
Treatments Part B: B1 = A/Sing MAP, 15 ug (FA) with biopsy; B2 = Placebo MAP (FA) with biopsy
B3 = A/Sing MAP, 15 ug (FA); B4 = A/Sing MAP, 10 ug (FA); B5 = A/Sing MAP, 5 ug (FA); B6 = A/Sing MAP, 2.5 ug (FA)
B7 = Placebo MAP (FA); B8 = A/Sing MAP, 15 ug (UA); B9 = A/Sing IM, 15 ug, as 2018 Afluria Quad (UA)

Listing 16.2.9.3
Numeric Pain Intensity per Site

Part B

Randomisation Number	Group	Visit	Time Point	Was Pain Assessment Performed?	Site Number	Treatment	Date of assessment	Time of Assessment	Score	Unscheduled Reason
B0013	Group B5	Day 1	1 min	Yes	F3	uncoated	12-MAY-2018	12:17	1	
B0013	Group B5	Day 1	10 min	Yes	F1	5 ug	12-MAY-2018	12:28	0	
B0013	Group B5	Day 1	10 min	Yes	F2	uncoated	12-MAY-2018	12:28	0	
B0013	Group B5	Day 1	10 min	Yes	F3	uncoated	12-MAY-2018	12:28	0	
B0013	Group B5	Day 1	1 hour	Yes	F1	5 ug	12-MAY-2018	13:18	0	
B0013	Group B5	Day 1	1 hour	Yes	F2	uncoated	12-MAY-2018	13:18	0	
B0013	Group B5	Day 1	1 hour	Yes	F3	uncoated	12-MAY-2018	13:18	0	
B0013	Group B5	Day 1	2 hour	Yes	F1	5 ug	12-MAY-2018	14:18	0	
B0013	Group B5	Day 1	2 hour	Yes	F2	uncoated	12-MAY-2018	14:18	0	
B0013	Group B5	Day 1	2 hour	Yes	F3	uncoated	12-MAY-2018	14:18	0	
B0013	Group B5	Day 2		Yes	F1	5 ug	13-MAY-2018	11:31	0	
B0013	Group B5	Day 2		Yes	F2	uncoated	13-MAY-2018	11:31	0	
B0013	Group B5	Day 2		Yes	F3	uncoated	13-MAY-2018	11:31	0	
B0013	Group B5	Day 4		Yes	F1	5 ug	15-MAY-2018	08:30	0	
B0013	Group B5	Day 4		Yes	F2	uncoated	15-MAY-2018	08:30	0	
B0013	Group B5	Day 4		Yes	F3	uncoated	15-MAY-2018	08:30	0	
B0013	Group B5	Day 8		Yes	F1	5 ug	19-MAY-2018	10:32	0	
B0013	Group B5	Day 8		Yes	F2	uncoated	19-MAY-2018	10:32	0	
B0013	Group B5	Day 8		Yes	F3	uncoated	19-MAY-2018	10:32	0	
B0013	Group B5	Day 22		Yes	F1	5 ug	02-JUN-2018	11:27	0	
B0013	Group B5	Day 22		Yes	F2	uncoated	02-JUN-2018	11:27	0	

Scale: 0= No Pain; 10= Worst Pain Possible
Administration Site Key: FA = Forearm; UA= Upper arm
Treatments Part A: A1 = A/Sing MAP, 15 ug (FA); A2 = Afluria Quad IM (UA), 15 ug; A3 = Placebo MAP (FA); A4 = A/Sing Mono IM, 15 ug (UA)
Treatments Part B: B1 = A/Sing MAP, 15 ug (FA) with biopsy; B2 = Placebo MAP (FA) with biopsy
B3 = A/Sing MAP, 15 ug (FA); B4 = A/Sing MAP, 10 ug (FA); B5 = A/Sing MAP, 5 ug (FA); B6 = A/Sing MAP, 2.5 ug (FA)
B7 = Placebo MAP (FA); B8 = A/Sing MAP, 15 ug (UA); B9 = A/Sing IM, 15 ug, as 2018 Afluria Quad (UA)

Listing 16.2.9.3
Numeric Pain Intensity per Site

Part B

Randomisation Number	Group	Visit	Time Point	Was Pain Assessment Performed?	Site Number	Treatment	Date of assessment	Time of Assessment	Score	Unscheduled Reason
B0013	Group B5	Day 22		Yes	F3	uncoated	02-JUN-2018	11:27	0	
B0013	Group B5	EOS/ET (Day 61)		Yes	F1	5 ug	11-JUL-2018	11:15	0	
B0013	Group B5	EOS/ET (Day 61)		Yes	F2	uncoated	11-JUL-2018	11:15	0	
B0013	Group B5	EOS/ET (Day 61)		Yes	F3	uncoated	11-JUL-2018	11:15	0	
B0014	Group B3	Day 1	1 min	Yes	F1	5 ug	12-MAY-2018	12:21	2	
B0014	Group B3	Day 1	1 min	Yes	F2	5 ug	12-MAY-2018	12:24	1	
B0014	Group B3	Day 1	1 min	Yes	F3	5 ug	12-MAY-2018	12:27	2	
B0014	Group B3	Day 1	10 min	Yes	F1	5 ug	12-MAY-2018	12:38	0	
B0014	Group B3	Day 1	10 min	Yes	F2	5 ug	12-MAY-2018	12:38	0	
B0014	Group B3	Day 1	10 min	Yes	F3	5 ug	12-MAY-2018	12:38	0	
B0014	Group B3	Day 1	1 hour	Yes	F1	5 ug	12-MAY-2018	13:28	0	
B0014	Group B3	Day 1	1 hour	Yes	F2	5 ug	12-MAY-2018	13:28	0	
B0014	Group B3	Day 1	1 hour	Yes	F3	5 ug	12-MAY-2018	13:28	0	
B0014	Group B3	Day 1	2 hour	Yes	F1	5 ug	12-MAY-2018	14:30	0	
B0014	Group B3	Day 1	2 hour	Yes	F2	5 ug	12-MAY-2018	14:30	0	
B0014	Group B3	Day 1	2 hour	Yes	F3	5 ug	12-MAY-2018	14:30	0	
B0014	Group B3	Day 2		Yes	F1	5 ug	13-MAY-2018	11:50	0	
B0014	Group B3	Day 2		Yes	F2	5 ug	13-MAY-2018	11:50	0	
B0014	Group B3	Day 2		Yes	F3	5 ug	13-MAY-2018	11:50	0	
B0014	Group B3	Day 4		Yes	F1	5 ug	15-MAY-2018	09:34	0	
B0014	Group B3	Day 4		Yes	F2	5 ug	15-MAY-2018	09:34	0	

Scale: 0= No Pain; 10= Worst Pain Possible
Administration Site Key: FA = Forearm; UA= Upper arm
Treatments Part A: A1 = A/Sing MAP, 15 ug (FA); A2 = Afluria Quad IM (UA), 15 ug; A3 = Placebo MAP (FA); A4 = A/Sing Mono IM, 15 ug (UA)
Treatments Part B: B1 = A/Sing MAP, 15 ug (FA) with biopsy; B2 = Placebo MAP (FA) with biopsy
B3 = A/Sing MAP, 15 ug (FA); B4 = A/Sing MAP, 10 ug (FA); B5 = A/Sing MAP, 5 ug (FA); B6 = A/Sing MAP, 2.5 ug (FA)
B7 = Placebo MAP (FA); B8 = A/Sing MAP, 15 ug (UA); B9 = A/Sing IM, 15 ug, as 2018 Afluria Quad (UA)

Listing 16.2.9.3
Numeric Pain Intensity per Site

Part B

Randomisation Number	Group	Visit	Time Point	Was Pain Assessment Performed?	Site Number	Treatment	Date of assessment	Time of Assessment	Score	Unscheduled Reason
B0014	Group B3	Day 4		Yes	F3	5 ug	15-MAY-2018	09:34	0	
B0014	Group B3	Day 8		Yes	F1	5 ug	19-MAY-2018	16:26	0	
B0014	Group B3	Day 8		Yes	F2	5 ug	19-MAY-2018	16:26	0	
B0014	Group B3	Day 8		Yes	F3	5 ug	19-MAY-2018	16:26	0	
B0014	Group B3	Day 22		Yes	F1	5 ug	02-JUN-2018	09:31	0	
B0014	Group B3	Day 22		Yes	F2	5 ug	02-JUN-2018	09:31	0	
B0014	Group B3	Day 22		Yes	F3	5 ug	02-JUN-2018	09:31	0	
B0014	Group B3	EOS/ET (Day 61)		Yes	F1	5 ug	12-JUL-2018	11:21	0	
B0014	Group B3	EOS/ET (Day 61)		Yes	F2	5 ug	12-JUL-2018	11:21	0	
B0014	Group B3	EOS/ET (Day 61)		Yes	F3	5 ug	12-JUL-2018	11:21	0	
B0015	Group B9	Day 1	1 min	Yes	D1	[IM]	12-MAY-2018	11:11	1	
B0015	Group B9	Day 1	10 min	Yes	D1	[IM]	12-MAY-2018	11:20	2	
B0015	Group B9	Day 1	1 hour	Yes	D1	[IM]	12-MAY-2018	12:10	1	
B0015	Group B9	Day 1	2 hour	Yes	D1	[IM]	12-MAY-2018	13:30	0	
B0015	Group B9	Day 2		Yes	D1	[IM]	13-MAY-2018	11:09	0	
B0015	Group B9	Day 4		Yes	D1	[IM]	15-MAY-2018	11:28	0	
B0015	Group B9	Day 8		Yes	D1	[IM]	19-MAY-2018	11:06	0	
B0015	Group B9	Day 22		Yes	D1	[IM]	02-JUN-2018	12:40	0	
B0015	Group B9	EOS/ET (Day 61)		Yes	D1	[IM]	11-JUL-2018	09:37	0	
B0016	Group B4	Day 1	1 min	Yes	F1	uncoated	12-MAY-2018	12:31	3	

Scale: 0= No Pain; 10= Worst Pain Possible
Administration Site Key: FA = Forearm; UA= Upper arm
Treatments Part A: A1 = A/Sing MAP, 15 ug (FA); A2 = Afluria Quad IM (UA), 15 ug; A3 = Placebo MAP (FA); A4 = A/Sing Mono IM, 15 ug (UA)
Treatments Part B: B1 = A/Sing MAP, 15 ug (FA) with biopsy; B2 = Placebo MAP (FA) with biopsy
B3 = A/Sing MAP, 15 ug (FA); B4 = A/Sing MAP, 10 ug (FA); B5 = A/Sing MAP, 5 ug (FA); B6 = A/Sing MAP, 2.5 ug (FA)
B7 = Placebo MAP (FA); B8 = A/Sing MAP, 15 ug (UA); B9 = A/Sing IM, 15 ug, as 2018 Afluria Quad (UA)

Listing 16.2.9.3
Numeric Pain Intensity per Site

Part B

Randomisation Number	Group	Visit	Time Point	Was Pain Assessment Performed?	Site Number	Treatment	Date of assessment	Time of Assessment	Score	Unscheduled Reason
B0016	Group B4	Day 1	1 min	Yes	F2	5 ug	12-MAY-2018	12:34	4	
B0016	Group B4	Day 1	1 min	Yes	F3	5 ug	12-MAY-2018	12:37	5	
B0016	Group B4	Day 1	10 min	Yes	F1	uncoated	12-MAY-2018	12:48	0	
B0016	Group B4	Day 1	10 min	Yes	F2	5 ug	12-MAY-2018	12:48	0	
B0016	Group B4	Day 1	10 min	Yes	F3	5 ug	12-MAY-2018	12:48	0	
B0016	Group B4	Day 1	1 hour	Yes	F1	uncoated	12-MAY-2018	13:38	1	
B0016	Group B4	Day 1	1 hour	Yes	F2	5 ug	12-MAY-2018	13:38	1	
B0016	Group B4	Day 1	1 hour	Yes	F3	5 ug	12-MAY-2018	13:38	1	
B0016	Group B4	Day 1	2 hour	Yes	F1	uncoated	12-MAY-2018	14:38	0	
B0016	Group B4	Day 1	2 hour	Yes	F2	5 ug	12-MAY-2018	14:38	0	
B0016	Group B4	Day 1	2 hour	Yes	F3	5 ug	12-MAY-2018	14:38	0	
B0016	Group B4	Day 2		Yes	F1	uncoated	13-MAY-2018	12:13	0	
B0016	Group B4	Day 2		Yes	F2	5 ug	13-MAY-2018	12:13	0	
B0016	Group B4	Day 2		Yes	F3	5 ug	13-MAY-2018	12:13	0	
B0016	Group B4	Day 4		Yes	F1	uncoated	15-MAY-2018	08:42	0	
B0016	Group B4	Day 4		Yes	F2	5 ug	15-MAY-2018	08:42	0	
B0016	Group B4	Day 4		Yes	F3	5 ug	15-MAY-2018	08:43	0	
B0016	Group B4	Day 8		Yes	F1	uncoated	19-MAY-2018	10:11	0	
B0016	Group B4	Day 8		Yes	F2	5 ug	19-MAY-2018	10:12	0	
B0016	Group B4	Day 8		Yes	F3	5 ug	19-MAY-2018	10:12	0	
B0016	Group B4	Day 22		Yes	F1	uncoated	02-JUN-2018	13:05	0	

Scale: 0= No Pain; 10= Worst Pain Possible
Administration Site Key: FA = Forearm; UA= Upper arm
Treatments Part A: A1 = A/Sing MAP, 15 ug (FA); A2 = Afluria Quad IM (UA), 15 ug; A3 = Placebo MAP (FA); A4 = A/Sing Mono IM, 15 ug (UA)
Treatments Part B: B1 = A/Sing MAP, 15 ug (FA) with biopsy; B2 = Placebo MAP (FA) with biopsy
B3 = A/Sing MAP, 15 ug (FA); B4 = A/Sing MAP, 10 ug (FA); B5 = A/Sing MAP, 5 ug (FA); B6 = A/Sing MAP, 2.5 ug (FA)
B7 = Placebo MAP (FA); B8 = A/Sing MAP, 15 ug (UA); B9 = A/Sing IM, 15 ug, as 2018 Afluria Quad (UA)

Listing 16.2.9.3
Numeric Pain Intensity per Site

Part B

Randomisation Number	Group	Visit	Time Point	Was Pain Assessment Performed?	Site Number	Treatment	Date of assessment	Time of Assessment	Score	Unscheduled Reason
B0016	Group B4	Day 22		Yes	F2	5 ug	02-JUN-2018	13:05	0	
B0016	Group B4	Day 22		Yes	F3	5 ug	02-JUN-2018	13:05	0	
B0016	Group B4	EOS/ET (Day 61)		Yes	F1	uncoated	11-JUL-2018	08:30	0	
B0016	Group B4	EOS/ET (Day 61)		Yes	F2	5 ug	11-JUL-2018	08:30	0	
B0016	Group B4	EOS/ET (Day 61)		Yes	F3	5 ug	11-JUL-2018	08:30	0	
B0017	Group B7	Day 1	1 min	Yes	F1	uncoated	12-MAY-2018	12:41	1	
B0017	Group B7	Day 1	1 min	Yes	F2	uncoated	12-MAY-2018	12:44	1	
B0017	Group B7	Day 1	1 min	Yes	F3	uncoated	12-MAY-2018	12:47	2	
B0017	Group B7	Day 1	10 min	Yes	F1	uncoated	12-MAY-2018	12:58	0	
B0017	Group B7	Day 1	10 min	Yes	F2	uncoated	12-MAY-2018	12:58	0	
B0017	Group B7	Day 1	10 min	Yes	F3	uncoated	12-MAY-2018	12:58	0	
B0017	Group B7	Day 1	1 hour	Yes	F1	uncoated	12-MAY-2018	13:48	0	
B0017	Group B7	Day 1	1 hour	Yes	F2	uncoated	12-MAY-2018	13:48	0	
B0017	Group B7	Day 1	1 hour	Yes	F3	uncoated	12-MAY-2018	13:48	0	
B0017	Group B7	Day 1	2 hour	Yes	F1	uncoated	12-MAY-2018	14:47	0	
B0017	Group B7	Day 1	2 hour	Yes	F2	uncoated	12-MAY-2018	14:47	0	
B0017	Group B7	Day 1	2 hour	Yes	F3	uncoated	12-MAY-2018	14:57	0	
B0017	Group B7	Day 2		Yes	F1	uncoated	13-MAY-2018	12:25	0	
B0017	Group B7	Day 2		Yes	F2	uncoated	13-MAY-2018	12:25	0	
B0017	Group B7	Day 2		Yes	F3	uncoated	13-MAY-2018	12:25	0	
B0017	Group B7	Day 4		Yes	F1	uncoated	15-MAY-2018	15:13	0	

Scale: 0= No Pain; 10= Worst Pain Possible
Administration Site Key: FA = Forearm; UA= Upper arm
Treatments Part A: A1 = A/Sing MAP, 15 ug (FA); A2 = Afluria Quad IM (UA), 15 ug; A3 = Placebo MAP (FA); A4 = A/Sing Mono IM, 15 ug (UA)
Treatments Part B: B1 = A/Sing MAP, 15 ug (FA) with biopsy; B2 = Placebo MAP (FA) with biopsy
B3 = A/Sing MAP, 15 ug (FA); B4 = A/Sing MAP, 10 ug (FA); B5 = A/Sing MAP, 5 ug (FA); B6 = A/Sing MAP, 2.5 ug (FA)
B7 = Placebo MAP (FA); B8 = A/Sing MAP, 15 ug (UA); B9 = A/Sing IM, 15 ug, as 2018 Afluria Quad (UA)

Listing 16.2.9.3
Numeric Pain Intensity per Site

Part B

Randomisation Number	Group	Visit	Time Point	Was Pain Assessment Performed?	Site Number	Treatment	Date of assessment	Time of Assessment	Score	Unscheduled Reason
B0017	Group B7	Day 4		Yes	F2	uncoated	15-MAY-2018	15:13	0	
B0017	Group B7	Day 4		Yes	F3	uncoated	15-MAY-2018	15:13	0	
B0017	Group B7	Day 8		Yes	F1	uncoated	19-MAY-2018	15:13	0	
B0017	Group B7	Day 8		Yes	F2	uncoated	19-MAY-2018	15:13	0	
B0017	Group B7	Day 8		Yes	F3	uncoated	19-MAY-2018	15:13	0	
B0017	Group B7	Day 22		Yes	F1	uncoated	04-JUN-2018	11:42	0	
B0017	Group B7	Day 22		Yes	F2	uncoated	04-JUN-2018	11:42	0	
B0017	Group B7	Day 22		Yes	F3	uncoated	04-JUN-2018	11:42	0	
B0017	Group B7	EOS/ET (Day 61)		Yes	F1	uncoated	07-JUL-2018	11:34	0	
B0017	Group B7	EOS/ET (Day 61)		Yes	F2	uncoated	07-JUL-2018	11:34	0	
B0017	Group B7	EOS/ET (Day 61)		Yes	F3	uncoated	07-JUL-2018	11:34	0	
B0018	Group B9	Day 1	1 min	Yes	D1	[IM]	12-MAY-2018	11:21	1	
B0018	Group B9	Day 1	10 min	Yes	D1	[IM]	12-MAY-2018	11:30	0	
B0018	Group B9	Day 1	1 hour	Yes	D1	[IM]	12-MAY-2018	12:20	0	
B0018	Group B9	Day 1	2 hour	Yes	D1	[IM]	12-MAY-2018	13:25	0	
B0018	Group B9	Day 2		Yes	D1	[IM]	13-MAY-2018	10:51	1	
B0018	Group B9	Day 4		Yes	D1	[IM]	15-MAY-2018	16:54	0	
B0018	Group B9	Day 8		Yes	D1	[IM]	19-MAY-2018	11:09	0	
B0018	Group B9	Day 22		Yes	D1	[IM]	02-JUN-2018	11:24	0	
B0018	Group B9	EOS/ET (Day 61)		Yes	D1	[IM]	11-JUL-2018	16:47	0	

Scale: 0= No Pain; 10= Worst Pain Possible
Administration Site Key: FA = Forearm; UA= Upper arm
Treatments Part A: A1 = A/Sing MAP, 15 ug (FA); A2 = Afluria Quad IM (UA), 15 ug; A3 = Placebo MAP (FA); A4 = A/Sing Mono IM, 15 ug (UA)
Treatments Part B: B1 = A/Sing MAP, 15 ug (FA) with biopsy; B2 = Placebo MAP (FA) with biopsy
B3 = A/Sing MAP, 15 ug (FA); B4 = A/Sing MAP, 10 ug (FA); B5 = A/Sing MAP, 5 ug (FA); B6 = A/Sing MAP, 2.5 ug (FA)
B7 = Placebo MAP (FA); B8 = A/Sing MAP, 15 ug (UA); B9 = A/Sing IM, 15 ug, as 2018 Afluria Quad (UA)

Listing 16.2.9.3
Numeric Pain Intensity per Site

Part B

Randomisation Number	Group	Visit	Time Point	Was Pain Assessment Performed?	Site Number	Treatment	Date of assessment	Time of Assessment	Score	Unscheduled Reason
B0020	Group B6	Day 1	1 min	Yes	F1	uncoated	12-MAY-2018	12:51	2	
B0020	Group B6	Day 1	1 min	Yes	F2	uncoated	12-MAY-2018	12:54	1	
B0020	Group B6	Day 1	1 min	Yes	F3	2.5 ug	12-MAY-2018	12:57	3	
B0020	Group B6	Day 1	10 min	Yes	F1	uncoated	12-MAY-2018	13:08	0	
B0020	Group B6	Day 1	10 min	Yes	F2	uncoated	12-MAY-2018	13:08	3	
B0020	Group B6	Day 1	10 min	Yes	F3	2.5 ug	12-MAY-2018	13:08	0	
B0020	Group B6	Day 1	1 hour	Yes	F1	uncoated	12-MAY-2018	13:58	4	
B0020	Group B6	Day 1	1 hour	Yes	F2	uncoated	12-MAY-2018	13:58	4	
B0020	Group B6	Day 1	1 hour	Yes	F3	2.5 ug	12-MAY-2018	13:58	5	
B0020	Group B6	Day 1	2 hour	Yes	F1	uncoated	12-MAY-2018	14:58	0	
B0020	Group B6	Day 1	2 hour	Yes	F2	uncoated	12-MAY-2018	14:58	0	
B0020	Group B6	Day 1	2 hour	Yes	F3	2.5 ug	12-MAY-2018	14:58	0	
B0020	Group B6	Day 2		Yes	F1	uncoated	13-MAY-2018	12:05	0	
B0020	Group B6	Day 2		Yes	F2	uncoated	13-MAY-2018	12:05	0	
B0020	Group B6	Day 2		Yes	F3	2.5 ug	13-MAY-2018	12:05	0	
B0020	Group B6	Day 4		Yes	F1	uncoated	15-MAY-2018	12:47	0	
B0020	Group B6	Day 4		Yes	F2	uncoated	15-MAY-2018	12:48	0	
B0020	Group B6	Day 4		Yes	F3	2.5 ug	15-MAY-2018	12:48	0	
B0020	Group B6	Day 8		Yes	F1	uncoated	19-MAY-2018	16:08	0	
B0020	Group B6	Day 8		Yes	F2	uncoated	19-MAY-2018	16:08	0	
B0020	Group B6	Day 8		Yes	F3	2.5 ug	19-MAY-2018	16:08	0	

Scale: 0= No Pain; 10= Worst Pain Possible
Administration Site Key: FA = Forearm; UA= Upper arm
Treatments Part A: A1 = A/Sing MAP, 15 ug (FA); A2 = Afluria Quad IM (UA), 15 ug; A3 = Placebo MAP (FA); A4 = A/Sing Mono IM, 15 ug (UA)
Treatments Part B: B1 = A/Sing MAP, 15 ug (FA) with biopsy; B2 = Placebo MAP (FA) with biopsy
B3 = A/Sing MAP, 15 ug (FA); B4 = A/Sing MAP, 10 ug (FA); B5 = A/Sing MAP, 5 ug (FA); B6 = A/Sing MAP, 2.5 ug (FA)
B7 = Placebo MAP (FA); B8 = A/Sing MAP, 15 ug (UA); B9 = A/Sing IM, 15 ug, as 2018 Afluria Quad (UA)

Listing 16.2.9.3
Numeric Pain Intensity per Site

Part B

Randomisation Number	Group	Visit	Time Point	Was Pain Assessment Performed?	Site Number	Treatment	Date of assessment	Time of Assessment	Score	Unscheduled Reason
B0020	Group B6	Day 22		Yes	F1	uncoated	13-JUN-2018	12:50	0	
B0020	Group B6	Day 22		Yes	F2	uncoated	13-JUN-2018	12:50	0	
B0020	Group B6	Day 22		Yes	F3	2.5 ug	13-JUN-2018	12:50	0	
B0020	Group B6	EOS/ET (Day 61)		Yes	F1	uncoated	11-JUL-2018	13:29	0	
B0020	Group B6	EOS/ET (Day 61)		Yes	F2	uncoated	11-JUL-2018	13:29	0	
B0020	Group B6	EOS/ET (Day 61)		Yes	F3	2.5 ug	11-JUL-2018	13:29	0	
B0021	Group B7	Day 1	1 min	Yes	F1	uncoated	12-MAY-2018	13:01	2	
B0021	Group B7	Day 1	1 min	Yes	F2	uncoated	12-MAY-2018	13:04	3	
B0021	Group B7	Day 1	1 min	Yes	F3	uncoated	12-MAY-2018	13:07	3	
B0021	Group B7	Day 1	10 min	Yes	F1	uncoated	12-MAY-2018	13:18	0	
B0021	Group B7	Day 1	10 min	Yes	F2	uncoated	12-MAY-2018	13:18	0	
B0021	Group B7	Day 1	10 min	Yes	F3	uncoated	12-MAY-2018	13:18	0	
B0021	Group B7	Day 1	1 hour	Yes	F1	uncoated	12-MAY-2018	14:08	0	
B0021	Group B7	Day 1	1 hour	Yes	F2	uncoated	12-MAY-2018	14:08	0	
B0021	Group B7	Day 1	1 hour	Yes	F3	uncoated	12-MAY-2018	14:08	0	
B0021	Group B7	Day 1	2 hour	Yes	F1	uncoated	12-MAY-2018	15:08	0	
B0021	Group B7	Day 1	2 hour	Yes	F2	uncoated	12-MAY-2018	15:08	0	
B0021	Group B7	Day 1	2 hour	Yes	F3	uncoated	12-MAY-2018	15:08	0	
B0021	Group B7	Day 2		Yes	F1	uncoated	13-MAY-2018	12:38	0	
B0021	Group B7	Day 2		Yes	F2	uncoated	13-MAY-2018	12:38	0	
B0021	Group B7	Day 2		Yes	F3	uncoated	13-MAY-2018	12:38	0	

Scale: 0= No Pain; 10= Worst Pain Possible
Administration Site Key: FA = Forearm; UA= Upper arm
Treatments Part A: A1 = A/Sing MAP, 15 ug (FA); A2 = Afluria Quad IM (UA), 15 ug; A3 = Placebo MAP (FA); A4 = A/Sing Mono IM, 15 ug (UA)
Treatments Part B: B1 = A/Sing MAP, 15 ug (FA) with biopsy; B2 = Placebo MAP (FA) with biopsy
B3 = A/Sing MAP, 15 ug (FA); B4 = A/Sing MAP, 10 ug (FA); B5 = A/Sing MAP, 5 ug (FA); B6 = A/Sing MAP, 2.5 ug (FA)
B7 = Placebo MAP (FA); B8 = A/Sing MAP, 15 ug (UA); B9 = A/Sing IM, 15 ug, as 2018 Afluria Quad (UA)

Listing 16.2.9.3
Numeric Pain Intensity per Site

Part B

Randomisation Number	Group	Visit	Time Point	Was Pain Assessment Performed?	Site Number	Treatment	Date of assessment	Time of Assessment	Score	Unscheduled Reason
B0021	Group B7	Day 4		Yes	F1	uncoated	15-MAY-2018	09:58	0	
B0021	Group B7	Day 4		Yes	F2	uncoated	15-MAY-2018	09:58	0	
B0021	Group B7	Day 4		Yes	F3	uncoated	15-MAY-2018	09:58	0	
B0021	Group B7	Day 8		Yes	F1	uncoated	19-MAY-2018	12:06	0	
B0021	Group B7	Day 8		Yes	F2	uncoated	19-MAY-2018	12:06	0	
B0021	Group B7	Day 8		Yes	F3	uncoated	19-MAY-2018	12:06	0	
B0021	Group B7	Day 22		Yes	F1	uncoated	02-JUN-2018	13:15	0	
B0021	Group B7	Day 22		Yes	F2	uncoated	02-JUN-2018	13:15	0	
B0021	Group B7	Day 22		Yes	F3	uncoated	02-JUN-2018	13:15	0	
B0021	Group B7	EOS/ET (Day 61)		Yes	F1	uncoated	11-JUL-2018	13:35	0	
B0021	Group B7	EOS/ET (Day 61)		Yes	F2	uncoated	11-JUL-2018	13:35	0	
B0021	Group B7	EOS/ET (Day 61)		Yes	F3	uncoated	11-JUL-2018	13:35	0	
B0022	Group B4	Day 1	1 min	Yes	F1	uncoated	12-MAY-2018	13:12	0	
B0022	Group B4	Day 1	1 min	Yes	F2	5 ug	12-MAY-2018	13:15	1	
B0022	Group B4	Day 1	1 min	Yes	F3	5 ug	12-MAY-2018	13:18	1	
B0022	Group B4	Day 1	10 min	Yes	F1	uncoated	12-MAY-2018	13:28	0	
B0022	Group B4	Day 1	10 min	Yes	F2	5 ug	12-MAY-2018	13:28	0	
B0022	Group B4	Day 1	10 min	Yes	F3	5 ug	12-MAY-2018	13:28	0	
B0022	Group B4	Day 1	1 hour	Yes	F1	uncoated	12-MAY-2018	14:19	0	
B0022	Group B4	Day 1	1 hour	Yes	F2	5 ug	12-MAY-2018	14:19	0	
B0022	Group B4	Day 1	1 hour	Yes	F3	5 ug	12-MAY-2018	14:19	0	

Scale: 0= No Pain; 10= Worst Pain Possible
Administration Site Key: FA = Forearm; UA= Upper arm
Treatments Part A: A1 = A/Sing MAP, 15 ug (FA); A2 = Afluria Quad IM (UA), 15 ug; A3 = Placebo MAP (FA); A4 = A/Sing Mono IM, 15 ug (UA)
Treatments Part B: B1 = A/Sing MAP, 15 ug (FA) with biopsy; B2 = Placebo MAP (FA) with biopsy
B3 = A/Sing MAP, 15 ug (FA); B4 = A/Sing MAP, 10 ug (FA); B5 = A/Sing MAP, 5 ug (FA); B6 = A/Sing MAP, 2.5 ug (FA)
B7 = Placebo MAP (FA); B8 = A/Sing MAP, 15 ug (UA); B9 = A/Sing IM, 15 ug, as 2018 Afluria Quad (UA)

Listing 16.2.9.3
Numeric Pain Intensity per Site

Part B

Randomisation Number	Group	Visit	Time Point	Was Pain Assessment Performed?	Site Number	Treatment	Date of assessment	Time of Assessment	Score	Unscheduled Reason
B0022	Group B4	Day 1	2 hour	Yes	F1	uncoated	12-MAY-2018	15:22	0	
B0022	Group B4	Day 1	2 hour	Yes	F2	5 ug	12-MAY-2018	15:22	0	
B0022	Group B4	Day 1	2 hour	Yes	F3	5 ug	12-MAY-2018	15:22	0	
B0022	Group B4	Day 2		Yes	F1	uncoated	13-MAY-2018	13:05	0	
B0022	Group B4	Day 2		Yes	F2	5 ug	13-MAY-2018	13:05	0	
B0022	Group B4	Day 2		Yes	F3	5 ug	13-MAY-2018	13:05	0	
B0022	Group B4	Day 4		Yes	F1	uncoated	15-MAY-2018	13:37	0	
B0022	Group B4	Day 4		Yes	F2	5 ug	15-MAY-2018	13:37	0	
B0022	Group B4	Day 4		Yes	F3	5 ug	15-MAY-2018	13:37	0	
B0022	Group B4	Day 8		Yes	F1	uncoated	19-MAY-2018	13:20	0	
B0022	Group B4	Day 8		Yes	F2	5 ug	19-MAY-2018	13:20	0	
B0022	Group B4	Day 8		Yes	F3	5 ug	19-MAY-2018	13:20	0	
B0022	Group B4	Day 22		Yes	F1	uncoated	02-JUN-2018	13:51	0	
B0022	Group B4	Day 22		Yes	F2	5 ug	02-JUN-2018	13:51	0	
B0022	Group B4	Day 22		Yes	F3	5 ug	02-JUN-2018	13:51	0	
B0022	Group B4	EOS/ET (Day 61)		Yes	F1	uncoated	12-JUL-2018	11:02	0	
B0022	Group B4	EOS/ET (Day 61)		Yes	F2	5 ug	12-JUL-2018	11:02	0	
B0022	Group B4	EOS/ET (Day 61)		Yes	F3	5 ug	12-JUL-2018	11:02	0	
B0023	Group B3	Day 1	1 min	Yes	F1	5 ug	12-MAY-2018	13:21	3	
B0023	Group B3	Day 1	1 min	Yes	F2	5 ug	12-MAY-2018	13:30	3	
B0023	Group B3	Day 1	1 min	Yes	F3	5 ug	12-MAY-2018	13:33	4	

Scale: 0= No Pain; 10= Worst Pain Possible
Administration Site Key: FA = Forearm; UA= Upper arm
Treatments Part A: A1 = A/Sing MAP, 15 ug (FA); A2 = Afluria Quad IM (UA), 15 ug; A3 = Placebo MAP (FA); A4 = A/Sing Mono IM, 15 ug (UA)
Treatments Part B: B1 = A/Sing MAP, 15 ug (FA) with biopsy; B2 = Placebo MAP (FA) with biopsy
B3 = A/Sing MAP, 15 ug (FA); B4 = A/Sing MAP, 10 ug (FA); B5 = A/Sing MAP, 5 ug (FA); B6 = A/Sing MAP, 2.5 ug (FA)
B7 = Placebo MAP (FA); B8 = A/Sing MAP, 15 ug (UA); B9 = A/Sing IM, 15 ug, as 2018 Afluria Quad (UA)

Listing 16.2.9.3
Numeric Pain Intensity per Site

Part B

Randomisation Number	Group	Visit	Time Point	Was Pain Assessment Performed?	Site Number	Treatment	Date of assessment	Time of Assessment	Score	Unscheduled Reason
B0023	Group B3	Day 1	10 min	Yes	F1	5 ug	12-MAY-2018	13:44	0	
B0023	Group B3	Day 1	10 min	Yes	F2	5 ug	12-MAY-2018	13:44	0	
B0023	Group B3	Day 1	10 min	Yes	F3	5 ug	12-MAY-2018	13:44	0	
B0023	Group B3	Day 1	1 hour	Yes	F1	5 ug	12-MAY-2018	14:34	1	
B0023	Group B3	Day 1	1 hour	Yes	F2	5 ug	12-MAY-2018	14:34	1	
B0023	Group B3	Day 1	1 hour	Yes	F3	5 ug	12-MAY-2018	14:34	1	
B0023	Group B3	Day 1	2 hour	Yes	F1	5 ug	12-MAY-2018	15:34	0	
B0023	Group B3	Day 1	2 hour	Yes	F2	5 ug	12-MAY-2018	15:34	0	
B0023	Group B3	Day 1	2 hour	Yes	F3	5 ug	12-MAY-2018	15:34	0	
B0023	Group B3	Day 2		Yes	F1	5 ug	13-MAY-2018	13:19	0	
B0023	Group B3	Day 2		Yes	F2	5 ug	13-MAY-2018	13:19	0	
B0023	Group B3	Day 2		Yes	F3	5 ug	13-MAY-2018	13:19	0	
B0023	Group B3	Day 4		Yes	F1	5 ug	15-MAY-2018	14:31	0	
B0023	Group B3	Day 4		Yes	F2	5 ug	15-MAY-2018	14:31	0	
B0023	Group B3	Day 4		Yes	F3	5 ug	15-MAY-2018	14:31	0	
B0023	Group B3	Day 8		Yes	F1	5 ug	19-MAY-2018	10:17	0	
B0023	Group B3	Day 8		Yes	F2	5 ug	19-MAY-2018	10:17	0	
B0023	Group B3	Day 8		Yes	F3	5 ug	19-MAY-2018	10:17	0	
B0023	Group B3	Day 22		Yes	F1	5 ug	02-JUN-2018	11:14	0	
B0023	Group B3	Day 22		Yes	F2	5 ug	02-JUN-2018	11:14	0	
B0023	Group B3	Day 22		Yes	F3	5 ug	02-JUN-2018	11:14	0	

Scale: 0= No Pain; 10= Worst Pain Possible
Administration Site Key: FA = Forearm; UA= Upper arm
Treatments Part A: A1 = A/Sing MAP, 15 ug (FA); A2 = Afluria Quad IM (UA), 15 ug; A3 = Placebo MAP (FA); A4 = A/Sing Mono IM, 15 ug (UA)
Treatments Part B: B1 = A/Sing MAP, 15 ug (FA) with biopsy; B2 = Placebo MAP (FA) with biopsy
B3 = A/Sing MAP, 15 ug (FA); B4 = A/Sing MAP, 10 ug (FA); B5 = A/Sing MAP, 5 ug (FA); B6 = A/Sing MAP, 2.5 ug (FA)
B7 = Placebo MAP (FA); B8 = A/Sing MAP, 15 ug (UA); B9 = A/Sing IM, 15 ug, as 2018 Afluria Quad (UA)

Listing 16.2.9.3
Numeric Pain Intensity per Site

Part B

Randomisation Number	Group	Visit	Time Point	Was Pain Assessment Performed?	Site Number	Treatment	Date of assessment	Time of Assessment	Score	Unscheduled Reason
B0023	Group B3	EOS/ET (Day 61)		Yes	F1	5 ug	11-JUL-2018	14:12	0	
B0023	Group B3	EOS/ET (Day 61)		Yes	F2	5 ug	11-JUL-2018	14:13	0	
B0023	Group B3	EOS/ET (Day 61)		Yes	F3	5 ug	11-JUL-2018	14:13	0	
B0024	Group B5	Day 1	1 min	Yes	F1	uncoated	12-MAY-2018	13:37	0	
B0024	Group B5	Day 1	1 min	Yes	F2	uncoated	12-MAY-2018	13:40	2	
B0024	Group B5	Day 1	1 min	Yes	F3	5 ug	12-MAY-2018	13:43	2	
B0024	Group B5	Day 1	10 min	Yes	F1	uncoated	12-MAY-2018	13:54	1	
B0024	Group B5	Day 1	10 min	Yes	F2	uncoated	12-MAY-2018	13:54	1	
B0024	Group B5	Day 1	10 min	Yes	F3	5 ug	12-MAY-2018	13:54	1	
B0024	Group B5	Day 1	1 hour	Yes	F1	uncoated	12-MAY-2018	14:48	0	
B0024	Group B5	Day 1	1 hour	Yes	F2	uncoated	12-MAY-2018	14:48	0	
B0024	Group B5	Day 1	1 hour	Yes	F3	5 ug	12-MAY-2018	14:48	0	
B0024	Group B5	Day 1	2 hour	Yes	F1	uncoated	12-MAY-2018	15:44	0	
B0024	Group B5	Day 1	2 hour	Yes	F2	uncoated	12-MAY-2018	15:44	0	
B0024	Group B5	Day 1	2 hour	Yes	F3	5 ug	12-MAY-2018	15:44	0	
B0024	Group B5	Day 2		Yes	F1	uncoated	13-MAY-2018	13:35	0	
B0024	Group B5	Day 2		Yes	F2	uncoated	13-MAY-2018	13:35	0	
B0024	Group B5	Day 2		Yes	F3	5 ug	13-MAY-2018	13:35	0	
B0024	Group B5	Day 4		Yes	F1	uncoated	15-MAY-2018	13:40	0	
B0024	Group B5	Day 4		Yes	F2	uncoated	15-MAY-2018	13:40	0	
B0024	Group B5	Day 4		Yes	F3	5 ug	15-MAY-2018	13:41	0	

Scale: 0= No Pain; 10= Worst Pain Possible
Administration Site Key: FA = Forearm; UA= Upper arm
Treatments Part A: A1 = A/Sing MAP, 15 ug (FA); A2 = Afluria Quad IM (UA), 15 ug; A3 = Placebo MAP (FA); A4 = A/Sing Mono IM, 15 ug (UA)
Treatments Part B: B1 = A/Sing MAP, 15 ug (FA) with biopsy; B2 = Placebo MAP (FA) with biopsy
B3 = A/Sing MAP, 15 ug (FA); B4 = A/Sing MAP, 10 ug (FA); B5 = A/Sing MAP, 5 ug (FA); B6 = A/Sing MAP, 2.5 ug (FA)
B7 = Placebo MAP (FA); B8 = A/Sing MAP, 15 ug (UA); B9 = A/Sing IM, 15 ug, as 2018 Afluria Quad (UA)

Listing 16.2.9.3
Numeric Pain Intensity per Site

Part B

Randomisation Number	Group	Visit	Time Point	Was Pain Assessment Performed?	Site Number	Treatment	Date of assessment	Time of Assessment	Score	Unscheduled Reason
B0024	Group B5	Day 8		Yes	F1	uncoated	19-MAY-2018	13:25	0	
B0024	Group B5	Day 8		Yes	F2	uncoated	19-MAY-2018	13:25	0	
B0024	Group B5	Day 8		Yes	F3	5 ug	19-MAY-2018	13:25	0	
B0024	Group B5	Day 22		Yes	F1	uncoated	02-JUN-2018	13:47	0	
B0024	Group B5	Day 22		Yes	F2	uncoated	02-JUN-2018	13:47	0	
B0024	Group B5	Day 22		Yes	F3	5 ug	02-JUN-2018	13:47	0	
B0024	Group B5	EOS/ET (Day 61)		Yes	F1	uncoated	12-JUL-2018	14:28	0	
B0024	Group B5	EOS/ET (Day 61)		Yes	F2	uncoated	12-JUL-2018	14:28	0	
B0024	Group B5	EOS/ET (Day 61)		Yes	F3	5 ug	12-JUL-2018	14:28	0	
B0025	Group B8	Day 1	1 min	Yes	D1	5 ug	12-MAY-2018	13:41	0	
B0025	Group B8	Day 1	1 min	Yes	D2	5 ug	12-MAY-2018	13:44	0	
B0025	Group B8	Day 1	1 min	Yes	D3	5 ug	12-MAY-2018	13:47	0	
B0025	Group B8	Day 1	10 min	Yes	D1	5 ug	12-MAY-2018	13:58	1	
B0025	Group B8	Day 1	10 min	Yes	D2	5 ug	12-MAY-2018	13:58	1	
B0025	Group B8	Day 1	10 min	Yes	D3	5 ug	12-MAY-2018	13:58	1	
B0025	Group B8	Day 1	1 hour	Yes	D1	5 ug	12-MAY-2018	14:48	0	
B0025	Group B8	Day 1	1 hour	Yes	D2	5 ug	12-MAY-2018	14:48	0	
B0025	Group B8	Day 1	1 hour	Yes	D3	5 ug	12-MAY-2018	14:48	0	
B0025	Group B8	Day 1	2 hour	Yes	D1	5 ug	12-MAY-2018	15:48	0	
B0025	Group B8	Day 1	2 hour	Yes	D2	5 ug	12-MAY-2018	15:48	0	
B0025	Group B8	Day 1	2 hour	Yes	D3	5 ug	12-MAY-2018	15:48	0	

Scale: 0= No Pain; 10= Worst Pain Possible
Administration Site Key: FA = Forearm; UA= Upper arm
Treatments Part A: A1 = A/Sing MAP, 15 ug (FA); A2 = Afluria Quad IM (UA), 15 ug; A3 = Placebo MAP (FA); A4 = A/Sing Mono IM, 15 ug (UA)
Treatments Part B: B1 = A/Sing MAP, 15 ug (FA) with biopsy; B2 = Placebo MAP (FA) with biopsy
B3 = A/Sing MAP, 15 ug (FA); B4 = A/Sing MAP, 10 ug (FA); B5 = A/Sing MAP, 5 ug (FA); B6 = A/Sing MAP, 2.5 ug (FA)
B7 = Placebo MAP (FA); B8 = A/Sing MAP, 15 ug (UA); B9 = A/Sing IM, 15 ug, as 2018 Afluria Quad (UA)

Listing 16.2.9.3
Numeric Pain Intensity per Site

Part B

Randomisation Number	Group	Visit	Time Point	Was Pain Assessment Performed?	Site Number	Treatment	Date of assessment	Time of Assessment	Score	Unscheduled Reason
B0025	Group B8	Day 2		Yes	D1	5 ug	13-MAY-2018	12:17	0	
B0025	Group B8	Day 2		Yes	D2	5 ug	13-MAY-2018	12:17	0	
B0025	Group B8	Day 2		Yes	D3	5 ug	13-MAY-2018	12:17	0	
B0025	Group B8	Day 4		Yes	D1	5 ug	15-MAY-2018	16:42	0	
B0025	Group B8	Day 4		Yes	D2	5 ug	15-MAY-2018	16:42	0	
B0025	Group B8	Day 4		Yes	D3	5 ug	15-MAY-2018	16:42	0	
B0025	Group B8	Day 8		Yes	D1	5 ug	19-MAY-2018	12:36	0	
B0025	Group B8	Day 8		Yes	D2	5 ug	19-MAY-2018	12:35	0	
B0025	Group B8	Day 8		Yes	D3	5 ug	19-MAY-2018	12:35	0	
B0025	Group B8	Day 22		Yes	D1	5 ug	04-JUN-2018	13:08	0	
B0025	Group B8	Day 22		Yes	D2	5 ug	04-JUN-2018	13:08	0	
B0025	Group B8	Day 22		Yes	D3	5 ug	04-JUN-2018	13:08	0	
B0025	Group B8	EOS/ET (Day 61)		Yes	D1	5 ug	13-JUL-2018	13:37	0	
B0025	Group B8	EOS/ET (Day 61)		Yes	D2	5 ug	13-JUL-2018	13:37	0	
B0025	Group B8	EOS/ET (Day 61)		Yes	D3	5 ug	13-JUL-2018	13:37	0	
B0026	Group B9	Day 1	1 min	Yes	D1	[IM]	12-MAY-2018	11:31	1	
B0026	Group B9	Day 1	10 min	Yes	D1	[IM]	12-MAY-2018	11:40	0	
B0026	Group B9	Day 1	1 hour	Yes	D1	[IM]	12-MAY-2018	12:30	0	
B0026	Group B9	Day 1	2 hour	Yes	D1	[IM]	12-MAY-2018	13:35	0	
B0026	Group B9	Day 2		Yes	D1	[IM]	13-MAY-2018	12:21	0	
B0026	Group B9	Day 4		Yes	D1	[IM]	15-MAY-2018	14:51	0	

Scale: 0= No Pain; 10= Worst Pain Possible
Administration Site Key: FA = Forearm; UA= Upper arm
Treatments Part A: A1 = A/Sing MAP, 15 ug (FA); A2 = Afluria Quad IM (UA), 15 ug; A3 = Placebo MAP (FA); A4 = A/Sing Mono IM, 15 ug (UA)
Treatments Part B: B1 = A/Sing MAP, 15 ug (FA) with biopsy; B2 = Placebo MAP (FA) with biopsy
B3 = A/Sing MAP, 15 ug (FA); B4 = A/Sing MAP, 10 ug (FA); B5 = A/Sing MAP, 5 ug (FA); B6 = A/Sing MAP, 2.5 ug (FA)
B7 = Placebo MAP (FA); B8 = A/Sing MAP, 15 ug (UA); B9 = A/Sing IM, 15 ug, as 2018 Afluria Quad (UA)

Listing 16.2.9.3
Numeric Pain Intensity per Site

Part B

Randomisation Number	Group	Visit	Time Point	Was Pain Assessment Performed?	Site Number	Treatment	Date of assessment	Time of Assessment	Score	Unscheduled Reason
B0026	Group B9	Day 8		Yes	D1	[IM]	19-MAY-2018	15:57	0	
B0026	Group B9	Day 22		Yes	D1	[IM]	02-JUN-2018	14:31	0	
B0026	Group B9	EOS/ET (Day 61)		Yes	D1	[IM]	11-JUL-2018	16:15	0	
B0027	Group B3	Day 1	1 min	Yes	F1	5 ug	12-MAY-2018	14:31	1	
B0027	Group B3	Day 1	1 min	Yes	F2	5 ug	12-MAY-2018	14:34	1	
B0027	Group B3	Day 1	1 min	Yes	F3	5 ug	12-MAY-2018	14:37	2	
B0027	Group B3	Day 1	10 min	Yes	F1	5 ug	12-MAY-2018	14:48	2	
B0027	Group B3	Day 1	10 min	Yes	F2	5 ug	12-MAY-2018	14:48	2	
B0027	Group B3	Day 1	10 min	Yes	F3	5 ug	12-MAY-2018	14:48	1	
B0027	Group B3	Day 1	1 hour	Yes	F1	5 ug	12-MAY-2018	15:38	1	
B0027	Group B3	Day 1	1 hour	Yes	F2	5 ug	12-MAY-2018	15:38	0	
B0027	Group B3	Day 1	1 hour	Yes	F3	5 ug	12-MAY-2018	15:38	0	
B0027	Group B3	Day 1	2 hour	Yes	F1	5 ug	12-MAY-2018	16:40	0	
B0027	Group B3	Day 1	2 hour	Yes	F2	5 ug	12-MAY-2018	16:40	0	
B0027	Group B3	Day 1	2 hour	Yes	F3	5 ug	12-MAY-2018	16:40	0	
B0027	Group B3	Day 2		Yes	F1	5 ug	13-MAY-2018	14:00	0	
B0027	Group B3	Day 2		Yes	F2	5 ug	13-MAY-2018	14:00	0	
B0027	Group B3	Day 2		Yes	F3	5 ug	13-MAY-2018	14:00	0	
B0027	Group B3	Day 4		Yes	F1	5 ug	15-MAY-2018	17:13	0	
B0027	Group B3	Day 4		Yes	F2	5 ug	15-MAY-2018	17:13	0	
B0027	Group B3	Day 4		Yes	F3	5 ug	15-MAY-2018	17:13	0	

Scale: 0= No Pain; 10= Worst Pain Possible
Administration Site Key: FA = Forearm; UA= Upper arm
Treatments Part A: A1 = A/Sing MAP, 15 ug (FA); A2 = Afluria Quad IM (UA), 15 ug; A3 = Placebo MAP (FA); A4 = A/Sing Mono IM, 15 ug (UA)
Treatments Part B: B1 = A/Sing MAP, 15 ug (FA) with biopsy; B2 = Placebo MAP (FA) with biopsy
B3 = A/Sing MAP, 15 ug (FA); B4 = A/Sing MAP, 10 ug (FA); B5 = A/Sing MAP, 5 ug (FA); B6 = A/Sing MAP, 2.5 ug (FA)
B7 = Placebo MAP (FA); B8 = A/Sing MAP, 15 ug (UA); B9 = A/Sing IM, 15 ug, as 2018 Afluria Quad (UA)

Listing 16.2.9.3
Numeric Pain Intensity per Site

Part B

Randomisation Number	Group	Visit	Time Point	Was Pain Assessment Performed?	Site Number	Treatment	Date of assessment	Time of Assessment	Score	Unscheduled Reason
B0027	Group B3	Day 8		Yes	F1	5 ug	19-MAY-2018	14:26	0	
B0027	Group B3	Day 8		Yes	F2	5 ug	19-MAY-2018	14:26	0	
B0027	Group B3	Day 8		Yes	F3	5 ug	19-MAY-2018	14:26	0	
B0027	Group B3	Day 22		Yes	F1	5 ug	02-JUN-2018	16:08	0	
B0027	Group B3	Day 22		Yes	F2	5 ug	02-JUN-2018	16:08	0	
B0027	Group B3	Day 22		Yes	F3	5 ug	02-JUN-2018	16:08	0	
B0027	Group B3	EOS/ET (Day 61)		Yes	F1	5 ug	11-JUL-2018	17:20	0	
B0027	Group B3	EOS/ET (Day 61)		Yes	F2	5 ug	11-JUL-2018	17:20	0	
B0027	Group B3	EOS/ET (Day 61)		Yes	F3	5 ug	11-JUL-2018	17:20	0	
B0028	Group B5	Day 1	1 min	Yes	F1	5 ug	12-MAY-2018	14:21	1	
B0028	Group B5	Day 1	1 min	Yes	F2	uncoated	12-MAY-2018	14:24	2	
B0028	Group B5	Day 1	1 min	Yes	F3	uncoated	12-MAY-2018	14:27	1	
B0028	Group B5	Day 1	10 min	Yes	F1	5 ug	12-MAY-2018	14:38	0	
B0028	Group B5	Day 1	10 min	Yes	F2	uncoated	12-MAY-2018	14:38	2	
B0028	Group B5	Day 1	10 min	Yes	F3	uncoated	12-MAY-2018	14:38	1	
B0028	Group B5	Day 1	1 hour	Yes	F1	5 ug	12-MAY-2018	15:28	0	
B0028	Group B5	Day 1	1 hour	Yes	F2	uncoated	12-MAY-2018	15:28	0	
B0028	Group B5	Day 1	1 hour	Yes	F3	uncoated	12-MAY-2018	15:28	0	
B0028	Group B5	Day 1	2 hour	Yes	F1	5 ug	12-MAY-2018	16:28	0	
B0028	Group B5	Day 1	2 hour	Yes	F2	uncoated	12-MAY-2018	16:28	0	
B0028	Group B5	Day 1	2 hour	Yes	F3	uncoated	12-MAY-2018	16:28	0	

Scale: 0= No Pain; 10= Worst Pain Possible
Administration Site Key: FA = Forearm; UA= Upper arm
Treatments Part A: A1 = A/Sing MAP, 15 ug (FA); A2 = Afluria Quad IM (UA), 15 ug; A3 = Placebo MAP (FA); A4 = A/Sing Mono IM, 15 ug (UA)
Treatments Part B: B1 = A/Sing MAP, 15 ug (FA) with biopsy; B2 = Placebo MAP (FA) with biopsy
B3 = A/Sing MAP, 15 ug (FA); B4 = A/Sing MAP, 10 ug (FA); B5 = A/Sing MAP, 5 ug (FA); B6 = A/Sing MAP, 2.5 ug (FA)
B7 = Placebo MAP (FA); B8 = A/Sing MAP, 15 ug (UA); B9 = A/Sing IM, 15 ug, as 2018 Afluria Quad (UA)

Listing 16.2.9.3
Numeric Pain Intensity per Site

Part B

Randomisation Number	Group	Visit	Time Point	Was Pain Assessment Performed?	Site Number	Treatment	Date of assessment	Time of Assessment	Score	Unscheduled Reason
B0028	Group B5	Day 2		Yes	F1	5 ug	13-MAY-2018	10:05	0	
B0028	Group B5	Day 2		Yes	F2	uncoated	13-MAY-2018	10:05	0	
B0028	Group B5	Day 2		Yes	F3	uncoated	13-MAY-2018	10:05	0	
B0028	Group B5	Day 4		Yes	F1	5 ug	16-MAY-2018	16:31	0	
B0028	Group B5	Day 4		Yes	F2	uncoated	16-MAY-2018	16:31	0	
B0028	Group B5	Day 4		Yes	F3	uncoated	16-MAY-2018	16:31	0	
B0028	Group B5	Day 8		Yes	F1	5 ug	19-MAY-2018	14:30	0	
B0028	Group B5	Day 8		Yes	F2	uncoated	19-MAY-2018	14:30	0	
B0028	Group B5	Day 8		Yes	F3	uncoated	19-MAY-2018	14:30	0	
B0028	Group B5	Day 22		Yes	F1	5 ug	02-JUN-2018	14:33	0	
B0028	Group B5	Day 22		Yes	F2	uncoated	02-JUN-2018	14:33	0	
B0028	Group B5	Day 22		Yes	F3	uncoated	02-JUN-2018	14:33	0	
B0028	Group B5	EOS/ET (Day 61)		Yes	F1	5 ug	11-JUL-2018	14:34	0	
B0028	Group B5	EOS/ET (Day 61)		Yes	F2	uncoated	11-JUL-2018	14:34	0	
B0028	Group B5	EOS/ET (Day 61)		Yes	F3	uncoated	11-JUL-2018	14:34	0	
B0029	Group B6	Day 1	1 min	Yes	F1	uncoated	12-MAY-2018	14:12	2	
B0029	Group B6	Day 1	1 min	Yes	F2	uncoated	12-MAY-2018	14:15	3	
B0029	Group B6	Day 1	1 min	Yes	F3	2.5 ug	12-MAY-2018	14:18	2	
B0029	Group B6	Day 1	10 min	Yes	F1	uncoated	12-MAY-2018	14:29	0	
B0029	Group B6	Day 1	10 min	Yes	F2	uncoated	12-MAY-2018	14:29	1	
B0029	Group B6	Day 1	10 min	Yes	F3	2.5 ug	12-MAY-2018	14:29	2	

Scale: 0= No Pain; 10= Worst Pain Possible
Administration Site Key: FA = Forearm; UA= Upper arm
Treatments Part A: A1 = A/Sing MAP, 15 ug (FA); A2 = Afluria Quad IM (UA), 15 ug; A3 = Placebo MAP (FA); A4 = A/Sing Mono IM, 15 ug (UA)
Treatments Part B: B1 = A/Sing MAP, 15 ug (FA) with biopsy; B2 = Placebo MAP (FA) with biopsy
B3 = A/Sing MAP, 15 ug (FA); B4 = A/Sing MAP, 10 ug (FA); B5 = A/Sing MAP, 5 ug (FA); B6 = A/Sing MAP, 2.5 ug (FA)
B7 = Placebo MAP (FA); B8 = A/Sing MAP, 15 ug (UA); B9 = A/Sing IM, 15 ug, as 2018 Afluria Quad (UA)

Listing 16.2.9.3
Numeric Pain Intensity per Site

Part B

Randomisation Number	Group	Visit	Time Point	Was Pain Assessment Performed?	Site Number	Treatment	Date of assessment	Time of Assessment	Score	Unscheduled Reason
B0029	Group B6	Day 1	1 hour	Yes	F1	uncoated	12-MAY-2018	15:19	0	
B0029	Group B6	Day 1	1 hour	Yes	F2	uncoated	12-MAY-2018	15:19	0	
B0029	Group B6	Day 1	1 hour	Yes	F3	2.5 ug	12-MAY-2018	15:19	0	
B0029	Group B6	Day 1	2 hour	Yes	F1	uncoated	12-MAY-2018	16:21	0	
B0029	Group B6	Day 1	2 hour	Yes	F2	uncoated	12-MAY-2018	16:21	0	
B0029	Group B6	Day 1	2 hour	Yes	F3	2.5 ug	12-MAY-2018	16:21	0	
B0029	Group B6	Day 2		Yes	F1	uncoated	13-MAY-2018	13:42	0	
B0029	Group B6	Day 2		Yes	F2	uncoated	13-MAY-2018	13:42	0	
B0029	Group B6	Day 2		Yes	F3	2.5 ug	13-MAY-2018	13:42	0	
B0029	Group B6	Day 4		Yes	F1	uncoated	15-MAY-2018	14:54	0	
B0029	Group B6	Day 4		Yes	F2	uncoated	15-MAY-2018	14:54	0	
B0029	Group B6	Day 4		Yes	F3	2.5 ug	15-MAY-2018	14:54	0	
B0029	Group B6	Day 8		Yes	F1	uncoated	19-MAY-2018	14:38	0	
B0029	Group B6	Day 8		Yes	F2	uncoated	19-MAY-2018	14:38	0	
B0029	Group B6	Day 8		Yes	F3	2.5 ug	19-MAY-2018	14:38	0	
B0029	Group B6	Day 22		Yes	F1	uncoated	02-JUN-2018	15:59	0	
B0029	Group B6	Day 22		Yes	F2	uncoated	02-JUN-2018	15:59	0	
B0029	Group B6	Day 22		Yes	F3	2.5 ug	02-JUN-2018	15:59	0	
B0029	Group B6	EOS/ET (Day 61)		Yes	F1	uncoated	11-JUL-2018	14:54	0	
B0029	Group B6	EOS/ET (Day 61)		Yes	F2	uncoated	11-JUL-2018	14:54	0	
B0029	Group B6	EOS/ET (Day 61)		Yes	F3	2.5 ug	11-JUL-2018	14:54	0	

Scale: 0= No Pain; 10= Worst Pain Possible
Administration Site Key: FA = Forearm; UA= Upper arm
Treatments Part A: A1 = A/Sing MAP, 15 ug (FA); A2 = Afluria Quad IM (UA), 15 ug; A3 = Placebo MAP (FA); A4 = A/Sing Mono IM, 15 ug (UA)
Treatments Part B: B1 = A/Sing MAP, 15 ug (FA) with biopsy; B2 = Placebo MAP (FA) with biopsy
B3 = A/Sing MAP, 15 ug (FA); B4 = A/Sing MAP, 10 ug (FA); B5 = A/Sing MAP, 5 ug (FA); B6 = A/Sing MAP, 2.5 ug (FA)
B7 = Placebo MAP (FA); B8 = A/Sing MAP, 15 ug (UA); B9 = A/Sing IM, 15 ug, as 2018 Afluria Quad (UA)

Listing 16.2.9.3
Numeric Pain Intensity per Site

Part B

Randomisation Number	Group	Visit	Time Point	Was Pain Assessment Performed?	Site Number	Treatment	Date of assessment	Time of Assessment	Score	Unscheduled Reason
B0030	Group B7	Day 1	1 min	Yes	F1	uncoated	12-MAY-2018	14:01	2	
B0030	Group B7	Day 1	1 min	Yes	F2	uncoated	12-MAY-2018	14:04	3	
B0030	Group B7	Day 1	1 min	Yes	F3	uncoated	12-MAY-2018	14:07	3	
B0030	Group B7	Day 1	10 min	Yes	F1	uncoated	12-MAY-2018	14:18	0	
B0030	Group B7	Day 1	10 min	Yes	F2	uncoated	12-MAY-2018	14:18	0	
B0030	Group B7	Day 1	10 min	Yes	F3	uncoated	12-MAY-2018	14:18	0	
B0030	Group B7	Day 1	1 hour	Yes	F1	uncoated	12-MAY-2018	15:08	0	
B0030	Group B7	Day 1	1 hour	Yes	F2	uncoated	12-MAY-2018	15:08	0	
B0030	Group B7	Day 1	1 hour	Yes	F3	uncoated	12-MAY-2018	15:08	0	
B0030	Group B7	Day 1	2 hour	Yes	F1	uncoated	12-MAY-2018	16:08	0	
B0030	Group B7	Day 1	2 hour	Yes	F2	uncoated	12-MAY-2018	16:08	0	
B0030	Group B7	Day 1	2 hour	Yes	F3	uncoated	12-MAY-2018	16:08	0	
B0030	Group B7	Day 2		Yes	F1	uncoated	13-MAY-2018	13:46	0	
B0030	Group B7	Day 2		Yes	F2	uncoated	13-MAY-2018	13:46	0	
B0030	Group B7	Day 2		Yes	F3	uncoated	13-MAY-2018	13:46	0	
B0030	Group B7	Day 4		Yes	F1	uncoated	15-MAY-2018	13:39	0	
B0030	Group B7	Day 4		Yes	F2	uncoated	15-MAY-2018	13:39	0	
B0030	Group B7	Day 4		Yes	F3	uncoated	15-MAY-2018	13:59	0	
B0030	Group B7	Day 8		Yes	F1	uncoated	19-MAY-2018	16:41	0	
B0030	Group B7	Day 8		Yes	F2	uncoated	19-MAY-2018	16:41	0	
B0030	Group B7	Day 8		Yes	F3	uncoated	19-MAY-2018	16:41	0	

Scale: 0= No Pain; 10= Worst Pain Possible
Administration Site Key: FA = Forearm; UA= Upper arm
Treatments Part A: A1 = A/Sing MAP, 15 ug (FA); A2 = Afluria Quad IM (UA), 15 ug; A3 = Placebo MAP (FA); A4 = A/Sing Mono IM, 15 ug (UA)
Treatments Part B: B1 = A/Sing MAP, 15 ug (FA) with biopsy; B2 = Placebo MAP (FA) with biopsy
B3 = A/Sing MAP, 15 ug (FA); B4 = A/Sing MAP, 10 ug (FA); B5 = A/Sing MAP, 5 ug (FA); B6 = A/Sing MAP, 2.5 ug (FA)
B7 = Placebo MAP (FA); B8 = A/Sing MAP, 15 ug (UA); B9 = A/Sing IM, 15 ug, as 2018 Afluria Quad (UA)

Listing 16.2.9.3
Numeric Pain Intensity per Site

Part B

Randomisation Number	Group	Visit	Time Point	Was Pain Assessment Performed?	Site Number	Treatment	Date of assessment	Time of Assessment	Score	Unscheduled Reason
B0030	Group B7	Day 22		Yes	F1	uncoated	02-JUN-2018	15:26	0	
B0030	Group B7	Day 22		Yes	F2	uncoated	02-JUN-2018	15:26	0	
B0030	Group B7	Day 22		Yes	F3	uncoated	02-JUN-2018	15:26	0	
B0030	Group B7	EOS/ET (Day 61)		Yes	F1	uncoated	13-JUL-2018	10:46	0	
B0030	Group B7	EOS/ET (Day 61)		Yes	F2	uncoated	13-JUL-2018	10:47	0	
B0030	Group B7	EOS/ET (Day 61)		Yes	F3	uncoated	13-JUL-2018	10:47	0	
B0031	Group B4	Day 1	1 min	Yes	F1	uncoated	19-MAY-2018	13:21	2	
B0031	Group B4	Day 1	1 min	Yes	F2	5 ug	19-MAY-2018	13:24	2	
B0031	Group B4	Day 1	1 min	Yes	F3	5 ug	19-MAY-2018	13:27	2	
B0031	Group B4	Day 1	10 min	Yes	F1	uncoated	19-MAY-2018	13:38	2	
B0031	Group B4	Day 1	10 min	Yes	F2	5 ug	19-MAY-2018	13:38	2	
B0031	Group B4	Day 1	10 min	Yes	F3	5 ug	19-MAY-2018	13:38	2	
B0031	Group B4	Day 1	1 hour	Yes	F1	uncoated	19-MAY-2018	14:28	1	
B0031	Group B4	Day 1	1 hour	Yes	F2	5 ug	19-MAY-2018	14:28	1	
B0031	Group B4	Day 1	1 hour	Yes	F3	5 ug	19-MAY-2018	14:28	1	
B0031	Group B4	Day 1	2 hour	Yes	F1	uncoated	19-MAY-2018	15:28	0	
B0031	Group B4	Day 1	2 hour	Yes	F2	5 ug	19-MAY-2018	15:28	0	
B0031	Group B4	Day 1	2 hour	Yes	F3	5 ug	19-MAY-2018	15:28	0	
B0031	Group B4	Day 2		Yes	F1	uncoated	20-MAY-2018	13:09	0	
B0031	Group B4	Day 2		Yes	F2	5 ug	20-MAY-2018	13:09	0	
B0031	Group B4	Day 2		Yes	F3	5 ug	20-MAY-2018	13:09	0	

Scale: 0= No Pain; 10= Worst Pain Possible
Administration Site Key: FA = Forearm; UA= Upper arm
Treatments Part A: A1 = A/Sing MAP, 15 ug (FA); A2 = Afluria Quad IM (UA), 15 ug; A3 = Placebo MAP (FA); A4 = A/Sing Mono IM, 15 ug (UA)
Treatments Part B: B1 = A/Sing MAP, 15 ug (FA) with biopsy; B2 = Placebo MAP (FA) with biopsy
B3 = A/Sing MAP, 15 ug (FA); B4 = A/Sing MAP, 10 ug (FA); B5 = A/Sing MAP, 5 ug (FA); B6 = A/Sing MAP, 2.5 ug (FA)
B7 = Placebo MAP (FA); B8 = A/Sing MAP, 15 ug (UA); B9 = A/Sing IM, 15 ug, as 2018 Afluria Quad (UA)

Listing 16.2.9.3
Numeric Pain Intensity per Site

Part B

Randomisation Number	Group	Visit	Time Point	Was Pain Assessment Performed?	Site Number	Treatment	Date of assessment	Time of Assessment	Score	Unscheduled Reason
B0031	Group B4	Day 4		Yes	F1	uncoated	22-MAY-2018	13:08	0	
B0031	Group B4	Day 4		Yes	F2	5 ug	22-MAY-2018	13:08	0	
B0031	Group B4	Day 4		Yes	F3	5 ug	22-MAY-2018	13:08	0	
B0031	Group B4	Day 8		Yes	F1	uncoated	26-MAY-2018	11:26	0	
B0031	Group B4	Day 8		Yes	F2	5 ug	26-MAY-2018	11:26	0	
B0031	Group B4	Day 8		Yes	F3	5 ug	26-MAY-2018	11:26	0	
B0031	Group B4	Day 22		Yes	F1	uncoated	08-JUN-2018	13:52	0	
B0031	Group B4	Day 22		Yes	F2	5 ug	08-JUN-2018	13:52	0	
B0031	Group B4	Day 22		Yes	F3	5 ug	08-JUN-2018	13:52	0	
B0031	Group B4	EOS/ET (Day 61)		Yes	F1	uncoated	18-JUL-2018	10:35	0	
B0031	Group B4	EOS/ET (Day 61)		Yes	F2	5 ug	18-JUL-2018	10:35	0	
B0031	Group B4	EOS/ET (Day 61)		Yes	F3	5 ug	18-JUL-2018	10:35	0	
B0032	Group B6	Day 1	1 min	Yes	F1	uncoated	19-MAY-2018	11:41	0	
B0032	Group B6	Day 1	1 min	Yes	F2	uncoated	19-MAY-2018	11:44	1	
B0032	Group B6	Day 1	1 min	Yes	F3	2.5 ug	19-MAY-2018	11:47	1	
B0032	Group B6	Day 1	10 min	Yes	F1	uncoated	19-MAY-2018	11:58	0	
B0032	Group B6	Day 1	10 min	Yes	F2	uncoated	19-MAY-2018	11:58	0	
B0032	Group B6	Day 1	10 min	Yes	F3	2.5 ug	19-MAY-2018	11:58	1	
B0032	Group B6	Day 1	1 hour	Yes	F1	uncoated	19-MAY-2018	12:48	0	
B0032	Group B6	Day 1	1 hour	Yes	F2	uncoated	19-MAY-2018	12:48	0	
B0032	Group B6	Day 1	1 hour	Yes	F3	2.5 ug	19-MAY-2018	12:48	0	

Scale: 0= No Pain; 10= Worst Pain Possible
Administration Site Key: FA = Forearm; UA= Upper arm
Treatments Part A: A1 = A/Sing MAP, 15 ug (FA); A2 = Afluria Quad IM (UA), 15 ug; A3 = Placebo MAP (FA); A4 = A/Sing Mono IM, 15 ug (UA)
Treatments Part B: B1 = A/Sing MAP, 15 ug (FA) with biopsy; B2 = Placebo MAP (FA) with biopsy
B3 = A/Sing MAP, 15 ug (FA); B4 = A/Sing MAP, 10 ug (FA); B5 = A/Sing MAP, 5 ug (FA); B6 = A/Sing MAP, 2.5 ug (FA)
B7 = Placebo MAP (FA); B8 = A/Sing MAP, 15 ug (UA); B9 = A/Sing IM, 15 ug, as 2018 Afluria Quad (UA)

Listing 16.2.9.3
Numeric Pain Intensity per Site

Part B

Randomisation Number	Group	Visit	Time Point	Was Pain Assessment Performed?	Site Number	Treatment	Date of assessment	Time of Assessment	Score	Unscheduled Reason
B0032	Group B6	Day 1	2 hour	Yes	F1	uncoated	19-MAY-2018	13:48	0	
B0032	Group B6	Day 1	2 hour	Yes	F2	uncoated	19-MAY-2018	13:48	0	
B0032	Group B6	Day 1	2 hour	Yes	F3	2.5 ug	19-MAY-2018	13:48	0	
B0032	Group B6	Day 2		Yes	F1	uncoated	20-MAY-2018	11:35	0	
B0032	Group B6	Day 2		Yes	F2	uncoated	20-MAY-2018	11:35	0	
B0032	Group B6	Day 2		Yes	F3	2.5 ug	20-MAY-2018	11:35	0	
B0032	Group B6	Day 4		Yes	F1	uncoated	22-MAY-2018	08:13	0	
B0032	Group B6	Day 4		Yes	F2	uncoated	22-MAY-2018	08:13	0	
B0032	Group B6	Day 4		Yes	F3	2.5 ug	22-MAY-2018	08:13	0	
B0032	Group B6	Day 8		Yes	F1	uncoated	26-MAY-2018	08:38	0	
B0032	Group B6	Day 8		Yes	F2	uncoated	26-MAY-2018	08:38	0	
B0032	Group B6	Day 8		Yes	F3	2.5 ug	26-MAY-2018	08:38	0	
B0032	Group B6	Day 22		Yes	F1	uncoated	12-JUN-2018	08:23	0	
B0032	Group B6	Day 22		Yes	F2	uncoated	12-JUN-2018	08:23	0	
B0032	Group B6	Day 22		Yes	F3	2.5 ug	12-JUN-2018	08:23	0	
B0032	Group B6	EOS/ET (Day 61)		Yes	F1	uncoated	18-JUL-2018	08:14	0	
B0032	Group B6	EOS/ET (Day 61)		Yes	F2	uncoated	18-JUL-2018	08:14	0	
B0032	Group B6	EOS/ET (Day 61)		Yes	F3	2.5 ug	18-JUL-2018	08:14	0	
B0033	Group B5	Day 1	1 min	Yes	F1	uncoated	19-MAY-2018	12:01	4	
B0033	Group B5	Day 1	1 min	Yes	F2	uncoated	19-MAY-2018	12:04	3	
B0033	Group B5	Day 1	1 min	Yes	F3	5 ug	19-MAY-2018	12:07	2	

Scale: 0= No Pain; 10= Worst Pain Possible
Administration Site Key: FA = Forearm; UA= Upper arm
Treatments Part A: A1 = A/Sing MAP, 15 ug (FA); A2 = Afluria Quad IM (UA), 15 ug; A3 = Placebo MAP (FA); A4 = A/Sing Mono IM, 15 ug (UA)
Treatments Part B: B1 = A/Sing MAP, 15 ug (FA) with biopsy; B2 = Placebo MAP (FA) with biopsy
B3 = A/Sing MAP, 15 ug (FA); B4 = A/Sing MAP, 10 ug (FA); B5 = A/Sing MAP, 5 ug (FA); B6 = A/Sing MAP, 2.5 ug (FA)
B7 = Placebo MAP (FA); B8 = A/Sing MAP, 15 ug (UA); B9 = A/Sing IM, 15 ug, as 2018 Afluria Quad (UA)

Listing 16.2.9.3
Numeric Pain Intensity per Site

Part B

Randomisation Number	Group	Visit	Time Point	Was Pain Assessment Performed?	Site Number	Treatment	Date of assessment	Time of Assessment	Score	Unscheduled Reason
B0033	Group B5	Day 1	10 min	Yes	F1	uncoated	19-MAY-2018	12:18	2	
B0033	Group B5	Day 1	10 min	Yes	F2	uncoated	19-MAY-2018	12:18	3	
B0033	Group B5	Day 1	10 min	Yes	F3	5 ug	19-MAY-2018	12:18	3	
B0033	Group B5	Day 1	1 hour	Yes	F1	uncoated	19-MAY-2018	13:08	1	
B0033	Group B5	Day 1	1 hour	Yes	F2	uncoated	19-MAY-2018	13:08	1	
B0033	Group B5	Day 1	1 hour	Yes	F3	5 ug	19-MAY-2018	13:08	1	
B0033	Group B5	Day 1	2 hour	Yes	F1	uncoated	19-MAY-2018	14:08	1	
B0033	Group B5	Day 1	2 hour	Yes	F2	uncoated	19-MAY-2018	14:08	1	
B0033	Group B5	Day 1	2 hour	Yes	F3	5 ug	19-MAY-2018	14:08	1	
B0033	Group B5	Day 2		Yes	F1	uncoated	20-MAY-2018	11:44	0	
B0033	Group B5	Day 2		Yes	F2	uncoated	20-MAY-2018	11:44	0	
B0033	Group B5	Day 2		Yes	F3	5 ug	20-MAY-2018	11:44	0	
B0033	Group B5	Day 4		Yes	F1	uncoated	22-MAY-2018	12:14	0	
B0033	Group B5	Day 4		Yes	F2	uncoated	22-MAY-2018	12:14	0	
B0033	Group B5	Day 4		Yes	F3	5 ug	22-MAY-2018	12:14	0	
B0033	Group B5	Day 8		Yes	F1	uncoated	26-MAY-2018	11:12	0	
B0033	Group B5	Day 8		Yes	F2	uncoated	26-MAY-2018	11:12	0	
B0033	Group B5	Day 8		Yes	F3	5 ug	26-MAY-2018	11:12	0	
B0033	Group B5	Day 22		Yes	F1	uncoated	09-JUN-2018	14:00	0	
B0033	Group B5	Day 22		Yes	F2	uncoated	09-JUN-2018	14:00	0	
B0033	Group B5	Day 22		Yes	F3	5 ug	09-JUN-2018	14:00		

Scale: 0= No Pain; 10= Worst Pain Possible
Administration Site Key: FA = Forearm; UA= Upper arm
Treatments Part A: A1 = A/Sing MAP, 15 ug (FA); A2 = Afluria Quad IM (UA), 15 ug; A3 = Placebo MAP (FA); A4 = A/Sing Mono IM, 15 ug (UA)
Treatments Part B: B1 = A/Sing MAP, 15 ug (FA) with biopsy; B2 = Placebo MAP (FA) with biopsy
B3 = A/Sing MAP, 15 ug (FA); B4 = A/Sing MAP, 10 ug (FA); B5 = A/Sing MAP, 5 ug (FA); B6 = A/Sing MAP, 2.5 ug (FA)
B7 = Placebo MAP (FA); B8 = A/Sing MAP, 15 ug (UA); B9 = A/Sing IM, 15 ug, as 2018 Afluria Quad (UA)

Listing 16.2.9.3
Numeric Pain Intensity per Site

Part B

Randomisation Number	Group	Visit	Time Point	Was Pain Assessment Performed?	Site Number	Treatment	Date of assessment	Time of Assessment	Score	Unscheduled Reason
B0033	Group B5	EOS/ET (Day 61)		Yes	F1	uncoated	18-JUL-2018	13:40	0	
B0033	Group B5	EOS/ET (Day 61)		Yes	F2	uncoated	18-JUL-2018	13:40	0	
B0033	Group B5	EOS/ET (Day 61)		Yes	F3	5 ug	18-JUL-2018	13:40	0	
B0034	Group B8	Day 1	1 min	Yes	D1	5 ug	19-MAY-2018	14:01	1	
B0034	Group B8	Day 1	1 min	Yes	D2	5 ug	19-MAY-2018	14:04	3	
B0034	Group B8	Day 1	1 min	Yes	D3	5 ug	19-MAY-2018	14:07	1	
B0034	Group B8	Day 1	10 min	Yes	D1	5 ug	19-MAY-2018	14:18	0	
B0034	Group B8	Day 1	10 min	Yes	D2	5 ug	19-MAY-2018	14:18	1	
B0034	Group B8	Day 1	10 min	Yes	D3	5 ug	19-MAY-2018	14:18	0	
B0034	Group B8	Day 1	1 hour	Yes	D1	5 ug	19-MAY-2018	15:08	0	
B0034	Group B8	Day 1	1 hour	Yes	D2	5 ug	19-MAY-2018	15:08	1	
B0034	Group B8	Day 1	1 hour	Yes	D3	5 ug	19-MAY-2018	15:08	1	
B0034	Group B8	Day 1	2 hour	Yes	D1	5 ug	19-MAY-2018	16:08	0	
B0034	Group B8	Day 1	2 hour	Yes	D2	5 ug	19-MAY-2018	16:08	0	
B0034	Group B8	Day 1	2 hour	Yes	D3	5 ug	19-MAY-2018	16:08	0	
B0034	Group B8	Day 2		Yes	D1	5 ug	20-MAY-2018	13:52	0	
B0034	Group B8	Day 2		Yes	D2	5 ug	20-MAY-2018	13:52	0	
B0034	Group B8	Day 2		Yes	D3	5 ug	20-MAY-2018	13:52	0	
B0034	Group B8	Day 4		Yes	D1	5 ug	22-MAY-2018	14:53	0	
B0034	Group B8	Day 4		Yes	D2	5 ug	22-MAY-2018	14:53	0	
B0034	Group B8	Day 4		Yes	D3	5 ug	22-MAY-2018	14:53	0	

Scale: 0= No Pain; 10= Worst Pain Possible
Administration Site Key: FA = Forearm; UA= Upper arm
Treatments Part A: A1 = A/Sing MAP, 15 ug (FA); A2 = Afluria Quad IM (UA), 15 ug; A3 = Placebo MAP (FA); A4 = A/Sing Mono IM, 15 ug (UA)
Treatments Part B: B1 = A/Sing MAP, 15 ug (FA) with biopsy; B2 = Placebo MAP (FA) with biopsy
B3 = A/Sing MAP, 15 ug (FA); B4 = A/Sing MAP, 10 ug (FA); B5 = A/Sing MAP, 5 ug (FA); B6 = A/Sing MAP, 2.5 ug (FA)
B7 = Placebo MAP (FA); B8 = A/Sing MAP, 15 ug (UA); B9 = A/Sing IM, 15 ug, as 2018 Afluria Quad (UA)

Listing 16.2.9.3
Numeric Pain Intensity per Site

Part B

Randomisation Number	Group	Visit	Time Point	Was Pain Assessment Performed?	Site Number	Treatment	Date of assessment	Time of Assessment	Score	Unscheduled Reason
B0034	Group B8	Day 8		Yes	D1	5 ug	26-MAY-2018	11:35	0	
B0034	Group B8	Day 8		Yes	D2	5 ug	26-MAY-2018	11:36	0	
B0034	Group B8	Day 8		Yes	D3	5 ug	26-MAY-2018	11:37	0	
B0034	Group B8	Day 22		Yes	D1	5 ug	09-JUN-2018	12:48	0	
B0034	Group B8	Day 22		Yes	D2	5 ug	09-JUN-2018	12:48	0	
B0034	Group B8	Day 22		Yes	D3	5 ug	09-JUN-2018	12:48	0	
B0034	Group B8	EOS/ET (Day 61)		Yes	D1	5 ug	18-JUL-2018	10:54	0	
B0034	Group B8	EOS/ET (Day 61)		Yes	D2	5 ug	18-JUL-2018	10:54	0	
B0034	Group B8	EOS/ET (Day 61)		Yes	D3	5 ug	18-JUL-2018	10:54	0	
B0035	Group B9	Day 1	1 min	Yes	D1	[IM]	19-MAY-2018	11:21	0	
B0035	Group B9	Day 1	10 min	Yes	D1	[IM]	19-MAY-2018	11:30	0	
B0035	Group B9	Day 1	1 hour	Yes	D1	[IM]	19-MAY-2018	12:15	0	
B0035	Group B9	Day 1	2 hour	Yes	D1	[IM]	19-MAY-2018	13:25	0	
B0035	Group B9	Day 2		Yes	D1	[IM]	20-MAY-2018	11:03	0	
B0035	Group B9	Day 4		Yes	D1	[IM]	22-MAY-2018	12:46	0	
B0035	Group B9	Day 8		Yes	D1	[IM]	26-MAY-2018	13:12	0	
B0035	Group B9	Day 22		Yes	D1	[IM]	09-JUN-2018	13:52	0	
B0035	Group B9	EOS/ET (Day 61)		Yes	D1	[IM]	18-JUL-2018	08:18	0	
B0036	Group B7	Day 1	1 min	Yes	F1	uncoated	19-MAY-2018	12:13	0	
B0036	Group B7	Day 1	1 min	Yes	F2	uncoated	19-MAY-2018	12:16	0	

Scale: 0= No Pain; 10= Worst Pain Possible
Administration Site Key: FA = Forearm; UA= Upper arm
Treatments Part A: A1 = A/Sing MAP, 15 ug (FA); A2 = Afluria Quad IM (UA), 15 ug; A3 = Placebo MAP (FA); A4 = A/Sing Mono IM, 15 ug (UA)
Treatments Part B: B1 = A/Sing MAP, 15 ug (FA) with biopsy; B2 = Placebo MAP (FA) with biopsy
B3 = A/Sing MAP, 15 ug (FA); B4 = A/Sing MAP, 10 ug (FA); B5 = A/Sing MAP, 5 ug (FA); B6 = A/Sing MAP, 2.5 ug (FA)
B7 = Placebo MAP (FA); B8 = A/Sing MAP, 15 ug (UA); B9 = A/Sing IM, 15 ug, as 2018 Afluria Quad (UA)

Listing 16.2.9.3
Numeric Pain Intensity per Site

Part B

Randomisation Number	Group	Visit	Time Point	Was Pain Assessment Performed?	Site Number	Treatment	Date of assessment	Time of Assessment	Score	Unscheduled Reason
B0036	Group B7	Day 1	1 min	Yes	F3	uncoated	19-MAY-2018	12:19	1	
B0036	Group B7	Day 1	10 min	Yes	F1	uncoated	19-MAY-2018	12:30	1	
B0036	Group B7	Day 1	10 min	Yes	F2	uncoated	19-MAY-2018	12:30	1	
B0036	Group B7	Day 1	10 min	Yes	F3	uncoated	19-MAY-2018	12:30	1	
B0036	Group B7	Day 1	1 hour	Yes	F1	uncoated	19-MAY-2018	13:20	0	
B0036	Group B7	Day 1	1 hour	Yes	F2	uncoated	19-MAY-2018		0	
B0036	Group B7	Day 1	1 hour	Yes	F3	uncoated	19-MAY-2018		0	
B0036	Group B7	Day 1	2 hour	Yes	F1	uncoated	19-MAY-2018	14:20	0	
B0036	Group B7	Day 1	2 hour	Yes	F2	uncoated	19-MAY-2018	14:20	0	
B0036	Group B7	Day 1	2 hour	Yes	F3	uncoated	19-MAY-2018	14:20	0	
B0036	Group B7	Day 2		Yes	F1	uncoated	20-MAY-2018	11:39	0	
B0036	Group B7	Day 2		Yes	F2	uncoated	20-MAY-2018	11:39	0	
B0036	Group B7	Day 2		Yes	F3	uncoated	20-MAY-2018	11:39	0	
B0036	Group B7	Day 4		Yes	F1	uncoated	22-MAY-2018	12:38	0	
B0036	Group B7	Day 4		Yes	F2	uncoated	22-MAY-2018	12:38	0	
B0036	Group B7	Day 4		Yes	F3	uncoated	22-MAY-2018	12:38	0	
B0036	Group B7	Day 8		Yes	F1	uncoated	26-MAY-2018	10:52	0	
B0036	Group B7	Day 8		Yes	F2	uncoated	26-MAY-2018	10:53	0	
B0036	Group B7	Day 8		Yes	F3	uncoated	26-MAY-2018	10:54	0	
B0036	Group B7	Day 22		Yes	F1	uncoated	09-JUN-2018	11:39	0	
B0036	Group B7	Day 22		Yes	F2	uncoated	09-JUN-2018	11:39	0	

Scale: 0= No Pain; 10= Worst Pain Possible
Administration Site Key: FA = Forearm; UA= Upper arm
Treatments Part A: A1 = A/Sing MAP, 15 ug (FA); A2 = Afluria Quad IM (UA), 15 ug; A3 = Placebo MAP (FA); A4 = A/Sing Mono IM, 15 ug (UA)
Treatments Part B: B1 = A/Sing MAP, 15 ug (FA) with biopsy; B2 = Placebo MAP (FA) with biopsy
B3 = A/Sing MAP, 15 ug (FA); B4 = A/Sing MAP, 10 ug (FA); B5 = A/Sing MAP, 5 ug (FA); B6 = A/Sing MAP, 2.5 ug (FA)
B7 = Placebo MAP (FA); B8 = A/Sing MAP, 15 ug (UA); B9 = A/Sing IM, 15 ug, as 2018 Afluria Quad (UA)

Listing 16.2.9.3
Numeric Pain Intensity per Site

Part B

Randomisation Number	Group	Visit	Time Point	Was Pain Assessment Performed?	Site Number	Treatment	Date of assessment	Time of Assessment	Score	Unscheduled Reason
B0036	Group B7	Day 22		Yes	F3	uncoated	09-JUN-2018	11:39	0	
B0036	Group B7	EOS/ET (Day 61)		Yes	F1	uncoated	18-JUL-2018	10:22	0	
B0036	Group B7	EOS/ET (Day 61)		Yes	F2	uncoated	18-JUL-2018	10:22	0	
B0036	Group B7	EOS/ET (Day 61)		Yes	F3	uncoated	18-JUL-2018	10:22	0	
B0037	Group B4	Day 1	1 min	Yes	F1	uncoated	19-MAY-2018	12:21	0	
B0037	Group B4	Day 1	1 min	Yes	F2	5 ug	19-MAY-2018	12:24	0	
B0037	Group B4	Day 1	1 min	Yes	F3	5 ug	19-MAY-2018	12:27	0	
B0037	Group B4	Day 1	10 min	Yes	F1	uncoated	19-MAY-2018	12:38	0	
B0037	Group B4	Day 1	10 min	Yes	F2	5 ug	19-MAY-2018	12:38	0	
B0037	Group B4	Day 1	10 min	Yes	F3	5 ug	19-MAY-2018	12:38	0	
B0037	Group B4	Day 1	1 hour	Yes	F1	uncoated	19-MAY-2018	13:31	0	
B0037	Group B4	Day 1	1 hour	Yes	F2	5 ug	19-MAY-2018	13:31	0	
B0037	Group B4	Day 1	1 hour	Yes	F3	5 ug	19-MAY-2018	13:31	0	
B0037	Group B4	Day 1	2 hour	Yes	F1	uncoated	19-MAY-2018	14:28	0	
B0037	Group B4	Day 1	2 hour	Yes	F2	5 ug	19-MAY-2018	14:28	0	
B0037	Group B4	Day 1	2 hour	Yes	F3	5 ug	19-MAY-2018	14:28	0	
B0037	Group B4	Day 2		Yes	F1	uncoated	20-MAY-2018	11:58	0	
B0037	Group B4	Day 2		Yes	F2	5 ug	20-MAY-2018	11:58	0	
B0037	Group B4	Day 2		Yes	F3	5 ug	20-MAY-2018	11:58	0	
B0037	Group B4	Day 4		Yes	F1	uncoated	22-MAY-2018	12:11	0	
B0037	Group B4	Day 4		Yes	F2	5 ug	22-MAY-2018	12:11	0	

Scale: 0= No Pain; 10= Worst Pain Possible
Administration Site Key: FA = Forearm; UA= Upper arm
Treatments Part A: A1 = A/Sing MAP, 15 ug (FA); A2 = Afluria Quad IM (UA), 15 ug; A3 = Placebo MAP (FA); A4 = A/Sing Mono IM, 15 ug (UA)
Treatments Part B: B1 = A/Sing MAP, 15 ug (FA) with biopsy; B2 = Placebo MAP (FA) with biopsy
B3 = A/Sing MAP, 15 ug (FA); B4 = A/Sing MAP, 10 ug (FA); B5 = A/Sing MAP, 5 ug (FA); B6 = A/Sing MAP, 2.5 ug (FA)
B7 = Placebo MAP (FA); B8 = A/Sing MAP, 15 ug (UA); B9 = A/Sing IM, 15 ug, as 2018 Afluria Quad (UA)

Listing 16.2.9.3
Numeric Pain Intensity per Site

Part B

Randomisation Number	Group	Visit	Time Point	Was Pain Assessment Performed?	Site Number	Treatment	Date of assessment	Time of Assessment	Score	Unscheduled Reason
B0037	Group B4	Day 4		Yes	F3	5 ug	22-MAY-2018	12:11	0	
B0037	Group B4	Day 8		Yes	F1	uncoated	26-MAY-2018	10:52	0	
B0037	Group B4	Day 8		Yes	F2	5 ug	26-MAY-2018	10:52	0	
B0037	Group B4	Day 8		Yes	F3	5 ug	26-MAY-2018	10:52	0	
B0037	Group B4	Day 22		Yes	F1	uncoated	09-JUN-2018	14:04	0	
B0037	Group B4	Day 22		Yes	F2	5 ug	09-JUN-2018	14:04	0	
B0037	Group B4	Day 22		Yes	F3	5 ug	09-JUN-2018	14:04	0	
B0037	Group B4	EOS/ET (Day 61)		Yes	F1	uncoated	18-JUL-2018	15:17	0	
B0037	Group B4	EOS/ET (Day 61)		Yes	F2	5 ug	18-JUL-2018	15:17	0	
B0037	Group B4	EOS/ET (Day 61)		Yes	F3	5 ug	18-JUL-2018	15:17	0	
B0038	Group B3	Day 1	1 min	Yes	F1	5 ug	19-MAY-2018	12:34	1	
B0038	Group B3	Day 1	1 min	Yes	F2	5 ug	19-MAY-2018	12:37	1	
B0038	Group B3	Day 1	1 min	Yes	F3	5 ug	19-MAY-2018	12:40	2	
B0038	Group B3	Day 1	10 min	Yes	F1	5 ug	19-MAY-2018	12:51	1	
B0038	Group B3	Day 1	10 min	Yes	F2	5 ug	19-MAY-2018	12:52	1	
B0038	Group B3	Day 1	10 min	Yes	F3	5 ug	19-MAY-2018	12:54	2	
B0038	Group B3	Day 1	1 hour	Yes	F1	5 ug	19-MAY-2018	13:45	0	
B0038	Group B3	Day 1	1 hour	Yes	F2	5 ug	19-MAY-2018	13:45	0	
B0038	Group B3	Day 1	1 hour	Yes	F3	5 ug	19-MAY-2018	13:45	0	
B0038	Group B3	Day 1	2 hour	Yes	F1	5 ug	19-MAY-2018	14:41	0	
B0038	Group B3	Day 1	2 hour	Yes	F2	5 ug	19-MAY-2018	14:41	0	

Scale: 0= No Pain; 10= Worst Pain Possible
Administration Site Key: FA = Forearm; UA= Upper arm
Treatments Part A: A1 = A/Sing MAP, 15 ug (FA); A2 = Afluria Quad IM (UA), 15 ug; A3 = Placebo MAP (FA); A4 = A/Sing Mono IM, 15 ug (UA)
Treatments Part B: B1 = A/Sing MAP, 15 ug (FA) with biopsy; B2 = Placebo MAP (FA) with biopsy
B3 = A/Sing MAP, 15 ug (FA); B4 = A/Sing MAP, 10 ug (FA); B5 = A/Sing MAP, 5 ug (FA); B6 = A/Sing MAP, 2.5 ug (FA)
B7 = Placebo MAP (FA); B8 = A/Sing MAP, 15 ug (UA); B9 = A/Sing IM, 15 ug, as 2018 Afluria Quad (UA)

Listing 16.2.9.3
Numeric Pain Intensity per Site

Part B

Randomisation Number	Group	Visit	Time Point	Was Pain Assessment Performed?	Site Number	Treatment	Date of assessment	Time of Assessment	Score	Unscheduled Reason
B0038	Group B3	Day 1	2 hour	Yes	F3	5 ug	19-MAY-2018	14:41	0	
B0038	Group B3	Day 2		Yes	F1	5 ug	20-MAY-2018	08:21	0	
B0038	Group B3	Day 2		Yes	F2	5 ug	20-MAY-2018	08:21	0	
B0038	Group B3	Day 2		Yes	F3	5 ug	20-MAY-2018	08:21	0	
B0038	Group B3	Day 4		Yes	F1	5 ug	22-MAY-2018	09:02	0	
B0038	Group B3	Day 4		Yes	F2	5 ug	22-MAY-2018	09:02	0	
B0038	Group B3	Day 4		Yes	F3	5 ug	22-MAY-2018	09:02	0	
B0038	Group B3	Day 8		Yes	F1	5 ug	26-MAY-2018	09:22	0	
B0038	Group B3	Day 8		Yes	F2	5 ug	26-MAY-2018	09:22	0	
B0038	Group B3	Day 8		Yes	F3	5 ug	26-MAY-2018	09:23	0	
B0038	Group B3	Day 22		Yes	F1	5 ug	09-JUN-2018	08:22	0	
B0038	Group B3	Day 22		Yes	F2	5 ug	09-JUN-2018	08:22	0	
B0038	Group B3	Day 22		Yes	F3	5 ug	09-JUN-2018	08:22	0	
B0038	Group B3	EOS/ET (Day 61)		Yes	F1	5 ug	21-JUL-2018	09:15	0	
B0038	Group B3	EOS/ET (Day 61)		Yes	F2	5 ug	21-JUL-2018	09:15	0	
B0038	Group B3	EOS/ET (Day 61)		Yes	F3	5 ug	21-JUL-2018	09:15	0	
B0039	Group B7	Day 1	1 min	Yes	F1	uncoated	19-MAY-2018	13:51	2	
B0039	Group B7	Day 1	1 min	Yes	F2	uncoated	19-MAY-2018	13:54	1	
B0039	Group B7	Day 1	1 min	Yes	F3	uncoated	19-MAY-2018	13:57	2	
B0039	Group B7	Day 1	10 min	Yes	F1	uncoated	19-MAY-2018	14:08	0	
B0039	Group B7	Day 1	10 min	Yes	F2	uncoated	19-MAY-2018	14:08	0	

Scale: 0= No Pain; 10= Worst Pain Possible
Administration Site Key: FA = Forearm; UA= Upper arm
Treatments Part A: A1 = A/Sing MAP, 15 ug (FA); A2 = Afluria Quad IM (UA), 15 ug; A3 = Placebo MAP (FA); A4 = A/Sing Mono IM, 15 ug (UA)
Treatments Part B: B1 = A/Sing MAP, 15 ug (FA) with biopsy; B2 = Placebo MAP (FA) with biopsy
B3 = A/Sing MAP, 15 ug (FA); B4 = A/Sing MAP, 10 ug (FA); B5 = A/Sing MAP, 5 ug (FA); B6 = A/Sing MAP, 2.5 ug (FA)
B7 = Placebo MAP (FA); B8 = A/Sing MAP, 15 ug (UA); B9 = A/Sing IM, 15 ug, as 2018 Afluria Quad (UA)

Listing 16.2.9.3
Numeric Pain Intensity per Site

Part B

Randomisation Number	Group	Visit	Time Point	Was Pain Assessment Performed?	Site Number	Treatment	Date of assessment	Time of Assessment	Score	Unscheduled Reason
B0039	Group B7	Day 1	10 min	Yes	F3	uncoated	19-MAY-2018	14:08	1	
B0039	Group B7	Day 1	1 hour	Yes	F1	uncoated	19-MAY-2018	14:58	0	
B0039	Group B7	Day 1	1 hour	Yes	F2	uncoated	19-MAY-2018	14:58	0	
B0039	Group B7	Day 1	1 hour	Yes	F3	uncoated	19-MAY-2018	14:58	0	
B0039	Group B7	Day 1	2 hour	Yes	F1	uncoated	19-MAY-2018	15:58	0	
B0039	Group B7	Day 1	2 hour	Yes	F2	uncoated	19-MAY-2018	15:58	0	
B0039	Group B7	Day 1	2 hour	Yes	F3	uncoated	19-MAY-2018	15:58	0	
B0039	Group B7	Day 2		Yes	F1	uncoated	20-MAY-2018	14:36	0	
B0039	Group B7	Day 2		Yes	F2	uncoated	20-MAY-2018	14:36	0	
B0039	Group B7	Day 2		Yes	F3	uncoated	20-MAY-2018	14:36	0	
B0039	Group B7	Day 4		Yes	F1	uncoated	22-MAY-2018	12:25	0	
B0039	Group B7	Day 4		Yes	F2	uncoated	22-MAY-2018	12:25	0	
B0039	Group B7	Day 4		Yes	F3	uncoated	22-MAY-2018	12:25	0	
B0039	Group B7	Day 8		Yes	F1	uncoated	26-MAY-2018	15:05	0	
B0039	Group B7	Day 8		Yes	F2	uncoated	26-MAY-2018	15:05	0	
B0039	Group B7	Day 8		Yes	F3	uncoated	26-MAY-2018	15:05	0	
B0039	Group B7	Day 22		Yes	F1	uncoated	09-JUN-2018	14:01	0	
B0039	Group B7	Day 22		Yes	F2	uncoated	09-JUN-2018	14:01	0	
B0039	Group B7	Day 22		Yes	F3	uncoated	09-JUN-2018	14:01	0	
B0039	Group B7	EOS/ET (Day 61)		Yes	F1	uncoated	18-JUL-2018	16:57	0	
B0039	Group B7	EOS/ET (Day 61)		Yes	F2	uncoated	18-JUL-2018	16:58	0	

Scale: 0= No Pain; 10= Worst Pain Possible
Administration Site Key: FA = Forearm; UA= Upper arm
Treatments Part A: A1 = A/Sing MAP, 15 ug (FA); A2 = Afluria Quad IM (UA), 15 ug; A3 = Placebo MAP (FA); A4 = A/Sing Mono IM, 15 ug (UA)
Treatments Part B: B1 = A/Sing MAP, 15 ug (FA) with biopsy; B2 = Placebo MAP (FA) with biopsy
B3 = A/Sing MAP, 15 ug (FA); B4 = A/Sing MAP, 10 ug (FA); B5 = A/Sing MAP, 5 ug (FA); B6 = A/Sing MAP, 2.5 ug (FA)
B7 = Placebo MAP (FA); B8 = A/Sing MAP, 15 ug (UA); B9 = A/Sing IM, 15 ug, as 2018 Afluria Quad (UA)

Listing 16.2.9.3
Numeric Pain Intensity per Site

Part B

Randomisation Number	Group	Visit	Time Point	Was Pain Assessment Performed?	Site Number	Treatment	Date of assessment	Time of Assessment	Score	Unscheduled Reason
B0039	Group B7	EOS/ET (Day 61)		Yes	F3	uncoated	18-JUL-2018	16:59	0	
B0040	Group B9	Day 1	1 min	Yes	D1	[IM]	19-MAY-2018	11:11	0	
B0040	Group B9	Day 1	10 min	Yes	D1	[IM]	19-MAY-2018	11:21	0	
B0040	Group B9	Day 1	1 hour	Yes	D1	[IM]	19-MAY-2018	12:12	0	
B0040	Group B9	Day 1	2 hour	Yes	D1	[IM]	19-MAY-2018	13:35	0	
B0040	Group B9	Day 2		Yes	D1	[IM]	20-MAY-2018	11:05	0	
B0040	Group B9	Day 4		Yes	D1	[IM]	22-MAY-2018	08:01	0	
B0040	Group B9	Day 8		Yes	D1	[IM]	26-MAY-2018	12:29	0	
B0040	Group B9	Day 22		Yes	D1	[IM]	09-JUN-2018	11:39	0	
B0040	Group B9	EOS/ET (Day 61)		Yes	D1	[IM]	18-JUL-2018	08:43	0	
B0041	Group B3	Day 1	1 min	Yes	F1	5 ug	19-MAY-2018	12:46	0	
B0041	Group B3	Day 1	1 min	Yes	F2	5 ug	19-MAY-2018	12:49	2	
B0041	Group B3	Day 1	1 min	Yes	F3	5 ug	19-MAY-2018	12:52	1	
B0041	Group B3	Day 1	10 min	Yes	F1	5 ug	19-MAY-2018	13:12	0	
B0041	Group B3	Day 1	10 min	Yes	F2	5 ug	19-MAY-2018	13:12	1	
B0041	Group B3	Day 1	10 min	Yes	F3	5 ug	19-MAY-2018	13:12	0	
B0041	Group B3	Day 1	1 hour	Yes	F1	5 ug	19-MAY-2018		0	
B0041	Group B3	Day 1	1 hour	Yes	F2	5 ug	19-MAY-2018		0	
B0041	Group B3	Day 1	1 hour	Yes	F3	5 ug	19-MAY-2018		0	
B0041	Group B3	Day 1	2 hour	Yes	F1	5 ug	19-MAY-2018	14:53	0	

Scale: 0= No Pain; 10= Worst Pain Possible
Administration Site Key: FA = Forearm; UA= Upper arm
Treatments Part A: A1 = A/Sing MAP, 15 ug (FA); A2 = Afluria Quad IM (UA), 15 ug; A3 = Placebo MAP (FA); A4 = A/Sing Mono IM, 15 ug (UA)
Treatments Part B: B1 = A/Sing MAP, 15 ug (FA) with biopsy; B2 = Placebo MAP (FA) with biopsy
B3 = A/Sing MAP, 15 ug (FA); B4 = A/Sing MAP, 10 ug (FA); B5 = A/Sing MAP, 5 ug (FA); B6 = A/Sing MAP, 2.5 ug (FA)
B7 = Placebo MAP (FA); B8 = A/Sing MAP, 15 ug (UA); B9 = A/Sing IM, 15 ug, as 2018 Afluria Quad (UA)

Listing 16.2.9.3
Numeric Pain Intensity per Site

Part B

Randomisation Number	Group	Visit	Time Point	Was Pain Assessment Performed?	Site Number	Treatment	Date of assessment	Time of Assessment	Score	Unscheduled Reason
B0041	Group B3	Day 1	2 hour	Yes	F2	5 ug	19-MAY-2018	14:53	0	
B0041	Group B3	Day 1	2 hour	Yes	F3	5 ug	19-MAY-2018	14:53	0	
B0041	Group B3	Day 2		Yes	F1	5 ug	20-MAY-2018	13:15	1	
B0041	Group B3	Day 2		Yes	F2	5 ug	20-MAY-2018	13:15	1	
B0041	Group B3	Day 2		Yes	F3	5 ug	20-MAY-2018	13:15	1	
B0041	Group B3	Day 4		Yes	F1	5 ug	22-MAY-2018	15:28	0	
B0041	Group B3	Day 4		Yes	F2	5 ug	22-MAY-2018	15:28	0	
B0041	Group B3	Day 4		Yes	F3	5 ug	22-MAY-2018	15:28	0	
B0041	Group B3	Day 8		Yes	F1	5 ug	26-MAY-2018	12:35	0	
B0041	Group B3	Day 8		Yes	F2	5 ug	26-MAY-2018	12:35	0	
B0041	Group B3	Day 8		Yes	F3	5 ug	26-MAY-2018	12:36	0	
B0041	Group B3	Day 22		Yes	F1	5 ug	09-JUN-2018	15:04	0	
B0041	Group B3	Day 22		Yes	F2	5 ug	09-JUN-2018	15:04	0	
B0041	Group B3	Day 22		Yes	F3	5 ug	09-JUN-2018	15:04	0	
B0041	Group B3	EOS/ET (Day 61)		Yes	F1	5 ug	20-JUL-2018	15:24	0	
B0041	Group B3	EOS/ET (Day 61)		Yes	F2	5 ug	20-JUL-2018	15:24	0	
B0041	Group B3	EOS/ET (Day 61)		Yes	F3	5 ug	20-JUL-2018	15:24	0	
B0042	Group B5	Day 1	1 min	Yes	F1	uncoated	19-MAY-2018	12:51	1	
B0042	Group B5	Day 1	1 min	Yes	F2	uncoated	19-MAY-2018	12:54	3	
B0042	Group B5	Day 1	1 min	Yes	F3	5 ug	19-MAY-2018	12:57	3	
B0042	Group B5	Day 1	10 min	Yes	F1	uncoated	19-MAY-2018	13:13	0	

Scale: 0= No Pain; 10= Worst Pain Possible
Administration Site Key: FA = Forearm; UA= Upper arm
Treatments Part A: A1 = A/Sing MAP, 15 ug (FA); A2 = Afluria Quad IM (UA), 15 ug; A3 = Placebo MAP (FA); A4 = A/Sing Mono IM, 15 ug (UA)
Treatments Part B: B1 = A/Sing MAP, 15 ug (FA) with biopsy; B2 = Placebo MAP (FA) with biopsy
B3 = A/Sing MAP, 15 ug (FA); B4 = A/Sing MAP, 10 ug (FA); B5 = A/Sing MAP, 5 ug (FA); B6 = A/Sing MAP, 2.5 ug (FA)
B7 = Placebo MAP (FA); B8 = A/Sing MAP, 15 ug (UA); B9 = A/Sing IM, 15 ug, as 2018 Afluria Quad (UA)

Listing 16.2.9.3
Numeric Pain Intensity per Site

Part B

Randomisation Number	Group	Visit	Time Point	Was Pain Assessment Performed?	Site Number	Treatment	Date of assessment	Time of Assessment	Score	Unscheduled Reason
B0042	Group B5	Day 1	10 min	Yes	F2	uncoated	19-MAY-2018	13:13	1	
B0042	Group B5	Day 1	10 min	Yes	F3	5 ug	19-MAY-2018	13:13	0	
B0042	Group B5	Day 1	1 hour	Yes	F1	uncoated	19-MAY-2018	13:56	0	
B0042	Group B5	Day 1	1 hour	No	F2	uncoated				
B0042	Group B5	Day 1	1 hour	No	F3	5 ug				
B0042	Group B5	Day 1	2 hour	Yes	F1	uncoated	19-MAY-2018	14:58	0	
B0042	Group B5	Day 1	2 hour	Yes	F2	uncoated	19-MAY-2018	14:58	0	
B0042	Group B5	Day 1	2 hour	Yes	F3	5 ug	19-MAY-2018	14:58	0	
B0042	Group B5	Day 2		Yes	F1	uncoated	20-MAY-2018	12:16	0	
B0042	Group B5	Day 2		Yes	F2	uncoated	20-MAY-2018	12:16	0	
B0042	Group B5	Day 2		Yes	F3	5 ug	20-MAY-2018	12:16	0	
B0042	Group B5	Day 4		Yes	F1	uncoated	22-MAY-2018	10:02	0	
B0042	Group B5	Day 4		Yes	F2	uncoated	22-MAY-2018	10:02	0	
B0042	Group B5	Day 4		Yes	F3	5 ug	22-MAY-2018	10:02		
B0042	Group B5	Day 8		Yes	F1	uncoated	26-MAY-2018	11:40	0	
B0042	Group B5	Day 8		Yes	F2	uncoated	26-MAY-2018	11:40	0	
B0042	Group B5	Day 8		Yes	F3	5 ug	26-MAY-2018	11:40	0	
B0042	Group B5	Day 22		Yes	F1	uncoated	09-JUN-2018	13:36	0	
B0042	Group B5	Day 22		Yes	F2	uncoated	09-JUN-2018	13:36	0	
B0042	Group B5	Day 22		Yes	F3	5 ug	09-JUN-2018	13:36	0	
B0042	Group B5	EOS/ET (Day 61)		Yes	F1	uncoated	18-JUL-2018	09:30	0	

Scale: 0= No Pain; 10= Worst Pain Possible
Administration Site Key: FA = Forearm; UA= Upper arm
Treatments Part A: A1 = A/Sing MAP, 15 ug (FA); A2 = Afluria Quad IM (UA), 15 ug; A3 = Placebo MAP (FA); A4 = A/Sing Mono IM, 15 ug (UA)
Treatments Part B: B1 = A/Sing MAP, 15 ug (FA) with biopsy; B2 = Placebo MAP (FA) with biopsy
B3 = A/Sing MAP, 15 ug (FA); B4 = A/Sing MAP, 10 ug (FA); B5 = A/Sing MAP, 5 ug (FA); B6 = A/Sing MAP, 2.5 ug (FA)
B7 = Placebo MAP (FA); B8 = A/Sing MAP, 15 ug (UA); B9 = A/Sing IM, 15 ug, as 2018 Afluria Quad (UA)

Listing 16.2.9.3
Numeric Pain Intensity per Site

Part B

Randomisation Number	Group	Visit	Time Point	Was Pain Assessment Performed?	Site Number	Treatment	Date of assessment	Time of Assessment	Score	Unscheduled Reason
B0042	Group B5	EOS/ET (Day 61)		Yes	F2	uncoated	18-JUL-2018	09:30	0	
B0042	Group B5	EOS/ET (Day 61)		Yes	F3	5 ug	18-JUL-2018	09:30	0	
B0043	Group B8	Day 1	1 min	Yes	D1	5 ug	19-MAY-2018	13:01	2	
B0043	Group B8	Day 1	1 min	Yes	D2	5 ug	19-MAY-2018	13:04	3	
B0043	Group B8	Day 1	1 min	Yes	D3	5 ug	19-MAY-2018	13:07	2	
B0043	Group B8	Day 1	10 min	Yes	D1	5 ug	19-MAY-2018	13:18	0	
B0043	Group B8	Day 1	10 min	Yes	D2	5 ug	19-MAY-2018	13:18	3	
B0043	Group B8	Day 1	10 min	Yes	D3	5 ug	19-MAY-2018	13:18	0	
B0043	Group B8	Day 1	1 hour	Yes	D1	5 ug	19-MAY-2018	14:15	0	
B0043	Group B8	Day 1	1 hour	Yes	D2	5 ug	19-MAY-2018	14:15	0	
B0043	Group B8	Day 1	1 hour	No	D3	5 ug				
B0043	Group B8	Day 1	2 hour	Yes	D1	5 ug	19-MAY-2018	15:08	0	
B0043	Group B8	Day 1	2 hour	Yes	D2	5 ug	19-MAY-2018	15:08	0	
B0043	Group B8	Day 1	2 hour	Yes	D3	5 ug	19-MAY-2018	15:08	0	
B0043	Group B8	Day 2		Yes	D1	5 ug	20-MAY-2018	12:26	0	
B0043	Group B8	Day 2		Yes	D2	5 ug	20-MAY-2018	12:26	0	
B0043	Group B8	Day 2		Yes	D3	5 ug	20-MAY-2018	12:26	0	
B0043	Group B8	Day 4		Yes	D1	5 ug	22-MAY-2018	14:01	0	
B0043	Group B8	Day 4		Yes	D2	5 ug	22-MAY-2018	14:01	0	
B0043	Group B8	Day 4		Yes	D3	5 ug	22-MAY-2018	14:01	0	
B0043	Group B8	Day 8		Yes	D1	5 ug	26-MAY-2018	08:12	0	

Scale: 0= No Pain; 10= Worst Pain Possible
Administration Site Key: FA = Forearm; UA= Upper arm
Treatments Part A: A1 = A/Sing MAP, 15 ug (FA); A2 = Afluria Quad IM (UA), 15 ug; A3 = Placebo MAP (FA); A4 = A/Sing Mono IM, 15 ug (UA)
Treatments Part B: B1 = A/Sing MAP, 15 ug (FA) with biopsy; B2 = Placebo MAP (FA) with biopsy
B3 = A/Sing MAP, 15 ug (FA); B4 = A/Sing MAP, 10 ug (FA); B5 = A/Sing MAP, 5 ug (FA); B6 = A/Sing MAP, 2.5 ug (FA)
B7 = Placebo MAP (FA); B8 = A/Sing MAP, 15 ug (UA); B9 = A/Sing IM, 15 ug, as 2018 Afluria Quad (UA)

Listing 16.2.9.3
Numeric Pain Intensity per Site

Part B

Randomisation Number	Group	Visit	Time Point	Was Pain Assessment Performed?	Site Number	Treatment	Date of assessment	Time of Assessment	Score	Unscheduled Reason
B0043	Group B8	Day 8		Yes	D2	5 ug	26-MAY-2018	08:12	0	
B0043	Group B8	Day 8		Yes	D3	5 ug	26-MAY-2018	08:12	0	
B0043	Group B8	Day 22		Yes	D1	5 ug	09-JUN-2018	10:13	0	
B0043	Group B8	Day 22		Yes	D2	5 ug	09-JUN-2018	10:15	0	
B0043	Group B8	Day 22		Yes	D3	5 ug	09-JUN-2018	10:15	0	
B0043	Group B8	EOS/ET (Day 61)		Yes	D1	5 ug	18-JUL-2018	09:25	0	
B0043	Group B8	EOS/ET (Day 61)		Yes	D2	5 ug	18-JUL-2018	09:25	0	
B0043	Group B8	EOS/ET (Day 61)		Yes	D3	5 ug	18-JUL-2018	09:25	0	
B0044	Group B6	Day 1	1 min	Yes	F1	uncoated	19-MAY-2018	13:11	2	
B0044	Group B6	Day 1	1 min	Yes	F2	uncoated	19-MAY-2018	13:14	2	
B0044	Group B6	Day 1	1 min	Yes	F3	2.5 ug	19-MAY-2018	13:17	2	
B0044	Group B6	Day 1	10 min	Yes	F1	uncoated	19-MAY-2018	13:28	0	
B0044	Group B6	Day 1	10 min	Yes	F2	uncoated	19-MAY-2018	13:28	1	
B0044	Group B6	Day 1	10 min	Yes	F3	2.5 ug	19-MAY-2018	13:28	1	
B0044	Group B6	Day 1	1 hour	Yes	F1	uncoated	19-MAY-2018	14:18	1	
B0044	Group B6	Day 1	1 hour	Yes	F2	uncoated	19-MAY-2018	14:18	1	
B0044	Group B6	Day 1	1 hour	Yes	F3	2.5 ug	19-MAY-2018	14:18	1	
B0044	Group B6	Day 1	2 hour	Yes	F1	uncoated	19-MAY-2018	15:18	0	
B0044	Group B6	Day 1	2 hour	Yes	F2	uncoated	19-MAY-2018	15:18	0	
B0044	Group B6	Day 1	2 hour	Yes	F3	2.5 ug	19-MAY-2018	15:18	0	
B0044	Group B6	Day 2		Yes	F1	uncoated	20-MAY-2018	12:30	0	

Scale: 0= No Pain; 10= Worst Pain Possible
Administration Site Key: FA = Forearm; UA= Upper arm
Treatments Part A: A1 = A/Sing MAP, 15 ug (FA); A2 = Afluria Quad IM (UA), 15 ug; A3 = Placebo MAP (FA); A4 = A/Sing Mono IM, 15 ug (UA)
Treatments Part B: B1 = A/Sing MAP, 15 ug (FA) with biopsy; B2 = Placebo MAP (FA) with biopsy
B3 = A/Sing MAP, 15 ug (FA); B4 = A/Sing MAP, 10 ug (FA); B5 = A/Sing MAP, 5 ug (FA); B6 = A/Sing MAP, 2.5 ug (FA)
B7 = Placebo MAP (FA); B8 = A/Sing MAP, 15 ug (UA); B9 = A/Sing IM, 15 ug, as 2018 Afluria Quad (UA)

Listing 16.2.9.3
Numeric Pain Intensity per Site

Part B

Randomisation Number	Group	Visit	Time Point	Was Pain Assessment Performed?	Site Number	Treatment	Date of assessment	Time of Assessment	Score	Unscheduled Reason
B0044	Group B6	Day 2		Yes	F2	uncoated	20-MAY-2018	12:30	0	
B0044	Group B6	Day 2		Yes	F3	2.5 ug	20-MAY-2018	12:30	0	
B0044	Group B6	Day 4		Yes	F1	uncoated	22-MAY-2018	18:21	0	
B0044	Group B6	Day 4		Yes	F2	uncoated	22-MAY-2018	18:21	0	
B0044	Group B6	Day 4		Yes	F3	2.5 ug	22-MAY-2018	18:21	0	
B0044	Group B6	Day 8		Yes	F1	uncoated	26-MAY-2018	14:40	0	
B0044	Group B6	Day 8		Yes	F2	uncoated	26-MAY-2018	14:40	0	
B0044	Group B6	Day 8		Yes	F3	2.5 ug	26-MAY-2018	14:40	0	
B0044	Group B6	Day 22		Yes	F1	uncoated	09-JUN-2018	13:47	0	
B0044	Group B6	Day 22		Yes	F2	uncoated	09-JUN-2018	13:47	0	
B0044	Group B6	Day 22		Yes	F3	2.5 ug	09-JUN-2018	13:47	0	
B0044	Group B6	EOS/ET (Day 61)		Yes	F1	uncoated	18-JUL-2018	13:19	0	
B0044	Group B6	EOS/ET (Day 61)		Yes	F2	uncoated	18-JUL-2018	13:19	0	
B0044	Group B6	EOS/ET (Day 61)		Yes	F3	2.5 ug	18-JUL-2018	13:19	0	
B0045	Group B4	Day 1	1 min	Yes	F1	5 ug	19-MAY-2018	11:51	3	
B0045	Group B4	Day 1	1 min	Yes	F2	5 ug	19-MAY-2018	11:54	3	
B0045	Group B4	Day 1	1 min	Yes	F3	uncoated	19-MAY-2018	11:57	5	
B0045	Group B4	Day 1	10 min	Yes	F1	5 ug	19-MAY-2018	12:08	0	
B0045	Group B4	Day 1	10 min	Yes	F2	5 ug	19-MAY-2018	12:08	1	
B0045	Group B4	Day 1	10 min	Yes	F3	uncoated	19-MAY-2018	12:08	2	
B0045	Group B4	Day 1	1 hour	Yes	F1	5 ug	19-MAY-2018	12:48	0	

Scale: 0= No Pain; 10= Worst Pain Possible
Administration Site Key: FA = Forearm; UA= Upper arm
Treatments Part A: A1 = A/Sing MAP, 15 ug (FA); A2 = Afluria Quad IM (UA), 15 ug; A3 = Placebo MAP (FA); A4 = A/Sing Mono IM, 15 ug (UA)
Treatments Part B: B1 = A/Sing MAP, 15 ug (FA) with biopsy; B2 = Placebo MAP (FA) with biopsy
B3 = A/Sing MAP, 15 ug (FA); B4 = A/Sing MAP, 10 ug (FA); B5 = A/Sing MAP, 5 ug (FA); B6 = A/Sing MAP, 2.5 ug (FA)
B7 = Placebo MAP (FA); B8 = A/Sing MAP, 15 ug (UA); B9 = A/Sing IM, 15 ug, as 2018 Afluria Quad (UA)

Listing 16.2.9.3
Numeric Pain Intensity per Site

Part B

Randomisation Number	Group	Visit	Time Point	Was Pain Assessment Performed?	Site Number	Treatment	Date of assessment	Time of Assessment	Score	Unscheduled Reason
B0045	Group B4	Day 1	1 hour	Yes	F2	5 ug	19-MAY-2018	12:58	0	
B0045	Group B4	Day 1	1 hour	Yes	F3	uncoated	19-MAY-2018	12:58	0	
B0045	Group B4	Day 1	2 hour	Yes	F1	5 ug	19-MAY-2018	13:58	0	
B0045	Group B4	Day 1	2 hour	Yes	F2	5 ug	19-MAY-2018	13:58	0	
B0045	Group B4	Day 1	2 hour	Yes	F3	uncoated	19-MAY-2018	13:58	0	
B0045	Group B4	Day 2		Yes	F1	5 ug	20-MAY-2018	12:37	0	
B0045	Group B4	Day 2		Yes	F2	5 ug	20-MAY-2018	12:37	0	
B0045	Group B4	Day 2		Yes	F3	uncoated	20-MAY-2018	12:37	0	
B0045	Group B4	Day 4		Yes	F1	5 ug	22-MAY-2018	10:41	0	
B0045	Group B4	Day 4		Yes	F2	5 ug	22-MAY-2018	10:41	0	
B0045	Group B4	Day 4		Yes	F3	uncoated	22-MAY-2018	10:41	0	
B0045	Group B4	Day 8		Yes	F1	5 ug	26-MAY-2018	12:20	0	
B0045	Group B4	Day 8		Yes	F2	5 ug	26-MAY-2018	12:20	0	
B0045	Group B4	Day 8		Yes	F3	uncoated	26-MAY-2018	12:20	0	
B0045	Group B4	Day 22		Yes	F1	5 ug	09-JUN-2018	10:17	0	
B0045	Group B4	Day 22		Yes	F2	5 ug	09-JUN-2018	10:17	0	
B0045	Group B4	Day 22		Yes	F3	uncoated	09-JUN-2018	10:17	0	
B0045	Group B4	EOS/ET (Day 61)		Yes	F1	5 ug	18-JUL-2018	11:47	0	
B0045	Group B4	EOS/ET (Day 61)		Yes	F2	5 ug	18-JUL-2018	11:47	0	
B0045	Group B4	EOS/ET (Day 61)		Yes	F3	uncoated	18-JUL-2018	11:47	0	
B0046	Group B7	Day 1	1 min	Yes	F1	uncoated	19-MAY-2018	13:31	2	

Scale: 0= No Pain; 10= Worst Pain Possible
Administration Site Key: FA = Forearm; UA= Upper arm
Treatments Part A: A1 = A/Sing MAP, 15 ug (FA); A2 = Afluria Quad IM (UA), 15 ug; A3 = Placebo MAP (FA); A4 = A/Sing Mono IM, 15 ug (UA)
Treatments Part B: B1 = A/Sing MAP, 15 ug (FA) with biopsy; B2 = Placebo MAP (FA) with biopsy
B3 = A/Sing MAP, 15 ug (FA); B4 = A/Sing MAP, 10 ug (FA); B5 = A/Sing MAP, 5 ug (FA); B6 = A/Sing MAP, 2.5 ug (FA)
B7 = Placebo MAP (FA); B8 = A/Sing MAP, 15 ug (UA); B9 = A/Sing IM, 15 ug, as 2018 Afluria Quad (UA)

Listing 16.2.9.3
Numeric Pain Intensity per Site

Part B

Randomisation Number	Group	Visit	Time Point	Was Pain Assessment Performed?	Site Number	Treatment	Date of assessment	Time of Assessment	Score	Unscheduled Reason
B0046	Group B7	Day 1	1 min	Yes	F2	uncoated	19-MAY-2018	13:34	3	
B0046	Group B7	Day 1	1 min	Yes	F3	uncoated	19-MAY-2018	13:37	3	
B0046	Group B7	Day 1	10 min	Yes	F1	uncoated	19-MAY-2018	13:48	1	
B0046	Group B7	Day 1	10 min	Yes	F2	uncoated	19-MAY-2018	13:48	1	
B0046	Group B7	Day 1	10 min	Yes	F3	uncoated	19-MAY-2018	13:48	3	
B0046	Group B7	Day 1	1 hour	Yes	F1	uncoated	19-MAY-2018	14:38	0	
B0046	Group B7	Day 1	1 hour	Yes	F2	uncoated	19-MAY-2018	14:38	0	
B0046	Group B7	Day 1	1 hour	Yes	F3	uncoated	19-MAY-2018	14:38	0	
B0046	Group B7	Day 1	2 hour	Yes	F1	uncoated	19-MAY-2018	15:38	0	
B0046	Group B7	Day 1	2 hour	Yes	F2	uncoated	19-MAY-2018	15:38	0	
B0046	Group B7	Day 1	2 hour	Yes	F3	uncoated	19-MAY-2018	15:38	0	
B0046	Group B7	Day 2		Yes	F1	uncoated	20-MAY-2018	13:29	0	
B0046	Group B7	Day 2		Yes	F2	uncoated	20-MAY-2018	13:29	0	
B0046	Group B7	Day 2		Yes	F3	uncoated	20-MAY-2018	13:29	0	
B0046	Group B7	Day 4		Yes	F1	uncoated	22-MAY-2018	13:44	0	
B0046	Group B7	Day 4		Yes	F2	uncoated	22-MAY-2018	13:44	0	
B0046	Group B7	Day 4		Yes	F3	uncoated	22-MAY-2018	13:44	0	
B0046	Group B7	Day 8		Yes	F1	uncoated	29-MAY-2018	13:26	0	
B0046	Group B7	Day 8		Yes	F2	uncoated	29-MAY-2018	13:26	0	
B0046	Group B7	Day 8		Yes	F3	uncoated	29-MAY-2018	13:26	0	
B0046	Group B7	Day 22		Yes	F1	uncoated	09-JUN-2018	13:22	0	

Scale: 0= No Pain; 10= Worst Pain Possible
Administration Site Key: FA = Forearm; UA= Upper arm
Treatments Part A: A1 = A/Sing MAP, 15 ug (FA); A2 = Afluria Quad IM (UA), 15 ug; A3 = Placebo MAP (FA); A4 = A/Sing Mono IM, 15 ug (UA)
Treatments Part B: B1 = A/Sing MAP, 15 ug (FA) with biopsy; B2 = Placebo MAP (FA) with biopsy
B3 = A/Sing MAP, 15 ug (FA); B4 = A/Sing MAP, 10 ug (FA); B5 = A/Sing MAP, 5 ug (FA); B6 = A/Sing MAP, 2.5 ug (FA)
B7 = Placebo MAP (FA); B8 = A/Sing MAP, 15 ug (UA); B9 = A/Sing IM, 15 ug, as 2018 Afluria Quad (UA)

Listing 16.2.9.3
Numeric Pain Intensity per Site

Part B

Randomisation Number	Group	Visit	Time Point	Was Pain Assessment Performed?	Site Number	Treatment	Date of assessment	Time of Assessment	Score	Unscheduled Reason
B0046	Group B7	Day 22		Yes	F2	uncoated	09-JUN-2018	13:22	0	
B0046	Group B7	Day 22		Yes	F3	uncoated	09-JUN-2018	13:22	0	
B0046	Group B7	EOS/ET (Day 61)		Yes	F1	uncoated	18-JUL-2018	13:00	0	
B0046	Group B7	EOS/ET (Day 61)		Yes	F2	uncoated	18-JUL-2018	13:00	0	
B0046	Group B7	EOS/ET (Day 61)		Yes	F3	uncoated	18-JUL-2018	13:00	0	
B0047	Group B9	Day 1	1 min	Yes	D1	[IM]	19-MAY-2018	11:31	0	
B0047	Group B9	Day 1	10 min	Yes	D1	[IM]	19-MAY-2018	11:40	0	
B0047	Group B9	Day 1	1 hour	Yes	D1	[IM]	19-MAY-2018	12:30	0	
B0047	Group B9	Day 1	2 hour	Yes	D1	[IM]	19-MAY-2018	13:42	0	
B0047	Group B9	Day 2		Yes	D1	[IM]	20-MAY-2018	10:57	0	
B0047	Group B9	Day 4		Yes	D1	[IM]	22-MAY-2018	08:24	0	
B0047	Group B9	Day 8		Yes	D1	[IM]	26-MAY-2018	12:24	0	
B0047	Group B9	Day 22		Yes	D1	[IM]	09-JUN-2018	13:35	0	
B0047	Group B9	EOS/ET (Day 61)		Yes	D1	[IM]	18-JUL-2018	08:43	0	
B0048	Group B6	Day 1	1 min	Yes	F1	2.5 ug	19-MAY-2018	13:41	2	
B0048	Group B6	Day 1	1 min	Yes	F2	uncoated	19-MAY-2018	13:44	2	
B0048	Group B6	Day 1	1 min	Yes	F3	uncoated	19-MAY-2018	13:47	1	
B0048	Group B6	Day 1	10 min	Yes	F1	2.5 ug	19-MAY-2018	13:58	0	
B0048	Group B6	Day 1	10 min	Yes	F2	uncoated	19-MAY-2018	13:58	0	
B0048	Group B6	Day 1	10 min	Yes	F3	uncoated	19-MAY-2018	13:58	0	

Scale: 0= No Pain; 10= Worst Pain Possible
Administration Site Key: FA = Forearm; UA= Upper arm
Treatments Part A: A1 = A/Sing MAP, 15 ug (FA); A2 = Afluria Quad IM (UA), 15 ug; A3 = Placebo MAP (FA); A4 = A/Sing Mono IM, 15 ug (UA)
Treatments Part B: B1 = A/Sing MAP, 15 ug (FA) with biopsy; B2 = Placebo MAP (FA) with biopsy
B3 = A/Sing MAP, 15 ug (FA); B4 = A/Sing MAP, 10 ug (FA); B5 = A/Sing MAP, 5 ug (FA); B6 = A/Sing MAP, 2.5 ug (FA)
B7 = Placebo MAP (FA); B8 = A/Sing MAP, 15 ug (UA); B9 = A/Sing IM, 15 ug, as 2018 Afluria Quad (UA)

Listing 16.2.9.3
Numeric Pain Intensity per Site

Part B

Randomisation Number	Group	Visit	Time Point	Was Pain Assessment Performed?	Site Number	Treatment	Date of assessment	Time of Assessment	Score	Unscheduled Reason
B0048	Group B6	Day 1	1 hour	Yes	F1	2.5 ug	19-MAY-2018	14:48	0	
B0048	Group B6	Day 1	1 hour	Yes	F2	uncoated	19-MAY-2018	14:48	0	
B0048	Group B6	Day 1	1 hour	Yes	F3	uncoated	19-MAY-2018	14:48	0	
B0048	Group B6	Day 1	2 hour	Yes	F1	2.5 ug	19-MAY-2018	15:48	0	
B0048	Group B6	Day 1	2 hour	Yes	F2	uncoated	19-MAY-2018	15:48	0	
B0048	Group B6	Day 1	2 hour	Yes	F3	uncoated	19-MAY-2018	15:48	0	
B0048	Group B6	Day 2		Yes	F1	2.5 ug	20-MAY-2018	13:17	0	
B0048	Group B6	Day 2		Yes	F2	uncoated	20-MAY-2018	13:17	0	
B0048	Group B6	Day 2		Yes	F3	uncoated	20-MAY-2018	13:17	0	
B0048	Group B6	Day 4		Yes	F1	2.5 ug	22-MAY-2018	10:49	0	
B0048	Group B6	Day 4		Yes	F2	uncoated	22-MAY-2018	10:49	0	
B0048	Group B6	Day 4		Yes	F3	uncoated	22-MAY-2018	10:49	0	
B0048	Group B6	Day 8		Yes	F1	2.5 ug	26-MAY-2018	10:38	0	
B0048	Group B6	Day 8		Yes	F2	uncoated	26-MAY-2018	10:39	0	
B0048	Group B6	Day 8		Yes	F3	uncoated	26-MAY-2018	10:40	0	
B0048	Group B6	Day 22		Yes	F1	2.5 ug	09-JUN-2018	12:14	0	
B0048	Group B6	Day 22		Yes	F2	uncoated	09-JUN-2018	12:14	0	
B0048	Group B6	Day 22		Yes	F3	uncoated	09-JUN-2018	12:14	0	
B0048	Group B6	EOS/ET (Day 61)		Yes	F1	2.5 ug	18-JUL-2018	15:54	0	
B0048	Group B6	EOS/ET (Day 61)		Yes	F2	uncoated	18-JUL-2018	15:54	0	
B0048	Group B6	EOS/ET (Day 61)		Yes	F3	uncoated	18-JUL-2018	15:54	0	

Scale: 0= No Pain; 10= Worst Pain Possible
Administration Site Key: FA = Forearm; UA= Upper arm
Treatments Part A: A1 = A/Sing MAP, 15 ug (FA); A2 = Afluria Quad IM (UA), 15 ug; A3 = Placebo MAP (FA); A4 = A/Sing Mono IM, 15 ug (UA)
Treatments Part B: B1 = A/Sing MAP, 15 ug (FA) with biopsy; B2 = Placebo MAP (FA) with biopsy
B3 = A/Sing MAP, 15 ug (FA); B4 = A/Sing MAP, 10 ug (FA); B5 = A/Sing MAP, 5 ug (FA); B6 = A/Sing MAP, 2.5 ug (FA)
B7 = Placebo MAP (FA); B8 = A/Sing MAP, 15 ug (UA); B9 = A/Sing IM, 15 ug, as 2018 Afluria Quad (UA)

Listing 16.2.9.3
Numeric Pain Intensity per Site

Part B

Randomisation Number	Group	Visit	Time Point	Was Pain Assessment Performed?	Site Number	Treatment	Date of assessment	Time of Assessment	Score	Unscheduled Reason
B0049	Group B4	Day 1	1 min	Yes	F1	uncoated	19-MAY-2018	14:11	4	
B0049	Group B4	Day 1	1 min	Yes	F2	5 ug	19-MAY-2018	14:14	0	
B0049	Group B4	Day 1	1 min	Yes	F3	5 ug	19-MAY-2018	14:17	3	
B0049	Group B4	Day 1	10 min	Yes	F1	uncoated	19-MAY-2018	14:28	2	
B0049	Group B4	Day 1	10 min	Yes	F2	5 ug	19-MAY-2018	14:28	3	
B0049	Group B4	Day 1	10 min	Yes	F3	5 ug	19-MAY-2018	14:28	2	
B0049	Group B4	Day 1	1 hour	Yes	F1	uncoated	19-MAY-2018	15:18	0	
B0049	Group B4	Day 1	1 hour	Yes	F2	5 ug	19-MAY-2018	15:18	1	
B0049	Group B4	Day 1	1 hour	Yes	F3	5 ug	19-MAY-2018	15:18	0	
B0049	Group B4	Day 1	2 hour	Yes	F1	uncoated	19-MAY-2018	16:18	0	
B0049	Group B4	Day 1	2 hour	Yes	F2	5 ug	19-MAY-2018	16:18	0	
B0049	Group B4	Day 1	2 hour	Yes	F3	5 ug	19-MAY-2018	16:18	0	
B0049	Group B4	Day 2		Yes	F1	uncoated	20-MAY-2018	14:02	0	
B0049	Group B4	Day 2		Yes	F2	5 ug	20-MAY-2018	14:02	0	
B0049	Group B4	Day 2		Yes	F3	5 ug	20-MAY-2018	14:02	0	
B0049	Group B4	Day 4		Yes	F1	uncoated	22-MAY-2018	15:08	0	
B0049	Group B4	Day 4		Yes	F2	5 ug	22-MAY-2018	15:08	0	
B0049	Group B4	Day 4		Yes	F3	5 ug	22-MAY-2018	15:08	0	
B0049	Group B4	Day 8		Yes	F1	uncoated	26-MAY-2018	10:58	0	
B0049	Group B4	Day 8		Yes	F2	5 ug	26-MAY-2018	10:59	0	
B0049	Group B4	Day 8		Yes	F3	5 ug	26-MAY-2018	11:00	0	

Scale: 0= No Pain; 10= Worst Pain Possible
Administration Site Key: FA = Forearm; UA= Upper arm
Treatments Part A: A1 = A/Sing MAP, 15 ug (FA); A2 = Afluria Quad IM (UA), 15 ug; A3 = Placebo MAP (FA); A4 = A/Sing Mono IM, 15 ug (UA)
Treatments Part B: B1 = A/Sing MAP, 15 ug (FA) with biopsy; B2 = Placebo MAP (FA) with biopsy
B3 = A/Sing MAP, 15 ug (FA); B4 = A/Sing MAP, 10 ug (FA); B5 = A/Sing MAP, 5 ug (FA); B6 = A/Sing MAP, 2.5 ug (FA)
B7 = Placebo MAP (FA); B8 = A/Sing MAP, 15 ug (UA); B9 = A/Sing IM, 15 ug, as 2018 Afluria Quad (UA)

Listing 16.2.9.3
Numeric Pain Intensity per Site

Part B

Randomisation Number	Group	Visit	Time Point	Was Pain Assessment Performed?	Site Number	Treatment	Date of assessment	Time of Assessment	Score	Unscheduled Reason
B0049	Group B4	Day 22		Yes	F1	uncoated	09-JUN-2018	12:22	0	
B0049	Group B4	Day 22		Yes	F2	5 ug	09-JUN-2018	12:22	0	
B0049	Group B4	Day 22		Yes	F3	5 ug	09-JUN-2018	12:22	0	
B0049	Group B4	EOS/ET (Day 61)		Yes	F1	uncoated	18-JUL-2018	10:49	0	
B0049	Group B4	EOS/ET (Day 61)		Yes	F2	5 ug	18-JUL-2018	10:49	0	
B0049	Group B4	EOS/ET (Day 61)		Yes	F3	5 ug	18-JUL-2018	10:49	0	
B0050	Group B8	Day 1	1 min	Yes	D1	5 ug	19-MAY-2018	14:21	1	
B0050	Group B8	Day 1	1 min	Yes	D2	5 ug	19-MAY-2018	14:24	1	
B0050	Group B8	Day 1	1 min	Yes	D3	5 ug	19-MAY-2018	14:27	1	
B0050	Group B8	Day 1	10 min	Yes	D1	5 ug	19-MAY-2018	14:38	0	
B0050	Group B8	Day 1	10 min	Yes	D2	5 ug	19-MAY-2018	14:38	1	
B0050	Group B8	Day 1	10 min	Yes	D3	5 ug	19-MAY-2018	14:38	0	
B0050	Group B8	Day 1	1 hour	Yes	D1	5 ug	19-MAY-2018	15:28	0	
B0050	Group B8	Day 1	1 hour	Yes	D2	5 ug	19-MAY-2018	15:28	1	
B0050	Group B8	Day 1	1 hour	Yes	D3	5 ug	19-MAY-2018	15:28	1	
B0050	Group B8	Day 1	2 hour	Yes	D1	5 ug	19-MAY-2018	16:28	0	
B0050	Group B8	Day 1	2 hour	Yes	D2	5 ug	19-MAY-2018	16:28	0	
B0050	Group B8	Day 1	2 hour	Yes	D3	5 ug	19-MAY-2018	16:28	0	
B0050	Group B8	Day 2		Yes	D1	5 ug	20-MAY-2018	14:45	0	
B0050	Group B8	Day 2		Yes	D2	5 ug	20-MAY-2018	14:45	0	
B0050	Group B8	Day 2		Yes	D3	5 ug	20-MAY-2018	14:45	0	

Scale: 0= No Pain; 10= Worst Pain Possible
Administration Site Key: FA = Forearm; UA= Upper arm
Treatments Part A: A1 = A/Sing MAP, 15 ug (FA); A2 = Afluria Quad IM (UA), 15 ug; A3 = Placebo MAP (FA); A4 = A/Sing Mono IM, 15 ug (UA)
Treatments Part B: B1 = A/Sing MAP, 15 ug (FA) with biopsy; B2 = Placebo MAP (FA) with biopsy
B3 = A/Sing MAP, 15 ug (FA); B4 = A/Sing MAP, 10 ug (FA); B5 = A/Sing MAP, 5 ug (FA); B6 = A/Sing MAP, 2.5 ug (FA)
B7 = Placebo MAP (FA); B8 = A/Sing MAP, 15 ug (UA); B9 = A/Sing IM, 15 ug, as 2018 Afluria Quad (UA)

Listing 16.2.9.3
Numeric Pain Intensity per Site

Part B

Randomisation Number	Group	Visit	Time Point	Was Pain Assessment Performed?	Site Number	Treatment	Date of assessment	Time of Assessment	Score	Unscheduled Reason
B0050	Group B8	Day 4		Yes	D1	5 ug	22-MAY-2018	11:48	0	
B0050	Group B8	Day 4		Yes	D2	5 ug	22-MAY-2018	11:48	0	
B0050	Group B8	Day 4		Yes	D3	5 ug	22-MAY-2018	11:48	0	
B0050	Group B8	Day 8		Yes	D1	5 ug	26-MAY-2018	11:43	0	
B0050	Group B8	Day 8		Yes	D2	5 ug	26-MAY-2018	11:43	0	
B0050	Group B8	Day 8		Yes	D3	5 ug	26-MAY-2018	11:43	0	
B0050	Group B8	Day 22		Yes	D1	5 ug	09-JUN-2018	12:41	0	
B0050	Group B8	Day 22		Yes	D2	5 ug	09-JUN-2018	12:41	0	
B0050	Group B8	Day 22		Yes	D3	5 ug	09-JUN-2018	12:41	0	
B0050	Group B8	EOS/ET (Day 61)		Yes	D1	5 ug	18-JUL-2018	14:38	0	
B0050	Group B8	EOS/ET (Day 61)		Yes	D2	5 ug	18-JUL-2018	14:38	0	
B0050	Group B8	EOS/ET (Day 61)		Yes	D3	5 ug	18-JUL-2018	14:38	0	
B0051	Group B3	Day 1	1 min	Yes	F1	5 ug	25-MAY-2018	12:51	1	
B0051	Group B3	Day 1	1 min	Yes	F2	5 ug	25-MAY-2018	12:54	2	
B0051	Group B3	Day 1	1 min	Yes	F3	5 ug	25-MAY-2018	12:57	1	
B0051	Group B3	Day 1	10 min	Yes	F1	5 ug	25-MAY-2018	13:08	0	
B0051	Group B3	Day 1	10 min	Yes	F2	5 ug	25-MAY-2018	13:08	0	
B0051	Group B3	Day 1	10 min	Yes	F3	5 ug	25-MAY-2018	13:08	0	
B0051	Group B3	Day 1	1 hour	Yes	F1	5 ug	25-MAY-2018	13:58	0	
B0051	Group B3	Day 1	1 hour	Yes	F2	5 ug	25-MAY-2018	13:58	0	
B0051	Group B3	Day 1	1 hour	Yes	F3	5 ug	25-MAY-2018	13:58	0	

Scale: 0= No Pain; 10= Worst Pain Possible
Administration Site Key: FA = Forearm; UA= Upper arm
Treatments Part A: A1 = A/Sing MAP, 15 ug (FA); A2 = Afluria Quad IM (UA), 15 ug; A3 = Placebo MAP (FA); A4 = A/Sing Mono IM, 15 ug (UA)
Treatments Part B: B1 = A/Sing MAP, 15 ug (FA) with biopsy; B2 = Placebo MAP (FA) with biopsy
B3 = A/Sing MAP, 15 ug (FA); B4 = A/Sing MAP, 10 ug (FA); B5 = A/Sing MAP, 5 ug (FA); B6 = A/Sing MAP, 2.5 ug (FA)
B7 = Placebo MAP (FA); B8 = A/Sing MAP, 15 ug (UA); B9 = A/Sing IM, 15 ug, as 2018 Afluria Quad (UA)

Listing 16.2.9.3
Numeric Pain Intensity per Site

Part B

Randomisation Number	Group	Visit	Time Point	Was Pain Assessment Performed?	Site Number	Treatment	Date of assessment	Time of Assessment	Score	Unscheduled Reason
B0051	Group B3	Day 1	2 hour	Yes	F1	5 ug	25-MAY-2018	14:58	0	
B0051	Group B3	Day 1	2 hour	Yes	F2	5 ug	25-MAY-2018	14:58	0	
B0051	Group B3	Day 1	2 hour	Yes	F3	5 ug	25-MAY-2018	14:58	0	
B0051	Group B3	Day 2		Yes	F1	5 ug	26-MAY-2018	10:24	0	
B0051	Group B3	Day 2		Yes	F2	5 ug	26-MAY-2018	10:24	0	
B0051	Group B3	Day 2		Yes	F3	5 ug	26-MAY-2018	10:24	0	
B0051	Group B3	Day 4		Yes	F1	5 ug	28-MAY-2018	08:50	0	
B0051	Group B3	Day 4		Yes	F2	5 ug	28-MAY-2018	08:50	0	
B0051	Group B3	Day 4		Yes	F3	5 ug	28-MAY-2018	08:51	0	
B0051	Group B3	Day 8		Yes	F1	5 ug	01-JUN-2018	10:46	0	
B0051	Group B3	Day 8		Yes	F2	5 ug	01-JUN-2018	10:46	0	
B0051	Group B3	Day 8		Yes	F3	5 ug	01-JUN-2018	10:46	0	
B0051	Group B3	Day 22		Yes	F1	5 ug	15-JUN-2018	08:10	0	
B0051	Group B3	Day 22		Yes	F2	5 ug	15-JUN-2018	08:10	0	
B0051	Group B3	Day 22		Yes	F3	5 ug	15-JUN-2018	08:10	0	
B0051	Group B3	EOS/ET (Day 61)		Yes	F1	5 ug	24-JUL-2018	08:36	0	
B0051	Group B3	EOS/ET (Day 61)		Yes	F2	5 ug	24-JUL-2018	08:37	0	
B0051	Group B3	EOS/ET (Day 61)		Yes	F3	5 ug	24-JUL-2018	08:37	0	
B0052	Group B5	Day 1	1 min	Yes	F1	5 ug	25-MAY-2018	13:01	0	
B0052	Group B5	Day 1	1 min	Yes	F2	uncoated	25-MAY-2018	13:11	1	
B0052	Group B5	Day 1	1 min	Yes	F3	uncoated	25-MAY-2018	13:06	0	

Scale: 0= No Pain; 10= Worst Pain Possible
Administration Site Key: FA = Forearm; UA= Upper arm
Treatments Part A: A1 = A/Sing MAP, 15 ug (FA); A2 = Afluria Quad IM (UA), 15 ug; A3 = Placebo MAP (FA); A4 = A/Sing Mono IM, 15 ug (UA)
Treatments Part B: B1 = A/Sing MAP, 15 ug (FA) with biopsy; B2 = Placebo MAP (FA) with biopsy
B3 = A/Sing MAP, 15 ug (FA); B4 = A/Sing MAP, 10 ug (FA); B5 = A/Sing MAP, 5 ug (FA); B6 = A/Sing MAP, 2.5 ug (FA)
B7 = Placebo MAP (FA); B8 = A/Sing MAP, 15 ug (UA); B9 = A/Sing IM, 15 ug, as 2018 Afluria Quad (UA)

Listing 16.2.9.3
Numeric Pain Intensity per Site

Part B

Randomisation Number	Group	Visit	Time Point	Was Pain Assessment Performed?	Site Number	Treatment	Date of assessment	Time of Assessment	Score	Unscheduled Reason
B0052	Group B5	Day 1	10 min	Yes	F1	5 ug	25-MAY-2018	13:22	0	
B0052	Group B5	Day 1	10 min	Yes	F2	uncoated	25-MAY-2018	13:22	1	
B0052	Group B5	Day 1	10 min	Yes	F3	uncoated	25-MAY-2018	13:22	0	
B0052	Group B5	Day 1	1 hour	Yes	F1	5 ug	25-MAY-2018	14:12	0	
B0052	Group B5	Day 1	1 hour	Yes	F2	uncoated	25-MAY-2018	14:12	0	
B0052	Group B5	Day 1	1 hour	Yes	F3	uncoated	25-MAY-2018	14:12	0	
B0052	Group B5	Day 1	2 hour	Yes	F1	5 ug	25-MAY-2018	15:12	0	
B0052	Group B5	Day 1	2 hour	Yes	F2	uncoated	25-MAY-2018	15:12	0	
B0052	Group B5	Day 1	2 hour	Yes	F3	uncoated	25-MAY-2018	15:12	0	
B0052	Group B5	Day 2		Yes	F1	5 ug	26-MAY-2018	09:37	1	
B0052	Group B5	Day 2		Yes	F2	uncoated	26-MAY-2018	09:37	1	
B0052	Group B5	Day 2		Yes	F3	uncoated	26-MAY-2018	09:37	1	
B0052	Group B5	Day 4		Yes	F1	5 ug	28-MAY-2018	13:05	0	
B0052	Group B5	Day 4		Yes	F2	uncoated	28-MAY-2018	13:05	0	
B0052	Group B5	Day 4		Yes	F3	uncoated	28-MAY-2018	13:06	0	
B0052	Group B5	Day 8		Yes	F1	5 ug	01-JUN-2018	13:55	0	
B0052	Group B5	Day 8		Yes	F2	uncoated	01-JUN-2018	13:55	0	
B0052	Group B5	Day 8		Yes	F3	uncoated	01-JUN-2018	13:55	0	
B0052	Group B5	Day 22		Yes	F1	5 ug	15-JUN-2018	13:22	0	
B0052	Group B5	Day 22		Yes	F2	uncoated	15-JUN-2018	13:22	0	
B0052	Group B5	Day 22		Yes	F3	uncoated	15-JUN-2018	13:22	0	

Scale: 0= No Pain; 10= Worst Pain Possible
Administration Site Key: FA = Forearm; UA= Upper arm
Treatments Part A: A1 = A/Sing MAP, 15 ug (FA); A2 = Afluria Quad IM (UA), 15 ug; A3 = Placebo MAP (FA); A4 = A/Sing Mono IM, 15 ug (UA)
Treatments Part B: B1 = A/Sing MAP, 15 ug (FA) with biopsy; B2 = Placebo MAP (FA) with biopsy
B3 = A/Sing MAP, 15 ug (FA); B4 = A/Sing MAP, 10 ug (FA); B5 = A/Sing MAP, 5 ug (FA); B6 = A/Sing MAP, 2.5 ug (FA)
B7 = Placebo MAP (FA); B8 = A/Sing MAP, 15 ug (UA); B9 = A/Sing IM, 15 ug, as 2018 Afluria Quad (UA)

Listing 16.2.9.3
Numeric Pain Intensity per Site

Part B

Randomisation Number	Group	Visit	Time Point	Was Pain Assessment Performed?	Site Number	Treatment	Date of assessment	Time of Assessment	Score	Unscheduled Reason
B0052	Group B5	EOS/ET (Day 61)		Yes	F1	5 ug	20-JUL-2018	14:10	0	
B0052	Group B5	EOS/ET (Day 61)		Yes	F2	uncoated	20-JUL-2018	14:10	0	
B0052	Group B5	EOS/ET (Day 61)		Yes	F3	uncoated	20-JUL-2018	14:10	0	
B0053	Group B6	Day 1	1 min	Yes	F1	uncoated	25-MAY-2018	13:11	0	
B0053	Group B6	Day 1	1 min	Yes	F2	uncoated	25-MAY-2018	13:14	0	
B0053	Group B6	Day 1	1 min	Yes	F3	2.5 ug	25-MAY-2018	13:17	1	
B0053	Group B6	Day 1	10 min	Yes	F1	uncoated	25-MAY-2018	13:28	0	
B0053	Group B6	Day 1	10 min	Yes	F2	uncoated	25-MAY-2018	13:28	0	
B0053	Group B6	Day 1	10 min	Yes	F3	2.5 ug	25-MAY-2018	13:28	0	
B0053	Group B6	Day 1	1 hour	Yes	F1	uncoated	25-MAY-2018	14:18	0	
B0053	Group B6	Day 1	1 hour	Yes	F2	uncoated	25-MAY-2018	14:18	0	
B0053	Group B6	Day 1	1 hour	Yes	F3	2.5 ug	25-MAY-2018	14:18	0	
B0053	Group B6	Day 1	2 hour	Yes	F1	uncoated	25-MAY-2018	15:18	0	
B0053	Group B6	Day 1	2 hour	Yes	F2	uncoated	25-MAY-2018	15:18	0	
B0053	Group B6	Day 1	2 hour	Yes	F3	2.5 ug	25-MAY-2018	15:18	0	
B0053	Group B6	Day 2		Yes	F1	uncoated	26-MAY-2018	10:38	0	
B0053	Group B6	Day 2		Yes	F2	uncoated	26-MAY-2018	10:38	0	
B0053	Group B6	Day 2		Yes	F3	2.5 ug	26-MAY-2018	10:38	0	
B0053	Group B6	Day 4		Yes	F1	uncoated	28-MAY-2018	12:27	0	
B0053	Group B6	Day 4		Yes	F2	uncoated	28-MAY-2018	12:28	0	
B0053	Group B6	Day 4		Yes	F3	2.5 ug	28-MAY-2018	12:29	0	

Scale: 0= No Pain; 10= Worst Pain Possible
Administration Site Key: FA = Forearm; UA= Upper arm
Treatments Part A: A1 = A/Sing MAP, 15 ug (FA); A2 = Afluria Quad IM (UA), 15 ug; A3 = Placebo MAP (FA); A4 = A/Sing Mono IM, 15 ug (UA)
Treatments Part B: B1 = A/Sing MAP, 15 ug (FA) with biopsy; B2 = Placebo MAP (FA) with biopsy
B3 = A/Sing MAP, 15 ug (FA); B4 = A/Sing MAP, 10 ug (FA); B5 = A/Sing MAP, 5 ug (FA); B6 = A/Sing MAP, 2.5 ug (FA)
B7 = Placebo MAP (FA); B8 = A/Sing MAP, 15 ug (UA); B9 = A/Sing IM, 15 ug, as 2018 Afluria Quad (UA)

Listing 16.2.9.3
Numeric Pain Intensity per Site

Part B

Randomisation Number	Group	Visit	Time Point	Was Pain Assessment Performed?	Site Number	Treatment	Date of assessment	Time of Assessment	Score	Unscheduled Reason
B0053	Group B6	Day 8		Yes	F1	uncoated	01-JUN-2018	11:10	0	
B0053	Group B6	Day 8		Yes	F2	uncoated	01-JUN-2018	11:10	0	
B0053	Group B6	Day 8		Yes	F3	2.5 ug	01-JUN-2018	11:10	0	
B0053	Group B6	Day 22		Yes	F1	uncoated	15-JUN-2018	13:02	0	
B0053	Group B6	Day 22		Yes	F2	uncoated	15-JUN-2018	13:02	0	
B0053	Group B6	Day 22		Yes	F3	2.5 ug	15-JUN-2018	13:02	0	
B0053	Group B6	EOS/ET (Day 61)		Yes	F1	uncoated	25-JUL-2018	12:49	0	
B0053	Group B6	EOS/ET (Day 61)		Yes	F2	uncoated	25-JUL-2018	12:49	0	
B0053	Group B6	EOS/ET (Day 61)		Yes	F3	2.5 ug	25-JUL-2018	12:49	0	
B0054	Group B5	Day 1	1 min	Yes	F1	uncoated	25-MAY-2018	13:21	0	
B0054	Group B5	Day 1	1 min	Yes	F2	uncoated	25-MAY-2018	13:24	0	
B0054	Group B5	Day 1	1 min	Yes	F3	5 ug	25-MAY-2018	13:27	1	
B0054	Group B5	Day 1	10 min	Yes	F1	uncoated	25-MAY-2018	13:38	0	
B0054	Group B5	Day 1	10 min	Yes	F2	uncoated	25-MAY-2018	13:38	0	
B0054	Group B5	Day 1	10 min	Yes	F3	5 ug	25-MAY-2018	13:38	0	
B0054	Group B5	Day 1	1 hour	Yes	F1	uncoated	25-MAY-2018	14:28	0	
B0054	Group B5	Day 1	1 hour	Yes	F2	uncoated	25-MAY-2018	14:28	0	
B0054	Group B5	Day 1	1 hour	No	F3	5 ug				
B0054	Group B5	Day 1	2 hour	Yes	F1	uncoated	25-MAY-2018	15:28	0	
B0054	Group B5	Day 1	2 hour	Yes	F2	uncoated	25-MAY-2018	15:28	0	
B0054	Group B5	Day 1	2 hour	Yes	F3	5 ug	25-MAY-2018	15:28	0	

Scale: 0= No Pain; 10= Worst Pain Possible
Administration Site Key: FA = Forearm; UA= Upper arm
Treatments Part A: A1 = A/Sing MAP, 15 ug (FA); A2 = Afluria Quad IM (UA), 15 ug; A3 = Placebo MAP (FA); A4 = A/Sing Mono IM, 15 ug (UA)
Treatments Part B: B1 = A/Sing MAP, 15 ug (FA) with biopsy; B2 = Placebo MAP (FA) with biopsy
B3 = A/Sing MAP, 15 ug (FA); B4 = A/Sing MAP, 10 ug (FA); B5 = A/Sing MAP, 5 ug (FA); B6 = A/Sing MAP, 2.5 ug (FA)
B7 = Placebo MAP (FA); B8 = A/Sing MAP, 15 ug (UA); B9 = A/Sing IM, 15 ug, as 2018 Afluria Quad (UA)

Listing 16.2.9.3
Numeric Pain Intensity per Site

Part B

Randomisation Number	Group	Visit	Time Point	Was Pain Assessment Performed?	Site Number	Treatment	Date of assessment	Time of Assessment	Score	Unscheduled Reason
B0054	Group B5	Day 2		Yes	F1	uncoated	26-MAY-2018	08:46	0	
B0054	Group B5	Day 2		Yes	F2	uncoated	26-MAY-2018	08:47	0	
B0054	Group B5	Day 2		Yes	F3	5 ug	26-MAY-2018	08:47	0	
B0054	Group B5	Day 4		Yes	F1	uncoated	28-MAY-2018	09:13	0	
B0054	Group B5	Day 4		Yes	F2	uncoated	28-MAY-2018	09:13	0	
B0054	Group B5	Day 4		Yes	F3	5 ug	28-MAY-2018	09:13	0	
B0054	Group B5	Day 8		Yes	F1	uncoated	01-JUN-2018	09:37	0	
B0054	Group B5	Day 8		Yes	F2	uncoated	01-JUN-2018	09:37	0	
B0054	Group B5	Day 8		Yes	F3	5 ug	01-JUN-2018	09:37	0	
B0054	Group B5	Day 22		Yes	F1	uncoated	15-JUN-2018	08:12	0	
B0054	Group B5	Day 22		Yes	F2	uncoated	15-JUN-2018	08:12	0	
B0054	Group B5	Day 22		Yes	F3	5 ug	15-JUN-2018	08:12	0	
B0054	Group B5	EOS/ET (Day 61)		Yes	F1	uncoated	24-JUL-2018	08:41	0	
B0054	Group B5	EOS/ET (Day 61)		Yes	F2	uncoated	24-JUL-2018	08:41	0	
B0054	Group B5	EOS/ET (Day 61)		Yes	F3	5 ug	24-JUL-2018	08:41	0	
B0055	Group B8	Day 1	1 min	Yes	D1	5 ug	25-MAY-2018	13:31	1	
B0055	Group B8	Day 1	1 min	Yes	D2	5 ug	25-MAY-2018	13:34	1	
B0055	Group B8	Day 1	1 min	Yes	D3	5 ug	25-MAY-2018	13:37	2	
B0055	Group B8	Day 1	10 min	Yes	D1	5 ug	25-MAY-2018	13:48	2	
B0055	Group B8	Day 1	10 min	Yes	D2	5 ug	25-MAY-2018	13:48	2	
B0055	Group B8	Day 1	10 min	Yes	D3	5 ug	25-MAY-2018	13:48	2	

Scale: 0= No Pain; 10= Worst Pain Possible
Administration Site Key: FA = Forearm; UA= Upper arm
Treatments Part A: A1 = A/Sing MAP, 15 ug (FA); A2 = Afluria Quad IM (UA), 15 ug; A3 = Placebo MAP (FA); A4 = A/Sing Mono IM, 15 ug (UA)
Treatments Part B: B1 = A/Sing MAP, 15 ug (FA) with biopsy; B2 = Placebo MAP (FA) with biopsy
B3 = A/Sing MAP, 15 ug (FA); B4 = A/Sing MAP, 10 ug (FA); B5 = A/Sing MAP, 5 ug (FA); B6 = A/Sing MAP, 2.5 ug (FA)
B7 = Placebo MAP (FA); B8 = A/Sing MAP, 15 ug (UA); B9 = A/Sing IM, 15 ug, as 2018 Afluria Quad (UA)

Listing 16.2.9.3
Numeric Pain Intensity per Site

Part B

Randomisation Number	Group	Visit	Time Point	Was Pain Assessment Performed?	Site Number	Treatment	Date of assessment	Time of Assessment	Score	Unscheduled Reason
B0055	Group B8	Day 1	1 hour	Yes	D1	5 ug	25-MAY-2018	14:38	0	
B0055	Group B8	Day 1	1 hour	Yes	D2	5 ug	25-MAY-2018	14:38	0	
B0055	Group B8	Day 1	1 hour	Yes	D3	5 ug	25-MAY-2018	14:38	0	
B0055	Group B8	Day 1	2 hour	Yes	D1	5 ug	25-MAY-2018	15:38	0	
B0055	Group B8	Day 1	2 hour	Yes	D2	5 ug	25-MAY-2018	15:38	0	
B0055	Group B8	Day 1	2 hour	Yes	D3	5 ug	25-MAY-2018	15:38	1	
B0055	Group B8	Day 2		Yes	D1	5 ug	26-MAY-2018	12:54	0	
B0055	Group B8	Day 2		Yes	D2	5 ug	26-MAY-2018	12:54	0	
B0055	Group B8	Day 2		Yes	D3	5 ug	26-MAY-2018	12:54	0	
B0055	Group B8	Day 4		Yes	D1	5 ug	28-MAY-2018	17:37	0	
B0055	Group B8	Day 4		Yes	D2	5 ug	28-MAY-2018	17:37	0	
B0055	Group B8	Day 4		Yes	D3	5 ug	28-MAY-2018	17:37	0	
B0055	Group B8	Day 8		Yes	D1	5 ug	01-JUN-2018	14:57	0	
B0055	Group B8	Day 8		Yes	D2	5 ug	01-JUN-2018	14:57	0	
B0055	Group B8	Day 8		Yes	D3	5 ug	01-JUN-2018	14:57	0	
B0055	Group B8	Day 22		Yes	D1	5 ug	15-JUN-2018	13:57	0	
B0055	Group B8	Day 22		Yes	D2	5 ug	15-JUN-2018	13:57	0	
B0055	Group B8	Day 22		Yes	D3	5 ug	15-JUN-2018	13:57	0	
B0055	Group B8	EOS/ET (Day 61)		Yes	D1	5 ug	24-JUL-2018	15:48	0	
B0055	Group B8	EOS/ET (Day 61)		Yes	D2	5 ug	24-JUL-2018	15:48	0	
B0055	Group B8	EOS/ET (Day 61)		Yes	D3	5 ug	24-JUL-2018	15:48	0	

Scale: 0= No Pain; 10= Worst Pain Possible
Administration Site Key: FA = Forearm; UA= Upper arm
Treatments Part A: A1 = A/Sing MAP, 15 ug (FA); A2 = Afluria Quad IM (UA), 15 ug; A3 = Placebo MAP (FA); A4 = A/Sing Mono IM, 15 ug (UA)
Treatments Part B: B1 = A/Sing MAP, 15 ug (FA) with biopsy; B2 = Placebo MAP (FA) with biopsy
B3 = A/Sing MAP, 15 ug (FA); B4 = A/Sing MAP, 10 ug (FA); B5 = A/Sing MAP, 5 ug (FA); B6 = A/Sing MAP, 2.5 ug (FA)
B7 = Placebo MAP (FA); B8 = A/Sing MAP, 15 ug (UA); B9 = A/Sing IM, 15 ug, as 2018 Afluria Quad (UA)

Listing 16.2.9.3
Numeric Pain Intensity per Site

Part B

Randomisation Number	Group	Visit	Time Point	Was Pain Assessment Performed?	Site Number	Treatment	Date of assessment	Time of Assessment	Score	Unscheduled Reason
B0056	Group B3	Day 1	1 min	Yes	F1	5 ug	25-MAY-2018	13:41	1	
B0056	Group B3	Day 1	1 min	Yes	F2	5 ug	25-MAY-2018	13:44	1	
B0056	Group B3	Day 1	1 min	Yes	F3	5 ug	25-MAY-2018	13:47	1	
B0056	Group B3	Day 1	10 min	Yes	F1	5 ug	25-MAY-2018	13:58	2	
B0056	Group B3	Day 1	10 min	Yes	F2	5 ug	25-MAY-2018	13:58	2	
B0056	Group B3	Day 1	10 min	Yes	F3	5 ug	25-MAY-2018	13:58	3	
B0056	Group B3	Day 1	1 hour	Yes	F1	5 ug	25-MAY-2018	14:48	0	
B0056	Group B3	Day 1	1 hour	Yes	F2	5 ug	25-MAY-2018	14:48	0	
B0056	Group B3	Day 1	1 hour	Yes	F3	5 ug	25-MAY-2018	14:48	0	
B0056	Group B3	Day 1	2 hour	Yes	F1	5 ug	25-MAY-2018	15:48	0	
B0056	Group B3	Day 1	2 hour	Yes	F2	5 ug	25-MAY-2018	15:48	0	
B0056	Group B3	Day 1	2 hour	Yes	F3	5 ug	25-MAY-2018	15:48	0	
B0056	Group B3	Day 2		Yes	F1	5 ug	26-MAY-2018	12:31	0	
B0056	Group B3	Day 2		Yes	F2	5 ug	26-MAY-2018	12:31	0	
B0056	Group B3	Day 2		Yes	F3	5 ug	26-MAY-2018	12:31	0	
B0056	Group B3	Day 4		Yes	F1	5 ug	28-MAY-2018	14:31	0	
B0056	Group B3	Day 4		Yes	F2	5 ug	28-MAY-2018	14:31	0	
B0056	Group B3	Day 4		Yes	F3	5 ug	28-MAY-2018	14:31	0	
B0056	Group B3	Day 8		Yes	F1	5 ug	01-JUN-2018	08:49	0	
B0056	Group B3	Day 8		Yes	F2	5 ug	01-JUN-2018	08:49	0	
B0056	Group B3	Day 8		Yes	F3	5 ug	01-JUN-2018	08:49	0	

Scale: 0= No Pain; 10= Worst Pain Possible
Administration Site Key: FA = Forearm; UA= Upper arm
Treatments Part A: A1 = A/Sing MAP, 15 ug (FA); A2 = Afluria Quad IM (UA), 15 ug; A3 = Placebo MAP (FA); A4 = A/Sing Mono IM, 15 ug (UA)
Treatments Part B: B1 = A/Sing MAP, 15 ug (FA) with biopsy; B2 = Placebo MAP (FA) with biopsy
B3 = A/Sing MAP, 15 ug (FA); B4 = A/Sing MAP, 10 ug (FA); B5 = A/Sing MAP, 5 ug (FA); B6 = A/Sing MAP, 2.5 ug (FA)
B7 = Placebo MAP (FA); B8 = A/Sing MAP, 15 ug (UA); B9 = A/Sing IM, 15 ug, as 2018 Afluria Quad (UA)

Listing 16.2.9.3
Numeric Pain Intensity per Site

Part B

Randomisation Number	Group	Visit	Time Point	Was Pain Assessment Performed?	Site Number	Treatment	Date of assessment	Time of Assessment	Score	Unscheduled Reason
B0056	Group B3	Day 22		Yes	F1	5 ug	15-JUN-2018	14:23	0	
B0056	Group B3	Day 22		Yes	F2	5 ug	15-JUN-2018	14:23	0	
B0056	Group B3	Day 22		Yes	F3	5 ug	15-JUN-2018	14:23	0	
B0056	Group B3	EOS/ET (Day 61)		Yes	F1	5 ug	24-JUL-2018	18:12	0	
B0056	Group B3	EOS/ET (Day 61)		Yes	F2	5 ug	24-JUL-2018	18:12	0	
B0056	Group B3	EOS/ET (Day 61)		Yes	F3	5 ug	24-JUL-2018	18:12	0	
B0057	Group B4	Day 1	1 min	Yes	F1	5 ug	25-MAY-2018	13:51	4	
B0057	Group B4	Day 1	1 min	Yes	F2	5 ug	25-MAY-2018	13:54	2	
B0057	Group B4	Day 1	1 min	Yes	F3	uncoated	25-MAY-2018	13:57	3	
B0057	Group B4	Day 1	10 min	Yes	F1	5 ug	25-MAY-2018	14:08	0	
B0057	Group B4	Day 1	10 min	Yes	F2	5 ug	25-MAY-2018	14:08	1	
B0057	Group B4	Day 1	10 min	Yes	F3	uncoated	25-MAY-2018	14:08	2	
B0057	Group B4	Day 1	1 hour	Yes	F1	5 ug	25-MAY-2018	15:00	0	
B0057	Group B4	Day 1	1 hour	Yes	F2	5 ug	25-MAY-2018	15:00	0	
B0057	Group B4	Day 1	1 hour	Yes	F3	uncoated	25-MAY-2018	15:00	0	
B0057	Group B4	Day 1	2 hour	Yes	F1	5 ug	25-MAY-2018	15:58	0	
B0057	Group B4	Day 1	2 hour	Yes	F2	5 ug	25-MAY-2018	15:58	0	
B0057	Group B4	Day 1	2 hour	Yes	F3	uncoated	25-MAY-2018	15:58	0	
B0057	Group B4	Day 2		Yes	F1	5 ug	26-MAY-2018	08:26	0	
B0057	Group B4	Day 2		Yes	F2	5 ug	26-MAY-2018	08:26	0	
B0057	Group B4	Day 2		Yes	F3	uncoated	26-MAY-2018	08:26	0	

Scale: 0= No Pain; 10= Worst Pain Possible
Administration Site Key: FA = Forearm; UA= Upper arm
Treatments Part A: A1 = A/Sing MAP, 15 ug (FA); A2 = Afluria Quad IM (UA), 15 ug; A3 = Placebo MAP (FA); A4 = A/Sing Mono IM, 15 ug (UA)
Treatments Part B: B1 = A/Sing MAP, 15 ug (FA) with biopsy; B2 = Placebo MAP (FA) with biopsy
B3 = A/Sing MAP, 15 ug (FA); B4 = A/Sing MAP, 10 ug (FA); B5 = A/Sing MAP, 5 ug (FA); B6 = A/Sing MAP, 2.5 ug (FA)
B7 = Placebo MAP (FA); B8 = A/Sing MAP, 15 ug (UA); B9 = A/Sing IM, 15 ug, as 2018 Afluria Quad (UA)

Listing 16.2.9.3
Numeric Pain Intensity per Site

Part B

Randomisation Number	Group	Visit	Time Point	Was Pain Assessment Performed?	Site Number	Treatment	Date of assessment	Time of Assessment	Score	Unscheduled Reason
B0057	Group B4	Day 4		Yes	F1	5 ug	28-MAY-2018	11:50	0	
B0057	Group B4	Day 4		Yes	F2	5 ug	28-MAY-2018	11:51	0	
B0057	Group B4	Day 4		Yes	F3	uncoated	28-MAY-2018	11:51	0	
B0057	Group B4	Day 8		Yes	F1	5 ug	01-JUN-2018	09:27	0	
B0057	Group B4	Day 8		Yes	F2	5 ug	01-JUN-2018	09:27	0	
B0057	Group B4	Day 8		Yes	F3	uncoated	01-JUN-2018	09:27	0	
B0057	Group B4	Day 22		Yes	F1	5 ug	15-JUN-2018	09:40	0	
B0057	Group B4	Day 22		Yes	F2	5 ug	15-JUN-2018	09:40	0	
B0057	Group B4	Day 22		Yes	F3	uncoated	15-JUN-2018	09:40	0	
B0057	Group B4	EOS/ET (Day 61)		Yes	F1	5 ug	24-JUL-2018	09:43	0	
B0057	Group B4	EOS/ET (Day 61)		Yes	F2	5 ug	24-JUL-2018	09:43	0	
B0057	Group B4	EOS/ET (Day 61)		Yes	F3	uncoated	24-JUL-2018	09:43	0	
B0058	Group B9	Day 2		No	D1	[IM]				
B0058	Group B9	Day 1	1 min	Yes	D1	[IM]	25-MAY-2018	12:31	1	
B0058	Group B9	Day 1	10 min	Yes	D1	[IM]	25-MAY-2018	12:40	0	
B0058	Group B9	Day 1	1 hour	Yes	D1	[IM]	25-MAY-2018	13:30	0	
B0058	Group B9	Day 1	2 hour	Yes	D1	[IM]	25-MAY-2018	14:35	0	
B0058	Group B9	Day 4		Yes	D1	[IM]	28-MAY-2018	17:33	0	
B0058	Group B9	Day 8		Yes	D1	[IM]	01-JUN-2018	17:35	0	
B0058	Group B9	Day 22		Yes	D1	[IM]	16-JUN-2018	13:03	0	
B0058	Group B9	EOS/ET (Day 61)		Yes	D1	[IM]	24-JUL-2018	10:22	0	

Scale: 0= No Pain; 10= Worst Pain Possible
Administration Site Key: FA = Forearm; UA= Upper arm
Treatments Part A: A1 = A/Sing MAP, 15 ug (FA); A2 = Afluria Quad IM (UA), 15 ug; A3 = Placebo MAP (FA); A4 = A/Sing Mono IM, 15 ug (UA)
Treatments Part B: B1 = A/Sing MAP, 15 ug (FA) with biopsy; B2 = Placebo MAP (FA) with biopsy
B3 = A/Sing MAP, 15 ug (FA); B4 = A/Sing MAP, 10 ug (FA); B5 = A/Sing MAP, 5 ug (FA); B6 = A/Sing MAP, 2.5 ug (FA)
B7 = Placebo MAP (FA); B8 = A/Sing MAP, 15 ug (UA); B9 = A/Sing IM, 15 ug, as 2018 Afluria Quad (UA)

Listing 16.2.9.3
Numeric Pain Intensity per Site

Part B

Randomisation Number	Group	Visit	Time Point	Was Pain Assessment Performed?	Site Number	Treatment	Date of assessment	Time of Assessment	Score	Unscheduled Reason
B0059	Group B7	Day 1	1 min	Yes	F1	uncoated	25-MAY-2018	14:11	2	
B0059	Group B7	Day 1	1 min	Yes	F2	uncoated	25-MAY-2018	14:14	2	
B0059	Group B7	Day 1	1 min	Yes	F3	uncoated	25-MAY-2018	14:17	2	
B0059	Group B7	Day 1	10 min	Yes	F1	uncoated	25-MAY-2018	14:28	0	
B0059	Group B7	Day 1	10 min	Yes	F2	uncoated	25-MAY-2018	14:28	0	
B0059	Group B7	Day 1	10 min	Yes	F3	uncoated	25-MAY-2018	14:28	0	
B0059	Group B7	Day 1	1 hour	Yes	F1	uncoated	25-MAY-2018	15:18	0	
B0059	Group B7	Day 1	1 hour	Yes	F2	uncoated	25-MAY-2018	15:08	0	
B0059	Group B7	Day 1	1 hour	Yes	F3	uncoated	25-MAY-2018	15:08	0	
B0059	Group B7	Day 1	2 hour	Yes	F1	uncoated	25-MAY-2018	16:18	0	
B0059	Group B7	Day 1	2 hour	Yes	F2	uncoated	25-MAY-2018	16:18	0	
B0059	Group B7	Day 1	2 hour	Yes	F3	uncoated	25-MAY-2018	16:18	0	
B0059	Group B7	Day 2		Yes	F1	uncoated	26-MAY-2018	11:02	0	
B0059	Group B7	Day 2		Yes	F2	uncoated	26-MAY-2018	11:02	0	
B0059	Group B7	Day 2		Yes	F3	uncoated	26-MAY-2018	11:02	0	
B0059	Group B7	Day 4		Yes	F1	uncoated	28-MAY-2018	14:23	0	
B0059	Group B7	Day 4		Yes	F2	uncoated	28-MAY-2018	14:23	0	
B0059	Group B7	Day 4		Yes	F3	uncoated	28-MAY-2018	14:23	0	
B0059	Group B7	Day 8		Yes	F1	uncoated	01-JUN-2018	13:52	0	
B0059	Group B7	Day 8		Yes	F2	uncoated	01-JUN-2018	13:52	0	
B0059	Group B7	Day 8		Yes	F3	uncoated	01-JUN-2018	13:52	0	

Scale: 0= No Pain; 10= Worst Pain Possible
Administration Site Key: FA = Forearm; UA= Upper arm
Treatments Part A: A1 = A/Sing MAP, 15 ug (FA); A2 = Afluria Quad IM (UA), 15 ug; A3 = Placebo MAP (FA); A4 = A/Sing Mono IM, 15 ug (UA)
Treatments Part B: B1 = A/Sing MAP, 15 ug (FA) with biopsy; B2 = Placebo MAP (FA) with biopsy
B3 = A/Sing MAP, 15 ug (FA); B4 = A/Sing MAP, 10 ug (FA); B5 = A/Sing MAP, 5 ug (FA); B6 = A/Sing MAP, 2.5 ug (FA)
B7 = Placebo MAP (FA); B8 = A/Sing MAP, 15 ug (UA); B9 = A/Sing IM, 15 ug, as 2018 Afluria Quad (UA)

Listing 16.2.9.3
Numeric Pain Intensity per Site

Part B

Randomisation Number	Group	Visit	Time Point	Was Pain Assessment Performed?	Site Number	Treatment	Date of assessment	Time of Assessment	Score	Unscheduled Reason
B0059	Group B7	Day 22		Yes	F1	uncoated	15-JUN-2018	14:57	0	
B0059	Group B7	Day 22		Yes	F2	uncoated	15-JUN-2018	14:57	0	
B0059	Group B7	Day 22		Yes	F3	uncoated	15-JUN-2018	14:57	0	
B0059	Group B7	EOS/ET (Day 61)		Yes	F1	uncoated	24-JUL-2018	16:22	0	
B0059	Group B7	EOS/ET (Day 61)		Yes	F2	uncoated	24-JUL-2018	16:22	0	
B0059	Group B7	EOS/ET (Day 61)		Yes	F3	uncoated	24-JUL-2018	16:22	0	
B0060	Group B3	Day 1	1 min	Yes	F1	5 ug	25-MAY-2018	14:01	0	
B0060	Group B3	Day 1	1 min	Yes	F2	5 ug	25-MAY-2018	14:04	0	
B0060	Group B3	Day 1	1 min	Yes	F3	5 ug	25-MAY-2018	14:07	1	
B0060	Group B3	Day 1	10 min	Yes	F1	5 ug	25-MAY-2018	14:18	0	
B0060	Group B3	Day 1	10 min	Yes	F2	5 ug	25-MAY-2018	14:18	0	
B0060	Group B3	Day 1	10 min	Yes	F3	5 ug	25-MAY-2018	14:18	0	
B0060	Group B3	Day 1	1 hour	Yes	F1	5 ug	25-MAY-2018	15:08	0	
B0060	Group B3	Day 1	1 hour	Yes	F2	5 ug	25-MAY-2018	15:08	0	
B0060	Group B3	Day 1	1 hour	Yes	F3	5 ug	25-MAY-2018	15:08	0	
B0060	Group B3	Day 1	2 hour	Yes	F1	5 ug	25-MAY-2018	16:08	0	
B0060	Group B3	Day 1	2 hour	Yes	F2	5 ug	25-MAY-2018	16:08	0	
B0060	Group B3	Day 1	2 hour	Yes	F3	5 ug	25-MAY-2018	16:08	0	
B0060	Group B3	Day 2		Yes	F1	5 ug	26-MAY-2018	11:32	0	
B0060	Group B3	Day 2		Yes	F2	5 ug	26-MAY-2018	11:33	0	
B0060	Group B3	Day 2		Yes	F3	5 ug	26-MAY-2018	11:34	0	

Scale: 0= No Pain; 10= Worst Pain Possible
Administration Site Key: FA = Forearm; UA= Upper arm
Treatments Part A: A1 = A/Sing MAP, 15 ug (FA); A2 = Afluria Quad IM (UA), 15 ug; A3 = Placebo MAP (FA); A4 = A/Sing Mono IM, 15 ug (UA)
Treatments Part B: B1 = A/Sing MAP, 15 ug (FA) with biopsy; B2 = Placebo MAP (FA) with biopsy
B3 = A/Sing MAP, 15 ug (FA); B4 = A/Sing MAP, 10 ug (FA); B5 = A/Sing MAP, 5 ug (FA); B6 = A/Sing MAP, 2.5 ug (FA)
B7 = Placebo MAP (FA); B8 = A/Sing MAP, 15 ug (UA); B9 = A/Sing IM, 15 ug, as 2018 Afluria Quad (UA)

Listing 16.2.9.3
Numeric Pain Intensity per Site

Part B

Randomisation Number	Group	Visit	Time Point	Was Pain Assessment Performed?	Site Number	Treatment	Date of assessment	Time of Assessment	Score	Unscheduled Reason
B0060	Group B3	Day 4		Yes	F1	5 ug	28-MAY-2018	14:38	0	
B0060	Group B3	Day 4		Yes	F2	5 ug	28-MAY-2018	14:38	0	
B0060	Group B3	Day 4		Yes	F3	5 ug	28-MAY-2018	14:38	0	
B0060	Group B3	Day 8		Yes	F1	5 ug	01-JUN-2018	15:52	0	
B0060	Group B3	Day 8		Yes	F2	5 ug	01-JUN-2018	15:52	0	
B0060	Group B3	Day 8		Yes	F3	5 ug	01-JUN-2018	15:52	0	
B0060	Group B3	Day 22		Yes	F1	5 ug	15-JUN-2018	12:13	0	
B0060	Group B3	Day 22		Yes	F2	5 ug	15-JUN-2018	12:13	0	
B0060	Group B3	Day 22		Yes	F3	5 ug	15-JUN-2018	12:13	0	
B0060	Group B3	EOS/ET (Day 61)		Yes	F1	5 ug	24-JUL-2018	13:48	0	
B0060	Group B3	EOS/ET (Day 61)		Yes	F2	5 ug	24-JUL-2018	13:48	0	
B0060	Group B3	EOS/ET (Day 61)		Yes	F3	5 ug	24-JUL-2018	13:49	0	
B0061	Group B8	Day 1	1 min	Yes	D1	5 ug	25-MAY-2018	14:21	0	
B0061	Group B8	Day 1	1 min	Yes	D2	5 ug	25-MAY-2018	14:24	0	
B0061	Group B8	Day 1	1 min	Yes	D3	5 ug	25-MAY-2018	14:27	1	
B0061	Group B8	Day 1	10 min	Yes	D1	5 ug	25-MAY-2018	14:38	0	
B0061	Group B8	Day 1	10 min	Yes	D2	5 ug	25-MAY-2018	14:38	0	
B0061	Group B8	Day 1	10 min	Yes	D3	5 ug	25-MAY-2018	14:38	0	
B0061	Group B8	Day 1	1 hour	Yes	D1	5 ug	25-MAY-2018	15:28	0	
B0061	Group B8	Day 1	1 hour	Yes	D2	5 ug	25-MAY-2018	15:28	0	
B0061	Group B8	Day 1	1 hour	Yes	D3	5 ug	25-MAY-2018	15:25	0	

Scale: 0= No Pain; 10= Worst Pain Possible
Administration Site Key: FA = Forearm; UA= Upper arm
Treatments Part A: A1 = A/Sing MAP, 15 ug (FA); A2 = Afluria Quad IM (UA), 15 ug; A3 = Placebo MAP (FA); A4 = A/Sing Mono IM, 15 ug (UA)
Treatments Part B: B1 = A/Sing MAP, 15 ug (FA) with biopsy; B2 = Placebo MAP (FA) with biopsy
B3 = A/Sing MAP, 15 ug (FA); B4 = A/Sing MAP, 10 ug (FA); B5 = A/Sing MAP, 5 ug (FA); B6 = A/Sing MAP, 2.5 ug (FA)
B7 = Placebo MAP (FA); B8 = A/Sing MAP, 15 ug (UA); B9 = A/Sing IM, 15 ug, as 2018 Afluria Quad (UA)

Listing 16.2.9.3
Numeric Pain Intensity per Site

Part B

Randomisation Number	Group	Visit	Time Point	Was Pain Assessment Performed?	Site Number	Treatment	Date of assessment	Time of Assessment	Score	Unscheduled Reason
B0061	Group B8	Day 1	2 hour	Yes	D1	5 ug	25-MAY-2018	16:28	0	
B0061	Group B8	Day 1	2 hour	Yes	D2	5 ug	25-MAY-2018	16:28	0	
B0061	Group B8	Day 1	2 hour	Yes	D3	5 ug	25-MAY-2018	16:28	0	
B0061	Group B8	Day 2		Yes	D1	5 ug	26-MAY-2018	10:46	0	
B0061	Group B8	Day 2		Yes	D2	5 ug	26-MAY-2018	10:47	0	
B0061	Group B8	Day 2		Yes	D3	5 ug	26-MAY-2018	10:48	0	
B0061	Group B8	Day 4		Yes	D1	5 ug	28-MAY-2018	13:35	0	
B0061	Group B8	Day 4		Yes	D2	5 ug	28-MAY-2018	13:35	0	
B0061	Group B8	Day 4		Yes	D3	5 ug	28-MAY-2018	13:35	0	
B0061	Group B8	Day 8		Yes	D1	5 ug	01-JUN-2018	11:45	0	
B0061	Group B8	Day 8		Yes	D2	5 ug	01-JUN-2018	11:45	0	
B0061	Group B8	Day 8		Yes	D3	5 ug	01-JUN-2018	11:45	0	
B0061	Group B8	Day 22		Yes	D1	5 ug	15-JUN-2018	12:10	0	
B0061	Group B8	Day 22		Yes	D2	5 ug	15-JUN-2018	12:10	0	
B0061	Group B8	Day 22		Yes	D3	5 ug	15-JUN-2018	12:10	0	
B0061	Group B8	EOS/ET (Day 61)		Yes	D1	5 ug	24-JUL-2018	14:41	0	
B0061	Group B8	EOS/ET (Day 61)		Yes	D2	5 ug	24-JUL-2018	14:41	0	
B0061	Group B8	EOS/ET (Day 61)		Yes	D3	5 ug	24-JUL-2018	14:41	0	
B0062	Group B5	Day 1	1 min	Yes	F1	uncoated	25-MAY-2018	14:31	1	
B0062	Group B5	Day 1	1 min	Yes	F2	5 ug	25-MAY-2018	14:34	2	
B0062	Group B5	Day 1	1 min	Yes	F3	uncoated	25-MAY-2018	14:37	0	

Scale: 0= No Pain; 10= Worst Pain Possible
Administration Site Key: FA = Forearm; UA= Upper arm
Treatments Part A: A1 = A/Sing MAP, 15 ug (FA); A2 = Afluria Quad IM (UA), 15 ug; A3 = Placebo MAP (FA); A4 = A/Sing Mono IM, 15 ug (UA)
Treatments Part B: B1 = A/Sing MAP, 15 ug (FA) with biopsy; B2 = Placebo MAP (FA) with biopsy
B3 = A/Sing MAP, 15 ug (FA); B4 = A/Sing MAP, 10 ug (FA); B5 = A/Sing MAP, 5 ug (FA); B6 = A/Sing MAP, 2.5 ug (FA)
B7 = Placebo MAP (FA); B8 = A/Sing MAP, 15 ug (UA); B9 = A/Sing IM, 15 ug, as 2018 Afluria Quad (UA)

Listing 16.2.9.3
Numeric Pain Intensity per Site

Part B

Randomisation Number	Group	Visit	Time Point	Was Pain Assessment Performed?	Site Number	Treatment	Date of assessment	Time of Assessment	Score	Unscheduled Reason
B0062	Group B5	Day 1	10 min	Yes	F1	uncoated	25-MAY-2018	14:48	0	
B0062	Group B5	Day 1	10 min	Yes	F2	5 ug	25-MAY-2018	14:48	0	
B0062	Group B5	Day 1	10 min	Yes	F3	uncoated	25-MAY-2018	14:48	0	
B0062	Group B5	Day 1	1 hour	Yes	F1	uncoated	25-MAY-2018	15:38	0	
B0062	Group B5	Day 1	1 hour	Yes	F2	5 ug	25-MAY-2018	15:38	0	
B0062	Group B5	Day 1	1 hour	Yes	F3	uncoated	25-MAY-2018	15:38	0	
B0062	Group B5	Day 1	2 hour	Yes	F1	uncoated	25-MAY-2018	16:38	0	
B0062	Group B5	Day 1	2 hour	Yes	F2	5 ug	25-MAY-2018	16:38	0	
B0062	Group B5	Day 1	2 hour	Yes	F3	uncoated	25-MAY-2018	16:38	0	
B0062	Group B5	Day 2		Yes	F1	uncoated	26-MAY-2018	11:30	0	
B0062	Group B5	Day 2		Yes	F2	5 ug	26-MAY-2018	11:30	0	
B0062	Group B5	Day 2		Yes	F3	uncoated	26-MAY-2018	11:30	0	
B0062	Group B5	Day 4		Yes	F1	uncoated	29-MAY-2018	14:07	0	
B0062	Group B5	Day 4		Yes	F2	5 ug	29-MAY-2018	14:07	0	
B0062	Group B5	Day 4		Yes	F3	uncoated	29-MAY-2018	14:07	0	
B0062	Group B5	Day 8		Yes	F1	uncoated	01-JUN-2018	12:46	0	
B0062	Group B5	Day 8		Yes	F2	5 ug	01-JUN-2018	12:46	0	
B0062	Group B5	Day 8		Yes	F3	uncoated	01-JUN-2018	12:46	0	
B0062	Group B5	Day 22		Yes	F1	uncoated	15-JUN-2018	10:59	0	
B0062	Group B5	Day 22		Yes	F2	5 ug	15-JUN-2018	10:59	0	
B0062	Group B5	Day 22		Yes	F3	uncoated	15-JUN-2018	10:59	0	

Scale: 0= No Pain; 10= Worst Pain Possible
Administration Site Key: FA = Forearm; UA= Upper arm
Treatments Part A: A1 = A/Sing MAP, 15 ug (FA); A2 = Afluria Quad IM (UA), 15 ug; A3 = Placebo MAP (FA); A4 = A/Sing Mono IM, 15 ug (UA)
Treatments Part B: B1 = A/Sing MAP, 15 ug (FA) with biopsy; B2 = Placebo MAP (FA) with biopsy
B3 = A/Sing MAP, 15 ug (FA); B4 = A/Sing MAP, 10 ug (FA); B5 = A/Sing MAP, 5 ug (FA); B6 = A/Sing MAP, 2.5 ug (FA)
B7 = Placebo MAP (FA); B8 = A/Sing MAP, 15 ug (UA); B9 = A/Sing IM, 15 ug, as 2018 Afluria Quad (UA)

Listing 16.2.9.3
Numeric Pain Intensity per Site

Part B

Randomisation Number	Group	Visit	Time Point	Was Pain Assessment Performed?	Site Number	Treatment	Date of assessment	Time of Assessment	Score	Unscheduled Reason
B0062	Group B5	EOS/ET (Day 61)		Yes	F1	uncoated	24-JUL-2018	15:51	0	
B0062	Group B5	EOS/ET (Day 61)		Yes	F2	5 ug	24-JUL-2018	15:51	0	
B0062	Group B5	EOS/ET (Day 61)		Yes	F3	uncoated	24-JUL-2018	15:51	0	
B0063	Group B7	Day 1	1 min	Yes	F1	uncoated	25-MAY-2018	14:41	2	
B0063	Group B7	Day 1	1 min	Yes	F2	uncoated	25-MAY-2018	14:44	2	
B0063	Group B7	Day 1	1 min	Yes	F3	uncoated	25-MAY-2018	14:47	1	
B0063	Group B7	Day 1	10 min	Yes	F1	uncoated	25-MAY-2018	14:58	0	
B0063	Group B7	Day 1	10 min	Yes	F2	uncoated	25-MAY-2018	14:58	0	
B0063	Group B7	Day 1	10 min	Yes	F3	uncoated	25-MAY-2018	14:58	0	
B0063	Group B7	Day 1	1 hour	Yes	F1	uncoated	25-MAY-2018	15:48	0	
B0063	Group B7	Day 1	1 hour	Yes	F2	uncoated	25-MAY-2018	15:48	0	
B0063	Group B7	Day 1	1 hour	Yes	F3	uncoated	25-MAY-2018	15:48	0	
B0063	Group B7	Day 1	2 hour	Yes	F1	uncoated	25-MAY-2018	16:48	0	
B0063	Group B7	Day 1	2 hour	Yes	F2	uncoated	25-MAY-2018	16:48	0	
B0063	Group B7	Day 1	2 hour	Yes	F3	uncoated	25-MAY-2018	16:48	0	
B0063	Group B7	Day 2		Yes	F1	uncoated	26-MAY-2018	11:18	0	
B0063	Group B7	Day 2		Yes	F2	uncoated	26-MAY-2018	11:18	0	
B0063	Group B7	Day 2		Yes	F3	uncoated	26-MAY-2018	11:18	0	
B0063	Group B7	Day 4		Yes	F1	uncoated	28-MAY-2018	12:44	0	
B0063	Group B7	Day 4		Yes	F2	uncoated	28-MAY-2018	12:45	0	
B0063	Group B7	Day 4		Yes	F3	uncoated	28-MAY-2018	12:46	0	

Scale: 0= No Pain; 10= Worst Pain Possible
Administration Site Key: FA = Forearm; UA= Upper arm
Treatments Part A: A1 = A/Sing MAP, 15 ug (FA); A2 = Afluria Quad IM (UA), 15 ug; A3 = Placebo MAP (FA); A4 = A/Sing Mono IM, 15 ug (UA)
Treatments Part B: B1 = A/Sing MAP, 15 ug (FA) with biopsy; B2 = Placebo MAP (FA) with biopsy
B3 = A/Sing MAP, 15 ug (FA); B4 = A/Sing MAP, 10 ug (FA); B5 = A/Sing MAP, 5 ug (FA); B6 = A/Sing MAP, 2.5 ug (FA)
B7 = Placebo MAP (FA); B8 = A/Sing MAP, 15 ug (UA); B9 = A/Sing IM, 15 ug, as 2018 Afluria Quad (UA)

Listing 16.2.9.3
Numeric Pain Intensity per Site

Part B

Randomisation Number	Group	Visit	Time Point	Was Pain Assessment Performed?	Site Number	Treatment	Date of assessment	Time of Assessment	Score	Unscheduled Reason
B0063	Group B7	Day 8		Yes	F1	uncoated	01-JUN-2018	14:01	0	
B0063	Group B7	Day 8		Yes	F2	uncoated	01-JUN-2018	14:01	0	
B0063	Group B7	Day 8		Yes	F3	uncoated	01-JUN-2018	14:01	0	
B0063	Group B7	Day 22		Yes	F1	uncoated	15-JUN-2018	13:20	0	
B0063	Group B7	Day 22		Yes	F2	uncoated	15-JUN-2018	13:20	0	
B0063	Group B7	Day 22		Yes	F3	uncoated	15-JUN-2018	13:20	0	
B0063	Group B7	EOS/ET (Day 61)		Yes	F1	uncoated	12-JUL-2018	14:10	0	
B0063	Group B7	EOS/ET (Day 61)		Yes	F2	uncoated	12-JUL-2018	14:10	0	
B0063	Group B7	EOS/ET (Day 61)		Yes	F3	uncoated	12-JUL-2018	14:10	0	
B0064	Group B9	Day 1	1 min	Yes	D1	[IM]	25-MAY-2018	12:41	1	
B0064	Group B9	Day 1	10 min	Yes	D1	[IM]	25-MAY-2018	12:50	0	
B0064	Group B9	Day 1	1 hour	Yes	D1	[IM]	25-MAY-2018	13:40	0	
B0064	Group B9	Day 1	2 hour	Yes	D1	[IM]	25-MAY-2018	14:45	0	
B0064	Group B9	Day 2		Yes	D1	[IM]	26-MAY-2018	12:00	2	
B0064	Group B9	Day 4		Yes	D1	[IM]	28-MAY-2018	13:29	0	
B0064	Group B9	Day 8		Yes	D1	[IM]	01-JUN-2018	09:52	0	
B0064	Group B9	Day 22		Yes	D1	[IM]	15-JUN-2018	10:08	0	
B0064	Group B9	EOS/ET (Day 61)		Yes	D1	[IM]	24-JUL-2018	12:59	0	
B0065	Group B4	Day 1	1 min	Yes	F1	5 ug	25-MAY-2018	14:51	1	
B0065	Group B4	Day 1	1 min	Yes	F2	uncoated	25-MAY-2018	14:54	1	

Scale: 0= No Pain; 10= Worst Pain Possible
Administration Site Key: FA = Forearm; UA= Upper arm
Treatments Part A: A1 = A/Sing MAP, 15 ug (FA); A2 = Afluria Quad IM (UA), 15 ug; A3 = Placebo MAP (FA); A4 = A/Sing Mono IM, 15 ug (UA)
Treatments Part B: B1 = A/Sing MAP, 15 ug (FA) with biopsy; B2 = Placebo MAP (FA) with biopsy
B3 = A/Sing MAP, 15 ug (FA); B4 = A/Sing MAP, 10 ug (FA); B5 = A/Sing MAP, 5 ug (FA); B6 = A/Sing MAP, 2.5 ug (FA)
B7 = Placebo MAP (FA); B8 = A/Sing MAP, 15 ug (UA); B9 = A/Sing IM, 15 ug, as 2018 Afluria Quad (UA)

Listing 16.2.9.3
Numeric Pain Intensity per Site

Part B

Randomisation Number	Group	Visit	Time Point	Was Pain Assessment Performed?	Site Number	Treatment	Date of assessment	Time of Assessment	Score	Unscheduled Reason
B0065	Group B4	Day 1	1 min	Yes	F3	5 ug	25-MAY-2018	14:57	2	
B0065	Group B4	Day 1	10 min	Yes	F1	5 ug	25-MAY-2018	15:08	0	
B0065	Group B4	Day 1	10 min	Yes	F2	uncoated	25-MAY-2018	15:08	1	
B0065	Group B4	Day 1	10 min	Yes	F3	5 ug	25-MAY-2018	15:08	0	
B0065	Group B4	Day 1	1 hour	Yes	F1	5 ug	25-MAY-2018	15:58	0	
B0065	Group B4	Day 1	1 hour	Yes	F2	uncoated	25-MAY-2018	15:58	0	
B0065	Group B4	Day 1	1 hour	Yes	F3	5 ug	25-MAY-2018	15:58	0	
B0065	Group B4	Day 1	2 hour	Yes	F1	5 ug	25-MAY-2018	16:58	0	
B0065	Group B4	Day 1	2 hour	Yes	F2	uncoated	25-MAY-2018	16:58	0	
B0065	Group B4	Day 1	2 hour	Yes	F3	5 ug	25-MAY-2018	16:58	0	
B0065	Group B4	Day 2		Yes	F1	5 ug	26-MAY-2018	12:10	2	
B0065	Group B4	Day 2		Yes	F2	uncoated	26-MAY-2018	12:10	0	
B0065	Group B4	Day 2		Yes	F3	5 ug	26-MAY-2018	12:10	1	
B0065	Group B4	Day 4		Yes	F1	5 ug	28-MAY-2018	13:32	0	
B0065	Group B4	Day 4		Yes	F2	uncoated	28-MAY-2018	13:32	0	
B0065	Group B4	Day 4		Yes	F3	5 ug	28-MAY-2018	13:32	0	
B0065	Group B4	Day 8		Yes	F1	5 ug	01-JUN-2018	09:53	0	
B0065	Group B4	Day 8		Yes	F2	uncoated	01-JUN-2018	09:53	0	
B0065	Group B4	Day 8		Yes	F3	5 ug	01-JUN-2018	09:53	0	
B0065	Group B4	Day 22		Yes	F1	5 ug	15-JUN-2018	09:59	0	
B0065	Group B4	Day 22		Yes	F2	uncoated	15-JUN-2018	09:59	0	

Scale: 0= No Pain; 10= Worst Pain Possible
Administration Site Key: FA = Forearm; UA= Upper arm
Treatments Part A: A1 = A/Sing MAP, 15 ug (FA); A2 = Afluria Quad IM (UA), 15 ug; A3 = Placebo MAP (FA); A4 = A/Sing Mono IM, 15 ug (UA)
Treatments Part B: B1 = A/Sing MAP, 15 ug (FA) with biopsy; B2 = Placebo MAP (FA) with biopsy
B3 = A/Sing MAP, 15 ug (FA); B4 = A/Sing MAP, 10 ug (FA); B5 = A/Sing MAP, 5 ug (FA); B6 = A/Sing MAP, 2.5 ug (FA)
B7 = Placebo MAP (FA); B8 = A/Sing MAP, 15 ug (UA); B9 = A/Sing IM, 15 ug, as 2018 Afluria Quad (UA)

Listing 16.2.9.3
Numeric Pain Intensity per Site

Part B

Randomisation Number	Group	Visit	Time Point	Was Pain Assessment Performed?	Site Number	Treatment	Date of assessment	Time of Assessment	Score	Unscheduled Reason
B0065	Group B4	Day 22		Yes	F3	5 ug	15-JUN-2018	09:59	0	
B0065	Group B4	EOS/ET (Day 61)		Yes	F1	5 ug	24-JUL-2018	13:03	0	
B0065	Group B4	EOS/ET (Day 61)		Yes	F2	uncoated	24-JUL-2018	13:03	0	
B0065	Group B4	EOS/ET (Day 61)		Yes	F3	5 ug	24-JUL-2018	13:04	0	
B0066	Group B6	Day 1	1 min	Yes	F1	2.5 ug	25-MAY-2018	15:01	1	
B0066	Group B6	Day 1	1 min	Yes	F2	uncoated	25-MAY-2018	15:04	1	
B0066	Group B6	Day 1	1 min	Yes	F3	uncoated	25-MAY-2018	15:07	1	
B0066	Group B6	Day 1	10 min	Yes	F1	2.5 ug	25-MAY-2018	15:18	0	
B0066	Group B6	Day 1	10 min	Yes	F2	uncoated	25-MAY-2018	15:18	0	
B0066	Group B6	Day 1	10 min	Yes	F3	uncoated	25-MAY-2018	15:18	0	
B0066	Group B6	Day 1	1 hour	Yes	F1	2.5 ug	25-MAY-2018	16:08	0	
B0066	Group B6	Day 1	1 hour	Yes	F2	uncoated	25-MAY-2018	16:08	0	
B0066	Group B6	Day 1	1 hour	Yes	F3	uncoated	25-MAY-2018	16:08	0	
B0066	Group B6	Day 1	2 hour	Yes	F1	2.5 ug	25-MAY-2018	17:08	0	
B0066	Group B6	Day 1	2 hour	Yes	F2	uncoated	25-MAY-2018	17:08	0	
B0066	Group B6	Day 1	2 hour	Yes	F3	uncoated	25-MAY-2018	17:08	0	
B0066	Group B6	Day 2		Yes	F1	2.5 ug	26-MAY-2018	10:35	0	
B0066	Group B6	Day 2		Yes	F2	uncoated	26-MAY-2018	10:35	0	
B0066	Group B6	Day 2		Yes	F3	uncoated	26-MAY-2018	10:35	0	
B0066	Group B6	Day 4		Yes	F1	2.5 ug	28-MAY-2018	10:12	0	
B0066	Group B6	Day 4		Yes	F2	uncoated	28-MAY-2018	10:13		

Scale: 0= No Pain; 10= Worst Pain Possible
Administration Site Key: FA = Forearm; UA= Upper arm
Treatments Part A: A1 = A/Sing MAP, 15 ug (FA); A2 = Afluria Quad IM (UA), 15 ug; A3 = Placebo MAP (FA); A4 = A/Sing Mono IM, 15 ug (UA)
Treatments Part B: B1 = A/Sing MAP, 15 ug (FA) with biopsy; B2 = Placebo MAP (FA) with biopsy
B3 = A/Sing MAP, 15 ug (FA); B4 = A/Sing MAP, 10 ug (FA); B5 = A/Sing MAP, 5 ug (FA); B6 = A/Sing MAP, 2.5 ug (FA)
B7 = Placebo MAP (FA); B8 = A/Sing MAP, 15 ug (UA); B9 = A/Sing IM, 15 ug, as 2018 Afluria Quad (UA)

Listing 16.2.9.3
Numeric Pain Intensity per Site

Part B

Randomisation Number	Group	Visit	Time Point	Was Pain Assessment Performed?	Site Number	Treatment	Date of assessment	Time of Assessment	Score	Unscheduled Reason
B0066	Group B6	Day 4		Yes	F3	uncoated	28-MAY-2018	10:14	0	
B0066	Group B6	Day 8		Yes	F1	2.5 ug	01-JUN-2018	10:20	0	
B0066	Group B6	Day 8		Yes	F2	uncoated	01-JUN-2018	10:20	0	
B0066	Group B6	Day 8		Yes	F3	uncoated	01-JUN-2018	10:20	0	
B0066	Group B6	Day 22		Yes	F1	2.5 ug	15-JUN-2018	10:56	0	
B0066	Group B6	Day 22		Yes	F2	uncoated	15-JUN-2018	10:56	0	
B0066	Group B6	Day 22		Yes	F3	uncoated	15-JUN-2018	10:56	0	
B0066	Group B6	EOS/ET (Day 61)		Yes	F1	2.5 ug	24-JUL-2018	10:49	0	
B0066	Group B6	EOS/ET (Day 61)		Yes	F2	uncoated	24-JUL-2018	10:49	0	
B0066	Group B6	EOS/ET (Day 61)		Yes	F3	uncoated	24-JUL-2018	10:50	0	
B0067	Group B8	Day 1	1 min	Yes	D1	5 ug	25-MAY-2018	15:11	0	
B0067	Group B8	Day 1	1 min	Yes	D2	5 ug	25-MAY-2018	15:14	1	
B0067	Group B8	Day 1	1 min	Yes	D3	5 ug	25-MAY-2018	15:17	1	
B0067	Group B8	Day 1	10 min	Yes	D1	5 ug	25-MAY-2018	15:28	0	
B0067	Group B8	Day 1	10 min	Yes	D2	5 ug	25-MAY-2018	15:28	0	
B0067	Group B8	Day 1	10 min	Yes	D3	5 ug	25-MAY-2018	15:28	0	
B0067	Group B8	Day 1	1 hour	Yes	D1	5 ug	25-MAY-2018	16:18	0	
B0067	Group B8	Day 1	1 hour	Yes	D2	5 ug	25-MAY-2018	16:18	0	
B0067	Group B8	Day 1	1 hour	Yes	D3	5 ug	25-MAY-2018	16:18	0	
B0067	Group B8	Day 1	2 hour	Yes	D1	5 ug	25-MAY-2018	17:18	0	
B0067	Group B8	Day 1	2 hour	Yes	D2	5 ug	25-MAY-2018	17:18	0	

Scale: 0= No Pain; 10= Worst Pain Possible
Administration Site Key: FA = Forearm; UA= Upper arm
Treatments Part A: A1 = A/Sing MAP, 15 ug (FA); A2 = Afluria Quad IM (UA), 15 ug; A3 = Placebo MAP (FA); A4 = A/Sing Mono IM, 15 ug (UA)
Treatments Part B: B1 = A/Sing MAP, 15 ug (FA) with biopsy; B2 = Placebo MAP (FA) with biopsy
B3 = A/Sing MAP, 15 ug (FA); B4 = A/Sing MAP, 10 ug (FA); B5 = A/Sing MAP, 5 ug (FA); B6 = A/Sing MAP, 2.5 ug (FA)
B7 = Placebo MAP (FA); B8 = A/Sing MAP, 15 ug (UA); B9 = A/Sing IM, 15 ug, as 2018 Afluria Quad (UA)

Listing 16.2.9.3
Numeric Pain Intensity per Site

Part B

Randomisation Number	Group	Visit	Time Point	Was Pain Assessment Performed?	Site Number	Treatment	Date of assessment	Time of Assessment	Score	Unscheduled Reason
B0067	Group B8	Day 1	2 hour	Yes	D3	5 ug	25-MAY-2018	17:18	0	
B0067	Group B8	Day 2		Yes	D1	5 ug	26-MAY-2018	08:55	0	
B0067	Group B8	Day 2		Yes	D2	5 ug	26-MAY-2018	08:55	1	
B0067	Group B8	Day 2		Yes	D3	5 ug	26-MAY-2018	08:55	1	
B0067	Group B8	Day 4		Yes	D1	5 ug	28-MAY-2018	17:10	0	
B0067	Group B8	Day 4		Yes	D2	5 ug	28-MAY-2018	17:10	0	
B0067	Group B8	Day 4		Yes	D3	5 ug	28-MAY-2018	17:10	0	
B0067	Group B8	Day 8		Yes	D1	5 ug	02-JUN-2018	08:34	0	
B0067	Group B8	Day 8		Yes	D2	5 ug	02-JUN-2018	08:34	0	
B0067	Group B8	Day 8		Yes	D3	5 ug	02-JUN-2018	08:34	0	
B0067	Group B8	Day 22		Yes	D1	5 ug	15-JUN-2018	14:05	0	
B0067	Group B8	Day 22		Yes	D2	5 ug	15-JUN-2018	14:05	0	
B0067	Group B8	Day 22		Yes	D3	5 ug	15-JUN-2018	14:05	0	
B0067	Group B8	EOS/ET (Day 61)		Yes	D1	5 ug	25-JUL-2018	17:51	0	
B0067	Group B8	EOS/ET (Day 61)		Yes	D2	5 ug	25-JUL-2018	17:51	0	
B0067	Group B8	EOS/ET (Day 61)		Yes	D3	5 ug	25-JUL-2018	17:51	0	
B0068	Group B5	Day 1	1 min	Yes	F1	uncoated	25-MAY-2018	15:21	1	
B0068	Group B5	Day 1	1 min	Yes	F2	5 ug	25-MAY-2018	15:59	1	
B0068	Group B5	Day 1	1 min	Yes	F3	uncoated	25-MAY-2018	15:25	1	
B0068	Group B5	Day 1	10 min	Yes	F1	uncoated	25-MAY-2018	15:36	1	
B0068	Group B5	Day 1	10 min	Yes	F2	5 ug	25-MAY-2018	16:10	1	

Scale: 0= No Pain; 10= Worst Pain Possible
Administration Site Key: FA = Forearm; UA= Upper arm
Treatments Part A: A1 = A/Sing MAP, 15 ug (FA); A2 = Afluria Quad IM (UA), 15 ug; A3 = Placebo MAP (FA); A4 = A/Sing Mono IM, 15 ug (UA)
Treatments Part B: B1 = A/Sing MAP, 15 ug (FA) with biopsy; B2 = Placebo MAP (FA) with biopsy
B3 = A/Sing MAP, 15 ug (FA); B4 = A/Sing MAP, 10 ug (FA); B5 = A/Sing MAP, 5 ug (FA); B6 = A/Sing MAP, 2.5 ug (FA)
B7 = Placebo MAP (FA); B8 = A/Sing MAP, 15 ug (UA); B9 = A/Sing IM, 15 ug, as 2018 Afluria Quad (UA)

Listing 16.2.9.3
Numeric Pain Intensity per Site

Part B

Randomisation Number	Group	Visit	Time Point	Was Pain Assessment Performed?	Site Number	Treatment	Date of assessment	Time of Assessment	Score	Unscheduled Reason
B0068	Group B5	Day 1	10 min	Yes	F3	uncoated	25-MAY-2018	15:36	1	
B0068	Group B5	Day 1	1 hour	Yes	F1	uncoated	25-MAY-2018	17:00	0	
B0068	Group B5	Day 1	1 hour	Yes	F2	5 ug	25-MAY-2018	17:00	0	
B0068	Group B5	Day 1	1 hour	Yes	F3	uncoated	25-MAY-2018	17:00	0	
B0068	Group B5	Day 1	2 hour	Yes	F1	uncoated	25-MAY-2018	18:00	0	
B0068	Group B5	Day 1	2 hour	Yes	F2	5 ug	25-MAY-2018	18:00	0	
B0068	Group B5	Day 1	2 hour	Yes	F3	uncoated	25-MAY-2018	18:00	0	
B0068	Group B5	Day 8		Yes	F1	uncoated	01-JUN-2018	09:58	0	
B0068	Group B5	Day 8		Yes	F2	5 ug	01-JUN-2018	09:58	0	
B0068	Group B5	Day 8		Yes	F3	uncoated	01-JUN-2018	09:58	0	
B0068	Group B5	Day 22		Yes	F1	uncoated	16-JUN-2018	08:13	0	
B0068	Group B5	Day 22		Yes	F2	5 ug	16-JUN-2018	08:13	0	
B0068	Group B5	Day 22		Yes	F3	uncoated	16-JUN-2018	08:13	0	
B0068	Group B5	EOS/ET (Day 61)		Yes	F1	uncoated	24-JUL-2018	10:42	0	
B0068	Group B5	EOS/ET (Day 61)		Yes	F2	5 ug	24-JUL-2018	10:42	0	
B0068	Group B5	EOS/ET (Day 61)		Yes	F3	uncoated	24-JUL-2018	10:43	0	
B0069	Group B7	Day 1	1 min	Yes	F1	uncoated	01-JUN-2018	12:01	0	
B0069	Group B7	Day 1	1 min	Yes	F2	uncoated	01-JUN-2018	12:04	0	
B0069	Group B7	Day 1	1 min	Yes	F3	uncoated	01-JUN-2018	12:07	0	
B0069	Group B7	Day 1	10 min	Yes	F1	uncoated	01-JUN-2018	12:16	0	
B0069	Group B7	Day 1	10 min	Yes	F2	uncoated	01-JUN-2018	12:16	0	

Scale: 0= No Pain; 10= Worst Pain Possible
Administration Site Key: FA = Forearm; UA= Upper arm
Treatments Part A: A1 = A/Sing MAP, 15 ug (FA); A2 = Afluria Quad IM (UA), 15 ug; A3 = Placebo MAP (FA); A4 = A/Sing Mono IM, 15 ug (UA)
Treatments Part B: B1 = A/Sing MAP, 15 ug (FA) with biopsy; B2 = Placebo MAP (FA) with biopsy
B3 = A/Sing MAP, 15 ug (FA); B4 = A/Sing MAP, 10 ug (FA); B5 = A/Sing MAP, 5 ug (FA); B6 = A/Sing MAP, 2.5 ug (FA)
B7 = Placebo MAP (FA); B8 = A/Sing MAP, 15 ug (UA); B9 = A/Sing IM, 15 ug, as 2018 Afluria Quad (UA)

Listing 16.2.9.3
Numeric Pain Intensity per Site

Part B

Randomisation Number	Group	Visit	Time Point	Was Pain Assessment Performed?	Site Number	Treatment	Date of assessment	Time of Assessment	Score	Unscheduled Reason
B0069	Group B7	Day 1	10 min	Yes	F3	uncoated	01-JUN-2018	12:16	0	
B0069	Group B7	Day 1	1 hour	Yes	F1	uncoated	01-JUN-2018	13:03	0	
B0069	Group B7	Day 1	1 hour	Yes	F2	uncoated	01-JUN-2018	13:08	0	
B0069	Group B7	Day 1	1 hour	Yes	F3	uncoated	01-JUN-2018	13:08	0	
B0069	Group B7	Day 1	2 hour	Yes	F1	uncoated	01-JUN-2018	14:02	0	
B0069	Group B7	Day 1	2 hour	Yes	F2	uncoated	01-JUN-2018	14:02	0	
B0069	Group B7	Day 1	2 hour	Yes	F3	uncoated	01-JUN-2018	14:02	0	
B0069	Group B7	Day 2		Yes	F1	uncoated	02-JUN-2018	14:27	0	
B0069	Group B7	Day 2		Yes	F2	uncoated	02-JUN-2018	14:27	0	
B0069	Group B7	Day 2		Yes	F3	uncoated	02-JUN-2018	14:27	0	
B0069	Group B7	Day 4		Yes	F1	uncoated	04-JUN-2018	11:03	0	
B0069	Group B7	Day 4		Yes	F2	uncoated	04-JUN-2018	11:03	0	
B0069	Group B7	Day 4		Yes	F3	uncoated	04-JUN-2018	11:03	0	
B0069	Group B7	Day 8		Yes	F1	uncoated	08-JUN-2018	13:53	0	
B0069	Group B7	Day 8		Yes	F2	uncoated	08-JUN-2018	13:53	0	
B0069	Group B7	Day 8		Yes	F3	uncoated	08-JUN-2018	13:53	0	
B0069	Group B7	Day 22		Yes	F1	uncoated	22-JUN-2018	10:11	0	
B0069	Group B7	Day 22		Yes	F2	uncoated	22-JUN-2018	10:11	0	
B0069	Group B7	Day 22		Yes	F3	uncoated	22-JUN-2018	10:11	0	
B0069	Group B7	EOS/ET (Day 61)		Yes	F1	uncoated	31-JUL-2018	10:44	0	
B0069	Group B7	EOS/ET (Day 61)		Yes	F2	uncoated	31-JUL-2018	10:44	0	

Scale: 0= No Pain; 10= Worst Pain Possible
Administration Site Key: FA = Forearm; UA= Upper arm
Treatments Part A: A1 = A/Sing MAP, 15 ug (FA); A2 = Afluria Quad IM (UA), 15 ug; A3 = Placebo MAP (FA); A4 = A/Sing Mono IM, 15 ug (UA)
Treatments Part B: B1 = A/Sing MAP, 15 ug (FA) with biopsy; B2 = Placebo MAP (FA) with biopsy
B3 = A/Sing MAP, 15 ug (FA); B4 = A/Sing MAP, 10 ug (FA); B5 = A/Sing MAP, 5 ug (FA); B6 = A/Sing MAP, 2.5 ug (FA)
B7 = Placebo MAP (FA); B8 = A/Sing MAP, 15 ug (UA); B9 = A/Sing IM, 15 ug, as 2018 Afluria Quad (UA)

Listing 16.2.9.3
Numeric Pain Intensity per Site

Part B

Randomisation Number	Group	Visit	Time Point	Was Pain Assessment Performed?	Site Number	Treatment	Date of assessment	Time of Assessment	Score	Unscheduled Reason
B0069	Group B7	EOS/ET (Day 61)		Yes	F3	uncoated	31-JUL-2018	10:44	0	
B0070	Group B9	Day 1	1 min	Yes	D1	[IM]	01-JUN-2018	11:21	1	
B0070	Group B9	Day 1	10 min	Yes	D1	[IM]	01-JUN-2018	11:30	1	
B0070	Group B9	Day 1	1 hour	Yes	D1	[IM]	01-JUN-2018	12:20	1	
B0070	Group B9	Day 1	2 hour	Yes	D1	[IM]	01-JUN-2018	13:22	1	
B0070	Group B9	Day 2		Yes	D1	[IM]	02-JUN-2018	11:53	0	
B0070	Group B9	Day 4		Yes	D1	[IM]	04-JUN-2018	12:21	0	
B0070	Group B9	Day 8		Yes	D1	[IM]	08-JUN-2018	10:13	0	
B0070	Group B9	Day 22		Yes	D1	[IM]	22-JUN-2018	10:14	0	
B0070	Group B9	EOS/ET (Day 61)		Yes	D1	[IM]	04-JUL-2018	12:02	0	
B0071	Group B4	Day 1	1 min	Yes	F1	5 ug	01-JUN-2018	11:31	0	
B0071	Group B4	Day 1	1 min	Yes	F2	uncoated	01-JUN-2018	11:34	1	
B0071	Group B4	Day 1	1 min	Yes	F3	5 ug	01-JUN-2018	11:37	1	
B0071	Group B4	Day 1	10 min	Yes	F1	5 ug	01-JUN-2018	11:46	0	
B0071	Group B4	Day 1	10 min	Yes	F2	uncoated	01-JUN-2018	11:46	0	
B0071	Group B4	Day 1	10 min	Yes	F3	5 ug	01-JUN-2018	11:46	0	
B0071	Group B4	Day 1	1 hour	Yes	F1	5 ug	01-JUN-2018	12:38	0	
B0071	Group B4	Day 1	1 hour	Yes	F2	uncoated	01-JUN-2018	12:38	0	
B0071	Group B4	Day 1	1 hour	Yes	F3	5 ug	01-JUN-2018	12:38	0	
B0071	Group B4	Day 1	2 hour	Yes	F1	5 ug	01-JUN-2018	13:38	0	

Scale: 0= No Pain; 10= Worst Pain Possible
Administration Site Key: FA = Forearm; UA= Upper arm
Treatments Part A: A1 = A/Sing MAP, 15 ug (FA); A2 = Afluria Quad IM (UA), 15 ug; A3 = Placebo MAP (FA); A4 = A/Sing Mono IM, 15 ug (UA)
Treatments Part B: B1 = A/Sing MAP, 15 ug (FA) with biopsy; B2 = Placebo MAP (FA) with biopsy
B3 = A/Sing MAP, 15 ug (FA); B4 = A/Sing MAP, 10 ug (FA); B5 = A/Sing MAP, 5 ug (FA); B6 = A/Sing MAP, 2.5 ug (FA)
B7 = Placebo MAP (FA); B8 = A/Sing MAP, 15 ug (UA); B9 = A/Sing IM, 15 ug, as 2018 Afluria Quad (UA)

Listing 16.2.9.3
Numeric Pain Intensity per Site

Part B

Randomisation Number	Group	Visit	Time Point	Was Pain Assessment Performed?	Site Number	Treatment	Date of assessment	Time of Assessment	Score	Unscheduled Reason
B0071	Group B4	Day 1	2 hour	Yes	F2	uncoated	01-JUN-2018	13:38	0	
B0071	Group B4	Day 1	2 hour	Yes	F3	5 ug	01-JUN-2018	13:38	0	
B0071	Group B4	Day 2		Yes	F1	5 ug	02-JUN-2018	12:03	0	
B0071	Group B4	Day 2		Yes	F2	uncoated	02-JUN-2018	12:03	0	
B0071	Group B4	Day 2		Yes	F3	5 ug	02-JUN-2018	12:03	0	
B0071	Group B4	Day 4		Yes	F1	5 ug	04-JUN-2018	12:06	0	
B0071	Group B4	Day 4		Yes	F2	uncoated	04-JUN-2018	12:06	0	
B0071	Group B4	Day 4		Yes	F3	5 ug	04-JUN-2018	12:06	0	
B0071	Group B4	Day 8		Yes	F1	5 ug	08-JUN-2018	08:17	0	
B0071	Group B4	Day 8		Yes	F2	uncoated	08-JUN-2018	08:17	0	
B0071	Group B4	Day 8		Yes	F3	5 ug	08-JUN-2018	08:18	0	
B0071	Group B4	Day 22		Yes	F1	5 ug	22-JUN-2018	13:30	0	
B0071	Group B4	Day 22		Yes	F2	uncoated	22-JUN-2018	13:30	0	
B0071	Group B4	Day 22		Yes	F3	5 ug	22-JUN-2018	13:30	0	
B0071	Group B4	EOS/ET (Day 61)		Yes	F1	5 ug	31-JUL-2018	09:33	0	
B0071	Group B4	EOS/ET (Day 61)		Yes	F2	uncoated	31-JUL-2018	09:33	0	
B0071	Group B4	EOS/ET (Day 61)		Yes	F3	5 ug	31-JUL-2018	09:33	0	
B0072	Group B3	Day 1	1 min	Yes	F1	5 ug	01-JUN-2018	11:41	2	
B0072	Group B3	Day 1	1 min	Yes	F2	5 ug	01-JUN-2018	11:44	3	
B0072	Group B3	Day 1	1 min	Yes	F3	5 ug	01-JUN-2018	11:47	3	
B0072	Group B3	Day 1	10 min	Yes	F1	5 ug	01-JUN-2018	11:58	1	

Scale: 0= No Pain; 10= Worst Pain Possible
Administration Site Key: FA = Forearm; UA= Upper arm
Treatments Part A: A1 = A/Sing MAP, 15 ug (FA); A2 = Afluria Quad IM (UA), 15 ug; A3 = Placebo MAP (FA); A4 = A/Sing Mono IM, 15 ug (UA)
Treatments Part B: B1 = A/Sing MAP, 15 ug (FA) with biopsy; B2 = Placebo MAP (FA) with biopsy
B3 = A/Sing MAP, 15 ug (FA); B4 = A/Sing MAP, 10 ug (FA); B5 = A/Sing MAP, 5 ug (FA); B6 = A/Sing MAP, 2.5 ug (FA)
B7 = Placebo MAP (FA); B8 = A/Sing MAP, 15 ug (UA); B9 = A/Sing IM, 15 ug, as 2018 Afluria Quad (UA)

Listing 16.2.9.3
Numeric Pain Intensity per Site

Part B

Randomisation Number	Group	Visit	Time Point	Was Pain Assessment Performed?	Site Number	Treatment	Date of assessment	Time of Assessment	Score	Unscheduled Reason
B0072	Group B3	Day 1	10 min	Yes	F2	5 ug	01-JUN-2018	11:58	1	
B0072	Group B3	Day 1	10 min	Yes	F3	5 ug	01-JUN-2018	11:58	1	
B0072	Group B3	Day 1	1 hour	Yes	F1	5 ug	01-JUN-2018	12:48	0	
B0072	Group B3	Day 1	1 hour	Yes	F2	5 ug	01-JUN-2018	12:48	0	
B0072	Group B3	Day 1	1 hour	Yes	F3	5 ug	01-JUN-2018	12:48	0	
B0072	Group B3	Day 1	2 hour	Yes	F1	5 ug	01-JUN-2018	13:45	0	
B0072	Group B3	Day 1	2 hour	Yes	F2	5 ug	01-JUN-2018	13:45	1	
B0072	Group B3	Day 1	2 hour	Yes	F3	5 ug	01-JUN-2018	13:45	1	
B0072	Group B3	Day 2		Yes	F1	5 ug	02-JUN-2018	12:10	0	
B0072	Group B3	Day 2		Yes	F2	5 ug	02-JUN-2018	12:10	0	
B0072	Group B3	Day 2		Yes	F3	5 ug	02-JUN-2018	12:10	0	
B0072	Group B3	Day 4		Yes	F1	5 ug	04-JUN-2018	12:23	0	
B0072	Group B3	Day 4		Yes	F2	5 ug	04-JUN-2018	12:23	0	
B0072	Group B3	Day 4		Yes	F3	5 ug	04-JUN-2018	12:23	0	
B0072	Group B3	Day 8		Yes	F1	5 ug	08-JUN-2018	13:46	0	
B0072	Group B3	Day 8		Yes	F2	5 ug	08-JUN-2018	13:46	0	
B0072	Group B3	Day 8		Yes	F3	5 ug	08-JUN-2018	13:46	0	
B0072	Group B3	Day 22		Yes	F1	5 ug	22-JUN-2018	12:36	0	
B0072	Group B3	Day 22		Yes	F2	5 ug	22-JUN-2018	12:36	0	
B0072	Group B3	Day 22		Yes	F3	5 ug	22-JUN-2018	12:36	0	
B0072	Group B3	EOS/ET (Day 61)		Yes	F1	5 ug	31-JUL-2018	12:11	0	

Scale: 0= No Pain; 10= Worst Pain Possible
Administration Site Key: FA = Forearm; UA= Upper arm
Treatments Part A: A1 = A/Sing MAP, 15 ug (FA); A2 = Afluria Quad IM (UA), 15 ug; A3 = Placebo MAP (FA); A4 = A/Sing Mono IM, 15 ug (UA)
Treatments Part B: B1 = A/Sing MAP, 15 ug (FA) with biopsy; B2 = Placebo MAP (FA) with biopsy
B3 = A/Sing MAP, 15 ug (FA); B4 = A/Sing MAP, 10 ug (FA); B5 = A/Sing MAP, 5 ug (FA); B6 = A/Sing MAP, 2.5 ug (FA)
B7 = Placebo MAP (FA); B8 = A/Sing MAP, 15 ug (UA); B9 = A/Sing IM, 15 ug, as 2018 Afluria Quad (UA)

Listing 16.2.9.3
Numeric Pain Intensity per Site

Part B

Randomisation Number	Group	Visit	Time Point	Was Pain Assessment Performed?	Site Number	Treatment	Date of assessment	Time of Assessment	Score	Unscheduled Reason
B0072	Group B3	EOS/ET (Day 61)		Yes	F2	5 ug	31-JUL-2018	12:12	0	
B0072	Group B3	EOS/ET (Day 61)		Yes	F3	5 ug	31-JUL-2018	12:12	0	
B0073	Group B6	Day 1	1 min	Yes	F1	uncoated	01-JUN-2018	11:51	1	
B0073	Group B6	Day 1	1 min	Yes	F2	2.5 ug	01-JUN-2018	11:54	1	
B0073	Group B6	Day 1	1 min	Yes	F3	uncoated	01-JUN-2018	11:57	1	
B0073	Group B6	Day 1	10 min	Yes	F1	uncoated	01-JUN-2018	12:05	1	
B0073	Group B6	Day 1	10 min	Yes	F2	2.5 ug	01-JUN-2018	12:05	1	
B0073	Group B6	Day 1	10 min	Yes	F3	uncoated	01-JUN-2018	12:05	1	
B0073	Group B6	Day 1	1 hour	Yes	F1	uncoated	01-JUN-2018	12:58	1	
B0073	Group B6	Day 1	1 hour	No	F2	2.5 ug				
B0073	Group B6	Day 1	1 hour	Yes	F3	uncoated	01-JUN-2018	12:58	1	
B0073	Group B6	Day 1	2 hour	Yes	F1	uncoated	01-JUN-2018	13:50	0	
B0073	Group B6	Day 1	2 hour	Yes	F2	2.5 ug	01-JUN-2018	13:50	0	
B0073	Group B6	Day 1	2 hour	Yes	F3	uncoated	01-JUN-2018	13:50	0	
B0073	Group B6	Day 2		Yes	F1	uncoated	02-JUN-2018	11:56	0	
B0073	Group B6	Day 2		Yes	F2	2.5 ug	02-JUN-2018	11:56	0	
B0073	Group B6	Day 2		Yes	F3	uncoated	02-JUN-2018	11:56	0	
B0073	Group B6	Day 4		Yes	F1	uncoated	04-JUN-2018	13:53	0	
B0073	Group B6	Day 4		Yes	F2	2.5 ug	04-JUN-2018	13:53	0	
B0073	Group B6	Day 4		Yes	F3	uncoated	04-JUN-2018	13:54	0	
B0073	Group B6	Day 8		Yes	F1	uncoated	08-JUN-2018	12:29	0	

Scale: 0= No Pain; 10= Worst Pain Possible
Administration Site Key: FA = Forearm; UA= Upper arm
Treatments Part A: A1 = A/Sing MAP, 15 ug (FA); A2 = Afluria Quad IM (UA), 15 ug; A3 = Placebo MAP (FA); A4 = A/Sing Mono IM, 15 ug (UA)
Treatments Part B: B1 = A/Sing MAP, 15 ug (FA) with biopsy; B2 = Placebo MAP (FA) with biopsy
B3 = A/Sing MAP, 15 ug (FA); B4 = A/Sing MAP, 10 ug (FA); B5 = A/Sing MAP, 5 ug (FA); B6 = A/Sing MAP, 2.5 ug (FA)
B7 = Placebo MAP (FA); B8 = A/Sing MAP, 15 ug (UA); B9 = A/Sing IM, 15 ug, as 2018 Afluria Quad (UA)

Listing 16.2.9.3
Numeric Pain Intensity per Site

Part B

Randomisation Number	Group	Visit	Time Point	Was Pain Assessment Performed?	Site Number	Treatment	Date of assessment	Time of Assessment	Score	Unscheduled Reason
B0073	Group B6	Day 8		Yes	F2	2.5 ug	08-JUN-2018	12:29	0	
B0073	Group B6	Day 8		Yes	F3	uncoated	08-JUN-2018	12:29	0	
B0073	Group B6	Day 22		Yes	F1	uncoated	22-JUN-2018	14:19	0	
B0073	Group B6	Day 22		Yes	F2	2.5 ug	22-JUN-2018	14:19	0	
B0073	Group B6	Day 22		Yes	F3	uncoated	22-JUN-2018	14:19	0	
B0073	Group B6	EOS/ET (Day 61)		Yes	F1	uncoated	31-JUL-2018	11:30	0	
B0073	Group B6	EOS/ET (Day 61)		Yes	F2	2.5 ug	31-JUL-2018	11:30	0	
B0073	Group B6	EOS/ET (Day 61)		Yes	F3	uncoated	31-JUL-2018	11:30	0	
B0074	Group B9	Day 1	1 min	Yes	D1	[IM]	01-JUN-2018	11:03	2	
B0074	Group B9	Day 1	10 min	Yes	D1	[IM]	01-JUN-2018	11:10	1	
B0074	Group B9	Day 1	1 hour	Yes	D1	[IM]	01-JUN-2018	12:02	0	
B0074	Group B9	Day 1	2 hour	Yes	D1	[IM]	01-JUN-2018	13:08	0	
B0074	Group B9	Day 2		No	D1	[IM]				
B0074	Group B9	Day 4		Yes	D1	[IM]	04-JUN-2018	15:02	0	
B0074	Group B9	Day 8		Yes	D1	[IM]	08-JUN-2018	12:07	0	
B0074	Group B9	Day 22		Yes	D1	[IM]	22-JUN-2018	11:56	0	
B0074	Group B9	EOS/ET (Day 61)		Yes	D1	[IM]	31-JUL-2018	16:42	0	
B0075	Group B3	Day 1	1 min	Yes	F1	5 ug	01-JUN-2018	12:11	2	
B0075	Group B3	Day 1	1 min	Yes	F2	5 ug	01-JUN-2018	12:14	2	
B0075	Group B3	Day 1	1 min	Yes	F3	5 ug	01-JUN-2018	12:17	2	

Scale: 0= No Pain; 10= Worst Pain Possible
Administration Site Key: FA = Forearm; UA= Upper arm
Treatments Part A: A1 = A/Sing MAP, 15 ug (FA); A2 = Afluria Quad IM (UA), 15 ug; A3 = Placebo MAP (FA); A4 = A/Sing Mono IM, 15 ug (UA)
Treatments Part B: B1 = A/Sing MAP, 15 ug (FA) with biopsy; B2 = Placebo MAP (FA) with biopsy
B3 = A/Sing MAP, 15 ug (FA); B4 = A/Sing MAP, 10 ug (FA); B5 = A/Sing MAP, 5 ug (FA); B6 = A/Sing MAP, 2.5 ug (FA)
B7 = Placebo MAP (FA); B8 = A/Sing MAP, 15 ug (UA); B9 = A/Sing IM, 15 ug, as 2018 Afluria Quad (UA)

Listing 16.2.9.3
Numeric Pain Intensity per Site

Part B

Randomisation Number	Group	Visit	Time Point	Was Pain Assessment Performed?	Site Number	Treatment	Date of assessment	Time of Assessment	Score	Unscheduled Reason
B0075	Group B3	Day 1	10 min	Yes	F1	5 ug	01-JUN-2018	12:25	2	
B0075	Group B3	Day 1	10 min	Yes	F2	5 ug	01-JUN-2018	12:25	3	
B0075	Group B3	Day 1	10 min	Yes	F3	5 ug	01-JUN-2018	12:25	3	
B0075	Group B3	Day 1	1 hour	Yes	F1	5 ug	01-JUN-2018	13:18	1	
B0075	Group B3	Day 1	1 hour	Yes	F2	5 ug	01-JUN-2018	13:18	2	
B0075	Group B3	Day 1	1 hour	Yes	F3	5 ug	01-JUN-2018	13:18	3	
B0075	Group B3	Day 1	2 hour	Yes	F1	5 ug	01-JUN-2018	14:02	0	
B0075	Group B3	Day 1	2 hour	Yes	F2	5 ug	01-JUN-2018	14:02	0	
B0075	Group B3	Day 1	2 hour	Yes	F3	5 ug	01-JUN-2018	14:02	1	
B0075	Group B3	Day 2		Yes	F1	5 ug	02-JUN-2018	08:24	0	
B0075	Group B3	Day 2		Yes	F2	5 ug	02-JUN-2018	08:24	0	
B0075	Group B3	Day 2		Yes	F3	5 ug	02-JUN-2018	08:24	1	
B0075	Group B3	Day 4		Yes	F1	5 ug	04-JUN-2018	08:51	0	
B0075	Group B3	Day 4		Yes	F2	5 ug	04-JUN-2018	08:51	0	
B0075	Group B3	Day 4		Yes	F3	5 ug	04-JUN-2018	08:51	0	
B0075	Group B3	Day 8		Yes	F1	5 ug	08-JUN-2018	12:38	0	
B0075	Group B3	Day 8		Yes	F2	5 ug	08-JUN-2018	12:38	0	
B0075	Group B3	Day 8		Yes	F3	5 ug	08-JUN-2018	12:38	0	
B0075	Group B3	Day 22		Yes	F1	5 ug	22-JUN-2018	13:31	0	
B0075	Group B3	Day 22		Yes	F2	5 ug	22-JUN-2018	13:31	0	
B0075	Group B3	Day 22		Yes	F3	5 ug	22-JUN-2018	13:31	0	

Scale: 0= No Pain; 10= Worst Pain Possible
Administration Site Key: FA = Forearm; UA= Upper arm
Treatments Part A: A1 = A/Sing MAP, 15 ug (FA); A2 = Afluria Quad IM (UA), 15 ug; A3 = Placebo MAP (FA); A4 = A/Sing Mono IM, 15 ug (UA)
Treatments Part B: B1 = A/Sing MAP, 15 ug (FA) with biopsy; B2 = Placebo MAP (FA) with biopsy
B3 = A/Sing MAP, 15 ug (FA); B4 = A/Sing MAP, 10 ug (FA); B5 = A/Sing MAP, 5 ug (FA); B6 = A/Sing MAP, 2.5 ug (FA)
B7 = Placebo MAP (FA); B8 = A/Sing MAP, 15 ug (UA); B9 = A/Sing IM, 15 ug, as 2018 Afluria Quad (UA)

Listing 16.2.9.3
Numeric Pain Intensity per Site

Part B

Randomisation Number	Group	Visit	Time Point	Was Pain Assessment Performed?	Site Number	Treatment	Date of assessment	Time of Assessment	Score	Unscheduled Reason
B0075	Group B3	EOS/ET (Day 61)		Yes	F1	5 ug	31-JUL-2018	08:27	0	
B0075	Group B3	EOS/ET (Day 61)		Yes	F2	5 ug	31-JUL-2018	08:27	0	
B0075	Group B3	EOS/ET (Day 61)		Yes	F3	5 ug	31-JUL-2018	08:27	0	
B0077	Group B4	Day 1	1 min	Yes	F1	5 ug	01-JUN-2018	12:21	2	
B0077	Group B4	Day 1	1 min	Yes	F2	uncoated	01-JUN-2018	12:24	2	
B0077	Group B4	Day 1	1 min	Yes	F3	5 ug	01-JUN-2018	12:27	3	
B0077	Group B4	Day 1	10 min	Yes	F1	5 ug	01-JUN-2018	12:37	0	
B0077	Group B4	Day 1	10 min	Yes	F2	uncoated	01-JUN-2018	12:37	0	
B0077	Group B4	Day 1	10 min	Yes	F3	5 ug	01-JUN-2018	12:37	0	
B0077	Group B4	Day 1	1 hour	Yes	F1	5 ug	01-JUN-2018	13:28	0	
B0077	Group B4	Day 1	1 hour	Yes	F2	uncoated	01-JUN-2018	13:28	0	
B0077	Group B4	Day 1	1 hour	Yes	F3	5 ug	01-JUN-2018		0	
B0077	Group B4	Day 1	2 hour	Yes	F1	5 ug	01-JUN-2018	14:21	0	
B0077	Group B4	Day 1	2 hour	Yes	F2	uncoated	01-JUN-2018	14:21	1	
B0077	Group B4	Day 1	2 hour	Yes	F3	5 ug	01-JUN-2018	14:21	0	
B0077	Group B4	Day 2		Yes	F1	5 ug	02-JUN-2018	14:42	0	
B0077	Group B4	Day 2		Yes	F2	uncoated	02-JUN-2018	14:42	0	
B0077	Group B4	Day 2		Yes	F3	5 ug	02-JUN-2018	14:42	0	
B0077	Group B4	Day 4		Yes	F1	5 ug	04-JUN-2018	08:45	0	
B0077	Group B4	Day 4		Yes	F2	uncoated	04-JUN-2018	08:45	0	
B0077	Group B4	Day 4		Yes	F3	5 ug	04-JUN-2018	08:45	0	

Scale: 0= No Pain; 10= Worst Pain Possible
Administration Site Key: FA = Forearm; UA= Upper arm
Treatments Part A: A1 = A/Sing MAP, 15 ug (FA); A2 = Afluria Quad IM (UA), 15 ug; A3 = Placebo MAP (FA); A4 = A/Sing Mono IM, 15 ug (UA)
Treatments Part B: B1 = A/Sing MAP, 15 ug (FA) with biopsy; B2 = Placebo MAP (FA) with biopsy
B3 = A/Sing MAP, 15 ug (FA); B4 = A/Sing MAP, 10 ug (FA); B5 = A/Sing MAP, 5 ug (FA); B6 = A/Sing MAP, 2.5 ug (FA)
B7 = Placebo MAP (FA); B8 = A/Sing MAP, 15 ug (UA); B9 = A/Sing IM, 15 ug, as 2018 Afluria Quad (UA)

Listing 16.2.9.3
Numeric Pain Intensity per Site

Part B

Randomisation Number	Group	Visit	Time Point	Was Pain Assessment Performed?	Site Number	Treatment	Date of assessment	Time of Assessment	Score	Unscheduled Reason
B0077	Group B4	Day 8		Yes	F1	5 ug	08-JUN-2018	18:57	0	
B0077	Group B4	Day 8		Yes	F2	uncoated	08-JUN-2018	18:57	0	
B0077	Group B4	Day 8		Yes	F3	5 ug	08-JUN-2018	18:57	0	
B0077	Group B4	Day 22		Yes	F1	5 ug	22-JUN-2018	14:42	0	
B0077	Group B4	Day 22		Yes	F2	uncoated	22-JUN-2018	14:42	0	
B0077	Group B4	Day 22		Yes	F3	5 ug	22-JUN-2018	14:42	0	
B0077	Group B4	EOS/ET (Day 61)		Yes	F1	5 ug	30-JUL-2018	15:30	0	
B0077	Group B4	EOS/ET (Day 61)		Yes	F2	uncoated	30-JUL-2018	15:30	0	
B0077	Group B4	EOS/ET (Day 61)		Yes	F3	5 ug	30-JUL-2018	15:30	0	
B0078	Group B8	Day 1	1 min	Yes	D1	5 ug	01-JUN-2018	12:31	0	
B0078	Group B8	Day 1	1 min	Yes	D2	5 ug	01-JUN-2018	12:34	1	
B0078	Group B8	Day 1	1 min	Yes	D3	5 ug	01-JUN-2018	12:37	1	
B0078	Group B8	Day 1	10 min	Yes	D1	5 ug	01-JUN-2018	12:46	0	
B0078	Group B8	Day 1	10 min	Yes	D2	5 ug	01-JUN-2018	12:46	0	
B0078	Group B8	Day 1	10 min	Yes	D3	5 ug	01-JUN-2018	12:46	1	
B0078	Group B8	Day 1	1 hour	Yes	D1	5 ug	01-JUN-2018	13:38	0	
B0078	Group B8	Day 1	1 hour	Yes	D2	5 ug	01-JUN-2018	13:38	0	
B0078	Group B8	Day 1	1 hour	Yes	D3	5 ug	01-JUN-2018	13:38	0	
B0078	Group B8	Day 1	2 hour	Yes	D1	5 ug	01-JUN-2018	14:30	0	
B0078	Group B8	Day 1	2 hour	Yes	D2	5 ug	01-JUN-2018	14:30	0	
B0078	Group B8	Day 1	2 hour	Yes	D3	5 ug	01-JUN-2018	14:30	0	

Scale: 0= No Pain; 10= Worst Pain Possible
Administration Site Key: FA = Forearm; UA= Upper arm
Treatments Part A: A1 = A/Sing MAP, 15 ug (FA); A2 = Afluria Quad IM (UA), 15 ug; A3 = Placebo MAP (FA); A4 = A/Sing Mono IM, 15 ug (UA)
Treatments Part B: B1 = A/Sing MAP, 15 ug (FA) with biopsy; B2 = Placebo MAP (FA) with biopsy
B3 = A/Sing MAP, 15 ug (FA); B4 = A/Sing MAP, 10 ug (FA); B5 = A/Sing MAP, 5 ug (FA); B6 = A/Sing MAP, 2.5 ug (FA)
B7 = Placebo MAP (FA); B8 = A/Sing MAP, 15 ug (UA); B9 = A/Sing IM, 15 ug, as 2018 Afluria Quad (UA)

Listing 16.2.9.3
Numeric Pain Intensity per Site

Part B

Randomisation Number	Group	Visit	Time Point	Was Pain Assessment Performed?	Site Number	Treatment	Date of assessment	Time of Assessment	Score	Unscheduled Reason
B0078	Group B8	Day 2		Yes	D1	5 ug	02-JUN-2018	10:10	0	
B0078	Group B8	Day 2		Yes	D2	5 ug	02-JUN-2018	10:10	0	
B0078	Group B8	Day 2		Yes	D3	5 ug	02-JUN-2018	10:10	0	
B0078	Group B8	Day 4		Yes	D1	5 ug	04-JUN-2018	11:31	0	
B0078	Group B8	Day 4		Yes	D2	5 ug	04-JUN-2018	11:31	0	
B0078	Group B8	Day 4		Yes	D3	5 ug	04-JUN-2018	11:31	0	
B0078	Group B8	Day 8		Yes	D1	5 ug	08-JUN-2018	10:57	0	
B0078	Group B8	Day 8		Yes	D2	5 ug	08-JUN-2018	10:57	0	
B0078	Group B8	Day 8		Yes	D3	5 ug	08-JUN-2018	10:58	0	
B0078	Group B8	Day 22		Yes	D1	5 ug	22-JUN-2018	12:09	0	
B0078	Group B8	Day 22		Yes	D2	5 ug	22-JUN-2018	12:09	0	
B0078	Group B8	Day 22		Yes	D3	5 ug	22-JUN-2018	12:09	0	
B0078	Group B8	EOS/ET (Day 61)		Yes	D1	5 ug	06-AUG-2018	18:01	0	
B0078	Group B8	EOS/ET (Day 61)		No	D2	5 ug				
B0078	Group B8	EOS/ET (Day 61)		No	D3	5 ug				
B0079	Group B6	Day 1	1 min	Yes	F1	uncoated	01-JUN-2018	12:41	1	
B0079	Group B6	Day 1	1 min	Yes	F2	2.5 ug	01-JUN-2018	12:44	1	
B0079	Group B6	Day 1	1 min	Yes	F3	uncoated	01-JUN-2018	12:47	1	
B0079	Group B6	Day 1	10 min	Yes	F1	uncoated	01-JUN-2018	12:55	0	
B0079	Group B6	Day 1	10 min	Yes	F2	2.5 ug	01-JUN-2018	12:55	0	
B0079	Group B6	Day 1	10 min	Yes	F3	uncoated	01-JUN-2018	12:55	0	

Scale: 0= No Pain; 10= Worst Pain Possible
Administration Site Key: FA = Forearm; UA= Upper arm
Treatments Part A: A1 = A/Sing MAP, 15 ug (FA); A2 = Afluria Quad IM (UA), 15 ug; A3 = Placebo MAP (FA); A4 = A/Sing Mono IM, 15 ug (UA)
Treatments Part B: B1 = A/Sing MAP, 15 ug (FA) with biopsy; B2 = Placebo MAP (FA) with biopsy
B3 = A/Sing MAP, 15 ug (FA); B4 = A/Sing MAP, 10 ug (FA); B5 = A/Sing MAP, 5 ug (FA); B6 = A/Sing MAP, 2.5 ug (FA)
B7 = Placebo MAP (FA); B8 = A/Sing MAP, 15 ug (UA); B9 = A/Sing IM, 15 ug, as 2018 Afluria Quad (UA)

Listing 16.2.9.3
Numeric Pain Intensity per Site

Part B

Randomisation Number	Group	Visit	Time Point	Was Pain Assessment Performed?	Site Number	Treatment	Date of assessment	Time of Assessment	Score	Unscheduled Reason
B0079	Group B6	Day 1	1 hour	Yes	F1	uncoated	01-JUN-2018	13:48	0	
B0079	Group B6	Day 1	1 hour	Yes	F2	2.5 ug	01-JUN-2018	13:48	0	
B0079	Group B6	Day 1	1 hour	Yes	F3	uncoated	01-JUN-2018	13:48	0	
B0079	Group B6	Day 1	2 hour	Yes	F1	uncoated	01-JUN-2018	14:40	0	
B0079	Group B6	Day 1	2 hour	Yes	F2	2.5 ug	01-JUN-2018	14:40	0	
B0079	Group B6	Day 1	2 hour	Yes	F3	uncoated	01-JUN-2018	14:40	0	
B0079	Group B6	Day 2		Yes	F1	uncoated	02-JUN-2018	11:47	0	
B0079	Group B6	Day 2		Yes	F2	2.5 ug	02-JUN-2018	11:47	0	
B0079	Group B6	Day 2		Yes	F3	uncoated	02-JUN-2018	11:47	0	
B0079	Group B6	Day 4		Yes	F1	uncoated	04-JUN-2018	11:23	0	
B0079	Group B6	Day 4		Yes	F2	2.5 ug	04-JUN-2018	11:23	0	
B0079	Group B6	Day 4		Yes	F3	uncoated	04-JUN-2018	11:23	0	
B0079	Group B6	Day 8		Yes	F1	uncoated	08-JUN-2018	11:51	0	
B0079	Group B6	Day 8		Yes	F2	2.5 ug	08-JUN-2018	11:51	0	
B0079	Group B6	Day 8		Yes	F3	uncoated	08-JUN-2018	11:51	0	
B0079	Group B6	Day 22		Yes	F1	uncoated	22-JUN-2018	12:00	0	
B0079	Group B6	Day 22		Yes	F2	2.5 ug	22-JUN-2018	12:00	0	
B0079	Group B6	Day 22		Yes	F3	uncoated	22-JUN-2018	12:00	0	
B0079	Group B6	EOS/ET (Day 61)		Yes	F1	uncoated	31-JUL-2018	12:18	0	
B0079	Group B6	EOS/ET (Day 61)		Yes	F2	2.5 ug	31-JUL-2018	12:18	0	
B0079	Group B6	EOS/ET (Day 61)		Yes	F3	uncoated	31-JUL-2018	12:18	0	

Scale: 0= No Pain; 10= Worst Pain Possible
Administration Site Key: FA = Forearm; UA= Upper arm
Treatments Part A: A1 = A/Sing MAP, 15 ug (FA); A2 = Afluria Quad IM (UA), 15 ug; A3 = Placebo MAP (FA); A4 = A/Sing Mono IM, 15 ug (UA)
Treatments Part B: B1 = A/Sing MAP, 15 ug (FA) with biopsy; B2 = Placebo MAP (FA) with biopsy
B3 = A/Sing MAP, 15 ug (FA); B4 = A/Sing MAP, 10 ug (FA); B5 = A/Sing MAP, 5 ug (FA); B6 = A/Sing MAP, 2.5 ug (FA)
B7 = Placebo MAP (FA); B8 = A/Sing MAP, 15 ug (UA); B9 = A/Sing IM, 15 ug, as 2018 Afluria Quad (UA)

Listing 16.2.9.3
Numeric Pain Intensity per Site

Part B

Randomisation Number	Group	Visit	Time Point	Was Pain Assessment Performed?	Site Number	Treatment	Date of assessment	Time of Assessment	Score	Unscheduled Reason
B0081	Group B6	Day 1	1 min	Yes	F1	2.5 ug	01-JUN-2018	12:51	2	
B0081	Group B6	Day 1	1 min	Yes	F2	uncoated	01-JUN-2018	12:54	1	
B0081	Group B6	Day 1	1 min	Yes	F3	uncoated	01-JUN-2018	12:57	2	
B0081	Group B6	Day 1	10 min	Yes	F1	2.5 ug	01-JUN-2018	13:06	0	
B0081	Group B6	Day 1	10 min	Yes	F2	uncoated	01-JUN-2018	13:06	0	
B0081	Group B6	Day 1	10 min	Yes	F3	uncoated	01-JUN-2018	13:06	0	
B0081	Group B6	Day 1	1 hour	Yes	F1	2.5 ug	01-JUN-2018	13:58	0	
B0081	Group B6	Day 1	1 hour	Yes	F2	uncoated	01-JUN-2018	13:58	0	
B0081	Group B6	Day 1	1 hour	Yes	F3	uncoated	01-JUN-2018	13:58	0	
B0081	Group B6	Day 1	2 hour	Yes	F1	2.5 ug	01-JUN-2018	14:50	0	
B0081	Group B6	Day 1	2 hour	Yes	F2	uncoated	01-JUN-2018	14:50	0	
B0081	Group B6	Day 1	2 hour	Yes	F3	uncoated	01-JUN-2018	14:50	0	
B0081	Group B6	Day 2		Yes	F1	2.5 ug	02-JUN-2018	13:49	0	
B0081	Group B6	Day 2		Yes	F2	uncoated	02-JUN-2018	13:49	0	
B0081	Group B6	Day 2		Yes	F3	uncoated	02-JUN-2018	13:49	0	
B0081	Group B6	Day 4		Yes	F1	2.5 ug	04-JUN-2018	14:20	0	
B0081	Group B6	Day 4		Yes	F2	uncoated	04-JUN-2018	14:20	0	
B0081	Group B6	Day 4		Yes	F3	uncoated	04-JUN-2018	14:20	0	
B0081	Group B6	Day 8		Yes	F1	2.5 ug	08-JUN-2018	15:15	0	
B0081	Group B6	Day 8		Yes	F2	uncoated	08-JUN-2018	15:15	0	
B0081	Group B6	Day 8		Yes	F3	uncoated	08-JUN-2018	15:15	0	

Scale: 0= No Pain; 10= Worst Pain Possible
Administration Site Key: FA = Forearm; UA= Upper arm
Treatments Part A: A1 = A/Sing MAP, 15 ug (FA); A2 = Afluria Quad IM (UA), 15 ug; A3 = Placebo MAP (FA); A4 = A/Sing Mono IM, 15 ug (UA)
Treatments Part B: B1 = A/Sing MAP, 15 ug (FA) with biopsy; B2 = Placebo MAP (FA) with biopsy
B3 = A/Sing MAP, 15 ug (FA); B4 = A/Sing MAP, 10 ug (FA); B5 = A/Sing MAP, 5 ug (FA); B6 = A/Sing MAP, 2.5 ug (FA)
B7 = Placebo MAP (FA); B8 = A/Sing MAP, 15 ug (UA); B9 = A/Sing IM, 15 ug, as 2018 Afluria Quad (UA)

Listing 16.2.9.3
Numeric Pain Intensity per Site

Part B

Randomisation Number	Group	Visit	Time Point	Was Pain Assessment Performed?	Site Number	Treatment	Date of assessment	Time of Assessment	Score	Unscheduled Reason
B0081	Group B6	Day 22		Yes	F1	2.5 ug	23-JUN-2018	10:05	0	
B0081	Group B6	Day 22		Yes	F2	uncoated	23-JUN-2018	10:05	0	
B0081	Group B6	Day 22		Yes	F3	uncoated	23-JUN-2018	10:05	0	
B0081	Group B6	EOS/ET (Day 61)		Yes	F1	2.5 ug	31-JUL-2018	13:51	0	
B0081	Group B6	EOS/ET (Day 61)		Yes	F2	uncoated	31-JUL-2018	13:51	0	
B0081	Group B6	EOS/ET (Day 61)		Yes	F3	uncoated	31-JUL-2018	13:51	0	
B0082	Group B3	Day 1	1 min	Yes	F1	5 ug	01-JUN-2018	13:21	1	
B0082	Group B3	Day 1	1 min	Yes	F2	5 ug	01-JUN-2018	13:24	1	
B0082	Group B3	Day 1	1 min	Yes	F3	5 ug	01-JUN-2018	13:27	1	
B0082	Group B3	Day 1	10 min	Yes	F1	5 ug	01-JUN-2018	13:36	0	
B0082	Group B3	Day 1	10 min	Yes	F2	5 ug	01-JUN-2018	13:36	0	
B0082	Group B3	Day 1	10 min	Yes	F3	5 ug	01-JUN-2018	13:36	0	
B0082	Group B3	Day 1	1 hour	Yes	F1	5 ug	01-JUN-2018	14:28	0	
B0082	Group B3	Day 1	1 hour	Yes	F2	5 ug	01-JUN-2018	14:28	0	
B0082	Group B3	Day 1	1 hour	Yes	F3	5 ug	01-JUN-2018	14:28	0	
B0082	Group B3	Day 1	2 hour	Yes	F1	5 ug	01-JUN-2018	15:25	0	
B0082	Group B3	Day 1	2 hour	Yes	F2	5 ug	01-JUN-2018	15:25	0	
B0082	Group B3	Day 1	2 hour	Yes	F3	5 ug	01-JUN-2018	15:25	0	
B0082	Group B3	Day 2		Yes	F1	5 ug	02-JUN-2018	11:46	0	
B0082	Group B3	Day 2		Yes	F2	5 ug	02-JUN-2018	11:46	0	
B0082	Group B3	Day 2		Yes	F3	5 ug	02-JUN-2018	11:46	0	

Scale: 0= No Pain; 10= Worst Pain Possible
Administration Site Key: FA = Forearm; UA= Upper arm
Treatments Part A: A1 = A/Sing MAP, 15 ug (FA); A2 = Afluria Quad IM (UA), 15 ug; A3 = Placebo MAP (FA); A4 = A/Sing Mono IM, 15 ug (UA)
Treatments Part B: B1 = A/Sing MAP, 15 ug (FA) with biopsy; B2 = Placebo MAP (FA) with biopsy
B3 = A/Sing MAP, 15 ug (FA); B4 = A/Sing MAP, 10 ug (FA); B5 = A/Sing MAP, 5 ug (FA); B6 = A/Sing MAP, 2.5 ug (FA)
B7 = Placebo MAP (FA); B8 = A/Sing MAP, 15 ug (UA); B9 = A/Sing IM, 15 ug, as 2018 Afluria Quad (UA)

Listing 16.2.9.3
Numeric Pain Intensity per Site

Part B

Randomisation Number	Group	Visit	Time Point	Was Pain Assessment Performed?	Site Number	Treatment	Date of assessment	Time of Assessment	Score	Unscheduled Reason
B0082	Group B3	Day 4		Yes	F1	5 ug	04-JUN-2018	16:32	0	
B0082	Group B3	Day 4		Yes	F2	5 ug	04-JUN-2018	16:32	0	
B0082	Group B3	Day 4		Yes	F3	5 ug	04-JUN-2018	16:32	0	
B0082	Group B3	Day 8		Yes	F1	5 ug	08-JUN-2018	14:22	0	
B0082	Group B3	Day 8		Yes	F2	5 ug	08-JUN-2018	14:22	0	
B0082	Group B3	Day 8		Yes	F3	5 ug	08-JUN-2018	14:23	0	
B0082	Group B3	Day 22		Yes	F1	5 ug	22-JUN-2018	13:45	0	
B0082	Group B3	Day 22		Yes	F2	5 ug	22-JUN-2018	13:45	0	
B0082	Group B3	Day 22		Yes	F3	5 ug	22-JUN-2018	13:45	0	
B0082	Group B3	EOS/ET (Day 61)		Yes	F1	5 ug	31-JUL-2018	15:17	0	
B0082	Group B3	EOS/ET (Day 61)		Yes	F2	5 ug	31-JUL-2018	15:17	0	
B0082	Group B3	EOS/ET (Day 61)		Yes	F3	5 ug	31-JUL-2018	15:17	0	
B0083	Group B8	Day 1	1 min	Yes	D1	5 ug	01-JUN-2018	13:41	1	
B0083	Group B8	Day 1	1 min	Yes	D2	5 ug	01-JUN-2018	13:44	1	
B0083	Group B8	Day 1	1 min	Yes	D3	5 ug	01-JUN-2018	13:47	1	
B0083	Group B8	Day 1	10 min	Yes	D1	5 ug	01-JUN-2018	13:56	0	
B0083	Group B8	Day 1	10 min	Yes	D2	5 ug	01-JUN-2018	13:56	0	
B0083	Group B8	Day 1	10 min	Yes	D3	5 ug	01-JUN-2018	13:56	0	
B0083	Group B8	Day 1	1 hour	Yes	D1	5 ug	01-JUN-2018	14:48	1	
B0083	Group B8	Day 1	1 hour	Yes	D2	5 ug	01-JUN-2018	14:48	1	
B0083	Group B8	Day 1	1 hour	Yes	D3	5 ug	01-JUN-2018	14:48	1	

Scale: 0= No Pain; 10= Worst Pain Possible
Administration Site Key: FA = Forearm; UA= Upper arm
Treatments Part A: A1 = A/Sing MAP, 15 ug (FA); A2 = Afluria Quad IM (UA), 15 ug; A3 = Placebo MAP (FA); A4 = A/Sing Mono IM, 15 ug (UA)
Treatments Part B: B1 = A/Sing MAP, 15 ug (FA) with biopsy; B2 = Placebo MAP (FA) with biopsy
B3 = A/Sing MAP, 15 ug (FA); B4 = A/Sing MAP, 10 ug (FA); B5 = A/Sing MAP, 5 ug (FA); B6 = A/Sing MAP, 2.5 ug (FA)
B7 = Placebo MAP (FA); B8 = A/Sing MAP, 15 ug (UA); B9 = A/Sing IM, 15 ug, as 2018 Afluria Quad (UA)

Listing 16.2.9.3
Numeric Pain Intensity per Site

Part B

Randomisation Number	Group	Visit	Time Point	Was Pain Assessment Performed?	Site Number	Treatment	Date of assessment	Time of Assessment	Score	Unscheduled Reason
B0083	Group B8	Day 1	2 hour	Yes	D1	5 ug	01-JUN-2018	15:40	0	
B0083	Group B8	Day 1	2 hour	Yes	D2	5 ug	01-JUN-2018	15:40	0	
B0083	Group B8	Day 1	2 hour	Yes	D3	5 ug	01-JUN-2018	15:40	0	
B0083	Group B8	Day 2		Yes	D1	5 ug	02-JUN-2018	09:52	0	
B0083	Group B8	Day 2		Yes	D2	5 ug	02-JUN-2018	09:52	0	
B0083	Group B8	Day 2		Yes	D3	5 ug	02-JUN-2018	09:52	0	
B0083	Group B8	Day 4		Yes	D1	5 ug	04-JUN-2018	14:02	0	
B0083	Group B8	Day 4		Yes	D2	5 ug	04-JUN-2018	14:02	0	
B0083	Group B8	Day 4		Yes	D3	5 ug	04-JUN-2018	14:02	0	
B0083	Group B8	Day 8		Yes	D1	5 ug	08-JUN-2018	14:03	0	
B0083	Group B8	Day 8		Yes	D2	5 ug	08-JUN-2018	14:03	0	
B0083	Group B8	Day 8		Yes	D3	5 ug	08-JUN-2018	14:03	0	
B0083	Group B8	Day 22		Yes	D1	5 ug	22-JUN-2018	14:35	0	
B0083	Group B8	Day 22		Yes	D2	5 ug	22-JUN-2018	14:35	0	
B0083	Group B8	Day 22		Yes	D3	5 ug	22-JUN-2018	14:35	0	
B0083	Group B8	EOS/ET (Day 61)		Yes	D1	5 ug	31-JUL-2018	08:33	0	
B0083	Group B8	EOS/ET (Day 61)		Yes	D2	5 ug	31-JUL-2018	08:33	0	
B0083	Group B8	EOS/ET (Day 61)		Yes	D3	5 ug	31-JUL-2018	08:33	0	
B0084	Group B5	Day 1	1 min	Yes	F1	uncoated	09-JUN-2018	11:51	1	
B0084	Group B5	Day 1	1 min	Yes	F2	uncoated	09-JUN-2018	11:54	2	
B0084	Group B5	Day 1	1 min	Yes	F3	5 ug	09-JUN-2018	11:57	1	

Scale: 0= No Pain; 10= Worst Pain Possible
Administration Site Key: FA = Forearm; UA= Upper arm
Treatments Part A: A1 = A/Sing MAP, 15 ug (FA); A2 = Afluria Quad IM (UA), 15 ug; A3 = Placebo MAP (FA); A4 = A/Sing Mono IM, 15 ug (UA)
Treatments Part B: B1 = A/Sing MAP, 15 ug (FA) with biopsy; B2 = Placebo MAP (FA) with biopsy
B3 = A/Sing MAP, 15 ug (FA); B4 = A/Sing MAP, 10 ug (FA); B5 = A/Sing MAP, 5 ug (FA); B6 = A/Sing MAP, 2.5 ug (FA)
B7 = Placebo MAP (FA); B8 = A/Sing MAP, 15 ug (UA); B9 = A/Sing IM, 15 ug, as 2018 Afluria Quad (UA)

Listing 16.2.9.3
Numeric Pain Intensity per Site

Part B

Randomisation Number	Group	Visit	Time Point	Was Pain Assessment Performed?	Site Number	Treatment	Date of assessment	Time of Assessment	Score	Unscheduled Reason
B0084	Group B5	Day 1	10 min	Yes	F1	uncoated	09-JUN-2018	12:07	0	
B0084	Group B5	Day 1	10 min	Yes	F2	uncoated	09-JUN-2018	12:07	0	
B0084	Group B5	Day 1	10 min	Yes	F3	5 ug	09-JUN-2018	12:07	0	
B0084	Group B5	Day 1	1 hour	Yes	F1	uncoated	09-JUN-2018	12:58	0	
B0084	Group B5	Day 1	1 hour	Yes	F2	uncoated	09-JUN-2018	12:58	0	
B0084	Group B5	Day 1	1 hour	Yes	F3	5 ug	09-JUN-2018	12:58	0	
B0084	Group B5	Day 1	2 hour	Yes	F1	uncoated	09-JUN-2018	13:58	0	
B0084	Group B5	Day 1	2 hour	Yes	F2	uncoated	09-JUN-2018	13:58	0	
B0084	Group B5	Day 1	2 hour	Yes	F3	5 ug	09-JUN-2018	13:58	0	
B0084	Group B5	Day 2		Yes	F1	uncoated	10-JUN-2018	12:02	0	
B0084	Group B5	Day 2		Yes	F2	uncoated	10-JUN-2018	12:02	0	
B0084	Group B5	Day 2		Yes	F3	5 ug	10-JUN-2018	12:02	0	
B0084	Group B5	Day 4		Yes	F1	uncoated	12-JUN-2018	15:19	0	
B0084	Group B5	Day 4		Yes	F2	uncoated	12-JUN-2018	15:19	0	
B0084	Group B5	Day 4		Yes	F3	5 ug	12-JUN-2018	15:19	0	
B0084	Group B5	Day 8		Yes	F1	uncoated	16-JUN-2018	13:10	0	
B0084	Group B5	Day 8		Yes	F2	uncoated	16-JUN-2018	13:10	0	
B0084	Group B5	Day 8		Yes	F3	5 ug	16-JUN-2018	13:10	0	
B0084	Group B5	Day 22		Yes	F1	uncoated	30-JUN-2018	14:22	0	
B0084	Group B5	Day 22		Yes	F2	uncoated	30-JUN-2018	14:22	0	
B0084	Group B5	Day 22		Yes	F3	5 ug	30-JUN-2018	14:22	0	

Scale: 0= No Pain; 10= Worst Pain Possible
Administration Site Key: FA = Forearm; UA= Upper arm
Treatments Part A: A1 = A/Sing MAP, 15 ug (FA); A2 = Afluria Quad IM (UA), 15 ug; A3 = Placebo MAP (FA); A4 = A/Sing Mono IM, 15 ug (UA)
Treatments Part B: B1 = A/Sing MAP, 15 ug (FA) with biopsy; B2 = Placebo MAP (FA) with biopsy
B3 = A/Sing MAP, 15 ug (FA); B4 = A/Sing MAP, 10 ug (FA); B5 = A/Sing MAP, 5 ug (FA); B6 = A/Sing MAP, 2.5 ug (FA)
B7 = Placebo MAP (FA); B8 = A/Sing MAP, 15 ug (UA); B9 = A/Sing IM, 15 ug, as 2018 Afluria Quad (UA)

Listing 16.2.9.3
Numeric Pain Intensity per Site

Part B

Randomisation Number	Group	Visit	Time Point	Was Pain Assessment Performed?	Site Number	Treatment	Date of assessment	Time of Assessment	Score	Unscheduled Reason
B0084	Group B5	EOS/ET (Day 61)		Yes	F1	uncoated	08-AUG-2018	16:31	0	
B0084	Group B5	EOS/ET (Day 61)		Yes	F2	uncoated	08-AUG-2018	16:31	0	
B0084	Group B5	EOS/ET (Day 61)		Yes	F3	5 ug	08-AUG-2018	16:31	0	
B0085	Group B4	Day 1	1 min	Yes	F1	uncoated	09-JUN-2018	12:01	1	
B0085	Group B4	Day 1	1 min	Yes	F2	5 ug	09-JUN-2018	12:04	0	
B0085	Group B4	Day 1	1 min	Yes	F3	5 ug	09-JUN-2018	12:07	1	
B0085	Group B4	Day 1	10 min	Yes	F1	uncoated	09-JUN-2018	12:15	0	
B0085	Group B4	Day 1	10 min	Yes	F2	5 ug	09-JUN-2018	12:15	0	
B0085	Group B4	Day 1	10 min	Yes	F3	5 ug	09-JUN-2018	12:15	0	
B0085	Group B4	Day 1	1 hour	Yes	F1	uncoated	09-JUN-2018	13:08	0	
B0085	Group B4	Day 1	1 hour	Yes	F2	5 ug	09-JUN-2018	13:08	0	
B0085	Group B4	Day 1	1 hour	Yes	F3	5 ug	09-JUN-2018	13:08	0	
B0085	Group B4	Day 1	2 hour	Yes	F1	uncoated	09-JUN-2018	14:08	0	
B0085	Group B4	Day 1	2 hour	Yes	F2	5 ug	09-JUN-2018	14:08	0	
B0085	Group B4	Day 1	2 hour	Yes	F3	5 ug	09-JUN-2018	14:08	0	
B0085	Group B4	Day 2		Yes	F1	uncoated	10-JUN-2018	12:16	0	
B0085	Group B4	Day 2		Yes	F2	5 ug	10-JUN-2018	12:16	0	
B0085	Group B4	Day 2		Yes	F3	5 ug	10-JUN-2018	12:16	0	
B0085	Group B4	Day 4		Yes	F1	uncoated	12-JUN-2018	16:34	0	
B0085	Group B4	Day 4		Yes	F2	5 ug	12-JUN-2018	16:34	0	
B0085	Group B4	Day 4		Yes	F3	5 ug	12-JUN-2018	16:34	0	

Scale: 0= No Pain; 10= Worst Pain Possible
Administration Site Key: FA = Forearm; UA= Upper arm
Treatments Part A: A1 = A/Sing MAP, 15 ug (FA); A2 = Afluria Quad IM (UA), 15 ug; A3 = Placebo MAP (FA); A4 = A/Sing Mono IM, 15 ug (UA)
Treatments Part B: B1 = A/Sing MAP, 15 ug (FA) with biopsy; B2 = Placebo MAP (FA) with biopsy
B3 = A/Sing MAP, 15 ug (FA); B4 = A/Sing MAP, 10 ug (FA); B5 = A/Sing MAP, 5 ug (FA); B6 = A/Sing MAP, 2.5 ug (FA)
B7 = Placebo MAP (FA); B8 = A/Sing MAP, 15 ug (UA); B9 = A/Sing IM, 15 ug, as 2018 Afluria Quad (UA)

Listing 16.2.9.3
Numeric Pain Intensity per Site

Part B

Randomisation Number	Group	Visit	Time Point	Was Pain Assessment Performed?	Site Number	Treatment	Date of assessment	Time of Assessment	Score	Unscheduled Reason
B0085	Group B4	Day 8		Yes	F1	uncoated	16-JUN-2018	13:24	0	
B0085	Group B4	Day 8		Yes	F2	5 ug	16-JUN-2018	13:25	0	
B0085	Group B4	Day 8		Yes	F3	5 ug	16-JUN-2018	13:26	0	
B0085	Group B4	Day 22		Yes	F1	uncoated	30-JUN-2018	12:29	0	
B0085	Group B4	Day 22		Yes	F2	5 ug	30-JUN-2018	12:29	0	
B0085	Group B4	Day 22		Yes	F3	5 ug	30-JUN-2018	12:29		
B0085	Group B4	EOS/ET (Day 61)		Yes	F1	uncoated	11-AUG-2018	10:33	0	
B0085	Group B4	EOS/ET (Day 61)		Yes	F2	5 ug	11-AUG-2018	10:33	0	
B0085	Group B4	EOS/ET (Day 61)		Yes	F3	5 ug	11-AUG-2018	10:33	0	
B0086	Group B9	Day 1	1 min	Yes	D1	[IM]	09-JUN-2018	11:21	2	
B0086	Group B9	Day 1	10 min	Yes	D1	[IM]	09-JUN-2018	11:30	0	
B0086	Group B9	Day 1	1 hour	Yes	D1	[IM]	09-JUN-2018	12:20	0	
B0086	Group B9	Day 1	2 hour	Yes	D1	[IM]	09-JUN-2018	13:25	0	
B0086	Group B9	Day 2		Yes	D1	[IM]	10-JUN-2018	09:28	0	
B0086	Group B9	Day 4		Yes	D1	[IM]	12-JUN-2018	14:56		
B0086	Group B9	Day 8		Yes	D1	[IM]	16-JUN-2018	09:57	0	
B0086	Group B9	Day 22		Yes	D1	[IM]	02-JUL-2018	14:21	0	
B0086	Group B9	EOS/ET (Day 61)		Yes	D1	[IM]	09-AUG-2018	13:37	0	
B0087	Group B7	Day 1	1 min	Yes	F1	uncoated	09-JUN-2018	12:11	2	
B0087	Group B7	Day 1	1 min	Yes	F2	uncoated	09-JUN-2018	12:14	1	

Scale: 0= No Pain; 10= Worst Pain Possible
Administration Site Key: FA = Forearm; UA= Upper arm
Treatments Part A: A1 = A/Sing MAP, 15 ug (FA); A2 = Afluria Quad IM (UA), 15 ug; A3 = Placebo MAP (FA); A4 = A/Sing Mono IM, 15 ug (UA)
Treatments Part B: B1 = A/Sing MAP, 15 ug (FA) with biopsy; B2 = Placebo MAP (FA) with biopsy
B3 = A/Sing MAP, 15 ug (FA); B4 = A/Sing MAP, 10 ug (FA); B5 = A/Sing MAP, 5 ug (FA); B6 = A/Sing MAP, 2.5 ug (FA)
B7 = Placebo MAP (FA); B8 = A/Sing MAP, 15 ug (UA); B9 = A/Sing IM, 15 ug, as 2018 Afluria Quad (UA)

Listing 16.2.9.3
Numeric Pain Intensity per Site

Part B

Randomisation Number	Group	Visit	Time Point	Was Pain Assessment Performed?	Site Number	Treatment	Date of assessment	Time of Assessment	Score	Unscheduled Reason
B0087	Group B7	Day 1	1 min	Yes	F3	uncoated	09-JUN-2018	12:17	2	
B0087	Group B7	Day 1	10 min	Yes	F1	uncoated	09-JUN-2018	12:25	0	
B0087	Group B7	Day 1	10 min	Yes	F2	uncoated	09-JUN-2018	12:25	0	
B0087	Group B7	Day 1	10 min	Yes	F3	uncoated	09-JUN-2018	12:25	0	
B0087	Group B7	Day 1	1 hour	Yes	F1	uncoated	09-JUN-2018	13:18	0	
B0087	Group B7	Day 1	1 hour	Yes	F2	uncoated	09-JUN-2018	13:18	0	
B0087	Group B7	Day 1	1 hour	Yes	F3	uncoated	09-JUN-2018	13:18	0	
B0087	Group B7	Day 1	2 hour	Yes	F1	uncoated	09-JUN-2018	14:18	0	
B0087	Group B7	Day 1	2 hour	Yes	F2	uncoated	09-JUN-2018	14:18	0	
B0087	Group B7	Day 1	2 hour	Yes	F3	uncoated	09-JUN-2018	14:18	0	
B0087	Group B7	Day 2		Yes	F1	uncoated	10-JUN-2018	09:42	0	
B0087	Group B7	Day 2		Yes	F2	uncoated	10-JUN-2018	09:42	0	
B0087	Group B7	Day 2		Yes	F3	uncoated	10-JUN-2018	09:42	0	
B0087	Group B7	Day 4		Yes	F1	uncoated	12-JUN-2018	08:58	0	
B0087	Group B7	Day 4		Yes	F2	uncoated	12-JUN-2018	08:58	0	
B0087	Group B7	Day 4		Yes	F3	uncoated	12-JUN-2018	08:58		
B0087	Group B7	Day 8		Yes	F1	uncoated	16-JUN-2018	09:45	0	
B0087	Group B7	Day 8		Yes	F2	uncoated	16-JUN-2018	09:46	0	
B0087	Group B7	Day 8		Yes	F3	uncoated	16-JUN-2018	09:47	0	
B0087	Group B7	Day 22		Yes	F1	uncoated	03-JUL-2018	09:27	0	
B0087	Group B7	Day 22		Yes	F2	uncoated	03-JUL-2018	09:27	0	

Scale: 0= No Pain; 10= Worst Pain Possible
Administration Site Key: FA = Forearm; UA= Upper arm
Treatments Part A: A1 = A/Sing MAP, 15 ug (FA); A2 = Afluria Quad IM (UA), 15 ug; A3 = Placebo MAP (FA); A4 = A/Sing Mono IM, 15 ug (UA)
Treatments Part B: B1 = A/Sing MAP, 15 ug (FA) with biopsy; B2 = Placebo MAP (FA) with biopsy
B3 = A/Sing MAP, 15 ug (FA); B4 = A/Sing MAP, 10 ug (FA); B5 = A/Sing MAP, 5 ug (FA); B6 = A/Sing MAP, 2.5 ug (FA)
B7 = Placebo MAP (FA); B8 = A/Sing MAP, 15 ug (UA); B9 = A/Sing IM, 15 ug, as 2018 Afluria Quad (UA)

Listing 16.2.9.3
Numeric Pain Intensity per Site

Part B

Randomisation Number	Group	Visit	Time Point	Was Pain Assessment Performed?	Site Number	Treatment	Date of assessment	Time of Assessment	Score	Unscheduled Reason
B0087	Group B7	Day 22		Yes	F3	uncoated	03-JUL-2018	09:27	0	
B0087	Group B7	EOS/ET (Day 61)		Yes	F1	uncoated	07-AUG-2018	09:02	0	
B0087	Group B7	EOS/ET (Day 61)		Yes	F2	uncoated	07-AUG-2018	09:02	0	
B0087	Group B7	EOS/ET (Day 61)		Yes	F3	uncoated	07-AUG-2018	09:02	0	
B0088	Group B9	Day 1	1 min	Yes	D1	[IM]	09-JUN-2018	11:11	0	
B0088	Group B9	Day 1	10 min	Yes	D1	[IM]	09-JUN-2018	11:20	0	
B0088	Group B9	Day 1	1 hour	Yes	D1	[IM]	09-JUN-2018	12:10	0	
B0088	Group B9	Day 1	2 hour	Yes	D1	[IM]	09-JUN-2018	13:15	0	
B0088	Group B9	Day 2		Yes	D1	[IM]	10-JUN-2018	11:14	0	
B0088	Group B9	Day 4		Yes	D1	[IM]	12-JUN-2018	13:52	0	
B0088	Group B9	Day 8		Yes	D1	[IM]	16-JUN-2018	17:27	0	
B0088	Group B9	Day 22		Yes	D1	[IM]	30-JUN-2018	13:17	0	
B0088	Group B9	EOS/ET (Day 61)		Yes	D1	[IM]	14-AUG-2018	14:44	0	
B0089	Group B5	Day 1	1 min	Yes	F1	uncoated	09-JUN-2018	12:21	1	
B0089	Group B5	Day 1	1 min	Yes	F2	5 ug	09-JUN-2018	12:24	0	
B0089	Group B5	Day 1	1 min	Yes	F3	uncoated	09-JUN-2018	12:27	0	
B0089	Group B5	Day 1	10 min	Yes	F1	uncoated	09-JUN-2018	12:35	0	
B0089	Group B5	Day 1	10 min	Yes	F2	5 ug	09-JUN-2018	12:35	0	
B0089	Group B5	Day 1	10 min	Yes	F3	uncoated	09-JUN-2018	12:35	0	
B0089	Group B5	Day 1	1 hour	Yes	F1	uncoated	09-JUN-2018	13:28	0	

Scale: 0= No Pain; 10= Worst Pain Possible
Administration Site Key: FA = Forearm; UA= Upper arm
Treatments Part A: A1 = A/Sing MAP, 15 ug (FA); A2 = Afluria Quad IM (UA), 15 ug; A3 = Placebo MAP (FA); A4 = A/Sing Mono IM, 15 ug (UA)
Treatments Part B: B1 = A/Sing MAP, 15 ug (FA) with biopsy; B2 = Placebo MAP (FA) with biopsy
B3 = A/Sing MAP, 15 ug (FA); B4 = A/Sing MAP, 10 ug (FA); B5 = A/Sing MAP, 5 ug (FA); B6 = A/Sing MAP, 2.5 ug (FA)
B7 = Placebo MAP (FA); B8 = A/Sing MAP, 15 ug (UA); B9 = A/Sing IM, 15 ug, as 2018 Afluria Quad (UA)

Listing 16.2.9.3
Numeric Pain Intensity per Site

Part B

Randomisation Number	Group	Visit	Time Point	Was Pain Assessment Performed?	Site Number	Treatment	Date of assessment	Time of Assessment	Score	Unscheduled Reason
B0089	Group B5	Day 1	1 hour	Yes	F2	5 ug	09-JUN-2018	13:28	0	
B0089	Group B5	Day 1	1 hour	Yes	F3	uncoated	09-JUN-2018	13:28	0	
B0089	Group B5	Day 1	2 hour	Yes	F1	uncoated	09-JUN-2018	14:28	0	
B0089	Group B5	Day 1	2 hour	Yes	F2	5 ug	09-JUN-2018	14:28	0	
B0089	Group B5	Day 1	2 hour	Yes	F3	uncoated	09-JUN-2018	14:28	0	
B0089	Group B5	Day 2		Yes	F1	uncoated	10-JUN-2018	10:45	0	
B0089	Group B5	Day 2		Yes	F2	5 ug	10-JUN-2018	10:45	0	
B0089	Group B5	Day 2		Yes	F3	uncoated	10-JUN-2018	10:45	0	
B0089	Group B5	Day 4		Yes	F1	uncoated	12-JUN-2018	08:55	0	
B0089	Group B5	Day 4		Yes	F2	5 ug	12-JUN-2018	08:55	0	
B0089	Group B5	Day 4		Yes	F3	uncoated	12-JUN-2018	08:55	0	
B0089	Group B5	Day 8		Yes	F1	uncoated	16-JUN-2018	12:00	0	
B0089	Group B5	Day 8		Yes	F2	5 ug	16-JUN-2018	12:01	0	
B0089	Group B5	Day 8		Yes	F3	uncoated	16-JUN-2018	12:01	0	
B0089	Group B5	Day 22		Yes	F1	uncoated	30-JUN-2018	08:22	0	
B0089	Group B5	Day 22		Yes	F2	5 ug	30-JUN-2018	08:22	0	
B0089	Group B5	Day 22		Yes	F3	uncoated	30-JUN-2018	08:22	0	
B0089	Group B5	EOS/ET (Day 61)		Yes	F1	uncoated	08-AUG-2018	12:28	0	
B0089	Group B5	EOS/ET (Day 61)		Yes	F2	5 ug	08-AUG-2018	12:29	0	
B0089	Group B5	EOS/ET (Day 61)		Yes	F3	uncoated	08-AUG-2018	12:29	0	
B0090	Group B6	Day 1	1 min	Yes	F1	2.5 ug	09-JUN-2018	12:31	2	

Scale: 0= No Pain; 10= Worst Pain Possible
Administration Site Key: FA = Forearm; UA= Upper arm
Treatments Part A: A1 = A/Sing MAP, 15 ug (FA); A2 = Afluria Quad IM (UA), 15 ug; A3 = Placebo MAP (FA); A4 = A/Sing Mono IM, 15 ug (UA)
Treatments Part B: B1 = A/Sing MAP, 15 ug (FA) with biopsy; B2 = Placebo MAP (FA) with biopsy
B3 = A/Sing MAP, 15 ug (FA); B4 = A/Sing MAP, 10 ug (FA); B5 = A/Sing MAP, 5 ug (FA); B6 = A/Sing MAP, 2.5 ug (FA)
B7 = Placebo MAP (FA); B8 = A/Sing MAP, 15 ug (UA); B9 = A/Sing IM, 15 ug, as 2018 Afluria Quad (UA)

Listing 16.2.9.3
Numeric Pain Intensity per Site

Part B

Randomisation Number	Group	Visit	Time Point	Was Pain Assessment Performed?	Site Number	Treatment	Date of assessment	Time of Assessment	Score	Unscheduled Reason
B0090	Group B6	Day 1	1 min	Yes	F2	uncoated	09-JUN-2018	12:34	3	
B0090	Group B6	Day 1	1 min	Yes	F3	uncoated	09-JUN-2018	12:37	1	
B0090	Group B6	Day 1	10 min	Yes	F1	2.5 ug	09-JUN-2018	12:45	3	
B0090	Group B6	Day 1	10 min	Yes	F2	uncoated	09-JUN-2018	12:45	3	
B0090	Group B6	Day 1	10 min	Yes	F3	uncoated	09-JUN-2018	12:45	3	
B0090	Group B6	Day 1	1 hour	Yes	F1	2.5 ug	09-JUN-2018	13:38	3	
B0090	Group B6	Day 1	1 hour	Yes	F2	uncoated	09-JUN-2018	13:38	3	
B0090	Group B6	Day 1	1 hour	Yes	F3	uncoated	09-JUN-2018	13:38	3	
B0090	Group B6	Day 1	2 hour	Yes	F1	2.5 ug	09-JUN-2018	14:38	0	
B0090	Group B6	Day 1	2 hour	Yes	F2	uncoated	09-JUN-2018	14:38	0	
B0090	Group B6	Day 1	2 hour	Yes	F3	uncoated	09-JUN-2018	14:38	0	
B0090	Group B6	Day 2		Yes	F1	2.5 ug	10-JUN-2018	11:46	0	
B0090	Group B6	Day 2		Yes	F2	uncoated	10-JUN-2018	11:46	0	
B0090	Group B6	Day 2		Yes	F3	uncoated	10-JUN-2018	11:46	0	
B0090	Group B6	Day 4		Yes	F1	2.5 ug	12-JUN-2018	10:55	0	
B0090	Group B6	Day 4		Yes	F2	uncoated	12-JUN-2018	10:55	0	
B0090	Group B6	Day 4		Yes	F3	uncoated	12-JUN-2018	10:55	0	
B0090	Group B6	Day 8		Yes	F1	2.5 ug	16-JUN-2018	11:39	0	
B0090	Group B6	Day 8		Yes	F2	uncoated	16-JUN-2018	11:39	0	
B0090	Group B6	Day 8		Yes	F3	uncoated	16-JUN-2018	11:40	0	
B0090	Group B6	Day 22		Yes	F1	2.5 ug	02-JUL-2018	10:35	0	

Scale: 0= No Pain; 10= Worst Pain Possible
Administration Site Key: FA = Forearm; UA= Upper arm
Treatments Part A: A1 = A/Sing MAP, 15 ug (FA); A2 = Afluria Quad IM (UA), 15 ug; A3 = Placebo MAP (FA); A4 = A/Sing Mono IM, 15 ug (UA)
Treatments Part B: B1 = A/Sing MAP, 15 ug (FA) with biopsy; B2 = Placebo MAP (FA) with biopsy
B3 = A/Sing MAP, 15 ug (FA); B4 = A/Sing MAP, 10 ug (FA); B5 = A/Sing MAP, 5 ug (FA); B6 = A/Sing MAP, 2.5 ug (FA)
B7 = Placebo MAP (FA); B8 = A/Sing MAP, 15 ug (UA); B9 = A/Sing IM, 15 ug, as 2018 Afluria Quad (UA)

Listing 16.2.9.3
Numeric Pain Intensity per Site

Part B

Randomisation Number	Group	Visit	Time Point	Was Pain Assessment Performed?	Site Number	Treatment	Date of assessment	Time of Assessment	Score	Unscheduled Reason
B0090	Group B6	Day 22		Yes	F2	uncoated	02-JUL-2018	10:35	0	
B0090	Group B6	Day 22		Yes	F3	uncoated	02-JUL-2018	10:35	0	
B0090	Group B6	EOS/ET (Day 61)		Yes	F1	2.5 ug	08-AUG-2018	10:21	0	
B0090	Group B6	EOS/ET (Day 61)		Yes	F2	uncoated	08-AUG-2018	10:21	0	
B0090	Group B6	EOS/ET (Day 61)		Yes	F3	uncoated	08-AUG-2018	10:21	0	
B0091	Group B8	Day 1	1 min	Yes	D1	5 ug	09-JUN-2018	12:41	2	
B0091	Group B8	Day 1	1 min	Yes	D2	5 ug	09-JUN-2018	12:44	0	
B0091	Group B8	Day 1	1 min	Yes	D3	5 ug	09-JUN-2018	12:47	0	
B0091	Group B8	Day 1	10 min	Yes	D1	5 ug	09-JUN-2018	12:56	0	
B0091	Group B8	Day 1	10 min	Yes	D2	5 ug	09-JUN-2018	12:56		
B0091	Group B8	Day 1	10 min	Yes	D3	5 ug	09-JUN-2018	12:56	0	
B0091	Group B8	Day 1	1 hour	Yes	D1	5 ug	09-JUN-2018	13:48	0	
B0091	Group B8	Day 1	1 hour	Yes	D2	5 ug	09-JUN-2018	13:48	0	
B0091	Group B8	Day 1	1 hour	Yes	D3	5 ug	09-JUN-2018	13:48	0	
B0091	Group B8	Day 1	2 hour	Yes	D1	5 ug	09-JUN-2018	14:48	0	
B0091	Group B8	Day 1	2 hour	Yes	D2	5 ug	09-JUN-2018	14:48	0	
B0091	Group B8	Day 1	2 hour	Yes	D3	5 ug	09-JUN-2018	14:48	0	
B0091	Group B8	Day 2		Yes	D1	5 ug	10-JUN-2018	14:37	0	
B0091	Group B8	Day 2		Yes	D2	5 ug	10-JUN-2018	14:37	0	
B0091	Group B8	Day 2		Yes	D3	5 ug	10-JUN-2018	14:37	0	
B0091	Group B8	Day 4		Yes	D1	5 ug	12-JUN-2018	13:06	0	

Scale: 0= No Pain; 10= Worst Pain Possible
Administration Site Key: FA = Forearm; UA= Upper arm
Treatments Part A: A1 = A/Sing MAP, 15 ug (FA); A2 = Afluria Quad IM (UA), 15 ug; A3 = Placebo MAP (FA); A4 = A/Sing Mono IM, 15 ug (UA)
Treatments Part B: B1 = A/Sing MAP, 15 ug (FA) with biopsy; B2 = Placebo MAP (FA) with biopsy
B3 = A/Sing MAP, 15 ug (FA); B4 = A/Sing MAP, 10 ug (FA); B5 = A/Sing MAP, 5 ug (FA); B6 = A/Sing MAP, 2.5 ug (FA)
B7 = Placebo MAP (FA); B8 = A/Sing MAP, 15 ug (UA); B9 = A/Sing IM, 15 ug, as 2018 Afluria Quad (UA)

Listing 16.2.9.3
Numeric Pain Intensity per Site

Part B

Randomisation Number	Group	Visit	Time Point	Was Pain Assessment Performed?	Site Number	Treatment	Date of assessment	Time of Assessment	Score	Unscheduled Reason
B0091	Group B8	Day 4		Yes	D2	5 ug	12-JUN-2018	13:06	0	
B0091	Group B8	Day 4		Yes	D3	5 ug	12-JUN-2018	13:06	0	
B0091	Group B8	Day 8		Yes	D1	5 ug	16-JUN-2018	10:02	0	
B0091	Group B8	Day 8		Yes	D2	5 ug	16-JUN-2018	10:02	0	
B0091	Group B8	Day 8		Yes	D3	5 ug	16-JUN-2018	10:02	0	
B0091	Group B8	Day 22		Yes	D1	5 ug	30-JUN-2018	09:35	0	
B0091	Group B8	Day 22		Yes	D2	5 ug	30-JUN-2018	09:35	0	
B0091	Group B8	Day 22		Yes	D3	5 ug	30-JUN-2018	09:35	0	
B0091	Group B8	EOS/ET (Day 61)		Yes	D1	5 ug	08-AUG-2018	10:05	0	
B0091	Group B8	EOS/ET (Day 61)		Yes	D2	5 ug	08-AUG-2018	10:05	0	
B0091	Group B8	EOS/ET (Day 61)		Yes	D3	5 ug	08-AUG-2018	10:05	0	
B0092	Group B4	Day 1	1 min	Yes	F1	5 ug	09-JUN-2018	12:51	2	
B0092	Group B4	Day 1	1 min	Yes	F2	uncoated	09-JUN-2018	12:54	2	
B0092	Group B4	Day 1	1 min	Yes	F3	5 ug	09-JUN-2018	12:57	3	
B0092	Group B4	Day 1	10 min	Yes	F1	5 ug	09-JUN-2018	13:05	0	
B0092	Group B4	Day 1	10 min	Yes	F2	uncoated	09-JUN-2018	13:05	2	
B0092	Group B4	Day 1	10 min	Yes	F3	5 ug	09-JUN-2018	13:05	3	
B0092	Group B4	Day 1	1 hour	Yes	F1	5 ug	09-JUN-2018	13:58	0	
B0092	Group B4	Day 1	1 hour	Yes	F2	uncoated	09-JUN-2018	13:58	0	
B0092	Group B4	Day 1	1 hour	Yes	F3	5 ug	09-JUN-2018	13:58	2	
B0092	Group B4	Day 1	2 hour	Yes	F1	5 ug	09-JUN-2018	14:58	0	

Scale: 0= No Pain; 10= Worst Pain Possible
Administration Site Key: FA = Forearm; UA= Upper arm
Treatments Part A: A1 = A/Sing MAP, 15 ug (FA); A2 = Afluria Quad IM (UA), 15 ug; A3 = Placebo MAP (FA); A4 = A/Sing Mono IM, 15 ug (UA)
Treatments Part B: B1 = A/Sing MAP, 15 ug (FA) with biopsy; B2 = Placebo MAP (FA) with biopsy
B3 = A/Sing MAP, 15 ug (FA); B4 = A/Sing MAP, 10 ug (FA); B5 = A/Sing MAP, 5 ug (FA); B6 = A/Sing MAP, 2.5 ug (FA)
B7 = Placebo MAP (FA); B8 = A/Sing MAP, 15 ug (UA); B9 = A/Sing IM, 15 ug, as 2018 Afluria Quad (UA)

Listing 16.2.9.3
Numeric Pain Intensity per Site

Part B

Randomisation Number	Group	Visit	Time Point	Was Pain Assessment Performed?	Site Number	Treatment	Date of assessment	Time of Assessment	Score	Unscheduled Reason
B0092	Group B4	Day 1	2 hour	Yes	F2	uncoated	09-JUN-2018	14:58	0	
B0092	Group B4	Day 1	2 hour	Yes	F3	5 ug	09-JUN-2018	14:58	1	
B0092	Group B4	Day 2		Yes	F1	5 ug	10-JUN-2018	09:13	0	
B0092	Group B4	Day 2		Yes	F2	uncoated	10-JUN-2018	09:13	0	
B0092	Group B4	Day 2		Yes	F3	5 ug	10-JUN-2018	09:13	0	
B0092	Group B4	Day 4		Yes	F1	5 ug	12-JUN-2018	10:16	0	
B0092	Group B4	Day 4		Yes	F2	uncoated	12-JUN-2018	10:16	0	
B0092	Group B4	Day 4		Yes	F3	5 ug	12-JUN-2018	10:16	0	
B0092	Group B4	Day 8		Yes	F1	5 ug	16-JUN-2018	13:39	0	
B0092	Group B4	Day 8		Yes	F2	uncoated	16-JUN-2018	13:40	0	
B0092	Group B4	Day 8		Yes	F3	5 ug	16-JUN-2018	13:40	0	
B0092	Group B4	Day 22		Yes	F1	5 ug	30-JUN-2018	12:05	0	
B0092	Group B4	Day 22		Yes	F2	uncoated	30-JUN-2018	12:05	0	
B0092	Group B4	Day 22		Yes	F3	5 ug	30-JUN-2018	12:05	0	
B0092	Group B4	EOS/ET (Day 61)		Yes	F1	5 ug	02-AUG-2018	08:12	0	
B0092	Group B4	EOS/ET (Day 61)		Yes	F2	uncoated	02-AUG-2018	08:12	0	
B0092	Group B4	EOS/ET (Day 61)		Yes	F3	5 ug	02-AUG-2018	08:12	0	
B0093	Group B7	Day 1	1 min	Yes	F1	uncoated	09-JUN-2018	13:01	1	
B0093	Group B7	Day 1	1 min	Yes	F2	uncoated	09-JUN-2018	13:04	1	
B0093	Group B7	Day 1	1 min	Yes	F3	uncoated	09-JUN-2018	13:07	1	
B0093	Group B7	Day 1	10 min	Yes	F1	uncoated	09-JUN-2018	13:16	0	

Scale: 0= No Pain; 10= Worst Pain Possible
Administration Site Key: FA = Forearm; UA= Upper arm
Treatments Part A: A1 = A/Sing MAP, 15 ug (FA); A2 = Afluria Quad IM (UA), 15 ug; A3 = Placebo MAP (FA); A4 = A/Sing Mono IM, 15 ug (UA)
Treatments Part B: B1 = A/Sing MAP, 15 ug (FA) with biopsy; B2 = Placebo MAP (FA) with biopsy
B3 = A/Sing MAP, 15 ug (FA); B4 = A/Sing MAP, 10 ug (FA); B5 = A/Sing MAP, 5 ug (FA); B6 = A/Sing MAP, 2.5 ug (FA)
B7 = Placebo MAP (FA); B8 = A/Sing MAP, 15 ug (UA); B9 = A/Sing IM, 15 ug, as 2018 Afluria Quad (UA)

Listing 16.2.9.3
Numeric Pain Intensity per Site

Part B

Randomisation Number	Group	Visit	Time Point	Was Pain Assessment Performed?	Site Number	Treatment	Date of assessment	Time of Assessment	Score	Unscheduled Reason
B0093	Group B7	Day 1	10 min	Yes	F2	uncoated	09-JUN-2018	13:16	0	
B0093	Group B7	Day 1	10 min	Yes	F3	uncoated	09-JUN-2018	13:16	0	
B0093	Group B7	Day 1	1 hour	Yes	F1	uncoated	09-JUN-2018	14:08	0	
B0093	Group B7	Day 1	1 hour	Yes	F2	uncoated	09-JUN-2018	14:08	0	
B0093	Group B7	Day 1	1 hour	Yes	F3	uncoated	09-JUN-2018	14:08	0	
B0093	Group B7	Day 1	2 hour	Yes	F1	uncoated	09-JUN-2018	15:08	0	
B0093	Group B7	Day 1	2 hour	Yes	F2	uncoated	09-JUN-2018	15:08	0	
B0093	Group B7	Day 1	2 hour	Yes	F3	uncoated	09-JUN-2018	15:08	0	
B0093	Group B7	Day 2		Yes	F1	uncoated	10-JUN-2018	10:07	0	
B0093	Group B7	Day 2		Yes	F2	uncoated	10-JUN-2018	10:07	0	
B0093	Group B7	Day 2		Yes	F3	uncoated	10-JUN-2018	10:07	0	
B0093	Group B7	Day 4		Yes	F1	uncoated	12-JUN-2018	09:25	0	
B0093	Group B7	Day 4		Yes	F2	uncoated	12-JUN-2018	09:25	0	
B0093	Group B7	Day 4		Yes	F3	uncoated	12-JUN-2018	09:25	0	
B0093	Group B7	Day 8		Yes	F1	uncoated	16-JUN-2018	09:57	0	
B0093	Group B7	Day 8		Yes	F2	uncoated	16-JUN-2018	09:58	0	
B0093	Group B7	Day 8		Yes	F3	uncoated	16-JUN-2018	09:59	0	
B0093	Group B7	Day 22		Yes	F1	uncoated	30-JUN-2018	09:05	0	
B0093	Group B7	Day 22		Yes	F2	uncoated	30-JUN-2018	09:05	0	
B0093	Group B7	Day 22		Yes	F3	uncoated	30-JUN-2018	09:05	0	
B0093	Group B7	EOS/ET (Day 61)		Yes	F1	uncoated	08-AUG-2018	09:27	0	

Scale: 0= No Pain; 10= Worst Pain Possible
Administration Site Key: FA = Forearm; UA= Upper arm
Treatments Part A: A1 = A/Sing MAP, 15 ug (FA); A2 = Afluria Quad IM (UA), 15 ug; A3 = Placebo MAP (FA); A4 = A/Sing Mono IM, 15 ug (UA)
Treatments Part B: B1 = A/Sing MAP, 15 ug (FA) with biopsy; B2 = Placebo MAP (FA) with biopsy
B3 = A/Sing MAP, 15 ug (FA); B4 = A/Sing MAP, 10 ug (FA); B5 = A/Sing MAP, 5 ug (FA); B6 = A/Sing MAP, 2.5 ug (FA)
B7 = Placebo MAP (FA); B8 = A/Sing MAP, 15 ug (UA); B9 = A/Sing IM, 15 ug, as 2018 Afluria Quad (UA)

Listing 16.2.9.3
Numeric Pain Intensity per Site

Part B

Randomisation Number	Group	Visit	Time Point	Was Pain Assessment Performed?	Site Number	Treatment	Date of assessment	Time of Assessment	Score	Unscheduled Reason
B0093	Group B7	EOS/ET (Day 61)		Yes	F2	uncoated	08-AUG-2018	09:27	0	
B0093	Group B7	EOS/ET (Day 61)		Yes	F3	uncoated	08-AUG-2018	09:27	0	
B0094	Group B3	Day 1	1 min	Yes	F1	5 ug	09-JUN-2018	13:11	0	
B0094	Group B3	Day 1	1 min	Yes	F2	5 ug	09-JUN-2018	13:14	0	
B0094	Group B3	Day 1	1 min	Yes	F3	5 ug	09-JUN-2018	13:17	0	
B0094	Group B3	Day 1	10 min	Yes	F1	5 ug	09-JUN-2018	13:26	0	
B0094	Group B3	Day 1	10 min	Yes	F2	5 ug	09-JUN-2018	13:26	0	
B0094	Group B3	Day 1	10 min	Yes	F3	5 ug	09-JUN-2018	13:27	0	
B0094	Group B3	Day 1	1 hour	Yes	F1	5 ug	09-JUN-2018	14:18	0	
B0094	Group B3	Day 1	1 hour	Yes	F2	5 ug	09-JUN-2018	14:18	0	
B0094	Group B3	Day 1	1 hour	Yes	F3	5 ug	09-JUN-2018	14:18	0	
B0094	Group B3	Day 1	2 hour	Yes	F1	5 ug	09-JUN-2018	15:18	0	
B0094	Group B3	Day 1	2 hour	Yes	F2	5 ug	09-JUN-2018	15:18	0	
B0094	Group B3	Day 1	2 hour	Yes	F3	5 ug	09-JUN-2018	15:18	0	
B0094	Group B3	Day 2		Yes	F1	5 ug	10-JUN-2018	13:04	0	
B0094	Group B3	Day 2		Yes	F2	5 ug	10-JUN-2018	13:05	0	
B0094	Group B3	Day 2		Yes	F3	5 ug	10-JUN-2018	13:05	0	
B0094	Group B3	Day 4		Yes	F1	5 ug	12-JUN-2018	13:25	0	
B0094	Group B3	Day 4		Yes	F2	5 ug	12-JUN-2018	13:25	0	
B0094	Group B3	Day 4		Yes	F3	5 ug	12-JUN-2018	13:25	0	
B0094	Group B3	Day 8		Yes	F1	5 ug	16-JUN-2018	13:50	0	

Scale: 0= No Pain; 10= Worst Pain Possible
Administration Site Key: FA = Forearm; UA= Upper arm
Treatments Part A: A1 = A/Sing MAP, 15 ug (FA); A2 = Afluria Quad IM (UA), 15 ug; A3 = Placebo MAP (FA); A4 = A/Sing Mono IM, 15 ug (UA)
Treatments Part B: B1 = A/Sing MAP, 15 ug (FA) with biopsy; B2 = Placebo MAP (FA) with biopsy
B3 = A/Sing MAP, 15 ug (FA); B4 = A/Sing MAP, 10 ug (FA); B5 = A/Sing MAP, 5 ug (FA); B6 = A/Sing MAP, 2.5 ug (FA)
B7 = Placebo MAP (FA); B8 = A/Sing MAP, 15 ug (UA); B9 = A/Sing IM, 15 ug, as 2018 Afluria Quad (UA)

Listing 16.2.9.3
Numeric Pain Intensity per Site

Part B

Randomisation Number	Group	Visit	Time Point	Was Pain Assessment Performed?	Site Number	Treatment	Date of assessment	Time of Assessment	Score	Unscheduled Reason
B0094	Group B3	Day 8		Yes	F2	5 ug	16-JUN-2018	13:50	0	
B0094	Group B3	Day 8		Yes	F3	5 ug	16-JUN-2018	13:51	0	
B0094	Group B3	Day 22		Yes	F1	5 ug	30-JUN-2018	13:48	0	
B0094	Group B3	Day 22		Yes	F2	5 ug	30-JUN-2018	13:48	0	
B0094	Group B3	Day 22		Yes	F3	5 ug	30-JUN-2018	13:48	0	
B0094	Group B3	EOS/ET (Day 61)		Yes	F1	5 ug	08-AUG-2018	10:13	0	
B0094	Group B3	EOS/ET (Day 61)		Yes	F2	5 ug	08-AUG-2018	10:13	0	
B0094	Group B3	EOS/ET (Day 61)		Yes	F3	5 ug	08-AUG-2018	10:13	0	
B0095	Group B9	Day 1	1 min	Yes	D1	[IM]	09-JUN-2018	11:41	3	
B0095	Group B9	Day 1	10 min	Yes	D1	[IM]	09-JUN-2018	11:50	0	
B0095	Group B9	Day 1	1 hour	Yes	D1	[IM]	09-JUN-2018	12:40	0	
B0095	Group B9	Day 1	2 hour	Yes	D1	[IM]	09-JUN-2018	13:45	0	
B0095	Group B9	Day 2		Yes	D1	[IM]	10-JUN-2018	12:59	0	
B0095	Group B9	Day 4		Yes	D1	[IM]	12-JUN-2018	13:13	0	
B0095	Group B9	Day 8		Yes	D1	[IM]	16-JUN-2018	13:22	0	
B0095	Group B9	Day 22		Yes	D1	[IM]	30-JUN-2018	13:35	0	
B0095	Group B9	EOS/ET (Day 61)		Yes	D1	[IM]	08-AUG-2018	12:34	0	
B0096	Group B6	Day 1	1 min	Yes	F1	2.5 ug	09-JUN-2018	13:21	1	
B0096	Group B6	Day 1	1 min	Yes	F2	uncoated	09-JUN-2018	13:24	2	
B0096	Group B6	Day 1	1 min	Yes	F3	uncoated	09-JUN-2018	13:27	2	

Scale: 0= No Pain; 10= Worst Pain Possible
Administration Site Key: FA = Forearm; UA= Upper arm
Treatments Part A: A1 = A/Sing MAP, 15 ug (FA); A2 = Afluria Quad IM (UA), 15 ug; A3 = Placebo MAP (FA); A4 = A/Sing Mono IM, 15 ug (UA)
Treatments Part B: B1 = A/Sing MAP, 15 ug (FA) with biopsy; B2 = Placebo MAP (FA) with biopsy
B3 = A/Sing MAP, 15 ug (FA); B4 = A/Sing MAP, 10 ug (FA); B5 = A/Sing MAP, 5 ug (FA); B6 = A/Sing MAP, 2.5 ug (FA)
B7 = Placebo MAP (FA); B8 = A/Sing MAP, 15 ug (UA); B9 = A/Sing IM, 15 ug, as 2018 Afluria Quad (UA)

Listing 16.2.9.3
Numeric Pain Intensity per Site

Part B

Randomisation Number	Group	Visit	Time Point	Was Pain Assessment Performed?	Site Number	Treatment	Date of assessment	Time of Assessment	Score	Unscheduled Reason
B0096	Group B6	Day 1	10 min	Yes	F1	2.5 ug	09-JUN-2018	13:35	0	
B0096	Group B6	Day 1	10 min	Yes	F2	uncoated	09-JUN-2018	13:35	0	
B0096	Group B6	Day 1	10 min	Yes	F3	uncoated	09-JUN-2018	13:35	0	
B0096	Group B6	Day 1	1 hour	Yes	F1	2.5 ug	09-JUN-2018	14:28	0	
B0096	Group B6	Day 1	1 hour	Yes	F2	uncoated	09-JUN-2018	14:28	0	
B0096	Group B6	Day 1	1 hour	Yes	F3	uncoated	09-JUN-2018	14:28	0	
B0096	Group B6	Day 1	2 hour	Yes	F1	2.5 ug	09-JUN-2018	15:28	0	
B0096	Group B6	Day 1	2 hour	Yes	F2	uncoated	09-JUN-2018	15:28	0	
B0096	Group B6	Day 1	2 hour	Yes	F3	uncoated	09-JUN-2018	15:28	0	
B0096	Group B6	Day 2		Yes	F1	2.5 ug	10-JUN-2018	13:46	0	
B0096	Group B6	Day 2		Yes	F2	uncoated	10-JUN-2018	13:46	0	
B0096	Group B6	Day 2		Yes	F3	uncoated	10-JUN-2018	13:46	0	
B0096	Group B6	Day 4		Yes	F1	2.5 ug	12-JUN-2018	12:29	0	
B0096	Group B6	Day 4		Yes	F2	uncoated	12-JUN-2018	12:29	0	
B0096	Group B6	Day 4		Yes	F3	uncoated	12-JUN-2018	12:29	0	
B0096	Group B6	Day 8		Yes	F1	2.5 ug	16-JUN-2018	13:32	0	
B0096	Group B6	Day 8		Yes	F2	uncoated	16-JUN-2018	13:33	0	
B0096	Group B6	Day 8		Yes	F3	uncoated	16-JUN-2018	13:33	0	
B0096	Group B6	Day 22		Yes	F1	2.5 ug	30-JUN-2018	13:40	0	
B0096	Group B6	Day 22		Yes	F2	uncoated	30-JUN-2018	13:40	0	
B0096	Group B6	Day 22		Yes	F3	uncoated	30-JUN-2018	13:40	0	

Scale: 0= No Pain; 10= Worst Pain Possible
Administration Site Key: FA = Forearm; UA= Upper arm
Treatments Part A: A1 = A/Sing MAP, 15 ug (FA); A2 = Afluria Quad IM (UA), 15 ug; A3 = Placebo MAP (FA); A4 = A/Sing Mono IM, 15 ug (UA)
Treatments Part B: B1 = A/Sing MAP, 15 ug (FA) with biopsy; B2 = Placebo MAP (FA) with biopsy
B3 = A/Sing MAP, 15 ug (FA); B4 = A/Sing MAP, 10 ug (FA); B5 = A/Sing MAP, 5 ug (FA); B6 = A/Sing MAP, 2.5 ug (FA)
B7 = Placebo MAP (FA); B8 = A/Sing MAP, 15 ug (UA); B9 = A/Sing IM, 15 ug, as 2018 Afluria Quad (UA)

Listing 16.2.9.3
Numeric Pain Intensity per Site

Part B

Randomisation Number	Group	Visit	Time Point	Was Pain Assessment Performed?	Site Number	Treatment	Date of assessment	Time of Assessment	Score	Unscheduled Reason
B0096	Group B6	EOS/ET (Day 61)		Yes	F1	2.5 ug	08-AUG-2018	13:48	0	
B0096	Group B6	EOS/ET (Day 61)		Yes	F2	uncoated	08-AUG-2018	13:48	0	
B0096	Group B6	EOS/ET (Day 61)		Yes	F3	uncoated	08-AUG-2018	13:48	0	
B0097	Group B5	Day 1	1 min	Yes	F1	5 ug	09-JUN-2018	13:31	0	
B0097	Group B5	Day 1	1 min	Yes	F2	uncoated	09-JUN-2018	13:34	1	
B0097	Group B5	Day 1	1 min	Yes	F3	uncoated	09-JUN-2018	13:37	0	
B0097	Group B5	Day 1	10 min	Yes	F1	5 ug	09-JUN-2018	13:46	0	
B0097	Group B5	Day 1	10 min	Yes	F2	uncoated	09-JUN-2018	13:46	0	
B0097	Group B5	Day 1	10 min	Yes	F3	uncoated	09-JUN-2018	13:46	0	
B0097	Group B5	Day 1	1 hour	Yes	F1	5 ug	09-JUN-2018	14:38	0	
B0097	Group B5	Day 1	1 hour	Yes	F2	uncoated	09-JUN-2018	14:38	0	
B0097	Group B5	Day 1	1 hour	Yes	F3	uncoated	09-JUN-2018	14:38	0	
B0097	Group B5	Day 1	2 hour	Yes	F1	5 ug	09-JUN-2018	15:38	0	
B0097	Group B5	Day 1	2 hour	Yes	F2	uncoated	09-JUN-2018	15:38	0	
B0097	Group B5	Day 1	2 hour	Yes	F3	uncoated	09-JUN-2018	15:38	0	
B0097	Group B5	Day 2		Yes	F1	5 ug	10-JUN-2018	13:40	0	
B0097	Group B5	Day 2		Yes	F2	uncoated	10-JUN-2018	13:40	0	
B0097	Group B5	Day 2		Yes	F3	uncoated	10-JUN-2018	13:41	0	
B0097	Group B5	Day 4		Yes	F1	5 ug	12-JUN-2018	16:39	0	
B0097	Group B5	Day 4		Yes	F2	uncoated	12-JUN-2018	16:39	0	
B0097	Group B5	Day 4		Yes	F3	uncoated	12-JUN-2018	16:40	0	

Scale: 0= No Pain; 10= Worst Pain Possible
Administration Site Key: FA = Forearm; UA= Upper arm
Treatments Part A: A1 = A/Sing MAP, 15 ug (FA); A2 = Afluria Quad IM (UA), 15 ug; A3 = Placebo MAP (FA); A4 = A/Sing Mono IM, 15 ug (UA)
Treatments Part B: B1 = A/Sing MAP, 15 ug (FA) with biopsy; B2 = Placebo MAP (FA) with biopsy
B3 = A/Sing MAP, 15 ug (FA); B4 = A/Sing MAP, 10 ug (FA); B5 = A/Sing MAP, 5 ug (FA); B6 = A/Sing MAP, 2.5 ug (FA)
B7 = Placebo MAP (FA); B8 = A/Sing MAP, 15 ug (UA); B9 = A/Sing IM, 15 ug, as 2018 Afluria Quad (UA)

Listing 16.2.9.3
Numeric Pain Intensity per Site

Part B

Randomisation Number	Group	Visit	Time Point	Was Pain Assessment Performed?	Site Number	Treatment	Date of assessment	Time of Assessment	Score	Unscheduled Reason
B0097	Group B5	Day 8		Yes	F1	5 ug	16-JUN-2018	14:33	0	
B0097	Group B5	Day 8		Yes	F2	uncoated	16-JUN-2018	14:36	0	
B0097	Group B5	Day 8		Yes	F3	uncoated	16-JUN-2018	14:37	0	
B0097	Group B5	Day 22		Yes	F1	5 ug	30-JUN-2018	14:58	0	
B0097	Group B5	Day 22		Yes	F2	uncoated	30-JUN-2018	14:58	0	
B0097	Group B5	Day 22		Yes	F3	uncoated	30-JUN-2018	14:58	0	
B0097	Group B5	EOS/ET (Day 61)		Yes	F1	5 ug	09-AUG-2018	17:18	0	
B0097	Group B5	EOS/ET (Day 61)		Yes	F2	uncoated	09-AUG-2018	17:18	0	
B0097	Group B5	EOS/ET (Day 61)		Yes	F3	uncoated	09-AUG-2018	17:18	0	
B0098	Group B7	Day 1	1 min	Yes	F1	uncoated	09-JUN-2018	13:41	2	
B0098	Group B7	Day 1	1 min	Yes	F2	uncoated	09-JUN-2018	13:44	2	
B0098	Group B7	Day 1	1 min	Yes	F3	uncoated	09-JUN-2018	13:47	2	
B0098	Group B7	Day 1	10 min	Yes	F1	uncoated	09-JUN-2018	13:56	2	
B0098	Group B7	Day 1	10 min	Yes	F2	uncoated	09-JUN-2018	13:56	2	
B0098	Group B7	Day 1	10 min	Yes	F3	uncoated	09-JUN-2018	13:56	2	
B0098	Group B7	Day 1	1 hour	Yes	F1	uncoated	09-JUN-2018	14:48	1	
B0098	Group B7	Day 1	1 hour	Yes	F2	uncoated	09-JUN-2018	14:48	1	
B0098	Group B7	Day 1	1 hour	Yes	F3	uncoated	09-JUN-2018	14:48	1	
B0098	Group B7	Day 1	2 hour	Yes	F1	uncoated	09-JUN-2018	15:48	0	
B0098	Group B7	Day 1	2 hour	Yes	F2	uncoated	09-JUN-2018	15:48	0	
B0098	Group B7	Day 1	2 hour	Yes	F3	uncoated	09-JUN-2018	15:48	0	

Scale: 0= No Pain; 10= Worst Pain Possible
Administration Site Key: FA = Forearm; UA= Upper arm
Treatments Part A: A1 = A/Sing MAP, 15 ug (FA); A2 = Afluria Quad IM (UA), 15 ug; A3 = Placebo MAP (FA); A4 = A/Sing Mono IM, 15 ug (UA)
Treatments Part B: B1 = A/Sing MAP, 15 ug (FA) with biopsy; B2 = Placebo MAP (FA) with biopsy
B3 = A/Sing MAP, 15 ug (FA); B4 = A/Sing MAP, 10 ug (FA); B5 = A/Sing MAP, 5 ug (FA); B6 = A/Sing MAP, 2.5 ug (FA)
B7 = Placebo MAP (FA); B8 = A/Sing MAP, 15 ug (UA); B9 = A/Sing IM, 15 ug, as 2018 Afluria Quad (UA)

Listing 16.2.9.3
Numeric Pain Intensity per Site

Part B

Randomisation Number	Group	Visit	Time Point	Was Pain Assessment Performed?	Site Number	Treatment	Date of assessment	Time of Assessment	Score	Unscheduled Reason
B0098	Group B7	Day 2		Yes	F1	uncoated	10-JUN-2018	14:03	0	
B0098	Group B7	Day 2		Yes	F2	uncoated	10-JUN-2018	14:03	0	
B0098	Group B7	Day 2		Yes	F3	uncoated	10-JUN-2018	14:03	0	
B0098	Group B7	Day 4		Yes	F1	uncoated	12-JUN-2018	13:53	0	
B0098	Group B7	Day 4		Yes	F2	uncoated	12-JUN-2018	13:53	0	
B0098	Group B7	Day 4		Yes	F3	uncoated	12-JUN-2018	13:53	0	
B0098	Group B7	Day 8		Yes	F1	uncoated	16-JUN-2018	14:15	0	
B0098	Group B7	Day 8		Yes	F2	uncoated	16-JUN-2018	14:16	0	
B0098	Group B7	Day 8		Yes	F3	uncoated	16-JUN-2018	14:17	0	
B0098	Group B7	Day 22		Yes	F1	uncoated	30-JUN-2018	15:00	0	
B0098	Group B7	Day 22		Yes	F2	uncoated	30-JUN-2018	15:00	0	
B0098	Group B7	Day 22		Yes	F3	uncoated	30-JUN-2018	15:00	0	
B0098	Group B7	EOS/ET (Day 61)		Yes	F1	uncoated	07-AUG-2018	09:13	0	
B0098	Group B7	EOS/ET (Day 61)		Yes	F2	uncoated	07-AUG-2018	09:13	0	
B0098	Group B7	EOS/ET (Day 61)		Yes	F3	uncoated	07-AUG-2018	09:13	0	
B0099	Group B4	Day 22		No	F1	5 ug				
B0099	Group B4	Day 22		No	F2	5 ug				
B0099	Group B4	Day 22		No	F3	uncoated				
B0099	Group B4	Day 1	1 min	Yes	F1	5 ug	09-JUN-2018	15:11	1	
B0099	Group B4	Day 1	1 min	Yes	F2	5 ug	09-JUN-2018	15:14	2	
B0099	Group B4	Day 1	1 min	Yes	F3	uncoated	09-JUN-2018	15:17	2	

Scale: 0= No Pain; 10= Worst Pain Possible
Administration Site Key: FA = Forearm; UA= Upper arm
Treatments Part A: A1 = A/Sing MAP, 15 ug (FA); A2 = Afluria Quad IM (UA), 15 ug; A3 = Placebo MAP (FA); A4 = A/Sing Mono IM, 15 ug (UA)
Treatments Part B: B1 = A/Sing MAP, 15 ug (FA) with biopsy; B2 = Placebo MAP (FA) with biopsy
B3 = A/Sing MAP, 15 ug (FA); B4 = A/Sing MAP, 10 ug (FA); B5 = A/Sing MAP, 5 ug (FA); B6 = A/Sing MAP, 2.5 ug (FA)
B7 = Placebo MAP (FA); B8 = A/Sing MAP, 15 ug (UA); B9 = A/Sing IM, 15 ug, as 2018 Afluria Quad (UA)

Listing 16.2.9.3
Numeric Pain Intensity per Site

Part B

Randomisation Number	Group	Visit	Time Point	Was Pain Assessment Performed?	Site Number	Treatment	Date of assessment	Time of Assessment	Score	Unscheduled Reason
B0099	Group B4	Day 1	10 min	Yes	F1	5 ug	09-JUN-2018	15:28	1	
B0099	Group B4	Day 1	10 min	Yes	F2	5 ug	09-JUN-2018	15:28	1	
B0099	Group B4	Day 1	10 min	Yes	F3	uncoated	09-JUN-2018	15:28	1	
B0099	Group B4	Day 1	1 hour	Yes	F1	5 ug	09-JUN-2018	16:18	1	
B0099	Group B4	Day 1	1 hour	Yes	F2	5 ug	09-JUN-2018	16:18	1	
B0099	Group B4	Day 1	1 hour	Yes	F3	uncoated	09-JUN-2018	16:18	1	
B0099	Group B4	Day 1	2 hour	Yes	F1	5 ug	09-JUN-2018	17:18	0	
B0099	Group B4	Day 1	2 hour	Yes	F2	5 ug	09-JUN-2018	17:18	0	
B0099	Group B4	Day 1	2 hour	Yes	F3	uncoated	09-JUN-2018	17:18	0	
B0099	Group B4	Day 2		Yes	F1	5 ug	10-JUN-2018	11:16	0	
B0099	Group B4	Day 2		Yes	F2	5 ug	10-JUN-2018	11:16	0	
B0099	Group B4	Day 2		Yes	F3	uncoated	10-JUN-2018	11:16	0	
B0099	Group B4	Day 4		Yes	F1	5 ug	12-JUN-2018	09:22	0	
B0099	Group B4	Day 4		Yes	F2	5 ug	12-JUN-2018	09:22	0	
B0099	Group B4	Day 4		Yes	F3	uncoated	12-JUN-2018	09:22	0	
B0099	Group B4	Day 8		Yes	F1	5 ug	16-JUN-2018	11:20	0	
B0099	Group B4	Day 8		Yes	F2	5 ug	16-JUN-2018	11:20	0	
B0099	Group B4	Day 8		Yes	F3	uncoated	16-JUN-2018	11:20	0	
B0099	Group B4	EOS/ET (Day 61)		Yes	F1	5 ug	06-AUG-2018	11:23	0	
B0099	Group B4	EOS/ET (Day 61)		Yes	F2	5 ug	06-AUG-2018	11:23	0	
B0099	Group B4	EOS/ET (Day 61)		Yes	F3	uncoated	06-AUG-2018	11:23	0	

Scale: 0= No Pain; 10= Worst Pain Possible
Administration Site Key: FA = Forearm; UA= Upper arm
Treatments Part A: A1 = A/Sing MAP, 15 ug (FA); A2 = Afluria Quad IM (UA), 15 ug; A3 = Placebo MAP (FA); A4 = A/Sing Mono IM, 15 ug (UA)
Treatments Part B: B1 = A/Sing MAP, 15 ug (FA) with biopsy; B2 = Placebo MAP (FA) with biopsy
B3 = A/Sing MAP, 15 ug (FA); B4 = A/Sing MAP, 10 ug (FA); B5 = A/Sing MAP, 5 ug (FA); B6 = A/Sing MAP, 2.5 ug (FA)
B7 = Placebo MAP (FA); B8 = A/Sing MAP, 15 ug (UA); B9 = A/Sing IM, 15 ug, as 2018 Afluria Quad (UA)

Listing 16.2.9.3
Numeric Pain Intensity per Site

Part B

Randomisation Number	Group	Visit	Time Point	Was Pain Assessment Performed?	Site Number	Treatment	Date of assessment	Time of Assessment	Score	Unscheduled Reason
B0100	Group B8	Day 1	1 min	Yes	D1	5 ug	09-JUN-2018	13:51	0	
B0100	Group B8	Day 1	1 min	Yes	D2	5 ug	09-JUN-2018	13:54	1	
B0100	Group B8	Day 1	1 min	Yes	D3	5 ug	09-JUN-2018	13:57	0	
B0100	Group B8	Day 1	10 min	Yes	D1	5 ug	09-JUN-2018	14:05	0	
B0100	Group B8	Day 1	10 min	Yes	D2	5 ug	09-JUN-2018	14:05	0	
B0100	Group B8	Day 1	10 min	Yes	D3	5 ug	09-JUN-2018	14:05	0	
B0100	Group B8	Day 1	1 hour	Yes	D1	5 ug	09-JUN-2018	14:58	0	
B0100	Group B8	Day 1	1 hour	Yes	D2	5 ug	09-JUN-2018	14:58	0	
B0100	Group B8	Day 1	1 hour	Yes	D3	5 ug	09-JUN-2018	14:58	0	
B0100	Group B8	Day 1	2 hour	Yes	D1	5 ug	09-JUN-2018	15:58	0	
B0100	Group B8	Day 1	2 hour	Yes	D2	5 ug	09-JUN-2018	15:58	0	
B0100	Group B8	Day 1	2 hour	Yes	D3	5 ug	09-JUN-2018	15:58	0	
B0100	Group B8	Day 2		Yes	D1	5 ug	10-JUN-2018	13:22	0	
B0100	Group B8	Day 2		Yes	D2	5 ug	10-JUN-2018	13:22	0	
B0100	Group B8	Day 2		Yes	D3	5 ug	10-JUN-2018	13:22	0	
B0100	Group B8	Day 4		Yes	D1	5 ug	12-JUN-2018	17:18	0	
B0100	Group B8	Day 4		Yes	D2	5 ug	12-JUN-2018	17:18	0	
B0100	Group B8	Day 4		Yes	D3	5 ug	12-JUN-2018	17:18	0	
B0100	Group B8	Day 8		Yes	D1	5 ug	16-JUN-2018	16:27	0	
B0100	Group B8	Day 8		Yes	D2	5 ug	16-JUN-2018	16:27	0	
B0100	Group B8	Day 8		Yes	D3	5 ug	16-JUN-2018	16:27	0	

Scale: 0= No Pain; 10= Worst Pain Possible
Administration Site Key: FA = Forearm; UA= Upper arm
Treatments Part A: A1 = A/Sing MAP, 15 ug (FA); A2 = Afluria Quad IM (UA), 15 ug; A3 = Placebo MAP (FA); A4 = A/Sing Mono IM, 15 ug (UA)
Treatments Part B: B1 = A/Sing MAP, 15 ug (FA) with biopsy; B2 = Placebo MAP (FA) with biopsy
B3 = A/Sing MAP, 15 ug (FA); B4 = A/Sing MAP, 10 ug (FA); B5 = A/Sing MAP, 5 ug (FA); B6 = A/Sing MAP, 2.5 ug (FA)
B7 = Placebo MAP (FA); B8 = A/Sing MAP, 15 ug (UA); B9 = A/Sing IM, 15 ug, as 2018 Afluria Quad (UA)

Listing 16.2.9.3
Numeric Pain Intensity per Site

Part B

Randomisation Number	Group	Visit	Time Point	Was Pain Assessment Performed?	Site Number	Treatment	Date of assessment	Time of Assessment	Score	Unscheduled Reason
B0100	Group B8	Day 22		Yes	D1	5 ug	30-JUN-2018	09:22	0	
B0100	Group B8	Day 22		Yes	D2	5 ug	30-JUN-2018	09:22	0	
B0100	Group B8	Day 22		Yes	D3	5 ug	30-JUN-2018	09:22	0	
B0100	Group B8	EOS/ET (Day 61)		Yes	D1	5 ug	08-AUG-2018	15:23	0	
B0100	Group B8	EOS/ET (Day 61)		Yes	D2	5 ug	08-AUG-2018	15:23	0	
B0100	Group B8	EOS/ET (Day 61)		Yes	D3	5 ug	08-AUG-2018	15:23	0	
B0101	Group B3	Day 1	1 min	Yes	F1	5 ug	09-JUN-2018	14:01	1	
B0101	Group B3	Day 1	1 min	Yes	F2	5 ug	09-JUN-2018	14:04	0	
B0101	Group B3	Day 1	1 min	Yes	F3	5 ug	09-JUN-2018	14:07	0	
B0101	Group B3	Day 1	10 min	Yes	F1	5 ug	09-JUN-2018	14:18	0	
B0101	Group B3	Day 1	10 min	Yes	F2	5 ug	09-JUN-2018	14:18	0	
B0101	Group B3	Day 1	10 min	Yes	F3	5 ug	09-JUN-2018	14:18	0	
B0101	Group B3	Day 1	1 hour	Yes	F1	5 ug	09-JUN-2018	15:08	0	
B0101	Group B3	Day 1	1 hour	Yes	F2	5 ug	09-JUN-2018	15:08	0	
B0101	Group B3	Day 1	1 hour	Yes	F3	5 ug	09-JUN-2018	15:08	0	
B0101	Group B3	Day 1	2 hour	Yes	F1	5 ug	09-JUN-2018	16:09		
B0101	Group B3	Day 1	2 hour	Yes	F2	5 ug	09-JUN-2018	16:09		
B0101	Group B3	Day 1	2 hour	Yes	F3	5 ug	09-JUN-2018	16:09		
B0101	Group B3	Day 2		Yes	F1	5 ug	10-JUN-2018	13:59	0	
B0101	Group B3	Day 2		Yes	F2	5 ug	10-JUN-2018	13:59	0	
B0101	Group B3	Day 2		Yes	F3	5 ug	10-JUN-2018	13:59	0	

Scale: 0= No Pain; 10= Worst Pain Possible
Administration Site Key: FA = Forearm; UA= Upper arm
Treatments Part A: A1 = A/Sing MAP, 15 ug (FA); A2 = Afluria Quad IM (UA), 15 ug; A3 = Placebo MAP (FA); A4 = A/Sing Mono IM, 15 ug (UA)
Treatments Part B: B1 = A/Sing MAP, 15 ug (FA) with biopsy; B2 = Placebo MAP (FA) with biopsy
B3 = A/Sing MAP, 15 ug (FA); B4 = A/Sing MAP, 10 ug (FA); B5 = A/Sing MAP, 5 ug (FA); B6 = A/Sing MAP, 2.5 ug (FA)
B7 = Placebo MAP (FA); B8 = A/Sing MAP, 15 ug (UA); B9 = A/Sing IM, 15 ug, as 2018 Afluria Quad (UA)

Listing 16.2.9.3
Numeric Pain Intensity per Site

Part B

Randomisation Number	Group	Visit	Time Point	Was Pain Assessment Performed?	Site Number	Treatment	Date of assessment	Time of Assessment	Score	Unscheduled Reason
B0101	Group B3	Day 4		Yes	F1	5 ug	12-JUN-2018	12:34	0	
B0101	Group B3	Day 4		Yes	F2	5 ug	12-JUN-2018	12:34	0	
B0101	Group B3	Day 4		Yes	F3	5 ug	12-JUN-2018	12:34	0	
B0101	Group B3	Day 8		Yes	F1	5 ug	16-JUN-2018	14:20	0	
B0101	Group B3	Day 8		Yes	F2	5 ug	16-JUN-2018	14:21	0	
B0101	Group B3	Day 8		Yes	F3	5 ug	16-JUN-2018	14:22	0	
B0101	Group B3	Day 22		Yes	F1	5 ug	30-JUN-2018	13:12	0	
B0101	Group B3	Day 22		Yes	F2	5 ug	30-JUN-2018	13:12	0	
B0101	Group B3	Day 22		Yes	F3	5 ug	30-JUN-2018	13:12	0	
B0101	Group B3	EOS/ET (Day 61)		Yes	F1	5 ug	08-AUG-2018	16:33	0	
B0101	Group B3	EOS/ET (Day 61)		Yes	F2	5 ug	08-AUG-2018	16:33	0	
B0101	Group B3	EOS/ET (Day 61)		Yes	F3	5 ug	08-AUG-2018	16:33	0	
B0102	Group B8	Day 1	1 min	Yes	D1	5 ug	09-JUN-2018	14:11	0	
B0102	Group B8	Day 1	1 min	Yes	D2	5 ug	09-JUN-2018	14:14	0	
B0102	Group B8	Day 1	1 min	Yes	D3	5 ug	09-JUN-2018	14:17	0	
B0102	Group B8	Day 1	10 min	Yes	D1	5 ug	09-JUN-2018	14:28	0	
B0102	Group B8	Day 1	10 min	Yes	D2	5 ug	09-JUN-2018	14:28	0	
B0102	Group B8	Day 1	10 min	Yes	D3	5 ug	09-JUN-2018	14:28	0	
B0102	Group B8	Day 1	1 hour	Yes	D1	5 ug	09-JUN-2018	15:18	0	
B0102	Group B8	Day 1	1 hour	Yes	D2	5 ug	09-JUN-2018	15:18	0	
B0102	Group B8	Day 1	1 hour	Yes	D3	5 ug	09-JUN-2018	15:18	0	

Scale: 0= No Pain; 10= Worst Pain Possible
Administration Site Key: FA = Forearm; UA= Upper arm
Treatments Part A: A1 = A/Sing MAP, 15 ug (FA); A2 = Afluria Quad IM (UA), 15 ug; A3 = Placebo MAP (FA); A4 = A/Sing Mono IM, 15 ug (UA)
Treatments Part B: B1 = A/Sing MAP, 15 ug (FA) with biopsy; B2 = Placebo MAP (FA) with biopsy
B3 = A/Sing MAP, 15 ug (FA); B4 = A/Sing MAP, 10 ug (FA); B5 = A/Sing MAP, 5 ug (FA); B6 = A/Sing MAP, 2.5 ug (FA)
B7 = Placebo MAP (FA); B8 = A/Sing MAP, 15 ug (UA); B9 = A/Sing IM, 15 ug, as 2018 Afluria Quad (UA)

Listing 16.2.9.3
Numeric Pain Intensity per Site

Part B

Randomisation Number	Group	Visit	Time Point	Was Pain Assessment Performed?	Site Number	Treatment	Date of assessment	Time of Assessment	Score	Unscheduled Reason
B0102	Group B8	Day 1	2 hour	Yes	D1	5 ug	09-JUN-2018	16:18	0	
B0102	Group B8	Day 1	2 hour	Yes	D2	5 ug	09-JUN-2018	16:18	0	
B0102	Group B8	Day 1	2 hour	Yes	D3	5 ug	09-JUN-2018	16:18	0	
B0102	Group B8	Day 2		Yes	D1	5 ug	10-JUN-2018	17:03	0	
B0102	Group B8	Day 2		Yes	D2	5 ug	10-JUN-2018	17:03	0	
B0102	Group B8	Day 2		Yes	D3	5 ug	10-JUN-2018	17:03	0	
B0102	Group B8	Day 4		Yes	D1	5 ug	12-JUN-2018	14:24	0	
B0102	Group B8	Day 4		Yes	D2	5 ug	12-JUN-2018	14:24	0	
B0102	Group B8	Day 4		Yes	D3	5 ug	12-JUN-2018	14:24	0	
B0102	Group B8	Day 8		Yes	D1	5 ug	16-JUN-2018	14:50	0	
B0102	Group B8	Day 8		Yes	D2	5 ug	16-JUN-2018	14:51	0	
B0102	Group B8	Day 8		Yes	D3	5 ug	16-JUN-2018	14:51	0	
B0102	Group B8	Day 22		Yes	D1	5 ug	30-JUN-2018	11:18	0	
B0102	Group B8	Day 22		Yes	D2	5 ug	30-JUN-2018	11:18	0	
B0102	Group B8	Day 22		Yes	D3	5 ug	30-JUN-2018	11:18	0	
B0102	Group B8	EOS/ET (Day 61)		Yes	D1	5 ug	08-AUG-2018	16:27	0	
B0102	Group B8	EOS/ET (Day 61)		Yes	D2	5 ug	08-AUG-2018	16:27	0	
B0102	Group B8	EOS/ET (Day 61)		Yes	D3	5 ug	08-AUG-2018	16:27	0	
B0103	Group B5	Day 1	1 min	Yes	F1	5 ug	09-JUN-2018	14:21	0	
B0103	Group B5	Day 1	1 min	Yes	F2	uncoated	09-JUN-2018	14:24	2	
B0103	Group B5	Day 1	1 min	Yes	F3	uncoated	09-JUN-2018	14:27	2	

Scale: 0= No Pain; 10= Worst Pain Possible
Administration Site Key: FA = Forearm; UA= Upper arm
Treatments Part A: A1 = A/Sing MAP, 15 ug (FA); A2 = Afluria Quad IM (UA), 15 ug; A3 = Placebo MAP (FA); A4 = A/Sing Mono IM, 15 ug (UA)
Treatments Part B: B1 = A/Sing MAP, 15 ug (FA) with biopsy; B2 = Placebo MAP (FA) with biopsy
B3 = A/Sing MAP, 15 ug (FA); B4 = A/Sing MAP, 10 ug (FA); B5 = A/Sing MAP, 5 ug (FA); B6 = A/Sing MAP, 2.5 ug (FA)
B7 = Placebo MAP (FA); B8 = A/Sing MAP, 15 ug (UA); B9 = A/Sing IM, 15 ug, as 2018 Afluria Quad (UA)

Listing 16.2.9.3
Numeric Pain Intensity per Site

Part B

Randomisation Number	Group	Visit	Time Point	Was Pain Assessment Performed?	Site Number	Treatment	Date of assessment	Time of Assessment	Score	Unscheduled Reason
B0103	Group B5	Day 1	10 min	Yes	F1	5 ug	09-JUN-2018	14:35	0	
B0103	Group B5	Day 1	10 min	Yes	F2	uncoated	09-JUN-2018	14:35	0	
B0103	Group B5	Day 1	10 min	Yes	F3	uncoated	09-JUN-2018	14:35	4	
B0103	Group B5	Day 1	1 hour	Yes	F1	5 ug	09-JUN-2018	15:28	0	
B0103	Group B5	Day 1	1 hour	Yes	F2	uncoated	09-JUN-2018	15:28	0	
B0103	Group B5	Day 1	1 hour	Yes	F3	uncoated	09-JUN-2018	15:28	0	
B0103	Group B5	Day 1	2 hour	Yes	F1	5 ug	09-JUN-2018	16:28	0	
B0103	Group B5	Day 1	2 hour	Yes	F2	uncoated	09-JUN-2018	16:28	0	
B0103	Group B5	Day 1	2 hour	Yes	F3	uncoated	09-JUN-2018	16:28	0	
B0103	Group B5	Day 2		Yes	F1	5 ug	10-JUN-2018	14:17	0	
B0103	Group B5	Day 2		Yes	F2	uncoated	10-JUN-2018	14:17	0	
B0103	Group B5	Day 2		Yes	F3	uncoated	10-JUN-2018	14:17	0	
B0103	Group B5	Day 4		Yes	F1	5 ug	12-JUN-2018	15:36	0	
B0103	Group B5	Day 4		Yes	F2	uncoated	12-JUN-2018	15:36	0	
B0103	Group B5	Day 4		Yes	F3	uncoated	12-JUN-2018	15:36	0	
B0103	Group B5	Day 8		Yes	F1	5 ug	16-JUN-2018	10:41	0	
B0103	Group B5	Day 8		Yes	F2	uncoated	16-JUN-2018	10:42	0	
B0103	Group B5	Day 8		Yes	F3	uncoated	16-JUN-2018	10:43	0	
B0103	Group B5	Day 22		Yes	F1	5 ug	29-JUN-2018	08:42	0	
B0103	Group B5	Day 22		Yes	F2	uncoated	29-JUN-2018	08:42	0	
B0103	Group B5	Day 22		Yes	F3	uncoated	29-JUN-2018	08:42	0	

Scale: 0= No Pain; 10= Worst Pain Possible
Administration Site Key: FA = Forearm; UA= Upper arm
Treatments Part A: A1 = A/Sing MAP, 15 ug (FA); A2 = Afluria Quad IM (UA), 15 ug; A3 = Placebo MAP (FA); A4 = A/Sing Mono IM, 15 ug (UA)
Treatments Part B: B1 = A/Sing MAP, 15 ug (FA) with biopsy; B2 = Placebo MAP (FA) with biopsy
B3 = A/Sing MAP, 15 ug (FA); B4 = A/Sing MAP, 10 ug (FA); B5 = A/Sing MAP, 5 ug (FA); B6 = A/Sing MAP, 2.5 ug (FA)
B7 = Placebo MAP (FA); B8 = A/Sing MAP, 15 ug (UA); B9 = A/Sing IM, 15 ug, as 2018 Afluria Quad (UA)

Listing 16.2.9.3
Numeric Pain Intensity per Site

Part B

Randomisation Number	Group	Visit	Time Point	Was Pain Assessment Performed?	Site Number	Treatment	Date of assessment	Time of Assessment	Score	Unscheduled Reason
B0103	Group B5	EOS/ET (Day 61)		Yes	F1	5 ug	08-AUG-2018	11:25	0	
B0103	Group B5	EOS/ET (Day 61)		Yes	F2	uncoated	08-AUG-2018	11:25	0	
B0103	Group B5	EOS/ET (Day 61)		Yes	F3	uncoated	08-AUG-2018	11:25	0	
B0104	Group B3	Day 1	1 min	Yes	F1	5 ug	09-JUN-2018	14:31	0	
B0104	Group B3	Day 1	1 min	Yes	F2	5 ug	09-JUN-2018	14:34	0	
B0104	Group B3	Day 1	1 min	Yes	F3	5 ug	09-JUN-2018	14:37	2	
B0104	Group B3	Day 1	10 min	Yes	F1	5 ug	09-JUN-2018	14:41	1	
B0104	Group B3	Day 1	10 min	Yes	F2	5 ug	09-JUN-2018	14:43	1	
B0104	Group B3	Day 1	10 min	Yes	F3	5 ug	09-JUN-2018	14:46	1	
B0104	Group B3	Day 1	1 hour	Yes	F1	5 ug	09-JUN-2018	15:38	0	
B0104	Group B3	Day 1	1 hour	Yes	F2	5 ug	09-JUN-2018	15:38	0	
B0104	Group B3	Day 1	1 hour	Yes	F3	5 ug	09-JUN-2018	15:38	0	
B0104	Group B3	Day 1	2 hour	Yes	F1	5 ug	09-JUN-2018	16:38	0	
B0104	Group B3	Day 1	2 hour	Yes	F2	5 ug	09-JUN-2018	16:38	0	
B0104	Group B3	Day 1	2 hour	Yes	F3	5 ug	09-JUN-2018	16:38	0	
B0104	Group B3	Day 2		Yes	F1	5 ug	10-JUN-2018	15:19	0	
B0104	Group B3	Day 2		Yes	F2	5 ug	10-JUN-2018	15:19	0	
B0104	Group B3	Day 2		Yes	F3	5 ug	10-JUN-2018	15:19	0	
B0104	Group B3	Day 4		Yes	F1	5 ug	12-JUN-2018	14:29	0	
B0104	Group B3	Day 4		Yes	F2	5 ug	12-JUN-2018	14:29	0	
B0104	Group B3	Day 4		Yes	F3	5 ug	12-JUN-2018	14:29	0	

Scale: 0= No Pain; 10= Worst Pain Possible
Administration Site Key: FA = Forearm; UA= Upper arm
Treatments Part A: A1 = A/Sing MAP, 15 ug (FA); A2 = Afluria Quad IM (UA), 15 ug; A3 = Placebo MAP (FA); A4 = A/Sing Mono IM, 15 ug (UA)
Treatments Part B: B1 = A/Sing MAP, 15 ug (FA) with biopsy; B2 = Placebo MAP (FA) with biopsy
B3 = A/Sing MAP, 15 ug (FA); B4 = A/Sing MAP, 10 ug (FA); B5 = A/Sing MAP, 5 ug (FA); B6 = A/Sing MAP, 2.5 ug (FA)
B7 = Placebo MAP (FA); B8 = A/Sing MAP, 15 ug (UA); B9 = A/Sing IM, 15 ug, as 2018 Afluria Quad (UA)

Listing 16.2.9.3
Numeric Pain Intensity per Site

Part B

Randomisation Number	Group	Visit	Time Point	Was Pain Assessment Performed?	Site Number	Treatment	Date of assessment	Time of Assessment	Score	Unscheduled Reason
B0104	Group B3	Day 8		Yes	F1	5 ug	16-JUN-2018	16:55	0	
B0104	Group B3	Day 8		Yes	F2	5 ug	16-JUN-2018	16:55	0	
B0104	Group B3	Day 8		Yes	F3	5 ug	16-JUN-2018	16:55	0	
B0104	Group B3	Day 22		Yes	F1	5 ug	30-JUN-2018	10:26	0	
B0104	Group B3	Day 22		Yes	F2	5 ug	30-JUN-2018	10:26	0	
B0104	Group B3	Day 22		Yes	F3	5 ug	30-JUN-2018	10:26	0	
B0104	Group B3	EOS/ET (Day 61)		Yes	F1	5 ug	10-AUG-2018	09:30	0	
B0104	Group B3	EOS/ET (Day 61)		Yes	F2	5 ug	10-AUG-2018	09:30	0	
B0104	Group B3	EOS/ET (Day 61)		Yes	F3	5 ug	10-AUG-2018	09:30	0	
B0105	Group B9	Day 1	1 min	Yes	D1	[IM]	09-JUN-2018	11:31	0	
B0105	Group B9	Day 1	10 min	Yes	D1	[IM]	09-JUN-2018	11:40	0	
B0105	Group B9	Day 1	1 hour	Yes	D1	[IM]	09-JUN-2018	12:30	0	
B0105	Group B9	Day 1	2 hour	Yes	D1	[IM]	09-JUN-2018	13:35	0	
B0105	Group B9	Day 2		Yes	D1	[IM]	10-JUN-2018	11:41	2	
B0105	Group B9	Day 4		Yes	D1	[IM]	12-JUN-2018	15:53	0	
B0105	Group B9	Day 8		Yes	D1	[IM]	16-JUN-2018	11:36	0	
B0105	Group B9	Day 22		Yes	D1	[IM]	30-JUN-2018	10:40	0	
B0105	Group B9	EOS/ET (Day 61)		Yes	D1	[IM]	08-AUG-2018	15:17	0	
B0106	Group B4	Day 1	1 min	Yes	F1	uncoated	09-JUN-2018	14:41	1	
B0106	Group B4	Day 1	1 min	Yes	F2	5 ug	09-JUN-2018	14:44	1	

Scale: 0= No Pain; 10= Worst Pain Possible
Administration Site Key: FA = Forearm; UA= Upper arm
Treatments Part A: A1 = A/Sing MAP, 15 ug (FA); A2 = Afluria Quad IM (UA), 15 ug; A3 = Placebo MAP (FA); A4 = A/Sing Mono IM, 15 ug (UA)
Treatments Part B: B1 = A/Sing MAP, 15 ug (FA) with biopsy; B2 = Placebo MAP (FA) with biopsy
B3 = A/Sing MAP, 15 ug (FA); B4 = A/Sing MAP, 10 ug (FA); B5 = A/Sing MAP, 5 ug (FA); B6 = A/Sing MAP, 2.5 ug (FA)
B7 = Placebo MAP (FA); B8 = A/Sing MAP, 15 ug (UA); B9 = A/Sing IM, 15 ug, as 2018 Afluria Quad (UA)

Listing 16.2.9.3
Numeric Pain Intensity per Site

Part B

Randomisation Number	Group	Visit	Time Point	Was Pain Assessment Performed?	Site Number	Treatment	Date of assessment	Time of Assessment	Score	Unscheduled Reason
B0106	Group B4	Day 1	1 min	Yes	F3	5 ug	09-JUN-2018	14:47	2	
B0106	Group B4	Day 1	10 min	Yes	F1	uncoated	09-JUN-2018	14:50	1	
B0106	Group B4	Day 1	10 min	Yes	F2	5 ug	09-JUN-2018	14:53	1	
B0106	Group B4	Day 1	10 min	Yes	F3	5 ug	09-JUN-2018	14:53	2	
B0106	Group B4	Day 1	1 hour	Yes	F1	uncoated	09-JUN-2018	15:48	0	
B0106	Group B4	Day 1	1 hour	Yes	F2	5 ug	09-JUN-2018	15:48	0	
B0106	Group B4	Day 1	1 hour	Yes	F3	5 ug	09-JUN-2018	15:48	0	
B0106	Group B4	Day 1	2 hour	Yes	F1	uncoated	09-JUN-2018	16:48	0	
B0106	Group B4	Day 1	2 hour	Yes	F2	5 ug	09-JUN-2018	16:48	0	
B0106	Group B4	Day 1	2 hour	Yes	F3	5 ug	09-JUN-2018	16:48	0	
B0106	Group B4	Day 2		Yes	F1	uncoated	10-JUN-2018	14:30	0	
B0106	Group B4	Day 2		Yes	F2	5 ug	10-JUN-2018	14:30	0	
B0106	Group B4	Day 2		Yes	F3	5 ug	10-JUN-2018	14:30	0	
B0106	Group B4	Day 4		Yes	F1	uncoated	12-JUN-2018	12:16	0	
B0106	Group B4	Day 4		Yes	F2	5 ug	12-JUN-2018	12:16	0	
B0106	Group B4	Day 4		Yes	F3	5 ug	12-JUN-2018	12:16	0	
B0106	Group B4	Day 8		Yes	F1	uncoated	16-JUN-2018	15:52	0	
B0106	Group B4	Day 8		Yes	F2	5 ug	16-JUN-2018	15:52	0	
B0106	Group B4	Day 8		Yes	F3	5 ug	16-JUN-2018	15:52	0	
B0106	Group B4	Day 22		Yes	F1	uncoated	30-JUN-2018	12:33	0	
B0106	Group B4	Day 22		Yes	F2	5 ug	30-JUN-2018	12:33	0	

Scale: 0= No Pain; 10= Worst Pain Possible
Administration Site Key: FA = Forearm; UA= Upper arm
Treatments Part A: A1 = A/Sing MAP, 15 ug (FA); A2 = Afluria Quad IM (UA), 15 ug; A3 = Placebo MAP (FA); A4 = A/Sing Mono IM, 15 ug (UA)
Treatments Part B: B1 = A/Sing MAP, 15 ug (FA) with biopsy; B2 = Placebo MAP (FA) with biopsy
B3 = A/Sing MAP, 15 ug (FA); B4 = A/Sing MAP, 10 ug (FA); B5 = A/Sing MAP, 5 ug (FA); B6 = A/Sing MAP, 2.5 ug (FA)
B7 = Placebo MAP (FA); B8 = A/Sing MAP, 15 ug (UA); B9 = A/Sing IM, 15 ug, as 2018 Afluria Quad (UA)

Listing 16.2.9.3
Numeric Pain Intensity per Site

Part B

Randomisation Number	Group	Visit	Time Point	Was Pain Assessment Performed?	Site Number	Treatment	Date of assessment	Time of Assessment	Score	Unscheduled Reason
B0106	Group B4	Day 22		Yes	F3	5 ug	30-JUN-2018	12:33	0	
B0106	Group B4	EOS/ET (Day 61)		Yes	F1	uncoated	08-AUG-2018	15:18	0	
B0106	Group B4	EOS/ET (Day 61)		Yes	F2	5 ug	08-AUG-2018	15:18	0	
B0106	Group B4	EOS/ET (Day 61)		Yes	F3	5 ug	08-AUG-2018	15:18	0	
B0107	Group B6	Day 1	1 min	Yes	F1	uncoated	09-JUN-2018	14:51	1	
B0107	Group B6	Day 1	1 min	Yes	F2	uncoated	09-JUN-2018	14:54	1	
B0107	Group B6	Day 1	1 min	Yes	F3	2.5 ug	09-JUN-2018	14:57	1	
B0107	Group B6	Day 1	10 min	Yes	F1	uncoated	09-JUN-2018	15:08	0	
B0107	Group B6	Day 1	10 min	Yes	F2	uncoated	09-JUN-2018	15:08	0	
B0107	Group B6	Day 1	10 min	Yes	F3	2.5 ug	09-JUN-2018	15:08	0	
B0107	Group B6	Day 1	1 hour	Yes	F1	uncoated	09-JUN-2018	15:58	0	
B0107	Group B6	Day 1	1 hour	Yes	F2	uncoated	09-JUN-2018	15:58	0	
B0107	Group B6	Day 1	1 hour	Yes	F3	2.5 ug	09-JUN-2018	15:58	0	
B0107	Group B6	Day 1	2 hour	Yes	F1	uncoated	09-JUN-2018	16:58	0	
B0107	Group B6	Day 1	2 hour	Yes	F2	uncoated	09-JUN-2018	16:58	0	
B0107	Group B6	Day 1	2 hour	Yes	F3	2.5 ug	09-JUN-2018	16:58	0	
B0107	Group B6	Day 2		Yes	F1	uncoated	10-JUN-2018	10:02	0	
B0107	Group B6	Day 2		Yes	F2	uncoated	10-JUN-2018	10:02	0	
B0107	Group B6	Day 2		Yes	F3	2.5 ug	10-JUN-2018	10:02	0	
B0107	Group B6	Day 4		Yes	F1	uncoated	12-JUN-2018	11:00	0	
B0107	Group B6	Day 4		Yes	F2	uncoated	12-JUN-2018	11:00	0	

Scale: 0= No Pain; 10= Worst Pain Possible
Administration Site Key: FA = Forearm; UA= Upper arm
Treatments Part A: A1 = A/Sing MAP, 15 ug (FA); A2 = Afluria Quad IM (UA), 15 ug; A3 = Placebo MAP (FA); A4 = A/Sing Mono IM, 15 ug (UA)
Treatments Part B: B1 = A/Sing MAP, 15 ug (FA) with biopsy; B2 = Placebo MAP (FA) with biopsy
B3 = A/Sing MAP, 15 ug (FA); B4 = A/Sing MAP, 10 ug (FA); B5 = A/Sing MAP, 5 ug (FA); B6 = A/Sing MAP, 2.5 ug (FA)
B7 = Placebo MAP (FA); B8 = A/Sing MAP, 15 ug (UA); B9 = A/Sing IM, 15 ug, as 2018 Afluria Quad (UA)

Listing 16.2.9.3
Numeric Pain Intensity per Site

Part B

Randomisation Number	Group	Visit	Time Point	Was Pain Assessment Performed?	Site Number	Treatment	Date of assessment	Time of Assessment	Score	Unscheduled Reason
B0107	Group B6	Day 4		Yes	F3	2.5 ug	12-JUN-2018	11:00	0	
B0107	Group B6	Day 8		Yes	F1	uncoated	16-JUN-2018	09:58	0	
B0107	Group B6	Day 8		Yes	F2	uncoated	16-JUN-2018	09:58	0	
B0107	Group B6	Day 8		Yes	F3	2.5 ug	16-JUN-2018	09:58	0	
B0107	Group B6	Day 22		Yes	F1	uncoated	29-JUN-2018	17:31	0	
B0107	Group B6	Day 22		Yes	F2	uncoated	29-JUN-2018	17:31	0	
B0107	Group B6	Day 22		Yes	F3	2.5 ug	29-JUN-2018	17:31	0	
B0107	Group B6	EOS/ET (Day 61)		Yes	F1	uncoated	08-AUG-2018	17:34	0	
B0107	Group B6	EOS/ET (Day 61)		Yes	F2	uncoated	08-AUG-2018	17:34	0	
B0107	Group B6	EOS/ET (Day 61)		Yes	F3	2.5 ug	08-AUG-2018	17:34	0	
B0108	Group B7	Day 1	1 min	Yes	F1	uncoated	09-JUN-2018	15:01	1	
B0108	Group B7	Day 1	1 min	Yes	F2	uncoated	09-JUN-2018	15:04	1	
B0108	Group B7	Day 1	1 min	Yes	F3	uncoated	09-JUN-2018	15:07	1	
B0108	Group B7	Day 1	10 min	Yes	F1	uncoated	09-JUN-2018	15:13	1	
B0108	Group B7	Day 1	10 min	Yes	F2	uncoated	09-JUN-2018	15:13	1	
B0108	Group B7	Day 1	10 min	Yes	F3	uncoated	09-JUN-2018	15:13	1	
B0108	Group B7	Day 1	1 hour	Yes	F1	uncoated	09-JUN-2018	16:08	0	
B0108	Group B7	Day 1	1 hour	Yes	F2	uncoated	09-JUN-2018	16:08	0	
B0108	Group B7	Day 1	1 hour	Yes	F3	uncoated	09-JUN-2018	16:08	0	
B0108	Group B7	Day 1	2 hour	Yes	F1	uncoated	09-JUN-2018	17:08	0	
B0108	Group B7	Day 1	2 hour	Yes	F2	uncoated	09-JUN-2018	17:08	0	

Scale: 0= No Pain; 10= Worst Pain Possible
Administration Site Key: FA = Forearm; UA= Upper arm
Treatments Part A: A1 = A/Sing MAP, 15 ug (FA); A2 = Afluria Quad IM (UA), 15 ug; A3 = Placebo MAP (FA); A4 = A/Sing Mono IM, 15 ug (UA)
Treatments Part B: B1 = A/Sing MAP, 15 ug (FA) with biopsy; B2 = Placebo MAP (FA) with biopsy
B3 = A/Sing MAP, 15 ug (FA); B4 = A/Sing MAP, 10 ug (FA); B5 = A/Sing MAP, 5 ug (FA); B6 = A/Sing MAP, 2.5 ug (FA)
B7 = Placebo MAP (FA); B8 = A/Sing MAP, 15 ug (UA); B9 = A/Sing IM, 15 ug, as 2018 Afluria Quad (UA)

Listing 16.2.9.3
Numeric Pain Intensity per Site

Part B

Randomisation Number	Group	Visit	Time Point	Was Pain Assessment Performed?	Site Number	Treatment	Date of assessment	Time of Assessment	Score	Unscheduled Reason
B0108	Group B7	Day 1	2 hour	Yes	F3	uncoated	09-JUN-2018	17:08	0	
B0108	Group B7	Day 2		Yes	F1	uncoated	10-JUN-2018	15:07	0	
B0108	Group B7	Day 2		Yes	F2	uncoated	10-JUN-2018	15:07	0	
B0108	Group B7	Day 2		Yes	F3	uncoated	10-JUN-2018	15:07	0	
B0108	Group B7	Day 4		Yes	F1	uncoated	12-JUN-2018	14:33	0	
B0108	Group B7	Day 4		Yes	F2	uncoated	12-JUN-2018	14:33	0	
B0108	Group B7	Day 4		Yes	F3	uncoated	12-JUN-2018	14:33	0	
B0108	Group B7	Day 8		Yes	F1	uncoated	16-JUN-2018	08:57	0	
B0108	Group B7	Day 8		Yes	F2	uncoated	16-JUN-2018	08:57	0	
B0108	Group B7	Day 8		Yes	F3	uncoated	16-JUN-2018	08:58	0	
B0108	Group B7	Day 22		Yes	F1	uncoated	30-JUN-2018	13:30	0	
B0108	Group B7	Day 22		Yes	F2	uncoated	30-JUN-2018	13:30	0	
B0108	Group B7	Day 22		Yes	F3	uncoated	30-JUN-2018	13:30	0	
B0108	Group B7	EOS/ET (Day 61)		Yes	F1	uncoated	28-AUG-2018	16:25	0	
B0108	Group B7	EOS/ET (Day 61)		Yes	F2	uncoated	28-AUG-2018	16:25	0	
B0108	Group B7	EOS/ET (Day 61)		Yes	F3	uncoated	28-AUG-2018	16:25	0	
B0109	Group B6	Day 1	1 min	Yes	F1	uncoated	16-JUN-2018	11:51	0	
B0109	Group B6	Day 1	1 min	Yes	F2	2.5 ug	16-JUN-2018	11:54	0	
B0109	Group B6	Day 1	1 min	Yes	F3	uncoated	16-JUN-2018	11:57	0	
B0109	Group B6	Day 1	10 min	Yes	F1	uncoated	16-JUN-2018	12:08	0	
B0109	Group B6	Day 1	10 min	Yes	F2	2.5 ug	16-JUN-2018	12:08	0	

Scale: 0= No Pain; 10= Worst Pain Possible
Administration Site Key: FA = Forearm; UA= Upper arm
Treatments Part A: A1 = A/Sing MAP, 15 ug (FA); A2 = Afluria Quad IM (UA), 15 ug; A3 = Placebo MAP (FA); A4 = A/Sing Mono IM, 15 ug (UA)
Treatments Part B: B1 = A/Sing MAP, 15 ug (FA) with biopsy; B2 = Placebo MAP (FA) with biopsy
B3 = A/Sing MAP, 15 ug (FA); B4 = A/Sing MAP, 10 ug (FA); B5 = A/Sing MAP, 5 ug (FA); B6 = A/Sing MAP, 2.5 ug (FA)
B7 = Placebo MAP (FA); B8 = A/Sing MAP, 15 ug (UA); B9 = A/Sing IM, 15 ug, as 2018 Afluria Quad (UA)

Listing 16.2.9.3
Numeric Pain Intensity per Site

Part B

Randomisation Number	Group	Visit	Time Point	Was Pain Assessment Performed?	Site Number	Treatment	Date of assessment	Time of Assessment	Score	Unscheduled Reason
B0109	Group B6	Day 1	10 min	Yes	F3	uncoated	16-JUN-2018	12:08	0	
B0109	Group B6	Day 1	1 hour	Yes	F1	uncoated	16-JUN-2018	12:50	0	
B0109	Group B6	Day 1	1 hour	Yes	F2	2.5 ug	16-JUN-2018	12:50	0	
B0109	Group B6	Day 1	1 hour	Yes	F3	uncoated	16-JUN-2018	12:50	0	
B0109	Group B6	Day 1	2 hour	Yes	F1	uncoated	16-JUN-2018	13:58	0	
B0109	Group B6	Day 1	2 hour	Yes	F2	2.5 ug	16-JUN-2018	13:58	0	
B0109	Group B6	Day 1	2 hour	Yes	F3	uncoated	16-JUN-2018	13:58	0	
B0109	Group B6	Day 2		Yes	F1	uncoated	17-JUN-2018	12:00	0	
B0109	Group B6	Day 2		Yes	F2	2.5 ug	17-JUN-2018	12:00	0	
B0109	Group B6	Day 2		Yes	F3	uncoated	17-JUN-2018	12:01	0	
B0109	Group B6	Day 4		Yes	F1	uncoated	19-JUN-2018	15:28	0	
B0109	Group B6	Day 4		Yes	F2	2.5 ug	19-JUN-2018	15:29	0	
B0109	Group B6	Day 4		Yes	F3	uncoated	19-JUN-2018	15:28	0	
B0109	Group B6	Day 8		Yes	F1	uncoated	23-JUN-2018	09:47	0	
B0109	Group B6	Day 8		Yes	F2	2.5 ug	23-JUN-2018	09:47	0	
B0109	Group B6	Day 8		Yes	F3	uncoated	23-JUN-2018	09:48	0	
B0109	Group B6	Day 22		Yes	F1	uncoated	07-JUL-2018	10:17	0	
B0109	Group B6	Day 22		Yes	F2	2.5 ug	07-JUL-2018	10:17	0	
B0109	Group B6	Day 22		Yes	F3	uncoated	07-JUL-2018	10:17	0	
B0109	Group B6	EOS/ET (Day 61)		Yes	F1	uncoated	15-AUG-2018	09:53	0	
B0109	Group B6	EOS/ET (Day 61)		Yes	F2	2.5 ug	15-AUG-2018	09:53	0	

Scale: 0= No Pain; 10= Worst Pain Possible
Administration Site Key: FA = Forearm; UA= Upper arm
Treatments Part A: A1 = A/Sing MAP, 15 ug (FA); A2 = Afluria Quad IM (UA), 15 ug; A3 = Placebo MAP (FA); A4 = A/Sing Mono IM, 15 ug (UA)
Treatments Part B: B1 = A/Sing MAP, 15 ug (FA) with biopsy; B2 = Placebo MAP (FA) with biopsy
B3 = A/Sing MAP, 15 ug (FA); B4 = A/Sing MAP, 10 ug (FA); B5 = A/Sing MAP, 5 ug (FA); B6 = A/Sing MAP, 2.5 ug (FA)
B7 = Placebo MAP (FA); B8 = A/Sing MAP, 15 ug (UA); B9 = A/Sing IM, 15 ug, as 2018 Afluria Quad (UA)

Listing 16.2.9.3
Numeric Pain Intensity per Site

Part B

Randomisation Number	Group	Visit	Time Point	Was Pain Assessment Performed?	Site Number	Treatment	Date of assessment	Time of Assessment	Score	Unscheduled Reason
B0109	Group B6	EOS/ET (Day 61)		Yes	F3	uncoated	15-AUG-2018	09:53	0	
B0110	Group B3	Day 1	1 min	Yes	F1	5 ug	16-JUN-2018	12:01	2	
B0110	Group B3	Day 1	1 min	Yes	F2	5 ug	16-JUN-2018	12:04	2	
B0110	Group B3	Day 1	1 min	Yes	F3	5 ug	16-JUN-2018	12:07	2	
B0110	Group B3	Day 1	10 min	Yes	F1	5 ug	16-JUN-2018	12:18	1	
B0110	Group B3	Day 1	10 min	Yes	F2	5 ug	16-JUN-2018	12:18	1	
B0110	Group B3	Day 1	10 min	Yes	F3	5 ug	16-JUN-2018	12:18	1	
B0110	Group B3	Day 1	1 hour	Yes	F1	5 ug	16-JUN-2018	13:08	0	
B0110	Group B3	Day 1	1 hour	Yes	F2	5 ug	16-JUN-2018	13:08	0	
B0110	Group B3	Day 1	1 hour	Yes	F3	5 ug	16-JUN-2018	13:08	0	
B0110	Group B3	Day 1	2 hour	Yes	F1	5 ug	16-JUN-2018	14:08	0	
B0110	Group B3	Day 1	2 hour	Yes	F2	5 ug	16-JUN-2018	14:08	0	
B0110	Group B3	Day 1	2 hour	Yes	F3	5 ug	16-JUN-2018	14:08	0	
B0110	Group B3	Day 2		Yes	F1	5 ug	17-JUN-2018	12:45	0	
B0110	Group B3	Day 2		Yes	F2	5 ug	17-JUN-2018	12:45	0	
B0110	Group B3	Day 2		Yes	F3	5 ug	17-JUN-2018	12:45	0	
B0110	Group B3	Day 4		Yes	F1	5 ug	19-JUN-2018	13:18	0	
B0110	Group B3	Day 4		Yes	F2	5 ug	19-JUN-2018	13:18	0	
B0110	Group B3	Day 4		Yes	F3	5 ug	19-JUN-2018	13:18	0	
B0110	Group B3	Day 8		Yes	F1	5 ug	23-JUN-2018	13:54	0	
B0110	Group B3	Day 8		Yes	F2	5 ug	23-JUN-2018	13:54	0	

Scale: 0= No Pain; 10= Worst Pain Possible
Administration Site Key: FA = Forearm; UA= Upper arm
Treatments Part A: A1 = A/Sing MAP, 15 ug (FA); A2 = Afluria Quad IM (UA), 15 ug; A3 = Placebo MAP (FA); A4 = A/Sing Mono IM, 15 ug (UA)
Treatments Part B: B1 = A/Sing MAP, 15 ug (FA) with biopsy; B2 = Placebo MAP (FA) with biopsy
B3 = A/Sing MAP, 15 ug (FA); B4 = A/Sing MAP, 10 ug (FA); B5 = A/Sing MAP, 5 ug (FA); B6 = A/Sing MAP, 2.5 ug (FA)
B7 = Placebo MAP (FA); B8 = A/Sing MAP, 15 ug (UA); B9 = A/Sing IM, 15 ug, as 2018 Afluria Quad (UA)

Listing 16.2.9.3
Numeric Pain Intensity per Site

Part B

Randomisation Number	Group	Visit	Time Point	Was Pain Assessment Performed?	Site Number	Treatment	Date of assessment	Time of Assessment	Score	Unscheduled Reason
B0110	Group B3	Day 8		Yes	F3	5 ug	23-JUN-2018	13:54	0	
B0110	Group B3	Day 22		Yes	F1	5 ug	07-JUL-2018	13:56	0	
B0110	Group B3	Day 22		Yes	F2	5 ug	07-JUL-2018	13:56	0	
B0110	Group B3	Day 22		Yes	F3	5 ug	07-JUL-2018	13:56	0	
B0110	Group B3	EOS/ET (Day 61)		Yes	F1	5 ug	30-AUG-2018	13:12	0	
B0110	Group B3	EOS/ET (Day 61)		Yes	F2	5 ug	30-AUG-2018	13:13	0	
B0110	Group B3	EOS/ET (Day 61)		Yes	F3	5 ug	30-AUG-2018	13:14	0	
B0111	Group B8	Day 1	1 min	Yes	D1	5 ug	16-JUN-2018	12:11	2	
B0111	Group B8	Day 1	1 min	Yes	D2	5 ug	16-JUN-2018	12:14	1	
B0111	Group B8	Day 1	1 min	Yes	D3	5 ug	16-JUN-2018	12:17	1	
B0111	Group B8	Day 1	10 min	Yes	D1	5 ug	16-JUN-2018	12:28	1	
B0111	Group B8	Day 1	10 min	Yes	D2	5 ug	16-JUN-2018	12:28	1	
B0111	Group B8	Day 1	10 min	Yes	D3	5 ug	16-JUN-2018	12:28	1	
B0111	Group B8	Day 1	1 hour	Yes	D1	5 ug	16-JUN-2018	13:18	0	
B0111	Group B8	Day 1	1 hour	Yes	D2	5 ug	16-JUN-2018	13:18	0	
B0111	Group B8	Day 1	1 hour	Yes	D3	5 ug	16-JUN-2018	13:18	0	
B0111	Group B8	Day 1	2 hour	Yes	D1	5 ug	16-JUN-2018	14:18	0	
B0111	Group B8	Day 1	2 hour	Yes	D2	5 ug	16-JUN-2018	14:18	0	
B0111	Group B8	Day 1	2 hour	Yes	D3	5 ug	16-JUN-2018	14:18	0	
B0111	Group B8	Day 2		Yes	D1	5 ug	17-JUN-2018	12:40	0	
B0111	Group B8	Day 2		Yes	D2	5 ug	17-JUN-2018	12:40	0	

Scale: 0= No Pain; 10= Worst Pain Possible
Administration Site Key: FA = Forearm; UA= Upper arm
Treatments Part A: A1 = A/Sing MAP, 15 ug (FA); A2 = Afluria Quad IM (UA), 15 ug; A3 = Placebo MAP (FA); A4 = A/Sing Mono IM, 15 ug (UA)
Treatments Part B: B1 = A/Sing MAP, 15 ug (FA) with biopsy; B2 = Placebo MAP (FA) with biopsy
B3 = A/Sing MAP, 15 ug (FA); B4 = A/Sing MAP, 10 ug (FA); B5 = A/Sing MAP, 5 ug (FA); B6 = A/Sing MAP, 2.5 ug (FA)
B7 = Placebo MAP (FA); B8 = A/Sing MAP, 15 ug (UA); B9 = A/Sing IM, 15 ug, as 2018 Afluria Quad (UA)

Listing 16.2.9.3
Numeric Pain Intensity per Site

Part B

Randomisation Number	Group	Visit	Time Point	Was Pain Assessment Performed?	Site Number	Treatment	Date of assessment	Time of Assessment	Score	Unscheduled Reason
B0111	Group B8	Day 2		Yes	D3	5 ug	17-JUN-2018	12:40	0	
B0111	Group B8	Day 4		Yes	D1	5 ug	19-JUN-2018	15:53	0	
B0111	Group B8	Day 4		Yes	D2	5 ug	19-JUN-2018	15:53	0	
B0111	Group B8	Day 4		Yes	D3	5 ug	19-JUN-2018	15:53	0	
B0111	Group B8	Day 8		Yes	D1	5 ug	23-JUN-2018	13:07	0	
B0111	Group B8	Day 8		Yes	D2	5 ug	23-JUN-2018	13:07	0	
B0111	Group B8	Day 8		Yes	D3	5 ug	23-JUN-2018	13:07	0	
B0111	Group B8	Day 22		Yes	D1	5 ug	07-JUL-2018	13:05	0	
B0111	Group B8	Day 22		Yes	D2	5 ug	07-JUL-2018	13:05	0	
B0111	Group B8	Day 22		Yes	D3	5 ug	07-JUL-2018	13:05	0	
B0111	Group B8	EOS/ET (Day 61)		Yes	D1	5 ug	15-AUG-2018	09:42	0	
B0111	Group B8	EOS/ET (Day 61)		Yes	D2	5 ug	15-AUG-2018	09:42	0	
B0111	Group B8	EOS/ET (Day 61)		Yes	D3	5 ug	15-AUG-2018	09:42	0	
B0112	Group B4	Day 1	1 min	Yes	F1	uncoated	16-JUN-2018	12:21	4	
B0112	Group B4	Day 1	1 min	Yes	F2	5 ug	16-JUN-2018	12:24	3	
B0112	Group B4	Day 1	1 min	Yes	F3	5 ug	16-JUN-2018	12:27	3	
B0112	Group B4	Day 1	10 min	Yes	F1	uncoated	16-JUN-2018	12:38	1	
B0112	Group B4	Day 1	10 min	Yes	F2	5 ug	16-JUN-2018	12:38	1	
B0112	Group B4	Day 1	10 min	Yes	F3	5 ug	16-JUN-2018	12:38	1	
B0112	Group B4	Day 1	1 hour	Yes	F1	uncoated	16-JUN-2018	13:28	0	
B0112	Group B4	Day 1	1 hour	Yes	F2	5 ug	16-JUN-2018	13:28	0	

Scale: 0= No Pain; 10= Worst Pain Possible
Administration Site Key: FA = Forearm; UA= Upper arm
Treatments Part A: A1 = A/Sing MAP, 15 ug (FA); A2 = Afluria Quad IM (UA), 15 ug; A3 = Placebo MAP (FA); A4 = A/Sing Mono IM, 15 ug (UA)
Treatments Part B: B1 = A/Sing MAP, 15 ug (FA) with biopsy; B2 = Placebo MAP (FA) with biopsy
B3 = A/Sing MAP, 15 ug (FA); B4 = A/Sing MAP, 10 ug (FA); B5 = A/Sing MAP, 5 ug (FA); B6 = A/Sing MAP, 2.5 ug (FA)
B7 = Placebo MAP (FA); B8 = A/Sing MAP, 15 ug (UA); B9 = A/Sing IM, 15 ug, as 2018 Afluria Quad (UA)

Listing 16.2.9.3
Numeric Pain Intensity per Site

Part B

Randomisation Number	Group	Visit	Time Point	Was Pain Assessment Performed?	Site Number	Treatment	Date of assessment	Time of Assessment	Score	Unscheduled Reason
B0112	Group B4	Day 1	1 hour	Yes	F3	5 ug	16-JUN-2018	13:28	0	
B0112	Group B4	Day 1	2 hour	Yes	F1	uncoated	16-JUN-2018	14:28	0	
B0112	Group B4	Day 1	2 hour	Yes	F2	5 ug	16-JUN-2018	14:28	0	
B0112	Group B4	Day 1	2 hour	Yes	F3	5 ug	16-JUN-2018	14:28	0	
B0112	Group B4	Day 2		Yes	F1	uncoated	17-JUN-2018	13:05	0	
B0112	Group B4	Day 2		Yes	F2	5 ug	17-JUN-2018	13:05	0	
B0112	Group B4	Day 2		Yes	F3	5 ug	17-JUN-2018	13:05	0	
B0112	Group B4	Day 4		Yes	F1	uncoated	19-JUN-2018	12:13	0	
B0112	Group B4	Day 4		Yes	F2	5 ug	19-JUN-2018	12:13	0	
B0112	Group B4	Day 4		Yes	F3	5 ug	19-JUN-2018	12:13	0	
B0112	Group B4	Day 8		Yes	F1	uncoated	23-JUN-2018	15:08	0	
B0112	Group B4	Day 8		Yes	F2	5 ug	23-JUN-2018	15:08	0	
B0112	Group B4	Day 8		Yes	F3	5 ug	23-JUN-2018	15:08	0	
B0112	Group B4	Day 22		Yes	F1	uncoated	07-JUL-2018	11:44	0	
B0112	Group B4	Day 22		Yes	F2	5 ug	07-JUL-2018	11:44	0	
B0112	Group B4	Day 22		Yes	F3	5 ug	07-JUL-2018	11:44	0	
B0112	Group B4	EOS/ET (Day 61)		Yes	F1	uncoated	15-AUG-2018	13:47	0	
B0112	Group B4	EOS/ET (Day 61)		Yes	F2	5 ug	15-AUG-2018	13:47	0	
B0112	Group B4	EOS/ET (Day 61)		Yes	F3	5 ug	15-AUG-2018	13:47	0	
B0113	Group B5	Day 1	1 min	Yes	F1	uncoated	16-JUN-2018	12:31	2	
B0113	Group B5	Day 1	1 min	Yes	F2	5 ug	16-JUN-2018	12:34	2	

Scale: 0= No Pain; 10= Worst Pain Possible
Administration Site Key: FA = Forearm; UA= Upper arm
Treatments Part A: A1 = A/Sing MAP, 15 ug (FA); A2 = Afluria Quad IM (UA), 15 ug; A3 = Placebo MAP (FA); A4 = A/Sing Mono IM, 15 ug (UA)
Treatments Part B: B1 = A/Sing MAP, 15 ug (FA) with biopsy; B2 = Placebo MAP (FA) with biopsy
B3 = A/Sing MAP, 15 ug (FA); B4 = A/Sing MAP, 10 ug (FA); B5 = A/Sing MAP, 5 ug (FA); B6 = A/Sing MAP, 2.5 ug (FA)
B7 = Placebo MAP (FA); B8 = A/Sing MAP, 15 ug (UA); B9 = A/Sing IM, 15 ug, as 2018 Afluria Quad (UA)

Listing 16.2.9.3
Numeric Pain Intensity per Site

Part B

Randomisation Number	Group	Visit	Time Point	Was Pain Assessment Performed?	Site Number	Treatment	Date of assessment	Time of Assessment	Score	Unscheduled Reason
B0113	Group B5	Day 1	1 min	Yes	F3	uncoated	16-JUN-2018	12:37	2	
B0113	Group B5	Day 1	10 min	Yes	F1	uncoated	16-JUN-2018	12:48	1	
B0113	Group B5	Day 1	10 min	Yes	F2	5 ug	16-JUN-2018	12:48	1	
B0113	Group B5	Day 1	10 min	Yes	F3	uncoated	16-JUN-2018	12:48	2	
B0113	Group B5	Day 1	1 hour	Yes	F1	uncoated	16-JUN-2018	13:38	0	
B0113	Group B5	Day 1	1 hour	Yes	F2	5 ug	16-JUN-2018	13:38	0	
B0113	Group B5	Day 1	1 hour	Yes	F3	uncoated	16-JUN-2018	13:38	0	
B0113	Group B5	Day 1	2 hour	Yes	F1	uncoated	16-JUN-2018	14:38	0	
B0113	Group B5	Day 1	2 hour	Yes	F2	5 ug	16-JUN-2018	14:38	0	
B0113	Group B5	Day 1	2 hour	Yes	F3	uncoated	16-JUN-2018	14:38	0	
B0113	Group B5	Day 2		Yes	F1	uncoated	17-JUN-2018	12:52	0	
B0113	Group B5	Day 2		Yes	F2	5 ug	17-JUN-2018	12:52	0	
B0113	Group B5	Day 2		Yes	F3	uncoated	17-JUN-2018	12:53	0	
B0113	Group B5	Day 4		Yes	F1	uncoated	19-JUN-2018	13:13	0	
B0113	Group B5	Day 4		Yes	F2	5 ug	19-JUN-2018	13:13	0	
B0113	Group B5	Day 4		Yes	F3	uncoated	19-JUN-2018	13:13	0	
B0113	Group B5	Day 8		Yes	F1	uncoated	23-JUN-2018	10:43	0	
B0113	Group B5	Day 8		Yes	F2	5 ug	23-JUN-2018	10:43	0	
B0113	Group B5	Day 8		Yes	F3	uncoated	23-JUN-2018	10:43	0	
B0113	Group B5	Day 22		Yes	F1	uncoated	07-JUL-2018	12:18	0	
B0113	Group B5	Day 22		Yes	F2	5 ug	07-JUL-2018	12:18	0	

Scale: 0= No Pain; 10= Worst Pain Possible
Administration Site Key: FA = Forearm; UA= Upper arm
Treatments Part A: A1 = A/Sing MAP, 15 ug (FA); A2 = Afluria Quad IM (UA), 15 ug; A3 = Placebo MAP (FA); A4 = A/Sing Mono IM, 15 ug (UA)
Treatments Part B: B1 = A/Sing MAP, 15 ug (FA) with biopsy; B2 = Placebo MAP (FA) with biopsy
B3 = A/Sing MAP, 15 ug (FA); B4 = A/Sing MAP, 10 ug (FA); B5 = A/Sing MAP, 5 ug (FA); B6 = A/Sing MAP, 2.5 ug (FA)
B7 = Placebo MAP (FA); B8 = A/Sing MAP, 15 ug (UA); B9 = A/Sing IM, 15 ug, as 2018 Afluria Quad (UA)

Listing 16.2.9.3
Numeric Pain Intensity per Site

Part B

Randomisation Number	Group	Visit	Time Point	Was Pain Assessment Performed?	Site Number	Treatment	Date of assessment	Time of Assessment	Score	Unscheduled Reason
B0113	Group B5	Day 22		Yes	F3	uncoated	07-JUL-2018	12:18	0	
B0113	Group B5	EOS/ET (Day 61)		Yes	F1	uncoated	15-AUG-2018	11:59	0	
B0113	Group B5	EOS/ET (Day 61)		Yes	F2	5 ug	15-AUG-2018	11:59	0	
B0113	Group B5	EOS/ET (Day 61)		Yes	F3	uncoated	15-AUG-2018	11:59	0	
B0114	Group B7	Day 1	1 min	Yes	F1	uncoated	16-JUN-2018	12:41	3	
B0114	Group B7	Day 1	1 min	Yes	F2	uncoated	16-JUN-2018	12:44	1	
B0114	Group B7	Day 1	1 min	Yes	F3	uncoated	16-JUN-2018	12:47	0	
B0114	Group B7	Day 1	10 min	Yes	F1	uncoated	16-JUN-2018	12:58	1	
B0114	Group B7	Day 1	10 min	Yes	F2	uncoated	16-JUN-2018	12:58	0	
B0114	Group B7	Day 1	10 min	Yes	F3	uncoated	16-JUN-2018	12:58	0	
B0114	Group B7	Day 1	1 hour	Yes	F1	uncoated	16-JUN-2018	13:48	0	
B0114	Group B7	Day 1	1 hour	Yes	F2	uncoated	16-JUN-2018	13:48	0	
B0114	Group B7	Day 1	1 hour	Yes	F3	uncoated	16-JUN-2018	13:48	0	
B0114	Group B7	Day 1	2 hour	Yes	F1	uncoated	16-JUN-2018	14:48	0	
B0114	Group B7	Day 1	2 hour	Yes	F2	uncoated	16-JUN-2018	14:48	0	
B0114	Group B7	Day 1	2 hour	Yes	F3	uncoated	16-JUN-2018	14:48	0	
B0114	Group B7	Day 2		Yes	F1	uncoated	17-JUN-2018	11:05	0	
B0114	Group B7	Day 2		Yes	F2	uncoated	17-JUN-2018	11:06	0	
B0114	Group B7	Day 2		Yes	F3	uncoated	17-JUN-2018	11:06	0	
B0114	Group B7	Day 4		Yes	F1	uncoated	19-JUN-2018	12:02	0	
B0114	Group B7	Day 4		Yes	F2	uncoated	19-JUN-2018	12:02	0	

Scale: 0= No Pain; 10= Worst Pain Possible
Administration Site Key: FA = Forearm; UA= Upper arm
Treatments Part A: A1 = A/Sing MAP, 15 ug (FA); A2 = Afluria Quad IM (UA), 15 ug; A3 = Placebo MAP (FA); A4 = A/Sing Mono IM, 15 ug (UA)
Treatments Part B: B1 = A/Sing MAP, 15 ug (FA) with biopsy; B2 = Placebo MAP (FA) with biopsy
B3 = A/Sing MAP, 15 ug (FA); B4 = A/Sing MAP, 10 ug (FA); B5 = A/Sing MAP, 5 ug (FA); B6 = A/Sing MAP, 2.5 ug (FA)
B7 = Placebo MAP (FA); B8 = A/Sing MAP, 15 ug (UA); B9 = A/Sing IM, 15 ug, as 2018 Afluria Quad (UA)

Listing 16.2.9.3
Numeric Pain Intensity per Site

Part B

Randomisation Number	Group	Visit	Time Point	Was Pain Assessment Performed?	Site Number	Treatment	Date of assessment	Time of Assessment	Score	Unscheduled Reason
B0114	Group B7	Day 4		Yes	F3	uncoated	19-JUN-2018	12:02	0	
B0114	Group B7	Day 8		Yes	F1	uncoated	23-JUN-2018	12:45	0	
B0114	Group B7	Day 8		Yes	F2	uncoated	23-JUN-2018	12:47	0	
B0114	Group B7	Day 8		Yes	F3	uncoated	23-JUN-2018	12:47	0	
B0114	Group B7	Day 22		Yes	F1	uncoated	09-JUL-2018	11:52	0	
B0114	Group B7	Day 22		Yes	F2	uncoated	09-JUL-2018	11:52	0	
B0114	Group B7	Day 22		Yes	F3	uncoated	09-JUL-2018	11:52	0	
B0114	Group B7	EOS/ET (Day 61)		Yes	F1	uncoated	15-AUG-2018	13:04	0	
B0114	Group B7	EOS/ET (Day 61)		Yes	F2	uncoated	15-AUG-2018	13:04	0	
B0114	Group B7	EOS/ET (Day 61)		Yes	F3	uncoated	15-AUG-2018	13:04	0	
B0115	Group B9	Day 1	1 min	Yes	D1	[IM]	16-JUN-2018	11:01	0	
B0115	Group B9	Day 1	10 min	Yes	D1	[IM]	16-JUN-2018	11:10	0	
B0115	Group B9	Day 1	1 hour	Yes	D1	[IM]	16-JUN-2018	11:55	0	
B0115	Group B9	Day 1	2 hour	Yes	D1	[IM]	16-JUN-2018	13:05	0	
B0115	Group B9	Day 2		Yes	D1	[IM]	17-JUN-2018	11:11	0	
B0115	Group B9	Day 4		Yes	D1	[IM]	19-JUN-2018	11:30	0	
B0115	Group B9	Day 8		Yes	D1	[IM]	23-JUN-2018	12:43	0	
B0115	Group B9	Day 22		Yes	D1	[IM]	07-JUL-2018	11:51	0	
B0115	Group B9	EOS/ET (Day 61)		Yes	D1	[IM]	17-AUG-2018	19:27	0	
B0116	Group B7	Day 1	1 min	Yes	F1	uncoated	16-JUN-2018	12:51	4	

Scale: 0= No Pain; 10= Worst Pain Possible
Administration Site Key: FA = Forearm; UA= Upper arm
Treatments Part A: A1 = A/Sing MAP, 15 ug (FA); A2 = Afluria Quad IM (UA), 15 ug; A3 = Placebo MAP (FA); A4 = A/Sing Mono IM, 15 ug (UA)
Treatments Part B: B1 = A/Sing MAP, 15 ug (FA) with biopsy; B2 = Placebo MAP (FA) with biopsy
B3 = A/Sing MAP, 15 ug (FA); B4 = A/Sing MAP, 10 ug (FA); B5 = A/Sing MAP, 5 ug (FA); B6 = A/Sing MAP, 2.5 ug (FA)
B7 = Placebo MAP (FA); B8 = A/Sing MAP, 15 ug (UA); B9 = A/Sing IM, 15 ug, as 2018 Afluria Quad (UA)

Listing 16.2.9.3
Numeric Pain Intensity per Site

Part B

Randomisation Number	Group	Visit	Time Point	Was Pain Assessment Performed?	Site Number	Treatment	Date of assessment	Time of Assessment	Score	Unscheduled Reason
B0116	Group B7	Day 1	1 min	Yes	F2	uncoated	16-JUN-2018	12:54	5	
B0116	Group B7	Day 1	1 min	Yes	F3	uncoated	16-JUN-2018	12:57	4	
B0116	Group B7	Day 1	10 min	Yes	F1	uncoated	16-JUN-2018	13:08	2	
B0116	Group B7	Day 1	10 min	Yes	F2	uncoated	16-JUN-2018	13:08	1	
B0116	Group B7	Day 1	10 min	Yes	F3	uncoated	16-JUN-2018	13:08	1	
B0116	Group B7	Day 1	1 hour	Yes	F1	uncoated	16-JUN-2018	13:58	0	
B0116	Group B7	Day 1	1 hour	Yes	F2	uncoated	16-JUN-2018	13:58	0	
B0116	Group B7	Day 1	1 hour	Yes	F3	uncoated	16-JUN-2018	13:58	0	
B0116	Group B7	Day 1	2 hour	Yes	F1	uncoated	16-JUN-2018	14:58	0	
B0116	Group B7	Day 1	2 hour	Yes	F2	uncoated	16-JUN-2018	14:58	0	
B0116	Group B7	Day 1	2 hour	Yes	F3	uncoated	16-JUN-2018	14:58	0	
B0116	Group B7	Day 2		Yes	F1	uncoated	17-JUN-2018	13:05	0	
B0116	Group B7	Day 2		Yes	F2	uncoated	17-JUN-2018	13:05	0	
B0116	Group B7	Day 2		Yes	F3	uncoated	17-JUN-2018	13:06	0	
B0116	Group B7	Day 4		Yes	F1	uncoated	19-JUN-2018	11:48	0	
B0116	Group B7	Day 4		Yes	F2	uncoated	19-JUN-2018	11:48	0	
B0116	Group B7	Day 4		Yes	F3	uncoated	19-JUN-2018	11:48	0	
B0116	Group B7	Day 8		Yes	F1	uncoated	23-JUN-2018	13:00	0	
B0116	Group B7	Day 8		Yes	F2	uncoated	23-JUN-2018	13:00	0	
B0116	Group B7	Day 8		Yes	F3	uncoated	23-JUN-2018	13:00	0	
B0116	Group B7	Day 22		Yes	F1	uncoated	07-JUL-2018	11:53	0	

Scale: 0= No Pain; 10= Worst Pain Possible
Administration Site Key: FA = Forearm; UA= Upper arm
Treatments Part A: A1 = A/Sing MAP, 15 ug (FA); A2 = Afluria Quad IM (UA), 15 ug; A3 = Placebo MAP (FA); A4 = A/Sing Mono IM, 15 ug (UA)
Treatments Part B: B1 = A/Sing MAP, 15 ug (FA) with biopsy; B2 = Placebo MAP (FA) with biopsy
B3 = A/Sing MAP, 15 ug (FA); B4 = A/Sing MAP, 10 ug (FA); B5 = A/Sing MAP, 5 ug (FA); B6 = A/Sing MAP, 2.5 ug (FA)
B7 = Placebo MAP (FA); B8 = A/Sing MAP, 15 ug (UA); B9 = A/Sing IM, 15 ug, as 2018 Afluria Quad (UA)

Listing 16.2.9.3
Numeric Pain Intensity per Site

Part B

Randomisation Number	Group	Visit	Time Point	Was Pain Assessment Performed?	Site Number	Treatment	Date of assessment	Time of Assessment	Score	Unscheduled Reason
B0116	Group B7	Day 22		Yes	F2	uncoated	07-JUL-2018	11:53	0	
B0116	Group B7	Day 22		Yes	F3	uncoated	07-JUL-2018	11:53	0	
B0116	Group B7	EOS/ET (Day 61)		Yes	F1	uncoated	15-AUG-2018	10:54	0	
B0116	Group B7	EOS/ET (Day 61)		Yes	F2	uncoated	15-AUG-2018	10:55	0	
B0116	Group B7	EOS/ET (Day 61)		Yes	F3	uncoated	15-AUG-2018	10:55	0	
B0117	Group B9	Day 1	1 min	Yes	D1	[IM]	16-JUN-2018	11:11	4	
B0117	Group B9	Day 1	10 min	Yes	D1	[IM]	16-JUN-2018	11:20	0	
B0117	Group B9	Day 1	1 hour	Yes	D1	[IM]	16-JUN-2018	12:10	1	
B0117	Group B9	Day 1	2 hour	Yes	D1	[IM]	16-JUN-2018	13:17	2	
B0117	Group B9	Day 2		Yes	D1	[IM]	18-JUN-2018	11:10	0	
B0117	Group B9	Day 4		Yes	D1	[IM]	19-JUN-2018	11:31	0	
B0117	Group B9	Day 8		Yes	D1	[IM]	23-JUN-2018	11:20	0	
B0117	Group B9	Day 22		Yes	D1	[IM]	07-JUL-2018	14:21	0	
B0117	Group B9	EOS/ET (Day 61)		Yes	D1	[IM]	16-AUG-2018	14:47	0	
B0118	Group B6	Day 1	1 min	Yes	F1	uncoated	16-JUN-2018		3	
B0118	Group B6	Day 1	1 min	Yes	F2	2.5 ug	16-JUN-2018	14:38	2	
B0118	Group B6	Day 1	1 min	Yes	F3	uncoated	16-JUN-2018	14:41	1	
B0118	Group B6	Day 1	10 min	Yes	F1	uncoated	16-JUN-2018	13:13	1	
B0118	Group B6	Day 1	10 min	Yes	F2	2.5 ug	16-JUN-2018	14:52	2	
B0118	Group B6	Day 1	10 min	Yes	F3	uncoated	16-JUN-2018	14:52	3	

Scale: 0= No Pain; 10= Worst Pain Possible
Administration Site Key: FA = Forearm; UA= Upper arm
Treatments Part A: A1 = A/Sing MAP, 15 ug (FA); A2 = Afluria Quad IM (UA), 15 ug; A3 = Placebo MAP (FA); A4 = A/Sing Mono IM, 15 ug (UA)
Treatments Part B: B1 = A/Sing MAP, 15 ug (FA) with biopsy; B2 = Placebo MAP (FA) with biopsy
B3 = A/Sing MAP, 15 ug (FA); B4 = A/Sing MAP, 10 ug (FA); B5 = A/Sing MAP, 5 ug (FA); B6 = A/Sing MAP, 2.5 ug (FA)
B7 = Placebo MAP (FA); B8 = A/Sing MAP, 15 ug (UA); B9 = A/Sing IM, 15 ug, as 2018 Afluria Quad (UA)

Listing 16.2.9.3
Numeric Pain Intensity per Site

Part B

Randomisation Number	Group	Visit	Time Point	Was Pain Assessment Performed?	Site Number	Treatment	Date of assessment	Time of Assessment	Score	Unscheduled Reason
B0118	Group B6	Day 1	1 hour	Yes	F1	uncoated	16-JUN-2018	14:03	0	
B0118	Group B6	Day 1	1 hour	Yes	F2	2.5 ug	16-JUN-2018	15:42	0	
B0118	Group B6	Day 1	1 hour	Yes	F3	uncoated	16-JUN-2018	15:42	0	
B0118	Group B6	Day 1	2 hour	Yes	F1	uncoated	16-JUN-2018	15:03	0	
B0118	Group B6	Day 1	2 hour	Yes	F2	2.5 ug	16-JUN-2018	16:42	0	
B0118	Group B6	Day 1	2 hour	Yes	F3	uncoated	16-JUN-2018	16:42	0	
B0118	Group B6	Day 2		Yes	F1	uncoated	17-JUN-2018	10:21	0	
B0118	Group B6	Day 2		Yes	F2	2.5 ug	17-JUN-2018	10:21	0	
B0118	Group B6	Day 2		Yes	F3	uncoated	17-JUN-2018	10:21	0	
B0118	Group B6	Day 4		Yes	F1	uncoated	19-JUN-2018	14:13	0	
B0118	Group B6	Day 4		Yes	F2	2.5 ug	19-JUN-2018	14:13	0	
B0118	Group B6	Day 4		Yes	F3	uncoated	19-JUN-2018	14:13	0	
B0118	Group B6	Day 8		Yes	F1	uncoated	23-JUN-2018	15:16	0	
B0118	Group B6	Day 8		Yes	F2	2.5 ug	23-JUN-2018	15:16	0	
B0118	Group B6	Day 8		Yes	F3	uncoated	23-JUN-2018	15:16	0	
B0118	Group B6	Day 22		Yes	F1	uncoated	07-JUL-2018	13:16	0	
B0118	Group B6	Day 22		Yes	F2	2.5 ug	07-JUL-2018	13:17	0	
B0118	Group B6	Day 22		Yes	F3	uncoated	07-JUL-2018	13:17	0	
B0118	Group B6	EOS/ET (Day 61)		Yes	F1	uncoated	15-AUG-2018	13:13	0	
B0118	Group B6	EOS/ET (Day 61)		Yes	F2	2.5 ug	15-AUG-2018	13:13	0	
B0118	Group B6	EOS/ET (Day 61)		Yes	F3	uncoated	15-AUG-2018	13:13	0	

Scale: 0= No Pain; 10= Worst Pain Possible
Administration Site Key: FA = Forearm; UA= Upper arm
Treatments Part A: A1 = A/Sing MAP, 15 ug (FA); A2 = Afluria Quad IM (UA), 15 ug; A3 = Placebo MAP (FA); A4 = A/Sing Mono IM, 15 ug (UA)
Treatments Part B: B1 = A/Sing MAP, 15 ug (FA) with biopsy; B2 = Placebo MAP (FA) with biopsy
B3 = A/Sing MAP, 15 ug (FA); B4 = A/Sing MAP, 10 ug (FA); B5 = A/Sing MAP, 5 ug (FA); B6 = A/Sing MAP, 2.5 ug (FA)
B7 = Placebo MAP (FA); B8 = A/Sing MAP, 15 ug (UA); B9 = A/Sing IM, 15 ug, as 2018 Afluria Quad (UA)

Listing 16.2.9.3
Numeric Pain Intensity per Site

Part B

Randomisation Number	Group	Visit	Time Point	Was Pain Assessment Performed?	Site Number	Treatment	Date of assessment	Time of Assessment	Score	Unscheduled Reason
B0119	Group B4	Day 1	1 min	Yes	F1	5 ug	16-JUN-2018	13:11	0	
B0119	Group B4	Day 1	1 min	Yes	F2	uncoated	16-JUN-2018	13:14	0	
B0119	Group B4	Day 1	1 min	Yes	F3	5 ug	16-JUN-2018	13:17	0	
B0119	Group B4	Day 1	10 min	Yes	F1	5 ug	16-JUN-2018	13:28	1	
B0119	Group B4	Day 1	10 min	Yes	F2	uncoated	16-JUN-2018	13:28	1	
B0119	Group B4	Day 1	10 min	Yes	F3	5 ug	16-JUN-2018	13:28	1	
B0119	Group B4	Day 1	1 hour	Yes	F1	5 ug	16-JUN-2018	14:18	1	
B0119	Group B4	Day 1	1 hour	Yes	F2	uncoated	16-JUN-2018	14:18	1	
B0119	Group B4	Day 1	1 hour	Yes	F3	5 ug	16-JUN-2018	14:18	1	
B0119	Group B4	Day 1	2 hour	Yes	F1	5 ug	16-JUN-2018	15:18	0	
B0119	Group B4	Day 1	2 hour	Yes	F2	uncoated	16-JUN-2018	15:18	0	
B0119	Group B4	Day 1	2 hour	Yes	F3	5 ug	16-JUN-2018	15:18	0	
B0119	Group B4	Day 2		Yes	F1	5 ug	17-JUN-2018	13:12	0	
B0119	Group B4	Day 2		Yes	F2	uncoated	17-JUN-2018	13:13	0	
B0119	Group B4	Day 2		Yes	F3	5 ug	17-JUN-2018	13:13	0	
B0119	Group B4	Day 4		Yes	F1	5 ug	19-JUN-2018	15:24	0	
B0119	Group B4	Day 4		Yes	F2	uncoated	19-JUN-2018	15:24	0	
B0119	Group B4	Day 4		Yes	F3	5 ug	19-JUN-2018	15:24	0	
B0119	Group B4	Day 8		Yes	F1	5 ug	26-JUN-2018	15:19	0	
B0119	Group B4	Day 8		Yes	F2	uncoated	26-JUN-2018	15:19	0	
B0119	Group B4	Day 8		Yes	F3	5 ug	26-JUN-2018	15:19	0	

Scale: 0= No Pain; 10= Worst Pain Possible
Administration Site Key: FA = Forearm; UA= Upper arm
Treatments Part A: A1 = A/Sing MAP, 15 ug (FA); A2 = Afluria Quad IM (UA), 15 ug; A3 = Placebo MAP (FA); A4 = A/Sing Mono IM, 15 ug (UA)
Treatments Part B: B1 = A/Sing MAP, 15 ug (FA) with biopsy; B2 = Placebo MAP (FA) with biopsy
B3 = A/Sing MAP, 15 ug (FA); B4 = A/Sing MAP, 10 ug (FA); B5 = A/Sing MAP, 5 ug (FA); B6 = A/Sing MAP, 2.5 ug (FA)
B7 = Placebo MAP (FA); B8 = A/Sing MAP, 15 ug (UA); B9 = A/Sing IM, 15 ug, as 2018 Afluria Quad (UA)

Listing 16.2.9.3
Numeric Pain Intensity per Site

Part B

Randomisation Number	Group	Visit	Time Point	Was Pain Assessment Performed?	Site Number	Treatment	Date of assessment	Time of Assessment	Score	Unscheduled Reason
B0119	Group B4	Day 22		Yes	F1	5 ug	07-JUL-2018	12:07	0	
B0119	Group B4	Day 22		Yes	F2	uncoated	07-JUL-2018	12:07	0	
B0119	Group B4	Day 22		Yes	F3	5 ug	07-JUL-2018	12:07	0	
B0119	Group B4	EOS/ET (Day 61)		Yes	F1	5 ug	15-AUG-2018	14:14	0	
B0119	Group B4	EOS/ET (Day 61)		Yes	F2	uncoated	15-AUG-2018	14:14	0	
B0119	Group B4	EOS/ET (Day 61)		Yes	F3	5 ug	15-AUG-2018	14:14	0	
B0120	Group B3	Day 1	1 min	Yes	F1	5 ug	16-JUN-2018	13:21	5	
B0120	Group B3	Day 1	1 min	Yes	F2	5 ug	16-JUN-2018	13:24	5	
B0120	Group B3	Day 1	1 min	Yes	F3	5 ug	16-JUN-2018	13:27	6	
B0120	Group B3	Day 1	10 min	Yes	F1	5 ug	16-JUN-2018	13:38	0	
B0120	Group B3	Day 1	10 min	Yes	F2	5 ug	16-JUN-2018	13:38	3	
B0120	Group B3	Day 1	10 min	Yes	F3	5 ug	16-JUN-2018	13:38	3	
B0120	Group B3	Day 1	1 hour	Yes	F1	5 ug	16-JUN-2018	14:28	1	
B0120	Group B3	Day 1	1 hour	Yes	F2	5 ug	16-JUN-2018	14:28	1	
B0120	Group B3	Day 1	1 hour	Yes	F3	5 ug	16-JUN-2018	14:28	1	
B0120	Group B3	Day 1	2 hour	Yes	F1	5 ug	16-JUN-2018	15:28	0	
B0120	Group B3	Day 1	2 hour	Yes	F2	5 ug	16-JUN-2018	15:28	0	
B0120	Group B3	Day 1	2 hour	Yes	F3	5 ug	16-JUN-2018	15:28	0	
B0120	Group B3	Day 2		Yes	F1	5 ug	17-JUN-2018	13:11	0	
B0120	Group B3	Day 2		Yes	F2	5 ug	17-JUN-2018	13:11	0	
B0120	Group B3	Day 2		Yes	F3	5 ug	17-JUN-2018	13:11	0	

Scale: 0= No Pain; 10= Worst Pain Possible
Administration Site Key: FA = Forearm; UA= Upper arm
Treatments Part A: A1 = A/Sing MAP, 15 ug (FA); A2 = Afluria Quad IM (UA), 15 ug; A3 = Placebo MAP (FA); A4 = A/Sing Mono IM, 15 ug (UA)
Treatments Part B: B1 = A/Sing MAP, 15 ug (FA) with biopsy; B2 = Placebo MAP (FA) with biopsy
B3 = A/Sing MAP, 15 ug (FA); B4 = A/Sing MAP, 10 ug (FA); B5 = A/Sing MAP, 5 ug (FA); B6 = A/Sing MAP, 2.5 ug (FA)
B7 = Placebo MAP (FA); B8 = A/Sing MAP, 15 ug (UA); B9 = A/Sing IM, 15 ug, as 2018 Afluria Quad (UA)

Listing 16.2.9.3
Numeric Pain Intensity per Site

Part B

Randomisation Number	Group	Visit	Time Point	Was Pain Assessment Performed?	Site Number	Treatment	Date of assessment	Time of Assessment	Score	Unscheduled Reason
B0120	Group B3	Day 4		Yes	F1	5 ug	19-JUN-2018	13:55	0	
B0120	Group B3	Day 4		Yes	F2	5 ug	19-JUN-2018	13:55	0	
B0120	Group B3	Day 4		Yes	F3	5 ug	19-JUN-2018	13:55	0	
B0120	Group B3	Day 8		Yes	F1	5 ug	23-JUN-2018	14:13	0	
B0120	Group B3	Day 8		Yes	F2	5 ug	23-JUN-2018	14:13	0	
B0120	Group B3	Day 8		Yes	F3	5 ug	23-JUN-2018	14:13	0	
B0120	Group B3	Day 22		Yes	F1	5 ug	07-JUL-2018	14:00	0	
B0120	Group B3	Day 22		Yes	F2	5 ug	07-JUL-2018	14:00	0	
B0120	Group B3	Day 22		Yes	F3	5 ug	07-JUL-2018	14:00	0	
B0120	Group B3	EOS/ET (Day 61)		Yes	F1	5 ug	15-AUG-2018	13:58	0	
B0120	Group B3	EOS/ET (Day 61)		Yes	F2	5 ug	15-AUG-2018	13:58	0	
B0120	Group B3	EOS/ET (Day 61)		Yes	F3	5 ug	15-AUG-2018	13:58	0	
B0121	Group B8	Day 1	1 min	Yes	D1	5 ug	16-JUN-2018	13:31	1	
B0121	Group B8	Day 1	1 min	Yes	D2	5 ug	16-JUN-2018	13:34	0	
B0121	Group B8	Day 1	1 min	Yes	D3	5 ug	16-JUN-2018	13:37	0	
B0121	Group B8	Day 1	10 min	Yes	D1	5 ug	16-JUN-2018	13:48	0	
B0121	Group B8	Day 1	10 min	Yes	D2	5 ug	16-JUN-2018	13:48	1	
B0121	Group B8	Day 1	10 min	Yes	D3	5 ug	16-JUN-2018	13:48	1	
B0121	Group B8	Day 1	1 hour	Yes	D1	5 ug	16-JUN-2018	14:38	2	
B0121	Group B8	Day 1	1 hour	Yes	D2	5 ug	16-JUN-2018	14:38	2	
B0121	Group B8	Day 1	1 hour	Yes	D3	5 ug	16-JUN-2018	14:38	2	

Scale: 0= No Pain; 10= Worst Pain Possible
Administration Site Key: FA = Forearm; UA= Upper arm
Treatments Part A: A1 = A/Sing MAP, 15 ug (FA); A2 = Afluria Quad IM (UA), 15 ug; A3 = Placebo MAP (FA); A4 = A/Sing Mono IM, 15 ug (UA)
Treatments Part B: B1 = A/Sing MAP, 15 ug (FA) with biopsy; B2 = Placebo MAP (FA) with biopsy
B3 = A/Sing MAP, 15 ug (FA); B4 = A/Sing MAP, 10 ug (FA); B5 = A/Sing MAP, 5 ug (FA); B6 = A/Sing MAP, 2.5 ug (FA)
B7 = Placebo MAP (FA); B8 = A/Sing MAP, 15 ug (UA); B9 = A/Sing IM, 15 ug, as 2018 Afluria Quad (UA)

Listing 16.2.9.3
Numeric Pain Intensity per Site

Part B

Randomisation Number	Group	Visit	Time Point	Was Pain Assessment Performed?	Site Number	Treatment	Date of assessment	Time of Assessment	Score	Unscheduled Reason
B0121	Group B8	Day 1	2 hour	Yes	D1	5 ug	16-JUN-2018	15:38	0	
B0121	Group B8	Day 1	2 hour	Yes	D2	5 ug	16-JUN-2018	15:38	0	
B0121	Group B8	Day 1	2 hour	Yes	D3	5 ug	16-JUN-2018	15:38	0	
B0121	Group B8	Day 2		Yes	D1	5 ug	17-JUN-2018	10:16	0	
B0121	Group B8	Day 2		Yes	D2	5 ug	17-JUN-2018	10:16	0	
B0121	Group B8	Day 2		Yes	D3	5 ug	17-JUN-2018	10:17	0	
B0121	Group B8	Day 4		Yes	D1	5 ug	19-JUN-2018	14:00	0	
B0121	Group B8	Day 4		Yes	D2	5 ug	19-JUN-2018	14:00	0	
B0121	Group B8	Day 4		Yes	D3	5 ug	19-JUN-2018	14:00	0	
B0121	Group B8	Day 8		Yes	D1	5 ug	23-JUN-2018	15:03	0	
B0121	Group B8	Day 8		Yes	D2	5 ug	23-JUN-2018	15:03	0	
B0121	Group B8	Day 8		Yes	D3	5 ug	23-JUN-2018	15:03	0	
B0121	Group B8	Day 22		Yes	D1	5 ug	09-JUL-2018	09:51	0	
B0121	Group B8	Day 22		Yes	D2	5 ug	09-JUL-2018	09:51	0	
B0121	Group B8	Day 22		Yes	D3	5 ug	09-JUL-2018	09:51	0	
B0121	Group B8	EOS/ET (Day 61)		Yes	D1	5 ug	08-AUG-2018	09:40	0	
B0121	Group B8	EOS/ET (Day 61)		Yes	D2	5 ug	08-AUG-2018	09:40	0	
B0121	Group B8	EOS/ET (Day 61)		Yes	D3	5 ug	08-AUG-2018	09:40	0	
B0122	Group B5	Day 1	1 min	Yes	F1	uncoated	16-JUN-2018	13:41	1	
B0122	Group B5	Day 1	1 min	Yes	F2	5 ug	16-JUN-2018	13:44	1	
B0122	Group B5	Day 1	1 min	Yes	F3	uncoated	16-JUN-2018	13:47	1	

Scale: 0= No Pain; 10= Worst Pain Possible
Administration Site Key: FA = Forearm; UA= Upper arm
Treatments Part A: A1 = A/Sing MAP, 15 ug (FA); A2 = Afluria Quad IM (UA), 15 ug; A3 = Placebo MAP (FA); A4 = A/Sing Mono IM, 15 ug (UA)
Treatments Part B: B1 = A/Sing MAP, 15 ug (FA) with biopsy; B2 = Placebo MAP (FA) with biopsy
B3 = A/Sing MAP, 15 ug (FA); B4 = A/Sing MAP, 10 ug (FA); B5 = A/Sing MAP, 5 ug (FA); B6 = A/Sing MAP, 2.5 ug (FA)
B7 = Placebo MAP (FA); B8 = A/Sing MAP, 15 ug (UA); B9 = A/Sing IM, 15 ug, as 2018 Afluria Quad (UA)

Listing 16.2.9.3
Numeric Pain Intensity per Site

Part B

Randomisation Number	Group	Visit	Time Point	Was Pain Assessment Performed?	Site Number	Treatment	Date of assessment	Time of Assessment	Score	Unscheduled Reason
B0122	Group B5	Day 1	10 min	Yes	F1	uncoated	16-JUN-2018	13:58	1	
B0122	Group B5	Day 1	10 min	Yes	F2	5 ug	16-JUN-2018	13:58	0	
B0122	Group B5	Day 1	10 min	Yes	F3	uncoated	16-JUN-2018	13:58	1	
B0122	Group B5	Day 1	1 hour	Yes	F1	uncoated	16-JUN-2018	14:48	1	
B0122	Group B5	Day 1	1 hour	Yes	F2	5 ug	16-JUN-2018	14:48	1	
B0122	Group B5	Day 1	1 hour	Yes	F3	uncoated	16-JUN-2018	14:48	1	
B0122	Group B5	Day 1	2 hour	Yes	F1	uncoated	16-JUN-2018	15:48	0	
B0122	Group B5	Day 1	2 hour	Yes	F2	5 ug	16-JUN-2018	15:48	0	
B0122	Group B5	Day 1	2 hour	Yes	F3	uncoated	16-JUN-2018	15:48	0	
B0122	Group B5	Day 2		Yes	F1	uncoated	17-JUN-2018	09:10		
B0122	Group B5	Day 2		Yes	F2	5 ug	17-JUN-2018	09:10	0	
B0122	Group B5	Day 2		Yes	F3	uncoated	17-JUN-2018	09:11	0	
B0122	Group B5	Day 4		Yes	F1	uncoated	19-JUN-2018	12:12	0	
B0122	Group B5	Day 4		Yes	F2	5 ug	19-JUN-2018	12:12	0	
B0122	Group B5	Day 4		Yes	F3	uncoated	19-JUN-2018	12:12	0	
B0122	Group B5	Day 8		Yes	F1	uncoated	23-JUN-2018	09:43	0	
B0122	Group B5	Day 8		Yes	F2	5 ug	23-JUN-2018	09:43	0	
B0122	Group B5	Day 8		Yes	F3	uncoated	23-JUN-2018	09:43	0	
B0122	Group B5	Day 22		Yes	F1	uncoated	07-JUL-2018	09:00	0	
B0122	Group B5	Day 22		Yes	F2	5 ug	07-JUL-2018	09:00	0	
B0122	Group B5	Day 22		Yes	F3	uncoated	07-JUL-2018	09:00	0	

Scale: 0= No Pain; 10= Worst Pain Possible
Administration Site Key: FA = Forearm; UA= Upper arm
Treatments Part A: A1 = A/Sing MAP, 15 ug (FA); A2 = Afluria Quad IM (UA), 15 ug; A3 = Placebo MAP (FA); A4 = A/Sing Mono IM, 15 ug (UA)
Treatments Part B: B1 = A/Sing MAP, 15 ug (FA) with biopsy; B2 = Placebo MAP (FA) with biopsy
B3 = A/Sing MAP, 15 ug (FA); B4 = A/Sing MAP, 10 ug (FA); B5 = A/Sing MAP, 5 ug (FA); B6 = A/Sing MAP, 2.5 ug (FA)
B7 = Placebo MAP (FA); B8 = A/Sing MAP, 15 ug (UA); B9 = A/Sing IM, 15 ug, as 2018 Afluria Quad (UA)

Listing 16.2.9.3
Numeric Pain Intensity per Site

Part B

Randomisation Number	Group	Visit	Time Point	Was Pain Assessment Performed?	Site Number	Treatment	Date of assessment	Time of Assessment	Score	Unscheduled Reason
B0122	Group B5	EOS/ET (Day 61)		Yes	F1	uncoated	15-AUG-2018	14:06	0	
B0122	Group B5	EOS/ET (Day 61)		Yes	F2	5 ug	15-AUG-2018	14:06	0	
B0122	Group B5	EOS/ET (Day 61)		Yes	F3	uncoated	15-AUG-2018	14:06	0	
B0123	Group B6	Day 1	1 min	Yes	F1	2.5 ug	16-JUN-2018	13:51	1	
B0123	Group B6	Day 1	1 min	Yes	F2	uncoated	16-JUN-2018	13:54	1	
B0123	Group B6	Day 1	1 min	Yes	F3	uncoated	16-JUN-2018	13:57	2	
B0123	Group B6	Day 1	10 min	Yes	F1	2.5 ug	16-JUN-2018	14:08	1	
B0123	Group B6	Day 1	10 min	Yes	F2	uncoated	16-JUN-2018	14:08	1	
B0123	Group B6	Day 1	10 min	Yes	F3	uncoated	16-JUN-2018	14:08	1	
B0123	Group B6	Day 1	1 hour	Yes	F1	2.5 ug	16-JUN-2018	14:58	0	
B0123	Group B6	Day 1	1 hour	Yes	F2	uncoated	16-JUN-2018	14:58	0	
B0123	Group B6	Day 1	1 hour	Yes	F3	uncoated	16-JUN-2018	14:58	0	
B0123	Group B6	Day 1	2 hour	Yes	F1	2.5 ug	16-JUN-2018	15:58	0	
B0123	Group B6	Day 1	2 hour	Yes	F2	uncoated	16-JUN-2018	15:58	0	
B0123	Group B6	Day 1	2 hour	Yes	F3	uncoated	16-JUN-2018	15:58	0	
B0123	Group B6	Day 2		Yes	F1	2.5 ug	17-JUN-2018	10:36	0	
B0123	Group B6	Day 2		Yes	F2	uncoated	17-JUN-2018	10:36	0	
B0123	Group B6	Day 2		Yes	F3	uncoated	17-JUN-2018	10:36	0	
B0123	Group B6	Day 4		Yes	F1	2.5 ug	19-JUN-2018	12:09	0	
B0123	Group B6	Day 4		Yes	F2	uncoated	19-JUN-2018	12:09	0	
B0123	Group B6	Day 4		Yes	F3	uncoated	19-JUN-2018	12:09	0	

Scale: 0= No Pain; 10= Worst Pain Possible
Administration Site Key: FA = Forearm; UA= Upper arm
Treatments Part A: A1 = A/Sing MAP, 15 ug (FA); A2 = Afluria Quad IM (UA), 15 ug; A3 = Placebo MAP (FA); A4 = A/Sing Mono IM, 15 ug (UA)
Treatments Part B: B1 = A/Sing MAP, 15 ug (FA) with biopsy; B2 = Placebo MAP (FA) with biopsy
B3 = A/Sing MAP, 15 ug (FA); B4 = A/Sing MAP, 10 ug (FA); B5 = A/Sing MAP, 5 ug (FA); B6 = A/Sing MAP, 2.5 ug (FA)
B7 = Placebo MAP (FA); B8 = A/Sing MAP, 15 ug (UA); B9 = A/Sing IM, 15 ug, as 2018 Afluria Quad (UA)

Listing 16.2.9.3
Numeric Pain Intensity per Site

Part B

Randomisation Number	Group	Visit	Time Point	Was Pain Assessment Performed?	Site Number	Treatment	Date of assessment	Time of Assessment	Score	Unscheduled Reason
B0123	Group B6	Day 8		Yes	F1	2.5 ug	23-JUN-2018	12:06	0	
B0123	Group B6	Day 8		Yes	F2	uncoated	23-JUN-2018	12:06	0	
B0123	Group B6	Day 8		Yes	F3	uncoated	23-JUN-2018	12:07	0	
B0123	Group B6	Day 22		Yes	F1	2.5 ug	06-JUL-2018	11:06	0	
B0123	Group B6	Day 22		Yes	F2	uncoated	06-JUL-2018	11:06	0	
B0123	Group B6	Day 22		Yes	F3	uncoated	06-JUL-2018	11:06	0	
B0123	Group B6	EOS/ET (Day 61)		Yes	F1	2.5 ug	15-AUG-2018	11:48	0	
B0123	Group B6	EOS/ET (Day 61)		Yes	F2	uncoated	15-AUG-2018	11:48	0	
B0123	Group B6	EOS/ET (Day 61)		Yes	F3	uncoated	15-AUG-2018	11:48	0	
B0124	Group B4	Day 1	1 min	Yes	F1	uncoated	16-JUN-2018	14:01	2	
B0124	Group B4	Day 1	1 min	Yes	F2	5 ug	16-JUN-2018	14:04	3	
B0124	Group B4	Day 1	1 min	Yes	F3	5 ug	16-JUN-2018	14:07	3	
B0124	Group B4	Day 1	10 min	Yes	F1	uncoated	16-JUN-2018	14:18	0	
B0124	Group B4	Day 1	10 min	Yes	F2	5 ug	16-JUN-2018	14:18	0	
B0124	Group B4	Day 1	10 min	Yes	F3	5 ug	16-JUN-2018	14:18	1	
B0124	Group B4	Day 1	1 hour	Yes	F1	uncoated	16-JUN-2018	15:08	1	
B0124	Group B4	Day 1	1 hour	Yes	F2	5 ug	16-JUN-2018	15:08	1	
B0124	Group B4	Day 1	1 hour	Yes	F3	5 ug	16-JUN-2018	15:08	1	
B0124	Group B4	Day 1	2 hour	Yes	F1	uncoated	16-JUN-2018	16:08	0	
B0124	Group B4	Day 1	2 hour	Yes	F2	5 ug	16-JUN-2018	16:08	1	
B0124	Group B4	Day 1	2 hour	Yes	F3	5 ug	16-JUN-2018	16:08	1	

Scale: 0= No Pain; 10= Worst Pain Possible
Administration Site Key: FA = Forearm; UA= Upper arm
Treatments Part A: A1 = A/Sing MAP, 15 ug (FA); A2 = Afluria Quad IM (UA), 15 ug; A3 = Placebo MAP (FA); A4 = A/Sing Mono IM, 15 ug (UA)
Treatments Part B: B1 = A/Sing MAP, 15 ug (FA) with biopsy; B2 = Placebo MAP (FA) with biopsy
B3 = A/Sing MAP, 15 ug (FA); B4 = A/Sing MAP, 10 ug (FA); B5 = A/Sing MAP, 5 ug (FA); B6 = A/Sing MAP, 2.5 ug (FA)
B7 = Placebo MAP (FA); B8 = A/Sing MAP, 15 ug (UA); B9 = A/Sing IM, 15 ug, as 2018 Afluria Quad (UA)

Listing 16.2.9.3
Numeric Pain Intensity per Site

Part B

Randomisation Number	Group	Visit	Time Point	Was Pain Assessment Performed?	Site Number	Treatment	Date of assessment	Time of Assessment	Score	Unscheduled Reason
B0124	Group B4	Day 2		Yes	F1	uncoated	17-JUN-2018	11:45	0	
B0124	Group B4	Day 2		Yes	F2	5 ug	17-JUN-2018	11:46	0	
B0124	Group B4	Day 2		Yes	F3	5 ug	17-JUN-2018	11:47	0	
B0124	Group B4	Day 4		Yes	F1	uncoated	19-JUN-2018	14:46	0	
B0124	Group B4	Day 4		Yes	F2	5 ug	19-JUN-2018	14:46	0	
B0124	Group B4	Day 4		Yes	F3	5 ug	19-JUN-2018	14:46	0	
B0124	Group B4	Day 8		Yes	F1	uncoated	23-JUN-2018	13:41	0	
B0124	Group B4	Day 8		Yes	F2	5 ug	23-JUN-2018	13:42	0	
B0124	Group B4	Day 8		Yes	F3	5 ug	23-JUN-2018	13:43	0	
B0124	Group B4	Day 22		Yes	F1	uncoated	07-JUL-2018	13:08	0	
B0124	Group B4	Day 22		Yes	F2	5 ug	07-JUL-2018	13:08	0	
B0124	Group B4	Day 22		Yes	F3	5 ug	07-JUL-2018	13:08	0	
B0124	Group B4	EOS/ET (Day 61)		Yes	F1	uncoated	15-AUG-2018	15:00	0	
B0124	Group B4	EOS/ET (Day 61)		Yes	F2	5 ug	15-AUG-2018	15:00	0	
B0124	Group B4	EOS/ET (Day 61)		Yes	F3	5 ug	15-AUG-2018	15:00	0	
B0125	Group B7	Day 1	1 min	Yes	F1	uncoated	16-JUN-2018	14:11	2	
B0125	Group B7	Day 1	1 min	Yes	F2	uncoated	16-JUN-2018	14:14	2	
B0125	Group B7	Day 1	1 min	Yes	F3	uncoated	16-JUN-2018	14:17	2	
B0125	Group B7	Day 1	10 min	Yes	F1	uncoated	16-JUN-2018	14:28	0	
B0125	Group B7	Day 1	10 min	Yes	F2	uncoated	16-JUN-2018	14:28	0	
B0125	Group B7	Day 1	10 min	Yes	F3	uncoated	16-JUN-2018	14:28	0	

Scale: 0= No Pain; 10= Worst Pain Possible
Administration Site Key: FA = Forearm; UA= Upper arm
Treatments Part A: A1 = A/Sing MAP, 15 ug (FA); A2 = Afluria Quad IM (UA), 15 ug; A3 = Placebo MAP (FA); A4 = A/Sing Mono IM, 15 ug (UA)
Treatments Part B: B1 = A/Sing MAP, 15 ug (FA) with biopsy; B2 = Placebo MAP (FA) with biopsy
B3 = A/Sing MAP, 15 ug (FA); B4 = A/Sing MAP, 10 ug (FA); B5 = A/Sing MAP, 5 ug (FA); B6 = A/Sing MAP, 2.5 ug (FA)
B7 = Placebo MAP (FA); B8 = A/Sing MAP, 15 ug (UA); B9 = A/Sing IM, 15 ug, as 2018 Afluria Quad (UA)

Listing 16.2.9.3
Numeric Pain Intensity per Site

Part B

Randomisation Number	Group	Visit	Time Point	Was Pain Assessment Performed?	Site Number	Treatment	Date of assessment	Time of Assessment	Score	Unscheduled Reason
B0125	Group B7	Day 1	1 hour	Yes	F1	uncoated	16-JUN-2018	15:18	0	
B0125	Group B7	Day 1	1 hour	Yes	F2	uncoated	16-JUN-2018	15:18	0	
B0125	Group B7	Day 1	1 hour	Yes	F3	uncoated	16-JUN-2018	15:18	0	
B0125	Group B7	Day 1	2 hour	Yes	F1	uncoated	16-JUN-2018	16:18	0	
B0125	Group B7	Day 1	2 hour	Yes	F2	uncoated	16-JUN-2018	16:18	0	
B0125	Group B7	Day 1	2 hour	Yes	F3	uncoated	16-JUN-2018	16:18	0	
B0125	Group B7	Day 2		Yes	F1	uncoated	17-JUN-2018	12:58	0	
B0125	Group B7	Day 2		Yes	F2	uncoated	17-JUN-2018	12:59	0	
B0125	Group B7	Day 2		Yes	F3	uncoated	17-JUN-2018	12:59	0	
B0125	Group B7	Day 4		Yes	F1	uncoated	19-JUN-2018	15:18	0	
B0125	Group B7	Day 4		Yes	F2	uncoated	19-JUN-2018	15:18	0	
B0125	Group B7	Day 4		Yes	F3	uncoated	19-JUN-2018	15:18	0	
B0125	Group B7	Day 8		Yes	F1	uncoated	22-JUN-2018	14:02	0	
B0125	Group B7	Day 8		Yes	F2	uncoated	22-JUN-2018	14:02	0	
B0125	Group B7	Day 8		Yes	F3	uncoated	22-JUN-2018	14:02	0	
B0125	Group B7	Day 22		Yes	F1	uncoated	07-JUL-2018	13:52	0	
B0125	Group B7	Day 22		Yes	F2	uncoated	07-JUL-2018	13:52	0	
B0125	Group B7	Day 22		Yes	F3	uncoated	07-JUL-2018	13:52	0	
B0125	Group B7	EOS/ET (Day 61)		Yes	F1	uncoated	15-AUG-2018	15:46	0	
B0125	Group B7	EOS/ET (Day 61)		Yes	F2	uncoated	15-AUG-2018	15:46	0	
B0125	Group B7	EOS/ET (Day 61)		Yes	F3	uncoated	15-AUG-2018	15:47	0	

Scale: 0= No Pain; 10= Worst Pain Possible
Administration Site Key: FA = Forearm; UA= Upper arm
Treatments Part A: A1 = A/Sing MAP, 15 ug (FA); A2 = Afluria Quad IM (UA), 15 ug; A3 = Placebo MAP (FA); A4 = A/Sing Mono IM, 15 ug (UA)
Treatments Part B: B1 = A/Sing MAP, 15 ug (FA) with biopsy; B2 = Placebo MAP (FA) with biopsy
B3 = A/Sing MAP, 15 ug (FA); B4 = A/Sing MAP, 10 ug (FA); B5 = A/Sing MAP, 5 ug (FA); B6 = A/Sing MAP, 2.5 ug (FA)
B7 = Placebo MAP (FA); B8 = A/Sing MAP, 15 ug (UA); B9 = A/Sing IM, 15 ug, as 2018 Afluria Quad (UA)

Listing 16.2.9.3
Numeric Pain Intensity per Site

Part B

Randomisation Number	Group	Visit	Time Point	Was Pain Assessment Performed?	Site Number	Treatment	Date of assessment	Time of Assessment	Score	Unscheduled Reason
B0126	Group B3	Day 1	1 min	Yes	F1	5 ug	16-JUN-2018	14:21	2	
B0126	Group B3	Day 1	1 min	Yes	F2	5 ug	16-JUN-2018	14:24	2	
B0126	Group B3	Day 1	1 min	Yes	F3	5 ug	16-JUN-2018	14:27	2	
B0126	Group B3	Day 1	10 min	Yes	F1	5 ug	16-JUN-2018	14:38	2	
B0126	Group B3	Day 1	10 min	Yes	F2	5 ug	16-JUN-2018	14:38	2	
B0126	Group B3	Day 1	10 min	Yes	F3	5 ug	16-JUN-2018	14:38	0	
B0126	Group B3	Day 1	1 hour	Yes	F1	5 ug	16-JUN-2018	15:28	0	
B0126	Group B3	Day 1	1 hour	Yes	F2	5 ug	16-JUN-2018	15:28	0	
B0126	Group B3	Day 1	1 hour	Yes	F3	5 ug	16-JUN-2018	15:28	0	
B0126	Group B3	Day 1	2 hour	Yes	F1	5 ug	16-JUN-2018	16:28	0	
B0126	Group B3	Day 1	2 hour	Yes	F2	5 ug	16-JUN-2018	16:28	0	
B0126	Group B3	Day 1	2 hour	Yes	F3	5 ug	16-JUN-2018	16:28	0	
B0126	Group B3	Day 2		Yes	F1	5 ug	17-JUN-2018	11:39	0	
B0126	Group B3	Day 2		Yes	F2	5 ug	17-JUN-2018	11:40	0	
B0126	Group B3	Day 2		Yes	F3	5 ug	17-JUN-2018	11:41	0	
B0126	Group B3	Day 4		Yes	F1	5 ug	19-JUN-2018	15:34	0	
B0126	Group B3	Day 4		Yes	F2	5 ug	19-JUN-2018	15:34	0	
B0126	Group B3	Day 4		Yes	F3	5 ug	19-JUN-2018	15:34	0	
B0126	Group B3	Day 8		Yes	F1	5 ug	23-JUN-2018	15:31	0	
B0126	Group B3	Day 8		Yes	F2	5 ug	23-JUN-2018	15:31	0	
B0126	Group B3	Day 8		Yes	F3	5 ug	23-JUN-2018	15:31	0	

Scale: 0= No Pain; 10= Worst Pain Possible
Administration Site Key: FA = Forearm; UA= Upper arm
Treatments Part A: A1 = A/Sing MAP, 15 ug (FA); A2 = Afluria Quad IM (UA), 15 ug; A3 = Placebo MAP (FA); A4 = A/Sing Mono IM, 15 ug (UA)
Treatments Part B: B1 = A/Sing MAP, 15 ug (FA) with biopsy; B2 = Placebo MAP (FA) with biopsy
B3 = A/Sing MAP, 15 ug (FA); B4 = A/Sing MAP, 10 ug (FA); B5 = A/Sing MAP, 5 ug (FA); B6 = A/Sing MAP, 2.5 ug (FA)
B7 = Placebo MAP (FA); B8 = A/Sing MAP, 15 ug (UA); B9 = A/Sing IM, 15 ug, as 2018 Afluria Quad (UA)

Listing 16.2.9.3
Numeric Pain Intensity per Site

Part B

Randomisation Number	Group	Visit	Time Point	Was Pain Assessment Performed?	Site Number	Treatment	Date of assessment	Time of Assessment	Score	Unscheduled Reason
B0126	Group B3	Day 22		Yes	F1	5 ug	06-JUL-2018	14:33	0	
B0126	Group B3	Day 22		Yes	F2	5 ug	06-JUL-2018	14:33	0	
B0126	Group B3	Day 22		Yes	F3	5 ug	06-JUL-2018	14:33	0	
B0126	Group B3	EOS/ET (Day 61)		Yes	F1	5 ug	15-AUG-2018	15:14	0	
B0126	Group B3	EOS/ET (Day 61)		Yes	F2	5 ug	15-AUG-2018	15:14	0	
B0126	Group B3	EOS/ET (Day 61)		Yes	F3	5 ug	15-AUG-2018	15:14	0	
B0127	Group B9	Day 1	1 min	Yes	D1	[IM]	16-JUN-2018	11:21	1	
B0127	Group B9	Day 1	10 min	Yes	D1	[IM]	16-JUN-2018	11:30	0	
B0127	Group B9	Day 1	1 hour	Yes	D1	[IM]	16-JUN-2018	12:15	0	
B0127	Group B9	Day 1	2 hour	Yes	D1	[IM]	16-JUN-2018	13:25	1	
B0127	Group B9	Day 2		Yes	D1	[IM]	17-JUN-2018	11:13	1	
B0127	Group B9	Day 4		Yes	D1	[IM]	19-JUN-2018	15:36	0	
B0127	Group B9	Day 8		Yes	D1	[IM]	23-JUN-2018	13:03	0	
B0127	Group B9	Day 22		Yes	D1	[IM]	13-JUL-2018	14:20	0	
B0127	Group B9	EOS/ET (Day 61)		Yes	D1	[IM]	20-AUG-2018	13:59	0	
B0128	Group B5	Day 1	1 min	Yes	F1	uncoated	23-JUN-2018	11:31	0	
B0128	Group B5	Day 1	1 min	Yes	F2	5 ug	23-JUN-2018	11:34	0	
B0128	Group B5	Day 1	1 min	Yes	F3	uncoated	23-JUN-2018	11:37	0	
B0128	Group B5	Day 1	10 min	Yes	F1	uncoated	23-JUN-2018	11:48	0	
B0128	Group B5	Day 1	10 min	Yes	F2	5 ug	23-JUN-2018	11:48	0	

Scale: 0= No Pain; 10= Worst Pain Possible
Administration Site Key: FA = Forearm; UA= Upper arm
Treatments Part A: A1 = A/Sing MAP, 15 ug (FA); A2 = Afluria Quad IM (UA), 15 ug; A3 = Placebo MAP (FA); A4 = A/Sing Mono IM, 15 ug (UA)
Treatments Part B: B1 = A/Sing MAP, 15 ug (FA) with biopsy; B2 = Placebo MAP (FA) with biopsy
B3 = A/Sing MAP, 15 ug (FA); B4 = A/Sing MAP, 10 ug (FA); B5 = A/Sing MAP, 5 ug (FA); B6 = A/Sing MAP, 2.5 ug (FA)
B7 = Placebo MAP (FA); B8 = A/Sing MAP, 15 ug (UA); B9 = A/Sing IM, 15 ug, as 2018 Afluria Quad (UA)

Listing 16.2.9.3
Numeric Pain Intensity per Site

Part B

Randomisation Number	Group	Visit	Time Point	Was Pain Assessment Performed?	Site Number	Treatment	Date of assessment	Time of Assessment	Score	Unscheduled Reason
B0128	Group B5	Day 1	10 min	Yes	F3	uncoated	23-JUN-2018	11:48	0	
B0128	Group B5	Day 1	1 hour	Yes	F1	uncoated	23-JUN-2018	12:38	0	
B0128	Group B5	Day 1	1 hour	Yes	F2	5 ug	23-JUN-2018	12:38	0	
B0128	Group B5	Day 1	1 hour	Yes	F3	uncoated	23-JUN-2018	12:38	0	
B0128	Group B5	Day 1	2 hour	Yes	F1	uncoated	23-JUN-2018	13:38	0	
B0128	Group B5	Day 1	2 hour	Yes	F2	5 ug	23-JUN-2018	13:38	0	
B0128	Group B5	Day 1	2 hour	Yes	F3	uncoated	23-JUN-2018	13:38	0	
B0128	Group B5	Day 2		Yes	F1	uncoated	24-JUN-2018	11:58	0	
B0128	Group B5	Day 2		Yes	F2	5 ug	24-JUN-2018	11:58	0	
B0128	Group B5	Day 2		Yes	F3	uncoated	24-JUN-2018	11:58	0	
B0128	Group B5	Day 4		Yes	F1	uncoated	26-JUN-2018	11:58	0	
B0128	Group B5	Day 4		Yes	F2	5 ug	26-JUN-2018	11:58	0	
B0128	Group B5	Day 4		Yes	F3	uncoated	26-JUN-2018	11:58	0	
B0128	Group B5	Day 8		Yes	F1	uncoated	30-JUN-2018	12:09	0	
B0128	Group B5	Day 8		Yes	F2	5 ug	30-JUN-2018	12:09	0	
B0128	Group B5	Day 8		Yes	F3	uncoated	30-JUN-2018	12:09	0	
B0128	Group B5	Day 22		Yes	F1	uncoated	14-JUL-2018	11:38	0	
B0128	Group B5	Day 22		Yes	F2	5 ug	14-JUL-2018	11:38	0	
B0128	Group B5	Day 22		Yes	F3	uncoated	14-JUL-2018	11:38	0	
B0128	Group B5	EOS/ET (Day 61)		Yes	F1	uncoated	22-AUG-2018	11:14	0	
B0128	Group B5	EOS/ET (Day 61)		Yes	F2	5 ug	22-AUG-2018	11:14	0	

Scale: 0= No Pain; 10= Worst Pain Possible
Administration Site Key: FA = Forearm; UA= Upper arm
Treatments Part A: A1 = A/Sing MAP, 15 ug (FA); A2 = Afluria Quad IM (UA), 15 ug; A3 = Placebo MAP (FA); A4 = A/Sing Mono IM, 15 ug (UA)
Treatments Part B: B1 = A/Sing MAP, 15 ug (FA) with biopsy; B2 = Placebo MAP (FA) with biopsy
B3 = A/Sing MAP, 15 ug (FA); B4 = A/Sing MAP, 10 ug (FA); B5 = A/Sing MAP, 5 ug (FA); B6 = A/Sing MAP, 2.5 ug (FA)
B7 = Placebo MAP (FA); B8 = A/Sing MAP, 15 ug (UA); B9 = A/Sing IM, 15 ug, as 2018 Afluria Quad (UA)

Listing 16.2.9.3
Numeric Pain Intensity per Site

Part B

Randomisation Number	Group	Visit	Time Point	Was Pain Assessment Performed?	Site Number	Treatment	Date of assessment	Time of Assessment	Score	Unscheduled Reason
B0128	Group B5	EOS/ET (Day 61)		Yes	F3	uncoated	22-AUG-2018	11:14	0	
B0129	Group B8	Day 1	1 min	Yes	D1	5 ug	23-JUN-2018	11:51	1	
B0129	Group B8	Day 1	1 min	Yes	D2	5 ug	23-JUN-2018	11:54	1	
B0129	Group B8	Day 1	1 min	Yes	D3	5 ug	23-JUN-2018	11:57	1	
B0129	Group B8	Day 1	10 min	Yes	D1	5 ug	23-JUN-2018	12:08	1	
B0129	Group B8	Day 1	10 min	Yes	D2	5 ug	23-JUN-2018	12:08	1	
B0129	Group B8	Day 1	10 min	Yes	D3	5 ug	23-JUN-2018	12:08	1	
B0129	Group B8	Day 1	1 hour	Yes	D1	5 ug	23-JUN-2018	12:58	0	
B0129	Group B8	Day 1	1 hour	Yes	D2	5 ug	23-JUN-2018	12:58	0	
B0129	Group B8	Day 1	1 hour	Yes	D3	5 ug	23-JUN-2018	12:58	0	
B0129	Group B8	Day 1	2 hour	Yes	D1	5 ug	23-JUN-2018	13:58	0	
B0129	Group B8	Day 1	2 hour	Yes	D2	5 ug	23-JUN-2018	13:58	0	
B0129	Group B8	Day 1	2 hour	Yes	D3	5 ug	23-JUN-2018	13:58	0	
B0129	Group B8	Day 2		Yes	D1	5 ug	24-JUN-2018	12:10	0	
B0129	Group B8	Day 2		Yes	D2	5 ug	24-JUN-2018	12:10	0	
B0129	Group B8	Day 2		Yes	D3	5 ug	24-JUN-2018	12:10	0	
B0129	Group B8	Day 4		Yes	D1	5 ug	26-JUN-2018	14:10	0	
B0129	Group B8	Day 4		Yes	D2	5 ug	26-JUN-2018	14:10	0	
B0129	Group B8	Day 4		Yes	D3	5 ug	26-JUN-2018	14:10	0	
B0129	Group B8	Day 8		Yes	D1	5 ug	30-JUN-2018	15:06	0	
B0129	Group B8	Day 8		Yes	D2	5 ug	30-JUN-2018	15:06	0	

Scale: 0= No Pain; 10= Worst Pain Possible
Administration Site Key: FA = Forearm; UA= Upper arm
Treatments Part A: A1 = A/Sing MAP, 15 ug (FA); A2 = Afluria Quad IM (UA), 15 ug; A3 = Placebo MAP (FA); A4 = A/Sing Mono IM, 15 ug (UA)
Treatments Part B: B1 = A/Sing MAP, 15 ug (FA) with biopsy; B2 = Placebo MAP (FA) with biopsy
B3 = A/Sing MAP, 15 ug (FA); B4 = A/Sing MAP, 10 ug (FA); B5 = A/Sing MAP, 5 ug (FA); B6 = A/Sing MAP, 2.5 ug (FA)
B7 = Placebo MAP (FA); B8 = A/Sing MAP, 15 ug (UA); B9 = A/Sing IM, 15 ug, as 2018 Afluria Quad (UA)

Listing 16.2.9.3
Numeric Pain Intensity per Site

Part B

Randomisation Number	Group	Visit	Time Point	Was Pain Assessment Performed?	Site Number	Treatment	Date of assessment	Time of Assessment	Score	Unscheduled Reason
B0129	Group B8	Day 8		Yes	D3	5 ug	30-JUN-2018	15:06	0	
B0129	Group B8	Day 22		Yes	D1	5 ug	14-JUL-2018	14:30	0	
B0129	Group B8	Day 22		Yes	D2	5 ug	14-JUL-2018	14:30	0	
B0129	Group B8	Day 22		Yes	D3	5 ug	14-JUL-2018	14:30	0	
B0129	Group B8	EOS/ET (Day 61)		Yes	D1	5 ug	22-AUG-2018	11:11	0	
B0129	Group B8	EOS/ET (Day 61)		Yes	D2	5 ug	22-AUG-2018	11:11	0	
B0129	Group B8	EOS/ET (Day 61)		Yes	D3	5 ug	22-AUG-2018	11:11	0	
B0130	Group B5	Day 1	1 min	Yes	F1	5 ug	23-JUN-2018	11:41	1	
B0130	Group B5	Day 1	1 min	Yes	F2	uncoated	23-JUN-2018	11:44	2	
B0130	Group B5	Day 1	1 min	Yes	F3	uncoated	23-JUN-2018	11:47	1	
B0130	Group B5	Day 1	10 min	Yes	F1	5 ug	23-JUN-2018	11:58	1	
B0130	Group B5	Day 1	10 min	Yes	F2	uncoated	23-JUN-2018	11:58	0	
B0130	Group B5	Day 1	10 min	Yes	F3	uncoated	23-JUN-2018	11:58	0	
B0130	Group B5	Day 1	1 hour	Yes	F1	5 ug	23-JUN-2018	12:48	0	
B0130	Group B5	Day 1	1 hour	Yes	F2	uncoated	23-JUN-2018	12:48	0	
B0130	Group B5	Day 1	1 hour	Yes	F3	uncoated	23-JUN-2018	12:48	0	
B0130	Group B5	Day 1	2 hour	Yes	F1	5 ug	23-JUN-2018	13:48	0	
B0130	Group B5	Day 1	2 hour	Yes	F2	uncoated	23-JUN-2018	13:48	0	
B0130	Group B5	Day 1	2 hour	Yes	F3	uncoated	23-JUN-2018	13:48	0	
B0130	Group B5	Day 2		Yes	F1	5 ug	24-JUN-2018	12:03	0	
B0130	Group B5	Day 2		Yes	F2	uncoated	24-JUN-2018	12:03	0	

Scale: 0= No Pain; 10= Worst Pain Possible
Administration Site Key: FA = Forearm; UA= Upper arm
Treatments Part A: A1 = A/Sing MAP, 15 ug (FA); A2 = Afluria Quad IM (UA), 15 ug; A3 = Placebo MAP (FA); A4 = A/Sing Mono IM, 15 ug (UA)
Treatments Part B: B1 = A/Sing MAP, 15 ug (FA) with biopsy; B2 = Placebo MAP (FA) with biopsy
B3 = A/Sing MAP, 15 ug (FA); B4 = A/Sing MAP, 10 ug (FA); B5 = A/Sing MAP, 5 ug (FA); B6 = A/Sing MAP, 2.5 ug (FA)
B7 = Placebo MAP (FA); B8 = A/Sing MAP, 15 ug (UA); B9 = A/Sing IM, 15 ug, as 2018 Afluria Quad (UA)

Listing 16.2.9.3
Numeric Pain Intensity per Site

Part B

Randomisation Number	Group	Visit	Time Point	Was Pain Assessment Performed?	Site Number	Treatment	Date of assessment	Time of Assessment	Score	Unscheduled Reason
B0130	Group B5	Day 2		Yes	F3	uncoated	24-JUN-2018	12:03	0	
B0130	Group B5	Day 4		Yes	F1	5 ug	26-JUN-2018	10:13	0	
B0130	Group B5	Day 4		Yes	F2	uncoated	26-JUN-2018	10:13	0	
B0130	Group B5	Day 4		Yes	F3	uncoated	26-JUN-2018	10:13	0	
B0130	Group B5	Day 8		Yes	F1	5 ug	30-JUN-2018	10:02	0	
B0130	Group B5	Day 8		Yes	F2	uncoated	30-JUN-2018	10:02	0	
B0130	Group B5	Day 8		Yes	F3	uncoated	30-JUN-2018	10:02	0	
B0130	Group B5	Day 22		Yes	F1	5 ug	14-JUL-2018	12:16	0	
B0130	Group B5	Day 22		Yes	F2	uncoated	14-JUL-2018	12:16	0	
B0130	Group B5	Day 22		Yes	F3	uncoated	14-JUL-2018	12:17	0	
B0130	Group B5	EOS/ET (Day 61)		Yes	F1	5 ug	22-AUG-2018	10:20	0	
B0130	Group B5	EOS/ET (Day 61)		Yes	F2	uncoated	22-AUG-2018	10:20	0	
B0130	Group B5	EOS/ET (Day 61)		Yes	F3	uncoated	22-AUG-2018	10:20	0	
B0131	Group B8	Day 1	1 min	Yes	D1	5 ug	23-JUN-2018	12:01	1	
B0131	Group B8	Day 1	1 min	Yes	D2	5 ug	23-JUN-2018	12:04	1	
B0131	Group B8	Day 1	1 min	Yes	D3	5 ug	23-JUN-2018	12:07	1	
B0131	Group B8	Day 1	10 min	Yes	D1	5 ug	23-JUN-2018	12:18	1	
B0131	Group B8	Day 1	10 min	Yes	D2	5 ug	23-JUN-2018	12:18	1	
B0131	Group B8	Day 1	10 min	Yes	D3	5 ug	23-JUN-2018	12:18	1	
B0131	Group B8	Day 1	1 hour	Yes	D1	5 ug	23-JUN-2018	13:08	0	
B0131	Group B8	Day 1	1 hour	Yes	D2	5 ug	23-JUN-2018	13:08	0	

Scale: 0= No Pain; 10= Worst Pain Possible
Administration Site Key: FA = Forearm; UA= Upper arm
Treatments Part A: A1 = A/Sing MAP, 15 ug (FA); A2 = Afluria Quad IM (UA), 15 ug; A3 = Placebo MAP (FA); A4 = A/Sing Mono IM, 15 ug (UA)
Treatments Part B: B1 = A/Sing MAP, 15 ug (FA) with biopsy; B2 = Placebo MAP (FA) with biopsy
B3 = A/Sing MAP, 15 ug (FA); B4 = A/Sing MAP, 10 ug (FA); B5 = A/Sing MAP, 5 ug (FA); B6 = A/Sing MAP, 2.5 ug (FA)
B7 = Placebo MAP (FA); B8 = A/Sing MAP, 15 ug (UA); B9 = A/Sing IM, 15 ug, as 2018 Afluria Quad (UA)

Listing 16.2.9.3
Numeric Pain Intensity per Site

Part B

Randomisation Number	Group	Visit	Time Point	Was Pain Assessment Performed?	Site Number	Treatment	Date of assessment	Time of Assessment	Score	Unscheduled Reason
B0131	Group B8	Day 1	1 hour	Yes	D3	5 ug	23-JUN-2018	13:08	0	
B0131	Group B8	Day 1	2 hour	Yes	D1	5 ug	23-JUN-2018	14:08	0	
B0131	Group B8	Day 1	2 hour	Yes	D2	5 ug	23-JUN-2018	14:08	1	
B0131	Group B8	Day 1	2 hour	Yes	D3	5 ug	23-JUN-2018	14:08	0	
B0131	Group B8	Day 2		Yes	D1	5 ug	24-JUN-2018	12:13	0	
B0131	Group B8	Day 2		Yes	D2	5 ug	24-JUN-2018	12:13	0	
B0131	Group B8	Day 2		Yes	D3	5 ug	24-JUN-2018	12:13	0	
B0131	Group B8	Day 4		Yes	D1	5 ug	26-JUN-2018	13:36	0	
B0131	Group B8	Day 4		Yes	D2	5 ug	26-JUN-2018	13:36	0	
B0131	Group B8	Day 4		Yes	D3	5 ug	26-JUN-2018	13:36	0	
B0131	Group B8	Day 8		Yes	D1	5 ug	30-JUN-2018	11:54	0	
B0131	Group B8	Day 8		Yes	D2	5 ug	30-JUN-2018	11:54	0	
B0131	Group B8	Day 8		Yes	D3	5 ug	30-JUN-2018	11:54	0	
B0131	Group B8	Day 22		Yes	D1	5 ug	14-JUL-2018	11:50	0	
B0131	Group B8	Day 22		Yes	D2	5 ug	14-JUL-2018	11:50	0	
B0131	Group B8	Day 22		Yes	D3	5 ug	14-JUL-2018	11:50	0	
B0131	Group B8	EOS/ET (Day 61)		Yes	D1	5 ug	22-AUG-2018	16:58	0	
B0131	Group B8	EOS/ET (Day 61)		Yes	D2	5 ug	22-AUG-2018	16:58	0	
B0131	Group B8	EOS/ET (Day 61)		Yes	D3	5 ug	22-AUG-2018	16:58	0	
B0132	Group B6	Day 1	1 min	Yes	F1	2.5 ug	23-JUN-2018	12:11	1	
B0132	Group B6	Day 1	1 min	Yes	F2	uncoated	23-JUN-2018	12:14	2	

Scale: 0= No Pain; 10= Worst Pain Possible
Administration Site Key: FA = Forearm; UA= Upper arm
Treatments Part A: A1 = A/Sing MAP, 15 ug (FA); A2 = Afluria Quad IM (UA), 15 ug; A3 = Placebo MAP (FA); A4 = A/Sing Mono IM, 15 ug (UA)
Treatments Part B: B1 = A/Sing MAP, 15 ug (FA) with biopsy; B2 = Placebo MAP (FA) with biopsy
B3 = A/Sing MAP, 15 ug (FA); B4 = A/Sing MAP, 10 ug (FA); B5 = A/Sing MAP, 5 ug (FA); B6 = A/Sing MAP, 2.5 ug (FA)
B7 = Placebo MAP (FA); B8 = A/Sing MAP, 15 ug (UA); B9 = A/Sing IM, 15 ug, as 2018 Afluria Quad (UA)

Listing 16.2.9.3
Numeric Pain Intensity per Site

Part B

Randomisation Number	Group	Visit	Time Point	Was Pain Assessment Performed?	Site Number	Treatment	Date of assessment	Time of Assessment	Score	Unscheduled Reason
B0132	Group B6	Day 1	1 min	Yes	F3	uncoated	23-JUN-2018	12:17	2	
B0132	Group B6	Day 1	10 min	Yes	F1	2.5 ug	23-JUN-2018	12:28	0	
B0132	Group B6	Day 1	10 min	Yes	F2	uncoated	23-JUN-2018	12:28	0	
B0132	Group B6	Day 1	10 min	Yes	F3	uncoated	23-JUN-2018	12:28	0	
B0132	Group B6	Day 1	1 hour	Yes	F1	2.5 ug	23-JUN-2018	13:18	0	
B0132	Group B6	Day 1	1 hour	Yes	F2	uncoated	23-JUN-2018	13:18	0	
B0132	Group B6	Day 1	1 hour	Yes	F3	uncoated	23-JUN-2018	13:18	0	
B0132	Group B6	Day 1	2 hour	Yes	F1	2.5 ug	23-JUN-2018	14:18	0	
B0132	Group B6	Day 1	2 hour	Yes	F2	uncoated	23-JUN-2018	14:18	0	
B0132	Group B6	Day 1	2 hour	Yes	F3	uncoated	23-JUN-2018	14:18	0	
B0132	Group B6	Day 2		Yes	F1	2.5 ug	24-JUN-2018	13:20	0	
B0132	Group B6	Day 2		Yes	F2	uncoated	24-JUN-2018	13:20	0	
B0132	Group B6	Day 2		Yes	F3	uncoated	24-JUN-2018	13:20	0	
B0132	Group B6	Day 4		Yes	F1	2.5 ug	26-JUN-2018	18:11	0	
B0132	Group B6	Day 4		Yes	F2	uncoated	26-JUN-2018	18:11	0	
B0132	Group B6	Day 4		Yes	F3	uncoated	26-JUN-2018	18:11	0	
B0132	Group B6	Day 8		Yes	F1	2.5 ug	30-JUN-2018	11:49	0	
B0132	Group B6	Day 8		Yes	F2	uncoated	30-JUN-2018	11:49	0	
B0132	Group B6	Day 8		Yes	F3	uncoated	30-JUN-2018	11:49	0	
B0132	Group B6	Day 22		Yes	F1	2.5 ug	14-JUL-2018	10:35	0	
B0132	Group B6	Day 22		Yes	F2	uncoated	14-JUL-2018	10:35	0	

Scale: 0= No Pain; 10= Worst Pain Possible
Administration Site Key: FA = Forearm; UA= Upper arm
Treatments Part A: A1 = A/Sing MAP, 15 ug (FA); A2 = Afluria Quad IM (UA), 15 ug; A3 = Placebo MAP (FA); A4 = A/Sing Mono IM, 15 ug (UA)
Treatments Part B: B1 = A/Sing MAP, 15 ug (FA) with biopsy; B2 = Placebo MAP (FA) with biopsy
B3 = A/Sing MAP, 15 ug (FA); B4 = A/Sing MAP, 10 ug (FA); B5 = A/Sing MAP, 5 ug (FA); B6 = A/Sing MAP, 2.5 ug (FA)
B7 = Placebo MAP (FA); B8 = A/Sing MAP, 15 ug (UA); B9 = A/Sing IM, 15 ug, as 2018 Afluria Quad (UA)

Listing 16.2.9.3
Numeric Pain Intensity per Site

Part B

Randomisation Number	Group	Visit	Time Point	Was Pain Assessment Performed?	Site Number	Treatment	Date of assessment	Time of Assessment	Score	Unscheduled Reason
B0132	Group B6	Day 22		Yes	F3	uncoated	14-JUL-2018	10:35	0	
B0132	Group B6	EOS/ET (Day 61)		Yes	F1	2.5 ug	23-AUG-2018	08:24	0	
B0132	Group B6	EOS/ET (Day 61)		Yes	F2	uncoated	23-AUG-2018	08:24	0	
B0132	Group B6	EOS/ET (Day 61)		Yes	F3	uncoated	23-AUG-2018	08:24	0	
B0133	Group B4	Day 1	1 min	Yes	F1	uncoated	23-JUN-2018	12:21	1	
B0133	Group B4	Day 1	1 min	Yes	F2	5 ug	23-JUN-2018	12:24	1	
B0133	Group B4	Day 1	1 min	Yes	F3	5 ug	23-JUN-2018	12:27	1	
B0133	Group B4	Day 1	10 min	Yes	F1	uncoated	23-JUN-2018	12:38	0	
B0133	Group B4	Day 1	10 min	Yes	F2	5 ug	23-JUN-2018	12:38	0	
B0133	Group B4	Day 1	10 min	Yes	F3	5 ug	23-JUN-2018	12:38	0	
B0133	Group B4	Day 1	1 hour	Yes	F1	uncoated	23-JUN-2018	13:28	0	
B0133	Group B4	Day 1	1 hour	Yes	F2	5 ug	23-JUN-2018	13:28	0	
B0133	Group B4	Day 1	1 hour	Yes	F3	5 ug	23-JUN-2018	13:28	0	
B0133	Group B4	Day 1	2 hour	Yes	F1	uncoated	23-JUN-2018	14:28	0	
B0133	Group B4	Day 1	2 hour	Yes	F2	5 ug	23-JUN-2018	14:28	0	
B0133	Group B4	Day 1	2 hour	Yes	F3	5 ug	23-JUN-2018	14:28	0	
B0133	Group B4	Day 2		Yes	F1	uncoated	24-JUN-2018	10:59	0	
B0133	Group B4	Day 2		Yes	F2	5 ug	24-JUN-2018	10:59	0	
B0133	Group B4	Day 2		Yes	F3	5 ug	24-JUN-2018	10:59	0	
B0133	Group B4	Day 4		Yes	F1	uncoated	26-JUN-2018	17:30	0	
B0133	Group B4	Day 4		Yes	F2	5 ug	26-JUN-2018	17:30	0	

Scale: 0= No Pain; 10= Worst Pain Possible
Administration Site Key: FA = Forearm; UA= Upper arm
Treatments Part A: A1 = A/Sing MAP, 15 ug (FA); A2 = Afluria Quad IM (UA), 15 ug; A3 = Placebo MAP (FA); A4 = A/Sing Mono IM, 15 ug (UA)
Treatments Part B: B1 = A/Sing MAP, 15 ug (FA) with biopsy; B2 = Placebo MAP (FA) with biopsy
B3 = A/Sing MAP, 15 ug (FA); B4 = A/Sing MAP, 10 ug (FA); B5 = A/Sing MAP, 5 ug (FA); B6 = A/Sing MAP, 2.5 ug (FA)
B7 = Placebo MAP (FA); B8 = A/Sing MAP, 15 ug (UA); B9 = A/Sing IM, 15 ug, as 2018 Afluria Quad (UA)

Listing 16.2.9.3
Numeric Pain Intensity per Site

Part B

Randomisation Number	Group	Visit	Time Point	Was Pain Assessment Performed?	Site Number	Treatment	Date of assessment	Time of Assessment	Score	Unscheduled Reason
B0133	Group B4	Day 4		Yes	F3	5 ug	26-JUN-2018	17:30	0	
B0133	Group B4	Day 8		Yes	F1	uncoated	30-JUN-2018	11:49	0	
B0133	Group B4	Day 8		Yes	F2	5 ug	30-JUN-2018	11:49	0	
B0133	Group B4	Day 8		Yes	F3	5 ug	30-JUN-2018	11:49	0	
B0133	Group B4	Day 22		Yes	F1	uncoated	14-JUL-2018	12:50	0	
B0133	Group B4	Day 22		Yes	F2	5 ug	14-JUL-2018	12:50	0	
B0133	Group B4	Day 22		Yes	F3	5 ug	14-JUL-2018	12:50	0	
B0133	Group B4	EOS/ET (Day 61)		Yes	F1	uncoated	22-AUG-2018	14:03	0	
B0133	Group B4	EOS/ET (Day 61)		Yes	F2	5 ug	22-AUG-2018	14:03	0	
B0133	Group B4	EOS/ET (Day 61)		Yes	F3	5 ug	22-AUG-2018	14:03	0	
B0134	Group B3	Day 1	1 min	Yes	F1	5 ug	23-JUN-2018	12:31	1	
B0134	Group B3	Day 1	1 min	Yes	F2	5 ug	23-JUN-2018	12:34	1	
B0134	Group B3	Day 1	1 min	Yes	F3	5 ug	23-JUN-2018	12:37	1	
B0134	Group B3	Day 1	10 min	Yes	F1	5 ug	23-JUN-2018	12:48	0	
B0134	Group B3	Day 1	10 min	Yes	F2	5 ug	23-JUN-2018	12:48	0	
B0134	Group B3	Day 1	10 min	Yes	F3	5 ug	23-JUN-2018	12:48	0	
B0134	Group B3	Day 1	1 hour	Yes	F1	5 ug	23-JUN-2018	13:38	0	
B0134	Group B3	Day 1	1 hour	Yes	F2	5 ug	23-JUN-2018	13:38	0	
B0134	Group B3	Day 1	1 hour	Yes	F3	5 ug	23-JUN-2018	13:38	0	
B0134	Group B3	Day 1	2 hour	Yes	F1	5 ug	23-JUN-2018	14:38	0	
B0134	Group B3	Day 1	2 hour	Yes	F2	5 ug	23-JUN-2018	14:38	0	

Scale: 0= No Pain; 10= Worst Pain Possible
Administration Site Key: FA = Forearm; UA= Upper arm
Treatments Part A: A1 = A/Sing MAP, 15 ug (FA); A2 = Afluria Quad IM (UA), 15 ug; A3 = Placebo MAP (FA); A4 = A/Sing Mono IM, 15 ug (UA)
Treatments Part B: B1 = A/Sing MAP, 15 ug (FA) with biopsy; B2 = Placebo MAP (FA) with biopsy
B3 = A/Sing MAP, 15 ug (FA); B4 = A/Sing MAP, 10 ug (FA); B5 = A/Sing MAP, 5 ug (FA); B6 = A/Sing MAP, 2.5 ug (FA)
B7 = Placebo MAP (FA); B8 = A/Sing MAP, 15 ug (UA); B9 = A/Sing IM, 15 ug, as 2018 Afluria Quad (UA)

Listing 16.2.9.3
Numeric Pain Intensity per Site

Part B

Randomisation Number	Group	Visit	Time Point	Was Pain Assessment Performed?	Site Number	Treatment	Date of assessment	Time of Assessment	Score	Unscheduled Reason
B0134	Group B3	Day 1	2 hour	Yes	F3	5 ug	23-JUN-2018	14:38	0	
B0134	Group B3	Day 2		Yes	F1	5 ug	24-JUN-2018	10:02	0	
B0134	Group B3	Day 2		Yes	F2	5 ug	24-JUN-2018	10:02	0	
B0134	Group B3	Day 2		Yes	F3	5 ug	24-JUN-2018	10:02	0	
B0134	Group B3	Day 4		Yes	F1	5 ug	26-JUN-2018	11:23	0	
B0134	Group B3	Day 4		Yes	F2	5 ug	26-JUN-2018	11:23	0	
B0134	Group B3	Day 4		Yes	F3	5 ug	26-JUN-2018	11:23	0	
B0134	Group B3	Day 8		Yes	F1	5 ug	30-JUN-2018	13:45	0	
B0134	Group B3	Day 8		Yes	F2	5 ug	30-JUN-2018	13:45	0	
B0134	Group B3	Day 8		Yes	F3	5 ug	30-JUN-2018	13:45	0	
B0134	Group B3	Day 22		Yes	F1	5 ug	14-JUL-2018	11:45	0	
B0134	Group B3	Day 22		Yes	F2	5 ug	14-JUL-2018	11:45	0	
B0134	Group B3	Day 22		Yes	F3	5 ug	14-JUL-2018	11:45	0	
B0134	Group B3	EOS/ET (Day 61)		Yes	F1	5 ug	23-AUG-2018	09:23	0	
B0134	Group B3	EOS/ET (Day 61)		Yes	F2	5 ug	23-AUG-2018	09:23	0	
B0134	Group B3	EOS/ET (Day 61)		Yes	F3	5 ug	23-AUG-2018	09:23	0	
B0135	Group B7	Day 1	1 min	Yes	F1	uncoated	23-JUN-2018	12:41	1	
B0135	Group B7	Day 1	1 min	Yes	F2	uncoated	23-JUN-2018	12:44	1	
B0135	Group B7	Day 1	1 min	Yes	F3	uncoated	23-JUN-2018	12:47	1	
B0135	Group B7	Day 1	10 min	Yes	F1	uncoated	23-JUN-2018	12:58	0	
B0135	Group B7	Day 1	10 min	Yes	F2	uncoated	23-JUN-2018	12:58	1	

Scale: 0= No Pain; 10= Worst Pain Possible
Administration Site Key: FA = Forearm; UA= Upper arm
Treatments Part A: A1 = A/Sing MAP, 15 ug (FA); A2 = Afluria Quad IM (UA), 15 ug; A3 = Placebo MAP (FA); A4 = A/Sing Mono IM, 15 ug (UA)
Treatments Part B: B1 = A/Sing MAP, 15 ug (FA) with biopsy; B2 = Placebo MAP (FA) with biopsy
B3 = A/Sing MAP, 15 ug (FA); B4 = A/Sing MAP, 10 ug (FA); B5 = A/Sing MAP, 5 ug (FA); B6 = A/Sing MAP, 2.5 ug (FA)
B7 = Placebo MAP (FA); B8 = A/Sing MAP, 15 ug (UA); B9 = A/Sing IM, 15 ug, as 2018 Afluria Quad (UA)

Listing 16.2.9.3
Numeric Pain Intensity per Site

Part B

Randomisation Number	Group	Visit	Time Point	Was Pain Assessment Performed?	Site Number	Treatment	Date of assessment	Time of Assessment	Score	Unscheduled Reason
B0135	Group B7	Day 1	10 min	Yes	F3	uncoated	23-JUN-2018	12:58	1	
B0135	Group B7	Day 1	1 hour	Yes	F1	uncoated	23-JUN-2018	13:48	0	
B0135	Group B7	Day 1	1 hour	Yes	F2	uncoated	23-JUN-2018	13:48	0	
B0135	Group B7	Day 1	1 hour	Yes	F3	uncoated	23-JUN-2018	13:48	0	
B0135	Group B7	Day 1	2 hour	Yes	F1	uncoated	23-JUN-2018	14:48	0	
B0135	Group B7	Day 1	2 hour	Yes	F2	uncoated	23-JUN-2018	14:48	0	
B0135	Group B7	Day 1	2 hour	Yes	F3	uncoated	23-JUN-2018	14:48	0	
B0135	Group B7	Day 2		Yes	F1	uncoated	24-JUN-2018	13:15	0	
B0135	Group B7	Day 2		Yes	F2	uncoated	24-JUN-2018	13:15	0	
B0135	Group B7	Day 2		Yes	F3	uncoated	24-JUN-2018	13:15	0	
B0135	Group B7	Day 4		Yes	F1	uncoated	26-JUN-2018	13:40	0	
B0135	Group B7	Day 4		Yes	F2	uncoated	26-JUN-2018	13:40	0	
B0135	Group B7	Day 4		Yes	F3	uncoated	26-JUN-2018	13:40	0	
B0135	Group B7	Day 8		Yes	F1	uncoated	30-JUN-2018	13:56	0	
B0135	Group B7	Day 8		Yes	F2	uncoated	30-JUN-2018	13:56	0	
B0135	Group B7	Day 8		Yes	F3	uncoated	30-JUN-2018	13:56	0	
B0135	Group B7	Day 22		Yes	F1	uncoated	14-JUL-2018	14:19	0	
B0135	Group B7	Day 22		Yes	F2	uncoated	14-JUL-2018	14:19	0	
B0135	Group B7	Day 22		Yes	F3	uncoated	14-JUL-2018	14:19	0	
B0135	Group B7	EOS/ET (Day 61)		Yes	F1	uncoated	22-AUG-2018	16:01	0	
B0135	Group B7	EOS/ET (Day 61)		Yes	F2	uncoated	22-AUG-2018	16:01	0	

Scale: 0= No Pain; 10= Worst Pain Possible
Administration Site Key: FA = Forearm; UA= Upper arm
Treatments Part A: A1 = A/Sing MAP, 15 ug (FA); A2 = Afluria Quad IM (UA), 15 ug; A3 = Placebo MAP (FA); A4 = A/Sing Mono IM, 15 ug (UA)
Treatments Part B: B1 = A/Sing MAP, 15 ug (FA) with biopsy; B2 = Placebo MAP (FA) with biopsy
B3 = A/Sing MAP, 15 ug (FA); B4 = A/Sing MAP, 10 ug (FA); B5 = A/Sing MAP, 5 ug (FA); B6 = A/Sing MAP, 2.5 ug (FA)
B7 = Placebo MAP (FA); B8 = A/Sing MAP, 15 ug (UA); B9 = A/Sing IM, 15 ug, as 2018 Afluria Quad (UA)

Listing 16.2.9.3
Numeric Pain Intensity per Site

Part B

Randomisation Number	Group	Visit	Time Point	Was Pain Assessment Performed?	Site Number	Treatment	Date of assessment	Time of Assessment	Score	Unscheduled Reason
B0135	Group B7	EOS/ET (Day 61)		Yes	F3	uncoated	22-AUG-2018	16:01	0	
B0136	Group B9	Day 1	1 min	Yes	D1	[IM]	23-JUN-2018	11:11	0	
B0136	Group B9	Day 1	10 min	Yes	D1	[IM]	23-JUN-2018	11:20	0	
B0136	Group B9	Day 1	1 hour	Yes	D1	[IM]	23-JUN-2018	12:10	0	
B0136	Group B9	Day 1	2 hour	Yes	D1	[IM]	23-JUN-2018	13:13	0	
B0136	Group B9	Day 2		Yes	D1	[IM]	24-JUN-2018	11:05	0	
B0136	Group B9	Day 4		Yes	D1	[IM]	26-JUN-2018	11:21	0	
B0136	Group B9	Day 8		Yes	D1	[IM]	30-JUN-2018	15:47	0	
B0136	Group B9	Day 22		Yes	D1	[IM]	14-JUL-2018	14:02	0	
B0136	Group B9	EOS/ET (Day 61)		Yes	D1	[IM]	22-AUG-2018	11:23	0	
B0137	Group B8	Day 1	1 min	Yes	D1	5 ug	23-JUN-2018	12:51	0	
B0137	Group B8	Day 1	1 min	Yes	D2	5 ug	23-JUN-2018	12:54	1	
B0137	Group B8	Day 1	1 min	Yes	D3	5 ug	23-JUN-2018	12:57	1	
B0137	Group B8	Day 1	10 min	Yes	D1	5 ug	23-JUN-2018	13:08	0	
B0137	Group B8	Day 1	10 min	Yes	D2	5 ug	23-JUN-2018	13:08	0	
B0137	Group B8	Day 1	10 min	Yes	D3	5 ug	23-JUN-2018	13:08	1	
B0137	Group B8	Day 1	1 hour	Yes	D1	5 ug	23-JUN-2018	13:58	0	
B0137	Group B8	Day 1	1 hour	Yes	D2	5 ug	23-JUN-2018	13:58	0	
B0137	Group B8	Day 1	1 hour	Yes	D3	5 ug	23-JUN-2018	13:58	0	
B0137	Group B8	Day 1	2 hour	Yes	D1	5 ug	23-JUN-2018	14:58	0	

Scale: 0= No Pain; 10= Worst Pain Possible
Administration Site Key: FA = Forearm; UA= Upper arm
Treatments Part A: A1 = A/Sing MAP, 15 ug (FA); A2 = Afluria Quad IM (UA), 15 ug; A3 = Placebo MAP (FA); A4 = A/Sing Mono IM, 15 ug (UA)
Treatments Part B: B1 = A/Sing MAP, 15 ug (FA) with biopsy; B2 = Placebo MAP (FA) with biopsy
B3 = A/Sing MAP, 15 ug (FA); B4 = A/Sing MAP, 10 ug (FA); B5 = A/Sing MAP, 5 ug (FA); B6 = A/Sing MAP, 2.5 ug (FA)
B7 = Placebo MAP (FA); B8 = A/Sing MAP, 15 ug (UA); B9 = A/Sing IM, 15 ug, as 2018 Afluria Quad (UA)

Listing 16.2.9.3
Numeric Pain Intensity per Site

Part B

Randomisation Number	Group	Visit	Time Point	Was Pain Assessment Performed?	Site Number	Treatment	Date of assessment	Time of Assessment	Score	Unscheduled Reason
B0137	Group B8	Day 1	2 hour	Yes	D2	5 ug	23-JUN-2018	14:58	0	
B0137	Group B8	Day 1	2 hour	Yes	D3	5 ug	23-JUN-2018	14:58	0	
B0137	Group B8	Day 2		Yes	D1	5 ug	24-JUN-2018	08:50	0	
B0137	Group B8	Day 2		Yes	D2	5 ug	24-JUN-2018	08:50	0	
B0137	Group B8	Day 2		Yes	D3	5 ug	24-JUN-2018	08:50	0	
B0137	Group B8	Day 4		Yes	D1	5 ug	26-JUN-2018	10:21	0	
B0137	Group B8	Day 4		Yes	D2	5 ug	26-JUN-2018	10:21	0	
B0137	Group B8	Day 4		Yes	D3	5 ug	26-JUN-2018	10:21	0	
B0137	Group B8	Day 8		Yes	D1	5 ug	30-JUN-2018	12:26	0	
B0137	Group B8	Day 8		Yes	D2	5 ug	30-JUN-2018	12:26	0	
B0137	Group B8	Day 8		Yes	D3	5 ug	30-JUN-2018	12:26	0	
B0137	Group B8	Day 22		Yes	D1	5 ug	13-JUL-2018	09:32	0	
B0137	Group B8	Day 22		Yes	D2	5 ug	13-JUL-2018	09:32	0	
B0137	Group B8	Day 22		Yes	D3	5 ug	13-JUL-2018	09:32	0	
B0137	Group B8	EOS/ET (Day 61)		Yes	D1	5 ug	30-AUG-2018	12:15	0	
B0137	Group B8	EOS/ET (Day 61)		Yes	D2	5 ug	30-AUG-2018	12:15	0	
B0137	Group B8	EOS/ET (Day 61)		Yes	D3	5 ug	30-AUG-2018	12:15	0	
B0138	Group B7	Day 1	1 min	Yes	F1	uncoated	23-JUN-2018	13:01	1	
B0138	Group B7	Day 1	1 min	Yes	F2	uncoated	23-JUN-2018	13:04	1	
B0138	Group B7	Day 1	1 min	Yes	F3	uncoated	23-JUN-2018	13:07	1	
B0138	Group B7	Day 1	10 min	Yes	F1	uncoated	23-JUN-2018	13:18	1	

Scale: 0= No Pain; 10= Worst Pain Possible
Administration Site Key: FA = Forearm; UA= Upper arm
Treatments Part A: A1 = A/Sing MAP, 15 ug (FA); A2 = Afluria Quad IM (UA), 15 ug; A3 = Placebo MAP (FA); A4 = A/Sing Mono IM, 15 ug (UA)
Treatments Part B: B1 = A/Sing MAP, 15 ug (FA) with biopsy; B2 = Placebo MAP (FA) with biopsy
B3 = A/Sing MAP, 15 ug (FA); B4 = A/Sing MAP, 10 ug (FA); B5 = A/Sing MAP, 5 ug (FA); B6 = A/Sing MAP, 2.5 ug (FA)
B7 = Placebo MAP (FA); B8 = A/Sing MAP, 15 ug (UA); B9 = A/Sing IM, 15 ug, as 2018 Afluria Quad (UA)

Listing 16.2.9.3
Numeric Pain Intensity per Site

Part B

Randomisation Number	Group	Visit	Time Point	Was Pain Assessment Performed?	Site Number	Treatment	Date of assessment	Time of Assessment	Score	Unscheduled Reason
B0138	Group B7	Day 1	10 min	Yes	F2	uncoated	23-JUN-2018	13:18	0	
B0138	Group B7	Day 1	10 min	Yes	F3	uncoated	23-JUN-2018	13:18	0	
B0138	Group B7	Day 1	1 hour	Yes	F1	uncoated	23-JUN-2018	14:08	0	
B0138	Group B7	Day 1	1 hour	Yes	F2	uncoated	23-JUN-2018	14:08	0	
B0138	Group B7	Day 1	1 hour	Yes	F3	uncoated	23-JUN-2018	14:08	0	
B0138	Group B7	Day 1	2 hour	Yes	F1	uncoated	23-JUN-2018	15:08	0	
B0138	Group B7	Day 1	2 hour	Yes	F2	uncoated	23-JUN-2018	15:08	0	
B0138	Group B7	Day 1	2 hour	Yes	F3	uncoated	23-JUN-2018	15:08	0	
B0138	Group B7	Day 2		Yes	F1	uncoated	24-JUN-2018	13:11	0	
B0138	Group B7	Day 2		Yes	F2	uncoated	24-JUN-2018	13:11	0	
B0138	Group B7	Day 2		Yes	F3	uncoated	24-JUN-2018	13:11	0	
B0138	Group B7	Day 4		Yes	F1	uncoated	26-JUN-2018	12:30	0	
B0138	Group B7	Day 4		Yes	F2	uncoated	26-JUN-2018	12:30	0	
B0138	Group B7	Day 4		Yes	F3	uncoated	26-JUN-2018	12:30	0	
B0138	Group B7	Day 8		Yes	F1	uncoated	30-JUN-2018	13:36	0	
B0138	Group B7	Day 8		Yes	F2	uncoated	30-JUN-2018	13:36	0	
B0138	Group B7	Day 8		Yes	F3	uncoated	30-JUN-2018	13:36	0	
B0138	Group B7	Day 22		Yes	F1	uncoated	14-JUL-2018	10:50	0	
B0138	Group B7	Day 22		Yes	F2	uncoated	14-JUL-2018	10:50	0	
B0138	Group B7	Day 22		Yes	F3	uncoated	14-JUL-2018	10:50	0	
B0138	Group B7	EOS/ET (Day 61)		Yes	F1	uncoated	22-AUG-2018	13:26	0	

Scale: 0= No Pain; 10= Worst Pain Possible
Administration Site Key: FA = Forearm; UA= Upper arm
Treatments Part A: A1 = A/Sing MAP, 15 ug (FA); A2 = Afluria Quad IM (UA), 15 ug; A3 = Placebo MAP (FA); A4 = A/Sing Mono IM, 15 ug (UA)
Treatments Part B: B1 = A/Sing MAP, 15 ug (FA) with biopsy; B2 = Placebo MAP (FA) with biopsy
B3 = A/Sing MAP, 15 ug (FA); B4 = A/Sing MAP, 10 ug (FA); B5 = A/Sing MAP, 5 ug (FA); B6 = A/Sing MAP, 2.5 ug (FA)
B7 = Placebo MAP (FA); B8 = A/Sing MAP, 15 ug (UA); B9 = A/Sing IM, 15 ug, as 2018 Afluria Quad (UA)

Listing 16.2.9.3
Numeric Pain Intensity per Site

Part B

Randomisation Number	Group	Visit	Time Point	Was Pain Assessment Performed?	Site Number	Treatment	Date of assessment	Time of Assessment	Score	Unscheduled Reason
B0138	Group B7	EOS/ET (Day 61)		Yes	F2	uncoated	22-AUG-2018	13:26	0	
B0138	Group B7	EOS/ET (Day 61)		Yes	F3	uncoated	22-AUG-2018	13:26	0	
B0139	Group B4	Day 1	1 min	Yes	F1	uncoated	23-JUN-2018	13:11	0	
B0139	Group B4	Day 1	1 min	Yes	F2	5 ug	23-JUN-2018	13:14	0	
B0139	Group B4	Day 1	1 min	Yes	F3	5 ug	23-JUN-2018	13:17	1	
B0139	Group B4	Day 1	10 min	Yes	F1	uncoated	23-JUN-2018	13:28	0	
B0139	Group B4	Day 1	10 min	Yes	F2	5 ug	23-JUN-2018	13:28	0	
B0139	Group B4	Day 1	10 min	Yes	F3	5 ug	23-JUN-2018	13:28	0	
B0139	Group B4	Day 1	1 hour	Yes	F1	uncoated	23-JUN-2018	14:18	0	
B0139	Group B4	Day 1	1 hour	Yes	F2	5 ug	23-JUN-2018	14:18	0	
B0139	Group B4	Day 1	1 hour	Yes	F3	5 ug	23-JUN-2018	14:18	0	
B0139	Group B4	Day 1	2 hour	Yes	F1	uncoated	23-JUN-2018	15:18	0	
B0139	Group B4	Day 1	2 hour	Yes	F2	5 ug	23-JUN-2018	15:18	0	
B0139	Group B4	Day 1	2 hour	Yes	F3	5 ug	23-JUN-2018	15:18	0	
B0139	Group B4	Day 2		Yes	F1	uncoated	24-JUN-2018	13:40	0	
B0139	Group B4	Day 2		Yes	F2	5 ug	24-JUN-2018	13:40	0	
B0139	Group B4	Day 2		Yes	F3	5 ug	24-JUN-2018	13:40	0	
B0139	Group B4	Day 4		Yes	F1	uncoated	26-JUN-2018	17:58	0	
B0139	Group B4	Day 4		Yes	F2	5 ug	26-JUN-2018	17:58	0	
B0139	Group B4	Day 4		Yes	F3	5 ug	26-JUN-2018	17:58	0	
B0139	Group B4	Day 8		Yes	F1	uncoated	30-JUN-2018	15:03	0	

Scale: 0= No Pain; 10= Worst Pain Possible
Administration Site Key: FA = Forearm; UA= Upper arm
Treatments Part A: A1 = A/Sing MAP, 15 ug (FA); A2 = Afluria Quad IM (UA), 15 ug; A3 = Placebo MAP (FA); A4 = A/Sing Mono IM, 15 ug (UA)
Treatments Part B: B1 = A/Sing MAP, 15 ug (FA) with biopsy; B2 = Placebo MAP (FA) with biopsy
B3 = A/Sing MAP, 15 ug (FA); B4 = A/Sing MAP, 10 ug (FA); B5 = A/Sing MAP, 5 ug (FA); B6 = A/Sing MAP, 2.5 ug (FA)
B7 = Placebo MAP (FA); B8 = A/Sing MAP, 15 ug (UA); B9 = A/Sing IM, 15 ug, as 2018 Afluria Quad (UA)

Listing 16.2.9.3
Numeric Pain Intensity per Site

Part B

Randomisation Number	Group	Visit	Time Point	Was Pain Assessment Performed?	Site Number	Treatment	Date of assessment	Time of Assessment	Score	Unscheduled Reason
B0139	Group B4	Day 8		Yes	F2	5 ug	30-JUN-2018	15:03	0	
B0139	Group B4	Day 8		Yes	F3	5 ug	30-JUN-2018	15:03	0	
B0139	Group B4	Day 22		Yes	F1	uncoated	14-JUL-2018	09:15	0	
B0139	Group B4	Day 22		Yes	F2	5 ug	14-JUL-2018	09:15	0	
B0139	Group B4	Day 22		Yes	F3	5 ug	14-JUL-2018	09:15	0	
B0139	Group B4	EOS/ET (Day 61)		Yes	F1	uncoated	22-AUG-2018	14:01	0	
B0139	Group B4	EOS/ET (Day 61)		Yes	F2	5 ug	22-AUG-2018	14:01	0	
B0139	Group B4	EOS/ET (Day 61)		Yes	F3	5 ug	22-AUG-2018	14:01	0	
B0140	Group B9	Day 1	1 min	Yes	D1	[IM]	23-JUN-2018	11:01	0	
B0140	Group B9	Day 1	10 min	Yes	D1	[IM]	23-JUN-2018	11:10	0	
B0140	Group B9	Day 1	1 hour	Yes	D1	[IM]	23-JUN-2018	12:00	0	
B0140	Group B9	Day 1	2 hour	Yes	D1	[IM]	23-JUN-2018	13:08	0	
B0140	Group B9	Day 2		Yes	D1	[IM]	24-JUN-2018	11:18	0	
B0140	Group B9	Day 4		Yes	D1	[IM]	26-JUN-2018	14:18	0	
B0140	Group B9	Day 8		Yes	D1	[IM]	01-JUL-2018	14:07	0	
B0140	Group B9	Day 22		Yes	D1	[IM]	14-JUL-2018	12:49	0	
B0140	Group B9	EOS/ET (Day 61)		Yes	D1	[IM]	22-AUG-2018	09:14	0	
B0141	Group B3	Day 1	1 min	Yes	F1	5 ug	23-JUN-2018	13:21	0	
B0141	Group B3	Day 1	1 min	Yes	F2	5 ug	23-JUN-2018	13:24	1	
B0141	Group B3	Day 1	1 min	Yes	F3	5 ug	23-JUN-2018	13:27	1	

Scale: 0= No Pain; 10= Worst Pain Possible
Administration Site Key: FA = Forearm; UA= Upper arm
Treatments Part A: A1 = A/Sing MAP, 15 ug (FA); A2 = Afluria Quad IM (UA), 15 ug; A3 = Placebo MAP (FA); A4 = A/Sing Mono IM, 15 ug (UA)
Treatments Part B: B1 = A/Sing MAP, 15 ug (FA) with biopsy; B2 = Placebo MAP (FA) with biopsy
B3 = A/Sing MAP, 15 ug (FA); B4 = A/Sing MAP, 10 ug (FA); B5 = A/Sing MAP, 5 ug (FA); B6 = A/Sing MAP, 2.5 ug (FA)
B7 = Placebo MAP (FA); B8 = A/Sing MAP, 15 ug (UA); B9 = A/Sing IM, 15 ug, as 2018 Afluria Quad (UA)

Listing 16.2.9.3
Numeric Pain Intensity per Site

Part B

Randomisation Number	Group	Visit	Time Point	Was Pain Assessment Performed?	Site Number	Treatment	Date of assessment	Time of Assessment	Score	Unscheduled Reason
B0141	Group B3	Day 1	10 min	Yes	F1	5 ug	23-JUN-2018	13:38	0	
B0141	Group B3	Day 1	10 min	Yes	F2	5 ug	23-JUN-2018	13:38	1	
B0141	Group B3	Day 1	10 min	Yes	F3	5 ug	23-JUN-2018	13:28	1	
B0141	Group B3	Day 1	1 hour	Yes	F1	5 ug	23-JUN-2018	14:28	0	
B0141	Group B3	Day 1	1 hour	Yes	F2	5 ug	23-JUN-2018	14:28	0	
B0141	Group B3	Day 1	1 hour	Yes	F3	5 ug	23-JUN-2018	14:28	0	
B0141	Group B3	Day 1	2 hour	Yes	F1	5 ug	23-JUN-2018	15:28	0	
B0141	Group B3	Day 1	2 hour	Yes	F2	5 ug	23-JUN-2018	15:28	0	
B0141	Group B3	Day 1	2 hour	Yes	F3	5 ug	23-JUN-2018	15:28	0	
B0141	Group B3	Day 2		Yes	F1	5 ug	24-JUN-2018	13:46	0	
B0141	Group B3	Day 2		Yes	F2	5 ug	24-JUN-2018	13:46	0	
B0141	Group B3	Day 2		Yes	F3	5 ug	24-JUN-2018	13:46	0	
B0141	Group B3	Day 4		Yes	F1	5 ug	26-JUN-2018	14:15	0	
B0141	Group B3	Day 4		Yes	F2	5 ug	26-JUN-2018	14:15	0	
B0141	Group B3	Day 4		Yes	F3	5 ug	26-JUN-2018	14:15	0	
B0141	Group B3	Day 8		Yes	F1	5 ug	30-JUN-2018	13:51	0	
B0141	Group B3	Day 8		Yes	F2	5 ug	30-JUN-2018	13:51	0	
B0141	Group B3	Day 8		Yes	F3	5 ug	30-JUN-2018	13:51	0	
B0141	Group B3	Day 22		Yes	F1	5 ug	14-JUL-2018	14:29	0	
B0141	Group B3	Day 22		Yes	F2	5 ug	14-JUL-2018	14:29	0	
B0141	Group B3	Day 22		Yes	F3	5 ug	14-JUL-2018	14:29	0	

Scale: 0= No Pain; 10= Worst Pain Possible
Administration Site Key: FA = Forearm; UA= Upper arm
Treatments Part A: A1 = A/Sing MAP, 15 ug (FA); A2 = Afluria Quad IM (UA), 15 ug; A3 = Placebo MAP (FA); A4 = A/Sing Mono IM, 15 ug (UA)
Treatments Part B: B1 = A/Sing MAP, 15 ug (FA) with biopsy; B2 = Placebo MAP (FA) with biopsy
B3 = A/Sing MAP, 15 ug (FA); B4 = A/Sing MAP, 10 ug (FA); B5 = A/Sing MAP, 5 ug (FA); B6 = A/Sing MAP, 2.5 ug (FA)
B7 = Placebo MAP (FA); B8 = A/Sing MAP, 15 ug (UA); B9 = A/Sing IM, 15 ug, as 2018 Afluria Quad (UA)

Listing 16.2.9.3
Numeric Pain Intensity per Site

Part B

Randomisation Number	Group	Visit	Time Point	Was Pain Assessment Performed?	Site Number	Treatment	Date of assessment	Time of Assessment	Score	Unscheduled Reason
B0141	Group B3	EOS/ET (Day 61)		Yes	F1	5 ug	23-AUG-2018	13:51	0	
B0141	Group B3	EOS/ET (Day 61)		Yes	F2	5 ug	23-AUG-2018	13:51	0	
B0141	Group B3	EOS/ET (Day 61)		Yes	F3	5 ug	23-AUG-2018	13:51	0	
B0142	Group B5	Day 1	1 min	Yes	F1	5 ug	23-JUN-2018	13:31	0	
B0142	Group B5	Day 1	1 min	Yes	F2	uncoated	23-JUN-2018	13:34	0	
B0142	Group B5	Day 1	1 min	Yes	F3	uncoated	23-JUN-2018	13:37	0	
B0142	Group B5	Day 1	10 min	Yes	F1	5 ug	23-JUN-2018	13:48	0	
B0142	Group B5	Day 1	10 min	Yes	F2	uncoated	23-JUN-2018	13:48	0	
B0142	Group B5	Day 1	10 min	Yes	F3	uncoated	23-JUN-2018	13:48	0	
B0142	Group B5	Day 1	1 hour	Yes	F1	5 ug	23-JUN-2018	14:38	0	
B0142	Group B5	Day 1	1 hour	Yes	F2	uncoated	23-JUN-2018	14:38	0	
B0142	Group B5	Day 1	1 hour	Yes	F3	uncoated	23-JUN-2018	14:38	0	
B0142	Group B5	Day 1	2 hour	Yes	F1	5 ug	23-JUN-2018	15:38	0	
B0142	Group B5	Day 1	2 hour	Yes	F2	uncoated	23-JUN-2018	15:38	0	
B0142	Group B5	Day 1	2 hour	Yes	F3	uncoated	23-JUN-2018	15:38	0	
B0142	Group B5	Day 2		Yes	F1	5 ug	24-JUN-2018	14:09	0	
B0142	Group B5	Day 2		Yes	F2	uncoated	24-JUN-2018	14:09	0	
B0142	Group B5	Day 2		Yes	F3	uncoated	24-JUN-2018	14:09	0	
B0142	Group B5	Day 4		Yes	F1	5 ug	26-JUN-2018	08:49	0	
B0142	Group B5	Day 4		Yes	F2	uncoated	26-JUN-2018	08:49	0	
B0142	Group B5	Day 4		Yes	F3	uncoated	26-JUN-2018	08:49	0	

Scale: 0= No Pain; 10= Worst Pain Possible
Administration Site Key: FA = Forearm; UA= Upper arm
Treatments Part A: A1 = A/Sing MAP, 15 ug (FA); A2 = Afluria Quad IM (UA), 15 ug; A3 = Placebo MAP (FA); A4 = A/Sing Mono IM, 15 ug (UA)
Treatments Part B: B1 = A/Sing MAP, 15 ug (FA) with biopsy; B2 = Placebo MAP (FA) with biopsy
B3 = A/Sing MAP, 15 ug (FA); B4 = A/Sing MAP, 10 ug (FA); B5 = A/Sing MAP, 5 ug (FA); B6 = A/Sing MAP, 2.5 ug (FA)
B7 = Placebo MAP (FA); B8 = A/Sing MAP, 15 ug (UA); B9 = A/Sing IM, 15 ug, as 2018 Afluria Quad (UA)

Listing 16.2.9.3
Numeric Pain Intensity per Site

Part B

Randomisation Number	Group	Visit	Time Point	Was Pain Assessment Performed?	Site Number	Treatment	Date of assessment	Time of Assessment	Score	Unscheduled Reason
B0142	Group B5	Day 8		Yes	F1	5 ug	30-JUN-2018	11:11	0	
B0142	Group B5	Day 8		Yes	F2	uncoated	30-JUN-2018	11:11	0	
B0142	Group B5	Day 8		Yes	F3	uncoated	30-JUN-2018	11:11	0	
B0142	Group B5	Day 22		Yes	F1	5 ug	14-JUL-2018	13:30	0	
B0142	Group B5	Day 22		Yes	F2	uncoated	14-JUL-2018	13:30	0	
B0142	Group B5	Day 22		Yes	F3	uncoated	14-JUL-2018	13:30	0	
B0142	Group B5	EOS/ET (Day 61)		Yes	F1	5 ug	25-AUG-2018	11:54	0	
B0142	Group B5	EOS/ET (Day 61)		Yes	F2	uncoated	25-AUG-2018	11:54	0	
B0142	Group B5	EOS/ET (Day 61)		Yes	F3	uncoated	25-AUG-2018	11:54	0	
B0143	Group B6	Day 1	1 min	Yes	F1	uncoated	23-JUN-2018	13:41	5	
B0143	Group B6	Day 1	1 min	Yes	F2	uncoated	23-JUN-2018	13:44	2	
B0143	Group B6	Day 1	1 min	Yes	F3	2.5 ug	23-JUN-2018	13:47	0	
B0143	Group B6	Day 1	10 min	Yes	F1	uncoated	23-JUN-2018	13:58	0	
B0143	Group B6	Day 1	10 min	Yes	F2	uncoated	23-JUN-2018	13:58	0	
B0143	Group B6	Day 1	10 min	Yes	F3	2.5 ug	23-JUN-2018	13:58	0	
B0143	Group B6	Day 1	1 hour	Yes	F1	uncoated	23-JUN-2018	14:48	0	
B0143	Group B6	Day 1	1 hour	Yes	F2	uncoated	23-JUN-2018	14:48	0	
B0143	Group B6	Day 1	1 hour	Yes	F3	2.5 ug	23-JUN-2018	14:48	1	
B0143	Group B6	Day 1	2 hour	Yes	F1	uncoated	23-JUN-2018	15:48	0	
B0143	Group B6	Day 1	2 hour	Yes	F2	uncoated	23-JUN-2018	15:48	0	
B0143	Group B6	Day 1	2 hour	Yes	F3	2.5 ug	23-JUN-2018	15:48	1	

Scale: 0= No Pain; 10= Worst Pain Possible
Administration Site Key: FA = Forearm; UA= Upper arm
Treatments Part A: A1 = A/Sing MAP, 15 ug (FA); A2 = Afluria Quad IM (UA), 15 ug; A3 = Placebo MAP (FA); A4 = A/Sing Mono IM, 15 ug (UA)
Treatments Part B: B1 = A/Sing MAP, 15 ug (FA) with biopsy; B2 = Placebo MAP (FA) with biopsy
B3 = A/Sing MAP, 15 ug (FA); B4 = A/Sing MAP, 10 ug (FA); B5 = A/Sing MAP, 5 ug (FA); B6 = A/Sing MAP, 2.5 ug (FA)
B7 = Placebo MAP (FA); B8 = A/Sing MAP, 15 ug (UA); B9 = A/Sing IM, 15 ug, as 2018 Afluria Quad (UA)

Listing 16.2.9.3
Numeric Pain Intensity per Site

Part B

Randomisation Number	Group	Visit	Time Point	Was Pain Assessment Performed?	Site Number	Treatment	Date of assessment	Time of Assessment	Score	Unscheduled Reason
B0143	Group B6	Day 2		Yes	F1	uncoated	24-JUN-2018	11:53	0	
B0143	Group B6	Day 2		Yes	F2	uncoated	24-JUN-2018	11:53	0	
B0143	Group B6	Day 2		Yes	F3	2.5 ug	24-JUN-2018	11:53	0	
B0143	Group B6	Day 4		Yes	F1	uncoated	26-JUN-2018	12:07	0	
B0143	Group B6	Day 4		Yes	F2	uncoated	26-JUN-2018	12:07	0	
B0143	Group B6	Day 4		Yes	F3	2.5 ug	26-JUN-2018	12:07	0	
B0143	Group B6	Day 8		Yes	F1	uncoated	30-JUN-2018	12:14	0	
B0143	Group B6	Day 8		Yes	F2	uncoated	30-JUN-2018	12:14	0	
B0143	Group B6	Day 8		Yes	F3	2.5 ug	30-JUN-2018	12:14	0	
B0143	Group B6	Day 22		Yes	F1	uncoated	14-JUL-2018	13:40	0	
B0143	Group B6	Day 22		Yes	F2	uncoated	14-JUL-2018	13:40	0	
B0143	Group B6	Day 22		Yes	F3	2.5 ug	14-JUL-2018	13:40	0	
B0143	Group B6	EOS/ET (Day 61)		Yes	F1	uncoated	22-AUG-2018	13:09	0	
B0143	Group B6	EOS/ET (Day 61)		Yes	F2	uncoated	22-AUG-2018	13:09	0	
B0143	Group B6	EOS/ET (Day 61)		Yes	F3	2.5 ug	22-AUG-2018	13:09	0	
B0144	Group B6	Day 1	1 min	Yes	F1	2.5 ug	23-JUN-2018	13:51	0	
B0144	Group B6	Day 1	1 min	Yes	F2	uncoated	23-JUN-2018	13:54	1	
B0144	Group B6	Day 1	1 min	Yes	F3	uncoated	23-JUN-2018	13:57	0	
B0144	Group B6	Day 1	10 min	Yes	F1	2.5 ug	23-JUN-2018	14:08	0	
B0144	Group B6	Day 1	10 min	Yes	F2	uncoated	23-JUN-2018	14:08	0	
B0144	Group B6	Day 1	10 min	Yes	F3	uncoated	23-JUN-2018	14:08	0	

Scale: 0= No Pain; 10= Worst Pain Possible
Administration Site Key: FA = Forearm; UA= Upper arm
Treatments Part A: A1 = A/Sing MAP, 15 ug (FA); A2 = Afluria Quad IM (UA), 15 ug; A3 = Placebo MAP (FA); A4 = A/Sing Mono IM, 15 ug (UA)
Treatments Part B: B1 = A/Sing MAP, 15 ug (FA) with biopsy; B2 = Placebo MAP (FA) with biopsy
B3 = A/Sing MAP, 15 ug (FA); B4 = A/Sing MAP, 10 ug (FA); B5 = A/Sing MAP, 5 ug (FA); B6 = A/Sing MAP, 2.5 ug (FA)
B7 = Placebo MAP (FA); B8 = A/Sing MAP, 15 ug (UA); B9 = A/Sing IM, 15 ug, as 2018 Afluria Quad (UA)

Listing 16.2.9.3
Numeric Pain Intensity per Site

Part B

Randomisation Number	Group	Visit	Time Point	Was Pain Assessment Performed?	Site Number	Treatment	Date of assessment	Time of Assessment	Score	Unscheduled Reason
B0144	Group B6	Day 1	1 hour	Yes	F1	2.5 ug	23-JUN-2018	14:58	0	
B0144	Group B6	Day 1	1 hour	Yes	F2	uncoated	23-JUN-2018	14:58	0	
B0144	Group B6	Day 1	1 hour	Yes	F3	uncoated	23-JUN-2018	14:58	0	
B0144	Group B6	Day 1	2 hour	Yes	F1	2.5 ug	23-JUN-2018	15:58	0	
B0144	Group B6	Day 1	2 hour	Yes	F2	uncoated	23-JUN-2018	15:58	0	
B0144	Group B6	Day 1	2 hour	Yes	F3	uncoated	23-JUN-2018	15:58	0	
B0144	Group B6	Day 2		Yes	F1	2.5 ug	24-JUN-2018	12:22	0	
B0144	Group B6	Day 2		Yes	F2	uncoated	24-JUN-2018	12:22	0	
B0144	Group B6	Day 2		Yes	F3	uncoated	24-JUN-2018	12:22	0	
B0144	Group B6	Day 4		Yes	F1	2.5 ug	26-JUN-2018	12:22	0	
B0144	Group B6	Day 4		Yes	F2	uncoated	26-JUN-2018	12:22	0	
B0144	Group B6	Day 4		Yes	F3	uncoated	26-JUN-2018	12:22	0	
B0144	Group B6	Day 8		Yes	F1	2.5 ug	30-JUN-2018	13:05	0	
B0144	Group B6	Day 8		Yes	F2	uncoated	30-JUN-2018	13:05	0	
B0144	Group B6	Day 8		Yes	F3	uncoated	30-JUN-2018	13:05	0	
B0144	Group B6	Day 22		Yes	F1	2.5 ug	16-JUL-2018	13:11	0	
B0144	Group B6	Day 22		Yes	F2	uncoated	16-JUL-2018	13:11	0	
B0144	Group B6	Day 22		Yes	F3	uncoated	16-JUL-2018	13:11	0	
B0144	Group B6	EOS/ET (Day 61)		Yes	F1	2.5 ug	23-AUG-2018	17:04	0	
B0144	Group B6	EOS/ET (Day 61)		Yes	F2	uncoated	23-AUG-2018	17:04	0	
B0144	Group B6	EOS/ET (Day 61)		Yes	F3	uncoated	23-AUG-2018	17:04	0	

Scale: 0= No Pain; 10= Worst Pain Possible
Administration Site Key: FA = Forearm; UA= Upper arm
Treatments Part A: A1 = A/Sing MAP, 15 ug (FA); A2 = Afluria Quad IM (UA), 15 ug; A3 = Placebo MAP (FA); A4 = A/Sing Mono IM, 15 ug (UA)
Treatments Part B: B1 = A/Sing MAP, 15 ug (FA) with biopsy; B2 = Placebo MAP (FA) with biopsy
B3 = A/Sing MAP, 15 ug (FA); B4 = A/Sing MAP, 10 ug (FA); B5 = A/Sing MAP, 5 ug (FA); B6 = A/Sing MAP, 2.5 ug (FA)
B7 = Placebo MAP (FA); B8 = A/Sing MAP, 15 ug (UA); B9 = A/Sing IM, 15 ug, as 2018 Afluria Quad (UA)

Listing 16.2.9.3
Numeric Pain Intensity per Site

Part B

Randomisation Number	Group	Visit	Time Point	Was Pain Assessment Performed?	Site Number	Treatment	Date of assessment	Time of Assessment	Score	Unscheduled Reason
B0145	Group B3	Day 1	1 min	Yes	F1	5 ug	23-JUN-2018	14:01	0	
B0145	Group B3	Day 1	1 min	Yes	F2	5 ug	23-JUN-2018	14:04	1	
B0145	Group B3	Day 1	1 min	Yes	F3	5 ug	23-JUN-2018	14:07	1	
B0145	Group B3	Day 1	10 min	Yes	F1	5 ug	23-JUN-2018	14:18	0	
B0145	Group B3	Day 1	10 min	Yes	F2	5 ug	23-JUN-2018	14:18	1	
B0145	Group B3	Day 1	10 min	Yes	F3	5 ug	23-JUN-2018	14:18	1	
B0145	Group B3	Day 1	1 hour	Yes	F1	5 ug	23-JUN-2018	15:08	0	
B0145	Group B3	Day 1	1 hour	Yes	F2	5 ug	23-JUN-2018	15:08	0	
B0145	Group B3	Day 1	1 hour	Yes	F3	5 ug	23-JUN-2018	15:08	0	
B0145	Group B3	Day 1	2 hour	Yes	F1	5 ug	23-JUN-2018	16:08	0	
B0145	Group B3	Day 1	2 hour	Yes	F2	5 ug	23-JUN-2018	16:08	0	
B0145	Group B3	Day 1	2 hour	Yes	F3	5 ug	23-JUN-2018	16:08	0	
B0145	Group B3	Day 2		Yes	F1	5 ug	24-JUN-2018	13:54	0	
B0145	Group B3	Day 2		Yes	F2	5 ug	24-JUN-2018	13:54	0	
B0145	Group B3	Day 2		Yes	F3	5 ug	24-JUN-2018	13:54	0	
B0145	Group B3	Day 4		Yes	F1	5 ug	26-JUN-2018	10:44	0	
B0145	Group B3	Day 4		Yes	F2	5 ug	26-JUN-2018	10:44	0	
B0145	Group B3	Day 4		Yes	F3	5 ug	26-JUN-2018	10:44	0	
B0145	Group B3	Day 8		Yes	F1	5 ug	30-JUN-2018	08:16	0	
B0145	Group B3	Day 8		Yes	F2	5 ug	30-JUN-2018	08:16	0	
B0145	Group B3	Day 8		Yes	F3	5 ug	30-JUN-2018	08:16	0	

Scale: 0= No Pain; 10= Worst Pain Possible
Administration Site Key: FA = Forearm; UA= Upper arm
Treatments Part A: A1 = A/Sing MAP, 15 ug (FA); A2 = Afluria Quad IM (UA), 15 ug; A3 = Placebo MAP (FA); A4 = A/Sing Mono IM, 15 ug (UA)
Treatments Part B: B1 = A/Sing MAP, 15 ug (FA) with biopsy; B2 = Placebo MAP (FA) with biopsy
B3 = A/Sing MAP, 15 ug (FA); B4 = A/Sing MAP, 10 ug (FA); B5 = A/Sing MAP, 5 ug (FA); B6 = A/Sing MAP, 2.5 ug (FA)
B7 = Placebo MAP (FA); B8 = A/Sing MAP, 15 ug (UA); B9 = A/Sing IM, 15 ug, as 2018 Afluria Quad (UA)

Listing 16.2.9.3
Numeric Pain Intensity per Site

Part B

Randomisation Number	Group	Visit	Time Point	Was Pain Assessment Performed?	Site Number	Treatment	Date of assessment	Time of Assessment	Score	Unscheduled Reason
B0145	Group B3	Day 22		Yes	F1	5 ug	14-JUL-2018	09:00	0	
B0145	Group B3	Day 22		Yes	F2	5 ug	14-JUL-2018	09:00	0	
B0145	Group B3	Day 22		Yes	F3	5 ug	14-JUL-2018	09:00	0	
B0145	Group B3	EOS/ET (Day 61)		Yes	F1	5 ug	22-AUG-2018	10:38	0	
B0145	Group B3	EOS/ET (Day 61)		Yes	F2	5 ug	22-AUG-2018	10:38	0	
B0145	Group B3	EOS/ET (Day 61)		Yes	F3	5 ug	22-AUG-2018	10:38	0	
B0146	Group B7	Day 1	1 min	Yes	F1	uncoated	28-JUN-2018	11:11	1	
B0146	Group B7	Day 1	1 min	Yes	F2	uncoated	28-JUN-2018	11:14	1	
B0146	Group B7	Day 1	1 min	Yes	F3	uncoated	28-JUN-2018	11:17	1	
B0146	Group B7	Day 1	10 min	Yes	F1	uncoated	28-JUN-2018	11:28	0	
B0146	Group B7	Day 1	10 min	Yes	F2	uncoated	28-JUN-2018	11:28	0	
B0146	Group B7	Day 1	10 min	Yes	F3	uncoated	28-JUN-2018	11:28	0	
B0146	Group B7	Day 1	1 hour	Yes	F1	uncoated	28-JUN-2018	12:18	0	
B0146	Group B7	Day 1	1 hour	Yes	F2	uncoated	28-JUN-2018	12:18	0	
B0146	Group B7	Day 1	1 hour	Yes	F3	uncoated	28-JUN-2018	12:18	0	
B0146	Group B7	Day 1	2 hour	Yes	F1	uncoated	28-JUN-2018	13:18	0	
B0146	Group B7	Day 1	2 hour	Yes	F2	uncoated	28-JUN-2018	13:18	0	
B0146	Group B7	Day 1	2 hour	Yes	F3	uncoated	28-JUN-2018	13:18	0	
B0146	Group B7	Day 2		Yes	F1	uncoated	29-JUN-2018	11:21	0	
B0146	Group B7	Day 2		Yes	F2	uncoated	29-JUN-2018	11:21	0	
B0146	Group B7	Day 2		Yes	F3	uncoated	29-JUN-2018	11:21	0	

Scale: 0= No Pain; 10= Worst Pain Possible
Administration Site Key: FA = Forearm; UA= Upper arm
Treatments Part A: A1 = A/Sing MAP, 15 ug (FA); A2 = Afluria Quad IM (UA), 15 ug; A3 = Placebo MAP (FA); A4 = A/Sing Mono IM, 15 ug (UA)
Treatments Part B: B1 = A/Sing MAP, 15 ug (FA) with biopsy; B2 = Placebo MAP (FA) with biopsy
B3 = A/Sing MAP, 15 ug (FA); B4 = A/Sing MAP, 10 ug (FA); B5 = A/Sing MAP, 5 ug (FA); B6 = A/Sing MAP, 2.5 ug (FA)
B7 = Placebo MAP (FA); B8 = A/Sing MAP, 15 ug (UA); B9 = A/Sing IM, 15 ug, as 2018 Afluria Quad (UA)

Listing 16.2.9.3
Numeric Pain Intensity per Site

Part B

Randomisation Number	Group	Visit	Time Point	Was Pain Assessment Performed?	Site Number	Treatment	Date of assessment	Time of Assessment	Score	Unscheduled Reason
B0146	Group B7	Day 4		Yes	F1	uncoated	01-JUL-2018	08:17	0	
B0146	Group B7	Day 4		Yes	F2	uncoated	01-JUL-2018	08:17	0	
B0146	Group B7	Day 4		Yes	F3	uncoated	01-JUL-2018	08:17	0	
B0146	Group B7	Day 8		Yes	F1	uncoated	05-JUL-2018	08:44	0	
B0146	Group B7	Day 8		Yes	F2	uncoated	05-JUL-2018	08:44	0	
B0146	Group B7	Day 8		Yes	F3	uncoated	05-JUL-2018	08:44	0	
B0146	Group B7	Day 22		Yes	F1	uncoated	19-JUL-2018	08:14	0	
B0146	Group B7	Day 22		Yes	F2	uncoated	19-JUL-2018	08:14	0	
B0146	Group B7	Day 22		Yes	F3	uncoated	19-JUL-2018	08:14	0	
B0146	Group B7	EOS/ET (Day 61)		Yes	F1	uncoated	28-AUG-2018	12:48	0	
B0146	Group B7	EOS/ET (Day 61)		Yes	F2	uncoated	28-AUG-2018	12:48	0	
B0146	Group B7	EOS/ET (Day 61)		Yes	F3	uncoated	28-AUG-2018	12:48	0	
B0147	Group B9	Day 1	1 min	Yes	D1	[IM]	28-JUN-2018	11:01	1	
B0147	Group B9	Day 1	10 min	Yes	D1	[IM]	28-JUN-2018	11:10	1	
B0147	Group B9	Day 1	1 hour	Yes	D1	[IM]	28-JUN-2018	12:03		
B0147	Group B9	Day 1	2 hour	Yes	D1	[IM]	28-JUN-2018	13:05	0	
B0147	Group B9	Day 2		Yes	D1	[IM]	29-JUN-2018	11:21	0	
B0147	Group B9	Day 4		Yes	D1	[IM]	01-JUL-2018	11:54	0	
B0147	Group B9	Day 8		Yes	D1	[IM]	05-JUL-2018	11:58	0	
B0147	Group B9	Day 22		Yes	D1	[IM]	19-JUL-2018	12:59	0	
B0147	Group B9	EOS/ET (Day 61)		Yes	D1	[IM]	27-AUG-2018	11:44	0	

Scale: 0= No Pain; 10= Worst Pain Possible
Administration Site Key: FA = Forearm; UA= Upper arm
Treatments Part A: A1 = A/Sing MAP, 15 ug (FA); A2 = Afluria Quad IM (UA), 15 ug; A3 = Placebo MAP (FA); A4 = A/Sing Mono IM, 15 ug (UA)
Treatments Part B: B1 = A/Sing MAP, 15 ug (FA) with biopsy; B2 = Placebo MAP (FA) with biopsy
B3 = A/Sing MAP, 15 ug (FA); B4 = A/Sing MAP, 10 ug (FA); B5 = A/Sing MAP, 5 ug (FA); B6 = A/Sing MAP, 2.5 ug (FA)
B7 = Placebo MAP (FA); B8 = A/Sing MAP, 15 ug (UA); B9 = A/Sing IM, 15 ug, as 2018 Afluria Quad (UA)

Listing 16.2.9.3
Numeric Pain Intensity per Site

Part B

Randomisation Number	Group	Visit	Time Point	Was Pain Assessment Performed?	Site Number	Treatment	Date of assessment	Time of Assessment	Score	Unscheduled Reason
B0148	Group B5	Day 1	1 min	Yes	F1	5 ug	28-JUN-2018	11:21	0	
B0148	Group B5	Day 1	1 min	Yes	F2	uncoated	28-JUN-2018	11:24	0	
B0148	Group B5	Day 1	1 min	Yes	F3	uncoated	28-JUN-2018	11:27	0	
B0148	Group B5	Day 1	10 min	Yes	F1	5 ug	28-JUN-2018	11:38	0	
B0148	Group B5	Day 1	10 min	Yes	F2	uncoated	28-JUN-2018	11:38	0	
B0148	Group B5	Day 1	10 min	Yes	F3	uncoated	28-JUN-2018	11:38	0	
B0148	Group B5	Day 1	1 hour	Yes	F1	5 ug	28-JUN-2018	12:28	0	
B0148	Group B5	Day 1	1 hour	Yes	F2	uncoated	28-JUN-2018	12:28	0	
B0148	Group B5	Day 1	1 hour	Yes	F3	uncoated	28-JUN-2018	12:28	0	
B0148	Group B5	Day 1	2 hour	Yes	F1	5 ug	28-JUN-2018	13:38	0	
B0148	Group B5	Day 1	2 hour	Yes	F2	uncoated	28-JUN-2018	13:38	0	
B0148	Group B5	Day 1	2 hour	Yes	F3	uncoated	28-JUN-2018	13:38	0	
B0148	Group B5	Day 2		Yes	F1	5 ug	29-JUN-2018	09:50	0	
B0148	Group B5	Day 2		Yes	F2	uncoated	29-JUN-2018	09:50	0	
B0148	Group B5	Day 2		Yes	F3	uncoated	29-JUN-2018	09:50	0	
B0148	Group B5	Day 4		Yes	F1	5 ug	01-JUL-2018	09:52	0	
B0148	Group B5	Day 4		Yes	F2	uncoated	01-JUL-2018	09:52	0	
B0148	Group B5	Day 4		Yes	F3	uncoated	01-JUL-2018	09:52	0	
B0148	Group B5	Day 8		Yes	F1	5 ug	05-JUL-2018	09:39	0	
B0148	Group B5	Day 8		Yes	F2	uncoated	05-JUL-2018	09:39	0	
B0148	Group B5	Day 8		Yes	F3	uncoated	05-JUL-2018	09:39	0	

Scale: 0= No Pain; 10= Worst Pain Possible
Administration Site Key: FA = Forearm; UA= Upper arm
Treatments Part A: A1 = A/Sing MAP, 15 ug (FA); A2 = Afluria Quad IM (UA), 15 ug; A3 = Placebo MAP (FA); A4 = A/Sing Mono IM, 15 ug (UA)
Treatments Part B: B1 = A/Sing MAP, 15 ug (FA) with biopsy; B2 = Placebo MAP (FA) with biopsy
B3 = A/Sing MAP, 15 ug (FA); B4 = A/Sing MAP, 10 ug (FA); B5 = A/Sing MAP, 5 ug (FA); B6 = A/Sing MAP, 2.5 ug (FA)
B7 = Placebo MAP (FA); B8 = A/Sing MAP, 15 ug (UA); B9 = A/Sing IM, 15 ug, as 2018 Afluria Quad (UA)

Listing 16.2.9.3
Numeric Pain Intensity per Site

Part B

Randomisation Number	Group	Visit	Time Point	Was Pain Assessment Performed?	Site Number	Treatment	Date of assessment	Time of Assessment	Score	Unscheduled Reason
B0148	Group B5	Day 22		Yes	F1	5 ug	18-JUL-2018	10:18	0	
B0148	Group B5	Day 22		Yes	F2	uncoated	18-JUL-2018	10:18	0	
B0148	Group B5	Day 22		Yes	F3	uncoated	18-JUL-2018	10:18	0	
B0148	Group B5	EOS/ET (Day 61)		Yes	F1	5 ug	27-AUG-2018	11:20	0	
B0148	Group B5	EOS/ET (Day 61)		Yes	F2	uncoated	27-AUG-2018	11:20	0	
B0148	Group B5	EOS/ET (Day 61)		Yes	F3	uncoated	27-AUG-2018	11:20	0	
B0149	Group B8	Day 1	1 min	Yes	D1	5 ug	28-JUN-2018	11:31	0	
B0149	Group B8	Day 1	1 min	Yes	D2	5 ug	28-JUN-2018	11:34	0	
B0149	Group B8	Day 1	1 min	Yes	D3	5 ug	28-JUN-2018	11:37	0	
B0149	Group B8	Day 1	10 min	Yes	D1	5 ug	28-JUN-2018	11:48	0	
B0149	Group B8	Day 1	10 min	Yes	D2	5 ug	28-JUN-2018	11:48	0	
B0149	Group B8	Day 1	10 min	Yes	D3	5 ug	28-JUN-2018	11:48	0	
B0149	Group B8	Day 1	1 hour	Yes	D1	5 ug	28-JUN-2018	12:38	0	
B0149	Group B8	Day 1	1 hour	Yes	D2	5 ug	28-JUN-2018	12:38	0	
B0149	Group B8	Day 1	1 hour	Yes	D3	5 ug	28-JUN-2018	12:38	0	
B0149	Group B8	Day 1	2 hour	Yes	D1	5 ug	28-JUN-2018	13:39	0	
B0149	Group B8	Day 1	2 hour	Yes	D2	5 ug	28-JUN-2018	13:39	0	
B0149	Group B8	Day 1	2 hour	Yes	D3	5 ug	28-JUN-2018	13:39	0	
B0149	Group B8	Day 2		Yes	D1	5 ug	29-JUN-2018	08:57	0	
B0149	Group B8	Day 2		Yes	D2	5 ug	29-JUN-2018	08:57	0	
B0149	Group B8	Day 2		Yes	D3	5 ug	29-JUN-2018	08:57	0	

Scale: 0= No Pain; 10= Worst Pain Possible
Administration Site Key: FA = Forearm; UA= Upper arm
Treatments Part A: A1 = A/Sing MAP, 15 ug (FA); A2 = Afluria Quad IM (UA), 15 ug; A3 = Placebo MAP (FA); A4 = A/Sing Mono IM, 15 ug (UA)
Treatments Part B: B1 = A/Sing MAP, 15 ug (FA) with biopsy; B2 = Placebo MAP (FA) with biopsy
B3 = A/Sing MAP, 15 ug (FA); B4 = A/Sing MAP, 10 ug (FA); B5 = A/Sing MAP, 5 ug (FA); B6 = A/Sing MAP, 2.5 ug (FA)
B7 = Placebo MAP (FA); B8 = A/Sing MAP, 15 ug (UA); B9 = A/Sing IM, 15 ug, as 2018 Afluria Quad (UA)

Listing 16.2.9.3
Numeric Pain Intensity per Site

Part B

Randomisation Number	Group	Visit	Time Point	Was Pain Assessment Performed?	Site Number	Treatment	Date of assessment	Time of Assessment	Score	Unscheduled Reason
B0149	Group B8	Day 4		Yes	D1	5 ug	01-JUL-2018	10:30	0	
B0149	Group B8	Day 4		Yes	D2	5 ug	01-JUL-2018	10:30	0	
B0149	Group B8	Day 4		Yes	D3	5 ug	01-JUL-2018	10:30	0	
B0149	Group B8	Day 8		Yes	D1	5 ug	05-JUL-2018	09:36	0	
B0149	Group B8	Day 8		Yes	D2	5 ug	05-JUL-2018	09:36	0	
B0149	Group B8	Day 8		Yes	D3	5 ug	05-JUL-2018	09:36	0	
B0149	Group B8	Day 22		Yes	D1	5 ug	19-JUL-2018	11:44	0	
B0149	Group B8	Day 22		Yes	D2	5 ug	19-JUL-2018	11:44	0	
B0149	Group B8	Day 22		Yes	D3	5 ug	19-JUL-2018	11:44	0	
B0149	Group B8	EOS/ET (Day 61)		Yes	D1	5 ug	27-AUG-2018	12:04	0	
B0149	Group B8	EOS/ET (Day 61)		Yes	D2	5 ug	27-AUG-2018	12:04	0	
B0149	Group B8	EOS/ET (Day 61)		Yes	D3	5 ug	27-AUG-2018	12:04	0	
B0150	Group B4	Day 1	1 min	Yes	F1	5 ug	28-JUN-2018	11:41	1	
B0150	Group B4	Day 1	1 min	Yes	F2	5 ug	28-JUN-2018	11:44	3	
B0150	Group B4	Day 1	1 min	Yes	F3	uncoated	28-JUN-2018	11:47	3	
B0150	Group B4	Day 1	10 min	Yes	F1	5 ug	28-JUN-2018	11:58	1	
B0150	Group B4	Day 1	10 min	Yes	F2	5 ug	28-JUN-2018	11:58	1	
B0150	Group B4	Day 1	10 min	Yes	F3	uncoated	28-JUN-2018	11:58	1	
B0150	Group B4	Day 1	1 hour	Yes	F1	5 ug	28-JUN-2018	12:48	0	
B0150	Group B4	Day 1	1 hour	Yes	F2	5 ug	28-JUN-2018	12:48	0	
B0150	Group B4	Day 1	1 hour	Yes	F3	uncoated	28-JUN-2018	12:48	0	

Scale: 0= No Pain; 10= Worst Pain Possible
Administration Site Key: FA = Forearm; UA= Upper arm
Treatments Part A: A1 = A/Sing MAP, 15 ug (FA); A2 = Afluria Quad IM (UA), 15 ug; A3 = Placebo MAP (FA); A4 = A/Sing Mono IM, 15 ug (UA)
Treatments Part B: B1 = A/Sing MAP, 15 ug (FA) with biopsy; B2 = Placebo MAP (FA) with biopsy
B3 = A/Sing MAP, 15 ug (FA); B4 = A/Sing MAP, 10 ug (FA); B5 = A/Sing MAP, 5 ug (FA); B6 = A/Sing MAP, 2.5 ug (FA)
B7 = Placebo MAP (FA); B8 = A/Sing MAP, 15 ug (UA); B9 = A/Sing IM, 15 ug, as 2018 Afluria Quad (UA)

Listing 16.2.9.3
Numeric Pain Intensity per Site

Part B

Randomisation Number	Group	Visit	Time Point	Was Pain Assessment Performed?	Site Number	Treatment	Date of assessment	Time of Assessment	Score	Unscheduled Reason
B0150	Group B4	Day 1	2 hour	Yes	F1	5 ug	28-JUN-2018	13:50	0	
B0150	Group B4	Day 1	2 hour	Yes	F2	5 ug	28-JUN-2018	13:50	0	
B0150	Group B4	Day 1	2 hour	Yes	F3	uncoated	28-JUN-2018	13:50	0	
B0150	Group B4	Day 2		Yes	F1	5 ug	29-JUN-2018	10:20	0	
B0150	Group B4	Day 2		Yes	F2	5 ug	29-JUN-2018	10:20	0	
B0150	Group B4	Day 2		Yes	F3	uncoated	29-JUN-2018	10:20	0	
B0150	Group B4	Day 4		Yes	F1	5 ug	01-JUL-2018	09:21	0	
B0150	Group B4	Day 4		Yes	F2	5 ug	01-JUL-2018	09:21	0	
B0150	Group B4	Day 4		Yes	F3	uncoated	01-JUL-2018	09:21	0	
B0150	Group B4	Day 8		Yes	F1	5 ug	05-JUL-2018	14:39	0	
B0150	Group B4	Day 8		Yes	F2	5 ug	05-JUL-2018	14:39	0	
B0150	Group B4	Day 8		Yes	F3	uncoated	05-JUL-2018	14:39	0	
B0150	Group B4	Day 22		Yes	F1	5 ug	19-JUL-2018	15:15	0	
B0150	Group B4	Day 22		Yes	F2	5 ug	19-JUL-2018	15:15	0	
B0150	Group B4	Day 22		Yes	F3	uncoated	19-JUL-2018	15:15	0	
B0150	Group B4	EOS/ET (Day 61)		Yes	F1	5 ug	28-AUG-2018	13:33	0	
B0150	Group B4	EOS/ET (Day 61)		Yes	F2	5 ug	28-AUG-2018	13:33	0	
B0150	Group B4	EOS/ET (Day 61)		Yes	F3	uncoated	28-AUG-2018	13:33	0	
B1019	Group B8	Day 1	1 min	Yes	D1	5 ug	19-MAY-2018	11:01	1	
B1019	Group B8	Day 1	1 min	Yes	D2	5 ug	19-MAY-2018	11:04	0	
B1019	Group B8	Day 1	1 min	Yes	D3	5 ug	19-MAY-2018	11:07	0	

Scale: 0= No Pain; 10= Worst Pain Possible
Administration Site Key: FA = Forearm; UA= Upper arm
Treatments Part A: A1 = A/Sing MAP, 15 ug (FA); A2 = Afluria Quad IM (UA), 15 ug; A3 = Placebo MAP (FA); A4 = A/Sing Mono IM, 15 ug (UA)
Treatments Part B: B1 = A/Sing MAP, 15 ug (FA) with biopsy; B2 = Placebo MAP (FA) with biopsy
B3 = A/Sing MAP, 15 ug (FA); B4 = A/Sing MAP, 10 ug (FA); B5 = A/Sing MAP, 5 ug (FA); B6 = A/Sing MAP, 2.5 ug (FA)
B7 = Placebo MAP (FA); B8 = A/Sing MAP, 15 ug (UA); B9 = A/Sing IM, 15 ug, as 2018 Afluria Quad (UA)

Listing 16.2.9.3
Numeric Pain Intensity per Site

Part B

Randomisation Number	Group	Visit	Time Point	Was Pain Assessment Performed?	Site Number	Treatment	Date of assessment	Time of Assessment	Score	Unscheduled Reason
B1019	Group B8	Day 1	10 min	Yes	D1	5 ug	19-MAY-2018	11:18	1	
B1019	Group B8	Day 1	10 min	Yes	D2	5 ug	19-MAY-2018	11:18	1	
B1019	Group B8	Day 1	10 min	Yes	D3	5 ug	19-MAY-2018	11:18	0	
B1019	Group B8	Day 1	1 hour	Yes	D1	5 ug	19-MAY-2018	12:08	0	
B1019	Group B8	Day 1	1 hour	Yes	D2	5 ug	19-MAY-2018	12:09	0	
B1019	Group B8	Day 1	1 hour	Yes	D3	5 ug	19-MAY-2018	12:09	0	
B1019	Group B8	Day 1	2 hour	Yes	D1	5 ug	19-MAY-2018	13:22	0	
B1019	Group B8	Day 1	2 hour	Yes	D2	5 ug	19-MAY-2018	13:22	0	
B1019	Group B8	Day 1	2 hour	Yes	D3	5 ug	19-MAY-2018	13:22	0	
B1019	Group B8	Day 2		Yes	D1	5 ug	20-MAY-2018	13:43	0	
B1019	Group B8	Day 2		Yes	D2	5 ug	20-MAY-2018	13:43	0	
B1019	Group B8	Day 2		Yes	D3	5 ug	20-MAY-2018	13:43	0	
B1019	Group B8	Day 4		Yes	D1	5 ug	22-MAY-2018	12:05	0	
B1019	Group B8	Day 4		Yes	D2	5 ug	22-MAY-2018	12:05	0	
B1019	Group B8	Day 4		Yes	D3	5 ug	22-MAY-2018	12:05	0	
B1019	Group B8	Day 8		Yes	D1	5 ug	28-MAY-2018	14:08	0	
B1019	Group B8	Day 8		Yes	D2	5 ug	28-MAY-2018	14:08	0	
B1019	Group B8	Day 8		Yes	D3	5 ug	28-MAY-2018	14:08	0	
B1019	Group B8	Day 22		Yes	D1	5 ug	13-JUN-2018	14:46	0	
B1019	Group B8	Day 22		Yes	D2	5 ug	13-JUN-2018	14:46	0	
B1019	Group B8	Day 22		Yes	D3	5 ug	13-JUN-2018	14:46	0	

Scale: 0= No Pain; 10= Worst Pain Possible
Administration Site Key: FA = Forearm; UA= Upper arm
Treatments Part A: A1 = A/Sing MAP, 15 ug (FA); A2 = Afluria Quad IM (UA), 15 ug; A3 = Placebo MAP (FA); A4 = A/Sing Mono IM, 15 ug (UA)
Treatments Part B: B1 = A/Sing MAP, 15 ug (FA) with biopsy; B2 = Placebo MAP (FA) with biopsy
B3 = A/Sing MAP, 15 ug (FA); B4 = A/Sing MAP, 10 ug (FA); B5 = A/Sing MAP, 5 ug (FA); B6 = A/Sing MAP, 2.5 ug (FA)
B7 = Placebo MAP (FA); B8 = A/Sing MAP, 15 ug (UA); B9 = A/Sing IM, 15 ug, as 2018 Afluria Quad (UA)

Listing 16.2.9.3
Numeric Pain Intensity per Site

Part B

Randomisation Number	Group	Visit	Time Point	Was Pain Assessment Performed?	Site Number	Treatment	Date of assessment	Time of Assessment	Score	Unscheduled Reason
B1019	Group B8	EOS/ET (Day 61)		Yes	D1	5 ug	20-JUL-2018	13:49	0	
B1019	Group B8	EOS/ET (Day 61)		Yes	D2	5 ug	20-JUL-2018	13:49	0	
B1019	Group B8	EOS/ET (Day 61)		Yes	D3	5 ug	20-JUL-2018	13:49	0	
B1076	Group B5	Day 1	1 min	Yes	F1	5 ug	16-JUN-2018	11:31	2	
B1076	Group B5	Day 1	1 min	Yes	F2	uncoated	16-JUN-2018	11:34	1	
B1076	Group B5	Day 1	1 min	Yes	F3	uncoated	16-JUN-2018	11:37	1	
B1076	Group B5	Day 1	10 min	Yes	F1	5 ug	16-JUN-2018	11:40	0	
B1076	Group B5	Day 1	10 min	Yes	F2	uncoated	16-JUN-2018	11:40	0	
B1076	Group B5	Day 1	10 min	No	F3	uncoated				
B1076	Group B5	Day 1	1 hour	Yes	F1	5 ug	16-JUN-2018	12:33	0	
B1076	Group B5	Day 1	1 hour	Yes	F2	uncoated	16-JUN-2018	12:33	0	
B1076	Group B5	Day 1	1 hour	Yes	F3	uncoated	16-JUN-2018	12:33	0	
B1076	Group B5	Day 1	2 hour	Yes	F1	5 ug	16-JUN-2018	13:38	0	
B1076	Group B5	Day 1	2 hour	Yes	F2	uncoated	16-JUN-2018	13:38	0	
B1076	Group B5	Day 1	2 hour	Yes	F3	uncoated	16-JUN-2018	13:38	0	
B1076	Group B5	Day 2		Yes	F1	5 ug	17-JUN-2018	11:34	0	
B1076	Group B5	Day 2		Yes	F2	uncoated	17-JUN-2018	11:34	0	
B1076	Group B5	Day 2		Yes	F3	uncoated	17-JUN-2018	11:35	0	
B1076	Group B5	Day 4		No	F1	5 ug				
B1076	Group B5	Day 4		Yes	F2	uncoated	19-JUN-2018	12:38	0	
B1076	Group B5	Day 4		Yes	F3	uncoated	19-JUN-2018	12:38	0	

Scale: 0= No Pain; 10= Worst Pain Possible
Administration Site Key: FA = Forearm; UA= Upper arm
Treatments Part A: A1 = A/Sing MAP, 15 ug (FA); A2 = Afluria Quad IM (UA), 15 ug; A3 = Placebo MAP (FA); A4 = A/Sing Mono IM, 15 ug (UA)
Treatments Part B: B1 = A/Sing MAP, 15 ug (FA) with biopsy; B2 = Placebo MAP (FA) with biopsy
B3 = A/Sing MAP, 15 ug (FA); B4 = A/Sing MAP, 10 ug (FA); B5 = A/Sing MAP, 5 ug (FA); B6 = A/Sing MAP, 2.5 ug (FA)
B7 = Placebo MAP (FA); B8 = A/Sing MAP, 15 ug (UA); B9 = A/Sing IM, 15 ug, as 2018 Afluria Quad (UA)

Listing 16.2.9.3
Numeric Pain Intensity per Site

Part B

Randomisation Number	Group	Visit	Time Point	Was Pain Assessment Performed?	Site Number	Treatment	Date of assessment	Time of Assessment	Score	Unscheduled Reason
B1076	Group B5	Day 8		Yes	F1	5 ug	23-JUN-2018	10:45	0	
B1076	Group B5	Day 8		Yes	F2	uncoated	23-JUN-2018	10:45	0	
B1076	Group B5	Day 8		Yes	F3	uncoated	23-JUN-2018	10:45	0	
B1076	Group B5	Day 22		Yes	F1	5 ug	07-JUL-2018	10:22	0	
B1076	Group B5	Day 22		Yes	F2	uncoated	07-JUL-2018	10:22	0	
B1076	Group B5	Day 22		Yes	F3	uncoated	07-JUL-2018	10:22	0	
B1076	Group B5	EOS/ET (Day 61)		Yes	F1	5 ug	15-AUG-2018	13:42	0	
B1076	Group B5	EOS/ET (Day 61)		Yes	F2	uncoated	15-AUG-2018	13:42	0	
B1076	Group B5	EOS/ET (Day 61)		Yes	F3	uncoated	15-AUG-2018	13:42	0	
B1080	Group B7	Day 1	1 min	Yes	F1	uncoated	16-JUN-2018	11:41	0	
B1080	Group B7	Day 1	1 min	Yes	F2	uncoated	16-JUN-2018	11:44	0	
B1080	Group B7	Day 1	1 min	Yes	F3	uncoated	16-JUN-2018	11:47	0	
B1080	Group B7	Day 1	10 min	Yes	F1	uncoated	16-JUN-2018	11:58	0	
B1080	Group B7	Day 1	10 min	Yes	F2	uncoated	16-JUN-2018	11:58	0	
B1080	Group B7	Day 1	10 min	Yes	F3	uncoated	16-JUN-2018	11:58	0	
B1080	Group B7	Day 1	1 hour	Yes	F1	uncoated	16-JUN-2018	12:49	0	
B1080	Group B7	Day 1	1 hour	Yes	F2	uncoated	16-JUN-2018	12:49	0	
B1080	Group B7	Day 1	1 hour	Yes	F3	uncoated	16-JUN-2018	12:49	0	
B1080	Group B7	Day 1	2 hour	Yes	F1	uncoated	16-JUN-2018	13:48	0	
B1080	Group B7	Day 1	2 hour	Yes	F2	uncoated	16-JUN-2018	13:48	0	
B1080	Group B7	Day 1	2 hour	Yes	F3	uncoated	16-JUN-2018	13:48	0	

Scale: 0= No Pain; 10= Worst Pain Possible
Administration Site Key: FA = Forearm; UA= Upper arm
Treatments Part A: A1 = A/Sing MAP, 15 ug (FA); A2 = Afluria Quad IM (UA), 15 ug; A3 = Placebo MAP (FA); A4 = A/Sing Mono IM, 15 ug (UA)
Treatments Part B: B1 = A/Sing MAP, 15 ug (FA) with biopsy; B2 = Placebo MAP (FA) with biopsy
B3 = A/Sing MAP, 15 ug (FA); B4 = A/Sing MAP, 10 ug (FA); B5 = A/Sing MAP, 5 ug (FA); B6 = A/Sing MAP, 2.5 ug (FA)
B7 = Placebo MAP (FA); B8 = A/Sing MAP, 15 ug (UA); B9 = A/Sing IM, 15 ug, as 2018 Afluria Quad (UA)

Listing 16.2.9.3
Numeric Pain Intensity per Site

Part B

Randomisation Number	Group	Visit	Time Point	Was Pain Assessment Performed?	Site Number	Treatment	Date of assessment	Time of Assessment	Score	Unscheduled Reason
B1080	Group B7	Day 2		Yes	F1	uncoated	17-JUN-2018	13:34	0	
B1080	Group B7	Day 2		Yes	F2	uncoated	17-JUN-2018	13:34	0	
B1080	Group B7	Day 2		Yes	F3	uncoated	17-JUN-2018	13:34	0	
B1080	Group B7	Day 4		Yes	F1	uncoated	19-JUN-2018	12:30	0	
B1080	Group B7	Day 4		Yes	F2	uncoated	19-JUN-2018	12:30	0	
B1080	Group B7	Day 4		Yes	F3	uncoated	19-JUN-2018	12:30	0	
B1080	Group B7	Day 8		Yes	F1	uncoated	23-JUN-2018	12:58	0	
B1080	Group B7	Day 8		Yes	F2	uncoated	23-JUN-2018	12:58	0	
B1080	Group B7	Day 8		Yes	F3	uncoated	23-JUN-2018	12:58	0	
B1080	Group B7	Day 22		Yes	F1	uncoated	07-JUL-2018	13:12	0	
B1080	Group B7	Day 22		Yes	F2	uncoated	07-JUL-2018	13:12	0	
B1080	Group B7	Day 22		Yes	F3	uncoated	07-JUL-2018	13:12	0	
B1080	Group B7	EOS/ET (Day 61)		Yes	F1	uncoated	15-AUG-2018	09:37	0	
B1080	Group B7	EOS/ET (Day 61)		Yes	F2	uncoated	15-AUG-2018	09:37	0	
B1080	Group B7	EOS/ET (Day 61)		Yes	F3	uncoated	15-AUG-2018	09:37	0	

Scale: 0= No Pain; 10= Worst Pain Possible
Administration Site Key: FA = Forearm; UA= Upper arm
Treatments Part A: A1 = A/Sing MAP, 15 ug (FA); A2 = Afluria Quad IM (UA), 15 ug; A3 = Placebo MAP (FA); A4 = A/Sing Mono IM, 15 ug (UA)
Treatments Part B: B1 = A/Sing MAP, 15 ug (FA) with biopsy; B2 = Placebo MAP (FA) with biopsy
B3 = A/Sing MAP, 15 ug (FA); B4 = A/Sing MAP, 10 ug (FA); B5 = A/Sing MAP, 5 ug (FA); B6 = A/Sing MAP, 2.5 ug (FA)
B7 = Placebo MAP (FA); B8 = A/Sing MAP, 15 ug (UA); B9 = A/Sing IM, 15 ug, as 2018 Afluria Quad (UA)
