# Supplementary material for: Safety, tolerability, and immunogenicity of influenza vaccination with a high-density microarray patch: Results from a randomized, controlled phase I clinical trial
Source: PLoS Med. 2020 Mar 17;17(3):e1003024. doi: 10.1371/journal.pmed.1003024 (PMC7077342; doi:10.1371/journal.pmed.1003024)
Supplement: S1 Data — Data 1: Informed Consent; Data 2: Analysis Sets; Data 3: Demographics; Data 4: Study Drug Administration; Data 5: Skin Hardness Assessment; Data 6: Immunogenicity (ELISA IgG); Data 7: Immunogenicity (ADCC); Data 8: Immunogenicity (MBCs); Data 9: Immunogenicity (CMI); Data 10: Immunogenicity (Mucosal IgA); Data 11: Immunogenicity Results (HAI, MN); Data 12: TEAE–CRF Data only; Data 13: TEAEs–MedDRA Coding; Data 15: Treatment Site Tolerability Assessment; Data 16: Numeric Pain Intensity; Data 17: SII; Data 18: Application Site Status at End of Study; Data 19: Individual Treatment Site Tolerability Assessment; Data 20: Numeric Pain Intensity by Treatment; Data 21: Individual SII by Treatment. ADCC, antibody-dependent cellular cytotoxicity; CMI, cell-mediated immunity; CRF, case report form; ELISA, enzyme-linked immunosorbent assay; HAI, haemagglutination inhibition; IgA, immunoglobulin A; IgG, immunoglobulin G; MBC, memory B cell; MedDRA, medical dictionary for regulatory activities; MN, microneutralisation; SII, Skin Irritation Index; TEA, treatment emergent adverse event. (ZIP) [file pmed.1003024.s008.zip › S1_Data/clinical supporting/S1_Data18 Application Site Status at End of Study.pdf]

Listing 16.2.9.6
Application Site Local Reaction Status at End of Study

Part A

Randomisation Number	Group	Visit	Is Any Application Site Still Visible?	Visible Sites	Treatment for Site 1	Treatment for Site 2	Treatment for Site 3
A0001	Group A3	EOS/ET (Day 61)	No		uncoated	uncoated	uncoated
A0002	Group A1	EOS/ET (Day 61)	No		5 ug	5 ug	5 ug
A0003	Group A1	EOS/ET (Day 61)	No		5 ug	5 ug	5 ug
A0004	Group A3	EOS/ET (Day 61)	No		uncoated	uncoated	uncoated
A0005	Group A1	EOS/ET (Day 61)	No		5 ug	5 ug	5 ug
A0006	Group A3	EOS/ET (Day 61)	Yes	F1,F2,F3	uncoated	uncoated	uncoated
A0007	Group A1	EOS/ET (Day 61)	No		5 ug	5 ug	5 ug
A0008	Group A3	EOS/ET (Day 61)	No		uncoated	uncoated	uncoated
A0009	Group A1	EOS/ET (Day 61)	Yes	F1,F2,F3	5 ug	5 ug	5 ug
A0010	Group A3	EOS/ET (Day 61)	No		uncoated	uncoated	uncoated
A0011	Group A3	EOS/ET (Day 61)	No		uncoated	uncoated	uncoated
A0012	Group A1	EOS/ET (Day 61)	No		5 ug	5 ug	5 ug
A0013	Group A1	EOS/ET (Day 61)	Yes	F1,F2,F3	5 ug	5 ug	5 ug
A0014	Group A3	EOS/ET (Day 61)	No		uncoated	uncoated	uncoated
A0015	Group A1	EOS/ET (Day 61)	Yes	F1,F2,F3	5 ug	5 ug	5 ug
A0016	Group A3	EOS/ET (Day 61)	No		uncoated	uncoated	uncoated
A0017	Group A3	EOS/ET (Day 61)	No		uncoated	uncoated	uncoated
A0018	Group A1	EOS/ET (Day 61)	No		5 ug	5 ug	5 ug
A0019	Group A3	EOS/ET (Day 61)	No		uncoated	uncoated	uncoated

Administration Site Key: FA = Forearm; UA= Upper arm
Treatments Part A: A1 = A/Sing MAP, 15 ug (FA); A2 = Afluria Quad IM (UA), 15 ug; A3 = Placebo MAP (FA); A4 = A/Sing Mono IM, 15 ug (UA)
Treatments Part B: B1 = A/Sing MAP, 15 ug (FA) with biopsy; B2 = Placebo MAP (FA) with biopsy
B3 = A/Sing MAP, 15 ug (FA); B4 = A/Sing MAP, 10 ug (FA); B5 = A/Sing MAP, 5 ug (FA); B6 = A/Sing MAP, 2.5 ug (FA)
B7 = Placebo MAP (FA); B8 = A/Sing MAP, 15 ug (UA); B9 = A/Sing IM, 15 ug, as 2018 Afluria Quad (UA)

Listing 16.2.9.6
Application Site Local Reaction Status at End of Study

Part A

Randomisation Number	Group	Visit	Is Any Application Site Still Visible?	Visible Sites	Treatment for Site 1	Treatment for Site 2	Treatment for Site 3
A0020	Group A1	EOS/ET (Day 61)	No		5 ug	5 ug	5 ug
A0021	Group A1	EOS/ET (Day 61)	No		5 ug	5 ug	5 ug
A0022	Group A3	EOS/ET (Day 61)	No		uncoated	uncoated	uncoated
A0023	Group A3	EOS/ET (Day 61)	No		uncoated	uncoated	uncoated
A0024	Group A1	EOS/ET (Day 61)	No		5 ug	5 ug	5 ug
A0025	Group A3	EOS/ET (Day 61)	No		uncoated	uncoated	uncoated
A0027	Group A3	EOS/ET (Day 61)	No		uncoated	uncoated	uncoated
A0028	Group A1	EOS/ET (Day 61)	No		5 ug	5 ug	5 ug
A0029	Group A1	EOS/ET (Day 61)	Yes	F1,F2,F3	5 ug	5 ug	5 ug
A0030	Group A3	EOS/ET (Day 61)	Yes	F1,F2,F3	uncoated	uncoated	uncoated
A0032	Group A4	EOS/ET (Day 61)	No		[IM]		
A0033	Group A2	EOS/ET (Day 61)	No		[IM]		
A0034	Group A4	EOS/ET (Day 61)	No		[IM]		
A0035	Group A4	EOS/ET (Day 61)	No		[IM]		
A0036	Group A2	EOS/ET (Day 61)	No		[IM]		
A0037	Group A4	EOS/ET (Day 61)	No		[IM]		
A0038	Group A2	EOS/ET (Day 61)	No		[IM]		
A0039	Group A2	EOS/ET (Day 61)	No		[IM]		
A0040	Group A4	EOS/ET (Day 61)	No		[IM]		

Administration Site Key: FA = Forearm; UA= Upper arm
Treatments Part A: A1 = A/Sing MAP, 15 ug (FA); A2 = Afluria Quad IM (UA), 15 ug; A3 = Placebo MAP (FA); A4 = A/Sing Mono IM, 15 ug (UA)
Treatments Part B: B1 = A/Sing MAP, 15 ug (FA) with biopsy; B2 = Placebo MAP (FA) with biopsy
B3 = A/Sing MAP, 15 ug (FA); B4 = A/Sing MAP, 10 ug (FA); B5 = A/Sing MAP, 5 ug (FA); B6 = A/Sing MAP, 2.5 ug (FA)
B7 = Placebo MAP (FA); B8 = A/Sing MAP, 15 ug (UA); B9 = A/Sing IM, 15 ug, as 2018 Afluria Quad (UA)

Listing 16.2.9.6
Application Site Local Reaction Status at End of Study

Part A

Randomisation Number	Group	Visit	Is Any Application Site Still Visible?	Visible Sites	Treatment for Site 1	Treatment for Site 2	Treatment for Site 3
A0041	Group A4	EOS/ET (Day 61)	No		[IM]		
A0042	Group A2	EOS/ET (Day 61)	No		[IM]		
A0043	Group A2	EOS/ET (Day 61)	No		[IM]		
A0044	Group A4	EOS/ET (Day 61)	No		[IM]		
A0045	Group A2	EOS/ET (Day 61)	No		[IM]		
A0046	Group A4	EOS/ET (Day 61)	No		[IM]		
A0047	Group A2	EOS/ET (Day 61)	No		[IM]		
A0048	Group A4	EOS/ET (Day 61)	No		[IM]		
A0049	Group A2	EOS/ET (Day 61)	No		[IM]		
A0050	Group A4	EOS/ET (Day 61)	No		[IM]		
A0051	Group A2	EOS/ET (Day 61)	No		[IM]		
A0052	Group A4	EOS/ET (Day 61)	No		[IM]		
A0053	Group A4	EOS/ET (Day 61)	No		[IM]		
A0054	Group A2	EOS/ET (Day 61)	No		[IM]		
A0055	Group A4	EOS/ET (Day 61)	No		[IM]		
A0056	Group A2	EOS/ET (Day 61)	No		[IM]		
A0057	Group A4	EOS/ET (Day 61)	No		[IM]		
A0058	Group A2	EOS/ET (Day 61)	No		[IM]		
A0059	Group A2	EOS/ET (Day 61)	No		[IM]		

Administration Site Key: FA = Forearm; UA= Upper arm
Treatments Part A: A1 = A/Sing MAP, 15 ug (FA); A2 = Afluria Quad IM (UA), 15 ug; A3 = Placebo MAP (FA); A4 = A/Sing Mono IM, 15 ug (UA)
Treatments Part B: B1 = A/Sing MAP, 15 ug (FA) with biopsy; B2 = Placebo MAP (FA) with biopsy
B3 = A/Sing MAP, 15 ug (FA); B4 = A/Sing MAP, 10 ug (FA); B5 = A/Sing MAP, 5 ug (FA); B6 = A/Sing MAP, 2.5 ug (FA)
B7 = Placebo MAP (FA); B8 = A/Sing MAP, 15 ug (UA); B9 = A/Sing IM, 15 ug, as 2018 Afluria Quad (UA)

Listing 16.2.9.6
Application Site Local Reaction Status at End of Study

Part A

Randomisation Number	Group	Visit	Is Any Application Site Still Visible?	Visible Sites	Treatment for Site 1	Treatment for Site 2	Treatment for Site 3
A0060	Group A4	EOS/ET (Day 61)	No		[IM]		
A1026	Group A1	EOS/ET (Day 61)	No		5 ug	5 ug	5 ug

Administration Site Key: FA = Forearm; UA= Upper arm
Treatments Part A: A1 = A/Sing MAP, 15 ug (FA); A2 = Afluria Quad IM (UA), 15 ug; A3 = Placebo MAP (FA); A4 = A/Sing Mono IM, 15 ug (UA)
Treatments Part B: B1 = A/Sing MAP, 15 ug (FA) with biopsy; B2 = Placebo MAP (FA) with biopsy
B3 = A/Sing MAP, 15 ug (FA); B4 = A/Sing MAP, 10 ug (FA); B5 = A/Sing MAP, 5 ug (FA); B6 = A/Sing MAP, 2.5 ug (FA)
B7 = Placebo MAP (FA); B8 = A/Sing MAP, 15 ug (UA); B9 = A/Sing IM, 15 ug, as 2018 Afluria Quad (UA)

Listing 16.2.9.6
Application Site Local Reaction Status at End of Study

Part B

Randomisation Number	Group	Visit	Is Any Application Site Still Visible?	Visible Sites	Treatment for Site 1	Treatment for Site 2	Treatment for Site 3
B0001	Group B1	EOS/ET (Day 61)	No		5 ug	5 ug	5 ug
B0002	Group B2	EOS/ET (Day 61)	No		uncoated	uncoated	uncoated
B0003	Group B2	EOS/ET (Day 61)	No		uncoated	uncoated	uncoated
B0004	Group B1	EOS/ET (Day 61)	No		5 ug	5 ug	5 ug
B0005	Group B1	EOS/ET (Day 61)	No		5 ug	5 ug	5 ug
B0006	Group B2	EOS/ET (Day 61)	No		uncoated	uncoated	uncoated
B0007	Group B2	EOS/ET (Day 61)	No		uncoated	uncoated	uncoated
B0008	Group B1	EOS/ET (Day 61)	No		5 ug	5 ug	5 ug
B0009	Group B1	EOS/ET (Day 61)	No		5 ug	5 ug	5 ug
B0010	Group B2	EOS/ET (Day 61)	No		uncoated	uncoated	uncoated
B0011	Group B8	EOS/ET (Day 61)	No		5 ug	5 ug	5 ug
B0012	Group B6	EOS/ET (Day 61)	No		2.5 ug	uncoated	uncoated
B0013	Group B5	EOS/ET (Day 61)	No		5 ug	uncoated	uncoated
B0014	Group B3	EOS/ET (Day 61)	Yes	F1,F2,F3	5 ug	5 ug	5 ug
B0015	Group B9	EOS/ET (Day 61)	No		[IM]		
B0016	Group B4	EOS/ET (Day 61)	No		uncoated	5 ug	5 ug
B0017	Group B7	EOS/ET (Day 61)	No		uncoated	uncoated	uncoated
B0018	Group B9	EOS/ET (Day 61)	No		[IM]		
B0020	Group B6	EOS/ET (Day 61)	No		uncoated	uncoated	2.5 ug

Administration Site Key: FA = Forearm; UA= Upper arm
Treatments Part A: A1 = A/Sing MAP, 15 ug (FA); A2 = Afluria Quad IM (UA), 15 ug; A3 = Placebo MAP (FA); A4 = A/Sing Mono IM, 15 ug (UA)
Treatments Part B: B1 = A/Sing MAP, 15 ug (FA) with biopsy; B2 = Placebo MAP (FA) with biopsy
B3 = A/Sing MAP, 15 ug (FA); B4 = A/Sing MAP, 10 ug (FA); B5 = A/Sing MAP, 5 ug (FA); B6 = A/Sing MAP, 2.5 ug (FA)
B7 = Placebo MAP (FA); B8 = A/Sing MAP, 15 ug (UA); B9 = A/Sing IM, 15 ug, as 2018 Afluria Quad (UA)

Listing 16.2.9.6
Application Site Local Reaction Status at End of Study

Part B

Randomisation Number	Group	Visit	Is Any Application Site Still Visible?	Visible Sites	Treatment for Site 1	Treatment for Site 2	Treatment for Site 3
B0021	Group B7	EOS/ET (Day 61)	No		uncoated	uncoated	uncoated
B0022	Group B4	EOS/ET (Day 61)	Yes	F1,F2,F3	uncoated	5 ug	5 ug
B0023	Group B3	EOS/ET (Day 61)	No		5 ug	5 ug	5 ug
B0024	Group B5	EOS/ET (Day 61)	Yes	F3	uncoated	uncoated	5 ug
B0025	Group B8	EOS/ET (Day 61)	Yes	D1,D2,D3	5 ug	5 ug	5 ug
B0026	Group B9	EOS/ET (Day 61)	No		[IM]		
B0027	Group B3	EOS/ET (Day 61)	Yes	F1,F3	5 ug	5 ug	5 ug
B0028	Group B5	EOS/ET (Day 61)	No		5 ug	uncoated	uncoated
B0029	Group B6	EOS/ET (Day 61)	Yes	F1,F2,F3	uncoated	uncoated	2.5 ug
B0030	Group B7	EOS/ET (Day 61)	No		uncoated	uncoated	uncoated
B0031	Group B4	EOS/ET (Day 61)	No		uncoated	5 ug	5 ug
B0032	Group B6	EOS/ET (Day 61)	No		uncoated	uncoated	2.5 ug
B0033	Group B5	EOS/ET (Day 61)	Yes	F3	uncoated	uncoated	5 ug
B0034	Group B8	EOS/ET (Day 61)	No		5 ug	5 ug	5 ug
B0035	Group B9	EOS/ET (Day 61)	No		[IM]		
B0036	Group B7	EOS/ET (Day 61)	No		uncoated	uncoated	uncoated
B0037	Group B4	EOS/ET (Day 61)	Yes	F2,F3	uncoated	5 ug	5 ug
B0038	Group B3	EOS/ET (Day 61)	No		5 ug	5 ug	5 ug
B0039	Group B7	EOS/ET (Day 61)	No		uncoated	uncoated	uncoated

Administration Site Key: FA = Forearm; UA= Upper arm
Treatments Part A: A1 = A/Sing MAP, 15 ug (FA); A2 = Afluria Quad IM (UA), 15 ug; A3 = Placebo MAP (FA); A4 = A/Sing Mono IM, 15 ug (UA)
Treatments Part B: B1 = A/Sing MAP, 15 ug (FA) with biopsy; B2 = Placebo MAP (FA) with biopsy
B3 = A/Sing MAP, 15 ug (FA); B4 = A/Sing MAP, 10 ug (FA); B5 = A/Sing MAP, 5 ug (FA); B6 = A/Sing MAP, 2.5 ug (FA)
B7 = Placebo MAP (FA); B8 = A/Sing MAP, 15 ug (UA); B9 = A/Sing IM, 15 ug, as 2018 Afluria Quad (UA)

Listing 16.2.9.6
Application Site Local Reaction Status at End of Study

Part B

Randomisation Number	Group	Visit	Is Any Application Site Still Visible?	Visible Sites	Treatment for Site 1	Treatment for Site 2	Treatment for Site 3
B0040	Group B9	EOS/ET (Day 61)	No		[IM]		
B0041	Group B3	EOS/ET (Day 61)	Yes	F1,F2,F3	5 ug	5 ug	5 ug
B0042	Group B5	EOS/ET (Day 61)	Yes	F3	uncoated	uncoated	5 ug
B0043	Group B8	EOS/ET (Day 61)	Yes	D1,D2,D3	5 ug	5 ug	5 ug
B0044	Group B6	EOS/ET (Day 61)	Yes	F3	uncoated	uncoated	2.5 ug
B0045	Group B4	EOS/ET (Day 61)	Yes	F1,F2	5 ug	5 ug	uncoated
B0046	Group B7	EOS/ET (Day 61)	No		uncoated	uncoated	uncoated
B0047	Group B9	EOS/ET (Day 61)	No		[IM]		
B0048	Group B6	EOS/ET (Day 61)	Yes	F1	2.5 ug	uncoated	uncoated
B0049	Group B4	EOS/ET (Day 61)	Yes	F1,F2,F3	uncoated	5 ug	5 ug
B0050	Group B8	EOS/ET (Day 61)	No		5 ug	5 ug	5 ug
B0051	Group B3	EOS/ET (Day 61)	No		5 ug	5 ug	5 ug
B0052	Group B5	EOS/ET (Day 61)	Yes	F1,F2,F3	5 ug	uncoated	uncoated
B0053	Group B6	EOS/ET (Day 61)	Yes	F1,F2,F3	uncoated	uncoated	2.5 ug
B0054	Group B5	EOS/ET (Day 61)	No		uncoated	uncoated	5 ug
B0055	Group B8	EOS/ET (Day 61)	No		5 ug	5 ug	5 ug
B0056	Group B3	EOS/ET (Day 61)	Yes	F1,F2,F3	5 ug	5 ug	5 ug
B0057	Group B4	EOS/ET (Day 61)	Yes	F1,F2,F3	5 ug	5 ug	uncoated
B0058	Group B9	EOS/ET (Day 61)	No		[IM]		

Administration Site Key: FA = Forearm; UA= Upper arm
Treatments Part A: A1 = A/Sing MAP, 15 ug (FA); A2 = Afluria Quad IM (UA), 15 ug; A3 = Placebo MAP (FA); A4 = A/Sing Mono IM, 15 ug (UA)
Treatments Part B: B1 = A/Sing MAP, 15 ug (FA) with biopsy; B2 = Placebo MAP (FA) with biopsy
B3 = A/Sing MAP, 15 ug (FA); B4 = A/Sing MAP, 10 ug (FA); B5 = A/Sing MAP, 5 ug (FA); B6 = A/Sing MAP, 2.5 ug (FA)
B7 = Placebo MAP (FA); B8 = A/Sing MAP, 15 ug (UA); B9 = A/Sing IM, 15 ug, as 2018 Afluria Quad (UA)

Listing 16.2.9.6
Application Site Local Reaction Status at End of Study

Part B

Randomisation Number	Group	Visit	Is Any Application Site Still Visible?	Visible Sites	Treatment for Site 1	Treatment for Site 2	Treatment for Site 3
B0059	Group B7	EOS/ET (Day 61)	No		uncoated	uncoated	uncoated
B0060	Group B3	EOS/ET (Day 61)	No		5 ug	5 ug	5 ug
B0061	Group B8	EOS/ET (Day 61)	No		5 ug	5 ug	5 ug
B0062	Group B5	EOS/ET (Day 61)	No		uncoated	5 ug	uncoated
B0063	Group B7	EOS/ET (Day 61)	No		uncoated	uncoated	uncoated
B0064	Group B9	EOS/ET (Day 61)	No		[IM]		
B0065	Group B4	EOS/ET (Day 61)	Yes	F1,F2,F3	5 ug	uncoated	5 ug
B0066	Group B6	EOS/ET (Day 61)	No		2.5 ug	uncoated	uncoated
B0067	Group B8	EOS/ET (Day 61)	No		5 ug	5 ug	5 ug
B0068	Group B5	EOS/ET (Day 61)	Yes	F2	uncoated	5 ug	uncoated
B0069	Group B7	EOS/ET (Day 61)	No		uncoated	uncoated	uncoated
B0070	Group B9	EOS/ET (Day 61)	No		[IM]		
B0071	Group B4	EOS/ET (Day 61)	No		5 ug	uncoated	5 ug
B0072	Group B3	EOS/ET (Day 61)	No		5 ug	5 ug	5 ug
B0073	Group B6	EOS/ET (Day 61)	No		uncoated	2.5 ug	uncoated
B0074	Group B9	EOS/ET (Day 61)	No		[IM]		
B0075	Group B3	EOS/ET (Day 61)	Yes	F2,F3	5 ug	5 ug	5 ug
B0077	Group B4	EOS/ET (Day 61)	No		5 ug	uncoated	5 ug
B0078	Group B8	EOS/ET (Day 61)	Yes	D1,D2,D3	5 ug	5 ug	5 ug

Administration Site Key: FA = Forearm; UA= Upper arm
Treatments Part A: A1 = A/Sing MAP, 15 ug (FA); A2 = Afluria Quad IM (UA), 15 ug; A3 = Placebo MAP (FA); A4 = A/Sing Mono IM, 15 ug (UA)
Treatments Part B: B1 = A/Sing MAP, 15 ug (FA) with biopsy; B2 = Placebo MAP (FA) with biopsy
B3 = A/Sing MAP, 15 ug (FA); B4 = A/Sing MAP, 10 ug (FA); B5 = A/Sing MAP, 5 ug (FA); B6 = A/Sing MAP, 2.5 ug (FA)
B7 = Placebo MAP (FA); B8 = A/Sing MAP, 15 ug (UA); B9 = A/Sing IM, 15 ug, as 2018 Afluria Quad (UA)

Listing 16.2.9.6
Application Site Local Reaction Status at End of Study

Part B

Randomisation Number	Group	Visit	Is Any Application Site Still Visible?	Visible Sites	Treatment for Site 1	Treatment for Site 2	Treatment for Site 3
B0079	Group B6	EOS/ET (Day 61)	Yes	F2	uncoated	2.5 ug	uncoated
B0081	Group B6	EOS/ET (Day 61)	No		2.5 ug	uncoated	uncoated
B0082	Group B3	EOS/ET (Day 61)	Yes	F1,F2,F3	5 ug	5 ug	5 ug
B0083	Group B8	EOS/ET (Day 61)	Yes	D1,D2,D3	5 ug	5 ug	5 ug
B0084	Group B5	EOS/ET (Day 61)	No		uncoated	uncoated	5 ug
B0085	Group B4	EOS/ET (Day 61)	Yes	F3	uncoated	5 ug	5 ug
B0086	Group B9	EOS/ET (Day 61)	No		[IM]		
B0087	Group B7	EOS/ET (Day 61)	No		uncoated	uncoated	uncoated
B0088	Group B9	EOS/ET (Day 61)	No		[IM]		
B0089	Group B5	EOS/ET (Day 61)	No		uncoated	5 ug	uncoated
B0090	Group B6	EOS/ET (Day 61)	No		2.5 ug	uncoated	uncoated
B0091	Group B8	EOS/ET (Day 61)	Yes	D1,D2,D3	5 ug	5 ug	5 ug
B0092	Group B4	EOS/ET (Day 61)	Yes	F1,F3	5 ug	uncoated	5 ug
B0093	Group B7	EOS/ET (Day 61)	No		uncoated	uncoated	uncoated
B0094	Group B3	EOS/ET (Day 61)	No		5 ug	5 ug	5 ug
B0095	Group B9	EOS/ET (Day 61)	No		[IM]		
B0096	Group B6	EOS/ET (Day 61)	Yes	F1	2.5 ug	uncoated	uncoated
B0097	Group B5	EOS/ET (Day 61)	No		5 ug	uncoated	uncoated
B0098	Group B7	EOS/ET (Day 61)	No		uncoated	uncoated	uncoated

Administration Site Key: FA = Forearm; UA= Upper arm
Treatments Part A: A1 = A/Sing MAP, 15 ug (FA); A2 = Afluria Quad IM (UA), 15 ug; A3 = Placebo MAP (FA); A4 = A/Sing Mono IM, 15 ug (UA)
Treatments Part B: B1 = A/Sing MAP, 15 ug (FA) with biopsy; B2 = Placebo MAP (FA) with biopsy
B3 = A/Sing MAP, 15 ug (FA); B4 = A/Sing MAP, 10 ug (FA); B5 = A/Sing MAP, 5 ug (FA); B6 = A/Sing MAP, 2.5 ug (FA)
B7 = Placebo MAP (FA); B8 = A/Sing MAP, 15 ug (UA); B9 = A/Sing IM, 15 ug, as 2018 Afluria Quad (UA)

Listing 16.2.9.6
Application Site Local Reaction Status at End of Study

Part B

Randomisation Number	Group	Visit	Is Any Application Site Still Visible?	Visible Sites	Treatment for Site 1	Treatment for Site 2	Treatment for Site 3
B0099	Group B4	EOS/ET (Day 61)	No		5 ug	5 ug	uncoated
B0100	Group B8	EOS/ET (Day 61)	No		5 ug	5 ug	5 ug
B0101	Group B3	EOS/ET (Day 61)	No		5 ug	5 ug	5 ug
B0102	Group B8	EOS/ET (Day 61)	No		5 ug	5 ug	5 ug
B0103	Group B5	EOS/ET (Day 61)	No		5 ug	uncoated	uncoated
B0104	Group B3	EOS/ET (Day 61)	Yes	F1,F2,F3	5 ug	5 ug	5 ug
B0105	Group B9	EOS/ET (Day 61)	No		[IM]		
B0106	Group B4	EOS/ET (Day 61)	No		uncoated	5 ug	5 ug
B0107	Group B6	EOS/ET (Day 61)	Yes	F1,F2,F3	uncoated	uncoated	2.5 ug
B0108	Group B7	EOS/ET (Day 61)	No		uncoated	uncoated	uncoated
B0109	Group B6	EOS/ET (Day 61)	Yes	F2,F3	uncoated	2.5 ug	uncoated
B0110	Group B3	EOS/ET (Day 61)	No		5 ug	5 ug	5 ug
B0111	Group B8	EOS/ET (Day 61)	No		5 ug	5 ug	5 ug
B0112	Group B4	EOS/ET (Day 61)	Yes	F2,F3	uncoated	5 ug	5 ug
B0113	Group B5	EOS/ET (Day 61)	No		uncoated	5 ug	uncoated
B0114	Group B7	EOS/ET (Day 61)	No		uncoated	uncoated	uncoated
B0115	Group B9	EOS/ET (Day 61)	No		[IM]		
B0116	Group B7	EOS/ET (Day 61)	No		uncoated	uncoated	uncoated
B0117	Group B9	EOS/ET (Day 61)	No		[IM]		

Administration Site Key: FA = Forearm; UA= Upper arm
Treatments Part A: A1 = A/Sing MAP, 15 ug (FA); A2 = Afluria Quad IM (UA), 15 ug; A3 = Placebo MAP (FA); A4 = A/Sing Mono IM, 15 ug (UA)
Treatments Part B: B1 = A/Sing MAP, 15 ug (FA) with biopsy; B2 = Placebo MAP (FA) with biopsy
B3 = A/Sing MAP, 15 ug (FA); B4 = A/Sing MAP, 10 ug (FA); B5 = A/Sing MAP, 5 ug (FA); B6 = A/Sing MAP, 2.5 ug (FA)
B7 = Placebo MAP (FA); B8 = A/Sing MAP, 15 ug (UA); B9 = A/Sing IM, 15 ug, as 2018 Afluria Quad (UA)

Listing 16.2.9.6
Application Site Local Reaction Status at End of Study

Part B

Randomisation Number	Group	Visit	Is Any Application Site Still Visible?	Visible Sites	Treatment for Site 1	Treatment for Site 2	Treatment for Site 3
B0118	Group B6	EOS/ET (Day 61)	Yes	F2	uncoated	2.5 ug	uncoated
B0119	Group B4	EOS/ET (Day 61)	Yes	F1,F3	5 ug	uncoated	5 ug
B0120	Group B3	EOS/ET (Day 61)	Yes	F1,F2,F3	5 ug	5 ug	5 ug
B0121	Group B8	EOS/ET (Day 61)	No		5 ug	5 ug	5 ug
B0122	Group B5	EOS/ET (Day 61)	Yes	F2	uncoated	5 ug	uncoated
B0123	Group B6	EOS/ET (Day 61)	No		2.5 ug	uncoated	uncoated
B0124	Group B4	EOS/ET (Day 61)	Yes	F2,F3	uncoated	5 ug	5 ug
B0125	Group B7	EOS/ET (Day 61)	No		uncoated	uncoated	uncoated
B0126	Group B3	EOS/ET (Day 61)	Yes	F1,F2,F3	5 ug	5 ug	5 ug
B0127	Group B9	EOS/ET (Day 61)	No		[IM]		
B0128	Group B5	EOS/ET (Day 61)	Yes	F2	uncoated	5 ug	uncoated
B0129	Group B8	EOS/ET (Day 61)	Yes	D1,D2,D3	5 ug	5 ug	5 ug
B0130	Group B5	EOS/ET (Day 61)	Yes	F1	5 ug	uncoated	uncoated
B0131	Group B8	EOS/ET (Day 61)	No		5 ug	5 ug	5 ug
B0132	Group B6	EOS/ET (Day 61)	No		2.5 ug	uncoated	uncoated
B0133	Group B4	EOS/ET (Day 61)	No		uncoated	5 ug	5 ug
B0134	Group B3	EOS/ET (Day 61)	Yes	F2,F3	5 ug	5 ug	5 ug
B0135	Group B7	EOS/ET (Day 61)	No		uncoated	uncoated	uncoated
B0136	Group B9	EOS/ET (Day 61)	Not Done		[IM]		

Administration Site Key: FA = Forearm; UA= Upper arm
Treatments Part A: A1 = A/Sing MAP, 15 ug (FA); A2 = Afluria Quad IM (UA), 15 ug; A3 = Placebo MAP (FA); A4 = A/Sing Mono IM, 15 ug (UA)
Treatments Part B: B1 = A/Sing MAP, 15 ug (FA) with biopsy; B2 = Placebo MAP (FA) with biopsy
B3 = A/Sing MAP, 15 ug (FA); B4 = A/Sing MAP, 10 ug (FA); B5 = A/Sing MAP, 5 ug (FA); B6 = A/Sing MAP, 2.5 ug (FA)
B7 = Placebo MAP (FA); B8 = A/Sing MAP, 15 ug (UA); B9 = A/Sing IM, 15 ug, as 2018 Afluria Quad (UA)

Listing 16.2.9.6
Application Site Local Reaction Status at End of Study

Part B

Randomisation Number	Group	Visit	Is Any Application Site Still Visible?	Visible Sites	Treatment for Site 1	Treatment for Site 2	Treatment for Site 3
B0137	Group B8	EOS/ET (Day 61)	No		5 ug	5 ug	5 ug
B0138	Group B7	EOS/ET (Day 61)	Yes	F1,F2,F3	uncoated	uncoated	uncoated
B0139	Group B4	EOS/ET (Day 61)	No		uncoated	5 ug	5 ug
B0140	Group B9	EOS/ET (Day 61)	Not Done		[IM]		
B0141	Group B3	EOS/ET (Day 61)	No		5 ug	5 ug	5 ug
B0142	Group B5	EOS/ET (Day 61)	Yes	F1	5 ug	uncoated	uncoated
B0143	Group B6	EOS/ET (Day 61)	Yes	F3	uncoated	uncoated	2.5 ug
B0144	Group B6	EOS/ET (Day 61)	No		2.5 ug	uncoated	uncoated
B0145	Group B3	EOS/ET (Day 61)	No		5 ug	5 ug	5 ug
B0146	Group B7	EOS/ET (Day 61)	No		uncoated	uncoated	uncoated
B0147	Group B9	EOS/ET (Day 61)	No		[IM]		
B0148	Group B5	EOS/ET (Day 61)	Yes	F1	5 ug	uncoated	uncoated
B0149	Group B8	EOS/ET (Day 61)	No		5 ug	5 ug	5 ug
B0150	Group B4	EOS/ET (Day 61)	Yes	F1,F2	5 ug	5 ug	uncoated
B1019	Group B8	EOS/ET (Day 61)	Yes	D1,D2,D3	5 ug	5 ug	5 ug
B1076	Group B5	EOS/ET (Day 61)	No		5 ug	uncoated	uncoated
B1080	Group B7	EOS/ET (Day 61)	No		uncoated	uncoated	uncoated

Administration Site Key: FA = Forearm; UA= Upper arm
Treatments Part A: A1 = A/Sing MAP, 15 ug (FA); A2 = Afluria Quad IM (UA), 15 ug; A3 = Placebo MAP (FA); A4 = A/Sing Mono IM, 15 ug (UA)
Treatments Part B: B1 = A/Sing MAP, 15 ug (FA) with biopsy; B2 = Placebo MAP (FA) with biopsy
B3 = A/Sing MAP, 15 ug (FA); B4 = A/Sing MAP, 10 ug (FA); B5 = A/Sing MAP, 5 ug (FA); B6 = A/Sing MAP, 2.5 ug (FA)
B7 = Placebo MAP (FA); B8 = A/Sing MAP, 15 ug (UA); B9 = A/Sing IM, 15 ug, as 2018 Afluria Quad (UA)
