# Supplementary material for: Safety, tolerability, and immunogenicity of influenza vaccination with a high-density microarray patch: Results from a randomized, controlled phase I clinical trial
Source: PLoS Med. 2020 Mar 17;17(3):e1003024. doi: 10.1371/journal.pmed.1003024 (PMC7077342; doi:10.1371/journal.pmed.1003024)
Supplement: S1 Data — Data 1: Informed Consent; Data 2: Analysis Sets; Data 3: Demographics; Data 4: Study Drug Administration; Data 5: Skin Hardness Assessment; Data 6: Immunogenicity (ELISA IgG); Data 7: Immunogenicity (ADCC); Data 8: Immunogenicity (MBCs); Data 9: Immunogenicity (CMI); Data 10: Immunogenicity (Mucosal IgA); Data 11: Immunogenicity Results (HAI, MN); Data 12: TEAE–CRF Data only; Data 13: TEAEs–MedDRA Coding; Data 15: Treatment Site Tolerability Assessment; Data 16: Numeric Pain Intensity; Data 17: SII; Data 18: Application Site Status at End of Study; Data 19: Individual Treatment Site Tolerability Assessment; Data 20: Numeric Pain Intensity by Treatment; Data 21: Individual SII by Treatment. ADCC, antibody-dependent cellular cytotoxicity; CMI, cell-mediated immunity; CRF, case report form; ELISA, enzyme-linked immunosorbent assay; HAI, haemagglutination inhibition; IgA, immunoglobulin A; IgG, immunoglobulin G; MBC, memory B cell; MedDRA, medical dictionary for regulatory activities; MN, microneutralisation; SII, Skin Irritation Index; TEA, treatment emergent adverse event. (ZIP) [file pmed.1003024.s008.zip › S1_Data/clinical supporting/S1_Data2 Analysis Sets.pdf]

Listing 16.2.3.1
Analysis Set Inclusion per Subject

Part A

Randomisation Number	Treatment Group	Safety	Biopsy	Immunogenicity	Exploratory	Immunogenicity Sub Set B	Reason for Exclusion From an Analysis Population
		Analysis Populations					
A0001	Group A3	Yes	No	Yes	No	No	NA
A0002	Group A1	Yes	No	Yes	No	No	NA
A0003	Group A1	Yes	No	Yes	No	No	NA
A0004	Group A3	Yes	No	Yes	No	No	NA
A0005	Group A1	Yes	No	Yes	No	No	NA
A0006	Group A3	Yes	No	Yes	No	No	NA
A0007	Group A1	Yes	No	Yes	No	No	NA
A0008	Group A3	Yes	No	Yes	No	No	NA
A0009	Group A1	Yes	No	Yes	No	No	NA
A0010	Group A3	Yes	No	Yes	No	No	NA
A0011	Group A3	Yes	No	Yes	No	No	NA
A0012	Group A1	Yes	No	Yes	No	No	NA
A0013	Group A1	Yes	No	Yes	No	No	NA
A0014	Group A3	Yes	No	Yes	No	No	NA
A0015	Group A1	Yes	No	Yes	No	No	NA
A0016	Group A3	Yes	No	Yes	No	No	NA
A0017	Group A3	Yes	No	Yes	No	No	NA
A0018	Group A1	Yes	No	Yes	No	No	NA
A0019	Group A3	Yes	No	Yes	No	No	NA
A0020	Group A1	Yes	No	Yes	No	No	NA
A0021	Group A1	Yes	No	Yes	No	No	NA

Administration Site Key: FA = Forearm; UA= Upper arm
Treatments Part A: A1 = A/Sing MAP, 15 ug (FA); A2 = Afluria Quad IM (UA), 15 ug; A3 = Placebo MAP (FA); A4 = A/Sing Mono IM, 15 ug (UA)
Treatments Part B: B1 = A/Sing MAP, 15 ug (FA) with biopsy; B2 = Placebo MAP (FA) with biopsy
B3 = A/Sing MAP, 15 ug (FA); B4 = A/Sing MAP, 10 ug (FA); B5 = A/Sing MAP, 5 ug (FA); B6 = A/Sing MAP, 2.5 ug (FA)
B7 = Placebo MAP (FA); B8 = A/Sing MAP, 15 ug (UA); B9 = A/Sing IM, 15 ug, as 2018 Afluria Quad (UA)

Listing 16.2.3.1
Analysis Set Inclusion per Subject

Part A

Randomisation Number	Treatment Group	Safety	Biopsy	Immunogenicity	Exploratory	Immunogenicity Sub Set B	Reason for Exclusion From an Analysis Population
		Analysis Populations					
A0022	Group A3	Yes	No	Yes	No	No	NA
A0023	Group A3	Yes	No	Yes	No	No	NA
A0024	Group A1	Yes	No	Yes	No	No	NA
A0025	Group A3	Yes	No	Yes	No	No	NA
A0027	Group A3	Yes	No	Yes	No	No	NA
A0028	Group A1	Yes	No	Yes	No	No	NA
A0029	Group A1	Yes	No	Yes	No	No	NA
A0030	Group A3	Yes	No	Yes	No	No	NA
A0031	Group A2	Yes	No	No	No	No	Subject Withdrawn
A0032	Group A4	Yes	No	Yes	No	No	NA
A0033	Group A2	Yes	No	Yes	No	No	NA
A0034	Group A4	Yes	No	Yes	No	No	NA
A0035	Group A4	Yes	No	Yes	No	No	NA
A0036	Group A2	Yes	No	Yes	No	No	NA
A0037	Group A4	Yes	No	Yes	No	No	NA
A0038	Group A2	Yes	No	Yes	No	No	NA
A0039	Group A2	Yes	No	Yes	No	No	NA
A0040	Group A4	Yes	No	Yes	No	No	NA
A0041	Group A4	Yes	No	Yes	No	No	NA
A0042	Group A2	Yes	No	Yes	No	No	NA
A0043	Group A2	Yes	No	Yes	No	No	NA

Administration Site Key: FA = Forearm; UA= Upper arm
Treatments Part A: A1 = A/Sing MAP, 15 ug (FA); A2 = Afluria Quad IM (UA), 15 ug; A3 = Placebo MAP (FA); A4 = A/Sing Mono IM, 15 ug (UA)
Treatments Part B: B1 = A/Sing MAP, 15 ug (FA) with biopsy; B2 = Placebo MAP (FA) with biopsy
B3 = A/Sing MAP, 15 ug (FA); B4 = A/Sing MAP, 10 ug (FA); B5 = A/Sing MAP, 5 ug (FA); B6 = A/Sing MAP, 2.5 ug (FA)
B7 = Placebo MAP (FA); B8 = A/Sing MAP, 15 ug (UA); B9 = A/Sing IM, 15 ug, as 2018 Afluria Quad (UA)

Listing 16.2.3.1
Analysis Set Inclusion per Subject

Part A

Randomisation Number	Treatment Group	Safety	Biopsy	Immunogenicity	Exploratory	Immunogenicity Sub Set B	Reason for Exclusion From an Analysis Population
		Analysis Populations					
A0044	Group A4	Yes	No	Yes	No	No	NA
A0045	Group A2	Yes	No	Yes	No	No	NA
A0046	Group A4	Yes	No	Yes	No	No	NA
A0047	Group A2	Yes	No	Yes	No	No	NA
A0048	Group A4	Yes	No	Yes	No	No	NA
A0049	Group A2	Yes	No	Yes	No	No	NA
A0050	Group A4	Yes	No	Yes	No	No	NA
A0051	Group A2	Yes	No	Yes	No	No	NA
A0052	Group A4	Yes	No	Yes	No	No	NA
A0053	Group A4	Yes	No	Yes	No	No	NA
A0054	Group A2	Yes	No	Yes	No	No	NA
A0055	Group A4	Yes	No	Yes	No	No	NA
A0056	Group A2	Yes	No	Yes	No	No	NA
A0057	Group A4	Yes	No	Yes	No	No	NA
A0058	Group A2	Yes	No	Yes	No	No	NA
A0059	Group A2	Yes	No	Yes	No	No	NA
A0060	Group A4	Yes	No	Yes	No	No	NA
A1026	Group A1	Yes	No	Yes	No	No	NA

Administration Site Key: FA = Forearm; UA= Upper arm
Treatments Part A: A1 = A/Sing MAP, 15 ug (FA); A2 = Afluria Quad IM (UA), 15 ug; A3 = Placebo MAP (FA); A4 = A/Sing Mono IM, 15 ug (UA)
Treatments Part B: B1 = A/Sing MAP, 15 ug (FA) with biopsy; B2 = Placebo MAP (FA) with biopsy
B3 = A/Sing MAP, 15 ug (FA); B4 = A/Sing MAP, 10 ug (FA); B5 = A/Sing MAP, 5 ug (FA); B6 = A/Sing MAP, 2.5 ug (FA)
B7 = Placebo MAP (FA); B8 = A/Sing MAP, 15 ug (UA); B9 = A/Sing IM, 15 ug, as 2018 Afluria Quad (UA)

Listing 16.2.3.1
Analysis Set Inclusion per Subject

Part B

Randomisation Number	Treatment Group	Safety	Biopsy	Immunogenicity	Exploratory	Immunogenicity Sub Set B	Reason for Exclusion From an Analysis Population
		Analysis Populations					
B0001	Group B1	Yes	Yes	No	No	No	NA
B0002	Group B2	Yes	Yes	No	No	No	NA
B0003	Group B2	Yes	Yes	No	No	No	NA
B0004	Group B1	Yes	Yes	No	No	No	NA
B0005	Group B1	Yes	Yes	No	No	No	NA
B0006	Group B2	Yes	Yes	No	No	No	NA
B0007	Group B2	Yes	Yes	No	No	No	NA
B0008	Group B1	Yes	Yes	No	No	No	NA
B0009	Group B1	Yes	Yes	No	No	No	NA
B0010	Group B2	Yes	Yes	No	No	No	NA
B0011	Group B8	Yes	No	Yes	Yes	Yes	NA
B0012	Group B6	Yes	No	Yes	No	Yes	NA
B0013	Group B5	Yes	No	Yes	No	Yes	NA
B0014	Group B3	Yes	No	Yes	Yes	Yes	NA
B0015	Group B9	Yes	No	Yes	Yes	Yes	NA
B0016	Group B4	Yes	No	Yes	No	Yes	NA
B0017	Group B7	Yes	No	Yes	Yes	Yes	NA
B0018	Group B9	Yes	No	Yes	Yes	Yes	NA
B0020	Group B6	Yes	No	Yes	No	Yes	NA
B0021	Group B7	Yes	No	Yes	Yes	Yes	NA
B0022	Group B4	Yes	No	Yes	No	Yes	NA

Administration Site Key: FA = Forearm; UA= Upper arm
Treatments Part A: A1 = A/Sing MAP, 15 ug (FA); A2 = Afluria Quad IM (UA), 15 ug; A3 = Placebo MAP (FA); A4 = A/Sing Mono IM, 15 ug (UA)
Treatments Part B: B1 = A/Sing MAP, 15 ug (FA) with biopsy; B2 = Placebo MAP (FA) with biopsy
B3 = A/Sing MAP, 15 ug (FA); B4 = A/Sing MAP, 10 ug (FA); B5 = A/Sing MAP, 5 ug (FA); B6 = A/Sing MAP, 2.5 ug (FA)
B7 = Placebo MAP (FA); B8 = A/Sing MAP, 15 ug (UA); B9 = A/Sing IM, 15 ug, as 2018 Afluria Quad (UA)

Listing 16.2.3.1
Analysis Set Inclusion per Subject

Part B

Randomisation Number	Treatment Group	Safety	Biopsy	Immunogenicity	Exploratory	Immunogenicity Sub Set B	Reason for Exclusion From an Analysis Population
		Analysis Populations					
B0023	Group B3	Yes	No	Yes	Yes	Yes	NA
B0024	Group B5	Yes	No	Yes	No	Yes	NA
B0025	Group B8	Yes	No	Yes	Yes	Yes	NA
B0026	Group B9	Yes	No	Yes	Yes	Yes	NA
B0027	Group B3	Yes	No	Yes	Yes	Yes	NA
B0028	Group B5	Yes	No	Yes	No	Yes	NA
B0029	Group B6	Yes	No	Yes	No	Yes	NA
B0030	Group B7	Yes	No	Yes	Yes	Yes	NA
B0031	Group B4	Yes	No	Yes	No	Yes	NA
B0032	Group B6	Yes	No	Yes	No	Yes	NA
B0033	Group B5	Yes	No	Yes	No	Yes	NA
B0034	Group B8	Yes	No	Yes	Yes	Yes	NA
B0035	Group B9	Yes	No	Yes	Yes	Yes	NA
B0036	Group B7	Yes	No	Yes	Yes	Yes	NA
B0037	Group B4	Yes	No	Yes	No	Yes	NA
B0038	Group B3	Yes	No	Yes	Yes	Yes	NA
B0039	Group B7	Yes	No	Yes	Yes	Yes	NA
B0040	Group B9	Yes	No	Yes	Yes	Yes	NA
B0041	Group B3	Yes	No	Yes	Yes	Yes	NA
B0042	Group B5	Yes	No	Yes	No	Yes	NA
B0043	Group B8	Yes	No	Yes	Yes	Yes	NA

Administration Site Key: FA = Forearm; UA= Upper arm
Treatments Part A: A1 = A/Sing MAP, 15 ug (FA); A2 = Afluria Quad IM (UA), 15 ug; A3 = Placebo MAP (FA); A4 = A/Sing Mono IM, 15 ug (UA)
Treatments Part B: B1 = A/Sing MAP, 15 ug (FA) with biopsy; B2 = Placebo MAP (FA) with biopsy
B3 = A/Sing MAP, 15 ug (FA); B4 = A/Sing MAP, 10 ug (FA); B5 = A/Sing MAP, 5 ug (FA); B6 = A/Sing MAP, 2.5 ug (FA)
B7 = Placebo MAP (FA); B8 = A/Sing MAP, 15 ug (UA); B9 = A/Sing IM, 15 ug, as 2018 Afluria Quad (UA)

Listing 16.2.3.1
Analysis Set Inclusion per Subject

Part B

Randomisation Number	Treatment Group	Safety	Biopsy	Immunogenicity	Exploratory	Immunogenicity Sub Set B	Reason for Exclusion From an Analysis Population
		Analysis Populations					
B0044	Group B6	Yes	No	Yes	No	Yes	NA
B0045	Group B4	Yes	No	Yes	No	Yes	NA
B0046	Group B7	Yes	No	Yes	Yes	Yes	NA
B0047	Group B9	Yes	No	Yes	Yes	Yes	NA
B0048	Group B6	Yes	No	Yes	No	Yes	NA
B0049	Group B4	Yes	No	Yes	No	Yes	NA
B0050	Group B8	Yes	No	Yes	Yes	Yes	NA
B0051	Group B3	Yes	No	Yes	Yes	Yes	NA
B0052	Group B5	Yes	No	Yes	No	Yes	NA
B0053	Group B6	Yes	No	Yes	No	Yes	NA
B0054	Group B5	Yes	No	Yes	No	Yes	NA
B0055	Group B8	Yes	No	Yes	Yes	Yes	NA
B0056	Group B3	Yes	No	Yes	Yes	Yes	NA
B0057	Group B4	Yes	No	Yes	No	Yes	NA
B0058	Group B9	Yes	No	Yes	Yes	Yes	NA
B0059	Group B7	Yes	No	Yes	Yes	Yes	NA
B0060	Group B3	Yes	No	Yes	Yes	Yes	NA
B0061	Group B8	Yes	No	Yes	Yes	Yes	NA
B0062	Group B5	Yes	No	Yes	No	Yes	NA
B0063	Group B7	Yes	No	Yes	Yes	Yes	NA
B0064	Group B9	Yes	No	Yes	Yes	Yes	NA

Administration Site Key: FA = Forearm; UA= Upper arm
Treatments Part A: A1 = A/Sing MAP, 15 ug (FA); A2 = Afluria Quad IM (UA), 15 ug; A3 = Placebo MAP (FA); A4 = A/Sing Mono IM, 15 ug (UA)
Treatments Part B: B1 = A/Sing MAP, 15 ug (FA) with biopsy; B2 = Placebo MAP (FA) with biopsy
B3 = A/Sing MAP, 15 ug (FA); B4 = A/Sing MAP, 10 ug (FA); B5 = A/Sing MAP, 5 ug (FA); B6 = A/Sing MAP, 2.5 ug (FA)
B7 = Placebo MAP (FA); B8 = A/Sing MAP, 15 ug (UA); B9 = A/Sing IM, 15 ug, as 2018 Afluria Quad (UA)

Listing 16.2.3.1
Analysis Set Inclusion per Subject

Part B

Randomisation Number	Treatment Group	Safety	Biopsy	Immunogenicity	Exploratory	Immunogenicity Sub Set B	Reason for Exclusion From an Analysis Population
		Analysis Populations					
B0065	Group B4	Yes	No	Yes	No	Yes	NA
B0066	Group B6	Yes	No	Yes	No	Yes	NA
B0067	Group B8	Yes	No	Yes	Yes	Yes	NA
B0068	Group B5	Yes	No	Yes	No	Yes	NA
B0069	Group B7	Yes	No	Yes	Yes	Yes	NA
B0070	Group B9	Yes	No	Yes	Yes	Yes	NA
B0071	Group B4	Yes	No	Yes	No	Yes	NA
B0072	Group B3	Yes	No	Yes	Yes	Yes	NA
B0073	Group B6	Yes	No	Yes	No	Yes	NA
B0074	Group B9	Yes	No	Yes	Yes	Yes	NA
B0075	Group B3	Yes	No	Yes	Yes	Yes	NA
B0077	Group B4	Yes	No	Yes	No	Yes	NA
B0078	Group B8	Yes	No	Yes	Yes	Yes	NA
B0079	Group B6	Yes	No	Yes	No	Yes	NA
B0081	Group B6	Yes	No	Yes	No	Yes	NA
B0082	Group B3	Yes	No	Yes	Yes	Yes	NA
B0083	Group B8	Yes	No	Yes	Yes	Yes	NA
B0084	Group B5	Yes	No	Yes	No	Yes	NA
B0085	Group B4	Yes	No	Yes	No	Yes	NA
B0086	Group B9	Yes	No	Yes	Yes	Yes	NA
B0087	Group B7	Yes	No	Yes	Yes	Yes	NA

Administration Site Key: FA = Forearm; UA= Upper arm
Treatments Part A: A1 = A/Sing MAP, 15 ug (FA); A2 = Afluria Quad IM (UA), 15 ug; A3 = Placebo MAP (FA); A4 = A/Sing Mono IM, 15 ug (UA)
Treatments Part B: B1 = A/Sing MAP, 15 ug (FA) with biopsy; B2 = Placebo MAP (FA) with biopsy
B3 = A/Sing MAP, 15 ug (FA); B4 = A/Sing MAP, 10 ug (FA); B5 = A/Sing MAP, 5 ug (FA); B6 = A/Sing MAP, 2.5 ug (FA)
B7 = Placebo MAP (FA); B8 = A/Sing MAP, 15 ug (UA); B9 = A/Sing IM, 15 ug, as 2018 Afluria Quad (UA)

Listing 16.2.3.1
Analysis Set Inclusion per Subject

Part B

Randomisation Number	Treatment Group	Safety	Biopsy	Immunogenicity	Exploratory	Immunogenicity Sub Set B	Reason for Exclusion From an Analysis Population
		Analysis Populations					
B0088	Group B9	Yes	No	Yes	Yes	Yes	NA
B0089	Group B5	Yes	No	Yes	No	Yes	NA
B0090	Group B6	Yes	No	Yes	No	Yes	NA
B0091	Group B8	Yes	No	Yes	Yes	Yes	NA
B0092	Group B4	Yes	No	Yes	No	Yes	NA
B0093	Group B7	Yes	No	Yes	Yes	Yes	NA
B0094	Group B3	Yes	No	Yes	Yes	Yes	NA
B0095	Group B9	Yes	No	Yes	Yes	Yes	NA
B0096	Group B6	Yes	No	Yes	No	Yes	NA
B0097	Group B5	Yes	No	Yes	No	Yes	NA
B0098	Group B7	Yes	No	Yes	Yes	Yes	NA
B0099	Group B4	Yes	No	Yes	No	Yes	NA
B0100	Group B8	Yes	No	Yes	Yes	Yes	NA
B0101	Group B3	Yes	No	Yes	Yes	Yes	NA
B0102	Group B8	Yes	No	Yes	Yes	Yes	NA
B0103	Group B5	Yes	No	Yes	No	Yes	NA
B0104	Group B3	Yes	No	Yes	Yes	Yes	NA
B0105	Group B9	Yes	No	Yes	Yes	Yes	NA
B0106	Group B4	Yes	No	Yes	No	Yes	NA
B0107	Group B6	Yes	No	Yes	No	Yes	NA
B0108	Group B7	Yes	No	Yes	Yes	Yes	NA

Administration Site Key: FA = Forearm; UA= Upper arm
Treatments Part A: A1 = A/Sing MAP, 15 ug (FA); A2 = Afluria Quad IM (UA), 15 ug; A3 = Placebo MAP (FA); A4 = A/Sing Mono IM, 15 ug (UA)
Treatments Part B: B1 = A/Sing MAP, 15 ug (FA) with biopsy; B2 = Placebo MAP (FA) with biopsy
B3 = A/Sing MAP, 15 ug (FA); B4 = A/Sing MAP, 10 ug (FA); B5 = A/Sing MAP, 5 ug (FA); B6 = A/Sing MAP, 2.5 ug (FA)
B7 = Placebo MAP (FA); B8 = A/Sing MAP, 15 ug (UA); B9 = A/Sing IM, 15 ug, as 2018 Afluria Quad (UA)

Listing 16.2.3.1
Analysis Set Inclusion per Subject

Part B

Randomisation Number	Treatment Group	Analysis Populations: Safety	Biopsy	Immunogenicity	Exploratory	Immunogenicity Sub Set B	Reason for Exclusion From an Analysis Population
B0109	Group B6	Yes	No	Yes	No	Yes	NA
B0110	Group B3	Yes	No	Yes	Yes	Yes	NA
B0111	Group B8	Yes	No	Yes	Yes	Yes	NA
B0112	Group B4	Yes	No	Yes	No	Yes	NA
B0113	Group B5	Yes	No	Yes	No	Yes	NA
B0114	Group B7	Yes	No	Yes	Yes	Yes	NA
B0115	Group B9	Yes	No	Yes	Yes	Yes	NA
B0116	Group B7	Yes	No	Yes	Yes	Yes	NA
B0117	Group B9	Yes	No	Yes	Yes	Yes	NA
B0118	Group B6	Yes	No	Yes	No	Yes	NA
B0119	Group B4	Yes	No	Yes	No	Yes	NA
B0120	Group B3	Yes	No	Yes	Yes	Yes	NA
B0121	Group B8	Yes	No	Yes	Yes	Yes	NA
B0122	Group B5	Yes	No	Yes	No	Yes	NA
B0123	Group B6	Yes	No	Yes	No	Yes	NA
B0124	Group B4	Yes	No	Yes	No	Yes	NA
B0125	Group B7	Yes	No	Yes	Yes	Yes	NA
B0126	Group B3	Yes	No	Yes	Yes	Yes	NA
B0127	Group B9	Yes	No	Yes	Yes	Yes	NA
B0128	Group B5	Yes	No	Yes	No	Yes	NA
B0129	Group B8	Yes	No	Yes	Yes	Yes	NA

Administration Site Key: FA = Forearm; UA= Upper arm
Treatments Part A: A1 = A/Sing MAP, 15 ug (FA); A2 = Afluria Quad IM (UA), 15 ug; A3 = Placebo MAP (FA); A4 = A/Sing Mono IM, 15 ug (UA)
Treatments Part B: B1 = A/Sing MAP, 15 ug (FA) with biopsy; B2 = Placebo MAP (FA) with biopsy
B3 = A/Sing MAP, 15 ug (FA); B4 = A/Sing MAP, 10 ug (FA); B5 = A/Sing MAP, 5 ug (FA); B6 = A/Sing MAP, 2.5 ug (FA)
B7 = Placebo MAP (FA); B8 = A/Sing MAP, 15 ug (UA); B9 = A/Sing IM, 15 ug, as 2018 Afluria Quad (UA)

Listing 16.2.3.1
Analysis Set Inclusion per Subject

Part B

Randomisation Number	Treatment Group	Safety	Biopsy	Immunogenicity	Exploratory	Immunogenicity Sub Set B	Reason for Exclusion From an Analysis Population
		Analysis Populations					
B0130	Group B5	Yes	No	Yes	No	Yes	NA
B0131	Group B8	Yes	No	Yes	Yes	Yes	NA
B0132	Group B6	Yes	No	Yes	No	Yes	NA
B0133	Group B4	Yes	No	Yes	No	Yes	NA
B0134	Group B3	Yes	No	Yes	Yes	Yes	NA
B0135	Group B7	Yes	No	Yes	Yes	Yes	NA
B0136	Group B9	Yes	No	Yes	Yes	Yes	NA
B0137	Group B8	Yes	No	Yes	Yes	Yes	NA
B0138	Group B7	Yes	No	Yes	Yes	Yes	NA
B0139	Group B4	Yes	No	Yes	No	Yes	NA
B0140	Group B9	Yes	No	Yes	Yes	Yes	NA
B0141	Group B3	Yes	No	Yes	Yes	Yes	NA
B0142	Group B5	Yes	No	Yes	No	Yes	NA
B0143	Group B6	Yes	No	Yes	No	Yes	NA
B0144	Group B6	Yes	No	Yes	No	Yes	NA
B0145	Group B3	Yes	No	Yes	Yes	Yes	NA
B0146	Group B7	Yes	No	Yes	Yes	Yes	NA
B0147	Group B9	Yes	No	Yes	Yes	Yes	NA
B0148	Group B5	Yes	No	Yes	No	Yes	NA
B0149	Group B8	Yes	No	Yes	Yes	Yes	NA
B0150	Group B4	Yes	No	Yes	No	Yes	NA

Administration Site Key: FA = Forearm; UA= Upper arm
Treatments Part A: A1 = A/Sing MAP, 15 ug (FA); A2 = Afluria Quad IM (UA), 15 ug; A3 = Placebo MAP (FA); A4 = A/Sing Mono IM, 15 ug (UA)
Treatments Part B: B1 = A/Sing MAP, 15 ug (FA) with biopsy; B2 = Placebo MAP (FA) with biopsy
B3 = A/Sing MAP, 15 ug (FA); B4 = A/Sing MAP, 10 ug (FA); B5 = A/Sing MAP, 5 ug (FA); B6 = A/Sing MAP, 2.5 ug (FA)
B7 = Placebo MAP (FA); B8 = A/Sing MAP, 15 ug (UA); B9 = A/Sing IM, 15 ug, as 2018 Afluria Quad (UA)

Listing 16.2.3.1
Analysis Set Inclusion per Subject

Part B

Randomisation Number	Treatment Group	Safety	Biopsy	Immunogenicity	Exploratory	Immunogenicity Sub Set B	Reason for Exclusion From an Analysis Population
		Analysis Populations					
B1019	Group B8	Yes	No	Yes	Yes	Yes	NA
B1076	Group B5	Yes	No	Yes	No	Yes	NA
B1080	Group B7	Yes	No	Yes	Yes	Yes	NA

Administration Site Key: FA = Forearm; UA= Upper arm
Treatments Part A: A1 = A/Sing MAP, 15 ug (FA); A2 = Afluria Quad IM (UA), 15 ug; A3 = Placebo MAP (FA); A4 = A/Sing Mono IM, 15 ug (UA)
Treatments Part B: B1 = A/Sing MAP, 15 ug (FA) with biopsy; B2 = Placebo MAP (FA) with biopsy
B3 = A/Sing MAP, 15 ug (FA); B4 = A/Sing MAP, 10 ug (FA); B5 = A/Sing MAP, 5 ug (FA); B6 = A/Sing MAP, 2.5 ug (FA)
B7 = Placebo MAP (FA); B8 = A/Sing MAP, 15 ug (UA); B9 = A/Sing IM, 15 ug, as 2018 Afluria Quad (UA)
