# Supplementary material for: Safety, tolerability, and immunogenicity of influenza vaccination with a high-density microarray patch: Results from a randomized, controlled phase I clinical trial
Source: PLoS Med. 2020 Mar 17;17(3):e1003024. doi: 10.1371/journal.pmed.1003024 (PMC7077342; doi:10.1371/journal.pmed.1003024)
Supplement: S1 Data — Data 1: Informed Consent; Data 2: Analysis Sets; Data 3: Demographics; Data 4: Study Drug Administration; Data 5: Skin Hardness Assessment; Data 6: Immunogenicity (ELISA IgG); Data 7: Immunogenicity (ADCC); Data 8: Immunogenicity (MBCs); Data 9: Immunogenicity (CMI); Data 10: Immunogenicity (Mucosal IgA); Data 11: Immunogenicity Results (HAI, MN); Data 12: TEAE–CRF Data only; Data 13: TEAEs–MedDRA Coding; Data 15: Treatment Site Tolerability Assessment; Data 16: Numeric Pain Intensity; Data 17: SII; Data 18: Application Site Status at End of Study; Data 19: Individual Treatment Site Tolerability Assessment; Data 20: Numeric Pain Intensity by Treatment; Data 21: Individual SII by Treatment. ADCC, antibody-dependent cellular cytotoxicity; CMI, cell-mediated immunity; CRF, case report form; ELISA, enzyme-linked immunosorbent assay; HAI, haemagglutination inhibition; IgA, immunoglobulin A; IgG, immunoglobulin G; MBC, memory B cell; MedDRA, medical dictionary for regulatory activities; MN, microneutralisation; SII, Skin Irritation Index; TEA, treatment emergent adverse event. (ZIP) [file pmed.1003024.s008.zip › S1_Data/clinical supporting/S1_Data20 Numeric Pain Intensity by Treatment.pdf]

Listing 16.2.9.10
Individual Characterisation of Numeric Pain Intensity by Treatment

Part A
A1= 3 x 5 ug A/Sing MAPs on volar forearm (15 ug)

| | | | -------Active------- | | | -------Placebo------- | | |
|---|---|---|---|---|---|---|---|---|
| Randomisation Number | Visit | Time Point | #> 0 | Maximum | Average | #> 0 | Maximum | Average |
| A0002 | Day 1 | 1 min | 3 | 1 | 1.0 | - | - | - |
| A0002 | Day 1 | 10 min | 3 | 1 | 1.0 | - | - | - |
| A0002 | Day 1 | 1 hour | 0 | 0 | 0.0 | - | - | - |
| A0002 | Day 1 | 2 hour | 0 | 0 | 0.0 | - | - | - |
| A0002 | Day 4 | | 0 | 0 | 0.0 | - | - | - |
| A0002 | Day 8 | | 0 | 0 | 0.0 | - | - | - |
| A0002 | Day 22 | | 0 | 0 | 0.0 | - | - | - |
| A0002 | EOS/ET (Day 61) | | 0 | 0 | 0.0 | - | - | - |
| A0003 | Day 1 | 1 min | 2 | 1 | 0.7 | - | - | - |
| A0003 | Day 1 | 10 min | 0 | 0 | 0.0 | - | - | - |
| A0003 | Day 1 | 1 hour | 0 | 0 | 0.0 | - | - | - |
| A0003 | Day 1 | 2 hour | 0 | 0 | 0.0 | - | - | - |
| A0003 | Day 4 | | 0 | 0 | 0.0 | - | - | - |
| A0003 | Day 8 | | 0 | 0 | 0.0 | - | - | - |
| A0003 | Day 22 | | 0 | 0 | 0.0 | - | - | - |
| A0003 | EOS/ET (Day 61) | | 0 | 0 | 0.0 | - | - | - |
| A0005 | Day 1 | 1 min | 0 | 0 | 0.0 | - | - | - |
| A0005 | Day 1 | 10 min | 0 | 0 | 0.0 | - | - | - |
| A0005 | Day 1 | 1 hour | 0 | 0 | 0.0 | - | - | - |


Source: Listing 16.2.9.3

Administration Site Key: FA = Forearm; UA= Upper arm
Treatments Part A: A1 = A/Sing MAP, 15 ug (FA); A2 = Afluria Quad IM (UA), 15 ug; A3 = Placebo MAP (FA); A4 = A/Sing Mono IM, 15 ug (UA)
Treatments Part B: B1 = A/Sing MAP, 15 ug (FA) with biopsy; B2 = Placebo MAP (FA) with biopsy
B3 = A/Sing MAP, 15 ug (FA); B4 = A/Sing MAP, 10 ug (FA); B5 = A/Sing MAP, 5 ug (FA); B6 = A/Sing MAP, 2.5 ug (FA)
B7 = Placebo MAP (FA); B8 = A/Sing MAP, 15 ug (UA); B9 = A/Sing IM, 15 ug, as 2018 Afluria Quad (UA)

Listing 16.2.9.10
Individual Characterisation of Numeric Pain Intensity by Treatment

Part A
A1= 3 x 5 ug A/Sing MAPs on volar forearm (15 ug)

| | | | -------Active------- | | | -------Placebo------- | | |
|---|---|---|---|---|---|---|---|---|
| Randomisation Number | Visit | Time Point | #> 0 | Maximum | Average | #> 0 | Maximum | Average |
| A0005 | Day 1 | 2 hour | 0 | 0 | 0.0 | - | - | - |
| A0005 | Day 4 | | 0 | 0 | 0.0 | - | - | - |
| A0005 | Day 8 | | 0 | 0 | 0.0 | - | - | - |
| A0005 | Day 22 | | 0 | 0 | 0.0 | - | - | - |
| A0005 | EOS/ET (Day 61) | | 0 | 0 | 0.0 | - | - | - |
| A0007 | Day 1 | 1 min | 3 | 4 | 3.3 | - | - | - |
| A0007 | Day 1 | 10 min | 3 | 2 | 2.0 | - | - | - |
| A0007 | Day 1 | 1 hour | 0 | 0 | 0.0 | - | - | - |
| A0007 | Day 1 | 2 hour | 0 | 0 | 0.0 | - | - | - |
| A0007 | Day 4 | | 0 | 0 | 0.0 | - | - | - |
| A0007 | Day 8 | | 0 | 0 | 0.0 | - | - | - |
| A0007 | Day 22 | | 0 | 0 | 0.0 | - | - | - |
| A0007 | EOS/ET (Day 61) | | 0 | 0 | 0.0 | - | - | - |
| A0009 | Day 1 | 1 min | 3 | 6 | 4.7 | - | - | - |
| A0009 | Day 1 | 10 min | 3 | 2 | 2.0 | - | - | - |
| A0009 | Day 1 | 1 hour | - | - | - | - | - | - |
| A0009 | Day 1 | 2 hour | 0 | 0 | 0.0 | - | - | - |
| A0009 | Day 4 | | 1 | 1 | 0.3 | - | - | - |
| A0009 | Day 8 | | 0 | 0 | 0.0 | - | - | - |


Source: Listing 16.2.9.3

Administration Site Key: FA = Forearm; UA= Upper arm
Treatments Part A: A1 = A/Sing MAP, 15 ug (FA); A2 = Afluria Quad IM (UA), 15 ug; A3 = Placebo MAP (FA); A4 = A/Sing Mono IM, 15 ug (UA)
Treatments Part B: B1 = A/Sing MAP, 15 ug (FA) with biopsy; B2 = Placebo MAP (FA) with biopsy
B3 = A/Sing MAP, 15 ug (FA); B4 = A/Sing MAP, 10 ug (FA); B5 = A/Sing MAP, 5 ug (FA); B6 = A/Sing MAP, 2.5 ug (FA)
B7 = Placebo MAP (FA); B8 = A/Sing MAP, 15 ug (UA); B9 = A/Sing IM, 15 ug, as 2018 Afluria Quad (UA)

Listing 16.2.9.10
Individual Characterisation of Numeric Pain Intensity by Treatment

Part A
A1= 3 x 5 ug A/Sing MAPs on volar forearm (15 ug)

| Randomisation Number | Visit | Time Point | Active #> 0 | Active Maximum | Active Average | Placebo #> 0 | Placebo Maximum | Placebo Average |
|---|---|---|---|---|---|---|---|---|
| A0009 | Day 22 | | 0 | 0 | 0.0 | - | - | - |
| A0009 | EOS/ET (Day 61) | | 0 | 0 | 0.0 | - | - | - |
| A0012 | Day 1 | 1 min | 3 | 1 | 1.0 | - | - | - |
| A0012 | Day 1 | 10 min | 3 | 1 | 1.0 | - | - | - |
| A0012 | Day 1 | 1 hour | 0 | 0 | 0.0 | - | - | - |
| A0012 | Day 1 | 2 hour | 0 | 0 | 0.0 | - | - | - |
| A0012 | Day 4 | | 0 | 0 | 0.0 | - | - | - |
| A0012 | Day 8 | | 0 | 0 | 0.0 | - | - | - |
| A0012 | Day 22 | | 0 | 0 | 0.0 | - | - | - |
| A0012 | EOS/ET (Day 61) | | 0 | 0 | 0.0 | - | - | - |
| A0013 | Day 1 | 1 min | 3 | 2 | 1.7 | - | - | - |
| A0013 | Day 1 | 10 min | 1 | 2 | 2.0 | - | - | - |
| A0013 | Day 1 | 1 hour | 0 | 0 | 0.0 | - | - | - |
| A0013 | Day 1 | 2 hour | 0 | 0 | 0.0 | - | - | - |
| A0013 | Day 4 | | 0 | 0 | 0.0 | - | - | - |
| A0013 | Day 8 | | 0 | 0 | 0.0 | - | - | - |
| A0013 | Day 22 | | 0 | 0 | 0.0 | - | - | - |
| A0013 | EOS/ET (Day 61) | | 0 | 0 | 0.0 | - | - | - |


Source: Listing 16.2.9.3

Administration Site Key: FA = Forearm; UA= Upper arm
Treatments Part A: A1 = A/Sing MAP, 15 ug (FA); A2 = Afluria Quad IM (UA), 15 ug; A3 = Placebo MAP (FA); A4 = A/Sing Mono IM, 15 ug (UA)
Treatments Part B: B1 = A/Sing MAP, 15 ug (FA) with biopsy; B2 = Placebo MAP (FA) with biopsy
B3 = A/Sing MAP, 15 ug (FA); B4 = A/Sing MAP, 10 ug (FA); B5 = A/Sing MAP, 5 ug (FA); B6 = A/Sing MAP, 2.5 ug (FA)
B7 = Placebo MAP (FA); B8 = A/Sing MAP, 15 ug (UA); B9 = A/Sing IM, 15 ug, as 2018 Afluria Quad (UA)

Listing 16.2.9.10
Individual Characterisation of Numeric Pain Intensity by Treatment

Part A
A1= 3 x 5 ug A/Sing MAPs on volar forearm (15 ug)

| | | | -------Active------- | | | -------Placebo------- | | |
|---|---|---|---|---|---|---|---|---|
| Randomisation Number | Visit | Time Point | #> 0 | Maximum | Average | #> 0 | Maximum | Average |
| A0015 | Day 1 | 1 min | 3 | 2 | 1.3 | - | - | - |
| A0015 | Day 1 | 10 min | 3 | 1 | 1.0 | - | - | - |
| A0015 | Day 1 | 1 hour | 0 | 0 | 0.0 | - | - | - |
| A0015 | Day 1 | 2 hour | 0 | 0 | 0.0 | - | - | - |
| A0015 | Day 4 | | 0 | 0 | 0.0 | - | - | - |
| A0015 | Day 8 | | 0 | 0 | 0.0 | - | - | - |
| A0015 | Day 22 | | 0 | 0 | 0.0 | - | - | - |
| A0015 | EOS/ET (Day 61) | | 0 | 0 | 0.0 | - | - | - |
| A0018 | Day 1 | 1 min | 3 | 1 | 1.0 | - | - | - |
| A0018 | Day 1 | 10 min | 3 | 1 | 1.0 | - | - | - |
| A0018 | Day 1 | 1 hour | 0 | 0 | 0.0 | - | - | - |
| A0018 | Day 1 | 2 hour | 0 | 0 | 0.0 | - | - | - |
| A0018 | Day 4 | | 0 | 0 | 0.0 | - | - | - |
| A0018 | Day 8 | | 0 | 0 | 0.0 | - | - | - |
| A0018 | Day 22 | | 0 | 0 | 0.0 | - | - | - |
| A0018 | EOS/ET (Day 61) | | 0 | 0 | 0.0 | - | - | - |
| A0020 | Day 1 | 1 min | 0 | 0 | 0.0 | - | - | - |
| A0020 | Day 1 | 10 min | 0 | 0 | 0.0 | - | - | - |
| A0020 | Day 1 | 1 hour | 0 | 0 | 0.0 | - | - | - |


Source: Listing 16.2.9.3

Administration Site Key: FA = Forearm; UA= Upper arm
Treatments Part A: A1 = A/Sing MAP, 15 ug (FA); A2 = Afluria Quad IM (UA), 15 ug; A3 = Placebo MAP (FA); A4 = A/Sing Mono IM, 15 ug (UA)
Treatments Part B: B1 = A/Sing MAP, 15 ug (FA) with biopsy; B2 = Placebo MAP (FA) with biopsy
B3 = A/Sing MAP, 15 ug (FA); B4 = A/Sing MAP, 10 ug (FA); B5 = A/Sing MAP, 5 ug (FA); B6 = A/Sing MAP, 2.5 ug (FA)
B7 = Placebo MAP (FA); B8 = A/Sing MAP, 15 ug (UA); B9 = A/Sing IM, 15 ug, as 2018 Afluria Quad (UA)

Listing 16.2.9.10
Individual Characterisation of Numeric Pain Intensity by Treatment

Part A
A1= 3 x 5 ug A/Sing MAPs on volar forearm (15 ug)

| | | | -------Active------- | | | -------Placebo------- | | |
|---|---|---|---|---|---|---|---|---|
| Randomisation Number | Visit | Time Point | #> 0 | Maximum | Average | #> 0 | Maximum | Average |
| A0020 | Day 1 | 2 hour | 0 | 0 | 0.0 | - | - | - |
| A0020 | Day 4 | | 0 | 0 | 0.0 | - | - | - |
| A0020 | Day 8 | | 0 | 0 | 0.0 | - | - | - |
| A0020 | Day 22 | | 0 | 0 | 0.0 | - | - | - |
| A0020 | EOS/ET (Day 61) | | 0 | 0 | 0.0 | - | - | - |
| A0021 | Day 1 | 1 min | 3 | 2 | 1.7 | - | - | - |
| A0021 | Day 1 | 10 min | 3 | 1 | 1.0 | - | - | - |
| A0021 | Day 1 | 1 hour | 0 | 0 | 0.0 | - | - | - |
| A0021 | Day 1 | 2 hour | 0 | 0 | 0.0 | - | - | - |
| A0021 | Day 4 | | 0 | 0 | 0.0 | - | - | - |
| A0021 | Day 8 | | 0 | 0 | 0.0 | - | - | - |
| A0021 | Day 22 | | 0 | 0 | 0.0 | - | - | - |
| A0021 | EOS/ET (Day 61) | | 0 | 0 | 0.0 | - | - | - |
| A0024 | Day 1 | 1 min | 3 | 2 | 1.3 | - | - | - |
| A0024 | Day 1 | 10 min | 1 | 1 | 0.3 | - | - | - |
| A0024 | Day 1 | 1 hour | 0 | 0 | 0.0 | - | - | - |
| A0024 | Day 1 | 2 hour | 0 | 0 | 0.0 | - | - | - |
| A0024 | Day 4 | | 0 | 0 | 0.0 | - | - | - |
| A0024 | Day 8 | | 0 | 0 | 0.0 | - | - | - |


Source: Listing 16.2.9.3

Administration Site Key: FA = Forearm; UA= Upper arm
Treatments Part A: A1 = A/Sing MAP, 15 ug (FA); A2 = Afluria Quad IM (UA), 15 ug; A3 = Placebo MAP (FA); A4 = A/Sing Mono IM, 15 ug (UA)
Treatments Part B: B1 = A/Sing MAP, 15 ug (FA) with biopsy; B2 = Placebo MAP (FA) with biopsy
B3 = A/Sing MAP, 15 ug (FA); B4 = A/Sing MAP, 10 ug (FA); B5 = A/Sing MAP, 5 ug (FA); B6 = A/Sing MAP, 2.5 ug (FA)
B7 = Placebo MAP (FA); B8 = A/Sing MAP, 15 ug (UA); B9 = A/Sing IM, 15 ug, as 2018 Afluria Quad (UA)

Listing 16.2.9.10
Individual Characterisation of Numeric Pain Intensity by Treatment

Part A
A1= 3 x 5 ug A/Sing MAPs on volar forearm (15 ug)

| | | | -------Active------- | | | -------Placebo------- | | |
|---|---|---|---|---|---|---|---|---|
| Randomisation Number | Visit | Time Point | #> 0 | Maximum | Average | #> 0 | Maximum | Average |
| A0024 | Day 22 | | 0 | 0 | 0.0 | - | - | - |
| A0024 | EOS/ET (Day 61) | | 0 | 0 | 0.0 | - | - | - |
| A0028 | Day 1 | 1 min | 2 | 1 | 0.7 | - | - | - |
| A0028 | Day 1 | 10 min | 0 | 0 | 0.0 | - | - | - |
| A0028 | Day 1 | 1 hour | 0 | 0 | 0.0 | - | - | - |
| A0028 | Day 1 | 2 hour | 0 | 0 | 0.0 | - | - | - |
| A0028 | Day 4 | | 0 | 0 | 0.0 | - | - | - |
| A0028 | Day 8 | | 0 | 0 | 0.0 | - | - | - |
| A0028 | Day 22 | | 0 | 0 | 0.0 | - | - | - |
| A0028 | EOS/ET (Day 61) | | 0 | 0 | 0.0 | - | - | - |
| A0029 | Day 1 | 1 min | 3 | 2 | 1.3 | - | - | - |
| A0029 | Day 1 | 10 min | 3 | 2 | 1.3 | - | - | - |
| A0029 | Day 1 | 1 hour | 0 | 0 | 0.0 | - | - | - |
| A0029 | Day 1 | 2 hour | 0 | 0 | 0.0 | - | - | - |
| A0029 | Day 4 | | 0 | 0 | 0.0 | - | - | - |
| A0029 | Day 8 | | 0 | 0 | 0.0 | - | - | - |
| A0029 | Day 22 | | 0 | 0 | 0.0 | - | - | - |
| A0029 | EOS/ET (Day 61) | | 0 | 0 | 0.0 | - | - | - |



Administration Site Key: FA = Forearm; UA= Upper arm
Treatments Part A: A1 = A/Sing MAP, 15 ug (FA); A2 = Afluria Quad IM (UA), 15 ug; A3 = Placebo MAP (FA); A4 = A/Sing Mono IM, 15 ug (UA)
Treatments Part B: B1 = A/Sing MAP, 15 ug (FA) with biopsy; B2 = Placebo MAP (FA) with biopsy
B3 = A/Sing MAP, 15 ug (FA); B4 = A/Sing MAP, 10 ug (FA); B5 = A/Sing MAP, 5 ug (FA); B6 = A/Sing MAP, 2.5 ug (FA)
B7 = Placebo MAP (FA); B8 = A/Sing MAP, 15 ug (UA); B9 = A/Sing IM, 15 ug, as 2018 Afluria Quad (UA)

Listing 16.2.9.10
Individual Characterisation of Numeric Pain Intensity by Treatment

Part A
A1= 3 x 5 ug A/Sing MAPs on volar forearm (15 ug)

| | | | -------Active------- | | | -------Placebo------- | | |
|---|---|---|---|---|---|---|---|---|
| Randomisation Number | Visit | Time Point | #> 0 | Maximum | Average | #> 0 | Maximum | Average |
| A1026 | Day 1 | 1 min | 0 | 0 | 0.0 | - | - | - |
| A1026 | Day 1 | 10 min | 0 | 0 | 0.0 | - | - | - |
| A1026 | Day 1 | 1 hour | 0 | 0 | 0.0 | - | - | - |
| A1026 | Day 1 | 2 hour | 0 | 0 | 0.0 | - | - | - |
| A1026 | Day 4 | | 0 | 0 | 0.0 | - | - | - |
| A1026 | Day 8 | | 0 | 0 | 0.0 | - | - | - |
| A1026 | Day 22 | | 0 | 0 | 0.0 | - | - | - |
| A1026 | EOS/ET (Day 61) | | 0 | 0 | 0.0 | - | - | - |


Source: Listing 16.2.9.3

Administration Site Key: FA = Forearm; UA= Upper arm
Treatments Part A: A1 = A/Sing MAP, 15 ug (FA); A2 = Afluria Quad IM (UA), 15 ug; A3 = Placebo MAP (FA); A4 = A/Sing Mono IM, 15 ug (UA)
Treatments Part B: B1 = A/Sing MAP, 15 ug (FA) with biopsy; B2 = Placebo MAP (FA) with biopsy
B3 = A/Sing MAP, 15 ug (FA); B4 = A/Sing MAP, 10 ug (FA); B5 = A/Sing MAP, 5 ug (FA); B6 = A/Sing MAP, 2.5 ug (FA)
B7 = Placebo MAP (FA); B8 = A/Sing MAP, 15 ug (UA); B9 = A/Sing IM, 15 ug, as 2018 Afluria Quad (UA)

Listing 16.2.9.10
Individual Characterisation of Numeric Pain Intensity by Treatment

Part A
A3= 3 x uncoated MAPs on volar forearm

| | | | -------Active------- | | | -------Placebo------- | | |
|---|---|---|---|---|---|---|---|---|
| Randomisation Number | Visit | Time Point | #> 0 | Maximum | Average | #> 0 | Maximum | Average |
| A0001 | Day 1 | 1 min | - | - | - | 3 | 1 | 1.0 |
| A0001 | Day 1 | 10 min | - | - | - | 0 | 0 | 0.0 |
| A0001 | Day 1 | 1 hour | - | - | - | 0 | 0 | 0.0 |
| A0001 | Day 1 | 2 hour | - | - | - | 0 | 0 | 0.0 |
| A0001 | Day 4 | | - | - | - | 0 | 0 | 0.0 |
| A0001 | Day 8 | | - | - | - | 0 | 0 | 0.0 |
| A0001 | Day 22 | | - | - | - | 0 | 0 | 0.0 |
| A0001 | EOS/ET (Day 61) | | - | - | - | 0 | 0 | 0.0 |
| A0004 | Day 1 | 1 min | - | - | - | 3 | 2 | 2.0 |
| A0004 | Day 1 | 10 min | - | - | - | 3 | 1 | 1.0 |
| A0004 | Day 1 | 1 hour | - | - | - | 0 | 0 | 0.0 |
| A0004 | Day 1 | 2 hour | - | - | - | 0 | 0 | 0.0 |
| A0004 | Day 4 | | - | - | - | 0 | 0 | 0.0 |
| A0004 | Day 8 | | - | - | - | 0 | 0 | 0.0 |
| A0004 | Day 22 | | - | - | - | 0 | 0 | 0.0 |
| A0004 | EOS/ET (Day 61) | | - | - | - | 0 | 0 | 0.0 |
| A0006 | Day 1 | 1 min | - | - | - | 0 | 0 | 0.0 |
| A0006 | Day 1 | 10 min | - | - | - | 3 | 1 | 1.0 |
| A0006 | Day 1 | 1 hour | - | - | - | 0 | 0 | 0.0 |


Source: Listing 16.2.9.3

Administration Site Key: FA = Forearm; UA= Upper arm
Treatments Part A: A1 = A/Sing MAP, 15 ug (FA); A2 = Afluria Quad IM (UA), 15 ug; A3 = Placebo MAP (FA); A4 = A/Sing Mono IM, 15 ug (UA)
Treatments Part B: B1 = A/Sing MAP, 15 ug (FA) with biopsy; B2 = Placebo MAP (FA) with biopsy
B3 = A/Sing MAP, 15 ug (FA); B4 = A/Sing MAP, 10 ug (FA); B5 = A/Sing MAP, 5 ug (FA); B6 = A/Sing MAP, 2.5 ug (FA)
B7 = Placebo MAP (FA); B8 = A/Sing MAP, 15 ug (UA); B9 = A/Sing IM, 15 ug, as 2018 Afluria Quad (UA)

Listing 16.2.9.10
Individual Characterisation of Numeric Pain Intensity by Treatment

Part A
A3= 3 x uncoated MAPs on volar forearm

| | | | -------Active------- | | | -------Placebo------- | | |
|---|---|---|---|---|---|---|---|---|
| Randomisation Number | Visit | Time Point | #> 0 | Maximum | Average | #> 0 | Maximum | Average |
| A0006 | Day 1 | 2 hour | - | - | - | 0 | 0 | 0.0 |
| A0006 | Day 4 | | - | - | - | 0 | 0 | 0.0 |
| A0006 | Day 8 | | - | - | - | 0 | 0 | 0.0 |
| A0006 | Day 22 | | - | - | - | 0 | 0 | 0.0 |
| A0006 | EOS/ET (Day 61) | | - | - | - | 0 | 0 | 0.0 |
| A0008 | Day 1 | 1 min | - | - | - | 3 | 3 | 2.3 |
| A0008 | Day 1 | 10 min | - | - | - | 2 | 2 | 1.3 |
| A0008 | Day 1 | 1 hour | - | - | - | 0 | 0 | 0.0 |
| A0008 | Day 1 | 2 hour | - | - | - | 0 | 0 | 0.0 |
| A0008 | Day 4 | | - | - | - | 0 | 0 | 0.0 |
| A0008 | Day 8 | | - | - | - | 0 | 0 | 0.0 |
| A0008 | Day 22 | | - | - | - | 0 | 0 | 0.0 |
| A0008 | EOS/ET (Day 61) | | - | - | - | 0 | 0 | 0.0 |
| A0010 | Day 1 | 1 min | - | - | - | 3 | 7 | 6.0 |
| A0010 | Day 1 | 10 min | - | - | - | 3 | 3 | 3.0 |
| A0010 | Day 1 | 1 hour | - | - | - | 0 | 0 | 0.0 |
| A0010 | Day 1 | 2 hour | - | - | - | 0 | 0 | 0.0 |
| A0010 | Day 4 | | - | - | - | 0 | 0 | 0.0 |
| A0010 | Day 8 | | - | - | - | 0 | 0 | 0.0 |


Source: Listing 16.2.9.3

Administration Site Key: FA = Forearm; UA= Upper arm
Treatments Part A: A1 = A/Sing MAP, 15 ug (FA); A2 = Afluria Quad IM (UA), 15 ug; A3 = Placebo MAP (FA); A4 = A/Sing Mono IM, 15 ug (UA)
Treatments Part B: B1 = A/Sing MAP, 15 ug (FA) with biopsy; B2 = Placebo MAP (FA) with biopsy
B3 = A/Sing MAP, 15 ug (FA); B4 = A/Sing MAP, 10 ug (FA); B5 = A/Sing MAP, 5 ug (FA); B6 = A/Sing MAP, 2.5 ug (FA)
B7 = Placebo MAP (FA); B8 = A/Sing MAP, 15 ug (UA); B9 = A/Sing IM, 15 ug, as 2018 Afluria Quad (UA)

Listing 16.2.9.10
Individual Characterisation of Numeric Pain Intensity by Treatment

Part A
A3= 3 x uncoated MAPs on volar forearm

| | | | -------Active------- | | | -------Placebo------- | | |
|---|---|---|---|---|---|---|---|---|
| Randomisation Number | Visit | Time Point | #> 0 | Maximum | Average | #> 0 | Maximum | Average |
| A0010 | Day 22 | | - | - | - | 0 | 0 | 0.0 |
| A0010 | EOS/ET (Day 61) | | - | - | - | 0 | 0 | 0.0 |
| A0011 | Day 1 | 1 min | - | - | - | 3 | 1 | 1.0 |
| A0011 | Day 1 | 10 min | - | - | - | 3 | 1 | 1.0 |
| A0011 | Day 1 | 1 hour | - | - | - | 0 | 0 | 0.0 |
| A0011 | Day 1 | 2 hour | - | - | - | 0 | 0 | 0.0 |
| A0011 | Day 4 | | - | - | - | 0 | 0 | 0.0 |
| A0011 | Day 8 | | - | - | - | 0 | 0 | 0.0 |
| A0011 | Day 22 | | - | - | - | 0 | 0 | 0.0 |
| A0011 | EOS/ET (Day 61) | | - | - | - | 0 | 0 | 0.0 |
| A0014 | Day 1 | 1 min | - | - | - | 3 | 3 | 3.0 |
| A0014 | Day 1 | 10 min | - | - | - | 3 | 2 | 2.0 |
| A0014 | Day 1 | 1 hour | - | - | - | 3 | 1 | 1.0 |
| A0014 | Day 1 | 2 hour | - | - | - | 0 | 0 | 0.0 |
| A0014 | Day 4 | | - | - | - | 0 | 0 | 0.0 |
| A0014 | Day 8 | | - | - | - | 0 | 0 | 0.0 |
| A0014 | Day 22 | | - | - | - | 0 | 0 | 0.0 |
| A0014 | EOS/ET (Day 61) | | - | - | - | 0 | 0 | 0.0 |


Source: Listing 16.2.9.3

Administration Site Key: FA = Forearm; UA= Upper arm
Treatments Part A: A1 = A/Sing MAP, 15 ug (FA); A2 = Afluria Quad IM (UA), 15 ug; A3 = Placebo MAP (FA); A4 = A/Sing Mono IM, 15 ug (UA)
Treatments Part B: B1 = A/Sing MAP, 15 ug (FA) with biopsy; B2 = Placebo MAP (FA) with biopsy
B3 = A/Sing MAP, 15 ug (FA); B4 = A/Sing MAP, 10 ug (FA); B5 = A/Sing MAP, 5 ug (FA); B6 = A/Sing MAP, 2.5 ug (FA)
B7 = Placebo MAP (FA); B8 = A/Sing MAP, 15 ug (UA); B9 = A/Sing IM, 15 ug, as 2018 Afluria Quad (UA)

Listing 16.2.9.10
Individual Characterisation of Numeric Pain Intensity by Treatment

Part A
A3= 3 x uncoated MAPs on volar forearm

| Randomisation Number | Visit | Time Point | Active #> 0 | Active Maximum | Active Average | Placebo #> 0 | Placebo Maximum | Placebo Average |
|---|---|---|---|---|---|---|---|---|
| A0016 | Day 1 | 1 min | - | - | - | 3 | 1 | 1.0 |
| A0016 | Day 1 | 10 min | - | - | - | 0 | 0 | 0.0 |
| A0016 | Day 1 | 1 hour | - | - | - | 0 | 0 | 0.0 |
| A0016 | Day 1 | 2 hour | - | - | - | 0 | 0 | 0.0 |
| A0016 | Day 4 | | - | - | - | 0 | 0 | 0.0 |
| A0016 | Day 8 | | - | - | - | 0 | 0 | 0.0 |
| A0016 | Day 22 | | - | - | - | 0 | 0 | 0.0 |
| A0016 | EOS/ET (Day 61) | | - | - | - | 0 | 0 | 0.0 |
| A0017 | Day 1 | 1 min | - | - | - | 0 | 0 | 0.0 |
| A0017 | Day 1 | 10 min | - | - | - | 0 | 0 | 0.0 |
| A0017 | Day 1 | 1 hour | - | - | - | 0 | 0 | 0.0 |
| A0017 | Day 1 | 2 hour | - | - | - | 0 | 0 | 0.0 |
| A0017 | Day 4 | | - | - | - | 0 | 0 | 0.0 |
| A0017 | Day 8 | | - | - | - | 0 | 0 | 0.0 |
| A0017 | Day 22 | | - | - | - | 0 | 0 | 0.0 |
| A0017 | EOS/ET (Day 61) | | - | - | - | 0 | 0 | 0.0 |
| A0019 | Day 1 | 1 min | - | - | - | 1 | 1 | 0.3 |
| A0019 | Day 1 | 10 min | - | - | - | 0 | 0 | 0.0 |
| A0019 | Day 1 | 1 hour | - | - | - | 0 | 0 | 0.0 |


Source: Listing 16.2.9.3

Administration Site Key: FA = Forearm; UA= Upper arm
Treatments Part A: A1 = A/Sing MAP, 15 ug (FA); A2 = Afluria Quad IM (UA), 15 ug; A3 = Placebo MAP (FA); A4 = A/Sing Mono IM, 15 ug (UA)
Treatments Part B: B1 = A/Sing MAP, 15 ug (FA) with biopsy; B2 = Placebo MAP (FA) with biopsy
B3 = A/Sing MAP, 15 ug (FA); B4 = A/Sing MAP, 10 ug (FA); B5 = A/Sing MAP, 5 ug (FA); B6 = A/Sing MAP, 2.5 ug (FA)
B7 = Placebo MAP (FA); B8 = A/Sing MAP, 15 ug (UA); B9 = A/Sing IM, 15 ug, as 2018 Afluria Quad (UA)

Listing 16.2.9.10
Individual Characterisation of Numeric Pain Intensity by Treatment

Part A
A3= 3 x uncoated MAPs on volar forearm

| | | | -------Active------- | | | -------Placebo------- | | |
|---|---|---|---|---|---|---|---|---|
| Randomisation Number | Visit | Time Point | #> 0 | Maximum | Average | #> 0 | Maximum | Average |
| A0019 | Day 1 | 2 hour | - | - | - | 0 | 0 | 0.0 |
| A0019 | Day 4 | | - | - | - | 0 | 0 | 0.0 |
| A0019 | Day 8 | | - | - | - | 0 | 0 | 0.0 |
| A0019 | Day 22 | | - | - | - | 0 | 0 | 0.0 |
| A0019 | EOS/ET (Day 61) | | - | - | - | 0 | 0 | 0.0 |
| A0022 | Day 1 | 1 min | - | - | - | 3 | 3 | 2.0 |
| A0022 | Day 1 | 10 min | - | - | - | 2 | 4 | 2.0 |
| A0022 | Day 1 | 1 hour | - | - | - | 0 | 0 | 0.0 |
| A0022 | Day 1 | 2 hour | - | - | - | 0 | 0 | 0.0 |
| A0022 | Day 4 | | - | - | - | 0 | 0 | 0.0 |
| A0022 | Day 8 | | - | - | - | 0 | 0 | 0.0 |
| A0022 | Day 22 | | - | - | - | 0 | 0 | 0.0 |
| A0022 | EOS/ET (Day 61) | | - | - | - | 0 | 0 | 0.0 |
| A0023 | Day 1 | 1 min | - | - | - | 3 | 3 | 2.0 |
| A0023 | Day 1 | 10 min | - | - | - | 2 | 1 | 0.7 |
| A0023 | Day 1 | 1 hour | - | - | - | 0 | 0 | 0.0 |
| A0023 | Day 1 | 2 hour | - | - | - | 0 | 0 | 0.0 |
| A0023 | Day 4 | | - | - | - | 0 | 0 | 0.0 |
| A0023 | Day 8 | | - | - | - | 0 | 0 | 0.0 |



Administration Site Key: FA = Forearm; UA= Upper arm
Treatments Part A: A1 = A/Sing MAP, 15 ug (FA); A2 = Afluria Quad IM (UA), 15 ug; A3 = Placebo MAP (FA); A4 = A/Sing Mono IM, 15 ug (UA)
Treatments Part B: B1 = A/Sing MAP, 15 ug (FA) with biopsy; B2 = Placebo MAP (FA) with biopsy
B3 = A/Sing MAP, 15 ug (FA); B4 = A/Sing MAP, 10 ug (FA); B5 = A/Sing MAP, 5 ug (FA); B6 = A/Sing MAP, 2.5 ug (FA)
B7 = Placebo MAP (FA); B8 = A/Sing MAP, 15 ug (UA); B9 = A/Sing IM, 15 ug, as 2018 Afluria Quad (UA)

Listing 16.2.9.10
Individual Characterisation of Numeric Pain Intensity by Treatment

Part A
A3= 3 x uncoated MAPs on volar forearm

| | | | -------Active------- | | | -------Placebo------- | | |
|---|---|---|---|---|---|---|---|---|
| Randomisation Number | Visit | Time Point | #> 0 | Maximum | Average | #> 0 | Maximum | Average |
| A0023 | Day 22 | | - | - | - | 0 | 0 | 0.0 |
| A0023 | EOS/ET (Day 61) | | - | - | - | 0 | 0 | 0.0 |
| A0025 | Day 1 | 1 min | - | - | - | 0 | 0 | 0.0 |
| A0025 | Day 1 | 10 min | - | - | - | 3 | 3 | 2.7 |
| A0025 | Day 1 | 1 hour | - | - | - | 0 | 0 | 0.0 |
| A0025 | Day 1 | 2 hour | - | - | - | 0 | 0 | 0.0 |
| A0025 | Day 4 | | - | - | - | 0 | 0 | 0.0 |
| A0025 | Day 8 | | - | - | - | 0 | 0 | 0.0 |
| A0025 | Day 22 | | - | - | - | 0 | 0 | 0.0 |
| A0025 | EOS/ET (Day 61) | | - | - | - | 0 | 0 | 0.0 |
| A0027 | Day 1 | 1 min | - | - | - | 3 | 1 | 1.0 |
| A0027 | Day 1 | 10 min | - | - | - | 3 | 1 | 1.0 |
| A0027 | Day 1 | 1 hour | - | - | - | 0 | 0 | 0.0 |
| A0027 | Day 1 | 2 hour | - | - | - | 0 | 0 | 0.0 |
| A0027 | Day 4 | | - | - | - | 0 | 0 | 0.0 |
| A0027 | Day 8 | | - | - | - | 0 | 0 | 0.0 |
| A0027 | Day 22 | | - | - | - | 0 | 0 | 0.0 |
| A0027 | EOS/ET (Day 61) | | - | - | - | 0 | 0 | 0.0 |


Source: Listing 16.2.9.3

Administration Site Key: FA = Forearm; UA= Upper arm
Treatments Part A: A1 = A/Sing MAP, 15 ug (FA); A2 = Afluria Quad IM (UA), 15 ug; A3 = Placebo MAP (FA); A4 = A/Sing Mono IM, 15 ug (UA)
Treatments Part B: B1 = A/Sing MAP, 15 ug (FA) with biopsy; B2 = Placebo MAP (FA) with biopsy
B3 = A/Sing MAP, 15 ug (FA); B4 = A/Sing MAP, 10 ug (FA); B5 = A/Sing MAP, 5 ug (FA); B6 = A/Sing MAP, 2.5 ug (FA)
B7 = Placebo MAP (FA); B8 = A/Sing MAP, 15 ug (UA); B9 = A/Sing IM, 15 ug, as 2018 Afluria Quad (UA)

Listing 16.2.9.10
Individual Characterisation of Numeric Pain Intensity by Treatment

Part A
A3= 3 x uncoated MAPs on volar forearm

| | | | -------Active------- | | | -------Placebo------- | | |
|---|---|---|---|---|---|---|---|---|
| Randomisation Number | Visit | Time Point | #> 0 | Maximum | Average | #> 0 | Maximum | Average |
| A0030 | Day 1 | 1 min | - | - | - | 3 | 1 | 1.0 |
| A0030 | Day 1 | 10 min | - | - | - | 0 | 0 | 0.0 |
| A0030 | Day 1 | 1 hour | - | - | - | 0 | 0 | 0.0 |
| A0030 | Day 1 | 2 hour | - | - | - | 0 | 0 | 0.0 |
| A0030 | Day 4 | | - | - | - | 0 | 0 | 0.0 |
| A0030 | Day 8 | | - | - | - | 0 | 0 | 0.0 |
| A0030 | Day 22 | | - | - | - | 0 | 0 | 0.0 |
| A0030 | EOS/ET (Day 61) | | - | - | - | 0 | 0 | 0.0 |


Source: Listing 16.2.9.3

Administration Site Key: FA = Forearm; UA= Upper arm
Treatments Part A: A1 = A/Sing MAP, 15 ug (FA); A2 = Afluria Quad IM (UA), 15 ug; A3 = Placebo MAP (FA); A4 = A/Sing Mono IM, 15 ug (UA)
Treatments Part B: B1 = A/Sing MAP, 15 ug (FA) with biopsy; B2 = Placebo MAP (FA) with biopsy
B3 = A/Sing MAP, 15 ug (FA); B4 = A/Sing MAP, 10 ug (FA); B5 = A/Sing MAP, 5 ug (FA); B6 = A/Sing MAP, 2.5 ug (FA)
B7 = Placebo MAP (FA); B8 = A/Sing MAP, 15 ug (UA); B9 = A/Sing IM, 15 ug, as 2018 Afluria Quad (UA)

Listing 16.2.9.10
Individual Characterisation of Numeric Pain Intensity by Treatment

Part B, Biopsy
B1= 3 x 5 ug A/Sing MAPs on volar forearm (15 ug)

| | | | -------Active------- | | | -------Placebo------- | | |
|---|---|---|---|---|---|---|---|---|
| Randomisation Number | Visit | Time Point | #> 0 | Maximum | Average | #> 0 | Maximum | Average |
| B0001 | Day 2 | | 0 | 0 | 0.0 | - | - | - |
| B0001 | Day 1 | 1 min | 3 | 3 | 2.7 | - | - | - |
| B0001 | Day 1 | 10 min | 3 | 1 | 1.0 | - | - | - |
| B0001 | Day 1 | 1 hour | 0 | 0 | 0.0 | - | - | - |
| B0001 | Day 1 | 2 hour | 0 | 0 | 0.0 | - | - | - |
| B0001 | Day 4 | | 0 | 0 | 0.0 | - | - | - |
| B0001 | Day 8 | | 0 | 0 | 0.0 | - | - | - |
| B0001 | Day 22 | | 0 | 0 | 0.0 | - | - | - |
| B0001 | EOS/ET (Day 61) | | 0 | 0 | 0.0 | - | - | - |
| B0004 | Day 2 | | 3 | 1 | 1.0 | - | - | - |
| B0004 | Day 1 | 1 min | 3 | 1 | 1.0 | - | - | - |
| B0004 | Day 1 | 10 min | 3 | 2 | 1.3 | - | - | - |
| B0004 | Day 1 | 1 hour | 0 | 0 | 0.0 | - | - | - |
| B0004 | Day 1 | 2 hour | 0 | 0 | 0.0 | - | - | - |
| B0004 | Day 4 | | 0 | 0 | 0.0 | - | - | - |
| B0004 | Day 8 | | 0 | 0 | 0.0 | - | - | - |
| B0004 | Day 22 | | 0 | 0 | 0.0 | - | - | - |
| B0004 | EOS/ET (Day 61) | | 0 | 0 | 0.0 | - | - | - |
| B0005 | Day 2 | | 0 | 0 | 0.0 | - | - | - |


Source: Listing 16.2.9.3

Administration Site Key: FA = Forearm; UA= Upper arm
Treatments Part A: A1 = A/Sing MAP, 15 ug (FA); A2 = Afluria Quad IM (UA), 15 ug; A3 = Placebo MAP (FA); A4 = A/Sing Mono IM, 15 ug (UA)
Treatments Part B: B1 = A/Sing MAP, 15 ug (FA) with biopsy; B2 = Placebo MAP (FA) with biopsy
B3 = A/Sing MAP, 15 ug (FA); B4 = A/Sing MAP, 10 ug (FA); B5 = A/Sing MAP, 5 ug (FA); B6 = A/Sing MAP, 2.5 ug (FA)
B7 = Placebo MAP (FA); B8 = A/Sing MAP, 15 ug (UA); B9 = A/Sing IM, 15 ug, as 2018 Afluria Quad (UA)

Listing 16.2.9.10
Individual Characterisation of Numeric Pain Intensity by Treatment

Part B, Biopsy
B1= 3 x 5 ug A/Sing MAPs on volar forearm (15 ug)

| | | | Active | | | Placebo | | |
|---|---|---|---|---|---|---|---|---|
| Randomisation Number | Visit | Time Point | #> 0 | Maximum | Average | #> 0 | Maximum | Average |
| B0005 | Day 1 | 1 min | 3 | 2 | 1.3 | - | - | - |
| B0005 | Day 1 | 10 min | 2 | 2 | 1.0 | - | - | - |
| B0005 | Day 1 | 1 hour | 0 | 0 | 0.0 | - | - | - |
| B0005 | Day 1 | 2 hour | 0 | 0 | 0.0 | - | - | - |
| B0005 | Day 4 | | 3 | 1 | 1.0 | - | - | - |
| B0005 | Day 8 | | 2 | 1 | 0.7 | - | - | - |
| B0005 | Day 22 | | 0 | 0 | 0.0 | - | - | - |
| B0005 | EOS/ET (Day 61) | | 0 | 0 | 0.0 | - | - | - |
| B0008 | Day 2 | | 0 | 0 | 0.0 | - | - | - |
| B0008 | Day 1 | 1 min | 0 | 0 | 0.0 | - | - | - |
| B0008 | Day 1 | 10 min | 0 | 0 | 0.0 | - | - | - |
| B0008 | Day 1 | 1 hour | 0 | 0 | 0.0 | - | - | - |
| B0008 | Day 1 | 2 hour | 0 | 0 | 0.0 | - | - | - |
| B0008 | Day 4 | | 0 | 0 | 0.0 | - | - | - |
| B0008 | Day 8 | | 0 | 0 | 0.0 | - | - | - |
| B0008 | Day 22 | | 0 | 0 | 0.0 | - | - | - |
| B0008 | EOS/ET (Day 61) | | 0 | 0 | 0.0 | - | - | - |
| B0009 | Day 2 | | 0 | 0 | 0.0 | - | - | - |
| B0009 | Day 1 | 1 min | 1 | 1 | 0.3 | - | - | - |


Source: Listing 16.2.9.3

Administration Site Key: FA = Forearm; UA= Upper arm
Treatments Part A: A1 = A/Sing MAP, 15 ug (FA); A2 = Afluria Quad IM (UA), 15 ug; A3 = Placebo MAP (FA); A4 = A/Sing Mono IM, 15 ug (UA)
Treatments Part B: B1 = A/Sing MAP, 15 ug (FA) with biopsy; B2 = Placebo MAP (FA) with biopsy
B3 = A/Sing MAP, 15 ug (FA); B4 = A/Sing MAP, 10 ug (FA); B5 = A/Sing MAP, 5 ug (FA); B6 = A/Sing MAP, 2.5 ug (FA)
B7 = Placebo MAP (FA); B8 = A/Sing MAP, 15 ug (UA); B9 = A/Sing IM, 15 ug, as 2018 Afluria Quad (UA)

Listing 16.2.9.10
Individual Characterisation of Numeric Pain Intensity by Treatment

Part B, Biopsy
B1= 3 x 5 ug A/Sing MAPs on volar forearm (15 ug)

| | | | -------Active------- | | | -------Placebo------- | | |
|---|---|---|---|---|---|---|---|---|
| Randomisation Number | Visit | Time Point | #> 0 | Maximum | Average | #> 0 | Maximum | Average |
| B0009 | Day 1 | 10 min | 1 | 1 | 0.3 | - | - | - |
| B0009 | Day 1 | 1 hour | 0 | 0 | 0.0 | - | - | - |
| B0009 | Day 1 | 2 hour | 0 | 0 | 0.0 | - | - | - |
| B0009 | Day 4 | | 0 | 0 | 0.0 | - | - | - |
| B0009 | Day 8 | | 1 | 1 | 0.3 | - | - | - |
| B0009 | Day 22 | | 0 | 0 | 0.0 | - | - | - |
| B0009 | EOS/ET (Day 61) | | 0 | 0 | 0.0 | - | - | - |



Administration Site Key: FA = Forearm; UA= Upper arm
Treatments Part A: A1 = A/Sing MAP, 15 ug (FA); A2 = Afluria Quad IM (UA), 15 ug; A3 = Placebo MAP (FA); A4 = A/Sing Mono IM, 15 ug (UA)
Treatments Part B: B1 = A/Sing MAP, 15 ug (FA) with biopsy; B2 = Placebo MAP (FA) with biopsy
B3 = A/Sing MAP, 15 ug (FA); B4 = A/Sing MAP, 10 ug (FA); B5 = A/Sing MAP, 5 ug (FA); B6 = A/Sing MAP, 2.5 ug (FA)
B7 = Placebo MAP (FA); B8 = A/Sing MAP, 15 ug (UA); B9 = A/Sing IM, 15 ug, as 2018 Afluria Quad (UA)

Listing 16.2.9.10
Individual Characterisation of Numeric Pain Intensity by Treatment

Part B, Biopsy
B2= 3 x uncoated MAPs on volar forearm

| Randomisation Number | Visit | Time Point | Active #> 0 | Active Maximum | Active Average | Placebo #> 0 | Placebo Maximum | Placebo Average |
|---|---|---|---|---|---|---|---|---|
| B0002 | Day 2 | | - | - | - | 0 | 0 | 0.0 |
| B0002 | Unscheduled | | - | - | - | 0 | 0 | 0.0 |
| B0002 | Day 1 | 1 min | - | - | - | 3 | 1 | 1.0 |
| B0002 | Day 1 | 10 min | - | - | - | 3 | 1 | 1.0 |
| B0002 | Day 1 | 1 hour | - | - | - | 0 | 0 | 0.0 |
| B0002 | Day 1 | 2 hour | - | - | - | 0 | 0 | 0.0 |
| B0002 | Day 4 | | - | - | - | 0 | 0 | 0.0 |
| B0002 | Day 8 | | - | - | - | 0 | 0 | 0.0 |
| B0002 | Day 22 | | - | - | - | 0 | 0 | 0.0 |
| B0002 | EOS/ET (Day 61) | | - | - | - | 0 | 0 | 0.0 |
| B0003 | Day 2 | | - | - | - | 0 | 0 | 0.0 |
| B0003 | Day 1 | 1 min | - | - | - | 3 | 3 | 2.7 |
| B0003 | Day 1 | 10 min | - | - | - | 3 | 3 | 2.3 |
| B0003 | Day 1 | 1 hour | - | - | - | 0 | 0 | 0.0 |
| B0003 | Day 1 | 2 hour | - | - | - | 0 | 0 | 0.0 |
| B0003 | Day 4 | | - | - | - | 0 | 0 | 0.0 |
| B0003 | Day 8 | | - | - | - | 0 | 0 | 0.0 |
| B0003 | Day 22 | | - | - | - | 0 | 0 | 0.0 |
| B0003 | EOS/ET (Day 61) | | - | - | - | 0 | 0 | 0.0 |


Source: Listing 16.2.9.3

Administration Site Key: FA = Forearm; UA= Upper arm
Treatments Part A: A1 = A/Sing MAP, 15 ug (FA); A2 = Afluria Quad IM (UA), 15 ug; A3 = Placebo MAP (FA); A4 = A/Sing Mono IM, 15 ug (UA)
Treatments Part B: B1 = A/Sing MAP, 15 ug (FA) with biopsy; B2 = Placebo MAP (FA) with biopsy
B3 = A/Sing MAP, 15 ug (FA); B4 = A/Sing MAP, 10 ug (FA); B5 = A/Sing MAP, 5 ug (FA); B6 = A/Sing MAP, 2.5 ug (FA)
B7 = Placebo MAP (FA); B8 = A/Sing MAP, 15 ug (UA); B9 = A/Sing IM, 15 ug, as 2018 Afluria Quad (UA)

Listing 16.2.9.10
Individual Characterisation of Numeric Pain Intensity by Treatment

Part B, Biopsy
B2= 3 x uncoated MAPs on volar forearm

| | | | -------Active------- | | | -------Placebo------- | | |
|---|---|---|---|---|---|---|---|---|
| Randomisation Number | Visit | Time Point | #> 0 | Maximum | Average | #> 0 | Maximum | Average |
| B0006 | Day 2 | | - | - | - | 0 | 0 | 0.0 |
| B0006 | Day 1 | 1 min | - | - | - | 1 | 1 | 0.3 |
| B0006 | Day 1 | 10 min | - | - | - | 3 | 1 | 1.0 |
| B0006 | Day 1 | 1 hour | - | - | - | 0 | 0 | 0.0 |
| B0006 | Day 1 | 2 hour | - | - | - | 3 | 1 | 1.0 |
| B0006 | Day 4 | | - | - | - | 0 | 0 | 0.0 |
| B0006 | Day 8 | | - | - | - | 3 | 3 | 3.0 |
| B0006 | Day 22 | | - | - | - | 0 | 0 | 0.0 |
| B0006 | EOS/ET (Day 61) | | - | - | - | 0 | 0 | 0.0 |
| B0007 | Day 2 | | - | - | - | 1 | 0 | 0.0 |
| B0007 | Day 1 | 1 min | - | - | - | 3 | 2 | 1.7 |
| B0007 | Day 1 | 10 min | - | - | - | 1 | 1 | 0.3 |
| B0007 | Day 1 | 1 hour | - | - | - | 0 | 0 | 0.0 |
| B0007 | Day 1 | 2 hour | - | - | - | 0 | 0 | 0.0 |
| B0007 | Day 4 | | - | - | - | 0 | 0 | 0.0 |
| B0007 | Day 8 | | - | - | - | 0 | 0 | 0.0 |
| B0007 | Day 22 | | - | - | - | 0 | 0 | 0.0 |
| B0007 | EOS/ET (Day 61) | | - | - | - | 0 | 0 | 0.0 |
| B0010 | Day 2 | | - | - | - | 0 | 0 | 0.0 |


Source: Listing 16.2.9.3

Administration Site Key: FA = Forearm; UA= Upper arm
Treatments Part A: A1 = A/Sing MAP, 15 ug (FA); A2 = Afluria Quad IM (UA), 15 ug; A3 = Placebo MAP (FA); A4 = A/Sing Mono IM, 15 ug (UA)
Treatments Part B: B1 = A/Sing MAP, 15 ug (FA) with biopsy; B2 = Placebo MAP (FA) with biopsy
B3 = A/Sing MAP, 15 ug (FA); B4 = A/Sing MAP, 10 ug (FA); B5 = A/Sing MAP, 5 ug (FA); B6 = A/Sing MAP, 2.5 ug (FA)
B7 = Placebo MAP (FA); B8 = A/Sing MAP, 15 ug (UA); B9 = A/Sing IM, 15 ug, as 2018 Afluria Quad (UA)

Listing 16.2.9.10
Individual Characterisation of Numeric Pain Intensity by Treatment

Part B, Biopsy
B2= 3 x uncoated MAPs on volar forearm

| | | | -------Active------- | | | -------Placebo------- | | |
|---|---|---|---|---|---|---|---|---|
| Randomisation Number | Visit | Time Point | #> 0 | Maximum | Average | #> 0 | Maximum | Average |
| B0010 | Day 1 | 1 min | - | - | - | 3 | 1 | 1.0 |
| B0010 | Day 1 | 10 min | - | - | - | 1 | 1 | 0.3 |
| B0010 | Day 1 | 1 hour | - | - | - | 0 | 0 | 0.0 |
| B0010 | Day 1 | 2 hour | - | - | - | 0 | 0 | 0.0 |
| B0010 | Day 4 | | - | - | - | 0 | 0 | 0.0 |
| B0010 | Day 8 | | - | - | - | 2 | 1 | 0.7 |
| B0010 | Day 22 | | - | - | - | 2 | 2 | 1.3 |
| B0010 | EOS/ET (Day 61) | | - | - | - | 0 | 0 | 0.0 |



Administration Site Key: FA = Forearm; UA= Upper arm
Treatments Part A: A1 = A/Sing MAP, 15 ug (FA); A2 = Afluria Quad IM (UA), 15 ug; A3 = Placebo MAP (FA); A4 = A/Sing Mono IM, 15 ug (UA)
Treatments Part B: B1 = A/Sing MAP, 15 ug (FA) with biopsy; B2 = Placebo MAP (FA) with biopsy
B3 = A/Sing MAP, 15 ug (FA); B4 = A/Sing MAP, 10 ug (FA); B5 = A/Sing MAP, 5 ug (FA); B6 = A/Sing MAP, 2.5 ug (FA)
B7 = Placebo MAP (FA); B8 = A/Sing MAP, 15 ug (UA); B9 = A/Sing IM, 15 ug, as 2018 Afluria Quad (UA)

Listing 16.2.9.10
Individual Characterisation of Numeric Pain Intensity by Treatment

Part B, Non-biopsy
B3= 3 x 5 ug A/Sing MAPs on volar forearm (15 ug)

| | | | -------Active------- | | | -------Placebo------- | | |
|---|---|---|---|---|---|---|---|---|
| Randomisation Number | Visit | Time Point | #> 0 | Maximum | Average | #> 0 | Maximum | Average |
| B0014 | Day 2 | | 0 | 0 | 0.0 | - | - | - |
| B0014 | Day 1 | 1 min | 3 | 2 | 1.7 | - | - | - |
| B0014 | Day 1 | 10 min | 0 | 0 | 0.0 | - | - | - |
| B0014 | Day 1 | 1 hour | 0 | 0 | 0.0 | - | - | - |
| B0014 | Day 1 | 2 hour | 0 | 0 | 0.0 | - | - | - |
| B0014 | Day 4 | | 0 | 0 | 0.0 | - | - | - |
| B0014 | Day 8 | | 0 | 0 | 0.0 | - | - | - |
| B0014 | Day 22 | | 0 | 0 | 0.0 | - | - | - |
| B0014 | EOS/ET (Day 61) | | 0 | 0 | 0.0 | - | - | - |
| B0023 | Day 2 | | 0 | 0 | 0.0 | - | - | - |
| B0023 | Day 1 | 1 min | 3 | 4 | 3.3 | - | - | - |
| B0023 | Day 1 | 10 min | 0 | 0 | 0.0 | - | - | - |
| B0023 | Day 1 | 1 hour | 3 | 1 | 1.0 | - | - | - |
| B0023 | Day 1 | 2 hour | 0 | 0 | 0.0 | - | - | - |
| B0023 | Day 4 | | 0 | 0 | 0.0 | - | - | - |
| B0023 | Day 8 | | 0 | 0 | 0.0 | - | - | - |
| B0023 | Day 22 | | 0 | 0 | 0.0 | - | - | - |
| B0023 | EOS/ET (Day 61) | | 0 | 0 | 0.0 | - | - | - |
| B0027 | Day 2 | | 0 | 0 | 0.0 | - | - | - |


Source: Listing 16.2.9.3

Administration Site Key: FA = Forearm; UA= Upper arm
Treatments Part A: A1 = A/Sing MAP, 15 ug (FA); A2 = Afluria Quad IM (UA), 15 ug; A3 = Placebo MAP (FA); A4 = A/Sing Mono IM, 15 ug (UA)
Treatments Part B: B1 = A/Sing MAP, 15 ug (FA) with biopsy; B2 = Placebo MAP (FA) with biopsy
B3 = A/Sing MAP, 15 ug (FA); B4 = A/Sing MAP, 10 ug (FA); B5 = A/Sing MAP, 5 ug (FA); B6 = A/Sing MAP, 2.5 ug (FA)
B7 = Placebo MAP (FA); B8 = A/Sing MAP, 15 ug (UA); B9 = A/Sing IM, 15 ug, as 2018 Afluria Quad (UA)

Listing 16.2.9.10
Individual Characterisation of Numeric Pain Intensity by Treatment

Part B, Non-biopsy
B3= 3 x 5 ug A/Sing MAPs on volar forearm (15 ug)

| | | | -------Active------- | | | -------Placebo------- | | |
|---|---|---|---|---|---|---|---|---|
| Randomisation Number | Visit | Time Point | #> 0 | Maximum | Average | #> 0 | Maximum | Average |
| B0027 | Day 1 | 1 min | 3 | 2 | 1.3 | - | - | - |
| B0027 | Day 1 | 10 min | 3 | 2 | 1.7 | - | - | - |
| B0027 | Day 1 | 1 hour | 1 | 1 | 0.3 | - | - | - |
| B0027 | Day 1 | 2 hour | 0 | 0 | 0.0 | - | - | - |
| B0027 | Day 4 | | 0 | 0 | 0.0 | - | - | - |
| B0027 | Day 8 | | 0 | 0 | 0.0 | - | - | - |
| B0027 | Day 22 | | 0 | 0 | 0.0 | - | - | - |
| B0027 | EOS/ET (Day 61) | | 0 | 0 | 0.0 | - | - | - |
| B0038 | Day 2 | | 0 | 0 | 0.0 | - | - | - |
| B0038 | Day 1 | 1 min | 3 | 2 | 1.3 | - | - | - |
| B0038 | Day 1 | 10 min | 3 | 2 | 1.3 | - | - | - |
| B0038 | Day 1 | 1 hour | 0 | 0 | 0.0 | - | - | - |
| B0038 | Day 1 | 2 hour | 0 | 0 | 0.0 | - | - | - |
| B0038 | Day 4 | | 0 | 0 | 0.0 | - | - | - |
| B0038 | Day 8 | | 0 | 0 | 0.0 | - | - | - |
| B0038 | Day 22 | | 0 | 0 | 0.0 | - | - | - |
| B0038 | EOS/ET (Day 61) | | 0 | 0 | 0.0 | - | - | - |
| B0041 | Day 2 | | 3 | 1 | 1.0 | - | - | - |
| B0041 | Day 1 | 1 min | 2 | 2 | 1.0 | - | - | - |


Source: Listing 16.2.9.3

Administration Site Key: FA = Forearm; UA= Upper arm
Treatments Part A: A1 = A/Sing MAP, 15 ug (FA); A2 = Afluria Quad IM (UA), 15 ug; A3 = Placebo MAP (FA); A4 = A/Sing Mono IM, 15 ug (UA)
Treatments Part B: B1 = A/Sing MAP, 15 ug (FA) with biopsy; B2 = Placebo MAP (FA) with biopsy
B3 = A/Sing MAP, 15 ug (FA); B4 = A/Sing MAP, 10 ug (FA); B5 = A/Sing MAP, 5 ug (FA); B6 = A/Sing MAP, 2.5 ug (FA)
B7 = Placebo MAP (FA); B8 = A/Sing MAP, 15 ug (UA); B9 = A/Sing IM, 15 ug, as 2018 Afluria Quad (UA)

Listing 16.2.9.10
Individual Characterisation of Numeric Pain Intensity by Treatment

Part B, Non-biopsy
B3= 3 x 5 ug A/Sing MAPs on volar forearm (15 ug)

| | | | -------Active------- | | | -------Placebo------- | | |
|---|---|---|---|---|---|---|---|---|
| Randomisation Number | Visit | Time Point | #> 0 | Maximum | Average | #> 0 | Maximum | Average |
| B0041 | Day 1 | 10 min | 1 | 1 | 0.3 | - | - | - |
| B0041 | Day 1 | 1 hour | 0 | 0 | 0.0 | - | - | - |
| B0041 | Day 1 | 2 hour | 0 | 0 | 0.0 | - | - | - |
| B0041 | Day 4 | | 0 | 0 | 0.0 | - | - | - |
| B0041 | Day 8 | | 0 | 0 | 0.0 | - | - | - |
| B0041 | Day 22 | | 0 | 0 | 0.0 | - | - | - |
| B0041 | EOS/ET (Day 61) | | 0 | 0 | 0.0 | - | - | - |
| B0051 | Day 2 | | 0 | 0 | 0.0 | - | - | - |
| B0051 | Day 1 | 1 min | 3 | 2 | 1.3 | - | - | - |
| B0051 | Day 1 | 10 min | 0 | 0 | 0.0 | - | - | - |
| B0051 | Day 1 | 1 hour | 0 | 0 | 0.0 | - | - | - |
| B0051 | Day 1 | 2 hour | 0 | 0 | 0.0 | - | - | - |
| B0051 | Day 4 | | 0 | 0 | 0.0 | - | - | - |
| B0051 | Day 8 | | 0 | 0 | 0.0 | - | - | - |
| B0051 | Day 22 | | 0 | 0 | 0.0 | - | - | - |
| B0051 | EOS/ET (Day 61) | | 0 | 0 | 0.0 | - | - | - |
| B0056 | Day 2 | | 0 | 0 | 0.0 | - | - | - |
| B0056 | Day 1 | 1 min | 3 | 1 | 1.0 | - | - | - |
| B0056 | Day 1 | 10 min | 3 | 3 | 2.3 | - | - | - |


Source: Listing 16.2.9.3

Administration Site Key: FA = Forearm; UA= Upper arm
Treatments Part A: A1 = A/Sing MAP, 15 ug (FA); A2 = Afluria Quad IM (UA), 15 ug; A3 = Placebo MAP (FA); A4 = A/Sing Mono IM, 15 ug (UA)
Treatments Part B: B1 = A/Sing MAP, 15 ug (FA) with biopsy; B2 = Placebo MAP (FA) with biopsy
B3 = A/Sing MAP, 15 ug (FA); B4 = A/Sing MAP, 10 ug (FA); B5 = A/Sing MAP, 5 ug (FA); B6 = A/Sing MAP, 2.5 ug (FA)
B7 = Placebo MAP (FA); B8 = A/Sing MAP, 15 ug (UA); B9 = A/Sing IM, 15 ug, as 2018 Afluria Quad (UA)

Listing 16.2.9.10
Individual Characterisation of Numeric Pain Intensity by Treatment

Part B, Non-biopsy
B3= 3 x 5 ug A/Sing MAPs on volar forearm (15 ug)

| | | | Active | | | Placebo | | |
|---|---|---|---|---|---|---|---|---|
| Randomisation Number | Visit | Time Point | #> 0 | Maximum | Average | #> 0 | Maximum | Average |
| B0056 | Day 1 | 1 hour | 0 | 0 | 0.0 | - | - | - |
| B0056 | Day 1 | 2 hour | 0 | 0 | 0.0 | - | - | - |
| B0056 | Day 4 | | 0 | 0 | 0.0 | - | - | - |
| B0056 | Day 8 | | 0 | 0 | 0.0 | - | - | - |
| B0056 | Day 22 | | 0 | 0 | 0.0 | - | - | - |
| B0056 | EOS/ET (Day 61) | | 0 | 0 | 0.0 | - | - | - |
| B0060 | Day 2 | | 0 | 0 | 0.0 | - | - | - |
| B0060 | Day 1 | 1 min | 1 | 1 | 0.3 | - | - | - |
| B0060 | Day 1 | 10 min | 0 | 0 | 0.0 | - | - | - |
| B0060 | Day 1 | 1 hour | 0 | 0 | 0.0 | - | - | - |
| B0060 | Day 1 | 2 hour | 0 | 0 | 0.0 | - | - | - |
| B0060 | Day 4 | | 0 | 0 | 0.0 | - | - | - |
| B0060 | Day 8 | | 0 | 0 | 0.0 | - | - | - |
| B0060 | Day 22 | | 0 | 0 | 0.0 | - | - | - |
| B0060 | EOS/ET (Day 61) | | 0 | 0 | 0.0 | - | - | - |
| B0072 | Day 2 | | 0 | 0 | 0.0 | - | - | - |
| B0072 | Day 1 | 1 min | 3 | 3 | 2.7 | - | - | - |
| B0072 | Day 1 | 10 min | 3 | 1 | 1.0 | - | - | - |
| B0072 | Day 1 | 1 hour | 0 | 0 | 0.0 | - | - | - |


Source: Listing 16.2.9.3

Administration Site Key: FA = Forearm; UA= Upper arm
Treatments Part A: A1 = A/Sing MAP, 15 ug (FA); A2 = Afluria Quad IM (UA), 15 ug; A3 = Placebo MAP (FA); A4 = A/Sing Mono IM, 15 ug (UA)
Treatments Part B: B1 = A/Sing MAP, 15 ug (FA) with biopsy; B2 = Placebo MAP (FA) with biopsy
B3 = A/Sing MAP, 15 ug (FA); B4 = A/Sing MAP, 10 ug (FA); B5 = A/Sing MAP, 5 ug (FA); B6 = A/Sing MAP, 2.5 ug (FA)
B7 = Placebo MAP (FA); B8 = A/Sing MAP, 15 ug (UA); B9 = A/Sing IM, 15 ug, as 2018 Afluria Quad (UA)

Listing 16.2.9.10
Individual Characterisation of Numeric Pain Intensity by Treatment

Part B, Non-biopsy
B3= 3 x 5 ug A/Sing MAPs on volar forearm (15 ug)

| | | | -------Active------- | | | -------Placebo------- | | |
|---|---|---|---|---|---|---|---|---|
| Randomisation Number | Visit | Time Point | #> 0 | Maximum | Average | #> 0 | Maximum | Average |
| B0072 | Day 1 | 2 hour | 2 | 1 | 0.7 | - | - | - |
| B0072 | Day 4 | | 0 | 0 | 0.0 | - | - | - |
| B0072 | Day 8 | | 0 | 0 | 0.0 | - | - | - |
| B0072 | Day 22 | | 0 | 0 | 0.0 | - | - | - |
| B0072 | EOS/ET (Day 61) | | 0 | 0 | 0.0 | - | - | - |
| B0075 | Day 2 | | 1 | 1 | 0.3 | - | - | - |
| B0075 | Day 1 | 1 min | 3 | 2 | 2.0 | - | - | - |
| B0075 | Day 1 | 10 min | 3 | 3 | 2.7 | - | - | - |
| B0075 | Day 1 | 1 hour | 3 | 3 | 2.0 | - | - | - |
| B0075 | Day 1 | 2 hour | 1 | 1 | 0.3 | - | - | - |
| B0075 | Day 4 | | 0 | 0 | 0.0 | - | - | - |
| B0075 | Day 8 | | 0 | 0 | 0.0 | - | - | - |
| B0075 | Day 22 | | 0 | 0 | 0.0 | - | - | - |
| B0075 | EOS/ET (Day 61) | | 0 | 0 | 0.0 | - | - | - |
| B0082 | Day 2 | | 0 | 0 | 0.0 | - | - | - |
| B0082 | Day 1 | 1 min | 3 | 1 | 1.0 | - | - | - |
| B0082 | Day 1 | 10 min | 0 | 0 | 0.0 | - | - | - |
| B0082 | Day 1 | 1 hour | 0 | 0 | 0.0 | - | - | - |
| B0082 | Day 1 | 2 hour | 0 | 0 | 0.0 | - | - | - |


Source: Listing 16.2.9.3

Administration Site Key: FA = Forearm; UA= Upper arm
Treatments Part A: A1 = A/Sing MAP, 15 ug (FA); A2 = Afluria Quad IM (UA), 15 ug; A3 = Placebo MAP (FA); A4 = A/Sing Mono IM, 15 ug (UA)
Treatments Part B: B1 = A/Sing MAP, 15 ug (FA) with biopsy; B2 = Placebo MAP (FA) with biopsy
B3 = A/Sing MAP, 15 ug (FA); B4 = A/Sing MAP, 10 ug (FA); B5 = A/Sing MAP, 5 ug (FA); B6 = A/Sing MAP, 2.5 ug (FA)
B7 = Placebo MAP (FA); B8 = A/Sing MAP, 15 ug (UA); B9 = A/Sing IM, 15 ug, as 2018 Afluria Quad (UA)

Listing 16.2.9.10
Individual Characterisation of Numeric Pain Intensity by Treatment

Part B, Non-biopsy
B3= 3 x 5 ug A/Sing MAPs on volar forearm (15 ug)

| | | | -------Active------- | | | -------Placebo------- | | |
|---|---|---|---|---|---|---|---|---|
| Randomisation Number | Visit | Time Point | #> 0 | Maximum | Average | #> 0 | Maximum | Average |
| B0082 | Day 4 | | 0 | 0 | 0.0 | - | - | - |
| B0082 | Day 8 | | 0 | 0 | 0.0 | - | - | - |
| B0082 | Day 22 | | 0 | 0 | 0.0 | - | - | - |
| B0082 | EOS/ET (Day 61) | | 0 | 0 | 0.0 | - | - | - |
| B0094 | Day 2 | | 0 | 0 | 0.0 | - | - | - |
| B0094 | Day 1 | 1 min | 0 | 0 | 0.0 | - | - | - |
| B0094 | Day 1 | 10 min | 0 | 0 | 0.0 | - | - | - |
| B0094 | Day 1 | 1 hour | 0 | 0 | 0.0 | - | - | - |
| B0094 | Day 1 | 2 hour | 0 | 0 | 0.0 | - | - | - |
| B0094 | Day 4 | | 0 | 0 | 0.0 | - | - | - |
| B0094 | Day 8 | | 0 | 0 | 0.0 | - | - | - |
| B0094 | Day 22 | | 0 | 0 | 0.0 | - | - | - |
| B0094 | EOS/ET (Day 61) | | 0 | 0 | 0.0 | - | - | - |
| B0101 | Day 2 | | 0 | 0 | 0.0 | - | - | - |
| B0101 | Day 1 | 1 min | 1 | 1 | 0.3 | - | - | - |
| B0101 | Day 1 | 10 min | 0 | 0 | 0.0 | - | - | - |
| B0101 | Day 1 | 1 hour | 0 | 0 | 0.0 | - | - | - |
| B0101 | Day 1 | 2 hour | 3 | - | - | - | - | - |
| B0101 | Day 4 | | 0 | 0 | 0.0 | - | - | - |


Source: Listing 16.2.9.3

Administration Site Key: FA = Forearm; UA= Upper arm
Treatments Part A: A1 = A/Sing MAP, 15 ug (FA); A2 = Afluria Quad IM (UA), 15 ug; A3 = Placebo MAP (FA); A4 = A/Sing Mono IM, 15 ug (UA)
Treatments Part B: B1 = A/Sing MAP, 15 ug (FA) with biopsy; B2 = Placebo MAP (FA) with biopsy
B3 = A/Sing MAP, 15 ug (FA); B4 = A/Sing MAP, 10 ug (FA); B5 = A/Sing MAP, 5 ug (FA); B6 = A/Sing MAP, 2.5 ug (FA)
B7 = Placebo MAP (FA); B8 = A/Sing MAP, 15 ug (UA); B9 = A/Sing IM, 15 ug, as 2018 Afluria Quad (UA)

Listing 16.2.9.10
Individual Characterisation of Numeric Pain Intensity by Treatment

Part B, Non-biopsy
B3= 3 x 5 ug A/Sing MAPs on volar forearm (15 ug)

| | | | -------Active------- | | | -------Placebo------- | | |
|---|---|---|---|---|---|---|---|---|
| Randomisation Number | Visit | Time Point | #> 0 | Maximum | Average | #> 0 | Maximum | Average |
| B0101 | Day 8 | | 0 | 0 | 0.0 | - | - | - |
| B0101 | Day 22 | | 0 | 0 | 0.0 | - | - | - |
| B0101 | EOS/ET (Day 61) | | 0 | 0 | 0.0 | - | - | - |
| B0104 | Day 2 | | 0 | 0 | 0.0 | - | - | - |
| B0104 | Day 1 | 1 min | 1 | 2 | 0.7 | - | - | - |
| B0104 | Day 1 | 10 min | 3 | 1 | 1.0 | - | - | - |
| B0104 | Day 1 | 1 hour | 0 | 0 | 0.0 | - | - | - |
| B0104 | Day 1 | 2 hour | 0 | 0 | 0.0 | - | - | - |
| B0104 | Day 4 | | 0 | 0 | 0.0 | - | - | - |
| B0104 | Day 8 | | 0 | 0 | 0.0 | - | - | - |
| B0104 | Day 22 | | 0 | 0 | 0.0 | - | - | - |
| B0104 | EOS/ET (Day 61) | | 0 | 0 | 0.0 | - | - | - |
| B0110 | Day 2 | | 0 | 0 | 0.0 | - | - | - |
| B0110 | Day 1 | 1 min | 3 | 2 | 2.0 | - | - | - |
| B0110 | Day 1 | 10 min | 3 | 1 | 1.0 | - | - | - |
| B0110 | Day 1 | 1 hour | 0 | 0 | 0.0 | - | - | - |
| B0110 | Day 1 | 2 hour | 0 | 0 | 0.0 | - | - | - |
| B0110 | Day 4 | | 0 | 0 | 0.0 | - | - | - |
| B0110 | Day 8 | | 0 | 0 | 0.0 | - | - | - |


Source: Listing 16.2.9.3

Administration Site Key: FA = Forearm; UA= Upper arm
Treatments Part A: A1 = A/Sing MAP, 15 ug (FA); A2 = Afluria Quad IM (UA), 15 ug; A3 = Placebo MAP (FA); A4 = A/Sing Mono IM, 15 ug (UA)
Treatments Part B: B1 = A/Sing MAP, 15 ug (FA) with biopsy; B2 = Placebo MAP (FA) with biopsy
B3 = A/Sing MAP, 15 ug (FA); B4 = A/Sing MAP, 10 ug (FA); B5 = A/Sing MAP, 5 ug (FA); B6 = A/Sing MAP, 2.5 ug (FA)
B7 = Placebo MAP (FA); B8 = A/Sing MAP, 15 ug (UA); B9 = A/Sing IM, 15 ug, as 2018 Afluria Quad (UA)

Listing 16.2.9.10
Individual Characterisation of Numeric Pain Intensity by Treatment

Part B, Non-biopsy
B3= 3 x 5 ug A/Sing MAPs on volar forearm (15 ug)

| | | | Active | | | Placebo | | |
|---|---|---|---|---|---|---|---|---|
| Randomisation Number | Visit | Time Point | #> 0 | Maximum | Average | #> 0 | Maximum | Average |
| B0110 | Day 22 | | 0 | 0 | 0.0 | - | - | - |
| B0110 | EOS/ET (Day 61) | | 0 | 0 | 0.0 | - | - | - |
| B0120 | Day 2 | | 0 | 0 | 0.0 | - | - | - |
| B0120 | Day 1 | 1 min | 3 | 6 | 5.3 | - | - | - |
| B0120 | Day 1 | 10 min | 2 | 3 | 2.0 | - | - | - |
| B0120 | Day 1 | 1 hour | 3 | 1 | 1.0 | - | - | - |
| B0120 | Day 1 | 2 hour | 0 | 0 | 0.0 | - | - | - |
| B0120 | Day 4 | | 0 | 0 | 0.0 | - | - | - |
| B0120 | Day 8 | | 0 | 0 | 0.0 | - | - | - |
| B0120 | Day 22 | | 0 | 0 | 0.0 | - | - | - |
| B0120 | EOS/ET (Day 61) | | 0 | 0 | 0.0 | - | - | - |
| B0126 | Day 2 | | 0 | 0 | 0.0 | - | - | - |
| B0126 | Day 1 | 1 min | 3 | 2 | 2.0 | - | - | - |
| B0126 | Day 1 | 10 min | 2 | 2 | 1.3 | - | - | - |
| B0126 | Day 1 | 1 hour | 0 | 0 | 0.0 | - | - | - |
| B0126 | Day 1 | 2 hour | 0 | 0 | 0.0 | - | - | - |
| B0126 | Day 4 | | 0 | 0 | 0.0 | - | - | - |
| B0126 | Day 8 | | 0 | 0 | 0.0 | - | - | - |
| B0126 | Day 22 | | 0 | 0 | 0.0 | - | - | - |



Administration Site Key: FA = Forearm; UA= Upper arm
Treatments Part A: A1 = A/Sing MAP, 15 ug (FA); A2 = Afluria Quad IM (UA), 15 ug; A3 = Placebo MAP (FA); A4 = A/Sing Mono IM, 15 ug (UA)
Treatments Part B: B1 = A/Sing MAP, 15 ug (FA) with biopsy; B2 = Placebo MAP (FA) with biopsy
B3 = A/Sing MAP, 15 ug (FA); B4 = A/Sing MAP, 10 ug (FA); B5 = A/Sing MAP, 5 ug (FA); B6 = A/Sing MAP, 2.5 ug (FA)
B7 = Placebo MAP (FA); B8 = A/Sing MAP, 15 ug (UA); B9 = A/Sing IM, 15 ug, as 2018 Afluria Quad (UA)

Listing 16.2.9.10
Individual Characterisation of Numeric Pain Intensity by Treatment

Part B, Non-biopsy
B3= 3 x 5 ug A/Sing MAPs on volar forearm (15 ug)

| | | | -------Active------- | | | -------Placebo------- | | |
|---|---|---|---|---|---|---|---|---|
| Randomisation Number | Visit | Time Point | #> 0 | Maximum | Average | #> 0 | Maximum | Average |
| B0126 | EOS/ET (Day 61) | | 0 | 0 | 0.0 | - | - | - |
| B0134 | Day 2 | | 0 | 0 | 0.0 | - | - | - |
| B0134 | Day 1 | 1 min | 3 | 1 | 1.0 | - | - | - |
| B0134 | Day 1 | 10 min | 0 | 0 | 0.0 | - | - | - |
| B0134 | Day 1 | 1 hour | 0 | 0 | 0.0 | - | - | - |
| B0134 | Day 1 | 2 hour | 0 | 0 | 0.0 | - | - | - |
| B0134 | Day 4 | | 0 | 0 | 0.0 | - | - | - |
| B0134 | Day 8 | | 0 | 0 | 0.0 | - | - | - |
| B0134 | Day 22 | | 0 | 0 | 0.0 | - | - | - |
| B0134 | EOS/ET (Day 61) | | 0 | 0 | 0.0 | - | - | - |
| B0141 | Day 2 | | 0 | 0 | 0.0 | - | - | - |
| B0141 | Day 1 | 1 min | 2 | 1 | 0.7 | - | - | - |
| B0141 | Day 1 | 10 min | 2 | 1 | 0.7 | - | - | - |
| B0141 | Day 1 | 1 hour | 0 | 0 | 0.0 | - | - | - |
| B0141 | Day 1 | 2 hour | 0 | 0 | 0.0 | - | - | - |
| B0141 | Day 4 | | 0 | 0 | 0.0 | - | - | - |
| B0141 | Day 8 | | 0 | 0 | 0.0 | - | - | - |
| B0141 | Day 22 | | 0 | 0 | 0.0 | - | - | - |
| B0141 | EOS/ET (Day 61) | | 0 | 0 | 0.0 | - | - | - |


Source: Listing 16.2.9.3

Administration Site Key: FA = Forearm; UA= Upper arm
Treatments Part A: A1 = A/Sing MAP, 15 ug (FA); A2 = Afluria Quad IM (UA), 15 ug; A3 = Placebo MAP (FA); A4 = A/Sing Mono IM, 15 ug (UA)
Treatments Part B: B1 = A/Sing MAP, 15 ug (FA) with biopsy; B2 = Placebo MAP (FA) with biopsy
B3 = A/Sing MAP, 15 ug (FA); B4 = A/Sing MAP, 10 ug (FA); B5 = A/Sing MAP, 5 ug (FA); B6 = A/Sing MAP, 2.5 ug (FA)
B7 = Placebo MAP (FA); B8 = A/Sing MAP, 15 ug (UA); B9 = A/Sing IM, 15 ug, as 2018 Afluria Quad (UA)

Listing 16.2.9.10
Individual Characterisation of Numeric Pain Intensity by Treatment

Part B, Non-biopsy
B3= 3 x 5 ug A/Sing MAPs on volar forearm (15 ug)

| | | | -------Active------- | | | -------Placebo------- | | |
|---|---|---|---|---|---|---|---|---|
| Randomisation Number | Visit | Time Point | #> 0 | Maximum | Average | #> 0 | Maximum | Average |
| B0145 | Day 2 | | 0 | 0 | 0.0 | - | - | - |
| B0145 | Day 1 | 1 min | 2 | 1 | 0.7 | - | - | - |
| B0145 | Day 1 | 10 min | 2 | 1 | 0.7 | - | - | - |
| B0145 | Day 1 | 1 hour | 0 | 0 | 0.0 | - | - | - |
| B0145 | Day 1 | 2 hour | 0 | 0 | 0.0 | - | - | - |
| B0145 | Day 4 | | 0 | 0 | 0.0 | - | - | - |
| B0145 | Day 8 | | 0 | 0 | 0.0 | - | - | - |
| B0145 | Day 22 | | 0 | 0 | 0.0 | - | - | - |
| B0145 | EOS/ET (Day 61) | | 0 | 0 | 0.0 | - | - | - |


Source: Listing 16.2.9.3

Administration Site Key: FA = Forearm; UA= Upper arm
Treatments Part A: A1 = A/Sing MAP, 15 ug (FA); A2 = Afluria Quad IM (UA), 15 ug; A3 = Placebo MAP (FA); A4 = A/Sing Mono IM, 15 ug (UA)
Treatments Part B: B1 = A/Sing MAP, 15 ug (FA) with biopsy; B2 = Placebo MAP (FA) with biopsy
B3 = A/Sing MAP, 15 ug (FA); B4 = A/Sing MAP, 10 ug (FA); B5 = A/Sing MAP, 5 ug (FA); B6 = A/Sing MAP, 2.5 ug (FA)
B7 = Placebo MAP (FA); B8 = A/Sing MAP, 15 ug (UA); B9 = A/Sing IM, 15 ug, as 2018 Afluria Quad (UA)

Listing 16.2.9.10
Individual Characterisation of Numeric Pain Intensity by Treatment

Part B, Non-biopsy
B4= 2 x 5 ug A/Sing MAPs + 1 uncoated MAP on volar forearm (10 ug)

| | | | -------Active------- | | | -------Placebo------- | | |
|---|---|---|---|---|---|---|---|---|
| Randomisation Number | Visit | Time Point | #> 0 | Maximum | Average | #> 0 | Maximum | Average |
| B0016 | Day 2 | | 0 | 0 | 0.0 | 0 | 0 | 0.0 |
| B0016 | Day 1 | 1 min | 2 | 5 | 4.5 | 1 | 3 | 3.0 |
| B0016 | Day 1 | 10 min | 0 | 0 | 0.0 | 0 | 0 | 0.0 |
| B0016 | Day 1 | 1 hour | 2 | 1 | 1.0 | 1 | 1 | 1.0 |
| B0016 | Day 1 | 2 hour | 0 | 0 | 0.0 | 0 | 0 | 0.0 |
| B0016 | Day 4 | | 0 | 0 | 0.0 | 0 | 0 | 0.0 |
| B0016 | Day 8 | | 0 | 0 | 0.0 | 0 | 0 | 0.0 |
| B0016 | Day 22 | | 0 | 0 | 0.0 | 0 | 0 | 0.0 |
| B0016 | EOS/ET (Day 61) | | 0 | 0 | 0.0 | 0 | 0 | 0.0 |
| B0022 | Day 2 | | 0 | 0 | 0.0 | 0 | 0 | 0.0 |
| B0022 | Day 1 | 1 min | 2 | 1 | 1.0 | 0 | 0 | 0.0 |
| B0022 | Day 1 | 10 min | 0 | 0 | 0.0 | 0 | 0 | 0.0 |
| B0022 | Day 1 | 1 hour | 0 | 0 | 0.0 | 0 | 0 | 0.0 |
| B0022 | Day 1 | 2 hour | 0 | 0 | 0.0 | 0 | 0 | 0.0 |
| B0022 | Day 4 | | 0 | 0 | 0.0 | 0 | 0 | 0.0 |
| B0022 | Day 8 | | 0 | 0 | 0.0 | 0 | 0 | 0.0 |
| B0022 | Day 22 | | 0 | 0 | 0.0 | 0 | 0 | 0.0 |
| B0022 | EOS/ET (Day 61) | | 0 | 0 | 0.0 | 0 | 0 | 0.0 |
| B0031 | Day 2 | | 0 | 0 | 0.0 | 0 | 0 | 0.0 |


Source: Listing 16.2.9.3

Administration Site Key: FA = Forearm; UA= Upper arm
Treatments Part A: A1 = A/Sing MAP, 15 ug (FA); A2 = Afluria Quad IM (UA), 15 ug; A3 = Placebo MAP (FA); A4 = A/Sing Mono IM, 15 ug (UA)
Treatments Part B: B1 = A/Sing MAP, 15 ug (FA) with biopsy; B2 = Placebo MAP (FA) with biopsy
B3 = A/Sing MAP, 15 ug (FA); B4 = A/Sing MAP, 10 ug (FA); B5 = A/Sing MAP, 5 ug (FA); B6 = A/Sing MAP, 2.5 ug (FA)
B7 = Placebo MAP (FA); B8 = A/Sing MAP, 15 ug (UA); B9 = A/Sing IM, 15 ug, as 2018 Afluria Quad (UA)

Listing 16.2.9.10
Individual Characterisation of Numeric Pain Intensity by Treatment

Part B, Non-biopsy
B4= 2 x 5 ug A/Sing MAPs + 1 uncoated MAP on volar forearm (10 ug)

| | | | -------Active------- | | | -------Placebo------- | | |
|---|---|---|---|---|---|---|---|---|
| Randomisation Number | Visit | Time Point | #> 0 | Maximum | Average | #> 0 | Maximum | Average |
| B0031 | Day 1 | 1 min | 2 | 2 | 2.0 | 1 | 2 | 2.0 |
| B0031 | Day 1 | 10 min | 2 | 2 | 2.0 | 1 | 2 | 2.0 |
| B0031 | Day 1 | 1 hour | 2 | 1 | 1.0 | 1 | 1 | 1.0 |
| B0031 | Day 1 | 2 hour | 0 | 0 | 0.0 | 0 | 0 | 0.0 |
| B0031 | Day 4 | | 0 | 0 | 0.0 | 0 | 0 | 0.0 |
| B0031 | Day 8 | | 0 | 0 | 0.0 | 0 | 0 | 0.0 |
| B0031 | Day 22 | | 0 | 0 | 0.0 | 0 | 0 | 0.0 |
| B0031 | EOS/ET (Day 61) | | 0 | 0 | 0.0 | 0 | 0 | 0.0 |
| | | | | | | | | |
| B0037 | Day 2 | | 0 | 0 | 0.0 | 0 | 0 | 0.0 |
| B0037 | Day 1 | 1 min | 0 | 0 | 0.0 | 0 | 0 | 0.0 |
| B0037 | Day 1 | 10 min | 0 | 0 | 0.0 | 0 | 0 | 0.0 |
| B0037 | Day 1 | 1 hour | 0 | 0 | 0.0 | 0 | 0 | 0.0 |
| B0037 | Day 1 | 2 hour | 0 | 0 | 0.0 | 0 | 0 | 0.0 |
| B0037 | Day 4 | | 0 | 0 | 0.0 | 0 | 0 | 0.0 |
| B0037 | Day 8 | | 0 | 0 | 0.0 | 0 | 0 | 0.0 |
| B0037 | Day 22 | | 0 | 0 | 0.0 | 0 | 0 | 0.0 |
| B0037 | EOS/ET (Day 61) | | 0 | 0 | 0.0 | 0 | 0 | 0.0 |
| | | | | | | | | |
| B0045 | Day 2 | | 0 | 0 | 0.0 | 0 | 0 | 0.0 |
| B0045 | Day 1 | 1 min | 2 | 3 | 3.0 | 1 | 5 | 5.0 |



Administration Site Key: FA = Forearm; UA= Upper arm
Treatments Part A: A1 = A/Sing MAP, 15 ug (FA); A2 = Afluria Quad IM (UA), 15 ug; A3 = Placebo MAP (FA); A4 = A/Sing Mono IM, 15 ug (UA)
Treatments Part B: B1 = A/Sing MAP, 15 ug (FA) with biopsy; B2 = Placebo MAP (FA) with biopsy
B3 = A/Sing MAP, 15 ug (FA); B4 = A/Sing MAP, 10 ug (FA); B5 = A/Sing MAP, 5 ug (FA); B6 = A/Sing MAP, 2.5 ug (FA)
B7 = Placebo MAP (FA); B8 = A/Sing MAP, 15 ug (UA); B9 = A/Sing IM, 15 ug, as 2018 Afluria Quad (UA)

Listing 16.2.9.10
Individual Characterisation of Numeric Pain Intensity by Treatment

Part B, Non-biopsy
B4= 2 x 5 ug A/Sing MAPs + 1 uncoated MAP on volar forearm (10 ug)

| | | | -------Active------- | | | -------Placebo------- | | |
|---|---|---|---|---|---|---|---|---|
| Randomisation Number | Visit | Time Point | #> 0 | Maximum | Average | #> 0 | Maximum | Average |
| B0045 | Day 1 | 10 min | 1 | 1 | 0.5 | 1 | 2 | 2.0 |
| B0045 | Day 1 | 1 hour | 0 | 0 | 0.0 | 0 | 0 | 0.0 |
| B0045 | Day 1 | 2 hour | 0 | 0 | 0.0 | 0 | 0 | 0.0 |
| B0045 | Day 4 | | 0 | 0 | 0.0 | 0 | 0 | 0.0 |
| B0045 | Day 8 | | 0 | 0 | 0.0 | 0 | 0 | 0.0 |
| B0045 | Day 22 | | 0 | 0 | 0.0 | 0 | 0 | 0.0 |
| B0045 | EOS/ET (Day 61) | | 0 | 0 | 0.0 | 0 | 0 | 0.0 |
| B0049 | Day 2 | | 0 | 0 | 0.0 | 0 | 0 | 0.0 |
| B0049 | Day 1 | 1 min | 1 | 3 | 1.5 | 1 | 4 | 4.0 |
| B0049 | Day 1 | 10 min | 2 | 3 | 2.5 | 1 | 2 | 2.0 |
| B0049 | Day 1 | 1 hour | 1 | 1 | 0.5 | 0 | 0 | 0.0 |
| B0049 | Day 1 | 2 hour | 0 | 0 | 0.0 | 0 | 0 | 0.0 |
| B0049 | Day 4 | | 0 | 0 | 0.0 | 0 | 0 | 0.0 |
| B0049 | Day 8 | | 0 | 0 | 0.0 | 0 | 0 | 0.0 |
| B0049 | Day 22 | | 0 | 0 | 0.0 | 0 | 0 | 0.0 |
| B0049 | EOS/ET (Day 61) | | 0 | 0 | 0.0 | 0 | 0 | 0.0 |
| B0057 | Day 2 | | 0 | 0 | 0.0 | 0 | 0 | 0.0 |
| B0057 | Day 1 | 1 min | 2 | 4 | 3.0 | 1 | 3 | 3.0 |
| B0057 | Day 1 | 10 min | 1 | 1 | 0.5 | 1 | 2 | 2.0 |



Administration Site Key: FA = Forearm; UA= Upper arm
Treatments Part A: A1 = A/Sing MAP, 15 ug (FA); A2 = Afluria Quad IM (UA), 15 ug; A3 = Placebo MAP (FA); A4 = A/Sing Mono IM, 15 ug (UA)
Treatments Part B: B1 = A/Sing MAP, 15 ug (FA) with biopsy; B2 = Placebo MAP (FA) with biopsy
B3 = A/Sing MAP, 15 ug (FA); B4 = A/Sing MAP, 10 ug (FA); B5 = A/Sing MAP, 5 ug (FA); B6 = A/Sing MAP, 2.5 ug (FA)
B7 = Placebo MAP (FA); B8 = A/Sing MAP, 15 ug (UA); B9 = A/Sing IM, 15 ug, as 2018 Afluria Quad (UA)

Listing 16.2.9.10
Individual Characterisation of Numeric Pain Intensity by Treatment

Part B, Non-biopsy
B4= 2 x 5 ug A/Sing MAPs + 1 uncoated MAP on volar forearm (10 ug)

| | | | -------Active------- | | | -------Placebo------- | | |
|---|---|---|---|---|---|---|---|---|
| Randomisation Number | Visit | Time Point | #> 0 | Maximum | Average | #> 0 | Maximum | Average |
| B0057 | Day 1 | 1 hour | 0 | 0 | 0.0 | 0 | 0 | 0.0 |
| B0057 | Day 1 | 2 hour | 0 | 0 | 0.0 | 0 | 0 | 0.0 |
| B0057 | Day 4 | | 0 | 0 | 0.0 | 0 | 0 | 0.0 |
| B0057 | Day 8 | | 0 | 0 | 0.0 | 0 | 0 | 0.0 |
| B0057 | Day 22 | | 0 | 0 | 0.0 | 0 | 0 | 0.0 |
| B0057 | EOS/ET (Day 61) | | 0 | 0 | 0.0 | 0 | 0 | 0.0 |
| B0065 | Day 2 | | 2 | 2 | 1.5 | 0 | 0 | 0.0 |
| B0065 | Day 1 | 1 min | 2 | 2 | 1.5 | 1 | 1 | 1.0 |
| B0065 | Day 1 | 10 min | 0 | 0 | 0.0 | 1 | 1 | 1.0 |
| B0065 | Day 1 | 1 hour | 0 | 0 | 0.0 | 0 | 0 | 0.0 |
| B0065 | Day 1 | 2 hour | 0 | 0 | 0.0 | 0 | 0 | 0.0 |
| B0065 | Day 4 | | 0 | 0 | 0.0 | 0 | 0 | 0.0 |
| B0065 | Day 8 | | 0 | 0 | 0.0 | 0 | 0 | 0.0 |
| B0065 | Day 22 | | 0 | 0 | 0.0 | 0 | 0 | 0.0 |
| B0065 | EOS/ET (Day 61) | | 0 | 0 | 0.0 | 0 | 0 | 0.0 |
| B0071 | Day 2 | | 0 | 0 | 0.0 | 0 | 0 | 0.0 |
| B0071 | Day 1 | 1 min | 1 | 1 | 0.5 | 1 | 1 | 1.0 |
| B0071 | Day 1 | 10 min | 0 | 0 | 0.0 | 0 | 0 | 0.0 |
| B0071 | Day 1 | 1 hour | 0 | 0 | 0.0 | 0 | 0 | 0.0 |


Source: Listing 16.2.9.3

Administration Site Key: FA = Forearm; UA= Upper arm
Treatments Part A: A1 = A/Sing MAP, 15 ug (FA); A2 = Afluria Quad IM (UA), 15 ug; A3 = Placebo MAP (FA); A4 = A/Sing Mono IM, 15 ug (UA)
Treatments Part B: B1 = A/Sing MAP, 15 ug (FA) with biopsy; B2 = Placebo MAP (FA) with biopsy
B3 = A/Sing MAP, 15 ug (FA); B4 = A/Sing MAP, 10 ug (FA); B5 = A/Sing MAP, 5 ug (FA); B6 = A/Sing MAP, 2.5 ug (FA)
B7 = Placebo MAP (FA); B8 = A/Sing MAP, 15 ug (UA); B9 = A/Sing IM, 15 ug, as 2018 Afluria Quad (UA)

Listing 16.2.9.10
Individual Characterisation of Numeric Pain Intensity by Treatment

Part B, Non-biopsy
B4= 2 x 5 ug A/Sing MAPs + 1 uncoated MAP on volar forearm (10 ug)

| | | | Active | | | Placebo | | |
|---|---|---|---|---|---|---|---|---|
| Randomisation Number | Visit | Time Point | #> 0 | Maximum | Average | #> 0 | Maximum | Average |
| B0071 | Day 1 | 2 hour | 0 | 0 | 0.0 | 0 | 0 | 0.0 |
| B0071 | Day 4 | | 0 | 0 | 0.0 | 0 | 0 | 0.0 |
| B0071 | Day 8 | | 0 | 0 | 0.0 | 0 | 0 | 0.0 |
| B0071 | Day 22 | | 0 | 0 | 0.0 | 0 | 0 | 0.0 |
| B0071 | EOS/ET (Day 61) | | 0 | 0 | 0.0 | 0 | 0 | 0.0 |
| B0077 | Day 2 | | 0 | 0 | 0.0 | 0 | 0 | 0.0 |
| B0077 | Day 1 | 1 min | 2 | 3 | 2.5 | 1 | 2 | 2.0 |
| B0077 | Day 1 | 10 min | 0 | 0 | 0.0 | 0 | 0 | 0.0 |
| B0077 | Day 1 | 1 hour | 0 | 0 | 0.0 | 0 | 0 | 0.0 |
| B0077 | Day 1 | 2 hour | 0 | 0 | 0.0 | 1 | 1 | 1.0 |
| B0077 | Day 4 | | 0 | 0 | 0.0 | 0 | 0 | 0.0 |
| B0077 | Day 8 | | 0 | 0 | 0.0 | 0 | 0 | 0.0 |
| B0077 | Day 22 | | 0 | 0 | 0.0 | 0 | 0 | 0.0 |
| B0077 | EOS/ET (Day 61) | | 0 | 0 | 0.0 | 0 | 0 | 0.0 |
| B0085 | Day 2 | | 0 | 0 | 0.0 | 0 | 0 | 0.0 |
| B0085 | Day 1 | 1 min | 1 | 1 | 0.5 | 1 | 1 | 1.0 |
| B0085 | Day 1 | 10 min | 0 | 0 | 0.0 | 0 | 0 | 0.0 |
| B0085 | Day 1 | 1 hour | 0 | 0 | 0.0 | 0 | 0 | 0.0 |
| B0085 | Day 1 | 2 hour | 0 | 0 | 0.0 | 0 | 0 | 0.0 |


Source: Listing 16.2.9.3

Administration Site Key: FA = Forearm; UA= Upper arm
Treatments Part A: A1 = A/Sing MAP, 15 ug (FA); A2 = Afluria Quad IM (UA), 15 ug; A3 = Placebo MAP (FA); A4 = A/Sing Mono IM, 15 ug (UA)
Treatments Part B: B1 = A/Sing MAP, 15 ug (FA) with biopsy; B2 = Placebo MAP (FA) with biopsy
B3 = A/Sing MAP, 15 ug (FA); B4 = A/Sing MAP, 10 ug (FA); B5 = A/Sing MAP, 5 ug (FA); B6 = A/Sing MAP, 2.5 ug (FA)
B7 = Placebo MAP (FA); B8 = A/Sing MAP, 15 ug (UA); B9 = A/Sing IM, 15 ug, as 2018 Afluria Quad (UA)

Listing 16.2.9.10
Individual Characterisation of Numeric Pain Intensity by Treatment

Part B, Non-biopsy
B4= 2 x 5 ug A/Sing MAPs + 1 uncoated MAP on volar forearm (10 ug)

| | | | -------Active------- | | | -------Placebo------- | | |
|---|---|---|---|---|---|---|---|---|
| Randomisation Number | Visit | Time Point | #> 0 | Maximum | Average | #> 0 | Maximum | Average |
| B0085 | Day 4 | | 0 | 0 | 0.0 | 0 | 0 | 0.0 |
| B0085 | Day 8 | | 0 | 0 | 0.0 | 0 | 0 | 0.0 |
| B0085 | Day 22 | | 1 | 0 | 0.0 | 0 | 0 | 0.0 |
| B0085 | EOS/ET (Day 61) | | 0 | 0 | 0.0 | 0 | 0 | 0.0 |
| B0092 | Day 2 | | 0 | 0 | 0.0 | 0 | 0 | 0.0 |
| B0092 | Day 1 | 1 min | 2 | 3 | 2.5 | 1 | 2 | 2.0 |
| B0092 | Day 1 | 10 min | 1 | 3 | 1.5 | 1 | 2 | 2.0 |
| B0092 | Day 1 | 1 hour | 1 | 2 | 1.0 | 0 | 0 | 0.0 |
| B0092 | Day 1 | 2 hour | 1 | 1 | 0.5 | 0 | 0 | 0.0 |
| B0092 | Day 4 | | 0 | 0 | 0.0 | 0 | 0 | 0.0 |
| B0092 | Day 8 | | 0 | 0 | 0.0 | 0 | 0 | 0.0 |
| B0092 | Day 22 | | 0 | 0 | 0.0 | 0 | 0 | 0.0 |
| B0092 | EOS/ET (Day 61) | | 0 | 0 | 0.0 | 0 | 0 | 0.0 |
| B0099 | Day 2 | | 0 | 0 | 0.0 | 0 | 0 | 0.0 |
| B0099 | Day 1 | 1 min | 2 | 2 | 1.5 | 1 | 2 | 2.0 |
| B0099 | Day 1 | 10 min | 2 | 1 | 1.0 | 1 | 1 | 1.0 |
| B0099 | Day 1 | 1 hour | 2 | 1 | 1.0 | 1 | 1 | 1.0 |
| B0099 | Day 1 | 2 hour | 0 | 0 | 0.0 | 0 | 0 | 0.0 |
| B0099 | Day 4 | | 0 | 0 | 0.0 | 0 | 0 | 0.0 |


Source: Listing 16.2.9.3

Administration Site Key: FA = Forearm; UA= Upper arm
Treatments Part A: A1 = A/Sing MAP, 15 ug (FA); A2 = Afluria Quad IM (UA), 15 ug; A3 = Placebo MAP (FA); A4 = A/Sing Mono IM, 15 ug (UA)
Treatments Part B: B1 = A/Sing MAP, 15 ug (FA) with biopsy; B2 = Placebo MAP (FA) with biopsy
B3 = A/Sing MAP, 15 ug (FA); B4 = A/Sing MAP, 10 ug (FA); B5 = A/Sing MAP, 5 ug (FA); B6 = A/Sing MAP, 2.5 ug (FA)
B7 = Placebo MAP (FA); B8 = A/Sing MAP, 15 ug (UA); B9 = A/Sing IM, 15 ug, as 2018 Afluria Quad (UA)

Listing 16.2.9.10
Individual Characterisation of Numeric Pain Intensity by Treatment

Part B, Non-biopsy
B4= 2 x 5 ug A/Sing MAPs + 1 uncoated MAP on volar forearm (10 ug)

| | | | -------Active------- | | | -------Placebo------- | | |
|---|---|---|---|---|---|---|---|---|
| Randomisation Number | Visit | Time Point | #> 0 | Maximum | Average | #> 0 | Maximum | Average |
| B0099 | Day 8 | | 0 | 0 | 0.0 | 0 | 0 | 0.0 |
| B0099 | Day 22 | | - | - | - | - | - | - |
| B0099 | EOS/ET (Day 61) | | 0 | 0 | 0.0 | 0 | 0 | 0.0 |
| B0106 | Day 2 | | 0 | 0 | 0.0 | 0 | 0 | 0.0 |
| B0106 | Day 1 | 1 min | 2 | 2 | 1.5 | 1 | 1 | 1.0 |
| B0106 | Day 1 | 10 min | 2 | 2 | 1.5 | 1 | 1 | 1.0 |
| B0106 | Day 1 | 1 hour | 0 | 0 | 0.0 | 0 | 0 | 0.0 |
| B0106 | Day 1 | 2 hour | 0 | 0 | 0.0 | 0 | 0 | 0.0 |
| B0106 | Day 4 | | 0 | 0 | 0.0 | 0 | 0 | 0.0 |
| B0106 | Day 8 | | 0 | 0 | 0.0 | 0 | 0 | 0.0 |
| B0106 | Day 22 | | 0 | 0 | 0.0 | 0 | 0 | 0.0 |
| B0106 | EOS/ET (Day 61) | | 0 | 0 | 0.0 | 0 | 0 | 0.0 |
| B0112 | Day 2 | | 0 | 0 | 0.0 | 0 | 0 | 0.0 |
| B0112 | Day 1 | 1 min | 2 | 3 | 3.0 | 1 | 4 | 4.0 |
| B0112 | Day 1 | 10 min | 2 | 1 | 1.0 | 1 | 1 | 1.0 |
| B0112 | Day 1 | 1 hour | 0 | 0 | 0.0 | 0 | 0 | 0.0 |
| B0112 | Day 1 | 2 hour | 0 | 0 | 0.0 | 0 | 0 | 0.0 |
| B0112 | Day 4 | | 0 | 0 | 0.0 | 0 | 0 | 0.0 |
| B0112 | Day 8 | | 0 | 0 | 0.0 | 0 | 0 | 0.0 |



Administration Site Key: FA = Forearm; UA= Upper arm
Treatments Part A: A1 = A/Sing MAP, 15 ug (FA); A2 = Afluria Quad IM (UA), 15 ug; A3 = Placebo MAP (FA); A4 = A/Sing Mono IM, 15 ug (UA)
Treatments Part B: B1 = A/Sing MAP, 15 ug (FA) with biopsy; B2 = Placebo MAP (FA) with biopsy
B3 = A/Sing MAP, 15 ug (FA); B4 = A/Sing MAP, 10 ug (FA); B5 = A/Sing MAP, 5 ug (FA); B6 = A/Sing MAP, 2.5 ug (FA)
B7 = Placebo MAP (FA); B8 = A/Sing MAP, 15 ug (UA); B9 = A/Sing IM, 15 ug, as 2018 Afluria Quad (UA)

Listing 16.2.9.10
Individual Characterisation of Numeric Pain Intensity by Treatment

Part B, Non-biopsy
B4= 2 x 5 ug A/Sing MAPs + 1 uncoated MAP on volar forearm (10 ug)

| | | | -------Active------- | | | -------Placebo------- | | |
|---|---|---|---|---|---|---|---|---|
| Randomisation Number | Visit | Time Point | #> 0 | Maximum | Average | #> 0 | Maximum | Average |
| B0112 | Day 22 | | 0 | 0 | 0.0 | 0 | 0 | 0.0 |
| B0112 | EOS/ET (Day 61) | | 0 | 0 | 0.0 | 0 | 0 | 0.0 |
| B0119 | Day 2 | | 0 | 0 | 0.0 | 0 | 0 | 0.0 |
| B0119 | Day 1 | 1 min | 0 | 0 | 0.0 | 0 | 0 | 0.0 |
| B0119 | Day 1 | 10 min | 2 | 1 | 1.0 | 1 | 1 | 1.0 |
| B0119 | Day 1 | 1 hour | 2 | 1 | 1.0 | 1 | 1 | 1.0 |
| B0119 | Day 1 | 2 hour | 0 | 0 | 0.0 | 0 | 0 | 0.0 |
| B0119 | Day 4 | | 0 | 0 | 0.0 | 0 | 0 | 0.0 |
| B0119 | Day 8 | | 0 | 0 | 0.0 | 0 | 0 | 0.0 |
| B0119 | Day 22 | | 0 | 0 | 0.0 | 0 | 0 | 0.0 |
| B0119 | EOS/ET (Day 61) | | 0 | 0 | 0.0 | 0 | 0 | 0.0 |
| B0124 | Day 2 | | 0 | 0 | 0.0 | 0 | 0 | 0.0 |
| B0124 | Day 1 | 1 min | 2 | 3 | 3.0 | 1 | 2 | 2.0 |
| B0124 | Day 1 | 10 min | 1 | 1 | 0.5 | 0 | 0 | 0.0 |
| B0124 | Day 1 | 1 hour | 2 | 1 | 1.0 | 1 | 1 | 1.0 |
| B0124 | Day 1 | 2 hour | 2 | 1 | 1.0 | 0 | 0 | 0.0 |
| B0124 | Day 4 | | 0 | 0 | 0.0 | 0 | 0 | 0.0 |
| B0124 | Day 8 | | 0 | 0 | 0.0 | 0 | 0 | 0.0 |
| B0124 | Day 22 | | 0 | 0 | 0.0 | 0 | 0 | 0.0 |



Administration Site Key: FA = Forearm; UA= Upper arm
Treatments Part A: A1 = A/Sing MAP, 15 ug (FA); A2 = Afluria Quad IM (UA), 15 ug; A3 = Placebo MAP (FA); A4 = A/Sing Mono IM, 15 ug (UA)
Treatments Part B: B1 = A/Sing MAP, 15 ug (FA) with biopsy; B2 = Placebo MAP (FA) with biopsy
B3 = A/Sing MAP, 15 ug (FA); B4 = A/Sing MAP, 10 ug (FA); B5 = A/Sing MAP, 5 ug (FA); B6 = A/Sing MAP, 2.5 ug (FA)
B7 = Placebo MAP (FA); B8 = A/Sing MAP, 15 ug (UA); B9 = A/Sing IM, 15 ug, as 2018 Afluria Quad (UA)

Listing 16.2.9.10
Individual Characterisation of Numeric Pain Intensity by Treatment

Part B, Non-biopsy
B4= 2 x 5 ug A/Sing MAPs + 1 uncoated MAP on volar forearm (10 ug)

| | | | -------Active------- | | | -------Placebo------- | | |
|---|---|---|---|---|---|---|---|---|
| Randomisation Number | Visit | Time Point | #> 0 | Maximum | Average | #> 0 | Maximum | Average |
| B0124 | EOS/ET (Day 61) | | 0 | 0 | 0.0 | 0 | 0 | 0.0 |
| B0133 | Day 2 | | 0 | 0 | 0.0 | 0 | 0 | 0.0 |
| B0133 | Day 1 | 1 min | 2 | 1 | 1.0 | 1 | 1 | 1.0 |
| B0133 | Day 1 | 10 min | 0 | 0 | 0.0 | 0 | 0 | 0.0 |
| B0133 | Day 1 | 1 hour | 0 | 0 | 0.0 | 0 | 0 | 0.0 |
| B0133 | Day 1 | 2 hour | 0 | 0 | 0.0 | 0 | 0 | 0.0 |
| B0133 | Day 4 | | 0 | 0 | 0.0 | 0 | 0 | 0.0 |
| B0133 | Day 8 | | 0 | 0 | 0.0 | 0 | 0 | 0.0 |
| B0133 | Day 22 | | 0 | 0 | 0.0 | 0 | 0 | 0.0 |
| B0133 | EOS/ET (Day 61) | | 0 | 0 | 0.0 | 0 | 0 | 0.0 |
| B0139 | Day 2 | | 0 | 0 | 0.0 | 0 | 0 | 0.0 |
| B0139 | Day 1 | 1 min | 1 | 1 | 0.5 | 0 | 0 | 0.0 |
| B0139 | Day 1 | 10 min | 0 | 0 | 0.0 | 0 | 0 | 0.0 |
| B0139 | Day 1 | 1 hour | 0 | 0 | 0.0 | 0 | 0 | 0.0 |
| B0139 | Day 1 | 2 hour | 0 | 0 | 0.0 | 0 | 0 | 0.0 |
| B0139 | Day 4 | | 0 | 0 | 0.0 | 0 | 0 | 0.0 |
| B0139 | Day 8 | | 0 | 0 | 0.0 | 0 | 0 | 0.0 |
| B0139 | Day 22 | | 0 | 0 | 0.0 | 0 | 0 | 0.0 |
| B0139 | EOS/ET (Day 61) | | 0 | 0 | 0.0 | 0 | 0 | 0.0 |



Administration Site Key: FA = Forearm; UA= Upper arm
Treatments Part A: A1 = A/Sing MAP, 15 ug (FA); A2 = Afluria Quad IM (UA), 15 ug; A3 = Placebo MAP (FA); A4 = A/Sing Mono IM, 15 ug (UA)
Treatments Part B: B1 = A/Sing MAP, 15 ug (FA) with biopsy; B2 = Placebo MAP (FA) with biopsy
B3 = A/Sing MAP, 15 ug (FA); B4 = A/Sing MAP, 10 ug (FA); B5 = A/Sing MAP, 5 ug (FA); B6 = A/Sing MAP, 2.5 ug (FA)
B7 = Placebo MAP (FA); B8 = A/Sing MAP, 15 ug (UA); B9 = A/Sing IM, 15 ug, as 2018 Afluria Quad (UA)

Listing 16.2.9.10
Individual Characterisation of Numeric Pain Intensity by Treatment

Part B, Non-biopsy
B4= 2 x 5 ug A/Sing MAPs + 1 uncoated MAP on volar forearm (10 ug)

| | | | -------Active------- | | | -------Placebo------- | | |
|---|---|---|---|---|---|---|---|---|
| Randomisation Number | Visit | Time Point | #> 0 | Maximum | Average | #> 0 | Maximum | Average |
| B0150 | Day 2 | | 0 | 0 | 0.0 | 0 | 0 | 0.0 |
| B0150 | Day 1 | 1 min | 2 | 3 | 2.0 | 1 | 3 | 3.0 |
| B0150 | Day 1 | 10 min | 2 | 1 | 1.0 | 1 | 1 | 1.0 |
| B0150 | Day 1 | 1 hour | 0 | 0 | 0.0 | 0 | 0 | 0.0 |
| B0150 | Day 1 | 2 hour | 0 | 0 | 0.0 | 0 | 0 | 0.0 |
| B0150 | Day 4 | | 0 | 0 | 0.0 | 0 | 0 | 0.0 |
| B0150 | Day 8 | | 0 | 0 | 0.0 | 0 | 0 | 0.0 |
| B0150 | Day 22 | | 0 | 0 | 0.0 | 0 | 0 | 0.0 |
| B0150 | EOS/ET (Day 61) | | 0 | 0 | 0.0 | 0 | 0 | 0.0 |


Source: Listing 16.2.9.3

Administration Site Key: FA = Forearm; UA= Upper arm
Treatments Part A: A1 = A/Sing MAP, 15 ug (FA); A2 = Afluria Quad IM (UA), 15 ug; A3 = Placebo MAP (FA); A4 = A/Sing Mono IM, 15 ug (UA)
Treatments Part B: B1 = A/Sing MAP, 15 ug (FA) with biopsy; B2 = Placebo MAP (FA) with biopsy
B3 = A/Sing MAP, 15 ug (FA); B4 = A/Sing MAP, 10 ug (FA); B5 = A/Sing MAP, 5 ug (FA); B6 = A/Sing MAP, 2.5 ug (FA)
B7 = Placebo MAP (FA); B8 = A/Sing MAP, 15 ug (UA); B9 = A/Sing IM, 15 ug, as 2018 Afluria Quad (UA)

Listing 16.2.9.10
Individual Characterisation of Numeric Pain Intensity by Treatment

Part B, Non-biopsy
B5= 1 x 5 ug A/Sing MAP + 2 uncoated MAPs on volar forearm (5 ug)

| | | | -------Active------- | | | -------Placebo------- | | |
|---|---|---|---|---|---|---|---|---|
| Randomisation Number | Visit | Time Point | #> 0 | Maximum | Average | #> 0 | Maximum | Average |
| B0013 | Day 2 | | 0 | 0 | 0.0 | 0 | 0 | 0.0 |
| B0013 | Day 1 | 1 min | 1 | 1 | 1.0 | 2 | 1 | 1.0 |
| B0013 | Day 1 | 10 min | 0 | 0 | 0.0 | 0 | 0 | 0.0 |
| B0013 | Day 1 | 1 hour | 0 | 0 | 0.0 | 0 | 0 | 0.0 |
| B0013 | Day 1 | 2 hour | 0 | 0 | 0.0 | 0 | 0 | 0.0 |
| B0013 | Day 4 | | 0 | 0 | 0.0 | 0 | 0 | 0.0 |
| B0013 | Day 8 | | 0 | 0 | 0.0 | 0 | 0 | 0.0 |
| B0013 | Day 22 | | 0 | 0 | 0.0 | 0 | 0 | 0.0 |
| B0013 | EOS/ET (Day 61) | | 0 | 0 | 0.0 | 0 | 0 | 0.0 |
| B0024 | Day 2 | | 0 | 0 | 0.0 | 0 | 0 | 0.0 |
| B0024 | Day 1 | 1 min | 1 | 2 | 2.0 | 1 | 2 | 1.0 |
| B0024 | Day 1 | 10 min | 1 | 1 | 1.0 | 2 | 1 | 1.0 |
| B0024 | Day 1 | 1 hour | 0 | 0 | 0.0 | 0 | 0 | 0.0 |
| B0024 | Day 1 | 2 hour | 0 | 0 | 0.0 | 0 | 0 | 0.0 |
| B0024 | Day 4 | | 0 | 0 | 0.0 | 0 | 0 | 0.0 |
| B0024 | Day 8 | | 0 | 0 | 0.0 | 0 | 0 | 0.0 |
| B0024 | Day 22 | | 0 | 0 | 0.0 | 0 | 0 | 0.0 |
| B0024 | EOS/ET (Day 61) | | 0 | 0 | 0.0 | 0 | 0 | 0.0 |
| B0028 | Day 2 | | 0 | 0 | 0.0 | 0 | 0 | 0.0 |


Source: Listing 16.2.9.3

Administration Site Key: FA = Forearm; UA= Upper arm
Treatments Part A: A1 = A/Sing MAP, 15 ug (FA); A2 = Afluria Quad IM (UA), 15 ug; A3 = Placebo MAP (FA); A4 = A/Sing Mono IM, 15 ug (UA)
Treatments Part B: B1 = A/Sing MAP, 15 ug (FA) with biopsy; B2 = Placebo MAP (FA) with biopsy
B3 = A/Sing MAP, 15 ug (FA); B4 = A/Sing MAP, 10 ug (FA); B5 = A/Sing MAP, 5 ug (FA); B6 = A/Sing MAP, 2.5 ug (FA)
B7 = Placebo MAP (FA); B8 = A/Sing MAP, 15 ug (UA); B9 = A/Sing IM, 15 ug, as 2018 Afluria Quad (UA)

Listing 16.2.9.10
Individual Characterisation of Numeric Pain Intensity by Treatment

Part B, Non-biopsy
B5= 1 x 5 ug A/Sing MAP + 2 uncoated MAPs on volar forearm (5 ug)

| | | | -------Active------- | | | -------Placebo------- | | |
|---|---|---|---|---|---|---|---|---|
| Randomisation Number | Visit | Time Point | #> 0 | Maximum | Average | #> 0 | Maximum | Average |
| B0028 | Day 1 | 1 min | 1 | 1 | 1.0 | 2 | 2 | 1.5 |
| B0028 | Day 1 | 10 min | 0 | 0 | 0.0 | 2 | 2 | 1.5 |
| B0028 | Day 1 | 1 hour | 0 | 0 | 0.0 | 0 | 0 | 0.0 |
| B0028 | Day 1 | 2 hour | 0 | 0 | 0.0 | 0 | 0 | 0.0 |
| B0028 | Day 4 | | 0 | 0 | 0.0 | 0 | 0 | 0.0 |
| B0028 | Day 8 | | 0 | 0 | 0.0 | 0 | 0 | 0.0 |
| B0028 | Day 22 | | 0 | 0 | 0.0 | 0 | 0 | 0.0 |
| B0028 | EOS/ET (Day 61) | | 0 | 0 | 0.0 | 0 | 0 | 0.0 |
| B0033 | Day 2 | | 0 | 0 | 0.0 | 0 | 0 | 0.0 |
| B0033 | Day 1 | 1 min | 1 | 2 | 2.0 | 2 | 4 | 3.5 |
| B0033 | Day 1 | 10 min | 1 | 3 | 3.0 | 2 | 3 | 2.5 |
| B0033 | Day 1 | 1 hour | 1 | 1 | 1.0 | 2 | 1 | 1.0 |
| B0033 | Day 1 | 2 hour | 1 | 1 | 1.0 | 2 | 1 | 1.0 |
| B0033 | Day 4 | | 0 | 0 | 0.0 | 0 | 0 | 0.0 |
| B0033 | Day 8 | | 0 | 0 | 0.0 | 0 | 0 | 0.0 |
| B0033 | Day 22 | | 1 | - | - | 0 | 0 | 0.0 |
| B0033 | EOS/ET (Day 61) | | 0 | 0 | 0.0 | 0 | 0 | 0.0 |
| B0042 | Day 2 | | 0 | 0 | 0.0 | 0 | 0 | 0.0 |
| B0042 | Day 1 | 1 min | 1 | 3 | 3.0 | 2 | 3 | 2.0 |


Source: Listing 16.2.9.3

Administration Site Key: FA = Forearm; UA= Upper arm
Treatments Part A: A1 = A/Sing MAP, 15 ug (FA); A2 = Afluria Quad IM (UA), 15 ug; A3 = Placebo MAP (FA); A4 = A/Sing Mono IM, 15 ug (UA)
Treatments Part B: B1 = A/Sing MAP, 15 ug (FA) with biopsy; B2 = Placebo MAP (FA) with biopsy
B3 = A/Sing MAP, 15 ug (FA); B4 = A/Sing MAP, 10 ug (FA); B5 = A/Sing MAP, 5 ug (FA); B6 = A/Sing MAP, 2.5 ug (FA)
B7 = Placebo MAP (FA); B8 = A/Sing MAP, 15 ug (UA); B9 = A/Sing IM, 15 ug, as 2018 Afluria Quad (UA)

Listing 16.2.9.10
Individual Characterisation of Numeric Pain Intensity by Treatment

Part B, Non-biopsy
B5= 1 x 5 ug A/Sing MAP + 2 uncoated MAPs on volar forearm (5 ug)

| | | | -------Active------- | | | -------Placebo------- | | |
|---|---|---|---|---|---|---|---|---|
| Randomisation Number | Visit | Time Point | #> 0 | Maximum | Average | #> 0 | Maximum | Average |
| B0042 | Day 1 | 10 min | 0 | 0 | 0.0 | 1 | 1 | 0.5 |
| B0042 | Day 1 | 1 hour | - | - | - | 0 | 0 | 0.0 |
| B0042 | Day 1 | 2 hour | 0 | 0 | 0.0 | 0 | 0 | 0.0 |
| B0042 | Day 4 | | 1 | - | - | 0 | 0 | 0.0 |
| B0042 | Day 8 | | 0 | 0 | 0.0 | 0 | 0 | 0.0 |
| B0042 | Day 22 | | 0 | 0 | 0.0 | 0 | 0 | 0.0 |
| B0042 | EOS/ET (Day 61) | | 0 | 0 | 0.0 | 0 | 0 | 0.0 |
| | | | | | | | | |
| B0052 | Day 2 | | 1 | 1 | 1.0 | 2 | 1 | 1.0 |
| B0052 | Day 1 | 1 min | 0 | 0 | 0.0 | 1 | 1 | 0.5 |
| B0052 | Day 1 | 10 min | 0 | 0 | 0.0 | 1 | 1 | 0.5 |
| B0052 | Day 1 | 1 hour | 0 | 0 | 0.0 | 0 | 0 | 0.0 |
| B0052 | Day 1 | 2 hour | 0 | 0 | 0.0 | 0 | 0 | 0.0 |
| B0052 | Day 4 | | 0 | 0 | 0.0 | 0 | 0 | 0.0 |
| B0052 | Day 8 | | 0 | 0 | 0.0 | 0 | 0 | 0.0 |
| B0052 | Day 22 | | 0 | 0 | 0.0 | 0 | 0 | 0.0 |
| B0052 | EOS/ET (Day 61) | | 0 | 0 | 0.0 | 0 | 0 | 0.0 |
| | | | | | | | | |
| B0054 | Day 2 | | 0 | 0 | 0.0 | 0 | 0 | 0.0 |
| B0054 | Day 1 | 1 min | 1 | 1 | 1.0 | 0 | 0 | 0.0 |
| B0054 | Day 1 | 10 min | 0 | 0 | 0.0 | 0 | 0 | 0.0 |


Source: Listing 16.2.9.3

Administration Site Key: FA = Forearm; UA= Upper arm
Treatments Part A: A1 = A/Sing MAP, 15 ug (FA); A2 = Afluria Quad IM (UA), 15 ug; A3 = Placebo MAP (FA); A4 = A/Sing Mono IM, 15 ug (UA)
Treatments Part B: B1 = A/Sing MAP, 15 ug (FA) with biopsy; B2 = Placebo MAP (FA) with biopsy
B3 = A/Sing MAP, 15 ug (FA); B4 = A/Sing MAP, 10 ug (FA); B5 = A/Sing MAP, 5 ug (FA); B6 = A/Sing MAP, 2.5 ug (FA)
B7 = Placebo MAP (FA); B8 = A/Sing MAP, 15 ug (UA); B9 = A/Sing IM, 15 ug, as 2018 Afluria Quad (UA)

Listing 16.2.9.10
Individual Characterisation of Numeric Pain Intensity by Treatment

Part B, Non-biopsy
B5= 1 x 5 ug A/Sing MAP + 2 uncoated MAPs on volar forearm (5 ug)

| | | | -------Active------- | | | -------Placebo------- | | |
|---|---|---|---|---|---|---|---|---|
| Randomisation Number | Visit | Time Point | #> 0 | Maximum | Average | #> 0 | Maximum | Average |
| B0054 | Day 1 | 1 hour | - | - | - | 0 | 0 | 0.0 |
| B0054 | Day 1 | 2 hour | 0 | 0 | 0.0 | 0 | 0 | 0.0 |
| B0054 | Day 4 | | 0 | 0 | 0.0 | 0 | 0 | 0.0 |
| B0054 | Day 8 | | 0 | 0 | 0.0 | 0 | 0 | 0.0 |
| B0054 | Day 22 | | 0 | 0 | 0.0 | 0 | 0 | 0.0 |
| B0054 | EOS/ET (Day 61) | | 0 | 0 | 0.0 | 0 | 0 | 0.0 |
| B0062 | Day 2 | | 0 | 0 | 0.0 | 0 | 0 | 0.0 |
| B0062 | Day 1 | 1 min | 1 | 2 | 2.0 | 1 | 1 | 0.5 |
| B0062 | Day 1 | 10 min | 0 | 0 | 0.0 | 0 | 0 | 0.0 |
| B0062 | Day 1 | 1 hour | 0 | 0 | 0.0 | 0 | 0 | 0.0 |
| B0062 | Day 1 | 2 hour | 0 | 0 | 0.0 | 0 | 0 | 0.0 |
| B0062 | Day 4 | | 0 | 0 | 0.0 | 0 | 0 | 0.0 |
| B0062 | Day 8 | | 0 | 0 | 0.0 | 0 | 0 | 0.0 |
| B0062 | Day 22 | | 0 | 0 | 0.0 | 0 | 0 | 0.0 |
| B0062 | EOS/ET (Day 61) | | 0 | 0 | 0.0 | 0 | 0 | 0.0 |
| B0068 | Day 1 | 1 min | 1 | 1 | 1.0 | 2 | 1 | 1.0 |
| B0068 | Day 1 | 10 min | 1 | 1 | 1.0 | 2 | 1 | 1.0 |
| B0068 | Day 1 | 1 hour | 0 | 0 | 0.0 | 0 | 0 | 0.0 |
| B0068 | Day 1 | 2 hour | 0 | 0 | 0.0 | 0 | 0 | 0.0 |


Source: Listing 16.2.9.3

Administration Site Key: FA = Forearm; UA= Upper arm
Treatments Part A: A1 = A/Sing MAP, 15 ug (FA); A2 = Afluria Quad IM (UA), 15 ug; A3 = Placebo MAP (FA); A4 = A/Sing Mono IM, 15 ug (UA)
Treatments Part B: B1 = A/Sing MAP, 15 ug (FA) with biopsy; B2 = Placebo MAP (FA) with biopsy
B3 = A/Sing MAP, 15 ug (FA); B4 = A/Sing MAP, 10 ug (FA); B5 = A/Sing MAP, 5 ug (FA); B6 = A/Sing MAP, 2.5 ug (FA)
B7 = Placebo MAP (FA); B8 = A/Sing MAP, 15 ug (UA); B9 = A/Sing IM, 15 ug, as 2018 Afluria Quad (UA)

Listing 16.2.9.10
Individual Characterisation of Numeric Pain Intensity by Treatment

Part B, Non-biopsy
B5= 1 x 5 ug A/Sing MAP + 2 uncoated MAPs on volar forearm (5 ug)

| | | | -------Active------- | | | -------Placebo------- | | |
|---|---|---|---|---|---|---|---|---|
| Randomisation Number | Visit | Time Point | #> 0 | Maximum | Average | #> 0 | Maximum | Average |
| B0068 | Day 8 | | 0 | 0 | 0.0 | 0 | 0 | 0.0 |
| B0068 | Day 22 | | 0 | 0 | 0.0 | 0 | 0 | 0.0 |
| B0068 | EOS/ET (Day 61) | | 0 | 0 | 0.0 | 0 | 0 | 0.0 |
| B0084 | Day 2 | | 0 | 0 | 0.0 | 0 | 0 | 0.0 |
| B0084 | Day 1 | 1 min | 1 | 1 | 1.0 | 2 | 2 | 1.5 |
| B0084 | Day 1 | 10 min | 0 | 0 | 0.0 | 0 | 0 | 0.0 |
| B0084 | Day 1 | 1 hour | 0 | 0 | 0.0 | 0 | 0 | 0.0 |
| B0084 | Day 1 | 2 hour | 0 | 0 | 0.0 | 0 | 0 | 0.0 |
| B0084 | Day 4 | | 0 | 0 | 0.0 | 0 | 0 | 0.0 |
| B0084 | Day 8 | | 0 | 0 | 0.0 | 0 | 0 | 0.0 |
| B0084 | Day 22 | | 0 | 0 | 0.0 | 0 | 0 | 0.0 |
| B0084 | EOS/ET (Day 61) | | 0 | 0 | 0.0 | 0 | 0 | 0.0 |
| B0089 | Day 2 | | 0 | 0 | 0.0 | 0 | 0 | 0.0 |
| B0089 | Day 1 | 1 min | 0 | 0 | 0.0 | 1 | 1 | 0.5 |
| B0089 | Day 1 | 10 min | 0 | 0 | 0.0 | 0 | 0 | 0.0 |
| B0089 | Day 1 | 1 hour | 0 | 0 | 0.0 | 0 | 0 | 0.0 |
| B0089 | Day 1 | 2 hour | 0 | 0 | 0.0 | 0 | 0 | 0.0 |
| B0089 | Day 4 | | 0 | 0 | 0.0 | 0 | 0 | 0.0 |
| B0089 | Day 8 | | 0 | 0 | 0.0 | 0 | 0 | 0.0 |


Source: Listing 16.2.9.3

Administration Site Key: FA = Forearm; UA= Upper arm
Treatments Part A: A1 = A/Sing MAP, 15 ug (FA); A2 = Afluria Quad IM (UA), 15 ug; A3 = Placebo MAP (FA); A4 = A/Sing Mono IM, 15 ug (UA)
Treatments Part B: B1 = A/Sing MAP, 15 ug (FA) with biopsy; B2 = Placebo MAP (FA) with biopsy
B3 = A/Sing MAP, 15 ug (FA); B4 = A/Sing MAP, 10 ug (FA); B5 = A/Sing MAP, 5 ug (FA); B6 = A/Sing MAP, 2.5 ug (FA)
B7 = Placebo MAP (FA); B8 = A/Sing MAP, 15 ug (UA); B9 = A/Sing IM, 15 ug, as 2018 Afluria Quad (UA)

Listing 16.2.9.10
Individual Characterisation of Numeric Pain Intensity by Treatment

Part B, Non-biopsy
B5= 1 x 5 ug A/Sing MAP + 2 uncoated MAPs on volar forearm (5 ug)

| | | | -------Active------- | | | -------Placebo------- | | |
|---|---|---|---|---|---|---|---|---|
| Randomisation Number | Visit | Time Point | #> 0 | Maximum | Average | #> 0 | Maximum | Average |
| B0089 | Day 22 | | 0 | 0 | 0.0 | 0 | 0 | 0.0 |
| B0089 | EOS/ET (Day 61) | | 0 | 0 | 0.0 | 0 | 0 | 0.0 |
| B0097 | Day 2 | | 0 | 0 | 0.0 | 0 | 0 | 0.0 |
| B0097 | Day 1 | 1 min | 0 | 0 | 0.0 | 1 | 1 | 0.5 |
| B0097 | Day 1 | 10 min | 0 | 0 | 0.0 | 0 | 0 | 0.0 |
| B0097 | Day 1 | 1 hour | 0 | 0 | 0.0 | 0 | 0 | 0.0 |
| B0097 | Day 1 | 2 hour | 0 | 0 | 0.0 | 0 | 0 | 0.0 |
| B0097 | Day 4 | | 0 | 0 | 0.0 | 0 | 0 | 0.0 |
| B0097 | Day 8 | | 0 | 0 | 0.0 | 0 | 0 | 0.0 |
| B0097 | Day 22 | | 0 | 0 | 0.0 | 0 | 0 | 0.0 |
| B0097 | EOS/ET (Day 61) | | 0 | 0 | 0.0 | 0 | 0 | 0.0 |
| B0103 | Day 2 | | 0 | 0 | 0.0 | 0 | 0 | 0.0 |
| B0103 | Day 1 | 1 min | 0 | 0 | 0.0 | 2 | 2 | 2.0 |
| B0103 | Day 1 | 10 min | 0 | 0 | 0.0 | 1 | 4 | 2.0 |
| B0103 | Day 1 | 1 hour | 0 | 0 | 0.0 | 0 | 0 | 0.0 |
| B0103 | Day 1 | 2 hour | 0 | 0 | 0.0 | 0 | 0 | 0.0 |
| B0103 | Day 4 | | 0 | 0 | 0.0 | 0 | 0 | 0.0 |
| B0103 | Day 8 | | 0 | 0 | 0.0 | 0 | 0 | 0.0 |
| B0103 | Day 22 | | 0 | 0 | 0.0 | 0 | 0 | 0.0 |


Source: Listing 16.2.9.3

Administration Site Key: FA = Forearm; UA= Upper arm
Treatments Part A: A1 = A/Sing MAP, 15 ug (FA); A2 = Afluria Quad IM (UA), 15 ug; A3 = Placebo MAP (FA); A4 = A/Sing Mono IM, 15 ug (UA)
Treatments Part B: B1 = A/Sing MAP, 15 ug (FA) with biopsy; B2 = Placebo MAP (FA) with biopsy
B3 = A/Sing MAP, 15 ug (FA); B4 = A/Sing MAP, 10 ug (FA); B5 = A/Sing MAP, 5 ug (FA); B6 = A/Sing MAP, 2.5 ug (FA)
B7 = Placebo MAP (FA); B8 = A/Sing MAP, 15 ug (UA); B9 = A/Sing IM, 15 ug, as 2018 Afluria Quad (UA)

Listing 16.2.9.10
Individual Characterisation of Numeric Pain Intensity by Treatment

Part B, Non-biopsy
B5= 1 x 5 ug A/Sing MAP + 2 uncoated MAPs on volar forearm (5 ug)

| | | | Active | | | Placebo | | |
|---|---|---|---|---|---|---|---|---|
| Randomisation Number | Visit | Time Point | #> 0 | Maximum | Average | #> 0 | Maximum | Average |
| B0103 | EOS/ET (Day 61) | | 0 | 0 | 0.0 | 0 | 0 | 0.0 |
| B0113 | Day 2 | | 0 | 0 | 0.0 | 0 | 0 | 0.0 |
| B0113 | Day 1 | 1 min | 1 | 2 | 2.0 | 2 | 2 | 2.0 |
| B0113 | Day 1 | 10 min | 1 | 1 | 1.0 | 2 | 2 | 1.5 |
| B0113 | Day 1 | 1 hour | 0 | 0 | 0.0 | 0 | 0 | 0.0 |
| B0113 | Day 1 | 2 hour | 0 | 0 | 0.0 | 0 | 0 | 0.0 |
| B0113 | Day 4 | | 0 | 0 | 0.0 | 0 | 0 | 0.0 |
| B0113 | Day 8 | | 0 | 0 | 0.0 | 0 | 0 | 0.0 |
| B0113 | Day 22 | | 0 | 0 | 0.0 | 0 | 0 | 0.0 |
| B0113 | EOS/ET (Day 61) | | 0 | 0 | 0.0 | 0 | 0 | 0.0 |
| B0122 | Day 2 | | 0 | 0 | 0.0 | 1 | 0 | 0.0 |
| B0122 | Day 1 | 1 min | 1 | 1 | 1.0 | 2 | 1 | 1.0 |
| B0122 | Day 1 | 10 min | 0 | 0 | 0.0 | 2 | 1 | 1.0 |
| B0122 | Day 1 | 1 hour | 1 | 1 | 1.0 | 2 | 1 | 1.0 |
| B0122 | Day 1 | 2 hour | 0 | 0 | 0.0 | 0 | 0 | 0.0 |
| B0122 | Day 4 | | 0 | 0 | 0.0 | 0 | 0 | 0.0 |
| B0122 | Day 8 | | 0 | 0 | 0.0 | 0 | 0 | 0.0 |
| B0122 | Day 22 | | 0 | 0 | 0.0 | 0 | 0 | 0.0 |
| B0122 | EOS/ET (Day 61) | | 0 | 0 | 0.0 | 0 | 0 | 0.0 |



Administration Site Key: FA = Forearm; UA= Upper arm
Treatments Part A: A1 = A/Sing MAP, 15 ug (FA); A2 = Afluria Quad IM (UA), 15 ug; A3 = Placebo MAP (FA); A4 = A/Sing Mono IM, 15 ug (UA)
Treatments Part B: B1 = A/Sing MAP, 15 ug (FA) with biopsy; B2 = Placebo MAP (FA) with biopsy
B3 = A/Sing MAP, 15 ug (FA); B4 = A/Sing MAP, 10 ug (FA); B5 = A/Sing MAP, 5 ug (FA); B6 = A/Sing MAP, 2.5 ug (FA)
B7 = Placebo MAP (FA); B8 = A/Sing MAP, 15 ug (UA); B9 = A/Sing IM, 15 ug, as 2018 Afluria Quad (UA)

Listing 16.2.9.10
Individual Characterisation of Numeric Pain Intensity by Treatment

Part B, Non-biopsy
B5= 1 x 5 ug A/Sing MAP + 2 uncoated MAPs on volar forearm (5 ug)

| | | | -------Active------- | | | -------Placebo------- | | |
|---|---|---|---|---|---|---|---|---|
| Randomisation Number | Visit | Time Point | #> 0 | Maximum | Average | #> 0 | Maximum | Average |
| B0128 | Day 2 | | 0 | 0 | 0.0 | 0 | 0 | 0.0 |
| B0128 | Day 1 | 1 min | 0 | 0 | 0.0 | 0 | 0 | 0.0 |
| B0128 | Day 1 | 10 min | 0 | 0 | 0.0 | 0 | 0 | 0.0 |
| B0128 | Day 1 | 1 hour | 0 | 0 | 0.0 | 0 | 0 | 0.0 |
| B0128 | Day 1 | 2 hour | 0 | 0 | 0.0 | 0 | 0 | 0.0 |
| B0128 | Day 4 | | 0 | 0 | 0.0 | 0 | 0 | 0.0 |
| B0128 | Day 8 | | 0 | 0 | 0.0 | 0 | 0 | 0.0 |
| B0128 | Day 22 | | 0 | 0 | 0.0 | 0 | 0 | 0.0 |
| B0128 | EOS/ET (Day 61) | | 0 | 0 | 0.0 | 0 | 0 | 0.0 |
| B0130 | Day 2 | | 0 | 0 | 0.0 | 0 | 0 | 0.0 |
| B0130 | Day 1 | 1 min | 1 | 1 | 1.0 | 2 | 2 | 1.5 |
| B0130 | Day 1 | 10 min | 1 | 1 | 1.0 | 0 | 0 | 0.0 |
| B0130 | Day 1 | 1 hour | 0 | 0 | 0.0 | 0 | 0 | 0.0 |
| B0130 | Day 1 | 2 hour | 0 | 0 | 0.0 | 0 | 0 | 0.0 |
| B0130 | Day 4 | | 0 | 0 | 0.0 | 0 | 0 | 0.0 |
| B0130 | Day 8 | | 0 | 0 | 0.0 | 0 | 0 | 0.0 |
| B0130 | Day 22 | | 0 | 0 | 0.0 | 0 | 0 | 0.0 |
| B0130 | EOS/ET (Day 61) | | 0 | 0 | 0.0 | 0 | 0 | 0.0 |


Source: Listing 16.2.9.3

Administration Site Key: FA = Forearm; UA= Upper arm
Treatments Part A: A1 = A/Sing MAP, 15 ug (FA); A2 = Afluria Quad IM (UA), 15 ug; A3 = Placebo MAP (FA); A4 = A/Sing Mono IM, 15 ug (UA)
Treatments Part B: B1 = A/Sing MAP, 15 ug (FA) with biopsy; B2 = Placebo MAP (FA) with biopsy
B3 = A/Sing MAP, 15 ug (FA); B4 = A/Sing MAP, 10 ug (FA); B5 = A/Sing MAP, 5 ug (FA); B6 = A/Sing MAP, 2.5 ug (FA)
B7 = Placebo MAP (FA); B8 = A/Sing MAP, 15 ug (UA); B9 = A/Sing IM, 15 ug, as 2018 Afluria Quad (UA)

Listing 16.2.9.10
Individual Characterisation of Numeric Pain Intensity by Treatment

Part B, Non-biopsy
B5= 1 x 5 ug A/Sing MAP + 2 uncoated MAPs on volar forearm (5 ug)

| | | | -------Active------- | | | -------Placebo------- | | |
|---|---|---|---|---|---|---|---|---|
| Randomisation Number | Visit | Time Point | #> 0 | Maximum | Average | #> 0 | Maximum | Average |
| B0142 | Day 2 | | 0 | 0 | 0.0 | 0 | 0 | 0.0 |
| B0142 | Day 1 | 1 min | 0 | 0 | 0.0 | 0 | 0 | 0.0 |
| B0142 | Day 1 | 10 min | 0 | 0 | 0.0 | 0 | 0 | 0.0 |
| B0142 | Day 1 | 1 hour | 0 | 0 | 0.0 | 0 | 0 | 0.0 |
| B0142 | Day 1 | 2 hour | 0 | 0 | 0.0 | 0 | 0 | 0.0 |
| B0142 | Day 4 | | 0 | 0 | 0.0 | 0 | 0 | 0.0 |
| B0142 | Day 8 | | 0 | 0 | 0.0 | 0 | 0 | 0.0 |
| B0142 | Day 22 | | 0 | 0 | 0.0 | 0 | 0 | 0.0 |
| B0142 | EOS/ET (Day 61) | | 0 | 0 | 0.0 | 0 | 0 | 0.0 |
| B0148 | Day 2 | | 0 | 0 | 0.0 | 0 | 0 | 0.0 |
| B0148 | Day 1 | 1 min | 0 | 0 | 0.0 | 0 | 0 | 0.0 |
| B0148 | Day 1 | 10 min | 0 | 0 | 0.0 | 0 | 0 | 0.0 |
| B0148 | Day 1 | 1 hour | 0 | 0 | 0.0 | 0 | 0 | 0.0 |
| B0148 | Day 1 | 2 hour | 0 | 0 | 0.0 | 0 | 0 | 0.0 |
| B0148 | Day 4 | | 0 | 0 | 0.0 | 0 | 0 | 0.0 |
| B0148 | Day 8 | | 0 | 0 | 0.0 | 0 | 0 | 0.0 |
| B0148 | Day 22 | | 0 | 0 | 0.0 | 0 | 0 | 0.0 |
| B0148 | EOS/ET (Day 61) | | 0 | 0 | 0.0 | 0 | 0 | 0.0 |
| B1076 | Day 2 | | 0 | 0 | 0.0 | 0 | 0 | 0.0 |


Source: Listing 16.2.9.3

Administration Site Key: FA = Forearm; UA= Upper arm
Treatments Part A: A1 = A/Sing MAP, 15 ug (FA); A2 = Afluria Quad IM (UA), 15 ug; A3 = Placebo MAP (FA); A4 = A/Sing Mono IM, 15 ug (UA)
Treatments Part B: B1 = A/Sing MAP, 15 ug (FA) with biopsy; B2 = Placebo MAP (FA) with biopsy
B3 = A/Sing MAP, 15 ug (FA); B4 = A/Sing MAP, 10 ug (FA); B5 = A/Sing MAP, 5 ug (FA); B6 = A/Sing MAP, 2.5 ug (FA)
B7 = Placebo MAP (FA); B8 = A/Sing MAP, 15 ug (UA); B9 = A/Sing IM, 15 ug, as 2018 Afluria Quad (UA)

Listing 16.2.9.10
Individual Characterisation of Numeric Pain Intensity by Treatment

Part B, Non-biopsy
B5= 1 x 5 ug A/Sing MAP + 2 uncoated MAPs on volar forearm (5 ug)

| | | | -------Active------- | | | -------Placebo------- | | |
|---|---|---|---|---|---|---|---|---|
| Randomisation Number | Visit | Time Point | #> 0 | Maximum | Average | #> 0 | Maximum | Average |
| B1076 | Day 1 | 1 min | 1 | 2 | 2.0 | 2 | 1 | 1.0 |
| B1076 | Day 1 | 10 min | 0 | 0 | 0.0 | 0 | 0 | 0.0 |
| B1076 | Day 1 | 1 hour | 0 | 0 | 0.0 | 0 | 0 | 0.0 |
| B1076 | Day 1 | 2 hour | 0 | 0 | 0.0 | 0 | 0 | 0.0 |
| B1076 | Day 4 | | - | - | - | 0 | 0 | 0.0 |
| B1076 | Day 8 | | 0 | 0 | 0.0 | 0 | 0 | 0.0 |
| B1076 | Day 22 | | 0 | 0 | 0.0 | 0 | 0 | 0.0 |
| B1076 | EOS/ET (Day 61) | | 0 | 0 | 0.0 | 0 | 0 | 0.0 |



Administration Site Key: FA = Forearm; UA= Upper arm
Treatments Part A: A1 = A/Sing MAP, 15 ug (FA); A2 = Afluria Quad IM (UA), 15 ug; A3 = Placebo MAP (FA); A4 = A/Sing Mono IM, 15 ug (UA)
Treatments Part B: B1 = A/Sing MAP, 15 ug (FA) with biopsy; B2 = Placebo MAP (FA) with biopsy
B3 = A/Sing MAP, 15 ug (FA); B4 = A/Sing MAP, 10 ug (FA); B5 = A/Sing MAP, 5 ug (FA); B6 = A/Sing MAP, 2.5 ug (FA)
B7 = Placebo MAP (FA); B8 = A/Sing MAP, 15 ug (UA); B9 = A/Sing IM, 15 ug, as 2018 Afluria Quad (UA)

## Listing 16.2.9.10
## Individual Characterisation of Numeric Pain Intensity by Treatment

### Part B, Non-biopsy
### B6= 1 x 2.5 ug A/Sing MAP + 2 uncoated MAPs on volar forearm (2.5 ug)

| | | | -------Active------- | | | -------Placebo------- | | |
|---|---|---|---|---|---|---|---|---|
| Randomisation Number | Visit | Time Point | #> 0 | Maximum | Average | #> 0 | Maximum | Average |
| B0012 | Day 2 | | 0 | 0 | 0.0 | 0 | 0 | 0.0 |
| B0012 | Unscheduled | | 1 | 2 | 2.0 | - | - | - |
| B0012 | Day 1 | 1 min | 0 | 0 | 0.0 | 1 | 3 | 1.5 |
| B0012 | Day 1 | 10 min | 1 | 2 | 2.0 | 0 | 0 | 0.0 |
| B0012 | Day 1 | 1 hour | 0 | 0 | 0.0 | 2 | 2 | 1.5 |
| B0012 | Day 1 | 2 hour | 0 | 0 | 0.0 | 0 | 0 | 0.0 |
| B0012 | Day 4 | | 0 | 0 | 0.0 | 0 | 0 | 0.0 |
| B0012 | Day 8 | | 0 | 0 | 0.0 | 0 | 0 | 0.0 |
| B0012 | Day 22 | | 0 | 0 | 0.0 | 0 | 0 | 0.0 |
| B0012 | EOS/ET (Day 61) | | 0 | 0 | 0.0 | 0 | 0 | 0.0 |
| B0020 | Day 2 | | 0 | 0 | 0.0 | 0 | 0 | 0.0 |
| B0020 | Day 1 | 1 min | 1 | 3 | 3.0 | 2 | 2 | 1.5 |
| B0020 | Day 1 | 10 min | 0 | 0 | 0.0 | 1 | 3 | 1.5 |
| B0020 | Day 1 | 1 hour | 1 | 5 | 5.0 | 2 | 4 | 4.0 |
| B0020 | Day 1 | 2 hour | 0 | 0 | 0.0 | 0 | 0 | 0.0 |
| B0020 | Day 4 | | 0 | 0 | 0.0 | 0 | 0 | 0.0 |
| B0020 | Day 8 | | 0 | 0 | 0.0 | 0 | 0 | 0.0 |
| B0020 | Day 22 | | 0 | 0 | 0.0 | 0 | 0 | 0.0 |
| B0020 | EOS/ET (Day 61) | | 0 | 0 | 0.0 | 0 | 0 | 0.0 |


Source: Listing 16.2.9.3

Administration Site Key: FA = Forearm; UA= Upper arm
Treatments Part A: A1 = A/Sing MAP, 15 ug (FA); A2 = Afluria Quad IM (UA), 15 ug; A3 = Placebo MAP (FA); A4 = A/Sing Mono IM, 15 ug (UA)
Treatments Part B: B1 = A/Sing MAP, 15 ug (FA) with biopsy; B2 = Placebo MAP (FA) with biopsy
B3 = A/Sing MAP, 15 ug (FA); B4 = A/Sing MAP, 10 ug (FA); B5 = A/Sing MAP, 5 ug (FA); B6 = A/Sing MAP, 2.5 ug (FA)
B7 = Placebo MAP (FA); B8 = A/Sing MAP, 15 ug (UA); B9 = A/Sing IM, 15 ug, as 2018 Afluria Quad (UA)

Listing 16.2.9.10
Individual Characterisation of Numeric Pain Intensity by Treatment

Part B, Non-biopsy
B6= 1 x 2.5 ug A/Sing MAP + 2 uncoated MAPs on volar forearm (2.5 ug)

| | | | -------Active------- | | | -------Placebo------- | | |
|---|---|---|---|---|---|---|---|---|
| Randomisation Number | Visit | Time Point | #> 0 | Maximum | Average | #> 0 | Maximum | Average |
| B0029 | Day 2 | | 0 | 0 | 0.0 | 0 | 0 | 0.0 |
| B0029 | Day 1 | 1 min | 1 | 2 | 2.0 | 2 | 3 | 2.5 |
| B0029 | Day 1 | 10 min | 1 | 2 | 2.0 | 1 | 1 | 0.5 |
| B0029 | Day 1 | 1 hour | 0 | 0 | 0.0 | 0 | 0 | 0.0 |
| B0029 | Day 1 | 2 hour | 0 | 0 | 0.0 | 0 | 0 | 0.0 |
| B0029 | Day 4 | | 0 | 0 | 0.0 | 0 | 0 | 0.0 |
| B0029 | Day 8 | | 0 | 0 | 0.0 | 0 | 0 | 0.0 |
| B0029 | Day 22 | | 0 | 0 | 0.0 | 0 | 0 | 0.0 |
| B0029 | EOS/ET (Day 61) | | 0 | 0 | 0.0 | 0 | 0 | 0.0 |
| B0032 | Day 2 | | 0 | 0 | 0.0 | 0 | 0 | 0.0 |
| B0032 | Day 1 | 1 min | 1 | 1 | 1.0 | 1 | 1 | 0.5 |
| B0032 | Day 1 | 10 min | 1 | 1 | 1.0 | 0 | 0 | 0.0 |
| B0032 | Day 1 | 1 hour | 0 | 0 | 0.0 | 0 | 0 | 0.0 |
| B0032 | Day 1 | 2 hour | 0 | 0 | 0.0 | 0 | 0 | 0.0 |
| B0032 | Day 4 | | 0 | 0 | 0.0 | 0 | 0 | 0.0 |
| B0032 | Day 8 | | 0 | 0 | 0.0 | 0 | 0 | 0.0 |
| B0032 | Day 22 | | 0 | 0 | 0.0 | 0 | 0 | 0.0 |
| B0032 | EOS/ET (Day 61) | | 0 | 0 | 0.0 | 0 | 0 | 0.0 |
| B0044 | Day 2 | | 0 | 0 | 0.0 | 0 | 0 | 0.0 |


Source: Listing 16.2.9.3

Administration Site Key: FA = Forearm; UA= Upper arm
Treatments Part A: A1 = A/Sing MAP, 15 ug (FA); A2 = Afluria Quad IM (UA), 15 ug; A3 = Placebo MAP (FA); A4 = A/Sing Mono IM, 15 ug (UA)
Treatments Part B: B1 = A/Sing MAP, 15 ug (FA) with biopsy; B2 = Placebo MAP (FA) with biopsy
B3 = A/Sing MAP, 15 ug (FA); B4 = A/Sing MAP, 10 ug (FA); B5 = A/Sing MAP, 5 ug (FA); B6 = A/Sing MAP, 2.5 ug (FA)
B7 = Placebo MAP (FA); B8 = A/Sing MAP, 15 ug (UA); B9 = A/Sing IM, 15 ug, as 2018 Afluria Quad (UA)

Listing 16.2.9.10
Individual Characterisation of Numeric Pain Intensity by Treatment

Part B, Non-biopsy
B6= 1 x 2.5 ug A/Sing MAP + 2 uncoated MAPs on volar forearm (2.5 ug)

| | | | -------Active------- | | | -------Placebo------- | | |
|---|---|---|---|---|---|---|---|---|
| Randomisation Number | Visit | Time Point | #> 0 | Maximum | Average | #> 0 | Maximum | Average |
| B0044 | Day 1 | 1 min | 1 | 2 | 2.0 | 2 | 2 | 2.0 |
| B0044 | Day 1 | 10 min | 1 | 1 | 1.0 | 1 | 1 | 0.5 |
| B0044 | Day 1 | 1 hour | 1 | 1 | 1.0 | 2 | 1 | 1.0 |
| B0044 | Day 1 | 2 hour | 0 | 0 | 0.0 | 0 | 0 | 0.0 |
| B0044 | Day 4 | | 0 | 0 | 0.0 | 0 | 0 | 0.0 |
| B0044 | Day 8 | | 0 | 0 | 0.0 | 0 | 0 | 0.0 |
| B0044 | Day 22 | | 0 | 0 | 0.0 | 0 | 0 | 0.0 |
| B0044 | EOS/ET (Day 61) | | 0 | 0 | 0.0 | 0 | 0 | 0.0 |
| B0048 | Day 2 | | 0 | 0 | 0.0 | 0 | 0 | 0.0 |
| B0048 | Day 1 | 1 min | 1 | 2 | 2.0 | 2 | 2 | 1.5 |
| B0048 | Day 1 | 10 min | 0 | 0 | 0.0 | 0 | 0 | 0.0 |
| B0048 | Day 1 | 1 hour | 0 | 0 | 0.0 | 0 | 0 | 0.0 |
| B0048 | Day 1 | 2 hour | 0 | 0 | 0.0 | 0 | 0 | 0.0 |
| B0048 | Day 4 | | 0 | 0 | 0.0 | 0 | 0 | 0.0 |
| B0048 | Day 8 | | 0 | 0 | 0.0 | 0 | 0 | 0.0 |
| B0048 | Day 22 | | 0 | 0 | 0.0 | 0 | 0 | 0.0 |
| B0048 | EOS/ET (Day 61) | | 0 | 0 | 0.0 | 0 | 0 | 0.0 |
| B0053 | Day 2 | | 0 | 0 | 0.0 | 0 | 0 | 0.0 |
| B0053 | Day 1 | 1 min | 1 | 1 | 1.0 | 0 | 0 | 0.0 |


Source: Listing 16.2.9.3

Administration Site Key: FA = Forearm; UA= Upper arm
Treatments Part A: A1 = A/Sing MAP, 15 ug (FA); A2 = Afluria Quad IM (UA), 15 ug; A3 = Placebo MAP (FA); A4 = A/Sing Mono IM, 15 ug (UA)
Treatments Part B: B1 = A/Sing MAP, 15 ug (FA) with biopsy; B2 = Placebo MAP (FA) with biopsy
B3 = A/Sing MAP, 15 ug (FA); B4 = A/Sing MAP, 10 ug (FA); B5 = A/Sing MAP, 5 ug (FA); B6 = A/Sing MAP, 2.5 ug (FA)
B7 = Placebo MAP (FA); B8 = A/Sing MAP, 15 ug (UA); B9 = A/Sing IM, 15 ug, as 2018 Afluria Quad (UA)

Listing 16.2.9.10

Individual Characterisation of Numeric Pain Intensity by Treatment

Part B, Non-biopsy

B6= 1 x 2.5 ug A/Sing MAP + 2 uncoated MAPs on volar forearm (2.5 ug)

| | | | -------Active------- | | | -------Placebo------- | | |
|---|---|---|---|---|---|---|---|---|
| Randomisation Number | Visit | Time Point | #> 0 | Maximum | Average | #> 0 | Maximum | Average |
| B0053 | Day 1 | 10 min | 0 | 0 | 0.0 | 0 | 0 | 0.0 |
| B0053 | Day 1 | 1 hour | 0 | 0 | 0.0 | 0 | 0 | 0.0 |
| B0053 | Day 1 | 2 hour | 0 | 0 | 0.0 | 0 | 0 | 0.0 |
| B0053 | Day 4 | | 0 | 0 | 0.0 | 0 | 0 | 0.0 |
| B0053 | Day 8 | | 0 | 0 | 0.0 | 0 | 0 | 0.0 |
| B0053 | Day 22 | | 0 | 0 | 0.0 | 0 | 0 | 0.0 |
| B0053 | EOS/ET (Day 61) | | 0 | 0 | 0.0 | 0 | 0 | 0.0 |
| B0066 | Day 2 | | 0 | 0 | 0.0 | 0 | 0 | 0.0 |
| B0066 | Day 1 | 1 min | 1 | 1 | 1.0 | 2 | 1 | 1.0 |
| B0066 | Day 1 | 10 min | 0 | 0 | 0.0 | 0 | 0 | 0.0 |
| B0066 | Day 1 | 1 hour | 0 | 0 | 0.0 | 0 | 0 | 0.0 |
| B0066 | Day 1 | 2 hour | 0 | 0 | 0.0 | 0 | 0 | 0.0 |
| B0066 | Day 4 | | 0 | 0 | 0.0 | 1 | 0 | 0.0 |
| B0066 | Day 8 | | 0 | 0 | 0.0 | 0 | 0 | 0.0 |
| B0066 | Day 22 | | 0 | 0 | 0.0 | 0 | 0 | 0.0 |
| B0066 | EOS/ET (Day 61) | | 0 | 0 | 0.0 | 0 | 0 | 0.0 |
| B0073 | Day 2 | | 0 | 0 | 0.0 | 0 | 0 | 0.0 |
| B0073 | Day 1 | 1 min | 1 | 1 | 1.0 | 2 | 1 | 1.0 |
| B0073 | Day 1 | 10 min | 1 | 1 | 1.0 | 2 | 1 | 1.0 |



Source: Listing 16.2.9.3

Administration Site Key: FA = Forearm; UA= Upper arm

Treatments Part A: A1 = A/Sing MAP, 15 ug (FA); A2 = Afluria Quad IM (UA), 15 ug; A3 = Placebo MAP (FA); A4 = A/Sing Mono IM, 15 ug (UA)

Treatments Part B: B1 = A/Sing MAP, 15 ug (FA) with biopsy; B2 = Placebo MAP (FA) with biopsy

B3 = A/Sing MAP, 15 ug (FA); B4 = A/Sing MAP, 10 ug (FA); B5 = A/Sing MAP, 5 ug (FA); B6 = A/Sing MAP, 2.5 ug (FA)

B7 = Placebo MAP (FA); B8 = A/Sing MAP, 15 ug (UA); B9 = A/Sing IM, 15 ug, as 2018 Afluria Quad (UA)

Listing 16.2.9.10
Individual Characterisation of Numeric Pain Intensity by Treatment

Part B, Non-biopsy
B6= 1 x 2.5 ug A/Sing MAP + 2 uncoated MAPs on volar forearm (2.5 ug)

| | | | -------Active------- | | | -------Placebo------- | | |
|---|---|---|---|---|---|---|---|---|
| Randomisation Number | Visit | Time Point | #> 0 | Maximum | Average | #> 0 | Maximum | Average |
| B0073 | Day 1 | 1 hour | - | - | - | 2 | 1 | 1.0 |
| B0073 | Day 1 | 2 hour | 0 | 0 | 0.0 | 0 | 0 | 0.0 |
| B0073 | Day 4 | | 0 | 0 | 0.0 | 0 | 0 | 0.0 |
| B0073 | Day 8 | | 0 | 0 | 0.0 | 0 | 0 | 0.0 |
| B0073 | Day 22 | | 0 | 0 | 0.0 | 0 | 0 | 0.0 |
| B0073 | EOS/ET (Day 61) | | 0 | 0 | 0.0 | 0 | 0 | 0.0 |
| B0079 | Day 2 | | 0 | 0 | 0.0 | 0 | 0 | 0.0 |
| B0079 | Day 1 | 1 min | 1 | 1 | 1.0 | 2 | 1 | 1.0 |
| B0079 | Day 1 | 10 min | 0 | 0 | 0.0 | 0 | 0 | 0.0 |
| B0079 | Day 1 | 1 hour | 0 | 0 | 0.0 | 0 | 0 | 0.0 |
| B0079 | Day 1 | 2 hour | 0 | 0 | 0.0 | 0 | 0 | 0.0 |
| B0079 | Day 4 | | 0 | 0 | 0.0 | 0 | 0 | 0.0 |
| B0079 | Day 8 | | 0 | 0 | 0.0 | 0 | 0 | 0.0 |
| B0079 | Day 22 | | 0 | 0 | 0.0 | 0 | 0 | 0.0 |
| B0079 | EOS/ET (Day 61) | | 0 | 0 | 0.0 | 0 | 0 | 0.0 |
| B0081 | Day 2 | | 0 | 0 | 0.0 | 0 | 0 | 0.0 |
| B0081 | Day 1 | 1 min | 1 | 2 | 2.0 | 2 | 2 | 1.5 |
| B0081 | Day 1 | 10 min | 0 | 0 | 0.0 | 0 | 0 | 0.0 |
| B0081 | Day 1 | 1 hour | 0 | 0 | 0.0 | 0 | 0 | 0.0 |


Source: Listing 16.2.9.3

Administration Site Key: FA = Forearm; UA= Upper arm
Treatments Part A: A1 = A/Sing MAP, 15 ug (FA); A2 = Afluria Quad IM (UA), 15 ug; A3 = Placebo MAP (FA); A4 = A/Sing Mono IM, 15 ug (UA)
Treatments Part B: B1 = A/Sing MAP, 15 ug (FA) with biopsy; B2 = Placebo MAP (FA) with biopsy
B3 = A/Sing MAP, 15 ug (FA); B4 = A/Sing MAP, 10 ug (FA); B5 = A/Sing MAP, 5 ug (FA); B6 = A/Sing MAP, 2.5 ug (FA)
B7 = Placebo MAP (FA); B8 = A/Sing MAP, 15 ug (UA); B9 = A/Sing IM, 15 ug, as 2018 Afluria Quad (UA)

Listing 16.2.9.10
Individual Characterisation of Numeric Pain Intensity by Treatment

Part B, Non-biopsy
B6= 1 x 2.5 ug A/Sing MAP + 2 uncoated MAPs on volar forearm (2.5 ug)

| | | | -------Active------- | | | -------Placebo------- | | |
|---|---|---|---|---|---|---|---|---|
| Randomisation Number | Visit | Time Point | #> 0 | Maximum | Average | #> 0 | Maximum | Average |
| B0081 | Day 1 | 2 hour | 0 | 0 | 0.0 | 0 | 0 | 0.0 |
| B0081 | Day 4 | | 0 | 0 | 0.0 | 0 | 0 | 0.0 |
| B0081 | Day 8 | | 0 | 0 | 0.0 | 0 | 0 | 0.0 |
| B0081 | Day 22 | | 0 | 0 | 0.0 | 0 | 0 | 0.0 |
| B0081 | EOS/ET (Day 61) | | 0 | 0 | 0.0 | 0 | 0 | 0.0 |
| B0090 | Day 2 | | 0 | 0 | 0.0 | 0 | 0 | 0.0 |
| B0090 | Day 1 | 1 min | 1 | 2 | 2.0 | 2 | 3 | 2.0 |
| B0090 | Day 1 | 10 min | 1 | 3 | 3.0 | 2 | 3 | 3.0 |
| B0090 | Day 1 | 1 hour | 1 | 3 | 3.0 | 2 | 3 | 3.0 |
| B0090 | Day 1 | 2 hour | 0 | 0 | 0.0 | 0 | 0 | 0.0 |
| B0090 | Day 4 | | 0 | 0 | 0.0 | 0 | 0 | 0.0 |
| B0090 | Day 8 | | 0 | 0 | 0.0 | 0 | 0 | 0.0 |
| B0090 | Day 22 | | 0 | 0 | 0.0 | 0 | 0 | 0.0 |
| B0090 | EOS/ET (Day 61) | | 0 | 0 | 0.0 | 0 | 0 | 0.0 |
| B0096 | Day 2 | | 0 | 0 | 0.0 | 0 | 0 | 0.0 |
| B0096 | Day 1 | 1 min | 1 | 1 | 1.0 | 2 | 2 | 2.0 |
| B0096 | Day 1 | 10 min | 0 | 0 | 0.0 | 0 | 0 | 0.0 |
| B0096 | Day 1 | 1 hour | 0 | 0 | 0.0 | 0 | 0 | 0.0 |
| B0096 | Day 1 | 2 hour | 0 | 0 | 0.0 | 0 | 0 | 0.0 |



Administration Site Key: FA = Forearm; UA= Upper arm
Treatments Part A: A1 = A/Sing MAP, 15 ug (FA); A2 = Afluria Quad IM (UA), 15 ug; A3 = Placebo MAP (FA); A4 = A/Sing Mono IM, 15 ug (UA)
Treatments Part B: B1 = A/Sing MAP, 15 ug (FA) with biopsy; B2 = Placebo MAP (FA) with biopsy
B3 = A/Sing MAP, 15 ug (FA); B4 = A/Sing MAP, 10 ug (FA); B5 = A/Sing MAP, 5 ug (FA); B6 = A/Sing MAP, 2.5 ug (FA)
B7 = Placebo MAP (FA); B8 = A/Sing MAP, 15 ug (UA); B9 = A/Sing IM, 15 ug, as 2018 Afluria Quad (UA)

Listing 16.2.9.10
Individual Characterisation of Numeric Pain Intensity by Treatment

Part B, Non-biopsy
B6= 1 x 2.5 ug A/Sing MAP + 2 uncoated MAPs on volar forearm (2.5 ug)

| | | | -------Active------- | | | -------Placebo------- | | |
|---|---|---|---|---|---|---|---|---|
| Randomisation Number | Visit | Time Point | #> 0 | Maximum | Average | #> 0 | Maximum | Average |
| B0096 | Day 4 | | 0 | 0 | 0.0 | 0 | 0 | 0.0 |
| B0096 | Day 8 | | 0 | 0 | 0.0 | 0 | 0 | 0.0 |
| B0096 | Day 22 | | 0 | 0 | 0.0 | 0 | 0 | 0.0 |
| B0096 | EOS/ET (Day 61) | | 0 | 0 | 0.0 | 0 | 0 | 0.0 |
| B0107 | Day 2 | | 0 | 0 | 0.0 | 0 | 0 | 0.0 |
| B0107 | Day 1 | 1 min | 1 | 1 | 1.0 | 2 | 1 | 1.0 |
| B0107 | Day 1 | 10 min | 0 | 0 | 0.0 | 0 | 0 | 0.0 |
| B0107 | Day 1 | 1 hour | 0 | 0 | 0.0 | 0 | 0 | 0.0 |
| B0107 | Day 1 | 2 hour | 0 | 0 | 0.0 | 0 | 0 | 0.0 |
| B0107 | Day 4 | | 0 | 0 | 0.0 | 0 | 0 | 0.0 |
| B0107 | Day 8 | | 0 | 0 | 0.0 | 0 | 0 | 0.0 |
| B0107 | Day 22 | | 0 | 0 | 0.0 | 0 | 0 | 0.0 |
| B0107 | EOS/ET (Day 61) | | 0 | 0 | 0.0 | 0 | 0 | 0.0 |
| B0109 | Day 2 | | 0 | 0 | 0.0 | 0 | 0 | 0.0 |
| B0109 | Day 1 | 1 min | 0 | 0 | 0.0 | 0 | 0 | 0.0 |
| B0109 | Day 1 | 10 min | 0 | 0 | 0.0 | 0 | 0 | 0.0 |
| B0109 | Day 1 | 1 hour | 0 | 0 | 0.0 | 0 | 0 | 0.0 |
| B0109 | Day 1 | 2 hour | 0 | 0 | 0.0 | 0 | 0 | 0.0 |
| B0109 | Day 4 | | 0 | 0 | 0.0 | 0 | 0 | 0.0 |


Source: Listing 16.2.9.3

Administration Site Key: FA = Forearm; UA= Upper arm
Treatments Part A: A1 = A/Sing MAP, 15 ug (FA); A2 = Afluria Quad IM (UA), 15 ug; A3 = Placebo MAP (FA); A4 = A/Sing Mono IM, 15 ug (UA)
Treatments Part B: B1 = A/Sing MAP, 15 ug (FA) with biopsy; B2 = Placebo MAP (FA) with biopsy
B3 = A/Sing MAP, 15 ug (FA); B4 = A/Sing MAP, 10 ug (FA); B5 = A/Sing MAP, 5 ug (FA); B6 = A/Sing MAP, 2.5 ug (FA)
B7 = Placebo MAP (FA); B8 = A/Sing MAP, 15 ug (UA); B9 = A/Sing IM, 15 ug, as 2018 Afluria Quad (UA)

Listing 16.2.9.10
Individual Characterisation of Numeric Pain Intensity by Treatment

Part B, Non-biopsy
B6= 1 x 2.5 ug A/Sing MAP + 2 uncoated MAPs on volar forearm (2.5 ug)

| | | | -------Active------- | | | -------Placebo------- | | |
|---|---|---|---|---|---|---|---|---|
| Randomisation Number | Visit | Time Point | #> 0 | Maximum | Average | #> 0 | Maximum | Average |
| B0109 | Day 8 | | 0 | 0 | 0.0 | 0 | 0 | 0.0 |
| B0109 | Day 22 | | 0 | 0 | 0.0 | 0 | 0 | 0.0 |
| B0109 | EOS/ET (Day 61) | | 0 | 0 | 0.0 | 0 | 0 | 0.0 |
| B0118 | Day 2 | | 0 | 0 | 0.0 | 0 | 0 | 0.0 |
| B0118 | Day 1 | 1 min | 1 | 2 | 2.0 | 2 | 3 | 2.0 |
| B0118 | Day 1 | 10 min | 1 | 2 | 2.0 | 2 | 3 | 2.0 |
| B0118 | Day 1 | 1 hour | 0 | 0 | 0.0 | 0 | 0 | 0.0 |
| B0118 | Day 1 | 2 hour | 0 | 0 | 0.0 | 0 | 0 | 0.0 |
| B0118 | Day 4 | | 0 | 0 | 0.0 | 0 | 0 | 0.0 |
| B0118 | Day 8 | | 0 | 0 | 0.0 | 0 | 0 | 0.0 |
| B0118 | Day 22 | | 0 | 0 | 0.0 | 0 | 0 | 0.0 |
| B0118 | EOS/ET (Day 61) | | 0 | 0 | 0.0 | 0 | 0 | 0.0 |
| B0123 | Day 2 | | 0 | 0 | 0.0 | 0 | 0 | 0.0 |
| B0123 | Day 1 | 1 min | 1 | 1 | 1.0 | 2 | 2 | 1.5 |
| B0123 | Day 1 | 10 min | 1 | 1 | 1.0 | 2 | 1 | 1.0 |
| B0123 | Day 1 | 1 hour | 0 | 0 | 0.0 | 0 | 0 | 0.0 |
| B0123 | Day 1 | 2 hour | 0 | 0 | 0.0 | 0 | 0 | 0.0 |
| B0123 | Day 4 | | 0 | 0 | 0.0 | 0 | 0 | 0.0 |
| B0123 | Day 8 | | 0 | 0 | 0.0 | 0 | 0 | 0.0 |



Administration Site Key: FA = Forearm; UA= Upper arm
Treatments Part A: A1 = A/Sing MAP, 15 ug (FA); A2 = Afluria Quad IM (UA), 15 ug; A3 = Placebo MAP (FA); A4 = A/Sing Mono IM, 15 ug (UA)
Treatments Part B: B1 = A/Sing MAP, 15 ug (FA) with biopsy; B2 = Placebo MAP (FA) with biopsy
B3 = A/Sing MAP, 15 ug (FA); B4 = A/Sing MAP, 10 ug (FA); B5 = A/Sing MAP, 5 ug (FA); B6 = A/Sing MAP, 2.5 ug (FA)
B7 = Placebo MAP (FA); B8 = A/Sing MAP, 15 ug (UA); B9 = A/Sing IM, 15 ug, as 2018 Afluria Quad (UA)

Listing 16.2.9.10
Individual Characterisation of Numeric Pain Intensity by Treatment

Part B, Non-biopsy
B6= 1 x 2.5 ug A/Sing MAP + 2 uncoated MAPs on volar forearm (2.5 ug)

| | | | -------Active------- | | | -------Placebo------- | | |
|---|---|---|---|---|---|---|---|---|
| Randomisation Number | Visit | Time Point | #> 0 | Maximum | Average | #> 0 | Maximum | Average |
| B0123 | Day 22 | | 0 | 0 | 0.0 | 0 | 0 | 0.0 |
| B0123 | EOS/ET (Day 61) | | 0 | 0 | 0.0 | 0 | 0 | 0.0 |
| B0132 | Day 2 | | 0 | 0 | 0.0 | 0 | 0 | 0.0 |
| B0132 | Day 1 | 1 min | 1 | 1 | 1.0 | 2 | 2 | 2.0 |
| B0132 | Day 1 | 10 min | 0 | 0 | 0.0 | 0 | 0 | 0.0 |
| B0132 | Day 1 | 1 hour | 0 | 0 | 0.0 | 0 | 0 | 0.0 |
| B0132 | Day 1 | 2 hour | 0 | 0 | 0.0 | 0 | 0 | 0.0 |
| B0132 | Day 4 | | 0 | 0 | 0.0 | 0 | 0 | 0.0 |
| B0132 | Day 8 | | 0 | 0 | 0.0 | 0 | 0 | 0.0 |
| B0132 | Day 22 | | 0 | 0 | 0.0 | 0 | 0 | 0.0 |
| B0132 | EOS/ET (Day 61) | | 0 | 0 | 0.0 | 0 | 0 | 0.0 |
| B0143 | Day 2 | | 0 | 0 | 0.0 | 0 | 0 | 0.0 |
| B0143 | Day 1 | 1 min | 0 | 0 | 0.0 | 2 | 5 | 3.5 |
| B0143 | Day 1 | 10 min | 0 | 0 | 0.0 | 0 | 0 | 0.0 |
| B0143 | Day 1 | 1 hour | 1 | 1 | 1.0 | 0 | 0 | 0.0 |
| B0143 | Day 1 | 2 hour | 1 | 1 | 1.0 | 0 | 0 | 0.0 |
| B0143 | Day 4 | | 0 | 0 | 0.0 | 0 | 0 | 0.0 |
| B0143 | Day 8 | | 0 | 0 | 0.0 | 0 | 0 | 0.0 |
| B0143 | Day 22 | | 0 | 0 | 0.0 | 0 | 0 | 0.0 |


Source: Listing 16.2.9.3

Administration Site Key: FA = Forearm; UA= Upper arm
Treatments Part A: A1 = A/Sing MAP, 15 ug (FA); A2 = Afluria Quad IM (UA), 15 ug; A3 = Placebo MAP (FA); A4 = A/Sing Mono IM, 15 ug (UA)
Treatments Part B: B1 = A/Sing MAP, 15 ug (FA) with biopsy; B2 = Placebo MAP (FA) with biopsy
B3 = A/Sing MAP, 15 ug (FA); B4 = A/Sing MAP, 10 ug (FA); B5 = A/Sing MAP, 5 ug (FA); B6 = A/Sing MAP, 2.5 ug (FA)
B7 = Placebo MAP (FA); B8 = A/Sing MAP, 15 ug (UA); B9 = A/Sing IM, 15 ug, as 2018 Afluria Quad (UA)

Listing 16.2.9.10
Individual Characterisation of Numeric Pain Intensity by Treatment

Part B, Non-biopsy
B6= 1 x 2.5 ug A/Sing MAP + 2 uncoated MAPs on volar forearm (2.5 ug)

| | | | -------Active------- | | | -------Placebo------- | | |
|---|---|---|---|---|---|---|---|---|
| Randomisation Number | Visit | Time Point | #> 0 | Maximum | Average | #> 0 | Maximum | Average |
| B0143 | EOS/ET (Day 61) | | 0 | 0 | 0.0 | 0 | 0 | 0.0 |
| B0144 | Day 2 | | 0 | 0 | 0.0 | 0 | 0 | 0.0 |
| B0144 | Day 1 | 1 min | 0 | 0 | 0.0 | 1 | 1 | 0.5 |
| B0144 | Day 1 | 10 min | 0 | 0 | 0.0 | 0 | 0 | 0.0 |
| B0144 | Day 1 | 1 hour | 0 | 0 | 0.0 | 0 | 0 | 0.0 |
| B0144 | Day 1 | 2 hour | 0 | 0 | 0.0 | 0 | 0 | 0.0 |
| B0144 | Day 4 | | 0 | 0 | 0.0 | 0 | 0 | 0.0 |
| B0144 | Day 8 | | 0 | 0 | 0.0 | 0 | 0 | 0.0 |
| B0144 | Day 22 | | 0 | 0 | 0.0 | 0 | 0 | 0.0 |
| B0144 | EOS/ET (Day 61) | | 0 | 0 | 0.0 | 0 | 0 | 0.0 |


Source: Listing 16.2.9.3

Administration Site Key: FA = Forearm; UA= Upper arm
Treatments Part A: A1 = A/Sing MAP, 15 ug (FA); A2 = Afluria Quad IM (UA), 15 ug; A3 = Placebo MAP (FA); A4 = A/Sing Mono IM, 15 ug (UA)
Treatments Part B: B1 = A/Sing MAP, 15 ug (FA) with biopsy; B2 = Placebo MAP (FA) with biopsy
B3 = A/Sing MAP, 15 ug (FA); B4 = A/Sing MAP, 10 ug (FA); B5 = A/Sing MAP, 5 ug (FA); B6 = A/Sing MAP, 2.5 ug (FA)
B7 = Placebo MAP (FA); B8 = A/Sing MAP, 15 ug (UA); B9 = A/Sing IM, 15 ug, as 2018 Afluria Quad (UA)

Listing 16.2.9.10
Individual Characterisation of Numeric Pain Intensity by Treatment

Part B, Non-biopsy
B7= 3 x uncoated MAPs on volar forearm

| | | | -------Active------- | | | -------Placebo------- | | |
|---|---|---|---|---|---|---|---|---|
| Randomisation Number | Visit | Time Point | #> 0 | Maximum | Average | #> 0 | Maximum | Average |
| B0017 | Day 2 | | - | - | - | 0 | 0 | 0.0 |
| B0017 | Day 1 | 1 min | - | - | - | 3 | 2 | 1.3 |
| B0017 | Day 1 | 10 min | - | - | - | 0 | 0 | 0.0 |
| B0017 | Day 1 | 1 hour | - | - | - | 0 | 0 | 0.0 |
| B0017 | Day 1 | 2 hour | - | - | - | 0 | 0 | 0.0 |
| B0017 | Day 4 | | - | - | - | 0 | 0 | 0.0 |
| B0017 | Day 8 | | - | - | - | 0 | 0 | 0.0 |
| B0017 | Day 22 | | - | - | - | 0 | 0 | 0.0 |
| B0017 | EOS/ET (Day 61) | | - | - | - | 0 | 0 | 0.0 |
| B0021 | Day 2 | | - | - | - | 0 | 0 | 0.0 |
| B0021 | Day 1 | 1 min | - | - | - | 3 | 3 | 2.7 |
| B0021 | Day 1 | 10 min | - | - | - | 0 | 0 | 0.0 |
| B0021 | Day 1 | 1 hour | - | - | - | 0 | 0 | 0.0 |
| B0021 | Day 1 | 2 hour | - | - | - | 0 | 0 | 0.0 |
| B0021 | Day 4 | | - | - | - | 0 | 0 | 0.0 |
| B0021 | Day 8 | | - | - | - | 0 | 0 | 0.0 |
| B0021 | Day 22 | | - | - | - | 0 | 0 | 0.0 |
| B0021 | EOS/ET (Day 61) | | - | - | - | 0 | 0 | 0.0 |
| B0030 | Day 2 | | - | - | - | 0 | 0 | 0.0 |



Administration Site Key: FA = Forearm; UA= Upper arm
Treatments Part A: A1 = A/Sing MAP, 15 ug (FA); A2 = Afluria Quad IM (UA), 15 ug; A3 = Placebo MAP (FA); A4 = A/Sing Mono IM, 15 ug (UA)
Treatments Part B: B1 = A/Sing MAP, 15 ug (FA) with biopsy; B2 = Placebo MAP (FA) with biopsy
B3 = A/Sing MAP, 15 ug (FA); B4 = A/Sing MAP, 10 ug (FA); B5 = A/Sing MAP, 5 ug (FA); B6 = A/Sing MAP, 2.5 ug (FA)
B7 = Placebo MAP (FA); B8 = A/Sing MAP, 15 ug (UA); B9 = A/Sing IM, 15 ug, as 2018 Afluria Quad (UA)

Listing 16.2.9.10
Individual Characterisation of Numeric Pain Intensity by Treatment

Part B, Non-biopsy
B7= 3 x uncoated MAPs on volar forearm

| | | | -------Active------- | | | -------Placebo------- | | |
|---|---|---|---|---|---|---|---|---|
| Randomisation Number | Visit | Time Point | #> 0 | Maximum | Average | #> 0 | Maximum | Average |
| B0030 | Day 1 | 1 min | - | - | - | 3 | 3 | 2.7 |
| B0030 | Day 1 | 10 min | - | - | - | 0 | 0 | 0.0 |
| B0030 | Day 1 | 1 hour | - | - | - | 0 | 0 | 0.0 |
| B0030 | Day 1 | 2 hour | - | - | - | 0 | 0 | 0.0 |
| B0030 | Day 4 | | - | - | - | 0 | 0 | 0.0 |
| B0030 | Day 8 | | - | - | - | 0 | 0 | 0.0 |
| B0030 | Day 22 | | - | - | - | 0 | 0 | 0.0 |
| B0030 | EOS/ET (Day 61) | | - | - | - | 0 | 0 | 0.0 |
| B0036 | Day 2 | | - | - | - | 0 | 0 | 0.0 |
| B0036 | Day 1 | 1 min | - | - | - | 1 | 1 | 0.3 |
| B0036 | Day 1 | 10 min | - | - | - | 3 | 1 | 1.0 |
| B0036 | Day 1 | 1 hour | - | - | - | 0 | 0 | 0.0 |
| B0036 | Day 1 | 2 hour | - | - | - | 0 | 0 | 0.0 |
| B0036 | Day 4 | | - | - | - | 0 | 0 | 0.0 |
| B0036 | Day 8 | | - | - | - | 0 | 0 | 0.0 |
| B0036 | Day 22 | | - | - | - | 0 | 0 | 0.0 |
| B0036 | EOS/ET (Day 61) | | - | - | - | 0 | 0 | 0.0 |
| B0039 | Day 2 | | - | - | - | 0 | 0 | 0.0 |
| B0039 | Day 1 | 1 min | - | - | - | 3 | 2 | 1.7 |


Source: Listing 16.2.9.3

Administration Site Key: FA = Forearm; UA= Upper arm
Treatments Part A: A1 = A/Sing MAP, 15 ug (FA); A2 = Afluria Quad IM (UA), 15 ug; A3 = Placebo MAP (FA); A4 = A/Sing Mono IM, 15 ug (UA)
Treatments Part B: B1 = A/Sing MAP, 15 ug (FA) with biopsy; B2 = Placebo MAP (FA) with biopsy
B3 = A/Sing MAP, 15 ug (FA); B4 = A/Sing MAP, 10 ug (FA); B5 = A/Sing MAP, 5 ug (FA); B6 = A/Sing MAP, 2.5 ug (FA)
B7 = Placebo MAP (FA); B8 = A/Sing MAP, 15 ug (UA); B9 = A/Sing IM, 15 ug, as 2018 Afluria Quad (UA)

Listing 16.2.9.10
Individual Characterisation of Numeric Pain Intensity by Treatment

Part B, Non-biopsy
B7= 3 x uncoated MAPs on volar forearm

| | | | -------Active------- | | | -------Placebo------- | | |
|---|---|---|---|---|---|---|---|---|
| Randomisation Number | Visit | Time Point | #> 0 | Maximum | Average | #> 0 | Maximum | Average |
| B0039 | Day 1 | 10 min | - | - | - | 1 | 1 | 0.3 |
| B0039 | Day 1 | 1 hour | - | - | - | 0 | 0 | 0.0 |
| B0039 | Day 1 | 2 hour | - | - | - | 0 | 0 | 0.0 |
| B0039 | Day 4 | | - | - | - | 0 | 0 | 0.0 |
| B0039 | Day 8 | | - | - | - | 0 | 0 | 0.0 |
| B0039 | Day 22 | | - | - | - | 0 | 0 | 0.0 |
| B0039 | EOS/ET (Day 61) | | - | - | - | 0 | 0 | 0.0 |
| B0046 | Day 2 | | - | - | - | 0 | 0 | 0.0 |
| B0046 | Day 1 | 1 min | - | - | - | 3 | 3 | 2.7 |
| B0046 | Day 1 | 10 min | - | - | - | 3 | 3 | 1.7 |
| B0046 | Day 1 | 1 hour | - | - | - | 0 | 0 | 0.0 |
| B0046 | Day 1 | 2 hour | - | - | - | 0 | 0 | 0.0 |
| B0046 | Day 4 | | - | - | - | 0 | 0 | 0.0 |
| B0046 | Day 8 | | - | - | - | 0 | 0 | 0.0 |
| B0046 | Day 22 | | - | - | - | 0 | 0 | 0.0 |
| B0046 | EOS/ET (Day 61) | | - | - | - | 0 | 0 | 0.0 |
| B0059 | Day 2 | | - | - | - | 0 | 0 | 0.0 |
| B0059 | Day 1 | 1 min | - | - | - | 3 | 2 | 2.0 |
| B0059 | Day 1 | 10 min | - | - | - | 0 | 0 | 0.0 |


Source: Listing 16.2.9.3

Administration Site Key: FA = Forearm; UA= Upper arm
Treatments Part A: A1 = A/Sing MAP, 15 ug (FA); A2 = Afluria Quad IM (UA), 15 ug; A3 = Placebo MAP (FA); A4 = A/Sing Mono IM, 15 ug (UA)
Treatments Part B: B1 = A/Sing MAP, 15 ug (FA) with biopsy; B2 = Placebo MAP (FA) with biopsy
B3 = A/Sing MAP, 15 ug (FA); B4 = A/Sing MAP, 10 ug (FA); B5 = A/Sing MAP, 5 ug (FA); B6 = A/Sing MAP, 2.5 ug (FA)
B7 = Placebo MAP (FA); B8 = A/Sing MAP, 15 ug (UA); B9 = A/Sing IM, 15 ug, as 2018 Afluria Quad (UA)

Listing 16.2.9.10
Individual Characterisation of Numeric Pain Intensity by Treatment

Part B, Non-biopsy
B7= 3 x uncoated MAPs on volar forearm

| Randomisation Number | Visit | Time Point | Active #> 0 | Active Maximum | Active Average | Placebo #> 0 | Placebo Maximum | Placebo Average |
|---|---|---|---|---|---|---|---|---|
| B0059 | Day 1 | 1 hour | - | - | - | 0 | 0 | 0.0 |
| B0059 | Day 1 | 2 hour | - | - | - | 0 | 0 | 0.0 |
| B0059 | Day 4 | | - | - | - | 0 | 0 | 0.0 |
| B0059 | Day 8 | | - | - | - | 0 | 0 | 0.0 |
| B0059 | Day 22 | | - | - | - | 0 | 0 | 0.0 |
| B0059 | EOS/ET (Day 61) | | - | - | - | 0 | 0 | 0.0 |
| B0063 | Day 2 | | - | - | - | 0 | 0 | 0.0 |
| B0063 | Day 1 | 1 min | - | - | - | 3 | 2 | 1.7 |
| B0063 | Day 1 | 10 min | - | - | - | 0 | 0 | 0.0 |
| B0063 | Day 1 | 1 hour | - | - | - | 0 | 0 | 0.0 |
| B0063 | Day 1 | 2 hour | - | - | - | 0 | 0 | 0.0 |
| B0063 | Day 4 | | - | - | - | 0 | 0 | 0.0 |
| B0063 | Day 8 | | - | - | - | 0 | 0 | 0.0 |
| B0063 | Day 22 | | - | - | - | 0 | 0 | 0.0 |
| B0063 | EOS/ET (Day 61) | | - | - | - | 0 | 0 | 0.0 |
| B0069 | Day 2 | | - | - | - | 0 | 0 | 0.0 |
| B0069 | Day 1 | 1 min | - | - | - | 0 | 0 | 0.0 |
| B0069 | Day 1 | 10 min | - | - | - | 0 | 0 | 0.0 |
| B0069 | Day 1 | 1 hour | - | - | - | 0 | 0 | 0.0 |


Source: Listing 16.2.9.3

Administration Site Key: FA = Forearm; UA= Upper arm
Treatments Part A: A1 = A/Sing MAP, 15 ug (FA); A2 = Afluria Quad IM (UA), 15 ug; A3 = Placebo MAP (FA); A4 = A/Sing Mono IM, 15 ug (UA)
Treatments Part B: B1 = A/Sing MAP, 15 ug (FA) with biopsy; B2 = Placebo MAP (FA) with biopsy
B3 = A/Sing MAP, 15 ug (FA); B4 = A/Sing MAP, 10 ug (FA); B5 = A/Sing MAP, 5 ug (FA); B6 = A/Sing MAP, 2.5 ug (FA)
B7 = Placebo MAP (FA); B8 = A/Sing MAP, 15 ug (UA); B9 = A/Sing IM, 15 ug, as 2018 Afluria Quad (UA)

Listing 16.2.9.10
Individual Characterisation of Numeric Pain Intensity by Treatment

Part B, Non-biopsy
B7= 3 x uncoated MAPs on volar forearm

| | | | -------Active------- | | | -------Placebo------- | | |
|---|---|---|---|---|---|---|---|---|
| Randomisation Number | Visit | Time Point | #> 0 | Maximum | Average | #> 0 | Maximum | Average |
| B0069 | Day 1 | 2 hour | - | - | - | 0 | 0 | 0.0 |
| B0069 | Day 4 | | - | - | - | 0 | 0 | 0.0 |
| B0069 | Day 8 | | - | - | - | 0 | 0 | 0.0 |
| B0069 | Day 22 | | - | - | - | 0 | 0 | 0.0 |
| B0069 | EOS/ET (Day 61) | | - | - | - | 0 | 0 | 0.0 |
| B0087 | Day 2 | | - | - | - | 0 | 0 | 0.0 |
| B0087 | Day 1 | 1 min | - | - | - | 3 | 2 | 1.7 |
| B0087 | Day 1 | 10 min | - | - | - | 0 | 0 | 0.0 |
| B0087 | Day 1 | 1 hour | - | - | - | 0 | 0 | 0.0 |
| B0087 | Day 1 | 2 hour | - | - | - | 0 | 0 | 0.0 |
| B0087 | Day 4 | | - | - | - | 1 | 0 | 0.0 |
| B0087 | Day 8 | | - | - | - | 0 | 0 | 0.0 |
| B0087 | Day 22 | | - | - | - | 0 | 0 | 0.0 |
| B0087 | EOS/ET (Day 61) | | - | - | - | 0 | 0 | 0.0 |
| B0093 | Day 2 | | - | - | - | 0 | 0 | 0.0 |
| B0093 | Day 1 | 1 min | - | - | - | 3 | 1 | 1.0 |
| B0093 | Day 1 | 10 min | - | - | - | 0 | 0 | 0.0 |
| B0093 | Day 1 | 1 hour | - | - | - | 0 | 0 | 0.0 |
| B0093 | Day 1 | 2 hour | - | - | - | 0 | 0 | 0.0 |


Source: Listing 16.2.9.3

Administration Site Key: FA = Forearm; UA= Upper arm
Treatments Part A: A1 = A/Sing MAP, 15 ug (FA); A2 = Afluria Quad IM (UA), 15 ug; A3 = Placebo MAP (FA); A4 = A/Sing Mono IM, 15 ug (UA)
Treatments Part B: B1 = A/Sing MAP, 15 ug (FA) with biopsy; B2 = Placebo MAP (FA) with biopsy
B3 = A/Sing MAP, 15 ug (FA); B4 = A/Sing MAP, 10 ug (FA); B5 = A/Sing MAP, 5 ug (FA); B6 = A/Sing MAP, 2.5 ug (FA)
B7 = Placebo MAP (FA); B8 = A/Sing MAP, 15 ug (UA); B9 = A/Sing IM, 15 ug, as 2018 Afluria Quad (UA)

Listing 16.2.9.10
Individual Characterisation of Numeric Pain Intensity by Treatment

Part B, Non-biopsy
B7= 3 x uncoated MAPs on volar forearm

| | | | Active | | | Placebo | | |
|---|---|---|---|---|---|---|---|---|
| Randomisation Number | Visit | Time Point | #> 0 | Maximum | Average | #> 0 | Maximum | Average |
| B0093 | Day 4 | | - | - | - | 0 | 0 | 0.0 |
| B0093 | Day 8 | | - | - | - | 0 | 0 | 0.0 |
| B0093 | Day 22 | | - | - | - | 0 | 0 | 0.0 |
| B0093 | EOS/ET (Day 61) | | - | - | - | 0 | 0 | 0.0 |
| B0098 | Day 2 | | - | - | - | 0 | 0 | 0.0 |
| B0098 | Day 1 | 1 min | - | - | - | 3 | 2 | 2.0 |
| B0098 | Day 1 | 10 min | - | - | - | 3 | 2 | 2.0 |
| B0098 | Day 1 | 1 hour | - | - | - | 3 | 1 | 1.0 |
| B0098 | Day 1 | 2 hour | - | - | - | 0 | 0 | 0.0 |
| B0098 | Day 4 | | - | - | - | 0 | 0 | 0.0 |
| B0098 | Day 8 | | - | - | - | 0 | 0 | 0.0 |
| B0098 | Day 22 | | - | - | - | 0 | 0 | 0.0 |
| B0098 | EOS/ET (Day 61) | | - | - | - | 0 | 0 | 0.0 |
| B0108 | Day 2 | | - | - | - | 0 | 0 | 0.0 |
| B0108 | Day 1 | 1 min | - | - | - | 3 | 1 | 1.0 |
| B0108 | Day 1 | 10 min | - | - | - | 3 | 1 | 1.0 |
| B0108 | Day 1 | 1 hour | - | - | - | 0 | 0 | 0.0 |
| B0108 | Day 1 | 2 hour | - | - | - | 0 | 0 | 0.0 |
| B0108 | Day 4 | | - | - | - | 0 | 0 | 0.0 |


Source: Listing 16.2.9.3

Administration Site Key: FA = Forearm; UA= Upper arm
Treatments Part A: A1 = A/Sing MAP, 15 ug (FA); A2 = Afluria Quad IM (UA), 15 ug; A3 = Placebo MAP (FA); A4 = A/Sing Mono IM, 15 ug (UA)
Treatments Part B: B1 = A/Sing MAP, 15 ug (FA) with biopsy; B2 = Placebo MAP (FA) with biopsy
B3 = A/Sing MAP, 15 ug (FA); B4 = A/Sing MAP, 10 ug (FA); B5 = A/Sing MAP, 5 ug (FA); B6 = A/Sing MAP, 2.5 ug (FA)
B7 = Placebo MAP (FA); B8 = A/Sing MAP, 15 ug (UA); B9 = A/Sing IM, 15 ug, as 2018 Afluria Quad (UA)

Listing 16.2.9.10
Individual Characterisation of Numeric Pain Intensity by Treatment

Part B, Non-biopsy
B7= 3 x uncoated MAPs on volar forearm

| | | | -------Active------- | | | -------Placebo------- | | |
|---|---|---|---|---|---|---|---|---|
| Randomisation Number | Visit | Time Point | #> 0 | Maximum | Average | #> 0 | Maximum | Average |
| B0108 | Day 8 | | - | - | - | 0 | 0 | 0.0 |
| B0108 | Day 22 | | - | - | - | 0 | 0 | 0.0 |
| B0108 | EOS/ET (Day 61) | | - | - | - | 0 | 0 | 0.0 |
| B0114 | Day 2 | | - | - | - | 0 | 0 | 0.0 |
| B0114 | Day 1 | 1 min | - | - | - | 2 | 3 | 1.3 |
| B0114 | Day 1 | 10 min | - | - | - | 1 | 1 | 0.3 |
| B0114 | Day 1 | 1 hour | - | - | - | 0 | 0 | 0.0 |
| B0114 | Day 1 | 2 hour | - | - | - | 0 | 0 | 0.0 |
| B0114 | Day 4 | | - | - | - | 0 | 0 | 0.0 |
| B0114 | Day 8 | | - | - | - | 0 | 0 | 0.0 |
| B0114 | Day 22 | | - | - | - | 0 | 0 | 0.0 |
| B0114 | EOS/ET (Day 61) | | - | - | - | 0 | 0 | 0.0 |
| B0116 | Day 2 | | - | - | - | 0 | 0 | 0.0 |
| B0116 | Day 1 | 1 min | - | - | - | 3 | 5 | 4.3 |
| B0116 | Day 1 | 10 min | - | - | - | 3 | 2 | 1.3 |
| B0116 | Day 1 | 1 hour | - | - | - | 0 | 0 | 0.0 |
| B0116 | Day 1 | 2 hour | - | - | - | 0 | 0 | 0.0 |
| B0116 | Day 4 | | - | - | - | 0 | 0 | 0.0 |
| B0116 | Day 8 | | - | - | - | 0 | 0 | 0.0 |


Source: Listing 16.2.9.3

Administration Site Key: FA = Forearm; UA= Upper arm
Treatments Part A: A1 = A/Sing MAP, 15 ug (FA); A2 = Afluria Quad IM (UA), 15 ug; A3 = Placebo MAP (FA); A4 = A/Sing Mono IM, 15 ug (UA)
Treatments Part B: B1 = A/Sing MAP, 15 ug (FA) with biopsy; B2 = Placebo MAP (FA) with biopsy
B3 = A/Sing MAP, 15 ug (FA); B4 = A/Sing MAP, 10 ug (FA); B5 = A/Sing MAP, 5 ug (FA); B6 = A/Sing MAP, 2.5 ug (FA)
B7 = Placebo MAP (FA); B8 = A/Sing MAP, 15 ug (UA); B9 = A/Sing IM, 15 ug, as 2018 Afluria Quad (UA)

Listing 16.2.9.10
Individual Characterisation of Numeric Pain Intensity by Treatment

Part B, Non-biopsy
B7= 3 x uncoated MAPs on volar forearm

| | | | -------Active------- | | | -------Placebo------- | | |
|---|---|---|---|---|---|---|---|---|
| Randomisation Number | Visit | Time Point | #> 0 | Maximum | Average | #> 0 | Maximum | Average |
| B0116 | Day 22 | | - | - | - | 0 | 0 | 0.0 |
| B0116 | EOS/ET (Day 61) | | - | - | - | 0 | 0 | 0.0 |
| | | | | | | | | |
| B0125 | Day 2 | | - | - | - | 0 | 0 | 0.0 |
| B0125 | Day 1 | 1 min | - | - | - | 3 | 2 | 2.0 |
| B0125 | Day 1 | 10 min | - | - | - | 0 | 0 | 0.0 |
| B0125 | Day 1 | 1 hour | - | - | - | 0 | 0 | 0.0 |
| B0125 | Day 1 | 2 hour | - | - | - | 0 | 0 | 0.0 |
| B0125 | Day 4 | | - | - | - | 0 | 0 | 0.0 |
| B0125 | Day 8 | | - | - | - | 0 | 0 | 0.0 |
| B0125 | Day 22 | | - | - | - | 0 | 0 | 0.0 |
| B0125 | EOS/ET (Day 61) | | - | - | - | 0 | 0 | 0.0 |
| | | | | | | | | |
| B0135 | Day 2 | | - | - | - | 0 | 0 | 0.0 |
| B0135 | Day 1 | 1 min | - | - | - | 3 | 1 | 1.0 |
| B0135 | Day 1 | 10 min | - | - | - | 2 | 1 | 0.7 |
| B0135 | Day 1 | 1 hour | - | - | - | 0 | 0 | 0.0 |
| B0135 | Day 1 | 2 hour | - | - | - | 0 | 0 | 0.0 |
| B0135 | Day 4 | | - | - | - | 0 | 0 | 0.0 |
| B0135 | Day 8 | | - | - | - | 0 | 0 | 0.0 |
| B0135 | Day 22 | | - | - | - | 0 | 0 | 0.0 |



Administration Site Key: FA = Forearm; UA= Upper arm
Treatments Part A: A1 = A/Sing MAP, 15 ug (FA); A2 = Afluria Quad IM (UA), 15 ug; A3 = Placebo MAP (FA); A4 = A/Sing Mono IM, 15 ug (UA)
Treatments Part B: B1 = A/Sing MAP, 15 ug (FA) with biopsy; B2 = Placebo MAP (FA) with biopsy
B3 = A/Sing MAP, 15 ug (FA); B4 = A/Sing MAP, 10 ug (FA); B5 = A/Sing MAP, 5 ug (FA); B6 = A/Sing MAP, 2.5 ug (FA)
B7 = Placebo MAP (FA); B8 = A/Sing MAP, 15 ug (UA); B9 = A/Sing IM, 15 ug, as 2018 Afluria Quad (UA)

Listing 16.2.9.10
Individual Characterisation of Numeric Pain Intensity by Treatment

Part B, Non-biopsy
B7= 3 x uncoated MAPs on volar forearm

| | | | -------Active------- | | | -------Placebo------- | | |
|---|---|---|---|---|---|---|---|---|
| Randomisation Number | Visit | Time Point | #> 0 | Maximum | Average | #> 0 | Maximum | Average |
| B0135 | EOS/ET (Day 61) | | - | - | - | 0 | 0 | 0.0 |
| B0138 | Day 2 | | - | - | - | 0 | 0 | 0.0 |
| B0138 | Day 1 | 1 min | - | - | - | 3 | 1 | 1.0 |
| B0138 | Day 1 | 10 min | - | - | - | 1 | 1 | 0.3 |
| B0138 | Day 1 | 1 hour | - | - | - | 0 | 0 | 0.0 |
| B0138 | Day 1 | 2 hour | - | - | - | 0 | 0 | 0.0 |
| B0138 | Day 4 | | - | - | - | 0 | 0 | 0.0 |
| B0138 | Day 8 | | - | - | - | 0 | 0 | 0.0 |
| B0138 | Day 22 | | - | - | - | 0 | 0 | 0.0 |
| B0138 | EOS/ET (Day 61) | | - | - | - | 0 | 0 | 0.0 |
| B0146 | Day 2 | | - | - | - | 0 | 0 | 0.0 |
| B0146 | Day 1 | 1 min | - | - | - | 3 | 1 | 1.0 |
| B0146 | Day 1 | 10 min | - | - | - | 0 | 0 | 0.0 |
| B0146 | Day 1 | 1 hour | - | - | - | 0 | 0 | 0.0 |
| B0146 | Day 1 | 2 hour | - | - | - | 0 | 0 | 0.0 |
| B0146 | Day 4 | | - | - | - | 0 | 0 | 0.0 |
| B0146 | Day 8 | | - | - | - | 0 | 0 | 0.0 |
| B0146 | Day 22 | | - | - | - | 0 | 0 | 0.0 |
| B0146 | EOS/ET (Day 61) | | - | - | - | 0 | 0 | 0.0 |


Source: Listing 16.2.9.3

Administration Site Key: FA = Forearm; UA= Upper arm
Treatments Part A: A1 = A/Sing MAP, 15 ug (FA); A2 = Afluria Quad IM (UA), 15 ug; A3 = Placebo MAP (FA); A4 = A/Sing Mono IM, 15 ug (UA)
Treatments Part B: B1 = A/Sing MAP, 15 ug (FA) with biopsy; B2 = Placebo MAP (FA) with biopsy
B3 = A/Sing MAP, 15 ug (FA); B4 = A/Sing MAP, 10 ug (FA); B5 = A/Sing MAP, 5 ug (FA); B6 = A/Sing MAP, 2.5 ug (FA)
B7 = Placebo MAP (FA); B8 = A/Sing MAP, 15 ug (UA); B9 = A/Sing IM, 15 ug, as 2018 Afluria Quad (UA)

Listing 16.2.9.10
Individual Characterisation of Numeric Pain Intensity by Treatment

Part B, Non-biopsy
B7= 3 x uncoated MAPs on volar forearm

| | | | -------Active------- | | | -------Placebo------- | | |
|---|---|---|---|---|---|---|---|---|
| Randomisation Number | Visit | Time Point | #> 0 | Maximum | Average | #> 0 | Maximum | Average |
| B1080 | Day 2 | | - | - | - | 0 | 0 | 0.0 |
| B1080 | Day 1 | 1 min | - | - | - | 0 | 0 | 0.0 |
| B1080 | Day 1 | 10 min | - | - | - | 0 | 0 | 0.0 |
| B1080 | Day 1 | 1 hour | - | - | - | 0 | 0 | 0.0 |
| B1080 | Day 1 | 2 hour | - | - | - | 0 | 0 | 0.0 |
| B1080 | Day 4 | | - | - | - | 0 | 0 | 0.0 |
| B1080 | Day 8 | | - | - | - | 0 | 0 | 0.0 |
| B1080 | Day 22 | | - | - | - | 0 | 0 | 0.0 |
| B1080 | EOS/ET (Day 61) | | - | - | - | 0 | 0 | 0.0 |



Administration Site Key: FA = Forearm; UA= Upper arm
Treatments Part A: A1 = A/Sing MAP, 15 ug (FA); A2 = Afluria Quad IM (UA), 15 ug; A3 = Placebo MAP (FA); A4 = A/Sing Mono IM, 15 ug (UA)
Treatments Part B: B1 = A/Sing MAP, 15 ug (FA) with biopsy; B2 = Placebo MAP (FA) with biopsy
B3 = A/Sing MAP, 15 ug (FA); B4 = A/Sing MAP, 10 ug (FA); B5 = A/Sing MAP, 5 ug (FA); B6 = A/Sing MAP, 2.5 ug (FA)
B7 = Placebo MAP (FA); B8 = A/Sing MAP, 15 ug (UA); B9 = A/Sing IM, 15 ug, as 2018 Afluria Quad (UA)

Listing 16.2.9.10
Individual Characterisation of Numeric Pain Intensity by Treatment

Part B, Non-biopsy
B8= 3 x 5 ug A/Sing MAPs on upper arm (15 ug)

| | | | -------Active------- | | | -------Placebo------- | | |
|---|---|---|---|---|---|---|---|---|
| Randomisation Number | Visit | Time Point | #> 0 | Maximum | Average | #> 0 | Maximum | Average |
| B0011 | Day 2 | | 0 | 0 | 0.0 | - | - | - |
| B0011 | Day 1 | 1 min | 1 | 1 | 0.3 | - | - | - |
| B0011 | Day 1 | 10 min | 0 | 0 | 0.0 | - | - | - |
| B0011 | Day 1 | 1 hour | 0 | 0 | 0.0 | - | - | - |
| B0011 | Day 1 | 2 hour | 0 | 0 | 0.0 | - | - | - |
| B0011 | Day 4 | | 0 | 0 | 0.0 | - | - | - |
| B0011 | Day 8 | | 0 | 0 | 0.0 | - | - | - |
| B0011 | Day 22 | | 0 | 0 | 0.0 | - | - | - |
| B0011 | EOS/ET (Day 61) | | 0 | 0 | 0.0 | - | - | - |
| B0025 | Day 2 | | 0 | 0 | 0.0 | - | - | - |
| B0025 | Day 1 | 1 min | 0 | 0 | 0.0 | - | - | - |
| B0025 | Day 1 | 10 min | 3 | 1 | 1.0 | - | - | - |
| B0025 | Day 1 | 1 hour | 0 | 0 | 0.0 | - | - | - |
| B0025 | Day 1 | 2 hour | 0 | 0 | 0.0 | - | - | - |
| B0025 | Day 4 | | 0 | 0 | 0.0 | - | - | - |
| B0025 | Day 8 | | 0 | 0 | 0.0 | - | - | - |
| B0025 | Day 22 | | 0 | 0 | 0.0 | - | - | - |
| B0025 | EOS/ET (Day 61) | | 0 | 0 | 0.0 | - | - | - |
| B0034 | Day 2 | | 0 | 0 | 0.0 | - | - | - |


Source: Listing 16.2.9.3

Administration Site Key: FA = Forearm; UA= Upper arm
Treatments Part A: A1 = A/Sing MAP, 15 ug (FA); A2 = Afluria Quad IM (UA), 15 ug; A3 = Placebo MAP (FA); A4 = A/Sing Mono IM, 15 ug (UA)
Treatments Part B: B1 = A/Sing MAP, 15 ug (FA) with biopsy; B2 = Placebo MAP (FA) with biopsy
B3 = A/Sing MAP, 15 ug (FA); B4 = A/Sing MAP, 10 ug (FA); B5 = A/Sing MAP, 5 ug (FA); B6 = A/Sing MAP, 2.5 ug (FA)
B7 = Placebo MAP (FA); B8 = A/Sing MAP, 15 ug (UA); B9 = A/Sing IM, 15 ug, as 2018 Afluria Quad (UA)

Listing 16.2.9.10
Individual Characterisation of Numeric Pain Intensity by Treatment

Part B, Non-biopsy
B8= 3 x 5 ug A/Sing MAPs on upper arm (15 ug)

| | | | -------Active------- | | | -------Placebo------- | | |
|---|---|---|---|---|---|---|---|---|
| Randomisation Number | Visit | Time Point | #> 0 | Maximum | Average | #> 0 | Maximum | Average |
| B0034 | Day 1 | 1 min | 3 | 3 | 1.7 | - | - | - |
| B0034 | Day 1 | 10 min | 1 | 1 | 0.3 | - | - | - |
| B0034 | Day 1 | 1 hour | 2 | 1 | 0.7 | - | - | - |
| B0034 | Day 1 | 2 hour | 0 | 0 | 0.0 | - | - | - |
| B0034 | Day 4 | | 0 | 0 | 0.0 | - | - | - |
| B0034 | Day 8 | | 0 | 0 | 0.0 | - | - | - |
| B0034 | Day 22 | | 0 | 0 | 0.0 | - | - | - |
| B0034 | EOS/ET (Day 61) | | 0 | 0 | 0.0 | - | - | - |
| B0043 | Day 2 | | 0 | 0 | 0.0 | - | - | - |
| B0043 | Day 1 | 1 min | 3 | 3 | 2.3 | - | - | - |
| B0043 | Day 1 | 10 min | 1 | 3 | 1.0 | - | - | - |
| B0043 | Day 1 | 1 hour | 0 | 0 | 0.0 | - | - | - |
| B0043 | Day 1 | 2 hour | 0 | 0 | 0.0 | - | - | - |
| B0043 | Day 4 | | 0 | 0 | 0.0 | - | - | - |
| B0043 | Day 8 | | 0 | 0 | 0.0 | - | - | - |
| B0043 | Day 22 | | 0 | 0 | 0.0 | - | - | - |
| B0043 | EOS/ET (Day 61) | | 0 | 0 | 0.0 | - | - | - |
| B0050 | Day 2 | | 0 | 0 | 0.0 | - | - | - |
| B0050 | Day 1 | 1 min | 3 | 1 | 1.0 | - | - | - |


Source: Listing 16.2.9.3

Administration Site Key: FA = Forearm; UA= Upper arm
Treatments Part A: A1 = A/Sing MAP, 15 ug (FA); A2 = Afluria Quad IM (UA), 15 ug; A3 = Placebo MAP (FA); A4 = A/Sing Mono IM, 15 ug (UA)
Treatments Part B: B1 = A/Sing MAP, 15 ug (FA) with biopsy; B2 = Placebo MAP (FA) with biopsy
B3 = A/Sing MAP, 15 ug (FA); B4 = A/Sing MAP, 10 ug (FA); B5 = A/Sing MAP, 5 ug (FA); B6 = A/Sing MAP, 2.5 ug (FA)
B7 = Placebo MAP (FA); B8 = A/Sing MAP, 15 ug (UA); B9 = A/Sing IM, 15 ug, as 2018 Afluria Quad (UA)

Listing 16.2.9.10
Individual Characterisation of Numeric Pain Intensity by Treatment

Part B, Non-biopsy
B8= 3 x 5 ug A/Sing MAPs on upper arm (15 ug)

| | | | -------Active------- | | | -------Placebo------- | | |
|---|---|---|---|---|---|---|---|---|
| Randomisation Number | Visit | Time Point | #> 0 | Maximum | Average | #> 0 | Maximum | Average |
| B0050 | Day 1 | 10 min | 1 | 1 | 0.3 | - | - | - |
| B0050 | Day 1 | 1 hour | 2 | 1 | 0.7 | - | - | - |
| B0050 | Day 1 | 2 hour | 0 | 0 | 0.0 | - | - | - |
| B0050 | Day 4 | | 0 | 0 | 0.0 | - | - | - |
| B0050 | Day 8 | | 0 | 0 | 0.0 | - | - | - |
| B0050 | Day 22 | | 0 | 0 | 0.0 | - | - | - |
| B0050 | EOS/ET (Day 61) | | 0 | 0 | 0.0 | - | - | - |
| B0055 | Day 2 | | 0 | 0 | 0.0 | - | - | - |
| B0055 | Day 1 | 1 min | 3 | 2 | 1.3 | - | - | - |
| B0055 | Day 1 | 10 min | 3 | 2 | 2.0 | - | - | - |
| B0055 | Day 1 | 1 hour | 0 | 0 | 0.0 | - | - | - |
| B0055 | Day 1 | 2 hour | 1 | 1 | 0.3 | - | - | - |
| B0055 | Day 4 | | 0 | 0 | 0.0 | - | - | - |
| B0055 | Day 8 | | 0 | 0 | 0.0 | - | - | - |
| B0055 | Day 22 | | 0 | 0 | 0.0 | - | - | - |
| B0055 | EOS/ET (Day 61) | | 0 | 0 | 0.0 | - | - | - |
| B0061 | Day 2 | | 0 | 0 | 0.0 | - | - | - |
| B0061 | Day 1 | 1 min | 1 | 1 | 0.3 | - | - | - |
| B0061 | Day 1 | 10 min | 0 | 0 | 0.0 | - | - | - |


Source: Listing 16.2.9.3

Administration Site Key: FA = Forearm; UA= Upper arm
Treatments Part A: A1 = A/Sing MAP, 15 ug (FA); A2 = Afluria Quad IM (UA), 15 ug; A3 = Placebo MAP (FA); A4 = A/Sing Mono IM, 15 ug (UA)
Treatments Part B: B1 = A/Sing MAP, 15 ug (FA) with biopsy; B2 = Placebo MAP (FA) with biopsy
B3 = A/Sing MAP, 15 ug (FA); B4 = A/Sing MAP, 10 ug (FA); B5 = A/Sing MAP, 5 ug (FA); B6 = A/Sing MAP, 2.5 ug (FA)
B7 = Placebo MAP (FA); B8 = A/Sing MAP, 15 ug (UA); B9 = A/Sing IM, 15 ug, as 2018 Afluria Quad (UA)

Listing 16.2.9.10
Individual Characterisation of Numeric Pain Intensity by Treatment

Part B, Non-biopsy
B8= 3 x 5 ug A/Sing MAPs on upper arm (15 ug)

| | | | -------Active------- | | | -------Placebo------- | | |
|---|---|---|---|---|---|---|---|---|
| Randomisation Number | Visit | Time Point | #> 0 | Maximum | Average | #> 0 | Maximum | Average |
| B0061 | Day 1 | 1 hour | 0 | 0 | 0.0 | - | - | - |
| B0061 | Day 1 | 2 hour | 0 | 0 | 0.0 | - | - | - |
| B0061 | Day 4 | | 0 | 0 | 0.0 | - | - | - |
| B0061 | Day 8 | | 0 | 0 | 0.0 | - | - | - |
| B0061 | Day 22 | | 0 | 0 | 0.0 | - | - | - |
| B0061 | EOS/ET (Day 61) | | 0 | 0 | 0.0 | - | - | - |
| B0067 | Day 2 | | 2 | 1 | 0.7 | - | - | - |
| B0067 | Day 1 | 1 min | 2 | 1 | 0.7 | - | - | - |
| B0067 | Day 1 | 10 min | 0 | 0 | 0.0 | - | - | - |
| B0067 | Day 1 | 1 hour | 0 | 0 | 0.0 | - | - | - |
| B0067 | Day 1 | 2 hour | 0 | 0 | 0.0 | - | - | - |
| B0067 | Day 4 | | 0 | 0 | 0.0 | - | - | - |
| B0067 | Day 8 | | 0 | 0 | 0.0 | - | - | - |
| B0067 | Day 22 | | 0 | 0 | 0.0 | - | - | - |
| B0067 | EOS/ET (Day 61) | | 0 | 0 | 0.0 | - | - | - |
| B0078 | Day 2 | | 0 | 0 | 0.0 | - | - | - |
| B0078 | Day 1 | 1 min | 2 | 1 | 0.7 | - | - | - |
| B0078 | Day 1 | 10 min | 1 | 1 | 0.3 | - | - | - |
| B0078 | Day 1 | 1 hour | 0 | 0 | 0.0 | - | - | - |


Source: Listing 16.2.9.3

Administration Site Key: FA = Forearm; UA= Upper arm
Treatments Part A: A1 = A/Sing MAP, 15 ug (FA); A2 = Afluria Quad IM (UA), 15 ug; A3 = Placebo MAP (FA); A4 = A/Sing Mono IM, 15 ug (UA)
Treatments Part B: B1 = A/Sing MAP, 15 ug (FA) with biopsy; B2 = Placebo MAP (FA) with biopsy
B3 = A/Sing MAP, 15 ug (FA); B4 = A/Sing MAP, 10 ug (FA); B5 = A/Sing MAP, 5 ug (FA); B6 = A/Sing MAP, 2.5 ug (FA)
B7 = Placebo MAP (FA); B8 = A/Sing MAP, 15 ug (UA); B9 = A/Sing IM, 15 ug, as 2018 Afluria Quad (UA)

Listing 16.2.9.10
Individual Characterisation of Numeric Pain Intensity by Treatment

Part B, Non-biopsy
B8= 3 x 5 ug A/Sing MAPs on upper arm (15 ug)

| | | | -------Active------- | | | -------Placebo------- | | |
|---|---|---|---|---|---|---|---|---|
| Randomisation Number | Visit | Time Point | #> 0 | Maximum | Average | #> 0 | Maximum | Average |
| B0078 | Day 1 | 2 hour | 0 | 0 | 0.0 | - | - | - |
| B0078 | Day 4 | | 0 | 0 | 0.0 | - | - | - |
| B0078 | Day 8 | | 0 | 0 | 0.0 | - | - | - |
| B0078 | Day 22 | | 0 | 0 | 0.0 | - | - | - |
| B0078 | EOS/ET (Day 61) | | 0 | 0 | 0.0 | - | - | - |
| B0083 | Day 2 | | 0 | 0 | 0.0 | - | - | - |
| B0083 | Day 1 | 1 min | 3 | 1 | 1.0 | - | - | - |
| B0083 | Day 1 | 10 min | 0 | 0 | 0.0 | - | - | - |
| B0083 | Day 1 | 1 hour | 3 | 1 | 1.0 | - | - | - |
| B0083 | Day 1 | 2 hour | 0 | 0 | 0.0 | - | - | - |
| B0083 | Day 4 | | 0 | 0 | 0.0 | - | - | - |
| B0083 | Day 8 | | 0 | 0 | 0.0 | - | - | - |
| B0083 | Day 22 | | 0 | 0 | 0.0 | - | - | - |
| B0083 | EOS/ET (Day 61) | | 0 | 0 | 0.0 | - | - | - |
| B0091 | Day 2 | | 0 | 0 | 0.0 | - | - | - |
| B0091 | Day 1 | 1 min | 1 | 2 | 0.7 | - | - | - |
| B0091 | Day 1 | 10 min | 1 | 0 | 0.0 | - | - | - |
| B0091 | Day 1 | 1 hour | 0 | 0 | 0.0 | - | - | - |
| B0091 | Day 1 | 2 hour | 0 | 0 | 0.0 | - | - | - |


Source: Listing 16.2.9.3

Administration Site Key: FA = Forearm; UA= Upper arm
Treatments Part A: A1 = A/Sing MAP, 15 ug (FA); A2 = Afluria Quad IM (UA), 15 ug; A3 = Placebo MAP (FA); A4 = A/Sing Mono IM, 15 ug (UA)
Treatments Part B: B1 = A/Sing MAP, 15 ug (FA) with biopsy; B2 = Placebo MAP (FA) with biopsy
B3 = A/Sing MAP, 15 ug (FA); B4 = A/Sing MAP, 10 ug (FA); B5 = A/Sing MAP, 5 ug (FA); B6 = A/Sing MAP, 2.5 ug (FA)
B7 = Placebo MAP (FA); B8 = A/Sing MAP, 15 ug (UA); B9 = A/Sing IM, 15 ug, as 2018 Afluria Quad (UA)

Listing 16.2.9.10
Individual Characterisation of Numeric Pain Intensity by Treatment

Part B, Non-biopsy
B8= 3 x 5 ug A/Sing MAPs on upper arm (15 ug)

| | | | Active | | | Placebo | | |
|---|---|---|---|---|---|---|---|---|
| Randomisation Number | Visit | Time Point | #> 0 | Maximum | Average | #> 0 | Maximum | Average |
| B0091 | Day 4 | | 0 | 0 | 0.0 | - | - | - |
| B0091 | Day 8 | | 0 | 0 | 0.0 | - | - | - |
| B0091 | Day 22 | | 0 | 0 | 0.0 | - | - | - |
| B0091 | EOS/ET (Day 61) | | 0 | 0 | 0.0 | - | - | - |
| B0100 | Day 2 | | 0 | 0 | 0.0 | - | - | - |
| B0100 | Day 1 | 1 min | 1 | 1 | 0.3 | - | - | - |
| B0100 | Day 1 | 10 min | 0 | 0 | 0.0 | - | - | - |
| B0100 | Day 1 | 1 hour | 0 | 0 | 0.0 | - | - | - |
| B0100 | Day 1 | 2 hour | 0 | 0 | 0.0 | - | - | - |
| B0100 | Day 4 | | 0 | 0 | 0.0 | - | - | - |
| B0100 | Day 8 | | 0 | 0 | 0.0 | - | - | - |
| B0100 | Day 22 | | 0 | 0 | 0.0 | - | - | - |
| B0100 | EOS/ET (Day 61) | | 0 | 0 | 0.0 | - | - | - |
| B0102 | Day 2 | | 0 | 0 | 0.0 | - | - | - |
| B0102 | Day 1 | 1 min | 0 | 0 | 0.0 | - | - | - |
| B0102 | Day 1 | 10 min | 0 | 0 | 0.0 | - | - | - |
| B0102 | Day 1 | 1 hour | 0 | 0 | 0.0 | - | - | - |
| B0102 | Day 1 | 2 hour | 0 | 0 | 0.0 | - | - | - |
| B0102 | Day 4 | | 0 | 0 | 0.0 | - | - | - |



Administration Site Key: FA = Forearm; UA= Upper arm
Treatments Part A: A1 = A/Sing MAP, 15 ug (FA); A2 = Afluria Quad IM (UA), 15 ug; A3 = Placebo MAP (FA); A4 = A/Sing Mono IM, 15 ug (UA)
Treatments Part B: B1 = A/Sing MAP, 15 ug (FA) with biopsy; B2 = Placebo MAP (FA) with biopsy
B3 = A/Sing MAP, 15 ug (FA); B4 = A/Sing MAP, 10 ug (FA); B5 = A/Sing MAP, 5 ug (FA); B6 = A/Sing MAP, 2.5 ug (FA)
B7 = Placebo MAP (FA); B8 = A/Sing MAP, 15 ug (UA); B9 = A/Sing IM, 15 ug, as 2018 Afluria Quad (UA)

Listing 16.2.9.10
Individual Characterisation of Numeric Pain Intensity by Treatment

Part B, Non-biopsy
B8= 3 x 5 ug A/Sing MAPs on upper arm (15 ug)

| | | | -------Active------- | | | -------Placebo------- | | |
|---|---|---|---|---|---|---|---|---|
| Randomisation Number | Visit | Time Point | #> 0 | Maximum | Average | #> 0 | Maximum | Average |
| B0102 | Day 8 | | 0 | 0 | 0.0 | - | - | - |
| B0102 | Day 22 | | 0 | 0 | 0.0 | - | - | - |
| B0102 | EOS/ET (Day 61) | | 0 | 0 | 0.0 | - | - | - |
| B0111 | Day 2 | | 0 | 0 | 0.0 | - | - | - |
| B0111 | Day 1 | 1 min | 3 | 2 | 1.3 | - | - | - |
| B0111 | Day 1 | 10 min | 3 | 1 | 1.0 | - | - | - |
| B0111 | Day 1 | 1 hour | 0 | 0 | 0.0 | - | - | - |
| B0111 | Day 1 | 2 hour | 0 | 0 | 0.0 | - | - | - |
| B0111 | Day 4 | | 0 | 0 | 0.0 | - | - | - |
| B0111 | Day 8 | | 0 | 0 | 0.0 | - | - | - |
| B0111 | Day 22 | | 0 | 0 | 0.0 | - | - | - |
| B0111 | EOS/ET (Day 61) | | 0 | 0 | 0.0 | - | - | - |
| B0121 | Day 2 | | 0 | 0 | 0.0 | - | - | - |
| B0121 | Day 1 | 1 min | 1 | 1 | 0.3 | - | - | - |
| B0121 | Day 1 | 10 min | 2 | 1 | 0.7 | - | - | - |
| B0121 | Day 1 | 1 hour | 3 | 2 | 2.0 | - | - | - |
| B0121 | Day 1 | 2 hour | 0 | 0 | 0.0 | - | - | - |
| B0121 | Day 4 | | 0 | 0 | 0.0 | - | - | - |
| B0121 | Day 8 | | 0 | 0 | 0.0 | - | - | - |


Source: Listing 16.2.9.3

Administration Site Key: FA = Forearm; UA= Upper arm
Treatments Part A: A1 = A/Sing MAP, 15 ug (FA); A2 = Afluria Quad IM (UA), 15 ug; A3 = Placebo MAP (FA); A4 = A/Sing Mono IM, 15 ug (UA)
Treatments Part B: B1 = A/Sing MAP, 15 ug (FA) with biopsy; B2 = Placebo MAP (FA) with biopsy
B3 = A/Sing MAP, 15 ug (FA); B4 = A/Sing MAP, 10 ug (FA); B5 = A/Sing MAP, 5 ug (FA); B6 = A/Sing MAP, 2.5 ug (FA)
B7 = Placebo MAP (FA); B8 = A/Sing MAP, 15 ug (UA); B9 = A/Sing IM, 15 ug, as 2018 Afluria Quad (UA)

Listing 16.2.9.10
Individual Characterisation of Numeric Pain Intensity by Treatment

Part B, Non-biopsy
B8= 3 x 5 ug A/Sing MAPs on upper arm (15 ug)

| | | | -------Active------- | | | -------Placebo------- | | |
|---|---|---|---|---|---|---|---|---|
| Randomisation Number | Visit | Time Point | #> 0 | Maximum | Average | #> 0 | Maximum | Average |
| B0121 | Day 22 | | 0 | 0 | 0.0 | - | - | - |
| B0121 | EOS/ET (Day 61) | | 0 | 0 | 0.0 | - | - | - |
| B0129 | Day 2 | | 0 | 0 | 0.0 | - | - | - |
| B0129 | Day 1 | 1 min | 3 | 1 | 1.0 | - | - | - |
| B0129 | Day 1 | 10 min | 3 | 1 | 1.0 | - | - | - |
| B0129 | Day 1 | 1 hour | 0 | 0 | 0.0 | - | - | - |
| B0129 | Day 1 | 2 hour | 0 | 0 | 0.0 | - | - | - |
| B0129 | Day 4 | | 0 | 0 | 0.0 | - | - | - |
| B0129 | Day 8 | | 0 | 0 | 0.0 | - | - | - |
| B0129 | Day 22 | | 0 | 0 | 0.0 | - | - | - |
| B0129 | EOS/ET (Day 61) | | 0 | 0 | 0.0 | - | - | - |
| B0131 | Day 2 | | 0 | 0 | 0.0 | - | - | - |
| B0131 | Day 1 | 1 min | 3 | 1 | 1.0 | - | - | - |
| B0131 | Day 1 | 10 min | 3 | 1 | 1.0 | - | - | - |
| B0131 | Day 1 | 1 hour | 0 | 0 | 0.0 | - | - | - |
| B0131 | Day 1 | 2 hour | 1 | 1 | 0.3 | - | - | - |
| B0131 | Day 4 | | 0 | 0 | 0.0 | - | - | - |
| B0131 | Day 8 | | 0 | 0 | 0.0 | - | - | - |
| B0131 | Day 22 | | 0 | 0 | 0.0 | - | - | - |


Source: Listing 16.2.9.3

Administration Site Key: FA = Forearm; UA= Upper arm
Treatments Part A: A1 = A/Sing MAP, 15 ug (FA); A2 = Afluria Quad IM (UA), 15 ug; A3 = Placebo MAP (FA); A4 = A/Sing Mono IM, 15 ug (UA)
Treatments Part B: B1 = A/Sing MAP, 15 ug (FA) with biopsy; B2 = Placebo MAP (FA) with biopsy
B3 = A/Sing MAP, 15 ug (FA); B4 = A/Sing MAP, 10 ug (FA); B5 = A/Sing MAP, 5 ug (FA); B6 = A/Sing MAP, 2.5 ug (FA)
B7 = Placebo MAP (FA); B8 = A/Sing MAP, 15 ug (UA); B9 = A/Sing IM, 15 ug, as 2018 Afluria Quad (UA)

Listing 16.2.9.10
Individual Characterisation of Numeric Pain Intensity by Treatment

Part B, Non-biopsy
B8= 3 x 5 ug A/Sing MAPs on upper arm (15 ug)

| | | | -------Active------- | | | -------Placebo------- | | |
|---|---|---|---|---|---|---|---|---|
| Randomisation Number | Visit | Time Point | #> 0 | Maximum | Average | #> 0 | Maximum | Average |
| B0131 | EOS/ET (Day 61) | | 0 | 0 | 0.0 | - | - | - |
| B0137 | Day 2 | | 0 | 0 | 0.0 | - | - | - |
| B0137 | Day 1 | 1 min | 2 | 1 | 0.7 | - | - | - |
| B0137 | Day 1 | 10 min | 1 | 1 | 0.3 | - | - | - |
| B0137 | Day 1 | 1 hour | 0 | 0 | 0.0 | - | - | - |
| B0137 | Day 1 | 2 hour | 0 | 0 | 0.0 | - | - | - |
| B0137 | Day 4 | | 0 | 0 | 0.0 | - | - | - |
| B0137 | Day 8 | | 0 | 0 | 0.0 | - | - | - |
| B0137 | Day 22 | | 0 | 0 | 0.0 | - | - | - |
| B0137 | EOS/ET (Day 61) | | 0 | 0 | 0.0 | - | - | - |
| B0149 | Day 2 | | 0 | 0 | 0.0 | - | - | - |
| B0149 | Day 1 | 1 min | 0 | 0 | 0.0 | - | - | - |
| B0149 | Day 1 | 10 min | 0 | 0 | 0.0 | - | - | - |
| B0149 | Day 1 | 1 hour | 0 | 0 | 0.0 | - | - | - |
| B0149 | Day 1 | 2 hour | 0 | 0 | 0.0 | - | - | - |
| B0149 | Day 4 | | 0 | 0 | 0.0 | - | - | - |
| B0149 | Day 8 | | 0 | 0 | 0.0 | - | - | - |
| B0149 | Day 22 | | 0 | 0 | 0.0 | - | - | - |
| B0149 | EOS/ET (Day 61) | | 0 | 0 | 0.0 | - | - | - |


Source: Listing 16.2.9.3

Administration Site Key: FA = Forearm; UA= Upper arm
Treatments Part A: A1 = A/Sing MAP, 15 ug (FA); A2 = Afluria Quad IM (UA), 15 ug; A3 = Placebo MAP (FA); A4 = A/Sing Mono IM, 15 ug (UA)
Treatments Part B: B1 = A/Sing MAP, 15 ug (FA) with biopsy; B2 = Placebo MAP (FA) with biopsy
B3 = A/Sing MAP, 15 ug (FA); B4 = A/Sing MAP, 10 ug (FA); B5 = A/Sing MAP, 5 ug (FA); B6 = A/Sing MAP, 2.5 ug (FA)
B7 = Placebo MAP (FA); B8 = A/Sing MAP, 15 ug (UA); B9 = A/Sing IM, 15 ug, as 2018 Afluria Quad (UA)

Listing 16.2.9.10
Individual Characterisation of Numeric Pain Intensity by Treatment

Part B, Non-biopsy
B8= 3 x 5 ug A/Sing MAPs on upper arm (15 ug)

| | | | -------Active------- | | | -------Placebo------- | | |
|---|---|---|---|---|---|---|---|---|
| Randomisation Number | Visit | Time Point | #> 0 | Maximum | Average | #> 0 | Maximum | Average |
| B1019 | Day 2 | | 0 | 0 | 0.0 | - | - | - |
| B1019 | Day 1 | 1 min | 1 | 1 | 0.3 | - | - | - |
| B1019 | Day 1 | 10 min | 2 | 1 | 0.7 | - | - | - |
| B1019 | Day 1 | 1 hour | 0 | 0 | 0.0 | - | - | - |
| B1019 | Day 1 | 2 hour | 0 | 0 | 0.0 | - | - | - |
| B1019 | Day 4 | | 0 | 0 | 0.0 | - | - | - |
| B1019 | Day 8 | | 0 | 0 | 0.0 | - | - | - |
| B1019 | Day 22 | | 0 | 0 | 0.0 | - | - | - |
| B1019 | EOS/ET (Day 61) | | 0 | 0 | 0.0 | - | - | - |


Source: Listing 16.2.9.3

Administration Site Key: FA = Forearm; UA= Upper arm
Treatments Part A: A1 = A/Sing MAP, 15 ug (FA); A2 = Afluria Quad IM (UA), 15 ug; A3 = Placebo MAP (FA); A4 = A/Sing Mono IM, 15 ug (UA)
Treatments Part B: B1 = A/Sing MAP, 15 ug (FA) with biopsy; B2 = Placebo MAP (FA) with biopsy
B3 = A/Sing MAP, 15 ug (FA); B4 = A/Sing MAP, 10 ug (FA); B5 = A/Sing MAP, 5 ug (FA); B6 = A/Sing MAP, 2.5 ug (FA)
B7 = Placebo MAP (FA); B8 = A/Sing MAP, 15 ug (UA); B9 = A/Sing IM, 15 ug, as 2018 Afluria Quad (UA)
