# Supplementary material for: Safety, tolerability, and immunogenicity of influenza vaccination with a high-density microarray patch: Results from a randomized, controlled phase I clinical trial
Source: PLoS Med. 2020 Mar 17;17(3):e1003024. doi: 10.1371/journal.pmed.1003024 (PMC7077342; doi:10.1371/journal.pmed.1003024)
Supplement: S1 Data — Data 1: Informed Consent; Data 2: Analysis Sets; Data 3: Demographics; Data 4: Study Drug Administration; Data 5: Skin Hardness Assessment; Data 6: Immunogenicity (ELISA IgG); Data 7: Immunogenicity (ADCC); Data 8: Immunogenicity (MBCs); Data 9: Immunogenicity (CMI); Data 10: Immunogenicity (Mucosal IgA); Data 11: Immunogenicity Results (HAI, MN); Data 12: TEAE–CRF Data only; Data 13: TEAEs–MedDRA Coding; Data 15: Treatment Site Tolerability Assessment; Data 16: Numeric Pain Intensity; Data 17: SII; Data 18: Application Site Status at End of Study; Data 19: Individual Treatment Site Tolerability Assessment; Data 20: Numeric Pain Intensity by Treatment; Data 21: Individual SII by Treatment. ADCC, antibody-dependent cellular cytotoxicity; CMI, cell-mediated immunity; CRF, case report form; ELISA, enzyme-linked immunosorbent assay; HAI, haemagglutination inhibition; IgA, immunoglobulin A; IgG, immunoglobulin G; MBC, memory B cell; MedDRA, medical dictionary for regulatory activities; MN, microneutralisation; SII, Skin Irritation Index; TEA, treatment emergent adverse event. (ZIP) [file pmed.1003024.s008.zip › S1_Data/clinical supporting/S1_Data21 Individual Skin Irritation Index by Treatment.pdf]

Listing 16.2.9.11
Individual Characterisation of Skin Irritation Index by Treatment
Erythema
Part A
A1= 3 x 5 ug A/Sing MAPs on volar forearm (15 ug)

Randomisation Number	Visit	Time Point	Active #> 0	Active Maximum	Active Average	Placebo #> 0	Placebo Maximum	Placebo Average
A0002	Day 4		3	2	2.0	-	-	-
A0002	Day 8		3	3	2.0	-	-	-
A0002	Day 22		0	0	0.0	-	-	-
A0002	EOS/ET (Day 61)		0	0	0.0	-	-	-
A0003	Day 4		3	2	2.0	-	-	-
A0003	Day 8		3	2	2.0	-	-	-
A0003	Day 22		1	1	0.3	-	-	-
A0003	EOS/ET (Day 61)		0	0	0.0	-	-	-
A0005	Day 4		3	2	2.0	-	-	-
A0005	Day 8		2	3	2.0	-	-	-
A0005	Day 22		0	0	0.0	-	-	-
A0005	EOS/ET (Day 61)		0	0	0.0	-	-	-
A0007	Day 4		3	2	2.0	-	-	-
A0007	Day 8		3	1	1.0	-	-	-
A0007	Day 22		3	1	1.0	-	-	-
A0007	EOS/ET (Day 61)		0	0	0.0	-	-	-
A0009	Day 4		3	2	2.0	-	-	-

Source: Listing 16.2.9.4

Administration Site Key: FA = Forearm; UA= Upper arm
Treatments Part A: A1 = A/Sing MAP, 15 ug (FA); A2 = Afluria Quad IM (UA), 15 ug; A3 = Placebo MAP (FA); A4 = A/Sing Mono IM, 15 ug (UA)
Treatments Part B: B1 = A/Sing MAP, 15 ug (FA) with biopsy; B2 = Placebo MAP (FA) with biopsy
B3 = A/Sing MAP, 15 ug (FA); B4 = A/Sing MAP, 10 ug (FA); B5 = A/Sing MAP, 5 ug (FA); B6 = A/Sing MAP, 2.5 ug (FA)
B7 = Placebo MAP (FA); B8 = A/Sing MAP, 15 ug (UA); B9 = A/Sing IM, 15 ug, as 2018 Afluria Quad (UA)

Listing 16.2.9.11
Individual Characterisation of Skin Irritation Index by Treatment
Erythema
Part A
A1= 3 x 5 ug A/Sing MAPs on volar forearm (15 ug)

			Active			Placebo		
Randomisation Number	Visit	Time Point	#> 0	Maximum	Average	#> 0	Maximum	Average
A0009	Day 8		0	0	0.0	-	-	-
A0009	Day 22		0	0	0.0	-	-	-
A0009	EOS/ET (Day 61)		0	0	0.0	-	-	-
A0012	Day 4		3	2	2.0	-	-	-
A0012	Day 8		0	0	0.0	-	-	-
A0012	Day 22		0	0	0.0	-	-	-
A0012	EOS/ET (Day 61)		0	0	0.0	-	-	-
A0013	Day 4		3	2	2.0	-	-	-
A0013	Day 8		0	0	0.0	-	-	-
A0013	Day 22		0	0	0.0	-	-	-
A0013	EOS/ET (Day 61)		0	0	0.0	-	-	-
A0015	Day 4		3	3	2.7	-	-	-
A0015	Day 8		0	0	0.0	-	-	-
A0015	Day 22		0	0	0.0	-	-	-
A0015	EOS/ET (Day 61)		0	0	0.0	-	-	-
A0018	Day 1	Pre-dose	-	-	-	-	-	-
A0018	Day 1	10 min	-	-	-	-	-	-

Source: Listing 16.2.9.4

Administration Site Key: FA = Forearm; UA= Upper arm
Treatments Part A: A1 = A/Sing MAP, 15 ug (FA); A2 = Afluria Quad IM (UA), 15 ug; A3 = Placebo MAP (FA); A4 = A/Sing Mono IM, 15 ug (UA)
Treatments Part B: B1 = A/Sing MAP, 15 ug (FA) with biopsy; B2 = Placebo MAP (FA) with biopsy
B3 = A/Sing MAP, 15 ug (FA); B4 = A/Sing MAP, 10 ug (FA); B5 = A/Sing MAP, 5 ug (FA); B6 = A/Sing MAP, 2.5 ug (FA)
B7 = Placebo MAP (FA); B8 = A/Sing MAP, 15 ug (UA); B9 = A/Sing IM, 15 ug, as 2018 Afluria Quad (UA)

Listing 16.2.9.11
Individual Characterisation of Skin Irritation Index by Treatment
Erythema
Part A
A1= 3 x 5 ug A/Sing MAPs on volar forearm (15 ug)

Randomisation Number	Visit	Time Point	Active #> 0	Active Maximum	Active Average	Placebo #> 0	Placebo Maximum	Placebo Average
A0018	Day 1	1 hour	-	-	-	-	-	-
A0018	Day 1	2 hour	3	1	1.0	-	-	-
A0018	Day 4		3	2	2.0	-	-	-
A0018	Day 8		0	0	0.0	-	-	-
A0018	Day 22		0	0	0.0	-	-	-
A0018	EOS/ET (Day 61)		0	0	0.0	-	-	-
A0020	Day 1	Pre-dose	-	-	-	-	-	-
A0020	Day 1	10 min	-	-	-	-	-	-
A0020	Day 1	1 hour	-	-	-	-	-	-
A0020	Day 1	2 hour	3	2	2.0	-	-	-
A0020	Day 4		3	2	2.0	-	-	-
A0020	Day 8		0	0	0.0	-	-	-
A0020	Day 22		0	0	0.0	-	-	-
A0020	EOS/ET (Day 61)		0	0	0.0	-	-	-
A0021	Day 1	Pre-dose	-	-	-	-	-	-
A0021	Day 1	10 min	-	-	-	-	-	-
A0021	Day 1	1 hour	3	2	2.0	-	-	-
A0021	Day 1	2 hour	3	2	2.0	-	-	-
A0021	Day 4		3	3	2.7	-	-	-

Source: Listing 16.2.9.4

Administration Site Key: FA = Forearm; UA= Upper arm
Treatments Part A: A1 = A/Sing MAP, 15 ug (FA); A2 = Afluria Quad IM (UA), 15 ug; A3 = Placebo MAP (FA); A4 = A/Sing Mono IM, 15 ug (UA)
Treatments Part B: B1 = A/Sing MAP, 15 ug (FA) with biopsy; B2 = Placebo MAP (FA) with biopsy
B3 = A/Sing MAP, 15 ug (FA); B4 = A/Sing MAP, 10 ug (FA); B5 = A/Sing MAP, 5 ug (FA); B6 = A/Sing MAP, 2.5 ug (FA)
B7 = Placebo MAP (FA); B8 = A/Sing MAP, 15 ug (UA); B9 = A/Sing IM, 15 ug, as 2018 Afluria Quad (UA)

Listing 16.2.9.11
Individual Characterisation of Skin Irritation Index by Treatment
Erythema
Part A
A1= 3 x 5 ug A/Sing MAPs on volar forearm (15 ug)

Randomisation Number	Visit	Time Point	Active #> 0	Active Maximum	Active Average	Placebo #> 0	Placebo Maximum	Placebo Average
A0021	Day 8		3	2	2.0	-	-	-
A0021	Day 22		0	0	0.0	-	-	-
A0021	EOS/ET (Day 61)		0	0	0.0	-	-	-
A0024	Day 1	Pre-dose	-	-	-	-	-	-
A0024	Day 1	10 min	-	-	-	-	-	-
A0024	Day 1	1 hour	3	3	2.3	-	-	-
A0024	Day 1	2 hour	3	2	2.0	-	-	-
A0024	Day 4		3	2	2.0	-	-	-
A0024	Day 8		0	0	0.0	-	-	-
A0024	Day 22		0	0	0.0	-	-	-
A0024	EOS/ET (Day 61)		0	0	0.0	-	-	-
A0028	Day 1	Pre-dose	-	-	-	-	-	-
A0028	Day 1	10 min	-	-	-	-	-	-
A0028	Day 1	1 hour	3	2	2.0	-	-	-
A0028	Day 1	2 hour	3	2	2.0	-	-	-
A0028	Day 4		0	0	0.0	-	-	-
A0028	Day 8		0	0	0.0	-	-	-
A0028	Day 22		0	0	0.0	-	-	-
A0028	EOS/ET (Day 61)		0	0	0.0	-	-	-

Source: Listing 16.2.9.4

Administration Site Key: FA = Forearm; UA= Upper arm
Treatments Part A: A1 = A/Sing MAP, 15 ug (FA); A2 = Afluria Quad IM (UA), 15 ug; A3 = Placebo MAP (FA); A4 = A/Sing Mono IM, 15 ug (UA)
Treatments Part B: B1 = A/Sing MAP, 15 ug (FA) with biopsy; B2 = Placebo MAP (FA) with biopsy
B3 = A/Sing MAP, 15 ug (FA); B4 = A/Sing MAP, 10 ug (FA); B5 = A/Sing MAP, 5 ug (FA); B6 = A/Sing MAP, 2.5 ug (FA)
B7 = Placebo MAP (FA); B8 = A/Sing MAP, 15 ug (UA); B9 = A/Sing IM, 15 ug, as 2018 Afluria Quad (UA)

Listing 16.2.9.11
Individual Characterisation of Skin Irritation Index by Treatment
Erythema
Part A
A1= 3 x 5 ug A/Sing MAPs on volar forearm (15 ug)

			Active			Placebo		
Randomisation Number	Visit	Time Point	#> 0	Maximum	Average	#> 0	Maximum	Average
A0029	Day 1	Pre-dose	-	-	-	-	-	-
A0029	Day 1	10 min	-	-	-	-	-	-
A0029	Day 1	1 hour	3	2	2.0	-	-	-
A0029	Day 1	2 hour	3	2	2.0	-	-	-
A0029	Day 4		3	2	2.0	-	-	-
A0029	Day 8		3	2	2.0	-	-	-
A0029	Day 22		3	1	1.0	-	-	-
A0029	EOS/ET (Day 61)		0	0	0.0	-	-	-
A1026	Day 1	Pre-dose	0	0	0.0	-	-	-
A1026	Day 1	10 min	3	2	2.0	-	-	-
A1026	Day 1	1 hour	3	2	2.0	-	-	-
A1026	Day 1	2 hour	3	2	2.0	-	-	-
A1026	Day 4		3	2	2.0	-	-	-
A1026	Day 8		0	0	0.0	-	-	-
A1026	Day 22		0	0	0.0	-	-	-
A1026	EOS/ET (Day 61)		0	0	0.0	-	-	-

Source: Listing 16.2.9.4

Administration Site Key: FA = Forearm; UA= Upper arm
Treatments Part A: A1 = A/Sing MAP, 15 ug (FA); A2 = Afluria Quad IM (UA), 15 ug; A3 = Placebo MAP (FA); A4 = A/Sing Mono IM, 15 ug (UA)
Treatments Part B: B1 = A/Sing MAP, 15 ug (FA) with biopsy; B2 = Placebo MAP (FA) with biopsy
B3 = A/Sing MAP, 15 ug (FA); B4 = A/Sing MAP, 10 ug (FA); B5 = A/Sing MAP, 5 ug (FA); B6 = A/Sing MAP, 2.5 ug (FA)
B7 = Placebo MAP (FA); B8 = A/Sing MAP, 15 ug (UA); B9 = A/Sing IM, 15 ug, as 2018 Afluria Quad (UA)

Listing 16.2.9.11
Individual Characterisation of Skin Irritation Index by Treatment
Erythema
Part A
A3= 3 x uncoated MAPs on volar forearm

Randomisation Number	Visit	Time Point	Active #> 0	Active Maximum	Active Average	Placebo #> 0	Placebo Maximum	Placebo Average
A0001	Day 4		-	-	-	3	1	1.0
A0001	Day 8		-	-	-	0	0	0.0
A0001	Day 22		-	-	-	0	0	0.0
A0001	EOS/ET (Day 61)		-	-	-	0	0	0.0
A0004	Day 4		-	-	-	3	1	1.0
A0004	Day 8		-	-	-	0	0	0.0
A0004	Day 22		-	-	-	0	0	0.0
A0004	EOS/ET (Day 61)		-	-	-	0	0	0.0
A0006	Day 4		-	-	-	3	1	1.0
A0006	Day 8		-	-	-	0	0	0.0
A0006	Day 22		-	-	-	0	0	0.0
A0006	EOS/ET (Day 61)		-	-	-	0	0	0.0
A0008	Day 4		-	-	-	0	0	0.0
A0008	Day 8		-	-	-	0	0	0.0
A0008	Day 22		-	-	-	0	0	0.0
A0008	EOS/ET (Day 61)		-	-	-	0	0	0.0
A0010	Day 4		-	-	-	3	1	1.0

Source: Listing 16.2.9.4

Administration Site Key: FA = Forearm; UA= Upper arm
Treatments Part A: A1 = A/Sing MAP, 15 ug (FA); A2 = Afluria Quad IM (UA), 15 ug; A3 = Placebo MAP (FA); A4 = A/Sing Mono IM, 15 ug (UA)
Treatments Part B: B1 = A/Sing MAP, 15 ug (FA) with biopsy; B2 = Placebo MAP (FA) with biopsy
B3 = A/Sing MAP, 15 ug (FA); B4 = A/Sing MAP, 10 ug (FA); B5 = A/Sing MAP, 5 ug (FA); B6 = A/Sing MAP, 2.5 ug (FA)
B7 = Placebo MAP (FA); B8 = A/Sing MAP, 15 ug (UA); B9 = A/Sing IM, 15 ug, as 2018 Afluria Quad (UA)

Listing 16.2.9.11
Individual Characterisation of Skin Irritation Index by Treatment
Erythema
Part A
A3= 3 x uncoated MAPs on volar forearm

Randomisation Number	Visit	Time Point	Active #> 0	Active Maximum	Active Average	Placebo #> 0	Placebo Maximum	Placebo Average
A0010	Day 8		-	-	-	0	0	0.0
A0010	Day 22		-	-	-	0	0	0.0
A0010	EOS/ET (Day 61)		-	-	-	0	0	0.0
A0011	Day 4		-	-	-	0	0	0.0
A0011	Day 8		-	-	-	0	0	0.0
A0011	Day 22		-	-	-	0	0	0.0
A0011	EOS/ET (Day 61)		-	-	-	0	0	0.0
A0014	Day 4		-	-	-	0	0	0.0
A0014	Day 8		-	-	-	0	0	0.0
A0014	Day 22		-	-	-	0	0	0.0
A0014	EOS/ET (Day 61)		-	-	-	0	0	0.0
A0016	Day 1	Pre-dose	-	-	-	-	-	-
A0016	Day 1	10 min	-	-	-	-	-	-
A0016	Day 1	1 hour	-	-	-	-	-	-
A0016	Day 1	2 hour	-	-	-	3	2	2.0
A0016	Day 4		-	-	-	0	0	0.0
A0016	Day 8		-	-	-	0	0	0.0
A0016	Day 22		-	-	-	0	0	0.0

Source: Listing 16.2.9.4

Administration Site Key: FA = Forearm; UA= Upper arm
Treatments Part A: A1 = A/Sing MAP, 15 ug (FA); A2 = Afluria Quad IM (UA), 15 ug; A3 = Placebo MAP (FA); A4 = A/Sing Mono IM, 15 ug (UA)
Treatments Part B: B1 = A/Sing MAP, 15 ug (FA) with biopsy; B2 = Placebo MAP (FA) with biopsy
B3 = A/Sing MAP, 15 ug (FA); B4 = A/Sing MAP, 10 ug (FA); B5 = A/Sing MAP, 5 ug (FA); B6 = A/Sing MAP, 2.5 ug (FA)
B7 = Placebo MAP (FA); B8 = A/Sing MAP, 15 ug (UA); B9 = A/Sing IM, 15 ug, as 2018 Afluria Quad (UA)

Listing 16.2.9.11
Individual Characterisation of Skin Irritation Index by Treatment
Erythema
Part A
A3= 3 x uncoated MAPs on volar forearm

Randomisation Number	Visit	Time Point	Active #> 0	Active Maximum	Active Average	Placebo #> 0	Placebo Maximum	Placebo Average
A0016	EOS/ET (Day 61)		-	-	-	0	0	0.0
A0017	Day 1	Pre-dose	-	-	-	-	-	-
A0017	Day 1	10 min	-	-	-	-	-	-
A0017	Day 1	1 hour	-	-	-	-	-	-
A0017	Day 1	2 hour	-	-	-	3	2	2.0
A0017	Day 4		-	-	-	0	0	0.0
A0017	Day 8		-	-	-	0	0	0.0
A0017	Day 22		-	-	-	0	0	0.0
A0017	EOS/ET (Day 61)		-	-	-	0	0	0.0
A0019	Day 1	Pre-dose	-	-	-	-	-	-
A0019	Day 1	10 min	-	-	-	-	-	-
A0019	Day 1	1 hour	-	-	-	-	-	-
A0019	Day 1	2 hour	-	-	-	3	2	2.0
A0019	Day 4		-	-	-	0	0	0.0
A0019	Day 8		-	-	-	0	0	0.0
A0019	Day 22		-	-	-	0	0	0.0
A0019	EOS/ET (Day 61)		-	-	-	0	0	0.0
A0022	Day 1	Pre-dose	-	-	-	-	-	-

Administration Site Key: FA = Forearm; UA= Upper arm
Treatments Part A: A1 = A/Sing MAP, 15 ug (FA); A2 = Afluria Quad IM (UA), 15 ug; A3 = Placebo MAP (FA); A4 = A/Sing Mono IM, 15 ug (UA)
Treatments Part B: B1 = A/Sing MAP, 15 ug (FA) with biopsy; B2 = Placebo MAP (FA) with biopsy
B3 = A/Sing MAP, 15 ug (FA); B4 = A/Sing MAP, 10 ug (FA); B5 = A/Sing MAP, 5 ug (FA); B6 = A/Sing MAP, 2.5 ug (FA)
B7 = Placebo MAP (FA); B8 = A/Sing MAP, 15 ug (UA); B9 = A/Sing IM, 15 ug, as 2018 Afluria Quad (UA)

Listing 16.2.9.11
Individual Characterisation of Skin Irritation Index by Treatment
Erythema
Part A
A3= 3 x uncoated MAPs on volar forearm

Randomisation Number	Visit	Time Point	Active #> 0	Active Maximum	Active Average	Placebo #> 0	Placebo Maximum	Placebo Average
A0022	Day 1	10 min	-	-	-	-	-	-
A0022	Day 1	1 hour	-	-	-	-	-	-
A0022	Day 1	2 hour	-	-	-	3	1	1.0
A0022	Day 4		-	-	-	0	0	0.0
A0022	Day 8		-	-	-	0	0	0.0
A0022	Day 22		-	-	-	0	0	0.0
A0022	EOS/ET (Day 61)		-	-	-	0	0	0.0
A0023	Day 1	Pre-dose	-	-	-	-	-	-
A0023	Day 1	10 min	-	-	-	-	-	-
A0023	Day 1	1 hour	-	-	-	3	2	2.0
A0023	Day 1	2 hour	-	-	-	3	3	2.3
A0023	Day 4		-	-	-	0	0	0.0
A0023	Day 8		-	-	-	0	0	0.0
A0023	Day 22		-	-	-	0	0	0.0
A0023	EOS/ET (Day 61)		-	-	-	0	0	0.0
A0025	Day 1	Pre-dose	-	-	-	-	-	-
A0025	Day 1	10 min	-	-	-	-	-	-
A0025	Day 1	1 hour	-	-	-	3	3	2.3
A0025	Day 1	2 hour	-	-	-	3	2	2.0

Administration Site Key: FA = Forearm; UA= Upper arm
Treatments Part A: A1 = A/Sing MAP, 15 ug (FA); A2 = Afluria Quad IM (UA), 15 ug; A3 = Placebo MAP (FA); A4 = A/Sing Mono IM, 15 ug (UA)
Treatments Part B: B1 = A/Sing MAP, 15 ug (FA) with biopsy; B2 = Placebo MAP (FA) with biopsy
B3 = A/Sing MAP, 15 ug (FA); B4 = A/Sing MAP, 10 ug (FA); B5 = A/Sing MAP, 5 ug (FA); B6 = A/Sing MAP, 2.5 ug (FA)
B7 = Placebo MAP (FA); B8 = A/Sing MAP, 15 ug (UA); B9 = A/Sing IM, 15 ug, as 2018 Afluria Quad (UA)

Listing 16.2.9.11
Individual Characterisation of Skin Irritation Index by Treatment
Erythema
Part A
A3= 3 x uncoated MAPs on volar forearm

Randomisation Number	Visit	Time Point	Active #> 0	Active Maximum	Active Average	Placebo #> 0	Placebo Maximum	Placebo Average
A0025	Day 4		-	-	-	3	2	2.0
A0025	Day 8		-	-	-	0	0	0.0
A0025	Day 22		-	-	-	0	0	0.0
A0025	EOS/ET (Day 61)		-	-	-	0	0	0.0
A0027	Day 1	Pre-dose	-	-	-	-	-	-
A0027	Day 1	10 min	-	-	-	-	-	-
A0027	Day 1	1 hour	-	-	-	3	2	2.0
A0027	Day 1	2 hour	-	-	-	3	2	2.0
A0027	Day 4		-	-	-	0	0	0.0
A0027	Day 8		-	-	-	0	0	0.0
A0027	Day 22		-	-	-	3	1	1.0
A0027	EOS/ET (Day 61)		-	-	-	0	0	0.0
A0030	Day 1	Pre-dose	-	-	-	0	0	0.0
A0030	Day 1	10 min	-	-	-	3	2	2.0
A0030	Day 1	1 hour	-	-	-	3	1	1.0
A0030	Day 1	2 hour	-	-	-	3	2	2.0
A0030	Day 4		-	-	-	0	0	0.0
A0030	Day 8		-	-	-	0	0	0.0
A0030	Day 22		-	-	-	0	0	0.0

Source: Listing 16.2.9.4

Administration Site Key: FA = Forearm; UA= Upper arm
Treatments Part A: A1 = A/Sing MAP, 15 ug (FA); A2 = Afluria Quad IM (UA), 15 ug; A3 = Placebo MAP (FA); A4 = A/Sing Mono IM, 15 ug (UA)
Treatments Part B: B1 = A/Sing MAP, 15 ug (FA) with biopsy; B2 = Placebo MAP (FA) with biopsy
B3 = A/Sing MAP, 15 ug (FA); B4 = A/Sing MAP, 10 ug (FA); B5 = A/Sing MAP, 5 ug (FA); B6 = A/Sing MAP, 2.5 ug (FA)
B7 = Placebo MAP (FA); B8 = A/Sing MAP, 15 ug (UA); B9 = A/Sing IM, 15 ug, as 2018 Afluria Quad (UA)

Listing 16.2.9.11
Individual Characterisation of Skin Irritation Index by Treatment
Erythema
Part A
A3= 3 x uncoated MAPs on volar forearm

Randomisation Number	Visit	Time Point	Active #> 0	Active Maximum	Active Average	Placebo #> 0	Placebo Maximum	Placebo Average
A0030	EOS/ET (Day 61)		-	-	-	0	0	0.0

Source: Listing 16.2.9.4

Administration Site Key: FA = Forearm; UA= Upper arm
Treatments Part A: A1 = A/Sing MAP, 15 ug (FA); A2 = Afluria Quad IM (UA), 15 ug; A3 = Placebo MAP (FA); A4 = A/Sing Mono IM, 15 ug (UA)
Treatments Part B: B1 = A/Sing MAP, 15 ug (FA) with biopsy; B2 = Placebo MAP (FA) with biopsy
B3 = A/Sing MAP, 15 ug (FA); B4 = A/Sing MAP, 10 ug (FA); B5 = A/Sing MAP, 5 ug (FA); B6 = A/Sing MAP, 2.5 ug (FA)
B7 = Placebo MAP (FA); B8 = A/Sing MAP, 15 ug (UA); B9 = A/Sing IM, 15 ug, as 2018 Afluria Quad (UA)

Listing 16.2.9.11
Individual Characterisation of Skin Irritation Index by Treatment
Erythema
Part B, Biopsy
B1= 3 x 5 ug A/Sing MAPs on volar forearm (15 ug)

			Active			Placebo		
Randomisation Number	Visit	Time Point	#> 0	Maximum	Average	#> 0	Maximum	Average
B0001	Day 1	Pre-dose	0	0	0.0	-	-	-
B0001	Day 1	10 min	3	1	1.0	-	-	-
B0001	Day 1	1 hour	3	1	1.0	-	-	-
B0001	Day 1	2 hour	3	1	1.0	-	-	-
B0001	Day 2		3	1	1.0	-	-	-
B0001	Day 4		3	3	3.0	-	-	-
B0001	Day 8		3	1	1.0	-	-	-
B0001	Day 22		2	2	1.3	-	-	-
B0001	EOS/ET (Day 61)		0	0	0.0	-	-	-
B0004	Day 1	Pre-dose	0	0	0.0	-	-	-
B0004	Day 1	10 min	3	1	1.0	-	-	-
B0004	Day 1	1 hour	3	1	1.0	-	-	-
B0004	Day 1	2 hour	3	2	2.0	-	-	-
B0004	Day 2		3	3	3.0	-	-	-
B0004	Day 4		3	3	3.0	-	-	-
B0004	Day 8		0	0	0.0	-	-	-
B0004	Day 22		2	3	2.0	-	-	-
B0004	EOS/ET (Day 61)		1	1	0.3	-	-	-
B0005	Day 1	Pre-dose	0	0	0.0	-	-	-

Source: Listing 16.2.9.4

Administration Site Key: FA = Forearm; UA= Upper arm
Treatments Part A: A1 = A/Sing MAP, 15 ug (FA); A2 = Afluria Quad IM (UA), 15 ug; A3 = Placebo MAP (FA); A4 = A/Sing Mono IM, 15 ug (UA)
Treatments Part B: B1 = A/Sing MAP, 15 ug (FA) with biopsy; B2 = Placebo MAP (FA) with biopsy
B3 = A/Sing MAP, 15 ug (FA); B4 = A/Sing MAP, 10 ug (FA); B5 = A/Sing MAP, 5 ug (FA); B6 = A/Sing MAP, 2.5 ug (FA)
B7 = Placebo MAP (FA); B8 = A/Sing MAP, 15 ug (UA); B9 = A/Sing IM, 15 ug, as 2018 Afluria Quad (UA)

Listing 16.2.9.11
Individual Characterisation of Skin Irritation Index by Treatment
Erythema
Part B, Biopsy
B1= 3 x 5 ug A/Sing MAPs on volar forearm (15 ug)

			Active			Placebo		
Randomisation Number	Visit	Time Point	#> 0	Maximum	Average	#> 0	Maximum	Average
B0005	Day 1	10 min	3	1	1.0	-	-	-
B0005	Day 1	1 hour	3	2	2.0	-	-	-
B0005	Day 1	2 hour	3	1	1.0	-	-	-
B0005	Day 2		3	1	1.0	-	-	-
B0005	Day 4		3	3	3.0	-	-	-
B0005	Day 8		3	2	1.3	-	-	-
B0005	Day 22		2	3	2.0	-	-	-
B0005	EOS/ET (Day 61)		0	0	0.0	-	-	-
B0008	Day 1	Pre-dose	0	0	0.0	-	-	-
B0008	Day 1	10 min	3	1	1.0	-	-	-
B0008	Day 1	1 hour	3	2	2.0	-	-	-
B0008	Day 1	2 hour	3	2	2.0	-	-	-
B0008	Day 2		3	2	2.0	-	-	-
B0008	Day 4		3	3	3.0	-	-	-
B0008	Day 8		2	2	1.3	-	-	-
B0008	Day 22		3	2	1.7	-	-	-
B0008	EOS/ET (Day 61)		0	0	0.0	-	-	-
B0009	Day 1	Pre-dose	0	0	0.0	-	-	-
B0009	Day 1	10 min	3	1	1.0	-	-	-

Source: Listing 16.2.9.4

Administration Site Key: FA = Forearm; UA= Upper arm
Treatments Part A: A1 = A/Sing MAP, 15 ug (FA); A2 = Afluria Quad IM (UA), 15 ug; A3 = Placebo MAP (FA); A4 = A/Sing Mono IM, 15 ug (UA)
Treatments Part B: B1 = A/Sing MAP, 15 ug (FA) with biopsy; B2 = Placebo MAP (FA) with biopsy
B3 = A/Sing MAP, 15 ug (FA); B4 = A/Sing MAP, 10 ug (FA); B5 = A/Sing MAP, 5 ug (FA); B6 = A/Sing MAP, 2.5 ug (FA)
B7 = Placebo MAP (FA); B8 = A/Sing MAP, 15 ug (UA); B9 = A/Sing IM, 15 ug, as 2018 Afluria Quad (UA)

Listing 16.2.9.11
Individual Characterisation of Skin Irritation Index by Treatment
Erythema
Part B, Biopsy
B1= 3 x 5 ug A/Sing MAPs on volar forearm (15 ug)

Randomisation Number	Visit	Time Point	Active #> 0	Active Maximum	Active Average	Placebo #> 0	Placebo Maximum	Placebo Average
B0009	Day 1	1 hour	3	2	2.0	-	-	-
B0009	Day 1	2 hour	3	2	2.0	-	-	-
B0009	Day 2		3	2	2.0	-	-	-
B0009	Day 4		3	3	3.0	-	-	-
B0009	Day 8		0	0	0.0	-	-	-
B0009	Day 22		3	2	1.7	-	-	-
B0009	EOS/ET (Day 61)		0	0	0.0	-	-	-

Source: Listing 16.2.9.4

Administration Site Key: FA = Forearm; UA= Upper arm
Treatments Part A: A1 = A/Sing MAP, 15 ug (FA); A2 = Afluria Quad IM (UA), 15 ug; A3 = Placebo MAP (FA); A4 = A/Sing Mono IM, 15 ug (UA)
Treatments Part B: B1 = A/Sing MAP, 15 ug (FA) with biopsy; B2 = Placebo MAP (FA) with biopsy
B3 = A/Sing MAP, 15 ug (FA); B4 = A/Sing MAP, 10 ug (FA); B5 = A/Sing MAP, 5 ug (FA); B6 = A/Sing MAP, 2.5 ug (FA)
B7 = Placebo MAP (FA); B8 = A/Sing MAP, 15 ug (UA); B9 = A/Sing IM, 15 ug, as 2018 Afluria Quad (UA)

Listing 16.2.9.11
Individual Characterisation of Skin Irritation Index by Treatment
Erythema
Part B, Biopsy
B2= 3 x uncoated MAPs on volar forearm

Randomisation Number	Visit	Time Point	Active #> 0	Active Maximum	Active Average	Placebo #> 0	Placebo Maximum	Placebo Average
B0002	Day 1	Pre-dose	-	-	-	0	0	0.0
B0002	Day 1	10 min	-	-	-	3	1	1.0
B0002	Day 1	1 hour	-	-	-	3	2	2.0
B0002	Day 1	2 hour	-	-	-	3	1	1.0
B0002	Day 2		-	-	-	3	2	2.0
B0002	Day 4		-	-	-	3	2	2.0
B0002	Day 8		-	-	-	1	1	0.3
B0002	Day 22		-	-	-	2	2	1.3
B0002	EOS/ET (Day 61)		-	-	-	0	0	0.0
B0003	Day 1	Pre-dose	-	-	-	0	0	0.0
B0003	Day 1	10 min	-	-	-	3	1	1.0
B0003	Day 1	1 hour	-	-	-	3	1	1.0
B0003	Day 1	2 hour	-	-	-	3	1	1.0
B0003	Day 2		-	-	-	3	1	1.0
B0003	Day 4		-	-	-	0	0	0.0
B0003	Day 8		-	-	-	0	0	0.0
B0003	Day 22		-	-	-	2	3	1.7
B0003	EOS/ET (Day 61)		-	-	-	0	0	0.0
B0006	Day 1	Pre-dose	-	-	-	0	0	0.0

Source: Listing 16.2.9.4

Administration Site Key: FA = Forearm; UA= Upper arm
Treatments Part A: A1 = A/Sing MAP, 15 ug (FA); A2 = Afluria Quad IM (UA), 15 ug; A3 = Placebo MAP (FA); A4 = A/Sing Mono IM, 15 ug (UA)
Treatments Part B: B1 = A/Sing MAP, 15 ug (FA) with biopsy; B2 = Placebo MAP (FA) with biopsy
B3 = A/Sing MAP, 15 ug (FA); B4 = A/Sing MAP, 10 ug (FA); B5 = A/Sing MAP, 5 ug (FA); B6 = A/Sing MAP, 2.5 ug (FA)
B7 = Placebo MAP (FA); B8 = A/Sing MAP, 15 ug (UA); B9 = A/Sing IM, 15 ug, as 2018 Afluria Quad (UA)

Listing 16.2.9.11
Individual Characterisation of Skin Irritation Index by Treatment
Erythema
Part B, Biopsy
B2= 3 x uncoated MAPs on volar forearm

Randomisation Number	Visit	Time Point	Active #> 0	Active Maximum	Active Average	Placebo #> 0	Placebo Maximum	Placebo Average
B0006	Day 1	10 min	-	-	-	3	1	1.0
B0006	Day 1	1 hour	-	-	-	3	2	2.0
B0006	Day 1	2 hour	-	-	-	3	1	1.0
B0006	Day 2		-	-	-	3	2	2.0
B0006	Day 4		-	-	-	3	2	2.0
B0006	Day 8		-	-	-	2	2	1.3
B0006	Day 22		-	-	-	2	2	1.3
B0006	EOS/ET (Day 61)		-	-	-	0	0	0.0
B0007	Day 1	Pre-dose	-	-	-	0	0	0.0
B0007	Day 1	10 min	-	-	-	3	1	1.0
B0007	Day 1	1 hour	-	-	-	3	2	2.0
B0007	Day 1	2 hour	-	-	-	3	1	1.0
B0007	Day 2		-	-	-	3	2	2.0
B0007	Day 4		-	-	-	3	2	2.0
B0007	Day 8		-	-	-	0	0	0.0
B0007	Day 22		-	-	-	2	2	1.3
B0007	EOS/ET (Day 61)		-	-	-	0	0	0.0
B0010	Day 1	Pre-dose	-	-	-	0	0	0.0
B0010	Day 1	10 min	-	-	-	3	1	1.0

Source: Listing 16.2.9.4

Administration Site Key: FA = Forearm; UA= Upper arm
Treatments Part A: A1 = A/Sing MAP, 15 ug (FA); A2 = Afluria Quad IM (UA), 15 ug; A3 = Placebo MAP (FA); A4 = A/Sing Mono IM, 15 ug (UA)
Treatments Part B: B1 = A/Sing MAP, 15 ug (FA) with biopsy; B2 = Placebo MAP (FA) with biopsy
B3 = A/Sing MAP, 15 ug (FA); B4 = A/Sing MAP, 10 ug (FA); B5 = A/Sing MAP, 5 ug (FA); B6 = A/Sing MAP, 2.5 ug (FA)
B7 = Placebo MAP (FA); B8 = A/Sing MAP, 15 ug (UA); B9 = A/Sing IM, 15 ug, as 2018 Afluria Quad (UA)

Listing 16.2.9.11
Individual Characterisation of Skin Irritation Index by Treatment
Erythema
Part B, Biopsy
B2= 3 x uncoated MAPs on volar forearm

			Active			Placebo		
Randomisation Number	Visit	Time Point	#> 0	Maximum	Average	#> 0	Maximum	Average
B0010	Day 1	1 hour	-	-	-	3	2	2.0
B0010	Day 1	2 hour	-	-	-	3	1	1.0
B0010	Day 2		-	-	-	3	2	2.0
B0010	Day 4		-	-	-	3	2	2.0
B0010	Day 8		-	-	-	2	2	1.0
B0010	Day 22		-	-	-	3	2	1.7
B0010	EOS/ET (Day 61)		-	-	-	0	0	0.0

Source: Listing 16.2.9.4

Administration Site Key: FA = Forearm; UA= Upper arm
Treatments Part A: A1 = A/Sing MAP, 15 ug (FA); A2 = Afluria Quad IM (UA), 15 ug; A3 = Placebo MAP (FA); A4 = A/Sing Mono IM, 15 ug (UA)
Treatments Part B: B1 = A/Sing MAP, 15 ug (FA) with biopsy; B2 = Placebo MAP (FA) with biopsy
B3 = A/Sing MAP, 15 ug (FA); B4 = A/Sing MAP, 10 ug (FA); B5 = A/Sing MAP, 5 ug (FA); B6 = A/Sing MAP, 2.5 ug (FA)
B7 = Placebo MAP (FA); B8 = A/Sing MAP, 15 ug (UA); B9 = A/Sing IM, 15 ug, as 2018 Afluria Quad (UA)

Listing 16.2.9.11
Individual Characterisation of Skin Irritation Index by Treatment
Erythema
Part B, Non-biopsy
B3= 3 x 5 ug A/Sing MAPs on volar forearm (15 ug)

			Active			Placebo		
Randomisation Number	Visit	Time Point	#> 0	Maximum	Average	#> 0	Maximum	Average
B0014	Day 1	Pre-dose	0	0	0.0	-	-	-
B0014	Day 1	10 min	3	1	1.0	-	-	-
B0014	Day 1	1 hour	3	1	1.0	-	-	-
B0014	Day 1	2 hour	3	1	1.0	-	-	-
B0014	Day 2		3	2	2.0	-	-	-
B0014	Day 4		3	3	3.0	-	-	-
B0014	Day 8		0	0	0.0	-	-	-
B0014	Day 22		0	0	0.0	-	-	-
B0014	EOS/ET (Day 61)		0	0	0.0	-	-	-
B0023	Day 1	Pre-dose	0	0	0.0	-	-	-
B0023	Day 1	10 min	3	2	2.0	-	-	-
B0023	Day 1	1 hour	3	2	2.0	-	-	-
B0023	Day 1	2 hour	3	3	3.0	-	-	-
B0023	Day 2		3	3	3.0	-	-	-
B0023	Day 4		3	3	3.0	-	-	-
B0023	Day 8		3	2	2.0	-	-	-
B0023	Day 22		0	0	0.0	-	-	-
B0023	EOS/ET (Day 61)		0	0	0.0	-	-	-
B0027	Day 1	Pre-dose	0	0	0.0	-	-	-

Administration Site Key: FA = Forearm; UA= Upper arm
Treatments Part A: A1 = A/Sing MAP, 15 ug (FA); A2 = Afluria Quad IM (UA), 15 ug; A3 = Placebo MAP (FA); A4 = A/Sing Mono IM, 15 ug (UA)
Treatments Part B: B1 = A/Sing MAP, 15 ug (FA) with biopsy; B2 = Placebo MAP (FA) with biopsy
B3 = A/Sing MAP, 15 ug (FA); B4 = A/Sing MAP, 10 ug (FA); B5 = A/Sing MAP, 5 ug (FA); B6 = A/Sing MAP, 2.5 ug (FA)
B7 = Placebo MAP (FA); B8 = A/Sing MAP, 15 ug (UA); B9 = A/Sing IM, 15 ug, as 2018 Afluria Quad (UA)

Listing 16.2.9.11
Individual Characterisation of Skin Irritation Index by Treatment
Erythema
Part B, Non-biopsy
B3= 3 x 5 ug A/Sing MAPs on volar forearm (15 ug)

			Active			Placebo		
Randomisation Number	Visit	Time Point	#> 0	Maximum	Average	#> 0	Maximum	Average
B0027	Day 1	10 min	3	2	2.0	-	-	-
B0027	Day 1	1 hour	3	2	2.0	-	-	-
B0027	Day 1	2 hour	3	2	2.0	-	-	-
B0027	Day 2		3	2	2.0	-	-	-
B0027	Day 4		3	3	2.3	-	-	-
B0027	Day 8		3	2	2.0	-	-	-
B0027	Day 22		0	0	0.0	-	-	-
B0027	EOS/ET (Day 61)		0	0	0.0	-	-	-
B0038	Day 1	Pre-dose	0	0	0.0	-	-	-
B0038	Day 1	10 min	3	2	2.0	-	-	-
B0038	Day 1	1 hour	3	2	2.0	-	-	-
B0038	Day 1	2 hour	3	2	2.0	-	-	-
B0038	Day 2		3	3	3.0	-	-	-
B0038	Day 4		3	3	3.0	-	-	-
B0038	Day 8		3	2	2.0	-	-	-
B0038	Day 22		3	1	1.0	-	-	-
B0038	EOS/ET (Day 61)		0	0	0.0	-	-	-
B0041	Day 1	Pre-dose	0	0	0.0	-	-	-
B0041	Day 1	10 min	3	1	1.0	-	-	-

Source: Listing 16.2.9.4

Administration Site Key: FA = Forearm; UA= Upper arm
Treatments Part A: A1 = A/Sing MAP, 15 ug (FA); A2 = Afluria Quad IM (UA), 15 ug; A3 = Placebo MAP (FA); A4 = A/Sing Mono IM, 15 ug (UA)
Treatments Part B: B1 = A/Sing MAP, 15 ug (FA) with biopsy; B2 = Placebo MAP (FA) with biopsy
B3 = A/Sing MAP, 15 ug (FA); B4 = A/Sing MAP, 10 ug (FA); B5 = A/Sing MAP, 5 ug (FA); B6 = A/Sing MAP, 2.5 ug (FA)
B7 = Placebo MAP (FA); B8 = A/Sing MAP, 15 ug (UA); B9 = A/Sing IM, 15 ug, as 2018 Afluria Quad (UA)

Listing 16.2.9.11
Individual Characterisation of Skin Irritation Index by Treatment
Erythema
Part B, Non-biopsy
B3= 3 x 5 ug A/Sing MAPs on volar forearm (15 ug)

			Active			Placebo		
Randomisation Number	Visit	Time Point	#> 0	Maximum	Average	#> 0	Maximum	Average
B0041	Day 1	1 hour	3	3	3.0	-	-	-
B0041	Day 1	2 hour	3	2	2.0	-	-	-
B0041	Day 2		3	3	3.0	-	-	-
B0041	Day 4		3	2	2.0	-	-	-
B0041	Day 8		0	0	0.0	-	-	-
B0041	Day 22		0	0	0.0	-	-	-
B0041	EOS/ET (Day 61)		0	0	0.0	-	-	-
B0051	Day 1	Pre-dose	0	0	0.0	-	-	-
B0051	Day 1	10 min	3	2	2.0	-	-	-
B0051	Day 1	1 hour	3	2	2.0	-	-	-
B0051	Day 1	2 hour	3	2	2.0	-	-	-
B0051	Day 2		3	3	3.0	-	-	-
B0051	Day 4		3	3	3.0	-	-	-
B0051	Day 8		3	2	2.0	-	-	-
B0051	Day 22		0	0	0.0	-	-	-
B0051	EOS/ET (Day 61)		0	0	0.0	-	-	-
B0056	Day 1	Pre-dose	0	0	0.0	-	-	-
B0056	Day 1	10 min	3	2	2.0	-	-	-
B0056	Day 1	1 hour	3	2	2.0	-	-	-

Source: Listing 16.2.9.4

Administration Site Key: FA = Forearm; UA= Upper arm
Treatments Part A: A1 = A/Sing MAP, 15 ug (FA); A2 = Afluria Quad IM (UA), 15 ug; A3 = Placebo MAP (FA); A4 = A/Sing Mono IM, 15 ug (UA)
Treatments Part B: B1 = A/Sing MAP, 15 ug (FA) with biopsy; B2 = Placebo MAP (FA) with biopsy
B3 = A/Sing MAP, 15 ug (FA); B4 = A/Sing MAP, 10 ug (FA); B5 = A/Sing MAP, 5 ug (FA); B6 = A/Sing MAP, 2.5 ug (FA)
B7 = Placebo MAP (FA); B8 = A/Sing MAP, 15 ug (UA); B9 = A/Sing IM, 15 ug, as 2018 Afluria Quad (UA)

Listing 16.2.9.11
Individual Characterisation of Skin Irritation Index by Treatment
Erythema
Part B, Non-biopsy
B3= 3 x 5 ug A/Sing MAPs on volar forearm (15 ug)

			Active			Placebo		
Randomisation Number	Visit	Time Point	#> 0	Maximum	Average	#> 0	Maximum	Average
B0056	Day 1	2 hour	3	2	2.0	-	-	-
B0056	Day 2		3	3	2.7	-	-	-
B0056	Day 4		0	0	0.0	-	-	-
B0056	Day 8		0	0	0.0	-	-	-
B0056	Day 22		0	0	0.0	-	-	-
B0056	EOS/ET (Day 61)		0	0	0.0	-	-	-
B0060	Day 1	Pre-dose	0	0	0.0	-	-	-
B0060	Day 1	10 min	3	2	2.0	-	-	-
B0060	Day 1	1 hour	3	2	2.0	-	-	-
B0060	Day 1	2 hour	3	2	2.0	-	-	-
B0060	Day 2		3	2	2.0	-	-	-
B0060	Day 4		3	3	3.0	-	-	-
B0060	Day 8		1	1	0.3	-	-	-
B0060	Day 22		0	0	0.0	-	-	-
B0060	EOS/ET (Day 61)		0	0	0.0	-	-	-
B0072	Day 1	Pre-dose	0	0	0.0	-	-	-
B0072	Day 1	10 min	3	2	2.0	-	-	-
B0072	Day 1	1 hour	3	1	1.0	-	-	-
B0072	Day 1	2 hour	3	2	2.0	-	-	-

Source: Listing 16.2.9.4

Administration Site Key: FA = Forearm; UA= Upper arm
Treatments Part A: A1 = A/Sing MAP, 15 ug (FA); A2 = Afluria Quad IM (UA), 15 ug; A3 = Placebo MAP (FA); A4 = A/Sing Mono IM, 15 ug (UA)
Treatments Part B: B1 = A/Sing MAP, 15 ug (FA) with biopsy; B2 = Placebo MAP (FA) with biopsy
B3 = A/Sing MAP, 15 ug (FA); B4 = A/Sing MAP, 10 ug (FA); B5 = A/Sing MAP, 5 ug (FA); B6 = A/Sing MAP, 2.5 ug (FA)
B7 = Placebo MAP (FA); B8 = A/Sing MAP, 15 ug (UA); B9 = A/Sing IM, 15 ug, as 2018 Afluria Quad (UA)

Listing 16.2.9.11
Individual Characterisation of Skin Irritation Index by Treatment
Erythema
Part B, Non-biopsy
B3= 3 x 5 ug A/Sing MAPs on volar forearm (15 ug)

			Active			Placebo		
Randomisation Number	Visit	Time Point	#> 0	Maximum	Average	#> 0	Maximum	Average
B0072	Day 2		3	3	3.0	-	-	-
B0072	Day 4		3	3	3.0	-	-	-
B0072	Day 8		0	0	0.0	-	-	-
B0072	Day 22		0	0	0.0	-	-	-
B0072	EOS/ET (Day 61)		0	0	0.0	-	-	-
B0075	Day 1	Pre-dose	0	0	0.0	-	-	-
B0075	Day 1	10 min	3	2	2.0	-	-	-
B0075	Day 1	1 hour	3	2	1.7	-	-	-
B0075	Day 1	2 hour	3	2	2.0	-	-	-
B0075	Day 2		3	3	3.0	-	-	-
B0075	Day 4		3	2	2.0	-	-	-
B0075	Day 8		0	0	0.0	-	-	-
B0075	Day 22		3	2	2.0	-	-	-
B0075	EOS/ET (Day 61)		0	0	0.0	-	-	-
B0082	Day 1	Pre-dose	0	0	0.0	-	-	-
B0082	Day 1	10 min	3	2	2.0	-	-	-
B0082	Day 1	1 hour	3	2	2.0	-	-	-
B0082	Day 1	2 hour	3	2	1.7	-	-	-
B0082	Day 2		3	3	3.0	-	-	-

Administration Site Key: FA = Forearm; UA= Upper arm
Treatments Part A: A1 = A/Sing MAP, 15 ug (FA); A2 = Afluria Quad IM (UA), 15 ug; A3 = Placebo MAP (FA); A4 = A/Sing Mono IM, 15 ug (UA)
Treatments Part B: B1 = A/Sing MAP, 15 ug (FA) with biopsy; B2 = Placebo MAP (FA) with biopsy
B3 = A/Sing MAP, 15 ug (FA); B4 = A/Sing MAP, 10 ug (FA); B5 = A/Sing MAP, 5 ug (FA); B6 = A/Sing MAP, 2.5 ug (FA)
B7 = Placebo MAP (FA); B8 = A/Sing MAP, 15 ug (UA); B9 = A/Sing IM, 15 ug, as 2018 Afluria Quad (UA)

Listing 16.2.9.11
Individual Characterisation of Skin Irritation Index by Treatment
Erythema
Part B, Non-biopsy
B3= 3 x 5 ug A/Sing MAPs on volar forearm (15 ug)

Randomisation Number	Visit	Time Point	Active #> 0	Active Maximum	Active Average	Placebo #> 0	Placebo Maximum	Placebo Average
B0082	Day 4		3	3	2.7	-	-	-
B0082	Day 8		0	0	0.0	-	-	-
B0082	Day 22		0	0	0.0	-	-	-
B0082	EOS/ET (Day 61)		0	0	0.0	-	-	-
B0094	Day 1	Pre-dose	0	0	0.0	-	-	-
B0094	Day 1	10 min	3	2	2.0	-	-	-
B0094	Day 1	1 hour	3	1	1.0	-	-	-
B0094	Day 1	2 hour	3	1	1.0	-	-	-
B0094	Day 2		3	3	3.0	-	-	-
B0094	Day 4		3	3	3.0	-	-	-
B0094	Day 8		0	0	0.0	-	-	-
B0094	Day 22		0	0	0.0	-	-	-
B0094	EOS/ET (Day 61)		0	0	0.0	-	-	-
B0101	Day 1	Pre-dose	0	0	0.0	-	-	-
B0101	Day 1	10 min	3	2	2.0	-	-	-
B0101	Day 1	1 hour	3	2	2.0	-	-	-
B0101	Day 1	2 hour	3	1	1.0	-	-	-
B0101	Day 2		3	2	2.0	-	-	-
B0101	Day 4		3	3	2.7	-	-	-

Administration Site Key: FA = Forearm; UA= Upper arm
Treatments Part A: A1 = A/Sing MAP, 15 ug (FA); A2 = Afluria Quad IM (UA), 15 ug; A3 = Placebo MAP (FA); A4 = A/Sing Mono IM, 15 ug (UA)
Treatments Part B: B1 = A/Sing MAP, 15 ug (FA) with biopsy; B2 = Placebo MAP (FA) with biopsy
B3 = A/Sing MAP, 15 ug (FA); B4 = A/Sing MAP, 10 ug (FA); B5 = A/Sing MAP, 5 ug (FA); B6 = A/Sing MAP, 2.5 ug (FA)
B7 = Placebo MAP (FA); B8 = A/Sing MAP, 15 ug (UA); B9 = A/Sing IM, 15 ug, as 2018 Afluria Quad (UA)

Listing 16.2.9.11
Individual Characterisation of Skin Irritation Index by Treatment
Erythema
Part B, Non-biopsy
B3= 3 x 5 ug A/Sing MAPs on volar forearm (15 ug)

			Active			Placebo		
Randomisation Number	Visit	Time Point	#> 0	Maximum	Average	#> 0	Maximum	Average
B0101	Day 8		3	3	2.3	-	-	-
B0101	Day 22		0	0	0.0	-	-	-
B0101	EOS/ET (Day 61)		0	0	0.0	-	-	-
B0104	Day 1	Pre-dose	0	0	0.0	-	-	-
B0104	Day 1	10 min	3	2	2.0	-	-	-
B0104	Day 1	1 hour	3	2	1.7	-	-	-
B0104	Day 1	2 hour	3	2	2.0	-	-	-
B0104	Day 2		3	3	2.7	-	-	-
B0104	Day 4		3	3	3.0	-	-	-
B0104	Day 8		0	0	0.0	-	-	-
B0104	Day 22		0	0	0.0	-	-	-
B0104	EOS/ET (Day 61)		0	0	0.0	-	-	-
B0110	Day 1	Pre-dose	0	0	0.0	-	-	-
B0110	Day 1	10 min	3	2	2.0	-	-	-
B0110	Day 1	1 hour	3	1	1.0	-	-	-
B0110	Day 1	2 hour	3	2	2.0	-	-	-
B0110	Day 2		3	2	2.0	-	-	-
B0110	Day 4		3	3	3.0	-	-	-
B0110	Day 8		3	3	3.0	-	-	-

Source: Listing 16.2.9.4

Administration Site Key: FA = Forearm; UA= Upper arm
Treatments Part A: A1 = A/Sing MAP, 15 ug (FA); A2 = Afluria Quad IM (UA), 15 ug; A3 = Placebo MAP (FA); A4 = A/Sing Mono IM, 15 ug (UA)
Treatments Part B: B1 = A/Sing MAP, 15 ug (FA) with biopsy; B2 = Placebo MAP (FA) with biopsy
B3 = A/Sing MAP, 15 ug (FA); B4 = A/Sing MAP, 10 ug (FA); B5 = A/Sing MAP, 5 ug (FA); B6 = A/Sing MAP, 2.5 ug (FA)
B7 = Placebo MAP (FA); B8 = A/Sing MAP, 15 ug (UA); B9 = A/Sing IM, 15 ug, as 2018 Afluria Quad (UA)

Listing 16.2.9.11
Individual Characterisation of Skin Irritation Index by Treatment
Erythema
Part B, Non-biopsy
B3= 3 x 5 ug A/Sing MAPs on volar forearm (15 ug)

Randomisation Number	Visit	Time Point	Active #> 0	Active Maximum	Active Average	Placebo #> 0	Placebo Maximum	Placebo Average
B0110	Day 22		0	0	0.0	-	-	-
B0110	EOS/ET (Day 61)		0	0	0.0	-	-	-
B0120	Day 1	Pre-dose	0	0	0.0	-	-	-
B0120	Day 1	10 min	3	2	2.0	-	-	-
B0120	Day 1	1 hour	3	2	2.0	-	-	-
B0120	Day 1	2 hour	3	2	2.0	-	-	-
B0120	Day 2		3	3	3.0	-	-	-
B0120	Day 4		3	3	3.0	-	-	-
B0120	Day 8		3	3	3.0	-	-	-
B0120	Day 22		0	0	0.0	-	-	-
B0120	EOS/ET (Day 61)		0	0	0.0	-	-	-
B0126	Day 1	Pre-dose	0	0	0.0	-	-	-
B0126	Day 1	10 min	3	2	2.0	-	-	-
B0126	Day 1	1 hour	3	2	2.0	-	-	-
B0126	Day 1	2 hour	3	2	2.0	-	-	-
B0126	Day 2		3	2	2.0	-	-	-
B0126	Day 4		3	2	2.0	-	-	-
B0126	Day 8		0	0	0.0	-	-	-
B0126	Day 22		0	0	0.0	-	-	-

Source: Listing 16.2.9.4

Administration Site Key: FA = Forearm; UA= Upper arm
Treatments Part A: A1 = A/Sing MAP, 15 ug (FA); A2 = Afluria Quad IM (UA), 15 ug; A3 = Placebo MAP (FA); A4 = A/Sing Mono IM, 15 ug (UA)
Treatments Part B: B1 = A/Sing MAP, 15 ug (FA) with biopsy; B2 = Placebo MAP (FA) with biopsy
B3 = A/Sing MAP, 15 ug (FA); B4 = A/Sing MAP, 10 ug (FA); B5 = A/Sing MAP, 5 ug (FA); B6 = A/Sing MAP, 2.5 ug (FA)
B7 = Placebo MAP (FA); B8 = A/Sing MAP, 15 ug (UA); B9 = A/Sing IM, 15 ug, as 2018 Afluria Quad (UA)

Listing 16.2.9.11
Individual Characterisation of Skin Irritation Index by Treatment
Erythema
Part B, Non-biopsy
B3= 3 x 5 ug A/Sing MAPs on volar forearm (15 ug)

			Active			Placebo		
Randomisation Number	Visit	Time Point	#> 0	Maximum	Average	#> 0	Maximum	Average
B0126	EOS/ET (Day 61)		0	0	0.0	-	-	-
B0134	Day 1	Pre-dose	0	0	0.0	-	-	-
B0134	Day 1	10 min	3	2	2.0	-	-	-
B0134	Day 1	1 hour	3	2	2.0	-	-	-
B0134	Day 1	2 hour	3	1	1.0	-	-	-
B0134	Day 2		3	2	2.0	-	-	-
B0134	Day 4		3	2	2.0	-	-	-
B0134	Day 8		0	0	0.0	-	-	-
B0134	Day 22		0	0	0.0	-	-	-
B0134	EOS/ET (Day 61)		0	0	0.0	-	-	-
B0141	Day 1	Pre-dose	0	0	0.0	-	-	-
B0141	Day 1	10 min	3	2	2.0	-	-	-
B0141	Day 1	1 hour	3	2	2.0	-	-	-
B0141	Day 1	2 hour	3	2	2.0	-	-	-
B0141	Day 2		3	2	2.0	-	-	-
B0141	Day 4		3	2	2.0	-	-	-
B0141	Day 8		0	0	0.0	-	-	-
B0141	Day 22		0	0	0.0	-	-	-
B0141	EOS/ET (Day 61)		0	0	0.0	-	-	-

Source: Listing 16.2.9.4

Administration Site Key: FA = Forearm; UA= Upper arm
Treatments Part A: A1 = A/Sing MAP, 15 ug (FA); A2 = Afluria Quad IM (UA), 15 ug; A3 = Placebo MAP (FA); A4 = A/Sing Mono IM, 15 ug (UA)
Treatments Part B: B1 = A/Sing MAP, 15 ug (FA) with biopsy; B2 = Placebo MAP (FA) with biopsy
B3 = A/Sing MAP, 15 ug (FA); B4 = A/Sing MAP, 10 ug (FA); B5 = A/Sing MAP, 5 ug (FA); B6 = A/Sing MAP, 2.5 ug (FA)
B7 = Placebo MAP (FA); B8 = A/Sing MAP, 15 ug (UA); B9 = A/Sing IM, 15 ug, as 2018 Afluria Quad (UA)

Listing 16.2.9.11
Individual Characterisation of Skin Irritation Index by Treatment
Erythema
Part B, Non-biopsy
B3= 3 x 5 ug A/Sing MAPs on volar forearm (15 ug)

			Active			Placebo		
Randomisation Number	Visit	Time Point	#> 0	Maximum	Average	#> 0	Maximum	Average
B0145	Day 1	Pre-dose	0	0	0.0	-	-	-
B0145	Day 1	10 min	3	2	2.0	-	-	-
B0145	Day 1	1 hour	3	2	2.0	-	-	-
B0145	Day 1	2 hour	3	2	2.0	-	-	-
B0145	Day 2		3	2	2.0	-	-	-
B0145	Day 4		3	3	2.7	-	-	-
B0145	Day 8		0	0	0.0	-	-	-
B0145	Day 22		3	1	1.0	-	-	-
B0145	EOS/ET (Day 61)		0	0	0.0	-	-	-

Source: Listing 16.2.9.4

Administration Site Key: FA = Forearm; UA= Upper arm
Treatments Part A: A1 = A/Sing MAP, 15 ug (FA); A2 = Afluria Quad IM (UA), 15 ug; A3 = Placebo MAP (FA); A4 = A/Sing Mono IM, 15 ug (UA)
Treatments Part B: B1 = A/Sing MAP, 15 ug (FA) with biopsy; B2 = Placebo MAP (FA) with biopsy
B3 = A/Sing MAP, 15 ug (FA); B4 = A/Sing MAP, 10 ug (FA); B5 = A/Sing MAP, 5 ug (FA); B6 = A/Sing MAP, 2.5 ug (FA)
B7 = Placebo MAP (FA); B8 = A/Sing MAP, 15 ug (UA); B9 = A/Sing IM, 15 ug, as 2018 Afluria Quad (UA)

Listing 16.2.9.11
Individual Characterisation of Skin Irritation Index by Treatment
Erythema
Part B, Non-biopsy
B4= 2 x 5 ug A/Sing MAPs + 1 uncoated MAP on volar forearm (10 ug)

			Active			Placebo		
Randomisation Number	Visit	Time Point	#> 0	Maximum	Average	#> 0	Maximum	Average
B0016	Day 1	Pre-dose	0	0	0.0	0	0	0.0
B0016	Day 1	10 min	2	2	2.0	1	3	3.0
B0016	Day 1	1 hour	2	1	1.0	1	1	1.0
B0016	Day 1	2 hour	2	2	2.0	1	2	2.0
B0016	Day 2		2	3	2.5	1	1	1.0
B0016	Day 4		2	3	2.5	1	1	1.0
B0016	Day 8		0	0	0.0	0	0	0.0
B0016	Day 22		0	0	0.0	0	0	0.0
B0016	EOS/ET (Day 61)		0	0	0.0	0	0	0.0
B0022	Day 1	Pre-dose	0	0	0.0	0	0	0.0
B0022	Day 1	10 min	2	2	2.0	1	2	2.0
B0022	Day 1	1 hour	2	1	1.0	1	1	1.0
B0022	Day 1	2 hour	2	1	1.0	1	1	1.0
B0022	Day 2		2	3	3.0	1	2	2.0
B0022	Day 4		2	3	3.0	1	2	2.0
B0022	Day 8		0	0	0.0	0	0	0.0
B0022	Day 22		0	0	0.0	0	0	0.0
B0022	EOS/ET (Day 61)		0	0	0.0	0	0	0.0
B0031	Day 1	Pre-dose	0	0	0.0	0	0	0.0

Source: Listing 16.2.9.4

Administration Site Key: FA = Forearm; UA= Upper arm
Treatments Part A: A1 = A/Sing MAP, 15 ug (FA); A2 = Afluria Quad IM (UA), 15 ug; A3 = Placebo MAP (FA); A4 = A/Sing Mono IM, 15 ug (UA)
Treatments Part B: B1 = A/Sing MAP, 15 ug (FA) with biopsy; B2 = Placebo MAP (FA) with biopsy
B3 = A/Sing MAP, 15 ug (FA); B4 = A/Sing MAP, 10 ug (FA); B5 = A/Sing MAP, 5 ug (FA); B6 = A/Sing MAP, 2.5 ug (FA)
B7 = Placebo MAP (FA); B8 = A/Sing MAP, 15 ug (UA); B9 = A/Sing IM, 15 ug, as 2018 Afluria Quad (UA)

Listing 16.2.9.11
Individual Characterisation of Skin Irritation Index by Treatment
Erythema
Part B, Non-biopsy
B4= 2 x 5 ug A/Sing MAPs + 1 uncoated MAP on volar forearm (10 ug)

			Active			Placebo		
Randomisation Number	Visit	Time Point	#> 0	Maximum	Average	#> 0	Maximum	Average
B0031	Day 1	10 min	2	2	2.0	1	2	2.0
B0031	Day 1	1 hour	2	1	1.0	1	3	3.0
B0031	Day 1	2 hour	2	2	2.0	1	2	2.0
B0031	Day 2		2	3	3.0	1	2	2.0
B0031	Day 4		2	3	3.0	1	1	1.0
B0031	Day 8		0	0	0.0	0	0	0.0
B0031	Day 22		0	0	0.0	0	0	0.0
B0031	EOS/ET (Day 61)		0	0	0.0	0	0	0.0
B0037	Day 1	Pre-dose	0	0	0.0	0	0	0.0
B0037	Day 1	10 min	2	2	2.0	1	2	2.0
B0037	Day 1	1 hour	2	1	1.0	1	1	1.0
B0037	Day 1	2 hour	2	2	1.5	1	2	2.0
B0037	Day 2		2	3	3.0	1	1	1.0
B0037	Day 4		2	3	3.0	0	0	0.0
B0037	Day 8		2	2	2.0	1	1	1.0
B0037	Day 22		0	0	0.0	0	0	0.0
B0037	EOS/ET (Day 61)		0	0	0.0	0	0	0.0
B0045	Day 1	Pre-dose	0	0	0.0	0	0	0.0
B0045	Day 1	10 min	2	2	2.0	1	2	2.0

Administration Site Key: FA = Forearm; UA= Upper arm
Treatments Part A: A1 = A/Sing MAP, 15 ug (FA); A2 = Afluria Quad IM (UA), 15 ug; A3 = Placebo MAP (FA); A4 = A/Sing Mono IM, 15 ug (UA)
Treatments Part B: B1 = A/Sing MAP, 15 ug (FA) with biopsy; B2 = Placebo MAP (FA) with biopsy
B3 = A/Sing MAP, 15 ug (FA); B4 = A/Sing MAP, 10 ug (FA); B5 = A/Sing MAP, 5 ug (FA); B6 = A/Sing MAP, 2.5 ug (FA)
B7 = Placebo MAP (FA); B8 = A/Sing MAP, 15 ug (UA); B9 = A/Sing IM, 15 ug, as 2018 Afluria Quad (UA)

Listing 16.2.9.11
Individual Characterisation of Skin Irritation Index by Treatment
Erythema
Part B, Non-biopsy
B4= 2 x 5 ug A/Sing MAPs + 1 uncoated MAP on volar forearm (10 ug)

			Active			Placebo		
Randomisation Number	Visit	Time Point	#> 0	Maximum	Average	#> 0	Maximum	Average
B0045	Day 1	1 hour	2	3	3.0	1	3	3.0
B0045	Day 1	2 hour	2	2	2.0	1	2	2.0
B0045	Day 2		2	3	2.5	1	2	2.0
B0045	Day 4		2	3	3.0	0	0	0.0
B0045	Day 8		0	0	0.0	0	0	0.0
B0045	Day 22		2	1	1.0	0	0	0.0
B0045	EOS/ET (Day 61)		0	0	0.0	0	0	0.0
B0049	Day 1	Pre-dose	0	0	0.0	0	0	0.0
B0049	Day 1	10 min	2	1	1.0	1	1	1.0
B0049	Day 1	1 hour	2	2	2.0	1	2	2.0
B0049	Day 1	2 hour	2	2	2.0	1	2	2.0
B0049	Day 2		2	3	3.0	1	1	1.0
B0049	Day 4		0	0	0.0	0	0	0.0
B0049	Day 8		0	0	0.0	0	0	0.0
B0049	Day 22		0	0	0.0	0	0	0.0
B0049	EOS/ET (Day 61)		0	0	0.0	0	0	0.0
B0057	Day 1	Pre-dose	0	0	0.0	0	0	0.0
B0057	Day 1	10 min	2	2	2.0	1	2	2.0
B0057	Day 1	1 hour	2	2	2.0	1	2	2.0

Administration Site Key: FA = Forearm; UA= Upper arm
Treatments Part A: A1 = A/Sing MAP, 15 ug (FA); A2 = Afluria Quad IM (UA), 15 ug; A3 = Placebo MAP (FA); A4 = A/Sing Mono IM, 15 ug (UA)
Treatments Part B: B1 = A/Sing MAP, 15 ug (FA) with biopsy; B2 = Placebo MAP (FA) with biopsy
B3 = A/Sing MAP, 15 ug (FA); B4 = A/Sing MAP, 10 ug (FA); B5 = A/Sing MAP, 5 ug (FA); B6 = A/Sing MAP, 2.5 ug (FA)
B7 = Placebo MAP (FA); B8 = A/Sing MAP, 15 ug (UA); B9 = A/Sing IM, 15 ug, as 2018 Afluria Quad (UA)

Listing 16.2.9.11
Individual Characterisation of Skin Irritation Index by Treatment
Erythema
Part B, Non-biopsy
B4= 2 x 5 ug A/Sing MAPs + 1 uncoated MAP on volar forearm (10 ug)

			Active			Placebo		
Randomisation Number	Visit	Time Point	#> 0	Maximum	Average	#> 0	Maximum	Average
B0057	Day 1	2 hour	2	2	2.0	1	2	2.0
B0057	Day 2		2	2	2.0	1	1	1.0
B0057	Day 4		2	2	2.0	1	1	1.0
B0057	Day 8		2	2	2.0	1	1	1.0
B0057	Day 22		0	0	0.0	0	0	0.0
B0057	EOS/ET (Day 61)		0	0	0.0	0	0	0.0
B0065	Day 1	Pre-dose	0	0	0.0	0	0	0.0
B0065	Day 1	10 min	2	2	2.0	1	2	2.0
B0065	Day 1	1 hour	2	2	2.0	1	2	2.0
B0065	Day 1	2 hour	2	2	2.0	1	2	2.0
B0065	Day 2		2	3	3.0	1	2	2.0
B0065	Day 4		2	3	2.5	0	0	0.0
B0065	Day 8		1	2	1.0	0	0	0.0
B0065	Day 22		0	0	0.0	0	0	0.0
B0065	EOS/ET (Day 61)		0	0	0.0	0	0	0.0
B0071	Day 1	Pre-dose	0	0	0.0	0	0	0.0
B0071	Day 1	10 min	2	2	2.0	1	2	2.0
B0071	Day 1	1 hour	2	1	1.0	1	1	1.0
B0071	Day 1	2 hour	2	1	1.0	1	1	1.0

Source: Listing 16.2.9.4

Administration Site Key: FA = Forearm; UA= Upper arm
Treatments Part A: A1 = A/Sing MAP, 15 ug (FA); A2 = Afluria Quad IM (UA), 15 ug; A3 = Placebo MAP (FA); A4 = A/Sing Mono IM, 15 ug (UA)
Treatments Part B: B1 = A/Sing MAP, 15 ug (FA) with biopsy; B2 = Placebo MAP (FA) with biopsy
B3 = A/Sing MAP, 15 ug (FA); B4 = A/Sing MAP, 10 ug (FA); B5 = A/Sing MAP, 5 ug (FA); B6 = A/Sing MAP, 2.5 ug (FA)
B7 = Placebo MAP (FA); B8 = A/Sing MAP, 15 ug (UA); B9 = A/Sing IM, 15 ug, as 2018 Afluria Quad (UA)

Listing 16.2.9.11
Individual Characterisation of Skin Irritation Index by Treatment
Erythema
Part B, Non-biopsy
B4= 2 x 5 ug A/Sing MAPs + 1 uncoated MAP on volar forearm (10 ug)

			Active			Placebo		
Randomisation Number	Visit	Time Point	#> 0	Maximum	Average	#> 0	Maximum	Average
B0071	Day 2		2	3	2.5	1	2	2.0
B0071	Day 4		2	2	2.0	0	0	0.0
B0071	Day 8		1	2	1.0	0	0	0.0
B0071	Day 22		0	0	0.0	0	0	0.0
B0071	EOS/ET (Day 61)		0	0	0.0	0	0	0.0
B0077	Day 1	Pre-dose	0	0	0.0	0	0	0.0
B0077	Day 1	10 min	2	2	2.0	1	2	2.0
B0077	Day 1	1 hour	2	1	1.0	1	1	1.0
B0077	Day 1	2 hour	2	2	2.0	1	2	2.0
B0077	Day 2		2	2	2.0	1	1	1.0
B0077	Day 4		2	3	3.0	1	2	2.0
B0077	Day 8		2	1	1.0	0	0	0.0
B0077	Day 22		0	0	0.0	0	0	0.0
B0077	EOS/ET (Day 61)		0	0	0.0	0	0	0.0
B0085	Day 1	Pre-dose	0	0	0.0	0	0	0.0
B0085	Day 1	10 min	2	2	2.0	1	2	2.0
B0085	Day 1	1 hour	2	2	2.0	1	2	2.0
B0085	Day 1	2 hour	2	2	2.0	1	2	2.0
B0085	Day 2		2	4	4.0	1	2	2.0

Administration Site Key: FA = Forearm; UA= Upper arm
Treatments Part A: A1 = A/Sing MAP, 15 ug (FA); A2 = Afluria Quad IM (UA), 15 ug; A3 = Placebo MAP (FA); A4 = A/Sing Mono IM, 15 ug (UA)
Treatments Part B: B1 = A/Sing MAP, 15 ug (FA) with biopsy; B2 = Placebo MAP (FA) with biopsy
B3 = A/Sing MAP, 15 ug (FA); B4 = A/Sing MAP, 10 ug (FA); B5 = A/Sing MAP, 5 ug (FA); B6 = A/Sing MAP, 2.5 ug (FA)
B7 = Placebo MAP (FA); B8 = A/Sing MAP, 15 ug (UA); B9 = A/Sing IM, 15 ug, as 2018 Afluria Quad (UA)

Listing 16.2.9.11
Individual Characterisation of Skin Irritation Index by Treatment
Erythema
Part B, Non-biopsy
B4= 2 x 5 ug A/Sing MAPs + 1 uncoated MAP on volar forearm (10 ug)

			Active			Placebo		
Randomisation Number	Visit	Time Point	#> 0	Maximum	Average	#> 0	Maximum	Average
B0085	Day 4		2	3	3.0	0	0	0.0
B0085	Day 8		0	0	0.0	0	0	0.0
B0085	Day 22		0	0	0.0	0	0	0.0
B0085	EOS/ET (Day 61)		0	0	0.0	0	0	0.0
B0092	Day 1	Pre-dose	0	0	0.0	0	0	0.0
B0092	Day 1	10 min	2	1	1.0	1	1	1.0
B0092	Day 1	1 hour	2	1	1.0	1	1	1.0
B0092	Day 1	2 hour	2	2	2.0	1	2	2.0
B0092	Day 2		2	2	2.0	1	1	1.0
B0092	Day 4		2	3	3.0	0	0	0.0
B0092	Day 8		1	2	1.0	0	0	0.0
B0092	Day 22		0	0	0.0	0	0	0.0
B0092	EOS/ET (Day 61)		0	0	0.0	0	0	0.0
B0099	Day 1	Pre-dose	0	0	0.0	0	0	0.0
B0099	Day 1	10 min	2	2	2.0	1	2	2.0
B0099	Day 1	1 hour	2	2	2.0	1	2	2.0
B0099	Day 1	2 hour	2	2	2.0	1	2	2.0
B0099	Day 2		2	3	2.5	1	2	2.0
B0099	Day 4		2	2	2.0	0	0	0.0

Administration Site Key: FA = Forearm; UA= Upper arm
Treatments Part A: A1 = A/Sing MAP, 15 ug (FA); A2 = Afluria Quad IM (UA), 15 ug; A3 = Placebo MAP (FA); A4 = A/Sing Mono IM, 15 ug (UA)
Treatments Part B: B1 = A/Sing MAP, 15 ug (FA) with biopsy; B2 = Placebo MAP (FA) with biopsy
B3 = A/Sing MAP, 15 ug (FA); B4 = A/Sing MAP, 10 ug (FA); B5 = A/Sing MAP, 5 ug (FA); B6 = A/Sing MAP, 2.5 ug (FA)
B7 = Placebo MAP (FA); B8 = A/Sing MAP, 15 ug (UA); B9 = A/Sing IM, 15 ug, as 2018 Afluria Quad (UA)

Listing 16.2.9.11
Individual Characterisation of Skin Irritation Index by Treatment
Erythema
Part B, Non-biopsy
B4= 2 x 5 ug A/Sing MAPs + 1 uncoated MAP on volar forearm (10 ug)

			Active			Placebo		
Randomisation Number	Visit	Time Point	#> 0	Maximum	Average	#> 0	Maximum	Average
B0099	Day 8		0	0	0.0	0	0	0.0
B0099	EOS/ET (Day 61)		0	0	0.0	0	0	0.0
B0106	Day 1	Pre-dose	0	0	0.0	0	0	0.0
B0106	Day 1	10 min	2	2	2.0	1	2	2.0
B0106	Day 1	1 hour	2	2	2.0	1	2	2.0
B0106	Day 1	2 hour	2	1	1.0	1	1	1.0
B0106	Day 2		2	2	2.0	1	1	1.0
B0106	Day 4		2	3	3.0	0	0	0.0
B0106	Day 8		0	0	0.0	0	0	0.0
B0106	Day 22		0	0	0.0	0	0	0.0
B0106	EOS/ET (Day 61)		0	0	0.0	0	0	0.0
B0112	Day 1	Pre-dose	0	0	0.0	0	0	0.0
B0112	Day 1	10 min	2	2	2.0	1	2	2.0
B0112	Day 1	1 hour	2	2	2.0	1	2	2.0
B0112	Day 1	2 hour	2	2	2.0	1	2	2.0
B0112	Day 2		2	2	2.0	1	2	2.0
B0112	Day 4		2	3	3.0	0	0	0.0
B0112	Day 8		2	2	2.0	1	1	1.0
B0112	Day 22		0	0	0.0	0	0	0.0

Administration Site Key: FA = Forearm; UA= Upper arm
Treatments Part A: A1 = A/Sing MAP, 15 ug (FA); A2 = Afluria Quad IM (UA), 15 ug; A3 = Placebo MAP (FA); A4 = A/Sing Mono IM, 15 ug (UA)
Treatments Part B: B1 = A/Sing MAP, 15 ug (FA) with biopsy; B2 = Placebo MAP (FA) with biopsy
B3 = A/Sing MAP, 15 ug (FA); B4 = A/Sing MAP, 10 ug (FA); B5 = A/Sing MAP, 5 ug (FA); B6 = A/Sing MAP, 2.5 ug (FA)
B7 = Placebo MAP (FA); B8 = A/Sing MAP, 15 ug (UA); B9 = A/Sing IM, 15 ug, as 2018 Afluria Quad (UA)

Listing 16.2.9.11
Individual Characterisation of Skin Irritation Index by Treatment
Erythema
Part B, Non-biopsy
B4= 2 x 5 ug A/Sing MAPs + 1 uncoated MAP on volar forearm (10 ug)

			Active			Placebo		
Randomisation Number	Visit	Time Point	#> 0	Maximum	Average	#> 0	Maximum	Average
B0112	EOS/ET (Day 61)		0	0	0.0	0	0	0.0
B0119	Day 1	Pre-dose	0	0	0.0	0	0	0.0
B0119	Day 1	10 min	2	2	2.0	1	2	2.0
B0119	Day 1	1 hour	2	2	2.0	1	2	2.0
B0119	Day 1	2 hour	2	2	2.0	1	2	2.0
B0119	Day 2		2	3	3.0	1	2	2.0
B0119	Day 4		2	3	2.5	0	0	0.0
B0119	Day 8		0	0	0.0	0	0	0.0
B0119	Day 22		0	0	0.0	0	0	0.0
B0119	EOS/ET (Day 61)		0	0	0.0	0	0	0.0
B0124	Day 1	Pre-dose	0	0	0.0	0	0	0.0
B0124	Day 1	10 min	2	2	2.0	1	2	2.0
B0124	Day 1	1 hour	2	2	2.0	1	2	2.0
B0124	Day 1	2 hour	2	2	2.0	1	2	2.0
B0124	Day 2		2	2	2.0	1	1	1.0
B0124	Day 4		2	3	3.0	0	0	0.0
B0124	Day 8		1	2	1.0	0	0	0.0
B0124	Day 22		0	0	0.0	0	0	0.0
B0124	EOS/ET (Day 61)		0	0	0.0	0	0	0.0

Source: Listing 16.2.9.4

Administration Site Key: FA = Forearm; UA= Upper arm
Treatments Part A: A1 = A/Sing MAP, 15 ug (FA); A2 = Afluria Quad IM (UA), 15 ug; A3 = Placebo MAP (FA); A4 = A/Sing Mono IM, 15 ug (UA)
Treatments Part B: B1 = A/Sing MAP, 15 ug (FA) with biopsy; B2 = Placebo MAP (FA) with biopsy
B3 = A/Sing MAP, 15 ug (FA); B4 = A/Sing MAP, 10 ug (FA); B5 = A/Sing MAP, 5 ug (FA); B6 = A/Sing MAP, 2.5 ug (FA)
B7 = Placebo MAP (FA); B8 = A/Sing MAP, 15 ug (UA); B9 = A/Sing IM, 15 ug, as 2018 Afluria Quad (UA)

Listing 16.2.9.11
Individual Characterisation of Skin Irritation Index by Treatment
Erythema
Part B, Non-biopsy
B4= 2 x 5 ug A/Sing MAPs + 1 uncoated MAP on volar forearm (10 ug)

			Active			Placebo		
Randomisation Number	Visit	Time Point	#> 0	Maximum	Average	#> 0	Maximum	Average
B0133	Day 1	Pre-dose	0	0	0.0	0	0	0.0
B0133	Day 1	10 min	2	2	2.0	1	2	2.0
B0133	Day 1	1 hour	2	2	2.0	1	2	2.0
B0133	Day 1	2 hour	2	2	2.0	1	2	2.0
B0133	Day 2		2	2	2.0	1	2	2.0
B0133	Day 4		2	2	2.0	1	1	1.0
B0133	Day 8		2	2	2.0	0	0	0.0
B0133	Day 22		2	1	1.0	0	0	0.0
B0133	EOS/ET (Day 61)		0	0	0.0	0	0	0.0
B0139	Day 1	Pre-dose	0	0	0.0	0	0	0.0
B0139	Day 1	10 min	2	2	2.0	1	2	2.0
B0139	Day 1	1 hour	2	2	2.0	1	2	2.0
B0139	Day 1	2 hour	2	2	2.0	1	2	2.0
B0139	Day 2		2	3	2.5	1	1	1.0
B0139	Day 4		2	2	2.0	1	1	1.0
B0139	Day 8		0	0	0.0	0	0	0.0
B0139	Day 22		0	0	0.0	0	0	0.0
B0139	EOS/ET (Day 61)		0	0	0.0	0	0	0.0

Administration Site Key: FA = Forearm; UA= Upper arm
Treatments Part A: A1 = A/Sing MAP, 15 ug (FA); A2 = Afluria Quad IM (UA), 15 ug; A3 = Placebo MAP (FA); A4 = A/Sing Mono IM, 15 ug (UA)
Treatments Part B: B1 = A/Sing MAP, 15 ug (FA) with biopsy; B2 = Placebo MAP (FA) with biopsy
B3 = A/Sing MAP, 15 ug (FA); B4 = A/Sing MAP, 10 ug (FA); B5 = A/Sing MAP, 5 ug (FA); B6 = A/Sing MAP, 2.5 ug (FA)
B7 = Placebo MAP (FA); B8 = A/Sing MAP, 15 ug (UA); B9 = A/Sing IM, 15 ug, as 2018 Afluria Quad (UA)

Listing 16.2.9.11
Individual Characterisation of Skin Irritation Index by Treatment
Erythema
Part B, Non-biopsy
B4= 2 x 5 ug A/Sing MAPs + 1 uncoated MAP on volar forearm (10 ug)

			Active			Placebo		
Randomisation Number	Visit	Time Point	#> 0	Maximum	Average	#> 0	Maximum	Average
B0150	Day 1	Pre-dose	0	0	0.0	0	0	0.0
B0150	Day 1	10 min	2	2	2.0	1	2	2.0
B0150	Day 1	1 hour	2	2	2.0	1	2	2.0
B0150	Day 1	2 hour	2	2	2.0	1	2	2.0
B0150	Day 2		2	3	2.5	1	2	2.0
B0150	Day 4		2	2	2.0	0	0	0.0
B0150	Day 8		2	2	2.0	0	0	0.0
B0150	Day 22		2	1	1.0	0	0	0.0
B0150	EOS/ET (Day 61)		0	0	0.0	0	0	0.0

Source: Listing 16.2.9.4

Administration Site Key: FA = Forearm; UA= Upper arm
Treatments Part A: A1 = A/Sing MAP, 15 ug (FA); A2 = Afluria Quad IM (UA), 15 ug; A3 = Placebo MAP (FA); A4 = A/Sing Mono IM, 15 ug (UA)
Treatments Part B: B1 = A/Sing MAP, 15 ug (FA) with biopsy; B2 = Placebo MAP (FA) with biopsy
B3 = A/Sing MAP, 15 ug (FA); B4 = A/Sing MAP, 10 ug (FA); B5 = A/Sing MAP, 5 ug (FA); B6 = A/Sing MAP, 2.5 ug (FA)
B7 = Placebo MAP (FA); B8 = A/Sing MAP, 15 ug (UA); B9 = A/Sing IM, 15 ug, as 2018 Afluria Quad (UA)

Listing 16.2.9.11
Individual Characterisation of Skin Irritation Index by Treatment
Erythema
Part B, Non-biopsy
B5= 1 x 5 ug A/Sing MAP + 2 uncoated MAPs on volar forearm (5 ug)

			Active			Placebo		
Randomisation Number	Visit	Time Point	#> 0	Maximum	Average	#> 0	Maximum	Average
B0013	Day 1	Pre-dose	0	0	0.0	0	0	0.0
B0013	Day 1	10 min	1	2	2.0	2	2	1.5
B0013	Day 1	1 hour	1	1	1.0	2	1	1.0
B0013	Day 1	2 hour	1	1	1.0	2	1	1.0
B0013	Day 2		1	2	2.0	2	1	1.0
B0013	Day 4		1	2	2.0	2	1	1.0
B0013	Day 8		1	2	2.0	0	0	0.0
B0013	Day 22		1	1	1.0	0	0	0.0
B0013	EOS/ET (Day 61)		0	0	0.0	0	0	0.0
B0024	Day 1	Pre-dose	0	0	0.0	0	0	0.0
B0024	Day 1	10 min	1	2	2.0	2	2	2.0
B0024	Day 1	1 hour	1	2	2.0	2	3	2.5
B0024	Day 1	2 hour	1	2	2.0	2	3	2.5
B0024	Day 2		1	3	3.0	2	1	1.0
B0024	Day 4		1	3	3.0	0	0	0.0
B0024	Day 8		0	0	0.0	0	0	0.0
B0024	Day 22		0	0	0.0	0	0	0.0
B0024	EOS/ET (Day 61)		0	0	0.0	0	0	0.0
B0028	Day 1	Pre-dose	0	0	0.0	0	0	0.0

Source: Listing 16.2.9.4

Administration Site Key: FA = Forearm; UA= Upper arm
Treatments Part A: A1 = A/Sing MAP, 15 ug (FA); A2 = Afluria Quad IM (UA), 15 ug; A3 = Placebo MAP (FA); A4 = A/Sing Mono IM, 15 ug (UA)
Treatments Part B: B1 = A/Sing MAP, 15 ug (FA) with biopsy; B2 = Placebo MAP (FA) with biopsy
B3 = A/Sing MAP, 15 ug (FA); B4 = A/Sing MAP, 10 ug (FA); B5 = A/Sing MAP, 5 ug (FA); B6 = A/Sing MAP, 2.5 ug (FA)
B7 = Placebo MAP (FA); B8 = A/Sing MAP, 15 ug (UA); B9 = A/Sing IM, 15 ug, as 2018 Afluria Quad (UA)

Listing 16.2.9.11
Individual Characterisation of Skin Irritation Index by Treatment
Erythema
Part B, Non-biopsy
B5= 1 x 5 ug A/Sing MAP + 2 uncoated MAPs on volar forearm (5 ug)

			Active			Placebo		
Randomisation Number	Visit	Time Point	#> 0	Maximum	Average	#> 0	Maximum	Average
B0028	Day 1	10 min	1	2	2.0	2	2	2.0
B0028	Day 1	1 hour	1	1	1.0	2	1	1.0
B0028	Day 1	2 hour	1	1	1.0	2	1	1.0
B0028	Day 2		1	3	3.0	2	1	1.0
B0028	Day 4		1	3	3.0	0	0	0.0
B0028	Day 8		1	2	2.0	0	0	0.0
B0028	Day 22		0	0	0.0	0	0	0.0
B0028	EOS/ET (Day 61)		0	0	0.0	0	0	0.0
B0033	Day 1	Pre-dose	0	0	0.0	0	0	0.0
B0033	Day 1	10 min	1	2	2.0	2	2	2.0
B0033	Day 1	1 hour	1	3	3.0	2	3	3.0
B0033	Day 1	2 hour	1	2	2.0	2	2	2.0
B0033	Day 2		1	3	3.0	2	2	2.0
B0033	Day 4		1	3	3.0	2	1	1.0
B0033	Day 8		1	2	2.0	2	1	1.0
B0033	Day 22		0	0	0.0	0	0	0.0
B0033	EOS/ET (Day 61)		0	0	0.0	0	0	0.0
B0042	Day 1	Pre-dose	0	0	0.0	0	0	0.0
B0042	Day 1	10 min	1	2	2.0	2	2	2.0

Source: Listing 16.2.9.4

Administration Site Key: FA = Forearm; UA= Upper arm
Treatments Part A: A1 = A/Sing MAP, 15 ug (FA); A2 = Afluria Quad IM (UA), 15 ug; A3 = Placebo MAP (FA); A4 = A/Sing Mono IM, 15 ug (UA)
Treatments Part B: B1 = A/Sing MAP, 15 ug (FA) with biopsy; B2 = Placebo MAP (FA) with biopsy
B3 = A/Sing MAP, 15 ug (FA); B4 = A/Sing MAP, 10 ug (FA); B5 = A/Sing MAP, 5 ug (FA); B6 = A/Sing MAP, 2.5 ug (FA)
B7 = Placebo MAP (FA); B8 = A/Sing MAP, 15 ug (UA); B9 = A/Sing IM, 15 ug, as 2018 Afluria Quad (UA)

Listing 16.2.9.11
Individual Characterisation of Skin Irritation Index by Treatment
Erythema
Part B, Non-biopsy
B5= 1 x 5 ug A/Sing MAP + 2 uncoated MAPs on volar forearm (5 ug)

			Active			Placebo		
Randomisation Number	Visit	Time Point	#> 0	Maximum	Average	#> 0	Maximum	Average
B0042	Day 1	1 hour	1	1	1.0	2	1	1.0
B0042	Day 1	2 hour	1	2	2.0	2	2	2.0
B0042	Day 2		1	3	3.0	2	1	1.0
B0042	Day 4		1	2	2.0	0	0	0.0
B0042	Day 8		1	2	2.0	0	0	0.0
B0042	Day 22		1	2	2.0	0	0	0.0
B0042	EOS/ET (Day 61)		0	0	0.0	0	0	0.0
B0052	Day 1	Pre-dose	0	0	0.0	0	0	0.0
B0052	Day 1	10 min	1	2	2.0	2	2	2.0
B0052	Day 1	1 hour	1	2	2.0	2	2	2.0
B0052	Day 1	2 hour	1	2	2.0	2	2	2.0
B0052	Day 2		1	3	3.0	2	2	2.0
B0052	Day 4		1	3	3.0	2	2	2.0
B0052	Day 8		0	0	0.0	0	0	0.0
B0052	Day 22		1	2	2.0	0	0	0.0
B0052	EOS/ET (Day 61)		0	0	0.0	0	0	0.0
B0054	Day 1	Pre-dose	0	0	0.0	0	0	0.0
B0054	Day 1	10 min	1	2	2.0	2	2	2.0
B0054	Day 1	1 hour	1	2	2.0	2	2	2.0

Administration Site Key: FA = Forearm; UA= Upper arm
Treatments Part A: A1 = A/Sing MAP, 15 ug (FA); A2 = Afluria Quad IM (UA), 15 ug; A3 = Placebo MAP (FA); A4 = A/Sing Mono IM, 15 ug (UA)
Treatments Part B: B1 = A/Sing MAP, 15 ug (FA) with biopsy; B2 = Placebo MAP (FA) with biopsy
B3 = A/Sing MAP, 15 ug (FA); B4 = A/Sing MAP, 10 ug (FA); B5 = A/Sing MAP, 5 ug (FA); B6 = A/Sing MAP, 2.5 ug (FA)
B7 = Placebo MAP (FA); B8 = A/Sing MAP, 15 ug (UA); B9 = A/Sing IM, 15 ug, as 2018 Afluria Quad (UA)

Listing 16.2.9.11
Individual Characterisation of Skin Irritation Index by Treatment
Erythema
Part B, Non-biopsy
B5= 1 x 5 ug A/Sing MAP + 2 uncoated MAPs on volar forearm (5 ug)

				Active			Placebo	
Randomisation Number	Visit	Time Point	#> 0	Maximum	Average	#> 0	Maximum	Average
B0054	Day 1	2 hour	1	1	1.0	2	2	1.5
B0054	Day 2		1	3	3.0	2	2	2.0
B0054	Day 4		1	3	3.0	2	2	2.0
B0054	Day 8		1	3	3.0	2	2	2.0
B0054	Day 22		0	0	0.0	0	0	0.0
B0054	EOS/ET (Day 61)		0	0	0.0	0	0	0.0
B0062	Day 1	Pre-dose	0	0	0.0	0	0	0.0
B0062	Day 1	10 min	1	2	2.0	2	2	2.0
B0062	Day 1	1 hour	1	2	2.0	2	2	2.0
B0062	Day 1	2 hour	1	2	2.0	2	2	2.0
B0062	Day 2		1	2	2.0	2	2	1.5
B0062	Day 4		1	2	2.0	0	0	0.0
B0062	Day 8		0	0	0.0	0	0	0.0
B0062	Day 22		0	0	0.0	0	0	0.0
B0062	EOS/ET (Day 61)		0	0	0.0	0	0	0.0
B0068	Day 1	Pre-dose	0	0	0.0	0	0	0.0
B0068	Day 1	10 min	1	2	2.0	2	2	2.0
B0068	Day 1	1 hour	1	2	2.0	2	2	2.0
B0068	Day 1	2 hour	1	2	2.0	2	2	2.0

Administration Site Key: FA = Forearm; UA= Upper arm
Treatments Part A: A1 = A/Sing MAP, 15 ug (FA); A2 = Afluria Quad IM (UA), 15 ug; A3 = Placebo MAP (FA); A4 = A/Sing Mono IM, 15 ug (UA)
Treatments Part B: B1 = A/Sing MAP, 15 ug (FA) with biopsy; B2 = Placebo MAP (FA) with biopsy
B3 = A/Sing MAP, 15 ug (FA); B4 = A/Sing MAP, 10 ug (FA); B5 = A/Sing MAP, 5 ug (FA); B6 = A/Sing MAP, 2.5 ug (FA)
B7 = Placebo MAP (FA); B8 = A/Sing MAP, 15 ug (UA); B9 = A/Sing IM, 15 ug, as 2018 Afluria Quad (UA)

Listing 16.2.9.11
Individual Characterisation of Skin Irritation Index by Treatment
Erythema
Part B, Non-biopsy
B5= 1 x 5 ug A/Sing MAP + 2 uncoated MAPs on volar forearm (5 ug)

			Active			Placebo		
Randomisation Number	Visit	Time Point	#> 0	Maximum	Average	#> 0	Maximum	Average
B0068	Day 8		1	3	3.0	0	0	0.0
B0068	Day 22		0	0	0.0	0	0	0.0
B0068	EOS/ET (Day 61)		0	0	0.0	0	0	0.0
B0084	Day 1	Pre-dose	0	0	0.0	0	0	0.0
B0084	Day 1	10 min	1	2	2.0	2	2	2.0
B0084	Day 1	1 hour	1	1	1.0	2	1	1.0
B0084	Day 1	2 hour	1	2	2.0	2	2	2.0
B0084	Day 2		1	3	3.0	2	1	1.0
B0084	Day 4		1	1	1.0	0	0	0.0
B0084	Day 8		1	2	2.0	0	0	0.0
B0084	Day 22		0	0	0.0	0	0	0.0
B0084	EOS/ET (Day 61)		0	0	0.0	0	0	0.0
B0089	Day 1	Pre-dose	0	0	0.0	0	0	0.0
B0089	Day 1	10 min	1	2	2.0	2	2	2.0
B0089	Day 1	1 hour	1	2	2.0	2	2	2.0
B0089	Day 1	2 hour	1	2	2.0	2	2	2.0
B0089	Day 2		1	2	2.0	2	2	2.0
B0089	Day 4		1	3	3.0	2	1	1.0
B0089	Day 8		0	0	0.0	0	0	0.0

Administration Site Key: FA = Forearm; UA= Upper arm
Treatments Part A: A1 = A/Sing MAP, 15 ug (FA); A2 = Afluria Quad IM (UA), 15 ug; A3 = Placebo MAP (FA); A4 = A/Sing Mono IM, 15 ug (UA)
Treatments Part B: B1 = A/Sing MAP, 15 ug (FA) with biopsy; B2 = Placebo MAP (FA) with biopsy
B3 = A/Sing MAP, 15 ug (FA); B4 = A/Sing MAP, 10 ug (FA); B5 = A/Sing MAP, 5 ug (FA); B6 = A/Sing MAP, 2.5 ug (FA)
B7 = Placebo MAP (FA); B8 = A/Sing MAP, 15 ug (UA); B9 = A/Sing IM, 15 ug, as 2018 Afluria Quad (UA)

Listing 16.2.9.11
Individual Characterisation of Skin Irritation Index by Treatment
Erythema
Part B, Non-biopsy
B5= 1 x 5 ug A/Sing MAP + 2 uncoated MAPs on volar forearm (5 ug)

			Active			Placebo		
Randomisation Number	Visit	Time Point	#> 0	Maximum	Average	#> 0	Maximum	Average
B0089	Day 22		0	0	0.0	0	0	0.0
B0089	EOS/ET (Day 61)		0	0	0.0	0	0	0.0
B0097	Day 1	Pre-dose	0	0	0.0	0	0	0.0
B0097	Day 1	10 min	1	2	2.0	2	2	2.0
B0097	Day 1	1 hour	1	2	2.0	2	2	2.0
B0097	Day 1	2 hour	1	1	1.0	2	1	1.0
B0097	Day 2		1	3	3.0	2	2	2.0
B0097	Day 4		1	2	2.0	2	2	2.0
B0097	Day 8		1	2	2.0	0	0	0.0
B0097	Day 22		0	0	0.0	0	0	0.0
B0097	EOS/ET (Day 61)		0	0	0.0	0	0	0.0
B0103	Day 1	Pre-dose	0	0	0.0	0	0	0.0
B0103	Day 1	10 min	1	2	2.0	2	2	2.0
B0103	Day 1	1 hour	1	2	2.0	2	2	2.0
B0103	Day 1	2 hour	1	2	2.0	2	2	2.0
B0103	Day 2		1	2	2.0	2	1	1.0
B0103	Day 4		1	2	2.0	0	0	0.0
B0103	Day 8		0	0	0.0	0	0	0.0
B0103	Day 22		0	0	0.0	0	0	0.0

Source: Listing 16.2.9.4

Administration Site Key: FA = Forearm; UA= Upper arm
Treatments Part A: A1 = A/Sing MAP, 15 ug (FA); A2 = Afluria Quad IM (UA), 15 ug; A3 = Placebo MAP (FA); A4 = A/Sing Mono IM, 15 ug (UA)
Treatments Part B: B1 = A/Sing MAP, 15 ug (FA) with biopsy; B2 = Placebo MAP (FA) with biopsy
B3 = A/Sing MAP, 15 ug (FA); B4 = A/Sing MAP, 10 ug (FA); B5 = A/Sing MAP, 5 ug (FA); B6 = A/Sing MAP, 2.5 ug (FA)
B7 = Placebo MAP (FA); B8 = A/Sing MAP, 15 ug (UA); B9 = A/Sing IM, 15 ug, as 2018 Afluria Quad (UA)

Listing 16.2.9.11
Individual Characterisation of Skin Irritation Index by Treatment
Erythema
Part B, Non-biopsy
B5= 1 x 5 ug A/Sing MAP + 2 uncoated MAPs on volar forearm (5 ug)

Randomisation Number	Visit	Time Point	Active #> 0	Active Maximum	Active Average	Placebo #> 0	Placebo Maximum	Placebo Average
B0103	EOS/ET (Day 61)		0	0	0.0	0	0	0.0
B0113	Day 1	Pre-dose	0	0	0.0	0	0	0.0
B0113	Day 1	10 min	1	2	2.0	2	2	2.0
B0113	Day 1	1 hour	1	2	2.0	2	2	2.0
B0113	Day 1	2 hour	1	2	2.0	2	2	2.0
B0113	Day 2		1	3	3.0	2	2	2.0
B0113	Day 4		0	0	0.0	0	0	0.0
B0113	Day 8		0	0	0.0	0	0	0.0
B0113	Day 22		0	0	0.0	0	0	0.0
B0113	EOS/ET (Day 61)		0	0	0.0	0	0	0.0
B0122	Day 1	Pre-dose	0	0	0.0	0	0	0.0
B0122	Day 1	10 min	1	2	2.0	2	2	2.0
B0122	Day 1	1 hour	1	2	2.0	2	2	2.0
B0122	Day 1	2 hour	1	2	2.0	2	2	2.0
B0122	Day 2		1	3	3.0	2	2	2.0
B0122	Day 4		1	3	3.0	1	2	1.0
B0122	Day 8		0	0	0.0	0	0	0.0
B0122	Day 22		1	2	2.0	0	0	0.0
B0122	EOS/ET (Day 61)		0	0	0.0	0	0	0.0

Administration Site Key: FA = Forearm; UA= Upper arm
Treatments Part A: A1 = A/Sing MAP, 15 ug (FA); A2 = Afluria Quad IM (UA), 15 ug; A3 = Placebo MAP (FA); A4 = A/Sing Mono IM, 15 ug (UA)
Treatments Part B: B1 = A/Sing MAP, 15 ug (FA) with biopsy; B2 = Placebo MAP (FA) with biopsy
B3 = A/Sing MAP, 15 ug (FA); B4 = A/Sing MAP, 10 ug (FA); B5 = A/Sing MAP, 5 ug (FA); B6 = A/Sing MAP, 2.5 ug (FA)
B7 = Placebo MAP (FA); B8 = A/Sing MAP, 15 ug (UA); B9 = A/Sing IM, 15 ug, as 2018 Afluria Quad (UA)

Listing 16.2.9.11
Individual Characterisation of Skin Irritation Index by Treatment
Erythema
Part B, Non-biopsy
B5= 1 x 5 ug A/Sing MAP + 2 uncoated MAPs on volar forearm (5 ug)

Randomisation Number	Visit	Time Point	Active #> 0	Active Maximum	Active Average	Placebo #> 0	Placebo Maximum	Placebo Average
B0128	Day 1	Pre-dose	0	0	0.0	0	0	0.0
B0128	Day 1	10 min	1	2	2.0	2	2	2.0
B0128	Day 1	1 hour	1	2	2.0	2	2	2.0
B0128	Day 1	2 hour	1	2	2.0	2	2	2.0
B0128	Day 2		1	2	2.0	2	1	1.0
B0128	Day 4		1	2	2.0	2	1	1.0
B0128	Day 8		0	0	0.0	0	0	0.0
B0128	Day 22		0	0	0.0	0	0	0.0
B0128	EOS/ET (Day 61)		0	0	0.0	0	0	0.0
B0130	Day 1	Pre-dose	0	0	0.0	0	0	0.0
B0130	Day 1	10 min	1	2	2.0	2	2	2.0
B0130	Day 1	1 hour	1	2	2.0	2	2	2.0
B0130	Day 1	2 hour	1	2	2.0	2	2	2.0
B0130	Day 2		1	2	2.0	2	2	2.0
B0130	Day 4		1	3	3.0	0	0	0.0
B0130	Day 8		1	3	3.0	0	0	0.0
B0130	Day 22		0	0	0.0	0	0	0.0
B0130	EOS/ET (Day 61)		0	0	0.0	0	0	0.0

Administration Site Key: FA = Forearm; UA= Upper arm
Treatments Part A: A1 = A/Sing MAP, 15 ug (FA); A2 = Afluria Quad IM (UA), 15 ug; A3 = Placebo MAP (FA); A4 = A/Sing Mono IM, 15 ug (UA)
Treatments Part B: B1 = A/Sing MAP, 15 ug (FA) with biopsy; B2 = Placebo MAP (FA) with biopsy
B3 = A/Sing MAP, 15 ug (FA); B4 = A/Sing MAP, 10 ug (FA); B5 = A/Sing MAP, 5 ug (FA); B6 = A/Sing MAP, 2.5 ug (FA)
B7 = Placebo MAP (FA); B8 = A/Sing MAP, 15 ug (UA); B9 = A/Sing IM, 15 ug, as 2018 Afluria Quad (UA)

Listing 16.2.9.11
Individual Characterisation of Skin Irritation Index by Treatment
Erythema
Part B, Non-biopsy
B5= 1 x 5 ug A/Sing MAP + 2 uncoated MAPs on volar forearm (5 ug)

			Active			Placebo		
Randomisation Number	Visit	Time Point	#> 0	Maximum	Average	#> 0	Maximum	Average
B0142	Day 1	Pre-dose	0	0	0.0	0	0	0.0
B0142	Day 1	10 min	1	2	2.0	2	2	2.0
B0142	Day 1	1 hour	1	3	3.0	2	3	3.0
B0142	Day 1	2 hour	1	2	2.0	2	2	2.0
B0142	Day 2		1	3	3.0	2	1	1.0
B0142	Day 4		1	2	2.0	0	0	0.0
B0142	Day 8		1	2	2.0	2	1	1.0
B0142	Day 22		0	0	0.0	0	0	0.0
B0142	EOS/ET (Day 61)		0	0	0.0	0	0	0.0
B0148	Day 1	Pre-dose	0	0	0.0	0	0	0.0
B0148	Day 1	10 min	1	2	2.0	2	2	2.0
B0148	Day 1	1 hour	1	2	2.0	2	2	2.0
B0148	Day 1	2 hour	1	1	1.0	2	1	1.0
B0148	Day 2		1	3	3.0	2	1	1.0
B0148	Day 4		1	4	4.0	0	0	0.0
B0148	Day 8		1	2	2.0	0	0	0.0
B0148	Day 22		0	0	0.0	0	0	0.0
B0148	EOS/ET (Day 61)		0	0	0.0	0	0	0.0
B1076	Day 1	Pre-dose	0	0	0.0	0	0	0.0

Source: Listing 16.2.9.4

Administration Site Key: FA = Forearm; UA= Upper arm
Treatments Part A: A1 = A/Sing MAP, 15 ug (FA); A2 = Afluria Quad IM (UA), 15 ug; A3 = Placebo MAP (FA); A4 = A/Sing Mono IM, 15 ug (UA)
Treatments Part B: B1 = A/Sing MAP, 15 ug (FA) with biopsy; B2 = Placebo MAP (FA) with biopsy
B3 = A/Sing MAP, 15 ug (FA); B4 = A/Sing MAP, 10 ug (FA); B5 = A/Sing MAP, 5 ug (FA); B6 = A/Sing MAP, 2.5 ug (FA)
B7 = Placebo MAP (FA); B8 = A/Sing MAP, 15 ug (UA); B9 = A/Sing IM, 15 ug, as 2018 Afluria Quad (UA)

Listing 16.2.9.11
Individual Characterisation of Skin Irritation Index by Treatment
Erythema
Part B, Non-biopsy
B5= 1 x 5 ug A/Sing MAP + 2 uncoated MAPs on volar forearm (5 ug)

			Active			Placebo		
Randomisation Number	Visit	Time Point	#> 0	Maximum	Average	#> 0	Maximum	Average
B1076	Day 1	10 min	1	2	2.0	2	2	2.0
B1076	Day 1	1 hour	1	2	2.0	2	2	2.0
B1076	Day 1	2 hour	1	2	2.0	2	2	2.0
B1076	Day 2		1	2	2.0	2	1	1.0
B1076	Day 4		1	3	3.0	0	0	0.0
B1076	Day 8		0	0	0.0	0	0	0.0
B1076	Day 22		0	0	0.0	0	0	0.0
B1076	EOS/ET (Day 61)		0	0	0.0	0	0	0.0

Administration Site Key: FA = Forearm; UA= Upper arm
Treatments Part A: A1 = A/Sing MAP, 15 ug (FA); A2 = Afluria Quad IM (UA), 15 ug; A3 = Placebo MAP (FA); A4 = A/Sing Mono IM, 15 ug (UA)
Treatments Part B: B1 = A/Sing MAP, 15 ug (FA) with biopsy; B2 = Placebo MAP (FA) with biopsy
B3 = A/Sing MAP, 15 ug (FA); B4 = A/Sing MAP, 10 ug (FA); B5 = A/Sing MAP, 5 ug (FA); B6 = A/Sing MAP, 2.5 ug (FA)
B7 = Placebo MAP (FA); B8 = A/Sing MAP, 15 ug (UA); B9 = A/Sing IM, 15 ug, as 2018 Afluria Quad (UA)

Listing 16.2.9.11
Individual Characterisation of Skin Irritation Index by Treatment
Erythema
Part B, Non-biopsy
B6= 1 x 2.5 ug A/Sing MAP + 2 uncoated MAPs on volar forearm (2.5 ug)

			Active			Placebo		
Randomisation Number	Visit	Time Point	#> 0	Maximum	Average	#> 0	Maximum	Average
B0012	Day 1	Pre-dose	0	0	0.0	0	0	0.0
B0012	Day 1	10 min	1	2	2.0	2	2	2.0
B0012	Day 1	1 hour	1	1	1.0	2	1	1.0
B0012	Day 1	2 hour	1	1	1.0	2	1	1.0
B0012	Day 2		1	3	3.0	2	2	2.0
B0012	Day 4		1	3	3.0	2	2	2.0
B0012	Day 8		0	0	0.0	0	0	0.0
B0012	Day 22		0	0	0.0	0	0	0.0
B0012	EOS/ET (Day 61)		0	0	0.0	0	0	0.0
B0020	Day 1	Pre-dose	0	0	0.0	0	0	0.0
B0020	Day 1	10 min	1	2	2.0	2	2	2.0
B0020	Day 1	1 hour	1	2	2.0	2	2	2.0
B0020	Day 1	2 hour	1	2	2.0	2	2	2.0
B0020	Day 2		1	3	3.0	2	2	2.0
B0020	Day 4		1	2	2.0	0	0	0.0
B0020	Day 8		0	0	0.0	0	0	0.0
B0020	Day 22		0	0	0.0	0	0	0.0
B0020	EOS/ET (Day 61)		0	0	0.0	0	0	0.0
B0029	Day 1	Pre-dose	0	0	0.0	0	0	0.0

Administration Site Key: FA = Forearm; UA= Upper arm
Treatments Part A: A1 = A/Sing MAP, 15 ug (FA); A2 = Afluria Quad IM (UA), 15 ug; A3 = Placebo MAP (FA); A4 = A/Sing Mono IM, 15 ug (UA)
Treatments Part B: B1 = A/Sing MAP, 15 ug (FA) with biopsy; B2 = Placebo MAP (FA) with biopsy
B3 = A/Sing MAP, 15 ug (FA); B4 = A/Sing MAP, 10 ug (FA); B5 = A/Sing MAP, 5 ug (FA); B6 = A/Sing MAP, 2.5 ug (FA)
B7 = Placebo MAP (FA); B8 = A/Sing MAP, 15 ug (UA); B9 = A/Sing IM, 15 ug, as 2018 Afluria Quad (UA)

Listing 16.2.9.11
Individual Characterisation of Skin Irritation Index by Treatment
Erythema
Part B, Non-biopsy
B6= 1 x 2.5 ug A/Sing MAP + 2 uncoated MAPs on volar forearm (2.5 ug)

			Active			Placebo		
Randomisation Number	Visit	Time Point	#> 0	Maximum	Average	#> 0	Maximum	Average
B0029	Day 1	10 min	1	1	1.0	2	1	1.0
B0029	Day 1	1 hour	1	1	1.0	2	1	1.0
B0029	Day 1	2 hour	0	0	0.0	0	0	0.0
B0029	Day 2		1	3	3.0	2	2	2.0
B0029	Day 4		1	3	3.0	0	0	0.0
B0029	Day 8		0	0	0.0	0	0	0.0
B0029	Day 22		0	0	0.0	0	0	0.0
B0029	EOS/ET (Day 61)		0	0	0.0	0	0	0.0
B0032	Day 1	Pre-dose	0	0	0.0	0	0	0.0
B0032	Day 1	10 min	1	2	2.0	2	2	2.0
B0032	Day 1	1 hour	1	3	3.0	2	3	3.0
B0032	Day 1	2 hour	1	2	2.0	2	2	2.0
B0032	Day 2		1	2	2.0	2	2	2.0
B0032	Day 4		1	3	3.0	2	2	2.0
B0032	Day 8		1	2	2.0	2	1	1.0
B0032	Day 22		0	0	0.0	0	0	0.0
B0032	EOS/ET (Day 61)		0	0	0.0	0	0	0.0
B0044	Day 1	Pre-dose	0	0	0.0	0	0	0.0
B0044	Day 1	10 min	1	2	2.0	2	2	2.0

Source: Listing 16.2.9.4

Administration Site Key: FA = Forearm; UA= Upper arm
Treatments Part A: A1 = A/Sing MAP, 15 ug (FA); A2 = Afluria Quad IM (UA), 15 ug; A3 = Placebo MAP (FA); A4 = A/Sing Mono IM, 15 ug (UA)
Treatments Part B: B1 = A/Sing MAP, 15 ug (FA) with biopsy; B2 = Placebo MAP (FA) with biopsy
B3 = A/Sing MAP, 15 ug (FA); B4 = A/Sing MAP, 10 ug (FA); B5 = A/Sing MAP, 5 ug (FA); B6 = A/Sing MAP, 2.5 ug (FA)
B7 = Placebo MAP (FA); B8 = A/Sing MAP, 15 ug (UA); B9 = A/Sing IM, 15 ug, as 2018 Afluria Quad (UA)

Listing 16.2.9.11
Individual Characterisation of Skin Irritation Index by Treatment
Erythema
Part B, Non-biopsy
B6= 1 x 2.5 ug A/Sing MAP + 2 uncoated MAPs on volar forearm (2.5 ug)

			Active			Placebo		
Randomisation Number	Visit	Time Point	#> 0	Maximum	Average	#> 0	Maximum	Average
B0044	Day 1	1 hour	1	2	2.0	2	2	2.0
B0044	Day 1	2 hour	1	2	2.0	2	2	2.0
B0044	Day 2		1	3	3.0	2	2	2.0
B0044	Day 4		1	2	2.0	2	1	1.0
B0044	Day 8		1	3	3.0	2	1	1.0
B0044	Day 22		1	1	1.0	0	0	0.0
B0044	EOS/ET (Day 61)		0	0	0.0	0	0	0.0
B0048	Day 1	Pre-dose	0	0	0.0	0	0	0.0
B0048	Day 1	10 min	1	2	2.0	2	2	2.0
B0048	Day 1	1 hour	1	2	2.0	2	2	2.0
B0048	Day 1	2 hour	1	2	2.0	2	2	2.0
B0048	Day 2		1	3	3.0	2	2	2.0
B0048	Day 4		1	2	2.0	0	0	0.0
B0048	Day 8		0	0	0.0	0	0	0.0
B0048	Day 22		0	0	0.0	0	0	0.0
B0048	EOS/ET (Day 61)		0	0	0.0	0	0	0.0
B0053	Day 1	Pre-dose	0	0	0.0	0	0	0.0
B0053	Day 1	10 min	1	2	2.0	2	2	2.0
B0053	Day 1	1 hour	1	2	2.0	2	2	2.0

Source: Listing 16.2.9.4

Administration Site Key: FA = Forearm; UA= Upper arm
Treatments Part A: A1 = A/Sing MAP, 15 ug (FA); A2 = Afluria Quad IM (UA), 15 ug; A3 = Placebo MAP (FA); A4 = A/Sing Mono IM, 15 ug (UA)
Treatments Part B: B1 = A/Sing MAP, 15 ug (FA) with biopsy; B2 = Placebo MAP (FA) with biopsy
B3 = A/Sing MAP, 15 ug (FA); B4 = A/Sing MAP, 10 ug (FA); B5 = A/Sing MAP, 5 ug (FA); B6 = A/Sing MAP, 2.5 ug (FA)
B7 = Placebo MAP (FA); B8 = A/Sing MAP, 15 ug (UA); B9 = A/Sing IM, 15 ug, as 2018 Afluria Quad (UA)

Listing 16.2.9.11
Individual Characterisation of Skin Irritation Index by Treatment
Erythema
Part B, Non-biopsy
B6= 1 x 2.5 ug A/Sing MAP + 2 uncoated MAPs on volar forearm (2.5 ug)

			Active			Placebo		
Randomisation Number	Visit	Time Point	#> 0	Maximum	Average	#> 0	Maximum	Average
B0053	Day 1	2 hour	1	2	2.0	2	2	2.0
B0053	Day 2		1	2	2.0	2	2	2.0
B0053	Day 4		1	2	2.0	2	2	2.0
B0053	Day 8		0	0	0.0	0	0	0.0
B0053	Day 22		0	0	0.0	0	0	0.0
B0053	EOS/ET (Day 61)		0	0	0.0	0	0	0.0
B0066	Day 1	Pre-dose	0	0	0.0	0	0	0.0
B0066	Day 1	10 min	1	2	2.0	2	2	2.0
B0066	Day 1	1 hour	1	2	2.0	2	2	2.0
B0066	Day 1	2 hour	1	2	2.0	2	2	2.0
B0066	Day 2		1	2	2.0	2	1	1.0
B0066	Day 4		1	3	3.0	2	2	2.0
B0066	Day 8		0	0	0.0	0	0	0.0
B0066	Day 22		0	0	0.0	0	0	0.0
B0066	EOS/ET (Day 61)		0	0	0.0	0	0	0.0
B0073	Day 1	Pre-dose	0	0	0.0	0	0	0.0
B0073	Day 1	10 min	1	2	2.0	2	2	2.0
B0073	Day 1	1 hour	1	2	2.0	2	2	1.5
B0073	Day 1	2 hour	1	2	2.0	2	2	2.0

Administration Site Key: FA = Forearm; UA= Upper arm
Treatments Part A: A1 = A/Sing MAP, 15 ug (FA); A2 = Afluria Quad IM (UA), 15 ug; A3 = Placebo MAP (FA); A4 = A/Sing Mono IM, 15 ug (UA)
Treatments Part B: B1 = A/Sing MAP, 15 ug (FA) with biopsy; B2 = Placebo MAP (FA) with biopsy
B3 = A/Sing MAP, 15 ug (FA); B4 = A/Sing MAP, 10 ug (FA); B5 = A/Sing MAP, 5 ug (FA); B6 = A/Sing MAP, 2.5 ug (FA)
B7 = Placebo MAP (FA); B8 = A/Sing MAP, 15 ug (UA); B9 = A/Sing IM, 15 ug, as 2018 Afluria Quad (UA)

Listing 16.2.9.11
Individual Characterisation of Skin Irritation Index by Treatment
Erythema
Part B, Non-biopsy
B6= 1 x 2.5 ug A/Sing MAP + 2 uncoated MAPs on volar forearm (2.5 ug)

Randomisation Number	Visit	Time Point	Active #> 0	Active Maximum	Active Average	Placebo #> 0	Placebo Maximum	Placebo Average
B0073	Day 2		1	3	3.0	2	3	3.0
B0073	Day 4		1	2	2.0	1	1	0.5
B0073	Day 8		0	0	0.0	0	0	0.0
B0073	Day 22		0	0	0.0	0	0	0.0
B0073	EOS/ET (Day 61)		0	0	0.0	0	0	0.0
B0079	Day 1	Pre-dose	0	0	0.0	0	0	0.0
B0079	Day 1	10 min	1	2	2.0	2	2	2.0
B0079	Day 1	1 hour	1	2	2.0	2	2	1.5
B0079	Day 1	2 hour	1	2	2.0	2	2	2.0
B0079	Day 2		1	2	2.0	2	2	2.0
B0079	Day 4		1	2	2.0	2	1	1.0
B0079	Day 8		1	3	3.0	1	2	1.0
B0079	Day 22		0	0	0.0	0	0	0.0
B0079	EOS/ET (Day 61)		0	0	0.0	0	0	0.0
B0081	Day 1	Pre-dose	0	0	0.0	0	0	0.0
B0081	Day 1	10 min	1	2	2.0	2	2	2.0
B0081	Day 1	1 hour	1	2	2.0	2	2	2.0
B0081	Day 1	2 hour	1	3	3.0	2	3	3.0
B0081	Day 2		1	3	3.0	2	3	3.0

Source: Listing 16.2.9.4

Administration Site Key: FA = Forearm; UA= Upper arm
Treatments Part A: A1 = A/Sing MAP, 15 ug (FA); A2 = Afluria Quad IM (UA), 15 ug; A3 = Placebo MAP (FA); A4 = A/Sing Mono IM, 15 ug (UA)
Treatments Part B: B1 = A/Sing MAP, 15 ug (FA) with biopsy; B2 = Placebo MAP (FA) with biopsy
B3 = A/Sing MAP, 15 ug (FA); B4 = A/Sing MAP, 10 ug (FA); B5 = A/Sing MAP, 5 ug (FA); B6 = A/Sing MAP, 2.5 ug (FA)
B7 = Placebo MAP (FA); B8 = A/Sing MAP, 15 ug (UA); B9 = A/Sing IM, 15 ug, as 2018 Afluria Quad (UA)

Listing 16.2.9.11
Individual Characterisation of Skin Irritation Index by Treatment
Erythema
Part B, Non-biopsy
B6= 1 x 2.5 ug A/Sing MAP + 2 uncoated MAPs on volar forearm (2.5 ug)

			Active			Placebo		
Randomisation Number	Visit	Time Point	#> 0	Maximum	Average	#> 0	Maximum	Average
B0081	Day 4		0	0	0.0	0	0	0.0
B0081	Day 8		0	0	0.0	0	0	0.0
B0081	Day 22		0	0	0.0	0	0	0.0
B0081	EOS/ET (Day 61)		0	0	0.0	0	0	0.0
B0090	Day 1	Pre-dose	0	0	0.0	0	0	0.0
B0090	Day 1	10 min	1	2	2.0	2	2	2.0
B0090	Day 1	1 hour	1	1	1.0	2	1	1.0
B0090	Day 1	2 hour	1	1	1.0	2	2	2.0
B0090	Day 2		1	2	2.0	2	2	2.0
B0090	Day 4		1	1	1.0	0	0	0.0
B0090	Day 8		0	0	0.0	0	0	0.0
B0090	Day 22		0	0	0.0	0	0	0.0
B0090	EOS/ET (Day 61)		0	0	0.0	0	0	0.0
B0096	Day 1	Pre-dose	0	0	0.0	0	0	0.0
B0096	Day 1	10 min	1	2	2.0	2	2	2.0
B0096	Day 1	1 hour	1	1	1.0	2	1	1.0
B0096	Day 1	2 hour	1	2	2.0	2	2	2.0
B0096	Day 2		1	3	3.0	2	2	2.0
B0096	Day 4		1	3	3.0	0	0	0.0

Source: Listing 16.2.9.4

Administration Site Key: FA = Forearm; UA= Upper arm
Treatments Part A: A1 = A/Sing MAP, 15 ug (FA); A2 = Afluria Quad IM (UA), 15 ug; A3 = Placebo MAP (FA); A4 = A/Sing Mono IM, 15 ug (UA)
Treatments Part B: B1 = A/Sing MAP, 15 ug (FA) with biopsy; B2 = Placebo MAP (FA) with biopsy
B3 = A/Sing MAP, 15 ug (FA); B4 = A/Sing MAP, 10 ug (FA); B5 = A/Sing MAP, 5 ug (FA); B6 = A/Sing MAP, 2.5 ug (FA)
B7 = Placebo MAP (FA); B8 = A/Sing MAP, 15 ug (UA); B9 = A/Sing IM, 15 ug, as 2018 Afluria Quad (UA)

Listing 16.2.9.11
Individual Characterisation of Skin Irritation Index by Treatment
Erythema
Part B, Non-biopsy
B6= 1 x 2.5 ug A/Sing MAP + 2 uncoated MAPs on volar forearm (2.5 ug)

			Active			Placebo		
Randomisation Number	Visit	Time Point	#> 0	Maximum	Average	#> 0	Maximum	Average
B0096	Day 8		0	0	0.0	0	0	0.0
B0096	Day 22		0	0	0.0	0	0	0.0
B0096	EOS/ET (Day 61)		0	0	0.0	0	0	0.0
B0107	Day 1	Pre-dose	0	0	0.0	0	0	0.0
B0107	Day 1	10 min	1	2	2.0	2	2	2.0
B0107	Day 1	1 hour	1	2	2.0	2	2	2.0
B0107	Day 1	2 hour	1	1	1.0	2	1	1.0
B0107	Day 2		1	2	2.0	2	2	2.0
B0107	Day 4		1	3	3.0	2	3	2.5
B0107	Day 8		0	0	0.0	0	0	0.0
B0107	Day 22		0	0	0.0	0	0	0.0
B0107	EOS/ET (Day 61)		0	0	0.0	0	0	0.0
B0109	Day 1	Pre-dose	0	0	0.0	0	0	0.0
B0109	Day 1	10 min	1	2	2.0	2	2	2.0
B0109	Day 1	1 hour	1	3	3.0	2	2	2.0
B0109	Day 1	2 hour	1	2	2.0	2	1	1.0
B0109	Day 2		1	2	2.0	2	1	1.0
B0109	Day 4		1	3	3.0	0	0	0.0
B0109	Day 8		0	0	0.0	0	0	0.0

Administration Site Key: FA = Forearm; UA= Upper arm
Treatments Part A: A1 = A/Sing MAP, 15 ug (FA); A2 = Afluria Quad IM (UA), 15 ug; A3 = Placebo MAP (FA); A4 = A/Sing Mono IM, 15 ug (UA)
Treatments Part B: B1 = A/Sing MAP, 15 ug (FA) with biopsy; B2 = Placebo MAP (FA) with biopsy
B3 = A/Sing MAP, 15 ug (FA); B4 = A/Sing MAP, 10 ug (FA); B5 = A/Sing MAP, 5 ug (FA); B6 = A/Sing MAP, 2.5 ug (FA)
B7 = Placebo MAP (FA); B8 = A/Sing MAP, 15 ug (UA); B9 = A/Sing IM, 15 ug, as 2018 Afluria Quad (UA)

Listing 16.2.9.11
Individual Characterisation of Skin Irritation Index by Treatment
Erythema
Part B, Non-biopsy
B6= 1 x 2.5 ug A/Sing MAP + 2 uncoated MAPs on volar forearm (2.5 ug)

			Active			Placebo		
Randomisation Number	Visit	Time Point	#> 0	Maximum	Average	#> 0	Maximum	Average
B0109	Day 22		0	0	0.0	0	0	0.0
B0109	EOS/ET (Day 61)		0	0	0.0	0	0	0.0
B0118	Day 1	Pre-dose	0	0	0.0	0	0	0.0
B0118	Day 1	10 min	1	2	2.0	2	2	2.0
B0118	Day 1	1 hour	1	2	2.0	2	2	2.0
B0118	Day 1	2 hour	1	2	2.0	2	2	2.0
B0118	Day 2		1	3	3.0	2	1	1.0
B0118	Day 4		1	2	2.0	0	0	0.0
B0118	Day 8		0	0	0.0	0	0	0.0
B0118	Day 22		0	0	0.0	0	0	0.0
B0118	EOS/ET (Day 61)		0	0	0.0	0	0	0.0
B0123	Day 1	Pre-dose	0	0	0.0	0	0	0.0
B0123	Day 1	10 min	1	2	2.0	2	2	2.0
B0123	Day 1	1 hour	1	2	2.0	2	2	2.0
B0123	Day 1	2 hour	1	2	2.0	2	2	2.0
B0123	Day 2		1	2	2.0	2	1	1.0
B0123	Day 4		1	2	2.0	0	0	0.0
B0123	Day 8		0	0	0.0	0	0	0.0
B0123	Day 22		1	2	2.0	0	0	0.0

Source: Listing 16.2.9.4

Administration Site Key: FA = Forearm; UA= Upper arm
Treatments Part A: A1 = A/Sing MAP, 15 ug (FA); A2 = Afluria Quad IM (UA), 15 ug; A3 = Placebo MAP (FA); A4 = A/Sing Mono IM, 15 ug (UA)
Treatments Part B: B1 = A/Sing MAP, 15 ug (FA) with biopsy; B2 = Placebo MAP (FA) with biopsy
B3 = A/Sing MAP, 15 ug (FA); B4 = A/Sing MAP, 10 ug (FA); B5 = A/Sing MAP, 5 ug (FA); B6 = A/Sing MAP, 2.5 ug (FA)
B7 = Placebo MAP (FA); B8 = A/Sing MAP, 15 ug (UA); B9 = A/Sing IM, 15 ug, as 2018 Afluria Quad (UA)

Listing 16.2.9.11
Individual Characterisation of Skin Irritation Index by Treatment
Erythema
Part B, Non-biopsy
B6= 1 x 2.5 ug A/Sing MAP + 2 uncoated MAPs on volar forearm (2.5 ug)

			Active			Placebo		
Randomisation Number	Visit	Time Point	#> 0	Maximum	Average	#> 0	Maximum	Average
B0123	EOS/ET (Day 61)		0	0	0.0	0	0	0.0
B0132	Day 1	Pre-dose	0	0	0.0	0	0	0.0
B0132	Day 1	10 min	1	2	2.0	2	2	2.0
B0132	Day 1	1 hour	1	2	2.0	2	3	3.0
B0132	Day 1	2 hour	1	2	2.0	2	2	2.0
B0132	Day 2		1	3	3.0	2	2	2.0
B0132	Day 4		1	1	1.0	0	0	0.0
B0132	Day 8		0	0	0.0	0	0	0.0
B0132	Day 22		1	1	1.0	0	0	0.0
B0132	EOS/ET (Day 61)		0	0	0.0	0	0	0.0
B0143	Day 1	Pre-dose	0	0	0.0	0	0	0.0
B0143	Day 1	10 min	1	2	2.0	2	2	2.0
B0143	Day 1	1 hour	1	3	3.0	2	3	3.0
B0143	Day 1	2 hour	1	2	2.0	2	2	2.0
B0143	Day 2		1	2	2.0	2	2	2.0
B0143	Day 4		1	3	3.0	2	2	2.0
B0143	Day 8		0	0	0.0	0	0	0.0
B0143	Day 22		0	0	0.0	0	0	0.0
B0143	EOS/ET (Day 61)		0	0	0.0	0	0	0.0

Source: Listing 16.2.9.4

Administration Site Key: FA = Forearm; UA= Upper arm
Treatments Part A: A1 = A/Sing MAP, 15 ug (FA); A2 = Afluria Quad IM (UA), 15 ug; A3 = Placebo MAP (FA); A4 = A/Sing Mono IM, 15 ug (UA)
Treatments Part B: B1 = A/Sing MAP, 15 ug (FA) with biopsy; B2 = Placebo MAP (FA) with biopsy
B3 = A/Sing MAP, 15 ug (FA); B4 = A/Sing MAP, 10 ug (FA); B5 = A/Sing MAP, 5 ug (FA); B6 = A/Sing MAP, 2.5 ug (FA)
B7 = Placebo MAP (FA); B8 = A/Sing MAP, 15 ug (UA); B9 = A/Sing IM, 15 ug, as 2018 Afluria Quad (UA)

Listing 16.2.9.11
Individual Characterisation of Skin Irritation Index by Treatment
Erythema
Part B, Non-biopsy
B6= 1 x 2.5 ug A/Sing MAP + 2 uncoated MAPs on volar forearm (2.5 ug)

			Active			Placebo		
Randomisation Number	Visit	Time Point	#> 0	Maximum	Average	#> 0	Maximum	Average
B0144	Day 1	Pre-dose	0	0	0.0	0	0	0.0
B0144	Day 1	10 min	1	2	2.0	2	2	2.0
B0144	Day 1	1 hour	1	2	2.0	2	2	2.0
B0144	Day 1	2 hour	1	2	2.0	2	2	2.0
B0144	Day 2		1	2	2.0	2	2	2.0
B0144	Day 4		1	2	2.0	0	0	0.0
B0144	Day 8		1	1	1.0	0	0	0.0
B0144	Day 22		1	1	1.0	0	0	0.0
B0144	EOS/ET (Day 61)		0	0	0.0	0	0	0.0

Source: Listing 16.2.9.4

Administration Site Key: FA = Forearm; UA= Upper arm
Treatments Part A: A1 = A/Sing MAP, 15 ug (FA); A2 = Afluria Quad IM (UA), 15 ug; A3 = Placebo MAP (FA); A4 = A/Sing Mono IM, 15 ug (UA)
Treatments Part B: B1 = A/Sing MAP, 15 ug (FA) with biopsy; B2 = Placebo MAP (FA) with biopsy
B3 = A/Sing MAP, 15 ug (FA); B4 = A/Sing MAP, 10 ug (FA); B5 = A/Sing MAP, 5 ug (FA); B6 = A/Sing MAP, 2.5 ug (FA)
B7 = Placebo MAP (FA); B8 = A/Sing MAP, 15 ug (UA); B9 = A/Sing IM, 15 ug, as 2018 Afluria Quad (UA)

Listing 16.2.9.11
Individual Characterisation of Skin Irritation Index by Treatment
Erythema
Part B, Non-biopsy
B7= 3 x uncoated MAPs on volar forearm

			Active			Placebo		
Randomisation Number	Visit	Time Point	#> 0	Maximum	Average	#> 0	Maximum	Average
B0017	Day 1	Pre-dose	-	-	-	0	0	0.0
B0017	Day 1	10 min	-	-	-	3	3	3.0
B0017	Day 1	1 hour	-	-	-	3	2	2.0
B0017	Day 1	2 hour	-	-	-	3	2	2.0
B0017	Day 2		-	-	-	3	2	2.0
B0017	Day 4		-	-	-	3	2	2.0
B0017	Day 8		-	-	-	0	0	0.0
B0017	Day 22		-	-	-	0	0	0.0
B0017	EOS/ET (Day 61)		-	-	-	0	0	0.0
B0021	Day 1	Pre-dose	-	-	-	0	0	0.0
B0021	Day 1	10 min	-	-	-	3	2	2.0
B0021	Day 1	1 hour	-	-	-	3	2	1.7
B0021	Day 1	2 hour	-	-	-	3	2	2.0
B0021	Day 2		-	-	-	3	2	2.0
B0021	Day 4		-	-	-	3	2	2.0
B0021	Day 8		-	-	-	0	0	0.0
B0021	Day 22		-	-	-	0	0	0.0
B0021	EOS/ET (Day 61)		-	-	-	0	0	0.0
B0030	Day 1	Pre-dose	-	-	-	0	0	0.0

Source: Listing 16.2.9.4

Administration Site Key: FA = Forearm; UA= Upper arm
Treatments Part A: A1 = A/Sing MAP, 15 ug (FA); A2 = Afluria Quad IM (UA), 15 ug; A3 = Placebo MAP (FA); A4 = A/Sing Mono IM, 15 ug (UA)
Treatments Part B: B1 = A/Sing MAP, 15 ug (FA) with biopsy; B2 = Placebo MAP (FA) with biopsy
B3 = A/Sing MAP, 15 ug (FA); B4 = A/Sing MAP, 10 ug (FA); B5 = A/Sing MAP, 5 ug (FA); B6 = A/Sing MAP, 2.5 ug (FA)
B7 = Placebo MAP (FA); B8 = A/Sing MAP, 15 ug (UA); B9 = A/Sing IM, 15 ug, as 2018 Afluria Quad (UA)

Listing 16.2.9.11
Individual Characterisation of Skin Irritation Index by Treatment
Erythema
Part B, Non-biopsy
B7= 3 x uncoated MAPs on volar forearm

Randomisation Number	Visit	Time Point	Active #> 0	Active Maximum	Active Average	Placebo #> 0	Placebo Maximum	Placebo Average
B0030	Day 1	10 min	-	-	-	3	2	2.0
B0030	Day 1	1 hour	-	-	-	3	1	1.0
B0030	Day 1	2 hour	-	-	-	3	2	2.0
B0030	Day 2		-	-	-	3	1	1.0
B0030	Day 4		-	-	-	0	0	0.0
B0030	Day 8		-	-	-	0	0	0.0
B0030	Day 22		-	-	-	0	0	0.0
B0030	EOS/ET (Day 61)		-	-	-	0	0	0.0
B0036	Day 1	Pre-dose	-	-	-	0	0	0.0
B0036	Day 1	10 min	-	-	-	3	2	2.0
B0036	Day 1	1 hour	-	-	-	3	3	3.0
B0036	Day 1	2 hour	-	-	-	3	2	2.0
B0036	Day 2		-	-	-	3	2	2.0
B0036	Day 4		-	-	-	2	1	0.7
B0036	Day 8		-	-	-	0	0	0.0
B0036	Day 22		-	-	-	0	0	0.0
B0036	EOS/ET (Day 61)		-	-	-	0	0	0.0
B0039	Day 1	Pre-dose	-	-	-	0	0	0.0
B0039	Day 1	10 min	-	-	-	3	2	2.0

Administration Site Key: FA = Forearm; UA= Upper arm
Treatments Part A: A1 = A/Sing MAP, 15 ug (FA); A2 = Afluria Quad IM (UA), 15 ug; A3 = Placebo MAP (FA); A4 = A/Sing Mono IM, 15 ug (UA)
Treatments Part B: B1 = A/Sing MAP, 15 ug (FA) with biopsy; B2 = Placebo MAP (FA) with biopsy
B3 = A/Sing MAP, 15 ug (FA); B4 = A/Sing MAP, 10 ug (FA); B5 = A/Sing MAP, 5 ug (FA); B6 = A/Sing MAP, 2.5 ug (FA)
B7 = Placebo MAP (FA); B8 = A/Sing MAP, 15 ug (UA); B9 = A/Sing IM, 15 ug, as 2018 Afluria Quad (UA)

Listing 16.2.9.11
Individual Characterisation of Skin Irritation Index by Treatment
Erythema
Part B, Non-biopsy
B7= 3 x uncoated MAPs on volar forearm

			Active			Placebo		
Randomisation Number	Visit	Time Point	#> 0	Maximum	Average	#> 0	Maximum	Average
B0039	Day 1	1 hour	-	-	-	3	2	2.0
B0039	Day 1	2 hour	-	-	-	3	2	2.0
B0039	Day 2		-	-	-	3	2	2.0
B0039	Day 4		-	-	-	0	0	0.0
B0039	Day 8		-	-	-	0	0	0.0
B0039	Day 22		-	-	-	0	0	0.0
B0039	EOS/ET (Day 61)		-	-	-	0	0	0.0
B0046	Day 1	Pre-dose	-	-	-	0	0	0.0
B0046	Day 1	10 min	-	-	-	3	2	2.0
B0046	Day 1	1 hour	-	-	-	3	3	2.7
B0046	Day 1	2 hour	-	-	-	3	2	2.0
B0046	Day 2		-	-	-	3	2	2.0
B0046	Day 4		-	-	-	3	1	1.0
B0046	Day 8		-	-	-	0	0	0.0
B0046	Day 22		-	-	-	0	0	0.0
B0046	EOS/ET (Day 61)		-	-	-	0	0	0.0
B0059	Day 1	Pre-dose	-	-	-	0	0	0.0
B0059	Day 1	10 min	-	-	-	3	2	2.0
B0059	Day 1	1 hour	-	-	-	3	2	2.0

Source: Listing 16.2.9.4

Administration Site Key: FA = Forearm; UA= Upper arm
Treatments Part A: A1 = A/Sing MAP, 15 ug (FA); A2 = Afluria Quad IM (UA), 15 ug; A3 = Placebo MAP (FA); A4 = A/Sing Mono IM, 15 ug (UA)
Treatments Part B: B1 = A/Sing MAP, 15 ug (FA) with biopsy; B2 = Placebo MAP (FA) with biopsy
B3 = A/Sing MAP, 15 ug (FA); B4 = A/Sing MAP, 10 ug (FA); B5 = A/Sing MAP, 5 ug (FA); B6 = A/Sing MAP, 2.5 ug (FA)
B7 = Placebo MAP (FA); B8 = A/Sing MAP, 15 ug (UA); B9 = A/Sing IM, 15 ug, as 2018 Afluria Quad (UA)

Listing 16.2.9.11
Individual Characterisation of Skin Irritation Index by Treatment
Erythema
Part B, Non-biopsy
B7= 3 x uncoated MAPs on volar forearm

			Active			Placebo		
Randomisation Number	Visit	Time Point	#> 0	Maximum	Average	#> 0	Maximum	Average
B0059	Day 1	2 hour	-	-	-	3	3	2.3
B0059	Day 2		-	-	-	3	2	2.0
B0059	Day 4		-	-	-	0	0	0.0
B0059	Day 8		-	-	-	0	0	0.0
B0059	Day 22		-	-	-	0	0	0.0
B0059	EOS/ET (Day 61)		-	-	-	0	0	0.0
B0063	Day 1	Pre-dose	-	-	-	0	0	0.0
B0063	Day 1	10 min	-	-	-	3	2	2.0
B0063	Day 1	1 hour	-	-	-	3	2	2.0
B0063	Day 1	2 hour	-	-	-	3	2	2.0
B0063	Day 2		-	-	-	3	2	2.0
B0063	Day 4		-	-	-	3	2	2.0
B0063	Day 8		-	-	-	0	0	0.0
B0063	Day 22		-	-	-	0	0	0.0
B0063	EOS/ET (Day 61)		-	-	-	0	0	0.0
B0069	Day 1	Pre-dose	-	-	-	0	0	0.0
B0069	Day 1	10 min	-	-	-	3	2	2.0
B0069	Day 1	1 hour	-	-	-	3	2	2.0
B0069	Day 1	2 hour	-	-	-	3	2	2.0

Source: Listing 16.2.9.4

Administration Site Key: FA = Forearm; UA= Upper arm
Treatments Part A: A1 = A/Sing MAP, 15 ug (FA); A2 = Afluria Quad IM (UA), 15 ug; A3 = Placebo MAP (FA); A4 = A/Sing Mono IM, 15 ug (UA)
Treatments Part B: B1 = A/Sing MAP, 15 ug (FA) with biopsy; B2 = Placebo MAP (FA) with biopsy
B3 = A/Sing MAP, 15 ug (FA); B4 = A/Sing MAP, 10 ug (FA); B5 = A/Sing MAP, 5 ug (FA); B6 = A/Sing MAP, 2.5 ug (FA)
B7 = Placebo MAP (FA); B8 = A/Sing MAP, 15 ug (UA); B9 = A/Sing IM, 15 ug, as 2018 Afluria Quad (UA)

Listing 16.2.9.11
Individual Characterisation of Skin Irritation Index by Treatment
Erythema
Part B, Non-biopsy
B7= 3 x uncoated MAPs on volar forearm

			Active			Placebo		
Randomisation Number	Visit	Time Point	#> 0	Maximum	Average	#> 0	Maximum	Average
B0069	Day 2		-	-	-	3	2	2.0
B0069	Day 4		-	-	-	3	2	2.0
B0069	Day 8		-	-	-	0	0	0.0
B0069	Day 22		-	-	-	0	0	0.0
B0069	EOS/ET (Day 61)		-	-	-	0	0	0.0
B0087	Day 1	Pre-dose	-	-	-	0	0	0.0
B0087	Day 1	10 min	-	-	-	3	2	2.0
B0087	Day 1	1 hour	-	-	-	3	2	2.0
B0087	Day 1	2 hour	-	-	-	3	2	2.0
B0087	Day 2		-	-	-	3	2	2.0
B0087	Day 4		-	-	-	1	1	0.3
B0087	Day 8		-	-	-	0	0	0.0
B0087	Day 22		-	-	-	0	0	0.0
B0087	EOS/ET (Day 61)		-	-	-	0	0	0.0
B0093	Day 1	Pre-dose	-	-	-	0	0	0.0
B0093	Day 1	10 min	-	-	-	3	2	2.0
B0093	Day 1	1 hour	-	-	-	3	2	2.0
B0093	Day 1	2 hour	-	-	-	3	2	2.0
B0093	Day 2		-	-	-	3	2	2.0

Source: Listing 16.2.9.4

Administration Site Key: FA = Forearm; UA= Upper arm
Treatments Part A: A1 = A/Sing MAP, 15 ug (FA); A2 = Afluria Quad IM (UA), 15 ug; A3 = Placebo MAP (FA); A4 = A/Sing Mono IM, 15 ug (UA)
Treatments Part B: B1 = A/Sing MAP, 15 ug (FA) with biopsy; B2 = Placebo MAP (FA) with biopsy
B3 = A/Sing MAP, 15 ug (FA); B4 = A/Sing MAP, 10 ug (FA); B5 = A/Sing MAP, 5 ug (FA); B6 = A/Sing MAP, 2.5 ug (FA)
B7 = Placebo MAP (FA); B8 = A/Sing MAP, 15 ug (UA); B9 = A/Sing IM, 15 ug, as 2018 Afluria Quad (UA)

Listing 16.2.9.11
Individual Characterisation of Skin Irritation Index by Treatment
Erythema
Part B, Non-biopsy
B7= 3 x uncoated MAPs on volar forearm

Randomisation Number	Visit	Time Point	Active #> 0	Active Maximum	Active Average	Placebo #> 0	Placebo Maximum	Placebo Average
B0093	Day 4		-	-	-	0	0	0.0
B0093	Day 8		-	-	-	0	0	0.0
B0093	Day 22		-	-	-	0	0	0.0
B0093	EOS/ET (Day 61)		-	-	-	0	0	0.0
B0098	Day 1	Pre-dose	-	-	-	0	0	0.0
B0098	Day 1	10 min	-	-	-	3	2	2.0
B0098	Day 1	1 hour	-	-	-	3	2	2.0
B0098	Day 1	2 hour	-	-	-	3	2	2.0
B0098	Day 2		-	-	-	3	1	1.0
B0098	Day 4		-	-	-	0	0	0.0
B0098	Day 8		-	-	-	0	0	0.0
B0098	Day 22		-	-	-	0	0	0.0
B0098	EOS/ET (Day 61)		-	-	-	0	0	0.0
B0108	Day 1	Pre-dose	-	-	-	0	0	0.0
B0108	Day 1	10 min	-	-	-	3	1	1.0
B0108	Day 1	1 hour	-	-	-	3	2	2.0
B0108	Day 1	2 hour	-	-	-	3	2	1.7
B0108	Day 2		-	-	-	3	2	2.0
B0108	Day 4		-	-	-	0	0	0.0

Source: Listing 16.2.9.4

Administration Site Key: FA = Forearm; UA= Upper arm
Treatments Part A: A1 = A/Sing MAP, 15 ug (FA); A2 = Afluria Quad IM (UA), 15 ug; A3 = Placebo MAP (FA); A4 = A/Sing Mono IM, 15 ug (UA)
Treatments Part B: B1 = A/Sing MAP, 15 ug (FA) with biopsy; B2 = Placebo MAP (FA) with biopsy
B3 = A/Sing MAP, 15 ug (FA); B4 = A/Sing MAP, 10 ug (FA); B5 = A/Sing MAP, 5 ug (FA); B6 = A/Sing MAP, 2.5 ug (FA)
B7 = Placebo MAP (FA); B8 = A/Sing MAP, 15 ug (UA); B9 = A/Sing IM, 15 ug, as 2018 Afluria Quad (UA)

Listing 16.2.9.11
Individual Characterisation of Skin Irritation Index by Treatment
Erythema
Part B, Non-biopsy
B7= 3 x uncoated MAPs on volar forearm

			Active			Placebo		
Randomisation Number	Visit	Time Point	#> 0	Maximum	Average	#> 0	Maximum	Average
B0108	Day 8		-	-	-	0	0	0.0
B0108	Day 22		-	-	-	0	0	0.0
B0108	EOS/ET (Day 61)		-	-	-	0	0	0.0
B0114	Day 1	Pre-dose	-	-	-	0	0	0.0
B0114	Day 1	10 min	-	-	-	3	2	2.0
B0114	Day 1	1 hour	-	-	-	3	2	2.0
B0114	Day 1	2 hour	-	-	-	3	2	2.0
B0114	Day 2		-	-	-	3	2	2.0
B0114	Day 4		-	-	-	2	2	1.0
B0114	Day 8		-	-	-	3	2	1.3
B0114	Day 22		-	-	-	0	0	0.0
B0114	EOS/ET (Day 61)		-	-	-	0	0	0.0
B0116	Day 1	Pre-dose	-	-	-	0	0	0.0
B0116	Day 1	10 min	-	-	-	3	2	2.0
B0116	Day 1	1 hour	-	-	-	3	2	2.0
B0116	Day 1	2 hour	-	-	-	3	2	2.0
B0116	Day 2		-	-	-	3	2	2.0
B0116	Day 4		-	-	-	0	0	0.0
B0116	Day 8		-	-	-	0	0	0.0

Source: Listing 16.2.9.4

Administration Site Key: FA = Forearm; UA= Upper arm
Treatments Part A: A1 = A/Sing MAP, 15 ug (FA); A2 = Afluria Quad IM (UA), 15 ug; A3 = Placebo MAP (FA); A4 = A/Sing Mono IM, 15 ug (UA)
Treatments Part B: B1 = A/Sing MAP, 15 ug (FA) with biopsy; B2 = Placebo MAP (FA) with biopsy
B3 = A/Sing MAP, 15 ug (FA); B4 = A/Sing MAP, 10 ug (FA); B5 = A/Sing MAP, 5 ug (FA); B6 = A/Sing MAP, 2.5 ug (FA)
B7 = Placebo MAP (FA); B8 = A/Sing MAP, 15 ug (UA); B9 = A/Sing IM, 15 ug, as 2018 Afluria Quad (UA)

Listing 16.2.9.11
Individual Characterisation of Skin Irritation Index by Treatment
Erythema
Part B, Non-biopsy
B7= 3 x uncoated MAPs on volar forearm

Randomisation Number	Visit	Time Point	Active #> 0	Active Maximum	Active Average	Placebo #> 0	Placebo Maximum	Placebo Average
B0116	Day 22		-	-	-	0	0	0.0
B0116	EOS/ET (Day 61)		-	-	-	0	0	0.0
B0125	Day 1	Pre-dose	-	-	-	0	0	0.0
B0125	Day 1	10 min	-	-	-	3	2	2.0
B0125	Day 1	1 hour	-	-	-	3	2	2.0
B0125	Day 1	2 hour	-	-	-	3	2	2.0
B0125	Day 2		-	-	-	3	2	2.0
B0125	Day 4		-	-	-	0	0	0.0
B0125	Day 8		-	-	-	0	0	0.0
B0125	Day 22		-	-	-	0	0	0.0
B0125	EOS/ET (Day 61)		-	-	-	0	0	0.0
B0135	Day 1	Pre-dose	-	-	-	0	0	0.0
B0135	Day 1	10 min	-	-	-	3	2	2.0
B0135	Day 1	1 hour	-	-	-	3	3	2.3
B0135	Day 1	2 hour	-	-	-	3	2	1.7
B0135	Day 2		-	-	-	3	2	2.0
B0135	Day 4		-	-	-	0	0	0.0
B0135	Day 8		-	-	-	0	0	0.0
B0135	Day 22		-	-	-	0	0	0.0

Administration Site Key: FA = Forearm; UA= Upper arm
Treatments Part A: A1 = A/Sing MAP, 15 ug (FA); A2 = Afluria Quad IM (UA), 15 ug; A3 = Placebo MAP (FA); A4 = A/Sing Mono IM, 15 ug (UA)
Treatments Part B: B1 = A/Sing MAP, 15 ug (FA) with biopsy; B2 = Placebo MAP (FA) with biopsy
B3 = A/Sing MAP, 15 ug (FA); B4 = A/Sing MAP, 10 ug (FA); B5 = A/Sing MAP, 5 ug (FA); B6 = A/Sing MAP, 2.5 ug (FA)
B7 = Placebo MAP (FA); B8 = A/Sing MAP, 15 ug (UA); B9 = A/Sing IM, 15 ug, as 2018 Afluria Quad (UA)

Listing 16.2.9.11
Individual Characterisation of Skin Irritation Index by Treatment
Erythema
Part B, Non-biopsy
B7= 3 x uncoated MAPs on volar forearm

			Active			Placebo		
Randomisation Number	Visit	Time Point	#> 0	Maximum	Average	#> 0	Maximum	Average
B0135	EOS/ET (Day 61)		-	-	-	0	0	0.0
B0138	Day 1	Pre-dose	-	-	-	0	0	0.0
B0138	Day 1	10 min	-	-	-	3	2	2.0
B0138	Day 1	1 hour	-	-	-	3	2	2.0
B0138	Day 1	2 hour	-	-	-	3	2	2.0
B0138	Day 2		-	-	-	3	2	2.0
B0138	Day 4		-	-	-	0	0	0.0
B0138	Day 8		-	-	-	0	0	0.0
B0138	Day 22		-	-	-	0	0	0.0
B0138	EOS/ET (Day 61)		-	-	-	0	0	0.0
B0146	Day 1	Pre-dose	-	-	-	0	0	0.0
B0146	Day 1	10 min	-	-	-	3	1	1.0
B0146	Day 1	1 hour	-	-	-	3	2	2.0
B0146	Day 1	2 hour	-	-	-	3	2	2.0
B0146	Day 2		-	-	-	3	1	1.0
B0146	Day 4		-	-	-	3	1	1.0
B0146	Day 8		-	-	-	0	0	0.0
B0146	Day 22		-	-	-	0	0	0.0
B0146	EOS/ET (Day 61)		-	-	-	0	0	0.0

Source: Listing 16.2.9.4

Administration Site Key: FA = Forearm; UA= Upper arm
Treatments Part A: A1 = A/Sing MAP, 15 ug (FA); A2 = Afluria Quad IM (UA), 15 ug; A3 = Placebo MAP (FA); A4 = A/Sing Mono IM, 15 ug (UA)
Treatments Part B: B1 = A/Sing MAP, 15 ug (FA) with biopsy; B2 = Placebo MAP (FA) with biopsy
B3 = A/Sing MAP, 15 ug (FA); B4 = A/Sing MAP, 10 ug (FA); B5 = A/Sing MAP, 5 ug (FA); B6 = A/Sing MAP, 2.5 ug (FA)
B7 = Placebo MAP (FA); B8 = A/Sing MAP, 15 ug (UA); B9 = A/Sing IM, 15 ug, as 2018 Afluria Quad (UA)

Listing 16.2.9.11
Individual Characterisation of Skin Irritation Index by Treatment
Erythema
Part B, Non-biopsy
B7= 3 x uncoated MAPs on volar forearm

Randomisation Number	Visit	Time Point	Active #> 0	Active Maximum	Active Average	Placebo #> 0	Placebo Maximum	Placebo Average
B1080	Day 1	Pre-dose	-	-	-	0	0	0.0
B1080	Day 1	10 min	-	-	-	3	2	2.0
B1080	Day 1	1 hour	-	-	-	3	2	2.0
B1080	Day 1	2 hour	-	-	-	3	2	2.0
B1080	Day 2		-	-	-	3	2	2.0
B1080	Day 4		-	-	-	0	0	0.0
B1080	Day 8		-	-	-	0	0	0.0
B1080	Day 22		-	-	-	0	0	0.0
B1080	EOS/ET (Day 61)		-	-	-	0	0	0.0

Source: Listing 16.2.9.4

Administration Site Key: FA = Forearm; UA= Upper arm
Treatments Part A: A1 = A/Sing MAP, 15 ug (FA); A2 = Afluria Quad IM (UA), 15 ug; A3 = Placebo MAP (FA); A4 = A/Sing Mono IM, 15 ug (UA)
Treatments Part B: B1 = A/Sing MAP, 15 ug (FA) with biopsy; B2 = Placebo MAP (FA) with biopsy
B3 = A/Sing MAP, 15 ug (FA); B4 = A/Sing MAP, 10 ug (FA); B5 = A/Sing MAP, 5 ug (FA); B6 = A/Sing MAP, 2.5 ug (FA)
B7 = Placebo MAP (FA); B8 = A/Sing MAP, 15 ug (UA); B9 = A/Sing IM, 15 ug, as 2018 Afluria Quad (UA)

Listing 16.2.9.11
Individual Characterisation of Skin Irritation Index by Treatment
Erythema
Part B, Non-biopsy
B8= 3 x 5 ug A/Sing MAPs on upper arm (15 ug)

			Active			Placebo		
Randomisation Number	Visit	Time Point	#> 0	Maximum	Average	#> 0	Maximum	Average
B0011	Day 1	Pre-dose	0	0	0.0	-	-	-
B0011	Day 1	10 min	3	2	2.0	-	-	-
B0011	Day 1	1 hour	3	2	2.0	-	-	-
B0011	Day 1	2 hour	3	2	2.0	-	-	-
B0011	Day 2		3	3	3.0	-	-	-
B0011	Day 4		3	3	3.0	-	-	-
B0011	Day 8		3	1	1.0	-	-	-
B0011	Day 22		0	0	0.0	-	-	-
B0011	EOS/ET (Day 61)		0	0	0.0	-	-	-
B0025	Day 1	Pre-dose	0	0	0.0	-	-	-
B0025	Day 1	10 min	3	2	2.0	-	-	-
B0025	Day 1	1 hour	3	1	1.0	-	-	-
B0025	Day 1	2 hour	3	2	2.0	-	-	-
B0025	Day 2		3	3	3.0	-	-	-
B0025	Day 4		3	2	2.0	-	-	-
B0025	Day 8		3	2	2.0	-	-	-
B0025	Day 22		0	0	0.0	-	-	-
B0025	EOS/ET (Day 61)		0	0	0.0	-	-	-
B0034	Day 1	Pre-dose	0	0	0.0	-	-	-

Source: Listing 16.2.9.4

Administration Site Key: FA = Forearm; UA= Upper arm
Treatments Part A: A1 = A/Sing MAP, 15 ug (FA); A2 = Afluria Quad IM (UA), 15 ug; A3 = Placebo MAP (FA); A4 = A/Sing Mono IM, 15 ug (UA)
Treatments Part B: B1 = A/Sing MAP, 15 ug (FA) with biopsy; B2 = Placebo MAP (FA) with biopsy
B3 = A/Sing MAP, 15 ug (FA); B4 = A/Sing MAP, 10 ug (FA); B5 = A/Sing MAP, 5 ug (FA); B6 = A/Sing MAP, 2.5 ug (FA)
B7 = Placebo MAP (FA); B8 = A/Sing MAP, 15 ug (UA); B9 = A/Sing IM, 15 ug, as 2018 Afluria Quad (UA)

Listing 16.2.9.11
Individual Characterisation of Skin Irritation Index by Treatment
Erythema
Part B, Non-biopsy
B8= 3 x 5 ug A/Sing MAPs on upper arm (15 ug)

			Active			Placebo		
Randomisation Number	Visit	Time Point	#> 0	Maximum	Average	#> 0	Maximum	Average
B0034	Day 1	10 min	3	2	2.0	-	-	-
B0034	Day 1	1 hour	3	2	2.0	-	-	-
B0034	Day 1	2 hour	3	2	2.0	-	-	-
B0034	Day 2		3	3	2.3	-	-	-
B0034	Day 4		3	3	3.0	-	-	-
B0034	Day 8		0	0	0.0	-	-	-
B0034	Day 22		0	0	0.0	-	-	-
B0034	EOS/ET (Day 61)		0	0	0.0	-	-	-
B0043	Day 1	Pre-dose	0	0	0.0	-	-	-
B0043	Day 1	10 min	3	2	2.0	-	-	-
B0043	Day 1	1 hour	3	2	2.0	-	-	-
B0043	Day 1	2 hour	3	2	2.0	-	-	-
B0043	Day 2		3	2	2.0	-	-	-
B0043	Day 4		3	2	2.0	-	-	-
B0043	Day 8		3	2	2.0	-	-	-
B0043	Day 22		3	1	1.0	-	-	-
B0043	EOS/ET (Day 61)		0	0	0.0	-	-	-
B0050	Day 1	Pre-dose	0	0	0.0	-	-	-
B0050	Day 1	10 min	3	1	1.0	-	-	-

Source: Listing 16.2.9.4

Administration Site Key: FA = Forearm; UA= Upper arm
Treatments Part A: A1 = A/Sing MAP, 15 ug (FA); A2 = Afluria Quad IM (UA), 15 ug; A3 = Placebo MAP (FA); A4 = A/Sing Mono IM, 15 ug (UA)
Treatments Part B: B1 = A/Sing MAP, 15 ug (FA) with biopsy; B2 = Placebo MAP (FA) with biopsy
B3 = A/Sing MAP, 15 ug (FA); B4 = A/Sing MAP, 10 ug (FA); B5 = A/Sing MAP, 5 ug (FA); B6 = A/Sing MAP, 2.5 ug (FA)
B7 = Placebo MAP (FA); B8 = A/Sing MAP, 15 ug (UA); B9 = A/Sing IM, 15 ug, as 2018 Afluria Quad (UA)

Listing 16.2.9.11
Individual Characterisation of Skin Irritation Index by Treatment
Erythema
Part B, Non-biopsy
B8= 3 x 5 ug A/Sing MAPs on upper arm (15 ug)

			Active			Placebo		
Randomisation Number	Visit	Time Point	#> 0	Maximum	Average	#> 0	Maximum	Average
B0050	Day 1	1 hour	3	2	2.0	-	-	-
B0050	Day 1	2 hour	3	2	2.0	-	-	-
B0050	Day 2		3	3	3.0	-	-	-
B0050	Day 4		3	3	2.3	-	-	-
B0050	Day 8		3	2	2.0	-	-	-
B0050	Day 22		0	0	0.0	-	-	-
B0050	EOS/ET (Day 61)		0	0	0.0	-	-	-
B0055	Day 1	Pre-dose	0	0	0.0	-	-	-
B0055	Day 1	10 min	3	2	2.0	-	-	-
B0055	Day 1	1 hour	3	2	2.0	-	-	-
B0055	Day 1	2 hour	3	2	2.0	-	-	-
B0055	Day 2		3	2	2.0	-	-	-
B0055	Day 4		3	2	1.3	-	-	-
B0055	Day 8		3	2	1.7	-	-	-
B0055	Day 22		0	0	0.0	-	-	-
B0055	EOS/ET (Day 61)		0	0	0.0	-	-	-
B0061	Day 1	Pre-dose	0	0	0.0	-	-	-
B0061	Day 1	10 min	3	2	2.0	-	-	-
B0061	Day 1	1 hour	3	2	2.0	-	-	-

Source: Listing 16.2.9.4

Administration Site Key: FA = Forearm; UA= Upper arm
Treatments Part A: A1 = A/Sing MAP, 15 ug (FA); A2 = Afluria Quad IM (UA), 15 ug; A3 = Placebo MAP (FA); A4 = A/Sing Mono IM, 15 ug (UA)
Treatments Part B: B1 = A/Sing MAP, 15 ug (FA) with biopsy; B2 = Placebo MAP (FA) with biopsy
B3 = A/Sing MAP, 15 ug (FA); B4 = A/Sing MAP, 10 ug (FA); B5 = A/Sing MAP, 5 ug (FA); B6 = A/Sing MAP, 2.5 ug (FA)
B7 = Placebo MAP (FA); B8 = A/Sing MAP, 15 ug (UA); B9 = A/Sing IM, 15 ug, as 2018 Afluria Quad (UA)

Listing 16.2.9.11
Individual Characterisation of Skin Irritation Index by Treatment
Erythema
Part B, Non-biopsy
B8= 3 x 5 ug A/Sing MAPs on upper arm (15 ug)

			Active			Placebo		
Randomisation Number	Visit	Time Point	#> 0	Maximum	Average	#> 0	Maximum	Average
B0061	Day 1	2 hour	3	2	2.0	-	-	-
B0061	Day 2		3	2	2.0	-	-	-
B0061	Day 4		3	3	2.7	-	-	-
B0061	Day 8		3	2	2.0	-	-	-
B0061	Day 22		0	0	0.0	-	-	-
B0061	EOS/ET (Day 61)		0	0	0.0	-	-	-
B0067	Day 1	Pre-dose	0	0	0.0	-	-	-
B0067	Day 1	10 min	3	2	2.0	-	-	-
B0067	Day 1	1 hour	3	2	2.0	-	-	-
B0067	Day 1	2 hour	3	2	2.0	-	-	-
B0067	Day 2		3	3	3.0	-	-	-
B0067	Day 4		3	2	2.0	-	-	-
B0067	Day 8		0	0	0.0	-	-	-
B0067	Day 22		0	0	0.0	-	-	-
B0067	EOS/ET (Day 61)		0	0	0.0	-	-	-
B0078	Day 1	Pre-dose	0	0	0.0	-	-	-
B0078	Day 1	10 min	3	2	2.0	-	-	-
B0078	Day 1	1 hour	3	1	1.0	-	-	-
B0078	Day 1	2 hour	3	2	2.0	-	-	-

Source: Listing 16.2.9.4

Administration Site Key: FA = Forearm; UA= Upper arm
Treatments Part A: A1 = A/Sing MAP, 15 ug (FA); A2 = Afluria Quad IM (UA), 15 ug; A3 = Placebo MAP (FA); A4 = A/Sing Mono IM, 15 ug (UA)
Treatments Part B: B1 = A/Sing MAP, 15 ug (FA) with biopsy; B2 = Placebo MAP (FA) with biopsy
B3 = A/Sing MAP, 15 ug (FA); B4 = A/Sing MAP, 10 ug (FA); B5 = A/Sing MAP, 5 ug (FA); B6 = A/Sing MAP, 2.5 ug (FA)
B7 = Placebo MAP (FA); B8 = A/Sing MAP, 15 ug (UA); B9 = A/Sing IM, 15 ug, as 2018 Afluria Quad (UA)

Listing 16.2.9.11
Individual Characterisation of Skin Irritation Index by Treatment
Erythema
Part B, Non-biopsy
B8= 3 x 5 ug A/Sing MAPs on upper arm (15 ug)

			Active			Placebo		
Randomisation Number	Visit	Time Point	#> 0	Maximum	Average	#> 0	Maximum	Average
B0078	Day 2		3	3	3.0	-	-	-
B0078	Day 4		3	2	2.0	-	-	-
B0078	Day 8		2	1	0.7	-	-	-
B0078	Day 22		0	0	0.0	-	-	-
B0078	EOS/ET (Day 61)		0	0	0.0	-	-	-
B0083	Day 1	Pre-dose	0	0	0.0	-	-	-
B0083	Day 1	10 min	3	2	2.0	-	-	-
B0083	Day 1	1 hour	3	1	1.0	-	-	-
B0083	Day 1	2 hour	3	2	2.0	-	-	-
B0083	Day 2		3	3	3.0	-	-	-
B0083	Day 4		3	2	2.0	-	-	-
B0083	Day 8		0	0	0.0	-	-	-
B0083	Day 22		0	0	0.0	-	-	-
B0083	EOS/ET (Day 61)		0	0	0.0	-	-	-
B0091	Day 1	Pre-dose	0	0	0.0	-	-	-
B0091	Day 1	10 min	3	2	2.0	-	-	-
B0091	Day 1	1 hour	3	2	2.0	-	-	-
B0091	Day 1	2 hour	3	2	2.0	-	-	-
B0091	Day 2		3	2	2.0	-	-	-

Source: Listing 16.2.9.4

Administration Site Key: FA = Forearm; UA= Upper arm
Treatments Part A: A1 = A/Sing MAP, 15 ug (FA); A2 = Afluria Quad IM (UA), 15 ug; A3 = Placebo MAP (FA); A4 = A/Sing Mono IM, 15 ug (UA)
Treatments Part B: B1 = A/Sing MAP, 15 ug (FA) with biopsy; B2 = Placebo MAP (FA) with biopsy
B3 = A/Sing MAP, 15 ug (FA); B4 = A/Sing MAP, 10 ug (FA); B5 = A/Sing MAP, 5 ug (FA); B6 = A/Sing MAP, 2.5 ug (FA)
B7 = Placebo MAP (FA); B8 = A/Sing MAP, 15 ug (UA); B9 = A/Sing IM, 15 ug, as 2018 Afluria Quad (UA)

Listing 16.2.9.11
Individual Characterisation of Skin Irritation Index by Treatment
Erythema
Part B, Non-biopsy
B8= 3 x 5 ug A/Sing MAPs on upper arm (15 ug)

			Active			Placebo		
Randomisation Number	Visit	Time Point	#> 0	Maximum	Average	#> 0	Maximum	Average
B0091	Day 4		3	3	3.0	-	-	-
B0091	Day 8		0	0	0.0	-	-	-
B0091	Day 22		0	0	0.0	-	-	-
B0091	EOS/ET (Day 61)		0	0	0.0	-	-	-
B0100	Day 1	Pre-dose	0	0	0.0	-	-	-
B0100	Day 1	10 min	3	2	2.0	-	-	-
B0100	Day 1	1 hour	3	2	2.0	-	-	-
B0100	Day 1	2 hour	3	2	2.0	-	-	-
B0100	Day 2		3	3	3.0	-	-	-
B0100	Day 4		3	3	2.3	-	-	-
B0100	Day 8		3	2	1.3	-	-	-
B0100	Day 22		0	0	0.0	-	-	-
B0100	EOS/ET (Day 61)		0	0	0.0	-	-	-
B0102	Day 1	Pre-dose	0	0	0.0	-	-	-
B0102	Day 1	10 min	3	2	2.0	-	-	-
B0102	Day 1	1 hour	3	1	1.0	-	-	-
B0102	Day 1	2 hour	3	2	2.0	-	-	-
B0102	Day 2		3	2	2.0	-	-	-
B0102	Day 4		3	3	3.0	-	-	-

Administration Site Key: FA = Forearm; UA= Upper arm
Treatments Part A: A1 = A/Sing MAP, 15 ug (FA); A2 = Afluria Quad IM (UA), 15 ug; A3 = Placebo MAP (FA); A4 = A/Sing Mono IM, 15 ug (UA)
Treatments Part B: B1 = A/Sing MAP, 15 ug (FA) with biopsy; B2 = Placebo MAP (FA) with biopsy
B3 = A/Sing MAP, 15 ug (FA); B4 = A/Sing MAP, 10 ug (FA); B5 = A/Sing MAP, 5 ug (FA); B6 = A/Sing MAP, 2.5 ug (FA)
B7 = Placebo MAP (FA); B8 = A/Sing MAP, 15 ug (UA); B9 = A/Sing IM, 15 ug, as 2018 Afluria Quad (UA)

Listing 16.2.9.11
Individual Characterisation of Skin Irritation Index by Treatment
Erythema
Part B, Non-biopsy
B8= 3 x 5 ug A/Sing MAPs on upper arm (15 ug)

			Active			Placebo		
Randomisation Number	Visit	Time Point	#> 0	Maximum	Average	#> 0	Maximum	Average
B0102	Day 8		3	2	2.0	-	-	-
B0102	Day 22		0	0	0.0	-	-	-
B0102	EOS/ET (Day 61)		0	0	0.0	-	-	-
B0111	Day 1	Pre-dose	0	0	0.0	-	-	-
B0111	Day 1	10 min	3	2	2.0	-	-	-
B0111	Day 1	1 hour	3	2	2.0	-	-	-
B0111	Day 1	2 hour	3	1	1.0	-	-	-
B0111	Day 2		3	3	3.0	-	-	-
B0111	Day 4		3	2	2.0	-	-	-
B0111	Day 8		3	2	2.0	-	-	-
B0111	Day 22		0	0	0.0	-	-	-
B0111	EOS/ET (Day 61)		0	0	0.0	-	-	-
B0121	Day 1	Pre-dose	0	0	0.0	-	-	-
B0121	Day 1	10 min	3	2	2.0	-	-	-
B0121	Day 1	1 hour	3	2	2.0	-	-	-
B0121	Day 1	2 hour	3	2	2.0	-	-	-
B0121	Day 2		3	2	2.0	-	-	-
B0121	Day 4		3	3	3.0	-	-	-
B0121	Day 8		3	2	1.7	-	-	-

Source: Listing 16.2.9.4

Administration Site Key: FA = Forearm; UA= Upper arm
Treatments Part A: A1 = A/Sing MAP, 15 ug (FA); A2 = Afluria Quad IM (UA), 15 ug; A3 = Placebo MAP (FA); A4 = A/Sing Mono IM, 15 ug (UA)
Treatments Part B: B1 = A/Sing MAP, 15 ug (FA) with biopsy; B2 = Placebo MAP (FA) with biopsy
B3 = A/Sing MAP, 15 ug (FA); B4 = A/Sing MAP, 10 ug (FA); B5 = A/Sing MAP, 5 ug (FA); B6 = A/Sing MAP, 2.5 ug (FA)
B7 = Placebo MAP (FA); B8 = A/Sing MAP, 15 ug (UA); B9 = A/Sing IM, 15 ug, as 2018 Afluria Quad (UA)

Listing 16.2.9.11
Individual Characterisation of Skin Irritation Index by Treatment
Erythema
Part B, Non-biopsy
B8= 3 x 5 ug A/Sing MAPs on upper arm (15 ug)

Randomisation Number	Visit	Time Point	Active #> 0	Active Maximum	Active Average	Placebo #> 0	Placebo Maximum	Placebo Average
B0121	Day 22		3	1	1.0	-	-	-
B0121	EOS/ET (Day 61)		0	0	0.0	-	-	-
B0129	Day 1	Pre-dose	0	0	0.0	-	-	-
B0129	Day 1	10 min	3	2	2.0	-	-	-
B0129	Day 1	1 hour	3	2	2.0	-	-	-
B0129	Day 1	2 hour	3	2	2.0	-	-	-
B0129	Day 2		3	3	3.0	-	-	-
B0129	Day 4		3	2	2.0	-	-	-
B0129	Day 8		3	2	2.0	-	-	-
B0129	Day 22		0	0	0.0	-	-	-
B0129	EOS/ET (Day 61)		0	0	0.0	-	-	-
B0131	Day 1	Pre-dose	0	0	0.0	-	-	-
B0131	Day 1	10 min	3	2	2.0	-	-	-
B0131	Day 1	1 hour	3	2	2.0	-	-	-
B0131	Day 1	2 hour	3	2	1.3	-	-	-
B0131	Day 2		3	2	2.0	-	-	-
B0131	Day 4		3	2	2.0	-	-	-
B0131	Day 8		3	1	1.0	-	-	-
B0131	Day 22		0	0	0.0	-	-	-

Source: Listing 16.2.9.4

Administration Site Key: FA = Forearm; UA= Upper arm
Treatments Part A: A1 = A/Sing MAP, 15 ug (FA); A2 = Afluria Quad IM (UA), 15 ug; A3 = Placebo MAP (FA); A4 = A/Sing Mono IM, 15 ug (UA)
Treatments Part B: B1 = A/Sing MAP, 15 ug (FA) with biopsy; B2 = Placebo MAP (FA) with biopsy
B3 = A/Sing MAP, 15 ug (FA); B4 = A/Sing MAP, 10 ug (FA); B5 = A/Sing MAP, 5 ug (FA); B6 = A/Sing MAP, 2.5 ug (FA)
B7 = Placebo MAP (FA); B8 = A/Sing MAP, 15 ug (UA); B9 = A/Sing IM, 15 ug, as 2018 Afluria Quad (UA)

Listing 16.2.9.11
Individual Characterisation of Skin Irritation Index by Treatment
Erythema
Part B, Non-biopsy
B8= 3 x 5 ug A/Sing MAPs on upper arm (15 ug)

			Active			Placebo		
Randomisation Number	Visit	Time Point	#> 0	Maximum	Average	#> 0	Maximum	Average
B0131	EOS/ET (Day 61)		0	0	0.0	-	-	-
B0137	Day 1	Pre-dose	0	0	0.0	-	-	-
B0137	Day 1	10 min	3	2	2.0	-	-	-
B0137	Day 1	1 hour	3	2	2.0	-	-	-
B0137	Day 1	2 hour	3	2	2.0	-	-	-
B0137	Day 2		3	2	2.0	-	-	-
B0137	Day 4		3	3	3.0	-	-	-
B0137	Day 8		3	2	2.0	-	-	-
B0137	Day 22		0	0	0.0	-	-	-
B0137	EOS/ET (Day 61)		0	0	0.0	-	-	-
B0149	Day 1	Pre-dose	0	0	0.0	-	-	-
B0149	Day 1	10 min	3	2	2.0	-	-	-
B0149	Day 1	1 hour	3	2	2.0	-	-	-
B0149	Day 1	2 hour	3	2	2.0	-	-	-
B0149	Day 2		3	2	2.0	-	-	-
B0149	Day 4		3	2	2.0	-	-	-
B0149	Day 8		3	2	2.0	-	-	-
B0149	Day 22		0	0	0.0	-	-	-
B0149	EOS/ET (Day 61)		0	0	0.0	-	-	-

Administration Site Key: FA = Forearm; UA= Upper arm
Treatments Part A: A1 = A/Sing MAP, 15 ug (FA); A2 = Afluria Quad IM (UA), 15 ug; A3 = Placebo MAP (FA); A4 = A/Sing Mono IM, 15 ug (UA)
Treatments Part B: B1 = A/Sing MAP, 15 ug (FA) with biopsy; B2 = Placebo MAP (FA) with biopsy
B3 = A/Sing MAP, 15 ug (FA); B4 = A/Sing MAP, 10 ug (FA); B5 = A/Sing MAP, 5 ug (FA); B6 = A/Sing MAP, 2.5 ug (FA)
B7 = Placebo MAP (FA); B8 = A/Sing MAP, 15 ug (UA); B9 = A/Sing IM, 15 ug, as 2018 Afluria Quad (UA)

Listing 16.2.9.11
Individual Characterisation of Skin Irritation Index by Treatment
Erythema
Part B, Non-biopsy
B8= 3 x 5 ug A/Sing MAPs on upper arm (15 ug)

Randomisation Number	Visit	Time Point	Active #> 0	Active Maximum	Active Average	Placebo #> 0	Placebo Maximum	Placebo Average
B1019	Day 1	Pre-dose	0	0	0.0	-	-	-
B1019	Day 1	10 min	0	0	0.0	-	-	-
B1019	Day 1	1 hour	0	0	0.0	-	-	-
B1019	Day 1	2 hour	3	1	1.0	-	-	-
B1019	Day 2		3	1	1.0	-	-	-
B1019	Day 4		0	0	0.0	-	-	-
B1019	Day 8		0	0	0.0	-	-	-
B1019	Day 22		0	0	0.0	-	-	-
B1019	EOS/ET (Day 61)		0	0	0.0	-	-	-

Source: Listing 16.2.9.4

Administration Site Key: FA = Forearm; UA= Upper arm
Treatments Part A: A1 = A/Sing MAP, 15 ug (FA); A2 = Afluria Quad IM (UA), 15 ug; A3 = Placebo MAP (FA); A4 = A/Sing Mono IM, 15 ug (UA)
Treatments Part B: B1 = A/Sing MAP, 15 ug (FA) with biopsy; B2 = Placebo MAP (FA) with biopsy
B3 = A/Sing MAP, 15 ug (FA); B4 = A/Sing MAP, 10 ug (FA); B5 = A/Sing MAP, 5 ug (FA); B6 = A/Sing MAP, 2.5 ug (FA)
B7 = Placebo MAP (FA); B8 = A/Sing MAP, 15 ug (UA); B9 = A/Sing IM, 15 ug, as 2018 Afluria Quad (UA)

Listing 16.2.9.11
Individual Characterisation of Skin Irritation Index by Treatment
Oedema
Part A
A1= 3 x 5 ug A/Sing MAPs on volar forearm (15 ug)

Randomisation Number	Visit	Time Point	Active #> 0	Active Maximum	Active Average	Placebo #> 0	Placebo Maximum	Placebo Average
A0002	Day 4		3	2	2.0	-	-	-
A0002	Day 8		0	0	0.0	-	-	-
A0002	Day 22		0	0	0.0	-	-	-
A0002	EOS/ET (Day 61)		0	0	0.0	-	-	-
A0003	Day 4		3	2	1.7	-	-	-
A0003	Day 8		0	0	0.0	-	-	-
A0003	Day 22		0	0	0.0	-	-	-
A0003	EOS/ET (Day 61)		0	0	0.0	-	-	-
A0005	Day 4		3	3	3.0	-	-	-
A0005	Day 8		0	0	0.0	-	-	-
A0005	Day 22		0	0	0.0	-	-	-
A0005	EOS/ET (Day 61)		0	0	0.0	-	-	-
A0007	Day 4		0	0	0.0	-	-	-
A0007	Day 8		0	0	0.0	-	-	-
A0007	Day 22		0	0	0.0	-	-	-
A0007	EOS/ET (Day 61)		0	0	0.0	-	-	-
A0009	Day 4		1	2	0.7	-	-	-

Source: Listing 16.2.9.4

Administration Site Key: FA = Forearm; UA= Upper arm
Treatments Part A: A1 = A/Sing MAP, 15 ug (FA); A2 = Afluria Quad IM (UA), 15 ug; A3 = Placebo MAP (FA); A4 = A/Sing Mono IM, 15 ug (UA)
Treatments Part B: B1 = A/Sing MAP, 15 ug (FA) with biopsy; B2 = Placebo MAP (FA) with biopsy
B3 = A/Sing MAP, 15 ug (FA); B4 = A/Sing MAP, 10 ug (FA); B5 = A/Sing MAP, 5 ug (FA); B6 = A/Sing MAP, 2.5 ug (FA)
B7 = Placebo MAP (FA); B8 = A/Sing MAP, 15 ug (UA); B9 = A/Sing IM, 15 ug, as 2018 Afluria Quad (UA)

Listing 16.2.9.11
Individual Characterisation of Skin Irritation Index by Treatment
Oedema
Part A
A1= 3 x 5 ug A/Sing MAPs on volar forearm (15 ug)

			Active			Placebo		
Randomisation Number	Visit	Time Point	#> 0	Maximum	Average	#> 0	Maximum	Average
A0009	Day 8		0	0	0.0	-	-	-
A0009	Day 22		0	0	0.0	-	-	-
A0009	EOS/ET (Day 61)		0	0	0.0	-	-	-
A0012	Day 4		3	2	2.0	-	-	-
A0012	Day 8		0	0	0.0	-	-	-
A0012	Day 22		0	0	0.0	-	-	-
A0012	EOS/ET (Day 61)		0	0	0.0	-	-	-
A0013	Day 4		3	2	1.7	-	-	-
A0013	Day 8		0	0	0.0	-	-	-
A0013	Day 22		0	0	0.0	-	-	-
A0013	EOS/ET (Day 61)		0	0	0.0	-	-	-
A0015	Day 4		1	1	0.3	-	-	-
A0015	Day 8		0	0	0.0	-	-	-
A0015	Day 22		0	0	0.0	-	-	-
A0015	EOS/ET (Day 61)		0	0	0.0	-	-	-
A0018	Day 1	Pre-dose	-	-	-	-	-	-
A0018	Day 1	10 min	-	-	-	-	-	-

Source: Listing 16.2.9.4

Administration Site Key: FA = Forearm; UA= Upper arm
Treatments Part A: A1 = A/Sing MAP, 15 ug (FA); A2 = Afluria Quad IM (UA), 15 ug; A3 = Placebo MAP (FA); A4 = A/Sing Mono IM, 15 ug (UA)
Treatments Part B: B1 = A/Sing MAP, 15 ug (FA) with biopsy; B2 = Placebo MAP (FA) with biopsy
B3 = A/Sing MAP, 15 ug (FA); B4 = A/Sing MAP, 10 ug (FA); B5 = A/Sing MAP, 5 ug (FA); B6 = A/Sing MAP, 2.5 ug (FA)
B7 = Placebo MAP (FA); B8 = A/Sing MAP, 15 ug (UA); B9 = A/Sing IM, 15 ug, as 2018 Afluria Quad (UA)

Listing 16.2.9.11
Individual Characterisation of Skin Irritation Index by Treatment
Oedema
Part A
A1= 3 x 5 ug A/Sing MAPs on volar forearm (15 ug)

			Active			Placebo		
Randomisation Number	Visit	Time Point	#> 0	Maximum	Average	#> 0	Maximum	Average
A0018	Day 1	1 hour	-	-	-	-	-	-
A0018	Day 1	2 hour	0	0	0.0	-	-	-
A0018	Day 4		0	0	0.0	-	-	-
A0018	Day 8		0	0	0.0	-	-	-
A0018	Day 22		0	0	0.0	-	-	-
A0018	EOS/ET (Day 61)		0	0	0.0	-	-	-
A0020	Day 1	Pre-dose	-	-	-	-	-	-
A0020	Day 1	10 min	-	-	-	-	-	-
A0020	Day 1	1 hour	-	-	-	-	-	-
A0020	Day 1	2 hour	3	2	1.7	-	-	-
A0020	Day 4		0	0	0.0	-	-	-
A0020	Day 8		0	0	0.0	-	-	-
A0020	Day 22		0	0	0.0	-	-	-
A0020	EOS/ET (Day 61)		0	0	0.0	-	-	-
A0021	Day 1	Pre-dose	-	-	-	-	-	-
A0021	Day 1	10 min	-	-	-	-	-	-
A0021	Day 1	1 hour	3	3	2.7	-	-	-
A0021	Day 1	2 hour	3	2	1.7	-	-	-
A0021	Day 4		0	0	0.0	-	-	-

Source: Listing 16.2.9.4

Administration Site Key: FA = Forearm; UA= Upper arm
Treatments Part A: A1 = A/Sing MAP, 15 ug (FA); A2 = Afluria Quad IM (UA), 15 ug; A3 = Placebo MAP (FA); A4 = A/Sing Mono IM, 15 ug (UA)
Treatments Part B: B1 = A/Sing MAP, 15 ug (FA) with biopsy; B2 = Placebo MAP (FA) with biopsy
B3 = A/Sing MAP, 15 ug (FA); B4 = A/Sing MAP, 10 ug (FA); B5 = A/Sing MAP, 5 ug (FA); B6 = A/Sing MAP, 2.5 ug (FA)
B7 = Placebo MAP (FA); B8 = A/Sing MAP, 15 ug (UA); B9 = A/Sing IM, 15 ug, as 2018 Afluria Quad (UA)

Listing 16.2.9.11
Individual Characterisation of Skin Irritation Index by Treatment
Oedema
Part A
A1= 3 x 5 ug A/Sing MAPs on volar forearm (15 ug)

			Active			Placebo		
Randomisation Number	Visit	Time Point	#> 0	Maximum	Average	#> 0	Maximum	Average
A0021	Day 8		3	2	2.0	-	-	-
A0021	Day 22		0	0	0.0	-	-	-
A0021	EOS/ET (Day 61)		0	0	0.0	-	-	-
A0024	Day 1	Pre-dose	-	-	-	-	-	-
A0024	Day 1	10 min	-	-	-	-	-	-
A0024	Day 1	1 hour	3	2	2.0	-	-	-
A0024	Day 1	2 hour	3	2	2.0	-	-	-
A0024	Day 4		0	0	0.0	-	-	-
A0024	Day 8		0	0	0.0	-	-	-
A0024	Day 22		0	0	0.0	-	-	-
A0024	EOS/ET (Day 61)		0	0	0.0	-	-	-
A0028	Day 1	Pre-dose	-	-	-	-	-	-
A0028	Day 1	10 min	-	-	-	-	-	-
A0028	Day 1	1 hour	3	2	2.0	-	-	-
A0028	Day 1	2 hour	3	2	1.7	-	-	-
A0028	Day 4		0	0	0.0	-	-	-
A0028	Day 8		3	2	2.0	-	-	-
A0028	Day 22		0	0	0.0	-	-	-
A0028	EOS/ET (Day 61)		0	0	0.0	-	-	-

Source: Listing 16.2.9.4

Administration Site Key: FA = Forearm; UA= Upper arm
Treatments Part A: A1 = A/Sing MAP, 15 ug (FA); A2 = Afluria Quad IM (UA), 15 ug; A3 = Placebo MAP (FA); A4 = A/Sing Mono IM, 15 ug (UA)
Treatments Part B: B1 = A/Sing MAP, 15 ug (FA) with biopsy; B2 = Placebo MAP (FA) with biopsy
B3 = A/Sing MAP, 15 ug (FA); B4 = A/Sing MAP, 10 ug (FA); B5 = A/Sing MAP, 5 ug (FA); B6 = A/Sing MAP, 2.5 ug (FA)
B7 = Placebo MAP (FA); B8 = A/Sing MAP, 15 ug (UA); B9 = A/Sing IM, 15 ug, as 2018 Afluria Quad (UA)

Listing 16.2.9.11
Individual Characterisation of Skin Irritation Index by Treatment
Oedema
Part A
A1= 3 x 5 ug A/Sing MAPs on volar forearm (15 ug)

Randomisation Number	Visit	Time Point	Active #> 0	Active Maximum	Active Average	Placebo #> 0	Placebo Maximum	Placebo Average
A0029	Day 1	Pre-dose	-	-	-	-	-	-
A0029	Day 1	10 min	-	-	-	-	-	-
A0029	Day 1	1 hour	3	3	2.7	-	-	-
A0029	Day 1	2 hour	3	2	1.7	-	-	-
A0029	Day 4		0	0	0.0	-	-	-
A0029	Day 8		0	0	0.0	-	-	-
A0029	Day 22		0	0	0.0	-	-	-
A0029	EOS/ET (Day 61)		0	0	0.0	-	-	-
A1026	Day 1	Pre-dose	0	0	0.0	-	-	-
A1026	Day 1	10 min	3	2	2.0	-	-	-
A1026	Day 1	1 hour	3	2	1.7	-	-	-
A1026	Day 1	2 hour	3	2	1.7	-	-	-
A1026	Day 4		3	3	2.0	-	-	-
A1026	Day 8		0	0	0.0	-	-	-
A1026	Day 22		0	0	0.0	-	-	-
A1026	EOS/ET (Day 61)		0	0	0.0	-	-	-

Source: Listing 16.2.9.4

Administration Site Key: FA = Forearm; UA= Upper arm
Treatments Part A: A1 = A/Sing MAP, 15 ug (FA); A2 = Afluria Quad IM (UA), 15 ug; A3 = Placebo MAP (FA); A4 = A/Sing Mono IM, 15 ug (UA)
Treatments Part B: B1 = A/Sing MAP, 15 ug (FA) with biopsy; B2 = Placebo MAP (FA) with biopsy
B3 = A/Sing MAP, 15 ug (FA); B4 = A/Sing MAP, 10 ug (FA); B5 = A/Sing MAP, 5 ug (FA); B6 = A/Sing MAP, 2.5 ug (FA)
B7 = Placebo MAP (FA); B8 = A/Sing MAP, 15 ug (UA); B9 = A/Sing IM, 15 ug, as 2018 Afluria Quad (UA)

Listing 16.2.9.11
Individual Characterisation of Skin Irritation Index by Treatment
Oedema
Part A
A3= 3 x uncoated MAPs on volar forearm

Randomisation Number	Visit	Time Point	Active #> 0	Active Maximum	Active Average	Placebo #> 0	Placebo Maximum	Placebo Average
A0001	Day 4		-	-	-	0	0	0.0
A0001	Day 8		-	-	-	0	0	0.0
A0001	Day 22		-	-	-	0	0	0.0
A0001	EOS/ET (Day 61)		-	-	-	0	0	0.0
A0004	Day 4		-	-	-	0	0	0.0
A0004	Day 8		-	-	-	0	0	0.0
A0004	Day 22		-	-	-	0	0	0.0
A0004	EOS/ET (Day 61)		-	-	-	0	0	0.0
A0006	Day 4		-	-	-	0	0	0.0
A0006	Day 8		-	-	-	0	0	0.0
A0006	Day 22		-	-	-	0	0	0.0
A0006	EOS/ET (Day 61)		-	-	-	0	0	0.0
A0008	Day 4		-	-	-	0	0	0.0
A0008	Day 8		-	-	-	0	0	0.0
A0008	Day 22		-	-	-	0	0	0.0
A0008	EOS/ET (Day 61)		-	-	-	0	0	0.0
A0010	Day 4		-	-	-	0	0	0.0

Source: Listing 16.2.9.4

Administration Site Key: FA = Forearm; UA= Upper arm
Treatments Part A: A1 = A/Sing MAP, 15 ug (FA); A2 = Afluria Quad IM (UA), 15 ug; A3 = Placebo MAP (FA); A4 = A/Sing Mono IM, 15 ug (UA)
Treatments Part B: B1 = A/Sing MAP, 15 ug (FA) with biopsy; B2 = Placebo MAP (FA) with biopsy
B3 = A/Sing MAP, 15 ug (FA); B4 = A/Sing MAP, 10 ug (FA); B5 = A/Sing MAP, 5 ug (FA); B6 = A/Sing MAP, 2.5 ug (FA)
B7 = Placebo MAP (FA); B8 = A/Sing MAP, 15 ug (UA); B9 = A/Sing IM, 15 ug, as 2018 Afluria Quad (UA)

Listing 16.2.9.11
Individual Characterisation of Skin Irritation Index by Treatment
Oedema
Part A
A3= 3 x uncoated MAPs on volar forearm

			Active			Placebo		
Randomisation Number	Visit	Time Point	#> 0	Maximum	Average	#> 0	Maximum	Average
A0010	Day 8		-	-	-	0	0	0.0
A0010	Day 22		-	-	-	0	0	0.0
A0010	EOS/ET (Day 61)		-	-	-	0	0	0.0
A0011	Day 4		-	-	-	0	0	0.0
A0011	Day 8		-	-	-	0	0	0.0
A0011	Day 22		-	-	-	1	1	0.5
A0011	EOS/ET (Day 61)		-	-	-	0	0	0.0
A0014	Day 4		-	-	-	0	0	0.0
A0014	Day 8		-	-	-	0	0	0.0
A0014	Day 22		-	-	-	0	0	0.0
A0014	EOS/ET (Day 61)		-	-	-	0	0	0.0
A0016	Day 1	Pre-dose	-	-	-	-	-	-
A0016	Day 1	10 min	-	-	-	-	-	-
A0016	Day 1	1 hour	-	-	-	-	-	-
A0016	Day 1	2 hour	-	-	-	3	1	1.0
A0016	Day 4		-	-	-	0	0	0.0
A0016	Day 8		-	-	-	0	0	0.0
A0016	Day 22		-	-	-	0	0	0.0

Source: Listing 16.2.9.4

Administration Site Key: FA = Forearm; UA= Upper arm
Treatments Part A: A1 = A/Sing MAP, 15 ug (FA); A2 = Afluria Quad IM (UA), 15 ug; A3 = Placebo MAP (FA); A4 = A/Sing Mono IM, 15 ug (UA)
Treatments Part B: B1 = A/Sing MAP, 15 ug (FA) with biopsy; B2 = Placebo MAP (FA) with biopsy
B3 = A/Sing MAP, 15 ug (FA); B4 = A/Sing MAP, 10 ug (FA); B5 = A/Sing MAP, 5 ug (FA); B6 = A/Sing MAP, 2.5 ug (FA)
B7 = Placebo MAP (FA); B8 = A/Sing MAP, 15 ug (UA); B9 = A/Sing IM, 15 ug, as 2018 Afluria Quad (UA)

Listing 16.2.9.11
Individual Characterisation of Skin Irritation Index by Treatment
Oedema
Part A
A3= 3 x uncoated MAPs on volar forearm

Randomisation Number	Visit	Time Point	Active #> 0	Active Maximum	Active Average	Placebo #> 0	Placebo Maximum	Placebo Average
A0016	EOS/ET (Day 61)		-	-	-	0	0	0.0
A0017	Day 1	Pre-dose	-	-	-	-	-	-
A0017	Day 1	10 min	-	-	-	-	-	-
A0017	Day 1	1 hour	-	-	-	-	-	-
A0017	Day 1	2 hour	-	-	-	3	1	1.0
A0017	Day 4		-	-	-	0	0	0.0
A0017	Day 8		-	-	-	0	0	0.0
A0017	Day 22		-	-	-	0	0	0.0
A0017	EOS/ET (Day 61)		-	-	-	0	0	0.0
A0019	Day 1	Pre-dose	-	-	-	-	-	-
A0019	Day 1	10 min	-	-	-	-	-	-
A0019	Day 1	1 hour	-	-	-	-	-	-
A0019	Day 1	2 hour	-	-	-	3	2	2.0
A0019	Day 4		-	-	-	0	0	0.0
A0019	Day 8		-	-	-	0	0	0.0
A0019	Day 22		-	-	-	0	0	0.0
A0019	EOS/ET (Day 61)		-	-	-	0	0	0.0
A0022	Day 1	Pre-dose	-	-	-	-	-	-

Administration Site Key: FA = Forearm; UA= Upper arm
Treatments Part A: A1 = A/Sing MAP, 15 ug (FA); A2 = Afluria Quad IM (UA), 15 ug; A3 = Placebo MAP (FA); A4 = A/Sing Mono IM, 15 ug (UA)
Treatments Part B: B1 = A/Sing MAP, 15 ug (FA) with biopsy; B2 = Placebo MAP (FA) with biopsy
B3 = A/Sing MAP, 15 ug (FA); B4 = A/Sing MAP, 10 ug (FA); B5 = A/Sing MAP, 5 ug (FA); B6 = A/Sing MAP, 2.5 ug (FA)
B7 = Placebo MAP (FA); B8 = A/Sing MAP, 15 ug (UA); B9 = A/Sing IM, 15 ug, as 2018 Afluria Quad (UA)

Listing 16.2.9.11
Individual Characterisation of Skin Irritation Index by Treatment
Oedema
Part A
A3= 3 x uncoated MAPs on volar forearm

Randomisation Number	Visit	Time Point	Active #> 0	Active Maximum	Active Average	Placebo #> 0	Placebo Maximum	Placebo Average
A0022	Day 1	10 min	-	-	-	-	-	-
A0022	Day 1	1 hour	-	-	-	-	-	-
A0022	Day 1	2 hour	-	-	-	3	2	2.0
A0022	Day 4		-	-	-	0	0	0.0
A0022	Day 8		-	-	-	0	0	0.0
A0022	Day 22		-	-	-	0	0	0.0
A0022	EOS/ET (Day 61)		-	-	-	0	0	0.0
A0023	Day 1	Pre-dose	-	-	-	-	-	-
A0023	Day 1	10 min	-	-	-	-	-	-
A0023	Day 1	1 hour	-	-	-	3	2	2.0
A0023	Day 1	2 hour	-	-	-	3	2	2.0
A0023	Day 4		-	-	-	0	0	0.0
A0023	Day 8		-	-	-	0	0	0.0
A0023	Day 22		-	-	-	0	0	0.0
A0023	EOS/ET (Day 61)		-	-	-	0	0	0.0
A0025	Day 1	Pre-dose	-	-	-	-	-	-
A0025	Day 1	10 min	-	-	-	-	-	-
A0025	Day 1	1 hour	-	-	-	3	2	1.7
A0025	Day 1	2 hour	-	-	-	3	2	1.7

Source: Listing 16.2.9.4

Administration Site Key: FA = Forearm; UA= Upper arm
Treatments Part A: A1 = A/Sing MAP, 15 ug (FA); A2 = Afluria Quad IM (UA), 15 ug; A3 = Placebo MAP (FA); A4 = A/Sing Mono IM, 15 ug (UA)
Treatments Part B: B1 = A/Sing MAP, 15 ug (FA) with biopsy; B2 = Placebo MAP (FA) with biopsy
B3 = A/Sing MAP, 15 ug (FA); B4 = A/Sing MAP, 10 ug (FA); B5 = A/Sing MAP, 5 ug (FA); B6 = A/Sing MAP, 2.5 ug (FA)
B7 = Placebo MAP (FA); B8 = A/Sing MAP, 15 ug (UA); B9 = A/Sing IM, 15 ug, as 2018 Afluria Quad (UA)

Listing 16.2.9.11
Individual Characterisation of Skin Irritation Index by Treatment
Oedema
Part A
A3= 3 x uncoated MAPs on volar forearm

Randomisation Number	Visit	Time Point	Active #> 0	Active Maximum	Active Average	Placebo #> 0	Placebo Maximum	Placebo Average
A0025	Day 4		-	-	-	0	0	0.0
A0025	Day 8		-	-	-	0	0	0.0
A0025	Day 22		-	-	-	0	0	0.0
A0025	EOS/ET (Day 61)		-	-	-	0	0	0.0
A0027	Day 1	Pre-dose	-	-	-	-	-	-
A0027	Day 1	10 min	-	-	-	-	-	-
A0027	Day 1	1 hour	-	-	-	3	2	1.7
A0027	Day 1	2 hour	-	-	-	3	2	1.7
A0027	Day 4		-	-	-	2	1	0.7
A0027	Day 8		-	-	-	0	0	0.0
A0027	Day 22		-	-	-	0	0	0.0
A0027	EOS/ET (Day 61)		-	-	-	0	0	0.0
A0030	Day 1	Pre-dose	-	-	-	0	0	0.0
A0030	Day 1	10 min	-	-	-	3	3	3.0
A0030	Day 1	1 hour	-	-	-	3	3	3.0
A0030	Day 1	2 hour	-	-	-	0	0	0.0
A0030	Day 4		-	-	-	0	0	0.0
A0030	Day 8		-	-	-	0	0	0.0
A0030	Day 22		-	-	-	0	0	0.0

Source: Listing 16.2.9.4

Administration Site Key: FA = Forearm; UA= Upper arm
Treatments Part A: A1 = A/Sing MAP, 15 ug (FA); A2 = Afluria Quad IM (UA), 15 ug; A3 = Placebo MAP (FA); A4 = A/Sing Mono IM, 15 ug (UA)
Treatments Part B: B1 = A/Sing MAP, 15 ug (FA) with biopsy; B2 = Placebo MAP (FA) with biopsy
B3 = A/Sing MAP, 15 ug (FA); B4 = A/Sing MAP, 10 ug (FA); B5 = A/Sing MAP, 5 ug (FA); B6 = A/Sing MAP, 2.5 ug (FA)
B7 = Placebo MAP (FA); B8 = A/Sing MAP, 15 ug (UA); B9 = A/Sing IM, 15 ug, as 2018 Afluria Quad (UA)

Listing 16.2.9.11
Individual Characterisation of Skin Irritation Index by Treatment
Oedema
Part A
A3= 3 x uncoated MAPs on volar forearm

			Active			Placebo		
Randomisation Number	Visit	Time Point	#> 0	Maximum	Average	#> 0	Maximum	Average
A0030	EOS/ET (Day 61)		-	-	-	0	0	0.0

Source: Listing 16.2.9.4

Administration Site Key: FA = Forearm; UA= Upper arm
Treatments Part A: A1 = A/Sing MAP, 15 ug (FA); A2 = Afluria Quad IM (UA), 15 ug; A3 = Placebo MAP (FA); A4 = A/Sing Mono IM, 15 ug (UA)
Treatments Part B: B1 = A/Sing MAP, 15 ug (FA) with biopsy; B2 = Placebo MAP (FA) with biopsy
B3 = A/Sing MAP, 15 ug (FA); B4 = A/Sing MAP, 10 ug (FA); B5 = A/Sing MAP, 5 ug (FA); B6 = A/Sing MAP, 2.5 ug (FA)
B7 = Placebo MAP (FA); B8 = A/Sing MAP, 15 ug (UA); B9 = A/Sing IM, 15 ug, as 2018 Afluria Quad (UA)

Listing 16.2.9.11
Individual Characterisation of Skin Irritation Index by Treatment
Oedema
Part B, Biopsy
B1= 3 x 5 ug A/Sing MAPs on volar forearm (15 ug)

			Active			Placebo		
Randomisation Number	Visit	Time Point	#> 0	Maximum	Average	#> 0	Maximum	Average
B0001	Day 1	Pre-dose	0	0	0.0	-	-	-
B0001	Day 1	10 min	0	0	0.0	-	-	-
B0001	Day 1	1 hour	3	1	1.0	-	-	-
B0001	Day 1	2 hour	3	1	1.0	-	-	-
B0001	Day 2		2	1	0.7	-	-	-
B0001	Day 4		0	0	0.0	-	-	-
B0001	Day 8		0	0	0.0	-	-	-
B0001	Day 22		0	0	0.0	-	-	-
B0001	EOS/ET (Day 61)		0	0	0.0	-	-	-
B0004	Day 1	Pre-dose	0	0	0.0	-	-	-
B0004	Day 1	10 min	3	2	2.0	-	-	-
B0004	Day 1	1 hour	3	2	2.0	-	-	-
B0004	Day 1	2 hour	3	2	2.0	-	-	-
B0004	Day 2		0	0	0.0	-	-	-
B0004	Day 4		1	1	0.3	-	-	-
B0004	Day 8		0	0	0.0	-	-	-
B0004	Day 22		0	0	0.0	-	-	-
B0004	EOS/ET (Day 61)		0	0	0.0	-	-	-
B0005	Day 1	Pre-dose	0	0	0.0	-	-	-

Administration Site Key: FA = Forearm; UA= Upper arm
Treatments Part A: A1 = A/Sing MAP, 15 ug (FA); A2 = Afluria Quad IM (UA), 15 ug; A3 = Placebo MAP (FA); A4 = A/Sing Mono IM, 15 ug (UA)
Treatments Part B: B1 = A/Sing MAP, 15 ug (FA) with biopsy; B2 = Placebo MAP (FA) with biopsy
B3 = A/Sing MAP, 15 ug (FA); B4 = A/Sing MAP, 10 ug (FA); B5 = A/Sing MAP, 5 ug (FA); B6 = A/Sing MAP, 2.5 ug (FA)
B7 = Placebo MAP (FA); B8 = A/Sing MAP, 15 ug (UA); B9 = A/Sing IM, 15 ug, as 2018 Afluria Quad (UA)

Listing 16.2.9.11
Individual Characterisation of Skin Irritation Index by Treatment
Oedema
Part B, Biopsy
B1= 3 x 5 ug A/Sing MAPs on volar forearm (15 ug)

			Active			Placebo		
Randomisation Number	Visit	Time Point	#> 0	Maximum	Average	#> 0	Maximum	Average
B0005	Day 1	10 min	3	2	2.0	-	-	-
B0005	Day 1	1 hour	3	3	3.0	-	-	-
B0005	Day 1	2 hour	3	2	1.7	-	-	-
B0005	Day 2		3	1	1.0	-	-	-
B0005	Day 4		3	1	1.0	-	-	-
B0005	Day 8		0	0	0.0	-	-	-
B0005	Day 22		3	2	1.3	-	-	-
B0005	EOS/ET (Day 61)		0	0	0.0	-	-	-
B0008	Day 1	Pre-dose	0	0	0.0	-	-	-
B0008	Day 1	10 min	3	3	2.7	-	-	-
B0008	Day 1	1 hour	3	2	2.0	-	-	-
B0008	Day 1	2 hour	3	2	1.7	-	-	-
B0008	Day 2		2	1	0.7	-	-	-
B0008	Day 4		0	0	0.0	-	-	-
B0008	Day 8		0	0	0.0	-	-	-
B0008	Day 22		0	0	0.0	-	-	-
B0008	EOS/ET (Day 61)		0	0	0.0	-	-	-
B0009	Day 1	Pre-dose	0	0	0.0	-	-	-
B0009	Day 1	10 min	3	2	1.7	-	-	-

Source: Listing 16.2.9.4

Administration Site Key: FA = Forearm; UA= Upper arm
Treatments Part A: A1 = A/Sing MAP, 15 ug (FA); A2 = Afluria Quad IM (UA), 15 ug; A3 = Placebo MAP (FA); A4 = A/Sing Mono IM, 15 ug (UA)
Treatments Part B: B1 = A/Sing MAP, 15 ug (FA) with biopsy; B2 = Placebo MAP (FA) with biopsy
B3 = A/Sing MAP, 15 ug (FA); B4 = A/Sing MAP, 10 ug (FA); B5 = A/Sing MAP, 5 ug (FA); B6 = A/Sing MAP, 2.5 ug (FA)
B7 = Placebo MAP (FA); B8 = A/Sing MAP, 15 ug (UA); B9 = A/Sing IM, 15 ug, as 2018 Afluria Quad (UA)

Listing 16.2.9.11
Individual Characterisation of Skin Irritation Index by Treatment
Oedema
Part B, Biopsy
B1= 3 x 5 ug A/Sing MAPs on volar forearm (15 ug)

			Active			Placebo		
Randomisation Number	Visit	Time Point	#> 0	Maximum	Average	#> 0	Maximum	Average
B0009	Day 1	1 hour	3	2	2.0	-	-	-
B0009	Day 1	2 hour	3	2	1.7	-	-	-
B0009	Day 2		2	1	0.7	-	-	-
B0009	Day 4		3	1	1.0	-	-	-
B0009	Day 8		0	0	0.0	-	-	-
B0009	Day 22		0	0	0.0	-	-	-
B0009	EOS/ET (Day 61)		0	0	0.0	-	-	-

Source: Listing 16.2.9.4

Administration Site Key: FA = Forearm; UA= Upper arm
Treatments Part A: A1 = A/Sing MAP, 15 ug (FA); A2 = Afluria Quad IM (UA), 15 ug; A3 = Placebo MAP (FA); A4 = A/Sing Mono IM, 15 ug (UA)
Treatments Part B: B1 = A/Sing MAP, 15 ug (FA) with biopsy; B2 = Placebo MAP (FA) with biopsy
B3 = A/Sing MAP, 15 ug (FA); B4 = A/Sing MAP, 10 ug (FA); B5 = A/Sing MAP, 5 ug (FA); B6 = A/Sing MAP, 2.5 ug (FA)
B7 = Placebo MAP (FA); B8 = A/Sing MAP, 15 ug (UA); B9 = A/Sing IM, 15 ug, as 2018 Afluria Quad (UA)

Listing 16.2.9.11
Individual Characterisation of Skin Irritation Index by Treatment
Oedema
Part B, Biopsy
B2= 3 x uncoated MAPs on volar forearm

Randomisation Number	Visit	Time Point	Active #> 0	Active Maximum	Active Average	Placebo #> 0	Placebo Maximum	Placebo Average
B0002	Day 1	Pre-dose	-	-	-	0	0	0.0
B0002	Day 1	10 min	-	-	-	3	4	3.3
B0002	Day 1	1 hour	-	-	-	3	1	1.0
B0002	Day 1	2 hour	-	-	-	3	2	2.0
B0002	Day 2		-	-	-	0	0	0.0
B0002	Day 4		-	-	-	0	0	0.0
B0002	Day 8		-	-	-	0	0	0.0
B0002	Day 22		-	-	-	0	0	0.0
B0002	EOS/ET (Day 61)		-	-	-	0	0	0.0
B0003	Day 1	Pre-dose	-	-	-	0	0	0.0
B0003	Day 1	10 min	-	-	-	1	2	0.7
B0003	Day 1	1 hour	-	-	-	3	1	1.0
B0003	Day 1	2 hour	-	-	-	3	1	1.0
B0003	Day 2		-	-	-	1	1	0.3
B0003	Day 4		-	-	-	0	0	0.0
B0003	Day 8		-	-	-	0	0	0.0
B0003	Day 22		-	-	-	2	4	2.0
B0003	EOS/ET (Day 61)		-	-	-	0	0	0.0
B0006	Day 1	Pre-dose	-	-	-	0	0	0.0

Source: Listing 16.2.9.4

Administration Site Key: FA = Forearm; UA= Upper arm
Treatments Part A: A1 = A/Sing MAP, 15 ug (FA); A2 = Afluria Quad IM (UA), 15 ug; A3 = Placebo MAP (FA); A4 = A/Sing Mono IM, 15 ug (UA)
Treatments Part B: B1 = A/Sing MAP, 15 ug (FA) with biopsy; B2 = Placebo MAP (FA) with biopsy
B3 = A/Sing MAP, 15 ug (FA); B4 = A/Sing MAP, 10 ug (FA); B5 = A/Sing MAP, 5 ug (FA); B6 = A/Sing MAP, 2.5 ug (FA)
B7 = Placebo MAP (FA); B8 = A/Sing MAP, 15 ug (UA); B9 = A/Sing IM, 15 ug, as 2018 Afluria Quad (UA)

Listing 16.2.9.11
Individual Characterisation of Skin Irritation Index by Treatment
Oedema
Part B, Biopsy
B2= 3 x uncoated MAPs on volar forearm

			Active			Placebo		
Randomisation Number	Visit	Time Point	#> 0	Maximum	Average	#> 0	Maximum	Average
B0006	Day 1	10 min	-	-	-	3	2	1.3
B0006	Day 1	1 hour	-	-	-	2	1	0.7
B0006	Day 1	2 hour	-	-	-	3	2	1.7
B0006	Day 2		-	-	-	0	0	0.0
B0006	Day 4		-	-	-	1	1	0.3
B0006	Day 8		-	-	-	2	2	1.0
B0006	Day 22		-	-	-	2	2	1.0
B0006	EOS/ET (Day 61)		-	-	-	0	0	0.0
B0007	Day 1	Pre-dose	-	-	-	0	0	0.0
B0007	Day 1	10 min	-	-	-	3	2	1.3
B0007	Day 1	1 hour	-	-	-	3	2	1.7
B0007	Day 1	2 hour	-	-	-	3	1	1.0
B0007	Day 2		-	-	-	0	0	0.0
B0007	Day 4		-	-	-	0	0	0.0
B0007	Day 8		-	-	-	0	0	0.0
B0007	Day 22		-	-	-	0	0	0.0
B0007	EOS/ET (Day 61)		-	-	-	0	0	0.0
B0010	Day 1	Pre-dose	-	-	-	0	0	0.0
B0010	Day 1	10 min	-	-	-	3	1	1.0

Source: Listing 16.2.9.4

Administration Site Key: FA = Forearm; UA= Upper arm
Treatments Part A: A1 = A/Sing MAP, 15 ug (FA); A2 = Afluria Quad IM (UA), 15 ug; A3 = Placebo MAP (FA); A4 = A/Sing Mono IM, 15 ug (UA)
Treatments Part B: B1 = A/Sing MAP, 15 ug (FA) with biopsy; B2 = Placebo MAP (FA) with biopsy
B3 = A/Sing MAP, 15 ug (FA); B4 = A/Sing MAP, 10 ug (FA); B5 = A/Sing MAP, 5 ug (FA); B6 = A/Sing MAP, 2.5 ug (FA)
B7 = Placebo MAP (FA); B8 = A/Sing MAP, 15 ug (UA); B9 = A/Sing IM, 15 ug, as 2018 Afluria Quad (UA)

Listing 16.2.9.11
Individual Characterisation of Skin Irritation Index by Treatment
Oedema
Part B, Biopsy
B2= 3 x uncoated MAPs on volar forearm

			Active			Placebo		
Randomisation Number	Visit	Time Point	#> 0	Maximum	Average	#> 0	Maximum	Average
B0010	Day 1	1 hour	-	-	-	3	1	1.0
B0010	Day 1	2 hour	-	-	-	3	1	1.0
B0010	Day 2		-	-	-	1	1	0.3
B0010	Day 4		-	-	-	0	0	0.0
B0010	Day 8		-	-	-	2	1	0.7
B0010	Day 22		-	-	-	0	0	0.0
B0010	EOS/ET (Day 61)		-	-	-	0	0	0.0

Source: Listing 16.2.9.4

Administration Site Key: FA = Forearm; UA= Upper arm
Treatments Part A: A1 = A/Sing MAP, 15 ug (FA); A2 = Afluria Quad IM (UA), 15 ug; A3 = Placebo MAP (FA); A4 = A/Sing Mono IM, 15 ug (UA)
Treatments Part B: B1 = A/Sing MAP, 15 ug (FA) with biopsy; B2 = Placebo MAP (FA) with biopsy
B3 = A/Sing MAP, 15 ug (FA); B4 = A/Sing MAP, 10 ug (FA); B5 = A/Sing MAP, 5 ug (FA); B6 = A/Sing MAP, 2.5 ug (FA)
B7 = Placebo MAP (FA); B8 = A/Sing MAP, 15 ug (UA); B9 = A/Sing IM, 15 ug, as 2018 Afluria Quad (UA)

Listing 16.2.9.11
Individual Characterisation of Skin Irritation Index by Treatment
Oedema
Part B, Non-biopsy
B3= 3 x 5 ug A/Sing MAPs on volar forearm (15 ug)

Randomisation Number	Visit	Time Point	Active #> 0	Active Maximum	Active Average	Placebo #> 0	Placebo Maximum	Placebo Average
B0014	Day 1	Pre-dose	0	0	0.0	-	-	-
B0014	Day 1	10 min	3	1	1.0	-	-	-
B0014	Day 1	1 hour	3	1	1.0	-	-	-
B0014	Day 1	2 hour	3	2	2.0	-	-	-
B0014	Day 2		3	1	1.0	-	-	-
B0014	Day 4		3	3	2.3	-	-	-
B0014	Day 8		0	0	0.0	-	-	-
B0014	Day 22		0	0	0.0	-	-	-
B0014	EOS/ET (Day 61)		0	0	0.0	-	-	-
B0023	Day 1	Pre-dose	0	0	0.0	-	-	-
B0023	Day 1	10 min	3	1	1.0	-	-	-
B0023	Day 1	1 hour	3	3	2.3	-	-	-
B0023	Day 1	2 hour	3	3	3.0	-	-	-
B0023	Day 2		3	2	1.3	-	-	-
B0023	Day 4		3	2	2.0	-	-	-
B0023	Day 8		0	0	0.0	-	-	-
B0023	Day 22		0	0	0.0	-	-	-
B0023	EOS/ET (Day 61)		0	0	0.0	-	-	-
B0027	Day 1	Pre-dose	0	0	0.0	-	-	-

Source: Listing 16.2.9.4

Administration Site Key: FA = Forearm; UA= Upper arm
Treatments Part A: A1 = A/Sing MAP, 15 ug (FA); A2 = Afluria Quad IM (UA), 15 ug; A3 = Placebo MAP (FA); A4 = A/Sing Mono IM, 15 ug (UA)
Treatments Part B: B1 = A/Sing MAP, 15 ug (FA) with biopsy; B2 = Placebo MAP (FA) with biopsy
B3 = A/Sing MAP, 15 ug (FA); B4 = A/Sing MAP, 10 ug (FA); B5 = A/Sing MAP, 5 ug (FA); B6 = A/Sing MAP, 2.5 ug (FA)
B7 = Placebo MAP (FA); B8 = A/Sing MAP, 15 ug (UA); B9 = A/Sing IM, 15 ug, as 2018 Afluria Quad (UA)

Listing 16.2.9.11
Individual Characterisation of Skin Irritation Index by Treatment
Oedema
Part B, Non-biopsy
B3= 3 x 5 ug A/Sing MAPs on volar forearm (15 ug)

			Active			Placebo		
Randomisation Number	Visit	Time Point	#> 0	Maximum	Average	#> 0	Maximum	Average
B0027	Day 1	10 min	3	1	1.0	-	-	-
B0027	Day 1	1 hour	3	2	1.3	-	-	-
B0027	Day 1	2 hour	3	3	3.0	-	-	-
B0027	Day 2		3	1	1.0	-	-	-
B0027	Day 4		2	1	0.7	-	-	-
B0027	Day 8		3	1	1.0	-	-	-
B0027	Day 22		0	0	0.0	-	-	-
B0027	EOS/ET (Day 61)		0	0	0.0	-	-	-
B0038	Day 1	Pre-dose	0	0	0.0	-	-	-
B0038	Day 1	10 min	3	2	1.7	-	-	-
B0038	Day 1	1 hour	3	2	2.0	-	-	-
B0038	Day 1	2 hour	3	2	2.0	-	-	-
B0038	Day 2		3	1	1.0	-	-	-
B0038	Day 4		0	0	0.0	-	-	-
B0038	Day 8		0	0	0.0	-	-	-
B0038	Day 22		0	0	0.0	-	-	-
B0038	EOS/ET (Day 61)		0	0	0.0	-	-	-
B0041	Day 1	Pre-dose	0	0	0.0	-	-	-
B0041	Day 1	10 min	0	0	0.0	-	-	-

Source: Listing 16.2.9.4

Administration Site Key: FA = Forearm; UA= Upper arm
Treatments Part A: A1 = A/Sing MAP, 15 ug (FA); A2 = Afluria Quad IM (UA), 15 ug; A3 = Placebo MAP (FA); A4 = A/Sing Mono IM, 15 ug (UA)
Treatments Part B: B1 = A/Sing MAP, 15 ug (FA) with biopsy; B2 = Placebo MAP (FA) with biopsy
B3 = A/Sing MAP, 15 ug (FA); B4 = A/Sing MAP, 10 ug (FA); B5 = A/Sing MAP, 5 ug (FA); B6 = A/Sing MAP, 2.5 ug (FA)
B7 = Placebo MAP (FA); B8 = A/Sing MAP, 15 ug (UA); B9 = A/Sing IM, 15 ug, as 2018 Afluria Quad (UA)

Listing 16.2.9.11
Individual Characterisation of Skin Irritation Index by Treatment
Oedema
Part B, Non-biopsy
B3= 3 x 5 ug A/Sing MAPs on volar forearm (15 ug)

Randomisation Number	Visit	Time Point	Active #> 0	Active Maximum	Active Average	Placebo #> 0	Placebo Maximum	Placebo Average
B0041	Day 1	1 hour	3	1	1.0	-	-	-
B0041	Day 1	2 hour	3	1	1.0	-	-	-
B0041	Day 2		3	1	1.0	-	-	-
B0041	Day 4		1	1	0.3	-	-	-
B0041	Day 8		0	0	0.0	-	-	-
B0041	Day 22		0	0	0.0	-	-	-
B0041	EOS/ET (Day 61)		0	0	0.0	-	-	-
B0051	Day 1	Pre-dose	0	0	0.0	-	-	-
B0051	Day 1	10 min	3	1	1.0	-	-	-
B0051	Day 1	1 hour	3	1	1.0	-	-	-
B0051	Day 1	2 hour	3	1	1.0	-	-	-
B0051	Day 2		3	2	2.0	-	-	-
B0051	Day 4		0	0	0.0	-	-	-
B0051	Day 8		0	0	0.0	-	-	-
B0051	Day 22		0	0	0.0	-	-	-
B0051	EOS/ET (Day 61)		0	0	0.0	-	-	-
B0056	Day 1	Pre-dose	0	0	0.0	-	-	-
B0056	Day 1	10 min	3	1	1.0	-	-	-
B0056	Day 1	1 hour	3	1	1.0	-	-	-

Source: Listing 16.2.9.4

Administration Site Key: FA = Forearm; UA= Upper arm
Treatments Part A: A1 = A/Sing MAP, 15 ug (FA); A2 = Afluria Quad IM (UA), 15 ug; A3 = Placebo MAP (FA); A4 = A/Sing Mono IM, 15 ug (UA)
Treatments Part B: B1 = A/Sing MAP, 15 ug (FA) with biopsy; B2 = Placebo MAP (FA) with biopsy
B3 = A/Sing MAP, 15 ug (FA); B4 = A/Sing MAP, 10 ug (FA); B5 = A/Sing MAP, 5 ug (FA); B6 = A/Sing MAP, 2.5 ug (FA)
B7 = Placebo MAP (FA); B8 = A/Sing MAP, 15 ug (UA); B9 = A/Sing IM, 15 ug, as 2018 Afluria Quad (UA)

Listing 16.2.9.11
Individual Characterisation of Skin Irritation Index by Treatment
Oedema
Part B, Non-biopsy
B3= 3 x 5 ug A/Sing MAPs on volar forearm (15 ug)

			Active			Placebo		
Randomisation Number	Visit	Time Point	#> 0	Maximum	Average	#> 0	Maximum	Average
B0056	Day 1	2 hour	3	1	1.0	-	-	-
B0056	Day 2		3	1	1.0	-	-	-
B0056	Day 4		2	1	0.7	-	-	-
B0056	Day 8		0	0	0.0	-	-	-
B0056	Day 22		0	0	0.0	-	-	-
B0056	EOS/ET (Day 61)		0	0	0.0	-	-	-
B0060	Day 1	Pre-dose	0	0	0.0	-	-	-
B0060	Day 1	10 min	3	1	1.0	-	-	-
B0060	Day 1	1 hour	3	1	1.0	-	-	-
B0060	Day 1	2 hour	3	1	1.0	-	-	-
B0060	Day 2		3	2	1.3	-	-	-
B0060	Day 4		3	3	2.7	-	-	-
B0060	Day 8		3	1	1.0	-	-	-
B0060	Day 22		0	0	0.0	-	-	-
B0060	EOS/ET (Day 61)		0	0	0.0	-	-	-
B0072	Day 1	Pre-dose	0	0	0.0	-	-	-
B0072	Day 1	10 min	2	1	0.7	-	-	-
B0072	Day 1	1 hour	3	2	1.3	-	-	-
B0072	Day 1	2 hour	3	3	2.7	-	-	-

Source: Listing 16.2.9.4

Administration Site Key: FA = Forearm; UA= Upper arm
Treatments Part A: A1 = A/Sing MAP, 15 ug (FA); A2 = Afluria Quad IM (UA), 15 ug; A3 = Placebo MAP (FA); A4 = A/Sing Mono IM, 15 ug (UA)
Treatments Part B: B1 = A/Sing MAP, 15 ug (FA) with biopsy; B2 = Placebo MAP (FA) with biopsy
B3 = A/Sing MAP, 15 ug (FA); B4 = A/Sing MAP, 10 ug (FA); B5 = A/Sing MAP, 5 ug (FA); B6 = A/Sing MAP, 2.5 ug (FA)
B7 = Placebo MAP (FA); B8 = A/Sing MAP, 15 ug (UA); B9 = A/Sing IM, 15 ug, as 2018 Afluria Quad (UA)

Listing 16.2.9.11
Individual Characterisation of Skin Irritation Index by Treatment
Oedema
Part B, Non-biopsy
B3= 3 x 5 ug A/Sing MAPs on volar forearm (15 ug)

			Active			Placebo		
Randomisation Number	Visit	Time Point	#> 0	Maximum	Average	#> 0	Maximum	Average
B0072	Day 2		3	2	1.7	-	-	-
B0072	Day 4		2	1	0.7	-	-	-
B0072	Day 8		0	0	0.0	-	-	-
B0072	Day 22		0	0	0.0	-	-	-
B0072	EOS/ET (Day 61)		0	0	0.0	-	-	-
B0075	Day 1	Pre-dose	0	0	0.0	-	-	-
B0075	Day 1	10 min	0	0	0.0	-	-	-
B0075	Day 1	1 hour	3	1	1.0	-	-	-
B0075	Day 1	2 hour	3	2	2.0	-	-	-
B0075	Day 2		3	1	1.0	-	-	-
B0075	Day 4		3	2	2.0	-	-	-
B0075	Day 8		1	1	0.3	-	-	-
B0075	Day 22		0	0	0.0	-	-	-
B0075	EOS/ET (Day 61)		0	0	0.0	-	-	-
B0082	Day 1	Pre-dose	0	0	0.0	-	-	-
B0082	Day 1	10 min	3	1	1.0	-	-	-
B0082	Day 1	1 hour	3	1	1.0	-	-	-
B0082	Day 1	2 hour	3	2	1.3	-	-	-
B0082	Day 2		3	4	4.0	-	-	-

Source: Listing 16.2.9.4

Administration Site Key: FA = Forearm; UA= Upper arm
Treatments Part A: A1 = A/Sing MAP, 15 ug (FA); A2 = Afluria Quad IM (UA), 15 ug; A3 = Placebo MAP (FA); A4 = A/Sing Mono IM, 15 ug (UA)
Treatments Part B: B1 = A/Sing MAP, 15 ug (FA) with biopsy; B2 = Placebo MAP (FA) with biopsy
B3 = A/Sing MAP, 15 ug (FA); B4 = A/Sing MAP, 10 ug (FA); B5 = A/Sing MAP, 5 ug (FA); B6 = A/Sing MAP, 2.5 ug (FA)
B7 = Placebo MAP (FA); B8 = A/Sing MAP, 15 ug (UA); B9 = A/Sing IM, 15 ug, as 2018 Afluria Quad (UA)

Listing 16.2.9.11
Individual Characterisation of Skin Irritation Index by Treatment
Oedema
Part B, Non-biopsy
B3= 3 x 5 ug A/Sing MAPs on volar forearm (15 ug)

			Active			Placebo		
Randomisation Number	Visit	Time Point	#> 0	Maximum	Average	#> 0	Maximum	Average
B0082	Day 4		3	1	1.0	-	-	-
B0082	Day 8		2	1	0.7	-	-	-
B0082	Day 22		1	1	0.3	-	-	-
B0082	EOS/ET (Day 61)		1	1	0.3	-	-	-
B0094	Day 1	Pre-dose	0	0	0.0	-	-	-
B0094	Day 1	10 min	3	2	2.0	-	-	-
B0094	Day 1	1 hour	3	2	2.0	-	-	-
B0094	Day 1	2 hour	3	2	1.3	-	-	-
B0094	Day 2		3	2	1.7	-	-	-
B0094	Day 4		0	0	0.0	-	-	-
B0094	Day 8		0	0	0.0	-	-	-
B0094	Day 22		0	0	0.0	-	-	-
B0094	EOS/ET (Day 61)		0	0	0.0	-	-	-
B0101	Day 1	Pre-dose	0	0	0.0	-	-	-
B0101	Day 1	10 min	3	1	1.0	-	-	-
B0101	Day 1	1 hour	3	2	2.0	-	-	-
B0101	Day 1	2 hour	3	1	1.0	-	-	-
B0101	Day 2		1	1	0.3	-	-	-
B0101	Day 4		0	0	0.0	-	-	-

Source: Listing 16.2.9.4

Administration Site Key: FA = Forearm; UA= Upper arm
Treatments Part A: A1 = A/Sing MAP, 15 ug (FA); A2 = Afluria Quad IM (UA), 15 ug; A3 = Placebo MAP (FA); A4 = A/Sing Mono IM, 15 ug (UA)
Treatments Part B: B1 = A/Sing MAP, 15 ug (FA) with biopsy; B2 = Placebo MAP (FA) with biopsy
B3 = A/Sing MAP, 15 ug (FA); B4 = A/Sing MAP, 10 ug (FA); B5 = A/Sing MAP, 5 ug (FA); B6 = A/Sing MAP, 2.5 ug (FA)
B7 = Placebo MAP (FA); B8 = A/Sing MAP, 15 ug (UA); B9 = A/Sing IM, 15 ug, as 2018 Afluria Quad (UA)

Listing 16.2.9.11
Individual Characterisation of Skin Irritation Index by Treatment
Oedema
Part B, Non-biopsy
B3= 3 x 5 ug A/Sing MAPs on volar forearm (15 ug)

			Active			Placebo		
Randomisation Number	Visit	Time Point	#> 0	Maximum	Average	#> 0	Maximum	Average
B0101	Day 8		0	0	0.0	-	-	-
B0101	Day 22		0	0	0.0	-	-	-
B0101	EOS/ET (Day 61)		0	0	0.0	-	-	-
B0104	Day 1	Pre-dose	0	0	0.0	-	-	-
B0104	Day 1	10 min	3	1	1.0	-	-	-
B0104	Day 1	1 hour	3	2	1.7	-	-	-
B0104	Day 1	2 hour	3	1	1.0	-	-	-
B0104	Day 2		2	2	1.0	-	-	-
B0104	Day 4		0	0	0.0	-	-	-
B0104	Day 8		0	0	0.0	-	-	-
B0104	Day 22		0	0	0.0	-	-	-
B0104	EOS/ET (Day 61)		0	0	0.0	-	-	-
B0110	Day 1	Pre-dose	0	0	0.0	-	-	-
B0110	Day 1	10 min	3	2	1.3	-	-	-
B0110	Day 1	1 hour	3	1	1.0	-	-	-
B0110	Day 1	2 hour	3	2	1.7	-	-	-
B0110	Day 2		3	2	2.0	-	-	-
B0110	Day 4		0	0	0.0	-	-	-
B0110	Day 8		0	0	0.0	-	-	-

Source: Listing 16.2.9.4

Administration Site Key: FA = Forearm; UA= Upper arm
Treatments Part A: A1 = A/Sing MAP, 15 ug (FA); A2 = Afluria Quad IM (UA), 15 ug; A3 = Placebo MAP (FA); A4 = A/Sing Mono IM, 15 ug (UA)
Treatments Part B: B1 = A/Sing MAP, 15 ug (FA) with biopsy; B2 = Placebo MAP (FA) with biopsy
B3 = A/Sing MAP, 15 ug (FA); B4 = A/Sing MAP, 10 ug (FA); B5 = A/Sing MAP, 5 ug (FA); B6 = A/Sing MAP, 2.5 ug (FA)
B7 = Placebo MAP (FA); B8 = A/Sing MAP, 15 ug (UA); B9 = A/Sing IM, 15 ug, as 2018 Afluria Quad (UA)

Listing 16.2.9.11
Individual Characterisation of Skin Irritation Index by Treatment
Oedema
Part B, Non-biopsy
B3= 3 x 5 ug A/Sing MAPs on volar forearm (15 ug)

			Active			Placebo		
Randomisation Number	Visit	Time Point	#> 0	Maximum	Average	#> 0	Maximum	Average
B0110	Day 22		0	0	0.0	-	-	-
B0110	EOS/ET (Day 61)		0	0	0.0	-	-	-
B0120	Day 1	Pre-dose	0	0	0.0	-	-	-
B0120	Day 1	10 min	3	2	1.7	-	-	-
B0120	Day 1	1 hour	3	3	3.0	-	-	-
B0120	Day 1	2 hour	3	3	3.0	-	-	-
B0120	Day 2		3	2	1.3	-	-	-
B0120	Day 4		0	0	0.0	-	-	-
B0120	Day 8		0	0	0.0	-	-	-
B0120	Day 22		0	0	0.0	-	-	-
B0120	EOS/ET (Day 61)		0	0	0.0	-	-	-
B0126	Day 1	Pre-dose	0	0	0.0	-	-	-
B0126	Day 1	10 min	2	1	0.7	-	-	-
B0126	Day 1	1 hour	3	2	2.0	-	-	-
B0126	Day 1	2 hour	3	3	3.0	-	-	-
B0126	Day 2		3	1	1.0	-	-	-
B0126	Day 4		0	0	0.0	-	-	-
B0126	Day 8		0	0	0.0	-	-	-
B0126	Day 22		0	0	0.0	-	-	-

Source: Listing 16.2.9.4

Administration Site Key: FA = Forearm; UA= Upper arm
Treatments Part A: A1 = A/Sing MAP, 15 ug (FA); A2 = Afluria Quad IM (UA), 15 ug; A3 = Placebo MAP (FA); A4 = A/Sing Mono IM, 15 ug (UA)
Treatments Part B: B1 = A/Sing MAP, 15 ug (FA) with biopsy; B2 = Placebo MAP (FA) with biopsy
B3 = A/Sing MAP, 15 ug (FA); B4 = A/Sing MAP, 10 ug (FA); B5 = A/Sing MAP, 5 ug (FA); B6 = A/Sing MAP, 2.5 ug (FA)
B7 = Placebo MAP (FA); B8 = A/Sing MAP, 15 ug (UA); B9 = A/Sing IM, 15 ug, as 2018 Afluria Quad (UA)

Listing 16.2.9.11
Individual Characterisation of Skin Irritation Index by Treatment
Oedema
Part B, Non-biopsy
B3= 3 x 5 ug A/Sing MAPs on volar forearm (15 ug)

			Active			Placebo		
Randomisation Number	Visit	Time Point	#> 0	Maximum	Average	#> 0	Maximum	Average
B0126	EOS/ET (Day 61)		0	0	0.0	-	-	-
B0134	Day 1	Pre-dose	0	0	0.0	-	-	-
B0134	Day 1	10 min	3	2	2.0	-	-	-
B0134	Day 1	1 hour	3	3	2.7	-	-	-
B0134	Day 1	2 hour	3	2	2.0	-	-	-
B0134	Day 2		3	2	2.0	-	-	-
B0134	Day 4		0	0	0.0	-	-	-
B0134	Day 8		0	0	0.0	-	-	-
B0134	Day 22		0	0	0.0	-	-	-
B0134	EOS/ET (Day 61)		0	0	0.0	-	-	-
B0141	Day 1	Pre-dose	0	0	0.0	-	-	-
B0141	Day 1	10 min	3	2	2.0	-	-	-
B0141	Day 1	1 hour	3	3	2.3	-	-	-
B0141	Day 1	2 hour	3	1	1.0	-	-	-
B0141	Day 2		3	1	1.0	-	-	-
B0141	Day 4		3	1	1.0	-	-	-
B0141	Day 8		0	0	0.0	-	-	-
B0141	Day 22		0	0	0.0	-	-	-
B0141	EOS/ET (Day 61)		0	0	0.0	-	-	-

Source: Listing 16.2.9.4

Administration Site Key: FA = Forearm; UA= Upper arm
Treatments Part A: A1 = A/Sing MAP, 15 ug (FA); A2 = Afluria Quad IM (UA), 15 ug; A3 = Placebo MAP (FA); A4 = A/Sing Mono IM, 15 ug (UA)
Treatments Part B: B1 = A/Sing MAP, 15 ug (FA) with biopsy; B2 = Placebo MAP (FA) with biopsy
B3 = A/Sing MAP, 15 ug (FA); B4 = A/Sing MAP, 10 ug (FA); B5 = A/Sing MAP, 5 ug (FA); B6 = A/Sing MAP, 2.5 ug (FA)
B7 = Placebo MAP (FA); B8 = A/Sing MAP, 15 ug (UA); B9 = A/Sing IM, 15 ug, as 2018 Afluria Quad (UA)

Listing 16.2.9.11
Individual Characterisation of Skin Irritation Index by Treatment
Oedema
Part B, Non-biopsy
B3= 3 x 5 ug A/Sing MAPs on volar forearm (15 ug)

			Active			Placebo		
Randomisation Number	Visit	Time Point	#> 0	Maximum	Average	#> 0	Maximum	Average
B0145	Day 1	Pre-dose	0	0	0.0	-	-	-
B0145	Day 1	10 min	3	1	1.0	-	-	-
B0145	Day 1	1 hour	3	3	3.0	-	-	-
B0145	Day 1	2 hour	3	2	1.7	-	-	-
B0145	Day 2		3	1	1.0	-	-	-
B0145	Day 4		0	0	0.0	-	-	-
B0145	Day 8		3	2	2.0	-	-	-
B0145	Day 22		0	0	0.0	-	-	-
B0145	EOS/ET (Day 61)		0	0	0.0	-	-	-

Source: Listing 16.2.9.4

Administration Site Key: FA = Forearm; UA= Upper arm
Treatments Part A: A1 = A/Sing MAP, 15 ug (FA); A2 = Afluria Quad IM (UA), 15 ug; A3 = Placebo MAP (FA); A4 = A/Sing Mono IM, 15 ug (UA)
Treatments Part B: B1 = A/Sing MAP, 15 ug (FA) with biopsy; B2 = Placebo MAP (FA) with biopsy
B3 = A/Sing MAP, 15 ug (FA); B4 = A/Sing MAP, 10 ug (FA); B5 = A/Sing MAP, 5 ug (FA); B6 = A/Sing MAP, 2.5 ug (FA)
B7 = Placebo MAP (FA); B8 = A/Sing MAP, 15 ug (UA); B9 = A/Sing IM, 15 ug, as 2018 Afluria Quad (UA)

Listing 16.2.9.11
Individual Characterisation of Skin Irritation Index by Treatment
Oedema
Part B, Non-biopsy
B4= 2 x 5 ug A/Sing MAPs + 1 uncoated MAP on volar forearm (10 ug)

			Active			Placebo		
Randomisation Number	Visit	Time Point	#> 0	Maximum	Average	#> 0	Maximum	Average
B0016	Day 1	Pre-dose	0	0	0.0	0	0	0.0
B0016	Day 1	10 min	2	1	1.0	1	1	1.0
B0016	Day 1	1 hour	2	1	1.0	1	1	1.0
B0016	Day 1	2 hour	2	1	1.0	1	1	1.0
B0016	Day 2		2	2	1.5	0	0	0.0
B0016	Day 4		2	3	2.5	0	0	0.0
B0016	Day 8		0	0	0.0	0	0	0.0
B0016	Day 22		0	0	0.0	0	0	0.0
B0016	EOS/ET (Day 61)		0	0	0.0	0	0	0.0
B0022	Day 1	Pre-dose	0	0	0.0	0	0	0.0
B0022	Day 1	10 min	2	1	1.0	1	1	1.0
B0022	Day 1	1 hour	2	1	1.0	1	1	1.0
B0022	Day 1	2 hour	2	1	1.0	1	1	1.0
B0022	Day 2		2	1	1.0	0	0	0.0
B0022	Day 4		2	3	3.0	1	1	1.0
B0022	Day 8		2	1	1.0	0	0	0.0
B0022	Day 22		0	0	0.0	0	0	0.0
B0022	EOS/ET (Day 61)		0	0	0.0	0	0	0.0
B0031	Day 1	Pre-dose	0	0	0.0	0	0	0.0

Source: Listing 16.2.9.4

Administration Site Key: FA = Forearm; UA= Upper arm
Treatments Part A: A1 = A/Sing MAP, 15 ug (FA); A2 = Afluria Quad IM (UA), 15 ug; A3 = Placebo MAP (FA); A4 = A/Sing Mono IM, 15 ug (UA)
Treatments Part B: B1 = A/Sing MAP, 15 ug (FA) with biopsy; B2 = Placebo MAP (FA) with biopsy
B3 = A/Sing MAP, 15 ug (FA); B4 = A/Sing MAP, 10 ug (FA); B5 = A/Sing MAP, 5 ug (FA); B6 = A/Sing MAP, 2.5 ug (FA)
B7 = Placebo MAP (FA); B8 = A/Sing MAP, 15 ug (UA); B9 = A/Sing IM, 15 ug, as 2018 Afluria Quad (UA)

Listing 16.2.9.11
Individual Characterisation of Skin Irritation Index by Treatment
Oedema
Part B, Non-biopsy
B4= 2 x 5 ug A/Sing MAPs + 1 uncoated MAP on volar forearm (10 ug)

			Active			Placebo		
Randomisation Number	Visit	Time Point	#> 0	Maximum	Average	#> 0	Maximum	Average
B0031	Day 1	10 min	2	2	2.0	0	0	0.0
B0031	Day 1	1 hour	2	2	2.0	0	0	0.0
B0031	Day 1	2 hour	2	3	2.5	1	1	1.0
B0031	Day 2		2	1	1.0	0	0	0.0
B0031	Day 4		1	1	0.5	0	0	0.0
B0031	Day 8		0	0	0.0	0	0	0.0
B0031	Day 22		0	0	0.0	0	0	0.0
B0031	EOS/ET (Day 61)		0	0	0.0	0	0	0.0
B0037	Day 1	Pre-dose	0	0	0.0	0	0	0.0
B0037	Day 1	10 min	0	0	0.0	0	0	0.0
B0037	Day 1	1 hour	0	0	0.0	0	0	0.0
B0037	Day 1	2 hour	2	1	1.0	1	1	1.0
B0037	Day 2		2	1	1.0	0	0	0.0
B0037	Day 4		0	0	0.0	0	0	0.0
B0037	Day 8		0	0	0.0	0	0	0.0
B0037	Day 22		0	0	0.0	0	0	0.0
B0037	EOS/ET (Day 61)		0	0	0.0	0	0	0.0
B0045	Day 1	Pre-dose	0	0	0.0	0	0	0.0
B0045	Day 1	10 min	1	1	0.5	0	0	0.0

Administration Site Key: FA = Forearm; UA= Upper arm
Treatments Part A: A1 = A/Sing MAP, 15 ug (FA); A2 = Afluria Quad IM (UA), 15 ug; A3 = Placebo MAP (FA); A4 = A/Sing Mono IM, 15 ug (UA)
Treatments Part B: B1 = A/Sing MAP, 15 ug (FA) with biopsy; B2 = Placebo MAP (FA) with biopsy
B3 = A/Sing MAP, 15 ug (FA); B4 = A/Sing MAP, 10 ug (FA); B5 = A/Sing MAP, 5 ug (FA); B6 = A/Sing MAP, 2.5 ug (FA)
B7 = Placebo MAP (FA); B8 = A/Sing MAP, 15 ug (UA); B9 = A/Sing IM, 15 ug, as 2018 Afluria Quad (UA)

Listing 16.2.9.11
Individual Characterisation of Skin Irritation Index by Treatment
Oedema
Part B, Non-biopsy
B4= 2 x 5 ug A/Sing MAPs + 1 uncoated MAP on volar forearm (10 ug)

			Active			Placebo		
Randomisation Number	Visit	Time Point	#> 0	Maximum	Average	#> 0	Maximum	Average
B0045	Day 1	1 hour	1	2	1.0	1	1	1.0
B0045	Day 1	2 hour	1	1	0.5	1	1	1.0
B0045	Day 2		1	4	2.0	0	0	0.0
B0045	Day 4		0	0	0.0	0	0	0.0
B0045	Day 8		0	0	0.0	0	0	0.0
B0045	Day 22		0	0	0.0	0	0	0.0
B0045	EOS/ET (Day 61)		0	0	0.0	0	0	0.0
B0049	Day 1	Pre-dose	0	0	0.0	0	0	0.0
B0049	Day 1	10 min	2	1	1.0	0	0	0.0
B0049	Day 1	1 hour	2	1	1.0	0	0	0.0
B0049	Day 1	2 hour	2	2	2.0	1	1	1.0
B0049	Day 2		2	1	1.0	0	0	0.0
B0049	Day 4		0	0	0.0	0	0	0.0
B0049	Day 8		0	0	0.0	0	0	0.0
B0049	Day 22		0	0	0.0	0	0	0.0
B0049	EOS/ET (Day 61)		0	0	0.0	0	0	0.0
B0057	Day 1	Pre-dose	0	0	0.0	0	0	0.0
B0057	Day 1	10 min	2	1	1.0	1	1	1.0
B0057	Day 1	1 hour	2	1	1.0	1	1	1.0

Source: Listing 16.2.9.4

Administration Site Key: FA = Forearm; UA= Upper arm
Treatments Part A: A1 = A/Sing MAP, 15 ug (FA); A2 = Afluria Quad IM (UA), 15 ug; A3 = Placebo MAP (FA); A4 = A/Sing Mono IM, 15 ug (UA)
Treatments Part B: B1 = A/Sing MAP, 15 ug (FA) with biopsy; B2 = Placebo MAP (FA) with biopsy
B3 = A/Sing MAP, 15 ug (FA); B4 = A/Sing MAP, 10 ug (FA); B5 = A/Sing MAP, 5 ug (FA); B6 = A/Sing MAP, 2.5 ug (FA)
B7 = Placebo MAP (FA); B8 = A/Sing MAP, 15 ug (UA); B9 = A/Sing IM, 15 ug, as 2018 Afluria Quad (UA)

Listing 16.2.9.11
Individual Characterisation of Skin Irritation Index by Treatment
Oedema
Part B, Non-biopsy
B4= 2 x 5 ug A/Sing MAPs + 1 uncoated MAP on volar forearm (10 ug)

Randomisation Number	Visit	Time Point	Active #> 0	Active Maximum	Active Average	Placebo #> 0	Placebo Maximum	Placebo Average
B0057	Day 1	2 hour	2	1	1.0	1	1	1.0
B0057	Day 2		2	2	1.5	0	0	0.0
B0057	Day 4		0	0	0.0	0	0	0.0
B0057	Day 8		0	0	0.0	0	0	0.0
B0057	Day 22		0	0	0.0	0	0	0.0
B0057	EOS/ET (Day 61)		0	0	0.0	0	0	0.0
B0065	Day 1	Pre-dose	0	0	0.0	0	0	0.0
B0065	Day 1	10 min	2	1	1.0	1	1	1.0
B0065	Day 1	1 hour	2	1	1.0	1	1	1.0
B0065	Day 1	2 hour	2	2	1.5	1	1	1.0
B0065	Day 2		2	2	2.0	0	0	0.0
B0065	Day 4		2	1	1.0	0	0	0.0
B0065	Day 8		1	1	0.5	1	1	1.0
B0065	Day 22		0	0	0.0	0	0	0.0
B0065	EOS/ET (Day 61)		0	0	0.0	0	0	0.0
B0071	Day 1	Pre-dose	0	0	0.0	0	0	0.0
B0071	Day 1	10 min	2	1	1.0	1	1	1.0
B0071	Day 1	1 hour	2	1	1.0	1	1	1.0
B0071	Day 1	2 hour	2	2	2.0	1	2	2.0

Source: Listing 16.2.9.4

Administration Site Key: FA = Forearm; UA= Upper arm
Treatments Part A: A1 = A/Sing MAP, 15 ug (FA); A2 = Afluria Quad IM (UA), 15 ug; A3 = Placebo MAP (FA); A4 = A/Sing Mono IM, 15 ug (UA)
Treatments Part B: B1 = A/Sing MAP, 15 ug (FA) with biopsy; B2 = Placebo MAP (FA) with biopsy
B3 = A/Sing MAP, 15 ug (FA); B4 = A/Sing MAP, 10 ug (FA); B5 = A/Sing MAP, 5 ug (FA); B6 = A/Sing MAP, 2.5 ug (FA)
B7 = Placebo MAP (FA); B8 = A/Sing MAP, 15 ug (UA); B9 = A/Sing IM, 15 ug, as 2018 Afluria Quad (UA)

Listing 16.2.9.11
Individual Characterisation of Skin Irritation Index by Treatment
Oedema
Part B, Non-biopsy
B4= 2 x 5 ug A/Sing MAPs + 1 uncoated MAP on volar forearm (10 ug)

			Active			Placebo		
Randomisation Number	Visit	Time Point	#> 0	Maximum	Average	#> 0	Maximum	Average
B0071	Day 2		2	2	1.5	1	1	1.0
B0071	Day 4		2	1	1.0	0	0	0.0
B0071	Day 8		0	0	0.0	0	0	0.0
B0071	Day 22		0	0	0.0	0	0	0.0
B0071	EOS/ET (Day 61)		0	0	0.0	0	0	0.0
B0077	Day 1	Pre-dose	0	0	0.0	0	0	0.0
B0077	Day 1	10 min	2	1	1.0	0	0	0.0
B0077	Day 1	1 hour	2	1	1.0	1	1	1.0
B0077	Day 1	2 hour	2	1	1.0	1	1	1.0
B0077	Day 2		1	1	0.5	0	0	0.0
B0077	Day 4		2	3	3.0	1	1	1.0
B0077	Day 8		0	0	0.0	0	0	0.0
B0077	Day 22		0	0	0.0	0	0	0.0
B0077	EOS/ET (Day 61)		0	0	0.0	0	0	0.0
B0085	Day 1	Pre-dose	0	0	0.0	0	0	0.0
B0085	Day 1	10 min	2	2	1.5	1	1	1.0
B0085	Day 1	1 hour	2	1	1.0	1	1	1.0
B0085	Day 1	2 hour	0	0	0.0	0	0	0.0
B0085	Day 2		2	3	3.0	0	0	0.0

Administration Site Key: FA = Forearm; UA= Upper arm
Treatments Part A: A1 = A/Sing MAP, 15 ug (FA); A2 = Afluria Quad IM (UA), 15 ug; A3 = Placebo MAP (FA); A4 = A/Sing Mono IM, 15 ug (UA)
Treatments Part B: B1 = A/Sing MAP, 15 ug (FA) with biopsy; B2 = Placebo MAP (FA) with biopsy
B3 = A/Sing MAP, 15 ug (FA); B4 = A/Sing MAP, 10 ug (FA); B5 = A/Sing MAP, 5 ug (FA); B6 = A/Sing MAP, 2.5 ug (FA)
B7 = Placebo MAP (FA); B8 = A/Sing MAP, 15 ug (UA); B9 = A/Sing IM, 15 ug, as 2018 Afluria Quad (UA)

Listing 16.2.9.11
Individual Characterisation of Skin Irritation Index by Treatment
Oedema
Part B, Non-biopsy
B4= 2 x 5 ug A/Sing MAPs + 1 uncoated MAP on volar forearm (10 ug)

			Active			Placebo		
Randomisation Number	Visit	Time Point	#> 0	Maximum	Average	#> 0	Maximum	Average
B0085	Day 4		1	3	1.5	0	0	0.0
B0085	Day 8		0	0	0.0	0	0	0.0
B0085	Day 22		0	0	0.0	0	0	0.0
B0085	EOS/ET (Day 61)		0	0	0.0	0	0	0.0
B0092	Day 1	Pre-dose	0	0	0.0	0	0	0.0
B0092	Day 1	10 min	2	2	1.5	1	2	2.0
B0092	Day 1	1 hour	2	2	2.0	1	1	1.0
B0092	Day 1	2 hour	2	2	1.5	1	1	1.0
B0092	Day 2		2	1	1.0	0	0	0.0
B0092	Day 4		0	0	0.0	0	0	0.0
B0092	Day 8		0	0	0.0	0	0	0.0
B0092	Day 22		0	0	0.0	0	0	0.0
B0092	EOS/ET (Day 61)		0	0	0.0	0	0	0.0
B0099	Day 1	Pre-dose	0	0	0.0	0	0	0.0
B0099	Day 1	10 min	0	0	0.0	0	0	0.0
B0099	Day 1	1 hour	2	2	1.5	1	2	2.0
B0099	Day 1	2 hour	1	1	0.5	1	1	1.0
B0099	Day 2		1	2	1.0	0	0	0.0
B0099	Day 4		0	0	0.0	0	0	0.0

Source: Listing 16.2.9.4

Administration Site Key: FA = Forearm; UA= Upper arm
Treatments Part A: A1 = A/Sing MAP, 15 ug (FA); A2 = Afluria Quad IM (UA), 15 ug; A3 = Placebo MAP (FA); A4 = A/Sing Mono IM, 15 ug (UA)
Treatments Part B: B1 = A/Sing MAP, 15 ug (FA) with biopsy; B2 = Placebo MAP (FA) with biopsy
B3 = A/Sing MAP, 15 ug (FA); B4 = A/Sing MAP, 10 ug (FA); B5 = A/Sing MAP, 5 ug (FA); B6 = A/Sing MAP, 2.5 ug (FA)
B7 = Placebo MAP (FA); B8 = A/Sing MAP, 15 ug (UA); B9 = A/Sing IM, 15 ug, as 2018 Afluria Quad (UA)

Listing 16.2.9.11
Individual Characterisation of Skin Irritation Index by Treatment
Oedema
Part B, Non-biopsy
B4= 2 x 5 ug A/Sing MAPs + 1 uncoated MAP on volar forearm (10 ug)

			Active			Placebo		
Randomisation Number	Visit	Time Point	#> 0	Maximum	Average	#> 0	Maximum	Average
B0099	Day 8		0	0	0.0	0	0	0.0
B0099	EOS/ET (Day 61)		0	0	0.0	0	0	0.0
B0106	Day 1	Pre-dose	0	0	0.0	0	0	0.0
B0106	Day 1	10 min	2	2	1.5	1	1	1.0
B0106	Day 1	1 hour	2	2	2.0	1	1	1.0
B0106	Day 1	2 hour	2	2	2.0	1	1	1.0
B0106	Day 2		2	1	1.0	0	0	0.0
B0106	Day 4		0	0	0.0	0	0	0.0
B0106	Day 8		0	0	0.0	0	0	0.0
B0106	Day 22		0	0	0.0	0	0	0.0
B0106	EOS/ET (Day 61)		0	0	0.0	0	0	0.0
B0112	Day 1	Pre-dose	0	0	0.0	0	0	0.0
B0112	Day 1	10 min	2	1	1.0	0	0	0.0
B0112	Day 1	1 hour	0	0	0.0	0	0	0.0
B0112	Day 1	2 hour	2	3	2.5	1	1	1.0
B0112	Day 2		2	1	1.0	0	0	0.0
B0112	Day 4		2	2	2.0	0	0	0.0
B0112	Day 8		0	0	0.0	0	0	0.0
B0112	Day 22		0	0	0.0	0	0	0.0

Source: Listing 16.2.9.4

Administration Site Key: FA = Forearm; UA= Upper arm
Treatments Part A: A1 = A/Sing MAP, 15 ug (FA); A2 = Afluria Quad IM (UA), 15 ug; A3 = Placebo MAP (FA); A4 = A/Sing Mono IM, 15 ug (UA)
Treatments Part B: B1 = A/Sing MAP, 15 ug (FA) with biopsy; B2 = Placebo MAP (FA) with biopsy
B3 = A/Sing MAP, 15 ug (FA); B4 = A/Sing MAP, 10 ug (FA); B5 = A/Sing MAP, 5 ug (FA); B6 = A/Sing MAP, 2.5 ug (FA)
B7 = Placebo MAP (FA); B8 = A/Sing MAP, 15 ug (UA); B9 = A/Sing IM, 15 ug, as 2018 Afluria Quad (UA)

Listing 16.2.9.11
Individual Characterisation of Skin Irritation Index by Treatment
Oedema
Part B, Non-biopsy
B4= 2 x 5 ug A/Sing MAPs + 1 uncoated MAP on volar forearm (10 ug)

			Active			Placebo		
Randomisation Number	Visit	Time Point	#> 0	Maximum	Average	#> 0	Maximum	Average
B0112	EOS/ET (Day 61)		0	0	0.0	0	0	0.0
B0119	Day 1	Pre-dose	0	0	0.0	0	0	0.0
B0119	Day 1	10 min	2	1	1.0	1	1	1.0
B0119	Day 1	1 hour	1	1	0.5	1	1	1.0
B0119	Day 1	2 hour	2	2	1.5	1	2	2.0
B0119	Day 2		2	1	1.0	0	0	0.0
B0119	Day 4		2	2	2.0	0	0	0.0
B0119	Day 8		0	0	0.0	0	0	0.0
B0119	Day 22		0	0	0.0	0	0	0.0
B0119	EOS/ET (Day 61)		0	0	0.0	0	0	0.0
B0124	Day 1	Pre-dose	0	0	0.0	0	0	0.0
B0124	Day 1	10 min	2	1	1.0	1	1	1.0
B0124	Day 1	1 hour	2	3	3.0	0	0	0.0
B0124	Day 1	2 hour	2	3	3.0	1	2	2.0
B0124	Day 2		2	2	2.0	0	0	0.0
B0124	Day 4		2	3	2.5	0	0	0.0
B0124	Day 8		1	2	1.0	0	0	0.0
B0124	Day 22		0	0	0.0	0	0	0.0
B0124	EOS/ET (Day 61)		0	0	0.0	0	0	0.0

Source: Listing 16.2.9.4

Administration Site Key: FA = Forearm; UA= Upper arm
Treatments Part A: A1 = A/Sing MAP, 15 ug (FA); A2 = Afluria Quad IM (UA), 15 ug; A3 = Placebo MAP (FA); A4 = A/Sing Mono IM, 15 ug (UA)
Treatments Part B: B1 = A/Sing MAP, 15 ug (FA) with biopsy; B2 = Placebo MAP (FA) with biopsy
B3 = A/Sing MAP, 15 ug (FA); B4 = A/Sing MAP, 10 ug (FA); B5 = A/Sing MAP, 5 ug (FA); B6 = A/Sing MAP, 2.5 ug (FA)
B7 = Placebo MAP (FA); B8 = A/Sing MAP, 15 ug (UA); B9 = A/Sing IM, 15 ug, as 2018 Afluria Quad (UA)

Listing 16.2.9.11
Individual Characterisation of Skin Irritation Index by Treatment
Oedema
Part B, Non-biopsy
B4= 2 x 5 ug A/Sing MAPs + 1 uncoated MAP on volar forearm (10 ug)

			Active			Placebo		
Randomisation Number	Visit	Time Point	#> 0	Maximum	Average	#> 0	Maximum	Average
B0133	Day 1	Pre-dose	0	0	0.0	0	0	0.0
B0133	Day 1	10 min	2	1	1.0	1	1	1.0
B0133	Day 1	1 hour	2	3	3.0	1	1	1.0
B0133	Day 1	2 hour	2	2	2.0	1	1	1.0
B0133	Day 2		2	1	1.0	0	0	0.0
B0133	Day 4		2	1	1.0	0	0	0.0
B0133	Day 8		0	0	0.0	0	0	0.0
B0133	Day 22		0	0	0.0	0	0	0.0
B0133	EOS/ET (Day 61)		0	0	0.0	0	0	0.0
B0139	Day 1	Pre-dose	0	0	0.0	0	0	0.0
B0139	Day 1	10 min	2	2	2.0	1	2	2.0
B0139	Day 1	1 hour	2	2	2.0	1	1	1.0
B0139	Day 1	2 hour	2	3	3.0	1	1	1.0
B0139	Day 2		2	1	1.0	0	0	0.0
B0139	Day 4		2	1	1.0	0	0	0.0
B0139	Day 8		0	0	0.0	0	0	0.0
B0139	Day 22		0	0	0.0	0	0	0.0
B0139	EOS/ET (Day 61)		0	0	0.0	0	0	0.0

Administration Site Key: FA = Forearm; UA= Upper arm
Treatments Part A: A1 = A/Sing MAP, 15 ug (FA); A2 = Afluria Quad IM (UA), 15 ug; A3 = Placebo MAP (FA); A4 = A/Sing Mono IM, 15 ug (UA)
Treatments Part B: B1 = A/Sing MAP, 15 ug (FA) with biopsy; B2 = Placebo MAP (FA) with biopsy
B3 = A/Sing MAP, 15 ug (FA); B4 = A/Sing MAP, 10 ug (FA); B5 = A/Sing MAP, 5 ug (FA); B6 = A/Sing MAP, 2.5 ug (FA)
B7 = Placebo MAP (FA); B8 = A/Sing MAP, 15 ug (UA); B9 = A/Sing IM, 15 ug, as 2018 Afluria Quad (UA)

Listing 16.2.9.11
Individual Characterisation of Skin Irritation Index by Treatment
Oedema
Part B, Non-biopsy
B4= 2 x 5 ug A/Sing MAPs + 1 uncoated MAP on volar forearm (10 ug)

			Active			Placebo		
Randomisation Number	Visit	Time Point	#> 0	Maximum	Average	#> 0	Maximum	Average
B0150	Day 1	Pre-dose	0	0	0.0	0	0	0.0
B0150	Day 1	10 min	2	1	1.0	1	1	1.0
B0150	Day 1	1 hour	2	2	2.0	1	1	1.0
B0150	Day 1	2 hour	2	2	2.0	1	2	2.0
B0150	Day 2		2	2	1.5	1	1	1.0
B0150	Day 4		2	1	1.0	0	0	0.0
B0150	Day 8		2	1	1.0	0	0	0.0
B0150	Day 22		0	0	0.0	0	0	0.0
B0150	EOS/ET (Day 61)		0	0	0.0	0	0	0.0

Source: Listing 16.2.9.4

Administration Site Key: FA = Forearm; UA= Upper arm
Treatments Part A: A1 = A/Sing MAP, 15 ug (FA); A2 = Afluria Quad IM (UA), 15 ug; A3 = Placebo MAP (FA); A4 = A/Sing Mono IM, 15 ug (UA)
Treatments Part B: B1 = A/Sing MAP, 15 ug (FA) with biopsy; B2 = Placebo MAP (FA) with biopsy
B3 = A/Sing MAP, 15 ug (FA); B4 = A/Sing MAP, 10 ug (FA); B5 = A/Sing MAP, 5 ug (FA); B6 = A/Sing MAP, 2.5 ug (FA)
B7 = Placebo MAP (FA); B8 = A/Sing MAP, 15 ug (UA); B9 = A/Sing IM, 15 ug, as 2018 Afluria Quad (UA)

Listing 16.2.9.11
Individual Characterisation of Skin Irritation Index by Treatment
Oedema
Part B, Non-biopsy
B5= 1 x 5 ug A/Sing MAP + 2 uncoated MAPs on volar forearm (5 ug)

				Active			Placebo	
Randomisation Number	Visit	Time Point	#> 0	Maximum	Average	#> 0	Maximum	Average
B0013	Day 1	Pre-dose	0	0	0.0	0	0	0.0
B0013	Day 1	10 min	0	0	0.0	0	0	0.0
B0013	Day 1	1 hour	1	2	2.0	2	2	1.5
B0013	Day 1	2 hour	1	1	1.0	2	1	1.0
B0013	Day 2		1	1	1.0	2	1	1.0
B0013	Day 4		1	2	2.0	0	0	0.0
B0013	Day 8		0	0	0.0	0	0	0.0
B0013	Day 22		0	0	0.0	0	0	0.0
B0013	EOS/ET (Day 61)		0	0	0.0	0	0	0.0
B0024	Day 1	Pre-dose	0	0	0.0	0	0	0.0
B0024	Day 1	10 min	1	1	1.0	2	1	1.0
B0024	Day 1	1 hour	1	2	2.0	2	1	1.0
B0024	Day 1	2 hour	1	3	3.0	2	2	2.0
B0024	Day 2		1	1	1.0	0	0	0.0
B0024	Day 4		1	1	1.0	0	0	0.0
B0024	Day 8		0	0	0.0	0	0	0.0
B0024	Day 22		0	0	0.0	0	0	0.0
B0024	EOS/ET (Day 61)		0	0	0.0	0	0	0.0
B0028	Day 1	Pre-dose	0	0	0.0	0	0	0.0

Administration Site Key: FA = Forearm; UA= Upper arm
Treatments Part A: A1 = A/Sing MAP, 15 ug (FA); A2 = Afluria Quad IM (UA), 15 ug; A3 = Placebo MAP (FA); A4 = A/Sing Mono IM, 15 ug (UA)
Treatments Part B: B1 = A/Sing MAP, 15 ug (FA) with biopsy; B2 = Placebo MAP (FA) with biopsy
B3 = A/Sing MAP, 15 ug (FA); B4 = A/Sing MAP, 10 ug (FA); B5 = A/Sing MAP, 5 ug (FA); B6 = A/Sing MAP, 2.5 ug (FA)
B7 = Placebo MAP (FA); B8 = A/Sing MAP, 15 ug (UA); B9 = A/Sing IM, 15 ug, as 2018 Afluria Quad (UA)

Listing 16.2.9.11
Individual Characterisation of Skin Irritation Index by Treatment
Oedema
Part B, Non-biopsy
B5= 1 x 5 ug A/Sing MAP + 2 uncoated MAPs on volar forearm (5 ug)

			Active			Placebo		
Randomisation Number	Visit	Time Point	#> 0	Maximum	Average	#> 0	Maximum	Average
B0028	Day 1	10 min	1	1	1.0	2	1	1.0
B0028	Day 1	1 hour	1	2	2.0	2	2	2.0
B0028	Day 1	2 hour	1	1	1.0	2	1	1.0
B0028	Day 2		1	1	1.0	2	1	1.0
B0028	Day 4		1	1	1.0	0	0	0.0
B0028	Day 8		1	1	1.0	0	0	0.0
B0028	Day 22		0	0	0.0	0	0	0.0
B0028	EOS/ET (Day 61)		0	0	0.0	0	0	0.0
B0033	Day 1	Pre-dose	0	0	0.0	0	0	0.0
B0033	Day 1	10 min	1	1	1.0	2	1	1.0
B0033	Day 1	1 hour	1	3	3.0	2	2	2.0
B0033	Day 1	2 hour	1	2	2.0	2	1	1.0
B0033	Day 2		1	1	1.0	0	0	0.0
B0033	Day 4		0	0	0.0	0	0	0.0
B0033	Day 8		1	1	1.0	0	0	0.0
B0033	Day 22		0	0	0.0	0	0	0.0
B0033	EOS/ET (Day 61)		0	0	0.0	0	0	0.0
B0042	Day 1	Pre-dose	0	0	0.0	0	0	0.0
B0042	Day 1	10 min	0	0	0.0	0	0	0.0

Source: Listing 16.2.9.4

Administration Site Key: FA = Forearm; UA= Upper arm
Treatments Part A: A1 = A/Sing MAP, 15 ug (FA); A2 = Afluria Quad IM (UA), 15 ug; A3 = Placebo MAP (FA); A4 = A/Sing Mono IM, 15 ug (UA)
Treatments Part B: B1 = A/Sing MAP, 15 ug (FA) with biopsy; B2 = Placebo MAP (FA) with biopsy
B3 = A/Sing MAP, 15 ug (FA); B4 = A/Sing MAP, 10 ug (FA); B5 = A/Sing MAP, 5 ug (FA); B6 = A/Sing MAP, 2.5 ug (FA)
B7 = Placebo MAP (FA); B8 = A/Sing MAP, 15 ug (UA); B9 = A/Sing IM, 15 ug, as 2018 Afluria Quad (UA)

Listing 16.2.9.11
Individual Characterisation of Skin Irritation Index by Treatment
Oedema
Part B, Non-biopsy
B5= 1 x 5 ug A/Sing MAP + 2 uncoated MAPs on volar forearm (5 ug)

Randomisation Number	Visit	Time Point	Active #> 0	Active Maximum	Active Average	Placebo #> 0	Placebo Maximum	Placebo Average
B0042	Day 1	1 hour	1	2	2.0	2	2	2.0
B0042	Day 1	2 hour	1	1	1.0	2	1	1.0
B0042	Day 2		1	1	1.0	0	0	0.0
B0042	Day 4		1	1	1.0	0	0	0.0
B0042	Day 8		0	0	0.0	0	0	0.0
B0042	Day 22		1	1	1.0	0	0	0.0
B0042	EOS/ET (Day 61)		0	0	0.0	0	0	0.0
B0052	Day 1	Pre-dose	0	0	0.0	0	0	0.0
B0052	Day 1	10 min	1	1	1.0	1	1	0.5
B0052	Day 1	1 hour	1	1	1.0	2	1	1.0
B0052	Day 1	2 hour	1	1	1.0	2	1	1.0
B0052	Day 2		1	1	1.0	0	0	0.0
B0052	Day 4		0	0	0.0	0	0	0.0
B0052	Day 8		0	0	0.0	0	0	0.0
B0052	Day 22		0	0	0.0	0	0	0.0
B0052	EOS/ET (Day 61)		0	0	0.0	0	0	0.0
B0054	Day 1	Pre-dose	0	0	0.0	0	0	0.0
B0054	Day 1	10 min	1	1	1.0	2	1	1.0
B0054	Day 1	1 hour	1	1	1.0	2	1	1.0

Source: Listing 16.2.9.4

Administration Site Key: FA = Forearm; UA= Upper arm
Treatments Part A: A1 = A/Sing MAP, 15 ug (FA); A2 = Afluria Quad IM (UA), 15 ug; A3 = Placebo MAP (FA); A4 = A/Sing Mono IM, 15 ug (UA)
Treatments Part B: B1 = A/Sing MAP, 15 ug (FA) with biopsy; B2 = Placebo MAP (FA) with biopsy
B3 = A/Sing MAP, 15 ug (FA); B4 = A/Sing MAP, 10 ug (FA); B5 = A/Sing MAP, 5 ug (FA); B6 = A/Sing MAP, 2.5 ug (FA)
B7 = Placebo MAP (FA); B8 = A/Sing MAP, 15 ug (UA); B9 = A/Sing IM, 15 ug, as 2018 Afluria Quad (UA)

Listing 16.2.9.11
Individual Characterisation of Skin Irritation Index by Treatment
Oedema
Part B, Non-biopsy
B5= 1 x 5 ug A/Sing MAP + 2 uncoated MAPs on volar forearm (5 ug)

			Active			Placebo		
Randomisation Number	Visit	Time Point	#> 0	Maximum	Average	#> 0	Maximum	Average
B0054	Day 1	2 hour	1	1	1.0	2	1	1.0
B0054	Day 2		1	2	2.0	0	0	0.0
B0054	Day 4		0	0	0.0	0	0	0.0
B0054	Day 8		0	0	0.0	0	0	0.0
B0054	Day 22		0	0	0.0	0	0	0.0
B0054	EOS/ET (Day 61)		0	0	0.0	0	0	0.0
B0062	Day 1	Pre-dose	0	0	0.0	0	0	0.0
B0062	Day 1	10 min	1	1	1.0	2	1	1.0
B0062	Day 1	1 hour	1	1	1.0	2	1	1.0
B0062	Day 1	2 hour	1	1	1.0	1	1	0.5
B0062	Day 2		1	2	2.0	0	0	0.0
B0062	Day 4		1	1	1.0	0	0	0.0
B0062	Day 8		0	0	0.0	0	0	0.0
B0062	Day 22		0	0	0.0	0	0	0.0
B0062	EOS/ET (Day 61)		0	0	0.0	0	0	0.0
B0068	Day 1	Pre-dose	0	0	0.0	0	0	0.0
B0068	Day 1	10 min	1	1	1.0	2	1	1.0
B0068	Day 1	1 hour	1	2	2.0	2	2	1.5
B0068	Day 1	2 hour	1	2	2.0	2	1	1.0

Source: Listing 16.2.9.4

Administration Site Key: FA = Forearm; UA= Upper arm
Treatments Part A: A1 = A/Sing MAP, 15 ug (FA); A2 = Afluria Quad IM (UA), 15 ug; A3 = Placebo MAP (FA); A4 = A/Sing Mono IM, 15 ug (UA)
Treatments Part B: B1 = A/Sing MAP, 15 ug (FA) with biopsy; B2 = Placebo MAP (FA) with biopsy
B3 = A/Sing MAP, 15 ug (FA); B4 = A/Sing MAP, 10 ug (FA); B5 = A/Sing MAP, 5 ug (FA); B6 = A/Sing MAP, 2.5 ug (FA)
B7 = Placebo MAP (FA); B8 = A/Sing MAP, 15 ug (UA); B9 = A/Sing IM, 15 ug, as 2018 Afluria Quad (UA)

Listing 16.2.9.11
Individual Characterisation of Skin Irritation Index by Treatment
Oedema
Part B, Non-biopsy
B5= 1 x 5 ug A/Sing MAP + 2 uncoated MAPs on volar forearm (5 ug)

Randomisation Number	Visit	Time Point	Active #> 0	Active Maximum	Active Average	Placebo #> 0	Placebo Maximum	Placebo Average
B0068	Day 8		1	1	1.0	0	0	0.0
B0068	Day 22		0	0	0.0	0	0	0.0
B0068	EOS/ET (Day 61)		0	0	0.0	0	0	0.0
B0084	Day 1	Pre-dose	0	0	0.0	0	0	0.0
B0084	Day 1	10 min	0	0	0.0	0	0	0.0
B0084	Day 1	1 hour	0	0	0.0	0	0	0.0
B0084	Day 1	2 hour	0	0	0.0	0	0	0.0
B0084	Day 2		1	2	2.0	1	1	0.5
B0084	Day 4		0	0	0.0	0	0	0.0
B0084	Day 8		1	1	1.0	0	0	0.0
B0084	Day 22		0	0	0.0	0	0	0.0
B0084	EOS/ET (Day 61)		0	0	0.0	0	0	0.0
B0089	Day 1	Pre-dose	0	0	0.0	0	0	0.0
B0089	Day 1	10 min	1	2	2.0	2	1	1.0
B0089	Day 1	1 hour	1	2	2.0	2	1	1.0
B0089	Day 1	2 hour	1	1	1.0	2	1	1.0
B0089	Day 2		1	1	1.0	1	1	0.5
B0089	Day 4		0	0	0.0	0	0	0.0
B0089	Day 8		0	0	0.0	0	0	0.0

Source: Listing 16.2.9.4

Administration Site Key: FA = Forearm; UA= Upper arm
Treatments Part A: A1 = A/Sing MAP, 15 ug (FA); A2 = Afluria Quad IM (UA), 15 ug; A3 = Placebo MAP (FA); A4 = A/Sing Mono IM, 15 ug (UA)
Treatments Part B: B1 = A/Sing MAP, 15 ug (FA) with biopsy; B2 = Placebo MAP (FA) with biopsy
B3 = A/Sing MAP, 15 ug (FA); B4 = A/Sing MAP, 10 ug (FA); B5 = A/Sing MAP, 5 ug (FA); B6 = A/Sing MAP, 2.5 ug (FA)
B7 = Placebo MAP (FA); B8 = A/Sing MAP, 15 ug (UA); B9 = A/Sing IM, 15 ug, as 2018 Afluria Quad (UA)

Listing 16.2.9.11
Individual Characterisation of Skin Irritation Index by Treatment
Oedema
Part B, Non-biopsy
B5= 1 x 5 ug A/Sing MAP + 2 uncoated MAPs on volar forearm (5 ug)

			Active			Placebo		
Randomisation Number	Visit	Time Point	#> 0	Maximum	Average	#> 0	Maximum	Average
B0089	Day 22		0	0	0.0	0	0	0.0
B0089	EOS/ET (Day 61)		0	0	0.0	0	0	0.0
B0097	Day 1	Pre-dose	0	0	0.0	0	0	0.0
B0097	Day 1	10 min	1	2	2.0	2	2	1.5
B0097	Day 1	1 hour	1	2	2.0	2	1	1.0
B0097	Day 1	2 hour	1	1	1.0	0	0	0.0
B0097	Day 2		1	2	2.0	2	1	1.0
B0097	Day 4		0	0	0.0	2	2	1.5
B0097	Day 8		0	0	0.0	0	0	0.0
B0097	Day 22		0	0	0.0	0	0	0.0
B0097	EOS/ET (Day 61)		0	0	0.0	0	0	0.0
B0103	Day 1	Pre-dose	0	0	0.0	0	0	0.0
B0103	Day 1	10 min	1	2	2.0	2	2	2.0
B0103	Day 1	1 hour	1	2	2.0	2	2	2.0
B0103	Day 1	2 hour	1	1	1.0	2	1	1.0
B0103	Day 2		0	0	0.0	0	0	0.0
B0103	Day 4		0	0	0.0	0	0	0.0
B0103	Day 8		0	0	0.0	0	0	0.0
B0103	Day 22		0	0	0.0	0	0	0.0

Source: Listing 16.2.9.4

Administration Site Key: FA = Forearm; UA= Upper arm
Treatments Part A: A1 = A/Sing MAP, 15 ug (FA); A2 = Afluria Quad IM (UA), 15 ug; A3 = Placebo MAP (FA); A4 = A/Sing Mono IM, 15 ug (UA)
Treatments Part B: B1 = A/Sing MAP, 15 ug (FA) with biopsy; B2 = Placebo MAP (FA) with biopsy
B3 = A/Sing MAP, 15 ug (FA); B4 = A/Sing MAP, 10 ug (FA); B5 = A/Sing MAP, 5 ug (FA); B6 = A/Sing MAP, 2.5 ug (FA)
B7 = Placebo MAP (FA); B8 = A/Sing MAP, 15 ug (UA); B9 = A/Sing IM, 15 ug, as 2018 Afluria Quad (UA)

Listing 16.2.9.11
Individual Characterisation of Skin Irritation Index by Treatment
Oedema
Part B, Non-biopsy
B5= 1 x 5 ug A/Sing MAP + 2 uncoated MAPs on volar forearm (5 ug)

			Active			Placebo		
Randomisation Number	Visit	Time Point	#> 0	Maximum	Average	#> 0	Maximum	Average
B0103	EOS/ET (Day 61)		0	0	0.0	0	0	0.0
B0113	Day 1	Pre-dose	0	0	0.0	0	0	0.0
B0113	Day 1	10 min	1	2	2.0	2	3	2.5
B0113	Day 1	1 hour	1	3	3.0	1	1	0.5
B0113	Day 1	2 hour	1	3	3.0	2	2	1.5
B0113	Day 2		1	2	2.0	0	0	0.0
B0113	Day 4		0	0	0.0	0	0	0.0
B0113	Day 8		0	0	0.0	0	0	0.0
B0113	Day 22		0	0	0.0	0	0	0.0
B0113	EOS/ET (Day 61)		0	0	0.0	0	0	0.0
B0122	Day 1	Pre-dose	0	0	0.0	0	0	0.0
B0122	Day 1	10 min	1	1	1.0	2	1	1.0
B0122	Day 1	1 hour	1	2	2.0	0	0	0.0
B0122	Day 1	2 hour	1	3	3.0	2	3	2.5
B0122	Day 2		1	1	1.0	0	0	0.0
B0122	Day 4		1	3	3.0	1	1	0.5
B0122	Day 8		0	0	0.0	0	0	0.0
B0122	Day 22		1	1	1.0	0	0	0.0
B0122	EOS/ET (Day 61)		0	0	0.0	0	0	0.0

Source: Listing 16.2.9.4

Administration Site Key: FA = Forearm; UA= Upper arm
Treatments Part A: A1 = A/Sing MAP, 15 ug (FA); A2 = Afluria Quad IM (UA), 15 ug; A3 = Placebo MAP (FA); A4 = A/Sing Mono IM, 15 ug (UA)
Treatments Part B: B1 = A/Sing MAP, 15 ug (FA) with biopsy; B2 = Placebo MAP (FA) with biopsy
B3 = A/Sing MAP, 15 ug (FA); B4 = A/Sing MAP, 10 ug (FA); B5 = A/Sing MAP, 5 ug (FA); B6 = A/Sing MAP, 2.5 ug (FA)
B7 = Placebo MAP (FA); B8 = A/Sing MAP, 15 ug (UA); B9 = A/Sing IM, 15 ug, as 2018 Afluria Quad (UA)

Listing 16.2.9.11
Individual Characterisation of Skin Irritation Index by Treatment
Oedema
Part B, Non-biopsy
B5= 1 x 5 ug A/Sing MAP + 2 uncoated MAPs on volar forearm (5 ug)

			Active			Placebo		
Randomisation Number	Visit	Time Point	#> 0	Maximum	Average	#> 0	Maximum	Average
B0128	Day 1	Pre-dose	0	0	0.0	0	0	0.0
B0128	Day 1	10 min	1	1	1.0	2	1	1.0
B0128	Day 1	1 hour	1	1	1.0	2	2	1.5
B0128	Day 1	2 hour	1	2	2.0	1	2	1.0
B0128	Day 2		1	1	1.0	0	0	0.0
B0128	Day 4		1	3	3.0	0	0	0.0
B0128	Day 8		0	0	0.0	0	0	0.0
B0128	Day 22		1	1	1.0	0	0	0.0
B0128	EOS/ET (Day 61)		0	0	0.0	0	0	0.0
B0130	Day 1	Pre-dose	0	0	0.0	0	0	0.0
B0130	Day 1	10 min	1	2	2.0	2	2	2.0
B0130	Day 1	1 hour	1	2	2.0	2	2	1.5
B0130	Day 1	2 hour	1	2	2.0	2	2	2.0
B0130	Day 2		1	2	2.0	2	1	1.0
B0130	Day 4		0	0	0.0	0	0	0.0
B0130	Day 8		1	1	1.0	0	0	0.0
B0130	Day 22		0	0	0.0	0	0	0.0
B0130	EOS/ET (Day 61)		0	0	0.0	0	0	0.0

Administration Site Key: FA = Forearm; UA= Upper arm
Treatments Part A: A1 = A/Sing MAP, 15 ug (FA); A2 = Afluria Quad IM (UA), 15 ug; A3 = Placebo MAP (FA); A4 = A/Sing Mono IM, 15 ug (UA)
Treatments Part B: B1 = A/Sing MAP, 15 ug (FA) with biopsy; B2 = Placebo MAP (FA) with biopsy
B3 = A/Sing MAP, 15 ug (FA); B4 = A/Sing MAP, 10 ug (FA); B5 = A/Sing MAP, 5 ug (FA); B6 = A/Sing MAP, 2.5 ug (FA)
B7 = Placebo MAP (FA); B8 = A/Sing MAP, 15 ug (UA); B9 = A/Sing IM, 15 ug, as 2018 Afluria Quad (UA)

Listing 16.2.9.11
Individual Characterisation of Skin Irritation Index by Treatment
Oedema
Part B, Non-biopsy
B5= 1 x 5 ug A/Sing MAP + 2 uncoated MAPs on volar forearm (5 ug)

			Active			Placebo		
Randomisation Number	Visit	Time Point	#> 0	Maximum	Average	#> 0	Maximum	Average
B0142	Day 1	Pre-dose	0	0	0.0	0	0	0.0
B0142	Day 1	10 min	1	1	1.0	2	1	1.0
B0142	Day 1	1 hour	1	1	1.0	2	1	1.0
B0142	Day 1	2 hour	1	1	1.0	2	1	1.0
B0142	Day 2		1	1	1.0	0	0	0.0
B0142	Day 4		0	0	0.0	0	0	0.0
B0142	Day 8		1	2	2.0	2	1	1.0
B0142	Day 22		0	0	0.0	0	0	0.0
B0142	EOS/ET (Day 61)		0	0	0.0	0	0	0.0
B0148	Day 1	Pre-dose	0	0	0.0	0	0	0.0
B0148	Day 1	10 min	1	2	2.0	2	1	1.0
B0148	Day 1	1 hour	1	1	1.0	2	1	1.0
B0148	Day 1	2 hour	1	2	2.0	2	2	1.5
B0148	Day 2		1	2	2.0	0	0	0.0
B0148	Day 4		0	0	0.0	0	0	0.0
B0148	Day 8		1	1	1.0	0	0	0.0
B0148	Day 22		0	0	0.0	0	0	0.0
B0148	EOS/ET (Day 61)		0	0	0.0	0	0	0.0
B1076	Day 1	Pre-dose	0	0	0.0	0	0	0.0

Administration Site Key: FA = Forearm; UA= Upper arm
Treatments Part A: A1 = A/Sing MAP, 15 ug (FA); A2 = Afluria Quad IM (UA), 15 ug; A3 = Placebo MAP (FA); A4 = A/Sing Mono IM, 15 ug (UA)
Treatments Part B: B1 = A/Sing MAP, 15 ug (FA) with biopsy; B2 = Placebo MAP (FA) with biopsy
B3 = A/Sing MAP, 15 ug (FA); B4 = A/Sing MAP, 10 ug (FA); B5 = A/Sing MAP, 5 ug (FA); B6 = A/Sing MAP, 2.5 ug (FA)
B7 = Placebo MAP (FA); B8 = A/Sing MAP, 15 ug (UA); B9 = A/Sing IM, 15 ug, as 2018 Afluria Quad (UA)

Listing 16.2.9.11
Individual Characterisation of Skin Irritation Index by Treatment
Oedema
Part B, Non-biopsy
B5= 1 x 5 ug A/Sing MAP + 2 uncoated MAPs on volar forearm (5 ug)

			Active			Placebo		
Randomisation Number	Visit	Time Point	#> 0	Maximum	Average	#> 0	Maximum	Average
B1076	Day 1	10 min	1	3	3.0	1	1	0.5
B1076	Day 1	1 hour	1	3	3.0	0	0	0.0
B1076	Day 1	2 hour	1	1	1.0	1	1	0.5
B1076	Day 2		1	1	1.0	0	0	0.0
B1076	Day 4		0	0	0.0	0	0	0.0
B1076	Day 8		0	0	0.0	0	0	0.0
B1076	Day 22		0	0	0.0	0	0	0.0
B1076	EOS/ET (Day 61)		0	0	0.0	0	0	0.0

Source: Listing 16.2.9.4

Administration Site Key: FA = Forearm; UA= Upper arm
Treatments Part A: A1 = A/Sing MAP, 15 ug (FA); A2 = Afluria Quad IM (UA), 15 ug; A3 = Placebo MAP (FA); A4 = A/Sing Mono IM, 15 ug (UA)
Treatments Part B: B1 = A/Sing MAP, 15 ug (FA) with biopsy; B2 = Placebo MAP (FA) with biopsy
B3 = A/Sing MAP, 15 ug (FA); B4 = A/Sing MAP, 10 ug (FA); B5 = A/Sing MAP, 5 ug (FA); B6 = A/Sing MAP, 2.5 ug (FA)
B7 = Placebo MAP (FA); B8 = A/Sing MAP, 15 ug (UA); B9 = A/Sing IM, 15 ug, as 2018 Afluria Quad (UA)

Listing 16.2.9.11
Individual Characterisation of Skin Irritation Index by Treatment
Oedema
Part B, Non-biopsy
B6= 1 x 2.5 ug A/Sing MAP + 2 uncoated MAPs on volar forearm (2.5 ug)

			Active			Placebo		
Randomisation Number	Visit	Time Point	#> 0	Maximum	Average	#> 0	Maximum	Average
B0012	Day 1	Pre-dose	0	0	0.0	0	0	0.0
B0012	Day 1	10 min	1	1	1.0	2	1	1.0
B0012	Day 1	1 hour	1	1	1.0	1	1	0.5
B0012	Day 1	2 hour	1	1	1.0	2	1	1.0
B0012	Day 2		1	1	1.0	2	1	1.0
B0012	Day 4		1	3	3.0	2	2	2.0
B0012	Day 8		0	0	0.0	0	0	0.0
B0012	Day 22		0	0	0.0	0	0	0.0
B0012	EOS/ET (Day 61)		0	0	0.0	0	0	0.0
B0020	Day 1	Pre-dose	0	0	0.0	0	0	0.0
B0020	Day 1	10 min	1	1	1.0	2	1	1.0
B0020	Day 1	1 hour	1	1	1.0	2	1	1.0
B0020	Day 1	2 hour	1	1	1.0	2	1	1.0
B0020	Day 2		1	1	1.0	0	0	0.0
B0020	Day 4		0	0	0.0	0	0	0.0
B0020	Day 8		0	0	0.0	0	0	0.0
B0020	Day 22		0	0	0.0	0	0	0.0
B0020	EOS/ET (Day 61)		0	0	0.0	0	0	0.0
B0029	Day 1	Pre-dose	0	0	0.0	0	0	0.0

Source: Listing 16.2.9.4

Administration Site Key: FA = Forearm; UA= Upper arm
Treatments Part A: A1 = A/Sing MAP, 15 ug (FA); A2 = Afluria Quad IM (UA), 15 ug; A3 = Placebo MAP (FA); A4 = A/Sing Mono IM, 15 ug (UA)
Treatments Part B: B1 = A/Sing MAP, 15 ug (FA) with biopsy; B2 = Placebo MAP (FA) with biopsy
B3 = A/Sing MAP, 15 ug (FA); B4 = A/Sing MAP, 10 ug (FA); B5 = A/Sing MAP, 5 ug (FA); B6 = A/Sing MAP, 2.5 ug (FA)
B7 = Placebo MAP (FA); B8 = A/Sing MAP, 15 ug (UA); B9 = A/Sing IM, 15 ug, as 2018 Afluria Quad (UA)

Listing 16.2.9.11
Individual Characterisation of Skin Irritation Index by Treatment
Oedema
Part B, Non-biopsy
B6= 1 x 2.5 ug A/Sing MAP + 2 uncoated MAPs on volar forearm (2.5 ug)

			Active			Placebo		
Randomisation Number	Visit	Time Point	#> 0	Maximum	Average	#> 0	Maximum	Average
B0029	Day 1	10 min	1	1	1.0	2	1	1.0
B0029	Day 1	1 hour	1	2	2.0	2	2	2.0
B0029	Day 1	2 hour	0	0	0.0	0	0	0.0
B0029	Day 2		1	1	1.0	0	0	0.0
B0029	Day 4		1	3	3.0	2	1	1.0
B0029	Day 8		0	0	0.0	0	0	0.0
B0029	Day 22		0	0	0.0	0	0	0.0
B0029	EOS/ET (Day 61)		0	0	0.0	0	0	0.0
B0032	Day 1	Pre-dose	0	0	0.0	0	0	0.0
B0032	Day 1	10 min	0	0	0.0	0	0	0.0
B0032	Day 1	1 hour	1	2	2.0	2	2	1.5
B0032	Day 1	2 hour	1	1	1.0	2	2	1.5
B0032	Day 2		1	2	2.0	2	1	1.0
B0032	Day 4		0	0	0.0	0	0	0.0
B0032	Day 8		0	0	0.0	0	0	0.0
B0032	Day 22		0	0	0.0	0	0	0.0
B0032	EOS/ET (Day 61)		0	0	0.0	0	0	0.0
B0044	Day 1	Pre-dose	0	0	0.0	0	0	0.0
B0044	Day 1	10 min	1	1	1.0	2	1	1.0

Source: Listing 16.2.9.4

Administration Site Key: FA = Forearm; UA= Upper arm
Treatments Part A: A1 = A/Sing MAP, 15 ug (FA); A2 = Afluria Quad IM (UA), 15 ug; A3 = Placebo MAP (FA); A4 = A/Sing Mono IM, 15 ug (UA)
Treatments Part B: B1 = A/Sing MAP, 15 ug (FA) with biopsy; B2 = Placebo MAP (FA) with biopsy
B3 = A/Sing MAP, 15 ug (FA); B4 = A/Sing MAP, 10 ug (FA); B5 = A/Sing MAP, 5 ug (FA); B6 = A/Sing MAP, 2.5 ug (FA)
B7 = Placebo MAP (FA); B8 = A/Sing MAP, 15 ug (UA); B9 = A/Sing IM, 15 ug, as 2018 Afluria Quad (UA)

Listing 16.2.9.11
Individual Characterisation of Skin Irritation Index by Treatment
Oedema
Part B, Non-biopsy
B6= 1 x 2.5 ug A/Sing MAP + 2 uncoated MAPs on volar forearm (2.5 ug)

Randomisation Number	Visit	Time Point	Active #> 0	Active Maximum	Active Average	Placebo #> 0	Placebo Maximum	Placebo Average
B0044	Day 1	1 hour	1	2	2.0	2	2	2.0
B0044	Day 1	2 hour	1	2	2.0	2	2	2.0
B0044	Day 2		1	1	1.0	0	0	0.0
B0044	Day 4		1	1	1.0	0	0	0.0
B0044	Day 8		0	0	0.0	0	0	0.0
B0044	Day 22		0	0	0.0	0	0	0.0
B0044	EOS/ET (Day 61)		0	0	0.0	0	0	0.0
B0048	Day 1	Pre-dose	0	0	0.0	0	0	0.0
B0048	Day 1	10 min	1	1	1.0	2	1	1.0
B0048	Day 1	1 hour	0	0	0.0	1	2	1.0
B0048	Day 1	2 hour	1	1	1.0	2	2	2.0
B0048	Day 2		1	1	1.0	0	0	0.0
B0048	Day 4		0	0	0.0	0	0	0.0
B0048	Day 8		0	0	0.0	0	0	0.0
B0048	Day 22		0	0	0.0	0	0	0.0
B0048	EOS/ET (Day 61)		0	0	0.0	0	0	0.0
B0053	Day 1	Pre-dose	0	0	0.0	0	0	0.0
B0053	Day 1	10 min	1	1	1.0	2	1	1.0
B0053	Day 1	1 hour	1	1	1.0	2	1	1.0

Source: Listing 16.2.9.4

Administration Site Key: FA = Forearm; UA= Upper arm
Treatments Part A: A1 = A/Sing MAP, 15 ug (FA); A2 = Afluria Quad IM (UA), 15 ug; A3 = Placebo MAP (FA); A4 = A/Sing Mono IM, 15 ug (UA)
Treatments Part B: B1 = A/Sing MAP, 15 ug (FA) with biopsy; B2 = Placebo MAP (FA) with biopsy
B3 = A/Sing MAP, 15 ug (FA); B4 = A/Sing MAP, 10 ug (FA); B5 = A/Sing MAP, 5 ug (FA); B6 = A/Sing MAP, 2.5 ug (FA)
B7 = Placebo MAP (FA); B8 = A/Sing MAP, 15 ug (UA); B9 = A/Sing IM, 15 ug, as 2018 Afluria Quad (UA)

Listing 16.2.9.11
Individual Characterisation of Skin Irritation Index by Treatment
Oedema
Part B, Non-biopsy
B6= 1 x 2.5 ug A/Sing MAP + 2 uncoated MAPs on volar forearm (2.5 ug)

			Active			Placebo		
Randomisation Number	Visit	Time Point	#> 0	Maximum	Average	#> 0	Maximum	Average
B0053	Day 1	2 hour	1	1	1.0	2	1	1.0
B0053	Day 2		1	1	1.0	0	0	0.0
B0053	Day 4		0	0	0.0	0	0	0.0
B0053	Day 8		1	1	1.0	0	0	0.0
B0053	Day 22		0	0	0.0	0	0	0.0
B0053	EOS/ET (Day 61)		0	0	0.0	0	0	0.0
B0066	Day 1	Pre-dose	0	0	0.0	0	0	0.0
B0066	Day 1	10 min	1	1	1.0	2	1	1.0
B0066	Day 1	1 hour	1	1	1.0	1	1	0.5
B0066	Day 1	2 hour	1	1	1.0	2	1	1.0
B0066	Day 2		1	1	1.0	0	0	0.0
B0066	Day 4		0	0	0.0	0	0	0.0
B0066	Day 8		0	0	0.0	0	0	0.0
B0066	Day 22		0	0	0.0	0	0	0.0
B0066	EOS/ET (Day 61)		0	0	0.0	0	0	0.0
B0073	Day 1	Pre-dose	0	0	0.0	0	0	0.0
B0073	Day 1	10 min	1	1	1.0	0	0	0.0
B0073	Day 1	1 hour	1	2	2.0	1	1	0.5
B0073	Day 1	2 hour	1	3	3.0	1	2	1.0

Administration Site Key: FA = Forearm; UA= Upper arm
Treatments Part A: A1 = A/Sing MAP, 15 ug (FA); A2 = Afluria Quad IM (UA), 15 ug; A3 = Placebo MAP (FA); A4 = A/Sing Mono IM, 15 ug (UA)
Treatments Part B: B1 = A/Sing MAP, 15 ug (FA) with biopsy; B2 = Placebo MAP (FA) with biopsy
B3 = A/Sing MAP, 15 ug (FA); B4 = A/Sing MAP, 10 ug (FA); B5 = A/Sing MAP, 5 ug (FA); B6 = A/Sing MAP, 2.5 ug (FA)
B7 = Placebo MAP (FA); B8 = A/Sing MAP, 15 ug (UA); B9 = A/Sing IM, 15 ug, as 2018 Afluria Quad (UA)

Listing 16.2.9.11
Individual Characterisation of Skin Irritation Index by Treatment
Oedema
Part B, Non-biopsy
B6= 1 x 2.5 ug A/Sing MAP + 2 uncoated MAPs on volar forearm (2.5 ug)

			Active			Placebo		
Randomisation Number	Visit	Time Point	#> 0	Maximum	Average	#> 0	Maximum	Average
B0073	Day 2		1	2	2.0	2	1	1.0
B0073	Day 4		1	1	1.0	1	1	0.5
B0073	Day 8		0	0	0.0	0	0	0.0
B0073	Day 22		0	0	0.0	0	0	0.0
B0073	EOS/ET (Day 61)		0	0	0.0	0	0	0.0
B0079	Day 1	Pre-dose	0	0	0.0	0	0	0.0
B0079	Day 1	10 min	1	1	1.0	1	1	0.5
B0079	Day 1	1 hour	1	1	1.0	2	1	1.0
B0079	Day 1	2 hour	1	3	3.0	2	3	2.5
B0079	Day 2		1	2	2.0	0	0	0.0
B0079	Day 4		1	3	3.0	2	2	1.5
B0079	Day 8		0	0	0.0	0	0	0.0
B0079	Day 22		0	0	0.0	0	0	0.0
B0079	EOS/ET (Day 61)		0	0	0.0	0	0	0.0
B0081	Day 1	Pre-dose	0	0	0.0	0	0	0.0
B0081	Day 1	10 min	1	1	1.0	2	1	1.0
B0081	Day 1	1 hour	1	1	1.0	2	1	1.0
B0081	Day 1	2 hour	1	3	3.0	2	3	3.0
B0081	Day 2		1	1	1.0	1	1	0.5

Administration Site Key: FA = Forearm; UA= Upper arm
Treatments Part A: A1 = A/Sing MAP, 15 ug (FA); A2 = Afluria Quad IM (UA), 15 ug; A3 = Placebo MAP (FA); A4 = A/Sing Mono IM, 15 ug (UA)
Treatments Part B: B1 = A/Sing MAP, 15 ug (FA) with biopsy; B2 = Placebo MAP (FA) with biopsy
B3 = A/Sing MAP, 15 ug (FA); B4 = A/Sing MAP, 10 ug (FA); B5 = A/Sing MAP, 5 ug (FA); B6 = A/Sing MAP, 2.5 ug (FA)
B7 = Placebo MAP (FA); B8 = A/Sing MAP, 15 ug (UA); B9 = A/Sing IM, 15 ug, as 2018 Afluria Quad (UA)

Listing 16.2.9.11
Individual Characterisation of Skin Irritation Index by Treatment
Oedema
Part B, Non-biopsy
B6= 1 x 2.5 ug A/Sing MAP + 2 uncoated MAPs on volar forearm (2.5 ug)

			Active			Placebo		
Randomisation Number	Visit	Time Point	#> 0	Maximum	Average	#> 0	Maximum	Average
B0081	Day 4		0	0	0.0	0	0	0.0
B0081	Day 8		0	0	0.0	0	0	0.0
B0081	Day 22		0	0	0.0	0	0	0.0
B0081	EOS/ET (Day 61)		0	0	0.0	0	0	0.0
B0090	Day 1	Pre-dose	0	0	0.0	0	0	0.0
B0090	Day 1	10 min	1	1	1.0	0	0	0.0
B0090	Day 1	1 hour	1	1	1.0	2	1	1.0
B0090	Day 1	2 hour	1	1	1.0	2	1	1.0
B0090	Day 2		1	1	1.0	0	0	0.0
B0090	Day 4		0	0	0.0	0	0	0.0
B0090	Day 8		0	0	0.0	0	0	0.0
B0090	Day 22		0	0	0.0	0	0	0.0
B0090	EOS/ET (Day 61)		0	0	0.0	0	0	0.0
B0096	Day 1	Pre-dose	0	0	0.0	0	0	0.0
B0096	Day 1	10 min	1	3	3.0	2	3	2.5
B0096	Day 1	1 hour	1	2	2.0	2	2	2.0
B0096	Day 1	2 hour	1	2	2.0	2	2	2.0
B0096	Day 2		1	1	1.0	2	1	1.0
B0096	Day 4		0	0	0.0	0	0	0.0

Source: Listing 16.2.9.4

Administration Site Key: FA = Forearm; UA= Upper arm
Treatments Part A: A1 = A/Sing MAP, 15 ug (FA); A2 = Afluria Quad IM (UA), 15 ug; A3 = Placebo MAP (FA); A4 = A/Sing Mono IM, 15 ug (UA)
Treatments Part B: B1 = A/Sing MAP, 15 ug (FA) with biopsy; B2 = Placebo MAP (FA) with biopsy
B3 = A/Sing MAP, 15 ug (FA); B4 = A/Sing MAP, 10 ug (FA); B5 = A/Sing MAP, 5 ug (FA); B6 = A/Sing MAP, 2.5 ug (FA)
B7 = Placebo MAP (FA); B8 = A/Sing MAP, 15 ug (UA); B9 = A/Sing IM, 15 ug, as 2018 Afluria Quad (UA)

Listing 16.2.9.11
Individual Characterisation of Skin Irritation Index by Treatment
Oedema
Part B, Non-biopsy
B6= 1 x 2.5 ug A/Sing MAP + 2 uncoated MAPs on volar forearm (2.5 ug)

			Active			Placebo		
Randomisation Number	Visit	Time Point	#> 0	Maximum	Average	#> 0	Maximum	Average
B0096	Day 8		0	0	0.0	0	0	0.0
B0096	Day 22		0	0	0.0	0	0	0.0
B0096	EOS/ET (Day 61)		0	0	0.0	0	0	0.0
B0107	Day 1	Pre-dose	0	0	0.0	0	0	0.0
B0107	Day 1	10 min	1	1	1.0	2	1	1.0
B0107	Day 1	1 hour	1	2	2.0	2	1	1.0
B0107	Day 1	2 hour	1	1	1.0	2	1	1.0
B0107	Day 2		1	1	1.0	2	1	1.0
B0107	Day 4		0	0	0.0	0	0	0.0
B0107	Day 8		0	0	0.0	0	0	0.0
B0107	Day 22		0	0	0.0	0	0	0.0
B0107	EOS/ET (Day 61)		0	0	0.0	0	0	0.0
B0109	Day 1	Pre-dose	0	0	0.0	0	0	0.0
B0109	Day 1	10 min	1	2	2.0	0	0	0.0
B0109	Day 1	1 hour	1	3	3.0	2	2	1.5
B0109	Day 1	2 hour	1	2	2.0	2	1	1.0
B0109	Day 2		1	1	1.0	0	0	0.0
B0109	Day 4		1	3	3.0	0	0	0.0
B0109	Day 8		0	0	0.0	0	0	0.0

Administration Site Key: FA = Forearm; UA= Upper arm
Treatments Part A: A1 = A/Sing MAP, 15 ug (FA); A2 = Afluria Quad IM (UA), 15 ug; A3 = Placebo MAP (FA); A4 = A/Sing Mono IM, 15 ug (UA)
Treatments Part B: B1 = A/Sing MAP, 15 ug (FA) with biopsy; B2 = Placebo MAP (FA) with biopsy
B3 = A/Sing MAP, 15 ug (FA); B4 = A/Sing MAP, 10 ug (FA); B5 = A/Sing MAP, 5 ug (FA); B6 = A/Sing MAP, 2.5 ug (FA)
B7 = Placebo MAP (FA); B8 = A/Sing MAP, 15 ug (UA); B9 = A/Sing IM, 15 ug, as 2018 Afluria Quad (UA)

Listing 16.2.9.11
Individual Characterisation of Skin Irritation Index by Treatment
Oedema
Part B, Non-biopsy
B6= 1 x 2.5 ug A/Sing MAP + 2 uncoated MAPs on volar forearm (2.5 ug)

			Active			Placebo		
Randomisation Number	Visit	Time Point	#> 0	Maximum	Average	#> 0	Maximum	Average
B0109	Day 22		0	0	0.0	0	0	0.0
B0109	EOS/ET (Day 61)		0	0	0.0	0	0	0.0
B0118	Day 1	Pre-dose	0	0	0.0	0	0	0.0
B0118	Day 1	10 min	1	1	1.0	2	1	1.0
B0118	Day 1	1 hour	1	1	1.0	2	1	1.0
B0118	Day 1	2 hour	1	1	1.0	2	2	1.5
B0118	Day 2		1	1	1.0	1	1	0.5
B0118	Day 4		0	0	0.0	0	0	0.0
B0118	Day 8		0	0	0.0	0	0	0.0
B0118	Day 22		0	0	0.0	0	0	0.0
B0118	EOS/ET (Day 61)		0	0	0.0	0	0	0.0
B0123	Day 1	Pre-dose	0	0	0.0	0	0	0.0
B0123	Day 1	10 min	1	1	1.0	1	1	0.5
B0123	Day 1	1 hour	0	0	0.0	2	1	1.0
B0123	Day 1	2 hour	1	1	1.0	2	2	1.5
B0123	Day 2		1	1	1.0	0	0	0.0
B0123	Day 4		0	0	0.0	0	0	0.0
B0123	Day 8		0	0	0.0	0	0	0.0
B0123	Day 22		0	0	0.0	0	0	0.0

Administration Site Key: FA = Forearm; UA= Upper arm
Treatments Part A: A1 = A/Sing MAP, 15 ug (FA); A2 = Afluria Quad IM (UA), 15 ug; A3 = Placebo MAP (FA); A4 = A/Sing Mono IM, 15 ug (UA)
Treatments Part B: B1 = A/Sing MAP, 15 ug (FA) with biopsy; B2 = Placebo MAP (FA) with biopsy
B3 = A/Sing MAP, 15 ug (FA); B4 = A/Sing MAP, 10 ug (FA); B5 = A/Sing MAP, 5 ug (FA); B6 = A/Sing MAP, 2.5 ug (FA)
B7 = Placebo MAP (FA); B8 = A/Sing MAP, 15 ug (UA); B9 = A/Sing IM, 15 ug, as 2018 Afluria Quad (UA)

Listing 16.2.9.11
Individual Characterisation of Skin Irritation Index by Treatment
Oedema
Part B, Non-biopsy
B6= 1 x 2.5 ug A/Sing MAP + 2 uncoated MAPs on volar forearm (2.5 ug)

			Active			Placebo		
Randomisation Number	Visit	Time Point	#> 0	Maximum	Average	#> 0	Maximum	Average
B0123	EOS/ET (Day 61)		0	0	0.0	0	0	0.0
B0132	Day 1	Pre-dose	0	0	0.0	0	0	0.0
B0132	Day 1	10 min	1	2	2.0	2	2	2.0
B0132	Day 1	1 hour	1	3	3.0	2	3	2.5
B0132	Day 1	2 hour	1	1	1.0	2	2	2.0
B0132	Day 2		0	0	0.0	0	0	0.0
B0132	Day 4		1	1	1.0	0	0	0.0
B0132	Day 8		0	0	0.0	0	0	0.0
B0132	Day 22		0	0	0.0	0	0	0.0
B0132	EOS/ET (Day 61)		0	0	0.0	0	0	0.0
B0143	Day 1	Pre-dose	0	0	0.0	0	0	0.0
B0143	Day 1	10 min	1	1	1.0	2	2	2.0
B0143	Day 1	1 hour	1	2	2.0	2	2	1.5
B0143	Day 1	2 hour	1	2	2.0	2	1	1.0
B0143	Day 2		1	2	2.0	0	0	0.0
B0143	Day 4		1	2	2.0	0	0	0.0
B0143	Day 8		0	0	0.0	0	0	0.0
B0143	Day 22		0	0	0.0	0	0	0.0
B0143	EOS/ET (Day 61)		0	0	0.0	0	0	0.0

Source: Listing 16.2.9.4

Administration Site Key: FA = Forearm; UA= Upper arm
Treatments Part A: A1 = A/Sing MAP, 15 ug (FA); A2 = Afluria Quad IM (UA), 15 ug; A3 = Placebo MAP (FA); A4 = A/Sing Mono IM, 15 ug (UA)
Treatments Part B: B1 = A/Sing MAP, 15 ug (FA) with biopsy; B2 = Placebo MAP (FA) with biopsy
B3 = A/Sing MAP, 15 ug (FA); B4 = A/Sing MAP, 10 ug (FA); B5 = A/Sing MAP, 5 ug (FA); B6 = A/Sing MAP, 2.5 ug (FA)
B7 = Placebo MAP (FA); B8 = A/Sing MAP, 15 ug (UA); B9 = A/Sing IM, 15 ug, as 2018 Afluria Quad (UA)

Listing 16.2.9.11
Individual Characterisation of Skin Irritation Index by Treatment
Oedema
Part B, Non-biopsy
B6= 1 x 2.5 ug A/Sing MAP + 2 uncoated MAPs on volar forearm (2.5 ug)

			Active			Placebo		
Randomisation Number	Visit	Time Point	#> 0	Maximum	Average	#> 0	Maximum	Average
B0144	Day 1	Pre-dose	0	0	0.0	0	0	0.0
B0144	Day 1	10 min	1	2	2.0	2	1	1.0
B0144	Day 1	1 hour	1	3	3.0	2	3	3.0
B0144	Day 1	2 hour	1	2	2.0	2	2	2.0
B0144	Day 2		1	1	1.0	0	0	0.0
B0144	Day 4		0	0	0.0	0	0	0.0
B0144	Day 8		0	0	0.0	0	0	0.0
B0144	Day 22		0	0	0.0	0	0	0.0
B0144	EOS/ET (Day 61)		0	0	0.0	0	0	0.0

Administration Site Key: FA = Forearm; UA= Upper arm
Treatments Part A: A1 = A/Sing MAP, 15 ug (FA); A2 = Afluria Quad IM (UA), 15 ug; A3 = Placebo MAP (FA); A4 = A/Sing Mono IM, 15 ug (UA)
Treatments Part B: B1 = A/Sing MAP, 15 ug (FA) with biopsy; B2 = Placebo MAP (FA) with biopsy
B3 = A/Sing MAP, 15 ug (FA); B4 = A/Sing MAP, 10 ug (FA); B5 = A/Sing MAP, 5 ug (FA); B6 = A/Sing MAP, 2.5 ug (FA)
B7 = Placebo MAP (FA); B8 = A/Sing MAP, 15 ug (UA); B9 = A/Sing IM, 15 ug, as 2018 Afluria Quad (UA)

Listing 16.2.9.11
Individual Characterisation of Skin Irritation Index by Treatment
Oedema
Part B, Non-biopsy
B7= 3 x uncoated MAPs on volar forearm

			Active			Placebo		
Randomisation Number	Visit	Time Point	#> 0	Maximum	Average	#> 0	Maximum	Average
B0017	Day 1	Pre-dose	-	-	-	0	0	0.0
B0017	Day 1	10 min	-	-	-	0	0	0.0
B0017	Day 1	1 hour	-	-	-	2	1	0.7
B0017	Day 1	2 hour	-	-	-	3	1	1.0
B0017	Day 2		-	-	-	2	1	0.7
B0017	Day 4		-	-	-	3	1	1.0
B0017	Day 8		-	-	-	0	0	0.0
B0017	Day 22		-	-	-	0	0	0.0
B0017	EOS/ET (Day 61)		-	-	-	0	0	0.0
B0021	Day 1	Pre-dose	-	-	-	0	0	0.0
B0021	Day 1	10 min	-	-	-	3	1	1.0
B0021	Day 1	1 hour	-	-	-	3	1	1.0
B0021	Day 1	2 hour	-	-	-	3	1	1.0
B0021	Day 2		-	-	-	0	0	0.0
B0021	Day 4		-	-	-	0	0	0.0
B0021	Day 8		-	-	-	0	0	0.0
B0021	Day 22		-	-	-	0	0	0.0
B0021	EOS/ET (Day 61)		-	-	-	0	0	0.0
B0030	Day 1	Pre-dose	-	-	-	0	0	0.0

Source: Listing 16.2.9.4

Administration Site Key: FA = Forearm; UA= Upper arm
Treatments Part A: A1 = A/Sing MAP, 15 ug (FA); A2 = Afluria Quad IM (UA), 15 ug; A3 = Placebo MAP (FA); A4 = A/Sing Mono IM, 15 ug (UA)
Treatments Part B: B1 = A/Sing MAP, 15 ug (FA) with biopsy; B2 = Placebo MAP (FA) with biopsy
B3 = A/Sing MAP, 15 ug (FA); B4 = A/Sing MAP, 10 ug (FA); B5 = A/Sing MAP, 5 ug (FA); B6 = A/Sing MAP, 2.5 ug (FA)
B7 = Placebo MAP (FA); B8 = A/Sing MAP, 15 ug (UA); B9 = A/Sing IM, 15 ug, as 2018 Afluria Quad (UA)

Listing 16.2.9.11
Individual Characterisation of Skin Irritation Index by Treatment
Oedema
Part B, Non-biopsy
B7= 3 x uncoated MAPs on volar forearm

			Active			Placebo		
Randomisation Number	Visit	Time Point	#> 0	Maximum	Average	#> 0	Maximum	Average
B0030	Day 1	10 min	-	-	-	3	3	2.7
B0030	Day 1	1 hour	-	-	-	3	3	3.0
B0030	Day 1	2 hour	-	-	-	3	2	2.0
B0030	Day 2		-	-	-	0	0	0.0
B0030	Day 4		-	-	-	0	0	0.0
B0030	Day 8		-	-	-	0	0	0.0
B0030	Day 22		-	-	-	0	0	0.0
B0030	EOS/ET (Day 61)		-	-	-	0	0	0.0
B0036	Day 1	Pre-dose	-	-	-	0	0	0.0
B0036	Day 1	10 min	-	-	-	0	0	0.0
B0036	Day 1	1 hour	-	-	-	0	0	0.0
B0036	Day 1	2 hour	-	-	-	1	1	0.3
B0036	Day 2		-	-	-	0	0	0.0
B0036	Day 4		-	-	-	0	0	0.0
B0036	Day 8		-	-	-	0	0	0.0
B0036	Day 22		-	-	-	0	0	0.0
B0036	EOS/ET (Day 61)		-	-	-	0	0	0.0
B0039	Day 1	Pre-dose	-	-	-	0	0	0.0
B0039	Day 1	10 min	-	-	-	1	1	0.3

Source: Listing 16.2.9.4

Administration Site Key: FA = Forearm; UA= Upper arm
Treatments Part A: A1 = A/Sing MAP, 15 ug (FA); A2 = Afluria Quad IM (UA), 15 ug; A3 = Placebo MAP (FA); A4 = A/Sing Mono IM, 15 ug (UA)
Treatments Part B: B1 = A/Sing MAP, 15 ug (FA) with biopsy; B2 = Placebo MAP (FA) with biopsy
B3 = A/Sing MAP, 15 ug (FA); B4 = A/Sing MAP, 10 ug (FA); B5 = A/Sing MAP, 5 ug (FA); B6 = A/Sing MAP, 2.5 ug (FA)
B7 = Placebo MAP (FA); B8 = A/Sing MAP, 15 ug (UA); B9 = A/Sing IM, 15 ug, as 2018 Afluria Quad (UA)

Listing 16.2.9.11
Individual Characterisation of Skin Irritation Index by Treatment
Oedema
Part B, Non-biopsy
B7= 3 x uncoated MAPs on volar forearm

			Active			Placebo		
Randomisation Number	Visit	Time Point	#> 0	Maximum	Average	#> 0	Maximum	Average
B0039	Day 1	1 hour	-	-	-	1	2	0.7
B0039	Day 1	2 hour	-	-	-	3	2	2.0
B0039	Day 2		-	-	-	0	0	0.0
B0039	Day 4		-	-	-	0	0	0.0
B0039	Day 8		-	-	-	0	0	0.0
B0039	Day 22		-	-	-	0	0	0.0
B0039	EOS/ET (Day 61)		-	-	-	0	0	0.0
B0046	Day 1	Pre-dose	-	-	-	0	0	0.0
B0046	Day 1	10 min	-	-	-	1	1	0.3
B0046	Day 1	1 hour	-	-	-	1	1	0.3
B0046	Day 1	2 hour	-	-	-	3	2	1.7
B0046	Day 2		-	-	-	0	0	0.0
B0046	Day 4		-	-	-	0	0	0.0
B0046	Day 8		-	-	-	0	0	0.0
B0046	Day 22		-	-	-	0	0	0.0
B0046	EOS/ET (Day 61)		-	-	-	0	0	0.0
B0059	Day 1	Pre-dose	-	-	-	0	0	0.0
B0059	Day 1	10 min	-	-	-	0	0	0.0
B0059	Day 1	1 hour	-	-	-	0	0	0.0

Source: Listing 16.2.9.4

Administration Site Key: FA = Forearm; UA= Upper arm
Treatments Part A: A1 = A/Sing MAP, 15 ug (FA); A2 = Afluria Quad IM (UA), 15 ug; A3 = Placebo MAP (FA); A4 = A/Sing Mono IM, 15 ug (UA)
Treatments Part B: B1 = A/Sing MAP, 15 ug (FA) with biopsy; B2 = Placebo MAP (FA) with biopsy
B3 = A/Sing MAP, 15 ug (FA); B4 = A/Sing MAP, 10 ug (FA); B5 = A/Sing MAP, 5 ug (FA); B6 = A/Sing MAP, 2.5 ug (FA)
B7 = Placebo MAP (FA); B8 = A/Sing MAP, 15 ug (UA); B9 = A/Sing IM, 15 ug, as 2018 Afluria Quad (UA)

Listing 16.2.9.11
Individual Characterisation of Skin Irritation Index by Treatment
Oedema
Part B, Non-biopsy
B7= 3 x uncoated MAPs on volar forearm

			Active			Placebo		
Randomisation Number	Visit	Time Point	#> 0	Maximum	Average	#> 0	Maximum	Average
B0059	Day 1	2 hour	-	-	-	0	0	0.0
B0059	Day 2		-	-	-	0	0	0.0
B0059	Day 4		-	-	-	0	0	0.0
B0059	Day 8		-	-	-	0	0	0.0
B0059	Day 22		-	-	-	0	0	0.0
B0059	EOS/ET (Day 61)		-	-	-	0	0	0.0
B0063	Day 1	Pre-dose	-	-	-	0	0	0.0
B0063	Day 1	10 min	-	-	-	3	1	1.0
B0063	Day 1	1 hour	-	-	-	0	0	0.0
B0063	Day 1	2 hour	-	-	-	2	1	0.7
B0063	Day 2		-	-	-	0	0	0.0
B0063	Day 4		-	-	-	0	0	0.0
B0063	Day 8		-	-	-	0	0	0.0
B0063	Day 22		-	-	-	0	0	0.0
B0063	EOS/ET (Day 61)		-	-	-	0	0	0.0
B0069	Day 1	Pre-dose	-	-	-	0	0	0.0
B0069	Day 1	10 min	-	-	-	1	1	0.3
B0069	Day 1	1 hour	-	-	-	3	1	1.0
B0069	Day 1	2 hour	-	-	-	2	1	0.7

Source: Listing 16.2.9.4

Administration Site Key: FA = Forearm; UA= Upper arm
Treatments Part A: A1 = A/Sing MAP, 15 ug (FA); A2 = Afluria Quad IM (UA), 15 ug; A3 = Placebo MAP (FA); A4 = A/Sing Mono IM, 15 ug (UA)
Treatments Part B: B1 = A/Sing MAP, 15 ug (FA) with biopsy; B2 = Placebo MAP (FA) with biopsy
B3 = A/Sing MAP, 15 ug (FA); B4 = A/Sing MAP, 10 ug (FA); B5 = A/Sing MAP, 5 ug (FA); B6 = A/Sing MAP, 2.5 ug (FA)
B7 = Placebo MAP (FA); B8 = A/Sing MAP, 15 ug (UA); B9 = A/Sing IM, 15 ug, as 2018 Afluria Quad (UA)

Listing 16.2.9.11
Individual Characterisation of Skin Irritation Index by Treatment
Oedema
Part B, Non-biopsy
B7= 3 x uncoated MAPs on volar forearm

			Active			Placebo		
Randomisation Number	Visit	Time Point	#> 0	Maximum	Average	#> 0	Maximum	Average
B0069	Day 2		-	-	-	0	0	0.0
B0069	Day 4		-	-	-	3	1	1.0
B0069	Day 8		-	-	-	0	0	0.0
B0069	Day 22		-	-	-	0	0	0.0
B0069	EOS/ET (Day 61)		-	-	-	0	0	0.0
B0087	Day 1	Pre-dose	-	-	-	0	0	0.0
B0087	Day 1	10 min	-	-	-	1	1	0.3
B0087	Day 1	1 hour	-	-	-	3	1	1.0
B0087	Day 1	2 hour	-	-	-	0	0	0.0
B0087	Day 2		-	-	-	3	1	1.0
B0087	Day 4		-	-	-	0	0	0.0
B0087	Day 8		-	-	-	0	0	0.0
B0087	Day 22		-	-	-	0	0	0.0
B0087	EOS/ET (Day 61)		-	-	-	0	0	0.0
B0093	Day 1	Pre-dose	-	-	-	0	0	0.0
B0093	Day 1	10 min	-	-	-	3	2	1.7
B0093	Day 1	1 hour	-	-	-	3	2	1.3
B0093	Day 1	2 hour	-	-	-	3	2	1.3
B0093	Day 2		-	-	-	2	1	0.7

Administration Site Key: FA = Forearm; UA= Upper arm
Treatments Part A: A1 = A/Sing MAP, 15 ug (FA); A2 = Afluria Quad IM (UA), 15 ug; A3 = Placebo MAP (FA); A4 = A/Sing Mono IM, 15 ug (UA)
Treatments Part B: B1 = A/Sing MAP, 15 ug (FA) with biopsy; B2 = Placebo MAP (FA) with biopsy
B3 = A/Sing MAP, 15 ug (FA); B4 = A/Sing MAP, 10 ug (FA); B5 = A/Sing MAP, 5 ug (FA); B6 = A/Sing MAP, 2.5 ug (FA)
B7 = Placebo MAP (FA); B8 = A/Sing MAP, 15 ug (UA); B9 = A/Sing IM, 15 ug, as 2018 Afluria Quad (UA)

Listing 16.2.9.11
Individual Characterisation of Skin Irritation Index by Treatment
Oedema
Part B, Non-biopsy
B7= 3 x uncoated MAPs on volar forearm

			Active			Placebo		
Randomisation Number	Visit	Time Point	#> 0	Maximum	Average	#> 0	Maximum	Average
B0093	Day 4		-	-	-	0	0	0.0
B0093	Day 8		-	-	-	0	0	0.0
B0093	Day 22		-	-	-	0	0	0.0
B0093	EOS/ET (Day 61)		-	-	-	0	0	0.0
B0098	Day 1	Pre-dose	-	-	-	0	0	0.0
B0098	Day 1	10 min	-	-	-	3	1	1.0
B0098	Day 1	1 hour	-	-	-	3	2	2.0
B0098	Day 1	2 hour	-	-	-	3	1	1.0
B0098	Day 2		-	-	-	0	0	0.0
B0098	Day 4		-	-	-	0	0	0.0
B0098	Day 8		-	-	-	0	0	0.0
B0098	Day 22		-	-	-	0	0	0.0
B0098	EOS/ET (Day 61)		-	-	-	0	0	0.0
B0108	Day 1	Pre-dose	-	-	-	0	0	0.0
B0108	Day 1	10 min	-	-	-	3	1	1.0
B0108	Day 1	1 hour	-	-	-	3	1	1.0
B0108	Day 1	2 hour	-	-	-	3	1	1.0
B0108	Day 2		-	-	-	2	1	0.7
B0108	Day 4		-	-	-	0	0	0.0

Source: Listing 16.2.9.4

Administration Site Key: FA = Forearm; UA= Upper arm
Treatments Part A: A1 = A/Sing MAP, 15 ug (FA); A2 = Afluria Quad IM (UA), 15 ug; A3 = Placebo MAP (FA); A4 = A/Sing Mono IM, 15 ug (UA)
Treatments Part B: B1 = A/Sing MAP, 15 ug (FA) with biopsy; B2 = Placebo MAP (FA) with biopsy
B3 = A/Sing MAP, 15 ug (FA); B4 = A/Sing MAP, 10 ug (FA); B5 = A/Sing MAP, 5 ug (FA); B6 = A/Sing MAP, 2.5 ug (FA)
B7 = Placebo MAP (FA); B8 = A/Sing MAP, 15 ug (UA); B9 = A/Sing IM, 15 ug, as 2018 Afluria Quad (UA)

Listing 16.2.9.11
Individual Characterisation of Skin Irritation Index by Treatment
Oedema
Part B, Non-biopsy
B7= 3 x uncoated MAPs on volar forearm

Randomisation Number	Visit	Time Point	Active #> 0	Active Maximum	Active Average	Placebo #> 0	Placebo Maximum	Placebo Average
B0108	Day 8		-	-	-	0	0	0.0
B0108	Day 22		-	-	-	0	0	0.0
B0108	EOS/ET (Day 61)		-	-	-	0	0	0.0
B0114	Day 1	Pre-dose	-	-	-	0	0	0.0
B0114	Day 1	10 min	-	-	-	3	2	2.0
B0114	Day 1	1 hour	-	-	-	1	1	0.3
B0114	Day 1	2 hour	-	-	-	3	3	2.3
B0114	Day 2		-	-	-	0	0	0.0
B0114	Day 4		-	-	-	1	1	0.3
B0114	Day 8		-	-	-	1	1	0.3
B0114	Day 22		-	-	-	0	0	0.0
B0114	EOS/ET (Day 61)		-	-	-	0	0	0.0
B0116	Day 1	Pre-dose	-	-	-	0	0	0.0
B0116	Day 1	10 min	-	-	-	3	3	3.0
B0116	Day 1	1 hour	-	-	-	3	3	3.0
B0116	Day 1	2 hour	-	-	-	3	3	2.3
B0116	Day 2		-	-	-	0	0	0.0
B0116	Day 4		-	-	-	0	0	0.0
B0116	Day 8		-	-	-	0	0	0.0

Source: Listing 16.2.9.4

Administration Site Key: FA = Forearm; UA= Upper arm
Treatments Part A: A1 = A/Sing MAP, 15 ug (FA); A2 = Afluria Quad IM (UA), 15 ug; A3 = Placebo MAP (FA); A4 = A/Sing Mono IM, 15 ug (UA)
Treatments Part B: B1 = A/Sing MAP, 15 ug (FA) with biopsy; B2 = Placebo MAP (FA) with biopsy
B3 = A/Sing MAP, 15 ug (FA); B4 = A/Sing MAP, 10 ug (FA); B5 = A/Sing MAP, 5 ug (FA); B6 = A/Sing MAP, 2.5 ug (FA)
B7 = Placebo MAP (FA); B8 = A/Sing MAP, 15 ug (UA); B9 = A/Sing IM, 15 ug, as 2018 Afluria Quad (UA)

Listing 16.2.9.11
Individual Characterisation of Skin Irritation Index by Treatment
Oedema
Part B, Non-biopsy
B7= 3 x uncoated MAPs on volar forearm

			Active			Placebo		
Randomisation Number	Visit	Time Point	#> 0	Maximum	Average	#> 0	Maximum	Average
B0116	Day 22		-	-	-	0	0	0.0
B0116	EOS/ET (Day 61)		-	-	-	0	0	0.0
B0125	Day 1	Pre-dose	-	-	-	0	0	0.0
B0125	Day 1	10 min	-	-	-	3	1	1.0
B0125	Day 1	1 hour	-	-	-	3	1	1.0
B0125	Day 1	2 hour	-	-	-	3	1	1.0
B0125	Day 2		-	-	-	0	0	0.0
B0125	Day 4		-	-	-	0	0	0.0
B0125	Day 8		-	-	-	0	0	0.0
B0125	Day 22		-	-	-	0	0	0.0
B0125	EOS/ET (Day 61)		-	-	-	0	0	0.0
B0135	Day 1	Pre-dose	-	-	-	0	0	0.0
B0135	Day 1	10 min	-	-	-	3	3	3.0
B0135	Day 1	1 hour	-	-	-	3	3	2.7
B0135	Day 1	2 hour	-	-	-	3	3	2.7
B0135	Day 2		-	-	-	0	0	0.0
B0135	Day 4		-	-	-	0	0	0.0
B0135	Day 8		-	-	-	0	0	0.0
B0135	Day 22		-	-	-	0	0	0.0

Source: Listing 16.2.9.4

Administration Site Key: FA = Forearm; UA= Upper arm
Treatments Part A: A1 = A/Sing MAP, 15 ug (FA); A2 = Afluria Quad IM (UA), 15 ug; A3 = Placebo MAP (FA); A4 = A/Sing Mono IM, 15 ug (UA)
Treatments Part B: B1 = A/Sing MAP, 15 ug (FA) with biopsy; B2 = Placebo MAP (FA) with biopsy
B3 = A/Sing MAP, 15 ug (FA); B4 = A/Sing MAP, 10 ug (FA); B5 = A/Sing MAP, 5 ug (FA); B6 = A/Sing MAP, 2.5 ug (FA)
B7 = Placebo MAP (FA); B8 = A/Sing MAP, 15 ug (UA); B9 = A/Sing IM, 15 ug, as 2018 Afluria Quad (UA)

Listing 16.2.9.11
Individual Characterisation of Skin Irritation Index by Treatment
Oedema
Part B, Non-biopsy
B7= 3 x uncoated MAPs on volar forearm

			Active			Placebo		
Randomisation Number	Visit	Time Point	#> 0	Maximum	Average	#> 0	Maximum	Average
B0135	EOS/ET (Day 61)		-	-	-	0	0	0.0
B0138	Day 1	Pre-dose	-	-	-	0	0	0.0
B0138	Day 1	10 min	-	-	-	3	2	1.7
B0138	Day 1	1 hour	-	-	-	3	3	2.7
B0138	Day 1	2 hour	-	-	-	3	2	2.0
B0138	Day 2		-	-	-	0	0	0.0
B0138	Day 4		-	-	-	0	0	0.0
B0138	Day 8		-	-	-	0	0	0.0
B0138	Day 22		-	-	-	0	0	0.0
B0138	EOS/ET (Day 61)		-	-	-	0	0	0.0
B0146	Day 1	Pre-dose	-	-	-	0	0	0.0
B0146	Day 1	10 min	-	-	-	1	1	0.3
B0146	Day 1	1 hour	-	-	-	3	2	1.3
B0146	Day 1	2 hour	-	-	-	3	2	1.3
B0146	Day 2		-	-	-	0	0	0.0
B0146	Day 4		-	-	-	0	0	0.0
B0146	Day 8		-	-	-	0	0	0.0
B0146	Day 22		-	-	-	0	0	0.0
B0146	EOS/ET (Day 61)		-	-	-	0	0	0.0

Administration Site Key: FA = Forearm; UA= Upper arm
Treatments Part A: A1 = A/Sing MAP, 15 ug (FA); A2 = Afluria Quad IM (UA), 15 ug; A3 = Placebo MAP (FA); A4 = A/Sing Mono IM, 15 ug (UA)
Treatments Part B: B1 = A/Sing MAP, 15 ug (FA) with biopsy; B2 = Placebo MAP (FA) with biopsy
B3 = A/Sing MAP, 15 ug (FA); B4 = A/Sing MAP, 10 ug (FA); B5 = A/Sing MAP, 5 ug (FA); B6 = A/Sing MAP, 2.5 ug (FA)
B7 = Placebo MAP (FA); B8 = A/Sing MAP, 15 ug (UA); B9 = A/Sing IM, 15 ug, as 2018 Afluria Quad (UA)

Listing 16.2.9.11
Individual Characterisation of Skin Irritation Index by Treatment
Oedema
Part B, Non-biopsy
B7= 3 x uncoated MAPs on volar forearm

			Active			Placebo		
Randomisation Number	Visit	Time Point	#> 0	Maximum	Average	#> 0	Maximum	Average
B1080	Day 1	Pre-dose	-	-	-	0	0	0.0
B1080	Day 1	10 min	-	-	-	0	0	0.0
B1080	Day 1	1 hour	-	-	-	2	1	0.7
B1080	Day 1	2 hour	-	-	-	2	1	0.7
B1080	Day 2		-	-	-	0	0	0.0
B1080	Day 4		-	-	-	0	0	0.0
B1080	Day 8		-	-	-	0	0	0.0
B1080	Day 22		-	-	-	0	0	0.0
B1080	EOS/ET (Day 61)		-	-	-	0	0	0.0

Source: Listing 16.2.9.4

Administration Site Key: FA = Forearm; UA= Upper arm
Treatments Part A: A1 = A/Sing MAP, 15 ug (FA); A2 = Afluria Quad IM (UA), 15 ug; A3 = Placebo MAP (FA); A4 = A/Sing Mono IM, 15 ug (UA)
Treatments Part B: B1 = A/Sing MAP, 15 ug (FA) with biopsy; B2 = Placebo MAP (FA) with biopsy
B3 = A/Sing MAP, 15 ug (FA); B4 = A/Sing MAP, 10 ug (FA); B5 = A/Sing MAP, 5 ug (FA); B6 = A/Sing MAP, 2.5 ug (FA)
B7 = Placebo MAP (FA); B8 = A/Sing MAP, 15 ug (UA); B9 = A/Sing IM, 15 ug, as 2018 Afluria Quad (UA)

Listing 16.2.9.11
Individual Characterisation of Skin Irritation Index by Treatment
Oedema
Part B, Non-biopsy
B8= 3 x 5 ug A/Sing MAPs on upper arm (15 ug)

			Active			Placebo		
Randomisation Number	Visit	Time Point	#> 0	Maximum	Average	#> 0	Maximum	Average
B0011	Day 1	Pre-dose	0	0	0.0	-	-	-
B0011	Day 1	10 min	0	0	0.0	-	-	-
B0011	Day 1	1 hour	3	2	2.0	-	-	-
B0011	Day 1	2 hour	3	2	2.0	-	-	-
B0011	Day 2		3	1	1.0	-	-	-
B0011	Day 4		3	2	2.0	-	-	-
B0011	Day 8		0	0	0.0	-	-	-
B0011	Day 22		3	1	1.0	-	-	-
B0011	EOS/ET (Day 61)		0	0	0.0	-	-	-
B0025	Day 1	Pre-dose	0	0	0.0	-	-	-
B0025	Day 1	10 min	3	1	1.0	-	-	-
B0025	Day 1	1 hour	3	2	2.0	-	-	-
B0025	Day 1	2 hour	3	2	2.0	-	-	-
B0025	Day 2		3	1	1.0	-	-	-
B0025	Day 4		0	0	0.0	-	-	-
B0025	Day 8		0	0	0.0	-	-	-
B0025	Day 22		0	0	0.0	-	-	-
B0025	EOS/ET (Day 61)		0	0	0.0	-	-	-
B0034	Day 1	Pre-dose	0	0	0.0	-	-	-

Source: Listing 16.2.9.4

Administration Site Key: FA = Forearm; UA= Upper arm
Treatments Part A: A1 = A/Sing MAP, 15 ug (FA); A2 = Afluria Quad IM (UA), 15 ug; A3 = Placebo MAP (FA); A4 = A/Sing Mono IM, 15 ug (UA)
Treatments Part B: B1 = A/Sing MAP, 15 ug (FA) with biopsy; B2 = Placebo MAP (FA) with biopsy
B3 = A/Sing MAP, 15 ug (FA); B4 = A/Sing MAP, 10 ug (FA); B5 = A/Sing MAP, 5 ug (FA); B6 = A/Sing MAP, 2.5 ug (FA)
B7 = Placebo MAP (FA); B8 = A/Sing MAP, 15 ug (UA); B9 = A/Sing IM, 15 ug, as 2018 Afluria Quad (UA)

Listing 16.2.9.11
Individual Characterisation of Skin Irritation Index by Treatment
Oedema
Part B, Non-biopsy
B8= 3 x 5 ug A/Sing MAPs on upper arm (15 ug)

			Active			Placebo		
Randomisation Number	Visit	Time Point	#> 0	Maximum	Average	#> 0	Maximum	Average
B0034	Day 1	10 min	3	2	2.0	-	-	-
B0034	Day 1	1 hour	3	2	1.7	-	-	-
B0034	Day 1	2 hour	3	2	2.0	-	-	-
B0034	Day 2		3	2	1.3	-	-	-
B0034	Day 4		3	2	1.7	-	-	-
B0034	Day 8		0	0	0.0	-	-	-
B0034	Day 22		1	1	0.3	-	-	-
B0034	EOS/ET (Day 61)		0	0	0.0	-	-	-
B0043	Day 1	Pre-dose	0	0	0.0	-	-	-
B0043	Day 1	10 min	3	2	1.3	-	-	-
B0043	Day 1	1 hour	3	1	1.0	-	-	-
B0043	Day 1	2 hour	3	3	3.0	-	-	-
B0043	Day 2		3	2	2.0	-	-	-
B0043	Day 4		3	3	2.3	-	-	-
B0043	Day 8		1	2	0.7	-	-	-
B0043	Day 22		0	0	0.0	-	-	-
B0043	EOS/ET (Day 61)		0	0	0.0	-	-	-
B0050	Day 1	Pre-dose	0	0	0.0	-	-	-
B0050	Day 1	10 min	3	2	2.0	-	-	-

Administration Site Key: FA = Forearm; UA= Upper arm
Treatments Part A: A1 = A/Sing MAP, 15 ug (FA); A2 = Afluria Quad IM (UA), 15 ug; A3 = Placebo MAP (FA); A4 = A/Sing Mono IM, 15 ug (UA)
Treatments Part B: B1 = A/Sing MAP, 15 ug (FA) with biopsy; B2 = Placebo MAP (FA) with biopsy
B3 = A/Sing MAP, 15 ug (FA); B4 = A/Sing MAP, 10 ug (FA); B5 = A/Sing MAP, 5 ug (FA); B6 = A/Sing MAP, 2.5 ug (FA)
B7 = Placebo MAP (FA); B8 = A/Sing MAP, 15 ug (UA); B9 = A/Sing IM, 15 ug, as 2018 Afluria Quad (UA)

Listing 16.2.9.11
Individual Characterisation of Skin Irritation Index by Treatment
Oedema
Part B, Non-biopsy
B8= 3 x 5 ug A/Sing MAPs on upper arm (15 ug)

Randomisation Number	Visit	Time Point	Active #> 0	Active Maximum	Active Average	Placebo #> 0	Placebo Maximum	Placebo Average
B0050	Day 1	1 hour	3	2	1.7	-	-	-
B0050	Day 1	2 hour	3	2	2.0	-	-	-
B0050	Day 2		3	1	1.0	-	-	-
B0050	Day 4		3	1	1.0	-	-	-
B0050	Day 8		3	1	1.0	-	-	-
B0050	Day 22		2	1	0.7	-	-	-
B0050	EOS/ET (Day 61)		0	0	0.0	-	-	-
B0055	Day 1	Pre-dose	0	0	0.0	-	-	-
B0055	Day 1	10 min	3	1	1.0	-	-	-
B0055	Day 1	1 hour	3	1	1.0	-	-	-
B0055	Day 1	2 hour	3	1	1.0	-	-	-
B0055	Day 2		3	1	1.0	-	-	-
B0055	Day 4		0	0	0.0	-	-	-
B0055	Day 8		0	0	0.0	-	-	-
B0055	Day 22		0	0	0.0	-	-	-
B0055	EOS/ET (Day 61)		0	0	0.0	-	-	-
B0061	Day 1	Pre-dose	0	0	0.0	-	-	-
B0061	Day 1	10 min	3	1	1.0	-	-	-
B0061	Day 1	1 hour	0	0	0.0	-	-	-

Source: Listing 16.2.9.4

Administration Site Key: FA = Forearm; UA= Upper arm
Treatments Part A: A1 = A/Sing MAP, 15 ug (FA); A2 = Afluria Quad IM (UA), 15 ug; A3 = Placebo MAP (FA); A4 = A/Sing Mono IM, 15 ug (UA)
Treatments Part B: B1 = A/Sing MAP, 15 ug (FA) with biopsy; B2 = Placebo MAP (FA) with biopsy
B3 = A/Sing MAP, 15 ug (FA); B4 = A/Sing MAP, 10 ug (FA); B5 = A/Sing MAP, 5 ug (FA); B6 = A/Sing MAP, 2.5 ug (FA)
B7 = Placebo MAP (FA); B8 = A/Sing MAP, 15 ug (UA); B9 = A/Sing IM, 15 ug, as 2018 Afluria Quad (UA)

Listing 16.2.9.11
Individual Characterisation of Skin Irritation Index by Treatment
Oedema
Part B, Non-biopsy
B8= 3 x 5 ug A/Sing MAPs on upper arm (15 ug)

			Active			Placebo		
Randomisation Number	Visit	Time Point	#> 0	Maximum	Average	#> 0	Maximum	Average
B0061	Day 1	2 hour	2	1	0.7	-	-	-
B0061	Day 2		0	0	0.0	-	-	-
B0061	Day 4		3	2	1.7	-	-	-
B0061	Day 8		0	0	0.0	-	-	-
B0061	Day 22		0	0	0.0	-	-	-
B0061	EOS/ET (Day 61)		0	0	0.0	-	-	-
B0067	Day 1	Pre-dose	0	0	0.0	-	-	-
B0067	Day 1	10 min	3	1	1.0	-	-	-
B0067	Day 1	1 hour	3	1	1.0	-	-	-
B0067	Day 1	2 hour	3	1	1.0	-	-	-
B0067	Day 2		3	2	1.3	-	-	-
B0067	Day 4		0	0	0.0	-	-	-
B0067	Day 8		0	0	0.0	-	-	-
B0067	Day 22		0	0	0.0	-	-	-
B0067	EOS/ET (Day 61)		0	0	0.0	-	-	-
B0078	Day 1	Pre-dose	0	0	0.0	-	-	-
B0078	Day 1	10 min	3	1	1.0	-	-	-
B0078	Day 1	1 hour	3	2	2.0	-	-	-
B0078	Day 1	2 hour	3	2	1.7	-	-	-

Source: Listing 16.2.9.4

Administration Site Key: FA = Forearm; UA= Upper arm
Treatments Part A: A1 = A/Sing MAP, 15 ug (FA); A2 = Afluria Quad IM (UA), 15 ug; A3 = Placebo MAP (FA); A4 = A/Sing Mono IM, 15 ug (UA)
Treatments Part B: B1 = A/Sing MAP, 15 ug (FA) with biopsy; B2 = Placebo MAP (FA) with biopsy
B3 = A/Sing MAP, 15 ug (FA); B4 = A/Sing MAP, 10 ug (FA); B5 = A/Sing MAP, 5 ug (FA); B6 = A/Sing MAP, 2.5 ug (FA)
B7 = Placebo MAP (FA); B8 = A/Sing MAP, 15 ug (UA); B9 = A/Sing IM, 15 ug, as 2018 Afluria Quad (UA)

Listing 16.2.9.11
Individual Characterisation of Skin Irritation Index by Treatment
Oedema
Part B, Non-biopsy
B8= 3 x 5 ug A/Sing MAPs on upper arm (15 ug)

			Active			Placebo		
Randomisation Number	Visit	Time Point	#> 0	Maximum	Average	#> 0	Maximum	Average
B0078	Day 2		3	1	1.0	-	-	-
B0078	Day 4		3	3	3.0	-	-	-
B0078	Day 8		0	0	0.0	-	-	-
B0078	Day 22		0	0	0.0	-	-	-
B0078	EOS/ET (Day 61)		0	0	0.0	-	-	-
B0083	Day 1	Pre-dose	0	0	0.0	-	-	-
B0083	Day 1	10 min	3	1	1.0	-	-	-
B0083	Day 1	1 hour	3	1	1.0	-	-	-
B0083	Day 1	2 hour	3	3	2.7	-	-	-
B0083	Day 2		3	4	4.0	-	-	-
B0083	Day 4		3	1	1.0	-	-	-
B0083	Day 8		0	0	0.0	-	-	-
B0083	Day 22		0	0	0.0	-	-	-
B0083	EOS/ET (Day 61)		0	0	0.0	-	-	-
B0091	Day 1	Pre-dose	0	0	0.0	-	-	-
B0091	Day 1	10 min	3	2	1.7	-	-	-
B0091	Day 1	1 hour	3	1	1.0	-	-	-
B0091	Day 1	2 hour	3	1	1.0	-	-	-
B0091	Day 2		3	2	2.0	-	-	-

Source: Listing 16.2.9.4

Administration Site Key: FA = Forearm; UA= Upper arm
Treatments Part A: A1 = A/Sing MAP, 15 ug (FA); A2 = Afluria Quad IM (UA), 15 ug; A3 = Placebo MAP (FA); A4 = A/Sing Mono IM, 15 ug (UA)
Treatments Part B: B1 = A/Sing MAP, 15 ug (FA) with biopsy; B2 = Placebo MAP (FA) with biopsy
B3 = A/Sing MAP, 15 ug (FA); B4 = A/Sing MAP, 10 ug (FA); B5 = A/Sing MAP, 5 ug (FA); B6 = A/Sing MAP, 2.5 ug (FA)
B7 = Placebo MAP (FA); B8 = A/Sing MAP, 15 ug (UA); B9 = A/Sing IM, 15 ug, as 2018 Afluria Quad (UA)

Listing 16.2.9.11
Individual Characterisation of Skin Irritation Index by Treatment
Oedema
Part B, Non-biopsy
B8= 3 x 5 ug A/Sing MAPs on upper arm (15 ug)

			Active			Placebo		
Randomisation Number	Visit	Time Point	#> 0	Maximum	Average	#> 0	Maximum	Average
B0091	Day 4		0	0	0.0	-	-	-
B0091	Day 8		0	0	0.0	-	-	-
B0091	Day 22		0	0	0.0	-	-	-
B0091	EOS/ET (Day 61)		0	0	0.0	-	-	-
B0100	Day 1	Pre-dose	0	0	0.0	-	-	-
B0100	Day 1	10 min	3	1	1.0	-	-	-
B0100	Day 1	1 hour	3	1	1.0	-	-	-
B0100	Day 1	2 hour	3	1	1.0	-	-	-
B0100	Day 2		3	3	2.7	-	-	-
B0100	Day 4		3	2	2.0	-	-	-
B0100	Day 8		2	1	0.7	-	-	-
B0100	Day 22		2	1	0.7	-	-	-
B0100	EOS/ET (Day 61)		0	0	0.0	-	-	-
B0102	Day 1	Pre-dose	0	0	0.0	-	-	-
B0102	Day 1	10 min	3	1	1.0	-	-	-
B0102	Day 1	1 hour	3	1	1.0	-	-	-
B0102	Day 1	2 hour	3	1	1.0	-	-	-
B0102	Day 2		3	2	1.3	-	-	-
B0102	Day 4		0	0	0.0	-	-	-

Source: Listing 16.2.9.4

Administration Site Key: FA = Forearm; UA= Upper arm
Treatments Part A: A1 = A/Sing MAP, 15 ug (FA); A2 = Afluria Quad IM (UA), 15 ug; A3 = Placebo MAP (FA); A4 = A/Sing Mono IM, 15 ug (UA)
Treatments Part B: B1 = A/Sing MAP, 15 ug (FA) with biopsy; B2 = Placebo MAP (FA) with biopsy
B3 = A/Sing MAP, 15 ug (FA); B4 = A/Sing MAP, 10 ug (FA); B5 = A/Sing MAP, 5 ug (FA); B6 = A/Sing MAP, 2.5 ug (FA)
B7 = Placebo MAP (FA); B8 = A/Sing MAP, 15 ug (UA); B9 = A/Sing IM, 15 ug, as 2018 Afluria Quad (UA)

Listing 16.2.9.11
Individual Characterisation of Skin Irritation Index by Treatment
Oedema
Part B, Non-biopsy
B8= 3 x 5 ug A/Sing MAPs on upper arm (15 ug)

			Active			Placebo		
Randomisation Number	Visit	Time Point	#> 0	Maximum	Average	#> 0	Maximum	Average
B0102	Day 8		0	0	0.0	-	-	-
B0102	Day 22		0	0	0.0	-	-	-
B0102	EOS/ET (Day 61)		0	0	0.0	-	-	-
B0111	Day 1	Pre-dose	0	0	0.0	-	-	-
B0111	Day 1	10 min	2	1	0.7	-	-	-
B0111	Day 1	1 hour	0	0	0.0	-	-	-
B0111	Day 1	2 hour	3	2	1.3	-	-	-
B0111	Day 2		3	2	2.0	-	-	-
B0111	Day 4		3	1	1.0	-	-	-
B0111	Day 8		3	2	2.0	-	-	-
B0111	Day 22		0	0	0.0	-	-	-
B0111	EOS/ET (Day 61)		0	0	0.0	-	-	-
B0121	Day 1	Pre-dose	0	0	0.0	-	-	-
B0121	Day 1	10 min	3	1	1.0	-	-	-
B0121	Day 1	1 hour	3	2	2.0	-	-	-
B0121	Day 1	2 hour	3	3	2.7	-	-	-
B0121	Day 2		3	1	1.0	-	-	-
B0121	Day 4		0	0	0.0	-	-	-
B0121	Day 8		0	0	0.0	-	-	-

Source: Listing 16.2.9.4

Administration Site Key: FA = Forearm; UA= Upper arm
Treatments Part A: A1 = A/Sing MAP, 15 ug (FA); A2 = Afluria Quad IM (UA), 15 ug; A3 = Placebo MAP (FA); A4 = A/Sing Mono IM, 15 ug (UA)
Treatments Part B: B1 = A/Sing MAP, 15 ug (FA) with biopsy; B2 = Placebo MAP (FA) with biopsy
B3 = A/Sing MAP, 15 ug (FA); B4 = A/Sing MAP, 10 ug (FA); B5 = A/Sing MAP, 5 ug (FA); B6 = A/Sing MAP, 2.5 ug (FA)
B7 = Placebo MAP (FA); B8 = A/Sing MAP, 15 ug (UA); B9 = A/Sing IM, 15 ug, as 2018 Afluria Quad (UA)

Listing 16.2.9.11
Individual Characterisation of Skin Irritation Index by Treatment
Oedema
Part B, Non-biopsy
B8= 3 x 5 ug A/Sing MAPs on upper arm (15 ug)

			Active			Placebo		
Randomisation Number	Visit	Time Point	#> 0	Maximum	Average	#> 0	Maximum	Average
B0121	Day 22		0	0	0.0	-	-	-
B0121	EOS/ET (Day 61)		0	0	0.0	-	-	-
B0129	Day 1	Pre-dose	0	0	0.0	-	-	-
B0129	Day 1	10 min	3	2	2.0	-	-	-
B0129	Day 1	1 hour	3	3	2.7	-	-	-
B0129	Day 1	2 hour	3	3	2.7	-	-	-
B0129	Day 2		3	2	2.0	-	-	-
B0129	Day 4		3	1	1.0	-	-	-
B0129	Day 8		0	0	0.0	-	-	-
B0129	Day 22		2	1	0.7	-	-	-
B0129	EOS/ET (Day 61)		0	0	0.0	-	-	-
B0131	Day 1	Pre-dose	0	0	0.0	-	-	-
B0131	Day 1	10 min	3	1	1.0	-	-	-
B0131	Day 1	1 hour	3	2	1.7	-	-	-
B0131	Day 1	2 hour	3	2	1.3	-	-	-
B0131	Day 2		3	1	1.0	-	-	-
B0131	Day 4		0	0	0.0	-	-	-
B0131	Day 8		1	1	0.3	-	-	-
B0131	Day 22		0	0	0.0	-	-	-

Administration Site Key: FA = Forearm; UA= Upper arm
Treatments Part A: A1 = A/Sing MAP, 15 ug (FA); A2 = Afluria Quad IM (UA), 15 ug; A3 = Placebo MAP (FA); A4 = A/Sing Mono IM, 15 ug (UA)
Treatments Part B: B1 = A/Sing MAP, 15 ug (FA) with biopsy; B2 = Placebo MAP (FA) with biopsy
B3 = A/Sing MAP, 15 ug (FA); B4 = A/Sing MAP, 10 ug (FA); B5 = A/Sing MAP, 5 ug (FA); B6 = A/Sing MAP, 2.5 ug (FA)
B7 = Placebo MAP (FA); B8 = A/Sing MAP, 15 ug (UA); B9 = A/Sing IM, 15 ug, as 2018 Afluria Quad (UA)

Listing 16.2.9.11
Individual Characterisation of Skin Irritation Index by Treatment
Oedema
Part B, Non-biopsy
B8= 3 x 5 ug A/Sing MAPs on upper arm (15 ug)

Randomisation Number	Visit	Time Point	Active #> 0	Active Maximum	Active Average	Placebo #> 0	Placebo Maximum	Placebo Average
B0131	EOS/ET (Day 61)		0	0	0.0	-	-	-
B0137	Day 1	Pre-dose	0	0	0.0	-	-	-
B0137	Day 1	10 min	3	2	1.7	-	-	-
B0137	Day 1	1 hour	3	3	2.7	-	-	-
B0137	Day 1	2 hour	3	3	2.7	-	-	-
B0137	Day 2		3	1	1.0	-	-	-
B0137	Day 4		0	0	0.0	-	-	-
B0137	Day 8		0	0	0.0	-	-	-
B0137	Day 22		1	1	0.3	-	-	-
B0137	EOS/ET (Day 61)		0	0	0.0	-	-	-
B0149	Day 1	Pre-dose	0	0	0.0	-	-	-
B0149	Day 1	10 min	3	1	1.0	-	-	-
B0149	Day 1	1 hour	3	2	2.0	-	-	-
B0149	Day 1	2 hour	3	2	1.7	-	-	-
B0149	Day 2		3	2	1.3	-	-	-
B0149	Day 4		0	0	0.0	-	-	-
B0149	Day 8		3	1	1.0	-	-	-
B0149	Day 22		0	0	0.0	-	-	-
B0149	EOS/ET (Day 61)		0	0	0.0	-	-	-

Administration Site Key: FA = Forearm; UA= Upper arm
Treatments Part A: A1 = A/Sing MAP, 15 ug (FA); A2 = Afluria Quad IM (UA), 15 ug; A3 = Placebo MAP (FA); A4 = A/Sing Mono IM, 15 ug (UA)
Treatments Part B: B1 = A/Sing MAP, 15 ug (FA) with biopsy; B2 = Placebo MAP (FA) with biopsy
B3 = A/Sing MAP, 15 ug (FA); B4 = A/Sing MAP, 10 ug (FA); B5 = A/Sing MAP, 5 ug (FA); B6 = A/Sing MAP, 2.5 ug (FA)
B7 = Placebo MAP (FA); B8 = A/Sing MAP, 15 ug (UA); B9 = A/Sing IM, 15 ug, as 2018 Afluria Quad (UA)

Listing 16.2.9.11
Individual Characterisation of Skin Irritation Index by Treatment
Oedema
Part B, Non-biopsy
B8= 3 x 5 ug A/Sing MAPs on upper arm (15 ug)

			Active			Placebo		
Randomisation Number	Visit	Time Point	#> 0	Maximum	Average	#> 0	Maximum	Average
B1019	Day 1	Pre-dose	0	0	0.0	-	-	-
B1019	Day 1	10 min	3	3	3.0	-	-	-
B1019	Day 1	1 hour	3	2	2.0	-	-	-
B1019	Day 1	2 hour	3	2	1.7	-	-	-
B1019	Day 2		2	2	1.0	-	-	-
B1019	Day 4		0	0	0.0	-	-	-
B1019	Day 8		0	0	0.0	-	-	-
B1019	Day 22		0	0	0.0	-	-	-
B1019	EOS/ET (Day 61)		0	0	0.0	-	-	-

Source: Listing 16.2.9.4

Administration Site Key: FA = Forearm; UA= Upper arm
Treatments Part A: A1 = A/Sing MAP, 15 ug (FA); A2 = Afluria Quad IM (UA), 15 ug; A3 = Placebo MAP (FA); A4 = A/Sing Mono IM, 15 ug (UA)
Treatments Part B: B1 = A/Sing MAP, 15 ug (FA) with biopsy; B2 = Placebo MAP (FA) with biopsy
B3 = A/Sing MAP, 15 ug (FA); B4 = A/Sing MAP, 10 ug (FA); B5 = A/Sing MAP, 5 ug (FA); B6 = A/Sing MAP, 2.5 ug (FA)
B7 = Placebo MAP (FA); B8 = A/Sing MAP, 15 ug (UA); B9 = A/Sing IM, 15 ug, as 2018 Afluria Quad (UA)

Listing 16.2.9.11
Individual Characterisation of Skin Irritation Index by Treatment
Total Skin Irritation Index
Part A
A1= 3 x 5 ug A/Sing MAPs on volar forearm (15 ug)

Randomisation Number	Visit	Time Point	Active #> 0	Active Maximum	Active Average	Placebo #> 0	Placebo Maximum	Placebo Average
A0002	Day 4		3	4	4.0	-	-	-
A0002	Day 8		3	3	2.0	-	-	-
A0002	Day 22		0	0	0.0	-	-	-
A0002	EOS/ET (Day 61)		0	0	0.0	-	-	-
A0003	Day 4		3	4	3.7	-	-	-
A0003	Day 8		3	2	2.0	-	-	-
A0003	Day 22		1	1	0.3	-	-	-
A0003	EOS/ET (Day 61)		0	0	0.0	-	-	-
A0005	Day 4		3	5	5.0	-	-	-
A0005	Day 8		2	3	2.0	-	-	-
A0005	Day 22		0	0	0.0	-	-	-
A0005	EOS/ET (Day 61)		0	0	0.0	-	-	-
A0007	Day 4		3	2	2.0	-	-	-
A0007	Day 8		3	1	1.0	-	-	-
A0007	Day 22		3	1	1.0	-	-	-
A0007	EOS/ET (Day 61)		0	0	0.0	-	-	-
A0009	Day 4		3	4	2.7	-	-	-

Source: Listing 16.2.9.4

Administration Site Key: FA = Forearm; UA= Upper arm
Treatments Part A: A1 = A/Sing MAP, 15 ug (FA); A2 = Afluria Quad IM (UA), 15 ug; A3 = Placebo MAP (FA); A4 = A/Sing Mono IM, 15 ug (UA)
Treatments Part B: B1 = A/Sing MAP, 15 ug (FA) with biopsy; B2 = Placebo MAP (FA) with biopsy
B3 = A/Sing MAP, 15 ug (FA); B4 = A/Sing MAP, 10 ug (FA); B5 = A/Sing MAP, 5 ug (FA); B6 = A/Sing MAP, 2.5 ug (FA)
B7 = Placebo MAP (FA); B8 = A/Sing MAP, 15 ug (UA); B9 = A/Sing IM, 15 ug, as 2018 Afluria Quad (UA)

Listing 16.2.9.11
Individual Characterisation of Skin Irritation Index by Treatment
Total Skin Irritation Index
Part A
A1= 3 x 5 ug A/Sing MAPs on volar forearm (15 ug)

			Active			Placebo		
Randomisation Number	Visit	Time Point	#> 0	Maximum	Average	#> 0	Maximum	Average
A0009	Day 8		0	0	0.0	-	-	-
A0009	Day 22		0	0	0.0	-	-	-
A0009	EOS/ET (Day 61)		0	0	0.0	-	-	-
A0012	Day 4		3	4	4.0	-	-	-
A0012	Day 8		0	0	0.0	-	-	-
A0012	Day 22		0	0	0.0	-	-	-
A0012	EOS/ET (Day 61)		0	0	0.0	-	-	-
A0013	Day 4		3	4	3.7	-	-	-
A0013	Day 8		0	0	0.0	-	-	-
A0013	Day 22		0	0	0.0	-	-	-
A0013	EOS/ET (Day 61)		0	0	0.0	-	-	-
A0015	Day 4		3	4	3.0	-	-	-
A0015	Day 8		0	0	0.0	-	-	-
A0015	Day 22		0	0	0.0	-	-	-
A0015	EOS/ET (Day 61)		0	0	0.0	-	-	-
A0018	Day 1	Pre-dose	-	-	-	-	-	-
A0018	Day 1	10 min	-	-	-	-	-	-

Administration Site Key: FA = Forearm; UA= Upper arm
Treatments Part A: A1 = A/Sing MAP, 15 ug (FA); A2 = Afluria Quad IM (UA), 15 ug; A3 = Placebo MAP (FA); A4 = A/Sing Mono IM, 15 ug (UA)
Treatments Part B: B1 = A/Sing MAP, 15 ug (FA) with biopsy; B2 = Placebo MAP (FA) with biopsy
B3 = A/Sing MAP, 15 ug (FA); B4 = A/Sing MAP, 10 ug (FA); B5 = A/Sing MAP, 5 ug (FA); B6 = A/Sing MAP, 2.5 ug (FA)
B7 = Placebo MAP (FA); B8 = A/Sing MAP, 15 ug (UA); B9 = A/Sing IM, 15 ug, as 2018 Afluria Quad (UA)

Listing 16.2.9.11
Individual Characterisation of Skin Irritation Index by Treatment
Total Skin Irritation Index
Part A
A1= 3 x 5 ug A/Sing MAPs on volar forearm (15 ug)

			Active			Placebo		
Randomisation Number	Visit	Time Point	#> 0	Maximum	Average	#> 0	Maximum	Average
A0018	Day 1	1 hour	-	-	-	-	-	-
A0018	Day 1	2 hour	3	1	1.0	-	-	-
A0018	Day 4		3	2	2.0	-	-	-
A0018	Day 8		0	0	0.0	-	-	-
A0018	Day 22		0	0	0.0	-	-	-
A0018	EOS/ET (Day 61)		0	0	0.0	-	-	-
A0020	Day 1	Pre-dose	-	-	-	-	-	-
A0020	Day 1	10 min	-	-	-	-	-	-
A0020	Day 1	1 hour	-	-	-	-	-	-
A0020	Day 1	2 hour	3	4	3.7	-	-	-
A0020	Day 4		3	2	2.0	-	-	-
A0020	Day 8		0	0	0.0	-	-	-
A0020	Day 22		0	0	0.0	-	-	-
A0020	EOS/ET (Day 61)		0	0	0.0	-	-	-
A0021	Day 1	Pre-dose	-	-	-	-	-	-
A0021	Day 1	10 min	-	-	-	-	-	-
A0021	Day 1	1 hour	3	5	4.7	-	-	-
A0021	Day 1	2 hour	3	4	3.7	-	-	-
A0021	Day 4		3	3	2.7	-	-	-

Source: Listing 16.2.9.4

Administration Site Key: FA = Forearm; UA= Upper arm
Treatments Part A: A1 = A/Sing MAP, 15 ug (FA); A2 = Afluria Quad IM (UA), 15 ug; A3 = Placebo MAP (FA); A4 = A/Sing Mono IM, 15 ug (UA)
Treatments Part B: B1 = A/Sing MAP, 15 ug (FA) with biopsy; B2 = Placebo MAP (FA) with biopsy
B3 = A/Sing MAP, 15 ug (FA); B4 = A/Sing MAP, 10 ug (FA); B5 = A/Sing MAP, 5 ug (FA); B6 = A/Sing MAP, 2.5 ug (FA)
B7 = Placebo MAP (FA); B8 = A/Sing MAP, 15 ug (UA); B9 = A/Sing IM, 15 ug, as 2018 Afluria Quad (UA)

Listing 16.2.9.11
Individual Characterisation of Skin Irritation Index by Treatment
Total Skin Irritation Index
Part A
A1= 3 x 5 ug A/Sing MAPs on volar forearm (15 ug)

Randomisation Number	Visit	Time Point	Active #> 0	Active Maximum	Active Average	Placebo #> 0	Placebo Maximum	Placebo Average
A0021	Day 8		3	4	4.0	-	-	-
A0021	Day 22		0	0	0.0	-	-	-
A0021	EOS/ET (Day 61)		0	0	0.0	-	-	-
A0024	Day 1	Pre-dose	-	-	-	-	-	-
A0024	Day 1	10 min	-	-	-	-	-	-
A0024	Day 1	1 hour	3	5	4.3	-	-	-
A0024	Day 1	2 hour	3	4	4.0	-	-	-
A0024	Day 4		3	2	2.0	-	-	-
A0024	Day 8		0	0	0.0	-	-	-
A0024	Day 22		0	0	0.0	-	-	-
A0024	EOS/ET (Day 61)		0	0	0.0	-	-	-
A0028	Day 1	Pre-dose	-	-	-	-	-	-
A0028	Day 1	10 min	-	-	-	-	-	-
A0028	Day 1	1 hour	3	4	4.0	-	-	-
A0028	Day 1	2 hour	3	4	3.7	-	-	-
A0028	Day 4		0	0	0.0	-	-	-
A0028	Day 8		3	2	2.0	-	-	-
A0028	Day 22		0	0	0.0	-	-	-
A0028	EOS/ET (Day 61)		0	0	0.0	-	-	-

Source: Listing 16.2.9.4

Administration Site Key: FA = Forearm; UA= Upper arm
Treatments Part A: A1 = A/Sing MAP, 15 ug (FA); A2 = Afluria Quad IM (UA), 15 ug; A3 = Placebo MAP (FA); A4 = A/Sing Mono IM, 15 ug (UA)
Treatments Part B: B1 = A/Sing MAP, 15 ug (FA) with biopsy; B2 = Placebo MAP (FA) with biopsy
B3 = A/Sing MAP, 15 ug (FA); B4 = A/Sing MAP, 10 ug (FA); B5 = A/Sing MAP, 5 ug (FA); B6 = A/Sing MAP, 2.5 ug (FA)
B7 = Placebo MAP (FA); B8 = A/Sing MAP, 15 ug (UA); B9 = A/Sing IM, 15 ug, as 2018 Afluria Quad (UA)

Listing 16.2.9.11
Individual Characterisation of Skin Irritation Index by Treatment
Total Skin Irritation Index
Part A
A1= 3 x 5 ug A/Sing MAPs on volar forearm (15 ug)

			Active			Placebo		
Randomisation Number	Visit	Time Point	#> 0	Maximum	Average	#> 0	Maximum	Average
A0029	Day 1	Pre-dose	-	-	-	-	-	-
A0029	Day 1	10 min	-	-	-	-	-	-
A0029	Day 1	1 hour	3	5	4.7	-	-	-
A0029	Day 1	2 hour	3	4	3.7	-	-	-
A0029	Day 4		3	2	2.0	-	-	-
A0029	Day 8		3	2	2.0	-	-	-
A0029	Day 22		3	1	1.0	-	-	-
A0029	EOS/ET (Day 61)		0	0	0.0	-	-	-
A1026	Day 1	Pre-dose	0	0	0.0	-	-	-
A1026	Day 1	10 min	3	4	4.0	-	-	-
A1026	Day 1	1 hour	3	4	3.7	-	-	-
A1026	Day 1	2 hour	3	4	3.7	-	-	-
A1026	Day 4		3	5	4.0	-	-	-
A1026	Day 8		0	0	0.0	-	-	-
A1026	Day 22		0	0	0.0	-	-	-
A1026	EOS/ET (Day 61)		0	0	0.0	-	-	-

Administration Site Key: FA = Forearm; UA= Upper arm
Treatments Part A: A1 = A/Sing MAP, 15 ug (FA); A2 = Afluria Quad IM (UA), 15 ug; A3 = Placebo MAP (FA); A4 = A/Sing Mono IM, 15 ug (UA)
Treatments Part B: B1 = A/Sing MAP, 15 ug (FA) with biopsy; B2 = Placebo MAP (FA) with biopsy
B3 = A/Sing MAP, 15 ug (FA); B4 = A/Sing MAP, 10 ug (FA); B5 = A/Sing MAP, 5 ug (FA); B6 = A/Sing MAP, 2.5 ug (FA)
B7 = Placebo MAP (FA); B8 = A/Sing MAP, 15 ug (UA); B9 = A/Sing IM, 15 ug, as 2018 Afluria Quad (UA)

Listing 16.2.9.11
Individual Characterisation of Skin Irritation Index by Treatment
Total Skin Irritation Index
Part A
A3= 3 x uncoated MAPs on volar forearm

			Active			Placebo		
Randomisation Number	Visit	Time Point	#> 0	Maximum	Average	#> 0	Maximum	Average
A0001	Day 4		-	-	-	3	1	1.0
A0001	Day 8		-	-	-	0	0	0.0
A0001	Day 22		-	-	-	0	0	0.0
A0001	EOS/ET (Day 61)		-	-	-	0	0	0.0
A0004	Day 4		-	-	-	3	1	1.0
A0004	Day 8		-	-	-	0	0	0.0
A0004	Day 22		-	-	-	0	0	0.0
A0004	EOS/ET (Day 61)		-	-	-	0	0	0.0
A0006	Day 4		-	-	-	3	1	1.0
A0006	Day 8		-	-	-	0	0	0.0
A0006	Day 22		-	-	-	0	0	0.0
A0006	EOS/ET (Day 61)		-	-	-	0	0	0.0
A0008	Day 4		-	-	-	0	0	0.0
A0008	Day 8		-	-	-	0	0	0.0
A0008	Day 22		-	-	-	0	0	0.0
A0008	EOS/ET (Day 61)		-	-	-	0	0	0.0
A0010	Day 4		-	-	-	3	1	1.0

Source: Listing 16.2.9.4

Administration Site Key: FA = Forearm; UA= Upper arm
Treatments Part A: A1 = A/Sing MAP, 15 ug (FA); A2 = Afluria Quad IM (UA), 15 ug; A3 = Placebo MAP (FA); A4 = A/Sing Mono IM, 15 ug (UA)
Treatments Part B: B1 = A/Sing MAP, 15 ug (FA) with biopsy; B2 = Placebo MAP (FA) with biopsy
B3 = A/Sing MAP, 15 ug (FA); B4 = A/Sing MAP, 10 ug (FA); B5 = A/Sing MAP, 5 ug (FA); B6 = A/Sing MAP, 2.5 ug (FA)
B7 = Placebo MAP (FA); B8 = A/Sing MAP, 15 ug (UA); B9 = A/Sing IM, 15 ug, as 2018 Afluria Quad (UA)

Listing 16.2.9.11
Individual Characterisation of Skin Irritation Index by Treatment
Total Skin Irritation Index
Part A
A3= 3 x uncoated MAPs on volar forearm

			Active			Placebo		
Randomisation Number	Visit	Time Point	#> 0	Maximum	Average	#> 0	Maximum	Average
A0010	Day 8		-	-	-	0	0	0.0
A0010	Day 22		-	-	-	0	0	0.0
A0010	EOS/ET (Day 61)		-	-	-	0	0	0.0
A0011	Day 4		-	-	-	0	0	0.0
A0011	Day 8		-	-	-	0	0	0.0
A0011	Day 22		-	-	-	1	1	0.5
A0011	EOS/ET (Day 61)		-	-	-	0	0	0.0
A0014	Day 4		-	-	-	0	0	0.0
A0014	Day 8		-	-	-	0	0	0.0
A0014	Day 22		-	-	-	0	0	0.0
A0014	EOS/ET (Day 61)		-	-	-	0	0	0.0
A0016	Day 1	Pre-dose	-	-	-	-	-	-
A0016	Day 1	10 min	-	-	-	-	-	-
A0016	Day 1	1 hour	-	-	-	-	-	-
A0016	Day 1	2 hour	-	-	-	3	3	3.0
A0016	Day 4		-	-	-	0	0	0.0
A0016	Day 8		-	-	-	0	0	0.0
A0016	Day 22		-	-	-	0	0	0.0

Administration Site Key: FA = Forearm; UA= Upper arm
Treatments Part A: A1 = A/Sing MAP, 15 ug (FA); A2 = Afluria Quad IM (UA), 15 ug; A3 = Placebo MAP (FA); A4 = A/Sing Mono IM, 15 ug (UA)
Treatments Part B: B1 = A/Sing MAP, 15 ug (FA) with biopsy; B2 = Placebo MAP (FA) with biopsy
B3 = A/Sing MAP, 15 ug (FA); B4 = A/Sing MAP, 10 ug (FA); B5 = A/Sing MAP, 5 ug (FA); B6 = A/Sing MAP, 2.5 ug (FA)
B7 = Placebo MAP (FA); B8 = A/Sing MAP, 15 ug (UA); B9 = A/Sing IM, 15 ug, as 2018 Afluria Quad (UA)

Listing 16.2.9.11
Individual Characterisation of Skin Irritation Index by Treatment
Total Skin Irritation Index
Part A
A3= 3 x uncoated MAPs on volar forearm

Randomisation Number	Visit	Time Point	Active #> 0	Active Maximum	Active Average	Placebo #> 0	Placebo Maximum	Placebo Average
A0016	EOS/ET (Day 61)		-	-	-	0	0	0.0
A0017	Day 1	Pre-dose	-	-	-	-	-	-
A0017	Day 1	10 min	-	-	-	-	-	-
A0017	Day 1	1 hour	-	-	-	-	-	-
A0017	Day 1	2 hour	-	-	-	3	3	3.0
A0017	Day 4		-	-	-	0	0	0.0
A0017	Day 8		-	-	-	0	0	0.0
A0017	Day 22		-	-	-	0	0	0.0
A0017	EOS/ET (Day 61)		-	-	-	0	0	0.0
A0019	Day 1	Pre-dose	-	-	-	-	-	-
A0019	Day 1	10 min	-	-	-	-	-	-
A0019	Day 1	1 hour	-	-	-	-	-	-
A0019	Day 1	2 hour	-	-	-	3	4	4.0
A0019	Day 4		-	-	-	0	0	0.0
A0019	Day 8		-	-	-	0	0	0.0
A0019	Day 22		-	-	-	0	0	0.0
A0019	EOS/ET (Day 61)		-	-	-	0	0	0.0
A0022	Day 1	Pre-dose	-	-	-	-	-	-

Source: Listing 16.2.9.4

Administration Site Key: FA = Forearm; UA= Upper arm
Treatments Part A: A1 = A/Sing MAP, 15 ug (FA); A2 = Afluria Quad IM (UA), 15 ug; A3 = Placebo MAP (FA); A4 = A/Sing Mono IM, 15 ug (UA)
Treatments Part B: B1 = A/Sing MAP, 15 ug (FA) with biopsy; B2 = Placebo MAP (FA) with biopsy
B3 = A/Sing MAP, 15 ug (FA); B4 = A/Sing MAP, 10 ug (FA); B5 = A/Sing MAP, 5 ug (FA); B6 = A/Sing MAP, 2.5 ug (FA)
B7 = Placebo MAP (FA); B8 = A/Sing MAP, 15 ug (UA); B9 = A/Sing IM, 15 ug, as 2018 Afluria Quad (UA)

Listing 16.2.9.11
Individual Characterisation of Skin Irritation Index by Treatment
Total Skin Irritation Index
Part A
A3= 3 x uncoated MAPs on volar forearm

			Active			Placebo		
Randomisation Number	Visit	Time Point	#> 0	Maximum	Average	#> 0	Maximum	Average
A0022	Day 1	10 min	-	-	-	-	-	-
A0022	Day 1	1 hour	-	-	-	-	-	-
A0022	Day 1	2 hour	-	-	-	3	3	3.0
A0022	Day 4		-	-	-	0	0	0.0
A0022	Day 8		-	-	-	0	0	0.0
A0022	Day 22		-	-	-	0	0	0.0
A0022	EOS/ET (Day 61)		-	-	-	0	0	0.0
A0023	Day 1	Pre-dose	-	-	-	-	-	-
A0023	Day 1	10 min	-	-	-	-	-	-
A0023	Day 1	1 hour	-	-	-	3	4	4.0
A0023	Day 1	2 hour	-	-	-	3	5	4.3
A0023	Day 4		-	-	-	0	0	0.0
A0023	Day 8		-	-	-	0	0	0.0
A0023	Day 22		-	-	-	0	0	0.0
A0023	EOS/ET (Day 61)		-	-	-	0	0	0.0
A0025	Day 1	Pre-dose	-	-	-	-	-	-
A0025	Day 1	10 min	-	-	-	-	-	-
A0025	Day 1	1 hour	-	-	-	3	4	4.0
A0025	Day 1	2 hour	-	-	-	3	4	3.7

Source: Listing 16.2.9.4

Administration Site Key: FA = Forearm; UA= Upper arm
Treatments Part A: A1 = A/Sing MAP, 15 ug (FA); A2 = Afluria Quad IM (UA), 15 ug; A3 = Placebo MAP (FA); A4 = A/Sing Mono IM, 15 ug (UA)
Treatments Part B: B1 = A/Sing MAP, 15 ug (FA) with biopsy; B2 = Placebo MAP (FA) with biopsy
B3 = A/Sing MAP, 15 ug (FA); B4 = A/Sing MAP, 10 ug (FA); B5 = A/Sing MAP, 5 ug (FA); B6 = A/Sing MAP, 2.5 ug (FA)
B7 = Placebo MAP (FA); B8 = A/Sing MAP, 15 ug (UA); B9 = A/Sing IM, 15 ug, as 2018 Afluria Quad (UA)

Listing 16.2.9.11
Individual Characterisation of Skin Irritation Index by Treatment
Total Skin Irritation Index
Part A
A3= 3 x uncoated MAPs on volar forearm

			Active			Placebo		
Randomisation Number	Visit	Time Point	#> 0	Maximum	Average	#> 0	Maximum	Average
A0025	Day 4		-	-	-	3	2	2.0
A0025	Day 8		-	-	-	0	0	0.0
A0025	Day 22		-	-	-	0	0	0.0
A0025	EOS/ET (Day 61)		-	-	-	0	0	0.0
A0027	Day 1	Pre-dose	-	-	-	-	-	-
A0027	Day 1	10 min	-	-	-	-	-	-
A0027	Day 1	1 hour	-	-	-	3	4	3.7
A0027	Day 1	2 hour	-	-	-	3	4	3.7
A0027	Day 4		-	-	-	2	1	0.7
A0027	Day 8		-	-	-	0	0	0.0
A0027	Day 22		-	-	-	3	1	1.0
A0027	EOS/ET (Day 61)		-	-	-	0	0	0.0
A0030	Day 1	Pre-dose	-	-	-	0	0	0.0
A0030	Day 1	10 min	-	-	-	3	5	5.0
A0030	Day 1	1 hour	-	-	-	3	4	4.0
A0030	Day 1	2 hour	-	-	-	3	2	2.0
A0030	Day 4		-	-	-	0	0	0.0
A0030	Day 8		-	-	-	0	0	0.0
A0030	Day 22		-	-	-	0	0	0.0

Source: Listing 16.2.9.4

Administration Site Key: FA = Forearm; UA= Upper arm
Treatments Part A: A1 = A/Sing MAP, 15 ug (FA); A2 = Afluria Quad IM (UA), 15 ug; A3 = Placebo MAP (FA); A4 = A/Sing Mono IM, 15 ug (UA)
Treatments Part B: B1 = A/Sing MAP, 15 ug (FA) with biopsy; B2 = Placebo MAP (FA) with biopsy
B3 = A/Sing MAP, 15 ug (FA); B4 = A/Sing MAP, 10 ug (FA); B5 = A/Sing MAP, 5 ug (FA); B6 = A/Sing MAP, 2.5 ug (FA)
B7 = Placebo MAP (FA); B8 = A/Sing MAP, 15 ug (UA); B9 = A/Sing IM, 15 ug, as 2018 Afluria Quad (UA)

Listing 16.2.9.11
Individual Characterisation of Skin Irritation Index by Treatment
Total Skin Irritation Index
Part A
A3= 3 x uncoated MAPs on volar forearm

Randomisation Number	Visit	Time Point	Active #> 0	Active Maximum	Active Average	Placebo #> 0	Placebo Maximum	Placebo Average
A0030	EOS/ET (Day 61)		-	-	-	0	0	0.0

Source: Listing 16.2.9.4

Administration Site Key: FA = Forearm; UA= Upper arm
Treatments Part A: A1 = A/Sing MAP, 15 ug (FA); A2 = Afluria Quad IM (UA), 15 ug; A3 = Placebo MAP (FA); A4 = A/Sing Mono IM, 15 ug (UA)
Treatments Part B: B1 = A/Sing MAP, 15 ug (FA) with biopsy; B2 = Placebo MAP (FA) with biopsy
B3 = A/Sing MAP, 15 ug (FA); B4 = A/Sing MAP, 10 ug (FA); B5 = A/Sing MAP, 5 ug (FA); B6 = A/Sing MAP, 2.5 ug (FA)
B7 = Placebo MAP (FA); B8 = A/Sing MAP, 15 ug (UA); B9 = A/Sing IM, 15 ug, as 2018 Afluria Quad (UA)

Listing 16.2.9.11
Individual Characterisation of Skin Irritation Index by Treatment
Total Skin Irritation Index
Part B, Biopsy
B1= 3 x 5 ug A/Sing MAPs on volar forearm (15 ug)

			Active			Placebo		
Randomisation Number	Visit	Time Point	#> 0	Maximum	Average	#> 0	Maximum	Average
B0001	Day 1	Pre-dose	0	0	0.0	-	-	-
B0001	Day 1	10 min	3	1	1.0	-	-	-
B0001	Day 1	1 hour	3	2	2.0	-	-	-
B0001	Day 1	2 hour	3	2	2.0	-	-	-
B0001	Day 2		3	2	1.7	-	-	-
B0001	Day 4		3	3	3.0	-	-	-
B0001	Day 8		3	1	1.0	-	-	-
B0001	Day 22		2	2	1.3	-	-	-
B0001	EOS/ET (Day 61)		0	0	0.0	-	-	-
B0004	Day 1	Pre-dose	0	0	0.0	-	-	-
B0004	Day 1	10 min	3	3	3.0	-	-	-
B0004	Day 1	1 hour	3	3	3.0	-	-	-
B0004	Day 1	2 hour	3	4	4.0	-	-	-
B0004	Day 2		3	3	3.0	-	-	-
B0004	Day 4		3	4	3.3	-	-	-
B0004	Day 8		0	0	0.0	-	-	-
B0004	Day 22		2	3	2.0	-	-	-
B0004	EOS/ET (Day 61)		1	1	0.3	-	-	-
B0005	Day 1	Pre-dose	0	0	0.0	-	-	-

Administration Site Key: FA = Forearm; UA= Upper arm
Treatments Part A: A1 = A/Sing MAP, 15 ug (FA); A2 = Afluria Quad IM (UA), 15 ug; A3 = Placebo MAP (FA); A4 = A/Sing Mono IM, 15 ug (UA)
Treatments Part B: B1 = A/Sing MAP, 15 ug (FA) with biopsy; B2 = Placebo MAP (FA) with biopsy
B3 = A/Sing MAP, 15 ug (FA); B4 = A/Sing MAP, 10 ug (FA); B5 = A/Sing MAP, 5 ug (FA); B6 = A/Sing MAP, 2.5 ug (FA)
B7 = Placebo MAP (FA); B8 = A/Sing MAP, 15 ug (UA); B9 = A/Sing IM, 15 ug, as 2018 Afluria Quad (UA)

Listing 16.2.9.11
Individual Characterisation of Skin Irritation Index by Treatment
Total Skin Irritation Index
Part B, Biopsy
B1= 3 x 5 ug A/Sing MAPs on volar forearm (15 ug)

			Active			Placebo		
Randomisation Number	Visit	Time Point	#> 0	Maximum	Average	#> 0	Maximum	Average
B0005	Day 1	10 min	3	3	3.0	-	-	-
B0005	Day 1	1 hour	3	5	5.0	-	-	-
B0005	Day 1	2 hour	3	3	2.7	-	-	-
B0005	Day 2		3	2	2.0	-	-	-
B0005	Day 4		3	4	4.0	-	-	-
B0005	Day 8		3	2	1.3	-	-	-
B0005	Day 22		3	4	3.3	-	-	-
B0005	EOS/ET (Day 61)		0	0	0.0	-	-	-
B0008	Day 1	Pre-dose	0	0	0.0	-	-	-
B0008	Day 1	10 min	3	4	3.7	-	-	-
B0008	Day 1	1 hour	3	4	4.0	-	-	-
B0008	Day 1	2 hour	3	4	3.7	-	-	-
B0008	Day 2		3	3	2.7	-	-	-
B0008	Day 4		3	3	3.0	-	-	-
B0008	Day 8		2	2	1.3	-	-	-
B0008	Day 22		3	2	1.7	-	-	-
B0008	EOS/ET (Day 61)		0	0	0.0	-	-	-
B0009	Day 1	Pre-dose	0	0	0.0	-	-	-
B0009	Day 1	10 min	3	3	2.7	-	-	-

Administration Site Key: FA = Forearm; UA= Upper arm
Treatments Part A: A1 = A/Sing MAP, 15 ug (FA); A2 = Afluria Quad IM (UA), 15 ug; A3 = Placebo MAP (FA); A4 = A/Sing Mono IM, 15 ug (UA)
Treatments Part B: B1 = A/Sing MAP, 15 ug (FA) with biopsy; B2 = Placebo MAP (FA) with biopsy
B3 = A/Sing MAP, 15 ug (FA); B4 = A/Sing MAP, 10 ug (FA); B5 = A/Sing MAP, 5 ug (FA); B6 = A/Sing MAP, 2.5 ug (FA)
B7 = Placebo MAP (FA); B8 = A/Sing MAP, 15 ug (UA); B9 = A/Sing IM, 15 ug, as 2018 Afluria Quad (UA)

Listing 16.2.9.11
Individual Characterisation of Skin Irritation Index by Treatment
Total Skin Irritation Index
Part B, Biopsy
B1= 3 x 5 ug A/Sing MAPs on volar forearm (15 ug)

			Active			Placebo		
Randomisation Number	Visit	Time Point	#> 0	Maximum	Average	#> 0	Maximum	Average
B0009	Day 1	1 hour	3	4	4.0	-	-	-
B0009	Day 1	2 hour	3	4	3.7	-	-	-
B0009	Day 2		3	3	2.7	-	-	-
B0009	Day 4		3	4	4.0	-	-	-
B0009	Day 8		0	0	0.0	-	-	-
B0009	Day 22		3	2	1.7	-	-	-
B0009	EOS/ET (Day 61)		0	0	0.0	-	-	-

Source: Listing 16.2.9.4

Administration Site Key: FA = Forearm; UA= Upper arm
Treatments Part A: A1 = A/Sing MAP, 15 ug (FA); A2 = Afluria Quad IM (UA), 15 ug; A3 = Placebo MAP (FA); A4 = A/Sing Mono IM, 15 ug (UA)
Treatments Part B: B1 = A/Sing MAP, 15 ug (FA) with biopsy; B2 = Placebo MAP (FA) with biopsy
B3 = A/Sing MAP, 15 ug (FA); B4 = A/Sing MAP, 10 ug (FA); B5 = A/Sing MAP, 5 ug (FA); B6 = A/Sing MAP, 2.5 ug (FA)
B7 = Placebo MAP (FA); B8 = A/Sing MAP, 15 ug (UA); B9 = A/Sing IM, 15 ug, as 2018 Afluria Quad (UA)

Listing 16.2.9.11
Individual Characterisation of Skin Irritation Index by Treatment
Total Skin Irritation Index
Part B, Biopsy
B2= 3 x uncoated MAPs on volar forearm

			Active			Placebo		
Randomisation Number	Visit	Time Point	#> 0	Maximum	Average	#> 0	Maximum	Average
B0002	Day 1	Pre-dose	-	-	-	0	0	0.0
B0002	Day 1	10 min	-	-	-	3	5	4.3
B0002	Day 1	1 hour	-	-	-	3	3	3.0
B0002	Day 1	2 hour	-	-	-	3	3	3.0
B0002	Day 2		-	-	-	3	2	2.0
B0002	Day 4		-	-	-	3	2	2.0
B0002	Day 8		-	-	-	1	1	0.3
B0002	Day 22		-	-	-	2	2	1.3
B0002	EOS/ET (Day 61)		-	-	-	0	0	0.0
B0003	Day 1	Pre-dose	-	-	-	0	0	0.0
B0003	Day 1	10 min	-	-	-	3	3	1.7
B0003	Day 1	1 hour	-	-	-	3	2	2.0
B0003	Day 1	2 hour	-	-	-	3	2	2.0
B0003	Day 2		-	-	-	3	2	1.3
B0003	Day 4		-	-	-	0	0	0.0
B0003	Day 8		-	-	-	0	0	0.0
B0003	Day 22		-	-	-	2	7	3.7
B0003	EOS/ET (Day 61)		-	-	-	0	0	0.0
B0006	Day 1	Pre-dose	-	-	-	0	0	0.0

Source: Listing 16.2.9.4

Administration Site Key: FA = Forearm; UA= Upper arm
Treatments Part A: A1 = A/Sing MAP, 15 ug (FA); A2 = Afluria Quad IM (UA), 15 ug; A3 = Placebo MAP (FA); A4 = A/Sing Mono IM, 15 ug (UA)
Treatments Part B: B1 = A/Sing MAP, 15 ug (FA) with biopsy; B2 = Placebo MAP (FA) with biopsy
B3 = A/Sing MAP, 15 ug (FA); B4 = A/Sing MAP, 10 ug (FA); B5 = A/Sing MAP, 5 ug (FA); B6 = A/Sing MAP, 2.5 ug (FA)
B7 = Placebo MAP (FA); B8 = A/Sing MAP, 15 ug (UA); B9 = A/Sing IM, 15 ug, as 2018 Afluria Quad (UA)

Listing 16.2.9.11
Individual Characterisation of Skin Irritation Index by Treatment
Total Skin Irritation Index
Part B, Biopsy
B2= 3 x uncoated MAPs on volar forearm

			Active			Placebo		
Randomisation Number	Visit	Time Point	#> 0	Maximum	Average	#> 0	Maximum	Average
B0006	Day 1	10 min	-	-	-	3	3	2.3
B0006	Day 1	1 hour	-	-	-	3	3	2.7
B0006	Day 1	2 hour	-	-	-	3	3	2.7
B0006	Day 2		-	-	-	3	2	2.0
B0006	Day 4		-	-	-	3	3	2.3
B0006	Day 8		-	-	-	2	4	2.3
B0006	Day 22		-	-	-	2	4	2.3
B0006	EOS/ET (Day 61)		-	-	-	0	0	0.0
B0007	Day 1	Pre-dose	-	-	-	0	0	0.0
B0007	Day 1	10 min	-	-	-	3	3	2.3
B0007	Day 1	1 hour	-	-	-	3	4	3.7
B0007	Day 1	2 hour	-	-	-	3	2	2.0
B0007	Day 2		-	-	-	3	2	2.0
B0007	Day 4		-	-	-	3	2	2.0
B0007	Day 8		-	-	-	0	0	0.0
B0007	Day 22		-	-	-	2	2	1.3
B0007	EOS/ET (Day 61)		-	-	-	0	0	0.0
B0010	Day 1	Pre-dose	-	-	-	0	0	0.0
B0010	Day 1	10 min	-	-	-	3	2	2.0

Source: Listing 16.2.9.4

Administration Site Key: FA = Forearm; UA= Upper arm
Treatments Part A: A1 = A/Sing MAP, 15 ug (FA); A2 = Afluria Quad IM (UA), 15 ug; A3 = Placebo MAP (FA); A4 = A/Sing Mono IM, 15 ug (UA)
Treatments Part B: B1 = A/Sing MAP, 15 ug (FA) with biopsy; B2 = Placebo MAP (FA) with biopsy
B3 = A/Sing MAP, 15 ug (FA); B4 = A/Sing MAP, 10 ug (FA); B5 = A/Sing MAP, 5 ug (FA); B6 = A/Sing MAP, 2.5 ug (FA)
B7 = Placebo MAP (FA); B8 = A/Sing MAP, 15 ug (UA); B9 = A/Sing IM, 15 ug, as 2018 Afluria Quad (UA)

Listing 16.2.9.11
Individual Characterisation of Skin Irritation Index by Treatment
Total Skin Irritation Index
Part B, Biopsy
B2= 3 x uncoated MAPs on volar forearm

			Active			Placebo		
Randomisation Number	Visit	Time Point	#> 0	Maximum	Average	#> 0	Maximum	Average
B0010	Day 1	1 hour	-	-	-	3	3	3.0
B0010	Day 1	2 hour	-	-	-	3	2	2.0
B0010	Day 2		-	-	-	3	3	2.3
B0010	Day 4		-	-	-	3	2	2.0
B0010	Day 8		-	-	-	2	3	1.7
B0010	Day 22		-	-	-	3	2	1.7
B0010	EOS/ET (Day 61)		-	-	-	0	0	0.0

Source: Listing 16.2.9.4

Administration Site Key: FA = Forearm; UA= Upper arm
Treatments Part A: A1 = A/Sing MAP, 15 ug (FA); A2 = Afluria Quad IM (UA), 15 ug; A3 = Placebo MAP (FA); A4 = A/Sing Mono IM, 15 ug (UA)
Treatments Part B: B1 = A/Sing MAP, 15 ug (FA) with biopsy; B2 = Placebo MAP (FA) with biopsy
B3 = A/Sing MAP, 15 ug (FA); B4 = A/Sing MAP, 10 ug (FA); B5 = A/Sing MAP, 5 ug (FA); B6 = A/Sing MAP, 2.5 ug (FA)
B7 = Placebo MAP (FA); B8 = A/Sing MAP, 15 ug (UA); B9 = A/Sing IM, 15 ug, as 2018 Afluria Quad (UA)

Listing 16.2.9.11
Individual Characterisation of Skin Irritation Index by Treatment
Total Skin Irritation Index
Part B, Non-biopsy
B3= 3 x 5 ug A/Sing MAPs on volar forearm (15 ug)

			Active			Placebo		
Randomisation Number	Visit	Time Point	#> 0	Maximum	Average	#> 0	Maximum	Average
B0014	Day 1	Pre-dose	0	0	0.0	-	-	-
B0014	Day 1	10 min	3	2	2.0	-	-	-
B0014	Day 1	1 hour	3	2	2.0	-	-	-
B0014	Day 1	2 hour	3	3	3.0	-	-	-
B0014	Day 2		3	3	3.0	-	-	-
B0014	Day 4		3	6	5.3	-	-	-
B0014	Day 8		0	0	0.0	-	-	-
B0014	Day 22		0	0	0.0	-	-	-
B0014	EOS/ET (Day 61)		0	0	0.0	-	-	-
B0023	Day 1	Pre-dose	0	0	0.0	-	-	-
B0023	Day 1	10 min	3	3	3.0	-	-	-
B0023	Day 1	1 hour	3	5	4.3	-	-	-
B0023	Day 1	2 hour	3	6	6.0	-	-	-
B0023	Day 2		3	5	4.3	-	-	-
B0023	Day 4		3	5	5.0	-	-	-
B0023	Day 8		3	2	2.0	-	-	-
B0023	Day 22		0	0	0.0	-	-	-
B0023	EOS/ET (Day 61)		0	0	0.0	-	-	-
B0027	Day 1	Pre-dose	0	0	0.0	-	-	-

Source: Listing 16.2.9.4

Administration Site Key: FA = Forearm; UA= Upper arm
Treatments Part A: A1 = A/Sing MAP, 15 ug (FA); A2 = Afluria Quad IM (UA), 15 ug; A3 = Placebo MAP (FA); A4 = A/Sing Mono IM, 15 ug (UA)
Treatments Part B: B1 = A/Sing MAP, 15 ug (FA) with biopsy; B2 = Placebo MAP (FA) with biopsy
B3 = A/Sing MAP, 15 ug (FA); B4 = A/Sing MAP, 10 ug (FA); B5 = A/Sing MAP, 5 ug (FA); B6 = A/Sing MAP, 2.5 ug (FA)
B7 = Placebo MAP (FA); B8 = A/Sing MAP, 15 ug (UA); B9 = A/Sing IM, 15 ug, as 2018 Afluria Quad (UA)

Listing 16.2.9.11
Individual Characterisation of Skin Irritation Index by Treatment
Total Skin Irritation Index
Part B, Non-biopsy
B3= 3 x 5 ug A/Sing MAPs on volar forearm (15 ug)

			Active			Placebo		
Randomisation Number	Visit	Time Point	#> 0	Maximum	Average	#> 0	Maximum	Average
B0027	Day 1	10 min	3	3	3.0	-	-	-
B0027	Day 1	1 hour	3	4	3.3	-	-	-
B0027	Day 1	2 hour	3	5	5.0	-	-	-
B0027	Day 2		3	3	3.0	-	-	-
B0027	Day 4		3	4	3.0	-	-	-
B0027	Day 8		3	3	3.0	-	-	-
B0027	Day 22		0	0	0.0	-	-	-
B0027	EOS/ET (Day 61)		0	0	0.0	-	-	-
B0038	Day 1	Pre-dose	0	0	0.0	-	-	-
B0038	Day 1	10 min	3	4	3.7	-	-	-
B0038	Day 1	1 hour	3	4	4.0	-	-	-
B0038	Day 1	2 hour	3	4	4.0	-	-	-
B0038	Day 2		3	4	4.0	-	-	-
B0038	Day 4		3	3	3.0	-	-	-
B0038	Day 8		3	2	2.0	-	-	-
B0038	Day 22		3	1	1.0	-	-	-
B0038	EOS/ET (Day 61)		0	0	0.0	-	-	-
B0041	Day 1	Pre-dose	0	0	0.0	-	-	-
B0041	Day 1	10 min	3	1	1.0	-	-	-

Administration Site Key: FA = Forearm; UA= Upper arm
Treatments Part A: A1 = A/Sing MAP, 15 ug (FA); A2 = Afluria Quad IM (UA), 15 ug; A3 = Placebo MAP (FA); A4 = A/Sing Mono IM, 15 ug (UA)
Treatments Part B: B1 = A/Sing MAP, 15 ug (FA) with biopsy; B2 = Placebo MAP (FA) with biopsy
B3 = A/Sing MAP, 15 ug (FA); B4 = A/Sing MAP, 10 ug (FA); B5 = A/Sing MAP, 5 ug (FA); B6 = A/Sing MAP, 2.5 ug (FA)
B7 = Placebo MAP (FA); B8 = A/Sing MAP, 15 ug (UA); B9 = A/Sing IM, 15 ug, as 2018 Afluria Quad (UA)

Listing 16.2.9.11
Individual Characterisation of Skin Irritation Index by Treatment
Total Skin Irritation Index
Part B, Non-biopsy
B3= 3 x 5 ug A/Sing MAPs on volar forearm (15 ug)

			Active			Placebo		
Randomisation Number	Visit	Time Point	#> 0	Maximum	Average	#> 0	Maximum	Average
B0041	Day 1	1 hour	3	4	4.0	-	-	-
B0041	Day 1	2 hour	3	3	3.0	-	-	-
B0041	Day 2		3	4	4.0	-	-	-
B0041	Day 4		3	3	2.3	-	-	-
B0041	Day 8		0	0	0.0	-	-	-
B0041	Day 22		0	0	0.0	-	-	-
B0041	EOS/ET (Day 61)		0	0	0.0	-	-	-
B0051	Day 1	Pre-dose	0	0	0.0	-	-	-
B0051	Day 1	10 min	3	3	3.0	-	-	-
B0051	Day 1	1 hour	3	3	3.0	-	-	-
B0051	Day 1	2 hour	3	3	3.0	-	-	-
B0051	Day 2		3	5	5.0	-	-	-
B0051	Day 4		3	3	3.0	-	-	-
B0051	Day 8		3	2	2.0	-	-	-
B0051	Day 22		0	0	0.0	-	-	-
B0051	EOS/ET (Day 61)		0	0	0.0	-	-	-
B0056	Day 1	Pre-dose	0	0	0.0	-	-	-
B0056	Day 1	10 min	3	3	3.0	-	-	-
B0056	Day 1	1 hour	3	3	3.0	-	-	-

Source: Listing 16.2.9.4

Administration Site Key: FA = Forearm; UA= Upper arm
Treatments Part A: A1 = A/Sing MAP, 15 ug (FA); A2 = Afluria Quad IM (UA), 15 ug; A3 = Placebo MAP (FA); A4 = A/Sing Mono IM, 15 ug (UA)
Treatments Part B: B1 = A/Sing MAP, 15 ug (FA) with biopsy; B2 = Placebo MAP (FA) with biopsy
B3 = A/Sing MAP, 15 ug (FA); B4 = A/Sing MAP, 10 ug (FA); B5 = A/Sing MAP, 5 ug (FA); B6 = A/Sing MAP, 2.5 ug (FA)
B7 = Placebo MAP (FA); B8 = A/Sing MAP, 15 ug (UA); B9 = A/Sing IM, 15 ug, as 2018 Afluria Quad (UA)

Listing 16.2.9.11
Individual Characterisation of Skin Irritation Index by Treatment
Total Skin Irritation Index
Part B, Non-biopsy
B3= 3 x 5 ug A/Sing MAPs on volar forearm (15 ug)

			Active			Placebo		
Randomisation Number	Visit	Time Point	#> 0	Maximum	Average	#> 0	Maximum	Average
B0056	Day 1	2 hour	3	3	3.0	-	-	-
B0056	Day 2		3	4	3.7	-	-	-
B0056	Day 4		2	1	0.7	-	-	-
B0056	Day 8		0	0	0.0	-	-	-
B0056	Day 22		0	0	0.0	-	-	-
B0056	EOS/ET (Day 61)		0	0	0.0	-	-	-
B0060	Day 1	Pre-dose	0	0	0.0	-	-	-
B0060	Day 1	10 min	3	3	3.0	-	-	-
B0060	Day 1	1 hour	3	3	3.0	-	-	-
B0060	Day 1	2 hour	3	3	3.0	-	-	-
B0060	Day 2		3	4	3.3	-	-	-
B0060	Day 4		3	6	5.7	-	-	-
B0060	Day 8		3	2	1.3	-	-	-
B0060	Day 22		0	0	0.0	-	-	-
B0060	EOS/ET (Day 61)		0	0	0.0	-	-	-
B0072	Day 1	Pre-dose	0	0	0.0	-	-	-
B0072	Day 1	10 min	3	3	2.7	-	-	-
B0072	Day 1	1 hour	3	3	2.3	-	-	-
B0072	Day 1	2 hour	3	5	4.7	-	-	-

Source: Listing 16.2.9.4

Administration Site Key: FA = Forearm; UA= Upper arm
Treatments Part A: A1 = A/Sing MAP, 15 ug (FA); A2 = Afluria Quad IM (UA), 15 ug; A3 = Placebo MAP (FA); A4 = A/Sing Mono IM, 15 ug (UA)
Treatments Part B: B1 = A/Sing MAP, 15 ug (FA) with biopsy; B2 = Placebo MAP (FA) with biopsy
B3 = A/Sing MAP, 15 ug (FA); B4 = A/Sing MAP, 10 ug (FA); B5 = A/Sing MAP, 5 ug (FA); B6 = A/Sing MAP, 2.5 ug (FA)
B7 = Placebo MAP (FA); B8 = A/Sing MAP, 15 ug (UA); B9 = A/Sing IM, 15 ug, as 2018 Afluria Quad (UA)

Listing 16.2.9.11
Individual Characterisation of Skin Irritation Index by Treatment
Total Skin Irritation Index
Part B, Non-biopsy
B3= 3 x 5 ug A/Sing MAPs on volar forearm (15 ug)

			Active			Placebo		
Randomisation Number	Visit	Time Point	#> 0	Maximum	Average	#> 0	Maximum	Average
B0072	Day 2		3	5	4.7	-	-	-
B0072	Day 4		3	4	3.7	-	-	-
B0072	Day 8		0	0	0.0	-	-	-
B0072	Day 22		0	0	0.0	-	-	-
B0072	EOS/ET (Day 61)		0	0	0.0	-	-	-
B0075	Day 1	Pre-dose	0	0	0.0	-	-	-
B0075	Day 1	10 min	3	2	2.0	-	-	-
B0075	Day 1	1 hour	3	3	2.7	-	-	-
B0075	Day 1	2 hour	3	4	4.0	-	-	-
B0075	Day 2		3	4	4.0	-	-	-
B0075	Day 4		3	4	4.0	-	-	-
B0075	Day 8		1	1	0.3	-	-	-
B0075	Day 22		3	2	2.0	-	-	-
B0075	EOS/ET (Day 61)		0	0	0.0	-	-	-
B0082	Day 1	Pre-dose	0	0	0.0	-	-	-
B0082	Day 1	10 min	3	3	3.0	-	-	-
B0082	Day 1	1 hour	3	3	3.0	-	-	-
B0082	Day 1	2 hour	3	4	3.0	-	-	-
B0082	Day 2		3	7	7.0	-	-	-

Administration Site Key: FA = Forearm; UA= Upper arm
Treatments Part A: A1 = A/Sing MAP, 15 ug (FA); A2 = Afluria Quad IM (UA), 15 ug; A3 = Placebo MAP (FA); A4 = A/Sing Mono IM, 15 ug (UA)
Treatments Part B: B1 = A/Sing MAP, 15 ug (FA) with biopsy; B2 = Placebo MAP (FA) with biopsy
B3 = A/Sing MAP, 15 ug (FA); B4 = A/Sing MAP, 10 ug (FA); B5 = A/Sing MAP, 5 ug (FA); B6 = A/Sing MAP, 2.5 ug (FA)
B7 = Placebo MAP (FA); B8 = A/Sing MAP, 15 ug (UA); B9 = A/Sing IM, 15 ug, as 2018 Afluria Quad (UA)

Listing 16.2.9.11
Individual Characterisation of Skin Irritation Index by Treatment
Total Skin Irritation Index
Part B, Non-biopsy
B3= 3 x 5 ug A/Sing MAPs on volar forearm (15 ug)

			Active			Placebo		
Randomisation Number	Visit	Time Point	#> 0	Maximum	Average	#> 0	Maximum	Average
B0082	Day 4		3	4	3.7	-	-	-
B0082	Day 8		2	1	0.7	-	-	-
B0082	Day 22		1	1	0.3	-	-	-
B0082	EOS/ET (Day 61)		1	1	0.3	-	-	-
B0094	Day 1	Pre-dose	0	0	0.0	-	-	-
B0094	Day 1	10 min	3	4	4.0	-	-	-
B0094	Day 1	1 hour	3	3	3.0	-	-	-
B0094	Day 1	2 hour	3	3	2.3	-	-	-
B0094	Day 2		3	5	4.7	-	-	-
B0094	Day 4		3	3	3.0	-	-	-
B0094	Day 8		0	0	0.0	-	-	-
B0094	Day 22		0	0	0.0	-	-	-
B0094	EOS/ET (Day 61)		0	0	0.0	-	-	-
B0101	Day 1	Pre-dose	0	0	0.0	-	-	-
B0101	Day 1	10 min	3	3	3.0	-	-	-
B0101	Day 1	1 hour	3	4	4.0	-	-	-
B0101	Day 1	2 hour	3	2	2.0	-	-	-
B0101	Day 2		3	3	2.3	-	-	-
B0101	Day 4		3	3	2.7	-	-	-

Source: Listing 16.2.9.4

Administration Site Key: FA = Forearm; UA= Upper arm
Treatments Part A: A1 = A/Sing MAP, 15 ug (FA); A2 = Afluria Quad IM (UA), 15 ug; A3 = Placebo MAP (FA); A4 = A/Sing Mono IM, 15 ug (UA)
Treatments Part B: B1 = A/Sing MAP, 15 ug (FA) with biopsy; B2 = Placebo MAP (FA) with biopsy
B3 = A/Sing MAP, 15 ug (FA); B4 = A/Sing MAP, 10 ug (FA); B5 = A/Sing MAP, 5 ug (FA); B6 = A/Sing MAP, 2.5 ug (FA)
B7 = Placebo MAP (FA); B8 = A/Sing MAP, 15 ug (UA); B9 = A/Sing IM, 15 ug, as 2018 Afluria Quad (UA)

Listing 16.2.9.11
Individual Characterisation of Skin Irritation Index by Treatment
Total Skin Irritation Index
Part B, Non-biopsy
B3= 3 x 5 ug A/Sing MAPs on volar forearm (15 ug)

			Active			Placebo		
Randomisation Number	Visit	Time Point	#> 0	Maximum	Average	#> 0	Maximum	Average
B0101	Day 8		3	3	2.3	-	-	-
B0101	Day 22		0	0	0.0	-	-	-
B0101	EOS/ET (Day 61)		0	0	0.0	-	-	-
B0104	Day 1	Pre-dose	0	0	0.0	-	-	-
B0104	Day 1	10 min	3	3	3.0	-	-	-
B0104	Day 1	1 hour	3	4	3.3	-	-	-
B0104	Day 1	2 hour	3	3	3.0	-	-	-
B0104	Day 2		3	5	3.7	-	-	-
B0104	Day 4		3	3	3.0	-	-	-
B0104	Day 8		0	0	0.0	-	-	-
B0104	Day 22		0	0	0.0	-	-	-
B0104	EOS/ET (Day 61)		0	0	0.0	-	-	-
B0110	Day 1	Pre-dose	0	0	0.0	-	-	-
B0110	Day 1	10 min	3	4	3.3	-	-	-
B0110	Day 1	1 hour	3	2	2.0	-	-	-
B0110	Day 1	2 hour	3	4	3.7	-	-	-
B0110	Day 2		3	4	4.0	-	-	-
B0110	Day 4		3	3	3.0	-	-	-
B0110	Day 8		3	3	3.0	-	-	-

Administration Site Key: FA = Forearm; UA= Upper arm
Treatments Part A: A1 = A/Sing MAP, 15 ug (FA); A2 = Afluria Quad IM (UA), 15 ug; A3 = Placebo MAP (FA); A4 = A/Sing Mono IM, 15 ug (UA)
Treatments Part B: B1 = A/Sing MAP, 15 ug (FA) with biopsy; B2 = Placebo MAP (FA) with biopsy
B3 = A/Sing MAP, 15 ug (FA); B4 = A/Sing MAP, 10 ug (FA); B5 = A/Sing MAP, 5 ug (FA); B6 = A/Sing MAP, 2.5 ug (FA)
B7 = Placebo MAP (FA); B8 = A/Sing MAP, 15 ug (UA); B9 = A/Sing IM, 15 ug, as 2018 Afluria Quad (UA)

Listing 16.2.9.11
Individual Characterisation of Skin Irritation Index by Treatment
Total Skin Irritation Index
Part B, Non-biopsy
B3= 3 x 5 ug A/Sing MAPs on volar forearm (15 ug)

			Active			Placebo		
Randomisation Number	Visit	Time Point	#> 0	Maximum	Average	#> 0	Maximum	Average
B0110	Day 22		0	0	0.0	-	-	-
B0110	EOS/ET (Day 61)		0	0	0.0	-	-	-
B0120	Day 1	Pre-dose	0	0	0.0	-	-	-
B0120	Day 1	10 min	3	4	3.7	-	-	-
B0120	Day 1	1 hour	3	5	5.0	-	-	-
B0120	Day 1	2 hour	3	5	5.0	-	-	-
B0120	Day 2		3	5	4.3	-	-	-
B0120	Day 4		3	3	3.0	-	-	-
B0120	Day 8		3	3	3.0	-	-	-
B0120	Day 22		0	0	0.0	-	-	-
B0120	EOS/ET (Day 61)		0	0	0.0	-	-	-
B0126	Day 1	Pre-dose	0	0	0.0	-	-	-
B0126	Day 1	10 min	3	3	2.7	-	-	-
B0126	Day 1	1 hour	3	4	4.0	-	-	-
B0126	Day 1	2 hour	3	5	5.0	-	-	-
B0126	Day 2		3	3	3.0	-	-	-
B0126	Day 4		3	2	2.0	-	-	-
B0126	Day 8		0	0	0.0	-	-	-
B0126	Day 22		0	0	0.0	-	-	-

Administration Site Key: FA = Forearm; UA= Upper arm
Treatments Part A: A1 = A/Sing MAP, 15 ug (FA); A2 = Afluria Quad IM (UA), 15 ug; A3 = Placebo MAP (FA); A4 = A/Sing Mono IM, 15 ug (UA)
Treatments Part B: B1 = A/Sing MAP, 15 ug (FA) with biopsy; B2 = Placebo MAP (FA) with biopsy
B3 = A/Sing MAP, 15 ug (FA); B4 = A/Sing MAP, 10 ug (FA); B5 = A/Sing MAP, 5 ug (FA); B6 = A/Sing MAP, 2.5 ug (FA)
B7 = Placebo MAP (FA); B8 = A/Sing MAP, 15 ug (UA); B9 = A/Sing IM, 15 ug, as 2018 Afluria Quad (UA)

Listing 16.2.9.11
Individual Characterisation of Skin Irritation Index by Treatment
Total Skin Irritation Index
Part B, Non-biopsy
B3= 3 x 5 ug A/Sing MAPs on volar forearm (15 ug)

			Active			Placebo		
Randomisation Number	Visit	Time Point	#> 0	Maximum	Average	#> 0	Maximum	Average
B0126	EOS/ET (Day 61)		0	0	0.0	-	-	-
B0134	Day 1	Pre-dose	0	0	0.0	-	-	-
B0134	Day 1	10 min	3	4	4.0	-	-	-
B0134	Day 1	1 hour	3	5	4.7	-	-	-
B0134	Day 1	2 hour	3	3	3.0	-	-	-
B0134	Day 2		3	4	4.0	-	-	-
B0134	Day 4		3	2	2.0	-	-	-
B0134	Day 8		0	0	0.0	-	-	-
B0134	Day 22		0	0	0.0	-	-	-
B0134	EOS/ET (Day 61)		0	0	0.0	-	-	-
B0141	Day 1	Pre-dose	0	0	0.0	-	-	-
B0141	Day 1	10 min	3	4	4.0	-	-	-
B0141	Day 1	1 hour	3	5	4.3	-	-	-
B0141	Day 1	2 hour	3	3	3.0	-	-	-
B0141	Day 2		3	3	3.0	-	-	-
B0141	Day 4		3	3	3.0	-	-	-
B0141	Day 8		0	0	0.0	-	-	-
B0141	Day 22		0	0	0.0	-	-	-
B0141	EOS/ET (Day 61)		0	0	0.0	-	-	-

Source: Listing 16.2.9.4

Administration Site Key: FA = Forearm; UA= Upper arm
Treatments Part A: A1 = A/Sing MAP, 15 ug (FA); A2 = Afluria Quad IM (UA), 15 ug; A3 = Placebo MAP (FA); A4 = A/Sing Mono IM, 15 ug (UA)
Treatments Part B: B1 = A/Sing MAP, 15 ug (FA) with biopsy; B2 = Placebo MAP (FA) with biopsy
B3 = A/Sing MAP, 15 ug (FA); B4 = A/Sing MAP, 10 ug (FA); B5 = A/Sing MAP, 5 ug (FA); B6 = A/Sing MAP, 2.5 ug (FA)
B7 = Placebo MAP (FA); B8 = A/Sing MAP, 15 ug (UA); B9 = A/Sing IM, 15 ug, as 2018 Afluria Quad (UA)

Listing 16.2.9.11
Individual Characterisation of Skin Irritation Index by Treatment
Total Skin Irritation Index
Part B, Non-biopsy
B3= 3 x 5 ug A/Sing MAPs on volar forearm (15 ug)

			Active			Placebo		
Randomisation Number	Visit	Time Point	#> 0	Maximum	Average	#> 0	Maximum	Average
B0145	Day 1	Pre-dose	0	0	0.0	-	-	-
B0145	Day 1	10 min	3	3	3.0	-	-	-
B0145	Day 1	1 hour	3	5	5.0	-	-	-
B0145	Day 1	2 hour	3	4	3.7	-	-	-
B0145	Day 2		3	3	3.0	-	-	-
B0145	Day 4		3	3	2.7	-	-	-
B0145	Day 8		3	2	2.0	-	-	-
B0145	Day 22		3	1	1.0	-	-	-
B0145	EOS/ET (Day 61)		0	0	0.0	-	-	-

Source: Listing 16.2.9.4

Administration Site Key: FA = Forearm; UA= Upper arm
Treatments Part A: A1 = A/Sing MAP, 15 ug (FA); A2 = Afluria Quad IM (UA), 15 ug; A3 = Placebo MAP (FA); A4 = A/Sing Mono IM, 15 ug (UA)
Treatments Part B: B1 = A/Sing MAP, 15 ug (FA) with biopsy; B2 = Placebo MAP (FA) with biopsy
B3 = A/Sing MAP, 15 ug (FA); B4 = A/Sing MAP, 10 ug (FA); B5 = A/Sing MAP, 5 ug (FA); B6 = A/Sing MAP, 2.5 ug (FA)
B7 = Placebo MAP (FA); B8 = A/Sing MAP, 15 ug (UA); B9 = A/Sing IM, 15 ug, as 2018 Afluria Quad (UA)

Listing 16.2.9.11
Individual Characterisation of Skin Irritation Index by Treatment
Total Skin Irritation Index
Part B, Non-biopsy
B4= 2 x 5 ug A/Sing MAPs + 1 uncoated MAP on volar forearm (10 ug)

			Active			Placebo		
Randomisation Number	Visit	Time Point	#> 0	Maximum	Average	#> 0	Maximum	Average
B0016	Day 1	Pre-dose	0	0	0.0	0	0	0.0
B0016	Day 1	10 min	2	3	3.0	1	4	4.0
B0016	Day 1	1 hour	2	2	2.0	1	2	2.0
B0016	Day 1	2 hour	2	3	3.0	1	3	3.0
B0016	Day 2		2	5	4.0	1	1	1.0
B0016	Day 4		2	6	5.0	1	1	1.0
B0016	Day 8		0	0	0.0	0	0	0.0
B0016	Day 22		0	0	0.0	0	0	0.0
B0016	EOS/ET (Day 61)		0	0	0.0	0	0	0.0
B0022	Day 1	Pre-dose	0	0	0.0	0	0	0.0
B0022	Day 1	10 min	2	3	3.0	1	3	3.0
B0022	Day 1	1 hour	2	2	2.0	1	2	2.0
B0022	Day 1	2 hour	2	2	2.0	1	2	2.0
B0022	Day 2		2	4	4.0	1	2	2.0
B0022	Day 4		2	6	6.0	1	3	3.0
B0022	Day 8		2	1	1.0	0	0	0.0
B0022	Day 22		0	0	0.0	0	0	0.0
B0022	EOS/ET (Day 61)		0	0	0.0	0	0	0.0
B0031	Day 1	Pre-dose	0	0	0.0	0	0	0.0

Source: Listing 16.2.9.4

Administration Site Key: FA = Forearm; UA= Upper arm
Treatments Part A: A1 = A/Sing MAP, 15 ug (FA); A2 = Afluria Quad IM (UA), 15 ug; A3 = Placebo MAP (FA); A4 = A/Sing Mono IM, 15 ug (UA)
Treatments Part B: B1 = A/Sing MAP, 15 ug (FA) with biopsy; B2 = Placebo MAP (FA) with biopsy
B3 = A/Sing MAP, 15 ug (FA); B4 = A/Sing MAP, 10 ug (FA); B5 = A/Sing MAP, 5 ug (FA); B6 = A/Sing MAP, 2.5 ug (FA)
B7 = Placebo MAP (FA); B8 = A/Sing MAP, 15 ug (UA); B9 = A/Sing IM, 15 ug, as 2018 Afluria Quad (UA)

Listing 16.2.9.11
Individual Characterisation of Skin Irritation Index by Treatment
Total Skin Irritation Index
Part B, Non-biopsy
B4= 2 x 5 ug A/Sing MAPs + 1 uncoated MAP on volar forearm (10 ug)

			Active			Placebo		
Randomisation Number	Visit	Time Point	#> 0	Maximum	Average	#> 0	Maximum	Average
B0031	Day 1	10 min	2	4	4.0	1	2	2.0
B0031	Day 1	1 hour	2	3	3.0	1	3	3.0
B0031	Day 1	2 hour	2	5	4.5	1	3	3.0
B0031	Day 2		2	4	4.0	1	2	2.0
B0031	Day 4		2	4	3.5	1	1	1.0
B0031	Day 8		0	0	0.0	0	0	0.0
B0031	Day 22		0	0	0.0	0	0	0.0
B0031	EOS/ET (Day 61)		0	0	0.0	0	0	0.0
B0037	Day 1	Pre-dose	0	0	0.0	0	0	0.0
B0037	Day 1	10 min	2	2	2.0	1	2	2.0
B0037	Day 1	1 hour	2	1	1.0	1	1	1.0
B0037	Day 1	2 hour	2	3	2.5	1	3	3.0
B0037	Day 2		2	4	4.0	1	1	1.0
B0037	Day 4		2	3	3.0	0	0	0.0
B0037	Day 8		2	2	2.0	1	1	1.0
B0037	Day 22		0	0	0.0	0	0	0.0
B0037	EOS/ET (Day 61)		0	0	0.0	0	0	0.0
B0045	Day 1	Pre-dose	0	0	0.0	0	0	0.0
B0045	Day 1	10 min	2	3	2.5	1	2	2.0

Administration Site Key: FA = Forearm; UA= Upper arm
Treatments Part A: A1 = A/Sing MAP, 15 ug (FA); A2 = Afluria Quad IM (UA), 15 ug; A3 = Placebo MAP (FA); A4 = A/Sing Mono IM, 15 ug (UA)
Treatments Part B: B1 = A/Sing MAP, 15 ug (FA) with biopsy; B2 = Placebo MAP (FA) with biopsy
B3 = A/Sing MAP, 15 ug (FA); B4 = A/Sing MAP, 10 ug (FA); B5 = A/Sing MAP, 5 ug (FA); B6 = A/Sing MAP, 2.5 ug (FA)
B7 = Placebo MAP (FA); B8 = A/Sing MAP, 15 ug (UA); B9 = A/Sing IM, 15 ug, as 2018 Afluria Quad (UA)

Listing 16.2.9.11
Individual Characterisation of Skin Irritation Index by Treatment
Total Skin Irritation Index
Part B, Non-biopsy
B4= 2 x 5 ug A/Sing MAPs + 1 uncoated MAP on volar forearm (10 ug)

			Active			Placebo		
Randomisation Number	Visit	Time Point	#> 0	Maximum	Average	#> 0	Maximum	Average
B0045	Day 1	1 hour	2	5	4.0	1	4	4.0
B0045	Day 1	2 hour	2	3	2.5	1	3	3.0
B0045	Day 2		2	7	4.5	1	2	2.0
B0045	Day 4		2	3	3.0	0	0	0.0
B0045	Day 8		0	0	0.0	0	0	0.0
B0045	Day 22		2	1	1.0	0	0	0.0
B0045	EOS/ET (Day 61)		0	0	0.0	0	0	0.0
B0049	Day 1	Pre-dose	0	0	0.0	0	0	0.0
B0049	Day 1	10 min	2	2	2.0	1	1	1.0
B0049	Day 1	1 hour	2	3	3.0	1	2	2.0
B0049	Day 1	2 hour	2	4	4.0	1	3	3.0
B0049	Day 2		2	4	4.0	1	1	1.0
B0049	Day 4		0	0	0.0	0	0	0.0
B0049	Day 8		0	0	0.0	0	0	0.0
B0049	Day 22		0	0	0.0	0	0	0.0
B0049	EOS/ET (Day 61)		0	0	0.0	0	0	0.0
B0057	Day 1	Pre-dose	0	0	0.0	0	0	0.0
B0057	Day 1	10 min	2	3	3.0	1	3	3.0
B0057	Day 1	1 hour	2	3	3.0	1	3	3.0

Source: Listing 16.2.9.4

Administration Site Key: FA = Forearm; UA= Upper arm
Treatments Part A: A1 = A/Sing MAP, 15 ug (FA); A2 = Afluria Quad IM (UA), 15 ug; A3 = Placebo MAP (FA); A4 = A/Sing Mono IM, 15 ug (UA)
Treatments Part B: B1 = A/Sing MAP, 15 ug (FA) with biopsy; B2 = Placebo MAP (FA) with biopsy
B3 = A/Sing MAP, 15 ug (FA); B4 = A/Sing MAP, 10 ug (FA); B5 = A/Sing MAP, 5 ug (FA); B6 = A/Sing MAP, 2.5 ug (FA)
B7 = Placebo MAP (FA); B8 = A/Sing MAP, 15 ug (UA); B9 = A/Sing IM, 15 ug, as 2018 Afluria Quad (UA)

Listing 16.2.9.11
Individual Characterisation of Skin Irritation Index by Treatment
Total Skin Irritation Index
Part B, Non-biopsy
B4= 2 x 5 ug A/Sing MAPs + 1 uncoated MAP on volar forearm (10 ug)

			Active			Placebo		
Randomisation Number	Visit	Time Point	#> 0	Maximum	Average	#> 0	Maximum	Average
B0057	Day 1	2 hour	2	3	3.0	1	3	3.0
B0057	Day 2		2	4	3.5	1	1	1.0
B0057	Day 4		2	2	2.0	1	1	1.0
B0057	Day 8		2	2	2.0	1	1	1.0
B0057	Day 22		0	0	0.0	0	0	0.0
B0057	EOS/ET (Day 61)		0	0	0.0	0	0	0.0
B0065	Day 1	Pre-dose	0	0	0.0	0	0	0.0
B0065	Day 1	10 min	2	3	3.0	1	3	3.0
B0065	Day 1	1 hour	2	3	3.0	1	3	3.0
B0065	Day 1	2 hour	2	4	3.5	1	3	3.0
B0065	Day 2		2	5	5.0	1	2	2.0
B0065	Day 4		2	4	3.5	0	0	0.0
B0065	Day 8		2	2	1.5	1	1	1.0
B0065	Day 22		0	0	0.0	0	0	0.0
B0065	EOS/ET (Day 61)		0	0	0.0	0	0	0.0
B0071	Day 1	Pre-dose	0	0	0.0	0	0	0.0
B0071	Day 1	10 min	2	3	3.0	1	3	3.0
B0071	Day 1	1 hour	2	2	2.0	1	2	2.0
B0071	Day 1	2 hour	2	3	3.0	1	3	3.0

Source: Listing 16.2.9.4

Administration Site Key: FA = Forearm; UA= Upper arm
Treatments Part A: A1 = A/Sing MAP, 15 ug (FA); A2 = Afluria Quad IM (UA), 15 ug; A3 = Placebo MAP (FA); A4 = A/Sing Mono IM, 15 ug (UA)
Treatments Part B: B1 = A/Sing MAP, 15 ug (FA) with biopsy; B2 = Placebo MAP (FA) with biopsy
B3 = A/Sing MAP, 15 ug (FA); B4 = A/Sing MAP, 10 ug (FA); B5 = A/Sing MAP, 5 ug (FA); B6 = A/Sing MAP, 2.5 ug (FA)
B7 = Placebo MAP (FA); B8 = A/Sing MAP, 15 ug (UA); B9 = A/Sing IM, 15 ug, as 2018 Afluria Quad (UA)

Listing 16.2.9.11
Individual Characterisation of Skin Irritation Index by Treatment
Total Skin Irritation Index
Part B, Non-biopsy
B4= 2 x 5 ug A/Sing MAPs + 1 uncoated MAP on volar forearm (10 ug)

			Active			Placebo		
Randomisation Number	Visit	Time Point	#> 0	Maximum	Average	#> 0	Maximum	Average
B0071	Day 2		2	4	4.0	1	3	3.0
B0071	Day 4		2	3	3.0	0	0	0.0
B0071	Day 8		1	2	1.0	0	0	0.0
B0071	Day 22		0	0	0.0	0	0	0.0
B0071	EOS/ET (Day 61)		0	0	0.0	0	0	0.0
B0077	Day 1	Pre-dose	0	0	0.0	0	0	0.0
B0077	Day 1	10 min	2	3	3.0	1	2	2.0
B0077	Day 1	1 hour	2	2	2.0	1	2	2.0
B0077	Day 1	2 hour	2	3	3.0	1	3	3.0
B0077	Day 2		2	3	2.5	1	1	1.0
B0077	Day 4		2	6	6.0	1	3	3.0
B0077	Day 8		2	1	1.0	0	0	0.0
B0077	Day 22		0	0	0.0	0	0	0.0
B0077	EOS/ET (Day 61)		0	0	0.0	0	0	0.0
B0085	Day 1	Pre-dose	0	0	0.0	0	0	0.0
B0085	Day 1	10 min	2	4	3.5	1	3	3.0
B0085	Day 1	1 hour	2	3	3.0	1	3	3.0
B0085	Day 1	2 hour	2	2	2.0	1	2	2.0
B0085	Day 2		2	7	7.0	1	2	2.0

Administration Site Key: FA = Forearm; UA= Upper arm
Treatments Part A: A1 = A/Sing MAP, 15 ug (FA); A2 = Afluria Quad IM (UA), 15 ug; A3 = Placebo MAP (FA); A4 = A/Sing Mono IM, 15 ug (UA)
Treatments Part B: B1 = A/Sing MAP, 15 ug (FA) with biopsy; B2 = Placebo MAP (FA) with biopsy
B3 = A/Sing MAP, 15 ug (FA); B4 = A/Sing MAP, 10 ug (FA); B5 = A/Sing MAP, 5 ug (FA); B6 = A/Sing MAP, 2.5 ug (FA)
B7 = Placebo MAP (FA); B8 = A/Sing MAP, 15 ug (UA); B9 = A/Sing IM, 15 ug, as 2018 Afluria Quad (UA)

Listing 16.2.9.11
Individual Characterisation of Skin Irritation Index by Treatment
Total Skin Irritation Index
Part B, Non-biopsy
B4= 2 x 5 ug A/Sing MAPs + 1 uncoated MAP on volar forearm (10 ug)

			Active			Placebo		
Randomisation Number	Visit	Time Point	#> 0	Maximum	Average	#> 0	Maximum	Average
B0085	Day 4		2	6	4.5	0	0	0.0
B0085	Day 8		0	0	0.0	0	0	0.0
B0085	Day 22		0	0	0.0	0	0	0.0
B0085	EOS/ET (Day 61)		0	0	0.0	0	0	0.0
B0092	Day 1	Pre-dose	0	0	0.0	0	0	0.0
B0092	Day 1	10 min	2	3	2.5	1	3	3.0
B0092	Day 1	1 hour	2	3	3.0	1	2	2.0
B0092	Day 1	2 hour	2	4	3.5	1	3	3.0
B0092	Day 2		2	3	3.0	1	1	1.0
B0092	Day 4		2	3	3.0	0	0	0.0
B0092	Day 8		1	2	1.0	0	0	0.0
B0092	Day 22		0	0	0.0	0	0	0.0
B0092	EOS/ET (Day 61)		0	0	0.0	0	0	0.0
B0099	Day 1	Pre-dose	0	0	0.0	0	0	0.0
B0099	Day 1	10 min	2	2	2.0	1	2	2.0
B0099	Day 1	1 hour	2	4	3.5	1	4	4.0
B0099	Day 1	2 hour	2	3	2.5	1	3	3.0
B0099	Day 2		2	5	3.5	1	2	2.0
B0099	Day 4		2	2	2.0	0	0	0.0

Administration Site Key: FA = Forearm; UA= Upper arm
Treatments Part A: A1 = A/Sing MAP, 15 ug (FA); A2 = Afluria Quad IM (UA), 15 ug; A3 = Placebo MAP (FA); A4 = A/Sing Mono IM, 15 ug (UA)
Treatments Part B: B1 = A/Sing MAP, 15 ug (FA) with biopsy; B2 = Placebo MAP (FA) with biopsy
B3 = A/Sing MAP, 15 ug (FA); B4 = A/Sing MAP, 10 ug (FA); B5 = A/Sing MAP, 5 ug (FA); B6 = A/Sing MAP, 2.5 ug (FA)
B7 = Placebo MAP (FA); B8 = A/Sing MAP, 15 ug (UA); B9 = A/Sing IM, 15 ug, as 2018 Afluria Quad (UA)

Listing 16.2.9.11
Individual Characterisation of Skin Irritation Index by Treatment
Total Skin Irritation Index
Part B, Non-biopsy
B4= 2 x 5 ug A/Sing MAPs + 1 uncoated MAP on volar forearm (10 ug)

Randomisation Number	Visit	Time Point	Active #> 0	Active Maximum	Active Average	Placebo #> 0	Placebo Maximum	Placebo Average
B0099	Day 8		0	0	0.0	0	0	0.0
B0099	EOS/ET (Day 61)		0	0	0.0	0	0	0.0
B0106	Day 1	Pre-dose	0	0	0.0	0	0	0.0
B0106	Day 1	10 min	2	4	3.5	1	3	3.0
B0106	Day 1	1 hour	2	4	4.0	1	3	3.0
B0106	Day 1	2 hour	2	3	3.0	1	2	2.0
B0106	Day 2		2	3	3.0	1	1	1.0
B0106	Day 4		2	3	3.0	0	0	0.0
B0106	Day 8		0	0	0.0	0	0	0.0
B0106	Day 22		0	0	0.0	0	0	0.0
B0106	EOS/ET (Day 61)		0	0	0.0	0	0	0.0
B0112	Day 1	Pre-dose	0	0	0.0	0	0	0.0
B0112	Day 1	10 min	2	3	3.0	1	2	2.0
B0112	Day 1	1 hour	2	2	2.0	1	2	2.0
B0112	Day 1	2 hour	2	5	4.5	1	3	3.0
B0112	Day 2		2	3	3.0	1	2	2.0
B0112	Day 4		2	5	5.0	0	0	0.0
B0112	Day 8		2	2	2.0	1	1	1.0
B0112	Day 22		0	0	0.0	0	0	0.0

Source: Listing 16.2.9.4

Administration Site Key: FA = Forearm; UA= Upper arm
Treatments Part A: A1 = A/Sing MAP, 15 ug (FA); A2 = Afluria Quad IM (UA), 15 ug; A3 = Placebo MAP (FA); A4 = A/Sing Mono IM, 15 ug (UA)
Treatments Part B: B1 = A/Sing MAP, 15 ug (FA) with biopsy; B2 = Placebo MAP (FA) with biopsy
B3 = A/Sing MAP, 15 ug (FA); B4 = A/Sing MAP, 10 ug (FA); B5 = A/Sing MAP, 5 ug (FA); B6 = A/Sing MAP, 2.5 ug (FA)
B7 = Placebo MAP (FA); B8 = A/Sing MAP, 15 ug (UA); B9 = A/Sing IM, 15 ug, as 2018 Afluria Quad (UA)

Listing 16.2.9.11
Individual Characterisation of Skin Irritation Index by Treatment
Total Skin Irritation Index
Part B, Non-biopsy
B4= 2 x 5 ug A/Sing MAPs + 1 uncoated MAP on volar forearm (10 ug)

			Active			Placebo		
Randomisation Number	Visit	Time Point	#> 0	Maximum	Average	#> 0	Maximum	Average
B0112	EOS/ET (Day 61)		0	0	0.0	0	0	0.0
B0119	Day 1	Pre-dose	0	0	0.0	0	0	0.0
B0119	Day 1	10 min	2	3	3.0	1	3	3.0
B0119	Day 1	1 hour	2	3	2.5	1	3	3.0
B0119	Day 1	2 hour	2	4	3.5	1	4	4.0
B0119	Day 2		2	4	4.0	1	2	2.0
B0119	Day 4		2	5	4.5	0	0	0.0
B0119	Day 8		0	0	0.0	0	0	0.0
B0119	Day 22		0	0	0.0	0	0	0.0
B0119	EOS/ET (Day 61)		0	0	0.0	0	0	0.0
B0124	Day 1	Pre-dose	0	0	0.0	0	0	0.0
B0124	Day 1	10 min	2	3	3.0	1	3	3.0
B0124	Day 1	1 hour	2	5	5.0	1	2	2.0
B0124	Day 1	2 hour	2	5	5.0	1	4	4.0
B0124	Day 2		2	4	4.0	1	1	1.0
B0124	Day 4		2	6	5.5	0	0	0.0
B0124	Day 8		1	4	2.0	0	0	0.0
B0124	Day 22		0	0	0.0	0	0	0.0
B0124	EOS/ET (Day 61)		0	0	0.0	0	0	0.0

Source: Listing 16.2.9.4

Administration Site Key: FA = Forearm; UA= Upper arm
Treatments Part A: A1 = A/Sing MAP, 15 ug (FA); A2 = Afluria Quad IM (UA), 15 ug; A3 = Placebo MAP (FA); A4 = A/Sing Mono IM, 15 ug (UA)
Treatments Part B: B1 = A/Sing MAP, 15 ug (FA) with biopsy; B2 = Placebo MAP (FA) with biopsy
B3 = A/Sing MAP, 15 ug (FA); B4 = A/Sing MAP, 10 ug (FA); B5 = A/Sing MAP, 5 ug (FA); B6 = A/Sing MAP, 2.5 ug (FA)
B7 = Placebo MAP (FA); B8 = A/Sing MAP, 15 ug (UA); B9 = A/Sing IM, 15 ug, as 2018 Afluria Quad (UA)

Listing 16.2.9.11
Individual Characterisation of Skin Irritation Index by Treatment
Total Skin Irritation Index
Part B, Non-biopsy
B4= 2 x 5 ug A/Sing MAPs + 1 uncoated MAP on volar forearm (10 ug)

			Active			Placebo		
Randomisation Number	Visit	Time Point	#> 0	Maximum	Average	#> 0	Maximum	Average
B0133	Day 1	Pre-dose	0	0	0.0	0	0	0.0
B0133	Day 1	10 min	2	3	3.0	1	3	3.0
B0133	Day 1	1 hour	2	5	5.0	1	3	3.0
B0133	Day 1	2 hour	2	4	4.0	1	3	3.0
B0133	Day 2		2	3	3.0	1	2	2.0
B0133	Day 4		2	3	3.0	1	1	1.0
B0133	Day 8		2	2	2.0	0	0	0.0
B0133	Day 22		2	1	1.0	0	0	0.0
B0133	EOS/ET (Day 61)		0	0	0.0	0	0	0.0
B0139	Day 1	Pre-dose	0	0	0.0	0	0	0.0
B0139	Day 1	10 min	2	4	4.0	1	4	4.0
B0139	Day 1	1 hour	2	4	4.0	1	3	3.0
B0139	Day 1	2 hour	2	5	5.0	1	3	3.0
B0139	Day 2		2	4	3.5	1	1	1.0
B0139	Day 4		2	3	3.0	1	1	1.0
B0139	Day 8		0	0	0.0	0	0	0.0
B0139	Day 22		0	0	0.0	0	0	0.0
B0139	EOS/ET (Day 61)		0	0	0.0	0	0	0.0

Source: Listing 16.2.9.4

Administration Site Key: FA = Forearm; UA= Upper arm
Treatments Part A: A1 = A/Sing MAP, 15 ug (FA); A2 = Afluria Quad IM (UA), 15 ug; A3 = Placebo MAP (FA); A4 = A/Sing Mono IM, 15 ug (UA)
Treatments Part B: B1 = A/Sing MAP, 15 ug (FA) with biopsy; B2 = Placebo MAP (FA) with biopsy
B3 = A/Sing MAP, 15 ug (FA); B4 = A/Sing MAP, 10 ug (FA); B5 = A/Sing MAP, 5 ug (FA); B6 = A/Sing MAP, 2.5 ug (FA)
B7 = Placebo MAP (FA); B8 = A/Sing MAP, 15 ug (UA); B9 = A/Sing IM, 15 ug, as 2018 Afluria Quad (UA)

Listing 16.2.9.11
Individual Characterisation of Skin Irritation Index by Treatment
Total Skin Irritation Index
Part B, Non-biopsy
B4= 2 x 5 ug A/Sing MAPs + 1 uncoated MAP on volar forearm (10 ug)

			Active			Placebo		
Randomisation Number	Visit	Time Point	#> 0	Maximum	Average	#> 0	Maximum	Average
B0150	Day 1	Pre-dose	0	0	0.0	0	0	0.0
B0150	Day 1	10 min	2	3	3.0	1	3	3.0
B0150	Day 1	1 hour	2	4	4.0	1	3	3.0
B0150	Day 1	2 hour	2	4	4.0	1	4	4.0
B0150	Day 2		2	5	4.0	1	3	3.0
B0150	Day 4		2	3	3.0	0	0	0.0
B0150	Day 8		2	3	3.0	0	0	0.0
B0150	Day 22		2	1	1.0	0	0	0.0
B0150	EOS/ET (Day 61)		0	0	0.0	0	0	0.0

Administration Site Key: FA = Forearm; UA= Upper arm
Treatments Part A: A1 = A/Sing MAP, 15 ug (FA); A2 = Afluria Quad IM (UA), 15 ug; A3 = Placebo MAP (FA); A4 = A/Sing Mono IM, 15 ug (UA)
Treatments Part B: B1 = A/Sing MAP, 15 ug (FA) with biopsy; B2 = Placebo MAP (FA) with biopsy
B3 = A/Sing MAP, 15 ug (FA); B4 = A/Sing MAP, 10 ug (FA); B5 = A/Sing MAP, 5 ug (FA); B6 = A/Sing MAP, 2.5 ug (FA)
B7 = Placebo MAP (FA); B8 = A/Sing MAP, 15 ug (UA); B9 = A/Sing IM, 15 ug, as 2018 Afluria Quad (UA)

Listing 16.2.9.11
Individual Characterisation of Skin Irritation Index by Treatment
Total Skin Irritation Index
Part B, Non-biopsy
B5= 1 x 5 ug A/Sing MAP + 2 uncoated MAPs on volar forearm (5 ug)

			Active			Placebo		
Randomisation Number	Visit	Time Point	#> 0	Maximum	Average	#> 0	Maximum	Average
B0013	Day 1	Pre-dose	0	0	0.0	0	0	0.0
B0013	Day 1	10 min	1	2	2.0	2	2	1.5
B0013	Day 1	1 hour	1	3	3.0	2	3	2.5
B0013	Day 1	2 hour	1	2	2.0	2	2	2.0
B0013	Day 2		1	3	3.0	2	2	2.0
B0013	Day 4		1	4	4.0	2	1	1.0
B0013	Day 8		1	2	2.0	0	0	0.0
B0013	Day 22		1	1	1.0	0	0	0.0
B0013	EOS/ET (Day 61)		0	0	0.0	0	0	0.0
B0024	Day 1	Pre-dose	0	0	0.0	0	0	0.0
B0024	Day 1	10 min	1	3	3.0	2	3	3.0
B0024	Day 1	1 hour	1	4	4.0	2	4	3.5
B0024	Day 1	2 hour	1	5	5.0	2	5	4.5
B0024	Day 2		1	4	4.0	2	1	1.0
B0024	Day 4		1	4	4.0	0	0	0.0
B0024	Day 8		0	0	0.0	0	0	0.0
B0024	Day 22		0	0	0.0	0	0	0.0
B0024	EOS/ET (Day 61)		0	0	0.0	0	0	0.0
B0028	Day 1	Pre-dose	0	0	0.0	0	0	0.0

Administration Site Key: FA = Forearm; UA= Upper arm
Treatments Part A: A1 = A/Sing MAP, 15 ug (FA); A2 = Afluria Quad IM (UA), 15 ug; A3 = Placebo MAP (FA); A4 = A/Sing Mono IM, 15 ug (UA)
Treatments Part B: B1 = A/Sing MAP, 15 ug (FA) with biopsy; B2 = Placebo MAP (FA) with biopsy
B3 = A/Sing MAP, 15 ug (FA); B4 = A/Sing MAP, 10 ug (FA); B5 = A/Sing MAP, 5 ug (FA); B6 = A/Sing MAP, 2.5 ug (FA)
B7 = Placebo MAP (FA); B8 = A/Sing MAP, 15 ug (UA); B9 = A/Sing IM, 15 ug, as 2018 Afluria Quad (UA)

Listing 16.2.9.11
Individual Characterisation of Skin Irritation Index by Treatment
Total Skin Irritation Index
Part B, Non-biopsy
B5= 1 x 5 ug A/Sing MAP + 2 uncoated MAPs on volar forearm (5 ug)

			Active			Placebo		
Randomisation Number	Visit	Time Point	#> 0	Maximum	Average	#> 0	Maximum	Average
B0028	Day 1	10 min	1	3	3.0	2	3	3.0
B0028	Day 1	1 hour	1	3	3.0	2	3	3.0
B0028	Day 1	2 hour	1	2	2.0	2	2	2.0
B0028	Day 2		1	4	4.0	2	2	2.0
B0028	Day 4		1	4	4.0	0	0	0.0
B0028	Day 8		1	3	3.0	0	0	0.0
B0028	Day 22		0	0	0.0	0	0	0.0
B0028	EOS/ET (Day 61)		0	0	0.0	0	0	0.0
B0033	Day 1	Pre-dose	0	0	0.0	0	0	0.0
B0033	Day 1	10 min	1	3	3.0	2	3	3.0
B0033	Day 1	1 hour	1	6	6.0	2	5	5.0
B0033	Day 1	2 hour	1	4	4.0	2	3	3.0
B0033	Day 2		1	4	4.0	2	2	2.0
B0033	Day 4		1	3	3.0	2	1	1.0
B0033	Day 8		1	3	3.0	2	1	1.0
B0033	Day 22		0	0	0.0	0	0	0.0
B0033	EOS/ET (Day 61)		0	0	0.0	0	0	0.0
B0042	Day 1	Pre-dose	0	0	0.0	0	0	0.0
B0042	Day 1	10 min	1	2	2.0	2	2	2.0

Source: Listing 16.2.9.4

Administration Site Key: FA = Forearm; UA= Upper arm
Treatments Part A: A1 = A/Sing MAP, 15 ug (FA); A2 = Afluria Quad IM (UA), 15 ug; A3 = Placebo MAP (FA); A4 = A/Sing Mono IM, 15 ug (UA)
Treatments Part B: B1 = A/Sing MAP, 15 ug (FA) with biopsy; B2 = Placebo MAP (FA) with biopsy
B3 = A/Sing MAP, 15 ug (FA); B4 = A/Sing MAP, 10 ug (FA); B5 = A/Sing MAP, 5 ug (FA); B6 = A/Sing MAP, 2.5 ug (FA)
B7 = Placebo MAP (FA); B8 = A/Sing MAP, 15 ug (UA); B9 = A/Sing IM, 15 ug, as 2018 Afluria Quad (UA)

Listing 16.2.9.11
Individual Characterisation of Skin Irritation Index by Treatment
Total Skin Irritation Index
Part B, Non-biopsy
B5= 1 x 5 ug A/Sing MAP + 2 uncoated MAPs on volar forearm (5 ug)

			Active			Placebo		
Randomisation Number	Visit	Time Point	#> 0	Maximum	Average	#> 0	Maximum	Average
B0042	Day 1	1 hour	1	3	3.0	2	3	3.0
B0042	Day 1	2 hour	1	3	3.0	2	3	3.0
B0042	Day 2		1	4	4.0	2	1	1.0
B0042	Day 4		1	3	3.0	0	0	0.0
B0042	Day 8		1	2	2.0	0	0	0.0
B0042	Day 22		1	3	3.0	0	0	0.0
B0042	EOS/ET (Day 61)		0	0	0.0	0	0	0.0
B0052	Day 1	Pre-dose	0	0	0.0	0	0	0.0
B0052	Day 1	10 min	1	3	3.0	2	3	2.5
B0052	Day 1	1 hour	1	3	3.0	2	3	3.0
B0052	Day 1	2 hour	1	3	3.0	2	3	3.0
B0052	Day 2		1	4	4.0	2	2	2.0
B0052	Day 4		1	3	3.0	2	2	2.0
B0052	Day 8		0	0	0.0	0	0	0.0
B0052	Day 22		1	2	2.0	0	0	0.0
B0052	EOS/ET (Day 61)		0	0	0.0	0	0	0.0
B0054	Day 1	Pre-dose	0	0	0.0	0	0	0.0
B0054	Day 1	10 min	1	3	3.0	2	3	3.0
B0054	Day 1	1 hour	1	3	3.0	2	3	3.0

Administration Site Key: FA = Forearm; UA= Upper arm
Treatments Part A: A1 = A/Sing MAP, 15 ug (FA); A2 = Afluria Quad IM (UA), 15 ug; A3 = Placebo MAP (FA); A4 = A/Sing Mono IM, 15 ug (UA)
Treatments Part B: B1 = A/Sing MAP, 15 ug (FA) with biopsy; B2 = Placebo MAP (FA) with biopsy
B3 = A/Sing MAP, 15 ug (FA); B4 = A/Sing MAP, 10 ug (FA); B5 = A/Sing MAP, 5 ug (FA); B6 = A/Sing MAP, 2.5 ug (FA)
B7 = Placebo MAP (FA); B8 = A/Sing MAP, 15 ug (UA); B9 = A/Sing IM, 15 ug, as 2018 Afluria Quad (UA)

Listing 16.2.9.11
Individual Characterisation of Skin Irritation Index by Treatment
Total Skin Irritation Index
Part B, Non-biopsy
B5= 1 x 5 ug A/Sing MAP + 2 uncoated MAPs on volar forearm (5 ug)

			Active			Placebo		
Randomisation Number	Visit	Time Point	#> 0	Maximum	Average	#> 0	Maximum	Average
B0054	Day 1	2 hour	1	2	2.0	2	3	2.5
B0054	Day 2		1	5	5.0	2	2	2.0
B0054	Day 4		1	3	3.0	2	2	2.0
B0054	Day 8		1	3	3.0	2	2	2.0
B0054	Day 22		0	0	0.0	0	0	0.0
B0054	EOS/ET (Day 61)		0	0	0.0	0	0	0.0
B0062	Day 1	Pre-dose	0	0	0.0	0	0	0.0
B0062	Day 1	10 min	1	3	3.0	2	3	3.0
B0062	Day 1	1 hour	1	3	3.0	2	3	3.0
B0062	Day 1	2 hour	1	3	3.0	2	3	2.5
B0062	Day 2		1	4	4.0	2	2	1.5
B0062	Day 4		1	3	3.0	0	0	0.0
B0062	Day 8		0	0	0.0	0	0	0.0
B0062	Day 22		0	0	0.0	0	0	0.0
B0062	EOS/ET (Day 61)		0	0	0.0	0	0	0.0
B0068	Day 1	Pre-dose	0	0	0.0	0	0	0.0
B0068	Day 1	10 min	1	3	3.0	2	3	3.0
B0068	Day 1	1 hour	1	4	4.0	2	4	3.5
B0068	Day 1	2 hour	1	4	4.0	2	3	3.0

Source: Listing 16.2.9.4

Administration Site Key: FA = Forearm; UA= Upper arm
Treatments Part A: A1 = A/Sing MAP, 15 ug (FA); A2 = Afluria Quad IM (UA), 15 ug; A3 = Placebo MAP (FA); A4 = A/Sing Mono IM, 15 ug (UA)
Treatments Part B: B1 = A/Sing MAP, 15 ug (FA) with biopsy; B2 = Placebo MAP (FA) with biopsy
B3 = A/Sing MAP, 15 ug (FA); B4 = A/Sing MAP, 10 ug (FA); B5 = A/Sing MAP, 5 ug (FA); B6 = A/Sing MAP, 2.5 ug (FA)
B7 = Placebo MAP (FA); B8 = A/Sing MAP, 15 ug (UA); B9 = A/Sing IM, 15 ug, as 2018 Afluria Quad (UA)

Listing 16.2.9.11
Individual Characterisation of Skin Irritation Index by Treatment
Total Skin Irritation Index
Part B, Non-biopsy
B5= 1 x 5 ug A/Sing MAP + 2 uncoated MAPs on volar forearm (5 ug)

			Active			Placebo		
Randomisation Number	Visit	Time Point	#> 0	Maximum	Average	#> 0	Maximum	Average
B0068	Day 8		1	4	4.0	0	0	0.0
B0068	Day 22		0	0	0.0	0	0	0.0
B0068	EOS/ET (Day 61)		0	0	0.0	0	0	0.0
B0084	Day 1	Pre-dose	0	0	0.0	0	0	0.0
B0084	Day 1	10 min	1	2	2.0	2	2	2.0
B0084	Day 1	1 hour	1	1	1.0	2	1	1.0
B0084	Day 1	2 hour	1	2	2.0	2	2	2.0
B0084	Day 2		1	5	5.0	2	2	1.5
B0084	Day 4		1	1	1.0	0	0	0.0
B0084	Day 8		1	3	3.0	0	0	0.0
B0084	Day 22		0	0	0.0	0	0	0.0
B0084	EOS/ET (Day 61)		0	0	0.0	0	0	0.0
B0089	Day 1	Pre-dose	0	0	0.0	0	0	0.0
B0089	Day 1	10 min	1	4	4.0	2	3	3.0
B0089	Day 1	1 hour	1	4	4.0	2	3	3.0
B0089	Day 1	2 hour	1	3	3.0	2	3	3.0
B0089	Day 2		1	3	3.0	2	3	2.5
B0089	Day 4		1	3	3.0	2	1	1.0
B0089	Day 8		0	0	0.0	0	0	0.0

Source: Listing 16.2.9.4

Administration Site Key: FA = Forearm; UA= Upper arm
Treatments Part A: A1 = A/Sing MAP, 15 ug (FA); A2 = Afluria Quad IM (UA), 15 ug; A3 = Placebo MAP (FA); A4 = A/Sing Mono IM, 15 ug (UA)
Treatments Part B: B1 = A/Sing MAP, 15 ug (FA) with biopsy; B2 = Placebo MAP (FA) with biopsy
B3 = A/Sing MAP, 15 ug (FA); B4 = A/Sing MAP, 10 ug (FA); B5 = A/Sing MAP, 5 ug (FA); B6 = A/Sing MAP, 2.5 ug (FA)
B7 = Placebo MAP (FA); B8 = A/Sing MAP, 15 ug (UA); B9 = A/Sing IM, 15 ug, as 2018 Afluria Quad (UA)

Listing 16.2.9.11
Individual Characterisation of Skin Irritation Index by Treatment
Total Skin Irritation Index
Part B, Non-biopsy
B5= 1 x 5 ug A/Sing MAP + 2 uncoated MAPs on volar forearm (5 ug)

			Active			Placebo		
Randomisation Number	Visit	Time Point	#> 0	Maximum	Average	#> 0	Maximum	Average
B0089	Day 22		0	0	0.0	0	0	0.0
B0089	EOS/ET (Day 61)		0	0	0.0	0	0	0.0
B0097	Day 1	Pre-dose	0	0	0.0	0	0	0.0
B0097	Day 1	10 min	1	4	4.0	2	4	3.5
B0097	Day 1	1 hour	1	4	4.0	2	3	3.0
B0097	Day 1	2 hour	1	2	2.0	2	1	1.0
B0097	Day 2		1	5	5.0	2	3	3.0
B0097	Day 4		1	2	2.0	2	4	3.5
B0097	Day 8		1	2	2.0	0	0	0.0
B0097	Day 22		0	0	0.0	0	0	0.0
B0097	EOS/ET (Day 61)		0	0	0.0	0	0	0.0
B0103	Day 1	Pre-dose	0	0	0.0	0	0	0.0
B0103	Day 1	10 min	1	4	4.0	2	4	4.0
B0103	Day 1	1 hour	1	4	4.0	2	4	4.0
B0103	Day 1	2 hour	1	3	3.0	2	3	3.0
B0103	Day 2		1	2	2.0	2	1	1.0
B0103	Day 4		1	2	2.0	0	0	0.0
B0103	Day 8		0	0	0.0	0	0	0.0
B0103	Day 22		0	0	0.0	0	0	0.0

Source: Listing 16.2.9.4

Administration Site Key: FA = Forearm; UA= Upper arm
Treatments Part A: A1 = A/Sing MAP, 15 ug (FA); A2 = Afluria Quad IM (UA), 15 ug; A3 = Placebo MAP (FA); A4 = A/Sing Mono IM, 15 ug (UA)
Treatments Part B: B1 = A/Sing MAP, 15 ug (FA) with biopsy; B2 = Placebo MAP (FA) with biopsy
B3 = A/Sing MAP, 15 ug (FA); B4 = A/Sing MAP, 10 ug (FA); B5 = A/Sing MAP, 5 ug (FA); B6 = A/Sing MAP, 2.5 ug (FA)
B7 = Placebo MAP (FA); B8 = A/Sing MAP, 15 ug (UA); B9 = A/Sing IM, 15 ug, as 2018 Afluria Quad (UA)

Listing 16.2.9.11
Individual Characterisation of Skin Irritation Index by Treatment
Total Skin Irritation Index
Part B, Non-biopsy
B5= 1 x 5 ug A/Sing MAP + 2 uncoated MAPs on volar forearm (5 ug)

			Active			Placebo		
Randomisation Number	Visit	Time Point	#> 0	Maximum	Average	#> 0	Maximum	Average
B0103	EOS/ET (Day 61)		0	0	0.0	0	0	0.0
B0113	Day 1	Pre-dose	0	0	0.0	0	0	0.0
B0113	Day 1	10 min	1	4	4.0	2	5	4.5
B0113	Day 1	1 hour	1	5	5.0	2	3	2.5
B0113	Day 1	2 hour	1	5	5.0	2	4	3.5
B0113	Day 2		1	5	5.0	2	2	2.0
B0113	Day 4		0	0	0.0	0	0	0.0
B0113	Day 8		0	0	0.0	0	0	0.0
B0113	Day 22		0	0	0.0	0	0	0.0
B0113	EOS/ET (Day 61)		0	0	0.0	0	0	0.0
B0122	Day 1	Pre-dose	0	0	0.0	0	0	0.0
B0122	Day 1	10 min	1	3	3.0	2	3	3.0
B0122	Day 1	1 hour	1	4	4.0	2	2	2.0
B0122	Day 1	2 hour	1	5	5.0	2	5	4.5
B0122	Day 2		1	4	4.0	2	2	2.0
B0122	Day 4		1	6	6.0	1	3	1.5
B0122	Day 8		0	0	0.0	0	0	0.0
B0122	Day 22		1	3	3.0	0	0	0.0
B0122	EOS/ET (Day 61)		0	0	0.0	0	0	0.0

Administration Site Key: FA = Forearm; UA= Upper arm
Treatments Part A: A1 = A/Sing MAP, 15 ug (FA); A2 = Afluria Quad IM (UA), 15 ug; A3 = Placebo MAP (FA); A4 = A/Sing Mono IM, 15 ug (UA)
Treatments Part B: B1 = A/Sing MAP, 15 ug (FA) with biopsy; B2 = Placebo MAP (FA) with biopsy
B3 = A/Sing MAP, 15 ug (FA); B4 = A/Sing MAP, 10 ug (FA); B5 = A/Sing MAP, 5 ug (FA); B6 = A/Sing MAP, 2.5 ug (FA)
B7 = Placebo MAP (FA); B8 = A/Sing MAP, 15 ug (UA); B9 = A/Sing IM, 15 ug, as 2018 Afluria Quad (UA)

Listing 16.2.9.11
Individual Characterisation of Skin Irritation Index by Treatment
Total Skin Irritation Index
Part B, Non-biopsy
B5= 1 x 5 ug A/Sing MAP + 2 uncoated MAPs on volar forearm (5 ug)

			Active			Placebo		
Randomisation Number	Visit	Time Point	#> 0	Maximum	Average	#> 0	Maximum	Average
B0128	Day 1	Pre-dose	0	0	0.0	0	0	0.0
B0128	Day 1	10 min	1	3	3.0	2	3	3.0
B0128	Day 1	1 hour	1	3	3.0	2	4	3.5
B0128	Day 1	2 hour	1	4	4.0	2	4	3.0
B0128	Day 2		1	3	3.0	2	1	1.0
B0128	Day 4		1	5	5.0	2	1	1.0
B0128	Day 8		0	0	0.0	0	0	0.0
B0128	Day 22		1	1	1.0	0	0	0.0
B0128	EOS/ET (Day 61)		0	0	0.0	0	0	0.0
B0130	Day 1	Pre-dose	0	0	0.0	0	0	0.0
B0130	Day 1	10 min	1	4	4.0	2	4	4.0
B0130	Day 1	1 hour	1	4	4.0	2	4	3.5
B0130	Day 1	2 hour	1	4	4.0	2	4	4.0
B0130	Day 2		1	4	4.0	2	3	3.0
B0130	Day 4		1	3	3.0	0	0	0.0
B0130	Day 8		1	4	4.0	0	0	0.0
B0130	Day 22		0	0	0.0	0	0	0.0
B0130	EOS/ET (Day 61)		0	0	0.0	0	0	0.0

Administration Site Key: FA = Forearm; UA= Upper arm
Treatments Part A: A1 = A/Sing MAP, 15 ug (FA); A2 = Afluria Quad IM (UA), 15 ug; A3 = Placebo MAP (FA); A4 = A/Sing Mono IM, 15 ug (UA)
Treatments Part B: B1 = A/Sing MAP, 15 ug (FA) with biopsy; B2 = Placebo MAP (FA) with biopsy
B3 = A/Sing MAP, 15 ug (FA); B4 = A/Sing MAP, 10 ug (FA); B5 = A/Sing MAP, 5 ug (FA); B6 = A/Sing MAP, 2.5 ug (FA)
B7 = Placebo MAP (FA); B8 = A/Sing MAP, 15 ug (UA); B9 = A/Sing IM, 15 ug, as 2018 Afluria Quad (UA)

Listing 16.2.9.11
Individual Characterisation of Skin Irritation Index by Treatment
Total Skin Irritation Index
Part B, Non-biopsy
B5= 1 x 5 ug A/Sing MAP + 2 uncoated MAPs on volar forearm (5 ug)

			Active			Placebo		
Randomisation Number	Visit	Time Point	#> 0	Maximum	Average	#> 0	Maximum	Average
B0142	Day 1	Pre-dose	0	0	0.0	0	0	0.0
B0142	Day 1	10 min	1	3	3.0	2	3	3.0
B0142	Day 1	1 hour	1	4	4.0	2	4	4.0
B0142	Day 1	2 hour	1	3	3.0	2	3	3.0
B0142	Day 2		1	4	4.0	2	1	1.0
B0142	Day 4		1	2	2.0	0	0	0.0
B0142	Day 8		1	4	4.0	2	2	2.0
B0142	Day 22		0	0	0.0	0	0	0.0
B0142	EOS/ET (Day 61)		0	0	0.0	0	0	0.0
B0148	Day 1	Pre-dose	0	0	0.0	0	0	0.0
B0148	Day 1	10 min	1	4	4.0	2	3	3.0
B0148	Day 1	1 hour	1	3	3.0	2	3	3.0
B0148	Day 1	2 hour	1	3	3.0	2	3	2.5
B0148	Day 2		1	5	5.0	2	1	1.0
B0148	Day 4		1	4	4.0	0	0	0.0
B0148	Day 8		1	3	3.0	0	0	0.0
B0148	Day 22		0	0	0.0	0	0	0.0
B0148	EOS/ET (Day 61)		0	0	0.0	0	0	0.0
B1076	Day 1	Pre-dose	0	0	0.0	0	0	0.0

Administration Site Key: FA = Forearm; UA= Upper arm
Treatments Part A: A1 = A/Sing MAP, 15 ug (FA); A2 = Afluria Quad IM (UA), 15 ug; A3 = Placebo MAP (FA); A4 = A/Sing Mono IM, 15 ug (UA)
Treatments Part B: B1 = A/Sing MAP, 15 ug (FA) with biopsy; B2 = Placebo MAP (FA) with biopsy
B3 = A/Sing MAP, 15 ug (FA); B4 = A/Sing MAP, 10 ug (FA); B5 = A/Sing MAP, 5 ug (FA); B6 = A/Sing MAP, 2.5 ug (FA)
B7 = Placebo MAP (FA); B8 = A/Sing MAP, 15 ug (UA); B9 = A/Sing IM, 15 ug, as 2018 Afluria Quad (UA)

Listing 16.2.9.11
Individual Characterisation of Skin Irritation Index by Treatment
Total Skin Irritation Index
Part B, Non-biopsy
B5= 1 x 5 ug A/Sing MAP + 2 uncoated MAPs on volar forearm (5 ug)

			Active			Placebo		
Randomisation Number	Visit	Time Point	#> 0	Maximum	Average	#> 0	Maximum	Average
B1076	Day 1	10 min	1	5	5.0	2	3	2.5
B1076	Day 1	1 hour	1	5	5.0	2	2	2.0
B1076	Day 1	2 hour	1	3	3.0	2	3	2.5
B1076	Day 2		1	3	3.0	2	1	1.0
B1076	Day 4		1	3	3.0	0	0	0.0
B1076	Day 8		0	0	0.0	0	0	0.0
B1076	Day 22		0	0	0.0	0	0	0.0
B1076	EOS/ET (Day 61)		0	0	0.0	0	0	0.0

Source: Listing 16.2.9.4

Administration Site Key: FA = Forearm; UA= Upper arm
Treatments Part A: A1 = A/Sing MAP, 15 ug (FA); A2 = Afluria Quad IM (UA), 15 ug; A3 = Placebo MAP (FA); A4 = A/Sing Mono IM, 15 ug (UA)
Treatments Part B: B1 = A/Sing MAP, 15 ug (FA) with biopsy; B2 = Placebo MAP (FA) with biopsy
B3 = A/Sing MAP, 15 ug (FA); B4 = A/Sing MAP, 10 ug (FA); B5 = A/Sing MAP, 5 ug (FA); B6 = A/Sing MAP, 2.5 ug (FA)
B7 = Placebo MAP (FA); B8 = A/Sing MAP, 15 ug (UA); B9 = A/Sing IM, 15 ug, as 2018 Afluria Quad (UA)

Listing 16.2.9.11
Individual Characterisation of Skin Irritation Index by Treatment
Total Skin Irritation Index
Part B, Non-biopsy
B6= 1 x 2.5 ug A/Sing MAP + 2 uncoated MAPs on volar forearm (2.5 ug)

			Active			Placebo		
Randomisation Number	Visit	Time Point	#> 0	Maximum	Average	#> 0	Maximum	Average
B0012	Day 1	Pre-dose	0	0	0.0	0	0	0.0
B0012	Day 1	10 min	1	3	3.0	2	3	3.0
B0012	Day 1	1 hour	1	2	2.0	2	2	1.5
B0012	Day 1	2 hour	1	2	2.0	2	2	2.0
B0012	Day 2		1	4	4.0	2	3	3.0
B0012	Day 4		1	6	6.0	2	4	4.0
B0012	Day 8		0	0	0.0	0	0	0.0
B0012	Day 22		0	0	0.0	0	0	0.0
B0012	EOS/ET (Day 61)		0	0	0.0	0	0	0.0
B0020	Day 1	Pre-dose	0	0	0.0	0	0	0.0
B0020	Day 1	10 min	1	3	3.0	2	3	3.0
B0020	Day 1	1 hour	1	3	3.0	2	3	3.0
B0020	Day 1	2 hour	1	3	3.0	2	3	3.0
B0020	Day 2		1	4	4.0	2	2	2.0
B0020	Day 4		1	2	2.0	0	0	0.0
B0020	Day 8		0	0	0.0	0	0	0.0
B0020	Day 22		0	0	0.0	0	0	0.0
B0020	EOS/ET (Day 61)		0	0	0.0	0	0	0.0
B0029	Day 1	Pre-dose	0	0	0.0	0	0	0.0

Source: Listing 16.2.9.4

Administration Site Key: FA = Forearm; UA= Upper arm
Treatments Part A: A1 = A/Sing MAP, 15 ug (FA); A2 = Afluria Quad IM (UA), 15 ug; A3 = Placebo MAP (FA); A4 = A/Sing Mono IM, 15 ug (UA)
Treatments Part B: B1 = A/Sing MAP, 15 ug (FA) with biopsy; B2 = Placebo MAP (FA) with biopsy
B3 = A/Sing MAP, 15 ug (FA); B4 = A/Sing MAP, 10 ug (FA); B5 = A/Sing MAP, 5 ug (FA); B6 = A/Sing MAP, 2.5 ug (FA)
B7 = Placebo MAP (FA); B8 = A/Sing MAP, 15 ug (UA); B9 = A/Sing IM, 15 ug, as 2018 Afluria Quad (UA)

Listing 16.2.9.11
Individual Characterisation of Skin Irritation Index by Treatment
Total Skin Irritation Index
Part B, Non-biopsy
B6= 1 x 2.5 ug A/Sing MAP + 2 uncoated MAPs on volar forearm (2.5 ug)

			Active			Placebo		
Randomisation Number	Visit	Time Point	#> 0	Maximum	Average	#> 0	Maximum	Average
B0029	Day 1	10 min	1	2	2.0	2	2	2.0
B0029	Day 1	1 hour	1	3	3.0	2	3	3.0
B0029	Day 1	2 hour	0	0	0.0	0	0	0.0
B0029	Day 2		1	4	4.0	2	2	2.0
B0029	Day 4		1	6	6.0	2	1	1.0
B0029	Day 8		0	0	0.0	0	0	0.0
B0029	Day 22		0	0	0.0	0	0	0.0
B0029	EOS/ET (Day 61)		0	0	0.0	0	0	0.0
B0032	Day 1	Pre-dose	0	0	0.0	0	0	0.0
B0032	Day 1	10 min	1	2	2.0	2	2	2.0
B0032	Day 1	1 hour	1	5	5.0	2	5	4.5
B0032	Day 1	2 hour	1	3	3.0	2	4	3.5
B0032	Day 2		1	4	4.0	2	3	3.0
B0032	Day 4		1	3	3.0	2	2	2.0
B0032	Day 8		1	2	2.0	2	1	1.0
B0032	Day 22		0	0	0.0	0	0	0.0
B0032	EOS/ET (Day 61)		0	0	0.0	0	0	0.0
B0044	Day 1	Pre-dose	0	0	0.0	0	0	0.0
B0044	Day 1	10 min	1	3	3.0	2	3	3.0

Administration Site Key: FA = Forearm; UA= Upper arm
Treatments Part A: A1 = A/Sing MAP, 15 ug (FA); A2 = Afluria Quad IM (UA), 15 ug; A3 = Placebo MAP (FA); A4 = A/Sing Mono IM, 15 ug (UA)
Treatments Part B: B1 = A/Sing MAP, 15 ug (FA) with biopsy; B2 = Placebo MAP (FA) with biopsy
B3 = A/Sing MAP, 15 ug (FA); B4 = A/Sing MAP, 10 ug (FA); B5 = A/Sing MAP, 5 ug (FA); B6 = A/Sing MAP, 2.5 ug (FA)
B7 = Placebo MAP (FA); B8 = A/Sing MAP, 15 ug (UA); B9 = A/Sing IM, 15 ug, as 2018 Afluria Quad (UA)

Listing 16.2.9.11
Individual Characterisation of Skin Irritation Index by Treatment
Total Skin Irritation Index
Part B, Non-biopsy
B6= 1 x 2.5 ug A/Sing MAP + 2 uncoated MAPs on volar forearm (2.5 ug)

			Active			Placebo		
Randomisation Number	Visit	Time Point	#> 0	Maximum	Average	#> 0	Maximum	Average
B0044	Day 1	1 hour	1	4	4.0	2	4	4.0
B0044	Day 1	2 hour	1	4	4.0	2	4	4.0
B0044	Day 2		1	4	4.0	2	2	2.0
B0044	Day 4		1	3	3.0	2	1	1.0
B0044	Day 8		1	3	3.0	2	1	1.0
B0044	Day 22		1	1	1.0	0	0	0.0
B0044	EOS/ET (Day 61)		0	0	0.0	0	0	0.0
B0048	Day 1	Pre-dose	0	0	0.0	0	0	0.0
B0048	Day 1	10 min	1	3	3.0	2	3	3.0
B0048	Day 1	1 hour	1	2	2.0	2	4	3.0
B0048	Day 1	2 hour	1	3	3.0	2	4	4.0
B0048	Day 2		1	4	4.0	2	2	2.0
B0048	Day 4		1	2	2.0	0	0	0.0
B0048	Day 8		0	0	0.0	0	0	0.0
B0048	Day 22		0	0	0.0	0	0	0.0
B0048	EOS/ET (Day 61)		0	0	0.0	0	0	0.0
B0053	Day 1	Pre-dose	0	0	0.0	0	0	0.0
B0053	Day 1	10 min	1	3	3.0	2	3	3.0
B0053	Day 1	1 hour	1	3	3.0	2	3	3.0

Source: Listing 16.2.9.4

Administration Site Key: FA = Forearm; UA= Upper arm
Treatments Part A: A1 = A/Sing MAP, 15 ug (FA); A2 = Afluria Quad IM (UA), 15 ug; A3 = Placebo MAP (FA); A4 = A/Sing Mono IM, 15 ug (UA)
Treatments Part B: B1 = A/Sing MAP, 15 ug (FA) with biopsy; B2 = Placebo MAP (FA) with biopsy
B3 = A/Sing MAP, 15 ug (FA); B4 = A/Sing MAP, 10 ug (FA); B5 = A/Sing MAP, 5 ug (FA); B6 = A/Sing MAP, 2.5 ug (FA)
B7 = Placebo MAP (FA); B8 = A/Sing MAP, 15 ug (UA); B9 = A/Sing IM, 15 ug, as 2018 Afluria Quad (UA)

Listing 16.2.9.11
Individual Characterisation of Skin Irritation Index by Treatment
Total Skin Irritation Index
Part B, Non-biopsy
B6= 1 x 2.5 ug A/Sing MAP + 2 uncoated MAPs on volar forearm (2.5 ug)

			Active			Placebo		
Randomisation Number	Visit	Time Point	#> 0	Maximum	Average	#> 0	Maximum	Average
B0053	Day 1	2 hour	1	3	3.0	2	3	3.0
B0053	Day 2		1	3	3.0	2	2	2.0
B0053	Day 4		1	2	2.0	2	2	2.0
B0053	Day 8		1	1	1.0	0	0	0.0
B0053	Day 22		0	0	0.0	0	0	0.0
B0053	EOS/ET (Day 61)		0	0	0.0	0	0	0.0
B0066	Day 1	Pre-dose	0	0	0.0	0	0	0.0
B0066	Day 1	10 min	1	3	3.0	2	3	3.0
B0066	Day 1	1 hour	1	3	3.0	2	3	2.5
B0066	Day 1	2 hour	1	3	3.0	2	3	3.0
B0066	Day 2		1	3	3.0	2	1	1.0
B0066	Day 4		1	3	3.0	2	2	2.0
B0066	Day 8		0	0	0.0	0	0	0.0
B0066	Day 22		0	0	0.0	0	0	0.0
B0066	EOS/ET (Day 61)		0	0	0.0	0	0	0.0
B0073	Day 1	Pre-dose	0	0	0.0	0	0	0.0
B0073	Day 1	10 min	1	3	3.0	2	2	2.0
B0073	Day 1	1 hour	1	4	4.0	2	3	2.0
B0073	Day 1	2 hour	1	5	5.0	2	4	3.0

Source: Listing 16.2.9.4

Administration Site Key: FA = Forearm; UA= Upper arm
Treatments Part A: A1 = A/Sing MAP, 15 ug (FA); A2 = Afluria Quad IM (UA), 15 ug; A3 = Placebo MAP (FA); A4 = A/Sing Mono IM, 15 ug (UA)
Treatments Part B: B1 = A/Sing MAP, 15 ug (FA) with biopsy; B2 = Placebo MAP (FA) with biopsy
B3 = A/Sing MAP, 15 ug (FA); B4 = A/Sing MAP, 10 ug (FA); B5 = A/Sing MAP, 5 ug (FA); B6 = A/Sing MAP, 2.5 ug (FA)
B7 = Placebo MAP (FA); B8 = A/Sing MAP, 15 ug (UA); B9 = A/Sing IM, 15 ug, as 2018 Afluria Quad (UA)

Listing 16.2.9.11
Individual Characterisation of Skin Irritation Index by Treatment
Total Skin Irritation Index
Part B, Non-biopsy
B6= 1 x 2.5 ug A/Sing MAP + 2 uncoated MAPs on volar forearm (2.5 ug)

			Active			Placebo		
Randomisation Number	Visit	Time Point	#> 0	Maximum	Average	#> 0	Maximum	Average
B0073	Day 2		1	5	5.0	2	4	4.0
B0073	Day 4		1	3	3.0	1	2	1.0
B0073	Day 8		0	0	0.0	0	0	0.0
B0073	Day 22		0	0	0.0	0	0	0.0
B0073	EOS/ET (Day 61)		0	0	0.0	0	0	0.0
B0079	Day 1	Pre-dose	0	0	0.0	0	0	0.0
B0079	Day 1	10 min	1	3	3.0	2	3	2.5
B0079	Day 1	1 hour	1	3	3.0	2	3	2.5
B0079	Day 1	2 hour	1	5	5.0	2	5	4.5
B0079	Day 2		1	4	4.0	2	2	2.0
B0079	Day 4		1	5	5.0	2	3	2.5
B0079	Day 8		1	3	3.0	1	2	1.0
B0079	Day 22		0	0	0.0	0	0	0.0
B0079	EOS/ET (Day 61)		0	0	0.0	0	0	0.0
B0081	Day 1	Pre-dose	0	0	0.0	0	0	0.0
B0081	Day 1	10 min	1	3	3.0	2	3	3.0
B0081	Day 1	1 hour	1	3	3.0	2	3	3.0
B0081	Day 1	2 hour	1	6	6.0	2	6	6.0
B0081	Day 2		1	4	4.0	2	4	3.5

Source: Listing 16.2.9.4

Administration Site Key: FA = Forearm; UA= Upper arm
Treatments Part A: A1 = A/Sing MAP, 15 ug (FA); A2 = Afluria Quad IM (UA), 15 ug; A3 = Placebo MAP (FA); A4 = A/Sing Mono IM, 15 ug (UA)
Treatments Part B: B1 = A/Sing MAP, 15 ug (FA) with biopsy; B2 = Placebo MAP (FA) with biopsy
B3 = A/Sing MAP, 15 ug (FA); B4 = A/Sing MAP, 10 ug (FA); B5 = A/Sing MAP, 5 ug (FA); B6 = A/Sing MAP, 2.5 ug (FA)
B7 = Placebo MAP (FA); B8 = A/Sing MAP, 15 ug (UA); B9 = A/Sing IM, 15 ug, as 2018 Afluria Quad (UA)

Listing 16.2.9.11
Individual Characterisation of Skin Irritation Index by Treatment
Total Skin Irritation Index
Part B, Non-biopsy
B6= 1 x 2.5 ug A/Sing MAP + 2 uncoated MAPs on volar forearm (2.5 ug)

			Active			Placebo		
Randomisation Number	Visit	Time Point	#> 0	Maximum	Average	#> 0	Maximum	Average
B0081	Day 4		0	0	0.0	0	0	0.0
B0081	Day 8		0	0	0.0	0	0	0.0
B0081	Day 22		0	0	0.0	0	0	0.0
B0081	EOS/ET (Day 61)		0	0	0.0	0	0	0.0
B0090	Day 1	Pre-dose	0	0	0.0	0	0	0.0
B0090	Day 1	10 min	1	3	3.0	2	2	2.0
B0090	Day 1	1 hour	1	2	2.0	2	2	2.0
B0090	Day 1	2 hour	1	2	2.0	2	3	3.0
B0090	Day 2		1	3	3.0	2	2	2.0
B0090	Day 4		1	1	1.0	0	0	0.0
B0090	Day 8		0	0	0.0	0	0	0.0
B0090	Day 22		0	0	0.0	0	0	0.0
B0090	EOS/ET (Day 61)		0	0	0.0	0	0	0.0
B0096	Day 1	Pre-dose	0	0	0.0	0	0	0.0
B0096	Day 1	10 min	1	5	5.0	2	5	4.5
B0096	Day 1	1 hour	1	3	3.0	2	3	3.0
B0096	Day 1	2 hour	1	4	4.0	2	4	4.0
B0096	Day 2		1	4	4.0	2	3	3.0
B0096	Day 4		1	3	3.0	0	0	0.0

Administration Site Key: FA = Forearm; UA= Upper arm
Treatments Part A: A1 = A/Sing MAP, 15 ug (FA); A2 = Afluria Quad IM (UA), 15 ug; A3 = Placebo MAP (FA); A4 = A/Sing Mono IM, 15 ug (UA)
Treatments Part B: B1 = A/Sing MAP, 15 ug (FA) with biopsy; B2 = Placebo MAP (FA) with biopsy
B3 = A/Sing MAP, 15 ug (FA); B4 = A/Sing MAP, 10 ug (FA); B5 = A/Sing MAP, 5 ug (FA); B6 = A/Sing MAP, 2.5 ug (FA)
B7 = Placebo MAP (FA); B8 = A/Sing MAP, 15 ug (UA); B9 = A/Sing IM, 15 ug, as 2018 Afluria Quad (UA)

Listing 16.2.9.11
Individual Characterisation of Skin Irritation Index by Treatment
Total Skin Irritation Index
Part B, Non-biopsy
B6= 1 x 2.5 ug A/Sing MAP + 2 uncoated MAPs on volar forearm (2.5 ug)

			Active			Placebo		
Randomisation Number	Visit	Time Point	#> 0	Maximum	Average	#> 0	Maximum	Average
B0096	Day 8		0	0	0.0	0	0	0.0
B0096	Day 22		0	0	0.0	0	0	0.0
B0096	EOS/ET (Day 61)		0	0	0.0	0	0	0.0
B0107	Day 1	Pre-dose	0	0	0.0	0	0	0.0
B0107	Day 1	10 min	1	3	3.0	2	3	3.0
B0107	Day 1	1 hour	1	4	4.0	2	3	3.0
B0107	Day 1	2 hour	1	2	2.0	2	2	2.0
B0107	Day 2		1	3	3.0	2	3	3.0
B0107	Day 4		1	3	3.0	2	3	2.5
B0107	Day 8		0	0	0.0	0	0	0.0
B0107	Day 22		0	0	0.0	0	0	0.0
B0107	EOS/ET (Day 61)		0	0	0.0	0	0	0.0
B0109	Day 1	Pre-dose	0	0	0.0	0	0	0.0
B0109	Day 1	10 min	1	4	4.0	2	2	2.0
B0109	Day 1	1 hour	1	6	6.0	2	4	3.5
B0109	Day 1	2 hour	1	4	4.0	2	2	2.0
B0109	Day 2		1	3	3.0	2	1	1.0
B0109	Day 4		1	6	6.0	0	0	0.0
B0109	Day 8		0	0	0.0	0	0	0.0

Source: Listing 16.2.9.4

Administration Site Key: FA = Forearm; UA= Upper arm
Treatments Part A: A1 = A/Sing MAP, 15 ug (FA); A2 = Afluria Quad IM (UA), 15 ug; A3 = Placebo MAP (FA); A4 = A/Sing Mono IM, 15 ug (UA)
Treatments Part B: B1 = A/Sing MAP, 15 ug (FA) with biopsy; B2 = Placebo MAP (FA) with biopsy
B3 = A/Sing MAP, 15 ug (FA); B4 = A/Sing MAP, 10 ug (FA); B5 = A/Sing MAP, 5 ug (FA); B6 = A/Sing MAP, 2.5 ug (FA)
B7 = Placebo MAP (FA); B8 = A/Sing MAP, 15 ug (UA); B9 = A/Sing IM, 15 ug, as 2018 Afluria Quad (UA)

Listing 16.2.9.11
Individual Characterisation of Skin Irritation Index by Treatment
Total Skin Irritation Index
Part B, Non-biopsy
B6= 1 x 2.5 ug A/Sing MAP + 2 uncoated MAPs on volar forearm (2.5 ug)

Randomisation Number	Visit	Time Point	Active #> 0	Active Maximum	Active Average	Placebo #> 0	Placebo Maximum	Placebo Average
B0109	Day 22		0	0	0.0	0	0	0.0
B0109	EOS/ET (Day 61)		0	0	0.0	0	0	0.0
B0118	Day 1	Pre-dose	0	0	0.0	0	0	0.0
B0118	Day 1	10 min	1	3	3.0	2	3	3.0
B0118	Day 1	1 hour	1	3	3.0	2	3	3.0
B0118	Day 1	2 hour	1	3	3.0	2	4	3.5
B0118	Day 2		1	4	4.0	2	2	1.5
B0118	Day 4		1	2	2.0	0	0	0.0
B0118	Day 8		0	0	0.0	0	0	0.0
B0118	Day 22		0	0	0.0	0	0	0.0
B0118	EOS/ET (Day 61)		0	0	0.0	0	0	0.0
B0123	Day 1	Pre-dose	0	0	0.0	0	0	0.0
B0123	Day 1	10 min	1	3	3.0	2	3	2.5
B0123	Day 1	1 hour	1	2	2.0	2	3	3.0
B0123	Day 1	2 hour	1	3	3.0	2	4	3.5
B0123	Day 2		1	3	3.0	2	1	1.0
B0123	Day 4		1	2	2.0	0	0	0.0
B0123	Day 8		0	0	0.0	0	0	0.0
B0123	Day 22		1	2	2.0	0	0	0.0

Administration Site Key: FA = Forearm; UA= Upper arm
Treatments Part A: A1 = A/Sing MAP, 15 ug (FA); A2 = Afluria Quad IM (UA), 15 ug; A3 = Placebo MAP (FA); A4 = A/Sing Mono IM, 15 ug (UA)
Treatments Part B: B1 = A/Sing MAP, 15 ug (FA) with biopsy; B2 = Placebo MAP (FA) with biopsy
B3 = A/Sing MAP, 15 ug (FA); B4 = A/Sing MAP, 10 ug (FA); B5 = A/Sing MAP, 5 ug (FA); B6 = A/Sing MAP, 2.5 ug (FA)
B7 = Placebo MAP (FA); B8 = A/Sing MAP, 15 ug (UA); B9 = A/Sing IM, 15 ug, as 2018 Afluria Quad (UA)

Listing 16.2.9.11
Individual Characterisation of Skin Irritation Index by Treatment
Total Skin Irritation Index
Part B, Non-biopsy
B6= 1 x 2.5 ug A/Sing MAP + 2 uncoated MAPs on volar forearm (2.5 ug)

			Active			Placebo		
Randomisation Number	Visit	Time Point	#> 0	Maximum	Average	#> 0	Maximum	Average
B0123	EOS/ET (Day 61)		0	0	0.0	0	0	0.0
B0132	Day 1	Pre-dose	0	0	0.0	0	0	0.0
B0132	Day 1	10 min	1	4	4.0	2	4	4.0
B0132	Day 1	1 hour	1	5	5.0	2	6	5.5
B0132	Day 1	2 hour	1	3	3.0	2	4	4.0
B0132	Day 2		1	3	3.0	2	2	2.0
B0132	Day 4		1	2	2.0	0	0	0.0
B0132	Day 8		0	0	0.0	0	0	0.0
B0132	Day 22		1	1	1.0	0	0	0.0
B0132	EOS/ET (Day 61)		0	0	0.0	0	0	0.0
B0143	Day 1	Pre-dose	0	0	0.0	0	0	0.0
B0143	Day 1	10 min	1	3	3.0	2	4	4.0
B0143	Day 1	1 hour	1	5	5.0	2	5	4.5
B0143	Day 1	2 hour	1	4	4.0	2	3	3.0
B0143	Day 2		1	4	4.0	2	2	2.0
B0143	Day 4		1	5	5.0	2	2	2.0
B0143	Day 8		0	0	0.0	0	0	0.0
B0143	Day 22		0	0	0.0	0	0	0.0
B0143	EOS/ET (Day 61)		0	0	0.0	0	0	0.0

Source: Listing 16.2.9.4

Administration Site Key: FA = Forearm; UA= Upper arm
Treatments Part A: A1 = A/Sing MAP, 15 ug (FA); A2 = Afluria Quad IM (UA), 15 ug; A3 = Placebo MAP (FA); A4 = A/Sing Mono IM, 15 ug (UA)
Treatments Part B: B1 = A/Sing MAP, 15 ug (FA) with biopsy; B2 = Placebo MAP (FA) with biopsy
B3 = A/Sing MAP, 15 ug (FA); B4 = A/Sing MAP, 10 ug (FA); B5 = A/Sing MAP, 5 ug (FA); B6 = A/Sing MAP, 2.5 ug (FA)
B7 = Placebo MAP (FA); B8 = A/Sing MAP, 15 ug (UA); B9 = A/Sing IM, 15 ug, as 2018 Afluria Quad (UA)

Listing 16.2.9.11
Individual Characterisation of Skin Irritation Index by Treatment
Total Skin Irritation Index
Part B, Non-biopsy
B6= 1 x 2.5 ug A/Sing MAP + 2 uncoated MAPs on volar forearm (2.5 ug)

			Active			Placebo		
Randomisation Number	Visit	Time Point	#> 0	Maximum	Average	#> 0	Maximum	Average
B0144	Day 1	Pre-dose	0	0	0.0	0	0	0.0
B0144	Day 1	10 min	1	4	4.0	2	3	3.0
B0144	Day 1	1 hour	1	5	5.0	2	5	5.0
B0144	Day 1	2 hour	1	4	4.0	2	4	4.0
B0144	Day 2		1	3	3.0	2	2	2.0
B0144	Day 4		1	2	2.0	0	0	0.0
B0144	Day 8		1	1	1.0	0	0	0.0
B0144	Day 22		1	1	1.0	0	0	0.0
B0144	EOS/ET (Day 61)		0	0	0.0	0	0	0.0

Source: Listing 16.2.9.4

Administration Site Key: FA = Forearm; UA= Upper arm
Treatments Part A: A1 = A/Sing MAP, 15 ug (FA); A2 = Afluria Quad IM (UA), 15 ug; A3 = Placebo MAP (FA); A4 = A/Sing Mono IM, 15 ug (UA)
Treatments Part B: B1 = A/Sing MAP, 15 ug (FA) with biopsy; B2 = Placebo MAP (FA) with biopsy
B3 = A/Sing MAP, 15 ug (FA); B4 = A/Sing MAP, 10 ug (FA); B5 = A/Sing MAP, 5 ug (FA); B6 = A/Sing MAP, 2.5 ug (FA)
B7 = Placebo MAP (FA); B8 = A/Sing MAP, 15 ug (UA); B9 = A/Sing IM, 15 ug, as 2018 Afluria Quad (UA)

Listing 16.2.9.11
Individual Characterisation of Skin Irritation Index by Treatment
Total Skin Irritation Index
Part B, Non-biopsy
B7= 3 x uncoated MAPs on volar forearm

Randomisation Number	Visit	Time Point	Active #> 0	Active Maximum	Active Average	Placebo #> 0	Placebo Maximum	Placebo Average
B0017	Day 1	Pre-dose	-	-	-	0	0	0.0
B0017	Day 1	10 min	-	-	-	3	3	3.0
B0017	Day 1	1 hour	-	-	-	3	3	2.7
B0017	Day 1	2 hour	-	-	-	3	3	3.0
B0017	Day 2		-	-	-	3	3	2.7
B0017	Day 4		-	-	-	3	3	3.0
B0017	Day 8		-	-	-	0	0	0.0
B0017	Day 22		-	-	-	0	0	0.0
B0017	EOS/ET (Day 61)		-	-	-	0	0	0.0
B0021	Day 1	Pre-dose	-	-	-	0	0	0.0
B0021	Day 1	10 min	-	-	-	3	3	3.0
B0021	Day 1	1 hour	-	-	-	3	3	2.7
B0021	Day 1	2 hour	-	-	-	3	3	3.0
B0021	Day 2		-	-	-	3	2	2.0
B0021	Day 4		-	-	-	3	2	2.0
B0021	Day 8		-	-	-	0	0	0.0
B0021	Day 22		-	-	-	0	0	0.0
B0021	EOS/ET (Day 61)		-	-	-	0	0	0.0
B0030	Day 1	Pre-dose	-	-	-	0	0	0.0

Administration Site Key: FA = Forearm; UA= Upper arm
Treatments Part A: A1 = A/Sing MAP, 15 ug (FA); A2 = Afluria Quad IM (UA), 15 ug; A3 = Placebo MAP (FA); A4 = A/Sing Mono IM, 15 ug (UA)
Treatments Part B: B1 = A/Sing MAP, 15 ug (FA) with biopsy; B2 = Placebo MAP (FA) with biopsy
B3 = A/Sing MAP, 15 ug (FA); B4 = A/Sing MAP, 10 ug (FA); B5 = A/Sing MAP, 5 ug (FA); B6 = A/Sing MAP, 2.5 ug (FA)
B7 = Placebo MAP (FA); B8 = A/Sing MAP, 15 ug (UA); B9 = A/Sing IM, 15 ug, as 2018 Afluria Quad (UA)

Listing 16.2.9.11
Individual Characterisation of Skin Irritation Index by Treatment
Total Skin Irritation Index
Part B, Non-biopsy
B7= 3 x uncoated MAPs on volar forearm

			Active			Placebo		
Randomisation Number	Visit	Time Point	#> 0	Maximum	Average	#> 0	Maximum	Average
B0030	Day 1	10 min	-	-	-	3	5	4.7
B0030	Day 1	1 hour	-	-	-	3	4	4.0
B0030	Day 1	2 hour	-	-	-	3	4	4.0
B0030	Day 2		-	-	-	3	1	1.0
B0030	Day 4		-	-	-	0	0	0.0
B0030	Day 8		-	-	-	0	0	0.0
B0030	Day 22		-	-	-	0	0	0.0
B0030	EOS/ET (Day 61)		-	-	-	0	0	0.0
B0036	Day 1	Pre-dose	-	-	-	0	0	0.0
B0036	Day 1	10 min	-	-	-	3	2	2.0
B0036	Day 1	1 hour	-	-	-	3	3	3.0
B0036	Day 1	2 hour	-	-	-	3	3	2.3
B0036	Day 2		-	-	-	3	2	2.0
B0036	Day 4		-	-	-	2	1	0.7
B0036	Day 8		-	-	-	0	0	0.0
B0036	Day 22		-	-	-	0	0	0.0
B0036	EOS/ET (Day 61)		-	-	-	0	0	0.0
B0039	Day 1	Pre-dose	-	-	-	0	0	0.0
B0039	Day 1	10 min	-	-	-	3	3	2.3

Administration Site Key: FA = Forearm; UA= Upper arm
Treatments Part A: A1 = A/Sing MAP, 15 ug (FA); A2 = Afluria Quad IM (UA), 15 ug; A3 = Placebo MAP (FA); A4 = A/Sing Mono IM, 15 ug (UA)
Treatments Part B: B1 = A/Sing MAP, 15 ug (FA) with biopsy; B2 = Placebo MAP (FA) with biopsy
B3 = A/Sing MAP, 15 ug (FA); B4 = A/Sing MAP, 10 ug (FA); B5 = A/Sing MAP, 5 ug (FA); B6 = A/Sing MAP, 2.5 ug (FA)
B7 = Placebo MAP (FA); B8 = A/Sing MAP, 15 ug (UA); B9 = A/Sing IM, 15 ug, as 2018 Afluria Quad (UA)

Listing 16.2.9.11
Individual Characterisation of Skin Irritation Index by Treatment
Total Skin Irritation Index
Part B, Non-biopsy
B7= 3 x uncoated MAPs on volar forearm

			Active			Placebo		
Randomisation Number	Visit	Time Point	#> 0	Maximum	Average	#> 0	Maximum	Average
B0039	Day 1	1 hour	-	-	-	3	4	2.7
B0039	Day 1	2 hour	-	-	-	3	4	4.0
B0039	Day 2		-	-	-	3	2	2.0
B0039	Day 4		-	-	-	0	0	0.0
B0039	Day 8		-	-	-	0	0	0.0
B0039	Day 22		-	-	-	0	0	0.0
B0039	EOS/ET (Day 61)		-	-	-	0	0	0.0
B0046	Day 1	Pre-dose	-	-	-	0	0	0.0
B0046	Day 1	10 min	-	-	-	3	3	2.3
B0046	Day 1	1 hour	-	-	-	3	3	3.0
B0046	Day 1	2 hour	-	-	-	3	4	3.7
B0046	Day 2		-	-	-	3	2	2.0
B0046	Day 4		-	-	-	3	1	1.0
B0046	Day 8		-	-	-	0	0	0.0
B0046	Day 22		-	-	-	0	0	0.0
B0046	EOS/ET (Day 61)		-	-	-	0	0	0.0
B0059	Day 1	Pre-dose	-	-	-	0	0	0.0
B0059	Day 1	10 min	-	-	-	3	2	2.0
B0059	Day 1	1 hour	-	-	-	3	2	2.0

Source: Listing 16.2.9.4

Administration Site Key: FA = Forearm; UA= Upper arm
Treatments Part A: A1 = A/Sing MAP, 15 ug (FA); A2 = Afluria Quad IM (UA), 15 ug; A3 = Placebo MAP (FA); A4 = A/Sing Mono IM, 15 ug (UA)
Treatments Part B: B1 = A/Sing MAP, 15 ug (FA) with biopsy; B2 = Placebo MAP (FA) with biopsy
B3 = A/Sing MAP, 15 ug (FA); B4 = A/Sing MAP, 10 ug (FA); B5 = A/Sing MAP, 5 ug (FA); B6 = A/Sing MAP, 2.5 ug (FA)
B7 = Placebo MAP (FA); B8 = A/Sing MAP, 15 ug (UA); B9 = A/Sing IM, 15 ug, as 2018 Afluria Quad (UA)

Listing 16.2.9.11
Individual Characterisation of Skin Irritation Index by Treatment
Total Skin Irritation Index
Part B, Non-biopsy
B7= 3 x uncoated MAPs on volar forearm

			Active			Placebo		
Randomisation Number	Visit	Time Point	#> 0	Maximum	Average	#> 0	Maximum	Average
B0059	Day 1	2 hour	-	-	-	3	3	2.3
B0059	Day 2		-	-	-	3	2	2.0
B0059	Day 4		-	-	-	0	0	0.0
B0059	Day 8		-	-	-	0	0	0.0
B0059	Day 22		-	-	-	0	0	0.0
B0059	EOS/ET (Day 61)		-	-	-	0	0	0.0
B0063	Day 1	Pre-dose	-	-	-	0	0	0.0
B0063	Day 1	10 min	-	-	-	3	3	3.0
B0063	Day 1	1 hour	-	-	-	3	2	2.0
B0063	Day 1	2 hour	-	-	-	3	3	2.7
B0063	Day 2		-	-	-	3	2	2.0
B0063	Day 4		-	-	-	3	2	2.0
B0063	Day 8		-	-	-	0	0	0.0
B0063	Day 22		-	-	-	0	0	0.0
B0063	EOS/ET (Day 61)		-	-	-	0	0	0.0
B0069	Day 1	Pre-dose	-	-	-	0	0	0.0
B0069	Day 1	10 min	-	-	-	3	3	2.3
B0069	Day 1	1 hour	-	-	-	3	3	3.0
B0069	Day 1	2 hour	-	-	-	3	3	2.7

Source: Listing 16.2.9.4

Administration Site Key: FA = Forearm; UA= Upper arm
Treatments Part A: A1 = A/Sing MAP, 15 ug (FA); A2 = Afluria Quad IM (UA), 15 ug; A3 = Placebo MAP (FA); A4 = A/Sing Mono IM, 15 ug (UA)
Treatments Part B: B1 = A/Sing MAP, 15 ug (FA) with biopsy; B2 = Placebo MAP (FA) with biopsy
B3 = A/Sing MAP, 15 ug (FA); B4 = A/Sing MAP, 10 ug (FA); B5 = A/Sing MAP, 5 ug (FA); B6 = A/Sing MAP, 2.5 ug (FA)
B7 = Placebo MAP (FA); B8 = A/Sing MAP, 15 ug (UA); B9 = A/Sing IM, 15 ug, as 2018 Afluria Quad (UA)

Listing 16.2.9.11
Individual Characterisation of Skin Irritation Index by Treatment
Total Skin Irritation Index
Part B, Non-biopsy
B7= 3 x uncoated MAPs on volar forearm

			Active			Placebo		
Randomisation Number	Visit	Time Point	#> 0	Maximum	Average	#> 0	Maximum	Average
B0069	Day 2		-	-	-	3	2	2.0
B0069	Day 4		-	-	-	3	3	3.0
B0069	Day 8		-	-	-	0	0	0.0
B0069	Day 22		-	-	-	0	0	0.0
B0069	EOS/ET (Day 61)		-	-	-	0	0	0.0
B0087	Day 1	Pre-dose	-	-	-	0	0	0.0
B0087	Day 1	10 min	-	-	-	3	3	2.3
B0087	Day 1	1 hour	-	-	-	3	3	3.0
B0087	Day 1	2 hour	-	-	-	3	2	2.0
B0087	Day 2		-	-	-	3	3	3.0
B0087	Day 4		-	-	-	1	1	0.3
B0087	Day 8		-	-	-	0	0	0.0
B0087	Day 22		-	-	-	0	0	0.0
B0087	EOS/ET (Day 61)		-	-	-	0	0	0.0
B0093	Day 1	Pre-dose	-	-	-	0	0	0.0
B0093	Day 1	10 min	-	-	-	3	4	3.7
B0093	Day 1	1 hour	-	-	-	3	4	3.3
B0093	Day 1	2 hour	-	-	-	3	4	3.3
B0093	Day 2		-	-	-	3	3	2.7

Source: Listing 16.2.9.4

Administration Site Key: FA = Forearm; UA= Upper arm
Treatments Part A: A1 = A/Sing MAP, 15 ug (FA); A2 = Afluria Quad IM (UA), 15 ug; A3 = Placebo MAP (FA); A4 = A/Sing Mono IM, 15 ug (UA)
Treatments Part B: B1 = A/Sing MAP, 15 ug (FA) with biopsy; B2 = Placebo MAP (FA) with biopsy
B3 = A/Sing MAP, 15 ug (FA); B4 = A/Sing MAP, 10 ug (FA); B5 = A/Sing MAP, 5 ug (FA); B6 = A/Sing MAP, 2.5 ug (FA)
B7 = Placebo MAP (FA); B8 = A/Sing MAP, 15 ug (UA); B9 = A/Sing IM, 15 ug, as 2018 Afluria Quad (UA)

Listing 16.2.9.11
Individual Characterisation of Skin Irritation Index by Treatment
Total Skin Irritation Index
Part B, Non-biopsy
B7= 3 x uncoated MAPs on volar forearm

			Active			Placebo		
Randomisation Number	Visit	Time Point	#> 0	Maximum	Average	#> 0	Maximum	Average
B0093	Day 4		-	-	-	0	0	0.0
B0093	Day 8		-	-	-	0	0	0.0
B0093	Day 22		-	-	-	0	0	0.0
B0093	EOS/ET (Day 61)		-	-	-	0	0	0.0
B0098	Day 1	Pre-dose	-	-	-	0	0	0.0
B0098	Day 1	10 min	-	-	-	3	3	3.0
B0098	Day 1	1 hour	-	-	-	3	4	4.0
B0098	Day 1	2 hour	-	-	-	3	3	3.0
B0098	Day 2		-	-	-	3	1	1.0
B0098	Day 4		-	-	-	0	0	0.0
B0098	Day 8		-	-	-	0	0	0.0
B0098	Day 22		-	-	-	0	0	0.0
B0098	EOS/ET (Day 61)		-	-	-	0	0	0.0
B0108	Day 1	Pre-dose	-	-	-	0	0	0.0
B0108	Day 1	10 min	-	-	-	3	2	2.0
B0108	Day 1	1 hour	-	-	-	3	3	3.0
B0108	Day 1	2 hour	-	-	-	3	3	2.7
B0108	Day 2		-	-	-	3	3	2.7
B0108	Day 4		-	-	-	0	0	0.0

Administration Site Key: FA = Forearm; UA= Upper arm
Treatments Part A: A1 = A/Sing MAP, 15 ug (FA); A2 = Afluria Quad IM (UA), 15 ug; A3 = Placebo MAP (FA); A4 = A/Sing Mono IM, 15 ug (UA)
Treatments Part B: B1 = A/Sing MAP, 15 ug (FA) with biopsy; B2 = Placebo MAP (FA) with biopsy
B3 = A/Sing MAP, 15 ug (FA); B4 = A/Sing MAP, 10 ug (FA); B5 = A/Sing MAP, 5 ug (FA); B6 = A/Sing MAP, 2.5 ug (FA)
B7 = Placebo MAP (FA); B8 = A/Sing MAP, 15 ug (UA); B9 = A/Sing IM, 15 ug, as 2018 Afluria Quad (UA)

Listing 16.2.9.11
Individual Characterisation of Skin Irritation Index by Treatment
Total Skin Irritation Index
Part B, Non-biopsy
B7= 3 x uncoated MAPs on volar forearm

			Active			Placebo		
Randomisation Number	Visit	Time Point	#> 0	Maximum	Average	#> 0	Maximum	Average
B0108	Day 8		-	-	-	0	0	0.0
B0108	Day 22		-	-	-	0	0	0.0
B0108	EOS/ET (Day 61)		-	-	-	0	0	0.0
B0114	Day 1	Pre-dose	-	-	-	0	0	0.0
B0114	Day 1	10 min	-	-	-	3	4	4.0
B0114	Day 1	1 hour	-	-	-	3	3	2.3
B0114	Day 1	2 hour	-	-	-	3	5	4.3
B0114	Day 2		-	-	-	3	2	2.0
B0114	Day 4		-	-	-	2	3	1.3
B0114	Day 8		-	-	-	3	3	1.7
B0114	Day 22		-	-	-	0	0	0.0
B0114	EOS/ET (Day 61)		-	-	-	0	0	0.0
B0116	Day 1	Pre-dose	-	-	-	0	0	0.0
B0116	Day 1	10 min	-	-	-	3	5	5.0
B0116	Day 1	1 hour	-	-	-	3	5	5.0
B0116	Day 1	2 hour	-	-	-	3	5	4.3
B0116	Day 2		-	-	-	3	2	2.0
B0116	Day 4		-	-	-	0	0	0.0
B0116	Day 8		-	-	-	0	0	0.0

Source: Listing 16.2.9.4

Administration Site Key: FA = Forearm; UA= Upper arm
Treatments Part A: A1 = A/Sing MAP, 15 ug (FA); A2 = Afluria Quad IM (UA), 15 ug; A3 = Placebo MAP (FA); A4 = A/Sing Mono IM, 15 ug (UA)
Treatments Part B: B1 = A/Sing MAP, 15 ug (FA) with biopsy; B2 = Placebo MAP (FA) with biopsy
B3 = A/Sing MAP, 15 ug (FA); B4 = A/Sing MAP, 10 ug (FA); B5 = A/Sing MAP, 5 ug (FA); B6 = A/Sing MAP, 2.5 ug (FA)
B7 = Placebo MAP (FA); B8 = A/Sing MAP, 15 ug (UA); B9 = A/Sing IM, 15 ug, as 2018 Afluria Quad (UA)

Listing 16.2.9.11
Individual Characterisation of Skin Irritation Index by Treatment
Total Skin Irritation Index
Part B, Non-biopsy
B7= 3 x uncoated MAPs on volar forearm

			Active			Placebo		
Randomisation Number	Visit	Time Point	#> 0	Maximum	Average	#> 0	Maximum	Average
B0116	Day 22		-	-	-	0	0	0.0
B0116	EOS/ET (Day 61)		-	-	-	0	0	0.0
B0125	Day 1	Pre-dose	-	-	-	0	0	0.0
B0125	Day 1	10 min	-	-	-	3	3	3.0
B0125	Day 1	1 hour	-	-	-	3	3	3.0
B0125	Day 1	2 hour	-	-	-	3	3	3.0
B0125	Day 2		-	-	-	3	2	2.0
B0125	Day 4		-	-	-	0	0	0.0
B0125	Day 8		-	-	-	0	0	0.0
B0125	Day 22		-	-	-	0	0	0.0
B0125	EOS/ET (Day 61)		-	-	-	0	0	0.0
B0135	Day 1	Pre-dose	-	-	-	0	0	0.0
B0135	Day 1	10 min	-	-	-	3	5	5.0
B0135	Day 1	1 hour	-	-	-	3	5	5.0
B0135	Day 1	2 hour	-	-	-	3	5	4.3
B0135	Day 2		-	-	-	3	2	2.0
B0135	Day 4		-	-	-	0	0	0.0
B0135	Day 8		-	-	-	0	0	0.0
B0135	Day 22		-	-	-	0	0	0.0

Source: Listing 16.2.9.4

Administration Site Key: FA = Forearm; UA= Upper arm
Treatments Part A: A1 = A/Sing MAP, 15 ug (FA); A2 = Afluria Quad IM (UA), 15 ug; A3 = Placebo MAP (FA); A4 = A/Sing Mono IM, 15 ug (UA)
Treatments Part B: B1 = A/Sing MAP, 15 ug (FA) with biopsy; B2 = Placebo MAP (FA) with biopsy
B3 = A/Sing MAP, 15 ug (FA); B4 = A/Sing MAP, 10 ug (FA); B5 = A/Sing MAP, 5 ug (FA); B6 = A/Sing MAP, 2.5 ug (FA)
B7 = Placebo MAP (FA); B8 = A/Sing MAP, 15 ug (UA); B9 = A/Sing IM, 15 ug, as 2018 Afluria Quad (UA)

Listing 16.2.9.11
Individual Characterisation of Skin Irritation Index by Treatment
Total Skin Irritation Index
Part B, Non-biopsy
B7= 3 x uncoated MAPs on volar forearm

			Active			Placebo		
Randomisation Number	Visit	Time Point	#> 0	Maximum	Average	#> 0	Maximum	Average
B0135	EOS/ET (Day 61)		-	-	-	0	0	0.0
B0138	Day 1	Pre-dose	-	-	-	0	0	0.0
B0138	Day 1	10 min	-	-	-	3	4	3.7
B0138	Day 1	1 hour	-	-	-	3	5	4.7
B0138	Day 1	2 hour	-	-	-	3	4	4.0
B0138	Day 2		-	-	-	3	2	2.0
B0138	Day 4		-	-	-	0	0	0.0
B0138	Day 8		-	-	-	0	0	0.0
B0138	Day 22		-	-	-	0	0	0.0
B0138	EOS/ET (Day 61)		-	-	-	0	0	0.0
B0146	Day 1	Pre-dose	-	-	-	0	0	0.0
B0146	Day 1	10 min	-	-	-	3	2	1.3
B0146	Day 1	1 hour	-	-	-	3	4	3.3
B0146	Day 1	2 hour	-	-	-	3	4	3.3
B0146	Day 2		-	-	-	3	1	1.0
B0146	Day 4		-	-	-	3	1	1.0
B0146	Day 8		-	-	-	0	0	0.0
B0146	Day 22		-	-	-	0	0	0.0
B0146	EOS/ET (Day 61)		-	-	-	0	0	0.0

Source: Listing 16.2.9.4

Administration Site Key: FA = Forearm; UA= Upper arm
Treatments Part A: A1 = A/Sing MAP, 15 ug (FA); A2 = Afluria Quad IM (UA), 15 ug; A3 = Placebo MAP (FA); A4 = A/Sing Mono IM, 15 ug (UA)
Treatments Part B: B1 = A/Sing MAP, 15 ug (FA) with biopsy; B2 = Placebo MAP (FA) with biopsy
B3 = A/Sing MAP, 15 ug (FA); B4 = A/Sing MAP, 10 ug (FA); B5 = A/Sing MAP, 5 ug (FA); B6 = A/Sing MAP, 2.5 ug (FA)
B7 = Placebo MAP (FA); B8 = A/Sing MAP, 15 ug (UA); B9 = A/Sing IM, 15 ug, as 2018 Afluria Quad (UA)

Listing 16.2.9.11
Individual Characterisation of Skin Irritation Index by Treatment
Total Skin Irritation Index
Part B, Non-biopsy
B7= 3 x uncoated MAPs on volar forearm

Randomisation Number	Visit	Time Point	Active #> 0	Active Maximum	Active Average	Placebo #> 0	Placebo Maximum	Placebo Average
B1080	Day 1	Pre-dose	-	-	-	0	0	0.0
B1080	Day 1	10 min	-	-	-	3	2	2.0
B1080	Day 1	1 hour	-	-	-	3	3	2.7
B1080	Day 1	2 hour	-	-	-	3	3	2.7
B1080	Day 2		-	-	-	3	2	2.0
B1080	Day 4		-	-	-	0	0	0.0
B1080	Day 8		-	-	-	0	0	0.0
B1080	Day 22		-	-	-	0	0	0.0
B1080	EOS/ET (Day 61)		-	-	-	0	0	0.0

Source: Listing 16.2.9.4

Administration Site Key: FA = Forearm; UA= Upper arm
Treatments Part A: A1 = A/Sing MAP, 15 ug (FA); A2 = Afluria Quad IM (UA), 15 ug; A3 = Placebo MAP (FA); A4 = A/Sing Mono IM, 15 ug (UA)
Treatments Part B: B1 = A/Sing MAP, 15 ug (FA) with biopsy; B2 = Placebo MAP (FA) with biopsy
B3 = A/Sing MAP, 15 ug (FA); B4 = A/Sing MAP, 10 ug (FA); B5 = A/Sing MAP, 5 ug (FA); B6 = A/Sing MAP, 2.5 ug (FA)
B7 = Placebo MAP (FA); B8 = A/Sing MAP, 15 ug (UA); B9 = A/Sing IM, 15 ug, as 2018 Afluria Quad (UA)

Listing 16.2.9.11
Individual Characterisation of Skin Irritation Index by Treatment
Total Skin Irritation Index
Part B, Non-biopsy
B8= 3 x 5 ug A/Sing MAPs on upper arm (15 ug)

Randomisation Number	Visit	Time Point	Active #> 0	Active Maximum	Active Average	Placebo #> 0	Placebo Maximum	Placebo Average
B0011	Day 1	Pre-dose	0	0	0.0	-	-	-
B0011	Day 1	10 min	3	2	2.0	-	-	-
B0011	Day 1	1 hour	3	4	4.0	-	-	-
B0011	Day 1	2 hour	3	4	4.0	-	-	-
B0011	Day 2		3	4	4.0	-	-	-
B0011	Day 4		3	5	5.0	-	-	-
B0011	Day 8		3	1	1.0	-	-	-
B0011	Day 22		3	1	1.0	-	-	-
B0011	EOS/ET (Day 61)		0	0	0.0	-	-	-
B0025	Day 1	Pre-dose	0	0	0.0	-	-	-
B0025	Day 1	10 min	3	3	3.0	-	-	-
B0025	Day 1	1 hour	3	3	3.0	-	-	-
B0025	Day 1	2 hour	3	4	4.0	-	-	-
B0025	Day 2		3	4	4.0	-	-	-
B0025	Day 4		3	2	2.0	-	-	-
B0025	Day 8		3	2	2.0	-	-	-
B0025	Day 22		0	0	0.0	-	-	-
B0025	EOS/ET (Day 61)		0	0	0.0	-	-	-
B0034	Day 1	Pre-dose	0	0	0.0	-	-	-

Source: Listing 16.2.9.4

Administration Site Key: FA = Forearm; UA= Upper arm
Treatments Part A: A1 = A/Sing MAP, 15 ug (FA); A2 = Afluria Quad IM (UA), 15 ug; A3 = Placebo MAP (FA); A4 = A/Sing Mono IM, 15 ug (UA)
Treatments Part B: B1 = A/Sing MAP, 15 ug (FA) with biopsy; B2 = Placebo MAP (FA) with biopsy
B3 = A/Sing MAP, 15 ug (FA); B4 = A/Sing MAP, 10 ug (FA); B5 = A/Sing MAP, 5 ug (FA); B6 = A/Sing MAP, 2.5 ug (FA)
B7 = Placebo MAP (FA); B8 = A/Sing MAP, 15 ug (UA); B9 = A/Sing IM, 15 ug, as 2018 Afluria Quad (UA)

Listing 16.2.9.11
Individual Characterisation of Skin Irritation Index by Treatment
Total Skin Irritation Index
Part B, Non-biopsy
B8= 3 x 5 ug A/Sing MAPs on upper arm (15 ug)

			Active			Placebo		
Randomisation Number	Visit	Time Point	#> 0	Maximum	Average	#> 0	Maximum	Average
B0034	Day 1	10 min	3	4	4.0	-	-	-
B0034	Day 1	1 hour	3	4	3.7	-	-	-
B0034	Day 1	2 hour	3	4	4.0	-	-	-
B0034	Day 2		3	4	3.7	-	-	-
B0034	Day 4		3	5	4.7	-	-	-
B0034	Day 8		0	0	0.0	-	-	-
B0034	Day 22		1	1	0.3	-	-	-
B0034	EOS/ET (Day 61)		0	0	0.0	-	-	-
B0043	Day 1	Pre-dose	0	0	0.0	-	-	-
B0043	Day 1	10 min	3	4	3.3	-	-	-
B0043	Day 1	1 hour	3	3	3.0	-	-	-
B0043	Day 1	2 hour	3	5	5.0	-	-	-
B0043	Day 2		3	4	4.0	-	-	-
B0043	Day 4		3	5	4.3	-	-	-
B0043	Day 8		3	4	2.7	-	-	-
B0043	Day 22		3	1	1.0	-	-	-
B0043	EOS/ET (Day 61)		0	0	0.0	-	-	-
B0050	Day 1	Pre-dose	0	0	0.0	-	-	-
B0050	Day 1	10 min	3	3	3.0	-	-	-

Administration Site Key: FA = Forearm; UA= Upper arm
Treatments Part A: A1 = A/Sing MAP, 15 ug (FA); A2 = Afluria Quad IM (UA), 15 ug; A3 = Placebo MAP (FA); A4 = A/Sing Mono IM, 15 ug (UA)
Treatments Part B: B1 = A/Sing MAP, 15 ug (FA) with biopsy; B2 = Placebo MAP (FA) with biopsy
B3 = A/Sing MAP, 15 ug (FA); B4 = A/Sing MAP, 10 ug (FA); B5 = A/Sing MAP, 5 ug (FA); B6 = A/Sing MAP, 2.5 ug (FA)
B7 = Placebo MAP (FA); B8 = A/Sing MAP, 15 ug (UA); B9 = A/Sing IM, 15 ug, as 2018 Afluria Quad (UA)

Listing 16.2.9.11
Individual Characterisation of Skin Irritation Index by Treatment
Total Skin Irritation Index
Part B, Non-biopsy
B8= 3 x 5 ug A/Sing MAPs on upper arm (15 ug)

			Active			Placebo		
Randomisation Number	Visit	Time Point	#> 0	Maximum	Average	#> 0	Maximum	Average
B0050	Day 1	1 hour	3	4	3.7	-	-	-
B0050	Day 1	2 hour	3	4	4.0	-	-	-
B0050	Day 2		3	4	4.0	-	-	-
B0050	Day 4		3	4	3.3	-	-	-
B0050	Day 8		3	3	3.0	-	-	-
B0050	Day 22		2	1	0.7	-	-	-
B0050	EOS/ET (Day 61)		0	0	0.0	-	-	-
B0055	Day 1	Pre-dose	0	0	0.0	-	-	-
B0055	Day 1	10 min	3	3	3.0	-	-	-
B0055	Day 1	1 hour	3	3	3.0	-	-	-
B0055	Day 1	2 hour	3	3	3.0	-	-	-
B0055	Day 2		3	3	3.0	-	-	-
B0055	Day 4		3	2	1.3	-	-	-
B0055	Day 8		3	2	1.7	-	-	-
B0055	Day 22		0	0	0.0	-	-	-
B0055	EOS/ET (Day 61)		0	0	0.0	-	-	-
B0061	Day 1	Pre-dose	0	0	0.0	-	-	-
B0061	Day 1	10 min	3	3	3.0	-	-	-
B0061	Day 1	1 hour	3	2	2.0	-	-	-

Source: Listing 16.2.9.4

Administration Site Key: FA = Forearm; UA= Upper arm
Treatments Part A: A1 = A/Sing MAP, 15 ug (FA); A2 = Afluria Quad IM (UA), 15 ug; A3 = Placebo MAP (FA); A4 = A/Sing Mono IM, 15 ug (UA)
Treatments Part B: B1 = A/Sing MAP, 15 ug (FA) with biopsy; B2 = Placebo MAP (FA) with biopsy
B3 = A/Sing MAP, 15 ug (FA); B4 = A/Sing MAP, 10 ug (FA); B5 = A/Sing MAP, 5 ug (FA); B6 = A/Sing MAP, 2.5 ug (FA)
B7 = Placebo MAP (FA); B8 = A/Sing MAP, 15 ug (UA); B9 = A/Sing IM, 15 ug, as 2018 Afluria Quad (UA)

Listing 16.2.9.11
Individual Characterisation of Skin Irritation Index by Treatment
Total Skin Irritation Index
Part B, Non-biopsy
B8= 3 x 5 ug A/Sing MAPs on upper arm (15 ug)

			Active			Placebo		
Randomisation Number	Visit	Time Point	#> 0	Maximum	Average	#> 0	Maximum	Average
B0061	Day 1	2 hour	3	3	2.7	-	-	-
B0061	Day 2		3	2	2.0	-	-	-
B0061	Day 4		3	5	4.3	-	-	-
B0061	Day 8		3	2	2.0	-	-	-
B0061	Day 22		0	0	0.0	-	-	-
B0061	EOS/ET (Day 61)		0	0	0.0	-	-	-
B0067	Day 1	Pre-dose	0	0	0.0	-	-	-
B0067	Day 1	10 min	3	3	3.0	-	-	-
B0067	Day 1	1 hour	3	3	3.0	-	-	-
B0067	Day 1	2 hour	3	3	3.0	-	-	-
B0067	Day 2		3	5	4.3	-	-	-
B0067	Day 4		3	2	2.0	-	-	-
B0067	Day 8		0	0	0.0	-	-	-
B0067	Day 22		0	0	0.0	-	-	-
B0067	EOS/ET (Day 61)		0	0	0.0	-	-	-
B0078	Day 1	Pre-dose	0	0	0.0	-	-	-
B0078	Day 1	10 min	3	3	3.0	-	-	-
B0078	Day 1	1 hour	3	3	3.0	-	-	-
B0078	Day 1	2 hour	3	4	3.7	-	-	-

Administration Site Key: FA = Forearm; UA= Upper arm
Treatments Part A: A1 = A/Sing MAP, 15 ug (FA); A2 = Afluria Quad IM (UA), 15 ug; A3 = Placebo MAP (FA); A4 = A/Sing Mono IM, 15 ug (UA)
Treatments Part B: B1 = A/Sing MAP, 15 ug (FA) with biopsy; B2 = Placebo MAP (FA) with biopsy
B3 = A/Sing MAP, 15 ug (FA); B4 = A/Sing MAP, 10 ug (FA); B5 = A/Sing MAP, 5 ug (FA); B6 = A/Sing MAP, 2.5 ug (FA)
B7 = Placebo MAP (FA); B8 = A/Sing MAP, 15 ug (UA); B9 = A/Sing IM, 15 ug, as 2018 Afluria Quad (UA)

Listing 16.2.9.11
Individual Characterisation of Skin Irritation Index by Treatment
Total Skin Irritation Index
Part B, Non-biopsy
B8= 3 x 5 ug A/Sing MAPs on upper arm (15 ug)

Randomisation Number	Visit	Time Point	Active #> 0	Active Maximum	Active Average	Placebo #> 0	Placebo Maximum	Placebo Average
B0078	Day 2		3	4	4.0	-	-	-
B0078	Day 4		3	5	5.0	-	-	-
B0078	Day 8		2	1	0.7	-	-	-
B0078	Day 22		0	0	0.0	-	-	-
B0078	EOS/ET (Day 61)		0	0	0.0	-	-	-
B0083	Day 1	Pre-dose	0	0	0.0	-	-	-
B0083	Day 1	10 min	3	3	3.0	-	-	-
B0083	Day 1	1 hour	3	2	2.0	-	-	-
B0083	Day 1	2 hour	3	5	4.7	-	-	-
B0083	Day 2		3	7	7.0	-	-	-
B0083	Day 4		3	3	3.0	-	-	-
B0083	Day 8		0	0	0.0	-	-	-
B0083	Day 22		0	0	0.0	-	-	-
B0083	EOS/ET (Day 61)		0	0	0.0	-	-	-
B0091	Day 1	Pre-dose	0	0	0.0	-	-	-
B0091	Day 1	10 min	3	4	3.7	-	-	-
B0091	Day 1	1 hour	3	3	3.0	-	-	-
B0091	Day 1	2 hour	3	3	3.0	-	-	-
B0091	Day 2		3	4	4.0	-	-	-

Source: Listing 16.2.9.4

Administration Site Key: FA = Forearm; UA= Upper arm
Treatments Part A: A1 = A/Sing MAP, 15 ug (FA); A2 = Afluria Quad IM (UA), 15 ug; A3 = Placebo MAP (FA); A4 = A/Sing Mono IM, 15 ug (UA)
Treatments Part B: B1 = A/Sing MAP, 15 ug (FA) with biopsy; B2 = Placebo MAP (FA) with biopsy
B3 = A/Sing MAP, 15 ug (FA); B4 = A/Sing MAP, 10 ug (FA); B5 = A/Sing MAP, 5 ug (FA); B6 = A/Sing MAP, 2.5 ug (FA)
B7 = Placebo MAP (FA); B8 = A/Sing MAP, 15 ug (UA); B9 = A/Sing IM, 15 ug, as 2018 Afluria Quad (UA)

Listing 16.2.9.11
Individual Characterisation of Skin Irritation Index by Treatment
Total Skin Irritation Index
Part B, Non-biopsy
B8= 3 x 5 ug A/Sing MAPs on upper arm (15 ug)

			Active			Placebo		
Randomisation Number	Visit	Time Point	#> 0	Maximum	Average	#> 0	Maximum	Average
B0091	Day 4		3	3	3.0	-	-	-
B0091	Day 8		0	0	0.0	-	-	-
B0091	Day 22		0	0	0.0	-	-	-
B0091	EOS/ET (Day 61)		0	0	0.0	-	-	-
B0100	Day 1	Pre-dose	0	0	0.0	-	-	-
B0100	Day 1	10 min	3	3	3.0	-	-	-
B0100	Day 1	1 hour	3	3	3.0	-	-	-
B0100	Day 1	2 hour	3	3	3.0	-	-	-
B0100	Day 2		3	6	5.7	-	-	-
B0100	Day 4		3	5	4.3	-	-	-
B0100	Day 8		3	3	2.0	-	-	-
B0100	Day 22		2	1	0.7	-	-	-
B0100	EOS/ET (Day 61)		0	0	0.0	-	-	-
B0102	Day 1	Pre-dose	0	0	0.0	-	-	-
B0102	Day 1	10 min	3	3	3.0	-	-	-
B0102	Day 1	1 hour	3	2	2.0	-	-	-
B0102	Day 1	2 hour	3	3	3.0	-	-	-
B0102	Day 2		3	4	3.3	-	-	-
B0102	Day 4		3	3	3.0	-	-	-

Administration Site Key: FA = Forearm; UA= Upper arm
Treatments Part A: A1 = A/Sing MAP, 15 ug (FA); A2 = Afluria Quad IM (UA), 15 ug; A3 = Placebo MAP (FA); A4 = A/Sing Mono IM, 15 ug (UA)
Treatments Part B: B1 = A/Sing MAP, 15 ug (FA) with biopsy; B2 = Placebo MAP (FA) with biopsy
B3 = A/Sing MAP, 15 ug (FA); B4 = A/Sing MAP, 10 ug (FA); B5 = A/Sing MAP, 5 ug (FA); B6 = A/Sing MAP, 2.5 ug (FA)
B7 = Placebo MAP (FA); B8 = A/Sing MAP, 15 ug (UA); B9 = A/Sing IM, 15 ug, as 2018 Afluria Quad (UA)

Listing 16.2.9.11
Individual Characterisation of Skin Irritation Index by Treatment
Total Skin Irritation Index
Part B, Non-biopsy
B8= 3 x 5 ug A/Sing MAPs on upper arm (15 ug)

			Active			Placebo		
Randomisation Number	Visit	Time Point	#> 0	Maximum	Average	#> 0	Maximum	Average
B0102	Day 8		3	2	2.0	-	-	-
B0102	Day 22		0	0	0.0	-	-	-
B0102	EOS/ET (Day 61)		0	0	0.0	-	-	-
B0111	Day 1	Pre-dose	0	0	0.0	-	-	-
B0111	Day 1	10 min	3	3	2.7	-	-	-
B0111	Day 1	1 hour	3	2	2.0	-	-	-
B0111	Day 1	2 hour	3	3	2.3	-	-	-
B0111	Day 2		3	5	5.0	-	-	-
B0111	Day 4		3	3	3.0	-	-	-
B0111	Day 8		3	4	4.0	-	-	-
B0111	Day 22		0	0	0.0	-	-	-
B0111	EOS/ET (Day 61)		0	0	0.0	-	-	-
B0121	Day 1	Pre-dose	0	0	0.0	-	-	-
B0121	Day 1	10 min	3	3	3.0	-	-	-
B0121	Day 1	1 hour	3	4	4.0	-	-	-
B0121	Day 1	2 hour	3	5	4.7	-	-	-
B0121	Day 2		3	3	3.0	-	-	-
B0121	Day 4		3	3	3.0	-	-	-
B0121	Day 8		3	2	1.7	-	-	-

Source: Listing 16.2.9.4

Administration Site Key: FA = Forearm; UA= Upper arm
Treatments Part A: A1 = A/Sing MAP, 15 ug (FA); A2 = Afluria Quad IM (UA), 15 ug; A3 = Placebo MAP (FA); A4 = A/Sing Mono IM, 15 ug (UA)
Treatments Part B: B1 = A/Sing MAP, 15 ug (FA) with biopsy; B2 = Placebo MAP (FA) with biopsy
B3 = A/Sing MAP, 15 ug (FA); B4 = A/Sing MAP, 10 ug (FA); B5 = A/Sing MAP, 5 ug (FA); B6 = A/Sing MAP, 2.5 ug (FA)
B7 = Placebo MAP (FA); B8 = A/Sing MAP, 15 ug (UA); B9 = A/Sing IM, 15 ug, as 2018 Afluria Quad (UA)

Listing 16.2.9.11
Individual Characterisation of Skin Irritation Index by Treatment
Total Skin Irritation Index
Part B, Non-biopsy
B8= 3 x 5 ug A/Sing MAPs on upper arm (15 ug)

			Active			Placebo		
Randomisation Number	Visit	Time Point	#> 0	Maximum	Average	#> 0	Maximum	Average
B0121	Day 22		3	1	1.0	-	-	-
B0121	EOS/ET (Day 61)		0	0	0.0	-	-	-
B0129	Day 1	Pre-dose	0	0	0.0	-	-	-
B0129	Day 1	10 min	3	4	4.0	-	-	-
B0129	Day 1	1 hour	3	5	4.7	-	-	-
B0129	Day 1	2 hour	3	5	4.7	-	-	-
B0129	Day 2		3	5	5.0	-	-	-
B0129	Day 4		3	3	3.0	-	-	-
B0129	Day 8		3	2	2.0	-	-	-
B0129	Day 22		2	1	0.7	-	-	-
B0129	EOS/ET (Day 61)		0	0	0.0	-	-	-
B0131	Day 1	Pre-dose	0	0	0.0	-	-	-
B0131	Day 1	10 min	3	3	3.0	-	-	-
B0131	Day 1	1 hour	3	4	3.7	-	-	-
B0131	Day 1	2 hour	3	4	2.7	-	-	-
B0131	Day 2		3	3	3.0	-	-	-
B0131	Day 4		3	2	2.0	-	-	-
B0131	Day 8		3	2	1.3	-	-	-
B0131	Day 22		0	0	0.0	-	-	-

Source: Listing 16.2.9.4

Administration Site Key: FA = Forearm; UA= Upper arm
Treatments Part A: A1 = A/Sing MAP, 15 ug (FA); A2 = Afluria Quad IM (UA), 15 ug; A3 = Placebo MAP (FA); A4 = A/Sing Mono IM, 15 ug (UA)
Treatments Part B: B1 = A/Sing MAP, 15 ug (FA) with biopsy; B2 = Placebo MAP (FA) with biopsy
B3 = A/Sing MAP, 15 ug (FA); B4 = A/Sing MAP, 10 ug (FA); B5 = A/Sing MAP, 5 ug (FA); B6 = A/Sing MAP, 2.5 ug (FA)
B7 = Placebo MAP (FA); B8 = A/Sing MAP, 15 ug (UA); B9 = A/Sing IM, 15 ug, as 2018 Afluria Quad (UA)

Listing 16.2.9.11
Individual Characterisation of Skin Irritation Index by Treatment
Total Skin Irritation Index
Part B, Non-biopsy
B8= 3 x 5 ug A/Sing MAPs on upper arm (15 ug)

Randomisation Number	Visit	Time Point	Active #> 0	Active Maximum	Active Average	Placebo #> 0	Placebo Maximum	Placebo Average
B0131	EOS/ET (Day 61)		0	0	0.0	-	-	-
B0137	Day 1	Pre-dose	0	0	0.0	-	-	-
B0137	Day 1	10 min	3	4	3.7	-	-	-
B0137	Day 1	1 hour	3	5	4.7	-	-	-
B0137	Day 1	2 hour	3	5	4.7	-	-	-
B0137	Day 2		3	3	3.0	-	-	-
B0137	Day 4		3	3	3.0	-	-	-
B0137	Day 8		3	2	2.0	-	-	-
B0137	Day 22		1	1	0.3	-	-	-
B0137	EOS/ET (Day 61)		0	0	0.0	-	-	-
B0149	Day 1	Pre-dose	0	0	0.0	-	-	-
B0149	Day 1	10 min	3	3	3.0	-	-	-
B0149	Day 1	1 hour	3	4	4.0	-	-	-
B0149	Day 1	2 hour	3	4	3.7	-	-	-
B0149	Day 2		3	4	3.3	-	-	-
B0149	Day 4		3	2	2.0	-	-	-
B0149	Day 8		3	3	3.0	-	-	-
B0149	Day 22		0	0	0.0	-	-	-
B0149	EOS/ET (Day 61)		0	0	0.0	-	-	-

Source: Listing 16.2.9.4

Administration Site Key: FA = Forearm; UA= Upper arm
Treatments Part A: A1 = A/Sing MAP, 15 ug (FA); A2 = Afluria Quad IM (UA), 15 ug; A3 = Placebo MAP (FA); A4 = A/Sing Mono IM, 15 ug (UA)
Treatments Part B: B1 = A/Sing MAP, 15 ug (FA) with biopsy; B2 = Placebo MAP (FA) with biopsy
B3 = A/Sing MAP, 15 ug (FA); B4 = A/Sing MAP, 10 ug (FA); B5 = A/Sing MAP, 5 ug (FA); B6 = A/Sing MAP, 2.5 ug (FA)
B7 = Placebo MAP (FA); B8 = A/Sing MAP, 15 ug (UA); B9 = A/Sing IM, 15 ug, as 2018 Afluria Quad (UA)

Listing 16.2.9.11
Individual Characterisation of Skin Irritation Index by Treatment
Total Skin Irritation Index
Part B, Non-biopsy
B8= 3 x 5 ug A/Sing MAPs on upper arm (15 ug)

			Active			Placebo		
Randomisation Number	Visit	Time Point	#> 0	Maximum	Average	#> 0	Maximum	Average
B1019	Day 1	Pre-dose	0	0	0.0	-	-	-
B1019	Day 1	10 min	3	3	3.0	-	-	-
B1019	Day 1	1 hour	3	2	2.0	-	-	-
B1019	Day 1	2 hour	3	3	2.7	-	-	-
B1019	Day 2		3	3	2.0	-	-	-
B1019	Day 4		0	0	0.0	-	-	-
B1019	Day 8		0	0	0.0	-	-	-
B1019	Day 22		0	0	0.0	-	-	-
B1019	EOS/ET (Day 61)		0	0	0.0	-	-	-

Source: Listing 16.2.9.4

Administration Site Key: FA = Forearm; UA= Upper arm
Treatments Part A: A1 = A/Sing MAP, 15 ug (FA); A2 = Afluria Quad IM (UA), 15 ug; A3 = Placebo MAP (FA); A4 = A/Sing Mono IM, 15 ug (UA)
Treatments Part B: B1 = A/Sing MAP, 15 ug (FA) with biopsy; B2 = Placebo MAP (FA) with biopsy
B3 = A/Sing MAP, 15 ug (FA); B4 = A/Sing MAP, 10 ug (FA); B5 = A/Sing MAP, 5 ug (FA); B6 = A/Sing MAP, 2.5 ug (FA)
B7 = Placebo MAP (FA); B8 = A/Sing MAP, 15 ug (UA); B9 = A/Sing IM, 15 ug, as 2018 Afluria Quad (UA)
