# Supplementary material for: Safety, tolerability, and immunogenicity of influenza vaccination with a high-density microarray patch: Results from a randomized, controlled phase I clinical trial
Source: PLoS Med. 2020 Mar 17;17(3):e1003024. doi: 10.1371/journal.pmed.1003024 (PMC7077342; doi:10.1371/journal.pmed.1003024)
Supplement: S1 Data — Data 1: Informed Consent; Data 2: Analysis Sets; Data 3: Demographics; Data 4: Study Drug Administration; Data 5: Skin Hardness Assessment; Data 6: Immunogenicity (ELISA IgG); Data 7: Immunogenicity (ADCC); Data 8: Immunogenicity (MBCs); Data 9: Immunogenicity (CMI); Data 10: Immunogenicity (Mucosal IgA); Data 11: Immunogenicity Results (HAI, MN); Data 12: TEAE–CRF Data only; Data 13: TEAEs–MedDRA Coding; Data 15: Treatment Site Tolerability Assessment; Data 16: Numeric Pain Intensity; Data 17: SII; Data 18: Application Site Status at End of Study; Data 19: Individual Treatment Site Tolerability Assessment; Data 20: Numeric Pain Intensity by Treatment; Data 21: Individual SII by Treatment. ADCC, antibody-dependent cellular cytotoxicity; CMI, cell-mediated immunity; CRF, case report form; ELISA, enzyme-linked immunosorbent assay; HAI, haemagglutination inhibition; IgA, immunoglobulin A; IgG, immunoglobulin G; MBC, memory B cell; MedDRA, medical dictionary for regulatory activities; MN, microneutralisation; SII, Skin Irritation Index; TEA, treatment emergent adverse event. (ZIP) [file pmed.1003024.s008.zip › S1_Data/clinical supporting/S1_Data3 Demographics.pdf]

Listing 16.2.4.1
Demographics

Part A

Randomisation Number	Group	Age (yrs) [C]	Gender	Race	Ethnicity
A0001	Group A3	31	Male	White	Non-Hispanic and Non-Latino
A0002	Group A1	21	Female	White	Non-Hispanic and Non-Latino
A0003	Group A1	30	Female	White	Non-Hispanic and Non-Latino
A0004	Group A3	29	Female	White	Non-Hispanic and Non-Latino
A0005	Group A1	29	Male	White	Hispanic or Latino
A0006	Group A3	42	Male	Asian	Non-Hispanic and Non-Latino
A0007	Group A1	22	Female	White	Non-Hispanic and Non-Latino
A0008	Group A3	22	Male	White	Non-Hispanic and Non-Latino
A0009	Group A1	21	Female	Black or African American	Non-Hispanic and Non-Latino
A0010	Group A3	20	Male	Asian	Non-Hispanic and Non-Latino
A0011	Group A3	26	Female	White,American Indian or Alaska Native	Non-Hispanic and Non-Latino
A0012	Group A1	20	Female	White	Non-Hispanic and Non-Latino
A0013	Group A1	18	Female	Asian	Non-Hispanic and Non-Latino
A0014	Group A3	32	Female	White	Non-Hispanic and Non-Latino
A0015	Group A1	19	Female	White	Non-Hispanic and Non-Latino
A0016	Group A3	24	Female	White	Non-Hispanic and Non-Latino
A0017	Group A3	28	Male	White	Non-Hispanic and Non-Latino
A0018	Group A1	28	Female	White	Non-Hispanic and Non-Latino
A0019	Group A3	25	Female	White	Non-Hispanic and Non-Latino
A0020	Group A1	44	Female	White	Non-Hispanic and Non-Latino
A0021	Group A1	22	Male	White	Non-Hispanic and Non-Latino

Key: [C] = Calculated
Note: Age calculated at Screening as age at last birthday in integer years

Administration Site Key: FA = Forearm; UA= Upper arm
Treatments Part A: A1 = A/Sing MAP, 15 ug (FA); A2 = Afluria Quad IM (UA), 15 ug; A3 = Placebo MAP (FA); A4 = A/Sing Mono IM, 15 ug (UA)
Treatments Part B: B1 = A/Sing MAP, 15 ug (FA) with biopsy; B2 = Placebo MAP (FA) with biopsy
B3 = A/Sing MAP, 15 ug (FA); B4 = A/Sing MAP, 10 ug (FA); B5 = A/Sing MAP, 5 ug (FA); B6 = A/Sing MAP, 2.5 ug (FA)
B7 = Placebo MAP (FA); B8 = A/Sing MAP, 15 ug (UA); B9 = A/Sing IM, 15 ug, as 2018 Afluria Quad (UA)

Listing 16.2.4.1
Demographics

Part A

Randomisation Number	Group	Age (yrs) [C]	Gender	Race	Ethnicity
A0022	Group A3	25	Female	Asian	Non-Hispanic and Non-Latino
A0023	Group A3	21	Female	White	Non-Hispanic and Non-Latino
A0024	Group A1	20	Female	White	Non-Hispanic and Non-Latino
A0025	Group A3	38	Female	White	Hispanic or Latino
A0027	Group A3	22	Female	White	Non-Hispanic and Non-Latino
A0028	Group A1	37	Male	White	Hispanic or Latino
A0029	Group A1	37	Female	White	Non-Hispanic and Non-Latino
A0030	Group A3	19	Female	Asian	Non-Hispanic and Non-Latino
A0031	Group A2	23	Male	White	Non-Hispanic and Non-Latino
A0032	Group A4	23	Male	Asian	Non-Hispanic and Non-Latino
A0033	Group A2	21	Female	White	Non-Hispanic and Non-Latino
A0034	Group A4	21	Female	White	Non-Hispanic and Non-Latino
A0035	Group A4	29	Female	White	Non-Hispanic and Non-Latino
A0036	Group A2	20	Female	White	Non-Hispanic and Non-Latino
A0037	Group A4	27	Female	Asian	Non-Hispanic and Non-Latino
A0038	Group A2	27	Male	Asian	Non-Hispanic and Non-Latino
A0039	Group A2	40	Female	White	Hispanic or Latino
A0040	Group A4	19	Female	Asian	Non-Hispanic and Non-Latino
A0041	Group A4	21	Male	White	Non-Hispanic and Non-Latino
A0042	Group A2	42	Male	White	Non-Hispanic and Non-Latino
A0043	Group A2	31	Female	White	Non-Hispanic and Non-Latino

Key: [C] = Calculated
Note: Age calculated at Screening as age at last birthday in integer years

Administration Site Key: FA = Forearm; UA= Upper arm
Treatments Part A: A1 = A/Sing MAP, 15 ug (FA); A2 = Afluria Quad IM (UA), 15 ug; A3 = Placebo MAP (FA); A4 = A/Sing Mono IM, 15 ug (UA)
Treatments Part B: B1 = A/Sing MAP, 15 ug (FA) with biopsy; B2 = Placebo MAP (FA) with biopsy
B3 = A/Sing MAP, 15 ug (FA); B4 = A/Sing MAP, 10 ug (FA); B5 = A/Sing MAP, 5 ug (FA); B6 = A/Sing MAP, 2.5 ug (FA)
B7 = Placebo MAP (FA); B8 = A/Sing MAP, 15 ug (UA); B9 = A/Sing IM, 15 ug, as 2018 Afluria Quad (UA)

Listing 16.2.4.1
Demographics

Part A

Randomisation Number	Group	Age (yrs) [C]	Gender	Race	Ethnicity
A0044	Group A4	22	Female	White	Non-Hispanic and Non-Latino
A0045	Group A2	22	Female	White,Asian	Non-Hispanic and Non-Latino
A0046	Group A4	38	Female	White	Non-Hispanic and Non-Latino
A0047	Group A2	30	Female	White	Non-Hispanic and Non-Latino
A0048	Group A4	23	Female	White	Non-Hispanic and Non-Latino
A0049	Group A2	25	Female	White	Non-Hispanic and Non-Latino
A0050	Group A4	34	Female	White	Hispanic or Latino
A0051	Group A2	20	Male	White	Non-Hispanic and Non-Latino
A0052	Group A4	19	Female	Black or African American	Non-Hispanic and Non-Latino
A0053	Group A4	30	Male	Asian	Non-Hispanic and Non-Latino
A0054	Group A2	20	Male	White	Non-Hispanic and Non-Latino
A0055	Group A4	18	Male	Asian	Non-Hispanic and Non-Latino
A0056	Group A2	28	Female	White	Non-Hispanic and Non-Latino
A0057	Group A4	37	Male	White	Non-Hispanic and Non-Latino
A0058	Group A2	33	Female	White	Non-Hispanic and Non-Latino
A0059	Group A2	22	Female	White	Non-Hispanic and Non-Latino
A0060	Group A4	19	Female	Asian	Non-Hispanic and Non-Latino
A1026	Group A1	32	Female	White	Non-Hispanic and Non-Latino

Key: [C] = Calculated
Note: Age calculated at Screening as age at last birthday in integer years

Administration Site Key: FA = Forearm; UA= Upper arm
Treatments Part A: A1 = A/Sing MAP, 15 ug (FA); A2 = Afluria Quad IM (UA), 15 ug; A3 = Placebo MAP (FA); A4 = A/Sing Mono IM, 15 ug (UA)
Treatments Part B: B1 = A/Sing MAP, 15 ug (FA) with biopsy; B2 = Placebo MAP (FA) with biopsy
B3 = A/Sing MAP, 15 ug (FA); B4 = A/Sing MAP, 10 ug (FA); B5 = A/Sing MAP, 5 ug (FA); B6 = A/Sing MAP, 2.5 ug (FA)
B7 = Placebo MAP (FA); B8 = A/Sing MAP, 15 ug (UA); B9 = A/Sing IM, 15 ug, as 2018 Afluria Quad (UA)

Listing 16.2.4.1
Demographics

Part B

Randomisation Number	Group	Age (yrs) [C]	Gender	Race	Ethnicity
B0001	Group B1	46	Female	White	Non-Hispanic and Non-Latino
B0002	Group B2	37	Male	White	Hispanic or Latino
B0003	Group B2	29	Female	White	Non-Hispanic and Non-Latino
B0004	Group B1	34	Female	White	Hispanic or Latino
B0005	Group B1	37	Female	White	Non-Hispanic and Non-Latino
B0006	Group B2	27	Male	White	Non-Hispanic and Non-Latino
B0007	Group B2	24	Female	White	Non-Hispanic and Non-Latino
B0008	Group B1	50	Male	White	Non-Hispanic and Non-Latino
B0009	Group B1	31	Female	White	Non-Hispanic and Non-Latino
B0010	Group B2	31	Female	White	Non-Hispanic and Non-Latino
B0011	Group B8	44	Female	White	Non-Hispanic and Non-Latino
B0012	Group B6	19	Female	White	Hispanic or Latino
B0013	Group B5	45	Female	White	Non-Hispanic and Non-Latino
B0014	Group B3	19	Female	White	Hispanic or Latino
B0015	Group B9	23	Male	White	Non-Hispanic and Non-Latino
B0016	Group B4	34	Female	Asian	Non-Hispanic and Non-Latino
B0017	Group B7	33	Male	Asian	Non-Hispanic and Non-Latino
B0018	Group B9	24	Female	White	Non-Hispanic and Non-Latino
B0020	Group B6	24	Female	White	Hispanic or Latino
B0021	Group B7	19	Female	White	Non-Hispanic and Non-Latino
B0022	Group B4	28	Female	Asian,Aboriginal/Torres Strait Islander	Non-Hispanic and Non-Latino

Key: [C] = Calculated
Note: Age calculated at Screening as age at last birthday in integer years

Administration Site Key: FA = Forearm; UA= Upper arm
Treatments Part A: A1 = A/Sing MAP, 15 ug (FA); A2 = Afluria Quad IM (UA), 15 ug; A3 = Placebo MAP (FA); A4 = A/Sing Mono IM, 15 ug (UA)
Treatments Part B: B1 = A/Sing MAP, 15 ug (FA) with biopsy; B2 = Placebo MAP (FA) with biopsy
B3 = A/Sing MAP, 15 ug (FA); B4 = A/Sing MAP, 10 ug (FA); B5 = A/Sing MAP, 5 ug (FA); B6 = A/Sing MAP, 2.5 ug (FA)
B7 = Placebo MAP (FA); B8 = A/Sing MAP, 15 ug (UA); B9 = A/Sing IM, 15 ug, as 2018 Afluria Quad (UA)

Listing 16.2.4.1
Demographics

Part B

Randomisation Number	Group	Age (yrs) [C]	Gender	Race	Ethnicity
B0023	Group B3	18	Female	White	Non-Hispanic and Non-Latino
B0024	Group B5	22	Female	Asian	Non-Hispanic and Non-Latino
B0025	Group B8	23	Female	White	Non-Hispanic and Non-Latino
B0026	Group B9	27	Female	Other: mixed race	Hispanic or Latino
B0027	Group B3	31	Female	White	Non-Hispanic and Non-Latino
B0028	Group B5	23	Female	White	Non-Hispanic and Non-Latino
B0029	Group B6	23	Male	White,Asian	Hispanic or Latino
B0030	Group B7	27	Female	Asian	Non-Hispanic and Non-Latino
B0031	Group B4	21	Male	Asian	Non-Hispanic and Non-Latino
B0032	Group B6	34	Male	White	Non-Hispanic and Non-Latino
B0033	Group B5	21	Female	Asian	Non-Hispanic and Non-Latino
B0034	Group B8	37	Female	Other: mixed race	Non-Hispanic and Non-Latino
B0035	Group B9	28	Female	Asian	Non-Hispanic and Non-Latino
B0036	Group B7	25	Female	White	Non-Hispanic and Non-Latino
B0037	Group B4	40	Female	White	Non-Hispanic and Non-Latino
B0038	Group B3	37	Female	White	Non-Hispanic and Non-Latino
B0039	Group B7	18	Female	Asian	Non-Hispanic and Non-Latino
B0040	Group B9	26	Female	White	Non-Hispanic and Non-Latino
B0041	Group B3	21	Male	Asian	Non-Hispanic and Non-Latino
B0042	Group B5	26	Female	White	Non-Hispanic and Non-Latino
B0043	Group B8	20	Female	White	Non-Hispanic and Non-Latino

Key: [C] = Calculated
Note: Age calculated at Screening as age at last birthday in integer years

Administration Site Key: FA = Forearm; UA= Upper arm
Treatments Part A: A1 = A/Sing MAP, 15 ug (FA); A2 = Afluria Quad IM (UA), 15 ug; A3 = Placebo MAP (FA); A4 = A/Sing Mono IM, 15 ug (UA)
Treatments Part B: B1 = A/Sing MAP, 15 ug (FA) with biopsy; B2 = Placebo MAP (FA) with biopsy
B3 = A/Sing MAP, 15 ug (FA); B4 = A/Sing MAP, 10 ug (FA); B5 = A/Sing MAP, 5 ug (FA); B6 = A/Sing MAP, 2.5 ug (FA)
B7 = Placebo MAP (FA); B8 = A/Sing MAP, 15 ug (UA); B9 = A/Sing IM, 15 ug, as 2018 Afluria Quad (UA)

Listing 16.2.4.1
Demographics

Part B

Randomisation Number	Group	Age (yrs) [C]	Gender	Race	Ethnicity
B0044	Group B6	31	Female	White	Non-Hispanic and Non-Latino
B0045	Group B4	22	Female	White	Non-Hispanic and Non-Latino
B0046	Group B7	22	Male	White	Non-Hispanic and Non-Latino
B0047	Group B9	28	Male	White	Non-Hispanic and Non-Latino
B0048	Group B6	22	Female	White	Non-Hispanic and Non-Latino
B0049	Group B4	20	Female	Asian	Non-Hispanic and Non-Latino
B0050	Group B8	28	Male	Asian,Native Hawaiian or Other Pacific Islander	Non-Hispanic and Non-Latino
B0051	Group B3	37	Female	White	Hispanic or Latino
B0052	Group B5	27	Female	White	Non-Hispanic and Non-Latino
B0053	Group B6	28	Female	Asian	Non-Hispanic and Non-Latino
B0054	Group B5	20	Female	Asian	Non-Hispanic and Non-Latino
B0055	Group B8	19	Female	White,Black or African American,Asian	Non-Hispanic and Non-Latino
B0056	Group B3	22	Male	White	Non-Hispanic and Non-Latino
B0057	Group B4	35	Female	Asian	Non-Hispanic and Non-Latino
B0058	Group B9	19	Female	Asian	Non-Hispanic and Non-Latino
B0059	Group B7	20	Female	White	Non-Hispanic and Non-Latino
B0060	Group B3	18	Male	White	Non-Hispanic and Non-Latino
B0061	Group B8	23	Female	White	Non-Hispanic and Non-Latino
B0062	Group B5	30	Male	White	Hispanic or Latino
B0063	Group B7	20	Female	White	Non-Hispanic and Non-Latino
B0064	Group B9	23	Male	Asian	Non-Hispanic and Non-Latino

Key: [C] = Calculated
Note: Age calculated at Screening as age at last birthday in integer years

Administration Site Key: FA = Forearm; UA= Upper arm
Treatments Part A: A1 = A/Sing MAP, 15 ug (FA); A2 = Afluria Quad IM (UA), 15 ug; A3 = Placebo MAP (FA); A4 = A/Sing Mono IM, 15 ug (UA)
Treatments Part B: B1 = A/Sing MAP, 15 ug (FA) with biopsy; B2 = Placebo MAP (FA) with biopsy
B3 = A/Sing MAP, 15 ug (FA); B4 = A/Sing MAP, 10 ug (FA); B5 = A/Sing MAP, 5 ug (FA); B6 = A/Sing MAP, 2.5 ug (FA)
B7 = Placebo MAP (FA); B8 = A/Sing MAP, 15 ug (UA); B9 = A/Sing IM, 15 ug, as 2018 Afluria Quad (UA)

Listing 16.2.4.1
Demographics

Part B

Randomisation Number	Group	Age (yrs) [C]	Gender	Race	Ethnicity
B0065	Group B4	20	Female	Asian	Non-Hispanic and Non-Latino
B0066	Group B6	23	Male	White	Non-Hispanic and Non-Latino
B0067	Group B8	30	Female	White,Asian	Non-Hispanic and Non-Latino
B0068	Group B5	21	Female	White	Non-Hispanic and Non-Latino
B0069	Group B7	30	Male	White	Hispanic or Latino
B0070	Group B9	26	Female	White	Non-Hispanic and Non-Latino
B0071	Group B4	18	Female	White	Non-Hispanic and Non-Latino
B0072	Group B3	29	Female	White	Non-Hispanic and Non-Latino
B0073	Group B6	22	Male	Asian	Non-Hispanic and Non-Latino
B0074	Group B9	20	Female	White	Non-Hispanic and Non-Latino
B0075	Group B3	22	Female	White	Non-Hispanic and Non-Latino
B0077	Group B4	30	Female	White	Hispanic or Latino
B0078	Group B8	31	Female	Asian	Non-Hispanic and Non-Latino
B0079	Group B6	21	Female	White	Non-Hispanic and Non-Latino
B0081	Group B6	18	Female	White	Non-Hispanic and Non-Latino
B0082	Group B3	26	Male	Asian	Non-Hispanic and Non-Latino
B0083	Group B8	21	Male	Asian	Non-Hispanic and Non-Latino
B0084	Group B5	28	Male	White	Non-Hispanic and Non-Latino
B0085	Group B4	32	Male	White	Non-Hispanic and Non-Latino
B0086	Group B9	23	Male	Asian	Non-Hispanic and Non-Latino
B0087	Group B7	31	Male	White	Hispanic or Latino

Key: [C] = Calculated

Note: Age calculated at Screening as age at last birthday in integer years

Administration Site Key: FA = Forearm; UA= Upper arm

Treatments Part A: A1 = A/Sing MAP, 15 ug (FA); A2 = Afluria Quad IM (UA), 15 ug; A3 = Placebo MAP (FA); A4 = A/Sing Mono IM, 15 ug (UA)

Treatments Part B: B1 = A/Sing MAP, 15 ug (FA) with biopsy; B2 = Placebo MAP (FA) with biopsy
B3 = A/Sing MAP, 15 ug (FA); B4 = A/Sing MAP, 10 ug (FA); B5 = A/Sing MAP, 5 ug (FA); B6 = A/Sing MAP, 2.5 ug (FA)
B7 = Placebo MAP (FA); B8 = A/Sing MAP, 15 ug (UA); B9 = A/Sing IM, 15 ug, as 2018 Afluria Quad (UA)

Listing 16.2.4.1
Demographics

Part B

Randomisation Number	Group	Age (yrs) [C]	Gender	Race	Ethnicity
B0088	Group B9	19	Male	White	Hispanic or Latino
B0089	Group B5	29	Male	Asian	Non-Hispanic and Non-Latino
B0090	Group B6	22	Female	White	Non-Hispanic and Non-Latino
B0091	Group B8	27	Female	White	Hispanic or Latino
B0092	Group B4	28	Female	White	Hispanic or Latino
B0093	Group B7	29	Female	White	Non-Hispanic and Non-Latino
B0094	Group B3	38	Male	White	Hispanic or Latino
B0095	Group B9	32	Female	Asian	Non-Hispanic and Non-Latino
B0096	Group B6	21	Female	Asian	Non-Hispanic and Non-Latino
B0097	Group B5	38	Female	Asian	Non-Hispanic and Non-Latino
B0098	Group B7	19	Female	White	Non-Hispanic and Non-Latino
B0099	Group B4	30	Female	White	Non-Hispanic and Non-Latino
B0100	Group B8	23	Male	Asian	Non-Hispanic and Non-Latino
B0101	Group B3	43	Female	White	Non-Hispanic and Non-Latino
B0102	Group B8	19	Male	Asian	Non-Hispanic and Non-Latino
B0103	Group B5	32	Female	Asian	Non-Hispanic and Non-Latino
B0104	Group B3	28	Female	White	Hispanic or Latino
B0105	Group B9	25	Female	White	Hispanic or Latino
B0106	Group B4	22	Female	White	Non-Hispanic and Non-Latino
B0107	Group B6	32	Female	Asian	Non-Hispanic and Non-Latino
B0108	Group B7	27	Male	White	Non-Hispanic and Non-Latino

Key: [C] = Calculated
Note: Age calculated at Screening as age at last birthday in integer years

Administration Site Key: FA = Forearm; UA= Upper arm
Treatments Part A: A1 = A/Sing MAP, 15 ug (FA); A2 = Afluria Quad IM (UA), 15 ug; A3 = Placebo MAP (FA); A4 = A/Sing Mono IM, 15 ug (UA)
Treatments Part B: B1 = A/Sing MAP, 15 ug (FA) with biopsy; B2 = Placebo MAP (FA) with biopsy
B3 = A/Sing MAP, 15 ug (FA); B4 = A/Sing MAP, 10 ug (FA); B5 = A/Sing MAP, 5 ug (FA); B6 = A/Sing MAP, 2.5 ug (FA)
B7 = Placebo MAP (FA); B8 = A/Sing MAP, 15 ug (UA); B9 = A/Sing IM, 15 ug, as 2018 Afluria Quad (UA)

Listing 16.2.4.1
Demographics

Part B

Randomisation Number	Group	Age (yrs) [C]	Gender	Race	Ethnicity
B0109	Group B6	25	Male	Asian	Non-Hispanic and Non-Latino
B0110	Group B3	24	Male	White	Non-Hispanic and Non-Latino
B0111	Group B8	48	Male	White	Non-Hispanic and Non-Latino
B0112	Group B4	22	Male	Asian	Non-Hispanic and Non-Latino
B0113	Group B5	22	Male	Asian	Non-Hispanic and Non-Latino
B0114	Group B7	43	Female	White	Non-Hispanic and Non-Latino
B0115	Group B9	25	Female	White	Non-Hispanic and Non-Latino
B0116	Group B7	22	Female	White	Non-Hispanic and Non-Latino
B0117	Group B9	22	Female	Asian	Non-Hispanic and Non-Latino
B0118	Group B6	18	Female	White	Non-Hispanic and Non-Latino
B0119	Group B4	20	Female	White,Asian	Hispanic or Latino
B0120	Group B3	24	Female	White	Non-Hispanic and Non-Latino
B0121	Group B8	48	Female	White,Aboriginal/Torres Strait Islander	Non-Hispanic and Non-Latino
B0122	Group B5	34	Female	White	Non-Hispanic and Non-Latino
B0123	Group B6	19	Female	White	Non-Hispanic and Non-Latino
B0124	Group B4	30	Female	White	Non-Hispanic and Non-Latino
B0125	Group B7	21	Female	White	Non-Hispanic and Non-Latino
B0126	Group B3	32	Male	Asian	Hispanic or Latino
B0127	Group B9	21	Female	White	Non-Hispanic and Non-Latino
B0128	Group B5	25	Male	White	Non-Hispanic and Non-Latino
B0129	Group B8	19	Male	White	Non-Hispanic and Non-Latino

Key: [C] = Calculated
Note: Age calculated at Screening as age at last birthday in integer years

Administration Site Key: FA = Forearm; UA= Upper arm
Treatments Part A: A1 = A/Sing MAP, 15 ug (FA); A2 = Afluria Quad IM (UA), 15 ug; A3 = Placebo MAP (FA); A4 = A/Sing Mono IM, 15 ug (UA)
Treatments Part B: B1 = A/Sing MAP, 15 ug (FA) with biopsy; B2 = Placebo MAP (FA) with biopsy
B3 = A/Sing MAP, 15 ug (FA); B4 = A/Sing MAP, 10 ug (FA); B5 = A/Sing MAP, 5 ug (FA); B6 = A/Sing MAP, 2.5 ug (FA)
B7 = Placebo MAP (FA); B8 = A/Sing MAP, 15 ug (UA); B9 = A/Sing IM, 15 ug, as 2018 Afluria Quad (UA)

Listing 16.2.4.1
Demographics

Part B

Randomisation Number	Group	Age (yrs) [C]	Gender	Race	Ethnicity
B0130	Group B5	19	Male	White	Non-Hispanic and Non-Latino
B0131	Group B8	22	Male	White	Hispanic or Latino
B0132	Group B6	30	Female	White	Non-Hispanic and Non-Latino
B0133	Group B4	26	Female	White	Non-Hispanic and Non-Latino
B0134	Group B3	21	Female	White	Non-Hispanic and Non-Latino
B0135	Group B7	25	Female	Asian	Non-Hispanic and Non-Latino
B0136	Group B9	23	Female	White	Non-Hispanic and Non-Latino
B0137	Group B8	21	Female	White	Non-Hispanic and Non-Latino
B0138	Group B7	23	Female	Asian	Non-Hispanic and Non-Latino
B0139	Group B4	24	Female	Asian	Non-Hispanic and Non-Latino
B0140	Group B9	26	Female	White	Non-Hispanic and Non-Latino
B0141	Group B3	33	Female	White	Non-Hispanic and Non-Latino
B0142	Group B5	25	Male	White	Non-Hispanic and Non-Latino
B0143	Group B6	36	Female	White	Non-Hispanic and Non-Latino
B0144	Group B6	25	Male	White	Non-Hispanic and Non-Latino
B0145	Group B3	24	Female	White	Non-Hispanic and Non-Latino
B0146	Group B7	42	Male	White	Non-Hispanic and Non-Latino
B0147	Group B9	22	Male	White	Non-Hispanic and Non-Latino
B0148	Group B5	21	Male	Asian	Non-Hispanic and Non-Latino
B0149	Group B8	19	Male	White	Non-Hispanic and Non-Latino
B0150	Group B4	20	Male	White	Non-Hispanic and Non-Latino

Key: [C] = Calculated
Note: Age calculated at Screening as age at last birthday in integer years

Administration Site Key: FA = Forearm; UA= Upper arm
Treatments Part A: A1 = A/Sing MAP, 15 ug (FA); A2 = Afluria Quad IM (UA), 15 ug; A3 = Placebo MAP (FA); A4 = A/Sing Mono IM, 15 ug (UA)
Treatments Part B: B1 = A/Sing MAP, 15 ug (FA) with biopsy; B2 = Placebo MAP (FA) with biopsy
B3 = A/Sing MAP, 15 ug (FA); B4 = A/Sing MAP, 10 ug (FA); B5 = A/Sing MAP, 5 ug (FA); B6 = A/Sing MAP, 2.5 ug (FA)
B7 = Placebo MAP (FA); B8 = A/Sing MAP, 15 ug (UA); B9 = A/Sing IM, 15 ug, as 2018 Afluria Quad (UA)

Listing 16.2.4.1
Demographics

Part B

Randomisation Number	Group	Age (yrs) [C]	Gender	Race	Ethnicity
B1019	Group B8	25	Male	Black or African American	Non-Hispanic and Non-Latino
B1076	Group B5	21	Male	White	Non-Hispanic and Non-Latino
B1080	Group B7	27	Male	White	Non-Hispanic and Non-Latino

Key: [C] = Calculated
Note: Age calculated at Screening as age at last birthday in integer years

Administration Site Key: FA = Forearm; UA= Upper arm
Treatments Part A: A1 = A/Sing MAP, 15 ug (FA); A2 = Afluria Quad IM (UA), 15 ug; A3 = Placebo MAP (FA); A4 = A/Sing Mono IM, 15 ug (UA)
Treatments Part B: B1 = A/Sing MAP, 15 ug (FA) with biopsy; B2 = Placebo MAP (FA) with biopsy
B3 = A/Sing MAP, 15 ug (FA); B4 = A/Sing MAP, 10 ug (FA); B5 = A/Sing MAP, 5 ug (FA); B6 = A/Sing MAP, 2.5 ug (FA)
B7 = Placebo MAP (FA); B8 = A/Sing MAP, 15 ug (UA); B9 = A/Sing IM, 15 ug, as 2018 Afluria Quad (UA)
