# Supplementary material for: Safety, tolerability, and immunogenicity of influenza vaccination with a high-density microarray patch: Results from a randomized, controlled phase I clinical trial
Source: PLoS Med. 2020 Mar 17;17(3):e1003024. doi: 10.1371/journal.pmed.1003024 (PMC7077342; doi:10.1371/journal.pmed.1003024)
Supplement: S1 Data — Data 1: Informed Consent; Data 2: Analysis Sets; Data 3: Demographics; Data 4: Study Drug Administration; Data 5: Skin Hardness Assessment; Data 6: Immunogenicity (ELISA IgG); Data 7: Immunogenicity (ADCC); Data 8: Immunogenicity (MBCs); Data 9: Immunogenicity (CMI); Data 10: Immunogenicity (Mucosal IgA); Data 11: Immunogenicity Results (HAI, MN); Data 12: TEAE–CRF Data only; Data 13: TEAEs–MedDRA Coding; Data 15: Treatment Site Tolerability Assessment; Data 16: Numeric Pain Intensity; Data 17: SII; Data 18: Application Site Status at End of Study; Data 19: Individual Treatment Site Tolerability Assessment; Data 20: Numeric Pain Intensity by Treatment; Data 21: Individual SII by Treatment. ADCC, antibody-dependent cellular cytotoxicity; CMI, cell-mediated immunity; CRF, case report form; ELISA, enzyme-linked immunosorbent assay; HAI, haemagglutination inhibition; IgA, immunoglobulin A; IgG, immunoglobulin G; MBC, memory B cell; MedDRA, medical dictionary for regulatory activities; MN, microneutralisation; SII, Skin Irritation Index; TEA, treatment emergent adverse event. (ZIP) [file pmed.1003024.s008.zip › S1_Data/clinical supporting/S1_Data4 Study Drug Administration.pdf]

Listing 16.2.5.1
Study Drug Administration

Part A

Randomi-sation Number	Group	Treatment	Study Day	Date of Dose	Time of MAP application	Time of MAP removal	Time of IM Injection	Was Dose Adminis-tered?	Dosed as per Protocol	Administra-tion Site	Site Number	Dose Administered at Site
A0001	Group A3	Placebo MAP	Day 1	09-MAR-2018	12:00	12:02		Yes	Yes	Forearm	F1	uncoated
A0001	Group A3	Placebo MAP	Day 1	09-MAR-2018	12:03	12:05		Yes	Yes	Forearm	F2	uncoated
A0001	Group A3	Placebo MAP	Day 1	09-MAR-2018	12:06	12:08		Yes	Yes	Forearm	F3	uncoated
A0002	Group A1	A/Sing MAP, 15 ug	Day 1	09-MAR-2018	12:11	12:13		Yes	Yes	Forearm	F1	5 ug
A0002	Group A1	A/Sing MAP, 15 ug	Day 1	09-MAR-2018	12:14	12:16		Yes	Yes	Forearm	F2	5 ug
A0002	Group A1	A/Sing MAP, 15 ug	Day 1	09-MAR-2018	12:17	12:19		Yes	Yes	Forearm	F3	5 ug
A0003	Group A1	A/Sing MAP, 15 ug	Day 1	09-MAR-2018	12:20	12:22		Yes	Yes	Forearm	F1	5 ug
A0003	Group A1	A/Sing MAP, 15 ug	Day 1	09-MAR-2018	12:23	12:25		Yes	Yes	Forearm	F2	5 ug
A0003	Group A1	A/Sing MAP, 15 ug	Day 1	09-MAR-2018	12:26	12:28		Yes	Yes	Forearm	F3	5 ug
A0004	Group A3	Placebo MAP	Day 1	09-MAR-2018	12:30	12:32		Yes	Yes	Forearm	F1	uncoated
A0004	Group A3	Placebo MAP	Day 1	09-MAR-2018	12:33	12:35		Yes	Yes	Forearm	F2	uncoated
A0004	Group A3	Placebo MAP	Day 1	09-MAR-2018	12:36	12:38		Yes	Yes	Forearm	F3	uncoated
A0005	Group A1	A/Sing MAP, 15 ug	Day 1	09-MAR-2018	12:50	12:52		Yes	Yes	Forearm	F1	5 ug
A0005	Group A1	A/Sing MAP, 15 ug	Day 1	09-MAR-2018	12:53	12:55		Yes	Yes	Forearm	F2	5 ug

Note: Delivered Dose=(Labelled Dose)/2 for MAP

Administration Site Key: FA = Forearm; UA= Upper arm
Treatments Part A: A1 = A/Sing MAP, 15 ug (FA); A2 = Afluria Quad IM (UA), 15 ug; A3 = Placebo MAP (FA); A4 = A/Sing Mono IM, 15 ug (UA)
Treatments Part B: B1 = A/Sing MAP, 15 ug (FA) with biopsy; B2 = Placebo MAP (FA) with biopsy
B3 = A/Sing MAP, 15 ug (FA); B4 = A/Sing MAP, 10 ug (FA); B5 = A/Sing MAP, 5 ug (FA); B6 = A/Sing MAP, 2.5 ug (FA)
B7 = Placebo MAP (FA); B8 = A/Sing MAP, 15 ug (UA); B9 = A/Sing IM, 15 ug, as 2018 Afluria Quad (UA)

Listing 16.2.5.1
Study Drug Administration

Part A

Randomi-sation Number	Group	Treatment	Study Day	Date of Dose	Time of MAP application	Time of MAP removal	Time of IM Injection	Was Dose Adminis-tered?	Dosed as per Protocol	Administra-tion Site	Site Number	Dose Administered at Site
A0005	Group A1	A/Sing MAP, 15 ug	Day 1	09-MAR-2018	12:56	12:58		Yes	Yes	Forearm	F3	5 ug
A0006	Group A3	Placebo MAP	Day 1	09-MAR-2018	12:40	12:42		Yes	No	Forearm	F1	uncoated
A0006	Group A3	Placebo MAP	Day 1	09-MAR-2018	12:43	12:45		Yes	No	Forearm	F2	uncoated
A0006	Group A3	Placebo MAP	Day 1	09-MAR-2018	12:46	12:47		Yes	No	Forearm	F3	uncoated
A0007	Group A1	A/Sing MAP, 15 ug	Day 1	09-MAR-2018	13:00	13:02		Yes	Yes	Forearm	F1	5 ug
A0007	Group A1	A/Sing MAP, 15 ug	Day 1	09-MAR-2018	13:03	13:05		Yes	Yes	Forearm	F2	5 ug
A0007	Group A1	A/Sing MAP, 15 ug	Day 1	09-MAR-2018	13:06	13:08		Yes	Yes	Forearm	F3	5 ug
A0008	Group A3	Placebo MAP	Day 1	09-MAR-2018	13:20	13:22		Yes	Yes	Forearm	F1	uncoated
A0008	Group A3	Placebo MAP	Day 1	09-MAR-2018	13:23	13:25		Yes	Yes	Forearm	F2	uncoated
A0008	Group A3	Placebo MAP	Day 1	09-MAR-2018	13:26	13:28		Yes	Yes	Forearm	F3	uncoated
A0009	Group A1	A/Sing MAP, 15 ug	Day 1	09-MAR-2018	13:10	13:12		Yes	Yes	Forearm	F1	5 ug
A0009	Group A1	A/Sing MAP, 15 ug	Day 1	09-MAR-2018	13:13	13:15		Yes	Yes	Forearm	F2	5 ug
A0009	Group A1	A/Sing MAP, 15 ug	Day 1	09-MAR-2018	13:16	13:18		Yes	Yes	Forearm	F3	5 ug

Note: Delivered Dose=(Labelled Dose)/2 for MAP

Administration Site Key: FA = Forearm; UA= Upper arm
Treatments Part A: A1 = A/Sing MAP, 15 ug (FA); A2 = Afluria Quad IM (UA), 15 ug; A3 = Placebo MAP (FA); A4 = A/Sing Mono IM, 15 ug (UA)
Treatments Part B: B1 = A/Sing MAP, 15 ug (FA) with biopsy; B2 = Placebo MAP (FA) with biopsy
B3 = A/Sing MAP, 15 ug (FA); B4 = A/Sing MAP, 10 ug (FA); B5 = A/Sing MAP, 5 ug (FA); B6 = A/Sing MAP, 2.5 ug (FA)
B7 = Placebo MAP (FA); B8 = A/Sing MAP, 15 ug (UA); B9 = A/Sing IM, 15 ug, as 2018 Afluria Quad (UA)

Listing 16.2.5.1
Study Drug Administration

Part A

Randomi-sation Number	Group	Treatment	Study Day	Date of Dose	Time of MAP application	Time of MAP removal	Time of IM Injection	Was Dose Adminis-tered?	Dosed as per Protocol	Administra-tion Site	Site Number	Dose Administered at Site
A0010	Group A3	Placebo MAP	Day 1	09-MAR-2018	13:30	13:32		Yes	Yes	Forearm	F1	uncoated
A0010	Group A3	Placebo MAP	Day 1	09-MAR-2018	13:33	13:35		Yes	Yes	Forearm	F2	uncoated
A0010	Group A3	Placebo MAP	Day 1	09-MAR-2018	13:36	13:38		Yes	Yes	Forearm	F3	uncoated
A0011	Group A3	Placebo MAP	Day 1	09-MAR-2018	13:40	13:42		Yes	Yes	Forearm	F1	uncoated
A0011	Group A3	Placebo MAP	Day 1	09-MAR-2018	13:43	13:45		Yes	Yes	Forearm	F2	uncoated
A0011	Group A3	Placebo MAP	Day 1	09-MAR-2018	13:46	13:48		Yes	Yes	Forearm	F3	uncoated
A0012	Group A1	A/Sing MAP, 15 ug	Day 1	09-MAR-2018	13:50	13:52		Yes	Yes	Forearm	F1	5 ug
A0012	Group A1	A/Sing MAP, 15 ug	Day 1	09-MAR-2018	13:53	13:55		Yes	Yes	Forearm	F2	5 ug
A0012	Group A1	A/Sing MAP, 15 ug	Day 1	09-MAR-2018	13:56	13:58		Yes	Yes	Forearm	F3	5 ug
A0013	Group A1	A/Sing MAP, 15 ug	Day 1	09-MAR-2018	14:00	14:02		Yes	Yes	Forearm	F1	5 ug
A0013	Group A1	A/Sing MAP, 15 ug	Day 1	09-MAR-2018	14:03	14:05		Yes	Yes	Forearm	F2	5 ug
A0013	Group A1	A/Sing MAP, 15 ug	Day 1	09-MAR-2018	14:06	14:08		Yes	Yes	Forearm	F3	5 ug
A0014	Group A3	Placebo MAP	Day 1	09-MAR-2018	14:10	14:12		Yes	Yes	Forearm	F1	uncoated
A0014	Group A3	Placebo MAP	Day 1	09-MAR-2018	14:13	14:15		Yes	Yes	Forearm	F2	uncoated

Note: Delivered Dose=(Labelled Dose)/2 for MAP

Administration Site Key: FA = Forearm; UA= Upper arm
Treatments Part A: A1 = A/Sing MAP, 15 ug (FA); A2 = Afluria Quad IM (UA), 15 ug; A3 = Placebo MAP (FA); A4 = A/Sing Mono IM, 15 ug (UA)
Treatments Part B: B1 = A/Sing MAP, 15 ug (FA) with biopsy; B2 = Placebo MAP (FA) with biopsy
B3 = A/Sing MAP, 15 ug (FA); B4 = A/Sing MAP, 10 ug (FA); B5 = A/Sing MAP, 5 ug (FA); B6 = A/Sing MAP, 2.5 ug (FA)
B7 = Placebo MAP (FA); B8 = A/Sing MAP, 15 ug (UA); B9 = A/Sing IM, 15 ug, as 2018 Afluria Quad (UA)

Listing 16.2.5.1
Study Drug Administration

Part A

Randomi-sation Number	Group	Treatment	Study Day	Date of Dose	Time of MAP application	Time of MAP removal	Time of IM Injection	Was Dose Adminis-tered?	Dosed as per Protocol	Administra-tion Site	Site Number	Dose Administered at Site
A0014	Group A3	Placebo MAP	Day 1	09-MAR-2018	14:16	14:18		Yes	Yes	Forearm	F3	uncoated
A0015	Group A1	A/Sing MAP, 15 ug	Day 1	09-MAR-2018	14:19	14:21		Yes	Yes	Forearm	F1	5 ug
A0015	Group A1	A/Sing MAP, 15 ug	Day 1	09-MAR-2018	14:23	14:25		Yes	Yes	Forearm	F2	5 ug
A0015	Group A1	A/Sing MAP, 15 ug	Day 1	09-MAR-2018	14:26	14:28		Yes	Yes	Forearm	F3	5 ug
A0016	Group A3	Placebo MAP	Day 1	10-MAR-2018	11:00	11:02		Yes	Yes	Forearm	F1	uncoated
A0016	Group A3	Placebo MAP	Day 1	10-MAR-2018	11:03	11:05		Yes	Yes	Forearm	F2	uncoated
A0016	Group A3	Placebo MAP	Day 1	10-MAR-2018	11:06	11:08		Yes	Yes	Forearm	F3	uncoated
A0017	Group A3	Placebo MAP	Day 1	10-MAR-2018	11:10	11:12		Yes	Yes	Forearm	F1	uncoated
A0017	Group A3	Placebo MAP	Day 1	10-MAR-2018	11:13	11:15		Yes	Yes	Forearm	F2	uncoated
A0017	Group A3	Placebo MAP	Day 1	10-MAR-2018	11:16	11:18		Yes	Yes	Forearm	F3	uncoated
A0018	Group A1	A/Sing MAP, 15 ug	Day 1	10-MAR-2018	11:20	11:22		Yes	Yes	Forearm	F1	5 ug
A0018	Group A1	A/Sing MAP, 15 ug	Day 1	10-MAR-2018	11:23	11:25		Yes	Yes	Forearm	F2	5 ug
A0018	Group A1	A/Sing MAP, 15 ug	Day 1	10-MAR-2018	11:26	11:28		Yes	Yes	Forearm	F3	5 ug

Note: Delivered Dose=(Labelled Dose)/2 for MAP

Administration Site Key: FA = Forearm; UA= Upper arm
Treatments Part A: A1 = A/Sing MAP, 15 ug (FA); A2 = Afluria Quad IM (UA), 15 ug; A3 = Placebo MAP (FA); A4 = A/Sing Mono IM, 15 ug (UA)
Treatments Part B: B1 = A/Sing MAP, 15 ug (FA) with biopsy; B2 = Placebo MAP (FA) with biopsy
B3 = A/Sing MAP, 15 ug (FA); B4 = A/Sing MAP, 10 ug (FA); B5 = A/Sing MAP, 5 ug (FA); B6 = A/Sing MAP, 2.5 ug (FA)
B7 = Placebo MAP (FA); B8 = A/Sing MAP, 15 ug (UA); B9 = A/Sing IM, 15 ug, as 2018 Afluria Quad (UA)

Listing 16.2.5.1
Study Drug Administration

Part A

Randomi-sation Number	Group	Treatment	Study Day	Date of Dose	Time of MAP application	Time of MAP removal	Time of IM Injection	Was Dose Adminis-tered?	Dosed as per Protocol	Administra-tion Site	Site Number	Dose Administered at Site
A0019	Group A3	Placebo MAP	Day 1	10-MAR-2018	11:30	11:32		Yes	Yes	Forearm	F1	uncoated
A0019	Group A3	Placebo MAP	Day 1	10-MAR-2018	11:33	11:35		Yes	Yes	Forearm	F2	uncoated
A0019	Group A3	Placebo MAP	Day 1	10-MAR-2018	11:36	11:38		Yes	Yes	Forearm	F3	uncoated
A0020	Group A1	A/Sing MAP, 15 ug	Day 1	10-MAR-2018	11:40	11:42		Yes	Yes	Forearm	F1	5 ug
A0020	Group A1	A/Sing MAP, 15 ug	Day 1	10-MAR-2018	11:43	11:45		Yes	Yes	Forearm	F2	5 ug
A0020	Group A1	A/Sing MAP, 15 ug	Day 1	10-MAR-2018	11:46	11:48		Yes	Yes	Forearm	F3	5 ug
A0021	Group A1	A/Sing MAP, 15 ug	Day 1	10-MAR-2018	12:10	12:12		Yes	Yes	Forearm	F1	5 ug
A0021	Group A1	A/Sing MAP, 15 ug	Day 1	10-MAR-2018	12:13	12:15		Yes	Yes	Forearm	F2	5 ug
A0021	Group A1	A/Sing MAP, 15 ug	Day 1	10-MAR-2018	12:16	12:18		Yes	Yes	Forearm	F3	5 ug
A0022	Group A3	Placebo MAP	Day 1	10-MAR-2018	11:50	11:52		Yes	Yes	Forearm	F1	uncoated
A0022	Group A3	Placebo MAP	Day 1	10-MAR-2018	11:53	11:55		Yes	Yes	Forearm	F2	uncoated
A0022	Group A3	Placebo MAP	Day 1	10-MAR-2018	11:56	11:58		Yes	Yes	Forearm	F3	uncoated
A0023	Group A3	Placebo MAP	Day 1	10-MAR-2018	12:00	12:02		Yes	Yes	Forearm	F1	uncoated
A0023	Group A3	Placebo MAP	Day 1	10-MAR-2018	12:03	12:05		Yes	Yes	Forearm	F2	uncoated

Note: Delivered Dose=(Labelled Dose)/2 for MAP

Administration Site Key: FA = Forearm; UA= Upper arm
Treatments Part A: A1 = A/Sing MAP, 15 ug (FA); A2 = Afluria Quad IM (UA), 15 ug; A3 = Placebo MAP (FA); A4 = A/Sing Mono IM, 15 ug (UA)
Treatments Part B: B1 = A/Sing MAP, 15 ug (FA) with biopsy; B2 = Placebo MAP (FA) with biopsy
B3 = A/Sing MAP, 15 ug (FA); B4 = A/Sing MAP, 10 ug (FA); B5 = A/Sing MAP, 5 ug (FA); B6 = A/Sing MAP, 2.5 ug (FA)
B7 = Placebo MAP (FA); B8 = A/Sing MAP, 15 ug (UA); B9 = A/Sing IM, 15 ug, as 2018 Afluria Quad (UA)

Listing 16.2.5.1
Study Drug Administration

Part A

Randomi-sation Number	Group	Treatment	Study Day	Date of Dose	Time of MAP application	Time of MAP removal	Time of IM Injection	Was Dose Adminis-tered?	Dosed as per Protocol	Administra-tion Site	Site Number	Dose Administered at Site
A0023	Group A3	Placebo MAP	Day 1	10-MAR-2018	12:06	12:08		Yes	Yes	Forearm	F3	uncoated
A0024	Group A1	A/Sing MAP, 15 ug	Day 1	10-MAR-2018	12:20	12:22		Yes	Yes	Forearm	F1	5 ug
A0024	Group A1	A/Sing MAP, 15 ug	Day 1	10-MAR-2018	12:23	12:25		Yes	Yes	Forearm	F2	5 ug
A0024	Group A1	A/Sing MAP, 15 ug	Day 1	10-MAR-2018	12:26	12:28		Yes	Yes	Forearm	F3	5 ug
A0025	Group A3	Placebo MAP	Day 1	10-MAR-2018	12:30	12:32		Yes	Yes	Forearm	F1	uncoated
A0025	Group A3	Placebo MAP	Day 1	10-MAR-2018	12:33	12:35		Yes	Yes	Forearm	F2	uncoated
A0025	Group A3	Placebo MAP	Day 1	10-MAR-2018	12:36	12:38		Yes	Yes	Forearm	F3	uncoated
A0027	Group A3	Placebo MAP	Day 1	10-MAR-2018	12:40	12:42		Yes	Yes	Forearm	F1	uncoated
A0027	Group A3	Placebo MAP	Day 1	10-MAR-2018	12:43	12:45		Yes	Yes	Forearm	F2	uncoated
A0027	Group A3	Placebo MAP	Day 1	10-MAR-2018	12:46	12:48		Yes	Yes	Forearm	F3	uncoated
A0028	Group A1	A/Sing MAP, 15 ug	Day 1	10-MAR-2018	12:50	12:52		Yes	Yes	Forearm	F1	5 ug
A0028	Group A1	A/Sing MAP, 15 ug	Day 1	10-MAR-2018	12:53	12:55		Yes	Yes	Forearm	F2	5 ug
A0028	Group A1	A/Sing MAP, 15 ug	Day 1	10-MAR-2018	12:56	12:58		Yes	Yes	Forearm	F3	5 ug

Note: Delivered Dose=(Labelled Dose)/2 for MAP

Administration Site Key: FA = Forearm; UA= Upper arm
Treatments Part A: A1 = A/Sing MAP, 15 ug (FA); A2 = Afluria Quad IM (UA), 15 ug; A3 = Placebo MAP (FA); A4 = A/Sing Mono IM, 15 ug (UA)
Treatments Part B: B1 = A/Sing MAP, 15 ug (FA) with biopsy; B2 = Placebo MAP (FA) with biopsy
B3 = A/Sing MAP, 15 ug (FA); B4 = A/Sing MAP, 10 ug (FA); B5 = A/Sing MAP, 5 ug (FA); B6 = A/Sing MAP, 2.5 ug (FA)
B7 = Placebo MAP (FA); B8 = A/Sing MAP, 15 ug (UA); B9 = A/Sing IM, 15 ug, as 2018 Afluria Quad (UA)

Listing 16.2.5.1
Study Drug Administration

Part A

Randomi-sation Number	Group	Treatment	Study Day	Date of Dose	Time of MAP application	Time of MAP removal	Time of IM Injection	Was Dose Adminis-tered?	Dosed as per Protocol	Administra-tion Site	Site Number	Dose Administered at Site
A0029	Group A1	A/Sing MAP, 15 ug	Day 1	10-MAR-2018	13:00	13:02		Yes	Yes	Forearm	F1	5 ug
A0029	Group A1	A/Sing MAP, 15 ug	Day 1	10-MAR-2018	13:03	13:05		Yes	Yes	Forearm	F2	5 ug
A0029	Group A1	A/Sing MAP, 15 ug	Day 1	10-MAR-2018	13:06	13:08		Yes	Yes	Forearm	F3	5 ug
A0030	Group A3	Placebo MAP	Day 1	16-MAR-2018	12:00	12:02		Yes	Yes	Forearm	F1	uncoated
A0030	Group A3	Placebo MAP	Day 1	16-MAR-2018	12:03	12:05		Yes	Yes	Forearm	F2	uncoated
A0030	Group A3	Placebo MAP	Day 1	16-MAR-2018	12:06	12:08		Yes	Yes	Forearm	F3	uncoated
A0031	Group A2	Afluria Quad IM, 15 ug	Day 1	16-MAR-2018			13:00	Yes	Yes	Upper Arm	D1	[IM]
A0032	Group A4	A/Sing Mono IM, 15 ug	Day 1	16-MAR-2018			13:10	Yes	Yes	Upper Arm	D1	[IM]
A0033	Group A2	Afluria Quad IM, 15 ug	Day 1	16-MAR-2018			13:20	Yes	Yes	Upper Arm	D1	[IM]
A0034	Group A4	A/Sing Mono IM, 15 ug	Day 1	16-MAR-2018			13:30	Yes	Yes	Upper Arm	D1	[IM]
A0035	Group A4	A/Sing Mono IM, 15 ug	Day 1	16-MAR-2018			13:40	Yes	Yes	Upper Arm	D1	[IM]

Note: Delivered Dose=(Labelled Dose)/2 for MAP

Administration Site Key: FA = Forearm; UA= Upper arm
Treatments Part A: A1 = A/Sing MAP, 15 ug (FA); A2 = Afluria Quad IM (UA), 15 ug; A3 = Placebo MAP (FA); A4 = A/Sing Mono IM, 15 ug (UA)
Treatments Part B: B1 = A/Sing MAP, 15 ug (FA) with biopsy; B2 = Placebo MAP (FA) with biopsy
B3 = A/Sing MAP, 15 ug (FA); B4 = A/Sing MAP, 10 ug (FA); B5 = A/Sing MAP, 5 ug (FA); B6 = A/Sing MAP, 2.5 ug (FA)
B7 = Placebo MAP (FA); B8 = A/Sing MAP, 15 ug (UA); B9 = A/Sing IM, 15 ug, as 2018 Afluria Quad (UA)

Listing 16.2.5.1
Study Drug Administration

Part A

Randomi-sation Number	Group	Treatment	Study Day	Date of Dose	Time of MAP application	Time of MAP removal	Time of IM Injection	Was Dose Adminis-tered?	Dosed as per Protocol	Administra-tion Site	Site Number	Dose Administered at Site
A0036	Group A2	Afluria Quad IM, 15 ug	Day 1	17-MAR-2018			11:30	Yes	Yes	Upper Arm	D1	[IM]
A0037	Group A4	A/Sing Mono IM, 15 ug	Day 1	17-MAR-2018			11:35	Yes	Yes	Upper Arm	D1	[IM]
A0038	Group A2	Afluria Quad IM, 15 ug	Day 1	17-MAR-2018			11:40	Yes	Yes	Upper Arm	D1	[IM]
A0039	Group A2	Afluria Quad IM, 15 ug	Day 1	17-MAR-2018			11:45	Yes	Yes	Upper Arm	D1	[IM]
A0040	Group A4	A/Sing Mono IM, 15 ug	Day 1	17-MAR-2018			11:50	Yes	Yes	Upper Arm	D1	[IM]
A0041	Group A4	A/Sing Mono IM, 15 ug	Day 1	17-MAR-2018			11:55	Yes	Yes	Upper Arm	D1	[IM]
A0042	Group A2	Afluria Quad IM, 15 ug	Day 1	17-MAR-2018			12:00	Yes	Yes	Upper Arm	D1	[IM]
A0043	Group A2	Afluria Quad IM, 15 ug	Day 1	17-MAR-2018			12:05	Yes	Yes	Upper Arm	D1	[IM]
A0044	Group A4	A/Sing Mono IM, 15 ug	Day 1	17-MAR-2018			12:10	Yes	Yes	Upper Arm	D1	[IM]
A0045	Group A2	Afluria Quad IM, 15 ug	Day 1	17-MAR-2018			12:15	Yes	Yes	Upper Arm	D1	[IM]

Note: Delivered Dose=(Labelled Dose)/2 for MAP

Administration Site Key: FA = Forearm; UA= Upper arm
Treatments Part A: A1 = A/Sing MAP, 15 ug (FA); A2 = Afluria Quad IM (UA), 15 ug; A3 = Placebo MAP (FA); A4 = A/Sing Mono IM, 15 ug (UA)
Treatments Part B: B1 = A/Sing MAP, 15 ug (FA) with biopsy; B2 = Placebo MAP (FA) with biopsy
B3 = A/Sing MAP, 15 ug (FA); B4 = A/Sing MAP, 10 ug (FA); B5 = A/Sing MAP, 5 ug (FA); B6 = A/Sing MAP, 2.5 ug (FA)
B7 = Placebo MAP (FA); B8 = A/Sing MAP, 15 ug (UA); B9 = A/Sing IM, 15 ug, as 2018 Afluria Quad (UA)

Listing 16.2.5.1
Study Drug Administration

Part A

Randomi-sation Number	Group	Treatment	Study Day	Date of Dose	Time of MAP application	Time of MAP removal	Time of IM Injection	Was Dose Adminis-tered?	Dosed as per Protocol	Administra-tion Site	Site Number	Dose Administered at Site
A0046	Group A4	A/Sing Mono IM, 15 ug	Day 1	17-MAR-2018			12:20	Yes	Yes	Upper Arm	D1	[IM]
A0047	Group A2	Afluria Quad IM, 15 ug	Day 1	17-MAR-2018			12:25	Yes	Yes	Upper Arm	D1	[IM]
A0048	Group A4	A/Sing Mono IM, 15 ug	Day 1	17-MAR-2018			13:00	Yes	Yes	Upper Arm	D1	[IM]
A0049	Group A2	Afluria Quad IM, 15 ug	Day 1	17-MAR-2018			12:35	Yes	Yes	Upper Arm	D1	[IM]
A0050	Group A4	A/Sing Mono IM, 15 ug	Day 1	17-MAR-2018			12:40	Yes	Yes	Upper Arm	D1	[IM]
A0051	Group A2	Afluria Quad IM, 15 ug	Day 1	17-MAR-2018			12:45	Yes	Yes	Upper Arm	D1	[IM]
A0052	Group A4	A/Sing Mono IM, 15 ug	Day 1	17-MAR-2018			12:50	Yes	Yes	Upper Arm	D1	[IM]
A0053	Group A4	A/Sing Mono IM, 15 ug	Day 1	17-MAR-2018			12:55	Yes	Yes	Upper Arm	D1	[IM]
A0054	Group A2	Afluria Quad IM, 15 ug	Day 1	17-MAR-2018			15:06	Yes	Yes	Upper Arm	D1	[IM]

Note: Delivered Dose=(Labelled Dose)/2 for MAP

Administration Site Key: FA = Forearm; UA= Upper arm
Treatments Part A: A1 = A/Sing MAP, 15 ug (FA); A2 = Afluria Quad IM (UA), 15 ug; A3 = Placebo MAP (FA); A4 = A/Sing Mono IM, 15 ug (UA)
Treatments Part B: B1 = A/Sing MAP, 15 ug (FA) with biopsy; B2 = Placebo MAP (FA) with biopsy
B3 = A/Sing MAP, 15 ug (FA); B4 = A/Sing MAP, 10 ug (FA); B5 = A/Sing MAP, 5 ug (FA); B6 = A/Sing MAP, 2.5 ug (FA)
B7 = Placebo MAP (FA); B8 = A/Sing MAP, 15 ug (UA); B9 = A/Sing IM, 15 ug, as 2018 Afluria Quad (UA)

Listing 16.2.5.1
Study Drug Administration

Part A

Randomi-sation Number	Group	Treatment	Study Day	Date of Dose	Time of MAP application	Time of MAP removal	Time of IM Injection	Was Dose Adminis-tered?	Dosed as per Protocol	Administra-tion Site	Site Number	Dose Administered at Site
A0055	Group A4	A/Sing Mono IM, 15 ug	Day 1	24-MAR-2018			12:30	Yes	Yes	Upper Arm	D1	[IM]
A0056	Group A2	Afluria Quad IM, 15 ug	Day 1	24-MAR-2018			12:40	Yes	Yes	Upper Arm	D1	[IM]
A0057	Group A4	A/Sing Mono IM, 15 ug	Day 1	24-MAR-2018			12:50	Yes	Yes	Upper Arm	D1	[IM]
A0058	Group A2	Afluria Quad IM, 15 ug	Day 1	24-MAR-2018			13:00	Yes	Yes	Upper Arm	D1	[IM]
A0059	Group A2	Afluria Quad IM, 15 ug	Day 1	24-MAR-2018			13:10	Yes	Yes	Upper Arm	D1	[IM]
A0060	Group A4	A/Sing Mono IM, 15 ug	Day 1	24-MAR-2018			13:20	Yes	Yes	Upper Arm	D1	[IM]
A1026	Group A1	A/Sing MAP, 15 ug	Day 1	10-MAR-2018	14:00	14:02		Yes	Yes	Forearm	F1	5 ug
A1026	Group A1	A/Sing MAP, 15 ug	Day 1	10-MAR-2018	14:03	14:05		Yes	Yes	Forearm	F2	5 ug
A1026	Group A1	A/Sing MAP, 15 ug	Day 1	10-MAR-2018	14:06	14:08		Yes	Yes	Forearm	F3	5 ug

Note: Delivered Dose=(Labelled Dose)/2 for MAP

Administration Site Key: FA = Forearm; UA= Upper arm
Treatments Part A: A1 = A/Sing MAP, 15 ug (FA); A2 = Afluria Quad IM (UA), 15 ug; A3 = Placebo MAP (FA); A4 = A/Sing Mono IM, 15 ug (UA)
Treatments Part B: B1 = A/Sing MAP, 15 ug (FA) with biopsy; B2 = Placebo MAP (FA) with biopsy
B3 = A/Sing MAP, 15 ug (FA); B4 = A/Sing MAP, 10 ug (FA); B5 = A/Sing MAP, 5 ug (FA); B6 = A/Sing MAP, 2.5 ug (FA)
B7 = Placebo MAP (FA); B8 = A/Sing MAP, 15 ug (UA); B9 = A/Sing IM, 15 ug, as 2018 Afluria Quad (UA)

Listing 16.2.5.1
Study Drug Administration

Part B

Randomi-sation Number	Group	Treatment	Study Day	Date of Dose	Time of MAP application	Time of MAP removal	Time of IM Injection	Was Dose Adminis-tered?	Dosed as per Protocol	Administra-tion Site	Site Number	Dose Administered at Site
B0001	Group B1	A/Sing MAP, 15 ug	Day 1	07-MAY-2018	11:00	11:02		Yes	Yes	Forearm	F1	5 ug
B0001	Group B1	A/Sing MAP, 15 ug	Day 1	07-MAY-2018	11:03	11:05		Yes	Yes	Forearm	F2	5 ug
B0001	Group B1	A/Sing MAP, 15 ug	Day 1	07-MAY-2018	11:06	11:08		Yes	Yes	Forearm	F3	5 ug
B0002	Group B2	Placebo MAP	Day 1	07-MAY-2018	11:10	11:12		Yes	Yes	Forearm	F1	uncoated
B0002	Group B2	Placebo MAP	Day 1	07-MAY-2018	11:13	11:15		Yes	Yes	Forearm	F2	uncoated
B0002	Group B2	Placebo MAP	Day 1	07-MAY-2018	11:16	11:18		Yes	Yes	Forearm	F3	uncoated
B0003	Group B2	Placebo MAP	Day 1	07-MAY-2018	11:20	11:22		Yes	Yes	Forearm	F1	uncoated
B0003	Group B2	Placebo MAP	Day 1	07-MAY-2018	11:23	11:25		Yes	Yes	Forearm	F2	uncoated
B0003	Group B2	Placebo MAP	Day 1	07-MAY-2018	11:26	11:28		Yes	Yes	Forearm	F3	uncoated
B0004	Group B1	A/Sing MAP, 15 ug	Day 1	07-MAY-2018	12:40	12:43		Yes	No	Forearm	F1	5 ug
B0004	Group B1	A/Sing MAP, 15 ug	Day 1	07-MAY-2018	12:44	12:46		Yes	No	Forearm	F2	5 ug
B0004	Group B1	A/Sing MAP, 15 ug	Day 1	07-MAY-2018	12:47	12:49		Yes	No	Forearm	F3	5 ug
B0005	Group B1	A/Sing MAP, 15 ug	Day 1	07-MAY-2018	11:40	11:42		Yes	Yes	Forearm	F1	5 ug
B0005	Group B1	A/Sing MAP, 15 ug	Day 1	07-MAY-2018	11:43	11:45		Yes	Yes	Forearm	F2	5 ug

Note: Delivered Dose=(Labelled Dose)/2 for MAP

Administration Site Key: FA = Forearm; UA= Upper arm
Treatments Part A: A1 = A/Sing MAP, 15 ug (FA); A2 = Afluria Quad IM (UA), 15 ug; A3 = Placebo MAP (FA); A4 = A/Sing Mono IM, 15 ug (UA)
Treatments Part B: B1 = A/Sing MAP, 15 ug (FA) with biopsy; B2 = Placebo MAP (FA) with biopsy
B3 = A/Sing MAP, 15 ug (FA); B4 = A/Sing MAP, 10 ug (FA); B5 = A/Sing MAP, 5 ug (FA); B6 = A/Sing MAP, 2.5 ug (FA)
B7 = Placebo MAP (FA); B8 = A/Sing MAP, 15 ug (UA); B9 = A/Sing IM, 15 ug, as 2018 Afluria Quad (UA)

Listing 16.2.5.1
Study Drug Administration

Part B

Randomi-sation Number	Group	Treatment	Study Day	Date of Dose	Time of MAP application	Time of MAP removal	Time of IM Injection	Was Dose Adminis-tered?	Dosed as per Protocol	Administra-tion Site	Site Number	Dose Administered at Site
B0005	Group B1	A/Sing MAP, 15 ug	Day 1	07-MAY-2018	11:46	11:48		Yes	Yes	Forearm	F3	5 ug
B0006	Group B2	Placebo MAP	Day 1	07-MAY-2018	11:50	11:52		Yes	Yes	Forearm	F1	uncoated
B0006	Group B2	Placebo MAP	Day 1	07-MAY-2018	11:53	11:55		Yes	Yes	Forearm	F2	uncoated
B0006	Group B2	Placebo MAP	Day 1	07-MAY-2018	11:56	11:58		Yes	Yes	Forearm	F3	uncoated
B0007	Group B2	Placebo MAP	Day 1	07-MAY-2018	12:00	12:02		Yes	Yes	Forearm	F1	uncoated
B0007	Group B2	Placebo MAP	Day 1	07-MAY-2018	12:03	12:05		Yes	Yes	Forearm	F2	uncoated
B0007	Group B2	Placebo MAP	Day 1	07-MAY-2018	12:06	12:08		Yes	Yes	Forearm	F3	uncoated
B0008	Group B1	A/Sing MAP, 15 ug	Day 1	07-MAY-2018	12:10	12:12		Yes	Yes	Forearm	F1	5 ug
B0008	Group B1	A/Sing MAP, 15 ug	Day 1	07-MAY-2018	12:13	12:15		Yes	Yes	Forearm	F2	5 ug
B0008	Group B1	A/Sing MAP, 15 ug	Day 1	07-MAY-2018	12:16	12:18		Yes	Yes	Forearm	F3	5 ug
B0009	Group B1	A/Sing MAP, 15 ug	Day 1	07-MAY-2018	12:20	12:22		Yes	Yes	Forearm	F1	5 ug
B0009	Group B1	A/Sing MAP, 15 ug	Day 1	07-MAY-2018	12:23	12:25		Yes	Yes	Forearm	F2	5 ug
B0009	Group B1	A/Sing MAP, 15 ug	Day 1	07-MAY-2018	12:26	12:28		Yes	Yes	Forearm	F3	5 ug

Note: Delivered Dose=(Labelled Dose)/2 for MAP

Administration Site Key: FA = Forearm; UA= Upper arm
Treatments Part A: A1 = A/Sing MAP, 15 ug (FA); A2 = Afluria Quad IM (UA), 15 ug; A3 = Placebo MAP (FA); A4 = A/Sing Mono IM, 15 ug (UA)
Treatments Part B: B1 = A/Sing MAP, 15 ug (FA) with biopsy; B2 = Placebo MAP (FA) with biopsy
B3 = A/Sing MAP, 15 ug (FA); B4 = A/Sing MAP, 10 ug (FA); B5 = A/Sing MAP, 5 ug (FA); B6 = A/Sing MAP, 2.5 ug (FA)
B7 = Placebo MAP (FA); B8 = A/Sing MAP, 15 ug (UA); B9 = A/Sing IM, 15 ug, as 2018 Afluria Quad (UA)

Listing 16.2.5.1
Study Drug Administration

Part B

Randomi-sation Number	Group	Treatment	Study Day	Date of Dose	Time of MAP application	Time of MAP removal	Time of IM Injection	Was Dose Adminis-tered?	Dosed as per Protocol	Administra-tion Site	Site Number	Dose Administered at Site
B0010	Group B2	Placebo MAP	Day 1	07-MAY-2018	12:30	12:32		Yes	Yes	Forearm	F1	uncoated
B0010	Group B2	Placebo MAP	Day 1	07-MAY-2018	12:33	12:35		Yes	Yes	Forearm	F2	uncoated
B0010	Group B2	Placebo MAP	Day 1	07-MAY-2018	12:36	12:38		Yes	Yes	Forearm	F3	uncoated
B0011	Group B8	A/Sing MAP, 15 ug	Day 1	12-MAY-2018	11:50	11:52		Yes	Yes	Upper Arm	D1	5 ug
B0011	Group B8	A/Sing MAP, 15 ug	Day 1	12-MAY-2018	11:53	11:55		Yes	Yes	Upper Arm	D2	5 ug
B0011	Group B8	A/Sing MAP, 15 ug	Day 1	12-MAY-2018	11:56	11:58		Yes	Yes	Upper Arm	D3	5 ug
B0012	Group B6	A/Sing MAP, 2.5 ug	Day 1	12-MAY-2018	12:00	12:02		Yes	Yes	Forearm	F1	2.5 ug
B0012	Group B6	A/Sing MAP, 2.5 ug	Day 1	12-MAY-2018	12:18	12:20		Yes	Yes	Forearm	F2	uncoated
B0012	Group B6	A/Sing MAP, 2.5 ug	Day 1	12-MAY-2018	12:21	12:23		Yes	Yes	Forearm	F3	uncoated
B0013	Group B5	A/Sing MAP, 5 ug	Day 1	12-MAY-2018	12:10	12:12		Yes	Yes	Forearm	F1	5 ug
B0013	Group B5	A/Sing MAP, 5 ug	Day 1	12-MAY-2018	12:13	12:15		Yes	Yes	Forearm	F2	uncoated
B0013	Group B5	A/Sing MAP, 5 ug	Day 1	12-MAY-2018	12:16	12:18		Yes	Yes	Forearm	F3	uncoated
B0014	Group B3	A/Sing MAP, 15 ug	Day 1	12-MAY-2018	12:20	12:22		Yes	Yes	Forearm	F1	5 ug
B0014	Group B3	A/Sing MAP, 15 ug	Day 1	12-MAY-2018	12:23	12:25		Yes	Yes	Forearm	F2	5 ug

Note: Delivered Dose=(Labelled Dose)/2 for MAP

Administration Site Key: FA = Forearm; UA= Upper arm
Treatments Part A: A1 = A/Sing MAP, 15 ug (FA); A2 = Afluria Quad IM (UA), 15 ug; A3 = Placebo MAP (FA); A4 = A/Sing Mono IM, 15 ug (UA)
Treatments Part B: B1 = A/Sing MAP, 15 ug (FA) with biopsy; B2 = Placebo MAP (FA) with biopsy
B3 = A/Sing MAP, 15 ug (FA); B4 = A/Sing MAP, 10 ug (FA); B5 = A/Sing MAP, 5 ug (FA); B6 = A/Sing MAP, 2.5 ug (FA)
B7 = Placebo MAP (FA); B8 = A/Sing MAP, 15 ug (UA); B9 = A/Sing IM, 15 ug, as 2018 Afluria Quad (UA)

Listing 16.2.5.1
Study Drug Administration

Part B

Randomi-sation Number	Group	Treatment	Study Day	Date of Dose	Time of MAP application	Time of MAP removal	Time of IM Injection	Was Dose Adminis-tered?	Dosed as per Protocol	Administra-tion Site	Site Number	Dose Administered at Site
B0014	Group B3	A/Sing MAP, 15 ug	Day 1	12-MAY-2018	12:26	12:28		Yes	Yes	Forearm	F3	5 ug
B0015	Group B9	A/Sing IM, 15 ug	Day 1	12-MAY-2018			11:10	Yes	Yes	Upper Arm	D1	[IM]
B0016	Group B4	A/Sing MAP, 10 ug	Day 1	12-MAY-2018	12:30	12:32		Yes	Yes	Forearm	F1	uncoated
B0016	Group B4	A/Sing MAP, 10 ug	Day 1	12-MAY-2018	12:33	12:35		Yes	Yes	Forearm	F2	5 ug
B0016	Group B4	A/Sing MAP, 10 ug	Day 1	12-MAY-2018	12:36	12:38		Yes	Yes	Forearm	F3	5 ug
B0017	Group B7	Placebo MAP	Day 1	12-MAY-2018	12:40	12:42		Yes	No	Forearm	F1	uncoated
B0017	Group B7	Placebo MAP	Day 1	12-MAY-2018	12:43	12:45		Yes	No	Forearm	F2	uncoated
B0017	Group B7	Placebo MAP	Day 1	12-MAY-2018	12:46	12:47		Yes	No	Forearm	F3	uncoated
B0018	Group B9	A/Sing IM, 15 ug	Day 1	12-MAY-2018			11:20	Yes	Yes	Upper Arm	D1	[IM]
B0020	Group B6	A/Sing MAP, 2.5 ug	Day 1	12-MAY-2018	12:50	12:52		Yes	Yes	Forearm	F1	uncoated
B0020	Group B6	A/Sing MAP, 2.5 ug	Day 1	12-MAY-2018	12:53	12:55		Yes	Yes	Forearm	F2	uncoated
B0020	Group B6	A/Sing MAP, 2.5 ug	Day 1	12-MAY-2018	12:56	12:58		Yes	Yes	Forearm	F3	2.5 ug

Note: Delivered Dose=(Labelled Dose)/2 for MAP

Administration Site Key: FA = Forearm; UA= Upper arm
Treatments Part A: A1 = A/Sing MAP, 15 ug (FA); A2 = Afluria Quad IM (UA), 15 ug; A3 = Placebo MAP (FA); A4 = A/Sing Mono IM, 15 ug (UA)
Treatments Part B: B1 = A/Sing MAP, 15 ug (FA) with biopsy; B2 = Placebo MAP (FA) with biopsy
B3 = A/Sing MAP, 15 ug (FA); B4 = A/Sing MAP, 10 ug (FA); B5 = A/Sing MAP, 5 ug (FA); B6 = A/Sing MAP, 2.5 ug (FA)
B7 = Placebo MAP (FA); B8 = A/Sing MAP, 15 ug (UA); B9 = A/Sing IM, 15 ug, as 2018 Afluria Quad (UA)

Listing 16.2.5.1
Study Drug Administration

Part B

Randomi-sation Number	Group	Treatment	Study Day	Date of Dose	Time of MAP application	Time of MAP removal	Time of IM Injection	Was Dose Adminis-tered?	Dosed as per Protocol	Administra-tion Site	Site Number	Dose Administered at Site
B0021	Group B7	Placebo MAP	Day 1	12-MAY-2018	13:00	13:02		Yes	Yes	Forearm	F1	uncoated
B0021	Group B7	Placebo MAP	Day 1	12-MAY-2018	13:03	13:05		Yes	Yes	Forearm	F2	uncoated
B0021	Group B7	Placebo MAP	Day 1	12-MAY-2018	13:06	13:08		Yes	Yes	Forearm	F3	uncoated
B0022	Group B4	A/Sing MAP, 10 ug	Day 1	12-MAY-2018	13:11	13:13		Yes	Yes	Forearm	F1	uncoated
B0022	Group B4	A/Sing MAP, 10 ug	Day 1	12-MAY-2018	13:14	13:16		Yes	Yes	Forearm	F2	5 ug
B0022	Group B4	A/Sing MAP, 10 ug	Day 1	12-MAY-2018	13:17	13:19		Yes	Yes	Forearm	F3	5 ug
B0023	Group B3	A/Sing MAP, 15 ug	Day 1	12-MAY-2018	13:20	13:22		Yes	Yes	Forearm	F1	5 ug
B0023	Group B3	A/Sing MAP, 15 ug	Day 1	12-MAY-2018	13:29	13:31		Yes	Yes	Forearm	F2	5 ug
B0023	Group B3	A/Sing MAP, 15 ug	Day 1	12-MAY-2018	13:32	13:34		Yes	Yes	Forearm	F3	5 ug
B0024	Group B5	A/Sing MAP, 5 ug	Day 1	12-MAY-2018	13:36	13:38		Yes	Yes	Forearm	F1	uncoated
B0024	Group B5	A/Sing MAP, 5 ug	Day 1	12-MAY-2018	13:39	13:41		Yes	Yes	Forearm	F2	uncoated
B0024	Group B5	A/Sing MAP, 5 ug	Day 1	12-MAY-2018	13:42	13:44		Yes	Yes	Forearm	F3	5 ug
B0025	Group B8	A/Sing MAP, 15 ug	Day 1	12-MAY-2018	13:40	13:42		Yes	Yes	Upper Arm	D1	5 ug
B0025	Group B8	A/Sing MAP, 15 ug	Day 1	12-MAY-2018	13:43	13:45		Yes	Yes	Upper Arm	D2	5 ug

Note: Delivered Dose=(Labelled Dose)/2 for MAP

Administration Site Key: FA = Forearm; UA= Upper arm
Treatments Part A: A1 = A/Sing MAP, 15 ug (FA); A2 = Afluria Quad IM (UA), 15 ug; A3 = Placebo MAP (FA); A4 = A/Sing Mono IM, 15 ug (UA)
Treatments Part B: B1 = A/Sing MAP, 15 ug (FA) with biopsy; B2 = Placebo MAP (FA) with biopsy
B3 = A/Sing MAP, 15 ug (FA); B4 = A/Sing MAP, 10 ug (FA); B5 = A/Sing MAP, 5 ug (FA); B6 = A/Sing MAP, 2.5 ug (FA)
B7 = Placebo MAP (FA); B8 = A/Sing MAP, 15 ug (UA); B9 = A/Sing IM, 15 ug, as 2018 Afluria Quad (UA)

Listing 16.2.5.1
Study Drug Administration

Part B

Randomi-sation Number	Group	Treatment	Study Day	Date of Dose	Time of MAP application	Time of MAP removal	Time of IM Injection	Was Dose Adminis-tered?	Dosed as per Protocol	Administra-tion Site	Site Number	Dose Administered at Site
B0025	Group B8	A/Sing MAP, 15 ug	Day 1	12-MAY-2018	13:46	13:48		Yes	Yes	Upper Arm	D3	5 ug
B0026	Group B9	A/Sing IM, 15 ug	Day 1	12-MAY-2018			11:30	Yes	Yes	Upper Arm	D1	[IM]
B0027	Group B3	A/Sing MAP, 15 ug	Day 1	12-MAY-2018	14:30	14:32		Yes	Yes	Forearm	F1	5 ug
B0027	Group B3	A/Sing MAP, 15 ug	Day 1	12-MAY-2018	14:33	14:35		Yes	Yes	Forearm	F2	5 ug
B0027	Group B3	A/Sing MAP, 15 ug	Day 1	12-MAY-2018	14:36	14:38		Yes	Yes	Forearm	F3	5 ug
B0028	Group B5	A/Sing MAP, 5 ug	Day 1	12-MAY-2018	14:20	14:22		Yes	Yes	Forearm	F1	5 ug
B0028	Group B5	A/Sing MAP, 5 ug	Day 1	12-MAY-2018	14:23	14:25		Yes	Yes	Forearm	F2	uncoated
B0028	Group B5	A/Sing MAP, 5 ug	Day 1	12-MAY-2018	14:26	14:28		Yes	Yes	Forearm	F3	uncoated
B0029	Group B6	A/Sing MAP, 2.5 ug	Day 1	12-MAY-2018	14:11	14:13		Yes	Yes	Forearm	F1	uncoated
B0029	Group B6	A/Sing MAP, 2.5 ug	Day 1	12-MAY-2018	14:14	14:16		Yes	Yes	Forearm	F2	uncoated
B0029	Group B6	A/Sing MAP, 2.5 ug	Day 1	12-MAY-2018	14:17	14:19		Yes	Yes	Forearm	F3	2.5 ug
B0030	Group B7	Placebo MAP	Day 1	12-MAY-2018	14:00	14:02		Yes	Yes	Forearm	F1	uncoated
B0030	Group B7	Placebo MAP	Day 1	12-MAY-2018	14:03	14:05		Yes	Yes	Forearm	F2	uncoated

Note: Delivered Dose=(Labelled Dose)/2 for MAP

Administration Site Key: FA = Forearm; UA= Upper arm
Treatments Part A: A1 = A/Sing MAP, 15 ug (FA); A2 = Afluria Quad IM (UA), 15 ug; A3 = Placebo MAP (FA); A4 = A/Sing Mono IM, 15 ug (UA)
Treatments Part B: B1 = A/Sing MAP, 15 ug (FA) with biopsy; B2 = Placebo MAP (FA) with biopsy
B3 = A/Sing MAP, 15 ug (FA); B4 = A/Sing MAP, 10 ug (FA); B5 = A/Sing MAP, 5 ug (FA); B6 = A/Sing MAP, 2.5 ug (FA)
B7 = Placebo MAP (FA); B8 = A/Sing MAP, 15 ug (UA); B9 = A/Sing IM, 15 ug, as 2018 Afluria Quad (UA)

Listing 16.2.5.1
Study Drug Administration

Part B

Randomi-sation Number	Group	Treatment	Study Day	Date of Dose	Time of MAP application	Time of MAP removal	Time of IM Injection	Was Dose Adminis-tered?	Dosed as per Protocol	Administra-tion Site	Site Number	Dose Administered at Site
B0030	Group B7	Placebo MAP	Day 1	12-MAY-2018	14:06	14:08		Yes	Yes	Forearm	F3	uncoated
B0031	Group B4	A/Sing MAP, 10 ug	Day 1	19-MAY-2018	13:20	13:22		Yes	Yes	Forearm	F1	uncoated
B0031	Group B4	A/Sing MAP, 10 ug	Day 1	19-MAY-2018	13:23	13:25		Yes	Yes	Forearm	F2	5 ug
B0031	Group B4	A/Sing MAP, 10 ug	Day 1	19-MAY-2018	13:26	13:28		Yes	Yes	Forearm	F3	5 ug
B0032	Group B6	A/Sing MAP, 2.5 ug	Day 1	19-MAY-2018	11:40	11:42		Yes	Yes	Forearm	F1	uncoated
B0032	Group B6	A/Sing MAP, 2.5 ug	Day 1	19-MAY-2018	11:43	11:45		Yes	Yes	Forearm	F2	uncoated
B0032	Group B6	A/Sing MAP, 2.5 ug	Day 1	19-MAY-2018	11:46	11:48		Yes	Yes	Forearm	F3	2.5 ug
B0033	Group B5	A/Sing MAP, 5 ug	Day 1	19-MAY-2018	12:00	12:02		Yes	Yes	Forearm	F1	uncoated
B0033	Group B5	A/Sing MAP, 5 ug	Day 1	19-MAY-2018	12:03	12:05		Yes	Yes	Forearm	F2	uncoated
B0033	Group B5	A/Sing MAP, 5 ug	Day 1	19-MAY-2018	12:06	12:08		Yes	Yes	Forearm	F3	5 ug
B0034	Group B8	A/Sing MAP, 15 ug	Day 1	19-MAY-2018	14:00	14:02		Yes	Yes	Upper Arm	D1	5 ug
B0034	Group B8	A/Sing MAP, 15 ug	Day 1	19-MAY-2018	14:03	14:05		Yes	Yes	Upper Arm	D2	5 ug
B0034	Group B8	A/Sing MAP, 15 ug	Day 1	19-MAY-2018	14:06	14:08		Yes	Yes	Upper Arm	D3	5 ug

Note: Delivered Dose=(Labelled Dose)/2 for MAP

Administration Site Key: FA = Forearm; UA= Upper arm
Treatments Part A: A1 = A/Sing MAP, 15 ug (FA); A2 = Afluria Quad IM (UA), 15 ug; A3 = Placebo MAP (FA); A4 = A/Sing Mono IM, 15 ug (UA)
Treatments Part B: B1 = A/Sing MAP, 15 ug (FA) with biopsy; B2 = Placebo MAP (FA) with biopsy
B3 = A/Sing MAP, 15 ug (FA); B4 = A/Sing MAP, 10 ug (FA); B5 = A/Sing MAP, 5 ug (FA); B6 = A/Sing MAP, 2.5 ug (FA)
B7 = Placebo MAP (FA); B8 = A/Sing MAP, 15 ug (UA); B9 = A/Sing IM, 15 ug, as 2018 Afluria Quad (UA)

Listing 16.2.5.1
Study Drug Administration

Part B

Randomi-sation Number	Group	Treatment	Study Day	Date of Dose	Time of MAP application	Time of MAP removal	Time of IM Injection	Was Dose Adminis-tered?	Dosed as per Protocol	Administra-tion Site	Site Number	Dose Administered at Site
B0035	Group B9	A/Sing IM, 15 ug	Day 1	19-MAY-2018			11:20	Yes	Yes	Upper Arm	D1	[IM]
B0036	Group B7	Placebo MAP	Day 1	19-MAY-2018	12:12	12:14		Yes	Yes	Forearm	F1	uncoated
B0036	Group B7	Placebo MAP	Day 1	19-MAY-2018	12:15	12:17		Yes	Yes	Forearm	F2	uncoated
B0036	Group B7	Placebo MAP	Day 1	19-MAY-2018	12:18	12:20		Yes	Yes	Forearm	F3	uncoated
B0037	Group B4	A/Sing MAP, 10 ug	Day 1	19-MAY-2018	12:20	12:22		Yes	Yes	Forearm	F1	uncoated
B0037	Group B4	A/Sing MAP, 10 ug	Day 1	19-MAY-2018	12:23	12:25		Yes	Yes	Forearm	F2	5 ug
B0037	Group B4	A/Sing MAP, 10 ug	Day 1	19-MAY-2018	12:26	12:28		Yes	Yes	Forearm	F3	5 ug
B0038	Group B3	A/Sing MAP, 15 ug	Day 1	19-MAY-2018	12:33	12:35		Yes	Yes	Forearm	F1	5 ug
B0038	Group B3	A/Sing MAP, 15 ug	Day 1	19-MAY-2018	12:36	12:38		Yes	Yes	Forearm	F2	5 ug
B0038	Group B3	A/Sing MAP, 15 ug	Day 1	19-MAY-2018	12:39	12:41		Yes	Yes	Forearm	F3	5 ug
B0039	Group B7	Placebo MAP	Day 1	19-MAY-2018	13:50	13:52		Yes	Yes	Forearm	F1	uncoated
B0039	Group B7	Placebo MAP	Day 1	19-MAY-2018	13:53	13:55		Yes	Yes	Forearm	F2	uncoated
B0039	Group B7	Placebo MAP	Day 1	19-MAY-2018	13:56	13:58		Yes	Yes	Forearm	F3	uncoated

Note: Delivered Dose=(Labelled Dose)/2 for MAP

Administration Site Key: FA = Forearm; UA= Upper arm
Treatments Part A: A1 = A/Sing MAP, 15 ug (FA); A2 = Afluria Quad IM (UA), 15 ug; A3 = Placebo MAP (FA); A4 = A/Sing Mono IM, 15 ug (UA)
Treatments Part B: B1 = A/Sing MAP, 15 ug (FA) with biopsy; B2 = Placebo MAP (FA) with biopsy
B3 = A/Sing MAP, 15 ug (FA); B4 = A/Sing MAP, 10 ug (FA); B5 = A/Sing MAP, 5 ug (FA); B6 = A/Sing MAP, 2.5 ug (FA)
B7 = Placebo MAP (FA); B8 = A/Sing MAP, 15 ug (UA); B9 = A/Sing IM, 15 ug, as 2018 Afluria Quad (UA)

Listing 16.2.5.1
Study Drug Administration

Part B

Randomi-sation Number	Group	Treatment	Study Day	Date of Dose	Time of MAP application	Time of MAP removal	Time of IM Injection	Was Dose Adminis-tered?	Dosed as per Protocol	Administra-tion Site	Site Number	Dose Administered at Site
B0040	Group B9	A/Sing IM, 15 ug	Day 1	19-MAY-2018			11:10	Yes	Yes	Upper Arm	D1	[IM]
B0041	Group B3	A/Sing MAP, 15 ug	Day 1	19-MAY-2018	12:45	12:47		Yes	Yes	Forearm	F1	5 ug
B0041	Group B3	A/Sing MAP, 15 ug	Day 1	19-MAY-2018	12:48	12:50		Yes	Yes	Forearm	F2	5 ug
B0041	Group B3	A/Sing MAP, 15 ug	Day 1	19-MAY-2018	12:51	12:53		Yes	Yes	Forearm	F3	5 ug
B0042	Group B5	A/Sing MAP, 5 ug	Day 1	19-MAY-2018	12:50	12:52		Yes	Yes	Forearm	F1	uncoated
B0042	Group B5	A/Sing MAP, 5 ug	Day 1	19-MAY-2018	12:53	12:55		Yes	Yes	Forearm	F2	uncoated
B0042	Group B5	A/Sing MAP, 5 ug	Day 1	19-MAY-2018	12:56	12:58		Yes	Yes	Forearm	F3	5 ug
B0043	Group B8	A/Sing MAP, 15 ug	Day 1	19-MAY-2018	13:00	13:02		Yes	Yes	Upper Arm	D1	5 ug
B0043	Group B8	A/Sing MAP, 15 ug	Day 1	19-MAY-2018	13:03	13:05		Yes	Yes	Upper Arm	D2	5 ug
B0043	Group B8	A/Sing MAP, 15 ug	Day 1	19-MAY-2018	13:06	13:08		Yes	Yes	Upper Arm	D3	5 ug
B0044	Group B6	A/Sing MAP, 2.5 ug	Day 1	19-MAY-2018	13:10	13:12		Yes	Yes	Forearm	F1	uncoated
B0044	Group B6	A/Sing MAP, 2.5 ug	Day 1	19-MAY-2018	13:13	13:15		Yes	Yes	Forearm	F2	uncoated
B0044	Group B6	A/Sing MAP, 2.5 ug	Day 1	19-MAY-2018	13:16	13:18		Yes	Yes	Forearm	F3	2.5 ug

Note: Delivered Dose=(Labelled Dose)/2 for MAP

Administration Site Key: FA = Forearm; UA= Upper arm
Treatments Part A: A1 = A/Sing MAP, 15 ug (FA); A2 = Afluria Quad IM (UA), 15 ug; A3 = Placebo MAP (FA); A4 = A/Sing Mono IM, 15 ug (UA)
Treatments Part B: B1 = A/Sing MAP, 15 ug (FA) with biopsy; B2 = Placebo MAP (FA) with biopsy
B3 = A/Sing MAP, 15 ug (FA); B4 = A/Sing MAP, 10 ug (FA); B5 = A/Sing MAP, 5 ug (FA); B6 = A/Sing MAP, 2.5 ug (FA)
B7 = Placebo MAP (FA); B8 = A/Sing MAP, 15 ug (UA); B9 = A/Sing IM, 15 ug, as 2018 Afluria Quad (UA)

Listing 16.2.5.1
Study Drug Administration

Part B

Randomi-sation Number	Group	Treatment	Study Day	Date of Dose	Time of MAP application	Time of MAP removal	Time of IM Injection	Was Dose Adminis-tered?	Dosed as per Protocol	Administra-tion Site	Site Number	Dose Administered at Site
B0045	Group B4	A/Sing MAP, 10 ug	Day 1	19-MAY-2018	11:50	11:52		Yes	Yes	Forearm	F1	5 ug
B0045	Group B4	A/Sing MAP, 10 ug	Day 1	19-MAY-2018	11:53	11:55		Yes	Yes	Forearm	F2	5 ug
B0045	Group B4	A/Sing MAP, 10 ug	Day 1	19-MAY-2018	11:56	11:58		Yes	Yes	Forearm	F3	uncoated
B0046	Group B7	Placebo MAP	Day 1	19-MAY-2018	13:30	13:32		Yes	Yes	Forearm	F1	uncoated
B0046	Group B7	Placebo MAP	Day 1	19-MAY-2018	13:33	13:35		Yes	Yes	Forearm	F2	uncoated
B0046	Group B7	Placebo MAP	Day 1	19-MAY-2018	13:36	13:38		Yes	Yes	Forearm	F3	uncoated
B0047	Group B9	A/Sing IM, 15 ug	Day 1	19-MAY-2018			11:30	Yes	Yes	Upper Arm	D1	[IM]
B0048	Group B6	A/Sing MAP, 2.5 ug	Day 1	19-MAY-2018	13:40	13:42		Yes	Yes	Forearm	F1	2.5 ug
B0048	Group B6	A/Sing MAP, 2.5 ug	Day 1	19-MAY-2018	13:43	13:45		Yes	Yes	Forearm	F2	uncoated
B0048	Group B6	A/Sing MAP, 2.5 ug	Day 1	19-MAY-2018	13:46	13:48		Yes	Yes	Forearm	F3	uncoated
B0049	Group B4	A/Sing MAP, 10 ug	Day 1	19-MAY-2018	14:10	14:12		Yes	Yes	Forearm	F1	uncoated
B0049	Group B4	A/Sing MAP, 10 ug	Day 1	19-MAY-2018	14:13	14:15		Yes	Yes	Forearm	F2	5 ug
B0049	Group B4	A/Sing MAP, 10 ug	Day 1	19-MAY-2018	14:16	14:18		Yes	Yes	Forearm	F3	5 ug

Note: Delivered Dose=(Labelled Dose)/2 for MAP

Administration Site Key: FA = Forearm; UA= Upper arm
Treatments Part A: A1 = A/Sing MAP, 15 ug (FA); A2 = Afluria Quad IM (UA), 15 ug; A3 = Placebo MAP (FA); A4 = A/Sing Mono IM, 15 ug (UA)
Treatments Part B: B1 = A/Sing MAP, 15 ug (FA) with biopsy; B2 = Placebo MAP (FA) with biopsy
B3 = A/Sing MAP, 15 ug (FA); B4 = A/Sing MAP, 10 ug (FA); B5 = A/Sing MAP, 5 ug (FA); B6 = A/Sing MAP, 2.5 ug (FA)
B7 = Placebo MAP (FA); B8 = A/Sing MAP, 15 ug (UA); B9 = A/Sing IM, 15 ug, as 2018 Afluria Quad (UA)

Listing 16.2.5.1
Study Drug Administration

Part B

Randomi-sation Number	Group	Treatment	Study Day	Date of Dose	Time of MAP application	Time of MAP removal	Time of IM Injection	Was Dose Adminis-tered?	Dosed as per Protocol	Administra-tion Site	Site Number	Dose Administered at Site
B0050	Group B8	A/Sing MAP, 15 ug	Day 1	19-MAY-2018	14:20	14:22		Yes	Yes	Upper Arm	D1	5 ug
B0050	Group B8	A/Sing MAP, 15 ug	Day 1	19-MAY-2018	14:23	14:25		Yes	Yes	Upper Arm	D2	5 ug
B0050	Group B8	A/Sing MAP, 15 ug	Day 1	19-MAY-2018	14:26	14:28		Yes	Yes	Upper Arm	D3	5 ug
B0051	Group B3	A/Sing MAP, 15 ug	Day 1	25-MAY-2018	12:50	12:52		Yes	Yes	Forearm	F1	5 ug
B0051	Group B3	A/Sing MAP, 15 ug	Day 1	25-MAY-2018	12:53	12:55		Yes	Yes	Forearm	F2	5 ug
B0051	Group B3	A/Sing MAP, 15 ug	Day 1	25-MAY-2018	12:56	12:58		Yes	Yes	Forearm	F3	5 ug
B0052	Group B5	A/Sing MAP, 5 ug	Day 1	25-MAY-2018	13:00	13:02		Yes	Yes	Forearm	F1	5 ug
B0052	Group B5	A/Sing MAP, 5 ug	Day 1	25-MAY-2018	13:05	13:07		Yes	Yes	Forearm	F3	uncoated
B0052	Group B5	A/Sing MAP, 5 ug	Day 1	25-MAY-2018	13:10	13:12		Yes	Yes	Forearm	F2	uncoated
B0053	Group B6	A/Sing MAP, 2.5 ug	Day 1	25-MAY-2018	13:10	13:12		Yes	Yes	Forearm	F1	uncoated
B0053	Group B6	A/Sing MAP, 2.5 ug	Day 1	25-MAY-2018	13:13	13:15		Yes	Yes	Forearm	F2	uncoated
B0053	Group B6	A/Sing MAP, 2.5 ug	Day 1	25-MAY-2018	13:16	13:18		Yes	Yes	Forearm	F3	2.5 ug
B0054	Group B5	A/Sing MAP, 5 ug	Day 1	25-MAY-2018	13:20	13:22		Yes	Yes	Forearm	F1	uncoated
B0054	Group B5	A/Sing MAP, 5 ug	Day 1	25-MAY-2018	13:23	13:25		Yes	Yes	Forearm	F2	uncoated

Note: Delivered Dose=(Labelled Dose)/2 for MAP

Administration Site Key: FA = Forearm; UA= Upper arm
Treatments Part A: A1 = A/Sing MAP, 15 ug (FA); A2 = Afluria Quad IM (UA), 15 ug; A3 = Placebo MAP (FA); A4 = A/Sing Mono IM, 15 ug (UA)
Treatments Part B: B1 = A/Sing MAP, 15 ug (FA) with biopsy; B2 = Placebo MAP (FA) with biopsy
B3 = A/Sing MAP, 15 ug (FA); B4 = A/Sing MAP, 10 ug (FA); B5 = A/Sing MAP, 5 ug (FA); B6 = A/Sing MAP, 2.5 ug (FA)
B7 = Placebo MAP (FA); B8 = A/Sing MAP, 15 ug (UA); B9 = A/Sing IM, 15 ug, as 2018 Afluria Quad (UA)

Listing 16.2.5.1
Study Drug Administration

Part B

Randomi-sation Number	Group	Treatment	Study Day	Date of Dose	Time of MAP application	Time of MAP removal	Time of IM Injection	Was Dose Adminis-tered?	Dosed as per Protocol	Administra-tion Site	Site Number	Dose Administered at Site
B0054	Group B5	A/Sing MAP, 5 ug	Day 1	25-MAY-2018	13:26	13:28		Yes	Yes	Forearm	F3	5 ug
B0055	Group B8	A/Sing MAP, 15 ug	Day 1	25-MAY-2018	13:30	13:32		Yes	Yes	Upper Arm	D1	5 ug
B0055	Group B8	A/Sing MAP, 15 ug	Day 1	25-MAY-2018	13:33	13:35		Yes	Yes	Upper Arm	D2	5 ug
B0055	Group B8	A/Sing MAP, 15 ug	Day 1	25-MAY-2018	13:36	13:38		Yes	Yes	Upper Arm	D3	5 ug
B0056	Group B3	A/Sing MAP, 15 ug	Day 1	25-MAY-2018	13:40	13:42		Yes	Yes	Forearm	F1	5 ug
B0056	Group B3	A/Sing MAP, 15 ug	Day 1	25-MAY-2018	13:43	13:45		Yes	Yes	Forearm	F2	5 ug
B0056	Group B3	A/Sing MAP, 15 ug	Day 1	25-MAY-2018	13:46	13:48		Yes	Yes	Forearm	F3	5 ug
B0057	Group B4	A/Sing MAP, 10 ug	Day 1	25-MAY-2018	13:50	13:52		Yes	Yes	Forearm	F1	5 ug
B0057	Group B4	A/Sing MAP, 10 ug	Day 1	25-MAY-2018	13:53	13:55		Yes	Yes	Forearm	F2	5 ug
B0057	Group B4	A/Sing MAP, 10 ug	Day 1	25-MAY-2018	13:56	13:58		Yes	Yes	Forearm	F3	uncoated
B0058	Group B9	A/Sing IM, 15 ug	Day 1	25-MAY-2018			12:30	Yes	Yes	Upper Arm	D1	[IM]
B0059	Group B7	Placebo MAP	Day 1	25-MAY-2018	14:10	14:12		Yes	Yes	Forearm	F1	uncoated
B0059	Group B7	Placebo MAP	Day 1	25-MAY-2018	14:13	14:15		Yes	Yes	Forearm	F2	uncoated

Note: Delivered Dose=(Labelled Dose)/2 for MAP

Administration Site Key: FA = Forearm; UA= Upper arm
Treatments Part A: A1 = A/Sing MAP, 15 ug (FA); A2 = Afluria Quad IM (UA), 15 ug; A3 = Placebo MAP (FA); A4 = A/Sing Mono IM, 15 ug (UA)
Treatments Part B: B1 = A/Sing MAP, 15 ug (FA) with biopsy; B2 = Placebo MAP (FA) with biopsy
B3 = A/Sing MAP, 15 ug (FA); B4 = A/Sing MAP, 10 ug (FA); B5 = A/Sing MAP, 5 ug (FA); B6 = A/Sing MAP, 2.5 ug (FA)
B7 = Placebo MAP (FA); B8 = A/Sing MAP, 15 ug (UA); B9 = A/Sing IM, 15 ug, as 2018 Afluria Quad (UA)

Listing 16.2.5.1
Study Drug Administration

Part B

Randomi-sation Number	Group	Treatment	Study Day	Date of Dose	Time of MAP application	Time of MAP removal	Time of IM Injection	Was Dose Adminis-tered?	Dosed as per Protocol	Administra-tion Site	Site Number	Dose Administered at Site
B0059	Group B7	Placebo MAP	Day 1	25-MAY-2018	14:16	14:18		Yes	Yes	Forearm	F3	uncoated
B0060	Group B3	A/Sing MAP, 15 ug	Day 1	25-MAY-2018	14:00	14:02		Yes	Yes	Forearm	F1	5 ug
B0060	Group B3	A/Sing MAP, 15 ug	Day 1	25-MAY-2018	14:03	14:05		Yes	Yes	Forearm	F2	5 ug
B0060	Group B3	A/Sing MAP, 15 ug	Day 1	25-MAY-2018	14:06	14:08		Yes	Yes	Forearm	F3	5 ug
B0061	Group B8	A/Sing MAP, 15 ug	Day 1	25-MAY-2018	14:20	14:22		Yes	Yes	Upper Arm	D1	5 ug
B0061	Group B8	A/Sing MAP, 15 ug	Day 1	25-MAY-2018	14:23	14:25		Yes	Yes	Upper Arm	D2	5 ug
B0061	Group B8	A/Sing MAP, 15 ug	Day 1	25-MAY-2018	14:26	14:28		Yes	Yes	Upper Arm	D3	5 ug
B0062	Group B5	A/Sing MAP, 5 ug	Day 1	25-MAY-2018	14:30	14:32		Yes	Yes	Forearm	F1	uncoated
B0062	Group B5	A/Sing MAP, 5 ug	Day 1	25-MAY-2018	14:33	14:35		Yes	Yes	Forearm	F2	5 ug
B0062	Group B5	A/Sing MAP, 5 ug	Day 1	25-MAY-2018	14:36	14:38		Yes	Yes	Forearm	F3	uncoated
B0063	Group B7	Placebo MAP	Day 1	25-MAY-2018	14:40	14:42		Yes	Yes	Forearm	F1	uncoated
B0063	Group B7	Placebo MAP	Day 1	25-MAY-2018	14:43	14:45		Yes	Yes	Forearm	F2	uncoated
B0063	Group B7	Placebo MAP	Day 1	25-MAY-2018	14:46	14:48		Yes	Yes	Forearm	F3	uncoated

Note: Delivered Dose=(Labelled Dose)/2 for MAP

Administration Site Key: FA = Forearm; UA= Upper arm
Treatments Part A: A1 = A/Sing MAP, 15 ug (FA); A2 = Afluria Quad IM (UA), 15 ug; A3 = Placebo MAP (FA); A4 = A/Sing Mono IM, 15 ug (UA)
Treatments Part B: B1 = A/Sing MAP, 15 ug (FA) with biopsy; B2 = Placebo MAP (FA) with biopsy
B3 = A/Sing MAP, 15 ug (FA); B4 = A/Sing MAP, 10 ug (FA); B5 = A/Sing MAP, 5 ug (FA); B6 = A/Sing MAP, 2.5 ug (FA)
B7 = Placebo MAP (FA); B8 = A/Sing MAP, 15 ug (UA); B9 = A/Sing IM, 15 ug, as 2018 Afluria Quad (UA)

Listing 16.2.5.1
Study Drug Administration

Part B

Randomi-sation Number	Group	Treatment	Study Day	Date of Dose	Time of MAP application	Time of MAP removal	Time of IM Injection	Was Dose Adminis-tered?	Dosed as per Protocol	Administra-tion Site	Site Number	Dose Administered at Site
B0064	Group B9	A/Sing IM, 15 ug	Day 1	25-MAY-2018			12:40	Yes	Yes	Upper Arm	D1	[IM]
B0065	Group B4	A/Sing MAP, 10 ug	Day 1	25-MAY-2018	14:50	14:52		Yes	Yes	Forearm	F1	5 ug
B0065	Group B4	A/Sing MAP, 10 ug	Day 1	25-MAY-2018	14:53	14:55		Yes	Yes	Forearm	F2	uncoated
B0065	Group B4	A/Sing MAP, 10 ug	Day 1	25-MAY-2018	14:56	14:58		Yes	Yes	Forearm	F3	5 ug
B0066	Group B6	A/Sing MAP, 2.5 ug	Day 1	25-MAY-2018	15:00	15:02		Yes	Yes	Forearm	F1	2.5 ug
B0066	Group B6	A/Sing MAP, 2.5 ug	Day 1	25-MAY-2018	15:03	15:05		Yes	Yes	Forearm	F2	uncoated
B0066	Group B6	A/Sing MAP, 2.5 ug	Day 1	25-MAY-2018	15:06	15:08		Yes	Yes	Forearm	F3	uncoated
B0067	Group B8	A/Sing MAP, 15 ug	Day 1	25-MAY-2018	15:10	15:12		Yes	Yes	Upper Arm	D1	5 ug
B0067	Group B8	A/Sing MAP, 15 ug	Day 1	25-MAY-2018	15:13	15:15		Yes	Yes	Upper Arm	D2	5 ug
B0067	Group B8	A/Sing MAP, 15 ug	Day 1	25-MAY-2018	15:16	15:18		Yes	Yes	Upper Arm	D3	5 ug
B0068	Group B5	A/Sing MAP, 5 ug	Day 1	25-MAY-2018	15:20	15:22		Yes	Yes	Forearm	F1	uncoated
B0068	Group B5	A/Sing MAP, 5 ug	Day 1	25-MAY-2018	15:24	15:26		Yes	Yes	Forearm	F3	uncoated
B0068	Group B5	A/Sing MAP, 5 ug	Day 1	25-MAY-2018	15:58	16:00		Yes	Yes	Forearm	F2	5 ug

Note: Delivered Dose=(Labelled Dose)/2 for MAP

Administration Site Key: FA = Forearm; UA= Upper arm
Treatments Part A: A1 = A/Sing MAP, 15 ug (FA); A2 = Afluria Quad IM (UA), 15 ug; A3 = Placebo MAP (FA); A4 = A/Sing Mono IM, 15 ug (UA)
Treatments Part B: B1 = A/Sing MAP, 15 ug (FA) with biopsy; B2 = Placebo MAP (FA) with biopsy
B3 = A/Sing MAP, 15 ug (FA); B4 = A/Sing MAP, 10 ug (FA); B5 = A/Sing MAP, 5 ug (FA); B6 = A/Sing MAP, 2.5 ug (FA)
B7 = Placebo MAP (FA); B8 = A/Sing MAP, 15 ug (UA); B9 = A/Sing IM, 15 ug, as 2018 Afluria Quad (UA)

Listing 16.2.5.1
Study Drug Administration

Part B

Randomi-sation Number	Group	Treatment	Study Day	Date of Dose	Time of MAP application	Time of MAP removal	Time of IM Injection	Was Dose Adminis-tered?	Dosed as per Protocol	Administra-tion Site	Site Number	Dose Administered at Site
B0069	Group B7	Placebo MAP	Day 1	01-JUN-2018	12:00	12:02		Yes	Yes	Forearm	F1	uncoated
B0069	Group B7	Placebo MAP	Day 1	01-JUN-2018	12:03	12:05		Yes	Yes	Forearm	F2	uncoated
B0069	Group B7	Placebo MAP	Day 1	01-JUN-2018	12:06	12:08		Yes	Yes	Forearm	F3	uncoated
B0070	Group B9	A/Sing IM, 15 ug	Day 1	01-JUN-2018			11:20	Yes	Yes	Upper Arm	D1	[IM]
B0071	Group B4	A/Sing MAP, 10 ug	Day 1	01-JUN-2018	11:30	11:32		Yes	Yes	Forearm	F1	5 ug
B0071	Group B4	A/Sing MAP, 10 ug	Day 1	01-JUN-2018	11:33	11:35		Yes	Yes	Forearm	F2	uncoated
B0071	Group B4	A/Sing MAP, 10 ug	Day 1	01-JUN-2018	11:36	11:38		Yes	Yes	Forearm	F3	5 ug
B0072	Group B3	A/Sing MAP, 15 ug	Day 1	01-JUN-2018	11:40	11:42		Yes	Yes	Forearm	F1	5 ug
B0072	Group B3	A/Sing MAP, 15 ug	Day 1	01-JUN-2018	11:43	11:45		Yes	Yes	Forearm	F2	5 ug
B0072	Group B3	A/Sing MAP, 15 ug	Day 1	01-JUN-2018	11:46	11:48		Yes	Yes	Forearm	F3	5 ug
B0073	Group B6	A/Sing MAP, 2.5 ug	Day 1	01-JUN-2018	11:50	11:52		Yes	Yes	Forearm	F1	uncoated
B0073	Group B6	A/Sing MAP, 2.5 ug	Day 1	01-JUN-2018	11:53	11:55		Yes	Yes	Forearm	F2	2.5 ug
B0073	Group B6	A/Sing MAP, 2.5 ug	Day 1	01-JUN-2018	11:56	11:58		Yes	Yes	Forearm	F3	uncoated

Note: Delivered Dose=(Labelled Dose)/2 for MAP

Administration Site Key: FA = Forearm; UA= Upper arm
Treatments Part A: A1 = A/Sing MAP, 15 ug (FA); A2 = Afluria Quad IM (UA), 15 ug; A3 = Placebo MAP (FA); A4 = A/Sing Mono IM, 15 ug (UA)
Treatments Part B: B1 = A/Sing MAP, 15 ug (FA) with biopsy; B2 = Placebo MAP (FA) with biopsy
B3 = A/Sing MAP, 15 ug (FA); B4 = A/Sing MAP, 10 ug (FA); B5 = A/Sing MAP, 5 ug (FA); B6 = A/Sing MAP, 2.5 ug (FA)
B7 = Placebo MAP (FA); B8 = A/Sing MAP, 15 ug (UA); B9 = A/Sing IM, 15 ug, as 2018 Afluria Quad (UA)

Listing 16.2.5.1
Study Drug Administration

Part B

Randomi-sation Number	Group	Treatment	Study Day	Date of Dose	Time of MAP application	Time of MAP removal	Time of IM Injection	Was Dose Adminis-tered?	Dosed as per Protocol	Administra-tion Site	Site Number	Dose Administered at Site
B0074	Group B9	A/Sing IM, 15 ug	Day 1	01-JUN-2018			11:02	Yes	Yes	Upper Arm	D1	[IM]
B0075	Group B3	A/Sing MAP, 15 ug	Day 1	01-JUN-2018	12:10	12:12		Yes	Yes	Forearm	F1	5 ug
B0075	Group B3	A/Sing MAP, 15 ug	Day 1	01-JUN-2018	12:13	12:15		Yes	Yes	Forearm	F2	5 ug
B0075	Group B3	A/Sing MAP, 15 ug	Day 1	01-JUN-2018	12:16	12:18		Yes	Yes	Forearm	F3	5 ug
B0077	Group B4	A/Sing MAP, 10 ug	Day 1	01-JUN-2018	12:20	12:22		Yes	Yes	Forearm	F1	5 ug
B0077	Group B4	A/Sing MAP, 10 ug	Day 1	01-JUN-2018	12:23	12:25		Yes	Yes	Forearm	F2	uncoated
B0077	Group B4	A/Sing MAP, 10 ug	Day 1	01-JUN-2018	12:26	12:28		Yes	Yes	Forearm	F3	5 ug
B0078	Group B8	A/Sing MAP, 15 ug	Day 1	01-JUN-2018	12:30	12:32		Yes	Yes	Upper Arm	D1	5 ug
B0078	Group B8	A/Sing MAP, 15 ug	Day 1	01-JUN-2018	12:33	12:35		Yes	Yes	Upper Arm	D2	5 ug
B0078	Group B8	A/Sing MAP, 15 ug	Day 1	01-JUN-2018	12:36	12:38		Yes	Yes	Upper Arm	D3	5 ug
B0079	Group B6	A/Sing MAP, 2.5 ug	Day 1	01-JUN-2018	12:40	12:42		Yes	Yes	Forearm	F1	uncoated
B0079	Group B6	A/Sing MAP, 2.5 ug	Day 1	01-JUN-2018	12:43	12:45		Yes	Yes	Forearm	F2	2.5 ug
B0079	Group B6	A/Sing MAP, 2.5 ug	Day 1	01-JUN-2018	12:46	12:48		Yes	Yes	Forearm	F3	uncoated

Note: Delivered Dose=(Labelled Dose)/2 for MAP

Administration Site Key: FA = Forearm; UA= Upper arm
Treatments Part A: A1 = A/Sing MAP, 15 ug (FA); A2 = Afluria Quad IM (UA), 15 ug; A3 = Placebo MAP (FA); A4 = A/Sing Mono IM, 15 ug (UA)
Treatments Part B: B1 = A/Sing MAP, 15 ug (FA) with biopsy; B2 = Placebo MAP (FA) with biopsy
B3 = A/Sing MAP, 15 ug (FA); B4 = A/Sing MAP, 10 ug (FA); B5 = A/Sing MAP, 5 ug (FA); B6 = A/Sing MAP, 2.5 ug (FA)
B7 = Placebo MAP (FA); B8 = A/Sing MAP, 15 ug (UA); B9 = A/Sing IM, 15 ug, as 2018 Afluria Quad (UA)

Listing 16.2.5.1
Study Drug Administration

Part B

Randomi-sation Number	Group	Treatment	Study Day	Date of Dose	Time of MAP application	Time of MAP removal	Time of IM Injection	Was Dose Adminis-tered?	Dosed as per Protocol	Administra-tion Site	Site Number	Dose Administered at Site
B0081	Group B6	A/Sing MAP, 2.5 ug	Day 1	01-JUN-2018	12:50	12:52		Yes	Yes	Forearm	F1	2.5 ug
B0081	Group B6	A/Sing MAP, 2.5 ug	Day 1	01-JUN-2018	12:53	12:55		Yes	Yes	Forearm	F2	uncoated
B0081	Group B6	A/Sing MAP, 2.5 ug	Day 1	01-JUN-2018	12:56	12:58		Yes	Yes	Forearm	F3	uncoated
B0082	Group B3	A/Sing MAP, 15 ug	Day 1	01-JUN-2018	13:20	13:22		Yes	Yes	Forearm	F1	5 ug
B0082	Group B3	A/Sing MAP, 15 ug	Day 1	01-JUN-2018	13:23	13:25		Yes	Yes	Forearm	F2	5 ug
B0082	Group B3	A/Sing MAP, 15 ug	Day 1	01-JUN-2018	13:26	13:28		Yes	Yes	Forearm	F3	5 ug
B0083	Group B8	A/Sing MAP, 15 ug	Day 1	01-JUN-2018	13:40	13:42		Yes	Yes	Upper Arm	D1	5 ug
B0083	Group B8	A/Sing MAP, 15 ug	Day 1	01-JUN-2018	13:43	13:45		Yes	Yes	Upper Arm	D2	5 ug
B0083	Group B8	A/Sing MAP, 15 ug	Day 1	01-JUN-2018	13:46	13:48		Yes	Yes	Upper Arm	D3	5 ug
B0084	Group B5	A/Sing MAP, 5 ug	Day 1	09-JUN-2018	11:50	11:52		Yes	Yes	Forearm	F1	uncoated
B0084	Group B5	A/Sing MAP, 5 ug	Day 1	09-JUN-2018	11:53	11:55		Yes	Yes	Forearm	F2	uncoated
B0084	Group B5	A/Sing MAP, 5 ug	Day 1	09-JUN-2018	11:56	11:58		Yes	Yes	Forearm	F3	5 ug
B0085	Group B4	A/Sing MAP, 10 ug	Day 1	09-JUN-2018	12:00	12:02		Yes	Yes	Forearm	F1	uncoated
B0085	Group B4	A/Sing MAP, 10 ug	Day 1	09-JUN-2018	12:03	12:05		Yes	Yes	Forearm	F2	5 ug

Note: Delivered Dose=(Labelled Dose)/2 for MAP

Administration Site Key: FA = Forearm; UA= Upper arm
Treatments Part A: A1 = A/Sing MAP, 15 ug (FA); A2 = Afluria Quad IM (UA), 15 ug; A3 = Placebo MAP (FA); A4 = A/Sing Mono IM, 15 ug (UA)
Treatments Part B: B1 = A/Sing MAP, 15 ug (FA) with biopsy; B2 = Placebo MAP (FA) with biopsy
B3 = A/Sing MAP, 15 ug (FA); B4 = A/Sing MAP, 10 ug (FA); B5 = A/Sing MAP, 5 ug (FA); B6 = A/Sing MAP, 2.5 ug (FA)
B7 = Placebo MAP (FA); B8 = A/Sing MAP, 15 ug (UA); B9 = A/Sing IM, 15 ug, as 2018 Afluria Quad (UA)

Listing 16.2.5.1
Study Drug Administration

Part B

Randomi-sation Number	Group	Treatment	Study Day	Date of Dose	Time of MAP application	Time of MAP removal	Time of IM Injection	Was Dose Adminis-tered?	Dosed as per Protocol	Administra-tion Site	Site Number	Dose Administered at Site
B0085	Group B4	A/Sing MAP, 10 ug	Day 1	09-JUN-2018	12:06	12:08		Yes	Yes	Forearm	F3	5 ug
B0086	Group B9	A/Sing IM, 15 ug	Day 1	09-JUN-2018			11:20	Yes	Yes	Upper Arm	D1	[IM]
B0087	Group B7	Placebo MAP	Day 1	09-JUN-2018	12:10	12:12		Yes	Yes	Forearm	F1	uncoated
B0087	Group B7	Placebo MAP	Day 1	09-JUN-2018	12:13	12:15		Yes	Yes	Forearm	F2	uncoated
B0087	Group B7	Placebo MAP	Day 1	09-JUN-2018	12:16	12:18		Yes	Yes	Forearm	F3	uncoated
B0088	Group B9	A/Sing IM, 15 ug	Day 1	09-JUN-2018			11:10	Yes	Yes	Upper Arm	D1	[IM]
B0089	Group B5	A/Sing MAP, 5 ug	Day 1	09-JUN-2018	12:20	12:22		Yes	Yes	Forearm	F1	uncoated
B0089	Group B5	A/Sing MAP, 5 ug	Day 1	09-JUN-2018	12:23	12:25		Yes	Yes	Forearm	F2	5 ug
B0089	Group B5	A/Sing MAP, 5 ug	Day 1	09-JUN-2018	12:26	12:28		Yes	Yes	Forearm	F3	uncoated
B0090	Group B6	A/Sing MAP, 2.5 ug	Day 1	09-JUN-2018	12:30	12:32		Yes	Yes	Forearm	F1	2.5 ug
B0090	Group B6	A/Sing MAP, 2.5 ug	Day 1	09-JUN-2018	12:33	12:35		Yes	Yes	Forearm	F2	uncoated
B0090	Group B6	A/Sing MAP, 2.5 ug	Day 1	09-JUN-2018	12:36	12:38		Yes	Yes	Forearm	F3	uncoated

Note: Delivered Dose=(Labelled Dose)/2 for MAP

Administration Site Key: FA = Forearm; UA= Upper arm
Treatments Part A: A1 = A/Sing MAP, 15 ug (FA); A2 = Afluria Quad IM (UA), 15 ug; A3 = Placebo MAP (FA); A4 = A/Sing Mono IM, 15 ug (UA)
Treatments Part B: B1 = A/Sing MAP, 15 ug (FA) with biopsy; B2 = Placebo MAP (FA) with biopsy
B3 = A/Sing MAP, 15 ug (FA); B4 = A/Sing MAP, 10 ug (FA); B5 = A/Sing MAP, 5 ug (FA); B6 = A/Sing MAP, 2.5 ug (FA)
B7 = Placebo MAP (FA); B8 = A/Sing MAP, 15 ug (UA); B9 = A/Sing IM, 15 ug, as 2018 Afluria Quad (UA)

Listing 16.2.5.1
Study Drug Administration

Part B

Randomi-sation Number	Group	Treatment	Study Day	Date of Dose	Time of MAP application	Time of MAP removal	Time of IM Injection	Was Dose Adminis-tered?	Dosed as per Protocol	Administra-tion Site	Site Number	Dose Administered at Site
B0091	Group B8	A/Sing MAP, 15 ug	Day 1	09-JUN-2018	12:40	12:42		Yes	Yes	Upper Arm	D1	5 ug
B0091	Group B8	A/Sing MAP, 15 ug	Day 1	09-JUN-2018	12:43	12:45		Yes	Yes	Upper Arm	D2	5 ug
B0091	Group B8	A/Sing MAP, 15 ug	Day 1	09-JUN-2018	12:46	12:48		Yes	Yes	Upper Arm	D3	5 ug
B0092	Group B4	A/Sing MAP, 10 ug	Day 1	09-JUN-2018	12:50	12:52		Yes	Yes	Forearm	F1	5 ug
B0092	Group B4	A/Sing MAP, 10 ug	Day 1	09-JUN-2018	12:53	12:55		Yes	Yes	Forearm	F2	uncoated
B0092	Group B4	A/Sing MAP, 10 ug	Day 1	09-JUN-2018	12:56	12:58		Yes	Yes	Forearm	F3	5 ug
B0093	Group B7	Placebo MAP	Day 1	09-JUN-2018	13:00	13:02		Yes	Yes	Forearm	F1	uncoated
B0093	Group B7	Placebo MAP	Day 1	09-JUN-2018	13:03	13:05		Yes	Yes	Forearm	F2	uncoated
B0093	Group B7	Placebo MAP	Day 1	09-JUN-2018	13:06	13:08		Yes	Yes	Forearm	F3	uncoated
B0094	Group B3	A/Sing MAP, 15 ug	Day 1	09-JUN-2018	13:10	13:12		Yes	Yes	Forearm	F1	5 ug
B0094	Group B3	A/Sing MAP, 15 ug	Day 1	09-JUN-2018	13:13	13:15		Yes	Yes	Forearm	F2	5 ug
B0094	Group B3	A/Sing MAP, 15 ug	Day 1	09-JUN-2018	13:16	13:18		Yes	Yes	Forearm	F3	5 ug
B0095	Group B9	A/Sing IM, 15 ug	Day 1	09-JUN-2018			11:40	Yes	Yes	Upper Arm	D1	[IM]

Note: Delivered Dose=(Labelled Dose)/2 for MAP

Administration Site Key: FA = Forearm; UA= Upper arm
Treatments Part A: A1 = A/Sing MAP, 15 ug (FA); A2 = Afluria Quad IM (UA), 15 ug; A3 = Placebo MAP (FA); A4 = A/Sing Mono IM, 15 ug (UA)
Treatments Part B: B1 = A/Sing MAP, 15 ug (FA) with biopsy; B2 = Placebo MAP (FA) with biopsy
B3 = A/Sing MAP, 15 ug (FA); B4 = A/Sing MAP, 10 ug (FA); B5 = A/Sing MAP, 5 ug (FA); B6 = A/Sing MAP, 2.5 ug (FA)
B7 = Placebo MAP (FA); B8 = A/Sing MAP, 15 ug (UA); B9 = A/Sing IM, 15 ug, as 2018 Afluria Quad (UA)

Listing 16.2.5.1
Study Drug Administration

Part B

Randomi-sation Number	Group	Treatment	Study Day	Date of Dose	Time of MAP application	Time of MAP removal	Time of IM Injection	Was Dose Adminis-tered?	Dosed as per Protocol	Administra-tion Site	Site Number	Dose Administered at Site
B0096	Group B6	A/Sing MAP, 2.5 ug	Day 1	09-JUN-2018	13:20	13:22		Yes	Yes	Forearm	F1	2.5 ug
B0096	Group B6	A/Sing MAP, 2.5 ug	Day 1	09-JUN-2018	13:23	13:25		Yes	Yes	Forearm	F2	uncoated
B0096	Group B6	A/Sing MAP, 2.5 ug	Day 1	09-JUN-2018	13:26	13:28		Yes	Yes	Forearm	F3	uncoated
B0097	Group B5	A/Sing MAP, 5 ug	Day 1	09-JUN-2018	13:30	13:32		Yes	Yes	Forearm	F1	5 ug
B0097	Group B5	A/Sing MAP, 5 ug	Day 1	09-JUN-2018	13:33	13:35		Yes	Yes	Forearm	F2	uncoated
B0097	Group B5	A/Sing MAP, 5 ug	Day 1	09-JUN-2018	13:36	13:38		Yes	Yes	Forearm	F3	uncoated
B0098	Group B7	Placebo MAP	Day 1	09-JUN-2018	13:40	13:42		Yes	Yes	Forearm	F1	uncoated
B0098	Group B7	Placebo MAP	Day 1	09-JUN-2018	13:43	13:45		Yes	Yes	Forearm	F2	uncoated
B0098	Group B7	Placebo MAP	Day 1	09-JUN-2018	13:46	13:48		Yes	Yes	Forearm	F3	uncoated
B0099	Group B4	A/Sing MAP, 10 ug	Day 1	09-JUN-2018	15:10	15:12		Yes	Yes	Forearm	F1	5 ug
B0099	Group B4	A/Sing MAP, 10 ug	Day 1	09-JUN-2018	15:13	15:15		Yes	Yes	Forearm	F2	5 ug
B0099	Group B4	A/Sing MAP, 10 ug	Day 1	09-JUN-2018	15:16	15:18		Yes	Yes	Forearm	F3	uncoated
B0100	Group B8	A/Sing MAP, 15 ug	Day 1	09-JUN-2018	13:50	13:52		Yes	Yes	Upper Arm	D1	5 ug
B0100	Group B8	A/Sing MAP, 15 ug	Day 1	09-JUN-2018	13:53	13:55		Yes	Yes	Upper Arm	D2	5 ug

Note: Delivered Dose=(Labelled Dose)/2 for MAP

Administration Site Key: FA = Forearm; UA= Upper arm
Treatments Part A: A1 = A/Sing MAP, 15 ug (FA); A2 = Afluria Quad IM (UA), 15 ug; A3 = Placebo MAP (FA); A4 = A/Sing Mono IM, 15 ug (UA)
Treatments Part B: B1 = A/Sing MAP, 15 ug (FA) with biopsy; B2 = Placebo MAP (FA) with biopsy
B3 = A/Sing MAP, 15 ug (FA); B4 = A/Sing MAP, 10 ug (FA); B5 = A/Sing MAP, 5 ug (FA); B6 = A/Sing MAP, 2.5 ug (FA)
B7 = Placebo MAP (FA); B8 = A/Sing MAP, 15 ug (UA); B9 = A/Sing IM, 15 ug, as 2018 Afluria Quad (UA)

Listing 16.2.5.1
Study Drug Administration

Part B

Randomi-sation Number	Group	Treatment	Study Day	Date of Dose	Time of MAP application	Time of MAP removal	Time of IM Injection	Was Dose Adminis-tered?	Dosed as per Protocol	Administra-tion Site	Site Number	Dose Administered at Site
B0100	Group B8	A/Sing MAP, 15 ug	Day 1	09-JUN-2018	13:56	13:58		Yes	Yes	Upper Arm	D3	5 ug
B0101	Group B3	A/Sing MAP, 15 ug	Day 1	09-JUN-2018	14:00	14:02		Yes	Yes	Forearm	F1	5 ug
B0101	Group B3	A/Sing MAP, 15 ug	Day 1	09-JUN-2018	14:03	14:05		Yes	Yes	Forearm	F2	5 ug
B0101	Group B3	A/Sing MAP, 15 ug	Day 1	09-JUN-2018	14:06	14:08		Yes	Yes	Forearm	F3	5 ug
B0102	Group B8	A/Sing MAP, 15 ug	Day 1	09-JUN-2018	14:10	14:12		Yes	Yes	Upper Arm	D1	5 ug
B0102	Group B8	A/Sing MAP, 15 ug	Day 1	09-JUN-2018	14:13	14:15		Yes	Yes	Upper Arm	D2	5 ug
B0102	Group B8	A/Sing MAP, 15 ug	Day 1	09-JUN-2018	14:16	14:18		Yes	Yes	Upper Arm	D3	5 ug
B0103	Group B5	A/Sing MAP, 5 ug	Day 1	09-JUN-2018	14:20	14:22		Yes	Yes	Forearm	F1	5 ug
B0103	Group B5	A/Sing MAP, 5 ug	Day 1	09-JUN-2018	14:23	14:25		Yes	Yes	Forearm	F2	uncoated
B0103	Group B5	A/Sing MAP, 5 ug	Day 1	09-JUN-2018	14:26	14:28		Yes	Yes	Forearm	F3	uncoated
B0104	Group B3	A/Sing MAP, 15 ug	Day 1	09-JUN-2018	14:30	14:32		Yes	Yes	Forearm	F1	5 ug
B0104	Group B3	A/Sing MAP, 15 ug	Day 1	09-JUN-2018	14:33	14:35		Yes	Yes	Forearm	F2	5 ug
B0104	Group B3	A/Sing MAP, 15 ug	Day 1	09-JUN-2018	14:36	14:38		Yes	Yes	Forearm	F3	5 ug

Note: Delivered Dose=(Labelled Dose)/2 for MAP

Administration Site Key: FA = Forearm; UA= Upper arm
Treatments Part A: A1 = A/Sing MAP, 15 ug (FA); A2 = Afluria Quad IM (UA), 15 ug; A3 = Placebo MAP (FA); A4 = A/Sing Mono IM, 15 ug (UA)
Treatments Part B: B1 = A/Sing MAP, 15 ug (FA) with biopsy; B2 = Placebo MAP (FA) with biopsy
B3 = A/Sing MAP, 15 ug (FA); B4 = A/Sing MAP, 10 ug (FA); B5 = A/Sing MAP, 5 ug (FA); B6 = A/Sing MAP, 2.5 ug (FA)
B7 = Placebo MAP (FA); B8 = A/Sing MAP, 15 ug (UA); B9 = A/Sing IM, 15 ug, as 2018 Afluria Quad (UA)

Listing 16.2.5.1
Study Drug Administration

Part B

Randomi-sation Number	Group	Treatment	Study Day	Date of Dose	Time of MAP application	Time of MAP removal	Time of IM Injection	Was Dose Adminis-tered?	Dosed as per Protocol	Administra-tion Site	Site Number	Dose Administered at Site
B0105	Group B9	A/Sing IM, 15 ug	Day 1	09-JUN-2018			11:30	Yes	Yes	Upper Arm	D1	[IM]
B0106	Group B4	A/Sing MAP, 10 ug	Day 1	09-JUN-2018	14:40	14:42		Yes	Yes	Forearm	F1	uncoated
B0106	Group B4	A/Sing MAP, 10 ug	Day 1	09-JUN-2018	14:43	14:45		Yes	Yes	Forearm	F2	5 ug
B0106	Group B4	A/Sing MAP, 10 ug	Day 1	09-JUN-2018	14:46	14:48		Yes	Yes	Forearm	F3	5 ug
B0107	Group B6	A/Sing MAP, 2.5 ug	Day 1	09-JUN-2018	14:50	14:52		Yes	Yes	Forearm	F1	uncoated
B0107	Group B6	A/Sing MAP, 2.5 ug	Day 1	09-JUN-2018	14:53	14:55		Yes	Yes	Forearm	F2	uncoated
B0107	Group B6	A/Sing MAP, 2.5 ug	Day 1	09-JUN-2018	14:56	14:58		Yes	Yes	Forearm	F3	2.5 ug
B0108	Group B7	Placebo MAP	Day 1	09-JUN-2018	15:00	15:02		Yes	Yes	Forearm	F1	uncoated
B0108	Group B7	Placebo MAP	Day 1	09-JUN-2018	15:03	15:05		Yes	Yes	Forearm	F2	uncoated
B0108	Group B7	Placebo MAP	Day 1	09-JUN-2018	15:06	15:08		Yes	Yes	Forearm	F3	uncoated
B0109	Group B6	A/Sing MAP, 2.5 ug	Day 1	16-JUN-2018	11:50	11:52		Yes	Yes	Forearm	F1	uncoated
B0109	Group B6	A/Sing MAP, 2.5 ug	Day 1	16-JUN-2018	11:53	11:55		Yes	Yes	Forearm	F2	2.5 ug
B0109	Group B6	A/Sing MAP, 2.5 ug	Day 1	16-JUN-2018	11:56	11:58		Yes	Yes	Forearm	F3	uncoated

Note: Delivered Dose=(Labelled Dose)/2 for MAP

Administration Site Key: FA = Forearm; UA= Upper arm
Treatments Part A: A1 = A/Sing MAP, 15 ug (FA); A2 = Afluria Quad IM (UA), 15 ug; A3 = Placebo MAP (FA); A4 = A/Sing Mono IM, 15 ug (UA)
Treatments Part B: B1 = A/Sing MAP, 15 ug (FA) with biopsy; B2 = Placebo MAP (FA) with biopsy
B3 = A/Sing MAP, 15 ug (FA); B4 = A/Sing MAP, 10 ug (FA); B5 = A/Sing MAP, 5 ug (FA); B6 = A/Sing MAP, 2.5 ug (FA)
B7 = Placebo MAP (FA); B8 = A/Sing MAP, 15 ug (UA); B9 = A/Sing IM, 15 ug, as 2018 Afluria Quad (UA)

Listing 16.2.5.1
Study Drug Administration

Part B

Randomi-sation Number	Group	Treatment	Study Day	Date of Dose	Time of MAP application	Time of MAP removal	Time of IM Injection	Was Dose Adminis-tered?	Dosed as per Protocol	Administra-tion Site	Site Number	Dose Administered at Site
B0110	Group B3	A/Sing MAP, 15 ug	Day 1	16-JUN-2018	12:00	12:02		Yes	Yes	Forearm	F1	5 ug
B0110	Group B3	A/Sing MAP, 15 ug	Day 1	16-JUN-2018	12:03	12:05		Yes	Yes	Forearm	F2	5 ug
B0110	Group B3	A/Sing MAP, 15 ug	Day 1	16-JUN-2018	12:06	12:08		Yes	Yes	Forearm	F3	5 ug
B0111	Group B8	A/Sing MAP, 15 ug	Day 1	16-JUN-2018	12:10	12:12		Yes	Yes	Upper Arm	D1	5 ug
B0111	Group B8	A/Sing MAP, 15 ug	Day 1	16-JUN-2018	12:13	12:15		Yes	Yes	Upper Arm	D2	5 ug
B0111	Group B8	A/Sing MAP, 15 ug	Day 1	16-JUN-2018	12:16	12:18		Yes	Yes	Upper Arm	D3	5 ug
B0112	Group B4	A/Sing MAP, 10 ug	Day 1	16-JUN-2018	12:20	12:22		Yes	Yes	Forearm	F1	uncoated
B0112	Group B4	A/Sing MAP, 10 ug	Day 1	16-JUN-2018	12:23	12:25		Yes	Yes	Forearm	F2	5 ug
B0112	Group B4	A/Sing MAP, 10 ug	Day 1	16-JUN-2018	12:26	12:28		Yes	Yes	Forearm	F3	5 ug
B0113	Group B5	A/Sing MAP, 5 ug	Day 1	16-JUN-2018	12:30	12:32		Yes	Yes	Forearm	F1	uncoated
B0113	Group B5	A/Sing MAP, 5 ug	Day 1	16-JUN-2018	12:33	12:35		Yes	Yes	Forearm	F2	5 ug
B0113	Group B5	A/Sing MAP, 5 ug	Day 1	16-JUN-2018	12:36	12:38		Yes	Yes	Forearm	F3	uncoated
B0114	Group B7	Placebo MAP	Day 1	16-JUN-2018	12:40	12:42		Yes	Yes	Forearm	F1	uncoated
B0114	Group B7	Placebo MAP	Day 1	16-JUN-2018	12:43	12:45		Yes	Yes	Forearm	F2	uncoated

Note: Delivered Dose=(Labelled Dose)/2 for MAP

Administration Site Key: FA = Forearm; UA= Upper arm
Treatments Part A: A1 = A/Sing MAP, 15 ug (FA); A2 = Afluria Quad IM (UA), 15 ug; A3 = Placebo MAP (FA); A4 = A/Sing Mono IM, 15 ug (UA)
Treatments Part B: B1 = A/Sing MAP, 15 ug (FA) with biopsy; B2 = Placebo MAP (FA) with biopsy
B3 = A/Sing MAP, 15 ug (FA); B4 = A/Sing MAP, 10 ug (FA); B5 = A/Sing MAP, 5 ug (FA); B6 = A/Sing MAP, 2.5 ug (FA)
B7 = Placebo MAP (FA); B8 = A/Sing MAP, 15 ug (UA); B9 = A/Sing IM, 15 ug, as 2018 Afluria Quad (UA)

Listing 16.2.5.1
Study Drug Administration

Part B

Randomi-sation Number	Group	Treatment	Study Day	Date of Dose	Time of MAP application	Time of MAP removal	Time of IM Injection	Was Dose Adminis-tered?	Dosed as per Protocol	Administra-tion Site	Site Number	Dose Administered at Site
B0114	Group B7	Placebo MAP	Day 1	16-JUN-2018	12:46	12:48		Yes	Yes	Forearm	F3	uncoated
B0115	Group B9	A/Sing IM, 15 ug	Day 1	16-JUN-2018			11:00	Yes	Yes	Upper Arm	D1	[IM]
B0116	Group B7	Placebo MAP	Day 1	16-JUN-2018	12:50	12:52		Yes	Yes	Forearm	F1	uncoated
B0116	Group B7	Placebo MAP	Day 1	16-JUN-2018	12:53	12:55		Yes	Yes	Forearm	F2	uncoated
B0116	Group B7	Placebo MAP	Day 1	16-JUN-2018	12:56	12:58		Yes	Yes	Forearm	F3	uncoated
B0117	Group B9	A/Sing IM, 15 ug	Day 1	16-JUN-2018			11:10	Yes	Yes	Upper Arm	D1	[IM]
B0118	Group B6	A/Sing MAP, 2.5 ug	Day 1	16-JUN-2018	13:01	13:03		Yes	Yes	Forearm	F1	uncoated
B0118	Group B6	A/Sing MAP, 2.5 ug	Day 1	16-JUN-2018	14:37	14:39		Yes	Yes	Forearm	F2	2.5 ug
B0118	Group B6	A/Sing MAP, 2.5 ug	Day 1	16-JUN-2018	14:40	14:42		Yes	Yes	Forearm	F3	uncoated
B0119	Group B4	A/Sing MAP, 10 ug	Day 1	16-JUN-2018	13:10	13:12		Yes	Yes	Forearm	F1	5 ug
B0119	Group B4	A/Sing MAP, 10 ug	Day 1	16-JUN-2018	13:13	13:15		Yes	Yes	Forearm	F2	uncoated
B0119	Group B4	A/Sing MAP, 10 ug	Day 1	16-JUN-2018	13:16	13:18		Yes	Yes	Forearm	F3	5 ug

Note: Delivered Dose=(Labelled Dose)/2 for MAP

Administration Site Key: FA = Forearm; UA= Upper arm
Treatments Part A: A1 = A/Sing MAP, 15 ug (FA); A2 = Afluria Quad IM (UA), 15 ug; A3 = Placebo MAP (FA); A4 = A/Sing Mono IM, 15 ug (UA)
Treatments Part B: B1 = A/Sing MAP, 15 ug (FA) with biopsy; B2 = Placebo MAP (FA) with biopsy
B3 = A/Sing MAP, 15 ug (FA); B4 = A/Sing MAP, 10 ug (FA); B5 = A/Sing MAP, 5 ug (FA); B6 = A/Sing MAP, 2.5 ug (FA)
B7 = Placebo MAP (FA); B8 = A/Sing MAP, 15 ug (UA); B9 = A/Sing IM, 15 ug, as 2018 Afluria Quad (UA)

Listing 16.2.5.1
Study Drug Administration

Part B

Randomi-sation Number	Group	Treatment	Study Day	Date of Dose	Time of MAP application	Time of MAP removal	Time of IM Injection	Was Dose Adminis-tered?	Dosed as per Protocol	Administra-tion Site	Site Number	Dose Administered at Site
B0120	Group B3	A/Sing MAP, 15 ug	Day 1	16-JUN-2018	13:20	13:22		Yes	Yes	Forearm	F1	5 ug
B0120	Group B3	A/Sing MAP, 15 ug	Day 1	16-JUN-2018	13:23	13:25		Yes	Yes	Forearm	F2	5 ug
B0120	Group B3	A/Sing MAP, 15 ug	Day 1	16-JUN-2018	13:26	13:28		Yes	Yes	Forearm	F3	5 ug
B0121	Group B8	A/Sing MAP, 15 ug	Day 1	16-JUN-2018	13:30	13:32		Yes	Yes	Upper Arm	D1	5 ug
B0121	Group B8	A/Sing MAP, 15 ug	Day 1	16-JUN-2018	13:33	13:35		Yes	Yes	Upper Arm	D2	5 ug
B0121	Group B8	A/Sing MAP, 15 ug	Day 1	16-JUN-2018	13:36	13:38		Yes	Yes	Upper Arm	D3	5 ug
B0122	Group B5	A/Sing MAP, 5 ug	Day 1	16-JUN-2018	13:40	13:42		Yes	Yes	Forearm	F1	uncoated
B0122	Group B5	A/Sing MAP, 5 ug	Day 1	16-JUN-2018	13:43	13:45		Yes	Yes	Forearm	F2	5 ug
B0122	Group B5	A/Sing MAP, 5 ug	Day 1	16-JUN-2018	13:46	13:48		Yes	Yes	Forearm	F3	uncoated
B0123	Group B6	A/Sing MAP, 2.5 ug	Day 1	16-JUN-2018	13:50	13:52		Yes	Yes	Forearm	F1	2.5 ug
B0123	Group B6	A/Sing MAP, 2.5 ug	Day 1	16-JUN-2018	13:53	13:55		Yes	Yes	Forearm	F2	uncoated
B0123	Group B6	A/Sing MAP, 2.5 ug	Day 1	16-JUN-2018	13:56	13:58		Yes	Yes	Forearm	F3	uncoated
B0124	Group B4	A/Sing MAP, 10 ug	Day 1	16-JUN-2018	14:00	14:02		Yes	Yes	Forearm	F1	uncoated
B0124	Group B4	A/Sing MAP, 10 ug	Day 1	16-JUN-2018	14:03	14:05		Yes	Yes	Forearm	F2	5 ug

Note: Delivered Dose=(Labelled Dose)/2 for MAP

Administration Site Key: FA = Forearm; UA= Upper arm
Treatments Part A: A1 = A/Sing MAP, 15 ug (FA); A2 = Afluria Quad IM (UA), 15 ug; A3 = Placebo MAP (FA); A4 = A/Sing Mono IM, 15 ug (UA)
Treatments Part B: B1 = A/Sing MAP, 15 ug (FA) with biopsy; B2 = Placebo MAP (FA) with biopsy
B3 = A/Sing MAP, 15 ug (FA); B4 = A/Sing MAP, 10 ug (FA); B5 = A/Sing MAP, 5 ug (FA); B6 = A/Sing MAP, 2.5 ug (FA)
B7 = Placebo MAP (FA); B8 = A/Sing MAP, 15 ug (UA); B9 = A/Sing IM, 15 ug, as 2018 Afluria Quad (UA)

Listing 16.2.5.1
Study Drug Administration

Part B

Randomi-sation Number	Group	Treatment	Study Day	Date of Dose	Time of MAP application	Time of MAP removal	Time of IM Injection	Was Dose Adminis-tered?	Dosed as per Protocol	Administra-tion Site	Site Number	Dose Administered at Site
B0124	Group B4	A/Sing MAP, 10 ug	Day 1	16-JUN-2018	14:06	14:08		Yes	Yes	Forearm	F3	5 ug
B0125	Group B7	Placebo MAP	Day 1	16-JUN-2018	14:10	14:12		Yes	Yes	Forearm	F1	uncoated
B0125	Group B7	Placebo MAP	Day 1	16-JUN-2018	14:13	14:15		Yes	Yes	Forearm	F2	uncoated
B0125	Group B7	Placebo MAP	Day 1	16-JUN-2018	14:16	14:18		Yes	Yes	Forearm	F3	uncoated
B0126	Group B3	A/Sing MAP, 15 ug	Day 1	16-JUN-2018	14:20	14:22		Yes	Yes	Forearm	F1	5 ug
B0126	Group B3	A/Sing MAP, 15 ug	Day 1	16-JUN-2018	14:23	14:25		Yes	Yes	Forearm	F2	5 ug
B0126	Group B3	A/Sing MAP, 15 ug	Day 1	16-JUN-2018	14:26	14:28		Yes	Yes	Forearm	F3	5 ug
B0127	Group B9	A/Sing IM, 15 ug	Day 1	16-JUN-2018			11:20	Yes	Yes	Upper Arm	D1	[IM]
B0128	Group B5	A/Sing MAP, 5 ug	Day 1	23-JUN-2018	11:30	11:32		Yes	Yes	Forearm	F1	uncoated
B0128	Group B5	A/Sing MAP, 5 ug	Day 1	23-JUN-2018	11:33	11:35		Yes	Yes	Forearm	F2	5 ug
B0128	Group B5	A/Sing MAP, 5 ug	Day 1	23-JUN-2018	11:36	11:38		Yes	Yes	Forearm	F3	uncoated
B0129	Group B8	A/Sing MAP, 15 ug	Day 1	23-JUN-2018	11:50	11:52		Yes	Yes	Upper Arm	D1	5 ug
B0129	Group B8	A/Sing MAP, 15 ug	Day 1	23-JUN-2018	11:53	11:55		Yes	Yes	Upper Arm	D2	5 ug

Note: Delivered Dose=(Labelled Dose)/2 for MAP

Administration Site Key: FA = Forearm; UA= Upper arm
Treatments Part A: A1 = A/Sing MAP, 15 ug (FA); A2 = Afluria Quad IM (UA), 15 ug; A3 = Placebo MAP (FA); A4 = A/Sing Mono IM, 15 ug (UA)
Treatments Part B: B1 = A/Sing MAP, 15 ug (FA) with biopsy; B2 = Placebo MAP (FA) with biopsy
B3 = A/Sing MAP, 15 ug (FA); B4 = A/Sing MAP, 10 ug (FA); B5 = A/Sing MAP, 5 ug (FA); B6 = A/Sing MAP, 2.5 ug (FA)
B7 = Placebo MAP (FA); B8 = A/Sing MAP, 15 ug (UA); B9 = A/Sing IM, 15 ug, as 2018 Afluria Quad (UA)

Listing 16.2.5.1
Study Drug Administration

Part B

Randomi-sation Number	Group	Treatment	Study Day	Date of Dose	Time of MAP application	Time of MAP removal	Time of IM Injection	Was Dose Adminis-tered?	Dosed as per Protocol	Administra-tion Site	Site Number	Dose Administered at Site
B0129	Group B8	A/Sing MAP, 15 ug	Day 1	23-JUN-2018	11:56	11:58		Yes	Yes	Upper Arm	D3	5 ug
B0130	Group B5	A/Sing MAP, 5 ug	Day 1	23-JUN-2018	11:40	11:42		Yes	Yes	Forearm	F1	5 ug
B0130	Group B5	A/Sing MAP, 5 ug	Day 1	23-JUN-2018	11:43	11:45		Yes	Yes	Forearm	F2	uncoated
B0130	Group B5	A/Sing MAP, 5 ug	Day 1	23-JUN-2018	11:46	11:48		Yes	Yes	Forearm	F3	uncoated
B0131	Group B8	A/Sing MAP, 15 ug	Day 1	23-JUN-2018	12:00	12:02		Yes	Yes	Upper Arm	D1	5 ug
B0131	Group B8	A/Sing MAP, 15 ug	Day 1	23-JUN-2018	12:03	12:05		Yes	Yes	Upper Arm	D2	5 ug
B0131	Group B8	A/Sing MAP, 15 ug	Day 1	23-JUN-2018	12:06	12:08		Yes	Yes	Upper Arm	D3	5 ug
B0132	Group B6	A/Sing MAP, 2.5 ug	Day 1	23-JUN-2018	12:10	12:12		Yes	Yes	Forearm	F1	2.5 ug
B0132	Group B6	A/Sing MAP, 2.5 ug	Day 1	23-JUN-2018	12:13	12:15		Yes	Yes	Forearm	F2	uncoated
B0132	Group B6	A/Sing MAP, 2.5 ug	Day 1	23-JUN-2018	12:16	12:18		Yes	Yes	Forearm	F3	uncoated
B0133	Group B4	A/Sing MAP, 10 ug	Day 1	23-JUN-2018	12:20	12:22		Yes	Yes	Forearm	F1	uncoated
B0133	Group B4	A/Sing MAP, 10 ug	Day 1	23-JUN-2018	12:23	12:25		Yes	Yes	Forearm	F2	5 ug
B0133	Group B4	A/Sing MAP, 10 ug	Day 1	23-JUN-2018	12:26	12:28		Yes	Yes	Forearm	F3	5 ug

Note: Delivered Dose=(Labelled Dose)/2 for MAP

Administration Site Key: FA = Forearm; UA= Upper arm
Treatments Part A: A1 = A/Sing MAP, 15 ug (FA); A2 = Afluria Quad IM (UA), 15 ug; A3 = Placebo MAP (FA); A4 = A/Sing Mono IM, 15 ug (UA)
Treatments Part B: B1 = A/Sing MAP, 15 ug (FA) with biopsy; B2 = Placebo MAP (FA) with biopsy
B3 = A/Sing MAP, 15 ug (FA); B4 = A/Sing MAP, 10 ug (FA); B5 = A/Sing MAP, 5 ug (FA); B6 = A/Sing MAP, 2.5 ug (FA)
B7 = Placebo MAP (FA); B8 = A/Sing MAP, 15 ug (UA); B9 = A/Sing IM, 15 ug, as 2018 Afluria Quad (UA)

Listing 16.2.5.1
Study Drug Administration

Part B

Randomi-sation Number	Group	Treatment	Study Day	Date of Dose	Time of MAP application	Time of MAP removal	Time of IM Injection	Was Dose Adminis-tered?	Dosed as per Protocol	Administra-tion Site	Site Number	Dose Administered at Site
B0134	Group B3	A/Sing MAP, 15 ug	Day 1	23-JUN-2018	12:30	12:32		Yes	Yes	Forearm	F1	5 ug
B0134	Group B3	A/Sing MAP, 15 ug	Day 1	23-JUN-2018	12:33	12:35		Yes	Yes	Forearm	F2	5 ug
B0134	Group B3	A/Sing MAP, 15 ug	Day 1	23-JUN-2018	12:36	12:38		Yes	Yes	Forearm	F3	5 ug
B0135	Group B7	Placebo MAP	Day 1	23-JUN-2018	12:40	12:42		Yes	Yes	Forearm	F1	uncoated
B0135	Group B7	Placebo MAP	Day 1	23-JUN-2018	12:43	12:45		Yes	Yes	Forearm	F2	uncoated
B0135	Group B7	Placebo MAP	Day 1	23-JUN-2018	12:46	12:48		Yes	Yes	Forearm	F3	uncoated
B0136	Group B9	A/Sing IM, 15 ug	Day 1	23-JUN-2018			11:10	Yes	Yes	Upper Arm	D1	[IM]
B0137	Group B8	A/Sing MAP, 15 ug	Day 1	23-JUN-2018	12:50	12:52		Yes	Yes	Upper Arm	D1	5 ug
B0137	Group B8	A/Sing MAP, 15 ug	Day 1	23-JUN-2018	12:53	12:55		Yes	Yes	Upper Arm	D2	5 ug
B0137	Group B8	A/Sing MAP, 15 ug	Day 1	23-JUN-2018	12:56	12:58		Yes	Yes	Upper Arm	D3	5 ug
B0138	Group B7	Placebo MAP	Day 1	23-JUN-2018	13:00	13:02		Yes	Yes	Forearm	F1	uncoated
B0138	Group B7	Placebo MAP	Day 1	23-JUN-2018	13:03	13:05		Yes	Yes	Forearm	F2	uncoated
B0138	Group B7	Placebo MAP	Day 1	23-JUN-2018	13:06	13:08		Yes	Yes	Forearm	F3	uncoated

Note: Delivered Dose=(Labelled Dose)/2 for MAP

Administration Site Key: FA = Forearm; UA= Upper arm
Treatments Part A: A1 = A/Sing MAP, 15 ug (FA); A2 = Afluria Quad IM (UA), 15 ug; A3 = Placebo MAP (FA); A4 = A/Sing Mono IM, 15 ug (UA)
Treatments Part B: B1 = A/Sing MAP, 15 ug (FA) with biopsy; B2 = Placebo MAP (FA) with biopsy
B3 = A/Sing MAP, 15 ug (FA); B4 = A/Sing MAP, 10 ug (FA); B5 = A/Sing MAP, 5 ug (FA); B6 = A/Sing MAP, 2.5 ug (FA)
B7 = Placebo MAP (FA); B8 = A/Sing MAP, 15 ug (UA); B9 = A/Sing IM, 15 ug, as 2018 Afluria Quad (UA)

Listing 16.2.5.1
Study Drug Administration

Part B

Randomi-sation Number	Group	Treatment	Study Day	Date of Dose	Time of MAP application	Time of MAP removal	Time of IM Injection	Was Dose Adminis-tered?	Dosed as per Protocol	Administra-tion Site	Site Number	Dose Administered at Site
B0139	Group B4	A/Sing MAP, 10 ug	Day 1	23-JUN-2018	13:10	13:12		Yes	Yes	Forearm	F1	uncoated
B0139	Group B4	A/Sing MAP, 10 ug	Day 1	23-JUN-2018	13:13	13:15		Yes	Yes	Forearm	F2	5 ug
B0139	Group B4	A/Sing MAP, 10 ug	Day 1	23-JUN-2018	13:16	13:18		Yes	Yes	Forearm	F3	5 ug
B0140	Group B9	A/Sing IM, 15 ug	Day 1	23-JUN-2018			11:00	Yes	Yes	Upper Arm	D1	[IM]
B0141	Group B3	A/Sing MAP, 15 ug	Day 1	23-JUN-2018	13:20	13:22		Yes	Yes	Forearm	F1	5 ug
B0141	Group B3	A/Sing MAP, 15 ug	Day 1	23-JUN-2018	13:23	13:25		Yes	Yes	Forearm	F2	5 ug
B0141	Group B3	A/Sing MAP, 15 ug	Day 1	23-JUN-2018	13:26	13:28		Yes	Yes	Forearm	F3	5 ug
B0142	Group B5	A/Sing MAP, 5 ug	Day 1	23-JUN-2018	13:30	13:32		Yes	Yes	Forearm	F1	5 ug
B0142	Group B5	A/Sing MAP, 5 ug	Day 1	23-JUN-2018	13:33	13:35		Yes	Yes	Forearm	F2	uncoated
B0142	Group B5	A/Sing MAP, 5 ug	Day 1	23-JUN-2018	13:36	13:38		Yes	Yes	Forearm	F3	uncoated
B0143	Group B6	A/Sing MAP, 2.5 ug	Day 1	23-JUN-2018	13:40	13:42		Yes	Yes	Forearm	F1	uncoated
B0143	Group B6	A/Sing MAP, 2.5 ug	Day 1	23-JUN-2018	13:43	13:45		Yes	Yes	Forearm	F2	uncoated
B0143	Group B6	A/Sing MAP, 2.5 ug	Day 1	23-JUN-2018	13:46	13:48		Yes	Yes	Forearm	F3	2.5 ug

Note: Delivered Dose=(Labelled Dose)/2 for MAP

Administration Site Key: FA = Forearm; UA= Upper arm
Treatments Part A: A1 = A/Sing MAP, 15 ug (FA); A2 = Afluria Quad IM (UA), 15 ug; A3 = Placebo MAP (FA); A4 = A/Sing Mono IM, 15 ug (UA)
Treatments Part B: B1 = A/Sing MAP, 15 ug (FA) with biopsy; B2 = Placebo MAP (FA) with biopsy
B3 = A/Sing MAP, 15 ug (FA); B4 = A/Sing MAP, 10 ug (FA); B5 = A/Sing MAP, 5 ug (FA); B6 = A/Sing MAP, 2.5 ug (FA)
B7 = Placebo MAP (FA); B8 = A/Sing MAP, 15 ug (UA); B9 = A/Sing IM, 15 ug, as 2018 Afluria Quad (UA)

Listing 16.2.5.1
Study Drug Administration

Part B

Randomi-sation Number	Group	Treatment	Study Day	Date of Dose	Time of MAP application	Time of MAP removal	Time of IM Injection	Was Dose Adminis-tered?	Dosed as per Protocol	Administra-tion Site	Site Number	Dose Administered at Site
B0144	Group B6	A/Sing MAP, 2.5 ug	Day 1	23-JUN-2018	13:50	13:52		Yes	Yes	Forearm	F1	2.5 ug
B0144	Group B6	A/Sing MAP, 2.5 ug	Day 1	23-JUN-2018	13:53	13:55		Yes	Yes	Forearm	F2	uncoated
B0144	Group B6	A/Sing MAP, 2.5 ug	Day 1	23-JUN-2018	13:56	13:58		Yes	Yes	Forearm	F3	uncoated
B0145	Group B3	A/Sing MAP, 15 ug	Day 1	23-JUN-2018	14:00	14:02		Yes	Yes	Forearm	F1	5 ug
B0145	Group B3	A/Sing MAP, 15 ug	Day 1	23-JUN-2018	14:03	14:05		Yes	Yes	Forearm	F2	5 ug
B0145	Group B3	A/Sing MAP, 15 ug	Day 1	23-JUN-2018	14:06	14:08		Yes	Yes	Forearm	F3	5 ug
B0146	Group B7	Placebo MAP	Day 1	28-JUN-2018	11:10	11:12		Yes	Yes	Forearm	F1	uncoated
B0146	Group B7	Placebo MAP	Day 1	28-JUN-2018	11:13	11:15		Yes	Yes	Forearm	F2	uncoated
B0146	Group B7	Placebo MAP	Day 1	28-JUN-2018	11:16	11:18		Yes	Yes	Forearm	F3	uncoated
B0147	Group B9	A/Sing IM, 15 ug	Day 1	28-JUN-2018			11:00	Yes	Yes	Upper Arm	D1	[IM]
B0148	Group B5	A/Sing MAP, 5 ug	Day 1	28-JUN-2018	11:20	11:22		Yes	Yes	Forearm	F1	5 ug
B0148	Group B5	A/Sing MAP, 5 ug	Day 1	28-JUN-2018	11:23	11:25		Yes	Yes	Forearm	F2	uncoated
B0148	Group B5	A/Sing MAP, 5 ug	Day 1	28-JUN-2018	11:26	11:28		Yes	Yes	Forearm	F3	uncoated

Note: Delivered Dose=(Labelled Dose)/2 for MAP

Administration Site Key: FA = Forearm; UA= Upper arm
Treatments Part A: A1 = A/Sing MAP, 15 ug (FA); A2 = Afluria Quad IM (UA), 15 ug; A3 = Placebo MAP (FA); A4 = A/Sing Mono IM, 15 ug (UA)
Treatments Part B: B1 = A/Sing MAP, 15 ug (FA) with biopsy; B2 = Placebo MAP (FA) with biopsy
B3 = A/Sing MAP, 15 ug (FA); B4 = A/Sing MAP, 10 ug (FA); B5 = A/Sing MAP, 5 ug (FA); B6 = A/Sing MAP, 2.5 ug (FA)
B7 = Placebo MAP (FA); B8 = A/Sing MAP, 15 ug (UA); B9 = A/Sing IM, 15 ug, as 2018 Afluria Quad (UA)

Listing 16.2.5.1
Study Drug Administration

Part B

Randomi-sation Number	Group	Treatment	Study Day	Date of Dose	Time of MAP application	Time of MAP removal	Time of IM Injection	Was Dose Adminis-tered?	Dosed as per Protocol	Administra-tion Site	Site Number	Dose Administered at Site
B0149	Group B8	A/Sing MAP, 15 ug	Day 1	28-JUN-2018	11:30	11:32		Yes	Yes	Upper Arm	D1	5 ug
B0149	Group B8	A/Sing MAP, 15 ug	Day 1	28-JUN-2018	11:33	11:35		Yes	Yes	Upper Arm	D2	5 ug
B0149	Group B8	A/Sing MAP, 15 ug	Day 1	28-JUN-2018	11:36	11:38		Yes	Yes	Upper Arm	D3	5 ug
B0150	Group B4	A/Sing MAP, 10 ug	Day 1	28-JUN-2018	11:40	11:42		Yes	Yes	Forearm	F1	5 ug
B0150	Group B4	A/Sing MAP, 10 ug	Day 1	28-JUN-2018	11:43	11:45		Yes	Yes	Forearm	F2	5 ug
B0150	Group B4	A/Sing MAP, 10 ug	Day 1	28-JUN-2018	11:46	11:48		Yes	Yes	Forearm	F3	uncoated
B1019	Group B8	A/Sing MAP, 15 ug	Day 1	19-MAY-2018	11:00	11:02		Yes	Yes	Upper Arm	D1	5 ug
B1019	Group B8	A/Sing MAP, 15 ug	Day 1	19-MAY-2018	11:03	11:05		Yes	Yes	Upper Arm	D2	5 ug
B1019	Group B8	A/Sing MAP, 15 ug	Day 1	19-MAY-2018	11:06	11:08		Yes	Yes	Upper Arm	D3	5 ug
B1076	Group B5	A/Sing MAP, 5 ug	Day 1	16-JUN-2018	11:30	11:32		Yes	Yes	Forearm	F1	5 ug
B1076	Group B5	A/Sing MAP, 5 ug	Day 1	16-JUN-2018	11:33	11:35		Yes	Yes	Forearm	F2	uncoated
B1076	Group B5	A/Sing MAP, 5 ug	Day 1	16-JUN-2018	11:36	11:38		Yes	Yes	Forearm	F3	uncoated
B1080	Group B7	Placebo MAP	Day 1	16-JUN-2018	11:40	11:42		Yes	Yes	Forearm	F1	uncoated
B1080	Group B7	Placebo MAP	Day 1	16-JUN-2018	11:43	11:45		Yes	Yes	Forearm	F2	uncoated

Note: Delivered Dose=(Labelled Dose)/2 for MAP

Administration Site Key: FA = Forearm; UA= Upper arm
Treatments Part A: A1 = A/Sing MAP, 15 ug (FA); A2 = Afluria Quad IM (UA), 15 ug; A3 = Placebo MAP (FA); A4 = A/Sing Mono IM, 15 ug (UA)
Treatments Part B: B1 = A/Sing MAP, 15 ug (FA) with biopsy; B2 = Placebo MAP (FA) with biopsy
B3 = A/Sing MAP, 15 ug (FA); B4 = A/Sing MAP, 10 ug (FA); B5 = A/Sing MAP, 5 ug (FA); B6 = A/Sing MAP, 2.5 ug (FA)
B7 = Placebo MAP (FA); B8 = A/Sing MAP, 15 ug (UA); B9 = A/Sing IM, 15 ug, as 2018 Afluria Quad (UA)

Listing 16.2.5.1
Study Drug Administration

Part B

Randomi-sation Number	Group	Treatment	Study Day	Date of Dose	Time of MAP application	Time of MAP removal	Time of IM Injection	Was Dose Adminis-tered?	Dosed as per Protocol	Administra-tion Site	Site Number	Dose Administered at Site
B1080	Group B7	Placebo MAP	Day 1	16-JUN-2018	11:46	11:48		Yes	Yes	Forearm	F3	uncoated

Note: Delivered Dose=(Labelled Dose)/2 for MAP

Administration Site Key: FA = Forearm; UA= Upper arm
Treatments Part A: A1 = A/Sing MAP, 15 ug (FA); A2 = Afluria Quad IM (UA), 15 ug; A3 = Placebo MAP (FA); A4 = A/Sing Mono IM, 15 ug (UA)
Treatments Part B: B1 = A/Sing MAP, 15 ug (FA) with biopsy; B2 = Placebo MAP (FA) with biopsy
B3 = A/Sing MAP, 15 ug (FA); B4 = A/Sing MAP, 10 ug (FA); B5 = A/Sing MAP, 5 ug (FA); B6 = A/Sing MAP, 2.5 ug (FA)
B7 = Placebo MAP (FA); B8 = A/Sing MAP, 15 ug (UA); B9 = A/Sing IM, 15 ug, as 2018 Afluria Quad (UA)
