# Supplementary material for: Safety, tolerability, and immunogenicity of influenza vaccination with a high-density microarray patch: Results from a randomized, controlled phase I clinical trial
Source: PLoS Med. 2020 Mar 17;17(3):e1003024. doi: 10.1371/journal.pmed.1003024 (PMC7077342; doi:10.1371/journal.pmed.1003024)
Supplement: S1 Data — Data 1: Informed Consent; Data 2: Analysis Sets; Data 3: Demographics; Data 4: Study Drug Administration; Data 5: Skin Hardness Assessment; Data 6: Immunogenicity (ELISA IgG); Data 7: Immunogenicity (ADCC); Data 8: Immunogenicity (MBCs); Data 9: Immunogenicity (CMI); Data 10: Immunogenicity (Mucosal IgA); Data 11: Immunogenicity Results (HAI, MN); Data 12: TEAE–CRF Data only; Data 13: TEAEs–MedDRA Coding; Data 15: Treatment Site Tolerability Assessment; Data 16: Numeric Pain Intensity; Data 17: SII; Data 18: Application Site Status at End of Study; Data 19: Individual Treatment Site Tolerability Assessment; Data 20: Numeric Pain Intensity by Treatment; Data 21: Individual SII by Treatment. ADCC, antibody-dependent cellular cytotoxicity; CMI, cell-mediated immunity; CRF, case report form; ELISA, enzyme-linked immunosorbent assay; HAI, haemagglutination inhibition; IgA, immunoglobulin A; IgG, immunoglobulin G; MBC, memory B cell; MedDRA, medical dictionary for regulatory activities; MN, microneutralisation; SII, Skin Irritation Index; TEA, treatment emergent adverse event. (ZIP) [file pmed.1003024.s008.zip › S1_Data/clinical supporting/S1_Data5 Skin Hardness Assessment.pdf]

Listing 16.2.5.3
Skin Hardness Assessment (MAP only)

Part A

Randomisation Number	Group	Visit	Time Point	Date of assessment	Time of Assessment	Site Number	Was assessment performed?	Reading 1 (Without compression)	Reading 2 (with skin conditioning ring pressed down)	Ratio R2/R1 Compression/ Without Compression
A0001	Group A3	Day 1	Pre-dose	09-MAR-2018	11:21	F1	Yes	23.3	31.7	1.36
A0001	Group A3	Day 1	Pre-dose	09-MAR-2018	11:21	F2	Yes	29.3	46.4	1.58
A0001	Group A3	Day 1	Pre-dose	09-MAR-2018	11:21	F3	Yes	28.8	36.3	1.26
A0002	Group A1	Day 1	Pre-dose	09-MAR-2018	11:24	F1	Yes	18.2	30.2	1.66
A0002	Group A1	Day 1	Pre-dose	09-MAR-2018	11:24	F2	Yes	26.0	38.0	1.46
A0002	Group A1	Day 1	Pre-dose	09-MAR-2018	11:24	F3	Yes	27.5	29.9	1.09
A0003	Group A1	Day 1	Pre-dose	09-MAR-2018	11:29	F1	Yes	27.7	41.4	1.49
A0003	Group A1	Day 1	Pre-dose	09-MAR-2018	11:29	F2	Yes	18.5	44.4	2.40
A0003	Group A1	Day 1	Pre-dose	09-MAR-2018	11:29	F3	Yes	26.6	35.9	1.35
A0004	Group A3	Day 1	Pre-dose	09-MAR-2018	11:36	F1	Yes	22.7	39.3	1.73
A0004	Group A3	Day 1	Pre-dose	09-MAR-2018	11:36	F2	Yes	30.3	35.8	1.18
A0004	Group A3	Day 1	Pre-dose	09-MAR-2018	11:36	F3	Yes	26.9	44.3	1.65
A0005	Group A1	Day 1	Pre-dose	09-MAR-2018	11:47	F1	Yes	40.0	41.3	1.03
A0005	Group A1	Day 1	Pre-dose	09-MAR-2018	11:47	F2	Yes	37.5	48.1	1.28

Administration Site Key: FA = Forearm; UA= Upper arm
Treatments Part A: A1 = A/Sing MAP, 15 ug (FA); A2 = Afluria Quad IM (UA), 15 ug; A3 = Placebo MAP (FA); A4 = A/Sing Mono IM, 15 ug (UA)
Treatments Part B: B1 = A/Sing MAP, 15 ug (FA) with biopsy; B2 = Placebo MAP (FA) with biopsy
B3 = A/Sing MAP, 15 ug (FA); B4 = A/Sing MAP, 10 ug (FA); B5 = A/Sing MAP, 5 ug (FA); B6 = A/Sing MAP, 2.5 ug (FA)
B7 = Placebo MAP (FA); B8 = A/Sing MAP, 15 ug (UA); B9 = A/Sing IM, 15 ug, as 2018 Afluria Quad (UA)

Listing 16.2.5.3
Skin Hardness Assessment (MAP only)

Part A

Randomisation Number	Group	Visit	Time Point	Date of assessment	Time of Assessment	Site Number	Was assessment performed?	Reading 1 (Without compression)	Reading 2 (with skin conditioning ring pressed down)	Ratio R2/R1 Compression/ Without Compression
A0005	Group A1	Day 1	Pre-dose	09-MAR-2018	11:47	F3	Yes	30.9	48.6	1.57
A0006	Group A3	Day 1	Pre-dose	09-MAR-2018	11:38	F1	Yes	24.6	36.9	1.50
A0006	Group A3	Day 1	Pre-dose	09-MAR-2018	11:38	F2	Yes	29.6	38.2	1.29
A0006	Group A3	Day 1	Pre-dose	09-MAR-2018	11:38	F3	Yes	30.1	34.3	1.14
A0007	Group A1	Day 1	Pre-dose	09-MAR-2018	11:58	F1	Yes	6.2	43.0	6.94
A0007	Group A1	Day 1	Pre-dose	09-MAR-2018	11:58	F2	Yes	7.0	43.2	6.17
A0007	Group A1	Day 1	Pre-dose	09-MAR-2018	11:58	F3	Yes	8.5	39.4	4.64
A0008	Group A3	Day 1	Pre-dose	09-MAR-2018	12:11	F1	Yes	15.2	20.3	1.34
A0008	Group A3	Day 1	Pre-dose	09-MAR-2018	12:11	F2	Yes	11.4	25.0	2.19
A0008	Group A3	Day 1	Pre-dose	09-MAR-2018	12:11	F3	Yes	14.3	27.0	1.89
A0009	Group A1	Day 1	Pre-dose	09-MAR-2018	12:04	F1	Yes	11.8	44.7	3.79
A0009	Group A1	Day 1	Pre-dose	09-MAR-2018	12:04	F2	Yes	14.0	39.8	2.84
A0009	Group A1	Day 1	Pre-dose	09-MAR-2018	12:04	F3	Yes	17.8	24.6	1.38

Administration Site Key: FA = Forearm; UA= Upper arm
Treatments Part A: A1 = A/Sing MAP, 15 ug (FA); A2 = Afluria Quad IM (UA), 15 ug; A3 = Placebo MAP (FA); A4 = A/Sing Mono IM, 15 ug (UA)
Treatments Part B: B1 = A/Sing MAP, 15 ug (FA) with biopsy; B2 = Placebo MAP (FA) with biopsy
B3 = A/Sing MAP, 15 ug (FA); B4 = A/Sing MAP, 10 ug (FA); B5 = A/Sing MAP, 5 ug (FA); B6 = A/Sing MAP, 2.5 ug (FA)
B7 = Placebo MAP (FA); B8 = A/Sing MAP, 15 ug (UA); B9 = A/Sing IM, 15 ug, as 2018 Afluria Quad (UA)

Listing 16.2.5.3
Skin Hardness Assessment (MAP only)

Part A

Randomisation Number	Group	Visit	Time Point	Date of assessment	Time of Assessment	Site Number	Was assessment performed?	Reading 1 (Without compression)	Reading 2 (with skin conditioning ring pressed down)	Ratio R2/R1 Compression/ Without Compression
A0010	Group A3	Day 1	Pre-dose	09-MAR-2018	12:31	F1	Yes	19.2	39.6	2.06
A0010	Group A3	Day 1	Pre-dose	09-MAR-2018	12:31	F2	Yes	27.5	36.3	1.32
A0010	Group A3	Day 1	Pre-dose	09-MAR-2018	12:31	F3	Yes	26.6	35.6	1.34
A0011	Group A3	Day 1	Pre-dose	09-MAR-2018	12:19	F1	Yes	13.9	38.5	2.77
A0011	Group A3	Day 1	Pre-dose	09-MAR-2018	12:19	F2	Yes	11.6	38.8	3.34
A0011	Group A3	Day 1	Pre-dose	09-MAR-2018	12:19	F3	Yes	15.0	47.0	3.13
A0012	Group A1	Day 1	Pre-dose	09-MAR-2018	12:49	F1	Yes	24.2	31.9	1.32
A0012	Group A1	Day 1	Pre-dose	09-MAR-2018	12:49	F2	Yes	21.5	30.7	1.43
A0012	Group A1	Day 1	Pre-dose	09-MAR-2018	12:49	F3	Yes	25.1	39.2	1.56
A0013	Group A1	Day 1	Pre-dose	09-MAR-2018	13:09	F1	Yes	12.1	32.7	2.70
A0013	Group A1	Day 1	Pre-dose	09-MAR-2018	13:09	F2	Yes	23.1	41.2	1.78
A0013	Group A1	Day 1	Pre-dose	09-MAR-2018	13:09	F3	Yes	20.8	31.3	1.50
A0014	Group A3	Day 1	Pre-dose	09-MAR-2018	13:20	F1	Yes	22.4	33.2	1.48
A0014	Group A3	Day 1	Pre-dose	09-MAR-2018	13:20	F2	Yes	29.3	39.7	1.35

Administration Site Key: FA = Forearm; UA= Upper arm
Treatments Part A: A1 = A/Sing MAP, 15 ug (FA); A2 = Afluria Quad IM (UA), 15 ug; A3 = Placebo MAP (FA); A4 = A/Sing Mono IM, 15 ug (UA)
Treatments Part B: B1 = A/Sing MAP, 15 ug (FA) with biopsy; B2 = Placebo MAP (FA) with biopsy
B3 = A/Sing MAP, 15 ug (FA); B4 = A/Sing MAP, 10 ug (FA); B5 = A/Sing MAP, 5 ug (FA); B6 = A/Sing MAP, 2.5 ug (FA)
B7 = Placebo MAP (FA); B8 = A/Sing MAP, 15 ug (UA); B9 = A/Sing IM, 15 ug, as 2018 Afluria Quad (UA)

Listing 16.2.5.3
Skin Hardness Assessment (MAP only)

Part A

Randomisation Number	Group	Visit	Time Point	Date of assessment	Time of Assessment	Site Number	Was assessment performed?	Reading 1 (Without compression)	Reading 2 (with skin conditioning ring pressed down)	Ratio R2/R1 Compression/ Without Compression
A0014	Group A3	Day 1	Pre-dose	09-MAR-2018	13:20	F3	Yes	36.7	45.3	1.23
A0015	Group A1	Day 1	Pre-dose	09-MAR-2018	13:28	F1	Yes	17.4	28.3	1.63
A0015	Group A1	Day 1	Pre-dose	09-MAR-2018	13:28	F2	Yes	22.0	38.3	1.74
A0015	Group A1	Day 1	Pre-dose	09-MAR-2018	13:28	F3	Yes	20.1	30.3	1.51
A0016	Group A3	Day 1	Pre-dose	10-MAR-2018	10:08	F1	Yes	17.8	39.2	2.20
A0016	Group A3	Day 1	Pre-dose	10-MAR-2018	10:08	F2	Yes	22.9	40.7	1.78
A0016	Group A3	Day 1	Pre-dose	10-MAR-2018	10:08	F3	Yes	24.1	41.7	1.73
A0017	Group A3	Day 1	Pre-dose	10-MAR-2018	10:17	F1	Yes	23.6	33.2	1.41
A0017	Group A3	Day 1	Pre-dose	10-MAR-2018	10:17	F2	Yes	27.7	32.1	1.16
A0017	Group A3	Day 1	Pre-dose	10-MAR-2018	10:17	F3	Yes	20.4	31.8	1.56
A0018	Group A1	Day 1	Pre-dose	10-MAR-2018	10:41	F1	Yes	16.8	25.3	1.51
A0018	Group A1	Day 1	Pre-dose	10-MAR-2018	10:41	F2	Yes	14.7	23.3	1.59
A0018	Group A1	Day 1	Pre-dose	10-MAR-2018	10:41	F3	Yes	18.4	23.3	1.27

Administration Site Key: FA = Forearm; UA= Upper arm
Treatments Part A: A1 = A/Sing MAP, 15 ug (FA); A2 = Afluria Quad IM (UA), 15 ug; A3 = Placebo MAP (FA); A4 = A/Sing Mono IM, 15 ug (UA)
Treatments Part B: B1 = A/Sing MAP, 15 ug (FA) with biopsy; B2 = Placebo MAP (FA) with biopsy
B3 = A/Sing MAP, 15 ug (FA); B4 = A/Sing MAP, 10 ug (FA); B5 = A/Sing MAP, 5 ug (FA); B6 = A/Sing MAP, 2.5 ug (FA)
B7 = Placebo MAP (FA); B8 = A/Sing MAP, 15 ug (UA); B9 = A/Sing IM, 15 ug, as 2018 Afluria Quad (UA)

Listing 16.2.5.3
Skin Hardness Assessment (MAP only)

Part A

Randomisation Number	Group	Visit	Time Point	Date of assessment	Time of Assessment	Site Number	Was assessment performed?	Reading 1 (Without compression)	Reading 2 (with skin conditioning ring pressed down)	Ratio R2/R1 Compression/ Without Compression
A0019	Group A3	Day 1	Pre-dose	10-MAR-2018	10:10	F1	Yes	14.2	31.6	2.23
A0019	Group A3	Day 1	Pre-dose	10-MAR-2018	10:10	F2	Yes	23.5	28.5	1.21
A0019	Group A3	Day 1	Pre-dose	10-MAR-2018	10:10	F3	Yes	26.3	30.6	1.16
A0020	Group A1	Day 1	Pre-dose	10-MAR-2018	11:01	F1	Yes	16.8	19.7	1.17
A0020	Group A1	Day 1	Pre-dose	10-MAR-2018	11:01	F2	Yes	17.4	25.5	1.47
A0020	Group A1	Day 1	Pre-dose	10-MAR-2018	11:01	F3	Yes	18.6	24.6	1.32
A0021	Group A1	Day 1	Pre-dose	10-MAR-2018	11:05	F1	Yes	17.5	19.9	1.14
A0021	Group A1	Day 1	Pre-dose	10-MAR-2018	11:05	F2	Yes	22.0	26.3	1.20
A0021	Group A1	Day 1	Pre-dose	10-MAR-2018	11:05	F3	Yes	21.4	32.6	1.52
A0022	Group A3	Day 1	Pre-dose	10-MAR-2018	10:57	F1	Yes	20.9	35.3	1.69
A0022	Group A3	Day 1	Pre-dose	10-MAR-2018	10:57	F2	Yes	19.5	33.5	1.72
A0022	Group A3	Day 1	Pre-dose	10-MAR-2018	10:57	F3	Yes	26.8	40.2	1.50
A0023	Group A3	Day 1	Pre-dose	10-MAR-2018	11:08	F1	Yes	9.7	14.0	1.44
A0023	Group A3	Day 1	Pre-dose	10-MAR-2018	11:08	F2	Yes	11.2	17.1	1.53

Administration Site Key: FA = Forearm; UA= Upper arm
Treatments Part A: A1 = A/Sing MAP, 15 ug (FA); A2 = Afluria Quad IM (UA), 15 ug; A3 = Placebo MAP (FA); A4 = A/Sing Mono IM, 15 ug (UA)
Treatments Part B: B1 = A/Sing MAP, 15 ug (FA) with biopsy; B2 = Placebo MAP (FA) with biopsy
B3 = A/Sing MAP, 15 ug (FA); B4 = A/Sing MAP, 10 ug (FA); B5 = A/Sing MAP, 5 ug (FA); B6 = A/Sing MAP, 2.5 ug (FA)
B7 = Placebo MAP (FA); B8 = A/Sing MAP, 15 ug (UA); B9 = A/Sing IM, 15 ug, as 2018 Afluria Quad (UA)

Listing 16.2.5.3
Skin Hardness Assessment (MAP only)

Part A

Randomisation Number	Group	Visit	Time Point	Date of assessment	Time of Assessment	Site Number	Was assessment performed?	Reading 1 (Without compression)	Reading 2 (with skin conditioning ring pressed down)	Ratio R2/R1 Compression/ Without Compression
A0023	Group A3	Day 1	Pre-dose	10-MAR-2018	11:08	F3	Yes	18.4	21.1	1.15
A0024	Group A1	Day 1	Pre-dose	10-MAR-2018	11:12	F1	Yes	14.0	19.5	1.39
A0024	Group A1	Day 1	Pre-dose	10-MAR-2018	11:12	F2	Yes	15.3	24.5	1.60
A0024	Group A1	Day 1	Pre-dose	10-MAR-2018	11:12	F3	Yes	17.4	21.8	1.25
A0025	Group A3	Day 1	Pre-dose	10-MAR-2018	11:16	F1	Yes	15.4	28.3	1.84
A0025	Group A3	Day 1	Pre-dose	10-MAR-2018	11:16	F2	Yes	22.3	42.0	1.88
A0025	Group A3	Day 1	Pre-dose	10-MAR-2018	11:16	F3	Yes	24.8	37.4	1.51
A0027	Group A3	Day 1	Pre-dose	10-MAR-2018	11:26	F1	Yes	11.7	13.5	1.15
A0027	Group A3	Day 1	Pre-dose	10-MAR-2018	11:26	F2	Yes	16.7	23.9	1.43
A0027	Group A3	Day 1	Pre-dose	10-MAR-2018	11:26	F3	Yes	20.6	25.5	1.24
A0028	Group A1	Day 1	Pre-dose	10-MAR-2018	11:37	F1	Yes	16.3	23.0	1.41
A0028	Group A1	Day 1	Pre-dose	10-MAR-2018	11:37	F2	Yes	30.2	39.3	1.30
A0028	Group A1	Day 1	Pre-dose	10-MAR-2018	11:37	F3	Yes	31.9	37.8	1.18

Administration Site Key: FA = Forearm; UA= Upper arm
Treatments Part A: A1 = A/Sing MAP, 15 ug (FA); A2 = Afluria Quad IM (UA), 15 ug; A3 = Placebo MAP (FA); A4 = A/Sing Mono IM, 15 ug (UA)
Treatments Part B: B1 = A/Sing MAP, 15 ug (FA) with biopsy; B2 = Placebo MAP (FA) with biopsy
B3 = A/Sing MAP, 15 ug (FA); B4 = A/Sing MAP, 10 ug (FA); B5 = A/Sing MAP, 5 ug (FA); B6 = A/Sing MAP, 2.5 ug (FA)
B7 = Placebo MAP (FA); B8 = A/Sing MAP, 15 ug (UA); B9 = A/Sing IM, 15 ug, as 2018 Afluria Quad (UA)

Listing 16.2.5.3
Skin Hardness Assessment (MAP only)

Part A

Randomisation Number	Group	Visit	Time Point	Date of assessment	Time of Assessment	Site Number	Was assessment performed?	Reading 1 (Without compression)	Reading 2 (with skin conditioning ring pressed down)	Ratio R2/R1 Compression/ Without Compression
A0029	Group A1	Day 1	Pre-dose	10-MAR-2018	11:42	F1	Yes	16.5	24.3	1.47
A0029	Group A1	Day 1	Pre-dose	10-MAR-2018	11:42	F2	Yes	20.0	36.2	1.81
A0029	Group A1	Day 1	Pre-dose	10-MAR-2018	11:42	F3	Yes	16.9	28.1	1.66
A0030	Group A3	Day 1	Pre-dose	16-MAR-2018	11:22	F1	Yes	21.1	32.5	1.54
A0030	Group A3	Day 1	Pre-dose	16-MAR-2018	11:22	F2	Yes	21.4	29.2	1.36
A0030	Group A3	Day 1	Pre-dose	16-MAR-2018	11:22	F3	Yes	24.5	29.7	1.21
A1026	Group A1	Day 1	Pre-dose	10-MAR-2018	13:27	F1	Yes	15.9	14.7	0.92
A1026	Group A1	Day 1	Pre-dose	10-MAR-2018	13:27	F2	Yes	25.7	42.5	1.65
A1026	Group A1	Day 1	Pre-dose	10-MAR-2018	13:27	F3	Yes	25.6	36.6	1.43

Administration Site Key: FA = Forearm; UA= Upper arm
Treatments Part A: A1 = A/Sing MAP, 15 ug (FA); A2 = Afluria Quad IM (UA), 15 ug; A3 = Placebo MAP (FA); A4 = A/Sing Mono IM, 15 ug (UA)
Treatments Part B: B1 = A/Sing MAP, 15 ug (FA) with biopsy; B2 = Placebo MAP (FA) with biopsy
B3 = A/Sing MAP, 15 ug (FA); B4 = A/Sing MAP, 10 ug (FA); B5 = A/Sing MAP, 5 ug (FA); B6 = A/Sing MAP, 2.5 ug (FA)
B7 = Placebo MAP (FA); B8 = A/Sing MAP, 15 ug (UA); B9 = A/Sing IM, 15 ug, as 2018 Afluria Quad (UA)

Listing 16.2.5.3
Skin Hardness Assessment (MAP only)

Part B

Randomisation Number	Group	Visit	Time Point	Date of assessment	Time of Assessment	Site Number	Was assessment performed?	Reading 1 (Without compression)	Reading 2 (with skin conditioning ring pressed down)	Ratio R2/R1 Compression/ Without Compression
B0001	Group B1	Day 1	Pre-dose	07-MAY-2018	08:55	F1	Yes	24.8	51.1	2.06
B0001	Group B1	Day 1	Pre-dose	07-MAY-2018	08:55	F2	Yes	15.3	53.2	3.48
B0001	Group B1	Day 1	Pre-dose	07-MAY-2018	08:55	F3	Yes	29.9	54.7	1.83
B0002	Group B2	Day 1	Pre-dose	07-MAY-2018	09:04	F1	Yes	31.7	39.9	1.26
B0002	Group B2	Day 1	Pre-dose	07-MAY-2018	09:04	F2	Yes	29.7	45.6	1.54
B0002	Group B2	Day 1	Pre-dose	07-MAY-2018	09:04	F3	Yes	25.6	41.3	1.61
B0003	Group B2	Day 1	Pre-dose	07-MAY-2018	09:12	F1	Yes	28.9	44.2	1.53
B0003	Group B2	Day 1	Pre-dose	07-MAY-2018	09:12	F2	Yes	29.3	43.8	1.49
B0003	Group B2	Day 1	Pre-dose	07-MAY-2018	09:12	F3	Yes	38.1	47.8	1.25
B0004	Group B1	Day 1	Pre-dose	07-MAY-2018	09:21	F1	Yes	26.0	41.1	1.58
B0004	Group B1	Day 1	Pre-dose	07-MAY-2018	09:21	F2	Yes	28.5	43.1	1.51
B0004	Group B1	Day 1	Pre-dose	07-MAY-2018	09:21	F3	Yes	28.7	46.8	1.63
B0005	Group B1	Day 1	Pre-dose	07-MAY-2018	09:35	F1	Yes	25.3	40.6	1.60
B0005	Group B1	Day 1	Pre-dose	07-MAY-2018	09:35	F2	Yes	20.2	37.6	1.86

Administration Site Key: FA = Forearm; UA= Upper arm
Treatments Part A: A1 = A/Sing MAP, 15 ug (FA); A2 = Afluria Quad IM (UA), 15 ug; A3 = Placebo MAP (FA); A4 = A/Sing Mono IM, 15 ug (UA)
Treatments Part B: B1 = A/Sing MAP, 15 ug (FA) with biopsy; B2 = Placebo MAP (FA) with biopsy
B3 = A/Sing MAP, 15 ug (FA); B4 = A/Sing MAP, 10 ug (FA); B5 = A/Sing MAP, 5 ug (FA); B6 = A/Sing MAP, 2.5 ug (FA)
B7 = Placebo MAP (FA); B8 = A/Sing MAP, 15 ug (UA); B9 = A/Sing IM, 15 ug, as 2018 Afluria Quad (UA)

Listing 16.2.5.3
Skin Hardness Assessment (MAP only)

Part B

Randomisation Number	Group	Visit	Time Point	Date of assessment	Time of Assessment	Site Number	Was assessment performed?	Reading 1 (Without compression)	Reading 2 (with skin conditioning ring pressed down)	Ratio R2/R1 Compression/ Without Compression
B0005	Group B1	Day 1	Pre-dose	07-MAY-2018	09:35	F3	Yes	37.7	41.1	1.09
B0006	Group B2	Day 1	Pre-dose	07-MAY-2018	09:50	F1	Yes	17.3	26.5	1.53
B0006	Group B2	Day 1	Pre-dose	07-MAY-2018	09:50	F2	Yes	21.4	33.6	1.57
B0006	Group B2	Day 1	Pre-dose	07-MAY-2018	09:50	F3	Yes	31.2	46.4	1.49
B0007	Group B2	Day 1	Pre-dose	07-MAY-2018	09:58	F1	Yes	26.9	44.7	1.66
B0007	Group B2	Day 1	Pre-dose	07-MAY-2018	09:58	F2	Yes	26.9	42.4	1.58
B0007	Group B2	Day 1	Pre-dose	07-MAY-2018	09:58	F3	Yes	25.5	39.6	1.55
B0008	Group B1	Day 1	Pre-dose	07-MAY-2018	10:09	F1	Yes	21.6	37.0	1.71
B0008	Group B1	Day 1	Pre-dose	07-MAY-2018	10:09	F2	Yes	25.4	42.3	1.67
B0008	Group B1	Day 1	Pre-dose	07-MAY-2018	10:09	F3	Yes	29.6	43.3	1.46
B0009	Group B1	Day 1	Pre-dose	07-MAY-2018	10:19	F1	Yes	23.8	35.0	1.47
B0009	Group B1	Day 1	Pre-dose	07-MAY-2018	10:19	F2	Yes	25.7	47.3	1.84
B0009	Group B1	Day 1	Pre-dose	07-MAY-2018	10:19	F3	Yes	28.0	53.6	1.91

Administration Site Key: FA = Forearm; UA= Upper arm
Treatments Part A: A1 = A/Sing MAP, 15 ug (FA); A2 = Afluria Quad IM (UA), 15 ug; A3 = Placebo MAP (FA); A4 = A/Sing Mono IM, 15 ug (UA)
Treatments Part B: B1 = A/Sing MAP, 15 ug (FA) with biopsy; B2 = Placebo MAP (FA) with biopsy
B3 = A/Sing MAP, 15 ug (FA); B4 = A/Sing MAP, 10 ug (FA); B5 = A/Sing MAP, 5 ug (FA); B6 = A/Sing MAP, 2.5 ug (FA)
B7 = Placebo MAP (FA); B8 = A/Sing MAP, 15 ug (UA); B9 = A/Sing IM, 15 ug, as 2018 Afluria Quad (UA)

Listing 16.2.5.3
Skin Hardness Assessment (MAP only)

Part B

Randomisation Number	Group	Visit	Time Point	Date of assessment	Time of Assessment	Site Number	Was assessment performed?	Reading 1 (Without compression)	Reading 2 (with skin conditioning ring pressed down)	Ratio R2/R1 Compression/ Without Compression
B0010	Group B2	Day 1	Pre-dose	07-MAY-2018	10:33	F1	Yes	19.3	42.5	2.20
B0010	Group B2	Day 1	Pre-dose	07-MAY-2018	10:33	F2	Yes	22.2	45.9	2.07
B0010	Group B2	Day 1	Pre-dose	07-MAY-2018	10:33	F3	Yes	32.4	48.2	1.49
B0011	Group B8	Day 1	Pre-dose	12-MAY-2018	11:20	D1	Yes	28.0	60.6	2.16
B0011	Group B8	Day 1	Pre-dose	12-MAY-2018	11:20	D2	Yes	30.9	56.0	1.81
B0011	Group B8	Day 1	Pre-dose	12-MAY-2018	11:20	D3	Yes	32.6	57.9	1.78
B0012	Group B6	Day 1	Pre-dose	12-MAY-2018	11:31	F1	Yes	21.3	39.7	1.86
B0012	Group B6	Day 1	Pre-dose	12-MAY-2018	11:31	F2	Yes	28.1	47.5	1.69
B0012	Group B6	Day 1	Pre-dose	12-MAY-2018	11:31	F3	Yes	28.7	49.0	1.71
B0013	Group B5	Day 1	Pre-dose	12-MAY-2018	11:38	F1	Yes	28.2	50.5	1.79
B0013	Group B5	Day 1	Pre-dose	12-MAY-2018	11:38	F2	Yes	33.8	46.1	1.36
B0013	Group B5	Day 1	Pre-dose	12-MAY-2018	11:38	F3	Yes	35.6	53.2	1.49
B0014	Group B3	Day 1	Pre-dose	12-MAY-2018	11:42	F1	Yes	23.1	39.2	1.70
B0014	Group B3	Day 1	Pre-dose	12-MAY-2018	11:42	F2	Yes	25.9	40.6	1.57

Administration Site Key: FA = Forearm; UA= Upper arm
Treatments Part A: A1 = A/Sing MAP, 15 ug (FA); A2 = Afluria Quad IM (UA), 15 ug; A3 = Placebo MAP (FA); A4 = A/Sing Mono IM, 15 ug (UA)
Treatments Part B: B1 = A/Sing MAP, 15 ug (FA) with biopsy; B2 = Placebo MAP (FA) with biopsy
B3 = A/Sing MAP, 15 ug (FA); B4 = A/Sing MAP, 10 ug (FA); B5 = A/Sing MAP, 5 ug (FA); B6 = A/Sing MAP, 2.5 ug (FA)
B7 = Placebo MAP (FA); B8 = A/Sing MAP, 15 ug (UA); B9 = A/Sing IM, 15 ug, as 2018 Afluria Quad (UA)

Listing 16.2.5.3
Skin Hardness Assessment (MAP only)

Part B

Randomisation Number	Group	Visit	Time Point	Date of assessment	Time of Assessment	Site Number	Was assessment performed?	Reading 1 (Without compression)	Reading 2 (with skin conditioning ring pressed down)	Ratio R2/R1 Compression/ Without Compression
B0014	Group B3	Day 1	Pre-dose	12-MAY-2018	11:42	F3	Yes	29.9	46.8	1.57
B0016	Group B4	Day 1	Pre-dose	12-MAY-2018	11:48	F1	Yes	16.1	30.3	1.88
B0016	Group B4	Day 1	Pre-dose	12-MAY-2018	11:48	F2	Yes	22.4	49.9	2.23
B0016	Group B4	Day 1	Pre-dose	12-MAY-2018	11:48	F3	Yes	24.8	39.8	1.60
B0017	Group B7	Day 1	Pre-dose	12-MAY-2018	12:00	F1	Yes	21.0	36.2	1.72
B0017	Group B7	Day 1	Pre-dose	12-MAY-2018	12:00	F2	Yes	33.8	48.2	1.43
B0017	Group B7	Day 1	Pre-dose	12-MAY-2018	12:00	F3	Yes	32.3	46.2	1.43
B0020	Group B6	Day 1	Pre-dose	12-MAY-2018	12:30	F1	Yes	20.7	44.9	2.17
B0020	Group B6	Day 1	Pre-dose	12-MAY-2018	12:30	F2	Yes	27.4	57.3	2.09
B0020	Group B6	Day 1	Pre-dose	12-MAY-2018	12:30	F3	Yes	29.4	56.4	1.92
B0021	Group B7	Day 1	Pre-dose	12-MAY-2018	12:33	F1	Yes	25.1	43.4	1.73
B0021	Group B7	Day 1	Pre-dose	12-MAY-2018	12:33	F2	Yes	26.3	50.3	1.91
B0021	Group B7	Day 1	Pre-dose	12-MAY-2018	12:33	F3	Yes	29.9	44.4	1.48

Administration Site Key: FA = Forearm; UA= Upper arm
Treatments Part A: A1 = A/Sing MAP, 15 ug (FA); A2 = Afluria Quad IM (UA), 15 ug; A3 = Placebo MAP (FA); A4 = A/Sing Mono IM, 15 ug (UA)
Treatments Part B: B1 = A/Sing MAP, 15 ug (FA) with biopsy; B2 = Placebo MAP (FA) with biopsy
B3 = A/Sing MAP, 15 ug (FA); B4 = A/Sing MAP, 10 ug (FA); B5 = A/Sing MAP, 5 ug (FA); B6 = A/Sing MAP, 2.5 ug (FA)
B7 = Placebo MAP (FA); B8 = A/Sing MAP, 15 ug (UA); B9 = A/Sing IM, 15 ug, as 2018 Afluria Quad (UA)

Listing 16.2.5.3
Skin Hardness Assessment (MAP only)

Part B

Randomisation Number	Group	Visit	Time Point	Date of assessment	Time of Assessment	Site Number	Was assessment performed?	Reading 1 (Without compression)	Reading 2 (with skin conditioning ring pressed down)	Ratio R2/R1 Compression/ Without Compression
B0022	Group B4	Day 1	Pre-dose	12-MAY-2018	12:39	F1	Yes	26.5	46.4	1.75
B0022	Group B4	Day 1	Pre-dose	12-MAY-2018	12:39	F2	Yes	35.3	49.4	1.40
B0022	Group B4	Day 1	Pre-dose	12-MAY-2018	12:39	F3	Yes	33.4	47.6	1.43
B0023	Group B3	Day 1	Pre-dose	12-MAY-2018	12:47	F1	Yes	21.5	28.2	1.31
B0023	Group B3	Day 1	Pre-dose	12-MAY-2018	12:47	F2	Yes	27.0	33.6	1.24
B0023	Group B3	Day 1	Pre-dose	12-MAY-2018	12:47	F3	Yes	27.0	29.5	1.09
B0024	Group B5	Day 1	Pre-dose	12-MAY-2018	13:00	F1	Yes	20.1	34.1	1.70
B0024	Group B5	Day 1	Pre-dose	12-MAY-2018	13:00	F2	Yes	22.9	36.7	1.60
B0024	Group B5	Day 1	Pre-dose	12-MAY-2018	13:00	F3	Yes	26.8	41.3	1.54
B0025	Group B8	Day 1	Pre-dose	12-MAY-2018	13:12	D1	Yes	35.6	49.2	1.38
B0025	Group B8	Day 1	Pre-dose	12-MAY-2018	13:12	D2	Yes	31.8	44.3	1.39
B0025	Group B8	Day 1	Pre-dose	12-MAY-2018	13:12	D3	Yes	29.3	40.5	1.38
B0027	Group B3	Day 1	Pre-dose	12-MAY-2018	13:34	F1	Yes	22.2	45.5	2.05
B0027	Group B3	Day 1	Pre-dose	12-MAY-2018	13:34	F2	Yes	26.5	43.7	1.65

Administration Site Key: FA = Forearm; UA= Upper arm
Treatments Part A: A1 = A/Sing MAP, 15 ug (FA); A2 = Afluria Quad IM (UA), 15 ug; A3 = Placebo MAP (FA); A4 = A/Sing Mono IM, 15 ug (UA)
Treatments Part B: B1 = A/Sing MAP, 15 ug (FA) with biopsy; B2 = Placebo MAP (FA) with biopsy
B3 = A/Sing MAP, 15 ug (FA); B4 = A/Sing MAP, 10 ug (FA); B5 = A/Sing MAP, 5 ug (FA); B6 = A/Sing MAP, 2.5 ug (FA)
B7 = Placebo MAP (FA); B8 = A/Sing MAP, 15 ug (UA); B9 = A/Sing IM, 15 ug, as 2018 Afluria Quad (UA)

Listing 16.2.5.3
Skin Hardness Assessment (MAP only)

Part B

Randomisation Number	Group	Visit	Time Point	Date of assessment	Time of Assessment	Site Number	Was assessment performed?	Reading 1 (Without compression)	Reading 2 (with skin conditioning ring pressed down)	Ratio R2/R1 Compression/ Without Compression
B0027	Group B3	Day 1	Pre-dose	12-MAY-2018	13:34	F3	Yes	32.6	46.9	1.44
B0028	Group B5	Day 1	Pre-dose	12-MAY-2018	13:58	F1	Yes	22.1	36.1	1.63
B0028	Group B5	Day 1	Pre-dose	12-MAY-2018	13:58	F2	Yes	26.6	38.4	1.44
B0028	Group B5	Day 1	Pre-dose	12-MAY-2018	13:58	F3	Yes	32.8	42.2	1.29
B0029	Group B6	Day 1	Pre-dose	12-MAY-2018	13:51	F1	Yes	40.1	51.8	1.29
B0029	Group B6	Day 1	Pre-dose	12-MAY-2018	13:51	F2	Yes	31.5	42.8	1.36
B0029	Group B6	Day 1	Pre-dose	12-MAY-2018	13:51	F3	Yes	31.7	51.3	1.62
B0030	Group B7	Day 1	Pre-dose	12-MAY-2018	13:56	F1	Yes	25.6	42.5	1.66
B0030	Group B7	Day 1	Pre-dose	12-MAY-2018	13:56	F2	Yes	27.2	43.7	1.61
B0030	Group B7	Day 1	Pre-dose	12-MAY-2018	13:56	F3	Yes	28.4	36.5	1.29
B0031	Group B4	Day 1	Pre-dose	19-MAY-2018	13:00	F1	Yes	24.2	35.1	1.45
B0031	Group B4	Day 1	Pre-dose	19-MAY-2018	13:00	F2	Yes	29.2	38.9	1.33
B0031	Group B4	Day 1	Pre-dose	19-MAY-2018	13:00	F3	Yes	35.6	42.3	1.19

Administration Site Key: FA = Forearm; UA= Upper arm
Treatments Part A: A1 = A/Sing MAP, 15 ug (FA); A2 = Afluria Quad IM (UA), 15 ug; A3 = Placebo MAP (FA); A4 = A/Sing Mono IM, 15 ug (UA)
Treatments Part B: B1 = A/Sing MAP, 15 ug (FA) with biopsy; B2 = Placebo MAP (FA) with biopsy
B3 = A/Sing MAP, 15 ug (FA); B4 = A/Sing MAP, 10 ug (FA); B5 = A/Sing MAP, 5 ug (FA); B6 = A/Sing MAP, 2.5 ug (FA)
B7 = Placebo MAP (FA); B8 = A/Sing MAP, 15 ug (UA); B9 = A/Sing IM, 15 ug, as 2018 Afluria Quad (UA)

Listing 16.2.5.3
Skin Hardness Assessment (MAP only)

Part B

Randomisation Number	Group	Visit	Time Point	Date of assessment	Time of Assessment	Site Number	Was assessment performed?	Reading 1 (Without compression)	Reading 2 (with skin conditioning ring pressed down)	Ratio R2/R1 Compression/ Without Compression
B0032	Group B6	Day 1	Pre-dose	19-MAY-2018	11:07	F1	Yes	24.0	40.3	1.68
B0032	Group B6	Day 1	Pre-dose	19-MAY-2018	11:07	F2	Yes	31.4	47.7	1.52
B0032	Group B6	Day 1	Pre-dose	19-MAY-2018	11:07	F3	Yes	43.1	45.5	1.06
B0033	Group B5	Day 1	Pre-dose	19-MAY-2018	11:14	F1	Yes	27.1	40.8	1.51
B0033	Group B5	Day 1	Pre-dose	19-MAY-2018	11:14	F2	Yes	25.6	43.3	1.69
B0033	Group B5	Day 1	Pre-dose	19-MAY-2018	11:14	F3	Yes	27.3	42.3	1.55
B0034	Group B8	Day 1	Pre-dose	19-MAY-2018	11:42	D1	Yes	27.4	31.8	1.16
B0034	Group B8	Day 1	Pre-dose	19-MAY-2018	11:42	D2	Yes	32.4	48.7	1.50
B0034	Group B8	Day 1	Pre-dose	19-MAY-2018	11:42	D3	Yes	35.6	57.1	1.60
B0036	Group B7	Day 1	Pre-dose	19-MAY-2018	11:50	F1	Yes	35.5	43.2	1.22
B0036	Group B7	Day 1	Pre-dose	19-MAY-2018	11:50	F2	Yes	38.1	46.3	1.22
B0036	Group B7	Day 1	Pre-dose	19-MAY-2018	11:50	F3	Yes	38.6	46.6	1.21
B0037	Group B4	Day 1	Pre-dose	19-MAY-2018	11:45	F1	Yes	31.3	45.1	1.44
B0037	Group B4	Day 1	Pre-dose	19-MAY-2018	11:45	F2	Yes	32.0	50.9	1.59

Administration Site Key: FA = Forearm; UA= Upper arm
Treatments Part A: A1 = A/Sing MAP, 15 ug (FA); A2 = Afluria Quad IM (UA), 15 ug; A3 = Placebo MAP (FA); A4 = A/Sing Mono IM, 15 ug (UA)
Treatments Part B: B1 = A/Sing MAP, 15 ug (FA) with biopsy; B2 = Placebo MAP (FA) with biopsy
B3 = A/Sing MAP, 15 ug (FA); B4 = A/Sing MAP, 10 ug (FA); B5 = A/Sing MAP, 5 ug (FA); B6 = A/Sing MAP, 2.5 ug (FA)
B7 = Placebo MAP (FA); B8 = A/Sing MAP, 15 ug (UA); B9 = A/Sing IM, 15 ug, as 2018 Afluria Quad (UA)

Listing 16.2.5.3
Skin Hardness Assessment (MAP only)

Part B

Randomisation Number	Group	Visit	Time Point	Date of assessment	Time of Assessment	Site Number	Was assessment performed?	Reading 1 (Without compression)	Reading 2 (with skin conditioning ring pressed down)	Ratio R2/R1 Compression/ Without Compression
B0037	Group B4	Day 1	Pre-dose	19-MAY-2018	11:45	F3	Yes	33.2	52.1	1.57
B0038	Group B3	Day 1	Pre-dose	19-MAY-2018	12:04	F1	Yes	19.6	32.8	1.67
B0038	Group B3	Day 1	Pre-dose	19-MAY-2018	12:04	F2	Yes	28.5	41.0	1.44
B0038	Group B3	Day 1	Pre-dose	19-MAY-2018	12:04	F3	Yes	28.9	33.6	1.16
B0039	Group B7	Day 1	Pre-dose	19-MAY-2018	12:13	F1	Yes	27.2	36.0	1.32
B0039	Group B7	Day 1	Pre-dose	19-MAY-2018	12:13	F2	Yes	34.2	46.7	1.37
B0039	Group B7	Day 1	Pre-dose	19-MAY-2018	12:13	F3	Yes	28.7	47.3	1.65
B0041	Group B3	Day 1	Pre-dose	19-MAY-2018	12:26	F1	Yes	23.2	43.9	1.89
B0041	Group B3	Day 1	Pre-dose	19-MAY-2018	12:26	F2	Yes	29.7	44.0	1.48
B0041	Group B3	Day 1	Pre-dose	19-MAY-2018	12:26	F3	Yes	36.3	43.4	1.20
B0042	Group B5	Day 1	Pre-dose	19-MAY-2018	12:28	F1	Yes	22.9	33.4	1.46
B0042	Group B5	Day 1	Pre-dose	19-MAY-2018	12:28	F2	Yes	22.7	35.8	1.58
B0042	Group B5	Day 1	Pre-dose	19-MAY-2018	12:28	F3	Yes	30.9	37.1	1.20

Administration Site Key: FA = Forearm; UA= Upper arm
Treatments Part A: A1 = A/Sing MAP, 15 ug (FA); A2 = Afluria Quad IM (UA), 15 ug; A3 = Placebo MAP (FA); A4 = A/Sing Mono IM, 15 ug (UA)
Treatments Part B: B1 = A/Sing MAP, 15 ug (FA) with biopsy; B2 = Placebo MAP (FA) with biopsy
B3 = A/Sing MAP, 15 ug (FA); B4 = A/Sing MAP, 10 ug (FA); B5 = A/Sing MAP, 5 ug (FA); B6 = A/Sing MAP, 2.5 ug (FA)
B7 = Placebo MAP (FA); B8 = A/Sing MAP, 15 ug (UA); B9 = A/Sing IM, 15 ug, as 2018 Afluria Quad (UA)

Listing 16.2.5.3
Skin Hardness Assessment (MAP only)

Part B

Randomisation Number	Group	Visit	Time Point	Date of assessment	Time of Assessment	Site Number	Was assessment performed?	Reading 1 (Without compression)	Reading 2 (with skin conditioning ring pressed down)	Ratio R2/R1 Compression/ Without Compression
B0043	Group B8	Day 1	Pre-dose	19-MAY-2018	12:34	D1	Yes	33.0	53.8	1.63
B0043	Group B8	Day 1	Pre-dose	19-MAY-2018	12:34	D2	Yes	35.7	53.9	1.51
B0043	Group B8	Day 1	Pre-dose	19-MAY-2018	12:34	D3	Yes	36.0	66.7	1.85
B0044	Group B6	Day 1	Pre-dose	19-MAY-2018	12:42	F1	Yes	31.8	42.7	1.34
B0044	Group B6	Day 1	Pre-dose	19-MAY-2018	12:42	F2	Yes	31.1	45.0	1.45
B0044	Group B6	Day 1	Pre-dose	19-MAY-2018	12:42	F3	Yes	28.0	43.0	1.54
B0045	Group B4	Day 1	Pre-dose	19-MAY-2018	11:01	F1	Yes	14.1	27.0	1.91
B0045	Group B4	Day 1	Pre-dose	19-MAY-2018	11:01	F2	Yes	22.9	38.8	1.69
B0045	Group B4	Day 1	Pre-dose	19-MAY-2018	11:01	F3	Yes	21.8	35.6	1.63
B0046	Group B7	Day 1	Pre-dose	19-MAY-2018	13:03	F1	Yes	31.4	46.8	1.49
B0046	Group B7	Day 1	Pre-dose	19-MAY-2018	13:03	F2	Yes	37.9	44.6	1.18
B0046	Group B7	Day 1	Pre-dose	19-MAY-2018	13:03	F3	Yes	34.3	48.3	1.41
B0048	Group B6	Day 1	Pre-dose	19-MAY-2018	12:53	F1	Yes	29.1	39.0	1.34
B0048	Group B6	Day 1	Pre-dose	19-MAY-2018	12:53	F2	Yes	28.5	43.5	1.53

Administration Site Key: FA = Forearm; UA= Upper arm
Treatments Part A: A1 = A/Sing MAP, 15 ug (FA); A2 = Afluria Quad IM (UA), 15 ug; A3 = Placebo MAP (FA); A4 = A/Sing Mono IM, 15 ug (UA)
Treatments Part B: B1 = A/Sing MAP, 15 ug (FA) with biopsy; B2 = Placebo MAP (FA) with biopsy
B3 = A/Sing MAP, 15 ug (FA); B4 = A/Sing MAP, 10 ug (FA); B5 = A/Sing MAP, 5 ug (FA); B6 = A/Sing MAP, 2.5 ug (FA)
B7 = Placebo MAP (FA); B8 = A/Sing MAP, 15 ug (UA); B9 = A/Sing IM, 15 ug, as 2018 Afluria Quad (UA)

Listing 16.2.5.3
Skin Hardness Assessment (MAP only)

Part B

Randomisation Number	Group	Visit	Time Point	Date of assessment	Time of Assessment	Site Number	Was assessment performed?	Reading 1 (Without compression)	Reading 2 (with skin conditioning ring pressed down)	Ratio R2/R1 Compression/ Without Compression
B0048	Group B6	Day 1	Pre-dose	19-MAY-2018	12:53	F3	Yes	33.2	40.5	1.22
B0049	Group B4	Day 1	Pre-dose	19-MAY-2018	14:03	F1	Yes	20.4	41.4	2.03
B0049	Group B4	Day 1	Pre-dose	19-MAY-2018	14:03	F2	Yes	23.5	44.5	1.89
B0049	Group B4	Day 1	Pre-dose	19-MAY-2018	14:03	F3	Yes	28.4	47.5	1.67
B0050	Group B8	Day 1	Pre-dose	19-MAY-2018	13:36	D1	Yes	34.8	39.8	1.14
B0050	Group B8	Day 1	Pre-dose	19-MAY-2018	13:36	D2	Yes	30.4	48.6	1.60
B0050	Group B8	Day 1	Pre-dose	19-MAY-2018	13:36	D3	Yes	29.7	37.8	1.27
B0051	Group B3	Day 1	Pre-dose	25-MAY-2018	10:58	F1	Yes	25.4	34.9	1.37
B0051	Group B3	Day 1	Pre-dose	25-MAY-2018	10:58	F2	Yes	25.4	41.7	1.64
B0051	Group B3	Day 1	Pre-dose	25-MAY-2018	10:58	F3	Yes	31.9	45.2	1.42
B0052	Group B5	Day 1	Pre-dose	25-MAY-2018	11:09	F1	Yes	18.7	43.9	2.35
B0052	Group B5	Day 1	Pre-dose	25-MAY-2018	11:09	F2	Yes	25.9	50.4	1.95
B0052	Group B5	Day 1	Pre-dose	25-MAY-2018	11:09	F3	Yes	26.4	49.6	1.88

Administration Site Key: FA = Forearm; UA= Upper arm
Treatments Part A: A1 = A/Sing MAP, 15 ug (FA); A2 = Afluria Quad IM (UA), 15 ug; A3 = Placebo MAP (FA); A4 = A/Sing Mono IM, 15 ug (UA)
Treatments Part B: B1 = A/Sing MAP, 15 ug (FA) with biopsy; B2 = Placebo MAP (FA) with biopsy
B3 = A/Sing MAP, 15 ug (FA); B4 = A/Sing MAP, 10 ug (FA); B5 = A/Sing MAP, 5 ug (FA); B6 = A/Sing MAP, 2.5 ug (FA)
B7 = Placebo MAP (FA); B8 = A/Sing MAP, 15 ug (UA); B9 = A/Sing IM, 15 ug, as 2018 Afluria Quad (UA)

Listing 16.2.5.3
Skin Hardness Assessment (MAP only)

Part B

Randomisation Number	Group	Visit	Time Point	Date of assessment	Time of Assessment	Site Number	Was assessment performed?	Reading 1 (Without compression)	Reading 2 (with skin conditioning ring pressed down)	Ratio R2/R1 Compression/ Without Compression
B0053	Group B6	Day 1	Pre-dose	25-MAY-2018	11:20	F1	Yes	27.0	38.2	1.41
B0053	Group B6	Day 1	Pre-dose	25-MAY-2018	11:20	F2	Yes	27.8	46.2	1.66
B0053	Group B6	Day 1	Pre-dose	25-MAY-2018	11:20	F3	Yes	38.0	49.0	1.29
B0054	Group B5	Day 1	Pre-dose	25-MAY-2018	11:26	F1	Yes	24.1	29.6	1.23
B0054	Group B5	Day 1	Pre-dose	25-MAY-2018	11:26	F2	Yes	24.4	34.1	1.40
B0054	Group B5	Day 1	Pre-dose	25-MAY-2018	11:26	F3	Yes	33.1	38.0	1.15
B0055	Group B8	Day 1	Pre-dose	25-MAY-2018	11:37	D1	Yes	30.9	47.4	1.53
B0055	Group B8	Day 1	Pre-dose	25-MAY-2018	11:37	D2	Yes	32.1	46.9	1.46
B0055	Group B8	Day 1	Pre-dose	25-MAY-2018	11:37	D3	Yes	36.0	52.5	1.46
B0056	Group B3	Day 1	Pre-dose	25-MAY-2018	11:48	F1	Yes	19.2	29.4	1.53
B0056	Group B3	Day 1	Pre-dose	25-MAY-2018	11:48	F2	Yes	19.5	30.7	1.57
B0056	Group B3	Day 1	Pre-dose	25-MAY-2018	11:48	F3	Yes	28.8	31.0	1.08
B0057	Group B4	Day 1	Pre-dose	25-MAY-2018	11:53	F1	Yes	22.5	38.1	1.69
B0057	Group B4	Day 1	Pre-dose	25-MAY-2018	11:53	F2	Yes	26.9	44.0	1.64

Administration Site Key: FA = Forearm; UA= Upper arm
Treatments Part A: A1 = A/Sing MAP, 15 ug (FA); A2 = Afluria Quad IM (UA), 15 ug; A3 = Placebo MAP (FA); A4 = A/Sing Mono IM, 15 ug (UA)
Treatments Part B: B1 = A/Sing MAP, 15 ug (FA) with biopsy; B2 = Placebo MAP (FA) with biopsy
B3 = A/Sing MAP, 15 ug (FA); B4 = A/Sing MAP, 10 ug (FA); B5 = A/Sing MAP, 5 ug (FA); B6 = A/Sing MAP, 2.5 ug (FA)
B7 = Placebo MAP (FA); B8 = A/Sing MAP, 15 ug (UA); B9 = A/Sing IM, 15 ug, as 2018 Afluria Quad (UA)

Listing 16.2.5.3
Skin Hardness Assessment (MAP only)

Part B

Randomisation Number	Group	Visit	Time Point	Date of assessment	Time of Assessment	Site Number	Was assessment performed?	Reading 1 (Without compression)	Reading 2 (with skin conditioning ring pressed down)	Ratio R2/R1 Compression/ Without Compression
B0057	Group B4	Day 1	Pre-dose	25-MAY-2018	11:53	F3	Yes	33.6	46.3	1.38
B0059	Group B7	Day 1	Pre-dose	25-MAY-2018	12:04	F1	Yes	21.3	34.4	1.62
B0059	Group B7	Day 1	Pre-dose	25-MAY-2018	12:04	F2	Yes	25.4	36.4	1.43
B0059	Group B7	Day 1	Pre-dose	25-MAY-2018	12:04	F3	Yes	29.9	38.3	1.28
B0060	Group B3	Day 1	Pre-dose	25-MAY-2018	12:16	F1	Yes	19.6	24.0	1.22
B0060	Group B3	Day 1	Pre-dose	25-MAY-2018	12:16	F2	Yes	21.1	24.7	1.17
B0060	Group B3	Day 1	Pre-dose	25-MAY-2018	12:16	F3	Yes	22.5	26.3	1.17
B0061	Group B8	Day 1	Pre-dose	25-MAY-2018	12:25	D1	Yes	24.9	34.1	1.37
B0061	Group B8	Day 1	Pre-dose	25-MAY-2018	12:25	D2	Yes	20.6	34.4	1.67
B0061	Group B8	Day 1	Pre-dose	25-MAY-2018	12:25	D3	Yes	22.9	38.9	1.70
B0062	Group B5	Day 1	Pre-dose	25-MAY-2018	12:34	F1	Yes	28.2	32.3	1.15
B0062	Group B5	Day 1	Pre-dose	25-MAY-2018	12:34	F2	Yes	31.0	36.1	1.16
B0062	Group B5	Day 1	Pre-dose	25-MAY-2018	12:34	F3	Yes	34.6	41.8	1.21

Administration Site Key: FA = Forearm; UA= Upper arm
Treatments Part A: A1 = A/Sing MAP, 15 ug (FA); A2 = Afluria Quad IM (UA), 15 ug; A3 = Placebo MAP (FA); A4 = A/Sing Mono IM, 15 ug (UA)
Treatments Part B: B1 = A/Sing MAP, 15 ug (FA) with biopsy; B2 = Placebo MAP (FA) with biopsy
B3 = A/Sing MAP, 15 ug (FA); B4 = A/Sing MAP, 10 ug (FA); B5 = A/Sing MAP, 5 ug (FA); B6 = A/Sing MAP, 2.5 ug (FA)
B7 = Placebo MAP (FA); B8 = A/Sing MAP, 15 ug (UA); B9 = A/Sing IM, 15 ug, as 2018 Afluria Quad (UA)

Listing 16.2.5.3
Skin Hardness Assessment (MAP only)

Part B

Randomisation Number	Group	Visit	Time Point	Date of assessment	Time of Assessment	Site Number	Was assessment performed?	Reading 1 (Without compression)	Reading 2 (with skin conditioning ring pressed down)	Ratio R2/R1 Compression/ Without Compression
B0063	Group B7	Day 1	Pre-dose	25-MAY-2018	12:45	F1	Yes	25.9	33.3	1.29
B0063	Group B7	Day 1	Pre-dose	25-MAY-2018	12:45	F2	Yes	26.5	36.6	1.38
B0063	Group B7	Day 1	Pre-dose	25-MAY-2018	12:45	F3	Yes	32.8	39.8	1.21
B0065	Group B4	Day 1	Pre-dose	25-MAY-2018	12:55	F1	Yes	26.2	38.0	1.45
B0065	Group B4	Day 1	Pre-dose	25-MAY-2018	12:55	F2	Yes	28.3	39.1	1.38
B0065	Group B4	Day 1	Pre-dose	25-MAY-2018	12:55	F3	Yes	30.0	47.5	1.58
B0066	Group B6	Day 1	Pre-dose	25-MAY-2018	13:57	F1	Yes	18.2	35.3	1.94
B0066	Group B6	Day 1	Pre-dose	25-MAY-2018	13:57	F2	Yes	22.9	37.9	1.66
B0066	Group B6	Day 1	Pre-dose	25-MAY-2018	13:57	F3	Yes	25.0	36.4	1.46
B0067	Group B8	Day 1	Pre-dose	25-MAY-2018	14:14	D1	Yes	34.0	41.6	1.22
B0067	Group B8	Day 1	Pre-dose	25-MAY-2018	14:14	D2	Yes	25.8	33.4	1.29
B0067	Group B8	Day 1	Pre-dose	25-MAY-2018	14:14	D3	Yes	29.4	36.5	1.24
B0068	Group B5	Day 1	Pre-dose	25-MAY-2018	14:24	F1	Yes	21.0	41.4	1.97
B0068	Group B5	Day 1	Pre-dose	25-MAY-2018	14:24	F2	Yes	25.8	51.1	1.98

Administration Site Key: FA = Forearm; UA= Upper arm
Treatments Part A: A1 = A/Sing MAP, 15 ug (FA); A2 = Afluria Quad IM (UA), 15 ug; A3 = Placebo MAP (FA); A4 = A/Sing Mono IM, 15 ug (UA)
Treatments Part B: B1 = A/Sing MAP, 15 ug (FA) with biopsy; B2 = Placebo MAP (FA) with biopsy
B3 = A/Sing MAP, 15 ug (FA); B4 = A/Sing MAP, 10 ug (FA); B5 = A/Sing MAP, 5 ug (FA); B6 = A/Sing MAP, 2.5 ug (FA)
B7 = Placebo MAP (FA); B8 = A/Sing MAP, 15 ug (UA); B9 = A/Sing IM, 15 ug, as 2018 Afluria Quad (UA)

Listing 16.2.5.3
Skin Hardness Assessment (MAP only)

Part B

Randomisation Number	Group	Visit	Time Point	Date of assessment	Time of Assessment	Site Number	Was assessment performed?	Reading 1 (Without compression)	Reading 2 (with skin conditioning ring pressed down)	Ratio R2/R1 Compression/ Without Compression
B0068	Group B5	Day 1	Pre-dose	25-MAY-2018	14:24	F3	Yes	32.0	46.8	1.46
B0069	Group B7	Day 1	Pre-dose	01-JUN-2018	11:13	F1	Yes	18.6	30.9	1.66
B0069	Group B7	Day 1	Pre-dose	01-JUN-2018	11:13	F2	Yes	25.2	29.6	1.17
B0069	Group B7	Day 1	Pre-dose	01-JUN-2018	11:13	F3	Yes	31.8	41.1	1.29
B0071	Group B4	Day 1	Pre-dose	01-JUN-2018	10:58	F1	Yes	19.5	43.2	2.22
B0071	Group B4	Day 1	Pre-dose	01-JUN-2018	10:58	F2	Yes	23.3	45.4	1.95
B0071	Group B4	Day 1	Pre-dose	01-JUN-2018	10:58	F3	Yes	29.9	51.3	1.72
B0072	Group B3	Day 1	Pre-dose	01-JUN-2018	11:02	F1	Yes	22.2	45.4	2.05
B0072	Group B3	Day 1	Pre-dose	01-JUN-2018	11:02	F2	Yes	26.4	48.5	1.84
B0072	Group B3	Day 1	Pre-dose	01-JUN-2018	11:02	F3	Yes	35.6	47.2	1.33
B0073	Group B6	Day 1	Pre-dose	01-JUN-2018	11:07	F1	Yes	26.5	27.7	1.05
B0073	Group B6	Day 1	Pre-dose	01-JUN-2018	11:07	F2	Yes	24.5	33.6	1.37
B0073	Group B6	Day 1	Pre-dose	01-JUN-2018	11:07	F3	Yes	30.0	39.7	1.32

Administration Site Key: FA = Forearm; UA= Upper arm
Treatments Part A: A1 = A/Sing MAP, 15 ug (FA); A2 = Afluria Quad IM (UA), 15 ug; A3 = Placebo MAP (FA); A4 = A/Sing Mono IM, 15 ug (UA)
Treatments Part B: B1 = A/Sing MAP, 15 ug (FA) with biopsy; B2 = Placebo MAP (FA) with biopsy
B3 = A/Sing MAP, 15 ug (FA); B4 = A/Sing MAP, 10 ug (FA); B5 = A/Sing MAP, 5 ug (FA); B6 = A/Sing MAP, 2.5 ug (FA)
B7 = Placebo MAP (FA); B8 = A/Sing MAP, 15 ug (UA); B9 = A/Sing IM, 15 ug, as 2018 Afluria Quad (UA)

Listing 16.2.5.3
Skin Hardness Assessment (MAP only)

Part B

Randomisation Number	Group	Visit	Time Point	Date of assessment	Time of Assessment	Site Number	Was assessment performed?	Reading 1 (Without compression)	Reading 2 (with skin conditioning ring pressed down)	Ratio R2/R1 Compression/ Without Compression
B0075	Group B3	Day 1	Pre-dose	01-JUN-2018	11:20	F1	Yes	24.7	44.2	1.79
B0075	Group B3	Day 1	Pre-dose	01-JUN-2018	11:20	F2	Yes	33.1	50.0	1.51
B0075	Group B3	Day 1	Pre-dose	01-JUN-2018	11:20	F3	Yes	30.9	53.4	1.73
B0077	Group B4	Day 1	Pre-dose	01-JUN-2018	11:30	F1	Yes	19.8	41.6	2.10
B0077	Group B4	Day 1	Pre-dose	01-JUN-2018	11:30	F2	Yes	30.0	47.5	1.58
B0077	Group B4	Day 1	Pre-dose	01-JUN-2018	11:30	F3	Yes	30.4	49.9	1.64
B0078	Group B8	Day 1	Pre-dose	01-JUN-2018	11:40	D1	Yes	32.8	50.0	1.52
B0078	Group B8	Day 1	Pre-dose	01-JUN-2018	11:40	D2	Yes	31.1	51.1	1.64
B0078	Group B8	Day 1	Pre-dose	01-JUN-2018	11:40	D3	Yes	34.7	56.5	1.63
B0079	Group B6	Day 1	Pre-dose	01-JUN-2018	11:46	F1	Yes	30.6	49.4	1.61
B0079	Group B6	Day 1	Pre-dose	01-JUN-2018	11:46	F2	Yes	35.6	51.0	1.43
B0079	Group B6	Day 1	Pre-dose	01-JUN-2018	11:46	F3	Yes	36.6	55.0	1.50
B0081	Group B6	Day 1	Pre-dose	01-JUN-2018	12:03	F1	Yes	21.9	39.9	1.82
B0081	Group B6	Day 1	Pre-dose	01-JUN-2018	12:03	F2	Yes	23.9	41.4	1.73

Administration Site Key: FA = Forearm; UA= Upper arm
Treatments Part A: A1 = A/Sing MAP, 15 ug (FA); A2 = Afluria Quad IM (UA), 15 ug; A3 = Placebo MAP (FA); A4 = A/Sing Mono IM, 15 ug (UA)
Treatments Part B: B1 = A/Sing MAP, 15 ug (FA) with biopsy; B2 = Placebo MAP (FA) with biopsy
B3 = A/Sing MAP, 15 ug (FA); B4 = A/Sing MAP, 10 ug (FA); B5 = A/Sing MAP, 5 ug (FA); B6 = A/Sing MAP, 2.5 ug (FA)
B7 = Placebo MAP (FA); B8 = A/Sing MAP, 15 ug (UA); B9 = A/Sing IM, 15 ug, as 2018 Afluria Quad (UA)

Listing 16.2.5.3
Skin Hardness Assessment (MAP only)

Part B

Randomisation Number	Group	Visit	Time Point	Date of assessment	Time of Assessment	Site Number	Was assessment performed?	Reading 1 (Without compression)	Reading 2 (with skin conditioning ring pressed down)	Ratio R2/R1 Compression/ Without Compression
B0081	Group B6	Day 1	Pre-dose	01-JUN-2018	12:03	F3	Yes	29.5	45.4	1.54
B0082	Group B3	Day 1	Pre-dose	01-JUN-2018	13:13	F1	Yes	20.3	31.9	1.57
B0082	Group B3	Day 1	Pre-dose	01-JUN-2018	13:13	F2	Yes	22.9	46.2	2.02
B0082	Group B3	Day 1	Pre-dose	01-JUN-2018	13:13	F3	Yes	28.0	46.8	1.67
B0083	Group B8	Day 1	Pre-dose	01-JUN-2018	13:18	D1	Yes	27.8	38.1	1.37
B0083	Group B8	Day 1	Pre-dose	01-JUN-2018	13:18	D2	Yes	26.5	47.3	1.78
B0083	Group B8	Day 1	Pre-dose	01-JUN-2018	13:18	D3	Yes	25.6	43.5	1.70
B0084	Group B5	Day 1	Pre-dose	09-JUN-2018	11:13	F1	Yes	27.9	43.3	1.55
B0084	Group B5	Day 1	Pre-dose	09-JUN-2018	11:13	F2	Yes	36.1	49.4	1.37
B0084	Group B5	Day 1	Pre-dose	09-JUN-2018	11:13	F3	Yes	36.1	47.3	1.31
B0085	Group B4	Day 1	Pre-dose	09-JUN-2018	11:19	F1	Yes	22.7	36.2	1.59
B0085	Group B4	Day 1	Pre-dose	09-JUN-2018	11:19	F2	Yes	29.4	41.6	1.41
B0085	Group B4	Day 1	Pre-dose	09-JUN-2018	11:19	F3	Yes	22.5	33.0	1.47

Administration Site Key: FA = Forearm; UA= Upper arm
Treatments Part A: A1 = A/Sing MAP, 15 ug (FA); A2 = Afluria Quad IM (UA), 15 ug; A3 = Placebo MAP (FA); A4 = A/Sing Mono IM, 15 ug (UA)
Treatments Part B: B1 = A/Sing MAP, 15 ug (FA) with biopsy; B2 = Placebo MAP (FA) with biopsy
B3 = A/Sing MAP, 15 ug (FA); B4 = A/Sing MAP, 10 ug (FA); B5 = A/Sing MAP, 5 ug (FA); B6 = A/Sing MAP, 2.5 ug (FA)
B7 = Placebo MAP (FA); B8 = A/Sing MAP, 15 ug (UA); B9 = A/Sing IM, 15 ug, as 2018 Afluria Quad (UA)

Listing 16.2.5.3
Skin Hardness Assessment (MAP only)

Part B

Randomisation Number	Group	Visit	Time Point	Date of assessment	Time of Assessment	Site Number	Was assessment performed?	Reading 1 (Without compression)	Reading 2 (with skin conditioning ring pressed down)	Ratio R2/R1 Compression/ Without Compression
B0087	Group B7	Day 1	Pre-dose	09-JUN-2018	11:33	F1	Yes	13.5	26.9	1.99
B0087	Group B7	Day 1	Pre-dose	09-JUN-2018	11:33	F2	Yes	29.6	42.5	1.44
B0087	Group B7	Day 1	Pre-dose	09-JUN-2018	11:33	F3	Yes	42.6	48.9	1.15
B0089	Group B5	Day 1	Pre-dose	09-JUN-2018	11:43	F1	Yes	27.1	45.3	1.67
B0089	Group B5	Day 1	Pre-dose	09-JUN-2018	11:43	F2	Yes	20.1	43.4	2.16
B0089	Group B5	Day 1	Pre-dose	09-JUN-2018	11:43	F3	Yes	38.8	49.2	1.27
B0090	Group B6	Day 1	Pre-dose	09-JUN-2018	11:55	F1	Yes	13.8	31.8	2.30
B0090	Group B6	Day 1	Pre-dose	09-JUN-2018	11:55	F2	Yes	32.1	45.5	1.42
B0090	Group B6	Day 1	Pre-dose	09-JUN-2018	11:55	F3	Yes	30.1	42.8	1.42
B0091	Group B8	Day 1	Pre-dose	09-JUN-2018	12:06	D1	Yes	34.5	52.4	1.52
B0091	Group B8	Day 1	Pre-dose	09-JUN-2018	12:06	D2	Yes	28.7	48.0	1.67
B0091	Group B8	Day 1	Pre-dose	09-JUN-2018	12:06	D3	Yes	29.9	42.2	1.41
B0092	Group B4	Day 1	Pre-dose	09-JUN-2018	12:14	F1	Yes	27.7	38.8	1.40
B0092	Group B4	Day 1	Pre-dose	09-JUN-2018	12:14	F2	Yes	26.5	45.5	1.72

Administration Site Key: FA = Forearm; UA= Upper arm
Treatments Part A: A1 = A/Sing MAP, 15 ug (FA); A2 = Afluria Quad IM (UA), 15 ug; A3 = Placebo MAP (FA); A4 = A/Sing Mono IM, 15 ug (UA)
Treatments Part B: B1 = A/Sing MAP, 15 ug (FA) with biopsy; B2 = Placebo MAP (FA) with biopsy
B3 = A/Sing MAP, 15 ug (FA); B4 = A/Sing MAP, 10 ug (FA); B5 = A/Sing MAP, 5 ug (FA); B6 = A/Sing MAP, 2.5 ug (FA)
B7 = Placebo MAP (FA); B8 = A/Sing MAP, 15 ug (UA); B9 = A/Sing IM, 15 ug, as 2018 Afluria Quad (UA)

Listing 16.2.5.3
Skin Hardness Assessment (MAP only)

Part B

Randomisation Number	Group	Visit	Time Point	Date of assessment	Time of Assessment	Site Number	Was assessment performed?	Reading 1 (Without compression)	Reading 2 (with skin conditioning ring pressed down)	Ratio R2/R1 Compression/ Without Compression
B0092	Group B4	Day 1	Pre-dose	09-JUN-2018	12:14	F3	Yes	21.9	41.0	1.87
B0093	Group B7	Day 1	Pre-dose	09-JUN-2018	12:19	F1	Yes	20.6	43.9	2.13
B0093	Group B7	Day 1	Pre-dose	09-JUN-2018	12:19	F2	Yes	19.1	47.6	2.49
B0093	Group B7	Day 1	Pre-dose	09-JUN-2018	12:19	F3	Yes	27.0	40.9	1.51
B0094	Group B3	Day 1	Pre-dose	09-JUN-2018	12:29	F1	Yes	22.6	30.4	1.35
B0094	Group B3	Day 1	Pre-dose	09-JUN-2018	12:29	F2	Yes	26.5	34.1	1.29
B0094	Group B3	Day 1	Pre-dose	09-JUN-2018	12:29	F3	Yes	26.9	32.6	1.21
B0096	Group B6	Day 1	Pre-dose	09-JUN-2018	12:44	F1	Yes	13.8	32.1	2.33
B0096	Group B6	Day 1	Pre-dose	09-JUN-2018	12:44	F2	Yes	26.4	42.7	1.62
B0096	Group B6	Day 1	Pre-dose	09-JUN-2018	12:44	F3	Yes	26.3	39.9	1.52
B0097	Group B5	Day 1	Pre-dose	09-JUN-2018	12:50	F1	Yes	12.4	27.9	2.25
B0097	Group B5	Day 1	Pre-dose	09-JUN-2018	12:50	F2	Yes	29.5	37.5	1.27
B0097	Group B5	Day 1	Pre-dose	09-JUN-2018	12:50	F3	Yes	38.5	39.0	1.01

Administration Site Key: FA = Forearm; UA= Upper arm
Treatments Part A: A1 = A/Sing MAP, 15 ug (FA); A2 = Afluria Quad IM (UA), 15 ug; A3 = Placebo MAP (FA); A4 = A/Sing Mono IM, 15 ug (UA)
Treatments Part B: B1 = A/Sing MAP, 15 ug (FA) with biopsy; B2 = Placebo MAP (FA) with biopsy
B3 = A/Sing MAP, 15 ug (FA); B4 = A/Sing MAP, 10 ug (FA); B5 = A/Sing MAP, 5 ug (FA); B6 = A/Sing MAP, 2.5 ug (FA)
B7 = Placebo MAP (FA); B8 = A/Sing MAP, 15 ug (UA); B9 = A/Sing IM, 15 ug, as 2018 Afluria Quad (UA)

Listing 16.2.5.3
Skin Hardness Assessment (MAP only)

Part B

Randomisation Number	Group	Visit	Time Point	Date of assessment	Time of Assessment	Site Number	Was assessment performed?	Reading 1 (Without compression)	Reading 2 (with skin conditioning ring pressed down)	Ratio R2/R1 Compression/ Without Compression
B0098	Group B7	Day 1	Pre-dose	09-JUN-2018	12:58	F1	Yes	19.8	41.2	2.08
B0098	Group B7	Day 1	Pre-dose	09-JUN-2018	12:58	F2	Yes	28.8	46.3	1.61
B0098	Group B7	Day 1	Pre-dose	09-JUN-2018	12:58	F3	Yes	23.6	34.4	1.46
B0099	Group B4	Day 1	Pre-dose	09-JUN-2018	13:09	F1	Yes	21.7	46.2	2.13
B0099	Group B4	Day 1	Pre-dose	09-JUN-2018	13:09	F2	Yes	31.8	46.3	1.46
B0099	Group B4	Day 1	Pre-dose	09-JUN-2018	13:09	F3	Yes	27.9	48.8	1.75
B0100	Group B8	Day 1	Pre-dose	09-JUN-2018	13:20	D1	Yes	27.0	38.0	1.41
B0100	Group B8	Day 1	Pre-dose	09-JUN-2018	13:20	D2	Yes	30.0	40.7	1.36
B0100	Group B8	Day 1	Pre-dose	09-JUN-2018	13:20	D3	Yes	25.3	45.0	1.78
B0101	Group B3	Day 1	Pre-dose	09-JUN-2018	13:30	F1	Yes	27.9	45.9	1.65
B0101	Group B3	Day 1	Pre-dose	09-JUN-2018	13:30	F2	Yes	29.9	49.9	1.67
B0101	Group B3	Day 1	Pre-dose	09-JUN-2018	13:30	F3	Yes	30.3	47.7	1.57
B0102	Group B8	Day 1	Pre-dose	09-JUN-2018	13:40	D1	Yes	23.0	52.8	2.30
B0102	Group B8	Day 1	Pre-dose	09-JUN-2018	13:40	D2	Yes	31.0	37.8	1.22

Administration Site Key: FA = Forearm; UA= Upper arm
Treatments Part A: A1 = A/Sing MAP, 15 ug (FA); A2 = Afluria Quad IM (UA), 15 ug; A3 = Placebo MAP (FA); A4 = A/Sing Mono IM, 15 ug (UA)
Treatments Part B: B1 = A/Sing MAP, 15 ug (FA) with biopsy; B2 = Placebo MAP (FA) with biopsy
B3 = A/Sing MAP, 15 ug (FA); B4 = A/Sing MAP, 10 ug (FA); B5 = A/Sing MAP, 5 ug (FA); B6 = A/Sing MAP, 2.5 ug (FA)
B7 = Placebo MAP (FA); B8 = A/Sing MAP, 15 ug (UA); B9 = A/Sing IM, 15 ug, as 2018 Afluria Quad (UA)

Listing 16.2.5.3
Skin Hardness Assessment (MAP only)

Part B

Randomisation Number	Group	Visit	Time Point	Date of assessment	Time of Assessment	Site Number	Was assessment performed?	Reading 1 (Without compression)	Reading 2 (with skin conditioning ring pressed down)	Ratio R2/R1 Compression/ Without Compression
B0102	Group B8	Day 1	Pre-dose	09-JUN-2018	13:40	D3	Yes	32.7	50.2	1.54
B0103	Group B5	Day 1	Pre-dose	09-JUN-2018	13:52	F1	Yes	25.1	37.2	1.48
B0103	Group B5	Day 1	Pre-dose	09-JUN-2018	13:52	F2	Yes	22.8	40.1	1.76
B0103	Group B5	Day 1	Pre-dose	09-JUN-2018	13:52	F3	Yes	26.1	38.3	1.47
B0104	Group B3	Day 1	Pre-dose	09-JUN-2018	14:01	F1	Yes	25.3	40.6	1.60
B0104	Group B3	Day 1	Pre-dose	09-JUN-2018	14:01	F2	Yes	28.3	41.4	1.46
B0104	Group B3	Day 1	Pre-dose	09-JUN-2018	14:01	F3	Yes	23.7	45.7	1.93
B0106	Group B4	Day 1	Pre-dose	09-JUN-2018	14:10	F1	Yes	28.6	32.3	1.13
B0106	Group B4	Day 1	Pre-dose	09-JUN-2018	14:10	F2	Yes	21.4	37.6	1.76
B0106	Group B4	Day 1	Pre-dose	09-JUN-2018	14:10	F3	Yes	19.0	33.7	1.77
B0107	Group B6	Day 1	Pre-dose	09-JUN-2018	14:20	F1	Yes	22.1	39.0	1.76
B0107	Group B6	Day 1	Pre-dose	09-JUN-2018	14:20	F2	Yes	27.8	43.1	1.55
B0107	Group B6	Day 1	Pre-dose	09-JUN-2018	14:20	F3	Yes	21.8	41.2	1.89

Administration Site Key: FA = Forearm; UA= Upper arm
Treatments Part A: A1 = A/Sing MAP, 15 ug (FA); A2 = Afluria Quad IM (UA), 15 ug; A3 = Placebo MAP (FA); A4 = A/Sing Mono IM, 15 ug (UA)
Treatments Part B: B1 = A/Sing MAP, 15 ug (FA) with biopsy; B2 = Placebo MAP (FA) with biopsy
B3 = A/Sing MAP, 15 ug (FA); B4 = A/Sing MAP, 10 ug (FA); B5 = A/Sing MAP, 5 ug (FA); B6 = A/Sing MAP, 2.5 ug (FA)
B7 = Placebo MAP (FA); B8 = A/Sing MAP, 15 ug (UA); B9 = A/Sing IM, 15 ug, as 2018 Afluria Quad (UA)

Listing 16.2.5.3
Skin Hardness Assessment (MAP only)

Part B

Randomisation Number	Group	Visit	Time Point	Date of assessment	Time of Assessment	Site Number	Was assessment performed?	Reading 1 (Without compression)	Reading 2 (with skin conditioning ring pressed down)	Ratio R2/R1 Compression/ Without Compression
B0108	Group B7	Day 1	Pre-dose	09-JUN-2018	14:28	F1	Yes	26.4	32.9	1.25
B0108	Group B7	Day 1	Pre-dose	09-JUN-2018	14:28	F2	Yes	27.3	40.2	1.47
B0108	Group B7	Day 1	Pre-dose	09-JUN-2018	14:28	F3	Yes	31.6	38.6	1.22
B0109	Group B6	Day 1	Pre-dose	16-JUN-2018	11:13	F1	Yes	28.5	31.2	1.09
B0109	Group B6	Day 1	Pre-dose	16-JUN-2018	11:13	F2	Yes	35.8	40.0	1.12
B0109	Group B6	Day 1	Pre-dose	16-JUN-2018	11:13	F3	Yes	37.1	38.4	1.04
B0110	Group B3	Day 1	Pre-dose	16-JUN-2018	11:23	F1	Yes	24.1	30.3	1.26
B0110	Group B3	Day 1	Pre-dose	16-JUN-2018	11:23	F2	Yes	26.8	35.8	1.34
B0110	Group B3	Day 1	Pre-dose	16-JUN-2018	11:23	F3	Yes	25.9	31.7	1.22
B0111	Group B8	Day 1	Pre-dose	16-JUN-2018	11:35	D1	Yes	27.7	36.4	1.31
B0111	Group B8	Day 1	Pre-dose	16-JUN-2018	11:35	D2	Yes	32.8	37.8	1.15
B0111	Group B8	Day 1	Pre-dose	16-JUN-2018	11:35	D3	Yes	30.2	43.4	1.44
B0112	Group B4	Day 1	Pre-dose	16-JUN-2018	11:46	F1	Yes	33.3	40.9	1.23
B0112	Group B4	Day 1	Pre-dose	16-JUN-2018	11:46	F2	Yes	30.4	36.9	1.21

Administration Site Key: FA = Forearm; UA= Upper arm
Treatments Part A: A1 = A/Sing MAP, 15 ug (FA); A2 = Afluria Quad IM (UA), 15 ug; A3 = Placebo MAP (FA); A4 = A/Sing Mono IM, 15 ug (UA)
Treatments Part B: B1 = A/Sing MAP, 15 ug (FA) with biopsy; B2 = Placebo MAP (FA) with biopsy
B3 = A/Sing MAP, 15 ug (FA); B4 = A/Sing MAP, 10 ug (FA); B5 = A/Sing MAP, 5 ug (FA); B6 = A/Sing MAP, 2.5 ug (FA)
B7 = Placebo MAP (FA); B8 = A/Sing MAP, 15 ug (UA); B9 = A/Sing IM, 15 ug, as 2018 Afluria Quad (UA)

Listing 16.2.5.3
Skin Hardness Assessment (MAP only)

Part B

Randomisation Number	Group	Visit	Time Point	Date of assessment	Time of Assessment	Site Number	Was assessment performed?	Reading 1 (Without compression)	Reading 2 (with skin conditioning ring pressed down)	Ratio R2/R1 Compression/ Without Compression
B0112	Group B4	Day 1	Pre-dose	16-JUN-2018	11:46	F3	Yes	32.4	38.0	1.17
B0113	Group B5	Day 1	Pre-dose	16-JUN-2018	11:58	F1	Yes	26.6	27.7	1.04
B0113	Group B5	Day 1	Pre-dose	16-JUN-2018	11:58	F2	Yes	27.9	34.8	1.25
B0113	Group B5	Day 1	Pre-dose	16-JUN-2018	11:58	F3	Yes	24.9	31.9	1.28
B0114	Group B7	Day 1	Pre-dose	16-JUN-2018	11:58	F1	Yes	26.5	37.4	1.41
B0114	Group B7	Day 1	Pre-dose	16-JUN-2018	11:58	F2	Yes	27.1	40.1	1.48
B0114	Group B7	Day 1	Pre-dose	16-JUN-2018	11:58	F3	Yes	30.1	40.5	1.35
B0116	Group B7	Day 1	Pre-dose	16-JUN-2018	12:04	F1	Yes	19.6	35.3	1.80
B0116	Group B7	Day 1	Pre-dose	16-JUN-2018	12:04	F2	Yes	24.2	38.8	1.60
B0116	Group B7	Day 1	Pre-dose	16-JUN-2018	12:04	F3	Yes	28.0	43.6	1.56
B0118	Group B6	Day 1	Pre-dose	16-JUN-2018	12:08	F1	Yes	22.8	31.3	1.37
B0118	Group B6	Day 1	Pre-dose	16-JUN-2018	12:08	F2	Yes	30.6	37.0	1.21
B0118	Group B6	Day 1	Pre-dose	16-JUN-2018	12:08	F3	Yes	26.9	28.2	1.05

Administration Site Key: FA = Forearm; UA= Upper arm
Treatments Part A: A1 = A/Sing MAP, 15 ug (FA); A2 = Afluria Quad IM (UA), 15 ug; A3 = Placebo MAP (FA); A4 = A/Sing Mono IM, 15 ug (UA)
Treatments Part B: B1 = A/Sing MAP, 15 ug (FA) with biopsy; B2 = Placebo MAP (FA) with biopsy
B3 = A/Sing MAP, 15 ug (FA); B4 = A/Sing MAP, 10 ug (FA); B5 = A/Sing MAP, 5 ug (FA); B6 = A/Sing MAP, 2.5 ug (FA)
B7 = Placebo MAP (FA); B8 = A/Sing MAP, 15 ug (UA); B9 = A/Sing IM, 15 ug, as 2018 Afluria Quad (UA)

Listing 16.2.5.3
Skin Hardness Assessment (MAP only)

Part B

Randomisation Number	Group	Visit	Time Point	Date of assessment	Time of Assessment	Site Number	Was assessment performed?	Reading 1 (Without compression)	Reading 2 (with skin conditioning ring pressed down)	Ratio R2/R1 Compression/ Without Compression
B0119	Group B4	Day 1	Pre-dose	16-JUN-2018	12:11	F1	Yes	26.7	46.2	1.73
B0119	Group B4	Day 1	Pre-dose	16-JUN-2018	12:11	F2	Yes	27.5	46.7	1.70
B0119	Group B4	Day 1	Pre-dose	16-JUN-2018	12:11	F3	Yes	33.7	49.2	1.46
B0120	Group B3	Day 1	Pre-dose	16-JUN-2018	12:19	F1	Yes	28.1	36.0	1.28
B0120	Group B3	Day 1	Pre-dose	16-JUN-2018	12:19	F2	Yes	31.7	34.4	1.09
B0120	Group B3	Day 1	Pre-dose	16-JUN-2018	12:19	F3	Yes	31.4	36.5	1.16
B0121	Group B8	Day 1	Pre-dose	16-JUN-2018	12:18	D1	Yes	42.9	51.2	1.19
B0121	Group B8	Day 1	Pre-dose	16-JUN-2018	12:18	D2	Yes	30.6	44.4	1.45
B0121	Group B8	Day 1	Pre-dose	16-JUN-2018	12:18	D3	Yes	29.7	47.9	1.61
B0122	Group B5	Day 1	Pre-dose	16-JUN-2018	12:27	F1	Yes	28.3	32.2	1.14
B0122	Group B5	Day 1	Pre-dose	16-JUN-2018	12:27	F2	Yes	32.2	37.4	1.16
B0122	Group B5	Day 1	Pre-dose	16-JUN-2018	12:27	F3	Yes	36.1	40.0	1.11
B0123	Group B6	Day 1	Pre-dose	16-JUN-2018	12:29	F1	Yes	25.2	37.8	1.50
B0123	Group B6	Day 1	Pre-dose	16-JUN-2018	12:29	F2	Yes	25.1	40.5	1.61

Administration Site Key: FA = Forearm; UA= Upper arm
Treatments Part A: A1 = A/Sing MAP, 15 ug (FA); A2 = Afluria Quad IM (UA), 15 ug; A3 = Placebo MAP (FA); A4 = A/Sing Mono IM, 15 ug (UA)
Treatments Part B: B1 = A/Sing MAP, 15 ug (FA) with biopsy; B2 = Placebo MAP (FA) with biopsy
B3 = A/Sing MAP, 15 ug (FA); B4 = A/Sing MAP, 10 ug (FA); B5 = A/Sing MAP, 5 ug (FA); B6 = A/Sing MAP, 2.5 ug (FA)
B7 = Placebo MAP (FA); B8 = A/Sing MAP, 15 ug (UA); B9 = A/Sing IM, 15 ug, as 2018 Afluria Quad (UA)

Listing 16.2.5.3
Skin Hardness Assessment (MAP only)

Part B

Randomisation Number	Group	Visit	Time Point	Date of assessment	Time of Assessment	Site Number	Was assessment performed?	Reading 1 (Without compression)	Reading 2 (with skin conditioning ring pressed down)	Ratio R2/R1 Compression/ Without Compression
B0123	Group B6	Day 1	Pre-dose	16-JUN-2018	12:29	F3	Yes	29.4	43.0	1.46
B0124	Group B4	Day 1	Pre-dose	16-JUN-2018	12:35	F1	Yes	25.2	40.8	1.62
B0124	Group B4	Day 1	Pre-dose	16-JUN-2018	12:35	F2	Yes	32.1	47.7	1.49
B0124	Group B4	Day 1	Pre-dose	16-JUN-2018	12:35	F3	Yes	32.9	46.5	1.41
B0125	Group B7	Day 1	Pre-dose	16-JUN-2018	12:39	F1	Yes	26.1	30.8	1.18
B0125	Group B7	Day 1	Pre-dose	16-JUN-2018	12:39	F2	Yes	25.2	28.2	1.12
B0125	Group B7	Day 1	Pre-dose	16-JUN-2018	12:39	F3	Yes	29.0	29.5	1.02
B0126	Group B3	Day 1	Pre-dose	16-JUN-2018	12:52	F1	Yes	22.5	26.1	1.16
B0126	Group B3	Day 1	Pre-dose	16-JUN-2018	12:52	F2	Yes	28.4	33.4	1.18
B0126	Group B3	Day 1	Pre-dose	16-JUN-2018	12:52	F3	Yes	28.8	34.4	1.19
B0128	Group B5	Day 1	Pre-dose	23-JUN-2018	10:49	F1	Yes	16.7	33.4	2.00
B0128	Group B5	Day 1	Pre-dose	23-JUN-2018	10:49	F2	Yes	30.8	37.8	1.23
B0128	Group B5	Day 1	Pre-dose	23-JUN-2018	10:49	F3	Yes	30.6	37.5	1.23

Administration Site Key: FA = Forearm; UA= Upper arm
Treatments Part A: A1 = A/Sing MAP, 15 ug (FA); A2 = Afluria Quad IM (UA), 15 ug; A3 = Placebo MAP (FA); A4 = A/Sing Mono IM, 15 ug (UA)
Treatments Part B: B1 = A/Sing MAP, 15 ug (FA) with biopsy; B2 = Placebo MAP (FA) with biopsy
B3 = A/Sing MAP, 15 ug (FA); B4 = A/Sing MAP, 10 ug (FA); B5 = A/Sing MAP, 5 ug (FA); B6 = A/Sing MAP, 2.5 ug (FA)
B7 = Placebo MAP (FA); B8 = A/Sing MAP, 15 ug (UA); B9 = A/Sing IM, 15 ug, as 2018 Afluria Quad (UA)

Listing 16.2.5.3
Skin Hardness Assessment (MAP only)

Part B

Randomisation Number	Group	Visit	Time Point	Date of assessment	Time of Assessment	Site Number	Was assessment performed?	Reading 1 (Without compression)	Reading 2 (with skin conditioning ring pressed down)	Ratio R2/R1 Compression/ Without Compression
B0129	Group B8	Day 1	Pre-dose	23-JUN-2018	11:04	D1	Yes	21.3	43.2	2.03
B0129	Group B8	Day 1	Pre-dose	23-JUN-2018	11:04	D2	Yes	32.6	47.1	1.44
B0129	Group B8	Day 1	Pre-dose	23-JUN-2018	11:04	D3	Yes	34.5	47.1	1.37
B0130	Group B5	Day 1	Pre-dose	23-JUN-2018	11:01	F1	Yes	20.5	37.4	1.82
B0130	Group B5	Day 1	Pre-dose	23-JUN-2018	11:01	F2	Yes	27.7	38.3	1.38
B0130	Group B5	Day 1	Pre-dose	23-JUN-2018	11:01	F3	Yes	28.6	38.3	1.34
B0131	Group B8	Day 1	Pre-dose	23-JUN-2018	11:18	D1	Yes	37.9	41.5	1.09
B0131	Group B8	Day 1	Pre-dose	23-JUN-2018	11:18	D2	Yes	36.3	41.6	1.15
B0131	Group B8	Day 1	Pre-dose	23-JUN-2018	11:18	D3	Yes	40.7	53.8	1.32
B0132	Group B6	Day 1	Pre-dose	23-JUN-2018	11:15	F1	Yes	23.0	41.3	1.80
B0132	Group B6	Day 1	Pre-dose	23-JUN-2018	11:15	F2	Yes	25.0	42.1	1.68
B0132	Group B6	Day 1	Pre-dose	23-JUN-2018	11:15	F3	Yes	26.7	41.1	1.54
B0133	Group B4	Day 1	Pre-dose	23-JUN-2018	11:22	F1	Yes	22.0	42.1	1.91
B0133	Group B4	Day 1	Pre-dose	23-JUN-2018	11:22	F2	Yes	24.5	41.1	1.68

Administration Site Key: FA = Forearm; UA= Upper arm
Treatments Part A: A1 = A/Sing MAP, 15 ug (FA); A2 = Afluria Quad IM (UA), 15 ug; A3 = Placebo MAP (FA); A4 = A/Sing Mono IM, 15 ug (UA)
Treatments Part B: B1 = A/Sing MAP, 15 ug (FA) with biopsy; B2 = Placebo MAP (FA) with biopsy
B3 = A/Sing MAP, 15 ug (FA); B4 = A/Sing MAP, 10 ug (FA); B5 = A/Sing MAP, 5 ug (FA); B6 = A/Sing MAP, 2.5 ug (FA)
B7 = Placebo MAP (FA); B8 = A/Sing MAP, 15 ug (UA); B9 = A/Sing IM, 15 ug, as 2018 Afluria Quad (UA)

Listing 16.2.5.3
Skin Hardness Assessment (MAP only)

Part B

Randomisation Number	Group	Visit	Time Point	Date of assessment	Time of Assessment	Site Number	Was assessment performed?	Reading 1 (Without compression)	Reading 2 (with skin conditioning ring pressed down)	Ratio R2/R1 Compression/ Without Compression
B0133	Group B4	Day 1	Pre-dose	23-JUN-2018	11:22	F3	Yes	22.6	41.0	1.81
B0134	Group B3	Day 1	Pre-dose	23-JUN-2018	11:26	F1	Yes	17.5	31.8	1.82
B0134	Group B3	Day 1	Pre-dose	23-JUN-2018	11:26	F2	Yes	21.2	35.0	1.65
B0134	Group B3	Day 1	Pre-dose	23-JUN-2018	11:26	F3	Yes	20.2	29.8	1.48
B0135	Group B7	Day 1	Pre-dose	23-JUN-2018	11:31	F1	Yes	21.1	40.4	1.91
B0135	Group B7	Day 1	Pre-dose	23-JUN-2018	11:31	F2	Yes	33.3	40.6	1.22
B0135	Group B7	Day 1	Pre-dose	23-JUN-2018	11:31	F3	Yes	33.1	43.4	1.31
B0137	Group B8	Day 1	Pre-dose	23-JUN-2018	11:34	D1	Yes	29.9	49.1	1.64
B0137	Group B8	Day 1	Pre-dose	23-JUN-2018	11:34	D2	Yes	28.8	48.5	1.68
B0137	Group B8	Day 1	Pre-dose	23-JUN-2018	11:34	D3	Yes	28.5	48.4	1.70
B0138	Group B7	Day 1	Pre-dose	23-JUN-2018	11:44	F1	Yes	15.6	25.2	1.62
B0138	Group B7	Day 1	Pre-dose	23-JUN-2018	11:44	F2	Yes	28.2	41.3	1.46
B0138	Group B7	Day 1	Pre-dose	23-JUN-2018	11:44	F3	Yes	35.4	44.4	1.25

Administration Site Key: FA = Forearm; UA= Upper arm
Treatments Part A: A1 = A/Sing MAP, 15 ug (FA); A2 = Afluria Quad IM (UA), 15 ug; A3 = Placebo MAP (FA); A4 = A/Sing Mono IM, 15 ug (UA)
Treatments Part B: B1 = A/Sing MAP, 15 ug (FA) with biopsy; B2 = Placebo MAP (FA) with biopsy
B3 = A/Sing MAP, 15 ug (FA); B4 = A/Sing MAP, 10 ug (FA); B5 = A/Sing MAP, 5 ug (FA); B6 = A/Sing MAP, 2.5 ug (FA)
B7 = Placebo MAP (FA); B8 = A/Sing MAP, 15 ug (UA); B9 = A/Sing IM, 15 ug, as 2018 Afluria Quad (UA)

Listing 16.2.5.3
Skin Hardness Assessment (MAP only)

Part B

Randomisation Number	Group	Visit	Time Point	Date of assessment	Time of Assessment	Site Number	Was assessment performed?	Reading 1 (Without compression)	Reading 2 (with skin conditioning ring pressed down)	Ratio R2/R1 Compression/ Without Compression
B0139	Group B4	Day 1	Pre-dose	23-JUN-2018	11:52	F1	Yes	18.9	41.6	2.20
B0139	Group B4	Day 1	Pre-dose	23-JUN-2018	11:52	F2	Yes	27.1	46.8	1.73
B0139	Group B4	Day 1	Pre-dose	23-JUN-2018	11:52	F3	Yes	27.9	46.6	1.67
B0141	Group B3	Day 1	Pre-dose	23-JUN-2018	11:58	F1	Yes	21.0	30.0	1.43
B0141	Group B3	Day 1	Pre-dose	23-JUN-2018	11:58	F2	Yes	24.6	34.9	1.42
B0141	Group B3	Day 1	Pre-dose	23-JUN-2018	11:58	F3	Yes	24.9	33.5	1.35
B0142	Group B5	Day 1	Pre-dose	23-JUN-2018	12:05	F1	Yes	24.2	28.8	1.19
B0142	Group B5	Day 1	Pre-dose	23-JUN-2018	12:05	F2	Yes	26.9	34.5	1.28
B0142	Group B5	Day 1	Pre-dose	23-JUN-2018	12:05	F3	Yes	27.2	34.1	1.25
B0143	Group B6	Day 1	Pre-dose	23-JUN-2018	12:13	F1	Yes	13.0	31.2	2.40
B0143	Group B6	Day 1	Pre-dose	23-JUN-2018	12:13	F2	Yes	19.7	32.4	1.64
B0143	Group B6	Day 1	Pre-dose	23-JUN-2018	12:13	F3	Yes	27.8	29.2	1.05
B0144	Group B6	Day 1	Pre-dose	23-JUN-2018	12:23	F1	Yes	22.6	31.3	1.38
B0144	Group B6	Day 1	Pre-dose	23-JUN-2018	12:23	F2	Yes	31.0	41.1	1.33

Administration Site Key: FA = Forearm; UA= Upper arm
Treatments Part A: A1 = A/Sing MAP, 15 ug (FA); A2 = Afluria Quad IM (UA), 15 ug; A3 = Placebo MAP (FA); A4 = A/Sing Mono IM, 15 ug (UA)
Treatments Part B: B1 = A/Sing MAP, 15 ug (FA) with biopsy; B2 = Placebo MAP (FA) with biopsy
B3 = A/Sing MAP, 15 ug (FA); B4 = A/Sing MAP, 10 ug (FA); B5 = A/Sing MAP, 5 ug (FA); B6 = A/Sing MAP, 2.5 ug (FA)
B7 = Placebo MAP (FA); B8 = A/Sing MAP, 15 ug (UA); B9 = A/Sing IM, 15 ug, as 2018 Afluria Quad (UA)

Listing 16.2.5.3
Skin Hardness Assessment (MAP only)

Part B

Randomisation Number	Group	Visit	Time Point	Date of assessment	Time of Assessment	Site Number	Was assessment performed?	Reading 1 (Without compression)	Reading 2 (with skin conditioning ring pressed down)	Ratio R2/R1 Compression/ Without Compression
B0144	Group B6	Day 1	Pre-dose	23-JUN-2018	12:23	F3	Yes	26.0	42.4	1.63
B0145	Group B3	Day 1	Pre-dose	23-JUN-2018	12:34	F1	Yes	21.1	37.4	1.77
B0145	Group B3	Day 1	Pre-dose	23-JUN-2018	12:34	F2	Yes	29.1	42.9	1.47
B0145	Group B3	Day 1	Pre-dose	23-JUN-2018	12:34	F3	Yes	27.2	43.3	1.59
B0146	Group B7	Day 1	Pre-dose	28-JUN-2018	09:11	F1	Yes	20.2	28.8	1.43
B0146	Group B7	Day 1	Pre-dose	28-JUN-2018	09:11	F2	Yes	30.0	34.2	1.14
B0146	Group B7	Day 1	Pre-dose	28-JUN-2018	09:11	F3	Yes	20.5	33.3	1.62
B0148	Group B5	Day 1	Pre-dose	28-JUN-2018	09:17	F1	Yes	21.7	28.7	1.32
B0148	Group B5	Day 1	Pre-dose	28-JUN-2018	09:17	F2	Yes	31.0	44.4	1.43
B0148	Group B5	Day 1	Pre-dose	28-JUN-2018	09:17	F3	Yes	34.5	49.5	1.43
B0149	Group B8	Day 1	Pre-dose	28-JUN-2018	09:35	D1	Yes	26.4	35.4	1.34
B0149	Group B8	Day 1	Pre-dose	28-JUN-2018	09:35	D2	Yes	20.9	40.5	1.94
B0149	Group B8	Day 1	Pre-dose	28-JUN-2018	09:35	D3	Yes	26.8	33.8	1.26

Administration Site Key: FA = Forearm; UA= Upper arm
Treatments Part A: A1 = A/Sing MAP, 15 ug (FA); A2 = Afluria Quad IM (UA), 15 ug; A3 = Placebo MAP (FA); A4 = A/Sing Mono IM, 15 ug (UA)
Treatments Part B: B1 = A/Sing MAP, 15 ug (FA) with biopsy; B2 = Placebo MAP (FA) with biopsy
B3 = A/Sing MAP, 15 ug (FA); B4 = A/Sing MAP, 10 ug (FA); B5 = A/Sing MAP, 5 ug (FA); B6 = A/Sing MAP, 2.5 ug (FA)
B7 = Placebo MAP (FA); B8 = A/Sing MAP, 15 ug (UA); B9 = A/Sing IM, 15 ug, as 2018 Afluria Quad (UA)

Listing 16.2.5.3
Skin Hardness Assessment (MAP only)

Part B

Randomisation Number	Group	Visit	Time Point	Date of assessment	Time of Assessment	Site Number	Was assessment performed?	Reading 1 (Without compression)	Reading 2 (with skin conditioning ring pressed down)	Ratio R2/R1 Compression/ Without Compression
B0150	Group B4	Day 1	Pre-dose	28-JUN-2018	09:37	F1	Yes	32.0	35.2	1.10
B0150	Group B4	Day 1	Pre-dose	28-JUN-2018	09:37	F2	Yes	33.9	38.5	1.14
B0150	Group B4	Day 1	Pre-dose	28-JUN-2018	09:37	F3	Yes	37.9	39.1	1.03
B1019	Group B8	Day 1	Pre-dose	19-MAY-2018	10:45	D1	Yes	28.8	43.3	1.50
B1019	Group B8	Day 1	Pre-dose	19-MAY-2018	10:45	D2	Yes	30.1	43.0	1.43
B1019	Group B8	Day 1	Pre-dose	19-MAY-2018	10:45	D3	Yes	36.0	41.7	1.16
B1076	Group B5	Day 1	Pre-dose	16-JUN-2018	10:50	F1	Yes	26.3	40.5	1.54
B1076	Group B5	Day 1	Pre-dose	16-JUN-2018	10:50	F2	Yes	32.0	45.2	1.41
B1076	Group B5	Day 1	Pre-dose	16-JUN-2018	10:50	F3	Yes	37.6	44.5	1.18
B1080	Group B7	Day 1	Pre-dose	16-JUN-2018	11:01	F1	Yes	27.5	36.1	1.31
B1080	Group B7	Day 1	Pre-dose	16-JUN-2018	11:01	F2	Yes	37.2	45.1	1.21
B1080	Group B7	Day 1	Pre-dose	16-JUN-2018	11:01	F3	Yes	39.5	49.2	1.25

Administration Site Key: FA = Forearm; UA= Upper arm
Treatments Part A: A1 = A/Sing MAP, 15 ug (FA); A2 = Afluria Quad IM (UA), 15 ug; A3 = Placebo MAP (FA); A4 = A/Sing Mono IM, 15 ug (UA)
Treatments Part B: B1 = A/Sing MAP, 15 ug (FA) with biopsy; B2 = Placebo MAP (FA) with biopsy
B3 = A/Sing MAP, 15 ug (FA); B4 = A/Sing MAP, 10 ug (FA); B5 = A/Sing MAP, 5 ug (FA); B6 = A/Sing MAP, 2.5 ug (FA)
B7 = Placebo MAP (FA); B8 = A/Sing MAP, 15 ug (UA); B9 = A/Sing IM, 15 ug, as 2018 Afluria Quad (UA)
