# Supplementary material for: Safety, tolerability, and immunogenicity of influenza vaccination with a high-density microarray patch: Results from a randomized, controlled phase I clinical trial
Source: PLoS Med. 2020 Mar 17;17(3):e1003024. doi: 10.1371/journal.pmed.1003024 (PMC7077342; doi:10.1371/journal.pmed.1003024)
Supplement: S1 Data — Data 1: Informed Consent; Data 2: Analysis Sets; Data 3: Demographics; Data 4: Study Drug Administration; Data 5: Skin Hardness Assessment; Data 6: Immunogenicity (ELISA IgG); Data 7: Immunogenicity (ADCC); Data 8: Immunogenicity (MBCs); Data 9: Immunogenicity (CMI); Data 10: Immunogenicity (Mucosal IgA); Data 11: Immunogenicity Results (HAI, MN); Data 12: TEAE–CRF Data only; Data 13: TEAEs–MedDRA Coding; Data 15: Treatment Site Tolerability Assessment; Data 16: Numeric Pain Intensity; Data 17: SII; Data 18: Application Site Status at End of Study; Data 19: Individual Treatment Site Tolerability Assessment; Data 20: Numeric Pain Intensity by Treatment; Data 21: Individual SII by Treatment. ADCC, antibody-dependent cellular cytotoxicity; CMI, cell-mediated immunity; CRF, case report form; ELISA, enzyme-linked immunosorbent assay; HAI, haemagglutination inhibition; IgA, immunoglobulin A; IgG, immunoglobulin G; MBC, memory B cell; MedDRA, medical dictionary for regulatory activities; MN, microneutralisation; SII, Skin Irritation Index; TEA, treatment emergent adverse event. (ZIP) [file pmed.1003024.s008.zip › S1_Data/clinical supporting/S1_Data6 Immunogenicity (ELISA IgG).pdf]

Listing 16.2.6.3
Immunogenicity Results (ELISA IgG)

Part A

Randomisation		Midpoint Titre	
Number	Group	Day 1	Day 22
A0001	Group A3	215.7	221.6
A0002	Group A1	15.94	1598
A0003	Group A1	121.5	1646
A0004	Group A3	49.74	70.63
A0005	Group A1	175.2	306.1
A0006	Group A3	76.94	71.22
A0007	Group A1	57.1	1899
A0008	Group A3	297.5	344.7
A0009	Group A1	462.1	1012
A0010	Group A3	144	220.6
A0011	Group A3	53.03	49.14
A0012	Group A1	16.58	1396
A0013	Group A1	191.5	2200
A0014	Group A3	155	146.2
A0015	Group A1	212.7	3711
A0016	Group A3	12.34	0.002016
A0017	Group A3	42.53	11.61
A0018	Group A1	84.68	1079
A0019	Group A3	443.7	464

Administration Site Key: FA = Forearm; UA= Upper arm
Treatments Part A: A1 = A/Sing MAP, 15 ug (FA); A2 = Afluria Quad IM (UA), 15 ug; A3 = Placebo MAP (FA); A4 = A/Sing Mono IM, 15 ug (UA)
Treatments Part B: B1 = A/Sing MAP, 15 ug (FA) with biopsy; B2 = Placebo MAP (FA) with biopsy
B3 = A/Sing MAP, 15 ug (FA); B4 = A/Sing MAP, 10 ug (FA); B5 = A/Sing MAP, 5 ug (FA); B6 = A/Sing MAP, 2.5 ug (FA)
B7 = Placebo MAP (FA); B8 = A/Sing MAP, 15 ug (UA); B9 = A/Sing IM, 15 ug, as 2018 Afluria Quad (UA)

Listing 16.2.6.3
Immunogenicity Results (ELISA IgG)

Part A

Randomisation		Midpoint Titre	
Number	Group	Day 1	Day 22
A0020	Group A1	488.2	734.9
A0021	Group A1	0.05196	273.5
A0022	Group A3	66.09	85.54
A0023	Group A3	280.3	342.7
A0024	Group A1	30.57	1917
A0025	Group A3	110.8	163.9
A0027	Group A3	54.52	32.02
A0028	Group A1	311.8	683.6
A0029	Group A1	113.4	1478
A0030	Group A3	177.9	222
A0031	Group A2	Not Tested	Not Tested
A0032	Group A4	567	673.2
A0033	Group A2	133.7	1591
A0034	Group A4	73.87	2093
A0035	Group A4	108.1	527.4
A0036	Group A2	295	536.9
A0037	Group A4	259.1	314.7
A0038	Group A2	1104	1394
A0039	Group A2	104	1127

Administration Site Key: FA = Forearm; UA= Upper arm
Treatments Part A: A1 = A/Sing MAP, 15 ug (FA); A2 = Afluria Quad IM (UA), 15 ug; A3 = Placebo MAP (FA); A4 = A/Sing Mono IM, 15 ug (UA)
Treatments Part B: B1 = A/Sing MAP, 15 ug (FA) with biopsy; B2 = Placebo MAP (FA) with biopsy
B3 = A/Sing MAP, 15 ug (FA); B4 = A/Sing MAP, 10 ug (FA); B5 = A/Sing MAP, 5 ug (FA); B6 = A/Sing MAP, 2.5 ug (FA)
B7 = Placebo MAP (FA); B8 = A/Sing MAP, 15 ug (UA); B9 = A/Sing IM, 15 ug, as 2018 Afluria Quad (UA)

Listing 16.2.6.3
Immunogenicity Results (ELISA IgG)

Part A

Randomisation Number	Group	Midpoint Titre Day 1	Day 22
A0040	Group A4	473.1	636.5
A0041	Group A4	265	643.5
A0042	Group A2	202.7	282.9
A0043	Group A2	38.24	450.9
A0044	Group A4	260.6	1054
A0045	Group A2	261.9	1658
A0046	Group A4	35.84	2012
A0047	Group A2	140.5	741.2
A0048	Group A4	755.5	1043
A0049	Group A2	125.3	501.9
A0050	Group A4	49.13	780.9
A0051	Group A2	160.2	815.8
A0052	Group A4	84.8	1682
A0053	Group A4	129.3	515.1
A0054	Group A2	81.33	1506
A0055	Group A4	117.1	1221
A0056	Group A2	285	2178
A0057	Group A4	181.8	315.9
A0058	Group A2	86.77	446.9

Administration Site Key: FA = Forearm; UA= Upper arm
Treatments Part A: A1 = A/Sing MAP, 15 ug (FA); A2 = Afluria Quad IM (UA), 15 ug; A3 = Placebo MAP (FA); A4 = A/Sing Mono IM, 15 ug (UA)
Treatments Part B: B1 = A/Sing MAP, 15 ug (FA) with biopsy; B2 = Placebo MAP (FA) with biopsy
B3 = A/Sing MAP, 15 ug (FA); B4 = A/Sing MAP, 10 ug (FA); B5 = A/Sing MAP, 5 ug (FA); B6 = A/Sing MAP, 2.5 ug (FA)
B7 = Placebo MAP (FA); B8 = A/Sing MAP, 15 ug (UA); B9 = A/Sing IM, 15 ug, as 2018 Afluria Quad (UA)

Listing 16.2.6.3
Immunogenicity Results (ELISA IgG)

Part A

Randomisation Number	Group	Midpoint Titre Day 1	Midpoint Titre Day 22
A0059	Group A2	Not Tested	Not Tested
A0060	Group A4	174.1	2403
A1026	Group A1	0.008147	836.7

Administration Site Key: FA = Forearm; UA= Upper arm
Treatments Part A: A1 = A/Sing MAP, 15 ug (FA); A2 = Afluria Quad IM (UA), 15 ug; A3 = Placebo MAP (FA); A4 = A/Sing Mono IM, 15 ug (UA)
Treatments Part B: B1 = A/Sing MAP, 15 ug (FA) with biopsy; B2 = Placebo MAP (FA) with biopsy
B3 = A/Sing MAP, 15 ug (FA); B4 = A/Sing MAP, 10 ug (FA); B5 = A/Sing MAP, 5 ug (FA); B6 = A/Sing MAP, 2.5 ug (FA)
B7 = Placebo MAP (FA); B8 = A/Sing MAP, 15 ug (UA); B9 = A/Sing IM, 15 ug, as 2018 Afluria Quad (UA)
