# Supplementary material for: Safety, tolerability, and immunogenicity of influenza vaccination with a high-density microarray patch: Results from a randomized, controlled phase I clinical trial
Source: PLoS Med. 2020 Mar 17;17(3):e1003024. doi: 10.1371/journal.pmed.1003024 (PMC7077342; doi:10.1371/journal.pmed.1003024)
Supplement: S1 Data — Data 1: Informed Consent; Data 2: Analysis Sets; Data 3: Demographics; Data 4: Study Drug Administration; Data 5: Skin Hardness Assessment; Data 6: Immunogenicity (ELISA IgG); Data 7: Immunogenicity (ADCC); Data 8: Immunogenicity (MBCs); Data 9: Immunogenicity (CMI); Data 10: Immunogenicity (Mucosal IgA); Data 11: Immunogenicity Results (HAI, MN); Data 12: TEAE–CRF Data only; Data 13: TEAEs–MedDRA Coding; Data 15: Treatment Site Tolerability Assessment; Data 16: Numeric Pain Intensity; Data 17: SII; Data 18: Application Site Status at End of Study; Data 19: Individual Treatment Site Tolerability Assessment; Data 20: Numeric Pain Intensity by Treatment; Data 21: Individual SII by Treatment. ADCC, antibody-dependent cellular cytotoxicity; CMI, cell-mediated immunity; CRF, case report form; ELISA, enzyme-linked immunosorbent assay; HAI, haemagglutination inhibition; IgA, immunoglobulin A; IgG, immunoglobulin G; MBC, memory B cell; MedDRA, medical dictionary for regulatory activities; MN, microneutralisation; SII, Skin Irritation Index; TEA, treatment emergent adverse event. (ZIP) [file pmed.1003024.s008.zip › S1_Data/clinical supporting/S1_Data7 Immunogenicity (ADCC).pdf]

Listing 16.2.6.4
Immunogenicity Results (ADCC)

Part B

Randomisation Number	Treatment Group	Day 1	Day 22	Fold Day 22/ Day 1
B0011	Group B8	65.0	206.9	3.18
B0014	Group B3	7.3	803.9	110.85
B0015	Group B9	17.3	92.3	5.32
B0017	Group B7	223.8	204.1	0.91
B0018	Group B9	149.3	518.9	3.48
B0021	Group B7	259.3	253.8	0.98
B0023	Group B3	75.1	2319.1	30.89
B0025	Group B8	78.1	267.7	3.43
B0026	Group B9	114.2	175.2	1.53
B0027	Group B3	35.3	342.6	9.72
B0030	Group B7	209.7	240.3	1.15
B0034	Group B8	44.4	149.3	3.36
B0035	Group B9	119.4	233.6	1.96
B0036	Group B7	36.8	33.8	0.92
B0038	Group B3	107.1	222.2	2.07
B0039	Group B7	17.8	14.1	0.79
B0040	Group B9	37.7	433.7	11.51
B0041	Group B3	932.0	821.7	0.88
B0043	Group B8	37.3	1075.8	28.84

Administration Site Key: FA = Forearm; UA= Upper arm
Treatments Part A: A1 = A/Sing MAP, 15 ug (FA); A2 = Afluria Quad IM (UA), 15 ug; A3 = Placebo MAP (FA); A4 = A/Sing Mono IM, 15 ug (UA)
Treatments Part B: B1 = A/Sing MAP, 15 ug (FA) with biopsy; B2 = Placebo MAP (FA) with biopsy
B3 = A/Sing MAP, 15 ug (FA); B4 = A/Sing MAP, 10 ug (FA); B5 = A/Sing MAP, 5 ug (FA); B6 = A/Sing MAP, 2.5 ug (FA)
B7 = Placebo MAP (FA); B8 = A/Sing MAP, 15 ug (UA); B9 = A/Sing IM, 15 ug, as 2018 Afluria Quad (UA)

Listing 16.2.6.4
Immunogenicity Results (ADCC)

Part B

Randomisation Number	Treatment Group	Day 1	Day 22	Fold Day 22/ Day 1
B0046	Group B7	33.7	49.6	1.47
B0047	Group B9	74.3	618.8	8.33
B0050	Group B8	238.2	980.4	4.12
B0051	Group B3	59.8	266.2	4.45
B0055	Group B8	115.2	1154.5	10.02
B0056	Group B3	31.2	1394.9	44.71
B0058	Group B9	144.3	436.9	3.03
B0059	Group B7	123.6	97.1	0.79
B0060	Group B3	106.6	876.4	8.22
B0061	Group B8	29.9	724.6	24.22
B0063	Group B7	5.2	5.6	1.08
B0064	Group B9	153.5	704.7	4.59
B0067	Group B8	83.7	457.2	5.46
B0069	Group B7	62.0	66.4	1.07
B0070	Group B9	302.9	2923.1	9.65
B0072	Group B3	52.7	298.8	5.67
B0074	Group B9	290.2	842.5	2.90
B0075	Group B3	82.0	1758.1	21.43
B0078	Group B8	222.8	2405.6	10.80

Administration Site Key: FA = Forearm; UA= Upper arm
Treatments Part A: A1 = A/Sing MAP, 15 ug (FA); A2 = Afluria Quad IM (UA), 15 ug; A3 = Placebo MAP (FA); A4 = A/Sing Mono IM, 15 ug (UA)
Treatments Part B: B1 = A/Sing MAP, 15 ug (FA) with biopsy; B2 = Placebo MAP (FA) with biopsy
B3 = A/Sing MAP, 15 ug (FA); B4 = A/Sing MAP, 10 ug (FA); B5 = A/Sing MAP, 5 ug (FA); B6 = A/Sing MAP, 2.5 ug (FA)
B7 = Placebo MAP (FA); B8 = A/Sing MAP, 15 ug (UA); B9 = A/Sing IM, 15 ug, as 2018 Afluria Quad (UA)

Listing 16.2.6.4
Immunogenicity Results (ADCC)

Part B

Randomisation Number	Treatment Group	Day 1	Day 22	Fold Day 22/ Day 1
B0082	Group B3	92.7	155.9	1.68
B0083	Group B8	64.2	1871.6	29.14
B0086	Group B9	17.3	408.3	23.65
B0087	Group B7	259.5	257.7	0.99
B0088	Group B9	44.9	2012.1	44.83
B0091	Group B8	13.5	3822.6	283.87
B0093	Group B7	17.0	13.6	0.80
B0094	Group B3	53.1	304.2	5.73
B0095	Group B9	90.7	183.3	2.02
B0098	Group B7	39.5	45.1	1.14
B0100	Group B8	165.8	567.2	3.42
B0101	Group B3	141.6	193.2	1.36
B0102	Group B8	40.0	1433.9	35.86
B0104	Group B3	119.8	678.4	5.66
B0105	Group B9	138.0	524.9	3.81
B0108	Group B7	108.2	129.2	1.19
B0110	Group B3	39.2	1177.7	30.01
B0111	Group B8	27.3	85.2	3.11
B0114	Group B7	35.5	34.5	0.97

Administration Site Key: FA = Forearm; UA= Upper arm
Treatments Part A: A1 = A/Sing MAP, 15 ug (FA); A2 = Afluria Quad IM (UA), 15 ug; A3 = Placebo MAP (FA); A4 = A/Sing Mono IM, 15 ug (UA)
Treatments Part B: B1 = A/Sing MAP, 15 ug (FA) with biopsy; B2 = Placebo MAP (FA) with biopsy
B3 = A/Sing MAP, 15 ug (FA); B4 = A/Sing MAP, 10 ug (FA); B5 = A/Sing MAP, 5 ug (FA); B6 = A/Sing MAP, 2.5 ug (FA)
B7 = Placebo MAP (FA); B8 = A/Sing MAP, 15 ug (UA); B9 = A/Sing IM, 15 ug, as 2018 Afluria Quad (UA)

Listing 16.2.6.4
Immunogenicity Results (ADCC)

Part B

Randomisation Number	Treatment Group	Day 1	Day 22	Fold Day 22/ Day 1
B0115	Group B9	19.0	194.3	10.23
B0116	Group B7	450.9	375.5	0.83
B0117	Group B9	237.0	285.6	1.21
B0120	Group B3	47.8	2388.9	50.02
B0121	Group B8	34.0	279.8	8.24
B0125	Group B7	75.9	72.6	0.96
B0126	Group B3	319.4	1560.5	4.89
B0127	Group B9	32.5	239.0	7.35
B0129	Group B8	176.9	5010.0	28.32
B0131	Group B8	152.8	2522.7	16.51
B0134	Group B3	469.3	1723.2	3.67
B0135	Group B7	168.5	145.8	0.87
B0136	Group B9	34.7	228.6	6.58
B0137	Group B8	38.4	1717.9	44.79
B0138	Group B7	85.8	90.8	1.06
B0140	Group B9	255.2	310.8	1.22
B0141	Group B3	24.0	130.1	5.43
B0145	Group B3	30.0	455.4	15.19
B0146	Group B7	163.9	155.7	0.95

Administration Site Key: FA = Forearm; UA= Upper arm
Treatments Part A: A1 = A/Sing MAP, 15 ug (FA); A2 = Afluria Quad IM (UA), 15 ug; A3 = Placebo MAP (FA); A4 = A/Sing Mono IM, 15 ug (UA)
Treatments Part B: B1 = A/Sing MAP, 15 ug (FA) with biopsy; B2 = Placebo MAP (FA) with biopsy
B3 = A/Sing MAP, 15 ug (FA); B4 = A/Sing MAP, 10 ug (FA); B5 = A/Sing MAP, 5 ug (FA); B6 = A/Sing MAP, 2.5 ug (FA)
B7 = Placebo MAP (FA); B8 = A/Sing MAP, 15 ug (UA); B9 = A/Sing IM, 15 ug, as 2018 Afluria Quad (UA)

Listing 16.2.6.4
Immunogenicity Results (ADCC)

Part B

Randomisation Number	Treatment Group	Day 1	Day 22	Fold Day 22/ Day 1
B0147	Group B9	4.6	169.0	36.65
B0149	Group B8	175.8	2818.5	16.03
B1019	Group B8	38.9	1109.1	28.55
B1080	Group B7	70.9	92.5	1.30

Administration Site Key: FA = Forearm; UA= Upper arm
Treatments Part A: A1 = A/Sing MAP, 15 ug (FA); A2 = Afluria Quad IM (UA), 15 ug; A3 = Placebo MAP (FA); A4 = A/Sing Mono IM, 15 ug (UA)
Treatments Part B: B1 = A/Sing MAP, 15 ug (FA) with biopsy; B2 = Placebo MAP (FA) with biopsy
B3 = A/Sing MAP, 15 ug (FA); B4 = A/Sing MAP, 10 ug (FA); B5 = A/Sing MAP, 5 ug (FA); B6 = A/Sing MAP, 2.5 ug (FA)
B7 = Placebo MAP (FA); B8 = A/Sing MAP, 15 ug (UA); B9 = A/Sing IM, 15 ug, as 2018 Afluria Quad (UA)
