# Supplementary material for: Safety, tolerability, and immunogenicity of influenza vaccination with a high-density microarray patch: Results from a randomized, controlled phase I clinical trial
Source: PLoS Med. 2020 Mar 17;17(3):e1003024. doi: 10.1371/journal.pmed.1003024 (PMC7077342; doi:10.1371/journal.pmed.1003024)
Supplement: S1 Data — Data 1: Informed Consent; Data 2: Analysis Sets; Data 3: Demographics; Data 4: Study Drug Administration; Data 5: Skin Hardness Assessment; Data 6: Immunogenicity (ELISA IgG); Data 7: Immunogenicity (ADCC); Data 8: Immunogenicity (MBCs); Data 9: Immunogenicity (CMI); Data 10: Immunogenicity (Mucosal IgA); Data 11: Immunogenicity Results (HAI, MN); Data 12: TEAE–CRF Data only; Data 13: TEAEs–MedDRA Coding; Data 15: Treatment Site Tolerability Assessment; Data 16: Numeric Pain Intensity; Data 17: SII; Data 18: Application Site Status at End of Study; Data 19: Individual Treatment Site Tolerability Assessment; Data 20: Numeric Pain Intensity by Treatment; Data 21: Individual SII by Treatment. ADCC, antibody-dependent cellular cytotoxicity; CMI, cell-mediated immunity; CRF, case report form; ELISA, enzyme-linked immunosorbent assay; HAI, haemagglutination inhibition; IgA, immunoglobulin A; IgG, immunoglobulin G; MBC, memory B cell; MedDRA, medical dictionary for regulatory activities; MN, microneutralisation; SII, Skin Irritation Index; TEA, treatment emergent adverse event. (ZIP) [file pmed.1003024.s008.zip › S1_Data/clinical supporting/S1_Data8 Immunogenicity (Memory B Cells).pdf]

Listing 16.2.6.5
Immunogenicity Results (Memory B Cells)

Part B

| Randomi-sation Number | Group | Visit | MI TOTAL IgD- % | IgG% | IgA% | IgM% | MI IgG+ | MI IgG+ stem | MI IgG+ NC99+ | %stem cross | %NC99 cross | %CD27+Cd21- | %stem | Delta (D22-D1) MI IgG+ | Fold (D22/D1) MI IgG+ |
|---|---|---|---|---|---|---|---|---|---|---|---|---|---|---|---|
| B0011 | Group B8 | Day 1 | 0.05 | 0.0 | 61.5 | 15.4 | 0.000 | 0.000 | 0.00 | | | | 0.16 | | |
| B0011 | Group B8 | Day 22 | 0.09 | 56.3 | 6.3 | 12.5 | 0.052 | 0.029 | 0.00 | 55.56 | 0.0 | 44.4 | 1.17 | 0.052 | |
| B0014 | Group B3 | Day 1 | 0.02 | 46.7 | 40.0 | 0.0 | 0.011 | 0.002 | 0.00 | 14.29 | 0.0 | 14.3 | 0.14 | | |
| B0014 | Group B3 | Day 22 | 0.11 | 74.1 | 10.3 | 1.7 | 0.083 | 0.027 | 0.04 | 32.56 | 48.8 | 55.8 | 0.31 | 0.072 | 7.332 |
| B0015 | Group B9 | Day 1 | 0.03 | 37.5 | 50.0 | 0.0 | 0.011 | 0.004 | 0.00 | 33.33 | 0.0 | 0.0 | 0.29 | | |
| B0015 | Group B9 | Day 22 | 0.42 | 96.7 | 1.6 | 0.8 | 0.402 | 0.068 | 0.00 | 16.81 | 0.0 | 27.7 | 0.52 | 0.391 | 36.902 |
| B0017 | Group B7 | Day 1 | 0.05 | 83.7 | 9.3 | 2.3 | 0.045 | 0.001 | 0.00 | 2.78 | 0.0 | 0.0 | 0.13 | | |
| B0017 | Group B7 | Day 22 | 0.07 | 69.6 | 23.9 | 0.0 | 0.047 | 0.003 | 0.00 | 6.25 | 0.0 | 9.4 | 0.14 | 0.001 | 1.031 |
| B0018 | Group B9 | Day 1 | 0.04 | 56.0 | 32.0 | 8.0 | 0.024 | 0.005 | 0.00 | 21.43 | 0.0 | 0.0 | 0.20 | | |
| B0018 | Group B9 | Day 22 | 0.69 | 80.5 | 8.4 | 1.3 | 0.553 | 0.023 | 0.01 | 4.18 | 1.7 | 35.6 | 0.19 | 0.529 | 22.983 |
| B0021 | Group B7 | Day 1 | 0.23 | 83.7 | 6.1 | 0.0 | 0.195 | 0.005 | 0.01 | 2.44 | 7.3 | 0.0 | 0.27 | | |
| B0021 | Group B7 | Day 22 | 0.22 | 67.3 | 9.6 | 0.0 | 0.150 | 0.017 | 0.00 | 11.43 | 2.9 | 2.9 | 0.22 | -0.046 | 0.766 |
| B0023 | Group B3 | Day 1 | 0.08 | 64.5 | 12.9 | 16.1 | 0.051 | 0.008 | 0.00 | 15.00 | 0.0 | 25.0 | 0.12 | | |
| B0023 | Group B3 | Day 22 | 7.45 | 50.7 | 3.3 | 0.6 | 3.777 | 0.063 | 0.02 | 1.67 | 0.6 | 67.2 | 0.33 | 3.726 | 73.615 |
| B0025 | Group B8 | Day 1 | 0.14 | 83.3 | 10.2 | 2.8 | 0.116 | 0.006 | 0.00 | 5.56 | 2.2 | 3.3 | 0.19 | | |
| B0025 | Group B8 | Day 22 | 0.50 | 91.3 | 5.7 | 1.7 | 0.456 | 0.023 | 0.01 | 5.15 | 2.2 | 27.2 | 0.21 | 0.339 | 3.913 |



Administration Site Key: FA = Forearm; UA= Upper arm
Treatments Part A: A1 = A/Sing MAP, 15 ug (FA); A2 = Afluria Quad IM (UA), 15 ug; A3 = Placebo MAP (FA); A4 = A/Sing Mono IM, 15 ug (UA)
Treatments Part B: B1 = A/Sing MAP, 15 ug (FA) with biopsy; B2 = Placebo MAP (FA) with biopsy
B3 = A/Sing MAP, 15 ug (FA); B4 = A/Sing MAP, 10 ug (FA); B5 = A/Sing MAP, 5 ug (FA); B6 = A/Sing MAP, 2.5 ug (FA)
B7 = Placebo MAP (FA); B8 = A/Sing MAP, 15 ug (UA); B9 = A/Sing IM, 15 ug, as 2018 Afluria Quad (UA)

Listing 16.2.6.5
Immunogenicity Results (Memory B Cells)

Part B

| Randomi-sation Number | Group | Visit | MI TOTAL IgD- % | IgG% | IgA% | IgM% | MI IgG+ | MI IgG+ stem | MI IgG+ NC99+ | %stem cross | %NC99 cross | %CD27+Cd21- | %stem | Delta (D22-D1) MI IgG+ | Fold (D22/D1) MI IgG+ |
|---|---|---|---|---|---|---|---|---|---|---|---|---|---|---|---|
| B0026 | Group B9 | Day 1 | 0.03 | 63.6 | 18.2 | 9.1 | 0.017 | 0.005 | 0.00 | 28.57 | 14.3 | 14.3 | 0.17 | | |
| B0026 | Group B9 | Day 22 | 0.50 | 87.7 | 0.9 | 3.2 | 0.439 | 0.302 | 0.03 | 68.91 | 7.8 | 51.8 | 1.10 | 0.422 | 26.124 |
| B0027 | Group B3 | Day 1 | 0.05 | 20.0 | 20.0 | 10.0 | 0.010 | 0.000 | 0.00 | 0.00 | 0.0 | 0.0 | 0.10 | | |
| B0027 | Group B3 | Day 22 | 0.96 | 81.4 | 4.9 | 0.5 | 0.781 | 0.084 | 0.02 | 10.74 | 2.7 | 55.7 | 0.70 | 0.771 | 79.688 |
| B0030 | Group B7 | Day 1 | 0.05 | 62.5 | 25.0 | 4.2 | 0.032 | 0.000 | 0.00 | 0.00 | 6.7 | 6.7 | 0.11 | | |
| B0030 | Group B7 | Day 22 | 0.08 | 65.2 | 13.0 | 4.3 | 0.055 | 0.011 | 0.00 | 20.00 | 0.0 | 20.0 | 0.08 | 0.023 | 1.728 |
| B0034 | Group B8 | Day 1 | 0.06 | 46.7 | 6.7 | 13.3 | 0.029 | 0.017 | 0.00 | 57.14 | 0.0 | 14.3 | 0.27 | | |
| B0034 | Group B8 | Day 22 | 0.29 | 86.3 | 6.3 | 7.5 | 0.251 | 0.113 | 0.00 | 44.93 | 1.4 | 44.9 | 0.71 | 0.222 | 8.623 |
| B0035 | Group B9 | Day 1 | 0.05 | 58.3 | 25.0 | 0.0 | 0.028 | 0.008 | 0.00 | 28.57 | 14.3 | 0.0 | 0.21 | | |
| B0035 | Group B9 | Day 22 | 0.13 | 92.3 | 5.8 | 1.9 | 0.118 | 0.015 | 0.00 | 12.50 | 0.0 | 45.8 | 0.41 | 0.090 | 4.211 |
| B0036 | Group B7 | Day 1 | 0.06 | 74.2 | 12.9 | 9.7 | 0.046 | 0.006 | 0.00 | 13.04 | 0.0 | 0.0 | 0.24 | | |
| B0036 | Group B7 | Day 22 | 0.08 | 55.9 | 14.7 | 26.5 | 0.042 | 0.007 | 0.00 | 15.79 | 10.5 | 15.8 | 0.19 | -0.004 | 0.911 |
| B0038 | Group B3 | Day 1 | 0.06 | 81.8 | 18.2 | 0.0 | 0.049 | 0.011 | 0.00 | 22.22 | 0.0 | 11.1 | 0.22 | | |
| B0038 | Group B3 | Day 22 | 0.44 | 53.3 | 2.9 | 42.9 | 0.234 | 0.050 | 0.00 | 21.43 | 0.0 | 78.6 | 0.53 | 0.185 | 4.740 |
| B0039 | Group B7 | Day 1 | 0.05 | 100.0 | 0.0 | 0.0 | 0.045 | 0.012 | 0.00 | 26.67 | 0.0 | 0.0 | 0.24 | | |



Administration Site Key: FA = Forearm; UA= Upper arm
Treatments Part A: A1 = A/Sing MAP, 15 ug (FA); A2 = Afluria Quad IM (UA), 15 ug; A3 = Placebo MAP (FA); A4 = A/Sing Mono IM, 15 ug (UA)
Treatments Part B: B1 = A/Sing MAP, 15 ug (FA) with biopsy; B2 = Placebo MAP (FA) with biopsy
B3 = A/Sing MAP, 15 ug (FA); B4 = A/Sing MAP, 10 ug (FA); B5 = A/Sing MAP, 5 ug (FA); B6 = A/Sing MAP, 2.5 ug (FA)
B7 = Placebo MAP (FA); B8 = A/Sing MAP, 15 ug (UA); B9 = A/Sing IM, 15 ug, as 2018 Afluria Quad (UA)

Listing 16.2.6.5
Immunogenicity Results (Memory B Cells)

Part B

| Randomi-sation Number | Group | Visit | MI TOTAL IgD- % | IgG% | IgA% | IgM% | MI IgG+ | MI IgG+ stem | MI IgG+ NC99+ | %stem cross | %NC99 cross | %CD27+Cd21- | %stem | Delta (D22-D1) MI IgG+ | Fold (D22/D1) MI IgG+ |
|---|---|---|---|---|---|---|---|---|---|---|---|---|---|---|---|
| B0039 | Group B7 | Day 22 | 0.05 | 82.4 | 5.9 | 0.0 | 0.037 | 0.008 | 0.00 | 21.43 | 0.0 | 7.1 | 0.14 | -0.008 | 0.826 |
| B0040 | Group B9 | Day 1 | 0.04 | 0.0 | 25.0 | 50.0 | 0.000 | 0.000 | 0.00 | | | | 0.25 | | |
| B0040 | Group B9 | Day 22 | 1.28 | 96.6 | 0.0 | 0.8 | 1.233 | 0.182 | 0.02 | 14.78 | 1.7 | 71.3 | 0.62 | 1.233 | |
| B0041 | Group B3 | Day 1 | 0.56 | 86.7 | 8.5 | 1.8 | 0.485 | 0.014 | 0.04 | 2.80 | 7.7 | 53.8 | 0.41 | | |
| B0041 | Group B3 | Day 22 | 0.47 | 81.8 | 13.1 | 1.1 | 0.388 | 0.030 | 0.02 | 7.64 | 4.9 | 47.9 | 0.37 | -0.097 | 0.801 |
| B0043 | Group B8 | Day 1 | 0.04 | 88.9 | 5.6 | 0.0 | 0.039 | 0.000 | 0.00 | 0.00 | 6.3 | 0.0 | 0.14 | | |
| B0043 | Group B8 | Day 22 | 2.32 | 89.9 | 7.3 | 0.3 | 2.082 | 0.078 | 0.03 | 3.76 | 1.2 | 65.8 | 0.22 | 2.044 | 53.966 |
| B0046 | Group B7 | Day 1 | 0.06 | 47.1 | 29.4 | 0.0 | 0.028 | 0.000 | 0.00 | 0.00 | 0.0 | 12.5 | 0.14 | | |
| B0046 | Group B7 | Day 22 | 0.06 | 85.0 | 5.0 | 5.0 | 0.054 | 0.003 | 0.00 | 5.88 | 0.0 | 0.0 | 0.13 | 0.026 | 1.916 |
| B0047 | Group B9 | Day 1 | 0.05 | 62.5 | 4.2 | 25.0 | 0.032 | 0.000 | 0.00 | 0.00 | 0.0 | 13.3 | 0.13 | | |
| B0047 | Group B9 | Day 22 | 0.43 | 92.7 | 2.2 | 3.2 | 0.397 | 0.026 | 0.00 | 6.55 | 0.3 | 57.2 | 0.26 | 0.365 | 12.264 |
| B0050 | Group B8 | Day 1 | 0.07 | 87.5 | 0.0 | 0.0 | 0.057 | 0.005 | 0.01 | 9.52 | 9.5 | 0.0 | 0.18 | | |
| B0050 | Group B8 | Day 22 | 1.82 | 98.2 | 1.4 | 0.2 | 1.783 | 0.083 | 0.03 | 4.67 | 1.6 | 54.2 | 0.41 | 1.726 | 31.135 |
| B0051 | Group B3 | Day 1 | 0.03 | 36.4 | 36.4 | 0.0 | 0.013 | 0.003 | 0.00 | 25.00 | 25.0 | 0.0 | 0.20 | | |
| B0051 | Group B3 | Day 22 | 0.35 | 91.9 | 5.4 | 0.9 | 0.321 | 0.057 | 0.01 | 17.65 | 3.9 | 71.6 | 0.38 | 0.308 | 25.425 |



Administration Site Key: FA = Forearm; UA= Upper arm
Treatments Part A: A1 = A/Sing MAP, 15 ug (FA); A2 = Afluria Quad IM (UA), 15 ug; A3 = Placebo MAP (FA); A4 = A/Sing Mono IM, 15 ug (UA)
Treatments Part B: B1 = A/Sing MAP, 15 ug (FA) with biopsy; B2 = Placebo MAP (FA) with biopsy
B3 = A/Sing MAP, 15 ug (FA); B4 = A/Sing MAP, 10 ug (FA); B5 = A/Sing MAP, 5 ug (FA); B6 = A/Sing MAP, 2.5 ug (FA)
B7 = Placebo MAP (FA); B8 = A/Sing MAP, 15 ug (UA); B9 = A/Sing IM, 15 ug, as 2018 Afluria Quad (UA)

Listing 16.2.6.5
Immunogenicity Results (Memory B Cells)

Part B

| Randomi- sation Number | Group | Visit | MI TOTAL IgD- % | IgG% | IgA% | IgM% | MI IgG+ | MI IgG+ stem | MI IgG+ NC99+ | %stem cross | %NC99 cross | %CD27+Cd2 1- | %stem | Delta (D22-D1) MI IgG+ | Fold (D22/D1) MI IgG+ |
|---|---|---|---|---|---|---|---|---|---|---|---|---|---|---|---|
| B0055 | Group B8 | Day 1 | 0.11 | 82.4 | 11.8 | 5.9 | 0.089 | 0.019 | 0.01 | 21.43 | 7.1 | 21.4 | 0.11 | | |
| B0055 | Group B8 | Day 22 | 1.06 | 85.3 | 6.8 | 2.6 | 0.908 | 0.033 | 0.14 | 3.68 | 15.3 | 77.3 | 0.19 | 0.819 | 10.228 |
| B0056 | Group B3 | Day 1 | 0.01 | 100.0 | 0.0 | 0.0 | 0.011 | 0.000 | 0.00 | 0.00 | 0.0 | 0.0 | 0.04 | | |
| B0056 | Group B3 | Day 22 | 2.75 | 86.0 | 9.8 | 2.5 | 2.368 | 0.139 | 0.03 | 5.86 | 1.4 | 74.0 | 0.47 | 2.357 | 211.085 |
| B0058 | Group B9 | Day 1 | 0.05 | 66.7 | 26.7 | 0.0 | 0.031 | 0.009 | 0.01 | 30.00 | 30.0 | 10.0 | 0.11 | | |
| B0058 | Group B9 | Day 22 | 1.59 | 82.1 | 16.7 | 0.2 | 1.305 | 0.079 | 0.05 | 6.02 | 4.2 | 66.0 | 0.31 | 1.274 | 42.634 |
| B0059 | Group B7 | Day 1 | 0.11 | 93.5 | 4.3 | 2.2 | 0.102 | 0.021 | 0.01 | 20.93 | 11.6 | 2.3 | 0.19 | | |
| B0059 | Group B7 | Day 22 | 0.08 | 88.1 | 4.8 | 2.4 | 0.066 | 0.004 | 0.01 | 5.41 | 10.8 | 5.4 | 0.12 | -0.036 | 0.650 |
| B0060 | Group B3 | Day 1 | 0.07 | 66.7 | 0.0 | 11.1 | 0.046 | 0.000 | 0.00 | 0.00 | 0.0 | 0.0 | 0.13 | | |
| B0060 | Group B3 | Day 22 | 2.73 | 81.0 | 17.1 | 0.3 | 2.214 | 0.115 | 0.05 | 5.18 | 2.4 | 54.2 | 0.26 | 2.167 | 47.814 |
| B0061 | Group B8 | Day 1 | 0.04 | 47.1 | 23.5 | 23.5 | 0.020 | 0.002 | 0.00 | 12.50 | 0.0 | 12.5 | 0.15 | | |
| B0061 | Group B8 | Day 22 | 2.16 | 95.7 | 3.9 | 0.4 | 2.065 | 0.357 | 0.20 | 17.31 | 9.7 | 79.6 | 1.19 | 2.045 | 104.888 |
| B0063 | Group B7 | Day 1 | 0.04 | 40.0 | 13.3 | 40.0 | 0.016 | 0.000 | 0.00 | 0.00 | 16.7 | 33.3 | 0.05 | | |
| B0063 | Group B7 | Day 22 | 0.03 | 41.7 | 8.3 | 25.0 | 0.014 | 0.000 | 0.01 | 0.00 | 60.0 | 0.0 | 0.09 | -0.001 | 0.908 |
| B0064 | Group B9 | Day 1 | 0.04 | 70.6 | 5.9 | 11.8 | 0.026 | 0.000 | 0.00 | 0.00 | 8.3 | 25.0 | 0.11 | | |
| B0064 | Group B9 | Day 22 | 0.54 | 92.9 | 4.1 | 0.0 | 0.499 | 0.011 | 0.00 | 2.19 | 0.5 | 38.8 | 0.08 | 0.473 | 19.038 |



Administration Site Key: FA = Forearm; UA= Upper arm
Treatments Part A: A1 = A/Sing MAP, 15 ug (FA); A2 = Afluria Quad IM (UA), 15 ug; A3 = Placebo MAP (FA); A4 = A/Sing Mono IM, 15 ug (UA)
Treatments Part B: B1 = A/Sing MAP, 15 ug (FA) with biopsy; B2 = Placebo MAP (FA) with biopsy
B3 = A/Sing MAP, 15 ug (FA); B4 = A/Sing MAP, 10 ug (FA); B5 = A/Sing MAP, 5 ug (FA); B6 = A/Sing MAP, 2.5 ug (FA)
B7 = Placebo MAP (FA); B8 = A/Sing MAP, 15 ug (UA); B9 = A/Sing IM, 15 ug, as 2018 Afluria Quad (UA)

Listing 16.2.6.5
Immunogenicity Results (Memory B Cells)

Part B

| Randomi-sation Number | Group | Visit | MI TOTAL IgD- % | IgG% | IgA% | IgM% | MI IgG+ | MI IgG+ stem | MI IgG+ NC99+ | %stem cross | %NC99 cross | %CD27+Cd21- | %stem | Delta (D22-D1) MI IgG+ | Fold (D22/D1) MI IgG+ |
|---|---|---|---|---|---|---|---|---|---|---|---|---|---|---|---|
| B0067 | Group B8 | Day 1 | 0.37 | 96.5 | 3.5 | 0.0 | 0.355 | 0.013 | 0.01 | 3.61 | 2.4 | 8.4 | 0.13 | | |
| B0067 | Group B8 | Day 22 | 0.92 | 98.6 | 1.4 | 0.0 | 0.907 | 0.047 | 0.01 | 5.21 | 1.4 | 48.3 | 0.10 | 0.552 | 2.554 |
| B0069 | Group B7 | Day 1 | 0.04 | 76.9 | 23.1 | 0.0 | 0.032 | 0.000 | 0.00 | 0.00 | 0.0 | 0.0 | 0.05 | | |
| B0069 | Group B7 | Day 22 | 0.05 | 71.4 | 14.3 | 0.0 | 0.036 | 0.007 | 0.01 | 20.00 | 30.0 | 30.0 | 0.10 | 0.004 | 1.123 |
| B0070 | Group B9 | Day 1 | 0.06 | 81.5 | 7.4 | 3.7 | 0.053 | 0.002 | 0.00 | 4.55 | 9.1 | 0.0 | 0.11 | | |
| B0070 | Group B9 | Day 22 | 2.10 | 93.4 | 5.3 | 0.6 | 1.957 | 0.070 | 0.06 | 3.60 | 2.9 | 69.3 | 0.26 | 1.904 | 37.253 |
| B0072 | Group B3 | Day 1 | 0.10 | 87.5 | 0.0 | 6.3 | 0.091 | 0.033 | 0.01 | 35.71 | 14.3 | 0.0 | 0.16 | | |
| B0072 | Group B3 | Day 22 | 1.51 | 92.9 | 6.8 | 0.4 | 1.406 | 0.373 | 0.15 | 26.54 | 10.4 | 73.1 | 1.61 | 1.314 | 15.375 |
| B0074 | Group B9 | Day 1 | 0.11 | 56.3 | 31.3 | 9.4 | 0.061 | 0.003 | 0.01 | 5.56 | 16.7 | 11.1 | 0.10 | | |
| B0074 | Group B9 | Day 22 | 1.12 | 78.0 | 17.4 | 2.6 | 0.876 | 0.020 | 0.02 | 2.23 | 2.6 | 52.4 | 0.12 | 0.815 | 14.333 |
| B0075 | Group B3 | Day 1 | 0.08 | 71.0 | 16.1 | 3.2 | 0.057 | 0.005 | 0.00 | 9.09 | 0.0 | 4.5 | 0.11 | | |
| B0075 | Group B3 | Day 22 | 2.74 | 84.7 | 10.2 | 1.3 | 2.319 | 0.129 | 0.14 | 5.56 | 6.0 | 63.5 | 0.49 | 2.262 | 40.468 |
| B0078 | Group B8 | Day 1 | 0.07 | 78.1 | 0.0 | 6.3 | 0.057 | 0.011 | 0.02 | 20.00 | 28.0 | 16.0 | 0.22 | | |
| B0078 | Group B8 | Day 22 | 3.39 | 95.6 | 2.3 | 0.1 | 3.245 | 0.327 | 0.11 | 10.09 | 3.5 | 67.6 | 1.38 | 3.187 | 56.698 |
| B0082 | Group B3 | Day 1 | 0.07 | 68.4 | 0.0 | 15.8 | 0.045 | 0.027 | 0.01 | 61.54 | 23.1 | 23.1 | 0.19 | | |



Administration Site Key: FA = Forearm; UA= Upper arm
Treatments Part A: A1 = A/Sing MAP, 15 ug (FA); A2 = Afluria Quad IM (UA), 15 ug; A3 = Placebo MAP (FA); A4 = A/Sing Mono IM, 15 ug (UA)
Treatments Part B: B1 = A/Sing MAP, 15 ug (FA) with biopsy; B2 = Placebo MAP (FA) with biopsy
B3 = A/Sing MAP, 15 ug (FA); B4 = A/Sing MAP, 10 ug (FA); B5 = A/Sing MAP, 5 ug (FA); B6 = A/Sing MAP, 2.5 ug (FA)
B7 = Placebo MAP (FA); B8 = A/Sing MAP, 15 ug (UA); B9 = A/Sing IM, 15 ug, as 2018 Afluria Quad (UA)

Listing 16.2.6.5
Immunogenicity Results (Memory B Cells)

Part B

| Randomi-sation Number | Group | Visit | MI TOTAL IgD- % | IgG% | IgA% | IgM% | MI IgG+ | MI IgG+ stem | MI IgG+ NC99+ | %stem cross | %NC99 cross | %CD27+Cd21- | %stem | Delta (D22-D1) MI IgG+ | Fold (D22/D1) MI IgG+ |
|---|---|---|---|---|---|---|---|---|---|---|---|---|---|---|---|
| B0082 | Group B3 | Day 22 | 0.04 | 64.3 | 14.3 | 21.4 | 0.028 | 0.013 | 0.01 | 44.44 | 22.2 | 22.2 | 0.11 | -0.016 | 0.638 |
| B0083 | Group B8 | Day 1 | 0.05 | 75.8 | 15.2 | 0.0 | 0.037 | 0.000 | 0.00 | 0.00 | 0.0 | 0.0 | 0.17 | | |
| B0083 | Group B8 | Day 22 | 0.95 | 93.0 | 4.2 | 0.8 | 0.887 | 0.039 | 0.04 | 4.38 | 4.0 | 56.8 | 0.24 | 0.850 | 23.864 |
| B0086 | Group B9 | Day 1 | 0.02 | 50.0 | 0.0 | 50.0 | 0.010 | 0.000 | 0.01 | 0.00 | 100.0 | 100.0 | 0.03 | | |
| B0086 | Group B9 | Day 22 | 0.69 | 87.7 | 1.5 | 3.1 | 0.602 | 0.549 | 0.02 | 91.23 | 3.5 | 64.9 | 1.65 | 0.592 | 63.160 |
| B0087 | Group B7 | Day 1 | 0.04 | 69.6 | 30.4 | 0.0 | 0.031 | 0.006 | 0.00 | 18.75 | 0.0 | 18.8 | 0.10 | | |
| B0087 | Group B7 | Day 22 | 0.04 | 60.9 | 34.8 | 4.3 | 0.026 | 0.002 | 0.00 | 7.14 | 14.3 | 28.6 | 0.11 | -0.005 | 0.852 |
| B0088 | Group B9 | Day 1 | 0.05 | 69.2 | 19.2 | 7.7 | 0.034 | 0.008 | 0.01 | 22.22 | 27.8 | 5.6 | 0.09 | | |
| B0088 | Group B9 | Day 22 | 0.81 | 95.9 | 3.2 | 0.5 | 0.773 | 0.022 | 0.14 | 2.86 | 17.9 | 72.6 | 0.11 | 0.739 | 22.672 |
| B0091 | Group B8 | Day 1 | 0.05 | 69.2 | 15.4 | 11.5 | 0.034 | 0.006 | 0.00 | 16.67 | 0.0 | 0.0 | 0.12 | | |
| B0091 | Group B8 | Day 22 | 5.65 | 78.8 | 10.3 | 7.9 | 4.452 | 0.405 | 0.02 | 9.09 | 0.5 | 52.7 | 1.03 | 4.418 | 130.309 |
| B0093 | Group B7 | Day 1 | 0.03 | 0.0 | 66.7 | 0.0 | 0.000 | 0.000 | 0.00 | | | | 0.11 | | |
| B0093 | Group B7 | Day 22 | 0.04 | 25.0 | 25.0 | 25.0 | 0.010 | 0.000 | 0.01 | 0.00 | 100.0 | 0.0 | 0.08 | 0.010 | |
| B0094 | Group B3 | Day 1 | 0.03 | 76.9 | 7.7 | 7.7 | 0.023 | 0.005 | 0.01 | 20.00 | 30.0 | 20.0 | 0.14 | | |
| B0094 | Group B3 | Day 22 | 0.56 | 95.0 | 3.4 | 0.0 | 0.535 | 0.136 | 0.07 | 25.41 | 13.4 | 79.2 | 0.69 | 0.512 | 23.022 |



Administration Site Key: FA = Forearm; UA= Upper arm
Treatments Part A: A1 = A/Sing MAP, 15 ug (FA); A2 = Afluria Quad IM (UA), 15 ug; A3 = Placebo MAP (FA); A4 = A/Sing Mono IM, 15 ug (UA)
Treatments Part B: B1 = A/Sing MAP, 15 ug (FA) with biopsy; B2 = Placebo MAP (FA) with biopsy
B3 = A/Sing MAP, 15 ug (FA); B4 = A/Sing MAP, 10 ug (FA); B5 = A/Sing MAP, 5 ug (FA); B6 = A/Sing MAP, 2.5 ug (FA)
B7 = Placebo MAP (FA); B8 = A/Sing MAP, 15 ug (UA); B9 = A/Sing IM, 15 ug, as 2018 Afluria Quad (UA)

Listing 16.2.6.5
Immunogenicity Results (Memory B Cells)

Part B

| Randomi-sation Number | Group | Visit | MI TOTAL IgD- % | IgG% | IgA% | IgM% | MI IgG+ | MI IgG+ stem | MI IgG+ NC99+ | %stem cross | %NC99 cross | %CD27+Cd21- | %stem | Delta (D22-D1) MI IgG+ | Fold (D22/D1) MI IgG+ |
|---|---|---|---|---|---|---|---|---|---|---|---|---|---|---|---|
| B0095 | Group B9 | Day 1 | 0.04 | 64.3 | 32.1 | 0.0 | 0.028 | 0.005 | 0.01 | 16.67 | 38.9 | 0.0 | 0.10 | | |
| B0095 | Group B9 | Day 22 | 0.16 | 78.2 | 9.7 | 4.0 | 0.127 | 0.021 | 0.02 | 16.49 | 19.6 | 34.0 | 0.15 | 0.098 | 4.494 |
| B0098 | Group B7 | Day 1 | 0.06 | 42.1 | 21.1 | 26.3 | 0.023 | 0.015 | 0.00 | 62.50 | 12.5 | 25.0 | 0.14 | | |
| B0098 | Group B7 | Day 22 | 0.08 | 50.0 | 30.0 | 20.0 | 0.039 | 0.000 | 0.00 | 0.00 | 0.0 | 0.0 | 0.05 | 0.016 | 1.681 |
| B0100 | Group B8 | Day 1 | 0.05 | 65.0 | 30.0 | 5.0 | 0.033 | 0.008 | 0.00 | 23.08 | 0.0 | 0.0 | 0.16 | | |
| B0100 | Group B8 | Day 22 | 0.35 | 76.1 | 13.0 | 0.7 | 0.268 | 0.020 | 0.01 | 7.62 | 4.8 | 40.0 | 0.12 | 0.234 | 8.050 |
| B0101 | Group B3 | Day 1 | 0.08 | 56.5 | 34.8 | 8.7 | 0.045 | 0.007 | 0.00 | 15.38 | 7.7 | 7.7 | 0.10 | | |
| B0101 | Group B3 | Day 22 | 0.09 | 64.5 | 29.0 | 6.5 | 0.058 | 0.023 | 0.01 | 40.00 | 10.0 | 15.0 | 0.16 | 0.014 | 1.313 |
| B0102 | Group B8 | Day 1 | 0.12 | 43.7 | 25.3 | 13.8 | 0.052 | 0.025 | 0.00 | 47.37 | 5.3 | 15.8 | 0.19 | | |
| B0102 | Group B8 | Day 22 | 2.98 | 80.7 | 17.3 | 0.9 | 2.404 | 0.150 | 0.10 | 6.26 | 4.3 | 41.4 | 0.32 | 2.351 | 45.875 |
| B0104 | Group B3 | Day 1 | 0.03 | 65.0 | 25.0 | 5.0 | 0.022 | 0.005 | 0.00 | 23.08 | 15.4 | 0.0 | 0.10 | | |
| B0104 | Group B3 | Day 22 | 1.05 | 93.8 | 5.7 | 0.1 | 0.983 | 0.058 | 0.15 | 5.85 | 14.8 | 67.2 | 0.19 | 0.962 | 45.215 |
| B0105 | Group B9 | Day 1 | 0.06 | 44.9 | 34.7 | 14.3 | 0.028 | 0.013 | 0.01 | 45.45 | 22.7 | 4.5 | 0.13 | | |
| B0105 | Group B9 | Day 22 | 0.18 | 74.2 | 19.4 | 1.6 | 0.134 | 0.032 | 0.03 | 23.91 | 19.6 | 58.7 | 0.17 | 0.106 | 4.821 |
| B0108 | Group B7 | Day 1 | 0.09 | 56.3 | 34.4 | 0.0 | 0.053 | 0.006 | 0.00 | 11.11 | 0.0 | 16.7 | 0.13 | | |
| B0108 | Group B7 | Day 22 | 0.86 | 2.6 | 11.3 | 2.3 | 0.022 | 0.008 | 0.00 | 37.50 | 12.5 | 0.0 | 0.34 | -0.031 | 0.417 |



Administration Site Key: FA = Forearm; UA= Upper arm
Treatments Part A: A1 = A/Sing MAP, 15 ug (FA); A2 = Afluria Quad IM (UA), 15 ug; A3 = Placebo MAP (FA); A4 = A/Sing Mono IM, 15 ug (UA)
Treatments Part B: B1 = A/Sing MAP, 15 ug (FA) with biopsy; B2 = Placebo MAP (FA) with biopsy
B3 = A/Sing MAP, 15 ug (FA); B4 = A/Sing MAP, 10 ug (FA); B5 = A/Sing MAP, 5 ug (FA); B6 = A/Sing MAP, 2.5 ug (FA)
B7 = Placebo MAP (FA); B8 = A/Sing MAP, 15 ug (UA); B9 = A/Sing IM, 15 ug, as 2018 Afluria Quad (UA)

Listing 16.2.6.5
Immunogenicity Results (Memory B Cells)

Part B

| Randomi-sation Number | Group | Visit | MI TOTAL IgD- % | IgG% | IgA% | IgM% | MI IgG+ | MI IgG+ stem | MI IgG+ NC99+ | %stem cross | %NC99 cross | %CD27+Cd2 1- | %stem | Delta (D22-D1) MI IgG+ | Fold (D22/D1) MI IgG+ |
|---|---|---|---|---|---|---|---|---|---|---|---|---|---|---|---|
| B0110 | Group B3 | Day 1 | 0.06 | 73.9 | 17.4 | 4.3 | 0.047 | 0.014 | 0.00 | 29.41 | 0.0 | 0.0 | 0.08 | | |
| B0110 | Group B3 | Day 22 | 1.04 | 93.7 | 1.5 | 0.3 | 0.972 | 0.034 | 0.02 | 3.50 | 1.9 | 42.0 | 0.10 | 0.925 | 20.488 |
| B0111 | Group B8 | Day 1 | 0.02 | 28.6 | 42.9 | 14.3 | 0.005 | 0.000 | 0.00 | 0.00 | 0.0 | 0.0 | 0.02 | | |
| B0111 | Group B8 | Day 22 | 0.16 | 93.3 | 3.3 | 0.0 | 0.150 | 0.003 | 0.02 | 1.79 | 10.7 | 32.1 | 0.09 | 0.145 | 31.369 |
| B0114 | Group B7 | Day 1 | 0.05 | 16.7 | 25.0 | 8.3 | 0.008 | 0.004 | 0.00 | 50.00 | 0.0 | 0.0 | 0.03 | | |
| B0114 | Group B7 | Day 22 | 0.04 | 71.4 | 42.9 | 0.0 | 0.031 | 0.006 | 0.00 | 20.00 | 0.0 | 0.0 | 0.04 | 0.023 | 3.892 |
| B0115 | Group B9 | Day 1 | 0.02 | 33.3 | 66.7 | 0.0 | 0.007 | 0.000 | 0.00 | 0.00 | 0.0 | 0.0 | 0.04 | | |
| B0115 | Group B9 | Day 22 | 0.87 | 57.7 | 1.8 | 10.8 | 0.504 | 0.118 | 0.02 | 23.44 | 3.1 | 42.2 | 0.34 | 0.497 | 68.286 |
| B0116 | Group B7 | Day 1 | 0.20 | 74.5 | 3.9 | 3.9 | 0.151 | 0.012 | 0.01 | 7.89 | 5.3 | 5.3 | 0.03 | | |
| B0116 | Group B7 | Day 22 | 0.32 | 85.9 | 7.8 | 1.6 | 0.278 | 0.015 | 0.02 | 5.45 | 5.5 | 21.8 | 0.05 | 0.127 | 1.836 |
| B0117 | Group B9 | Day 1 | 0.13 | 78.5 | 15.4 | 0.8 | 0.099 | 0.023 | 0.01 | 23.53 | 5.9 | 2.0 | 0.11 | | |
| B0117 | Group B9 | Day 22 | 0.39 | 91.3 | 6.7 | 0.3 | 0.353 | 0.034 | 0.03 | 9.52 | 9.5 | 23.4 | 0.14 | 0.254 | 3.561 |
| B0120 | Group B3 | Day 1 | 0.05 | 31.6 | 42.1 | 21.1 | 0.016 | 0.000 | 0.00 | 0.00 | 0.0 | 0.0 | 0.09 | | |
| B0120 | Group B3 | Day 22 | 7.95 | 61.9 | 28.4 | 2.5 | 4.924 | 0.279 | 0.04 | 5.66 | 0.8 | 37.0 | 1.72 | 4.908 | 303.780 |
| B0121 | Group B8 | Day 1 | 0.00 | | | | 0.000 | 0.000 | 0.00 | | | | 0.03 | | |



Administration Site Key: FA = Forearm; UA= Upper arm
Treatments Part A: A1 = A/Sing MAP, 15 ug (FA); A2 = Afluria Quad IM (UA), 15 ug; A3 = Placebo MAP (FA); A4 = A/Sing Mono IM, 15 ug (UA)
Treatments Part B: B1 = A/Sing MAP, 15 ug (FA) with biopsy; B2 = Placebo MAP (FA) with biopsy
B3 = A/Sing MAP, 15 ug (FA); B4 = A/Sing MAP, 10 ug (FA); B5 = A/Sing MAP, 5 ug (FA); B6 = A/Sing MAP, 2.5 ug (FA)
B7 = Placebo MAP (FA); B8 = A/Sing MAP, 15 ug (UA); B9 = A/Sing IM, 15 ug, as 2018 Afluria Quad (UA)

Listing 16.2.6.5
Immunogenicity Results (Memory B Cells)

Part B

| Randomi-sation Number | Group | Visit | MI TOTAL IgD- % | IgG% | IgA% | IgM% | MI IgG+ | MI IgG+ stem | MI IgG+ NC99+ | %stem cross | %NC99 cross | %CD27+Cd21- | %stem | Delta (D22-D1) MI IgG+ | Fold (D22/D1) MI IgG+ |
|---|---|---|---|---|---|---|---|---|---|---|---|---|---|---|---|
| B0121 | Group B8 | Day 22 | 1.81 | 94.0 | 1.7 | 0.9 | 1.700 | 0.530 | 0.00 | 31.19 | 0.0 | 86.2 | 3.18 | 1.700 | |
| B0125 | Group B7 | Day 1 | 0.11 | 80.8 | 7.7 | 7.7 | 0.085 | 0.008 | 0.01 | 9.52 | 14.3 | 0.0 | 0.08 | | |
| B0125 | Group B7 | Day 22 | 0.14 | 63.6 | 21.2 | 3.0 | 0.087 | 0.017 | 0.02 | 19.05 | 19.0 | 0.0 | 0.18 | 0.002 | 1.027 |
| B0126 | Group B3 | Day 1 | 0.06 | 67.6 | 20.6 | 0.0 | 0.043 | 0.002 | 0.00 | 4.35 | 4.3 | 0.0 | 0.04 | | |
| B0126 | Group B3 | Day 22 | 1.01 | 85.4 | 11.1 | 0.0 | 0.866 | 0.079 | 0.06 | 9.09 | 7.2 | 25.3 | 0.25 | 0.823 | 20.035 |
| B0127 | Group B9 | Day 1 | 0.19 | 69.0 | 26.8 | 0.0 | 0.133 | 0.008 | 0.01 | 6.12 | 4.1 | 4.1 | 0.07 | | |
| B0127 | Group B9 | Day 22 | 1.10 | 68.9 | 28.1 | 0.0 | 0.759 | 0.052 | 0.03 | 6.85 | 3.4 | 37.7 | 0.16 | 0.626 | 5.697 |
| B0129 | Group B8 | Day 1 | 0.06 | 50.0 | 50.0 | 0.0 | 0.029 | 0.000 | 0.00 | 0.00 | 0.0 | 0.0 | 0.03 | | |
| B0129 | Group B8 | Day 22 | 3.15 | 92.9 | 3.1 | 0.4 | 2.922 | 0.161 | 0.01 | 5.51 | 0.4 | 76.3 | 0.35 | 2.893 | 101.577 |
| B0131 | Group B8 | Day 1 | 0.06 | 65.4 | 15.4 | 3.8 | 0.038 | 0.009 | 0.00 | 23.53 | 0.0 | 17.6 | 0.04 | | |
| B0131 | Group B8 | Day 22 | 0.90 | 95.7 | 3.7 | 0.2 | 0.859 | 0.041 | 0.01 | 4.76 | 1.1 | 74.8 | 0.06 | 0.821 | 22.573 |
| B0134 | Group B3 | Day 1 | 0.20 | 75.0 | 16.7 | 4.2 | 0.150 | 0.050 | 0.02 | 33.33 | 11.1 | 27.8 | 0.08 | | |
| B0134 | Group B3 | Day 22 | 1.45 | 94.2 | 2.9 | 0.6 | 1.362 | 0.084 | 0.08 | 6.17 | 6.2 | 66.0 | 0.12 | 1.211 | 9.054 |
| B0135 | Group B7 | Day 1 | 0.06 | 70.4 | 11.1 | 7.4 | 0.042 | 0.004 | 0.00 | 10.53 | 0.0 | 5.3 | 0.04 | | |
| B0135 | Group B7 | Day 22 | 0.07 | 84.8 | 9.1 | 0.0 | 0.059 | 0.008 | 0.00 | 14.29 | 3.6 | 10.7 | 0.04 | 0.018 | 1.427 |



Administration Site Key: FA = Forearm; UA= Upper arm
Treatments Part A: A1 = A/Sing MAP, 15 ug (FA); A2 = Afluria Quad IM (UA), 15 ug; A3 = Placebo MAP (FA); A4 = A/Sing Mono IM, 15 ug (UA)
Treatments Part B: B1 = A/Sing MAP, 15 ug (FA) with biopsy; B2 = Placebo MAP (FA) with biopsy
B3 = A/Sing MAP, 15 ug (FA); B4 = A/Sing MAP, 10 ug (FA); B5 = A/Sing MAP, 5 ug (FA); B6 = A/Sing MAP, 2.5 ug (FA)
B7 = Placebo MAP (FA); B8 = A/Sing MAP, 15 ug (UA); B9 = A/Sing IM, 15 ug, as 2018 Afluria Quad (UA)

Listing 16.2.6.5
Immunogenicity Results (Memory B Cells)

Part B

| Randomi-sation Number | Group | Visit | MI TOTAL IgD- % | IgG% | IgA% | IgM% | MI IgG+ | MI IgG+ stem | MI IgG+ NC99+ | %stem cross | %NC99 cross | %CD27+Cd21- | %stem | Delta (D22-D1) MI IgG+ | Fold (D22/D1) MI IgG+ |
|---|---|---|---|---|---|---|---|---|---|---|---|---|---|---|---|
| B0136 | Group B9 | Day 1 | 0.19 | 53.8 | 0.0 | 0.0 | 0.105 | 0.015 | 0.00 | 14.29 | 0.0 | 0.0 | 0.00 | | |
| B0136 | Group B9 | Day 22 | 0.41 | 65.9 | 34.1 | 0.0 | 0.269 | 0.037 | 0.00 | 13.79 | 0.0 | 41.4 | 0.23 | 0.164 | 2.562 |
| B0137 | Group B8 | Day 1 | 0.05 | 28.6 | 57.1 | 14.3 | 0.014 | 0.000 | 0.00 | 0.00 | 0.0 | 50.0 | 0.04 | | |
| B0137 | Group B8 | Day 22 | 1.91 | 86.6 | 3.6 | 0.2 | 1.655 | 0.048 | 0.05 | 2.90 | 2.9 | 48.8 | 0.09 | 1.640 | 114.197 |
| B0138 | Group B7 | Day 1 | 0.18 | 12.1 | 3.0 | 66.7 | 0.022 | 0.000 | 0.01 | 0.00 | 25.0 | 0.0 | 0.03 | | |
| B0138 | Group B7 | Day 22 | 0.13 | 38.7 | 0.0 | 45.2 | 0.051 | 0.004 | 0.00 | 8.33 | 8.3 | 16.7 | 0.04 | 0.029 | 2.313 |
| B0140 | Group B9 | Day 1 | 0.22 | 81.8 | 4.5 | 0.0 | 0.176 | 0.049 | 0.00 | 27.78 | 0.0 | 11.1 | 0.08 | | |
| B0140 | Group B9 | Day 22 | 0.35 | 65.1 | 16.3 | 0.0 | 0.227 | 0.032 | 0.01 | 14.29 | 3.6 | 42.9 | 0.11 | 0.050 | 1.286 |
| B0141 | Group B3 | Day 1 | 0.04 | 33.3 | 0.0 | 0.0 | 0.013 | 0.000 | 0.00 | 0.00 | 0.0 | 0.0 | 0.03 | | |
| B0141 | Group B3 | Day 22 | 0.95 | 77.7 | 12.8 | 2.1 | 0.739 | 0.486 | 0.01 | 65.75 | 1.4 | 69.9 | 1.07 | 0.726 | 56.992 |
| B0145 | Group B3 | Day 1 | 0.07 | 71.4 | 14.3 | 0.0 | 0.052 | 0.000 | 0.01 | 0.00 | 10.0 | 0.0 | 0.05 | | |
| B0145 | Group B3 | Day 22 | 1.18 | 74.9 | 4.3 | 0.8 | 0.884 | 0.139 | 0.03 | 15.72 | 3.0 | 61.9 | 0.29 | 0.832 | 16.964 |
| B0146 | Group B7 | Day 1 | 0.02 | 40.0 | 60.0 | 0.0 | 0.008 | 0.000 | 0.00 | 0.00 | 0.0 | 0.0 | 0.01 | | |
| B0146 | Group B7 | Day 22 | 0.05 | 15.4 | 30.8 | 7.7 | 0.008 | 0.000 | 0.00 | 0.00 | 0.0 | 0.0 | 0.00 | 0.001 | 1.072 |
| B0147 | Group B9 | Day 1 | 0.07 | 50.0 | 30.0 | 10.0 | 0.033 | 0.007 | 0.00 | 20.00 | 0.0 | 20.0 | 0.23 | | |
| B0147 | Group B9 | Day 22 | 0.32 | 75.8 | 9.1 | 3.0 | 0.239 | 0.172 | 0.03 | 72.00 | 12.0 | 44.0 | 0.43 | 0.206 | 7.272 |



Administration Site Key: FA = Forearm; UA= Upper arm
Treatments Part A: A1 = A/Sing MAP, 15 ug (FA); A2 = Afluria Quad IM (UA), 15 ug; A3 = Placebo MAP (FA); A4 = A/Sing Mono IM, 15 ug (UA)
Treatments Part B: B1 = A/Sing MAP, 15 ug (FA) with biopsy; B2 = Placebo MAP (FA) with biopsy
B3 = A/Sing MAP, 15 ug (FA); B4 = A/Sing MAP, 10 ug (FA); B5 = A/Sing MAP, 5 ug (FA); B6 = A/Sing MAP, 2.5 ug (FA)
B7 = Placebo MAP (FA); B8 = A/Sing MAP, 15 ug (UA); B9 = A/Sing IM, 15 ug, as 2018 Afluria Quad (UA)

Listing 16.2.6.5
Immunogenicity Results (Memory B Cells)

Part B

| Randomi-sation Number | Group | Visit | MI TOTAL IgD- % | IgG% | IgA% | IgM% | MI IgG+ | MI IgG+ stem | MI IgG+ NC99+ | %stem cross | %NC99 cross | %CD27+Cd21- | %stem | Delta (D22-D1) MI IgG+ | Fold (D22/D1) MI IgG+ |
|---|---|---|---|---|---|---|---|---|---|---|---|---|---|---|---|
| B0149 | Group B8 | Day 1 | 0.09 | 62.5 | 6.3 | 0.0 | 0.056 | 0.000 | 0.00 | 0.00 | 0.0 | 10.0 | 0.02 | | |
| B0149 | Group B8 | Day 22 | 4.78 | 88.6 | 5.0 | 0.2 | 4.233 | 0.034 | 0.09 | 0.81 | 2.1 | 73.0 | 0.08 | 4.177 | 75.694 |
| B1019 | Group B8 | Day 1 | 0.04 | 83.3 | 4.2 | 0.0 | 0.035 | 0.002 | 0.00 | 5.00 | 5.0 | 15.0 | 0.19 | | |
| B1019 | Group B8 | Day 22 | 0.96 | 95.2 | 3.6 | 0.5 | 0.911 | 0.043 | 0.05 | 4.76 | 5.9 | 52.1 | 0.22 | 0.876 | 26.289 |
| B1080 | Group B7 | Day 1 | 0.08 | 78.7 | 8.5 | 8.5 | 0.064 | 0.021 | 0.01 | 32.43 | 8.1 | 5.4 | 0.10 | | |
| B1080 | Group B7 | Day 22 | 0.12 | 60.4 | 20.8 | 2.1 | 0.073 | 0.008 | 0.00 | 10.34 | 0.0 | 6.9 | 0.11 | 0.009 | 1.137 |



Administration Site Key: FA = Forearm; UA= Upper arm
Treatments Part A: A1 = A/Sing MAP, 15 ug (FA); A2 = Afluria Quad IM (UA), 15 ug; A3 = Placebo MAP (FA); A4 = A/Sing Mono IM, 15 ug (UA)
Treatments Part B: B1 = A/Sing MAP, 15 ug (FA) with biopsy; B2 = Placebo MAP (FA) with biopsy
B3 = A/Sing MAP, 15 ug (FA); B4 = A/Sing MAP, 10 ug (FA); B5 = A/Sing MAP, 5 ug (FA); B6 = A/Sing MAP, 2.5 ug (FA)
B7 = Placebo MAP (FA); B8 = A/Sing MAP, 15 ug (UA); B9 = A/Sing IM, 15 ug, as 2018 Afluria Quad (UA)
