# Supplementary material for: Safety, tolerability, and immunogenicity of influenza vaccination with a high-density microarray patch: Results from a randomized, controlled phase I clinical trial
Source: PLoS Med. 2020 Mar 17;17(3):e1003024. doi: 10.1371/journal.pmed.1003024 (PMC7077342; doi:10.1371/journal.pmed.1003024)
Supplement: S1 Data — Data 1: Informed Consent; Data 2: Analysis Sets; Data 3: Demographics; Data 4: Study Drug Administration; Data 5: Skin Hardness Assessment; Data 6: Immunogenicity (ELISA IgG); Data 7: Immunogenicity (ADCC); Data 8: Immunogenicity (MBCs); Data 9: Immunogenicity (CMI); Data 10: Immunogenicity (Mucosal IgA); Data 11: Immunogenicity Results (HAI, MN); Data 12: TEAE–CRF Data only; Data 13: TEAEs–MedDRA Coding; Data 15: Treatment Site Tolerability Assessment; Data 16: Numeric Pain Intensity; Data 17: SII; Data 18: Application Site Status at End of Study; Data 19: Individual Treatment Site Tolerability Assessment; Data 20: Numeric Pain Intensity by Treatment; Data 21: Individual SII by Treatment. ADCC, antibody-dependent cellular cytotoxicity; CMI, cell-mediated immunity; CRF, case report form; ELISA, enzyme-linked immunosorbent assay; HAI, haemagglutination inhibition; IgA, immunoglobulin A; IgG, immunoglobulin G; MBC, memory B cell; MedDRA, medical dictionary for regulatory activities; MN, microneutralisation; SII, Skin Irritation Index; TEA, treatment emergent adverse event. (ZIP) [file pmed.1003024.s008.zip › S1_Data/clinical supporting/S1_Data9 Immunogenicity (CMI).pdf]

Listing 16.2.6.6
Immunogenicity Results (CMI)

Part B

Randomisation Number	Group	Visit	Test	Subset	IL2	TNFa	IFNg	Value
B0011	Group B8	Day 1	Peptide	CD4	+	+	+	0.01199%
B0011	Group B8	Day 1	Peptide	CD4	+	+	-	0.009%
B0011	Group B8	Day 1	Peptide	CD4	+	-	+	-0.000553%
B0011	Group B8	Day 1	Peptide	CD4	+	-	-	-0.062%
B0011	Group B8	Day 1	Peptide	CD4	-	+	+	0.00416%
B0011	Group B8	Day 1	Peptide	CD4	-	+	-	0.09%
B0011	Group B8	Day 1	Peptide	CD4	-	-	+	-0.019%
B0011	Group B8	Day 22	Peptide	CD4	+	+	+	0.00564%
B0011	Group B8	Day 22	Peptide	CD4	+	+	-	0.01585%
B0011	Group B8	Day 22	Peptide	CD4	+	-	+	0.000925%
B0011	Group B8	Day 22	Peptide	CD4	+	-	-	-0.011%
B0011	Group B8	Day 22	Peptide	CD4	-	+	+	0.00209%
B0011	Group B8	Day 22	Peptide	CD4	-	+	-	0.008%
B0011	Group B8	Day 22	Peptide	CD4	-	-	+	-0.00967%
B0011	Group B8	Day 1	Peptide	CD8	+	+	+	0%
B0011	Group B8	Day 1	Peptide	CD8	+	+	-	0.00124%
B0011	Group B8	Day 1	Peptide	CD8	+	-	+	-0.00115%
B0011	Group B8	Day 1	Peptide	CD8	+	-	-	-0.053%
B0011	Group B8	Day 1	Peptide	CD8	-	+	+	-0.007%

Administration Site Key: FA = Forearm; UA= Upper arm
Treatments Part A: A1 = A/Sing MAP, 15 ug (FA); A2 = Afluria Quad IM (UA), 15 ug; A3 = Placebo MAP (FA); A4 = A/Sing Mono IM, 15 ug (UA)
Treatments Part B: B1 = A/Sing MAP, 15 ug (FA) with biopsy; B2 = Placebo MAP (FA) with biopsy
B3 = A/Sing MAP, 15 ug (FA); B4 = A/Sing MAP, 10 ug (FA); B5 = A/Sing MAP, 5 ug (FA); B6 = A/Sing MAP, 2.5 ug (FA)
B7 = Placebo MAP (FA); B8 = A/Sing MAP, 15 ug (UA); B9 = A/Sing IM, 15 ug, as 2018 Afluria Quad (UA)

Listing 16.2.6.6
Immunogenicity Results (CMI)

Part B

Randomisation Number	Group	Visit	Test	Subset	IL2	TNFa	IFNg	Value
B0011	Group B8	Day 1	Peptide	CD8	-	+	-	0%
B0011	Group B8	Day 1	Peptide	CD8	-	-	+	-0.6%
B0011	Group B8	Day 22	Peptide	CD8	+	+	+	0%
B0011	Group B8	Day 22	Peptide	CD8	+	+	-	0%
B0011	Group B8	Day 22	Peptide	CD8	+	-	+	0%
B0011	Group B8	Day 22	Peptide	CD8	+	-	-	-0.003%
B0011	Group B8	Day 22	Peptide	CD8	-	+	+	0.00024%
B0011	Group B8	Day 22	Peptide	CD8	-	+	-	0.012%
B0011	Group B8	Day 22	Peptide	CD8	-	-	+	-0.01%
B0011	Group B8	Day 1	MPH	CD4	+	+	+	0.09099%
B0011	Group B8	Day 1	MPH	CD4	+	+	-	0.071%
B0011	Group B8	Day 1	MPH	CD4	+	-	+	0.0178%
B0011	Group B8	Day 1	MPH	CD4	+	-	-	0.07%
B0011	Group B8	Day 1	MPH	CD4	-	+	+	0.06939%
B0011	Group B8	Day 1	MPH	CD4	-	+	-	0.31%
B0011	Group B8	Day 1	MPH	CD4	-	-	+	0.066%
B0011	Group B8	Day 22	MPH	CD4	+	+	+	0.14824%
B0011	Group B8	Day 22	MPH	CD4	+	+	-	0.09085%
B0011	Group B8	Day 22	MPH	CD4	+	-	+	0.06%

Administration Site Key: FA = Forearm; UA= Upper arm
Treatments Part A: A1 = A/Sing MAP, 15 ug (FA); A2 = Afluria Quad IM (UA), 15 ug; A3 = Placebo MAP (FA); A4 = A/Sing Mono IM, 15 ug (UA)
Treatments Part B: B1 = A/Sing MAP, 15 ug (FA) with biopsy; B2 = Placebo MAP (FA) with biopsy
B3 = A/Sing MAP, 15 ug (FA); B4 = A/Sing MAP, 10 ug (FA); B5 = A/Sing MAP, 5 ug (FA); B6 = A/Sing MAP, 2.5 ug (FA)
B7 = Placebo MAP (FA); B8 = A/Sing MAP, 15 ug (UA); B9 = A/Sing IM, 15 ug, as 2018 Afluria Quad (UA)

Listing 16.2.6.6
Immunogenicity Results (CMI)

Part B

Randomisation Number	Group	Visit	Test	Subset	IL2	TNFa	IFNg	Value
B0011	Group B8	Day 22	MPH	CD4	+	-	-	0.058%
B0011	Group B8	Day 22	MPH	CD4	-	+	+	0.06061%
B0011	Group B8	Day 22	MPH	CD4	-	+	-	0.279%
B0011	Group B8	Day 22	MPH	CD4	-	-	+	0.092%
B0011	Group B8	Day 1	MPH	CD8	+	+	+	0.021%
B0011	Group B8	Day 1	MPH	CD8	+	+	-	0.00784%
B0011	Group B8	Day 1	MPH	CD8	+	-	+	0.00221%
B0011	Group B8	Day 1	MPH	CD8	+	-	-	0.148%
B0011	Group B8	Day 1	MPH	CD8	-	+	+	0.074%
B0011	Group B8	Day 1	MPH	CD8	-	+	-	0.27%
B0011	Group B8	Day 1	MPH	CD8	-	-	+	-0.73%
B0011	Group B8	Day 22	MPH	CD8	+	+	+	0.015%
B0011	Group B8	Day 22	MPH	CD8	+	+	-	0.014%
B0011	Group B8	Day 22	MPH	CD8	+	-	+	0.00417%
B0011	Group B8	Day 22	MPH	CD8	+	-	-	0.053%
B0011	Group B8	Day 22	MPH	CD8	-	+	+	0.11682%
B0011	Group B8	Day 22	MPH	CD8	-	+	-	0.292%
B0011	Group B8	Day 22	MPH	CD8	-	-	+	-0.09%

Administration Site Key: FA = Forearm; UA= Upper arm
Treatments Part A: A1 = A/Sing MAP, 15 ug (FA); A2 = Afluria Quad IM (UA), 15 ug; A3 = Placebo MAP (FA); A4 = A/Sing Mono IM, 15 ug (UA)
Treatments Part B: B1 = A/Sing MAP, 15 ug (FA) with biopsy; B2 = Placebo MAP (FA) with biopsy
B3 = A/Sing MAP, 15 ug (FA); B4 = A/Sing MAP, 10 ug (FA); B5 = A/Sing MAP, 5 ug (FA); B6 = A/Sing MAP, 2.5 ug (FA)
B7 = Placebo MAP (FA); B8 = A/Sing MAP, 15 ug (UA); B9 = A/Sing IM, 15 ug, as 2018 Afluria Quad (UA)

Listing 16.2.6.6
Immunogenicity Results (CMI)

Part B

Randomisation Number	Group	Visit	Test	Subset	IL2	TNFa	IFNg	Value
B0014	Group B3	Day 1	Peptide	CD4	+	+	+	0.006081%
B0014	Group B3	Day 1	Peptide	CD4	+	+	-	0.003%
B0014	Group B3	Day 1	Peptide	CD4	+	-	+	-0.000779%
B0014	Group B3	Day 1	Peptide	CD4	+	-	-	-0.009%
B0014	Group B3	Day 1	Peptide	CD4	-	+	+	0.0015%
B0014	Group B3	Day 1	Peptide	CD4	-	+	-	-0.008%
B0014	Group B3	Day 1	Peptide	CD4	-	-	+	-0.00415%
B0014	Group B3	Day 22	Peptide	CD4	+	+	+	0.00753%
B0014	Group B3	Day 22	Peptide	CD4	+	+	-	-0.003%
B0014	Group B3	Day 22	Peptide	CD4	+	-	+	0.00005%
B0014	Group B3	Day 22	Peptide	CD4	+	-	-	-0.029%
B0014	Group B3	Day 22	Peptide	CD4	-	+	+	0.00511%
B0014	Group B3	Day 22	Peptide	CD4	-	+	-	-0.066%
B0014	Group B3	Day 22	Peptide	CD4	-	-	+	-0.01%
B0014	Group B3	Day 1	Peptide	CD8	+	+	+	0.00102%
B0014	Group B3	Day 1	Peptide	CD8	+	+	-	0.00102%
B0014	Group B3	Day 1	Peptide	CD8	+	-	+	0%
B0014	Group B3	Day 1	Peptide	CD8	+	-	-	0.002%
B0014	Group B3	Day 1	Peptide	CD8	-	+	+	-0.00097%

Administration Site Key: FA = Forearm; UA= Upper arm
Treatments Part A: A1 = A/Sing MAP, 15 ug (FA); A2 = Afluria Quad IM (UA), 15 ug; A3 = Placebo MAP (FA); A4 = A/Sing Mono IM, 15 ug (UA)
Treatments Part B: B1 = A/Sing MAP, 15 ug (FA) with biopsy; B2 = Placebo MAP (FA) with biopsy
B3 = A/Sing MAP, 15 ug (FA); B4 = A/Sing MAP, 10 ug (FA); B5 = A/Sing MAP, 5 ug (FA); B6 = A/Sing MAP, 2.5 ug (FA)
B7 = Placebo MAP (FA); B8 = A/Sing MAP, 15 ug (UA); B9 = A/Sing IM, 15 ug, as 2018 Afluria Quad (UA)

Listing 16.2.6.6
Immunogenicity Results (CMI)

Part B

Randomisation Number	Group	Visit	Test	Subset	IL2	TNFa	IFNg	Value
B0014	Group B3	Day 1	Peptide	CD8	-	+	-	-0.01%
B0014	Group B3	Day 1	Peptide	CD8	-	-	+	-0.051%
B0014	Group B3	Day 22	Peptide	CD8	+	+	+	-0.000969%
B0014	Group B3	Day 22	Peptide	CD8	+	+	-	-0.00969%
B0014	Group B3	Day 22	Peptide	CD8	+	-	+	0%
B0014	Group B3	Day 22	Peptide	CD8	+	-	-	-0.011%
B0014	Group B3	Day 22	Peptide	CD8	-	+	+	-0.00491%
B0014	Group B3	Day 22	Peptide	CD8	-	+	-	-0.06%
B0014	Group B3	Day 22	Peptide	CD8	-	-	+	-0.07%
B0014	Group B3	Day 1	MPH	CD4	+	+	+	0.071231%
B0014	Group B3	Day 1	MPH	CD4	+	+	-	0.068%
B0014	Group B3	Day 1	MPH	CD4	+	-	+	0.01146%
B0014	Group B3	Day 1	MPH	CD4	+	-	-	0.024%
B0014	Group B3	Day 1	MPH	CD4	-	+	+	0.05246%
B0014	Group B3	Day 1	MPH	CD4	-	+	-	0.242%
B0014	Group B3	Day 1	MPH	CD4	-	-	+	0.039%
B0014	Group B3	Day 22	MPH	CD4	+	+	+	0.10653%
B0014	Group B3	Day 22	MPH	CD4	+	+	-	0.094%
B0014	Group B3	Day 22	MPH	CD4	+	-	+	0.03561%

Administration Site Key: FA = Forearm; UA= Upper arm
Treatments Part A: A1 = A/Sing MAP, 15 ug (FA); A2 = Afluria Quad IM (UA), 15 ug; A3 = Placebo MAP (FA); A4 = A/Sing Mono IM, 15 ug (UA)
Treatments Part B: B1 = A/Sing MAP, 15 ug (FA) with biopsy; B2 = Placebo MAP (FA) with biopsy
B3 = A/Sing MAP, 15 ug (FA); B4 = A/Sing MAP, 10 ug (FA); B5 = A/Sing MAP, 5 ug (FA); B6 = A/Sing MAP, 2.5 ug (FA)
B7 = Placebo MAP (FA); B8 = A/Sing MAP, 15 ug (UA); B9 = A/Sing IM, 15 ug, as 2018 Afluria Quad (UA)

Listing 16.2.6.6
Immunogenicity Results (CMI)

Part B

Randomisation Number	Group	Visit	Test	Subset	IL2	TNFa	IFNg	Value
B0014	Group B3	Day 22	MPH	CD4	+	-	-	0.019%
B0014	Group B3	Day 22	MPH	CD4	-	+	+	0.06592%
B0014	Group B3	Day 22	MPH	CD4	-	+	-	0.17%
B0014	Group B3	Day 22	MPH	CD4	-	-	+	0.06%
B0014	Group B3	Day 1	MPH	CD8	+	+	+	0.00746%
B0014	Group B3	Day 1	MPH	CD8	+	+	-	0.00373%
B0014	Group B3	Day 1	MPH	CD8	+	-	+	0.00186%
B0014	Group B3	Day 1	MPH	CD8	+	-	-	0.019%
B0014	Group B3	Day 1	MPH	CD8	-	+	+	0.03891%
B0014	Group B3	Day 1	MPH	CD8	-	+	-	0.28%
B0014	Group B3	Day 1	MPH	CD8	-	-	+	0.004%
B0014	Group B3	Day 22	MPH	CD8	+	+	+	0.000871%
B0014	Group B3	Day 22	MPH	CD8	+	+	-	-0.00418%
B0014	Group B3	Day 22	MPH	CD8	+	-	+	0.00459%
B0014	Group B3	Day 22	MPH	CD8	+	-	-	0.03%
B0014	Group B3	Day 22	MPH	CD8	-	+	+	0.032%
B0014	Group B3	Day 22	MPH	CD8	-	+	-	0.25%
B0014	Group B3	Day 22	MPH	CD8	-	-	+	-0.1%

Administration Site Key: FA = Forearm; UA= Upper arm
Treatments Part A: A1 = A/Sing MAP, 15 ug (FA); A2 = Afluria Quad IM (UA), 15 ug; A3 = Placebo MAP (FA); A4 = A/Sing Mono IM, 15 ug (UA)
Treatments Part B: B1 = A/Sing MAP, 15 ug (FA) with biopsy; B2 = Placebo MAP (FA) with biopsy
B3 = A/Sing MAP, 15 ug (FA); B4 = A/Sing MAP, 10 ug (FA); B5 = A/Sing MAP, 5 ug (FA); B6 = A/Sing MAP, 2.5 ug (FA)
B7 = Placebo MAP (FA); B8 = A/Sing MAP, 15 ug (UA); B9 = A/Sing IM, 15 ug, as 2018 Afluria Quad (UA)

Listing 16.2.6.6
Immunogenicity Results (CMI)

Part B

Randomisation Number	Group	Visit	Test	Subset	IL2	TNFa	IFNg	Value
B0015	Group B9	Day 1	Peptide	CD4	+	+	+	0.00191%
B0015	Group B9	Day 1	Peptide	CD4	+	+	-	-0.001%
B0015	Group B9	Day 1	Peptide	CD4	+	-	+	-0.000646%
B0015	Group B9	Day 1	Peptide	CD4	+	-	-	-0.00134%
B0015	Group B9	Day 1	Peptide	CD4	-	+	+	0.001274%
B0015	Group B9	Day 1	Peptide	CD4	-	+	-	0.022%
B0015	Group B9	Day 1	Peptide	CD4	-	-	+	0.00061%
B0015	Group B9	Day 22	Peptide	CD4	+	+	+	-0.00228%
B0015	Group B9	Day 22	Peptide	CD4	+	+	-	0.008%
B0015	Group B9	Day 22	Peptide	CD4	+	-	+	-0.001181%
B0015	Group B9	Day 22	Peptide	CD4	+	-	-	-0.003%
B0015	Group B9	Day 22	Peptide	CD4	-	+	+	-0.0017%
B0015	Group B9	Day 22	Peptide	CD4	-	+	-	-0.001%
B0015	Group B9	Day 22	Peptide	CD4	-	-	+	0.00196%
B0015	Group B9	Day 1	Peptide	CD8	+	+	+	0%
B0015	Group B9	Day 1	Peptide	CD8	+	+	-	0%
B0015	Group B9	Day 1	Peptide	CD8	+	-	+	0%
B0015	Group B9	Day 1	Peptide	CD8	+	-	-	0.00439%
B0015	Group B9	Day 1	Peptide	CD8	-	+	+	-0.0027%

Administration Site Key: FA = Forearm; UA= Upper arm
Treatments Part A: A1 = A/Sing MAP, 15 ug (FA); A2 = Afluria Quad IM (UA), 15 ug; A3 = Placebo MAP (FA); A4 = A/Sing Mono IM, 15 ug (UA)
Treatments Part B: B1 = A/Sing MAP, 15 ug (FA) with biopsy; B2 = Placebo MAP (FA) with biopsy
B3 = A/Sing MAP, 15 ug (FA); B4 = A/Sing MAP, 10 ug (FA); B5 = A/Sing MAP, 5 ug (FA); B6 = A/Sing MAP, 2.5 ug (FA)
B7 = Placebo MAP (FA); B8 = A/Sing MAP, 15 ug (UA); B9 = A/Sing IM, 15 ug, as 2018 Afluria Quad (UA)

Listing 16.2.6.6
Immunogenicity Results (CMI)

Part B

Randomisation Number	Group	Visit	Test	Subset	IL2	TNFa	IFNg	Value
B0015	Group B9	Day 1	Peptide	CD8	-	+	-	0.001%
B0015	Group B9	Day 1	Peptide	CD8	-	-	+	-0.03%
B0015	Group B9	Day 22	Peptide	CD8	+	+	+	0%
B0015	Group B9	Day 22	Peptide	CD8	+	+	-	0%
B0015	Group B9	Day 22	Peptide	CD8	+	-	+	0%
B0015	Group B9	Day 22	Peptide	CD8	+	-	-	-0.00274%
B0015	Group B9	Day 22	Peptide	CD8	-	+	+	-0.00183%
B0015	Group B9	Day 22	Peptide	CD8	-	+	-	-0.018%
B0015	Group B9	Day 22	Peptide	CD8	-	-	+	-0.09%
B0015	Group B9	Day 1	MPH	CD4	+	+	+	0.07671%
B0015	Group B9	Day 1	MPH	CD4	+	+	-	0.129%
B0015	Group B9	Day 1	MPH	CD4	+	-	+	0.009284%
B0015	Group B9	Day 1	MPH	CD4	+	-	-	0.01954%
B0015	Group B9	Day 1	MPH	CD4	-	+	+	0.055354%
B0015	Group B9	Day 1	MPH	CD4	-	+	-	0.323%
B0015	Group B9	Day 1	MPH	CD4	-	-	+	0.04777%
B0015	Group B9	Day 22	MPH	CD4	+	+	+	0.09301%
B0015	Group B9	Day 22	MPH	CD4	+	+	-	0.131%
B0015	Group B9	Day 22	MPH	CD4	+	-	+	0.0182%

Administration Site Key: FA = Forearm; UA= Upper arm
Treatments Part A: A1 = A/Sing MAP, 15 ug (FA); A2 = Afluria Quad IM (UA), 15 ug; A3 = Placebo MAP (FA); A4 = A/Sing Mono IM, 15 ug (UA)
Treatments Part B: B1 = A/Sing MAP, 15 ug (FA) with biopsy; B2 = Placebo MAP (FA) with biopsy
B3 = A/Sing MAP, 15 ug (FA); B4 = A/Sing MAP, 10 ug (FA); B5 = A/Sing MAP, 5 ug (FA); B6 = A/Sing MAP, 2.5 ug (FA)
B7 = Placebo MAP (FA); B8 = A/Sing MAP, 15 ug (UA); B9 = A/Sing IM, 15 ug, as 2018 Afluria Quad (UA)

Listing 16.2.6.6
Immunogenicity Results (CMI)

Part B

Randomisation Number	Group	Visit	Test	Subset	IL2	TNFa	IFNg	Value
B0015	Group B9	Day 22	MPH	CD4	+	-	-	0.047%
B0015	Group B9	Day 22	MPH	CD4	-	+	+	0.07821%
B0015	Group B9	Day 22	MPH	CD4	-	+	-	0.356%
B0015	Group B9	Day 22	MPH	CD4	-	-	+	0.11701%
B0015	Group B9	Day 1	MPH	CD8	+	+	+	0.00509%
B0015	Group B9	Day 1	MPH	CD8	+	+	-	0.00594%
B0015	Group B9	Day 1	MPH	CD8	+	-	+	0.00424%
B0015	Group B9	Day 1	MPH	CD8	+	-	-	0.0172%
B0015	Group B9	Day 1	MPH	CD8	-	+	+	0.0263%
B0015	Group B9	Day 1	MPH	CD8	-	+	-	0.049%
B0015	Group B9	Day 1	MPH	CD8	-	-	+	-0.22%
B0015	Group B9	Day 22	MPH	CD8	+	+	+	0.011%
B0015	Group B9	Day 22	MPH	CD8	+	+	-	0.00504%
B0015	Group B9	Day 22	MPH	CD8	+	-	+	0.0042%
B0015	Group B9	Day 22	MPH	CD8	+	-	-	0.01326%
B0015	Group B9	Day 22	MPH	CD8	-	+	+	0.03117%
B0015	Group B9	Day 22	MPH	CD8	-	+	-	0.049%
B0015	Group B9	Day 22	MPH	CD8	-	-	+	-0.03%

Administration Site Key: FA = Forearm; UA= Upper arm
Treatments Part A: A1 = A/Sing MAP, 15 ug (FA); A2 = Afluria Quad IM (UA), 15 ug; A3 = Placebo MAP (FA); A4 = A/Sing Mono IM, 15 ug (UA)
Treatments Part B: B1 = A/Sing MAP, 15 ug (FA) with biopsy; B2 = Placebo MAP (FA) with biopsy
B3 = A/Sing MAP, 15 ug (FA); B4 = A/Sing MAP, 10 ug (FA); B5 = A/Sing MAP, 5 ug (FA); B6 = A/Sing MAP, 2.5 ug (FA)
B7 = Placebo MAP (FA); B8 = A/Sing MAP, 15 ug (UA); B9 = A/Sing IM, 15 ug, as 2018 Afluria Quad (UA)

Listing 16.2.6.6
Immunogenicity Results (CMI)

Part B

Randomisation Number	Group	Visit	Test	Subset	IL2	TNFa	IFNg	Value
B0017	Group B7	Day 1	Peptide	CD4	+	+	+	0.01048%
B0017	Group B7	Day 1	Peptide	CD4	+	+	-	0.006%
B0017	Group B7	Day 1	Peptide	CD4	+	-	+	-0.000503%
B0017	Group B7	Day 1	Peptide	CD4	+	-	-	0.019%
B0017	Group B7	Day 1	Peptide	CD4	-	+	+	0.00255%
B0017	Group B7	Day 1	Peptide	CD4	-	+	-	0.021%
B0017	Group B7	Day 1	Peptide	CD4	-	-	+	0.002%
B0017	Group B7	Day 22	Peptide	CD4	+	+	+	-0.00047%
B0017	Group B7	Day 22	Peptide	CD4	+	+	-	0.005%
B0017	Group B7	Day 22	Peptide	CD4	+	-	+	0.00055%
B0017	Group B7	Day 22	Peptide	CD4	+	-	-	0.001%
B0017	Group B7	Day 22	Peptide	CD4	-	+	+	0.00057%
B0017	Group B7	Day 22	Peptide	CD4	-	+	-	0.003%
B0017	Group B7	Day 22	Peptide	CD4	-	-	+	0.00298%
B0017	Group B7	Day 1	Peptide	CD8	+	+	+	0%
B0017	Group B7	Day 1	Peptide	CD8	+	+	-	0.00001%
B0017	Group B7	Day 1	Peptide	CD8	+	-	+	0%
B0017	Group B7	Day 1	Peptide	CD8	+	-	-	0.013%
B0017	Group B7	Day 1	Peptide	CD8	-	+	+	0.00286%

Administration Site Key: FA = Forearm; UA= Upper arm
Treatments Part A: A1 = A/Sing MAP, 15 ug (FA); A2 = Afluria Quad IM (UA), 15 ug; A3 = Placebo MAP (FA); A4 = A/Sing Mono IM, 15 ug (UA)
Treatments Part B: B1 = A/Sing MAP, 15 ug (FA) with biopsy; B2 = Placebo MAP (FA) with biopsy
B3 = A/Sing MAP, 15 ug (FA); B4 = A/Sing MAP, 10 ug (FA); B5 = A/Sing MAP, 5 ug (FA); B6 = A/Sing MAP, 2.5 ug (FA)
B7 = Placebo MAP (FA); B8 = A/Sing MAP, 15 ug (UA); B9 = A/Sing IM, 15 ug, as 2018 Afluria Quad (UA)

Listing 16.2.6.6
Immunogenicity Results (CMI)

Part B

Randomisation Number	Group	Visit	Test	Subset	IL2	TNFa	IFNg	Value
B0017	Group B7	Day 1	Peptide	CD8	-	+	-	0.054%
B0017	Group B7	Day 1	Peptide	CD8	-	-	+	0.172%
B0017	Group B7	Day 22	Peptide	CD8	+	+	+	0%
B0017	Group B7	Day 22	Peptide	CD8	+	+	-	0%
B0017	Group B7	Day 22	Peptide	CD8	+	-	+	0%
B0017	Group B7	Day 22	Peptide	CD8	+	-	-	0.00502%
B0017	Group B7	Day 22	Peptide	CD8	-	+	+	0%
B0017	Group B7	Day 22	Peptide	CD8	-	+	-	0%
B0017	Group B7	Day 22	Peptide	CD8	-	-	+	0.035%
B0017	Group B7	Day 1	MPH	CD4	+	+	+	0.09648%
B0017	Group B7	Day 1	MPH	CD4	+	+	-	0.116%
B0017	Group B7	Day 1	MPH	CD4	+	-	+	0.013497%
B0017	Group B7	Day 1	MPH	CD4	+	-	-	0.062%
B0017	Group B7	Day 1	MPH	CD4	-	+	+	0.04249%
B0017	Group B7	Day 1	MPH	CD4	-	+	-	0.221%
B0017	Group B7	Day 1	MPH	CD4	-	-	+	0.065%
B0017	Group B7	Day 22	MPH	CD4	+	+	+	0.09406%
B0017	Group B7	Day 22	MPH	CD4	+	+	-	0.083%
B0017	Group B7	Day 22	MPH	CD4	+	-	+	0.02046%

Administration Site Key: FA = Forearm; UA= Upper arm
Treatments Part A: A1 = A/Sing MAP, 15 ug (FA); A2 = Afluria Quad IM (UA), 15 ug; A3 = Placebo MAP (FA); A4 = A/Sing Mono IM, 15 ug (UA)
Treatments Part B: B1 = A/Sing MAP, 15 ug (FA) with biopsy; B2 = Placebo MAP (FA) with biopsy
B3 = A/Sing MAP, 15 ug (FA); B4 = A/Sing MAP, 10 ug (FA); B5 = A/Sing MAP, 5 ug (FA); B6 = A/Sing MAP, 2.5 ug (FA)
B7 = Placebo MAP (FA); B8 = A/Sing MAP, 15 ug (UA); B9 = A/Sing IM, 15 ug, as 2018 Afluria Quad (UA)

Listing 16.2.6.6
Immunogenicity Results (CMI)

Part B

Randomisation Number	Group	Visit	Test	Subset	IL2	TNFa	IFNg	Value
B0017	Group B7	Day 22	MPH	CD4	+	-	-	0.054%
B0017	Group B7	Day 22	MPH	CD4	-	+	+	0.04184%
B0017	Group B7	Day 22	MPH	CD4	-	+	-	0.16%
B0017	Group B7	Day 22	MPH	CD4	-	-	+	0.05898%
B0017	Group B7	Day 1	MPH	CD8	+	+	+	0.015%
B0017	Group B7	Day 1	MPH	CD8	+	+	-	0.00414%
B0017	Group B7	Day 1	MPH	CD8	+	-	+	0.00139%
B0017	Group B7	Day 1	MPH	CD8	+	-	-	0.025%
B0017	Group B7	Day 1	MPH	CD8	-	+	+	0.04%
B0017	Group B7	Day 1	MPH	CD8	-	+	-	0.174%
B0017	Group B7	Day 1	MPH	CD8	-	-	+	0.062%
B0017	Group B7	Day 22	MPH	CD8	+	+	+	0.014%
B0017	Group B7	Day 22	MPH	CD8	+	+	-	0.00159%
B0017	Group B7	Day 22	MPH	CD8	+	-	+	0.00793%
B0017	Group B7	Day 22	MPH	CD8	+	-	-	0.02702%
B0017	Group B7	Day 22	MPH	CD8	-	+	+	0.021%
B0017	Group B7	Day 22	MPH	CD8	-	+	-	0.06%
B0017	Group B7	Day 22	MPH	CD8	-	-	+	0.029%

Administration Site Key: FA = Forearm; UA= Upper arm
Treatments Part A: A1 = A/Sing MAP, 15 ug (FA); A2 = Afluria Quad IM (UA), 15 ug; A3 = Placebo MAP (FA); A4 = A/Sing Mono IM, 15 ug (UA)
Treatments Part B: B1 = A/Sing MAP, 15 ug (FA) with biopsy; B2 = Placebo MAP (FA) with biopsy
B3 = A/Sing MAP, 15 ug (FA); B4 = A/Sing MAP, 10 ug (FA); B5 = A/Sing MAP, 5 ug (FA); B6 = A/Sing MAP, 2.5 ug (FA)
B7 = Placebo MAP (FA); B8 = A/Sing MAP, 15 ug (UA); B9 = A/Sing IM, 15 ug, as 2018 Afluria Quad (UA)

Listing 16.2.6.6
Immunogenicity Results (CMI)

Part B

Randomisation Number	Group	Visit	Test	Subset	IL2	TNFa	IFNg	Value
B0018	Group B9	Day 1	Peptide	CD4	+	+	+	0.00208%
B0018	Group B9	Day 1	Peptide	CD4	+	+	-	0.002%
B0018	Group B9	Day 1	Peptide	CD4	+	-	+	0%
B0018	Group B9	Day 1	Peptide	CD4	+	-	-	-0.007%
B0018	Group B9	Day 1	Peptide	CD4	-	+	+	-0.000083%
B0018	Group B9	Day 1	Peptide	CD4	-	+	-	0%
B0018	Group B9	Day 1	Peptide	CD4	-	-	+	-0.002%
B0018	Group B9	Day 22	Peptide	CD4	+	+	+	0.01543%
B0018	Group B9	Day 22	Peptide	CD4	+	+	-	0.011%
B0018	Group B9	Day 22	Peptide	CD4	+	-	+	0.000798%
B0018	Group B9	Day 22	Peptide	CD4	+	-	-	-0.045%
B0018	Group B9	Day 22	Peptide	CD4	-	+	+	0.001675%
B0018	Group B9	Day 22	Peptide	CD4	-	+	-	0.02%
B0018	Group B9	Day 22	Peptide	CD4	-	-	+	-0.01%
B0018	Group B9	Day 1	Peptide	CD8	+	+	+	0%
B0018	Group B9	Day 1	Peptide	CD8	+	+	-	-0.00209%
B0018	Group B9	Day 1	Peptide	CD8	+	-	+	0%
B0018	Group B9	Day 1	Peptide	CD8	+	-	-	0.002%
B0018	Group B9	Day 1	Peptide	CD8	-	+	+	0.00192%

Administration Site Key: FA = Forearm; UA= Upper arm
Treatments Part A: A1 = A/Sing MAP, 15 ug (FA); A2 = Afluria Quad IM (UA), 15 ug; A3 = Placebo MAP (FA); A4 = A/Sing Mono IM, 15 ug (UA)
Treatments Part B: B1 = A/Sing MAP, 15 ug (FA) with biopsy; B2 = Placebo MAP (FA) with biopsy
B3 = A/Sing MAP, 15 ug (FA); B4 = A/Sing MAP, 10 ug (FA); B5 = A/Sing MAP, 5 ug (FA); B6 = A/Sing MAP, 2.5 ug (FA)
B7 = Placebo MAP (FA); B8 = A/Sing MAP, 15 ug (UA); B9 = A/Sing IM, 15 ug, as 2018 Afluria Quad (UA)

Listing 16.2.6.6
Immunogenicity Results (CMI)

Part B

Randomisation Number	Group	Visit	Test	Subset	IL2	TNFa	IFNg	Value
B0018	Group B9	Day 1	Peptide	CD8	-	+	-	-0.021%
B0018	Group B9	Day 1	Peptide	CD8	-	-	+	0.16%
B0018	Group B9	Day 22	Peptide	CD8	+	+	+	0%
B0018	Group B9	Day 22	Peptide	CD8	+	+	-	0%
B0018	Group B9	Day 22	Peptide	CD8	+	-	+	0%
B0018	Group B9	Day 22	Peptide	CD8	+	-	-	-0.006%
B0018	Group B9	Day 22	Peptide	CD8	-	+	+	0.00456%
B0018	Group B9	Day 22	Peptide	CD8	-	+	-	-0.112%
B0018	Group B9	Day 22	Peptide	CD8	-	-	+	-0.02%
B0018	Group B9	Day 1	MPH	CD4	+	+	+	0.04443%
B0018	Group B9	Day 1	MPH	CD4	+	+	-	0.061%
B0018	Group B9	Day 1	MPH	CD4	+	-	+	0.00776%
B0018	Group B9	Day 1	MPH	CD4	+	-	-	0.033%
B0018	Group B9	Day 1	MPH	CD4	-	+	+	0.014143%
B0018	Group B9	Day 1	MPH	CD4	-	+	-	0.189%
B0018	Group B9	Day 1	MPH	CD4	-	-	+	0.022%
B0018	Group B9	Day 22	MPH	CD4	+	+	+	0.07843%
B0018	Group B9	Day 22	MPH	CD4	+	+	-	0.086%
B0018	Group B9	Day 22	MPH	CD4	+	-	+	0.053%

Administration Site Key: FA = Forearm; UA= Upper arm
Treatments Part A: A1 = A/Sing MAP, 15 ug (FA); A2 = Afluria Quad IM (UA), 15 ug; A3 = Placebo MAP (FA); A4 = A/Sing Mono IM, 15 ug (UA)
Treatments Part B: B1 = A/Sing MAP, 15 ug (FA) with biopsy; B2 = Placebo MAP (FA) with biopsy
B3 = A/Sing MAP, 15 ug (FA); B4 = A/Sing MAP, 10 ug (FA); B5 = A/Sing MAP, 5 ug (FA); B6 = A/Sing MAP, 2.5 ug (FA)
B7 = Placebo MAP (FA); B8 = A/Sing MAP, 15 ug (UA); B9 = A/Sing IM, 15 ug, as 2018 Afluria Quad (UA)

Listing 16.2.6.6
Immunogenicity Results (CMI)

Part B

Randomisation Number	Group	Visit	Test	Subset	IL2	TNFa	IFNg	Value
B0018	Group B9	Day 22	MPH	CD4	+	-	-	0.001%
B0018	Group B9	Day 22	MPH	CD4	-	+	+	0.036285%
B0018	Group B9	Day 22	MPH	CD4	-	+	-	0.19%
B0018	Group B9	Day 22	MPH	CD4	-	-	+	0.022%
B0018	Group B9	Day 1	MPH	CD8	+	+	+	0.00228%
B0018	Group B9	Day 1	MPH	CD8	+	+	-	-0.00209%
B0018	Group B9	Day 1	MPH	CD8	+	-	+	0.00457%
B0018	Group B9	Day 1	MPH	CD8	+	-	-	0.012%
B0018	Group B9	Day 1	MPH	CD8	-	+	+	0.016%
B0018	Group B9	Day 1	MPH	CD8	-	+	-	0.107%
B0018	Group B9	Day 1	MPH	CD8	-	-	+	-0.061%
B0018	Group B9	Day 22	MPH	CD8	+	+	+	0.011%
B0018	Group B9	Day 22	MPH	CD8	+	+	-	0.00358%
B0018	Group B9	Day 22	MPH	CD8	+	-	+	0.00179%
B0018	Group B9	Day 22	MPH	CD8	+	-	-	-0.009%
B0018	Group B9	Day 22	MPH	CD8	-	+	+	0.02685%
B0018	Group B9	Day 22	MPH	CD8	-	+	-	0.13%
B0018	Group B9	Day 22	MPH	CD8	-	-	+	-0.129%

Administration Site Key: FA = Forearm; UA= Upper arm
Treatments Part A: A1 = A/Sing MAP, 15 ug (FA); A2 = Afluria Quad IM (UA), 15 ug; A3 = Placebo MAP (FA); A4 = A/Sing Mono IM, 15 ug (UA)
Treatments Part B: B1 = A/Sing MAP, 15 ug (FA) with biopsy; B2 = Placebo MAP (FA) with biopsy
B3 = A/Sing MAP, 15 ug (FA); B4 = A/Sing MAP, 10 ug (FA); B5 = A/Sing MAP, 5 ug (FA); B6 = A/Sing MAP, 2.5 ug (FA)
B7 = Placebo MAP (FA); B8 = A/Sing MAP, 15 ug (UA); B9 = A/Sing IM, 15 ug, as 2018 Afluria Quad (UA)

Listing 16.2.6.6
Immunogenicity Results (CMI)

Part B

Randomisation Number	Group	Visit	Test	Subset	IL2	TNFa	IFNg	Value
B0021	Group B7	Day 1	Peptide	CD4	+	+	+	0.00834%
B0021	Group B7	Day 1	Peptide	CD4	+	+	-	-0.001%
B0021	Group B7	Day 1	Peptide	CD4	+	-	+	0.00111%
B0021	Group B7	Day 1	Peptide	CD4	+	-	-	-0.013%
B0021	Group B7	Day 1	Peptide	CD4	-	+	+	0.00235%
B0021	Group B7	Day 1	Peptide	CD4	-	+	-	-0.001%
B0021	Group B7	Day 1	Peptide	CD4	-	-	+	0.001%
B0021	Group B7	Day 22	Peptide	CD4	+	+	+	0.00678%
B0021	Group B7	Day 22	Peptide	CD4	+	+	-	0.009%
B0021	Group B7	Day 22	Peptide	CD4	+	-	+	0.00056%
B0021	Group B7	Day 22	Peptide	CD4	+	-	-	-0.008%
B0021	Group B7	Day 22	Peptide	CD4	-	+	+	0.001698%
B0021	Group B7	Day 22	Peptide	CD4	-	+	-	-0.021%
B0021	Group B7	Day 22	Peptide	CD4	-	-	+	0%
B0021	Group B7	Day 1	Peptide	CD8	+	+	+	0%
B0021	Group B7	Day 1	Peptide	CD8	+	+	-	0.00385%
B0021	Group B7	Day 1	Peptide	CD8	+	-	+	0%
B0021	Group B7	Day 1	Peptide	CD8	+	-	-	-0.012%
B0021	Group B7	Day 1	Peptide	CD8	-	+	+	-0.00341%

Administration Site Key: FA = Forearm; UA= Upper arm
Treatments Part A: A1 = A/Sing MAP, 15 ug (FA); A2 = Afluria Quad IM (UA), 15 ug; A3 = Placebo MAP (FA); A4 = A/Sing Mono IM, 15 ug (UA)
Treatments Part B: B1 = A/Sing MAP, 15 ug (FA) with biopsy; B2 = Placebo MAP (FA) with biopsy
B3 = A/Sing MAP, 15 ug (FA); B4 = A/Sing MAP, 10 ug (FA); B5 = A/Sing MAP, 5 ug (FA); B6 = A/Sing MAP, 2.5 ug (FA)
B7 = Placebo MAP (FA); B8 = A/Sing MAP, 15 ug (UA); B9 = A/Sing IM, 15 ug, as 2018 Afluria Quad (UA)

Listing 16.2.6.6
Immunogenicity Results (CMI)

Part B

Randomisation Number	Group	Visit	Test	Subset	IL2	TNFa	IFNg	Value
B0021	Group B7	Day 1	Peptide	CD8	-	+	-	-0.03%
B0021	Group B7	Day 1	Peptide	CD8	-	-	+	0%
B0021	Group B7	Day 22	Peptide	CD8	+	+	+	0.00199%
B0021	Group B7	Day 22	Peptide	CD8	+	+	-	0.00199%
B0021	Group B7	Day 22	Peptide	CD8	+	-	+	0%
B0021	Group B7	Day 22	Peptide	CD8	+	-	-	-0.00405%
B0021	Group B7	Day 22	Peptide	CD8	-	+	+	0.00598%
B0021	Group B7	Day 22	Peptide	CD8	-	+	-	-0.05%
B0021	Group B7	Day 22	Peptide	CD8	-	-	+	0.007%
B0021	Group B7	Day 1	MPH	CD4	+	+	+	0.05934%
B0021	Group B7	Day 1	MPH	CD4	+	+	-	0.072%
B0021	Group B7	Day 1	MPH	CD4	+	-	+	0.00959%
B0021	Group B7	Day 1	MPH	CD4	+	-	-	0.022%
B0021	Group B7	Day 1	MPH	CD4	-	+	+	0.04893%
B0021	Group B7	Day 1	MPH	CD4	-	+	-	0.292%
B0021	Group B7	Day 1	MPH	CD4	-	-	+	0.056%
B0021	Group B7	Day 22	MPH	CD4	+	+	+	0.05437%
B0021	Group B7	Day 22	MPH	CD4	+	+	-	0.051%
B0021	Group B7	Day 22	MPH	CD4	+	-	+	0.012%

Administration Site Key: FA = Forearm; UA= Upper arm
Treatments Part A: A1 = A/Sing MAP, 15 ug (FA); A2 = Afluria Quad IM (UA), 15 ug; A3 = Placebo MAP (FA); A4 = A/Sing Mono IM, 15 ug (UA)
Treatments Part B: B1 = A/Sing MAP, 15 ug (FA) with biopsy; B2 = Placebo MAP (FA) with biopsy
B3 = A/Sing MAP, 15 ug (FA); B4 = A/Sing MAP, 10 ug (FA); B5 = A/Sing MAP, 5 ug (FA); B6 = A/Sing MAP, 2.5 ug (FA)
B7 = Placebo MAP (FA); B8 = A/Sing MAP, 15 ug (UA); B9 = A/Sing IM, 15 ug, as 2018 Afluria Quad (UA)

Listing 16.2.6.6
Immunogenicity Results (CMI)

Part B

Randomisation Number	Group	Visit	Test	Subset	IL2	TNFa	IFNg	Value
B0021	Group B7	Day 22	MPH	CD4	+	-	-	0.018%
B0021	Group B7	Day 22	MPH	CD4	-	+	+	0.067458%
B0021	Group B7	Day 22	MPH	CD4	-	+	-	0.17%
B0021	Group B7	Day 22	MPH	CD4	-	-	+	0.048%
B0021	Group B7	Day 1	MPH	CD8	+	+	+	0.06%
B0021	Group B7	Day 1	MPH	CD8	+	+	-	0.019%
B0021	Group B7	Day 1	MPH	CD8	+	-	+	0.00683%
B0021	Group B7	Day 1	MPH	CD8	+	-	-	0.017%
B0021	Group B7	Day 1	MPH	CD8	-	+	+	0.16274%
B0021	Group B7	Day 1	MPH	CD8	-	+	-	0.81%
B0021	Group B7	Day 1	MPH	CD8	-	-	+	0.14%
B0021	Group B7	Day 22	MPH	CD8	+	+	+	0.03%
B0021	Group B7	Day 22	MPH	CD8	+	+	-	0.00358%
B0021	Group B7	Day 22	MPH	CD8	+	-	+	0.00179%
B0021	Group B7	Day 22	MPH	CD8	+	-	-	0.011%
B0021	Group B7	Day 22	MPH	CD8	-	+	+	0.12803%
B0021	Group B7	Day 22	MPH	CD8	-	+	-	0.53%
B0021	Group B7	Day 22	MPH	CD8	-	-	+	0.071%

Administration Site Key: FA = Forearm; UA= Upper arm
Treatments Part A: A1 = A/Sing MAP, 15 ug (FA); A2 = Afluria Quad IM (UA), 15 ug; A3 = Placebo MAP (FA); A4 = A/Sing Mono IM, 15 ug (UA)
Treatments Part B: B1 = A/Sing MAP, 15 ug (FA) with biopsy; B2 = Placebo MAP (FA) with biopsy
B3 = A/Sing MAP, 15 ug (FA); B4 = A/Sing MAP, 10 ug (FA); B5 = A/Sing MAP, 5 ug (FA); B6 = A/Sing MAP, 2.5 ug (FA)
B7 = Placebo MAP (FA); B8 = A/Sing MAP, 15 ug (UA); B9 = A/Sing IM, 15 ug, as 2018 Afluria Quad (UA)

Listing 16.2.6.6
Immunogenicity Results (CMI)

Part B

Randomisation Number	Group	Visit	Test	Subset	IL2	TNFa	IFNg	Value
B0023	Group B3	Day 1	Peptide	CD4	+	+	+	0.0094%
B0023	Group B3	Day 1	Peptide	CD4	+	+	-	0.00661%
B0023	Group B3	Day 1	Peptide	CD4	+	-	+	-0.0012%
B0023	Group B3	Day 1	Peptide	CD4	+	-	-	-0.011%
B0023	Group B3	Day 1	Peptide	CD4	-	+	+	0.00479%
B0023	Group B3	Day 1	Peptide	CD4	-	+	-	-0.01%
B0023	Group B3	Day 1	Peptide	CD4	-	-	+	-0.006%
B0023	Group B3	Day 22	Peptide	CD4	+	+	+	0.027335%
B0023	Group B3	Day 22	Peptide	CD4	+	+	-	0.02235%
B0023	Group B3	Day 22	Peptide	CD4	+	-	+	-0.000033%
B0023	Group B3	Day 22	Peptide	CD4	+	-	-	-0.007%
B0023	Group B3	Day 22	Peptide	CD4	-	+	+	0.01067%
B0023	Group B3	Day 22	Peptide	CD4	-	+	-	0.032%
B0023	Group B3	Day 22	Peptide	CD4	-	-	+	-0.00492%
B0023	Group B3	Day 1	Peptide	CD8	+	+	+	0%
B0023	Group B3	Day 1	Peptide	CD8	+	+	-	1E-5%
B0023	Group B3	Day 1	Peptide	CD8	+	-	+	-0.001%
B0023	Group B3	Day 1	Peptide	CD8	+	-	-	-0.022%
B0023	Group B3	Day 1	Peptide	CD8	-	+	+	-0.00995%

Administration Site Key: FA = Forearm; UA= Upper arm
Treatments Part A: A1 = A/Sing MAP, 15 ug (FA); A2 = Afluria Quad IM (UA), 15 ug; A3 = Placebo MAP (FA); A4 = A/Sing Mono IM, 15 ug (UA)
Treatments Part B: B1 = A/Sing MAP, 15 ug (FA) with biopsy; B2 = Placebo MAP (FA) with biopsy
B3 = A/Sing MAP, 15 ug (FA); B4 = A/Sing MAP, 10 ug (FA); B5 = A/Sing MAP, 5 ug (FA); B6 = A/Sing MAP, 2.5 ug (FA)
B7 = Placebo MAP (FA); B8 = A/Sing MAP, 15 ug (UA); B9 = A/Sing IM, 15 ug, as 2018 Afluria Quad (UA)

Listing 16.2.6.6
Immunogenicity Results (CMI)

Part B

Randomisation Number	Group	Visit	Test	Subset	IL2	TNFa	IFNg	Value
B0023	Group B3	Day 1	Peptide	CD8	-	+	-	-0.03%
B0023	Group B3	Day 1	Peptide	CD8	-	-	+	-0.59%
B0023	Group B3	Day 22	Peptide	CD8	+	+	+	0%
B0023	Group B3	Day 22	Peptide	CD8	+	+	-	-0.00005%
B0023	Group B3	Day 22	Peptide	CD8	+	-	+	0%
B0023	Group B3	Day 22	Peptide	CD8	+	-	-	0.00749%
B0023	Group B3	Day 22	Peptide	CD8	-	+	+	-0.00143%
B0023	Group B3	Day 22	Peptide	CD8	-	+	-	-0.004%
B0023	Group B3	Day 22	Peptide	CD8	-	-	+	-0.03%
B0023	Group B3	Day 1	MPH	CD4	+	+	+	0.0674%
B0023	Group B3	Day 1	MPH	CD4	+	+	-	0.04061%
B0023	Group B3	Day 1	MPH	CD4	+	-	+	0.00516%
B0023	Group B3	Day 1	MPH	CD4	+	-	-	0.018%
B0023	Group B3	Day 1	MPH	CD4	-	+	+	0.0634%
B0023	Group B3	Day 1	MPH	CD4	-	+	-	0.21%
B0023	Group B3	Day 1	MPH	CD4	-	-	+	0.028%
B0023	Group B3	Day 22	MPH	CD4	+	+	+	0.068335%
B0023	Group B3	Day 22	MPH	CD4	+	+	-	0.04935%
B0023	Group B3	Day 22	MPH	CD4	+	-	+	0.023335%

Administration Site Key: FA = Forearm; UA= Upper arm
Treatments Part A: A1 = A/Sing MAP, 15 ug (FA); A2 = Afluria Quad IM (UA), 15 ug; A3 = Placebo MAP (FA); A4 = A/Sing Mono IM, 15 ug (UA)
Treatments Part B: B1 = A/Sing MAP, 15 ug (FA) with biopsy; B2 = Placebo MAP (FA) with biopsy
B3 = A/Sing MAP, 15 ug (FA); B4 = A/Sing MAP, 10 ug (FA); B5 = A/Sing MAP, 5 ug (FA); B6 = A/Sing MAP, 2.5 ug (FA)
B7 = Placebo MAP (FA); B8 = A/Sing MAP, 15 ug (UA); B9 = A/Sing IM, 15 ug, as 2018 Afluria Quad (UA)

Listing 16.2.6.6
Immunogenicity Results (CMI)

Part B

Randomisation Number	Group	Visit	Test	Subset	IL2	TNFa	IFNg	Value
B0023	Group B3	Day 22	MPH	CD4	+	-	-	0.028%
B0023	Group B3	Day 22	MPH	CD4	-	+	+	0.07467%
B0023	Group B3	Day 22	MPH	CD4	-	+	-	0.217%
B0023	Group B3	Day 22	MPH	CD4	-	-	+	0.07302%
B0023	Group B3	Day 1	MPH	CD8	+	+	+	0.026%
B0023	Group B3	Day 1	MPH	CD8	+	+	-	0.00468%
B0023	Group B3	Day 1	MPH	CD8	+	-	+	0.011%
B0023	Group B3	Day 1	MPH	CD8	+	-	-	0.006%
B0023	Group B3	Day 1	MPH	CD8	-	+	+	0.079%
B0023	Group B3	Day 1	MPH	CD8	-	+	-	0.07%
B0023	Group B3	Day 1	MPH	CD8	-	-	+	-0.91%
B0023	Group B3	Day 22	MPH	CD8	+	+	+	0.022%
B0023	Group B3	Day 22	MPH	CD8	+	+	-	0.00306%
B0023	Group B3	Day 22	MPH	CD8	+	-	+	0.00212%
B0023	Group B3	Day 22	MPH	CD8	+	-	-	0.02849%
B0023	Group B3	Day 22	MPH	CD8	-	+	+	0.12287%
B0023	Group B3	Day 22	MPH	CD8	-	+	-	0.17%
B0023	Group B3	Day 22	MPH	CD8	-	-	+	-0.47%

Administration Site Key: FA = Forearm; UA= Upper arm
Treatments Part A: A1 = A/Sing MAP, 15 ug (FA); A2 = Afluria Quad IM (UA), 15 ug; A3 = Placebo MAP (FA); A4 = A/Sing Mono IM, 15 ug (UA)
Treatments Part B: B1 = A/Sing MAP, 15 ug (FA) with biopsy; B2 = Placebo MAP (FA) with biopsy
B3 = A/Sing MAP, 15 ug (FA); B4 = A/Sing MAP, 10 ug (FA); B5 = A/Sing MAP, 5 ug (FA); B6 = A/Sing MAP, 2.5 ug (FA)
B7 = Placebo MAP (FA); B8 = A/Sing MAP, 15 ug (UA); B9 = A/Sing IM, 15 ug, as 2018 Afluria Quad (UA)

Listing 16.2.6.6
Immunogenicity Results (CMI)

Part B

Randomisation Number	Group	Visit	Test	Subset	IL2	TNFa	IFNg	Value
B0025	Group B8	Day 1	Peptide	CD4	+	+	+	0.00212%
B0025	Group B8	Day 1	Peptide	CD4	+	+	-	0.005%
B0025	Group B8	Day 1	Peptide	CD4	+	-	+	-0.000441%
B0025	Group B8	Day 1	Peptide	CD4	+	-	-	0%
B0025	Group B8	Day 1	Peptide	CD4	-	+	+	0.0027%
B0025	Group B8	Day 1	Peptide	CD4	-	+	-	-0.01%
B0025	Group B8	Day 1	Peptide	CD4	-	-	+	-0.00461%
B0025	Group B8	Day 22	Peptide	CD4	+	+	+	0.00556%
B0025	Group B8	Day 22	Peptide	CD4	+	+	-	0.007%
B0025	Group B8	Day 22	Peptide	CD4	+	-	+	0.000503%
B0025	Group B8	Day 22	Peptide	CD4	+	-	-	-0.003%
B0025	Group B8	Day 22	Peptide	CD4	-	+	+	0.000511%
B0025	Group B8	Day 22	Peptide	CD4	-	+	-	-0.006%
B0025	Group B8	Day 22	Peptide	CD4	-	-	+	0.00007%
B0025	Group B8	Day 1	Peptide	CD8	+	+	+	0.00272%
B0025	Group B8	Day 1	Peptide	CD8	+	+	-	-0.00133%
B0025	Group B8	Day 1	Peptide	CD8	+	-	+	0%
B0025	Group B8	Day 1	Peptide	CD8	+	-	-	0.002%
B0025	Group B8	Day 1	Peptide	CD8	-	+	+	-0.00425%

Administration Site Key: FA = Forearm; UA= Upper arm
Treatments Part A: A1 = A/Sing MAP, 15 ug (FA); A2 = Afluria Quad IM (UA), 15 ug; A3 = Placebo MAP (FA); A4 = A/Sing Mono IM, 15 ug (UA)
Treatments Part B: B1 = A/Sing MAP, 15 ug (FA) with biopsy; B2 = Placebo MAP (FA) with biopsy
B3 = A/Sing MAP, 15 ug (FA); B4 = A/Sing MAP, 10 ug (FA); B5 = A/Sing MAP, 5 ug (FA); B6 = A/Sing MAP, 2.5 ug (FA)
B7 = Placebo MAP (FA); B8 = A/Sing MAP, 15 ug (UA); B9 = A/Sing IM, 15 ug, as 2018 Afluria Quad (UA)

Listing 16.2.6.6
Immunogenicity Results (CMI)

Part B

Randomisation Number	Group	Visit	Test	Subset	IL2	TNFa	IFNg	Value
B0025	Group B8	Day 1	Peptide	CD8	-	+	-	0.01%
B0025	Group B8	Day 1	Peptide	CD8	-	-	+	-0.09%
B0025	Group B8	Day 22	Peptide	CD8	+	+	+	0.00297%
B0025	Group B8	Day 22	Peptide	CD8	+	+	-	0.00293%
B0025	Group B8	Day 22	Peptide	CD8	+	-	+	0%
B0025	Group B8	Day 22	Peptide	CD8	+	-	-	0.006%
B0025	Group B8	Day 22	Peptide	CD8	-	+	+	-0.00285%
B0025	Group B8	Day 22	Peptide	CD8	-	+	-	-0.03%
B0025	Group B8	Day 22	Peptide	CD8	-	-	+	0.02%
B0025	Group B8	Day 1	MPH	CD4	+	+	+	0.03512%
B0025	Group B8	Day 1	MPH	CD4	+	+	-	0.078%
B0025	Group B8	Day 1	MPH	CD4	+	-	+	0.006612%
B0025	Group B8	Day 1	MPH	CD4	+	-	-	0.044%
B0025	Group B8	Day 1	MPH	CD4	-	+	+	0.04178%
B0025	Group B8	Day 1	MPH	CD4	-	+	-	0.24%
B0025	Group B8	Day 1	MPH	CD4	-	-	+	0.06%
B0025	Group B8	Day 22	MPH	CD4	+	+	+	0.03656%
B0025	Group B8	Day 22	MPH	CD4	+	+	-	0.074%
B0025	Group B8	Day 22	MPH	CD4	+	-	+	0.020505%

Administration Site Key: FA = Forearm; UA= Upper arm
Treatments Part A: A1 = A/Sing MAP, 15 ug (FA); A2 = Afluria Quad IM (UA), 15 ug; A3 = Placebo MAP (FA); A4 = A/Sing Mono IM, 15 ug (UA)
Treatments Part B: B1 = A/Sing MAP, 15 ug (FA) with biopsy; B2 = Placebo MAP (FA) with biopsy
B3 = A/Sing MAP, 15 ug (FA); B4 = A/Sing MAP, 10 ug (FA); B5 = A/Sing MAP, 5 ug (FA); B6 = A/Sing MAP, 2.5 ug (FA)
B7 = Placebo MAP (FA); B8 = A/Sing MAP, 15 ug (UA); B9 = A/Sing IM, 15 ug, as 2018 Afluria Quad (UA)

Listing 16.2.6.6
Immunogenicity Results (CMI)

Part B

Randomisation Number	Group	Visit	Test	Subset	IL2	TNFa	IFNg	Value
B0025	Group B8	Day 22	MPH	CD4	+	-	-	0.083%
B0025	Group B8	Day 22	MPH	CD4	-	+	+	0.021011%
B0025	Group B8	Day 22	MPH	CD4	-	+	-	0.158%
B0025	Group B8	Day 22	MPH	CD4	-	-	+	0.06858%
B0025	Group B8	Day 1	MPH	CD8	+	+	+	0.02167%
B0025	Group B8	Day 1	MPH	CD8	+	+	-	0.01867%
B0025	Group B8	Day 1	MPH	CD8	+	-	+	0.00894%
B0025	Group B8	Day 1	MPH	CD8	+	-	-	0.054%
B0025	Group B8	Day 1	MPH	CD8	-	+	+	0.072%
B0025	Group B8	Day 1	MPH	CD8	-	+	-	0.27%
B0025	Group B8	Day 1	MPH	CD8	-	-	+	0.19%
B0025	Group B8	Day 22	MPH	CD8	+	+	+	0.01758%
B0025	Group B8	Day 22	MPH	CD8	+	+	-	0.00971%
B0025	Group B8	Day 22	MPH	CD8	+	-	+	0.00139%
B0025	Group B8	Day 22	MPH	CD8	+	-	-	0.052%
B0025	Group B8	Day 22	MPH	CD8	-	+	+	0.06115%
B0025	Group B8	Day 22	MPH	CD8	-	+	-	0.28%
B0025	Group B8	Day 22	MPH	CD8	-	-	+	-0.16%

Administration Site Key: FA = Forearm; UA= Upper arm
Treatments Part A: A1 = A/Sing MAP, 15 ug (FA); A2 = Afluria Quad IM (UA), 15 ug; A3 = Placebo MAP (FA); A4 = A/Sing Mono IM, 15 ug (UA)
Treatments Part B: B1 = A/Sing MAP, 15 ug (FA) with biopsy; B2 = Placebo MAP (FA) with biopsy
B3 = A/Sing MAP, 15 ug (FA); B4 = A/Sing MAP, 10 ug (FA); B5 = A/Sing MAP, 5 ug (FA); B6 = A/Sing MAP, 2.5 ug (FA)
B7 = Placebo MAP (FA); B8 = A/Sing MAP, 15 ug (UA); B9 = A/Sing IM, 15 ug, as 2018 Afluria Quad (UA)

Listing 16.2.6.6
Immunogenicity Results (CMI)

Part B

Randomisation Number	Group	Visit	Test	Subset	IL2	TNFa	IFNg	Value
B0026	Group B9	Day 1	Peptide	CD4	+	+	+	0.00133%
B0026	Group B9	Day 1	Peptide	CD4	+	+	-	0.003%
B0026	Group B9	Day 1	Peptide	CD4	+	-	+	-0.000702%
B0026	Group B9	Day 1	Peptide	CD4	+	-	-	0.00191%
B0026	Group B9	Day 1	Peptide	CD4	-	+	+	0.00199%
B0026	Group B9	Day 1	Peptide	CD4	-	+	-	0.008%
B0026	Group B9	Day 1	Peptide	CD4	-	-	+	-0.00215%
B0026	Group B9	Day 22	Peptide	CD4	+	+	+	0.00556%
B0026	Group B9	Day 22	Peptide	CD4	+	+	-	0.016%
B0026	Group B9	Day 22	Peptide	CD4	+	-	+	-2E-6%
B0026	Group B9	Day 22	Peptide	CD4	+	-	-	-0.001%
B0026	Group B9	Day 22	Peptide	CD4	-	+	+	0.00482%
B0026	Group B9	Day 22	Peptide	CD4	-	+	-	-0.002%
B0026	Group B9	Day 22	Peptide	CD4	-	-	+	-0.00122%
B0026	Group B9	Day 1	Peptide	CD8	+	+	+	-0.00005%
B0026	Group B9	Day 1	Peptide	CD8	+	+	-	0.00142%
B0026	Group B9	Day 1	Peptide	CD8	+	-	+	0%
B0026	Group B9	Day 1	Peptide	CD8	+	-	-	0.0028%
B0026	Group B9	Day 1	Peptide	CD8	-	+	+	-0.00773%

Administration Site Key: FA = Forearm; UA= Upper arm
Treatments Part A: A1 = A/Sing MAP, 15 ug (FA); A2 = Afluria Quad IM (UA), 15 ug; A3 = Placebo MAP (FA); A4 = A/Sing Mono IM, 15 ug (UA)
Treatments Part B: B1 = A/Sing MAP, 15 ug (FA) with biopsy; B2 = Placebo MAP (FA) with biopsy
B3 = A/Sing MAP, 15 ug (FA); B4 = A/Sing MAP, 10 ug (FA); B5 = A/Sing MAP, 5 ug (FA); B6 = A/Sing MAP, 2.5 ug (FA)
B7 = Placebo MAP (FA); B8 = A/Sing MAP, 15 ug (UA); B9 = A/Sing IM, 15 ug, as 2018 Afluria Quad (UA)

Listing 16.2.6.6
Immunogenicity Results (CMI)

Part B

Randomisation Number	Group	Visit	Test	Subset	IL2	TNFa	IFNg	Value
B0026	Group B9	Day 1	Peptide	CD8	-	+	-	-0.006%
B0026	Group B9	Day 1	Peptide	CD8	-	-	+	-0.056%
B0026	Group B9	Day 22	Peptide	CD8	+	+	+	0.00125%
B0026	Group B9	Day 22	Peptide	CD8	+	+	-	0.00249%
B0026	Group B9	Day 22	Peptide	CD8	+	-	+	0%
B0026	Group B9	Day 22	Peptide	CD8	+	-	-	-0.00505%
B0026	Group B9	Day 22	Peptide	CD8	-	+	+	-0.0013%
B0026	Group B9	Day 22	Peptide	CD8	-	+	-	0.002%
B0026	Group B9	Day 22	Peptide	CD8	-	-	+	-0.018%
B0026	Group B9	Day 1	MPH	CD4	+	+	+	0.0806%
B0026	Group B9	Day 1	MPH	CD4	+	+	-	0.072%
B0026	Group B9	Day 1	MPH	CD4	+	-	+	0.004338%
B0026	Group B9	Day 1	MPH	CD4	+	-	-	0.02509%
B0026	Group B9	Day 1	MPH	CD4	-	+	+	0.0649%
B0026	Group B9	Day 1	MPH	CD4	-	+	-	0.24%
B0026	Group B9	Day 1	MPH	CD4	-	-	+	0.03649%
B0026	Group B9	Day 22	MPH	CD4	+	+	+	0.11456%
B0026	Group B9	Day 22	MPH	CD4	+	+	-	0.066%
B0026	Group B9	Day 22	MPH	CD4	+	-	+	0.017395%

Administration Site Key: FA = Forearm; UA= Upper arm
Treatments Part A: A1 = A/Sing MAP, 15 ug (FA); A2 = Afluria Quad IM (UA), 15 ug; A3 = Placebo MAP (FA); A4 = A/Sing Mono IM, 15 ug (UA)
Treatments Part B: B1 = A/Sing MAP, 15 ug (FA) with biopsy; B2 = Placebo MAP (FA) with biopsy
B3 = A/Sing MAP, 15 ug (FA); B4 = A/Sing MAP, 10 ug (FA); B5 = A/Sing MAP, 5 ug (FA); B6 = A/Sing MAP, 2.5 ug (FA)
B7 = Placebo MAP (FA); B8 = A/Sing MAP, 15 ug (UA); B9 = A/Sing IM, 15 ug, as 2018 Afluria Quad (UA)

Listing 16.2.6.6
Immunogenicity Results (CMI)

Part B

Randomisation Number	Group	Visit	Test	Subset	IL2	TNFa	IFNg	Value
B0026	Group B9	Day 22	MPH	CD4	+	-	-	0.025%
B0026	Group B9	Day 22	MPH	CD4	-	+	+	0.05858%
B0026	Group B9	Day 22	MPH	CD4	-	+	-	0.22%
B0026	Group B9	Day 22	MPH	CD4	-	-	+	0.04216%
B0026	Group B9	Day 1	MPH	CD8	+	+	+	0.00668%
B0026	Group B9	Day 1	MPH	CD8	+	+	-	0.00408%
B0026	Group B9	Day 1	MPH	CD8	+	-	+	0.00543%
B0026	Group B9	Day 1	MPH	CD8	+	-	-	0.03153%
B0026	Group B9	Day 1	MPH	CD8	-	+	+	0.012%
B0026	Group B9	Day 1	MPH	CD8	-	+	-	0.173%
B0026	Group B9	Day 1	MPH	CD8	-	-	+	0.07%
B0026	Group B9	Day 22	MPH	CD8	+	+	+	0.00769%
B0026	Group B9	Day 22	MPH	CD8	+	+	-	0.00259%
B0026	Group B9	Day 22	MPH	CD8	+	-	+	0.00128%
B0026	Group B9	Day 22	MPH	CD8	+	-	-	0.03995%
B0026	Group B9	Day 22	MPH	CD8	-	+	+	0.01795%
B0026	Group B9	Day 22	MPH	CD8	-	+	-	0.116%
B0026	Group B9	Day 22	MPH	CD8	-	-	+	0.008%

Administration Site Key: FA = Forearm; UA= Upper arm
Treatments Part A: A1 = A/Sing MAP, 15 ug (FA); A2 = Afluria Quad IM (UA), 15 ug; A3 = Placebo MAP (FA); A4 = A/Sing Mono IM, 15 ug (UA)
Treatments Part B: B1 = A/Sing MAP, 15 ug (FA) with biopsy; B2 = Placebo MAP (FA) with biopsy
B3 = A/Sing MAP, 15 ug (FA); B4 = A/Sing MAP, 10 ug (FA); B5 = A/Sing MAP, 5 ug (FA); B6 = A/Sing MAP, 2.5 ug (FA)
B7 = Placebo MAP (FA); B8 = A/Sing MAP, 15 ug (UA); B9 = A/Sing IM, 15 ug, as 2018 Afluria Quad (UA)

Listing 16.2.6.6
Immunogenicity Results (CMI)

Part B

Randomisation Number	Group	Visit	Test	Subset	IL2	TNFa	IFNg	Value
B0027	Group B3	Day 1	Peptide	CD4	+	+	+	0.00012%
B0027	Group B3	Day 1	Peptide	CD4	+	+	-	-0.00027%
B0027	Group B3	Day 1	Peptide	CD4	+	-	+	0.001173%
B0027	Group B3	Day 1	Peptide	CD4	+	-	-	0.00017%
B0027	Group B3	Day 1	Peptide	CD4	-	+	+	-0.00111%
B0027	Group B3	Day 1	Peptide	CD4	-	+	-	-0.006%
B0027	Group B3	Day 1	Peptide	CD4	-	-	+	-0.00044%
B0027	Group B3	Day 22	Peptide	CD4	+	+	+	0.0088%
B0027	Group B3	Day 22	Peptide	CD4	+	+	-	0.00565%
B0027	Group B3	Day 22	Peptide	CD4	+	-	+	-0.00055%
B0027	Group B3	Day 22	Peptide	CD4	+	-	-	-0.00448%
B0027	Group B3	Day 22	Peptide	CD4	-	+	+	-0.00055%
B0027	Group B3	Day 22	Peptide	CD4	-	+	-	0.012%
B0027	Group B3	Day 22	Peptide	CD4	-	-	+	-0.0006%
B0027	Group B3	Day 1	Peptide	CD8	+	+	+	0.00636%
B0027	Group B3	Day 1	Peptide	CD8	+	+	-	-0.000826%
B0027	Group B3	Day 1	Peptide	CD8	+	-	+	0%
B0027	Group B3	Day 1	Peptide	CD8	+	-	-	-0.00121%
B0027	Group B3	Day 1	Peptide	CD8	-	+	+	0.00382%

Administration Site Key: FA = Forearm; UA= Upper arm
Treatments Part A: A1 = A/Sing MAP, 15 ug (FA); A2 = Afluria Quad IM (UA), 15 ug; A3 = Placebo MAP (FA); A4 = A/Sing Mono IM, 15 ug (UA)
Treatments Part B: B1 = A/Sing MAP, 15 ug (FA) with biopsy; B2 = Placebo MAP (FA) with biopsy
B3 = A/Sing MAP, 15 ug (FA); B4 = A/Sing MAP, 10 ug (FA); B5 = A/Sing MAP, 5 ug (FA); B6 = A/Sing MAP, 2.5 ug (FA)
B7 = Placebo MAP (FA); B8 = A/Sing MAP, 15 ug (UA); B9 = A/Sing IM, 15 ug, as 2018 Afluria Quad (UA)

Listing 16.2.6.6
Immunogenicity Results (CMI)

Part B

Randomisation Number	Group	Visit	Test	Subset	IL2	TNFa	IFNg	Value
B0027	Group B3	Day 1	Peptide	CD8	-	+	-	-0.014%
B0027	Group B3	Day 1	Peptide	CD8	-	-	+	-0.199%
B0027	Group B3	Day 22	Peptide	CD8	+	+	+	0.00872%
B0027	Group B3	Day 22	Peptide	CD8	+	+	-	0.00174%
B0027	Group B3	Day 22	Peptide	CD8	+	-	+	0%
B0027	Group B3	Day 22	Peptide	CD8	+	-	-	-0.00362%
B0027	Group B3	Day 22	Peptide	CD8	-	+	+	0.003468%
B0027	Group B3	Day 22	Peptide	CD8	-	+	-	0.008%
B0027	Group B3	Day 22	Peptide	CD8	-	-	+	-0.06%
B0027	Group B3	Day 1	MPH	CD4	+	+	+	0.05566%
B0027	Group B3	Day 1	MPH	CD4	+	+	-	0.06509%
B0027	Group B3	Day 1	MPH	CD4	+	-	+	0.003313%
B0027	Group B3	Day 1	MPH	CD4	+	-	-	0.00899%
B0027	Group B3	Day 1	MPH	CD4	-	+	+	0.02089%
B0027	Group B3	Day 1	MPH	CD4	-	+	-	0.185%
B0027	Group B3	Day 1	MPH	CD4	-	-	+	0.0281%
B0027	Group B3	Day 22	MPH	CD4	+	+	+	0.0828%
B0027	Group B3	Day 22	MPH	CD4	+	+	-	0.07765%
B0027	Group B3	Day 22	MPH	CD4	+	-	+	0.01145%

Administration Site Key: FA = Forearm; UA= Upper arm
Treatments Part A: A1 = A/Sing MAP, 15 ug (FA); A2 = Afluria Quad IM (UA), 15 ug; A3 = Placebo MAP (FA); A4 = A/Sing Mono IM, 15 ug (UA)
Treatments Part B: B1 = A/Sing MAP, 15 ug (FA) with biopsy; B2 = Placebo MAP (FA) with biopsy
B3 = A/Sing MAP, 15 ug (FA); B4 = A/Sing MAP, 10 ug (FA); B5 = A/Sing MAP, 5 ug (FA); B6 = A/Sing MAP, 2.5 ug (FA)
B7 = Placebo MAP (FA); B8 = A/Sing MAP, 15 ug (UA); B9 = A/Sing IM, 15 ug, as 2018 Afluria Quad (UA)

Listing 16.2.6.6
Immunogenicity Results (CMI)

Part B

Randomisation Number	Group	Visit	Test	Subset	IL2	TNFa	IFNg	Value
B0027	Group B3	Day 22	MPH	CD4	+	-	-	0.01465%
B0027	Group B3	Day 22	MPH	CD4	-	+	+	0.03645%
B0027	Group B3	Day 22	MPH	CD4	-	+	-	0.22%
B0027	Group B3	Day 22	MPH	CD4	-	-	+	0.0317%
B0027	Group B3	Day 1	MPH	CD8	+	+	+	0.00359%
B0027	Group B3	Day 1	MPH	CD8	+	+	-	0.002764%
B0027	Group B3	Day 1	MPH	CD8	+	-	+	0%
B0027	Group B3	Day 1	MPH	CD8	+	-	-	0.01052%
B0027	Group B3	Day 1	MPH	CD8	-	+	+	0.00808%
B0027	Group B3	Day 1	MPH	CD8	-	+	-	0.025%
B0027	Group B3	Day 1	MPH	CD8	-	-	+	-0.15%
B0027	Group B3	Day 22	MPH	CD8	+	+	+	0.00169%
B0027	Group B3	Day 22	MPH	CD8	+	+	-	0.00422%
B0027	Group B3	Day 22	MPH	CD8	+	-	+	0.00253%
B0027	Group B3	Day 22	MPH	CD8	+	-	-	0.00976%
B0027	Group B3	Day 22	MPH	CD8	-	+	+	0.016108%
B0027	Group B3	Day 22	MPH	CD8	-	+	-	0.077%
B0027	Group B3	Day 22	MPH	CD8	-	-	+	-0.133%

Administration Site Key: FA = Forearm; UA= Upper arm
Treatments Part A: A1 = A/Sing MAP, 15 ug (FA); A2 = Afluria Quad IM (UA), 15 ug; A3 = Placebo MAP (FA); A4 = A/Sing Mono IM, 15 ug (UA)
Treatments Part B: B1 = A/Sing MAP, 15 ug (FA) with biopsy; B2 = Placebo MAP (FA) with biopsy
B3 = A/Sing MAP, 15 ug (FA); B4 = A/Sing MAP, 10 ug (FA); B5 = A/Sing MAP, 5 ug (FA); B6 = A/Sing MAP, 2.5 ug (FA)
B7 = Placebo MAP (FA); B8 = A/Sing MAP, 15 ug (UA); B9 = A/Sing IM, 15 ug, as 2018 Afluria Quad (UA)

Listing 16.2.6.6
Immunogenicity Results (CMI)

Part B

Randomisation Number	Group	Visit	Test	Subset	IL2	TNFa	IFNg	Value
B0030	Group B7	Day 1	Peptide	CD4	+	+	+	0.002213%
B0030	Group B7	Day 1	Peptide	CD4	+	+	-	0.01433%
B0030	Group B7	Day 1	Peptide	CD4	+	-	+	0.000723%
B0030	Group B7	Day 1	Peptide	CD4	+	-	-	0.006%
B0030	Group B7	Day 1	Peptide	CD4	-	+	+	0.00068%
B0030	Group B7	Day 1	Peptide	CD4	-	+	-	0.023%
B0030	Group B7	Day 1	Peptide	CD4	-	-	+	0.0006%
B0030	Group B7	Day 22	Peptide	CD4	+	+	+	0.00277%
B0030	Group B7	Day 22	Peptide	CD4	+	+	-	0.00688%
B0030	Group B7	Day 22	Peptide	CD4	+	-	+	0.000043%
B0030	Group B7	Day 22	Peptide	CD4	+	-	-	0.033%
B0030	Group B7	Day 22	Peptide	CD4	-	+	+	0.00102%
B0030	Group B7	Day 22	Peptide	CD4	-	+	-	0.001%
B0030	Group B7	Day 22	Peptide	CD4	-	-	+	0.008%
B0030	Group B7	Day 1	Peptide	CD8	+	+	+	0.000908%
B0030	Group B7	Day 1	Peptide	CD8	+	+	-	0.000908%
B0030	Group B7	Day 1	Peptide	CD8	+	-	+	0%
B0030	Group B7	Day 1	Peptide	CD8	+	-	-	0.00551%
B0030	Group B7	Day 1	Peptide	CD8	-	+	+	-0.000962%

Administration Site Key: FA = Forearm; UA= Upper arm
Treatments Part A: A1 = A/Sing MAP, 15 ug (FA); A2 = Afluria Quad IM (UA), 15 ug; A3 = Placebo MAP (FA); A4 = A/Sing Mono IM, 15 ug (UA)
Treatments Part B: B1 = A/Sing MAP, 15 ug (FA) with biopsy; B2 = Placebo MAP (FA) with biopsy
B3 = A/Sing MAP, 15 ug (FA); B4 = A/Sing MAP, 10 ug (FA); B5 = A/Sing MAP, 5 ug (FA); B6 = A/Sing MAP, 2.5 ug (FA)
B7 = Placebo MAP (FA); B8 = A/Sing MAP, 15 ug (UA); B9 = A/Sing IM, 15 ug, as 2018 Afluria Quad (UA)

Listing 16.2.6.6
Immunogenicity Results (CMI)

Part B

Randomisation Number	Group	Visit	Test	Subset	IL2	TNFa	IFNg	Value
B0030	Group B7	Day 1	Peptide	CD8	-	+	-	-0.026%
B0030	Group B7	Day 1	Peptide	CD8	-	-	+	0.018%
B0030	Group B7	Day 22	Peptide	CD8	+	+	+	0.00127%
B0030	Group B7	Day 22	Peptide	CD8	+	+	-	-0.00105%
B0030	Group B7	Day 22	Peptide	CD8	+	-	+	0%
B0030	Group B7	Day 22	Peptide	CD8	+	-	-	0.015%
B0030	Group B7	Day 22	Peptide	CD8	-	+	+	-0.00684%
B0030	Group B7	Day 22	Peptide	CD8	-	+	-	0.02%
B0030	Group B7	Day 22	Peptide	CD8	-	-	+	-0.035%
B0030	Group B7	Day 1	MPH	CD4	+	+	+	0.059233%
B0030	Group B7	Day 1	MPH	CD4	+	+	-	0.06233%
B0030	Group B7	Day 1	MPH	CD4	+	-	+	0.002993%
B0030	Group B7	Day 1	MPH	CD4	+	-	-	0.027%
B0030	Group B7	Day 1	MPH	CD4	-	+	+	0.0267%
B0030	Group B7	Day 1	MPH	CD4	-	+	-	0.257%
B0030	Group B7	Day 1	MPH	CD4	-	-	+	0.03463%
B0030	Group B7	Day 22	MPH	CD4	+	+	+	0.09129%
B0030	Group B7	Day 22	MPH	CD4	+	+	-	0.09488%
B0030	Group B7	Day 22	MPH	CD4	+	-	+	0.020147%

Administration Site Key: FA = Forearm; UA= Upper arm
Treatments Part A: A1 = A/Sing MAP, 15 ug (FA); A2 = Afluria Quad IM (UA), 15 ug; A3 = Placebo MAP (FA); A4 = A/Sing Mono IM, 15 ug (UA)
Treatments Part B: B1 = A/Sing MAP, 15 ug (FA) with biopsy; B2 = Placebo MAP (FA) with biopsy
B3 = A/Sing MAP, 15 ug (FA); B4 = A/Sing MAP, 10 ug (FA); B5 = A/Sing MAP, 5 ug (FA); B6 = A/Sing MAP, 2.5 ug (FA)
B7 = Placebo MAP (FA); B8 = A/Sing MAP, 15 ug (UA); B9 = A/Sing IM, 15 ug, as 2018 Afluria Quad (UA)

Listing 16.2.6.6
Immunogenicity Results (CMI)

Part B

Randomisation Number	Group	Visit	Test	Subset	IL2	TNFa	IFNg	Value
B0030	Group B7	Day 22	MPH	CD4	+	-	-	0.054%
B0030	Group B7	Day 22	MPH	CD4	-	+	+	0.04644%
B0030	Group B7	Day 22	MPH	CD4	-	+	-	0.3%
B0030	Group B7	Day 22	MPH	CD4	-	-	+	0.043%
B0030	Group B7	Day 1	MPH	CD8	+	+	+	0.017%
B0030	Group B7	Day 1	MPH	CD8	+	+	-	0.00349%
B0030	Group B7	Day 1	MPH	CD8	+	-	+	0.00175%
B0030	Group B7	Day 1	MPH	CD8	+	-	-	0.01151%
B0030	Group B7	Day 1	MPH	CD8	-	+	+	0.04613%
B0030	Group B7	Day 1	MPH	CD8	-	+	-	0.13%
B0030	Group B7	Day 1	MPH	CD8	-	-	+	0.046%
B0030	Group B7	Day 22	MPH	CD8	+	+	+	0.013%
B0030	Group B7	Day 22	MPH	CD8	+	+	-	0.00317%
B0030	Group B7	Day 22	MPH	CD8	+	-	+	0.00659%
B0030	Group B7	Day 22	MPH	CD8	+	-	-	0.023%
B0030	Group B7	Day 22	MPH	CD8	-	+	+	0.04589%
B0030	Group B7	Day 22	MPH	CD8	-	+	-	0.27%
B0030	Group B7	Day 22	MPH	CD8	-	-	+	-0.047%

Administration Site Key: FA = Forearm; UA= Upper arm
Treatments Part A: A1 = A/Sing MAP, 15 ug (FA); A2 = Afluria Quad IM (UA), 15 ug; A3 = Placebo MAP (FA); A4 = A/Sing Mono IM, 15 ug (UA)
Treatments Part B: B1 = A/Sing MAP, 15 ug (FA) with biopsy; B2 = Placebo MAP (FA) with biopsy
B3 = A/Sing MAP, 15 ug (FA); B4 = A/Sing MAP, 10 ug (FA); B5 = A/Sing MAP, 5 ug (FA); B6 = A/Sing MAP, 2.5 ug (FA)
B7 = Placebo MAP (FA); B8 = A/Sing MAP, 15 ug (UA); B9 = A/Sing IM, 15 ug, as 2018 Afluria Quad (UA)

Listing 16.2.6.6
Immunogenicity Results (CMI)

Part B

Randomisation Number	Group	Visit	Test	Subset	IL2	TNFa	IFNg	Value
B0034	Group B8	Day 1	Peptide	CD4	+	+	+	0.0079%
B0034	Group B8	Day 1	Peptide	CD4	+	+	-	0.007%
B0034	Group B8	Day 1	Peptide	CD4	+	-	+	0.002029%
B0034	Group B8	Day 1	Peptide	CD4	+	-	-	-0.00339%
B0034	Group B8	Day 1	Peptide	CD4	-	+	+	0.00541%
B0034	Group B8	Day 1	Peptide	CD4	-	+	-	0.006%
B0034	Group B8	Day 1	Peptide	CD4	-	-	+	-0.003969%
B0034	Group B8	Day 22	Peptide	CD4	+	+	+	0.01002%
B0034	Group B8	Day 22	Peptide	CD4	+	+	-	0.01509%
B0034	Group B8	Day 22	Peptide	CD4	+	-	+	-0.000659%
B0034	Group B8	Day 22	Peptide	CD4	+	-	-	-0.0012%
B0034	Group B8	Day 22	Peptide	CD4	-	+	+	0.00421%
B0034	Group B8	Day 22	Peptide	CD4	-	+	-	0.015%
B0034	Group B8	Day 22	Peptide	CD4	-	-	+	-0.00132%
B0034	Group B8	Day 1	Peptide	CD8	+	+	+	0.00278%
B0034	Group B8	Day 1	Peptide	CD8	+	+	-	-0.00238%
B0034	Group B8	Day 1	Peptide	CD8	+	-	+	0%
B0034	Group B8	Day 1	Peptide	CD8	+	-	-	0.0004%
B0034	Group B8	Day 1	Peptide	CD8	-	+	+	0.00685%

Administration Site Key: FA = Forearm; UA= Upper arm
Treatments Part A: A1 = A/Sing MAP, 15 ug (FA); A2 = Afluria Quad IM (UA), 15 ug; A3 = Placebo MAP (FA); A4 = A/Sing Mono IM, 15 ug (UA)
Treatments Part B: B1 = A/Sing MAP, 15 ug (FA) with biopsy; B2 = Placebo MAP (FA) with biopsy
B3 = A/Sing MAP, 15 ug (FA); B4 = A/Sing MAP, 10 ug (FA); B5 = A/Sing MAP, 5 ug (FA); B6 = A/Sing MAP, 2.5 ug (FA)
B7 = Placebo MAP (FA); B8 = A/Sing MAP, 15 ug (UA); B9 = A/Sing IM, 15 ug, as 2018 Afluria Quad (UA)

Listing 16.2.6.6
Immunogenicity Results (CMI)

Part B

Randomisation Number	Group	Visit	Test	Subset	IL2	TNFa	IFNg	Value
B0034	Group B8	Day 1	Peptide	CD8	-	+	-	0.026%
B0034	Group B8	Day 1	Peptide	CD8	-	-	+	-2.02%
B0034	Group B8	Day 22	Peptide	CD8	+	+	+	0%
B0034	Group B8	Day 22	Peptide	CD8	+	+	-	0%
B0034	Group B8	Day 22	Peptide	CD8	+	-	+	0%
B0034	Group B8	Day 22	Peptide	CD8	+	-	-	0%
B0034	Group B8	Day 22	Peptide	CD8	-	+	+	0.00422%
B0034	Group B8	Day 22	Peptide	CD8	-	+	-	0.036%
B0034	Group B8	Day 22	Peptide	CD8	-	-	+	-0.41%
B0034	Group B8	Day 1	MPH	CD4	+	+	+	0.0539%
B0034	Group B8	Day 1	MPH	CD4	+	+	-	0.104%
B0034	Group B8	Day 1	MPH	CD4	+	-	+	0.007159%
B0034	Group B8	Day 1	MPH	CD4	+	-	-	0.03%
B0034	Group B8	Day 1	MPH	CD4	-	+	+	0.0459%
B0034	Group B8	Day 1	MPH	CD4	-	+	-	0.296%
B0034	Group B8	Day 1	MPH	CD4	-	-	+	0.08408%
B0034	Group B8	Day 22	MPH	CD4	+	+	+	0.07802%
B0034	Group B8	Day 22	MPH	CD4	+	+	-	0.10209%
B0034	Group B8	Day 22	MPH	CD4	+	-	+	0.017341%

Administration Site Key: FA = Forearm; UA= Upper arm
Treatments Part A: A1 = A/Sing MAP, 15 ug (FA); A2 = Afluria Quad IM (UA), 15 ug; A3 = Placebo MAP (FA); A4 = A/Sing Mono IM, 15 ug (UA)
Treatments Part B: B1 = A/Sing MAP, 15 ug (FA) with biopsy; B2 = Placebo MAP (FA) with biopsy
B3 = A/Sing MAP, 15 ug (FA); B4 = A/Sing MAP, 10 ug (FA); B5 = A/Sing MAP, 5 ug (FA); B6 = A/Sing MAP, 2.5 ug (FA)
B7 = Placebo MAP (FA); B8 = A/Sing MAP, 15 ug (UA); B9 = A/Sing IM, 15 ug, as 2018 Afluria Quad (UA)

Listing 16.2.6.6
Immunogenicity Results (CMI)

Part B

Randomisation Number	Group	Visit	Test	Subset	IL2	TNFa	IFNg	Value
B0034	Group B8	Day 22	MPH	CD4	+	-	-	0.03043%
B0034	Group B8	Day 22	MPH	CD4	-	+	+	0.04468%
B0034	Group B8	Day 22	MPH	CD4	-	+	-	0.311%
B0034	Group B8	Day 22	MPH	CD4	-	-	+	0.04068%
B0034	Group B8	Day 1	MPH	CD8	+	+	+	0.011%
B0034	Group B8	Day 1	MPH	CD8	+	+	-	0.00603%
B0034	Group B8	Day 1	MPH	CD8	+	-	+	0.00841%
B0034	Group B8	Day 1	MPH	CD8	+	-	-	0.02862%
B0034	Group B8	Day 1	MPH	CD8	-	+	+	0.12285%
B0034	Group B8	Day 1	MPH	CD8	-	+	-	0.249%
B0034	Group B8	Day 1	MPH	CD8	-	-	+	0.29%
B0034	Group B8	Day 22	MPH	CD8	+	+	+	0.00743%
B0034	Group B8	Day 22	MPH	CD8	+	+	-	0.00743%
B0034	Group B8	Day 22	MPH	CD8	+	-	+	0%
B0034	Group B8	Day 22	MPH	CD8	+	-	-	0.032%
B0034	Group B8	Day 22	MPH	CD8	-	+	+	0.11322%
B0034	Group B8	Day 22	MPH	CD8	-	+	-	0.268%
B0034	Group B8	Day 22	MPH	CD8	-	-	+	-0.2%

Administration Site Key: FA = Forearm; UA= Upper arm
Treatments Part A: A1 = A/Sing MAP, 15 ug (FA); A2 = Afluria Quad IM (UA), 15 ug; A3 = Placebo MAP (FA); A4 = A/Sing Mono IM, 15 ug (UA)
Treatments Part B: B1 = A/Sing MAP, 15 ug (FA) with biopsy; B2 = Placebo MAP (FA) with biopsy
B3 = A/Sing MAP, 15 ug (FA); B4 = A/Sing MAP, 10 ug (FA); B5 = A/Sing MAP, 5 ug (FA); B6 = A/Sing MAP, 2.5 ug (FA)
B7 = Placebo MAP (FA); B8 = A/Sing MAP, 15 ug (UA); B9 = A/Sing IM, 15 ug, as 2018 Afluria Quad (UA)

Listing 16.2.6.6
Immunogenicity Results (CMI)

Part B

Randomisation Number	Group	Visit	Test	Subset	IL2	TNFa	IFNg	Value
B0035	Group B9	Day 1	Peptide	CD4	+	+	+	0.01213%
B0035	Group B9	Day 1	Peptide	CD4	+	+	-	0.00212%
B0035	Group B9	Day 1	Peptide	CD4	+	-	+	-0.00382%
B0035	Group B9	Day 1	Peptide	CD4	+	-	-	0.00268%
B0035	Group B9	Day 1	Peptide	CD4	-	+	+	0.000813%
B0035	Group B9	Day 1	Peptide	CD4	-	+	-	0.006%
B0035	Group B9	Day 1	Peptide	CD4	-	-	+	0.00335%
B0035	Group B9	Day 22	Peptide	CD4	+	+	+	0.01858%
B0035	Group B9	Day 22	Peptide	CD4	+	+	-	0.01988%
B0035	Group B9	Day 22	Peptide	CD4	+	-	+	0%
B0035	Group B9	Day 22	Peptide	CD4	+	-	-	0.00069%
B0035	Group B9	Day 22	Peptide	CD4	-	+	+	-0.00044%
B0035	Group B9	Day 22	Peptide	CD4	-	+	-	0.033%
B0035	Group B9	Day 22	Peptide	CD4	-	-	+	0.00028%
B0035	Group B9	Day 1	Peptide	CD8	+	+	+	0.014%
B0035	Group B9	Day 1	Peptide	CD8	+	+	-	0%
B0035	Group B9	Day 1	Peptide	CD8	+	-	+	0%
B0035	Group B9	Day 1	Peptide	CD8	+	-	-	0.00964%
B0035	Group B9	Day 1	Peptide	CD8	-	+	+	0.014%

Administration Site Key: FA = Forearm; UA= Upper arm
Treatments Part A: A1 = A/Sing MAP, 15 ug (FA); A2 = Afluria Quad IM (UA), 15 ug; A3 = Placebo MAP (FA); A4 = A/Sing Mono IM, 15 ug (UA)
Treatments Part B: B1 = A/Sing MAP, 15 ug (FA) with biopsy; B2 = Placebo MAP (FA) with biopsy
B3 = A/Sing MAP, 15 ug (FA); B4 = A/Sing MAP, 10 ug (FA); B5 = A/Sing MAP, 5 ug (FA); B6 = A/Sing MAP, 2.5 ug (FA)
B7 = Placebo MAP (FA); B8 = A/Sing MAP, 15 ug (UA); B9 = A/Sing IM, 15 ug, as 2018 Afluria Quad (UA)

Listing 16.2.6.6
Immunogenicity Results (CMI)

Part B

Randomisation Number	Group	Visit	Test	Subset	IL2	TNFa	IFNg	Value
B0035	Group B9	Day 1	Peptide	CD8	-	+	-	0.003%
B0035	Group B9	Day 1	Peptide	CD8	-	-	+	0.68%
B0035	Group B9	Day 22	Peptide	CD8	+	+	+	0.014%
B0035	Group B9	Day 22	Peptide	CD8	+	+	-	0.00478%
B0035	Group B9	Day 22	Peptide	CD8	+	-	+	0%
B0035	Group B9	Day 22	Peptide	CD8	+	-	-	0.00239%
B0035	Group B9	Day 22	Peptide	CD8	-	+	+	0.00557%
B0035	Group B9	Day 22	Peptide	CD8	-	+	-	-0.002%
B0035	Group B9	Day 22	Peptide	CD8	-	-	+	-0.05%
B0035	Group B9	Day 1	MPH	CD4	+	+	+	0.05513%
B0035	Group B9	Day 1	MPH	CD4	+	+	-	0.07136%
B0035	Group B9	Day 1	MPH	CD4	+	-	+	0.00718%
B0035	Group B9	Day 1	MPH	CD4	+	-	-	0.01618%
B0035	Group B9	Day 1	MPH	CD4	-	+	+	0.04%
B0035	Group B9	Day 1	MPH	CD4	-	+	-	0.188%
B0035	Group B9	Day 1	MPH	CD4	-	-	+	0.02622%
B0035	Group B9	Day 22	MPH	CD4	+	+	+	0.09258%
B0035	Group B9	Day 22	MPH	CD4	+	+	-	0.06488%
B0035	Group B9	Day 22	MPH	CD4	+	-	+	0.016%

Administration Site Key: FA = Forearm; UA= Upper arm
Treatments Part A: A1 = A/Sing MAP, 15 ug (FA); A2 = Afluria Quad IM (UA), 15 ug; A3 = Placebo MAP (FA); A4 = A/Sing Mono IM, 15 ug (UA)
Treatments Part B: B1 = A/Sing MAP, 15 ug (FA) with biopsy; B2 = Placebo MAP (FA) with biopsy
B3 = A/Sing MAP, 15 ug (FA); B4 = A/Sing MAP, 10 ug (FA); B5 = A/Sing MAP, 5 ug (FA); B6 = A/Sing MAP, 2.5 ug (FA)
B7 = Placebo MAP (FA); B8 = A/Sing MAP, 15 ug (UA); B9 = A/Sing IM, 15 ug, as 2018 Afluria Quad (UA)

Listing 16.2.6.6
Immunogenicity Results (CMI)

Part B

Randomisation Number	Group	Visit	Test	Subset	IL2	TNFa	IFNg	Value
B0035	Group B9	Day 22	MPH	CD4	+	-	-	0.04144%
B0035	Group B9	Day 22	MPH	CD4	-	+	+	0.03386%
B0035	Group B9	Day 22	MPH	CD4	-	+	-	0.179%
B0035	Group B9	Day 22	MPH	CD4	-	-	+	0.04958%
B0035	Group B9	Day 1	MPH	CD8	+	+	+	0.019%
B0035	Group B9	Day 1	MPH	CD8	+	+	-	0.00729%
B0035	Group B9	Day 1	MPH	CD8	+	-	+	0.00972%
B0035	Group B9	Day 1	MPH	CD8	+	-	-	0.015%
B0035	Group B9	Day 1	MPH	CD8	-	+	+	0.049%
B0035	Group B9	Day 1	MPH	CD8	-	+	-	0.117%
B0035	Group B9	Day 1	MPH	CD8	-	-	+	0.01%
B0035	Group B9	Day 22	MPH	CD8	+	+	+	0.019%
B0035	Group B9	Day 22	MPH	CD8	+	+	-	0%
B0035	Group B9	Day 22	MPH	CD8	+	-	+	0.00751%
B0035	Group B9	Day 22	MPH	CD8	+	-	-	0.017%
B0035	Group B9	Day 22	MPH	CD8	-	+	+	0.03001%
B0035	Group B9	Day 22	MPH	CD8	-	+	-	0.194%
B0035	Group B9	Day 22	MPH	CD8	-	-	+	-0.05%

Administration Site Key: FA = Forearm; UA= Upper arm
Treatments Part A: A1 = A/Sing MAP, 15 ug (FA); A2 = Afluria Quad IM (UA), 15 ug; A3 = Placebo MAP (FA); A4 = A/Sing Mono IM, 15 ug (UA)
Treatments Part B: B1 = A/Sing MAP, 15 ug (FA) with biopsy; B2 = Placebo MAP (FA) with biopsy
B3 = A/Sing MAP, 15 ug (FA); B4 = A/Sing MAP, 10 ug (FA); B5 = A/Sing MAP, 5 ug (FA); B6 = A/Sing MAP, 2.5 ug (FA)
B7 = Placebo MAP (FA); B8 = A/Sing MAP, 15 ug (UA); B9 = A/Sing IM, 15 ug, as 2018 Afluria Quad (UA)

Listing 16.2.6.6
Immunogenicity Results (CMI)

Part B

Randomisation Number	Group	Visit	Test	Subset	IL2	TNFa	IFNg	Value
B0036	Group B7	Day 1	Peptide	CD4	+	+	+	0.00989%
B0036	Group B7	Day 1	Peptide	CD4	+	+	-	0.003%
B0036	Group B7	Day 1	Peptide	CD4	+	-	+	-0.001548%
B0036	Group B7	Day 1	Peptide	CD4	+	-	-	-0.039%
B0036	Group B7	Day 1	Peptide	CD4	-	+	+	0.00222%
B0036	Group B7	Day 1	Peptide	CD4	-	+	-	-0.014%
B0036	Group B7	Day 1	Peptide	CD4	-	-	+	-0.01809%
B0036	Group B7	Day 22	Peptide	CD4	+	+	+	0.002587%
B0036	Group B7	Day 22	Peptide	CD4	+	+	-	-0.00273%
B0036	Group B7	Day 22	Peptide	CD4	+	-	+	-0.000663%
B0036	Group B7	Day 22	Peptide	CD4	+	-	-	-0.12349%
B0036	Group B7	Day 22	Peptide	CD4	-	+	+	-0.001999%
B0036	Group B7	Day 22	Peptide	CD4	-	+	-	-0.019%
B0036	Group B7	Day 22	Peptide	CD4	-	-	+	-0.0344%
B0036	Group B7	Day 1	Peptide	CD8	+	+	+	0%
B0036	Group B7	Day 1	Peptide	CD8	+	+	-	0.00137%
B0036	Group B7	Day 1	Peptide	CD8	+	-	+	0%
B0036	Group B7	Day 1	Peptide	CD8	+	-	-	-0.022%
B0036	Group B7	Day 1	Peptide	CD8	-	+	+	-0.00135%

Administration Site Key: FA = Forearm; UA= Upper arm
Treatments Part A: A1 = A/Sing MAP, 15 ug (FA); A2 = Afluria Quad IM (UA), 15 ug; A3 = Placebo MAP (FA); A4 = A/Sing Mono IM, 15 ug (UA)
Treatments Part B: B1 = A/Sing MAP, 15 ug (FA) with biopsy; B2 = Placebo MAP (FA) with biopsy
B3 = A/Sing MAP, 15 ug (FA); B4 = A/Sing MAP, 10 ug (FA); B5 = A/Sing MAP, 5 ug (FA); B6 = A/Sing MAP, 2.5 ug (FA)
B7 = Placebo MAP (FA); B8 = A/Sing MAP, 15 ug (UA); B9 = A/Sing IM, 15 ug, as 2018 Afluria Quad (UA)

Listing 16.2.6.6
Immunogenicity Results (CMI)

Part B

Randomisation Number	Group	Visit	Test	Subset	IL2	TNFa	IFNg	Value
B0036	Group B7	Day 1	Peptide	CD8	-	+	-	-0.047%
B0036	Group B7	Day 1	Peptide	CD8	-	-	+	0.45%
B0036	Group B7	Day 22	Peptide	CD8	+	+	+	0%
B0036	Group B7	Day 22	Peptide	CD8	+	+	-	1E-5%
B0036	Group B7	Day 22	Peptide	CD8	+	-	+	0%
B0036	Group B7	Day 22	Peptide	CD8	+	-	-	-0.079%
B0036	Group B7	Day 22	Peptide	CD8	-	+	+	-0.01969%
B0036	Group B7	Day 22	Peptide	CD8	-	+	-	-0.131%
B0036	Group B7	Day 22	Peptide	CD8	-	-	+	-0.75%
B0036	Group B7	Day 1	MPH	CD4	+	+	+	0.08489%
B0036	Group B7	Day 1	MPH	CD4	+	+	-	0.084%
B0036	Group B7	Day 1	MPH	CD4	+	-	+	0.01192%
B0036	Group B7	Day 1	MPH	CD4	+	-	-	-0.019%
B0036	Group B7	Day 1	MPH	CD4	-	+	+	0.04637%
B0036	Group B7	Day 1	MPH	CD4	-	+	-	0.233%
B0036	Group B7	Day 1	MPH	CD4	-	-	+	0.026%
B0036	Group B7	Day 22	MPH	CD4	+	+	+	0.109337%
B0036	Group B7	Day 22	MPH	CD4	+	+	-	0.08571%
B0036	Group B7	Day 22	MPH	CD4	+	-	+	0.012337%

Administration Site Key: FA = Forearm; UA= Upper arm
Treatments Part A: A1 = A/Sing MAP, 15 ug (FA); A2 = Afluria Quad IM (UA), 15 ug; A3 = Placebo MAP (FA); A4 = A/Sing Mono IM, 15 ug (UA)
Treatments Part B: B1 = A/Sing MAP, 15 ug (FA) with biopsy; B2 = Placebo MAP (FA) with biopsy
B3 = A/Sing MAP, 15 ug (FA); B4 = A/Sing MAP, 10 ug (FA); B5 = A/Sing MAP, 5 ug (FA); B6 = A/Sing MAP, 2.5 ug (FA)
B7 = Placebo MAP (FA); B8 = A/Sing MAP, 15 ug (UA); B9 = A/Sing IM, 15 ug, as 2018 Afluria Quad (UA)

Listing 16.2.6.6
Immunogenicity Results (CMI)

Part B

Randomisation Number	Group	Visit	Test	Subset	IL2	TNFa	IFNg	Value
B0036	Group B7	Day 22	MPH	CD4	+	-	-	-0.103%
B0036	Group B7	Day 22	MPH	CD4	-	+	+	0.04735%
B0036	Group B7	Day 22	MPH	CD4	-	+	-	0.202%
B0036	Group B7	Day 22	MPH	CD4	-	-	+	-0.005%
B0036	Group B7	Day 1	MPH	CD8	+	+	+	0.032%
B0036	Group B7	Day 1	MPH	CD8	+	+	-	0.012%
B0036	Group B7	Day 1	MPH	CD8	+	-	+	0.01563%
B0036	Group B7	Day 1	MPH	CD8	+	-	-	-0.017%
B0036	Group B7	Day 1	MPH	CD8	-	+	+	0.0609%
B0036	Group B7	Day 1	MPH	CD8	-	+	-	0.05%
B0036	Group B7	Day 1	MPH	CD8	-	-	+	0.2%
B0036	Group B7	Day 22	MPH	CD8	+	+	+	0.041%
B0036	Group B7	Day 22	MPH	CD8	+	+	-	0.01724%
B0036	Group B7	Day 22	MPH	CD8	+	-	+	0.019%
B0036	Group B7	Day 22	MPH	CD8	+	-	-	-0.07218%
B0036	Group B7	Day 22	MPH	CD8	-	+	+	0.03%
B0036	Group B7	Day 22	MPH	CD8	-	+	-	-0.06%
B0036	Group B7	Day 22	MPH	CD8	-	-	+	0.07%

Administration Site Key: FA = Forearm; UA= Upper arm
Treatments Part A: A1 = A/Sing MAP, 15 ug (FA); A2 = Afluria Quad IM (UA), 15 ug; A3 = Placebo MAP (FA); A4 = A/Sing Mono IM, 15 ug (UA)
Treatments Part B: B1 = A/Sing MAP, 15 ug (FA) with biopsy; B2 = Placebo MAP (FA) with biopsy
B3 = A/Sing MAP, 15 ug (FA); B4 = A/Sing MAP, 10 ug (FA); B5 = A/Sing MAP, 5 ug (FA); B6 = A/Sing MAP, 2.5 ug (FA)
B7 = Placebo MAP (FA); B8 = A/Sing MAP, 15 ug (UA); B9 = A/Sing IM, 15 ug, as 2018 Afluria Quad (UA)

Listing 16.2.6.6
Immunogenicity Results (CMI)

Part B

Randomisation Number	Group	Visit	Test	Subset	IL2	TNFa	IFNg	Value
B0038	Group B3	Day 1	Peptide	CD4	+	+	+	0.00114%
B0038	Group B3	Day 1	Peptide	CD4	+	+	-	0.007%
B0038	Group B3	Day 1	Peptide	CD4	+	-	+	8E-6%
B0038	Group B3	Day 1	Peptide	CD4	+	-	-	-0.00151%
B0038	Group B3	Day 1	Peptide	CD4	-	+	+	0.00006%
B0038	Group B3	Day 1	Peptide	CD4	-	+	-	0.006%
B0038	Group B3	Day 1	Peptide	CD4	-	-	+	0.00009%
B0038	Group B3	Day 22	Peptide	CD4	+	+	+	0.00977%
B0038	Group B3	Day 22	Peptide	CD4	+	+	-	0.005%
B0038	Group B3	Day 22	Peptide	CD4	+	-	+	0.00163%
B0038	Group B3	Day 22	Peptide	CD4	+	-	-	-0.00304%
B0038	Group B3	Day 22	Peptide	CD4	-	+	+	0.00514%
B0038	Group B3	Day 22	Peptide	CD4	-	+	-	-0.002%
B0038	Group B3	Day 22	Peptide	CD4	-	-	+	-0.00267%
B0038	Group B3	Day 1	Peptide	CD8	+	+	+	-0.00661%
B0038	Group B3	Day 1	Peptide	CD8	+	+	-	-0.00162%
B0038	Group B3	Day 1	Peptide	CD8	+	-	+	0%
B0038	Group B3	Day 1	Peptide	CD8	+	-	-	-0.00493%
B0038	Group B3	Day 1	Peptide	CD8	-	+	+	-0.00326%

Administration Site Key: FA = Forearm; UA= Upper arm
Treatments Part A: A1 = A/Sing MAP, 15 ug (FA); A2 = Afluria Quad IM (UA), 15 ug; A3 = Placebo MAP (FA); A4 = A/Sing Mono IM, 15 ug (UA)
Treatments Part B: B1 = A/Sing MAP, 15 ug (FA) with biopsy; B2 = Placebo MAP (FA) with biopsy
B3 = A/Sing MAP, 15 ug (FA); B4 = A/Sing MAP, 10 ug (FA); B5 = A/Sing MAP, 5 ug (FA); B6 = A/Sing MAP, 2.5 ug (FA)
B7 = Placebo MAP (FA); B8 = A/Sing MAP, 15 ug (UA); B9 = A/Sing IM, 15 ug, as 2018 Afluria Quad (UA)

Listing 16.2.6.6
Immunogenicity Results (CMI)

Part B

Randomisation Number	Group	Visit	Test	Subset	IL2	TNFa	IFNg	Value
B0038	Group B3	Day 1	Peptide	CD8	-	+	-	0.007%
B0038	Group B3	Day 1	Peptide	CD8	-	-	+	-0.6%
B0038	Group B3	Day 22	Peptide	CD8	+	+	+	0%
B0038	Group B3	Day 22	Peptide	CD8	+	+	-	0%
B0038	Group B3	Day 22	Peptide	CD8	+	-	+	0%
B0038	Group B3	Day 22	Peptide	CD8	+	-	-	0.00022%
B0038	Group B3	Day 22	Peptide	CD8	-	+	+	0.00044%
B0038	Group B3	Day 22	Peptide	CD8	-	+	-	-0.017%
B0038	Group B3	Day 22	Peptide	CD8	-	-	+	-0.66%
B0038	Group B3	Day 1	MPH	CD4	+	+	+	0.06028%
B0038	Group B3	Day 1	MPH	CD4	+	+	-	0.098%
B0038	Group B3	Day 1	MPH	CD4	+	-	+	0.009135%
B0038	Group B3	Day 1	MPH	CD4	+	-	-	0.0297%
B0038	Group B3	Day 1	MPH	CD4	-	+	+	0.0428%
B0038	Group B3	Day 1	MPH	CD4	-	+	-	0.276%
B0038	Group B3	Day 1	MPH	CD4	-	-	+	0.03223%
B0038	Group B3	Day 22	MPH	CD4	+	+	+	0.094%
B0038	Group B3	Day 22	MPH	CD4	+	+	-	0.067%
B0038	Group B3	Day 22	MPH	CD4	+	-	+	0.022%

Administration Site Key: FA = Forearm; UA= Upper arm
Treatments Part A: A1 = A/Sing MAP, 15 ug (FA); A2 = Afluria Quad IM (UA), 15 ug; A3 = Placebo MAP (FA); A4 = A/Sing Mono IM, 15 ug (UA)
Treatments Part B: B1 = A/Sing MAP, 15 ug (FA) with biopsy; B2 = Placebo MAP (FA) with biopsy
B3 = A/Sing MAP, 15 ug (FA); B4 = A/Sing MAP, 10 ug (FA); B5 = A/Sing MAP, 5 ug (FA); B6 = A/Sing MAP, 2.5 ug (FA)
B7 = Placebo MAP (FA); B8 = A/Sing MAP, 15 ug (UA); B9 = A/Sing IM, 15 ug, as 2018 Afluria Quad (UA)

Listing 16.2.6.6
Immunogenicity Results (CMI)

Part B

Randomisation Number	Group	Visit	Test	Subset	IL2	TNFa	IFNg	Value
B0038	Group B3	Day 22	MPH	CD4	+	-	-	0.018%
B0038	Group B3	Day 22	MPH	CD4	-	+	+	0.04681%
B0038	Group B3	Day 22	MPH	CD4	-	+	-	0.22%
B0038	Group B3	Day 22	MPH	CD4	-	-	+	0.03489%
B0038	Group B3	Day 1	MPH	CD8	+	+	+	0.01839%
B0038	Group B3	Day 1	MPH	CD8	+	+	-	-0.0016%
B0038	Group B3	Day 1	MPH	CD8	+	-	+	0.027%
B0038	Group B3	Day 1	MPH	CD8	+	-	-	0.00739%
B0038	Group B3	Day 1	MPH	CD8	-	+	+	0.04239%
B0038	Group B3	Day 1	MPH	CD8	-	+	-	0.102%
B0038	Group B3	Day 1	MPH	CD8	-	-	+	-0.73%
B0038	Group B3	Day 22	MPH	CD8	+	+	+	0.028%
B0038	Group B3	Day 22	MPH	CD8	+	+	-	0.00504%
B0038	Group B3	Day 22	MPH	CD8	+	-	+	0.00504%
B0038	Group B3	Day 22	MPH	CD8	+	-	-	0.02084%
B0038	Group B3	Day 22	MPH	CD8	-	+	+	0.03368%
B0038	Group B3	Day 22	MPH	CD8	-	+	-	0.052%
B0038	Group B3	Day 22	MPH	CD8	-	-	+	-0.02%

Administration Site Key: FA = Forearm; UA= Upper arm
Treatments Part A: A1 = A/Sing MAP, 15 ug (FA); A2 = Afluria Quad IM (UA), 15 ug; A3 = Placebo MAP (FA); A4 = A/Sing Mono IM, 15 ug (UA)
Treatments Part B: B1 = A/Sing MAP, 15 ug (FA) with biopsy; B2 = Placebo MAP (FA) with biopsy
B3 = A/Sing MAP, 15 ug (FA); B4 = A/Sing MAP, 10 ug (FA); B5 = A/Sing MAP, 5 ug (FA); B6 = A/Sing MAP, 2.5 ug (FA)
B7 = Placebo MAP (FA); B8 = A/Sing MAP, 15 ug (UA); B9 = A/Sing IM, 15 ug, as 2018 Afluria Quad (UA)

Listing 16.2.6.6
Immunogenicity Results (CMI)

Part B

Randomisation Number	Group	Visit	Test	Subset	IL2	TNFa	IFNg	Value
B0039	Group B7	Day 1	Peptide	CD4	+	+	+	-0.001344%
B0039	Group B7	Day 1	Peptide	CD4	+	+	-	-0.00061%
B0039	Group B7	Day 1	Peptide	CD4	+	-	+	0%
B0039	Group B7	Day 1	Peptide	CD4	+	-	-	-0.00263%
B0039	Group B7	Day 1	Peptide	CD4	-	+	+	0.000708%
B0039	Group B7	Day 1	Peptide	CD4	-	+	-	-0.005%
B0039	Group B7	Day 1	Peptide	CD4	-	-	+	-0.00131%
B0039	Group B7	Day 22	Peptide	CD4	+	+	+	-0.000593%
B0039	Group B7	Day 22	Peptide	CD4	+	+	-	-0.00357%
B0039	Group B7	Day 22	Peptide	CD4	+	-	+	0%
B0039	Group B7	Day 22	Peptide	CD4	+	-	-	-0.00606%
B0039	Group B7	Day 22	Peptide	CD4	-	+	+	0.00072%
B0039	Group B7	Day 22	Peptide	CD4	-	+	-	0.006%
B0039	Group B7	Day 22	Peptide	CD4	-	-	+	0.0014%
B0039	Group B7	Day 1	Peptide	CD8	+	+	+	-0.00144%
B0039	Group B7	Day 1	Peptide	CD8	+	+	-	0.00007%
B0039	Group B7	Day 1	Peptide	CD8	+	-	+	0%
B0039	Group B7	Day 1	Peptide	CD8	+	-	-	0.00166%
B0039	Group B7	Day 1	Peptide	CD8	-	+	+	0.00302%

Administration Site Key: FA = Forearm; UA= Upper arm
Treatments Part A: A1 = A/Sing MAP, 15 ug (FA); A2 = Afluria Quad IM (UA), 15 ug; A3 = Placebo MAP (FA); A4 = A/Sing Mono IM, 15 ug (UA)
Treatments Part B: B1 = A/Sing MAP, 15 ug (FA) with biopsy; B2 = Placebo MAP (FA) with biopsy
B3 = A/Sing MAP, 15 ug (FA); B4 = A/Sing MAP, 10 ug (FA); B5 = A/Sing MAP, 5 ug (FA); B6 = A/Sing MAP, 2.5 ug (FA)
B7 = Placebo MAP (FA); B8 = A/Sing MAP, 15 ug (UA); B9 = A/Sing IM, 15 ug, as 2018 Afluria Quad (UA)

Listing 16.2.6.6
Immunogenicity Results (CMI)

Part B

Randomisation Number	Group	Visit	Test	Subset	IL2	TNFa	IFNg	Value
B0039	Group B7	Day 1	Peptide	CD8	-	+	-	-0.022%
B0039	Group B7	Day 1	Peptide	CD8	-	-	+	-0.02%
B0039	Group B7	Day 22	Peptide	CD8	+	+	+	0.00148%
B0039	Group B7	Day 22	Peptide	CD8	+	+	-	0%
B0039	Group B7	Day 22	Peptide	CD8	+	-	+	-0.00144%
B0039	Group B7	Day 22	Peptide	CD8	+	-	-	-0.00285%
B0039	Group B7	Day 22	Peptide	CD8	-	+	+	-0.00141%
B0039	Group B7	Day 22	Peptide	CD8	-	+	-	-0.004%
B0039	Group B7	Day 22	Peptide	CD8	-	-	+	0.029%
B0039	Group B7	Day 1	MPH	CD4	+	+	+	0.02896%
B0039	Group B7	Day 1	MPH	CD4	+	+	-	0.08691%
B0039	Group B7	Day 1	MPH	CD4	+	-	+	0.00591%
B0039	Group B7	Day 1	MPH	CD4	+	-	-	0.0175%
B0039	Group B7	Day 1	MPH	CD4	-	+	+	0.013318%
B0039	Group B7	Day 1	MPH	CD4	-	+	-	0.147%
B0039	Group B7	Day 1	MPH	CD4	-	-	+	0.01191%
B0039	Group B7	Day 22	MPH	CD4	+	+	+	0.03075%
B0039	Group B7	Day 22	MPH	CD4	+	+	-	0.07649%
B0039	Group B7	Day 22	MPH	CD4	+	-	+	0.00573%

Administration Site Key: FA = Forearm; UA= Upper arm
Treatments Part A: A1 = A/Sing MAP, 15 ug (FA); A2 = Afluria Quad IM (UA), 15 ug; A3 = Placebo MAP (FA); A4 = A/Sing Mono IM, 15 ug (UA)
Treatments Part B: B1 = A/Sing MAP, 15 ug (FA) with biopsy; B2 = Placebo MAP (FA) with biopsy
B3 = A/Sing MAP, 15 ug (FA); B4 = A/Sing MAP, 10 ug (FA); B5 = A/Sing MAP, 5 ug (FA); B6 = A/Sing MAP, 2.5 ug (FA)
B7 = Placebo MAP (FA); B8 = A/Sing MAP, 15 ug (UA); B9 = A/Sing IM, 15 ug, as 2018 Afluria Quad (UA)

Listing 16.2.6.6
Immunogenicity Results (CMI)

Part B

Randomisation Number	Group	Visit	Test	Subset	IL2	TNFa	IFNg	Value
B0039	Group B7	Day 22	MPH	CD4	+	-	-	0.014%
B0039	Group B7	Day 22	MPH	CD4	-	+	+	0.01875%
B0039	Group B7	Day 22	MPH	CD4	-	+	-	0.174%
B0039	Group B7	Day 22	MPH	CD4	-	-	+	0.00812%
B0039	Group B7	Day 1	MPH	CD8	+	+	+	0.00365%
B0039	Group B7	Day 1	MPH	CD8	+	+	-	0.0062%
B0039	Group B7	Day 1	MPH	CD8	+	-	+	0.00255%
B0039	Group B7	Day 1	MPH	CD8	+	-	-	0.00813%
B0039	Group B7	Day 1	MPH	CD8	-	+	+	0.011%
B0039	Group B7	Day 1	MPH	CD8	-	+	-	0.08%
B0039	Group B7	Day 1	MPH	CD8	-	-	+	0.012%
B0039	Group B7	Day 22	MPH	CD8	+	+	+	0.00897%
B0039	Group B7	Day 22	MPH	CD8	+	+	-	0.00385%
B0039	Group B7	Day 22	MPH	CD8	+	-	+	0.00241%
B0039	Group B7	Day 22	MPH	CD8	+	-	-	0.00567%
B0039	Group B7	Day 22	MPH	CD8	-	+	+	0.01911%
B0039	Group B7	Day 22	MPH	CD8	-	+	-	0.119%
B0039	Group B7	Day 22	MPH	CD8	-	-	+	0.036%

Administration Site Key: FA = Forearm; UA= Upper arm
Treatments Part A: A1 = A/Sing MAP, 15 ug (FA); A2 = Afluria Quad IM (UA), 15 ug; A3 = Placebo MAP (FA); A4 = A/Sing Mono IM, 15 ug (UA)
Treatments Part B: B1 = A/Sing MAP, 15 ug (FA) with biopsy; B2 = Placebo MAP (FA) with biopsy
B3 = A/Sing MAP, 15 ug (FA); B4 = A/Sing MAP, 10 ug (FA); B5 = A/Sing MAP, 5 ug (FA); B6 = A/Sing MAP, 2.5 ug (FA)
B7 = Placebo MAP (FA); B8 = A/Sing MAP, 15 ug (UA); B9 = A/Sing IM, 15 ug, as 2018 Afluria Quad (UA)

Listing 16.2.6.6
Immunogenicity Results (CMI)

Part B

Randomisation Number	Group	Visit	Test	Subset	IL2	TNFa	IFNg	Value
B0040	Group B9	Day 1	Peptide	CD4	+	+	+	0.00612%
B0040	Group B9	Day 1	Peptide	CD4	+	+	-	0.00718%
B0040	Group B9	Day 1	Peptide	CD4	+	-	+	0.00154%
B0040	Group B9	Day 1	Peptide	CD4	+	-	-	-0.00474%
B0040	Group B9	Day 1	Peptide	CD4	-	+	+	0.00154%
B0040	Group B9	Day 1	Peptide	CD4	-	+	-	-0.016%
B0040	Group B9	Day 1	Peptide	CD4	-	-	+	-0.00002%
B0040	Group B9	Day 22	Peptide	CD4	+	+	+	0.02%
B0040	Group B9	Day 22	Peptide	CD4	+	+	-	0.04808%
B0040	Group B9	Day 22	Peptide	CD4	+	-	+	-0.00007%
B0040	Group B9	Day 22	Peptide	CD4	+	-	-	0.0055%
B0040	Group B9	Day 22	Peptide	CD4	-	+	+	0.00144%
B0040	Group B9	Day 22	Peptide	CD4	-	+	-	0.011%
B0040	Group B9	Day 22	Peptide	CD4	-	-	+	-0.00158%
B0040	Group B9	Day 1	Peptide	CD8	+	+	+	0%
B0040	Group B9	Day 1	Peptide	CD8	+	+	-	0%
B0040	Group B9	Day 1	Peptide	CD8	+	-	+	0%
B0040	Group B9	Day 1	Peptide	CD8	+	-	-	-0.00497%
B0040	Group B9	Day 1	Peptide	CD8	-	+	+	-0.0121%

Administration Site Key: FA = Forearm; UA= Upper arm
Treatments Part A: A1 = A/Sing MAP, 15 ug (FA); A2 = Afluria Quad IM (UA), 15 ug; A3 = Placebo MAP (FA); A4 = A/Sing Mono IM, 15 ug (UA)
Treatments Part B: B1 = A/Sing MAP, 15 ug (FA) with biopsy; B2 = Placebo MAP (FA) with biopsy
B3 = A/Sing MAP, 15 ug (FA); B4 = A/Sing MAP, 10 ug (FA); B5 = A/Sing MAP, 5 ug (FA); B6 = A/Sing MAP, 2.5 ug (FA)
B7 = Placebo MAP (FA); B8 = A/Sing MAP, 15 ug (UA); B9 = A/Sing IM, 15 ug, as 2018 Afluria Quad (UA)

Listing 16.2.6.6
Immunogenicity Results (CMI)

Part B

Randomisation Number	Group	Visit	Test	Subset	IL2	TNFa	IFNg	Value
B0040	Group B9	Day 1	Peptide	CD8	-	+	-	-0.038%
B0040	Group B9	Day 1	Peptide	CD8	-	-	+	-0.93%
B0040	Group B9	Day 22	Peptide	CD8	+	+	+	0%
B0040	Group B9	Day 22	Peptide	CD8	+	+	-	0%
B0040	Group B9	Day 22	Peptide	CD8	+	-	+	0%
B0040	Group B9	Day 22	Peptide	CD8	+	-	-	0.00957%
B0040	Group B9	Day 22	Peptide	CD8	-	+	+	-0.00248%
B0040	Group B9	Day 22	Peptide	CD8	-	+	-	-0.028%
B0040	Group B9	Day 22	Peptide	CD8	-	-	+	0.69%
B0040	Group B9	Day 1	MPH	CD4	+	+	+	0.05787%
B0040	Group B9	Day 1	MPH	CD4	+	+	-	0.08518%
B0040	Group B9	Day 1	MPH	CD4	+	-	+	0.00645%
B0040	Group B9	Day 1	MPH	CD4	+	-	-	0.01818%
B0040	Group B9	Day 1	MPH	CD4	-	+	+	0.016%
B0040	Group B9	Day 1	MPH	CD4	-	+	-	0.152%
B0040	Group B9	Day 1	MPH	CD4	-	-	+	0.01644%
B0040	Group B9	Day 22	MPH	CD4	+	+	+	0.12%
B0040	Group B9	Day 22	MPH	CD4	+	+	-	0.10208%
B0040	Group B9	Day 22	MPH	CD4	+	-	+	0.01142%

Administration Site Key: FA = Forearm; UA= Upper arm
Treatments Part A: A1 = A/Sing MAP, 15 ug (FA); A2 = Afluria Quad IM (UA), 15 ug; A3 = Placebo MAP (FA); A4 = A/Sing Mono IM, 15 ug (UA)
Treatments Part B: B1 = A/Sing MAP, 15 ug (FA) with biopsy; B2 = Placebo MAP (FA) with biopsy
B3 = A/Sing MAP, 15 ug (FA); B4 = A/Sing MAP, 10 ug (FA); B5 = A/Sing MAP, 5 ug (FA); B6 = A/Sing MAP, 2.5 ug (FA)
B7 = Placebo MAP (FA); B8 = A/Sing MAP, 15 ug (UA); B9 = A/Sing IM, 15 ug, as 2018 Afluria Quad (UA)

Listing 16.2.6.6
Immunogenicity Results (CMI)

Part B

Randomisation Number	Group	Visit	Test	Subset	IL2	TNFa	IFNg	Value
B0040	Group B9	Day 22	MPH	CD4	+	-	-	0.0395%
B0040	Group B9	Day 22	MPH	CD4	-	+	+	0.02242%
B0040	Group B9	Day 22	MPH	CD4	-	+	-	0.181%
B0040	Group B9	Day 22	MPH	CD4	-	-	+	0.02442%
B0040	Group B9	Day 1	MPH	CD8	+	+	+	0.019%
B0040	Group B9	Day 1	MPH	CD8	+	+	-	0.00236%
B0040	Group B9	Day 1	MPH	CD8	+	-	+	0.00236%
B0040	Group B9	Day 1	MPH	CD8	+	-	-	0.00448%
B0040	Group B9	Day 1	MPH	CD8	-	+	+	0.018%
B0040	Group B9	Day 1	MPH	CD8	-	+	-	0.044%
B0040	Group B9	Day 1	MPH	CD8	-	-	+	-2.06%
B0040	Group B9	Day 22	MPH	CD8	+	+	+	0.017%
B0040	Group B9	Day 22	MPH	CD8	+	+	-	0%
B0040	Group B9	Day 22	MPH	CD8	+	-	+	0.011%
B0040	Group B9	Day 22	MPH	CD8	+	-	-	0.025%
B0040	Group B9	Day 22	MPH	CD8	-	+	+	0.02252%
B0040	Group B9	Day 22	MPH	CD8	-	+	-	0.024%
B0040	Group B9	Day 22	MPH	CD8	-	-	+	0.49%

Administration Site Key: FA = Forearm; UA= Upper arm
Treatments Part A: A1 = A/Sing MAP, 15 ug (FA); A2 = Afluria Quad IM (UA), 15 ug; A3 = Placebo MAP (FA); A4 = A/Sing Mono IM, 15 ug (UA)
Treatments Part B: B1 = A/Sing MAP, 15 ug (FA) with biopsy; B2 = Placebo MAP (FA) with biopsy
B3 = A/Sing MAP, 15 ug (FA); B4 = A/Sing MAP, 10 ug (FA); B5 = A/Sing MAP, 5 ug (FA); B6 = A/Sing MAP, 2.5 ug (FA)
B7 = Placebo MAP (FA); B8 = A/Sing MAP, 15 ug (UA); B9 = A/Sing IM, 15 ug, as 2018 Afluria Quad (UA)

Listing 16.2.6.6
Immunogenicity Results (CMI)

Part B

Randomisation Number	Group	Visit	Test	Subset	IL2	TNFa	IFNg	Value
B0041	Group B3	Day 1	Peptide	CD4	+	+	+	0.011%
B0041	Group B3	Day 1	Peptide	CD4	+	+	-	0.01489%
B0041	Group B3	Day 1	Peptide	CD4	+	-	+	0%
B0041	Group B3	Day 1	Peptide	CD4	+	-	-	0.00394%
B0041	Group B3	Day 1	Peptide	CD4	-	+	+	0%
B0041	Group B3	Day 1	Peptide	CD4	-	+	-	-0.012%
B0041	Group B3	Day 1	Peptide	CD4	-	-	+	0.00013%
B0041	Group B3	Day 22	Peptide	CD4	+	+	+	0.007494%
B0041	Group B3	Day 22	Peptide	CD4	+	+	-	0.01862%
B0041	Group B3	Day 22	Peptide	CD4	+	-	+	0%
B0041	Group B3	Day 22	Peptide	CD4	+	-	-	0.00494%
B0041	Group B3	Day 22	Peptide	CD4	-	+	+	0.00502%
B0041	Group B3	Day 22	Peptide	CD4	-	+	-	0.031%
B0041	Group B3	Day 22	Peptide	CD4	-	-	+	0%
B0041	Group B3	Day 1	Peptide	CD8	+	+	+	0%
B0041	Group B3	Day 1	Peptide	CD8	+	+	-	0%
B0041	Group B3	Day 1	Peptide	CD8	+	-	+	0%
B0041	Group B3	Day 1	Peptide	CD8	+	-	-	-0.00142%
B0041	Group B3	Day 1	Peptide	CD8	-	+	+	-0.0011%

Administration Site Key: FA = Forearm; UA= Upper arm
Treatments Part A: A1 = A/Sing MAP, 15 ug (FA); A2 = Afluria Quad IM (UA), 15 ug; A3 = Placebo MAP (FA); A4 = A/Sing Mono IM, 15 ug (UA)
Treatments Part B: B1 = A/Sing MAP, 15 ug (FA) with biopsy; B2 = Placebo MAP (FA) with biopsy
B3 = A/Sing MAP, 15 ug (FA); B4 = A/Sing MAP, 10 ug (FA); B5 = A/Sing MAP, 5 ug (FA); B6 = A/Sing MAP, 2.5 ug (FA)
B7 = Placebo MAP (FA); B8 = A/Sing MAP, 15 ug (UA); B9 = A/Sing IM, 15 ug, as 2018 Afluria Quad (UA)

Listing 16.2.6.6
Immunogenicity Results (CMI)

Part B

Randomisation Number	Group	Visit	Test	Subset	IL2	TNFa	IFNg	Value
B0041	Group B3	Day 1	Peptide	CD8	-	+	-	-0.005%
B0041	Group B3	Day 1	Peptide	CD8	-	-	+	-1.41%
B0041	Group B3	Day 22	Peptide	CD8	+	+	+	0%
B0041	Group B3	Day 22	Peptide	CD8	+	+	-	-0.00143%
B0041	Group B3	Day 22	Peptide	CD8	+	-	+	0%
B0041	Group B3	Day 22	Peptide	CD8	+	-	-	0.00135%
B0041	Group B3	Day 22	Peptide	CD8	-	+	+	-0.00435%
B0041	Group B3	Day 22	Peptide	CD8	-	+	-	0.016%
B0041	Group B3	Day 22	Peptide	CD8	-	-	+	-0.71%
B0041	Group B3	Day 1	MPH	CD4	+	+	+	0.089%
B0041	Group B3	Day 1	MPH	CD4	+	+	-	0.14789%
B0041	Group B3	Day 1	MPH	CD4	+	-	+	0.038%
B0041	Group B3	Day 1	MPH	CD4	+	-	-	0.08483%
B0041	Group B3	Day 1	MPH	CD4	-	+	+	0.031%
B0041	Group B3	Day 1	MPH	CD4	-	+	-	0.192%
B0041	Group B3	Day 1	MPH	CD4	-	-	+	0.04694%
B0041	Group B3	Day 22	MPH	CD4	+	+	+	0.099124%
B0041	Group B3	Day 22	MPH	CD4	+	+	-	0.13562%
B0041	Group B3	Day 22	MPH	CD4	+	-	+	0.036%

Administration Site Key: FA = Forearm; UA= Upper arm
Treatments Part A: A1 = A/Sing MAP, 15 ug (FA); A2 = Afluria Quad IM (UA), 15 ug; A3 = Placebo MAP (FA); A4 = A/Sing Mono IM, 15 ug (UA)
Treatments Part B: B1 = A/Sing MAP, 15 ug (FA) with biopsy; B2 = Placebo MAP (FA) with biopsy
B3 = A/Sing MAP, 15 ug (FA); B4 = A/Sing MAP, 10 ug (FA); B5 = A/Sing MAP, 5 ug (FA); B6 = A/Sing MAP, 2.5 ug (FA)
B7 = Placebo MAP (FA); B8 = A/Sing MAP, 15 ug (UA); B9 = A/Sing IM, 15 ug, as 2018 Afluria Quad (UA)

Listing 16.2.6.6
Immunogenicity Results (CMI)

Part B

Randomisation Number	Group	Visit	Test	Subset	IL2	TNFa	IFNg	Value
B0041	Group B3	Day 22	MPH	CD4	+	-	-	0.09425%
B0041	Group B3	Day 22	MPH	CD4	-	+	+	0.04%
B0041	Group B3	Day 22	MPH	CD4	-	+	-	0.185%
B0041	Group B3	Day 22	MPH	CD4	-	-	+	0.049%
B0041	Group B3	Day 1	MPH	CD8	+	+	+	0.00703%
B0041	Group B3	Day 1	MPH	CD8	+	+	-	0.00176%
B0041	Group B3	Day 1	MPH	CD8	+	-	+	0.00528%
B0041	Group B3	Day 1	MPH	CD8	+	-	-	0.00884%
B0041	Group B3	Day 1	MPH	CD8	-	+	+	0.02569%
B0041	Group B3	Day 1	MPH	CD8	-	+	-	0.074%
B0041	Group B3	Day 1	MPH	CD8	-	-	+	-1.57%
B0041	Group B3	Day 22	MPH	CD8	+	+	+	0.012%
B0041	Group B3	Day 22	MPH	CD8	+	+	-	0.00586%
B0041	Group B3	Day 22	MPH	CD8	+	-	+	0.00875%
B0041	Group B3	Day 22	MPH	CD8	+	-	-	0.016%
B0041	Group B3	Day 22	MPH	CD8	-	+	+	0.0193%
B0041	Group B3	Day 22	MPH	CD8	-	+	-	0.045%
B0041	Group B3	Day 22	MPH	CD8	-	-	+	-0.39%

Administration Site Key: FA = Forearm; UA= Upper arm
Treatments Part A: A1 = A/Sing MAP, 15 ug (FA); A2 = Afluria Quad IM (UA), 15 ug; A3 = Placebo MAP (FA); A4 = A/Sing Mono IM, 15 ug (UA)
Treatments Part B: B1 = A/Sing MAP, 15 ug (FA) with biopsy; B2 = Placebo MAP (FA) with biopsy
B3 = A/Sing MAP, 15 ug (FA); B4 = A/Sing MAP, 10 ug (FA); B5 = A/Sing MAP, 5 ug (FA); B6 = A/Sing MAP, 2.5 ug (FA)
B7 = Placebo MAP (FA); B8 = A/Sing MAP, 15 ug (UA); B9 = A/Sing IM, 15 ug, as 2018 Afluria Quad (UA)

Listing 16.2.6.6
Immunogenicity Results (CMI)

Part B

Randomisation Number	Group	Visit	Test	Subset	IL2	TNFa	IFNg	Value
B0043	Group B8	Day 1	Peptide	CD4	+	+	+	0.00251%
B0043	Group B8	Day 1	Peptide	CD4	+	+	-	0.00008%
B0043	Group B8	Day 1	Peptide	CD4	+	-	+	0%
B0043	Group B8	Day 1	Peptide	CD4	+	-	-	0.00088%
B0043	Group B8	Day 1	Peptide	CD4	-	+	+	0.000627%
B0043	Group B8	Day 1	Peptide	CD4	-	+	-	0.011%
B0043	Group B8	Day 1	Peptide	CD4	-	-	+	0.00251%
B0043	Group B8	Day 22	Peptide	CD4	+	+	+	0.00974%
B0043	Group B8	Day 22	Peptide	CD4	+	+	-	0.00717%
B0043	Group B8	Day 22	Peptide	CD4	+	-	+	0%
B0043	Group B8	Day 22	Peptide	CD4	+	-	-	-0.00091%
B0043	Group B8	Day 22	Peptide	CD4	-	+	+	0.00199%
B0043	Group B8	Day 22	Peptide	CD4	-	+	-	-0.002%
B0043	Group B8	Day 22	Peptide	CD4	-	-	+	0.00281%
B0043	Group B8	Day 1	Peptide	CD8	+	+	+	0%
B0043	Group B8	Day 1	Peptide	CD8	+	+	-	0%
B0043	Group B8	Day 1	Peptide	CD8	+	-	+	0%
B0043	Group B8	Day 1	Peptide	CD8	+	-	-	0.00149%
B0043	Group B8	Day 1	Peptide	CD8	-	+	+	0.00173%

Administration Site Key: FA = Forearm; UA= Upper arm
Treatments Part A: A1 = A/Sing MAP, 15 ug (FA); A2 = Afluria Quad IM (UA), 15 ug; A3 = Placebo MAP (FA); A4 = A/Sing Mono IM, 15 ug (UA)
Treatments Part B: B1 = A/Sing MAP, 15 ug (FA) with biopsy; B2 = Placebo MAP (FA) with biopsy
B3 = A/Sing MAP, 15 ug (FA); B4 = A/Sing MAP, 10 ug (FA); B5 = A/Sing MAP, 5 ug (FA); B6 = A/Sing MAP, 2.5 ug (FA)
B7 = Placebo MAP (FA); B8 = A/Sing MAP, 15 ug (UA); B9 = A/Sing IM, 15 ug, as 2018 Afluria Quad (UA)

Listing 16.2.6.6
Immunogenicity Results (CMI)

Part B

Randomisation Number	Group	Visit	Test	Subset	IL2	TNFa	IFNg	Value
B0043	Group B8	Day 1	Peptide	CD8	-	+	-	0.015%
B0043	Group B8	Day 1	Peptide	CD8	-	-	+	-0.14%
B0043	Group B8	Day 22	Peptide	CD8	+	+	+	0%
B0043	Group B8	Day 22	Peptide	CD8	+	+	-	0%
B0043	Group B8	Day 22	Peptide	CD8	+	-	+	0%
B0043	Group B8	Day 22	Peptide	CD8	+	-	-	0.00272%
B0043	Group B8	Day 22	Peptide	CD8	-	+	+	-0.0088%
B0043	Group B8	Day 22	Peptide	CD8	-	+	-	-0.014%
B0043	Group B8	Day 22	Peptide	CD8	-	-	+	-0.03%
B0043	Group B8	Day 1	MPH	CD4	+	+	+	0.022%
B0043	Group B8	Day 1	MPH	CD4	+	+	-	0.0502%
B0043	Group B8	Day 1	MPH	CD4	+	-	+	0.00497%
B0043	Group B8	Day 1	MPH	CD4	+	-	-	0.01661%
B0043	Group B8	Day 1	MPH	CD4	-	+	+	0.019%
B0043	Group B8	Day 1	MPH	CD4	-	+	-	0.105%
B0043	Group B8	Day 1	MPH	CD4	-	-	+	0.012%
B0043	Group B8	Day 22	MPH	CD4	+	+	+	0.06974%
B0043	Group B8	Day 22	MPH	CD4	+	+	-	0.07117%
B0043	Group B8	Day 22	MPH	CD4	+	-	+	0.023%

Administration Site Key: FA = Forearm; UA= Upper arm
Treatments Part A: A1 = A/Sing MAP, 15 ug (FA); A2 = Afluria Quad IM (UA), 15 ug; A3 = Placebo MAP (FA); A4 = A/Sing Mono IM, 15 ug (UA)
Treatments Part B: B1 = A/Sing MAP, 15 ug (FA) with biopsy; B2 = Placebo MAP (FA) with biopsy
B3 = A/Sing MAP, 15 ug (FA); B4 = A/Sing MAP, 10 ug (FA); B5 = A/Sing MAP, 5 ug (FA); B6 = A/Sing MAP, 2.5 ug (FA)
B7 = Placebo MAP (FA); B8 = A/Sing MAP, 15 ug (UA); B9 = A/Sing IM, 15 ug, as 2018 Afluria Quad (UA)

Listing 16.2.6.6
Immunogenicity Results (CMI)

Part B

Randomisation Number	Group	Visit	Test	Subset	IL2	TNFa	IFNg	Value
B0043	Group B8	Day 22	MPH	CD4	+	-	-	0.0348%
B0043	Group B8	Day 22	MPH	CD4	-	+	+	0.032%
B0043	Group B8	Day 22	MPH	CD4	-	+	-	0.198%
B0043	Group B8	Day 22	MPH	CD4	-	-	+	0.03085%
B0043	Group B8	Day 1	MPH	CD8	+	+	+	0.00285%
B0043	Group B8	Day 1	MPH	CD8	+	+	-	0%
B0043	Group B8	Day 1	MPH	CD8	+	-	+	0.00142%
B0043	Group B8	Day 1	MPH	CD8	+	-	-	0.0057%
B0043	Group B8	Day 1	MPH	CD8	-	+	+	0.00273%
B0043	Group B8	Day 1	MPH	CD8	-	+	-	0.044%
B0043	Group B8	Day 1	MPH	CD8	-	-	+	-0.36%
B0043	Group B8	Day 22	MPH	CD8	+	+	+	0.00667%
B0043	Group B8	Day 22	MPH	CD8	+	+	-	0.00333%
B0043	Group B8	Day 22	MPH	CD8	+	-	+	0.00167%
B0043	Group B8	Day 22	MPH	CD8	+	-	-	0.00925%
B0043	Group B8	Day 22	MPH	CD8	-	+	+	0.006%
B0043	Group B8	Day 22	MPH	CD8	-	+	-	0.13%
B0043	Group B8	Day 22	MPH	CD8	-	-	+	-0.07%

Administration Site Key: FA = Forearm; UA= Upper arm
Treatments Part A: A1 = A/Sing MAP, 15 ug (FA); A2 = Afluria Quad IM (UA), 15 ug; A3 = Placebo MAP (FA); A4 = A/Sing Mono IM, 15 ug (UA)
Treatments Part B: B1 = A/Sing MAP, 15 ug (FA) with biopsy; B2 = Placebo MAP (FA) with biopsy
B3 = A/Sing MAP, 15 ug (FA); B4 = A/Sing MAP, 10 ug (FA); B5 = A/Sing MAP, 5 ug (FA); B6 = A/Sing MAP, 2.5 ug (FA)
B7 = Placebo MAP (FA); B8 = A/Sing MAP, 15 ug (UA); B9 = A/Sing IM, 15 ug, as 2018 Afluria Quad (UA)

Listing 16.2.6.6
Immunogenicity Results (CMI)

Part B

Randomisation Number	Group	Visit	Test	Subset	IL2	TNFa	IFNg	Value
B0046	Group B7	Day 1	Peptide	CD4	+	+	+	-0.00083%
B0046	Group B7	Day 1	Peptide	CD4	+	+	-	0.00368%
B0046	Group B7	Day 1	Peptide	CD4	+	-	+	-0.00155%
B0046	Group B7	Day 1	Peptide	CD4	+	-	-	-0.00601%
B0046	Group B7	Day 1	Peptide	CD4	-	+	+	-0.00035%
B0046	Group B7	Day 1	Peptide	CD4	-	+	-	-0.011%
B0046	Group B7	Day 1	Peptide	CD4	-	-	+	-0.00294%
B0046	Group B7	Day 22	Peptide	CD4	+	+	+	-0.00286%
B0046	Group B7	Day 22	Peptide	CD4	+	+	-	0.00015%
B0046	Group B7	Day 22	Peptide	CD4	+	-	+	0.000012%
B0046	Group B7	Day 22	Peptide	CD4	+	-	-	0.00012%
B0046	Group B7	Day 22	Peptide	CD4	-	+	+	0.002009%
B0046	Group B7	Day 22	Peptide	CD4	-	+	-	-0.015%
B0046	Group B7	Day 22	Peptide	CD4	-	-	+	-0.00045%
B0046	Group B7	Day 1	Peptide	CD8	+	+	+	0.00184%
B0046	Group B7	Day 1	Peptide	CD8	+	+	-	0%
B0046	Group B7	Day 1	Peptide	CD8	+	-	+	0%
B0046	Group B7	Day 1	Peptide	CD8	+	-	-	-0.00175%
B0046	Group B7	Day 1	Peptide	CD8	-	+	+	-0.00465%

Administration Site Key: FA = Forearm; UA= Upper arm
Treatments Part A: A1 = A/Sing MAP, 15 ug (FA); A2 = Afluria Quad IM (UA), 15 ug; A3 = Placebo MAP (FA); A4 = A/Sing Mono IM, 15 ug (UA)
Treatments Part B: B1 = A/Sing MAP, 15 ug (FA) with biopsy; B2 = Placebo MAP (FA) with biopsy
B3 = A/Sing MAP, 15 ug (FA); B4 = A/Sing MAP, 10 ug (FA); B5 = A/Sing MAP, 5 ug (FA); B6 = A/Sing MAP, 2.5 ug (FA)
B7 = Placebo MAP (FA); B8 = A/Sing MAP, 15 ug (UA); B9 = A/Sing IM, 15 ug, as 2018 Afluria Quad (UA)

Listing 16.2.6.6
Immunogenicity Results (CMI)

Part B

Randomisation Number	Group	Visit	Test	Subset	IL2	TNFa	IFNg	Value
B0046	Group B7	Day 1	Peptide	CD8	-	+	-	-0.012%
B0046	Group B7	Day 1	Peptide	CD8	-	-	+	0.01%
B0046	Group B7	Day 22	Peptide	CD8	+	+	+	0%
B0046	Group B7	Day 22	Peptide	CD8	+	+	-	0.00389%
B0046	Group B7	Day 22	Peptide	CD8	+	-	+	0%
B0046	Group B7	Day 22	Peptide	CD8	+	-	-	0.00004%
B0046	Group B7	Day 22	Peptide	CD8	-	+	+	0.00203%
B0046	Group B7	Day 22	Peptide	CD8	-	+	-	0.011%
B0046	Group B7	Day 22	Peptide	CD8	-	-	+	0%
B0046	Group B7	Day 1	MPH	CD4	+	+	+	0.04034%
B0046	Group B7	Day 1	MPH	CD4	+	+	-	0.10068%
B0046	Group B7	Day 1	MPH	CD4	+	-	+	0.00174%
B0046	Group B7	Day 1	MPH	CD4	+	-	-	0.02016%
B0046	Group B7	Day 1	MPH	CD4	-	+	+	0.02637%
B0046	Group B7	Day 1	MPH	CD4	-	+	-	0.219%
B0046	Group B7	Day 1	MPH	CD4	-	-	+	0.01578%
B0046	Group B7	Day 22	MPH	CD4	+	+	+	0.04215%
B0046	Group B7	Day 22	MPH	CD4	+	+	-	0.09369%
B0046	Group B7	Day 22	MPH	CD4	+	-	+	0.004825%

Administration Site Key: FA = Forearm; UA= Upper arm
Treatments Part A: A1 = A/Sing MAP, 15 ug (FA); A2 = Afluria Quad IM (UA), 15 ug; A3 = Placebo MAP (FA); A4 = A/Sing Mono IM, 15 ug (UA)
Treatments Part B: B1 = A/Sing MAP, 15 ug (FA) with biopsy; B2 = Placebo MAP (FA) with biopsy
B3 = A/Sing MAP, 15 ug (FA); B4 = A/Sing MAP, 10 ug (FA); B5 = A/Sing MAP, 5 ug (FA); B6 = A/Sing MAP, 2.5 ug (FA)
B7 = Placebo MAP (FA); B8 = A/Sing MAP, 15 ug (UA); B9 = A/Sing IM, 15 ug, as 2018 Afluria Quad (UA)

Listing 16.2.6.6
Immunogenicity Results (CMI)

Part B

Randomisation Number	Group	Visit	Test	Subset	IL2	TNFa	IFNg	Value
B0046	Group B7	Day 22	MPH	CD4	+	-	-	0.02066%
B0046	Group B7	Day 22	MPH	CD4	-	+	+	0.037029%
B0046	Group B7	Day 22	MPH	CD4	-	+	-	0.35%
B0046	Group B7	Day 22	MPH	CD4	-	-	+	0.04406%
B0046	Group B7	Day 1	MPH	CD8	+	+	+	0.035%
B0046	Group B7	Day 1	MPH	CD8	+	+	-	0.00871%
B0046	Group B7	Day 1	MPH	CD8	+	-	+	0.014%
B0046	Group B7	Day 1	MPH	CD8	+	-	-	0.01225%
B0046	Group B7	Day 1	MPH	CD8	-	+	+	0.072%
B0046	Group B7	Day 1	MPH	CD8	-	+	-	0.136%
B0046	Group B7	Day 1	MPH	CD8	-	-	+	-0.27%
B0046	Group B7	Day 22	MPH	CD8	+	+	+	0.058%
B0046	Group B7	Day 22	MPH	CD8	+	+	-	0.00698%
B0046	Group B7	Day 22	MPH	CD8	+	-	+	0.016%
B0046	Group B7	Day 22	MPH	CD8	+	-	-	0.0101%
B0046	Group B7	Day 22	MPH	CD8	-	+	+	0.0832%
B0046	Group B7	Day 22	MPH	CD8	-	+	-	0.169%
B0046	Group B7	Day 22	MPH	CD8	-	-	+	0.02%

Administration Site Key: FA = Forearm; UA= Upper arm
Treatments Part A: A1 = A/Sing MAP, 15 ug (FA); A2 = Afluria Quad IM (UA), 15 ug; A3 = Placebo MAP (FA); A4 = A/Sing Mono IM, 15 ug (UA)
Treatments Part B: B1 = A/Sing MAP, 15 ug (FA) with biopsy; B2 = Placebo MAP (FA) with biopsy
B3 = A/Sing MAP, 15 ug (FA); B4 = A/Sing MAP, 10 ug (FA); B5 = A/Sing MAP, 5 ug (FA); B6 = A/Sing MAP, 2.5 ug (FA)
B7 = Placebo MAP (FA); B8 = A/Sing MAP, 15 ug (UA); B9 = A/Sing IM, 15 ug, as 2018 Afluria Quad (UA)

Listing 16.2.6.6
Immunogenicity Results (CMI)

Part B

Randomisation Number	Group	Visit	Test	Subset	IL2	TNFa	IFNg	Value
B0047	Group B9	Day 1	Peptide	CD4	+	+	+	0.00033%
B0047	Group B9	Day 1	Peptide	CD4	+	+	-	0.01428%
B0047	Group B9	Day 1	Peptide	CD4	+	-	+	0.00278%
B0047	Group B9	Day 1	Peptide	CD4	+	-	-	-0.00116%
B0047	Group B9	Day 1	Peptide	CD4	-	+	+	-0.0009%
B0047	Group B9	Day 1	Peptide	CD4	-	+	-	0.026%
B0047	Group B9	Day 1	Peptide	CD4	-	-	+	0.00278%
B0047	Group B9	Day 22	Peptide	CD4	+	+	+	0.00838%
B0047	Group B9	Day 22	Peptide	CD4	+	+	-	0.02179%
B0047	Group B9	Day 22	Peptide	CD4	+	-	+	-0.000656%
B0047	Group B9	Day 22	Peptide	CD4	+	-	-	0.00037%
B0047	Group B9	Day 22	Peptide	CD4	-	+	+	0.00215%
B0047	Group B9	Day 22	Peptide	CD4	-	+	-	0.012%
B0047	Group B9	Day 22	Peptide	CD4	-	-	+	0.00077%
B0047	Group B9	Day 1	Peptide	CD8	+	+	+	0%
B0047	Group B9	Day 1	Peptide	CD8	+	+	-	0%
B0047	Group B9	Day 1	Peptide	CD8	+	-	+	0%
B0047	Group B9	Day 1	Peptide	CD8	+	-	-	-0.00376%
B0047	Group B9	Day 1	Peptide	CD8	-	+	+	0.00161%

Administration Site Key: FA = Forearm; UA= Upper arm
Treatments Part A: A1 = A/Sing MAP, 15 ug (FA); A2 = Afluria Quad IM (UA), 15 ug; A3 = Placebo MAP (FA); A4 = A/Sing Mono IM, 15 ug (UA)
Treatments Part B: B1 = A/Sing MAP, 15 ug (FA) with biopsy; B2 = Placebo MAP (FA) with biopsy
B3 = A/Sing MAP, 15 ug (FA); B4 = A/Sing MAP, 10 ug (FA); B5 = A/Sing MAP, 5 ug (FA); B6 = A/Sing MAP, 2.5 ug (FA)
B7 = Placebo MAP (FA); B8 = A/Sing MAP, 15 ug (UA); B9 = A/Sing IM, 15 ug, as 2018 Afluria Quad (UA)

Listing 16.2.6.6
Immunogenicity Results (CMI)

Part B

Randomisation Number	Group	Visit	Test	Subset	IL2	TNFa	IFNg	Value
B0047	Group B9	Day 1	Peptide	CD8	-	+	-	-0.002%
B0047	Group B9	Day 1	Peptide	CD8	-	-	+	0.086%
B0047	Group B9	Day 22	Peptide	CD8	+	+	+	-0.0017%
B0047	Group B9	Day 22	Peptide	CD8	+	+	-	-0.0017%
B0047	Group B9	Day 22	Peptide	CD8	+	-	+	0%
B0047	Group B9	Day 22	Peptide	CD8	+	-	-	-0.00325%
B0047	Group B9	Day 22	Peptide	CD8	-	+	+	-0.0017%
B0047	Group B9	Day 22	Peptide	CD8	-	+	-	0.023%
B0047	Group B9	Day 22	Peptide	CD8	-	-	+	-0.029%
B0047	Group B9	Day 1	MPH	CD4	+	+	+	0.07577%
B0047	Group B9	Day 1	MPH	CD4	+	+	-	0.07228%
B0047	Group B9	Day 1	MPH	CD4	+	-	+	0.00991%
B0047	Group B9	Day 1	MPH	CD4	+	-	-	0.01928%
B0047	Group B9	Day 1	MPH	CD4	-	+	+	0.02701%
B0047	Group B9	Day 1	MPH	CD4	-	+	-	0.182%
B0047	Group B9	Day 1	MPH	CD4	-	-	+	0.029%
B0047	Group B9	Day 22	MPH	CD4	+	+	+	0.07538%
B0047	Group B9	Day 22	MPH	CD4	+	+	-	0.07879%
B0047	Group B9	Day 22	MPH	CD4	+	-	+	0.026344%

Administration Site Key: FA = Forearm; UA= Upper arm
Treatments Part A: A1 = A/Sing MAP, 15 ug (FA); A2 = Afluria Quad IM (UA), 15 ug; A3 = Placebo MAP (FA); A4 = A/Sing Mono IM, 15 ug (UA)
Treatments Part B: B1 = A/Sing MAP, 15 ug (FA) with biopsy; B2 = Placebo MAP (FA) with biopsy
B3 = A/Sing MAP, 15 ug (FA); B4 = A/Sing MAP, 10 ug (FA); B5 = A/Sing MAP, 5 ug (FA); B6 = A/Sing MAP, 2.5 ug (FA)
B7 = Placebo MAP (FA); B8 = A/Sing MAP, 15 ug (UA); B9 = A/Sing IM, 15 ug, as 2018 Afluria Quad (UA)

Listing 16.2.6.6
Immunogenicity Results (CMI)

Part B

Randomisation Number	Group	Visit	Test	Subset	IL2	TNFa	IFNg	Value
B0047	Group B9	Day 22	MPH	CD4	+	-	-	0.02044%
B0047	Group B9	Day 22	MPH	CD4	-	+	+	0.02969%
B0047	Group B9	Day 22	MPH	CD4	-	+	-	0.173%
B0047	Group B9	Day 22	MPH	CD4	-	-	+	0.04169%
B0047	Group B9	Day 1	MPH	CD8	+	+	+	0.026%
B0047	Group B9	Day 1	MPH	CD8	+	+	-	0.00455%
B0047	Group B9	Day 1	MPH	CD8	+	-	+	0.011%
B0047	Group B9	Day 1	MPH	CD8	+	-	-	0.01624%
B0047	Group B9	Day 1	MPH	CD8	-	+	+	0.026%
B0047	Group B9	Day 1	MPH	CD8	-	+	-	0.13%
B0047	Group B9	Day 1	MPH	CD8	-	-	+	0.065%
B0047	Group B9	Day 22	MPH	CD8	+	+	+	0.0173%
B0047	Group B9	Day 22	MPH	CD8	+	+	-	0.00311%
B0047	Group B9	Day 22	MPH	CD8	+	-	+	0.0016%
B0047	Group B9	Day 22	MPH	CD8	+	-	-	0.01391%
B0047	Group B9	Day 22	MPH	CD8	-	+	+	0.0413%
B0047	Group B9	Day 22	MPH	CD8	-	+	-	0.103%
B0047	Group B9	Day 22	MPH	CD8	-	-	+	0.01%

Administration Site Key: FA = Forearm; UA= Upper arm
Treatments Part A: A1 = A/Sing MAP, 15 ug (FA); A2 = Afluria Quad IM (UA), 15 ug; A3 = Placebo MAP (FA); A4 = A/Sing Mono IM, 15 ug (UA)
Treatments Part B: B1 = A/Sing MAP, 15 ug (FA) with biopsy; B2 = Placebo MAP (FA) with biopsy
B3 = A/Sing MAP, 15 ug (FA); B4 = A/Sing MAP, 10 ug (FA); B5 = A/Sing MAP, 5 ug (FA); B6 = A/Sing MAP, 2.5 ug (FA)
B7 = Placebo MAP (FA); B8 = A/Sing MAP, 15 ug (UA); B9 = A/Sing IM, 15 ug, as 2018 Afluria Quad (UA)

Listing 16.2.6.6
Immunogenicity Results (CMI)

Part B

Randomisation Number	Group	Visit	Test	Subset	IL2	TNFa	IFNg	Value
B0050	Group B8	Day 1	Peptide	CD4	+	+	+	0.003182%
B0050	Group B8	Day 1	Peptide	CD4	+	+	-	0.00069%
B0050	Group B8	Day 1	Peptide	CD4	+	-	+	0.000636%
B0050	Group B8	Day 1	Peptide	CD4	+	-	-	-0.00246%
B0050	Group B8	Day 1	Peptide	CD4	-	+	+	0.00001%
B0050	Group B8	Day 1	Peptide	CD4	-	+	-	-0.001%
B0050	Group B8	Day 1	Peptide	CD4	-	-	+	-0.00059%
B0050	Group B8	Day 22	Peptide	CD4	+	+	+	0.01508%
B0050	Group B8	Day 22	Peptide	CD4	+	+	-	0.02451%
B0050	Group B8	Day 22	Peptide	CD4	+	-	+	0.00131%
B0050	Group B8	Day 22	Peptide	CD4	+	-	-	-0.00052%
B0050	Group B8	Day 22	Peptide	CD4	-	+	+	0.00522%
B0050	Group B8	Day 22	Peptide	CD4	-	+	-	0%
B0050	Group B8	Day 22	Peptide	CD4	-	-	+	-0.0019%
B0050	Group B8	Day 1	Peptide	CD8	+	+	+	0%
B0050	Group B8	Day 1	Peptide	CD8	+	+	-	0%
B0050	Group B8	Day 1	Peptide	CD8	+	-	+	0%
B0050	Group B8	Day 1	Peptide	CD8	+	-	-	0.00281%
B0050	Group B8	Day 1	Peptide	CD8	-	+	+	0.00139%

Administration Site Key: FA = Forearm; UA= Upper arm
Treatments Part A: A1 = A/Sing MAP, 15 ug (FA); A2 = Afluria Quad IM (UA), 15 ug; A3 = Placebo MAP (FA); A4 = A/Sing Mono IM, 15 ug (UA)
Treatments Part B: B1 = A/Sing MAP, 15 ug (FA) with biopsy; B2 = Placebo MAP (FA) with biopsy
B3 = A/Sing MAP, 15 ug (FA); B4 = A/Sing MAP, 10 ug (FA); B5 = A/Sing MAP, 5 ug (FA); B6 = A/Sing MAP, 2.5 ug (FA)
B7 = Placebo MAP (FA); B8 = A/Sing MAP, 15 ug (UA); B9 = A/Sing IM, 15 ug, as 2018 Afluria Quad (UA)

Listing 16.2.6.6
Immunogenicity Results (CMI)

Part B

Randomisation Number	Group	Visit	Test	Subset	IL2	TNFa	IFNg	Value
B0050	Group B8	Day 1	Peptide	CD8	-	+	-	-0.032%
B0050	Group B8	Day 1	Peptide	CD8	-	-	+	0.04%
B0050	Group B8	Day 22	Peptide	CD8	+	+	+	0.00278%
B0050	Group B8	Day 22	Peptide	CD8	+	+	-	0%
B0050	Group B8	Day 22	Peptide	CD8	+	-	+	0%
B0050	Group B8	Day 22	Peptide	CD8	+	-	-	0.00004%
B0050	Group B8	Day 22	Peptide	CD8	-	+	+	0.00417%
B0050	Group B8	Day 22	Peptide	CD8	-	+	-	-0.038%
B0050	Group B8	Day 22	Peptide	CD8	-	-	+	0.47%
B0050	Group B8	Day 1	MPH	CD4	+	+	+	0.054372%
B0050	Group B8	Day 1	MPH	CD4	+	+	-	0.07197%
B0050	Group B8	Day 1	MPH	CD4	+	-	+	0.00652%
B0050	Group B8	Day 1	MPH	CD4	+	-	-	0.01809%
B0050	Group B8	Day 1	MPH	CD4	-	+	+	0.02274%
B0050	Group B8	Day 1	MPH	CD4	-	+	-	0.162%
B0050	Group B8	Day 1	MPH	CD4	-	-	+	0.04423%
B0050	Group B8	Day 22	MPH	CD4	+	+	+	0.08208%
B0050	Group B8	Day 22	MPH	CD4	+	+	-	0.09551%
B0050	Group B8	Day 22	MPH	CD4	+	-	+	0.025%

Administration Site Key: FA = Forearm; UA= Upper arm
Treatments Part A: A1 = A/Sing MAP, 15 ug (FA); A2 = Afluria Quad IM (UA), 15 ug; A3 = Placebo MAP (FA); A4 = A/Sing Mono IM, 15 ug (UA)
Treatments Part B: B1 = A/Sing MAP, 15 ug (FA) with biopsy; B2 = Placebo MAP (FA) with biopsy
B3 = A/Sing MAP, 15 ug (FA); B4 = A/Sing MAP, 10 ug (FA); B5 = A/Sing MAP, 5 ug (FA); B6 = A/Sing MAP, 2.5 ug (FA)
B7 = Placebo MAP (FA); B8 = A/Sing MAP, 15 ug (UA); B9 = A/Sing IM, 15 ug, as 2018 Afluria Quad (UA)

Listing 16.2.6.6
Immunogenicity Results (CMI)

Part B

Randomisation Number	Group	Visit	Test	Subset	IL2	TNFa	IFNg	Value
B0050	Group B8	Day 22	MPH	CD4	+	-	-	0.05395%
B0050	Group B8	Day 22	MPH	CD4	-	+	+	0.035%
B0050	Group B8	Day 22	MPH	CD4	-	+	-	0.108%
B0050	Group B8	Day 22	MPH	CD4	-	-	+	0.05979%
B0050	Group B8	Day 1	MPH	CD8	+	+	+	0.038%
B0050	Group B8	Day 1	MPH	CD8	+	+	-	0.012%
B0050	Group B8	Day 1	MPH	CD8	+	-	+	0.018%
B0050	Group B8	Day 1	MPH	CD8	+	-	-	0.01925%
B0050	Group B8	Day 1	MPH	CD8	-	+	+	0.12%
B0050	Group B8	Day 1	MPH	CD8	-	+	-	0.13%
B0050	Group B8	Day 1	MPH	CD8	-	-	+	0.49%
B0050	Group B8	Day 22	MPH	CD8	+	+	+	0.022%
B0050	Group B8	Day 22	MPH	CD8	+	+	-	0.013%
B0050	Group B8	Day 22	MPH	CD8	+	-	+	0.011%
B0050	Group B8	Day 22	MPH	CD8	+	-	-	0.04065%
B0050	Group B8	Day 22	MPH	CD8	-	+	+	0.059%
B0050	Group B8	Day 22	MPH	CD8	-	+	-	0.1%
B0050	Group B8	Day 22	MPH	CD8	-	-	+	0.05%

Administration Site Key: FA = Forearm; UA= Upper arm
Treatments Part A: A1 = A/Sing MAP, 15 ug (FA); A2 = Afluria Quad IM (UA), 15 ug; A3 = Placebo MAP (FA); A4 = A/Sing Mono IM, 15 ug (UA)
Treatments Part B: B1 = A/Sing MAP, 15 ug (FA) with biopsy; B2 = Placebo MAP (FA) with biopsy
B3 = A/Sing MAP, 15 ug (FA); B4 = A/Sing MAP, 10 ug (FA); B5 = A/Sing MAP, 5 ug (FA); B6 = A/Sing MAP, 2.5 ug (FA)
B7 = Placebo MAP (FA); B8 = A/Sing MAP, 15 ug (UA); B9 = A/Sing IM, 15 ug, as 2018 Afluria Quad (UA)

Listing 16.2.6.6
Immunogenicity Results (CMI)

Part B

Randomisation Number	Group	Visit	Test	Subset	IL2	TNFa	IFNg	Value
B0051	Group B3	Day 1	Peptide	CD4	+	+	+	0.0044%
B0051	Group B3	Day 1	Peptide	CD4	+	+	-	0.0001%
B0051	Group B3	Day 1	Peptide	CD4	+	-	+	0.000631%
B0051	Group B3	Day 1	Peptide	CD4	+	-	-	-0.016%
B0051	Group B3	Day 1	Peptide	CD4	-	+	+	0.000631%
B0051	Group B3	Day 1	Peptide	CD4	-	+	-	0.014%
B0051	Group B3	Day 1	Peptide	CD4	-	-	+	0%
B0051	Group B3	Day 22	Peptide	CD4	+	+	+	0.01155%
B0051	Group B3	Day 22	Peptide	CD4	+	+	-	0.006%
B0051	Group B3	Day 22	Peptide	CD4	+	-	+	0.00123%
B0051	Group B3	Day 22	Peptide	CD4	+	-	-	-0.00717%
B0051	Group B3	Day 22	Peptide	CD4	-	+	+	0.006758%
B0051	Group B3	Day 22	Peptide	CD4	-	+	-	0.003%
B0051	Group B3	Day 22	Peptide	CD4	-	-	+	-0.00182%
B0051	Group B3	Day 1	Peptide	CD8	+	+	+	0%
B0051	Group B3	Day 1	Peptide	CD8	+	+	-	0%
B0051	Group B3	Day 1	Peptide	CD8	+	-	+	-0.00237%
B0051	Group B3	Day 1	Peptide	CD8	+	-	-	-0.00346%
B0051	Group B3	Day 1	Peptide	CD8	-	+	+	0.001%

Administration Site Key: FA = Forearm; UA= Upper arm
Treatments Part A: A1 = A/Sing MAP, 15 ug (FA); A2 = Afluria Quad IM (UA), 15 ug; A3 = Placebo MAP (FA); A4 = A/Sing Mono IM, 15 ug (UA)
Treatments Part B: B1 = A/Sing MAP, 15 ug (FA) with biopsy; B2 = Placebo MAP (FA) with biopsy
B3 = A/Sing MAP, 15 ug (FA); B4 = A/Sing MAP, 10 ug (FA); B5 = A/Sing MAP, 5 ug (FA); B6 = A/Sing MAP, 2.5 ug (FA)
B7 = Placebo MAP (FA); B8 = A/Sing MAP, 15 ug (UA); B9 = A/Sing IM, 15 ug, as 2018 Afluria Quad (UA)

Listing 16.2.6.6
Immunogenicity Results (CMI)

Part B

Randomisation Number	Group	Visit	Test	Subset	IL2	TNFa	IFNg	Value
B0051	Group B3	Day 1	Peptide	CD8	-	+	-	-0.016%
B0051	Group B3	Day 1	Peptide	CD8	-	-	+	4.04%
B0051	Group B3	Day 22	Peptide	CD8	+	+	+	0%
B0051	Group B3	Day 22	Peptide	CD8	+	+	-	0%
B0051	Group B3	Day 22	Peptide	CD8	+	-	+	-0.0011%
B0051	Group B3	Day 22	Peptide	CD8	+	-	-	1E-5%
B0051	Group B3	Day 22	Peptide	CD8	-	+	+	-0.007%
B0051	Group B3	Day 22	Peptide	CD8	-	+	-	-0.024%
B0051	Group B3	Day 22	Peptide	CD8	-	-	+	-1.98%
B0051	Group B3	Day 1	MPH	CD4	+	+	+	0.09295%
B0051	Group B3	Day 1	MPH	CD4	+	+	-	0.07213%
B0051	Group B3	Day 1	MPH	CD4	+	-	+	0.016391%
B0051	Group B3	Day 1	MPH	CD4	+	-	-	0.028%
B0051	Group B3	Day 1	MPH	CD4	-	+	+	0.057391%
B0051	Group B3	Day 1	MPH	CD4	-	+	-	0.343%
B0051	Group B3	Day 1	MPH	CD4	-	-	+	0.069%
B0051	Group B3	Day 22	MPH	CD4	+	+	+	0.08955%
B0051	Group B3	Day 22	MPH	CD4	+	+	-	0.082%
B0051	Group B3	Day 22	MPH	CD4	+	-	+	0.023%

Administration Site Key: FA = Forearm; UA= Upper arm
Treatments Part A: A1 = A/Sing MAP, 15 ug (FA); A2 = Afluria Quad IM (UA), 15 ug; A3 = Placebo MAP (FA); A4 = A/Sing Mono IM, 15 ug (UA)
Treatments Part B: B1 = A/Sing MAP, 15 ug (FA) with biopsy; B2 = Placebo MAP (FA) with biopsy
B3 = A/Sing MAP, 15 ug (FA); B4 = A/Sing MAP, 10 ug (FA); B5 = A/Sing MAP, 5 ug (FA); B6 = A/Sing MAP, 2.5 ug (FA)
B7 = Placebo MAP (FA); B8 = A/Sing MAP, 15 ug (UA); B9 = A/Sing IM, 15 ug, as 2018 Afluria Quad (UA)

Listing 16.2.6.6
Immunogenicity Results (CMI)

Part B

Randomisation Number	Group	Visit	Test	Subset	IL2	TNFa	IFNg	Value
B0051	Group B3	Day 22	MPH	CD4	+	-	-	0.053%
B0051	Group B3	Day 22	MPH	CD4	-	+	+	0.062388%
B0051	Group B3	Day 22	MPH	CD4	-	+	-	0.239%
B0051	Group B3	Day 22	MPH	CD4	-	-	+	0.08788%
B0051	Group B3	Day 1	MPH	CD8	+	+	+	0.011%
B0051	Group B3	Day 1	MPH	CD8	+	+	-	0.0014%
B0051	Group B3	Day 1	MPH	CD8	+	-	+	0.00603%
B0051	Group B3	Day 1	MPH	CD8	+	-	-	-0.00271%
B0051	Group B3	Day 1	MPH	CD8	-	+	+	0.01%
B0051	Group B3	Day 1	MPH	CD8	-	+	-	0.072%
B0051	Group B3	Day 1	MPH	CD8	-	-	+	-2.26%
B0051	Group B3	Day 22	MPH	CD8	+	+	+	0.00481%
B0051	Group B3	Day 22	MPH	CD8	+	+	-	0%
B0051	Group B3	Day 22	MPH	CD8	+	-	+	0.00611%
B0051	Group B3	Day 22	MPH	CD8	+	-	-	0.01261%
B0051	Group B3	Day 22	MPH	CD8	-	+	+	0.003%
B0051	Group B3	Day 22	MPH	CD8	-	+	-	0.092%
B0051	Group B3	Day 22	MPH	CD8	-	-	+	-2.35%

Administration Site Key: FA = Forearm; UA= Upper arm
Treatments Part A: A1 = A/Sing MAP, 15 ug (FA); A2 = Afluria Quad IM (UA), 15 ug; A3 = Placebo MAP (FA); A4 = A/Sing Mono IM, 15 ug (UA)
Treatments Part B: B1 = A/Sing MAP, 15 ug (FA) with biopsy; B2 = Placebo MAP (FA) with biopsy
B3 = A/Sing MAP, 15 ug (FA); B4 = A/Sing MAP, 10 ug (FA); B5 = A/Sing MAP, 5 ug (FA); B6 = A/Sing MAP, 2.5 ug (FA)
B7 = Placebo MAP (FA); B8 = A/Sing MAP, 15 ug (UA); B9 = A/Sing IM, 15 ug, as 2018 Afluria Quad (UA)

Listing 16.2.6.6
Immunogenicity Results (CMI)

Part B

Randomisation Number	Group	Visit	Test	Subset	IL2	TNFa	IFNg	Value
B0055	Group B8	Day 1	Peptide	CD4	+	+	+	0.00474%
B0055	Group B8	Day 1	Peptide	CD4	+	+	-	0.00878%
B0055	Group B8	Day 1	Peptide	CD4	+	-	+	-0.001165%
B0055	Group B8	Day 1	Peptide	CD4	+	-	-	-0.009%
B0055	Group B8	Day 1	Peptide	CD4	-	+	+	0.00194%
B0055	Group B8	Day 1	Peptide	CD4	-	+	-	-0.003%
B0055	Group B8	Day 1	Peptide	CD4	-	-	+	0.00371%
B0055	Group B8	Day 22	Peptide	CD4	+	+	+	0.01235%
B0055	Group B8	Day 22	Peptide	CD4	+	+	-	0.016%
B0055	Group B8	Day 22	Peptide	CD4	+	-	+	0.00272%
B0055	Group B8	Day 22	Peptide	CD4	+	-	-	-0.015%
B0055	Group B8	Day 22	Peptide	CD4	-	+	+	-0.00195%
B0055	Group B8	Day 22	Peptide	CD4	-	+	-	-0.001%
B0055	Group B8	Day 22	Peptide	CD4	-	-	+	-0.00381%
B0055	Group B8	Day 1	Peptide	CD8	+	+	+	0%
B0055	Group B8	Day 1	Peptide	CD8	+	+	-	0%
B0055	Group B8	Day 1	Peptide	CD8	+	-	+	-0.00117%
B0055	Group B8	Day 1	Peptide	CD8	+	-	-	-0.0056%
B0055	Group B8	Day 1	Peptide	CD8	-	+	+	-0.001%

Administration Site Key: FA = Forearm; UA= Upper arm
Treatments Part A: A1 = A/Sing MAP, 15 ug (FA); A2 = Afluria Quad IM (UA), 15 ug; A3 = Placebo MAP (FA); A4 = A/Sing Mono IM, 15 ug (UA)
Treatments Part B: B1 = A/Sing MAP, 15 ug (FA) with biopsy; B2 = Placebo MAP (FA) with biopsy
B3 = A/Sing MAP, 15 ug (FA); B4 = A/Sing MAP, 10 ug (FA); B5 = A/Sing MAP, 5 ug (FA); B6 = A/Sing MAP, 2.5 ug (FA)
B7 = Placebo MAP (FA); B8 = A/Sing MAP, 15 ug (UA); B9 = A/Sing IM, 15 ug, as 2018 Afluria Quad (UA)

Listing 16.2.6.6
Immunogenicity Results (CMI)

Part B

Randomisation Number	Group	Visit	Test	Subset	IL2	TNFa	IFNg	Value
B0055	Group B8	Day 1	Peptide	CD8	-	+	-	-0.006%
B0055	Group B8	Day 1	Peptide	CD8	-	-	+	-1.46%
B0055	Group B8	Day 22	Peptide	CD8	+	+	+	0%
B0055	Group B8	Day 22	Peptide	CD8	+	+	-	-0.00096%
B0055	Group B8	Day 22	Peptide	CD8	+	-	+	0%
B0055	Group B8	Day 22	Peptide	CD8	+	-	-	-0.00022%
B0055	Group B8	Day 22	Peptide	CD8	-	+	+	-0.02362%
B0055	Group B8	Day 22	Peptide	CD8	-	+	-	-0.001%
B0055	Group B8	Day 22	Peptide	CD8	-	-	+	-3.33%
B0055	Group B8	Day 1	MPH	CD4	+	+	+	0.04789%
B0055	Group B8	Day 1	MPH	CD4	+	+	-	0.09178%
B0055	Group B8	Day 1	MPH	CD4	+	-	+	0.00621%
B0055	Group B8	Day 1	MPH	CD4	+	-	-	0.008%
B0055	Group B8	Day 1	MPH	CD4	-	+	+	0.01586%
B0055	Group B8	Day 1	MPH	CD4	-	+	-	0.173%
B0055	Group B8	Day 1	MPH	CD4	-	-	+	0.01386%
B0055	Group B8	Day 22	MPH	CD4	+	+	+	0.07235%
B0055	Group B8	Day 22	MPH	CD4	+	+	-	0.089%
B0055	Group B8	Day 22	MPH	CD4	+	-	+	0.03307%

Administration Site Key: FA = Forearm; UA= Upper arm
Treatments Part A: A1 = A/Sing MAP, 15 ug (FA); A2 = Afluria Quad IM (UA), 15 ug; A3 = Placebo MAP (FA); A4 = A/Sing Mono IM, 15 ug (UA)
Treatments Part B: B1 = A/Sing MAP, 15 ug (FA) with biopsy; B2 = Placebo MAP (FA) with biopsy
B3 = A/Sing MAP, 15 ug (FA); B4 = A/Sing MAP, 10 ug (FA); B5 = A/Sing MAP, 5 ug (FA); B6 = A/Sing MAP, 2.5 ug (FA)
B7 = Placebo MAP (FA); B8 = A/Sing MAP, 15 ug (UA); B9 = A/Sing IM, 15 ug, as 2018 Afluria Quad (UA)

Listing 16.2.6.6
Immunogenicity Results (CMI)

Part B

Randomisation Number	Group	Visit	Test	Subset	IL2	TNFa	IFNg	Value
B0055	Group B8	Day 22	MPH	CD4	+	-	-	0.03%
B0055	Group B8	Day 22	MPH	CD4	-	+	+	0.02449%
B0055	Group B8	Day 22	MPH	CD4	-	+	-	0.224%
B0055	Group B8	Day 22	MPH	CD4	-	-	+	0.02963%
B0055	Group B8	Day 1	MPH	CD8	+	+	+	0%
B0055	Group B8	Day 1	MPH	CD8	+	+	-	0.00602%
B0055	Group B8	Day 1	MPH	CD8	+	-	+	0.00084%
B0055	Group B8	Day 1	MPH	CD8	+	-	-	0.014%
B0055	Group B8	Day 1	MPH	CD8	-	+	+	0.004%
B0055	Group B8	Day 1	MPH	CD8	-	+	-	0.092%
B0055	Group B8	Day 1	MPH	CD8	-	-	+	-2.61%
B0055	Group B8	Day 22	MPH	CD8	+	+	+	0.00334%
B0055	Group B8	Day 22	MPH	CD8	+	+	-	0.01504%
B0055	Group B8	Day 22	MPH	CD8	+	-	+	0.00167%
B0055	Group B8	Day 22	MPH	CD8	+	-	-	0.07324%
B0055	Group B8	Day 22	MPH	CD8	-	+	+	0.006%
B0055	Group B8	Day 22	MPH	CD8	-	+	-	0.237%
B0055	Group B8	Day 22	MPH	CD8	-	-	+	-3.88%

Administration Site Key: FA = Forearm; UA= Upper arm
Treatments Part A: A1 = A/Sing MAP, 15 ug (FA); A2 = Afluria Quad IM (UA), 15 ug; A3 = Placebo MAP (FA); A4 = A/Sing Mono IM, 15 ug (UA)
Treatments Part B: B1 = A/Sing MAP, 15 ug (FA) with biopsy; B2 = Placebo MAP (FA) with biopsy
B3 = A/Sing MAP, 15 ug (FA); B4 = A/Sing MAP, 10 ug (FA); B5 = A/Sing MAP, 5 ug (FA); B6 = A/Sing MAP, 2.5 ug (FA)
B7 = Placebo MAP (FA); B8 = A/Sing MAP, 15 ug (UA); B9 = A/Sing IM, 15 ug, as 2018 Afluria Quad (UA)

Listing 16.2.6.6
Immunogenicity Results (CMI)

Part B

Randomisation Number	Group	Visit	Test	Subset	IL2	TNFa	IFNg	Value
B0056	Group B3	Day 1	Peptide	CD4	+	+	+	0.00449%
B0056	Group B3	Day 1	Peptide	CD4	+	+	-	0.01923%
B0056	Group B3	Day 1	Peptide	CD4	+	-	+	-0.001014%
B0056	Group B3	Day 1	Peptide	CD4	+	-	-	0.00123%
B0056	Group B3	Day 1	Peptide	CD4	-	+	+	-0.00011%
B0056	Group B3	Day 1	Peptide	CD4	-	+	-	0.031%
B0056	Group B3	Day 1	Peptide	CD4	-	-	+	-0.00304%
B0056	Group B3	Day 22	Peptide	CD4	+	+	+	0.00889%
B0056	Group B3	Day 22	Peptide	CD4	+	+	-	0.01%
B0056	Group B3	Day 22	Peptide	CD4	+	-	+	0.00169%
B0056	Group B3	Day 22	Peptide	CD4	+	-	-	0.00265%
B0056	Group B3	Day 22	Peptide	CD4	-	+	+	0.00894%
B0056	Group B3	Day 22	Peptide	CD4	-	+	-	0.113%
B0056	Group B3	Day 22	Peptide	CD4	-	-	+	0.00159%
B0056	Group B3	Day 1	Peptide	CD8	+	+	+	0%
B0056	Group B3	Day 1	Peptide	CD8	+	+	-	0.00272%
B0056	Group B3	Day 1	Peptide	CD8	+	-	+	0%
B0056	Group B3	Day 1	Peptide	CD8	+	-	-	-0.00015%
B0056	Group B3	Day 1	Peptide	CD8	-	+	+	-0.00173%

Administration Site Key: FA = Forearm; UA= Upper arm
Treatments Part A: A1 = A/Sing MAP, 15 ug (FA); A2 = Afluria Quad IM (UA), 15 ug; A3 = Placebo MAP (FA); A4 = A/Sing Mono IM, 15 ug (UA)
Treatments Part B: B1 = A/Sing MAP, 15 ug (FA) with biopsy; B2 = Placebo MAP (FA) with biopsy
B3 = A/Sing MAP, 15 ug (FA); B4 = A/Sing MAP, 10 ug (FA); B5 = A/Sing MAP, 5 ug (FA); B6 = A/Sing MAP, 2.5 ug (FA)
B7 = Placebo MAP (FA); B8 = A/Sing MAP, 15 ug (UA); B9 = A/Sing IM, 15 ug, as 2018 Afluria Quad (UA)

Listing 16.2.6.6
Immunogenicity Results (CMI)

Part B

Randomisation Number	Group	Visit	Test	Subset	IL2	TNFa	IFNg	Value
B0056	Group B3	Day 1	Peptide	CD8	-	+	-	-0.013%
B0056	Group B3	Day 1	Peptide	CD8	-	-	+	0.97%
B0056	Group B3	Day 22	Peptide	CD8	+	+	+	0.00152%
B0056	Group B3	Day 22	Peptide	CD8	+	+	-	0%
B0056	Group B3	Day 22	Peptide	CD8	+	-	+	0.00152%
B0056	Group B3	Day 22	Peptide	CD8	+	-	-	-0.00222%
B0056	Group B3	Day 22	Peptide	CD8	-	+	+	0.11626%
B0056	Group B3	Day 22	Peptide	CD8	-	+	-	-0.006%
B0056	Group B3	Day 22	Peptide	CD8	-	-	+	0.45%
B0056	Group B3	Day 1	MPH	CD4	+	+	+	0.10513%
B0056	Group B3	Day 1	MPH	CD4	+	+	-	0.07023%
B0056	Group B3	Day 1	MPH	CD4	+	-	+	0.01405%
B0056	Group B3	Day 1	MPH	CD4	+	-	-	0.02423%
B0056	Group B3	Day 1	MPH	CD4	-	+	+	0.08908%
B0056	Group B3	Day 1	MPH	CD4	-	+	-	0.31%
B0056	Group B3	Day 1	MPH	CD4	-	-	+	0.06715%
B0056	Group B3	Day 22	MPH	CD4	+	+	+	0.12789%
B0056	Group B3	Day 22	MPH	CD4	+	+	-	0.066%
B0056	Group B3	Day 22	MPH	CD4	+	-	+	0.05%

Administration Site Key: FA = Forearm; UA= Upper arm
Treatments Part A: A1 = A/Sing MAP, 15 ug (FA); A2 = Afluria Quad IM (UA), 15 ug; A3 = Placebo MAP (FA); A4 = A/Sing Mono IM, 15 ug (UA)
Treatments Part B: B1 = A/Sing MAP, 15 ug (FA) with biopsy; B2 = Placebo MAP (FA) with biopsy
B3 = A/Sing MAP, 15 ug (FA); B4 = A/Sing MAP, 10 ug (FA); B5 = A/Sing MAP, 5 ug (FA); B6 = A/Sing MAP, 2.5 ug (FA)
B7 = Placebo MAP (FA); B8 = A/Sing MAP, 15 ug (UA); B9 = A/Sing IM, 15 ug, as 2018 Afluria Quad (UA)

Listing 16.2.6.6
Immunogenicity Results (CMI)

Part B

Randomisation Number	Group	Visit	Test	Subset	IL2	TNFa	IFNg	Value
B0056	Group B3	Day 22	MPH	CD4	+	-	-	0.04719%
B0056	Group B3	Day 22	MPH	CD4	-	+	+	0.09894%
B0056	Group B3	Day 22	MPH	CD4	-	+	-	0.453%
B0056	Group B3	Day 22	MPH	CD4	-	-	+	0.13736%
B0056	Group B3	Day 1	MPH	CD8	+	+	+	0.014%
B0056	Group B3	Day 1	MPH	CD8	+	+	-	0.0041%
B0056	Group B3	Day 1	MPH	CD8	+	-	+	0.00137%
B0056	Group B3	Day 1	MPH	CD8	+	-	-	0.01613%
B0056	Group B3	Day 1	MPH	CD8	-	+	+	0.11283%
B0056	Group B3	Day 1	MPH	CD8	-	+	-	0.073%
B0056	Group B3	Day 1	MPH	CD8	-	-	+	2.53%
B0056	Group B3	Day 22	MPH	CD8	+	+	+	0.018%
B0056	Group B3	Day 22	MPH	CD8	+	+	-	0.0033%
B0056	Group B3	Day 22	MPH	CD8	+	-	+	0.00549%
B0056	Group B3	Day 22	MPH	CD8	+	-	-	0.01826%
B0056	Group B3	Day 22	MPH	CD8	-	+	+	0.23626%
B0056	Group B3	Day 22	MPH	CD8	-	+	-	0.484%
B0056	Group B3	Day 22	MPH	CD8	-	-	+	0.07%

Administration Site Key: FA = Forearm; UA= Upper arm
Treatments Part A: A1 = A/Sing MAP, 15 ug (FA); A2 = Afluria Quad IM (UA), 15 ug; A3 = Placebo MAP (FA); A4 = A/Sing Mono IM, 15 ug (UA)
Treatments Part B: B1 = A/Sing MAP, 15 ug (FA) with biopsy; B2 = Placebo MAP (FA) with biopsy
B3 = A/Sing MAP, 15 ug (FA); B4 = A/Sing MAP, 10 ug (FA); B5 = A/Sing MAP, 5 ug (FA); B6 = A/Sing MAP, 2.5 ug (FA)
B7 = Placebo MAP (FA); B8 = A/Sing MAP, 15 ug (UA); B9 = A/Sing IM, 15 ug, as 2018 Afluria Quad (UA)

Listing 16.2.6.6
Immunogenicity Results (CMI)

Part B

Randomisation Number	Group	Visit	Test	Subset	IL2	TNFa	IFNg	Value
B0058	Group B9	Day 1	Peptide	CD4	+	+	+	0.01008%
B0058	Group B9	Day 1	Peptide	CD4	+	+	-	0.00564%
B0058	Group B9	Day 1	Peptide	CD4	+	-	+	0.00016%
B0058	Group B9	Day 1	Peptide	CD4	+	-	-	0.00516%
B0058	Group B9	Day 1	Peptide	CD4	-	+	+	0.005227%
B0058	Group B9	Day 1	Peptide	CD4	-	+	-	0.04%
B0058	Group B9	Day 1	Peptide	CD4	-	-	+	-0.00114%
B0058	Group B9	Day 22	Peptide	CD4	+	+	+	0.03808%
B0058	Group B9	Day 22	Peptide	CD4	+	+	-	0.02829%
B0058	Group B9	Day 22	Peptide	CD4	+	-	+	0.00102%
B0058	Group B9	Day 22	Peptide	CD4	+	-	-	-0.00693%
B0058	Group B9	Day 22	Peptide	CD4	-	+	+	0.00526%
B0058	Group B9	Day 22	Peptide	CD4	-	+	-	0.029%
B0058	Group B9	Day 22	Peptide	CD4	-	-	+	0.00113%
B0058	Group B9	Day 1	Peptide	CD8	+	+	+	0%
B0058	Group B9	Day 1	Peptide	CD8	+	+	-	-0.00163%
B0058	Group B9	Day 1	Peptide	CD8	+	-	+	0%
B0058	Group B9	Day 1	Peptide	CD8	+	-	-	0.00545%
B0058	Group B9	Day 1	Peptide	CD8	-	+	+	0.00545%

Administration Site Key: FA = Forearm; UA= Upper arm
Treatments Part A: A1 = A/Sing MAP, 15 ug (FA); A2 = Afluria Quad IM (UA), 15 ug; A3 = Placebo MAP (FA); A4 = A/Sing Mono IM, 15 ug (UA)
Treatments Part B: B1 = A/Sing MAP, 15 ug (FA) with biopsy; B2 = Placebo MAP (FA) with biopsy
B3 = A/Sing MAP, 15 ug (FA); B4 = A/Sing MAP, 10 ug (FA); B5 = A/Sing MAP, 5 ug (FA); B6 = A/Sing MAP, 2.5 ug (FA)
B7 = Placebo MAP (FA); B8 = A/Sing MAP, 15 ug (UA); B9 = A/Sing IM, 15 ug, as 2018 Afluria Quad (UA)

Listing 16.2.6.6
Immunogenicity Results (CMI)

Part B

Randomisation Number	Group	Visit	Test	Subset	IL2	TNFa	IFNg	Value
B0058	Group B9	Day 1	Peptide	CD8	-	+	-	-0.006%
B0058	Group B9	Day 1	Peptide	CD8	-	-	+	1.86%
B0058	Group B9	Day 22	Peptide	CD8	+	+	+	0%
B0058	Group B9	Day 22	Peptide	CD8	+	+	-	-0.00208%
B0058	Group B9	Day 22	Peptide	CD8	+	-	+	0%
B0058	Group B9	Day 22	Peptide	CD8	+	-	-	-0.00193%
B0058	Group B9	Day 22	Peptide	CD8	-	+	+	0.00239%
B0058	Group B9	Day 22	Peptide	CD8	-	+	-	0.023%
B0058	Group B9	Day 22	Peptide	CD8	-	-	+	1.64%
B0058	Group B9	Day 1	MPH	CD4	+	+	+	0.10608%
B0058	Group B9	Day 1	MPH	CD4	+	+	-	0.04108%
B0058	Group B9	Day 1	MPH	CD4	+	-	+	0.01069%
B0058	Group B9	Day 1	MPH	CD4	+	-	-	0.02516%
B0058	Group B9	Day 1	MPH	CD4	-	+	+	0.043347%
B0058	Group B9	Day 1	MPH	CD4	-	+	-	0.141%
B0058	Group B9	Day 1	MPH	CD4	-	-	+	0.06239%
B0058	Group B9	Day 22	MPH	CD4	+	+	+	0.11808%
B0058	Group B9	Day 22	MPH	CD4	+	+	-	0.04529%
B0058	Group B9	Day 22	MPH	CD4	+	-	+	0.06%

Administration Site Key: FA = Forearm; UA= Upper arm
Treatments Part A: A1 = A/Sing MAP, 15 ug (FA); A2 = Afluria Quad IM (UA), 15 ug; A3 = Placebo MAP (FA); A4 = A/Sing Mono IM, 15 ug (UA)
Treatments Part B: B1 = A/Sing MAP, 15 ug (FA) with biopsy; B2 = Placebo MAP (FA) with biopsy
B3 = A/Sing MAP, 15 ug (FA); B4 = A/Sing MAP, 10 ug (FA); B5 = A/Sing MAP, 5 ug (FA); B6 = A/Sing MAP, 2.5 ug (FA)
B7 = Placebo MAP (FA); B8 = A/Sing MAP, 15 ug (UA); B9 = A/Sing IM, 15 ug, as 2018 Afluria Quad (UA)

Listing 16.2.6.6
Immunogenicity Results (CMI)

Part B

Randomisation Number	Group	Visit	Test	Subset	IL2	TNFa	IFNg	Value
B0058	Group B9	Day 22	MPH	CD4	+	-	-	0.043%
B0058	Group B9	Day 22	MPH	CD4	-	+	+	0.08512%
B0058	Group B9	Day 22	MPH	CD4	-	+	-	0.147%
B0058	Group B9	Day 22	MPH	CD4	-	-	+	0.07408%
B0058	Group B9	Day 1	MPH	CD8	+	+	+	0.013%
B0058	Group B9	Day 1	MPH	CD8	+	+	-	0.00645%
B0058	Group B9	Day 1	MPH	CD8	+	-	+	0.00485%
B0058	Group B9	Day 1	MPH	CD8	+	-	-	0.011%
B0058	Group B9	Day 1	MPH	CD8	-	+	+	0.04%
B0058	Group B9	Day 1	MPH	CD8	-	+	-	0.152%
B0058	Group B9	Day 1	MPH	CD8	-	-	+	-0.19%
B0058	Group B9	Day 22	MPH	CD8	+	+	+	0.00219%
B0058	Group B9	Day 22	MPH	CD8	+	+	-	0.00011%
B0058	Group B9	Day 22	MPH	CD8	+	-	+	0.00219%
B0058	Group B9	Day 22	MPH	CD8	+	-	-	0.00684%
B0058	Group B9	Day 22	MPH	CD8	-	+	+	0.00892%
B0058	Group B9	Day 22	MPH	CD8	-	+	-	0.117%
B0058	Group B9	Day 22	MPH	CD8	-	-	+	-0.09%

Administration Site Key: FA = Forearm; UA= Upper arm
Treatments Part A: A1 = A/Sing MAP, 15 ug (FA); A2 = Afluria Quad IM (UA), 15 ug; A3 = Placebo MAP (FA); A4 = A/Sing Mono IM, 15 ug (UA)
Treatments Part B: B1 = A/Sing MAP, 15 ug (FA) with biopsy; B2 = Placebo MAP (FA) with biopsy
B3 = A/Sing MAP, 15 ug (FA); B4 = A/Sing MAP, 10 ug (FA); B5 = A/Sing MAP, 5 ug (FA); B6 = A/Sing MAP, 2.5 ug (FA)
B7 = Placebo MAP (FA); B8 = A/Sing MAP, 15 ug (UA); B9 = A/Sing IM, 15 ug, as 2018 Afluria Quad (UA)

Listing 16.2.6.6
Immunogenicity Results (CMI)

Part B

Randomisation Number	Group	Visit	Test	Subset	IL2	TNFa	IFNg	Value
B0059	Group B7	Day 1	Peptide	CD4	+	+	+	-0.0004%
B0059	Group B7	Day 1	Peptide	CD4	+	+	-	0.00369%
B0059	Group B7	Day 1	Peptide	CD4	+	-	+	-0.000639%
B0059	Group B7	Day 1	Peptide	CD4	+	-	-	-0.01013%
B0059	Group B7	Day 1	Peptide	CD4	-	+	+	0.001081%
B0059	Group B7	Day 1	Peptide	CD4	-	+	-	0.007%
B0059	Group B7	Day 1	Peptide	CD4	-	-	+	0.00196%
B0059	Group B7	Day 22	Peptide	CD4	+	+	+	0.00156%
B0059	Group B7	Day 22	Peptide	CD4	+	+	-	0.007%
B0059	Group B7	Day 22	Peptide	CD4	+	-	+	0.000651%
B0059	Group B7	Day 22	Peptide	CD4	+	-	-	-0.0017%
B0059	Group B7	Day 22	Peptide	CD4	-	+	+	0.001221%
B0059	Group B7	Day 22	Peptide	CD4	-	+	-	-0.003%
B0059	Group B7	Day 22	Peptide	CD4	-	-	+	0.00098%
B0059	Group B7	Day 1	Peptide	CD8	+	+	+	0.00414%
B0059	Group B7	Day 1	Peptide	CD8	+	+	-	0%
B0059	Group B7	Day 1	Peptide	CD8	+	-	+	0.00047%
B0059	Group B7	Day 1	Peptide	CD8	+	-	-	-0.00636%
B0059	Group B7	Day 1	Peptide	CD8	-	+	+	0.069%

Administration Site Key: FA = Forearm; UA= Upper arm
Treatments Part A: A1 = A/Sing MAP, 15 ug (FA); A2 = Afluria Quad IM (UA), 15 ug; A3 = Placebo MAP (FA); A4 = A/Sing Mono IM, 15 ug (UA)
Treatments Part B: B1 = A/Sing MAP, 15 ug (FA) with biopsy; B2 = Placebo MAP (FA) with biopsy
B3 = A/Sing MAP, 15 ug (FA); B4 = A/Sing MAP, 10 ug (FA); B5 = A/Sing MAP, 5 ug (FA); B6 = A/Sing MAP, 2.5 ug (FA)
B7 = Placebo MAP (FA); B8 = A/Sing MAP, 15 ug (UA); B9 = A/Sing IM, 15 ug, as 2018 Afluria Quad (UA)

Listing 16.2.6.6
Immunogenicity Results (CMI)

Part B

Randomisation Number	Group	Visit	Test	Subset	IL2	TNFa	IFNg	Value
B0059	Group B7	Day 1	Peptide	CD8	-	+	-	-0.02%
B0059	Group B7	Day 1	Peptide	CD8	-	-	+	2.04%
B0059	Group B7	Day 22	Peptide	CD8	+	+	+	-0.00113%
B0059	Group B7	Day 22	Peptide	CD8	+	+	-	-0.00113%
B0059	Group B7	Day 22	Peptide	CD8	+	-	+	0%
B0059	Group B7	Day 22	Peptide	CD8	+	-	-	-0.00207%
B0059	Group B7	Day 22	Peptide	CD8	-	+	+	0.02%
B0059	Group B7	Day 22	Peptide	CD8	-	+	-	0.04%
B0059	Group B7	Day 22	Peptide	CD8	-	-	+	-1.21%
B0059	Group B7	Day 1	MPH	CD4	+	+	+	0.05816%
B0059	Group B7	Day 1	MPH	CD4	+	+	-	0.09169%
B0059	Group B7	Day 1	MPH	CD4	+	-	+	0.006891%
B0059	Group B7	Day 1	MPH	CD4	+	-	-	0.04%
B0059	Group B7	Day 1	MPH	CD4	-	+	+	0.029361%
B0059	Group B7	Day 1	MPH	CD4	-	+	-	0.193%
B0059	Group B7	Day 1	MPH	CD4	-	-	+	0.0408%
B0059	Group B7	Day 22	MPH	CD4	+	+	+	0.05656%
B0059	Group B7	Day 22	MPH	CD4	+	+	-	0.08%
B0059	Group B7	Day 22	MPH	CD4	+	-	+	0.004821%

Administration Site Key: FA = Forearm; UA= Upper arm
Treatments Part A: A1 = A/Sing MAP, 15 ug (FA); A2 = Afluria Quad IM (UA), 15 ug; A3 = Placebo MAP (FA); A4 = A/Sing Mono IM, 15 ug (UA)
Treatments Part B: B1 = A/Sing MAP, 15 ug (FA) with biopsy; B2 = Placebo MAP (FA) with biopsy
B3 = A/Sing MAP, 15 ug (FA); B4 = A/Sing MAP, 10 ug (FA); B5 = A/Sing MAP, 5 ug (FA); B6 = A/Sing MAP, 2.5 ug (FA)
B7 = Placebo MAP (FA); B8 = A/Sing MAP, 15 ug (UA); B9 = A/Sing IM, 15 ug, as 2018 Afluria Quad (UA)

Listing 16.2.6.6
Immunogenicity Results (CMI)

Part B

Randomisation Number	Group	Visit	Test	Subset	IL2	TNFa	IFNg	Value
B0059	Group B7	Day 22	MPH	CD4	+	-	-	0.02016%
B0059	Group B7	Day 22	MPH	CD4	-	+	+	0.024511%
B0059	Group B7	Day 22	MPH	CD4	-	+	-	0.201%
B0059	Group B7	Day 22	MPH	CD4	-	-	+	0.03256%
B0059	Group B7	Day 1	MPH	CD8	+	+	+	0.00747%
B0059	Group B7	Day 1	MPH	CD8	+	+	-	0.00632%
B0059	Group B7	Day 1	MPH	CD8	+	-	+	0.01263%
B0059	Group B7	Day 1	MPH	CD8	+	-	-	0.0268%
B0059	Group B7	Day 1	MPH	CD8	-	+	+	0.02%
B0059	Group B7	Day 1	MPH	CD8	-	+	-	0.05%
B0059	Group B7	Day 1	MPH	CD8	-	-	+	-1.99%
B0059	Group B7	Day 22	MPH	CD8	+	+	+	0.00887%
B0059	Group B7	Day 22	MPH	CD8	+	+	-	0.00003%
B0059	Group B7	Day 22	MPH	CD8	+	-	+	0.00927%
B0059	Group B7	Day 22	MPH	CD8	+	-	-	0.01362%
B0059	Group B7	Day 22	MPH	CD8	-	+	+	0.015%
B0059	Group B7	Day 22	MPH	CD8	-	+	-	0%
B0059	Group B7	Day 22	MPH	CD8	-	-	+	-1.29%

Administration Site Key: FA = Forearm; UA= Upper arm
Treatments Part A: A1 = A/Sing MAP, 15 ug (FA); A2 = Afluria Quad IM (UA), 15 ug; A3 = Placebo MAP (FA); A4 = A/Sing Mono IM, 15 ug (UA)
Treatments Part B: B1 = A/Sing MAP, 15 ug (FA) with biopsy; B2 = Placebo MAP (FA) with biopsy
B3 = A/Sing MAP, 15 ug (FA); B4 = A/Sing MAP, 10 ug (FA); B5 = A/Sing MAP, 5 ug (FA); B6 = A/Sing MAP, 2.5 ug (FA)
B7 = Placebo MAP (FA); B8 = A/Sing MAP, 15 ug (UA); B9 = A/Sing IM, 15 ug, as 2018 Afluria Quad (UA)

Listing 16.2.6.6
Immunogenicity Results (CMI)

Part B

Randomisation Number	Group	Visit	Test	Subset	IL2	TNFa	IFNg	Value
B0060	Group B3	Day 1	Peptide	CD4	+	+	+	-0.00068%
B0060	Group B3	Day 1	Peptide	CD4	+	+	-	0.00116%
B0060	Group B3	Day 1	Peptide	CD4	+	-	+	-0.000641%
B0060	Group B3	Day 1	Peptide	CD4	+	-	-	0.008%
B0060	Group B3	Day 1	Peptide	CD4	-	+	+	0.003119%
B0060	Group B3	Day 1	Peptide	CD4	-	+	-	0.004%
B0060	Group B3	Day 1	Peptide	CD4	-	-	+	-0.00061%
B0060	Group B3	Day 22	Peptide	CD4	+	+	+	0.049494%
B0060	Group B3	Day 22	Peptide	CD4	+	+	-	0.09191%
B0060	Group B3	Day 22	Peptide	CD4	+	-	+	0.01997%
B0060	Group B3	Day 22	Peptide	CD4	+	-	-	0.061%
B0060	Group B3	Day 22	Peptide	CD4	-	+	+	0.04699%
B0060	Group B3	Day 22	Peptide	CD4	-	+	-	0.193%
B0060	Group B3	Day 22	Peptide	CD4	-	-	+	0.03439%
B0060	Group B3	Day 1	Peptide	CD8	+	+	+	0%
B0060	Group B3	Day 1	Peptide	CD8	+	+	-	0%
B0060	Group B3	Day 1	Peptide	CD8	+	-	+	-0.00003%
B0060	Group B3	Day 1	Peptide	CD8	+	-	-	0.002%
B0060	Group B3	Day 1	Peptide	CD8	-	+	+	-0.00553%

Administration Site Key: FA = Forearm; UA= Upper arm
Treatments Part A: A1 = A/Sing MAP, 15 ug (FA); A2 = Afluria Quad IM (UA), 15 ug; A3 = Placebo MAP (FA); A4 = A/Sing Mono IM, 15 ug (UA)
Treatments Part B: B1 = A/Sing MAP, 15 ug (FA) with biopsy; B2 = Placebo MAP (FA) with biopsy
B3 = A/Sing MAP, 15 ug (FA); B4 = A/Sing MAP, 10 ug (FA); B5 = A/Sing MAP, 5 ug (FA); B6 = A/Sing MAP, 2.5 ug (FA)
B7 = Placebo MAP (FA); B8 = A/Sing MAP, 15 ug (UA); B9 = A/Sing IM, 15 ug, as 2018 Afluria Quad (UA)

Listing 16.2.6.6
Immunogenicity Results (CMI)

Part B

Randomisation Number	Group	Visit	Test	Subset	IL2	TNFa	IFNg	Value
B0060	Group B3	Day 1	Peptide	CD8	-	+	-	0.047%
B0060	Group B3	Day 1	Peptide	CD8	-	-	+	-0.25%
B0060	Group B3	Day 22	Peptide	CD8	+	+	+	0%
B0060	Group B3	Day 22	Peptide	CD8	+	+	-	0%
B0060	Group B3	Day 22	Peptide	CD8	+	-	+	0%
B0060	Group B3	Day 22	Peptide	CD8	+	-	-	0.008%
B0060	Group B3	Day 22	Peptide	CD8	-	+	+	-0.00558%
B0060	Group B3	Day 22	Peptide	CD8	-	+	-	-0.042%
B0060	Group B3	Day 22	Peptide	CD8	-	-	+	-0.58%
B0060	Group B3	Day 1	MPH	CD4	+	+	+	0.04244%
B0060	Group B3	Day 1	MPH	CD4	+	+	-	0.08652%
B0060	Group B3	Day 1	MPH	CD4	+	-	+	0.007719%
B0060	Group B3	Day 1	MPH	CD4	+	-	-	0.042%
B0060	Group B3	Day 1	MPH	CD4	-	+	+	0.056359%
B0060	Group B3	Day 1	MPH	CD4	-	+	-	0.201%
B0060	Group B3	Day 1	MPH	CD4	-	-	+	0.047%
B0060	Group B3	Day 22	MPH	CD4	+	+	+	0.049494%
B0060	Group B3	Day 22	MPH	CD4	+	+	-	0.09191%
B0060	Group B3	Day 22	MPH	CD4	+	-	+	0.01997%

Administration Site Key: FA = Forearm; UA= Upper arm
Treatments Part A: A1 = A/Sing MAP, 15 ug (FA); A2 = Afluria Quad IM (UA), 15 ug; A3 = Placebo MAP (FA); A4 = A/Sing Mono IM, 15 ug (UA)
Treatments Part B: B1 = A/Sing MAP, 15 ug (FA) with biopsy; B2 = Placebo MAP (FA) with biopsy
B3 = A/Sing MAP, 15 ug (FA); B4 = A/Sing MAP, 10 ug (FA); B5 = A/Sing MAP, 5 ug (FA); B6 = A/Sing MAP, 2.5 ug (FA)
B7 = Placebo MAP (FA); B8 = A/Sing MAP, 15 ug (UA); B9 = A/Sing IM, 15 ug, as 2018 Afluria Quad (UA)

Listing 16.2.6.6
Immunogenicity Results (CMI)

Part B

Randomisation Number	Group	Visit	Test	Subset	IL2	TNFa	IFNg	Value
B0060	Group B3	Day 22	MPH	CD4	+	-	-	0.061%
B0060	Group B3	Day 22	MPH	CD4	-	+	+	0.04699%
B0060	Group B3	Day 22	MPH	CD4	-	+	-	0.193%
B0060	Group B3	Day 22	MPH	CD4	-	-	+	0.03439%
B0060	Group B3	Day 1	MPH	CD8	+	+	+	0.032%
B0060	Group B3	Day 1	MPH	CD8	+	+	-	0.00191%
B0060	Group B3	Day 1	MPH	CD8	+	-	+	0.01491%
B0060	Group B3	Day 1	MPH	CD8	+	-	-	0.042%
B0060	Group B3	Day 1	MPH	CD8	-	+	+	0.07129%
B0060	Group B3	Day 1	MPH	CD8	-	+	-	0.107%
B0060	Group B3	Day 1	MPH	CD8	-	-	+	-0.51%
B0060	Group B3	Day 22	MPH	CD8	+	+	+	0.024%
B0060	Group B3	Day 22	MPH	CD8	+	+	-	0.00344%
B0060	Group B3	Day 22	MPH	CD8	+	-	+	0.014%
B0060	Group B3	Day 22	MPH	CD8	+	-	-	0.042%
B0060	Group B3	Day 22	MPH	CD8	-	+	+	0.07742%
B0060	Group B3	Day 22	MPH	CD8	-	+	-	0.09%
B0060	Group B3	Day 22	MPH	CD8	-	-	+	-0.75%

Administration Site Key: FA = Forearm; UA= Upper arm
Treatments Part A: A1 = A/Sing MAP, 15 ug (FA); A2 = Afluria Quad IM (UA), 15 ug; A3 = Placebo MAP (FA); A4 = A/Sing Mono IM, 15 ug (UA)
Treatments Part B: B1 = A/Sing MAP, 15 ug (FA) with biopsy; B2 = Placebo MAP (FA) with biopsy
B3 = A/Sing MAP, 15 ug (FA); B4 = A/Sing MAP, 10 ug (FA); B5 = A/Sing MAP, 5 ug (FA); B6 = A/Sing MAP, 2.5 ug (FA)
B7 = Placebo MAP (FA); B8 = A/Sing MAP, 15 ug (UA); B9 = A/Sing IM, 15 ug, as 2018 Afluria Quad (UA)

Listing 16.2.6.6
Immunogenicity Results (CMI)

Part B

Randomisation Number	Group	Visit	Test	Subset	IL2	TNFa	IFNg	Value
B0061	Group B8	Day 1	Peptide	CD4	+	+	+	0.00966%
B0061	Group B8	Day 1	Peptide	CD4	+	+	-	0.014%
B0061	Group B8	Day 1	Peptide	CD4	+	-	+	1E-5%
B0061	Group B8	Day 1	Peptide	CD4	+	-	-	-0.002%
B0061	Group B8	Day 1	Peptide	CD4	-	+	+	0.00484%
B0061	Group B8	Day 1	Peptide	CD4	-	+	-	0.007%
B0061	Group B8	Day 1	Peptide	CD4	-	-	+	-0.00134%
B0061	Group B8	Day 22	Peptide	CD4	+	+	+	0.01556%
B0061	Group B8	Day 22	Peptide	CD4	+	+	-	0.00951%
B0061	Group B8	Day 22	Peptide	CD4	+	-	+	0.00052%
B0061	Group B8	Day 22	Peptide	CD4	+	-	-	0.005%
B0061	Group B8	Day 22	Peptide	CD4	-	+	+	0.00825%
B0061	Group B8	Day 22	Peptide	CD4	-	+	-	0.023%
B0061	Group B8	Day 22	Peptide	CD4	-	-	+	0.00823%
B0061	Group B8	Day 1	Peptide	CD8	+	+	+	-0.00296%
B0061	Group B8	Day 1	Peptide	CD8	+	+	-	0%
B0061	Group B8	Day 1	Peptide	CD8	+	-	+	0%
B0061	Group B8	Day 1	Peptide	CD8	+	-	-	0.023%
B0061	Group B8	Day 1	Peptide	CD8	-	+	+	-0.01212%

Administration Site Key: FA = Forearm; UA= Upper arm
Treatments Part A: A1 = A/Sing MAP, 15 ug (FA); A2 = Afluria Quad IM (UA), 15 ug; A3 = Placebo MAP (FA); A4 = A/Sing Mono IM, 15 ug (UA)
Treatments Part B: B1 = A/Sing MAP, 15 ug (FA) with biopsy; B2 = Placebo MAP (FA) with biopsy
B3 = A/Sing MAP, 15 ug (FA); B4 = A/Sing MAP, 10 ug (FA); B5 = A/Sing MAP, 5 ug (FA); B6 = A/Sing MAP, 2.5 ug (FA)
B7 = Placebo MAP (FA); B8 = A/Sing MAP, 15 ug (UA); B9 = A/Sing IM, 15 ug, as 2018 Afluria Quad (UA)

Listing 16.2.6.6
Immunogenicity Results (CMI)

Part B

Randomisation Number	Group	Visit	Test	Subset	IL2	TNFa	IFNg	Value
B0061	Group B8	Day 1	Peptide	CD8	-	+	-	0.01%
B0061	Group B8	Day 1	Peptide	CD8	-	-	+	0.025%
B0061	Group B8	Day 22	Peptide	CD8	+	+	+	0%
B0061	Group B8	Day 22	Peptide	CD8	+	+	-	0.00161%
B0061	Group B8	Day 22	Peptide	CD8	+	-	+	0%
B0061	Group B8	Day 22	Peptide	CD8	+	-	-	0.002%
B0061	Group B8	Day 22	Peptide	CD8	-	+	+	-0.00322%
B0061	Group B8	Day 22	Peptide	CD8	-	+	-	-0.005%
B0061	Group B8	Day 22	Peptide	CD8	-	-	+	0.02%
B0061	Group B8	Day 1	MPH	CD4	+	+	+	0.099%
B0061	Group B8	Day 1	MPH	CD4	+	+	-	0.079%
B0061	Group B8	Day 1	MPH	CD4	+	-	+	0.0248%
B0061	Group B8	Day 1	MPH	CD4	+	-	-	0.039%
B0061	Group B8	Day 1	MPH	CD4	-	+	+	0.06659%
B0061	Group B8	Day 1	MPH	CD4	-	+	-	0.198%
B0061	Group B8	Day 1	MPH	CD4	-	-	+	0.046%
B0061	Group B8	Day 22	MPH	CD4	+	+	+	0.10756%
B0061	Group B8	Day 22	MPH	CD4	+	+	-	0.08251%
B0061	Group B8	Day 22	MPH	CD4	+	-	+	0.03756%

Administration Site Key: FA = Forearm; UA= Upper arm
Treatments Part A: A1 = A/Sing MAP, 15 ug (FA); A2 = Afluria Quad IM (UA), 15 ug; A3 = Placebo MAP (FA); A4 = A/Sing Mono IM, 15 ug (UA)
Treatments Part B: B1 = A/Sing MAP, 15 ug (FA) with biopsy; B2 = Placebo MAP (FA) with biopsy
B3 = A/Sing MAP, 15 ug (FA); B4 = A/Sing MAP, 10 ug (FA); B5 = A/Sing MAP, 5 ug (FA); B6 = A/Sing MAP, 2.5 ug (FA)
B7 = Placebo MAP (FA); B8 = A/Sing MAP, 15 ug (UA); B9 = A/Sing IM, 15 ug, as 2018 Afluria Quad (UA)

Listing 16.2.6.6
Immunogenicity Results (CMI)

Part B

Randomisation Number	Group	Visit	Test	Subset	IL2	TNFa	IFNg	Value
B0061	Group B8	Day 22	MPH	CD4	+	-	-	0.058%
B0061	Group B8	Day 22	MPH	CD4	-	+	+	0.05978%
B0061	Group B8	Day 22	MPH	CD4	-	+	-	0.2%
B0061	Group B8	Day 22	MPH	CD4	-	-	+	0.07423%
B0061	Group B8	Day 1	MPH	CD8	+	+	+	0.00524%
B0061	Group B8	Day 1	MPH	CD8	+	+	-	0.016%
B0061	Group B8	Day 1	MPH	CD8	+	-	+	0%
B0061	Group B8	Day 1	MPH	CD8	+	-	-	0.054%
B0061	Group B8	Day 1	MPH	CD8	-	+	+	0.185%
B0061	Group B8	Day 1	MPH	CD8	-	+	-	0.77%
B0061	Group B8	Day 1	MPH	CD8	-	-	+	0.125%
B0061	Group B8	Day 22	MPH	CD8	+	+	+	0.021%
B0061	Group B8	Day 22	MPH	CD8	+	+	-	0.00458%
B0061	Group B8	Day 22	MPH	CD8	+	-	+	0.00916%
B0061	Group B8	Day 22	MPH	CD8	+	-	-	0.062%
B0061	Group B8	Day 22	MPH	CD8	-	+	+	0.19678%
B0061	Group B8	Day 22	MPH	CD8	-	+	-	0.747%
B0061	Group B8	Day 22	MPH	CD8	-	-	+	0.08%

Administration Site Key: FA = Forearm; UA= Upper arm
Treatments Part A: A1 = A/Sing MAP, 15 ug (FA); A2 = Afluria Quad IM (UA), 15 ug; A3 = Placebo MAP (FA); A4 = A/Sing Mono IM, 15 ug (UA)
Treatments Part B: B1 = A/Sing MAP, 15 ug (FA) with biopsy; B2 = Placebo MAP (FA) with biopsy
B3 = A/Sing MAP, 15 ug (FA); B4 = A/Sing MAP, 10 ug (FA); B5 = A/Sing MAP, 5 ug (FA); B6 = A/Sing MAP, 2.5 ug (FA)
B7 = Placebo MAP (FA); B8 = A/Sing MAP, 15 ug (UA); B9 = A/Sing IM, 15 ug, as 2018 Afluria Quad (UA)

Listing 16.2.6.6
Immunogenicity Results (CMI)

Part B

Randomisation Number	Group	Visit	Test	Subset	IL2	TNFa	IFNg	Value
B0063	Group B7	Day 1	Peptide	CD4	+	+	+	-0.00086%
B0063	Group B7	Day 1	Peptide	CD4	+	+	-	-0.00377%
B0063	Group B7	Day 1	Peptide	CD4	+	-	+	0.00056%
B0063	Group B7	Day 1	Peptide	CD4	+	-	-	-0.004%
B0063	Group B7	Day 1	Peptide	CD4	-	+	+	-0.000734%
B0063	Group B7	Day 1	Peptide	CD4	-	+	-	-0.016%
B0063	Group B7	Day 1	Peptide	CD4	-	-	+	-0.00731%
B0063	Group B7	Day 22	Peptide	CD4	+	+	+	-0.00538%
B0063	Group B7	Day 22	Peptide	CD4	+	+	-	-0.00327%
B0063	Group B7	Day 22	Peptide	CD4	+	-	+	0.00158%
B0063	Group B7	Day 22	Peptide	CD4	+	-	-	-0.001%
B0063	Group B7	Day 22	Peptide	CD4	-	+	+	-0.00072%
B0063	Group B7	Day 22	Peptide	CD4	-	+	-	0.031%
B0063	Group B7	Day 22	Peptide	CD4	-	-	+	0.00601%
B0063	Group B7	Day 1	Peptide	CD8	+	+	+	0%
B0063	Group B7	Day 1	Peptide	CD8	+	+	-	-0.000915%
B0063	Group B7	Day 1	Peptide	CD8	+	-	+	0%
B0063	Group B7	Day 1	Peptide	CD8	+	-	-	-0.004%
B0063	Group B7	Day 1	Peptide	CD8	-	+	+	-0.00205%

Administration Site Key: FA = Forearm; UA= Upper arm
Treatments Part A: A1 = A/Sing MAP, 15 ug (FA); A2 = Afluria Quad IM (UA), 15 ug; A3 = Placebo MAP (FA); A4 = A/Sing Mono IM, 15 ug (UA)
Treatments Part B: B1 = A/Sing MAP, 15 ug (FA) with biopsy; B2 = Placebo MAP (FA) with biopsy
B3 = A/Sing MAP, 15 ug (FA); B4 = A/Sing MAP, 10 ug (FA); B5 = A/Sing MAP, 5 ug (FA); B6 = A/Sing MAP, 2.5 ug (FA)
B7 = Placebo MAP (FA); B8 = A/Sing MAP, 15 ug (UA); B9 = A/Sing IM, 15 ug, as 2018 Afluria Quad (UA)

Listing 16.2.6.6
Immunogenicity Results (CMI)

Part B

Randomisation Number	Group	Visit	Test	Subset	IL2	TNFa	IFNg	Value
B0063	Group B7	Day 1	Peptide	CD8	-	+	-	0.017%
B0063	Group B7	Day 1	Peptide	CD8	-	-	+	0.16%
B0063	Group B7	Day 22	Peptide	CD8	+	+	+	0%
B0063	Group B7	Day 22	Peptide	CD8	+	+	-	0%
B0063	Group B7	Day 22	Peptide	CD8	+	-	+	0%
B0063	Group B7	Day 22	Peptide	CD8	+	-	-	0.001%
B0063	Group B7	Day 22	Peptide	CD8	-	+	+	0.00303%
B0063	Group B7	Day 22	Peptide	CD8	-	+	-	0.046%
B0063	Group B7	Day 22	Peptide	CD8	-	-	+	-0.01%
B0063	Group B7	Day 1	MPH	CD4	+	+	+	0.03556%
B0063	Group B7	Day 1	MPH	CD4	+	+	-	0.06606%
B0063	Group B7	Day 1	MPH	CD4	+	-	+	0.00623%
B0063	Group B7	Day 1	MPH	CD4	+	-	-	0.024%
B0063	Group B7	Day 1	MPH	CD4	-	+	+	0.02262%
B0063	Group B7	Day 1	MPH	CD4	-	+	-	0.135%
B0063	Group B7	Day 1	MPH	CD4	-	-	+	0.022%
B0063	Group B7	Day 22	MPH	CD4	+	+	+	0.04268%
B0063	Group B7	Day 22	MPH	CD4	+	+	-	0.075%
B0063	Group B7	Day 22	MPH	CD4	+	-	+	0.01067%

Administration Site Key: FA = Forearm; UA= Upper arm
Treatments Part A: A1 = A/Sing MAP, 15 ug (FA); A2 = Afluria Quad IM (UA), 15 ug; A3 = Placebo MAP (FA); A4 = A/Sing Mono IM, 15 ug (UA)
Treatments Part B: B1 = A/Sing MAP, 15 ug (FA) with biopsy; B2 = Placebo MAP (FA) with biopsy
B3 = A/Sing MAP, 15 ug (FA); B4 = A/Sing MAP, 10 ug (FA); B5 = A/Sing MAP, 5 ug (FA); B6 = A/Sing MAP, 2.5 ug (FA)
B7 = Placebo MAP (FA); B8 = A/Sing MAP, 15 ug (UA); B9 = A/Sing IM, 15 ug, as 2018 Afluria Quad (UA)

Listing 16.2.6.6
Immunogenicity Results (CMI)

Part B

Randomisation Number	Group	Visit	Test	Subset	IL2	TNFa	IFNg	Value
B0063	Group B7	Day 22	MPH	CD4	+	-	-	0.075%
B0063	Group B7	Day 22	MPH	CD4	-	+	+	0.02834%
B0063	Group B7	Day 22	MPH	CD4	-	+	-	0.266%
B0063	Group B7	Day 22	MPH	CD4	-	-	+	0.04301%
B0063	Group B7	Day 1	MPH	CD8	+	+	+	0.00527%
B0063	Group B7	Day 1	MPH	CD8	+	+	-	0.001725%
B0063	Group B7	Day 1	MPH	CD8	+	-	+	0.00352%
B0063	Group B7	Day 1	MPH	CD8	+	-	-	0.009%
B0063	Group B7	Day 1	MPH	CD8	-	+	+	0.00959%
B0063	Group B7	Day 1	MPH	CD8	-	+	-	0.035%
B0063	Group B7	Day 1	MPH	CD8	-	-	+	-0.2%
B0063	Group B7	Day 22	MPH	CD8	+	+	+	0.00194%
B0063	Group B7	Day 22	MPH	CD8	+	+	-	0.0029%
B0063	Group B7	Day 22	MPH	CD8	+	-	+	0.00194%
B0063	Group B7	Day 22	MPH	CD8	+	-	-	0.073%
B0063	Group B7	Day 22	MPH	CD8	-	+	+	0.02034%
B0063	Group B7	Day 22	MPH	CD8	-	+	-	0.196%
B0063	Group B7	Day 22	MPH	CD8	-	-	+	-0.08%

Administration Site Key: FA = Forearm; UA= Upper arm
Treatments Part A: A1 = A/Sing MAP, 15 ug (FA); A2 = Afluria Quad IM (UA), 15 ug; A3 = Placebo MAP (FA); A4 = A/Sing Mono IM, 15 ug (UA)
Treatments Part B: B1 = A/Sing MAP, 15 ug (FA) with biopsy; B2 = Placebo MAP (FA) with biopsy
B3 = A/Sing MAP, 15 ug (FA); B4 = A/Sing MAP, 10 ug (FA); B5 = A/Sing MAP, 5 ug (FA); B6 = A/Sing MAP, 2.5 ug (FA)
B7 = Placebo MAP (FA); B8 = A/Sing MAP, 15 ug (UA); B9 = A/Sing IM, 15 ug, as 2018 Afluria Quad (UA)

Listing 16.2.6.6
Immunogenicity Results (CMI)

Part B

Randomisation Number	Group	Visit	Test	Subset	IL2	TNFa	IFNg	Value
B0064	Group B9	Day 1	Peptide	CD4	+	+	+	0.00457%
B0064	Group B9	Day 1	Peptide	CD4	+	+	-	-0.00103%
B0064	Group B9	Day 1	Peptide	CD4	+	-	+	0.00229%
B0064	Group B9	Day 1	Peptide	CD4	+	-	-	-0.00552%
B0064	Group B9	Day 1	Peptide	CD4	-	+	+	0.00002%
B0064	Group B9	Day 1	Peptide	CD4	-	+	-	0.004%
B0064	Group B9	Day 1	Peptide	CD4	-	-	+	0.00231%
B0064	Group B9	Day 22	Peptide	CD4	+	+	+	0.00708%
B0064	Group B9	Day 22	Peptide	CD4	+	+	-	0.0155%
B0064	Group B9	Day 22	Peptide	CD4	+	-	+	0.000778%
B0064	Group B9	Day 22	Peptide	CD4	+	-	-	0.00369%
B0064	Group B9	Day 22	Peptide	CD4	-	+	+	0.000778%
B0064	Group B9	Day 22	Peptide	CD4	-	+	-	0.006%
B0064	Group B9	Day 22	Peptide	CD4	-	-	+	0.00153%
B0064	Group B9	Day 1	Peptide	CD8	+	+	+	0%
B0064	Group B9	Day 1	Peptide	CD8	+	+	-	0.0014%
B0064	Group B9	Day 1	Peptide	CD8	+	-	+	0%
B0064	Group B9	Day 1	Peptide	CD8	+	-	-	-0.00394%
B0064	Group B9	Day 1	Peptide	CD8	-	+	+	0.00009%

Administration Site Key: FA = Forearm; UA= Upper arm
Treatments Part A: A1 = A/Sing MAP, 15 ug (FA); A2 = Afluria Quad IM (UA), 15 ug; A3 = Placebo MAP (FA); A4 = A/Sing Mono IM, 15 ug (UA)
Treatments Part B: B1 = A/Sing MAP, 15 ug (FA) with biopsy; B2 = Placebo MAP (FA) with biopsy
B3 = A/Sing MAP, 15 ug (FA); B4 = A/Sing MAP, 10 ug (FA); B5 = A/Sing MAP, 5 ug (FA); B6 = A/Sing MAP, 2.5 ug (FA)
B7 = Placebo MAP (FA); B8 = A/Sing MAP, 15 ug (UA); B9 = A/Sing IM, 15 ug, as 2018 Afluria Quad (UA)

Listing 16.2.6.6
Immunogenicity Results (CMI)

Part B

Randomisation Number	Group	Visit	Test	Subset	IL2	TNFa	IFNg	Value
B0064	Group B9	Day 1	Peptide	CD8	-	+	-	-0.001%
B0064	Group B9	Day 1	Peptide	CD8	-	-	+	-0.11%
B0064	Group B9	Day 22	Peptide	CD8	+	+	+	0%
B0064	Group B9	Day 22	Peptide	CD8	+	+	-	0%
B0064	Group B9	Day 22	Peptide	CD8	+	-	+	0%
B0064	Group B9	Day 22	Peptide	CD8	+	-	-	0.0052%
B0064	Group B9	Day 22	Peptide	CD8	-	+	+	-0.00011%
B0064	Group B9	Day 22	Peptide	CD8	-	+	-	0.019%
B0064	Group B9	Day 22	Peptide	CD8	-	-	+	0.25%
B0064	Group B9	Day 1	MPH	CD4	+	+	+	0.032%
B0064	Group B9	Day 1	MPH	CD4	+	+	-	0.09054%
B0064	Group B9	Day 1	MPH	CD4	+	-	+	0.013%
B0064	Group B9	Day 1	MPH	CD4	+	-	-	0.02819%
B0064	Group B9	Day 1	MPH	CD4	-	+	+	0.02188%
B0064	Group B9	Day 1	MPH	CD4	-	+	-	0.212%
B0064	Group B9	Day 1	MPH	CD4	-	-	+	0.02388%
B0064	Group B9	Day 22	MPH	CD4	+	+	+	0.05756%
B0064	Group B9	Day 22	MPH	CD4	+	+	-	0.0855%
B0064	Group B9	Day 22	MPH	CD4	+	-	+	0.006588%

Administration Site Key: FA = Forearm; UA= Upper arm
Treatments Part A: A1 = A/Sing MAP, 15 ug (FA); A2 = Afluria Quad IM (UA), 15 ug; A3 = Placebo MAP (FA); A4 = A/Sing Mono IM, 15 ug (UA)
Treatments Part B: B1 = A/Sing MAP, 15 ug (FA) with biopsy; B2 = Placebo MAP (FA) with biopsy
B3 = A/Sing MAP, 15 ug (FA); B4 = A/Sing MAP, 10 ug (FA); B5 = A/Sing MAP, 5 ug (FA); B6 = A/Sing MAP, 2.5 ug (FA)
B7 = Placebo MAP (FA); B8 = A/Sing MAP, 15 ug (UA); B9 = A/Sing IM, 15 ug, as 2018 Afluria Quad (UA)

Listing 16.2.6.6
Immunogenicity Results (CMI)

Part B

Randomisation Number	Group	Visit	Test	Subset	IL2	TNFa	IFNg	Value
B0064	Group B9	Day 22	MPH	CD4	+	-	-	0.03869%
B0064	Group B9	Day 22	MPH	CD4	-	+	+	0.034188%
B0064	Group B9	Day 22	MPH	CD4	-	+	-	0.196%
B0064	Group B9	Day 22	MPH	CD4	-	-	+	0.02856%
B0064	Group B9	Day 1	MPH	CD8	+	+	+	0.015%
B0064	Group B9	Day 1	MPH	CD8	+	+	-	0.00296%
B0064	Group B9	Day 1	MPH	CD8	+	-	+	0.00739%
B0064	Group B9	Day 1	MPH	CD8	+	-	-	0.00493%
B0064	Group B9	Day 1	MPH	CD8	-	+	+	0.04869%
B0064	Group B9	Day 1	MPH	CD8	-	+	-	0.091%
B0064	Group B9	Day 1	MPH	CD8	-	-	+	-0.55%
B0064	Group B9	Day 22	MPH	CD8	+	+	+	0.028%
B0064	Group B9	Day 22	MPH	CD8	+	+	-	0.0012%
B0064	Group B9	Day 22	MPH	CD8	+	-	+	0.00599%
B0064	Group B9	Day 22	MPH	CD8	+	-	-	0.0169%
B0064	Group B9	Day 22	MPH	CD8	-	+	+	0.09479%
B0064	Group B9	Day 22	MPH	CD8	-	+	-	0.233%
B0064	Group B9	Day 22	MPH	CD8	-	-	+	0.2%

Administration Site Key: FA = Forearm; UA= Upper arm
Treatments Part A: A1 = A/Sing MAP, 15 ug (FA); A2 = Afluria Quad IM (UA), 15 ug; A3 = Placebo MAP (FA); A4 = A/Sing Mono IM, 15 ug (UA)
Treatments Part B: B1 = A/Sing MAP, 15 ug (FA) with biopsy; B2 = Placebo MAP (FA) with biopsy
B3 = A/Sing MAP, 15 ug (FA); B4 = A/Sing MAP, 10 ug (FA); B5 = A/Sing MAP, 5 ug (FA); B6 = A/Sing MAP, 2.5 ug (FA)
B7 = Placebo MAP (FA); B8 = A/Sing MAP, 15 ug (UA); B9 = A/Sing IM, 15 ug, as 2018 Afluria Quad (UA)

Listing 16.2.6.6
Immunogenicity Results (CMI)

Part B

Randomisation Number	Group	Visit	Test	Subset	IL2	TNFa	IFNg	Value
B0067	Group B8	Day 1	Peptide	CD4	+	+	+	0.004%
B0067	Group B8	Day 1	Peptide	CD4	+	+	-	0%
B0067	Group B8	Day 1	Peptide	CD4	+	-	+	-0.001618%
B0067	Group B8	Day 1	Peptide	CD4	+	-	-	-0.01%
B0067	Group B8	Day 1	Peptide	CD4	-	+	+	0.00207%
B0067	Group B8	Day 1	Peptide	CD4	-	+	-	-0.012%
B0067	Group B8	Day 1	Peptide	CD4	-	-	+	-0.09095%
B0067	Group B8	Day 22	Peptide	CD4	+	+	+	0.00516%
B0067	Group B8	Day 22	Peptide	CD4	+	+	-	0.016%
B0067	Group B8	Day 22	Peptide	CD4	+	-	+	0.00055%
B0067	Group B8	Day 22	Peptide	CD4	+	-	-	-0.002%
B0067	Group B8	Day 22	Peptide	CD4	-	+	+	0.0042%
B0067	Group B8	Day 22	Peptide	CD4	-	+	-	0%
B0067	Group B8	Day 22	Peptide	CD4	-	-	+	0.00314%
B0067	Group B8	Day 1	Peptide	CD8	+	+	+	-0.00137%
B0067	Group B8	Day 1	Peptide	CD8	+	+	-	0.00137%
B0067	Group B8	Day 1	Peptide	CD8	+	-	+	0%
B0067	Group B8	Day 1	Peptide	CD8	+	-	-	0%
B0067	Group B8	Day 1	Peptide	CD8	-	+	+	-0.016%

Administration Site Key: FA = Forearm; UA= Upper arm
Treatments Part A: A1 = A/Sing MAP, 15 ug (FA); A2 = Afluria Quad IM (UA), 15 ug; A3 = Placebo MAP (FA); A4 = A/Sing Mono IM, 15 ug (UA)
Treatments Part B: B1 = A/Sing MAP, 15 ug (FA) with biopsy; B2 = Placebo MAP (FA) with biopsy
B3 = A/Sing MAP, 15 ug (FA); B4 = A/Sing MAP, 10 ug (FA); B5 = A/Sing MAP, 5 ug (FA); B6 = A/Sing MAP, 2.5 ug (FA)
B7 = Placebo MAP (FA); B8 = A/Sing MAP, 15 ug (UA); B9 = A/Sing IM, 15 ug, as 2018 Afluria Quad (UA)

Listing 16.2.6.6
Immunogenicity Results (CMI)

Part B

Randomisation Number	Group	Visit	Test	Subset	IL2	TNFa	IFNg	Value
B0067	Group B8	Day 1	Peptide	CD8	-	+	-	0.002%
B0067	Group B8	Day 1	Peptide	CD8	-	-	+	-2.67%
B0067	Group B8	Day 22	Peptide	CD8	+	+	+	0%
B0067	Group B8	Day 22	Peptide	CD8	+	+	-	0%
B0067	Group B8	Day 22	Peptide	CD8	+	-	+	0%
B0067	Group B8	Day 22	Peptide	CD8	+	-	-	-0.00349%
B0067	Group B8	Day 22	Peptide	CD8	-	+	+	-0.00492%
B0067	Group B8	Day 22	Peptide	CD8	-	+	-	0.008%
B0067	Group B8	Day 22	Peptide	CD8	-	-	+	-1.49%
B0067	Group B8	Day 1	MPH	CD4	+	+	+	0.1%
B0067	Group B8	Day 1	MPH	CD4	+	+	-	0.058%
B0067	Group B8	Day 1	MPH	CD4	+	-	+	0.00785%
B0067	Group B8	Day 1	MPH	CD4	+	-	-	0.016%
B0067	Group B8	Day 1	MPH	CD4	-	+	+	0.09056%
B0067	Group B8	Day 1	MPH	CD4	-	+	-	0.238%
B0067	Group B8	Day 1	MPH	CD4	-	-	+	0.03%
B0067	Group B8	Day 22	MPH	CD4	+	+	+	0.08316%
B0067	Group B8	Day 22	MPH	CD4	+	+	-	0.064%
B0067	Group B8	Day 22	MPH	CD4	+	-	+	0.019%

Administration Site Key: FA = Forearm; UA= Upper arm
Treatments Part A: A1 = A/Sing MAP, 15 ug (FA); A2 = Afluria Quad IM (UA), 15 ug; A3 = Placebo MAP (FA); A4 = A/Sing Mono IM, 15 ug (UA)
Treatments Part B: B1 = A/Sing MAP, 15 ug (FA) with biopsy; B2 = Placebo MAP (FA) with biopsy
B3 = A/Sing MAP, 15 ug (FA); B4 = A/Sing MAP, 10 ug (FA); B5 = A/Sing MAP, 5 ug (FA); B6 = A/Sing MAP, 2.5 ug (FA)
B7 = Placebo MAP (FA); B8 = A/Sing MAP, 15 ug (UA); B9 = A/Sing IM, 15 ug, as 2018 Afluria Quad (UA)

Listing 16.2.6.6
Immunogenicity Results (CMI)

Part B

Randomisation Number	Group	Visit	Test	Subset	IL2	TNFa	IFNg	Value
B0067	Group B8	Day 22	MPH	CD4	+	-	-	0.023%
B0067	Group B8	Day 22	MPH	CD4	-	+	+	0.08805%
B0067	Group B8	Day 22	MPH	CD4	-	+	-	0.214%
B0067	Group B8	Day 22	MPH	CD4	-	-	+	0.06064%
B0067	Group B8	Day 1	MPH	CD8	+	+	+	0.00002%
B0067	Group B8	Day 1	MPH	CD8	+	+	-	0.00418%
B0067	Group B8	Day 1	MPH	CD8	+	-	+	0.00142%
B0067	Group B8	Day 1	MPH	CD8	+	-	-	0.02126%
B0067	Group B8	Day 1	MPH	CD8	-	+	+	0.034%
B0067	Group B8	Day 1	MPH	CD8	-	+	-	0.145%
B0067	Group B8	Day 1	MPH	CD8	-	-	+	-5.2%
B0067	Group B8	Day 22	MPH	CD8	+	+	+	0.00759%
B0067	Group B8	Day 22	MPH	CD8	+	+	-	0.00304%
B0067	Group B8	Day 22	MPH	CD8	+	-	+	0%
B0067	Group B8	Day 22	MPH	CD8	+	-	-	0.02647%
B0067	Group B8	Day 22	MPH	CD8	-	+	+	0.027%
B0067	Group B8	Day 22	MPH	CD8	-	+	-	0.171%
B0067	Group B8	Day 22	MPH	CD8	-	-	+	-2.5%

Administration Site Key: FA = Forearm; UA= Upper arm
Treatments Part A: A1 = A/Sing MAP, 15 ug (FA); A2 = Afluria Quad IM (UA), 15 ug; A3 = Placebo MAP (FA); A4 = A/Sing Mono IM, 15 ug (UA)
Treatments Part B: B1 = A/Sing MAP, 15 ug (FA) with biopsy; B2 = Placebo MAP (FA) with biopsy
B3 = A/Sing MAP, 15 ug (FA); B4 = A/Sing MAP, 10 ug (FA); B5 = A/Sing MAP, 5 ug (FA); B6 = A/Sing MAP, 2.5 ug (FA)
B7 = Placebo MAP (FA); B8 = A/Sing MAP, 15 ug (UA); B9 = A/Sing IM, 15 ug, as 2018 Afluria Quad (UA)

Listing 16.2.6.6
Immunogenicity Results (CMI)

Part B

Randomisation Number	Group	Visit	Test	Subset	IL2	TNFa	IFNg	Value
B0069	Group B7	Day 1	Peptide	CD4	+	+	+	0.00147%
B0069	Group B7	Day 1	Peptide	CD4	+	+	-	0.00161%
B0069	Group B7	Day 1	Peptide	CD4	+	-	+	0.00135%
B0069	Group B7	Day 1	Peptide	CD4	+	-	-	-0.007%
B0069	Group B7	Day 1	Peptide	CD4	-	+	+	0.00404%
B0069	Group B7	Day 1	Peptide	CD4	-	+	-	0.005%
B0069	Group B7	Day 1	Peptide	CD4	-	-	+	0.00228%
B0069	Group B7	Day 22	Peptide	CD4	+	+	+	0%
B0069	Group B7	Day 22	Peptide	CD4	+	+	-	0.00522%
B0069	Group B7	Day 22	Peptide	CD4	+	-	+	0.00106%
B0069	Group B7	Day 22	Peptide	CD4	+	-	-	-0.004%
B0069	Group B7	Day 22	Peptide	CD4	-	+	+	-0.00111%
B0069	Group B7	Day 22	Peptide	CD4	-	+	-	-0.017%
B0069	Group B7	Day 22	Peptide	CD4	-	-	+	-0.00572%
B0069	Group B7	Day 1	Peptide	CD8	+	+	+	0.00306%
B0069	Group B7	Day 1	Peptide	CD8	+	+	-	0%
B0069	Group B7	Day 1	Peptide	CD8	+	-	+	-0.00132%
B0069	Group B7	Day 1	Peptide	CD8	+	-	-	-0.004%
B0069	Group B7	Day 1	Peptide	CD8	-	+	+	-0.002%

Administration Site Key: FA = Forearm; UA= Upper arm
Treatments Part A: A1 = A/Sing MAP, 15 ug (FA); A2 = Afluria Quad IM (UA), 15 ug; A3 = Placebo MAP (FA); A4 = A/Sing Mono IM, 15 ug (UA)
Treatments Part B: B1 = A/Sing MAP, 15 ug (FA) with biopsy; B2 = Placebo MAP (FA) with biopsy
B3 = A/Sing MAP, 15 ug (FA); B4 = A/Sing MAP, 10 ug (FA); B5 = A/Sing MAP, 5 ug (FA); B6 = A/Sing MAP, 2.5 ug (FA)
B7 = Placebo MAP (FA); B8 = A/Sing MAP, 15 ug (UA); B9 = A/Sing IM, 15 ug, as 2018 Afluria Quad (UA)

Listing 16.2.6.6
Immunogenicity Results (CMI)

Part B

Randomisation Number	Group	Visit	Test	Subset	IL2	TNFa	IFNg	Value
B0069	Group B7	Day 1	Peptide	CD8	-	+	-	-0.026%
B0069	Group B7	Day 1	Peptide	CD8	-	-	+	-0.03%
B0069	Group B7	Day 22	Peptide	CD8	+	+	+	0%
B0069	Group B7	Day 22	Peptide	CD8	+	+	-	0%
B0069	Group B7	Day 22	Peptide	CD8	+	-	+	0.0012%
B0069	Group B7	Day 22	Peptide	CD8	+	-	-	-0.003%
B0069	Group B7	Day 22	Peptide	CD8	-	+	+	0.009%
B0069	Group B7	Day 22	Peptide	CD8	-	+	-	-0.018%
B0069	Group B7	Day 22	Peptide	CD8	-	-	+	-0.15%
B0069	Group B7	Day 1	MPH	CD4	+	+	+	0.06378%
B0069	Group B7	Day 1	MPH	CD4	+	+	-	0.07757%
B0069	Group B7	Day 1	MPH	CD4	+	-	+	0.012%
B0069	Group B7	Day 1	MPH	CD4	+	-	-	0.011%
B0069	Group B7	Day 1	MPH	CD4	-	+	+	0.031%
B0069	Group B7	Day 1	MPH	CD4	-	+	-	0.236%
B0069	Group B7	Day 1	MPH	CD4	-	-	+	0.02528%
B0069	Group B7	Day 22	MPH	CD4	+	+	+	0.041%
B0069	Group B7	Day 22	MPH	CD4	+	+	-	0.08789%
B0069	Group B7	Day 22	MPH	CD4	+	-	+	0.00603%

Administration Site Key: FA = Forearm; UA= Upper arm
Treatments Part A: A1 = A/Sing MAP, 15 ug (FA); A2 = Afluria Quad IM (UA), 15 ug; A3 = Placebo MAP (FA); A4 = A/Sing Mono IM, 15 ug (UA)
Treatments Part B: B1 = A/Sing MAP, 15 ug (FA) with biopsy; B2 = Placebo MAP (FA) with biopsy
B3 = A/Sing MAP, 15 ug (FA); B4 = A/Sing MAP, 10 ug (FA); B5 = A/Sing MAP, 5 ug (FA); B6 = A/Sing MAP, 2.5 ug (FA)
B7 = Placebo MAP (FA); B8 = A/Sing MAP, 15 ug (UA); B9 = A/Sing IM, 15 ug, as 2018 Afluria Quad (UA)

Listing 16.2.6.6
Immunogenicity Results (CMI)

Part B

Randomisation Number	Group	Visit	Test	Subset	IL2	TNFa	IFNg	Value
B0069	Group B7	Day 22	MPH	CD4	+	-	-	0.029%
B0069	Group B7	Day 22	MPH	CD4	-	+	+	0.02889%
B0069	Group B7	Day 22	MPH	CD4	-	+	-	0.218%
B0069	Group B7	Day 22	MPH	CD4	-	-	+	0.017%
B0069	Group B7	Day 1	MPH	CD8	+	+	+	0.0052%
B0069	Group B7	Day 1	MPH	CD8	+	+	-	0.0013%
B0069	Group B7	Day 1	MPH	CD8	+	-	+	0.00648%
B0069	Group B7	Day 1	MPH	CD8	+	-	-	0.023%
B0069	Group B7	Day 1	MPH	CD8	-	+	+	-0.011%
B0069	Group B7	Day 1	MPH	CD8	-	+	-	0.05%
B0069	Group B7	Day 1	MPH	CD8	-	-	+	-1.01%
B0069	Group B7	Day 22	MPH	CD8	+	+	+	0.00291%
B0069	Group B7	Day 22	MPH	CD8	+	+	-	0.00194%
B0069	Group B7	Day 22	MPH	CD8	+	-	+	0.00194%
B0069	Group B7	Day 22	MPH	CD8	+	-	-	0.012%
B0069	Group B7	Day 22	MPH	CD8	-	+	+	-0.018%
B0069	Group B7	Day 22	MPH	CD8	-	+	-	0.055%
B0069	Group B7	Day 22	MPH	CD8	-	-	+	-0.61%

Administration Site Key: FA = Forearm; UA= Upper arm
Treatments Part A: A1 = A/Sing MAP, 15 ug (FA); A2 = Afluria Quad IM (UA), 15 ug; A3 = Placebo MAP (FA); A4 = A/Sing Mono IM, 15 ug (UA)
Treatments Part B: B1 = A/Sing MAP, 15 ug (FA) with biopsy; B2 = Placebo MAP (FA) with biopsy
B3 = A/Sing MAP, 15 ug (FA); B4 = A/Sing MAP, 10 ug (FA); B5 = A/Sing MAP, 5 ug (FA); B6 = A/Sing MAP, 2.5 ug (FA)
B7 = Placebo MAP (FA); B8 = A/Sing MAP, 15 ug (UA); B9 = A/Sing IM, 15 ug, as 2018 Afluria Quad (UA)

Listing 16.2.6.6
Immunogenicity Results (CMI)

Part B

Randomisation Number	Group	Visit	Test	Subset	IL2	TNFa	IFNg	Value
B0070	Group B9	Day 1	Peptide	CD4	+	+	+	0.01052%
B0070	Group B9	Day 1	Peptide	CD4	+	+	-	0.02896%
B0070	Group B9	Day 1	Peptide	CD4	+	-	+	-0.00157%
B0070	Group B9	Day 1	Peptide	CD4	+	-	-	0.04674%
B0070	Group B9	Day 1	Peptide	CD4	-	+	+	0.00501%
B0070	Group B9	Day 1	Peptide	CD4	-	+	-	0.001%
B0070	Group B9	Day 1	Peptide	CD4	-	-	+	-0.00149%
B0070	Group B9	Day 22	Peptide	CD4	+	+	+	0.05596%
B0070	Group B9	Day 22	Peptide	CD4	+	+	-	0.036%
B0070	Group B9	Day 22	Peptide	CD4	+	-	+	0.00018%
B0070	Group B9	Day 22	Peptide	CD4	+	-	-	-0.016%
B0070	Group B9	Day 22	Peptide	CD4	-	+	+	0.00752%
B0070	Group B9	Day 22	Peptide	CD4	-	+	-	0.049%
B0070	Group B9	Day 22	Peptide	CD4	-	-	+	-0.00182%
B0070	Group B9	Day 1	Peptide	CD8	+	+	+	0.0011%
B0070	Group B9	Day 1	Peptide	CD8	+	+	-	-0.00102%
B0070	Group B9	Day 1	Peptide	CD8	+	-	+	0%
B0070	Group B9	Day 1	Peptide	CD8	+	-	-	0.04796%
B0070	Group B9	Day 1	Peptide	CD8	-	+	+	-0.00306%

Administration Site Key: FA = Forearm; UA= Upper arm
Treatments Part A: A1 = A/Sing MAP, 15 ug (FA); A2 = Afluria Quad IM (UA), 15 ug; A3 = Placebo MAP (FA); A4 = A/Sing Mono IM, 15 ug (UA)
Treatments Part B: B1 = A/Sing MAP, 15 ug (FA) with biopsy; B2 = Placebo MAP (FA) with biopsy
B3 = A/Sing MAP, 15 ug (FA); B4 = A/Sing MAP, 10 ug (FA); B5 = A/Sing MAP, 5 ug (FA); B6 = A/Sing MAP, 2.5 ug (FA)
B7 = Placebo MAP (FA); B8 = A/Sing MAP, 15 ug (UA); B9 = A/Sing IM, 15 ug, as 2018 Afluria Quad (UA)

Listing 16.2.6.6
Immunogenicity Results (CMI)

Part B

Randomisation Number	Group	Visit	Test	Subset	IL2	TNFa	IFNg	Value
B0070	Group B9	Day 1	Peptide	CD8	-	+	-	0.002%
B0070	Group B9	Day 1	Peptide	CD8	-	-	+	-0.09%
B0070	Group B9	Day 22	Peptide	CD8	+	+	+	0.00135%
B0070	Group B9	Day 22	Peptide	CD8	+	+	-	0.00135%
B0070	Group B9	Day 22	Peptide	CD8	+	-	+	0%
B0070	Group B9	Day 22	Peptide	CD8	+	-	-	-0.022%
B0070	Group B9	Day 22	Peptide	CD8	-	+	+	0.00011%
B0070	Group B9	Day 22	Peptide	CD8	-	+	-	-0.009%
B0070	Group B9	Day 22	Peptide	CD8	-	-	+	-0.12%
B0070	Group B9	Day 1	MPH	CD4	+	+	+	0.13452%
B0070	Group B9	Day 1	MPH	CD4	+	+	-	0.11296%
B0070	Group B9	Day 1	MPH	CD4	+	-	+	0.01443%
B0070	Group B9	Day 1	MPH	CD4	+	-	-	0.04174%
B0070	Group B9	Day 1	MPH	CD4	-	+	+	0.04143%
B0070	Group B9	Day 1	MPH	CD4	-	+	-	0.259%
B0070	Group B9	Day 1	MPH	CD4	-	-	+	0.09387%
B0070	Group B9	Day 22	MPH	CD4	+	+	+	0.15296%
B0070	Group B9	Day 22	MPH	CD4	+	+	-	0.145%
B0070	Group B9	Day 22	MPH	CD4	+	-	+	0.03244%

Administration Site Key: FA = Forearm; UA= Upper arm
Treatments Part A: A1 = A/Sing MAP, 15 ug (FA); A2 = Afluria Quad IM (UA), 15 ug; A3 = Placebo MAP (FA); A4 = A/Sing Mono IM, 15 ug (UA)
Treatments Part B: B1 = A/Sing MAP, 15 ug (FA) with biopsy; B2 = Placebo MAP (FA) with biopsy
B3 = A/Sing MAP, 15 ug (FA); B4 = A/Sing MAP, 10 ug (FA); B5 = A/Sing MAP, 5 ug (FA); B6 = A/Sing MAP, 2.5 ug (FA)
B7 = Placebo MAP (FA); B8 = A/Sing MAP, 15 ug (UA); B9 = A/Sing IM, 15 ug, as 2018 Afluria Quad (UA)

Listing 16.2.6.6
Immunogenicity Results (CMI)

Part B

Randomisation Number	Group	Visit	Test	Subset	IL2	TNFa	IFNg	Value
B0070	Group B9	Day 22	MPH	CD4	+	-	-	0.006%
B0070	Group B9	Day 22	MPH	CD4	-	+	+	0.04252%
B0070	Group B9	Day 22	MPH	CD4	-	+	-	0.279%
B0070	Group B9	Day 22	MPH	CD4	-	-	+	0.13296%
B0070	Group B9	Day 1	MPH	CD8	+	+	+	0.04%
B0070	Group B9	Day 1	MPH	CD8	+	+	-	0.0019%
B0070	Group B9	Day 1	MPH	CD8	+	-	+	0.015%
B0070	Group B9	Day 1	MPH	CD8	+	-	-	0.04296%
B0070	Group B9	Day 1	MPH	CD8	-	+	+	0.05594%
B0070	Group B9	Day 1	MPH	CD8	-	+	-	0.135%
B0070	Group B9	Day 1	MPH	CD8	-	-	+	-0.2%
B0070	Group B9	Day 22	MPH	CD8	+	+	+	0.034%
B0070	Group B9	Day 22	MPH	CD8	+	+	-	0.0042%
B0070	Group B9	Day 22	MPH	CD8	+	-	+	0.0098%
B0070	Group B9	Day 22	MPH	CD8	+	-	-	-0.023%
B0070	Group B9	Day 22	MPH	CD8	-	+	+	0.05776%
B0070	Group B9	Day 22	MPH	CD8	-	+	-	0.125%
B0070	Group B9	Day 22	MPH	CD8	-	-	+	-0.11%

Administration Site Key: FA = Forearm; UA= Upper arm
Treatments Part A: A1 = A/Sing MAP, 15 ug (FA); A2 = Afluria Quad IM (UA), 15 ug; A3 = Placebo MAP (FA); A4 = A/Sing Mono IM, 15 ug (UA)
Treatments Part B: B1 = A/Sing MAP, 15 ug (FA) with biopsy; B2 = Placebo MAP (FA) with biopsy
B3 = A/Sing MAP, 15 ug (FA); B4 = A/Sing MAP, 10 ug (FA); B5 = A/Sing MAP, 5 ug (FA); B6 = A/Sing MAP, 2.5 ug (FA)
B7 = Placebo MAP (FA); B8 = A/Sing MAP, 15 ug (UA); B9 = A/Sing IM, 15 ug, as 2018 Afluria Quad (UA)

Listing 16.2.6.6
Immunogenicity Results (CMI)

Part B

Randomisation Number	Group	Visit	Test	Subset	IL2	TNFa	IFNg	Value
B0072	Group B3	Day 1	Peptide	CD4	+	+	+	0.0008%
B0072	Group B3	Day 1	Peptide	CD4	+	+	-	-0.00336%
B0072	Group B3	Day 1	Peptide	CD4	+	-	+	0%
B0072	Group B3	Day 1	Peptide	CD4	+	-	-	-0.00416%
B0072	Group B3	Day 1	Peptide	CD4	-	+	+	0.000736%
B0072	Group B3	Day 1	Peptide	CD4	-	+	-	0.002%
B0072	Group B3	Day 1	Peptide	CD4	-	-	+	-0.00202%
B0072	Group B3	Day 22	Peptide	CD4	+	+	+	0.00285%
B0072	Group B3	Day 22	Peptide	CD4	+	+	-	0.00685%
B0072	Group B3	Day 22	Peptide	CD4	+	-	+	0.000571%
B0072	Group B3	Day 22	Peptide	CD4	+	-	-	-0.02101%
B0072	Group B3	Day 22	Peptide	CD4	-	+	+	-0.00039%
B0072	Group B3	Day 22	Peptide	CD4	-	+	-	-0.01%
B0072	Group B3	Day 22	Peptide	CD4	-	-	+	-0.00447%
B0072	Group B3	Day 1	Peptide	CD8	+	+	+	0.00172%
B0072	Group B3	Day 1	Peptide	CD8	+	+	-	0%
B0072	Group B3	Day 1	Peptide	CD8	+	-	+	-0.00161%
B0072	Group B3	Day 1	Peptide	CD8	+	-	-	0.00011%
B0072	Group B3	Day 1	Peptide	CD8	-	+	+	-0.0015%

Administration Site Key: FA = Forearm; UA= Upper arm
Treatments Part A: A1 = A/Sing MAP, 15 ug (FA); A2 = Afluria Quad IM (UA), 15 ug; A3 = Placebo MAP (FA); A4 = A/Sing Mono IM, 15 ug (UA)
Treatments Part B: B1 = A/Sing MAP, 15 ug (FA) with biopsy; B2 = Placebo MAP (FA) with biopsy
B3 = A/Sing MAP, 15 ug (FA); B4 = A/Sing MAP, 10 ug (FA); B5 = A/Sing MAP, 5 ug (FA); B6 = A/Sing MAP, 2.5 ug (FA)
B7 = Placebo MAP (FA); B8 = A/Sing MAP, 15 ug (UA); B9 = A/Sing IM, 15 ug, as 2018 Afluria Quad (UA)

Listing 16.2.6.6
Immunogenicity Results (CMI)

Part B

Randomisation Number	Group	Visit	Test	Subset	IL2	TNFa	IFNg	Value
B0072	Group B3	Day 1	Peptide	CD8	-	+	-	0%
B0072	Group B3	Day 1	Peptide	CD8	-	-	+	-2.21%
B0072	Group B3	Day 22	Peptide	CD8	+	+	+	0%
B0072	Group B3	Day 22	Peptide	CD8	+	+	-	0%
B0072	Group B3	Day 22	Peptide	CD8	+	-	+	-0.00274%
B0072	Group B3	Day 22	Peptide	CD8	+	-	-	-0.012%
B0072	Group B3	Day 22	Peptide	CD8	-	+	+	-0.01996%
B0072	Group B3	Day 22	Peptide	CD8	-	+	-	-0.035%
B0072	Group B3	Day 22	Peptide	CD8	-	-	+	-2.98%
B0072	Group B3	Day 1	MPH	CD4	+	+	+	0.03459%
B0072	Group B3	Day 1	MPH	CD4	+	+	-	0.07196%
B0072	Group B3	Day 1	MPH	CD4	+	-	+	0.00351%
B0072	Group B3	Day 1	MPH	CD4	+	-	-	0.02337%
B0072	Group B3	Day 1	MPH	CD4	-	+	+	0.012%
B0072	Group B3	Day 1	MPH	CD4	-	+	-	0.146%
B0072	Group B3	Day 1	MPH	CD4	-	-	+	0.01377%
B0072	Group B3	Day 22	MPH	CD4	+	+	+	0.059%
B0072	Group B3	Day 22	MPH	CD4	+	+	-	0.10285%
B0072	Group B3	Day 22	MPH	CD4	+	-	+	0.029%

Administration Site Key: FA = Forearm; UA= Upper arm
Treatments Part A: A1 = A/Sing MAP, 15 ug (FA); A2 = Afluria Quad IM (UA), 15 ug; A3 = Placebo MAP (FA); A4 = A/Sing Mono IM, 15 ug (UA)
Treatments Part B: B1 = A/Sing MAP, 15 ug (FA) with biopsy; B2 = Placebo MAP (FA) with biopsy
B3 = A/Sing MAP, 15 ug (FA); B4 = A/Sing MAP, 10 ug (FA); B5 = A/Sing MAP, 5 ug (FA); B6 = A/Sing MAP, 2.5 ug (FA)
B7 = Placebo MAP (FA); B8 = A/Sing MAP, 15 ug (UA); B9 = A/Sing IM, 15 ug, as 2018 Afluria Quad (UA)

Listing 16.2.6.6
Immunogenicity Results (CMI)

Part B

Randomisation Number	Group	Visit	Test	Subset	IL2	TNFa	IFNg	Value
B0072	Group B3	Day 22	MPH	CD4	+	-	-	0.052%
B0072	Group B3	Day 22	MPH	CD4	-	+	+	0.01947%
B0072	Group B3	Day 22	MPH	CD4	-	+	-	0.17%
B0072	Group B3	Day 22	MPH	CD4	-	-	+	0.03039%
B0072	Group B3	Day 1	MPH	CD8	+	+	+	0.014%
B0072	Group B3	Day 1	MPH	CD8	+	+	-	0.0077%
B0072	Group B3	Day 1	MPH	CD8	+	-	+	-0.00007%
B0072	Group B3	Day 1	MPH	CD8	+	-	-	0.00939%
B0072	Group B3	Day 1	MPH	CD8	-	+	+	0.03678%
B0072	Group B3	Day 1	MPH	CD8	-	+	-	0.35%
B0072	Group B3	Day 1	MPH	CD8	-	-	+	-2.28%
B0072	Group B3	Day 22	MPH	CD8	+	+	+	0.013%
B0072	Group B3	Day 22	MPH	CD8	+	+	-	0.00538%
B0072	Group B3	Day 22	MPH	CD8	+	-	+	0.00826%
B0072	Group B3	Day 22	MPH	CD8	+	-	-	0.118%
B0072	Group B3	Day 22	MPH	CD8	-	+	+	0.007%
B0072	Group B3	Day 22	MPH	CD8	-	+	-	0.22%
B0072	Group B3	Day 22	MPH	CD8	-	-	+	-3.71%

Administration Site Key: FA = Forearm; UA= Upper arm
Treatments Part A: A1 = A/Sing MAP, 15 ug (FA); A2 = Afluria Quad IM (UA), 15 ug; A3 = Placebo MAP (FA); A4 = A/Sing Mono IM, 15 ug (UA)
Treatments Part B: B1 = A/Sing MAP, 15 ug (FA) with biopsy; B2 = Placebo MAP (FA) with biopsy
B3 = A/Sing MAP, 15 ug (FA); B4 = A/Sing MAP, 10 ug (FA); B5 = A/Sing MAP, 5 ug (FA); B6 = A/Sing MAP, 2.5 ug (FA)
B7 = Placebo MAP (FA); B8 = A/Sing MAP, 15 ug (UA); B9 = A/Sing IM, 15 ug, as 2018 Afluria Quad (UA)

Listing 16.2.6.6
Immunogenicity Results (CMI)

Part B

Randomisation Number	Group	Visit	Test	Subset	IL2	TNFa	IFNg	Value
B0074	Group B9	Day 1	Peptide	CD4	+	+	+	-0.00062%
B0074	Group B9	Day 1	Peptide	CD4	+	+	-	0.00534%
B0074	Group B9	Day 1	Peptide	CD4	+	-	+	-0.00197%
B0074	Group B9	Day 1	Peptide	CD4	+	-	-	0.00114%
B0074	Group B9	Day 1	Peptide	CD4	-	+	+	-0.00197%
B0074	Group B9	Day 1	Peptide	CD4	-	+	-	0.005%
B0074	Group B9	Day 1	Peptide	CD4	-	-	+	0.00135%
B0074	Group B9	Day 22	Peptide	CD4	+	+	+	0.00347%
B0074	Group B9	Day 22	Peptide	CD4	+	+	-	0.02824%
B0074	Group B9	Day 22	Peptide	CD4	+	-	+	0.000838%
B0074	Group B9	Day 22	Peptide	CD4	+	-	-	-0.00275%
B0074	Group B9	Day 22	Peptide	CD4	-	+	+	-0.00063%
B0074	Group B9	Day 22	Peptide	CD4	-	+	-	0.008%
B0074	Group B9	Day 22	Peptide	CD4	-	-	+	0.004018%
B0074	Group B9	Day 1	Peptide	CD8	+	+	+	0%
B0074	Group B9	Day 1	Peptide	CD8	+	+	-	0%
B0074	Group B9	Day 1	Peptide	CD8	+	-	+	0%
B0074	Group B9	Day 1	Peptide	CD8	+	-	-	0.00463%
B0074	Group B9	Day 1	Peptide	CD8	-	+	+	0%

Administration Site Key: FA = Forearm; UA= Upper arm
Treatments Part A: A1 = A/Sing MAP, 15 ug (FA); A2 = Afluria Quad IM (UA), 15 ug; A3 = Placebo MAP (FA); A4 = A/Sing Mono IM, 15 ug (UA)
Treatments Part B: B1 = A/Sing MAP, 15 ug (FA) with biopsy; B2 = Placebo MAP (FA) with biopsy
B3 = A/Sing MAP, 15 ug (FA); B4 = A/Sing MAP, 10 ug (FA); B5 = A/Sing MAP, 5 ug (FA); B6 = A/Sing MAP, 2.5 ug (FA)
B7 = Placebo MAP (FA); B8 = A/Sing MAP, 15 ug (UA); B9 = A/Sing IM, 15 ug, as 2018 Afluria Quad (UA)

Listing 16.2.6.6
Immunogenicity Results (CMI)

Part B

Randomisation Number	Group	Visit	Test	Subset	IL2	TNFa	IFNg	Value
B0074	Group B9	Day 1	Peptide	CD8	-	+	-	0.005%
B0074	Group B9	Day 1	Peptide	CD8	-	-	+	-0.08%
B0074	Group B9	Day 22	Peptide	CD8	+	+	+	0%
B0074	Group B9	Day 22	Peptide	CD8	+	+	-	0%
B0074	Group B9	Day 22	Peptide	CD8	+	-	+	0%
B0074	Group B9	Day 22	Peptide	CD8	+	-	-	0.00573%
B0074	Group B9	Day 22	Peptide	CD8	-	+	+	-0.00345%
B0074	Group B9	Day 22	Peptide	CD8	-	+	-	-0.003%
B0074	Group B9	Day 22	Peptide	CD8	-	-	+	-0.02%
B0074	Group B9	Day 1	MPH	CD4	+	+	+	0.0724%
B0074	Group B9	Day 1	MPH	CD4	+	+	-	0.07106%
B0074	Group B9	Day 1	MPH	CD4	+	-	+	0.01003%
B0074	Group B9	Day 1	MPH	CD4	+	-	-	0.03614%
B0074	Group B9	Day 1	MPH	CD4	-	+	+	0.03503%
B0074	Group B9	Day 1	MPH	CD4	-	+	-	0.248%
B0074	Group B9	Day 1	MPH	CD4	-	-	+	0.04337%
B0074	Group B9	Day 22	MPH	CD4	+	+	+	0.07173%
B0074	Group B9	Day 22	MPH	CD4	+	+	-	0.09624%
B0074	Group B9	Day 22	MPH	CD4	+	-	+	0.015248%

Administration Site Key: FA = Forearm; UA= Upper arm
Treatments Part A: A1 = A/Sing MAP, 15 ug (FA); A2 = Afluria Quad IM (UA), 15 ug; A3 = Placebo MAP (FA); A4 = A/Sing Mono IM, 15 ug (UA)
Treatments Part B: B1 = A/Sing MAP, 15 ug (FA) with biopsy; B2 = Placebo MAP (FA) with biopsy
B3 = A/Sing MAP, 15 ug (FA); B4 = A/Sing MAP, 10 ug (FA); B5 = A/Sing MAP, 5 ug (FA); B6 = A/Sing MAP, 2.5 ug (FA)
B7 = Placebo MAP (FA); B8 = A/Sing MAP, 15 ug (UA); B9 = A/Sing IM, 15 ug, as 2018 Afluria Quad (UA)

Listing 16.2.6.6
Immunogenicity Results (CMI)

Part B

Randomisation Number	Group	Visit	Test	Subset	IL2	TNFa	IFNg	Value
B0074	Group B9	Day 22	MPH	CD4	+	-	-	0.05048%
B0074	Group B9	Day 22	MPH	CD4	-	+	+	0.03699%
B0074	Group B9	Day 22	MPH	CD4	-	+	-	0.191%
B0074	Group B9	Day 22	MPH	CD4	-	-	+	0.064248%
B0074	Group B9	Day 1	MPH	CD8	+	+	+	0.024%
B0074	Group B9	Day 1	MPH	CD8	+	+	-	0.00569%
B0074	Group B9	Day 1	MPH	CD8	+	-	+	0.00711%
B0074	Group B9	Day 1	MPH	CD8	+	-	-	0.01949%
B0074	Group B9	Day 1	MPH	CD8	-	+	+	0.098%
B0074	Group B9	Day 1	MPH	CD8	-	+	-	0.877%
B0074	Group B9	Day 1	MPH	CD8	-	-	+	-0.12%
B0074	Group B9	Day 22	MPH	CD8	+	+	+	0.01%
B0074	Group B9	Day 22	MPH	CD8	+	+	-	0.012%
B0074	Group B9	Day 22	MPH	CD8	+	-	+	0.00338%
B0074	Group B9	Day 22	MPH	CD8	+	-	-	0.02522%
B0074	Group B9	Day 22	MPH	CD8	-	+	+	0.07267%
B0074	Group B9	Day 22	MPH	CD8	-	+	-	0.379%
B0074	Group B9	Day 22	MPH	CD8	-	-	+	-0.03%

Administration Site Key: FA = Forearm; UA= Upper arm
Treatments Part A: A1 = A/Sing MAP, 15 ug (FA); A2 = Afluria Quad IM (UA), 15 ug; A3 = Placebo MAP (FA); A4 = A/Sing Mono IM, 15 ug (UA)
Treatments Part B: B1 = A/Sing MAP, 15 ug (FA) with biopsy; B2 = Placebo MAP (FA) with biopsy
B3 = A/Sing MAP, 15 ug (FA); B4 = A/Sing MAP, 10 ug (FA); B5 = A/Sing MAP, 5 ug (FA); B6 = A/Sing MAP, 2.5 ug (FA)
B7 = Placebo MAP (FA); B8 = A/Sing MAP, 15 ug (UA); B9 = A/Sing IM, 15 ug, as 2018 Afluria Quad (UA)

Listing 16.2.6.6
Immunogenicity Results (CMI)

Part B

Randomisation Number	Group	Visit	Test	Subset	IL2	TNFa	IFNg	Value
B0075	Group B3	Day 1	Peptide	CD4	+	+	+	-0.00335%
B0075	Group B3	Day 1	Peptide	CD4	+	+	-	0%
B0075	Group B3	Day 1	Peptide	CD4	+	-	+	-7E-6%
B0075	Group B3	Day 1	Peptide	CD4	+	-	-	-0.00607%
B0075	Group B3	Day 1	Peptide	CD4	-	+	+	-0.00002%
B0075	Group B3	Day 1	Peptide	CD4	-	+	-	-0.014%
B0075	Group B3	Day 1	Peptide	CD4	-	-	+	-0.00168%
B0075	Group B3	Day 22	Peptide	CD4	+	+	+	0.02106%
B0075	Group B3	Day 22	Peptide	CD4	+	+	-	0.02452%
B0075	Group B3	Day 22	Peptide	CD4	+	-	+	0.000631%
B0075	Group B3	Day 22	Peptide	CD4	+	-	-	0.0024%
B0075	Group B3	Day 22	Peptide	CD4	-	+	+	0.00626%
B0075	Group B3	Day 22	Peptide	CD4	-	+	-	0.039%
B0075	Group B3	Day 22	Peptide	CD4	-	-	+	-0.00137%
B0075	Group B3	Day 1	Peptide	CD8	+	+	+	0%
B0075	Group B3	Day 1	Peptide	CD8	+	+	-	-0.00244%
B0075	Group B3	Day 1	Peptide	CD8	+	-	+	0%
B0075	Group B3	Day 1	Peptide	CD8	+	-	-	0.00493%
B0075	Group B3	Day 1	Peptide	CD8	-	+	+	0%

Administration Site Key: FA = Forearm; UA= Upper arm
Treatments Part A: A1 = A/Sing MAP, 15 ug (FA); A2 = Afluria Quad IM (UA), 15 ug; A3 = Placebo MAP (FA); A4 = A/Sing Mono IM, 15 ug (UA)
Treatments Part B: B1 = A/Sing MAP, 15 ug (FA) with biopsy; B2 = Placebo MAP (FA) with biopsy
B3 = A/Sing MAP, 15 ug (FA); B4 = A/Sing MAP, 10 ug (FA); B5 = A/Sing MAP, 5 ug (FA); B6 = A/Sing MAP, 2.5 ug (FA)
B7 = Placebo MAP (FA); B8 = A/Sing MAP, 15 ug (UA); B9 = A/Sing IM, 15 ug, as 2018 Afluria Quad (UA)

Listing 16.2.6.6
Immunogenicity Results (CMI)

Part B

Randomisation Number	Group	Visit	Test	Subset	IL2	TNFa	IFNg	Value
B0075	Group B3	Day 1	Peptide	CD8	-	+	-	-0.021%
B0075	Group B3	Day 1	Peptide	CD8	-	-	+	-0.36%
B0075	Group B3	Day 22	Peptide	CD8	+	+	+	0%
B0075	Group B3	Day 22	Peptide	CD8	+	+	-	0%
B0075	Group B3	Day 22	Peptide	CD8	+	-	+	0%
B0075	Group B3	Day 22	Peptide	CD8	+	-	-	0.00176%
B0075	Group B3	Day 22	Peptide	CD8	-	+	+	0.00173%
B0075	Group B3	Day 22	Peptide	CD8	-	+	-	-0.022%
B0075	Group B3	Day 22	Peptide	CD8	-	-	+	-0.03%
B0075	Group B3	Day 1	MPH	CD4	+	+	+	0.105%
B0075	Group B3	Day 1	MPH	CD4	+	+	-	0.094%
B0075	Group B3	Day 1	MPH	CD4	+	-	+	0.009056%
B0075	Group B3	Day 1	MPH	CD4	+	-	-	0.012%
B0075	Group B3	Day 1	MPH	CD4	-	+	+	0.0455%
B0075	Group B3	Day 1	MPH	CD4	-	+	-	0.342%
B0075	Group B3	Day 1	MPH	CD4	-	-	+	0.05167%
B0075	Group B3	Day 22	MPH	CD4	+	+	+	0.13806%
B0075	Group B3	Day 22	MPH	CD4	+	+	-	0.12352%
B0075	Group B3	Day 22	MPH	CD4	+	-	+	0.023%

Administration Site Key: FA = Forearm; UA= Upper arm
Treatments Part A: A1 = A/Sing MAP, 15 ug (FA); A2 = Afluria Quad IM (UA), 15 ug; A3 = Placebo MAP (FA); A4 = A/Sing Mono IM, 15 ug (UA)
Treatments Part B: B1 = A/Sing MAP, 15 ug (FA) with biopsy; B2 = Placebo MAP (FA) with biopsy
B3 = A/Sing MAP, 15 ug (FA); B4 = A/Sing MAP, 10 ug (FA); B5 = A/Sing MAP, 5 ug (FA); B6 = A/Sing MAP, 2.5 ug (FA)
B7 = Placebo MAP (FA); B8 = A/Sing MAP, 15 ug (UA); B9 = A/Sing IM, 15 ug, as 2018 Afluria Quad (UA)

Listing 16.2.6.6
Immunogenicity Results (CMI)

Part B

Randomisation Number	Group	Visit	Test	Subset	IL2	TNFa	IFNg	Value
B0075	Group B3	Day 22	MPH	CD4	+	-	-	0.03146%
B0075	Group B3	Day 22	MPH	CD4	-	+	+	0.06206%
B0075	Group B3	Day 22	MPH	CD4	-	+	-	0.345%
B0075	Group B3	Day 22	MPH	CD4	-	-	+	0.09611%
B0075	Group B3	Day 1	MPH	CD8	+	+	+	0.026%
B0075	Group B3	Day 1	MPH	CD8	+	+	-	0.01556%
B0075	Group B3	Day 1	MPH	CD8	+	-	+	0.01%
B0075	Group B3	Day 1	MPH	CD8	+	-	-	0.018%
B0075	Group B3	Day 1	MPH	CD8	-	+	+	0.071%
B0075	Group B3	Day 1	MPH	CD8	-	+	-	0.112%
B0075	Group B3	Day 1	MPH	CD8	-	-	+	-0.63%
B0075	Group B3	Day 22	MPH	CD8	+	+	+	0.022%
B0075	Group B3	Day 22	MPH	CD8	+	+	-	0.00361%
B0075	Group B3	Day 22	MPH	CD8	+	-	+	0.00361%
B0075	Group B3	Day 22	MPH	CD8	+	-	-	0.034%
B0075	Group B3	Day 22	MPH	CD8	-	+	+	0.11821%
B0075	Group B3	Day 22	MPH	CD8	-	+	-	0.3%
B0075	Group B3	Day 22	MPH	CD8	-	-	+	0.11%

Administration Site Key: FA = Forearm; UA= Upper arm
Treatments Part A: A1 = A/Sing MAP, 15 ug (FA); A2 = Afluria Quad IM (UA), 15 ug; A3 = Placebo MAP (FA); A4 = A/Sing Mono IM, 15 ug (UA)
Treatments Part B: B1 = A/Sing MAP, 15 ug (FA) with biopsy; B2 = Placebo MAP (FA) with biopsy
B3 = A/Sing MAP, 15 ug (FA); B4 = A/Sing MAP, 10 ug (FA); B5 = A/Sing MAP, 5 ug (FA); B6 = A/Sing MAP, 2.5 ug (FA)
B7 = Placebo MAP (FA); B8 = A/Sing MAP, 15 ug (UA); B9 = A/Sing IM, 15 ug, as 2018 Afluria Quad (UA)

Listing 16.2.6.6
Immunogenicity Results (CMI)

Part B

Randomisation Number	Group	Visit	Test	Subset	IL2	TNFa	IFNg	Value
B0078	Group B8	Day 1	Peptide	CD4	+	+	+	0.00242%
B0078	Group B8	Day 1	Peptide	CD4	+	+	-	0.00328%
B0078	Group B8	Day 1	Peptide	CD4	+	-	+	0%
B0078	Group B8	Day 1	Peptide	CD4	+	-	-	0.0023%
B0078	Group B8	Day 1	Peptide	CD4	-	+	+	0.000969%
B0078	Group B8	Day 1	Peptide	CD4	-	+	-	0.011%
B0078	Group B8	Day 1	Peptide	CD4	-	-	+	0.00339%
B0078	Group B8	Day 22	Peptide	CD4	+	+	+	0.01296%
B0078	Group B8	Day 22	Peptide	CD4	+	+	-	0.01742%
B0078	Group B8	Day 22	Peptide	CD4	+	-	+	0.00051%
B0078	Group B8	Day 22	Peptide	CD4	+	-	-	0.00257%
B0078	Group B8	Day 22	Peptide	CD4	-	+	+	0.00153%
B0078	Group B8	Day 22	Peptide	CD4	-	+	-	0.003%
B0078	Group B8	Day 22	Peptide	CD4	-	-	+	-0.00051%
B0078	Group B8	Day 1	Peptide	CD8	+	+	+	0%
B0078	Group B8	Day 1	Peptide	CD8	+	+	-	0%
B0078	Group B8	Day 1	Peptide	CD8	+	-	+	0%
B0078	Group B8	Day 1	Peptide	CD8	+	-	-	0.00783%
B0078	Group B8	Day 1	Peptide	CD8	-	+	+	-0.00318%

Administration Site Key: FA = Forearm; UA= Upper arm
Treatments Part A: A1 = A/Sing MAP, 15 ug (FA); A2 = Afluria Quad IM (UA), 15 ug; A3 = Placebo MAP (FA); A4 = A/Sing Mono IM, 15 ug (UA)
Treatments Part B: B1 = A/Sing MAP, 15 ug (FA) with biopsy; B2 = Placebo MAP (FA) with biopsy
B3 = A/Sing MAP, 15 ug (FA); B4 = A/Sing MAP, 10 ug (FA); B5 = A/Sing MAP, 5 ug (FA); B6 = A/Sing MAP, 2.5 ug (FA)
B7 = Placebo MAP (FA); B8 = A/Sing MAP, 15 ug (UA); B9 = A/Sing IM, 15 ug, as 2018 Afluria Quad (UA)

Listing 16.2.6.6
Immunogenicity Results (CMI)

Part B

Randomisation Number	Group	Visit	Test	Subset	IL2	TNFa	IFNg	Value
B0078	Group B8	Day 1	Peptide	CD8	-	+	-	-0.015%
B0078	Group B8	Day 1	Peptide	CD8	-	-	+	0.76%
B0078	Group B8	Day 22	Peptide	CD8	+	+	+	0%
B0078	Group B8	Day 22	Peptide	CD8	+	+	-	0%
B0078	Group B8	Day 22	Peptide	CD8	+	-	+	0%
B0078	Group B8	Day 22	Peptide	CD8	+	-	-	1E-5%
B0078	Group B8	Day 22	Peptide	CD8	-	+	+	1E-5%
B0078	Group B8	Day 22	Peptide	CD8	-	+	-	-0.015%
B0078	Group B8	Day 22	Peptide	CD8	-	-	+	0.24%
B0078	Group B8	Day 1	MPH	CD4	+	+	+	0.035%
B0078	Group B8	Day 1	MPH	CD4	+	+	-	0.07008%
B0078	Group B8	Day 1	MPH	CD4	+	-	+	0.00616%
B0078	Group B8	Day 1	MPH	CD4	+	-	-	0.03661%
B0078	Group B8	Day 1	MPH	CD4	-	+	+	0.03%
B0078	Group B8	Day 1	MPH	CD4	-	+	-	0.211%
B0078	Group B8	Day 1	MPH	CD4	-	-	+	0.018%
B0078	Group B8	Day 22	MPH	CD4	+	+	+	0.09796%
B0078	Group B8	Day 22	MPH	CD4	+	+	-	0.08042%
B0078	Group B8	Day 22	MPH	CD4	+	-	+	0.027%

Administration Site Key: FA = Forearm; UA= Upper arm
Treatments Part A: A1 = A/Sing MAP, 15 ug (FA); A2 = Afluria Quad IM (UA), 15 ug; A3 = Placebo MAP (FA); A4 = A/Sing Mono IM, 15 ug (UA)
Treatments Part B: B1 = A/Sing MAP, 15 ug (FA) with biopsy; B2 = Placebo MAP (FA) with biopsy
B3 = A/Sing MAP, 15 ug (FA); B4 = A/Sing MAP, 10 ug (FA); B5 = A/Sing MAP, 5 ug (FA); B6 = A/Sing MAP, 2.5 ug (FA)
B7 = Placebo MAP (FA); B8 = A/Sing MAP, 15 ug (UA); B9 = A/Sing IM, 15 ug, as 2018 Afluria Quad (UA)

Listing 16.2.6.6
Immunogenicity Results (CMI)

Part B

Randomisation Number	Group	Visit	Test	Subset	IL2	TNFa	IFNg	Value
B0078	Group B8	Day 22	MPH	CD4	+	-	-	0.04591%
B0078	Group B8	Day 22	MPH	CD4	-	+	+	0.05847%
B0078	Group B8	Day 22	MPH	CD4	-	+	-	0.23%
B0078	Group B8	Day 22	MPH	CD4	-	-	+	0.09394%
B0078	Group B8	Day 1	MPH	CD8	+	+	+	0.011%
B0078	Group B8	Day 1	MPH	CD8	+	+	-	0.00537%
B0078	Group B8	Day 1	MPH	CD8	+	-	+	0.00358%
B0078	Group B8	Day 1	MPH	CD8	+	-	-	0.016%
B0078	Group B8	Day 1	MPH	CD8	-	+	+	0.02882%
B0078	Group B8	Day 1	MPH	CD8	-	+	-	0.077%
B0078	Group B8	Day 1	MPH	CD8	-	-	+	-0.39%
B0078	Group B8	Day 22	MPH	CD8	+	+	+	0.013%
B0078	Group B8	Day 22	MPH	CD8	+	+	-	0.013%
B0078	Group B8	Day 22	MPH	CD8	+	-	+	0.00593%
B0078	Group B8	Day 22	MPH	CD8	+	-	-	0.02987%
B0078	Group B8	Day 22	MPH	CD8	-	+	+	0.07687%
B0078	Group B8	Day 22	MPH	CD8	-	+	-	0.76%
B0078	Group B8	Day 22	MPH	CD8	-	-	+	-0.02%

Administration Site Key: FA = Forearm; UA= Upper arm
Treatments Part A: A1 = A/Sing MAP, 15 ug (FA); A2 = Afluria Quad IM (UA), 15 ug; A3 = Placebo MAP (FA); A4 = A/Sing Mono IM, 15 ug (UA)
Treatments Part B: B1 = A/Sing MAP, 15 ug (FA) with biopsy; B2 = Placebo MAP (FA) with biopsy
B3 = A/Sing MAP, 15 ug (FA); B4 = A/Sing MAP, 10 ug (FA); B5 = A/Sing MAP, 5 ug (FA); B6 = A/Sing MAP, 2.5 ug (FA)
B7 = Placebo MAP (FA); B8 = A/Sing MAP, 15 ug (UA); B9 = A/Sing IM, 15 ug, as 2018 Afluria Quad (UA)

Listing 16.2.6.6
Immunogenicity Results (CMI)

Part B

Randomisation Number	Group	Visit	Test	Subset	IL2	TNFa	IFNg	Value
B0082	Group B3	Day 1	Peptide	CD4	+	+	+	0.01%
B0082	Group B3	Day 1	Peptide	CD4	+	+	-	0.01666%
B0082	Group B3	Day 1	Peptide	CD4	+	-	+	0%
B0082	Group B3	Day 1	Peptide	CD4	+	-	-	0.00154%
B0082	Group B3	Day 1	Peptide	CD4	-	+	+	-0.00258%
B0082	Group B3	Day 1	Peptide	CD4	-	+	-	-1.331%
B0082	Group B3	Day 1	Peptide	CD4	-	-	+	-0.00351%
B0082	Group B3	Day 22	Peptide	CD4	+	+	+	0.01731%
B0082	Group B3	Day 22	Peptide	CD4	+	+	-	0.013%
B0082	Group B3	Day 22	Peptide	CD4	+	-	+	-0.000844%
B0082	Group B3	Day 22	Peptide	CD4	+	-	-	-0.00425%
B0082	Group B3	Day 22	Peptide	CD4	-	+	+	0.003346%
B0082	Group B3	Day 22	Peptide	CD4	-	+	-	0.022%
B0082	Group B3	Day 22	Peptide	CD4	-	-	+	-0.0017%
B0082	Group B3	Day 1	Peptide	CD8	+	+	+	0%
B0082	Group B3	Day 1	Peptide	CD8	+	+	-	-0.00172%
B0082	Group B3	Day 1	Peptide	CD8	+	-	+	0%
B0082	Group B3	Day 1	Peptide	CD8	+	-	-	-0.00257%
B0082	Group B3	Day 1	Peptide	CD8	-	+	+	-1.12741%

Administration Site Key: FA = Forearm; UA= Upper arm
Treatments Part A: A1 = A/Sing MAP, 15 ug (FA); A2 = Afluria Quad IM (UA), 15 ug; A3 = Placebo MAP (FA); A4 = A/Sing Mono IM, 15 ug (UA)
Treatments Part B: B1 = A/Sing MAP, 15 ug (FA) with biopsy; B2 = Placebo MAP (FA) with biopsy
B3 = A/Sing MAP, 15 ug (FA); B4 = A/Sing MAP, 10 ug (FA); B5 = A/Sing MAP, 5 ug (FA); B6 = A/Sing MAP, 2.5 ug (FA)
B7 = Placebo MAP (FA); B8 = A/Sing MAP, 15 ug (UA); B9 = A/Sing IM, 15 ug, as 2018 Afluria Quad (UA)

Listing 16.2.6.6
Immunogenicity Results (CMI)

Part B

Randomisation Number	Group	Visit	Test	Subset	IL2	TNFa	IFNg	Value
B0082	Group B3	Day 1	Peptide	CD8	-	+	-	-0.025%
B0082	Group B3	Day 1	Peptide	CD8	-	-	+	-3.19%
B0082	Group B3	Day 22	Peptide	CD8	+	+	+	0%
B0082	Group B3	Day 22	Peptide	CD8	+	+	-	-0.00119%
B0082	Group B3	Day 22	Peptide	CD8	+	-	+	0.00117%
B0082	Group B3	Day 22	Peptide	CD8	+	-	-	0.00115%
B0082	Group B3	Day 22	Peptide	CD8	-	+	+	0.00343%
B0082	Group B3	Day 22	Peptide	CD8	-	+	-	-0.01%
B0082	Group B3	Day 22	Peptide	CD8	-	-	+	1.52%
B0082	Group B3	Day 1	MPH	CD4	+	+	+	0.097%
B0082	Group B3	Day 1	MPH	CD4	+	+	-	0.09866%
B0082	Group B3	Day 1	MPH	CD4	+	-	+	0.00727%
B0082	Group B3	Day 1	MPH	CD4	+	-	-	0.01398%
B0082	Group B3	Day 1	MPH	CD4	-	+	+	0.06664%
B0082	Group B3	Day 1	MPH	CD4	-	+	-	-1.14%
B0082	Group B3	Day 1	MPH	CD4	-	-	+	0.03564%
B0082	Group B3	Day 22	MPH	CD4	+	+	+	0.10831%
B0082	Group B3	Day 22	MPH	CD4	+	+	-	0.09%
B0082	Group B3	Day 22	MPH	CD4	+	-	+	0.015156%

Administration Site Key: FA = Forearm; UA= Upper arm
Treatments Part A: A1 = A/Sing MAP, 15 ug (FA); A2 = Afluria Quad IM (UA), 15 ug; A3 = Placebo MAP (FA); A4 = A/Sing Mono IM, 15 ug (UA)
Treatments Part B: B1 = A/Sing MAP, 15 ug (FA) with biopsy; B2 = Placebo MAP (FA) with biopsy
B3 = A/Sing MAP, 15 ug (FA); B4 = A/Sing MAP, 10 ug (FA); B5 = A/Sing MAP, 5 ug (FA); B6 = A/Sing MAP, 2.5 ug (FA)
B7 = Placebo MAP (FA); B8 = A/Sing MAP, 15 ug (UA); B9 = A/Sing IM, 15 ug, as 2018 Afluria Quad (UA)

Listing 16.2.6.6
Immunogenicity Results (CMI)

Part B

Randomisation Number	Group	Visit	Test	Subset	IL2	TNFa	IFNg	Value
B0082	Group B3	Day 22	MPH	CD4	+	-	-	0.03056%
B0082	Group B3	Day 22	MPH	CD4	-	+	+	0.067156%
B0082	Group B3	Day 22	MPH	CD4	-	+	-	0.183%
B0082	Group B3	Day 22	MPH	CD4	-	-	+	0.03562%
B0082	Group B3	Day 1	MPH	CD8	+	+	+	0.012%
B0082	Group B3	Day 1	MPH	CD8	+	+	-	0.00375%
B0082	Group B3	Day 1	MPH	CD8	+	-	+	0.00273%
B0082	Group B3	Day 1	MPH	CD8	+	-	-	0.00984%
B0082	Group B3	Day 1	MPH	CD8	-	+	+	-1.081%
B0082	Group B3	Day 1	MPH	CD8	-	+	-	0.089%
B0082	Group B3	Day 1	MPH	CD8	-	-	+	-3.367%
B0082	Group B3	Day 22	MPH	CD8	+	+	+	0.01%
B0082	Group B3	Day 22	MPH	CD8	+	+	-	0.00881%
B0082	Group B3	Day 22	MPH	CD8	+	-	+	0.00577%
B0082	Group B3	Day 22	MPH	CD8	+	-	-	0.03581%
B0082	Group B3	Day 22	MPH	CD8	-	+	+	0.02306%
B0082	Group B3	Day 22	MPH	CD8	-	+	-	0.113%
B0082	Group B3	Day 22	MPH	CD8	-	-	+	-2.56%

Administration Site Key: FA = Forearm; UA= Upper arm
Treatments Part A: A1 = A/Sing MAP, 15 ug (FA); A2 = Afluria Quad IM (UA), 15 ug; A3 = Placebo MAP (FA); A4 = A/Sing Mono IM, 15 ug (UA)
Treatments Part B: B1 = A/Sing MAP, 15 ug (FA) with biopsy; B2 = Placebo MAP (FA) with biopsy
B3 = A/Sing MAP, 15 ug (FA); B4 = A/Sing MAP, 10 ug (FA); B5 = A/Sing MAP, 5 ug (FA); B6 = A/Sing MAP, 2.5 ug (FA)
B7 = Placebo MAP (FA); B8 = A/Sing MAP, 15 ug (UA); B9 = A/Sing IM, 15 ug, as 2018 Afluria Quad (UA)

Listing 16.2.6.6
Immunogenicity Results (CMI)

Part B

Randomisation Number	Group	Visit	Test	Subset	IL2	TNFa	IFNg	Value
B0083	Group B8	Day 1	Peptide	CD4	+	+	+	0%
B0083	Group B8	Day 1	Peptide	CD4	+	+	-	0.00271%
B0083	Group B8	Day 1	Peptide	CD4	+	-	+	0%
B0083	Group B8	Day 1	Peptide	CD4	+	-	-	-0.00199%
B0083	Group B8	Day 1	Peptide	CD4	-	+	+	-0.000045%
B0083	Group B8	Day 1	Peptide	CD4	-	+	-	0.008%
B0083	Group B8	Day 1	Peptide	CD4	-	-	+	-0.00009%
B0083	Group B8	Day 22	Peptide	CD4	+	+	+	0.001869%
B0083	Group B8	Day 22	Peptide	CD4	+	+	-	0.01765%
B0083	Group B8	Day 22	Peptide	CD4	+	-	+	0%
B0083	Group B8	Day 22	Peptide	CD4	+	-	-	0.00381%
B0083	Group B8	Day 22	Peptide	CD4	-	+	+	0.00553%
B0083	Group B8	Day 22	Peptide	CD4	-	+	-	0.017%
B0083	Group B8	Day 22	Peptide	CD4	-	-	+	0.003709%
B0083	Group B8	Day 1	Peptide	CD8	+	+	+	0.00404%
B0083	Group B8	Day 1	Peptide	CD8	+	+	-	0.00606%
B0083	Group B8	Day 1	Peptide	CD8	+	-	+	0%
B0083	Group B8	Day 1	Peptide	CD8	+	-	-	-0.00329%
B0083	Group B8	Day 1	Peptide	CD8	-	+	+	0.00069%

Administration Site Key: FA = Forearm; UA= Upper arm
Treatments Part A: A1 = A/Sing MAP, 15 ug (FA); A2 = Afluria Quad IM (UA), 15 ug; A3 = Placebo MAP (FA); A4 = A/Sing Mono IM, 15 ug (UA)
Treatments Part B: B1 = A/Sing MAP, 15 ug (FA) with biopsy; B2 = Placebo MAP (FA) with biopsy
B3 = A/Sing MAP, 15 ug (FA); B4 = A/Sing MAP, 10 ug (FA); B5 = A/Sing MAP, 5 ug (FA); B6 = A/Sing MAP, 2.5 ug (FA)
B7 = Placebo MAP (FA); B8 = A/Sing MAP, 15 ug (UA); B9 = A/Sing IM, 15 ug, as 2018 Afluria Quad (UA)

Listing 16.2.6.6
Immunogenicity Results (CMI)

Part B

Randomisation Number	Group	Visit	Test	Subset	IL2	TNFa	IFNg	Value
B0083	Group B8	Day 1	Peptide	CD8	-	+	-	0.011%
B0083	Group B8	Day 1	Peptide	CD8	-	-	+	-0.06%
B0083	Group B8	Day 22	Peptide	CD8	+	+	+	0.00522%
B0083	Group B8	Day 22	Peptide	CD8	+	+	-	0%
B0083	Group B8	Day 22	Peptide	CD8	+	-	+	0%
B0083	Group B8	Day 22	Peptide	CD8	+	-	-	0.00626%
B0083	Group B8	Day 22	Peptide	CD8	-	+	+	0.01283%
B0083	Group B8	Day 22	Peptide	CD8	-	+	-	0.038%
B0083	Group B8	Day 22	Peptide	CD8	-	-	+	0.108%
B0083	Group B8	Day 1	MPH	CD4	+	+	+	0.052%
B0083	Group B8	Day 1	MPH	CD4	+	+	-	0.05002%
B0083	Group B8	Day 1	MPH	CD4	+	-	+	0.00391%
B0083	Group B8	Day 1	MPH	CD4	+	-	-	0.02101%
B0083	Group B8	Day 1	MPH	CD4	-	+	+	0.033006%
B0083	Group B8	Day 1	MPH	CD4	-	+	-	0.182%
B0083	Group B8	Day 1	MPH	CD4	-	-	+	0.02001%
B0083	Group B8	Day 22	MPH	CD4	+	+	+	0.109109%
B0083	Group B8	Day 22	MPH	CD4	+	+	-	0.05665%
B0083	Group B8	Day 22	MPH	CD4	+	-	+	0.026%

Administration Site Key: FA = Forearm; UA= Upper arm
Treatments Part A: A1 = A/Sing MAP, 15 ug (FA); A2 = Afluria Quad IM (UA), 15 ug; A3 = Placebo MAP (FA); A4 = A/Sing Mono IM, 15 ug (UA)
Treatments Part B: B1 = A/Sing MAP, 15 ug (FA) with biopsy; B2 = Placebo MAP (FA) with biopsy
B3 = A/Sing MAP, 15 ug (FA); B4 = A/Sing MAP, 10 ug (FA); B5 = A/Sing MAP, 5 ug (FA); B6 = A/Sing MAP, 2.5 ug (FA)
B7 = Placebo MAP (FA); B8 = A/Sing MAP, 15 ug (UA); B9 = A/Sing IM, 15 ug, as 2018 Afluria Quad (UA)

Listing 16.2.6.6
Immunogenicity Results (CMI)

Part B

Randomisation Number	Group	Visit	Test	Subset	IL2	TNFa	IFNg	Value
B0083	Group B8	Day 22	MPH	CD4	+	-	-	0.04044%
B0083	Group B8	Day 22	MPH	CD4	-	+	+	0.063%
B0083	Group B8	Day 22	MPH	CD4	-	+	-	0.155%
B0083	Group B8	Day 22	MPH	CD4	-	-	+	0.075109%
B0083	Group B8	Day 1	MPH	CD8	+	+	+	0.00414%
B0083	Group B8	Day 1	MPH	CD8	+	+	-	0.00104%
B0083	Group B8	Day 1	MPH	CD8	+	-	+	0%
B0083	Group B8	Day 1	MPH	CD8	+	-	-	0.0107%
B0083	Group B8	Day 1	MPH	CD8	-	+	+	0.00763%
B0083	Group B8	Day 1	MPH	CD8	-	+	-	0.07%
B0083	Group B8	Day 1	MPH	CD8	-	-	+	-0.03%
B0083	Group B8	Day 22	MPH	CD8	+	+	+	0.001%
B0083	Group B8	Day 22	MPH	CD8	+	+	-	0.001%
B0083	Group B8	Day 22	MPH	CD8	+	-	+	0%
B0083	Group B8	Day 22	MPH	CD8	+	-	-	0.02596%
B0083	Group B8	Day 22	MPH	CD8	-	+	+	0.02383%
B0083	Group B8	Day 22	MPH	CD8	-	+	-	0.097%
B0083	Group B8	Day 22	MPH	CD8	-	-	+	0.038%

Administration Site Key: FA = Forearm; UA= Upper arm
Treatments Part A: A1 = A/Sing MAP, 15 ug (FA); A2 = Afluria Quad IM (UA), 15 ug; A3 = Placebo MAP (FA); A4 = A/Sing Mono IM, 15 ug (UA)
Treatments Part B: B1 = A/Sing MAP, 15 ug (FA) with biopsy; B2 = Placebo MAP (FA) with biopsy
B3 = A/Sing MAP, 15 ug (FA); B4 = A/Sing MAP, 10 ug (FA); B5 = A/Sing MAP, 5 ug (FA); B6 = A/Sing MAP, 2.5 ug (FA)
B7 = Placebo MAP (FA); B8 = A/Sing MAP, 15 ug (UA); B9 = A/Sing IM, 15 ug, as 2018 Afluria Quad (UA)

Listing 16.2.6.6
Immunogenicity Results (CMI)

Part B

Randomisation Number	Group	Visit	Test	Subset	IL2	TNFa	IFNg	Value
B0086	Group B9	Day 1	Peptide	CD4	+	+	+	0.00005%
B0086	Group B9	Day 1	Peptide	CD4	+	+	-	0.00794%
B0086	Group B9	Day 1	Peptide	CD4	+	-	+	0.00002%
B0086	Group B9	Day 1	Peptide	CD4	+	-	-	0.00428%
B0086	Group B9	Day 1	Peptide	CD4	-	+	+	0.00002%
B0086	Group B9	Day 1	Peptide	CD4	-	+	-	0.032%
B0086	Group B9	Day 1	Peptide	CD4	-	-	+	0.00276%
B0086	Group B9	Day 22	Peptide	CD4	+	+	+	0.01372%
B0086	Group B9	Day 22	Peptide	CD4	+	+	-	0.01733%
B0086	Group B9	Day 22	Peptide	CD4	+	-	+	0.0025%
B0086	Group B9	Day 22	Peptide	CD4	+	-	-	0%
B0086	Group B9	Day 22	Peptide	CD4	-	+	+	0.00375%
B0086	Group B9	Day 22	Peptide	CD4	-	+	-	-0.009%
B0086	Group B9	Day 22	Peptide	CD4	-	-	+	-0.00136%
B0086	Group B9	Day 1	Peptide	CD8	+	+	+	0%
B0086	Group B9	Day 1	Peptide	CD8	+	+	-	0%
B0086	Group B9	Day 1	Peptide	CD8	+	-	+	0%
B0086	Group B9	Day 1	Peptide	CD8	+	-	-	0.00119%
B0086	Group B9	Day 1	Peptide	CD8	-	+	+	0%

Administration Site Key: FA = Forearm; UA= Upper arm
Treatments Part A: A1 = A/Sing MAP, 15 ug (FA); A2 = Afluria Quad IM (UA), 15 ug; A3 = Placebo MAP (FA); A4 = A/Sing Mono IM, 15 ug (UA)
Treatments Part B: B1 = A/Sing MAP, 15 ug (FA) with biopsy; B2 = Placebo MAP (FA) with biopsy
B3 = A/Sing MAP, 15 ug (FA); B4 = A/Sing MAP, 10 ug (FA); B5 = A/Sing MAP, 5 ug (FA); B6 = A/Sing MAP, 2.5 ug (FA)
B7 = Placebo MAP (FA); B8 = A/Sing MAP, 15 ug (UA); B9 = A/Sing IM, 15 ug, as 2018 Afluria Quad (UA)

Listing 16.2.6.6
Immunogenicity Results (CMI)

Part B

Randomisation Number	Group	Visit	Test	Subset	IL2	TNFa	IFNg	Value
B0086	Group B9	Day 1	Peptide	CD8	-	+	-	-0.024%
B0086	Group B9	Day 1	Peptide	CD8	-	-	+	0.12%
B0086	Group B9	Day 22	Peptide	CD8	+	+	+	-0.00125%
B0086	Group B9	Day 22	Peptide	CD8	+	+	-	0%
B0086	Group B9	Day 22	Peptide	CD8	+	-	+	0%
B0086	Group B9	Day 22	Peptide	CD8	+	-	-	-0.00125%
B0086	Group B9	Day 22	Peptide	CD8	-	+	+	0%
B0086	Group B9	Day 22	Peptide	CD8	-	+	-	-0.018%
B0086	Group B9	Day 22	Peptide	CD8	-	-	+	-0.016%
B0086	Group B9	Day 1	MPH	CD4	+	+	+	0.06197%
B0086	Group B9	Day 1	MPH	CD4	+	+	-	0.08194%
B0086	Group B9	Day 1	MPH	CD4	+	-	+	0.01266%
B0086	Group B9	Day 1	MPH	CD4	+	-	-	0.01828%
B0086	Group B9	Day 1	MPH	CD4	-	+	+	0.02066%
B0086	Group B9	Day 1	MPH	CD4	-	+	-	0.174%
B0086	Group B9	Day 1	MPH	CD4	-	-	+	0.01997%
B0086	Group B9	Day 22	MPH	CD4	+	+	+	0.17872%
B0086	Group B9	Day 22	MPH	CD4	+	+	-	0.13233%
B0086	Group B9	Day 22	MPH	CD4	+	-	+	0.039%

Administration Site Key: FA = Forearm; UA= Upper arm
Treatments Part A: A1 = A/Sing MAP, 15 ug (FA); A2 = Afluria Quad IM (UA), 15 ug; A3 = Placebo MAP (FA); A4 = A/Sing Mono IM, 15 ug (UA)
Treatments Part B: B1 = A/Sing MAP, 15 ug (FA) with biopsy; B2 = Placebo MAP (FA) with biopsy
B3 = A/Sing MAP, 15 ug (FA); B4 = A/Sing MAP, 10 ug (FA); B5 = A/Sing MAP, 5 ug (FA); B6 = A/Sing MAP, 2.5 ug (FA)
B7 = Placebo MAP (FA); B8 = A/Sing MAP, 15 ug (UA); B9 = A/Sing IM, 15 ug, as 2018 Afluria Quad (UA)

Listing 16.2.6.6
Immunogenicity Results (CMI)

Part B

Randomisation Number	Group	Visit	Test	Subset	IL2	TNFa	IFNg	Value
B0086	Group B9	Day 22	MPH	CD4	+	-	-	0.04%
B0086	Group B9	Day 22	MPH	CD4	-	+	+	0.054%
B0086	Group B9	Day 22	MPH	CD4	-	+	-	0.115%
B0086	Group B9	Day 22	MPH	CD4	-	-	+	0.05989%
B0086	Group B9	Day 1	MPH	CD8	+	+	+	0.00846%
B0086	Group B9	Day 1	MPH	CD8	+	+	-	0.00483%
B0086	Group B9	Day 1	MPH	CD8	+	-	+	0.00483%
B0086	Group B9	Day 1	MPH	CD8	+	-	-	0.00978%
B0086	Group B9	Day 1	MPH	CD8	-	+	+	0.00846%
B0086	Group B9	Day 1	MPH	CD8	-	+	-	0.059%
B0086	Group B9	Day 1	MPH	CD8	-	-	+	-0.033%
B0086	Group B9	Day 22	MPH	CD8	+	+	+	0.01875%
B0086	Group B9	Day 22	MPH	CD8	+	+	-	0.00815%
B0086	Group B9	Day 22	MPH	CD8	+	-	+	0.00466%
B0086	Group B9	Day 22	MPH	CD8	+	-	-	0.02475%
B0086	Group B9	Day 22	MPH	CD8	-	+	+	0.034%
B0086	Group B9	Day 22	MPH	CD8	-	+	-	0.089%
B0086	Group B9	Day 22	MPH	CD8	-	-	+	0.03%

Administration Site Key: FA = Forearm; UA= Upper arm
Treatments Part A: A1 = A/Sing MAP, 15 ug (FA); A2 = Afluria Quad IM (UA), 15 ug; A3 = Placebo MAP (FA); A4 = A/Sing Mono IM, 15 ug (UA)
Treatments Part B: B1 = A/Sing MAP, 15 ug (FA) with biopsy; B2 = Placebo MAP (FA) with biopsy
B3 = A/Sing MAP, 15 ug (FA); B4 = A/Sing MAP, 10 ug (FA); B5 = A/Sing MAP, 5 ug (FA); B6 = A/Sing MAP, 2.5 ug (FA)
B7 = Placebo MAP (FA); B8 = A/Sing MAP, 15 ug (UA); B9 = A/Sing IM, 15 ug, as 2018 Afluria Quad (UA)

Listing 16.2.6.6
Immunogenicity Results (CMI)

Part B

Randomisation Number	Group	Visit	Test	Subset	IL2	TNFa	IFNg	Value
B0087	Group B7	Day 1	Peptide	CD4	+	+	+	0.01078%
B0087	Group B7	Day 1	Peptide	CD4	+	+	-	0.00907%
B0087	Group B7	Day 1	Peptide	CD4	+	-	+	0.001026%
B0087	Group B7	Day 1	Peptide	CD4	+	-	-	0.00526%
B0087	Group B7	Day 1	Peptide	CD4	-	+	+	0.00552%
B0087	Group B7	Day 1	Peptide	CD4	-	+	-	0.013%
B0087	Group B7	Day 1	Peptide	CD4	-	-	+	-0.00373%
B0087	Group B7	Day 22	Peptide	CD4	+	+	+	-0.00023%
B0087	Group B7	Day 22	Peptide	CD4	+	+	-	-0.00026%
B0087	Group B7	Day 22	Peptide	CD4	+	-	+	0.000734%
B0087	Group B7	Day 22	Peptide	CD4	+	-	-	0.00055%
B0087	Group B7	Day 22	Peptide	CD4	-	+	+	-0.00312%
B0087	Group B7	Day 22	Peptide	CD4	-	+	-	0.004%
B0087	Group B7	Day 22	Peptide	CD4	-	-	+	0.00283%
B0087	Group B7	Day 1	Peptide	CD8	+	+	+	0.00478%
B0087	Group B7	Day 1	Peptide	CD8	+	+	-	0.00159%
B0087	Group B7	Day 1	Peptide	CD8	+	-	+	0%
B0087	Group B7	Day 1	Peptide	CD8	+	-	-	-0.00228%
B0087	Group B7	Day 1	Peptide	CD8	-	+	+	0.00379%

Administration Site Key: FA = Forearm; UA= Upper arm
Treatments Part A: A1 = A/Sing MAP, 15 ug (FA); A2 = Afluria Quad IM (UA), 15 ug; A3 = Placebo MAP (FA); A4 = A/Sing Mono IM, 15 ug (UA)
Treatments Part B: B1 = A/Sing MAP, 15 ug (FA) with biopsy; B2 = Placebo MAP (FA) with biopsy
B3 = A/Sing MAP, 15 ug (FA); B4 = A/Sing MAP, 10 ug (FA); B5 = A/Sing MAP, 5 ug (FA); B6 = A/Sing MAP, 2.5 ug (FA)
B7 = Placebo MAP (FA); B8 = A/Sing MAP, 15 ug (UA); B9 = A/Sing IM, 15 ug, as 2018 Afluria Quad (UA)

Listing 16.2.6.6
Immunogenicity Results (CMI)

Part B

Randomisation Number	Group	Visit	Test	Subset	IL2	TNFa	IFNg	Value
B0087	Group B7	Day 1	Peptide	CD8	-	+	-	0.002%
B0087	Group B7	Day 1	Peptide	CD8	-	-	+	0.41%
B0087	Group B7	Day 22	Peptide	CD8	+	+	+	0.00245%
B0087	Group B7	Day 22	Peptide	CD8	+	+	-	0%
B0087	Group B7	Day 22	Peptide	CD8	+	-	+	0%
B0087	Group B7	Day 22	Peptide	CD8	+	-	-	-0.00004%
B0087	Group B7	Day 22	Peptide	CD8	-	+	+	-0.00253%
B0087	Group B7	Day 22	Peptide	CD8	-	+	-	-0.026%
B0087	Group B7	Day 22	Peptide	CD8	-	-	+	-0.021%
B0087	Group B7	Day 1	MPH	CD4	+	+	+	0.11578%
B0087	Group B7	Day 1	MPH	CD4	+	+	-	0.10507%
B0087	Group B7	Day 1	MPH	CD4	+	-	+	0.008606%
B0087	Group B7	Day 1	MPH	CD4	+	-	-	0.01726%
B0087	Group B7	Day 1	MPH	CD4	-	+	+	0.05059%
B0087	Group B7	Day 1	MPH	CD4	-	+	-	0.318%
B0087	Group B7	Day 1	MPH	CD4	-	-	+	0.04967%
B0087	Group B7	Day 22	MPH	CD4	+	+	+	0.10316%
B0087	Group B7	Day 22	MPH	CD4	+	+	-	0.1124%
B0087	Group B7	Day 22	MPH	CD4	+	-	+	0.00954%

Administration Site Key: FA = Forearm; UA= Upper arm
Treatments Part A: A1 = A/Sing MAP, 15 ug (FA); A2 = Afluria Quad IM (UA), 15 ug; A3 = Placebo MAP (FA); A4 = A/Sing Mono IM, 15 ug (UA)
Treatments Part B: B1 = A/Sing MAP, 15 ug (FA) with biopsy; B2 = Placebo MAP (FA) with biopsy
B3 = A/Sing MAP, 15 ug (FA); B4 = A/Sing MAP, 10 ug (FA); B5 = A/Sing MAP, 5 ug (FA); B6 = A/Sing MAP, 2.5 ug (FA)
B7 = Placebo MAP (FA); B8 = A/Sing MAP, 15 ug (UA); B9 = A/Sing IM, 15 ug, as 2018 Afluria Quad (UA)

Listing 16.2.6.6
Immunogenicity Results (CMI)

Part B

Randomisation Number	Group	Visit	Test	Subset	IL2	TNFa	IFNg	Value
B0087	Group B7	Day 22	MPH	CD4	+	-	-	0.01468%
B0087	Group B7	Day 22	MPH	CD4	-	+	+	0.06768%
B0087	Group B7	Day 22	MPH	CD4	-	+	-	0.34%
B0087	Group B7	Day 22	MPH	CD4	-	-	+	0.07196%
B0087	Group B7	Day 1	MPH	CD8	+	+	+	0.025%
B0087	Group B7	Day 1	MPH	CD8	+	+	-	0.00292%
B0087	Group B7	Day 1	MPH	CD8	+	-	+	0.015%
B0087	Group B7	Day 1	MPH	CD8	+	-	-	0.00613%
B0087	Group B7	Day 1	MPH	CD8	-	+	+	0.05542%
B0087	Group B7	Day 1	MPH	CD8	-	+	-	0.193%
B0087	Group B7	Day 1	MPH	CD8	-	-	+	-0.01%
B0087	Group B7	Day 22	MPH	CD8	+	+	+	0.019%
B0087	Group B7	Day 22	MPH	CD8	+	+	-	0.00412%
B0087	Group B7	Day 22	MPH	CD8	+	-	+	0.014%
B0087	Group B7	Day 22	MPH	CD8	+	-	-	0.01074%
B0087	Group B7	Day 22	MPH	CD8	-	+	+	0.05647%
B0087	Group B7	Day 22	MPH	CD8	-	+	-	0.192%
B0087	Group B7	Day 22	MPH	CD8	-	-	+	0.061%

Administration Site Key: FA = Forearm; UA= Upper arm
Treatments Part A: A1 = A/Sing MAP, 15 ug (FA); A2 = Afluria Quad IM (UA), 15 ug; A3 = Placebo MAP (FA); A4 = A/Sing Mono IM, 15 ug (UA)
Treatments Part B: B1 = A/Sing MAP, 15 ug (FA) with biopsy; B2 = Placebo MAP (FA) with biopsy
B3 = A/Sing MAP, 15 ug (FA); B4 = A/Sing MAP, 10 ug (FA); B5 = A/Sing MAP, 5 ug (FA); B6 = A/Sing MAP, 2.5 ug (FA)
B7 = Placebo MAP (FA); B8 = A/Sing MAP, 15 ug (UA); B9 = A/Sing IM, 15 ug, as 2018 Afluria Quad (UA)

Listing 16.2.6.6
Immunogenicity Results (CMI)

Part B

Randomisation Number	Group	Visit	Test	Subset	IL2	TNFa	IFNg	Value
B0088	Group B9	Day 1	Peptide	CD4	+	+	+	-0.00157%
B0088	Group B9	Day 1	Peptide	CD4	+	+	-	0.01025%
B0088	Group B9	Day 1	Peptide	CD4	+	-	+	0.00147%
B0088	Group B9	Day 1	Peptide	CD4	+	-	-	0.00826%
B0088	Group B9	Day 1	Peptide	CD4	-	+	+	0.002191%
B0088	Group B9	Day 1	Peptide	CD4	-	+	-	0.021%
B0088	Group B9	Day 1	Peptide	CD4	-	-	+	-0.00081%
B0088	Group B9	Day 22	Peptide	CD4	+	+	+	0.024293%
B0088	Group B9	Day 22	Peptide	CD4	+	+	-	0.01622%
B0088	Group B9	Day 22	Peptide	CD4	+	-	+	0.000722%
B0088	Group B9	Day 22	Peptide	CD4	+	-	-	-0.00333%
B0088	Group B9	Day 22	Peptide	CD4	-	+	+	0.003623%
B0088	Group B9	Day 22	Peptide	CD4	-	+	-	0%
B0088	Group B9	Day 22	Peptide	CD4	-	-	+	0.00298%
B0088	Group B9	Day 1	Peptide	CD8	+	+	+	0.00652%
B0088	Group B9	Day 1	Peptide	CD8	+	+	-	0.00159%
B0088	Group B9	Day 1	Peptide	CD8	+	-	+	0.00163%
B0088	Group B9	Day 1	Peptide	CD8	+	-	-	0.0048%
B0088	Group B9	Day 1	Peptide	CD8	-	+	+	0.008%

Administration Site Key: FA = Forearm; UA= Upper arm
Treatments Part A: A1 = A/Sing MAP, 15 ug (FA); A2 = Afluria Quad IM (UA), 15 ug; A3 = Placebo MAP (FA); A4 = A/Sing Mono IM, 15 ug (UA)
Treatments Part B: B1 = A/Sing MAP, 15 ug (FA) with biopsy; B2 = Placebo MAP (FA) with biopsy
B3 = A/Sing MAP, 15 ug (FA); B4 = A/Sing MAP, 10 ug (FA); B5 = A/Sing MAP, 5 ug (FA); B6 = A/Sing MAP, 2.5 ug (FA)
B7 = Placebo MAP (FA); B8 = A/Sing MAP, 15 ug (UA); B9 = A/Sing IM, 15 ug, as 2018 Afluria Quad (UA)

Listing 16.2.6.6
Immunogenicity Results (CMI)

Part B

Randomisation Number	Group	Visit	Test	Subset	IL2	TNFa	IFNg	Value
B0088	Group B9	Day 1	Peptide	CD8	-	+	-	0.18%
B0088	Group B9	Day 1	Peptide	CD8	-	-	+	0.09%
B0088	Group B9	Day 22	Peptide	CD8	+	+	+	0.00918%
B0088	Group B9	Day 22	Peptide	CD8	+	+	-	0.00735%
B0088	Group B9	Day 22	Peptide	CD8	+	-	+	0.00367%
B0088	Group B9	Day 22	Peptide	CD8	+	-	-	0.00011%
B0088	Group B9	Day 22	Peptide	CD8	-	+	+	0.012%
B0088	Group B9	Day 22	Peptide	CD8	-	+	-	0%
B0088	Group B9	Day 22	Peptide	CD8	-	-	+	-0.329%
B0088	Group B9	Day 1	MPH	CD4	+	+	+	0.04776%
B0088	Group B9	Day 1	MPH	CD4	+	+	-	0.09025%
B0088	Group B9	Day 1	MPH	CD4	+	-	+	0.00452%
B0088	Group B9	Day 1	MPH	CD4	+	-	-	0.02426%
B0088	Group B9	Day 1	MPH	CD4	-	+	+	0.030251%
B0088	Group B9	Day 1	MPH	CD4	-	+	-	0.243%
B0088	Group B9	Day 1	MPH	CD4	-	-	+	0.05325%
B0088	Group B9	Day 22	MPH	CD4	+	+	+	0.076293%
B0088	Group B9	Day 22	MPH	CD4	+	+	-	0.10222%
B0088	Group B9	Day 22	MPH	CD4	+	-	+	0.02%

Administration Site Key: FA = Forearm; UA= Upper arm
Treatments Part A: A1 = A/Sing MAP, 15 ug (FA); A2 = Afluria Quad IM (UA), 15 ug; A3 = Placebo MAP (FA); A4 = A/Sing Mono IM, 15 ug (UA)
Treatments Part B: B1 = A/Sing MAP, 15 ug (FA) with biopsy; B2 = Placebo MAP (FA) with biopsy
B3 = A/Sing MAP, 15 ug (FA); B4 = A/Sing MAP, 10 ug (FA); B5 = A/Sing MAP, 5 ug (FA); B6 = A/Sing MAP, 2.5 ug (FA)
B7 = Placebo MAP (FA); B8 = A/Sing MAP, 15 ug (UA); B9 = A/Sing IM, 15 ug, as 2018 Afluria Quad (UA)

Listing 16.2.6.6
Immunogenicity Results (CMI)

Part B

Randomisation Number	Group	Visit	Test	Subset	IL2	TNFa	IFNg	Value
B0088	Group B9	Day 22	MPH	CD4	+	-	-	0.04%
B0088	Group B9	Day 22	MPH	CD4	-	+	+	0.050293%
B0088	Group B9	Day 22	MPH	CD4	-	+	-	0.264%
B0088	Group B9	Day 22	MPH	CD4	-	-	+	0.18576%
B0088	Group B9	Day 1	MPH	CD8	+	+	+	0.00652%
B0088	Group B9	Day 1	MPH	CD8	+	+	-	0.00159%
B0088	Group B9	Day 1	MPH	CD8	+	-	+	0.00163%
B0088	Group B9	Day 1	MPH	CD8	+	-	-	0.0048%
B0088	Group B9	Day 1	MPH	CD8	-	+	+	0.008%
B0088	Group B9	Day 1	MPH	CD8	-	+	-	0.18%
B0088	Group B9	Day 1	MPH	CD8	-	-	+	0.09%
B0088	Group B9	Day 22	MPH	CD8	+	+	+	0.00918%
B0088	Group B9	Day 22	MPH	CD8	+	+	-	0.00735%
B0088	Group B9	Day 22	MPH	CD8	+	-	+	0.00367%
B0088	Group B9	Day 22	MPH	CD8	+	-	-	0.00011%
B0088	Group B9	Day 22	MPH	CD8	-	+	+	0.012%
B0088	Group B9	Day 22	MPH	CD8	-	+	-	0%
B0088	Group B9	Day 22	MPH	CD8	-	-	+	-0.329%

Administration Site Key: FA = Forearm; UA= Upper arm
Treatments Part A: A1 = A/Sing MAP, 15 ug (FA); A2 = Afluria Quad IM (UA), 15 ug; A3 = Placebo MAP (FA); A4 = A/Sing Mono IM, 15 ug (UA)
Treatments Part B: B1 = A/Sing MAP, 15 ug (FA) with biopsy; B2 = Placebo MAP (FA) with biopsy
B3 = A/Sing MAP, 15 ug (FA); B4 = A/Sing MAP, 10 ug (FA); B5 = A/Sing MAP, 5 ug (FA); B6 = A/Sing MAP, 2.5 ug (FA)
B7 = Placebo MAP (FA); B8 = A/Sing MAP, 15 ug (UA); B9 = A/Sing IM, 15 ug, as 2018 Afluria Quad (UA)

Listing 16.2.6.6
Immunogenicity Results (CMI)

Part B

Randomisation Number	Group	Visit	Test	Subset	IL2	TNFa	IFNg	Value
B0091	Group B8	Day 1	Peptide	CD4	+	+	+	0.007%
B0091	Group B8	Day 1	Peptide	CD4	+	+	-	0.007%
B0091	Group B8	Day 1	Peptide	CD4	+	-	+	0.00063%
B0091	Group B8	Day 1	Peptide	CD4	+	-	-	-0.002%
B0091	Group B8	Day 1	Peptide	CD4	-	+	+	0.00233%
B0091	Group B8	Day 1	Peptide	CD4	-	+	-	0.03%
B0091	Group B8	Day 1	Peptide	CD4	-	-	+	0.00616%
B0091	Group B8	Day 22	Peptide	CD4	+	+	+	0.041%
B0091	Group B8	Day 22	Peptide	CD4	+	+	-	0.017%
B0091	Group B8	Day 22	Peptide	CD4	+	-	+	-0.00005%
B0091	Group B8	Day 22	Peptide	CD4	+	-	-	0.002%
B0091	Group B8	Day 22	Peptide	CD4	-	+	+	0.01149%
B0091	Group B8	Day 22	Peptide	CD4	-	+	-	0.036%
B0091	Group B8	Day 22	Peptide	CD4	-	-	+	-0.00059%
B0091	Group B8	Day 1	Peptide	CD8	+	+	+	0%
B0091	Group B8	Day 1	Peptide	CD8	+	+	-	0.00151%
B0091	Group B8	Day 1	Peptide	CD8	+	-	+	0%
B0091	Group B8	Day 1	Peptide	CD8	+	-	-	-0.00899%
B0091	Group B8	Day 1	Peptide	CD8	-	+	+	-0.00546%

Administration Site Key: FA = Forearm; UA= Upper arm
Treatments Part A: A1 = A/Sing MAP, 15 ug (FA); A2 = Afluria Quad IM (UA), 15 ug; A3 = Placebo MAP (FA); A4 = A/Sing Mono IM, 15 ug (UA)
Treatments Part B: B1 = A/Sing MAP, 15 ug (FA) with biopsy; B2 = Placebo MAP (FA) with biopsy
B3 = A/Sing MAP, 15 ug (FA); B4 = A/Sing MAP, 10 ug (FA); B5 = A/Sing MAP, 5 ug (FA); B6 = A/Sing MAP, 2.5 ug (FA)
B7 = Placebo MAP (FA); B8 = A/Sing MAP, 15 ug (UA); B9 = A/Sing IM, 15 ug, as 2018 Afluria Quad (UA)

Listing 16.2.6.6
Immunogenicity Results (CMI)

Part B

Randomisation Number	Group	Visit	Test	Subset	IL2	TNFa	IFNg	Value
B0091	Group B8	Day 1	Peptide	CD8	-	+	-	0.03%
B0091	Group B8	Day 1	Peptide	CD8	-	-	+	-0.21%
B0091	Group B8	Day 22	Peptide	CD8	+	+	+	-0.00141%
B0091	Group B8	Day 22	Peptide	CD8	+	+	-	0%
B0091	Group B8	Day 22	Peptide	CD8	+	-	+	0%
B0091	Group B8	Day 22	Peptide	CD8	+	-	-	0.00129%
B0091	Group B8	Day 22	Peptide	CD8	-	+	+	0.004%
B0091	Group B8	Day 22	Peptide	CD8	-	+	-	0.03%
B0091	Group B8	Day 22	Peptide	CD8	-	-	+	0.02%
B0091	Group B8	Day 1	MPH	CD4	+	+	+	0.069%
B0091	Group B8	Day 1	MPH	CD4	+	+	-	0.05%
B0091	Group B8	Day 1	MPH	CD4	+	-	+	0.01935%
B0091	Group B8	Day 1	MPH	CD4	+	-	-	0.058%
B0091	Group B8	Day 1	MPH	CD4	-	+	+	0.05076%
B0091	Group B8	Day 1	MPH	CD4	-	+	-	0.253%
B0091	Group B8	Day 1	MPH	CD4	-	-	+	0.13416%
B0091	Group B8	Day 22	MPH	CD4	+	+	+	0.098%
B0091	Group B8	Day 22	MPH	CD4	+	+	-	0.08%
B0091	Group B8	Day 22	MPH	CD4	+	-	+	0.05549%

Administration Site Key: FA = Forearm; UA= Upper arm
Treatments Part A: A1 = A/Sing MAP, 15 ug (FA); A2 = Afluria Quad IM (UA), 15 ug; A3 = Placebo MAP (FA); A4 = A/Sing Mono IM, 15 ug (UA)
Treatments Part B: B1 = A/Sing MAP, 15 ug (FA) with biopsy; B2 = Placebo MAP (FA) with biopsy
B3 = A/Sing MAP, 15 ug (FA); B4 = A/Sing MAP, 10 ug (FA); B5 = A/Sing MAP, 5 ug (FA); B6 = A/Sing MAP, 2.5 ug (FA)
B7 = Placebo MAP (FA); B8 = A/Sing MAP, 15 ug (UA); B9 = A/Sing IM, 15 ug, as 2018 Afluria Quad (UA)

Listing 16.2.6.6
Immunogenicity Results (CMI)

Part B

Randomisation Number	Group	Visit	Test	Subset	IL2	TNFa	IFNg	Value
B0091	Group B8	Day 22	MPH	CD4	+	-	-	0.091%
B0091	Group B8	Day 22	MPH	CD4	-	+	+	0.07949%
B0091	Group B8	Day 22	MPH	CD4	-	+	-	0.226%
B0091	Group B8	Day 22	MPH	CD4	-	-	+	0.11099%
B0091	Group B8	Day 1	MPH	CD8	+	+	+	0.012%
B0091	Group B8	Day 1	MPH	CD8	+	+	-	0.00152%
B0091	Group B8	Day 1	MPH	CD8	+	-	+	0.00456%
B0091	Group B8	Day 1	MPH	CD8	+	-	-	0.041%
B0091	Group B8	Day 1	MPH	CD8	-	+	+	0.06%
B0091	Group B8	Day 1	MPH	CD8	-	+	-	-0.01%
B0091	Group B8	Day 1	MPH	CD8	-	-	+	-1.06%
B0091	Group B8	Day 22	MPH	CD8	+	+	+	0.00521%
B0091	Group B8	Day 22	MPH	CD8	+	+	-	0.013%
B0091	Group B8	Day 22	MPH	CD8	+	-	+	0.00397%
B0091	Group B8	Day 22	MPH	CD8	+	-	-	0.04837%
B0091	Group B8	Day 22	MPH	CD8	-	+	+	0.048%
B0091	Group B8	Day 22	MPH	CD8	-	+	-	0.17%
B0091	Group B8	Day 22	MPH	CD8	-	-	+	-1.05%

Administration Site Key: FA = Forearm; UA= Upper arm
Treatments Part A: A1 = A/Sing MAP, 15 ug (FA); A2 = Afluria Quad IM (UA), 15 ug; A3 = Placebo MAP (FA); A4 = A/Sing Mono IM, 15 ug (UA)
Treatments Part B: B1 = A/Sing MAP, 15 ug (FA) with biopsy; B2 = Placebo MAP (FA) with biopsy
B3 = A/Sing MAP, 15 ug (FA); B4 = A/Sing MAP, 10 ug (FA); B5 = A/Sing MAP, 5 ug (FA); B6 = A/Sing MAP, 2.5 ug (FA)
B7 = Placebo MAP (FA); B8 = A/Sing MAP, 15 ug (UA); B9 = A/Sing IM, 15 ug, as 2018 Afluria Quad (UA)

Listing 16.2.6.6
Immunogenicity Results (CMI)

Part B

Randomisation Number	Group	Visit	Test	Subset	IL2	TNFa	IFNg	Value
B0093	Group B7	Day 1	Peptide	CD4	+	+	+	0.00261%
B0093	Group B7	Day 1	Peptide	CD4	+	+	-	0.00859%
B0093	Group B7	Day 1	Peptide	CD4	+	-	+	0%
B0093	Group B7	Day 1	Peptide	CD4	+	-	-	0.001%
B0093	Group B7	Day 1	Peptide	CD4	-	+	+	0%
B0093	Group B7	Day 1	Peptide	CD4	-	+	-	0.001%
B0093	Group B7	Day 1	Peptide	CD4	-	-	+	-0.00676%
B0093	Group B7	Day 22	Peptide	CD4	+	+	+	0.001369%
B0093	Group B7	Day 22	Peptide	CD4	+	+	-	0.001%
B0093	Group B7	Day 22	Peptide	CD4	+	-	+	-0.000158%
B0093	Group B7	Day 22	Peptide	CD4	+	-	-	-0.007%
B0093	Group B7	Day 22	Peptide	CD4	-	+	+	-0.000158%
B0093	Group B7	Day 22	Peptide	CD4	-	+	-	-0.004%
B0093	Group B7	Day 22	Peptide	CD4	-	-	+	0.000609%
B0093	Group B7	Day 1	Peptide	CD8	+	+	+	0%
B0093	Group B7	Day 1	Peptide	CD8	+	+	-	0.00296%
B0093	Group B7	Day 1	Peptide	CD8	+	-	+	-0.00382%
B0093	Group B7	Day 1	Peptide	CD8	+	-	-	-0.00086%
B0093	Group B7	Day 1	Peptide	CD8	-	+	+	0.007%

Administration Site Key: FA = Forearm; UA= Upper arm
Treatments Part A: A1 = A/Sing MAP, 15 ug (FA); A2 = Afluria Quad IM (UA), 15 ug; A3 = Placebo MAP (FA); A4 = A/Sing Mono IM, 15 ug (UA)
Treatments Part B: B1 = A/Sing MAP, 15 ug (FA) with biopsy; B2 = Placebo MAP (FA) with biopsy
B3 = A/Sing MAP, 15 ug (FA); B4 = A/Sing MAP, 10 ug (FA); B5 = A/Sing MAP, 5 ug (FA); B6 = A/Sing MAP, 2.5 ug (FA)
B7 = Placebo MAP (FA); B8 = A/Sing MAP, 15 ug (UA); B9 = A/Sing IM, 15 ug, as 2018 Afluria Quad (UA)

Listing 16.2.6.6
Immunogenicity Results (CMI)

Part B

Randomisation Number	Group	Visit	Test	Subset	IL2	TNFa	IFNg	Value
B0093	Group B7	Day 1	Peptide	CD8	-	+	-	-0.024%
B0093	Group B7	Day 1	Peptide	CD8	-	-	+	-0.53%
B0093	Group B7	Day 22	Peptide	CD8	+	+	+	0%
B0093	Group B7	Day 22	Peptide	CD8	+	+	-	-0.00294%
B0093	Group B7	Day 22	Peptide	CD8	+	-	+	0%
B0093	Group B7	Day 22	Peptide	CD8	+	-	-	0.0047%
B0093	Group B7	Day 22	Peptide	CD8	-	+	+	-0.00294%
B0093	Group B7	Day 22	Peptide	CD8	-	+	-	0.002%
B0093	Group B7	Day 22	Peptide	CD8	-	-	+	-0.23%
B0093	Group B7	Day 1	MPH	CD4	+	+	+	0.0758%
B0093	Group B7	Day 1	MPH	CD4	+	+	-	0.11159%
B0093	Group B7	Day 1	MPH	CD4	+	-	+	0.00188%
B0093	Group B7	Day 1	MPH	CD4	+	-	-	0.026%
B0093	Group B7	Day 1	MPH	CD4	-	+	+	0.025%
B0093	Group B7	Day 1	MPH	CD4	-	+	-	0.238%
B0093	Group B7	Day 1	MPH	CD4	-	-	+	-0.00023%
B0093	Group B7	Day 22	MPH	CD4	+	+	+	0.073079%
B0093	Group B7	Day 22	MPH	CD4	+	+	-	0.11%
B0093	Group B7	Day 22	MPH	CD4	+	-	+	-0.000921%

Administration Site Key: FA = Forearm; UA= Upper arm
Treatments Part A: A1 = A/Sing MAP, 15 ug (FA); A2 = Afluria Quad IM (UA), 15 ug; A3 = Placebo MAP (FA); A4 = A/Sing Mono IM, 15 ug (UA)
Treatments Part B: B1 = A/Sing MAP, 15 ug (FA) with biopsy; B2 = Placebo MAP (FA) with biopsy
B3 = A/Sing MAP, 15 ug (FA); B4 = A/Sing MAP, 10 ug (FA); B5 = A/Sing MAP, 5 ug (FA); B6 = A/Sing MAP, 2.5 ug (FA)
B7 = Placebo MAP (FA); B8 = A/Sing MAP, 15 ug (UA); B9 = A/Sing IM, 15 ug, as 2018 Afluria Quad (UA)

Listing 16.2.6.6
Immunogenicity Results (CMI)

Part B

Randomisation Number	Group	Visit	Test	Subset	IL2	TNFa	IFNg	Value
B0093	Group B7	Day 22	MPH	CD4	+	-	-	0.011%
B0093	Group B7	Day 22	MPH	CD4	-	+	+	0.019079%
B0093	Group B7	Day 22	MPH	CD4	-	+	-	0.221%
B0093	Group B7	Day 22	MPH	CD4	-	-	+	0.014079%
B0093	Group B7	Day 1	MPH	CD8	+	+	+	0.014%
B0093	Group B7	Day 1	MPH	CD8	+	+	-	0.014%
B0093	Group B7	Day 1	MPH	CD8	+	-	+	-0.00382%
B0093	Group B7	Day 1	MPH	CD8	+	-	-	0.00818%
B0093	Group B7	Day 1	MPH	CD8	-	+	+	0.009%
B0093	Group B7	Day 1	MPH	CD8	-	+	-	0.086%
B0093	Group B7	Day 1	MPH	CD8	-	-	+	-2.43%
B0093	Group B7	Day 22	MPH	CD8	+	+	+	0.013%
B0093	Group B7	Day 22	MPH	CD8	+	+	-	0.00262%
B0093	Group B7	Day 22	MPH	CD8	+	-	+	0%
B0093	Group B7	Day 22	MPH	CD8	+	-	-	0.01%
B0093	Group B7	Day 22	MPH	CD8	-	+	+	0.026%
B0093	Group B7	Day 22	MPH	CD8	-	+	-	0.084%
B0093	Group B7	Day 22	MPH	CD8	-	-	+	0.17%

Administration Site Key: FA = Forearm; UA= Upper arm
Treatments Part A: A1 = A/Sing MAP, 15 ug (FA); A2 = Afluria Quad IM (UA), 15 ug; A3 = Placebo MAP (FA); A4 = A/Sing Mono IM, 15 ug (UA)
Treatments Part B: B1 = A/Sing MAP, 15 ug (FA) with biopsy; B2 = Placebo MAP (FA) with biopsy
B3 = A/Sing MAP, 15 ug (FA); B4 = A/Sing MAP, 10 ug (FA); B5 = A/Sing MAP, 5 ug (FA); B6 = A/Sing MAP, 2.5 ug (FA)
B7 = Placebo MAP (FA); B8 = A/Sing MAP, 15 ug (UA); B9 = A/Sing IM, 15 ug, as 2018 Afluria Quad (UA)

Listing 16.2.6.6
Immunogenicity Results (CMI)

Part B

Randomisation Number	Group	Visit	Test	Subset	IL2	TNFa	IFNg	Value
B0094	Group B3	Day 1	Peptide	CD4	+	+	+	-0.00308%
B0094	Group B3	Day 1	Peptide	CD4	+	+	-	0.00186%
B0094	Group B3	Day 1	Peptide	CD4	+	-	+	0%
B0094	Group B3	Day 1	Peptide	CD4	+	-	-	0.00391%
B0094	Group B3	Day 1	Peptide	CD4	-	+	+	-0.00079%
B0094	Group B3	Day 1	Peptide	CD4	-	+	-	0.004%
B0094	Group B3	Day 1	Peptide	CD4	-	-	+	-0.0016%
B0094	Group B3	Day 22	Peptide	CD4	+	+	+	0.00863%
B0094	Group B3	Day 22	Peptide	CD4	+	+	-	0.00983%
B0094	Group B3	Day 22	Peptide	CD4	+	-	+	0.00136%
B0094	Group B3	Day 22	Peptide	CD4	+	-	-	-0.00141%
B0094	Group B3	Day 22	Peptide	CD4	-	+	+	0.00746%
B0094	Group B3	Day 22	Peptide	CD4	-	+	-	0.021%
B0094	Group B3	Day 22	Peptide	CD4	-	-	+	-0.00072%
B0094	Group B3	Day 1	Peptide	CD8	+	+	+	0%
B0094	Group B3	Day 1	Peptide	CD8	+	+	-	0.00108%
B0094	Group B3	Day 1	Peptide	CD8	+	-	+	0%
B0094	Group B3	Day 1	Peptide	CD8	+	-	-	0.00108%
B0094	Group B3	Day 1	Peptide	CD8	-	+	+	0.00325%

Administration Site Key: FA = Forearm; UA= Upper arm
Treatments Part A: A1 = A/Sing MAP, 15 ug (FA); A2 = Afluria Quad IM (UA), 15 ug; A3 = Placebo MAP (FA); A4 = A/Sing Mono IM, 15 ug (UA)
Treatments Part B: B1 = A/Sing MAP, 15 ug (FA) with biopsy; B2 = Placebo MAP (FA) with biopsy
B3 = A/Sing MAP, 15 ug (FA); B4 = A/Sing MAP, 10 ug (FA); B5 = A/Sing MAP, 5 ug (FA); B6 = A/Sing MAP, 2.5 ug (FA)
B7 = Placebo MAP (FA); B8 = A/Sing MAP, 15 ug (UA); B9 = A/Sing IM, 15 ug, as 2018 Afluria Quad (UA)

Listing 16.2.6.6
Immunogenicity Results (CMI)

Part B

Randomisation Number	Group	Visit	Test	Subset	IL2	TNFa	IFNg	Value
B0094	Group B3	Day 1	Peptide	CD8	-	+	-	-0.015%
B0094	Group B3	Day 1	Peptide	CD8	-	-	+	-0.09%
B0094	Group B3	Day 22	Peptide	CD8	+	+	+	0%
B0094	Group B3	Day 22	Peptide	CD8	+	+	-	0%
B0094	Group B3	Day 22	Peptide	CD8	+	-	+	0%
B0094	Group B3	Day 22	Peptide	CD8	+	-	-	-0.0011%
B0094	Group B3	Day 22	Peptide	CD8	-	+	+	0.00199%
B0094	Group B3	Day 22	Peptide	CD8	-	+	-	0.017%
B0094	Group B3	Day 22	Peptide	CD8	-	-	+	-0.09%
B0094	Group B3	Day 1	MPH	CD4	+	+	+	0.04543%
B0094	Group B3	Day 1	MPH	CD4	+	+	-	0.09086%
B0094	Group B3	Day 1	MPH	CD4	+	-	+	0.00687%
B0094	Group B3	Day 1	MPH	CD4	+	-	-	0.00891%
B0094	Group B3	Day 1	MPH	CD4	-	+	+	0.01172%
B0094	Group B3	Day 1	MPH	CD4	-	+	-	0.195%
B0094	Group B3	Day 1	MPH	CD4	-	-	+	0.02243%
B0094	Group B3	Day 22	MPH	CD4	+	+	+	0.07663%
B0094	Group B3	Day 22	MPH	CD4	+	+	-	0.11383%
B0094	Group B3	Day 22	MPH	CD4	+	-	+	0.052%

Administration Site Key: FA = Forearm; UA= Upper arm
Treatments Part A: A1 = A/Sing MAP, 15 ug (FA); A2 = Afluria Quad IM (UA), 15 ug; A3 = Placebo MAP (FA); A4 = A/Sing Mono IM, 15 ug (UA)
Treatments Part B: B1 = A/Sing MAP, 15 ug (FA) with biopsy; B2 = Placebo MAP (FA) with biopsy
B3 = A/Sing MAP, 15 ug (FA); B4 = A/Sing MAP, 10 ug (FA); B5 = A/Sing MAP, 5 ug (FA); B6 = A/Sing MAP, 2.5 ug (FA)
B7 = Placebo MAP (FA); B8 = A/Sing MAP, 15 ug (UA); B9 = A/Sing IM, 15 ug, as 2018 Afluria Quad (UA)

Listing 16.2.6.6
Immunogenicity Results (CMI)

Part B

Randomisation Number	Group	Visit	Test	Subset	IL2	TNFa	IFNg	Value
B0094	Group B3	Day 22	MPH	CD4	+	-	-	0.0522%
B0094	Group B3	Day 22	MPH	CD4	-	+	+	0.044%
B0094	Group B3	Day 22	MPH	CD4	-	+	-	0.196%
B0094	Group B3	Day 22	MPH	CD4	-	-	+	0.09589%
B0094	Group B3	Day 1	MPH	CD8	+	+	+	0.01%
B0094	Group B3	Day 1	MPH	CD8	+	+	-	0.0079%
B0094	Group B3	Day 1	MPH	CD8	+	-	+	0.00564%
B0094	Group B3	Day 1	MPH	CD8	+	-	-	0.016%
B0094	Group B3	Day 1	MPH	CD8	-	+	+	0.04092%
B0094	Group B3	Day 1	MPH	CD8	-	+	-	0.315%
B0094	Group B3	Day 1	MPH	CD8	-	-	+	-0.34%
B0094	Group B3	Day 22	MPH	CD8	+	+	+	0.00618%
B0094	Group B3	Day 22	MPH	CD8	+	+	-	0.00825%
B0094	Group B3	Day 22	MPH	CD8	+	-	+	0%
B0094	Group B3	Day 22	MPH	CD8	+	-	-	0.02087%
B0094	Group B3	Day 22	MPH	CD8	-	+	+	0.02287%
B0094	Group B3	Day 22	MPH	CD8	-	+	-	0.199%
B0094	Group B3	Day 22	MPH	CD8	-	-	+	-0.27%

Administration Site Key: FA = Forearm; UA= Upper arm
Treatments Part A: A1 = A/Sing MAP, 15 ug (FA); A2 = Afluria Quad IM (UA), 15 ug; A3 = Placebo MAP (FA); A4 = A/Sing Mono IM, 15 ug (UA)
Treatments Part B: B1 = A/Sing MAP, 15 ug (FA) with biopsy; B2 = Placebo MAP (FA) with biopsy
B3 = A/Sing MAP, 15 ug (FA); B4 = A/Sing MAP, 10 ug (FA); B5 = A/Sing MAP, 5 ug (FA); B6 = A/Sing MAP, 2.5 ug (FA)
B7 = Placebo MAP (FA); B8 = A/Sing MAP, 15 ug (UA); B9 = A/Sing IM, 15 ug, as 2018 Afluria Quad (UA)

Listing 16.2.6.6
Immunogenicity Results (CMI)

Part B

Randomisation Number	Group	Visit	Test	Subset	IL2	TNFa	IFNg	Value
B0095	Group B9	Day 1	Peptide	CD4	+	+	+	0.051%
B0095	Group B9	Day 1	Peptide	CD4	+	+	-	0.013%
B0095	Group B9	Day 1	Peptide	CD4	+	-	+	-0.00004%
B0095	Group B9	Day 1	Peptide	CD4	+	-	-	-0.006%
B0095	Group B9	Day 1	Peptide	CD4	-	+	+	0.00109%
B0095	Group B9	Day 1	Peptide	CD4	-	+	-	0.018%
B0095	Group B9	Day 1	Peptide	CD4	-	-	+	-0.00465%
B0095	Group B9	Day 22	Peptide	CD4	+	+	+	0.04%
B0095	Group B9	Day 22	Peptide	CD4	+	+	-	0.017%
B0095	Group B9	Day 22	Peptide	CD4	+	-	+	-0.00203%
B0095	Group B9	Day 22	Peptide	CD4	+	-	-	-0.015%
B0095	Group B9	Day 22	Peptide	CD4	-	+	+	0.01294%
B0095	Group B9	Day 22	Peptide	CD4	-	+	-	0.024%
B0095	Group B9	Day 22	Peptide	CD4	-	-	+	-0.004%
B0095	Group B9	Day 1	Peptide	CD8	+	+	+	0%
B0095	Group B9	Day 1	Peptide	CD8	+	+	-	-0.000023%
B0095	Group B9	Day 1	Peptide	CD8	+	-	+	0%
B0095	Group B9	Day 1	Peptide	CD8	+	-	-	0.00155%
B0095	Group B9	Day 1	Peptide	CD8	-	+	+	0.00228%

Administration Site Key: FA = Forearm; UA= Upper arm
Treatments Part A: A1 = A/Sing MAP, 15 ug (FA); A2 = Afluria Quad IM (UA), 15 ug; A3 = Placebo MAP (FA); A4 = A/Sing Mono IM, 15 ug (UA)
Treatments Part B: B1 = A/Sing MAP, 15 ug (FA) with biopsy; B2 = Placebo MAP (FA) with biopsy
B3 = A/Sing MAP, 15 ug (FA); B4 = A/Sing MAP, 10 ug (FA); B5 = A/Sing MAP, 5 ug (FA); B6 = A/Sing MAP, 2.5 ug (FA)
B7 = Placebo MAP (FA); B8 = A/Sing MAP, 15 ug (UA); B9 = A/Sing IM, 15 ug, as 2018 Afluria Quad (UA)

Listing 16.2.6.6
Immunogenicity Results (CMI)

Part B

Randomisation Number	Group	Visit	Test	Subset	IL2	TNFa	IFNg	Value
B0095	Group B9	Day 1	Peptide	CD8	-	+	-	-0.006%
B0095	Group B9	Day 1	Peptide	CD8	-	-	+	0.35%
B0095	Group B9	Day 22	Peptide	CD8	+	+	+	-0.00184%
B0095	Group B9	Day 22	Peptide	CD8	+	+	-	-0.000901%
B0095	Group B9	Day 22	Peptide	CD8	+	-	+	0%
B0095	Group B9	Day 22	Peptide	CD8	+	-	-	-0.0063%
B0095	Group B9	Day 22	Peptide	CD8	-	+	+	-0.00549%
B0095	Group B9	Day 22	Peptide	CD8	-	+	-	0.009%
B0095	Group B9	Day 22	Peptide	CD8	-	-	+	-0.02%
B0095	Group B9	Day 1	MPH	CD4	+	+	+	0.129%
B0095	Group B9	Day 1	MPH	CD4	+	+	-	0.05%
B0095	Group B9	Day 1	MPH	CD4	+	-	+	0.04203%
B0095	Group B9	Day 1	MPH	CD4	+	-	-	0.051%
B0095	Group B9	Day 1	MPH	CD4	-	+	+	0.1021%
B0095	Group B9	Day 1	MPH	CD4	-	+	-	0.231%
B0095	Group B9	Day 1	MPH	CD4	-	-	+	0.23342%
B0095	Group B9	Day 22	MPH	CD4	+	+	+	0.145%
B0095	Group B9	Day 22	MPH	CD4	+	+	-	0.058%
B0095	Group B9	Day 22	MPH	CD4	+	-	+	0.05527%

Administration Site Key: FA = Forearm; UA= Upper arm
Treatments Part A: A1 = A/Sing MAP, 15 ug (FA); A2 = Afluria Quad IM (UA), 15 ug; A3 = Placebo MAP (FA); A4 = A/Sing Mono IM, 15 ug (UA)
Treatments Part B: B1 = A/Sing MAP, 15 ug (FA) with biopsy; B2 = Placebo MAP (FA) with biopsy
B3 = A/Sing MAP, 15 ug (FA); B4 = A/Sing MAP, 10 ug (FA); B5 = A/Sing MAP, 5 ug (FA); B6 = A/Sing MAP, 2.5 ug (FA)
B7 = Placebo MAP (FA); B8 = A/Sing MAP, 15 ug (UA); B9 = A/Sing IM, 15 ug, as 2018 Afluria Quad (UA)

Listing 16.2.6.6
Immunogenicity Results (CMI)

Part B

Randomisation Number	Group	Visit	Test	Subset	IL2	TNFa	IFNg	Value
B0095	Group B9	Day 22	MPH	CD4	+	-	-	0.051%
B0095	Group B9	Day 22	MPH	CD4	-	+	+	0.11394%
B0095	Group B9	Day 22	MPH	CD4	-	+	-	0.304%
B0095	Group B9	Day 22	MPH	CD4	-	-	+	0.183%
B0095	Group B9	Day 1	MPH	CD8	+	+	+	0.00565%
B0095	Group B9	Day 1	MPH	CD8	+	+	-	0.002402%
B0095	Group B9	Day 1	MPH	CD8	+	-	+	0.00484%
B0095	Group B9	Day 1	MPH	CD8	+	-	-	0.03152%
B0095	Group B9	Day 1	MPH	CD8	-	+	+	0.08803%
B0095	Group B9	Day 1	MPH	CD8	-	+	-	0.219%
B0095	Group B9	Day 1	MPH	CD8	-	-	+	-0.21%
B0095	Group B9	Day 22	MPH	CD8	+	+	+	0.00772%
B0095	Group B9	Day 22	MPH	CD8	+	+	-	0.00511%
B0095	Group B9	Day 22	MPH	CD8	+	-	+	0.00348%
B0095	Group B9	Day 22	MPH	CD8	+	-	-	0.038%
B0095	Group B9	Day 22	MPH	CD8	-	+	+	0.09263%
B0095	Group B9	Day 22	MPH	CD8	-	+	-	0.781%
B0095	Group B9	Day 22	MPH	CD8	-	-	+	-0.38%

Administration Site Key: FA = Forearm; UA= Upper arm
Treatments Part A: A1 = A/Sing MAP, 15 ug (FA); A2 = Afluria Quad IM (UA), 15 ug; A3 = Placebo MAP (FA); A4 = A/Sing Mono IM, 15 ug (UA)
Treatments Part B: B1 = A/Sing MAP, 15 ug (FA) with biopsy; B2 = Placebo MAP (FA) with biopsy
B3 = A/Sing MAP, 15 ug (FA); B4 = A/Sing MAP, 10 ug (FA); B5 = A/Sing MAP, 5 ug (FA); B6 = A/Sing MAP, 2.5 ug (FA)
B7 = Placebo MAP (FA); B8 = A/Sing MAP, 15 ug (UA); B9 = A/Sing IM, 15 ug, as 2018 Afluria Quad (UA)

Listing 16.2.6.6
Immunogenicity Results (CMI)

Part B

Randomisation Number	Group	Visit	Test	Subset	IL2	TNFa	IFNg	Value
B0098	Group B7	Day 1	Peptide	CD4	+	+	+	0.00061%
B0098	Group B7	Day 1	Peptide	CD4	+	+	-	0.00869%
B0098	Group B7	Day 1	Peptide	CD4	+	-	+	-0.0028%
B0098	Group B7	Day 1	Peptide	CD4	+	-	-	0.00277%
B0098	Group B7	Day 1	Peptide	CD4	-	+	+	0.001609%
B0098	Group B7	Day 1	Peptide	CD4	-	+	-	0.013%
B0098	Group B7	Day 1	Peptide	CD4	-	-	+	0.00187%
B0098	Group B7	Day 22	Peptide	CD4	+	+	+	0.00266%
B0098	Group B7	Day 22	Peptide	CD4	+	+	-	-0.00034%
B0098	Group B7	Day 22	Peptide	CD4	+	-	+	-0.000571%
B0098	Group B7	Day 22	Peptide	CD4	+	-	-	-0.0016%
B0098	Group B7	Day 22	Peptide	CD4	-	+	+	0.003115%
B0098	Group B7	Day 22	Peptide	CD4	-	+	-	0%
B0098	Group B7	Day 22	Peptide	CD4	-	-	+	0.00014%
B0098	Group B7	Day 1	Peptide	CD8	+	+	+	0%
B0098	Group B7	Day 1	Peptide	CD8	+	+	-	0.00216%
B0098	Group B7	Day 1	Peptide	CD8	+	-	+	0%
B0098	Group B7	Day 1	Peptide	CD8	+	-	-	0.00216%
B0098	Group B7	Day 1	Peptide	CD8	-	+	+	0.00315%

Administration Site Key: FA = Forearm; UA= Upper arm
Treatments Part A: A1 = A/Sing MAP, 15 ug (FA); A2 = Afluria Quad IM (UA), 15 ug; A3 = Placebo MAP (FA); A4 = A/Sing Mono IM, 15 ug (UA)
Treatments Part B: B1 = A/Sing MAP, 15 ug (FA) with biopsy; B2 = Placebo MAP (FA) with biopsy
B3 = A/Sing MAP, 15 ug (FA); B4 = A/Sing MAP, 10 ug (FA); B5 = A/Sing MAP, 5 ug (FA); B6 = A/Sing MAP, 2.5 ug (FA)
B7 = Placebo MAP (FA); B8 = A/Sing MAP, 15 ug (UA); B9 = A/Sing IM, 15 ug, as 2018 Afluria Quad (UA)

Listing 16.2.6.6
Immunogenicity Results (CMI)

Part B

Randomisation Number	Group	Visit	Test	Subset	IL2	TNFa	IFNg	Value
B0098	Group B7	Day 1	Peptide	CD8	-	+	-	0.022%
B0098	Group B7	Day 1	Peptide	CD8	-	-	+	0.046%
B0098	Group B7	Day 22	Peptide	CD8	+	+	+	0%
B0098	Group B7	Day 22	Peptide	CD8	+	+	-	0%
B0098	Group B7	Day 22	Peptide	CD8	+	-	+	0%
B0098	Group B7	Day 22	Peptide	CD8	+	-	-	-0.00322%
B0098	Group B7	Day 22	Peptide	CD8	-	+	+	0.00163%
B0098	Group B7	Day 22	Peptide	CD8	-	+	-	0.001%
B0098	Group B7	Day 22	Peptide	CD8	-	-	+	-0.316%
B0098	Group B7	Day 1	MPH	CD4	+	+	+	0.10369%
B0098	Group B7	Day 1	MPH	CD4	+	+	-	0.08669%
B0098	Group B7	Day 1	MPH	CD4	+	-	+	0.0182%
B0098	Group B7	Day 1	MPH	CD4	+	-	-	0.02809%
B0098	Group B7	Day 1	MPH	CD4	-	+	+	0.068299%
B0098	Group B7	Day 1	MPH	CD4	-	+	-	0.308%
B0098	Group B7	Day 1	MPH	CD4	-	-	+	0.10649%
B0098	Group B7	Day 22	MPH	CD4	+	+	+	0.05224%
B0098	Group B7	Day 22	MPH	CD4	+	+	-	0.06886%
B0098	Group B7	Day 22	MPH	CD4	+	-	+	0.00635%

Administration Site Key: FA = Forearm; UA= Upper arm
Treatments Part A: A1 = A/Sing MAP, 15 ug (FA); A2 = Afluria Quad IM (UA), 15 ug; A3 = Placebo MAP (FA); A4 = A/Sing Mono IM, 15 ug (UA)
Treatments Part B: B1 = A/Sing MAP, 15 ug (FA) with biopsy; B2 = Placebo MAP (FA) with biopsy
B3 = A/Sing MAP, 15 ug (FA); B4 = A/Sing MAP, 10 ug (FA); B5 = A/Sing MAP, 5 ug (FA); B6 = A/Sing MAP, 2.5 ug (FA)
B7 = Placebo MAP (FA); B8 = A/Sing MAP, 15 ug (UA); B9 = A/Sing IM, 15 ug, as 2018 Afluria Quad (UA)

Listing 16.2.6.6
Immunogenicity Results (CMI)

Part B

Randomisation Number	Group	Visit	Test	Subset	IL2	TNFa	IFNg	Value
B0098	Group B7	Day 22	MPH	CD4	+	-	-	0.02745%
B0098	Group B7	Day 22	MPH	CD4	-	+	+	0.028405%
B0098	Group B7	Day 22	MPH	CD4	-	+	-	0.19%
B0098	Group B7	Day 22	MPH	CD4	-	-	+	0.04443%
B0098	Group B7	Day 1	MPH	CD8	+	+	+	0.03%
B0098	Group B7	Day 1	MPH	CD8	+	+	-	0.0093%
B0098	Group B7	Day 1	MPH	CD8	+	-	+	0.013%
B0098	Group B7	Day 1	MPH	CD8	+	-	-	0.032%
B0098	Group B7	Day 1	MPH	CD8	-	+	+	0.04615%
B0098	Group B7	Day 1	MPH	CD8	-	+	-	0.162%
B0098	Group B7	Day 1	MPH	CD8	-	-	+	0.086%
B0098	Group B7	Day 22	MPH	CD8	+	+	+	0.022%
B0098	Group B7	Day 22	MPH	CD8	+	+	-	0.00596%
B0098	Group B7	Day 22	MPH	CD8	+	-	+	0.00894%
B0098	Group B7	Day 22	MPH	CD8	+	-	-	0.01278%
B0098	Group B7	Day 22	MPH	CD8	-	+	+	0.051%
B0098	Group B7	Day 22	MPH	CD8	-	+	-	0.175%
B0098	Group B7	Day 22	MPH	CD8	-	-	+	-0.25%

Administration Site Key: FA = Forearm; UA= Upper arm
Treatments Part A: A1 = A/Sing MAP, 15 ug (FA); A2 = Afluria Quad IM (UA), 15 ug; A3 = Placebo MAP (FA); A4 = A/Sing Mono IM, 15 ug (UA)
Treatments Part B: B1 = A/Sing MAP, 15 ug (FA) with biopsy; B2 = Placebo MAP (FA) with biopsy
B3 = A/Sing MAP, 15 ug (FA); B4 = A/Sing MAP, 10 ug (FA); B5 = A/Sing MAP, 5 ug (FA); B6 = A/Sing MAP, 2.5 ug (FA)
B7 = Placebo MAP (FA); B8 = A/Sing MAP, 15 ug (UA); B9 = A/Sing IM, 15 ug, as 2018 Afluria Quad (UA)

Listing 16.2.6.6
Immunogenicity Results (CMI)

Part B

Randomisation Number	Group	Visit	Test	Subset	IL2	TNFa	IFNg	Value
B0100	Group B8	Day 1	Peptide	CD4	+	+	+	-0.00269%
B0100	Group B8	Day 1	Peptide	CD4	+	+	-	0.003%
B0100	Group B8	Day 1	Peptide	CD4	+	-	+	-0.00187%
B0100	Group B8	Day 1	Peptide	CD4	+	-	-	-0.00082%
B0100	Group B8	Day 1	Peptide	CD4	-	+	+	0.00003%
B0100	Group B8	Day 1	Peptide	CD4	-	+	-	-0.002%
B0100	Group B8	Day 1	Peptide	CD4	-	-	+	-0.00368%
B0100	Group B8	Day 22	Peptide	CD4	+	+	+	0.00659%
B0100	Group B8	Day 22	Peptide	CD4	+	+	-	0.009%
B0100	Group B8	Day 22	Peptide	CD4	+	-	+	0.000029%
B0100	Group B8	Day 22	Peptide	CD4	+	-	-	-0.007%
B0100	Group B8	Day 22	Peptide	CD4	-	+	+	0.001895%
B0100	Group B8	Day 22	Peptide	CD4	-	+	-	0.015%
B0100	Group B8	Day 22	Peptide	CD4	-	-	+	-0.00257%
B0100	Group B8	Day 1	Peptide	CD8	+	+	+	0%
B0100	Group B8	Day 1	Peptide	CD8	+	+	-	-0.000902%
B0100	Group B8	Day 1	Peptide	CD8	+	-	+	0%
B0100	Group B8	Day 1	Peptide	CD8	+	-	-	0.00293%
B0100	Group B8	Day 1	Peptide	CD8	-	+	+	-0.00093%

Administration Site Key: FA = Forearm; UA= Upper arm
Treatments Part A: A1 = A/Sing MAP, 15 ug (FA); A2 = Afluria Quad IM (UA), 15 ug; A3 = Placebo MAP (FA); A4 = A/Sing Mono IM, 15 ug (UA)
Treatments Part B: B1 = A/Sing MAP, 15 ug (FA) with biopsy; B2 = Placebo MAP (FA) with biopsy
B3 = A/Sing MAP, 15 ug (FA); B4 = A/Sing MAP, 10 ug (FA); B5 = A/Sing MAP, 5 ug (FA); B6 = A/Sing MAP, 2.5 ug (FA)
B7 = Placebo MAP (FA); B8 = A/Sing MAP, 15 ug (UA); B9 = A/Sing IM, 15 ug, as 2018 Afluria Quad (UA)

Listing 16.2.6.6
Immunogenicity Results (CMI)

Part B

Randomisation Number	Group	Visit	Test	Subset	IL2	TNFa	IFNg	Value
B0100	Group B8	Day 1	Peptide	CD8	-	+	-	0.007%
B0100	Group B8	Day 1	Peptide	CD8	-	-	+	0.03%
B0100	Group B8	Day 22	Peptide	CD8	+	+	+	0%
B0100	Group B8	Day 22	Peptide	CD8	+	+	-	0.00001%
B0100	Group B8	Day 22	Peptide	CD8	+	-	+	0%
B0100	Group B8	Day 22	Peptide	CD8	+	-	-	-0.00082%
B0100	Group B8	Day 22	Peptide	CD8	-	+	+	-0.002593%
B0100	Group B8	Day 22	Peptide	CD8	-	+	-	-0.004%
B0100	Group B8	Day 22	Peptide	CD8	-	-	+	-0.78%
B0100	Group B8	Day 1	MPH	CD4	+	+	+	0.07965%
B0100	Group B8	Day 1	MPH	CD4	+	+	-	0.072%
B0100	Group B8	Day 1	MPH	CD4	+	-	+	0.00775%
B0100	Group B8	Day 1	MPH	CD4	+	-	-	0.02852%
B0100	Group B8	Day 1	MPH	CD4	-	+	+	0.04313%
B0100	Group B8	Day 1	MPH	CD4	-	+	-	0.244%
B0100	Group B8	Day 1	MPH	CD4	-	-	+	0.05346%
B0100	Group B8	Day 22	MPH	CD4	+	+	+	0.16819%
B0100	Group B8	Day 22	MPH	CD4	+	+	-	0.116%
B0100	Group B8	Day 22	MPH	CD4	+	-	+	0.031095%

Administration Site Key: FA = Forearm; UA= Upper arm
Treatments Part A: A1 = A/Sing MAP, 15 ug (FA); A2 = Afluria Quad IM (UA), 15 ug; A3 = Placebo MAP (FA); A4 = A/Sing Mono IM, 15 ug (UA)
Treatments Part B: B1 = A/Sing MAP, 15 ug (FA) with biopsy; B2 = Placebo MAP (FA) with biopsy
B3 = A/Sing MAP, 15 ug (FA); B4 = A/Sing MAP, 10 ug (FA); B5 = A/Sing MAP, 5 ug (FA); B6 = A/Sing MAP, 2.5 ug (FA)
B7 = Placebo MAP (FA); B8 = A/Sing MAP, 15 ug (UA); B9 = A/Sing IM, 15 ug, as 2018 Afluria Quad (UA)

Listing 16.2.6.6
Immunogenicity Results (CMI)

Part B

Randomisation Number	Group	Visit	Test	Subset	IL2	TNFa	IFNg	Value
B0100	Group B8	Day 22	MPH	CD4	+	-	-	0.02%
B0100	Group B8	Day 22	MPH	CD4	-	+	+	0.043095%
B0100	Group B8	Day 22	MPH	CD4	-	+	-	0.287%
B0100	Group B8	Day 22	MPH	CD4	-	-	+	0.07176%
B0100	Group B8	Day 1	MPH	CD8	+	+	+	0.073%
B0100	Group B8	Day 1	MPH	CD8	+	+	-	0.0032%
B0100	Group B8	Day 1	MPH	CD8	+	-	+	0.026%
B0100	Group B8	Day 1	MPH	CD8	+	-	-	0.03314%
B0100	Group B8	Day 1	MPH	CD8	-	+	+	0.03607%
B0100	Group B8	Day 1	MPH	CD8	-	+	-	0.074%
B0100	Group B8	Day 1	MPH	CD8	-	-	+	0.07%
B0100	Group B8	Day 22	MPH	CD8	+	+	+	0.064%
B0100	Group B8	Day 22	MPH	CD8	+	+	-	0.005693%
B0100	Group B8	Day 22	MPH	CD8	+	-	+	0.027%
B0100	Group B8	Day 22	MPH	CD8	+	-	-	0.0408%
B0100	Group B8	Day 22	MPH	CD8	-	+	+	0.04653%
B0100	Group B8	Day 22	MPH	CD8	-	+	-	0.045%
B0100	Group B8	Day 22	MPH	CD8	-	-	+	-0.76%

Administration Site Key: FA = Forearm; UA= Upper arm
Treatments Part A: A1 = A/Sing MAP, 15 ug (FA); A2 = Afluria Quad IM (UA), 15 ug; A3 = Placebo MAP (FA); A4 = A/Sing Mono IM, 15 ug (UA)
Treatments Part B: B1 = A/Sing MAP, 15 ug (FA) with biopsy; B2 = Placebo MAP (FA) with biopsy
B3 = A/Sing MAP, 15 ug (FA); B4 = A/Sing MAP, 10 ug (FA); B5 = A/Sing MAP, 5 ug (FA); B6 = A/Sing MAP, 2.5 ug (FA)
B7 = Placebo MAP (FA); B8 = A/Sing MAP, 15 ug (UA); B9 = A/Sing IM, 15 ug, as 2018 Afluria Quad (UA)

Listing 16.2.6.6
Immunogenicity Results (CMI)

Part B

Randomisation Number	Group	Visit	Test	Subset	IL2	TNFa	IFNg	Value
B0101	Group B3	Day 1	Peptide	CD4	+	+	+	0.01883%
B0101	Group B3	Day 1	Peptide	CD4	+	+	-	0.01145%
B0101	Group B3	Day 1	Peptide	CD4	+	-	+	-0.001609%
B0101	Group B3	Day 1	Peptide	CD4	+	-	-	-0.00383%
B0101	Group B3	Day 1	Peptide	CD4	-	+	+	0.003%
B0101	Group B3	Day 1	Peptide	CD4	-	+	-	-0.002%
B0101	Group B3	Day 1	Peptide	CD4	-	-	+	-0.00168%
B0101	Group B3	Day 22	Peptide	CD4	+	+	+	0.027%
B0101	Group B3	Day 22	Peptide	CD4	+	+	-	0.018%
B0101	Group B3	Day 22	Peptide	CD4	+	-	+	-0.002398%
B0101	Group B3	Day 22	Peptide	CD4	+	-	-	0.016%
B0101	Group B3	Day 22	Peptide	CD4	-	+	+	-0.00013%
B0101	Group B3	Day 22	Peptide	CD4	-	+	-	0.021%
B0101	Group B3	Day 22	Peptide	CD4	-	-	+	-0.00277%
B0101	Group B3	Day 1	Peptide	CD8	+	+	+	0%
B0101	Group B3	Day 1	Peptide	CD8	+	+	-	0%
B0101	Group B3	Day 1	Peptide	CD8	+	-	+	0%
B0101	Group B3	Day 1	Peptide	CD8	+	-	-	-0.00208%
B0101	Group B3	Day 1	Peptide	CD8	-	+	+	0.00525%

Administration Site Key: FA = Forearm; UA= Upper arm
Treatments Part A: A1 = A/Sing MAP, 15 ug (FA); A2 = Afluria Quad IM (UA), 15 ug; A3 = Placebo MAP (FA); A4 = A/Sing Mono IM, 15 ug (UA)
Treatments Part B: B1 = A/Sing MAP, 15 ug (FA) with biopsy; B2 = Placebo MAP (FA) with biopsy
B3 = A/Sing MAP, 15 ug (FA); B4 = A/Sing MAP, 10 ug (FA); B5 = A/Sing MAP, 5 ug (FA); B6 = A/Sing MAP, 2.5 ug (FA)
B7 = Placebo MAP (FA); B8 = A/Sing MAP, 15 ug (UA); B9 = A/Sing IM, 15 ug, as 2018 Afluria Quad (UA)

Listing 16.2.6.6
Immunogenicity Results (CMI)

Part B

Randomisation Number	Group	Visit	Test	Subset	IL2	TNFa	IFNg	Value
B0101	Group B3	Day 1	Peptide	CD8	-	+	-	0.027%
B0101	Group B3	Day 1	Peptide	CD8	-	-	+	-0.43%
B0101	Group B3	Day 22	Peptide	CD8	+	+	+	0%
B0101	Group B3	Day 22	Peptide	CD8	+	+	-	0.00212%
B0101	Group B3	Day 22	Peptide	CD8	+	-	+	0.00212%
B0101	Group B3	Day 22	Peptide	CD8	+	-	-	0.01111%
B0101	Group B3	Day 22	Peptide	CD8	-	+	+	-0.00763%
B0101	Group B3	Day 22	Peptide	CD8	-	+	-	0.012%
B0101	Group B3	Day 22	Peptide	CD8	-	-	+	0.7%
B0101	Group B3	Day 1	MPH	CD4	+	+	+	0.08883%
B0101	Group B3	Day 1	MPH	CD4	+	+	-	0.12445%
B0101	Group B3	Day 1	MPH	CD4	+	-	+	0.01762%
B0101	Group B3	Day 1	MPH	CD4	+	-	-	0.019%
B0101	Group B3	Day 1	MPH	CD4	-	+	+	0.03583%
B0101	Group B3	Day 1	MPH	CD4	-	+	-	0.287%
B0101	Group B3	Day 1	MPH	CD4	-	-	+	0.04824%
B0101	Group B3	Day 22	MPH	CD4	+	+	+	0.116%
B0101	Group B3	Day 22	MPH	CD4	+	+	-	0.154%
B0101	Group B3	Day 22	MPH	CD4	+	-	+	0.0159%

Administration Site Key: FA = Forearm; UA= Upper arm
Treatments Part A: A1 = A/Sing MAP, 15 ug (FA); A2 = Afluria Quad IM (UA), 15 ug; A3 = Placebo MAP (FA); A4 = A/Sing Mono IM, 15 ug (UA)
Treatments Part B: B1 = A/Sing MAP, 15 ug (FA) with biopsy; B2 = Placebo MAP (FA) with biopsy
B3 = A/Sing MAP, 15 ug (FA); B4 = A/Sing MAP, 10 ug (FA); B5 = A/Sing MAP, 5 ug (FA); B6 = A/Sing MAP, 2.5 ug (FA)
B7 = Placebo MAP (FA); B8 = A/Sing MAP, 15 ug (UA); B9 = A/Sing IM, 15 ug, as 2018 Afluria Quad (UA)

Listing 16.2.6.6
Immunogenicity Results (CMI)

Part B

Randomisation Number	Group	Visit	Test	Subset	IL2	TNFa	IFNg	Value
B0101	Group B3	Day 22	MPH	CD4	+	-	-	0.039%
B0101	Group B3	Day 22	MPH	CD4	-	+	+	0.06866%
B0101	Group B3	Day 22	MPH	CD4	-	+	-	0.405%
B0101	Group B3	Day 22	MPH	CD4	-	-	+	0.07942%
B0101	Group B3	Day 1	MPH	CD8	+	+	+	0.022%
B0101	Group B3	Day 1	MPH	CD8	+	+	-	0.00816%
B0101	Group B3	Day 1	MPH	CD8	+	-	+	0.012%
B0101	Group B3	Day 1	MPH	CD8	+	-	-	0.02194%
B0101	Group B3	Day 1	MPH	CD8	-	+	+	0.04429%
B0101	Group B3	Day 1	MPH	CD8	-	+	-	0.077%
B0101	Group B3	Day 1	MPH	CD8	-	-	+	-0.61%
B0101	Group B3	Day 22	MPH	CD8	+	+	+	0.031%
B0101	Group B3	Day 22	MPH	CD8	+	+	-	0.00445%
B0101	Group B3	Day 22	MPH	CD8	+	-	+	0.013%
B0101	Group B3	Day 22	MPH	CD8	+	-	-	0.03811%
B0101	Group B3	Day 22	MPH	CD8	-	+	+	0.126%
B0101	Group B3	Day 22	MPH	CD8	-	+	-	0.127%
B0101	Group B3	Day 22	MPH	CD8	-	-	+	-1.06%

Administration Site Key: FA = Forearm; UA= Upper arm
Treatments Part A: A1 = A/Sing MAP, 15 ug (FA); A2 = Afluria Quad IM (UA), 15 ug; A3 = Placebo MAP (FA); A4 = A/Sing Mono IM, 15 ug (UA)
Treatments Part B: B1 = A/Sing MAP, 15 ug (FA) with biopsy; B2 = Placebo MAP (FA) with biopsy
B3 = A/Sing MAP, 15 ug (FA); B4 = A/Sing MAP, 10 ug (FA); B5 = A/Sing MAP, 5 ug (FA); B6 = A/Sing MAP, 2.5 ug (FA)
B7 = Placebo MAP (FA); B8 = A/Sing MAP, 15 ug (UA); B9 = A/Sing IM, 15 ug, as 2018 Afluria Quad (UA)

Listing 16.2.6.6
Immunogenicity Results (CMI)

Part B

Randomisation Number	Group	Visit	Test	Subset	IL2	TNFa	IFNg	Value
B0102	Group B8	Day 1	Peptide	CD4	+	+	+	0.07821%
B0102	Group B8	Day 1	Peptide	CD4	+	+	-	0.016%
B0102	Group B8	Day 1	Peptide	CD4	+	-	+	0.000883%
B0102	Group B8	Day 1	Peptide	CD4	+	-	-	0.00124%
B0102	Group B8	Day 1	Peptide	CD4	-	+	+	0.00595%
B0102	Group B8	Day 1	Peptide	CD4	-	+	-	0.004%
B0102	Group B8	Day 1	Peptide	CD4	-	-	+	0.00621%
B0102	Group B8	Day 22	Peptide	CD4	+	+	+	0.08664%
B0102	Group B8	Day 22	Peptide	CD4	+	+	-	0.03458%
B0102	Group B8	Day 22	Peptide	CD4	+	-	+	0.000763%
B0102	Group B8	Day 22	Peptide	CD4	+	-	-	0.00458%
B0102	Group B8	Day 22	Peptide	CD4	-	+	+	0.00835%
B0102	Group B8	Day 22	Peptide	CD4	-	+	-	0.025%
B0102	Group B8	Day 22	Peptide	CD4	-	-	+	0.00065%
B0102	Group B8	Day 1	Peptide	CD8	+	+	+	0.00422%
B0102	Group B8	Day 1	Peptide	CD8	+	+	-	0.00105%
B0102	Group B8	Day 1	Peptide	CD8	+	-	+	0%
B0102	Group B8	Day 1	Peptide	CD8	+	-	-	0.00412%
B0102	Group B8	Day 1	Peptide	CD8	-	+	+	0.00517%

Administration Site Key: FA = Forearm; UA= Upper arm
Treatments Part A: A1 = A/Sing MAP, 15 ug (FA); A2 = Afluria Quad IM (UA), 15 ug; A3 = Placebo MAP (FA); A4 = A/Sing Mono IM, 15 ug (UA)
Treatments Part B: B1 = A/Sing MAP, 15 ug (FA) with biopsy; B2 = Placebo MAP (FA) with biopsy
B3 = A/Sing MAP, 15 ug (FA); B4 = A/Sing MAP, 10 ug (FA); B5 = A/Sing MAP, 5 ug (FA); B6 = A/Sing MAP, 2.5 ug (FA)
B7 = Placebo MAP (FA); B8 = A/Sing MAP, 15 ug (UA); B9 = A/Sing IM, 15 ug, as 2018 Afluria Quad (UA)

Listing 16.2.6.6
Immunogenicity Results (CMI)

Part B

Randomisation Number	Group	Visit	Test	Subset	IL2	TNFa	IFNg	Value
B0102	Group B8	Day 1	Peptide	CD8	-	+	-	0.009%
B0102	Group B8	Day 1	Peptide	CD8	-	-	+	-0.1%
B0102	Group B8	Day 22	Peptide	CD8	+	+	+	0.00105%
B0102	Group B8	Day 22	Peptide	CD8	+	+	-	0.00105%
B0102	Group B8	Day 22	Peptide	CD8	+	-	+	0%
B0102	Group B8	Day 22	Peptide	CD8	+	-	-	0.00314%
B0102	Group B8	Day 22	Peptide	CD8	-	+	+	0.00103%
B0102	Group B8	Day 22	Peptide	CD8	-	+	-	0.019%
B0102	Group B8	Day 22	Peptide	CD8	-	-	+	0.115%
B0102	Group B8	Day 1	MPH	CD4	+	+	+	0.08521%
B0102	Group B8	Day 1	MPH	CD4	+	+	-	0.06%
B0102	Group B8	Day 1	MPH	CD4	+	-	+	0.014%
B0102	Group B8	Day 1	MPH	CD4	+	-	-	0.04429%
B0102	Group B8	Day 1	MPH	CD4	-	+	+	0.06712%
B0102	Group B8	Day 1	MPH	CD4	-	+	-	0.179%
B0102	Group B8	Day 1	MPH	CD4	-	-	+	0.08121%
B0102	Group B8	Day 22	MPH	CD4	+	+	+	0.13764%
B0102	Group B8	Day 22	MPH	CD4	+	+	-	0.13058%
B0102	Group B8	Day 22	MPH	CD4	+	-	+	0.033%

Administration Site Key: FA = Forearm; UA= Upper arm
Treatments Part A: A1 = A/Sing MAP, 15 ug (FA); A2 = Afluria Quad IM (UA), 15 ug; A3 = Placebo MAP (FA); A4 = A/Sing Mono IM, 15 ug (UA)
Treatments Part B: B1 = A/Sing MAP, 15 ug (FA) with biopsy; B2 = Placebo MAP (FA) with biopsy
B3 = A/Sing MAP, 15 ug (FA); B4 = A/Sing MAP, 10 ug (FA); B5 = A/Sing MAP, 5 ug (FA); B6 = A/Sing MAP, 2.5 ug (FA)
B7 = Placebo MAP (FA); B8 = A/Sing MAP, 15 ug (UA); B9 = A/Sing IM, 15 ug, as 2018 Afluria Quad (UA)

Listing 16.2.6.6
Immunogenicity Results (CMI)

Part B

Randomisation Number	Group	Visit	Test	Subset	IL2	TNFa	IFNg	Value
B0102	Group B8	Day 22	MPH	CD4	+	-	-	0.07158%
B0102	Group B8	Day 22	MPH	CD4	-	+	+	0.08543%
B0102	Group B8	Day 22	MPH	CD4	-	+	-	0.189%
B0102	Group B8	Day 22	MPH	CD4	-	-	+	0.06707%
B0102	Group B8	Day 1	MPH	CD8	+	+	+	0.03%
B0102	Group B8	Day 1	MPH	CD8	+	+	-	0.00637%
B0102	Group B8	Day 1	MPH	CD8	+	-	+	0.014%
B0102	Group B8	Day 1	MPH	CD8	+	-	-	0.02185%
B0102	Group B8	Day 1	MPH	CD8	-	+	+	0.06585%
B0102	Group B8	Day 1	MPH	CD8	-	+	-	0.077%
B0102	Group B8	Day 1	MPH	CD8	-	-	+	-0.6%
B0102	Group B8	Day 22	MPH	CD8	+	+	+	0.018%
B0102	Group B8	Day 22	MPH	CD8	+	+	-	0.00415%
B0102	Group B8	Day 22	MPH	CD8	+	-	+	0.01%
B0102	Group B8	Day 22	MPH	CD8	+	-	-	0.03792%
B0102	Group B8	Day 22	MPH	CD8	-	+	+	0.05592%
B0102	Group B8	Day 22	MPH	CD8	-	+	-	0.112%
B0102	Group B8	Day 22	MPH	CD8	-	-	+	0.125%

Administration Site Key: FA = Forearm; UA= Upper arm
Treatments Part A: A1 = A/Sing MAP, 15 ug (FA); A2 = Afluria Quad IM (UA), 15 ug; A3 = Placebo MAP (FA); A4 = A/Sing Mono IM, 15 ug (UA)
Treatments Part B: B1 = A/Sing MAP, 15 ug (FA) with biopsy; B2 = Placebo MAP (FA) with biopsy
B3 = A/Sing MAP, 15 ug (FA); B4 = A/Sing MAP, 10 ug (FA); B5 = A/Sing MAP, 5 ug (FA); B6 = A/Sing MAP, 2.5 ug (FA)
B7 = Placebo MAP (FA); B8 = A/Sing MAP, 15 ug (UA); B9 = A/Sing IM, 15 ug, as 2018 Afluria Quad (UA)

Listing 16.2.6.6
Immunogenicity Results (CMI)

Part B

Randomisation Number	Group	Visit	Test	Subset	IL2	TNFa	IFNg	Value
B0104	Group B3	Day 1	Peptide	CD4	+	+	+	0.02683%
B0104	Group B3	Day 1	Peptide	CD4	+	+	-	0.00496%
B0104	Group B3	Day 1	Peptide	CD4	+	-	+	-0.000527%
B0104	Group B3	Day 1	Peptide	CD4	+	-	-	-0.00042%
B0104	Group B3	Day 1	Peptide	CD4	-	+	+	0.006088%
B0104	Group B3	Day 1	Peptide	CD4	-	+	-	0.013%
B0104	Group B3	Day 1	Peptide	CD4	-	-	+	0.00282%
B0104	Group B3	Day 22	Peptide	CD4	+	+	+	0.08306%
B0104	Group B3	Day 22	Peptide	CD4	+	+	-	0.0596%
B0104	Group B3	Day 22	Peptide	CD4	+	-	+	0.06406%
B0104	Group B3	Day 22	Peptide	CD4	+	-	-	0.028%
B0104	Group B3	Day 22	Peptide	CD4	-	+	+	0.07577%
B0104	Group B3	Day 22	Peptide	CD4	-	+	-	0.146%
B0104	Group B3	Day 22	Peptide	CD4	-	-	+	0.138%
B0104	Group B3	Day 1	Peptide	CD8	+	+	+	0%
B0104	Group B3	Day 1	Peptide	CD8	+	+	-	0%
B0104	Group B3	Day 1	Peptide	CD8	+	-	+	0%
B0104	Group B3	Day 1	Peptide	CD8	+	-	-	-0.00248%
B0104	Group B3	Day 1	Peptide	CD8	-	+	+	0%

Administration Site Key: FA = Forearm; UA= Upper arm
Treatments Part A: A1 = A/Sing MAP, 15 ug (FA); A2 = Afluria Quad IM (UA), 15 ug; A3 = Placebo MAP (FA); A4 = A/Sing Mono IM, 15 ug (UA)
Treatments Part B: B1 = A/Sing MAP, 15 ug (FA) with biopsy; B2 = Placebo MAP (FA) with biopsy
B3 = A/Sing MAP, 15 ug (FA); B4 = A/Sing MAP, 10 ug (FA); B5 = A/Sing MAP, 5 ug (FA); B6 = A/Sing MAP, 2.5 ug (FA)
B7 = Placebo MAP (FA); B8 = A/Sing MAP, 15 ug (UA); B9 = A/Sing IM, 15 ug, as 2018 Afluria Quad (UA)

Listing 16.2.6.6
Immunogenicity Results (CMI)

Part B

Randomisation Number	Group	Visit	Test	Subset	IL2	TNFa	IFNg	Value
B0104	Group B3	Day 1	Peptide	CD8	-	+	-	0.011%
B0104	Group B3	Day 1	Peptide	CD8	-	-	+	0.22%
B0104	Group B3	Day 22	Peptide	CD8	+	+	+	0%
B0104	Group B3	Day 22	Peptide	CD8	+	+	-	0%
B0104	Group B3	Day 22	Peptide	CD8	+	-	+	0%
B0104	Group B3	Day 22	Peptide	CD8	+	-	-	-0.00312%
B0104	Group B3	Day 22	Peptide	CD8	-	+	+	0.00111%
B0104	Group B3	Day 22	Peptide	CD8	-	+	-	-0.004%
B0104	Group B3	Day 22	Peptide	CD8	-	-	+	-0.09%
B0104	Group B3	Day 1	MPH	CD4	+	+	+	0.07183%
B0104	Group B3	Day 1	MPH	CD4	+	+	-	0.05296%
B0104	Group B3	Day 1	MPH	CD4	+	-	+	0.01592%
B0104	Group B3	Day 1	MPH	CD4	+	-	-	0.0225%
B0104	Group B3	Day 1	MPH	CD4	-	+	+	0.048458%
B0104	Group B3	Day 1	MPH	CD4	-	+	-	0.145%
B0104	Group B3	Day 1	MPH	CD4	-	-	+	0.08229%
B0104	Group B3	Day 22	MPH	CD4	+	+	+	0.08306%
B0104	Group B3	Day 22	MPH	CD4	+	+	-	0.0596%
B0104	Group B3	Day 22	MPH	CD4	+	-	+	0.06406%

Administration Site Key: FA = Forearm; UA= Upper arm
Treatments Part A: A1 = A/Sing MAP, 15 ug (FA); A2 = Afluria Quad IM (UA), 15 ug; A3 = Placebo MAP (FA); A4 = A/Sing Mono IM, 15 ug (UA)
Treatments Part B: B1 = A/Sing MAP, 15 ug (FA) with biopsy; B2 = Placebo MAP (FA) with biopsy
B3 = A/Sing MAP, 15 ug (FA); B4 = A/Sing MAP, 10 ug (FA); B5 = A/Sing MAP, 5 ug (FA); B6 = A/Sing MAP, 2.5 ug (FA)
B7 = Placebo MAP (FA); B8 = A/Sing MAP, 15 ug (UA); B9 = A/Sing IM, 15 ug, as 2018 Afluria Quad (UA)

Listing 16.2.6.6
Immunogenicity Results (CMI)

Part B

Randomisation Number	Group	Visit	Test	Subset	IL2	TNFa	IFNg	Value
B0104	Group B3	Day 22	MPH	CD4	+	-	-	0.028%
B0104	Group B3	Day 22	MPH	CD4	-	+	+	0.07577%
B0104	Group B3	Day 22	MPH	CD4	-	+	-	0.146%
B0104	Group B3	Day 22	MPH	CD4	-	-	+	0.138%
B0104	Group B3	Day 1	MPH	CD8	+	+	+	0.012%
B0104	Group B3	Day 1	MPH	CD8	+	+	-	0.00368%
B0104	Group B3	Day 1	MPH	CD8	+	-	+	0.00368%
B0104	Group B3	Day 1	MPH	CD8	+	-	-	0.00852%
B0104	Group B3	Day 1	MPH	CD8	-	+	+	0.023%
B0104	Group B3	Day 1	MPH	CD8	-	+	-	0.089%
B0104	Group B3	Day 1	MPH	CD8	-	-	+	0.03%
B0104	Group B3	Day 22	MPH	CD8	+	+	+	0.014%
B0104	Group B3	Day 22	MPH	CD8	+	+	-	0.00107%
B0104	Group B3	Day 22	MPH	CD8	+	-	+	0.00537%
B0104	Group B3	Day 22	MPH	CD8	+	-	-	0.01966%
B0104	Group B3	Day 22	MPH	CD8	-	+	+	0.035%
B0104	Group B3	Day 22	MPH	CD8	-	+	-	0.241%
B0104	Group B3	Day 22	MPH	CD8	-	-	+	-0.04%

Administration Site Key: FA = Forearm; UA= Upper arm
Treatments Part A: A1 = A/Sing MAP, 15 ug (FA); A2 = Afluria Quad IM (UA), 15 ug; A3 = Placebo MAP (FA); A4 = A/Sing Mono IM, 15 ug (UA)
Treatments Part B: B1 = A/Sing MAP, 15 ug (FA) with biopsy; B2 = Placebo MAP (FA) with biopsy
B3 = A/Sing MAP, 15 ug (FA); B4 = A/Sing MAP, 10 ug (FA); B5 = A/Sing MAP, 5 ug (FA); B6 = A/Sing MAP, 2.5 ug (FA)
B7 = Placebo MAP (FA); B8 = A/Sing MAP, 15 ug (UA); B9 = A/Sing IM, 15 ug, as 2018 Afluria Quad (UA)

Listing 16.2.6.6
Immunogenicity Results (CMI)

Part B

Randomisation Number	Group	Visit	Test	Subset	IL2	TNFa	IFNg	Value
B0105	Group B9	Day 1	Peptide	CD4	+	+	+	0.00528%
B0105	Group B9	Day 1	Peptide	CD4	+	+	-	0.00607%
B0105	Group B9	Day 1	Peptide	CD4	+	-	+	0%
B0105	Group B9	Day 1	Peptide	CD4	+	-	-	0.00231%
B0105	Group B9	Day 1	Peptide	CD4	-	+	+	0.00002%
B0105	Group B9	Day 1	Peptide	CD4	-	+	-	0.011%
B0105	Group B9	Day 1	Peptide	CD4	-	-	+	0.00233%
B0105	Group B9	Day 22	Peptide	CD4	+	+	+	0.00302%
B0105	Group B9	Day 22	Peptide	CD4	+	+	-	0.01524%
B0105	Group B9	Day 22	Peptide	CD4	+	-	+	0%
B0105	Group B9	Day 22	Peptide	CD4	+	-	-	0.00206%
B0105	Group B9	Day 22	Peptide	CD4	-	+	+	0.00102%
B0105	Group B9	Day 22	Peptide	CD4	-	+	-	0.002%
B0105	Group B9	Day 22	Peptide	CD4	-	-	+	-0.00181%
B0105	Group B9	Day 1	Peptide	CD8	+	+	+	0%
B0105	Group B9	Day 1	Peptide	CD8	+	+	-	-0.00173%
B0105	Group B9	Day 1	Peptide	CD8	+	-	+	0%
B0105	Group B9	Day 1	Peptide	CD8	+	-	-	-0.0034%
B0105	Group B9	Day 1	Peptide	CD8	-	+	+	-0.00168%

Administration Site Key: FA = Forearm; UA= Upper arm
Treatments Part A: A1 = A/Sing MAP, 15 ug (FA); A2 = Afluria Quad IM (UA), 15 ug; A3 = Placebo MAP (FA); A4 = A/Sing Mono IM, 15 ug (UA)
Treatments Part B: B1 = A/Sing MAP, 15 ug (FA) with biopsy; B2 = Placebo MAP (FA) with biopsy
B3 = A/Sing MAP, 15 ug (FA); B4 = A/Sing MAP, 10 ug (FA); B5 = A/Sing MAP, 5 ug (FA); B6 = A/Sing MAP, 2.5 ug (FA)
B7 = Placebo MAP (FA); B8 = A/Sing MAP, 15 ug (UA); B9 = A/Sing IM, 15 ug, as 2018 Afluria Quad (UA)

Listing 16.2.6.6
Immunogenicity Results (CMI)

Part B

Randomisation Number	Group	Visit	Test	Subset	IL2	TNFa	IFNg	Value
B0105	Group B9	Day 1	Peptide	CD8	-	+	-	-0.02%
B0105	Group B9	Day 1	Peptide	CD8	-	-	+	-0.029%
B0105	Group B9	Day 22	Peptide	CD8	+	+	+	0%
B0105	Group B9	Day 22	Peptide	CD8	+	+	-	0.00252%
B0105	Group B9	Day 22	Peptide	CD8	+	-	+	0%
B0105	Group B9	Day 22	Peptide	CD8	+	-	-	-0.00491%
B0105	Group B9	Day 22	Peptide	CD8	-	+	+	0.00757%
B0105	Group B9	Day 22	Peptide	CD8	-	+	-	0.026%
B0105	Group B9	Day 22	Peptide	CD8	-	-	+	0.026%
B0105	Group B9	Day 1	MPH	CD4	+	+	+	0.075%
B0105	Group B9	Day 1	MPH	CD4	+	+	-	0.06202%
B0105	Group B9	Day 1	MPH	CD4	+	-	+	0.014%
B0105	Group B9	Day 1	MPH	CD4	+	-	-	0.01631%
B0105	Group B9	Day 1	MPH	CD4	-	+	+	0.04651%
B0105	Group B9	Day 1	MPH	CD4	-	+	-	0.212%
B0105	Group B9	Day 1	MPH	CD4	-	-	+	0.06804%
B0105	Group B9	Day 22	MPH	CD4	+	+	+	0.10808%
B0105	Group B9	Day 22	MPH	CD4	+	+	-	0.10424%
B0105	Group B9	Day 22	MPH	CD4	+	-	+	0.023%

Administration Site Key: FA = Forearm; UA= Upper arm
Treatments Part A: A1 = A/Sing MAP, 15 ug (FA); A2 = Afluria Quad IM (UA), 15 ug; A3 = Placebo MAP (FA); A4 = A/Sing Mono IM, 15 ug (UA)
Treatments Part B: B1 = A/Sing MAP, 15 ug (FA) with biopsy; B2 = Placebo MAP (FA) with biopsy
B3 = A/Sing MAP, 15 ug (FA); B4 = A/Sing MAP, 10 ug (FA); B5 = A/Sing MAP, 5 ug (FA); B6 = A/Sing MAP, 2.5 ug (FA)
B7 = Placebo MAP (FA); B8 = A/Sing MAP, 15 ug (UA); B9 = A/Sing IM, 15 ug, as 2018 Afluria Quad (UA)

Listing 16.2.6.6
Immunogenicity Results (CMI)

Part B

Randomisation Number	Group	Visit	Test	Subset	IL2	TNFa	IFNg	Value
B0105	Group B9	Day 22	MPH	CD4	+	-	-	0.02912%
B0105	Group B9	Day 22	MPH	CD4	-	+	+	0.03504%
B0105	Group B9	Day 22	MPH	CD4	-	+	-	0.318%
B0105	Group B9	Day 22	MPH	CD4	-	-	+	0.06824%
B0105	Group B9	Day 1	MPH	CD8	+	+	+	0.00935%
B0105	Group B9	Day 1	MPH	CD8	+	+	-	0.00762%
B0105	Group B9	Day 1	MPH	CD8	+	-	+	0.00156%
B0105	Group B9	Day 1	MPH	CD8	+	-	-	0.02309%
B0105	Group B9	Day 1	MPH	CD8	-	+	+	0.05881%
B0105	Group B9	Day 1	MPH	CD8	-	+	-	0.579%
B0105	Group B9	Day 1	MPH	CD8	-	-	+	0.044%
B0105	Group B9	Day 22	MPH	CD8	+	+	+	0.019%
B0105	Group B9	Day 22	MPH	CD8	+	+	-	0.00843%
B0105	Group B9	Day 22	MPH	CD8	+	-	+	0.00211%
B0105	Group B9	Day 22	MPH	CD8	+	-	-	0.01209%
B0105	Group B9	Day 22	MPH	CD8	-	+	+	0.051%
B0105	Group B9	Day 22	MPH	CD8	-	+	-	0.333%
B0105	Group B9	Day 22	MPH	CD8	-	-	+	0.063%

Administration Site Key: FA = Forearm; UA= Upper arm
Treatments Part A: A1 = A/Sing MAP, 15 ug (FA); A2 = Afluria Quad IM (UA), 15 ug; A3 = Placebo MAP (FA); A4 = A/Sing Mono IM, 15 ug (UA)
Treatments Part B: B1 = A/Sing MAP, 15 ug (FA) with biopsy; B2 = Placebo MAP (FA) with biopsy
B3 = A/Sing MAP, 15 ug (FA); B4 = A/Sing MAP, 10 ug (FA); B5 = A/Sing MAP, 5 ug (FA); B6 = A/Sing MAP, 2.5 ug (FA)
B7 = Placebo MAP (FA); B8 = A/Sing MAP, 15 ug (UA); B9 = A/Sing IM, 15 ug, as 2018 Afluria Quad (UA)

Listing 16.2.6.6
Immunogenicity Results (CMI)

Part B

Randomisation Number	Group	Visit	Test	Subset	IL2	TNFa	IFNg	Value
B0108	Group B7	Day 1	Peptide	CD4	+	+	+	-0.00155%
B0108	Group B7	Day 1	Peptide	CD4	+	+	-	0.00958%
B0108	Group B7	Day 1	Peptide	CD4	+	-	+	0.00083%
B0108	Group B7	Day 1	Peptide	CD4	+	-	-	-0.00072%
B0108	Group B7	Day 1	Peptide	CD4	-	+	+	0.003343%
B0108	Group B7	Day 1	Peptide	CD4	-	+	-	-0.003%
B0108	Group B7	Day 1	Peptide	CD4	-	-	+	0.00016%
B0108	Group B7	Day 22	Peptide	CD4	+	+	+	0.00204%
B0108	Group B7	Day 22	Peptide	CD4	+	+	-	-0.00507%
B0108	Group B7	Day 22	Peptide	CD4	+	-	+	-0.001938%
B0108	Group B7	Day 22	Peptide	CD4	+	-	-	-0.00246%
B0108	Group B7	Day 22	Peptide	CD4	-	+	+	0.00004%
B0108	Group B7	Day 22	Peptide	CD4	-	+	-	0.004%
B0108	Group B7	Day 22	Peptide	CD4	-	-	+	0.00007%
B0108	Group B7	Day 1	Peptide	CD8	+	+	+	0%
B0108	Group B7	Day 1	Peptide	CD8	+	+	-	0%
B0108	Group B7	Day 1	Peptide	CD8	+	-	+	0%
B0108	Group B7	Day 1	Peptide	CD8	+	-	-	0.00396%
B0108	Group B7	Day 1	Peptide	CD8	-	+	+	0.00006%

Administration Site Key: FA = Forearm; UA= Upper arm
Treatments Part A: A1 = A/Sing MAP, 15 ug (FA); A2 = Afluria Quad IM (UA), 15 ug; A3 = Placebo MAP (FA); A4 = A/Sing Mono IM, 15 ug (UA)
Treatments Part B: B1 = A/Sing MAP, 15 ug (FA) with biopsy; B2 = Placebo MAP (FA) with biopsy
B3 = A/Sing MAP, 15 ug (FA); B4 = A/Sing MAP, 10 ug (FA); B5 = A/Sing MAP, 5 ug (FA); B6 = A/Sing MAP, 2.5 ug (FA)
B7 = Placebo MAP (FA); B8 = A/Sing MAP, 15 ug (UA); B9 = A/Sing IM, 15 ug, as 2018 Afluria Quad (UA)

Listing 16.2.6.6
Immunogenicity Results (CMI)

Part B

Randomisation Number	Group	Visit	Test	Subset	IL2	TNFa	IFNg	Value
B0108	Group B7	Day 1	Peptide	CD8	-	+	-	0.009%
B0108	Group B7	Day 1	Peptide	CD8	-	-	+	-0.02%
B0108	Group B7	Day 22	Peptide	CD8	+	+	+	0%
B0108	Group B7	Day 22	Peptide	CD8	+	+	-	0.00155%
B0108	Group B7	Day 22	Peptide	CD8	+	-	+	0%
B0108	Group B7	Day 22	Peptide	CD8	+	-	-	0.00458%
B0108	Group B7	Day 22	Peptide	CD8	-	+	+	0.00306%
B0108	Group B7	Day 22	Peptide	CD8	-	+	-	-0.02%
B0108	Group B7	Day 22	Peptide	CD8	-	-	+	-0.01%
B0108	Group B7	Day 1	MPH	CD4	+	+	+	0.05496%
B0108	Group B7	Day 1	MPH	CD4	+	+	-	0.06358%
B0108	Group B7	Day 1	MPH	CD4	+	-	+	0.00575%
B0108	Group B7	Day 1	MPH	CD4	+	-	-	0.03596%
B0108	Group B7	Day 1	MPH	CD4	-	+	+	0.023193%
B0108	Group B7	Day 1	MPH	CD4	-	+	-	0.155%
B0108	Group B7	Day 1	MPH	CD4	-	-	+	0.01635%
B0108	Group B7	Day 22	MPH	CD4	+	+	+	0.1074%
B0108	Group B7	Day 22	MPH	CD4	+	+	-	0.062%
B0108	Group B7	Day 22	MPH	CD4	+	-	+	0.0224%

Administration Site Key: FA = Forearm; UA= Upper arm
Treatments Part A: A1 = A/Sing MAP, 15 ug (FA); A2 = Afluria Quad IM (UA), 15 ug; A3 = Placebo MAP (FA); A4 = A/Sing Mono IM, 15 ug (UA)
Treatments Part B: B1 = A/Sing MAP, 15 ug (FA) with biopsy; B2 = Placebo MAP (FA) with biopsy
B3 = A/Sing MAP, 15 ug (FA); B4 = A/Sing MAP, 10 ug (FA); B5 = A/Sing MAP, 5 ug (FA); B6 = A/Sing MAP, 2.5 ug (FA)
B7 = Placebo MAP (FA); B8 = A/Sing MAP, 15 ug (UA); B9 = A/Sing IM, 15 ug, as 2018 Afluria Quad (UA)

Listing 16.2.6.6
Immunogenicity Results (CMI)

Part B

Randomisation Number	Group	Visit	Test	Subset	IL2	TNFa	IFNg	Value
B0108	Group B7	Day 22	MPH	CD4	+	-	-	0.05425%
B0108	Group B7	Day 22	MPH	CD4	-	+	+	0.08605%
B0108	Group B7	Day 22	MPH	CD4	-	+	-	0.3%
B0108	Group B7	Day 22	MPH	CD4	-	-	+	0.0771%
B0108	Group B7	Day 1	MPH	CD8	+	+	+	0.018%
B0108	Group B7	Day 1	MPH	CD8	+	+	-	0.00391%
B0108	Group B7	Day 1	MPH	CD8	+	-	+	0.00978%
B0108	Group B7	Day 1	MPH	CD8	+	-	-	0.016%
B0108	Group B7	Day 1	MPH	CD8	-	+	+	0.04908%
B0108	Group B7	Day 1	MPH	CD8	-	+	-	0.125%
B0108	Group B7	Day 1	MPH	CD8	-	-	+	-0.11%
B0108	Group B7	Day 22	MPH	CD8	+	+	+	0.019%
B0108	Group B7	Day 22	MPH	CD8	+	+	-	0.00435%
B0108	Group B7	Day 22	MPH	CD8	+	-	+	0.0087%
B0108	Group B7	Day 22	MPH	CD8	+	-	-	0.03884%
B0108	Group B7	Day 22	MPH	CD8	-	+	+	0.11842%
B0108	Group B7	Day 22	MPH	CD8	-	+	-	0.41%
B0108	Group B7	Day 22	MPH	CD8	-	-	+	0.06%

Administration Site Key: FA = Forearm; UA= Upper arm
Treatments Part A: A1 = A/Sing MAP, 15 ug (FA); A2 = Afluria Quad IM (UA), 15 ug; A3 = Placebo MAP (FA); A4 = A/Sing Mono IM, 15 ug (UA)
Treatments Part B: B1 = A/Sing MAP, 15 ug (FA) with biopsy; B2 = Placebo MAP (FA) with biopsy
B3 = A/Sing MAP, 15 ug (FA); B4 = A/Sing MAP, 10 ug (FA); B5 = A/Sing MAP, 5 ug (FA); B6 = A/Sing MAP, 2.5 ug (FA)
B7 = Placebo MAP (FA); B8 = A/Sing MAP, 15 ug (UA); B9 = A/Sing IM, 15 ug, as 2018 Afluria Quad (UA)

Listing 16.2.6.6
Immunogenicity Results (CMI)

Part B

Randomisation Number	Group	Visit	Test	Subset	IL2	TNFa	IFNg	Value
B0110	Group B3	Day 1	Peptide	CD4	+	+	+	-6E-6%
B0110	Group B3	Day 1	Peptide	CD4	+	+	-	0.00637%
B0110	Group B3	Day 1	Peptide	CD4	+	-	+	0%
B0110	Group B3	Day 1	Peptide	CD4	+	-	-	-0.00002%
B0110	Group B3	Day 1	Peptide	CD4	-	+	+	0.000922%
B0110	Group B3	Day 1	Peptide	CD4	-	+	-	-0.008%
B0110	Group B3	Day 1	Peptide	CD4	-	-	+	-0.0019%
B0110	Group B3	Day 22	Peptide	CD4	+	+	+	0.01384%
B0110	Group B3	Day 22	Peptide	CD4	+	+	-	0.015%
B0110	Group B3	Day 22	Peptide	CD4	+	-	+	9E-6%
B0110	Group B3	Day 22	Peptide	CD4	+	-	-	0.0001%
B0110	Group B3	Day 22	Peptide	CD4	-	+	+	0.00437%
B0110	Group B3	Day 22	Peptide	CD4	-	+	-	-0.002%
B0110	Group B3	Day 22	Peptide	CD4	-	-	+	-0.00356%
B0110	Group B3	Day 1	Peptide	CD8	+	+	+	0%
B0110	Group B3	Day 1	Peptide	CD8	+	+	-	0%
B0110	Group B3	Day 1	Peptide	CD8	+	-	+	0%
B0110	Group B3	Day 1	Peptide	CD8	+	-	-	0.00103%
B0110	Group B3	Day 1	Peptide	CD8	-	+	+	-0.00413%

Administration Site Key: FA = Forearm; UA= Upper arm
Treatments Part A: A1 = A/Sing MAP, 15 ug (FA); A2 = Afluria Quad IM (UA), 15 ug; A3 = Placebo MAP (FA); A4 = A/Sing Mono IM, 15 ug (UA)
Treatments Part B: B1 = A/Sing MAP, 15 ug (FA) with biopsy; B2 = Placebo MAP (FA) with biopsy
B3 = A/Sing MAP, 15 ug (FA); B4 = A/Sing MAP, 10 ug (FA); B5 = A/Sing MAP, 5 ug (FA); B6 = A/Sing MAP, 2.5 ug (FA)
B7 = Placebo MAP (FA); B8 = A/Sing MAP, 15 ug (UA); B9 = A/Sing IM, 15 ug, as 2018 Afluria Quad (UA)

Listing 16.2.6.6
Immunogenicity Results (CMI)

Part B

Randomisation Number	Group	Visit	Test	Subset	IL2	TNFa	IFNg	Value
B0110	Group B3	Day 1	Peptide	CD8	-	+	-	0.013%
B0110	Group B3	Day 1	Peptide	CD8	-	-	+	-0.04%
B0110	Group B3	Day 22	Peptide	CD8	+	+	+	0%
B0110	Group B3	Day 22	Peptide	CD8	+	+	-	0%
B0110	Group B3	Day 22	Peptide	CD8	+	-	+	0%
B0110	Group B3	Day 22	Peptide	CD8	+	-	-	-0.00226%
B0110	Group B3	Day 22	Peptide	CD8	-	+	+	-0.00107%
B0110	Group B3	Day 22	Peptide	CD8	-	+	-	-0.05%
B0110	Group B3	Day 22	Peptide	CD8	-	-	+	-0.08%
B0110	Group B3	Day 1	MPH	CD4	+	+	+	0.052062%
B0110	Group B3	Day 1	MPH	CD4	+	+	-	0.07937%
B0110	Group B3	Day 1	MPH	CD4	+	-	+	0.00631%
B0110	Group B3	Day 1	MPH	CD4	+	-	-	0.02712%
B0110	Group B3	Day 1	MPH	CD4	-	+	+	0.034062%
B0110	Group B3	Day 1	MPH	CD4	-	+	-	0.252%
B0110	Group B3	Day 1	MPH	CD4	-	-	+	0.02637%
B0110	Group B3	Day 22	MPH	CD4	+	+	+	0.11784%
B0110	Group B3	Day 22	MPH	CD4	+	+	-	0.086%
B0110	Group B3	Day 22	MPH	CD4	+	-	+	0.02828%

Administration Site Key: FA = Forearm; UA= Upper arm
Treatments Part A: A1 = A/Sing MAP, 15 ug (FA); A2 = Afluria Quad IM (UA), 15 ug; A3 = Placebo MAP (FA); A4 = A/Sing Mono IM, 15 ug (UA)
Treatments Part B: B1 = A/Sing MAP, 15 ug (FA) with biopsy; B2 = Placebo MAP (FA) with biopsy
B3 = A/Sing MAP, 15 ug (FA); B4 = A/Sing MAP, 10 ug (FA); B5 = A/Sing MAP, 5 ug (FA); B6 = A/Sing MAP, 2.5 ug (FA)
B7 = Placebo MAP (FA); B8 = A/Sing MAP, 15 ug (UA); B9 = A/Sing IM, 15 ug, as 2018 Afluria Quad (UA)

Listing 16.2.6.6
Immunogenicity Results (CMI)

Part B

Randomisation Number	Group	Visit	Test	Subset	IL2	TNFa	IFNg	Value
B0110	Group B3	Day 22	MPH	CD4	+	-	-	0.06408%
B0110	Group B3	Day 22	MPH	CD4	-	+	+	0.041%
B0110	Group B3	Day 22	MPH	CD4	-	+	-	0.279%
B0110	Group B3	Day 22	MPH	CD4	-	-	+	0.07852%
B0110	Group B3	Day 1	MPH	CD8	+	+	+	0.013%
B0110	Group B3	Day 1	MPH	CD8	+	+	-	0.00291%
B0110	Group B3	Day 1	MPH	CD8	+	-	+	0.00775%
B0110	Group B3	Day 1	MPH	CD8	+	-	-	0.00484%
B0110	Group B3	Day 1	MPH	CD8	-	+	+	0.02384%
B0110	Group B3	Day 1	MPH	CD8	-	+	-	0.11%
B0110	Group B3	Day 1	MPH	CD8	-	-	+	-0.132%
B0110	Group B3	Day 22	MPH	CD8	+	+	+	0.00854%
B0110	Group B3	Day 22	MPH	CD8	+	+	-	0.0032%
B0110	Group B3	Day 22	MPH	CD8	+	-	+	0.0096%
B0110	Group B3	Day 22	MPH	CD8	+	-	-	0.04474%
B0110	Group B3	Day 22	MPH	CD8	-	+	+	0.05661%
B0110	Group B3	Day 22	MPH	CD8	-	+	-	0.14%
B0110	Group B3	Day 22	MPH	CD8	-	-	+	-0.15%

Administration Site Key: FA = Forearm; UA= Upper arm
Treatments Part A: A1 = A/Sing MAP, 15 ug (FA); A2 = Afluria Quad IM (UA), 15 ug; A3 = Placebo MAP (FA); A4 = A/Sing Mono IM, 15 ug (UA)
Treatments Part B: B1 = A/Sing MAP, 15 ug (FA) with biopsy; B2 = Placebo MAP (FA) with biopsy
B3 = A/Sing MAP, 15 ug (FA); B4 = A/Sing MAP, 10 ug (FA); B5 = A/Sing MAP, 5 ug (FA); B6 = A/Sing MAP, 2.5 ug (FA)
B7 = Placebo MAP (FA); B8 = A/Sing MAP, 15 ug (UA); B9 = A/Sing IM, 15 ug, as 2018 Afluria Quad (UA)

Listing 16.2.6.6
Immunogenicity Results (CMI)

Part B

Randomisation Number	Group	Visit	Test	Subset	IL2	TNFa	IFNg	Value
B0111	Group B8	Day 1	Peptide	CD4	+	+	+	-0.00122%
B0111	Group B8	Day 1	Peptide	CD4	+	+	-	-0.006%
B0111	Group B8	Day 1	Peptide	CD4	+	-	+	0.000075%
B0111	Group B8	Day 1	Peptide	CD4	+	-	-	-0.00964%
B0111	Group B8	Day 1	Peptide	CD4	-	+	+	0.00626%
B0111	Group B8	Day 1	Peptide	CD4	-	+	-	0.013%
B0111	Group B8	Day 1	Peptide	CD4	-	-	+	0.002693%
B0111	Group B8	Day 22	Peptide	CD4	+	+	+	-0.00059%
B0111	Group B8	Day 22	Peptide	CD4	+	+	-	0.01424%
B0111	Group B8	Day 22	Peptide	CD4	+	-	+	0.00153%
B0111	Group B8	Day 22	Peptide	CD4	+	-	-	-0.00235%
B0111	Group B8	Day 22	Peptide	CD4	-	+	+	0.00317%
B0111	Group B8	Day 22	Peptide	CD4	-	+	-	0.019%
B0111	Group B8	Day 22	Peptide	CD4	-	-	+	-0.00358%
B0111	Group B8	Day 1	Peptide	CD8	+	+	+	0%
B0111	Group B8	Day 1	Peptide	CD8	+	+	-	0%
B0111	Group B8	Day 1	Peptide	CD8	+	-	+	0%
B0111	Group B8	Day 1	Peptide	CD8	+	-	-	-0.0024%
B0111	Group B8	Day 1	Peptide	CD8	-	+	+	0.001%

Administration Site Key: FA = Forearm; UA= Upper arm
Treatments Part A: A1 = A/Sing MAP, 15 ug (FA); A2 = Afluria Quad IM (UA), 15 ug; A3 = Placebo MAP (FA); A4 = A/Sing Mono IM, 15 ug (UA)
Treatments Part B: B1 = A/Sing MAP, 15 ug (FA) with biopsy; B2 = Placebo MAP (FA) with biopsy
B3 = A/Sing MAP, 15 ug (FA); B4 = A/Sing MAP, 10 ug (FA); B5 = A/Sing MAP, 5 ug (FA); B6 = A/Sing MAP, 2.5 ug (FA)
B7 = Placebo MAP (FA); B8 = A/Sing MAP, 15 ug (UA); B9 = A/Sing IM, 15 ug, as 2018 Afluria Quad (UA)

Listing 16.2.6.6
Immunogenicity Results (CMI)

Part B

Randomisation Number	Group	Visit	Test	Subset	IL2	TNFa	IFNg	Value
B0111	Group B8	Day 1	Peptide	CD8	-	+	-	0.028%
B0111	Group B8	Day 1	Peptide	CD8	-	-	+	-0.3%
B0111	Group B8	Day 22	Peptide	CD8	+	+	+	0%
B0111	Group B8	Day 22	Peptide	CD8	+	+	-	-0.0011%
B0111	Group B8	Day 22	Peptide	CD8	+	-	+	0%
B0111	Group B8	Day 22	Peptide	CD8	+	-	-	-0.0011%
B0111	Group B8	Day 22	Peptide	CD8	-	+	+	-0.0009%
B0111	Group B8	Day 22	Peptide	CD8	-	+	-	0.024%
B0111	Group B8	Day 22	Peptide	CD8	-	-	+	-0.13%
B0111	Group B8	Day 1	MPH	CD4	+	+	+	0.08042%
B0111	Group B8	Day 1	MPH	CD4	+	+	-	0.074%
B0111	Group B8	Day 1	MPH	CD4	+	-	+	0.006903%
B0111	Group B8	Day 1	MPH	CD4	+	-	-	0.019%
B0111	Group B8	Day 1	MPH	CD4	-	+	+	0.05741%
B0111	Group B8	Day 1	MPH	CD4	-	+	-	0.275%
B0111	Group B8	Day 1	MPH	CD4	-	-	+	0.050203%
B0111	Group B8	Day 22	MPH	CD4	+	+	+	0.07635%
B0111	Group B8	Day 22	MPH	CD4	+	+	-	0.05024%
B0111	Group B8	Day 22	MPH	CD4	+	-	+	0.01%

Administration Site Key: FA = Forearm; UA= Upper arm
Treatments Part A: A1 = A/Sing MAP, 15 ug (FA); A2 = Afluria Quad IM (UA), 15 ug; A3 = Placebo MAP (FA); A4 = A/Sing Mono IM, 15 ug (UA)
Treatments Part B: B1 = A/Sing MAP, 15 ug (FA) with biopsy; B2 = Placebo MAP (FA) with biopsy
B3 = A/Sing MAP, 15 ug (FA); B4 = A/Sing MAP, 10 ug (FA); B5 = A/Sing MAP, 5 ug (FA); B6 = A/Sing MAP, 2.5 ug (FA)
B7 = Placebo MAP (FA); B8 = A/Sing MAP, 15 ug (UA); B9 = A/Sing IM, 15 ug, as 2018 Afluria Quad (UA)

Listing 16.2.6.6
Immunogenicity Results (CMI)

Part B

Randomisation Number	Group	Visit	Test	Subset	IL2	TNFa	IFNg	Value
B0111	Group B8	Day 22	MPH	CD4	+	-	-	0.03%
B0111	Group B8	Day 22	MPH	CD4	-	+	+	0.06181%
B0111	Group B8	Day 22	MPH	CD4	-	+	-	0.291%
B0111	Group B8	Day 22	MPH	CD4	-	-	+	0.09489%
B0111	Group B8	Day 1	MPH	CD8	+	+	+	0.058%
B0111	Group B8	Day 1	MPH	CD8	+	+	-	0.013%
B0111	Group B8	Day 1	MPH	CD8	+	-	+	0.00504%
B0111	Group B8	Day 1	MPH	CD8	+	-	-	0.0456%
B0111	Group B8	Day 1	MPH	CD8	-	+	+	0.058%
B0111	Group B8	Day 1	MPH	CD8	-	+	-	0.268%
B0111	Group B8	Day 1	MPH	CD8	-	-	+	-0.45%
B0111	Group B8	Day 22	MPH	CD8	+	+	+	0.041%
B0111	Group B8	Day 22	MPH	CD8	+	+	-	0.00634%
B0111	Group B8	Day 22	MPH	CD8	+	-	+	0.00648%
B0111	Group B8	Day 22	MPH	CD8	+	-	-	0.0437%
B0111	Group B8	Day 22	MPH	CD8	-	+	+	0.04909%
B0111	Group B8	Day 22	MPH	CD8	-	+	-	0.254%
B0111	Group B8	Day 22	MPH	CD8	-	-	+	-0.24%

Administration Site Key: FA = Forearm; UA= Upper arm
Treatments Part A: A1 = A/Sing MAP, 15 ug (FA); A2 = Afluria Quad IM (UA), 15 ug; A3 = Placebo MAP (FA); A4 = A/Sing Mono IM, 15 ug (UA)
Treatments Part B: B1 = A/Sing MAP, 15 ug (FA) with biopsy; B2 = Placebo MAP (FA) with biopsy
B3 = A/Sing MAP, 15 ug (FA); B4 = A/Sing MAP, 10 ug (FA); B5 = A/Sing MAP, 5 ug (FA); B6 = A/Sing MAP, 2.5 ug (FA)
B7 = Placebo MAP (FA); B8 = A/Sing MAP, 15 ug (UA); B9 = A/Sing IM, 15 ug, as 2018 Afluria Quad (UA)

Listing 16.2.6.6
Immunogenicity Results (CMI)

Part B

Randomisation Number	Group	Visit	Test	Subset	IL2	TNFa	IFNg	Value
B0114	Group B7	Day 1	Peptide	CD4	+	+	+	0.00707%
B0114	Group B7	Day 1	Peptide	CD4	+	+	-	0.004%
B0114	Group B7	Day 1	Peptide	CD4	+	-	+	0.001024%
B0114	Group B7	Day 1	Peptide	CD4	+	-	-	-0.0044%
B0114	Group B7	Day 1	Peptide	CD4	-	+	+	0.00162%
B0114	Group B7	Day 1	Peptide	CD4	-	+	-	0.002%
B0114	Group B7	Day 1	Peptide	CD4	-	-	+	-0.00339%
B0114	Group B7	Day 22	Peptide	CD4	+	+	+	-0.00227%
B0114	Group B7	Day 22	Peptide	CD4	+	+	-	0.001%
B0114	Group B7	Day 22	Peptide	CD4	+	-	+	0%
B0114	Group B7	Day 22	Peptide	CD4	+	-	-	0.00055%
B0114	Group B7	Day 22	Peptide	CD4	-	+	+	0.00338%
B0114	Group B7	Day 22	Peptide	CD4	-	+	-	0.08%
B0114	Group B7	Day 22	Peptide	CD4	-	-	+	-0.00057%
B0114	Group B7	Day 1	Peptide	CD8	+	+	+	-0.00119%
B0114	Group B7	Day 1	Peptide	CD8	+	+	-	0.00137%
B0114	Group B7	Day 1	Peptide	CD8	+	-	+	0.00006%
B0114	Group B7	Day 1	Peptide	CD8	+	-	-	-0.00263%
B0114	Group B7	Day 1	Peptide	CD8	-	+	+	0.00012%

Administration Site Key: FA = Forearm; UA= Upper arm
Treatments Part A: A1 = A/Sing MAP, 15 ug (FA); A2 = Afluria Quad IM (UA), 15 ug; A3 = Placebo MAP (FA); A4 = A/Sing Mono IM, 15 ug (UA)
Treatments Part B: B1 = A/Sing MAP, 15 ug (FA) with biopsy; B2 = Placebo MAP (FA) with biopsy
B3 = A/Sing MAP, 15 ug (FA); B4 = A/Sing MAP, 10 ug (FA); B5 = A/Sing MAP, 5 ug (FA); B6 = A/Sing MAP, 2.5 ug (FA)
B7 = Placebo MAP (FA); B8 = A/Sing MAP, 15 ug (UA); B9 = A/Sing IM, 15 ug, as 2018 Afluria Quad (UA)

Listing 16.2.6.6
Immunogenicity Results (CMI)

Part B

Randomisation Number	Group	Visit	Test	Subset	IL2	TNFa	IFNg	Value
B0114	Group B7	Day 1	Peptide	CD8	-	+	-	-0.002%
B0114	Group B7	Day 1	Peptide	CD8	-	-	+	-0.29%
B0114	Group B7	Day 22	Peptide	CD8	+	+	+	-1E-5%
B0114	Group B7	Day 22	Peptide	CD8	+	+	-	0%
B0114	Group B7	Day 22	Peptide	CD8	+	-	+	0%
B0114	Group B7	Day 22	Peptide	CD8	+	-	-	-0.00146%
B0114	Group B7	Day 22	Peptide	CD8	-	+	+	-0.00724%
B0114	Group B7	Day 22	Peptide	CD8	-	+	-	0.068%
B0114	Group B7	Day 22	Peptide	CD8	-	-	+	0.11%
B0114	Group B7	Day 1	MPH	CD4	+	+	+	0.04507%
B0114	Group B7	Day 1	MPH	CD4	+	+	-	0.064%
B0114	Group B7	Day 1	MPH	CD4	+	-	+	0.008954%
B0114	Group B7	Day 1	MPH	CD4	+	-	-	0.032%
B0114	Group B7	Day 1	MPH	CD4	-	+	+	0.02904%
B0114	Group B7	Day 1	MPH	CD4	-	+	-	0.299%
B0114	Group B7	Day 1	MPH	CD4	-	-	+	0.04456%
B0114	Group B7	Day 22	MPH	CD4	+	+	+	0.05066%
B0114	Group B7	Day 22	MPH	CD4	+	+	-	0.088%
B0114	Group B7	Day 22	MPH	CD4	+	-	+	0.00769%

Administration Site Key: FA = Forearm; UA= Upper arm
Treatments Part A: A1 = A/Sing MAP, 15 ug (FA); A2 = Afluria Quad IM (UA), 15 ug; A3 = Placebo MAP (FA); A4 = A/Sing Mono IM, 15 ug (UA)
Treatments Part B: B1 = A/Sing MAP, 15 ug (FA) with biopsy; B2 = Placebo MAP (FA) with biopsy
B3 = A/Sing MAP, 15 ug (FA); B4 = A/Sing MAP, 10 ug (FA); B5 = A/Sing MAP, 5 ug (FA); B6 = A/Sing MAP, 2.5 ug (FA)
B7 = Placebo MAP (FA); B8 = A/Sing MAP, 15 ug (UA); B9 = A/Sing IM, 15 ug, as 2018 Afluria Quad (UA)

Listing 16.2.6.6
Immunogenicity Results (CMI)

Part B

Randomisation Number	Group	Visit	Test	Subset	IL2	TNFa	IFNg	Value
B0114	Group B7	Day 22	MPH	CD4	+	-	-	0.02822%
B0114	Group B7	Day 22	MPH	CD4	-	+	+	0.03318%
B0114	Group B7	Day 22	MPH	CD4	-	+	-	0.23%
B0114	Group B7	Day 22	MPH	CD4	-	-	+	0.03505%
B0114	Group B7	Day 1	MPH	CD8	+	+	+	0.02206%
B0114	Group B7	Day 1	MPH	CD8	+	+	-	0.0013%
B0114	Group B7	Day 1	MPH	CD8	+	-	+	0.01469%
B0114	Group B7	Day 1	MPH	CD8	+	-	-	0.01637%
B0114	Group B7	Day 1	MPH	CD8	-	+	+	0.04637%
B0114	Group B7	Day 1	MPH	CD8	-	+	-	0.144%
B0114	Group B7	Day 1	MPH	CD8	-	-	+	-0.67%
B0114	Group B7	Day 22	MPH	CD8	+	+	+	0.01656%
B0114	Group B7	Day 22	MPH	CD8	+	+	-	0.00825%
B0114	Group B7	Day 22	MPH	CD8	+	-	+	0.014%
B0114	Group B7	Day 22	MPH	CD8	+	-	-	0.00967%
B0114	Group B7	Day 22	MPH	CD8	-	+	+	0.02833%
B0114	Group B7	Day 22	MPH	CD8	-	+	-	0.148%
B0114	Group B7	Day 22	MPH	CD8	-	-	+	-0.76%

Administration Site Key: FA = Forearm; UA= Upper arm
Treatments Part A: A1 = A/Sing MAP, 15 ug (FA); A2 = Afluria Quad IM (UA), 15 ug; A3 = Placebo MAP (FA); A4 = A/Sing Mono IM, 15 ug (UA)
Treatments Part B: B1 = A/Sing MAP, 15 ug (FA) with biopsy; B2 = Placebo MAP (FA) with biopsy
B3 = A/Sing MAP, 15 ug (FA); B4 = A/Sing MAP, 10 ug (FA); B5 = A/Sing MAP, 5 ug (FA); B6 = A/Sing MAP, 2.5 ug (FA)
B7 = Placebo MAP (FA); B8 = A/Sing MAP, 15 ug (UA); B9 = A/Sing IM, 15 ug, as 2018 Afluria Quad (UA)

Listing 16.2.6.6
Immunogenicity Results (CMI)

Part B

Randomisation Number	Group	Visit	Test	Subset	IL2	TNFa	IFNg	Value
B0115	Group B9	Day 1	Peptide	CD4	+	+	+	0.00283%
B0115	Group B9	Day 1	Peptide	CD4	+	+	-	0.00168%
B0115	Group B9	Day 1	Peptide	CD4	+	-	+	0.0001%
B0115	Group B9	Day 1	Peptide	CD4	+	-	-	0.00283%
B0115	Group B9	Day 1	Peptide	CD4	-	+	+	-0.000778%
B0115	Group B9	Day 1	Peptide	CD4	-	+	-	-0.003%
B0115	Group B9	Day 1	Peptide	CD4	-	-	+	0.0005%
B0115	Group B9	Day 22	Peptide	CD4	+	+	+	0.00744%
B0115	Group B9	Day 22	Peptide	CD4	+	+	-	0.00796%
B0115	Group B9	Day 22	Peptide	CD4	+	-	+	0.0015%
B0115	Group B9	Day 22	Peptide	CD4	+	-	-	-0.00154%
B0115	Group B9	Day 22	Peptide	CD4	-	+	+	0.00224%
B0115	Group B9	Day 22	Peptide	CD4	-	+	-	-0.007%
B0115	Group B9	Day 22	Peptide	CD4	-	-	+	-0.00003%
B0115	Group B9	Day 1	Peptide	CD8	+	+	+	0%
B0115	Group B9	Day 1	Peptide	CD8	+	+	-	0.00222%
B0115	Group B9	Day 1	Peptide	CD8	+	-	+	0%
B0115	Group B9	Day 1	Peptide	CD8	+	-	-	0.00222%
B0115	Group B9	Day 1	Peptide	CD8	-	+	+	-0.00171%

Administration Site Key: FA = Forearm; UA= Upper arm
Treatments Part A: A1 = A/Sing MAP, 15 ug (FA); A2 = Afluria Quad IM (UA), 15 ug; A3 = Placebo MAP (FA); A4 = A/Sing Mono IM, 15 ug (UA)
Treatments Part B: B1 = A/Sing MAP, 15 ug (FA) with biopsy; B2 = Placebo MAP (FA) with biopsy
B3 = A/Sing MAP, 15 ug (FA); B4 = A/Sing MAP, 10 ug (FA); B5 = A/Sing MAP, 5 ug (FA); B6 = A/Sing MAP, 2.5 ug (FA)
B7 = Placebo MAP (FA); B8 = A/Sing MAP, 15 ug (UA); B9 = A/Sing IM, 15 ug, as 2018 Afluria Quad (UA)

Listing 16.2.6.6
Immunogenicity Results (CMI)

Part B

Randomisation Number	Group	Visit	Test	Subset	IL2	TNFa	IFNg	Value
B0115	Group B9	Day 1	Peptide	CD8	-	+	-	-0.019%
B0115	Group B9	Day 1	Peptide	CD8	-	-	+	0.044%
B0115	Group B9	Day 22	Peptide	CD8	+	+	+	0%
B0115	Group B9	Day 22	Peptide	CD8	+	+	-	0%
B0115	Group B9	Day 22	Peptide	CD8	+	-	+	0%
B0115	Group B9	Day 22	Peptide	CD8	+	-	-	0.0038%
B0115	Group B9	Day 22	Peptide	CD8	-	+	+	0.0019%
B0115	Group B9	Day 22	Peptide	CD8	-	+	-	-0.008%
B0115	Group B9	Day 22	Peptide	CD8	-	-	+	0.032%
B0115	Group B9	Day 1	MPH	CD4	+	+	+	0.04744%
B0115	Group B9	Day 1	MPH	CD4	+	+	-	0.05678%
B0115	Group B9	Day 1	MPH	CD4	+	-	+	0.001722%
B0115	Group B9	Day 1	MPH	CD4	+	-	-	0.02144%
B0115	Group B9	Day 1	MPH	CD4	-	+	+	0.032222%
B0115	Group B9	Day 1	MPH	CD4	-	+	-	0.17%
B0115	Group B9	Day 1	MPH	CD4	-	-	+	0.03211%
B0115	Group B9	Day 22	MPH	CD4	+	+	+	0.05444%
B0115	Group B9	Day 22	MPH	CD4	+	+	-	0.05196%
B0115	Group B9	Day 22	MPH	CD4	+	-	+	0.01624%

Administration Site Key: FA = Forearm; UA= Upper arm
Treatments Part A: A1 = A/Sing MAP, 15 ug (FA); A2 = Afluria Quad IM (UA), 15 ug; A3 = Placebo MAP (FA); A4 = A/Sing Mono IM, 15 ug (UA)
Treatments Part B: B1 = A/Sing MAP, 15 ug (FA) with biopsy; B2 = Placebo MAP (FA) with biopsy
B3 = A/Sing MAP, 15 ug (FA); B4 = A/Sing MAP, 10 ug (FA); B5 = A/Sing MAP, 5 ug (FA); B6 = A/Sing MAP, 2.5 ug (FA)
B7 = Placebo MAP (FA); B8 = A/Sing MAP, 15 ug (UA); B9 = A/Sing IM, 15 ug, as 2018 Afluria Quad (UA)

Listing 16.2.6.6
Immunogenicity Results (CMI)

Part B

Randomisation Number	Group	Visit	Test	Subset	IL2	TNFa	IFNg	Value
B0115	Group B9	Day 22	MPH	CD4	+	-	-	0.0222%
B0115	Group B9	Day 22	MPH	CD4	-	+	+	0.02948%
B0115	Group B9	Day 22	MPH	CD4	-	+	-	0.143%
B0115	Group B9	Day 22	MPH	CD4	-	-	+	0.07396%
B0115	Group B9	Day 1	MPH	CD8	+	+	+	0.026%
B0115	Group B9	Day 1	MPH	CD8	+	+	-	0.00607%
B0115	Group B9	Day 1	MPH	CD8	+	-	+	0.00607%
B0115	Group B9	Day 1	MPH	CD8	+	-	-	0.014%
B0115	Group B9	Day 1	MPH	CD8	-	+	+	0.02607%
B0115	Group B9	Day 1	MPH	CD8	-	+	-	0.04%
B0115	Group B9	Day 1	MPH	CD8	-	-	+	0.02%
B0115	Group B9	Day 22	MPH	CD8	+	+	+	0.00918%
B0115	Group B9	Day 22	MPH	CD8	+	+	-	0.00551%
B0115	Group B9	Day 22	MPH	CD8	+	-	+	0.013%
B0115	Group B9	Day 22	MPH	CD8	+	-	-	0.017%
B0115	Group B9	Day 22	MPH	CD8	-	+	+	0.037%
B0115	Group B9	Day 22	MPH	CD8	-	+	-	0.234%
B0115	Group B9	Day 22	MPH	CD8	-	-	+	0.053%

Administration Site Key: FA = Forearm; UA= Upper arm
Treatments Part A: A1 = A/Sing MAP, 15 ug (FA); A2 = Afluria Quad IM (UA), 15 ug; A3 = Placebo MAP (FA); A4 = A/Sing Mono IM, 15 ug (UA)
Treatments Part B: B1 = A/Sing MAP, 15 ug (FA) with biopsy; B2 = Placebo MAP (FA) with biopsy
B3 = A/Sing MAP, 15 ug (FA); B4 = A/Sing MAP, 10 ug (FA); B5 = A/Sing MAP, 5 ug (FA); B6 = A/Sing MAP, 2.5 ug (FA)
B7 = Placebo MAP (FA); B8 = A/Sing MAP, 15 ug (UA); B9 = A/Sing IM, 15 ug, as 2018 Afluria Quad (UA)

Listing 16.2.6.6
Immunogenicity Results (CMI)

Part B

Randomisation Number	Group	Visit	Test	Subset	IL2	TNFa	IFNg	Value
B0116	Group B7	Day 1	Peptide	CD4	+	+	+	0.00815%
B0116	Group B7	Day 1	Peptide	CD4	+	+	-	0.006%
B0116	Group B7	Day 1	Peptide	CD4	+	-	+	-0.000219%
B0116	Group B7	Day 1	Peptide	CD4	+	-	-	-0.01124%
B0116	Group B7	Day 1	Peptide	CD4	-	+	+	-0.00035%
B0116	Group B7	Day 1	Peptide	CD4	-	+	-	0.012%
B0116	Group B7	Day 1	Peptide	CD4	-	-	+	-0.0085%
B0116	Group B7	Day 22	Peptide	CD4	+	+	+	0.01082%
B0116	Group B7	Day 22	Peptide	CD4	+	+	-	0.00908%
B0116	Group B7	Day 22	Peptide	CD4	+	-	+	-0.000829%
B0116	Group B7	Day 22	Peptide	CD4	+	-	-	-0.00058%
B0116	Group B7	Day 22	Peptide	CD4	-	+	+	-0.00058%
B0116	Group B7	Day 22	Peptide	CD4	-	+	-	0.019%
B0116	Group B7	Day 22	Peptide	CD4	-	-	+	0.00232%
B0116	Group B7	Day 1	Peptide	CD8	+	+	+	0%
B0116	Group B7	Day 1	Peptide	CD8	+	+	-	-0.00194%
B0116	Group B7	Day 1	Peptide	CD8	+	-	+	0%
B0116	Group B7	Day 1	Peptide	CD8	+	-	-	0%
B0116	Group B7	Day 1	Peptide	CD8	-	+	+	0.00074%

Administration Site Key: FA = Forearm; UA= Upper arm
Treatments Part A: A1 = A/Sing MAP, 15 ug (FA); A2 = Afluria Quad IM (UA), 15 ug; A3 = Placebo MAP (FA); A4 = A/Sing Mono IM, 15 ug (UA)
Treatments Part B: B1 = A/Sing MAP, 15 ug (FA) with biopsy; B2 = Placebo MAP (FA) with biopsy
B3 = A/Sing MAP, 15 ug (FA); B4 = A/Sing MAP, 10 ug (FA); B5 = A/Sing MAP, 5 ug (FA); B6 = A/Sing MAP, 2.5 ug (FA)
B7 = Placebo MAP (FA); B8 = A/Sing MAP, 15 ug (UA); B9 = A/Sing IM, 15 ug, as 2018 Afluria Quad (UA)

Listing 16.2.6.6
Immunogenicity Results (CMI)

Part B

Randomisation Number	Group	Visit	Test	Subset	IL2	TNFa	IFNg	Value
B0116	Group B7	Day 1	Peptide	CD8	-	+	-	0.012%
B0116	Group B7	Day 1	Peptide	CD8	-	-	+	-0.46%
B0116	Group B7	Day 22	Peptide	CD8	+	+	+	0.00145%
B0116	Group B7	Day 22	Peptide	CD8	+	+	-	0%
B0116	Group B7	Day 22	Peptide	CD8	+	-	+	0%
B0116	Group B7	Day 22	Peptide	CD8	+	-	-	0%
B0116	Group B7	Day 22	Peptide	CD8	-	+	+	-0.00352%
B0116	Group B7	Day 22	Peptide	CD8	-	+	-	0.012%
B0116	Group B7	Day 22	Peptide	CD8	-	-	+	0.18%
B0116	Group B7	Day 1	MPH	CD4	+	+	+	0.08715%
B0116	Group B7	Day 1	MPH	CD4	+	+	-	0.043%
B0116	Group B7	Day 1	MPH	CD4	+	-	+	0.021031%
B0116	Group B7	Day 1	MPH	CD4	+	-	-	0.015%
B0116	Group B7	Day 1	MPH	CD4	-	+	+	0.03815%
B0116	Group B7	Day 1	MPH	CD4	-	+	-	0.202%
B0116	Group B7	Day 1	MPH	CD4	-	-	+	0.107%
B0116	Group B7	Day 22	MPH	CD4	+	+	+	0.09182%
B0116	Group B7	Day 22	MPH	CD4	+	+	-	0.06508%
B0116	Group B7	Day 22	MPH	CD4	+	-	+	0.00951%

Administration Site Key: FA = Forearm; UA= Upper arm
Treatments Part A: A1 = A/Sing MAP, 15 ug (FA); A2 = Afluria Quad IM (UA), 15 ug; A3 = Placebo MAP (FA); A4 = A/Sing Mono IM, 15 ug (UA)
Treatments Part B: B1 = A/Sing MAP, 15 ug (FA) with biopsy; B2 = Placebo MAP (FA) with biopsy
B3 = A/Sing MAP, 15 ug (FA); B4 = A/Sing MAP, 10 ug (FA); B5 = A/Sing MAP, 5 ug (FA); B6 = A/Sing MAP, 2.5 ug (FA)
B7 = Placebo MAP (FA); B8 = A/Sing MAP, 15 ug (UA); B9 = A/Sing IM, 15 ug, as 2018 Afluria Quad (UA)

Listing 16.2.6.6
Immunogenicity Results (CMI)

Part B

Randomisation Number	Group	Visit	Test	Subset	IL2	TNFa	IFNg	Value
B0116	Group B7	Day 22	MPH	CD4	+	-	-	0.01979%
B0116	Group B7	Day 22	MPH	CD4	-	+	+	0.06279%
B0116	Group B7	Day 22	MPH	CD4	-	+	-	0.309%
B0116	Group B7	Day 22	MPH	CD4	-	-	+	0.08103%
B0116	Group B7	Day 1	MPH	CD8	+	+	+	0.021%
B0116	Group B7	Day 1	MPH	CD8	+	+	-	0.00511%
B0116	Group B7	Day 1	MPH	CD8	+	-	+	0.00564%
B0116	Group B7	Day 1	MPH	CD8	+	-	-	0.024%
B0116	Group B7	Day 1	MPH	CD8	-	+	+	0.02811%
B0116	Group B7	Day 1	MPH	CD8	-	+	-	0.139%
B0116	Group B7	Day 1	MPH	CD8	-	-	+	-0.9%
B0116	Group B7	Day 22	MPH	CD8	+	+	+	0.024%
B0116	Group B7	Day 22	MPH	CD8	+	+	-	0.00757%
B0116	Group B7	Day 22	MPH	CD8	+	-	+	0.00757%
B0116	Group B7	Day 22	MPH	CD8	+	-	-	0.023%
B0116	Group B7	Day 22	MPH	CD8	-	+	+	0.03157%
B0116	Group B7	Day 22	MPH	CD8	-	+	-	0.235%
B0116	Group B7	Day 22	MPH	CD8	-	-	+	-0.33%

Administration Site Key: FA = Forearm; UA= Upper arm
Treatments Part A: A1 = A/Sing MAP, 15 ug (FA); A2 = Afluria Quad IM (UA), 15 ug; A3 = Placebo MAP (FA); A4 = A/Sing Mono IM, 15 ug (UA)
Treatments Part B: B1 = A/Sing MAP, 15 ug (FA) with biopsy; B2 = Placebo MAP (FA) with biopsy
B3 = A/Sing MAP, 15 ug (FA); B4 = A/Sing MAP, 10 ug (FA); B5 = A/Sing MAP, 5 ug (FA); B6 = A/Sing MAP, 2.5 ug (FA)
B7 = Placebo MAP (FA); B8 = A/Sing MAP, 15 ug (UA); B9 = A/Sing IM, 15 ug, as 2018 Afluria Quad (UA)

Listing 16.2.6.6
Immunogenicity Results (CMI)

Part B

Randomisation Number	Group	Visit	Test	Subset	IL2	TNFa	IFNg	Value
B0117	Group B9	Day 1	Peptide	CD4	+	+	+	-0.00086%
B0117	Group B9	Day 1	Peptide	CD4	+	+	-	0.003%
B0117	Group B9	Day 1	Peptide	CD4	+	-	+	-0.000019%
B0117	Group B9	Day 1	Peptide	CD4	+	-	-	0.0006%
B0117	Group B9	Day 1	Peptide	CD4	-	+	+	0%
B0117	Group B9	Day 1	Peptide	CD4	-	+	-	0.01%
B0117	Group B9	Day 1	Peptide	CD4	-	-	+	-0.00007%
B0117	Group B9	Day 22	Peptide	CD4	+	+	+	0.001%
B0117	Group B9	Day 22	Peptide	CD4	+	+	-	0.005%
B0117	Group B9	Day 22	Peptide	CD4	+	-	+	0.00005%
B0117	Group B9	Day 22	Peptide	CD4	+	-	-	-0.00259%
B0117	Group B9	Day 22	Peptide	CD4	-	+	+	0.0016%
B0117	Group B9	Day 22	Peptide	CD4	-	+	-	0.003%
B0117	Group B9	Day 22	Peptide	CD4	-	-	+	0.00083%
B0117	Group B9	Day 1	Peptide	CD8	+	+	+	0%
B0117	Group B9	Day 1	Peptide	CD8	+	+	-	0.00305%
B0117	Group B9	Day 1	Peptide	CD8	+	-	+	0%
B0117	Group B9	Day 1	Peptide	CD8	+	-	-	-0.00004%
B0117	Group B9	Day 1	Peptide	CD8	-	+	+	-0.0099%

Administration Site Key: FA = Forearm; UA= Upper arm
Treatments Part A: A1 = A/Sing MAP, 15 ug (FA); A2 = Afluria Quad IM (UA), 15 ug; A3 = Placebo MAP (FA); A4 = A/Sing Mono IM, 15 ug (UA)
Treatments Part B: B1 = A/Sing MAP, 15 ug (FA) with biopsy; B2 = Placebo MAP (FA) with biopsy
B3 = A/Sing MAP, 15 ug (FA); B4 = A/Sing MAP, 10 ug (FA); B5 = A/Sing MAP, 5 ug (FA); B6 = A/Sing MAP, 2.5 ug (FA)
B7 = Placebo MAP (FA); B8 = A/Sing MAP, 15 ug (UA); B9 = A/Sing IM, 15 ug, as 2018 Afluria Quad (UA)

Listing 16.2.6.6
Immunogenicity Results (CMI)

Part B

Randomisation Number	Group	Visit	Test	Subset	IL2	TNFa	IFNg	Value
B0117	Group B9	Day 1	Peptide	CD8	-	+	-	-0.09%
B0117	Group B9	Day 1	Peptide	CD8	-	-	+	0.003%
B0117	Group B9	Day 22	Peptide	CD8	+	+	+	-0.0015%
B0117	Group B9	Day 22	Peptide	CD8	+	+	-	-0.00288%
B0117	Group B9	Day 22	Peptide	CD8	+	-	+	0%
B0117	Group B9	Day 22	Peptide	CD8	+	-	-	0.00163%
B0117	Group B9	Day 22	Peptide	CD8	-	+	+	0.00198%
B0117	Group B9	Day 22	Peptide	CD8	-	+	-	-0.11%
B0117	Group B9	Day 22	Peptide	CD8	-	-	+	0.017%
B0117	Group B9	Day 1	MPH	CD4	+	+	+	0.0388%
B0117	Group B9	Day 1	MPH	CD4	+	+	-	0.055%
B0117	Group B9	Day 1	MPH	CD4	+	-	+	0.0112%
B0117	Group B9	Day 1	MPH	CD4	+	-	-	0.022%
B0117	Group B9	Day 1	MPH	CD4	-	+	+	0.03%
B0117	Group B9	Day 1	MPH	CD4	-	+	-	0.199%
B0117	Group B9	Day 1	MPH	CD4	-	-	+	0.0248%
B0117	Group B9	Day 22	MPH	CD4	+	+	+	0.04958%
B0117	Group B9	Day 22	MPH	CD4	+	+	-	0.059%
B0117	Group B9	Day 22	MPH	CD4	+	-	+	0.01757%

Administration Site Key: FA = Forearm; UA= Upper arm
Treatments Part A: A1 = A/Sing MAP, 15 ug (FA); A2 = Afluria Quad IM (UA), 15 ug; A3 = Placebo MAP (FA); A4 = A/Sing Mono IM, 15 ug (UA)
Treatments Part B: B1 = A/Sing MAP, 15 ug (FA) with biopsy; B2 = Placebo MAP (FA) with biopsy
B3 = A/Sing MAP, 15 ug (FA); B4 = A/Sing MAP, 10 ug (FA); B5 = A/Sing MAP, 5 ug (FA); B6 = A/Sing MAP, 2.5 ug (FA)
B7 = Placebo MAP (FA); B8 = A/Sing MAP, 15 ug (UA); B9 = A/Sing IM, 15 ug, as 2018 Afluria Quad (UA)

Listing 16.2.6.6
Immunogenicity Results (CMI)

Part B

Randomisation Number	Group	Visit	Test	Subset	IL2	TNFa	IFNg	Value
B0117	Group B9	Day 22	MPH	CD4	+	-	-	0.03673%
B0117	Group B9	Day 22	MPH	CD4	-	+	+	0.02315%
B0117	Group B9	Day 22	MPH	CD4	-	+	-	0.201%
B0117	Group B9	Day 22	MPH	CD4	-	-	+	0.04886%
B0117	Group B9	Day 1	MPH	CD8	+	+	+	0.017%
B0117	Group B9	Day 1	MPH	CD8	+	+	-	0.04%
B0117	Group B9	Day 1	MPH	CD8	+	-	+	0.00154%
B0117	Group B9	Day 1	MPH	CD8	+	-	-	0.02344%
B0117	Group B9	Day 1	MPH	CD8	-	+	+	0.058%
B0117	Group B9	Day 1	MPH	CD8	-	+	-	0.39%
B0117	Group B9	Day 1	MPH	CD8	-	-	+	0.021%
B0117	Group B9	Day 22	MPH	CD8	+	+	+	0.0245%
B0117	Group B9	Day 22	MPH	CD8	+	+	-	0.07299%
B0117	Group B9	Day 22	MPH	CD8	+	-	+	0.00292%
B0117	Group B9	Day 22	MPH	CD8	+	-	-	0.0205%
B0117	Group B9	Day 22	MPH	CD8	-	+	+	0.12098%
B0117	Group B9	Day 22	MPH	CD8	-	+	-	0.96%
B0117	Group B9	Day 22	MPH	CD8	-	-	+	0.038%

Administration Site Key: FA = Forearm; UA= Upper arm
Treatments Part A: A1 = A/Sing MAP, 15 ug (FA); A2 = Afluria Quad IM (UA), 15 ug; A3 = Placebo MAP (FA); A4 = A/Sing Mono IM, 15 ug (UA)
Treatments Part B: B1 = A/Sing MAP, 15 ug (FA) with biopsy; B2 = Placebo MAP (FA) with biopsy
B3 = A/Sing MAP, 15 ug (FA); B4 = A/Sing MAP, 10 ug (FA); B5 = A/Sing MAP, 5 ug (FA); B6 = A/Sing MAP, 2.5 ug (FA)
B7 = Placebo MAP (FA); B8 = A/Sing MAP, 15 ug (UA); B9 = A/Sing IM, 15 ug, as 2018 Afluria Quad (UA)

Listing 16.2.6.6
Immunogenicity Results (CMI)

Part B

Randomisation Number	Group	Visit	Test	Subset	IL2	TNFa	IFNg	Value
B0120	Group B3	Day 1	Peptide	CD4	+	+	+	0.00896%
B0120	Group B3	Day 1	Peptide	CD4	+	+	-	0.00469%
B0120	Group B3	Day 1	Peptide	CD4	+	-	+	-0.00104%
B0120	Group B3	Day 1	Peptide	CD4	+	-	-	0.00413%
B0120	Group B3	Day 1	Peptide	CD4	-	+	+	0.00104%
B0120	Group B3	Day 1	Peptide	CD4	-	+	-	0.034%
B0120	Group B3	Day 1	Peptide	CD4	-	-	+	-0.00157%
B0120	Group B3	Day 22	Peptide	CD4	+	+	+	0.04358%
B0120	Group B3	Day 22	Peptide	CD4	+	+	-	0.03573%
B0120	Group B3	Day 22	Peptide	CD4	+	-	+	0.00179%
B0120	Group B3	Day 22	Peptide	CD4	+	-	-	-0.00205%
B0120	Group B3	Day 22	Peptide	CD4	-	+	+	0.01%
B0120	Group B3	Day 22	Peptide	CD4	-	+	-	0.019%
B0120	Group B3	Day 22	Peptide	CD4	-	-	+	0.00024%
B0120	Group B3	Day 1	Peptide	CD8	+	+	+	0.00808%
B0120	Group B3	Day 1	Peptide	CD8	+	+	-	0.00404%
B0120	Group B3	Day 1	Peptide	CD8	+	-	+	0%
B0120	Group B3	Day 1	Peptide	CD8	+	-	-	-0.00203%
B0120	Group B3	Day 1	Peptide	CD8	-	+	+	0.01397%

Administration Site Key: FA = Forearm; UA= Upper arm
Treatments Part A: A1 = A/Sing MAP, 15 ug (FA); A2 = Afluria Quad IM (UA), 15 ug; A3 = Placebo MAP (FA); A4 = A/Sing Mono IM, 15 ug (UA)
Treatments Part B: B1 = A/Sing MAP, 15 ug (FA) with biopsy; B2 = Placebo MAP (FA) with biopsy
B3 = A/Sing MAP, 15 ug (FA); B4 = A/Sing MAP, 10 ug (FA); B5 = A/Sing MAP, 5 ug (FA); B6 = A/Sing MAP, 2.5 ug (FA)
B7 = Placebo MAP (FA); B8 = A/Sing MAP, 15 ug (UA); B9 = A/Sing IM, 15 ug, as 2018 Afluria Quad (UA)

Listing 16.2.6.6
Immunogenicity Results (CMI)

Part B

Randomisation Number	Group	Visit	Test	Subset	IL2	TNFa	IFNg	Value
B0120	Group B3	Day 1	Peptide	CD8	-	+	-	0.044%
B0120	Group B3	Day 1	Peptide	CD8	-	-	+	-0.61%
B0120	Group B3	Day 22	Peptide	CD8	+	+	+	0.00667%
B0120	Group B3	Day 22	Peptide	CD8	+	+	-	0.00222%
B0120	Group B3	Day 22	Peptide	CD8	+	-	+	0%
B0120	Group B3	Day 22	Peptide	CD8	+	-	-	0.00222%
B0120	Group B3	Day 22	Peptide	CD8	-	+	+	0.00876%
B0120	Group B3	Day 22	Peptide	CD8	-	+	-	0.016%
B0120	Group B3	Day 22	Peptide	CD8	-	-	+	-0.19%
B0120	Group B3	Day 1	MPH	CD4	+	+	+	0.06296%
B0120	Group B3	Day 1	MPH	CD4	+	+	-	0.08369%
B0120	Group B3	Day 1	MPH	CD4	+	-	+	0.00702%
B0120	Group B3	Day 1	MPH	CD4	+	-	-	0.05326%
B0120	Group B3	Day 1	MPH	CD4	-	+	+	0.04596%
B0120	Group B3	Day 1	MPH	CD4	-	+	-	0.292%
B0120	Group B3	Day 1	MPH	CD4	-	-	+	0.06235%
B0120	Group B3	Day 22	MPH	CD4	+	+	+	0.09158%
B0120	Group B3	Day 22	MPH	CD4	+	+	-	0.08873%
B0120	Group B3	Day 22	MPH	CD4	+	-	+	0.033%

Administration Site Key: FA = Forearm; UA= Upper arm
Treatments Part A: A1 = A/Sing MAP, 15 ug (FA); A2 = Afluria Quad IM (UA), 15 ug; A3 = Placebo MAP (FA); A4 = A/Sing Mono IM, 15 ug (UA)
Treatments Part B: B1 = A/Sing MAP, 15 ug (FA) with biopsy; B2 = Placebo MAP (FA) with biopsy
B3 = A/Sing MAP, 15 ug (FA); B4 = A/Sing MAP, 10 ug (FA); B5 = A/Sing MAP, 5 ug (FA); B6 = A/Sing MAP, 2.5 ug (FA)
B7 = Placebo MAP (FA); B8 = A/Sing MAP, 15 ug (UA); B9 = A/Sing IM, 15 ug, as 2018 Afluria Quad (UA)

Listing 16.2.6.6
Immunogenicity Results (CMI)

Part B

Randomisation Number	Group	Visit	Test	Subset	IL2	TNFa	IFNg	Value
B0120	Group B3	Day 22	MPH	CD4	+	-	-	0.059%
B0120	Group B3	Day 22	MPH	CD4	-	+	+	0.062%
B0120	Group B3	Day 22	MPH	CD4	-	+	-	0.236%
B0120	Group B3	Day 22	MPH	CD4	-	-	+	0.13487%
B0120	Group B3	Day 1	MPH	CD8	+	+	+	0.084%
B0120	Group B3	Day 1	MPH	CD8	+	+	-	0.00617%
B0120	Group B3	Day 1	MPH	CD8	+	-	+	0.016%
B0120	Group B3	Day 1	MPH	CD8	+	-	-	0.02097%
B0120	Group B3	Day 1	MPH	CD8	-	+	+	0.10797%
B0120	Group B3	Day 1	MPH	CD8	-	+	-	0.251%
B0120	Group B3	Day 1	MPH	CD8	-	-	+	-1.21%
B0120	Group B3	Day 22	MPH	CD8	+	+	+	0.072%
B0120	Group B3	Day 22	MPH	CD8	+	+	-	0.011%
B0120	Group B3	Day 22	MPH	CD8	+	-	+	0.00873%
B0120	Group B3	Day 22	MPH	CD8	+	-	-	0.094%
B0120	Group B3	Day 22	MPH	CD8	-	+	+	0.16576%
B0120	Group B3	Day 22	MPH	CD8	-	+	-	0.518%
B0120	Group B3	Day 22	MPH	CD8	-	-	+	-0.59%

Administration Site Key: FA = Forearm; UA= Upper arm
Treatments Part A: A1 = A/Sing MAP, 15 ug (FA); A2 = Afluria Quad IM (UA), 15 ug; A3 = Placebo MAP (FA); A4 = A/Sing Mono IM, 15 ug (UA)
Treatments Part B: B1 = A/Sing MAP, 15 ug (FA) with biopsy; B2 = Placebo MAP (FA) with biopsy
B3 = A/Sing MAP, 15 ug (FA); B4 = A/Sing MAP, 10 ug (FA); B5 = A/Sing MAP, 5 ug (FA); B6 = A/Sing MAP, 2.5 ug (FA)
B7 = Placebo MAP (FA); B8 = A/Sing MAP, 15 ug (UA); B9 = A/Sing IM, 15 ug, as 2018 Afluria Quad (UA)

Listing 16.2.6.6
Immunogenicity Results (CMI)

Part B

Randomisation Number	Group	Visit	Test	Subset	IL2	TNFa	IFNg	Value
B0121	Group B8	Day 1	Peptide	CD4	+	+	+	0.00816%
B0121	Group B8	Day 1	Peptide	CD4	+	+	-	-0.006%
B0121	Group B8	Day 1	Peptide	CD4	+	-	+	-0.001893%
B0121	Group B8	Day 1	Peptide	CD4	+	-	-	-0.001%
B0121	Group B8	Day 1	Peptide	CD4	-	+	+	-0.00077%
B0121	Group B8	Day 1	Peptide	CD4	-	+	-	0.018%
B0121	Group B8	Day 1	Peptide	CD4	-	-	+	0.00078%
B0121	Group B8	Day 22	Peptide	CD4	+	+	+	0.0101%
B0121	Group B8	Day 22	Peptide	CD4	+	+	-	0.005%
B0121	Group B8	Day 22	Peptide	CD4	+	-	+	0%
B0121	Group B8	Day 22	Peptide	CD4	+	-	-	-0.00009%
B0121	Group B8	Day 22	Peptide	CD4	-	+	+	-0.000481%
B0121	Group B8	Day 22	Peptide	CD4	-	+	-	0.003%
B0121	Group B8	Day 22	Peptide	CD4	-	-	+	-0.00145%
B0121	Group B8	Day 1	Peptide	CD8	+	+	+	0%
B0121	Group B8	Day 1	Peptide	CD8	+	+	-	0%
B0121	Group B8	Day 1	Peptide	CD8	+	-	+	-0.00118%
B0121	Group B8	Day 1	Peptide	CD8	+	-	-	-0.00724%
B0121	Group B8	Day 1	Peptide	CD8	-	+	+	0.00409%

Administration Site Key: FA = Forearm; UA= Upper arm
Treatments Part A: A1 = A/Sing MAP, 15 ug (FA); A2 = Afluria Quad IM (UA), 15 ug; A3 = Placebo MAP (FA); A4 = A/Sing Mono IM, 15 ug (UA)
Treatments Part B: B1 = A/Sing MAP, 15 ug (FA) with biopsy; B2 = Placebo MAP (FA) with biopsy
B3 = A/Sing MAP, 15 ug (FA); B4 = A/Sing MAP, 10 ug (FA); B5 = A/Sing MAP, 5 ug (FA); B6 = A/Sing MAP, 2.5 ug (FA)
B7 = Placebo MAP (FA); B8 = A/Sing MAP, 15 ug (UA); B9 = A/Sing IM, 15 ug, as 2018 Afluria Quad (UA)

Listing 16.2.6.6
Immunogenicity Results (CMI)

Part B

Randomisation Number	Group	Visit	Test	Subset	IL2	TNFa	IFNg	Value
B0121	Group B8	Day 1	Peptide	CD8	-	+	-	0.032%
B0121	Group B8	Day 1	Peptide	CD8	-	-	+	-0.24%
B0121	Group B8	Day 22	Peptide	CD8	+	+	+	0%
B0121	Group B8	Day 22	Peptide	CD8	+	+	-	0%
B0121	Group B8	Day 22	Peptide	CD8	+	-	+	0%
B0121	Group B8	Day 22	Peptide	CD8	+	-	-	-0.00003%
B0121	Group B8	Day 22	Peptide	CD8	-	+	+	0.00565%
B0121	Group B8	Day 22	Peptide	CD8	-	+	-	-0.023%
B0121	Group B8	Day 22	Peptide	CD8	-	-	+	-0.2%
B0121	Group B8	Day 1	MPH	CD4	+	+	+	0.05416%
B0121	Group B8	Day 1	MPH	CD4	+	+	-	0.081%
B0121	Group B8	Day 1	MPH	CD4	+	-	+	0.00064%
B0121	Group B8	Day 1	MPH	CD4	+	-	-	0.038%
B0121	Group B8	Day 1	MPH	CD4	-	+	+	0.04868%
B0121	Group B8	Day 1	MPH	CD4	-	+	-	0.231%
B0121	Group B8	Day 1	MPH	CD4	-	-	+	0.0372%
B0121	Group B8	Day 22	MPH	CD4	+	+	+	0.0891%
B0121	Group B8	Day 22	MPH	CD4	+	+	-	0.121%
B0121	Group B8	Day 22	MPH	CD4	+	-	+	0.028%

Administration Site Key: FA = Forearm; UA= Upper arm
Treatments Part A: A1 = A/Sing MAP, 15 ug (FA); A2 = Afluria Quad IM (UA), 15 ug; A3 = Placebo MAP (FA); A4 = A/Sing Mono IM, 15 ug (UA)
Treatments Part B: B1 = A/Sing MAP, 15 ug (FA) with biopsy; B2 = Placebo MAP (FA) with biopsy
B3 = A/Sing MAP, 15 ug (FA); B4 = A/Sing MAP, 10 ug (FA); B5 = A/Sing MAP, 5 ug (FA); B6 = A/Sing MAP, 2.5 ug (FA)
B7 = Placebo MAP (FA); B8 = A/Sing MAP, 15 ug (UA); B9 = A/Sing IM, 15 ug, as 2018 Afluria Quad (UA)

Listing 16.2.6.6
Immunogenicity Results (CMI)

Part B

Randomisation Number	Group	Visit	Test	Subset	IL2	TNFa	IFNg	Value
B0121	Group B8	Day 22	MPH	CD4	+	-	-	0.04501%
B0121	Group B8	Day 22	MPH	CD4	-	+	+	0.054048%
B0121	Group B8	Day 22	MPH	CD4	-	+	-	0.264%
B0121	Group B8	Day 22	MPH	CD4	-	-	+	0.07672%
B0121	Group B8	Day 1	MPH	CD8	+	+	+	0.038%
B0121	Group B8	Day 1	MPH	CD8	+	+	-	0.015%
B0121	Group B8	Day 1	MPH	CD8	+	-	+	0.00882%
B0121	Group B8	Day 1	MPH	CD8	+	-	-	0.012%
B0121	Group B8	Day 1	MPH	CD8	-	+	+	0.05109%
B0121	Group B8	Day 1	MPH	CD8	-	+	-	0.114%
B0121	Group B8	Day 1	MPH	CD8	-	-	+	-1.2%
B0121	Group B8	Day 22	MPH	CD8	+	+	+	0.027%
B0121	Group B8	Day 22	MPH	CD8	+	+	-	0.00135%
B0121	Group B8	Day 22	MPH	CD8	+	-	+	0.00541%
B0121	Group B8	Day 22	MPH	CD8	+	-	-	0.02514%
B0121	Group B8	Day 22	MPH	CD8	-	+	+	0.07557%
B0121	Group B8	Day 22	MPH	CD8	-	+	-	0.149%
B0121	Group B8	Day 22	MPH	CD8	-	-	+	-0.47%

Administration Site Key: FA = Forearm; UA= Upper arm
Treatments Part A: A1 = A/Sing MAP, 15 ug (FA); A2 = Afluria Quad IM (UA), 15 ug; A3 = Placebo MAP (FA); A4 = A/Sing Mono IM, 15 ug (UA)
Treatments Part B: B1 = A/Sing MAP, 15 ug (FA) with biopsy; B2 = Placebo MAP (FA) with biopsy
B3 = A/Sing MAP, 15 ug (FA); B4 = A/Sing MAP, 10 ug (FA); B5 = A/Sing MAP, 5 ug (FA); B6 = A/Sing MAP, 2.5 ug (FA)
B7 = Placebo MAP (FA); B8 = A/Sing MAP, 15 ug (UA); B9 = A/Sing IM, 15 ug, as 2018 Afluria Quad (UA)

Listing 16.2.6.6
Immunogenicity Results (CMI)

Part B

Randomisation Number	Group	Visit	Test	Subset	IL2	TNFa	IFNg	Value
B0125	Group B7	Day 1	Peptide	CD4	+	+	+	0.00071%
B0125	Group B7	Day 1	Peptide	CD4	+	+	-	0.002%
B0125	Group B7	Day 1	Peptide	CD4	+	-	+	0%
B0125	Group B7	Day 1	Peptide	CD4	+	-	-	-0.00182%
B0125	Group B7	Day 1	Peptide	CD4	-	+	+	0.000034%
B0125	Group B7	Day 1	Peptide	CD4	-	+	-	0.006%
B0125	Group B7	Day 1	Peptide	CD4	-	-	+	0.00017%
B0125	Group B7	Day 22	Peptide	CD4	+	+	+	0.005175%
B0125	Group B7	Day 22	Peptide	CD4	+	+	-	0.01409%
B0125	Group B7	Day 22	Peptide	CD4	+	-	+	0%
B0125	Group B7	Day 22	Peptide	CD4	+	-	-	-0.00109%
B0125	Group B7	Day 22	Peptide	CD4	-	+	+	0.00172%
B0125	Group B7	Day 22	Peptide	CD4	-	+	-	0.016%
B0125	Group B7	Day 22	Peptide	CD4	-	-	+	0.00004%
B0125	Group B7	Day 1	Peptide	CD8	+	+	+	0%
B0125	Group B7	Day 1	Peptide	CD8	+	+	-	0%
B0125	Group B7	Day 1	Peptide	CD8	+	-	+	0%
B0125	Group B7	Day 1	Peptide	CD8	+	-	-	-0.00214%
B0125	Group B7	Day 1	Peptide	CD8	-	+	+	-0.00093%

Administration Site Key: FA = Forearm; UA= Upper arm
Treatments Part A: A1 = A/Sing MAP, 15 ug (FA); A2 = Afluria Quad IM (UA), 15 ug; A3 = Placebo MAP (FA); A4 = A/Sing Mono IM, 15 ug (UA)
Treatments Part B: B1 = A/Sing MAP, 15 ug (FA) with biopsy; B2 = Placebo MAP (FA) with biopsy
B3 = A/Sing MAP, 15 ug (FA); B4 = A/Sing MAP, 10 ug (FA); B5 = A/Sing MAP, 5 ug (FA); B6 = A/Sing MAP, 2.5 ug (FA)
B7 = Placebo MAP (FA); B8 = A/Sing MAP, 15 ug (UA); B9 = A/Sing IM, 15 ug, as 2018 Afluria Quad (UA)

Listing 16.2.6.6
Immunogenicity Results (CMI)

Part B

Randomisation Number	Group	Visit	Test	Subset	IL2	TNFa	IFNg	Value
B0125	Group B7	Day 1	Peptide	CD8	-	+	-	-0.009%
B0125	Group B7	Day 1	Peptide	CD8	-	-	+	-0.06%
B0125	Group B7	Day 22	Peptide	CD8	+	+	+	0%
B0125	Group B7	Day 22	Peptide	CD8	+	+	-	-0.00127%
B0125	Group B7	Day 22	Peptide	CD8	+	-	+	0%
B0125	Group B7	Day 22	Peptide	CD8	+	-	-	0.00135%
B0125	Group B7	Day 22	Peptide	CD8	-	+	+	-0.00127%
B0125	Group B7	Day 22	Peptide	CD8	-	+	-	0%
B0125	Group B7	Day 22	Peptide	CD8	-	-	+	-0.14%
B0125	Group B7	Day 1	MPH	CD4	+	+	+	0.07747%
B0125	Group B7	Day 1	MPH	CD4	+	+	-	0.084%
B0125	Group B7	Day 1	MPH	CD4	+	-	+	0.013%
B0125	Group B7	Day 1	MPH	CD4	+	-	-	0.01694%
B0125	Group B7	Day 1	MPH	CD4	-	+	+	0.032494%
B0125	Group B7	Day 1	MPH	CD4	-	+	-	0.281%
B0125	Group B7	Day 1	MPH	CD4	-	-	+	0.06147%
B0125	Group B7	Day 22	MPH	CD4	+	+	+	0.091435%
B0125	Group B7	Day 22	MPH	CD4	+	+	-	0.10209%
B0125	Group B7	Day 22	MPH	CD4	+	-	+	0.00834%

Administration Site Key: FA = Forearm; UA= Upper arm
Treatments Part A: A1 = A/Sing MAP, 15 ug (FA); A2 = Afluria Quad IM (UA), 15 ug; A3 = Placebo MAP (FA); A4 = A/Sing Mono IM, 15 ug (UA)
Treatments Part B: B1 = A/Sing MAP, 15 ug (FA) with biopsy; B2 = Placebo MAP (FA) with biopsy
B3 = A/Sing MAP, 15 ug (FA); B4 = A/Sing MAP, 10 ug (FA); B5 = A/Sing MAP, 5 ug (FA); B6 = A/Sing MAP, 2.5 ug (FA)
B7 = Placebo MAP (FA); B8 = A/Sing MAP, 15 ug (UA); B9 = A/Sing IM, 15 ug, as 2018 Afluria Quad (UA)

Listing 16.2.6.6
Immunogenicity Results (CMI)

Part B

Randomisation Number	Group	Visit	Test	Subset	IL2	TNFa	IFNg	Value
B0125	Group B7	Day 22	MPH	CD4	+	-	-	0.02804%
B0125	Group B7	Day 22	MPH	CD4	-	+	+	0.036%
B0125	Group B7	Day 22	MPH	CD4	-	+	-	0.324%
B0125	Group B7	Day 22	MPH	CD4	-	-	+	0.05674%
B0125	Group B7	Day 1	MPH	CD8	+	+	+	0.049%
B0125	Group B7	Day 1	MPH	CD8	+	+	-	0.00455%
B0125	Group B7	Day 1	MPH	CD8	+	-	+	0.02%
B0125	Group B7	Day 1	MPH	CD8	+	-	-	0.04145%
B0125	Group B7	Day 1	MPH	CD8	-	+	+	0.10545%
B0125	Group B7	Day 1	MPH	CD8	-	+	-	0.16%
B0125	Group B7	Day 1	MPH	CD8	-	-	+	-0.18%
B0125	Group B7	Day 22	MPH	CD8	+	+	+	0.059%
B0125	Group B7	Day 22	MPH	CD8	+	+	-	0.00716%
B0125	Group B7	Day 22	MPH	CD8	+	-	+	0.012%
B0125	Group B7	Day 22	MPH	CD8	+	-	-	0.04545%
B0125	Group B7	Day 22	MPH	CD8	-	+	+	0.09873%
B0125	Group B7	Day 22	MPH	CD8	-	+	-	0.252%
B0125	Group B7	Day 22	MPH	CD8	-	-	+	-0.21%

Administration Site Key: FA = Forearm; UA= Upper arm
Treatments Part A: A1 = A/Sing MAP, 15 ug (FA); A2 = Afluria Quad IM (UA), 15 ug; A3 = Placebo MAP (FA); A4 = A/Sing Mono IM, 15 ug (UA)
Treatments Part B: B1 = A/Sing MAP, 15 ug (FA) with biopsy; B2 = Placebo MAP (FA) with biopsy
B3 = A/Sing MAP, 15 ug (FA); B4 = A/Sing MAP, 10 ug (FA); B5 = A/Sing MAP, 5 ug (FA); B6 = A/Sing MAP, 2.5 ug (FA)
B7 = Placebo MAP (FA); B8 = A/Sing MAP, 15 ug (UA); B9 = A/Sing IM, 15 ug, as 2018 Afluria Quad (UA)

Listing 16.2.6.6
Immunogenicity Results (CMI)

Part B

Randomisation Number	Group	Visit	Test	Subset	IL2	TNFa	IFNg	Value
B0126	Group B3	Day 1	Peptide	CD4	+	+	+	0.00158%
B0126	Group B3	Day 1	Peptide	CD4	+	+	-	0.00266%
B0126	Group B3	Day 1	Peptide	CD4	+	-	+	-0.000834%
B0126	Group B3	Day 1	Peptide	CD4	+	-	-	0.001%
B0126	Group B3	Day 1	Peptide	CD4	-	+	+	0%
B0126	Group B3	Day 1	Peptide	CD4	-	+	-	0.001%
B0126	Group B3	Day 1	Peptide	CD4	-	-	+	-0.00009%
B0126	Group B3	Day 22	Peptide	CD4	+	+	+	0.00753%
B0126	Group B3	Day 22	Peptide	CD4	+	+	-	0.00878%
B0126	Group B3	Day 22	Peptide	CD4	+	-	+	-0.00103%
B0126	Group B3	Day 22	Peptide	CD4	+	-	-	0.00476%
B0126	Group B3	Day 22	Peptide	CD4	-	+	+	-0.00197%
B0126	Group B3	Day 22	Peptide	CD4	-	+	-	0.002%
B0126	Group B3	Day 22	Peptide	CD4	-	-	+	-0.00197%
B0126	Group B3	Day 1	Peptide	CD8	+	+	+	0%
B0126	Group B3	Day 1	Peptide	CD8	+	+	-	0.000844%
B0126	Group B3	Day 1	Peptide	CD8	+	-	+	0%
B0126	Group B3	Day 1	Peptide	CD8	+	-	-	-0.00434%
B0126	Group B3	Day 1	Peptide	CD8	-	+	+	-0.00259%

Administration Site Key: FA = Forearm; UA= Upper arm
Treatments Part A: A1 = A/Sing MAP, 15 ug (FA); A2 = Afluria Quad IM (UA), 15 ug; A3 = Placebo MAP (FA); A4 = A/Sing Mono IM, 15 ug (UA)
Treatments Part B: B1 = A/Sing MAP, 15 ug (FA) with biopsy; B2 = Placebo MAP (FA) with biopsy
B3 = A/Sing MAP, 15 ug (FA); B4 = A/Sing MAP, 10 ug (FA); B5 = A/Sing MAP, 5 ug (FA); B6 = A/Sing MAP, 2.5 ug (FA)
B7 = Placebo MAP (FA); B8 = A/Sing MAP, 15 ug (UA); B9 = A/Sing IM, 15 ug, as 2018 Afluria Quad (UA)

Listing 16.2.6.6
Immunogenicity Results (CMI)

Part B

Randomisation Number	Group	Visit	Test	Subset	IL2	TNFa	IFNg	Value
B0126	Group B3	Day 1	Peptide	CD8	-	+	-	-0.017%
B0126	Group B3	Day 1	Peptide	CD8	-	-	+	-0.03%
B0126	Group B3	Day 22	Peptide	CD8	+	+	+	0%
B0126	Group B3	Day 22	Peptide	CD8	+	+	-	0%
B0126	Group B3	Day 22	Peptide	CD8	+	-	+	0%
B0126	Group B3	Day 22	Peptide	CD8	+	-	-	0.001857%
B0126	Group B3	Day 22	Peptide	CD8	-	+	+	-0.000857%
B0126	Group B3	Day 22	Peptide	CD8	-	+	-	0.013%
B0126	Group B3	Day 22	Peptide	CD8	-	-	+	-0.023%
B0126	Group B3	Day 1	MPH	CD4	+	+	+	0.02533%
B0126	Group B3	Day 1	MPH	CD4	+	+	-	0.07666%
B0126	Group B3	Day 1	MPH	CD4	+	-	+	0.003306%
B0126	Group B3	Day 1	MPH	CD4	+	-	-	0.029%
B0126	Group B3	Day 1	MPH	CD4	-	+	+	0.034%
B0126	Group B3	Day 1	MPH	CD4	-	+	-	0.174%
B0126	Group B3	Day 1	MPH	CD4	-	-	+	0.02166%
B0126	Group B3	Day 22	MPH	CD4	+	+	+	0.05997%
B0126	Group B3	Day 22	MPH	CD4	+	+	-	0.08178%
B0126	Group B3	Day 22	MPH	CD4	+	-	+	0.01797%

Administration Site Key: FA = Forearm; UA= Upper arm
Treatments Part A: A1 = A/Sing MAP, 15 ug (FA); A2 = Afluria Quad IM (UA), 15 ug; A3 = Placebo MAP (FA); A4 = A/Sing Mono IM, 15 ug (UA)
Treatments Part B: B1 = A/Sing MAP, 15 ug (FA) with biopsy; B2 = Placebo MAP (FA) with biopsy
B3 = A/Sing MAP, 15 ug (FA); B4 = A/Sing MAP, 10 ug (FA); B5 = A/Sing MAP, 5 ug (FA); B6 = A/Sing MAP, 2.5 ug (FA)
B7 = Placebo MAP (FA); B8 = A/Sing MAP, 15 ug (UA); B9 = A/Sing IM, 15 ug, as 2018 Afluria Quad (UA)

Listing 16.2.6.6
Immunogenicity Results (CMI)

Part B

Randomisation Number	Group	Visit	Test	Subset	IL2	TNFa	IFNg	Value
B0126	Group B3	Day 22	MPH	CD4	+	-	-	0.05776%
B0126	Group B3	Day 22	MPH	CD4	-	+	+	0.02389%
B0126	Group B3	Day 22	MPH	CD4	-	+	-	0.201%
B0126	Group B3	Day 22	MPH	CD4	-	-	+	0.03889%
B0126	Group B3	Day 1	MPH	CD8	+	+	+	0.01%
B0126	Group B3	Day 1	MPH	CD8	+	+	-	0.00159%
B0126	Group B3	Day 1	MPH	CD8	+	-	+	0.00238%
B0126	Group B3	Day 1	MPH	CD8	+	-	-	-0.00126%
B0126	Group B3	Day 1	MPH	CD8	-	+	+	0.00841%
B0126	Group B3	Day 1	MPH	CD8	-	+	-	0.093%
B0126	Group B3	Day 1	MPH	CD8	-	-	+	-0.009%
B0126	Group B3	Day 22	MPH	CD8	+	+	+	0.00335%
B0126	Group B3	Day 22	MPH	CD8	+	+	-	0.00168%
B0126	Group B3	Day 22	MPH	CD8	+	-	+	0.00335%
B0126	Group B3	Day 22	MPH	CD8	+	-	-	0.018117%
B0126	Group B3	Day 22	MPH	CD8	-	+	+	0.01023%
B0126	Group B3	Day 22	MPH	CD8	-	+	-	0.098%
B0126	Group B3	Day 22	MPH	CD8	-	-	+	-0.04%

Administration Site Key: FA = Forearm; UA= Upper arm
Treatments Part A: A1 = A/Sing MAP, 15 ug (FA); A2 = Afluria Quad IM (UA), 15 ug; A3 = Placebo MAP (FA); A4 = A/Sing Mono IM, 15 ug (UA)
Treatments Part B: B1 = A/Sing MAP, 15 ug (FA) with biopsy; B2 = Placebo MAP (FA) with biopsy
B3 = A/Sing MAP, 15 ug (FA); B4 = A/Sing MAP, 10 ug (FA); B5 = A/Sing MAP, 5 ug (FA); B6 = A/Sing MAP, 2.5 ug (FA)
B7 = Placebo MAP (FA); B8 = A/Sing MAP, 15 ug (UA); B9 = A/Sing IM, 15 ug, as 2018 Afluria Quad (UA)

Listing 16.2.6.6
Immunogenicity Results (CMI)

Part B

Randomisation Number	Group	Visit	Test	Subset	IL2	TNFa	IFNg	Value
B0127	Group B9	Day 1	Peptide	CD4	+	+	+	0.00229%
B0127	Group B9	Day 1	Peptide	CD4	+	+	-	-0.00003%
B0127	Group B9	Day 1	Peptide	CD4	+	-	+	0.000576%
B0127	Group B9	Day 1	Peptide	CD4	+	-	-	0.00055%
B0127	Group B9	Day 1	Peptide	CD4	-	+	+	0.00173%
B0127	Group B9	Day 1	Peptide	CD4	-	+	-	0.022%
B0127	Group B9	Day 1	Peptide	CD4	-	-	+	-0.00175%
B0127	Group B9	Day 22	Peptide	CD4	+	+	+	0.00553%
B0127	Group B9	Day 22	Peptide	CD4	+	+	-	0.00622%
B0127	Group B9	Day 22	Peptide	CD4	+	-	+	0.00125%
B0127	Group B9	Day 22	Peptide	CD4	+	-	-	0.00111%
B0127	Group B9	Day 22	Peptide	CD4	-	+	+	0.00125%
B0127	Group B9	Day 22	Peptide	CD4	-	+	-	0.014%
B0127	Group B9	Day 22	Peptide	CD4	-	-	+	0.00175%
B0127	Group B9	Day 1	Peptide	CD8	+	+	+	0%
B0127	Group B9	Day 1	Peptide	CD8	+	+	-	0%
B0127	Group B9	Day 1	Peptide	CD8	+	-	+	0%
B0127	Group B9	Day 1	Peptide	CD8	+	-	-	-0.0024%
B0127	Group B9	Day 1	Peptide	CD8	-	+	+	-0.0012%

Administration Site Key: FA = Forearm; UA= Upper arm
Treatments Part A: A1 = A/Sing MAP, 15 ug (FA); A2 = Afluria Quad IM (UA), 15 ug; A3 = Placebo MAP (FA); A4 = A/Sing Mono IM, 15 ug (UA)
Treatments Part B: B1 = A/Sing MAP, 15 ug (FA) with biopsy; B2 = Placebo MAP (FA) with biopsy
B3 = A/Sing MAP, 15 ug (FA); B4 = A/Sing MAP, 10 ug (FA); B5 = A/Sing MAP, 5 ug (FA); B6 = A/Sing MAP, 2.5 ug (FA)
B7 = Placebo MAP (FA); B8 = A/Sing MAP, 15 ug (UA); B9 = A/Sing IM, 15 ug, as 2018 Afluria Quad (UA)

Listing 16.2.6.6
Immunogenicity Results (CMI)

Part B

Randomisation Number	Group	Visit	Test	Subset	IL2	TNFa	IFNg	Value
B0127	Group B9	Day 1	Peptide	CD8	-	+	-	-0.038%
B0127	Group B9	Day 1	Peptide	CD8	-	-	+	-0.043%
B0127	Group B9	Day 22	Peptide	CD8	+	+	+	-0.00001%
B0127	Group B9	Day 22	Peptide	CD8	+	+	-	0%
B0127	Group B9	Day 22	Peptide	CD8	+	-	+	0%
B0127	Group B9	Day 22	Peptide	CD8	+	-	-	-0.00003%
B0127	Group B9	Day 22	Peptide	CD8	-	+	+	0.00122%
B0127	Group B9	Day 22	Peptide	CD8	-	+	-	-0.036%
B0127	Group B9	Day 22	Peptide	CD8	-	-	+	-0.005%
B0127	Group B9	Day 1	MPH	CD4	+	+	+	0.04926%
B0127	Group B9	Day 1	MPH	CD4	+	+	-	0.06036%
B0127	Group B9	Day 1	MPH	CD4	+	-	+	0.00446%
B0127	Group B9	Day 1	MPH	CD4	+	-	-	0.0181%
B0127	Group B9	Day 1	MPH	CD4	-	+	+	0.046%
B0127	Group B9	Day 1	MPH	CD4	-	+	-	0.315%
B0127	Group B9	Day 1	MPH	CD4	-	-	+	0.0941%
B0127	Group B9	Day 22	MPH	CD4	+	+	+	0.07243%
B0127	Group B9	Day 22	MPH	CD4	+	+	-	0.06822%
B0127	Group B9	Day 22	MPH	CD4	+	-	+	0.021%

Administration Site Key: FA = Forearm; UA= Upper arm
Treatments Part A: A1 = A/Sing MAP, 15 ug (FA); A2 = Afluria Quad IM (UA), 15 ug; A3 = Placebo MAP (FA); A4 = A/Sing Mono IM, 15 ug (UA)
Treatments Part B: B1 = A/Sing MAP, 15 ug (FA) with biopsy; B2 = Placebo MAP (FA) with biopsy
B3 = A/Sing MAP, 15 ug (FA); B4 = A/Sing MAP, 10 ug (FA); B5 = A/Sing MAP, 5 ug (FA); B6 = A/Sing MAP, 2.5 ug (FA)
B7 = Placebo MAP (FA); B8 = A/Sing MAP, 15 ug (UA); B9 = A/Sing IM, 15 ug, as 2018 Afluria Quad (UA)

Listing 16.2.6.6
Immunogenicity Results (CMI)

Part B

Randomisation Number	Group	Visit	Test	Subset	IL2	TNFa	IFNg	Value
B0127	Group B9	Day 22	MPH	CD4	+	-	-	0.0185%
B0127	Group B9	Day 22	MPH	CD4	-	+	+	0.048%
B0127	Group B9	Day 22	MPH	CD4	-	+	-	0.293%
B0127	Group B9	Day 22	MPH	CD4	-	-	+	0.07814%
B0127	Group B9	Day 1	MPH	CD8	+	+	+	0.059%
B0127	Group B9	Day 1	MPH	CD8	+	+	-	0.011%
B0127	Group B9	Day 1	MPH	CD8	+	-	+	0.01%
B0127	Group B9	Day 1	MPH	CD8	+	-	-	0.00941%
B0127	Group B9	Day 1	MPH	CD8	-	+	+	0.13761%
B0127	Group B9	Day 1	MPH	CD8	-	+	-	0.32%
B0127	Group B9	Day 1	MPH	CD8	-	-	+	0.03%
B0127	Group B9	Day 22	MPH	CD8	+	+	+	0.09877%
B0127	Group B9	Day 22	MPH	CD8	+	+	-	0.016%
B0127	Group B9	Day 22	MPH	CD8	+	-	+	0.025%
B0127	Group B9	Day 22	MPH	CD8	+	-	-	0.01553%
B0127	Group B9	Day 22	MPH	CD8	-	+	+	0.22%
B0127	Group B9	Day 22	MPH	CD8	-	+	-	0.37%
B0127	Group B9	Day 22	MPH	CD8	-	-	+	0.099%

Administration Site Key: FA = Forearm; UA= Upper arm
Treatments Part A: A1 = A/Sing MAP, 15 ug (FA); A2 = Afluria Quad IM (UA), 15 ug; A3 = Placebo MAP (FA); A4 = A/Sing Mono IM, 15 ug (UA)
Treatments Part B: B1 = A/Sing MAP, 15 ug (FA) with biopsy; B2 = Placebo MAP (FA) with biopsy
B3 = A/Sing MAP, 15 ug (FA); B4 = A/Sing MAP, 10 ug (FA); B5 = A/Sing MAP, 5 ug (FA); B6 = A/Sing MAP, 2.5 ug (FA)
B7 = Placebo MAP (FA); B8 = A/Sing MAP, 15 ug (UA); B9 = A/Sing IM, 15 ug, as 2018 Afluria Quad (UA)

Listing 16.2.6.6
Immunogenicity Results (CMI)

Part B

Randomisation Number	Group	Visit	Test	Subset	IL2	TNFa	IFNg	Value
B0129	Group B8	Day 1	Peptide	CD4	+	+	+	0.005%
B0129	Group B8	Day 1	Peptide	CD4	+	+	-	0.012%
B0129	Group B8	Day 1	Peptide	CD4	+	-	+	0.000845%
B0129	Group B8	Day 1	Peptide	CD4	+	-	-	-0.013%
B0129	Group B8	Day 1	Peptide	CD4	-	+	+	0.00213%
B0129	Group B8	Day 1	Peptide	CD4	-	+	-	0.013%
B0129	Group B8	Day 1	Peptide	CD4	-	-	+	-0.00281%
B0129	Group B8	Day 22	Peptide	CD4	+	+	+	0.04345%
B0129	Group B8	Day 22	Peptide	CD4	+	+	-	0.028%
B0129	Group B8	Day 22	Peptide	CD4	+	-	+	-0.00067%
B0129	Group B8	Day 22	Peptide	CD4	+	-	-	-0.00043%
B0129	Group B8	Day 22	Peptide	CD4	-	+	+	0.01148%
B0129	Group B8	Day 22	Peptide	CD4	-	+	-	-0.006%
B0129	Group B8	Day 22	Peptide	CD4	-	-	+	0.00185%
B0129	Group B8	Day 1	Peptide	CD8	+	+	+	0%
B0129	Group B8	Day 1	Peptide	CD8	+	+	-	-0.00444%
B0129	Group B8	Day 1	Peptide	CD8	+	-	+	0%
B0129	Group B8	Day 1	Peptide	CD8	+	-	-	0.00012%
B0129	Group B8	Day 1	Peptide	CD8	-	+	+	-0.00592%

Administration Site Key: FA = Forearm; UA= Upper arm
Treatments Part A: A1 = A/Sing MAP, 15 ug (FA); A2 = Afluria Quad IM (UA), 15 ug; A3 = Placebo MAP (FA); A4 = A/Sing Mono IM, 15 ug (UA)
Treatments Part B: B1 = A/Sing MAP, 15 ug (FA) with biopsy; B2 = Placebo MAP (FA) with biopsy
B3 = A/Sing MAP, 15 ug (FA); B4 = A/Sing MAP, 10 ug (FA); B5 = A/Sing MAP, 5 ug (FA); B6 = A/Sing MAP, 2.5 ug (FA)
B7 = Placebo MAP (FA); B8 = A/Sing MAP, 15 ug (UA); B9 = A/Sing IM, 15 ug, as 2018 Afluria Quad (UA)

Listing 16.2.6.6
Immunogenicity Results (CMI)

Part B

Randomisation Number	Group	Visit	Test	Subset	IL2	TNFa	IFNg	Value
B0129	Group B8	Day 1	Peptide	CD8	-	+	-	0.001%
B0129	Group B8	Day 1	Peptide	CD8	-	-	+	-0.03%
B0129	Group B8	Day 22	Peptide	CD8	+	+	+	0.00145%
B0129	Group B8	Day 22	Peptide	CD8	+	+	-	0%
B0129	Group B8	Day 22	Peptide	CD8	+	-	+	0%
B0129	Group B8	Day 22	Peptide	CD8	+	-	-	-0.00142%
B0129	Group B8	Day 22	Peptide	CD8	-	+	+	-0.0028%
B0129	Group B8	Day 22	Peptide	CD8	-	+	-	-0.002%
B0129	Group B8	Day 22	Peptide	CD8	-	-	+	-0.047%
B0129	Group B8	Day 1	MPH	CD4	+	+	+	0.082%
B0129	Group B8	Day 1	MPH	CD4	+	+	-	0.103%
B0129	Group B8	Day 1	MPH	CD4	+	-	+	0.015%
B0129	Group B8	Day 1	MPH	CD4	+	-	-	0.006%
B0129	Group B8	Day 1	MPH	CD4	-	+	+	0.04752%
B0129	Group B8	Day 1	MPH	CD4	-	+	-	0.218%
B0129	Group B8	Day 1	MPH	CD4	-	-	+	0.06596%
B0129	Group B8	Day 22	MPH	CD4	+	+	+	0.10545%
B0129	Group B8	Day 22	MPH	CD4	+	+	-	0.095%
B0129	Group B8	Day 22	MPH	CD4	+	-	+	0.02796%

Administration Site Key: FA = Forearm; UA= Upper arm
Treatments Part A: A1 = A/Sing MAP, 15 ug (FA); A2 = Afluria Quad IM (UA), 15 ug; A3 = Placebo MAP (FA); A4 = A/Sing Mono IM, 15 ug (UA)
Treatments Part B: B1 = A/Sing MAP, 15 ug (FA) with biopsy; B2 = Placebo MAP (FA) with biopsy
B3 = A/Sing MAP, 15 ug (FA); B4 = A/Sing MAP, 10 ug (FA); B5 = A/Sing MAP, 5 ug (FA); B6 = A/Sing MAP, 2.5 ug (FA)
B7 = Placebo MAP (FA); B8 = A/Sing MAP, 15 ug (UA); B9 = A/Sing IM, 15 ug, as 2018 Afluria Quad (UA)

Listing 16.2.6.6
Immunogenicity Results (CMI)

Part B

Randomisation Number	Group	Visit	Test	Subset	IL2	TNFa	IFNg	Value
B0129	Group B8	Day 22	MPH	CD4	+	-	-	0.03889%
B0129	Group B8	Day 22	MPH	CD4	-	+	+	0.05448%
B0129	Group B8	Day 22	MPH	CD4	-	+	-	0.14%
B0129	Group B8	Day 22	MPH	CD4	-	-	+	0.09317%
B0129	Group B8	Day 1	MPH	CD8	+	+	+	0.031%
B0129	Group B8	Day 1	MPH	CD8	+	+	-	0.01056%
B0129	Group B8	Day 1	MPH	CD8	+	-	+	0%
B0129	Group B8	Day 1	MPH	CD8	+	-	-	0.03652%
B0129	Group B8	Day 1	MPH	CD8	-	+	+	0.08108%
B0129	Group B8	Day 1	MPH	CD8	-	+	-	0.37%
B0129	Group B8	Day 1	MPH	CD8	-	-	+	-0.07%
B0129	Group B8	Day 22	MPH	CD8	+	+	+	0.019%
B0129	Group B8	Day 22	MPH	CD8	+	+	-	0.00865%
B0129	Group B8	Day 22	MPH	CD8	+	-	+	0.00144%
B0129	Group B8	Day 22	MPH	CD8	+	-	-	0.03358%
B0129	Group B8	Day 22	MPH	CD8	-	+	+	0.07475%
B0129	Group B8	Day 22	MPH	CD8	-	+	-	0.366%
B0129	Group B8	Day 22	MPH	CD8	-	-	+	0.038%

Administration Site Key: FA = Forearm; UA= Upper arm
Treatments Part A: A1 = A/Sing MAP, 15 ug (FA); A2 = Afluria Quad IM (UA), 15 ug; A3 = Placebo MAP (FA); A4 = A/Sing Mono IM, 15 ug (UA)
Treatments Part B: B1 = A/Sing MAP, 15 ug (FA) with biopsy; B2 = Placebo MAP (FA) with biopsy
B3 = A/Sing MAP, 15 ug (FA); B4 = A/Sing MAP, 10 ug (FA); B5 = A/Sing MAP, 5 ug (FA); B6 = A/Sing MAP, 2.5 ug (FA)
B7 = Placebo MAP (FA); B8 = A/Sing MAP, 15 ug (UA); B9 = A/Sing IM, 15 ug, as 2018 Afluria Quad (UA)

Listing 16.2.6.6
Immunogenicity Results (CMI)

Part B

Randomisation Number	Group	Visit	Test	Subset	IL2	TNFa	IFNg	Value
B0131	Group B8	Day 1	Peptide	CD4	+	+	+	0.00885%
B0131	Group B8	Day 1	Peptide	CD4	+	+	-	0.002%
B0131	Group B8	Day 1	Peptide	CD4	+	-	+	0%
B0131	Group B8	Day 1	Peptide	CD4	+	-	-	-0.00285%
B0131	Group B8	Day 1	Peptide	CD4	-	+	+	0.003034%
B0131	Group B8	Day 1	Peptide	CD4	-	+	-	0.014%
B0131	Group B8	Day 1	Peptide	CD4	-	-	+	-0.00364%
B0131	Group B8	Day 22	Peptide	CD4	+	+	+	0.01958%
B0131	Group B8	Day 22	Peptide	CD4	+	+	-	0.013%
B0131	Group B8	Day 22	Peptide	CD4	+	-	+	0.000903%
B0131	Group B8	Day 22	Peptide	CD4	+	-	-	0.00195%
B0131	Group B8	Day 22	Peptide	CD4	-	+	+	0.00451%
B0131	Group B8	Day 22	Peptide	CD4	-	+	-	0.013%
B0131	Group B8	Day 22	Peptide	CD4	-	-	+	-0.00084%
B0131	Group B8	Day 1	Peptide	CD8	+	+	+	0.00116%
B0131	Group B8	Day 1	Peptide	CD8	+	+	-	0.00116%
B0131	Group B8	Day 1	Peptide	CD8	+	-	+	0%
B0131	Group B8	Day 1	Peptide	CD8	+	-	-	-0.0011%
B0131	Group B8	Day 1	Peptide	CD8	-	+	+	-0.00445%

Administration Site Key: FA = Forearm; UA= Upper arm
Treatments Part A: A1 = A/Sing MAP, 15 ug (FA); A2 = Afluria Quad IM (UA), 15 ug; A3 = Placebo MAP (FA); A4 = A/Sing Mono IM, 15 ug (UA)
Treatments Part B: B1 = A/Sing MAP, 15 ug (FA) with biopsy; B2 = Placebo MAP (FA) with biopsy
B3 = A/Sing MAP, 15 ug (FA); B4 = A/Sing MAP, 10 ug (FA); B5 = A/Sing MAP, 5 ug (FA); B6 = A/Sing MAP, 2.5 ug (FA)
B7 = Placebo MAP (FA); B8 = A/Sing MAP, 15 ug (UA); B9 = A/Sing IM, 15 ug, as 2018 Afluria Quad (UA)

Listing 16.2.6.6
Immunogenicity Results (CMI)

Part B

Randomisation Number	Group	Visit	Test	Subset	IL2	TNFa	IFNg	Value
B0131	Group B8	Day 1	Peptide	CD8	-	+	-	-0.02%
B0131	Group B8	Day 1	Peptide	CD8	-	-	+	-0.31%
B0131	Group B8	Day 22	Peptide	CD8	+	+	+	-0.00125%
B0131	Group B8	Day 22	Peptide	CD8	+	+	-	-0.00125%
B0131	Group B8	Day 22	Peptide	CD8	+	-	+	0%
B0131	Group B8	Day 22	Peptide	CD8	+	-	-	-0.00372%
B0131	Group B8	Day 22	Peptide	CD8	-	+	+	0.00387%
B0131	Group B8	Day 22	Peptide	CD8	-	+	-	-0.026%
B0131	Group B8	Day 22	Peptide	CD8	-	-	+	-0.12%
B0131	Group B8	Day 1	MPH	CD4	+	+	+	0.09085%
B0131	Group B8	Day 1	MPH	CD4	+	+	-	0.071%
B0131	Group B8	Day 1	MPH	CD4	+	-	+	0.0097%
B0131	Group B8	Day 1	MPH	CD4	+	-	-	0.02238%
B0131	Group B8	Day 1	MPH	CD4	-	+	+	0.030264%
B0131	Group B8	Day 1	MPH	CD4	-	+	-	0.287%
B0131	Group B8	Day 1	MPH	CD4	-	-	+	0.02685%
B0131	Group B8	Day 22	MPH	CD4	+	+	+	0.08658%
B0131	Group B8	Day 22	MPH	CD4	+	+	-	0.081%
B0131	Group B8	Day 22	MPH	CD4	+	-	+	0.03%

Administration Site Key: FA = Forearm; UA= Upper arm
Treatments Part A: A1 = A/Sing MAP, 15 ug (FA); A2 = Afluria Quad IM (UA), 15 ug; A3 = Placebo MAP (FA); A4 = A/Sing Mono IM, 15 ug (UA)
Treatments Part B: B1 = A/Sing MAP, 15 ug (FA) with biopsy; B2 = Placebo MAP (FA) with biopsy
B3 = A/Sing MAP, 15 ug (FA); B4 = A/Sing MAP, 10 ug (FA); B5 = A/Sing MAP, 5 ug (FA); B6 = A/Sing MAP, 2.5 ug (FA)
B7 = Placebo MAP (FA); B8 = A/Sing MAP, 15 ug (UA); B9 = A/Sing IM, 15 ug, as 2018 Afluria Quad (UA)

Listing 16.2.6.6
Immunogenicity Results (CMI)

Part B

Randomisation Number	Group	Visit	Test	Subset	IL2	TNFa	IFNg	Value
B0131	Group B8	Day 22	MPH	CD4	+	-	-	0.04392%
B0131	Group B8	Day 22	MPH	CD4	-	+	+	0.041%
B0131	Group B8	Day 22	MPH	CD4	-	+	-	0.235%
B0131	Group B8	Day 22	MPH	CD4	-	-	+	0.06835%
B0131	Group B8	Day 1	MPH	CD8	+	+	+	0.00369%
B0131	Group B8	Day 1	MPH	CD8	+	+	-	0.00246%
B0131	Group B8	Day 1	MPH	CD8	+	-	+	0.00492%
B0131	Group B8	Day 1	MPH	CD8	+	-	-	0.00389%
B0131	Group B8	Day 1	MPH	CD8	-	+	+	0.01508%
B0131	Group B8	Day 1	MPH	CD8	-	+	-	0.12%
B0131	Group B8	Day 1	MPH	CD8	-	-	+	-0.86%
B0131	Group B8	Day 22	MPH	CD8	+	+	+	0.00403%
B0131	Group B8	Day 22	MPH	CD8	+	+	-	0.00403%
B0131	Group B8	Day 22	MPH	CD8	+	-	+	0.00528%
B0131	Group B8	Day 22	MPH	CD8	+	-	-	0.01501%
B0131	Group B8	Day 22	MPH	CD8	-	+	+	0.0315%
B0131	Group B8	Day 22	MPH	CD8	-	+	-	0.185%
B0131	Group B8	Day 22	MPH	CD8	-	-	+	-0.09%

Administration Site Key: FA = Forearm; UA= Upper arm
Treatments Part A: A1 = A/Sing MAP, 15 ug (FA); A2 = Afluria Quad IM (UA), 15 ug; A3 = Placebo MAP (FA); A4 = A/Sing Mono IM, 15 ug (UA)
Treatments Part B: B1 = A/Sing MAP, 15 ug (FA) with biopsy; B2 = Placebo MAP (FA) with biopsy
B3 = A/Sing MAP, 15 ug (FA); B4 = A/Sing MAP, 10 ug (FA); B5 = A/Sing MAP, 5 ug (FA); B6 = A/Sing MAP, 2.5 ug (FA)
B7 = Placebo MAP (FA); B8 = A/Sing MAP, 15 ug (UA); B9 = A/Sing IM, 15 ug, as 2018 Afluria Quad (UA)

Listing 16.2.6.6
Immunogenicity Results (CMI)

Part B

Randomisation Number	Group	Visit	Test	Subset	IL2	TNFa	IFNg	Value
B0134	Group B3	Day 1	Peptide	CD4	+	+	+	0.0042%
B0134	Group B3	Day 1	Peptide	CD4	+	+	-	0.01055%
B0134	Group B3	Day 1	Peptide	CD4	+	-	+	-7E-6%
B0134	Group B3	Day 1	Peptide	CD4	+	-	-	-0.00276%
B0134	Group B3	Day 1	Peptide	CD4	-	+	+	0.00052%
B0134	Group B3	Day 1	Peptide	CD4	-	+	-	-0.005%
B0134	Group B3	Day 1	Peptide	CD4	-	-	+	-0.00004%
B0134	Group B3	Day 22	Peptide	CD4	+	+	+	0.01418%
B0134	Group B3	Day 22	Peptide	CD4	+	+	-	0.0161%
B0134	Group B3	Day 22	Peptide	CD4	+	-	+	0.000664%
B0134	Group B3	Day 22	Peptide	CD4	+	-	-	0.00166%
B0134	Group B3	Day 22	Peptide	CD4	-	+	+	0.001382%
B0134	Group B3	Day 22	Peptide	CD4	-	+	-	0.013%
B0134	Group B3	Day 22	Peptide	CD4	-	-	+	0.00266%
B0134	Group B3	Day 1	Peptide	CD8	+	+	+	0.00113%
B0134	Group B3	Day 1	Peptide	CD8	+	+	-	0.00225%
B0134	Group B3	Day 1	Peptide	CD8	+	-	+	0%
B0134	Group B3	Day 1	Peptide	CD8	+	-	-	-0.0023%
B0134	Group B3	Day 1	Peptide	CD8	-	+	+	0.00225%

Administration Site Key: FA = Forearm; UA= Upper arm
Treatments Part A: A1 = A/Sing MAP, 15 ug (FA); A2 = Afluria Quad IM (UA), 15 ug; A3 = Placebo MAP (FA); A4 = A/Sing Mono IM, 15 ug (UA)
Treatments Part B: B1 = A/Sing MAP, 15 ug (FA) with biopsy; B2 = Placebo MAP (FA) with biopsy
B3 = A/Sing MAP, 15 ug (FA); B4 = A/Sing MAP, 10 ug (FA); B5 = A/Sing MAP, 5 ug (FA); B6 = A/Sing MAP, 2.5 ug (FA)
B7 = Placebo MAP (FA); B8 = A/Sing MAP, 15 ug (UA); B9 = A/Sing IM, 15 ug, as 2018 Afluria Quad (UA)

Listing 16.2.6.6
Immunogenicity Results (CMI)

Part B

Randomisation Number	Group	Visit	Test	Subset	IL2	TNFa	IFNg	Value
B0134	Group B3	Day 1	Peptide	CD8	-	+	-	0.012%
B0134	Group B3	Day 1	Peptide	CD8	-	-	+	0.017%
B0134	Group B3	Day 22	Peptide	CD8	+	+	+	0.00123%
B0134	Group B3	Day 22	Peptide	CD8	+	+	-	0%
B0134	Group B3	Day 22	Peptide	CD8	+	-	+	0%
B0134	Group B3	Day 22	Peptide	CD8	+	-	-	-0.00215%
B0134	Group B3	Day 22	Peptide	CD8	-	+	+	-0.00113%
B0134	Group B3	Day 22	Peptide	CD8	-	+	-	0.001%
B0134	Group B3	Day 22	Peptide	CD8	-	-	+	-0.15%
B0134	Group B3	Day 1	MPH	CD4	+	+	+	0.07885%
B0134	Group B3	Day 1	MPH	CD4	+	+	-	0.11355%
B0134	Group B3	Day 1	MPH	CD4	+	-	+	0.007683%
B0134	Group B3	Day 1	MPH	CD4	+	-	-	0.01694%
B0134	Group B3	Day 1	MPH	CD4	-	+	+	0.02293%
B0134	Group B3	Day 1	MPH	CD4	-	+	-	0.263%
B0134	Group B3	Day 1	MPH	CD4	-	-	+	0.06031%
B0134	Group B3	Day 22	MPH	CD4	+	+	+	0.11818%
B0134	Group B3	Day 22	MPH	CD4	+	+	-	0.1021%
B0134	Group B3	Day 22	MPH	CD4	+	-	+	0.023%

Administration Site Key: FA = Forearm; UA= Upper arm
Treatments Part A: A1 = A/Sing MAP, 15 ug (FA); A2 = Afluria Quad IM (UA), 15 ug; A3 = Placebo MAP (FA); A4 = A/Sing Mono IM, 15 ug (UA)
Treatments Part B: B1 = A/Sing MAP, 15 ug (FA) with biopsy; B2 = Placebo MAP (FA) with biopsy
B3 = A/Sing MAP, 15 ug (FA); B4 = A/Sing MAP, 10 ug (FA); B5 = A/Sing MAP, 5 ug (FA); B6 = A/Sing MAP, 2.5 ug (FA)
B7 = Placebo MAP (FA); B8 = A/Sing MAP, 15 ug (UA); B9 = A/Sing IM, 15 ug, as 2018 Afluria Quad (UA)

Listing 16.2.6.6
Immunogenicity Results (CMI)

Part B

Randomisation Number	Group	Visit	Test	Subset	IL2	TNFa	IFNg	Value
B0134	Group B3	Day 22	MPH	CD4	+	-	-	0.04335%
B0134	Group B3	Day 22	MPH	CD4	-	+	+	0.032392%
B0134	Group B3	Day 22	MPH	CD4	-	+	-	0.181%
B0134	Group B3	Day 22	MPH	CD4	-	-	+	0.05%
B0134	Group B3	Day 1	MPH	CD8	+	+	+	0.11%
B0134	Group B3	Day 1	MPH	CD8	+	+	-	0.02%
B0134	Group B3	Day 1	MPH	CD8	+	-	+	0.041%
B0134	Group B3	Day 1	MPH	CD8	+	-	-	0.0447%
B0134	Group B3	Day 1	MPH	CD8	-	+	+	0.16%
B0134	Group B3	Day 1	MPH	CD8	-	+	-	0.171%
B0134	Group B3	Day 1	MPH	CD8	-	-	+	0.127%
B0134	Group B3	Day 22	MPH	CD8	+	+	+	0.056%
B0134	Group B3	Day 22	MPH	CD8	+	+	-	0.017%
B0134	Group B3	Day 22	MPH	CD8	+	-	+	0.032%
B0134	Group B3	Day 22	MPH	CD8	+	-	-	0.05362%
B0134	Group B3	Day 22	MPH	CD8	-	+	+	0.12887%
B0134	Group B3	Day 22	MPH	CD8	-	+	-	0.134%
B0134	Group B3	Day 22	MPH	CD8	-	-	+	-0.1%

Administration Site Key: FA = Forearm; UA= Upper arm
Treatments Part A: A1 = A/Sing MAP, 15 ug (FA); A2 = Afluria Quad IM (UA), 15 ug; A3 = Placebo MAP (FA); A4 = A/Sing Mono IM, 15 ug (UA)
Treatments Part B: B1 = A/Sing MAP, 15 ug (FA) with biopsy; B2 = Placebo MAP (FA) with biopsy
B3 = A/Sing MAP, 15 ug (FA); B4 = A/Sing MAP, 10 ug (FA); B5 = A/Sing MAP, 5 ug (FA); B6 = A/Sing MAP, 2.5 ug (FA)
B7 = Placebo MAP (FA); B8 = A/Sing MAP, 15 ug (UA); B9 = A/Sing IM, 15 ug, as 2018 Afluria Quad (UA)

Listing 16.2.6.6
Immunogenicity Results (CMI)

Part B

Randomisation Number	Group	Visit	Test	Subset	IL2	TNFa	IFNg	Value
B0135	Group B7	Day 1	Peptide	CD4	+	+	+	0.00268%
B0135	Group B7	Day 1	Peptide	CD4	+	+	-	0.003%
B0135	Group B7	Day 1	Peptide	CD4	+	-	+	-0.001562%
B0135	Group B7	Day 1	Peptide	CD4	+	-	-	-0.00067%
B0135	Group B7	Day 1	Peptide	CD4	-	+	+	-0.00476%
B0135	Group B7	Day 1	Peptide	CD4	-	+	-	0.004%
B0135	Group B7	Day 1	Peptide	CD4	-	-	+	-0.01173%
B0135	Group B7	Day 22	Peptide	CD4	+	+	+	0.003%
B0135	Group B7	Day 22	Peptide	CD4	+	+	-	-0.009%
B0135	Group B7	Day 22	Peptide	CD4	+	-	+	0.00147%
B0135	Group B7	Day 22	Peptide	CD4	+	-	-	-0.00347%
B0135	Group B7	Day 22	Peptide	CD4	-	+	+	-0.00022%
B0135	Group B7	Day 22	Peptide	CD4	-	+	-	0.003%
B0135	Group B7	Day 22	Peptide	CD4	-	-	+	0.00047%
B0135	Group B7	Day 1	Peptide	CD8	+	+	+	0%
B0135	Group B7	Day 1	Peptide	CD8	+	+	-	0.00315%
B0135	Group B7	Day 1	Peptide	CD8	+	-	+	0%
B0135	Group B7	Day 1	Peptide	CD8	+	-	-	0.00003%
B0135	Group B7	Day 1	Peptide	CD8	-	+	+	0.00318%

Administration Site Key: FA = Forearm; UA= Upper arm
Treatments Part A: A1 = A/Sing MAP, 15 ug (FA); A2 = Afluria Quad IM (UA), 15 ug; A3 = Placebo MAP (FA); A4 = A/Sing Mono IM, 15 ug (UA)
Treatments Part B: B1 = A/Sing MAP, 15 ug (FA) with biopsy; B2 = Placebo MAP (FA) with biopsy
B3 = A/Sing MAP, 15 ug (FA); B4 = A/Sing MAP, 10 ug (FA); B5 = A/Sing MAP, 5 ug (FA); B6 = A/Sing MAP, 2.5 ug (FA)
B7 = Placebo MAP (FA); B8 = A/Sing MAP, 15 ug (UA); B9 = A/Sing IM, 15 ug, as 2018 Afluria Quad (UA)

Listing 16.2.6.6
Immunogenicity Results (CMI)

Part B

Randomisation Number	Group	Visit	Test	Subset	IL2	TNFa	IFNg	Value
B0135	Group B7	Day 1	Peptide	CD8	-	+	-	-0.004%
B0135	Group B7	Day 1	Peptide	CD8	-	-	+	0.053%
B0135	Group B7	Day 22	Peptide	CD8	+	+	+	0.00139%
B0135	Group B7	Day 22	Peptide	CD8	+	+	-	0.00278%
B0135	Group B7	Day 22	Peptide	CD8	+	-	+	0%
B0135	Group B7	Day 22	Peptide	CD8	+	-	-	-0.00302%
B0135	Group B7	Day 22	Peptide	CD8	-	+	+	0.00139%
B0135	Group B7	Day 22	Peptide	CD8	-	+	-	-0.011%
B0135	Group B7	Day 22	Peptide	CD8	-	-	+	0.019%
B0135	Group B7	Day 1	MPH	CD4	+	+	+	0.08486%
B0135	Group B7	Day 1	MPH	CD4	+	+	-	0.054%
B0135	Group B7	Day 1	MPH	CD4	+	-	+	0.01962%
B0135	Group B7	Day 1	MPH	CD4	+	-	-	0.04224%
B0135	Group B7	Day 1	MPH	CD4	-	+	+	0.04324%
B0135	Group B7	Day 1	MPH	CD4	-	+	-	0.138%
B0135	Group B7	Day 1	MPH	CD4	-	-	+	0.044%
B0135	Group B7	Day 22	MPH	CD4	+	+	+	0.109%
B0135	Group B7	Day 22	MPH	CD4	+	+	-	0.052%
B0135	Group B7	Day 22	MPH	CD4	+	-	+	0.026%

Administration Site Key: FA = Forearm; UA= Upper arm
Treatments Part A: A1 = A/Sing MAP, 15 ug (FA); A2 = Afluria Quad IM (UA), 15 ug; A3 = Placebo MAP (FA); A4 = A/Sing Mono IM, 15 ug (UA)
Treatments Part B: B1 = A/Sing MAP, 15 ug (FA) with biopsy; B2 = Placebo MAP (FA) with biopsy
B3 = A/Sing MAP, 15 ug (FA); B4 = A/Sing MAP, 10 ug (FA); B5 = A/Sing MAP, 5 ug (FA); B6 = A/Sing MAP, 2.5 ug (FA)
B7 = Placebo MAP (FA); B8 = A/Sing MAP, 15 ug (UA); B9 = A/Sing IM, 15 ug, as 2018 Afluria Quad (UA)

Listing 16.2.6.6
Immunogenicity Results (CMI)

Part B

Randomisation Number	Group	Visit	Test	Subset	IL2	TNFa	IFNg	Value
B0135	Group B7	Day 22	MPH	CD4	+	-	-	0.02%
B0135	Group B7	Day 22	MPH	CD4	-	+	+	0.04712%
B0135	Group B7	Day 22	MPH	CD4	-	+	-	0.173%
B0135	Group B7	Day 22	MPH	CD4	-	-	+	0.03934%
B0135	Group B7	Day 1	MPH	CD8	+	+	+	0.00763%
B0135	Group B7	Day 1	MPH	CD8	+	+	-	0.00458%
B0135	Group B7	Day 1	MPH	CD8	+	-	+	0%
B0135	Group B7	Day 1	MPH	CD8	+	-	-	0.01246%
B0135	Group B7	Day 1	MPH	CD8	-	+	+	0.01346%
B0135	Group B7	Day 1	MPH	CD8	-	+	-	0.033%
B0135	Group B7	Day 1	MPH	CD8	-	-	+	-0.008%
B0135	Group B7	Day 22	MPH	CD8	+	+	+	0.00715%
B0135	Group B7	Day 22	MPH	CD8	+	+	-	0.00572%
B0135	Group B7	Day 22	MPH	CD8	+	-	+	0.00143%
B0135	Group B7	Day 22	MPH	CD8	+	-	-	0.00859%
B0135	Group B7	Day 22	MPH	CD8	-	+	+	0.016%
B0135	Group B7	Day 22	MPH	CD8	-	+	-	0.03%
B0135	Group B7	Day 22	MPH	CD8	-	-	+	0.034%

Administration Site Key: FA = Forearm; UA= Upper arm
Treatments Part A: A1 = A/Sing MAP, 15 ug (FA); A2 = Afluria Quad IM (UA), 15 ug; A3 = Placebo MAP (FA); A4 = A/Sing Mono IM, 15 ug (UA)
Treatments Part B: B1 = A/Sing MAP, 15 ug (FA) with biopsy; B2 = Placebo MAP (FA) with biopsy
B3 = A/Sing MAP, 15 ug (FA); B4 = A/Sing MAP, 10 ug (FA); B5 = A/Sing MAP, 5 ug (FA); B6 = A/Sing MAP, 2.5 ug (FA)
B7 = Placebo MAP (FA); B8 = A/Sing MAP, 15 ug (UA); B9 = A/Sing IM, 15 ug, as 2018 Afluria Quad (UA)

Listing 16.2.6.6
Immunogenicity Results (CMI)

Part B

Randomisation Number	Group	Visit	Test	Subset	IL2	TNFa	IFNg	Value
B0136	Group B9	Day 1	Peptide	CD4	+	+	+	0.03088%
B0136	Group B9	Day 1	Peptide	CD4	+	+	-	0.021%
B0136	Group B9	Day 1	Peptide	CD4	+	-	+	-0.00082%
B0136	Group B9	Day 1	Peptide	CD4	+	-	-	-0.00599%
B0136	Group B9	Day 1	Peptide	CD4	-	+	+	0.00503%
B0136	Group B9	Day 1	Peptide	CD4	-	+	-	0.056%
B0136	Group B9	Day 1	Peptide	CD4	-	-	+	0.00502%
B0136	Group B9	Day 22	Peptide	CD4	+	+	+	0.031%
B0136	Group B9	Day 22	Peptide	CD4	+	+	-	-0.001%
B0136	Group B9	Day 22	Peptide	CD4	+	-	+	0.00339%
B0136	Group B9	Day 22	Peptide	CD4	+	-	-	-0.00253%
B0136	Group B9	Day 22	Peptide	CD4	-	+	+	0.00334%
B0136	Group B9	Day 22	Peptide	CD4	-	+	-	-0.036%
B0136	Group B9	Day 22	Peptide	CD4	-	-	+	0.0004%
B0136	Group B9	Day 1	Peptide	CD8	+	+	+	0%
B0136	Group B9	Day 1	Peptide	CD8	+	+	-	0%
B0136	Group B9	Day 1	Peptide	CD8	+	-	+	0%
B0136	Group B9	Day 1	Peptide	CD8	+	-	-	0%
B0136	Group B9	Day 1	Peptide	CD8	-	+	+	0.00387%

Administration Site Key: FA = Forearm; UA= Upper arm
Treatments Part A: A1 = A/Sing MAP, 15 ug (FA); A2 = Afluria Quad IM (UA), 15 ug; A3 = Placebo MAP (FA); A4 = A/Sing Mono IM, 15 ug (UA)
Treatments Part B: B1 = A/Sing MAP, 15 ug (FA) with biopsy; B2 = Placebo MAP (FA) with biopsy
B3 = A/Sing MAP, 15 ug (FA); B4 = A/Sing MAP, 10 ug (FA); B5 = A/Sing MAP, 5 ug (FA); B6 = A/Sing MAP, 2.5 ug (FA)
B7 = Placebo MAP (FA); B8 = A/Sing MAP, 15 ug (UA); B9 = A/Sing IM, 15 ug, as 2018 Afluria Quad (UA)

Listing 16.2.6.6
Immunogenicity Results (CMI)

Part B

Randomisation Number	Group	Visit	Test	Subset	IL2	TNFa	IFNg	Value
B0136	Group B9	Day 1	Peptide	CD8	-	+	-	0.006%
B0136	Group B9	Day 1	Peptide	CD8	-	-	+	0.006%
B0136	Group B9	Day 22	Peptide	CD8	+	+	+	0%
B0136	Group B9	Day 22	Peptide	CD8	+	+	-	-0.00181%
B0136	Group B9	Day 22	Peptide	CD8	+	-	+	0%
B0136	Group B9	Day 22	Peptide	CD8	+	-	-	0.00216%
B0136	Group B9	Day 22	Peptide	CD8	-	+	+	-0.047%
B0136	Group B9	Day 22	Peptide	CD8	-	+	-	-0.021%
B0136	Group B9	Day 22	Peptide	CD8	-	-	+	-0.2%
B0136	Group B9	Day 1	MPH	CD4	+	+	+	0.13088%
B0136	Group B9	Day 1	MPH	CD4	+	+	-	0.068%
B0136	Group B9	Day 1	MPH	CD4	+	-	+	0.00968%
B0136	Group B9	Day 1	MPH	CD4	+	-	-	0.012%
B0136	Group B9	Day 1	MPH	CD4	-	+	+	0.04768%
B0136	Group B9	Day 1	MPH	CD4	-	+	-	0.296%
B0136	Group B9	Day 1	MPH	CD4	-	-	+	0.04951%
B0136	Group B9	Day 22	MPH	CD4	+	+	+	0.159%
B0136	Group B9	Day 22	MPH	CD4	+	+	-	0.084%
B0136	Group B9	Day 22	MPH	CD4	+	-	+	0.042%

Administration Site Key: FA = Forearm; UA= Upper arm
Treatments Part A: A1 = A/Sing MAP, 15 ug (FA); A2 = Afluria Quad IM (UA), 15 ug; A3 = Placebo MAP (FA); A4 = A/Sing Mono IM, 15 ug (UA)
Treatments Part B: B1 = A/Sing MAP, 15 ug (FA) with biopsy; B2 = Placebo MAP (FA) with biopsy
B3 = A/Sing MAP, 15 ug (FA); B4 = A/Sing MAP, 10 ug (FA); B5 = A/Sing MAP, 5 ug (FA); B6 = A/Sing MAP, 2.5 ug (FA)
B7 = Placebo MAP (FA); B8 = A/Sing MAP, 15 ug (UA); B9 = A/Sing IM, 15 ug, as 2018 Afluria Quad (UA)

Listing 16.2.6.6
Immunogenicity Results (CMI)

Part B

Randomisation Number	Group	Visit	Test	Subset	IL2	TNFa	IFNg	Value
B0136	Group B9	Day 22	MPH	CD4	+	-	-	0.02%
B0136	Group B9	Day 22	MPH	CD4	-	+	+	0.08772%
B0136	Group B9	Day 22	MPH	CD4	-	+	-	0.176%
B0136	Group B9	Day 22	MPH	CD4	-	-	+	0.08186%
B0136	Group B9	Day 1	MPH	CD8	+	+	+	0.0041%
B0136	Group B9	Day 1	MPH	CD8	+	+	-	0.00615%
B0136	Group B9	Day 1	MPH	CD8	+	-	+	0.0041%
B0136	Group B9	Day 1	MPH	CD8	+	-	-	0.0041%
B0136	Group B9	Day 1	MPH	CD8	-	+	+	0.014%
B0136	Group B9	Day 1	MPH	CD8	-	+	-	0.086%
B0136	Group B9	Day 1	MPH	CD8	-	-	+	0.019%
B0136	Group B9	Day 22	MPH	CD8	+	+	+	0.00844%
B0136	Group B9	Day 22	MPH	CD8	+	+	-	0.00241%
B0136	Group B9	Day 22	MPH	CD8	+	-	+	0.00211%
B0136	Group B9	Day 22	MPH	CD8	+	-	-	0.017%
B0136	Group B9	Day 22	MPH	CD8	-	+	+	-0.015%
B0136	Group B9	Day 22	MPH	CD8	-	+	-	0.06%
B0136	Group B9	Day 22	MPH	CD8	-	-	+	-0.13%

Administration Site Key: FA = Forearm; UA= Upper arm
Treatments Part A: A1 = A/Sing MAP, 15 ug (FA); A2 = Afluria Quad IM (UA), 15 ug; A3 = Placebo MAP (FA); A4 = A/Sing Mono IM, 15 ug (UA)
Treatments Part B: B1 = A/Sing MAP, 15 ug (FA) with biopsy; B2 = Placebo MAP (FA) with biopsy
B3 = A/Sing MAP, 15 ug (FA); B4 = A/Sing MAP, 10 ug (FA); B5 = A/Sing MAP, 5 ug (FA); B6 = A/Sing MAP, 2.5 ug (FA)
B7 = Placebo MAP (FA); B8 = A/Sing MAP, 15 ug (UA); B9 = A/Sing IM, 15 ug, as 2018 Afluria Quad (UA)

Listing 16.2.6.6
Immunogenicity Results (CMI)

Part B

Randomisation Number	Group	Visit	Test	Subset	IL2	TNFa	IFNg	Value
B0137	Group B8	Day 1	Peptide	CD4	+	+	+	0.002903%
B0137	Group B8	Day 1	Peptide	CD4	+	+	-	-0.00064%
B0137	Group B8	Day 1	Peptide	CD4	+	-	+	-0.00123%
B0137	Group B8	Day 1	Peptide	CD4	+	-	-	-0.0008%
B0137	Group B8	Day 1	Peptide	CD4	-	+	+	0.00018%
B0137	Group B8	Day 1	Peptide	CD4	-	+	-	0.002%
B0137	Group B8	Day 1	Peptide	CD4	-	-	+	0.00183%
B0137	Group B8	Day 22	Peptide	CD4	+	+	+	0.008273%
B0137	Group B8	Day 22	Peptide	CD4	+	+	-	0.00258%
B0137	Group B8	Day 22	Peptide	CD4	+	-	+	-0.000787%
B0137	Group B8	Day 22	Peptide	CD4	+	-	-	-0.00065%
B0137	Group B8	Day 22	Peptide	CD4	-	+	+	0.004153%
B0137	Group B8	Day 22	Peptide	CD4	-	+	-	-0.007%
B0137	Group B8	Day 22	Peptide	CD4	-	-	+	0.00011%
B0137	Group B8	Day 1	Peptide	CD8	+	+	+	0.00224%
B0137	Group B8	Day 1	Peptide	CD8	+	+	-	-0.00198%
B0137	Group B8	Day 1	Peptide	CD8	+	-	+	0%
B0137	Group B8	Day 1	Peptide	CD8	+	-	-	0.00448%
B0137	Group B8	Day 1	Peptide	CD8	-	+	+	-0.00504%

Administration Site Key: FA = Forearm; UA= Upper arm
Treatments Part A: A1 = A/Sing MAP, 15 ug (FA); A2 = Afluria Quad IM (UA), 15 ug; A3 = Placebo MAP (FA); A4 = A/Sing Mono IM, 15 ug (UA)
Treatments Part B: B1 = A/Sing MAP, 15 ug (FA) with biopsy; B2 = Placebo MAP (FA) with biopsy
B3 = A/Sing MAP, 15 ug (FA); B4 = A/Sing MAP, 10 ug (FA); B5 = A/Sing MAP, 5 ug (FA); B6 = A/Sing MAP, 2.5 ug (FA)
B7 = Placebo MAP (FA); B8 = A/Sing MAP, 15 ug (UA); B9 = A/Sing IM, 15 ug, as 2018 Afluria Quad (UA)

Listing 16.2.6.6
Immunogenicity Results (CMI)

Part B

Randomisation Number	Group	Visit	Test	Subset	IL2	TNFa	IFNg	Value
B0137	Group B8	Day 1	Peptide	CD8	-	+	-	-0.027%
B0137	Group B8	Day 1	Peptide	CD8	-	-	+	-0.125%
B0137	Group B8	Day 22	Peptide	CD8	+	+	+	0%
B0137	Group B8	Day 22	Peptide	CD8	+	+	-	0.0052%
B0137	Group B8	Day 22	Peptide	CD8	+	-	+	0%
B0137	Group B8	Day 22	Peptide	CD8	+	-	-	0.00013%
B0137	Group B8	Day 22	Peptide	CD8	-	+	+	-0.00741%
B0137	Group B8	Day 22	Peptide	CD8	-	+	-	-0.032%
B0137	Group B8	Day 22	Peptide	CD8	-	-	+	-0.027%
B0137	Group B8	Day 1	MPH	CD4	+	+	+	0.066383%
B0137	Group B8	Day 1	MPH	CD4	+	+	-	0.07744%
B0137	Group B8	Day 1	MPH	CD4	+	-	+	0.01177%
B0137	Group B8	Day 1	MPH	CD4	+	-	-	0.03168%
B0137	Group B8	Day 1	MPH	CD4	-	+	+	0.02877%
B0137	Group B8	Day 1	MPH	CD4	-	+	-	0.205%
B0137	Group B8	Day 1	MPH	CD4	-	-	+	0.04191%
B0137	Group B8	Day 22	MPH	CD4	+	+	+	0.074213%
B0137	Group B8	Day 22	MPH	CD4	+	+	-	0.10764%
B0137	Group B8	Day 22	MPH	CD4	+	-	+	0.014213%

Administration Site Key: FA = Forearm; UA= Upper arm
Treatments Part A: A1 = A/Sing MAP, 15 ug (FA); A2 = Afluria Quad IM (UA), 15 ug; A3 = Placebo MAP (FA); A4 = A/Sing Mono IM, 15 ug (UA)
Treatments Part B: B1 = A/Sing MAP, 15 ug (FA) with biopsy; B2 = Placebo MAP (FA) with biopsy
B3 = A/Sing MAP, 15 ug (FA); B4 = A/Sing MAP, 10 ug (FA); B5 = A/Sing MAP, 5 ug (FA); B6 = A/Sing MAP, 2.5 ug (FA)
B7 = Placebo MAP (FA); B8 = A/Sing MAP, 15 ug (UA); B9 = A/Sing IM, 15 ug, as 2018 Afluria Quad (UA)

Listing 16.2.6.6
Immunogenicity Results (CMI)

Part B

Randomisation Number	Group	Visit	Test	Subset	IL2	TNFa	IFNg	Value
B0137	Group B8	Day 22	MPH	CD4	+	-	-	0.04006%
B0137	Group B8	Day 22	MPH	CD4	-	+	+	0.030213%
B0137	Group B8	Day 22	MPH	CD4	-	+	-	0.216%
B0137	Group B8	Day 22	MPH	CD4	-	-	+	0.03864%
B0137	Group B8	Day 1	MPH	CD8	+	+	+	0.00978%
B0137	Group B8	Day 1	MPH	CD8	+	+	-	0.01202%
B0137	Group B8	Day 1	MPH	CD8	+	-	+	0.00196%
B0137	Group B8	Day 1	MPH	CD8	+	-	-	0.029%
B0137	Group B8	Day 1	MPH	CD8	-	+	+	0.008%
B0137	Group B8	Day 1	MPH	CD8	-	+	-	0.085%
B0137	Group B8	Day 1	MPH	CD8	-	-	+	-0.09%
B0137	Group B8	Day 22	MPH	CD8	+	+	+	0.015%
B0137	Group B8	Day 22	MPH	CD8	+	+	-	0.00633%
B0137	Group B8	Day 22	MPH	CD8	+	-	+	0.011%
B0137	Group B8	Day 22	MPH	CD8	+	-	-	0.03753%
B0137	Group B8	Day 22	MPH	CD8	-	+	+	0.01959%
B0137	Group B8	Day 22	MPH	CD8	-	+	-	0.059%
B0137	Group B8	Day 22	MPH	CD8	-	-	+	-0.013%

Administration Site Key: FA = Forearm; UA= Upper arm
Treatments Part A: A1 = A/Sing MAP, 15 ug (FA); A2 = Afluria Quad IM (UA), 15 ug; A3 = Placebo MAP (FA); A4 = A/Sing Mono IM, 15 ug (UA)
Treatments Part B: B1 = A/Sing MAP, 15 ug (FA) with biopsy; B2 = Placebo MAP (FA) with biopsy
B3 = A/Sing MAP, 15 ug (FA); B4 = A/Sing MAP, 10 ug (FA); B5 = A/Sing MAP, 5 ug (FA); B6 = A/Sing MAP, 2.5 ug (FA)
B7 = Placebo MAP (FA); B8 = A/Sing MAP, 15 ug (UA); B9 = A/Sing IM, 15 ug, as 2018 Afluria Quad (UA)

Listing 16.2.6.6
Immunogenicity Results (CMI)

Part B

Randomisation Number	Group	Visit	Test	Subset	IL2	TNFa	IFNg	Value
B0138	Group B7	Day 1	Peptide	CD4	+	+	+	-0.001584%
B0138	Group B7	Day 1	Peptide	CD4	+	+	-	0.00679%
B0138	Group B7	Day 1	Peptide	CD4	+	-	+	-0.000524%
B0138	Group B7	Day 1	Peptide	CD4	+	-	-	0.00053%
B0138	Group B7	Day 1	Peptide	CD4	-	+	+	0.000524%
B0138	Group B7	Day 1	Peptide	CD4	-	+	-	0.009%
B0138	Group B7	Day 1	Peptide	CD4	-	-	+	0.0021%
B0138	Group B7	Day 22	Peptide	CD4	+	+	+	-0.00005%
B0138	Group B7	Day 22	Peptide	CD4	+	+	-	0.00192%
B0138	Group B7	Day 22	Peptide	CD4	+	-	+	-0.000016%
B0138	Group B7	Day 22	Peptide	CD4	+	-	-	0.00142%
B0138	Group B7	Day 22	Peptide	CD4	-	+	+	0.000509%
B0138	Group B7	Day 22	Peptide	CD4	-	+	-	-0.01%
B0138	Group B7	Day 22	Peptide	CD4	-	-	+	-0.00384%
B0138	Group B7	Day 1	Peptide	CD8	+	+	+	0%
B0138	Group B7	Day 1	Peptide	CD8	+	+	-	0.00183%
B0138	Group B7	Day 1	Peptide	CD8	+	-	+	0%
B0138	Group B7	Day 1	Peptide	CD8	+	-	-	0.00001%
B0138	Group B7	Day 1	Peptide	CD8	-	+	+	-0.00364%

Administration Site Key: FA = Forearm; UA= Upper arm
Treatments Part A: A1 = A/Sing MAP, 15 ug (FA); A2 = Afluria Quad IM (UA), 15 ug; A3 = Placebo MAP (FA); A4 = A/Sing Mono IM, 15 ug (UA)
Treatments Part B: B1 = A/Sing MAP, 15 ug (FA) with biopsy; B2 = Placebo MAP (FA) with biopsy
B3 = A/Sing MAP, 15 ug (FA); B4 = A/Sing MAP, 10 ug (FA); B5 = A/Sing MAP, 5 ug (FA); B6 = A/Sing MAP, 2.5 ug (FA)
B7 = Placebo MAP (FA); B8 = A/Sing MAP, 15 ug (UA); B9 = A/Sing IM, 15 ug, as 2018 Afluria Quad (UA)

Listing 16.2.6.6
Immunogenicity Results (CMI)

Part B

Randomisation Number	Group	Visit	Test	Subset	IL2	TNFa	IFNg	Value
B0138	Group B7	Day 1	Peptide	CD8	-	+	-	0.011%
B0138	Group B7	Day 1	Peptide	CD8	-	-	+	-0.016%
B0138	Group B7	Day 22	Peptide	CD8	+	+	+	0%
B0138	Group B7	Day 22	Peptide	CD8	+	+	-	0%
B0138	Group B7	Day 22	Peptide	CD8	+	-	+	0%
B0138	Group B7	Day 22	Peptide	CD8	+	-	-	0%
B0138	Group B7	Day 22	Peptide	CD8	-	+	+	0.00184%
B0138	Group B7	Day 22	Peptide	CD8	-	+	-	0.01%
B0138	Group B7	Day 22	Peptide	CD8	-	-	+	-0.011%
B0138	Group B7	Day 1	MPH	CD4	+	+	+	0.02489%
B0138	Group B7	Day 1	MPH	CD4	+	+	-	0.07279%
B0138	Group B7	Day 1	MPH	CD4	+	-	+	0.00209%
B0138	Group B7	Day 1	MPH	CD4	+	-	-	0.01774%
B0138	Group B7	Day 1	MPH	CD4	-	+	+	0.016474%
B0138	Group B7	Day 1	MPH	CD4	-	+	-	0.177%
B0138	Group B7	Day 1	MPH	CD4	-	-	+	0.01284%
B0138	Group B7	Day 22	MPH	CD4	+	+	+	0.02038%
B0138	Group B7	Day 22	MPH	CD4	+	+	-	0.05805%
B0138	Group B7	Day 22	MPH	CD4	+	-	+	0.003129%

Administration Site Key: FA = Forearm; UA= Upper arm
Treatments Part A: A1 = A/Sing MAP, 15 ug (FA); A2 = Afluria Quad IM (UA), 15 ug; A3 = Placebo MAP (FA); A4 = A/Sing Mono IM, 15 ug (UA)
Treatments Part B: B1 = A/Sing MAP, 15 ug (FA) with biopsy; B2 = Placebo MAP (FA) with biopsy
B3 = A/Sing MAP, 15 ug (FA); B4 = A/Sing MAP, 10 ug (FA); B5 = A/Sing MAP, 5 ug (FA); B6 = A/Sing MAP, 2.5 ug (FA)
B7 = Placebo MAP (FA); B8 = A/Sing MAP, 15 ug (UA); B9 = A/Sing IM, 15 ug, as 2018 Afluria Quad (UA)

Listing 16.2.6.6
Immunogenicity Results (CMI)

Part B

Randomisation Number	Group	Visit	Test	Subset	IL2	TNFa	IFNg	Value
B0138	Group B7	Day 22	MPH	CD4	+	-	-	0.03213%
B0138	Group B7	Day 22	MPH	CD4	-	+	+	0.008889%
B0138	Group B7	Day 22	MPH	CD4	-	+	-	0.131%
B0138	Group B7	Day 22	MPH	CD4	-	-	+	0.01559%
B0138	Group B7	Day 1	MPH	CD8	+	+	+	0.011%
B0138	Group B7	Day 1	MPH	CD8	+	+	-	0.00177%
B0138	Group B7	Day 1	MPH	CD8	+	-	+	0.00353%
B0138	Group B7	Day 1	MPH	CD8	+	-	-	0.00701%
B0138	Group B7	Day 1	MPH	CD8	-	+	+	0.03036%
B0138	Group B7	Day 1	MPH	CD8	-	+	-	0.109%
B0138	Group B7	Day 1	MPH	CD8	-	-	+	0.01%
B0138	Group B7	Day 22	MPH	CD8	+	+	+	0.00385%
B0138	Group B7	Day 22	MPH	CD8	+	+	-	0.00385%
B0138	Group B7	Day 22	MPH	CD8	+	-	+	0.00192%
B0138	Group B7	Day 22	MPH	CD8	+	-	-	0.00577%
B0138	Group B7	Day 22	MPH	CD8	-	+	+	0.01492%
B0138	Group B7	Day 22	MPH	CD8	-	+	-	0.119%
B0138	Group B7	Day 22	MPH	CD8	-	-	+	0.019%

Administration Site Key: FA = Forearm; UA= Upper arm
Treatments Part A: A1 = A/Sing MAP, 15 ug (FA); A2 = Afluria Quad IM (UA), 15 ug; A3 = Placebo MAP (FA); A4 = A/Sing Mono IM, 15 ug (UA)
Treatments Part B: B1 = A/Sing MAP, 15 ug (FA) with biopsy; B2 = Placebo MAP (FA) with biopsy
B3 = A/Sing MAP, 15 ug (FA); B4 = A/Sing MAP, 10 ug (FA); B5 = A/Sing MAP, 5 ug (FA); B6 = A/Sing MAP, 2.5 ug (FA)
B7 = Placebo MAP (FA); B8 = A/Sing MAP, 15 ug (UA); B9 = A/Sing IM, 15 ug, as 2018 Afluria Quad (UA)

Listing 16.2.6.6
Immunogenicity Results (CMI)

Part B

Randomisation Number	Group	Visit	Test	Subset	IL2	TNFa	IFNg	Value
B0140	Group B9	Day 1	Peptide	CD4	+	+	+	0.00068%
B0140	Group B9	Day 1	Peptide	CD4	+	+	-	0.00575%
B0140	Group B9	Day 1	Peptide	CD4	+	-	+	0.000053%
B0140	Group B9	Day 1	Peptide	CD4	+	-	-	0.00419%
B0140	Group B9	Day 1	Peptide	CD4	-	+	+	0.00136%
B0140	Group B9	Day 1	Peptide	CD4	-	+	-	-0.01%
B0140	Group B9	Day 1	Peptide	CD4	-	-	+	-0.00104%
B0140	Group B9	Day 22	Peptide	CD4	+	+	+	0%
B0140	Group B9	Day 22	Peptide	CD4	+	+	-	0.00788%
B0140	Group B9	Day 22	Peptide	CD4	+	-	+	0.00108%
B0140	Group B9	Day 22	Peptide	CD4	+	-	-	0.00268%
B0140	Group B9	Day 22	Peptide	CD4	-	+	+	0.00054%
B0140	Group B9	Day 22	Peptide	CD4	-	+	-	0.012%
B0140	Group B9	Day 22	Peptide	CD4	-	-	+	-0.00164%
B0140	Group B9	Day 1	Peptide	CD8	+	+	+	0%
B0140	Group B9	Day 1	Peptide	CD8	+	+	-	0%
B0140	Group B9	Day 1	Peptide	CD8	+	-	+	0%
B0140	Group B9	Day 1	Peptide	CD8	+	-	-	-0.00324%
B0140	Group B9	Day 1	Peptide	CD8	-	+	+	-0.00337%

Administration Site Key: FA = Forearm; UA= Upper arm
Treatments Part A: A1 = A/Sing MAP, 15 ug (FA); A2 = Afluria Quad IM (UA), 15 ug; A3 = Placebo MAP (FA); A4 = A/Sing Mono IM, 15 ug (UA)
Treatments Part B: B1 = A/Sing MAP, 15 ug (FA) with biopsy; B2 = Placebo MAP (FA) with biopsy
B3 = A/Sing MAP, 15 ug (FA); B4 = A/Sing MAP, 10 ug (FA); B5 = A/Sing MAP, 5 ug (FA); B6 = A/Sing MAP, 2.5 ug (FA)
B7 = Placebo MAP (FA); B8 = A/Sing MAP, 15 ug (UA); B9 = A/Sing IM, 15 ug, as 2018 Afluria Quad (UA)

Listing 16.2.6.6
Immunogenicity Results (CMI)

Part B

Randomisation Number	Group	Visit	Test	Subset	IL2	TNFa	IFNg	Value
B0140	Group B9	Day 1	Peptide	CD8	-	+	-	-0.003%
B0140	Group B9	Day 1	Peptide	CD8	-	-	+	-0.69%
B0140	Group B9	Day 22	Peptide	CD8	+	+	+	0%
B0140	Group B9	Day 22	Peptide	CD8	+	+	-	0.00161%
B0140	Group B9	Day 22	Peptide	CD8	+	-	+	0%
B0140	Group B9	Day 22	Peptide	CD8	+	-	-	-0.00002%
B0140	Group B9	Day 22	Peptide	CD8	-	+	+	0.00161%
B0140	Group B9	Day 22	Peptide	CD8	-	+	-	0.006%
B0140	Group B9	Day 22	Peptide	CD8	-	-	+	0.036%
B0140	Group B9	Day 1	MPH	CD4	+	+	+	0.087%
B0140	Group B9	Day 1	MPH	CD4	+	+	-	0.09324%
B0140	Group B9	Day 1	MPH	CD4	+	-	+	0.018373%
B0140	Group B9	Day 1	MPH	CD4	+	-	-	0.02375%
B0140	Group B9	Day 1	MPH	CD4	-	+	+	0.038%
B0140	Group B9	Day 1	MPH	CD4	-	+	-	0.202%
B0140	Group B9	Day 1	MPH	CD4	-	-	+	0.06324%
B0140	Group B9	Day 22	MPH	CD4	+	+	+	0.08692%
B0140	Group B9	Day 22	MPH	CD4	+	+	-	0.09188%
B0140	Group B9	Day 22	MPH	CD4	+	-	+	0.02%

Administration Site Key: FA = Forearm; UA= Upper arm
Treatments Part A: A1 = A/Sing MAP, 15 ug (FA); A2 = Afluria Quad IM (UA), 15 ug; A3 = Placebo MAP (FA); A4 = A/Sing Mono IM, 15 ug (UA)
Treatments Part B: B1 = A/Sing MAP, 15 ug (FA) with biopsy; B2 = Placebo MAP (FA) with biopsy
B3 = A/Sing MAP, 15 ug (FA); B4 = A/Sing MAP, 10 ug (FA); B5 = A/Sing MAP, 5 ug (FA); B6 = A/Sing MAP, 2.5 ug (FA)
B7 = Placebo MAP (FA); B8 = A/Sing MAP, 15 ug (UA); B9 = A/Sing IM, 15 ug, as 2018 Afluria Quad (UA)

Listing 16.2.6.6
Immunogenicity Results (CMI)

Part B

Randomisation Number	Group	Visit	Test	Subset	IL2	TNFa	IFNg	Value
B0140	Group B9	Day 22	MPH	CD4	+	-	-	0.02829%
B0140	Group B9	Day 22	MPH	CD4	-	+	+	0.02892%
B0140	Group B9	Day 22	MPH	CD4	-	+	-	0.194%
B0140	Group B9	Day 22	MPH	CD4	-	-	+	0.04213%
B0140	Group B9	Day 1	MPH	CD8	+	+	+	0.016%
B0140	Group B9	Day 1	MPH	CD8	+	+	-	0.00658%
B0140	Group B9	Day 1	MPH	CD8	+	-	+	0.00494%
B0140	Group B9	Day 1	MPH	CD8	+	-	-	0.02495%
B0140	Group B9	Day 1	MPH	CD8	-	+	+	0.03963%
B0140	Group B9	Day 1	MPH	CD8	-	+	-	0.235%
B0140	Group B9	Day 1	MPH	CD8	-	-	+	-0.829%
B0140	Group B9	Day 22	MPH	CD8	+	+	+	0.015%
B0140	Group B9	Day 22	MPH	CD8	+	+	-	0.00586%
B0140	Group B9	Day 22	MPH	CD8	+	-	+	0.013%
B0140	Group B9	Day 22	MPH	CD8	+	-	-	0.01777%
B0140	Group B9	Day 22	MPH	CD8	-	+	+	0.041%
B0140	Group B9	Day 22	MPH	CD8	-	+	-	0.143%
B0140	Group B9	Day 22	MPH	CD8	-	-	+	0.026%

Administration Site Key: FA = Forearm; UA= Upper arm
Treatments Part A: A1 = A/Sing MAP, 15 ug (FA); A2 = Afluria Quad IM (UA), 15 ug; A3 = Placebo MAP (FA); A4 = A/Sing Mono IM, 15 ug (UA)
Treatments Part B: B1 = A/Sing MAP, 15 ug (FA) with biopsy; B2 = Placebo MAP (FA) with biopsy
B3 = A/Sing MAP, 15 ug (FA); B4 = A/Sing MAP, 10 ug (FA); B5 = A/Sing MAP, 5 ug (FA); B6 = A/Sing MAP, 2.5 ug (FA)
B7 = Placebo MAP (FA); B8 = A/Sing MAP, 15 ug (UA); B9 = A/Sing IM, 15 ug, as 2018 Afluria Quad (UA)

Listing 16.2.6.6
Immunogenicity Results (CMI)

Part B

Randomisation Number	Group	Visit	Test	Subset	IL2	TNFa	IFNg	Value
B0141	Group B3	Day 1	Peptide	CD4	+	+	+	0.00651%
B0141	Group B3	Day 1	Peptide	CD4	+	+	-	0.008%
B0141	Group B3	Day 1	Peptide	CD4	+	-	+	-0.000534%
B0141	Group B3	Day 1	Peptide	CD4	+	-	-	-0.00101%
B0141	Group B3	Day 1	Peptide	CD4	-	+	+	0.00311%
B0141	Group B3	Day 1	Peptide	CD4	-	+	-	0.012%
B0141	Group B3	Day 1	Peptide	CD4	-	-	+	-0.00562%
B0141	Group B3	Day 22	Peptide	CD4	+	+	+	0.02682%
B0141	Group B3	Day 22	Peptide	CD4	+	+	-	0.028%
B0141	Group B3	Day 22	Peptide	CD4	+	-	+	0.000619%
B0141	Group B3	Day 22	Peptide	CD4	+	-	-	-0.00516%
B0141	Group B3	Day 22	Peptide	CD4	-	+	+	0.00836%
B0141	Group B3	Day 22	Peptide	CD4	-	+	-	0.015%
B0141	Group B3	Day 22	Peptide	CD4	-	-	+	0.00038%
B0141	Group B3	Day 1	Peptide	CD8	+	+	+	0.0013%
B0141	Group B3	Day 1	Peptide	CD8	+	+	-	0.0013%
B0141	Group B3	Day 1	Peptide	CD8	+	-	+	0%
B0141	Group B3	Day 1	Peptide	CD8	+	-	-	-0.00008%
B0141	Group B3	Day 1	Peptide	CD8	-	+	+	0.00433%

Administration Site Key: FA = Forearm; UA= Upper arm
Treatments Part A: A1 = A/Sing MAP, 15 ug (FA); A2 = Afluria Quad IM (UA), 15 ug; A3 = Placebo MAP (FA); A4 = A/Sing Mono IM, 15 ug (UA)
Treatments Part B: B1 = A/Sing MAP, 15 ug (FA) with biopsy; B2 = Placebo MAP (FA) with biopsy
B3 = A/Sing MAP, 15 ug (FA); B4 = A/Sing MAP, 10 ug (FA); B5 = A/Sing MAP, 5 ug (FA); B6 = A/Sing MAP, 2.5 ug (FA)
B7 = Placebo MAP (FA); B8 = A/Sing MAP, 15 ug (UA); B9 = A/Sing IM, 15 ug, as 2018 Afluria Quad (UA)

Listing 16.2.6.6
Immunogenicity Results (CMI)

Part B

Randomisation Number	Group	Visit	Test	Subset	IL2	TNFa	IFNg	Value
B0141	Group B3	Day 1	Peptide	CD8	-	+	-	0.04%
B0141	Group B3	Day 1	Peptide	CD8	-	-	+	-0.03%
B0141	Group B3	Day 22	Peptide	CD8	+	+	+	0%
B0141	Group B3	Day 22	Peptide	CD8	+	+	-	-0.00157%
B0141	Group B3	Day 22	Peptide	CD8	+	-	+	-0.00157%
B0141	Group B3	Day 22	Peptide	CD8	+	-	-	0.00032%
B0141	Group B3	Day 22	Peptide	CD8	-	+	+	-0.00251%
B0141	Group B3	Day 22	Peptide	CD8	-	+	-	0%
B0141	Group B3	Day 22	Peptide	CD8	-	-	+	-0.15%
B0141	Group B3	Day 1	MPH	CD4	+	+	+	0.05951%
B0141	Group B3	Day 1	MPH	CD4	+	+	-	0.074%
B0141	Group B3	Day 1	MPH	CD4	+	-	+	0.006392%
B0141	Group B3	Day 1	MPH	CD4	+	-	-	0.02602%
B0141	Group B3	Day 1	MPH	CD4	-	+	+	0.03%
B0141	Group B3	Day 1	MPH	CD4	-	+	-	0.257%
B0141	Group B3	Day 1	MPH	CD4	-	-	+	0.02552%
B0141	Group B3	Day 22	MPH	CD4	+	+	+	0.13382%
B0141	Group B3	Day 22	MPH	CD4	+	+	-	0.089%
B0141	Group B3	Day 22	MPH	CD4	+	-	+	0.065049%

Administration Site Key: FA = Forearm; UA= Upper arm
Treatments Part A: A1 = A/Sing MAP, 15 ug (FA); A2 = Afluria Quad IM (UA), 15 ug; A3 = Placebo MAP (FA); A4 = A/Sing Mono IM, 15 ug (UA)
Treatments Part B: B1 = A/Sing MAP, 15 ug (FA) with biopsy; B2 = Placebo MAP (FA) with biopsy
B3 = A/Sing MAP, 15 ug (FA); B4 = A/Sing MAP, 10 ug (FA); B5 = A/Sing MAP, 5 ug (FA); B6 = A/Sing MAP, 2.5 ug (FA)
B7 = Placebo MAP (FA); B8 = A/Sing MAP, 15 ug (UA); B9 = A/Sing IM, 15 ug, as 2018 Afluria Quad (UA)

Listing 16.2.6.6
Immunogenicity Results (CMI)

Part B

Randomisation Number	Group	Visit	Test	Subset	IL2	TNFa	IFNg	Value
B0141	Group B3	Day 22	MPH	CD4	+	-	-	0.071%
B0141	Group B3	Day 22	MPH	CD4	-	+	+	0.067%
B0141	Group B3	Day 22	MPH	CD4	-	+	-	0.241%
B0141	Group B3	Day 22	MPH	CD4	-	-	+	0.0732%
B0141	Group B3	Day 1	MPH	CD8	+	+	+	0.012%
B0141	Group B3	Day 1	MPH	CD8	+	+	-	0.024%
B0141	Group B3	Day 1	MPH	CD8	+	-	+	0.00149%
B0141	Group B3	Day 1	MPH	CD8	+	-	-	0.01762%
B0141	Group B3	Day 1	MPH	CD8	-	+	+	0.04833%
B0141	Group B3	Day 1	MPH	CD8	-	+	-	0.35%
B0141	Group B3	Day 1	MPH	CD8	-	-	+	-0.71%
B0141	Group B3	Day 22	MPH	CD8	+	+	+	0.019%
B0141	Group B3	Day 22	MPH	CD8	+	+	-	0.01743%
B0141	Group B3	Day 22	MPH	CD8	+	-	+	0.00216%
B0141	Group B3	Day 22	MPH	CD8	+	-	-	0.08486%
B0141	Group B3	Day 22	MPH	CD8	-	+	+	0.11057%
B0141	Group B3	Day 22	MPH	CD8	-	+	-	0.5%
B0141	Group B3	Day 22	MPH	CD8	-	-	+	-0.74%

Administration Site Key: FA = Forearm; UA= Upper arm
Treatments Part A: A1 = A/Sing MAP, 15 ug (FA); A2 = Afluria Quad IM (UA), 15 ug; A3 = Placebo MAP (FA); A4 = A/Sing Mono IM, 15 ug (UA)
Treatments Part B: B1 = A/Sing MAP, 15 ug (FA) with biopsy; B2 = Placebo MAP (FA) with biopsy
B3 = A/Sing MAP, 15 ug (FA); B4 = A/Sing MAP, 10 ug (FA); B5 = A/Sing MAP, 5 ug (FA); B6 = A/Sing MAP, 2.5 ug (FA)
B7 = Placebo MAP (FA); B8 = A/Sing MAP, 15 ug (UA); B9 = A/Sing IM, 15 ug, as 2018 Afluria Quad (UA)

Listing 16.2.6.6
Immunogenicity Results (CMI)

Part B

Randomisation Number	Group	Visit	Test	Subset	IL2	TNFa	IFNg	Value
B0145	Group B3	Day 1	Peptide	CD4	+	+	+	0.00306%
B0145	Group B3	Day 1	Peptide	CD4	+	+	-	0.00465%
B0145	Group B3	Day 1	Peptide	CD4	+	-	+	0.000618%
B0145	Group B3	Day 1	Peptide	CD4	+	-	-	0.00055%
B0145	Group B3	Day 1	Peptide	CD4	-	+	+	-0.00074%
B0145	Group B3	Day 1	Peptide	CD4	-	+	-	0%
B0145	Group B3	Day 1	Peptide	CD4	-	-	+	0.00301%
B0145	Group B3	Day 22	Peptide	CD4	+	+	+	0.025198%
B0145	Group B3	Day 22	Peptide	CD4	+	+	-	0.02218%
B0145	Group B3	Day 22	Peptide	CD4	+	-	+	0.000898%
B0145	Group B3	Day 22	Peptide	CD4	+	-	-	-0.00472%
B0145	Group B3	Day 22	Peptide	CD4	-	+	+	0.00606%
B0145	Group B3	Day 22	Peptide	CD4	-	+	-	0.064%
B0145	Group B3	Day 22	Peptide	CD4	-	-	+	0.00035%
B0145	Group B3	Day 1	Peptide	CD8	+	+	+	0%
B0145	Group B3	Day 1	Peptide	CD8	+	+	-	-0.00111%
B0145	Group B3	Day 1	Peptide	CD8	+	-	+	0%
B0145	Group B3	Day 1	Peptide	CD8	+	-	-	0.00216%
B0145	Group B3	Day 1	Peptide	CD8	-	+	+	0.00098%

Administration Site Key: FA = Forearm; UA= Upper arm
Treatments Part A: A1 = A/Sing MAP, 15 ug (FA); A2 = Afluria Quad IM (UA), 15 ug; A3 = Placebo MAP (FA); A4 = A/Sing Mono IM, 15 ug (UA)
Treatments Part B: B1 = A/Sing MAP, 15 ug (FA) with biopsy; B2 = Placebo MAP (FA) with biopsy
B3 = A/Sing MAP, 15 ug (FA); B4 = A/Sing MAP, 10 ug (FA); B5 = A/Sing MAP, 5 ug (FA); B6 = A/Sing MAP, 2.5 ug (FA)
B7 = Placebo MAP (FA); B8 = A/Sing MAP, 15 ug (UA); B9 = A/Sing IM, 15 ug, as 2018 Afluria Quad (UA)

Listing 16.2.6.6
Immunogenicity Results (CMI)

Part B

Randomisation Number	Group	Visit	Test	Subset	IL2	TNFa	IFNg	Value
B0145	Group B3	Day 1	Peptide	CD8	-	+	-	-0.025%
B0145	Group B3	Day 1	Peptide	CD8	-	-	+	0.78%
B0145	Group B3	Day 22	Peptide	CD8	+	+	+	0%
B0145	Group B3	Day 22	Peptide	CD8	+	+	-	-0.00129%
B0145	Group B3	Day 22	Peptide	CD8	+	-	+	0%
B0145	Group B3	Day 22	Peptide	CD8	+	-	-	-0.00113%
B0145	Group B3	Day 22	Peptide	CD8	-	+	+	-0.00516%
B0145	Group B3	Day 22	Peptide	CD8	-	+	-	-0.014%
B0145	Group B3	Day 22	Peptide	CD8	-	-	+	-0.08%
B0145	Group B3	Day 1	MPH	CD4	+	+	+	0.08715%
B0145	Group B3	Day 1	MPH	CD4	+	+	-	0.08865%
B0145	Group B3	Day 1	MPH	CD4	+	-	+	0.014358%
B0145	Group B3	Day 1	MPH	CD4	+	-	-	0.07115%
B0145	Group B3	Day 1	MPH	CD4	-	+	+	0.04586%
B0145	Group B3	Day 1	MPH	CD4	-	+	-	0.228%
B0145	Group B3	Day 1	MPH	CD4	-	-	+	0.06858%
B0145	Group B3	Day 22	MPH	CD4	+	+	+	0.199198%
B0145	Group B3	Day 22	MPH	CD4	+	+	-	0.14118%
B0145	Group B3	Day 22	MPH	CD4	+	-	+	0.035198%

Administration Site Key: FA = Forearm; UA= Upper arm
Treatments Part A: A1 = A/Sing MAP, 15 ug (FA); A2 = Afluria Quad IM (UA), 15 ug; A3 = Placebo MAP (FA); A4 = A/Sing Mono IM, 15 ug (UA)
Treatments Part B: B1 = A/Sing MAP, 15 ug (FA) with biopsy; B2 = Placebo MAP (FA) with biopsy
B3 = A/Sing MAP, 15 ug (FA); B4 = A/Sing MAP, 10 ug (FA); B5 = A/Sing MAP, 5 ug (FA); B6 = A/Sing MAP, 2.5 ug (FA)
B7 = Placebo MAP (FA); B8 = A/Sing MAP, 15 ug (UA); B9 = A/Sing IM, 15 ug, as 2018 Afluria Quad (UA)

Listing 16.2.6.6
Immunogenicity Results (CMI)

Part B

Randomisation Number	Group	Visit	Test	Subset	IL2	TNFa	IFNg	Value
B0145	Group B3	Day 22	MPH	CD4	+	-	-	0.02658%
B0145	Group B3	Day 22	MPH	CD4	-	+	+	0.0824%
B0145	Group B3	Day 22	MPH	CD4	-	+	-	0.31%
B0145	Group B3	Day 22	MPH	CD4	-	-	+	0.11439%
B0145	Group B3	Day 1	MPH	CD8	+	+	+	0.058%
B0145	Group B3	Day 1	MPH	CD8	+	+	-	0.00726%
B0145	Group B3	Day 1	MPH	CD8	+	-	+	0.012%
B0145	Group B3	Day 1	MPH	CD8	+	-	-	0.04689%
B0145	Group B3	Day 1	MPH	CD8	-	+	+	0.08744%
B0145	Group B3	Day 1	MPH	CD8	-	+	-	0.175%
B0145	Group B3	Day 1	MPH	CD8	-	-	+	-0.2%
B0145	Group B3	Day 22	MPH	CD8	+	+	+	0.05%
B0145	Group B3	Day 22	MPH	CD8	+	+	-	0.01171%
B0145	Group B3	Day 22	MPH	CD8	+	-	+	0.032%
B0145	Group B3	Day 22	MPH	CD8	+	-	-	0.02613%
B0145	Group B3	Day 22	MPH	CD8	-	+	+	0.14484%
B0145	Group B3	Day 22	MPH	CD8	-	+	-	0.223%
B0145	Group B3	Day 22	MPH	CD8	-	-	+	-0.06%

Administration Site Key: FA = Forearm; UA= Upper arm
Treatments Part A: A1 = A/Sing MAP, 15 ug (FA); A2 = Afluria Quad IM (UA), 15 ug; A3 = Placebo MAP (FA); A4 = A/Sing Mono IM, 15 ug (UA)
Treatments Part B: B1 = A/Sing MAP, 15 ug (FA) with biopsy; B2 = Placebo MAP (FA) with biopsy
B3 = A/Sing MAP, 15 ug (FA); B4 = A/Sing MAP, 10 ug (FA); B5 = A/Sing MAP, 5 ug (FA); B6 = A/Sing MAP, 2.5 ug (FA)
B7 = Placebo MAP (FA); B8 = A/Sing MAP, 15 ug (UA); B9 = A/Sing IM, 15 ug, as 2018 Afluria Quad (UA)

Listing 16.2.6.6
Immunogenicity Results (CMI)

Part B

Randomisation Number	Group	Visit	Test	Subset	IL2	TNFa	IFNg	Value
B0146	Group B7	Day 1	Peptide	CD4	+	+	+	0.01801%
B0146	Group B7	Day 1	Peptide	CD4	+	+	-	0.024%
B0146	Group B7	Day 1	Peptide	CD4	+	-	+	-0.0008%
B0146	Group B7	Day 1	Peptide	CD4	+	-	-	-0.00357%
B0146	Group B7	Day 1	Peptide	CD4	-	+	+	0.00651%
B0146	Group B7	Day 1	Peptide	CD4	-	+	-	0.033%
B0146	Group B7	Day 1	Peptide	CD4	-	-	+	0%
B0146	Group B7	Day 22	Peptide	CD4	+	+	+	0.00126%
B0146	Group B7	Day 22	Peptide	CD4	+	+	-	0.00407%
B0146	Group B7	Day 22	Peptide	CD4	+	-	+	1E-5%
B0146	Group B7	Day 22	Peptide	CD4	+	-	-	-0.0029%
B0146	Group B7	Day 22	Peptide	CD4	-	+	+	0.00426%
B0146	Group B7	Day 22	Peptide	CD4	-	+	-	0.004%
B0146	Group B7	Day 22	Peptide	CD4	-	-	+	-0.007%
B0146	Group B7	Day 1	Peptide	CD8	+	+	+	0%
B0146	Group B7	Day 1	Peptide	CD8	+	+	-	-0.00114%
B0146	Group B7	Day 1	Peptide	CD8	+	-	+	0%
B0146	Group B7	Day 1	Peptide	CD8	+	-	-	0%
B0146	Group B7	Day 1	Peptide	CD8	-	+	+	0.00614%

Administration Site Key: FA = Forearm; UA= Upper arm
Treatments Part A: A1 = A/Sing MAP, 15 ug (FA); A2 = Afluria Quad IM (UA), 15 ug; A3 = Placebo MAP (FA); A4 = A/Sing Mono IM, 15 ug (UA)
Treatments Part B: B1 = A/Sing MAP, 15 ug (FA) with biopsy; B2 = Placebo MAP (FA) with biopsy
B3 = A/Sing MAP, 15 ug (FA); B4 = A/Sing MAP, 10 ug (FA); B5 = A/Sing MAP, 5 ug (FA); B6 = A/Sing MAP, 2.5 ug (FA)
B7 = Placebo MAP (FA); B8 = A/Sing MAP, 15 ug (UA); B9 = A/Sing IM, 15 ug, as 2018 Afluria Quad (UA)

Listing 16.2.6.6
Immunogenicity Results (CMI)

Part B

Randomisation Number	Group	Visit	Test	Subset	IL2	TNFa	IFNg	Value
B0146	Group B7	Day 1	Peptide	CD8	-	+	-	-0.005%
B0146	Group B7	Day 1	Peptide	CD8	-	-	+	0.04%
B0146	Group B7	Day 22	Peptide	CD8	+	+	+	0%
B0146	Group B7	Day 22	Peptide	CD8	+	+	-	0.00001%
B0146	Group B7	Day 22	Peptide	CD8	+	-	+	0%
B0146	Group B7	Day 22	Peptide	CD8	+	-	-	-0.00102%
B0146	Group B7	Day 22	Peptide	CD8	-	+	+	0.00212%
B0146	Group B7	Day 22	Peptide	CD8	-	+	-	-0.011%
B0146	Group B7	Day 22	Peptide	CD8	-	-	+	-0.05%
B0146	Group B7	Day 1	MPH	CD4	+	+	+	0.21101%
B0146	Group B7	Day 1	MPH	CD4	+	+	-	0.137%
B0146	Group B7	Day 1	MPH	CD4	+	-	+	0.00979%
B0146	Group B7	Day 1	MPH	CD4	+	-	-	0.011%
B0146	Group B7	Day 1	MPH	CD4	-	+	+	0.17551%
B0146	Group B7	Day 1	MPH	CD4	-	+	-	0.741%
B0146	Group B7	Day 1	MPH	CD4	-	-	+	0.206%
B0146	Group B7	Day 22	MPH	CD4	+	+	+	0.12702%
B0146	Group B7	Day 22	MPH	CD4	+	+	-	0.13107%
B0146	Group B7	Day 22	MPH	CD4	+	-	+	0.011405%

Administration Site Key: FA = Forearm; UA= Upper arm
Treatments Part A: A1 = A/Sing MAP, 15 ug (FA); A2 = Afluria Quad IM (UA), 15 ug; A3 = Placebo MAP (FA); A4 = A/Sing Mono IM, 15 ug (UA)
Treatments Part B: B1 = A/Sing MAP, 15 ug (FA) with biopsy; B2 = Placebo MAP (FA) with biopsy
B3 = A/Sing MAP, 15 ug (FA); B4 = A/Sing MAP, 10 ug (FA); B5 = A/Sing MAP, 5 ug (FA); B6 = A/Sing MAP, 2.5 ug (FA)
B7 = Placebo MAP (FA); B8 = A/Sing MAP, 15 ug (UA); B9 = A/Sing IM, 15 ug, as 2018 Afluria Quad (UA)

Listing 16.2.6.6
Immunogenicity Results (CMI)

Part B

Randomisation Number	Group	Visit	Test	Subset	IL2	TNFa	IFNg	Value
B0146	Group B7	Day 22	MPH	CD4	+	-	-	0.02026%
B0146	Group B7	Day 22	MPH	CD4	-	+	+	0.06881%
B0146	Group B7	Day 22	MPH	CD4	-	+	-	0.411%
B0146	Group B7	Day 22	MPH	CD4	-	-	+	0.123%
B0146	Group B7	Day 1	MPH	CD8	+	+	+	0.04%
B0146	Group B7	Day 1	MPH	CD8	+	+	-	0.01886%
B0146	Group B7	Day 1	MPH	CD8	+	-	+	0.00205%
B0146	Group B7	Day 1	MPH	CD8	+	-	-	0.024%
B0146	Group B7	Day 1	MPH	CD8	-	+	+	0.15658%
B0146	Group B7	Day 1	MPH	CD8	-	+	-	1.053%
B0146	Group B7	Day 1	MPH	CD8	-	-	+	0%
B0146	Group B7	Day 22	MPH	CD8	+	+	+	0.024%
B0146	Group B7	Day 22	MPH	CD8	+	+	-	0.01195%
B0146	Group B7	Day 22	MPH	CD8	+	-	+	0.00673%
B0146	Group B7	Day 22	MPH	CD8	+	-	-	0.01186%
B0146	Group B7	Day 22	MPH	CD8	-	+	+	0.069%
B0146	Group B7	Day 22	MPH	CD8	-	+	-	0.297%
B0146	Group B7	Day 22	MPH	CD8	-	-	+	-0.11%

Administration Site Key: FA = Forearm; UA= Upper arm
Treatments Part A: A1 = A/Sing MAP, 15 ug (FA); A2 = Afluria Quad IM (UA), 15 ug; A3 = Placebo MAP (FA); A4 = A/Sing Mono IM, 15 ug (UA)
Treatments Part B: B1 = A/Sing MAP, 15 ug (FA) with biopsy; B2 = Placebo MAP (FA) with biopsy
B3 = A/Sing MAP, 15 ug (FA); B4 = A/Sing MAP, 10 ug (FA); B5 = A/Sing MAP, 5 ug (FA); B6 = A/Sing MAP, 2.5 ug (FA)
B7 = Placebo MAP (FA); B8 = A/Sing MAP, 15 ug (UA); B9 = A/Sing IM, 15 ug, as 2018 Afluria Quad (UA)

Listing 16.2.6.6
Immunogenicity Results (CMI)

Part B

Randomisation Number	Group	Visit	Test	Subset	IL2	TNFa	IFNg	Value
B0147	Group B9	Day 1	Peptide	CD4	+	+	+	-0.0026%
B0147	Group B9	Day 1	Peptide	CD4	+	+	-	0.00546%
B0147	Group B9	Day 1	Peptide	CD4	+	-	+	0.000738%
B0147	Group B9	Day 1	Peptide	CD4	+	-	-	-0.00342%
B0147	Group B9	Day 1	Peptide	CD4	-	+	+	0.003598%
B0147	Group B9	Day 1	Peptide	CD4	-	+	-	0%
B0147	Group B9	Day 1	Peptide	CD4	-	-	+	0.00018%
B0147	Group B9	Day 22	Peptide	CD4	+	+	+	0.01166%
B0147	Group B9	Day 22	Peptide	CD4	+	+	-	0.00896%
B0147	Group B9	Day 22	Peptide	CD4	+	-	+	0.00095%
B0147	Group B9	Day 22	Peptide	CD4	+	-	-	0.002%
B0147	Group B9	Day 22	Peptide	CD4	-	+	+	0.00665%
B0147	Group B9	Day 22	Peptide	CD4	-	+	-	0.009%
B0147	Group B9	Day 22	Peptide	CD4	-	-	+	0.0057%
B0147	Group B9	Day 1	Peptide	CD8	+	+	+	0.00005%
B0147	Group B9	Day 1	Peptide	CD8	+	+	-	-0.00117%
B0147	Group B9	Day 1	Peptide	CD8	+	-	+	0%
B0147	Group B9	Day 1	Peptide	CD8	+	-	-	0.00249%
B0147	Group B9	Day 1	Peptide	CD8	-	+	+	-0.001%

Administration Site Key: FA = Forearm; UA= Upper arm
Treatments Part A: A1 = A/Sing MAP, 15 ug (FA); A2 = Afluria Quad IM (UA), 15 ug; A3 = Placebo MAP (FA); A4 = A/Sing Mono IM, 15 ug (UA)
Treatments Part B: B1 = A/Sing MAP, 15 ug (FA) with biopsy; B2 = Placebo MAP (FA) with biopsy
B3 = A/Sing MAP, 15 ug (FA); B4 = A/Sing MAP, 10 ug (FA); B5 = A/Sing MAP, 5 ug (FA); B6 = A/Sing MAP, 2.5 ug (FA)
B7 = Placebo MAP (FA); B8 = A/Sing MAP, 15 ug (UA); B9 = A/Sing IM, 15 ug, as 2018 Afluria Quad (UA)

Listing 16.2.6.6
Immunogenicity Results (CMI)

Part B

Randomisation Number	Group	Visit	Test	Subset	IL2	TNFa	IFNg	Value
B0147	Group B9	Day 1	Peptide	CD8	-	+	-	-0.03%
B0147	Group B9	Day 1	Peptide	CD8	-	-	+	0.03%
B0147	Group B9	Day 22	Peptide	CD8	+	+	+	0%
B0147	Group B9	Day 22	Peptide	CD8	+	+	-	0%
B0147	Group B9	Day 22	Peptide	CD8	+	-	+	0%
B0147	Group B9	Day 22	Peptide	CD8	+	-	-	0%
B0147	Group B9	Day 22	Peptide	CD8	-	+	+	-0.02489%
B0147	Group B9	Day 22	Peptide	CD8	-	+	-	-0.03%
B0147	Group B9	Day 22	Peptide	CD8	-	-	+	-0.89%
B0147	Group B9	Day 1	MPH	CD4	+	+	+	0.11239%
B0147	Group B9	Day 1	MPH	CD4	+	+	-	0.10446%
B0147	Group B9	Day 1	MPH	CD4	+	-	+	0.007188%
B0147	Group B9	Day 1	MPH	CD4	+	-	-	0.032%
B0147	Group B9	Day 1	MPH	CD4	-	+	+	0.063308%
B0147	Group B9	Day 1	MPH	CD4	-	+	-	0.292%
B0147	Group B9	Day 1	MPH	CD4	-	-	+	0.04146%
B0147	Group B9	Day 22	MPH	CD4	+	+	+	0.12866%
B0147	Group B9	Day 22	MPH	CD4	+	+	-	0.11196%
B0147	Group B9	Day 22	MPH	CD4	+	-	+	0.021%

Administration Site Key: FA = Forearm; UA= Upper arm
Treatments Part A: A1 = A/Sing MAP, 15 ug (FA); A2 = Afluria Quad IM (UA), 15 ug; A3 = Placebo MAP (FA); A4 = A/Sing Mono IM, 15 ug (UA)
Treatments Part B: B1 = A/Sing MAP, 15 ug (FA) with biopsy; B2 = Placebo MAP (FA) with biopsy
B3 = A/Sing MAP, 15 ug (FA); B4 = A/Sing MAP, 10 ug (FA); B5 = A/Sing MAP, 5 ug (FA); B6 = A/Sing MAP, 2.5 ug (FA)
B7 = Placebo MAP (FA); B8 = A/Sing MAP, 15 ug (UA); B9 = A/Sing IM, 15 ug, as 2018 Afluria Quad (UA)

Listing 16.2.6.6
Immunogenicity Results (CMI)

Part B

Randomisation Number	Group	Visit	Test	Subset	IL2	TNFa	IFNg	Value
B0147	Group B9	Day 22	MPH	CD4	+	-	-	0.033%
B0147	Group B9	Day 22	MPH	CD4	-	+	+	0.064%
B0147	Group B9	Day 22	MPH	CD4	-	+	-	0.218%
B0147	Group B9	Day 22	MPH	CD4	-	-	+	0.047%
B0147	Group B9	Day 1	MPH	CD8	+	+	+	0.01683%
B0147	Group B9	Day 1	MPH	CD8	+	+	-	0.03183%
B0147	Group B9	Day 1	MPH	CD8	+	-	+	0.00783%
B0147	Group B9	Day 1	MPH	CD8	+	-	-	0.06183%
B0147	Group B9	Day 1	MPH	CD8	-	+	+	0.073%
B0147	Group B9	Day 1	MPH	CD8	-	+	-	0.27%
B0147	Group B9	Day 1	MPH	CD8	-	-	+	-2.1%
B0147	Group B9	Day 22	MPH	CD8	+	+	+	0.019%
B0147	Group B9	Day 22	MPH	CD8	+	+	-	0.019%
B0147	Group B9	Day 22	MPH	CD8	+	-	+	0.00515%
B0147	Group B9	Day 22	MPH	CD8	+	-	-	0.028%
B0147	Group B9	Day 22	MPH	CD8	-	+	+	0.025%
B0147	Group B9	Day 22	MPH	CD8	-	+	-	0.095%
B0147	Group B9	Day 22	MPH	CD8	-	-	+	-1.28%

Administration Site Key: FA = Forearm; UA= Upper arm
Treatments Part A: A1 = A/Sing MAP, 15 ug (FA); A2 = Afluria Quad IM (UA), 15 ug; A3 = Placebo MAP (FA); A4 = A/Sing Mono IM, 15 ug (UA)
Treatments Part B: B1 = A/Sing MAP, 15 ug (FA) with biopsy; B2 = Placebo MAP (FA) with biopsy
B3 = A/Sing MAP, 15 ug (FA); B4 = A/Sing MAP, 10 ug (FA); B5 = A/Sing MAP, 5 ug (FA); B6 = A/Sing MAP, 2.5 ug (FA)
B7 = Placebo MAP (FA); B8 = A/Sing MAP, 15 ug (UA); B9 = A/Sing IM, 15 ug, as 2018 Afluria Quad (UA)

Listing 16.2.6.6
Immunogenicity Results (CMI)

Part B

Randomisation Number	Group	Visit	Test	Subset	IL2	TNFa	IFNg	Value
B0149	Group B8	Day 1	Peptide	CD4	+	+	+	0.00674%
B0149	Group B8	Day 1	Peptide	CD4	+	+	-	0.00916%
B0149	Group B8	Day 1	Peptide	CD4	+	-	+	-0.000692%
B0149	Group B8	Day 1	Peptide	CD4	+	-	-	0.00458%
B0149	Group B8	Day 1	Peptide	CD4	-	+	+	0.00441%
B0149	Group B8	Day 1	Peptide	CD4	-	+	-	0.02%
B0149	Group B8	Day 1	Peptide	CD4	-	-	+	0.00021%
B0149	Group B8	Day 22	Peptide	CD4	+	+	+	0.02253%
B0149	Group B8	Day 22	Peptide	CD4	+	+	-	0.0204%
B0149	Group B8	Day 22	Peptide	CD4	+	-	+	0.00058%
B0149	Group B8	Day 22	Peptide	CD4	+	-	-	-0.00109%
B0149	Group B8	Day 22	Peptide	CD4	-	+	+	0.00627%
B0149	Group B8	Day 22	Peptide	CD4	-	+	-	0.027%
B0149	Group B8	Day 22	Peptide	CD4	-	-	+	-0.00038%
B0149	Group B8	Day 1	Peptide	CD8	+	+	+	0.00501%
B0149	Group B8	Day 1	Peptide	CD8	+	+	-	0.00164%
B0149	Group B8	Day 1	Peptide	CD8	+	-	+	0%
B0149	Group B8	Day 1	Peptide	CD8	+	-	-	0.00185%
B0149	Group B8	Day 1	Peptide	CD8	-	+	+	0%

Administration Site Key: FA = Forearm; UA= Upper arm
Treatments Part A: A1 = A/Sing MAP, 15 ug (FA); A2 = Afluria Quad IM (UA), 15 ug; A3 = Placebo MAP (FA); A4 = A/Sing Mono IM, 15 ug (UA)
Treatments Part B: B1 = A/Sing MAP, 15 ug (FA) with biopsy; B2 = Placebo MAP (FA) with biopsy
B3 = A/Sing MAP, 15 ug (FA); B4 = A/Sing MAP, 10 ug (FA); B5 = A/Sing MAP, 5 ug (FA); B6 = A/Sing MAP, 2.5 ug (FA)
B7 = Placebo MAP (FA); B8 = A/Sing MAP, 15 ug (UA); B9 = A/Sing IM, 15 ug, as 2018 Afluria Quad (UA)

Listing 16.2.6.6
Immunogenicity Results (CMI)

Part B

Randomisation Number	Group	Visit	Test	Subset	IL2	TNFa	IFNg	Value
B0149	Group B8	Day 1	Peptide	CD8	-	+	-	0.004%
B0149	Group B8	Day 1	Peptide	CD8	-	-	+	-0.07%
B0149	Group B8	Day 22	Peptide	CD8	+	+	+	0.00907%
B0149	Group B8	Day 22	Peptide	CD8	+	+	-	0.00254%
B0149	Group B8	Day 22	Peptide	CD8	+	-	+	0.0013%
B0149	Group B8	Day 22	Peptide	CD8	+	-	-	0.00259%
B0149	Group B8	Day 22	Peptide	CD8	-	+	+	0.006%
B0149	Group B8	Day 22	Peptide	CD8	-	+	-	0.025%
B0149	Group B8	Day 22	Peptide	CD8	-	-	+	0.12%
B0149	Group B8	Day 1	MPH	CD4	+	+	+	0.06292%
B0149	Group B8	Day 1	MPH	CD4	+	+	-	0.06116%
B0149	Group B8	Day 1	MPH	CD4	+	-	+	0.008678%
B0149	Group B8	Day 1	MPH	CD4	+	-	-	0.04023%
B0149	Group B8	Day 1	MPH	CD4	-	+	+	0.035%
B0149	Group B8	Day 1	MPH	CD4	-	+	-	0.233%
B0149	Group B8	Day 1	MPH	CD4	-	-	+	0.02654%
B0149	Group B8	Day 22	MPH	CD4	+	+	+	0.09253%
B0149	Group B8	Day 22	MPH	CD4	+	+	-	0.0894%
B0149	Group B8	Day 22	MPH	CD4	+	-	+	0.017%

Administration Site Key: FA = Forearm; UA= Upper arm
Treatments Part A: A1 = A/Sing MAP, 15 ug (FA); A2 = Afluria Quad IM (UA), 15 ug; A3 = Placebo MAP (FA); A4 = A/Sing Mono IM, 15 ug (UA)
Treatments Part B: B1 = A/Sing MAP, 15 ug (FA) with biopsy; B2 = Placebo MAP (FA) with biopsy
B3 = A/Sing MAP, 15 ug (FA); B4 = A/Sing MAP, 10 ug (FA); B5 = A/Sing MAP, 5 ug (FA); B6 = A/Sing MAP, 2.5 ug (FA)
B7 = Placebo MAP (FA); B8 = A/Sing MAP, 15 ug (UA); B9 = A/Sing IM, 15 ug, as 2018 Afluria Quad (UA)

Listing 16.2.6.6
Immunogenicity Results (CMI)

Part B

Randomisation Number	Group	Visit	Test	Subset	IL2	TNFa	IFNg	Value
B0149	Group B8	Day 22	MPH	CD4	+	-	-	0.03953%
B0149	Group B8	Day 22	MPH	CD4	-	+	+	0.04727%
B0149	Group B8	Day 22	MPH	CD4	-	+	-	0.258%
B0149	Group B8	Day 22	MPH	CD4	-	-	+	0.0614%
B0149	Group B8	Day 1	MPH	CD8	+	+	+	0.00309%
B0149	Group B8	Day 1	MPH	CD8	+	+	-	0.00769%
B0149	Group B8	Day 1	MPH	CD8	+	-	+	0%
B0149	Group B8	Day 1	MPH	CD8	+	-	-	0.01494%
B0149	Group B8	Day 1	MPH	CD8	-	+	+	0.015%
B0149	Group B8	Day 1	MPH	CD8	-	+	-	0.222%
B0149	Group B8	Day 1	MPH	CD8	-	-	+	-0.55%
B0149	Group B8	Day 22	MPH	CD8	+	+	+	0.00511%
B0149	Group B8	Day 22	MPH	CD8	+	+	-	0.00376%
B0149	Group B8	Day 22	MPH	CD8	+	-	+	0.00383%
B0149	Group B8	Day 22	MPH	CD8	+	-	-	0.024%
B0149	Group B8	Day 22	MPH	CD8	-	+	+	0.024%
B0149	Group B8	Day 22	MPH	CD8	-	+	-	0.227%
B0149	Group B8	Day 22	MPH	CD8	-	-	+	-0.55%

Administration Site Key: FA = Forearm; UA= Upper arm
Treatments Part A: A1 = A/Sing MAP, 15 ug (FA); A2 = Afluria Quad IM (UA), 15 ug; A3 = Placebo MAP (FA); A4 = A/Sing Mono IM, 15 ug (UA)
Treatments Part B: B1 = A/Sing MAP, 15 ug (FA) with biopsy; B2 = Placebo MAP (FA) with biopsy
B3 = A/Sing MAP, 15 ug (FA); B4 = A/Sing MAP, 10 ug (FA); B5 = A/Sing MAP, 5 ug (FA); B6 = A/Sing MAP, 2.5 ug (FA)
B7 = Placebo MAP (FA); B8 = A/Sing MAP, 15 ug (UA); B9 = A/Sing IM, 15 ug, as 2018 Afluria Quad (UA)

Listing 16.2.6.6
Immunogenicity Results (CMI)

Part B

Randomisation Number	Group	Visit	Test	Subset	IL2	TNFa	IFNg	Value
B1019	Group B8	Day 1	Peptide	CD4	+	+	+	0.01015%
B1019	Group B8	Day 1	Peptide	CD4	+	+	-	0.06412%
B1019	Group B8	Day 1	Peptide	CD4	+	-	+	0.00307%
B1019	Group B8	Day 1	Peptide	CD4	+	-	-	0.01712%
B1019	Group B8	Day 1	Peptide	CD4	-	+	+	0.02631%
B1019	Group B8	Day 1	Peptide	CD4	-	+	-	0.224%
B1019	Group B8	Day 1	Peptide	CD4	-	-	+	0.04909%
B1019	Group B8	Day 22	Peptide	CD4	+	+	+	0.02556%
B1019	Group B8	Day 22	Peptide	CD4	+	+	-	0.083%
B1019	Group B8	Day 22	Peptide	CD4	+	-	+	0.005002%
B1019	Group B8	Day 22	Peptide	CD4	+	-	-	0.129%
B1019	Group B8	Day 22	Peptide	CD4	-	+	+	0.03226%
B1019	Group B8	Day 22	Peptide	CD4	-	+	-	0.305%
B1019	Group B8	Day 22	Peptide	CD4	-	-	+	0.07554%
B1019	Group B8	Day 1	Peptide	CD8	+	+	+	0%
B1019	Group B8	Day 1	Peptide	CD8	+	+	-	0%
B1019	Group B8	Day 1	Peptide	CD8	+	-	+	0%
B1019	Group B8	Day 1	Peptide	CD8	+	-	-	0.00151%
B1019	Group B8	Day 1	Peptide	CD8	-	+	+	0.00151%

Administration Site Key: FA = Forearm; UA= Upper arm
Treatments Part A: A1 = A/Sing MAP, 15 ug (FA); A2 = Afluria Quad IM (UA), 15 ug; A3 = Placebo MAP (FA); A4 = A/Sing Mono IM, 15 ug (UA)
Treatments Part B: B1 = A/Sing MAP, 15 ug (FA) with biopsy; B2 = Placebo MAP (FA) with biopsy
B3 = A/Sing MAP, 15 ug (FA); B4 = A/Sing MAP, 10 ug (FA); B5 = A/Sing MAP, 5 ug (FA); B6 = A/Sing MAP, 2.5 ug (FA)
B7 = Placebo MAP (FA); B8 = A/Sing MAP, 15 ug (UA); B9 = A/Sing IM, 15 ug, as 2018 Afluria Quad (UA)

Listing 16.2.6.6
Immunogenicity Results (CMI)

Part B

Randomisation Number	Group	Visit	Test	Subset	IL2	TNFa	IFNg	Value
B1019	Group B8	Day 1	Peptide	CD8	-	+	-	-0.033%
B1019	Group B8	Day 1	Peptide	CD8	-	-	+	0.38%
B1019	Group B8	Day 22	Peptide	CD8	+	+	+	0%
B1019	Group B8	Day 22	Peptide	CD8	+	+	-	0%
B1019	Group B8	Day 22	Peptide	CD8	+	-	+	0%
B1019	Group B8	Day 22	Peptide	CD8	+	-	-	0.00448%
B1019	Group B8	Day 22	Peptide	CD8	-	+	+	-0.00925%
B1019	Group B8	Day 22	Peptide	CD8	-	+	-	0.022%
B1019	Group B8	Day 22	Peptide	CD8	-	-	+	0.17%
B1019	Group B8	Day 1	MPH	CD4	+	+	+	0.01015%
B1019	Group B8	Day 1	MPH	CD4	+	+	-	0.06412%
B1019	Group B8	Day 1	MPH	CD4	+	-	+	0.00307%
B1019	Group B8	Day 1	MPH	CD4	+	-	-	0.01712%
B1019	Group B8	Day 1	MPH	CD4	-	+	+	0.02631%
B1019	Group B8	Day 1	MPH	CD4	-	+	-	0.224%
B1019	Group B8	Day 1	MPH	CD4	-	-	+	0.04909%
B1019	Group B8	Day 22	MPH	CD4	+	+	+	0.02556%
B1019	Group B8	Day 22	MPH	CD4	+	+	-	0.083%
B1019	Group B8	Day 22	MPH	CD4	+	-	+	0.005002%

Administration Site Key: FA = Forearm; UA= Upper arm
Treatments Part A: A1 = A/Sing MAP, 15 ug (FA); A2 = Afluria Quad IM (UA), 15 ug; A3 = Placebo MAP (FA); A4 = A/Sing Mono IM, 15 ug (UA)
Treatments Part B: B1 = A/Sing MAP, 15 ug (FA) with biopsy; B2 = Placebo MAP (FA) with biopsy
B3 = A/Sing MAP, 15 ug (FA); B4 = A/Sing MAP, 10 ug (FA); B5 = A/Sing MAP, 5 ug (FA); B6 = A/Sing MAP, 2.5 ug (FA)
B7 = Placebo MAP (FA); B8 = A/Sing MAP, 15 ug (UA); B9 = A/Sing IM, 15 ug, as 2018 Afluria Quad (UA)

Listing 16.2.6.6
Immunogenicity Results (CMI)

Part B

Randomisation Number	Group	Visit	Test	Subset	IL2	TNFa	IFNg	Value
B1019	Group B8	Day 22	MPH	CD4	+	-	-	0.129%
B1019	Group B8	Day 22	MPH	CD4	-	+	+	0.03226%
B1019	Group B8	Day 22	MPH	CD4	-	+	-	0.305%
B1019	Group B8	Day 22	MPH	CD4	-	-	+	0.07554%
B1019	Group B8	Day 1	MPH	CD8	+	+	+	0.017%
B1019	Group B8	Day 1	MPH	CD8	+	+	-	0.00643%
B1019	Group B8	Day 1	MPH	CD8	+	-	+	0.00429%
B1019	Group B8	Day 1	MPH	CD8	+	-	-	0.00155%
B1019	Group B8	Day 1	MPH	CD8	-	+	+	0.03626%
B1019	Group B8	Day 1	MPH	CD8	-	+	-	0.098%
B1019	Group B8	Day 1	MPH	CD8	-	-	+	-0.45%
B1019	Group B8	Day 22	MPH	CD8	+	+	+	0.018%
B1019	Group B8	Day 22	MPH	CD8	+	+	-	0.011%
B1019	Group B8	Day 22	MPH	CD8	+	-	+	0.00881%
B1019	Group B8	Day 22	MPH	CD8	+	-	-	0.13%
B1019	Group B8	Day 22	MPH	CD8	-	+	+	0.08575%
B1019	Group B8	Day 22	MPH	CD8	-	+	-	0.28%
B1019	Group B8	Day 22	MPH	CD8	-	-	+	0.77%

Administration Site Key: FA = Forearm; UA= Upper arm
Treatments Part A: A1 = A/Sing MAP, 15 ug (FA); A2 = Afluria Quad IM (UA), 15 ug; A3 = Placebo MAP (FA); A4 = A/Sing Mono IM, 15 ug (UA)
Treatments Part B: B1 = A/Sing MAP, 15 ug (FA) with biopsy; B2 = Placebo MAP (FA) with biopsy
B3 = A/Sing MAP, 15 ug (FA); B4 = A/Sing MAP, 10 ug (FA); B5 = A/Sing MAP, 5 ug (FA); B6 = A/Sing MAP, 2.5 ug (FA)
B7 = Placebo MAP (FA); B8 = A/Sing MAP, 15 ug (UA); B9 = A/Sing IM, 15 ug, as 2018 Afluria Quad (UA)

Listing 16.2.6.6
Immunogenicity Results (CMI)

Part B

Randomisation Number	Group	Visit	Test	Subset	IL2	TNFa	IFNg	Value
B1080	Group B7	Day 1	Peptide	CD4	+	+	+	0.00652%
B1080	Group B7	Day 1	Peptide	CD4	+	+	-	0.009%
B1080	Group B7	Day 1	Peptide	CD4	+	-	+	1E-5%
B1080	Group B7	Day 1	Peptide	CD4	+	-	-	0.00614%
B1080	Group B7	Day 1	Peptide	CD4	-	+	+	0.0033%
B1080	Group B7	Day 1	Peptide	CD4	-	+	-	0.013%
B1080	Group B7	Day 1	Peptide	CD4	-	-	+	-0.00159%
B1080	Group B7	Day 22	Peptide	CD4	+	+	+	0.00662%
B1080	Group B7	Day 22	Peptide	CD4	+	+	-	0.009%
B1080	Group B7	Day 22	Peptide	CD4	+	-	+	-0.00074%
B1080	Group B7	Day 22	Peptide	CD4	+	-	-	0.00774%
B1080	Group B7	Day 22	Peptide	CD4	-	+	+	0.00192%
B1080	Group B7	Day 22	Peptide	CD4	-	+	-	0.01%
B1080	Group B7	Day 22	Peptide	CD4	-	-	+	0.0026%
B1080	Group B7	Day 1	Peptide	CD8	+	+	+	0%
B1080	Group B7	Day 1	Peptide	CD8	+	+	-	0%
B1080	Group B7	Day 1	Peptide	CD8	+	-	+	0%
B1080	Group B7	Day 1	Peptide	CD8	+	-	-	-0.0011%
B1080	Group B7	Day 1	Peptide	CD8	-	+	+	0.00353%

Administration Site Key: FA = Forearm; UA= Upper arm
Treatments Part A: A1 = A/Sing MAP, 15 ug (FA); A2 = Afluria Quad IM (UA), 15 ug; A3 = Placebo MAP (FA); A4 = A/Sing Mono IM, 15 ug (UA)
Treatments Part B: B1 = A/Sing MAP, 15 ug (FA) with biopsy; B2 = Placebo MAP (FA) with biopsy
B3 = A/Sing MAP, 15 ug (FA); B4 = A/Sing MAP, 10 ug (FA); B5 = A/Sing MAP, 5 ug (FA); B6 = A/Sing MAP, 2.5 ug (FA)
B7 = Placebo MAP (FA); B8 = A/Sing MAP, 15 ug (UA); B9 = A/Sing IM, 15 ug, as 2018 Afluria Quad (UA)

Listing 16.2.6.6
Immunogenicity Results (CMI)

Part B

Randomisation Number	Group	Visit	Test	Subset	IL2	TNFa	IFNg	Value
B1080	Group B7	Day 1	Peptide	CD8	-	+	-	-0.003%
B1080	Group B7	Day 1	Peptide	CD8	-	-	+	-0.23%
B1080	Group B7	Day 22	Peptide	CD8	+	+	+	0.0011%
B1080	Group B7	Day 22	Peptide	CD8	+	+	-	0%
B1080	Group B7	Day 22	Peptide	CD8	+	-	+	0%
B1080	Group B7	Day 22	Peptide	CD8	+	-	-	0.00103%
B1080	Group B7	Day 22	Peptide	CD8	-	+	+	-0.00008%
B1080	Group B7	Day 22	Peptide	CD8	-	+	-	-0.008%
B1080	Group B7	Day 22	Peptide	CD8	-	-	+	0.26%
B1080	Group B7	Day 1	MPH	CD4	+	+	+	0.16352%
B1080	Group B7	Day 1	MPH	CD4	+	+	-	0.065%
B1080	Group B7	Day 1	MPH	CD4	+	-	+	0.022189%
B1080	Group B7	Day 1	MPH	CD4	+	-	-	0.02214%
B1080	Group B7	Day 1	MPH	CD4	-	+	+	0.06038%
B1080	Group B7	Day 1	MPH	CD4	-	+	-	0.212%
B1080	Group B7	Day 1	MPH	CD4	-	-	+	0.21595%
B1080	Group B7	Day 22	MPH	CD4	+	+	+	0.13362%
B1080	Group B7	Day 22	MPH	CD4	+	+	-	0.068%
B1080	Group B7	Day 22	MPH	CD4	+	-	+	0.01658%

Administration Site Key: FA = Forearm; UA= Upper arm
Treatments Part A: A1 = A/Sing MAP, 15 ug (FA); A2 = Afluria Quad IM (UA), 15 ug; A3 = Placebo MAP (FA); A4 = A/Sing Mono IM, 15 ug (UA)
Treatments Part B: B1 = A/Sing MAP, 15 ug (FA) with biopsy; B2 = Placebo MAP (FA) with biopsy
B3 = A/Sing MAP, 15 ug (FA); B4 = A/Sing MAP, 10 ug (FA); B5 = A/Sing MAP, 5 ug (FA); B6 = A/Sing MAP, 2.5 ug (FA)
B7 = Placebo MAP (FA); B8 = A/Sing MAP, 15 ug (UA); B9 = A/Sing IM, 15 ug, as 2018 Afluria Quad (UA)

Listing 16.2.6.6
Immunogenicity Results (CMI)

Part B

Randomisation Number	Group	Visit	Test	Subset	IL2	TNFa	IFNg	Value
B1080	Group B7	Day 22	MPH	CD4	+	-	-	0.02474%
B1080	Group B7	Day 22	MPH	CD4	-	+	+	0.05516%
B1080	Group B7	Day 22	MPH	CD4	-	+	-	0.244%
B1080	Group B7	Day 22	MPH	CD4	-	-	+	0.14716%
B1080	Group B7	Day 1	MPH	CD8	+	+	+	0.083%
B1080	Group B7	Day 1	MPH	CD8	+	+	-	0.016%
B1080	Group B7	Day 1	MPH	CD8	+	-	+	0.014%
B1080	Group B7	Day 1	MPH	CD8	+	-	-	0.00957%
B1080	Group B7	Day 1	MPH	CD8	-	+	+	0.29771%
B1080	Group B7	Day 1	MPH	CD8	-	+	-	0.411%
B1080	Group B7	Day 1	MPH	CD8	-	-	+	-0.14%
B1080	Group B7	Day 22	MPH	CD8	+	+	+	0.08%
B1080	Group B7	Day 22	MPH	CD8	+	+	-	0.00105%
B1080	Group B7	Day 22	MPH	CD8	+	-	+	0.012%
B1080	Group B7	Day 22	MPH	CD8	+	-	-	0.01172%
B1080	Group B7	Day 22	MPH	CD8	-	+	+	0.29772%
B1080	Group B7	Day 22	MPH	CD8	-	+	-	0.39%
B1080	Group B7	Day 22	MPH	CD8	-	-	+	0.02%

Administration Site Key: FA = Forearm; UA= Upper arm
Treatments Part A: A1 = A/Sing MAP, 15 ug (FA); A2 = Afluria Quad IM (UA), 15 ug; A3 = Placebo MAP (FA); A4 = A/Sing Mono IM, 15 ug (UA)
Treatments Part B: B1 = A/Sing MAP, 15 ug (FA) with biopsy; B2 = Placebo MAP (FA) with biopsy
B3 = A/Sing MAP, 15 ug (FA); B4 = A/Sing MAP, 10 ug (FA); B5 = A/Sing MAP, 5 ug (FA); B6 = A/Sing MAP, 2.5 ug (FA)
B7 = Placebo MAP (FA); B8 = A/Sing MAP, 15 ug (UA); B9 = A/Sing IM, 15 ug, as 2018 Afluria Quad (UA)

Listing 16.2.6.6
Immunogenicity Results (CMI)

Part B

Administration Site Key: FA = Forearm; UA= Upper arm
Treatments Part A: A1 = A/Sing MAP, 15 ug (FA); A2 = Afluria Quad IM (UA), 15 ug; A3 = Placebo MAP (FA); A4 = A/Sing Mono IM, 15 ug (UA)
Treatments Part B: B1 = A/Sing MAP, 15 ug (FA) with biopsy; B2 = Placebo MAP (FA) with biopsy
B3 = A/Sing MAP, 15 ug (FA); B4 = A/Sing MAP, 10 ug (FA); B5 = A/Sing MAP, 5 ug (FA); B6 = A/Sing MAP, 2.5 ug (FA)
B7 = Placebo MAP (FA); B8 = A/Sing MAP, 15 ug (UA); B9 = A/Sing IM, 15 ug, as 2018 Afluria Quad (UA)
